# Supplementary material for: Taxonomic Re-Evaluation and Genomic Comparison of Novel Extracellular Electron Uptake-Capable Rhodovulum visakhapatnamense and Rhodovulum sulfidophilum Isolates
Source: Microorganisms. 2022 Jun 16;10(6):1235. doi: 10.3390/microorganisms10061235 (PMC9230146; doi:10.3390/microorganisms10061235)
Supplement: Supplementary file 1 [file Supplemental_Materials_File S2.pdf › Supplemental_Materials_File S2.pdf]

>OLS42237.1 transposase [Rhodovulum sulfidophilum]AB14
MSKTMDDSIKRWTAKRKTALVIEIIQGKTTVSEASRSFDLTPSEIEGWVEDAKRGMENSLRANPLDIREQYEKQLRDLQE
AYGEAMLELRARKKLQALLDAQDGN
>OLS42238.1 transposase [Rhodovulum sulfidophilum]AB14
MIRRTQQGLIAEGVVVSVAKLCAWFGIPRRTVYYKPVKAAPKVEARFSEPIKKLIEEEPSFGYRTVAWLLGFNKNTVQRI
FQIKGWQVRKRAVGMRPRIQAVPSVAAAPNERWSTDLARIWTGKDGWASLALVIDCHTRELLGWHLSKSGKATTAS
AALE
HALIARFGTLGKVEKEFLLRSDNGLVFTSHHFTKIARSYGLKQEFITPHCPQQNGMVERFIRTLKEQCVHRHRFETIQHA
MRVIGDWISFYNNRRPHQALAMRTPAEAFTLAA
>OLS42239.1 hypothetical protein BV379_20765 [Rhodovulum sulfidophilum]AB14
MTPSEACDLLQRMFAAFMNPNTKQDDLAEFLTPDYVQRVDGKQLDYSEFLMHHQALKGAISSGSVNFEHFVTDGVS
AATV
HIAEAVKRNGERIRLRVIAYYQFCGNRISLVDELTQLLDGDQEDRDLGSRTVPA
>OLS42304.1 hypothetical protein BV379_20740 [Rhodovulum sulfidophilum]AB14
MSGFFNQELRIGRISSALGEGALALCHFESADQLNVFSASRSGDRMRHAAAGTALYAGLRSRSGLSGMDLLK
>OLS42305.1 hypothetical protein BV379_20745 [Rhodovulum sulfidophilum]AB14
MTADFWMDLSGGKPGGQMSSDGLSALHIETLTNFRPLDSQFGKWPTRTVPEDLMPHLFPAGDDGDSVLALIDAARIP
MLP
EVLEASGLDHRCLFKGDARDEWGDAAPWLVRLEQESDLTRKLFTGGKSAGQYWNNAAAIFIRSSRSLDMIWSHLRKFT
KF
QDAGGRWIFFRFWAPPYLARYLKGHDEAATPFLENFLLDMTFILPEPVRGMVSIAHVSHPDLHRDIRTGNSPGRWPGF
RR
DIARIRLDLFVEKLDRKLTKVIPSTTSVSEDRRLTSLHRMARSAISLGLREERAVERYCLSRLMLGFPPEQDRRFDGILQ
GSAHDLDRSRDMLALAISIGKEA
>OLS42306.1 hypothetical protein BV379_20750 [Rhodovulum sulfidophilum]AB14
MTTVNCDDPLARCEMGNLFRVNSPSGGLLMEGVVNGEATGILPSGERMILPNTLTGTTNSLDVILNGTTYPVCSRLQEF
T
YSRP
>OLS42307.1 hypothetical protein BV379_20755, partial [Rhodovulum sulfidophilum]AB14
MLQAPPPLSLDEGEIDVDTAPDDVLLAIARAILVTLEIIDEISGAQDLYESSGHDIDLRMTGTDGAPAIAEVVAAVRSMN
GRRAIRALVDGGKGALRVAR
>OLS42308.1 hypothetical protein BV379_20715 [Rhodovulum sulfidophilum]AB14
MADPALFPDDPIATYFLALTPGLFKALHLPAVWLGIDVGAWNLLVVSPLSIALFLAAANTFSARMQLARPERHALVLLLL
AAVYSDVTSGLPRGFGPTIILGLLGLFMARRRIALGIAMAAGANIYPIAALVGGGAIGFNAVLRSVSVRRIDRNDAILVL
IAAAAGIVGLSPFLASTDGAGATFTLAEARDIPIFGPGGRTSFFRDSWVDMLLCSSQRSGLLPTCAGGPGWPPLALTAAV
FIMGALLLRRRDVPRDVGTALISLVLSGLVLTALAYVFAFKGHLPGRYSRLSLEQVYAFSWVFAVTSGIVWAARRTGLAE
RRLFAGLVVLLLAAALVGTRPSAEFIRDPAPLISAALRSQPPETVVAGFSRYVDSVPALTGRKVFASLELTVPYKKPYYY
RIEQKMADMGALYRGPVSSGWRALLDRSGIDFFILDSEASARARQWYVSFPVLKGSREQSVFVSNPDAVRSCTFEDQG
SL
KMISAGCFADRALE
>OLS42309.1 hypothetical protein BV379_20720 [Rhodovulum sulfidophilum]AB14
MKRLVSVLVSLIFLAVVYSLLDVDAIIGALRRTDPGMLGLSLILLVGLIFVSALRLSILSRTGGLPLGRRKAIQATFAAN
ALNMFLPGKLGDILKASLMAEDDSAQLAKATSLGIWEKISDLAALFLMASVPLALTSPSSLAWLPLGLLGLCGLAALLAP
RILQLPFGALRATRPLARDWGAVVRRLRSRPGSLTLCLALTALLWLGHLVQISLMAASLGVSGDASFWAHFMALMPIAI
V
AGLIPLTFAGVGTRDAAIVMLIGAQAGPETAAALGVLFWLRYLVPGLIGSPLLPRFLRTAAAHRARFAK
>OLS42310.1 hypothetical protein BV379_20725 [Rhodovulum sulfidophilum]AB14

MTGSHPLRKRLERLGTRSLPKLLTLQDRNPHSPTYGCFDRNFWHLRITDFPSGMAQEFVLPLALAYSNRLEGNDFFGDP
M
IREWVRAGISYAARSAHPDGSCDDYYPFEKAAGAAAFSLYACIEAIPAARLDPAPFLPFLTQRAEWLARQHESGRLSNHE
ALIANALYRLSALVGEPRFAELGRRRLDRLLTWQSEEGWFFEYQGCDPGYLSLTIANLAELAELRPELPLKAPLLRAIGF
LHSLQPPDGWLGGEWTSRNTNNFFPHGFELLGKTIPEALDINTRAVGAMDPAPEYDDDHIIGHHCWSYIKTALSWRDT
RP
EPSLSPDRDVVFPEAGLSLLRKGPMTLLTAEKKGGAFRLYRGDRLIHADTGVSLQLRRGDRTRTYVCHLWSDDNEIERCP
ASVTVSGAMGRAKSSQMTPLKNLVLRSLMLTLGRFNPDLIRAILQRLLITGKPRSGLRFARHLALDDSGLTVRDRIEGGG
EIVDCGIGPAQTSIYTVMSRVYHPVQRQPWCDLSAEIMPGSALRLDHTRRIGEAE
>OLS42311.1 hypothetical protein BV379_20730 [Rhodovulum sulfidophilum]AB14
MSTNVDSRIVVQIPCYNEEATLEATIAEIRKATAAFRNCLILVVDDGSTDGTVEVARKAKADFIARHSSNRGLARAYMTG
LAASLNLGADIIVNTDADNQYRAAGIPALIRAISEEQADVAVGARPIEQTEHFSLLKRRLQRLGTRVVRGLSGTDVRDAT
SGFRAITREAALRLNTFSDYTYTLETLIQAGRSGLRVTSVDIETNPPTRPSRLMRSMGQYILRSIGDMLRISTIYSPLRS
YLTVAAPPLAVAIFLGLRYVFLVSLVDPSRSHAPSLILAGILTGLAFLLCGLGIIGEIMTVNRRILEELRINQRRQLAES
GSLGGRVDFSLIEISHGTSK
>OLS42312.1 hypothetical protein BV379_20735 [Rhodovulum sulfidophilum]AB14
MSSPTSRSCKTLNWLSYNGALKRRDSLTIWFDLEVSWEAVPTGKRGQQQTYSDAAIQTYLAMEGEGRPANTGARNG
ASGE
RSTSGSTSKFWRTEPSSSPAVASATPPCCRNCSIRSHPNGSVAQIG
>OLS42315.1 hypothetical protein BV379_20665 [Rhodovulum sulfidophilum]AB14
MSSKTEAARLVTDRSIAVSRSARDLDLSESVLRRWMRELTIAQAAAFPVGQMRADLAEIAVLKKEVVRLRAERDILKKAA
AFSRARRYEIRLRPRGPPPAATP
>OLS42316.1 esterase [Rhodovulum sulfidophilum]AB14
MPVEHYSVKTRDGLQLRLTRKSAKSKAAMPVIMVHGAGVRSRIFCPPREGAPNLDDYLQAAGFDVWNLDWRASIDLA
PNP
WTLDKAAVHDYPAAVQAIAEVTGASEVRAMVHCQGATSFMLSLVAGLLPQVSTVVSNAVSLHPVVPRLARLKALYAV
PSL
GGLFPYLNPQWGISAPPGWPRVFDAYVRAIHHECNNPVCKWSSFTFGSGFPTLWSHDMLDDPTHDWLAGEFAYVP
MTFFR
QMGRCIRAGHLVTTGEFDDLPADVVSTAPRTDARIVLLTGARNACFVPESQTRSFDFLDRHAPGRHAFHELPGYGHLD
VF
IGKNAPGDIWPLITAELGKGCA
>OLS42317.1 acetoacetate decarboxylase [Rhodovulum sulfidophilum]AB14
MGIPKRQREIEGRHAYVDGIRYVMPINSWEASAMIAAFPCDYDAAAALLPDAYVHPFRYFGRALLVVTVIDYRQTDIGS
Y
IEYSIAIACTKGRDKAPPLLPALFMKSFGTGQFVVDLPVSSEVSVKGGRGIWGMPKHQANLDFKTGLSWVSSQYDLDG
RM
VSRFDVRRPKKVWAPVNMGATNYCQFRGMIYRSFIYFKGKAGVHLMHPDSARFILGDHPNADPIRALEHRPTPLFAAF
IP
DVQGVLDDYFDCWFVTPNDLPEKPLGAGLEVTYPLGYSEDWLKPPQRDATFDLDKE
>OLS42318.1 hypothetical protein BV379_20680 [Rhodovulum sulfidophilum]AB14
MPRLLALNNAVLFLCCSIYLGTGVSLIFFQFPVEPQLTPENYYLIFVDPVRRATEFFTSMTLVMLATGALMLATEWFSGL
RWVPILVLGGLIASTLLTLYGIFPHNKALAAGIDDPRQLAETFAVWANLNRIRVSLWCVQWTAMMIYFYKLAIRARADR
>OLS42319.1 hypothetical protein BV379_20685 [Rhodovulum sulfidophilum]AB14
MNDAFLRLCLIAIAAITVISGAAQMIAGQTVMAIIATSATPTEVHLFATIGMFMVITGAMFLQSLLTRSPESAIPLWIAV
QKLAAAILVAMAFANGYFRWLALGVAGFDALSAALAFLFWQRMPR
>OLS42320.1 hypothetical protein BV379_20690 [Rhodovulum sulfidophilum]AB14
MIGPIATALQLIYALFLVAVGAYGTVCLQWELASFYGIDTATLTGRDGATLLNQFRFLKAIELSFGLFCLLFRRDIMAGG

QACTLFLSGLGLGVFSRGLSMVLDGRPHGVFVFFLLAEILIFVCVWLNARHVAGSR
>OLS42321.1 hypothetical protein BV379_20695 [Rhodovulum sulfidophilum]AB14
MTARPAIASARAGRRALILPGGGMRVAYQAGAIQRLHEEGLRFSYGDGTSGGIMNLGALLSGVTPEDLALRWRRLRPT
AF
VSPLSLRGYLKFPRLAAFGDFDGITGTIFPHLGIDPDKIRASRGVTAQFNICNFRRKAVEAIPHTRITLNHMLAGMSLPM
FTPAVVEGGETWTDAVWIQDSNLLKTAEAGANELWVVWCIGNTPAWQTGMLNQYVHMIEMSAVAALNAELAAIA
QLNNRI
AAGEAPLGHKTPIKVHLIRPDLPIPLDPDYVAGKVSGNALVDQGYMDASRYLAARAAGGIALDESATMTARPKRGVSFR
E
RMAGRISFDTTDPVTGGVDRNAVPVVLNATINLRDVAGFVRDPQHRGEMAAHLYSPRLGFIRPGTDSNFSLFTPSDQP
DL
TYMTYELGVLLDGKPHWFSGRKYIRRGWFWRLWRETTTLHVTLHEGHDRTGPVVAAGILRLGLIDFIGLMTTLTARDC
PG
LGARLGTTGQFIEYFAGSLVRTYLRKGRAR
>OLS42322.1 hypothetical protein BV379_20700 [Rhodovulum sulfidophilum]AB14
MQRPAVGWFSLRLMLRAGVKALVSALAGTRTARREVLAALDLVRQHGHAGGRIRPAAAEPGQDGAIWVDYVADLG
DGFAA
THSIAWLVGRDYLGLGAPGQVVAQPVPDDATHEVGKGDFPAGLTLLPAGQITVFGGDQVYPYATQSDYADRCTGVYY
AAR
PWDRHPDRPEGRRLYTIPGNHDWYDGLSAYVQSFCQPGRWHGAWQVQQARSYFALDLPHGVSIWGLDLAIENDFD
APQLD
YFTERAGRLKDGDTVILCVPTPAWIKRDARLAPADAGAATWDAWSKIEMIQQLVKHRNGSAQVRAVISGDLHHYARY
HAG
RDGERNSCQYITCGGGGAFMLGTNSVPDEVRVLDGKTATMAATFPSCAVSRAIRNGVYKLVYRHKLFCLALAVVLFGL
VW
VFQAASYVMKATGSSAAMGPVWTGDTALDMLQSTIRNWVGLIDFWHVLLKSMIHNPLLSLITVGIAAGFIAFAASGRS
IY
SQNWAVPVAGFAHFMVQICVAFLIAAVIRAVLGPLQDWILVYITLMIALCLFFGWMVCGTLFALYLRIANATTGLHEQE
L
CSSQAIEDWKCFLRIKVAADGLTIHPIGLRHAVRDWRSAPGTQAPKTTGARGLMQALRVSANDFEVPDGTTHLLDPVA
PL
APQLIEAPIFIPTIRGGRL
>OLS42323.1 choline dehydrogenase [Rhodovulum sulfidophilum]AB14
MTPDVIIIGSGFGGAVHACRQAVAGRSVLVLERGRRWSPQDYPRGPYDAWIFDVDEPHRQNGWLDLRILDDMWVA
QGAGV
GGGSLIYASVSIDARPQMLDAGWPDPISARELAPYYEAVADMLRPEVLPDNQRTPRFALMQEAAQKTGAADRFRKLD
LAV
SFDPDGRFPDTRPASEEAQKTFTNSQGRQQGYCVHAGNCDIGCKAQAKNTLDLNYLAAAEDAGAEIRPLSLVSHIVAE
GA
GYAVVYTDISGGQRRERVVRAAQVVLAAGSLGSTEILFRSRDQFGTLPNISPALGQSWSSNGDFLTPAFYDNRILSPTIG
PTITSAIDYLDGSDGGMRYFVEDGGFPDVLGNALKARSRTGRRFGMANRALMSFAKRLYDGDPLEGMMPWFGQAM
DGGDG
RLYLGRDRLRPWKKRLKLDWDAARSERGVGGLIAAHDRLSRATGGTPKVPPNWTIFRNLVTPHPLGGCKMGSARISGV
VD
HRGEVFGHQGLYVSDGSVFPRPIGLNPSKTIAALAERTAAGILAR
>OLS42324.1 hypothetical protein BV379_20710, partial [Rhodovulum sulfidophilum]AB14
AAPIAEPVFKATGLYLRGLAPLSATNRLSASGGKLHLVETGSRSLITVCPEKDRTESDHFRRTQLHGKTP
>OLS42421.1 hypothetical protein BV379_20380, partial [Rhodovulum sulfidophilum]AB14

MRRRRTYRLTGGLAAAGLVLLGIPPVAAQEQGGLETQLRFSQRLTQEDGTLSTTTGLGVTLSSRTRTEAFLFSADSGLEQ
DFDGSALQQKDPRLILSYSRESLSSALAADFSYRQSDVDALSEDDDISDGVLRLDEGLLKRYSGNLSLQLGRDAPFGATV
DLGYSAKTYSETSDPDLLDEESVNATVALRFDLDPRISAGLRSSFSETDRDDPGQDTDSASIGGTLDFGVTKTLSTNAYL
GYSRVTKSTPGFPEDVQKGLAYTLSFTELRPNGDLSGSLGSDIDEAGRR
>OLS42422.1 sugar transporter [Rhodovulum sulfidophilum]AB14
MLYLKRILSGIVLILAATAAMAQQEYRVRPGDTLAIEVLEDTTLNRTAVVLPDGRFSFPFAGTIPAAGRTVGQIQNAVTQ
AIASNFAVSPNVFVSVQPGLASPYEGTMTDAIDVFFMGEVNTPGLVQVATGTTFLQALAQSGGLTRFAATKRIQLRRTD
P
RTNVQKVYQIDYKSLSRGAVLAKDIRLLDGDVILVPERRLFE
>OLS42423.1 sugar transferase [Rhodovulum sulfidophilum]AB14
MTLYLTDLNADASARPVLGAFSVRSAAAGWGAYRKVFKRVVDLTLVLLSAPFSVPLIALLALLVALDGHNPFYTQLRVGR
NGRYFRIWKLRSMVPDADRILEAYLASNPAARREWNATQKLRHDPRITRIGRVLRKLSLDELPQLFNVANGTMSLVGPR
P
MMVKQQLFYPGQAYYRLRPGITGLWQVSDRNDCDFVGRVDYDEIYDRDMSLRTDLDVLFKTVSVVVRATGC
>OLS42424.1 ATPase [Rhodovulum sulfidophilum]AB14
MNTSLYNAHFGFHERPFSLSPDPDFLFWSKAHTRAFAVLEFGLVSRSPLTVVTGEVGTGKTTLIQALLRQVEDDATIGLI
SNAQGGRGDLLRWVLNALDVEAPEGADYVSLFQTFQSFVLQEYAAGRHVILIVDEAQNLGSETLEELRMLTNINSGKD
DL
LQIVLVGQPELRDLIRHPSLRQFAQRVSAVYHLEPMDLPTTRAYILHRLKHAGGSGEEFSASAIRHIHEEAGGIPRMVNK
LCDLALVYASSADAPAVDEATIRELIDDGLILKPFRDPLLLSGAIDPFGKAAE
>OLS42425.1 chain length-determining protein [Rhodovulum sulfidophilum]AB14
MTIDLPLYWRLFLRRLPVMLIFIICGTAAGVVAALRMPETYSTSARLLVEAPQIPDSMVSSMVQTDAVEQLDIIEQRLMT
RANMIDIANRFKVFPNIRQMEPDEVVAAMRSGTRIRRSAGRNQATLMTISFSSEYPRVAANVVNEYVTLVLQTNAEFR
KS
RAENTLQFFEQEVERLGQELDRQSSAIASFKSQNAEALPDDQSYRMGRQNLLQERLGGLMRDLKAGQSQRADVVRIF
EAT
GRVKQGAFAERRSPEEERLNAARAELDNAKAIYSETSPRVISLQAVVDRLEQVVTEQKEDGTAAAEAGDVSPEQAMLD
AT
LSEMDSRLTTLQEEIDTTNSEIDKLQGAIAKSAANGIQLASLERDYEIIQTRYNAAVSNLNQARMSERIETTSKGQRISV
IENASVPQLPSGPGRTKVMALGAAAGLGLAGAYFVLLELLNRTIRSPAELSNRFDITPITTIPYMKPPKERGSRLRRRNR
KEPPPSGSIMPG
>OLS42426.1 hypothetical protein BV379_20415 [Rhodovulum sulfidophilum]AB14
MKITFVIAVASLAGGNRVVATYASRLKARGHEVTVISRPWPHPSPMRRLRARLRGRKLPSYTSSPLFDALGDSHKVLDRR
RPVVAEDLPDADVVIATWWETAEWVAALPASKGRKFYLLQDVELFDHLPVDRVRATYRLPLRKLAVSDYIRDRVRDEA
PG
SKIDLLPNAVDHAVFDAPPRARGTPFTVGFLHSIAPRKRIDLAFDTIGKARLRLPDLRVVSFGTTPATENFPRDLIYRRA
PPQQEIPKLYAACDLWLFTSAHEGFGLPLLEAMACRTPVLATRAGAAPDLIDGRNGRLLPPDPEAFSAEIETISRMDPAE
WRAMSDAAHATAAAHSWETATDRLLDYLGEEARS
>OLS42427.1 hypothetical protein BV379_20420 [Rhodovulum sulfidophilum]AB14
MADLFKLHTQGLDSPPSRLTEVMPDDSAELPFASRALNVASSGTVRVTTVDGDTGTVFVASGIALPLRVRRVWATGTS
AT
GIVALF
>OLS42428.1 hypothetical protein BV379_20425 [Rhodovulum sulfidophilum]AB14
MIGLSISIGSGQNGGQRSGNRSLTLSGLIDGAARPGSASGITAAFSDGFPIQSLSWGMTPGGTEFGTGASPQDFSSADT
G
LFDAPGLLHVTAGAAGRSYSLSVPIRTAPPVFLTPPAFAAPLYKIGEAVEVSLGTVDAAPATLSVASFTLDGEDRRSGLA
GLAWSSAGETAWGRAGVLRLQARAESSGGVVLSEPVTARLGDAPRAFSAADWSVDETGVRIAALPDDGNVPITNIRY
RID

GGPDRSVNGVTDFALADYGDIQIRAVNALGIGAWSDTKTRIAQTPTLSIEAAPLLAGVAAVEGNTLTATAGPVSGGPSS
R
TWQWYRGTAAISGATGESYMLQPADIGRRIKVRQIETPSGGSPVTRDSAASAAVVALNFRAEENDDGTVGVSYADGA
RST
VEFELTRPAAYAGSFNLDLGALETGPVCLVRPALSGTPAVGATLSATPFFWVYPEALGALSVSSGWRRNGTVLAGETGS
T
HTVISADAGTDLQYIETGTQSGAAAPVTAASPAVRIGDAAPTSYSPQTSTSALAWWDASDEATITHEAGTVSQLADKIG
T
VHLIGSGTTGVGASKINGRNTVVMDGLSDVLTAHGLIYPASGDWVMTIVAVIGTVNNITDGLIDGRGGDSSINIVSYKN
D
AFNGHLRVSDTSSHFATPPYSGLHVVQIVADLTDTGTLKVLIDGEEVASVSYDTPIQTDLNRFFRFGESKHLGGQFCEVF
IDSDKNVQAATYTYAARKWGLS
>OLS42429.1 hypothetical protein BV379_20430 [Rhodovulum sulfidophilum]AB14
MVYARGSYGSGAPFEHWKGRERDIAISWGEQTYNPDGLDLSDVNDATIFRWYDAKRGNAPDTTTDIMEIYWWLREH
EGPE
IQALVRDFIAFMQAPLGIAPAWRTSAGPCSFVPDLAEDGCRWDNPNNWGGDLIPGSFPADTAALGGHKVFFQDHTLT
VAG
LDLGAGGHVHAVNGRLNVSAAPTCTGGGAITTDESGQVWIEGYAGTAPLALTVKGGRFANTGTFSGPADIRIDGSTDP
HG
VAEFLCAYGEASMTIRSGRSMEIMGGGPRVGFDGLDGAQARLSFEGGAELRFVAENGRLATIREFRSGVNGTQATDV
VSS
VDLGGAALVLDLTGVTAGSHVLIAADSVTGSFGSVSGAIDPGLDATVTVSATGVSLTVTAGSGLIALA
>OLS42430.1 hypothetical protein BV379_20435 [Rhodovulum sulfidophilum]AB14
MPAMPSASPFIPTILEIRAIFVHVPKTAGTSVKIEFYGQPGGGHRRIIEFAAHDPGRTDDFFKFSFVRNPWDRLLSAHAY
LTDGRKATGRDRRFANEVLALHPGFEAFVLALEDPGFRRQVMAYDHFRAQTYWLCMPGARDHAMDYLGRFERFDED
METL
RHRLGRPGRPTVRARPSHHPPYRDSYSTRMRDIAGDIYARDAALFGYAF
>OLS42431.1 hypothetical protein BV379_20440 [Rhodovulum sulfidophilum]AB14
MRSGTHVMIDLLLNNLPAYRNRPLYIDLDQHLRRGEMPVDPQAGYVVKTHYPFTMPGGKFPEDRLMGLFQNSLVITI
QRD
QAEILRSLARWHAATSDQILSRFGDGFDRFNAFWPGRDRMAIDHRTLFDRDAMQMLIRQIAERTGTEPARRFVPPPP
RDA
TQRIYLMKATTRLLGARAPRINTTIFTLK
>OLS42432.1 colanic acid biosynthesis glycosyltransferase WcaL [Rhodovulum sulfidophilum]AB14
MTANVRIAYLTGEYPAVSHTFILREIEALRSHGLEVITASIRRTGPEHHRGPSEKAAAASTFYVLDAARRPGAVARALLM
ALRRPGALPGVLRLAWQTRRPGLRGALYQLFYLAEALILAAHLRRAGATHLHNHFAQASATVAMLAAELAGLPFSFTLH
G
PADFLDPLGWRLDEKIRRARFVACISHFCRSQAMIHARPADWPKLAIVHCGVEPGRYRRDPPPATGPATARELLFVGRL
A
AVKGVPLLLEAMARLGPEHPGARLTLIGDGPERARIEAEAARLGLGDRVVFAGYRSQEEVAGALARSDLFVLPSFAEGVP
VVLMEAMAAGLPVIATRIAGIPELVEDGVSGRVVDPGSATALTAAIGAVLADPEGAARMGAAGRERVCTGFDVRAEAL
RL
ATLFRNGAAAGEDR
>OLS42433.1 hypothetical protein BV379_20450 [Rhodovulum sulfidophilum]AB14
MPLFFFCAGITASFAVHRAPRDFWHKRLLPMVWVLAIFTALVPLLIRLNGFTVIAVFLGLAVLWHGGLLRYERTLIYPKD
IKAVLNNAPFYMAGLYFAPRVIALLAHPRRSRLVLAGTAAVFAGLVLGAALMPSTAEILGKAARFAGVAFGAALAAEIAL
FAVAGSVALKAMLRAARMEVFYAPPRLLAHRPARTAQV
>OLS42434.1 hypothetical protein BV379_20455 [Rhodovulum sulfidophilum]AB14

MRILKTLAASAALALSAGALHASTLSFAGGTGYNVAFGSGHYGSDCSGGKITCYDPSGSAGALTEDLRAFDRNNAGPGL
L
LSRAARLTVTFLGKEANSLNWAFTVAGGMIDSSEGVGASYSAIVNAGTLDFTFGSVAGGGALAGNDGSFIGSAAIAFSKI
YGNKVYAYFDNSGAKDDRDFDDMVVEISIAPIPLPAGALLLGTALGGLGLARRRKAVKA
>OLS42435.1 hypothetical protein BV379_20460 [Rhodovulum sulfidophilum]AB14
MAPGSAAPPAGDRGAEARTVVLFTLAAAAVQLTLIGNNSFTGDEQVTLLFLRADWSELWGPLWGADTHRPYYYMLLK
AWS
GIFGESRVALRSLGALLHAASLLFIWILARRLGGRRAAVWTLAFSVLSPLAIFYGRDIRNYPLVAFSIFGSLAALSALAA
AERAGATALPARRRMLLWAGVTLGAASAFYAHSISVLYPILVCLWALGSAVAGLVSWRFVGQLVLAGLALALISVPAVL
P
MLHHAETTLKEFWIPLTTPGRLVSQMRDAYPYPAPLLPVVLGLLGLGLWRLRGSGAAGLLIPAMVIGQPAALALLGLVE
P
VFITRAMVWPAMLASLLLGLGLAGLPGRKLRLGAAALVLLAQAIAARPDYVPHYQPSEPERLAAFFDRIDPAETELILGR
QNVEHLLRWSYPALFEGPVFAFNKDDEKIVYDAANRSVRVLRAEAGKLPLARPRFLIVQQIRPPAPSGAEVTPALDRIAA
RGRLIAEAKGAWWRAELYERTGPE
>OLS42436.1 dTDP-4-dehydrorhamnose 3,5-epimerase [Rhodovulum sulfidophilum]AB14
MQIEHTALSDILILTPARHGDARGFFCETWNRERLAGHGIETEFVQDNHSFSQARNTLRGLHFQAPPDAQAKLVRCGR
GA
LFDVAVDIRKGSPTYGHWVGVELSFENGRQLLIPEGFLHGFVTLTDETEIVYKCSDYYAPQSDGAVRWDSCGIDWPLSD
A
PVLSAKDSAAPPLAEFDSPFIWTRT
>OLS42437.1 dTDP-glucose 4,6-dehydratase [Rhodovulum sulfidophilum]AB14
MKLLVTGGAGFIGSAVVRQALADGHEVVNLDALTYAACLENLAPVAGHPGYAFVKADIRDQAVLETVFAEHAPDAVM
HLA
AESHVDRSIDGPGAFIETNVLGTYALLQAARGYWQDKGRPESFRFHHISTDEVFGSLGPEGKFTETTPYDPRSPYSASKA
SSDHLVRAWHETYGLPVLLTNCSNNYGPYHFPEKLIPVVILNALAGRPIPVYGAGENVRDWLYVEDHAAALLLVMQKG
RV
GRSYNIGGENEARNIDLVRMICALLDEMKPADRPYSSLISFVADRPGHDLRYAIDPTRIREELGWRPSVTLEEGLRRTVR
WYLDNEAWWRPLLGREGVGVRMGCFLENTSVKGN
>OLS42438.1 hypothetical protein BV379_20480 [Rhodovulum sulfidophilum]AB14
MLRTRTSDGVPIEVDITDHDGRILWMCGSNDFKVSRVANALLSQNMDFLDIGANYGTIGFSAACRMKPGDTVHLFEP
QPR
LSGQLRKAIEASGLDAVIKLHEVALYDRDVKIPFSVPLQHSGKGSLVNSAPANESLTVEARSTASYVEPLIGDRPFGVKI
DVEGAEAVILPDLIALKDQMRFCIFEGSSNQVALYEMFNSRGFLVFGLCRTVLKVNLSFISSQEELHSFHDFLAIRLSTR
PTQKRMTLDNLVKCVRSPS
>OLS42439.1 dTDP-4-dehydrorhamnose reductase [Rhodovulum sulfidophilum]AB14
MLLVFGSTGQVATELRARAPGARFLSRAEADLEDPRACAAAIAASGAEAVINAAAYTAVDRAEEEEDLATTINAAAPAA
M
AWACAKKGIPFVHLSTDYVFDGRGDDPRETDAATGPLGAYGRSKLLGEEAVREAGGAFVILRTSWVFSAHGSNFVKTM
LR
LGAERAQMKIVADQVGGPTAAGDIAGACLKIAAHLQDTPEHRGTFHFAGAPDVSWADFAREIFRQAGLSTAVEDIPSS
AY
PTPAKRPANSRLDCTGTEVVFDLPRPDWRQSLARVLSDLEAAKA
>OLS42440.1 glucose-1-phosphate thymidylyltransferase [Rhodovulum sulfidophilum]AB14
MSNRKGIILAGGSGTRLYPITMGVSKQLLPLYDKPMIYYPLSVLMLAGIREIAVITTPEDQAQFLRLMGDGSKWGLSLTW
ITQPSPDGLAQAYLLAEDFLDGAPSAMVLGDNVFFGHGLPEILARADARPEGGTVFGYQVADPERYGVVSFDASGTKA
VE
IVEKPQYPASDYAVTGLYFLDGSAPERARRITPSARGELEITSLLETYLAEGSLTVEKMGRGYAWLDTGTHASLLDAANF

VRTLQDRQGLQISAPEEIAYRQGWIDRAALLAHAAFFGKTRYGAFLARLAEED
>OLS42441.1 hypothetical protein BV379_20495 [Rhodovulum sulfidophilum]AB14
MQVSSMTPTAIAYLVPEFPGQTHAFFWREIQALEEAGLRVRTLSTRRPAPGACPHDFAAAAAARTVYLFPPPLAAAARL
A
GRPVRTLRALAYVLGLSETPWRRRLALVGLIASASALGRVCRRERLGHVHIHSCATAAHLGALAAILDDLSYSLTLHGDL
PVYGTDHAAKMARAGFVAAVTCPLAAQIAAVSPGTPAPVIWMGVDTDRFHPPASARPGGEALEMVTVARLNHTKG
HVHLL
NAMARLRAEGVAIRYRMAGDGPERAALEAHVARLGLQDEVRFLGPLSEAGVIDLLHSADVLALTSFGAGEAAPVTVME
AM
ATGLPTVVSAIGGTPDMIEDGVDGLLVPQRDEEAIAAALRRLAADPALRARIGQAARKTALARFDYRAQARALLEALTV
G
R
>OLS42442.1 hypothetical protein BV379_20500 [Rhodovulum sulfidophilum]AB14
MPIEALRVLAVLLLVAYHVIGAGPDAGLNIEYPHPLRLFAEFLVDARMPLFAFVAGFVYAFRPPVLAGYGGFLTGKIRRL
IIPGWIAALLFSVAGMVVGNVYSLTVQGPVRVLFHHYAHYWFLQSIMVIFVVFGLVDALLRHRHTALLFLAAVAMTLAG
I
TLRTSFLSIGGAFYLLPYFLFGTLCHRHADQLLRHRIWLAALALAAILAAGWWNIGLYLETGRLSGYRGDLQSLGFGLGI
ALAMLLLMPRIGWLDALRPFSFTIYLYHVLATSATRRALLALDVTDVWVLFGCGLSTGLLVPVLLHLVIDRIPALRQPVL
GLRR
>OLS42443.1 phosphomannomutase [Rhodovulum sulfidophilum]AB14
MAPKFGTSGLRGLVSELTSDLVGAYVRAFLVACPAGPWVLVGRDLRASSPRIAEDAIVAIRAAGREAVDCGALPTPALA
L
AAMGQGAAAIMVTGSHIPADRNGLKFYLPSGEISKADEAAIGQALERPVATPAPAAGQGARSFASEAGAAYVARYVQ
AFG
DAALAGLRVGVYQHSSVARETLVEILGALGAEPVPLAASDSFVPVDTEAVDAGTRAMLAEWCAGQGLDALVSTDGDA
DRP
MVTDAAGRVVPGDILGAITARALGAGAVCTPVSSNSLVTRLPEFETVRLTRIGSPFVIAAMEEILAADPAARVAGFEANG
GFLLGFAARGPAGPLAPLMTRDAMLPLVTVLAAARAAGSGVAGLVAALPARHTAADRLQQVAPEAAAALLARLDASA
GAR
AAFFAPLGREAGCDRTDGLRVSFASGEILHLRPSGNAPEFRVYAEADSPERAAAVLKLGLERVGEALYA
>OLS42444.1 hypothetical protein BV379_20520 [Rhodovulum sulfidophilum]AB14
MTLPDFFIIGAAKAGTTSIFALLEQHPDIFMPTPKEPEFFARDDLYSAGIETYSALFAGARPGQRLGEASTLYSHAPLFP
DTAARIARHAPGAQFIYVLREPVARAYSFYVQLVKNYQNGTRDYAVHRCFEDFVLPERHALAAPREKALAPFNAHFPDT
P
ELCLAGSDYLLQIETYLACFPRDRFLFLTFEAFRDDPRATLRRITGFLGLDPLPETAFGGGAEARNVSRAHFDQLEAAAG
AQRLRGRVGPLWALRRLLPDPLRSALRRRVLPTGAGRAHEPPPMEPATRQLLAERFHPQIPRLEQLTGLSLASWQAGL
G
>OLS42445.1 glycosyl transferase family 2 [Rhodovulum sulfidophilum]AB14
MSDPSRAYLLISPCRDEAAHMRRTLDSVLAQSLRPAKWVIVDDGSTDETPQILADYAAAHPWIEIVAKPDRGRRAVGP
GV
IEAFYAGLDRVEPGAFPYLCKLDLDLDLPPRYFETLIERMEAEPRIGTCSGKPYIRRGGALVSERRGDEMSVGMTKFYRS
ACFRQIGGFMRAVMWDAIDCHKARELGWIAVSWDHPDLNFEHLRPMGSSQSSIYAGRRRHGFGQYYMGSDPLYFA
ATCVF
RGLEPPYLLGGLAMMQGYLGAWFGGAPRHPDAELVAFIRAYQRRALRVGRARAVAGIEAERAAIFDPDRAV
>OLS42446.1 hypothetical protein BV379_20530 [Rhodovulum sulfidophilum]AB14
MRPDFLLIGSMKCGTSTVSAYLEDHPEVFMLPGQEPNFFSHDKVHDRGAEWYERLFDGAAGARRIGEGSNDYASGLM
YPD

TAARIAAYSPEMRLIYMVRDPVARLASAWVQNRVDQGDQVPPTLERAVREMPDLYLGQSLYWHNLQLYRTHFPDDRI
FIG
FMEDLKTDRFAFFARLCGFLGIEPMNEIPRGHINPSRGKAVPGQLYSALNALPGIGRLKALAPKGLRSTVKTRLLSRRIG
ETPEIPAALRRELTERLGPDTRAFLAHCGRPTDFWGL
>OLS42447.1 hypothetical protein BV379_20535 [Rhodovulum sulfidophilum]AB14
MSVGEDLARIRINTGISVIVPTYREAENIPHLLVELDRLRREYDVTLEVLFMDDDSRDGSVEAVAASGFDWARIIVRTGD
RGLSRAVIDGFRAARHPVLICMDCDLSHPVDAIPQMILGLASGQQFAIGSRYVPGGSTDDDWGLFRWLNSVVATLLAR
PL
TKVRDPMSGFFALRKADFDAAQGLNPVGYKIALELIVKCRMENVGEVPIDFSDRRFGESKLTLKEQLKYIQHLRRLYLFR
FANAMYLLQFLVVGATGAVVNLAVLSGLQLAGLAPWICLAGGIGVSLVTNFLLNRRFTFSYARRGHAGRQFLGFLGASL
V
GLTVNYAVALGLSHKVLTGAYGLQVSAMIGIAAGMVFNFLGNRYIVFRKRYIRD
>OLS42448.1 hypothetical protein BV379_20540 [Rhodovulum sulfidophilum]AB14
MARRKRLSPITGPAEDTGPVPPGPIPAIARPPVAEIAAASASQAALEQLADEVAVARREGRMVVSLPLEAIEAGHLLRDR
MHHDAEDMAALKASLAARGQQTPIEVTDLGQGRFGLISGARRLAALSELAAETREPRFGKVLCLLRSPEGAAEAYLAM
VE
ENEIRTDLSFYERGRLVAEAVRMGLFADPPAAVKGLFGNAPPARRSKILSFVALHRALGTALRFPTAIPEKLGLAIVAGL
KADPKLADRLGATLAKAQPADAEAERRLIERALRKAPAAAAPRGREVAPGIRLEARKGRLVLSGAGVGPELAEALEAFLA
ARDPLE
>OLS42449.1 chromosome partitioning protein [Rhodovulum sulfidophilum]AB14
MSRDDIPPYLNISPDRAAELLGPPADTPGFASVAEACRRGRQDLASRGLDETGERQLRLFSTWEITRYLIPVAPAHFRRV
LRQNPDLPQGQSESAGGAKWFTLDEVLRLRAHFGAEGSKAKNYLPYRPKGLPAKIVAVANFKGGVGKTSTCAHLAMS
AAL
DGYRVLVIDLDSQGSMTSIFGGQVEDEWQTVFPLLARHYATTLRADNQRRLDRGEPPLPFDDTLTEALKLKAPDLIRKT
H
WPNIDLIGAQLNLYWSEFQIPVWRMQSRGWRLWDALTDVLAEDGVLDDYDLVFLDTPPALGYLTINGLAAADILLVPF
GA
SFLEFDSTGRFFDMLHATFASIEEGENVAARALGREELHFEWDAVRAVMTRYDAGQQAEMAGLIQAYMGQVLSPHR
QDFT
ALIGQAGEQVNGIYEADYRDFNRETYARGRATFDATYAAFKMLLYGIWRREETARTAAAE
>OLS42450.1 mannose-1-phosphate guanylyltransferase/mannose-6-phosphate isomerase
[Rhodovulum sulfidophilum]AB14
MIHPVILCGGSGTRLWPSSRKAYPKQFAPLIGRESLYQRTLRRFSGGGFAAPLVMTGNEFRFMATEQAAAIGLSDARVV
V
EPLGRDTAPAILAAALMLERDDPEALMLVAPSDHVIADLPAFLAAVEAGCPPARDGALVTFGVAPDRPETGYGYLELTA
P
PAGNTPVPLASFREKPDLATAREMLEAGTYLWNAGIFLFRAADAIAAFETHAPELLAPCRAAIAEGQTDLGFFRLAEAPY
AGARAISFDYAVMEKASRVMAVPLEGGWSDLGSWDALWAAAGPDADGVVADGAVTAIDCRDSYLRSEDESMQLVG
LGLEG
VVAVAMRDAVLVADKSRAQEVKSVVETLRADGVAQADQYPRFHRPWGWYETLCLGTRFQVKRIMVRPGGVLSLQS
HHHRS
EHWIVVSGTAEVTIGEEARLVTENQSVYIPLGTVHRMANPGKLPMYLIEVQTGAYLGEDDIVRYQDIYDRS
>OLS42451.1 hypothetical protein BV379_20560 [Rhodovulum sulfidophilum]AB14
MSRIAFYRLAGLVGLLFAAGTLYRLFPIVTGRPALSEFFMTEDGYLMLTVARNMAMGLGMSVSDGTIPTNGVQPLATFL
F
TLPYLQTGGDKVTSLVGIHLIMAAWSLAGALLVRALAARVLDPGRQAPALPWIAALLWYLSPLLVLHMMNGLETGLYS
AA
VLAVLLMFLRLLRREGPLGWGWHLGFGLMGGIAFLARIDAVFLLLAVYLVWAIDGLFLRRDPIRTVAARIVPSGLISILV

AAPWLLNNQIRFGSIMPISGTAQSMAAEFGQNLGVLPVKIFEYVMPMLPVPNPLEKTLPVILLASAVSLVVLAWFLIGVF
TRASRPVRLMVLAYLLFGAMVSGYYGLWFGAPHFMSRYLAPLAPLCIIASLWVLADLSRWLFPARGLRPAIWTGSAAIG
L
CVLLLGRLLMPGVQDQGHMQVVDWVERNVPDSAWVAAVQTGTLGYWHDRTINLDGKVNPEALEARRRAGNVLAY
VVNSRI
DYIADWYGVGGWATREDGGFAEAFELIEHRKSPSLAVMRRRVLHEPG
>OLS42452.1 hypothetical protein BV379_20570 [Rhodovulum sulfidophilum]AB14
MKIYIHCGFHKTASTYLQRVLRDNAERLSSYLTIINRIELDTYDLRMACLAYNSGRAEASTVRAELDRLVQRVAGSEHPV
LITDEAFFGPHVGQDGETRLFPRAHEVTQMMVEAFAPHPVEILLYTRDEFSWLKSVHNQSVKQDRYAGSFEEFLAEIGG
G
ADLDAVATRIAATIGPEQVRVFRLEDDHHHPFGSGGALLDHVGIPREVQQDLKRASGINQSLDARHLRIMRRLNGLALP
D
FLYSRLRRGVLRLQRRARS
>OLS42453.1 hypothetical protein BV379_20575 [Rhodovulum sulfidophilum]AB14
MGDPLAFGQPRPPKEVFVPFASALRRRVRQGLGTALWALRDLPGAPTFEIFARRLDRMNRAHVAEIRSRGGEPMTPR
APG
TIPKIVWIYWAQGLDEAPPVVQRCIASWRAANPDWDLRVLDRDAAGALVDLSDLPAALPRRHEADFLRLRLLERFGGI
WA
DATVFCHRPLQDWLPLLAGSGFFVFRDPGPDRWLSTWFIAAAPGHPLIAAWARQYGEYLIARRRPPGPYFMVMYAFQ
FAV
SRSPELMREWRCCSGLWAVPSFRLMAALDGQVGAEEAAVESLRRGLPVSKLNWKKPFDQVRFDTILGAAGVKRDVG
>OLS42454.1 glycosyl transferase [Rhodovulum sulfidophilum]AB14
MTPPAPSAPRDLAAIVIGRNEGARLIRCLDSLAGRVARIVYVDSGSTDGSLEAARQRGAEVVELDRTRPFTAARARNAGL
ARLTETGPLPRHVQMVDGDCELREGWIETARAFLDATPAAAVACGRRRERFPEATLWNGLIDAEWDTPVGRARACG
GDAM
MRLEALVAVGFYNPALIAGEEPELCVRLRRAGWEIWRLEAEMTWHDAAMTRFGQWWTRARRAGHAYAEGAHLHG
APPERH
NVAQMRRALIWGPGVPLAAALAALVLGPWALALLLVWPLQVLRLGLRGTGWRRALFLTLGKLPEALGVAQFHLNRLR
GRN
ARLIEYK
>OLS42455.1 sulfotransferase [Rhodovulum sulfidophilum]AB14
MTHPDFIIIGAMKSGTTTLAAQLAAQPGLFLTDPKEPNFFSNDEIHARGPDWYAGLFAAAPPDALKGEASTHYTKLPTY
P
DTVRRLAAAGPAPKLIYMIRNPLERAVSHYIHEWTTGEIRTDLETALERHPTLTDYGRYGMQIAPFAETFGPGRILLTSL
EGLKADPEGELGRISAFIGLETPPVWQSDLGARNVSAERFRKLPFQRILVDNPLARSLRHALVPKALRERVRRARSITRR
PELSEAARARLIPIFAADRDALARVFPDDPALGLCYPFLAP
>OLS42456.1 glycosyl transferase [Rhodovulum sulfidophilum]AB14
MTHSLVVSIINYRTADMTLTCLASVLEDLDGIDGHVVIVDNASGDGSETVLQDWLDAHPGAPVTLVRSAANTGFSGGH
NQ
GMAARPARFYLLLNSDAVLRPGFCRTILAAAEEEPEAGLFAPRILRDSGETQDSCFRFHSPMSELIRGANTGLVTRALRR
FEVSLGTEPVPAQIDWASFACILLRAEMTTAIGPMDEGYFLYYEDAEYCLRARRAGWRIARVPEAVAVHFRGGSGPVKT
L
RTARKRLPPYYYASRARFLYQAHGLMGLWAANLMWHTGRGLALLRRLAARPGRQPPLEGEARDLWINAADPLGPRL
APHE
T
>OLS42457.1 glycosyl transferase family 1 [Rhodovulum sulfidophilum]AB14
MRKPTVLVIAEAANPEWVSVPLIGWSLASALGKVAETHLVTQIRNREAILRAGLVEGRDFTAIDTEPLMRPVWKLASLLR

MGKGKGWTTTTAIAALAYPYFEHLVWQRFGAGIQAGQFDIVHRVTPLTPTTVSPLAARTAAAGVPFVLGPLNGGVPW
PRG
FDAARRREREWLSYVRGAYRMFPGRRRMLTATAAVLAGSHHTASEFPDACADRVVYLPENAIDPARFNRLAAQDISG
PLR
AVFIGRLVPYKGPDMAIEAAAPLLASGQLQLDMIGDGPMQAELEAQVAALGLGGSVRFHGRLDHARVQDVAAAANL
LLFP
SIREFGGGVVLEAMSLGVVPLVVDYAGPGELVTAETGYKVPIGPRAEIVRDLRARLEALTEDPSELPQLGRAARDRVLTG
FTWAAKAHQVGEVYDWVLGRRPDKPAFAF
>OLS42458.1 VPLPA-CTERM-specific exosortase XrtD [Rhodovulum sulfidophilum]AB14
MSIFNEAFALRRQSRAFPGLGLFWLGLAVLGAGLFFRPGLDALLVAWQKPEYSHGPLIPLLSGLLFLRQLKTVPERPGEI
PHRWPGVLVVLLALMLGMVGRVSNIDDIVAYALIVWVGGLLLISFGWSTGKHFWPPVLHLVYMLPLPDTLYYKATTWL
QF
ISSELGVWFLGLLAVPVYLEGNIIDLGVYKLHVAEACSGLRYLFPILSFSYIFAVLYRGPVWHKAVLLLSAAPITVLMNA
VRIAIAGWMVDEFGVEWVEGFTHFFEGWVIFIICVAILFALARIMLFFHPAKMSLTEALDLDTEGLVPQLMRLRLVQPSL
ALIAAAGLMFAGVGVLQMLPDRSATAVARDSFVTFPRRFGDWQQSGPPAVLAPDIARALQADDYHSITLTSPDTPAYV
GV
FLAWYADQSHGGVHPPEVCLPAGGWEIAWLERTDVASELGSAEPFHLNKAIIQKGEVRMMVYYWFDQKGRKVAWD
FAAKL
WLIVDALRTGRTDGALVRLTTPILPEESDADAAARLDAILENMAPQLPRFIPED
>OLS42459.1 hypothetical protein BV379_20615 [Rhodovulum sulfidophilum]AB14
MRVVFVQHGNYAEAYRRMCAGEAETYRDQFRSVSLVAGLAAAGPVVTVALGDAHHDAQLAPNLRSVGLPRADIDRA
GSLL
DDLAPDRLVCRTPHTRLLAAAAARGIATLPCFADMMRPPSPLGLWRHWRLRKALRARTVPCVANHSLNASRSLVEAGL
VS
AGHVVPWDWSRLVPNPDPKPGMPDPQNPTILFAGKMTVEKGLGDVLAAMSVLAGQGLNARLTVAGGGDVRPWQ
AEAARLG
LAEQVDFLGLVPNTEIRHRMRAYDIVVVPSRHDYPEGLPNTIYEGLASRSVLMLSDHPAFRGRVEPGRHCLMFRAGNP
AA
LGAEIRKLCAAPELYAHLSRQAPEALDSLYVGLAWGDLVTYFLDDPANLTGWVERHSLCALGLSG
>OLS42460.1 hypothetical protein BV379_20620 [Rhodovulum sulfidophilum]AB14
MQDIYSCVFGTWQPGIGDPTWQGWTTVAVYLAVAALALRAACRAVFPAPSARRERIFWSLVALVLLGLAINKQLDLQ
SAL
TATGRCLAKLQGWYGQRRAVQTEFIAAIGALSLLSLIGLLWLLRGTLRRSAPAIVGLCFVSGFVLIRAAGFHHFDHLLGM
PTLLSVRANMVLELTGPLLIALAALRLPRLRHSQGHGIAGKP
>OLS42461.1 hypothetical protein BV379_20625 [Rhodovulum sulfidophilum]AB14
MKRFLQLVMAFSGAAGLFGGSTAGALVLRGIATSDEEVEKAGPEAPPGGAETAELAAQSAEADDIGMDETGAEEDPA
SAA
AGDETGDVAAGGSGATGRNASDGDAFDAAHADAVALPDTTTEPGSFVASAPSEGSALSPTIVKFASLLGVDPWSLVSS
SG
GSGSGSGGQTPPDDGVGSPPPGDGDPGDGPDDGPGEGPGGDLGGEPPDQGPDEGQDGSGTAVPLDGEVEVTTGR
VTTLTL
PDAEDAVSIEIVAGPGFGNVTVNPDLSLALVLTGETQTGALSFDYAVTHADGTVTTHGASLSAVPGEQGGGWGAGDF
YML
ETAADGSLVIEHGDVHETVYVTASEDGLSRADIAAMEGMKVEQVNDYWLADQTTYGFDEGLAVDQDVGSAIWRSLT
GPEP
SSHWLLLERGYSYGDFKGLVDPSIRGESELHPIYVGAYGEGAPPEITEEMRSFAQTKENVVVEGLTFSDGFSMFNSKNIL
LSDLTITGDSLIIKTADGFTLRNSSVYDIWREAPANDGDTWAQNLNRVSGFFLTQSDGVLVENNVFDHNGWQDGYDY
ARS

VEAGQPPSMYSHNIYMTATNGDVTLRDNIIMRAASYGAQFRMGGVVEDNVFIDNNGAINPAKGGSDASGNYSLVLG
NVVT
SGGNKTVDDSEGALSQGINVQGWDESLVDNIITHLADPNNAADQAEKEKGHFALAINGSLYYNDTVIYNWTGAKNAD
KAG
EIEANVDGLDRAVLDQTTIQNFAADLLGKPSATISDLADYLRMQADGALDGMVDADLITAFFRTGFGMDTGLRAEETV
LR
FIPDDRGEGMRWDNRLNWSTDDLPGTQDGDSVDLGGNWVSYGSTTTALADLDLGQGGWLNVAQGRLDIEGAIAV
GSSGGR
LTLDGAGQFWTDGYADSDLLSISVMGGRFANTGVFMGNADLTVGENGQAILATDGAGFLLQAGRTLTVMGDDAKV
GFDGE
GGLSLLRLDDDATLKFVAEDGAMGTIGEFRSGRFETSDVVSGVDLGEATLAIDLSGMAGAPSQTVLLEADELIGRFGDLD
FTGLGENRNATVTIDYASDQVRLTLSASGTGTGQVTLDILGDESDGSGMTHYQEILEALQADYGTPITDPIAAPPTGASA
SILDW
>OLS42462.1 transposase [Rhodovulum sulfidophilum]AB14
MSIAKLCTWFGIPRRTVYYKPVKAAPKVDPRFADPIKKIIEEEPSFGYRTVAWLLGFNKNTVQRIFQIKGWQVRKRAVG
M
RPRIQAVPSVAAAPNERWSTDLARIWTGKDGWASLASVVDCHTRELLGWHLSKSGKATTASAALEHALIARFGTLGRV
EE
EFLLRSDNGLVFTSHHFTAIVRSYGLKQEFIIPHCPQQNGMVERFIRTLKEQCVHRHRFESIQHAMRVIGDWIAFYNHRR
PHQALAMRTPVEAFRLAA
>OLS42463.1 transposase [Rhodovulum sulfidophilum]AB14
MSMTMDDSIKRWTAKRKTALVIEIIQGKTTVSEASRSFDLTPSEIEGWVEDAKRSMENSLRANPLDIREQYEKQLRDLQ
E
AYGEAMLELGARNKLQALLDTQDGT
>OLS42464.1 rhomboid family intramembrane serine protease [Rhodovulum sulfidophilum]AB14
MAVLRRGRAVLIGLIALCTLLELTLSLSDAGLLAPRLRTLAYDYGGFWPGLLGNWKPNYPAQPYAMFVTYSILHGGPAHL
AVNMITLWSLGSAVIDRVGAWRFGLLYGASILGGAAGFALLAPDLRPMVGASGALFGLAGGLIAWASLDRVTYRETLW
PV
IRAVLLLLGLNVVLWWAMHGMLAWQTHLGGFLVGALCALALDRRALGPEDA
>OLS42465.1 hypothetical protein BV379_20660, partial [Rhodovulum sulfidophilum]AB14
AARLDRSLELPSGALRAGVGLSTSEDDDLRPLLSFSYDRGLKRGTFGVKLDQAFSTDSDGEEALNSDFRVTYRQELTPLS
SLQAGVVLRDTDFVDDANTDSRKLQLDLVYRHSLAEDWNLVGGYSHSISDKSDEDRDHSSKVFIGLEKATRWRF
>OLS42466.1 hypothetical protein BV379_20390 [Rhodovulum sulfidophilum]AB14
MLSACQSSEEKAEAHYQNALTLLQEGDVDRAIVELRNVFHEDGRHRDARQKLAELLLQKGNRQQAYSQYLRLAEQYPD
DL
EVRIALAEIAFSSGRWDEVDRHGKAAEKLAPDDPRVKAISIARAYRDAAASDDASARREAAARAEALLTEQPDNALLHV
T
LLDNAVRDQDTTRALAETDWLIAHDPSDIRPYQQRLQILVSLGDMKGAEALLREMVAKFPKDKENKAALIRFYLAQKDL
D
GAEAFLRELAAASAPDNPGPTIDLIRFLAELRGPEAARAAIDEAIAERPDPLPFQVIGAGLDFEAGRRDQAISTLETILN
GAEPSEQTRNIKVALARMLLATGNEVGARQRVEEVLAEDAAQPEALKMQAAWQIQSDETDAAIAGLRTALDHAPND
SQAM
TLMAEAYIRAGRPELANDYLSLAVDASGNAPAETLRYARKLIDDDRLRPAEDLLLAALRRAPDNSDLLNTLGRLYLQMQ
D
YGRARHVADTLRRLGDEASVAAADRIEAERLNLQSGTDEALSYLEEIAGTADASLSAKIALIRARLATGDVDAALAQAQE
LRQDHPDDDTVAVVLAATYGAQGALDRAAEIYQGLLRNDPNRPGIWLELSNLQMRQGDRAAAAASIGKGLEQTPDD
PRLL

WAKASMLERDGDIDGAIGIYESLYARNSSSLVVANNLASLLATYRDDAESLDRAWTVARRFRDTKVPAMQDTYGWIAL
RR
GESAEAVTYLEAAAKGLPEDPLVHYHLGKAYMALSRQADALEQFRKTVEIAGPADRRPQIEEARAQIEALATAAPAEN
>OLS42467.1 exopolysaccharide biosynthesis protein [Rhodovulum sulfidophilum]AB14
MPTRQQLRVTPASDADGAWQALAPADLDEAALSQHLIVTREAGVEATPFDILRTKALLQMRQNGWKRLAITSPVPNS
GKT
TVACNLALALGRQRDLRAMLFDLDLRDPSVHDFLNVTPEHGIGEMLSGRVPFAAQGLRHGENVAFSISLHPELDPTRLL
L
AEETAERLDEIEAVYKPDVMIFDLPSVLVNDDTRAFLKNVDCALIVIRAETTRHAQFDTCEREIAEHTNVLGVVLNAYRQ
ADAKG
>OLS42468.1 prenyltransferase [Rhodovulum sulfidophilum]AB14
MQHATCQDRVLVVDLDGTLIRSDMLYESLWGACRADWKAGLAVAAALPRGRAAVKRLLAGRAPLDPAALPYDAAVL
ERVR
SWRAAGGRCVLVTATDQELADRIAAHLGLFDEVHGSDGTTNLKGPEKAAFLTRRFGAGRYAYIGDSAADLAVWRDAA
HAI
TVNAPGTLRRQVEALGHETEHLGTEARPLRPYLRALRPHQWLKNVLVFLPMVAGHQLQAGVLLQSLLAFLAFSLVASSV
Y
VLNDLLDLAADRAHPRKRNRPLASGALSIAHGTAMAPALLGLGFLIAAALGPAFLGVMAVYYLLTTVYSFDLKRRPIVDI
CALACLYTLRIVAGAAATGIVLSVWLLAFAIFFFFALAAVKRQAELVDIAARGALGASGRGYQVGDLAVVSQMSVASGY
V
SVLVMALYVNSPAVSELYSFPAALWGICLILLYWISRMIMTTHRGNMHDDPVVYAAKDRVSQVCGLLIFALALIATMG
>OLS42469.1 plasmid replication initiator RepA [Rhodovulum sulfidophilum]AB14
MEAAAIGDFFVCDFLGPSPKHDIAAMEHPLFSLSTRPDRRILDYEHNGVRVTVTPSVRGRATIFDADILIFCISQLMAAL
NAGKPTSRHLTLTAHDLLLATNRETSGDSYKRLRDAFERLVGTRITTNIATGDVETTTGFGLIDSWEIVRRTRAGRMVSV
SVTLSEWLYRAVLAKSVLTLSRDYFHLRKPLERRIYELARKHCGRQPEWRISIPVLWKKTGSASPVRVFRAMVREILRED
RLPEYRLVEEPGDILCVLRRDGVIERGTGEPPPVLSPEALEAARARMPGADVYALEAQWRGVWAASGRPRIRQPDRAF
LA
WVEKRRTED
>OLS42470.1 hypothetical protein BV379_20565 [Rhodovulum sulfidophilum]AB14
MRGTVFSLAEFGAANLLRLVSNLILTRLLFPEAFGLMAIVQVFMTGLQMFSDVGIRTSITLSKSGDDPDYLNTAWTVQIL
RGAILWLAAFAISGPVADFYDEPLLAQLLPVVALNALFAGFMSTNMATANRHLRLGRQTALNIGSQVLALALNIVLAFW
L
RSVWALIWGGLASTLFMVAASHLLMPGIRNRLRWHRASLGELIRFGKWIFLSTIAGFAMNQADRAILGKFITLDQLGVY
T
IAFFFGNLPILLSRATLGKILGPLYRMRHPSKSEANRRNVIKARRLAVGGLVAMTVPLAYGGGLLIELLYDPRYVLAGPM
VVLFAFALPPQLVSVAYGGILLAVGDSRRQFYLTALTAGLQVALMLVGVMTVGIAGVILAPALAAMLIYPLRSRYLNLYN
ADDPVSDMMLLAVSLALNGYACWLHKDGLLQLLS
>OLS42471.1 hypothetical protein BV379_20595 [Rhodovulum sulfidophilum]AB14
MLALWSWVIVTVILFRVLRPAGALVVCLLGAYLLLPTRTSFDLPLMPTLNKHSLPAITAAVMTMILATGGQRPRPVGTES
AGRILPGWIPGNIWIRLLFLVAVLSVGLSALVNNDTLVYGPTVRRGLGPYDAGSMVLTMAVTLLPFVLARRLLADEAGR
R
ALLWGLCLSASAYAFLALFEVRMSPQLNMMVYGFRPSAWIQHLRGNGYRPLVFLEHGLRLGIFLTMATLVACGLSRIDR
R
RRIVCLLAAAWLFGTLVLSKNLGALAIGLLLVPVALFLSTRLQIVAAAVLAGTVLFYPLLRSADLIPVDQVVGLFEKYSS
ANRIGSLNYRLKNEDILLDKAMQRPALGWSTFARNRVFDPETGRDISVTDGVWVIVIGESGLIGYMAQFGLLTVPILLLV
RHPRQVEPSTALLALALAANLADMIPNSSLTPISWLIAGTLAGRLEQARAPAAATAGPTTTDPPRPPRLRGGQAAEEEPP
LPAMRTTPYTRQTTLHSRSRRG
>OLS42472.1 glycosyltransferase [Rhodovulum sulfidophilum]AB14

MGWSPTGRGTDRAVTVTVASMAGLLADLEARMTRGEGFSVATLNLDHVVKLTRDPAFRTAYLAQSHVTADGNPVV
WLSRL
AGQRVELVPGSDLIGPVFALAARHALPVAFLGSTAETLERAREVVEADYPGLRVVAALAPPMGFDPDGAAADEMIEELR
R
SGARICLVALGAPKQERFAARAAPRLPGTGFLSIGASLDFVAGRQRRAPRWVRRIASEWLWRMLSDPTRLGRRYLDCLL
I
LPGLTRRALCQRLRRGG
>OLS42478.1 hypothetical protein BV379_19955, partial [Rhodovulum sulfidophilum]AB14
AGTLRLSGTTTVAGDVTNDGNIETAGGKDTALNLTDDGGFTNNGSITAEDGALTVAADTITISDGASMKGDIDFVGAV
VN
KGELDLEN
>OLS42479.1 hypothetical protein BV379_19960, partial [Rhodovulum sulfidophilum]AB14
SVSGGSLSSSGSIGGKLDVSEGEARIDGSVSGAVDVSGGSLEAGRDSALKSGLGVSGGSAETAGTVSGGVSVTGGRYDN
S
GSVSDGLDLADGSVGNTGTIGGAVTVGGGTLTNGGTSSISRTISGTRSGPTRTG
>OLS42480.1 hypothetical protein BV379_19965, partial [Rhodovulum sulfidophilum]AB14
MTRIVASLDGAGNDVENAGRLELGADLSFTGVGKVSNSGTVEIGANGRLQAERIGNAGRLNLGAGAALEGTGNTLEN
TGK
ITVGDGGSVTDAGAIENRETGLIEFTGGGTIASDSDASGDEPIRNAGRIVTTDDGDDTITLGGANPNVLENLASGSLEIG
DSDSVIGSATTLDNAGSVTIGSGSTLELAALATAAGGSVTNDGRLKAGSVTSAGSLTNNAGAEIASDVTSSGSLSNAGTI
SGDLSVTGGTATSSGSLQDVSVSGGSLSSSGSIGGKLDVSEGEARIDGSVSGAVDVSGGSLEAGKDSALKGGLGVSGGS
A
ETAGTVSGGVSVTGGRYDNSGSVSDGLDLADGSVGNTGTIGGAVTVGGGELTNGGTLSDDVSVSGGVLSSSGSIGGKL
DV
SEGEARIDGSVTGAVDVSGGSLEAGKDSALKGGLAVSGGSAETAGTVSGGVSVTGGRYDNSGSVSGGLGLADGAVGN
TGT
IGGAVTVGGGTLTNGGTLSDDVSVSGGVLSSSGSIGGALDVTGGTATNSGRVGGKSSVGSGGVLVSEGGRFGDDLTIE
GD
TGKGAGTLRL
>OLS42481.1 hypothetical protein BV379_19970, partial [Rhodovulum sulfidophilum]AB14
ELDLENDLRDTLRTDEDGVTRIVASLDGAGNDVENAGRLELGTDLSFTGVGEVSNSGTVEIGANGRLQAERIGNSGTLTL
GAGAALEGTGNTLENTGTITVGDGGSVTDAGAIENRETGLIEFTGGGTIASDSDASGDEPIRNAGRIVTTDDGDDTITLG
GANPNVLENLASGSLEIGDSDSVIGSATTLDNAGSVTIGSGSTLELAALATAAGGSVTNDGRLKAGSVTSAGSLTNNAGA
EIASDVTSSGSLSNAGTISGDLSVTGGTATNSGSLQDVSVSGGSLRSRAGR
>OLS42482.1 hypothetical protein BV379_19975 [Rhodovulum sulfidophilum]AB14
MTAGSLTGLSVSGGAADVDVAVDGTADVTGGRLTIGADGSVAELTTVGSAGSLDTAGTLASLTSAGQVENSGTITGDV
AV
TGGRTENTGTIGGDVAASGGTFLSSGTVSGDVAATGGALASVSGRIDGALTAGSGSEARVTGDLALGGGLGIDAGGKF
DL
QSGTVTMAEDTDAVNDGTAIIGDGTLKGGTGGSFRNRDGGQLSVTGTGRLETDLVNAEGAQAEIDGTVAGAVENDG
EIAL
GGIIDGPLGNDGTVTVAADDTAGITGAIAGLGSYLVAGRLDFGGSFDLGREGETEGRLALGENATVSGDLVTSYGTVTL
A
DGSRLEADLVSSGRLTASGSATVDGDLTLSDGAVLSLSDGTAGDRLDVTGDARLNGTIGMDIDLTDGAQSADAIYIDGV
A
SGNVLLAFNDLTTGDYGQIRDRLDLLSYGSDGGLSVRSSGLPTSGSILYRLDRNAAGDGWGLISGANPAFGGLSSGLALT
QSLIGSVVNRPTSPYVSGLASTEGSPCGWGSWGRAMGGKATASGNSHTALGSFSTEIDASYGGVQAGLDYSCFDGGE
DGW

NLSFGTIFGLNDGSIKQPVYLFDPDTGTVNQGIQTSRNHTDFQQLYGGAYIGASRGRFFADLQVTVNETEFDLENKAAA
G
AYGERLGVDDQSYDSTGHTVSGTVGYAFPLGEESGFSLIPSLGFSVSRTKSDDLYFSNDPGDDADDGVLKFDPINNEVGF
ASVTLSRSRVLPSGVSVLNGFATATAYHDFSDRSVAKYYELDSSGAPLGAPLVSENDGLGNYGELSAGFNYTRILGKGQA
GAARQMDASVRVDSRFGDDLDGWGVTAQMRFQF
>OLS42483.1 type VI secretion protein [Rhodovulum sulfidophilum]AB14
MSRFSKVAWKEGLFLQPHHLQQADRHLEHLIRAQVAALSPYPWGLAELRFDRDMLQQGRIGLLAAAGTMPDGTAFE
APGA
SPLPVAAEVPEDGAGLDVWLTLPDAPVDGQEVAPAAADAATRYVLGRETVADNAAAARSEQVLEVAHPRLELALRKT
PRP
GYQCLRLGRVLDLRDGIVTLDDRVPPTALRLAVHPAYDGYLGRVIGWIESKLDTLARYAADPSSGGGMQAGDYLMLML
LN
RELPVLRHLKSMASVHPERLYEKLVGLAGELATFDQGERRAPAYEAYRHDDPKASFPPVVADIQRLLARDVGRAVRLPL
Q
EVRANSYAALINDRNLFAQATFVLEVSSGLPLAEVQQQFPQLCKVGPSTQMKTIINNNLPGIGLVHLPNPPRQIRVVSTN
VYFLLDRSTPLWSEFSSAPAIGMHFAGNWPDLKLELWAVPEGA
>OLS42484.1 hypothetical protein BV379_19985 [Rhodovulum sulfidophilum]AB14
MADNDDDDDKTVFGQPLPPPPLPPHPLTYPPGSGWNVYPQNPSGTERTAFGQTASTPGTGVPPTPRSPGPHPFYQP
GTGQ
RSAAAGIFPEIRRPEAAPRPAAQARIALDDALRAADAGRGAASNPLLAEATQLLILLGRLRTGLVEMQAGPLIDHVTREI
DDIERRCLAAGLPASEVRDAKYALSATADDIVQNLPGADRAMWLQYSMVARFFGERSSGVGFFRKMDEAMKAPGQR
YHLL
ELMLVCLSLGFEGQYRAMPSGAAELARIRSAIYETLRRISPRPDDDVSPHWTAVPLDARRRGGQTPLWVIAAIAAAMLV
A
LFATLSTLLTRQGATVRDDILALHAGQPEIAIERTEPVIAPYQAPETTQMDRIRTALAEPLETGRVEVDQVNDWVLVRLR
EALRFATGSADLGNGSGPLLAEIAAMLDGEDGPVRVVGHTDSVPMSGRGRFRSNHDLSLARAETVADRLGALVADPA
RLS
AEGKGATDPVAGNDTPEGRAMNRRVEIMIPRED
>OLS42485.1 type VI secretion protein [Rhodovulum sulfidophilum]AB14
MRRALRAVGAAFAAMWRARWMRVLLIVLGLLALGAAIWFGLPMTGSALLASVWLRAGLIVLIAGTLGLVYGLGWLRR
RRR
AEALEDSLMAEPAGDGRVLAERMQEALAKLRRSGGKSALYDLPWYVIIGPPGAGKTTALANSGIGFPGRDGPGSGIEG
FG
GTRNCDWWFAEEAVLIDTAGRYTTQDSDAEADRASWTSFLQLLKKGRPDQPVNGVILAFSVADMMAASEADLARHA
ETVR
TRLAELHETLKIDVPVYVLFTKADLISGFREYFGSFSQSRRRLVWGTTFQTRDRSARTCELVPAEFDALVARLSDEVSDR
MTEEPDGAARIAIFGLPGQMAMLRDTVSDFLRRVFAPTRYQSNAILRGFYFTSGTQEGTPIDQVLGAMAKGGDEAAF
QPA
FLSGKGRSYFLHDLLKKVIFEERDWVGFDRRAVRRRQILRSAAMVLIATVTLGGMAGFGYSFWQNASLLAAAEADADA
YR
DAARNELARTVIDDSDASVVLPQLQQLRQMTAGYVDPRRPGLFEGLGLSRHDELHAAADRAYSDGLERMLRPRLILAL
EN
RLPQLIADDDTAAIYRALKVYLLLGGQGGTRGAEDDAAIAGYFDEIWRARFAAPGEFRLREELTQHLQAMLDLDGDRRI
T
IGIDPEIVRKARDAIVTLPLAEQAYASIRDRALTSGLPAFAPAEAIGGQVERVLATTDGTPLDRLSVPGLYTFEGYWGFF
LDELTHARSRLKDDRWVLGEAADRVDYDRQLGSLERDLHRRYRQEFDAAWTGLLSRLTMARMSADPPRYDALAAAAS
PVD

SPLARLVEEIGRQTRLSRLYDEIDAMAPEDAAQAAAGGDLAGNMGDAAFSRIYSRSGVFQRVVLDAVQARGKSQVRA
GAV
SEDSQRAQVQRISDDFADWHRLLTGEPGARPIDAVLANLAALRENRRNAALAPSPADETLLRQTLSALTMNNSALPPD
LG
RMLSAVEAEFRSEAQDASLGQIERAMNDEVGAFCREFIEPYFPFTEGGRHVSPAIFGQFFAPGGRADRFFTTYLQPHVT
R
GPDGLEPIPGSSIGERLSPDLLKAFDRIEAIRMAFFASGASEPRVDMAVTHISSSPSVNLAVLAINGSELRTQPGSTPGE
LSWPGPGSGVSLALFPQQSGRPNGIRFDAGRWDIVTFLRSGRSRVNGTSVEVTQDVGGRSITYRFSFDSTTVPFLMPEL
S
QFSCPVSVEARP
>OLS42486.1 type VI secretion-associated protein [Rhodovulum sulfidophilum]AB14
MTVAGRASIGLLGKHPGYGDFLRAGLSDEVADGLTGWIDATLAELRDELGQDWGPFWDGAQELRFRVGRAVLGRTL
AGVL
RPSCDRVGRRYPLLLLTEGAEVAPPTASDASQDLWEALSAHLDGAEPGHGARALLRGLDLAIEAEPVGRAAEGPVVWA
HR
PDGDLGALLRAARPVEAERAGLLRAQFWTPGNGPCAAQWLAGTGLPEARALAWMLAGVPCDRGDAGAQQ
>OLS42487.1 hypothetical protein BV379_20010 [Rhodovulum sulfidophilum]AB14
MPAPLLLARYRALDRLAAEVGGQVAMRAPDHPEFRAHVAEALLGQGYRMTAFEPLDAQSLSVDPALNLRLGRGRPVF
LDT
PAPSRERPQASGLRGLDLAECGWPALFEGPSPAWALIDPVCWPDALKLLRDSAQGALCLYATPDAERQAAAPWLVPLS
GR
DPVSRALMALPPDRHAGICFQSQAGAMALRAHFRRYTMAWLPGREAAPLYFRFHDPRVLSDMAQALEPARFAGFAS
PVQR
FFLPLSTELSLAAHLPGGTVGPLDDPATLQGRLLRLAPRGRAVAPGNLHVTEAEFARFDRLKRQRTAQSVARGLYRSLGQ
DKGPEACLEAAGHAMRLGELHDMPSRAQVGALARAVLFFGADFAERDSRAAEILEDRRALPFQRKDRLLRWLALALGE
RG
GAALAAPALRTGQG
>OLS42488.1 hypothetical protein BV379_20015 [Rhodovulum sulfidophilum]AB14
MAGTTMNANEALLWAVGSDMPKPPMMLNTLTMYPPMIEAESSPGRRRMLQQQLDQVVEQANGLRAREEAEVFD
LANGGAA
CDCAPGEPCCLKEFVIEDSEDASRKVTWPVVENGPRTLFLTCKDPYGGQPSAKAKLKITSKEDCKQGREGVPSLQPSSFA
DDNSVIPDSDERIVTTPFQMPALSGKILPPEVITAVYILGLAIQASTYRGRSLPSAGPCQCVGMGTGGLLVCPLPHAKLE
AELEGSLGITIFSSRLPSMSAKLTGKITGEVGNQTLEFEKSGEAEEKDAPETRAEEAAREGSPLITFIEDIQDAMNGLAG
ESGSADALRGPQQDEFGSSIGIELRLGLKIGNLELKGKEASPDLSLALSGCEVTYGLAVTGKMDLLDLFLSRFPRGPEIR
RRLANSDNAVSASAQCAIEVGGEGDLSYGIGDIASLTIGNPDDWEARFEEIEQTFTAQAKITGKVYAEARIEAETWFFKA
EAAAGASVSTGWHFGGRVVRSAGSSRKIERLYHFEGIILRAYATVSVSAAGSKSDSSSDVFGDYASSRSVSGGVTSTSNL
AEPDYTVEIMPPEGEEDGWA
>OLS42489.1 hypothetical protein BV379_20020 [Rhodovulum sulfidophilum]AB14
MTDYPKNAYSLICMGHHAVLEVYLNGAPTLLHASPEDLVFSFPATELLRDGANRLDVVYEPLDVEKRSYTPTPEVAVQIQ
LSQGGVGEATLLNARYGMEEERLVPQPRTVFSGRPVQPDLGTISRPRPEPERPFTIWFGDGKPSREFTRIVPVEFRLDDP
PLGDVAWAGTEPLEDDEDTRDALWQAMARLHGAVERRDRNAFVEIARPYLARMARIWGKPDAGAAADTILAKAPW
ASERA
AGMVPLLDRQEARSATLAFGSDRRLVEFSDRRVAATDAGGEVLSALPLYFARRPGEPFLACYSRDMNIGL
>OLS42490.1 tricarboxylate transporter [Rhodovulum sulfidophilum]AB14
MLADLAQGFLALAHPATFLHLIGGFLLGLVFGAIPGLTATLAIALLLPFTFGMEVTDALVMVAGIYMAGIYSGSITGITL
NIPGAPSGAITTLDGHALMKRGEGPQALGMSAFSSALGGLIGAVVLIALAQPVSSLALLFQTPDKFSLVLMAIVTVTIVS
SGSLSKAIAAMVLGLMISTVGIDPMVPEGRFDFGSYHLIEGIGLLPAVIGLFAVCELIAQAATPSAMTRADRVAQVRAPR

RRDFLPARARICEVGPVAYAKSAVIGVVIGMLPGGGASMASFIAYAEAKRTARRPEAFGKGSYEGLAASETANNAMCG
GA
VIPLLTMGIPGDAVTAIIFGVLLLHGLVPGPALLGSNFIEIAPMFAALFVSSILVFLSVLAFGPVYLRLSQINRGLLYTF
IALVSMVGVFASSWSVFQMWMALAIGVLAFLMRRFGYPVVPALMGVILGPYFEEFLRRSLIVSEGDPTIFLTSPASAALL
VLTGVFVWFLRLRPALRDRQSARESGEGE
>OLS42491.1 hypothetical protein BV379_20030 [Rhodovulum sulfidophilum]AB14
MTGPRNGQILFALGLALANTVYGWQVLQMSRPFAAGEPGPAFLPALLCLALYGLLARVLISEFRARRAEASGEAADTGL
L
PVLGPLFAIGATALFVIGFVWLGYVISAALYSFAIAFYFNVEDSGRWGRSVLVALPVAAGVTGFGWLFFDRLFGLTLPVW
GF
>OLS42492.1 hypothetical protein BV379_20040 [Rhodovulum sulfidophilum]AB14
MNGAQVILKMLELHGVTHVFGLPGETTIGWYKEWRAHSDIEFVLTRDERTASYAAEAYAKVTGRPAVLEAPSPGVTHC
TP
GIAEAWLSSVPVIFFSSDIPVNQDKKHGLTGIDQTALYASICKESFYLTSVKDIPILMRRAFRVATSGRPAPVHIRVPIN
IFHDTAEIADLYAQPEYSEYPAHRPVADHGRIRAAIDVLLAAEKPLIVCGQGALISKASAEVQDLAELLQIPVATTTPGK
GLMAETHPLALRVIGARGGMGYANDYAKSADTVFFVGTNTDSSSTDHWTLYGDPATKTFLHLDIAEAHVGNNFPLKV
GMV
GDARATLAYMAETIRAEHPGLDRPRLDLTEMKRTALDAVFNSNTPMPEGTISPVKLTRALDALLPPGAIVTAEPGVAAIY
PSALLSVREPGRRYLTNYSMGALGYSVPAGIGAAFAAEGPVISLTGDGSLAFVLGDFETVRRSGKNITVILTRNDTYGWI
RGEAILLDDVDEPWSTDFGAVDYVKVAEGFGFQTARIESDADIEPALRAAIAHEGASFIEIRVPSQDRIVPFVPSWVRAA
KKKKLPYFT
>OLS42493.1 GntR family transcriptional regulator [Rhodovulum sulfidophilum]AB14
MTKKTACSVERAYRMLKEMAATYAFKPDSRLNEGVLARQLETSRTPLREALNRLAAEGFLVFRPGQGFFCRSLTPGEIM
D
LYEARAAIECEAAKLAARRADPQDLDALEAFLEASCADYQPGTSPVELVRLDEDFHMRLTALSGNAELSRLLENVNGRIH
FVRLIDLRTLSQRDGPEVVTTAPHRRILEAVRARDPDAAQNEMRGHIERRLEAITENVRNAFAELYAP
>OLS42494.1 hypothetical protein BV379_20050 [Rhodovulum sulfidophilum]AB14
MSIRRLKTLVAISRHGSLSAAAASEHLTRSAVSLQMKQFEEDLGVEIFDRRRRIPELNARGRLMVPKAIEVLAAYDELRR
TARGEATITGTLNIGAMFTAMTGAVPHAMKRMRALYPDLHIQAAPAHSANLVAPVDRGALDAAFIGRPPVLGSRLLFH
TV
TEEPFHLLTAEDCPLDDPREILETYPFIRISRSLWSGQLIDDWLVREDITVNEAMELDGIQMISTMVYHGLGVAVVPRRC
VPAPNPVAVREIAIPSLAGRPVGILARDDSPKASLIDAFCTQMRLVVAEIGRRDAPGEPVGPDQGA
>OLS42495.1 hypothetical protein BV379_20055 [Rhodovulum sulfidophilum]AB14
MRDTKPSASLEAAQLARDLRGQGRRVISLATGEPDFPTPDHIKAACVTALAENRTGYPPVQGIPALREAICQKFHLDND
L
DYRASEVIVANGVKQIIFNALAASLEPGQEVLIPAPGWVSYSEMARLNGGVPVWLETRWDDNFRLRPEALEAAITENTR
W
LILNNPCNPTGTILTEAEIRALAEVLERHPQVMVLSDDIYEHLRYGSAPYFTIAQTSEAMKARTLTANGVSKAWCMTGW
R
VGFAAGPQALIDAMRTYQGQTTGGVMHPAQFGALAALTGSRCFMGPNLEAFRARRALGVQLINETEGLSCHAPDGA
FYLF
VNCAPLIGATRPDGGTIASDADLTRYFVEEAEVVCVPGAAFGLSPFFRISFACGIEVLEEACLKLRAAVARLTPAKEGPS
A
>OLS42496.1 hypothetical protein BV379_20065 [Rhodovulum sulfidophilum]AB14
MKRITASLAAIGLLAGTAALADYPEKPITIVVPYGAGGGSDIMVRTVAPYIEKYLGDDAKIVVENRTGASGIIGWQAVHD
AKPDGYTIGVITTPSIVTKPIEGKAEFTWQDFTIIANMVTDPAALNVKKGSQFEDVTGLVDYARENPKVVTMATGNFGG
D

DHLAGLQLEKLTGAEFTFVPFPGVAARNATMGGHVAVGSFNLGEAVNYQESIDILGIMADERSELAPDVPTLKEQGFD
VI
ASSQRGFAGPKGMPDEAVEKLSAAIAQAVNDPDFRKDAQNQGVVLDFADSADFTAQLDAFDKAMRKIWAESPWLQ
>OLS42497.1 hypothetical protein BV379_20070 [Rhodovulum sulfidophilum]AB14
MKSIHNDVVLGTSVAILAALGIHRALGFGADARVFPLLILGPTVVLGLVIAVRGHLKQRRTGDSPDFFASPGRFALVAIG
MALALVGLRYLGFLTTSAVTIPVLAAALGYRRPLPILATTAGFIAMVYIVFIQILSRPLPAEIWTRLGGS
>OLS42498.1 LysR family transcriptional regulator [Rhodovulum sulfidophilum]AB14
MKLGSENLNITLRHLRALHAIRAHGSFARAAEALGVVPSALTETVRQAEAEAGAPLFDRRMRPPQPTPLGLAFLEETAP
V
LADLDRALGRMRAQAGLSEGRLAVGAAPSAIGDLVGPALARFRAAHPGISCLLHDDVAERLAGLVSDGTLDLAVAGRA
RQ
SPDLTQTELRRDPFGLACGAAHRLAGRDAVALSEIDPAEVITLSAETGTRQLLAGAAALPEALKETRLQAHSTIAQLCMI
RAGLGVALMPKAAVGLFGDPAIRFVPVADLGLWRSLYLLEPARRARSHVATAFLGQLETGAEAT
>OLS42499.1 FAD-binding oxidoreductase [Rhodovulum sulfidophilum]AB14
MTDQSPPALTTALRTAGFRGEIETDRALCSAMSTDNSVYQVMPDLVVAPMDAVDMVTLERVMAEPRFAHLPVTARG
GGTG
TNGQSLSRGIIVDTRRHMTRLLDFDAAAGWADVEPGMVLDDLNERLRPHGVFFAPETSTSTRCTIGGMVSTDASGKG
SRV
YGKTSDNILGLEIARPEGLLSSLAPAPDWARPMLDAAETAARSGRAAFIAATPRLNRRFTGYDLERACPEPGGFEWWRL
F
LGAEGTLGLVTKIRVALRPLAREKRLLVAGFGSFRDALASAMPLAEFEPTAIEVMDERVQKLADGAGILTRLPPALRASE
GQPLAYVFIEFNGDDPDDLQTRLSACETAIKALPGARAIHMARDAAEIRELWAVRSAGVGLLGKVDGRARPVAFVEDC
VV
PPENLPAFLDDFLAVLDAHGLDFGIYGHVDVGCLHVRPALNIDTDRDRLVAVSDAVFALTRKHGGIFWGEHGKGVRGA
YL
RDWIGPEAYAALQGVKAAFDPAGRCNPGKLVGLDAPVPAIDEIPFRAFNAPEGDALSRAFRCNGNAQCLSYAAATPM
CPS
FKATADLRQSPKGRADALRAWRQARLKGRVDAALEADLTATLDACLGCKACASSCPVQVDIPAMRAAFYADLYARTR
RPL
ADRLMLLAERESVRVQALAPFMRPLWPLLSRIGGALTGTVDLPKALAPALPRADRIGAADLARPLSEGTVILVEDWFSAL
FDGAARADALAGLKALGYRPRMLDMRPAGKTAQSLGDPRFAAMATRLRRHLEAAAATGAPLIGLDPALVMQLRQDY
PQAG
IALPPVLLPQEFLAREIAAGRRFPDFATAKGPAPALFSHCTEATGAAQAAADWRRVFAALGVEIDTPATGCCGMAGLFG
H
KTRHQTVSKRLFEMSWAPKIGTGAPVLATGFSCRCQTERLAKLPARHPLGLIADLAA
>OLS42500.1 GntR family transcriptional regulator [Rhodovulum sulfidophilum]AB14
MNGADPAAWIRPVEKQSLSEQAYRGLRNALMRGELQPGTVLTLRPLSQRFGISATPMREALMRLVAENALRLDSRGR
TCV
PHLTRGILMEIRGIREHLEGNAAAEAARVAVPGDIDALEAMHRRMIAAQDAGDFAEAIGENTHFHLMLCEMAGQPVA
LDL
VSNLWMRCGPILSHLYDAGIPPNWEPHPHRRIVAAIRAGDPEAARDALIYDIRGNGEGLLAHVAEGLPD
>OLS42501.1 NAD(P)H quinone oxidoreductase [Rhodovulum sulfidophilum]AB14
MTGVLSVTAPVPGGPEALTLETRTLPAPGPGEMRVRVHGAGVNRPDAMQREGNYPPPPGASDVLGLELSGEVLECGP
GVT
RFAPGDRVMALVAGGAYAEEAIVQADVALPVPEGLSMTEAAGIPETYFTVWSNLFIRAGLQPGETALIHGGTSGIGVTA
T
LLARAIGAEVITTCGSDEKCRASEKLGARASVNYRDTDFVAAVRELTGGKGPEVIVDMVGGPYMQRNLDLVAEDGRIA
QI

AFQQGSRAELDMGPVLFKRLTVCGSTLRARPLAMKAELAREIEAKVLPLILEKGARPIIDSTFPLNRVQDAHARLEASAH
TGKIVLLTSAD
>OLS42502.1 amino acid-binding protein [Rhodovulum sulfidophilum]AB14
MTTRSILLGTALGLTLAGSAMADVKIGSLYPFSGPLALLGEESARGLDLAVDRINSEGGIHGEQIVLERGDAVDNNQAIG
EARRLISLEGVKAVFGTYSSSRVIAASQVTELAGIPYFELGAVADEVTGRGMTYLFRTNPTAENMGIAIIDMLADQIAPA
AGKAPEDLKIGIIYEDSSYGTSVAGHEVAHAKERGLNVVLKSGYPANIVDMSSLVLDLKGKGVDVVLQTSYQNDTVLFLQ
QANEAGYEPMAIIGGGGGYSMQPTADAVGDDVIDGIYDVDFTQYAINPDFTPGIETFLDAYRAKYGEEPRSGHSLTNY
VG
ALAVLQAIGEGEGVEPDQIVASVKAMDIPDGSTAAGYGVKFDDTNQNERARMMGMQWQDGKLVTVYPSDAAVAEI
RLPE
>OLS42503.1 branched-chain amino acid ABC transporter permease [Rhodovulum sulfidophilum]AB14
MTTFLQILLNGLMLGGLFAIVSIGLTLIFGIIKVVNFAHGEFLMGGMFITWLITTQLGLHPYAAVIVVVPVLFVMGALTQ
RLLIQPLMSARDDHIQIFATVGLSTALINLALLIFGADIANTPPHGLRAPLEFGDLRLLSGQLMIFGAAIVLVVGLELFL
KYSQTGRAIRAVAQNRGAAELMGINVNHIYVLTFGLGAACVGLAAVLIAPLYPTSSNIGTYFVLTAFVVVVLGGLGSIPG
AFLGALIIGEVDALSGYYIGSDLREAVVFGLFLLILILRPSGLFGARQNLSHLS
>OLS42504.1 ABC transporter ATP-binding protein [Rhodovulum sulfidophilum]AB14
MLTVENVHAGYGPTSILQDVSLDVGEGEIVTIVGANGAGKTTTLKTIAGLIAPTSGRITFCGEDITRLRGDQVVDRGITL
IPEGRQLFPDMTVRENLLMGAYRRAARDFQDERLEEVLDIFPRVRERLNQTASSLSGGEQQMVAIARGMMANPRLL
MFDE
PSLGLAPIIVSQVFEVVQQIAAKGTTVLIVEQNVYSTLKVAQRGYVLENGRIVLSESAEDLLQDDYVRQAYLGI
>OLS42505.1 shikimate dehydrogenase [Rhodovulum sulfidophilum]AB14
MITGKTRVWSILADPIGHVKTPQEINALCAAQGIDGVMVPMQVHPENLATAVAGLKAIESFGGFVVTVPHKTRIVALC
DR
LEGDAARIGAVNVVRREADGTLTGAILDGLGFVEGLRSEGIEPGGMDACLLGAGGAGAAIAFALAEAGVRRLTIVNRSQ
D
KAEDLAARVGQAYPDVPVAAGAPETASRDLIVNATSLGLKPDDPLPLDDTAFHAGQVVADAIMDPVETRLLAAARAAG
AR
VHPGLPMLRQQIRLMAEHLGALPRPPQQD
>OLS42506.1 glucose-methanol-choline oxidoreductase [Rhodovulum sulfidophilum]AB14
MPQDLPDRSRFDYVIVGGGTAGCILAETLSRSGRASVLLCEAGGEARSPWITIPAGFYKLLTNPAYNWGFWSEAEPATL
N
RRIAIPRGKGLGGSTLINGMIYVRGQPQDYDLWAERGCTGWGWDDVLPHFRALERWAGPDPDGMRGQDGPMPISE
VVEKN
PVGQAFIDAARAQGQPVNPDYNGARQEGVGWYQVNQERGERHSAARAWLARARRRPNLTVLTHARATRILLEGRR
ATGVT
LARPSGAVRVDAGEVILAAGAVQTPQLLELSGIGDAERLRGLGIEAVHDLPGIGENYLDHFCTRMNWRVSQPVTLNELT
R
FPKLLGEVAKYALNRRGVLTYGTGLAHGFLRSRDGLDRPDVQYFFMHASYANAAERKLDRFPGMTLGVTQLRPQSRGS
IH
AVSPDLAVQPEIRPNFLDHPEDRRAMVDGMKLGREIVGQSPMDAFRVAELSPGPDCRDDEDWLQFARANGQTIYHA
AGTC
RMGTDIAAVVDPELKVRGIDGLRVADASVMPEMVSGNTQAAVMMIARKAAQGIISAG
>OLS42507.1 aldehyde-activating protein [Rhodovulum sulfidophilum]AB14
MSGVHEGGCLCGAVRFRVTGDFGHFFLCHCARCRHDSGSAFAATLFSGAARLDWLEGREQVRTFRLEGTRHGRAFCG
RCG
SALPLETEGAGLAVPAGCLDGDPGIAPGAHICWADRAVWEDVLEHAPRIAGLPGAE
>OLS42508.1 GntR family transcriptional regulator [Rhodovulum sulfidophilum]AB14

MGRDLPDDIAARLGRRLAGGEFGAGERLPSERALAAEYGVSRAVVREALSHLKSDGLVEARAGSGVFVTENREARSFR
MT
GLDLDGTEALAQVMELLEAVEVAATRSAALHRDAADLKAIRRALIGMEYAIASDELGDEQDFAFHQAIVEATHNPHFVS
L
CKHLEFGARDVIRKARANTRANLSDLLDAVQAEHQAIYRAIAEGDPEAAAAAAACHLRNAAERTQLYRHRERGA
>OLS42509.1 3-oxoacyl-ACP reductase [Rhodovulum sulfidophilum]AB14
MPDRDSTPPARVPVTFDFTGRSLILTGANGGIGRAVARIFAQGGAHLLLADRDLAPLEDFAATLPGPGARICQQIDAAD
P
DAAQALIDEAVAEFGGIDHVVPSAGIYLAEPFGEMTDAAWRRTLSINLDGVFYLLRRAVPHLREGSSIVTLSSMAAYRGA
LTNAHYGASKGAMVSLTRSLANELGPKTRVNGVAPGIIETPMIGDYMKTRAEATLANTPLGRLGQAEEVAAAIAFLCSD
A
AGFITGETLQVNGGLHMA
>OLS42510.1 3-oxoacyl-ACP reductase [Rhodovulum sulfidophilum]AB14
MTDFTGKRVLVTGAGRGLGAAIAEGFAEAGAETLLTDIDPETLSATAEALGARGLTARAIPLDVTDRAAVEALAATLDGL
DVLVSNAGVAGRATFDDPAQAEVWDRVIAVNLGGAFNIARALTPALIARRGNVVQLSSVAGFVAGGSTAGYVTSKGAI
RS
LTQAMARDLAPHGVRVNAVAPGIMDSEMATAQLARPGGADWFLNRAMLGRIGTQAELVAPILFLASDRASFITGTVL
PVD
GGFLAA
>OLS42511.1 MFS transporter [Rhodovulum sulfidophilum]AB14
MPDITDLTPLAPWREIRATEADRTALDPETGRTMLAQLCLIRAFEEKVLDLAGHGLVHGPAHSAIGQEGGAVGSALAM
RP
TDQVNGSHRAHHQFLAKALAYVAEAGIDPEAEFDGEIRDLSKKTLAEIMGLSQGFCRGRGGSMHLRWAEAGNLGTNA
IVG
GGVPMAAGAAWAHQQAGTGDVVYTYFGDGAVNIGSVLETMNLAAAWKLPICFFIENNRYAVSTHVNEATAEPRLSA
RGLA
FGIPAFKVDGMDALSVLIASHEANRIMRSGEGPVVIEADLYRYFHQNGPLPGSAFGYRDKAEEAEWRARDPIEALAGLL
Q
TRQIIGEAAVAGLRARCETMMEEIAGELTETEGGKTRVIPSLWPEESFRDAGLRGDLSEMDGARYEEEHSFSGALDPNA
K
FIDAVAAVMHRRMDTDERVVVLGEDVHRLKGGTNGATRGLSQDFPDRCLGTPIAENAFTGLAAGMAADGRVRPVIE
FMYP
DFMWVAADQVFNQIGKARHMFGGDSPMPLVLRTKVAMGTGYGSQHSMDPAGIFATAPGWRIVAPSTPFDYVGLM
NSALRC
DDPVLVIEHVDLYASKGAGPVDDLDYCIPLGKAKVVRPGARLTILTYLAMVEKTRQVVEDLGVDAEIIDLRSLDRASLDW
ETIEASIRKTNNVLIVEQGARGTSYGGMLADELQRRVFDWLDQPVQRVTGGEASPSISKVLEAAACARPADIAGAIRAA
M
AAQGLPLAAE
>OLS42512.1 pyruvate dehydrogenase complex dihydrolipoamide acetyltransferase [Rhodovulum sulfidophilum]AB14
MATELTLPALSAGMEDAVIAAWLVGPGDRVEKGQPLAEVETDKATMELEAEAPGRVGRLAVAAGARADVGQVIAVL
LAEG
EALPAPDTEAAAPASASAPAPAPAPAAMAPTMGPAAEPDRPRASPLARRLAAQAGLALDGLSGSGPKGRIVRVDIERLI
A
SAPTQTTPAPAPAPEPAPEAAAIPPGLGPYEARPVSQMRRTIARRLSEAKQTIPHFYLEAEADLEALMALRAQVNEGRE
G
PARISVNDFIVKAAARALARLPEANVIWRGDEIWQLSDIDIAVAVATDGGLMTPILRGADRMSLGTLSAEMKDLAARA
RA

GTLRPEDYQGGGFSVSNLGMYGVRSFSAIVNPPQSCILAAGAAERRPVGRGDQIVLAPVMSLTLSVDHRSVDGALGAQ
LL
AEIRQGLEDPMSLLL
>OLS42513.1 AraC family transcriptional regulator [Rhodovulum sulfidophilum]AB14
MQRNRYHHISWLGGIELFEAAFSTQTFPRHAHEGFAIGAISEGAGGYLCRGENMVLPAGSLSLMNPEEAHTGHAAVGL
VR
YNMVYASEGAVRDVLGLRQLRGFAEIAPQDRDFALTRALARLAVCLNDARAADRKLATEEAVHDVLAQAFSHYGRAEL
RK
PGREPAAIRAMLDRIEAAVAEGDDLSLTDLAGAVGLTPSYLIRSARRATGLTPHGHVMRIRVDRARRMLLDGVAAAEA
AA
EAGFCDQAHLIRQFRRHYGVTPGALIRH
>OLS42514.1 hypothetical protein BV379_20180 [Rhodovulum sulfidophilum]AB14
MPPLQTVLAMAGFALAASATPGPVNIIGAMTGARHGARRALRFVTGATASFLALFVVFGTGILAGTGWILAIARPMTVI
G
ALYLLWMAWSLLRDDGRISGDDIRQAPGFWSGAVVQGLNPKAWLAAVSSLSTFVLPLEDRATGLAVFALLFGAICWLS
LA
AWAWGGARIGHGQMRAFNRIMALILALSVIWMLSRSLA
>OLS42515.1 Cro/Cl family transcriptional regulator [Rhodovulum sulfidophilum]AB14
MKKAFLGGRLRQLRETRGLTQAALAERLGLSPSYLNQIERNQRPLTVQVLLKINAAFGLDIQVFSEEGDARLLAELREAV
QGGEPAVGASEIRDLIDNAPALARRVVALHRHLREAEDRAAALALAQGAPDAMPVPAPFEEVRDFFYDNRNFFDRLDA
QA
EALAETLSGSAAGLTPGLVPGDPLPGLAAHLKTRHGIVLRRNEADGGDLRHFDPDRRELSLSRDLSPGQMAFQAATQIG
L
IEAAGEIDRLVGDGALSGEEARRLARIGLANYFAGAAVMPYGAFWRAAEESGYDIGWLGHRFGMGFEATCHRLSTLQR
PG
MQGVPFFFLRVDRAGNISKRQSATDFHFSKVGGSCPLWRVHSAFDRPGEILTQIAEMPEGRRYFWLTRMVQSRRGRH
GSP
GKAFAVALGCDIAHAGRLVYSKGLDLADPAAATPIGAGCKICPRNGCPQRAFPMLGRPLAADPARAPFAPYARAAGPD
EP
PR
>OLS42516.1 hypothetical protein BV379_20190 [Rhodovulum sulfidophilum]AB14
MMKKLALPLLAATVLATAAHADEREAAAISAFESYCLASGGDLGKAIEALDASDSFEDGRKSGSGSFVYASYLGPDGINA
SVVIGGSMSDDKCSIILQNVADPMALADEMSLDMAKAAGAEPMKWEGFGDYGKGAYGYQRDDGDVLVAPMTTGIS
NDIVH
INFYPT
>OLS42517.1 hypothetical protein BV379_20195 [Rhodovulum sulfidophilum]AB14
MSPLRATLTCLRRFARFGGRAARAEYWWFVPACLLALLGAAALDMVLFPADPDRGLPDRRPLSVFVSFVLTLPALAAG
WR
RLHDVGRSGWWLLLPGAALLAFLVPAVVIRGMAAQATGGSALDVILLGAPLVSLLSCIWLIVQLARPGEPHENRFGPVP
E
D
>OLS42518.1 hypothetical protein BV379_20200 [Rhodovulum sulfidophilum]AB14
MIRGTATALALLLAVPGLAAPETGAPPAEAGVAADGQAVDVRTEHWPSGKLRSQRHFVDGREVGLSRRWSREGALIR
ETR
FSDDGKVLHDRHWKDGKLVHERLPATVGGHGAGEKIVERYGSITETDIRAEGYRLTTRHRGDELIDRSEVIDGQIQGRYI
LTGRIDNDVSRVTYVDNQPHGLFTRTWRGETLERGMYDHGTRIGEWLRTERYGTAIHEVYDPEGRLDGPRREIGANG
QLR

RLETYVHGTLDGPWEERDREGRLLSGGAYSAGQKTGPWREGGPHGEEYWQGSYVAGLRNGPWERLDAQGYRLERA
RYEDG
ARDGLRYLFRPDGAVGEVQSWKDGERDGPTTRYEHGLPVGRDLWRDGRPVATGLSPEAE
>OLS42519.1 hypothetical protein BV379_20205 [Rhodovulum sulfidophilum]AB14
MTYKTAGYGLILVLAANVAGAETLTFAPAEGSERSYRVELSVRSVEPDSQTVPLDPERVSFLARIRAAERDGDNVDLTLN
PLWLKTRLGSQTMTTAAPLPDRVRQGFEAGFGARLDLATGRLGEVRPLGETEGPAPMLGGLREQFAAAFPPVPIELAE
GW
STTVPEYGGVRDVTITVDHLTADRVFLRYEGEDFIARLAGLVVVDRDEGWVERAAITTDRRLVPGTDENGSLRETLAIAP
RGYPVTVHADNSMRPAEWRDMPERIEPPAPLASEAEVFPGGPGRLVEDFGGLRLEFDHRAAQWRNIGRFALSDLRLF
AGD
RALDVPLTAEPPLTLPNWRDPEGVPQISRSLLDATTLRDVSQDLEAATELRADLDWYPVAPFVMSLVPDETGHAEASRD
G
ARAELAPAPEGYVLTLSGAETDVFSWALPEGSGAQGMIFAGDRGPDWLTPGESLARRIASPDYSAIRVLLRMEAPPEEL
T
VRVDRYAEAPAASREMRFLTEEGRRLDPDTAPERRPLFRDGPPPAADEVAPEGLESGALRLPLAPAQAAHCTASLAPPA
T
EGAETLVFVRRKAEDADVLELQTGDGQRRHFYGLGSRQVTLSCDATLAWTEAGVQPSSDRPWRIDPAALDVADDMT
VAEV
TEQLRFVDAQGVPLALSAPGGGALSPDDGIREAMFPDGTLQVAGPPAKILRAELKPEPLNRSFAVRFPDLPVPEAAAQ
>OLS42520.1 hypothetical protein BV379_20210 [Rhodovulum sulfidophilum]AB14
MRFGYLMSEERGAASLVLAETATLLSAQGLRLAGAVQFSAADPDGHPCDGDLKVLPDGPVLQLSQPLGQGARGCRM
DAGA
VETAAAEAARHLGDAALLIVNKFGKLEAGGHGFVPLIAEALERDIPVLVGVNGLNRSAFESFAAGLAEPVLAETGAVLA
W
LGYAPAIASANVPNTPRARASA
>OLS42521.1 trimethylamine N-oxide reductase I catalytic subunit [Rhodovulum sulfidophilum]AB14
MTSTSNSSLTLSKRSFLKGSLATGLGFAGLPLFGRGAAAQAASKGVVSGCHWGVFYGMVEDGRAVEFKPWEGDPAPS
PQL
PGVLDSLYSESRIRYPMVRRAWLEQGPGADPDGRGSGDFVRVSWEKAIELVAGEITRVRETYGHPAVFAGSYGWKSSG
KL
HNCQTLLRRMLNLTGSYTNSVGDYSTGAAQVILPYVSGSIEVYEQCTTWDNLAENCELMVFWGANPLNNSEISWQVA
DHG
SFSGVDQIKEAGVESICIDPVRTETAQHLDSEWIAPAPQTDVAMMLGIMYALQDEGLHDQAFLDRYTVGFDKFLPYLLG
D
TDGTPKTPEWAAEICGVPADKLRDLARRFSSKRTMLALGYSTQRQHHGEQIHWALITLASMLGQIGLPGGGYGLSYHY
SS
GGSPTHTTPILKAVDDASGVKVEGAAWLAASGAVSIPVSRLVETLLNPGKVMQFNGHEVELPEIKLAYWAGGNPFSHH
QD
RNEMLRAWRRLDTFIVHDFQWTASARHADIVLPATTSYERNDIEQVGDYALSHIVPMKKIVEPVFEARSDYDIFADIAA
K
LGKGYEFTQGLSEMDWIRGFYEAAKVEARAKSMEMPVFDAFWDSNKPLAFPLAEAQKDFVRHADFRADPLLNALGT
ASGK
FELYSTAIEKYGYDDCPPHATWMEPIERLGGPTTLYPLAVASNHPDLRLHSQLCGTKIRETYEVQGREPCWMHPEDAEA
R
GLSDGEVVRVFNQRGQILAGLVITDVIRPGVIRVSEGGWFDPVEPRKIGSLCAYGDINNLTTGVATSRLAQANCGQTA
MA
EVERFEGPLPPVTVFSQPA
>OLS42522.1 hypothetical protein BV379_20220 [Rhodovulum sulfidophilum]AB14

MTEFLVDMSEEEAADLAFMAEFLASLLLTPPDRDCLQALRSMQGQVSLGRIGGYLGEETAVDAIRQILTAGSAEEVAVS
L
ERRHTALFEGIFRQRSLPPYASAWDGTGRLCGPAVGRMQQILRDLDIHLEGSSEMPDHIGIQLATLAEALRRGRPDIAA
R
LLDELDWCARFGAALIKADDTGFYGALARLVLAFKTLMTADEAAAQPLHDPATARI
>OLS42523.1 cytochrome C [Rhodovulum sulfidophilum]AB14
MTVKARTLVKLAGSLLVLGGAGFLVVTSPWTWSAVNGSRELPALEGADLENGRTVFLASDCATCHASPDAEDETHLGG
GR
SLDTAFGIFHMPNISPDAETGIGSWTLAEFDRALREGVGPEGVMPDGQNLYPAFPYTSYQRLTGEDSRDLYAYLMSLPA
V
RSDVPDHELKFPYNLRRGVGIWRLAFLDGDALIPGEVPDGVDPDVYHRGEYLVEGAGHCAECHSPRGLMGEVIADKRY
GG
GPNPEGTGWFPNISPDPTGIGYWSTARIANYLHTGKNPIGRMADGDMAEVVANTSQLPFSDVQAMAVYLKSVPPVE
NRAP
GQPAPNYADHVVMLDQAVGKAPQLPVSAASAIAAGDSATVVETKDVWLGADMVATENQPDGKILGGAAAEVTARD
GDKVQ
LTLRGWQMEDAPTVLYQAKGQRVMMAVFDDAAAAAVTRGAPEVAAATGQSWVPAEIALWSDAGGMNTDRGAV
WAYGQDTF
QTACAACHVLPQKTHFTANQWIGTLKAMRRFTSFSDDQYRLILAYLQNHSKDLNVSKETVQ
>OLS42524.1 hybrid sensor histidine kinase/response regulator [Rhodovulum sulfidophilum]AB14
MPPPPAPSSGPPCWPADILTDLLSGASDALVDAILRLGAANGFTDQTSSMRAAWVESIEIINASLIASLSAPNQESGPNG
HNDYATDPRFAGLREVAWRHRRSGVPLWLNHGLLKDYRHGYVAFFRELFRDEKRAPALDDPEALIERLGLFFDEVELA
ML
APWSEPGTEEAAMADRLRRLTRERDQLFGVLESLRGPVFIADEDNRLISANRAAMQTFLGLDETGALAYRLALQSRRSE
L
QPIVDEILGTEGSQWSAIWLQTQSGSRCFDIRLETVEDTAYKLDRWRIVLMNDVTDHQQAIEQAREAERTMSLFLAAM
SH
EIRTPLHSVLGAAELLRDVPPQDVEERTRLTELLDISARGLSATLENVLSFSRFEQQAPKPRPGPVVLAEVLENLVRSRD
ILARQQGVPLMLSLAADLPDEVGLDWSMVQQILGNLIQNAIRHDDGRGVALELSRDTGASGDELVFAVTDHGPGLPA
PIR
EMLTAAPTALRPRPTGKSGSGLGLAIAQRMTLAHGGTISGGSAAGPGGGARIEVRLPLVAAPAPAKAPPRPDGALSSKS
C
LLIDDDPVGVLVTLAMLERMGLSVDHARSLRQAGALCQAHPENYDLFIVDYRLPDGNGVDFARSLRQGGTLGERPVFL
LS
ANVDLVRQTDGQAALFTALLEKPLDAGALGRAIRSGTRPIRASAMLDGLSPAAQRRMAEAFAEGWRAFGARLETCTD
RAP
DAELAALAHKLANGAAIFGRTELHAALRTFEQAFDSPAATERMRAVARARVLATAPEDMTAPNEAPDA
>OLS42525.1 DNA-binding response regulator [Rhodovulum sulfidophilum]AB14
MSATADILLVDDDILVQVMVEDIVTQLGHRFHAAGDGETAREAIGRNGFDLVILDRRLPDTDGLLLAPLLQQDRKIPFIV
FSSLDAPTDQVLGLGMGASDYVCKPVEPAVLRARIQARLTARRTPEEQTTFRLGDALRLDAVSRRLWIADRVEVLSPAE
S
RLLICLLRNIGKPRDRMQISMATCGREWVYGDRTIDVLISRLRRRLKGSEVRITTVHGLGYVLTLDN
>OLS42526.1 monooxygenase [Rhodovulum sulfidophilum]AB14
MKKIGFLSFGHWSPAPGSGTRSAADALIQSIELAEAAEDLGADGAYFRVHHFARQLASPFPLLAAVAARTRTIEIGTGVI
DMRYENPFYMVEDAGAADLISGGRLQLGISRGSPEQVIEGWRHFGHAPEAGQSDADMGREHGRIFFDLLKGEGFAQ
PNPH
PMFPNPPGLLRIEPHSEGLRDRIWWGSGSNATAIWAAEMGMNLQSSTLKDDETGEPFHVQQARQIRAYREAWARA
GHSRK

PRVSVSRSIFALVDDRDRAYFGRGVQSRDSVGQLDETTRAIFGRSYAAEPDRLVAELEKDEAIAEADTLLLTVPNQLGVD
YNAHVIEAILTHVAPQLGWR
>OLS42527.1 prevent-host-death protein [Rhodovulum sulfidophilum]AB14
MWTLQDAKNRFSAVVEAALAGVPQEVTRRGKPAVVVLSADDYRRLLKGAAAARESFAEHLMAFPAPEIEATGIGRAE
VRP
RDVRF
>OLS42528.1 VapC toxin family PIN domain ribonuclease [Rhodovulum sulfidophilum]AB14
MYLIDTNVISAVRRPDRAPQVRAWLAARPEPELFLSVITLGEIERGIRQQETRDPGFARDLRVWLDRTMLVFADRLLPFG
AAEARIWGRLSAEIGHPGADLMIAATALQHGATVVTGNVPDFEPTGVPIENPF
>OLS42529.1 replication protein RepB [Rhodovulum sulfidophilum]AB14
MDDIPRDRLTGPLRRGAVKKHVAAIHVSGKLTLLQRKLSNVLLLNAYDTLISQSRHHIDARTLCLMIGYNSNDMETLKQS
LRGLAETVAEWDMLDAEGRQEWGVSALLSYAKLKGGICEYAYSPALAEKLHDPKVFALINLNIQRRFTSGHALALYENC
Y
RFIRTGSTGWWSLDLFRRLMGVADSPYYEVFKHLNAKVIKPAVAEVNKTSNIVVTPELRKMGRSVTDIRFRIAENPQLAI
LDLDDGEGLRNSEVYARLRALGVSDRLARQWIAEHGQDYVRRKLDYVAGQGGVKNAVGYLTAALREDFDGSAPEPGV
SPR
NPAPPRSFRARRLERIRDLAAARKPTQRDADKRLFMARLDAAARDDFERHGWMSALNAEAIAAFWEELHPGAFDGLD
>OLS42530.1 ATPase [Rhodovulum sulfidophilum]AB14
MAKKTGAPSKPDLPPYFNIDPAAAAAKLSAPIDTPRFAKAAAFAARGRDDLARRGYAPDGKKRLRKFSTWEVCRYLIPV
N
PAHLRRVLRQNPDLPQGEGEGGAKWYTLDEVLRLREHFAEEGAKDREYQAWRPEGLPAKVVAVANFKGGVGKTSTC
AHLA
MSAALDGYRVLVIDLDSQGSMTSIMGGQVEDEWQTVFPMLARDYARHLQAENRVREAAGEAPFGFDETLTAALEVS
PRNL
VQPTHWPNIDLIGAQLNLYWAEFQVPVWRMQMRSWPLWDSLQNALEEGGMLDDYDIVLLDTPPALGYLTINALSA
ADILL
VPLGASFLEFDSTGRFFDMIYSTFASIEEGENRARRADGLPEIRFEWDAVRALVTRFDAAQQTDLANVIQAYFGDFMTT
Y
RQELTAMVGQAGEQVSGIYETDYRDFNRDTYVRGRETFDRTWAEVKEVILGTWWRDARMADAATEEV
>OLS42531.1 chromosome partitioning protein ParB [Rhodovulum sulfidophilum]AB14
MAKRRKLQAPSAEELSRIEEEFRRETGPGVAARPMAPIAQVAAESAGAHEPRPAEERADTARDRTDARRLREAEGRGL
VM
LELPLDEIDADALVRDRVVLDAEELEELKSSIAKNGLRLPVEVFELASDGRGFRYGLLSGYRRLKAFRDLKALTNDSRYD
SIKAVLRDPEEMGGTFAAMIEENEIRATLSHFERGRIAVIAVQQGAFVNTEAAVNALYPMASKAKRSKIRSFALIFEELG
DMLSFPDLLREKDGLKLAAALRDGAEARLREALAERVPADPADEAEIIAEALSKVETGTDPRRGGRPPKRARQGAVRRIS
GGLRMQAGEDARGWYIRLAGTGVDRELVERAMEELERLLERPEG
>OLS42532.1 type IV secretion protein Rhs [Rhodovulum sulfidophilum]AB14
MAAFFDQKARMGRLETALGENELVLQRVEGSERVNGLYDFEVGCLAARADLDFDALLGTHATVTLVAHDGAERDFDG
LVT
EARWLGAGENGHRYRLRLRPWLWLAGLRRNQRIFHNRTVVEILEELFAPYASAGRLSVELSADYPELEYTVQYGETDLA
F
ACRMMERHGISYHFRHARGSHEMVLTDTAEAHGAIGARPFRPVDGHHQEDVEHFWEWRPARRITTGAIRLTDYNFK
TPVA
AMETDRAGDAAFEHGQIESFDWPGDYLDQGRGQQVAELRLGAERGQDRRFEAVGDIPALAAGMRVTLEGDPVPGS
GEDYL
CLVATHSYTSDNYGSGGEGGDGYAYTGHYVLMPAEAPLLPELKTPRADVRGPQTATVAGEGEVDCDEYGRILVRFHW
DLD

EDLSMRCRVSQSWAGPGWGGMVIPRVGMEVVVEFLDGDPDKPLVTGCVYNGRNAPPAALPEHKSRSVFKTKSHEG
EGFNE
LTFEDQAGEEFIYMHAQKNLDVHVENSAQRRVEFDDSVSVGNDSMLAVAANRTETVEGRMDVTVTGAVSEKTEAAR
GMDV
AADLAVRAGGDLTLKAGGEIVIDASKITLVAGGAALTLEGGAVNAVPVLNVGSASPGAAAVPAIPEVLKAAAGEGTPFV
S
HCPLADA
>OLS42533.1 hypothetical protein BV379_20275 [Rhodovulum sulfidophilum]AB14
MKPVARLGDLHVCPKHGKGEIVTASGKTFCDGRPVALVGDKISCGAVIVDGSDVAFCDGRPVARIGSRTDHGGQITTG
SD
LKTC
>OLS42534.1 hypothetical protein BV379_20280 [Rhodovulum sulfidophilum]AB14
MMLILRIENAERLTDGSPAWIGLDGRDFCVGRRRGMDWVLPDPARHVSGHHFDLKFSEGRYWLVDLSTNGTFLEGH
PHRL
QAPHPLVPGDRFRVGPYVIAVAEGSDGAAAGEAGGGPLPAEDDPWDFGALAAAPVASPGPRPAPAMSGPLAGSGR
DDGLQ
GFVPLAPSAPAVPGAPVPAGTGAVSGAVSGALSGGPAPRRQPSGAPPVAPEPQPFHPPGRDPAAAQAAPSPAGPDR
DEAF
LRAFCEGAGLDPDLAKGADAEALARDLGRVARVATGGVMRMLRDRASVKQFTRSGERTMRSASGNNPMKFLPDTDS
ALEV
LFLVPRDGFLDGAGGFETALADLARHQQGIFAAIQPALAAVLDGLAPEAVEAGATGGNLLAGSRKARAWDTYVTRWD
ALA
GKGENGMLDAFLEAFSEAYARAQGEMPD
>OLS42535.1 hypothetical protein BV379_20285 [Rhodovulum sulfidophilum]AB14
MGLDSLAEPMADDGPCGPDLNARMDPDFDAYYFAALGRLPDAYARPGVTRPDGSVSPDRVFDPGTLDLAGELRQID
ALLE
RSRDLRLLALRVQWLALAGRVPALAETLETVALLLERFPREVHPGAESDPAERREALAELAAPATVIQPLAWAGLAGTAE
VTLRKLRVAAGQATPLQGESGLSTESLTAVLADPGNGARLTRTLAALDQATAAVTRIVAACAADPEAPFTPHLDPLTAVL
DEMRGAIVAVRPDLAAPVPMPETAAPEPVPHSAPVQVPPPTRELPAGAPEVRDQDHARRLLEACEAYYRRAEPSSPAL
LL
VTQARLLIGRPLIEALEVLMPGRAAEAQIGFGPETGFVLDSARLRSLTEEGGPGDPAPVRACSTLRPPPPAIDSAEHAAG
TLRAVEDHFRRTERSSPVPMLLERAQAYLGKDFPAILRDLLPSAEGGR
>OLS42536.1 type VI secretion system-associated protein [Rhodovulum sulfidophilum]AB14
MSSNSGSGFIKRNRPPRVQIQYQDPYDSEKMVELPFVMGVMSDLSGNNPGVEKPAVEDRSFTDVTKDTLDDYMASV
TPGV
TFTVDNRLQPGAGEKLAVNLQFEKMEDLEPAAVARQVPALRQLLEAREQLANLQRYMGTKPRAQEHIKRLLADPELM
AAL
ADSAARDDDASSDDA
>OLS42537.1 EvpB family type VI secretion protein [Rhodovulum sulfidophilum]AB14
MTQNAERQAGAAEGALDELSEFQDILKQTIKPRSDIAAREVDNAVTALAREALGSSELIAEDVIDTLDAMIARLDEKLSD
QVNEILHAEEFQQIESSWRGLAYTLNNAETDASLRVKVLNISKSELQAMMRRYPGAKWDKSPLHTMIYEQNLGTLGGK
PF
GALVGDYYFDHSSPDVNLLQSVARISEASLAPFISGTAPELLGLDSWNEISVPPDLSEIFETPEYAQWNSLRDSENSRFV
ALVAPRVLAREPYGPNSNAVVEEFNFVEETDGHGGRNYAWMNAAHAMAANINRAFKEYGWTVRIRGVTSGGEVAN
LPTHL
FDTGDGAKDMKCPTEVSITDRREGELSKAGLIGLIHRQHTDKAAFIGAQSLYRPKKYVDDLATASDNMSSRIPYIFAVSR
FAHYLKVMVRDKIGQSPDRLQLEKELQAWVNKYVSGNPESASEMEKARKPLAGAKVEVIEDELNPGYYQGKFYLRPHF
QL

EGMDIGMSLVSRLPVGK
>OLS42538.1 Hcp1 family type VI secretion system effector [Rhodovulum sulfidophilum]AB14
MAIDCFLKLENNIKGESLDDKHKDWIDVLSWNWGLSQSGTTHMGHGGGGGKVDVQDITVTKYVDAATHDLIKRCCS
GEHI
TAGQLVVRKSGGSAPVDYLKIDLKDVMITSYVTGGSKDGLDRVQETLTLNFRAFQVTYTMQEETGGAGPESTAGWEIA
EN
KEWAA
>OLS42539.1 hypothetical protein BV379_20305 [Rhodovulum sulfidophilum]AB14
MAGPPQETGPGAREAWRDRDRAKISIFQIFRSAHEEHDARRANRPAENGETEVSARAKPRREGVSASILRAHLQADLD
GL
LNTIRLDAAIDLSTAPHVANSILNYGFRDLSSVSPAEFHSPAIVESIRESLLRHEPRFLPGTLEVRVVDANGDSGQRLSV
TVEGELTGDPVDLSVGFDAEVDLGAGKMDMSDLRMRS
>OLS42540.1 type VI secretion protein [Rhodovulum sulfidophilum]AB14
MNPALRDTYNRELALLKERTAEFARDYPGLADRLGGLMADNLDPTVAGLLEGSAFLAARVQLKLQDEFRGFTEALLDQ
VF
PDALAPTPSAMLVEASVPAGNSDIVKGLRFDPGAYMDARFRDADKRIACRFSLAAPLTIWPLKLAGLRYLSGAGPVGAL
G
QEIAEGTKAGLVLDLARIAPSGLADGSAPLAELELDRLDLHLTGPMAEAVALYEQIFARSLRVSLRWLDPQGDPVFARLP
PDCLEQVGFDPEERLFPHHARLFDGFALLREYFVFPRKFLGFRIKGLADHLPRIRGSEVQVIVEFDRADPRLAERLSEGD
VALNCAPAVNLFEEMSSTVRIDGKQHEYVVTPNATPVTHYELLDILDVQAHYATHRSKERVQPLYALPEGGQDPRRTLY
Y
TARRKPRRLTAREHRFGPARNRYRGTETFISIYEPDGGDDIQRLQVRALCSNRHLPAELPIAQSEGDFHMTDDVTVSLAC
RAGPTPPRNPLGRSDPGAGPRAGAGDVYWRLISYLSLNQFGLGGRQEGDSAAALREMLALFADFSDSAAEAGVRGLR
AVE
TRPVTRSVAMADGYHLARGTEISLSFDESDYESTGVMLIGAVLDRFLSEYAAVNSFTQVRVASVQRGAIRSWPPRSGSG
P
IL
>OLS42541.1 ClpV1 family T6SS ATPase [Rhodovulum sulfidophilum]AB14
MTEITIETYAGKMNRAGYDAFLQGMRHARGEKNRHVDLSHWLFHSVSNQNADISVTLNELGLDRGRVLRDLDAAMA
ALQK
NVTETPGISEVLSDALNHAWTYATLFFGEAQIRTGHVLTALLNDPGLKRHLLQLSKVFAEISVDRLGQDARSLWAASEEE
DMRPMDGSGLAARGEGAEGAAGRGGGQSALGRFAQDMTAEAEAGRLDRVVGRDDEIRQVIDALLRRRQNNPILTG
EAGVG
KTAVVEGFAQKLAAGEVPPKLKGTRLYMLDIQAMQAGASMRGEFEQRLKSVIDEVQASDVPIILFIDEAHTLIGAGGQA
G
TGDAANLLKPALARGTLRTIAATTWSEYRQHIEKDPALTRRFQPVTVDEPDVGTCCAMLRGILGPMEAHHGVRISDEA
VV
AAVQLSQRYIPARQLPDKAVSLLDTAAARVAVSQASVPARIEDLKARIDALDSEIAAKRGAQDLGEDAGERIAELEAERA
EAGQDLARLEELHDEQKALVEQILGLRARIAGAADGAAAEDMAGEEMAGEAGTDHGEPGEGEEPLDIDALRAEMHER
LAT
LEAGDPETRMVEPHVGAQTVAQVISDWTGIPTGRMVRDELQAVLTLKDHLRERVVGQDHALAAIARRIETSRAGLAN
PEK
PIGVFMLCGPSGVGKTETAIALAEALYGGAQNMITINMSEFQEAHTVSLLKGAPPGYVGYGEGGRLTEAVRRKPYSVVL
L
DEVEKAHPDVHEIFFQVFDKGWMEDGNGRRIDFRNTLILLTSNVGTDTIMAMAEDGRTRPDLDEVTAALKPRQLEVFP
PA
LLGRVVSIPYLPLSAEGLAAIVRLKLSAVEARMRGAHGAELVYGDAVMDHIVDQCHDPDSGGRMIDNIITNTILPDLSRE
VLGRMVSGEGIARVEILLQDGALAYSFEGENA

>OLS42542.1 hypothetical protein BV379_20325 [Rhodovulum sulfidophilum]AB14
MTQCFVDDAGVSWSYDQQAALRLIAHYARLRAMNETTRVVTSRDSLIQKLYLARELANVETDVNRMLARKTEIEEVEG
RR
FATLMLGAAGDAQGYLIGLRRETDRLMSANQSIYRSAQDINQAAIDHAETAIKVAKGVRDSSVLTLGVIATVATGGTTIA
VASGAGAGLSATATYQDTGSASKAAIKGGLFLLPLGTRQITGAATAAGASPTMTAAMSLAIDTGAETFEGVYVNDATLE
R
ALSAALINQVSGRYVGALAKENTRWMGQRLRGAVRLLDNHGGQMSNRAYPFVSEMGGKLTQEAIKVSPGETPAGTG
PGTG
PGSAARAAQSQARDTASLWVMREVMRRL
>OLS42543.1 hypothetical protein BV379_20330 [Rhodovulum sulfidophilum]AB14
MKPPGFGIDPAFRAAAARARARRRRRLVRPVLLGLLALALATAGWRLTRDRPETPPAIATGDDLAMVPEVEPDAETPA
AP
DPSLALRGLPRDPMVLRLSAGPSAPARTLPGPPDFEPGRAGPPAPDRLTLIEVGLGPGSGPRALDPPSSREDLALYQARR
SRALAQARRSSSAPGDLALGLLPEAARHPFFADSLLRLGPGADLTTALTGAGLGAEAASAATGALPTLADPPRLAALRLR
PGGTRSGTGPEPEAELLQLSLYGAEGYLTTLARLGAGRYARAADPWADRDLASEGRTPPKAPPRLLDAVYGAALGAGL
GP
DLTGELIVLLAQRMDLGRPAAPDQRLRVLYATRPGPGGAARLFYVGLDGRSGCYVLPMPAGGHACAGTGSLAGATAG
IDD
DAAVETLVARIVHVESGGRADAKNPLSSALGLGQFISSTWLRMMREHHPELAARMDRAALLALRTDPVLSVEMVYAL
ARE
NAAYLAARSHGLTPGRLYLAHFLGAEGANRALSADPAAGVAEIMGTAVVSANPFLTGKTIGELLAWADRKMTGSAAP
AVV
SPEIRRYRETVDAILATG
>OLS42544.1 hypothetical protein BV379_20335 [Rhodovulum sulfidophilum]AB14
MRIGGQARTRWPAAAGLALWLLPAIALASETPEQAETAPAPRRVAPVRVAPAASAAPVLSARPEAPAVEIFTPPPRQSL
A
ALPRAEGLPEPSVIVGLLNPDTEGLPVWSDLATRTRLRQDDPEGFARLVAAGALDPPEDALPRAIQTELARMNCYRGGI
D
GLWGEGSRAAVARYFAERGLGRAPDAPSPGLFRRIVAAEDVACPAPPAPTTARAPTPRRQTAPERSDAPPPQIRLAPSL
G
VFR
>OLS42545.1 hypothetical protein BV379_20340 [Rhodovulum sulfidophilum]AB14
MPRAPLACPALLCALLAAAPALAGFSVCNRSLDEVNLALGQPGENGFGTSGWWLIGPGECASPLARPLDARFLYVFAL
DA
FGRVLLEGATPMCVGPGRFEIRGATDCLTRGYLEARFREIDTGNAADFTLTLHPDAE
>OLS42546.1 hypothetical protein BV379_20345 [Rhodovulum sulfidophilum]AB14
MIRATALGSLLVGLTVAGGIALALGHGLVSERRAAVSAPTPAVPAATPVAGARIASPAPADPTLPVFRRRTVPAIPAGAT
EAARPDPALPVPAAPRGSALLPQRDGPRLVSIEAPRPGPGHGPRIGGRLAPRFDAAADMAPPPRPPFRQDVAAGAGLP
EQ
VDSENYLIGVYR
>OLS42547.1 hypothetical protein BV379_20350 [Rhodovulum sulfidophilum]AB14
MIRIPRFVPVLTALALAPVGPALAQGVPDTASGMLVELNAAAETPAGACRLTYVATNRSETGLSEIAYEVAVFDAEGIVS
QLLVLDFGAMPPGKTRVVQFDMPDQPCEAISRIVVNAAPGCVRAEDGAQSPVCLDGLETRARGAIQFGL
>OLS42548.1 biopolymer transporter ExbD [Rhodovulum sulfidophilum]AB14
MARPPLLGAPKRARRPRFALTALADTMFQLLIFFMLSSGLTPYALLPLTPAAGNRLAETRAPGGEGAETAGAVLPAGTR
L
WQIGPGSLTAGGQRFGFDEIGALARALAARDPVPPVVLLAGPGARVQDLTTALDRLRAAGLTEVRLGLGGEG
>OLS42549.1 hypothetical protein BV379_20365 [Rhodovulum sulfidophilum]AB14

MRRGTILPVRRRRDGPDTAIAIVNLVLLLILFFLVSGGLWNRDAPTGLRLAETRAFDPARLPRPLLELTPDGRLALDGQA
VALAGLPEALAGASRVHLLIDRSAPARQLLHLLGSAGFDGREVVLVTVRAGGGGAGRGAGQ
>OLS42550.1 hypothetical protein BV379_20370 [Rhodovulum sulfidophilum]AB14
MSYLPYGPEPRRGWALAGLAALAAHGLLGAAVALWIGQDPVSPGPPAPRFEVTLERLDSDTLAGLRLSEGRANAADPA
LG
PGKDSEESEDGPAPATGPDSRPEPRVEARVEARAEMREGTAGETPSSSAPLSAALPQPVPSHRLSPVMARSGAAAVAP
DR
IAPVGTGAGAGAGTGTASGTGIARRPASASSEPRARDLAAAALIRRIRETPAPDCLLALPRRDAEGGAGLDLMAASEAG
M
ADYAGRLLTGDLAGTAQRRRLIDPRQCPVLELLRDSPDYPATRLGLGLDRTEIVSGGRLTGRLRGVGGRYVLLLLIDNNG
VVQDLQRFLSFAGNRAKFEVPVSLAGPARDTSQLLLAVAADGPVSGLRAQIGRRADEVFSSGRRTALGAAALALAGFDL
R
>OLS42551.1 hypothetical protein BV379_20375, partial [Rhodovulum sulfidophilum]AB14
MAAGLTLGTAAAGADTFHWTGTAEDGFWATPENWADAMGDPIEGDAPGLDGMPAGIVLGEAATGPIGLEAGRATL
SALEL
TDNSGTTLTDGTLAFSLPDGASATAARLTVAGSGDFMGESLAFDLEDDLLVEASVDTGLSGNISGTGALRLRAAGDSQIT
LSGTNSQTGGTVIEAGTVVATGGEALSEAGTVTLAGGIDGRAASVLRIEQDETVGGLASAEGARGGLELADGSLTVSGG
P
ASSFGLGISGSGGLTVSDGADLTLGAANSYEGGTVVSGGALSVMTGGSLGSGDVSVADGSLLTHGAALGADQTVTLGD
GG
LWALAGDETVRGLAGTGTADLGGNRLDIALPGTEPLAFGGSLKGGADSLLALTGGGEYMLTGSLSDYRGGFRIEAGRLG
L
SDAGGALNDNALTLAGGTLVADLDNAFGGTITTEAGSASTITAATGTTLSLTGGMALAGDLSFTAEGEDAEIGLKMASV
T
LQEGVSLGFDRARFKIGSEAAAGMFAADGGTRLGAEAVLDLAGYDVGIAALTGSGRILTGSGDAVLGLGDGTDFGGVL
AD
GAAGRLSLETDGTVRLTGDNSLTGGTSVNGGSLTLAGGGTLGDIEIASAGRFVLEAGSAGSVVNSGTGSSNAGTIAALT
Q
KAGSFGNSGTVSGATSVEGGDLSNTGTLSDDVSVSGGVLSSSGSIGGDLDVTGGTATNSGRVGGKSSVGSGGVLVSEG
GR
FGDDLTIEGDTGKGAGTLRL
>OLS42552.1 serine/threonine protein phosphatase [Rhodovulum sulfidophilum]AB14
MATDVGCRREINEDDMIARPDLGLWAVADGMGGHAAGEFASQAIVAELSEAGLPASAVDLQARFMERLIRANDRIR
AHSA
ELAAGPIGSTVAALLVHEDSYGVVWCGDSRGYLLRDGHLVQQSRDHTELRAALEAGRITPEEARTWPRRNVITRAIGVG
P
DPECDIVSGRIAPGDLFLLCSDGLTEHLDDTEIADILLDLPPQAACEALIAETVARGARDNVTAVVLACRSGDDDDEKTA
PPL
>OLS42553.1 hypothetical protein BV379_20005 [Rhodovulum sulfidophilum]AB14
MSVPPDAPGDAGDERTAFMAPGGGEDATRISVASAPSGPDASPAAEEARVPILSAGTVINNNYRIAGVLSSGGMGAV
YHG
VEIGTGDPVAIKAILPNLAADPRAGALFRSEARMLRQLADDAIVRYYNYVHDHALERYFLVMEFIEGITLSDHMARSGPL
PPAAAEALIRRLARALSRAHALGVIHRDLSPDNVMLPGGAVAEARLIDFGIARSARLSEEEAPGTFAGKLKYVAPEQLGH
FGGHVGPETDIYGLALLVGAALSGRALPMGNSIEEAAERRRRVPDLSGVSSGLRPLLTHMLEPDPAARPASMEEVLRLIE
HPHLLPPRYGGPSAAVPGGLTLPPGLGRPGAARPAGAGQGAGPAPDPGPEPPRDRAALRLIGGLAAVFLALLGAAGW
YGW
TAGLIGPGKAPEGGIEARLPPRQEAGRAGFLAAWQSGPCSYLTRVETGPRAGVVERYGRQRGDAADDNAALAEAYEA
RFG

SRPAVLDRPVAEAQCAVLDFARSLQARPGPLPVLSATPEQVASGEEVRAAVAAPGPARLWVALVAPDGAVYDLTDRIG
PD
GRLRFGLSLPEGASAAPQLLLAVAATRPLASAATATDGTPAARLLPAVLEEIAGRGGGAGVAVAHLELLPPGPAEDG
>OLS42554.1 hypothetical protein BV379_20035 [Rhodovulum sulfidophilum]AB14
MLRRPILRRTMLSRAVLAAAALALPLAAPAAAEFPEKDLTHIMPWSAGGGTDTVMRQFMSFAEKTLGTGINTQNVTG
AQS
GIGTLRLMKARPDGYTIGSLTWDSVITVPYYELVPGYDTDQLAYLGSVTVHPTALIVRADAPYATLDDFVAAAKSAPGTL
KIANVGTGGVWHLPALDFAKEAGIDVQHIPYPKGSGPEREALLSGEVDAMSSSISAAYAAVQAGQARVLAVMSEERDP
KF
PEVPTFRDSGYDVVWGSFRLVATQARVDADKRAALEAGFAAVFDLPEFQQAAEKSAMGAHWMDAEATAAYVKAAQ
AKAFA
LIDELVAEGVLQK
>OLS42555.1 hypothetical protein BV379_20060 [Rhodovulum sulfidophilum]AB14
MSAEFAAGFNDIPAPVANDCYGRRNIMDAGIKPIARGMHLCGRAVTVAMPPDDNLMLHAALHAAEPGDIIVASTSN
DRHS
GVWGELMTRAAMARGLGGLVLDGMCRDSAWIAESGFQLFARGTCARGSRKLGPGHVNIPVAVGNVVVHHGDIIIG
DDDGV
MVVPAADAAALLPLCRDKLAKEGARIEAIASGSPKPGWLMPALEKWNVA
>OLS42556.1 C4-dicarboxylate ABC transporter permease [Rhodovulum sulfidophilum]AB14
MFSPLLDALPAVLTFPNLLAMIGGVLVGIVVGSLPGLTATMSIAVLLPLTFTMDPLVALGMMAGIYNGAMYGGAIPAIL
L
RIPGTPAAIVTTFDGYPLAQQGRAGYALQVALLSSAFGGMMSAIAMMILAPPLSLVTLAFGPPEVFWVGIFGMAAVAV
FL
GEDMPKGFLSALIGLFIGMIGLDPVMSTDRFTFGRLELESGIDIVVLMVGLYAIPPCLEMAEDTAKAMGARMKLEFQPF
L
KTIREIPRYFKIWSWAYLYGIFVGLLPGASGSFVSFLAYERAKRISKDPDSFGKGNPEGIAASEFGNNADNAAALIPTVT
LGIPGSSVAAVIMGGLLVQGLQPGPDLFRNSADIIYGFMIQMFLTSLILIPLGGSFATNLFAQVLRIPRPLLIPLIICLS
IVGAYCVQNSLFDVWLMFGFGLLGYVMSKTGLPLAPVALGVILGPLIESNLRLSLLLDVRNEHILFTRPICMVLIALLVL
TTVLPPVWKHIRARRAREA
>OLS42557.1 3-oxoacyl-ACP reductase [Rhodovulum sulfidophilum]AB14
MTRSETVRPEFRNRLSHQGRRVLVTGGARGIGLAIAEAFAARGATVALCDLDPEAVAGATAQIAASDAAGSVLPVTCD
VS
DYAALSAALEAAGGNFDTVINNAGISPKHDGVAHKVWEMDPAEFRHVLDVNLTGSFNTVRALAPGMIAAKRGWVV
NMSSV
AGKTFMPVVACHYAATKSAILGFTRHLAGELGPHGIRVNALAPGRIETPMVLGVAPEVNAAQVEMTPLARLGEPAEVA
EA
ALWLTDAESSFVTGQTIDVAGGLLMT
>OLS42558.1 branched-chain amino acid ABC transporter ATP-binding protein [Rhodovulum sulfidophilum]AB14
MPAPSLPWPAIGVALVLASLLLIPLGIGNAFVTHIFVTICLMAALSSAWNLVGGFAGQLSLGHATFYGIGAYTAVILVNL
GITPWIGMFAGAGIAAIVGMAISYPCFRLKGPFFSLASIAFLEVFRVLTLHFSDFTGGATGKMIPLKLGWTWMVFRERW
P
SLLIIFGMLVAVLVVVWAVRRSRLGFYLIATRERESAAKAAGVKTVQVRLVAVAISSGLCAMLGTFHAMYLTFIEPSAMF
SLTFSIQIAMFALIGGLGTVSGPLLGAVLLVPLTEWARASLGASALGLHGFVYGVVLLLAVLFMPNGIMGVLQKFRRAPH
HDIDEDEKTTQTDSIKAPAQAATPPSPDREGIGRDVMRVQNLNKRFGGLHVTKNVNFTLREGEVLGLIGPNGAGKTTLF
N
MLSGFLTPNEGSVTVRGPDGGYHTPVNPADFAALGVGRTFQIVQPFAAMTVEENIMVGAFYRHHDEHDAREAARQT
AWRM

GLGPLLQDEARGLTIGGLKRLEVARVMAMEPRILLLDEVMAGINQTDVRRAIDLMLSIRDSGVSIIAIEHVMQAVMSLS
D
RVLVLAAGEVIAEGDPHEVVRDPHVIEAYLGKEFANAHR
>OLS42559.1 NAD-dependent succinate-semialdehyde dehydrogenase [Rhodovulum sulfidophilum]AB14
MYPDLTLILGGEARATASEGTMTVSNPANGQDLATIPRAGRAEAEEAARLSLSGFAEWSALSPYERSKVMRRAADLMR
ER
ADRAARIMTLEQGKPLAEARGEWMGSADLLDWFAEEGRRVYGRIVPSRAPSIHISVLKKPAGPVACFTPWNFPAWNT
MQK
VAPALGAGCSVVIKPASETPGTAWMIGQALLDAGLPPAAISVVWGKTSDISDALIAAPEIRKVSLTGSTRVGRIVAAQAG
QHLKKVTMELGGHGPVIVAGDADLDRLVPMAVQWKYRNAGQVCVSPTRFIVDDAVHDEFVTRLAEGAAALKLGDGL
EEGT
QMGPLTQKAQLNQVAEMVADAESRGAKVETGGARVGNIGNFFAPTVVSGMTPEMLAMNEEPFGPIALVIRSTGLEA
ALTE
ANRLPVGLGAYLFTRSMEVSQEVQDRLKSGMLGINHFALALPETPFGGVLDSGYGSEGGIEGIEAYLSTMTVTAAI
>OLS42560.1 hypothetical protein BV379_20175 [Rhodovulum sulfidophilum]AB14
MKPVIILDETLPTGLKANFAAVLGMSLGKTRPDLVGADTPTQDDVALAGITSVALPVLGAPAADLPALFDAAADLPLRLA
YMRAAFEARDYADYSARIAAAPLPDHAPQAILIAGPRKAVDRICGRLPLLR
>OLS42561.1 hypothetical protein BV379_20315 [Rhodovulum sulfidophilum]AB14
MGLFAALRALERAAPDAPRIGRNARVAEAVVRLGQDPFLAFPVSDLSRAGRDGRARPVFRAQFLGFYGAFGALPLAWT
EE
VRRWFDAGDESFTAFTDIFTERFQELFFRAWSDARPITQFDHPDDRFQGYLLGFAGTATPAFRDRCRVPDTALARLVPL
A
AGRVKSPVRLRQMLGLHFEGRARVEIEEMVPGWLDFEPGTETRLGLGAASLGRDAHLGARARTISDRIRVHLHVPDM
AAF
DRFLPGGPDNRELADLCRWYLGETLEIEAALWLPQPKVVPAVLGRSARLGWMACLAPDPENPEQMVHIARFDLNRED
DAP
RPAAAAPAAIAA
>OLS42562.1 flagellar motor protein MotA [Rhodovulum sulfidophilum]AB14
MPATLAVFAALGVLSVMALTISIWKAWQFRRMGVGRRALAETVLDCWLSGRVEEAMERAVAGRAVLGRVLRAVMS
AAQAR
PGETGYAEELGRQTALIELARMGERMRALELIVSSAPMLGLLGTVVGMIDAFSALSLAAEAADPASLAAGIWTALTTTA
A
GLALALVANALTTWFEGRIEAERTLLEAAISAAIYGRVDSDGGV
>OLS42578.1 hypothetical protein BV379_19425, partial [Rhodovulum sulfidophilum]AB14
MVVQLALPMALFGMIVMLGAPLVIWVIGGRLDTIGTGSAVTALLLLFSMYAPLLALLGVMEAVRMADA
>OLS42579.1 hypothetical protein BV379_19430 [Rhodovulum sulfidophilum]AB14
MPEVPNLVGLSQDKARNVLRDRGLRMGGTATRESGSVAPGTVLAQNPGEGAELAPGSAVAVVLAAAPRVTVDEDCI
RHNP
GNIAAKQIGGRWKIVDGSHWMMDFGTDADSAKKVLAVLKAYGADRICYVSRPQPPMMYILNGDSAPSATAATRQRL
AAAG
LGREDCIGFDPATLDLESGGAGVTLVSDRSRMIRFRNMDEAKKAMLVIRKHGFTRQCFVGRPNPEFKYFLR
>OLS42580.1 hypothetical protein BV379_19435 [Rhodovulum sulfidophilum]AB14
MALALPKTMKKAVHIIGILCAAAAGLALIIHGALITSAPMEIAWGAIFLAGSLLAVLGGATASPRGGIDPPSPGAKFTRI
ADWAWYCIAALVVVGIVLSFVI
>OLS42581.1 hypothetical protein BV379_19440 [Rhodovulum sulfidophilum]AB14
MGVKGAVALLLVFGLVFYLTLGAGLMTGRGGDPPEADSFSAPAWTGALERIVAPFRPRLKLSRTRFVFTEAGFIEVPRIS
GDAMRRASFALEGRGQLVYSCPGCDGDQVWPDEGTRDFGKVSFAIFPSGGRIAVRPRSRQLVLTLRDGR

>OLS42582.1 acyl-CoA dehydrogenase [Rhodovulum sulfidophilum]AB14
MMLTEEQDQIREAARAFAQGRLAPGAAARDRDHAFPRAELTELGELGFMGMLVPEAHGGSDTGMLAYALALEEIAA
ADGA
CSTIVSVHSSVGCMPILRYGTEEQKARFLPKMAAGEWIGGFALTEPQAGSDASALKTTARREGDHYVIDGAKQFITSGK
N
GAVVIVFAVTDKAAGKKGISAFIVPTDIQGYKVVSVERKLGQHATDTCALAFDGMRVPAENLLGAEGEGYRIALSNLEG
G
RIGIAAQAVGMARAAHEAALAYARERIAFGRPIAEHQAVAFKLADMATRIDAARLLVLRAAELREAGKPCLTEASMAKL
F
ASEIAEEVCSAAIQIHGGYGYLADYPVERIYRDVRVCQIYEGTSEVQRMVIARGL
>OLS42583.1 acetyl-CoA acetyltransferase [Rhodovulum sulfidophilum]AB14
MTSDPIVITGARRSPMGGFQGDLAAADAPALGATAIRAALGGLDPEAVEEILMGCVLPAGQGQAPARQAALGAGLPL
GAG
ATTVNKMCGSGMKAAMLAHDLIAAGSAQIAVAGGMESMSNAPYLLPKARGGYRMGHGEVKDHMFLDGLEDAYDR
GRLMGT
FAEDCAEAYQFTREAQDAYAIASLDRANAAINEGRFRAEIAPVTLATRKGEQVIDTDEQPGKARPEKIPSLKPAFREGGT
VTAANSSSISDGAAALVLMRASEAERRGLTPLARILGHATHADKPSLFPTAPIGSIRKLMERTGLTLSEIDLFEVNEAFA
VVAMAAIRDLGLSHDIVNVHGGACALGHPIGASGARVLVTLLAALETHGLKRGVASLCIGGGEATAMAVERFA
>OLS42584.1 3-hydroxyacyl-CoA dehydrogenase [Rhodovulum sulfidophilum]AB14
MQIENRVFLVTGAASGLGAAVAAMAVAAGGRVLLADINAEAGEAMAQELGEAARFVQTDVTSGEDGQAAVEAAVE
AFGGL
HVLVNCAGIAPGERLLGRNGPHGLDSFQRAITINLGGSFNMMRLAAEAMAGNEGEERGVIVNTASVAAFDGQIGQIA
YAA
SKGGIAAMTLPAARELARHAIRVMAIAPGIFATPMMAGLPQEVQDSLGATVPYPPRLGRPEEYARMVRSIVENQMLN
GEV
IRLDGALRMAPK
>OLS42585.1 AMP-binding protein [Rhodovulum sulfidophilum]AB14
MKLDRIARRDYADAHARFSIEDEIASLAGDLEAGLNAAVECCDRHCGRDLVALRTVSAGGSLAEYTFEDLRALSARTANV
LKAHGVGPGDVVAGLLPRTLDLIATILGVWRLGAVYQPLFTAFGPKAIEHRLTCAGTRMIVTTAAQRAKLDEIPDCPPVA
LIRDADTPLPAGDIDLCAERAAAPAEFAPVLRDKDAPFMVMSTSGTTGLPKALMVPQRAMLAFAVYMRDGIGLHEDD
TFW
NLADAGWAYGLYYAVCGPLLLGAATTLHEGGFSAETTIRLIDELGITSLAGSPTAFRMLIAAGPEAVAPIKGRLRTVSSA
GEPLNPEIIHWFEKHLSVAIHDHYGQTELGMCVNNHHGLEHDVRPGSAGYAMPGYRIAVLDDAGNEQPPNSPGELAV
DIA
NSPLLWFTGYYATETPAIAGGYYRTGDSVEIEPDGSLSFIGRADDVITSSGYRIGPFDVESALIEHPAVIEAAVVGIPDP
ERTELVKAFVILGKGHEASDALCEELQQHVKKRLSAHAYPRLIDFVDSLPKTPSGKIQRFLLRKAEVEKQNVR
>OLS42586.1 AraC family transcriptional regulator [Rhodovulum sulfidophilum]AB14
MQRRIVSAGFVADALEPLARRGIDPAPLLAGAGIAAADGPVTNQEYGRLWLAVAEALDDEFFGLGARPMRPGGFVFL
GHA
VLHAATLNVALRRALRFLGVLLEDPSGSLRVADGQAEIVLSDRRGLRSAFAYRTYWLVLMGLSCWLIGRRIPLTRLDFAC
AAPPNRADYQRFFGAPVQFGQPESRLCFAARYLELPTIRDERALKLFLAGAPANILVRYRHDQGLSARIRARLRGTDPAL
WPDLEQVARELALSPATLRRRLRAEGQGWQAIRDDLRFAESRRLLAGTGESVAAIALRIGYAEPSAFHRAFLKWSGETP
A
AYRAARAG
>OLS42587.1 hypothetical protein BV379_19475 [Rhodovulum sulfidophilum]AB14
MIDVETELAFPGAETAHAALSAHAKSHDIPPEIDAPGHLAFSFQGNRLEFLTVPGRMRIRLSAVSPNTLYFLKEAVAHHL
EEADPEAARKLRWPGDEGADSHRTPPNFRLLTVLERSRPVEGMTRLHLAGEDLEPLTRDGIHVKLMLPADRSRSPVWP
GV

AANGATRWPKGADRLHVRYFTLLELDAEAGRVAIDFVDHPDGQISDWARAAAPGDRIGIMGPGGGLPPATDRPLILM
GDA
TALPALLRILEALPASAEGCVIAPLRPDDAGYLPASPLETLLLGPEGFAAGAAGCFAEALAARPEARVWVGAEHALVHET
RRIAAALPAEQKEIGTYWRRGRRGDARRDDHDD
>OLS42588.1 hypothetical protein BV379_19480 [Rhodovulum sulfidophilum]AB14
MVGRRRFLTGAAAALAAPRLARAGAGIEARDVLGNRVALPAPPERIVLLDATDLISMAALIPEPGARLAGWASVPRLDL
G
RADWLAPEGLPVVGKLSPDTVSAEGILALSPDLVVASAYMLPPGGSVLESVLRAAGIPLAWTGGHDRALPPGDKLDRA
MG
FWGAVLDRPQTARELAAWGRARFDAVRAATSGPRPRTYMEIMSTYDTCCWAAGHAFWGGIFEIAGGHLLEASDGW
GAKLG
PEGLIAFDPEVYIATGGSFAPELQPGIAPGLDPEQGREGLARAAARPALKDSAATRAGRVHGIWSGLATAPLMAPVLAD
C
LGAWLHPETGANLDPGRTLAALNDRFASPLPGPLWLSLDAT
>OLS42589.1 TonB-dependent siderophore receptor [Rhodovulum sulfidophilum]AB14
MDRGTLGRTTAALAAALGTAATAAAAQDDAYALEAIVLQADVADTSSSYALPDMRSATKTDTPVVETPQALTVLTRKQ
FD
DQNTQTVGQALRYTSGVLSEIDASTRYDSVFLRGFGGFGISSQFVSSLDGLRLPRGQAFAQPAVDPFLLDRVDVLKGPS
A
LLYGPSSPGGLVNMVSRAPDGSTGGEARLEYGTHDRVQAGFEQHGTLDAAGTLQYSLTAIGRSANTRYRDVDEKRYAI
AP
VLVWQPSADTRLTFGGYWQDDPEGGYFNSIYPKGAAPAAYARYLDRDFNIGDPDFDSFERTQWALNAGLEHAFSTDL
TLR
SKLRYGRIDADMKGLQMGAPLDASGMLARNALISDETARALNWDTNLEYRFQTGAVSHRLLAGADLLWNDSDWQY
SYTSA
APLDVTDPVYGGVSGPFTTMTDSHQTTRQAGIYLTDQLSFGRFRAVLGLRHDRIETDSLNRLSGTTTEQSSDTTSYRAAL
LYLFDNGAAPYVSYSTSLEPTSGVDAAGNPFVPTESEQWELGIKYQPPGMDALFTMAAFSIDQTDVLTPGDVPGYFIQT
G
KIRSRGVEFEGRGKLTERLELIGALTLLDTEVRASSNAAIIGNRPQAVPDYFGSLWLNRSFGGRLDGLELGGGVRFVGSS
YGDDANSLKSGSYTLADLAIRYDLGARNPSLAGLKATLNVRNLFDETYYSSCSYDYFCQYGEGRIVTVGLRKTW
>OLS42590.1 hypothetical protein BV379_19490 [Rhodovulum sulfidophilum]AB14
MTTAKTPRPARAGRPTKHQSERIAAEIVARARELFIAQGFAATTMEQVIAACGIGKDTLYRRFATKEELFAAVTQKALER
TEAWFEAVAAECPERPLDRVRHLARWFLDANLDPELLALKREAFIEAMRARPLVGADPFTPKLAEAIRAAAAAGELAAP
D
ADFVADQLIAALVLGPSNAALMGDDALADPAARGVWFDRAWRLFVEGAAPGRG
>OLS42591.1 exopolysaccharide biosynthesis protein exod [Rhodovulum sulfidophilum]AB14
MNRNRGSRRRVSLSLPILRTSRDQHDAGNLTLGSLLSGLGETSFGWAVVVFALMTMLPLPPGSSLVTALPVLVTTAQM
ML
GYRHVRLPGPLARLRLDHDKLRRTVLRLRPVTRRLERVLRPRNPALFRRRNERALGAALFVIAFTLFLPVPGSGWFPAIS
LFVAGVGMVEHDGRVTIWGLVMGAASVLLTVAIVVSLAASAEAVIP
>OLS42592.1 EamA family transporter [Rhodovulum sulfidophilum]AB14
MTGTAATVAGRDPMAGIAAMCAGVACLCVNDAFAKALTEGYSPVQILFLRSLIALPFAAVIAVGTGGPRALVSRRPLA
HL
ARGGLVIVAALMFFTSFRYLGLAEATGLIFVAPIFITALSALVLREPVGWRRWLAVAAGFAGVLIVVRPGAAAFQPASVL
PVLAALFYAVLMLSARWLDARESVWTMQLWLVGSSVLLSGLAVGFVWTPVPAGDLWAFGCIALFGTLGMTLITQAFR
MAP
AVVIAPLDYTALLWATGFGWLFWQEVPDRATAVGAAVIVASGVFIMIRERRAAG
>OLS42593.1 short-chain dehydrogenase [Rhodovulum sulfidophilum]AB14

MTRTLFITGASSGIGAATARAAVARGWSVGLMARSADKLDALATELGAAALALPGDATDPAAQEEAVARTVEHFGAL
HAA
FANAGRGLERPGTEAGDPEDWRGMVDLNVMGVLFTARAALPHLRRTKGHLVLTGSAAGRTHIKGSIYSATKWFVHG
YGGN
MSEEMREWGGRCTVISPGMVDTPFFDSPKPDKIRPEDVADAVLYALEQNPRAELREIHIMPTG
>OLS42594.1 VapC toxin family PIN domain ribonuclease [Rhodovulum sulfidophilum]AB14
MPADFFDTNVILYLLDDGPKADRAEALLAQGGTISVQVLNEALVNCLRKARMSHDEAGEFLFGIRDLCETVELTVDTHD
V
GRALAARYKLSVYDSMIVAAALLSGCTRLWSEDMQDGLTVEGRLKIVNPFADAA
>OLS42595.1 MazF family transcriptional regulator [Rhodovulum sulfidophilum]AB14
MQIARWGNSLAVRLPADLVRTLGLREGDRIELEPSGEGRLAVSRHPRADEVLSGLRRFRGRLASEDRLSRDAANAR
>OLS42596.1 LacI family transcriptional regulator [Rhodovulum sulfidophilum]AB14
MTGPATGKPDRSRRPPTLEDVARAAGVSTATVSRSLNAPERVRAETRMAVEAAVRDLGYTPNFGGQALASNRTNTIG
AII
PTMENAIFARALQALQETLTESRVTLLVATSHYDPAREEAQIRALIARGVDGLALIGSQRTAAADALLETRGIPFVILWS
APSETPHPAIGFDNAKAAAQLTAQVLARGHRRIAMISGPLAHNDRAQARLAGVRETLCATGLELAPGDLRECPYTLAD
GE
AAAAALLGRAEPPTAIICGNDVLAAGALRAARRLGVDVPGALSVTGFDDIELATAVHPPLATIRVPHRGMGEAAGACL
MD
WIDSGRRPVSRLLPTEFVARESLGPVG
>OLS42597.1 hypothetical protein BV379_19525 [Rhodovulum sulfidophilum]AB14
MKFFGMAAFGASALLLAVFAINLIGARAGAALIGTAAETVLLFLAAACFGLGTLSCEARRDACDADGPEVSRRS
>OLS42598.1 C4-dicarboxylate ABC transporter [Rhodovulum sulfidophilum]AB14
MRNENDDTGWKAAASRRNFLRLAGTGSFTAAVMASAGGMLWSDKAAAQTVNEERDRKRAAEHVMTLATAYVMS
ADRSYPI
MQLDLKENIQNASEGRVYVQLAPAGQLGAGNQLVQKVQSGTIQAAQHSIANFAPFAPVVDLINLPYLCGSNQRFVNL
TRS
DVWTGTVHPMVEARGFKPLFYVVIDPRVVALRKGGPGPVLTPSDLAGVKFRVPGSEMLQTYYRLVGANPTPVAWGET
PSA
IQQGVADALDPAVGALYVYGFKEMLSHVTFTRAVPDAQVYSCNLEWYNGLPGDIREAIDTAADITAQQNLAKVPAAR
GFA
MSELKNAGVEFHRLSDDQLGLWQEAGGYQRSEWDDYKTGLAGSMAAFERMAEAAATEVRAYVDDA
>OLS42599.1 TRAP transporter small permease protein [Rhodovulum sulfidophilum]AB14
MLNALDRNAERWALLIFYIMLVATMAIEVIRREVFAYSSIWGEEVVRYCFIYLCWIGAAAAVRERAHIRIDLLLNWAPPR
GRALLYMFGDLVTLALAVLALWLSAQTVSVSWTFGSVSHGLRVSMVWFLMAVPVGFSLMIFRLLQSLVRDFADLRAG
RPV
YEGERLFE
>OLS42600.1 C4-dicarboxylate ABC transporter [Rhodovulum sulfidophilum]AB14
MLWQQMQQGAVALDWTFYLPVAAFLLLVALAVPVWVAIGAASVMLLTASQVLPLSLVGEGLFSGIDHFALTAIPLFILT
G
DVLVRTGLSRKFLDVAEALTNWTRGGFGSATVLVCGMFSAISGSDAAGAAAVGRMTIERLVESGYPRPYACALVAAGA
CT
GILIPPSIAYIVIGLVLGISASTLFKAALIPGVLILAAILATNTVLNRRHAWEQGHALSLSEWLANLGRALARGWYAFIV
PGVIFWGIFSGRLTPTEAGASAVTITIAMGFVMGTLKLADFPSMMVSSAKVNGVIVPIVAMSLPLAQALSAMGVPQSF
VY
TMTGFVSEPWMLILVMIAILVAAGCVMETTPNIVILAPLLKPLADEIGMNEIQFCVMMITALGVGFITPPLGLNLFVVSG
LTGESVLRISARAVPFVLFMLTVVLLIAFAPAVSTLMLS
>OLS42601.1 histidinol dehydrogenase [Rhodovulum sulfidophilum]AB14

MTVSYLKKAPRRAVSDATETRETVQKILTEIEAGGEPAALDYAAKFDRYEGNVVLTRDEIDAAAASLDQRVKDDIRFAHD
NVRRFAEAQLKTVGDFQTEIRPGLTLGQKAIPCRAAGCYAPGGRYSHVASALMTVTTARVAGCGHVSVCSPPRPGVGV
HP
AIVYTADLCGADQILALGGVQGIAAMAFGLFGMPAADILVGPGNQFVAEAKRLLFGRVGIDMFAGPTDSMVLADASA
DPE
IVAADLVGQAEHGYNSPVWLVTNHRPLAEAVMARVPELIAALPPVNAENAAAAWRDYAEVILCDSREEIAALSDSYAP
EH
LTVQAADLDWWLGRLSCYGSLFLGEETTVAFGDKAAGTNHVLPTSGAARYTGGLSVHKYLKLVTWQRATRDGARPIA
EAT
ARISRLEGMEGHARTADIRLAKYFPDRSFDLGADPAAVQGSAKVSA
>OLS42602.1 gluconate 5-dehydrogenase [Rhodovulum sulfidophilum]AB14
MTAATPLEGKVACVTGASAGLGRAIAEALVGAGAKVVAVARRRSDWCDGTNAVALSADLSDPGRAKGVARAVSDPF
GPPD
ILVNAAGVNTREAADEVTLEGWTLTQNLNLAMPFFLAQALVPAMRERGWGRIVNFASLQSLRAFPGGIAYGASKGGI
VQL
TRAMAEAWSRHGITANALAPGFFPTELTGPVFDDPERAARNAAQTCIGRNGRLPDIVGPALFLCSAASDYVTGQVLPV
DG
GFTAK
>OLS42603.1 galactitol-1-phosphate 5-dehydrogenase [Rhodovulum sulfidophilum]AB14
MKALIYTGPRLLELRELPDPEPGPGEALIRVEAAGICGSDMHAWAGHDDRRPAPLILGHEAAGIVVSTPPGGPAAGSRV
T
VNPLVTCGHCRFCLAGRDNLCETRQILSMPPREGAFAGLLAVPARNLTPVPQGISPVAAALTEPLACGWHAVGLAERA
LE
LPLAEARCLVIGGGAIGLGAALVLRARGAAEIWLAEPSGPRRDIAAAEGGIRTFDPTGPGTGPGGVDLVIDGVGFGATR
A
AASAALRPGGAMVHIGLGDAGPGLDIRRATLQELTFIGSYTYTAQEFRDTAAALFAGRLGGLGWVRTMPLAEGAAAFA
SI
AGGGEPAPKIALLPDG
>OLS42604.1 hypothetical protein BV379_19560 [Rhodovulum sulfidophilum]AB14
MAGKALNKKNLLELGADTLADLLLEAVKGDAARQRRVRMALSADQGPEAITADLLKRFASIRRGRSFISRKAQKKLAREL
AELTRLVETRIAPDAPDAAFDLLWAELQLAPGIHERTDDSQGTIGEVMGEVMAAIERLAPRLGKDPAALAETVFEAILED
GYGAFDDALPALAEALGATGLARLKALAEAARAAPPTEADLARYAFISDRDARADRAVAGRNRSLDMILRDVADAEGD
VD
SWLARYTPEQLTYSTIAPDAATRLLAAGRAGDALRLIEAALPGESDDPWFDDPELDAAHFACLEALGRKDDLRAALWQ
RF
ERRLCPDALRRHLRLLPDFEDIEAEDRARGIVLDYEPVEKALAYCLEAPDLSLAAELIEARSEEIDGNAYEILTPLAEAL
APAYPLAAVLLWRAMIDFALGRRRAGRYGHAARHLSDCAAADAEIRDYGPHLSHADYLDGLRKSHARKSAFWERLRT
G
>OLS42605.1 NADP-dependent oxidoreductase [Rhodovulum sulfidophilum]AB14
MAQTDTVNRRYVLARRPKGAPDENTLRLETAEVPGPGPGQMLLRNEYLSLDPYMRGRMSDAPSYAAPVEIGEVMVG
GTVA
EVVTSDVDGFETGDRVVAFGGWQDYALSDGTGVINMGKSPEHPSWALGVLGMPGLTAWAGLTRIGQPKAGETLVV
AGASG
PVGATVGQIGKILGLRVVGIAGGGEKCRHVTETLGFDACIDYKAKGFPGALEAAVPDGIDIYFENVGGAVFDAVLPLLNA
SARIPVCGLISQYNATSLPDGPDRMSLLMGTILRKRMTMRGFIVFDDFGHLYPEFARQMGDWVAQGKIRYREEMIEGL
EQ
APAAFVGLLNGEAFGKRVVRLGQ

>OLS42606.1 type 1 glutamine amidotransferase domain-containing protein [Rhodovulum sulfidophilum]AB14
MKILMVLTSHDKLGDTGNRTGFWLEEFAAPYYVFKDAGAEITLASPKGGQPPLDPASDSDDAGTEATRRFKADDAAQ
KHL
ASSEVLSSVSADGFDAIFFPGGHGPLWDLAEDKDAIALIEAFTARDRPVGAVCHAPGVFRHPKGPDGKPLVAGRTVTGF
T
NSEEDGVGLTDVVPFLVEDMLKSNGGQYRKGEDWASFVVIDGKLVTGQNPASSEEAARNLLSLL
>OLS42607.1 hypothetical protein BV379_19575 [Rhodovulum sulfidophilum]AB14
MTGSIRAVVPVIRDSRFGAEACGTAARGDVAAGLAQAIDMPGRVGKTALMPPAFGQTPMPLRSRNGQILPSEIFAMR
SGI
RLTALRCLAREGYGSQIR
>OLS42608.1 hypothetical protein BV379_19580 [Rhodovulum sulfidophilum]AB14
MHRSDPQEGAIYAEFRLAGLISAVFLNVCLCNDTVAANIRMARSDAGEAGLRISVDIAHSKGRGPRPAIPAGGLREASG
K
GSRSRPLPKDALIVIHDAPVAILAAESELSVQLGIDAVVPDTTVVMIAHRLSTVEGATGCWCRRTVGGRIRAVMPNCPS
A
RAATGGCGRRICGPEI
>OLS42609.1 hypothetical protein BV379_19585 [Rhodovulum sulfidophilum]AB14
MPELHLGATRPVSEFEQDDPADPMNDPSRAPSFPSSSAPDPTRTRRRFATAEERSYLRRFNRLRRLRRLIFATTFVWPGL
AIGVLAWIGIEARALDGYVVSALVFLALCVLVGVFVFSGWGAALVGVLRMRPALRVPDDLQVEVSGDSLRTIVHPTRDS
T
NIFTLRRSRDLVEIPFHWERDLVADGLLPGHDQHIEVARLPDASGEVLRLPMSPRHGRARIDPTLPDLGVFLLRLGDRSI
AREMRAGLPLLRAQDTILRLGFGAGLLGLLACLVCWIAVDDRLDEMAGLRSTVTRIVGTYGAGAEIPAEALAARGLDDL
R
PDPEFGNRLLHAGPVSFRTVSLSPDADPFLMSDEEIVAVNRIGRVFPFKLVGVASAEPALIADYRDQLMKRARSLSAVM
P
DLPDRITALSDTLIASQLRAMSFGSPPDSDFVEAVLPRPTIRTPRRSSFLISVEPSCLMQDFLCGQRDPAEAYENPVFIS
GPRGIVLRDGADLDRLAEARDRLREMDAETWPSIATAVCAISVLLPVFCILAWVEANRRIRRWYRSGGPVR
>OLS42610.1 hypothetical protein BV379_19590 [Rhodovulum sulfidophilum]AB14
MGAAARADVVLDQHSDAADRFAAQLLMPGLLIVCGYPIWRQTRSSVLTTFSPRQATRGSAAGQEREWVSTMV
>OLS42611.1 hypothetical protein BV379_19600 [Rhodovulum sulfidophilum]AB14
MNPYRFALASFCVLPTALPVSAQQEAATGAFTLDPIYLDVQFRQETEQEVPISVTSVQSDELKARGADRLDKVLPLVPNA
NFATVRGGIEASSVALRGISTTAYGADPSVAFYMDGVYVGADSAFNAEIPDLTAVNVMQGPQGTLSGHNAIGGAVQ
MRTA
DPEFGVNSTKLTFGVAEHGERSGEIVGNAALGERAALRYVLKRTYSDGWLVNEAGGHNLDSKDTLTGRIKLKAQVTDT
WT
SLLSYDHSKDKGRRYGFGPLDEVADTRRADSDQPLDETVENEGLSWTNTWDLEAGRLESITAWRHSDSEMHPGAHST
VSP
ILRYSSRDYEQLSQELRLTGQTERLDWTVGLFAMKSSDKRVEGIALRDDYVYGGTFTIPAGYGERSRAKIESETLAAYFD
GAWALTDRIGILGGLRVSHDKKSIDYNHADTAGYGYSLLALTQDLDLSTSGTFASPRIGMTWDVTPEMTAYGMITAGY
KP
AGFVSSFAETSDLEYDRETAINYELGLKGTLGGDMWRYALSAFYMDIKDQQVYSMKGSSTVFSIANAPKSRSKGLELSF
D
GQATRKLRLGAAFGYADATFVDYPGSKIGDAGGNRQPLSSKYTAALTAEYRLPLAGDSDLVVNADYTWRSKFYFDAEN
TL
PQSAYGLANASVAIETGTWDLQLYCDNLFDKHYLRSAANLTEGDFGYAGEERTFGIRFTRTF
>OLS42612.1 hypothetical protein BV379_19605 [Rhodovulum sulfidophilum]AB14
MALRPESPDLQKRLAAEVARPQGLIAGIDALDPGIRGAVLLRARFRPDRYRPELFRELGLDRPEQLARAVAKRCCEYLAG

RLLARAALAALGHPGGPVLPGPGRAPVWPAGAAGSISHSADICACLALHMAGKPVHPGVDVETVADARNLDAIFKLCC
TP
VDRALIAADPDRTALRATLLFSAKEALYKALYPVFREVAPFEAAELTALPSDGLLHLRLTAELHPSLPAGRVFALRYLTW
AAAAQDSGQGHGDRQVLTWILHEPASVRLGPET
>OLS42613.1 hypothetical protein BV379_19610 [Rhodovulum sulfidophilum]AB14
MQDASPAPRRRGRKPGPRPRLSRAAIARAALDIGLDTVTLKALAAHLGADHSSLYRHVKSRADIVSAAADLALSELDWY
R
PEDGWHEMLDRLCTALWDLFERHPGLAAAYRDMEITPAEGIHTFADAVAHLQRCGFRLEDAIIAVDTLVDGLTDCFSG
WH
RMTRPGAGGQSLAGILATRLQSASVAAPDHAPQIEGMISAMEAGPRAWWDKRKALILAGIEALCVPPANGRTVCPAA
EGP
AGRDGPK
>OLS42614.1 ABC transporter [Rhodovulum sulfidophilum]AB14
MSDTEIRSDLESLRRAWTLAGPLKGRVARGVGFRFLQSMMLGMSFAVVVWTVTGLAAGRPPTLAFIAGATGLMALSL
AGQ
LLFGWLSVRDSWLSSYEVAGHLRLRILDHLRHLPLGFHQARHRGDTVSVLTGDMQMLEAFFSDGLPRIAQALGLPVAV
FL
VLLVQDWPLALAAAASVGLAIPVFAATSRHLSRLGIRRQDMQAEAGARMIEYVQGMAVIRAFNRVAAGQESFRRAID
DFR
DISIRMLAQLTAPMTLFGMIVMAGVPVAIWAAWLRSAETGPATAITALILVLSIYAPITALLGVMELVRMADASLTRMD
R
ILTAPPLPAPAREAHPEGFELRFEQVSFGYHPDRPVLHGINFTVPERSMTAIVGPSGSGKSTILNLIPRFWDAGAGRVSI
GGANVADMREKTLSDLVTVVFQDVYLFAGTIRENIALGRPGAGQAEIEQAARAAQAHAFIAALPEGYETQVGEGGAAL
SG
GERQRISIARAILKDAPIVLLDEATAAIDPTNERAIQMALARLVEGRTLIVVAHKLSTIRSADQILVLENGRIAERGRHD
ELLAATGLYARLWSHRAQAAGWQIAG
>OLS42615.1 hypothetical protein BV379_19625 [Rhodovulum sulfidophilum]AB14
MVDNMHDEGALSKLRRKSVSYELLCWLAELYDDPERLEAEALRESCLARVQRQITSCPGEIKSIDALARKNGLSASKLKR
DYQLSYGISVGTAVRNARLETAHRLIEKGQAISLASYETGYSHASSFSAAFRKYFGYSPRDLKRRHALASRSQVAMHPA
>OLS42616.1 hypothetical protein BV379_19630 [Rhodovulum sulfidophilum]AB14
MQRSTLRKQGQAAICEQTVIRLSLLQQITLREWAWARIGELKVTDPSMTTDNTMQGSCSISMEKGSEKLALNKFKTKP
GA
ISSVL
>OLS42617.1 hypothetical protein BV379_19635 [Rhodovulum sulfidophilum]AB14
MLRFLAGYRARLLLSLYVSQYIGMGFVFAAIPAIARSKGLSLDDIAIFYSLAMIWTLKVLWAPLMDRYQPFGGPAYRGWL
IGAQLCLAAATLACSFFDPVDHLGAVAACVAAMCFTSSVQDVAADAVAVRLLPPEDRGIGNAVQTAGGLIGALVGAG
GAV
LAYEQVGWQATLWGLAALTLLPSILILQMRSGQQQEETPAEKVTYGLIWQFLLRPGTPRWLAVLVVAEFGLGAQIALV
NP
MLVDHGWNLTKIALGTALWGALFGVSGAGMAAVLFQRFTRRAALIGALLLSAALSVPIAFYHAAAMADWQMVVVLA
LYYT
GWGMTTTALATVMMDHCRARSAGSDYSSQFSIGALIGLMGSSLALSMAETYGYTAALAAGACSCLLAAILAKGKGAV
KAP
>OLS42618.1 crotonyl-CoA carboxylase/reductase [Rhodovulum sulfidophilum]AB14
MPLPPMAGDEVLIAVMAAGLNYNSVWSSMFMPGSPFDYLRRFGSFSPRNGTHVHDYMVLGSDAAGVVLRVGSQVT
NCKPG
DEVCVHPGVVNDHAPECHEDDLLSPQTRAWGFETNFGAFADFTVVRASQILPKPRHLSWAEAASLPLVNGTVYRMLI
GSN

GARMHLGESVLIWGAAGGLGLLACQYVLRGGGQPVCVVSSEDRAEILRRIGVQAVVNRSAAGISLWTRDGAPNERGI
ARL
RGRIRAAAGDPSIDIVFEHPGAETFWASVALLRHGGRVVTCGSTSGHEHRYDNRRLWMHVKSIIGSHGANYHEAWQA
NRL
VCRGLIHPVLTDTGQLADGVGLVRRLRDNLHIGKIAMLCQAAAPKDGIRDPGLRRAAGVDPFLQKLMVS
>OLS42619.1 hypothetical protein BV379_19645 [Rhodovulum sulfidophilum]AB14
MKKQFDQASEGVRAQAQEMGLGDDIVAQGSCAGGMARAGSDMDITIRASPDRFDEIINDPNIVPNRILHPNPGSAA
ERTR
NLIIQ
>OLS42620.1 hypothetical protein BV379_19650 [Rhodovulum sulfidophilum]AB14
MMIDLTCRLYIEGPESLDTLVEHVGTLTGGARTGRTISTPSLEIDLELSEDADAKQAQTADGFLYWLYGAYVEPGRQPM
A
EADYIALVAGLIVALLRDGWHGVASCDFEDEIARLTGWNWSELTTAHP
>OLS42621.1 hypothetical protein BV379_19655 [Rhodovulum sulfidophilum]AB14
MGSLLRQLCKILRGLARPDLRIHGIRRCRTIFAPFARDLLRGDDVDQPVREDTNRKSFPGASSQHIGNISFTIDEGSSCH
EAKTRAAKVMEVARELAGHVRDWLDRAADRIFDRLEPARFAGPEQSELALAGGREVEEHHDAQEQTRQQGIEKEGAS
IAN
RDGVHGHSL
>OLS42622.1 iron ABC transporter substrate-binding protein [Rhodovulum sulfidophilum]AB14
MVQRRVARLFGGASIVALCAVSPTATKAQDLSDDAYSLGTLFIIGDTLIRSFQKTAASVTAVEEADIEAAQGKDSVKDIV
ADMANVTYAASGGQGGAPTIRGQDGEGPNSGAPAFFGGSVPRIAVNLDGHYLSYNELVYSSTSIWDVDHIELFRGPQ
TIS
QGANSIAGALVVKTKDPTFEREGAVRLEYGSRGKRRASLMASGAVAPDLAARIALDYSARDNYIDYVNPAFSLGGTDQ
DH
LSQTARFKLLWTPAAMPGFEAMLTFSHTDGNRPTWEAATVPFSDYDSTVTQNPSWKQFTNTTILDLKQEFDNGATLT
NQS
QFSSLYTRRTTSPVTAGSAVIDQDSYSNETRLTFGNEATALSGVAGLYLSHTDSDETLNLRGATAFDDTKDSLGIFAGLD
YDFADRWTISGSVRYQRDRVQREGSSSFATNDLDYDETFDDWLPKLSLSYDVNDTTTIGAMVSKGYNPGGVTLGLTSG
EW
VAFDPETSTDYELFARTRVLDDRLGLSANLFYTDFKNMQRYVTSEAVAGYFEAVTVNAEEANAYGLELGFDYEASQTLR
L
YGSLGLLSTEIEKFSSAGADYSGNEFGRAPGQTISLGADWKIRPDLTLSGNVRYSGSYFSDDDNLSASFVDSYTVANTRI
AYDLQDNLQLYGYVNNLFDEDAVTYLRASRAVVGGYEATLVEPREIGIGVKMTF
>OLS42623.1 2,3-dihydro-2,3-dihydroxybenzoate synthetase [Rhodovulum sulfidophilum]AB14
MALPEIASYPLPAPDALPRSRAPWRPDPDRAALLIHDMQRYFCAPYPREAAPLPGVIANIAALARAARAAGVPVFYTAQ
E
GDQLRADRGLQADLWGPGMSRRPDHQDILSELAPAEGDIVLVKHRYSAFQRSNLDSLMRARGRDQLMIAGVYAHIG
CLAT
AAEAFQRDIQPFALADAQADFDAARHDLALRWVAGCCGVVIATGDALAALPAEAACA
>OLS42624.1 2,3-dihydro-2,3-dihydroxybenzoate dehydrogenase [Rhodovulum sulfidophilum]AB14
MRLSGFDGRTALVTGAAGGIGRACVELLTGAGARVVATDTPAAIEALAERPRAAAWLPCDLRDPEAVGAMVAEAETR
AGP
LSLGLHAAGVLATGALLEMSPDDWRRVQGINLDGSFHLLQTLGRRLVRRPGAALVVVSSNGGGIPRLDMGAYCASKA
GLT
MLTRCLGLELARHGVRCNVIAPGSTLTPMQTGMWKDGEGARRVIAGDPGSYRAGIPLGKLATPEDIARAAMFLLSEEA
GH
VTMADLYVDGGATLRG
>OLS42625.1 hypothetical protein BV379_19680 [Rhodovulum sulfidophilum]AB14

MAECLRGPAGLEAALARLGAALPDCRAAVVPVARTEGPKTRRAAEFRAGRLAAAAASASLTGMPCLAGRGADGAPL
WPAG
LTGSISHSGGQALALVGPSARRAGLGVDIEGPLSPRVAAEIAPVALTPEERACFGTDPVWVGLIFSAKESLFKALSPRIG
RRFDFDAARLLPGGATRPPLLCLTCDLAPGWEAGRRIEPVLIAHQQGVMTAVALQPRKVAPPST
>OLS42626.1 hypothetical protein BV379_19685 [Rhodovulum sulfidophilum]AB14
MTLTTTSTAPDRHWLSARLNSLLEDEGPIDPAESLIYYGLDSLRVMKLAAELKAHGIHVGFEDLAREPTLDAWTALIDAR
RDG
>OLS42627.1 2,3-dihydroxybenzoate-AMP ligase [Rhodovulum sulfidophilum]AB14
MDDFTPWPEDLARRYRERGYWRGVPLSEGVRAQAAARPGACAILCGNRSFSYGALDRMSSVMAGRLIAAGLRPGDH
AVVQ
LPNRAEFYITFLALLKAGIRPVNALYSHRAHEMRAYVEQLEPALLVGSRAHPLFAEDGFLDALRRDGLAPRLGLWLDGSE
PGQDLAQWMDPEAGLPEAPLPAPDPAGVAFFQLSGGSTGTSKLIPRTHDDYDYSVRASVEICGVDAETRFLVALPAAH
NY
LLSSPGALGVFRAGGTVVMAESPEPHLCFGIMRRHRVTMAALVPSAVALWFEAVRDGLPVPESLRLVQVGGASFAEA
QAR
QVPELLGCALQQVFGMAEGLVNYTRLDDPPERVFATQGRPISPDDEVRVLDRDGRPCAPGQTGRLWVRGPYTFRGYF
RSP
GHNATAFDAEGFYHSGDLVRRDADGYLSVVGRVKDQINRGGEKIAAEEVEGLLLRHPDIIQAALVAQPDARLGERGCA
VL
VARKKLRAVELRRHLIALGVAEYKLPDRFAFAETIPLTPVGKPDKKALRAAMLHPR
>OLS42628.1 hypothetical protein BV379_19700 [Rhodovulum sulfidophilum]AB14
MTAEPRGAAWLELTLAQLDFWEEFRFHPDRPLSTVAHCIEIEGAAEEIALVAALSRLAREAQVLALRFRDGPGGPRQRV
D
PGGVPAVRVQDLRGAADPEARARRMMADDIARPIDLERDPLAALWLLRVGPRRWLWYLRGHHIVLDGYGVSLIERRA
GAL
YAEALGQCAGPGPFLPFSRYLEEETVYRAGPRHDRDRAHWAEVLRDIPDLIVLPKGAEDYAPTPVAAAPDLPSGLDGQL
Q
RAARAVGMGWPDLLTLLSAAWIVRDRPEGAPGLPVWLPYQSRLGSVAAAIPAMVVNILPLIVSHRHGETLGAYLERSG
RA
LRGLRRHGRYRVEQLAADRGLRAGERFFFSPLVNVLPFDPPEFPGCTARSEVLAAGPGDGFNLTWSARTDGSDLALSID
A
DPAAAEEVAAAARTLVPFLARACAPGARDRPLADLLAERPAGPAACHTPRTARPIASRQEKT
>OLS42629.1 Fe2+-enterobactin ABC transporter substrate-binding protein [Rhodovulum
sulfidophilum]AB14
MNRRSFLTLATCAAALGSSAGAVTGWPRRLTNADGSETALPRAPARILSTSVTLTGSLLAIGAPVIASATTVRGAFFDQ
W
QAVAKARGVEKLWRAGSIDLEAAWALEPDLIVVSAGGADSALAQRAALAEVAPVLVLDYGAMAWEDLTRRLARATG
REAE
AEALLTGFAETLDATRATLTLPEGRANIVSYNGPGAPNPIARAQGAHGRLLTGLGFDLEDPPPGWQSASMPASADVVR
AS
YEHLTQLSAETTFLLAGTKADAARFMADPILANLPSVQRGQVYGLGKNSFRIDYYSAREVVDGVARQFGGA
>OLS42630.1 ABC transporter permease [Rhodovulum sulfidophilum]AB14
MARTRRLWPTLGLLVAVTLASLFFGPREITPDVTLEALLRFDPTDGAHLLVREQRLPRALLALVVGAALGAAGAMMQTL
T
RNPLADPGLLGVSAGATLAIVCLIALTGRIDIGGSLWAGIGGAGLGGAAVFALGGMARTRDPVRLVLAGAALSIVLLTLT
RLVTVNVEAHVFDQFRHWAVGSLQGRDWQVLGPAAALVALGALAALALARPLDALALGQDFGQSLGADPRRIWLAA
AGVI
VVLSGVATAAAGPITFVGLSAPHLARHIVGPAHRGLLLASMILGGALVGIADLAGRVIGGPTQIDVGIMAALIGGPVFVA

LARRMRMNAL
>OLS42631.1 ABC transporter permease [Rhodovulum sulfidophilum]AB14
MSGAAHIVWRWRGLSLRSGRRALWVNLALALLIAAGALVSLRLGRIAIGPAEIWQVLAGEAPSRMLDHVVMDIRLPRLLV
TLGAGAALGASGAIFQTVSRNPLGSPDVIGFTAGAASGALVWIIGLGGSGAGVVPAALAGALLTGICVYLLARKDGRVGA
YRLVLTGIGVGAILSAFNALLLVRGDLDAAISGNLWLAGSVEGRSWSHVWPLWLGLGLLMPPLLLGMRPLGLMAMGDDLA
GPLGVPVERARRRMLAGAVTLAALATAAAGPIAFVALAAPQLVRRLARMPDQPLIGAALMGAALMLGADLLSRVAPWGAI
LPIGQVTGVIGGLYLIWLLTRKRRRG
>OLS42632.1 hypothetical protein BV379_19720 [Rhodovulum sulfidophilum]AB14
MTTHGPEQALPSRPSRPGEGRAWLAHQQSEDGLAPRIAQVSLPDHLEPGAVVVAIAEALAGRAELTRLYRFDENCGLLVG
PGRGRPDLRLRWAASAEGIGPVIDSCAAAPWDLSRQPPLRVVLVLAPGAARLALIRHPVADDLPDSATLLAGLIPGAPPA
LAGETGTGDDITRLILAEFRSALEAPQMTADCDFFDMGGHSLTATRIIGRLAATHGIELRLNDLFLHPTAAALAPLARRS
GTGLAAAPDRSAAPADRAPLSLAQASLWKIYEALGFGPIFNIPFVLRFLDLVDETVFATAFRDVMERHPALRSRFADSPD
GVLQEAVPMAELDCMGWFMPSGDVEDPAPVLAAEAAHDFDLARELPIRLRFVRTGEGQLLSFLFHHIVLDEWSVNLMMDE
LARAYAARAAGRPPLWAAPAAPFHAFARRQAARGPDPRALSYWLDALRDAPPPCPIRRPDAPPLPRDPDAANDAGGAWCE
IALEPHVSDGLYALSKACSASLFNTVYAAISVALQRLAGLDALTVGTSASGRTDPDYFDSIGYFTTVTAHCLRAPGHLTP
RAVIGTVRDIINGSLPHCEIPIDLVEERLSDGTAAPGAHMFEVFIQIHARNALNGALEGPDGPVRFRQVDPDKTDSVLGL
QFEVVEDVIGGKRGLRMMMSYRYAHYSADDVAELVAAVTRTCTAFAREGAADRPLDALLS
>OLS42633.1 ABC transporter permease [Rhodovulum sulfidophilum]AB14
MNATAKAGAAPRKTGKTGATSFATTFRQLADSVGPEDAPGLRRALAGLIAAAALQGFGLAAIAPLFTAAATGPGLGEALF
WFGVMVVLMGAASALRWVAQGFDFDGRMVRATWRLRSRLGAKLREIPLEQLQDRRAGELNSMVLGNVDENLLYALTVLNL
VFTAVVTPFCAALGILLFDWRLGLLLLLVFPAIVPLYRWRRPALGRGKRILGQAHQEASADVLEYTQGLPILRAVGRAGE
KAERLSATFRKLEQIQIFGHRKGAKPNIIVASVVELGLVAVLGVGVWLVVAGSLHPAALGAVFVIVVRLSEPLSTFILYT
AMLEVMEAALERIAALMAIRPAPQMDPPAMPESHEIRFEGVGFTYANADSPTIRDFDAVLPERSLTALVGPSGCGKTTLT
RLLLRHFDPQKGRVTIGGVDLRQIPADRIDGLVSVVFQDVYLFNDTVLANIRMGRPEAGDDEVRAAAAAAQCLDFIERLP
QGWHTPLGEIGGRLSGGERQRISIARALLKDAPIVVLDEPTAALDTESELAVQSALDALVAEKTVIVIAHRLSTIVGADQ
ILVIEDGRLAQAGRHGALIGQGGRYARMWAAQGARKRWSVAG
>OLS42634.1 plasmid partitioning protein RepA [Rhodovulum sulfidophilum]AB14
MVRRQERIDLLVGEHAQKLSERLQAHRAQLFPPDAKRTLRRFTSGEVAQLLGVKDAYLRKLHLDGKGPSPEIRANGRRYY
SPENIQELRKFLEAGTKTPGTYLPGRREGDHLQVITVINFKGGSGKTTTAAHLAQKMALDGYRVLGVDLDPQASFSALHG
FQPEFDLLDGGTLYDAIRYDDPVPLPRVIQKTYFTNLDIIPGNLDLMEFEHETPRALTNRSGSLFFTRVGDALAQVEGDY
DVVVMDCPPQLGFLTMSALSAATAVLVTVHPQMLDVMSMCQFLLMVSNLLGVVSEAGADMSYDWLRYLITRYEPGDGPQN
QMVSFMRSMFGDHVLNHPMLKSTAISDAGITKQTLYEVERSQFTRSTYERAMESLNNVNAEIEALVRQAWGR
>OLS42635.1 plasmid partitioning protein RepB [Rhodovulum sulfidophilum]AB14

MARKDLLKGLMDPGKPAPATEAGAEPRPTRVDAARPRYSKGAIGAVSQSIAELKSRSIVEVDPRMIDDAGFRDRLDAE
DP
GLEQLIESIRVYGQQVPVLLRPNPDDTERYQVVYGRRRVAALKALHLPIKAMVRELDDCGLVIAQGQENAARRDLTFIER
ANFARQMRDAGYDRKIICDALHVDKTLISRMLSVADRVPIELIEVIGSAPGIGRDRWLALADKLKGRDLTDRAVGESSDA
RFEAVMAALAQPRPPAPRPRIVTAADGRALAEVARKRGRTVLSVDNGVSAGFEEWLVENLARLHGEWQDGRED
>OLS42636.1 hypothetical protein BV379_19750 [Rhodovulum sulfidophilum]AB14
MQVLTTTPFGQRPMTTALLDRARDAQRPASVPEFDKWELFRELCAGRAAYGIGDRELTVLNALLSFHPGKVLAEADPLI
V
FPSNAALSERAHGMAESTLRRHLASLVAAGLIARHDSPNGKRYAARGADGGIARAFGFDLRPLLVEARRIASAAAEARA
A
AEALRRLREDLTLLKRDAVKLTVYGRESGLPGDWEGIEARLLDIHRQMRRRLDRAELEILKAVLSGLLSEIRVLLVETEN
PSGSDGEIGRHYQNSNPDLNESEPCQEMAKETGMPFSGTASMTGTGAELAPGSGTWTDSGSEPATDPGSAPSVASIR
SAG
SKTPDLSAIPGGTDAPPVLPLALVLKACPDILDYAPSPPRHWHELVALAGFVRGMMGISADAWAEAERAMGAETAAIT
VA
AILQRVSEIQSPGGYLRALSRKAAQGGFSPGPMVMALLRPMAA
>OLS42637.1 hypothetical protein BV379_19760 [Rhodovulum sulfidophilum]AB14
MGADFNAVFGDPNVGPRTVTISGGGTLNAAVENWAVVAIGWTAGDTESIMTVTGEGSKLVTSAVGSQAEGVGWAT
IGYED
GTTGVLFIQDGGALEAGADIAVGHGVNSTARIDVDAGFLETKGNAQLRVAYERGSRGEVNLSNGSTATLGGNVLMGR
NTG
ATAAFNILSGSEATSNGRVVVGETDGSNATFTVDGEGSTYISNGAFDLGYASGATGKLVVGNRAEATFNDDLRVGLMG
NG
AIEVNTGGKVTANTDVHIGGTEGERKGGKGSLIIDGAGSSFTVNDKGLFIGFSTPGTESNPSGSVTVSNGGTLTLKAEAT
EPPGNVTVIPDSTERAGNLTLGYGDGTYGSLDVKGAGSSVAVGGDLYAGAFSTRVSDDASFVVDGKTYMGTDGGVLG
YFN
VSGGDVTLNDETYIGDGGTVIMAIDQGGTVSANSAVFLGRANGTGRISVKGDESKLNMSGSRLAVGYEGIGEQQSTN
GQV
YILEGGAVSVGELMIGELSGSIGQISVSGTGINGNNEQIASRLDVTGNATIGGAGTGYLQVDTGAVADIGGSLILGDTGS
GTVYVRSSDIMTRLDVDEEVVVAAGAQSQGTLDLYYSSAMSVGGDFTVAKGDESEGAVSLGSSNSSIDIGGGLAIGVN
DG
SNGTVRVFGGATVSVGTEDGEGNYDGTISVAQNSGSTGTLIIGGDRTAGGDYDRARAGRVNAGTLTFGDGNGSLVFS
VTD
TDYTFGLDVSGGGAIEAYNGTVSLTGDYSEYDGDTNIHGGTLSVDTGTFTHSAIKVMGGATLAGSGTVSDVTLQDGST
VA
PGGAGISTLTVSGGDLSFADGASYAVTVSSDGSSDLLAVKSTEEDEAPGIAVNNIGGAGGTVTLGGSSSLSVSRLASDTA
GDFKVNTWYTILTADQGISGEFSEVSDDFAYLDTSVDYDDDTKVLLALTRNDTDFEDVVGSSNTNARNAARAVGSMDE
EN
PLYEKVLFLEEGDVDRTYQALSGETHAGMAGALVQSTAPTRRLVVNRMRQLTPGAGAAPAAVTRNATSYGAEAGSFF
RDP
EVWGQAYGSWSKLDGSDGASGMESTTGGVVIGADAETDGGWRTGVFGSFGMVDTDARDGSADSDADSYQLGVYT
ARSFGQ
MALRMGASYSWDDVSADRTVTVGGVRQDLEADYWARTAQAFVELGYQTWAGSTSLEPFIGQAILYQEIDSFTETGGS
AAL
SVDGEDLTQGITTVGLRFGRDLEGLGAGMSNLHGAVAWQHVTGDLNAESTMRFASGGDAFSVTGSALDRNQAVLSL
GLRT
ELTERSVLDIGYEGTLSENSQSHGLQATYSIRF
>OLS42638.1 hypothetical protein BV379_19765 [Rhodovulum sulfidophilum]AB14

MPAFLAGLLSLGACASITNDANVPVALSFSTGESGSCKLSNKRQAQVTEIPAVVSVRRSDDALRYDCKTRRGREAVGAIP
STMGGKIVASAVFLDFGIVDSITDKHREYPASYVIPVER
>OLS42639.1 hypothetical protein BV379_19775 [Rhodovulum sulfidophilum]AB14
MPRFSVTPEPPALLTRSVLFVALMIAAMVSRGDAQPLHPGMAPPERVPAVMVGDRVVNVAWHLGVVPEAMSVRG
DLWAFG
RTLANTSSMILGCPGYIVMKDPDIVPRILREKGLERVLVERSDPFDQTRPHRDPMNLLPILEETGIAADPGGRITVIDFT
AGLEAAIRQIGAVLGREAQAEDLIAARRAQLDAVMAGLPLDRSGLNVLILDGRVEDETAQAFVRAHLPGGYADDFLLTP
L
GVQNAAGPLADQGLNGFAALAGLDGLEALRPDVIVMTGESDAVQRQLAEALARHPDLASSVPALANHALVSLPAYYD
SAV
VDYPRILGRWAAAFTSLIEG
>OLS42640.1 hypothetical protein BV379_19780 [Rhodovulum sulfidophilum]AB14
MDTATTLENDLTNAERDILHDVGFADETTRAGTSVLADHRMRMALSNDPDVVILPLAEALKTYDFVQGLMFGLVDPE
END
HIRLVAEHMHDPLGHFIWVRPGARVRLPVQSFTLLETPQARQFTHDITLIDEGAEVEMVSGSGVPSDLQKGRHISIGESY
LRKGAKCTSVTIEHWGPEMEVYSYGYSQLDATATSTSTAIMMSPIRHHSSRSVSTLGAGASAVSQSIVFAPEGTERVLVS
ETRLAAAGAKSKDVTRMVSAGGTIVNEALLIGDAAGSEGFLGCDGLKLKDTGEIRAVPSLRALAEGSQLSHEASVGMID
G
EKLNYLMASGMAEDAARDLIVQGFLALSDEQIPDSLRRRVTEMVAEAKSGGM
>OLS42641.1 ABC transporter ATP-binding protein [Rhodovulum sulfidophilum]AB14
MLEISDLCVEVAGQPVLHDINLSLGEGELHVLLGPNGSGKSSLLATILGLPGYRITRGGISFDGAPLRDLSLQERAELGI
GMAFQRPPSMGGVSVSAFAAALGASDILESEAAALDLSDFVTRDMNVGFSGGEIKRWEILKLFLQDPRLMLFDEPESG
VD
LEHVSAIGQAIRRITRSNDRKGRPRAGLVITHTGLILNYVEADRAHIMKGGRLLHSGEPEALFRHIQSHGYTVPAAA
>OLS42642.1 short-chain dehydrogenase [Rhodovulum sulfidophilum]AB14
MSTIPRPALFSYGFRPFFLLAGLFAAAVIPLWLAIWAGHAALAGPFGPRDWHIHEMLFGYVPAVLAGFLFTAIPNWTGR
M
PRRGLPLVALSGLWLLARLAVAGALALPAWAVMVLDLSFLLAVAAISVVEIVAGRNWRNLAVIVPVGLLAAANATFHIE
A
MTRGAADIGMRAGFACILFLILLIGGRIVPSFTRNWLSRQGPGPLPAPVGRFDRLALMASAAVLALWSLAPGLPGLSAA
F
AGLAGLHLLRLARWRGWRCGRAPILWILHIAYLFLPLGFAALALDAPVAGLHLLGIGAIGGMTLAVMMRASMGHTGR
PLE
AGRVLTASFLLLCAAALIRALLPDQGLGGISGPGLSGALWTAAFAGFVLKVGPWLTLPSVARRQPNPAARPRA
>OLS42643.1 hypothetical protein BV379_19795 [Rhodovulum sulfidophilum]AB14
MRHPVSLSLAAAPTARAASATPLDMEMPDRGEARGMVCAPAFARAGPDDTSRVAPTGTGPGIDGMQPAGRAAVG
GAPGRE
IEPGVTGAGLSGTKGPPPVASGMAPPRIGAPALPHRGRTAGPFGDLVAQVVQ
>OLS42644.1 Crp/Fnr family transcriptional regulator [Rhodovulum sulfidophilum]AB14
MVGKLDESLLQDVGPFRRLDRGQIREILDLATARRFDEGAAVFREGHPAERFHLLMDGVIRVMRITAEGDAVTALHIPS
G
QLFGIARALRRETYPATAVCARECLTLSWPMSLWDGFVDSYDGFAEEGYRTVGTRVGEMNTRLIEMATQQVEQRVAG
ALL
RLIDQSGRKGKEGIEIAFPVTRQDISEMTGTTLHSVSRLLSAWEKAGIVASRRRHVTVTDPQRLELIGDGR
>OLS42645.1 5-methyltetrahydropteroyltriglutamate--homocysteine S-methyltransferase [Rhodovulum sulfidophilum]AB14
MTTEDTSSLRPGLPRADHVGSLLRPQAIAGARMQGLKGAELRAVEDREIAALVAMQKEAGLRVFTDGEARRQFWHYD
FMG

MLTGLDMKPSTRSLPFQTKHKTPPIEAHLTGPLDFPADHPMLRHFTYVRDLVAPGEGIAKISIPGPSACHYRMVPENIEH
KPYLDQELMFHDIAATYRKAVQAFYEAGCRYLQLDDIFFAYLGDEKQREERRAMGQDPDELVRKYAWMLEEAIKDRPA
DM
VIGMHMCRGNFRSSHVAEGGYHGAADAIFNTTSVDIYLMEYDTERAGGLEPLASLPKGRKRVMPGFITTKTGKLESLD
WI
RAKYDEASKYVDIDQLGVAPQCGFASTEHGNEITEDDQKRKLELVVKTAEAIWGES
>OLS42646.1 transcriptional repressor [Rhodovulum sulfidophilum]AB14
MTKANHESLLDICRRHGLRITGQRRVILEVLDASGDHPDVEELHRRVAAIEPGINIATVYRTMNKLAEKGLIERHMFEDG
RLRYEPAPEQHHDHFINLETGEVVEFRSDEIERLQEEIARKHGFEIVSHKLELYVRARKPR
>OLS42647.1 iron ABC transporter substrate-binding protein [Rhodovulum sulfidophilum]AB14
MSLKMTRLMGMAATAGLMAGPALADEDRMKVVTTFTVLADMARHVAGDAADVVSITKPGAEIHGYEPTPRDIVRA
QDADL
ILSNGLNLELWFQQFLDHLGDVPSVTLTDGIDPIPIAEGAYQGKPNPHAWMGLDNALVYIDNILEAFAEHDPGNAATYV
R
NASAYKDELRATLEPLKAEIATIPEAKRWLVTCEGAFSYLARDFGMKELYLWPMNADQMGTPQQVRAVIDGVRQNHV
PVV
FCESTVNTAPAEQVARETGARYGGVLYVDSLSEPGGPVPSYLDLLRVTSQTVADGLTATTN
>OLS42648.1 manganese/iron transporter ATP-binding protein [Rhodovulum sulfidophilum]AB14
MKDELQKTGAARPDAADPGSGIEARGVTVTYRNGHTALRNASFRIPRGTVTALVGVNGAGKSTLFKAIMGFVPVAQG
DIR
LLGLGVKEALRRNLISYVPQSEEVDWSFPVLVEDVVMMGRYGHMGFLRRPSKADRAAVDAALGRVGMREFRHRQIG
ELSG
GQKKRVFLARALAQDGQVILLDEPFTGVDVKTEEQIIALLRALKQEGRVMLVSTHNLGSVPEFCDRTILVKGTVLAHGPT
ETTFTRPNLEAAFGGVLRHFTLGGPDLHEDDDMRQVSIFTDDERPLVQYGERTRKAERRQ
>OLS42649.1 hypothetical protein BV379_19825 [Rhodovulum sulfidophilum]AB14
MSVLLEPFAYGYMTNAMWVSALVGGVCAFLSCYLMLKGWSLIGDALSHSVVPGVAGAYILGLPFALGAFLSGGLAAG
AML
FLSDRSGLKTDVVIGIIFTAFFGLGLFMASVNPMSVSIQTITMGNILAITPGDTLQLALIGIVSLTVLVLKWKDLMVTFF
DESHARSIGLRPGLLKAVFFVLLSASVVAAMQTVGAFLVIAMVVTPGATAYLLCDRFPRLILTSVAIGASTSFAGAYISY
FLDGATGGVIVVLQTLVFLATFLMAPKHGLLAARRKAAAALTEEPA
>OLS42650.1 hypothetical protein BV379_19830 [Rhodovulum sulfidophilum]AB14
MLETLLMPFQFGFMQSAFAIALIVSVPTALLSCFLVLKGWALMGDAVSHAVLPGIVLAYVAGLPLILGAFAAGMLTAVA
T
GFLSENSRIKQDTVMGVVFSGMFGLGIVMFVALRTNVHLDHVLFGNMLGVGPADLWTAGAISVCVSLVLVVKWKDL
MLYA
FDPAQARASGLPVGLLHYGLLAILSLTIVATLSATGLVLAVGLLISPGAIAFLIVRSFGRMLAVSVLVCAVAMLGGTYAS
FFLDSAPAATIILVLTGLFVLAFLRRLHLTRSASRRAATA
>OLS42651.1 XRE family transcriptional regulator [Rhodovulum sulfidophilum]AB14
MAKRGEKLVIGQRLKVLRKSLGLTQAQMAAELGISPSYITLIEADQRPASARLLMRLAEVYDLNVSELAPGIDAQLASDF
AAALKDPALEARGVGRAEIEAVLQSAPGIAAALVRLQAKYTHAAMQAQAEDNPLTDRNKVELLGQIARPVEIVRDWFY
ET
QNYIDRVDRAAEAVAEEMALRRTEPHLALTERLADHGIRIRILPAPVMGGSLRRFDLHRRELLLSELLGQSSRRFQIGVL
LARLEQQELIDAVIRSAGFEDGLEDGDACRLARVSLANYFAAALMMPYGRFLAACESERYDVELLSHRFGTSYEQTAHR
M
STLQRPEARGIPFFFVRVDRAGNVSKRFSAGRFPFSRFGGTCPLWNIHGAFEAPGRVHTQVIRMPEGASYFSIARTVTR
A
GGTHSAPAPQLAIGLGCDIAYAPRLVYADQLDLGRVKPVDIGLNCFLCERRDCASRAQAPINRKLAVNELERSLAVYRFE
GE

>OLS42652.1 malate synthase A [Rhodovulum sulfidophilum]AB14
MSLDHHVPTASTPLVLREAPGAERVLTAEALEFIAALQRRFGMNLHALMIARQRRQERNDNGRLPDYLEDTQAIRQGV
WQ
AAPVPEALADRRVEITGPVDRKMMINALNSGARVFMADFEDATAPSFANIVAGHANLLDYRDGTLTHDDPKTGKCYR
VGE
NPALLIARPRGLHMEEANVLLAGRPVSAALFDFGLTLFHCGRALAATGRGPFYYLPKIESHREAAFWNSVFEFAQDRVG
L
PRGTIKATVLIETLPAAFEMDEIIWELRDHIAGLNCGRWDYIFSYIKTLRAHADYVLPDRAEVVMDEAFLAAYAARLVKV
CHRRGVHAMGGMAAQIPVRNDPEANEAAFARVRADKLREVRLGHDGTWVAHPDLVPVALEVFDAEMSGPNQIRTP
RQEYR
LLPEMLLRPHEGRITEEGVRTNISVAIEYLAQWLSGRGAVPIMNLMEDAATAEISRAQIWQWIRHGARVAARDGTERR
MD
AAWLDALVQEEIAAILGRTGPGGFHRGHYASAARIFQEAATAETLPDFITTPAYAVLNALD
>OLS42653.1 isocitrate lyase [Rhodovulum sulfidophilum]AB14
MSQRKTYSELRSDVLSRYPSGRSSSGVSVDDIVQLKLQNTYATHLDIAREMASVMRADMAAYDADPRNSTQSLGCWS
GFH
AQQMIKSVKRLRGSARGVYVYLSGWMVAGLRNRWGHLPDQSMHEKTAVAELIQEIYTSLRQADEVALNDLFKALDA
ARAA
GDTAAEAAAIEAIDGFESHVVPIIADIDAGFGNEHATYLLAKELIKAGACCLQIENQVSDAKQCGHQDGKVTVPREDFIE
KLRACRLAFEELGVDDGVIVARTDSLGAGLTQKVPVSRAPGDLASQYIKWLKTEPITEENPIREGELALYRDGEFMRPAR
LPNGLFPFMEGTGRARVVEDCIASLTQGGADLLWIETDTPNVDEIASMVSEIRAAVPDAKLTYNNSPSFNWTLNLRKQ
VR
AQWLAEGKISEDSYPDGNELMKPDYDETDLGREADARLRAFQTDISTRAGVFHNLITLPTFHLTAKAMDELSRGYFGED
R
MLAYVATVQREEIRRGISAVRHQHEVGSDLGDTFKEMVGGERALKAGGSANTMNQFAAE
>OLS42654.1 hypothetical protein BV379_19850 [Rhodovulum sulfidophilum]AB14
MLKESPKDGTAEGRLFRIAYKSRAHPGCDLASAGAMAAEDALRNRDRKVTGVLLFERGWFLQWLEGPARTVLSLASAI
RT
DPRHGEMQILSAGATDERRFAGWPMRLAPPSGSLADRADFGAAAAALAFDRAAALYREECGGEADWRDLADGFVR
DLARA
PEEAVPDLPPSALETLSARAGFVDALCAALAEAWHASALSGFEVTLATVRLNRLLLRAGRPATSLAPKGRVLVLAPYGCT
EIAGAIAKTDLLRQAGYSVRMVPWASGAALDDAVGETGSCPVIVYAGRVGIGTKDAMRGAALAERLSARMPGRTVLL
GGR
DAGPLCDWSHRLAFLAQQLPTGADPAADPALRTRQRV
>OLS42655.1 hypothetical protein BV379_19860 [Rhodovulum sulfidophilum]AB14
MPALALVGLYAAQSITGSMVQSALPVVLRDAGVGLDRIGLLSLLFIPWVLKVFWSPLVDRFATPRRWILTCQGLLCLCFL
VGAAFAPERHLGALLPVVFAMAVLAATQDIATDAAGVYATTRRTRSLASGASTVGGYAGFLIGGGLWLWIYARLGWEI
SM
IFLAVAMLLLTLPVRALRIGRPEPAARPPSPLLSLKNPVLMRGAGLLLLWQGGVRLGLAMTGPMLVDAGFSMELIGWL
RG
AGGMAAGLIAAVAGTVLCRRFGPRPVLLAAGGGIGTCCAGLALWAAAPGALWQLAALQLGLSALTAVSFVALYAEM
MNWC
APAQVATDFALLQSLDAALAVATGILAGQLAQHMGYAPVFGLAVVLLISALPLVRRLAGTCPASEIPLKNMEIAE
>OLS42656.1 hypothetical protein BV379_19865 [Rhodovulum sulfidophilum]AB14
MMRSLLNTACVAALAFGSLAFVARPLEARDRSADGELDLGTIFVRGAKRPQEALSFPGAVAEADASQLQAREVRNIAD
LD
RVFPGLTVDTRSARVYSNFTLRGQSSLDYYNPSVQVYVDGLPQDPAVLAQMFPGALENVEVLYGPQGSLYGRGAVGG
VIN

VTTTKPGEGAPFSWSAGGSDQGYQAQMRGQVQLGDGLWADLSLARDDAEDDLDVMGTGEDVGGTVEDRAQLRLR
YAPEGS
PLDIMLSAARNRIDSSEEQFVKGTELSGRKAVPFPSDYIQDTDSYGLTADYDLGWGKLSLLTGYQDRSLDRTIFGSDTPE
WQKTLTQELRLSSATGGPVDYVVGLFYSHTKFHRDAYGASTLQKTDSYAAFGDLTWHATDRLDVTAGARWDYEKTDA
TAT
GAVSLDGSDDWSALSPKLGVSYALAPDWQAYGIVSSGFKAGGFTRTLTPSNISYTYDPATTWNGEAGLKYRSGDGAVE
AS
VAAYYSVTDDYQMFVGPAASQYLQNVGEVTAKGIDARLRVNRGGWGVTGGATWGSSRFTGYDDASGTDHDGNDV
PYAPDF
TARLAVDYTHDLGHGRGSLIPRMGLSYQGKVWFDETNSLGQGGYWLVDAGLAWDMGQDRVLDLYATNLTDETYAS
YGFDA
SGYGLGDVYQLGRGREVGIRFTQSF
>OLS42657.1 addiction module protein [Rhodovulum sulfidophilum]AB14
MKIVFSDNAWEDYLWWQSPGHEKELERINTLIKAATRTPFKGLGKPEPLKGDLRGWWSRRITDEHRFVYRVSGKGDE
QSL
QIAQLRYHY
>OLS42658.1 prevent-host-death protein [Rhodovulum sulfidophilum]AB14
MHVISYTDARASLKDVMDRVIHDRDEVIVTRKKNEAVVMIGLDEYNAIQETLHLLKSPENAARLRSSIAQLEAGEGTERD
IDL
>OLS42659.1 type IV secretion protein Rhs [Rhodovulum sulfidophilum]AB14
MSGFFRQDARMGRISTVLGQDVLVLRRFEGTDHLNALFDYSADCLAATADVDFDRLIGTHATVTLTTKEGERPFDGIVT
E
ARWLGSGDNGHRYRLRLRPWAFLASLRRNQRIFHNKTVVEILTELLSAYADAGALTVELAQDYPELEYTVQYRESDLAFA
CRMMERHGISYHFRHADGGHEMVLTDLAEAHDSIGARPFRPAEGHHQEEVSHFRAWRPARRITTGAIRLTDYNFKTP
VAA
METDHLGDAAYEQGQIESFDWPGDYLDQGRGRVVAELRAAGERGQDRRFEAEGDIVALGAGLRVTLSGDPVPGTGG
EYIC
LSARHSYTADNYGSGGSDDGDGRAYDGQYVLMPVEAPLLPEQKTARADVRGPQTATVVGEGEIDCDEYGRILVRFHW
DLD
EAWSMRCRVSQSWAGAGWGGMVIPRIGMEVVVEFLDGDPDKPLVTGCVYNGRNPVPYDLPANKTVSTFKSDTHQ
GAGYNE
FRFEDEKDREEVFMHAQKDHNTIIENDESHSIGHDRSKSVGNDQSEAIGHDKTISVGNDHRETIGQDMYYTVGRNQQ
EDY
GKDHIHRVGNIHKQSVWADHLYEVGRNYQGEVAGKYVLDVGTSITNNTGKHTLMAFEKFQIKGPGGKITIDGSGITLEA
A
NIRLKGNVSVGGSGSAQVPTLKLAAKEGLPLCEECAAIKEDV
>OLS42660.1 hypothetical protein BV379_19895 [Rhodovulum sulfidophilum]AB14
MPPIARVGDTHVCPVKGHGSNVIVTGGSALIDGRQVARVGDKCACGCVIVSGASQMTCDERPVAILGSKTSGGGTIVS
GS
PNHVTL
>OLS42661.1 hypothetical protein BV379_19900 [Rhodovulum sulfidophilum]AB14
MQGAPLPSVRFGTASELRAALVGWIGATVPGCRVGAPEEEAGEETGEDGLRAVLRLGDIAPVSHGEGRDLVLARAIYRL
D
LAADGIAEAEQALVDLILEGDGHPAFAVEAVARLDAPLPGLAIRACLERRRAAPTAPPVKIHNVELRPGRRSERQRTG
>OLS42662.1 hypothetical protein BV379_19910 [Rhodovulum sulfidophilum]AB14
MPENATAARPEPLRNYQFSLQIQNREIAAFTACTNLGMRIAPIRYREGGAHEAVRWLTGDTEYGEVCLRFGVTNSTEL
WD
WIMTAAAGAVERRNVAIILYTPRGDEALRYNLTAAWPCEWAGAHLDALDQGVAYEYVKLVFEQIAAVPRAAAAT

>OLS42663.1 hypothetical protein BV379_19915 [Rhodovulum sulfidophilum]AB14
MTGRRGFLAGTVSALWRLLGRIAALLSGRGGTVEAGQEDLPPAHWLAAIRAAAPDKLDGTRLIADFAGRAPDPSAGEC
RA
ETGTGFVPPSFPARSVGVEKTATASRAAPGSLRSVSPRPLAAPLAIVAPKTIAPEIVESVIAGPGPIIPSPVGRRGGLRP
RVSVLSPASRAAEAAGPQSVEQSPEQRPERNGGSGISRPRRIPAVRVLSGSGLAAARSGAAEREAAFAWPDEAPGKGA
SQ
GWEGPHRPLSGAGQSEGQQDEHGDSHGGDQKDDDQRDGQTRLRPGPLVRPVAPVPLPDAAGLHPSPATVPQGPE
GEVAFA
GVAPTPWRPPDVDLVAASGTALAEGWSRRGFAPTPAPTGAGLSLVADAAGRFPAMDPVREAEPLPFFERRLDGPEAR
RAR
FAREQGGELWTV
>OLS42664.1 hypothetical protein BV379_19920 [Rhodovulum sulfidophilum]AB14
MDRVTLLVDETGDQISALLNPASLSFRRQSGMRPRRRIGTAVAGGTGRDAPLVFAGDAVTELDLELLFDADFAGSATPA
P
SGPPRDSLDVRGLTAPIWRLSDAAEAHGEALDDLPQVFLIWGRATKLRCRLLGLAERLERFDAVGIPRRSWLALRFRVF
Q
EAEEDPRGYLPLPAPVESFLDEVPVPGALAGPQDPDLPDLLDQSPGVTVIGGERLDEIAARWLGSPAAWRWLAFVNDL
AD
PLRLEPGTVLSLPPPGSGPGAPTTGAAP
>OLS42665.1 hypothetical protein BV379_19925 [Rhodovulum sulfidophilum]AB14
MTEMLRPGRARERKLTVRLDGVPLAAGAVDALTGIELRQTVSAPALCELCFEAGGALPLDPGALAGRRLEIAAGPDAVL
F
EGSIGAVAPGRARPNRRSLRIRAHDPLAALYRSALLARHEADGLDALARALLGRHGLELRGESGAPCPAVLLQHGLDDLR
FLARQAAKLGFHLAAEGKAVDLFGAEGRGAPLPLSFEGGLLEADIEENALAGFAATRQTLWDTGLNRPLAGESPGAVP
GR
AGELTLAAGLYEPPVAPERLTEARAAQHGAAARRISGIALGDAALRPGRRIALELEPGRTARGTIAETLHRVEPGLGWVT
EFRTGLPAHLPCEGMLAAPGTVAEVSGGDDPLLRLKLDLCDGLETDWLAVALPGAGADKGMIALPETGDRVLALVFAE
NP
AHGFVLGGLYGEQEVPRLGRGPGGVRPYLLRTPRGQQLLLSDRGARVELRASGGSRLTAGPSRTELHSAGDLVIEAPGR
A
ITIRAASIGFEEG
>OLS42666.1 hypothetical protein BV379_19930 [Rhodovulum sulfidophilum]AB14
MRVLVEPATIVCAHGGTIPQRASQGFVTVMGAPVLIEPDPVPRRVDHCPLPPPQKKCLTTITVAAGYSRFVTIGGRPVCL
DTLAGLTEGSPVGTWRVLASGQALVECSG
>OLS42667.1 hypothetical protein BV379_19940 [Rhodovulum sulfidophilum]AB14
MSLPLPQLDDRDFAALVEEALARARQTCPEWTDMSPADPGRALIEAFAHLTDVMIFRLNRLPRKLYVAFLNLLGGEMR
PP
SAATAMLEFRREAAGVADAAPLALTIPAGTRIGGRGEGAPVFVTLDQAVLAADAQSVAVRAIHAAWVEGELLSEGVEG
PG
AVLALARGPVIAPLPYETGILLGVEDDAGGRPLRRFGGRDFALWPGLETPFELFSDGPGFYCARGVGRIFLPRAAPGSAG
VPRRDAQIRAWYLTGGGAAGNVAAGTLTDLRDPIPGLAVRNPERAAGGRDAEPLEAALERIPREVFSDRRAVTARDFE
AI
ATGEGGVARAQARTGVQLWAHAEPGSVELLLVPEPAPGQSDAVGRRAGIEALQTEELRGRIARLIAARKPLGVTVNVA
WA
RLKPLSIRVDVEIEPDAPEDAVLRRIEAALDRLISPFRDWPPGRPIRPADVYGTVLAEPGVRSARSVVFEVAEAPDADVR
DAVPDPHQPRTWFVAAGDRIFRSLNDGASWESLARQEGCPATAVRPHRRRPGLVAAVWAEGGNHTEDGSRLAVSF
DCGET

WQSDAIRFDSWIYDLAWLGTAADPVLLIATRAGLYSWQVRSEALQRRLPVAAGVDAVGLYAVLATGTALGRPVVAVA
ARE
QRGVWISYDPLEPGSFVATGLDGSDIRVLDAADLAGGRYIWAGARATGGSAGEGAFRLNLPERPQDLDAWQPLRAR
WRGG
SCLGFAFLGTRAWAGSFDAGVLEADLGADPIAWTAPEVDIGLPFRSRENLFEPVAALAARAAGPGGRGLMMAAGPRG
LYA
RGADGGPFAATSQTLFENEVRLPAPWLFASEPHLIRVIGSDDTP
>OLS42668.1 hypothetical protein BV379_19945 [Rhodovulum sulfidophilum]AB14
MTRHEIAALLPEILREADRPASPFRALLEAMAGLADPVDREIDGLDAQIDPRRCRDRMLGMLLGWLDLGPVAGAVARH
VR
DPALRDARLRRLALAAPALLPRRGTQAGLRDMLAVASGLEALRLRESLPGPGFRPFHVVIEYPRPPRPVRALIRDVAELE
KPVFVSFEIVELSAPEDSEEGA
>OLS42669.1 hypothetical protein BV379_19950, partial [Rhodovulum sulfidophilum]AB14
MPSFEIDTPQLVIEPSGSLQVGISNSANQARTCRLEIASEDAEAAGWIAISPWQERNLQPNERSEITLSLTLPPGATAGP
RRFRLLAVEVSNPDEDVARSPDFRFEVPGSGGGGKLPLWPFIAAGVAALLLVIGVGSYLFWPAGTAQVPGLSGLTLAAA
E
ARLEAEDLAVGQVDRRESGSAVPGTVLAQDPGEGAELAPGSEVALTVATEPARAEVPNLVGLSQDRARNVLRDRGLR
MGG
TATRESGRVAPGTVLAQDPGEGAELALGSAVSLTVATEPARAGVPNLVGLSQDRARDVLRDRGLRMGGTATRESGSV
APG
TVLAQNPGEGVELAPGSAVALTVATEPARAEVPNLVG
>OLS42670.1 hypothetical protein BV379_19445 [Rhodovulum sulfidophilum]AB14
METLRMPFFVLALVLLAFAVSFEIGLGLIWPALVPPPGGQLPDVSPGFGVPYLAAIDLILVVYATAAIALAFLWPALAGRV
QAAIGILLALIGGLLVLALILAAFAALMLMLTLLLAVPFGTLAYFAAFADFPTAKAAAALAAIMAFKLGFAICLILAHQR
FLKMKILMLVIASSLALTWLTALLHALVPGFLAAITDALAALITSVLALVWIAVLFIGNLPGLVRAIRSLLTARPAT
>OLS42671.1 ABC transporter [Rhodovulum sulfidophilum]AB14
MVPVWAIWRAVTALAADEAGAVLFARLALLTLAAVVSGYTLLGLAMALSHLAAYRLIHALRLALARHLARLPMGWFLG
KR
SGGAKKLIVDEPERMELIVAHGLPEGVSSLGTWLAVTIWLFVVDWRMALAAVALTPASFALISMAMARTTVMAGEYQ
RLS
ERLNGAVVEYLAGMPVVKVFNRTGAAQSETAEAVRAYADIETGMARRFVPLGAAFYTLVVANVVTILPAGLLMMKAG
WID
LPTLCFFLILGANYSQPLLKLFNLFHNMAHISMASTLISDVLDTPAQPDTGRRVALPSHDIAFEDVRFGYGPHEVLHGIS
FTAAAGEVTALVGPSGSGKSTAASLIPRLYDVDSGRVTLGGVDIREIGLDQLMEETAFVFQQTFLFSGTIAENIRFGKPG
ATASELYAAAKAARAHDFILALPQGYETRLGEGGASLSGGERQRIAIARAILKDAPVIVLDEATAFADPDSEAAIQEALS
ALSRGRTLIVVAHRLHTVMGAGRIVVLDKGRVAETGRHDDLVAKGGLYARLWDDYVAARAIPLRPGAPETQKPGATPE
KR
SVPRMETAQ
>OLS42672.1 isochorismate synthase [Rhodovulum sulfidophilum]AB14
MPFPDATLTEAPSLPPDQPFSFRNARDQVRGQVCATGPVRPLDRGGADTLADRVAPFFGAARDAGDDGALIGGALPF
DKA
RPDCLWQVAGGDGSWPGATAGAPARPVPEQRPARPCPQPPANGYMDSVARALEIMRTERGLPDALTKVVLARSLLV
EDRM
AFALPGLMARLAEDPAVTVFQVALPQDADGSAPRQLVGATPELLLRKRGDSIVSHPLAGSARRLDDPEEDARNANALA
RS
EKDRREHGLVVEYILDMLAPVCKRLGTPEGTALTTTRSMWHIGTRIEGRLKDPETPAILLAAALHPTPAVCGLPCDRAAR
LIRDLEPVAREFYAGAVGWCDRRGDGDWYVAIRCAELCGARARLFAGAGIVLGSDPRAEASETGAKFGAFLSALGLPP
DT

ALSDPSLHNQPNG
>OLS42673.1 hypothetical protein BV379_19695 [Rhodovulum sulfidophilum]AB14
MQAASWVNGHAGARAAQGPSAHLYVELDRSDGALDPDRLERAVAALIARHANLRLAVAPDGTPRILPPGPGPFLVRQ
DLR
RLGTLDCQLALERIREDQSHQRLALERGEAVAFSLSLLPGGGCRLHVDLDMIAADPSCFPDLMDDLARLYQDGPGCDLP
P
AGDFAAHLGVRHSDPARPEHEAAARAWWQDRIAALPEAPDLPRPPGRREDTPRTARLAERMDPARTRALMLRARE
MRVTP
TALTLAVFAQELARACGQPALRLTVPMFHRPEGETTVIGDFSDFALVGVAGAEPDLSVLAGRVQADMAGAIGHSAHP
GPG
QMRDLARHLGHVPEAPVVFTAGFDHPRGSILSDRARQCLGDLVWSVSQGPGVALDAQIARLGDGLLINWDIRLDLVD
RDW
IESLFKAFMDRLCALSDAAPAPRSWPLGALQRAYLAGREDVLPLGGVAMHEARLFRGSLDEAALRARLQALCAAHPAL
GL
RIDASAGRQYLARRPGLPLLTRDLRAAPDAEARLQALWQDFARSPCPLDGLLWQVRALLLPEGQEDALAVKFDALALD
GP
AIAQLCAELFAKDPPPARPASAPLPVPQPVAAAERAADAAYWAGALARVESAPRLPWRRPPDRPGPARYRRASRILAP
EL
IRPLRRAAAREGLFLNTVLGFAILEVLARFTPDLRICAGLPTAPALDREDLGNRSSFIAIDHDATSGTARERAAALQAQT
MAGLGHMGFSGVDLARQLMGQTGGPLALPVVLTNGLDWQAPPVGAPMRQVDGLTQTPQVALDMRLMRAPGGG
IEIAADHA
EGVLDPATVKAILDASLRALETMARTGELSLPDPLVPVELPPQSPHVADPAPHLLRIVDALHRARGPALICGEERLDYPA
LRARVTAALAGLAAQGMGPGDVLALHLPRGIDHVVLQLAAALAGIVWVPIDAAAPPARREYLLDRAAPGLVVSGEDLP
GR
PCLRPGDLPRPGGALPDEASLRARSLDTGPGYYLFTSGTTGAPKCVVLSNRATANVLDQTLSAWKPGPEDVLISVTPLH
H
DMSLFDIFGGLAAGACVVLPAAGHEKDAMEWARLVARHRVTLWVSVPAIVEMLLACARPGQIDSLRLIAQGGDYIKP
ATV
AELRRLCPAARLISLGGPTETTIWSIWHEIGPEIVSAADDMIASGTAPVPYGRPLDGAGYRICNPLGEPCPPGVTGRIHT
TGDCLSLGYLEAGGLSQDGFVTLPDPDGTPRRAFRTGDLGQWSPDRPGQILFAGRVGGYVKVRGVRVSLGEIEAALAD
HP
ALSQTMVVDLAPGDGRETTLAALYAARDGQPPGAAELRAWLRGRLPQSHLPDRFLAVAALPLSSNGKPDRAAARALA
LSP
AAPCPDGLGREVLDIYLRQTGPDPEATIDSPLIELGLLPGHLIPVAAALNVRFGTALRPSRLIPARTARQAAQLIPPRPE
PEDLG
>OLS42674.1 iron-dicitrate transporter ATP-binding subunit [Rhodovulum sulfidophilum]AB14
MSADIPATGPRLTAHALGLSYAGKPVCRDITLEVPEGRVSVILGPNGCGKSTLLRGLCNLLPAQSGQVLLDGRNIARMPA
RALACQVGLLPQSAQAPAGITVGDLVARGRYPHQGPFRQWSERDERIVAEAMAATGIADLAGRDVDALSGGQRQRV
WIAL
VLAQETPILLLDEPTTYLDIAHQLDLLRLLQRLNRERGRTIVAVLHDLNQACRHADHLIVMAEGALVAEGTPRDLMTPEL
LRRVFGLDALILDDPVSHTPLIVAR
>OLS42675.1 ABC transporter ATP-binding protein [Rhodovulum sulfidophilum]AB14
MDGQALGPLQDTSRQDTSPQDAPPARRRRVSPWGILTPVRPRIWLAMAISAGSALFTLAVMVLLMAAVGRLAAEPG
SLPW
GLFAGVVICTIGAYVARLGAFNQAHYAAFRLERLLRNGLAARLARVPLGYVETQGAGALAKVIHDDVKALHVFVADSTP
L
YARAYVTPVVTLAVLLWLDWRLAMVTLAVLGLGYGILTRAQRRRRDMVRAYNAAREKVSAAVVEYVQAMPVVRNFD
SGQQ

SFGRYQAALDGYLKVMLDWYRAAGFPARFSWLVLSPMPTLTLVLGAGLILTAADDLEMSRWLGVLLICTGMAEAMM
PMMM
LRHMIEKVGLSVARIEEVMQAPELKQRADLPVAAPEGAPALSFEGVRFAYGPDLAPALQDVSFEVARGSVTALVGPSG
AG
KTTVARLIPRFWDVTAGRICLDGTDIRALAPDDLLEKVAFVFQESFLFADTIAGNIRIGRPDASDEQVIAAARAAQADGF
IRELPQGYDTPVGERGVFLSGGQRQRIAIARALLQDRPLLVLDEATAFADPENEAELIAALSELMRGRTVIMVAHRLATI
RDADQILVFERGRIAERGSHDALLEAGGTYARLWQNYEKAQSWSLGTARAPVTEEVAR
>OLS42676.1 heme iron utilization protein [Rhodovulum sulfidophilum]AB14
MTPSPDTVAAIRADLAEHPGATLEDAAARTGTTVAEVLACLPEGEATLVPGAAFEEVMAEIARWGDITLVMNTGDVIL
EA
KGPLPEGRVAQGFFNLQGRPIGGHIRVGACAQVAFVARSLFGTDTRSVQFLDTRGGVLFKIYLGRDAARALIPAQLVAF
D
ALRARLAQGQMA
>OLS42677.1 hypothetical protein BV379_19855 [Rhodovulum sulfidophilum]AB14
MGLQIETRQLAHAEPHEVAVLGPQPLSSSFGGMPESGSYRILGLRKGVTISLFDIETGTGMAADMHTAPCVALSLLFEA
A
GTGWITEGEGQFGPIPLRPGIYCMIAPAGATGQDRFKPASRLRGIDIRLEPSFWSALGGPSSIDALGAGHPHAAMASPS
V
WVGLLPLTPRLMADAQALFRSGMSGTADLTVEARSLYIIDEAIRALAAGVNMATAIAPAPLLARDRRAVQRVIALMEG
DL
GRAWTVPELAQAAGISHNRLKQSFRAETGCAVYAFLQERRLGEARRLLLEGKGSVTDVALSVGYGSLSHFTALFRRRFG
Q
TPSDVLSLSKTARRTG
>OLS42678.1 ABC transporter [Rhodovulum sulfidophilum]AB14
MAGAPPGNEASKPPDFFASLKLLMRHGGKAGPLLSISAAMATCAVALELVPVWAVWRLVTELVSGTATPAIFVTCAVA
AL
VAVVLGYLLMGLALGLSHVAAFRLIHALRLALARHLARLPMGWFAGRRSGGAKKLIVDEPERLELLVAHGLPEGISAGAT
WLAVTIWLFAVDWRMALASIVLTPLAFGAMFSAMGRNARMAGAYQTASERLNGAIVEYLAGMAVVKVFNRSGESL
GETAG
AVRDYAAIETAMGRAFVPRGALFYTLVGANICVILPVGMVLMQTGSLDLERLALFVILGANYSLPLMKLFNLFHNMAHI
S
MASTLIEDVLATPAQPDTGARIALPDHEIVFDRVRFGYEAGTEVIHGVSFTARDGKVTALVGPSGSGKSTLAGLIPRLHD
VGAGRITLGGRDIREIGLDQLMEEIAFVFQDTVLFSGTIAENLRFGKPGATEAELQAAATAARAHDFIAALPEGYDTRLG
EGGAALSGGERQRLAIARAILKDAPVIVLDEATAFADPDSEAAIQAALSELSRGRTLIVVAHRLHTIMGADRIVVMDEGS
VAETGRHEDLLAQGGLYARLWADYTAAQAIPLRGGRTMETTR
>OLS42679.1 ABC transporter [Rhodovulum sulfidophilum]AB14
MKRAWHLAGPLKRQVSLGVLYRFLQSMMLGLGFGVVVLVVTGLADGRPLSTGWIWQTCGLMALSLAGQLLFGYLSV
RASW
LSSYEMAGHLRLRILEKLRHLPLGFHQARHRGDTVTVLTSDMQMLESFFSDGLPRIAQALGLPSAIFLVLLARDPLLALA
TAVSVVVALPVFLATSRQLSKLGIRRQDMQARAGARMIEFVQGMGVIRAFNHMAQGQQGFRRAIDDFRDISIRMVV
QLAL
PMALFGMIVMLGAPLVIWVIGGRLDTIGTGSAVTALLLLFSMYAPLLALLGVMEAVRMADASLIRMDRILAAPDLPEPA
D
ARPRGSEVRFEKVDFCYRPGQRVLRDISFTVPERSMTAIVGPSGSGKSTILNLIPRFWDVEAGRITIGGADVARIGEAGL
SDLVTVVFQQVYLFSGTIRDNIAAGRPGASQAEIEEAARAAQAHEFISRLPRGYETEAGEGGAALSGGERQRISIARAIL
KDAPIVLMDEATAAIDPTNERAIQTALARLVEGRTLIVVAHKLSTIRSADQILVLEGGGIVERGRHEALLAQEGLYARLW
SHRARAAGWQIGG
>OLS42680.1 hypothetical protein BV379_19905 [Rhodovulum sulfidophilum]AB14

MMTVAAPGVYVFEEKLGPRTIEAVGTSVAALVGRVPLRGVAAGTPVPVNGWMEFRRRFADEDSSSTDLSNAAFGAFANGL
RRLWVMPTEAGSEISDDDLRPLEGIDEIAIVAAPGYASKASHDALATHATRCGDRFVLFDLPKDAPVEAMTEVGGAARRG
TATEDATPSPAPAKAVRPSSIDCGACYAPWLLMQDPLKPGDRVLAPPSGHMAGIYARVDATRGVHKAPANEPVLAAIDAE
RRITRQQQELLNPRGVNAIRFMSGGLRVWGARTLSEDSLNRYVPVVRLVLMIKESIEQGTGWVVFEPNSYPLWEAIKLDV
RAFMTRLWRSGALVGRTPEEAFFVKCDEETNPPELRDAGRVETLIGFAPVRPAEFVIFRLAQVTAGAEPA
>OLS42681.1 hypothetical protein BV379_19935 [Rhodovulum sulfidophilum]AB14
MQGLDFRLGAGVGASRASGPGAGHTGALALLPEAACIRQDLLLLLLTVPGERVMRPAYGCHLDRLLFQPNDETLAGLAIH
YVRQAVARFEPRIEILRLDAGRPAEAGAGNLLEIVLSYRIRRSRTEDGLVLQVDLDAGGTP
>OLS46830.1 hypothetical protein BV379_00005 [Rhodovulum sulfidophilum]AB14
MTPERLFGAMAFRHPWRSYQARILADLEAHLDDRKLHVVAAPGAGKTVLGLEIIRRIGLRCLVLAPSLLVRDQWIERLKS
DFVQDRLPDLAGLVSTDLTADVPLRVSTYQGIHQKRNETFPAFELLCLDEAHHLRRSWWHTLTGMADRHDCVTLSLTATP
PYDAAAREWANYDALCGPIDAEIPAPELVKSGDLCPHQDLVFAAVTRHAADYVRNREEEAGSLDMLRNDPEMLAMIEALP
WIADCRAHAAEILEAAELFSAMLIYLQDAGRPLPSYARRLLQIRRGDIPPQSWDWLQVLCDGLKARLPSSTLQALTRMGA
LRNDRILLPPPKFTDRIEILRDDDTRLVACLEIHALERAARGADLRMAILVDRIGRLSLTSEATATGFNAVHLFRRLTAD
GGCSDAAIMTGQVVVLPRALAEGLAAQPLPGRPDHVLIEGRDFDAAIARANAAFHAGDVRVIVGTQAFLGQGWDAPGLNS
LVLGTNLASFVAVNQLRGRALRIDRRPPGKCSNIWHIAIVPDEHVSGEDLEALQRRFACFARIDREALEIRSWFAPEASC
TAQNEKARRLAATHGDLARDWDSAMQVGEDIGGRLMQETGIARRQSQVILPRGSGLASLWRRLFDRPVPEHEIRRTLGRM
GHLVLESLRQLGDIGTARDITPYILEGPEGHRLGLDRATRYEETLFHETLRQVLDPVTNPRYLIVVPAGFLRGRAQLFAV
PPRFDGNKRRAEIFWKNWQAMLGRGQLVYTRTVAGRGLLQSARLRTSRNRTTHNLQWK
>OLS46831.1 hypothetical protein BV379_00010 [Rhodovulum sulfidophilum]AB14
MSAERFRLAAEALPAPQARIFWDLAAASTRLRDEISKAPDLITPSRRLVFFYLPKMSELSLRWARLAARDPFKPGTPADQ
ADFQAYLDILQGAVSSCRARRDDELAQSMEAFRIQLERLAK
>OLS46832.1 hypothetical protein BV379_00015 [Rhodovulum sulfidophilum]AB14
MNFSDKIEADQDCLPCQHGNLFLSDMSAEMQSRLRFSGAVNGKTTETLPVGRVFQLENSADRTINGRSGGAAPRALTILE
SLALGHPGRPAGLTEEVSDEVRASGAVNDDRAGDRHHDVISALSKSMRGPDPDATLYRLARQIPAGADPRVIAQRTLSHV
SEYKSSGEGGVRQNRMFCNTAHRGPRETTVFARPARNRT
>OLS46833.1 flotillin [Rhodovulum sulfidophilum]AB14
MSSFAIPVIMGAFVAFLLLIGLVLARLYKRATREVSLVRTGAGGKRIVLDGGCVVIPLLHEVSRVNMRTLRLEVRRSGEG
ALITQDRMRVDVGVEFYVCVAPTEDGIAKAAQTLGDRTFETEGLREMIEGKLIDGLRSVAARMTMDGLHENRADFVQEVQ
NAVSEDLTKNGLSLESVSLTALDQTPFEDLDENNAFNAEGMKILAEVIANSKKRRAEIEKDADVAVARSKMEGEREKISI
EQDEEQARIEQNQRLARMRAEQEAQIAADQEKSSEEKERARIVREQAIRAAEIASARELEVAEQDRQITVSRKSEEESQA
RASADTARAEAIGAEESVKTAREVAEAERAKQIALIGAAREAEERATEIRVAALAEKEAAADRAAALREQAQAEADAARL
RAEAQKAEMLAEAEGRRAMVESENGLNQSVIDMKIALARLEAFPAILAEAVRPVEKIDGIRIHQVTGLGSASFGHGAPGT

ETAAGPESPVNQALNGLMGMAVQMPALRRLGEQAGLSMDAGLTGLVDAALSDSVPGHEGVPVNGAAARGGPAET
AGSLAQ
>OLS46834.1 IS5 family transposase [Rhodovulum sulfidophilum]AB14
MHAICDSQGRPINLFLTAGSVSDYIGARALVSGLPNVKWLLGDRGYDADWFREALQDKKIRPVRGRFDPLDQILSPLTP
G
RKKRKTAVRYDKCRYKRRNRIEIMFGRLKDWRRVATGYDRCPKVFLSTIDLTATAIFWL
>OLS46835.1 hypothetical protein BV379_00040 [Rhodovulum sulfidophilum]AB14
MPPRPVAMEACDGAHFRGRELTKPGHDIRLIPPAHMEPFIKHRKNDAADAEAICEAVQRPNMRFVPMRNEKTQGKA
SAFR
VRELLIRQRTPAPNALRGHLAEYGRVAPTGHAECPAPGAHCRGR
>OLS46836.1 hypothetical protein BV379_00045 [Rhodovulum sulfidophilum]AB14
MLTTVYFDPLDSQFGQFPRRTLPAPLERHLAQTEAGALYALIDSARLGDCDPLLDAGGLCLFEQDAQEMLGDVAPYLVA
L
DHAPELLRRLFTRSDQTTALWGRETAVILRSDAGIVSLRRHLRRFTQIPDMEGKPRFLRFYDPVVMGHILTGLKDDEARL
ARWFWHRQVQVVREWIVPQERLDRALLATAPQIVPEEGSMPIPPFRIDARYSLIFDGIVTDRLARKISRAVVHSVGNPF
D
LPPEDWAQLMEAVTALCRRAGMVTPQAIGHVASVAVWRNGLPAPQELPKWVPDAGHPARELAQAKAFHLAQKRS
HDAWRN
GAVSPHSAGA
>OLS46837.1 hypothetical protein BV379_00050 [Rhodovulum sulfidophilum]AB14
MGSPKTSYDPADTEGLDGLSASAQGSDACVPCESPYLIAVYPLRIAWADLLGEVDDGFTYPDSLSKANGGNGFVPRRLR
K
SKANAGNGFTLRRLRKGYLYIRDTAAAVWSVFEFTPDETATGGKFQRLEFDGEAENGPWMKASEAPVDLPYLPDSADE
IE
LCYASHLWSPQTFARAEADTDGFRAHVMTRLTLSAPADGTFSAPLADLARHAEEFSGRGIVNPHEDWTALSDQPLVPL
SA
DAVIRRGAMMGAKSKTGPVLVALHDPEGVAAEICMCHANRVGIRTRYLEVNSYAILSGQASKVLQNAGRARLDEGGL
GWL
ETRNLNKWMSSIRPERDVVLDEIEARLASYDDALEGILNAWRKFFDMGRSAPLSTPGSLQTHSLQFDPDDSQGSDIAG
RI
RFAQISVAAITGSKAGIAMLRDTVFAQEAWAEAQNPVASAIRLVTGSASMANLASAHVKEIREAASSLMTDISMPLALE
I
VTVERVDMLKEINRFSNGIVNHRLTRVTTPISDVYAEIAAQSRTVQSGKLIARGHQLNTVRTPTNVYKVDGIVHVATDRA
LDMFGTAGAGFAIFGNVLNIVALSSTSGPSLASGRAGRFANNPGMGIALNSAEALSKAFELSRNALMADMPATRSFVI
NG
QLDRTKLAKLVNKVQGADDLVRVMVNDGKMVQTGQMHPEGSRLARNVGRIGSVVGLATAVMDALNAWEAYKRSD
MVAIGA
YTASAAGGLILGSLLFIGVPVAGQIVGIILLIIGGVLALFIDDTVTKWLKNSYWGTSAGYLYWKDGSRSALAKRFDKEFG
HAAQVEAIRQQDPEYYEPRFMERELQEFHQIVYWPRNARDPAALSALRDNGVGFFRRTTTHTESSKKYNSVSFRLPNYI
D
GLSQFDGKIFAYVQLKQKHGGDITFQQIDVTDQFKASMELTASDVVTGGFTINAAQNIDDNLLNNVYWDVIQVKIVEG
WS
YEPTADIAIPMNYSDFIFKEGYSEDELGGKNSIEILG
>OLS46838.1 hypothetical protein BV379_00055 [Rhodovulum sulfidophilum]AB14
MFAELAFYGSFLAFLVMLLFKAPAPIVVDRKRRLVYTWHRGRIWAARWDDVAITRVEGHLRHTALAFALHDTKGKGGT
RW
FILSGAPAWGEKDDPRDRLLSDARDFNRWQGMRLWLHEWVEGRREPSQADLSRRFAVMHLLVPLQRKIPEAAIRQL
DGI

>OLS46839.1 hypothetical protein BV379_00060 [Rhodovulum sulfidophilum]AB14
MFCDYAEWQSGISGPQPLRKVAPRIAPILQSATRDRFFLQAGYHVILRRAIITFALMVIMGGMALPAIRDVGSYISYDNS
LIYFVKDAQGETERILIKTEPSFFGYFHKLWNEVSLAFAPNAFESRGGLGPTFLSIFSSKLDLILFYGSFLAFLVMLLFK
APAPIVVDRKRRLVYTWHRGRIWAARWDDVAITRVEGHLRHTALAFALHDTKGKGGTRWFILSGAPAWGEKDDPRD
RLLS
DARDFNRWQGMRLWLHEWVEGRREPSQADLSRRFAVMHLLVPRQRKIPEAAIRQLDGM
>OLS46840.1 hypothetical protein BV379_00065 [Rhodovulum sulfidophilum]AB14
MTERGVSVSQACRDLELAESVLRRWMRETTSAPASAFPGNGQQRAELAEIAALKKEVAKLKAERDILKKGRGLSAIGPR
T
MPSAFFAREVT
>OLS46841.1 hypothetical protein BV379_00070 [Rhodovulum sulfidophilum]AB14
MPKHYDLAVLCAKCDELEQLQAVFRKEFTKVSGDRLVDQIYKFDTPNGTKRLIVMTCGEMGHTSAAVQTGRLIIQHHP
KV
IFFVGTAASLCPNEVQLGDVLVPRKAVYRMYDKISEKGQSDYERRMNGMGSKEQFFGENVLCADTATTQISPDMLGA
LSG
VRLDERALKSGETKEIELEGRRIQLRAPKIFKDEDIFSCGMVVDSLSYREFISGIAKEEFRKLKIIDMESFGFYSAIDHA
KKSGVGSDTEGVMIRGVSDYAGRKQQTETRPEDWKSVCVTNAAIVAAQIIKDIYTPHDDEEDSPT
>OLS46842.1 hypothetical protein BV379_00075 [Rhodovulum sulfidophilum]AB14
MRRQSLSLPNTLSIRSLRLQRRLSYVTGWPRDLGTVLGRGAIEGADGSSRDFALRIVTFRFECAVVVRWGR
>OLS46843.1 hypothetical protein BV379_00080 [Rhodovulum sulfidophilum]AB14
MAAAAFARSIGLRDYSAFTALIERGHIAASHVKSPRTARLQWLMSEAEIADFRKRFVMPTMITQETGRHRNTIFAVFSA
A
GVKPFQPEGLEAGPIYLREVAMRATSNHLKKR
>OLS46844.1 hypothetical protein BV379_00085 [Rhodovulum sulfidophilum]AB14
MQCVQGEKFNDDTMTTEDLRPFSSLNTSFSDRADNAADIKARPLAGPLNGLPSRGLGGQKDCMMLQLLD
>OLS46845.1 hypothetical protein BV379_00090 [Rhodovulum sulfidophilum]AB14
MIRIWPLAAGDMVLGQEVQVRQVHSLLTASEETGITTALLDSLLTEAGVFRQDETRSPSGKIFDAEVHAGLLHEIATRVG
PNAMCEAIGATLSEFRALVEEGVLELRSRLPKIKNP
>OLS46846.1 transcriptional regulator [Rhodovulum sulfidophilum]AB14
MNDMVTIPRGEYERLRAAAEDLADIQAANQVKADLAAGKEELIPLEFADRLIDGESPVRVYRALRGLTQAALAEKAGVN
R
VTVAELETGRKQGSAQTLKKIADALGVTVDDLI
>OLS46847.1 cytotoxic translational repressor of toxin-antitoxin stability system [Rhodovulum sulfidophilum]AB14
MKQVAYTRDAIKTLKRMPKNTADRIRGKIAEYATDPASQANNVKAFKGSDYIRLRGGDWRVIMDDQGNVLAILTIGAR
GG
VYG
>OLS46848.1 hypothetical protein BV379_00110 [Rhodovulum sulfidophilum]AB14
MSNTFDVRVTLPWWRVAIAFGAAHMTGLVAAQMPSRWVEAAITNLARWAVRWVTLRMVL
>OLS46849.1 hypothetical protein BV379_00115 [Rhodovulum sulfidophilum]AB14
MGHPGRQKAARQARHRGYGLSHGRLAQIDAYRYGNGLQMTGAFLGDMASAFATGGDKMVKISRIFGAAQALISTYV
GAAK
AMELPFPANIAAAAKVIATGMSFVSAIKSGSKSGAGGASSAGAAAQAAAPAPQRTANVTVQGDVIGRQSGEALVKAL
NEA
FGDGYRLNLDWQGAAG
>OLS46850.1 hypothetical protein BV379_00120 [Rhodovulum sulfidophilum]AB14
MRLVILALPVSLAACIAVPPPMVSDYNGHMVKIVDHPYALGDKYKQSPIYTLAKETCGKEAVYQGMRQINEYQGEHVF
LC

R
>OLS46851.1 amino acid transporter [Rhodovulum sulfidophilum]AB14
MTTIRDEQTKLTANYVNGLALALFAVGGLASLFSNAYSEHGLTTQVVLASAVCFVASLLLHLLARLILKGLKS
>OLS46852.1 hypothetical protein BV379_00130 [Rhodovulum sulfidophilum]AB14
MPPDLKARVKVAAEANNRSMNAEIVAALEEKYPDPTVVWVDELARKIMAKLAAGREPKATKARLTKVVEEFFAENPD
AAE
ALERGRGRSS
>OLS46853.1 hypothetical protein BV379_00140 [Rhodovulum sulfidophilum]AB14
MQRLENGKTAGAATPTVSMNPNHYEGSLPMNMNHAHPEINQPLAALTAGAEVTMSSREIAELSEKRHDHVLRDIEK
ML
>OLS46854.1 hypothetical protein BV379_00145 [Rhodovulum sulfidophilum]AB14
MFGEKLDQNHRVGQVRQGVALILQLLKPLDLPIAQIGPVAGDQRDGQVLRQAVFLEAPPGAVGVVGQ
>OLS46855.1 hypothetical protein BV379_00150 [Rhodovulum sulfidophilum]AB14
MYTGKALGMPRMGYTNLGPTDLGRTAVLTSYISEGGQLMGRFIQRAGLSGAFEWQNLPEDWYRQIFDPFTRAARTEP
FFI
AATREPGRRRDPCTTP
>OLS46856.1 hypothetical protein BV379_00155 [Rhodovulum sulfidophilum]AB14
MFEDLGRLLPAVGQSWRRLLAQRLSKEGLSDAAALPILVLLRARKGASRQNFLAHQLGLETSGVVRLLDALSKRGLLRRT
EDPTDRRAKLVELTDEGIAMGERADRIARALRSELLAELDRQDLIATIRILRSLSAVLDASEERQKGR
>OLS46857.1 hypothetical protein BV379_00160 [Rhodovulum sulfidophilum]AB14
MMIPGGIKLEHVLFSLKTFGAGMLAYWIAIRFGLSDPYWAAGTVYVIANPLAGAIASRALYRLAGTVLGAVLIVLLVPNL
VFSPLLLSAAIGLTCGACLFVSMLDRSPRSYAFMLAGYTVSLTGFPLVNAPGTSFDVSVERVEEIAIGILCAAITVQVVF
PWHIGPMLAKRVEGWLDNAAAMTRSVLTRNTNRAKLQDERHKLAADVISLRSFTDQVAHEGVRGWNRAERMGAL
QQRMIA
VLPLLSEIEDLLNTLERNGPDVSGSAALTGKVLQWLDKETSATRDETDALLFDIDAEQRRLDDQPHSWEGLLRQRLTGKL
RDLVEVWADCQSLRTDISSGETHELRQHLAARHRRARDLHTDYGAALRSAVSVFILIMVACAIWIWTGWTYGAGLAQF
GS
VFCCVLATMDNATPVLRKVAPLMLVAFVIALLYQFVFMPALGDFYALVALLGLVLIPAGVLMAVPATWMTGFQVSVNL
IY
MMTLGNQVSTDFTAFANASLSTFGALALATIIMSTVRAVSAEHSAMRLLRSGWRVVSQIAAGRRQVAPELIAGRMTD
RLG
LIVPRLTLLPPDAALLENDLLRDLRAGLCVLELRNLRPEMPDAFRLGLDRLLDQVSEHYRNDKSGPARAERESAMAGMV
D
ECLNQALATSGTTARRFGNALAGLRSAICPGQPAPVLNFSDRGTLA
>OLS46858.1 DUF1656 domain-containing protein [Rhodovulum sulfidophilum]AB14
MKPDIDLFGVFVPTLLVLALGSYIALRLLSAVLARLGVYRHVWHPALFNLCLYFTVLGATVLFMEQLKS
>OLS46859.1 alcohol dehydrogenase AdhP [Rhodovulum sulfidophilum]AB14
MAKTMKAAVVREFGKPLSLDEVPVPEPGPGQIQVAIRASGICHTDLHAAEGDWPVKPTPPFIPGHEGVGYVSGVGAG
VTR
VKEGDRVGIPWLYTACGHCEHCVGGWETLCESQQNTGYSVNGGFAEYVVADPNYVGHLPDNVGFTEIAPILCAGVTV
YKG
LKTTDARPGNWVVISGIGGLGHLAVQYAKAMGFNVAAVDIDDHKLELARKLGATLTANAATEDAAAKIKKETDGGAH
GVL
VTAVSLKAFEQAENMVRRGGTIAMNGLPPGSFPISVFDMVLAGITVRGSIVGTRLDLQEALDFAGHGKVKSIINPVKLE
D
VNSVFEKMHKGQIDGRAVIEIAD
>OLS46860.1 aldehyde dehydrogenase [Rhodovulum sulfidophilum]AB14

MNQLERSFDQTRPPFAARYDNYIGGKWVAPKSERYFDNVTPITGKTLCEVARSDAADIEAALDAAHAAKDAWGKTNV
AQR
AFILNRIADRMEENLDLLATAETWDNGKPIRETKAADLPLAIDHFRYFASCVRAQEGTLSEIDHETIAYHFHEPLGVVGQ
IIPWNFPILMAVWKLAPALAAGNCVVLKPAEQTPASIMVLMDVIGDLLPDGVLNVVNGFGVEAGKPLASSNRISKIAFT
G
ETSTGRLIMQYASQNLIPVTLELGGKSPNIFFDDVCAEDDEFFDKAVEGFVMFALNNGEVCTCPSRALIQHSIYDRFMER
ALERVKAIKQGNPLDPETMIGAQASSEQLEKILSYIDIGQQEGAEMLVGGARSHLGGELEGGYFVEPTVFHGNNRMRIF
Q
EEIFGPVVSVTTFKDEEEALHIANDTLYGLGSGVWSRDMNRCYHFGRNIQAGRVWTNCYHAYPAHAAFGGYKQSGIG
RET
HSMMLDHYQQTKNLLVSYNPKKLGFF
>OLS46861.1 Fis family transcriptional regulator [Rhodovulum sulfidophilum]AB14
MTAAEFANVRERMEPWIRAAGASLDRLFGAVGAAGCCVLLADSDGVPVERRGVAGDDATFDDWGLWPGALWSEA
TEGTNG
IGTCVIERRPVTVHRDQHFLARNGALGCIAAPIHDHDGELVGVLDVSSCRTDLTATFAGLIALSVREAARRIEAEAFRRS
FAHARILALPGLEHNPAALVAVTADDLAIGASHSARVALGIKGNLAKAPVPASDLLSVPGVESLEEAERAVLARALARTG
GNVSAAARILGISRATFHRKLGHRKSGAKRPEAATQDPAAPCRIPATDAPSFPRGGG
>OLS46862.1 hypothetical protein BV379_00200 [Rhodovulum sulfidophilum]AB14
MLRADDIGGLGCWIKANPLLGVTITSAYLALQVKQARDIPAKANGIRRPHFCEWADAESA
>OLS46863.1 hypothetical protein BV379_00205 [Rhodovulum sulfidophilum]AB14
MLAIWFDLEMSWNAMPTGRLGHQQTYSDTAIRSCLTMKGEGRPASMAAPNGGSGARPTLASTRKRWRSRLPRSPA
SIPEG
FVAQSKKSAAAAAYTKGFLRVKQRNVLPRKPLGDARSFDYATKPFRRTEKLVVPRRMAPTTRASAMTRLQTVGTMRSG
IP
SELWQSSPHSRPARTRSPG
>OLS46864.1 5-dehydro-2-deoxygluconokinase [Rhodovulum sulfidophilum]AB14
MSSQPSPIYDIITIGRTCADLYADQVGCSMEDARSFSKYVGGCPTNIAIGGARLGLKTALLSRVGNDQMGRFVRQTLEA
E
GVETGGLATDPERLTALAFLGLEDDRNFPLLFVRTDCADAALSEADIDPDFIRSAAAIVVTGTHFSKPHLEAASFKAMRI
AREAGRKVVFDIDYRPNLWGLLGAGDGDIRFVADAAVTARVQSILPGCDVVVGTDDEVRIAGGSTDTREALVNIRKVT
AA
MIVLKTGPKGCLVFPGPIPEDLEEGISSQGFPVEVFNVLGAGDGFMAGFLRGYVRGLPLEECCRIANACGAFAVSRHGC
A
PAYPSWEELQFFLDRGVVNPRLREDADLEHVHWATTRRGHWDNVCAFAIDHRVQLEDMAAQCGADPARIARLKDLA
LDAM
LAARRPEIMLGTLLDSTHGRQALFRAEKEALWIGRPVELPGSRPLKFEGAASLGAEVASWPRDHVIKCLVFYNPDDPEAL
RRIQLDQLHRLASAARLSRHEYLIEVIGTADGKRGETATAEAMQQIYDEGIRPDWWKLPDQSAAGWAEVSTVIERNDR
WC
RGVLLLGLDAPAEVLTRSIARAAACAHVRGFAVGRTIFGDVARSWLAGDIDDAAATDRMQARFEQLIDCWQAARQAK
AA
>OLS46865.1 hypothetical protein BV379_00220 [Rhodovulum sulfidophilum]AB14
MTTDGEDPAPAVDFDSLREAIADAHGDLSKRLKLIADFVLRDPSSVAFRTAATLAEEIGVAPSAIVRFAQHFGYSGFSEM
QAVFRAQLQASSTPYQDRIRDLAKSGAARDTAEMTDRFVEAGIASLRALRQGAPVERVEAAARMLAEARIVHVMAHK
RTF
TAATYLAYNLSRLEVPTNLLDGSGGTLDQQMAMVSPEDTFVAFSFNPSAEETVRTFERARATGARTIAITDLARAPFIHA
DCWFQLSEASVDGFRSLNATISLALILAIRAGTLCAELRSDNED
>OLS46866.1 L-idonate 5-dehydrogenase [Rhodovulum sulfidophilum]AB14

MKACVIHGAERVLIEEAPRPAPARGQIRLRFAFGGICGSDIHYFQHGRVGDSILRGPMVLGHEFSAVVDLPGEGVEGFA
P
GDKVAVTPARPCLNCDQCRRGLTNLCTDMAFMGSAARFPHCDGGFASELVVEARQCIRLPQKADLRHVAMAEPYAV
ALHA
AAMAGDLTGATVLVTGAGVIGQMCATAARAAGATRILMSDIAPAALARARELGVDEVFDAGDPAAMAALTDSPRCD
AVFE
ASGAAPALGTAIAAAKPRGTVVQVGFLPPKAPVDLAKLLTREIRLVGTYRFIDEFAEAVRQIVEDEVDLRPMISADMPLD
RPEEAFARALDKTAALKVMVHFP
>OLS46867.1 hypothetical protein BV379_00230 [Rhodovulum sulfidophilum]AB14
MTSALTYTPGALRPRAAHLGVGAFHRAHQQSYYDDLARAGGDWGVVGINLTPPDLAAAHAAQDGIYAVLTDDGQTR
ACRG
IGTLIGLHDARSAPDVDWSGIDFATLTITEKGYCHRSGSFELDPAAVAADLAAPEAPVTAIGYLAWMLERRRRAGGGPIT
LASCDNVADNGRLLGGVLRDYAARVFPGLPDWIDRNVAFPTSMVDRIVPAMSASSRARLEAACGGPDALGVVAEPFR
QWV
IENRFAGPRPALERAGVQIVDDLAPHEHMKHRLLNGLQSAYAELGRLCGHESSHDAATDPVLSDWAGLFLRAQAETLA
CP
EGEDLTAYGRTSLARLQNPTIHHPLNQIASDASFKLPQRIATPAAERLARGAPAELQAMVLAGWALQAGDTRPDSTGV
PT
RDPMGEEMSRRRAAARDAAGFAREVLALPLWPARLGQDAGFVADMARWLRAFAQGDPRQRRAQIDDFVRERANA
>OLS46868.1 C4-dicarboxylate ABC transporter permease [Rhodovulum sulfidophilum]AB14
MDPTLLMALSFVLLLLIGVPVGISMGLAAVLAMTSANMPMSYLVQTAYSAVDEFTIIAVPMFILAGTLMEKGGLTRRLI
G
FSRAIVGPSPGGLATVTIVACTFFAAISGSGPATTAAIGSIMIPAMIRDGYDRGFGAAITSSAGGIGVVIPPSIPMIIYG
ITSETSISALFVAGVVPGLLLAVVLWLTSRFISVRRGYSSGDVEPRSLGNIGRAALEAKWALAAPVLILGGIYSGVFTVT
EASVVAVLYALFVGIFVYREIRVPVFIDAVVYAVRITGTVMLVLVTGRIIGRMLSMFQVPQLVSGFLIETVSDPIMLIVL
VLLMLVFIGMWMETLTQIIILTPLLLPVVANVGIDPIQFGIIFVIACEIGYSTPPLGVNLFVASEIGETTVEKVSKASIP
FLFAEIAVMALVAFVPQLTLWLPGLLGFVN
>OLS46869.1 C4-dicarboxylate ABC transporter substrate-binding protein [Rhodovulum
sulfidophilum]AB14
MLKPFIRTLATAALAMCVALPAAAADYTMRLSHGDNENNPTHKGALRFQELVEKYSDGRIEVQIFPSNQLGSEQEVAQ
AL
RMGSIESEILYTGNLVPLAPSVGVMMLPYAFTSNEEAHAAMDALKGPLNERLPKEAGVRVLGYFEKGFRVLTNDRREV
KT
LDDLQGLKIRVSKNNIAIDTFKSWGLDPIPMAWAEVFPALQQGVIDGQENPYTTAISSKFYEVQKYITEIHYLIWSGPII
VSERFYQGLPEDLQQALDRAGEEAAQYERQVSAEETEAAKKKLVELGMTLTGAPEDEAKWQEAAKALWPGFADDIGG
AAW
VEEATGIMNDAIAAQ
>OLS46870.1 universal stress protein UspA [Rhodovulum sulfidophilum]AB14
MTMTAMLMALVDGSAYSESVCRHAAWIAKRTGAQVKLYHVLDPRRAQGRRDLSGSIRLGARSRLMEQLSDLDEQHA
KLSQ
AQGRAILEDARALIEGEGVADVITRLRQDDIVNTVEQKEAAAEMIVIGKRGEAAGLESPHLGSNFERIVRASQKPVFVSN
RSFAPIDRVLIAYDGGVSSMKAVDYVARDPMFAGLKVIVATAGQETADMRRALENARATLNAGGHEAEAKILKGAPDK
AL
ADLVETDGIDLLVMGAYGHSRIRTLVIGSTTTEMIRSCKVPVVLMR
>OLS46871.1 sodium-independent anion transporter [Rhodovulum sulfidophilum]AB14
MTALYAYRDQWIGNVRGDILAGLVVALALIPEAIAFSIIAGVDPKVGLYASFSIAVIVAITGGRPGMISAATAATAVLMV
TLVKEHGLQYLLAATVLAGLIQIGAGLARLGHLMRFVSRSVITGFVNALAILIFLAQVPELIGVTWVTYVMVAAGLAIIY
LFPLLTTAVPSPLVTIVVLTALTMAFGIDVRSVSDMGALPDTLPIFLIPDIPLNLETLKIILPYSVGVAAVGLLESLMTQ

TIVDDLTDTSSDRNQECIGQGIANTCTGFIGGMAGCAMIGQSMINVKSGGRGRLSTFTAGAMLLFLIVVLGDLVGQIP
MP
ALVAIMIMVSIGTFSWSSIRNLRDHPRSSSIVMLATVFFVVYTHNLAIGVLVGVLLSGIFFSAKIAQLFKVRSEISADGR
ERTYVVEGQLFFASTEDFMRSFDFKEALERVTIDVSRAHIWDVSSVAALDMAVLKFRREGAEVEIEGMNAASETIVDKL
A
VHDKPGAMDDLLGH
>OLS46872.1 phosphoserine phosphatase SerB [Rhodovulum sulfidophilum]AB14
MFVVTLLTSPAQPMLERGTVESLRDAWGGGEAVWLAPGIAAEFPVASLPGNFREVWSDLQTLGIDLAAQPAEGRRKR
LLI
ADMDSTMIRQECIDELAAEAGVGPRVAEITARAMNGELDFHGALRERVGLLAGLDAHVIDKVLAERISFMPGGRTLVA
TM
KANGAHALLVSGGFTNFAAKVGAALGFDEYRANTLLIEDGRLTGTVAEPILGREAKVEALREVTERLGIGLEDALAVGDG
ANDLGMLGLAGAGVALHAKPSVAAECELRVNHGDLTALLYLQGYTRNDFVDA
>OLS46873.1 aminoacyl-tRNA hydrolase [Rhodovulum sulfidophilum]AB14
MLRITDTLALEDWEITEVFSRSSGPGGQNVNKVSTAVDLRFEAERSPSLPGPVKARLKRLAGRRWTQEGAVLIRVEETRS
QARNREIARERLAELIRKALEKPKRRVPTRPTLGSQRRRIAAKKERGAVKALRGPVGETD
>OLS46874.1 hypothetical protein BV379_00270 [Rhodovulum sulfidophilum]AB14
MTRLIPGILAMAAIVVASNILVQFLFGQWLTWGAFTYPLAFLVTDVMNRVYGVAAARRVVLAGFVVGVICSFIGTQIQG
E
FGPLVTLRIALGSGLAFLGAQLLDVAVFDRLRAGRWWRAPLASTLIGSSVDTLVFFTVAFSGTLSVLEPGNDVSWAGEM
L
PLLGVGPQVPLWVSLALADWSVKLALSLIALVPFRLIVRRLTAQLA
>OLS46875.1 penicillin-insensitive murein endopeptidase [Rhodovulum sulfidophilum]AB14
MARTLIGTCLAVLLAVSLAAPALAEPTAASLFGAEKRGSRQAPQPIGTYARGCAAGLVQLPETGPTWQAMRLSRNRN
WGH
PETIAFIQRLGARAAAIGWKGIYVGDISQPRGGPMTSGHASHQIGLDVDIWMLPPKRLTLSRAERESISSISVRSADQRR
VNGNYTAAHAALLRAAAMDPAVDRIFVTAPVKVEMCRRATRADRDWLQKIRPYYGHHYHFHVRLKCPRGARNCVTQ
RPTV
AELSKGGDGCDETLNWWVTDYLDPPKKAARPKPPSKPAPKKRGARDYTMDDLPRECRRVLAAD
>OLS46876.1 MFS transporter [Rhodovulum sulfidophilum]AB14
MTAVTEAAAERRIRGWWFYDWASQPYATLLLTFIFAPYVKDLLGDGSAAQAAWGYGVGAAGLVVALLAPALGAVAD
RSGY
RMRWIVLFSGLYVIGAAGLWCADPTGFSLPLVLCFFGLGLVGMEFSAIFVNAMLPELGPRAELGRISGSGWAFGYVGG
LI
ALVITLAFLAENETGRTLIGQPPVFGLDPATREGTRAVGPFVAIWYAVSMIPFFLWLRLPRRPRTATVGRALAEALPELG
RTLRRLPRTPSLLAYLMSSMFYRDALNGMYVFGGIYAAGVLGWSVVDVGVFGILAVIAAAAFAWLGGRADRRFGPKP
VIA
VCLVTLIAAAVLAISISREAVLWLPVAQGSALPDIAYYAVGAIIGAAGGAMQSASRTMMVRQANPARMTESFGLYALSG
K
ATSFLAPMSIGLVTDLSGSQPAGIVPLVVLFLLGLGLLGFVRAEGERTERWHAP
>OLS46877.1 thioeseterase [Rhodovulum sulfidophilum]AB14
MYPFFRLAKELYLHRKAEPLPLDGIHVSRHVCWPWDLDMWMELNNGRTLTLLDLGRLPLAQRVGLIGVLKRERWALT
MAG
VSVRYRRRVKMFDSFEIRSRCVGWDDKFLYIEQGMWRHGDCANHALYRAAVTDRNGIVPTARMMAAMGLAPETPP
LPDWV
QAWIAAEAERPWPPMTD
>OLS46878.1 YggT family protein [Rhodovulum sulfidophilum]AB14
MLSLFQILLLILDIVWFIIIVQIIMSWLISFNVLNIYQPLVRQIWSGLNRLLEPLYSPVRRILPDTGGLDLAPLIVLIAI

YALRIIIANNMMAFA
>OLS46879.1 DNA helicase RecQ [Rhodovulum sulfidophilum]AB14
MDRPHALLQSIFGFDAFRPGQQDIVEAVASGRNTLAIMPTGGGKSLCFQLPALLRDGVTVVISPLIALMRDQVRGLTEA
G
VPAGALTSGNTDEETRAVFEALEAGRLKLLYMAPERLAAGGTAAMLRRIGVGLIAVDEAHCVSQWGHDFRPDYLRIGE
LR
RALNVPLAAFTATADAETRAEIVTRLFDGEAPETFLRGFDRPNIRLAFSPKDQPRRQILAYAAARKGQSGIVYCGTRAKT
ESLARALEAEGHSACSYHGGMEAQDRRAVEARFQTEDGLIVVATVAFGMGIDKPDIRWVAHADLPKSIEAYYQEIGRA
GR
DGAPADTLTLYGPEDIRLRRSQIDEGAAPPERRAADHARLNALLGLAEAMDCRRQTLLGYFGEEAAPCGHCDLCETPPE
R
FDATEAVRKALSAMLRTGEYFGTGHLIDILTGADTAKIRERGHDKLPTYGVGREFDKRGWQAVFRQMMGRDLVRPDP
ERH
GALRMMEPARPILKGEATIELRRDTIAAAAAARPAVRMLVSEEDAPLLSALKAKRRALAEAAGVPAYIIFTDRTLIEMAE
TRPASLDAMARISGVGATKLERHGAEFLAVITGAETDLPHPARRKLAGGTAATLYDRLMETQSRLARGADGCEKPLSCT
A
SQVAKLAEIRPADMGTLTRLLGERRADRFGAAFLDVLAEG
>OLS46880.1 hypothetical protein BV379_00300 [Rhodovulum sulfidophilum]AB14
MLVVISPAKRLDEKPGPEMAGTTRPEFQAEATHLAGVAAQLSPDALKRLMHISDSLASLNAARFAGFQADPAPEAVKP
AV
HLFDGDTYAGLDAASLDADALRFAQSHLRILSGLYGLLRPLDAIQPHRLEMGSRLATERGSCLYDFWGDRVAEALNRAA
E
AAGTDVLVNCASKEYFTAADRAALKPKVVTPVFFEERPSGPKIVSFYAKKARGAMARFIVEHGLTDPEALKEFDAGGYRF
RPDVSDDSSLVFLRGSDAD
>OLS46881.1 hybrid sensor histidine kinase/response regulator [Rhodovulum sulfidophilum]AB14
MDLHDKLARERRARLAAERLLERKSRELFDANRRLALQARALSEEVDLTRQAADELRGCNDKVMTELAEVSKAFTVAK
QR
LWDSLETVRDGFALFGADDRLVIGNKAFFDLFEGLEEVIPGIRYDEIIRLAVEEGVFDIGQERPQEYAETLLSRWHLDPV
PPRVVRLWTGEYLKLTDRRTPSGDMVCLAINITDMMRMWAALEAIPDAFVLYDAEDRLVMCNDRYREFYAESAQAM
VPGA
RFEDILRQGLANGQYPEAVGREEDWLTERLVNHQKASSPVEQRLADGRWLRIFEQRTPDGGIVGLRVDITEQKRQQEA
LE
QARVAAEAANRAKSAFLANMSHELRTPMNGVVGMAEILCETEMSEEQRLYAHTIRESGEALLGLLNDVLDYSKIEADKL
T
LNPAPFDLERIIHEVVTLLQAPARDKRLDMLIDYDIFLPTRFIGDGARLRQILTNLVGNAVKFTEEGHVLVRVVGFEDAS
GRYQIHLIVEDSGIGIAPDLQEHIFGEFNQVDDQENRKFEGTGLGLAITKRLIEMMEGEIWLVSGKGEGACFGIRLTLPV
CEEADALPAAPATRARRVLVVDDQEVNRTILERQLGQLGLEVVSHICGELALQETGRFDLVVTDHRMPGMDGMSLAR
ALR
DGGYDGPILMLSSNPTRMRAADDPVASGVDLVLQKPLLRRALYEAVAQLAPPAAGQPARLPAPPAPAGAPVAPDAVP
MRI
LAAEDNRTNRLVLEKMLDGLALDIRFVKDGDEAVAAFSEARPDLILMDISMPGCDGKEATRRIRALEAGSDLAPVPVVA
L
TAHAMIDDVDAILAAGLNGCLTKPLRKAEIVETLEAYRPDGCLPIRMPGTG
>OLS46882.1 penicillin-binding protein 1C [Rhodovulum sulfidophilum]AB14
MRALLPVLAAVLFLGAAARDAVDAWIDATMLPPLAAETSVEVLDRNGTLLRAYTVEDGRWRLAASPDRVDPLYLRML
VAY
EDRRFRSHPGIDALALLRAAGQALAHGRIVSGGSTLSMQTARLLEDSGTGRWAGKLRQMRVALALERRIGKDGILALYL
A

RAPFGGNLEGIAAGSLAWFGKPPARLSPAEAALLVALPQSPETRRPDRAADAARAARDRVLARMAEAGVLEPETAARA
AR
SPVPATRRPFPELAPHLADRARAAEPGARLHRLTLDAGLQARLETLVRDTVRPMGARLSAALIVADHQSGEILAEIGSPD
YTDTARDGFVDMARAPRSPGSTLKPLIYGLAFAEGLAHPETLIEDRPTAFGTYRPSNFDGRFRGTLPVRAALQASLNIPA
VALTEALGPARLLARLKRAGASPALPDGPPGLAIALGGLGLTLEDLARLYAALAEGGRRVPLHWRIGAAPVAEGAPVLPP
EAAWQVGDILRGVPAPASAPQGRIAFKTGTSYGHRDAWAVGFDGRHVVAVWIGRADGTPVPGAFGTDRAAPLLFDA
FARL
GPPEALPPPPPGTLIATNSALPAPLRHFARPGASQGADPLRLAFPPDGADLALDGATLTVKLAGGTPPFTLLADGRPIAT
GLRHRTAALPAPGPGFVTLSAIDATGHAARARLHLID
>OLS46883.1 acetyl-CoA acetyltransferase [Rhodovulum sulfidophilum]AB14
MSGVVILGAARTAIGTFGGSLAGVPPIALGATVSKAALARARLPADRVGQVAFGHVINTEPRDMYLGRVAAMEAGVP
DTV
PAMNVNRLCGSGAQAIVSVMQALILNEADFGLAGGAECMSRSPHILADARWGRKMGDTPAADMMLGALTCPFGS
GHMGVT
AENVAEAHGVSRADQDAFAMQSQERAAWAIAEGRFDDQIVPVEMQHRRETVAFRVDEHPKATTVDGLAGLRPAFR
KDGSV
TAGNASGINDGAAAVVLAREEAAEAADLMPLARILGYAHAGVAPEVMGIGPVPAVEALCARTGLTVGDFDVIESNEAF
AA
QALAVSRMLGFDPAKVNPNGGAIALGHPVGATGAILTVKALYELQRTGGRRALVTMCIGGGQGIALALERI
>OLS46884.1 hypothetical protein BV379_00325 [Rhodovulum sulfidophilum]AB14
MAYDDKPLPYSAAPGLSGPEPRHRVVVVGAGPVGLTMALNLARLGIASVVLEDKSAITVGSRAIVWSQRSLQFFARLGI
V
DRIMAQAVPWSIGRISHGDVEIFSMDMAGTDADRFPPFANLQQYHVEDILIERALAEPLIDLRFRNRLTGVDQTGDGV
EL
SVKTPDGSYRLTADYLLACDGARSLTRALLDLDLEDGAFHEERFMVADIEMEADFPAERWFWFDPSFHSGQSAMLMP
QAD
NIYRIDLQLGRDANVAEEVRPDRVIPRIQAVVGPDFDLEWVSVYTFQSRRLAEFVHGRVIFLGDAAHLVPPFSARGANA
G
LRDVENLGWKLAAVLEGRADEGLLDSYNRERVQAADENLRDSARSADFISPAPGVERLFRDQVLALARKTAFAREWVN
AG
RLAVPCIYRTGAPDDTLLPAEARPGAAAPEARIGNGWLLDELGTGPMVLAINCAAPEGLPVPALALRADPTLRSRYLGD
A
ERALYLIRPDRVVAARWIAAEPRDITAALRAMWEGRE
>OLS46885.1 DUF2783 domain-containing protein [Rhodovulum sulfidophilum]AB14
MKLITDPNLPDPDGFYAELLAAHRGLSDPESQALNARLILILANHVGDREALTEALALARGR
>OLS46886.1 1-deoxy-D-xylulose-5-phosphate synthase [Rhodovulum sulfidophilum]AB14
MTVKPPTPTSTPILDRVAGPADLKGMSQADLALLARDLREEVISVVARTGGHLGSSLGVVELTVALHAVFNTPFDKLVW
D
VSHQCYPHKILTGRRDRMHTLRQGGGLAGFCKRSESEYDAFGAGHSSTSISAALGFAVGRDMGQPTGDSIAVIGDGSIS
A
GMAYEALNNAGHEKRRLFVILNDNEMSIAPPVGAMSKYLSGLYHETPLGKLKDMAEDFESALPGPIREGARRARQLVT
GF
PGGGTLFEELGFDYIGPIDGHDMSQLLTVLRAARARASGPVLIHVCTVKGKGYKPAETSADRGHGVSKFDVASGTQKKS
S
GNAPSYTSVFGRTLTDEAARDAKIVGVTAAMPSGTGLDILQDRFPKRVFDVGIAEQHAVTFAGGMAASGLKPFVAIYST
F
LQRGYDQIVHDVVLQKLPVRFCVDRAGLVGADGSTHAGAFDVAFLSNLPGMVVMGAGDEAELRHMVTTAAAYDEG
PISLR

YPRGEGMGVEMPERGQILEIGKGRVVREGSKVALLSFGAPLVDCLAAADALEARGVSVTVADARFAKPLDTDLIARLVS
E
HEALITVEHGAEGGFGALVLHWLARTGRLDGGVSVRTMTLPDAFIEQASPAEMYAEAGLTAQDIAATALQALGVAPA
QFG
QASA
>OLS46887.1 photosynthetic reaction center subunit M [Rhodovulum sulfidophilum]AB14
MAEYQNIFTQVQVQGPSDWGMDNENNMMEERAGMGHFSILGWLGNAQLGPIYLGYTGVVSLIAGCFAFLIIGLNM
LAQVD
WSLVQLLRQGFWLALEPPSPEYGLRIPPLKEGGWYMVASFFLLISVWAWWARTYMLAVEHKMGKHIAWAFLSAIWL
FMVL
GFFRPILMGSWSEMVPYGIFPHLDWTTAFSIRYGNLYYNPFHALSIAFLYGSALLFAMHGGTILAVTRFGGDRELEQIYD
RGTASERAALFWRWTMGFNATMEGIHRWAWWFAVLTPLTGGIGILLTGTVVDNWYIWAQEHNFVTEYTQPYGVD
AYVGQG
G
>OLS46888.1 photosynthetic reaction center subunit L [Rhodovulum sulfidophilum]AB14
MALLSFERKYRVRGGTLIGGDLFDFWVGPFFVGFFGVTTIFFATLGTAMIFWGAALQGTFNPLKIHIAPPDLSYGLSIAP
LAEGGLWQFITLCAIGAFVSWALREVEICRKLGIGYHIPIAFSVAIFAYLTLEVFRPMLMGAWGHAFPYGIFTHLDWVSN
TGYHYLHFHYNPFHMLGVSLFFATCLALALHGGLILSACNPEKGEEAKTPDHEDSFFRDLIGYSIGTLGIHRAGWLLAIN
AVFFSAVCIIISGPLWTEGWPEWWTWWSNLPIWPEAIPAVQGSM
>OLS46889.1 light-harvesting protein [Rhodovulum sulfidophilum]AB14
MAKFYKIWMIFDPRRVLVAQGVFLFLLAVMIHLVLLSTDYFNWLTIAAEKAAGA
>OLS46890.1 light-harvesting protein [Rhodovulum sulfidophilum]AB14
MAESDVSFTGLTDEQAQEIHAVYMSGLWLFSAVAVLAHLAVYIWRPWL
>OLS46891.1 protein PufQ [Rhodovulum sulfidophilum]AB14
MTDQTSDVHMVRGHRPPKAEFMVYFTIIFIAALPLAFIASFLAMVRQGDLKTKGPIARAWSQARIITPMIFSA
>OLS46892.1 chlorophyllide reductase subunit Z [Rhodovulum sulfidophilum]AB14
MLVLDHDRAGGYWGAVYAFCAVKGLQVVIDGPVGCENLPVTSVLHYTDALPPHELPIVVTGLGEEELGREGTEGAMK
RAW
STLDPVLPAVVVTGSIAEMIGGGVTPQGTNIQRFLPRTIDEDQWEAADRAMTWIFTEFGMTKGRMPPEKPREEGAKP
RVN
ILGPMYGVFNMPSDLAEIRRLIEGIGAEVNMVMPLGAHVAEMRNLVNADANVVMYREFGRGLAEVLGKPYLQAPIGI
DST
TKFLRKLGEMLGLDPEPFIAREKHSTIKPVWDLWRSVTQDFFATASFAIVANETYARGVRHFLETDLGLPCAFAVARQR
G
AKTNNAEVRALMGQKRPLVVFGSINEKIYLAELSAGHGPKPAFIPAGFPGAAIRRATGTPFMGYAGATYLVQEVCNSLF
D
ALFHILPLGTEMDSASATPTRLRRDFPWDEDAQALLDRIVAEHPVLTRISAAKSLRDAAEKAALEQGAERVVLETIEALA
PAGFARPSSKGETK
>OLS46893.1 chlorophyllide a reductase subunit Y [Rhodovulum sulfidophilum]AB14
MTEEVKYDEAHTAEVIEGTPREAGNPPEGADPRLGDGMGCHAGAAEMEKAARAAGASEILDQYAKDYPQGPHDRP
QSMCP
AFGSLRVGLRMRRVATVLSGSACCVYGLSFVSHFYGARRSIGYVPFSSETLVTGKLFEDIRDAVHELADPDRYDAVVVTN
LCVPTASGVPLRLLPDEINGVRIVGIDVPGFGVPTHAEAKDVLAGAMLNYARKEIEAGPVAAPEGGVSDRPTVTLLGEM
F
PADPIVIGQMLDPLGLACGPVVPAREWRELYAALDCGAVAAIHPFYTAAMREFDAAGRPIVGSAPVGHDGTAAWLAN
IGD
AFGLSADKVAKAQNMFLPTISEALAGAPVKGTITLSGYEGSELLVARLLIESGANIPYVGTACPRTKQAEADRDWLESKG

VKVVFRASLEDDKAAVDAIRPDLAVGTTPVVQHAKEQGIPSLYFTNLISARPLMGSAGAGSLAQVVNAAIANKGRMASMK
EFFEGVGEGDTAGVWAGDPNLHPEFRALQQKKLDKKRRAEKAQEMI
>OLS46894.1 chlorophyllide reductase iron protein subunit X [Rhodovulum sulfidophilum]AB14
MTLDVPNIKDIDQRLRDEANAPLDDEIPVTEPTKKTQIIAIYGKGGIGKSFTLANLSNMMAEQGKRVLLIGCDPKSDTTS
LLFGGKACPTIIETSTKKKLAGEEVAIGDVCFKRNGVFAMELGGPEVGRGCGGRGIIHGFELLEKLGFHDWDFDYVLLDF
LGDVVCGGFGLPIARDMAQKVILVGSNDLQSLYVANNVCSAVEYFRKLGGNVGVAGLVINKDDGTGEAQAFAEAVDIPVL
AAIPQDEDLRRKSANYQIVGTHEGPWGDLFAGLASAVAEAPPIRPAPLTNDGLLGLFAPEDVGADFELTPATDADMRGKN
AKPKKSLEVVYDDV
>OLS46895.1 chlorophyll synthesis pathway protein BchC [Rhodovulum sulfidophilum]AB14
METTAVILRGPREIGLGKLGLSAPGPRDLVVEISHSGISTGTEKLFYTGEMPPFPGMGYPLVPGYESAGEVIEAGSETNF
KVGEHIFVPGANCYEGARGLFGGSAKRLVTDASRVTRIDPGLGPEGALLALAATARHALAGVDKKMPELIVGHGVLGRLL
ARLTIAAGAPAPTVWEIDPDRQSGAMGYEVIHPDDDPRRDYRTIYDASGVGSLLDTLITRIARGGEVVLAGFYTAPISFA
FPPAFMKEARLRIAAEWEREDLIATRQLVESGALSLGGLITHSVPAEKAAEAYETAFNDPACLKMILNWGAVHDA
>OLS46896.1 SAM-dependent methyltransferase [Rhodovulum sulfidophilum]AB14
MTEILSGPGPEPRRGSGWRRWRNRKIASPGFQSWASRFFLTRRHVRREGERMFDLVSGFVYSQVLWAVVELDLLEATCDE
AQSLAALAHRAAVPVDRMEALMQAGTALGLFDRVGGGYQLSSLGAALMGAPGVVGMIRHHDVFYRDMQDPVAILRGEKET
ELARFWPYVFGAGAAENPEVARRYSKLMADSQALVAEETLRSGALDGVARLMDVGGGTGAFLAAAGTARPDLKMVLFDLP
AVVPAADERFRALGMADRVTVAGGSFRDDPLPEGADAVSLVRVCYDHADDTVLALMRKILATLPAGGRLIVSEPMAGGAR
PNRAGDAYFAFYCMAMRTGRARSPERLAEMMKEAGFQGVEMPRTHRPFIASVVVGCKPGAT
>OLS46897.1 geranylgeranyl pyrophosphate synthase [Rhodovulum sulfidophilum]AB14
MDISARIEAAMRQAIAEGQAGAAPGKLASAFEYALTPGGARIRPTICLNVARACGDDRPALADAAAISIELIHCASLVHD
DMPCFDDADIRRGKPALHKAYGQPLALLTGDSLIVAGFEVIARAAHQDPARAVELIRILGQRTGMPYGICAGQGWESEET
IDLSAYHRAKTGALFIAATSMGAVAAGQPAEPWEELGDRIGEAFQVADDLKDALLDAEAMGKPAGQDEAHGRPSAVTELG
VEGAVQRFRDILSGAIASIPSCPGEADLAQMVRHMAERLIPEPARQPAE
>OLS46898.1 methoxyneurosporene dehydrogenase [Rhodovulum sulfidophilum]AB14
MAGSAGKVVVIGAGMGGLSAAIRLAHAGYEVEVVDRAPVPGGKMRTFASDAGPIDAGPTVMTLRPVFEELFDSVGAKLSD
YVTLHREEVLARHWWPDGSTLDLFASREASADAVGRFAGARAEKEFRAFCRRAERLYRAFDAPMMRAAEPSQGALTRHVV
THPWLALDMAPHQSLAKALGKGFSDRRLAQLFGRYATYVGGSPFQSPALLALIWHAEEQGVWRVEGGMHRLARALEQLAH
DRGVRFSFGSRATRVEIQKGAAAAVHLDSGTRLAADAVVFNGDPAALAAGLLGEGLRETMAPEAIRPRSLSAYVWSYAAV
PHGPEMVHHNVFFCDDPHTEFDPIARGAMPEDPTLYICAQDRGTGRSPEGLERFEIIMNGPPVDDGTDPTDEEQETCRTR
TFQTLARFGLTFDPRPGVQALTTPRQFAKLFPASDGSLYGRSPHGLTAALKRPTARTKVAGLYLAGGGTHPGAGIPMATL
SGRHAAEAILTDRALLSTSPRTAMPGGISTA

>OLS46899.1 hypothetical protein BV379_00410 [Rhodovulum sulfidophilum]AB14
MTDAVQADRKMLESLVCPMTQGPLAYDAERQELVSKAAGLAFPIRGGIPIMLVDEARRLD
>OLS46900.1 ATP-dependent protease [Rhodovulum sulfidophilum]AB14
MFSAVDLPDTLPLFPLPGALLLPRARLPLHIFEPRYLAMLDDTLKTTHRLIGMVQPREVPGSDEQKLHAIGCAGRVTAFS
ETEDGRYMLTLSGISRFRIARDQAGFMPYIRADVVWDGFGRDLGPGEDDPGFDREGFLKLLGRFFSSRGLSTDWDSLR
DA
EDELLINSLSMLCPFAPEDKQALLEAPSLSTRRETLVTLIEFALRSGDDEEKMQ
>OLS46901.1 co-chaperone YbbN [Rhodovulum sulfidophilum]AB14
MLELGGKTTETGGAGAALIKDVTEASFMADVIEASQEVPVIVDFWATWCGPCKTLGPMLEQAVTAAGGKVRMAKVD
VDQN
QTIAAQLRIQSIPTVYAFYQGQPVDGFQGALPASEIQAFVERVAAMGGDGGLAEAVAAAEELLAEGAAVEAAQTFAAIL
G
EDPKNAAAYGGLARAHLALGEVDQAEQFLAAVLPEIAEAPEVEAARAQVALARQAAEAGPVAELRAAVDHDPDDHAA
RFE
LAKALHAGGEVEAAVDELLELFRRDREWNDGAAKAQLFTIFEALRPQDPIVLKGRRKLSSMIFA
>OLS46902.1 exodeoxyribonuclease III [Rhodovulum sulfidophilum]AB14
MPFTLATWNINSVRLRAPLVARLMEEEAPDILCLQECKSPVEKLPLEVFAPLGYTHVVARGQKGYNGVAILSKLPLEDAG
SVDFAGLDQARHVAARLANGVLLHNFYVPAGGDVPDREVNVKFGQKLDFLTGMRDFFRESRPERSILVGDLNIAPGEE
DV
WSHKQLLKIVSHTPVEVEHLGAAQEAGGWVDVTRAHIPEGKLYSWWSYRARDWDSADKGRRLDHLWATPDIAGAA
ADSRI
LRPVRGWEKPSDHVPVFARFDL
>OLS46903.1 hypothetical protein BV379_00430 [Rhodovulum sulfidophilum]AB14
MTCFFGILCALCQIGPAAEAAADIPGYWSVLPGWQSWQVTSAYQVGFEGSAELAVACGPEGQPLMGVLTDGAEPPD
GDFE
RPAKIGERHFLIAWTGQAGLALTRPDAEFLAAMRWGSTLVLYDYLGAPVDFRLSGAADALDSALAGCPPPLAVRPDPA
AM
RRTFDETLKARIDRECRALGRSGAEIGSGAVRERLLPGSAYTEVTVDFSYVSCRTGPRLHRDFCTEAGCARQRYLPGASA
YVLIEDGDPE
>OLS46904.1 DNA-binding response regulator [Rhodovulum sulfidophilum]AB14
MSSLKKILLVDDDTDLREALSEQLVMTEDFDVFEAGSGVEAMEKARSAIYELVILDVGLPDTDGRELCRLMRKQGVKCPI
LMLTGHDSDADTILGLDSGANDYVVKPFKFPVLLARIRAQLRQHEQSEDAVFQLGPYTFKPAMKMLITEDDRKIRLTEKE
TNILKFLYRASEGVVARDVLLHEVWGYNAGVTTHTLETHIYRLRQKIEPDPSNARLLVTESGGYRLVA
>OLS46905.1 GTP cyclohydrolase II [Rhodovulum sulfidophilum]AB14
MRLGPGSTERLARARGDLRMGVPVVVGPLVVVAAETLTPERLTALRALGDAPVLAITARRAETLKARSYDGDIARLLLPG
EVGTDWVRAVADPADDLDKPMLGPFASARGGDAEAHRAAIALAKSARLLPAVLAIAPDAPEALAEAEGLTRVSLEEAA
LA
RAEGTTLAEVVSARVPLEVSKAGRLHVFRPADGSEEHYAVEIGTPPRGEPVLARLHSACFTGDLIGSLKCDCGAQLRAAL
AQMGAEGAGVLLYLNQEGRGIGLANKMRAYSLQDQGFDTVEANHRLGFEDDERDFRIGAAILREMGFSAVRLMTNN
PAKI
EMMRARGVEVTERVPLKVGHTPENARYLAVKAQKSGHLL
>OLS46906.1 hypothetical protein BV379_00445 [Rhodovulum sulfidophilum]AB14
METAKVMDYARSLLAARGDKAQAEAAQKAAALEKDGRAEDARTWRAIESALREMRAPHQG
>OLS46907.1 hypothetical protein BV379_00450 [Rhodovulum sulfidophilum]AB14
MPTACDLVLTPTHLRFAGRRFPCSIGRGGLTADKREGDGATPRGLHRIVGMLYRPDRLLPPAPWASPIGPADLWSDDP
AD
PAYNTRVRAPHGFGHERLRRADPLYDLVLLTDWNWPDARPGRGSAIFLHQWRRPHYPTAGCIAFSRRDLLWIAARIAP
GS

RLVVP
>OLS46908.1 ATP-dependent RNA helicase [Rhodovulum sulfidophilum]AB14
MIPSLSEALAERGFDTLTDVQKAVTAPELAGADLLVSAQTGSGKTVGFGLAIAPTLMGEENRMGPPAAPLALVVAPTRE
L
ALQVQRELVWLYARTGAKVVSCIGGMDARDERRALDRGAHIVVGTPGRLRDHITRGALDLSDIRAVVLDEADEMLDL
GFR
EDLEFMLGAAPAGRRTLLFSATVPPAIAELAKAYQRDAVRVTTLSRGSQHADIAYKAVQVTAADAEHAIINLLRYHDAPT
AIVFANTRAAVSRLAARFANRGLSAVSLSGELSQDERSHALQAMRDGRARVCVATDVAARGIDLPNLDLVIHADLPSNT
E
ALLHRSGRTGRAGRKGISALIVPDKLAKKAGRLLKWAKIEAEWILAPTPEEILALDETRLLADPIWEAAPSEAEAAFAAR
LLAERSPELVAAACLRLYRAGLSAPEELNAPAAPGARPERPPFGPSVWFELSVGRDRRAEPRWLLPLVCRAGNLERSQIG
RIQLRAETSLVEIAEAAVPGFVAGIGPDGLLEEDITARRFDGPVEDTRGSRPKSDRPKADRLKSDRPRSERSRPDARPDR
APRPPRPAPAEAATGASTGTAAPWGRPDAPPPRGDAAEAPRPKPEFEARPTPRPTPGPERREVLSAEAEAPKPGAPRR
EG
DAAPVERPRKPRPGAERSGPRSEAGGPRPKEGRKDGWKDARKDDRKDGRKDDRKDGRKDGWKDGPARGPKTGPKT
GPKGG
SRDWPKDKRPAGNDAAPAERRRADAADTSKRFERPGAPKGKGAKPTGGGKRGAPKSGRSSGNAPPRRRPR
>OLS46909.1 alkylphosphonate utilization protein [Rhodovulum sulfidophilum]AB14
MTDTLPPCPECASSFTYQVDALLTCPECGHEWAPGSDDPSEEAGSGIRDSVGNLLQDGDTVTVIKSLKVKGASAPLKVG
T
KVRGIRLVAGDHDIDCTIPGFGKMGLKSQFVKKVQD
>OLS46910.1 hypothetical protein BV379_00465 [Rhodovulum sulfidophilum]AB14
MLVLYLLGLFGVLIGPWFSLLALVLVAVAAACILYMRHPEKMERWLSALPRRARPAGQPAPAPQPAMAAAASTGTEIS
DK
PVQLPALREPDPSDEAAEGDSKLSMLDALGWKSPAAMARDFALLGLAGLVMVVVGRILPEWPLLSWIGGVLEIVGVLC
VL
FSSGVLYSFGREFFWRGRG
>OLS46911.1 chorismate synthase [Rhodovulum sulfidophilum]AB14
MSLNTFGHLFRVTTWGESHGPALGATVDGCPPGIAVDAAMLQHWLDRRRPGQNRFTTQRREPDAVEILSGVFEGQS
TGTP
IQLMIRNTDQRSKDYGEIARTFRPGHADITYWQKYGIRDYRGGGRSSARETAARVAAGGVARAALAALAPDLRISGYM
VQ
IGPHAIDRARFDADEIERNPFWAPDRLAAAEWEGHIDALRKAGNSVGAVVEVVASGVPAGLGAPVYGKLDTDLAAAM
MSI
NAVKGVEIGDGMAVAALSGVENADEITMGPDGPVYASNHAGGILGGISTGQDVVVRFAVKPTSSILTPRRTITRTGEET
E
IVTKGRHDPCVGIRAVPVGEAMMACVLLDHLLLHRGQIGESRGRIG
>OLS46912.1 thiamine ABC transporter substrate binding subunit [Rhodovulum sulfidophilum]AB14
MRIPFLAAALAATALPASAAADRPVLTVYTYESFVAEWGPGPIVEAAFEKTCGCDLNFVAAGDGAALLARLRLEGERTR
A
DLVLGLDTNLTAAAAETGLFAPHDQDPDRLDLPVAWNDPLFLPYDWSYFAFVYDKTRLPEPPASFEALIAAPEDLKIVIQ
DPRSSTPGLGLLLWVRAAYGERAPEIWEGLAPHILTVTKGWTESYGLFIDGEADMALSYTTSPAYHLIAEDDDTKAAARF
DEGHYLQIEVAGLLASSDQPELARQFLDFMLTEPFQTAIPTTNWMYPAVTPEAGLPAGFETLIQPDTALLFPADEAARV
R
DEALAEWRNALSR
>OLS46913.1 thiamine/thiamine pyrophosphate ABC transporter permease ThiP [Rhodovulum sulfidophilum]AB14

MAQRAQPLGIATGLGIGAAALALTLALGTIAAVAWRAEPGGAGLGAADLAAIRFTLLQALASAALSTLLAVPVARALAR
R
RFPGRGVLITLLGAPFILPVIVAVMGLLAVFGRSGLLSHALAPLGLGPVDIYGPFGVVLAHVFFNLPLATRLVLQGWLAI
PAERFRLAAALGFGPAERFRHLERPMLREVLPGAFLVVFLICLTSFAVALTLGGGPRATTIELAIYQAFRFEFDLGRAAW
LALIQVGVSITAALAAFAIAVPGAFGPGLDRPVAQIGPDGRGLRLQDMACLGLAALFLILPLAMVALRGLPGLADLPASV
YAAALRSIAVALGSATLTLALALPMAIAIDRLDGLAGRTIQAAGTLTIAASPLVMGTGLFILLFPLADPRLLALPVTATV
NAAMSLPFALRALVPAVAEAERSHGRLADSLGMTGRARLRLMLLPRLRRPIGFTLGLSAALSMGDLGVIALFSDPGHAT
L
PLQLYGLMAAYQMDSAAAAGLLLLVLSLGLFWIFDRGGRARAEP
>OLS46914.1 thiamine ABC transporter ATP-binding protein [Rhodovulum sulfidophilum]AB14
MLNLEGLAIRQGNFVLEADLTIAPGRIVALLGPSGAGKSTLLDSIAGFLAPDRGRVLWQGSDLGPLAPGDRPLSVLFQDN
NLFPHLTAARNVGLGLRPDLRLTPAQNRAVEAALDRVGLGGLGDRKPAALSGGQRGRVALARMLLRNRPLMLLDEPF
AAL
GPALKAEMLALVAELARETGATLLFVTHDPTDARAIADETVLVAEGRAHPPRPTERLLADPPPALRAYLG
>OLS46915.1 cytochrome c1 [Rhodovulum sulfidophilum]AB14
MFKKLTITALTALTLGAGGAWAAEGGHVTDYKFSFEGPFGKYDQAQLQRGLQIYTQICSVCHGLKFVPLRSLSEPGGPSL
SDDAMRAYAETFEVFDPELDDYRTAKPTDHFPEGTYPGAPDLSLMAKGRAGFHGPLGTGISQFFKGMGGPEYITSYLLA
F
TDETKEEAGTLYYQNPVFSTGWSAMPPQLFGDDVEYADGTEATMEQEAKDIAAFLMWTAEPKLMARKEAGVISVLFL
GVL
TVLLYLTNKRLWAPYKPKKQI
>OLS46916.1 cytochrome b [Rhodovulum sulfidophilum]AB14
MSRIPHDHYEPGSAPEKWLERRLPVIGLLYDTLMIPTPKNLNWMWIWGVVLFFTLVLQIVTGIVLAMHYAAHVDIAFA
SV
EHIMRNVNGGHFLRYLHSNGASLFFVAVYLHIFRGLYYGSYKSPREVTWIIGILIYLAMMATAFMGYVLPWGQMSFWG
AT
VITGLFGAIPWIGHDIQQWLLGGPAVGNATLNRFFSLHYLIPFVILGLSIVHVWAFHTTGNSNPTGVEVRRGSKDEAEKD
TLPFWPYFVMKDLFALGVVLLVFFAIVGFMPNYLGHPDNYIEANPLQTPTHIVPEWYFLPFYAILRAFTGDVWVVMIAD
W
LTFGVVNAKFFGVLAMFGSILVMALVPWLDTSNVRSGRFRPQFRIAFWLFVVDFVVLTWAGGMPAEAVYANISLAGA
AYW
FAYFLVILPVLGVIEKPEPLPETIEEDFNAHYGDGAAAGTATPAE
>OLS46917.1 ubiquinol-cytochrome c reductase iron-sulfur subunit [Rhodovulum sulfidophilum]AB14
MSHAEDHDGTRRDFLYYATAGAGAVTAGAAIWPLVNQMNPSADVQALSSIRVDVSSVSEGQQITVKFLGKPVFVRLR
TPE
DIELARSIPLADLRDNNSRNPNKPGEATDENRTMDEEGKWLVMIGVCTHLGCVPIGNQSGDFGGWFCPCHGSHYDS
AGRI
RKGPAPENMYIPVASFVDETTIQLG
>OLS46918.1 hypothetical protein BV379_00505 [Rhodovulum sulfidophilum]AB14
MPLPLAPIATVALRYGLRYGAVALAAYALSKSQNGLRRDQRHEDAMDELGEGLAFRRDQEQVSGGARMRRTFRLGDT
GPG
VEIDASALGRLRVRRV
>OLS46919.1 alpha/beta hydrolase [Rhodovulum sulfidophilum]AB14
MRRHSFEHDGLVFSFLDAGEEGRPLLALHGHWMGGSDFSGLAADLGPGWRLIALDQRGFGETDHNAPHDIPAYLSD
AIAL
LDHLSIDRPVPVLGHSFGGVVACHLAAARPDRVSALIIEDIGVNIDDDSSAHVANWGGVFRLREDLEDRLGPRLSPYLQN
SIRRVADGWTLNFEPAEFLLSERAVNGDHRGVWLQSRCPALVVSGSESRVCDPAELQAMAQSRENTAFIQLRAGHSV
HID

APAEFARHLRAFLEDSCA
>OLS46920.1 tRNA (N(6)-L-threonylcarbamoyladenosine(37)-C(2))-methylthiotransferase MtaB [Rhodovulum sulfidophilum]AB14
MDGAAPRFTTLGCRLNAYETEAMKELVAEAGLENALIVNTCAVTSEAVRKARQEIRRLRRENPEARIVVTGCAAQTEPDR
FAGMDEVDLVIGNAEKMRPETWAGIAKGPDFIGETEKVMVDDIMSVRETAGHLIDGFGTRSRAFVQVQNGCDHRCTFCII
PFGRGNSRSVPAGVLVEQIRRLVGAGYNEVVLTGVDLTSWGADLPGAPRLGDLVTRILRLVPELPRLRISSIDSIEADDA
LMRAIAGEERLMPHLHLSLQAGDDMILKRMKRRHSRADAIRFCEEARRLRPDMTFGADIIAGFPTETEEMFANSLRLVEE
CDLTWLHVFPYSPRPGTPAARMPQVEGGAIRDRAARLRAAGEAQVARHLAAQAGRSHRILMESPRMGRTEQFTEVDFGSD
QPEGQIVTATISGRSDTRLRAD
>OLS46921.1 diaminopimelate epimerase [Rhodovulum sulfidophilum]AB14
MARMDDTERLPFMKMHGLGNDFVVIDRRQGGRPLSAGLVAAIADRHRGVGFDQLAVIEPAEDADARLVFYNADGSLSATC
GNATRCIARHLIDQTGRRELTLVTERGRLSARDAGEGLTSVNMGPPILDWRGVPLADDVDLDRLPIAGEPVATGMGNPHC
TFFVADAEAVDLEAFGPEHEHHPLFPERTNVQVAHLAGPDRVRMRVWERGTGLTLASGSSSCAVAVAAARRGLTGRSVTI
LLDGGELRIDWREDGVWMTGPTAHVFDGVFTAAFLAEVG
>OLS46922.1 DEAD/DEAH box helicase [Rhodovulum sulfidophilum]AB14
MVDFKKRLNKKNSSKPTDPIKLYDSLDRASDKGPLRPAQETVLTDWASRSAQRRDVIVKLHTGQGKTLVGLLMLQSRLNA
KAGPAVYLCPDHFLIAQTCEQAEQFGISTCTAENSLPDTFLDGKAILVTSVQKLFNGLTQFGIGNRSVDVGTILLDDAHA
CADRIRESCAIKIPKGEEAYSSLKTLFSSDLEQQGVGTFADVENGKREAILPVPYWAWTERESEVATILSKCSDRKSVKF
AWPLLKDMLSHCQCVFSGASVEIEPRLAPLDMFGSFAKAGHRIFMSATVTDDAFLIKGLKLTPKTITTPLTDASETWSGE
KMVIIPSLIHENLDSHAIQKALGKPRDGAKFGMVALVPSFARAEPWVAQGAALADKKSIEEAISGLISGDRDETVVLANR
YDGIDLPDASCRILIFDSRPYSENLADLLAEQVRPHSETTLMRTVRTVEQGLGRSVRGEKDYSVIVLTGSDLVRLVRESG
SRKFLSPQVAKQIEIGLEVSELAREDVLDNGETPTEAFKTLVNQCLRRDEDWKAFYAEQMDGIKPKGANDALLELYAGEL
KAETLFVGGDYQGASKHLQKIMDSGLVNSDDKAWFLQERARYHYEADRGESQKLQLAAHRKNRMLLKPASGVTVTQLHIV
SHGRAERIIAWLSSFADYDELNVAVTDILGRLRFGVKAEPFEAALNELATALGFAGERPDREWKEGPDNLWALDDRSYLV
IEAKSEVDVTRAEINKGETEQMNKSAAWFDKHYKGMLATRVIIHPAKKIGSAAAFTHDVQGIVEHDLRKLEKACRVFFKS
FEGQDLKSLSSGNIQTLIDAHRLSVSDIQTGYSRKLRDIRRS
>OLS46923.1 hypothetical protein BV379_00540 [Rhodovulum sulfidophilum]AB14
MHFMHYNAHSSIQTVYAKSEAGYVGRDLPSADQQLSINSLVWGLAEEIGSPAYWAAQAWMWEVEAPAHYKLGRSLEEELI
ACLLGGYGIPAEVGLAAYERLRVANTEDPNRLLNEESVLELLADPLELNGKFVRYRFAKQKSAYLAGSMRALRSLDREAP
DRELRDALTTLPGVGPKTASWIVRNWRDSDCVSILDIHILRAGRMLQIFPEGKSVERHYLELEAAFLDFAEAISVKASIL
DSVMWMNMRQIPAAILRRLADPSAEVFSPKAEPVQLSLAL
>OLS46924.1 hypothetical protein BV379_00545 [Rhodovulum sulfidophilum]AB14
MITIAGGVYRESCIEPHWDDIFGSAGRAAVALADTGETIALHTVRAKSLSAGIENLSAAFGIDATGPEVEEGVEFEYMHS
LGTPRITPRPDAIPQYPPFEVVGDVVLRFGMLEGSARVTGGRVVYDPQSAFDPRPYHENGSSAETLAVILNRLEGRRLSG
RTEPSEIVEHILDEWKADVVVLKMGGHGAMVHVRGETPVPVPSYRSENVWKVGSGDVFSAAFAYYWGKLERPAAEAADLA

SRATSYYCGTRSLPLAPEETLREVVKDAVEPRGGKVYLAAPFFNLAERWIVEEVREQLLHMEVEVFSPLHDVGVGPGDV
V
AQQDLAGLDDCSVVLAILNGGDAGTIFEIGYAVAKGIPVIALAQNVRPEDLKMPLGSGCRVYEDLVTAIYQTVWAEP
>OLS46925.1 7-cyano-7-deazaguanine synthase [Rhodovulum sulfidophilum]AB14
MKALLLSGGIDSIAVAFWQHPEIAITIDYGQRAAEAEITAASQVAKEIGMQHEIIAIDCRAIGSGDMAGNDALDVAPVPE
WWPFRNQLLVTFAAARGIVLGVTELMTGSVSSDGSHADGRPEFYDAMDRVTAVQEGGIRISAPALTMTTAELVRHSG
VPR
EILAWAHSCHTGNLACGQCRGCVKHYQVTKEIYGVAY
>OLS46926.1 hypothetical protein BV379_00555 [Rhodovulum sulfidophilum]AB14
MSSVVFDNDRGLARSDPAVKEALDAFKSTGGNAHKAGYRESRSEMLETICNEIPIAGIFLVSVEQHIYELTSELAHGSFF
GFLEVYSGGDFGNVEAWEKNAELVHFAAVLSSAAMAIAVRSTFGSCECSDEIVLAGEDFIRDLATNIESLDGSFTIRTHS
E
>OLS46927.1 hypothetical protein BV379_00560 [Rhodovulum sulfidophilum]AB14
MWLEAASQMIPDTLPKTACLPYVDERFSVGITDSIDYEPVNFGDIQLIHKLSDTISLCCLF
>OLS46928.1 transposase [Rhodovulum sulfidophilum]AB14
MAEKAVATKGVSIALACRAFGVSETCFRYSPKRDDENEMIADLLVGLTNAHKTWGFGLCFLHLRNVKGHVWNHKRVH
RIY
CELELNLRIKPRRRLKREKPEELAVPEVPNLVWSMDFMVDRLADGRQFRLLNVLDDFNREGLGIEVDFSLPAERVVRSL
N
QVIEWRGKPLAIRVDNGPEYVSSTLMTWAEQQGIALTYIQPGKPQQNAYVERYNRTVRHEWLDLHIFDTIEEVQQIAT
EW
LWTYNNERPNMGIGGVTPAMKLKMAA
>OLS46929.1 transposase [Rhodovulum sulfidophilum]AB14
MDEEIPMKKSRFTEAQIMGALRQAEGGMPVAELCREHGVSSATFYKWRAKYGGMDASMMSQMKALEDENRRLKR
MFADLS
MQADLLREALGKK
>OLS46930.1 hypothetical protein BV379_00585 [Rhodovulum sulfidophilum]AB14
MKLDIRLHKQLRAELRARGVSLNQIAVEIDRSPALVTAVCQGRCKSHLAQSAIARHLERPPEEIWPDRYGKEETA
>OLS46931.1 hypothetical protein BV379_00590 [Rhodovulum sulfidophilum]AB14
MYLGSHRQIVVYESLLELMCILLIISRGDVIDVWDQPPAIHFSRPDGSHHEHTPDYLVELADGTRFAVAVKPIEQVNRRD
FDIELSYVAACMPQSYADGLVLMSEADFTRAQALNAQRYHEFNKTPDALADQALAAVIDQQNEPTTLELLVEQVGLAG
RG
FRAAFRAIIEGRLRQVAHGKIGLQTLVTAEGTA
>OLS46932.1 hypothetical protein BV379_00595 [Rhodovulum sulfidophilum]AB14
MLEENNRRAELGQRPLQVPSERTIERRIDFLDPFEVAVYRDGIDQARMDFGVSSGGLDLLFPLERVEIDEWKADIRTLFQ
RLGIWNELPADLREEVPSGRRWVYAAIDCSTRCILALAVCDRPNADTARRVLGQILEDKTEAARAAGAEGVWDFFGTP
HL
ICADTGSAFSADMFVTAVNFLGSAILFPPVKIPELRSRIERLFGTMTRQITPWLPGRVFENPKARGDYPSEARTILTDEE
LTRILTIWVVDHYHQQPHRGLPGGQSPASRWRELVQKYGVTEPPDLNTRRVALGYEFERVPSSLGVTFLSNQYSCPEM
VQ
FWRRNKGKPIKLRIEPSNIGAISVQIETEWFLAPALNGEELEGMTLTNWTKVMRELRSTYAAEATATLPQRNRAIQKIKA
IVDEARLREGLIDPEYTAEHLEYLDKNLCGGLLFDHPDQPPVSAEGAPFGRLITPSTKVANPGPLKSQDEHPRPTSAAAP
IGEPTWKLQD
>OLS46933.1 hypothetical protein BV379_00600 [Rhodovulum sulfidophilum]AB14
MNINAQISPAIHRELARLRNRFVPRGCYVQLTEAMSVLEDRRRAELTEGLNPEARGVAVIGNSGSGKSTAVAHFFRGHE
T
LRLLSHDAETADVASFLVPSPATLKGVGISCLNGLGYPLMRDRTAGIIWELVQSHLRELQIMFLHLDEAQDLMGSQSERE

MLAVVNTLKSVMQNRYWPVSIVLSGLPTLKTLLNLDPQLGRRFTPISFGQIDDIQDGQNVLELIQQYRQAADLESSGSV
E
TDDFAARLIHAAAHEFGILIEVIIGAIEICMRRSDSTLQLQHFTAEFLRRTGCAADLNPFIRRDFTALNPRLLLPGNDDM
QPSHVPPRRGR
>OLS46934.1 hypothetical protein BV379_00605 [Rhodovulum sulfidophilum]AB14
MPRLKLTVSPREGETTLSVVSRLALRNGASFVQDFCADMGVSWRDAANGEAGEIHRLADLAGLDVDGLAAHSVVRVA
HPG
YRVNKQYLTSKTLVRRELRVCPRCLCEDDRQGGALARHGRLEWLVATYRTCHIHQVPMLTLPDADYPRCRHDFSQRVR
DH
WRCISRAERELVPSSGLGDLETDIASRIRGLGGHDHARFLDLDLMCRLSEDLGVVLRFGASTYPSKLNRTELARAAEAGF
QAIAGGPDALTVAFGEIKVASSSPTPGFQADFGVLARRIERVDHGSPRYAHFLGRLTEFAFNNYPYGKGDVLFGRTCPER
RVHNLASAASAHGLNYSRMYRIAVGMGLGTTEQAERIEFSTADHDDQLADYAACLSPKKSADHLGIRGEMLNRLVSAG
LI
APRFDLPGLNPIYHPEDLRLLIEPLVGQAALMDRLPSGYASLVSIGGHAKCRFETVMLLACDGKLTSLSRLNASPKLDGL
FVNLDDLRDQLEEPAPSGITRNEAKRLLRINSSTVAWLIRQGWLPATTVKHHRHRRPVALISREALEVFLADYATLGMM
A
ATAHTQAIHVARRLEDARIFPLDLDCRLSKLYPRTSRLEELFDPGRIPEPTPS
>OLS46935.1 NAD-dependent succinate-semialdehyde dehydrogenase [Rhodovulum
sulfidophilum]AB14
MTGNTIADRTGLSDRDLLRDRSRIGGDWVGSETRFEVRDPADGAVLGNVAAMSAEQSRAAIDSAQTAFPGWAGTLP
QDRS
AILRRWFELVTEAREDLARIMTVEQGKPLSEARGEIDYAASFIEFYAEEAKRPNIEGVTSHLPDAEVELWLEPVGVVALI
TPWNFPAAMLTRKAAAALAAGCSVLAHPSAETPHSALALAELAARAGVPPGVFNVVTGDAATIVGPWTEDPRVRALS
FTG
STEIGRLLYRQSAATVKRLVLELGGHAPVIVFSGAELDHAVAETIKAKFATSGQDCLGANRIYVERPVYDEFCRRFTAAT
RALTVGRGMDDPDIGPLMNEAALTKQEDHVADALAKGARLACGGSRLPLGDLFYAPTVLTDVPHDAKIMREETFGPV
AAL
TPFDTEDEVIARANDSEFGLVAYVHSLNPRRIYRVSRALQYGMVAVNRTKVTGAPIPFGGMKQSGIGREGARRGMEAF
ME
IKYVCRDWH
>OLS46936.1 C4-dicarboxylate ABC transporter permease [Rhodovulum sulfidophilum]AB14
MAGDLIGAMALIATPDTILSIVIASFFGFVVGALPGLTATMAVALLIPITFLLSPVAAIASIVSAAAMSIFAGDIPGALL
RIPGTPASAAYMTDANALVRKGEAGFVIGASAVFGMIGGVIGTVILIAAAPLLADFALSFSSFEFFWLGCLGLSCAVLVA
GDDLLKGMGALMIGLLIACVGMDYTTGYPRFTFGSWALLEGISFIPALIGMFALPEILRLLSDRSPRHQLPEAEPGSALM
KIARAGRRHWWPSLRGSVMGTAIGSLPGAGADIAAWISVAVTRRATREPEKWGKGHIEPIVAGGSANNAALAGAWIP
ALV
FAIPGDTITAIAIGVLYLKGVNPGPMVFVENGQIVYAVFLVFFLANLMLLPFGWIAIRMAGLVLKVPTAVIAPTLLVLAC
LGAFAINNDMSGLLVMLGLGALAVGMNAAGLPLAPAVLGIVMGRIVEQNLMQSLISTQGSLAGFFERPASAVLGALTI
GL
WSIALLRQLARMLRGARRAG
>OLS46937.1 hypothetical protein BV379_00625 [Rhodovulum sulfidophilum]AB14
MRIHDALAGALLAALGGAVIWYVAGFPRVAGQIYGPDLFPRLIGIGLVLFGAGLVLRGARAAGGLPALLDLPSNGEIRRA
LLATLYIVASVLAVVFLAERLGSQILVFVILISGLLAVYRRPVLSLTLAVGLTVAFDLIFRVLLRVPLPSGPLTGVL
>OLS46938.1 twin-arginine translocation pathway signal [Rhodovulum sulfidophilum]AB14
MTRTKALIAGLSLTLAAGAAWAEFPDRPVQLIVPYPAGGGTDIVARTLAVELEKELGQPVNVVNRAGGGGIPGQTAIA
HA
RPDGYTIGLIASDISLYKPQGLTDLTHADMTALGQTNELPGSVTVAADAPYRTMDDLVTAIRDNPATIKGTGAAPGASW
H

AGFLGLMFALDMAPDQVIWVPTQGGTKGHLDVAAGNSDFSTASLVEARALIEAGKLRPLATMAAERVAIFPEVPTLQE
QG
IDYTYGLWHGVTGPKDMDPAALEVLSAAVEKAANSDGFVATLEERGFKHVWRPGPEFEAFMAEDLAKMQAIFDRLN
D
>OLS46939.1 thioesterase [Rhodovulum sulfidophilum]AB14
MTRRAGAETVQIVDAHCHMWDLSLGKHPWLAEGVLHPHRYGDYSAVKRDYLPADYRAATRPWTIAAAVHMEAEW
TPDDPL
GEARWIADLHAKTGFPAAMTAQVWLDRPEARDLIAAQAAFPLVRSLRHKPRAFARAADWHEGHALPGSMRCPEFRR
GYAG
LAAHGLHFELQTPFWHLPDAAELARDFPDTMIVINHAGVPGSRDPAVLAQWRRALSVAAARPNIRIKVSGLGVPGEA
WTT
EAQREVILTCIETFGAGRAMFASNMPVDGMFRSFPDLIADFMAVTSDLPEADRRAFFAGTAADTYGLDLPGLT
>OLS46940.1 GntR family transcriptional regulator [Rhodovulum sulfidophilum]AB14
MSEEVAAWPRGQRSILVTQFIRDLIVSGEIPPGERINERVLTERLGISRTPLREAFKILDGEGLVSIRPNAGATVVRLSP
EDVEDSLEILIGLESIGAERAVEHATPEMIEEIAELDARMGEAFGRRALMEYFHINQAIHQKIIDAAQNEALSRVYASES
ARIRRFRFAGNRDHGRWARAVQEHAQILDALQRREGPLLRELLRAHHRAGWKVARHALEAVIPNH
>OLS46941.1 flagellar biosynthesis protein FlgA [Rhodovulum sulfidophilum]AB14
MNYHTYFSAGDRMVEACVIGTGGFGRSFLAQGRKVPALSVRIGVDVSAEIAAEAMRDVGIPEDRIRIVTGAAEALAAFE
A
GAHVAAGDFAHVAGLPFEVAVEATGNPVHGARHARLAIEAGKHVAMVSKEVDSVVGPGLAAMAAEKGLIVTPVDGD
QPSL
LIGLITWAETLGLEIVAAGKSSEYDFVFDRGTGTILCNGQTYPAADFAPYARMGDMEAAALVAARAEAARTLPQRAVPD
L
CEMTVVANACTMGVDTPELHCPIARIDEVPSILCPVADGGILGRAGVLDVFHCLREPGEISFAGGVFVVIRCDHADTWE
V
LREKGHIVSRNGRTAMVFLPRHLLGLEAATSILEVGLRGVSSGTSRPAHHADLVAHADRDLAAGHVLKMGGHHHSIDA
VS
ARILPGGPLSEDSPAPFYLASDCRLLRDVPAGEPIRMRDIEIPEDSPLAALRALQDSHFAPSGQTT
>OLS46942.1 hypothetical protein BV379_00650 [Rhodovulum sulfidophilum]AB14
MTLNRRSFIAAAGALLAAPSILGAAGKITLRMGHPHPETDSWQDTALWMGETLAEKSGGEIGLQVFPNGLLGNDPTM
INS
VRAGALDIMVTGNPFFTSLAPELNVLDLPYLFHDRDHVARVLDGEIGDRLRSDFEGTGLYALALWETGWRHVTNSRRP
VE
TPDDIKGLKIRTTPNPAHIEAFRLLGAVPTPMAFTELFTALEMGAVDGQENPTTLILNSRFYEAQKYLSLTQHAFTAAPL
VTNAAKLDAMDEDMRALLIETAQDGARRQRALNVEREVTSLAKLREEGMEVVETPDRKAFRAIVAEPVQASFGEEFGS
EL
IEQINAAA
>OLS46943.1 cyclase [Rhodovulum sulfidophilum]AB14
MTIVDLSMTVGPHFRWSPEISVTGDKSAGDQFRVTRLSTTCHGFSHVDAQAHFIENGPTIEATPLDRVVGPCRIFDLSA
V
APDEEIGPETLAAADPGGAEGEILLLAAQWDLKRDAGSREFWTESPWLNRAAAEYLLAQKPSAVAVDFPQDYPIRLLLD
G
IAVPDAEHVTHDVLLRAGVTLIEYVVNTAALKGPRTFLCAAPLKIGNADGAPARIFAIEDMG
>OLS46944.1 hypothetical protein BV379_00660 [Rhodovulum sulfidophilum]AB14
MRPARLAVLAVRVADRVLAAVEAVLVLVLAAMTLLVFFNVVLRYGFDTGITVTDEVSRMMFVWIAFAGGIAVSRRQQ
QLG
IDVLVAALPPLPRRLAHVAGNLAVLVCCLVLVSGAWTQMQLNWRNTAPVSGLPTAITYAAPWFAGIGIGCVALANALG
AA

FGLTGGIMASGKEDPGA
>OLS46945.1 L-dehydroascorbate transporter large permease subunit [Rhodovulum sulfidophilum]AB14
MTTILIFVGVLLVTAIAGIPIAFGLMLAGIAMLWQLGMLDWQSIALQMTNSADSFPLLAIPFFLLAGEAMNAGGLSRRL
V
NLGLALVGHLRGGLGYVAIVTAVLLASLSGSAIADTAVLAGMLVPIMRQSGYPMGRASGLIASGGIIAPVIPPSIGYIVF
GVAGNVSITRLFLSGIVPGVAMALALTATWWLMCRRENMATLPRRTGPEIRSALVDAVVAMMLPAIIVGGLKFGIFTP
TE
AGVVACIYALLAGTVIYREITLGVLYECMLNAVRTTAAVMLLIAAAGITAYAITIAGVPQQLAVLLGPLAQHQTMLMLAI
VVIVILVGTALDFIPTLLVLTPVLLPIAKQAGIDPVYFGVIFMMSAAIGLLTPPVGAVLNVVCAVARQQYADVVRGVMPF
IVVQVLLLLLLVLFPQIVILPGRWLY
>OLS46946.1 hypothetical protein BV379_00670 [Rhodovulum sulfidophilum]AB14
MILGAIADDLTGATDLSLILARNGMRVVQTIGLPDDLGAVAAEADAVVVALKSRTIPAEEAVAMSLDAARALQAAGAR
RF
FFKYCSTFDSTDEGNIGPVASALMSHFDMECSVACPSFPENGRTVYQGHLFVHDRLISDSPMKDHPLTPMRDPDLVRV
LS
RQTERPVHLVPLATMRGAPGAIAAQVTATPGIFILDAIDDSDLRAIARDLADLPLITGGSAVAMGLPDIYRARGWLSAAA
AQAPFEAPAGKGVILAGSCSEMTRKQIDAARRAGLPMLQIDAEGIAEGRITAGTVVDFVEKAAGQGPLPPVVYSSADPE
T
VARAQKALGRMRAGELVETLLGEVAASLAGRGFTRIVSAGGETSGAVAGALGASALRIGPEIDPGVPWVMTLDGERPL
CL
ALKSGNFGSEDFFLKAWELLA
>OLS46947.1 aldolase [Rhodovulum sulfidophilum]AB14
MTAILSEATAARDRICAAGKSIFDRGLTFGSTGNISVRLSDGSMLMTPTNACLGALDPARLSRFSADGQFLDGDRPTKES
FLHMCMYCKRPHSNAVVHLHSTHSVAVSILADIDEEDVLPPLTAYYVMRVGRLPLIPYFAPGDEKLARAVEAQAGSHHA
V
LLANHGPVVAGGSLEEAQYATEELEETARLFLMLQGHRIRPLGEEEAKALRSRG
>OLS46948.1 GntR family transcriptional regulator [Rhodovulum sulfidophilum]AB14
MATPLYQTVIDTIVARIASGELPSGALLPSETQLGAELGVSQGTARKALSELEKRGLVQRAQGRGTFVTLRTPEMSLFNF
FRLRSPDGTLTAPELVSETVIRRQATAEERAALFGRPEEVIEIRRVRSVGGRRTTHERSCLPPALYPGIVDRAPLPNALY
VLYQRAYSIIIVRADEKLRAVGAPADVAAALELGEGAPVLLMRRAAVDVLDRVVELRVCHLVSEVLDYFVTLS
>OLS46949.1 flagellar biosynthesis protein FlgA [Rhodovulum sulfidophilum]AB14
MGKPHLLKHDHSDNVGVVVVEGLTAGTEMLCVVTADNSDFTVTAKADIPIGHKVALSDLKEGDTVLKYGEDIGRMVG
AAN
VGEHVHVHNCKTKRW
>OLS46950.1 D-galactarate dehydratase [Rhodovulum sulfidophilum]AB14
MPFDFKSMTVNAWRRENGRVGVRNHVLILPVDDISNAACEAVANNVKGTMAIPHAYGRLQFGEDLDLHFRTIIGTGA
NPN
VAAVVVIGIEPEWTQVIVDGIAKTGKPVTGFSIEQKGDFETIRAASWKAKEYVQWATELQKEACPVSDLWISTKCGESD
T
TTGLSSCPTVGNMYDKLLPEGIYGCFGETSEITGAEHICEKRAATPEAGAAFRKIFDAYQDEVIEAHKTSDLSDSQPTKG
NILGGLTTIEEKALGNLEKIGRSSTYIDAMGPAVAPEKGPGLYFMDTSSAAAECVTLMAAAGYVIHTFPTGQGNVIGNPI
VPVIKISGNPRTLRTMSEHIDVDVTGVLTREQTIDQAGDALIEMILRTANGRNTAAEALGHREFSLTKLYRSA
>OLS46951.1 3-phosphoglycerate dehydrogenase [Rhodovulum sulfidophilum]AB14
MRVVVSEFMDTEALAGFGEEVEVVHDPGLVDDRGALIAALPGTHGLIVRNRTQVDAALLEAAPDLRVVGRLGVGLDNI
DL
ASCKARGVKVCPATGANTLSVAEYVISTALSLLRGAYGSKAAMIAGEWPRNALIGGEASGRVMGLVGYGGIARAVAER
AR
ALGMRIAAHDPFLDAGDPAWQGVENCTLPELLARADILSLHVPLSEGTRNLIGPEAISAMKPGAIVINTARGGVIDEPAL

ARALTEGRLGGAALDVFATEPLTAEAGKIFEGVPNLILTPHIAGVTDEGNVRVSTVTVRNVKEALADAPA
>OLS46952.1 sulfolactate dehydrogenase [Rhodovulum sulfidophilum]AB14
MPRPDADITRTEAEALIARILEARGTSARNAQSVARALVAAEIDGQAGHGFSRVASYAAQAGSGKVTGAACPVALRSG
AR
VDIDARNGFAYPAIDLAISELTEMAPEHGIAMAAIRRSHHCGQLGAHVERLAEAGFAALMVANTPKAMAPWGGSSAL
FGT
NPIAFAAPRADEPPLVIDLSLSKVARGKVMAASKSGEPIPEGWALDAEGQPTTDPRAALGGTMVPAGDAKGAALALVV
EI
LAATLTGANRSDEASSFFEAEGAPPGVGQTILAFDVARAPGFGERLEGLLDAMLAQEGTRLPGERRLRGRSDSAGRVRV
P
GHLMEELSTLPS
>OLS46953.1 C4-dicarboxylate ABC transporter substrate-binding protein [Rhodovulum sulfidophilum]AB14
MTKFTLYAAAGAIAAALFAAPASAGDFPPGQVEYIIPFGPGGESDITARLQQPYFEKLYGEQMIVSYKPGGGGAVGWS
QL
NGMAADGSVIMGVNLPHTIIKPLQGNVGFETDDIVNAYIFHYTPDAIVVRADSPYQSLDDLIAAAKETPRKITFSGSGKG
TANHLVSVRFDELAGIETTYVSFKGTGAAVTAMLGGQVSAEWGYTTVGGSQGDKVRMLAVAMEERHPAFPDVPTFR
ELGY
DIVSGAYRGVAVPKDTPEETRQAVSDAIAAINADPEFRQKMIDGGFALIDVPYGPEMDAWMKARAEEYIPAARAAGVI
D
>OLS46954.1 C4-dicarboxylate ABC transporter permease [Rhodovulum sulfidophilum]AB14
MLDFFLAALTPLNLLLALAGVAAGTIIGSVPGLTATMAVAVLVPLTFGMPPESALILLGAIYTGAIYGGAYAAILLNTPG
TPSAIGTTFDGYPMAKRGDGDLAVTIACLSSLVGGIVGAGALLLLAPPLAEAALAFGPVEYFWLAIFGLSLIAALSTRDF
LKGLIGAAFGILIAMVGISETSAEVRFTFGSNTLLGGIETVSALIGLYCVPVLIELAATSGRHLKEDTGGTRGFRLPEAF
STLMRHKVNVIRSSVIGTLVGALPGAGGSIAGLVAYSEARRTDRRAPAYGQGNPGGIVATESANNATVGGGFIPTLVLGI
PGTPPDAVILGALLVQGVRTGPTLFADGSSIVYTFIFGLFLATLLMLPVGLLIGRFAYRSIVRAPKAGLVPVVAFMTIIG
SFAIRNSLSDVGIMLALGVVGWVANRRGFSVSPIVLGLILGRIAEEGFVQSWTIGDAMGNVWGQFFGRPLSLVIIALTLL
SFVYPFLPALTRTLRRRGTAPPAPSDKREPADPAAILRDLAGIGVMAAIAGVALWQCAGLNPEAAVFPRTIATGMLVLC
G
LAALETVLTRRVVEDRSYGSTGRRAGVVLSMAAGIALAPVLGFATAGIAMALGLMLSSEHNNRSLRGWGLLALSVAAVI
I
VFTIGFREALSVPLPGGLIF
>OLS46955.1 hypothetical protein BV379_00715 [Rhodovulum sulfidophilum]AB14
MTDHPATMPRLRGILGRADALWPGLLVCGLAALAAQFLSDHYGAPAMLMALLLGLSLHFLVEDGDRCAAGIGLAART
VLR
IGVALLGARISFELFTELGLPVVGLVIGALAATMGFGWLAARMLGRGWRLAILTAGAVSICGASAAMAIAAVLPKNELSE
RNLSFTVFGVTLLSTVAMVLYPVIATLLGLSDLQSGVFLGGTIHDVAQVVGAGFSVSDAVGETATTLKLIRVSMLAPFVL
ILTLALRGAGAQDQSGAKRPPILPGFVIAFLILALINSLGWIPAALQALLWALSKWAMLTAIAAVGMKTDLKRILSVPPQ
SVVLIFLETLFIAGVVLAGVMVLL
>OLS46956.1 hypothetical protein BV379_00720 [Rhodovulum sulfidophilum]AB14
MGIALILLFLLIALGLPIAAVLGWLAVLLDWLLTPFPLQRAFGEIAWSHSIEFTLIAIPMFIMMGEILLRSGIADRMYAG
VSAWLGWLPGGLMHANIGASALFAATSGSSVATAATIGTTAMPQVDRHGYSERLFLGSLAAGGTLGILIPPSINMIIYGF
LTDTSVPRLFMAGLVPGIVLAAIFSMTILVLCILRPSLDGDRSPPTARALLHSLSDLAPPIGLFGLVIGSIYAGWATPTE
AASVGVVAALGLTALYRRLSWPMMVEVFLGTVRTSSMMILITLLAFLLNFVIGATGMAGTVNDLVRGLNWSPLATML
VIL
SVYLVLGMFMDTLAMIVLTIPIVTPIVVGLGYDPIWFGVMMVLIAETSVLTPPIGMLCYVVHGVRGRGELTDVFIGVTPF
ILALMAMIALMLATPQLALWLPNLLF
>OLS46957.1 hypothetical protein BV379_00725 [Rhodovulum sulfidophilum]AB14

MDAPLISRKLARLARFAAIGSGLLLLASAGLMTAEILMRRLFHHSLVGVDELAGYAFAVAMAWGFAQALFMRAHIRVD
LV
YLRLPSRLQRCLDVLALAAFAGIIGIVVTHAWGTLAESLRLGARASTPLATPLWMPQAAWLAGLIFFLVCLIALLFDLVR
ALARGDWDLAADLGASESASRRI
>OLS46958.1 hypothetical protein BV379_00730 [Rhodovulum sulfidophilum]AB14
MTTLSRLFGAAALAGLAPLAVQAGDLPATELSVIGSPVNSPNWTNVLQPFWTETVPADSDGRVTAEAVSMTDLGVKG
PEL
MRLAGAGIADIIAGSTTLISGELPENDSIDLAGLAQDIDTLQQVIDAYRPVLEERYRDRLGVVPLGFWPTGAQVLWCAAP
VEDLDDLAGKKVRVFSTTTASLTAALDATPVTMAFNEVVPALQRGVIDCAFTGANSGNNAKWTDVTTHLVDLTVGW
GVNF
IVANSRTWDGLDPAVQAFLRDEIRDRLEPAGWEMARVATRQGIWCSVGDDRCEPEATAPRNLTKTDLTLVELSDDDN
ERL
RRIVAEQVMPEFTRACGADCVARWNETVGQVVDIQLDPAG
>OLS46959.1 hypothetical protein BV379_00735 [Rhodovulum sulfidophilum]AB14
MPDIIGEYVDRLVTVEMRNRGMNHNIIAPIYDEARREGGGRPITARAAEALVEHVGEGDVVLIVTGAGYLPEVPHGESD
G
PPGAVSLARALYWGLKAVPVYVSEVCHAPPIKASSEAAGLMVRDYDLAKGRRMGAAQVTAPEDQAEVDDWARDLLE
RMQP
KAIIAIERLGPNETGIVHYSTGVAPKNCVNVAPLFDQAAAKGVLSVGVGDNGNEIGFGRIHSFMQDFHPYGRETQTDKP
G
GAITVTATDIFLPASISNWGAYGIAAMLGFLLKDRELVQDPETEQRMIRACLDAGGWEMRYCTSRFIVDGAEGESSMAI
V
QLLRDMVRLNIAPPDRGLSHGKMKEAAG
>OLS46960.1 LysR family transcriptional regulator [Rhodovulum sulfidophilum]AB14
MRSLNHRQMLAFRAVMISGSMTAAGRILHVSQPAVTRLVRDLEAELGLVLFVRKWSSVRPTAEAVALFREVERYFESM
ER
IRDNARQMREKQDGRLRIAAMSTLSSGALPEAIRRFRTRQPETDFFIHSDNSVHILDALQHDEFSVGLCRVPPERADIDH
VEMPVSSAICLLPRDHPLARQDSVSVADLAGQPFISLGISSLLRMQIEASMEAAGVQPGRTVQTLYSNTVPSYVSAGLGI
SVADIFSVMAIDPEKIAVKPFVPAIEFRFSAIFPAHARSPGAEIFSRVFFDVVRDQIAEVEQLFRT
>OLS46961.1 hypothetical protein BV379_00745 [Rhodovulum sulfidophilum]AB14
MTARDERRLRSLIREGGGIADFAHCRVLFHRMKKGLLRKGSGPDGRLHPDGGVRAERSVYVRKSCSTSAI
>OLS46962.1 adenylyltransferase [Rhodovulum sulfidophilum]AB14
MQTAPHQNEQIGSALGILRRRLGDALLAVYLHGSAVSGGLRPQSDIDLLAVVGSGLTESQRNGLLTELLCLSGRHPAVP
G
GLRCLEVMVFCRSDLARGNYPARAEFVYGEWLREAFEAGESPVPEGDPEYSLVLAQARQEAIALCGPPGNEVPPEHVR
QA
MRDALPDLLDGLHGDARNVLLTLARMWHTARTGKFVTKDTAATWAMRRLPGPDAMTLDHARGAYLGELADDWQS
RAGAAR
QVAGRMAEYVRRSL
>OLS46963.1 hypothetical protein BV379_00760 [Rhodovulum sulfidophilum]AB14
MISWSVSPVLRIGVMSFSSGKEASSLFTTSEHVAKWSGQAATWGAICQDIPWMHSRQGRGIRLDVLRLMTFRFRRQP
SSW
VAAPLGGVTVIP
>OLS46964.1 hypothetical protein BV379_00765 [Rhodovulum sulfidophilum]AB14
MMREAMRALPVLGVIEIRPGGGAWVSALDASSLLSQMNVFLGLSEISVDQLCQARRLIGGELAARQAKAADLEALDPP
AA
SDASRRPWSPQ
>OLS46965.1 hypothetical protein BV379_00770 [Rhodovulum sulfidophilum]AB14

MSRRLVVETDDTLPERAAAEVSSAEFLAHQCQGPADVAKAIRSADMAVVQFAPDDDAEVITGIADGGALIRYGIGFGNID
VTAANDRGLPASHVPSCCIDEVAGPSVSAPLRKPPALAPSARGGGGDGMADTAMMRGGMTEAQLEGFRPAIPIGSQATSG
ALAAPTAFLLSTRPGTSPAPGCM
>OLS46966.1 2-hydroxyhepta-2,4-diene-1,7-dioate isomerase [Rhodovulum sulfidophilum]AB14
MKFLRYGPRGNERPGLLDAEGTIRDLSGLIGDLGGEVLGDLGRFADLDISTLPTVEGDPRIGACVAGTRKFMGIGLNYAD
HAAESGMAVPEEPVLFMKATSAICGPDDPIIIPRGSEKTDWEVELGVVIGKRAKYVSEEAALDHVAGYCVINDLSERAFQ
IERGGQWVKGKSCDNFGPAGPWLVTPDEAGDPQDLGLWLCVNGEKVQDGSTRTMVFGVAHLISYLSRFMTLEPGDIISTG
TPPGVGLGMTPPRYLKPGDRVKLGVDGLGIQRQVCVADI
>OLS46967.1 hypothetical protein BV379_00780 [Rhodovulum sulfidophilum]AB14
MEEARQITPLCPDQLALEIRQRDLRCRTTSYARIISRSASVRSAFRPVRSAESPSITQAAACVTLGAVAAAFAPADKTVS
PEIRTMQTEPVRW
>OLS46968.1 3-keto-5-aminohexanoate cleavage protein [Rhodovulum sulfidophilum]AB14
MAKPRKVIITCAVTGAIHTPSMSEFLPVTPAEIADAAIGAAKAGAAVVHLHARDPGDGRPDQSPEAFAPFLGVIKQASDC
VVNITTGGAPTMSIEERVRPAETHRPEIASLNMGSMNFGLFPMLKRFPDLKHQWERDYVGNKNIIFNNAFGQIEYILTTL
GPLGTKFEFECYDTAHLYNLKHFLDEGLVEGPLFIQTVFGLMGGIGAHPDDVMHMKRTADRLFGDRYIWSVLGAGKNQLP
IAAMAAAMGGNIRVGLEDSLWIGPGQFAQSNAEQVAKACQIIEGLGLAPATPDEAREMLALKGAERVGF
>OLS46969.1 3-hydroxyacyl-CoA dehydrogenase [Rhodovulum sulfidophilum]AB14
MTETVAIIGAGLIGRSWSALFARGGYEVRVWDPDAKVLDRFRADTETLCETLSAEGMLDDPAATLARVRVCPTLEEAVAE
AVHVQENGPERIEVKQELFARLDAACPEGAVIASSTSAIVPSRFTADLAGRARCLVAHPVNPPHLIPVVELCPAPWTAAE
ALDRAEAIYARIGQVPVRMTGERDGFVLNRLQGALLGEALRLLGEGVVGVEGLDATIRHGLGLRWALMGPIETIDLNAPD
GLVDYVARYGDFYADIVAEPLGREVFSVETASRIAASWAQDRDPQDIRRRQDRRDRRLAALRRHLASETTQTSGA
>OLS46970.1 ABC transporter [Rhodovulum sulfidophilum]AB14
MTRIHPLLIAGAALIALPFAIQAAGLTFTSATEVVVYGLACMGLNVLAGRTGLFSFGHGAWFGLGAYVAGLSSLAMGGAF
WLPLLIAVGATALIAAGFGSVMLRRSGVYFSLMTLALSALGFSIAFRWTEVTGGENGLGGIDRPSFAGLDFEMSEPYYWL
VAAIAFAMLVLLWRVNNSPLGTVLRAIRENEQRTRFLGYRVDRYKLAAFVLSASITALAGVLLLYKNRMTSAEPMSVVFS
GELLAMVVIGGMRGFLGPALGALFYILFREYLSIYTDNWLFWFGLVFVGFVLFAHDGLIGIPGQIRRHLRPPPETHAAMA
GRKAETHPLPAFLKPSDHVDGPLLVADGISKYFGGFKAVDEVSIAVRDRTLHALIGPNGAGKTTAFNLLSGLYEPSSGSL
TLAGAPLSGRTPEEVSAAGLGRSFQITNLFPSLSVKENIRLAVQAASPRRMNPLIRAEGLEEVNDQTAQILDYAGLAGME
TAEAASLSYGGQRLLDLGLALGNHPRILLADEPLAGLSVAERERIGRLLKALSADIPVLLVEHDIDRVFELADRVTVMNE
GHVLLDGTVEETRSDPRVQEVYIGSGTSAVAARPRVSAAQDKVLLSLDGVNVSYGKSHILHDVSCALRQGEILALLGRNG
AGKSTLLKALIGIAPVGSGEIRLDGTVVSGLSSAEMARSGISYVPQGRGLFAGMSVGENLELGRIKRRKGHGVHWTDERI
FSYFPRLKERIDTPADYLSGGEQQMAAVARALAGDTRVLLLDEPFEGLSPAVVEQLFETFDRLRQEVSIVIVDHNLDLAL
TLSDRTVALERGSIIHEGPSAALATDIDLRRKVLWL
>OLS46971.1 branched-chain amino acid ABC transporter permease [Rhodovulum sulfidophilum]AB14
MLGLFVAQVLNGLLDGTYYLLIALGLSLIFSLGGIINLAHGAFFAIGAYLAITIAPFAGFFGALVLVPLLVAGLGMATER
TLFTRFYRADPLYSLLLTFGLAMVIEQGLRWIFGASPQSFNMPEILRGQVFFGDFIYSRYRIFLIVVAVVTVGALWVLLN
RTAFGRIVRAGVQNPEIVGALGISLRPYLSVVAGIGIGLAGLAGVLLAPIYTIHPAMGAEIITLAFVVVVIGGLGSFWGV
IIAAVLVGLTKGLLVAVGLSSWSTAAIYLLMFVVLMLRPRGLLGERILRFE

>OLS46972.1 ABC transporter substrate-binding protein [Rhodovulum sulfidophilum]AB14
MTTTNSTRRGFLRTGAAGLALGVAAPTYLRAQSAPLKIGHLTPTTGFLGPLGEYAQMGIKLAVQHINDNGGAGGRQVE
LI
MEDSVNPQTASTKAERMFERDRVDMIIGEISSASCLTISQVAARYKKCFVNTGGNSDSLRGKDCNRYMFHVETQNSM
YVN
AEGQFFDGKGLVDGKNWYTMTADYAFGHDLLAAAKAFLDANGGNLIGDELVPTDATDFSSYLLKIRQAEPDVVALNL
AGT
QITNFLKQYGEFGLDFTLGGFGFDTVSAWAAGARNFRGTWPNVWNHLVPNDASQAFVRAFVDAYGKPPENQAWG
DYNAGL
IMARAVAEAGDAGGDALADYLESPEAQFDLMKTRKGYFRPSDHQLIQEIYAITALPADEVQNKWDIFTTSDPLPGPDQ
PL
ETLATSVTGGTCSL
>OLS46973.1 hypothetical protein BV379_00810 [Rhodovulum sulfidophilum]AB14
MTQPLDRIRPVRRKTLSDQAFDTLSEMLLSGALRPRDRLSMRDLADRLEVSMMPVREAVSRLAASGALEVSPKRAVM
VPL
MTAAEFADLTLVRMLNEGQAARLAAERSSKTEIAGIIALAERFEEVLDRAYGSAAAVAANKDLHFAVYRAAGSETLMQL
I
TMLWLKAGPIINFDIGVEAGLPAEQQSAHSRGHHSRRHHRMLCEALLARDGDTAAQAISEDIRFASEFIRAHAGMDQ
QDS
RRRIIS
>OLS46974.1 3-oxoacyl-[acyl-carrier-protein] reductase [Rhodovulum sulfidophilum]AB14
MKLDLEGARVVVTAGAQGIGRAIVEAYLAEGARVAACDIDAAALETLPAAVFRQRTDMGDAGQIADFMTAALDHLG
GLDV
LVNNAGIAGPTGPVEDIDPADWETCLSVCLTSQFHCVRHAVPALRQSANASIINLSSLAGRVGFALRTPYSAAKWGVIG
F
TKSLAIELGRDGIRCNAILPGIVAGDRQRRVLEAKAQRRGIAFAQVEEEAFSHTSLKEYVQPEQIADQILFLTSPRGRTV
SGQAVSVCGDCQMLT
>OLS46975.1 hypothetical protein BV379_00820 [Rhodovulum sulfidophilum]AB14
MESFNGRLRDECLSEHLFANLRHARDLISAWRDDYNHHRPHTSLDGLTPWEYHQRSVEGQSLNRANQRTRTLLGAGH
RAR
WIRVLRRAERRSTAEGIRPCRRTSGSARAPVRSASGSRRRPIPPVRKPCAPAGWSGTGSDRRSVRAEHTP
>OLS46976.1 hypothetical protein BV379_00845 [Rhodovulum sulfidophilum]AB14
MKVQATSPSTRTKSLGSSVFVRIAGLIALTIAIVAALLITLSVRSTLQLVDRSVDRLMTQMTQASSEALGGHIAFRNEAE
IEAEMAQYVDGAGEELVLVEVTAADGTVLAETRDDRSADRGLLEVLSGRAIKSGAVVYSDDGLTIAVPVRYGKSGRVVG
A
FAATWSQKAMITHFLEEMAPAMAMIAVLFLLLLVGATLLLRRMIGRPLDEVSDAMAMVADGVLDRQVPDTERKDEIG
VIA
RSLEALRGTLLDAKHAEAERDIDLTDQKRAVDLLGRGLTALASGDLTFTIEQEFAGGRDQLRSDYNATVQTLNALMGAV
V
ENATEITTRSEEISGASDNLSRRTENQAATLEETAAALDEMTTSVRAAADSAAQVETVVRDARGNAEQSGRVVKEAIGA
M
SEIKNSSDGIGQIIGVIDDIAFQTNLLALNAGVEAARAGEAGRGFAVVASEVRALAQRSSEAAKEIKTLISKSSDQVESG
VALVNRAGDALTDIVDRVGNIAELISGIASGAQGQSAGLGEINVGMSELDKVTQQNAAMVEEVTAAATTLKQEAQSL
QTQ
VARFQLQSGPSGGGSRRAERMSVRDASVASDRSPAPRAPQKRAVKDAGWQDF
>OLS46977.1 hypothetical protein BV379_00850 [Rhodovulum sulfidophilum]AB14
MLALRVVCLALLGGGYACVAQAGEFDSQMRAFYEASVTSWSGAPQILNAVAAQNARTADMAQDDIDRLDRQWVAE
IGAVS

EPLIESVTRNPVSDFLRDQLMALEGAVTEIIVMDARGLNVATSSVPSDYWQGDEAKYQKTFGVGPDGMHFGEVEFDE
SSM
KYQAQISFTLVDPASGRPIGAMTVAVDAEEFL
>OLS46978.1 hypothetical protein BV379_00860 [Rhodovulum sulfidophilum]AB14
MPDARPRLKPEDWTVNRWGRHAKTADAATLGIEGWQPIPRRNRKGVIVQDAVRFTPCVWDPTPARIAAARRAYLD
WWGYL
LAMQAALMGADLIRIKVTRDLPPMRPWNQI
>OLS46979.1 hypothetical protein BV379_00865 [Rhodovulum sulfidophilum]AB14
MAAWLLLGDPEAGQPPAEPRICPGCGELVTDGLGCGGGICRASDAFPKPVEDPADKAFREAVAELVDCAMREGKVD
G
>OLS46980.1 hypothetical protein BV379_00870 [Rhodovulum sulfidophilum]AB14
MGENSKIEWCAHTFNPWIGCTKISPACDNCYAAAWDARWGNSWGPHVRRRTSAANWRKPLAWDKAAAAAGERH
RVFCASL
ADVFDNDRSITSGWRGDLWHLFHRTPNLDWLLLTKRTQNIARYLRPE
>OLS46981.1 hypothetical protein BV379_00875 [Rhodovulum sulfidophilum]AB14
MPKWGDGWPNVWLGTTVENQEEADRRIPQLLAMPAAVRFVSAEPLLEPVDLTPHLCPKQWSCDFGAESGGGSARG
TIPKL
DWVIVGGESGPRARPMHPDWVRGLRDQCQAWPSSSSSGRNGGRQNLTRYQLSHA
>OLS46982.1 hypothetical protein BV379_00880 [Rhodovulum sulfidophilum]AB14
MSRTLGYCLLLGSYDAWIGFSCLAKSLLTARERASLAYAALRSLEPEQAEMTAATVLRAAGAPMPPFLRGMEEARFWA
SL
ANRAELKAFALASFEAMAPQDQAAFFRHISEIEVAA
>OLS46983.1 hypothetical protein BV379_00885 [Rhodovulum sulfidophilum]AB14
MKMLVHRQSDSTELPFVMNDLLKFVALSEDDADFFSELEPIGKAAAEEIEKFAQIALLNQTVRVTIFDPVKEYGLSLPIG
PVAEDKTPIVTIDGEVFTGFDFVGGNRPYIRWFAKFYDLTPSRMTIEYEAGFGDTAADIPADLYQALMDQAALHFGWR
SP
LDAKSLTNSPHMARIAARYRGVQV
>OLS46984.1 hypothetical protein BV379_00890 [Rhodovulum sulfidophilum]AB14
MRPHEVDFLFYAMTRVARLGRIEADRAFARKMADAAQDRRFKPSRQQIERMTEVEARLYREGGAA
>OLS46985.1 hypothetical protein BV379_00895 [Rhodovulum sulfidophilum]AB14
MTEQELDEILTLRWPLVMRRVMADGSDAWLKGFVRSVARHGRRPGWRPSFKQEQIMRRLLAEIGRPPKPGRPPEPD
FELI
EE
>OLS46986.1 endonuclease [Rhodovulum sulfidophilum]AB14
MSVRRDHHRFSKKVTRTKRWQALRAEILERDRYRCRSCGCGGRLEVDHIKPVRTHPELSYDPGNLQALCPSCHTRKTRI
E
CGHPPPRKDRQDWLSMVASIERPTKPKPQIEQE
>OLS46987.1 major capsid protein [Rhodovulum sulfidophilum]AB14
MLESVKIARRQSEIRQSLAELAGKDSPSEDEIRSMDELDREYRQNETRYRAALIAEDTERREAGADLETRSAQDWANLM
A
GFELRQVALALDEGRQLDGQTAEIVSELRSAGGFRGIPVPWQALEARAGETVSADTPSATQLRPIIDRLFPDSVAARMG
A
QMLSIESGAVEWPVTTSAVSAGWADGEGGTVAGPTAFTTANRAMKPDHNLGVQMRITRKTLKQSGAALEQAVRRD
MSGAM
GMELDRAAFLGTGEGGQPLGVIAGAAAYGIRQTAVGAVASWGAFRSAVTRFMTANTAGSPGAVRALIRPELWDFLD
GALI
EGTAVSEWDRLVKNIPAGNIAMTHNALAAPSGNPLATASLLTTAAGGVAPIFIGAWGAVDMIRDPFSDAQSGGLRITA
LA

TMDVTVARPAQLEVLTGLKLGGA
>OLS46988.1 hypothetical protein BV379_00915 [Rhodovulum sulfidophilum]AB14
MLLGATLGALELRSEGGTTRLRACFPYGAETELAAGRREIIAPRAFADRIERGEDIHLLAGHSYDRPLASRGSGSLTLRD
SDEGVELVAEIDPGTSWARDFLAAHAAGLIRGLSPGFRVQPGGERIERRGADLVRTVTSAAVFEFSAVTRPAYQQAQIE
A
RNWQPVGEVIRRVLDRPAACRWR
>OLS46989.1 hypothetical protein BV379_00920 [Rhodovulum sulfidophilum]AB14
MFGWIKDRLRPVEERSSGAGYTAQVMAARDSYISGQRGVAELTATVQSCVSLWEGGFAMADVAGTGLLTRQTMARI
ARSA
ALRGEAVYLIGDEGLVPVTDWDLTTRLGRPRAYRLSLPEAGGGRSLTALAAEVLHLRIGSDALTPWIGTAPLRRASLTGA
MLHAVEASLAETFENAPLGSLIVPLPDTGSEDMATMRSAFQGRRGSTVVVEGVAQATAAGMNPQLGQKPDQLSPDL
SKSM
TAETLAAAREGILMAYGVLPSLLNRAATGPVVREAQRQLAIWTLQPVAQLLAEEASEKLGQPVEIDTIRPLQAFDAGGR
A
RALSAIIKTLAEAKAAGIPPGDVSGAMRMVDWKE
>OLS46990.1 hypothetical protein BV379_00925 [Rhodovulum sulfidophilum]AB14
MKIRDVKKNIKKISDRIYNLEIEWEQKGTLPGETDVDIESLFDPEADKKREEIYEAAIEEAKLDKYYWTTIYIEMELAKF
EYPLPDREAHPEKWWACFPGGYCLTPKAIEEAFTILRQEKIQAHKMMMSRLSMVIGVIGASTGLLSVLIQSWINFKN
>OLS46991.1 transcriptional regulator [Rhodovulum sulfidophilum]AB14
MTPSQCRAARALVAMSQGRLAEASGVAKRTIASFEKEERQPYERTISALRYALEAAGVDFIPGNGSGVGVRLKK
>OLS46992.1 hypothetical protein BV379_00935 [Rhodovulum sulfidophilum]AB14
MARPKHDNPADFIVSEVMAATGFDRRTVQLIRDRRAGPFGNVGVMERAKTGLYDESTVSELAMASGVHHAGFPLLQ
ALAI
VRAYLDENSNHGPAVFSNLDEFEDPRRSRETLSWFHAACKTREARGGKDSGAPVSGDALLVIADRKYVLEDHHQPRFG
RP
AVPKGVDDTLPLPLCRIVSFDGNQADVVSVPDDFPAGPDFDFREAYEVYHRAYLRAVGLIRVNISLAIRNAFDRVQDMR
I
ERGGPMFGTGGE
>OLS46993.1 hypothetical protein BV379_00940 [Rhodovulum sulfidophilum]AB14
MGNANLNISVIEKRMLNQTESAAYTGMPVKHFKTTCPVQPLEMGRGIRCWDKRDLDRWIDDMKQGTEMTTRDAIL
GKL
>OLS46994.1 hypothetical protein BV379_00945 [Rhodovulum sulfidophilum]AB14
MTRVRVKGFKIFEDRHGKPRCYHRTTGHKIDLEKTPLGSAGFFAECARIEAIAQARKVQEPKPGTLGGLINVYFETEHFQ
NRAAATKRDYRKCADFLYPIRDTPVSAINTPLVAGIHDKAAQKIGWRRANMVRTFLGQVFRFAIPKGLIDRDYSSGVIPK
PRPKDRPYANRPWTVAERAVVLERAADHVRVTIALIMNTGLDPSDALKLMRNQIEGNTIWGVRGKTGEEIAIPIGPTLQ
K
ALDAAPGHDAVTILANSKGKPWTYDGFSTVWHRLKKQLEAEGVVQPGLTLKGLRHTVATTLREAGMEERQIADLLGQ
KTP
AMARHYSRSANLAERNRLTLEVLEKENERRSETVKPLKKSVKP
>OLS46995.1 DNA gyrase inhibitor YacG [Rhodovulum sulfidophilum]AB14
MTCPVCKKPTDPAYRPFCSRRCADVDLGRWMTGGYALPGDPAEPTEPDDEPPPSPSWRH
>OLS46996.1 ribonuclease G [Rhodovulum sulfidophilum]AB14
MNGRSILLDEIGGRKAAALLEDGRLADLLIDPPDERPMPGAILRGVVDRQMKGQGGVFLRLPDGARAFLKQARGLRPG
QG
LLVQVTGYAEESKAVPVTPRFLFKSRYAILTPEAPGLNVSRAIRDEDARDRLLELLHDGMAGAPDGLGAILRSAAELADP
EAVAEDIAAQLALATAILAEPADGAPELFLDGPDAHTLAWRDWPGPDELDDSAGCFAARGVLEAIDALSGPEALPGGG
SV

HVEPTRALTAVDVNTGSDSSPAAGLKANIAAARALPRLLRLRGLAGQVVVDFAPMPKKDRVALEQVMKAAFRADAVE
TTF
VGWTPLGHAELTRKRERLPLTETLPR
>OLS46997.1 septum formation protein Maf [Rhodovulum sulfidophilum]AB14
MRLILGSQSPRRKQLLAQIGVIPDDVRPADIDETPHRGELPRPYCIRLAAEKARAVVAAGDELVLSADTTVALGRRILGK
PADAGEAAAFLYAMSGRRHRVVTAVALRLGDRIWERDVVTQVRMKQLTDAEINAYLASGEWQGKAGGYGIQGRAG
AFVPW
IGGSFTAVVGLPLAETATLLRAAGYPLDAERI
>OLS46998.1 translation initiation factor IF-1 [Rhodovulum sulfidophilum]AB14
MAKEEMLEFPGVVKELLPNATFRVELENGHEIIAHTAGKMRKNRIRVLAGDRVQVEMTPYDLTKGRINYRFK
>OLS46999.1 GNAT family N-acetyltransferase [Rhodovulum sulfidophilum]AB14
MSLGIRTLTGAGIEAALDDLARLRIAVFRDWPYLYDGDRDYERRYMASYRDNPRAILVGAFAGEQLVGAATGTPMEDH
AE
GFAGAFAGSGLDLSDIFYCAESVLLPDWRGQGAGHAFFEAREAQGRRLGRRYSAFCSVIRPEDHPRRPEGYRPLDGFW
RK
RGYAPLPGVVAEFDWKDIDDDAPTRHRLQFWIREL
>OLS47000.1 isopropylmalate/homocitrate/citramalate synthase [Rhodovulum sulfidophilum]AB14
MTRDVIRDTIARYFAAFNAGDTEAMLAELTEDVEHHVNEGRIRRGKALFSEFCAHMSRCYRESLTDLVIFTGDEGDRAA
A
EFVVNGTYLETDAGLPEARGQSYRLPAGSFFTLRQGRIARITTYYNLADWLGQVR
>OLS47001.1 low molecular weight phosphatase family protein [Rhodovulum sulfidophilum]AB14
MTGQLPSSVLFCCDHNSVRSPMAEGLMKKFYGQRAYVQSVGVHNDREIDGFTVSVCDEMGVKLDRHRVRSFDEME
DWGDD
LSGFDLIIALSPASLRRAQELTRYYHLEVEYWPVLDPTGLGETRPDKLAAYRQTRDQIVDRLTGRFGPPRRGDAP
>OLS47002.1 histidinol dehydrogenase [Rhodovulum sulfidophilum]AB14
MPVFLDTTSADFEAQFSALLGAKREDSPDVDDAVAAIIADVKARGDAAVIELTAKFDRLELTPETLAFTSDEIDAACAAV
SREERAALELAAERIRAYHVRQMPENAQWTDPEGATLGWRWTPVSAAGLYVPGGLASYPSSVLMNAIPAQVAGVER
LVIC
APTPDGKSNPLVLMAARLSGVEKVYRIGGAQAIAAMAHGTETIAPVDKITGPGNAYVAAAKRRVFGKVGIDMIAGPSEI
L
VIADRDNDPDWIAIDLLSQAEHDESAQALLLTDDAEFGRAVAAAVERRLETLERRAIAGASWRDFGAVITVRDLDEAAC
L
SNRVAPEHLELCVADPDALARKITHAGAIFLGQWTPEAIGDYIGGPNHVLPTARSARFSSGLGVHDFIKRTTLAKMTPQ
A
LKAIGPAAERLAISESLEAHGLSIRTRLDRLNEDAP
>OLS47003.1 hypothetical protein BV379_01005 [Rhodovulum sulfidophilum]AB14
MTEDARFEDGAEAPLHLMAVTEADLEVISTLAQDAVLPATEMRWDRPRRRFAALVNRFRWEDRDAAERRGRPFERV
QAVL
TVEDVTHVSSQGIDRTDRDLVLSLLSVGFEPGADGTGRVVLTLAGDGAVALDVECLEVTLTDVTRPYAAPSGKAPRHS
>OLS47004.1 UDP-N-acetylglucosamine 1-carboxyvinyltransferase [Rhodovulum sulfidophilum]AB14
MDQIMIRGGAPLTGEIPIAGAKNACLALMPATLLTDEPLTLTNAPRLSDIRTMTTLLQSLGAEVSALHRGKVLALSSHRL
TSHRADYDIVRKMRASILVLGPLLARHGRAEVSLPGGCAIGARPVDLHLRALEAMGAQLELREGYVHAEAPGGLKGAVF
E
FPKVSVGATENALMAATLARGTTVLKNAAREPEIVDLARCLRAMGARIEGEGSDTITIEGVTALGGATHRVVTDRIELGT
YMLAPAITGGDVTCLGGRIELLPAFAETLDAAGITVEETGRGLRVARKNGRVSAVDVTTEPFPGFPTDLQAQMMALLCT
A
EGTSRLEETIFENRFMHAPELIRMGARIDVSGGTATVTGVERLRGAPVMATDLRASVSLILAGLAAEGETVVSRVYHLDR
GYEKVEQKLSACGAHIERMSAE

>OLS47005.1 chemotaxis protein MotB [Rhodovulum sulfidophilum]AB14
MAVGTNAPVIIKRKKVVGGDGHHGGAWKVAYADFVTAMMAFFLLMWLLNATTEKQRKGIADYFNPTIPVNRVSGG
GEGAF
GGDSIFSEDTLVQQGTGATRPSPSPDRQAKGEIGSGPQVQKDDEGGSSANAEAEIQALQQVAEMLNGKSGETLLSEQ
MMR
HIVTRLTDEGLVIELFDIEGVPLFDDRDRPTEILDELMEVIGAAFSIVRNKVSVAGHVSARPVVLVDNPVWDISTLHAQI
ARKMLEDIGVAPARIERVTGYADRKPAISRPMAVRNNRLELTLLRSDLRN
>OLS47006.1 flagellar biosynthesis protein FlgE [Rhodovulum sulfidophilum]AB14
MTISSSLNASVAGLSANATRLATISDNIANSSTYGYKTANADFHSMVINSSRGTYSAGGVRTTTTRMIDQPGALLTTANA
TDLAVRGRGFLPVMTQSELISKSGNYTMYMTTTGSFRTDEDGYLKTSTGLVLMGWPADADGSIPSYSRDTTDDLVPIQI
Q
TNQFATEPTTEMTIGVNLPATETADTASGTALPLSVEYFDNLGTSQSIEISYTPDTSAPGLSNSWNMTLTDSATGAVVG
D
YDLVFSDTQQYGGTLESVAANSGGAWDPATGILTVQVGPNGDTMSINIGAQHSRSVTVQLSDSYGPTTISKNGFPVG
NMT
SIEIDANGMVRANFDTGITRTLYQVPIVDVSNPNGLYALDNQCYSPSDTSGSFFMWDAGDGPTGDVVSFALEESATDV
AG
ELTELIQTQRAYSSNAKVIQTVDEMLQETTNIKR
>OLS47007.1 flagellar hook-associated protein FlgK [Rhodovulum sulfidophilum]AB14
MSLSASLANALSGLNAASRGAQVVSSNVSNALTEGYGRRVLETTPNTVSTSGSGVKVVGVRRDVNHQLIADRRLADAA
SA
EAETRADGLSKLEAAFGISDSADSISGAISAFESALVAAASRPDSDARLADVLATTQTLAGRINEAADDIQQSRMDAEAG
IVSNVQILNETLQQVSELNTEIRKQRSYNNDASALVDQRQILIDKIAGIVPVREIARDNGVVALYTENGAALIDGPPAEF
EFVSAGVIVPEMTVEGGALSGLTMNGRPVRVSGTYAAMGGGSLEALFGQRDIAAVDAQTDLDALARDLIERFQDPAA
DAS
LGPGDAGLFTNFGAAFDLVSDPDAETGLSTRIGVNLLADPDQGGALWRLRSGLMAGASGPVGDASLLNSMGDALTAS
RTP
SSGSYTTQAFTMADLASEVVSGISVRAQTAIEQSSFSSARASALSEAELAEGVDTDYELQMLMLFEEAYAANAKVMKTI
D
ELINMLLEI
>OLS47008.1 hypothetical protein BV379_01035 [Rhodovulum sulfidophilum]AB14
MSLISMGDLAQGLGLRIRNAEVKAQFTRLTEELVSGVTADPAARVRGDFRPLGAIDRSLTLLDAYDLATSETKSTAQVM
Q
AALGVVSDTAKEIGSSLINGSSGQHGNMIAAAGQEAEEQFRQVVAALNTRFAGRSLFAGEATDSPALSSADTILSALGTT
VSGLTSVTDIETAIDDWFNTPGGFDTVAYQGSTASVGPVRLSETETVRLDITALDPAVRDVLKSLATGALLADETVLSGN
PVLRSLLAESAGEGLLTARSSVTELQALLGVRQEKIETIETENGANRKMLVLAREGVLGVDPYETVTEMDAVEAQIETLY
KVTARLARLSFTNYMP
>OLS47009.1 flagellar biosynthesis protein FlgA [Rhodovulum sulfidophilum]AB14
MIRPLTLLLAVLLVAFPAFAAPIRIKDLVEFDGVRGNDLVGYGLVVGLNGTGDGIRNAPFTEEIMSNILERLGVNVTGEQ
FRPKNVAAVLVTASLPPFARSGSQIDVTVSAIGDAKSLLGGTLVMTPLNAADGQIYAVSQGTVIAGGASAEGDGARVVE
G
VPTSGVIPSGARVEREIDFDFSSLRRLRLALRNPDFTTAGRIEAAINNDYGRGVARMLDSGTVMLDIAATRAPSPAHALQ
RIEGIMVEPQQKARVVVDQRSGTIVMGEDVRISRVAVSQGNLTLRVQEEPLVIQPNPFAQGQTVVVPRTRAAIEEEPG
TG
LAEINEGTSLSEVVAGLNALGVSPRDMIDILKSIKAAGALHADFLVR
>OLS47010.1 heme biosynthesis protein HemY [Rhodovulum sulfidophilum]AB14
MLWSLLKILFFVVLVAALAYGAGLLMETGSGLRVSVADMEFVLGPVQAVILALLTLVGVWLLLRVAGFLVALVRFLNGD
E

TAISRYFSRSRERRGFQALADGMLALASGEARLAMSNAAKAERFLRRPDLTNLLSAQAAEMLGDKGRARDIYKKLLGDD
R
TRFVGVRGLMKQKLAEGDSETALKLAEKAFALKPRHAETQTALLQLQASAEDWKGARKTLNAKLRHGNIPRDLHRRRD
AI
LALCEARDLMVDGDEPRARDLAMESNKLSPDLVPAAVMAARAYNVQGRDKQAVRVLRRAWEAQPHPDLAAAFAEL
VPEET
PQERIQRFVALTEVRPDDPETRMLSAELAIAAEDFPAARKALGDLAERAPTARALALMAAIERGLGADDEVVRGYLAKAI
GAPQGPQWVCESCGTVHSAWTPVCGHCSGFDTLAWKPAAAGPVGLGGASDMLPLLVGGGFQKSAE
>OLS47011.1 hypothetical protein BV379_01050 [Rhodovulum sulfidophilum]AB14
MAKTEDETDSEDTRTSQPEGGGETGEGRLETAIRTARDRDETDEDVNTEPPRDETPTEDDTPVEEVSETGPSEAAVSPA
P
AAVPPKRGLVGAVAPVLGGVLAAGIGFATAQYIQPEGWSFPGTSAGIGDKGMAVLTETGARLDRIEAALAERPSGTGA
DD
SLAPQISALQEQIGKIADQLAGIDARVTELAEAPAAAASGEAAAALKAYEDQIAQMREQNAELSASVQEVAQKAQADIG
Q
AMDRAAQVEARAALMRIDAALADGAPFDSALGQFGAIEVPEALTAVAGKGVETLPELQDSFPAAARDALETALKEQA
AGG
VGDKFAAFLRSQLGVRSLTPKEGDDADAVLSRAEAALREGDLRAALSELDGLDDGARAEMAGWIDAARARADAVEAA
QSL
EQTLNSN
>OLS47012.1 hypothetical protein BV379_01055 [Rhodovulum sulfidophilum]AB14
MTQHPPLLLLTRPRVQSARFAGDFVKRFGSGIAIRTAPVLDIVPRPDPVSLDGFAGLLFTSENGVAALAAVNERRDLPAY
CVGERTADAARSVGFRATAAEGTAASMIAHFSADPPKGPLLHLRGEHARVELAEALSAIGIPTEERIVYAQEALPFPSGI
AAEIEAAPLTLLPLFSPRSALLVAEWLRGLGGRFALVTMSAAVTASWDGPRPEILIEAERPDAPAMMDGLAAVMGAVG
VP
>OLS47013.1 tRNA (adenosine(37)-N6)-threonylcarbamoyltransferase complex transferase subunit TsaD [Rhodovulum sulfidophilum]AB14
MAETLTILGLESSCDDTAAALVRQNADGRPEILASVVEGQTALHAAYGGVVPEIAARAHAERLDLCVERAMAEAGLGFP
D
LDAVAVTAGPGLIGGVLSGVMVAKGIAAATGLPLVGVNHLAGHALTPRLTDGLDFPYLMLLASGGHCQFLAVSGPEDF
VR
IGGTIDDAPGEAFDKTAKLLALPQPGGPAVERTAETGDPARFAFPRPLMDRPGCDMSFSGLKTALLRARDRIIAEKGGIT
VQDRADLCAGFQAAVRDVLAGKAARALAACPEATALAVAGGVAANRAIRAALEDLASASGLPFVAPPLKLCTDNAAMI
AW
AGLERFRLGARDDMALAARPRWPLDSRSPALIGAGKKGAKA
>OLS47014.1 glycerol-3-phosphate dehydrogenase [Rhodovulum sulfidophilum]AB14
MTGIGIAGAGAFGTALGVVLAQSGRKATIWAHQAAFADRIRNSNENPRLPGVALPDNLSVTSEIADIAECDLVLLTTPM
Q
ALASVLEAHGPVLKARHMVACCKGVDLATGRGPSAIIAESCTGVVPAVLTGPSFAADIARGLPTALTLACADAKAGIALQ
ERLSTPTLRLYRSSDTVGAELGGALKNVMAIAAGIVIGTGLGESARAALITRGFAEMQRFAQSYGAEPETLAGLSGFGDL
VLTCTSQQSRNFGYGVALGRNAWFDPEITVEGAATARAVAAIARARGIDMPVTQSVAQVIDGHRSIEEAVKILLSRPLK
E
E
>OLS47015.1 hypothetical protein BV379_01070 [Rhodovulum sulfidophilum]AB14
MYFALICRDKPGHLEIRKANREAHLAYVAETGVVTQAGPFLDEAGEMCGSLVILDVEDKAAAEAWAANDPYAGAGLFE
SV
SVQAWKKVVG
>OLS47016.1 ubiquinol-cytochrome C reductase [Rhodovulum sulfidophilum]AB14

MAFWLFKSEPNTWGWDDQVAKGAAGEEWDGVRNYQARNNMRAMKIGDRGFFYHSLSEKAVVGIVEVIAEAHPDS
KADDPR
WDCVDIKAVAPLPRPVTLDMCKAEPRLADMVLVNNSRLSVQPVTEEEWRIVCEMGGL
>OLS47017.1 hypothetical protein BV379_01085 [Rhodovulum sulfidophilum]AB14
MGKRDALIEKYAQDLRTKCGMEPDMDLLRKVTIGCGPAIYDADAELVAAHQPGEIETVRTNFLIRKLGLPDSPDLTEAIH
AAIETYGVSNPHKYRAVLYYMLTRHFGKEHIYG
>OLS47018.1 tryptophan synthase subunit beta [Rhodovulum sulfidophilum]AB14
MTYLKTTPTRDGFFGSYGGAMLPPPLEPHFAEIREAYDRISKSADFIRELRYIRTHFQGRPTPVSYLRNLSELCGGAQIY
AKREDLNHTGAHKLNHCMGEGLLARFMGKTKLMAETGAGQHGVALATAAAYFGMECEIHMGEIDIAKEAPNVTRM
KLLGA
EVVPVGFGGRSLKEAVDSCFESYIAQADHALFAIGSVVGPHPFPMMVRDFQHVVGVEARAQFLDMTGELPDMVAAC
VGGG
SNAMGIFSGFLEDDVALYGVEPAGTSSNLGDHAASIAFGEDGDIHGFRTIVLKDANGEPAPVHTVASGLDYPGVGPEH
AH
LHRTGRVTYTTADDKEALNAFFELSRREGIIPALESAHAVAFAMREARANPGKSILVNMSGRGDKDIDYVTETFGFGEAL
>OLS47019.1 adenosylhomocysteinase [Rhodovulum sulfidophilum]AB14
MAPDYIVKDIALAGFGRKELDIAETEMPGLMALREEYGEAKPLKGARIAGSLHMTVQTAVLIETLVALGADVRWASCNI
F
STQDHAAAAIAEAGVPVFAIKGETLPEYWDYADRIFQFAEGTANMILDDGGDATLYILLGARVENGEEDLIAVPTSEEEE
ALFAQIRKRMAETPGWFTKQRDAIQGVSEETTTGVHRLYDLHKNGLLPFPAFNVNDSVTKSKFDNKYGCKESLVDGIRR
A
TDVMMAGKVAVVCGYGDVGKGSAASLRGAGARVKVTEVDPICALQAAMDGFEVVVLEDVVSSADIFITTTGNRDIIRI
EH
MREMKDMAIVGNIGHFDNEIQVAALKNHKWTNIKDQVDMIEMPSGARIILLSEGRLLNLGNATGHPSFVMSASFTNQ
VLA
QIELFTKGEQYENQVYVLPKHLDEKVARLHLDRIGVKLSTLTPEQAAYIGVTPEGPFKPEHYRY
>OLS47020.1 hypothetical protein BV379_01100 [Rhodovulum sulfidophilum]AB14
MKQARAWQRMLSGRRLDLLDPTPVDIEIEDIAHGLAFVARWNGQTRGDYPYSVAEHSLLVEEIFCRIQPEAPVRWRLA
AL
LHDAPEYVIGDMISPVKAAVGPSYGELDKRLASAIHIRFGLPAAVPKTVKARIKRADSLSAWLEATQIAGFAPAEANRFF
GRPRPDLIEGLEIRLRPPVEVRTAYTARHAALMARL
>OLS47021.1 DNA-binding response regulator [Rhodovulum sulfidophilum]AB14
MAEQEYEIGEDPSLLIVDDDEPFLRRLARAMEKRGFQPEMAETVAAGKAIASARPPAYAVVDLRLEDGTGLDVVETLRE
K
RPDAKIVVLTGYGAIATAVAAVKVGATDYLSKPADANDVTAALLSNGEALPPPPENPMSADRVRWEHIQRVYEQCDR
NVS
ETARRLNMHRRTLQRILAKRSPR
>OLS47022.1 SCO family protein [Rhodovulum sulfidophilum]AB14
MKRIVLAVAAAVALVAVAGPALFIWGPWAPEDRFADCRSGGPRPGLSELGGPFTLTDQTGTIMTEADVIAGPTLIYFGY
T
FCPDVCPFDNARNADAVDLLEGRGYEVRPVFISVDPARDTPEALAAFASMMHPRMLALTGTPEQVQAASRAYRTFYRI
RD
PDDAYYLIDHSTFTYLAFPGAGVVEAFERDTTPEDMAARVACFVDRARIRP
>OLS47023.1 two-component sensor histidine kinase [Rhodovulum sulfidophilum]AB14
MSDIATGLLPPTERSNWIRLRTLILLRWTAIAGQVVAIAVANLYFQIQLDLGLCLLVIGASVLANVIAVAIHPENRRLSE
KEAMLTLLFDVVQLSALLFLTGGLNNPFALMLLTPVTISATALGRMSTLFIAHTAFALITMMTFYYVPLVDASGEIQQLP
HLFLFGFWAALVIGISFLGLYADRITSEMNKMSDALLATQMALAREQKLTDLGGVVAAAAHELGTPLATIKLISSELMDE
VDDKPGLREDIETLREQADRCRDILQSMGRAGKDDLHMHTAPLKAVIDEAAEPHLDRGKEIIFSFEPIHGGSPRMPVIYR

RPEVIHGLRNLIQNAVDFAESRVWVMGQWTQGEISVRIMDDGPGYPPHLLGRIGDPFVRLRKSPEQQKKRPEYEGMG
LGL
FIAKTMLERSGATLSFANGRDPMLTSERSDQRGAVVEVEWPASKIGVSAEAQLAALGENTLIEA
>OLS47024.1 hypothetical protein BV379_01125 [Rhodovulum sulfidophilum]AB14
MDIDWGFALVLSGISLTSAVAAVFVLPFLPRRARNATAETQGDMVLLFDGEHLVDATPDGRRFLAGGPKGLTDWQRF
CAL
IVPRFPGFPERMERLPKDGSALLAEAGGEAQLTAVWLDGVIRLTLQMPRFSDPAGGLTDAQSLAALTAELETLRDVVEA
M
PTPAWQRDEKGGVAWANSAYLDIVRGRHDDGQIVWPLPVLFSQDQGETGSPRRQAIDIGAERDRWFDCVSVETHG
ASLHF
ALPADATVRAETALRNFVQTLTKTFAELPIGLAVFNRTRELVLFNPAMSDLFNLEPEFLTGRPTLLNLLDRLRETRMMPE
PKNFRSWREQILRLERDACDGVYSDTWTLPGGQVYRVTGRPHPDGAVAFLFEDISTEMSMTRRFRSEIETGQAVIDSLP
E
AMVVFSSAGILTLSNSAYAELWGEDPRLVMGTTDIADATAQWRAQCLPSPSEAGAELPRRIRQREPWSCELVRRDGQ
RLD
CHLKPIAGGCMLVEFRPSARTEASDTPAVSRASSG
>OLS47025.1 tRNA (adenosine(37)-N6)-threonylcarbamoyltransferase complex ATPase subunit type 1
TsaE [Rhodovulum sulfidophilum]AB14
MSGTRLSLALASEAQTRALAERLAARLGAGDTLLLDGPIGAGKTAFARALIQAAQARAGRDPEDVPSPTFTLVQTYEAG
P
LEIWHADLYRLGHPQEVMELGLDEAFETALCLIEWPDRLGDLAPAGALRLTLAPGEAETLRHASFVSDRADWTGRLSPIL
TEIQTGTAPDA
>OLS47026.1 aminoglycoside phosphotransferase [Rhodovulum sulfidophilum]AB14
MRDAAIADFLARAGWAEARRMSLAGDASNRRYDRLARDNGETAVLMDADPATGEDVRPFLAIARHLSSLGFSAPAIL
AED
SARGLLLLEDLGDDLFARLAARAPQLEEPLYAAATDLLARLHRAPLPEGLTPYGSAEMAELGCLPFRWYLPGQGLAAPEE
EIAAVRAGLETLLAEAAPPSVLILRDYHAENLLWLPERDGVARVGLLDFQDARIGHPAYDLVSLLQDARRDVSPEVGAR
M
RARYVAATGTDAAAFDRACAVAGAQRNLRIIGVFARLCLRDGKPGYLSMLPRVWVHLMRDLDHPALAGLRKTLLRLLP
PP
EPDRLDRIASRCAPRR
>OLS47027.1 nucleotidyltransferase [Rhodovulum sulfidophilum]AB14
MRPASLMIFGAGFGTRMRPLTDDRPKPLIEVASRPLIDHALALAPDAGIRRIAVNAHYRADQLAAHLADRPGLTVLRET
D
ALLDTGGGLRNALPVLGEGPVFTLNSDAVWTGPNPLCTLTNAWDPAQMDGLLLLIPPGHATGHSGKGDFLIGPYGRLR
RG
PGAIYSGAGILKTDGLAEIPERIFSLNRLWDGMLSKGRLYGILHSGGWCDVGHPGGIAEAEALLAGQGDA
>OLS47028.1 double-strand break repair protein AddB [Rhodovulum sulfidophilum]AB14
MLEGPAPRLFGLPPGADFPRELLIGLEQRLSGAPPEAWAEVEIWVNTERMRRRIVDLFQAGPPRLLPRIRLVTDLARHPI
LADLPAPVSPLRRRLELGQLVRALLDRSPDLAARSSAFDLADSLAALMDEMQAEGVPPRALHDLDVGNLSAHWARAR
QFV
TLVETWLGPDLSGRPGSGARQRLAVERLTERWRADPPRHPVIVAGTTGSRGATALFLREAARLPQGAAILPGYDFDMP
AA
IWDGLDDALAAEDHPQYRFRHILNDMGLGPGDVRPWCREAAPPCPERTRLVSLALRPAPVTDQWMEDGPRLTGLDR
ATDA
LTLIEAPSPRAEALAIALRLRRAAEDGQSAALVSPDRTLTRQVTAALDRWGIRPDDSAGTPLPLSPPGRFLRQVAALFGR
ALTVEALLSLLKHPLTASSPGLRANHLRWTRELELHLRRNGPAFPDGPALTRWAEGHEDGREAWAAWLAEALDGTDR
AGT

EPLSTHVARHRQLAETLAAGPETEGSGALWLEEAGLQARAALDLLEAEAEAGGEMSPADFSTLLDSVLRDGSVRSAVEP
H
PGVMIWGTLEARVQGADLVILGGLNEGTWPETPPPDPWLNRQMRAEAGLLLPERQIGLAAHDFQQAVCAPEVVISR
AIRT
AEAQTVPARWLNRLTNLMDGLGGNGGPEALAAMRARGRNWLDMAQALDRPATPLPAMRRPSPRPPLDRRPERLS
VTAVER
LIRDPYAVYARHILRLKRLDPLVRSADAPLRGTVLHKVMERFLGRPLDPDPDRAQADLMAIADEVLATEAPWPAARAL
WR
ARLGRAAESFLRDEARRQAEAACIARECSGAARLDSGFTLTAQADRIDRRPDGRLILYDYKTGTPPSLKAMEAFDKQLLL
EACIAERGGFEEIPAAPVDHVTYIGLGQPPKTVTNWLDDDLVARSWAELGRLIDRYARPEQGYTARNRIQKRSDITDYDL
LSRFGEWDETDPPEPEDVG
>OLS47029.1 double-strand break repair helicase AddA [Rhodovulum sulfidophilum]AB14
MTRNEASARQIQAADPSGSVWLSANAGSGKTRVLTDRVARLLLAGVPPQRILCLTYTKAAAGEMQNRLFDRLGGWA
MLAD
ADLRQALHELGVETAIGEDTLREARRLFARAIEAPGGLKIQTIHSFCAGILRRFPLEAGVSPQFVEMDDRSARRLRAELV
DALAEGPEAGRFADLARFHTDAELDALTAEIASHRDRFPATADPGAIWKSFGLSADEDSAVLMRGVFTGTEEGLLSRLK
S
ALAGGGKTDQTAAAKLGALNLGGADRALLLGLEDILLTGAAAKQPFSAKTGSFPTKAVRAALGPDLAPLEDLMVRVETA
R
PRRLSLDAAERTLALHRFAEAFLPAYAARKQLQGWLDFDDLILKARDLLTDPGVAQWVLFRLDGGLDHILVDEAQDTSP
V
QWQVIELLAQEFTAGEGARSGVDRTIFVVGDKKQSIYSFQGADPEGFDRMRALFRDRLENVDRGLLNLTLEYSFRSSEAI
LGAVDRTFPESARAGLDREMRHRAFHADLPGRVDIWPVVEDSEAADPGHWADPVDMLGASHHSVRLARQVASEIRA
MIDR
GETIAVKGGGRRPIHEGDILILVQSRTDLFHEIIRACKAERLEVAGADRLKIGGELAVRDLTALLSFLALPEDDLSLASA
LRSPLFGWSEDALFRLAAPRKGYLWQALRHADRAGTETVPMLQALLDEADFLRPYDLLERILTRHDGRRRLVARLGQEA
E
DGIDALLTQALAYETTEVPSLTGFLAWLSSGEVEIKRQLDAAGRRIRVMTVHGAKGLEAPVVILPHTAQRTVTIRDQIYT
LEDGLPAWKTGSDARPEALAAAHAARVQREEEERSRLLYVAMTRAEQWLIVAAAGKLAKDGSDWYSRIRRGTEALGP
VPR
DFGFGEGLRYERGLWPEDRVLAAPETAPPDTALPDWALRDAPRPERPPQPLSPSDLGGAKALPGDPLGPAEAEDVRAR
GT
WMHLLLEHLPGTDPADRPALARALLTAADPPAPAAAIPALAAEAEALMARPDLAPVFAPGSLAEVPLTAALGQGRLSG
TV
DRLIVSPTRITAVDFKTNAVVPDRPEDTPEGILRQLGAYAMALAQIYPGHEIDTAILWTRTGALMTIPRELGHAALARAG
LA
>OLS47030.1 thiol reductase thioredoxin [Rhodovulum sulfidophilum]AB14
MPTHAVTDDTFDAEVRQSDIPVVVDFWAEWCGPCKQIGPALEELSAELEGKVKIVKINVDDNPTSPAQLGVRGIPALFL
F
KDGQVISSKIGAAPKAALQNWIQESV
>OLS47031.1 HslU--HslV peptidase proteolytic subunit [Rhodovulum sulfidophilum]AB14
MSKSEFPGWHGTTIIGVRRGGQVVIAGDGQVSLGQTVIKGSARKVRRLSPGGYDVVAGFAGSTADAFTLLERLEAKLQ
AT
PGQLQRAAVELAKDWRTDKYLQKLEAMLIVSDGAELYVITGAGDVLAPEHDVAAIGSGGNYALAAARGLIENTELSAG
DI
ARKAMAIAADICVYTNGNLTVESITR
>OLS47032.1 HslU--HslV peptidase ATPase subunit [Rhodovulum sulfidophilum]AB14
MTDLTPREIVSELDRFIIGQAEAKRAVAVALRNRWRRKQLADDIREEVYPKNILMIGPTGVGKTEISRRLARLARAPFIK

VEATKFTEVGYVGRDVEQIVRDLVDTALVQTRDWMREDVKAAAYKAAEDRVIAAIAGADAREQTREMFRAKLKKGEL
DDT
LIELEIADTSNPLQGMEIPGMAPGQGQQGMMSLGDLFGKAFGGRTVKKRLTVADSYEILISEEADKLLDDETVNRTALE
A
VQENGIVFLDEIDKVCARSDMRGADVSREGVQRDLLPLIEGTTVSTKYGPVKTDHILFIASGAFHIAKPSDLLPELQGRL
PIRVELRALTEDDFVRILTETDNALTRQYTALMGTEEVAVEFTDDGIAALARVAAGVNESIENIGARRLYTVMERVFEEL
SFTAPDRAGDTVRVDADFVDQNLGELTKSADVTRYIL
>OLS47033.1 hypothetical protein BV379_01170 [Rhodovulum sulfidophilum]AB14
MRRRRLSREDRALWDEVARRVRPMRDAPPDAVSGPPPEKTLPEKTPPAPVPAAPLPLKPFRLGAKAPAAAPPTPSAPP
AI
RMDRKAFLRMKGGKLDPEARIDLHGMTLAEAHPALIGFILRSQAEARRLVLVITGKGSGAEWDPAFADRRGVLRRQVP
HW
LHQAPLSGAVMQVAPAHRRHGGEGAFYVYLRRSR
>OLS47034.1 hypothetical protein BV379_01180 [Rhodovulum sulfidophilum]AB14
MSAALVLSVSACAKRPDAIAPAPIPAGMYDGMSCAKARKTLIDVNAEIQKLSRRQSGAATADAIGVFLTLVSAVTGGDR
A
GRIAAAKGRKLALEARLESCG
>OLS47035.1 EF-P lysine aminoacylase GenX [Rhodovulum sulfidophilum]AB14
MTDTPWWSPERHADRRPALLARGRIQAAIRGWFAEEDFVEVDPPALVTSPGNEAHLHAFATDAIGNDGVARRMYLH
TSPE
FAMKKLLAAGEPRIAAFCHVWRNRERGPRHSPEFTMLEWYRAGEDYAVLMEDTVRMLQLACEAAGTRHLGHAGQD
CDPFA
PVERVSVAEAFAAHAGIDLLSTLAPGGAPDRAALAVQAEAAGIGFAADDGWSDIFSRVLVERVEPHLGRGRITVLDRYP
A
AEAALARTCADDPREAERFELYACGVELANGFGELTDPVEQRRRFEAEMDEKERLYGERYPLDEEFLAALAFMPPASGI
A
LGFDRLAMLATAAPRIEDVLWVPVA
>OLS47036.1 elongation factor P [Rhodovulum sulfidophilum]AB14
MKVIASSLRKGNVVEKDGQLYVVLKAENFHPGKGTPTTSVDMRRISDGVKTTERWKTTEQVEKANVDEREYDFLYDDA
EG
FHFMNPESFDQVAAQPDVVGDAAAYLEPGMRVFLQTFEGNPISIELPQKVVVEVTETEPVVKGQTASSSYKPAMTNIG
VR
VMVPPHIDVGTRIVINTADNTYVERSKD
>OLS47037.1 exlusion protein FxsA [Rhodovulum sulfidophilum]AB14
MWLFLLFLSIPLIEIALFIQVGGLIGLWPTLCIVLATAAAGTWLMRQQGALALSQVRSSFNELRDPTEPLAHGAMILFAG
AMLLTPGFFTDTMGLLLLIQPVRAAAYRWLSKRVVVHSAGFGPAGGPARQARPARDDIIDGDFEDLTSRNGHGDEDG
TLP
PSGWTRH
>OLS47038.1 protein-export chaperone SecB [Rhodovulum sulfidophilum]AB14
MSETEGNGAAAQQPQVRMQILGQYIRDMSFENILVQKGASGEVTPDVTVQVALDARKRPADHQYEIITKFTVSSKNK
ADQ
SQLFLMELDYAGLFHIENVPEDQLHPFLLIECPRMLFPYVRRIVSDVTRDGGFPALNLEPVDFVALYRQEIARRAADIQK
QETPSGTA
>OLS47039.1 DNA polymerase III subunit epsilon [Rhodovulum sulfidophilum]AB14
MREIVLDTETTGFEPETGDRIVEIGAVVLWNHLPTGETFHVYTNPGRDMPDEAFGVHGIGPDILANPRPAKPGEVTLRD
K
PPFSQVGQKFLDFVQDSKLVIHNASFDMKFLNAELGWLGLPKLPMSQAIDTLAMARKKFPGSPATLDALCRRFGIDNT
MR

DKHGALLDSEILADVYLELIGGRQPDFMLATRSRASRGTSGTETEAWRPRPRPTPLPSRLTEEEAAAHRAFVETMGDDA
L
WKSFD
>OLS47040.1 dephospho-CoA kinase [Rhodovulum sulfidophilum]AB14
MSRPRLLGLTGSIGMGKSTTAALFREKGVPVWDADAAVHALYAEGGGAVRPLAALCPEAVRHGAVDRDALKAWIARE
PEA
LMRIEAVVHPLVAADRQAFIDRHRDAPLLVLDIPLLFETGADKWLDAVLVVTAAPEVQRARVLDRPGMTEAQLDAILAR
Q
MPDAEKRARADHVIETLTPDHAGAEVAALIESLGTANA
>OLS47041.1 shikimate dehydrogenase [Rhodovulum sulfidophilum]AB14
MSDRIPLAGVIGSPIAHSKSPLLHRHWLERYGLRGFYVPMDVAHADLKQVLEALPRMGFVGVNVTIPHKETILSLADSVS
DRAALIGSANTLIFRADGKVYADNTDGIGFIENLRQGAPGWDPTSGPAAVLGAGGAARAVVAALSEQGVREIRVSNRT
RS
RAEALRAEFGPKIVIHDWVQAGAMIDGAATVVNTTSLGMVGKAEFKVPLDALSPDAVVSDLVYVPLETPFLREARARG
CV
AVDGLGMLLHQAAPGFERWFGRRPEVDEAARAVLLEQ
>OLS47042.1 septum formation protein Maf [Rhodovulum sulfidophilum]AB14
MSDRVILASGSAIRRQLLTRAGLVFEAMPARVDEDAIRAALEAEGAEPGDIAEELAAFKAKRVAEKAPGALVIGCDQVLA
HRGKVLAKAETREEAHSQLRALRGGQHQLLSAVVVYDDMRPLWRHVGKVSLTMRPFSDRYLDEYLDRNWPAMRDA
VGAYK
LEEEGVRLFSAIEGDYFDVLGLPLLPLLTFLTTRGVLMA
>OLS47043.1 hypothetical protein BV379_01230 [Rhodovulum sulfidophilum]AB14
MSEFLANMYPWTKSLHIIAVLTWMAGLFYLPRIFVYHTEKVTPGHETDALFQVMEMKLYRYIMNPSIIAVWIFGLALVL
T
PNVVYWDTIWPYTKGAAVIAMTWFHMWLGARRKDFVAGRNTRTGRTFRMMNELPTILMLVIVFSVVVKF
>OLS47044.1 transcription termination factor Rho [Rhodovulum sulfidophilum]AB14
MTQECLNLADLKAQSPKALLSMAEELEIENASAMRKGEMMFSILKERAEDGWEISGDGVLEVLQDGFGFLRSPEANYL
PG
PDDIYVSPDMIRLHALRTGDTVEGVMQAPRENERYFAITKVTKINFQDPEQARHKINFDNLTPLYPDERLKMEIEDPTIR
DRSARIIDLVSPIGKGQRALIVAPPRTGKTVILQNIANSIETNHPDTYLIVLLIDERPEEVTDMQRSVKGEVISSTFDEP
ATRHVAVAEMVIEKAKRLVEHKRDVVILLDSITRLGRAYNTVVPSSGKVLTGGVDANALQRPKRFFGAARNIEEGGSLTI
IATALIDTGSRMDEVIFEEFKGTGNSEIVLDRKVADKRVFPAMDILKSGTRKEELLVDKSDLQKTFVLRRILNPMGTTDA
IEFLISKLKQTKTNSEFFDSMNT
>OLS47045.1 tRNA uridine-5-carboxymethylaminomethyl(34) synthesis GTPase MnmE [Rhodovulum sulfidophilum]AB14
MDTIFAQATARGKAGVAVIRISGPRAHSAALALAGSLPAPRQAGLRRLADADGVPLDEALVLCFAKGASFTGEDVVEFQ
C
HGSPATVSALLSALGRIDGLRLAEPGEFTRRALENGVLDLAQVEGLGDLLEAETEAQRRQALRVLSGALGAKVDRWREA
L
IRAAALLEATIDFADEDVPVDVTPEVTGLLDEVAASLRSEIEGVSVAERIRDGFEVAIVGPPNAGKSTLLNHLAGREAAI
TSEHAGTTRDVIEVRMDLDGLPVTFLDTAGLRESADEVETIGIARAVVRAEAADLRVFLLDDTGKTQGLVPGPEDIVLRG
KADLADGAGHGVSGLTGAGVADLVAEISTTLEGRASRTVTATRERHAEAMRRAGGAIAEAQNQLSLGMETAELAAED
LRR
AVRSLETLLGRVDVENLLDEIFSRFCLGK
>OLS47046.1 tRNA uridine-5-carboxymethylaminomethyl(34) synthesis enzyme MnmG [Rhodovulum sulfidophilum]AB14
MFHVKHRAFDVIVVGGGHAGAEAAHAAARLGARTALVTLRRDGIGVMSCNPAIGGLGKGHLVREVDALDGIMGRAA
DRAG

IQFRLLNRRKGPAVQGPRAQADRKLYRQAVQTALSAVPGLEIVEGEAADFLQRDGRIAGVVLADGSELSAGAVVLTTGT
F
LRGVIHIGDRQSPGGRIGDRPSVRLAERIDEFGLPLGRLKTGTPPRLDGRTIDWDRLDHQPGDDEPVLFSFLSSHVEARQ
VACGITHTNAQTHDIIRKNLSRSAMYGGHIEGVGPRYCPSIEDKVVRFAEKESHQIFLEPEGLDDPTVYPNGISTSLPEE
VQEAYVHSIKGLENATILQPGYAIEYDYVDPRALRPTLELRDVPGLFLAGQINGTTGYEEAAAQGLVAGLNAARDVAGQ
G
PVIFSRTTSYIGVMIDDLITRGVSEPYRMFTSRAEFRLSLRADNADQRLTPVGLEIGCVGERRRTSFEEKMARLSAGRAR
LESMSVTPKQVAAAGIKVNADGTRRSGIDLLAFPDVSFEDLIPLDSELEEVDRESRVQLARDALYANYIARQTLDVEAMR
RDEAQRIPSGFDYEKLDGLSAELKGKLSRVQPETLGQAARIDGMTPAAATLILARLRQMERQSA
>OLS47047.1 chromosome partitioning protein ParA [Rhodovulum sulfidophilum]AB14
MSDPTRPKGPKIIAVANQKGGVGKTTTAINLGAALAELGKTVLIVDIDPQGNASTGLGVAPEARVRTTYDLILDDAPLVD
IVVKSPKGNLYIAPSTTDLSSADIEMVANEKRSFLLHDALRQPAMDAYGFDFVLIDCPPALSLLTINAMVAAHSVLVPLQ
SEFFALEGLSQLMLTIRELRQTANSNLRIEGVVLTMADRRNNLSQAVENDARAYLGDLVFRTVIPRNVRVSEAPSYAMP
V
LDYDPNSRGAEAYRALAQEMLEPKVPVGA
>OLS47048.1 chromosome partitioning protein ParB [Rhodovulum sulfidophilum]AB14
MKQPEKRGLGRGLSALMADVRIEPEAERDETPRSKADQMIALDLLEPNPDQPRRLFSEEALKELSDSIREKGVIQPLIVR
PSKRSEGRYEIVAGERRWRASQLAQKHEVPVIVRELDDTEVLEIAIIENIQRADLNPLEEAMGYRQLMDRFGHTQDKVA
E
ALGKSRSHIANLLRLLQLPDEVQGYLREGRLSAGHARALITTTDPATMARQVIKGRLSVRETEKLAKSGSTTKKRRPPPP
VAKDADTRALEQDLAANLGMDVSIEHRTDGSGDLVVSYRSLDDLDRLCAVLSAPSGGSGGED
>OLS47049.1 coproporphyrinogen III oxidase [Rhodovulum sulfidophilum]AB14
MTAEDWQEGGFGLYVHWPFCLSKCPYCDFNSHVSAEIDTARWQEAYLSEIRRLGAETGPRVLNTVFFGGGTPSLMPP
ELV
GAILETARSVWTPANDIEVTLEANPTSVEAGRFTGFAEAGVNRVSLGIQALNDADLRRLGRTHSVAEARAAFDIARSRFP
RVSFDLIYARQSQSLAAWRDELGEALAMAVDHLSLYQLTVEAGTAFGDRLARGGLKGLPDDDLGADLYELTQELCEAA
GM
PAYEVSNHARPGAESRHNLIYWQAGDYAGIGPGAHGRLTLGGRRTATDTPLPPMSWLERVEREGTGDLSRSTVAPAD
QGA
EYLMMGLRLAQGLSLRRYERIANSQIDGLKIKELSDLGLIETEADHLRATASGRAVLNAVIRALLPD
>OLS47050.1 non-canonical purine NTP pyrophosphatase, RdgB/HAM1 family [Rhodovulum
sulfidophilum]AB14
MRRFDGKTLLVATHNSGKLEEIADLLKPFGISVTGAAEHDLPEPEETEDTFVGNARIKAHAAAQATGLPALADDSGLAV
D
ALDGAPGVYTADWAETGSGRDFPMAMEKVWTRLEEANAPEPRNAQFCCTFVLAWPDGHDEVFEGRMPGRIVWP
MRGDQGH
GFDPIFQPDGHDITFGEMDRWEKNRISHRARAFEKLVSLFDR
>OLS47051.1 ribonuclease PH [Rhodovulum sulfidophilum]AB14
MRPSRRDVSELRPVSIETGVTKHAEGSCLIRMGDTHVLCTATLEERVPPFLKNSGLGWVTAEYGMLPRATTTRMRREA
AQ
GKQGGRTVEIQRLIGRSLRAGVDRVALGERQITIDCDVIQADGGTRCASITGGWVALRLAVNKLMKTGEVISDPLVDPV
A
AVSCGIYAGQPVLDLDYPEDSEAGVDGNFVMTGSGKLIEVQMSAEGSTYTRAQMNALLDLAEKGVAELVAAQKAAIG
A
>OLS47052.1 nucleotide exchange factor GrpE [Rhodovulum sulfidophilum]AB14
MAEPEKTEFDDVATETAAETETPEITPEEVEALKAERDELRDRFMRALADAENARKRADRDRREAEDYGGSKLARDML
PV

YDNLSRALEVVTDEQREANAALLEGVELTLRELLNVFAKHGIERVAPEAGDKFDPQLHQAMFEAPVPETRAGDIIQVM
AV
GFMLHDRLLRPAQVGVSSTPAA
>OLS47053.1 hypothetical protein BV379_01295 [Rhodovulum sulfidophilum]AB14
MLLNRKPLVLRDKPQVARGNKRALFLIDGYPDVVVKVVLPREGGTNMAGPKRLLRRFLPSTNYRFLFREYKCYLAAKLA
Q
AGQSGQSGALPIIELRGLVQTDLGPGMIEELIKDGEGNPGTSLRQMVQRKVFEEHHLELLNDFVRRIFAWRVRANDLN
PS
NILFGQRAGISQFVLVDGLGDSHLIPVRTWSGHLNERSLCKRFEDTSRVTGLTWDAKDRVFGLPRR
>OLS47054.1 NADP-dependent malic enzyme [Rhodovulum sulfidophilum]AB14
MSKKSRPTPEEALSYHIDPVPGKYEIVATKPMTTQRDLSLAYSPGVAVPVQAIADAPETAYDYTCKGNMVAVISNGTAIL
GMGNLGALAAKPVMEGKSVLFKRFADVNAVDIELDTEDPEEIIRAVRLMGPAFGGINLEDIKAPECFIIEQRLKEEMDIP
VFHDDQHGTAVICAAGLLNALHLSGKKIEDCRIVLNGAGAAGIACLELIKAMGARHDNCIACDTKGVIYQGRTDGMNQ
WK
SAHAADTEARTLEEAMVGADVFLGVSAKGAVTPEMVASMADNPVIFAMANPDPEITPEEAQEVRPDAIVATGRSDYP
NQV
NNVLGFPYLFRGALDIHARAINDEMKIACADALAALAREDVPDEVAMAYGKKLTFGRDYIIPTPFDPRLIYTIPPAVARA
GMDTGVARRPIVDIEDYEHRLKARMDPTASVLSGIAARAKAAQARMIFAEGDDERVLRAAVSYHRAGYGQALVVGRE
AEV
REKLEQAGLGDAYREIEVVNAANTRHLEAYRSFLYQRLQRKGFDTHDINRLAARDRHVFSALMLAHGHGDALVTGATR
KS
AHVLELINHVFDARAQDGAVGITALLHKGKIVFIADTLVHEWPEEEDLANIAVHAAEVARSLGIEPRVAFLSFSTFGYPV
SERATKMHRASEVLAKRGVDFEFDGEMTVDVALNPTAMARYPFCRLSGPANVLVVPARHSASISVKLMQEMAGATVI
GPI
LAGVEKPIQICSTVSTVNDILNMSLLAACEVG
>OLS47055.1 peptide-methionine (S)-S-oxide reductase [Rhodovulum sulfidophilum]AB14
MAMSLKTIKPVLLICALVLGYMAEGAKARAAEAETAIFAGGCFWCVEADFESVPGVSGVVSGYTGGDVEDPSYRQVAR
GG
TGHVEAVEITYDPAVISYEKLVSLFFRSVDPTDAGGQFCDRGETYTTAIFVSDPAERRVAEQAKANAGQALGAPVATEIR
PAATFWPAEAYHQDYYRSRALILTRFGPKTKAEAYKLYRKACGRDERVRALWGDAATFAGS
>OLS47056.1 argininosuccinate synthase [Rhodovulum sulfidophilum]AB14
MSTPKTAPKKVVLAYSGGLDTSIILKWLQTEYGCEVVTFTADLGQGEELEPARKKAEMMGAKEIFIEDLREEFVRDFVFP
MFRANAVYEGLYLLGTSIARPLISKRLIEIAEATGADAVAHGATGKGNDQVRFELAAYALNPDIKVIAPWREWDLTSRTK
LLDFAEQNQIPIAKDKRGEAPFSVDANLLHTSSEGKVLENPAEDAPDYVYQRTVNPEDAPNEPEYIEVTFEKGDAVAING
EAMSPATVLTKLNELGGKHGIGRLDLVEGRFVGMKSRGIYETPGGTVLLEAHRGIEQITLDRGAAHLKDELMPKYAELIY
NGFWFSPEREMLQALIDKSQEHVSGTVRLKLYKGSARTVGRWSEASLYSESHVTFEDDAGAYDQKDAAGFIKLNALRL
RL
LAMRNRKLKG
>OLS47057.1 hypothetical protein BV379_01325 [Rhodovulum sulfidophilum]AB14
MIDWTRVDELRCEVGEDAFEEVLELFLEEVDEVIGRLDPERDPADLAADLHFVRGSALNLGFRDFCMRCQGIEHQLAQ
RE
SIDLAPLLAAYAGSKAELLTRARTRRDVA
>OLS47058.1 fused response regulator/phosphatase [Rhodovulum sulfidophilum]AB14
MNAQPDTHPLDPTGSSLVRLVLLVDDSRLQRRILRALLERWGYDVAECGSGEEALEICRAQPVDLIISDWMMPGMDG
PEF
CRAFRALPRESYGYFILLTSKSETVEMIHGLDVGADEFLTKPVNSSELRARIASGERILRMEHQLRDKNRQLTDALAELR
TLYDALDRDLAEARKLQQSLVPERHRRFGASEVSLLLRPCGHVGGDLVGFFPAGPGEVGLFSADVSGHGISSAMLTARL
A

GLLGSRSPDQNIALEPAPCGQYRARPPEEVAAHLNRLFLEDIETEHYFTLLLAIVTLDTGRMRFVQAGHPHPVLCRADGS
LECLGDGGLPLGLIPGAGFEGCEVGLRPGDRLLLVSDGITECPAPDGAQLGDDGLRLLIETHAATRGPAFLDAIQAGLTF
FAGRDEFPDDVSAVLFEFG
>OLS47059.1 NUDIX hydrolase [Rhodovulum sulfidophilum]AB14
MIRRYGEAVQRDRRYRFRPGAYALLERDGMLLLTHQQAPVPEYQLPGGGIDPGESPIQALHREVFEETGWRIALRRRL
GA
FRRFTFMPDYEIWAEKLCTVYLGRPVRPHGAPGEPGHTAVWMPPDLALGRLGNAGDRAMLHRWLGQIR
>OLS47060.1 molecular chaperone Hsp33 [Rhodovulum sulfidophilum]AB14
MSIGSQIAWDDTVLPFQLDRSDVRGRVARLDGVLDRVLAQHDYPPAIEALVAETALLTALIGQTIKLRWKLSLQVRGDG
P
ARLIATDYYAPTEDGAPARIRAYASFDEDRLDAGADPFSQIGQGYFAVLLDQGAGTVPYQGITPISGGSLSACAETYFAQ
SEQLPTRFALSFGRAILPGADKDEHWRAGGVMLQMMPKASIPQTPEGGSGDEGLLQAEDMLEGDQAENWSRANILL
DTVE
EIELVGPRVHPTDLLVRLFHEERPRVFDPQRVQFGCTCSEDRVRQSLSIYSAKDIGHMTTPEGIVTADCQFCGAHYELDP
ATLGFEAEKDPDAE
>OLS47061.1 CoA pyrophosphatase [Rhodovulum sulfidophilum]AB14
MPSDPFDALAAALARPGCETSDHDLNPGFVLPEGRVLRPAAVLVAFWPAPGGTRLVLTKRSSRLKHHPGQIAFPGGKL
EP
GEDAPQAALREAREEIGLPEAGVEILGTMPPHETVTGFTVTPVLGRIAEDFPRIPEAGEVDEVFDVPFAHVSDPARFSIQ
SRMWRGQRRYYYTVPYGPYYIWGATARILRALAERVSA
>OLS47062.1 CCA tRNA nucleotidyltransferase [Rhodovulum sulfidophilum]AB14
MTRVSGDPFFADHTQAVCALLTDAGYRALFVGGCVRNALLGVAVSDIDIATDARPGKVMELAQAAGLKAVPTGFDHG
TVT
VVAAHVPHEVTTFRRDVETFGRRAVVAFADSPEEDARRRDFTMNALYATPAGEVLDPLGGMRDLRARRLRFIDDADA
RIR
EDYLRILRFFRFHAWYGDPSNGLDPEGLAACAANSAGIETLSRERIGAELKKLLAAADPGPSIAAMAATGVLARTLPGAD
PRVLPVMIHLEAALGLAPDPIRRLAALGGEDVAGRLRLSKAEARRLGRLVEGAAAGEGPAVLAYRVGPEVARDAVLLRA
A
SLGADLPEGFGTEIARGAQARFPVAAADLMPRFSGPALGQMLKRLEADWIASDFRLDRAALIALAGRGD
>OLS47063.1 multidrug ABC transporter ATP-binding protein [Rhodovulum sulfidophilum]AB14
MFRYFENLIDPYAPHAQTDTPPNRLWPFLAAYMRPFRKVFAIAAVLSLLSAFVEIWLIDVMGRLVDILSSGAPEQVWHE
H
GAALIGLALFILILRPLIQALDAAVLNNAIMPNFGALIRWRAHSHVLRQSVGWFENDFAGRIANRQMQTPPAAGEAVF
QV
FDAIAYALAYFGGALVLLGQADPRLMLPLLIWLGLYAALVRWTMKNVGVASKASSDARSTLTGRVVDAYTNIHSVKMF
AH
HDRELTYAREAIEGLRKAFGTEMRIITTMDVALVVLNGLLIVGVVGWAIALWMQGQASVGVVAAATALTLRLNSMTG
WIM
WAMSSFFRSLGVVSEGMETIAQPIALVDAPDATDLEFREGLVEFRSLTHHYGRGSGGLEGVELALKPGEKVGLVGRSGA
G
KSTLVKLLLRFYDPEAGQILIDGQDIARVTQESLRRQIGMVGQDASLMHRSVRENILYGRPEAGEEEMIAAAKRAEAHD
F
ILDLKDPEGRRGYDAHVGERGVKLSGGQRQRVALARAILKDAPILVLDEATSALDSEVEAVIQQTLYRVMEGKTVIAIAH
RLSTIAHMDRIVVLEDGRIAEDGSHSELLAKDGLYARFWARQSGGFIGTEAAE
>OLS47064.1 RNA methyltransferase [Rhodovulum sulfidophilum]AB14
MSHLVERLSLRGEGLTGDGLTVALALPGEEIEGVPEEGRIARPRIVTPVVERVRPPCRHFSSCGGCALQHGSDAFVAGW
K
TQVVERALTGQGLGASVRPILTSPSASRRRAVLSGRRTKKGALVGFHARASEVLVEIPDCRLLHPDLMAALPACEAATLL

GASRKGALSIALTRSEDGVDMAVEEARPLDTGLRMDLAALAETHDLARLSWNGEQLAERRPPRQAMGRAHVVPPAG
AFLQ
ATEAGQAALTDAVGAALGGVRSVADLFAGCGTFTLPLAATAEVHAVEGSAPMLAALDRGWRGTPGLKRVTTEARDLF
RRP
LLASELARFEAVAIDPPRAGAEAQCAEIARSDLARVAMVSCNPVTFARDAKRLCSGGFRIDWVQPVDQFRWSGHVELV
AQ
FSR
>OLS47065.1 hypothetical protein BV379_01370 [Rhodovulum sulfidophilum]AB14
MIRSALGLLAVLFALSACSTGNEVRIGPDGKPLPQVYRISRADQGKIQYRMLDSVNALRQARGIAPLELDSRLNAAAATH
SRDMSVQNRPWHFGSDGSSPLERVARAGYQGHLRGENISETYETELETLAAWMEESDTRNIILDPQATDLGFAFYQE
MNG
KIWWTLVTGQRDAAAPDA
>OLS47066.1 hypothetical protein BV379_01380 [Rhodovulum sulfidophilum]AB14
MKKLALAAAFAATASTAFAGGYAEPVIEPEVIVQDTSSSAQGVIVPLLALILVAAVASN
>OLS47067.1 SAM-dependent methyltransferase [Rhodovulum sulfidophilum]AB14
MTRPPDLTDRQALALHRARALKAGPALFLHEAAADEIKERLIEVNRTFTAPAVVTPFAEAWQGLLPGARLVPAEETLDL
A
PGAHDLVIHALALHWANDPVGQLIQCRRALKPDGLFLGVMFGGRSLQELRAVLAEAEAATLGGLSPRVLPMAEIRDLG
AL
LQRAGLALPVADCAEFSVTYQSAYHLMRDLRAMGETNALAARHRRTAPRRLFSEAARRYAEFHTMPDGRIPATVEMIY
LT
GWAPAETQPQALKPGSARMRLADALNRAAPQPGERNDGGSD
>OLS47068.1 amidophosphoribosyltransferase [Rhodovulum sulfidophilum]AB14
MLQSAMRLIYPPRCVACGEPVERDHALCARCWGELDFVTGLVCDACGLPLPGEEADDPVWCDDCLLAPRPWARGRA
ALLY
RGRARGLVLAFKHGDRTDLARAASGWMLRAGRPILVPGMLVAPVPLHRWRLLRRRYNQSALLSRGVAEGAGAEHCP
DLLV
RLRATRPQEGMNAEARAANQAGAIAVGPRHRGRVAGRTVLLIDDVMTSGATLGAAAGACLAAGAAEVRVLVLARVA
KAD
>OLS47069.1 glutaredoxin 3 [Rhodovulum sulfidophilum]AB14
MQTVEIYTTSSCGYCHAAKRLLDGKGVSYAEFDVTADPGLRQQMMERADGGYTVPQIFIGETHVGGCDDLYDLERAG
KLD
PLLAG
>OLS47070.1 amidohydrolase [Rhodovulum sulfidophilum]AB14
MRAALIQMTSSDLPEENIGQVRELVREAAAGGAGMVLTPEVTNCLSTSRDWQRNVLTEEAEDPTLAALRQEAASLGI
WLL
IGSLGLKTTDPDGRFANRSILIAPGGGIVARYDKIHMFDVEISATETFRESDGYRPGARAVLAPTPFGAVGMAICYDLRF
AHLFRALAKAGAEILTVPAAFSPVTGAAHWEPLLRARAIETGCYVLAPAQCGLHPASQGQSRRTHGHSLAVSPWGEVL
AD
GGEAPGISYVELDREEVARARARVPSITHDRGFEGP
>OLS47071.1 MarR family transcriptional regulator [Rhodovulum sulfidophilum]AB14
MNEISESLLVTLFSEIFMADQLARNRISKALPKGMEISHFSVLNHLARLNEERSPAQLAAAFHVTRGAMTNTLTRLEWA
G
HVHIRPDWDDARRKMVSISPSGRSAREVAIQAVAPVLAEAVADIGEDRLKAALTVLRMLRQKLEG
>OLS47072.1 bifunctional 3-demethylubiquinol 3-O-methyltransferase/2-polyprenyl-6-hydroxyphenol methylase [Rhodovulum sulfidophilum]AB14
MQAAETTVDPSEVAKFEAMAAEWWDPAGKFKPLHMLNPCRLDYITRQIAAEFDRDLAAEAPFKGLRLLDIGCGGGLL
SEP

MARLGAEVVGADAAERNIPVARIHAEQSGLQIDYRHTTAEALAEAGEQFDVVLNMEVVEHVADPLAYLTACRALLKPG
GL
HLCSTINRNPKAFMMTILGAEYVMRWLPKGTHEYQKFITPDELCSLLEQAGLKLLDRSGFVFNPLTWDWSISSRDLSVN
Y
VTAARKPA
>OLS47073.1 prolyl aminopeptidase [Rhodovulum sulfidophilum]AB14
MDKSAGQKRASQHLYPPIDPFDQRMLDVGDGHRIYVEQCGHPQGIPVIVLHGGPGGGCSPAMRRYFDPTEYRIVLFD
QRG
CGRSRPHASVENNTTWHLIADIEKIREMLGIGRWVLFGGSWGATLALAYAETHPDRVAYMALRGVFLMTGAELDWFY
GGG
AGQFWPDLWARFSALIPADERSDMIAAYHWRLFSGDLLTETRHARAWAAWENALASIQHEGPAGEAPADYARAFAR
LENH
YFMNRGFLERDGQLIEDVPRISHVPATIVQGRYDMVCPPGSAYRLAERWGRAELKMVPMAGHALSEPGISAELVRTM
RDV
QRLAVALGL
>OLS47074.1 hypothetical protein BV379_01425 [Rhodovulum sulfidophilum]AB14
MHVNARFWDRLAPRYARMQLRDPEAYEKKLAMTRAWLGPEAQVFEFGCGTGTTALKHAPHAGHVTAIDCAPAMIG
IARQK
AAEAGIGNVAFRVGDLADMPDSPAAFDMVMAHNVLHLLPDRAEALARVYRMLKPGGVFVSSTACAAGAPRLFRWVI
AAGH
ALRLLPLFNPLTADALRAEIAATGLGIVEDWRPEGGRVPVLFLIAQKPG
>OLS47075.1 transcription termination/antitermination protein NusA [Rhodovulum sulfidophilum]AB14
MAITSANQLELLQTAEAVAREKMIDPDLVVQAMEESLARAAKSRYGAEMDIRVAIDRKTGRATFTRVRTVVEDELLENY
Q
AELTVKQAKQYLADPQIGDTVVDEVPPVELGRIAAQSAKQVILQKVREAERDRQYEEFKDRAGTIINGQVKREEYGNVI
V
DIGRGEGILRRNEKIGRESYRPNDRIRCYIKDVRRETRGPQIFLSRTAPEFMAELFKMEVPEIYDGIIEIKAVARDPGSR
AKIAVISYDNSIDPVGACVGMRGSRVQAVVNELQGEKIDIIPWNEDQATFLVNALQPAEVSKVVIDEEAGKIEVVVPDE
Q
LSLAIGRRGQNVRLASQLTALDIDIMTEAEESQRRQAEFEQRTKLFMDTLDLDEFFAQLLVSEGFTNLEEVAYVDLDELL
VIDGVDEDTAGELQARARDHIEAMNRKALEHARELGVEDSLVDFGGLTPQMIEALAEDGVKTLEDFATCADWELAGG
WTT
VNGERQKDEGLLEKFDVSLEEAQTLVMTARVQLGWVDPSELEPEAEPEEDEGGEEEAGA
>OLS47076.1 hypothetical protein BV379_01440 [Rhodovulum sulfidophilum]AB14
MARGGAGRPADEPERRCIATGEVTPKAGLIRFVVGPDDAAVPDLLGKLPGRGMWVAADRDALEKAVKKRLFSRAAKR
PVT
VPEDLLEQVEAGLLRRVVDSLSLARKAGRAVVGYEKAKGWLDSGQAAVLIQASDGSERGKTKLRPPREDDGFIGCLTAR
E
LGLAFGREHVIHGALAAGGLTERVVEDAAKLSGVRKKDGGGTPGKG
>OLS47077.1 translation initiation factor IF-2 [Rhodovulum sulfidophilum]AB14
MDDTDGKKTLGLRGGSRSGQVKQSFSHGRTKNVVVETKRKRVVVPKPGAAKSGGAGGGRAGGDPSRRPAGISDSEM
ERRL
KALQAAKLREADETRQREEDERQREAERQRRREEAEAKEREQREIEEAARRKAEEEERKKREEEDAAKKKAAAAERAAS
A
PAKPAPSAGPEETRGPAKTSSPRKVDREREGGADRPNRGKTSPGKGDRRSGKLTVSQALTGGEGGRQRSLAAMKRKQ
ERM
RQKAMGGADKHEKVVRDVQVPETILVQELANRMAERSADVVKSLMQMGMMVTQNQPIDADTAELIVEEFGHKVV
RVSDAD

VEQVIDSVEDKAEDLKPRPPVITIMGHVDHGKTSLLDAIRNAKVVAGEAGGITQHIGAYQVKTDSGAVLSFLDTPGHAA
F
TSMRARGAQVTDIVVLVVAADDSVMPQTIEAINHAKAAGVPMIVAINKCDKPAADAQRVRTELLQHEVVVEAMSGE
VQDV
EVSAKTGQGLDTLLEAIALQAEILELKANPERAAMGAVIEAQLDVGRGPVATVLVQNGTLKQGDIFVVGEQWGKVRAL
VN
DKGERVQSAGPSVPVEVLGLNGTPEAGDVLNVVDTEAQAREIAEYREHAAKDKRAAAGAATTLDQLLAKAKEDANVA
ELP
ILMKADVQGSAEAIVQAMEKIGNDEVRVRVLHAGVGAITETDVGLAEASGAPIIGFNVRANAPARNAANQKGVEIRYY
SV
IYDLVDDVKAAASGLLSNEIRENFIGYAQIKEVFKVSGVGKVAGCLVTEGVARRSAGVRLLRDDVVVHEGTLKTLKRFKD
EVAEVQSGQECGMAFENYDDIRAGDVIEIFERQEVVRTLT
>OLS47078.1 NTP pyrophosphohydrolase [Rhodovulum sulfidophilum]AB14
MKIVLVAAVALIDADGRVLLAQRPAGKSMAGLWEFPGGKIEPGETPEAALKRELKEELGIDTWESCMAPLTFASHSYDD
F
HLLMPLYACRKWQGQPQPREGQVLKWAHARDLRDFPMPPADIPLIPVLQDLL
>OLS47079.1 peptidylprolyl isomerase [Rhodovulum sulfidophilum]AB14
MSFALRPLAVLALGATLALPALADEIGPDTVVARVGDQEITIGHMIVLRAQLPAEYQQLPDDVLYQAVLDQLIRQTAVG
E
AIGKELSTGARLALENERRSFVAGEALSRLAEAAVTDEAVEAAYNATYGEADPTTEYHAAHILVETEDEAQAIEAQLRDG
ADFAQLAKEKSTGPSGPNGGDLGWFSEGMMVKPFEEAVTVLEPGEVSAPVQTQFGWHVIKLYETRLKDAPPLADVRG
ELV
QQIQRDAMEEALEKYTEEADVSRPEIEVDPAILKDQSVLDR
>OLS47080.1 preprotein translocase subunit SecA [Rhodovulum sulfidophilum]AB14
MLGIGTLTRKVFGTRNDRLVKKVRPLVARINALEPEFQALDDDGLKAKTVEFRRRIAEGEALDDILPEAFANCREGARRA
LGLRAFDTQLVGGIFLHRGNIAEMKTGEGKTLVATFPAYLNALTGLGVHIVTVNDYLAKRDAEWMGKVYGMLGMTTG
VVY
PWQPEPEKRAAYRADITYGTNNELGFDYLRDNMKSELKDMAQRGHYYAIVDEVDSILIDEARTPLIISGPSQDRSELYM
T
LDKVVPDLTDEHYTLDEKMRNVTFTEEGNEFMEKRLHEMGILPEGQSLYDPESTTIVHHAQQALKAHTMFLKDKEYIVR
D
GEVMLIDEFTGRMMRGRRLSDGLHQAIEAKEGVQIQPENVTLASVTFQNYFRLYDKLAGMTGTAATEAEEFMQIYGL
GVV
EIPTNRPIARIDEHDAVYRTAAEKYAAIVESIKEAHDRGQPVLVGTTSIEKSEMLSEMLKKQGIPHNVLNARHHEQEAQI
VADAGRFGAVTIATNMAGRGTDIQLGGNAEIKVMEALDADPTANPDEVRARIEAEHAEEKQKVLEAGGLYVLATERH
ESR
RIDNQLRGRSGRQGDPGRSYFFLSLEDDLMRIFGSERLDKVLSSLGMKEGEAIIHPWVNKSLEKAQAKVEGRNFDIRKQ
L
LKFDDVMNDQRKVIFGQRREIMEADDLSDIVQDMRHEVIEDLVEAHIPPRSYADQWDAEGLQRAVAETLSLDLPVVD
WAE
EEGVDDEIISERIEKASDEAMAAKAAQFGPEAMRNIEKQMLLQTIDTKWREHLLKLEHLRSVIGFRGYAQRDPLNEYKTE
SFALFESMLNSLREDVTQKLAQIRPLTEDERNDMIRQLAEQQAQLAEATAKAQPSHAEAPADGTEAAPAAAPLAAAQS
GF
DESRPETWGNPSRNDPCPCGSGKKFKHCHGQLI
>OLS47081.1 hypothetical protein BV379_01470 [Rhodovulum sulfidophilum]AB14
MRFLTGAVLFCAALFLSATRPASAGEVTLVPREDGAVEIGGRLLGFDGEVYRLDTIYGPLAVNAAGVTCAGAACPGAQV
T
RIVLSGSPALGDMLIPALIEGFAAARGLVVERGAEGRDRLFRLRLPDGGPLRAEIALASGSTGEGFADLLTGAAQIVMAD

REILPEEIDRAREAGLGDLSAPFRRRIVALDAIVPVVAPDTRLGDPADGLSQAALLGLLTGEIADRSGPGGPEAPVTVHL
PGQAGQGLRQLPEAQVLGGRRPVASARRHGRAAAVATAVAADPTGLGLARVSALGDAVQVPLADGCGTRLLASHQS
VKTG
DYPYVAPLSLYLGRPRLPRLARDFLDYLATPAAQIAIRRGGFVDLLRDEIPLGRQGDRLALAIAEARPGNGLRELKRLVA
TLGGAVRLSETFRSGPRGPDPRWRANVAALAEALEAGHFDGRRLILAGFSEGSGGSEASAARARREAEAVRDAIRAAAT
A
ADYDRVRLEVAGFGDLLPVMCDGGSGKAGPEQSRDRPSGDWARRLNRRVEVWLR
>OLS47082.1 molecular chaperone DnaJ [Rhodovulum sulfidophilum]AB14
MAKRDFYEVLGIARGASAEEIKKAYRKKAKELHPDRNADNPKAEGQFKEVNEAYETLKDPDKKAAYDRFGHAAFEQGG
GR
GPGGMGGMGGGFGGQGDFASAFSNVFDDLFGDFMGGAGPGGGRQRATRGSDLRYNLRITLEEAYSGLQKTIKVPSS
VACD
VCNGTGAAGGAEPATCPTCSGMGKVRAQQGFFTVERTCPTCGGMGQIVKNPCKSCGGAGRVERERSLSVNIPAGVE
TGTR
IRLAGEGEAGLRGGPAGDLYIFIEVGGHPLFERDGLNLFCRVPVSMTAAALGGEIEVPTIDGGRSRVKVPGGSQSGKQM
R
LRSKGMPALRGNAQGDMFIELAVETPVNLTTRQKELLREFEELSEENNPEGSSFFTKVKSFWDGMKS
>OLS47083.1 molecular chaperone DnaK [Rhodovulum sulfidophilum]AB14
MGKVIGIDLGTTNSCVAIMDGAQPRVIENAEGARTTPSIVAFTDNERLAGQPAKRQAVTNPENTVFAVKRLIGRRVDD
AE
VEKDKKHVPYSIVDGGNGDAWVSVRGEKYSPSQISAFILQKMKETAESYLGEDVTQAVITVPAYFNDAQRQATKDAGK
IA
GLEVLRIINEPTAAALAYGLDKKETRTIAVYDLGGGTFDITILEIDDGLFEVKSTNGDTFLGGEDFDMRIVAYLADEFKK
EHGVDLAKDKMALQRLKEAAEKAKIELSSSSQTEINQPFISMDPQAGTPLHLVMKLTRAKLESLVGELIKKSIKPCQDAL
KDAGLSTSDIDEVVLVGGMTRMPRVIQEVTKFFGKEPHKGVNPDEVVAMGAAIQAGVLQGDVKDVVLLDVTPLSLGIE
TL
GGVFTRLIDRNTTIPTKKSQIFSTAEDNQSAVTIRVFQGEREMAADNKMLGQFNLEEIPPAPRGMPQIEVTFDIDANGIV
SVSAKDKGTGREQKITIQASGGLSDDEIDKMVREAETNAEADKQRRELVETKNQAESLIHSTEKSIEEHGDKVDPSTVEA
IELAVAALKDVLETEDAGKIKGGIQNVSEAAMKLGEAIYKAQQEDAPDSGSEPEDQRDEPSGVDDEIVDAEFEDLDDDR
K
RS
>OLS47084.1 alkylated DNA repair dioxygenase [Rhodovulum sulfidophilum]AB14
MSETERPGRPVPGLVVRGVEIFPGLLDSAAQAGIMAEIAAVMSAAPPVQPETRFGRKMSVRMTSAGKYGWVSDRRG
YRYE
TRHPSGVAWPPIPAPVLALWHRVTGLARAPDCCLVNLYRDGAKMGLHQDRDEADFSWPILSVSLGDEARFRIGDVTR
GGP
TESLWLKSGDVLAMRGAARLIHHGIDRVRGGSSTLIEGGGRINLTLRVVD
>OLS47085.1 3'(2'),5'-bisphosphate nucleotidase [Rhodovulum sulfidophilum]AB14
MRKTALLAGARIMEIYGAPDFEVRAKADSSPVTEADEAADALITAELKQAFPDLLIVTEEQADTHAERAADFLIVDPLDG
TKEFVKRRGDFTVNIAWVEAGVPVRGVVYAPARGRMFYTRADGSTVEETGDLELDTVGPVTPLSVSTPDNDALMVVA
SKS
HRDQATDDYIAKYAVRDMTSAGSSLKFCLVATGEADLYPRLGRTMEWDTAAGHAVLAGAGGTVVRFDDHSPLSYGKP
GYE
NPFFIALAPGVDLKGA
>OLS47086.1 3-deoxy-manno-octulosonate cytidylyltransferase [Rhodovulum sulfidophilum]AB14
MSVVIVIPARHASSRYPGKPLALLKGASGQEKSLIQRSWEAAQAVSGVDRVVVATDDDRIRAAAEAFGAEVVMTSETC
AN

GTERVAEALDLLGGEVEIAVNLQGDAPLTPAWFVEDLVAGLRADPGADLATPVLRCDGQALTALLEDRRAGRVGGTTA
VF
GAGNRALYFSKEVIPYTGQSYADRAPTPVFHHVGVYAYRPAALETYLRLAPGPLEGLEGLEQLRFLEHGRPVLCVEVEAR
GRQFWELNNPTDVPRIEAMMAAMGLD
>OLS47087.1 UTP--glucose-1-phosphate uridylyltransferase [Rhodovulum sulfidophilum]AB14
MFPVAGLGTRFLPATKSIPKEIMTLVDRPLIQYAIDEARAAGITDFIFVTSRGKSALEDYFDHAPHLEDSLRSKGKDDLL
EILRSTDMESGAIAYIRQKRAMGLGHAVWCARRLIGNEPFAVILPDDVIAAETPCLQQMTEAYRETGANMVAAMEVP
PEK
ASAYGILDIARDMGDRVLVKGMVEKPALEEAPSNLAVIGRYILTPKVLHNLDQNLRHERFGAGGELQLTDAIAQEIDGQ
G
VYGYRFGGQRFDCGSKAGFLQATVSFALARPELKGEFEDYLRDMFARPEAAE
>OLS47088.1 UDP-glucose 4-epimerase GalE [Rhodovulum sulfidophilum]AB14
MAHVLVTGGAGYIGSHACKALARAGHVPVCYDNLSTGWAEAVKFGPFEQGDLADRARLDAVFAAYRPVGVMHFAA
FSQVG
EAMRDPGKYWRNNVGGSLALIESAVAAGCLNMVFSSTCAVYGDQDGVKLDEDSAQAPLNAYGASKRAIEDMLRDFE
ASHG
LKSVIFRYFNVAGADPEAEIGEAHDPETHLIPVMLEAIEGRRPALTIHGTDYPTPDGTCIRDYIHVSDLVAAHVLGLDRL
LAGKGSRVFNLGSGTGYSVREVIEAAQAVTGRAVPHSEGPRRGGDAVRLVSGSARAETELGWVPTRSDLPTMIEDAW
KWF
RSGGYKS
>OLS47089.1 PTS lactose transporter subunit IIC [Rhodovulum sulfidophilum]AB14
MQLSTLITPEAVRVFGTMSSKKRLFQELGEVAHAAYTLPPGDAVDALQERESLGPTGVGQGVALPHARLSGIDRVRGA
FI
RLEKAIDFGAVDRQPVDLIFALFAPQDSGVDHLKALALVSRTLRDNTICAKLRANTDPATLHAVLTEAAASQAA
>OLS47090.1 ribosomal subunit interface protein [Rhodovulum sulfidophilum]AB14
MLYQISGKQIDIGEALQTHVKDELTVAVSKYADRATDANVVFSRNAHQFVCEATVHLSTGLTAQASAQATEIYAAFDSC
C
DKMEKQLRRYKRRLKDHHRERSEPVELSGGSAYILASTEEGDESDSLQPMIIAEMETQIPSISVGEAVMQMELRGAPLL
V
FRNEKTGKINVVHAREDGNVGWIEP
>OLS47091.1 hypothetical protein BV379_01530 [Rhodovulum sulfidophilum]AB14
MSLETSGKDLVLSVRAGLAALALVLTIPAGAGAQGAEIAFGSLNHDSSLPVEVTAESLSVDQGDGTAVFTGDVVVGQG
QM
RLAAGRMLVVYGAGGAGGGRISRLEASGSVVLTLDAEAAEADKAVYDIGAATIVMTGNVLLTQDGNAIEGQRLVVDLT
RG
TGSMEGRVRTVLTPSDKP
>OLS47092.1 hypothetical protein BV379_01535 [Rhodovulum sulfidophilum]AB14
MQAHDNAYSRFIAAAKIVLPLAALGLLSTLFMISRDHGGGDDGLPPRTQVEIEGLLREQRLGAPNYAGVTRDGTAISMR
A
DTARTDPDNHGRSEASNIQARLDLPDGSSAEIEARTGAIDTDDQAAVLEGDTRISTSTGYRIETDRLRSAFADTRVEADG
PVDAEGPPGHIEAGSMTLGPDPEHEGRYLLVFKDGVRLVYEPRN
>OLS47093.1 D-arabinose 5-phosphate [Rhodovulum sulfidophilum]AB14
MTPETCLSIGRRVLRIEADAVHRLAEGLDQEFIRAVETILGATGRVIVSGMGKSGHVARKIAATFASTGTPAHFVHPAEA
SHGDLGMVARGDVCLVLSNSGETPELADIVAYTRRFGIPLIGVAGRPRSTLLRQADVALLLPSADEACPMGLAPTTSTT
M
TLALGDALAVALMEHRQFTPEHFREFHPGGKLGARLSKVADLMHKGEDIPLVATGTGMAEALIVMSRKSFGVVGVTTP
DG

RLAGVVTDGDLRRHMAGLLDRSVDEVMTANPLTISPEVLAEKAVALMNERKITCLFAVGADGRPGGILHIHDCLRAGV
A
>OLS47094.1 ribonuclease D [Rhodovulum sulfidophilum]AB14
MANFLHKNDLPDDLDLGPMVAIDCETMGLNPHRDRLCLVQMSAGDGDAHMVQIAQGQTEAPNLTRMLADPDTLK
LFHFGR
FDIAVLEHTFGVTTAPVYCTKIASKLVRTYTDRHGLKYLLQELLAIDISKQQQSSDWGAAALSPAQLDYAASDVLYLHRL
KEVLDGMLAREGRSEIARACFDFLPTRARLDLSGWPETDIFAH
>OLS47095.1 complex I NDUFA9 subunit family protein [Rhodovulum sulfidophilum]AB14
MSKLVTIFGGSGFVGRYVARRMARAGWRVRVAVRRPNEALFVRTYGAVGQVEPVLCNVRDRESVRRALQGAEAAVN
CVGI
LAETGRNTFGLVQHYGALHIAEEARAAGVERLVQISAIGADIESDSGYAETKGRGEAAVRESFPDAVILRPSLVFGPEDQ
FFNRFAQMARFSPVLPVVGCYTRFQPVYVDDVAAAAAKAAMGEAAPGIYELGGPEIRTFGELMEEMFTVIGRPNRIVL
PL
PLALGAAMGTVFDLVQSLSLGLLENKVVTRDQVRNLGRDNVVGEGLPGLAELGIQPTAMEAVLPEYLWRYRPAGQFY
DVR
EAAKRLKS
>OLS47096.1 undecaprenyl-diphosphatase [Rhodovulum sulfidophilum]AB14
MTLFHLFLIAVIQGITEFLPISSSGHLILLPGLTGMADQGHIIDVAVHVGTLAAVILYFRKDAMLALGGLTRVARGQIDT
PGAFLALCLIIATIPVMAVGLVLELTGLEDMMRSIAVIGWTMLIFGLVLWVADTRFPATRKANSWTLKHALIMGLWQV
LA
LIPGTSRSGITITGARMLGYDRVGAARLSMLMSIPTILASAVLVGGKAATEADMAVLRDGAIAAGFTFVAALAALAVM
MR
LARSVSFTPYVIYRVILGLVLLWIAYT
>OLS47097.1 dihydropyrimidine dehydrogenase [Rhodovulum sulfidophilum]AB14
MAQQKMLQFVRVAKETPEKREADDRAQDFGEIYAEFAAEKAAEQASRCSQCGVPFCQSHCPLHNNIPDWLRLTAEG
RLEE
AYAMSQATNTFPEICGRICPQDRLCEGNCVIEQSGHGTVTIGAVEKYITDTAWEEGWVPPIAPVEERSESVAIIGAGPG
G
LAAADRLRRAGLQVTVYDRHDRAGGLLTYGIPGFKLEKDVVMRRVEQLEKGGVEFVLNCNVGTDISFDAIRGKHDAVLI
A
TGVYKGRDLTGPGAGAQGIVAALDYLTASNRVSFGDEVPEYLSGALNAEGKRVIVVGGGDTAMDCVRTAIRQGAVSV
KCL
YRRDRANMPGSQREVQNAEEEGVDFVWLSAPNGFAGDPVSSVMVQKMRLGPPDASGRQSPEVIEGADYAEPADLV
IKALG
FEPEDLPALWGEEALEVTRWGTVKADFRSHETSLPGVFAVGDIVRGASLVVWAIRDGREAADSILDFLAQPAQVAAE
>OLS47098.1 hypothetical protein BV379_01570 [Rhodovulum sulfidophilum]AB14
MSDTTLKGHCLCGAVTVEIDLAHRVLGACHCGMCRKWTSGPLMAVHTTQPIRAEGPVKTYASSQWAERAFCSECGSS
LWY
AFTAEGAHKGHGFVSAGLFEGTLDLPLNSEVYIESKPAGYAFAGDHVRLTEAQMMASAADGMSMGSTGSEGEQQ
>OLS47099.1 glutamate synthase large subunit [Rhodovulum sulfidophilum]AB14
MTKYDAAWVAREEAKRAFVAENGLYSPEDEHSSCGVGLVVSIDGKPSRKVVENGIDALKAVWHRGAVDADGKTGDG
AGIH
VQIPVPFFYDQIRRTGHEPHADRLIAVGQVFLPRTDFGAQEVCRTIVETEVLRMGYYIYGWRHVPVNTDVLGEKANATR
P
EIEQILISNSKDVDEETFERELYVIRRRIEKAAAAAHVPSLYICSLSCRSVIYKGMMLAEQVAEFYPDLKDDRFESAFAI
YHQRYSTNTFPQWWLAQPFRMLAHNGEINTLKGNLNWLKSHEIRMASSAFGELAEDIKPIVAQGSSDSAALDSVFEVL
VR

AGRNAPMAKTMLVPEAYSKQTTDEMKPAWRDMYAYVNSVMEPWDGPAALAMTDGRWVCAGLDRNGLRPLRYV
VTGDGLLI
AGSESGMVPTEESAVLEKGALGPGQMLGVDMATGKLFRDSEIKDMMAASQPFGEWLDTVSEIDEVLGDLPENVLFD
GADL
RRRQIAAGYTIEEIESILMPMAEDGKEALASMGDDTPSAVLSEKYRPLSHFFRQNFSQVTNPPIDSLREYRVMSLKTRFG
NLRNVLDEDGGQTRIVILESPFLGNAQFNELVKHFSVPMAEIDCTFPPGAGPEALRLGLQRIRNEAEDAVRSGAGHIVLT
DHHQGEDKVAMPMILATSAVHSWLTRKGLRTFCSLNVRSAECVDPHYFAVLIGCGATTVNAYLAEDTLADRIKRGLLD
CS
LTEAVSRYRKAIDLGLLKIMSKMGISVISSYRGGLNFEAVGLSRAMCAEYFPGMHSRISGIGVTGIQRKLEEVHRLGWKG
GQDVLPIGGFYKARRTGERHAWEASNMHMLQQACNTASFEVWKQYSAAMRSTPPIHLRDLLDIKSLGKPVPLEEVESI
TS
IRKRFVTPGMSLGALSPEAHKTLNVAMNRIGAKSDSGEGGEDPAHFYPEPNGDNPSAKIKQVASGRFGVTAEYLNHCE
EL
EIKVAQGAKPGEGGQLPGMKVTDLIARLRHSTKGVTLISPPPHHDIYSIEDLAQLIYDLKQINPRCKVTVKLVASSGVGT
IAAGVAKAKADVILISGHNGGTGASPATSIKYAGLPWEMGLTEAHQVLSMNNLRERVTLRTDGGLRTGRDIVMAAM
MGAE
EYGIGTAALIAMGCIMVRQCQSNTCPVGVCTQDEKLRGKFTGNADKVVNLITFYAQEVRELLSQIGARSMDEIIGRSDLL
NQISRGSAHLDDLDLNPLLIQVDANDRPTLDRTRSRTEVPDTLDAEILRDAAPFFEDGEKMQVSYAVRNTDRTIGTRVSS
LIVQRFGMRNKLQPDHLTVKLAGSAGQSLGAFGAPGLKIIVAGDANDYVGKGLSGATIVVRPPLSSPLVASENTIIGNTV
LYGATEGYLFAKGRAGERFAVRNSGARVVVEGCGSNGCEYMTGGIAVILGTIGANFGAGMTGGMAYLYDPEGAAAEK
INP
ETLVTCRIAVQHWEAELKGLISRHFDETGSPKARDLLTHWELEKGNFLQVCPKEMLPHLSHPLGIEERAVPAE
>OLS47100.1 hypothetical protein BV379_01580 [Rhodovulum sulfidophilum]AB14
MADLIAVAFDDESKAFDMRGEMVRMQKDYLLDMEDVVVVTRDAEGKVKLHQAVNLTAAGAVGGSFWGLLIGLLFL
NPLLG
LAVGAGAGAISGALSDIGIDDGFMKELGETLIPGSSALFVLVRKATGDRVIERLREVNVEGRILRTSLSKEDEATLREVL
EKSRAMAE
>OLS47101.1 glutathione S-transferase [Rhodovulum sulfidophilum]AB14
MNRLYHMPLSPFCRKVRLVLAEKRIEVELVEERYWERDADFMRRNPAGKVPVLRMDGRIMAESQAICEYLDESFPDPC
LL
PGSPAERCEARRLVFWFDDKFHHEVTSKLLYERVNKKVTKSGYPDSRNVKAGATAIKYHLDYMGWLLDQRRWLAGDV
MTL
ADFAAAAHLSCLDYISDVDWNRNAAVKDWYAKIKSRPAFRNILADQVPGFPPPRHYANLDF
>OLS47102.1 tRNA epoxyqueuosine(34) reductase QueG [Rhodovulum sulfidophilum]AB14
MTAALKDRLAARAREEGLAAMGVCRPDAIPEAAGRLAEFVAKERHGQMGWMAERMSWRGNPAALWPEARSVVM
FAEPYTQ
AHDPLAALDRPDRAAISVYAQNRDYHDVLKKRLKRLGRWFLEQAPGHEIKVFVDTAPVMEKPLAQAAGLGWQGKHT
NLLG
RRLGNWFFLGVIFTTVELPADAPETGHCGNCTACLDACPTGAFPAPFQLDARRCVSYLTIEHKGPVDPELRPLLGNRIYG
CDDCLGICPWNKFAVAATETKYAARPDLIEPPLAELAGLDDAAFRARFSGSPIKRIGRGRFVRNVCYAIGNSGDSRLRDA
VARLLEDADPVVAEAARWAVARLSRG
>OLS47103.1 EamA family transporter [Rhodovulum sulfidophilum]AB14
MNPSRAIGLKLTSVTLFVVMSALIKAASEQGVPPGETVFFRSFFALPVILVWLVWTGQLGAGLRTKDPLGQFWRGLVG
GT
AMVLNFAALGILPLPEATAISYSSPLLVVIFAAMFLGEKVRLFRLSAVALGLLGVLVVISPRLSSFGADAVDARATLGAV
VALGGASCAALAQVFVRKLVQTERTSAIVFWFSVVATVLSLFTIPFGWVMPSPGAAAMLILAGLVGGLAQICLTSAYRH
A
EAGLVAPFDYASILLALVIGYFVFAEVPTPMVLIGAGLIISAGVFIIWRERQLGLERGRARKGITPQG

>OLS47104.1 branched-chain amino acid aminotransferase [Rhodovulum sulfidophilum]AB14
MTGYDDRDGVIWMDGKLVPWRDANVHILTHAMHYASAVFEGERAYGGKIFKSHEHSERLKRSANMIDFEIPYSVEQI
DAA
KAEVMKANGLTDCYVRPLAWRGSGPEMGVAAAQNPVRMAIAAWEWGAYYGAAKMKGAKLDIAKWKRPDPATAP
SQAKASG
LYMICTMSKHAAQAKDCSDAMMFDYRGYVAEATGANIFFVKDGEVHTPKADCFLNGITRQTVIGLLKDKQIKVHERHI
EP
AELEGFQQCWLTGTAAEVTPVAEIGDYNFEVGALTHDIAETYETFVRA
>OLS47105.1 MarR family transcriptional regulator [Rhodovulum sulfidophilum]AB14
MAQIAPAVTQKSGESLLFLTDEQLRKGIEAMFFAYRGFTADPDRILEERGYGRAHHRAVHFIHQAPGTTVNNLLSILGVT
KQSLNRVLRALIEDGLVESRVGHRDKRERHLYLTEAGLDLEHHLSDAQRARMRDAYRAAGPAAVAGFRQVLEAMMDP
EMR
RHFHRITERGA
>OLS47106.1 acetoin utilization protein [Rhodovulum sulfidophilum]AB14
MTTAFLTHPDGLDHATPPGHAECADRLRAVTGALTTEGFAGLLREEAPLADEAELLRAHPQDYIDAVRAAIPAEGTVPL
D
PDTHVSSGSFRAALRALGGTLRAVDMVMAGAAANAFVAMRPPGHHAEAARAMGFCLFSNIAIAAQYALDRHGLARV
AVVD
FDVHHGNGTQDLLWSEPRTLFVSSHQMPLYPGTGEAAETGAADNVLNLPLPPRSDGRAFRAAFEAHALPRLGAFAPEL
VL
VSAGFDAHAADPLAELAWRTEDFAWITHRLCDIAETHSGGRLVSTLEGGYDLEALGASAAAHVAVLMERGA
>OLS47107.1 exodeoxyribonuclease VII small subunit [Rhodovulum sulfidophilum]AB14
MTEKPIGEMTFEDAMRALEEAVAALERGEVPLEESIALSERAMKLRQHCEAKLRDAEAKIEAMTLDGAGNPTGTKPAE
GL
>OLS47108.1 farnesyl-diphosphate synthase [Rhodovulum sulfidophilum]AB14
MFQSSLKSAAERIETRLTEALAGAADQPVTQAMRYAVSGGKRLRGFLVLESAALYDIAPEAAVDAAAAVEALHAYSLVH
D
DMPCMDDDDLRRGQPTVHVKWDDGTALLTGDALQTLAFELLSAPACHPDPAVRIDLVASLARASGIDGMVLGQAQD
IAAE
TAGRSLTLDEITELQANKTGALIRWSAEAGARLARADIAPLTAYSTALGLAFQIADDILDVEGDAAKTGKRVHKDAEAGK
ATFVSLLGLDGARARALALVEEAEAALDPFGGRADPLKAAARFVISRES
>OLS47109.1 1-deoxy-D-xylulose-5-phosphate synthase [Rhodovulum sulfidophilum]AB14
MNNRPNTPLLDGIVSPSDLKALSDAQLGQVAQELRQEVVSAVSETGGHLGAGLGVVELTVALHAVFDTPRDKLIWDVS
HQ
CYPHKILTGRRDRIRTLRQQGGLSGFCKRSESEYDPFGAGHSSTSISAALGFAVARDLGTAPEAGVGDAIAVIGDGSLSA
GMAYEALNHAGHLGKRLIVILNDNEMSIAPPVGAMSRYLNRLYAGAPFQEFKSIAKGAIRHLPEPFQEGAKRAKDLVKQ
V
TVGGTLFEELGFSYVGPVDGHDLDQLLAILRTVKTRATGPMLIHAITKKGKGYAPAENAPDCGHGVSKFDIETGVQKKTP
ANAPSYTKVFAEALIREARTERKIVAVTAAMPDGTGLNLFAERFPERIFDVGIAEQHAVTFSAGLAAGGQKPFCAIYSTF
LQRGYDQIVHDVAIQRLPVRFAIDRAGLVGADGATHAGAFDTAFLANLPGMVVMAAADEAELVHMVATAVAHDEG
PIAFR
YPRGEGVGVELPEKGVPLEIGKGRIIQEGKRVALLSFGTRLKEVREAAQALAAEGIEPTIADARFAKPLDEELILRLARE
HEALITIEEGAIGGFGSHVAQLLAERGVFDNGLKFRSMVLPDTFIDHASAEAMYADARLNAADIEAKLREVLAGGAAQA
A
LRA
>OLS47110.1 NAD(P)-dependent oxidoreductase [Rhodovulum sulfidophilum]AB14
MTGTLLSIGHGYSARELAQSLEPGWRVIGTSRSAEGAEALRRTGIEARIWPGDDLGPDLAAATHLMTSVPPGASGDPVL
A

ALGETLRAARHLRWVGYLSTTGVYGDHGGDWVDETAPLTPATSRGEARVAAEAGWAALGLPLHIFRLAGIYGPGRSPL
DR
VREGRAQRIVKEGQVFSRIHVADIAQVLAASMAAPDPGAIYNLADDEPAPPDEVVAFAAEMLGLPVPPAIPFEKAELSP
M
ARSFYAESKRVRNDRIKTELGVHLRHPDYRAGLAALLETERAAMIR
>OLS47111.1 AMP nucleosidase [Rhodovulum sulfidophilum]AB14
MPDQSDLPLSSPSLPEAEAFTDAAAAVGRLVELYEAATGFLRARFAEVLSGSAPHARFRAVYPEIRFTTVSHALVDSRLS
FGHVAEPGTYVTTVTRPDLFANYLRQQIGLLLENHGQPVVIGPSTTPIPIHFAMGSAGVTVPQDGALEVPLRDLFDVPDL
TTTNDDIVNGTWTMPEGAARPLAPFTAQRVDYSLARLAHYTATAPEHFQNHVLFTNYQFYVEEFEALARRALADPDSG
YT
ALVGPGNAEIRAPETQLPVPLKLPQMPSYHLKRADGQGITLVNIGVGPSNAKTATDHIAVLRPHAWLMVGHCAGLRN
SQR
LGDFVLAHGYLREDHVLDNDLPLWVPIPALAEIQVALEDAVEEVTQLEGYGLKRIMRTGTVATIDNRNWELSDQTGPV
QR
LSQSRAIALDMESATIAANGFRFRVPYGTLLCVSDRPLHGELKLPGMASDFYKTQVARHLAIGVRAMELLRDMPLERIHS
RKLRSFEETAFL
>OLS47112.1 DNA-binding protein [Rhodovulum sulfidophilum]AB14
MAKPMTKTQLVAALAEEMEADKKTASAALEAMTAIITREVAAGGSVTLPSVGKIYCRERPERMVRNPATGEQFKKDA
DKV
VKVTIAKALKDSVNG
>OLS47113.1 DeoR family transcriptional regulator [Rhodovulum sulfidophilum]AB14
MQPYERHAKILERLGHDRRVFTAQLARMFDVSHETVRRDLLEMEQSGSLTRVHGGAVAADREILPEPAFSERRVVHAD
KK
AAIGTLAATLIPPGSTVFIDAGTTTCAFARALAREGEMRIITNSIEIAQLAGPARDCDTLLLGGRPHTDVPATYGEMTLS
EIDRFLADYAVLSPVGLHADRGATDYALHEAEVARAMMRRSRDCIMLCHSEKIGTESRVSICRLDEIDHLVTDDAADRSL
RLPRGEIHYAETRLDL
>OLS47114.1 histidinol-phosphatase [Rhodovulum sulfidophilum]AB14
MSELGDALRFANDTADEAGRVALKHFRQALDVESKADDSPVTLADRAVEALMRDRIMARFPGHGIFGEEQAPLRPDS
DHL
WVVDPIDGTKSYVTGNPLFGGLMALLRDGAPCLGQIDMPALGERWCGVEGQATTLNGRPCRTSTCTDPAEAFAYTTD
PML
FSGTDSEVLEMLRRSVRMLRFGGDCYAYALLASGHCDLVLETGLQPYDYLPLVQVIRGAGGVITDWQGQALGVGSAGE
VL
AAATPELHAAMLDRIARLRAGQAA
>OLS47115.1 diguanylate cyclase [Rhodovulum sulfidophilum]AB14
MDRRSFLRYGALAGTALGAARINPDFFFSSAFAQEARPLVFLSAENITGNWDPTAHTTLSQTNIEGFVMGYLTRAPMR
PD
DPEKVVYELATEITELDAHRLQIKLRDGITFHDGKPFTAEDVKATFEYGAQLDRPKQVYPGGPDTFAVETPDDHTVIVDT
SKGGYGASLFIFLASYLPILSAKDVAGGPKGPLSQRLNGTGPFRFVEQRGNDTVMEAYEGYFRGAPKVTGVTFSFVGDA
T
TRMLSLMNGQADVIERLEPEQVETLEARDDIKISRLVSVENKYLWFRCSKPPFDDWRVRRAACHAIDRSMIMEIMGSA
GE
ASSNFISPIKFGYIDLENYPEYDPEECQRLLAEAGYPGGEGLPELEYITSTGFYPKTKEYGELIAALLQEQGFPVKLNVM
EVAAWNERLYDRPGGGPGHMVDCGWSTGSPEPDLVLRTHFHSSAKRICGIVDPEIDAALDAERDAPSLEARKESLQTN
LM
PMLADKAPALSLFTSVLIHGMRSNVDGLFIYPDGQSDASRTTLS
>OLS47116.1 peptide ABC transporter permease [Rhodovulum sulfidophilum]AB14
MFILRFLARRLGQGAIIIFLVSALIFTLLRVVPGDPVRLMVGGMAPDTLVEEIAAEMGLRDPIYVQFGRYMGNVLKGDLG

ESFVRPASGAAVGGASFDDSTRGERAKVLDLIAETAPMTLQLALLAMVFALMIGVPIGVWGGLYPGRLPDRLALYTSSV
F
VSLPNFWLGIVLALLLSVKLNLLPAIGYKGFSYTILPALVLAVEIAPFIIRTLTVSVAGVMGQSFIDIARVRGLSRNQIV
FRHALKNSAVPLLNLLGVQFSMLLGGVLVIEFIFDYPGLGLLTINAVMQRDFPLIQGIAIVTAAVFVLINIAVDLLATAI
DPRLDY
>OLS47117.1 peptide ABC transporter permease [Rhodovulum sulfidophilum]AB14
MTSTDMPLAMTGPETGRRVSTRIWQTAFASVEFRIGLTVFVLLLLAALLYPELSGIDPTKMNIRAKLTPPVFMGEGWS
WA
HPLGTDQLGRDMLSRALVGLRYSLLIGVSTTVLMLLAGAMIGLFSGYFGGRTDTVLMRLTDAQLSIPMIILAITILGVSR
PTIPSIILVLGLAGWPVYARVMRSTVMSERKKEYVRGAMVLGATDLRIMFTLLLPLVLPPMAFVAVLDIARMMIFESVLG
FLGLGVQPPTPTFGNVIADGRKYLMNAWWIATMPGVFLVLTLTSINLMGASLERARNAIYGGA
>OLS47118.1 glutathione ABC transporter ATP-binding protein [Rhodovulum sulfidophilum]AB14
MALDMTATPETLLPDTLLSVRGLNVVAGDRPILTDISFDLAPRSILSVIGATGSGKTVLARALASWLSAPLRVTGGEIRF
RGRDLVADPAHARNLAGREIAYVGGNPAGALDPTMTVGAQLVEKLRAVRPDISADAARQKAIRLLEAVHIPSAARRFH
EY
PFQYSGGMMQRAMIVDALISDPALLIADNITQPLDVTVAAQILKLMRELNSEFSTAILFICSSLPVACDASDEVMVLHEG
RVVERQTPAALVAGPEHSYTRALIAELPTLWQDGQTGAGRDIDRNRRAIMSVRGLAKAYEVRARGQAGVSRVQAVRN
VEF
DVFPGDNFGVVGESGCGKSSLMRLLTGLERPDAGSVFFEGENVAAASPRRLRELRRRFQLVLQDPYGCLPPQSAIGAILE
EPLKIHRIGNAAERRDRARAVMAEVGLDDSLYRSLPTGLSAGQRQRLNVARAMILEPRLLIMDETLSALDQTEQGKLLD
L
FDKLQAQHGFTYIFISHDLTMVRQVCSRIAVMYLGETVEVAPNERLFFDPGHPYSRALLSAAPTLEERRYRPEDCLLEGE
PPSPIDLPPGCAFASRCPQAFERCRAENPALRARGGHALAACFLNPEPDIALPENALHETATETPHA
>OLS47119.1 Zn-dependent hydrolase [Rhodovulum sulfidophilum]AB14
MPEMPSLARIDADLLHGLMEKVSEFGSTGDGGVDRPVLTDAHKAARDWFRSELTARGYSVLVDAIGNLFGRIDLAGPD
AP
LVMIGSHLDSQPKGGRFDGAYGVMAALAAVESFRKGGGTPRCNYVIADWMNEEGARFQPSLLGSSVFAGLVDLDWA
LARR
DRDGKSVGEELVRTGYKGTDTAPRPDLYLEIHIEGDARMETAGARIAPFLRHWGALKVRIEVTGEQNHTGPTPMEDRK
DA
VLGAAHIIAEIRRLADIAEDTLYTSVARVDISPNSPNIVPGKAVLFCELRAPEPAMLDWSEASLRAALPELAAKAATHAE
IVSIDRRPAGKFDPRLARLTERVADDFGLPRMQLDTIGGHDAVAVNAILPSLVFAVPSVGGVIHRNDEYTSPEDLAAGG
D
VLTDMVRRIDRAGGDLDLALGANA
>OLS47120.1 aminoglycoside phosphotransferase [Rhodovulum sulfidophilum]AB14
MTGALVTDQMMAAACAAAGLSDADARPGPAPLASPSYLALESASILTAGGVFLKVMHPEMREGFDLDAAMTLARQA
GAAG
VGPRVLWSDAGQGAIAMEALTEAGGWRRAMQKDLQAPPVLAGAMAALKALHATPALANRFDPFAQIDALIAEYRRL
GASL
PDDIDWLRRLIGAAEPVLRAGATLAPCRNDGSASNLMLGPDGAVLLVDYDRAGMNDPLYEVGALLAETTDFERDMQA
GFA
AYAEGFDETGFARARLWSFVDDMLHALWSRLKARTSVRGGVEWLKYGEWRLMRLRMALNHPGFEQKIRLAGGAA
>OLS47121.1 ethanolamine ammonia lyase large subunit [Rhodovulum sulfidophilum]AB14
MPRYSATAGGERFVFGSLARLLAAATPERSGDQLAGIAAESATERVAARRALADLPLAEFLTEAVVPYEEDEVTRLILDT
HDRAAFAPFAAMTLGDFRAWLLSPAADAARLAEAAPGLTPEMVAAVSKLMRNQDLIRVARKVEVVTAFRNTIGRAGT
LST
RLQPNHPADDARGIAVATLDGLLFGAGDAVIGINPASDNLENCAGLLSALDEMVAGLAIPTQTCVLTHVTNALRLIEGG
A

PVDLIFQSVAGTEAANEGFGVSLSVLAEAHEAGRALGRGTVGDNVMYFETGQGAALSAEAHHGVDQQTLEARAYGV
ARAF
RPLLVNTVVGFIGPEYLYDGKQIIRAGLEDHFCGKLLGLPMGVDVCYTNHVEADQDDMDVLMTLLTRAGVTFLITVPGA
D
DVMLGYQSLAFEDIGYLREVTGLRPAPEFEAWLARMGLAQGAAALPDPAALGKRMGILT
>OLS47122.1 hypothetical protein BV379_01705 [Rhodovulum sulfidophilum]AB14
MSDSVSTGFGRLRAMTPARVRLDAAAGPTPLGALLDFQEAHAGARDAIHARPDWDALAAALAPLATIRVQSQAVERS
IYL
RRPDLGRRLAEPVDLPRAGHPLVVVVGDGLSAPALAHAAPVIAALRPALPELDATPVILAEGARVALGDDIARRMGAA
MC
LMLIGERPGLSVSDSLGAYLTADPAPGTRDSARNCVSNIHRMGGLSPEAAAARIAWLIGAARKLGRTGVALKDESGTRP
A
GALAP
>OLS47123.1 ABC transporter ATP-binding protein [Rhodovulum sulfidophilum]AB14
MTRVLTIEGLSVSAAARPLLRDLSLEIAQGRTLALVGASGSGKSTACAAALGVQAGNLEVSGQVCLDGNPVPPDRLRGR
V
VASILQNPRSGFNPLRTMRDHMRETCRALERPWAEDTARHLLAEVGLEDPDRVLGLYAFEMSGGMLQRVAIALALLG
GAP
FLFADEPTTDLDLVVQARILDLLERSRDRARLGLLLITHDMGVVARLADEVAVLEAGIVVERGPVGQIFHAPRHPVTRML
VAAHLALYGKELPA
>OLS47124.1 nickel ABC transporter permease subunit NikC [Rhodovulum sulfidophilum]AB14
MSLALNRRQAGPVWRRLRLGAACLIVALLVLAALVGPALAPFEPDAVDIVHRLQPPGEGHLLGTDHLGRDIFSRLIAGTR
TSLGCVAATLVLILIMGLGLGGLSGYAGGRIDQAIMRVADIFMTFPTLILALFMIGMLGTGLVNVIIAIALSHWAWYARI
ARGIVMSMRHRDFLLAARLAGASRARIFIEHLLPATFSQMIVLATLDVGHMMLHVSGLSFLGLGVQAPTAEWGVMIA
DAR
QFVWTAPMLIFWPGLALFLTVMAFNMLGDALRDRLDPGLVGEAAH
>OLS47125.1 nickel ABC transporter permease subunit NikB [Rhodovulum sulfidophilum]AB14
MKAFILRRLLLLIPMLFGASLVIFLMLRLGPSDPALDYLRLSKLPPTPQALADAREMLGLDRPLATQYLDWIGRALRGDF
GTSYALQRPVLPDILHFLPATMQLAGTALAFTLLVSVPLGIWAARYRDRWPDHAVRLIAFLGVSVPNFWLGFLLVLIFSV
HLGWLPPMGREGPASLVMPVIAVALMSLAVNARLLRASMLEVAGQRHVRYARLRGLPPRMVERRHVLRNAWLPVIT
ATGM
HIGELLGGTLVIESIFGWPGLGRYAVSAILNRDYPVIQCFTLLMVVIFVLCNLIVDILYALADPRIRQATQAPQ
>OLS47126.1 Maebl [Rhodovulum sulfidophilum]AB14
MPETKPPLLAVLIDADNIPARHAEPILKEITGFGEPALRRVYGDWSSDRLKPWADKVLTLGLVAHQETANTKGKNASDI
G
LVIDAMDILHSGRFDGFVLVSSDSDFTRLASRIREHGLDVIGIGEAKTPVSLRNVCNRFVLIENIVEEPAPAAPRDKAQP
APTAAGKRPPQEVSELIIRAMDKINQEDDWFTLGQIGQFITADNPDFDTRTYGKRKLSDLVRELKRFETRQVGNQLLVR
R
LD
>OLS47127.1 hypothetical protein BV379_01745 [Rhodovulum sulfidophilum]AB14
MTRVTIFALAAAAATLAHAADATDPAVKARQEAMETIGKNMKSIADMAKGKADFDAQAAGAAAAVIAETADKVPGL
FETR
ADDPESEAKPAIWDDFGDFTEKSEALQAAAGSADMASLDALRNSVGEIGRTCKSCHDDYRVKN
>OLS47128.1 hypothetical protein BV379_01750 [Rhodovulum sulfidophilum]AB14
MSVIPPMPTPAERPAEQVAFHRSELGVILPLYGRMVAAGEWRDYGISCLRDVAIFSIFRRTAEHPLYRIEKRPRLRSRQG
MYSVIGMDGRILKRGHDLPGVLRVLERKLIRPVD
>OLS47129.1 NLP/P60 hydrolase [Rhodovulum sulfidophilum]AB14

MSGPAGRDRRETPANDRVAAARLRGVIEAPRYAEPERWRVTRPVTDLLRAPEGPRERQLVLGEAFELLEMHDGVAFG
WAA
RDGFVGYVAEAALAADGPAPTHRVAVPASHAYSAPDLKTPELMGLSFGSELRVVSAAGGFFETAEGWFVPRPHLRPLN
VP
FRDPVTVAQLFFGVPYLWGGNSVWGIDCSGLVQAAWRAAGLDCPGDSDQQERVLGEALPEGTPFQRGDLLFWRGH
VALAV
DAEVLIHANGHTMSVAYERIADAISRIEAQGEGRPTAHRRMPVGAD
>OLS47130.1 leucyl aminopeptidase [Rhodovulum sulfidophilum]AB14
MCPRFADPAAPSRPLFVVDEAAFETWRSGRPAAEAAWLEATGFAAGLGELQLLPGPDGSVAGAVAGHGTAAARARG
RFHL
AAIAGRLPEGAWHLETALTGADLEAEALGLLLAGYRFDRYRTQSGAPRASFKAPAGVDAARLEAMAAGEVLTRDLINTP
A
SDMGPEALEEAVAALAARHGATLEAIRGEALLARNFPMIHAVGRAAAQPPRLLDLHWGRSGPRLTLVGKGVCFDTGG
LNV
KPASSMGLMKKDMGGAATVLGLASMIMALDLPLRLRVLIPAVENSIAGNAMRPQDILTARNGLTVEINNTDAEGRLVL
AD
ALAYAAEEESDALISMATLTGAARVAVGPDIAPFYTDDEAVAGALARAGGLLADPVWRMPFHAPYETMIEPGIADLDN
AP
KGGFAGSITAALFLRRFVGAARYTHFDIYGWQPADAPARPKGGVGQGARAILGALPGVLKL
>OLS47131.1 hypothetical protein BV379_01765 [Rhodovulum sulfidophilum]AB14
MRQDEPLRLDSERLADMFVDLGEVRASHLIVLSIDRLETLIEGLHLAIGQMPPGDCIDLTGQIRALGDAMGMISLSSAAR
NVSRAAQSGDPVAIDATLARLKRVAVRSFRLAEELQHRSG
>OLS47132.1 carbonic anhydrase [Rhodovulum sulfidophilum]AB14
MKNARPLPSYLVQRYHGWKATTHAENKAWYRRLAEEGQRPRAMIISCCDSRIHVTSIFGADEGEFFIHRNIAGLVPPYA
P
DGDHHGTSAALEYAVTVLKVANLLVVGHSNCGGIRGCHDMCAGLAPQLEEKTSFIGRWMDLLRPSYERQAGIADETE
RLR
AMEKEAVLVSIENLMTFPFVQAAVESESLTLHGLWNDIGEGVVECYDPDSARFLPV
>OLS47133.1 hypothetical protein BV379_01775 [Rhodovulum sulfidophilum]AB14
MRALALCLILVPLPGWADDILVSSRVEAVTLFPDGAAVTRSADFELPAGTHRLILADLPRTTPLATVRVAVEGATLGSVS
ARRDFVLPRGAETPPEVAEAEAEVERRRTALDAAMAEISRIRAGAQGAEARAAFLGRLGHEGAAAMGVEDLQALARM
IGA
ETETALTDSIAARDRAAAAERGLEPLREALAEARQALAALVPEEEARAMLAITVTAADGPTEGKVTVGYTVNTARWMP
VY
DIRLDRAESRVTIERGALVSQETGESWTEVALGLSTARPGGQTGPSELRSLLRRISDPGEEAEASVVRGDRMRLAPARGA
IAAAAAPVRARATAFDGLAVHYDYPVAVDIATGADNLRLALGDLEMPADLLAEAVPERDDNAYLMASIVNGSDEIVLPS
A
QAMFYLDGRFVGQQPIELIPAGGKALLPFGPIDGLRLTRTVQDRKEGDRGVLTKSNERSEAVRIAVENLTGETWPLRLLD
RVPYSEQEDLKISWRADPAPSETDPDGRRGILEWRFELAPGATREVALSHRMVWPEGKVLD
>OLS47134.1 aspartate-semialdehyde dehydrogenase [Rhodovulum sulfidophilum]AB14
MGYKVAVVGATGNVGREMLNILAEREFPVDEIAALASRRSLGTECSFGDKIVKTQDLATFDFTGWDIALFAIGSDATKEY
APKAAKAGCIVIDNSSLYRYDPDVPLIVPEVNADAIEGYSKKNIIANPNCSTAQMVVALKPLHDRAKIKRVVVSTYQSVS
GSGKEAMDELWNQTKGMYVPGQEVAPSVYPKQIAFNVIPHIDVFLDDGSTKEEWKMVAETKKIVDTAIKVTATCVRV
PVF
VGHAEAINIEFEEFLDEDEARDILREAPGIMVVDKREDGGYVTPAECVGDFATFISRIRQDSTIENGLNLWCVSDNLRKG
AALNAVQIAEVLGQRVLKKG
>OLS47135.1 capsular biosynthesis protein [Rhodovulum sulfidophilum]AB14

MIAYLAGWTRRKDAIFRAIAAAAGGGHRLPLIGLSFRGFPETRARTAEALAVAKRQPKGRLGRALKRALIAAQYNWSRR
Y
FSRHPDRLAVCWNGLTGSRRAFMEGAADAGAGRIFAELAPFPGRVTLDPVGVNARNALPRDPGFYLDWAAADPSRQ
GEAW
RGLGRGLVARASRRADVGQAGAEALGDTGPFLFCPLQVPNDSQITMFAGWVESVEGMIAALARASAALPPGWHIRLK
EHP
SAKTSLAEPLAAALADAGGRLVIDNATDSFAQLAASQGVITINSSMGLQAFFHDKPVIVLGEAFFAIPGLTAPADSAAAL
AALLAAPGDLGFDPALRAAFMTYLDRVYYPKIEEAPDGVHIDAAALARILASARS
>OLS47136.1 hypothetical protein BV379_01790 [Rhodovulum sulfidophilum]AB14
MWRSAWAVLALGVTISGLAGAASSEGVREGGRSEQAIVGASAMRWLAPEPKSKPSRSSKSGRKAAALALDRRQGRGS
HIC
SASGFGQRPRCVAR
>OLS47137.1 beta-mannosidase [Rhodovulum sulfidophilum]AB14
MRPDPAIAAPSLVALSLAALILATAAPAEPVEAPPGEMPFGVYDPDGAFADDPEVSIEHLFLPWEDVYLPSLIDADAYAR
ARGRSVLATIEPWTWNRSERNTPDMLQEGIASGDYDRYMASICAVLNLFESPVTVRWAQEMEDDSGQFIWAGWDP
ATYKA
AYRRMVDICRETAPEVAYMWSPLGEEGLEAYYPGDDYVDIVGLSVFGLQPWETAILGEEQSFRSILAPRYARVAAFGKP
V
VVAELGYSGREDYVAKWTSEVRQIFPEFPNLVAVIYFNQIEVYPWPDGYGLPDWKVGDRIIAP
>OLS47138.1 UDP-glucose 4-epimerase GalE [Rhodovulum sulfidophilum]AB14
MSKPVPVLVTGGAGYIGSHTCRRLSDAGYLPVVFDNLSTGHADAVRWGPLVEGDTRDRAALVRTIRSHAPVAAIHFAA
AA
YVGQSMRDPGFYYDNNVGGMTGLLSACAETGLGRVIFSSSCASYGLPETLPITEATPQVPINPYGRTKLIGEQMLADFG
R
AHGLRHVALRYFNACGADPAAGLSERHDPETHLLPLALFAAAGRGPALKIYGTDYPTPDGTCIRDYIHVADLAEGHLLAL
RHLERGGGDLQVNLGTGRGQSIREVLAAVERVTGRPVPVSLHPRRPGDPPALVADPGRARAALGFEARHRDLDGILRD
AA
LAFGLEVADAGSA
>OLS47139.1 alpha/beta hydrolase [Rhodovulum sulfidophilum]AB14
MACYEVKGRNNYYMEFGQGRPILLLHGISNSGRAWGPQIPALVAAGYRVIVPDHAGHGASDPLTAPFGVADIADDTE
ELL
ARLGIETLDVVGLSLGGMVALELALRRPTGIGRLVVANSFDNTATPEFRSMSEGWASVFEQPHGPVTRLEQNWLVSVS
ET
FRNTPEGMRTYQVWHGIAATSHGPSLAHVARGIAGFDISGRLAELAMPTLFIAGELDRMSPPDLSRRMAEKTPQAKLC
VI
EGAAHISNADSPEDFTARLLRFLSSQSE
>OLS47140.1 MFS transporter [Rhodovulum sulfidophilum]AB14
MEGGNLSRFSERFLFAAAILVVGINLRPTMAAIGPMLDVIQRDTGLSDTGASLLTTLPVALMGICLLFTSRLKTLFGARN
GIILGLALILIGNLLRWPWSSAALLLVTAILGGIGIAMVQALLPIVIRHRAGASAAGLMGLYSTAIMAGAFLSGTFSPWI
AQASDWQPALGIWAVPALLGCLIWWRVTDPRASVPEDRPHFPVSRAPRAWLLLAFFGLGTGAYTLVLAWLPPFYTGL
GRS
AETAGLMLGVVTLAEVVAGLVVSCWVSRSTDRRPAIFTAIGALFAGLLGLIFAPLALAWPSAVFAGLGIGALFPLSLIVA
MDHGDNASAAGAIAGFVQGGGYLVAAALPLIAGLLREYLSDLTLAWALMAGLCVVLWLIAIRLRPGQRITF
>OLS47141.1 MarR family transcriptional regulator [Rhodovulum sulfidophilum]AB14
MDRARKAAGQWAREKPHLDIGPMVLLGRLGEAALVISRDRLNPVFAEFDLQPGEFDVLATLRRSGAPYAMTPTHLYE
AAM
ISSGSMTNRLNRLEQAGWVKRQPNPEDKRGTLVALTPGGLALIEKAVESHAANMRTIVSGLTEREQDQLSQLLEKLLAS
Q

GS
>OLS47142.1 hypothetical protein BV379_01830 [Rhodovulum sulfidophilum]AB14
MSCLESHSETQDPSIVQQSHLAGETLVTCPVARERIEIYTQVLVPAGALPGRHRTVETTDLILRLVASARAVSATPDRLL
QDEPDRRGIAPGSGLPRSIHPGLRTGERPEYFSGFIHLARRVRG
>OLS47143.1 class 1b ribonucleoside-diphosphate reductase subunit beta [Rhodovulum sulfidophilum]AB14
MKDMINQTRAVPRAINWNRLQDEKDLEVWNRLTVNFWLPEKVPLSNDVQSWSTLRPEERQLTIRVFTGLTLLDTIQG
AVG
APAMMRDALTPHEEAVLTNIAFMESVHARSYSSVFSTLCQTREVDEAFRWSEENPHLQDKARLILGKYDAAGDPLKRKI
A
SVFLESFLFYSGFYLPMYWSSRGKLTNTADLIRLIIRDEAIHGYYIGYKFQRAAEHLPEAERAALKDFAFALMFDLYEIE
AKYTEELYDGIGLTEDVKVFLHYNANKALQNLGFEALFPPAACEGNAAILAALSPDSENHDFFSGSGSSYVIGKAVATED
EDWDF
>OLS47144.1 ribonucleotide-diphosphate reductase subunit alpha [Rhodovulum sulfidophilum]AB14
MLDRVTDAKPAKPDSTLDYHALNAMLNLYDEDGAIRFEADRMAARQYFLQHVNRNTVFFHSLDEKLGYLVDEGYYEA
DVL
DQYPRAFLHRIWDAAYARKFRFPTFLGAFKYYTSYTLKTRDGQRYLERYEDRVVMVALALARGDEALAMSFMDEIISGR
F
QPATPTFLNAGKKSRGELISCFLLRVEDNMESIGRAINSALQLSKRGGGVALMLTNIREHGAPIKGIENQSSGVIPVMKL
LEDSFSYANQLGARQGAGAVYLNAHHPDILRFLDTKRENADEKIRIKTLSLGVVIPDVTFELAKQNEEMYLFSPHDVEKV
YGMPFSDISVTEKYDEMVGDKRIRKQKINAREFFQTLAEIQFESGYPYIMFEDTVNRANPVAGRIAMSNLCSEILQVNEA
SAFNDDLSYRHLGTDISCNLGSLNIAATMDGPDFGGTVEAAIRALTAVSEMSAIDSVPSIRRGNDEAHAVGLGQMNLH
GF
LARERIHYGSPEGIDFTRAYFAAVAYHAIRASNALAREKGQSFKGFEASKYADGAFFEKYVTRDWRPETDTVRDLFARFG
VDLPSREDWAQLKEAVSKDGLYNRNLQAVPPTGSISYINNSTSSIHPIVSKIEIRKEGKIGRVYYPAPFMTNETLEFYRD
AYEIGPEALIETYAAATEHVDQGLSLTLFFPAEATTRDINRAQILAWKKGIKTIYYVRLRQTALEGTEVQGCVSCTL
>OLS47145.1 ribonucleotide reductase assembly protein NrdI [Rhodovulum sulfidophilum]AB14
MAGLVYFSSRSGNTARFVERLGLPALRIPIAGDDLPRPDTPFALICPTYADGEGRGAVPKQVIRFLNDPARRALLRGVIA
SGNRNFGATYALSGEVISRKCKVPVLYRFELAGTDTDIARVRDGLDKFWEMECLTV
>OLS47146.1 NrdH-redoxin [Rhodovulum sulfidophilum]AB14
MSIIVYSKPACVQCTATTRALEARGLPYELIDLTEDDKAFAHVSELGYRQLPVVVAGKDHWAGFRPDLIGRLA
>OLS47147.1 rubrerythrin family protein [Rhodovulum sulfidophilum]AB14
MISGLSQRRRFSDLSEQEVLALAISSEEDDARIYRSYAEQLRADYPDSARVFDAMAEEEDGHRRRLIDTHRARFGEAIPL
LRREHVAGYYARRPVWLMRNLGIERIREEAERMEREAHDFYMRAAGRATDAATRKLLGDLAAAEAGHEAAADRLVET
QLD
TDAVERERRTAHRQFVLTWVQPGLAGLMDGSVSTLAPIFATAFATHDSGTTFLVGLAASVGAGISMGFTEAASDDGAI
SG
RGSPLKRGIASGVMTALGGLGHALPYLIPHFWTATGIAMAVVFCELWAIAWIQKKYMDTPFFRAAFQVVLGGALVFAA
GV
LIGSG
>OLS47148.1 S-formylglutathione hydrolase [Rhodovulum sulfidophilum]AB14
METVSENRCYNGVQGVYRHASEACGCDMTFGLFLPEEATLGPVPVLWYLSGLTCTHENAMTKAGAQQWAAEQGIAL
VFPD
TSPRGEGIADDPMYNLGTGAGFYVNATEEPWSKNYRMWDYIAEELPRVLTANFAIDAERQSICGHSMGGHGALTLA
MAYP
ERYAAVSAFAPITHPSQSDWARKPIHAYLGEGKVDWTRHDACLSLRANGYAGPMLIDQGTGDQFIDALMPEALAAA
MTET
RHPGFFRMQKGYDHSYFFISTFMEDHVAFHAEALYR

>OLS47149.1 hypothetical protein BV379_01865 [Rhodovulum sulfidophilum]AB14
MTVYVDADACPVKAEALRVAERHGAEVLYVANGGLRPVDHPLARMVYVPEGPDEADKWIAERAGPGDVVITADIPLA
AKA
VAAGARVLNHTGEAFTQANIGQKLATRDLMADLRAADPFRQGGGKGFGKADRSRFLDALERELSAATRR
>OLS47150.1 haloacid dehalogenase [Rhodovulum sulfidophilum]AB14
MPIDAVVFDIGRVLIDWQPEAFYDRRIGSDRRRALFAEVDLYGMNEGVDRGDDFGAAVAALAARHPDWEAEIRMW
QDDWL
EMASPALPESVAVLKAVKAAGLPVFALSNFGNGPFEIARRAYPFFELFDRLYVSARLRVLKPDPRIYEILETDSGIAPER
LIFTDDKAENIAAAAARGWKTHLFEGGRGWAARLVAEGVLDAAVLADGPLAHA
>OLS47151.1 cell division protein ZapE [Rhodovulum sulfidophilum]AB14
MSETLKDLYDARAAGGDLRPDPAQIEALVPLERIRQALENPPPAPRGGFFRKAPPPEPVRGLYLWGGVGRGKSMLMD
LFF
EAVAIEGKRRVHFHAFMQEVHRGLHVARAKGADDALKPVAAGIAEGLKLLCFDEMQIVDITDAMIVGRLFEFLFAAGV
TV
VTTSNRVPDELYKDGLQRERFVPFIKMLEERLEVMELGSATDYRQNRLTGEGVYFSPSDRAARARMAAIWEDFTHGAG
RP
LVLRVQGRQVELPLFHNGVARATFWDLCARPLGPADYLAVAQAVRVLILEDIPRLSSENYNEARRFVTLIDALYEGRVRL
IASAAAAPEMLYLEGEGSFEFERTASRLREMQAADWAKG
>OLS47152.1 bifunctional folylpolyglutamate synthase/dihydrofolate synthase [Rhodovulum sulfidophilum]AB14
MSAASSDILLERMMALHPKVIDLVLDRVWRLLAALDHPETRLAPVVHVAGTNGKGSTLTMIRAGLEAAGAQVQAYTS
PHL
ARFHERVRMADGPIAEAALAAVLGECERANAGAPITYFEITTCAALLAFSREAADYCLLEVGLGGRLDATNVIEAPRLTV
IAPVSVDHQQYLGETLAEIAAEKAGILKRGVPCVVGPQEEAAMEVIEARAARLGAPLIAHGQHWHVWEERGRLVFQDE
RG
LLDLPMPRLIGAHQVVNAGAALAALRALGQDETACEAAVTRAEWPARLQRLRHGPLVETAGPAELWLDGGHNPAAG
IALA
EAVRRLPPRPLHLVCGMLNTKDVTGFMRPLAAVAESLRAVSIPGEAATLLAAETCSAARGAGLAAETAGDVQEAVSRIA
A
RTPGARILICGSLYLAGRVLRENG
>OLS47153.1 acetyl-CoA carboxylase subunit beta [Rhodovulum sulfidophilum]AB14
MNWINNYVVRPKINSLFSRREMPENLWSKCDACGTMLFHRELSDNLNVCTSCGHHMAILPRDRFTTLFDGGVFSEVGV
PVP
VADPLQFRDQKRYPDRMRSAQRMTGEKEAMLVAEGEIGRTPVVAAAQDFSFMAGSMGMYVGNAIIAGAERAVALG
RPFIL
FSAAGGARMQEGILSLMQMPRTTVAVQMLKEAGLPYIVVLTHPTTGGVTASYAMLGDVHIAEPGALICFAGPRVIEQT
IR
EKLPEGFQRAEYLLDHGMLDRVTPRGKLREELITIIRMLMGLPPAVIADLPPPASFSHGPARDDGDNGAETQPDLPNRS
P
APPDAVPPAGGAKP
>OLS47154.1 hypothetical protein BV379_01900 [Rhodovulum sulfidophilum]AB14
MRSPLFEAYIEPARLYPETWRLIVGLLVVLLVYVGVFSTMLVAAYPALGPMAYFGWIMGLAAPATPGQTLFVLTSFLGM
G
LGVLIATPALHYREPGSLFGPARDWARGFFGALKVLVPAYALLFGLAFATMPLEANLDPGTWARMLPLALLLILIQTGSE
ELLFRGYLQQQLAARFAWRAVWMGLPALIFTALHYNPAAGMNNAIGLVGILAFALAAADLTERSGSLGTAMGWHFV
NNVS
SLLFTSVKGTITGLALFVTPFDVTDTATAPWAMALDLIAILLLWRLLRALI
>OLS47155.1 hypothetical protein BV379_01905 [Rhodovulum sulfidophilum]AB14

MRYDPHETLVSAARGQPALWRLAAGLAVIAAVQIGLTYTVYGLISALRGPGIAEAAFRGVFETTATASDTLWLLASFLCL
ICGTAVAAQLMQGRSLRSLTGPPGAALGQFVRVLRAVALLYLVLWIVLPSGPEKVAHRPVGAWLALLPLALPALAIQTF
A
EEMLFRGYLQQQLAARFRSPLLWIGMPALMFALAHYSPGAAGDNALPAALWAAAFSVAASDLTARTGTLGAAVALHF
VNN
AMAVLIVALPGPVSGLALYTYGFGADDPALGPLIAVDLAVLGLSWLAARVALRV
>OLS47156.1 hypothetical protein BV379_01910 [Rhodovulum sulfidophilum]AB14
MMPEQTPDIFRKILPPRRAVARALPCWLFAAVLLALPGCVTEGGGVHRTIVADGRVAIVAPYGFCVDESASRDDGDGA
FV
LLASCAALARGAEAAVPAEDAILTVSVAPSPGPGGSPEDRALVLQRYFASELGRAALARSGDPADVEVLETDTRGALFLV
HSLDRGPGLAKGLRSDTWRAVFEANGRIVTAAATGFASRPIGDQAARALLQDLAGRSRAATENLPDPTMAQP
>OLS47157.1 hypothetical protein BV379_01915 [Rhodovulum sulfidophilum]AB14
MPILLKRDSLADRSLADRLLFRRALAAWGRAGGAAERLDIPDLTALPGRAWRLRHRRDRAIHGADARRAAFHAAAETA
RL
PLGSDWGFRPAPFADRVKPCAATDIGTALRLGPQVTLLHDAARAGIAFRQVAKSHGEDGAPFGLALEVSGFDGGFLSV
VF
ELPSRAVEGLGRHHLIRLDAALGTEAPVDVFARISIRHGPNLAQLVRAVPPDESRIALDFDLAASDLHGHRADRMWLDL
L
FDGPGMNRILLSDIVLSRRPRAEA
>OLS47158.1 LysR family transcriptional regulator [Rhodovulum sulfidophilum]AB14
MDRLTEMEAFANVVDQGGFTDAARKMGISKSAVSKHVSSLEARLGARLLNRTTRRVSPTEIGLAYYDRARRVLNDAGE
AD
ALVSAMQSAPSGLLRISVATDFGVNHLSPLLGAFLQKYPDITVNMVLNNRFVELISEGFDLAIRVGELEDSTLRARKLAD
TTRRMIGSPEYFRKFGRPQKIDDLNGHKLLHYSNQASGNVWKITAPSGEKRQVRTAGWLTVNDGQSLLNAAVAGLGI
AYL
PSFLFRDALEKGLVEEAIPGLPMERLGIYAVYPPGRFTQPKVRAFIDFLLTHFEGDGIMI
>OLS47159.1 hypothetical protein BV379_01930 [Rhodovulum sulfidophilum]AB14
MKARRPLLALALLWSGLLIWPGLAGAMVPEIPEMATVAADETEAASSLRIPTGPYDGQVVPSQRAEGAIRRLVWQAPL
DG
RSTLQVIDPLKAELIAGGYRIAFQCAAAGCGGFDFRFATDTLPEPAMHVDLGDFRFLAAEKARPDGTDHVTLMVSRSEE
R
AFVQVTQVGRPDPAPAVQSTKSPGDSLRIETADGRSASAQDEATLADRLARDGHAVLAGIDFATAAATLGTGPFPALE
AL
ADYLRAHPGSKVVLVGHTDTQGGLAGNIALSKKRAEAVRDHLVTTLGIPAAQVAAEGVGFLAPLSSNATDRGRTENRR
VE
AVLDTSQ
>OLS47160.1 alkylhydroperoxidase [Rhodovulum sulfidophilum]AB14
MTHDKTRPTALDLPMVDPLPPETRKYFDLCAERLGLVPNVLAAYAFDIGKLNAFTAMYNDLMLGESGLSKLEREMIAV
VV
SSINRCWYCQVAHGATVRALSGDPALGEALVMNYRVAPLDPRQRAMLDFAAKLTEASHRVEEPDRQALREAGFSDRD
IWD
IAAVAGFFNMTNRVASASGMEPNPDYHGSHR
>OLS47161.1 GNAT family N-acetyltransferase [Rhodovulum sulfidophilum]AB14
MKIDRLFEGQEATWPPAARHVTGPWCLRDGQAGGKRVSAATLEGGFEPEAIAEAETGMARLGQTPLFMVRPGEGAL
DDTL
EARGYLRVDPVTILSGPAAAIAADPPAPLSTFTIWEPLAIMTELWAENGIGPARMAVMDRVQGPKTAVLGRRKDRAAG
TA

FVALDRDMAFVHAVAVSPEHRRQGTAVNMMRAAAQWALDGGASTLNVLVTEDNHAALALYASLNMTNVGQYHYR
MMAPQK
A
>OLS47162.1 competence/damage-inducible protein A [Rhodovulum sulfidophilum]AB14
MSNPTAAMLVIGDEILSGRTRDANMHYLAGEAIKHGIRLSEVRIVPDRQDRIVAALNALRTDYDIVFTSGGIGPTHDDIT
ADAVAAAFGIGIGVREDARALLAEHCARMGRDLNEARLRMARIPDGATLIANPISAAPGFVLGNVHVMAGVPSVFQA
MVA
AVLPGLASGDPLMSQTLCVERPEGDIAGELRDLADRFPELSIGSYPFSRDGVYGTNIVVRGTEARRIDAAMAELCDLFPE
AMA
>OLS47163.1 type I methionyl aminopeptidase [Rhodovulum sulfidophilum]AB14
MKEWALDEKTRGRRTKDNIRIYEPEDFAGMHAAGRIAAEILDEIAPLVFPGQTTGEIDRVITELVEAKGSVSATIGYKGY
KHASCISVNHVVCHGIPGSKVLKDGDILNVDVTVIVDGWYGDTSRMYVAGKLSRKAERLIQVTHDSLLKGIEAVRPGNT
F
GDIGAAIQAYVESHRMSVVRDFCGHGLGRVFHAPPNVLHYAPFRLNAEGQKVTISGNPTRDPTPVLEEGMFFTIEPMV
NL
GRPETKILADDWTAVTRDKSLSAQFEHSIGVTADGCEIFTLSPGGLFHPTYG
>OLS47164.1 alpha/beta hydrolase [Rhodovulum sulfidophilum]AB14
MIELFARLTPMRWIVGIAALVAIAVSVWQLEAERRGVVIAPVPGTGTTPATVYRLKDAPPAPVVIVSHGFAGSRQLMQ
AW
SLTLARAGYAAVAFDYEGHGRNPVPMSGDLDSPEGTALLLMAETRRVTDAALAQPWADGRVAILGHSMGSYIAVREA
RED
PRIAATIGIAAANDSITATEPPNLLMINGAWETRLAEAPLEALRLADPEAQLGDTVGDPATGTGRRALLAPDVEHIGVIW
SPTALRETRAWLDATFGRTSGGPVVEIGGWIALLLVGIVALAWPLARMRGQTEAPRPAPLPRRRFLIVALLPAVAVPLIL
APFRTQFLPVLVADYLAVHFALYGAMVLALLAWWGEIRWAGWREIGRILALAALVALYTIGVFGLAMDRYVTSFYPIPI
R
GLIIAAMAVGAVPYMLSDALMVEGGRAPVWRSLVSRGAFLVSLGIATALDFHRLFFLLLILPIILIFYLLFGTMGGWIGR
ATNRPAGVGLGLGLFLAWALGVTFPMFQAV
>OLS47165.1 hypothetical protein BV379_01960 [Rhodovulum sulfidophilum]AB14
MTEPLPRDADAALVDAARSYFAARRARVPAFARRRFGLAGTLALHRHALGWDVLRAPLNVLLSPVLILTRLGAAAAARS
R
APRLAAWLGTRRILLPTDVATAAERAVVVDLLELPWEGPGGTSSKDALARAILARHDLAALVEARSDAPALSARALGDY
G
GTRSAVAEMTTAFGTMGAGALAFQSLTPGMVSFAPGLAAIVAHQAAIAAFPLGGLLGTAWYGIFPAAAPAWMTLAIT
LPL
IGLGALVAAFAGVIADPVQVMLGMHQRRLNRLIDAMEDAFTGSGRRGFAAREHYFARLLDLSDAGLAAIRLIRS
>OLS47166.1 16S rRNA (guanine(966)-N(2))-methyltransferase RsmD [Rhodovulum sulfidophilum]AB14
MRIVAGRFRGRALASVGKGDPAAHLRPTTDRVRESLFNLLLNGGYGDPVGDARVLDLFAGTGALGLEALSRGAAHVTF
LE
DGPKALALIRENVALCRAEAETAILRRNATRPGPAPGAPATLVFLDPPYGKGLGEKALAASLAHGWIAPGALIVWEEGA
A
VTPPEGLALLDSRRYGETAISILEAP
>OLS47167.1 pyridine nucleotide-disulfide oxidoreductase [Rhodovulum sulfidophilum]AB14
MSHIVIIGAGQAGAALAAKLRALGHDGPLTLIGDEAAPPYQRPPLSKKYLLGAMPVDRLYLRPPTFYAEAGIDLWLGKTV
TGIDRDRRRVLLGDREIGYDMLALATGAAPRRLPAEAGGALGGVHVVRRLADVDAMAPEFRPGARALIVGGGYIGLEA
AA
VAATQGLQVTLTEAAPRILQRVAAPETSDWFRALHAAHGVQIREATGLVRLLGGTRVSGAELADGSVIGADFVIAGIGV
T

PATALAEAAGLRTEAGIAVDAMGRTADPAIWAAGDCTSLPYRGGRIRLESVQNAIDQAEAVAANMLGAETPYVPKPW
FWS
DQYDVKLQIAGLNSGYDRVVIRPGDRDGSVSHWYYRGAELLALDAMNDPRAYMTAKRLIEAGRSPAPESVAAGDLKA
LMA
A
>OLS47168.1 peroxiredoxin [Rhodovulum sulfidophilum]AB14
MAISEGDRLPEANFTEMGADGPGAVSLSDKLKDRKVVIFAVPGAFTPTCHSAHMPSFIKAKDKLAAKGVDEIVCVAVN
DP
FVMKAWGEATGAHAAGITMLADADASFTKAIGMEFDAPPAGLISRSKRYAMLVEDGVVKKLNVEASPGECSISAGETL
LE
QI
>OLS47169.1 4a-hydroxytetrahydrobiopterin dehydratase [Rhodovulum sulfidophilum]AB14
MTAKLTEDQRRLELSSLSAHGWAETQGGDAIIKTFEFGDFVSAFGFMTRAALWAEKMDHHPDWKNVYKTVEVTLSTH
DAG
GLTMKDVELARKMDALA
>OLS47170.1 phosphodiesterase [Rhodovulum sulfidophilum]AB14
MSPLPPAFLTAPLAHRALHDRAAGRPENSVEAVRAAIAGGYGIEIDIQPSADGVPMVFHDYDLARLTGETGPVAGRSA
AE
LGAIPLSGGTEGIPTLGRVLEEVAGRVPLLIEIKDQDGAMGAAVGPLERAVAAGLGGYDGPVALMSFNPHSVAVLAEAA
P
ERPRGLTTCAYEARDWPTLQAPMRDMLREIPDYDRVGASFISHLWRDLARPRVAELKARGAAILCWTVRSPVEERIARS
V
AANVTFEGYVPAHPAPPAA
>OLS47171.1 hypothetical protein BV379_01995 [Rhodovulum sulfidophilum]AB14
MSGKYEARLTELGVTLPDAPAPAANYVPYVISGNLVFVSGQVSAADGALIPGKLGADLDVAAGAAAARACAISLLAQVK
A
ACGGDLDRLVRVVKLTGFVNSTPDFGDQPKVINGASDFFVEALGDAGRHSRSAVSAASLPFGVAVEIEGIFEIA
>OLS47172.1 ABC transporter [Rhodovulum sulfidophilum]AB14
MPSKAAASAPTRRSLLLTGLAAGAALTVTGLTSGRAFALTTDEASQLIDRVIGDINRVINSGKSEAQMFPEFERIFSRYA
DVPVIARSVLGVAARQASQSELNAFTQAFQGYIARKYGRRFREFIGGEIKVNEARQVKAFYEVKTTALLRGQSPVEVVFL
VSDRSGKDKFFNIFIEGVNMLASERTEIGAMLDKRRGDIGALTRDLRSAG
>OLS47173.1 glycosyl transferase [Rhodovulum sulfidophilum]AB14
MGSTGRRPPPLVADRRYPKKTKRKPQPRNRKPRSRNGGGGGGRRGGVRRGNPILRAIRAFFRRLFRSILGTGLRLALLG
A
LALGCAVFYFYQQLPEMTALLDGRNRGSVTLLDRDGKVFAWRGEQFGGQITATTVAPALKNAVVATEDRRFYRHFGIS
PR
GIAGAIRINLSEGRGPLSGNGGSTITQQTAKLLCLGVPYDSKTWKNETAYEADCRRTTLGRKIKEALYAMAMEAKYSKDE
ILTIYLNRAYLGAGTRGFEAAAQRYFGKSANQVNISEAAMLAGLLKAPTTYAPTNNLKRSRNRANLIVGLMQDQGYITP
K
QAALAKANPAQLSEAAEARAGGYFADWVMGAGPDFLTRDTTEDVIIRTTFDPSIQKAAETALKEVFENKVKEGSKAEA
AI
IVMSADGAVRAMVGGRKTAIGGFNRATMALRQTGSAFKPFVYAAALDLGYTPNATVVDEPYTINVPGSGPYSPENYD
HKF
RGRVTLTEALQHSLNIPAVKVSEAVGRENVRRIAHDFGLKADLADGPALALGASESTLINMTGAYAGILNGGSSVTPYGL
TELKLKGDREPLMGQEGGMGERVISERAARSLVYMMWRAVEAGTGHRAKLAGRQVAGKTGTTSAARDAWFIGFSA
DYVAG
VWMGYDDNTPLRGVTGGGLPAEIWHETMQRVQEGVPPHPLPMLVPQEPSSAVAAREGGDRGGNVAENILRSVLGA
FTGRN

>OLS47174.1 transcriptional regulator [Rhodovulum sulfidophilum]AB14
MKLIIAAIKPFKLEEVREALTAIGVRGMMVTEIKGFGSQSGHTEIYRGAEYAVNFVPKVKLEIVVTSAMADQVVETIANT
AKTDKIGDGKIFVLDVNQAVRVRTGETNEDAV
>OLS47175.1 ammonia channel protein [Rhodovulum sulfidophilum]AB14
MANIKHILGLAALTATAAALPALAQEVAEAAPIMDKGDTAWMMTATILVLFMTMPGIALFYGGLVRQKNMLSVLMQ
STLI
AGMMMVIWVFWGYSFAFGGGSSPFWGGFGKLFLSGVTVDSTAATFTDGVVIPEYVFIAFQMTFAVITPALIVGAFAE
RMK
FSAVLLFVGLWTTFCYFPIAHMVWDGAGLLYNLGALDFAGGTVVHINAGIAALVGAILIGPRIGLGKENMAPHSMTLT
MV
GASMLWIGWFGFNAGSNLEANAFTGLPFINTFVATAAAILSWSAVEGLTRGKASMLGAASGMVAGLVAITPACGQLG
PVG
AIILGFVVSPICYFFVSTVKTKFGYDDSLDVFGVHGVGGIVGALGTGLLGGEALGGQGFAEGVGTIGQTIIQAEAVLVTI
VWCGVVSLIAYKIVDMIVGLRVDADSERQGLDLTSHGEAAYHS
>OLS47176.1 aromatic amino acid aminotransferase [Rhodovulum sulfidophilum]AB14
MLSRLSPQSPDKIIGLMQQFRADPRADKIDLGVGVYRDARGRTPVMRAVKAAEETLWRTQDSKGYVGIEGDPAFLEAI
GT
LVLGDAVPAERIAAVGTPGGTGAVRQALELVRLADAGARVFIPAPSWPNHAAIVNFLGLGEAAYRYYDAAAHALDLPG
LL
ADLETTGPGDVVILHGCCHNPTGAELTLADWAAIAEVLGRTGALPLVDLAYLGFGDGLEADAAGLRLLAGALPEMLVSV
S
CSKNFGLYRERVGAVLALSPDPAARDLVRGNLSNLNRQNFAFPPDHGARVVTGILGDAALRADWAAELETMRSRIETL
RR
TLADRLRDTSGSDRFAFLAGQKGLFSLIGATPDQVIAMRETHGVYLIGDGRINLAGLSETNIPKVAEAMLAAGL
>OLS47177.1 3-mercaptopyruvate sulfurtransferase [Rhodovulum sulfidophilum]AB14
MSYDDPKTLVSTDWLAAHLNDPDLRILDASCRPVFPTDTPRDPVAEYQAAHIPGARFFDIDEISDQRSDLPHMAPPPEK
F
ISRLRAMGVGDGHQVVVYDSAGLFSAARVWWTFRLMGKTDIAVLDGGLPKWRAEERPLEDMPPVVRDRHMTVQR
QAGLIK
DVTQVAAASKLGDYEIVDARGASRFEGTDPEPRPGLRSGHIPGSKNVPFATLLNADGTMKSPEALKAVLEAAGVDLRKP
V
ITSCGSGVTAAILSLALEVIGHRNHSLYDGSWSEWGMYGDLPVETGPARC
>OLS47178.1 rod shape-determining protein RodA [Rhodovulum sulfidophilum]AB14
MQARSAASFAVPSLAFAALTALALPALAEDLRTPSPEGARVFIISPEDGATVSSPVTVVFGIEGMEVVPAGTDTPASGHH
HLIVDLPQDEVDVAHAIPADDHHIHFGGGQTETTLELAPGTHSLWLLLGDANHVPHDPPVMSEPIEVTVE
>OLS47179.1 hypothetical protein BV379_02045 [Rhodovulum sulfidophilum]AB14
MRPGELAAGDLGTADGPALRVIGRARTQWGPDDCPKNIGRARERGAPATLEIDPVFAPALRGLSVGQPVIVLYWMDR
ARR
DLLVQAPRHVDGPRGSFVLRSPNRPNTISQSTVRITALDADTGRVEVDALDCYDGTPLIDIKPWLPSIDLPPGEPG
>OLS47180.1 4-hydroxy-tetrahydrodipicolinate synthase [Rhodovulum sulfidophilum]AB14
MIQGSLPALVTPFKDGAVDFDTLKRLVEWQLAEGSHGFVPVGTTGESPTLSHAEHEAVIEAVVKTVDGRVPVVAGAGS
NN
TAEAVRFMQYAEEVGADAALVVTPYYNKPTQAGMIRHFTELHDCCNLPIIIYNIPGRSVVDMSPETMGELAKLPRIVGV
K
DATGDLARVPKQRITCGPDFLQLSGEDGTALGFNAHGGRGCISVTANVAPRLCAEFQKATLAGDYAKALELQDRLMPL
HI
AIFLEPGVSAAKYALSRLGKCSDEVRSPLVGLTQGTRDRIDAALRHAGLLN
>OLS47181.1 hypothetical protein BV379_02055 [Rhodovulum sulfidophilum]AB14

MRVLILAFLISLASPVCAADQSALAKAMVAVRADRWAEARQLARPAGQVALDIVEWRRLRAGEGRLEEYRAFLARNPD
WP
GLGKVRSEAEGVIATGDDPAAIVAFFGSRDPVTGEGALRLAEAEAALGRRGKAEAVAVSAWRRLPLAEPVQSAFLARYG
R
ALAGHHTARLDALLWDGHDISAGRMLSLVPEGWQKLAAARIALRNQTPGVDALIAAVPASLRDDPGLAYERFMWRT
RKGR
GEDAIALLDARSGSAESLGRPARWAGWRRYYAHQVLREGNPKLAYRLAARHHLRAGASYAELEWLSGFIALRFLDRPET
A
LKHFRAFEAAVRTPISLGRAGYWEGRALEAMGHPQEARAAYAMGAQFQTSFYGLLSAERGGIAMDPSLTGKDHYPD
WRAQ
PFARSSVIAAALLLHEAGEQNLVEMFLTHLAESVDPAGQRALAGLALELNDHHIALRIAKTAAATGNVMVEAYFPVTGI
A
RSRHPVPTELVLSIVRRESEFDPSVASGAGALGLMQLMPTTAKAMADELSVRYSSSALTADPAYNARLGAAYLSHLVEEF
GPNPVLVAAAYNAGPSRAHRWIAEQGDPRAAGVDVIDWIELIPFTETRNYVMRVTESLPIYRARLAGRTGALSLSKELK
S
N
>OLS47182.1 EamA family transporter [Rhodovulum sulfidophilum]AB14
MTQQNTRLGILLMIATTFVFSAQDVLSRHLADHYNIPMVVMIRYWFFAAFVMVLARRNAGSLTAAARTRMPVLQALR
GLV
LVAEIWVMIAAFTHLGLIESHSVFASYPLMIAALSGPVLGEKVGWRRWCAIGVGFAGVLVILAPGLRVFSPLALIPLAAA
TLFAIYGLLTRYVAREDNAATSFFWTGTTGAVAATLAGVWSWEPMTGPDWGFMAGLCISGALGHFLLIKTYEVAEASA
VQ
PFAYLQLVFASAFGLAVFHETLRANVVAGGAIVVAAGLFTAWRERVRARRAAGSGPS
>OLS47183.1 oxidoreductase [Rhodovulum sulfidophilum]AB14
MARVDVTVMGAGVFGLSSAYACARRGARVRVVEWSHPGAGASGTPVGALSPHVPEQWNPKKQFQFESLIASAAFW
AEVEA
ESGHSTGYGRVGRYQPIAGEKALARARDRALEAGTLWQGQASWQVIRADEARGLVPETPSGWLVHDTLSARIAPRDA
CAA
LAEAVRAQGGEVSCGAEGPLEGRVIWATGYPGLAELSADLGHQVGSGVKGQAAILAHDAGAVPQVYADGVLIVPHAD
GTV
AVGSTSEREFEDPYSTDARLDAVIDRARAVCPVLAGAEVIERWAGVRPRARTIAPMLGGWPGRPGHFVANGGFKIGIG
LA
PKVGELMAALVLEGRDDIPEGFRVEASL
>OLS47184.1 hypothetical protein BV379_02075 [Rhodovulum sulfidophilum]AB14
MGIVFLLIVGAAAGFLATRIMRVEADILTTVALGVFGALIGGLLIRILLTITGALAGLVGAVLGAMALIWAYQTYIGRR
>OLS47185.1 phosphonate ABC transporter, permease protein PhnE [Rhodovulum sulfidophilum]AB14
MTEQDLTTRPAPLREAFPEDWHRYTPRQQMARYLGLLFAALVIGWSITSIDVIWEWVWDAPVQMADLFGRMVPPD
TTNLG
TILLVLWETVNIATIATLFAVLISLPVAWLAAQNTTPNRATLWLGRIILVSSRSINTIIWALLFVAIFGPGIVAGIAAIM
LRSVGFVGKLLGEAIEEIDPRPVEALQATGASRTKVILYAIVPQVMPTFWAVAILRWDINLRESTVLGLVGAGGIGLILQ
SAIDTFKWQEAATVLLCILGLVLLGEIVSAWLRRRIL
>OLS47186.1 phosphonate ABC transporter, permease protein PhnE [Rhodovulum sulfidophilum]AB14
MSAPAGPAALPDRWSKPRMIRNPVLRWALWLVVAAYIVGTIATLPIDWTRVSEGFSRAARIFGGSIPPNFERSGLLVDG
F
LESLKIAVLASFGGVLISVPAAFMAARNIAPLPVFYLGRALIIVARSFHPVIVAILFVKAVGFGPLAGVLTLIVYSIGFV
AKMLAERIEEIDFGQVEALRATGAPYLSVLFYAIFPQILPRLIGLSIYQLDSNLRASAMIGIVGAGGIGATLANAFGRYD
YDFALAITIVIVGAILVSEAISGQIRKRI
>OLS47187.1 phosphonate ABC transporter ATP-binding protein [Rhodovulum sulfidophilum]AB14

MLKITDLVKRYGTGDPVLKDLNLTIEGETVVSVIGSSGAGKSTLLRCINKLVAPTSGSIVLNGTELTGLTGRQLREARRK
IGMVFQGFNLVDRLTVMENVQSGRLGYLSTWAALTRRYPREEIRHAYELMERVGIAHYADKRADELSGGERQRVGVV
RAL
MQRPEILLADEPTASLDPKTSEQIMQLLRDLAGELKLPVLINIHNVNEARLYTDRIVGMRYGRIIFDSVPGDLTDAAMDE
IYSGIPSQDRAQDRAADAAAAPRLAT
>OLS47188.1 hypothetical protein BV379_02105 [Rhodovulum sulfidophilum]AB14
MPPPSRRLRKAERHERILMELKLRSHLRISDLARDFHVATETIRRDVAELQATGQVTKAFGGVIPVGPGQRPDVDARSR
D
RQQERAEIARQAATLVPPGATIMLDAGSTTQELARRLSLGGTPVTVLTNSLNVAMILGRSDAATVRLCPGDFLPREAAVI
GPDTVDFLRRFHVGRAFIGASAVSCNGVTEAVPGFAAVKRAMMAQSDELVVMTDQSKIGRTDCDLVTSFRPGLTLVT
DAS
LPRELGGAISKGGAQVL
>OLS47189.1 methionyl-tRNA formyltransferase [Rhodovulum sulfidophilum]AB14
MRVIFMGTPDFSVPVLDALVAAGHEIACVYTQPPRPAGRGRKDRPSPVQARAEELGLMVRHPKSLRAAPEQTAFAAL
DPD
VAVVVAYGLILPREVLEAPCRGCLNIHASLLPRWRGAAPIHRAIMAGDTQTGVCIMEMEEGLDTGPVLLRETTAIGPED
T
TGALHDRLSAMGARLVVEALERLRDLPREPQPAEGVTYAAKIDKAEAKVDWTRPAPEVARHINGLSPFPGAWCDVAG
ERL
KLLRARAVEARMVEDSAAPGTVLDGLTIACGEGAVEILEAQRQGKRPMSAAEILRGFPLPERLD
>OLS47190.1 peptide deformylase [Rhodovulum sulfidophilum]AB14
MIRPILPWPDPRLATPAAPVEAVTDEIRAIWADMIDTMEAMPGHGVGLAANQIGVLLRLAVLDCNETRGAAVRLANP
EIV
AVSDDLSAHDEASPCLPGVDATITRPARVTLRFLNEQGQPETREFSGLWATSAQHQLDHLAGRMYFDNLSKLKRDRLL
KK
ARKLGAR
>OLS47191.1 peptide deformylase [Rhodovulum sulfidophilum]AB14
MSVLPILRWPDPRLATACAPIGQVTPEIARLAQEMLDTMYEAPGRGLAAPQVGVLKRLFVMDVNGAAGPRNPRIFIDP
VI
EERASETMVGPEGCLSLPGIVAEVQRAIEITLRWTDLDGTRRREVLSGISAVCAQHEIDHLDGIVTLNRVDAGQRSELEA
AYGA
>OLS47192.1 peptide deformylase [Rhodovulum sulfidophilum]AB14
MSIRPILIHPDPRLKTVADAVPDISDTLQKLAEDMLETMYDAPGIGLAAPQIGVLSRLIVMDCAKDEGARPQPYAMFNP
E
VVWASDDKNIHEEGCLSIPETYAEVTRPAEVIVRWTDPSGDPKEDRFSGLWATCVQHEIDHLDGKLFIDYLKPLKRQLIT
RKMVKLKRARSRS
>OLS47193.1 aminotransferase [Rhodovulum sulfidophilum]AB14
MNFDEIIDRRGTHSSKWDMMEPIYDVSPEEGLAMWVADMDFRPPACVSQALQSMVDHGIYGYFGDDAAYKAAIG
WWMKSR
HGWEVDPAAIFTTHGLVNATALCIEAFTAPGDGVVLFTPVYHAFARVIRAAGREVVECPLATREGRYEMDFAAYDAQ
MTG
REKVVILCSPHNPGGRVWTREELQAVADFARRHDLVLISDEIHHDLVYPGQRHTVMDLVDDRIRDRLVILSATTKSFNIA
GVHNGNVIIHDPGLRAKFAARFAAMGMSANSFGMIMATAAYSPEGAEWIDALMGYLDGNRKLLDEGIAAIPGVSSM
PLEA
TYLSWVDFSGTGMAADEFTRRVEKEAKIAVNHGASFGTGGESFLRFNIATPRARISEAVERLQKAFADLQ
>OLS47194.1 lytic transglycosylase [Rhodovulum sulfidophilum]AB14
MRVSPSLSALCLLSLALASGVSDRALAACGGNFGGFVAGLRDEAVRRGHDAGAARNFFAGVRQDPAVIRADRAQGVF
QRD

FIDFARRLISRSRLDNGQRNAGRYSAVFDEIERRYGVSRGVLLAFWALETDFGAVQGDFNTRDALVTLAHDCRRPELFRP
QIFSALTLYERGDFDPARTTGAWAGEIGQVQMLPADILENGVDGDGDGHVRLKASAPDALMSAGRMLKGLGWRAN
EPWLQ
EIVIPGNLDWAETGLDHGKRVSDWARLGVRPRSGSLGPGNLRASVLLPMGRNGPAFLAYPNFRVFFEWNQSLVYVTT
AAY
LATRFEGAPIFDPRAPDPGLAGAEMKALQRKLVGRGHDVGGVDGILGEKTREAVQKEQIRLGFPADSWPTRALLDRL
>OLS47195.1 YbjN domain-containing protein [Rhodovulum sulfidophilum]AB14
MKRLVHALWALPLAAQAASADVIASDPDSVLKVLRDQGLAATMETDDLGDPKIVSHLTGDSNTEFVVLFYGCTDNVD
CSS
IQFYVAYDMKDPVTALKMNQWNREWLFTKATLDDEGDPGLEMDVILADPGIDRKLFKTTVDIWQQSIDAFEDYIDW
>OLS47196.1 chromosome segregation protein SMC, partial [Rhodovulum sulfidophilum]AB14
ERRAGEAREARAVAQARAEAARETVAQAAARIAEDLETTPADLLEKLGDDPDRMPSAEAIEHEVTRLKRQRESLGAVNL
R
AEEDARAVQEEHDTLVAEKSDLDEAIRTLRAGIAGLNREGRERLLTAFEQVNANFALLFTNLFRGGEARLVLVESDDPLE
AGLEIMCQPPGKKLSTLSLLSGGEQTLTALALIFGVFLANPAPICVLDEVDAPLDDANVTRMCDMLDEMTRRTDTRFLII
THHALTMSRMDRLFGVTMAEQGVSQLVSVDLKKAEALVA
>OLS47197.1 chromosome segregation protein SMC, partial [Rhodovulum sulfidophilum]AB14
MHFTRLRLNGFKSFVDPTDLVIADGLTGVVGPNGCGKSNLLEALRWVMGESRPTAMRGGGMEDVIFAGTSSRSARNF
AEV
SLQIENADRLAPAGFNDSDLIDVIRRITRDAGSAYKLNGKDVRARDVQMLFADASTGSTSPALVRQGQISELINAKPRAR
RRILEDAAGIAGLYQRRHEAELRLNAAEQNLARVDDVLEQLAQQLTQLARQAKQAARYRAIAGDLRRAEGMLLFRRW
QEA
DTGRAEAEAALTEHAREAGRAETAAREADRLRTETETALPALREGEAIAAAVLQRLGAQRDALVQEEERARQTIRTLTG
R
IEQMSRDIEREAALNRDAGETLERLEAEASELTAEHEGHDAALDEAAEAAHAAAEVLRDREEAQSQMTEDLARLVARH
QS
AQRLREDAARTLQRNEDEAAKARATVAAAEANLSTARSGMDEAAARQDAAQDMAEEAEARLIAADEARAEIQSREA
EARA
QKSSAEGEAGALRAEVAALARLVERDTAEGGQILDLLGVDKGYEKALGAALADDLRAPLVQDGTASGWLALPPYPAEQ
PL
PEGSAPLSDHVAVPEALIRRIGQIGLVPDAGTGARLQPALGPGQRLVSPDGDLWRWDGFRASAADAPSAAALRLQQL
NRL
ADLRRDLDEAEIRAETASEAHEALSRELQAATEADRAAREARREADRALAELGRALSRSEAEREMAESRLETARMSVSR
H
EDEATGARLRLSEAESQLASLGDLDAARAQLEDVRTTVEAARMTMIARRAAHDELKRDGEARSKRLTQIARDAQTWR
QRL
DTAGTRVAELEDRKAASEEELDRAAAMPEEIAEQREALAGQIETAGTRRAAAAETLAAA
>OLS47198.1 hypothetical protein BV379_02160 [Rhodovulum sulfidophilum]AB14
MAVAFAQRQGARMGLSIVIEEPTPAEGRARISGSAATLPDVRPPEERLPMTAEDLASPGSGSWGRVSAAGLSDVWLL
GAT
AMAIKRLYVEERTRSGRRPRAGPRGRLETGPELAAAMRLSRGFGHTDCGGFCGSVDIAPTLVMERRLPLQDPVQVSLA
>OLS47199.1 hypothetical protein BV379_02165 [Rhodovulum sulfidophilum]AB14
MLRILALALSLALSACDSPGMDYAGIPPVRIEVDGSTFAVRRKGPEAQAIRLNREYRKGAMQRAFRAIETGTGCVIRPDT
LSGDPAMAYAKLTCTGS
>OLS47200.1 aspartate racemase [Rhodovulum sulfidophilum]AB14
MKTIGILGGMSAASTALYYDVLNRGARARLGGLHSADLLIRSLDFALVEEMQAAGDWDAAGQMLNREARRLEAGGA
DFLM

LATNTMHKLVGPMLEGVSIPFLHIADATGHQIGAAGLKRPGLMATAYTMEQSFYTDRLRAAGLEPMLPGPGDRAETHRII
YDELCIGVLREESRQSYVEIAGRLAKRGADCVILGCTEVGLLLNAANAPLPVFDTTEIHCAAALEMALAA
>OLS47201.1 thiamine pyrophosphate-binding protein [Rhodovulum sulfidophilum]AB14
MRRHGGKILADQLAIQGVERVFSVPGESFLAALDGLYDVGIPNVVCRHEGGAAMMAEAHAKLTGRPGVLFVTRGPGSCNA
ASGLHVARQDSTPMVVFVGQIARRDRDREAFQELDYRSFFGPLVKWVAEVDETRRLPEYVSRAFQAAQSGRPGPVVLALP
EDMLSGIAEVPDIPARIWPEPDPTPAQVAAVLAALNEAERPLVVVGGPHWSAQAAADLARFAESHDLPVAVSFRRQDRLD
NRHPNYVGDLGVGMNPALGKRLGDADLVIALGTRLGDTATAGYSLMDPAVQDKRVVHIHPDPDELGRIWRPDPGIALSAP
DVVAALARSGPPATPPRWSAWTRAARADYEAWTAPKPTPGAVRMEEVVAWLSETLPETAVLTNGAGNYAAFLHRYFRYRG
PGTQLAPTSGSMGYGFPAAIAAKLARPELPVICLAGDGCFQMTLNEMSTAVQHGANVVVIVANNGRYGTIRMHQERKYPG
RVSGTDLVNPDYAALARAYGGEGETVTEQAGFAPALERALASDRPFVIELRLDPEALATGLTLSGARAMGEAAQSED
>OLS47202.1 NADPH-dependent FMN reductase [Rhodovulum sulfidophilum]AB14
MSDHKLLGIAGSLRKGSYNRMLLREAARIFDPAEFVEADLRLPLFDQDLEDAAGIPPAVQRLADQIAAADAVVIAGPEYN
KSITGVLKNALDWVSRTGGAPWADKPVAIVSATGGRAGGERTQFALRLCLVPFRPRVLAGPEVLVGLAPDQFDAAGRLTN
AINLEALEALMQALKAEISR
>OLS47203.1 ribonuclease HI [Rhodovulum sulfidophilum]AB14
MAELFAYTDGACSGNPGPGGWGALLQAREGDSVVKQRELSGGEAATTNNRMELLAAINALESLSRPSEITVVTDSAYVKN
GVTGWIHGWKKNGWKTAAKKPVKNVELWQRLDAAQARHKVTWTWVKGHAGHPENERADELARAGMAPYKR
>OLS47204.1 hypothetical protein BV379_02195 [Rhodovulum sulfidophilum]AB14
MSDADTLAVYAARAEAYSAIHHGRVAQDRLAAFIARLPAGGTALDLGCGPGWAAAGMAAAGLCVTALDACPEMAVAARAR
YGIKVEVAGFADLEAEAAFDGIWAHFSLLHAPRAALPAHLAAIARALRPGGVFAVAMKQGDGEARDRLGRFYTYVTPKGF
RAELERAGLTVEAEETEPGTGFDGSPFASLYLLARRAG
>OLS47205.1 DUF3429 domain-containing protein [Rhodovulum sulfidophilum]AB14
MTDIPRTPLLLGLAGLGPLLWGALSATMPGLFDIGIETVGPRFMGIYLLNFYGTVLLAFMSGILWGFAGRADGPVAGPAY
GLSVLPAIWAFLMVGGGPESSIVALIAGYLGLLLLDFFFWKLGLAPGWWLRLRLPLTAIATGCFALPLLL
>OLS47206.1 lysine transporter LysE [Rhodovulum sulfidophilum]AB14
MISAQFLLTALVVVLAPGTGVVYTLMMSLGQGRRAALPAALGCTLGILPHLAAAMLGLAALLHSSALLFQIVKFAGVAYL
MYLAWQAVRQGGALQIGARDGHDGAFRIGLRGALINILNPKLSLFFLALLPPFLSGNPVTATAEMLALGGVFMALTLAVF
LGYGLFAAAARDRLLASARAMTWLNRGFAAVFAGLGLKLAFEKA
>OLS47207.1 4-hydroxy-3-methylbut-2-enyl diphosphate reductase [Rhodovulum sulfidophilum]AB14
MTKPPLTLFLAAPRGFCAGVDRAIKIVEAALEKWGAPVYVRHEIVHNKFVVDRLRDMGAVFVEELEDCPPDRPVIFSAHG
VPKAVPAEARRRDMVYVDATCPLVSKVHLEAQRHHENGRQMVMIGHRGHPETIGTMGQLPEGEVLLIETADEVAGLEVRD
PDRLAYITQTTLSIDDTEGVVAALRARFPAIEGPHKEDICYATTNRQGAVKTVAPKVQALLVVGAPNSSNSRRLVEVAAN

AGCDYAQLVQRAADIDWRALEGVSAIGLTAGASAPDVLIEEVVQAFRDRFDLTVEEVVTADESIEFRLPRIVRELA
>OLS47208.1 hypothetical protein BV379_02215 [Rhodovulum sulfidophilum]AB14
MPASSLLLQLFALFAAAFGAATVLPFQSEVVFVGLQLAGQIGIGWLILVASIGNVLGAVVNYLLGRGIERFRHRRWFPAS
EAQLDRAQRWYRRWGVWTLLLSWAPFGDAFTVVAGMMRTPVWLFVLLVTLAKTGRYAALAWATAAASG
>OLS47209.1 NYN domain-containing protein [Rhodovulum sulfidophilum]AB14
MFYRDERLALFIDGSNLYAAAKALGFDIDYKLLRQEFMRRGKLLRAFYYTALLENDDYSPIRPLVDWLNYNGFTMVTKP
A
KEYTDSQGRRKVKGNMDIELTVDAMELAPRVDHVVLFSGDGDFKPLIESLQRQGVRVSVVSTIRSQPPMIADELRRQA
DN
FIELDELKEVIGRPPREPRAEPRSEAAQND
>OLS47210.1 2-amino-4-hydroxy-6-hydroxymethyldihydropteridine diphosphokinase [Rhodovulum sulfidophilum]AB14
MPQPEKSTPHRPKGLLALGGNVGSGAGAPEATLAAGLVALDSESVRVTKISKFYHTPSMPEGSGPEFVNAVVEIATDLP
A
NALLARLHEIEARFGRERRVRWAPRTLDLDLLDLDGAVLPDAAIQARWMGLAPEAQAREIPDRTILPHPRIQDRGFVLV
P
LAEIAPDWRHPVSGRTAQEMLAALPAAEIACISPFAGPWAGVSALVKSYPSP
>OLS47211.1 DNA-directed RNA polymerase subunit omega [Rhodovulum sulfidophilum]AB14
MARVTVEDCVDKVPNRFELVMLAAHRAREVTSGASLTVDRDNDKNPVVALREIADETQLADDLRERMIEAHQTQIEV
DEP
EEDSMALLMGGAASEPDRPADDDMSEEKLLRALMEAQGQR
>OLS47212.1 bifunctional (p)ppGpp synthetase/guanosine-3',5'-bis(diphosphate) 3'-pyrophosphohydrolase [Rhodovulum sulfidophilum]AB14
MIDVEDLISLVRNYNPRTDEDLIRRAYAYGREMHAGQRRRSGEEYFMHPVAVAAILTEQRLDDAAIVTALLHDTVEDTK
S
TFSEIERLFGREVAELVDGVTKLTNLQLASNETKQAENFRKLFMAMSKDLRVILVKLADRLHNMRTIKSLPIEKQVRKSH
ETMDIYAPLAGRMGMQWMREELEDLAFRVLNPEARNSIIRRFITLQRESGDVIQKITQDIRKELDREGIEADVFGRAKK
P
YSIWRKMEEKGQGFSRLSDIYGFRVITQSETQSEGDCYRVLGAIHQRWRAVPGRFKDYISQPKSNGYRSIQTTVSGRDG
K
RVEVQIRTRQMHEVAESGVAAHWSYRDGVRAENPFAVDPAKWLASLTERFETEDHDEFLEHVKLEMYSDQVFCFTPK
GDV
IKLPRGATPLDYAYAIHTRIGDSCVGAKVDGIRVPLWTRLKNGQSVEIITAEGQRPQATWIDIVVTGRAKAAIRKSLREE
DRERYIKLGRELARVAFEHLGKKSTDKALRTAAKTFGLANANELLARLGSAEMRAQDVVEALYPELANTTGDEIDATRAV
IGLSPDESFTRAPCCQPVPGERIVGITYRGRGVVVHAIDCPVLEKFEEQPDRWIDLHWHSGKHPAVHNVGFLLTIRNDA
G
VLGRICTLIGEQKANISDMEFVDKKPDFYRLLVQVDLRDVEHLHTVMTALEAESDVAELERYRDPARTM
>OLS47213.1 hypothetical protein BV379_02240 [Rhodovulum sulfidophilum]AB14
MVFKRRDKRSVGKLLLDSVYPRGGWGRAAYYISHRLRRLPDTPRKIGRGIFAGVFAVFTPFYGLHFLIAALLAKAMRGNI
LASLLSTLVGNPLTYVPIGVASLKLGHFLLGTRFDENVDETLVAKFTGAASDLKNNFLALFTDAVPHWARLSTFYHEVFL
PYMVGGILLGLVAALISYYVSVPLLSAYQNRRRGRLKKKLSELRASAAAKAEDRG
>OLS47214.1 pyridoxine 5'-phosphate synthase [Rhodovulum sulfidophilum]AB14
MTATGCGLRLGVNIDHVATLRNARGAAYPDPLRAALLAEAAGADGITAHLREDRRHIRDADIDALMAGLSVPLNLEMA
AT
DEMQAIALRHRPHAVCLVPERREERTTEGGLDVAGDAARLARFIAPLAGAGCRVSLFIAADPAQVEAAAEVGAAVVEL
HT
GAYCDLHHEGRLAERDAELARLRAMARHAQGLGLEVHAGHGLTYETVAPVAALPEIRELNIGHFLIGEAVFLGLEASIAG
MRRRMEAARG

>OLS47215.1 GTPase Era [Rhodovulum sulfidophilum]AB14
MSTRSGFVALIGEPNAGKSTLLNRMVGAKVSIVTHKVQTTRARIRGVAMEGDAQIVFVDTPGLFRPRRRLDRAMVAA
AWG
GAADADIVVLLIEAHRGRTEGVDSILETLHERAAAGQTVALAINKIDRVKAEALLALSADLNAAYPFVRTFMISAEKGHG
VDDLRGWLAETLPEGPWLYPEDQIADLPMRMIAAEMTREKLTLRLHQELPYELTVETERWEDRKDGSTRIDQIIYVARD
G
HKGIVLGHKGETVKAIGQAARAEIAEFLGRNVHLFLQVKVRPNWLDEAERYSEMGLDFRDGN
>OLS47216.1 bacterioferritin [Rhodovulum sulfidophilum]AB14
MSNDQTIKDLQTALEMEMTAGHQYQLHAQVLDDWGLDLLAAKMREEMQEEIGHSDLFMQRIIFLGGTPEIRFQKQP
VRAE
SLEQMFELDAADEQEAIDVYSKAAQRAMEVGDIGSRTLFEKIVLDEEGHKDWLDTQLALIRRLGEQAYSAKQVSGPGA
GE
D
>OLS47217.1 S26 family signal peptidase [Rhodovulum sulfidophilum]AB14
MAKTDEKAGGGLWETVKTIFWALLIAGVFRTLFFQPFWIPSGSMKDTLLIGDFLFVNKMAYGYSRYSCPFGICPFSGRIL
SSEPERGDVVVFRHPVSNSDYIKRLVGLPGDTVQMREGVLYINGAPVEVEPAGIFKEPYQKQGPMGYYPECENAPVGI
GG
TCMKGRSIETLPNGVSHDILNITDRGARDTTPVYTVPEGEYFVLGDNRDNSRDSRYAQANGGVGFVPAENLIGRADRI
MF
SSAGRSLFFIWTWRPDRFFKAIE
>OLS47218.1 holo-ACP synthase [Rhodovulum sulfidophilum]AB14
MILGIGTDLANIDRIAATLERFGDRFRTRVFTEAEQAKAERRADAAGTYAKRWAAKEACSKALGTGLRMGISWKDMAV
TN
LPTGQPVMALTGWAAERLAEMTPPGHEALVHVSLTDDHPWAQAFVVIEARPRNAAPPPR
>OLS47219.1 hypothetical protein BV379_02275 [Rhodovulum sulfidophilum]AB14
MHNAARDQRQILAIALPVGGARQIARCDDDALDCGPAIAAQHRPFGPNLASRPRARPPTAS
>OLS47220.1 alkene reductase [Rhodovulum sulfidophilum]AB14
MTEPKLFTPFVAGDIALANRVVMAPLTRNRAEHETLAPTDLHVTYYTQRAGAGLIVTEGAQISPEGQGYLDTPGIYSDL
Q
VERWRKVTDAVHAAGGRIAIQLWHVGRVSHVSLLPGGQAPVAPSAVPADGVKTFTAEGFTDVSAPRALETAEISRILG
DY
RKAAANAMAAGFDGVEIHGANGYLIDQFLRDGTNRRTDGYGGPIENRCRFLFEVVETVAGEIGAGRTGLRLSPFSNAN
GI
SDSDPFAVFSQAISGLNRFGLAFLHMVEGQTGGPRDWPEGRGIPELRAMFEGLYIANNGYDRASALAAVESGAADMV
AFG
RPFISNPDLVERLRQGAGLAEPNRETMYGGGAEGLIDYPALDAARPA
>OLS47221.1 hypothetical protein BV379_02285 [Rhodovulum sulfidophilum]AB14
MKRIIAALSLGLFATAPAGAASLYNTLDGNAPLIVAHRGASGYLPEHTLGGYELALEMGADVIEPDLQMTADGVLVAM
HD
ATLARTTNVEALFAPRNGAYRVSDFTLAEIKSLTVEPTGATASTDYPGFAPTMADPYKVPTFQEVMDLVAAHNAATGD
GI
GIYPEAKTPNSLAMNRQILEEMKSNGFDGSVPNTFVQTFSHEGARQLAALQTEIGMDNAIAALGYALDLGDGFGVYDA
TT
GSVTALEDLAGFADGLGLYLGYDLDATFIAAAHELGLSVHGWTFRPRTDEAAWEMFLPWIEAGMDGFFTDYAQRGV
DVLD
RYEAAEVPVPATLPLMLAGLGGLAALRRRRAAA
>OLS47222.1 acyl-CoA synthetase [Rhodovulum sulfidophilum]AB14

MSMESARSEWQAIAAEAPWNERDRPKTIYSFVARTAATFPDRPAVSFQIFSGPKDPAETLSWTEFQGRITQAANLFRG
LG
IGPDDVVAYILPNCNETAICFLGGAVAGIVNPINPLLEAEQIAAILRETGAKVVVTLKSFPKTDVAQKVSEAVRHAPRVQ
TVLEVDLVRYLTGAKRLIVPLIRRKSPGHYHADVLDFHRALAAEPADRLTFEDPEEDRIAAYFHTGGTTGMPKVAQHRVS
GMIYNGWLGHRLLFRENDTLICPLPLFHVFAAYPVLMSVVAAGAHFVMPTPAGYRGEGVFDNFWKLIERWGVTFVIT
VPT
AIAALMQRKVDADVSTLRYAFSGSAPLPVELYNRFEAATGVTICEGYGLTEATCLVSINPPDAQKKIGSVGLPFPYTEVR
ILKCEGDGTVQRECETDEVGEICISNPGVVPGSTYTEEAKNLGLFADGYFLRTGDLGRIDSDGYLFITGRAKDVIIRGGH
NIDPAEIEEALLSHHDVAFAGAIGQPDSFAGELPCAYVELVAGATATPEDLMAHARVHIHERAAIPKHVEILDDLPKTAV
GKVFKPALRKLAIQRIYDTALAEAGHMVRVCEVVEDRKRGLVARLERQGPVDDEAVVHLLGNFTRPWEWSDL
>OLS47223.1 ABC transporter [Rhodovulum sulfidophilum]AB14
MPPAPQPQDDRARSKRIGALAALWPFLAPYRRMLAAALAALVLTSIVYLAMPVAVRHVVDAFDTETAALLDRYFGFALI
I
VALLAVGTALRYYLVTRLGERVVADIRSAVFDRMIGMSPSFYEKTMTGEVLSRITTDTTLILSVLSSSASFALRNILVFF
GALVLMLATATKLSGLVLLLVPAVIVPIVLLGRRLRQLSRENQDWIAASSGNASEALLSVQTVQAFTNEARARAQFRGVT
EQSFQSARQRIGTRAVMTVIVIFLVFAGIVGVLWVGAHDVRAGEMSVGQLVQFVIYSVMVAGAVAAMTEIWGELQR
AAGA
TERLVELLTAQDSVTDPERPAPLPEPARGEIRFEDVRFSYPTRPDSPALNGIDLTVRAGETVALVGPSGAGKTTVMQMIL
RFYDPQQGRVLFDGADLRSLGREAFRSRLALVPQDPAIFAASARENIRFGRPGATDAEVEAAARAAAAHDFLSALPQGY
D
TEVGERGLMLSGGQKQRVAIARAILRDAPVLLLDEATSALDAESERAVQQAVDRMAATRTTIIIAHRLATVKKADRIVVL
DRGRIVAEGTHDALVAEDGLYARLARLQFTAGVETAA
>OLS47224.1 hypothetical protein BV379_02305 [Rhodovulum sulfidophilum]AB14
MTVPFLRPILASTGLAPTCLALACLAAIAGRPAAAWTLWEAEPPGTALEQHLDRLIEGGEDLTELHDRIEDSRERSGFRL
LGRSTKGLERDVDALVLEIEKALLQNGNVGAIEEIRRLRAEIAGLGREIEVLRRDLYLARQAAAPDPDETDRIEGRIARR
EAEAAALEGEVAARKQALQQSFAEAGAALGPEEIDSLTSRADGDDLLRMFAVFDITRKITDELKALMAETSPSPAMQIQ
Y
YGVFVVMAELQVMVHREYMRRLDEEYLPALETIKGEIDATVAFARERMTTATPEQQDIYAKNIAANALSLDVIARYERIL
DRQKARSAEAMEKARSWLDVTYSTYDTAVNSMNVLNFIDETDRLFGEIMTRQLPDLEPFDDTVLRQRFEEISDRVAEIV
G
NS
>OLS47225.1 nucleoside-diphosphate kinase [Rhodovulum sulfidophilum]AB14
MTEQRPTSRGTGRRPKVVIADDCLDKLEALAEGAIHRNPDLADRLLGELGRARIVAAAKLPANVVAIGRAVTYRDESTG
Q
EKTVTPVFPEAADISRGRVSVLTPIGIALIGLAEGASLPWDTRDGKRRVLTVLRVAPQEATEGLEAG
>OLS47226.1 hypothetical protein BV379_02320 [Rhodovulum sulfidophilum]AB14
MTITRITHLPPGSDFRPLFPRSAVETGFLLTVEDRRGMERLLRHDGTRNAANPALLNGFLRHKLRISREAPEPPPPGLVM
AGCHVTYMISGEGARSGVLSMSPAPVPGHLSIISLLGATLMGMRKLQKVPLLRDDGRIETVVVLDVGLPPDRHAA
>OLS47227.1 hypothetical protein BV379_02330 [Rhodovulum sulfidophilum]AB14
MSYHVEALCDHRRFGSAARKQIAMHLAKKASDDGGGIWCSKHTIARHTELSLASVKRTIRDFPDEGLRVETGELRPCDR
G
HTVVYRLVLAAVSRLEPLLPEQEPKETMGTVNPRRGSPCPPKP
>OLS47228.1 hypothetical protein BV379_02335 [Rhodovulum sulfidophilum]AB14
MTQTHLDFIVPADSDLARLLWHMPTVVSHARSEWAEGFAKSIIRQSRRRNWRPSEKQLGTMRRLVSELFIHPREESEF
QV
IED
>OLS47229.1 hypothetical protein BV379_02340 [Rhodovulum sulfidophilum]AB14

MLTAALMLEDFDAWAAASAVWVARLTDRERAALAWAALRSLDEDQAADMAETVIGGAGAPLTTLLSEMSDARWW
ARIASR
RELKSYALASFEAMSPRDRAAFLRHVAPEIGRAVA
>OLS47230.1 hypothetical protein BV379_02345 [Rhodovulum sulfidophilum]AB14
MSLKLFQRNGAWYVRGTVAGRKFYQSTKTTDRKIAERIKAETEAQAWQRRFDGPGAGLTMAQVFTAYLDADKPARFL
LKL
AEYWKDTLVEDVTSELIRQAAKKIYPNANEPTWNRQVIKPTQAAINHAAGFGWCQRISVKRYTETPEVKTPATLEWVR
AF
SSQAEEDGLAHLGALCLFMFGTAARVGEACNLTWRYVDLSAARVELHLFKPTPWKRIAHLPPEVVVSLANIPSNRNLDE
P
VFGYAGRGSVRGPWNNVCKRAKIERLTPHCCRHGFATSMLQAGIDVKTVADMGGWKDATTVLRTYAHAMKDPSVT
NVLFG
AKTVQETSKKRARSYQPFEKEGENRNDSAPRWEGCPAAGHTPAQKKQQVSIGPATQILAACGGTMRKQTRIARANSV
QLV
PLMCRDMTKENAAAAGPRNGAENACEASQLPEHYRLKAGV
>OLS47231.1 hypothetical protein BV379_02350 [Rhodovulum sulfidophilum]AB14
MEFANQRFTVSEVASLTETNLNTVQSWRRKGFFDLGASEGWHRFTLSELFSVAVFAEVSGGTGNQDVASSACSFAVQ
MLA
EIIESNAAPYFVGASRKGSDPVMEIAYGSLNVGATLGKLISEGADAGAYIVVDYSAIFSRLLKRLHAGGYAMENPNSNV
>OLS47232.1 hypothetical protein BV379_02355 [Rhodovulum sulfidophilum]AB14
MKRRTFLAAAPLAVPTMTKAEAEQSQILRLFHRHRAILDAARRHVCNATGRGEDAELDLLFYRHSNKIEDEMMSLPSTC
A
ADFAAKVIVETVCGQCGADWETSMIWKEARLLVGVAPSAW
>OLS47233.1 hypothetical protein BV379_02360 [Rhodovulum sulfidophilum]AB14
MPNPFPAAVTALPATRLYEIHDCLALALDATERPGRYSQSEREARSYLRTALRHTLHLMETRA
>OLS47234.1 transcriptional regulator [Rhodovulum sulfidophilum]AB14
MARSGLSMGVRELAEAAGVSTNTITRLEKGEALKPRTLEAIQSAFEAAGVEFIPENGGGPGVRLKK
>OLS47235.1 hypothetical protein BV379_02370 [Rhodovulum sulfidophilum]AB14
MRRRAMTLEAEIEKAARQVSTDAFSLTIGEVINLYRDGDIKINPDFQRLFRWDTERKSRLIESILVRIPLPPIFVFELGN
SKWEVIDGLQRLSTILEFVGELKNPDTGDLEPPAPLIGTQCLKSLRGAFWSEELLELQRGKDQARIDKGDPTFVDNRNSY
FVLESDIQRAIKRTKIGVQLLEKKSDPKSKYDLFQRLNSGGLAANDQELRNCALVMVSPEFFNKMKKFADGEMFSKMIP
L
GPTSIRKSNHLENLCKIMAFAFRDYSVGSDIEEFVTNAMVNIASNPSEFDEIFEKLDDALNLLTNACGQGALRPFRNGDF
AGRIGRTSIEIILVGVIRCLHNIRSKQDPEGFVKGQIIAFWESEESKKYTAAGVAGTDRVQYTIPRGVEIFSK
>OLS47236.1 hypothetical protein BV379_02375 [Rhodovulum sulfidophilum]AB14
MIAEDIRGAFASNPAQHSALTSRRGAGIARAEGSPSGAPANGSASVARNGQPSARHLAQMRGAAFKAGLSAEFADLM
AET
GVSAAQAETVASGLAEFQKVSAPFVQGEQLDHAALMSIAKRAAMAGLALPETDAAAITKEIARVAANEAMALMASNE
PPI
RPCAIGIGQEAPSRADAMADGLLARMQSGHVPTHGREFANMRLDDLARLSAGSSFLRGQRHAVMWGGAMGADDF
KTALAI
ASNKVLLAAYEAAESQIKKASREVSAPDFRPINTVRVSGGVELAKVLENGEFGQGQITDAGEAFVLETYGKIFTLTRQAI
VNDDLRAFEQSSRLFGQGAALTEARIFAGLLEANGGAGPAMSDGKTLFHADHGNLAETGAALSIGSLSLARTAMRRQK
GL
AGEAIRVDPALLIVPPELETQAERLVAEISAASADDVNPFQQKLTVLADANLTDPAAWYIAALPGRPDALQHAYLDGAS
G
PQIFTRDGFETDASEFKVRLDFGAGFVDHRAWYKNPGA
>OLS47237.1 hypothetical protein BV379_02380 [Rhodovulum sulfidophilum]AB14

MTKDPTKRTEGASEGAVSHLYGNRARLKPRALSLLGATTWAADLSPCLDRPYLVKGWLDQSALSVLYGPSNSGKSFLAL
D
LAHHVAKGRSWGNRRVNKGRVLYIAAEGGGGFANRVAALDEPEFFVLSIPVTLTGPDSAAAPLTEVLQHLAAVGGAGF
DM
IVIDTMARVMGGRDENAAPDIADLVRNLDLIRRVTGAHVMLVHHTGKDTGRGARGHSSLRAAIDTEIELSRDDLGQIT
AE
VTKQRDGPTGYRFCYQLRQVELGLDQDGDPVTTCLVDPAVPAEAGRAGVSEAARKALSLLNRLIEERGEAHRKPQYPS
HA
GVSFDLWRDACMTEGELSSSENRDTRKRAFNRCREELETAREIVVRDDLVWVVQ
>OLS47238.1 hypothetical protein BV379_02385 [Rhodovulum sulfidophilum]AB14
MTETLHIYGPIGEGENTPPRIGAFLDDNAGVPVVIAVNSYGGIASDGAAILAQLDRHGQVRVEVLGVAASAASLLVMGA
A
EIAMHRAAHLMIHEPSNLAWGTADDLRREAGMLDKISETYAQAYARTTGHPVERIRAWMAAETWMTADEALALNF
CDGLF
GDDDAPQSEPVAAFDYTRFKAPPPELVRLAVRNGWTGNAA
>OLS47239.1 hypothetical protein BV379_02390 [Rhodovulum sulfidophilum]AB14
MLAAALTLEDFDAWAAASAVWAARLSARERASLAYAALRSLEPEQAEMTAATVLRAAGAPMPPFLRGMEEARFWAS
HANR
AELKAFALASFEAMAPRDQTAFLRHISEIEVAA
>OLS47240.1 phosphoserine aminotransferase [Rhodovulum sulfidophilum]AB14
MVTATPATRPVNPRFSSGPCAKLPDFSLDMLADAALGRSHRAAIGKAKLKAAIETTREILGVPADYRIGIVPASDTGAVE
MAMWSMLGARGVEMLAWESFGAGWVTDVVKQLKLDATVREAPYGEIVDFGAVDFDKDVVFTWNGTTSGVRVAD
GDAIPAE
RAGLTICDATSAAFAMDLPWDKLDVVTFSWQKVMGGEGAHGMLILSPRAVERLESYSPAWPLPKIFRMTKGGKLNA
GIFE
GATINTPSMLAVEDYLVALDWAKSIGGLPKLIERSTANFRAVQDFVDTKPWIENLAVDPATRSNTSVCLKFDDARIKDG
A
AFAKAVAKRLEKEGVAYDIGAYRDAPAGLRIWCGATVETSDVAALMPWIEWAFEAEIEAQTAAA
>OLS47241.1 phosphoglycerate dehydrogenase [Rhodovulum sulfidophilum]AB14
MAPKVLVSDKLSETAVQIFRDRGIDVDFLPDLGKDKDKLLEVIGQYDGLAIRSATKVTEKILEAATNLKVVGRAGIGVDN
VDIPAASKKGVIVMNTPFGNSITTAEHAISMMMAVARQIPEANASTHAGKWEKSRFMGVELTGKTLGLIGCGNIGSIV
AD
RAIGLKMKVLAYDPFLSEERADKLGVTKVELEELLPKADFITLHVPMTEKTANILSAENLAKTKPGVRIVNCARGGLVDE
AALAELLQSGHVAGAGFDVFAVEPAKDNVLFNLPNVVCTPHLGASTTEAQENVALQVAEQMSDYLLTGAVTNAINMP
SIT
AEEAKVMGPWIKLAGHIGAFIGQMTDEPIKAINVLYDGFAAEMNTAALNASAIAGVMKQANPVVNMVSAPVVAKER
GIAV
STTTQGKSGTFDGYVKMTVVTAKRERSVAGTVFSDGKPRFIQIKGINIDAEIGEHMLYTTNRDVPGIIGALGAAMGAHG
V
NIANFTLGRTTVGEDAIALMYLDQPMPVHVVEELDKTGLFQQVRPLEFEIG
>OLS47242.1 serine/threonine protein phosphatase [Rhodovulum sulfidophilum]AB14
MSLSYAIGDIHGQRARLEAAHALIAEDRRQVGDTGAPVVHIGDYVDRGPDSRGVLDLLIEGRAAGRPWICLLGNHDRW
MQ
HFLRPGCEPEPARPDMLWLEDAVGGRATLASYGVDADPHRALGAIHAEARAAVPSAHLDFLAELLPSFRHGEAFFCHA
GI
RPGVPLEAQSVTDLIWIRGEFHRDTREHGALIVHGHTPVDRVTHFGNRVNLDTGAGYGRPLSVVAIEGRNVYLLTPGGR
V
PVPPEMPPEARCEAG

>OLS47243.1 hypothetical protein BV379_02415 [Rhodovulum sulfidophilum]AB14
MTAEGHSRRATRALQKLAEQDPAFAALALWCSHRDSDARADAAWTDGRAIRYGPGFEALSLPEQIGLAAHHILHVAF
RHA
ARMQAMQARFGDRFDAELFNLATDSIVNETLMLTGYILPRPAVRLTGLLKDAAREEMGGEEAVTRYDAERLYLRLFEAE
A
PPPRSRLRLVDRSPPGETGQGGGEAEGKGRGTAGDSRPRPGGAVAEAARDYGRRQGFAPDIEAEGDEGDQGAGSEE
EAEW
RQRVARAMEAGRVAGFGLGMLGHRLADLPEVHTPWERHLRALVSRAVMIRPAPQTTRPARRWIARDSDARATGAP
EPAFE
FAWSRAREIPRIVVGIDSSGSVEPVLLAKFAAEVAAISKRTGAETHVLVFDTRVHGRHVMGGGIWEGRITDVLFSREGGT
SFIEVMEEALALSPSAVVMLTDLMGPFGPPPPRRLPVIWAVPGEPPPAGAPFGQVLSLAR
>OLS47244.1 ATPase [Rhodovulum sulfidophilum]AB14
MSVNAMTVPAGEALEVLRAGWEAQKAGHITASYMLHGRPGVGKTQVVEQLAREIGARLFDLRLTTIEPQDLRGLPYY
DHE
TRKTRWYRPEDLPDDPDEPSILFLDELTAASPMLQPTVYGLLQERRVGRHVLPRTVFIVAAGNTVEDGAIAYQMNTALS
D
RLIHLHVQAAAADWLDNYAVPKGLDASVVAFIKTRPDLLDTTERAVAEGHLIATTPRSWERVSRIMGAVAERRVRNVLI
A
GTVGEAVAAEFALIADDIAATVQVQEMLEADRKARIALYPASMHGLNALIYGLIGVLDARNVQEVIDCMAEIRNLARHR
P
EEAFARMPLGELCTYGFEGLIRKGLEIGLGEHFLGNEAYRAYAAERKAAGL
>OLS47245.1 LysR family transcriptional regulator [Rhodovulum sulfidophilum]AB14
MTLDQLRIFIAVAEREHVTQAAAFLNLTQSATSAAIAALEERHAVQLFDRVGRRIVLTDAGRLFLDEARAVLARAEQATR
LLGDLAGMKRGRLRLAASQTIANYWLPPRMQRFHREHPGIVLELAVGNTSDVAQMVHAATVDLGIVEGEIDDTALAIH
SL
SGDRLALVVAPGHDWADRPPSGPDDLKAGPWVMREAGSGTRAVCETALARRGLHIEDVEVAFELPSNEAVRVAVES
GAGA
AILSTLVVAPAIDAGRLVPVAFDLPPRRFHMLRHKERHLSLAEQRFLELA
>OLS47246.1 ubiquinol-cytochrome C reductase [Rhodovulum sulfidophilum]AB14
MSDSKFESRPLSVPSGRFARMAKFGGLASNLAGTAAINGMQQLAQGHRPKARDLFLTPANMRKVADRLAHMRGAA
MKVGQ
LVSMDAGDMLPPELAEIMARLRADAHYMPGGQLKQVLNANWGEGWLKRFARFDVRPVAAASIGQVHRAQTKDGR
DLAIKV
QYPGVRRSIDSDVDNVAALLRLSGAMPKEMDIAPLLDEAKRQLHEEADYAREGHYLGRFADLLAGAPDFVVPGRHEDL
TT
ENVLAMDFVEGVGIETLTDAPQAERDRVMGLLVSLLFRELFEFRLMQTDPNFANYRYQPETGRVVLLDFGASREFPAA
MT
EAFRGLMRAGLAGDRAAIRQAILEIGFFAEDTAPRHQQLMIDMFEMSMEPLHRPGAFDFAENDVALRMRDAGMAL
GQDRD
FWHVPPMDTLFMQRKFGGMYLLASRLRARIDLREILAPYV
>OLS47247.1 tRNA pseudouridine(55) synthase TruB [Rhodovulum sulfidophilum]AB14
MGRSRKGRDISGWLVVDKPEGLTSTAVVNKVRWAFNAKKAGHAGTLDPAATGVLAVALGEATKTVPFVTDALKAYRF
TMR
FGAATNTDDAEGEVIATSEKRPSDDEIHAALPAFIGDIEQVPPQFSAVKIDGERAYALARAGEEVEIAARPLYVESLEMV
ERPDADHAVLEMICGKGGYVRSIARDLGQALGCHGHVVSLRRVWAGPFEIEDGVTFDLIEKLARSPELDTYLRPLEEGLA
DLPELRTTPDGATKLRNGNPGIVLASDVEYGDEAWASLDGEAVAVGIYKAGELHPSRVFVKG
>OLS47248.1 ribosome-binding factor A [Rhodovulum sulfidophilum]AB14
MSRSSAPSQRQLRVGELIRRSLSEVLARGDVHDPELTALSITVGEVRASPDLKVATAFVLPLGGNHAEEALAALRRNRGE

LRRAVGKKTQLKFTPELRFTLDETFDRMDETRRLLADETVRRDLATPGATPDGDED
>OLS47249.1 isochorismatase [Rhodovulum sulfidophilum]AB14
MKALVIIDMQMEMQHRIGAGRDCVNADAPERIAALAAAFRGKGLPVVHIRHVDADPRSPLAVDAEGCRPMPCAEAQ
GGEP
VFVKRTSSAFASTDLAAWLHENRISELVVTGAVAGFCVNSTVRNGADLGFDMTVVRDAVIGFDLPGPGLSARGIFDVT
MA
HLEADFARLIDTPGIIAMIG
>OLS47250.1 hypothetical protein BV379_02470 [Rhodovulum sulfidophilum]AB14
MRRQEWRETRLAFSRPDLLDQAISRLRAEQTGLWHRNADGSELERLAPPKAARYAAAIRHHMAAQDALDAAWGGW
LRQEG
LKPLRLRYDVLSTGPKQILAQVLSALGLDPAQGRSVESPTAKLADAESRKWRCRCERET
>OLS47251.1 hypothetical protein BV379_02475 [Rhodovulum sulfidophilum]AB14
MTGLERTVRVYRHWHLSVAVAGNFLFLVGSVLFLPTLSSWETAGVWMFIVGSFLMLIGAFGEVAKAVYEKHERDRM
>OLS47252.1 DUF1674 domain-containing protein [Rhodovulum sulfidophilum]AB14
MSDDAENTARKDLPPAARRALAEAAERRKAAEAAEAARPVELGGRDGPDPARYGDWEKKGIAIDF
>OLS47253.1 16S rRNA methyltransferase [Rhodovulum sulfidophilum]AB14
MDEARERGTERTRAAALELIRAVLEDRRLIAELAGPEGPLEPLEPSERARAQRLAVSTLRHMGRADAVLKPFLRRPPPPP
VRAILRLATVEMLDGGAAAHGAVNAAVALARADRKTEALAGLVNAVLRKVADLPASDWQALPPPRLPGWLRGRLDS
AWGR
AAVVGIEAAHAAGAPLDLTPKDGDAVTLAAATGGVALPTGSVRIAGPVQVSALPGYGEGAWWVQDAAAALPARLLA
ARPG
ERVLDLCAAPGGKTMQLAAAGAQVTALDISGQRMARLEENLARTELEAELVVADALDWGDDRRFDAILLDAPCSATG
TIR
RHPDLPLAKDGAAVKPLFPLQSALIDRALGLLAPGGRLIFCTCSLLPEEGEAQLAAALERHPDLSPIVFEAPWIEPGWRA
AHGALRLRPDFWTESGGMDGFFIAGLQIPA
>OLS47254.1 heparinase [Rhodovulum sulfidophilum]AB14
MAGQTEIEFPPLRRGAWRNRLHARLSALGRPASRLVFQPEPRSVGSFARGRQLCAGRFLFDGGAVEAPGLPILDVDSP
GL
RFEAARQGFGWLDDLAAAADGKARTVAQDWVLGWIDRFGRGGGPGWTPELAGRRTIRLIHHARFLEAGLDAARCD
RLHLA
LARQTAFLARRWRNAPPGLARFEALAGLIYGALCLEGMGRHLPGAQAALERACDTEIADDGALSSRNPEELLEVLTLLG
W
VESAMAAAKRPAWQGHRAAIQRIAPVLRALRHADGGLARFHGGGHGTEGRLDQALAAAGVKPRPLRGLAMGYARL
ASGRT
TVIVDAARPPSGPASAEAHASTLAFELTSGRRPVIVNCGAGGAFGPGWRLAGRASSSHSTLAIEGFSSSRLAPRRRGRPQ
LLAEIPGSVDWRAETGQGGHALTAWHDGYAATHGLIHVRQLNLAYDGRALSGEDTLAAFTPEERAAFERALERAGGA
GIA
FSIRFHLHPEIDAALDLGGSAVSLAPKSGEIWVFRHDGAASLSLVPSVYLETGRAEPRASQAILLSARMTDTACVVGWTL
AKAHEGAQAPRDLEREDEFDDTAAPWTRPHSMTKD
>OLS47255.1 bifunctional phosphoribosylaminoimidazolecarboxamide formyltransferase/IMP
cyclohydrolase [Rhodovulum sulfidophilum]AB14
MTELVPVRRALLSVSDKTGLIDLGRSLAARGVELLSTGGTAKALREAGLEVKDVSEVTGFPEMMDGRVKTLHPMVHGG
LL
ALRDNEAHVMAMEEHGIGPIDLLVVNLYPFEATVAKGADYDDCIENIDIGGPAMIRAAAKNHAFVNVVVDTEDYARLL
GE
LDRHDGATCLKFRRKLAQIAYARTGAYDAAVSTWMAKALGDAQPRRRVFAGALAQGLRYGENPHQQAAFYIDGSER
PGIA

TAKQWQGKELSYNNINDTDAAFELVSEFLPANGPACAIIKHANPCGVARGETLEQAYRRAFDCDRTSAFGGIVALNMK
LD
EPTAKAISEIFTEVVIAPAADDAAKEVFAAKKNLRLLTTGGLANPAAAGLTYRQVSGGLLVQDKDNGRIEASDLKVVTKR
APTEAEIADMLFAWTVAKHVKSNAIVYVRDGATVGVGAGQMSRVDSCRIAARKAEDMAEALGLDAPLTKGCAVASD
AFFP
FADGLMAAVEAGATAVIQPGGSIRDEDVIAAADEAGLAMVMTGMRHFRH
>OLS47256.1 signal peptidase II [Rhodovulum sulfidophilum]AB14
MRRLGLVAALVFLIDQVSKVYVVFHMGLALDQRIDVWPPYLTFRMAWNRGVNFGLLAHDADLARWLLIAVSLAISGG
VAI
WMARGRHRPAALVSAGLLIGGALGNALDRVLYGAVADFLNMSCCGIDNPYAFNIADVAIFAGALGLVLYTGKDKTP
>OLS47257.1 pyruvate/2-oxoglutarate dehydrogenase complex, dihydrolipoamide acyltransferase (E2) component [Rhodovulum sulfidophilum]AB14
MRSARILVLGAAVTAALAGCSRGDDYQPKLMHLRGAERGPDEFSVVPNKPLQMPKDMAALPAPTPGGSNLVDPTPE
ADAV
AALGGNIARGTPGDAGLMAQVSRFGVDPSIRQTLAQEDLDYRRKHDGRLLERWFNVTVYYNAYSRQSLDQYAELERW
RAR
GVRNVSAPPKD
>OLS47258.1 peptidase M16 [Rhodovulum sulfidophilum]AB14
MIRAVFSSLAFVLLAALPLRAEVDIQEVTSPGGIHAWLVESHEVPFVSLEIRFQGGASLDAPGKRGAINLMTGLLEEGAG
EMDARAFARERDALAASYRFDVGADSLAVSAQMLTENRDEAVGLLRQALVAPRFDEEAIERVRAQVISGIRSDATDPN
AI
AGETFDRMVFGDHPYATPISGTEDSVNALTRDDLIAARDRVIARDRLFVAAAGDISAEDLGKLLDTLLGDLPETGAPMP
G
PAPLNLEGGVTVVPFDTPQSVATFGQRGLDRDDPDFFAAFVMNQILGGGGFGARLMEEVRVKRGLTYGVYSYLMPM
DHAA
LIMGGVASGNDKIAEAVEVIRAEWARMAEAGATQEELDKVKTYLTGAYPLRFDGNAPIARILVGMQMEGLTPDYVKT
RND
RIRAVTLDDVRRVAAELLDPAHLQFVVVGQPQGLRTTN
>OLS47259.1 DNA mismatch repair protein MutL [Rhodovulum sulfidophilum]AB14
MTSPAPNIHRDIPRIRQLDEAAINRIAAGEVVERPASAVKELVENAIDAGARRIEVAYAQGGKGLIRVTDDGWGMAAE
DL
PLALSRHATSKIDGSDLLDIRSFGFRGEALPSLGAVGRLTITSRREGAEAVRIAVTGGAPGPVTPAALNRGTVVELRDLF
FATPARLKFLRSDRAEAQAIGDVVRRLSLAEPLVGFTLRDVSDGGEGRVLFRADAGTGDLFDALYGRVVSVLGRDFAEN
A
LRIEAERDGFALTGFAALPTYSRGAAVAQFFFVNGRPVRDKQLLGALRAAYMDVLSRDRHPAAALFIDCDPHLVDVNV
HP
AKAEVRFREPGIVRGLIVSALRHALAGAGHRASSTVAGATLGAMRPEPSGPPRVYQMDRPSPRALSMSHAAQAPATPF
EA
PSQAPPEAPLAEGFADPSARFEAVEPAPGAEALPLGAARAQLHENYIIAQTERGMVIVDQHAAHERLVYEKLKRQMAE
RG
VAAQALLIPEIVELSEGDIARLMEHEADLTRMGLSVEPFGPGAIAVRETPALLGEVDAGALIRDILDELADQGDSQTLQA
RLDAILSRVACHGSIRSGRRMRADEMNALLREMEATPLSGQCNHGRPTYVELRLSDIERLFGRT
>OLS47260.1 recombinase RmuC [Rhodovulum sulfidophilum]AB14
MIEIFGRAHAWDDPMVLAAGGAAALILLVLGLLISAVRRAGRSAEMAAPLIYQMDRLTRRVDQLGEGQQRLAGGLTH
VSE
AQAASQANMLRLMESRLAEVGEQMNRNLEGASRRTALSLGELQQRLATIDRAQENIEKLSGNVLSLQDILSNKQTRGA
FG
EIQLADIVGKALPRDSYVLQATLSNGRRADCLIRLPKPPGPIVVDSKFPLEAYEALRRAETQWDRQEAVRAFKTSVRAHI

RAISQKYIIAEETADGALMFLPSEAVYAELHANFPDLVRESFEARVWIVSPTTCMATLNTMRAILKDARMRDQAGAIRK
T
LKLLHRDVELVAERVDRLTTHFGQARADLEGIGLAAERAGKRAARLDNLDFEDLGADQDEMMPRLPGAAE
>OLS47261.1 transcriptional regulator [Rhodovulum sulfidophilum]AB14
MTTRITHHLNDALLMSYAAGSLPEAFSLVVATHVSLCDECRARLAGFEAVGGAVLEAEEPAAAMAEDSLAATLRRIAA
MP
GETHITAPEPDAAVDPVFPMPLRDYVGGDAQDVHWRALGGGVRQAVLPTEKDATVRLLYIPAGMAMPDHGHGGM
ELTMVL
QGAFEDAGGRYGRGDVEVAGEDLDHTPVAAPGPDCICLAATDAPLKFRGILPRLAQPFIGI
>OLS47262.1 cyclopropane-fatty-acyl-phospholipid synthase [Rhodovulum sulfidophilum]AB14
MPIDSAGGRRRIAVIGGGISGMGAAHLLAETARVTLFEAEGRLGGHARTVLAGRNGDRPVDTGFIVFNQVNYPHMVR
LFE
KLGVPVAKSDMSFGASFDGGAFEYGLKNLRAVFAQPANALNPRFWGMLRDVTRFNARALDEARDPAVSLGDFLAAL
GTGD
WFRDRYLLPLSGAIWSTPSQGILDFPAQALIRFFDNHALLSTSGQHQWYTVEGGSVEYVRRLEAALAAADVEIRKAAPV
E
AVRRGAEGAEIRARGGAWESFDEVVFATHSDDSLAMLADASPEERAALSAVRYQPNEAVLHADPSLMPKRRAVWSS
WNYT
EESGALADRIDLTYWMNSLQPIPEDDPLFVTLNSRRPVRDELIYDTATFRHPVYDRGALEAQQAVRRMNGTNRTWFC
GAW
MRNGFHEDGLASAVDVAEAIQRGGPEAVAA
>OLS47263.1 cyclopropane-fatty-acyl-phospholipid synthase [Rhodovulum sulfidophilum]AB14
MTAVVEHIRGKTFHQRRGRVGHKLNHTVDYVLFDAEAQELKAPGWFGRVPGKPMAVLDSDHGGPKGQGRGAAWV
REVLKA
RDLFAIRGKIELMTLPRVLGHEFNPVSFWLCHDTEDQLRAVIAEVNNTFGDRHCYLCCHPDQRPIEPSDILTATKILHVS
PFLPIAGGYRFRFDIRPDKVGIWIDYSADAEEGLYATLTGPRRPLSRWSMLKSALRRPFGSRRVLVLIHWHALVLFLKGA
KYRARPEPPAEELS
>OLS47264.1 sugar:cation symporter [Rhodovulum sulfidophilum]AB14
MRAAPLSGYAVFAAMLTAAGLPIYIHAPKFYVDQYGVSLAALGSVLFGLRLLDVVQDPALGWLSERLHRWRSLAVLAA
VA
LLVASMAALFAVVPPVAPLLWFALSLTGLFTAFSFLTITFYAQGVSRAGRMGPGGHVRLAAWRETGGLLGVSVAAVAP
TV
LMTVTAAPFAAFALGFGGLAAVAALAMWRDWSLPAAPETTGWREVLADAVARRLLLLALINATPVAVTSTLFLFFVES
RL
GLPGWEGPLLVLFFLSAAASAPGWGRAARRFGEKPALLAGMGLSILAFGFATLLGTGDGLAFAAICVASGAALGADMT
LL
PAIFAGRMARIAPNAGQAFGLWSFVSKFTLALAAVAVLPLLQAAGFEPGETASPERALVLLSLLYAALPCALKLVALALL
AATPLNRETIG
>OLS47265.1 hypothetical protein BV379_02555 [Rhodovulum sulfidophilum]AB14
MIAFVLGLALALALVAAKVRYLGFPAQKPADYSGQGPEFDLRRHLDGPILCEGVVYGPTGRVVSRFTGDFLAQWEGNS
GT
MTEEFRYDSGATQSRRWSFKLANDGSIAADAPDLVGSGHGMQQGSGVNLRYRIRLPEDSGGHVLAVTDWMYLMEN
GAIIN
RSQFRKFGFLVAELVATMRPKEAA
>OLS47266.1 short-chain dehydrogenase [Rhodovulum sulfidophilum]AB14
MTTDWIGKRYWIIGASEGIGRALAERLGALGVNLVLSARSEDRLKDLAASLPRKADVVPVDVTDAAAVKAAVEAVGEID
G

VIYVSGTYWPMTAGDWNAEQVETMLDVNLMGAARVLGHVVPWFVARDRGHIVLTGSLAGYRGLPGAIGYGASKAG
LMSLG
ESLQVDLRGTGVKVQIASPGFVKTRLTDKNDFKMPFIMSPEDVAHEYVELMMSDAMSRSFPTLFSWIMRLSQLLPDW
LYF
PLFGRKS
>OLS47267.1 hypothetical protein BV379_02565 [Rhodovulum sulfidophilum]AB14
MEELLEISPSKVVQVIFQAREKRLGEAELRGFIAAMNDDEKASLVALTWIGRETFEADELEEAITTAKAEATSPTEDYLV
NMPLFADYLESGLEAIGISPADVEEEFFDR
>OLS47268.1 long-chain fatty acid--CoA ligase [Rhodovulum sulfidophilum]AB14
MATRGVSADGLCSIPALLARNVALYGDRPAYREKEFGIWQAWTWAEASDEIRAMALGFLALGMAPGDHVAVIGRNR
PAHY
WAMVAAQMCGAVPVPLYQDAVAEEMAYVLDHCGARFVVCGDQEQVDKVIEIRDRAPGVEHVLYNDKRGMRKYDH
TRLNWL
HDIGEEGRAAHLRLEPELDARIAGQSWDTTCVMLYTSGTTGKPKGVVLSNRNIIETAKASVAFDHLGQDEEVLAYLPMA
W
VGDFIFSIGQAMVAGFCVNCPESPDTMMTDLREIGPTYFFAPPRIFETMLTSVMIRMEDAGDLKHALFRHFMEFAKGT
GE
RFGMPRYRRRQSRPTLRRRLRWIYRYAMGIGFAVETLAENAVRGFFVRLRHPLEARKYFRARGPLLNLYPRPLREYFEYR
TGEVLIYGPLKNTLGLSRIRVAYTAGEAIGPEIFEFYRSLGINLKQLYGQTEASVFITQQPDGQVRSDTVGVPSPGVEVK
IGESGEVYYRSPGTFVEYYRNPDSTASTKDPEGWVATGDAGFFEEASGHLRIIDRAKDVGRMADGALFAPKYVENKLKF
F
PDILEAVVFGAGRKICTAFINIDLTAVGNWAERNNIAYASYQELAGHPRVLETIKSHVEEVNRSLAGDEMLSGCQVRRFL
VLHKELDADDGELTRTRKVRRRIIEEKYADLVAALYDGSAEQYTETEVTYEDGRKGRIRATLKIRDARSFPVHSHDKVAA
E
>OLS47269.1 hypothetical protein BV379_02575 [Rhodovulum sulfidophilum]AB14
MTENRPTIPMPSLDWQIPFGRAQKDVLWASFALLTAMVALGAGFWALAAVFSLALAVFAQIRVPQEPPTPEGGRLAP
LSH
ALRYGLANLVTMVFVYGAATVLGSGLRMAVTKLLAVV
>OLS47270.1 ABC transporter ATP-binding protein [Rhodovulum sulfidophilum]AB14
MNRMAPPQPDEGLPTEDGRAIGPVLMELRNITLRFGGVVAIKDISFDIREGEIRAIIGPNGAGKSSMLNVISGFYRPSEG
EVWFEGARRPQMRPYKVARQGIARTFQNIALFSGMSTLDNIMTGRITRMKSSLMSQALWWGPAEREESEHRAAVEK
IIDF
LEIQHIRKTPVGRLPYGLQKRVELGRALAAEPRLLLLDEPMAGMNVEEKEDMSRFILDVNDEFGTTIVLIEHDMGVVMD
L
SDRVVVMDYGRKIGDGPPEEVRASKDVIDAYLGVAHD
>OLS47271.1 branched-chain amino acid ABC transporter permease [Rhodovulum sulfidophilum]AB14
MTDLLYAVEVMINGLMAGVLYALVALGFVLIFKASGIFNYAQGVLALFAALTLVGLQEGQVPFAHLINAALGTDLHHFG
W
QLPAVVAIVLTVGVMVLLAIAIERLILKHLVNQAPIILFMATIGLAYFLEGFGDIMWGADIKTLDVGLPQGGSAWLEQAT
AGWGGEDFYGFFIDRLDIVATLVAAVLVISLTLFSQYSKQGRALRAVADDHQAALSVGISLRFIWVLVWSIAGIVALVAG
IMWGAKSGVQFSLSLIALKALPVLMLGGFTSIPGAIVGGLIIGVGEKLFEFTVGPMIGGATENWFAYVLALVFLVFRPQG
LFGERIIERV
>OLS47272.1 hypothetical protein BV379_02590 [Rhodovulum sulfidophilum]AB14
MQKLFAITCVIGWGVFYVFAYLALASVNDAQWMSVAYGLLAFAGFVAGMLSWVRLVRGKRPVIRTVPKGAARVRRPL
>OLS47273.1 hypothetical protein BV379_02595 [Rhodovulum sulfidophilum]AB14
MSHRTRIGGFCIDCRTEDPGPALAFRSAASGRERALIEDVRSARPSGPPAAPQVILHDPRVPLDIEAECARPTGLGAVEV
TRVRDRGVRQAPGGHRFCPVPPEGDFPKDVMEVD
>OLS47274.1 branched-chain amino acid ABC transporter permease [Rhodovulum sulfidophilum]AB14

MFYREAGDFKTSYRDDSQTFPIGFDRYRYYAVLVLAFAVVPFAIDDYWASAIFVPFLIYAIAAIGLNILVGYCGQLSLGT
GGFMAVGAYACYKLMTAFPDLNIVFCILGGGLVTAAVGVLFGLPSLRIKGFYLAVATLAAQFFLVWLFNKVPWFYNHSA
S
GQISAPARTVFGQPVTGAGVEAWAQYMICLVFAVVLAWAARNLTRGTVGRSWMAIRDMDIAAEIIGVNPLKAKLTAF
AVS
SFYIGIAGALFFAVYLGAVEVGEAFGIQKSFLILFMIIIGGLGSIFGSFAGAAFLVLLPVLLKNVLVGTLGWPTDLTAHV
QLMILGALIIGFLIAEPHGLAQLWRVGKEKLRLWPFPH
>OLS47275.1 ABC transporter permease [Rhodovulum sulfidophilum]AB14
MTNRLAPLALGVLLAAGPAAADLVFPDLSYRTGPFAAGGVPFSDGYQDYFTLVNERDGGIGGVKARVPECETAYNTEK
GV
ECYEATKGDGALIYQPLSTGITYQLIPKVEADHIPLHTMGYGRTSAANGKVFEWVFNYPANYWDGASVIVKYLMDENG
GS
LEGKKITLLYHNSAYGKEPIPTLEALSKKHGFKLTLIPVDSPGQSQKSQWLQIRRERPDVVVMWGWGVMNQVAIQEAA
NI
NFPMDHFIGNWWAGAEHDVAPAGMAADGYKALNMNRVVDYPVFADIRSHVVDKGLAAGDGSYVGSVLYTRGMY
AAMLAVE
AAKTAQEMTGTAQIDAAMIRDGMEHLEMTDARMEALGMPGIGPEFTVSCADHGGSGMAIVQQWNAAEKTWVPL
TDFIAPD
AEVIAPLIEADSTAFAAENGIAPRCD
>OLS47276.1 ABC transporter ATP-binding protein [Rhodovulum sulfidophilum]AB14
MFDTAPQDETLLELNNIEVIYNHVILVLKGVSLSVPKGGITALLGGNGAGKTTTLKAISNLLRSERGEVTKGSILYRGAR
VQDLNPADLVKAGVIQVMEGRHCFEHLTVEENLLTGAYTRSDGRAALQADLEMVYGYFPRLKERRKSQAGYTSGGEQ
QMV
AIGRALMSRPQMILLDEPSMGLAPQLVQQIFEIVKSVNENEGVTFLLAEQNTNVALRYAHYGYILESGRIVMDGPAADL
R
ENPDVKEFYLGMSDQGRKSFRDVRSYRRRKRWLS
>OLS47277.1 AMP-dependent synthetase [Rhodovulum sulfidophilum]AB14
MSVHFDDLETRSEDERAASLAECLPALVAEARALPGYAGALAEVNPLTLTTREALAALPVLRKSGLVAAQSAEAPFGGYT
AWEAGQFDHVFQSPGPIYEPGCVSTNWWRLGRFLHAAGIGAGDIVQNCFGYHLTPAGMMFENGARAVGAAVLPA
GTGQTE
LQVRAARDVGVTAYAGTPDFLKVLLDRADALGMDLSRLRRAVVSGGALFPSLRQDYADRGIACLQCYATADLGNIAYES
P
AMEGMIVEEGVIVEIVRPGTGDPLPDGEVGEVVVTTLNPDYPLIRFATGDLSAVLPGKSPCGRTNMRIRGWMGRADQ
TAK
IKGMFVRPEQVADLVARHPEVAKARVVAGRAGERDTMTVRLETEATDPAPFAASVAEVLKLKGTVELVAPGSLPNDGL
VI
EDTRSYD
>OLS47278.1 hypothetical protein BV379_02620 [Rhodovulum sulfidophilum]AB14
MKPLGNTRALKLAGLVYLAVLSLFLVLNSYTRNHHNWDVIGYTAAAYQLMGETGTDRLSARTYDDIRRATAPETFEKLT
T
EGYRAAVYQDPEALRQQLPFYSIRPLYVALSIAGSKLTGTIAGGTALVSALCGGLLLILGWAAVSPRSIALAALLPFALY
EAGLGSLARLSTPDGLAALVALAALLLCHRHPRPAMLLFALLPLARTDFVILLPFAALLLHGRVPRLWIAAMLGVGAAAV
VGLNAFSGNYGYLRIFDFTLVSGPQPYPAEMEETLGMTDILDAYARGLQRLVSKDTGMIALFIGTAGFLAAKAMRGRLL
P
FDKAVLAALGFVLLHFLAFPSGFVRHYFVAACLCLILWARLIDGWSARRHHRSPDDAERQAAS
>OLS47279.1 poly-beta-hydroxybutyrate polymerase [Rhodovulum sulfidophilum]AB14
MKRQAMTAPNGHPKSDHPYETADRAGRAIQARMTSGASPNAAVAAWADWASHLARAPGRQMELAERAMRNGA
KLAAHAAG

WSARNPPFRPRAEDHRFTHPDWSRPPFSLWQQAFLAAQDWWDAAAEPARGTDPHSSDRVAFMARQMLDVLSPS
NMPLSNP
EILQETLRTGGANLMQGLQHLAEDMGAQAAGNPAPAPEGYAVGRNLAVTKGEVVFRNDLFELIQYAPRTETVHPEPV
LIV
PAWIMKYYILDLSPENSLISYLVEQGFTVFAMSWVNPTADYREVSLDDYRRDGVMAALEAVGRIVPEQKIHACGYCLG
GT
ILSIAAATMARDGDERLGSITLLAAQTDFTEAGELMLFLDESQVAFLEDMMWDQGYLAQEQMAGAFRALRDEDLVW
TRAV
RRYLMGKEDEAFDISAWNADATRMPARMHSDYLRALFLDNRLTSGRFAVDGRVIALKDIRAPMFVIGTEKDHIAPWH
SVY
KVALFTQNELTFVLTKGGHNGGILSEPGHKGRYYRIGCRTPDTRYMDPDSWYARHPAQEGSWWQEWAGWLAARTG
PAEAA
PPAMGAPDAGLPPLCPAPGTYVFMT
>OLS47280.1 50S ribosomal protein L36 [Rhodovulum sulfidophilum]AB14
MKVRNSLRSLKQRHRDCRVVRRKGRVYVINKTQRRFKARQG
>OLS47281.1 N-formylglutamate amidohydrolase [Rhodovulum sulfidophilum]AB14
MSGGGSGGVQAFKLFRPDWPTTSVVFSSPHSGRDYPLSFLRKTVLDDRTIRSSEDAFIDILFSDAPGQGAPLLAASVPRA
FIDLNRAAEELDPAVISGARMSGHNPRISSGLGVIPRVVANGRGIYRGKLAMTEAESRIETCWRPYHRQLESLVEEALAR
FGQSILIDCHSMPREAIESARAGGRQRPEVVLGDRFGTSAGAEIVDRIEQAFSGAGLRVGRNAPFAGAYITQRYGLPSRR
RHAVQVEIDRSLYMDETEIRPNAGFDPLRRVLGEVVASLSAIGRPDDVPLAAE
>OLS47282.1 hypothetical protein BV379_02645 [Rhodovulum sulfidophilum]AB14
MTENTDPVVLRHRHLEQIAMTALQAGVLLTETGGKSLVVRDGMRKIALGLGAEDVHVRVGFASLALTVTHGGNSITR
MTG
TGAHGVNMRLNHAIRDLCVAVERDGATAEEVRDALARLRAHTPHHPRLLIALAAGIACAAFGRLLGVDWAAFGPVLA
AGT
AGQWVRVSLVRHGANPFVLTAIVACLAATLAGVMAIPAGSHMVETAMTAAVLLLVPGVPAMNAQTDIMEGHPTTG
SARFV
TVGMILVFITVGISAARLLVPEAPDAVFEPQRSVLHQAVFGAVAAAGFGVLFNFDWLRLAWAAAAGALALAVRTLGLD
LG
WSLEAASFAAAASVALGVEVLSLPRLRVARMGTALAVAGCIPMIPGGAATQCIIGLLNLTAQDPATAAQTLGMTASAG
LR
VAFTIGAIGAGLTIVRSLFRRSDFV
>OLS47283.1 hypothetical protein BV379_02650 [Rhodovulum sulfidophilum]AB14
MSIAHPAKIGLGTWDRDSRGSALDDLGKLGLAWYYTWSDWALYDADPSPDPAEFVGMVWDEQLVSQAALDRILAA
GADTL
LGFNEPDLARQAGVSVEQALDLWGVLQATGLRLVSPAASQEQTLGDSSWLGRFMAGVETRGMGVDVIAVHYYSDSG
DIAA
FETWLKAVHDQYGKPVWVTEWVLADWTGARSFSAAEQAAFARAGAEMMDALDFVERHAWFAAYEGGDGWYLNS
GLFDAAG
NLTEVGRVFADLAAPVAPPTVAGSDGNDRLSGGAAAELMLGGAGDDLLTGGGADTLCGDTGNDTLRGSAAGDLLKG
GAGD
DLYMVVSTASAVLEEPGAGRDRVHATVGWTLGADLEDLTLGGGALEGTGNGLGNRLSGNAADNRLDGLGGADTLQG
GEGN
DWLLGGEGADRLWGGAGGDLMEGGTGDDLYAVDTSSDRILERPGEGWDRVCAGIDWTLGDNLEHLTLTGTAALEG
TGNGL
ANVLSGNPGQNLLYGLDGDDTLLGGAGDDGLIGGAGEDRLWGGEGNDWLDGGEGDDVFDGGAGSDLMIGGAGA
DLFIFGG
GWDRIADFEDDIDTLRLDQALWAGAAPGPDAVPDLAYATAEGVFLDFGDGNGILIEGLSAVGALIDDLQIV

>OLS47284.1 glucan biosynthesis protein D [Rhodovulum sulfidophilum]AB14
MPTVRPTAHRFLTSILFAAATSFAAAPFALADQVSAPAIAGPELGAPTAFSFDMLADRAAALAARPHVPTPVHQPDVLE
K
IDYDAHWKIRFRRDAALMLGNTPAQFFHLGTYFREPVKIFAVENGTAREVVYSTDYFDMPEDSPAHALKSDAGFAGFR
LM
RPDMKTDWISFLGAAYFRSDGALRQYGLSARGIALNTGMSVPEEFPRFSEFYLEPGPDGRTIVNALLDGPSVAGAYRM
VL
GDRPGQGQVMDITARLFFRAPVERLGIAPLTSMFWYSESNRFQSADWRPEVHDTDGLMIETGAGEHIWRPLNNPDR
VVTS
SYFDRDIRGFGLIQRDREFENYQDDGVFYDKRPAVWVEPTSPWGAGAVQLIEIPTDDEIFDNIVAFWNPETKPQAGDE
ME
FSYRLHWTESPPVEMKLARTVATRIGNGGVPGQPRPEDQIKVVVDFEGPALDGLTRKDAILPQVSAPEGVEIVTPFVLP
V
VGTDRWRMVFDLKAPPGTETVDLRAFLTRNGKPLTETWLGQIHPDQIERLR
>OLS47285.1 2-keto-gluconate dehydrogenase [Rhodovulum sulfidophilum]AB14
MAAKFDKTDDSVVVIIGTGAGGGVLANELAQKGIKVVALEAGGRYLPEDYINDEWESFGQLAWTDPRSTSGDWRVAR
DFP
ALPAWIVKAVGGTTTHWAGASLRFQEHEWKAKTTYGPVQGANLHDWPIDAAEMEKWYDLAEKKMGVTRTNGLPG
LPGNNN
YKVFEAGARALGYKEVSTGRMAINSVERDGRLSCQQTGFCFQGCKWGAKWSTAYTEIPNGEATGNLEVREKAHAARV
LHD
AKGKVTGVEYFDAEGNLQMQKARIVCVAGNSFESPRLLLNSASSMFPDGLANSSGQVGRNYMRHMTGSVYASFEQP
VRMW
RGTTMAGIIRDEVRHDPSRGFVGGYEFETISLGLPFMAAFLDPGGWGREFTSALDAYETMAGMWIVGEDMPQETNRI
TLN
HDVTDQHGLPVVDVHFDDHPNDRAMRAHAYAQGRAIYEAVGATRTFPTPPYPSTHNLGTNRMSENPRDGVVNRW
GQTHDV
TNLFVSDGSQFTTGAAENPTLTIVALAIRQADHIAREMSARTI
>OLS47286.1 Twin-arginine translocation pathway signal [Rhodovulum sulfidophilum]AB14
MTDQQAVRALTRRQLLARASAAGATLMVGAGFVAAPDAAWALETTALKPETFATLVQMARDIYPHDRIGTEYYVVAV
KGY
DTAEAAPAIEAGIAALDELARGHGFPDYLGTGWERDRAEILREIEDSVFFQTIRGGLVTGLYNQKAVWPLFGYEGESYSK
GGYLHRGFDDIAWL
>OLS47287.1 lactoylglutathione lyase [Rhodovulum sulfidophilum]AB14
MAKAIHSMIRVLDEERSVAFYDKAFGLKVAERLDFPDFTLIYMSNPETEFELELTVNKGRTEPYDLGDGYGHLAVSVEDL
DAEHARFEAEGLAPRKLVEFAPAGELVGRFFFVADPDGYQIEVLERSGRFK
>OLS47288.1 aryl-sulfate sulfotransferase [Rhodovulum sulfidophilum]AB14
MCQIFAGQDPERYQSTTRRLRLNGQSTSIRLENSFWAILDEIAASEGATTPAFISKLHAEVLELHGEPSNFSSLLRCTCL
IFRETAPAAGAMAMAAE
>OLS47289.1 bifunctional adenosylcobinamide kinase/adenosylcobinamide-phosphate
guanylyltransferase [Rhodovulum sulfidophilum]AB14
MGRIILVTGGARSGKSSHAEARVRAMPGAPVYIATSEPQDDEMRDRVARHREARGPGWTLIEEPLDLVGALDRSDGA
GPR
LVDCLTLWLANHMFAERDWEPEAARLTEALLRQKSPVVLVSNEVGMGIVPENALARAFRDAQGWVNQRVAKVADE
VEFVV
SGLPLRVK
>OLS47290.1 transcriptional regulator MntR [Rhodovulum sulfidophilum]AB14

MPPDTPPPADADRFLRAREAQAVALLEDYVEMIGDLIAEHGEARVADIAQRMGVAHPTATKAVSRLKREGLAVSRPYR
GV
FLTEDGAALAERVRQRHRVVVDMLVAVGVPREAAELDAEGIEHHVSETTLHAFERYLTRSG
>OLS47291.1 divalent metal cation transporter [Rhodovulum sulfidophilum]AB14
MSPSDRTRLEMSAFLSGKRHGLRARLLFVGPAVIASVAYMDPGNYATNIQAGAGYGYALLWVVLLANLIAMLFQGLSA
RL
GIVTGKNLAELCRDHFPRPLVGVMWAVSEVAAMATDLAEFLGGAIGLSLLFELPLIVGMAITAIVTYAILLVQGRGFRPI
ELIIGTLVGVIGLCYLAEILIAPIDWGAAGLGLVTPAMPDAAALTIAVGIIGATVMPHAIYLHSGLTQSRAEIHSEAERA
KVLRFSDIEVVAALAVAGIVNMAMVMMAASAFHSGHSEVSEIETAYHTLTPLLGAAAAGVFLLSLIASGISSSVVGTMA
G
QMIMQGFLRIRIPIWLRRLVTMLPAFAVVAAGVNATQALVVSQVILSIALPVPMIALIVFAGRRDIMGAYATGPLLRALA
IAGAAMVLGLNAVLLAGAFGVDLPFLGPMSLGA
>OLS47292.1 hypothetical protein BV379_02695 [Rhodovulum sulfidophilum]AB14
MPKPDRTEPSCRSLCTLPPVQALPILAVALAASVFLWIGLGAGLLWLGSGGGGHPALPDLCSLGLRGLAICGPALAALVL
LARRPQH
>OLS47293.1 ABC transporter ATP-binding protein [Rhodovulum sulfidophilum]AB14
MAGARGTPLVEMKDISVSFGGIKAVDHVSVDLYPGEVVGLLGHNGAGKSTLIKCLSGAYRRDAGEILIDGRPATISNPRD
ARRYNIETIYQTLALADNLDAASNLFLGRELTTPLGLMNDAAMEAETRRIMARLNPNFRKFAAPVSALSGGQRQSVAIA
R
AVYFNARILIMDEPTAALGPQETQMVAELIGELKRQGLGIFLIDHDVHQVKALCDRAAVMKNGRLVGIVRVDEVSTDDI
L
AMIIAGKTPAHLAA
>OLS47294.1 sugar ABC transporter permease [Rhodovulum sulfidophilum]AB14
MDNAQTRGDARARKSILDTLDLDTRLLGMIGAFVVLCLVFNFVTDGRFLTPRNIFNLTIQTVSVAIMATGMVFVIVTRNI
DLSVGALLATCSAVMAMAQTVVLPTWLGLGLGHWAIAPLAIAAGLATGALIGGVQGWLVGFLGIPAFIVTLGGLLVW
RNV
AWYLTNGQTIGPLDPTYQLLGGINGTLGESLSWGFGLAATVFALVYMATNRRNRRAHDFPVKPRWAEALLGLVAAGA
ILG
FVAVLNAYDVPARRLARIFEARGEVLPEGYSAGYGLPFSVILLVLIATGMTVIARRTRFGRYVFATGGNPDAAALSGINT
RWLTVKVFALMGVLCALSAVVASARLTFHSNDIGTLDELRVIAAAVIGGTALAGGSGTIYGAILGALIMQSLQSGMAMV
G
VDAPFQNIVVGAVLVLAVLVDILYRKRTGER
>OLS47295.1 D-xylose ABC transporter substrate-binding protein [Rhodovulum sulfidophilum]AB14
MQGRMLIAGLLAATCLGGPLRAQDLTVGVSWSNFQEERWKTDEAAIRAALGAAGAEYVSTDAQSSSAKQLSDVESLI
AQG
VDALLILAQDTSAIIPAVQAADDEGIPVIAYDRLIEDPRAFYLTFDNLEVGRMQARAVLEKAPEGNYVMIKGSPTDPNAD
FLRAGQQEVLQPAIDAGKVRIVGEAYTDGWLPANAQRNMEQILTANDNEVDAVVASNDGTAGGAVAALAAQGMA
GIPVSG
QDGDHAGLNRVAKGTQTVSVWKDARELGRTAAEIAIELADGTPMSDIAGAAQWTSPAGTTLWARFLEPIPVTQDNLT
TVT
DAGWIGVEALCQGVTDGPAPCN
>OLS47296.1 xylose isomerase [Rhodovulum sulfidophilum]AB14
MTDFFHGIDKIAYEGPDSTNPLAFRHYDPDALVLGKRMEDHLRFAAAYWHSLAYEGLDPFGGRTFERPWYGDGMER
AFLK
ADVAFELFSKLGVPYFCFHDADVRPEGKTLVDTVTNLNRVAEVFAHKMEATGIRLLWGTANLFSHRRYMAGAATNPD
PEV
FAYAAASIKAMMDVTHRLGGENYVLWGGREGYETLLNTDIGQEMDQMGRMLSMVVEYKHKIGFKGTILIEPKPQEPT
KHQ

YDYDVATCYGFLRRYGLEDEVKLNIEVGHAFLAGHSFEHELALASALGVLGSIDMNRNDPQCGWDTDHFPNDPQSVAL
AY
REILKAGGFTTGGTNFDARLRRQSIDPADLFHGHILGMDTCARGLKAAAAMIEEGSLDAALAERYEGWQAPEAQAML
KGE
RSLDQIAERVLAGEREPEPRSGRQEYLEGLVNRFV
>OLS47297.1 mannitol dehydrogenase [Rhodovulum sulfidophilum]AB14
MEAGLPADRLTRTAKAPEAGIVHLGLGAFFRAHGAIYTEEAVARSGGAWGIIGVSLKSPGTRDALAPQGCAYTALELGP
D
GAVAHPVEIVREVLVAPEDPQAVLEAMADPAIRIVTLTITEKGYCHDPATGALNLDHPDILHDLGSAHPVSAPGYLVRAL
QLRRDRGLRPFTVLTCDNLPDNGRLVRGVVLALASRIDPALAGWIGSEGRFPATMVDRIVPATSAEDIARVEELTGCHD
A
APVMHEPFRQWAIEDDFVDGARPDWGAVGAELVADVTPYEHMKLRMLNGAHSALAYLGYLAGHETISETVADPALG
GFVR
HLWAREIAPVLTAPPGVSLSAYAEALAARFANPAIRHRTWQIAMDGSQKLPQRILATLSDNLVEGRASPGLILTVAAW
MR
YVGGIDEAGQPIDLRDPLADRLRALSDGAETPEGKVEALLSVREVFPEALALTLKRQLAEAYSRLLSEGARATAEAVCRE
TAQAD
>OLS47298.1 RNA pyrophosphohydrolase [Rhodovulum sulfidophilum]AB14
MTREEIEKLPYRPGVGIMLVNGEGKVFVGQRKDWAEPAWQMPQGGIDKGEEPETALLRELWEETGVTADLVEILART
DDW
IHYDLPSELVGRVWKGKYRGQKQLWYLLRFLGSDDQVNIATEHPEFEAWTWIDAEALVAHIVPFKRALYERVVAELAPR
I
PSQQGAA
>OLS47299.1 phosphoglycerate mutase (2,3-diphosphoglycerate-independent) [Rhodovulum sulfidophilum]AB14
MTTPKPVVLCILDGWGIGPTHDDNAPLLAETPNFDRLMQSCPHSVLTTFGPDVGLPSGQMGNSEVGHTNIGAGRVV
AMDL
GQIDLAIEDGSFFARPALTDFIGKMKASGGTAHLIGLVSDGGVHGHISHILAAARCIAEAGVPVAIHALTDGRDVLPQSA
DRFIPELAKGLPEGARIATVIGRYYAMDRDNRWERVKLAYHAMANGLGEEVASAAEAVQKAYDRGETDEFIHPSVVAG
HD
GMKDGDGLFCLNFRADRAREILRAFVQPDFDNFDTGTRPDFAAALGMVEYSVEHNAWMTTVFPKRKIVNTLGEWVA
KQGL
RQFRLAETEKYPHVTFFLNGGKEAPETGEDRFMPPSPKVATYDLAPEMSAEAVADSLVEAIEARYDLIVVNFANPDMV
GH
TGIVAAAMKACEAVDKGLGRALAALEEVGGAMIVTADHGNCETMIDPATGGPHTAHTLNPVPVILFGGPEGARLRDG
RLA
DLAPTVLALMNLPKPPEMTGETLIGA
>OLS47300.1 ribosome silencing factor RsfS [Rhodovulum sulfidophilum]AB14
MSGLLPDVSSEELLERILASLDDDKAEDVVRIDLRGKSSVADYMVICSGRSTRQVSAIAEKLKDRLKEELGRGCKVEGKD
QGDWVLVDIGDVIVHVFRPEVREFYQLEKMWMPAAAMAPARA
>OLS47301.1 hypothetical protein BV379_02755 [Rhodovulum sulfidophilum]AB14
MRLSAFPTGLVLAAALLAGGLARAETYRGYPEVPYSIERQTGAFELRRYPARTIAEVVVDGTRFAAINRGFSELAHYIFG
ENAAGEKIAMTVPVGQTRTDLGWAIRFTMPEGSAPETLPEPTESWVRLRRLPPETLLVARFSGRATAEKLNAAGRELVA
E
ARAQGLRLSGPARLMFYDDPFTMPWNRRNEVAYPVVAR
>OLS47302.1 3-isopropylmalate dehydratase large subunit [Rhodovulum sulfidophilum]AB14
MTGKTLYDKLWDAHLVDQAEDGTCLLYIDRHLVHEVTSPQAFEGLRMTGRTVRAPEKTIAVPDHNVPTTEGRDTKIDN
EE

SRIQVEALDRNAREFGVNYYPVSDIRQGIVHIVGPEQGWTLPGMTVVCGDSHTATHGAFGALAHGIGTSEVEHVLATQ
TL
IQKKSKNMKVEITGTLPPGVTAKDITLAVIGRTGTAGGTGYVIEYCGEAIRALSMEGRMTVCNMAIEGGARAGLIAPDET
TFDYVKGRPHAPKGAQWEAALAWWKTLKSDDDAHWDEVVTIRGEDIAPVVTWGTSPEDVLPITGTVPAPEDFEGGK
VEAA
RRSLDYMGLKPGTPLTEIKVDAVFIGSCTNGRIEDLRAAAEILKGKKLAPGVRGMVVPGSGLVRAQAEEEGLDKVFTEAG
FEWRLAGCSMCLGMNPDQLSPGERCAATSNRNFEGRMGRGGRTHLMSPVMAAAAAVAGHLTDVRELLAETV
>OLS47303.1 hypothetical protein BV379_02770 [Rhodovulum sulfidophilum]AB14
MKNWLIYTLIGLALLIGGVVALANPFAATIAATQIVGLVFLIAGLLQMITVMRGGPDSHRWVNGFVGILFLLAGLSILIN
PLVGAAWMTLLLGLLFVAMGVMRAMLSFTFRHTSLFWLLLLTGAASILIGVLIFSNFFAAAETLLGLLLGIELIVDGIGL
IAFGIVARRPRHVETPA
>OLS47304.1 3-isopropylmalate dehydratase small subunit [Rhodovulum sulfidophilum]AB14
MDKFEKITGIAAPLPLVNIDTDMIIPKQYLKTIKREGLGVNLFDEMRYDQDGNEIPDFVLNKPAYRETQILVAGENFGCG
SSREHAPWAIKDFGIRCVIAPSFADIFFNNCFKNGILPIPLPQEQVDILMKDAEKGANARMTVDLESQVITTSDGIEIAF
EVDPFRKHCLLEGLDDIGLTLNKAPAIDGFESKMAQERPWV
>OLS47305.1 endonuclease [Rhodovulum sulfidophilum]AB14
MRRPAALVPGLLALCLAAPALADIRVASFNTDLSRKGPGLLLRDILSGKDSQVAAVLDVIAVARPDIIALQGIDYDLDGL
ALTALADRLRARDLDYPHLFALRPNTGLRTGLDLDGDGRVGGPGDAQGFGYFAGQDGMAILSRFPVLTDRVRDFSALV
WR
ELPGATLPEMDGAPFPSAEAQAVQRLSTTGHWDVPVDLGGSILHLLTFHASPPVFDGPEDRNGLRNRDELRLWTLYLD
GA
LDVPPPRGPFVVLGDANLDPEDGDGRSAAMRAFLADPRLTDPLPESPGGAAAPQTGASAAQRGEPARDTAEWDDTP
EDEA
PGNLRVDYVLPSSGIEVIGAGVFWPADGPLAETVRTASRHRLVWADLALP
>OLS47306.1 3-isopropylmalate dehydrogenase [Rhodovulum sulfidophilum]AB14
MNNTLLILPGDGIGPEVMAEVTRIIDWFGDKRGLKFDVTEDLVGGAAYDAHGKPLSDATMAKAQEVDAVLLGAVGGP
KYD
TLDFSLKPERGLLRLRKEMDLFANLRPAQCFDALADFSSLKRDVVSGLDIMIVRELTSGIYFGEPRGIVEEGGQRVGLNT
QRYTEAEIDRVARSAFELARKRGNKVCSMEKANVMESGILWREVVQKVHDESYPDVELSHMYADAGGMQLCRWPK
QFDVI
VTDNLFGDLLSDVAAMLTGSLGMLPSASLGAPMENGRPKAMYEPVHGSAPDIAGQGKANPIACILSFAMALRYSFDQ
GAE
ATRLEKAVEQVLADGVRTADLMQSDGGSPVSTAQMGDAILAALDASL
>OLS47307.1 EamA family transporter [Rhodovulum sulfidophilum]AB14
MAASTVRGAGFALAGFAVYSGHDAVVKGLGVHYDAIQILFFSALLGFPLIALILIREGDGGSLWPRHPGWIALRTVAVM
M
NILMAFYAFSAIPLAETYAIIFSTPLIITALSVPILGERVGLRRWAAVVLGLIGVLIVLRPWGASELSFGHLAALISAGS
SALVSVIVRKIGNEERSVVLLLYPLLANVVLMGCLLPVVYRPMPVAHLGGMALVAIGALTAMWLMINAYRASEAATVA
PM
QYSQMIWAVLIGSLFFDEIPDATTLIGATVIAASGSILFLREGRAQVSENQPALHATDRAGVAPGPVLPGVEESPE
>OLS47308.1 hypothetical protein BV379_02800 [Rhodovulum sulfidophilum]AB14
MQQSPNRGEKARRAARSHPKSRHLAPFILVALYTGSRKEAILALRFAPHTAGGWIDTERGVLYRKAEDMRETKKRRPPV
R
MPQKLLAHARRWKRNRTWVVEYEGARVGSIKTAWRRAIRDADLPDVTPHTLKHTAVTWAMHKGVPLADAAGFFGT
TVATL
ESVYLHHHPSFQEATAKALDGWK
>OLS47309.1 sodium:proline symporter [Rhodovulum sulfidophilum]AB14
MSIGAIISLTTYFILMIGIGIYAYRKSTATSADYILGGRNLPPAVAALSAGASDMSGWLLLGLPGALYAAGLVEAWIGIG

LFLGALVNWIVVAPRLREQTERYDNALTIPAFLANRFPSRGVMLRMVSAVIIVVFFAVYTASGLVAGGKLFQSAFDGSY
Q
FGVWLTLGVVLAYTVVGGFLAVSLTDFCQGLIMMLALIIMPAIVLYSGKGAGFAQVQETLSGVEGFSLDLWTPDMTLIG
F
ISLLAWGLGYFGQPHIIVRFMAVKSVAAVATARNIGMAWMGIALIGAVGVGIFGRAYVERNGVVIEDSETIFIYLADNLF
PGLITGFFLAALLAAIMSTVSSQLLVSSSSLAEDFYKLVLNRGASEKTLVAIGRISVVAVGAVAALIAGDPESEVLDLVA
NAWAGFGAAFGPLVILSLTWPRMNGAGAVAGLVVGAATVGLWIFTGLKAATGLYEIIPGFVLAWLAIYVVSLATKDRG
EF
RSTISAPAE
>OLS47310.1 hypothetical protein BV379_02815 [Rhodovulum sulfidophilum]AB14
MSNGVDGERVRVFLDQLYEATDSLEDLFPGRKFTLDGHLVGSVGEVVASYMFGLNLNPASTKGHDAVSPSGQLVEIKL
TQ
RERIALRHQPEHLLVLHRPKGSEISVVFNGPGAVAWESAGAMQKNGQRCIGIRKLAELDALVDDAARLQLLRHAPV
>OLS47311.1 acid-CoA ligase [Rhodovulum sulfidophilum]AB14
MTDPRPITFGARLRQLARERGAAEAVRFLTADGQEEAIGWDALDASVDRIAHVLAAQGVGRGDVVGIGLANTPTHLA
TSL
AVWRLGATTLVADPHLTPELAAALKARIGARLWISETPGGGDLDRARLERLAAFAPATPVADAVSCPGKIVLSGGSTGL
P
KMMADDRPYMRIPGASWGRIAPALGFRADQVQLVAGALAHNAPQTWAQNGLFEGNRLILMEKFDADRALLAIDRF
RVGFA
MVVPTMMVRMLDRLEATGAHLDSLHAFYHTGAPCPAWLKAAWIERLGPDRVFEMYGSGENTGQTVLTGPEWLARK
GTVGR
GFETEIRIYGPDGARLGPRARGEIFMRPEDLSGRSRYLGPEAPRPRRDADGFQSIGDMGWLDDEGYLYLAGRSDDVINT
G
GVKVHPEVVEAELLRHPQVADAVVLGISDRDWGQRVLACIVPAEGFSPDPAALHDFCAAHLAPAEVPKRFELRRTLPR
DG
FGKIRRKALRAELEPAGDKDGPGSPPSGD
>OLS47312.1 cyclophilin-like superfamily protein [Rhodovulum sulfidophilum]AB14
MTKLKVTIAGIEFDARFEDAAPKTVAAFRAKMPFKGKVVHVRWSGEGVWIPLGETDFGLGYENHTSYPVPGQIILYPG
GI
SETEILLAYGGVHFASKLGQLAGNHFITLTSNTDKLADLGPRVLYEGAQEILFEEVDA
>OLS47313.1 C4-dicarboxylate ABC transporter [Rhodovulum sulfidophilum]AB14
MLVIAVAFVLLLLAGVPVVFVLGASAVLALVTTTDVPVAIVSQRVFAGLNTFTLMSIPFFVFAGVIMDAGGISRRIVEFA
SAVIGWVVGSLLQVSCLAAAGLAAISGSGSADTAAISAIMQPQLRRRGYDVDFGAAIIASAGSIAQVIPPSLTMVILAVV
SNVSIGALFLSGVVPGLMCIAILMVIAYVHARKGGPAYRETEPFTFTRLLRTGWAALPAAGMPVLILGGILGGVFTPTEA
AAVSGFYGLLVGAFLYRDLDLRRLPDLILRTVSLSSAVMLIIGTASVFSWLIANANVPQLLGNWIAGVSSSQLMFLLIAN
VLFLFVGMFLETIAAILIIVPVLSPIAAQMGVDPIHFSLLIILNCAIGTVTPPYGVSLFVASSVAGRPVLSVARKLALPL
AGLFAVLIAVTLLPDLPLFLPRLAGLID
>OLS47314.1 C4-dicarboxylate ABC transporter permease [Rhodovulum sulfidophilum]AB14
MLPLIERALLMLRRLVSAVVVLLFSVMMAAVLIQIAGRYIFNYSIAQASEIATFCQIWLVLLGAGVAMARGQHVAIDMV
P
ARFSLPWARAALIFIALVSAAFLAVLAWGSQPLLRMGAFQTSPSLQIPMAWMYLCLPVGAAYIALELLLSVILRWNDPF
P
PPSLDHEEEAI
>OLS47315.1 C4-dicarboxylate transporter [Rhodovulum sulfidophilum]AB14
MKLTNLALCLALGASAALPAFAQEIVLKASHNANAEEPYGVGMRKMAEILEDRTGGKATIQVFDNAQLGDEMESIQGT
QM
GTVGLAVTANDTLANFVPDLAVFSLPFLFRDADQMDRTLSDPAVIAKAQEILNQKGFHLVTFFSAGTRHIMTKTPIETLD

DLAGLKIRTMQNPAHVDTFKAFGANPTPLAYTELYGALETGVVDGAEAANTNYYAKKFYEVSPDWAVIGWLELVAPVI
MG
EAAYQALPADVQAALDEAGTEAGRFQRKTYRDSDAARFADLEAVGVNISHPDDAAFRAAAAPIQDKYIRTDAQKELFE
LL
QAVN
>OLS47316.1 4-hydroxy-tetrahydrodipicolinate synthase [Rhodovulum sulfidophilum]AB14
MKFRGSYTVCVTAFDGEGRIDLPAMRSYTDWQIAEGAHGLVPLGSTGEFLSLTREERGQVAEAVIGQAAGRVPVLIGT
AA
EWTEEAIALSRDAEALGADGVMLVPPYYSSPTDDELFTHFRRVAEALSIPVMLYNNPFTANVDLTAPLVARLSEIDNIVA
IKDTSKTVHRVTELVELCGDRLTVFAGYYPWESYRCGAQGYSSVMANISPRLSAQMFTETLDGDAAKGREIYVKCLPLIN
ALAGDLYVSATKVAMAMIGRPMGDPRPPRLPLPQDRQHELRRILDDLGLLAEA
>OLS47317.1 hypothetical protein BV379_02850 [Rhodovulum sulfidophilum]AB14
MPFIEVFDREPVQETRAVATSLMTDGLCAAFGIKPEIVTVYYFSTPENSYGHAGKYDENAEIFRIFAKVHAFPRSAEAKA
EAARALTGALCRAYGAQPRDVIVYFFDRDPGDAFHGGVASA
>OLS47318.1 acyl-CoA dehydrogenase [Rhodovulum sulfidophilum]AB14
MDQFFTEEQRMVRDTARKLSDQVIAPAAAAIDRDDIFPRAIYETLAEQGLFGVALPEEAGGVGLDAMAACIVMEEIAR
GS
GAVGNAFAIPVEAALFLNHHGSEETRKWIPGIVEGRVIPATAVTEPDCGSDVAAMKTTARRDGDDYVIKGTKAWVTFG
EI
ADVVMVFAKTDPEAGHRGISCILVETDRPGVHRGKSEDLLGMHGLADCMLSFDEVRVPVANRIGPEHGAFKMAMEN
FNFS
RLMMSSMALGMAQAAMEDAVAYANDRRQFGKPIFDFQAIQFMIADMSKDIAAARLLIHHAAKLYDAGHPIALEAAQ
AKLF
TTDMAQVHISNALQIHGGNGYSREFRIERLFRDVRLSQIYEGTNQIQRMIIARQVQKACA
>OLS47319.1 monoamine oxidase [Rhodovulum sulfidophilum]AB14
MSLYYEDIEIGARFTTASHVITEADIADFCRLTRDHHPLHTDAAYARQAGFDGIIAHGLYGLALMEGLKTGLKLYEDSSI
ASLGWNAVRFVRPMVVGDEVHVEMAFTDRRESRSRPAGVVTEAVDLILADGRPAITAEHVSLIRKRG
>OLS47320.1 hypothetical protein BV379_02865 [Rhodovulum sulfidophilum]AB14
MVAPGPGSLKDRKLEPAANSVDQATRQIREAILAGRFAPGERLKVAELAASLGFSTMPLREALRKLEGEGLVEIEPNRGA
TVRRLDRAYISDLFELNTELRIFALRRGMRNLTLERLKELEALAETYAAHLAAGAEVEALEINRRFNARLVEFGGNREAL
RMFLRGWELIFAFRRSFGYGPGRQAVLAEEMTLMVEALRRQDLRAAEAILRMQNAAMMEDLAARVDPDGSRA
>OLS47321.1 C4-dicarboxylate transporter [Rhodovulum sulfidophilum]AB14
MSNRALPAIALMAMLTGLPALAADFTFQVAHANAADEVQDKGLQKMRDLLEEKTDGAATLEIYPSGQLGDEAQSIEG
VML
GSLDMSMTSNSLLSNYIEDFRVFDMPFLFPSISALGEKIDANWELMEDTANGSGFQLLGVFSSGIRHLMTSKPVESIEDV
AGLKIRTMQNPVHVEAWRAYGANPTPMTYSELYGALQSGVVDGAEAASTGYDGMKFYEVAPDFALVGWLNMTAPV
VMQKA
AFDALPEDIRTALMEAGQEAAQWQRGYVEAQEKPLLEGFEAEGVTITTPDTEPFREASLPLYESELKTPGQKALLEALTG
K
>OLS47322.1 C4-dicarboxylate ABC transporter [Rhodovulum sulfidophilum]AB14
MIWAVGGFLLLLISGIPIVLALGIAALAAIELSTSAPISIVAQRVYGGIQSFTLMAIPFFVASGLLMEAGGIARRFVNLA
GAMVGWITGSLYMVSVVTGTGLAAISGSGSADTAAISAVMVPEMKKRRYDIDLAAAIIAASGSLASIIPPSIVMIVIAVT
SSQSIGAMFLGGIVPGLIVMVGLMLGAWLFARRGGEAYRDGAPFTFARLAHAFRDAAPGMIVPVVIIGGIVGGVFTAT
EA
ACVALFTTLIVTMFIYKELKPRDLPRILLKAISLSATVLIVIATASVFTWIIATEGLPAMLSGWLTGITDNPIAFLLIVN
LLLLVVGMFMESISAVLILLPILLPIARSYGIDPVQFGIITALNLSIGLITPPYGICLYVAASVAGRRIEQVSRRIWLPF
LCMLVALMLVTFVPAVSLWLPALMMN
>OLS47323.1 C4-dicarboxylate ABC transporter permease [Rhodovulum sulfidophilum]AB14

MRRIVDGAALLLLGYMALAVLVQIVGRYVFNYSIAWSEETATFAQIWLALLGAGIAMRHNQHVGVDFLILKAPLQVQR
VF
NLAAFALGAWFLGVVVVGSVSMVGIGLIVKSPALRMPMAIPYSALPVGFAYFLLEFALSSLPRILRPGHDPLEETGI
>OLS47324.1 transcriptional regulator [Rhodovulum sulfidophilum]AB14
MSVPARGTARPMLNLRQLEILRAVVQYRTTVGAAERLGMSQPSVSNTIRHIETVLGFPLFERVSNRLVPTEEALILLEEA
EPLFLLRDAVNQRAADLRRGRIGRIRIASTAELSEAVLPGVIADYAGAYPKVQISLETRPLDGVLQVVENGLADVAFVIA
AYERQALDYETLVELRAVCLCPADDPLARLEAVTPRDLIGRQMVGPQVSNRIGLMIADSFRASGHVYSPAIETRFMNAA
A
RVVREGCGVTLIDELSAHTVLRPGLVVRAYEPALSFELTAATSRAKTTSRQTRRLITAFAEKVRAQIAEVRAEARRDPS
>OLS47325.1 hypothetical protein BV379_02900 [Rhodovulum sulfidophilum]AB14
MTEPRQTHMVFVDIDPEEEAAFDAWYDNTHLPQILACPGWLEARREKCLEGGPRHVAIYTITGPEAYETPEFDAIKGFG
P
FTDKIRNFRRVRLTKS
>OLS47326.1 dihydrodipicolinate synthase family protein [Rhodovulum sulfidophilum]AB14
MAAHPHGGLHAATICPMHADGALDLDSLEAHFAAVLATEGLDGLLLNGHAGEGIFLSRDEAATVVRHAKAIAPAKRVL
AA
VSAEATAEAAADARAARAAGADGIMVFAPFSWALGADPRAILAHHKGIAEAAEGPVYLFQGSVGSGGLHYAPDTLAA
LLD
IEAVVGIKEGSWETRAYEDTLRQTRALRPDVAVMASGDEHLFACFQLGSDGSAVSLAALVPELIVALDAATRAKDTDAA
L
AIHRKLYPLAQAIYARPGHLAAARLKACLARLGRIDSPACRAPTPPIDPAEAERLAAALAPCLEPGDD
>OLS47327.1 dihydrodipicolinate synthase family protein [Rhodovulum sulfidophilum]AB14
MSLTGKDLRGITVATVLPFDETGAIDWEGYAAVLEHCARPETTDCVFVNGHAGEATALTGAERDEVIRRTRDQIGKDRL
L
LAGVVPTGYPDAVMQAEAAREAGADVAVIFPPEALGGGNAATKAPVAFMEKLARELAMPISLFQFPIASGFGYSTEAL
AE
IAQIPGIVAIKEGSATMLAYDENRRAVKAAAPDVAILPSNFHWFLPQLALGGDGILSGLASLTPGLLAELWQASEAQDLA
RMRAVNDRLYPVVRAIYGPAPVIDMHTRMKDGLKMMGVIKCATPRLPLLPPSGAVVQGVRRALIEAELL
>OLS47328.1 acyl dehydratase [Rhodovulum sulfidophilum]AB14
MYDIRQIDELSVGDRISVTKTVTEADGAMYIAATGDFGPVHVDEDYAAGTRFGERLAPGILVAGICTSVLTAELVGTLGV
SIRDSFWFTGAVRYGDTLTFEIWISAKDEENRTVDWQASARNEEGTEVLKVDASLKFPRKKKVTA
>OLS47329.1 hypothetical protein BV379_02920 [Rhodovulum sulfidophilum]AB14
MESAMTIERGQSVTFRKTLTVAEQGFFTGISGNLGAAHVDRVKARAMGLPDMAVFELAAGALFSTALSRLAGPDYRIG
AI
SFSFHRAVTVGETLAATATSGGPEGRSLICHLAGEIDGQTVVTGEAVLVPRES
>OLS47330.1 acyl-CoA dehydrogenase [Rhodovulum sulfidophilum]AB14
MDFTISDEMKMVTEAVGRFVREKVQPLEQETEANAQIPPEKLAAVRAEAQALGFYALNMSEEVGGGGLGILDMCLVE
EQL
GQTSDALIRRIFGQVYPMLEQFKGDLRDRYLYPTVRGEKICAMAITEPGAGSDAAAIRTRAVPEGDHWVLNGTKHFISD
G
DIADYIIVMAVTDPEKRARGGITLLIVDKDMPGFKVTRNQPMMGHRGYGHAELTFDDVRVPKEQILGEVGEGFRVMM
ANV
GGIRLGHIGARAVGMASRVLDMMREHAATRNQFGQPIGEFQMVQQMIADSAMEIFATRMMVLNTAWEIDRGMD
PRDKVSM
VKVQASEMLGRVADRGIQTFGGYGFTKELPLERIYRDARVTRIYDGTSEIHRMLIARSVIKRGLTL
>OLS47331.1 CoA transferase [Rhodovulum sulfidophilum]AB14
MGALDGIRILSFNHFLSGPAAAQHLADMGADVIAIEPIGGAFQRNWAVANRFVDETSVNHITTGRNKRSLALDLKAPA
AL

DAVKRLIATADVVMENFRPGTMDKLGLGEAELRRINPRIIYAATTGYGADGPYAKRPGQDVLLQAMTGLAAHTGSLEG
GP
VAIGTVPIDHHAAALTAGGIVAALFERERTGTARKVEVNMLQAALDLQGESITAWMNGANRKGPRGEAGLANWFSP
APYG
IHAVKDGHVMISMCKPADLAGPLDLPELAAFTEEDGFSRRGEIATLVQRGMARLTMAEALPLLEAAGVWHTPVEDYD
ALP
ENPQVKHLGAFVEAETRGGASMVMLAHPVRYDGETPAIRMTPPALGQHSRELLAEAGLGQDEIDALIEAGAAAQTDR
MRG
>OLS47332.1 MerR family transcriptional regulator [Rhodovulum sulfidophilum]AB14
MLTIGKLGKAAGVKVPTIRYYEQIGLLAAPERSNGNQRLYPKAALHRLAFIRHARELGFPLDAIRELLDLADHPEQPCAA
ADAVARRQLTAVEARLARLTALKTELERMIEQCAGGSAAECRVLESIGDHALCSTDHPRIP
>OLS47333.1 paraslipin [Rhodovulum sulfidophilum]AB14
MSLPELLTALVGGNLVVLLLGGLILLTVFLGVRIVPQSEKHVVERFGRLRSVLGPGINFIVPYLDRVAHKISILERQLPN
ASQDAITADNVLVQVETSVFYRILEPEKTVYRIRDIDAAIATTVAGIVRAEIGKMELDQVQSNRSELISTIKESVEDAVV
NWGIEVTRAEILDVNLDAATREAMLQQLNAERARRAQVTEAEGTKRAVELQADAELYAARQAAEARRVSADAEAYAT
EVV
AKAIAENGLEAAQYQVALKQVEALTKVGEGAGKQTIILPAHAMDAFADAFKMLKGRG
>OLS47334.1 hypothetical protein BV379_02950 [Rhodovulum sulfidophilum]AB14
MMLETWWVWLAGAAVLAILEILAPVQVFFGVAVGAAAVGIALWLGLAVAWPWLLVIWGLVAGLSWLVLRRALGVR
KGQVR
IWDDDINEG
>OLS47335.1 DNA mismatch repair protein MutT [Rhodovulum sulfidophilum]AB14
MTTSTRDEPFGGAKLALLTGRGLVSILRDDVPHIAWPGHWDFAGGAREGRESPAACVLRETREELGLVLEPARLIWRQ
TY
PNHRGRAVWFFVARIGADEIARIRFGDEGQGWAVMPVGDYLAHPKGIGQLKARLAEYLAARPGG
>OLS47336.1 orotidine 5'-phosphate decarboxylase [Rhodovulum sulfidophilum]AB14
MTDDRLIVALDVPDAHAGLTLATRIGDAAAFYKIGLGMLTGGGLALARELKDEHGKRIFLDMKLFDIGATVEAAVRGLA
R
FDLDFLTVHGDPHVVRAACEGAAGSRTKILAVTILTSLDRADLDASLIRAGDLPDLVAERAGRAFGAGADGVIASPQEAA
MIRALPEAAGKLIVTPGVRPAGAALGDQKRVATPARAVADGADHIVVGRPVWQAEDPRAAALAIQAELASPEAGRA
>OLS47337.1 ATP-dependent chaperone ClpB [Rhodovulum sulfidophilum]AB14
MNLEKFTERSRGFLQAAQTIAMRESHQRLAPEHLLKALMDDEEGMASNLIRRAGGAPDRVLEAVEAALSKMPKVTGD
AGQ
VYMDQATGRVLDEAESLAKKAGDSFVPVERMLMALCMVPSKAKDALDAGAVTAQNLNAAINDIRKGRTADTASAED
GYEA
LKKYARDLTEMAEEGRIDPIIGRDEEIRRAMQVLSRRTKNNPVLIGEPGVGKTAIAEGLALRIVNGDVPESLKDKRLMAL
DMGALIAGAKYRGEFEERLKSILNEISAAAGEVILFIDEMHTLVGAGKSEGAMDAANLIKPALARGELHCIGATTLDEYR
KHVEKDAALARRFQPVFVDEPTVEDTISILRGIKEKYELHHGVRISDAALVAAATLSHRYITDRFLPDKAIDLVDEAASR
LRMEVDSKPEELDALDRDILQKQIEAEALRKEDDEASKERLEKLEAELAEMQQQSTGMTAKWQAERDKLDRSRQIKEQ
LD
RARAELDQAKREGNLARAGELSYGIIPQLEKALSEAESREDDVMVAEAVRPEQIAQVVERWTGIPTSKMLEGERDKLLR
M
EEELGRRVIGQTEAVKAVANAVRRARAGLNDEHRPLGSFLFLGPTGVGKTELTKAVAEYLFDDDQAMVRIDMSEFMEK
HA
VARLIGAPPGYVGYDEGGVLTEAVRRRPYQVILFDEVEKAHPDVFNVLLQVLDDGVLTDGQGRRVDFKQTLIVLTSNLG
S
QALSQLPDGADAEAARREVMEAVRDHFRPEFLNRLDETVIFHRLTREDMDGIVTIQLRQLQKRLDARGILLDLDEGARK
W

LADAGYDPVYGARPLKRVIQRSLQDPLAEALLAGEIRDGGNIGVTVGPEGLIIGDRVVGSNRPKPEDAVLH
>OLS47338.1 DUF302 domain-containing protein [Rhodovulum sulfidophilum]AB14
MKALLTATTLAAFLAAGAVAAAPVTYTTSQSFEDVAFELETAITGRGLVIDHVSHPGEMLERTRADLGSDKTIFLNADIY
SFCPVALSRKVMEIDPLNIRFCPYGIFVLQYPETPGKTTIGYSTYPEGPMKEVEELLDGIVREVVGLD
>OLS47339.1 FMN-binding glutamate synthase family protein [Rhodovulum sulfidophilum]AB14
MGWAYYALQVLAGAFALVIGTLLMVSLALFIIDRTQTGDAIRRNYPVIGRFRNIFTKLGEFFRQYFFAMDREEMPFNRA
Q
RDWVARAGAGKSNTVAFGSTRNLSIPGTPIFVNAAFPPLEGQFASTAPMKIGPFARIPYVTSSFFHISGMSYGAISKPAV
QALSKGAAEAGIWLNTGEGGLSPYHLEGGCDIVYQIGTAKYGVRDAHGHLSDEKLAALAAIPQVKMFEIKLSQGAKPGK
G
GILPAAKINEEIARIRGIPLGEDSYSPNRHAEVGNWEDMLDMIGHVRKVTGKPVGFKAVVGGPEPFEQLFDLVRARGAT
S
APDFITIDGGEGGTGAAPMPLIDLVGLPIREALLMMSDLRDAAGLHDRIRLVASGKLVNPADVAWAIAAGADVVVSAR
GF
MFALGCIQALKCNRNTCPTGITTHQPHLQKGLVVEEKYHRVARYARSVISEVETIAHSVGVSEPRQMRRRHVRLVLPDG
S
TRPMSQIYPRSDLVNELVR
>OLS47340.1 mononuclear molybdenum enzyme YedY [Rhodovulum sulfidophilum]AB14
MAGRWRNRLNWSEVTAERVWLDRRQVMSGLAAAGFGTLAGTASAGAEDLTPNSWDEITSYNNFYEFGTGKDDPSR
KAGAL
TTDPWAVEIGGMVERPGRYDLGDILAKVSIEDRLYRLRCVEAWSMVVPWQGFELADLLTIAGVQPSAKYVAFETLYRP
EE
MPGQQSRILDWPYVEGLRLDEAMHPLTIMATGLYGKPLPNQNGAPLRLVVPWKYGFKSIKSVVRLTLTEKQPPTSWN
RYA
PREYGFYANVNPTVDHPRWSQATERRIGGGFFAPRVPTLMFNGYGEEVAGLYAGMDLAARF
>OLS47341.1 sulfoxide reductase heme-binding subunit YedZ [Rhodovulum sulfidophilum]AB14
MTGLQRVNLALARLPTWPVYVVGLLPAFWAIWLGATGGLGVEPINALENRLGLLALQFLIAGLAVTPLRRWTGLNLIRF
R
RALGLVAMGHLTLHLAVWLLLDLRDWGLIWADILKRPYITVGMLAFALLLPLAATSSNLAIRRLGPPRWRRLHRLVYLAV
PLGGLHFVMQSKGWQMEPLVYLATILGLLALRFRSRRGRVAT
>OLS47342.1 hypothetical protein BV379_02995 [Rhodovulum sulfidophilum]AB14
MTEHPIWNPATGITDLDADPAQLSASEIPGIKAVWTDQRKRLKGSTQLSDFTEKLSREWAIETGVIENLYEIERGVTQTL
IERGFQAELLSHGSTNKPRDYVIQLLRDQKDALDGVFDFVKSDRALSASYVKELHAVLLRSQDTTEGLDAQGRHVEVPLI
KGAWKTLANYPVRDGITFTYCSPEHVGSEMDQLIKMHAEHVAKSVPSEVQAAWLHHRFTQIHPFQDGNGRVARAIAS
LVL
VKDGLFPLVVTRDDKPTYLDALEAADSGNLKPLIDLIAKLQISQFRKASAISESLLVEEDVQAALGGLLKAADKVAADQL
AALRGVFDLAHILEDDLCDRLAAIAPTVSTALQRVSDGATAFVTKSNSETDHYFRAQIIDNAKNHIGYFADTGEYRSWVA
LNMRWSRRGQLVFANHGIGRPFNGSLICAPFLEFKDTDEEGQMRATLVPLAEEGFVFFYNEDRERLLSRFRPWRESVLK
V
ALKELTQNL
>OLS47343.1 hypothetical protein BV379_03000 [Rhodovulum sulfidophilum]AB14
MKFTSLASILSARDKAITRANLPDFSNFPWFKFSTFERVKDLAVVSRRIGYPCVVKPSSATGSILAYPLYGEEDAKWMID
HCHSISYLQDERAGWMLGSGWICETLIDAPIITVFVVADAAGIRAVALARNARQDINLCVGFGSVLPVEISASMVSLLEF
AENACRGLGLDLGFFDVEIFLTNAGPILIEVNARPPGGEMLRALDASTSEDLFWEILRIYGGEKSNLRRDSFYGCMSVYK
IMAAEAVCLRKSVCSVIQKLFSEFDILFYVEQECCQILELEPLDLIARIVSPTDNPMVTFSDMTRRTELASRDLGFDLIL
GNLPDLPELYKS
>OLS47344.1 hypothetical protein BV379_03005 [Rhodovulum sulfidophilum]AB14
MASALMKNSKISQADLLSTCMNKGFIDTLRSQLSFWSDLGIKEFEISKIYIVGSGPLPQTQIYLSSLLGSKIVCVERDAR

SAEISAALQERMGYTNVETICADGSNVRYSDAEHVILATMVDDKKEVAAQVYDSCPDDCFLTIRTPVGAHALWRRPVD
LK
RISEIGWEQRGTLSPEGSTVQSLIFQK
>OLS47345.1 hypothetical protein BV379_03010 [Rhodovulum sulfidophilum]AB14
MTAIEDGMHDPSMKMRMEELKIERTRLASYLARSPEPPTLRLHPGLAESYRAKVAGLASALSDPELQLQASETFRGLISE
MRLVPDAEARNGHHIELAGDLASILSLQGAECRKPADCGARFRC
>OLS47346.1 hypothetical protein BV379_03015 [Rhodovulum sulfidophilum]AB14
MPVSPQTFSRLSDVNLAHGTLLPAADAGWETERLHQGLKVILLQSGEIHCKLPAHRQLSFDAPCLCLIWNQAQHEGM
QRF
VPGTEISHTQVTLPPETFSGRDIPDQLRQQPGNGPSVQSLPPSPGILSLATRLRHCDLTGPERRLFLCSKALEFAASALC
ILQERAEIPGPPRRPRDDAAVQRAAELLEQEIAAPPSLAEMALRVGINRRKLSAGFQRMFGQSFGEYIAALRLDRAYQM
L
EAGNLQVAEVAYNVGYTPAHLSVAFRKRFGISPREMRR
>OLS47347.1 TonB-dependent siderophore receptor [Rhodovulum sulfidophilum]AB14
MVRPFLERIRGKSGVYVTTIAALSAPVLATAETAREGDAVILDPIMLFSEREDGPVSGYVAQSSSTAGKTGTPISRTAQT
VHVIGRQEMEDRGAETVTDVLSYMPGVFATNGAASQRFDYFSQRGFSVGPTGTLLDGLRSTTQQSHVRYQPFGLERV
ETL
QGPASVLYGANSPGGVMNAISKRPTSEPRREVGLSFGSFDKAEARLDVSGPLNADNTLRGRLVALRRDSGTQYDEVPD
DT
TYFAPSLTWAPSDDTSLTFLGSYSKDKIGPPRTFLPIQGTLLDNPNGDLARNTYLDGVGLENTNEQTNLGLEFNHRFTDV
WSLEARARYTRNELMTQTLSGMSLAPDMRRLNRTAYEFGIDGDVFASDINLRADWGQGRVTGTSVIGLSYRKTEEDYY
LN
YGPAAGIDIYDPVHSAPFSATTPYASTDQTGEEIGLYAQSTITLDDRFVLDFGARQDWSDIDTDNRLAGTSTHQEDDAFT
YRIGATWLAGNGLAPYASYSTSFQPVAGTDFYGNAYKPTEGKQAEIGIKYAPEGMDALFSAALYHLEQTNVQTTDPDQ
PL
NRIQTGEVTSRGLELSASANISPAFRLTASYSANDLEVTSSTDPLARGNRPTGSPEHLASLWGRYEVQHGPLQGASLGAG
LRYVGETYADASNTITVPDFTLVDLALSYDFGARNPRLDGVAFQVNVSNLFDKEYYSGCSARSCTEGFERRVSAALTYQ
W
>OLS47348.1 siderophore ABC transporter substrate-binding protein [Rhodovulum sulfidophilum]AB14
MTPLSMPQGLRLLLLMLIMAVPLGARAEPITVTDMTGREVTLQAPAQRIVLAEGRHVLTLALVAPDPVARLVGWGND
LKR
YSPETYDALREAVPAIEDVTDLGEPMAGNFPMETVIAQRPDLVILTLYGPPPAGLEKLDAAGVPYVFVDFFQKPLERTVP
SLRILGQLLGEQDRTERFIGFYEAHMGRISERLAGLQDYPEVFFHLNPGGGACCYTSGPGNMSDFIAAAGGRNIGADVV
P
GAIGQMNPEAVIAADPDIYLAGGGSTVAPDGLLIGPGVPADTARRTLRHVLQAPALSHLRSVQEGRASGIWLFFFDNPL
F
VLGVEEMARAFHPELFADLDPEASLDELNRRFLPFDLTGSFAVTPAVQGQ
>OLS47349.1 iron ABC transporter permease [Rhodovulum sulfidophilum]AB14
MSTDAVSGAVAPAGGLFAAHDGAVRRRCVRLALLSLLLVAGVLADVLTGPSGLSVGDVLAALSGMGDTDAAVEVIVR
QVR
LPTAILAVLVGASLSLAGAEMQTILRNPLASPFTLGLSSAASFGAALVIVLGVALPYVPDRFAVPAMAFAFAFSTVLVIQ
LIGNRRSMPPGVLILFGIVMVFGFNALVAVIQFVAPADALQQLVFWTMGSLGRADMWSNLLLGGVLLAVMPFSFRAA
RAL
TVLELGRERAESLGVSFARLRFGSLLRASLLAATAVAFSGTIGFVGLVGPHVARLLVGDHHRFLLPAAILCGATVMSFAS
IAAKLILPGVLLPIGIVTTLVGLPVFLLLLLRQWSR
>OLS47350.1 hypothetical protein BV379_03035 [Rhodovulum sulfidophilum]AB14
MPGPVDSKGLPVQLEVSGVSVRFGRRTVLCDVSLPALRAGEIMAFVGPNGAGKSTLLKAIAGVVASEGEMRLGEDELR
AL

PPASRMCRVGYMPQSQDARTGLSVLEAVLAGLDLLMPGLSRAERRARAAAALVRLGLEPLAGTSLCDLSGGQRQLAVL
AQ
SLSYDPVALLLDEPTAALDIRHQYEVMEALRGIAAGGRIVALVLHDLALAAQWADRIVFVKDGGVYASGRTRDCLTAENI
RNVFEVEAEVLRDGGHGVHINVLGRPRPA
>OLS47351.1 methylisocitrate lyase [Rhodovulum sulfidophilum]AB14
MPYLVANDLPDRPAPARFRAALARPGIYRLPGAHNGMAALQARAAGFGGLYLSGAAMTAQMGLPDLGIITVDEVAFF
IRQ
IARASGLPLLVDGDTGYGEALNVMHMVRTFEEAGAAAVHLEDQLLPKKCGHLNDKKLARPEDMAAKIAAAARARRDL
FII
ARTDAAASEGIEGAVARAKLYVEAGAEAIFPEALTSVEMFREVRARLPGVKLLANMTEFGRTPSLTATEFQELGYDMVI
W
PVSSLRVANKAQERLYAELVRSGSTARQIPEMQTRAELYETIGLNAYETLDRSIVASVAPEPV
>OLS47352.1 2-methylcitrate synthase [Rhodovulum sulfidophilum]AB14
MTGIIRKPKKSVALSGIVAGNTALCSVGQTGNDLHYRGYDILDIAEACDFEEIAHLLIHGHLPSTAELNAYKAKLRRLRG
LPAAVRQTLEAIPAATHPMDVLRSGVSALGCVLPEPADQNPAAARDIADRLIAVQGSMLLYWMHYARNGRRIEVETE
DET
VAGHFLHLLHGAAPSQEHVRAMHTTLILYAEHEFNASTFTARSIAGTGADIYSAITGAIGALRGPKHGGANEAAFEIQKR
YATPDQAEEDIRRRVAAKEVIIGFGHPVYTVSDPRNVVIKRVARHLAEHEGATRRFDIAERIEEVMADAKGMFPNLDWF
S
AVSYHLLGVPTELFTPIFVIARTAGWGAHVVEQRADGKIIRPSANYIGPENRRFLPLDQRGGLLTDQTEA
>OLS47353.1 acyl-CoA thioesterase [Rhodovulum sulfidophilum]AB14
MTALIFPETVNPHGTLFAGAGLAMMSKAAFLVASRRARRTVVMAGSEKIDFRTPVLPGEALELTAVVTRIGRSSMSVEV
T
GEAETLTTGARHLAMTGRFEMVAVDENGRPTPIAIPKEEKTQ
>OLS47354.1 pseudoazurin [Rhodovulum sulfidophilum]AB14
MRRLALSVLLPAMILSAPFAAAKTVEVKMLNRNASGPMVYEPDYLVMEPGDTVRFLPTAPGHNAGTIDEMLPAGAEP
FKG
QINEEIEVTLTEPGIYGIKCSPHFAMGMVMLIQVGTQPAAEADLPAGLPTRADKRLREIIARQDTGSEQAETHP
>OLS47355.1 histidinol phosphatase [Rhodovulum sulfidophilum]AB14
MILSGEGLGWRVQGRAIVSDVTLSIRRGERFGLIGPNGSGKSSLLRMLAGIRAPSEGEVRLEGQPLSRLPRRDVAQRVAL
VEQQAETTERITVRDAVELGRTPWLSALHPFSDEDEAAVQAALVAVGMQDFAARYWHTLSGGERQRVHIARALAQR
PGVL
ILDEPTNHLDIGSRLSIMALVEGLPVTTIAALHDLNEAMACDRLAVLQDGQLIALGPPDEILVPPLLRKVFGVEAHLLTD
PADGVRILRFKPI
>OLS47356.1 ABC transporter permease [Rhodovulum sulfidophilum]AB14
MRSRARRARWHWGWIAPPVLLAALIAGTAIGETRIPASTVLQVLGNKLFGTVYPVARIDEGIIWNYRLARAVVAAACG
AG
LALSGVVLQALLRNALADPYLLGISAGASTGAVAVTVAGFGAGALSLSTGAFAGALLAFVMVAALARAAGGGTGLQAA
GV
IVLAGIAGAQLFNAMTAFLIAKSANAEQARGIMFWLLGNLSGARWQDSLVAVPAAVLGLGVCLWHVRSLDAFTFGAD
SAA
SLGINVRRVQAVLIGAAALVTALIVSLVGAIGFVGLVIPHAMRFLVGGRHRLLVPASALAGAVFLIAADIVSRILIPGQV
LPIGVITALIGAPAFAAILIRGRARK
>OLS47357.1 ABC transporter substrate-binding protein [Rhodovulum sulfidophilum]AB14
MNTKTALLSAALLVCAGTAQATDYPLTLENCGQNLTFDAAPARTVTVGQATTEILYLLGLGPTVTGTSVWFSNVLPQFA
E
LNAGIPRLADNDPSFESVAAETPDLVTVQHEWHVGPQGIVATREMFHDIGVPTYVLPSDCASKDNSRGSDGTRSEMFS
TA

SIHRSIEELARIYDVEDRGTELVDELKAREAAAIEHAQALDLPEGVSAVFWYSSAEMDIDPYVAGRKGAPGYMMQELGI
E
NVVASDEEWPTVGWESIAKADPTVIVLAEMTRRRFPADDVEKKLEFLRTDPVAKEMTAVRENRIVVMDLQAMEATVR
AVY
GLEELSAALAGLDLQG
>OLS47358.1 EamA family transporter [Rhodovulum sulfidophilum]AB14
MTARSSLAAPLHPVRNERLGIALRVLSTLAFAMMGVCVKALGDAVPLGQVVFFRSAVALLPLIAFLWWRGEWPRGLA
TSR
PMGHVGRCLMGAVAMFTSFATIRLLPLAEATMLAYLAPVMLALLGWALLGERPSAGRIGGVVLGLAGAAAFCLPAFA
GAL
PDSGALGVVLGLVTAALTAGALIQVRRLTLLGEGAGAIAFWFAVVSALIGLATLPWGWVWPGPATLLLLIGTGLAGGIA
H
ILMTLSFSHADASALAPFEYLSVLWAVALGFAFFAELPGLAFLLAGPLILTGAFIARPTKPK
>OLS47359.1 histidinol phosphatase [Rhodovulum sulfidophilum]AB14
MTILAENLTWGVKRRTILSDVSLRVAPGETLGLIGPNGSGKSSLLRLLAGLRRPISGRVRINGQDIAHVSRKALSRQVAF
VQQSAATDTNVTVSDVVRLGRTPHRSALAGWSETDEAAVTQALERVEMAAHRKQAWQTLSGGERQRVHIARALAQ
TPQVM
FLDEPTNHLDIHHQIEILAMVRDLDLTSVIALHDLNLAAMFCDRIVVLERGVMRACGTPDAVLTRDILRDVFRVTAHVCR
LQDASRPHIRFSAK
>OLS47360.1 ABC transporter permease [Rhodovulum sulfidophilum]AB14
MSFEVSEEGSARLVVFLSLSLIALIFAITAATAFGDYNIGLRTVFLSITNELGLTGAEITPIEQSVVWNLRLSRALVAAL
SGAGLAICGAILQALLRNALAEPFVLGVSAGASTGAVCVIVLGIGAGGLSLSLGAFAGAFAAFALVALLSNGATSGPNHT
ILAGVAASQLFNALTSYIVTTSGNAQQARDVMFWLLGSFGGVRWPDVQLLLAVVIVSLAICIWMARSLDAFTFGDEDA
AA
LGVPVARIRLVLFGVTALLTATIVSMVGAIGFVGLVVPHAARHIVGPLHMRLLPACAVVGAVFMVIADIASRMIATQQT
V
PIGVVTALVGVPFFAIILYRARPQA
>OLS47361.1 ABC transporter substrate-binding protein [Rhodovulum sulfidophilum]AB14
MSKKAILALGSAMLAVSGVAVQAATDYPLTIENCGHSVTFSQAPQNAVALGQNSAEIMLLLGLEDRMAATAFWPNAV
LPE
LAEANDGVEVLTVEFPTLESVLSKQPDFVAAMLTTLLGADSKVASRSDFEELGIPTYLSPSACATTLDAGDAYGSRDKLW
NMDLLYKEIEDLSRIFDVTDRGQAVIADFKAREAALRAEFARNEDLTFLFWFSSPSPADDAFLGGGNGPSGYIADLLGGS
NAIRTEADWPALGWEGIIAADPTVFVATQVDRNRWDLDAAQNKIDFLTSDPTVSQMPAVRNGRIVVMSGAAMNPG
IRTLY
GAEQVAEQLKSLDLP
>OLS47362.1 LysR family transcriptional regulator [Rhodovulum sulfidophilum]AB14
MDRPDLPLNALRVFEVAMRQGSFTKAAIELRVTQAAVSHQIARLEDLLGVVLFLRTSQGLVPTDEGRLLFPVLEHGFDA
M
ARVLGRLGGRRDVEVLKVGVNTTFALGWLMPRLAAFAQAHPEIDLRISTNNNRVEILREGLDMAIRFGTGGWTGHDAI
PL
IEAPLTPLCAPAMAAHLLHPADLGQMTLLRSYRSAEWPGWFEAAGVACPPITGPVFDSSIALSELAASGAGTALLPITMF
ESYIAQGRLAQPFGTTVSAGRYYLAWPSDRPATPAMRIFSSWLSGKSAL
>OLS47363.1 class A beta-lactamase [Rhodovulum sulfidophilum]AB14
MRIPASVLSRVAAGLSLGLCLASASLAQTPLETLADTVERVESRLGARIGVSLVDTGSGLSWTHRENERFLMNSTMKVPL
CGAVLARRDAGDLSLQTELPVRKSDLLAYAPVTEKRAGGEMTIAELCLAAIDMSDNTAANLLIARLGGPQAVTRFFRSA
G
DTISRLDRNEPELNTFNAADLRDTTTPVAMTETLRGLLLGDVLSRSSRGQLAEWMSHGGVTGNLLRAEAPEGWVILDK
SG

SGTHTRNLVALVTPVDGSPWIVTIFVSDVDADSQTRNRALQDVGRAAMAVMRD
>OLS47364.1 anti-anti-sigma factor [Rhodovulum sulfidophilum]AB14
MKLQSQLRGDALHIVVDEERIDSAVAIRFKEAVREQAADGPGRVVLDLGRVDFLDSSGLGAVVSVMKLIGPGGKLELCN
L
TPNVDKVFRLTRMDRVFTIHDSLDSAFPERQGIA
>OLS47365.1 phenylalanine--tRNA ligase subunit beta [Rhodovulum sulfidophilum]AB14
MKFTLSWLKEHLETEATLDEILYALTDLGLEVEGVENPARALQEFTIGKVLKAEQHPDADRLRVCQVATGQGEKQIVCG
A
PNAREGITVVIAHPGMYIPGIDITIQVGKIRGVESHGMMCSEREMELSDEHDGIIELPSGEVGQSFVDWLAANDPAKVD
P
VIEIAITPNRPDALGIHGVARDLAARGVGRLKPVEVAPVAGSFPCPVDVTIDPETQERACPVFYGRAIRGVKNGPSPQWL
QQRLKAIGLRPISALVDITNYFTYDMNRPLHVFDIAKLGGGLRVHAAAGGEEFLALDGKTYVIEPGMTVISDATGVESLG
GVMGGETTGCTEETVDVFLEAAYFDPIRTAMTGRALKINSDARYRFERGIDPAWTPEGIEAATRMILDLCGGEASELVV
A
GAVPDMARAYRLDTDRVESLVGMEIPAETQRATLTALGFRMEGDMAHVPSWRPDVLGEADLVEEVARVASLTKLEG
KPMP
RPQPGVPTAILTPMQRRSSAARRTCAALGYNECVTYSFVDRASAALFGGGSDAMALENPISSEMSHMRPDLLPGLLQA
AA
RNQARGFADLALFEVGPAFQGAEPGEQHIQATGLLVGAAAPRDPYGSRRPVDLYDAKADCEAVLAAIGAPAKAQILRA
AP
DWWHPGRSGRVCLGPKKVLASFGELHPKVLAEMNVKGPAVAFTVFLEETPMPRRKGATRAALTVSDLQAVERDFAFV
LEE
GVEAMTVVNAAAGADKALIEEVRVFDEFVGGALGEGKKSLAITVRLQPSDKTLTEKDIEAVGQKIVAKVEKATGGTLRR
>OLS47366.1 hypothetical protein BV379_03125 [Rhodovulum sulfidophilum]AB14
MFQSREAAGRLLAEKVLAADPENPLVLALGSAGVPVAAPVARALGCPLDPIFVKGIEAPGTPALAVGAVVDGAAREVLF
E
ADSLSAAGLTQADCAAGVEACLAETAAARCRLLGGRAPQRVSGRTAILVADGLATGASARAALRALHNRGPLEIWLAV
PV
GPQEVVWKMRGMTDRLIFLEAPRDFRGAAGHYTDFVPVGDDEAVRLLDELLARR
>OLS47367.1 phenylalanine--tRNA ligase subunit alpha [Rhodovulum sulfidophilum]AB14
MDELRQKYLGAIADAADEPALEELRLQAVGKKGEVSLKMRELGRMTPEERQEMGPKLNALKDEINSALAAKKAALADA
AL
NERLRAEWLDVTLPGRPRRQGTIHPVSQVTDEITAIFADMGFSVAEGPQIESDWFNFDALNIPPEHPARQEHDTFFMS
RA
EGDNRPPHVLRTHTSPVQIRAMQETGAPIRVIAPGRVYRCDYDQTHTPMFHQVEGLAIGRDISMANLKWVLEEFCRAF
FE
VEGVELRFRASHFPFTEPSAEVDIRCSWADGSLKIGEGDDWMEILGSGMVHPKVLSAAGVDPSQWQGFAFGMGIDRI
AML
KYGIPDLRAFFDSDLRWLRHYGFSALDVPTLQGGLSA
>OLS47368.1 GTP-binding protein Era [Rhodovulum sulfidophilum]AB14
MRLTAEFWVQAYLARLRLEGIPAFVVAHGDDTAGAVLVKLNTLDGQARAYQRAFDLNSGERRWMELAEGGEAEVDA
AIGR
QRGFDPDLWVIEVEDRDGRHLLDADGLA
>OLS47369.1 hypothetical protein BV379_03145 [Rhodovulum sulfidophilum]AB14
MKAIRHIPPFFLMIFGVGILLNLIVLTGLSHAAILVILQLFATLAMCVALYVLIIRHFMTRIGGASGTGDSDA
>OLS47370.1 RNA pseudouridine synthase [Rhodovulum sulfidophilum]AB14
MDSYAPPDTPLDILHHDHELLAVNKPPGLLSVPGKGPELADCLLTRLQAVFPETLLVHRLDRDTSGVMVFALTPHAQRH
L

GLQFEKRQTKKTYVARVWGRLEPKTGTVDLPLIVDWPNRPRQMVDHENGRPAVTDWRVVRYEETATRVRLFPRTGR
SHQL
RVHMRELGHPILGDPFYAEGPARAAPRLMLHAEALRLRHPDGGAGMNFTSRAPF
>OLS47371.1 peroxidase [Rhodovulum sulfidophilum]AB14
MSLRINDIAPNFTAESTDGPIDFHDWIGDSYAVLFSHPKDFTPVCTTEFAAAARLSGEFAKRNAKIIGLSVDPVEEHLKW
KADIETHAGCDASFPIIADDGLKVAKLYDMLPAEAYLPDGRTANDSATVRTTFIINPDKKIELMLIYPMTVGRNFDEILR
VLDAIRGNKTGPFATPANWRPGEDVIVGLSVSTEEARAQFGEVEEIFPYLRKVKQAG
>OLS47372.1 hypothetical protein BV379_03160 [Rhodovulum sulfidophilum]AB14
MRSELILSTYNNPQSLALCLESVACQRVAPGSICVADDGSGPETKAVIEAFAARHPDLTVRHLWHEDRGFEKGAILNKAI
ASSGADIIVFIDGDVLIHPDFVGRHLALTRPGRFATGSLIRLDAEATASVTPELVQDGTVFDRRWLTEHRAIRKLSTWLK
ARPFPVPVMAVLDRLSPVTRALCGANASVWRADLLKINGYDETIKYGGQDKHLGVRLNNAGVKGVHLRYSAPLVHLD
HPR
GYADPEKMRRHKALIRAARKSHLFWTPDGIVKGEAGAEGCGGKA
>OLS47373.1 lysine transporter LysE [Rhodovulum sulfidophilum]AB14
MSLEAWAVFALFWALFVTTPGPNAVNCITNGMAHGLPRALWGVTGILTQAALFLTLSAAGITALIAASPTVFAWAKLF
GA
AVLIWLGLRGWRNAAIAVAPRPASGASIYGRAFLIATVNAKSVAGYLAAFSQFVEPGVPIAQQMTAIVPTALTLTALSYS
TYTALGAWLGGLALGAVMNTRLRQVLAACFILYGILLGASAL
>OLS47374.1 diguanylate cyclase [Rhodovulum sulfidophilum]AB14
MRIDYDALSQTLAALTEGETDPVALMATMACELHHADDRFDWTGFYRNVGAEMLKIGPYQGGHGCLTIPFSRGVCG
AAAR
SGEVQIIEDVLAFPGHIACSSTTRSEIVLPVHDGTGALLGVLDIDSDRPAAFDATDAEGLARLLAQTFAR
>OLS47375.1 polyribonucleotide nucleotidyltransferase [Rhodovulum sulfidophilum]AB14
MFNVTTKSMQWGEETLTLETGKVARQADGSVIATLGETTVMANVTFAKEQKEGQDFFPLTVHYQEKYYAAGKVPGG
FFKR
EARPSEKETLTARLIDRPIRPLFVPGFKNEVLVMCTVLSHDLVNDPDIVAMIAASAALTISGVPFMGPIAGCRVGFVDGE
YVLNPDVDDMQGLRTNPDQRLDLVVAGTRDAVMMVESEAYELTEAEMLGAVTFAHDNIKPVCDLIISLAEAAAKEPF
DFT
PPDYSALYEAVKAVGEEKMRAAYAITDKQARVAAVSAAKDEIKASLSEEQLADTNLGSAMKKLESTVLRSDVVKHGRRI
D
GRALTQVRPIECEVGLLPRTHGSALFTRGETQGLIVTTLGTGDDEQIIDALHGNFRSNFLLHYNFPPYSVGEVGRVGSPG
RREIGHGKLAWRALQAVLPAPTDFPYTIRLVSEITESNGSSSMATVCGGSLSMMDAGVPLKAPVAGVAMGLVLEDDG
EFA
VLTDILGDEDHLGDMDFKVAGTEHGITSLQMDIKVAGITPAIMEKALAQAKDGRMHILGEMAKALTEAGDFSAYAPRI
ET
MTIPTDKIREVIGSGGKVIREIVEVSGAKVDINDDGVIKIASANGDAIKKAYDMIYSIVAEPEEGKIYKGTVVKIVDFGA
FVNFFGKRDGLVHVSQIENRRLNHPSDVLKEGQEVWVKLLGFDDRGKVRLSMKSIDQATGEEAPKEKEAEADE
>OLS47376.1 hypothetical protein BV379_03180 [Rhodovulum sulfidophilum]AB14
METKAFGTIPLEDGQATLPVGPGIVRPASLDALATLKDREAMAALSQFLDRLPRHIEKAREHFLDNYEAVAANVPAWLH
E
NEPEVFRAMFGDAAPSEIDAEALWTALDLSSVWTRDDTAVVLDLGFRETPMTHAFAAEFSYDGTLTGLALAS
>OLS47377.1 30S ribosomal protein S15 [Rhodovulum sulfidophilum]AB14
MSITPEEKTRLIKEFGSKDGDTGSPEVQIAILTSRITTLTEHFKTHKKDNHSRRGLLKLVAQRRKLLDYLKGKEVARYQD
LIKRLGIRR
>OLS47378.1 hypothetical protein BV379_03190 [Rhodovulum sulfidophilum]AB14
MITMVDLLGVMAVGASADPDDSTRSHAAGGTGSGDGPAPDHATDDLMAVMDLHAALAEAGPAIDGMSRADELLF
SWLSGG

PDSMDADDLPFPTDHDHDKDLPVAETCLVDAALGAGDETFSVQDLFEDGAALPTVIDDYDQTADALFVVYDSTTHPAPVL
SIDATETGAGDALIRIDGQPLAIVTGAAATLRLGHLSLVPDTMLSDALAARA
>OLS47379.1 hypothetical protein BV379_03195 [Rhodovulum sulfidophilum]AB14
MLHLVTPSTVTNRAIMRIRKVPRQLIGHGMGLLPPGSFGRAIFWRMATEISFLRYCAALTPFPVAMLLFPEAALPIGQFP
AFMFLVVYLVESRVLSVDNAERRHRLMPEEEAERGADIARARGRQILTRIAARRGLRSGDLHLVIEQSSLARIPPITLVS
LQTAMPEPQILELDDEERQLIRDTLFDAEFTEDRMHVTSLALGRFLHDVTLDARSVSAHARLEALATA
>OLS47380.1 nicotinamidase [Rhodovulum sulfidophilum]AB14
MRPANEALIVVDIQNDFCTGGALAVAGGEDIIPRVNALLDEFQLRVFTQDWHPAGHSSFAESREGCKPYEVVEMPYGSQI
LWPVHCVQDSAGAAFHPDLDVSKADHIVRKGSNPEVDSYSAFFENDHETPTGLDDYLASKGIEKITLVGLATDFCVLYSA
LDANHLGYAVTVLEGACRGIDLNGSLEDARTTMLDANIVLEP
>OLS47381.1 nicotinate phosphoribosyltransferase [Rhodovulum sulfidophilum]AB14
MADIATRVYNHKWKIDPIVRSLLDTDFYKLLMCQSVYRNHPETQVRFSLINRTTDIRLAELIDEGELRQQLDHIRTLRLT
RGESTWLRGNTFYGKRQMFRPDFMEWFEQLRLPPYTLEKRNGQYELTFEGRWPDVMMWEIPALAVLMELRSRAVLETLGK
FELQVLYARGMTKLWEKIERLRGLDGLKLADFGTRRRHSFLWQDWCVQAMIEGLGDRFTGTSNCLIAMRHDIEAIGTNAH
ELPMVQAALAGTDAELARAPYKVLADWHEEHDGNLRIMLPDTFGTRGFLENAPDWLAGWTGIRIDSGDPAEGAETAIAWW
KARGEDPSQKLIIFSDGLDVDKIVELHHRFAGRAKISFGWGTLLTNDFRGLVPGDGLAPFSLVCKAVSANGRPTVKLSDN
PNKAMGPAAEIERYKRVFAVGAQAPMEVVV
>OLS47382.1 hypothetical protein BV379_03210 [Rhodovulum sulfidophilum]AB14
MTEAETLSLAEEAIAHWEGCTAPRLLSHRENAVFAIALSGGRRGVLRLHRRGYQSADAIRSELWWMQALAGAGVAVPCPE
PTRDGALLAHLDDGRVASVLSWITGTPLASGAGPLDGGAAEQLRRFAELGHAIGKLHVATDRLTLPQGFSRPHWDIDGLL
GPAPFWGRFWDHPAASDAEAILLQEARRFARERLSAYAAAGGDHGLIHADLMRENVLFDGPRAHLIDFDDCGFGFRIYDL
GTALVPNLEEPELDAIATGLLEGYADERGLNDDDLAMLPVFVMLRTLASVGWTMPRLTSDDPRIRAYLNRAVRTAHAVME
GRPLFRIG
>OLS47383.1 aspartate aminotransferase family protein [Rhodovulum sulfidophilum]AB14
MTDPLIERRARLLGPNVPTFYDEPVHIVRGEGVWLWDAAGRRYLDAYNNVPHVGHCHPKVVEAIAAQAARLNTHTRYLHE
TILDYVERLTATLGHDLSQAVMVCTGSEANDVALRMAQAATGKTGLIATDNTYHGNTAAVSALSTRRPPIGGYPPHVRLV
PAPDSLAPLGGSAEAQPEAFAANVARAIAELEEAGHGFAGFMLCPFFANEGFPTLAPGFLDPTVEAVRKAGGLILADEVQ
PGFGRLGTAFWGHEVLGFAPDVVTLGKPMANGHPVGAAVTRPEIMAAFRGAFGYFNTFGGNPVSMAAALATLEVIEEEGL
MENARAVGAYALERLGALRHPWLAETRGRGLFFGAEFVDETGAPATEFAIRLVEELRAEGILTGRIGRAMNTLKFRPPMP
FSREHADLALDSLDRVLARTEVPR
>OLS47384.1 L-asparaginase [Rhodovulum sulfidophilum]AB14
MTGAVGMVEVLRGGMVESRHRGHAAICDSTGLIAAWGDPEAVILPRSSCKMVQALPLVESGAADAAGLGSEQLALACASH

QGAPLHVAKVTAWLAETGRREADLLCGVQPTRDRDERARMIRDGESACQLHNNCSGKHAGFLTLARHLGAGPDYVD
PDHP
VQRAVRAAFEEVTGMTSPGFGIDGCSAPNFATTVTGLARAMAVFAAARPDAGDARTRAAGRLSEAMRRHPDLVAGE
GRAC
TELMRATEGRVAVKTGAEGVFVAFLPERGLGVAVKIEDGATRAAEAAIAGLLVYLGALDPEHPAARKRMVGPIVNWRG
IE
TGAMRLAPGFA
>OLS47385.1 hypothetical protein BV379_03225 [Rhodovulum sulfidophilum]AB14
MTSLFRAAIAGGALALAVTAAHAQEESTNNVAVKTDWNVFVETDPTECWSVSPPKKTVNTRGGQVVSARRGEILLFVT
FR
PGDGIKGELSFSGGYPFKSGSTVALTVGDSNFELFTEGEWAWPASKADDAKIIEAMKRGAEAQVVGMSSRGTKTTDTF
SL
YGFTAAMDEAARRCSAN
>OLS47386.1 hypothetical protein BV379_03230 [Rhodovulum sulfidophilum]AB14
MRLLFTTAAGILAAGSVHAATWTLASHADYAFLDDNQTGLQMTQGVAWNAEANEWVTSWQYGLARFTEDWQFIE
ASGRFD
LETGRIVSGIPAALADRGFDHIGDFEIRDGIVHVPLDSANDHYQNGHVALFDAATLSYTGALHEMMGAPTNRRDDVAS
WT
AIDHETGLGYGKEWALGDTLNVYDTTDWAYLGTLQMDQELKRVQGAKVLDGMMYLSTDIGTKSVYSLDLATGHVEEL
FQL
PIFEGDIRFETEGLALRELADGGLEMFVEMVIDPDAVEGQFDGYTRLYRYVLENDPAPVPVPATLPLVLSGLAAFGLIRR
RA
>OLS47387.1 23S rRNA (adenine(2503)-C(2))-methyltransferase RlmN [Rhodovulum sulfidophilum]AB14
MSAPIVQDVLPLPRKLPEPDRINLVGLTRPRLREALIAAGTPEAQAKMRVNQLWQWIYQKGVRDFDAMTNLAKDYRA
LLK
AHFAIEVPEIVTRSVSADGTRKYLVRIAGGHEVEVVYIPEDGRGTLCISSQVGCTLTCTFCHTGTQRLVRNLTAAEIVGQ
VMLARDDLGEWPKPGRHPKDETRLISNLVLMGMGEPLYNFEAVRDAMLIAMDGEGISLSRRRITLSTSGVVPEIARTG
AE
IGCMLAISLHATTDELRNKIVPINRKYSLAQLFEALRAYPKLSNSERITFEYVMLKDVNDSDADARRLVRLIAGIPAKIN
LIPFNEWPGAPYQRSDWDRIEAFADIVYKAGYASPIRTPRGEDIMAACGQLKSATERARKNRFQIAAEAGLPEPERIVRR
PDAPDVH
>OLS47388.1 lysine decarboxylase [Rhodovulum sulfidophilum]AB14
MHDDARRPLRDAFRDIESARETPETSQTGSPSYRLAFADPDFLCRDEMRPVRVQLELLKPDILMREYGIESTIVLFGGAR
IPEPSQRHTAPTETLADLSKYYDEAREFARLMTLKGQRGKRLDHVIATGGGPGVMEAGNRGAADAGGVSIGLNIVLPH
EQ
MPNAYVTPELCFNFHYFAMRKMHFLLRASAIAIFPGGFGTLDEMFEALTLIQTHRMDPVPFLLFGRDFWETVINWKAL
AE
AGTISPEDLKLFSFVETAEEAVRIIETWTPPS
>OLS47389.1 2,3,4,5-tetrahydropyridine-2,6-dicarboxylate N-succinyltransferase [Rhodovulum sulfidophilum]AB14
MSNAQLETAIEAAWEARDTITPATRGETRDAIEATLEALDKGALRVAEKRDGGDWHVNQWAKKAVLLGFRIKDMECH
EGG
PQDGGWWDKVDSKFKGWGQTDWRAAGFRAVPNCVVRKSAYIAPGVVLMPSFVNLGAYVDEGTMVDTWATVGSC
AQIGKGV
HLSGGVGIGGVLEPMQAGPTIIEDNCFIGARSEVVEGCIVREGSVLGMGVFIGKSTKIVDRETGEVMYGEVPPYSVVVA
G
SMPSKGGVSLYCAVIVKRVDAQTRSKTGINELLRD
>OLS47390.1 hypothetical protein BV379_03255 [Rhodovulum sulfidophilum]AB14

MRKPGPRPASTSSCVTEKPSRRQQVYTLLVEVGRKPDDGLPEGATGAGLMCYASGIDEPEAVRETVAVLKQAGLAPLD
VT
GYGTLAERRDQGHEIGPEEEALMARALAENAVIVAQMEPFFGEGTGTA
>OLS47391.1 succinyl-diaminopimelate desuccinylase [Rhodovulum sulfidophilum]AB14
MTQPTDPVALTAELIRCRSVTPTEGGALVLLAERLAAAGFDCRRVDRNGTANLFARWGAKGHEKTFGFNGHTDVVPA
GDT
AAWTADPFGAEIRDGQLWGRGATDMKSGVAAFVAAAIDLVTDTPPDGAVVLALTGDEEGDATDGTIALLDWMQTE
GEHMA
CCLVGEPTCRETMGDMMKIGRRGSMTAYFTARGVQGHAAYPQRAQNPVPALARLVQRLDAYSLDQGTRHFDPSTLA
VTTF
DTGNPATNVIPGQCRATVNIRFNEAHSSDSLADWLAAEAARVTEETGIEIGIEIKVSGESFVTPPGPLSTLVADAVEAET
GRRPEFSTSGGTSDARFVKDLCPVVEFGLVGKTMHQVDERVETVQIEELKRIYARILGGYFG
>OLS47392.1 ribonuclease R [Rhodovulum sulfidophilum]AB14
MTRIPAKTEILDWIAAHPGQTSKRDIAKAFGLKGADRIDLKRLLKELEDEGHLEKRKRSYRDPERLPPVTVLQVLAPNAD
GDLFARPLEWHGEGAAPRVLYLPRQSDAALGEGDRILAKLQEVRAEDHQYEARLIRKIGSNPKRILGIFRKATEGGRLVP
IDKGADREWMVAASLTHGARDGELVEAELAGPRGRMGLPKARIVERLGDPTAPRAVSLIAIHQHGIPDDFPDEVIAEA
DR
AKPAGLGKREDLRALPLVTIDPADARDHDDAVLAIPDDDPANPGGHILWVAIADVAHYVTPGSRLDREARRRGNSSYF
PD
RVVPMLPDRLSGDLCSLHEGVPRACMAVRMKIDASGHKLSHRFTRGLMQSAGAFAYEEVQAAEEGKPSDRTAPLLDE
VIH
PLYAAYRALIRAREARQPLDLDLPERRIELSDEGKVASVKFRDRLDAHRLIEEFMVLANVAAAEELSRLHRPLLFRVHEE
PSPEKLDALREVAGASGFTLAKGQVLHARQLNDLLDQAMGTEFSELINLSTLRSMTQAYYFPENFGHFGLALKSYAHFTS
PIRRYADLIVHRALISAHGWGRDGLSPEDVDELEATAKHISETERRSMAAERDTTDRYLAAYLSDRVGNEFGGRISGIAR
FGVFVSLDETGADGLIPIRELGNEYFHHDRDAQTLMGSNSGLVIGIGQRVTVRLAEAVPVTGGLTFELLSIEDRALPRGA
ARGRGKPVRRKVVRAKTKSDKVKRKVKRTRR
>OLS47393.1 murein transglycosylase [Rhodovulum sulfidophilum]AB14
MTRYRSLAPGLGVALGVALGLGGPADAMLERVPRPLPRPDTLEMRQSPRPEIRSAVVAALARAGAPDTVADEARGFP
GWI
AGFRGRALAQGIAPGVFDRAFEDARFQPDVIRRDRNQSEFVTPIWDYLDNAVSEARVRNGRAALARYGAVLERIEARY
GV
DKEIVAAIWGMETSYGSYRGSAPIISSLATLAYEGRRAAFFEAQLVAALKIIQSGDTDARHMTGSWAGAMGHTQFMPT
SY
LEHAVDFTGDGRRDIWSEDPTDALASTAAYLAHFGWTRGQPWGVEVQLPRGFDHRLADRSVKRMPSDWARLGVRD
MNGGA
VRDYGRAAILQPAGARGAAFMIFDNFRVIERYNPADAYVIGVGHLADRLKGGGPIRGSWPRDDRALASAERKELQERL
TR
AGFDTGGVDGRIGPNTVSALRAYQSAVGMIPDGYASMAVLRRLR
>OLS47394.1 CidA/LrgA family protein [Rhodovulum sulfidophilum]AB14
MTTRLLARRLRVAVRRSLTVQIGILVGFWLAGEAAVRLSGLSLPGGLVGMAAALALLGSGLLRAGTLRRGANWFLAEM
LL
FFVPAVLAVLNHREFLGLLGLQILLIIVLGTLAVMAVTALTVELCCRWRIGGDGQPSALD
>OLS47395.1 murein hydrolase effector protein LrgB [Rhodovulum sulfidophilum]AB14
MDSLLHWTEPFWPVFGWSLLTVGFYLLAKRVYRRWPRWWLMPLALAPALLTITVLALHETYRDYIRGAGWLMLMLG
PATV
AFAVPIYEQRAMIRRYWPVLLIGMVTGSATAIAVSWGLAGLFGLDRSLQLSLLPRSISTPFAMEVSADIGGLPDLTAVFV
VVTGVLGAALGDLVLAWLPVRSALARGALLGVAAHGAGTARAHQIGRAEGSVAGLVMVLVGLLNVCLAPLVVSAVRV
VSD

>OLS47396.1 alpha,alpha-trehalose-phosphate synthase [Rhodovulum sulfidophilum]AB14
MNVATAPHPPLSDRRFVLATDLDGTFLGGTEAERRRLYDWIEANRVSVGLIFVTGRDPDFIMEMCAERGLPWPEYVV
GDV
GTSIAEVHPGEAIRPIPALEEDISRRWGDSGARVRAALDGHPGLTLQPTEFRYRVSFDMDAETYDPSAEAKVEAMGLD
WL
ISADCYFDVLPKGVSKGPSLRRLVAHLTIPEGRVLAAGDTLNDLSMLECGLNAVAVGNSEPALVERVRDLDHLHIARAHG
AAGIMEAIAAFDLHTVPTGA
>OLS47397.1 glucosylglycerol-phosphate synthase [Rhodovulum sulfidophilum]AB14
MPSDLVIVYHRQPYEEVEEDGKIVLRENKSPNGIVPTLKSFFGRFDKGAWIAWKQAEDPANPDFERVIEVDDAFGQYTV
S
RLPLTAEQVSSFYHVTSKEAFWPILHSFKERYNYDPVDWPTFREVNWIFAEAAAAEAAPGAVVWIHDYNLWLVPGYLR
KL
RPDVKISFFHHTPFPSADMFNVLPWRKEILQSLLACDVVGFHIPRYVSNFVSAARSLLDVEVTGRQKVEPEFAAEVTALS
ERSVPTELEYEDRRITVQASPVGVDADYIDAVARREETAERAEEIRAEMGGAKLLLSVGRTDYTKGGIDQLLSYERLLEA
QPDLCGQVRLMHVSVPANRNMTVYENIQNDLEQMAGRINGRFGSLDWQPIVLISRAIPFEDLVAYYRAADVAWITPL
ADG
MNLVCKEFCAARVDGDGVLVLSEFAGAAVEMGAALLTNPFSHRSMDSAIAQALEMPKAERRDRMAALRDVVHRHD
VRFWA
NHQMEALSGSAPGAAAMPAA
>OLS47398.1 DNA topoisomerase (ATP-hydrolyzing) subunit B [Rhodovulum sulfidophilum]AB14
MVENARAAEEYGADSIKVLKGLEAVRKRPGMYIGDTDDGSGLHHMVYEVVDNGIDEALAGHADAVTVKIHADSSVSV
SDN
GRGIPVGIHEEEGVSAAEVIMTQLHAGGKFDQNSYKVSGGLHGVGVSVVNALSDWLELRIWREGHEHVARFEHGDTA
EHL
RVVGPSEGRSGTEVRFMASTETFSNLEYSFKTLENRLRELAFLNSGVRIVLEDERGAEPQRVELCYEGGVREFVRYIDKL
KTPVMAEPIFMTGERDGIGVEVAMWWNDGYHETVLPFTNNIPQRDGGTHMAGFRGALTRTINAYAMSSGIAKREKV
NFTG
DDAREGLTCVLSVKVPDPKFSSQTKDKLVSSEVRPAVESLVNEKLAEWFEEHPQEARSIVGKIVEAALAREAARKARELT
RRKTAMDVASLPGKLADCQERDPSKSEIFLVEGDSAGGSAKQGRARANQAVLPLRGKILNVERARFDRMLSSQEIGTLI
T
ALGTGIGRDEFDISKLRYHKIVIMTDADVDGAHIRTLLLTFFFRQMPEVIEGGFLYIAQPPLYKVARGKSEVYLKDQAAL
EDYLVEMGIDGAVLRLGDGEEIVAKDLARVIDEARQMRRVLQAFPTHYPAHILEQAAIAGAFRPGQIDADLQGTADAV
AQ
RLDLIALEYERGWLGRPTQDKGMRFSRVVRGVEEVRVLDGQVLRSAEAARLGTFTAKLQEIYQHPARLIRKDRDQPIHA
P
SELLQAILTEGEKGLSLQRYKGLGEMNPEQLWETTLDPEARTLLQVRVEDVAEADNIFTKLMGDVVEPRREFIQQNALS
V
ANLDF
>OLS47399.1 DNA replication/repair protein RecF [Rhodovulum sulfidophilum]AB14
MPGLLLTELTLSQFRSHERARLVLDGRPVAVFGPNGAGKTNLLEAVSLLSPGRGLRRAAAEDLARAPGRLGWKVTAVLS
G
PGSGHEIETWAMPGGNRQVRLDGKTAAQVALARIARVLWLVPSMDRLWIEGAEGRRRFLDRIALSFEPGHAEATLAY
EKA
MRERNRLLKDEVRDPHWFGALEAQMAEAGAVLGANRRAALMRLEAAQAEAETAFPRATLALAGPEGSDPEDAGAL
AGALS
AGRGRDMAAGRTLAGPHRADLGAVFDAKGIEARHCSTGEQKALLIALILANARALAQDFGAPPLLLLDEVAAHLDAGR
RA
ALYDEISALKAQAFMTGTGAELFAGLGARAQYLEVRETDGTSQIERMDGPCTRSA

>OLS47400.1 DNA polymerase III subunit beta [Rhodovulum sulfidophilum]AB14
MKFSIERGALLKAVGQAQSVVERRNTIPILANLLIEAEDATVSFRATDLDIEVVDKAPATVERAGATTVPAVTLHEIVRK
LPDGALVELSDDGASGRLTVVAGRSHFTLATLPREDFPVMASSEYTSNFTAKAPVLRRLFDKSKFAISTEETRYYLNGVY
MHVAAGEDGQALRCVATDGHRLARIDATLPPGAETMPGVIVPRKTVGELRKLLEDDEVEIAVSVSETKVRFATPEITLTS
KVIDGTFPDYSRVIPVNNARRLEVDAAEFAKAVDRVATVSSERSRAVKLALEEDRLTLSVTSPDSGAAEEELVVAYGDER
LEIGFNAKYLLEIAGQVDRENAVFLFNSSGDPTLMREGDDASAVYVVMPMRV
>OLS47401.1 chromosomal replication initiation protein DnaA [Rhodovulum sulfidophilum]AB14
MTDDTWGRIRDDLEKSIGKNNFVTWIAPLEFRALEDGVARFGVPTSFVGDWVNRNFGDKIIEHLARSGVEVDRLEFAV
AA
PKRVAPAAAAAPEAAAAPARRAAAEPRDEGDMPGAPLDARFTFDSFVTGKPNELAHAAARRVGEGGPVAFNPLFLYG
GVG
LGKTHLMHAIAWDLLHRAPEKKVVYLSAEQFMYRFVQALRERDMMGFKSLFRSVDVLMVDDVQFIAGKDSTQEEFFH
TFN
ALVDQNKQIVISADRAPGEIKDLEDRIRSRLQCGLVVDLHPTDYELRLGILQQKVELYRMQYPGLVIADGVLEFLAYRIT
TNVRVLEGALTRLFAFASLVGKEITLDLAQDCLADILRASDRKVTIEEIQRKVSEHFNMRLSDMIGPKRHRTIARPRQIA
MYLAKQLTQRSLPEIGRRFGGRDHTTVMHAVKRIEDLRSKDSQIDEDLELLRRMLEA
>OLS47402.1 30S ribosomal protein S20 [Rhodovulum sulfidophilum]AB14
MANSPQAKKRARQNERRYAVNKARRSRIRTFLRKVEEAIASGDQAAAATALREAQPELMRGVTKGVLHKNTASRKMS
RLA
SRVKALNA
>OLS47403.1 enoyl-CoA hydratase [Rhodovulum sulfidophilum]AB14
MAYKTLIVEIEDHVALVRLNRPEALNALNGELMGELAEAVSEANANEKVRCIVLTGTEKAFAAGADVREMSEKSFVDVF
A
GDLFTPETERLLACRKPIIAAVAGYALGGGCELAMMCDFIIAADTAKFGQPEINLGVVAGIGGTQRLTRAVGKSKAMD
MH
LTGRFMDAEEAERAGLVSRVVPAKKLIEEAMAAAQKIAEKSALTTMAVKECVNRSFETTLREGLLFERRMFHMMFATE
DR
AEGMQAFVEKREPQFRDK
>OLS47404.1 DNA-formamidopyrimidine glycosylase [Rhodovulum sulfidophilum]AB14
MPELPEVETVRRGLTPAMEGRRIVATRVNRPDLRWPLPERMAERLTGAQVLRLGRRSKYILADLSTGETLILHLGMSGR
V
LISGVALGQFVHPHPAPQKHDHVVIELEGGARVTFNDPRRFGAMDLAPTEAVADHRLLAGLGPEPLGNGFDETYLAGR
LK
GRQTPIKAALLDQHVVAGLGNIYVCEALHRAGISPLRKAGRIAAARIATLVPAIRTVLSEAIEAGGSSLRDYRQADGELG
YFQHNFRAYGREGAPCPSPGCTGRIHRVVQSGRSTFYCPQCQR
>OLS47405.1 bifunctional demethylmenaquinone methyltransferase/2-methoxy-6-polyprenyl-1,4-
benzoquinol methylase [Rhodovulum sulfidophilum]AB14
MTESAQKTTHFGFQTVAEEEKAGLVHGVFTRVASRYDLMNDLMSGGVHRVWKDAMMDWLAPQPHQRLLDVAGG
TGDIAFR
FLGRAPGATAVVCDMTESMLVEGRKRAEADNLAASLDWVVGDGMALPFADNSFDVFTNSFGTRNITRIQDALNEAY
RVLK
PGGRMMILEFSQMPNPLLQWAYDRYSFNVIPPLGQFVVNDRDSYQYLVESIRKFPDQETFASMIRQAGFEQVKYRNLS
MG
IAALHSGWKI
>OLS47406.1 2-polyprenylphenol 6-hydroxylase [Rhodovulum sulfidophilum]AB14
MRGPHNIWRLVQTLATMERTGANRFVLEQLGAPASVRAAARVMGWPFKWLGENGDEALPPATRAITALGPAYIKFG
QILS
TRPDVVGDELAVQLRVLQDKLPPFPTEIAKETVAQELGEPADELFSEFSEPIAAASIAQVHRARIARTGELVAVKVLRPG

VERNFRKDIDAFYFAASTIELLAPFSRRLRPTDVIAHFDGVVTGELDLRIETAQAAEFVANTENDKGFTVPRVIWPLSSR
RVMTMSWCEGVPIGDNAALDAAGHDRRAIGERVLSMFLRHALRDGYFHADMHQGNMKVGASGEIQCFDFGIMGR
IDEYTR
RVYAEILYGFIRRDYRRVAEVHFEAGYVPADREIDEFAQALRSVGEPIFGMDATHISMAKLLAYLFEVTERFGMATRTEL
ILLQRTMVVVEGVARSLNPSINIWEVARPIVEEYIKENIGPKAVVRDLGRTVRVLSRFGPRLPELAEAALIRQSQTPPEP
RRRSWMQPLLWMGAGAALTWIGAGLALLLS
>OLS47407.1 hypothetical protein BV379_03355 [Rhodovulum sulfidophilum]AB14
MTDSVTAATAATNASSSSSGNSKTLISNDFETFLRMLTTQLENQDPLNPMESSEYALQIATFSGVEQQVLTNDLLTNLT
ENLGGGGLSQYGSWVGMEAAAPVAVKYDGSTQLTILPEIEADASFAKLVVRNSEGSIVESREIDLPAETVTWPGESEAK
A
DTYSFIVESYAADGSQMSETLGKVYAPIAEVRTEAGKVSVVFESGSIVNSNDVSALRDPV
>OLS47408.1 hypothetical protein BV379_03360 [Rhodovulum sulfidophilum]AB14
MQVPFPIDTLGASASSRPDRPASTAGATDPDGSDFRSAFDETRPSDRNETVREAARRDTPKTSAASDRAEETPSSAPPG
A
RKNSEDRAEEVDETAETKAGSDTVKNDPADASDAIIAELPAVAGFSTTPQPGTSEATKTQALPTETAAAGAGTTAASAD
S
DMARTTSVPLTADPRQQSGESQPNSALPASSSMADARPAIATGGTTAQQTAAQQAEDTGPHSDIAPFGSKDAKAAA
ASSS
TAAAQANAAPLLSGTSPEALQAGNKEGAALNSATPKDSTTNRSSPEITDATPEAVAPKEGSTGQTASRQGDGMLQGR
EAA
QSQAVAARTIASDDPATDPKGDQKGHASAHRENTSTTPTTPTAPHATAAQPDATASAPPIAGTAIDIQTAIDGSRGDPT
A
ATDSGASERGGSDQIRGSETARHTTAPALADTPRTAIRQIAKSLHRASDGSVHLTLSPEELGRVRLSLTPGDHGITVNIT
ADRADTLDLMRRHGDTLANAMRDLGYGEVVLDFTGRQNNAPGQGRGFAAAGPAPNEEGTETGLLSANRTSPKTAG
GGLDL
RL
>OLS47409.1 hypothetical protein BV379_03370 [Rhodovulum sulfidophilum]AB14
MTPIEMIADLLEREYQALRHSDFAALRALTDEKEQLVARLQMPSEADREALDGLRSKARRNDRLLAAVEKGLESARVSL
K
RLREPAAALDTYTQRGERTRIGATPSALTRRA
>OLS47410.1 hypothetical protein BV379_03375 [Rhodovulum sulfidophilum]AB14
MSSILTNSSAMVALQTLKGINSSLEKTQSEISTGKSVGSAKDNSAVWAISKVMESDVSGFDAVSDSLATGQAMIGVART
A
TETVTDLLKDIKTKVVAARDATSDTTKLQNETDELISLVESTIGAAQFNGVNLIDSGTGTSVVGSIDRSGGTVTANSITI
DAQNLNTGAAATAAGQLAAGTTSGTNLVVVGNNNDGAVSIASGAGESFALSAVAQDETITVTLGDQNFSYTVTADDV
ASG
NDANEIALSNLRDLINAAGITGVTALYDPVGSAGELTIDNQGTDDINMTVRVRADGAGALAGLNGFSITGGTALADIET
M
INSSIDAAAAFGAAESRLETQSDFLSKLTDALKTGIGALVDADMEEASARLQALQVQQQLGTQALSIANQSPQNLLSLFR
>OLS47411.1 flagellar biosynthesis repressor FlbT [Rhodovulum sulfidophilum]AB14
MSGGLVLKLGPKERVLVNGAVIENGDRRTRLSIKTPNANILRLRDAIHPEEVNTPVRRVCYVAQLVLSGDSTPAEARPQL
LRGIEQLSQALTDPDSRAQLAAATDAVLQDQYYQVLKALRGLLPREERLLTGLRDQ
>OLS47412.1 hypothetical protein BV379_03390 [Rhodovulum sulfidophilum]AB14
MSFQPVLPLTGYAGWRFLERTMERQQEAFNKSPATQKELDYFADNIGKVKSADELVSDYRLLKVALGAFGLQDEIGNK
YF
IKKVLTDGIEDKGALANKLADKSYYKLAEAFRFVSSGAAAATVEKDFANELSLKIEGAGYLAVTTPTGEVAYTRNVGLKR
AEDGQIVTAEGYTVASIADTSLLRGSDDEGDTNNEKIVFSGITIPDLAQSISVNDKGQVYGYFEDGGQGLLLGRLALSSF

ADDESLAVLGADQMDEGADAKPVDRETAYFVATEESGEPISGRPLQDGFGGFVRGEAVEAQEVFDTEMIAAAFLTRSF
EV
AVGEQDDSMRLALNIDRELAMLTADNMSEDAKWFSIMGSEPMRAVFDTAFGLPSSFASLDLDRQLDVYKTKARAMF
GETS
VVQFADDAIRDELIKLYMIRSEANTGAGYSSGQIALRLLGG
>OLS47413.1 flagellum-specific ATP synthase FliI [Rhodovulum sulfidophilum]AB14
MPQNPFSSIIARISALTPVRTIGRVVAAGSGTVTVAGLSESAGLGDLVEFRHGRGTRRRGEVLALAQGTVTVLPDGTPEG
LSVGDRAVLLGPGEIAPHSSWLGRVVDPFGQPLDGRPLMPGSVPRPLRSAPPPATARRPLGTRLGTGLAAFDTLLPIVR
G
QRIGLFAGSGVGKSSLLSKFARGVEADMTVIALVGERGREVGEFVSQVLGPEGMARAVVIAATSDQSPMVRRRAAWA
AMA
VAEHFRDQGNHVLFLADSVTRFAEAHRELALAADEPAALRGYPPSTAQAIMSLAERAGPGVADSGDITAVFSVLVAGS
DM
EEPIADILRGVLDGHIVLDRRIAERGRFPAVDLLRSVSRSLPRAATGEENAIIAQARRLLGAYDRAEMMIQAGLYAAGSD
PEIDAAIAVWPGLDAFLSEDAPGGVAESFARLAAILDSAKPSTAPVKPGLRTIQG
>OLS47414.1 hypothetical protein BV379_03400 [Rhodovulum sulfidophilum]AB14
MFEKLEIFRMASGLAKHAGTRQTVIARNIANSDTPGYRAEDVADFAKTYRRSDTGTQMRATRPGHMVGRETAYAARN
TDS
ADPEEPNGNTVSVEDQMVKAVETKRQHDLALAVYRSSLGILRTSLGRQR
>OLS47415.1 flagellar basal body rod protein FlgC [Rhodovulum sulfidophilum]AB14
MNDLSQSFGLSASGLKAQATRLRLVSENIANADTPGYRRKLTTFETELTSHGPTGEVRTGRVSLDQSDLTKVHDPSHPLA
DATGNYDGSNVDLVVEIADAREAQRSYEANLKMFDQARQMSRGLLDLLRR
>OLS47416.1 flagellar hook-basal body protein FliE [Rhodovulum sulfidophilum]AB14
MDLKSSTAALGYTTARPATLPQPGDGGVEKTFAAAAQGLSDTIRNAEQTAMSSMTGGADPHALVQALAQTELAVETA
ITI
RDKVVEAYQEILRMPV
>OLS47417.1 flagellar biosynthetic protein FliQ [Rhodovulum sulfidophilum]AB14
MNELVFYDTLRQGLWISVVISMPILTAALVTGVAIGLFQALTSVQEMTLTFVPKLAAILIVFWISMAFMTETLVGFFTGR
IVPMIAGI
>OLS47418.1 flagellar basal-body rod protein FlgF [Rhodovulum sulfidophilum]AB14
MQTTGYTTLTRQSGLMREMQSIANNIANSSTIGYRREGLIFSEYIVDVDGPGGSLSMAEANVRNTDFGQAPLTQTKGT
FD
FAIEGEGFFLLDTPGGQRLTRAGSFTPDAAGNLVSPDGYPLLDAGGAPVFAPPDAASVAVSADGTISADGRPLTQIGVYL
PADPNAMTHVAGTLFDPGGATKPVLESSILQGFVEQSNVDPMTEISRMIEVQHAYELGQKFLEREDERVRDVIRTLGSS
S
>OLS47419.1 flagellar basal-body rod protein FlgG [Rhodovulum sulfidophilum]AB14
MRALQIAATGMSAQQMRVEVISNNLANMSTTGYNARRADFADLHYQQVTRPGTINANDGTVLPTGVQLGLGVRPA
AVSVV
LAQGSLSQTNGDLDVAIDGRGYLEVTLPSGQAAYTRDGALKRTGDGLIVTSDGYTVSPGITIPDDARSLSVNSEGELYAY
FEGEVQAQLLGQFNLSMFSNDKGLEAIGSNLFVETEASGAALVGTPGVDGFGTLRQGYLEDSSVDAVREITDLIEAQRG
Y
ELNAKVITAADQMMSATTQVR
>OLS47420.1 flagella basal body P-ring formation protein FlgA [Rhodovulum sulfidophilum]AB14
MRLLVLLAVLGGALPVVADADILVATRSVRGQTILGPGDVAVVAGEAVGTLVAPEEAIGQEARINLYAGRPIRIGDVGPP
AIIERNQIVTLRYQSRGLSIAADARALNRAGVGDALRVMNLASRNTVTGFVAPDGSVVVGASLKP
>OLS47421.1 flagellar basal body L-ring protein [Rhodovulum sulfidophilum]AB14
MRPLVLVLCATALLFPAACDRLKEVGRPPSFKPVEGSNSYYAMTDSATLPMEVERRSPLDNSSLWSHGRQSLLGDHRA
GR

RGDILTVVIEIDEKAEISNTTARSRSGATSMGIPQLLGIPQRINKALPDGVTMDNAVDADSSSSHSGNGSVKRKEKLTLR
IAATVTDVLNNGVMKIEGSQEVRVNYEMRELLVSGYVRPEDISRQNEITYDKIAAARISYGGRGQIMDVQQPRIGHQIA
D
IILPF
>OLS47422.1 flagellar basal body-associated protein FliL [Rhodovulum sulfidophilum]AB14
MGKLLPVIIALVGLVIGGGAGFLLKPAPPPSDSTDHSASQGQAEDGSHMMADGHAADAASAAGHGDDPADNSEYVK
LSNQ
FVVPVVHGGRVGALVVMSISLETPTGSSEFVFEREPRLRDAFLQVLFDHANAGGFDGAFTESGNLTLLRRALLEASQSVL
SPIVSDILITDLVRQDA
>OLS47423.1 hypothetical protein BV379_03445 [Rhodovulum sulfidophilum]AB14
MNTRDDRPERLVRIGTILKDAALSRLRQAAQECRRIEAAIAELDDEAQRTAIDDDIAMRAVFDCSRTLRHRERRTALNAE
LARARAQEALLMREAARAFGRDEAIRGLFDRKHR
>OLS47424.1 flagellar biosynthesis protein FlhB [Rhodovulum sulfidophilum]AB14
MSDEDPGDKQFEPTQKKLDDARRKGEIARSTDINTAAVYAGFALTALALGGTSLIKMGELGAGMIGQADRLAPLLLEG
HG
SAPVGGLMAQFGLVVLPWLVIPALAALASVLAQRSLVVAPEKLMPKLSRINPIETAKNKFGRSGLFEFTKSTVKLALYTI
LLALYLSHHVEEMLGTMYLSPAMAAAKLVRMSAEFLTVVVAIAASIGVVDYLWQIAEHRRRHRMSLEELKEEIKQSEGD
P
HIKQQRRQRAMDIATNRMLRDVPDADVVIVNPTHYAVALKWDRARRGAPFCLAKGVDEVATRIREVAVEAGVPMRS
DPPT
ARAIYGSVEIGDEIKPEHYKAVAAAIRFAEEMRTRAKGL
>OLS47425.1 flagellar biosynthesis protein FliR [Rhodovulum sulfidophilum]AB14
MTLELAPALDIWRPLAWAGFLVFLRVGAIMAMLPAFGERAVPIRVRLGLALAFTAVVSPAVSPGLIETPPSLALAFATET
VIGLALGLVLRLMILALQMAGEIAAQATSLSQILGAASMDPQPAMGRLMLMAGLALAVMSGLHVRAAGAMILSYDLL
PAG
RFPDAGDLADWGVGGIAKGFALAATLAMPFVIASLIYNVALGVINKAMPQLMVAFVGAPAITAGGLIILFLSLPPMLTF
W
QDRLLTLFAAPFGAGG
>OLS47426.1 flagellar biosynthesis protein FlhA [Rhodovulum sulfidophilum]AB14
MPGFSVSELFRPTILMALALMAIIIMMVLPMPAWVLDIGLAASFSIAILIFTVTLFIERPLDFSSFPTILLASLMLRLSL
NVSSTKLIIGQGHTGTGAAGDVIEGFAMFVMGGNVFLGLVVFGVLMIVNFIVITKGAGRMAEVGARFALDGMPGKQL
AID
SDMAAGAIDHAEAKTRREREQAETTFFGSLDGASKFVKGDAVAGLLITLLNLLMGLAIGVIMHGMPLGGAFETYAILTV
G
DGLVSQIPAVIISIAAALLLSRGGTTGTTDLAVFTQLGKHPAALGTVSVLLCLFALVPGLPFVPFIVGSLVLGTAAFNRW
RSLQHEAEKAAQPEAAEKAEPANRMGDLLDLDDIHLEFAPDLVGMVLDPSTGIEARIVSMRRHVAESFGLILPEMRLTD
N
GALPPGTYVVRIQGVEQARDRLRPDRVLALQAGDITLLPPGEDVREPVYGAPARWVSADRREELALQGVTVVNASEVL
AT
HLLEVVKNNFARLLTNKALRQIFDELKNLSDQTRAQANRRMIDDLVPEKVPPDLLLAVLRLLLEERVSIRNMPLILEAIA
EARPSHAGAEAICEHVRRRLGFQLVAEHRREDGTLPLLQLSPEWESRFSTYQVGGEAGVSDVALPPEEFNRLASAVSEKL
AKAGENGIFPVIATSGRRRRFLQMALSAKGIAAPVLSFEEIGTNTRPALVGMVAA
>OLS47427.1 hypothetical protein BV379_03465 [Rhodovulum sulfidophilum]AB14
MNARHIFLALALFTCQPALAQQVEIRSGEHPDYSRLVFETEAPQGWTLGRGPDGYLLRFDNRALRLDLGDVFDRIPRRR
L
TDAALVAPGLVSLRIGQNAHVEAFELRPGLLVLDFRTGPAPEDSPFETALQSAADTPAEEEVPPAPVEPVRPDTPPAADI
AAQSPSPSPESSPVRLPIDLGLKSALNPSAGMKPPAAMVEKMLEPEIPPAETDPRVAAMRSELMKQIGRATAQGLLEPA
I

DLPTQPDPGTAETVSNAQDALPDPDQRAEENGQAETQIAEDHTPSDWSNMRVETSMDRGLDILSDDSTLAADGTRC
LSDD
NFDVIGWGGDRAPGDLLAEKRRALLDMRDATDPSGVIGLARAYIAMGFGAEARAVLAATATETPVVPLLREMAAIVD
TGQ
ANHPELFDSQISCPTRAALWAALARPKLGDLSDFNRTALLQSFSELPIHLRRHLGPRLAERFLAAGDQATALQIRNAVAR
TGAKGQTPDLTIVEAQIELARGSDARADRKIDEVLASGGSGTADALALRIETGLSRGQPPSEDSLALAESLAFERRGTAS
GSWLLTLAIRGHGARGDFETAFAMLAHHGLEGQTELSSELLGELARRGSDEEFMREVYLGALTLPALSADSTARLAVAD
R
LTAMGFQDRAATVLQPLAPQGTPRERLVRARLALAQGSAGEAQQYIAGIDTPEADSLRALIAQRQGDLKSAADYLGAV
GD
AEAQSAMAWRARDWSGVESLGDPNQRAFVKTRRDREPADLTMEPSLAVARDALGTSEIMRERLRTLLDRPSTQP
>OLS47428.1 flagellar motor stator protein MotA [Rhodovulum sulfidophilum]AB14
MIGIVGIILIFVMVFGGYVMAGGKMGIILHSLPFEFAMIGGAAAGAFVISNDMAAIKHTLKDISKVFKGPHWKPEDYRD
L
LCLLFELIRVARQNPLELETHIEAPGDSVIFGRYPKIVADHEAVDLICDTLRAAGMNYDDPHQVEEVLEKRLEAHLHHSM
HSSHALQSVADALPALGIVAAVLGVIKTMASIDQPPEILGKMIGGALVGTFLGVFLAYGFMGPFASKLKVVVEEDAHFYL
LIREVLVANLHNHPPAICIEVGRQNTPGGSRPSFSELEEALKASKQEAA
>OLS47429.1 hypothetical protein BV379_03475 [Rhodovulum sulfidophilum]AB14
MKGSSRSKRASGAVLPVIAGLFLASGLIRFGDGTAQALAQELQEMTAPVGDSGTDGGHDTATDPCPPPADIADLLEAL
KT
RAAELDAREAALNDRAATLDIAGEEIAQQMASLQAAEDSLKSTLALADQAAENDVARLTAVYESMKPEEASALFAQM
DPE
FAAGFLGRMRPDLAAAVMSGLEPNVAYSISVILAGRNARAPKE
>OLS47430.1 hypothetical protein BV379_03480 [Rhodovulum sulfidophilum]AB14
MALISDILLIAGALGATLYCYVLARRLRRFNDLEKGMGGAIAVLSAQVDDMTKALKGAQFSAKSSTDSLESLTGRAEDVA
RRLELLVAAMHDLPEDTVQTAASQTPKPAATAAPETAPAAQPPTATADGEPMVLFRRTAREAAE
>OLS47431.1 flagellar basal body protein FliL [Rhodovulum sulfidophilum]AB14
MAATDMAEAEEPAKSSKKPLIIGAALALVLGGGAFFALYSGLILGGDPTDETAAGTHGGPGKAAESGPLDVAFVPIEKMI
ISLGPESASRHLRFEAQLEVTPAYQEDVTLLMPRVLDVLNSYLRAVDMAELEDPTTLITLRAQMLRRVQLVTGNGRVRD
L
LITEFVLN
>OLS47432.1 flagellar M-ring protein FliF [Rhodovulum sulfidophilum]AB14
MQQLLSMWTGLSPRRRVVVAIATVAMFAAVLALSRVATSPRMALLYSGLDTAAAGDVIRALDQSGVAYEVKGPAIYV
DSS
QRDALRMTLASDGLPAIGSAGYELLDGLSGFGTTSQMFDAAYWRAKEGELARTIAGNSQFRSARVHIATPSSRPFARD
VR
PSAAVTVAAAGGSVSGAQAKALRYLVAASVSGMRPEDVSVIDANSGTVVADDSSLSAAGEDRAEILKARLERLLSARLG
P
GRSIVEVSVDTETQRESIRERIIDPEGRVAISENNQQRQSSSSDTNGGAVTVASNLPNGDGESDRSSQHKESETQEVVNY
EVSQTEREILRAPGAVKRLSIAVLVEGVTSTAADGTETWQPLPDADLESLRELIASAAGFDAERGDALTIKSMRFEPLDA
GGSGPQSSLFGEFTLDPLTLIQLAVLSVVALILGLFVVRPILAGQGKAAVAGLPAPAGGGQDQTWLNGEIDPTDGPQDG
M
EDDLGLPAMTMAAIDPADFSDQPLGMDGFGGGGGDSPVDRLRDLIESRREESIAILRGWMEDRQEKA
>OLS47433.1 hypothetical protein BV379_03495 [Rhodovulum sulfidophilum]AB14
MSRLFLEDFSDHEAGPQIAPEPPAIDTEEIRLNAYEEGYRAGWDDASAAEAAARTRIGADFEKTLQELSFGYHEARVHVL
EALEPLLSVMVERVLPELARWGLAPKVVEIAREMAETEADRPLQLQVRPENREALEELVGADPGLPLRIVDDPALGPGQ
A
VLRGGAAEREIDIDGMLAAIRSALNDFMTTEEEARRHG

>OLS47434.1 hypothetical protein BV379_03500 [Rhodovulum sulfidophilum]AB14
MADTSSDAPGEGLGAKFLDQVPIEITVSVGKARPLLRDLLQIDRGGILPLDRRIDDPVELYIGDRLIARGELQEVEGAQA
GQIAVRLTEIIDTVSGA
>OLS47435.1 flagellar biosynthetic protein FliP [Rhodovulum sulfidophilum]AB14
MSLRLVPAVLFLFALALPAQAQEISISLGEGGSLAGRGVQMILLVTVLSLAPGLAIMLTCFPFIVTVLSLLRQAIGLQQS
PPNMLIVSLALFLTYFVMEPTFTQAWQAGVVPMMNETLPIGEAVPRILDPFRNFMAGRADAETFERLLSLRPQADPTA
AI
TDAPLSLLVPSFLLSEIQRAFEIGFLIFLPFLIIDLVVAAILMSMGMMMVPPAVVAMPFKLAFFVVADGWSLISEALVRG
YFPGG
>OLS47436.1 S-(hydroxymethyl)glutathione dehydrogenase/class III alcohol dehydrogenase
[Rhodovulum sulfidophilum]AB14
MRTRAAVAFQAGKPLEVTEVELDGPREGEVLVEIKATGICHTDEFTLSGADPEGLFPAILGHEGAGVVVDVGPGVKSVK
K
GDHVIPLYTAECRECEYCLNPKTNLCQAIRTTQGQGLMPDGTSRFRTLDGDPILHYMGCSTFSNYTVLPEIAVAKIREDA
PFDKVCYIGCGVTTGVGAVVNTAKAEPGCRAVIFGLGGIGLNVIQGLRMIGADQIVGVDLNPAKKAMAERFGMTDFV
NPS
EVEGDLVPYLVDLTKGGADYSFDCTGNVNVMRTALECAHKGWGESIIIGVAPAGAEISTRPFQLVTGRTWKGTAFGGA
RG
RTDVPKIVEWYMQGKIEIDPLITHTLSLDEINKGFDLMHSGESIRSVVLY
>OLS47437.1 S49 family peptidase [Rhodovulum sulfidophilum]AB14
MKRWLPFVKSDPLVAVVRLSGTIAASSRGSALSDAGLAPVIERAFKKGKPDAVALIVNSPGGSPAQSALIAARIRRLADE
HQTPVYSFVEDVAASGGYWLACAGDEIWVDASSIMGSIGVIYAGFGFQDLIARHGVERRVHAAGESKSFLDPFKPEKPE
D
VARLMQLQEPIHAAFKDHVRERRGARLVSDRDLFTGDVWVGQQSVEVGLADGIGHLVPKMKERFGDKVRFSLHASR
RGLW
SRLGAPGVVGIADELEARLLWSRYGL
>OLS47438.1 hypothetical protein BV379_03520 [Rhodovulum sulfidophilum]AB14
MLLKVALLFLIGMAALGMFGKLRLPRPRGGLLPGRRRPAKCPHCGRFRIGRGPCPCGKG
>OLS47439.1 sodium:proton exchanger [Rhodovulum sulfidophilum]AB14
MASDLLHAGLGLALLLLAGDSLVKGAVNLSLRLGVPALIVSLTIVAFGTSAPELLISIKAVLDDSPGIAMGNVVGSNIAN
VLMVLGIPALMTGIRTDECDTRRTYALMIGATLIFLFVCLIGPITWIHGIALMLVLGFVLVDAFLSARKHRRDAAEAARC
AAPDATEDEVEGADPSLGWFKICMFLVLGLLGLPLGADLLVDASISIAREIGVSETVIGLTLVAVGTSLPELATTVMAAL
RRQADVAMGNVIGSNMFNLLAIIGMASFFGDIAVPPEMLRFDLWVMLGAALLLIPFVVWRVALGRAWGAVFFLLYA
AYAG
VLLL
>OLS47440.1 short chain dehydrogenase [Rhodovulum sulfidophilum]AB14
MSGRALVTGAAQRLGRAMALHLAERGHDVAVHYAGSAEAAAETVAEIRALGRRAVALRADLLDEDETQALVPRAAEA
LGG
PLTVLVNNASIFEYDTIRTATRESWDRHLGSNLRAPFVLMQGLAAGVTEPARDAAGEALATGLVVNVIDQRVLKPTPEF
M
TYTLAKMGLWALTRTAAQALAPAIRVNAIGPGPTLKGARQSEDHFARQRASTVLERGADPGDICAALDYLLTARAVTG
QL
ICVDGGQHLGWRTPDVLGVE
>OLS47441.1 excinuclease ABC subunit C [Rhodovulum sulfidophilum]AB14
MTPTEDSEAAPRPRGHACIQSYLATLDNSPGVYRMLDAESRVLYVGKARNLRKRVSNYAKPSGHSGRIARMISETASM
MF
LTTRTETEALLLEQNLIKQLKPRYNVLLRDDKSFPNILVAKDHRFPQIRKHRGAKTGKGAYFGPFASAGAVNRTLNQLQK

AFLLRNCSDAMFESRTRPCLLYQIKRCSGPCVGRISEAGYAAAVRDAEDFLKGKSSGLQEALAAEMQAASEAMEFERAA
G
LRDRIRALTQVQSVQGINPRGVAEADVIALHMEGGQACVQVFFIRAHQNWGNRDYYPKTGSGAEPPELLQGFLGQFY
DNK
EPPRLLLLSHDIEDRELMAEALSQKAGRKVELAVPQRGEKAELVENAARNARESLGRRLAESAAQGKLLAGLADSFGLEA
PPKRVEIYDNSHIQGSHAVGAMVVAGPEGFLKSQYRKFNIRGDSLTPGDDFGMMKEVLTRRFQRLLKEDPDRQGETW
PDL
LLIDGGAGQVGAVAEIMGDLGVEDLPVVGVAKGIDRDAGKEEFHRPGERPFALRHNDPVLYFVQRLRDEAHRFAIGTH
RA
KRAKAMGATPLDEVPGVGATRKRALLTHFGSAKAVSRAGLADLKAVEGISDSLAETIYGFFHERG
>OLS47442.1 CDP-diacylglycerol--glycerol-3-phosphate 3-phosphatidyltransferase [Rhodovulum sulfidophilum]AB14
MRWTLPNILSVLRLAAAPLFPLVYFLWDSPVADWVALVLFIGASVTDWIDGYVARRWHLTSRFGAMIDPIADKAMVL
AAL
LVVVAKFGMPTWLTLAATVIVFREVFVSGLREFLGAQAGRLKVTKLAKWKTTVQMVAISVLIAHGILPHAAIEWLGTAL
L
LAAAALTLITGADYFAKSWPYLKEEKVP
>OLS47443.1 molybdopterin converting factor subunit 1 [Rhodovulum sulfidophilum]AB14
MIDLLYFAWVRERIGLPKERIETGAPTVAALVDELAAREERYAAAFADLSALRVAVDQELADFDAPLAGVREVAFFPPM
T
GG
>OLS47444.1 molybdenum cofactor biosynthesis protein MoaE [Rhodovulum sulfidophilum]AB14
MRIRVQTAPFDFGAECAAFSKGCGNAGAVVTFAGLVRDDGGLARMELEHYPGMTERALEAISAEAMTRFGLLDCLVI
HRH
GSLTPGETIMMVATAAPHRADAFAAAEFLMDYLKSRAPFWKKEFGTDGAAWVAAREADEDALARWSDS
>OLS47445.1 phosphoenolpyruvate carboxykinase (ATP) [Rhodovulum sulfidophilum]AB14
MEFGRVNPNQRLEAQGIDGLGKVYYNLLEPALIEEALKRNEGTLGKGGSLLVATGVHTGRSPKDKHVVRTPEVVEHIW
WE
NNRPMEPEAFDRLHADMLAHLKGREVFVEDLYAGADPDNRLDVRIVTEMAWHGLFSRHLLRRPELDQLDSFAPEFTII
NC
PSFKADPERHGCRSKTVVALNFAKKLILIGDTEYAGEIKKSVFTLMNYILPGKGIMAMHCSANHAKDNPVDTAIFFGLSG
TGKTTLSADPARTLIGDDEHGWSDNGTFNFEGGCYAKTISLNPEAEPEIYATTSKFSTVIENMVFDPHTKELDFEDDSLT
ANMRCAYPLHYISNASATSLGGHPKNIIMLTCDAFGVLPPIARLTPAQAMYHFLSGFTSKVAGTEKGVTEPEPTFSTCFG
APFMPRRPEAYGNLLRDKIAKHGATCWLVNTGWTGGAYGTGSRMPIKATRALLTAALDGSLNDAEFRKDPNFGFEVP
LAL
HGVSEELLDPRRTWAEPAEYDAQAQKLVRMFSENFAQYLPFIDDDVKEAAIG
>OLS47446.1 DNA-binding response regulator [Rhodovulum sulfidophilum]AB14
MSRIALVDDDRNILTSVSMTLEAEGFEVETYNDGQAALDAFNKRMPDMAVFDIKMPRMDGMDLLQRVRRKSAMPV
IFLTS
KDDEIDEVLGLRMGADDYVKKPFSQRLLVERIRALLRRQEVIAADAGPSPEESKTMVRGQLTMDPLRHSVTWKGLDVS
LT
VTEFLLLQALAQRPGFVKSRDQLMDVAYDDQVYVDDRTIDSHIKRLRKKMRQVDPEFSAIETLYGIGYRYNEE
>OLS47447.1 serine kinase [Rhodovulum sulfidophilum]AB14
MPDASSCILHASTVAHRGRAVLIRGASGAGKSALALQLMALGAGLVADDRTILTRTEGGLLASAPETIRGLIEARRVGLL
RAEAVASAPVVLAVDLDTSETERLPPVRVTEILGESLPLLRRVDRPYFAAAILQYLTAGPSD
>OLS47448.1 RNase adaptor protein RapZ [Rhodovulum sulfidophilum]AB14
MSNPLEIDPPAPRQRIVLVTGASGAGRSTALGVLEDLGFEAIDNLPISLIPRLLEGPVPGPSLALGVDIRTRDFSARALA
DLILGLSAAPGFEADVLYLDCRPDVLLRRFSETRRRHPMAPAESPAEGVARELDILGPIRELASSLIDTSGLTIHEFRAE

MTARFAPGGAAPLAVSLHSFSYKRGLPLGIDMAFDCRFLRNPYWDPSLRALDGRDPAVAAHIAADPRHAPFLDRISDL
AA
LVLPAHRDEGRSHISIGFGCTGGQHRSVAMAEAVAKRLAATEWQVSIRHRELERRAQGVSAMRTGTSA
>OLS47449.1 PTS fructose transporter subunit IIA [Rhodovulum sulfidophilum]AB14
MIGIVIVAHGGLAREYLAAMEHVVGPQPGIRAIAIAPDDDRAAKQDEICRAADEVDEGGGVVVVTDMFGGSPSNLSIR
AC
APGDRKILYGANLPMLIKLAKSRRMSVSDATASALAAGRKYIDSFDAGGL
>OLS47450.1 HPr family phosphocarrier protein [Rhodovulum sulfidophilum]AB14
MTSNRVTRIFEIVNEKGLHARASAKFVECVEEHDAAAEVLRDGMSASGDSIMGLLMLAASKGTAIEVSTHGPEALKLAD
A
LERLVATRFGEDM
>OLS47451.1 acyltransferase [Rhodovulum sulfidophilum]AB14
MHDRPAAEPPHDARELSYARTFPDPLRAQTIRAIEILTGKLTLLRLLRDFEARGVPRGQAFWRQALDVMGIRVATPPEQ
V
ARIPREGPLVIVANHPHGLVDGMVLAELVGRVRRDYKILTRSLLTDVDEVAEFMIPVPFPHAEDARERSLEMRRAAMEH
L
GQGRAVVLFPAGVVASSQTFFGPAVEALWNPFTAKLIQRSGAQVLPVRFQGQNSRWYQIANRLSPTLRQGLLLHEVV
HTL
NRPQAPVVGAPIGRDEIEPWARDPRGFMAWLRARTLALT
>OLS47452.1 3-hydroxybutyryl-CoA dehydrogenase [Rhodovulum sulfidophilum]AB14
MDVKTVGIVGAGQMGNGIAHVFALAGYEVRLNDISADALDRALDTIGHNLDRQVSREKITETEKRDALGRIAITTALTD
L
GACDLIIEAATEREAVKTAIFEELQPHLTPTTILTSNTSSISITRLASRTDRPEKFMGFHFMNPVPVMQLVELIRGIATD
EATYRSLLKVVESLGKTAASAEDFPAFIVNRILMPMINEAVYTLYEGVGSVRSIDESMRLGANHPMGPLELADFIGLDTC
LAIMNVLHEGLADTKYRPCPLLTKYVEAGWLGRKTHRGFYDYRGETPVPTR
>OLS47453.1 hypothetical protein BV379_03600 [Rhodovulum sulfidophilum]AB14
MIFDNPGSGSLAYSPCRYGNSKLEFRGPRRPLEQPYCAAVGGTETYGKFVPKPYPALIEEATGLQMVNLGCINAGLDVFL
NDASLMDVVAGAQVTIFQALGAHNMSNRFYVVHPRRNDRFLRASAMLKALYPGVDFTEFHFTRHMLSVLKQKSPSSF
EIV
VAELQMAWQARMRKLAGRVPGDKILLWLPGRPPGYGQPCDGLGEDPLFVTQHMLDTVRSYFTRVVEFEPSPEALAR
GAEG
MVHGEADEAAAASLPGPAVHEELAEVLSGTLRELHAI
>OLS47454.1 electron transfer flavoprotein subunit alpha [Rhodovulum sulfidophilum]AB14
MAVLLLAEVTGGELQLDATAKAVSAAKTLGEVTALCVGEHARAAGEAAARIEGVAKVLVAEDAIYGHRLAEPTADLIVSL
AGAYDHIVAPATTDAKNIMPRVAALLDVMVISDVVAVVDADTFDRPIYAGNAIQTVKSSDAKKVMTIRTSTFEAAGEG
DA
APVEDIAATGNPSLSEWVGDTAAESDRPELTSAGVIVSGGRGVGSEENFKLIEALADKLGAAVGASRAAVDSGFAPND
WQ
VGQTGKVVAPELYIAVGISGAIQHLAGMKDSKVIVAINKDEEAPIFQVADYGLVADLFDAVPDLTAKL
>OLS47455.1 electron transfer flavoprotein subunit beta [Rhodovulum sulfidophilum]AB14
MKVIVPVKRVIDYNVKVRVKADGTGVDLANVKMSMNPFDEIAVEEAIRLKEKGQATEIVAVSIGVKQAQETLRTALAM
GA
DRAILVVAAEDIHDDIEPLAVAKILKAVVDEEQPGLVLCGKQAIDNDMNATGQMLSALLGWSQGTFASKLEIDGDTAQ
VT
REVDGGLQTIKVKLPAIVSVDLRLNEPRYASLPNIMKAKKKPLDEKTPADYGVDTAPRLTVLKTAEPRERKAGVMVASVD
ELLTKLKDEAGVL
>OLS47456.1 short-chain dehydrogenase [Rhodovulum sulfidophilum]AB14

MTGKSVLITGCASGIGHDAAHALKARGWRVFATCRQAADCDRLAAEGLESLPLDYASEDSIATALDEVLSRTGGRLDAL
Y
NNGAFACPGAVEDLPRDALREIFETNLFGVHDLTRRVIPTMRAQGHGRIVNCSSVLGLVPLKWRGAYSATKYALEGLTD
T
LRLEMRDTPIRVILIEPGPVTSKIRVNSIPHFERWIDWQNSARADQYRNTLLTRLYEDRGRDFFELPPSAVSRKLIHALE
SPRPRPRYFVTAPTHLMNLARRLLPTPALDWLIDKG
>OLS47457.1 DNA topoisomerase IV subunit A [Rhodovulum sulfidophilum]AB14
MSDATEDPKHDALSEPLRRAIGERYLTYALSTIMDRALPDARDGMKPVHRRILFAMRELKLTATGGFRKSAKISGDVMG
N
YHPHGDAAIYDAMARLAQDFNVRYPLVDGQGNFGNIDGDNPAASRYTEARLTAAAEALMEGLSENAVDFRPNYDGT
LEEP
AVLPAAFPNLLANGSSGIAVGMATNIPPHNIDDLVGACLHLIKAPDAQDDTLLQYVPGPDFPTGGVIVEPKDSIAQAYRT
GRGSFRLRARWEIEDLGRGQWQVVVTEIPYQVQKSKLIERIADLIQTKKIPILADVRDESAEDVRLVLEPRSRNVDPEVL
MSTLFRNSDLEIRFSLNMNVLIDGRTPKVCSLKEVLRAFLDHRREVLIRRSQHRLDKIDHRLEVLAGLLIAFLNLDRVID
IIRYDDDPKAALMAEDWTRPHARAMSERDYVSPLPAAGPGGLTEVQAEAILNMRLRSLRRLEEMELTAERDRLTEERA
GL
LELLGQEEIQWKRIAEELREVRKNFGARAPGGARRTVFAEAAEVAEVPIEAMIEREPVTVVCSKMGWIRAMKGHLADG
SA
LKFKDGDEGRFLIQAETTDKLILFGANGRFYTLPVVQLPGGRGMGEPVRLMVDLPNESEIVALFIHRPGEKLLVASSAGD
GFLVAGDDVLAQTRAGKQALNVRAPAVARVCVPVHGDHVACVGQNRKVLVFPLEELPEMGRGKGVRLQKYKDGGL
ADATT
FTLADGLSWHDPAGRTRTETDLTDWLGKRAGSGRMAPRGFPRDNRFT
>OLS47458.1 hypothetical protein BV379_03625 [Rhodovulum sulfidophilum]AB14
MRGLFTTLRLLLFVAGLALVAGCAGKDDLDEPPVPLGDFRLGYTIVVAKNAKDIPPSRQATAEEWEAVLKEELQRRFGRY
DGDRIYHFGIAVDGYALAVPGVPVILAPKSMLVLSANLWDDAAGKKLNAESEQLSVGEGLNGATLVGTGLIRTKDEQM
RA
LARNAAKRIERWLVEHREEWLHYDPDAVPPLDAMAGTPSDEASAPASVPGAEVVTTPLDDPLDAPPGPSAAKPGEAP
SRA
N
>OLS47459.1 translation elongation factor Tu [Rhodovulum sulfidophilum]AB14
MGKAKFERTKPHVNIGTIGHVDHGKTTLTAAITKYFGDFRAYDQIDGAPEEKARGITISTAHVEYETEARHYAHVDCPGH
ADYVKNMITGAAQMDGAILVVNAADGPMPQTREHILLARQVGVPALVVFMNKVDQVDDAELLELVEMEIRELLSSYD
FPG
DDIPIIAGSALAAMEGRDPEIGENKIRELMAAVDEFIPTPERPVDLPFLMPIEDVFSISGRGTVVTGRVERGVVNVGDEL
EIVGIRDTKKTTCTGVEMFRKLLDRGEAGDNIGALLRGIDRDQVERGQVLCKPGSVTPHTVFEAEAYILTKEEGGRHTPF
FANYRPQFYFRTTDVTGTVELPEGTEMVMPGDNLKFKVELIAPIAMEEKLRFAIREGGRTVGAGVVAKIIK
>OLS47460.1 AsnC family transcriptional regulator [Rhodovulum sulfidophilum]AB14
MDIMDQKILRELRHDGRISNVALAERVSLSLSACLRRVQELERTGVILGYRVVTSPERVGRGFAAYVMVGLSSHTKAAQ
E
GFERAVAAAPEVVECHNIAGAFEYLLRVESAYLSEYKRFHTEVLGTQSHVNAITSCIVMEPPKDERAT
>OLS47461.1 hypothetical protein BV379_03640 [Rhodovulum sulfidophilum]AB14
MSFAILSALLGFAFVTTVTPGPNNLMLMASGANFGFRRTLPHMMGIVGGMSVMALLLGAGLMALFDAVPALSLVLKV
ISA
GYLLWLVVKIATAAPLEERDADGRPMTFLQAATFQWVNPKAWAMCLSAVTLYAPDRSLLSVAIVAAAFTLVCFPAISV
WA
WLETVVCQWLSNATRLRVFNITMAALLVASLYPVLGLGA
>OLS47462.1 preprotein translocase subunit SecE [Rhodovulum sulfidophilum]AB14
MANPLQFIQQTRSEISKVVWPTRREVFLTTIMVFVMASLTAVFFFLVDSLIRVGLQALLAFFG

>OLS47463.1 50S ribosomal protein L11 [Rhodovulum sulfidophilum]AB14
MAKKVIGKLKLQVKAGQANPSPPVGPALGQRGINIMEFCKAFNAKTQDMEPGAPCPTVITYYQDKSFSMDIKTPPASY
YL
KKAAGLKPIGKRNRPKGSEKPGRETAGTVTVAQVREIAEAKMKDLNASDVEGAMQIILGSARSMGIEVKG
>OLS47464.1 50S ribosomal protein L1 [Rhodovulum sulfidophilum]AB14
MAKHGKRVRAAREAFAGKDNLSVEEAVSLVKENASAKFDETLEIALNLGVDPRHADQMVRGVVTLPNGTGKTVRVAV
FAR
GAKADEAQAAGADIVGAEDLMETIQGGAIEFDRCIATPDMMPIVGRLGKILGPRNLMPNPKVGTVTMDVTSAVQAA
KGGE
VQFRVEKAGVIHAGVGKTSFDAAKLAENVRAFIDAVVKAKPAGAKGTYMKKVSLSSTMGPGVSIDVTSATGN
>OLS47465.1 50S ribosomal protein L10 [Rhodovulum sulfidophilum]AB14
MDRAQKEKVVEELGQIFESSGVVVVAHYAGLTVAEMQDLRARMREAGGSVRVAKNRLAKIALEGKPCASLATLLTGM
TVL
TYSEDPVAAAKVAEGFAKDNSKFEILGGAMGETALDRAGVKAVSEMPSREELIASIAGCIGAPAANIAGAIGAPASNIAS
ILSTIEERAEAA
>OLS47466.1 50S ribosomal protein L7/L12 [Rhodovulum sulfidophilum]AB14
MADLKKLAEEIVNLTLLEAQELKTILKDEYGIEPAAGGAVMMAGPAAGGEAAAAEEQTEFDVILKSAGANKINVIKEVRA
ITGLGLKEAKDLVEAGGKAVKEGVDKAEAEDIKGKLEAAGAEVELK
>OLS47467.1 DNA-directed RNA polymerase subunit beta [Rhodovulum sulfidophilum]AB14
MAQSYVGHKRLRKYYGKIHEVLEMPNLIEVQKSSYDLFLNSGDGPAPADGEGIQGVFQSVFPIKDFNETAVLEFVRYELE
KPKYDVEECQQRDMTFSAPLKVTLRLIVFDVDEDTGAKSVKDIKEQDVFMGDMPLMTQNGTFIVNGTERVIVSQMHR
SPG
VFFDHDKGKTHSSGKLLFACRIIPYRGSWLDFEFDAKDIVFARIDRRRKLPVTTLLYALGLDQEGIMDAYYDTVSFRLER
NRGWVTRFFPERVRGTRPTYDLVDADTGEVICKAGDKVTPRQVKKLIEEGRVTELMVPFDHIVGRYVAKDIINEETGAIY
VEAGDELTWIVDKDGEVTGGSLKELLDAGITDIPVLDIDNINVGPYIRNTMAADKNMGRDTALMDIYRVMRPGEPPTV
EA
ATNLFESLFFDSERYDLSAVGRVKMNMRLNLDAPDTQRTLRREDIISCIKALVELRDGKGEVDDIDHLGNRRVRSVGEL
M
ENQYRVGLLRMERAIKERMSSVEIDTVMPQDLINAKPAAAAVREFFGSSQLSQFMDQTNPLSEVTHKRRLSALGPGGL
TR
ERAGFEVRDVHPTHYGRMCPIETPEGPNIGLINSLATYARVNKYGFIETPYRRVEDGQVTDDVHYMSATEEMRHTVAQ
AN
ANLDGEGKFINDLVNTRQAGEYTMAPRESVDLIDVSPKQLVSVGASLIPFLENDDANRALMGANMQRQAVPLLQADA
PFV
GTGIEQVVAQDSGAAIMARRGGIIDQVDAQRIVIRATEDLEPGDPGVDIYRLRKFQRSNQNTCINQRPLVKVGDTVQK
GE
VVADGPSTDMGELALGKNVIVAFMPWNGYNYEDSILISERIARDDVFTSIHIEEFEVAARDTKLGPEEITRDIPNVGEEA
LRNLDEAGIVYIGAEVGPGDILVGKITPKGESPMTPEEKLLRAIFGEKASDVRDTSLRLPPGDYGTVVEVRVFNRHGVDK
DERALQIEREEVERLARDRDDEQVILERNIYARLKSMVLGRVAVKGPKGVKPNSEITEELLETLSKGQWWQLALEDEAD
A
GQVEALNAQFEAQKRALEHRFEDKVEKVRRGDDLPPGVMKMVKVFIAVKRKLQPGDKMAGRHGNKGVVSRVVPME
DMPFL
ADGTPVDLVMNPLGVPSRMNVGQILETHMGWAARGLGQQVEEALHEYKRSGDLTPVRDAMHHAYGDDVYDEAIR
DMDEAH
LVESASSVTRGVPIATPVFDGAKEQDINDALIRAGFDTSGQSELFDGRTGERFARKVTVGVKYLLKLHHLVDDKIHARST
GPYSLVTQQPLGGKAQFGGQRFGEMEVWALEAYGAAYTLQEMLTVKSDDVAGRTKVYESIVKGEDNFEAGVPESFN
VLVK
EVRGLGLNMELLDAEDEE

>OLS47468.1 DNA-directed RNA polymerase subunit beta' [Rhodovulum sulfidophilum]AB14
MNQELTTNPFNPLASPKQFDEIKVSLASPERILSWSYGEIKKPETINYRTFKPERDGLFCARIFGPIKDYECLCGKYKRM
KYRGVVCEKCGVEVTLQKVRRERMGHIELAAPVAHIWFLKSLPSRIGLMLDMTLRDLERVLYFENYVVIEPGLTDLSYGQ
MLTEEEYLDAQDQFGTDAFTAGIGAEAIREMLSQIDLEAEAERLREELKEATGELKPKKIIKRLKVVESFIESGNRPEWM
VLTVIPVIPPELRPLVPLDGGRFATSDLNDLYRRVINRNNRLKRLIELRAPDIIVRNEKRMLQESVDALFDNGRRGRVIT
GANKRPLKSLSDMLKGKQGRFRQNLLGKRVDFSGRSVIVTGPELKLHQCGLPKKMALELFKPFIYSRLEAKGLSSTVKQA
KKLVEKERPEVWDILDEVIREHPVMLNRAPTLHRLGIQAFEPTLIEGKAIQLHPLVCSAFNADFDGDQMAVHVPLSLEA
Q
LEARVLMMSTNNVLSPANGAPIIVPSQDMILGLYYITMERKGMKGEGMAFADVEEVQHALDAGEVHLHAKIIARIKQI
DE
HGNEVLQRFETTPGRVRLGALLPMNAKAPFELVNRLLRKKEVQQVIDTVYRYCGQKESVIFCDQIMGLGFREAFKAGIS
F
GKDDMVIPERKWTIVEGVRQQVKEFEQQYMDGLITQGEKYNKVVDAWSKCSDEVAAAMMSEISAVRVDDAGAEM
EPNSVY
MMAHSGARGSPAQMKQLGGMRGLMAKPSGEIIETPIISNFKEGLTVLEYFNSTHGARKGLADTALKTANSGYLTRRLV
DV
AQDCIVRRDDCGTENAITCEAAINDGEVVATLAERLLGRVAADDVLKPGTDEVLVTRNELIDERKADAIEKAGVATVRIR
SPLTCEAEEGVCAACYGRDLARGTKVNIGEAVGIIAAQSIGEPGTQLTMRTFHIGGIAQGGQQSFQETSQEGRIEFRNA
N
LLTNAAGDQIVMGRNMQLAIIDDNDVERASFKLGYGTKVFVVEGARVARGDKLFEWDPYTLPIIAEKAGRARLVDLVT
GI
SVREDTDDATGMTQKIVSDWRTAPKGNELKPEIIIVDPETGEPVRNEAGNPVTYPMSVDAILSVEDQQEIRAGDVVARI
P
REGAKTKDITGGLPRVAELFEARRPKDHAIIAEIDGYVRFGRDYKNKRRITIEPADETIQPVEYMVPKGKHIPVQEGDFV
QKGDYIMDGNPAPHDILAIMGVEALANYMIDEVQDVYRLQGVKINDKHIEVIVRQMLQKWEISDSGDTTLLKGEHVD
KAE
FDEANDKVLARGGRPAEGQPILLGITKASLQTRSFISAASFQETTRVLTEASVQGKRDKLVGLKENVIVGRLIPAGTGGA
SQRVRRIAAERDQKVIDARQAEAVSAAALAAPMEDFGASDDDDLGLVETPESRE
>OLS47469.1 hypothetical protein BV379_03690 [Rhodovulum sulfidophilum]AB14
MQSKPFEKEWRKCAKIGAGGASGARPAALATQGVFILPLSDAGPVLEVLGVAHGPTRPAALLIGPVRHARSALRVTALA
D
RRRGVFGMSGDMTTILQVIGSSGTWWDRQSMVARSRRVGPHARVAADFVILRDIDTRGFNGVAVWLCRRTPVLGM
SPGEM
ARVAARGLAAPVACASATGDAVPGEASSGAGLSGQTGLGAVIAAAWPLWC
>OLS47470.1 30S ribosomal protein S12 [Rhodovulum sulfidophilum]AB14
MPTIQQLIRKPRQPKVKRQKSQHLQACPQKRGVCTRVYTTTPKKPNSAMRKVAKVRLTNGFEVISYIPGESHNLQEHSV
V
LIRGGRVKDLPGVRYHILRGVLDTQGVKDRKQRRSKYGAKRPK
>OLS47471.1 30S ribosomal protein S7 [Rhodovulum sulfidophilum]AB14
MSRRHAAEKREILPDAKFGDRVLTKFMNNLMVDGKKSAAESIVYNALDRVEGRLKRAPVEVFHEALDNVKPAVEVRSR
RV
GGATYQVPVEVRPERREALAIRWLIDASRKRNENTMEERLAGELMDAVQSRGTAVKKREDTHKMADANKAFSHYRW
>OLS47472.1 translation elongation factor G [Rhodovulum sulfidophilum]AB14
MARAYPLQRYRNFGIMAHIDAGKTTTTERILFYTGKSHKLGEVHDGAATMDWMEQEQERGITITSAATTTFWQRQED
PTA
EGTSDTKYRFNIIDTPGHVDFTIEVERSLAVLDGAICLLDANAGVEPQTETVWRQADRYKVPRIVFVNKMDKIGADFFNC
VKMIKDRTGATPCPIALPIGAEDKLEGIIDLVSMEEWTWKGEDLGASWTRQEIRAELKDVADEWRSNLVELAVEQDDE
AM

EAYLEGNEPDTDTLHQLIRKGTLALAFVPVTAGSSFKNKGVQPLLNAVIDYLPSPLDVPAYMGFAPDDETEERNIARHAN
DDEAFSALAFKIMNDPFVGSLTFTRIYSGVMRKGESILNATKGKKERVGRMMMMHSNNREEIDEAFAGDIIALAGLKE
TT
TGDTLCDASKPVVLETMSFPDPVIEIAVEPKSKADQEKMALALQRLAAEDPSFRVETNIESGQTIMKGMGELHLDILVDR
MKREFKVEANIGAPQVAYRETISHKADVDYTHKKQTGGTGQFARVKLEIMPTEPGEGYSFESRIVGGAVPKEYIPGVEK
G
IKSVMDSGPLAGFPVIDLKVALVDGAFHDVDSSVLAFEIATRAGMREGLKKAGAKLLEPIMKVEVVTPEEYTGSIIGDLT
SRRGMVQGQDTRGNANVISAMVPLANMFGYINTLRSMSSGRAVFTMQFDHYEPVPQNISDEIQAKYA
>OLS47473.1 translation elongation factor Tu [Rhodovulum sulfidophilum]AB14
MGKAKFERTKPHVNIGTIGHVDHGKTTLTAAITKYFGDFRAYDQIDGAPEEKARGITISTAHVEYETEARHYAHVDCPGH
ADYVKNMITGAAQMDGAILVVNAADGPMPQTREHILLARQVGVPALVVFMNKVDQVDDAELLELVEMEIRELLSSYD
FPG
DDIPIIAGSALAAMEGRDPEIGENKIRELMAAVDEFIPTPERPVDLPFLMPIEDVFSISGRGTVVTGRVERGVVNVGDEL
EIVGIRDTKKTTCTGVEMFRKLLDRGEAGDNIGALLRGIDRDQVERGQVLCKPGSVTPHTVFEAEAYILTKEEGGRHTPF
FANYRPQFYFRTTDVTGTVELPEGTEMVMPGDNLKFKVELIAPIAMEEKLRFAIREGGRTVGAGVVAKIIK
>OLS47474.1 30S ribosomal protein S10 [Rhodovulum sulfidophilum]AB14
MQSQNIRIRLKAFDYRVLDASTQEIVNTAKRTGAQVRGPIPLPNKIEKFTVLRGPHIDKKSRDQWEIRTHKRLLDIIDPT
PQTVDALMKLDLAAGVDVEIKV
>OLS47475.1 50S ribosomal protein L3 [Rhodovulum sulfidophilum]AB14
MRSGVIAKKLGMTRLFMDDGRQVPVTVLQLDGCQVVAQRTVETDGYTAVQLGSGSAKAKRVSAAMRGHFAKASVA
PKRKV
AEFRVAPENLIGVGEEITADHYFEGQFVDVSGTSIGKGFAGAMKRHNFGGLRASHGVSISHRSHGSTGQCQDPGRVFK
GK
KMAGHMGAARVTTQNLQVVKTDADRGLIMIKGAVPGAKGGWVTIKDAVKKATPDNLVLPAALKSAQAEATAAEAP
AEGGE
E
>OLS47476.1 50S ribosomal protein L4 [Rhodovulum sulfidophilum]AB14
MKLDVIKLDGAKAGTVDLGDEIFGLEPRTDILHRVVRWQRARTQAGTHKVKTRSETSYSTKKIYRQKGTGGARHGDRN
AP
IFRKGGVYKGPTPRSHAHDLPKKFRKLGLKHALSAKLKAGELIVLETADMAAPKTSELAKSVKNLGWKRVLIIDGAEVNE
NFAMAARNIEGLDVLPSMGANVYDILRRDTLVLTKAGVEALEARLK
>OLS47477.1 50S ribosomal protein L23 [Rhodovulum sulfidophilum]AB14
MSVKPEHYDVIRKPIITEKATMASEANAVVFEVAMDANKPEIKEAVENLFGVKVKAVNTTITKGKVKRFKGILGRRKNVK
KAYVTLEEGNTIDVTTGL
>OLS47478.1 50S ribosomal protein L2 [Rhodovulum sulfidophilum]AB14
MALKSYKPTTPGQRGLVLIDRSELWKGRPVKTLTEGLTKSGGRNNAGRITARRRGGGAKRLYRIVDFKRTKLDVGGAVE
R
IEYDPNRTAFIALIRYDDGELSYILAPQRLAVGDKVVASKKADVKPGNAMPFTGLPIGTIVHNVELKPGKGGQIARAAGT
YAQFVGRDGGYAQIRLSSGELRLVRQECMATVGAVSNPDNSNQNIGKAGRNRHLGKRPSVRGVVMNPIDHPHGGGE
GRTS
GGRHPVTPWGKPTKGAKTRRNKTTDKYILRSRHARKKGR
>OLS47479.1 30S ribosomal protein S19 [Rhodovulum sulfidophilum]AB14
MSRSVWKGPFVDSYVLKKAEKARESGRNEVIKIWSRRSTILPQFVGLTFGVYNGHKHIPVSVSEDMIGQKFGEYSPTRTY
YGHAADKKAKKR
>OLS47480.1 50S ribosomal protein L22 [Rhodovulum sulfidophilum]AB14
MGKAKNPRRVADNEAMAKVRMLRTSPQKLNLVAAMIRGKKVDRALADLTFSKKRISEDVKKCLQSAIANAEFNHRLDI
DE
LIVAEAYVGKNLTLKRGRPRARGRFGKIMKPFSELTIKVRQVEEQA

>OLS47481.1 30S ribosomal protein S3 [Rhodovulum sulfidophilum]AB14
MGHKVNPIGMRLQVNRTWDSRWYADTKDYGDLLLEDVKMRDFIRAECKQAGVSRIIIERPHKKCRVTIHTARPGVIIG
KK
GADIETLRKKLANMTKSELHLNIVEVRKPELDGQLVAESIAQQLERRVSFRRAMKRSVQNAMRMGSLGIRVNVSGRLG
GA
EIARTEWYREGRVPLHTLRADIDYATAEATTPYGIIGIKVWIFKGEIMEHDPSARDRRQAELQEGGSGPSRPRR
>OLS47482.1 50S ribosomal protein L16 [Rhodovulum sulfidophilum]AB14
MLQPKRTKFRKMHKGRIHGQAKGGFELNFGHFGLKATEPERVTARQIEAARRALTRHMKRQGRVWIRIFPDTPVSSKP
TE
VRMGKGKGSVDFWAAKIKPGRIMFEIDGVNEAIAREALRLAAMKLPIKTRIVEREDW
>OLS47483.1 50S ribosomal protein L29 [Rhodovulum sulfidophilum]AB14
MNAQELRDKTPDQLREALASLKKEAFNLRFQQATNALENTARFREVRRDVARVKTVLNEKAAKASE
>OLS47484.1 30S ribosomal protein S17 [Rhodovulum sulfidophilum]AB14
MPKRILQGTVTSDLNDQTVTVLVERRFTHPVLKKTIRKSKKYRAHDENNTFKVGDRVRIQECAPKSKTKRWEVVQA
>OLS47485.1 50S ribosomal protein L14 [Rhodovulum sulfidophilum]AB14
MIQMQTNLDVADNSGARRVQCIKVLGGSKRKYASVGDIIVVSVKEAIPRGRVKKGDVRKAVVVRTAKEVRREDGTAIR
FD
RNAAVILNNNNEPVGTRIFGPVVRELRAKSFMKIISLAPEVL
>OLS47486.1 50S ribosomal protein L24 [Rhodovulum sulfidophilum]AB14
MAAKLKKDDKVVVLAGKDKGREGTILSVDPKAGKAVVEGLNMAVRHQRQTQNSQGGRVPKAMPIDLSNLAIVDPKE
GGPT
RVGFRVEDGKKVRFAKKSGEVIDA
>OLS47487.1 50S ribosomal protein L5 [Rhodovulum sulfidophilum]AB14
MLDAATYTPRLKSLYRETIRAALKEEFGYKNDMMIPRLDKIVLNIGCGAEAVRDSKKSKSAQEDLSQIAGQKAVVTKAKK
SIAGFRVREDMPLGTKVTLRGDRMYEFLDRLITIAMPRIRDFRGVSGKAFDGRGNYAMGLKEHIVFPEIEYDKVDEVWG
M
DIVIATTASTDAEAKALLKHFNMPFNS
>OLS47488.1 30S ribosomal protein S14 [Rhodovulum sulfidophilum]AB14
MAKKSMVQRELKRQRLVEKYATKRAKLKEIATNEDLPMEERFKARLKLAKLPRNSSATRLHNRCQLTGRPHAYYRKLKIS
RIMLRDLGSMGQIPGMVKSSW
>OLS47489.1 50S ribosomal protein L6 [Rhodovulum sulfidophilum]AB14
MSRIGKKPVELPSGVSASVSGQTVEVKGPKGTRIFTATDDVTIAVEDNTVSITPRGTSKRARQQWGMSRSMVANLVKG
VS
EGFKRELEIQGVGYRAAMQGNVLKLNLGYSHDVNFEVPQGVTVTAPKPTEIVVEGIDQQQVGQVAANIREWRGPEPY
KGK
GIRYKGEFIFRKEGKKK
>OLS47490.1 50S ribosomal protein L18 [Rhodovulum sulfidophilum]AB14
MANSKRHLFLKRRLRVRNKLRKMNAGRLRLSVHRSNKNISVQLIDDVQGTTLAAASSLEKELGVIGKNNVEAAAKVGSV
I
AERAKKAGVEECYFDRGGFLFHGSVKALADAAREGGLKF
>OLS47491.1 30S ribosomal protein S5 [Rhodovulum sulfidophilum]AB14
MAEREHRRDRRDRDENPEFADRLVAINRVSKTVKGGKRFGFAALVVVGDQKGRVGFGKGKAKEVPEAIRKATEQAKR
SLM
RVPLREGRTLHHDIEGRHGAGRVVMRTAPQGTGIIAGGPMRAVFEMLGVQDVVAKSVGSQNPYNMIRATLDGLSRE
SSPR
QVAQRRGKKVAEILKKDDAPVAEAAEA
>OLS47492.1 50S ribosomal protein L30 [Rhodovulum sulfidophilum]AB14
MAKTIVVKQIGSPIRRPDIQRRTLIGLGLNKMHKTRELEDTPAIRGMVSKIPHLVEIIEERG

>OLS47493.1 50S ribosomal protein L15 [Rhodovulum sulfidophilum]AB14
MKLNELRDNDGAAKKAKRIGRGPGSGKGKMGGRGIKGQKSRSGVAINGYEGGQMPLYQRLPKRGFNKPNRKSYAVV
NLGL
IQKFVEAGKLDASAPITEDALLASGLVRRKLDGVRVLAKGEVTSKLLIEVTGASKAAIEAVEKAGGSLKVATVAAEAA
>OLS47494.1 preprotein translocase subunit SecY [Rhodovulum sulfidophilum]AB14
MASAAEQMAANMSWGAFGKATELRQRILFTLGLLIVYRLGTYIPVPGIDGAALRDFMERASTGLGGILSMFTGGALGR
MG
IFALGIMPYISASIIVQLLAAMVPQLEALKKEGEQGRKKINQYTRYGTVFLAAFQAYGLAVSLEAGSLATDPGWFFRAST
VITIVGGTMFLMWLGEQITARGVGNGISLIIFVGIIAQAPAALAQFLSQGRSGAIHPGVIIGVLIMVVAVIAFVVFMERA
LRKIHIQYPRRQVGMKMYDGGSSHLPVKVNPAGVIPAIFASSLLLLPTTISTFSGAQTGPVMSVILAYFGPGQPLYLLFF
SAMIVFFTYFYTFNVAFKTEDVADNLKNQNGFVPGIRPGKRTQDYLDYVVNRVLVLGSAYLALVCLLPEILRSQLSIPFY
FGGTSVLIVVSVTMDTIQQIQSHLLAHQYEGLIEKSQLRRKRKGARKGTARR
>OLS47495.1 adenylate kinase [Rhodovulum sulfidophilum]AB14
MNIILLGPPGAGKGTQARRLVEERGMTQLSTGDMLREARSSGTEMGRKVAEVMDRGELVTDEIVIGLIAEKLAGDAG
GGF
IFDGFPRTLGQADALGALLDRMGQKLDAVIEMRVDDEALVARITARSTCGNCGEVYNDLTKPIPADGKCSKCGGTEFKR
R
ADDNEDSLRQRLMEYYKKTSPLIGYYYAKGDLTGVDGLGEIDEVSGAIKAVLGA
>OLS47496.1 30S ribosomal protein S13 [Rhodovulum sulfidophilum]AB14
MARIAGVNIPTGKRVPIALTYIHGIGHSAAEAICEAVGIEKGRRVNELSDAEVLSIREHIDANLTVEGDLRREIQMNIKR
LMDLGCYRGLRHRRNLPVRGQRTHTNARTRKGPAKPIAGKKK
>OLS47497.1 30S ribosomal protein S11 [Rhodovulum sulfidophilum]AB14
MARDKTRVRRKERKNIAAGVAHVNSSFNNTKILISDVQGNAISWSSAGTMGFKGSRKSTPYAAQMAAEDAGRKAQE
HGVK
TLEVEVQGPGSGRESALRALAAVGFNITSIRDVTPIAHNGCRPPKRRRV
>OLS47498.1 DNA-directed RNA polymerase subunit alpha [Rhodovulum sulfidophilum]AB14
MIHKNWQELIKPTQLEIKPGNDPARQATVVAEPLERGFGLTLGNALRRVLMSSLQGAAITSVQIDNVLHEFSSVAGVRE
D
VTDIVLNLKGVALRMDVEGPKRLSISAKGPGVVTAGEISESAGIEVLNREHVICHLDEGADLFMELTVNTGKGYVAADK
N
RPEDAPIGLIPIDAIYSPVKKVAYDVQPTREGQVLDYDKLVLKIDTDGSVSPDDAVAFAARILQDQLSIFVNFDEPESAS
READDDGLEFNPLLLKKVDELELSVRSANCLKNDNIVYIGDLIQKTEAEMLRTPNFGRKSLNEIKEVLSGMGLHLGMDVE
EWPPENIEELAKKFEDQF
>OLS47499.1 50S ribosomal protein L17 [Rhodovulum sulfidophilum]AB14
MRHARGYRRLNRTHEHRKALFANMAGSLIEHEQIKTTLPKAKELKRIADKLITLGKRGDLHARRQAASRLKQDAHVAKL
F
DVLGPRYAERQGGYVRVLKAGFRYGDMAPMAIVEFVDRDVDAKGAADRSRLEAEDSVED
>OLS47500.1 hypothetical protein BV379_03850 [Rhodovulum sulfidophilum]AB14
MHPILFNRVMRLPSSLRSEVLEYAGAASLADDQIKSLLDDICDMIEKEGQTGTGTCLPERLYAA
>OLS47501.1 LuxR family transcriptional regulator [Rhodovulum sulfidophilum]AB14
MFEKSIFDRELRILDALAPQGYNIGLHIRFTSPLIAFQTYAAEWLAHYTEHGYLLRDPTVAWGFSTTGATRWSNDRIPDP
FGIFLNAARFGLKYGATISWGPIASRTIASVARADREFEDAEIAQVEALVRRLHDMTEPPQELTKAQIEALKCIADGDRH
AAAACKLGISESALKARLSSARQRLMARTTAEAIQRAKDYRLL
>OLS47502.1 N-acyl-L-homoserine lactone synthetase [Rhodovulum sulfidophilum]AB14
MQTTTLSFENIHNHGELFANMLRARRDLFIERNKWDLPEALGMEYDQYDTPASRWVVVHDELGHVLAGNRLTPTTAR
CGI
YSYMIRDAQKGLLDTIPSELLYDEAPVGETVWESSRGFVTQKVPLHQRRPIYFNLVQELARAARSLGASQCLALLNANW
P

LWNKRVGIDMTAMGPVMEIDGIRNQVISMNFATNLH
>OLS47503.1 serine protease [Rhodovulum sulfidophilum]AB14
MPRLATLLCLLLIAGPLAAENRLPESRAEIQLSFSPVVREAAPAVVNVFTSRVVADRPTPFAADPFFSDLFRNFGRAQPR
VENALGSGVIVSPDGMVVSNYHVVGRADQIRVVLADRREFEAEVLLADEESDLAVLRLEGAHDLPSLPLRDSDEIEVGDL
VLAIGNPFGIGQTVSSGIVSGLARSGLSIGGGRGYFIQTDAAINPGNSGGALVDMAGRLVGINTAILSRSGGSNGIGFAI
PANLVSRFIEQAEAGARRFQRPWAGLSGQPVDAALAEALGMDSPRGVVLNALHPESPFRAAGLGVGDVVLALGGDP
VNTP
QEMLFRLSAEGIGRQIEVSYRHRGAARPQTASVDLVAPPETPPRDPLTIRSQSVLAGLSVVNLNPAVVQENALPAGLSG
V
LVTDPGAIGGRGGLAPGDILLQINDREIRSTADVAAAARKGGRRWYVEYLRNGRRGVLRFRV
>OLS47504.1 AAA family ATPase [Rhodovulum sulfidophilum]AB14
MTDLFDRTPSAASDRAGPRPLADRLRPRRLAEVIGQEHVLGPEAPLGVMLAAGTLSSLIFWGPPGVGKTTIARLLADET
D
LAFVQISAIFSGVAELKKVFEAARLRHANGQGTLLFVDEIHRFNKAQQDGFLPHMEDGTILLVGATTENPSFELNAALLS
RSQVLVLERLDPAHLELLAQRAEHELGRPLPLTGEARDDLISMADGDGRALLNLIEQLMAWKVTGPLAPADLARRLQKR
A
AQYDKSGDGHYNLISALHKSVRGSDPDAALYWLTRMLEGGEDPRFLARRIVRMSVEDIGLADPQAQTVCLDAWSVYE
RLG
SPEGELALAQAVVYLALAPKSNAGYSAYKAARQAAQKTGSEPPPRHILNAPTQLMKEQGFGAGYQYDHDAEDAFSGQ
NYF
PESMKRGVYYTPVERGFERELKRRLDYFSKLRERRQDG
>OLS47505.1 protein CrcB [Rhodovulum sulfidophilum]AB14
MTGATLLQVALGGAIGSVGRYLSGVAATSLLGRGFPWGTIFVNVLGSFLMGVLIVVLAKKGGTSFAPFFQIGLLGGFTTF
SSFSLDAMTLFERGQAELAIAYVAGSLILSLSAIALAFWSMRSLLA
>OLS47506.1 RNA pseudouridine synthase [Rhodovulum sulfidophilum]AB14
MSGVQTLTVARGDGDQRLDRWFRRLFPHVSQGRIEKLCRKGEIRVDGGRVKPATRLEEGQQVRVPPLPDPGEIEAAPK
PK
VSEADAEMIRSCVIFRDAHVIALNKPPGLPVQGGSGQARHVDGMGEALRFGYDENPRLVHRLDRDTSGVLILARTRAV
AR
DLTAAFRARETRKIYWAVVAGVPHPRMGTIRFGLVKAPGHGSKGEGEKMLCIHPAKVDQTEGAKRATSDYAVLSALGT
RA
SWCALVPVTGRTHQLRAHMAEIGHPIVGDGKYGGSGQENLGEGWGAQLGGEISRKLHLHARHLSLTHPVTGAQLKLT
APL
PPHMKRTWDMLGWSEADVPLDPFEDLE
>OLS47507.1 HAD family hydrolase [Rhodovulum sulfidophilum]AB14
MTARRLAIFDVDGTLIDSQAHIAGAMEAAFAAEGLPVPAHAEVLGVVGLSLPLALEALAPDAPAERLDRMVEAYRQAF
FS
LRVGQAASPLFPGAAEALDALLAVDGMELGIATGKSRRGLDAILASHELGGYFATRQVSDDHPSKPHPSMVLAALAET
GV
APERAVMIGDTEFDMEMGRAAGVRTLAVAWGYHPAERLGAADAVVERFADLPAAVLALVGETA
>OLS47508.1 ATPase [Rhodovulum sulfidophilum]AB14
MSEWALKRFWTEAGVAPAEVGFAVLLDGRPVKTPAKRPLAVPTRAMAEAVAAEWDAQEDQVAPLSMPVTRAANA
AIDKVM
EQRAEVAELIAAYGESDLLCHRAESPAELVRRQAESWDPLLAWAASTLGAPLEPTPGVIPRPQPSESLSRLRSRVAGLDA
FELTALHDLVGLSGSLVIGLAAAEGLEPPERLWELSRIDETWQQEQWGLDDEAAEVAAARQRDFLQARHFLDLARASL
>OLS47509.1 amino acid ABC transporter substrate-binding protein [Rhodovulum sulfidophilum]AB14
MKKSIIPGALTMVACAAASAGAATLDDVKARGKLNCGTNTGLVGFAAPDANGDWQGFDVAVCRAVAAAVLGDSSA
VEFVP

TTGKTRFTALASGEIDMLVRNTTWTFSRDVDLKFTFVGVNYYDGQGFMVPKALGVSSAKELDGATVCIQTGTTTELNL
AD
FFRVNNISYEPVPIETNEQAQQQYLAGACDVYTTDASGLAATRATFEDPSAHVVLPEIISKEPLGPLVRHGDDEWADIVR
WTLNALIAAEEYGVTSANIDELTATVTTNPEVNRLLGTEGALGGMLGLSPDWAVKAIKAGGNYGELFEKYIGQNTPIGL
A
RGLNAQWTDGGLMYAPPFR
>OLS47510.1 amino acid ABC transporter permease [Rhodovulum sulfidophilum]AB14
MTSHPDLPRETFRLRMLIFDTRYRSLTIQIVTLAGFMLLVSWMISNTVQNLAELGKPIEFGFLGNQAQYDINQTLIAYTS
RDTHLRAAVVGLLNTLLVAFLGCALATVMGVAIGVLRLSRNWLVARLMTVYVELFRNVPVLLWILFFMAILIESLPPPNA
FRGEDATASMLLGDSVALTNRGFYIPEPLFSRGLGDLSVFGAFEISLDLVAFLAVLAAGLWVSARIRARADRIQNRTGLR
PTTWYLRLGVVVLPLLILLAALGFHLGYPELKGFNFRGGIYMRNSLIALWLALSFYTAAFIAEIVRGGILAISKGQTEAA
AALGLRPNRIMSLVILPQALRVIIPPLISQYLNLTKNSSLAIAVGYMDITGTLGGITMGQTGRELECVLLMMLFYLAISL
VISTAMNWYDARVRLVER
>OLS47511.1 amino acid ABC transporter permease [Rhodovulum sulfidophilum]AB14
MSDAPSHDTAFVRDAMLPPAPAPASSAGPLGWLRRNLFSSWLNAALTLLSLALVAWLLAAVLPWIVHSSWTASSLSEC
RA
QIKEAYGPEASGACWAVIRERWAQLIFGFYPSELYWRPILAFGIALGGMAPVLFPGVPRKVLYLSVAAPFLVPWLLWGG
T
VWLPVAVALGFVLGYAAFRLVTPFGGAILGTIAAALVPVMWWFWLAGPASGQLQAVLPLGIEPVSSRSFGGFMLSITI
GV
TAITLSLPFGILLALGRQSRMLIVKSICVGVIEFVRGVPLITLLFVAWLLLNYFMPPDTNFDIVLRVIIMVTLFASAYVA
EVIRGGLAALPRGQYEAADALGLDYWKAQRLIIMPQALKISIPGIVSTFIGVFKDTTLVSIIGLLDPVGFSNSIRADVNW
NGIVWELYGFIAALFFVFCFSMSRYSMYLERRLRTEHR
>OLS47512.1 ABC transporter ATP-binding protein [Rhodovulum sulfidophilum]AB14
MSDPVIDPSRPTVSEEVAIDILGMNKWYGAFHVLRDINLTIYKGERIVVCGPSGSGKSTLIRCINRLEDHQAGRIVVEGI
ELTSDLKHIDQVRSEVGMVFQQFNLFPHLTILENCTLAPIWVRKVPRREAVDIAMSYLEKVRIPEQANKYPGQLSGGQQ
Q
RVAIARALCMRPNIMLFDEPTSALDPEMIKEVLDTMIALAEEGMTMICVTHEMGFARQVANRVIFMDQGQIVEQNAP
EAF
FNAPRSDRTRLFLSQILGH
>OLS47513.1 ferredoxin [Rhodovulum sulfidophilum]AB14
MAIITFTSPQMHKDKTVYGVAGDTKTILSLAEDNRIPIPFDCRDGNCGSCLIEVSYDNPADKKAIMLTEKEKQKLKELGK
LTQQEIDDAEVRDMPPRYRLACQFIARDEDVTVSFSGMPGGSD
>OLS47514.1 acetylglutamate kinase [Rhodovulum sulfidophilum]AB14
MNRDWIATARTLSAALPYLQRYTGAIVVIKFGGHAMGDDAEMESFARDVVLMRQVGVNPVIVHGGGPMINEMLAK
LGVVS
EFVKGKRVTDAATVEVVEMVLSGRVNKRIAQAINDQGGKAVGLSGKDAGLIVCDQADPSLGFVGAPTEVNPEVLHQLF
AA
GMIPVVAPLGAGRAGETFNINGDTAAGAIAAALKADRLLLLTDVSGVKDASGRVMTELTPDNIREMTASGVIAGGMIP
KT
ETALSAIEGGVRAVVILDGRAPNACLLELFTDHGAGSLIRAPRP
>OLS47515.1 oxidoreductase [Rhodovulum sulfidophilum]AB14
MEQRVMLLTGASRGIGHATVKRFSAEGWRVLTCSRHPFDSRCPWPGGAENHVQIDLADPAMTKRAIEVVRGKLTGG
KLHA
LVNNAGISPKTEEGGRLNTLETDMRTWGQVFNVNFFASIVLARGLQDELAAARGAIVNVTSIAGSRVHPFAGAAYSTSK
A
ALAALTREMAHDFGPLGVRVNAIAPGEVETSILSPGTEKIVEGLPLRRLGQPEEVASVIHFLCSEEASYVTGTEIEVNAG
QHV

>OLS47516.1 YihA family ribosome biogenesis GTP-binding protein [Rhodovulum sulfidophilum]AB14
MTPLPFPLTPEPDAEAAERGRKLFAGPVEFLKGVVAMDGLPPADRPEICFAGRSNVGKSSLINALTGRKALARASNTPG
R
TQEINYFTLGESHYLVDLPGYGYAEAPLKVVEKWQRLLKAYLAGRQTLRRAFVLIDARHGVKPVDDEIMKLLDGAAVTF
Q
TVLTKADKVKVAERNKVLDQVRASLTRHPAAFPEIILSSSEKGDGIATLRATVEGIA
>OLS47517.1 molybdenum cofactor biosysynthesis protein [Rhodovulum sulfidophilum]AB14
MTARLARIYRHPIKGHGAEALEAVDLQAGRTLPWDRTWAVAHEASRADGTGWAPCANFSRGAKAPQLMAIGARLDE
AAGQ
VTLTHPDRPELSFSPDSEAAALIDWVAPLMPADRAASTRIVRAPGRGMTDSAFPSVSLLGLASLEALSGQLGQALSPLRF
RGNFWIEGLAPFAEFDWVGRKIRLGRAVIEVRERITRCMATAANPETGERDADTLGALQAGWGHKQFGVYAEVIEGG
RVA
LGDALEAL
>OLS47518.1 membrane protein insertase YidC [Rhodovulum sulfidophilum]AB14
MDDQNKNLILATALSFLVILGWFLLFPPEQQAPVEEQDAVTTTEQTSSGATAPDLAVAPPAPQGSGAASTPETAAPSLA
A
EAPRLSIETPRLTGSISLLGGRIDRLSLKDYHETLDPDSPLVSLLEPVGTERPYYALYGWAPAGSLGYEDVPGANTLWTL
DGNDALSVDSPVSMHWNNGKGLTFHRTISIDENYMFTVEQSVENATGEAVRLAPYGIVARHGQPEGSRFYILHEGVV
RQT
DSELEEIKYKKMPDMDYVERERANAEVTDVQENGWIGFTDKYWMTTLIPEPGQPFTSVAKYVQGPDIYQVEARLPVV
DVA
AGATESTSTMLFAGAKEWETIHHYQKDDGIFRFIDSIDWGWFFFFTKPIFRVLHWLNGAMSGLGVPGSMGWSIILLTVI
I
KAIVLPLAYKSYSSMAKMKELQPKMEELKERCGDDRQKLQQEMMTLYKKEKVNPAAGCLPLFVQIPIFFSLYKVIFVTIE
LRHAPWIGWIHDLSAPDPSSILNLFGLLPYAPPEPGSFFAILSLGVLPILLGISMWLQQKLNPTPTDATQAMVFAWLPW
V
FMFMLGNFASGLVLYWICNNTITFIQQYVIMRIHGFHPDVLGNIRSSFRKKKPEPEHK
>OLS47519.1 tRNA 2-thiocytidine(32) synthetase TtcA [Rhodovulum sulfidophilum]AB14
MSDETDDIHPLFHGAPSTTEFKKLRKRIIRDTREAIEQYGMIERGARWLVCLSGGKDSYTLLAALYELKWRGLLPVELLA
CNLDQGQPGFPATVLPAFLERMGVPHRIEYQDTYSIVMDKVPQGRTYCALCSRLRRGNLYRIAREEGCSAVVLGHHRD
DI
LETFLLNLFHGGRLATMPPKLVNEEGDLFVYRPLAHVAEADCARFARAMDYPIIPCDLCGSQDGLQRMQVKAMLDQ
WETN
HPGRRQKMFRALMHVRPSHLLDPTLFDFAGLLRETGAPSDIPPGKTPGG
>OLS47520.1 membrane protein insertion efficiency factor YidD [Rhodovulum sulfidophilum]AB14
MSPLARLLALPIRAYRIVLSPWIGFNCRYQPTCSAYALEALETHGALRGGALAFWRILRCNPWGGCGYDPVPPKPDPKR
>OLS47521.1 50S ribosomal protein L34 [Rhodovulum sulfidophilum]AB14
MKRTYQPSNLVRKKRHGFRARMATKAGRKILNARRARGRKSLCA
>OLS47522.1 hypothetical protein BV379_03975 [Rhodovulum sulfidophilum]AB14
MARTRQQRPRRRGLARYLLLAAIAAVALIGAVTLNDRLSFEALAAHREALIALRDANYALAVLGFMLAYVLIVAFSLPGA
TVATLTGGFLFGLFPGVFFNVTAATLGALLIFLAARWGLGDRLSARLDAGEGRIAAFRDGLRENEIPVLFLIRLVPAVPF
FVANLLPAMVGVRAGRFVFTTFFGIMPGALVYTWVGAGLGEVFARGARPDLGIIFAPYVLGPLLGLAALAALPIAIRVAR
RKG
>OLS47523.1 dihydrolipoamide dehydrogenase [Rhodovulum sulfidophilum]AB14
MAPMKTDLCIIGGGAAGLSLAAGAAQMGARVVLVEGHRMGGDCLNYGCVPSKALLAAAARAQAMREGGCGIAGV
APKIDH
AAVMAHVRGAIAAIAPHDSVERFEGLGVRVIRDWARFVSPREIQAGGETIRARRFVIATGSRPAVPPVPGLDRLPYLTN
E

TLFELDARPDRLLILGGGPIGIEMAQAHRRLGAEVAVIEAKRALGREDPEAAALVLDRLRAEGVEIFEHAEVRRVAGRAG
AIAVETADGLLEGSHLLVAAGRHPALERLELPAAGVEADAAGVRVDSGLRSTNRRIYAIGDAAGQGQFTHLAGYHAGLV
I
RPILLGLPARVRRDHVPRVTYSDPELAQIGPTEAEARAAHGDAIEVVRADYAGNDRAVTGGRTAGFVKVVLRRGRPLG
AT
LVGAEAGELVGPWALAIASGQRMTAMAGMILPYPTLGELNKRVAGAYFSPKLFENPWLKRGVRAIQRLLP
>OLS47524.1 sensor histidine kinase [Rhodovulum sulfidophilum]AB14
MFLNSLSGRFLLLTIVFVMLAEVLIFVPSVARFRVDFLLARLERSQIASLALLASDGMIGDELERELLQNAGVYNVVLRR
DQARELALSSPIPEPVYRSFDLRDASVLALIPDALIRLVDPKTRVIRVIGQPVQRAGLLIEITMSTAELRKAMIDYGLRI
LVLSAVISVLSALLLFLAARRLLVVPIRHVVRAIKLYAEAPEDNRRIITPSARVGELYEAETALRVLQEELTGALRQKDR
LAQLGAAVAKISHDLRNILTTAQLFADRMEASDDPAVKRAAPKIVGAISRAVHLCESTLAFGRAQEPPPRLERVALAPIV
NEVVDGERLAAGDYDISFSEDVPAGLTVRADVEQLMRVLTNLVRNARQAILASGKPGEIGIRADETEQDWVIQVTDTG
PG
LPPKAREHLFQPFHGGTRKDGTGLGLAISMELIRGHGGALELSRSDAEGTAFEIRLPKAAAALPGEDGIPN
>OLS47525.1 hypothetical protein BV379_03995 [Rhodovulum sulfidophilum]AB14
MRRGIRGTTVTLTNLMPVRRGNRTDWYFRKKGQKLVRLPDLPHDHPDFLAAYAAARKAAEAATRPLRLPPGTLGALIE
AT
VHSDRFLSRSAVYRATLRRHFEAIRRDGGEAPAKGLRDRHIRANVSTAESPVDRRKAWRFLCAFGVDAGLLETDPSLTVT
LPRAPKTIGHPAWTAADIDAFRTRWPIGTSKRAAFELLHWTGFRIGDAVMIGPGHVDRAGVLIYTQSKVKQPAFVPWT
AR
LPAYAECYAGDRDAMHDALAALGSRHMTFLATAAGAPRSEKGLGNMIRAAAAEAGIEKSAHGLRKTRGVALAEAGAS
AHQ
IMSWLGHQTLKEAEHYTRQASRRRAVMGEEQEQNVATHAAQVATFPKKS
>OLS47526.1 hypothetical protein BV379_04000 [Rhodovulum sulfidophilum]AB14
MGAPRFTEAELRRAVNVARAAGADIEITRGTIRILAGPSRLPVSFEEIDGDEAQCDAAFGGPR
>OLS47527.1 DNA methyltransferase [Rhodovulum sulfidophilum]AB14
MADGSSLLFDFAAERPADPRPMIIDSFAGGGGASTGIEMALGRSPDVAINHSGPALALHQANHPETLHLNSNIWDAD
PLS
VTGGRHVGLLWASPDCKHFSKAKGAALCDRNIRDLAWVVVRWAEEARPDVICMENVEEFVTWGPVGEDGRPIREFA
GMTY
ELWVKRLRKAGYKVKWRELRACDYGAPTIRKRWFCVARRDGRPIVWPAPTHGDPASPAVRKGRLLPWRAAADCIDW
SLPC
PSIFDSSAEIMARHGLRAVRPLAKNTLARIARGMRRYVLDAERPFIVNLTHGARTEDLARPLRTITGANRGEKALVAPSL
TRFNGGATGADLRDPMPTVTANSWIKRPGGAMPLGIVTPHLLSLKGSARRDGAATAPHPTVLAGGGHSAVVAPVLTY
AQQ
GGGNRSAEAPHHTICASKKDQNSLLAATMIHVGNGERPGQAPRALDIGAPLNTVVAGGVKQYPAAAFLAQQNTGHV
GRDA
REPMSTILKEGCHQTPVAAWFAKYYGTGDGARMDAPCHTVTVKDRLGHMQADLAAPPFAPEHEARAREVAEFLRAH
GAWD
GGEFVTLEIDGQSFVVIDIGMRMLSPRELFNAQGFPPDYVIEGAWEGLDSDDPTFRPFSKDVQISCCGNSVCPPVAAAII
AANCPHLAASRQEQEAHDAV
>OLS47528.1 hypothetical protein BV379_04010 [Rhodovulum sulfidophilum]AB14
MSEEAQTDATYRVTAGELRQFIERFERLEAEKKDLADQQKEVMAEAKARGYDTGVMRKVIALRKRDKDDIAEEEAILE
MY
KDALGMG
>OLS47529.1 hypothetical protein BV379_04015 [Rhodovulum sulfidophilum]AB14
MGHGARGMTDLSARGPLGLKAPRVPDDPGHLARIRALPCCICAEWGLPQLSPTQAHHCIHGRFSARRAPDCMAIPLC
EGH

HQGLLDGSKLALHQAPSRWRRLYGNDTDWISWAEDRLREREEQTI
>OLS47530.1 hypothetical protein BV379_04020 [Rhodovulum sulfidophilum]AB14
MLMQNPPAILIQKTPSGLMPYGPHSGPMLDELVMGQVLTAKPRKGRTIPRNAAYWAGLTTAIENAEAWPTTRHLHD
DLKR
LCGYVDRYHNPLTGRDEIRVQSTAFNRMGESEFAAYFRLAQMRFAQQMGFDPWAMGRAA
>OLS47531.1 hypothetical protein BV379_04025 [Rhodovulum sulfidophilum]AB14
MTRNTLAELAREYERRERAAGGLAGGADPEMIFAEMAEDHALSAAEIAEVVRASWVGLGAC
>OLS47532.1 hypothetical protein BV379_04035 [Rhodovulum sulfidophilum]AB14
MGELTKIPQEDAATPASFGAITPMGMISQAVANGASIETVERLIALHERWESGRARKAFDAAISAARAEIKPIVKGSTVD
YVSKKTSDRTNYSYETLDAIAREVDPILAQHGLSYRFRSQQGDRGGLAVTCIVAHCDGYSEETTLSGPPDQSGNKNAYQ
A
IGSAATYLQRYTLKLALGLSAAKDDDAQGAGRAAEDREPPRFDPRAAAGRIRRSLERAASLDDLGAVWMHETPVLKDIK
A
AEPGLYSELERSKDARKLTLTRAASDIGDDEIPY
>OLS47533.1 hypothetical protein BV379_04040 [Rhodovulum sulfidophilum]AB14
MIGPLRTAEALDPISTPQGRAEARDRRRAILHEAARAARWLAWTSLGLLAIGLIGHVADKAIGQVIHDMHGSEVSQWA
N
>OLS47534.1 hypothetical protein BV379_04045 [Rhodovulum sulfidophilum]AB14
MLALNGPHWLVMTDFGPHGVGAPDPAATWDDALDQLAAAADEDRPARVFRLDFNSPAMTEVTDEAWRGLEGRLA
GMAA
>OLS47535.1 hypothetical protein BV379_04050 [Rhodovulum sulfidophilum]AB14
MERPEWHAQLLSADLEVKMNRALAAFALLSAGVLSACQTTQVGGDGGSSSKYSRVVARDLQGGFMPNETLEFWPAV
VPGE
PVSAARVGIDPVSGGQYTYFSRGKSELLLPSGETWILGCRKDTIRGTTLCNMNFEPMDQGLIMNVTTSGAVRSICVGGH
D
FPGRRALVRIDQHPAISSDRNGCFHGGSAQRVASQLNGAQVLQTEAVEWPYDSGIQRQFALSGSYPAAKRLFRYALNA
DS
TLFASPGE
>OLS47536.1 hypothetical protein BV379_04055 [Rhodovulum sulfidophilum]AB14
MQSKDALDLAFKHADVATKSLQFFVVASVALGGWIISSDQLRQTEPFSSPRLAWAALYLCIGTLTWFIHAQIMRRLDAC
F
RLAQRLADDELNQDPDFSRITKVPPRIGSHLGLPTLIVIVEILILGFGDTGLFAPSSP
>OLS47537.1 hypothetical protein BV379_04060 [Rhodovulum sulfidophilum]AB14
MTITLTLTGPATPEVRAAAAELLETLSGQSPTERPSPGSGEERRDLGLGIAIVGVVLAVPGAVLATLDVRDRLRKRRELA
PKVEAVKRVLVETRADGTIRFEETVISLTGLDTDQILDRLLSDRRK
>OLS47538.1 hypothetical protein BV379_04065 [Rhodovulum sulfidophilum]AB14
MKLCIEARDGQVWRHVGDHSAPLPNCLQPPRDGAPPETPLGLLEDWARRYDRAVRFGDQDQLSAIGSELLAWLDQG
GALQ
AWISDGHRELEVLGTGGDAVAEALLAAPWEILHDKTAFLALDRMKNFLPLRRVVPEGPGAAPGHRDLSMLFMAAAPE
GQH
ELDYEAEEAAILEATRDRNTGRPLVHLAVEESGELEVLGDRHKTAGPFEILHLSCHGDIGKVGGETRPVLLLETATGGED
IVTPEKLTDALGQTPPPLLFLSACRTGQRGTASDGLPALDGRREGLAEAAASFAAAPRREAAAPAPGLTEPFARQMALR
V
PHVLGWDGSVYDADAAAFAQALYAGLARGETVAYAAARARHGVTDAATQGRAPGQHWHLARVYLGPGGGGRLCD
PAKPAR
PAPPPAAPRFLDAEKRIRVAGRGEFVGRRRQLQRLRRGFAGEAPGALIHGLGNLGKSSLAARLADRMEGHQLAVVVDR
YD
GLSILGELERVATPLLGKVFKSFKDRTAFQKEFAAMREAVRGDETHLAEALRFLFDDVFAAQPVLLVIDDFEQALEDPAP

DRPKVLPRAALRPALGALLDAFREHQGPSRLLITSRYDFAVPDGAGGDLAAWLIRVPLVGMRPRERQKQWQAKARAA
ATE
ERIGTADPELVDAALAAAAGNPGLQDVLTRPILGGEAEAARAAIEAIMAFRKTGTAPEEGNAALAFFTRMTFEKYETALT
DTERLVLAAASLFEEEEVPVPRAAVAAAASALGVAAPEPALDRLLALGLLDDFGAMAGWDGMPKVPHLAANPLARPL
ALP
LDPATAATAADAALPELARTWRDAEGDFPFDGRGIAACRLALAAAAPDPKVLEAAALAAVVFLFARHRLAPQALALAIP
A
LNRLGELGHTPGSLLFGHTINAAGQAGEVVLQEQLLEQALGSNRLDDNQRAQLLGLRADVLQARGDLDEALRIYREELI
P
AFEALGDRRALAVTKGNIADVLQARGDLDEALRIRREEEIPVYEALGDRRSLAVTKGQIADVLQARGDLDEALRIRREEE
IPVYEALGDRRSLALTKGQIADVLQARGDLDEALRIHREEQIPVYEALGDRRELAVTKGKIADVLQARGDLDEALRIRRE
EQIPVYEALGDRRALAVTKGKIADVLQARGDLDEALRIRREEQIPVFDALGDRRELAVTKGKIADVLQDRGDLDEALRIR
REEQIPVFDALGDRRSLAVTKGNIAAGMAVQGDVDGALDMHLSRFPDATAMGDLDSLVHIRFSCAQLRLDRGDHETG
GIQ
TIHDELAEAWTGANRLGRPDFIGAIGYLLGQVLAMGGHRNEATEVLETAAAAWDRLGQAASANQCRELIEMIKGMPS
>OLS47539.1 hypothetical protein BV379_04070 [Rhodovulum sulfidophilum]AB14
MMIGVADMLFADMAPRTPISAPLNEWLIQALDHSGLSQSALAELLDARVQKKIDRSIINKMTLGKRTISAEEMLLISEIT
GFDLPSDTARIATIAIAGKVGAGARVPVFDTYEKGDGPQVECPPGLSPHNIVAVEIEGDSMEPVYSAGDLLFYTRWSDG
S
VPSEAVGKRCVCECEEGLGWVKVVRPGRDHGTFDLHSFNDQTPTMYGVRLKWAAPIKLHWPAELARKV
>OLS47540.1 hypothetical protein BV379_04075 [Rhodovulum sulfidophilum]AB14
MEPASTIISALGGPSAVAKVIGIHRTRVSMWQASRERGGTDGLVPYRHIPKLLELAAERGVPLSPADFIPTPTGDAA
>OLS47541.1 hypothetical protein BV379_04080 [Rhodovulum sulfidophilum]AB14
MEESVEITSFRYQDRSDKGLPPVMMVKFRTNTQGAIERTAESAEGHVAIDLAPSADYATMEREALMEVARYLRQVADY
AE
SSARKR
>OLS47542.1 hypothetical protein BV379_04085 [Rhodovulum sulfidophilum]AB14
MDKRLFMHMARATIARVGGVDAACAAIEAEYGEPVSRGTISKIQNGHLDITFAQVVALQKATGDIAFANFLRRANEHC
GA
VPAVTHVHTLKEATEAVMAQAEAEQSGDADSQLRAVKETLEAVDIMRDWLAGKAASLKTGTTA
>OLS47543.1 hypothetical protein BV379_04090 [Rhodovulum sulfidophilum]AB14
MSNSIHVSVRDGRAHIDLPLDEVHGLRVALHLRLAGEPESNSTKAIREAFDKALARAQIRAGRV
>OLS47544.1 hypothetical protein BV379_04095 [Rhodovulum sulfidophilum]AB14
MVVYRFDRRPIRCALPLGAIRAAFGDGGAAPPEEWVECSLDGLAYLAREIMAGPGAAKIEIRGTVR
>OLS47545.1 hypothetical protein BV379_04100 [Rhodovulum sulfidophilum]AB14
MTTDLAALDPRHPAEVDRGLAYLTRVLRSLRVAPAEREAAAMIAAQLSRPGDPVRTMARVGALLTPYFDKETPRALREI
E
MEDWADALTDFPDWAIERAAKWWKSADNPQRRKRPLEGDIAERCRVEMRAVRAARVRARSGWAGTEAPEDRERC
SPEAAA
RAMRAAGFSPAEDFTGPKRFGGAADQRGAA
>OLS47546.1 single-stranded DNA-binding protein [Rhodovulum sulfidophilum]AB14
MAGTVNKVILIGHLGRDPEVRSFQSGGKVCNLRIATSETWKDRNSGERRERTEWHQVSIWAEGLVRLAEQYLRKGSKV
YV
EGKLETRKWQDQTGQDRYTTEIAVRPFAGEIAFLDRRESDRGGSGAGGHGYGQGPSGGYGGPDLDDEIPF
>OLS47547.1 hypothetical protein BV379_04110 [Rhodovulum sulfidophilum]AB14
MSIHEIKGKGVNRARVVCDGCGREEVVTCNYLHRPGRVWVPDAGQINRKMTGQGWAEVKGKLHCPACEAKRKATG
MTKTT

TAPAAKPAEVLRQPSREQRREIVDMLREVYDPEAERYRRNDTDATVADVLGVMPGWVAEIREAFFGPDGGNEGIEAA
TER
LAALEHEIRAISDLAGKQQEAASKKLAEVSAMRAELGRIKGAVGPRALRAAGVK
>OLS47548.1 hypothetical protein BV379_04115 [Rhodovulum sulfidophilum]AB14
MGKGSKTRARAVKLAKKAELAGVPGLAPVERREKDGRIRRSTKPGYERTKDDVALRARCRQMGWKARPEDMAKARA
QSLE
CDAGRVLHLLCDRHDEVARLWSAFAGLCGAHAAYAKRYLGVRLHPKTAKIEMLIERFEISGDDDLDLRSEEEKARAAVT
N
WMRWQGYLGQLDRWQQAAIWGAVREQTALIRDRQATDEGRGYVQALRKLADVVERR
>OLS47549.1 hypothetical protein BV379_04120 [Rhodovulum sulfidophilum]AB14
MSRKFPLRILIAALLAATLAEPAAAMQTQISGSVRLRDGDTPVVGGVPVRLSGLTCDERGSQMGDRATARLWDIIGRQ
HL
TCVLNGETTYDRQVGRCYLPDGRDIAEILISEGYCGRCARYDPQRTYVKAQRHAGRYRGAMPGYCRPR
>OLS47550.1 putative addiction module antidote protein [Rhodovulum sulfidophilum]AB14
MPIETTRFDAAEYLDTPETQAMLLSDAFETGDAAYIKHAIGIVARARGIKDTAEAAGITREALHRALGEKGNPRLSTILG
MTKALGLTLKVEASA
>OLS47551.1 transcriptional regulator [Rhodovulum sulfidophilum]AB14
MSDMVTIPRDEYERLREAAEDLEDILAYDRAKADLAAGRDELVPAEFADRIIDGESPVRVYRDLRGLTQAALAEKAGINR
VIVAELETGRKQGSAQTLKKIADALGVTVDDLI
>OLS47552.1 hypothetical protein BV379_04135 [Rhodovulum sulfidophilum]AB14
MADTLSLHIKSDGVDMGVYDQHRRRVEGVTALEARMEVDGLTHITMTVVSGTPARKGGKDEAHVRLHWI
>OLS47553.1 hypothetical protein BV379_04140 [Rhodovulum sulfidophilum]AB14
MSGSTGSESFGVPAAAGWRNRPSASERGYGRGWKALRKRVMQRDSWLCQPCLRTGRVTPATECDHVVPKAQGGT
DDEGNL
QAICADCHKAKTEREAAEAQGRRLRPSFGEDGWPIGPE
>OLS47554.1 hypothetical protein BV379_04145 [Rhodovulum sulfidophilum]AB14
MANPRLPADVAAVTGAAAKNPQRHKGRAAPMVKGLGDPPAYLGEIEQEAWRMFADEMPWLGASDRAITETASRLR
ARMMA
DPAMGVNALAQFRLCLSAMGGTPSDRSKVAAPEEPDDDPADAYFN
>OLS47555.1 terminase [Rhodovulum sulfidophilum]AB14
MTTRPTPTSTDPATAYALDVAAGRIVAGPHVRDACRRHLRDLEEGPERGLTWDGAAAERFYGFCRDVLRLSEGQFDGT
PF
ELEPSQKFICGSLFGWKRPDGFRRFRRAYIEQGKGNGKSPMVGAIGLYGLVADGEAGAQIYAAGATKEQASILFRDAVN
M
LDKAPRLSARVRRSGGPGREYNLAHLKSGSFFRPVSRETKKTGSGPRPQFALCDEVHEHPDGGVIEILERGFKFRTQPLL
IMITNSGSDRKSICWQERKHAVAVAAGDVEDDTTFSYVCALDEGDDPFTDPDCWIKANPLLGVTITPAYLALQVKQAR
DI
PAKANGIRRLHFCEWTDAESAWISRAAWEAIEDHSLDLEDFAGQRCCAGLDLSAKADLTAKALIFEDGFAEDGKPLFAA
F
VHGYTPADTLRARAERDGAPYDLWVDAGFLTATPGKKTRLDFVAQDLLDDVERFDLDFVAYDAFLIADFEAIIGDMGA
TL
PMLDHPQGWNKRKRETEDGEEITLWMPGSVDELETLILEKRLRVHVNPALRAAVMSATFDRSPADLRRFTKHKATARI
DM
AVALAMAVGAATARSVGEKGGNMDDFLAALEAS
>OLS47556.1 phage portal protein [Rhodovulum sulfidophilum]AB14
MGIGQRIAGWLRPVEQKADTVNLLPKVEDGALAAALGGGAGHAGEIVTAQSSMALSAVWACANLISGTISSLPFEVRR
RG

GNGVDGLADLHPLHSVIYDSPNFDQTALDFWDYLNLSIELWGNGYAQVERNGSRVVALYPIHPEAVTVRRLSSGALEY
RW
TRDGKAHVGADRDVLHVRGPGGDPLGGMSTLQFGRQAFSSALAADRAAAGMFKNGLRPSGVLKFDKWLTAEQRDL
VEAKL
PEKYLGAMNAGRPFIAEGGFDYQPISISPEDAQMIETRQFSVEEICRYFAVPPVMIGHAGASTAWPTSVEQQGLIFQKF
T
LRRRIKRIEQAVAKQLLSAAERAAGLSVRVNMEALLRGSSAERATYYQTMTQIGAMTINQVRGLEGMPPVAGGDVPR
MQM
QNVPITEAETNNGAQ
>OLS47557.1 hypothetical protein BV379_04160 [Rhodovulum sulfidophilum]AB14
MEHSDFALELKALTDDGTVEGYAAIFGNVDNGGDKIAPGAFVGGMTKARQSGRSIKMLWNHDPYQPIGVWEDLAED
AKGL
WGKGRLVLDVPKAREIHALMKAHVISGLSIGYRTREAEPDGNVRVIKKADLFEISPVTFPMNERARISAVKSDGMDDVV
N
KLRAGDRLTEREFELMVKGLGLSNSQAERAARIHLKGPGEPAEAAKDGAAFMRAFLGLSA
>OLS47558.1 capsid protein [Rhodovulum sulfidophilum]AB14
MADENKTAAELAVEMKAALDKSLDGVKALAEEALGKAKAGEALSETLKGTADEALTGMNELKAQLGEIEQKIARGAGE
DA
PAEAETAGQSFVDSDEFKRFAEGGFARGDRARIETKATLTTSTAASAGSIGPALAPTRLPGVIELPQRPLTIRALLAQGN
MDGQSIEFVKEHSRTDGAGMVAEGTAKPSSDFRLEVVSTSAKVIAHWMKASRQVLSDVSAVRSMIDSRLAYGLDLVEE
NQ
LLNGDGTGQNLLGIIPQATPYVSPLTGADTTSIDKIRLMMLQASLALLPADGVVMHPADWAWIELLKDTTGRYIIGQPQ
G
TIGATMWSLPVVPSMAMTLDKVLVGAFRTGAQIFDRWSTTIETGYENDDFTKNLVTILAEKRLALATYRPGAFIYGDFG
R
EA
>OLS47559.1 hypothetical protein BV379_04170 [Rhodovulum sulfidophilum]AB14
MPKFKVLRQHLGERMYREGEMRDAAASEVAHLVAAGVLAAPESIPAPDPKPSRARKGTGE
>OLS47560.1 hypothetical protein BV379_04175 [Rhodovulum sulfidophilum]AB14
MTAIPVLVTPPSAPVVSLEDAKMHLRIDHADQDAMIEGLVLAATAHLDGWRGVLGRAIMPQVWQETHVGPGPYLLA
MPDV
SEVSASAGGAIRVETTALGPVVTLAEPVEGVTLSYTCGLPAAQRRLAEVAVKLYIAHLYDGSDPSPAFGAMVEALRWRA
V
>OLS47561.1 hypothetical protein BV379_04180 [Rhodovulum sulfidophilum]AB14
MARGLRVTALDQRVAIERVSRMPDGMGGTTESWAPIAKVWAQVVPVRGAEKREAMRISPEARMKVRIHWQGDAA
GQPFCT
PADRLVWRGRTYNIESAVPWGGRERFIEIGISEGVS
>OLS47562.1 hypothetical protein BV379_04185 [Rhodovulum sulfidophilum]AB14
MKVKVELRGFEALQDMLRDLLADGEDALLETIRDLADETAEIARREMKAAAGAAPAGSYPHQQSGRLAESVTATHEAR
QS
RVGSDEIHGYHLEFGTESMEARPWLVPSFEEATAKAEQKLAAKLQEKAG
>OLS47563.1 hypothetical protein BV379_04190 [Rhodovulum sulfidophilum]AB14
MSFETVAQTAIFAALDGALSCPVYDTPPALPEGMPESGFPYVVIGDDTLVADEADDVIGAQATITIHFWSRAPGNRQVK
A
LMGEAFGLLHRAALTAPGFKLLDCLREFSTASVGPDGKTRHGVQRYRIIMHKE
>OLS47564.1 hypothetical protein BV379_04195 [Rhodovulum sulfidophilum]AB14
MATVGFSGRKLTIEWDGTIIKGTKNKTASFSREGIDITSDDDQGFQTFLAEPGVISAEWSVEGITGDETLIAACLAGAPY
TTPPATINLPTGGTVEITMFLSQLEITGNTDGAFEFSATMMSSGIPVYTPAGA

>OLS47565.1 hypothetical protein BV379_04200 [Rhodovulum sulfidophilum]AB14
MMMKRSMTAHLAGEEIDLAATFKAASEIGQKIADPLLIAREIAVTAMLESQGVERDPKFRFTVDNIAPILLIGARAAGDT
RDLAAMQEIVFDAGFFAAAAVARDYINLLCIPQTEESAPGEDAPAGE
>OLS47566.1 hypothetical protein BV379_04205 [Rhodovulum sulfidophilum]AB14
MTWAAFERQAYQAARDWGVQPSEFWAMSPAEWWAEADAKIRESRAAVGGPRVKWTAEEIERVKRGGRA
>OLS47567.1 hypothetical protein BV379_04210 [Rhodovulum sulfidophilum]AB14
MTKVASVEAIISGEAGGLKASIAEAVGLVGKFKSDAEKSARAIERAYAQQEKSVDRLRKSLDPLYASSKRYESALAQLDK
ALKHGVISQAEYNRTLGLAEKAYLEGGKQAAAAGGALGVLGNMSDQTRYRVQNMGYQVQDIAVQLQMGAKASSVF
AAQGS
QIASMFGPVGAVVGTLAAVGLPLLSMAFTSAAKDGRAFSEVLDDLESSVAALDAVTRSYSAKGLVELKERYGEVNAEILT
LIERQKQVAIADAVRDADAAAQGFADQLERISDLVARAEYQFSPIEKLNEEFGLTLGTAQQLKIAFDQLAAAKTFDGQA
D
AMARILSLMEGTKLETDEVFKELVRAEGAMRQLAASAPQAGWMSGAIAEVKTLATALWDAAKARAAAEGAANTDAA
GNPI
QPGVSRTSRPRAAPPGTGGVEWGTPPQSGGGGGGANKVQTDLDALRQSLMTQEELQIASYDRQRETLLQARQQELI
DQQE
YDALIEQTRQQHQDRMAQIDAYRYGNGLQMTEAFLGDMASAFATGGDKMVKISRIFGAAQALISTYVGAAEALKLPF
PEN
IAAAAKVIATGMSFVSAIKSGSKSAAGAGRASSAGAAAQAAAPAPQRTANVTVQGDVIGRQSGEALVKALNEAFGDG
YRL
NLDWQGAAG
>OLS47568.1 hypothetical protein BV379_04215 [Rhodovulum sulfidophilum]AB14
MAVTYTLGHAAASPYPMIHARILWDKAAGSVSATSEAEGFEAELADTVETNSWWKPEAVPASWQITYPFSRVVDAIGI
GA
HTLGTTGASARAEYLPGEIHENRIWYSQDFPLWPTIVNVDLVMAEGPDGSSDARFLTETGPGQHYIQAGFRDAEPSQP
YT
FSLYVLPRSMSPQRNLRMAFVGLAYPVPCGCDLGRDGQIISTFGTATAVSVPVGNTGWHRVSLTSPAQVADNANIRIAI
V
KDDGTLSYEGTGEFVQIFGAQAEARGTSSPYIPTLSGGATSSWWAVSDDWLLPSDDSAILYLFDPVETDGIRVSVSQPA
R
IGVVYTGKALGMPRMGYTNLGPADLGRTAVLTSYISEGGQLMGRFIQRAGLSGAFEWQNLPEDWYRQTFDPFARAA
RTEP
FFIAARPEGYPTDCAYAWVDDPIVPARQGMRNFVSVGFTATGHADAAA
>OLS47569.1 hypothetical protein BV379_04220 [Rhodovulum sulfidophilum]AB14
MPMPPREPIQIVELWQPRCAERFGVAPCRATLADGPRCYNCWGTCLDREHYRGGGSIAWRFVKPTQALVPLYERTGE
HVA
TNPFPMLRSVSVRSSKINVGSIRDGEKPLGVTGGVTVSLTDAPFDDYVGDWYRAARGARSGSFWAKWCARNPFYAN
MFLR
VYEGGAGDALADMERRLYVLDSVDGPDSNGRVTLKGVDPLRLTDQSRAQFPRETEMRLSGDIAADATDIVIEAAREAD
LS
DQFGNTATRYLCIGSEIIGYTGYSGAEGVWTLSGVTRAALGTKTASHSDDASAQRTGRYELMDGYLIAHDLLTGHTPVP
A
EFVDFDAWQTEASTYLNGYAFSRTVHKPTAVNKLLGELVRDGTFYIWWNEREQLIDFKAVRPEQSSVAITEDAHIVAGS
L
AQAREPDERISRIFIYYNPENPTKSDDPDNYRSMRGRIEPEFEMEAGGADVRSKTIYSRWITTDAQAYELCQRLLSRFRT
TPRYLTATLADGALQIGEVADLTTRLDVDTEGQPVTRRWQVIKAQQVKPGEVTEYLLQQFIYQATRYLVWMDDDAPEF
GS
ATDDEKLNGFWWADEDGLMPDGSEGYFWQ

>OLS47570.1 hypothetical protein BV379_04225 [Rhodovulum sulfidophilum]AB14
MTDWTTIPNDVIEAGKPGRAVDGRALRDNPVAIAEGAPGAPGISSVAFIDPIEVRSFSRDNTQVITGLEGVKLLEVDGAF
NITSGGRFYLYTSDDGGSTWPNRTEPFFFAVGDHRSRMLLDFEAGKLYLIGTVFPQTGDPKTESRDISLSLPAGPVNAIR
IIMENTTGQMFITKLCGYKAA
>OLS47571.1 hypothetical protein BV379_04230 [Rhodovulum sulfidophilum]AB14
MQELTTQIRDWWAVMLSLLGLAVWSVRLEARARTNTAALDRETARLAEEIRALEARWQRQRAEDLAARQRDREETN
ALLR
ELRADIKTVLQRVPR
>OLS47572.1 hypothetical protein BV379_04235 [Rhodovulum sulfidophilum]AB14
MHLVPQWRALIRRAWSIRLILLAGLLSGCEAGLSLASPDAIGLPRGLFAGLSALVTAAAFAARLIAQRME
>OLS47573.1 hypothetical protein BV379_04245 [Rhodovulum sulfidophilum]AB14
MRALLAALLLAGCGGLPGPLGPSVAANVQAGAENVQGQKVENAPSILRPRAREIRQEQSENRLRADRVETVIVNVIPP
WI
VLVALIGWIAPSPGEIGRRIGEAVTRRRA
>OLS47574.1 hypothetical protein BV379_04255 [Rhodovulum sulfidophilum]AB14
MTKSNSLRTNSRPRQIVRNSLQVNVIAIVDVARAIFSETLDDAVIFVDNSVTPEGKPSPGRGTTALKTQCLEGMVINWIV
YPVGPYGVPVPVSIADITFDEPICVKLQSYGSIVTAHSPNVIPGVTPSYDYWAGMVRPEVEPNTYGYRIEFQIGTQRMGI
STPAVEISTIPAPLQASGDRGKAQYVLG
>OLS47575.1 hypothetical protein BV379_04260 [Rhodovulum sulfidophilum]AB14
MGEVDTATARSGSDANAKRVITVIGLVDVVGALAEDALGRDLYLFDDNKAGGSSGHGTSCLRTCADAEDLVVWEVMP
LEC
EAFARIDDVLIDEKYAPYIDLESGVYDPSSVVYWAGRILKPLPPEGIPYRLAFRLGSRPEPLIAEASSLLLAATPEDEAG
LPPEPGTEIAETASDAKPAPSAAAASKASASKKSPATSEADDVGSSKGARK
>OLS47576.1 hypothetical protein BV379_04265 [Rhodovulum sulfidophilum]AB14
MVDIAAAIEANDLRGHLWLVDNGRWAGSTGEGTGNLASALDVTDSALAGAPILNWFQIGIGSIPITVPQTFFLHDEDR
KA
LAQTALSAKHRSRFSARLRNILGEEINIRNSPRKMAASTEPTRALPHAGQGFLEPGRARFRNTRKSRVTRTDDQDLIHYP
DPVIARIDGEAVEKKVIFPAQYGSPQLFSDGLYWSASVNPAMLGVYSYTLWITLYYARRDKDGEVTDHSVTLPHDAWIS
V
STGAIRSGFSNSTLDIIPA
>OLS47577.1 hypothetical protein BV379_04270 [Rhodovulum sulfidophilum]AB14
MQPVNILMTVDSITALSLGTLTGNLYLTDDAWCSQGKGTEALKTACQRGQTVNWRMLAVDVQSPAVIVDIRFLDDAA
TLN
NSVATSSEKAIDPDTSRYIRPFWQTWSGIIPCNLQPGLYHYHLAFQIGAGPNSVLFTTSPALQLTA
>OLS47578.1 hypothetical protein BV379_04275 [Rhodovulum sulfidophilum]AB14
MSTDTALRPVADTNTIAGLIAADELQPGSPAAIRGLESFLSKIRRAEQLTGRRFAEFAPDLQDALILRRLQQMIGTLQSN
PSWAARLAAAGITGAVRDFDQWQSIPLTDKTVMRDLYMGERQGLAVPLSFGGFEIVASGGTSSGQPVETVYSLRELQ
DTY
EFAGEFMGRYQLAPFFDEDAPKWVMTTLADYNLWSSGTMVGGVLQKIPGTNYLGIGPVTKDVFGHLMRYIGPKAFM
SITA
SVAALPELGSELSDDARRSLRVALYGSGVLNARHREELKAAYPNVSILSYFAATQAEAIALQLDPASPWLATVPGLHLVE
IVGPDGRWVKDGEEGELVVTRLHAHEAPLPRLLIGDRVIRRPGVAGAGLNTMQFEYVGRSGDVIHLGDTQFSAKRVLD
GL
GHGLAAIGIDLNRAAREVQFVNDRRNRRLTLLVAADEPEIINRQVGLIGAHGTYQMFGDALIRSLSLFNQGEANFHYLA
K
TGYHLELRFVPPGAPEINRTAVGKTPLLCDKQQ
>OLS47579.1 hypothetical protein BV379_04280 [Rhodovulum sulfidophilum]AB14
MLSRLLPDRDMSEIPENFLPIESIGIEDIDNLFESKRFSQHASQAIDQLITYLGDRSTRGLALTDPAGIRAAVTRLTLGG

QTSDTAEHDRKLAEIVDLYLQTGIQVYSPGYMGRQFSGPFPVTAVVDMVSAIATQPASFYEAGALPCVVERVMSEEFN
RF
LGFPSDRFTMFTTSGGSLANLTALLAARGRRYPQAWGEGMGETDCCPAIAVSSDVHYSITRAAGILGIGENRLVRLPLD
H
ARRITPAGAREALDAAAARGLDVFCIVANAGSTSFGAIDPLDELADMAAERGIWLHVDGSHGGSLIVSDRLRPRLAGLN
R
ADSLTLDAHKTMFVPAACTLLFYRDRDQATAAFRQEASYVFENTSDVYSEYNSADRNFECTKRPMIMSLWALWAMYG
RTA
IAEKLEALCDMTRKAHGILSAAPDFVPLHLPETNILCFRYLPNEQLSPKALGALQTEIRSRVRARGRFFISKVDIDGVSA
LRLVLMNHRITTDHIRQMLDEVRAVALTIREEFPQSEEI
>OLS47580.1 hypothetical protein BV379_04285, partial [Rhodovulum sulfidophilum]AB14
VAISLVAFETMRVALGTAALICISLAALSLAVRNARHDRMVVLAQERIGTAEDVPYLRPVSLAAEPVDLLPPPNLAVTFL
EYGMVVLILVFGPGFWIAAITDDLSER
>OLS47581.1 hypothetical protein BV379_04290, partial [Rhodovulum sulfidophilum]AB14
TLTVDGIALAISAIAAEAVISAVTIAFGGAAALTLGTAAIVVAAGIGTGLAVGMVLTWIANRWDLGTKCDQLYTAIGNGL
EAIAQGAVEVTGDFADYLTLKYDAARQWADDMAAEAGRQMERAADWTAKKAGEAADWASEAAQDTADWASESA
HDVADWA
VETAEGVEDWANETADDIREGWNVLQNRAAKAVPEGWF
>OLS47582.1 hypothetical protein BV379_04295 [Rhodovulum sulfidophilum]AB14
MSVLRSLWSVRLVLGSAPECWDGKLRSAALLGAVLFGVFYFAALLQLNDPLDMFRPEAVSWRAWVSWAAICLPATIFL
SS
WLLVVLRRAVGYIVFSVSMIYLGPMITAVAISLVAFETMRVALGTAALICISLAALSLAVRNARHDRMVVLAQERIGTAE
DSSLIWFVMVALFLASRRSVARTLLLARAMALYTPAVGCPAAGSLDTRIRSRP
>OLS47583.1 hypothetical protein BV379_04300 [Rhodovulum sulfidophilum]AB14
MKHDLPRLLAERKPGYALERPFYTDPAIFEADLEAIWYRDWLFAIPACELEKPGSFVTRP
>OLS47584.1 hypothetical protein BV379_04305 [Rhodovulum sulfidophilum]AB14
MTLREDPRLAEARALPIGEVAERLGIAGLKPPQAIERVGPCPVCGGRDRFGINTARNVFNCRHCGGGDAIALVGLVKGC
D
FRAALDWLMGAREVETDPAELARRKAAREKEARQRSRQAEAARRNAIAQARAIWHDSRPAEDSPVRDYLARRGLTRA
RLP
ELPACLRFHPDLPYMVPAEGNRGRWREIHRGPAMVALVQAPGGRGSGIHRTWLDLDQPKGKAVIAHEGETLAAKKTL
GSV
KGG
>OLS47585.1 hypothetical protein BV379_04310 [Rhodovulum sulfidophilum]AB14
MAEAPLTRADQVLIAAAAALAVPPVMDRDLTARRMAMALDVIPHVAPDGPTCGLAFEIEAMDRARRAGDGPTFSDC
HWRL
RSAVARFFETRAAHAYERWRRETGRDRNTERPEQEELRWQRLLKKGPGATPAT
>OLS47586.1 hypothetical protein BV379_04315 [Rhodovulum sulfidophilum]AB14
MTVRAEVAAEFQATALAMQSRQRPVSIRDRLRLAMAGLGSGDEELRAAIARFLADHDTAPAAAGAALQETILRRCTAA
PT
RHSWQDRADLDG
>OLS47587.1 hypothetical protein BV379_04320 [Rhodovulum sulfidophilum]AB14
MQKSFQKFRDPQERERKWFASLLWRSFPEATSEAQLADLVAEALNTAQRPVNPRTVRNWLRSENAPGLHYVVAVLAL
AGA
ETVFELFDPEQTA
>OLS47588.1 hypothetical protein BV379_04325 [Rhodovulum sulfidophilum]AB14
MDSGQMIAERLKALIETRGTNNRAVALAAGMGPTGVRDIISRKTKNPTYANLLKIADVLGVDVQEITGGAHERPTIAIA
G

KVGAGARVPVFDVYEKGDGPQVECPPGLSPHNIVAVEIEGDSMEPVYSAGDLLFYTRWSAEGVPSEAIGKRCVCECED
GF
GWVKLIRQGREPGLFDLHSFNDQTPPMYGVKLKWAAPIKLHWPAELARRV
>OLS47589.1 hypothetical protein BV379_04330 [Rhodovulum sulfidophilum]AB14
MAESKKHTAPDLSDDAPDLSAPEWQQKFAAAKVRRGRPKAEKTKISTTIRLSPEVIEYFKAGGPGWQSRIDEALRKAAG
L
>OLS47590.1 hypothetical protein BV379_04335 [Rhodovulum sulfidophilum]AB14
MRIEFDQSKRDNTLEARGLDMADAAEVFDGPHMTVEDDRIDYGEARFITIGFMSGRMVVLVWTERGRARRIISMRKA
NGR
EQKAYGPRFE
>OLS47591.1 hypothetical protein BV379_04340 [Rhodovulum sulfidophilum]AB14
MKTILTPTALALLAAPALAVTETTEDLRAIRQPSSDSQGCFAPIQGTNAFQRSNAPGCVYYTAPTGNDRDPAASGEGEG
E
GGESSDT
>OLS47592.1 hypothetical protein BV379_04345 [Rhodovulum sulfidophilum]AB14
MAAILAIAPQIADRLAAEAAAARAQANTARVAAKSSARDGKPLLARAAFARAERLQGRAVTLGALAAQARAGGAA
>OLS47593.1 hypothetical protein BV379_04350 [Rhodovulum sulfidophilum]AB14
MSVFDSIAPPPDALAAFVAEWRGAAPDVRLAAIRGALRRHGIEIPALPGRVAFYEIQLFGLVATGRDEAEAAQNWMEA
AI
RLCPFD
>OLS47594.1 hypothetical protein BV379_04355 [Rhodovulum sulfidophilum]AB14
MHPLRILIGCETSGVMRRAFAARGHDVWSCDLLPAEDATNRHMICDIREVLDLGWDLLAVMHPPCTRLCNSGVRWL
HVPP
TNAPAEAHPDERAAWPDLSEDTRRAIMWRLLDEGAALFSACWRAPIPRVAVENPVMHRHGRARLPADLPKPQIVQP
WWFG
EPVFKATGLYLRGLAPLSATNRLTPPAPGTGAHKRWSAVHRAPPGPDRWKIRSRTFEGLAAAAADQWGENARKEAA
>OLS47595.1 hypothetical protein BV379_04360 [Rhodovulum sulfidophilum]AB14
MMAETQWPREIWAAQSDPDDADGRHVFSVHATIPRFEGDSERDCAFHRYVDGDIVDSADRYHREMLTALRARAETA
EAER
DRLRAEVDQLREGARRYRWLRSRDLETIHAGGVFAGKTPENIALNLEDLDRAIDIASHHEDRSDG
>OLS47596.1 hypothetical protein BV379_04365 [Rhodovulum sulfidophilum]AB14
MAETFTRSFGRFEAEATPAEARLTIRRHGKDMIRLFGRDLIRSSLLGGSWTPDIERALRWGLDRIEAELGEDLRYRAGVM
AAVKLCWRLRLANDPRRAERLLLGDPEAGQPPAEPRICPACGELVTEGQGCGGGICRASDAFPKPVEDPADKAFREAV
AE
LVDCAMKEGKPDGYHS
>OLS47597.1 DNA-binding protein [Rhodovulum sulfidophilum]AB14
MAITRDFAPRLMPAPQAAHYLGVSESTLRTLDLPRRVVGAKRLFDRLDLDAFADSLATEGAAAEVNTCHGKFGRRAS
>OLS47598.1 hypothetical protein BV379_04375 [Rhodovulum sulfidophilum]AB14
MSWQVRAASVVTLRHIQTIHKGGRVYRYLRAPGGKRVRLPDLPMDDPAFLAAYASALASAPKITRAKPGTIAMLIEAFL
G
GRDYLSRSESYRRTIRRHAEAIKAQADDALARDLREQDIEADLSPLCPNAAADRLKAWRMICRWGKTNGYLTRDPSLAV
K
RKPIPKTIGHPAWTDAEIDRFRAHWALGSVQRLCFELVFWTGARISDAVKIGPGMAGRDGVLQFTQKKTGEPAFVPW
TCP
LPAYAVRMEDDRKLLFAALEARAERHMTFLATAYGTTRSEKALGHVISNAASKLGIAKSAHGLRKSRAKLLAEAGATVH
Q
IAAWTGHITLEEVEHYTREASRRAAVRGEQMKKPVNR
>OLS47599.1 single-stranded-DNA-specific exonuclease RecJ [Rhodovulum sulfidophilum]AB14

MTRAYLGVETSATGRRWVGPSEEEMRQAEALAQSTLLPAAVAAVLARREVTAETAAGFLDPTLKDLLPDPRALRDMG
RAG
ERILAALTGRQRIAVFADYDVDGGASAALLLDWLRAMGHRATLYIPDRIDEGYGPNIPAMQALARDHDLIVCVDCGTLS
H
DAIAAATGADVVVLDHHLGAETLPPALAVVNPNRQDEDGSLGHLCAAGVVFLLLVEINRQMRAAGMRGPELLQSLDL
VAL
ATVADVAPLIGVNRALVRQGLKIMARRQRPGLVALSDVARMDSAPTPYHLGFLLGPRVNAGGRIGKADLGARLLSTAD
PD
EAASLAERLDQLNTERREIEAAVRAAALAQAEERGTDGPLVWAAGEGWHPGVVGIVASRLKEATNRPAVVIGFDGDE
GKG
SGRSVSGVDLGAAIQRLASEGLLLKGGGHRMAAGLTVARDRLEPAMARLGELLARQGAGTMGPADLRLDALLMPGA
ATVE
LIDEIERAGPFGAGAPAPRFAFPDCTILHVRRVGDAHLKLSFGDGLGARIDAIAFGAFDGPLGPLIEAHGGARFHLAGRL
EINSWNGRQSVQLRLEDAARAI
>OLS47600.1 fructose-bisphosphatase, class II [Rhodovulum sulfidophilum]AB14
MTKNPPVDFNDRMLSLGLARVSEAAAMASAKLIGHGDEKAADQAAVDAMRRRLNELDIKGVVVIGEGERDEAPMLFI
GEE
VGTGEGPAVDIALDPLEGTTLTAKDMPNALAVVAMGPRGTMLHAPDVYMEKLAIGPGYPENLVTLDMSPTERVRVLA
EAK
GGGADDITVCILERPRHEDLIDEVRATGAAIRLITDGDVAGIMHCADRGRTGIDMYMGSGGAPEGVLAAAALKCMGG
QMW
GKLLFRNDDERGRAAKAGITDLDRVYARDDLVRADVIFAATGVTDGSLLPGIKREVGYLTSETLLMRSKTGSVRRMFYR
N
PTK
>OLS47601.1 DUF159 family protein [Rhodovulum sulfidophilum]AB14
MCGRFAITMPAEAMAEPFEAVPGNDLPGGPDFNICPTDMVAAVTSAGGLRRLRPMRWGFLPHWYKTPTDGPLLINA
RADT
LAHKPAFAAAARQRRCLIPASGFYEWQRGPQGARLPWYISRSDGAPLAFAAIWQDWERDGHSLTTCAIVTTWANAR
MAEI
HTRMPVILEPADWPLWLGEAGHGAALLMRPADEGVVQLHRVGTAVNSTRADGPELIEPI
>OLS47602.1 hypothetical protein BV379_04400 [Rhodovulum sulfidophilum]AB14
MDLLVAAASIAAGEPDPGLPPDRLRLTLEYRSPPDCTVERGRMAKLLKGERFSLEPLPDDLLPCFLVLQFPGVRRRMAPES
LFAAAGALADEMGLVSAVPDIGASFVVAPDAPGRSESVADAFLDLTCLVAPDETLADDWAIDAINVKPVWARGITGRG
VL
VAQPDTGVADHPELAGMLDLGQAWNTLTATADPTDPLSADMSSPGHGTATSSTLASRAPGRVFGAAPGAEVVPIRCI
DAV
VLGLDPTPVARAILHAVHIGADVISMSLGGVLYSRAMAAALERAAAAGIVIVSAAGNCLQPITVYPARDPNAVGMAGT
NH
LDQPWKGTSRGSRIAAAAPAENVRVARRRPDDRGQGTTGPSQGTSFAAALTAGVAALWIEHHGRQNLRDEAARVGI
TVND
LFRTALKASARRPADWPSGMGAGIVDADALLRLELSRIQPLSAPESAAPPPEDAILAALDESYEEIETGAGDWPRIGAEA
IYLLGDAWLRENRAAGVPVETTACPRPSPGLVRAMPEPVMRGLAARRDAGAFTPPLIAATGPSAQYAKLLAAGQPGTA
ES
SAKLSEPEAKARLSGEGAGRLMARTRTCFDRLQACGLGDRDAQARVLEGAEGVIRMAAEDRLHQLDLPGRIALEALVRI
Q
DRPAFRVTGDSVDIDDPLAGDWAPFLGLVANLPDWTRSVGRIDLDGIHIGSGFLIGGGQVMTNRHVLEACADELTGP
GGT

LWRFGRGAVTIDFSETADGSQCHALTGVVMAGPDPIGGVERLGHLDMALLSLDAAAPGLPRALPLARQLDDAAEMV
VIGY
PARPGTSAFVDPATGAISREIGTRLREIFGTDYGRKYVAPGHVMQGPGRLPGDSEGWAASHDCTTLGGNSGSVLVQFT
AT
PAVAGLHFSGAPLTANKAHALGQVDRSRFGPIPGATWL
>OLS47603.1 hsp70 family protein [Rhodovulum sulfidophilum]AB14
MSSPSAMLAVDFGTSNSAAAVLDGGQVRRLAIEPGAQTLPTAVFFPDERGAPLRIGSAAAEALIAGEEGRYMRALKSVL
G
TALFHEARPIGGRRRTLAEIVTGFLAELKARAEADAGTRFDRVLSGRPVHFHSADPARDAAAEADLRGCYEAAGFREVG
F
RFEPEAAALAARADARSDELGLIVDIGGGTSDFSVFRAAPGGIEILASHGIRLGGTDFDHAVSMAHAMPLLGLGGELRRS
FGEGLLPVPKALYVDLSTWAMIPFLYTRETERAVADMERHAVDRAAMERLGHVIGARLGHELAFAVEAGKIAANGGG
ARA
RIEMGQIEPGLGAPITAGSLNAALAPYREALRMAIYETLMLAKAGPDRIGAVVLVGGSSLMGLVEAEARAVCPAAAIRR
S
EAFTAVVDGLALATADA
>OLS47604.1 two-component sensor histidine kinase [Rhodovulum sulfidophilum]AB14
MRAPRSLQGRLALAVGLAVTVLWIAAAGATLRELRHEMDEVFDSALRDAAHRLLPLAVRAHEGRHRDDDRHDGRRV
RHAR
DWSERIRQFDGEVEDLSYVVRDRNGTVLLISDDADPEDFPAFRKEGFRRTATHRLYYDRARDEDVTIAVAEPLSHRAEV
A
REIAANLALPLVVVIPLSLLAIVIAVGTSLRPVRALRQSLAERGARNLSALGEGALPTELRPIVGSINELLTRLRAAFEA
ERSFAANAAHELRTPVAGAIAQAQRLRAETTDSDAAARASEIEATLKRLNSLSEKLMQLARAEGARLTAAEPSDLRQVLE
IVSGDLERLGRAGRIALSLPEGPVLSDLDPDAFGILCRNLIENALKHGRGTVDVALDARGVLSVANGGDPVPAADLARLT
ARFARSNGGAEGAGLGLAIVRTIAERSSGRIELVSPRPGRSDGFEARFTLPGSR
>OLS47605.1 DNA-binding response regulator [Rhodovulum sulfidophilum]AB14
MRILLIEDDGVLGAAVADQLRADGHGADWAKRLDEAADTLAVGAFDLVLLDLMLPDGRGIPFLRAMRARGDATPVIIL
TA
LDQVSDRIEGLNAGADDYMVKPFDLDELSARIGSVARRYSGNPNPLIHLGPLEIDLAAKAVRRDGRPVTLTHREWALFE
A
FVQAPGRLLSKAQLEERLYSFDDSIESNTIEVHVSRLRKKLGRSVIDTERGLGYRLGSP
>OLS47606.1 hypothetical protein BV379_04420 [Rhodovulum sulfidophilum]AB14
MTRISLPLLAACLALGLSGTAFAQSAGAAPTNSVPTSSGPSRIMAQQAGSGACLAGSARHESHRGAWHRGDDHHGR
GGRY
RDYDWRQDRSRDRSAACSRDCDRHNGGGQALRVPAPGTAPANGFFDNGTAPRARSN
>OLS47607.1 hypothetical protein BV379_04430 [Rhodovulum sulfidophilum]AB14
MAGVEAEMLIRCSDSDLMRRNRARGGIWGDRPARLVTVLRAGLAIVRGSGEACEIELGDATAA
>OLS47608.1 hypothetical protein BV379_04435 [Rhodovulum sulfidophilum]AB14
MQYRQGHGVDSLAEVARGFGAENTLQALGAWAVASLGDYRHWFDLGGRRLSHIMNQRRRAPLADSPASVSRCWR
GAAVEA
DARAATGAFGALSRA
>OLS47609.1 DUF4405 domain-containing protein [Rhodovulum sulfidophilum]AB14
MRSILMRYATPSTIGLFLVSLITGVALFFHVGPSAFHGIHEWLSMVLILAFGLHLWRNWRPMLHYMKGRPLALSLVITLL
ASAAFFFLPAGGKGGGRPPAFGLAMQVMAQPPATVAPALGTTAEELTARLAAAGFAVTDPARPLSEVARASGKSPMA
LAR
VLIDRPR
>OLS47610.1 VWA domain-containing protein [Rhodovulum sulfidophilum]AB14

MATLADYAELLEDLDAPQRETLEQAWPETVRLLSPRGLDNWLKGTAALSHMGRGDHIVRTWIETVPAVARDIGEDIIP
DL
AQACLGFASRTSGAVIERVLATAPVASRRLSDPDLFRAYLQLLNQLLAQAPRGLRPMLDHLEELLGVLTLGGLRRWASW
G
AEAHRTDYEELTRYFGLESAESQAILQRERKGTLFVDIHRRIGMYLRALWGRDFLMRPTAGDFEAREGIRPHIAEYFLHL
PDAYDDWEGVPGLDLYRAAAAHAAAHVMATTEPLKGDELNALQRACIGLIEDARIEALAIARFPRLRDLWLQFHRPTE
DG
SMTARFGRIARAVLDPAAPARDEIADWARTAFAGADMATARTSFDLGLQLAHRLRNEPYSAWRDGQAAPYRDDNRY
IWEF
EETVDWEKGPAPEQQVRKHVSVSEMVNEVEVETAGDDAQEIWVAETELFDDDGVSFNEKEGKAPTAPPVPYDEFDY
RIQM
HRPAWATVQEKRPRLGDPADIETILSENRKLTQRMRHLLDAMQPQGVQRIRRLEDGDEIDLNAATQALIDIRMGRQP
DPR
VMMRSVRKTRDIAVMALLDLSESTNDPVAGQDRTVLDLTRTATVLLAEAIAKVGDAFALHGFCSDGRHNVFYARYKDF
DQ
PWGEVPKARLAGMEGHLSTRMGAAIRHAGAHLGKVAAAKKLMLVITDGAPSDIDERDPQYLRQDARAAVQEVAKTG
VIPF
CLTLDPRADRYVAQIFGQRNFLVLENIARLPERLPQLYAGLTR
>OLS47611.1 AAA family ATPase [Rhodovulum sulfidophilum]AB14
MKDLLDQYRVADEPYYRPVADEVALYEAAYAARLPVMLKGPTGCGKTRFVEHMAWRLGKPLVTVACNEDMTASDIV
GRYL
LDAEGTRWQDGPLAFAARHGAICYLDEIVEARQDTTVVIHPLTDNRRVLPLEKKGELLRAHEDFNLVISYNPGYQSLMK
D
LKQSTKQRFGALDFSYPDHATEVEIVTHEAGVDATDADKLVSIAERARNLKGHGLDEGISTRMLIHAGSLMARGIEAPA
A
CRMALVRPITDDPDMRDALDAAVATYF
>OLS47612.1 ribulose bisphosphate carboxylase small subunit [Rhodovulum sulfidophilum]AB14
MSKVQDYTSRLSDPASRRMGTFSYLPEMDDAQIRKQVEWIVKHGWNPAIEHTEPQFAKSNYWYMWKLPMFGETDV
DAILA
ELKACHDANPDNHVRLIGYNNYSQSQGANMVVYRGTPV
>OLS47613.1 ribulose-bisphosphate carboxylase large subunit [Rhodovulum sulfidophilum]AB14
MAKTYDAGVKEYRSTYWEPDYTLKDSDILAVFKVIPQPGVSREEAAAAVAAESSTGTWTTVWTDLLTDLDYYKGRAYAI
E
DVPGSDEAFYAFIAYPMDLFEEGSVVNVFTSIVGNVFGFKAVRSLRLEDVRFPLWFVATCFGPPHGIQVERDKMDKYGR
P
LLGCTIKPKLGLSAKNYGRAVYECLRGGLDFTKDDENVNSQPFMRWRDRFLFCQEAIDKAEAETGERKGHYMNVTAPT
ME
EVYKRAEFAKELGTPIIMSDYLTLGWAAHNSLSKWCRDNGLLLHVHRAMHAVLDRNPNHGINFRVLAKMLRLLGGDH
LHS
GTVVGKLEGDRAATLGWIDLLRDRHIKEDRSRGIFFDQDWGAMPGVFPVASGGIHVWHMPALVSIFGNDSVLQFGG
GTLG
HPWGNAAGAAANRVALEACVQARNEGRELEKEGKEILTEAAKHSPELKVAMETWKEIKFEFDTVDKLDTQHR
>OLS47614.1 hypothetical protein BV379_04475 [Rhodovulum sulfidophilum]AB14
MLQVFVLYLVFSLGVLLGAAELERRAIVARRLGPNGRAMLVALAASALGALVVVVISTFAEGWIYMLHVLGGAILYHGF
M
GAFLVHGLQEVSARAAGHDIG
>OLS47615.1 ATPase [Rhodovulum sulfidophilum]AB14

MAHIIVVGNEKGGAGKSTVSMHVATALARQGFRVGALDLDVRQRSLGRFFDNRAGYVAQSGVNLASPEFRELPEIDP
GAL
DEGENLHDRRLSTAIAELEREKDFILIDCPGSHTRLSQVAHTLADTLITPLNDSFIDFDLLARHDGATGKILGPSIYSEM
VWNARQMRAQAGLKPIDWVVLRNRLGAQQMHNKRKMGEALKNLSRRIGFRVAPGFSERVIFRELFPNGLTLLDLKD
TGVT
SLNISNVAARQELRDLMAALKLPEVEPVA
>OLS47616.1 50S ribosomal protein L31 [Rhodovulum sulfidophilum]AB14
MRKDIHPDYHAIEVKLTDGTVVTMKSTYGKEGDTLSLDIDPSVHPAWTGGSSRLMDTGGRVSKFKKKYEGLGF
>OLS47617.1 50S ribosomal protein L19 [Rhodovulum sulfidophilum]AB14
MNLIAQLEAEQIAALGKTIPDFKPGDTVRVGYKVTEGTRTRVQNYEGVCISRKNGHGIAGSFTVRKISFGEGVERVFPLF
STNIDSIEVVRRGRVRRAKLYYLRSRRGKSARIAENSNYKPKA
>OLS47618.1 tRNA (guanosine(37)-N1)-methyltransferase TrmD [Rhodovulum sulfidophilum]AB14
MTDRPKSHGRLSISASLRPGELMTDRPRLKGAWTARVITLFPDAFPGILGLSLTGKALDQGLWALDPIDLRPFGEGKHR
N
VDDTPAGGGAGMVLRADVVAKALDQAALGTPGDRSRWPVIYVSPRGAPFTQATARRFAAAEGLTVLCGRFEGVDER
VLEE
YGVEEVSLGDFVLTGGEIAAEAMIDATVRLLPGVLGNAASTEEESHSGGLLEHPHYTRPAEWRGRAIPDVLLSGNHGAV
E
RWRREMAERLTEARRPDLWAAYRRGTD
>OLS47619.1 hypothetical protein BV379_04500 [Rhodovulum sulfidophilum]AB14
MNLPQILYAVILFGAFLAGAAGIDAWVIVVIAALAVVANVFDPTARQKRADEGKTLTKALPMIVVNQVIWTNLAFLIGL
G
VAWPFGGRIVALPVLVPLIVSAIGCAGALAAGMKPGPKA
>OLS47620.1 ribosome maturation factor RimM [Rhodovulum sulfidophilum]AB14
MTDRVCVGAISGAFGVRGEVRLKSFCADPEAIADYAPLYSEDGSRSFGLQITRAVKEGFAARMSGIDSKEEADALKGVRL
YADRDRLPSLPDDEFYHADLIGLTVVDPGGKVLGTVRAVHDHGAGDLLEIHGPGLTSTVLLPFTDACVPTVDLAAGRLIA
DPPEGVFP
>OLS47621.1 5,6-dimethylbenzimidazole synthase [Rhodovulum sulfidophilum]AB14
MASGTARADRFAPAFRDELFELMRWRRDVRHFRTDPVAPALLDRCLSAFRLAPSVGLSEPWRLIRVESPAARAAARAN
FE
DANAAALKGYEGEKAALYPRLKLSGMDRAPVQLAVFCDETTAQGAGLGAATMPEMRRYSVVAAISMFWLAVRAEGL
GLGW
VSVLDPDRLARDLGAPEGWSLVAYLCLGWPEVERDSPELEEVGWETRAAGLIVEER
>OLS47622.1 30S ribosomal protein S16 [Rhodovulum sulfidophilum]AB14
MALKLRLARGGSKKRPFYRIVVTDSRMPRDGRFIEKLGLYNPLLAKDSEERVKMDIERAQYWIGQGAQPTDRVARMLE
AA
GVLTKTERSNPKKGEPGDKAKKRAEEKAAKAAEATEAE
>OLS47623.1 chorismate mutase [Rhodovulum sulfidophilum]AB14
MTDDASRAAELLKEHRASIDRLDAILVYTLGERFKHTQAVGRLKAAHGLAPSDPAREDQQIARLEDLAHQADLDPEFAK
K
FLNFIIQEVIRHHIEHQK
>OLS47624.1 GNAT family N-acetyltransferase [Rhodovulum sulfidophilum]AB14
MAHSAIPVLETERLVLKGPAPEDYPNFEATFTSFRARFMGGPLSEYESWMLYAAEIGHWQIHGFGMWMIHDRDTDA
TLGM
AGGWYPKGWPEREIAWVIWPRVEGRGIALEAVHAARGWCYDRLGWDTAVTYVDPKNVSSVRLCERLGARRDDSAR
TIDSH
DIVYRHPAPAELRATPLVARIEAELAGHIDPSCQPKGAPLD
>OLS47625.1 GNAT family N-acetyltransferase [Rhodovulum sulfidophilum]AB14

MKITIDCPEIETERLILRGPKAADWEAHAAFAGSERARYIGGPFSRRDAWTKFASRWGHWALHGYGMFTVTRKGSDE
AIG
VIGPHFPEGWAEPELGWILYPGAEGQGVAREAALACRRFAYDTLGWTTAISYIDTRNDRSRALAERLGCSLDPSVPHPF
E
EDCVVYRHPGPEAAA
>OLS47626.1 GNAT family N-acetyltransferase [Rhodovulum sulfidophilum]AB14
MTAAPIIDTARLRLRPHRVEDFGPYAALFASERARFMGGPLSRRQAWQVFAADSGQWALMGFGAWGLEHREDGVA
VGQVA
LNKPDHFPERELGWLVFDGHEGRGYATEAAEAARDHAFGALGFDTLVSYVSPENRRSAALAERLGARPDATADRPDP
GDL
VYRHPRPEAAA
>OLS47627.1 signal recognition particle protein [Rhodovulum sulfidophilum]AB14
MFESLSDRLSGVFDRLTRQGALSEEDVATALREVRVALLEADVSLPVARDFVKSVQEKATGQEVTKSVTPGQQVVKIVH
D
ALVDVLTGEGEPGYMKIDNPPAPILMVGLQGGGKTTTTAKLAKRLKEINGKRVLMASLDVNRPAAMEQLAVLGTQIGV
DT
LPIVKGQKPVDIAKRAKQQAAMGGYDIYMLDTAGRLSIDDELMAEVEAVRDVATPRETLLVVDGLTGQDAVHTAENF
DER
IGITGVVLTRMDGDGRGGAALSMRAVTGKPIKYVGLGEKLDAIEEFHPERIAGRILGMGDIVSLVEKAQATIEAEQAER
M
MKRMQKGQFNMNDLKMQLEQMLKMGGMEGIMGMMPGMGKMQKQAAEAGFDDSVLRRQIALINSMTKRERA
NPQLLQASRK
KRIAAGAGLEVPELNKLLKMHRQMSDMMKKLGQKGGRAMLKKAMGGMFGKGGMPDMANMDQAQMEEAARK
MGAGLPGGMG
GGMGGMGGGMGLPPGLGGLGGFGKKK
>OLS47628.1 LysR family transcriptional regulator [Rhodovulum sulfidophilum]AB14
MDVDNWDEIRTAFHVARLGTVSGAAEALGVHHATVIRHVDALEDRLGVKLFQRHARGYTPTEAGEELLRVAQVTADQ
FSQ
LAGRLRGRGAAMTGELTVTTVDSLTPLLVPVLSGFQAEHPELTLRLMADSRLYRLEYGEAHVAVRAGSQPDEPDNIVQR
L
YTLRYALYANAGYVAAHGRLGEDLAAHCYVGPSDEDHRAPFNRWLKANVPAGRVGFRASDARAQVEAVAAGAGIGF
VPVW
WAERLPGLEQMHPPLAEWDVPLWLVTHVDLHRTAKVQSLTTRLKDAAKEWP
>OLS47629.1 EamA family transporter [Rhodovulum sulfidophilum]AB14
MALSLAAVPRGEALRGHLAMLSVSALVAGSFSFGSLIANDIDPVALTALRFLLAGVLIGAAAALGPGLRRHQFRAPWRF
V
LLGAIFALYFVLMFEGLKTAAPVPTAAVFTLTPMLSALFGWLLLRQRVTLRILAALVLGGAGALWVIFRGDPRAFLRFEP
GRGEAIFFIGCVAHAAYTPLVRKLNRGEPALVFTFGMMLAALAVLTLYGAPALVATDWGALPPLVWAVLAYLALCASA
MT
FVLLQYAALRLPAARVMAYTYLTPSWVILWEIALGHGAPRALVLGGVALSVLALVMLLKDG
>OLS47630.1 F0F1 ATP synthase subunit I [Rhodovulum sulfidophilum]AB14
MMDPAEKERLARLEARLAELRKAEAAPERSHMDSHYSQAQLAWRMVIELVSGLGIGFGIGYGLDSWLGTKPIFLVLFIL
L
GLAAGIRVMLRTAQEVQGQQAAAAAEDEKRD
>OLS47631.1 F0F1 ATP synthase subunit A [Rhodovulum sulfidophilum]AB14
MASEDATGGVNIDTMHQFMVTPLFGDGAVHWYTPTNVTLWMGIAVLCTVLLLVMGTRGRAIVPSRIQSVAELAYGF
VYKM
VEDVSGREGLKYFPYIMSLFIFIVFANFLGLIPGAFTTTSHLAVTATMALAVFFGVTILGFVKHGASFLSLFWITAAPLA

LRPILAMIEVISYFVRPVSHSIRLMGNMMAGHAVMKVFAGFAGVLGVGAVFPVVAIGAIYALEFLVAFIQAYVFAILTCV
YLRDALHPHH
>OLS47632.1 ATP synthase subunit C [Rhodovulum sulfidophilum]AB14
MEGDIVQMGAYIGAGLACTGMGGASVGIGHVVGNYISGALRNPSAAASQTATMFIGIAFAEALGIFSFLVALLLMFAV
>OLS47633.1 ATP F0F1 synthase subunit B' [Rhodovulum sulfidophilum]AB14
MATELEAAEAAGHAADAVGMPQLDFSTYPNQIFWLVVTLFAIYFVLSRIALPRIGAVLAERQGTISNDIAVAEELKLQAQ
EAERAYEKALADARAEAQRIVAEAKAEIKADLDAATAKADAEIAARSAEAEAQIAEIRAGALESVTEVARTTAVDVVAA
M
GFRADAGTIEAAVDSKLKG
>OLS47634.1 ATP F0F1 synthase subunit B [Rhodovulum sulfidophilum]AB14
MKKLAVALTFVASPALAAEGPFFSLHNAEFVVTISFIAFLALLVYLRVPGKLMAMLDKRAAGIKADLDEARALREEAQTV
LASYERKQKEVQVQAERIVETAKAEAETAAAKAKDDLKDAIARRLAAADDQIESAKAGAVKEVRDRAIAVAVEAARDVI
V
RQMTEQTSDKLVQDAIGEVRGKLHLH
>OLS47635.1 hypothetical protein BV379_04590 [Rhodovulum sulfidophilum]AB14
MPGEAKPASAPESRALFRPRTFSALLLGTGLVALLALTGVGPARRLLENHSDGIRAAQHAAAIEAVMAEDPRRQRRQDL
T
GRARRHAGDAVEAGFFVRTPTDEKLACITFRGGDMHAPEPYLVRIDLDTLGRMLAVRDELDFTTYWALYKRCRTHANL
TN
SVN
>OLS47636.1 GntR family transcriptional regulator [Rhodovulum sulfidophilum]AB14
MPFQKVQPEKLSAAVVRQIELLILRGVLRPGDRLPAERDLSERLGVSRPSLREAIEALEARGLLVRRAGAGVFVAEMLGA
AFSDALVRLFATHDEALFDYIAFRRDLEGLAADRAARTGSDTDLAVVDTLFTRMEAAHARRSPAEEARLDAEFHLSIIEA
SHNVVMTHMMRAMFDLLREGVFYNRQIMFRQRTTRRMLLDQHRAINTALQARDGAATRAAVERHLDYVVEALRAD
QDVER
NEAVARQRLEQALNKGGTGG
>OLS47637.1 hypothetical protein BV379_04600 [Rhodovulum sulfidophilum]AB14
MIRPSKPLLLTIAAVLALGACTDPSVQTGDARQRTKEGAAIGAGLGAVTGLIASNRGGNDAKSALIGAAVGAAAGAAIG
N
NLDKQAAELRQNFDSNRIGVVNTGNALVVTMPQDILFATDSAAIQSGLRADLQTLAYSLNDYPDTTVDVIGHTDNTGS
AS
YNQQLSTRRASAVASVLTGAGVNSSRVRAYGRGEEQPIASNLSPEGRAQNRRVEIVIRPVS
>OLS47638.1 6-O-methylguanine DNA methyltransferase [Rhodovulum sulfidophilum]AB14
MPKDTDRDENQAYHFAVIRRAIDRIDAAGGESLPLETLAAEMNMSPAHFQRVFSRWAGVSPKRYQQYLTLGHARTL
MRER
FTTLETAGALGLSGAGRLHDMFLRWEAMSPGDYAKGGAGLKIRWGWVDSPFGPALVMATARGICGIGFAAETGSDA
VMAD
LTSRWPHASFVEDPAALAPWAEAAFGRQDGKAAFGRRGGEARLHLIGAPFQIKVWEALLSVPPGQVTTYSEIARAIGH
PR
AVRAVGTAVGRNPIGYLIPCHRALRKTGALGGYHWGLPVKRALLGWEAARADAASGADTRVG
>OLS47639.1 endonuclease III [Rhodovulum sulfidophilum]AB14
MAQALPYHTIHEIFSRFQAAEPEPKGELEHVNAYTLLVAVALSAQATDAGVNRATRALFARVDTPEKMLALGEEGLIEHI
RTIGLFRNKAKNVIKLSRILVEEYGGEVPSSRAALQALPGVGRKTANVVLNMWWGLPAQAVDTHIFRVGNRTLIAPGKT
V
EAVERAIEDNVPAEFQRHAHHWLILHGRYLCKARKPMCGTCPIRDLCPYEDKTV
>OLS47640.1 adenosine kinase [Rhodovulum sulfidophilum]AB14
MTKTYHVVGIGNALVDVLAHAGEDFLAEHGVEKGIMQLIDTERAVELYGRIGPAQEISGGSAANTIAGIAHLGGRTAYV
G

KVKDDQLGTIFAHDLRAQGADFATPMAPGSDEGETGRCIILVTPDGERSMNTYLGLSERLSAADIDAAQIADAEWIYLE
G
YRFDGDESHAAFAKAIAAAKAAGGKVALTLSDPFCVDRHRDAFRRMIREDVDLLFANRAEMLSMYQTEVFDEALAQA
AAE
IELVACTDSADGAHVLSGEGRWHAPAVATRVVDATGAGDLFAGAFLWGLTQGHDLESCGRMGCVAASEIISHIGARA
EAD
LSALFRSHGLI
>OLS47641.1 hypothetical protein BV379_04620 [Rhodovulum sulfidophilum]AB14
MATIAYLLLCRGGPAATVALAERLSAAGDLVALHVDRRARPADTAAIRAAIAGNPSVVLARSVRCDRGEWSRVQAVLN
AA
RAALAAFPQATHLYLLTDECLPIKPAARLRAALDAEGCDHIESLDFQAVGGGGQMALQPICRQDVAAPWRRAGIRAAV
AT
GKPPGRAPPGDLRGMTGSRWWCLRRGTVEAVLAFLAGRPDVRRFFRAGGWAEAPLSATLFQTLVRHLVPGDEIRSRA
PTF
GLVAENGRPVTFYNDHFDLLTVQEGFFAHRISAGAGQLRARLEALHAAPGPGPAVSGGGAEIYRFLTGRGRVGQRFGD
RA
WERGASLGPGHDLMLAICKKRGVARRLAGRVRRTIGMPAVEFPFDDDAADLPDLGGIERGLAKRSRHHGAFLRLVFDS
YG
TDRLLLCLDPSDLDVMTDLHGDGARVRMLEIACPFSQADLLGHARGLGLLSQSSPPGLAALVLPVLGQQIRDESARLRA
A
GFPALYRLEPGLALSEAALVLAKFLEIPYDTARGIADTATLFDD
>OLS47642.1 histidine triad nucleotide-binding protein [Rhodovulum sulfidophilum]AB14
MTYAYDDQNVFARILRGEIPNATVLETEHSLAFNDIAPQAPVHVLVIPKGPYVCYDHFAAEASDAEIVDFTRTIAKVCEM
KELAADGFRMISNAGAHGVQEVPHLHVHILGGRVLGRMLEKA
>OLS47643.1 pseudouridine synthase [Rhodovulum sulfidophilum]AB14
MSRVILFNKPWGVLSQFTDDGAPRPDRRTLAEFIDRPGVYPAGRLDRDSEGLLVLTDDGRLQARIANPKAKTEKTYWA
QV
EGLPPDAALEALARGVTLKDGPTRPARVRRIDEPPGLWPRDPPVRVRKSVPDCWIELTISEGRNRQVRRMTAAMGHP
TLR
LIRYRVGDWTLDGIETGTWRPA
>OLS47644.1 multidrug ABC transporter ATP-binding protein [Rhodovulum sulfidophilum]AB14
MTGNAIEIEGLTKTYAASGSQPPKEALKGIDLDIPAGSIFGLLGPNGAGKSTLINIMAGLVLKTRGKVSIWGFDQDVNPR
QSRASIGVMPQELNIDPFFTPRAALEVQAGLYGVPKSQRITDRVLKAIGLEDKADAYARNLSGGMRRRLLLGKALVHRP
Q
VLVLDEPTAGVDIELRQMLWANIRRLNEEMGTTIILTTHYLEEAQEMCGEIAIINHGELIARDSTSALIGRMDAKTLVIQ
PEGEVASLPEMPGPVESCLRPDGSLCFTYRRSEIGTGAVIEAVRNAGISIGDVATEEPNLEDVFVALTSSKTAA
>OLS47645.1 zinc-finger domain-containing protein [Rhodovulum sulfidophilum]AB14
MAPDAPGQEKDTTPPETTVTAEWRVCCDGSGPGLGHPRVWLSIPRDKGFVECGYCDRKFVHESFADKLKT
>OLS47646.1 DNA polymerase I [Rhodovulum sulfidophilum]AB14
MTEGFGKGCHLHLIDGSAFIFRAFHALPPLTRKSDGLPIGAVAGFCNMIHKYVQDNRGPDAPTHVAVIFDKGSHTFRND
L
YDLYKANRDEMPENLRPQIPLTREATRAFNIACIEKENYEADDIIATYARQAVEKGGRVTIVSSDKDLMQLVGNGVEML
D
AMKNRRIGVEEVEAKFGVGPERVVDVQALAGDSVDNIPGAPGIGIKTAAQLIQEFGDLETLLDRAEEIKQPKRRQTLIEN
RAQIELSMKLVTLDAETPLDETLDDLEVKEPDAGTLLGFLAEMEFRTLLKRVADGLGAEPPALPETSPAAADPADGTAPA
ALPFEPEAYECVRAAAALEDWIGRARARGWVAVDTETTGLDEMRVDLVGISLAVEPGQACYIPLGHREGEGDLFGSAA
LA

EGQMPMDEALALLKPLLEDPAVMKIGQNMKYDAKILARHGIAVAPIDDTMLMSYAMHAGLHGHSMDALSERYLGH
QPIPI
KTLLGSGKSQITFDKVPVAEAVKYAAEDADVTLRLAQLFKPQLHRAKVATVYETLERPLVPVLAEMEMTGIKVDRDTLSR
MSNAFAQKMAALEAEIHELAGESFNVGSPKQLGEILFDKMSLSGGKKGKTGAYGTGADVLEDLAAEGHDLPARVLDW
RQL
SKLKSTYTDALQDHINPETGRVHTSYVITGAVTGRLSSTDPNLQNIPVRSEEGRRIREAFVAEEGKVLLSLDYSQIELRI
LAHVAGIDALKAAFREGQDIHAMTASEMFDVPMAEMTPEIRRRAKAINFGVIYGISGFGLARNLRIPRSEAQGFIDRYFE
RFPGIRDYMEATVAFAKEHGHVETLFGRRIHTPEIGAKGPQAGFAKRAAINAPIQGTAADIIRRAMIRMPAAIAGLPAK
M
LLQVHDELIFEVEAGAAEDVAVRVREVMEGAALPVVALDVPLVVDAGQGPNWAAAH
>OLS47647.1 proteophosphoglycan precursor [Rhodovulum sulfidophilum]AB14
MAKNRSGQMIVKPVTESLAEAARAASGKGLPPVHLWDPPFCGDIDMRIARDGTWFYLGTPIGRAPLVRLFSTILKHED
GK
FYLVTPVEKVGITVDDAPFVAVDFERGEEAGEPVLTFTTNVGDRVTAGPDHEIRVSRDPETGEPTPYLHVRAGLEALIDR
KSFYRLAEIGETRPHEGQDWFGFRSQGAFFPAIPAAELS
>OLS47648.1 AAA family ATPase [Rhodovulum sulfidophilum]AB14
MPDETELVAAIEALGEKLAEAKASITRRFIGQERVVDLTLSALLCGGHGLLIGLPGLGKTRLVSTLSTVMGLNGARIQFT
PDLMPADILGSEVLETAPDGSRAFRFIEGPIFCQLLMADEINRASPRTQSALLQAMQEKSVTIAGEAHPLSPPFHVLATQ
NPIEQEGTYPLPEAQLDRFLVRIDVPYPDRRTEHDILLATTGVAEDEAHAVFTAAELIAAQKIVRRMPVGEGVVEMILDL
VRACRPAEPEAPPIVREAVAWGPGPRAAQALMLTVRAQALLEGRLAPSAEDVLKMAGPVLIHRMALSFAARARGEDL
PRI
IETVARETVRVEAAA
>OLS47649.1 hypothetical protein BV379_04660 [Rhodovulum sulfidophilum]AB14
MISAAALRAHAEGLAEALPPLLAEAEHLAQAVLLGEHGRRRAGLGDEFWQYRPTHSGDEARMIDWRRSARSDAHFVR
EKE
WQAAQSVLLWVDDAASMGFSGASDHPPKVQRARVLALALAVLLTRCGERVGLLGSDLPPRRGQHQILRLTEALGREL
SPS
DYGVPATRGMPPHSRAVFLSDFLGDLAPIETALAEAADRGVKGALVQVLDPAEEAFPFDGRTIFESMGGSLSHETLKAG
D
LRDRYLARLAERKARLADLARVTGWHYSAHHTGEGAQGALLWIYRALERGR
>OLS47650.1 LytTR family transcriptional regulator [Rhodovulum sulfidophilum]AB14
MAGLGALGFSAPLLLWGLVLLPTLWFLLRAVPPAPIRRRFPGVALLLGLADDEAQADRTPWWLLLLRMLAVAALILGFA
G
PVLNPQERTPGQGPLLVLLDGSWADARDWPRRIDRLEAVLDEAGRDGRPVAVVLATDLPGDGLPFQAAELWAARLA
GVQP
AAWLPDPEAVAAWADGLEGRFDTVWLSGGLDWPGRTALLSALERHGRVGVLESQRPLMALRPAAFSDGTIALSVLRA
RPG
PETEAMLAAAGRDPAGVERVLARLPVTFAPGETRAGAELVLPPELRNRISRFAIEGVRSAGAVSLTDDSLKRREVALIAG
NENREGLQLLSPLHYLHQALAPTADLIEGTLGDVVLAHPDVIVLADVATLSPAESEALQGWVEDGGMLLRFAGSRLAAS
D
VSRAEEDPLMPVRLRAGGRTVGGAMSWGEPKTLRPFGEGSPFFGLPVPDDVRVNAQVMAQPDPSLADRSIAALSDG
TPLV
TRKKLGKGEVVLFHVTANAEWSTLPLSGLFVQMLERLAVSTRPARPDAVELAGTTWVPETVLDAFGALGEAGTLPGVP
GE
ALAGARPGPGLPPGLYAGEDRRLAVNVIGADTVLSPAAWPARIAVEGFAVARETVLKGWVLALALALLAVDILASLWLS
G
RLRGARVPSRAGGAMVLALALLLPGGLRAEEAMPDDALALRATSEVVLAHVRTGDAEVDRIADAGLRGLSRKLAERTSI
E

PAAPIEVDLETDELSFFPFLYWPVTANQPMPSPAAYARLNRYLRTGGMILFDTRDADVAGYGGGSSEGRRLQQLARPL
DI
PPLEPIPPDHVLTRTFYLLQDFPGRNAGREVWVEAAPADAERVDGMPFRDLNDGVTPVVIGGNDWASAWAVDGQG
VAMFP
IGRGYAGERQREIAFRFGINLIMHVLTGNYKSDQVHVPALLDRLGQ
>OLS47651.1 hypothetical protein BV379_04670 [Rhodovulum sulfidophilum]AB14
MTGEVIFDPLLPLPLIWALAVLAAGIVGFALWRGLAGWWLRGLAALAVLAALANPSLQTELRTPLSDIVVLVVDESASQ
R
LSDRPEQTARAVERVRAEVAALDDTELRILRLGDGEGDEGTLLMTALSEALAEVPRARLAGAILLTDGQLHDLERAPALP
APLHALLTGRKDDWDRRLLVQNAPAFAILGEEVTLTLKIEDQGAVPEAMGDTTSVTVSIDGGAPQSYRVPVGQTLDVP
VV
LDHAGQNVIQFSVAAEEGELTERNNAAVVQINGVRDRLRVLLVSGEPHPGERTWRNLLKSDSSVDLVHFTILRPPDKHD
G
VPVSELSLIAFPTRELFLDKIDEFDLIIFDRYKRRGILPSIYFDNVRNYVEKGGALLIAAGPDFAGAESIYRSPLADILP
AEPSARVIEQGYRPEITDLGRRHPVTEGLKAEAGGAWGRWFRLIDLLPKQGEVVMSGIDDRPLLVLDRVGEGRVALLAS
D
HAWLWSRGFEGGGPQLELLRRLAHWMMKEPELEEEALSATAEGQVMTIIRRSLSQEPRSVEIARPDGGTDRLDLVETA
PG
RFAATYEGPEMGLYRLTDGTLETVIALGPAAPREFEQTIADGARLEGPVSATRGGIVALSDGMPDLRRVREGRTAHGRG
W
LGITPRAAYLTSDVTIRPLAPAWVFLLLAALLSLGGWLREGRR
>OLS47652.1 FAD-binding oxidoreductase [Rhodovulum sulfidophilum]AB14
MQMPAPDARVMAKKDQVLRRLQAVLPAGAVIHDPTETRAYECDALTAYRCPPLAAVLPASTAEVSAVLRICHEEGVPV
VP
RGSGTSLAGGALPTADCVILGVARLNEVLEVDYPNRFIRVQAGRTNLSVTAAVEGDGWFYAPDPSSQLACAIAGNIAM
NS
GGAHCLKYGVTTNNLLGVTLVLMDGTVVEIGGAHLDAAGYDWLGVICGSEGQLGVVTEATLRILPKPEGARPVLMGF
GSS
EVAGACVADIIRAGVLPVAIEFMDRPCIEATEAFAGAGYPDCEALLIVEVEGSPAEIDEQLALILRIARANDPVELRESG
SAEESARIWLGRKSAFGAMGQMGDYMCLDGTIPVSALPDVLRRIGELSAEVGLRVANVFHAGDGNMHPLILFDANAP
GDL
ERCEALGAEILKLCVEAGGCLTGEHGVGVEKRDLMGVQYAPADLEAQMRVKDVFDPGWLLNPAKVFPLQASAARRA
G
>OLS47653.1 2-hydroxy-acid oxidase [Rhodovulum sulfidophilum]AB14
MRPETEAELAGMVAAAEAPLRIEGGGTRGLGGEGAGLSLSALSGVVLYEPGALTLVVKAGTALAEVEAVLAAEGQRLAF
E
PMDHRRLLGREGVPSIGGVAAANVSGPRRVQAGACRDAMLGVRFVDGRGEVIRNGGRVMKNVTGYDLVKLMAGSR
GTLGV
LTEIAFKVLPVPEAATTLVLEGLSDDAAVAAMAAALGAPFDVSGAAHRPETGETLIRIEGFAASVDYRAGRLAELLGRFG
GARVESDPVAGAALWRRVRDVEDFVDRPGDVWRISVRPSDGPRAAQAARAEAAVYDWGGGLVWVLVGEGRDIRP
DLAGIS
GHATRVRGAGPLASAEPAPLAAMAARLKAAFDPKGILNPGAMG
>OLS47654.1 glycolate oxidase iron-sulfur subunit [Rhodovulum sulfidophilum]AB14
MQTNFTEAQLQDPALARANEILRACVHCGFCTATCPTYQVLGDELDSPRGRIYLIKDMLESGRPADEKTVRHVDRCLSC
L
ACMTTCPSGVHYMHLVDQARAHIEATYRRPWRDRALRWLVAKTLPFPGRFRAALRLAQLGRPFARLIPDPRLRAMLQ
MAP

PDLRPPGESDRPQVFPARGARRMRVALLTGCAQKALDPDINDATIRVLTRHGVEVVVARDMGCCGALTHHMGRADE
AHAQ
AAQAIRAWSAEIAGEGLDAIVVNTSGCGTTVKDYGHMFRNDPLAEEAAAVAARAVDISELLERLELPPGRGGLRVAYH
AA
CSLQHGQKVKDAPKAVLRRAGFEVVEPVDAHLCCGSAGTYNLLQPEIAGQLKTRKVRTLEAKAADVIAAGNIGCIAQIG
S
GTGVPVLHTVELVDWATGGPRPRALATG
>OLS47655.1 heat-shock protein [Rhodovulum sulfidophilum]AB14
MRHYDFAPLYRATVGFDRVADLMDRVMASDMAQPTYPPYNIEKTSDQGYRISVAVAGFAADELSVEVKDSALVIAAR
KAA
EEAERTFLHRGIATRAFERRFALADHVRVTGATHADGMLHVDLVREVPEALKPRRIEISSDAARSIEAREDTSQPEGTAV
>OLS47656.1 hypothetical protein BV379_04700 [Rhodovulum sulfidophilum]AB14
MDPSPRFKKTLQSTWSWYLAQSIFVRALLLVPIVCGLLMTFLIGNMGLALMGTAYAINAFLVGSIGGAIAVVVGKALQII
ARDGRQSR
>OLS47657.1 elongation factor G [Rhodovulum sulfidophilum]AB14
MRCFTILGPTQSGKSTLLKALGELEGRPVTAAFTDALTVSQFSYLGEPWAGFDIAGGPDHLGHAGEALAASDAAILCVPP
DPDTAVLAAPYLRLIEQSGVPCFIFINKMDQDEARIRDIVGALQAYANHHIVLRQVPMRDADGHVTGSVDLVSERAFQY
R
EGQSSKLIEIPAAIQDREQEARGELLEHLADFDDGLMEQLIEDRAPASEEVYTILAEAHRDNAIMAAYLGAASHGNGLSR
LMKALRHEAPGREAIGLRLGIEAPLALGVASDTRKHVGKSTLIRALGQGIRHGEALAGAALGTLTGIDGKAVAEPLAPGA
LAVAVKSDHLDPGMAYDAEASLPLPRWTRGRPPTYTRLLLPAKERDDARLSAALGRMAAADPGMETSQDGETGHAV
VHLQ
GPIHLRRVLAKLAEDFGIEAVERPVSGIYRETVTGPVETHYRHRKQSGGAGQFADVVLTIRPQGRGAGFAFDETVKGGT
V
PRNYIPAVEAGAREAMERGPLGFPVVDVAVTLLDGKTHAVDSSDHAFRTAARAGVREAMERAGTVVLQPIERVEIHVP
NI
HSGSLVALVSSLKGQVLGFAAHGRARGWDVFQALLPAAAREELFQALGGLSHGTAWFESHFDHYEELHGKDAERIREA
RA
TELA
>OLS47658.1 succinate-semialdehyde dehydrogenase (NADP(+)) [Rhodovulum sulfidophilum]AB14
MPAPDLKSLLKDPDLLETRAYLAGDWAGADDGATFAVTNPANGETICEVPEMGRAETARAIEAAAAAQKSWAAKTAK
ERA
NAMRKWYDLMMAAQDDLAMILTAEMGKPLAEAKGEIGYGASFVEWFAEEAKRIYGETIPGHQPDKRLSVIRQPVGV
VAAI
TPWNFPNAMITRKAAPALAAGCAIVIKPAAETPLSALAMAVLAERAGLPKGLFSVVTSTQSPDIGLEFSENPVVRKITFT
GSTGVGRILLRQAADQVKKCSMELGGNAPFIVFDDADLDAAVLGAIACKFRNNGQTCVCANRIYVQSGVYDAFAAKLA
EA
VGKLKVGDGMEEGTDLGPLISDAAREKVETHLADATAKGARVILGGKPHQRGGTFFEPTIVTGATREMQFATDETFGP
LA
PLFRFETEDDVIAMANDTIFGLASYFYARDLSRVYKVAEALEYGIVGVNTGVISTEVAPFGGVKQSGLGREGSRHGIEDY
LEMKYICMSV
>OLS47659.1 alpha/beta hydrolase [Rhodovulum sulfidophilum]AB14
MVMQAETVDWDDAYANAPHIPDSETYPARLTAAAEAFRAVAPGELDCPYGEHPRQRFDLFLPEAEPKGLMVFVHGG
YWMR
FDKSFWSHMAAAALARGFAAVLPSYRLAPEVTIPEITADIRAAVAAAAARVPEGPLVLAGHSAGGHLVARMLCADAAP
GA
AVDRIARVMPISLISDLRPLLKTQMAGPLGLDADSAAAESPALMAPRADVPVHVWVGADERPAFVDQARWLAEAWT
GAAL

TVDPGHHHFNVIDGLAQADSPLATALFDGL
>OLS47660.1 peptidase T4 [Rhodovulum sulfidophilum]AB14
MRPGPRNLVTDVAGLRVGQAGDARVKTGVTVLLGEAPFVAGVHVMGGAPGTRETDLLSPDRLVQEVDALVLSGGSA
FGLD
AAAGVTEGLRAMGRGFEVAGQRVPIVPGAILFDLANGGDKDWDEAHYRALGRAALAVASEEIELGSAGAGLGATTAD
LKG
GLGSASLVLDSGITVGALVAVNALGSAVMGGGPEFWAAPFEIEAEFGGRGVGRHDPCAEPRPEAALGQSTTIAIVATD
AA
LTQAQATRMAIAAHDGMARALVPSHTPFDGDLVFAAATGEKPLPGDGFEALRLGHAAAICLARAIARGVYLARPAPGD
PK
PCWQDRFGA
>OLS47661.1 hypothetical protein BV379_04725 [Rhodovulum sulfidophilum]AB14
MAETVSVTELTPADLSETPLLRDHMAPPEARDARYEANYDRTTLWYDAFWREDRGEVTVIAPLLLNLARPLSQARILLD
G
RPVRLRRRRFQRHEIWRLPAPAHPERLRVEGQGWAVEASVGRARPERFAGRNVLLTISRNNDLQWVRDYALFHRKTQ
GAE
AILFIDNGSDAYGPGDIARVIGETGLAAHVISAPVPYGPVIRGISNPHRGKFFRSAMMNLARLRYLYGARAVLNSDIDEL
IWSVGRSVFDMAAAHPIGFAPFRGRWRVPGPDAEPPLRHVDHFVLPEKNMPARSNTRSRPDGLRAGSTGMCTGSSG
CRSS
TASCGGIPVTGTARRFRPDGNPTGG
>OLS47662.1 aldehyde dehydrogenase [Rhodovulum sulfidophilum]AB14
MRELWFETDRCFIGGVWQPPASRDFLAVENPSTGAPIGSIARGKVADIDAAVTAADEARQGPWGRMTATERGRVLT
RIGQ
FVRGRAETLARIEATDVGKPLTQARSDAMTLARYLEFYGGAADKVTGETLPYMEGYTVYTLREPHGVTGHIVPWSHPM
QI
IGRSVGAALAMGNACVLKPAEEACLTALAFAKLAEEAGLPDGALNVVPGLGDEAGAALASHPGVDHISFTGSLSVGRKV
Q
AEAARHVAPVTLELGGKSPQIVFADADLEAALPFLVNGGLQNAGQTCSASSRILVERRLYDELVERMAGAYRGLWVGP
AL
EDLNLGPLISSRRKEIVLDYLKKGQDLETAAEGRIIGNAPAGGYYVAPRLLTRVPQTHRLAQEEIFGPVQVVIPFGDEEE
AVAIANGTGYGLVAAVWTRDGARQMRMARSLRAGQVYLNNYGAGGGVELPFGGIGLSGHGREKGFQALYGFSVLKT
VAAR
HG
>OLS47663.1 peptide ABC transporter permease [Rhodovulum sulfidophilum]AB14
MSARPNLIAGGVLSGTVLALAALSLVWVPHDVAALNIAERLRPPGGPHPLGTDHLGRDMLSMLMVGARSSIAVALVA
VGI
GMGAGVPLGLMAAAARGSWLDELVMRMNDLIFAFPALVIAILITAILGPSALNAILAIGIFNIPVFARVTRGAALSLWTL
DYIRAAELAGKGRVRISAEHVLPNIVNLLIVQATIQFSLGILAEAGLSYVGLGAQPPTPSWGRMLAEAQTMIYSAPRLAI
LPGLAIVLTVLGLNLLGDGLRDALDPRLRRTTR
>OLS47664.1 peptide ABC transporter [Rhodovulum sulfidophilum]AB14
MLRYALTRLLSLSLSLVAASLAIFAAIEVIPGDPAAYMLGLNAAPETVAALRDELGLSGTLWTRYLDWISGLATGDMGLS
YTYRVPVADLVTERLAVSLPLAAYALALSTAIALPVGLWAAARRGRAADLGIMAATQLGIAVPNFWFAMLLVLVFAVKL
R
WVSAGGFPGWEAGLGHGLKALTLPAVALALPQAAILARVMRSALIETLGQDYIRSARAKGLSRAQALRRHAFRNALIPV
L
TILGLQFSFLLAGAIIIENVFFLPGLGRLIFQAITQRDLIVVESVVMLLVFAVILVTFLVDLAYAAVDPRLRRR
>OLS47665.1 creatininase [Rhodovulum sulfidophilum]AB14

MTRSDPRPAFYRFPGEKKAPLPFSSEEYDTRLMGLRSAMDATGAEVAVLTSMQNVAYYSGFLYCSFGRPYALVVTASG
SV
LIGAGIDAGQPWRRCHGEALTYTDWARDNFWRAVAHVAGQGRRLGIEADHLTLARREELESFLAPAALVDLAPATME
QRM
RKSPAELDLIRAGAAVADLGGHAIRAAVRAGTREIDVAMAGRDAMELEIARRFPEAEYRDTWVWFQSGPNTDGAHTP
LTG
RVLKRGDLLSLNAFPMISGYYTALERTMVLGEPDPAKLALWEANLAAHEYGMSLLKPGASCAEITHRINDFLAERDLLQY
RSFGYGHSFGILSHYYGREAGLELREDVETVLEPGMVISMEPMLTIPEGRPGAGGYREHDILIVTGDGAENITGYPYGPG
FNVID
>OLS47666.1 ABC transporter substrate-binding protein [Rhodovulum sulfidophilum]AB14
MRPIRTTALNLALALPFALALTIAPTAGPAQAAPPDTITIGMVLEPPNLDPTSGAAAAIDEVVYANLFEGLTRFAPDGTV
VPGLAASWEANEAATVYTFHLREGVRFHDGTAMEADDVVFTLDRARGPDSTNAQKALFEGIESVAAPDPLTVTVTLA
GPD
GAFPFKMAWGDAVIVAPETAGTEASAPVGTGPFRFERWVHGDRVELLRNPDYWGPAPALSRATFKFIPDPNAAFAA
MMAG
DVDAFPNFPAPETLPQFEADPRFRVIVGTTEGETILALNNRRPPLDNIRVREAIAHAVNRQDIIDGAMFGYGTPIGTHFA
PHHPDYVDLTDLSRHDPKKARALLAEAGQTDLTLRLALPPPSYARRGGEIVASELRAVGIETEIVNLEWAQWLDQVFRR
H
DFDLTIVSHTEPMDIGIYARPDYYFGYDRPDFPALMDRLAGATDPAERSEILKAAQRMISEDYVNVYLFQLAKTGVANA
K
LEGLWENAPTQANDLTAVHWTD
>OLS47667.1 3-methyl-2-oxobutanoate hydroxymethyltransferase [Rhodovulum sulfidophilum]AB14
MSASAESRQMTVAAVRARKGGTPLVCLTAYTTPMAEAVDAACDVVLVGDSVGMVLHGLPSTLGVTMEMMLMHGR
AVARGL
AHALMVVDMPFGSYEEGPQQAFRNAARLMAETGCGAVKLEGGAAMAETIRFLTARGIPVMGHVGLTPQGVMGFG
GYKVQG
RGADGARVLAEAQAVAEAGAFAVVLEKVPAALADRITAAVPIPTIGIGASVGCDGQILVVDDMLGVFTGFRPKFVKRYA
E
LGDAARASVEAYAEEVRSRRFPGPEHVFADEIGGGA
>OLS47668.1 pantoate--beta-alanine ligase [Rhodovulum sulfidophilum]AB14
MSAPILRSLAELRALRAAWRAEGARVAVVPTMGALHAGHLSLAAAAKAAADRVIVTIFVNPRQFNDPGDLARYPRTEA
RD
AEKLAPLGVDAIYVPAPDQVYPPGFATSISVTGVSEGLCGAHRPGHFDGVATVVTKLLLQTGADCAFFGEKDYQQLMV
VR
RLVRDLDIPVEIVGCPTVREPDGLALSSRNALLPGDARAVAPALYRALAGAGQALEAGAGAGEALAGARATILDAGFDE
V
EYLELRAAADLAPLDAAVVPARLLVAAHLGGVRLIDNIPVAAG
>OLS47669.1 succinyl-diaminopimelate desuccinylase [Rhodovulum sulfidophilum]AB14
MDGSLEHGLERAVAARRDEVAALTRDLIRIPTLNPPGRNYLEICEFLGARLAAKGFDISYVRARGAPADSDAHPRWNMI
A
RHEGGPGGDCVHFNGHHDVVEVGHGWSVDPFGGEIRDGRVWGRGACDMKGGIAAAVLAAETFVETCPGFRGAVEI
SATAD
EESGGFGGVAWLAERGWFAPERVQHVIIPEPLNKNRICLGHRGVWWAEIETKGRIAHGSMPFLGDCAVRHMGAVLA
EMEA
TLFPLLAGRHTAMPVVPEGARAATLNINAVHGGEPEQEEGYDGLPAPCVPDRCRITIDRRVLIEEDPEEVKREIRALMER
VRARRPGFDYEIRELFEVRPSMTARDAPVVRSVERAIGRVLGAAPDFVVSPGTYDQKHIDRIGRLRNCIAYGPGILDLAH
QPDEWVGIDDMVEAAQVMALTLSDLLGG
>OLS47670.1 hypothetical protein BV379_04775 [Rhodovulum sulfidophilum]AB14

MFAFLRFALAGFVILTVIYVAVSLYSRAVRRGKLGREWDEEVRIGDRQAYVDEGLRQYDRSLRRKLILGIYVVPAIVVGT
IIYITNYL
>OLS47671.1 hypothetical protein BV379_04780 [Rhodovulum sulfidophilum]AB14
MAYVKWTIVTIVILVIAGFLHYTLPQHDVVRITATKERQINFGENRIFFASNGAGNSEGDNTRFVNFIETFNRDGKPIVF
RNEDTSWGWPPYFKFDSSNLQAKASDMISDKENPEWVVVTHYGWRNEYLSIYPNAVELRPVGSPDDSPIPWFNILFL
GAL
AALVIWIWQLLARFKEDRIEPMLDEMGEQFDSVGDRAEGFWRRVFSR
>OLS47672.1 hypothetical protein BV379_04785 [Rhodovulum sulfidophilum]AB14
MAPNALDRGPGRAPDAEARAWIDQHDGAFGPWISGSLRPGADATAICSPASGAELARLADATSADLAEARAAAATA
QPLW
AALPGVKRARKLNDWTEEIAARETCLTQLLALETGRPVRACRRGLSAGLNALRQAADSLLAGAADRPGHVPLGVVAPG
LS
FGLSLGRIAPALAAGNAVILAPGPADGKLAVLCLAEAAATAGLPPGLLAMLPGAAGALSDLAQPPVATLRWPCLHLVLE
D
ADLDAAADGIAGMLGDPQPGTAGHWLLVEESVAERVHARLAGRMARLIIGDPLDPATDLGPLSGPSLRDRLTAALAET
RG
VTIQPLPRLPDRGAFMAPALITDLAPAMPEMETSLPGPVLLSMTFRTPAEAIRLANATPPAPAASLWSETLSTALDLATR
LEAGTVCVNGTKLEPAPDPLALWQRPEGLAPARPVVRPVAAAPVPSAALTTARRAQPDWAGRGTAERAAILTRAATRL
AE
SAEAPAARFASAAGAAATASAGWAETPGRATLTRHRPLGVIGVLCPEFCRQATPVLAMASILGPVLAAGNGALLHPGN
AA
EAEAEALIAALEAGGMPKGLVGIVSEPRAALACAFAARVDLDALWCFGAGDLPEDLPARAAATGTALWLSEGGASET
WTG
PGGASDLFRIAAGRRQTLRLSWGA
>OLS47673.1 deoxyribose-phosphate aldolase [Rhodovulum sulfidophilum]AB14
MTRRENKTPATTPARPASPALPRLAHARNGGLPLDTGWIASTRVNRPAAERHAAGLAARRPADPATEAAWLLRAVGL
IDL
TTLSGDDTPGRVCQLCAKARAPLRADLRKALGAGPVTTAAVCVYHEMIAPALAALEGSGLPVAAVSTGFPAGLSPFSTR
L
AEIEASVAAGASEIDIVISRRHVLLADWQALYDEVAAFRAACGPAHMKAILATGELGTLTNVARASLVAMMAGADFIKT
S
TGKEPVNATLPVGLTMCRMIRAYREATGVDVGFKPAGGISTAPDALDWLVLVGEELGPRWQEPDLFRFGTSSLLGAIE
RR
LDEIAFGAGSTPRRHSRG
>OLS47674.1 hypothetical protein BV379_04795 [Rhodovulum sulfidophilum]AB14
MPESSVSVPKADPAERLTEFALEALLASPEGWRSFARDSVFDCPDAPPLALIFALVNASAQIEAIFSEGSPARTAAQNGF
RLAGLLSADLYAMQSLGLPHARAADFSDYWHSSDPYFLTL
>OLS47675.1 efflux transporter periplasmic adaptor subunit [Rhodovulum sulfidophilum]AB14
MGRRLALIFALLAALPAAAAETVLAPVTITEWKPVYGEVETRNRVPARSRIGGTVVELKVTEGDPVEAGQEIARIEDDKL
SFQMRALDATLKALTAQLDTARRDLDRGRALRERGVVTQQRLDQLMTQVAELEGQIDSTAARRRVIEQQVAEGAVLS
PGS
GLVLSVPISPGSVLVAGDTVAEIGGGGLFLRLAIPERHALALRPGDRIAVGDSTGRDGDSGRLVKIYPQIEGGRVLADVE
VEGLDGRFVGRRVPVRLPVGTRQALLVPPGAVTHRGGLDFVAVAGPDGPVERVVVPGERLRHDGRDRVEILTGLEPG
DRV
VSPDE
>OLS47676.1 multidrug transporter AcrB [Rhodovulum sulfidophilum]AB14
MSDETRPAARRLGLAGYLTKGFIRSPLTPLLLLAALALGLIALIALPREEEPQISVPLVDIHVRADGLRAVDAVKLVTEP
LETIVKGIDEVEHVYSETRDDRAMVTARFKVGLSQDTAILRVRDKILANMDRIPVGIPEPLVVGRGINDVAIVTLTFTPV

FGQSAVTAADLTRIAREAETELVRIDDVGLTYLVGETEEALRVAPDPQKLALYGITLQELSQKVAGANAAAPAGLVRDAG
QQVGLVLGRTLATPAEIGNLELTARDGRTVYLRDLAEIGFVSDLDERHVASLARGPEGEILRRPAVTLALAKRAGANAVV
VGEQVLQRLEEMKGGLIPDTVAVEVTRNYGHTASEKANELLFHLGLATVSIVALVLFAIGWREALVVAIVIPVTILLTLF
AAHAMGYTLNRVSLFALIFSIGILVDDAIVVIENIARHWGMMKPGDSRVRAAVEAVAEVGNPTIVATLTVVVALLPMLF
V
SGMMGPYMSPIPANASAAMIFSFFVAVTITPWLMLKIAGRAPLHAGGAGAGHPGGRLGRLYAHLVRPILSRKWISLVF
LL
GVATVSFGSMAALYTKDVTVKLLPFDNKSELAIVIDLPEGASVEATDAVAQAAARVALAMPEAVSAQTHAGTAAPFNF
NG
LVRHYFLRESPEQGDVQVNLSPKDARDRTSHAIALDLRQRLGGLDLPAGTVLKVVEPPPGPPVIATLLAEIYGPDGDTRR
AVAARVRQAFEDTPFIVDVDDSFGTRARRIRAETDPSDLAFFDVAEADALDTLRMLNGATTVGYSHRGAGRAPIPIVVA
R
DKADRVLDERALATPVPANVLPGGRGVVELGDVVRLTEEQASFPVFRHNGRYAEMVQADLAGDFEAPLYGMLAVGD
RLDR
VDWQALGLTAPEIRLHGQPLSEDRPVLLWDGEWEVTWVTFRDMGAAFGVALVGIYILVVAQFGSFRLPLVVLTPVPLT
FL
GIMLGHWLFHAPFTATSMIGFIALAGIIVRNSILLVDFIRHADPEGRITVETLVEAGATRVKPILLTALAAMIGAAVILA
DPIFQGLALSLLFGLASSTMLTVLVIPAIYRIFKT
>OLS47677.1 hypothetical protein BV379_04810 [Rhodovulum sulfidophilum]AB14
MTADDAYAGLMAGAARSDQDAADLHATASILALALSEAASPEDLTAHCGLAAGALAELLGALFPAAPPEFRHGRGAAP
RG
VEEDSVRALLLAHSGGTALARPLAAMIARRAMEPDHLWQDLGLRNRHELSAMLTRHFGPLAAKNTANMKWKRFFYR
RICE
DEGFVLCATPVCTDCADFDMCFGEESGEARLARAGRAAAPAPAQVLKMR
>OLS47678.1 NUDIX hydrolase [Rhodovulum sulfidophilum]AB14
MGLDADLAIERQHPLGSQIAALPLRLDRNGRLGVLMVTSRGTGRWVVPKGWTMEGHSPWGAAGIEALEEAGAEGR
VASHS
IGSYEYDKRLDDGTVLRCRVEVFPMLVCKLRRNWKERDERIRRWFTPKAAAQRVDEPELAALLRGLSQKPEKLPVIRDLL
GSD
>OLS47679.1 hypothetical protein BV379_04820 [Rhodovulum sulfidophilum]AB14
MLSGDDLHGSVPTLGQTATALPGGARPFCNIDVILGQECNRKASSVAELPQQGRSLAGLPCKRLALLSRQGLWMRRFG
DI
HPPGTDRPREPLRDQPCWGAGLRETAR
>OLS47680.1 inorganic phosphate transporter [Rhodovulum sulfidophilum]AB14
MSETYKDRQWKTLDKDLKRLSRVEIAAQTVSRPLLGVGVSLAFIVIAALGAALVTGNTPGMLFVVAAAAFGAYMALNIG
A
NDVANNMGPAVGANALSMAGAIAIAAICESAGALIAGGDVVSTISKGIIAPESVADSATFTWAMMAALLAAAVWVNI
ATW
IGAPVSTTHSVVGGVLGAGVAAVGIAAVNWSTMGAIALSWVISPVLGGVIAAGMLAFIKWRVIYRDDKIAAARTWVP
VLI
GVMAATFATYLALKGLKHLFDVTIGHAVAIGVVVGIASGLGAVPLIRRQSRGLENRNRSLKLLFAAPLVISAGLLSFAHG
ANDVANAVGPLAAIVHAQQAGEIAGKVAIPVWVMVIGAFGISFGLVLFGPKLIRMVGSQITKLNPMRAYCVALSAAM
TVI
VASWLGLPVSSTHIAVGGVFGVGFFREWHAERRLRRANLTKPGAKRLGPEERRRRKLVRRSHFMTVVAAWGVTVPAT
AVL
SGGMFLAISALQ
>OLS47681.1 malonyl-CoA synthase [Rhodovulum sulfidophilum]AB14

MTNPLYDRLFGRHAGRETPFLHLPDGGSLTHAAFLALAARHAHALNAFGLGPGDRLAVQVEKSPEALALYAACLQAGV
IF
LPLNTAYTPAELSYFIGDSGARLVICDGRRMGALTPVAEAAGAHIATLNTDGSGSFAVIAATKSATYPTVARDPDDLAAL
LYTSGTTGRSKGAMLSQRNLLSNAEVLTEAWRFTEDDVLLHALPIFHTHGLFVATNIMLLAGGAMILLPKFDIDAVIAAL
PRATAMMGVPTFYTRLLDDPRFDRALAAHIRLFVSGSAPLLAETHRAFEARTGHRILERYGMTETNMSTSNPYDGERIA
G
TVGLPLPGVELRIADPATRAECSPGETGQIEVRGPNVFQGYWQMPGKTREELCEDGFFLTGDLGTIDAKGYVRIVGRAK
D
LIISGGLNVYPKEIEEVLDAQPGVLESAVIGVPHPDLGEAVLAVLVPAPGETPELQAIAGSVATQLAGFKRPKRLELIEA
LPRNAMGKVQKAALRESYRDSFA
>OLS47682.1 potassium transporter [Rhodovulum sulfidophilum]AB14
MEGFLLNATLYLLCIVLAVPLASRLGLGSVLGYLLIGLMLGPVLGLVGAEMVTLRHVSEIGVVMMLFLIGLELDPRALWA
MRNKLVGMGGLQVLITVALVTAAAIAFGQTWQSALAMGMIFSLSSTAIVLQTLTEKKLMNTPGGRSAFSVLLMQDIAV
IP
MLAIMPFLASPEIRAAGTEGGHGAMHLIGSLPAWGVTLVTLGAVAGTVLAGHYLVRPLYRYVQAARLRELNTAMALVI
VA
GIASLMLMVGLSPALGTFLAGVVLANSEFRHELESSIEPFKGLLLGLFFITVGAGMDFSVFWDAPILLPVYVTGLVMIKV
AVLYGLAMAFRLDRQSRWLFTLGLAQAGEFGFVLVGFASTQRIMPPDIVTMSSLVIALSMLITPALFILHDVIAKVLQGR
GGARPAADPIDETHPVIIAGIDRFGLTVNRMIALTGGKTTAIDDDLRAVRRVRKLGFKAYYGDATRPALLASAGIDTAEV
LVVCIGDAKAATTLVRYARRIRPDLFIIARARDREHVFELYRAGADETVREVYDSALRCGRQVLERSGLSAYEAENAERT
LVTFDRTALRDLAKVWRPGVPLEDNADYIELNRRLQADLEHAFAETLLHEVPPPRSQTEATSPADILAEADGNPERQTG
R
TQT
>OLS47683.1 hypothetical protein BV379_04840 [Rhodovulum sulfidophilum]AB14
MLQKILRKLPWYIYTILGILVLCGTYPTFQQARQEAADVAAISGEPAPTMQPLDSYIATRSNEAFHQAEFEANIDDYYFT
LDKSRRRGPDTHIVVMPIFTGDAAQEAKRTSGVLYFDSEEDMKAWARNHSDEASQSRNGHWHQQIHGYEDRHPDLV
GMVR
DTLIDDGYTLERPFLHVRPYSGSREAALAALATPDYVPVYVLLGVGVVLLVVSFGKRISARQARDATDGAA
>OLS47684.1 hypothetical protein BV379_04845 [Rhodovulum sulfidophilum]AB14
MSKPEAARYRTTNWKSYNEALTPSMPASRPRFSILKDLGFQAGRSNTGGKAMSFDSMADLVDPVVGPRHASQNITCL
RRS
KT
>OLS47685.1 hypothetical protein BV379_04850 [Rhodovulum sulfidophilum]AB14
MIGPDEILPLQCLLPLQVMPQLFALGALYLLLNFGTADGEVSDTDIAALRDYLGSAEAADAQRGPLLVETIPDPAAAQQV
ADLERQILSDLRVFASRMETVIQPWIDDWERSGWFGALGTFLGGLRDGAAAWVDGESEFWAGMWEWVSGLPDML
GEAWDD
LSAGVRALWDNRHRIVELLQSLAEGAVDAFERGFAALLDALDGLPGIEEIVELLRSLVIRSAEWAGAMYDHGPVAWRCC
R
TLGRQDSPLESGSLRGFS
>OLS47686.1 aldolase [Rhodovulum sulfidophilum]AB14
MSFRDKLLAGEVLIGTFVKTPSPHVVEILGLAGLDFAVIDQEHAPIGLAEMDMMAMAGRAVGLPLLSRRWGKGTDWI
APL
MDLGMTGVMVPHVLDPEGAEAVCDAVKFARLKRGLSPSPRAGDYGGMSIPSYREKSDAESVVMVQIEDECALAHLD
AIAA
IKDVDLVFIGPADLSQSMGVGFPSPELDDAIMTIVEACKHAGCAVGLFVGDATAIPAWVEKGVGVFVCGSDQALLRKG
AA
GIMAAR
>OLS47687.1 acetyl-CoA acetyltransferase [Rhodovulum sulfidophilum]AB14

MQDIYILGAARTPIGSFGGALSSLGPDALGRIAAEAAIARAEVSARMIDNAVAGNVIPTQPRDMYLARAVAMEAGMPA
TS
QALTLNRLCGSGAQAIATAATMIRAGESRLALAFGAEAMSRAPHTVGGMRDGVRMGDTSVTDWLTGALSDPFDIGH
MGVT
AENVAGLHGITRQEQDAFAAESQRRAGIAIDEGRFDTQICPVTIKGRRGDTVVSQDEYPKPGTDAARLAGLRPAFRKDG
T
VTAGNASGINDGAAALVLADEDAARAAHPIARLLSWSVAGVPPEVMGLGPVEAVPLALSRAGLSLADIDVIESNEAFAA
Q
AIAVNRLLGLDPEKVNPNGGAIALGHPLGATGAILAVKAIHELQRMAGRYALVTMCIGGGQGIALVIERLEDTA
>OLS47688.1 hypothetical protein BV379_04880 [Rhodovulum sulfidophilum]AB14
MQGTMPLEHEARISELLVGAVDPHVHSGPSIAPRALDHLELARELSEAGFSAVVTKDHDYSGVICATMIAHHHPDLTTR
V
FSGIALNNVVGGLNPYGVEHTAAMGGKIVWLPTLAAENHLSWQATSGWSHPASTSRIRAATAVRLWDDDGKMLPEL
HDIL
DVCAATGLALASGHVHVSETKKIFAEARKRGVERLIFTHPEDIVGASMEDTREIADMGAMVEHSLAFFLEGSKFRNHTT
E
QLGEYIELVGPERTILCSDLGQVGTLTPLQGFRRAVSDCLSLGYDDAAVRAMVSGNAAQVIGLDR
>OLS47689.1 hypothetical protein BV379_04885 [Rhodovulum sulfidophilum]AB14
MENDKKMGEGLQSVILTMQIVEHVSRTGKGVGVTSLATALGTSKSRIHRHLQTLVQQGYVFQHEDSERYEVGHQLVSL
AQ
AVLEGTGLIAATRDALLALRDRLGHSTVASQLTPDGMLVIATVSGRSPIEIGVRVGSLLSFHGSAQGKVAAAFSSEAFRN
RVLGGTLTAFTKATITDPDRLQAEFELIRERGWATAPDQAALGLNTLASPILDGANMVCGTVGCVNLTQSLGAEPSPEQ
I
EALLETARQVSVLMGHSGDQLLR
>OLS47690.1 hypothetical protein BV379_04890 [Rhodovulum sulfidophilum]AB14
MYDKSDPRAALASTPADSMIGVTGLVAEEQVATFYDSPPQLDDDTGQTWLYRGHNFLVALSHAASGASFDRVAQAD
EYCV
LLPKGGATIEWNGAVTEVPGHSIAFVPAGDSRVTAEGGEIVRLFTTANEDLLALCGNAGGYDRPRSHVKPLTAWPAPK
GG
FKVRHYSLDVAPEPGRFGRIFRCTTLMVNVLEPFEGPRDPSKMSPHHHDDFEQGSLALSGEFTHYLRWSWTSDMAD
WKDD
KVLKMAAPSLLVIPPPVIHTTRATGPGDNLLADIFSPPREDFSLKPGWVLNAEDYPMPEQA
>OLS47691.1 hypothetical protein BV379_04895 [Rhodovulum sulfidophilum]AB14
MLTRGAAFTTVLAAPNIARASGRRMRFAHAANEVHPGHIMATSFAAALEELRPGGFDVRIFPNRQLGDDKQLLEGAV
AGT
MDVVSVSGVLVPLVTGRQAMNAWQMPFLIRDYDHFGKLAHGPVGQQILDDLRPAGLIGLSIANTGQRHFLSIDRNVV
SIE
DFAGLKTRIVPVPLHQQIWETVGTAPAGLPYGEVYGALETGVIDAVEINVSSMLGENLWEVGKHFTLTGHYPWHMTTL
MS
ERFFEGLDEEERDVVVEAGKMSTAATIEYAKQQDLEGREELATRDVEIAELTDLSEMRARVEGLTADWSSKDPLIAAMV
D
AAAKSA
>OLS47692.1 hypothetical protein BV379_04900 [Rhodovulum sulfidophilum]AB14
MAAIVAIMLAQVFWRYVLGGSLIWAEELCRYLLIWQTFLVLGLAFSKGEFVSLDFVPSLLSARLRWGLRAVMAVPILAFL
AVMTWYGWDFAERMGPQTIPALDFIWSAVTGQALGLSIRWVYISVSVGMVLLAGHILTGLVVDFHRQVILNLGEEDA
ALA
QTSEMT
>OLS47693.1 C4-dicarboxylate ABC transporter permease [Rhodovulum sulfidophilum]AB14

MSSFFLLFAAFLATGVPIALTLGIAGTAYLYLSGNGAMALMLPQRMIAGIDQFVLLTIPLFLLAGALMNVGGVTTRIVTF
ARAMVGHRPGGMSSVSILSSGFFAGISGSATAEASALGSILIPSMSKQGMPPAYAAALIAVSSVMGPIIPPSITMIIYGV
LSGASIGQLFLAGIMPGILIASGLLIYATWRARRDGFPVIPRMTARERFASTRRTLPALIQPLIILVGIKAGIFTPTEAA
AVAVGYALIIGFIYRELTPSRVWEALIATALVSTSILFITSMASIVSFVFTLEQVPQLIADGMLSLTENPLLILLLLNVF
LLVLGMFLEPISILILTMPILLKFQDLIGMDSVQFGTVVVLNVVIGMATPPVGILLFIGAAISGQPVTRVIREALPLIAI
CLLVLAIIALVPQVALFIPRFMV
>OLS47694.1 hypothetical protein BV379_04910 [Rhodovulum sulfidophilum]AB14
MTHVLIVIAHPDPASFNHALAFATAEALRAAGHEVTISDLAAEGFRADLSPADMTERADPARFHVQAEQANAARTGTY
AP
DIAREQARVAQADAVILQFPIWWGGPPALLKGWIDRVLSYGFGYVDGRRFDTGLFKGRRAMISVTTGGTPERFSEQG
VYG
PIDPLLMPIRKLALEYMGFEVEPLQVAYAVSRISEPERKKHIDRLIAAACAMSSRPSHRSDAWKTALDDVPEGAWARRG
>OLS47695.1 hypothetical protein BV379_04920 [Rhodovulum sulfidophilum]AB14
MDMPFKSASEITAMAASCFPLARAAATGIDQDVLDLVAPKPKQEAKKLAGLLRSGKIFDKGVLAMLHELVGVLDAEIAE
G
TYIETRYDHSPENVSDYKTWGQERLTPEADRLSNALAVLIELYATLSAVHDILRAEKALEGLRSAA
>OLS47696.1 hypothetical protein BV379_04925 [Rhodovulum sulfidophilum]AB14
MYHHPQPFLPASNSEHLNRGDIVLFRFPLAEEDGEHGAPKRRPCLVLDTLTNGNERFVKIAYGTSAETRANRGYEVIVK
Q
SAARETAGLSKPTRFVCARRIIVHASHSGFDGRTKGPHIIGRLDVPLLERLNAVRARIQAEADIAAYYREEQRQEEQARE
DRGFQ
>OLS47697.1 hypothetical protein BV379_04930 [Rhodovulum sulfidophilum]AB14
MTVEELISLIKKSGQHDCVYHFTDESNFEKIDELGLVSKQRMRNENWWPNATGGNQWSHDQDRVRGIDPYVSLCFTS
NHP
MQYLAHNDGRLPNPRYLVIDPEVLRIPGVMVAFGVANANETEILPLADALHWIGSTWR
>OLS47698.1 hypothetical protein BV379_04935 [Rhodovulum sulfidophilum]AB14
MASRPVFLPVFNGPRIVQERQFDFTWAPGFAEVQKKKNISALHEAARKHGIEKILEISSKSENELGRRLSAFSLKVRVGE
CCYPLESVYQGSKVFEDAGPFPDIFEMPPRDAKRFIREKKAGNLVAFELEQKRFPLSPKNAFYDWLYIRALSDHAGWIKR
NVDYDAYADIEFNPAKQVNCQARAFAEYLSLMRQGKLHEAVGDFNSFSKMISGL
>OLS47699.1 hypothetical protein BV379_04940 [Rhodovulum sulfidophilum]AB14
MTKSRFEIAFEGDPFDDGEIDVRDLAPTLLAFGNVVQQANRALNGDRADARLKVAATEKGSFVAALTVDVAWLIDML
DSV
SAHPDRVVAADQLMDLLIKAGTIIGAPVGLFATLKKLKGKRPEKVEPKGDGTTEITINQTTFIVDDQTVDLLQDLPTREA
IENLGQKARRVDGLERLRIGSDEPDHKAVRLERDDFPSLSVPPETDEPDVEVTRRDAWLKIVSVHFRDGYKWRFSDGGE
R
PFTAEMEDTDFQNKVQEGYIAPNANDAIRCRLREEQSLSASNLSKIVYVEEVLEHRPGARQLNLL
>OLS47700.1 hypothetical protein BV379_04945 [Rhodovulum sulfidophilum]AB14
MLMRALVECTSDKAFASLHRLLMTCAHLFACHDSVRQMLETCHGQITALAELGEFSDIAEGRTTRPADDARAPLTDAC
LP
NRLAAPCTRALRARHGPAFLGGLRYEHRSARFPRPYPSPTRSGRLADP
>OLS47701.1 hypothetical protein BV379_04950 [Rhodovulum sulfidophilum]AB14
MCNPDERHYGGYRAPCEHELRRQFELVGRIAAGECKHRCDACQHEKEEAEAKARGWSLIGAAPEGGAGYRRYRHDW
SAMT
VAGRV
>OLS47702.1 hypothetical protein BV379_04960 [Rhodovulum sulfidophilum]AB14
MAGDDLSTAQGRTFHAFRFAYETAMRAGEIAELEWDRIDLEARVAHLQMTKNGRARDVPLSFEAVLLLEALPQISPGF
GL
TPRQIDALWRKVRDKAAVEGLRFHDSRAFALTRISATIDVLTLAKI

>OLS47703.1 hypothetical protein BV379_04965 [Rhodovulum sulfidophilum]AB14
MGNSGGEYNPVQGWRTQIALDGIRRPKIFETKREAQDWAARQEYLIKNAEKEASAQPFDDVLLRYA
>OLS47704.1 hypothetical protein BV379_04975 [Rhodovulum sulfidophilum]AB14
MHEHLKAKGWKPDSVRSLGDASSEVVSLLGNPNEETFACRGLVVGHVQSGKTANMTAVMAKAVDAGYNLAILLGGV
TNKL
RQQTQDRFERGGSGIETAGGYIPQVTAASFSRPTAGSPCRPRDMHNLSS
>OLS47705.1 hypothetical protein BV379_04980 [Rhodovulum sulfidophilum]AB14
MHNYLKMNNSFECCFTDQDRRNLRITSPQVESFAPQLRQRHVNYGSLEETADAIRSELSIWRPFDSRNADLTAAIGMV
RG
ELQDVEILRPFSIVDKRPEWYEGPIACDRH
>OLS47706.1 hypothetical protein BV379_04985 [Rhodovulum sulfidophilum]AB14
MKLYAITAVLFLFIDAVMLSLVMKPLFTRHIGDAMRDSPMMAPAALFYLAYVAGLVFLVAAPALRDAVPERAALQGAV
LG
AMAYGTYEFTSMSIMKNWSWTMVATDTIWGAVLTGFSAWAGVMIMMRLQG
>OLS47707.1 hypothetical protein BV379_04990 [Rhodovulum sulfidophilum]AB14
MLPRATKVIAAFAGLLSVTACSILGSTAAPEPDYSVNLAEPPFELRDYGELVVVKTAVNDGSRAAFGRLFDYISGANSGA
REIAMTAPVLNTDTAKGAKIAMTVPVLQTQEGSREMIFILPDTLTLDSAPAPTDPRVSLARIPPRRVAVVRYSGSMSNRA
SAEETRLRDWIASKELTPTGPAEVAGYNPPWTLPTYRRNEVLIPVEKD
>OLS47708.1 hypothetical protein BV379_04995 [Rhodovulum sulfidophilum]AB14
MRYLASKKPSAQAAAVSDFFDYATTSPNHPMLKSAAISDAGITKQAFCEVEKNQSTRATYGRAIERDVRPGWGLQRA
MRA
AQRGLKVPRGGRLIVAGTVPPMIGRRPAVFSIRAMLRSSRTRVSGRSGSPGTSPTDTVSTSPT
>OLS47709.1 hypothetical protein BV379_05000 [Rhodovulum sulfidophilum]AB14
MRVLALPEIGFGEAHLFQHLPDGHTSLGAGQGRGGGDGIYLAVLLGGLHFVMQSKGWQMEPLVYLAAILGLLALRFRS
SR
GSVAG
>OLS47710.1 hypothetical protein BV379_05030 [Rhodovulum sulfidophilum]AB14
MSFLRPQKSLPRHPVVTGMIVFLTVLAVANGAITIEKRALLEAERDLVAQEVMTFAARLQTEINSNVYLANGLIAFIAVS
KAPAPQEMKAAMASLHGAGRNIRNIGLAPGNIIEYVFPNTGNSSVIGLYYPDLKDQWPAVKRAIDRKTTIVAGPINLKQ
G
GVGLISRTPVFLEDGSYWGILSLVLDAESVFAQAGILPLQNNLNIALRLKADDGEPDRVFKGQEELFAPTSIIRDIVLPG
LVWQIAATPREGWTQSLGHLQLLRGLSLLLALFLGGLAAGRQRQRLDLARTEKRLASVTKAIRDAIVVVDGSGDIVELNA
AAEIMFGRPTAQAKSMPVAEMFRVETVLANTSVQTVVQGWRADGTELCLEVSSSTVDMMGNLKTIYSIRDVGDRMA
VENA
LRKSEERFRNITSQVPGVIYQWQEGPGDRRGFTYVSPRSHEILGLEADALVEDWSLFQIHPDEIDAWVASISHSISTGED
WQFEGRFLRGDETRWWRAFSRPQRFVDDPDGTVVFTGVIFDVTSVKAAQQALIAKERELAVVLESALDGIISIDTRGVIL
SANAAAAQIFQYSAEELVGQNVSMLMPDAIASRTERYLGSEAVSVIRKVVGMSRTVMGRRRNGEEFPVELSVAQTMA
GCG
STYTGVLRDITDRVAAERELEAARFGLEQKANDLEILADERDRARRDAERASEAKSSFLAVMSHELRTPMTGILGIADLL
LSETQSARHRKWINTLRRSGVSLLSLLDDILDFSKIEAGKLTLESIAFLPDVVMADVCELLSSTAQEKGLDFKMEQRISP
SPTLIGDPMRLQQILLNLTTNALKFTDKGGVTLSLGDAEKLPDGRWRMEFEVADTGIGIPFELQANLFQPFTQAEASTTR
RFGGTGLGLAISGRLAEAMNGEIDVSSVEGQGSVFRLTIVLPEAAEMTGNISHPARLALGPAHDRALNLLIAEDNETNRL
LLNDALEMCGHSVTTVENGAEAVAVVKKNDFDAVIMDMQMPVMDGMTAARLIQTEDENGPPIILLTADALARKSFE
KENV
FSAVLTKPIDWKQLFSALNEFARRDGEASADAQQHDKPSNFIGASKNNSDPGEEASSTEPPTLSLEWLDVLRQTIPPDR
V
NSIFASLIEKLDLQSRDLRARLSAGDLEGAERTLHSLLGLSRQFGLGKVAQCCQNISITSESGDVAHQSPLLERFDIEIS
SARRALKTFI

>OLS47711.1 hypothetical protein BV379_05035 [Rhodovulum sulfidophilum]AB14
MPGRFILKYDFLEAPRVWDAISSEVSASYPQDDLIRIRLGLTEIINNIVEHASNGQDDARFSLRILKLESGLFLCLSYAG
LAFVPPPDALDAEKVLASERTRGRGLWLISQCFTQVKYRDRGLTQRVIARL
>OLS47712.1 hypothetical protein BV379_05040 [Rhodovulum sulfidophilum]AB14
MIKTETSAGVTIFVIETQRLDASNATAFHSQLAPLIKGHDRIILDFTSVEFMDSTGIGALVGVTKGKSPDARIAMCGLTP
AVETIFKLLRMDMFFLVAAGRSDAIALLESA
>OLS47713.1 hypothetical protein BV379_05045 [Rhodovulum sulfidophilum]AB14
MKILVVDDDDLVRELILHSIGQLGHDLHSADSGETALEQIKAGLDVDLVISDWMMPGMTGVDLCAEVRRKTRRRHIFFL
L
LTARESREDYLSAMNAGADDFLVKPFDPEMLAAKVRAAERIFSLQTQLALSNTQLTQSNQELDDALRKMKNDIHAAAK
LQ
LGLLPPEKVRFGRFRMSAALLPSSAVSGDIYNYFNLGDETFGLYSLDIAGHGARAAMMSFAISRFIIDLGVVGNFERTRT
LSSYVQELNEIFQSSPPDNDYFTMLLATISSDEKMRVCQAGHPHPIATPIDGGPPFPLGKGGFPVGLQEGISYEEFLVDL
TQIHRVVFYTDGITECSGPNGELFGVERFQTLLHEAKGKPLDDVVPAVKQAMISWHGGDEFDDDISIIVLENCEATPEAP
>OLS47714.1 hypothetical protein BV379_05050 [Rhodovulum sulfidophilum]AB14
MVDIAPGFTVDSTASLSRIVVTLADDPLFAEDMLALSDETASDPVISDALSGGVLSLRFENDGRQAILEGDAPAEFFEQL
VRGLQLHTSSDQGSRTVSIDVYDTAGELLPLDSVNLTLNPNDPAASTDPGDLLAADTSFDAETDTTSLDWDAGSDALDP
P
TTDDPAPYQEMST
>OLS47715.1 hypothetical protein BV379_05055 [Rhodovulum sulfidophilum]AB14
MIAGVASDGSTTWSLGAADLAGLTLIPPPGFTGDFELEIAAIASDPDGGVATTNNLLPVTVLAEEPSLPPSSQVIELNGL
ITPEDTAVALELGQAFLEATGFSNLTFASISGLPEGTMLSAGDLQPDGSYLVSAADLEGLLLTPPNDSDDDMRIAVSFEL
TDGAQVNAAFDLQVDAVADMPTITISGASSDDVSVIPFNIGGGLSDVDGSETLTRFVSGLPEGARLSAGTYAGDGLWSL
T
PEDLTGLTLRPPTDFFGSFGFTTTVVATEASNGDVAVWNQNVTVTVSGGQEGDVWQSYDARGDEDTAVDLGTFLLLF
DLA
GFASIAVEGVPEGGTLSGGYLDENGDWIIAVGLLQDLTYTPPEHDASDVTLTVVGRILSFPITLGRIDVLVDAVADAPSL
SASAVDGIEDQALSLTINGALVDTDGSETLSFAVLGLPEGAVLSAGTRDPASGTWILTPEDLSGLTLTPPPDFSGQLTFD
VRANSLEDEGNAASTTETITINILPVTDAASLIVQAAAGHEDQPIPLNITLSAGDADGSENVTAIVISGLPDGAVLSAGT
ALGAGAHALQPEDLAGLTITPPANFSGQFDLQVTATLQDGTADPVTVSSSLTVTAAPVADAGAGRGRAGPECDRRSRR
GR
RTGGSEHIGRHGRGGWDRGALHRSERAAGRRLPHRRAQYGRRGMVARCAATRRIAASSTARLVRRIRPDRRCPCDDP
RDW
RHRQQYSGHRRAGAGARPHRHRGRGRYRARLHRRQHRLPVPDRRHPGR
>OLS47716.1 hypothetical protein BV379_05060 [Rhodovulum sulfidophilum]AB14
MSEGKKIAEPPLGTENDALSAQLTPPGRERVEVIERPVDANAAIRAVDDRSMSLIHRGEVDEPTLLSARAPDAVGGPSA
E
TMEVWRAEETTTMSVSDFGPMPSLSSSAPVSGAVFGTTALPSGVSNVVGGLDVRDAISFDVAELAVQSILAVDTIPPDT
S
ADGADFPNQDVTAPLAVTVTPVTDPPRLEVASATGAEDSAIALEAPPSRPA
>OLS47717.1 hypothetical protein BV379_05065 [Rhodovulum sulfidophilum]AB14
MSDEKNASAGIKDGFAVEDFVRDTGIGPLTHAMIWMIAGGLLAFLAWAAVTPVDEIAKGTGEFVPAGAVRALDHLEG
GIV
EEVLVKEGEVVAQGQPLLSLTSTATISNRDQLIARRTTLQLQAERFAAFAERRRADFHRFTNDEKLIAEQERLLNAQIGV
RVSQEKIFQEQVTGLEAQIDAVSLQKETLAEALALIDRQAAIREGLVGKGLNPLLQLLEIQRERTMAVAQLRELDSSIWS
LRNDISQVHTRISEFHGRLLEEALNQLETANAELAEIREQLTAQNDRIKRLLIVAPAPGIVQELAVRTVGGVLKPGETAA
RLVPLDDELIAEVQISPRDIGFVQVGQAVKLKVMAFDFSRYGHVYGKISSISPSTFLDANKVPYYLCRIHLDSSQIGSEG
ETRHFLPGMTIHADILTGKKTILQYFLKPLYTIVDGTLGER

>OLS47718.1 hypothetical protein BV379_05070 [Rhodovulum sulfidophilum]AB14
MWDAVDETLAVCLYDALRDLGWQGELRDLRLSFALDLSGDRVLDTLNAAARLGFAGHVRRVRAFSDKGLARTALGPG
GTT
QGLLFLPLRGEPRFLRMRAADARPSMRLGAGSLIRFERVETPASEIGKSWLGQRLRAQLPLVLRALLLTFFANLAAVANP
LFVMAVYDKVVVSESTALLAYLAAGVVAAAWMEMLMRRLRGRIMNRASLRLGYIVGNAVFGRILALPSRTTERAGLSS
QI
ARVRDVDRIRDLLRGPLEQALLDTPFTLLFVAAVAIVGGWLALIPLCFMILFAALAVIGERHMRGRGAEVAKASAQRQLV
TLEMMHRMRAIRAAGGETVWLERYRDSAAKAARASERNTTSAQILAAIGQSLGTGAALATLFAGIFLVLADAMTTGGL
IA
SMMLVWRLIAPIQNAFLAATRIGQLVASARQIDRLMETEAEIAPGEGDGERPPIQGEIAFNRVTFRYGRETEPILGGVSF
TAQPGEIVAIVGRNGSGKSTLLKLVAGLYEAQAGNIRLDGRDLRQFDPTHLRRAISYVPQLPSFHYGTLRDNLLMAEPEA
EEQDIAEALQLADAATAIAALPEGLDTFYDAQLVTLPVGLLSRLSLARMYLRKAPVVLLDEPATGIDFMGEFAFIGGLEL
LRARGATVLIVTHNRQHLGVVDKILMLEEGTVRFFGSAEKVRDRIPKGLI
>OLS47719.1 hypothetical protein BV379_05075 [Rhodovulum sulfidophilum]AB14
MRHALKRNGALPYVSIGAASLCINTLALAAPLFILQVYDRIIPNHSENTAALIGLGVLCALALETALKIARSKVLAPLAI
DYEVRASCGAARHLFHADLAAFERVSPGAHMERLAAVAQRRFNGTGQTLLAICDFPFLFVFMWMIWILGGPVIIAPAL
GA
IALAVAAAPANRSAREALARALSLDDQRYDFLLSTLSSLPSLKVANLERPVLRRYEGLQKRRLAAQGEAFTADQRLTDLS
QLVVQLSIIGVIVLGSLLILQGRMSIGGLSASTLLAGRFLALIRNLILLASRLQTSSIGREQMDAIFKIPVRSASSGILD
ASQAPPRLSLRGVVFDDNDGKRVLDGVDLDLREGHTVGLVGDNGSGKSILLALAAGLYSPTSGEVLLGGHPLSDYTDQS
L
RRTIALVSQQETLFAGSILENISMFAPERVDAALRAAERTGLTELIKPLPKGFATIVGDTAADGLPRGVVQRIALCRAMA
LDPQVLLFDDANSAIDDQGDEKVAELFADIRERCAILFVSHRPSVLGRADAIVRLANGRISTVEGTF
>OLS47720.1 hypothetical protein BV379_05090 [Rhodovulum sulfidophilum]AB14
MKMALFIDGEQEAITLCYRQETRILEILLSGVVTLGKIEGIRPHGAENTAFGKQLANHGEDYHPPQPLEVA
>OLS47721.1 ATPase [Rhodovulum sulfidophilum]AB14
MSDHFFVVTGGPGAGKTSLITELARRGFHTIPESGRAIIREEMQSGGDALPWADRAAYAERMLQRDLRSYEAAQAHSG
PV
IFDRGIPDILGYLTLCSLPVPPHVAAAAKAARYNARVFLAPYWDEIFTQDTERKQTRSEAEATCAVMFDTYIALGYEIIE
LPRTDIARRADFVCEQLAI
>OLS47722.1 TIGR02300 family protein [Rhodovulum sulfidophilum]AB14
MPKEEWGVKRVCPQCSTRFYDLQRDPMTCPSCGHSFPADSLAPGKQRPLIAEKAAPAKARNDEELDSDDAIIDDDDD
GND
VDLGDDVLEDDDDDNVSLDDIADMSSEDDDET
>OLS47723.1 zinc metalloprotease [Rhodovulum sulfidophilum]AB14
MSIIRIPGDPPLEVTLRRSARARRYSLRVSQLDGRVTLTLPQRAPEADGLAFVREKEAWIRRHLSDRPEDHVVRAGGTVP
YEGRALPLVPGTGRTPQLREGGLAVPGPAEDVPPRVLAFLKTRARARLAAAADGHAAALGIGYRRLTVREARSRWGSC
SS
RGDLMFAWRLIMAPPEVLDYVAAHEVAHLVEMNHSNRFWAVVGGLMPGYEAPRAWLRANGSALHRVRFGD
>OLS47724.1 GntR family transcriptional regulator [Rhodovulum sulfidophilum]AB14
MLIQTRSGDTVSATHERLYRTLRTQIMHGELTPGAALTLRGIGKSFGVSMTPAREAVRRLVAEGALTLSSSGRVSTPELS
NERLEELASLRGLLEPELASRALPRAHFALIERMQKINAEIGEVVSRGDAVAYIQKNLEFHRALYLRAQAPAMLAMAETV
WLQLGPTMRSFYGRLGRTEPPQHHRIILAALRAGDEPSLRLAVRTDVTQSLKFLAT
>OLS47725.1 dihydrolipoyl dehydrogenase [Rhodovulum sulfidophilum]AB14
MASYDVIVIGSGPGGYVCAIRCAQLGLKTACVEGRDTLGGTCLNVGCIPSKALLHASHQLHEAQHNFEKMGLKGKPPS
VD
WTQMLSYKDEVIGQNTKGIEFLFKKNKIDWLKGWGSIPEAGLVKVGDETHKAKHIVIASGSEASSLPGIEIDEKTIVTST
GALALPKVPKKMIVIGAGVIGLELGSVYARLGAEVTVLEYLDQITPGIDTEVARSFRALLKKQGLNIVTGAAVQSAEALK

TKAKVSYKLRKDDSEHQIDADVVLVATGRKPYTDKLGLDALGVEMTKRGQIATDAHYNTSADGIHAIGDAIEGPMLAH
KA
EDEGMAVAEILAGQAGHVNYDVIPGVIYTHPEVASVGRTEDQLKEAGRAYKVGKFSFMGNGRAKANFAADGFVKILV
DAE
TDRILGAHILGPSAGDLIHEICVAMEFGAAAEDIARTCHAHPTYSEAVREAALACGDGAIHS
>OLS47726.1 hypothetical protein BV379_05125 [Rhodovulum sulfidophilum]AB14
MTPELTALALAILVQAVQFVLMAIPANMELGTEYTTSPRDTPPDQQLSRRTGRMQRALNNHFEGLILFTAAVVLVTVSG
Q
SSWFTALCAWIYLIARIAYVPAYVRGWVPGRSMIWAAGFAATVLMTLAALI
>OLS47727.1 hypothetical protein BV379_05130 [Rhodovulum sulfidophilum]AB14
MTPELAALAATAVIHLAAVLWSQRALEADVGREGNTGPRDDIGDRLSPLTLRLRRALANHVENTGLFVIAVVLVQFTGS
N
GWFTALCAWIFVAARALYLPAYAFGWVPWRSVIFMAGLLACFAMILASIF
>OLS47728.1 dihydrolipoamide succinyltransferase [Rhodovulum sulfidophilum]AB14
MSTEVRVPTLGESVTEATVATWFKKPGDAIEADEMLCELETDKVTVEVPAPAAGTLSEIVAAEGETVGVDALLATIAEG
A
AAPAPKANGGGTPAPAPADDAGTEDLPVMVPTLGESVTEATVSTWFKQAGDKVERDEMLCELETDKVSVEVPAPAA
GVLK
EILAAEGATVEAGGQLAVLSSGEGAAASATDDKPAAETSKASSGKDVEDAPSAKKLMAEKGLTAAEVKGSGRDGRIMK
ED
VLKAASAPKPAASRASEPPKPAAIPDAARAPIPAEDAEREERVKMTRLRQTIARRLKDAQNSAAMLTTYNEVDMSGVM
AL
RKEYKDLFEKKHGVKLGFMSFFTKACCHALSEVPEVNAEIDGTDIVYKRFVHMGIAVGTPSGLVVPVLRNADSMSFAEIE
KRIAEMGTRARDGKLSMAEMQGGTFTISNGGVYGSLMSSPILNPPQSGILGMHKIQDRPMVEGGQIVIRPMMYLALS
YDH
RIVDGKGAVTFLVRVKEALEDPRRLLMDL
>OLS47729.1 2-oxoglutarate dehydrogenase E1 component [Rhodovulum sulfidophilum]AB14
MTEQSANDIFHSSSFLQGHNAEYVEQLYARYANDPNAVDEAWQRFFAEMGDDEVSVKREAAGPSWARRDWPPAP
NDDLTA
ALDGQWPTPPAAREAKAAEEKIREKAEEKGIAVTDDEVKRAVLDSIRALMLIRAYRIRGHLVADLDPLGLRNPTPHPELD
PKSYGFTDADMERPIFIDNVLGLQVASMREILAIVQRTYCGTFALQYMHISDPEQSSWLKERIEGLGKEIKFTREGRRAI
LNKLVEAEGFEKFLHVKYMGTKRFGLDGGEALIPAMEQIIKRGGALGLKEIAIGMPHRGRLSVLANVMGKPYRAIFNEF
Q
GGSFKPEDVDGSGDVKYHLGASSDREFDGNTVHLSLTANPSHLEAVNPVVLGKTRAKQAQMNDHDRKQVLSVLLHG
DAAF
AGQGVVAECFGLSGLTGHRTGGTIHIVVNNQIGFTTAPHFSRSSPYPTDIALMVEAPIFHVNGDDPEAVVHAAKVATEF
R
QKFGKDAVLDIFCYRRFGHNEGDEPMFTNPLMYQKIKKHKTTLQLYTERLVEDGLIPEGEIEDMKAAFQSHLNEEFDAS
K
EFKPNKADWLDGRWSHIQRGGGDDYQRGDTAIGEETLQELGQALTKPPEGFDLHRTVGRLLDAKKEMFATGKGFDW
ATAE
AMAFGSLLTEGYPVRLSGQDSARGTFSQRHSALINQDTEERYYPLNHIRKGQAKYEVIDSMLSEYAVLGFEYGYSLSEPN
ALVMWEGQFGDFANGAQIMFDQFISSGERKWLRMSGLVVLLPHGYEGQGPEHSSARLERFLQMSAEDNWIVANCS
TPANY
FHILRRQLHRSFRKPLILMTPKSLLRHRLAMSVASEFLTGSSFHRVLWDDAETGHMSETKLRPDAEIKRVVLCSGKVYYD
LLEERDARGIDDIYLMRLEQFYPFPALSLVKELDRFRDAEIVWCQEEPKNQGGWTFIEPNLEWVLTRINAKHGRPRYAG
R
PAAASPATGLASTHKAQQAALVNDALTIEG

>OLS47730.1 DUF805 domain-containing protein [Rhodovulum sulfidophilum]AB14
MGFAAAVKVCFRKYFTFSGRASRPEYWWFVLFTLLGSIATGLLDLLLFPSAIHTGPGSIEAESDGPLVTLFTLAVFVPSV
SAGWRRMHDSGRSGLYYIYPLIVLVGILGFAQFVGAFGLAGSGDLAEAFTGIAGLVLILSFLVLMISPLLVIWWLTRPSQ
SGPNQYGPTPHEVTP
>OLS47731.1 succinate--CoA ligase subunit alpha [Rhodovulum sulfidophilum]AB14
MAVLVDENTKVICQGFTGSQGTFHSEQAIAYGTKMVGGVTPGKGGQSHLELPVFNSCHEAVARTGANASVIYVPPPF
AAD
SILEAIDAQIPLIVCITEGIPVMDMMTVKRALEGSNSTLIGPNCPGVITPDACKIGIMPGHIHKRGSVGVVSRSGTLTYE
AVKQTTDVGLGQSTCVGIGGDPIKGTEHIDVLEWFLADDETQSIIMIGEIGGSAEEEAAQFIKDEAKRGRKKPVAGFIAG
RTAPPGRRMGHAGAIVAGGKGGAEDKIEAMKSAGIVVADSPAHLGEAVLKAIG
>OLS47732.1 succinate--CoA ligase subunit beta [Rhodovulum sulfidophilum]AB14
MNIHEYQAKALLRSYGAPVSKGHVVTKAEEAKSIAADIDGPVWVVKAQIHAGGRGKGRFVEEEAGEKGGVRLTKSVGE
AA
EEAKKMLGRTLVTHQTGPAGKQVGRVYIEEGAGIDRELYLALLVDRQTSRVAFVCSTEGGMDIEEVAAKTPDRILSFSVD
PVTGYQGYHGRRIAFALGLTGNQIKQCVRLMGQLYKLFTEKDCELLEINPLIVTDQGDLRCLDAKMSFDSNALYRQEDIL
ALRDETEEDPKEHQASKYDLNYIALDGEIGCMVNGAGLAMATMDIIKLYGSEPANFLDVGGGATKEKVTEAFKIITSDP
N
VKGILVNIFGGIMRCDVIAEGVVAAVKEVGLKVPLVVRLEGTNVEKGKAIINNSGLDVIAADDLSDAAQKIVKAVKG
>OLS47733.1 peptidase M3 [Rhodovulum sulfidophilum]AB14
MTNSLLAPRMAPFELPDFAAISDGDFAPAFDQALTEARAEIAAIAEASEPPDFANTIEALERSGQALSDVLSVFDLLAST
SSTPAREALQRAFSPKLADFSSDVVLNRALFERIDAIWQARETLDLSAEEARLLDRKHRDFVRAGAALPEDERGRLRAIN
TRLAELSTAFGQNLLADERDWTMPLAEADLAGLPGFVVEATRAAGAERGADGPVVTLSRSVIVPFLQFSPRRDLRKTAY
E
AWTSRGARGGATDNRGIVAETLKLRAERAALLGYDSYSAYALETQMAKTPGNVRDLLMQVWAPARARAETDAAILAE
MLV
ADGGSAPLEPWDWHYYSEKRRRALHDLNEAELKPYLQLDRMIEAAFDCANRLFGLDFAPLDVPLHHADARAWEVTRD
GRH
MGVFIGDYFARAGKESGAWCTALRSQQKLAGEIRPHVVNVCNFSKPAEGRPALLSYDDARTLFHEFGHALHQMLSDV
TYE
SLSGTSVPRDFVELPSQLYEHWLEVPEVLERFATHAETGQPMPEALRERVLAAATYDMGFQTVEFIASALVDLEFHDGP
A
PADPMQKQAELLEAIGMPHAIRMRHATPHFAHVFAGGYASAYYSYMWSEVMDADAFAAFEEAGDAFDPATARALE
ENVLS
TGGTAEPDALYTAFRGRLPGVEALLKGRGLDLEPA
>OLS47734.1 DEAD/DEAH box helicase [Rhodovulum sulfidophilum]AB14
MTTFDKMGLPKRLVTRLAETGITEPTPIQARAIPPALEGRDVIGLAQTGTGKTAAFGLPLLTALAESRGKPTPKTARALV
LAPTRELVKQIAESLRPLVAGTPVSVETVVGGASLSAQEKRMARGADLLVATPGRLIDLMGRGAVVLAETRFLVLDEAD
Q
MLDMGFIHALRRIAAKLPKERQTMLFSATMPKQMDEIAASYLTNPVRVEVSPPGTAVDKITQSVHFLEKAEKTGRLIELL
DGHRDDRAIVFGRTKHGAERLSKQLERAGFATASIHGNKSQGQRDRAIKAFRAGDVRVLVATDVAARGIDIPEVRHVY
NF
DLPNVPDNYVHRIGRTARAGADGAAVAFCAPEEISELRAIHKVMGCAIPVASGTPWEGLEPKQSRGGPRGAGKGAGK
PPA
KARARRRPRNKNRAAA
>OLS47735.1 hypothetical protein BV379_05170 [Rhodovulum sulfidophilum]AB14
MAGGWARDGAVNDQIDASIEDELNRMKAKRAPMGESLTHCAECGEPIPEARRRALPGVKLCIDCQGDRDRRPVTRG
GINR
RGSKDSQLK

>OLS47736.1 MFS transporter [Rhodovulum sulfidophilum]AB14
MTQTQRAAQAPLVTPVLIAGSVILLIGFAIRASFGVFQIPIAEEFGWARTEFSLAIAIQNLAWGIGQPLFGAMAEKIGDR
KAIVAGALLYALGLVLSAFAIQPWQHQGLEVLVGFGVAGTGFGVILAIVGRAAAPEHRSMALGVATAAGSAGQIVGPP
SA
ELLLRLLPWQGVFMVFAGVVLASLLALPFIRSPQIASRAELEQSLGQVLGRAFRDPSFVMIFMGFFSCGYQLAFITAHFP
PFVAELCGPIVPGGTLSAIGITTTSALGAVAISVIGLFNILGTVTAGWLGNRYSRKYLLAGIYTARTLAAAAFILMPITP
GTVLVFSMVMGALWLATVPLTSGLVAHIYGLRYMGTLYGFVFLSHQIGGFLGVWLGGRLYDLYGNYDLVWWVGVGV
GAFS
ALVHLPIRETPIPARAAA
>OLS47737.1 nitroreductase [Rhodovulum sulfidophilum]AB14
MSNKTLELLQKRRTQYALGKDLPLSADMVEGLIKAAIRHAPSSFNSQSSRAVILFGAESEKAWGLIEAELRKIVPAEDFG
PTEAKMKGFAAGAGTVLFYEDEDTIKGLQEQFPLYADAFPRFSQHSAGMAQLAVWTALAEEGIGASLQHYNPVADAA
LAE
AFDVPASWTLVAQMPFGGHAGEIGDKTFMEDAARFRTHGK
>OLS47738.1 alpha-D-glucose phosphate-specific phosphoglucomutase, partial [Rhodovulum sulfidophilum]AB14
AAGLKGVARSMPTSRAVDRVAAAAGLDCYETPTGWKFFGNLLDAGRATLCGEESAGTGSDHVREKDGLWAVLLWLN
ILAE
TGLSVAETLRAHWAAHGRNYYTRHDYEAVDKDRAEAMLEALRGRLDSLAGQGFAGRKVEMADEFSYDDPVDGSKST
GQGL
RIHFEGGARIVYRLSGTGTVGATLRVYLEEYTDDPARLELPVEEVLADVIAAADEITGIRDHTGRTEPDVRT
>OLS47739.1 phosphoglucomutase, partial [Rhodovulum sulfidophilum]AB14
VADYAELMQSLFDFDAIRRLIAGGFTLRFDAMHAVTGPYAKAILEDLLGAPAGSVINAVPQPDFGGGHPDPNPIWARP
LV
DLAMGPDAPDFAAASDGDGDRNMILGRGIVVTPSDSLAVLAANATRAPGYAAGLKG
>OLS47740.1 phosphoglucomutase, partial [Rhodovulum sulfidophilum]AB14
MLRPADIKATGEPIFMQKITVETKPIEGQKPGTSGLRKKTPVFMAPHYLENFVQSIFNAIGGVAGKSLVLGGDGRYFNER
AAQVILRMAAAGGAARVIVGQNALLSTPAASHLIRKRGTDGGIVLSASHNPGGPDEDFGVKFNTPNGGPAPEEVTNRI
FE
ATKTITCYEIVEARDLDLSAPGETTLGGTVVEIVDPVADYAE
>OLS47741.1 hybrid-cluster NAD(P)-dependent oxidoreductase [Rhodovulum sulfidophilum]AB14
MNDLSTLPDRAIWSDNEMLECVSVIPEVPNTATFTFRAPSGALFDFLPGQFLTLEIPMPGGTVHRTYTISSSPSRPRSIS
ITTKAQPDSLGTRWLLDNLTPGTRVRALGPAGVFSHLNHPAEKYLFISAGSGITPVMSMTTYMFDLGLEPDIVFVNCAR
R
PGEIIFRERLEHMASRVPGIDLKWVVERPDRYAPWIGFQGQFNQLMLGLMAPDYLEREVFCCGPEPFMRAVREALQG
LGY
DMDRYHQESFQAPATTEADLTELDDVVPDESAQVELTFAASGVTVKAAETDTILAAARANGLNIPSGCTFGVCGTCKIR
K
LSGEVHMVHNGGISEDDIEAGYILACCSNPIGKVSVDV
>OLS47742.1 hypothetical protein BV379_05210 [Rhodovulum sulfidophilum]AB14
MTTRPRNHGRPAYNENGSADAVTVTDNEATAILQGAGLSIKRVTRSPSGQLTAQWRKEPTDADKGEALKANTPAPCF
RYL
RFADLHAWRSERPQSAHSRPRHARCRARYVLRSQLM
>OLS47743.1 GNAT family N-acetyltransferase [Rhodovulum sulfidophilum]AB14
MLIRNEVGSDATMIRRIVTEAMQLLPQATGAEADIIDRLRRDDALILSLVAEDRGEAVGYLAVSMSRIGAESGWGLIGPL
AVLPSRHGEGFGGALMAEAIRRLRSTCKGAALVGDPGYYARFGFRSYPGLRVGDCPPQFVQALPFDAAEPEGELHHHA
AF
GLTQAE

>OLS47744.1 hypothetical protein BV379_05220 [Rhodovulum sulfidophilum]AB14
MNISRHEQRALHVLALGGRILHEREYGSKITSVTCVTREGMILDGFDMTVFHRLRRKRLIESRCGSAYRISKRGRLSVRA
QLDNQGA
>OLS47745.1 glutamate decarboxylase [Rhodovulum sulfidophilum]AB14
MPIHTVRDHSHSDIEDIYSTRLSEHGLPKYRMPEGTSEPHAAYALVRDELLLDGNARQNLATFCTTWVEDEVKQLMTD
AV
DKNMIDKDEYPQTAEIESRCVHILADLWHAEKSWDTIGCSTTGSSEAAMLGGLALKWKWRARREAEGKDTSKPNIVT
GPV
QICWKKFARYFDVEIREVPLKGDALGLRPEDLRDYCDENTIGVVATLGVTFTGVYEPVEALARELDAIERDLGLDIPIHV
DAASGGFIAPFIQRDLVWDFQLDRVRSINASGHKYGLAPLGVGWVIWASKEDLPDDLVFYVDYLGGNMPTFALNFSRP
GG
EIIAQYYNFLRLGREGYTQIQQACADTAQWLGEQLAEIGQFDMVYDGHGGLPAVAYKLTAGDHGFTLYDLSERVRMR
GWQ
IASYPLPADREDTVVQRILIRHGVSRDLAALLLEDIKRELDHLEKNPVPNSTAKPGFHHG
>OLS47746.1 ArsR family transcriptional regulator [Rhodovulum sulfidophilum]AB14
MAESSDDPLRLDNQVCFPLYAATNLLQRLYRPLLAPLGLTYSQYLVMLILWERAPVTVGEIGSCLHLDNGTLTPLLKRME
RGGFVTRSRDPRDERRVLVAPTPHGRELRAQALKIPEDLTRKITLSTEEIEALRSSVVSLVHLLADDIDRRKESDARVAT
Q
>OLS47747.1 Ohr subfamily peroxiredoxin [Rhodovulum sulfidophilum]AB14
MSTIYSTTVTAVGGRAGTVKSDDGLLELGLSLPKKLGGKEDATNPEQLFAAGYAACFGNAVIHVTRNKDNRIKDDDVAV
I
ATVGMTPNGSGGFGLTVALDVTIAGVDQASAEEIVADAHKACPYSNAVRGNIDVELSVNTR
>OLS47748.1 hypothetical protein BV379_05255 [Rhodovulum sulfidophilum]AB14
MTTLTHVAAPNAAFIARLSALAEDIRQAGRRYGIRRQTIRELSALSDRDLKDLGILRCDIGAIATEASRNA
>OLS47749.1 N-acetyltransferase [Rhodovulum sulfidophilum]AB14
MHANRSSWSFRIEPGLRPEHRERAAQGYWRAFLRKLRYPLGPETKAAVFIERVLDPTHAVSAMSEDGGFLGAAGYKSP
DG
AFIGGGFKDLAAVYGRTSAAIRGLLIGLLERNCADDTLLMDGIFVEPEARGRGVGKALLNAMEERAERSALRRVRLDVLD
TNPRARALYEREGFPEASTMSLGPLSVVFDFSHATEMTKSVGG
>OLS47750.1 alpha/beta hydrolase [Rhodovulum sulfidophilum]AB14
MKFRFGVIVALVVAMVALGLAVWRLADQDLSWRNSEPFDFVSGGNAITGTLWLPDDDPQAAIVLVHGDGAQDRTS
DDGYA
PLINAFLDHGIAVAAWDKPGVGPSGGNWLHQSMTDRTDETRAALRNLHKRFDRVALGAVGFSQAGWVLPGLTRDD
ADFLV
LIGAAVSWHDQGDYYTRVRLGREGMDREAIAQVIADQHRADDRAFGPDAQASDAPAGMTPDRWQFLRRNHGADA
RAALGR
LDLPLLAIWGEDDLNVDARRNAAIYREILAGHDARSRIVIWPDATHGLLKANAYNWQLIEDWPLSAELRFVLEGRYAYA
P
GALDAIMDWIKDRSQDGKASWR
>OLS47751.1 BMP family ABC transporter substrate-binding protein [Rhodovulum sulfidophilum]AB14
MATTRDGIAIGRRGLLLGGAGAAAGLGLGLWPRRGLAAEPVSVAGIYTVPVEQQWVSRLHKAADAASAAGEITYAFSE
NT
TNTDYPRVMREYAEQGVKLIMGEIFGVEQEAREVVLDYPETAFLMGSSFKPDPDYPNLAVFDNYIQDASYLSGIVAGA
MT
KTGNIGMVGGFPIPEVNRLMHAFMAGVREVKPDATFQVSFIGSWFDPPKAKETAFAMIENGADLLYAERFGVSDAAQ
EKG
VLAIGNVIDTQPDYPETVVASAIWDFAPTLAAALKNVSAGSFKADDYGVYSFMKEGGCSLAPLGTFAGKVPQSAMDLIA
Q

KEAAIKAGDFTVEINDKEPKSS
>OLS47752.1 sugar ABC transporter ATP-binding protein [Rhodovulum sulfidophilum]AB14
MSGNRPVVLKLEGITKSFGALRANDNIGFALHRGEVLALLGENGAGKTTLMNILFGHYTADAGTVEVMGQALPPGQP
GAA
LAAGVGMVHQHFTLAGNLTVTENILLGTRPLWRAGLGRSAARARIAALSRDFGLAVRPDAKVRDLSVGERQRVEILKAL
Y
REARVLILDEPTAVLTPQETDALLATLRKAVARGLSVIFISHKLHEVMAAADRVIVLRHGRVAGEVATAETDRHRLAEM
M
VGEEVRLPQPSTATPGPALMSLRQVTCHPEGSRSGLNRVTFDLRAGQITGLAGVSGNGQGALADLVSGMTAPDEGRL
EVH
GAEVSVWSPRAALAAGVGRIPEDRHATGSIADFTLTENAVLEDYARPPYSRHGWMNWRAARTHAEKIIAAYDLRCPG
PEA
RIRLLSGGNMQKLILGRALEAGPRIILANQPVRGLDIGALTYVQECLIAARDAGAAILLISEDLDEVLALSDVIHVMSEG
RLSPGFPRGSKTPAELGAWMAGQGFDHAA
>OLS47753.1 ABC transporter permease [Rhodovulum sulfidophilum]AB14
MRLDPVHNPTLTRRLGLPLAALAFTVAVASGLAKLAGADPFATLGLIVKGAAGSKFALLETLNRATPLIFTGLAVAVAFR
ARLWNIGAEAQLYAGALLTVLVGTQLGLPGAVAIPAMALAAILAGALVLLGPAVLKLRLGVDEVVTTLLLNFIMLLFISM
LLEGPLKDPMGMGWPKSPPIPRDARLPRLIEGLRLHWGFGLALIAAVSVWLIQIRTTLGYEIRAVGLNAQAARFAGIPV
N
RVIVKTALLSGGLAALAGFSEVAGLKGNLTLDLSPGFGYTGIVVAMLALLHPLGVVASAIFVAGVFVGANSMSRAADVP
T
YIADISVAVALLSMVLAILLTKFRVVRG
>OLS47754.1 ABC transporter permease [Rhodovulum sulfidophilum]AB14
MEILDILLSASFWAAVLRIASPLIFATMGELICERAGVLNLGIEGIMVAGAFAGWMAVYAGLGLWPGLLVALLTGMAFG
A
LHGVLTVPFGLSQHVVGLGITMLATSSAYYAYRLSLPEVTSPPRIEPFQPLHIPGLSDLPFFGEALFSQTALTYLAFACV
IAVALVLYRTPLGLALRAAGENPAAVEAQGLSVAAIRIGAVMAGSGLMAVGGAFLTMSAFSSFFFEMVNGRGWICIAL
VV
FGAWRPGKALIGALLFAAFDALQIRLQQTPFGADIPYQVFLMVPYALSILALVLMSRRAEVPAALMTPFNRGER
>OLS47755.1 cytosine deaminase [Rhodovulum sulfidophilum]AB14
MAEFDLLIVDGNLPDGQVGDIGISKDRIAAIGDLSGSTAGRVIDAAGDLVSPPFVDPHFHMDATLSYGTPRINASGTLLE
GIALWGELKEIATVDQMVERALAYCDWAVSMGLLAIRSHVDTCDDSLKGVEAMLEVREKVRPYLDLQLVAFPQDGLLR
DP
TAKANTLRALDMGVDVVGGIPHFERTMEDGRASVRELCEIAAERGLLVDLHCDETDDPLSRHVETLAYETQRLGLQGR
VN
GSHLTSMHSMDNYYVSKLLPLMAEAGLSAVPNPLINIVIQGRHDSFPKRRGLTRVKEMQAHGITVGWGQDCVRDPW
YPLG
TADMLDVAFMGLHVAQMTSPAEMRQCFEMATTESAKILHLDGYGLHEGAQASLVILDAGDAIEAIRLRPARLAVISKG
RV
VSETPRADARLDLPGRPTGVRRRHHVN
>OLS47756.1 PAS domain-containing sensor histidine kinase [Rhodovulum sulfidophilum]AB14
MARDITRGLVSGEGEFSDAVWADVLSAVDRTYVDLVDYQEQLEAKNAELETMRSFLGSILASVSDVLIVVARGGRVEEV
S
ASTALRTGAPREGFAGRPVTELFSEADRPALEAAMTRAGQGRCPVMLEAGLIAAEGETPLELSLSPRMDERGRVTGHV
LT
GRPLGELRQAYAELAASHDELKAAQALLVRNEKLASLGRLLAGVAHELNNPISFVYANAHALERYAGKFESYFERVQSGA
DRAELVRLREELRLDREIRNLRDAVAGARDGAERVRDIVEDLRRLSSEGSGERVAFDLVETARVAASWVMRGSRGDVEI
A

FAGADRVMALGRPGHVQQVVMNLVQNARDAAGPGGRMELWFGAEAEAAVLKVSDDGPGVPDAVRTLIFDPFFTT
KEVGQG
TGLGLAISAKIAAEHGGQLSLCPVGTGACFRLDLPLAGDRP
>OLS47757.1 HupU protein [Rhodovulum sulfidophilum]AB14
MNVLWLQASGCGGCTMSLLCAESPTLFDLFEGAGIRFLWHPSLSEATGAEARALLEAVAAGDIPLDVLCVEGAIARGPK
G
TGRFQMLSGTGRSMLDWVGRLVPRARHVVAVGSCAAFGGVTSAGGNPSDAAGLQYEGAHPGGILPPEFRDPTGLPV
VNVA
GCPTHPDWVTETLMLLAAGAMGPEDLDALARPRFYAEHLVHHGCPRNEFYEYKASATALCEMGCMMEHLGCVGTQ
AVGDC
NIRPWNGQGSCTRAGYPCISCTAPEFEEPRHAFTETPKVAGIPVGLPSDMPKAWFMALASLSKAATPERLAKNATADRI
E
VLPRPGKPRG
>OLS47758.1 HupV protein [Rhodovulum sulfidophilum]AB14
MTGTRLIVGPFNRVEGDLEVHLDIAEGRVRSARVNAPLYRGFERMLTGRPASDALTITPRICGICSISQSAAAARALADA
GGHEPAPAGAAMAALIHAIENVSDHLVHFNLFFMPDFARPAYAGRSWHARTVARFTAEQGAAPRAAIAMRAELMH
VLGLM
AGKWPHTLAIQPGGVTRAPGPRDKVRIGASLAAFRRYLEAELFGAPLEDFVALGTREGLMGWDRGDAGLFLEIAADLD
LD
ALGRGSGRYLSFGAYPLAEGHGFAPGIWMDGLLPLDLGGLCEDLSHAWMLGGAAHPSEGQTLPDEDMRAEAYSWCK
APRL
DGLGYETGALARQVMAGHPLAVALTGGGVMARVAGRLLDLARTQIFMEGLLRWLDPAAEFMRPAPKIREGEGVGLV
EAAR
GALGHWLRVENGRIASYQIVAPTTWNFSPRDASGTPGPLEAALEGVPVGEGETTPLAVQHVVRSFDPCMVCTVH
>OLS47759.1 uptake hydrogenase small subunit [Rhodovulum sulfidophilum]AB14
MAEIETFYEVMRRQGITRRSFMKYCSLTAAALGLGPAFVPKIAHAMETKPRTPVIWVHGLECTCCSESFIRSAHPLAKDV
VLSMISLDYDDTLMAAAGHAAEAALEDTIEKYKGNYILAVEGNPPLNEDGMYCIIGGKPFVDQLRHAAEHAKAIISWGA
C
ASYGCVQAAAPNPTRATPVHKVITDKPIIKVPGCPPIAEVMTGVITYMLTFDRMPELDRQGRPKMFYSQRIHDKCYRRP
H
FDAGQFVETWDDEYARKGYCLYKMGCKGPTTYNACSTVRWNEGVSFPIQSGHGCIGCSEDGFWDQGSFYDRLTTIKQ
FGI
EATADQIGFAAAGAVGAGIAAHIAASALKRAQKKSQDSTQKEEV
>OLS47760.1 hydrogenase 2 large subunit [Rhodovulum sulfidophilum]AB14
MVATPNGFDLDNTGRRIVVDPVTRIEGHMRCEVNVDDEGIIRNAVSTGTMWRGLEVILKGRDPRDAWAFTERICGVC
TGT
HALTSVRAVEDALGISIPQNANSIRNIMQLNLQIHDHIVHFYHLHALDWVNPVNALRADPKATSELQQAVSPSHPLSSP
G
YFRDVQNRLKKFVESGQLGLFKNGYWDNPAYRLPPEADLMATTHYLEALDLQKEVVKIHTIFGGKNPHPNWLVGGVP
CPI
NIDGTGAVGAINMERLNMVSSIIDLCMTFVKNVYIPDVVAIGGFYRNWLYGGGLSGQAVMAYGDIPENPNDFSAEQL
HLP
RGAIINGNMNEVLDVDPRDPEQVQEFVDHSWYTYGTPGKGLHPWDGVTEPKFELGPNAKGTKTNIQELDEAAKYSWI
KAP
RWKGHAMEVGPLARYVVGYAKGHEEIRGQVDGLLRTMDLPVEALFSTLGRTAARALEADYCARLQKHFFDKLITTCRN
GD
TATANVEKWEPKTWPKEAKGVGMTEAPRGALGHWIKIKDGRIENYQCVVPTTWNGSPRDTAGNIGAFEAALMDTK
MERPD

EPVEILRTLHSFDPCLACSTHVMAPDGQELTRVKVR
>OLS47761.1 urease accessory protein UreJ [Rhodovulum sulfidophilum]AB14
MRKYLLAGALAALAGPALAHTGQGAASGLMHGMLHPVTGPDHLLAMLAVGLWSGFALRRRVWAGAATFMGAMIA
GAALSW
AGVPIPGVEGMIAASVAVFGLLALFARQDQPRWLTGGALAAIALFGACHGHAHASETTGAALAYLGGFLLSTAALHLIG
I
AVARGVADLRAARMLRGAAGGTLLASGAWLLAG
>OLS47762.1 Ni/Fe-hydrogenase, b-type cytochrome subunit [Rhodovulum sulfidophilum]AB14
MSSKDRTDIPIHIPNPEAPTPLEAARLTGGARPEDIASLQLRTAVYVYEMPVRIWHWVNALAITVLIVTGYLIGSPPPTM
EISEATNQFVFGYIRFAHFAAGMVMTFAFLGRIYFAFIGNRHARQLFYIPFWRKRWWKELLFEARWYAFLEKEPKKYIG
H
NPLAHLAMFTFITLGMSFMIVTGFALYAEGAGQGSIYDRMFGWVLGLVGNSQRLHTLHHLGMWAITLFAIFHIYAAIR
ED
IMSRQSMITTMISGWRTFKDNRVE
>OLS47763.1 hydrogenase expression/formation protein [Rhodovulum sulfidophilum]AB14
MPAATDRLTLILGIGNVLWADEGFGPRCLEALAESHAFDAGVRLLDGGTQGLYLLPFLEEADDLLIFDAVDYGLPPGTLK
IVRDADVPAFMGAKKMSLHQTGFQDVLATAALVGCLPSRMTLIGCQPAELEDYGGGLTPTVAARLPEALSEALAELDG
WA
VAARPGRTVSADLADPSIRRDAYEAGRPPAELACRSGDDRFFPGADDTGRAG
>OLS47764.1 hydrogenase [Rhodovulum sulfidophilum]AB14
MCVGEPMRLSTVDGIVGRTGDGTLIDLSLTPDLRPGDWVLTFLGAARERLDPDEAQKILAALEGLRAVMAGGSAGDAF
AD
IDAREPTLPPHLQAALAAGAAQG
>OLS47765.1 hydrogenase accessory protein [Rhodovulum sulfidophilum]AB14
MTHPLIARLETDLGWPRLATIAEIEAFTHAPGLHCLFVPGDPVRNLETADAAVILPELRQAFQDAFDCAIVDDAIEARLR
EATRALKTPGFVFYREGRLLGDIQKIRDWDDYLTRIAHILSLTEA
>OLS47766.1 hydrogenase expression/formation protein [Rhodovulum sulfidophilum]AB14
MVSNFHLPPTGFGPGSQPADEDMGYMQMPSGMRTYAPHLPEIADADAAGPALALLTRVAAACDRAAATGEGEAFR
LSDLP
PEARRLLAETLGQGEVSVRIGGIAPVAAQESVFAGVWCVLGEGIDRIEVGAVPACARMACAPEGAALGLAAPKGPDVF
NA
PALLVELLDHAQAWQAGQPPHVVNLTLLPHTEGDLLWLDAALGTGAVTILSRGYGNCRVAATATPNVWRVQFFNSM
DSLI
LDTFEVTDMPEVALAAPEDLTDSAARLREVLEAIR
>OLS47767.1 rubredoxin [Rhodovulum sulfidophilum]AB14
MSAGFEGSYGGRADRISSAAVMECKICWTVYDPALGDEFRQILPGTPFTALPEDWTCPTCDAPAAQFMVRDDPGAA
DAAS
RPGARDAASWLAGRVAALEAEFREIWHGKMRDVPLVNASLHVEAVGFALYDGRPLGVLVAPWFMNLFLLPAEDED
WSDLT
PGAKEVLDFPSGSYEFIHNVRPMVGGYKACSLFSPMTDFASQLQAVDVARAVTVELFKAENLEETDRAAEIRSTREAEL
A
AAEPACEAEEGAADEAPAVPTRRAVISGGLATEAPGE
>OLS47768.1 hypothetical protein BV379_05360 [Rhodovulum sulfidophilum]AB14
MTTALHIALGAAGPRIVPPAPVPVEALLLNRKVEEAALLLPRIFGLCRAAQSLGARAALGLPVRPEDRAALADEILREHL
MRFFVFWPRRLGLPSTPLPAQGRMAEAVFGPVARCPGPGALDDWLASGAGVAPVLAAIAVRFGPGEAVADLPAATA
ESLA
SGAAQENSPWLRHRDSSLLAGIAARFGRGPLWRAAGRLVDLEACLTGRLAPEADSRQGPVLVPTARGICALSARVREG
RV

SSFRRWTPTDHLLARGGVLERALASLPRARVELAPLVVDILDPCLPVHFEEAADA
>OLS47769.1 hydrogenase maturation nickel metallochaperone HypA [Rhodovulum sulfidophilum]AB14
MHEMSLCEGIRGIVEDQARAHGFATVRVLRLEIGRFAGVEKPALEFAFDVVMRGSPAEGARLEMIDLPGRAMCYDCA
EEV
EIENRLDPCPRCGGGKLMPVAGDEMRIKDMEVV
>OLS47770.1 hydrogenase accessory protein HypB [Rhodovulum sulfidophilum]AB14
MCTVCGCGGATVEGQAEGQSHGSHHHAHGHGSHHHHDHAHDHHAHHHHGEHGDLHYGEGAAGVSVPGMSQSR
LIEIETDI
LSKNDAIASGNRRVLRALGAFAVNLVSSPGSGKTTLLCRTIEMLGDRPLAVIEGDQQTSNDADRIRATGARAIQVNTGK
G
CHLDAHMVGHAMDHLELERGTLLFIENVGNLVCPAGFDLGENAKVAILSVTEGEDKPLKYPDMFTVARLAILNKTDLAP
H
CDVDLDLYEANLRRVNPDIEILRVSARTGEGMPAWLDWLDAHSAGKAAGAAV
>OLS47771.1 sigma-54-dependent Fis family transcriptional regulator [Rhodovulum sulfidophilum]AB14
MAPASPRPAVLLVDDEPHSLAAMRMALEDEFEVLTAADAAEALAILEDEWVQVIFCDQRMPGRSGVEFLTEVRERWP
ETL
RLIVTGYTDSAAMVAAINEAGIHQILTKPWHPDQLLIAARNAARLFLLSRENERMALEMRLLATTAESRVEKRRKALREG
MGFETVLRAANSPMGAVVQMAGRFAGFDLPVVLTGEPGTGKAQLARAMHYASLRSDKPFYDLNLAGLPEDLAAIEL
MGAK
RGVLAGGVARVGLMQKADRGTLYLGGIETAPPGLQLTLLRVLTEGAFTPVGGQETVATNLRLICGAGTDLKALVAAGRF
R
GDLYYALAAGEIAVPPLRARRGDIALLAQAKLAELSQAHGKPVHGLDEAALEFLENYDWPGNLRELRNEIARMLVMAR
DS
VLGADLISRHILQAPPGEEGADRSADAVLTAAGTLKDRVELMEMRILRETLTRHRWNKSRAAAELGLSRVGLRAKLERY
G
IAGPAGRVAQDEED
>OLS47772.1 hydrogenase formation protein HypC [Rhodovulum sulfidophilum]AB14
MCLGIPGRIVAVTDEHRMMALAEVSGVRREVNVACILDGAIEDLLGEWALIHVGFAMARIDPDEAAKTLQALQDLGEA
QE
AAEAMAEGAKALEGQG
>OLS47773.1 hydrogenase formation protein HypD [Rhodovulum sulfidophilum]AB14
MKYATEFRDPKAAKAVLARIAELADEIGATRAKPVHIMEICGGHTHAIFRYGLDKLVHDGIEFIHGPGCPVCVLPMSRVD
ECVEIALRPGVIFTTFGDAMRVPGTKMSLMQAKAAGADIRMVYSPLDALELARRNPDRQVVFFGLGFETTTPSTALSIQ
Q
AAREGLENFSVFCNHITVPEPIRALLDDPHMVLDGFVGPGHVSMVIGTHPYDFIARDYGKPIVVAGFEPLDLLQSVLMV
L
IQIRDGRAEVENQYARVVPEHGNPVSLAAIADVYEKRPSFEWRGLGEIDHSGLRIRAKYRAYDAEETFGIGYAAGPRFVA
EPEGCACGQVMTGRIKPTACTQFGSGCTPEMPLGALMVSSEGACAAYWQYGGARATEATE
>OLS47774.1 hydrogenase expression/formation protein HypE [Rhodovulum sulfidophilum]AB14
MNVPNRVRLRDTHVTLSHGGGGRAMRDLIDEVFASAFRPDAMEDQARLSDAALAEPGARLAMTTDGFVVTPLEFPG
GDIG
KLAICGTVNDLAVGGARPLWLSAAFIIEEGTEVALLRRIAATMATEAEAAGVRIVTGDTKVVGRGAADGVFVTTTGVGV
I
PPGRDLAATHIRPGDVAIVNGVLGDHGATILAARGDLALSTDIRSDCQALGHLMEAVIAAAPGTRAARDATRGGLAAA
LN
EMAETAGVGVEIEEEALPLRDEVKGLCEILGLDPLYLANEGTLVLFVPEAEAEAALAAMRDRPEGRGAVIVGRATGDHP
G
QVRMRTVFGGARIVDMLVGEQLPRIC

>OLS47775.1 hypothetical protein BV379_05395 [Rhodovulum sulfidophilum]AB14
MRPLFDRSAGRARTALAERTGTCLATLRVFARDWDTAFAETAEGVLFELFRGGWRGAPGGRSLPGARPPHRF
>OLS47776.1 hypothetical protein BV379_05400 [Rhodovulum sulfidophilum]AB14
MADYNTIRAGAAGVRTAQIDEGLRAHMNKVYGTMSVGMLITALAAWAISGLAVTSDPSGAVAQIGADKYLTGLGQA
LYAS
PLKWLVMFAPLLFVFGFSAGINKMSAATAQVVFYAFAAVMGLSISSIFLVFTGMSIVQTFLVTAIAFAGLSLWGYTTKKD
ISGWGSFLIMGVIGLVVASIVNIFLASPAIMFAVSALGVLIFAGLTAYDTQRIKTDYLQHAQSMDSEWLGKSAIMGALSL
YLDFINLFMFLLQFLGNRD
>OLS47777.1 hypothetical protein BV379_05405 [Rhodovulum sulfidophilum]AB14
MTQVSTNIRAARDVGRAGLAKRIFLGLSVMRQRRQLVELDDRQLRDIGLTAEQARAEARRSAWDVPQSWLR
>OLS47778.1 LysR family transcriptional regulator [Rhodovulum sulfidophilum]AB14
MRNLDMTALRSFVAVSDAGGVTRAAGFLNLTQSAVSMQIKRLEEALDMQLLDRSERKVKLTGAGEQLLSYARRILSLN
DE
VYARMTAQEYEGEVVVGVPHDIVYPAIPVVLNRFARDFPRVKVQLISSYTMRLREMFRRGDVDIILTTEDRCGKTGETLT
ELPLVWVGAIGGSAWKQRPLRLAYEHTCIFRKGVQAALDTAGIPWVMTVDSDSSRTVEASVSADLAVHTSVDGALPPY
VE
CIQHGGALPDLMTTQINLYVSDLADGPVIGALAELIRQAYRAP
>OLS47779.1 zinc-binding dehydrogenase [Rhodovulum sulfidophilum]AB14
MRLFQLERIGEPPRLTETACPAPAAGEVRVRIAACGLNFADLLTIEGRYQERPALPATLGMELAGTVEALGDGVDPGLA
G
TRVAVFAGAGGLAEAGCFPAARCLPLPDGMSFVEAAGFQIAYGTSHVALSHKARLAPGETLLVTGAAGGVGLTAVEIG
KA
MGATVIACARGADRLEVARAAGADHLVDSDQGDLRAAVLALGGADVVYDPVGGPLFAAAMRACNPDGRLLTIGFAS
GEVP
QIKANHLLVKNLSVIGLYWGGYLSFRPEILTDSLATLFRWHAEGRLHPHVSHVLPLERAAEGLDLLRRRKATGKVVIAME
>OLS47780.1 transcriptional regulator [Rhodovulum sulfidophilum]AB14
MKHPVDVHVGKRIRHRRWMVGMTQQQLAERVGIKFQQIQKYETGMNRVSASRLWDIADALEVPVSFFFEGLDGRS
AEQST
NGMPDDLLSDKEALELVRSYYAIPENQRRRLFELARVLSDVA
>OLS47781.1 histidinol-phosphatase [Rhodovulum sulfidophilum]AB14
MTDYDARCLIETAHALADAAREATLMYFRSAALGVENKAGQGFDPVTRADRACEAAMRAILAERRPDDGILGEELDNI
PS
RSGLTWVLDPIDGTRGFMCGTPTWGVLIGLADATGPILGVIDQPYIGERFLGGFGRAEFTGPHGGRALATAAPRPLSEA
S
VFSTFPEVGSPEEGRAFAALAGQARLVRYGMDCYAYALVAMGHVDLVVEAGLHPYDIQAPIAVVEASGGIVTDWQGG
PAH
GGGRVIAAANPRVHAEALAILSAAA
>OLS47782.1 8-oxoguanine deaminase [Rhodovulum sulfidophilum]AB14
MGHVLIRGAELIVTMDDDRRELAGADLRLRDGCVAEIGCGLAPDGAEIVLAEGCLVTPGLVNTHHHLSQCLTRAVPGA
QD
ALLFGWLRTLYPIWSRFGPEEFRVSALVGLSELALSGCSMSSDHQYLYPNGARLDDTIAAAREVGLRFHATRGAMSIGES
KGGLPPDGLVEAEPAILEDCIRVIDAFHDPDPGAMIRVGIAPCSPFTVSPELMRDAALLARDKGVRLHTHLAENDEDIAY
SLERFGCRPGQYAEDLGWTGDDVWHAHCVKLDAQEISLFAQSGTGVAHCPCSNCRLGSGIAPVRAMRDAGVRVGLG
VDGS
ASNDGGSLIGEARQAMLLQRVAQGADAMSAREALEIATRGGADVLGRPDCGRLAPGKRADLAIWDMTGIEAAGLW
DRAAI
LLAGPSRVRDLFVEGRQVVRDGHIVTHDLGAAVTAQTRIARRLAGL
>OLS47783.1 magnesium transporter [Rhodovulum sulfidophilum]AB14

MEDEVIDRTDPEEEGYGLDNRTVAAIRLAVAAGDRARLGELLEPLHPADIADLLEQVDSGHRREILLLGSGFFDGEVLSE
LDESLREEVIAFLPREVLSEAVRELESDDVVDLLEDLDEPQQAVILNALEDADRLAVEQALTYPEYSAGRLMQRKVVRAP
EHWTVGETIDHLRAQKDALPDQFYHVIVVDPGIRPVGYVTLGKLLASRRSVRLSELYEESFRPIPATQEESEVAYAFNQY
HLISAPVVDDAGRLVGIITIDDAMAVLDEEHEEDILRLAGVGDSSLNDRVIETSRQRVPWLLVNLCTAMVSALVISQFEA
AITQLVALAVLMPVVASMGGNAGTQALTVAVRALATKDLTGSNVWRVVRREAAVGAINGLLFALLMGLVGVLWFHS
AGLA
LVIAAAMIINLGVAALAGILVPVMLDRFGVDPALASGTFVTTVTDVVGFFAFLGLASMVLL
>OLS47784.1 5-formyltetrahydrofolate cyclo-ligase [Rhodovulum sulfidophilum]AB14
MIAEAKAALRAEAQARRAEAHDPERGAAAIARLEAVLAPHRGRPAAGYLPIRTELDPRPAMAGLAASGPVGVPVIEGR
GQ
PLGFRLWAPDCALEAGPFGVAVPAGARPMVPEVLIVPLLAFDRRGIRLGYGGGFYDRTLELLRARGPVLAVGLAYAAQE
M
PDLPAEPTDLPLDAIVTETEVILP
>OLS47785.1 metallophosphoesterase [Rhodovulum sulfidophilum]AB14
MKLLFLGDVMGRSGRSAVADRLAALREAWGLDFVVVNGENASSGAGLTPAHAKGLLEAGADCVTLGDHAFDQKDM
LSFIE
QEPRILRPLNFARGAPGKGARLFNARGGRKVLVAQILGQVFMKRPFDDPFSAIETVLKAHPLGGLAQAAVVDVHCEAT
SE
KMALGHYCDGRASLVVGTHTHVPTGDAQILPDGTAYLTDAGMCGDYNSVIGMDRAEPMRRFVTGMSKTRFTPAMG
EATLS
GVYVETDDRTGRATRVRMIRQGGRLEQAAP
>OLS47786.1 dATP pyrophosphohydrolase [Rhodovulum sulfidophilum]AB14
MIPISLPPTGMALLALAVVAAMFVAFVRERYPTEVIAICGASVMLVLGILPYEDALRVLSNPAPWTIAAMFLVMGALVR
T
GALDWVIHAAERSGKHKPRRAIATLMAFVVISSAFVSNTPVVVVMLPVFVQLARSLGVSASKLLIPLSYAAILGGTLTLI
GTSTNLLVDGVARTQGLEPFSIFEITPLALVLILWGMIYLRVFGPRLLPARDSMADLLSDKSKMKFFTEAVIPPESNLIG
REVTGVQLFKREGVRLIDVVRGDVSLRRDLKGVTLQVGDRVVLRTQVTELLSLQRNKSLRRVDQLSAVETSTVEVLISPG
CKMIGQTLSALRLRRKYGVYVLAAHRRNQNIGRQLDDLVVRVGDTLLLEGAPGDIKRLAADMGLVDITHPSGRAYRRT
QA
PIAIGALVAIVALAALGVAPILMLAVVAVALVLATRCIDAEEAFTFVEGRLLALIFAMLAVGAALEASGAVQLIVDGLAP
YLGQLPPFFVVWAMYLLTSVLTELVSNNAVAVVVTPIAVGLGHALGVDPRPLVVAVMIAASASFATPIGYQTNMMVY
GPG
GYRFTDFLRVGIPLNLTMGLLSSALIPLLWPL
>OLS47787.1 histidinol-phosphate aminotransferase [Rhodovulum sulfidophilum]AB14
MTGPRFTPLADSLPATVPFIGPETLERDRGRPFRARLGANESLFGPSPKAVAAMQAAVREVWKYGDAQNHELRLALAE
AH
GVTPDCLMVGEGIDGLLGYLVRLVVGPGDPVVTSEGAYPTFNYHVAGFGGALHKVPYRDDREDPEALIARAAELKAKL
VY
LSNPDNPMGTWLPAGQVQAMIEAVPEGTLMVLDEAYIEFAPEDTAPAIDPEDPRVIRFRTFSKAHGLAGARVGYAIAA
AP
LITAFEKVRNHFGMCRISQAGALASLADTAWLAEVKSQVAEGRAMIARIAEANGLSALPSGTNFVTVDCGADGDFARR
VL
AGLAERDVFVRMPGVAPLDRCIRIASGPEDQLTVLAEELPKALAAARG
>OLS47788.1 alpha/beta hydrolase [Rhodovulum sulfidophilum]AB14
MTEPQMLTTPEGRRLAYHRTEGAGPGIAFLGGFMSDMEGTKATHLEAWARAQGRAFLRFDYSGHGQSSGRFDEGAI
GDWA
ADARAALGALSEGPQVLVGSSMGGWIALLLARAQPERVAGLVGIAAAPDFTEDSMWAGFSDAQRAELTETGRLELPS
AYA

DSPYVITRRLIEDGRRNLVLRDPLAFGFPVRLLQGTADEDVETRVALRLLERIEAEDLRLTLVKGADHRFSTPETLALIE
SAILEIG
>OLS47789.1 polyhydroxyalkanoate depolymerase [Rhodovulum sulfidophilum]AB14
MKFMTTYDLMETARNTNQWFGATARALGSYPAVAMVPSPLFRMVAAWGEVTERAFARMVAKPDWNINTVVGAD
GRDHIVS
VEKSVTRPFGDLIHFKVHGRPQMRRRVMLVAPMSGHYATLLRSTVASLLPDCDVYITDWHNARDIPVSEGKFDIEDYTL
Y
LVDFLKHLGPDTHVIAVCQPAPLALAATAYLAEEEPAAQPRSLTLIGGPIDPDAAATDVTDFGRRVTMGQLEHLVIQRV
G
FKYAGVGRLVYPGMLQLASFMSMNGDRHTRAFSEQILRAAKGEAGDHDKHNRFYDEYLAVMDMTSEFYLTTVDRIFK
RGE
IARNAFSVNGRKVDIAAITDVAVKTVEGANDDISAPGQCIAALDLLTGLPDHKKASHLEPGAGHYGIFAGKSWRNNIRPL
VLDFIDANSGDPQPARAHLKTV
>OLS47790.1 class I poly(R)-hydroxyalkanoic acid synthase [Rhodovulum sulfidophilum]AB14
MTIKDDAPATNLERLNENLVKLEELTERLVSAMSKKRRIDPNLQAPGQDLYMKATAAYMAEMMQNPSKLIEHQVSY
WGKA
LKHFVEAQEALARGKLQAPPDSTPKDPRFSNPLWQTHPYFNYIKQQYLMSAEAISSAVKDIDGLNPRDARRLEYFTQQI
V
DLMSPANFLGTNPEALERAAATDGQSLVDGLENLVRDIEANDGEILVTLADREAFAVGRNLATAPGKVVFRNRMMELI
QY
SPTTETVHATPIMIFPPWINKFYILDLTPQKSLIRWLVGQGYTVFVVSWVNPDAGYADVGLDQYVEEGYLTAIAEVKAIT
GEEKINAVGYCIAGTTLALTLALLQKRGDSSIRSATFFTTLTDFSDQGEVGVFLDNDFVDGIEREVTGKGYLDSFFMSRT
FSFLRSNDLIYRPAIRNYMMGEAPPAFDLLYWNGDSTNLPARMAVEYLRGLCQQDRFATEGFEICGERVHISEVRLPLC
A
VACETDHIAAWRSSYEGVRKMGSTSKTFILSQSGHIAGIVNPPDRKKYGHYVNDAPMKDLAADRWREDAAFTEGSW
WPRW
DSWLKRRSGRQVPAREPGADGRPLLGDAPGTYVMGKKAT
>OLS47791.1 Phasin [Rhodovulum sulfidophilum]AB14
MADAQDFTKMMQDMMSAFSMDPTTMQDAFKTQAALAEKMSKVALEAAEKSTEISSKWTKDTLSKVGEVATLKDEP
TDYTK
AMTDFASSSAEMAAENMAAFAEVAKKVQMETVELMLAAGKDFSEDATSAMKKATATATKTAKKATTVASEKA
>OLS47792.1 polyhydroxyalkanoate synthesis repressor PhaR [Rhodovulum sulfidophilum]AB14
MSDVKEPLLIKRYASRRLYNTETSDYVTLEDIAKVIRTGRDVKIVDLKSGDDLTRQYLLQIIAEHESKGESVLPINVLND
LVRSYTSSAQSVVPQFLAASFEMLRDSQSKMMENFGRINPMAMPGLGAIQAQQEAFLKAMTGGWSGAPGTEGEAG
EGEER
ELDEIKRQLAELQQKLSKMNG
>OLS47793.1 L-serine ammonia-lyase [Rhodovulum sulfidophilum]AB14
MDLSVFDIFKIGIGPSSSHTMGPMLAAARFLDLLRGSPFHPVGLRASLHGSLAFTGVGHATDRAVILGLDGFTPETYDRD
AAEEALRRIGTEAEVRPKGLPPLRFDPASDLVFDHDRALPGHANGMILSAIDAEGDILTSETYYSIGGGFVRTEAELAAG
ETDRGGGTRPLPYPFASAADLLELARTSGRSIAAMKRANEIARMGPEALETGLARIWSAMSDCIERGLATDGQLPGGLG
I
RRRAHAIHNALLAERGMNLTAPHTINDWMSVYAMAVNEENAAGGQVVTAPTNGAAGVVPATIRYWLTHVPAAQPS
KVGDF
LLTAAAIGGLIKKNASISGAECGCQAEVGSASAMAAAGLAAVMGGSPEQVENAAEIALEHHLGMTCDPVKGLVQVPCI
ER
NGLGAIKAVSAASLALRGDGRHFVPLDACIETMRQTGRDMNEKYKETALGGLAVNIPNC
>OLS47794.1 hypothetical protein BV379_05490 [Rhodovulum sulfidophilum]AB14
MTTIPTEKHRNPGLHAAAGALGLALIASFWLSTLGAEFGTTAQIVAVKTAIPYGIVLLVPALIVAGAGGTRLARDRRGP

LVTRKSRRMRIVALNGLLILVPSALFLAKKAAAQDFDTAFVLVQLAELAAGAVNLTLLGANLRDGLAMRDRRLALR
>OLS47795.1 MarR family transcriptional regulator [Rhodovulum sulfidophilum]AB14
MSFDKDDSAGYLANHMARLFAAALTRRIAPLGLMPGQFMVLLELWREDGLTQRDLVERLDVEQATIANTLNRMERD
GLIL
REPDAADRRARRVRLTDRARALEAPATEAATEVNGIALARLAPEDRARFAALLQTVIAGLREGR
>OLS47796.1 ribose 5-phosphate isomerase A [Rhodovulum sulfidophilum]AB14
MAAELSPIELAKYVAGRRAADLVEDGMRVGLGTGSTAAWLVKSLGARVRNEGLSITAVPTSDRTAALAREVGIDVVSLE
D
ARWLDLTIDGADEFDPAFNLIKGGGGAHLQEKIVATASDRMVVIADPAKAVAQLGAFPLPVEVIGFGWSASRKLIAEAL
E
GLDVLGREITLRMAGETPFRTDEGNLVLDLHLGRIGNPRQLGLVLNQIPGVVENGLFIDICDAVVIGHPDGRAELRDAEG
SEEARVDLPASDNLFADL
>OLS47797.1 glutathione-disulfide reductase [Rhodovulum sulfidophilum]AB14
MSFDYDLFVIGGGSGGVRAARLAASEAGARVGLAEEYRMGGTCVIRGCVPKKLMVYASGFAHAVEDARAYGWQVH
GGEFD
WSLFKARLDDELTRLEGVYRGNLERAGVEIFDSRAVLTDPHTVRLADGTEKTARHILIASGGRPFVPEFPGSELAITSND
VFLFESLPKRILIVGGGYIACEFACILNGLGVEVTQFYRGAQILRGFDDEARGHIAGAMCAQGVDLRVGCNVQRIDRVED
GLWVKATDGGDAVFDAVLFATGRRPNTDGMGLEEAGIALGANGAVAVDEWSQSSVPSVYAVGDVTDRIALTPVAIR
EGAA
FVETVFKGNPTRADHDLVPGAVFTRPEYGSVGLTEEEARGRGQIEVYSTAFRPMQSGFAGRPDRVMMKLIVAAESRKV
LG
CHIVADQAGEMIQLAAVAIKMGATKEDFDRTVAVHPTMAEELVTMKVPVRTG
>OLS47798.1 HflK protein [Rhodovulum sulfidophilum]AB14
MAGNSGGPWGSGGSGQGGGGNRGGGGGRGPGDEPPQIPEIDELMRRGQEQLRVLMGGRGGRGGGPKFTRTGVA
LAVLAAG
VAWAWASFYTVKPEEQSVELFLGRYLDTGNPGLNFAPWPLVSAEVIPVTREQTENMGVGRAGVDAGLMLTGDENIV
DVDF
QVVWNINDPAKFLFNLRDPRQTIRAVSESAMREIIAQSELAPILNRDRGLIASRLTDLIQQTLNSYDSGMNVVRVNFDKA
DPPKEVIDSFREVQAAEQERDRLEKQADAYANRVVAGARGEAAQILEEAEGYRARVVNEAEGEASRFSAVLDEYTKAPE
V
TRKRLYLETMEEVLGKVDMIMLDKDLTGTGGVVPYLPLNDLRRSEAGGRN
>OLS47799.1 protease modulator HflC [Rhodovulum sulfidophilum]AB14
MKKSALLIPLAILVGATALSSMFIVDERERALVMQFGQVKGVKSEPGLAFKIPLIQEVVRYDDRILSRDIDPLEVTPSDD
RRLVVDAFARYRIADVVQFRQAVGVGGIRAAEDRLDSVLRANTREVLGSVSSNDILSSDRAALMMRIRNAAAEEAQGL
GL
EIVDVRLKRTDLPEQNLEATFARMRAEREREAADEIARGDEAAQRIRAQSDRTVVELVSDARRESEIIRGQADAQRNRV
F
AEAFGADREFFEFYRSLNAYKAAFGKGNASMVLSPDSEFFEYLERADRPVVPAPSAPAAAAGPAAD
>OLS47800.1 hypothetical protein BV379_05520 [Rhodovulum sulfidophilum]AB14
MEKIVLGLGMVLVIEGLVFALAPSRLEEALELLRRIPAEARRLIGLAAITLGTGLVWLAQSLWG
>OLS47801.1 serine protease [Rhodovulum sulfidophilum]AB14
MTSQAISLSVRSASWSSSERLRPLWALMLGLLLLMAQTSLALARAAPESFADLAEKVSPAVVNITTSTTVEGRTGPNPLV
PEGSPFEDFFRDFMNPDGNPNMPPRRSQALGSGFVISEDGYIVTNNHVIEKADEIQVELFQGGTLEAEVVGTDPNTDIA
L
LKVKSDKPLPFVPFGKSEAMRVGDWVMAMGNPLGQGFSVSAGIISARGRALSGTYDDFLQTDAAINKGNSGGPLFN
MNGE
VVGVNTAILSPTGGSIGIGFAMSSEVVANVVSQLKEFGETRRGWLGVRIQDVTPDMAEALGLEQARGALVTDVPEGPA
AE

AGMQSGDVILSFDGIEVEDTHGLVRQVGNAPVGKAVRVVVFREGKTQTLLVTLGRRETAEGAGAGGSSDGSSAPEPE
AAE
ILGMTLVPLTDELRDQLGVGNLADGLAVSDIDETGEAYSKGLRAGDVITEAGQRQVATAADLQERIEEARDAGRKSLLLL
VRRQGEPRFVALSVEE
>OLS47802.1 aryl-sulfate sulfotransferase [Rhodovulum sulfidophilum]AB14
MSGRPVKRSLTLRGHRTSVSLEDEFWRALREIAAERGIAVNALAAEIDESRGMDVGLASALRVHVLTHFRRRQDER
>OLS47803.1 DUF4169 domain-containing protein [Rhodovulum sulfidophilum]AB14
MTGGVVNLRQARKARERAAKKAQGDENAARFGRTKGQKAREELDATRARDALDGHRRTPDDEA
>OLS47804.1 fumarate hydratase, class II [Rhodovulum sulfidophilum]AB14
MTETRTETDSFGPLDVPADKYWGAQTQRSIQNFPIGWERQPVAIIRALGVIKKACALVNIDQGGLDPKLGEAIAAAAQE
V
IEGRFDDNFPLVVWQTGSGTQSNMNANEVISNRAIEMLGGVKGSKDPVHPNDHCNMGQSSNDTFPTAMHVAIGM
VARDVL
LPGLEKLHEALSAKSDEFADIIKIGRTHTQDATPLTLGQEFSGYAKQVEKGIARVKAALPDIYELAQGGTAVGTGLNTKK
GWDGRVADRIAEITGLPFVTAGNKFEALAAHDAMVMFSGALKTVAASLFKIANDMRFLGSGPRSGLGELILPENEPGSS
I
MPGKVNPTQAEAMTMVCAHVMGNDAAVGFAGSQGHFELNVYNPMMSYNVLQSMQLLGDAASSFTDNMVAGTK
ANTARIDK
LMKESLMLVTALAPTIGYDNATKVAKTAHRNGTTLREEAIALGFVDAETFDRVVRPEDMIGPKD
>OLS47805.1 hypothetical protein BV379_05545 [Rhodovulum sulfidophilum]AB14
MSDRIEYGNLMHAAMRSLIRTVLSDVAADGLPGEHHFFITFDTTHPEAQLADWLRQRYPSEMTVVLQHWYDHLSVG
DDGF
SVTLNFGESPEPLFIPFDAIRTFVDPSVEFGLRFETQDNDEDEDEEDDASDLPETEPHDNDTGNGDAQIVSLDKFRK
>OLS47806.1 hypothetical protein BV379_05555 [Rhodovulum sulfidophilum]AB14
MSFIDLNRVNASVALQRGHYTFPRAAKLREAFENCLHDYYVKASIGGHFEARGLLVTGESRVGKTSETARLVEEFNASM
T
AMPNGKPGKIIYCKLDGSISWKDLGHKTLEALDYPANPRRTQGELWKMVRHQCREQDVIGLYYDECQHAFTSGPKSN
EKL
LDRFKALMKEPEWPLMLILSGVPVLASHVNSEEQIAHLLSHIHFDEIHLGRFADPTRDPDMIELNKLVYTYAERVDIDVD
DLVDVDFLQRLDFACASRWGLVIELLIRAFGLCRLRSQKSATVKIFSEAYAQNSRLPQGLCPFTAPDYRDMIDGDKLME
M
MLDE
>OLS47807.1 hypothetical protein BV379_05560 [Rhodovulum sulfidophilum]AB14
MVANKGERYARNKRGRGHSDVRALMIGEFCEMDECRASLITSAKAKGYWHTLSEGMKKAMEWADEEIRSRLSILVMI
DVA
TRMPLAWVVSDQPKAEATMQLLRMATRDKTRERHIYGCEGEAMPAMGLGMVRNDNGTGLRNAEVKSALMGIASA
NTDVRT
HASADKPYVERMFGTTESILLKLLHGYTGRKAGELPGYDAKKSGVLDIDLLYEILTKFFIDEYPSLKHTGVGMSGRRPAE
VYQELNETRETFRLLDEYLRRVHLDWPFKLQPNDEGVRVLEGLFYSSDKLQAALEHHQGKVTVYIDPDDLNFATAVIPR
D
PKRYRLALQTTVFADLTVSEFLELMTQYRRENPELTTLYEDRIAKVRLERHEQLKKIGVERRLPRNYVNVGEARKKARSL
FASSRLVTTHVPIDTVAPGTLATAISGPGILPLGGVNLIDHAPAETTSTPAIPARKSKPKSTKPSSEPPVRLGRPAQKGE
LL
>OLS47808.1 transposase, partial [Rhodovulum sulfidophilum]AB14
MSKTMDDSIKRWTAKRKTALVK
>OLS47809.1 hypothetical protein BV379_05575 [Rhodovulum sulfidophilum]AB14
MTEKMKAWTKEDVDRLLQNIDYEAAKKDTYAVNYYRDENDECDHLEVAVFVVLEWVREGHDDNIKSSTVRGTKRMG
WLWG

AFNARKRREEYGDLGNHGLISEDEARETLKEMGLALEEAKRYLVDSFTKTNGYAPVMLPVDPRWKNRRDAERKRLGLP
SK
E
>OLS47810.1 hypothetical protein BV379_05580 [Rhodovulum sulfidophilum]AB14
MTWDAAPTGRRGRQRIYSDAAIQSCLTMKVLFGMALRQTTGFVESLLRLVGLDWSVPDFSTLSRRQQTLAVNIGICAT
GP
FQSRRGNEGRSPLHDHFPRPDPCRRCTSHTRRAEQAGVLRSEVQEHLGRSRIQGYQRSLPDSGRVQVRTSVPYVGQLP
SK
GNRNTRTVRAAASTRARFKGVAEA
>OLS47811.1 IS5 family transposase [Rhodovulum sulfidophilum]AB14
MHAICDSKVRPIDLFVVAGQVSDYIGARSLLSGLPNVKWLLGDRGHDADWFREALQDKGIRACIPGRKKRKTPVKYDK
RS
YKRRNRIEIMFGRLKDWRRVATRYDRCPKVFLSAIAFAALVIYWL
>OLS47812.1 chemotaxis protein [Rhodovulum sulfidophilum]AB14
MANSTSSLEIAQAKGLRLIGYLNVLLTPVPAIGAWLAGLSPWPFLAMSFAIAAVTMGLQRRGGGIARIAVSLGAMGQL
PL
LTAALAGNPWQLDSHLIYFAVLAVLVILQDQRALLSASGIVVVHHALLTFFLPTLIYPSGGLLIDLGRLALHGTVWVAET
AGLFYLLRMQLSQRDQIEQEAARLSDALKSSQELQMNLAQKEAEQLNVMEQFSAALTKLAKGDLATRIETVFPDQFEPL
R
NEFNSAVNSLSVAMASVQENADSIKADTRNIASASEKLAKRTEDQAHALGSVTSRIDEISEVMSSASEDARQVAVTTAE
M
RADAENGMKVVDGALDAMGQIKSSSGQILSVVDLIEDVAFQTNLLALNAGVEAARAGSAGQGFAVVASEVRALAGRS
SDA
AREISALINDSARQVQNGVELVDNAGTVLRGVLEAVRKASDSMQNIASTVGTHSQGIVDLKHTMGELDQLTQQNAA
MFEE
TSAATTALTAATNLLSEVVAEFQNASEPDDPSLRLRHIA
>OLS47813.1 amidase [Rhodovulum sulfidophilum]AB14
MSDATDPADMGALEARRLIGRGQLSAEELARACIARIEAIDPAVNAVVSCDPDALLAGARQADADRAAGRPLGPLHGL
PV
AIKDMNDVAGLPTTYGSQIFRDNIPSADDALVAGLRAAGALPFCKTNVPEWSAGANTRNRVHGTTANPHDLTRNCGG
SSG
GSAVALACGFAPLASGSDLGGSLRTPAAYCGVVGFRPGFGLVPGPDRATALLPQPTAGPMARSVADCALMLSVLARPD
AR
DPFTTVIGGRTPWDAADFARLPSRDLSSLRVAVTEDFGFAPVSAATRQTFRRVLAPLLPFLGASEESTPDCTGADRIFSV
LRAVMFQATHARFLDETPDLVGALVADNVREGRRYSATDVAQALVSQGAYARRWLSFFDEFDVLISPAVTVQPRDWH
EAF
PAEIDGQPLQSYYHWLALAYAATIPGHPAIILPCGRDAQGLPFGVQLIGRRHGDLGLLAIAAELEAVIAGLPDLTPCAPD
IPTLSAAPPLRTAPGFLDAP
>OLS47814.1 carboxypeptidase [Rhodovulum sulfidophilum]AB14
MTDPLSLPFDTDEMLAGLKPWIETESPTFDAAAVNRMMDLVQHELAALGARVERIPGRMGLGDSLRATMPHPRAGE
GGIL
LLGHMDTVHPIGTLEKLPFRVEGDVCYGPGLMDMKGGNYVYLDALRKLQAAGVETPLPVTVLFTPDEEIGTPSTRELIE
A
EARRHKFILVPEPARPDGGAVIGRYAIARFDLQTRGKPSHAGWQLSDGRSAIAAMARKVAEIEGMTTDDCTFSVGVFR
AG
QWVNCVSSVCDAEVLSMAKTQDLLDEGVARMMALNSDAGEVVMEVRRGVTRPVWEPDQPGTMAMLALAQKIAG
EIGFEIS
GASAGGGSDGNFTGALGIPTLDSIGVCGKDLHTLNEHIHIDSLLERAKLAAALYCRLGA

>OLS47815.1 amidohydrolase [Rhodovulum sulfidophilum]AB14
MTVLPRIQDFAAELTAIFQDFHTHPELGFEETRTAGIVADKLRGFGVDEVHEGIGGTGVVGLIKGKGGGNRRVGLRAD
MD
ALPIVEASGLPYASQEAGRMHACGHDGHTTMLLGAARYLAETRDFDGTVVLIFQPAEEGLGGARRMIAEGLFERFPCD
EI
YGMHNDPNAEPGKVTVTPGPAMAGASFFDITVRGTGSHAAMPHQSKDPIVIGTALVQQLQSVVSRNTPPTKPVVISV
TTF
NAGSAYNVVPDTAVITGTIRYFHDEVAGMAHDRIRALCAGLGAAYGVEIGVDLRNVFDVLMNDPELSAAYIAAAADVV
GP
DMATLTEDQMTGSEDFADMLKVVPGAYCRVGHAGSVPLHNPAFVLDTGILPVGASVYARLVETRLAPTG
>OLS47816.1 microcin ABC transporter ATP-binding protein [Rhodovulum sulfidophilum]AB14
MEFKSRDFSEAPRVLKIRGLRVGLTGRPDAKPVLDGIDLDIRAGETHCLVGESGSGKSVTSLATMGLLPKGALELQAGSI
DLEGFEITQASASQMRELRALRLSMIFQEPMTALNPVLRVGAQIEEVLNMHTDLSAAEARKRTLDIMDQVHLPDVERIY
Q
SFPHQLSGGQRQRIMIAMALVLDPHLLIADEPTTALDVTTQQQILKLVAELQERKGTALLFITHDMGVVAEIADTVSVM
Q
NGRIVEHAPIRELLTNPREDYTRKLLNAVPNLAPRPARPGSAEPDVLKVEALEMTYGGGGKLFRKTGGVKAATDVSFGI
G
PGQTLGIVGESGSGKSTVARCIMRLIDPTGGQVHVAGAEISRLNRRALRPHRKHLQIVFQDPYRSLNPRWTVARSLCEG
P
VNYGASRAEALERAAELMELVGLPRDALDRYPHQFSGGQRQRIAIARAVAMKPDLLVADEAVSALDVSVQAQVLELLD
DV
QARFGIAMLFITHDLRVAAQICDEVLVMQKGRVVEHGPAANVLSDPHHAYTRSLIEAAPGRHWDFANFRALETAPGT
KGE
TA
>OLS47817.1 peptide ABC transporter permease [Rhodovulum sulfidophilum]AB14
MAKDISSRPSAFHVLTGLFSLPAGARIGPGPMIAAAILLAIVLASICAPLYVPYDPMDMNSLARLKPPSDAHLLGTDQYG
RDIFSRVMTGGRVSLLIGVGAACVSVLTGLLIGLVAGFFRAVDGIVMRIMDSLMAIPAILLAIALVALNGPSLGSVIIAI
TIPEVPRVVRLVRSVVLGAREEPYVEAAIALGSSMPKILIQHLMPNTLAPLIVQGTYICASAILIEAILSFLGAGVSTEI
PTWGNIMAEGRTFFQIKPGMIFWPGLLLSLCILAINLLGDTARDLLDPRLKKREG
>OLS47818.1 peptide ABC transporter [Rhodovulum sulfidophilum]AB14
MLGYILRRVLATIPVMIIVAIFVFLLLRLTPGDPAAILAGDAATPAQLERIRDRLGLNDPLYVQFLTWAGQTLRGDFGTS
LISNTPVSGMIASRLGPTINIAMMTIVISVLLAVPMGVIAAWRHRSWVDFLVMSFSVLGFSVPVFVIGYILIHIFAIDLR
WVPVQGYKPLSDGLGPFLARAILPSLTLATIYVALIARMTRASMLEVLGEDYIRTARAKGVRENVVLFRHALRNAAVPIL
TIIGTGFALLISGVVVTESVFNIPGIGRLTVDAILARDYPVIQAMILLTAGIYVFVNLLVDISYSIFDPRIRY
>OLS47819.1 ABC transporter substrate-binding protein [Rhodovulum sulfidophilum]AB14
MLTLRPNIRGTRRLAAALLAGGALLAPAASFADTTITAVMHSSLRVLDPVITTAHITRDHAYMIYDVLTAVDENFQPQPQ
MADWTVSDDKLTYTFTLRDGLKFHDGAPVTATDVVASLERWGKRDSGGQMIFDVTASLTAADDKTVVWTLASPFPPL
LNV
ISKQSALPPFIMPARIAATSPDETITEYVGSGPFVFNTDAYEPGVSATYDKFEDYVPRDEPPSWMAGGKVVKVDHVVW
TT
MPDNLTAINALANGEIDYIEQIQVDLKPILDASPDVTVEMRDPLGYVTIGRPNFLYPPFDNKKVRQAALLALSQEDVLAT
MMGSPEYYTICGAIFGCGTPLADETGSEPITSGAHPDEARALLEEAGYDGTPVVLMQPTDVVSLTNQPVVAAQALRNA
GF
NVDMQAMDWQSVVQRRAQQSPPDEGGWNLFFTNWMIPEISDPLINVMVNGRGDDAWFGWPDDPEIEALRSDFA
QAPDDAA
RKAAASKIQAHVMDNVNYIHLGEYAMPQARRDVIDNMIQSPVPVFWNITKDGE
>OLS47820.1 microcystin degradation protein MlrC [Rhodovulum sulfidophilum]AB14

MRVALAGFLHETNTFAPSPADLAAFRQGGGYIPMSRGAEMAAAARGVNLGISGALAVAEELGWDAVPILWTGAIPSA
HVT
REAFEIIADEIIAGIAGAGTLDGVFLDLHGAMVAEHLDDGEAEIARRVRDLVGPDVPVVAALDLHGNISAPFVESLDACV
GFRTYPHVDMAATGAAAARLLDRIVRTGTRPAKAFRQMSYLVPIPFQTTMMAPADALYAGLDVEDAAGAWSVSLFM
GFPA
ADIPDCGPSAVAYAATQAEADRAADRIAAAYEAAETRFDNRTFQPGEAVAEAVRLAAQATGPVVIADTQDNPGAGGV
SAT
TGLLQALIAADAQNAAIGLMVDPAAAAAAHAAGIGATARFRIGGHPGLPDDRPVEVEAVVETLSDGSLQATGPYYGGT
RL
DLGPSACLRIGGVRVAVTSHIAQMADREMFRFVGIVPEEQSVLVVKSSTHFRADFAPIAAEILVACAPGPMPFNPADLP
F
TRLRKGLRLSPNGPAFDPAGAGTGAAQGHAEPATAIARP
>OLS47821.1 crossover junction endodeoxyribonuclease RuvC [Rhodovulum sulfidophilum]AB14
MRVLGIDPGLRNTGWGMIEQRGSRLGHIANGVCHSSGETLAERLLSLHIQLTEIVARHAPEAAAVEQTFVNRDAAGTL
KL
GQARGIALLVPAQAGLEVGEYAPNAVKKAIVGVGHADKRQIDHMVRLQFPGIEIAGPDAADALAIALCHAHHLAGGAR
LA
AALARAGA
>OLS47822.1 Holliday junction branch migration protein RuvA [Rhodovulum sulfidophilum]AB14
MIGRLAGRLIHKGADQVMIDVGGVGYVVHVSDRTLMGLPGLGEAVALYTDLLVREDLLQLYGFPTLHDREWHRLLMG
VQG
IGAKASMAILGTLGAEGVGRAIALGDWGAIKAAPGVGPKIAQRVVNELKEKAPAVMALGGHLSPDPAAGEVIAPADPA
PK
AGNKTRTKAPAAAPVSSSVSAQADALSALGNLGYAPSEAAQAVARAAGETPEADTTALIRAALKLLAPRAD
>OLS47823.1 Holliday junction branch migration DNA helicase RuvB [Rhodovulum sulfidophilum]AB14
MTGPDPLLRPEAHPEDSDRALRPETLDAFIGQAEARANLRVFLQSARQRGEAMDHALFHGPPGLGKTTLAQIVARELG
VN
FRMTSGPVLAKAGDLAAILTNLEKRDVLFIDEIHRLSPVVEEVLYPAMEDFELDLVIGEGPAARTVRIELQPFTLVGATT
RLGLLTTPLRDRFGIPVRLQFYTVPELEEIVARGSRLMGIPADPEGIREIARRARGTPRIAGRLLRRVVDFALVEGDGRI
TRKVADHALTRMEVDAVGLDGADRRYLQLIADNYAGGPVGIETLSAALSESRDALEEVIEPYLLQQGLIQRTPRGRMLA
Q
KAWSHLGLAPPRVPGQTAQTDLFDEGA
>OLS47824.1 hypothetical protein BV379_05665 [Rhodovulum sulfidophilum]AB14
MTPEEVTALFTRSEGDYLFARWGRPIAPVVFGVDDATLSIFKGACEAVVGLAGHEMAETDADLGSNLIVFFFEDWTELV
R
VPHLDRLVPDLVRRVARLQAAGANQYRFFRFDEQGAIKAAFVFIRMDPDLSQMPAESIALNQIVQTILLWSDTAFAERS
P
LARAPDGMLVLRPEIAAVIQAGYDPAMPTVARDSSHALRLAARVGHTLGQIQILGQEP
>OLS47825.1 tol-pal system-associated acyl-CoA thioesterase [Rhodovulum sulfidophilum]AB14
MTHRLTVRVYYEDTDLAGIVYYANYLKFIERARSEWVRGLGLDQVRLKAESGIVFAVRRVEADYLSPAKFDDLLTVETDL
QRFTPARLVLDQAVLRGETRLFGARVTLAALGAGGRPVRLPAEFRRSLETG
>OLS47826.1 protein TolQ [Rhodovulum sulfidophilum]AB14
METDTLAMAQEIDFSLLALFLRATITVKLVMLLLIVASFWSWSIIVQKIITYRKARAEAARFDEEFWSGEPLDELFDKIG
PEPQAAPERIFAAGMMEWRRSHRQDGKLIAGAAARIDRSMDVAIAKETDSLNAGLSFLATVGSTAPFVGLFGTVWGIK
HA
FEQIALQQNTNLAVVAPGIAEALVATALGLLAAIPAVVFYNKLSADSDRLGGGYESFADEFSTILSRQLDA
>OLS47827.1 protein TolR [Rhodovulum sulfidophilum]AB14

MGSSLTPRKSSGGRRVRGHRRHPAAMSEINVTPMVDVMLVLLIIFMVAAPLLTAGVPVELPKTAASALPAEQEAPLTV
TL
TADGKIALQETELADEALIPKLKAVAAEREDDKVYLRADGAIPYERVVQVMGALNAAGFRNIGLVTDTGGPSLDGRDE
>OLS47828.1 hypothetical protein BV379_05685 [Rhodovulum sulfidophilum]AB14
MNTGTYISGGAHLGLIAWVMLAGLFEAPPPKPMEVSDVTILSGDEFAALTAPAAAPKPSQEIVPPEAPSLPSSPRPAPAP
EARPQRPAPPVPQEAAEPDPAPEALEPPAEADVAETVTAPEAPEIPDAPEAPVTDTAPPRPSTRIAPEPSAPPPEDAVLA
EQPSQATRPDPAAESPQPEADEAAPEEAATQTVTEAEEQPETGLALAASPRPRTRPNRPAAPPPEEAAAAPEAPEEPAE
P
ASEEDDLADAVADAVADAMSDPAAAPEGTGGAGRAASGPPLTGGERDALRVAVSRCWNVGSLSTDALQTSVVVGV
DMEET
GKPDIGSIRMVSWSGGSEAAARQTYESARRAIVMCGSSGFPLPVEKYDQWRQIEMTFDPGKMRIR
>OLS47829.1 Tol-Pal system beta propeller repeat protein TolB [Rhodovulum sulfidophilum]AB14
MMLRLLTAILALALGHAALPAQAQNGPLRIVIDEGVIEPLPFAVPEFVAENAAAAEYARNITRVIAADLEGSGLFREIPR
EAFLSTVTSFASPVQYSDWKAINAQALITGAVSVDGSGRLSVKFRLYDVFSQAPLGDGLQFGGAAASWRRVAHKVADQ
VY
SRITGEGGYFDSRVVFVAERGPKNERAKQLAIMDYDGANMQPLTDANSIVIAPRFSPTGDRVLYTSYESGFPRIYLLDVA
TGQRRVLDDQPGTMTFAPRFAPDGRRVLFSLSQGGNTDLYELDLQSGRRTRLSDAPSIETAPSFSPDGSQIVFESDRSG
K
QQLYVMPASGGEPRRISFGQGRYGTPVWSPRGDYIAFTKQNKGRFHIGVMRTDGSEERLLTASFLDEGPTWAPNGRV
IMF
TRETAGTAGAPGLYTVDITGRNLRKVPYQGQGSDPSWSPLQP
>OLS47830.1 peptidoglycan-associated lipoprotein [Rhodovulum sulfidophilum]AB14
MNRIATSLLLVTALAVSACSNADRFGSANGGAAGAYGVNGSPSDPTSVAYFNQTIGDRVLFAVDQSSLSPDATVTLDA
QA
DWLNQNTAYSATIEGHADEQGTREYNLALGARRANAVKEYLVSRGVSPNRLQTVTYGKERPLAICSAESCYAKNRRAV
SV
LAAGAGA
>OLS47831.1 tol-pal system protein YbgF [Rhodovulum sulfidophilum]AB14
MMRRIAAFALTVALGLAAGGPVSAQSRDETLADIRQELTVLNVTVQGLKRELSTTGGAMGRAAGGSALQRLDAIEEQL
QR
LTSKTEELEHRIRQVVDDGTRRIGDLEFRLVELEGGDVSKLGQTSTLGGGAGPVMPAPAITPAPDSTGSTDGMELAVGE
R
ADFDAGKAALASGENEKAAEILARFGETYPGSPLSGEAQFLRGEALSAAGETSQAARAYLESFSGSPSGPRAPVALLKLG
LALKDLGQSREACVTLGEVRNRFSGSAEAQQAASAMGAAGCN
>OLS47832.1 cell division protein FtsH [Rhodovulum sulfidophilum]AB14
MGNARQIAFWVVLFLLILALFNLFSGGQGTTSSQTISYSEFLRNVEAGEVSSVKLDGERALIRETDGTTVTAIVPRGTDV
TEQLLAKNVDVTAEPQEQNGLLAYIGTLLPFIILIGIWIFLMNRMQGGGRGGAMGFGKSKAKLLTEKHGRVTFDDVAGI
D
EAKDELEEIVEFLRNPQKFSRLGGKIPKGALLVGPPGTGKTLLARAIAGEAGVPFFTISGSDFVEMFVGVGASRVRDMFE
QAKKNAPCIVFIDEIDAVGRSRGVGYGGGNDEREQTLNQLLVEMDGFEANEGIIIVAATNRPDVLDPALLRPGRFDRQV
Q
VPNPDIKGREKILGVHARKVPLGPDVDLRIIARGTPGFSGADLANLVNEAALMAARVGRRFVTMEDFENAKDKVMMG
AER
RSMVMTEDEKKLTAYHEGGHAIVGLNVPQHDPIHKATIIPRGRALGLVLSLPERDQLSVSYTKYKSKIAMAMGGRVAEE
L
IFGKENVTSGASSDIQQATRIARAMVTQFGFDEELGYVDYANEQDSYLGSYNSGPKHSTATQKIIDDKVKALVDEGYRT
A
TQILTDKREDLERLAQGLLEYETLTGSEIAKVIAGEALSRGDDDPPSAGGDTPSVTAIPKTKPRPKSGFEPEPMA

>OLS47833.1 formate--tetrahydrofolate ligase [Rhodovulum sulfidophilum]AB14
MAPKTDIEIARAADKRPIEEIGARLGIPAEALLPYGRDKAKVGAGFIAGLEERPEGRLILVTAINPTPAGEGKTTTTVGL
GDGLSRLGHNTAICIREASLGPCFGMKGGAAGGGYAQIVPMEDMNLHFTGDFHAITSAHNLLAAMIDNHIYWGNEL
DIDQ
RRVTWRRVMDMNDRALRDIVCSLGGASNGYPRETGFDITVASEVMAILCLATDLADLQRRLGDIIIGYTRDKAPVHAR
DL
KADGAMTALLREAMQPNLVQTLEHTPAFVHGGPFANIAHGCNSVIATRTALRLADYVVTEAGFGADLGAEKFMNIKC
RKA
GLAPSAVVVVATVKALKMNGGVARDDLKAEDVAAVKAGCANLGRHIENMRSFGVPAIVAINHFAGDTEAEIAAVQDY
VRG
QGTEAVLCRHWAEGGAGIESLAEKVVALAETDHHSFAPLYPDEMGLFHKIETIARRIYRADHVVADQKIRDQLTAWEE
AG
YGHLPVCMAKTQYSFTADPERIGAPEGFDVPIREVRLAAGAGFVVAICGRIMTMPGLPRHPAAETIGLNAEGQVEGLF
>OLS47834.1 chorismate mutase [Rhodovulum sulfidophilum]AB14
MIAPDQCRSMDELRIQIDALDARLVALLAERARYIDRAAQLKSGLGLPARIDDRVEEVVANVRTRASAEGLDPGLAEML
W
RDLIEWSIGREEMALGAGKEPR
>OLS47835.1 bifunctional 5,10-methylene-tetrahydrofolate dehydrogenase/5,10-methylene-tetrahydrofolate cyclohydrolase [Rhodovulum sulfidophilum]AB14
MNATLIDGKVFAASIREKVAAHVARLQAEHGLTPGLAVVLVGEDPASQVYVRNKGQQTKEVGMASFEHRLAAETSEA
DLL
ALIARLNADPAVHGILVQLPVPDHIDEAKVINAIAPEKDVDGFHISNVGLLATGQKSMVPCTPLGCLLMLRDTLGSLSGL
NAVVVGRSNIVGKPMAQLLLKESCTVTVAHSRTRDLAGLCRSADILVAAVGRPQMIPGDWVKPGATVIDVGINRIPAP
EK
GEGKTRLVGDVDFAGAAEVAGAITPVPGGVGPMTIACLLANTVTACCRANDLPEPEGLTV
>OLS47836.1 metallophosphoesterase [Rhodovulum sulfidophilum]AB14
MTGHDFTLAGARLTARASGALWWQDDSLLVVADLHLGKAGRMARQGGALLPPYDSDETLARLSAEIDILAPRCVICLG
DS
FDDDEASRALPDPAARRLSALMAGRRWIWIAGNHDPAPAVPGGSALAETRIGPLRFRHIAIPGEKNEISGHYHPKSRISL
GGRRLARPCFLIDAARAILPAFGAYTGGLATSDPTLDRLMGPEALAVLTGQKALAFPMRS
>OLS47837.1 DNA ligase-associated DEXH box helicase [Rhodovulum sulfidophilum]AB14
MGLPAPFADWFATRGWQMHPHQSAMLDRAETPALLLVAPTGGGKTLAGFLPTLVDLAENPRDGLHSLYISPLKALTA
DIR
RNLGRPIEEMALPIRVEDRTGDTPQSRRRRQRADPPHILLTTPESLALLLSYEDAPRIFAGLRRVIVDEIHALADSKRGD
QMTLALAWLERLCPGLRRVGLSATVEDPPALGRFLAPAGMPCEILTADPGPPPDIAMLETGTPPPWAGGGARHAIPA
VLG
QIAEHRTTLIFHNTRAQAELFFRDLWLANEANLPIAIHHGSLSREARLKVEAAMAEGRLRAVVCTGSLDLGIDWGDVDL
V
IQVGAPKNVKRLVQRIGRANHRYSAPSKALLVPANRFEVVECLAALEAVRARDLDGAPRGPGARDVLCKHILACAAAGP
F
EADALFDEIRSAGPYAGLTRAGFDACLDFAATGGYALRAYDRWQRLKQATDGRWALRDPRAARRIRMNLGTIVEEERL
KV
RLKPSRRGAPLGEIEEAFAATLTQGDSFLIGGQVVRFEGLREMVVEVSPSPGRMPKIAVFAGTKFATSTRLCGRILDLLT
APDWPGLPAHTADWLKLQRRLSRLPDPGRLLVETFAFEGRHHLCVYGFAGRNAQQTLGLLLTQRMEAAGLAPLGFVA
ADH
ATLIWGLDEVEAPSDLLDSNSLHSAFADWLAENAVMKRSFRTSAVIAGLIERNLPGQRKTGRQATFSSDILYDTLLRHDP
GHLLLDITREEAMRGLVDFGRIEEMLARVRGRIDHMRLDRVSPLAAPLLLEAGRVPVRGAAEQRLLDAAAARLMTEAG
LA

>OLS47838.1 D-tyrosyl-tRNA(Tyr) deacylase [Rhodovulum sulfidophilum]AB14
MRALVQRASEAAVRVDGTAIGEIGPGLLVLVCAMAGDTETEADKLARRIARMRIFRDEAGKMNRALLDTGGAALVVS
QFT
LAADTASGNRPGFSRAAAPDLGERLYLRFAEALAAEGIKVATGRFGADMKVALVNDGPVTIWIET
>OLS47839.1 carbohydrate kinase [Rhodovulum sulfidophilum]AB14
MILCAGEALIDMLPRTSTEGEPAFVPHPGGAVFNTAIALGRLGVPTGFLAGLSTDLFGTRLAEALAASGVETGLCPRPAR
PTTLAFVTLVEGQARYAFHDEATAGRMLTPADLPALPASVRALFCGGISLMAEPCGSAYETLFLREAGRRVTMLDPNIR
P
GFIADEPAYRARIARMIAAADIVKVSDDDLHWLEGPGEPCDLARGLLGRGPRLVLLTRGAAGAHALSHTAERFMPAPA
VQ
AVDTVGAGDSFDAGILAGLAKAGALERKALAEALPDSVLEAALSLAIRAAALTVSRAGATPPWMEELT
>OLS47840.1 primosomal protein N' (replication factor Y) - superfamily II helicase [Rhodovulum sulfidophilum]AB14
MTASDHRFPCDQCGADLRYSPDADQLVCDHCGHREPVEDDARRTETLVELDYQATLDAMLPESEIEEIRQTRCTNCGA
EF
EMDEATHAAECPFCATPVVTGTGLRRRIKPRGVLPFALSEAEAHKAMTDWLGRLWFAPNGLKDYARKGRRMEGIYVP
YWT
FDADTTTNYRGQRGTVYYETRRVTRNGKTETVRVQRTSWRSVSGRVARVFDDVLVLASKSLPKRYTYALEPWDLSLLQ
PY
QPQFLAGFRSEGYTIDLEDGLIEARKIMEQVIRRDAKFDIGGDRQRVDWLETRISTLTFKHVLLPVWLAAYRYRGKTYRF
VVNGRNGRVQGERPWSAWKIAFAVGLVLAVVGGLAAGIGYLSALQQ
>OLS47841.1 antifreeze protein [Rhodovulum sulfidophilum]AB14
MGIFDFLSGEFIDVIHWVEDRRDTMVWRFEREGHAIKYGAKLTVREGQAAVFVHEGQLADVFRPGLYMLETNNMPV
LTTL
QHWDHGFRSPFLSDVYFVDTTRFTDLKWGTKNPVMLRDPEFGPTRIRAFGSYAIRVTDPARFMVEIVGTDGEFTMDEI
GF
QIRNIIVQEFSRVIAGSGIPVLDMAANTADLGALIAAEVSKTLDAYGLTMPEFYIENISLPPEVEKALDRRTSMGIAGDL
DRYTRYSAAEAMASAATSPAGGGMAAGLGAGLGMAMGSGMAGATGPWGARPEPAAAPVPPPPPPAEHVWHIAE
NGQTSGP
FSMARLGRMAAEGSFRRDSYVWTQGQDGWKRAEDVIELARLFTVAPPPPPPGV
>OLS47842.1 hypothetical protein BV379_05760 [Rhodovulum sulfidophilum]AB14
MNFGSLLSPETFEFFARYLLAGYVVIFVRSAFVMGLRPKPAELLIEAIILSLIVQFIVYVLAAVYGSVVVASASGHDLPE
WIAAPPDILKLVAIVIVVPTGLGWTLGRFLKSDWRNGLLRRLSLPVIHPVQRAHDFAFGDNRAPGLVEVTFNDGTKIGG
W
FGEKSLAASDDGRSDLYLERAYIIDTSGTWREPSHSRAILINLKDVRSIEFLMEAKAEDGPEGENDR
>OLS47843.1 toxic anion resistance protein [Rhodovulum sulfidophilum]AB14
MTSDSSRHMAAQDIAEVEKVTATALPEPAQDLVALDRADPPVADAIRARMAELDMSDSNSIIGFGSAAQSELQTISQA
ML
ADVRNKDVGPAGDSLREIVTTIRGFSVSELDVRREASWWEKLMGRTAPFAKFVARFEMVQGQIDSITDRLLTHEHRLLK
D
IKSLDKLYEKTLDFYDELALYIAAGEQKLTQLDSRDIPAKEAEIAAAPEADQVIRAQELRDLRAARDDLERRVHDLRLTR
QVTMQSLPSIRLVQENDKSLVSKINSTLVNTVPLWETQLAQAVTIQRSKEAAEAVRGANDLTNELLTANAANLRQANK
TV
REEMERGAFDIEAVKKANADLVATIQESLTIADEGKARRAQAEEDLDKMEAELRDTLASAKSRAPGAGAATGV
>OLS47844.1 hypothetical protein BV379_05775 [Rhodovulum sulfidophilum]AB14
MAHRFGGRYSPGRPAPTEPAPPRHPFQGRRPARAAGRANAMFLAPLPLVPRAFFSDPAGLALSLTAFCLLMLAAWLT
REG
LRAEDAWAARKIARRPAVPRKILGSVLTGAGLFLAGIGGEGAAPVIFGALGLVLHSLAFGIDPLSDKGMEGIDSFQNSRV

ARAVEEAEKHLATMSEAIRHTGERSLVYRVERFQATVREMLRTVEEDPRDLTAARRYLVVYLMGARDATVKFADLYPRS
R
DASARQDYETLLADLETHFAARTRALMQDDRTDLDVEIEVLRDRLHREGIAPVPVTPKTGETA
>OLS47845.1 pseudouridine synthase [Rhodovulum sulfidophilum]AB14
MDKTGQEGERIAKVLARAGIASRREAERLIEAGRVTVNGRRIASPALNVGPADRIAVDGAPVAAPDRPRLWLYHKPAG
LV
TTARDEKGRETVFDKLPEDMPRVMPVGRLDLNSEGLLLLTNDGAIKRRLELPSTGWLRKYRVRVKGTPEDATLAPLRTG
L
VIAGETFRPMEVTLDRQQGANAWLTVGLREGRNREIRRTMEAVGLVVNRLIRISYGPFQLGDLKPGEVEELRGRVLRD
QL
GLAAPEGEATAKPKPTRRKLRNRPAEAPEPRPARKPPRGTGPGPASPPRGARRGKAPPG
>OLS47846.1 tRNA-specific adenosine deaminase [Rhodovulum sulfidophilum]AB14
MSPFRSHMDQALAEARAAADRDEVPVGAVVVAADGRVVAAAGNRTRELSDPTAHAEILALRAACSAAGSERLPGFDL
YVT
LEPCAMCAAAIGQARIARLYYGAADPKSGGVAHGAQVFRHPQSHHVPEIYDGIGASEAEALLRAFFAGRR
>OLS47847.1 metal-dependent hydrolase [Rhodovulum sulfidophilum]AB14
MKIVWLGHSGFRIEIEDQVLLIDPWLAGNPLFPEERRAEAIGDATHVLVTHGHFDHAADTIAIARELSIPTVGIFDLMSW
WESREGISVIGFNKGGTVRLGNVSVTMVNATHSSSIQGPDGPVYGGTESGYMIAGEGHVIYVSGDTDVMADMEIFND
LHA
PDIGLLCAGGHFTMDMKRAAYAARTFFNFKTVIPCHYRTFPLLEQSAEALKQDLPGVRVIEPEVLVPIEI
>OLS47848.1 hypothetical protein BV379_05795 [Rhodovulum sulfidophilum]AB14
MDWTRQIDAYCERTDPAFWSEPVNAATNAAFLLAAVIMWRRTGGLPLGRALAAVLGLIGIGSFLFHTLATEWASVAD
VGP
ILGFILLYIFVANRAYWGLGLWPSLGLTALFIPYAVVTGSLFARLDWLGSSAGYAPVALLILVYALALARRLPQVAAGLG
IGVGLLAISLAFRTLDQPLCAALPLGTHFLWHLLNAAMLGWMIEVYRRHEAGRRSSI
>OLS47849.1 hypothetical protein BV379_05800 [Rhodovulum sulfidophilum]AB14
MDVVERIRGCRIPLAIIAIEADVPEAEIAAALEGSQALRFLQESRIARYLEGQDQRALACPPQPGHKARHRP
>OLS47850.1 formyltetrahydrofolate deformylase [Rhodovulum sulfidophilum]AB14
MASTDFILTLTCDDQPGIVATVANAVTALGGNIQDSAQFRDPESNLFFMRVAFHTAAPAEAEAVRARLAPAINGYGM
DAE
VVDAARRPKIILMVSKFDHALLHILYQIRVGWMEAEVAAVVSNHEDSRATAEAAGIPYYCWPVTKETKAEQEKQVLAL
VE
ETGAELVVLARYMQVLSDKLSRALEGRVINIHHSFLPSFKGAKPYHQAHERGVKLIGATAHFVTADLDEGPIIEQETERV
NHALSAEDYVAVGRDIESRVLARAVRLHLQRRVLLNGHKTVVFMPG
>OLS47851.1 asparaginyl/glutamyl-tRNA amidotransferase subunit C [Rhodovulum sulfidophilum]AB14
MSIDIDTARRVAHLARIAVQEDDLPALAQELSGILTFMEELNEVDVTGIEPMTGVTPMRLKRRADVVTDGEMQDRILS
NA
PDAREGFFAVPKVVE
>OLS47852.1 aspartyl/glutamyl-tRNA amidotransferase subunit A [Rhodovulum sulfidophilum]AB14
MSDLTRLTIADAREALRKGETTSVALTEAFLAEIEGADALNAFVHKTPEIALEQAKAADARIAAGDAPALCGMPLGIKDL
FCTRGVASQAASKILDGFRPEYESTVSQNLWDAGAVMLGKLNMDEFAMGSSNETSTYGNAVNPWKVDDRQLTPGG
SSGGS
ASAVAADLCMAATGTDTGGSIRQPAAFTGIVGIKPTYGRCSRWGIVAFASSLDQAGPMTRTVRDAAIMLGAMSGHDP
KDS
TSVDIPVPDFEAMLTGDIRGKTIGIPKEYRMEGMPAEIEKLWSDGAEMLRDAGAEIVEISLPHTKYALPAYYVIAPAEAS
SNLARYDGVRYGRRARLGQGDGIVEMYEKTRAEGFGKEVQRRVMIGTYVLSAGFYDAYYNRARKVRALIKRDFDEVFA
GG

VDAILTPATPSAAFGLGEMADADPVAMYLNDVFTVTVNLAGLPGISVPAGLDAQGLPLGLQLIGQPWGEGELLNTAHV
LE
RAAGFVSKPAKWW
>OLS47853.1 hypothetical protein BV379_05820 [Rhodovulum sulfidophilum]AB14
MRAVTSAIAVLALAACNTQLPDSAAGEGFDSYADYSTHREGVLQGAVSAEPITAAPSQDVVTEVTGAPISAVESSDGTE
P
PSAQGESHAGISDEQSFAAVTARETIASDRERLEQQREQYTVIPPEPLPPRPGGDGAGIIEYALSTTNPLGQQVYKRGRA
VAPETLARRCAKYGSDDHAQEAFLKNGGPERDRFGIDPDGDGFACYWDPSPFRLAER
>OLS47854.1 N-acetylmuramoyl-L-alanine amidase [Rhodovulum sulfidophilum]AB14
MVVLHFTAMTDAASALDRLCDPAAGVSAHYLIARDGRVFQLVDEAGRAWHAGAGCWGGVEDVNSRSIGIELDNPG
DRPFS
APLMAALEALLPGILARWSIRPERVIGHSDMAPDRKWDPGPRFDWRRLARQGLAVWPEVRVSAGPAPDETAFRKAA
TAFG
YGAGWDTGHVLDALRQRFRPWATGPLAAADMAVIADLARRFPVDPGSAGA
>OLS47855.1 hypothetical protein BV379_05835 [Rhodovulum sulfidophilum]AB14
MSFVRTMATLAVGFAAARGLETFQKMGGGAGIADSVKSAATRSGIGSQIGPLLERMQVPGGAAALKTNAKWLGAKG
RETG
DHALVGLGGLMAALGGAAWAGSVNARDILNAMGEVAPPDVSMEKNARLLIRSMVMAAKSDGEIDAAERAAIFDVL
GDATE
EERAFVEAEMARPIDIEALAAETGTHQRTAVYAAGVVTTRLDSPAEIAHLDRLADALHLGRAARQRIHKAMRLPQVLA
>OLS47856.1 50S ribosomal protein L33 [Rhodovulum sulfidophilum]AB14
MAKPTTIKIRLNSTAGTGHFYVTKKNARTMTEKMVVKKYDPVVRQHVEYKEGKIK
>OLS47857.1 hypothetical protein BV379_05845 [Rhodovulum sulfidophilum]AB14
MERSMGISHLGAAAAAGLLVSSLGAAAQEAGPHLSIELNAVAPAETGCLISFLARNGYETGIDRAVFETVLIDGQGQVE
R
LTLFDFGALPAGRPRMRQFSVPGLDCGDLGQILINGTTACEAEALDEEACAEGLGLSSRTDVELIG
>OLS47858.1 flagellar motor protein MotA [Rhodovulum sulfidophilum]AB14
MIGAVIELLRRVADLGGPVVLILIGISVLTVAVVLYKLWQFAASGVGRHRALSEAVAHWDRGERARARAALERSRSYLRP
VLDLAMSGQTDSERLEAEAEARFARLERGFRLLDSVAQLSPLLGLFGTVLGMIAAFQALQAAGTQVDPSILAGGIWVAL
L
TTAVGLAVAMPTALILSWFESRMEAERVLAETAIHTVLAPGNALGEDLPVVSGGRDHG
>OLS47859.1 biopolymer transporter ExbD [Rhodovulum sulfidophilum]AB14
MARPRRRRLSLTPLVDVIFLLLLFFMLSSTFTRFAEVDLVAAGQGGAPAAPDARPLFLQLEPDRLRLNGRELALDGLAEG
LDPVEPGQVVLVALRGAVTAQRLTDLLVALRAIPGLGVTVLGAT
>OLS47860.1 biopolymer transporter ExbD [Rhodovulum sulfidophilum]AB14
MTARKAAIRAEKVRRAGREPTIALINIVFLLLIFFLVAGTLAAPLDATLKLVDTEGLEGAAPAEALVLHADGRMTLQEQE
TTPEAFVAGLEGTEEEAATVARVVPDRDVPAADLVALGQALRAAGAERVVIVTERGLK
>OLS47861.1 hypothetical protein BV379_05865 [Rhodovulum sulfidophilum]AB14
MTIPSSRLGKVLALGLALGAHSALALVLVPQTETRMEGRSGAAEAAIGSSFAEMAAGRISAESVEDVVEPEAPVEPVRP
D
MPEPVETVVPDPAEALAPVPEPEVAEQPEPETVTAEPEPQPEPKPEPKPVSRGNSDRNARVGQAAGSERAQAASSGR
GGA
SAESGNAESSNYPGLVMRALSRVPKPRIRARGATVVAFRVATDGRLASASVARSSGSTALDRAALRIVERAAPFPAPPR
G
AQRSFSIRIEGR
>OLS47862.1 hypothetical protein BV379_05870 [Rhodovulum sulfidophilum]AB14
MTETGDEAAFRVAAIPLSPEQTVDALLATVEATLRAEGVRLAGFRQGRGADGGMAVEDLTGGGVFSISQQLGAGSQG
CRL

DPQGLAEAACAALAALEAGPDLLILPRFGKAEVEGHGFRAVIERACERQVPVLVAVKPDCVAAWEAFTGGAADRLDPD
RG
ALLEWCRAGLRARA
>OLS47863.1 molybdenum-dependent transcriptional regulator [Rhodovulum sulfidophilum]AB14
MPVGLRSALTLERPGARMGADRVALLAAVGRTGSISAAAREVGLSYKAAWDGVQAMNNIFASPLVSAAPGGRAGGG
ATLT
PAGEKVIAAFTAIQEGLERVVSSLDSQIDLDPGDILWSLMMKTSARNTYRATVTAVTESPVSAEVRMDIGGGQTLTSVIT
GQSAAGMGLAPGIEVFALVKSSFVILARGEELGALSVRNRLSGTVTGRTDGPVNSEIVLDLGGDKTLAATITRESAEALA
LAPGDSATALIKSSHVILALP
>OLS47864.1 tRNA (N6-threonylcarbamoyladenosine(37)-N6)-methyltransferase TrmO [Rhodovulum sulfidophilum]AB14
MEKVMAAIRPGEVCTAVPERNDAALWFVGRLRTPWQSPRDCPRQGDAVAGPVCRIEIDPRWTEALAGIERKERLLLLY
WM
DGARRDLVRQSPRNDGATTGTFSLRSPNRPNPVASSVVTLLGREGDVLLVRGLDCVDGTPLIDLKPDYGGRPCPEH
>OLS47865.1 transporter [Rhodovulum sulfidophilum]AB14
MKLSARNILAGTVSAVETGTVTTHVKIDIGGATVTAAITNEAATDLDLKVGDKASAIIKSSDVLVGKD
>OLS47866.1 hydrolase [Rhodovulum sulfidophilum]AB14
MTHPSPDLLVDGATILTSDPARPFLKTGWVEVTAGRITAIRDTRPERIAPATRIVPGEGRVLTPGFVNVHTHAILSMVRG
VAEDMGFAPAYTMGVPHGHDVRPDEAHALAQLGALEALSFGSTLINDSFTHQEIALPAMAGTGLRAFGCGRIHDVDF
TRV
HLGEWRHDDAIGDWTLGLASDLIAAFHDPEGLRTGVVLAPHAPDTCSRALLGRVRELRDAHGLKINTHVSQSRVEVDFI
R
ERDGMTPPQLLDEVGLLDESLIGAHCIHLTEDDIARLGRGRATLAHVAKGNQTHGCLAPTAKLRQAGMNLALATDNM
HAD
MVEVMRWALATGRLQEGGVSETWQPAAMFEAATMGGARALGLEAEIGSIETAKRADLVLFDFRRPHLRPLTDVLGTL
VHT
GQGRDVETVIVGGEVVIEGGEPLRVDRMAVLEAAEAAAAALWDRARAEAARPR
>OLS47867.1 C4-dicarboxylate ABC transporter permease [Rhodovulum sulfidophilum]AB14
MSALAVSVFAVLALIGAPLAFAIGLGAVAALYMGGFDLVVLPQRMMFAVNSFPLMAIPLFMLAGELMVKAGIVDRLVS
FA
NSLVGRVPGGVAHVTIVAGMVFASVSGAAVASASALGSTLVPALRKTYPDGYSSAVIASAANLGPVIPPSNAMIVYALM
A
GSSVSVGGMFMAGIVPGIILALGFMALATLIAKRRGYALSGTPFSVRNALIETRRAGLILMMPVIVVGGIVGGIFTATEG
AAIAVVYALVVGLFVTRRLRVADLPGCLFRAAVMAAMVGALIAFASVVTFVFTLDLVPMRLSSWIRDLTSDPMTFILLV
M
AMLLVVGMLIESNAAYIMLVPLFAPMAISYGIDPLFFGFLFMYNLVVGMMTPPVGVLLFVMSGITRVPMAQLTREVAP
FV
ALQFGVLALCVAFPPLVLWLPSLFGF
>OLS47868.1 hypothetical protein BV379_05900 [Rhodovulum sulfidophilum]AB14
MSDPGHPSPGPAAAGPLALLRRAVDLTDALSRWIIVGAMAAMTLLISAQVFHRYVLSSSIDAADELSRLFFVWSIFLAIP
HGIRRGVHVGIDIVLRLMPGRAQDLLQRASALAGLLLMAVVFVTALGAAGDKWGELMPTLPVTAALYYIPVCICALHSA
L
HLLLFALGGPEIWGDDAWKEAAS
>OLS47869.1 C4-dicarboxylate ABC transporter substrate-binding protein [Rhodovulum sulfidophilum]AB14
MKPLLPRAFGLCLGLSLLAGAASAQTVLRVAGNFPEEHSATGAMNVFKEEVERLSGGDLVVQNFPAMQLGGAQENV
DQVR
SGAIFAVFTSIAYFTRSVPELEAVSLPFLFDSREKAFEVMDGPVGKLMDKALLEQGFVNLGYGELGFRHVTNSVRPLTRL

EDFHGLKIRLQPNEVHLETFRALGANPQSLDISELYSALQQKVLDGEENPYNIIYTRRFFEVQKYLSDTGHFFDFINVVA
NKDAFDALSPENQEAVRSAMRSAIEWQRAEAARVDLEFREKLVAEGMQFDEIPAAERARLREATASIAENLKERVDPE
VI
DLVLEQAAQ
>OLS47870.1 hydrogenase expression protein HupH [Rhodovulum sulfidophilum]AB14
MRLLLVNPNMTGAMTDRMAGIARDVASPGTEIVPLTAGRGFPYIASRAEAQIGGALACEMIADHAEGADAAIIAAFG
DPG
LAGARELFDLPVVGMAEAALVSAAMLGERISIVTFSPVMRRWYLDSVRDAGLSARFAGVRTPDGHAPDLGNVQHSLR
EDL
IRLCNRAVREDGADVVVLGGAPLAGLAPEIRGEVEAPVVDPISAATLQAQALAVLAPQAGFAGRASKPLPKTATGLPPA
L
TRQFACG
>OLS47871.1 GntR family transcriptional regulator [Rhodovulum sulfidophilum]AB14
MSDTSLDIQASAERVADCLRDRIVKGECPPGSRLVERRLSAELNVSRTPIREALKLLRADGLVEISLHRGAQVVAYTAEE
AENLFDLIAAIESLAAERLAGRIGAEVLARLETLHERMLKQYAAREASAYFDTNTEIHDSIVREAGNPVLIDSHRRIAVR
ARRGRFMAIFDAERWRQSVEEHEGVMAAFRARDTAAAARIWRQHLRHSGESVAETLRRQRG
>OLS47872.1 IS5 family transposase [Rhodovulum sulfidophilum]AB14
MCDSQGRPIDLFVTAGQVSDYIGARALRSGLPSVKCMLGDRGYDAGWFRESLQDKVIRACIPGRKQRKMTVKYGKRR
YKR
CNRIEIMFGRLKDCRRVWTRYYRCPKVFLSATALAVVVIY
>OLS47873.1 hypothetical protein BV379_05945 [Rhodovulum sulfidophilum]AB14
MPIGPRLTMMIAAIMVVSMSSMGGFLYNRAYESSYNNAVTDLEGMARDQATRVSEWFADQRHALAAQAANPFIAR
GLSEF
RTALEATPGGFTPIIAAYGSDNPYPSGEGQKLSDAGDGSDYSRIHARMHPGFERMLESFGFYDVFLIAPDGQVIYSVVKE
ADFGEDVATGRYSGTALGKVFQRANKAPAGTSVVSDIEEYAPSANAPAAFMAAPVFAEDGTRLGVIAIQVPIGKLSAM
VT
MASALGRSGDMYIVGGDGRARTYSRYENRFDVLSPLPERPFITALETGQSGLFSPVEGITGTDSIVFTTPVGVDFADWTL
VVELDRSEFLAALIAFRNSVIVFLLIGLAAGLGVSVLAARTITTPLKGFIASMNEVSAGNYAKKIDVADRLDEIGAMGRD
LSAFRDRLAEADRLSQEQEERRAEQKKVVDRLGSAIRALADGDLTETVQEKFPDEYEALRHDFNSSMATMGDLIGAVQ
VN
ARAVQKLSEEISDSSENLARRTESQAATLEETAAAMDELTTSVSSTAEGATEVSRYVGEAQNRAQQSGQVVDEATGAM
SE
IQRSSEGITQIIGVIDDIAFQTNLLALNAGVEAARAGEAGRGFAVVASEVRALAQRSSEAAKEIKTLIDTSSTQVETGVS
LVNRTGAALQDIASRVSRISEHIEGIATGAQEQSVGLGEINVGVTQLDKVTQQNAAMVEEATAACVTLKQEADKLLTLV
A
RFRLGDDVTATTAAKPRRPSAPDALERALNERPAPQTSMSAGWTEF
>OLS47874.1 anaerobic ribonucleoside-triphosphate reductase activating protein [Rhodovulum sulfidophilum]AB14
MAELAISGLVRHSSVDWPGELVATVFCQGCPWACRYCHNTELLAAGPGRIGWDTVLAFLDRRRGLLDGVVISGGEPLL
QK
GLPEALAELRAMGFRTGLHTGGAYPARFARVLPLLDWIGFDVKAPFDAYDRITGAPGSGTKARESLGLLRDSGVDADLR
C
TVHPALLSPADLDRMDDDLSALGLPPARRQPFRAEGCTDPALCRTP
>OLS47875.1 hypothetical protein BV379_05955 [Rhodovulum sulfidophilum]AB14
MTMIDKDMTTRDAEIVLTDEERTRCEVWTRVMGYHRPMASFNTGKKGEAAERVYFDEAKAQLG
>OLS47876.1 anaerobic ribonucleoside triphosphate reductase [Rhodovulum sulfidophilum]AB14
MTAPSPRSVANRDGQIVPFDAGRIARAIARAGEATGEFGAEEAAHLTAQVVRVLSFRHADGTPDIEEIQDAVEQALIGA
N

RIATVRAYAVYRAQHARLREDRQVLVDVESSINEYLDRSDWRVNANANQGYSLGGMILNTSGKVTANYWLSHIYPPEVGQ
AHRDGDIHIHDLDMFAGYCAGWSLRTLLREGLNGVPGSIESGPPMHLTSALGQIVNFLGTLQNEWAGAQAFSSFDTYLAP
YVRIDGLDYAAVRQAMQEFIYNLNVPSRWGTQTPFTNLTFDWTCPDDLKDQVPVIGGREADFAYGALQSEMDMINRAYME
VMTAGDAKGRVFTFPIPTYNITPDFPWESENAGYLFEMTAKYGLPYFQNFVNSELEPHMVRSMCCRLQLDLTELLKRGNG
LFGSAEQTGSLGVVTINCARLGHLHKGDEAGLLAALDRLAAIARTSLELKRKVIERHIEAGLFPYTKRYLGTLRNHFSTL
GVNGVNEMIRNFTDDREDISTEAGQGLAIRLMDHLRARMVEYQEETGHMYNLEATPAEGTTYRFAREDRKRFPGILQAGT
DDQPYYTNSTQLPVGYTSDPFEALEHQEPLQARYTGGTVLHLYMGERISSAEACKKLVRRSLSSFRLPYITVTPTFSICP
KHGYLAGEHEFCPKCDAELIAAKRAKAS
>OLS47877.1 carbamoyl phosphate synthase large subunit [Rhodovulum sulfidophilum]AB14
MPKRTDIKSIMIIGAGPIVIGQACEFDYSGAQACKALREEGYRVILVNSNPATIMTDPGLADATYIEPITPDVVARIIEK
ERPDALLPTMGGQTGLNTSLKLADMGVLHKYGVELIGANRAAIEMAEDRKLFREAMDRIGLENPKATIANNMEECMAALE
HVGLPAIIRPAFTLGGTGGGVAYNRQEFEYYCKSGLDASPVSQILIDESLLGWKEFEMEVVRDKADNAIIVCAIENVDPM
GVHTGDSITVAPALTLTDKEYQIMRNGSIAVLREIGVETGGSNVQWAINPEDGRMVVIEMNPRVSRSSALASKATGFPIA
KIAAKLAVGYTLDELDNDITKVTPASFEPTIDYVVTKIPRFAFEKFPGSKPELTTAMKSVGEAMAIGRTIHESLQKALAS
LETGLTGFDEIDIPGAPDPAQIVAALTRQTPDRLRVVAQAMRHGLSDDEIHAATSYDPWFLARIREIVETEAEIRKAGLP
VTEDGLRHLKMMGFTDARLAMLTGRDEGQVRRARDNLGVHAVFKRIDTCAAEFEAQTPYMYSTYESPAMNETECEARPSA
RKKVVILGGGPNRIGQGIEFDYCCCHACFALTEAGYETIMINCNPETVSTDYDTSDRLYFEPLTFEHVMEILRVEKMAGT
LHGVIVQFGGQTPLKLANDLEAAGIPILGTTPDAIDLAEDRERFQALVNKLDLKQPRNAIAASAEAAMKAAQEIGYPLVI
RPSYVLGGRAMEIVRDTPQLERYINEAVVVSGKSPVLLDSYLSGATEVDVDALCDGEKVHVAGIMEHIEEAGVHSGDSAC
CLPPHSLPEEIVAELIRQAEALALGLGVVGLMNVQFAVKDGEIYLIEVNPRASRTVPFVAKATDSAIASIAARLMAGEKL
SAFPMRAPYPADTSPDTPLPLADQMSLADPIMPWFSVKEAVMPFARFPGVDTLLGPEMRSTGEVMGWDRSFPRAFLKAQL
GAGTDLPREGQVFLSIKEADKTPALVETAKILVDLGFGILATRGTARFLAESGIETTLVNKAYEGGRTIVDVMKDGLVQL
VMNTTEGNRAVEDSRSIRNVALMDRIPYFTTAAGCHAAALAMKSREEGEIGVRSLQG
>OLS47878.1 magnesium-protoporphyrin IX monomethyl ester cyclase [Rhodovulum sulfidophilum]AB14
MRIVFIHPNYKSGGAEIAGNWPPAWVAYLTGSLRVAGYDDITFIDAMTNDISEDEVRKQLKALQPDVIATTSITPSIYAA
ERLMEIAKEEVPNAVRVLGGIHATFMFKQVLSEAPWIDVIVRGEGEEIVVNLMNAIRDGKWPESRRDIKGLAFLDGDEIV
ATAAAPTIKDVDSIKPDWTILEWEKYIYIPLNCRVAIPNMARGCPFTCSFCSQWKFWRDYRIRDPKKVVDEIEDLVENHK
VGFFILADEEPTINRKKFIAFCQELIDRGLPDKVKWGINTRVTDILRDEELLPFYRQAGLVHISLGTEAAAQLKLDQFNK
ETRVEDNKRAIQLLREADILTEAQFIVGMDNETEETLEETFQLCWDWQPDLANWAMYTPWPFTPLFQELHNKVEVFDYSR
YNFVNPIMKPEAMDRAQLLDRVMHNYRRFYMRKALFHYPWRGNGFRRRYLLGCLKAFLKAGVGRSFYDLGKNNYWGPQSK
DKVHFDFDMSRKPAAAQIEDWESNADRAAQAAARRKSKDTSFKMPADDPSQVKACGGGDQQMEEV
>OLS47879.1 bacteriochlorophyll 4-vinyl reductase [Rhodovulum sulfidophilum]AB14
MKDDLSGRAGRIGPNAVLQLLPVLEAAGGAALRDEMLDAAGLSTPPSDTGLMDEGPAAAMHRALRRRLPGRAPDLLREAG

ARTGDYILAHRIPAMAQRVLKLLPPALAAPILAKAIAKHSWTFAGSGAFRVVSTRPLVFELTDNPLIRGETAPHPICVWH
EAVFDRLFQTLVDRRLRTREQSCSASGGAVCRFEIARS
>OLS47880.1 aspartate--tRNA ligase [Rhodovulum sulfidophilum]AB14
MHAYRSHTCADLNKSHVGETVRLSGWVHRVRDHGGILFIDLRDHYGITQVLADPDSPAFADVEKVRSEWCIRIDGVVK
AR
SDDLVNPKLPTGEIEVFVRELEVLGQSEELPLMVFGDQEYPEETRLRYRYLDLRREAMQANMTLRSDVVASIRRRMWD
KG
FREYQTPIITASSPEGARDFLVPSRLHPGKFYALPQAPQIFKQLIMVSGFDRYFQIAPCFRDEDPRADRSPTDFYQLDME
MSFVQQQDVFDTVQPVIAGIFEEFGGGRKVDADWPQIAYADAMKWYGTDKPDLRNPIRMQDVSEHFRGSGFAIFAK
LLEQ
DGTEIRAIPAPKGGSRKFCDRMNSFAQREGLPGMGYIFWRDKDGETEAAGPLAKNIGPERTEAIRQQLGLGVGDAAFF
LG
GKASSFQTVAGKARTVIGEELGLTETDRFAFCWIVDFPMYEKDAETGAVDFEHNPFSMPQGGMEALDGDPLTVKGFQ
YDL
ACNGYELVSGAIRNHRLDIMFKAFSLAGYSEDEVRRRFPSLVNAFRFGPPPHGGCAAGIDRIVMLLADQHNIREVIMFP
M
NQRAEDLMMGAPSEPGNDQLRELSLRVILQE
>OLS47881.1 hypothetical protein BV379_05995 [Rhodovulum sulfidophilum]AB14
MTVKLFATAAVLAGLSAMTAQAGQIERACLSSDRPGVTRGLCGCIQHAADITLTRGDQSRAAGFFRDPHQAQVVRQS
DRR
RDAEFWRRYKLFGETAAAYCS
>OLS47882.1 acylphosphatase [Rhodovulum sulfidophilum]AB14
MTDRIAVRVRIAGRVQGVGFRAWTEAQAAALGLSGWVRNEADGAVLALLAGPEDRVARMLDTLQEGPAPARVETV
ETTPG
DWPAEAGFRISR
>OLS47883.1 hypothetical protein BV379_06010 [Rhodovulum sulfidophilum]AB14
MDLTTVHAGLTRQIVSEDLPPSEENLALQTVCEEGARAVRATHPEIAENRRILSRQAWKEMTPEARATVEAALPVLTAIS
EDDLADQFRKDIPELVNDAIGPVPDWAPKLPGADPATRIFGRVSRMSIILRSSVETLDAVAARLGMTRGEVIGIFVGLVS
LGISLIS
>OLS47884.1 NADH:ubiquinone oxidoreductase subunit NDUFA12 [Rhodovulum sulfidophilum]AB14
MGVVDSFLSYVTWWNGQTVNMRLWTARNGQKVGEDAQGNVFYRSKDGKRRWVIYNGEIEASRISPDWHGWLHH
TYDAPPT
EAPLPHRAWEKPHQENLTGTPLAYAPPGSIRRPEPAPRRDYEAWQPE
>OLS47885.1 outer membrane lipid asymmetry maintenance protein MlaD [Rhodovulum
sulfidophilum]AB14
MAENSPTEILTGAAVLALAAGFFVYASNATGMIGGAGQQHYELSASFRSAEGVNLGTDVRLAGVKVGTVTGLELNPQT
FR
ADTRFTVRNGIEIPDDSTASVSSEGLLGGTFLELVPGGSPFNLEPGAEVIDTQGAVGLISLLMKFVGGGGDGGGSDTGTG
GGDDTK
>OLS47886.1 leucyl/phenylalanyl-tRNA--protein transferase [Rhodovulum sulfidophilum]AB14
MDAERLTPLTLLRAYAMGIFPMAESRHDPRIFWVNPRKRGIFPLEGFHISKSLARTIRRGRFRIHTDSAFEHVVECCADR
PETWINAEIFRLYRQLFDLGHAHSIEVWQDDILAGGVYGVTLGSAFFGESMFSRRTDASKVALAYLVDRLRIGGFTLFDT
QFLTDHLASLGAIEIEREDYLSRLERAVNRDANFDRAWPIPEPQLLLQRSTQTS
>OLS47887.1 acetyl-CoA carboxylase biotin carboxylase subunit [Rhodovulum sulfidophilum]AB14
MFDKILIANRGEIALRVIRACREMGIHSVAVHSTADADAMHVRMADESVCIGPPSGTESYLSIPSIISACEITGAQAIHP
GYGFLSENARFVQIVEDHGLTFIGPSAEHIRMMGDKITAKETMRALGVPCVPGSDGGVPDLETAKTVAAGIGYPVIIKAT
AGGGGRGMKLAKTPEDLEQAFQTARSEAKSAFGNDEVYIEKYLGRPRHIEIQVFGDGKGNAVHLGERDCSLQRRHQK
VLE

EAPGPVIDAETRARIGKVCADAVARIGYAGAGTIEFLYEDGEFYFIEMNTRLQVEHPVTEAIFGVDLVREQIRVAEGLPL
SFAQDELQLNGHALEVRINAERLPNFAPCPGKISAYHAPGGLGVRMDSALYDGYSIPPYYDSLIGKLIVHGRDRTEALAR
LDRALGELIVDGIETTAPLFRALLREPDIQRGNYNIHWLERWLESDPEL
>OLS47888.1 acetyl-CoA carboxylase biotin carboxyl carrier protein subunit [Rhodovulum sulfidophilum]AB14
MTKTSHEADVAFIQALAELLREHDLTELQVKREYGEDDSLDVRVSRSQPASVVAQAPTTMQIPAYAPAPAAGGGAPAP
GT
NAEAGVEDPAQHPGAVTSPMVGTAYLQPEPGSDAFVKVGDSVTEGQTLLIVEAMKTMNHIPAPRAGTVKRILVEDAS
PVE
YGTPLMILE
>OLS47889.1 dihydropyrimidine dehydrogenase [Rhodovulum sulfidophilum]AB14
MANSPLSPGVVPGRLKPEDYIRNFADKAPPLSAHEAAVAAERCYFCHDAPCVTACPTAIDIPLFIREIATGAPAAAARTI
FEQNILGGMCARVCPTEQLCEEACVRMTSEARPVEIGRLQRFATDTLMNREGHPFLRATPTGRRIAVVGAGPAGLACA
HR
LAMKGHEVQLFDARPKAGGLNEYGIAAYKTVDDYAQAEVAWLLRIGGIAVTNGKALGRDMTLDTLRAGHDGVFLAIG
LGG
VNALRAEGEEKAQVVDAVDFIARLRQSDDLSAIPVGRHVVVIGGGMTAVDAAVQSKLLGAESVTMVYRRGRAAMGA
SEEE
QHLASSKGVRIVTGAAPLRLLGDGAVEAVEFAYTRETGTGLDLTDETFRLRADQVFKAIGQKLEGAPEGVALAGRKIAVT
GPGRTSLAGVWAGGDCASGGDDLTVTAVAEGRDAAEDIHATLMAEG
>OLS47890.1 hypothetical protein BV379_06055 [Rhodovulum sulfidophilum]AB14
MADPSAREKAALERIAILDDPEKLRKFIDNARRLGAVAVEQAAFRRLCEILPSARRGTVEHDVWCSIHALEEMLKTERGT
TVRLSRTRQKISKDGEIKTVMDLVLRATPSEGFQMLIDRGHPELLFEAVVLRHPDAFGDDVRQAAKERLGNAGGAAAA
LA
EG
>OLS47891.1 dihydropyrimidine dehydrogenase subunit B [Rhodovulum sulfidophilum]AB14
MADLSCDFVGIKSPNPFWLASAPPTDKEYNVRRAFQAGWGGVVWKTLGEDGAPVVNVNGPRYGAIYGADRRLLGLN
NIEL
ITDRPLEVNLREITTVKRDYPDRALVVSLMVPCEEEAWKRILARVEETGADGVELNFGCPHGMSERGMGAAVGQVPD
YVE
MVTRWCKQNTRMPVIVKLTPNITDIRLASRAAHKGGADAVSLINTINSITSVDLDLMAPEPTIDGKGTHGGYCGPAVKP
I
ALNMVAEIARDPATRGLPISGIGGVTTWKDAAEFMALGAGNVQVCAAAMTYGFRIVQEMISGLSDWMDRKGHARP
EDFVG
KAVPNVTDWQYLNLNYIAKARIDPETCIKCGRCYAACEDTSHQAIEMTADRSFLVRDEDCVACNLCVDVCPVEGCIDM
RR
LSPGEVDPRTGKEVPANYGNWTTHPNNPGRKSVAEQGLE
>OLS47892.1 TetR family transcriptional regulator [Rhodovulum sulfidophilum]AB14
MKPSHPRTRIQRRNRETILEAALDVFSQQGFRGATLDQIARKAGLSKPNLLYYFPSKEAMHETLLAGLLDTWLAPLRALD
PEGEPLDEILGYVHRKLLMSRDFPRESRLFANEILQGAERIRPTLEGELKSLVDETAAVIAGWARTGRIAPVDPYHLIFS
IWALTQHYADFDVQVRAVLGKDKEDPFEGADRHLAMLFRRLLAP
>OLS47893.1 aspartate aminotransferase family protein [Rhodovulum sulfidophilum]AB14
MALDRNSSPNDLSAFWMPFTANRQFKAAPRMLVGAEGMYYRTADGRQVLDGTAGLWCCNAGHCRPKITEAVQKQ
VAELDY
APAFQMGHPKAFELASALRDMAPEGMEHVFFTNSGSESVETALKIAIAYHRARGEGARTRLIGRERGYHGVNFGGISV
GG
IGPNRKMFGSLLTGVDHLPHTHLPDRNAFTRGQPEHGANLADDLERLVALHGADTIAAVIVEPMSGSTGVLLPPKGYLE
R

LREITKKHGILLIFDEVITGFGRLGSSFAAEHFGVLPDLMTTAKGLTNGVIPMGAVLTTAGVHDAFMQGPEHMIEFFHGY
TYSGNPIASAAGLATLETYREEGLFENAAALAPYWEEALHSLKGLPHVIDIRNMGLIGAVELEPIAGEPTKRAFTAFLNA
FEKDLMIRTTGDIIAMSPPLMINKAQIDELFGKLTDVLKALD
>OLS47894.1 Zn-dependent hydrolase [Rhodovulum sulfidophilum]AB14
MTSPGENLRINGDRLWDSLMEMAKIGPGVAGGNNRQTLTDADAEGRALFSRWCDEAGLSMGVDQMGTMFATRP
GEDPDAL
PVYVGSHLDTQPTGGKYDGVLGVLGALEAVRTMNDLGIRTRHPIVVTNWANEEGARFAPAMLASGVFAGVHSLDYAY
SRQ
DLEGLSYGDELARIGWKGEEEVGARKMHAYYELHIEQGPILEAEGKEIGVVTHCQGLWWLEFTLTGREAHTGSTPMA
MRV
NAGLAAARIFEMVQEVAMAAQPNAVGGVGQMQFSPNSRNVLPGTVVFTVDIRTPDQAKLDRMRAEIETRAAAICAD
LGVG
CSVEPVGHFDPVTFTPDLVGAVRNAAGKLGYSHMDIVSGAGHDACWAAKVAPTTMIMCPCVGGLSHNEAEEISKDW
AAAG
ADVLCHAVLETAGIVD
>OLS47895.1 dihydropyrimidinase [Rhodovulum sulfidophilum]AB14
MTTVIKNGTIVTADLSYEADVLIEGGKIAAIGPGLSGDEVLDATGCYVMPGGIDPHTHLEMPFMGTYSSDDFESGTRAA
L
AGGTTMVVDFALPSPGQGLLDALKMWDNKSTRAHCDYSFHMAITWWDRQVFDEMETVVKERGITTFKHFMAYKG
SLMVND
DEMFASFRRCAELGAIPLVHAENGDVVAELSAKLLAEGNTGPEAHAYSRPPQVEGEATNRAIMIADMAGVPLYVVHVS
CE
ESHEAIRRARMQGKRVWGEPLIQHLTLDESEYFNADWDHAARRVMSPPFRNRQHQDSLWAGLASGSLSCVATDHCA
FSTD
QKRYGIGDFTKIPNGTGGLEDRLPMLWTHGVGTGRITPNEFVAVTSTNIAKILNLYPRKGAVLVGSDADLVVWDPEKTK
T
ISAKGQQSAIDYNVFEGKPVRGLPRYTLSRGRVALADGEVTPQEGWGEFVKRSPNGAVNKALSSWKALTAPRAIERAGI
P
ASGV
>OLS47896.1 hypothetical protein BV379_06085 [Rhodovulum sulfidophilum]AB14
MSPGNARKQRSYGYVFQAAGLYAWQSIAWNIRLALEIMGYPKAEQAAGVRVYLASFCNKYT
>OLS47897.1 IS481 family transposase [Rhodovulum sulfidophilum]AB14
MLISLHKQATTTPRIRAAIQASTDPAWMVAERYGISEQTVWKWRGRDDVHDRSHTPHRLQTTLTPAQVAVALRKALL
LPL
DDLLAVVREFLTPHVSRSGLDRCLRRHGLGNLRALKLKDPKPAHGSFKAYEPGYLHIDIKYLPQMADEDRRRYLFVAIDR
ATRWVFVRIYPAQTAANARRFLRDLERAAPMKITRVLTDNGKAFTDRLFGLRKRAATGQHEFDLLCTDLGIEHRLEPPM
R
PQTNGMVERFNGRIEDVLQSHRFHSGEDLEQTILRYVHLYNSQLPQSALKARTPIDALKDWQCQRPELFRKRVYNHAG
CD
S
>OLS47898.1 hypothetical protein BV379_06105 [Rhodovulum sulfidophilum]AB14
MWFGFDWQFEAVVVAMVFFPVLVNTPQGLTAADPMQCDQMEIRSENYLLTLTGLRLPAATPFIFNGLKIAPTLALMG
VLA
GLLLGTGGARLPTRSCCDSNG
>OLS47899.1 hypothetical protein BV379_06110 [Rhodovulum sulfidophilum]AB14
MMLPCLKETGVESPEDFPGRSFGVRFFVKEYPLLSWMAYPGMLTDGSHEAMTLLKQGFNVAPLLQEKADCISTMNCD
EYW
QLLDTGSASEELISFKYEDRGVAKL

>OLS47900.1 transposase [Rhodovulum sulfidophilum]AB14
MGQRLMARNFDSQVAELQVRIAVLNGYTALGTPITEAAG
>OLS47901.1 IclR family transcriptional regulator [Rhodovulum sulfidophilum]AB14
MDFAQQKTVRSVERTLQILELMNRVPVVRVQHLAQETGLPAPTVVRLLETLMSAGYVRKIGRQAGYCVTGKVAALGA
GHH
GLPQVFDVAREAAEALTRRTLWPAALATLDVDALVIRFSTIPMSPLSHYQSTINRRMTLLDYAHGRAYLGFCPTEERTHL
VALLQQSAASPAEAEAIAQRADAVVALTRASGFGERTGNIQPETHSIAVTLRLGAKLVGTLGITYFARANVDVAALKAEL
RAAADACEKGEPYM
>OLS47902.1 NAD(P)H oxidoreductase [Rhodovulum sulfidophilum]AB14
MTRTLVILAHPDMGRSRISRRWAEAIRAADDITLHDLYARYPEGQIDGEAERQLVTDHDRIVLQFPLTWYSCPPLLSVW
Q
TEIMKRGWAYGPGGRALRLKTLGIAFSTGSNGRDYQPDGRYGRTLDEVTVPFELMAVHTGMHYLPPFTLTGARECDD
AAI
AASAQAYLHHLRHAEPRLIASGKDDDRARTVYPEDAAAHM
>OLS47903.1 hypothetical protein BV379_06130 [Rhodovulum sulfidophilum]AB14
MFDDSDPRKALTSMSKERRFSGPFCGPEVGNFSQEPPQQAGDGWQAWMMRCTNLVVRLVRLEGTVTFTRTGQMD
EYMVFS
TDADLRFDIETAMGAVTDHPGDHLFVVPPGDSRVTVRGTGQVSFLYTTRSADLVAQCANPEIPANPNIPPFQQWPDP
VGG
FRLRAYDLDVPEQDGRFGRIFRCTTMMVNVLPRFTAPRDTSKVSPHYHASFEQISLTLEGDFEHFLRWPWTSDMADW
LPD
MHIRCASPSVAVLPPPAIHTTLWTSETTQIVDIFSPPRMDFSTKNGWVLNAADYPLARTEIE
>OLS47904.1 TetR family transcriptional regulator [Rhodovulum sulfidophilum]AB14
MSENTENADEAIVRMGYLTARNGRRAEIGAARRAKSRNAILTAAFACYGRADGRIVRIEDICKAAGVARGTFYNHFDDL
E
ALRYQLLEEMTGEFDRAVHHMFDGLENAAEQNAVAIRYYLHAAEKNPAWGWAMIHSSAPGHTFGEMVWHNSLVTI
RRGVE
EGLFQIPAAEIGRDILMGAVAAAMVSITSGTTPGDYPEQISEHILMAFGMSKAAARELSRRPLPTLPPIAHDTIVIASMP
ALGDIAD
>OLS47905.1 catechol 1,2-dioxygenase [Rhodovulum sulfidophilum]AB14
MGINRIDHAAIRVGDLGRALAWYEDVLGLTVLSRDADTVHLACRGKAADITLVKSTQSLENFTFGVDDQEDLDRVESIL
K
KEGVAAERLNGDSRPGEGATLKFHLPSGHEMRLCTGGAERRAGISDFDSKGSHVPTDMDHLNLLGEVDPAVMRDFL
MMIG
FKSSLNISVGGQLAGIWLRGSAYDHDIAYMRAVRPSDRLHHVAFAVEDGNHYFRLSDRLMDTGHRWEFGPGRHNAG
LGKS
TGFGTNNYAYAFDPFGNRNEFSSGMEQFADDTPPLDADIAPEQMTEVMNGWGYEMAETFMTVGS
>OLS47906.1 hypothetical protein BV379_06145 [Rhodovulum sulfidophilum]AB14
MSMSAETFLTAQDLVLRFWAVVDEVDDRPGSELFTPDGVLVIESFRAEGTAELSRYFAARRALSTERQRGTRHLCTNLR
V
LPGAGIRLAATVTVFSGTGARPLPLAAPSSLADFTFDCVETPDGWRFREVRGALVFAGADTPDLKKAAQAAKGGPA
>OLS47907.1 alpha/beta hydrolase [Rhodovulum sulfidophilum]AB14
MKRVALVHGWGGSYAATWQADGWDEALRDAGFEPVGVDIAGHGPQGGPHDPRAYADLASDLATKLPTGLHGAIG
FSLGTK
LLLELEARKPGANGRLVLGGIGDNLFAPETAGPAIVAALRGETAEHPPQVAALLAYAAKSGSDPVCLAAVLERPGNPVLT
ESRLAAAQAPTLIFNSTNDAVAQPDDRLRAALPGALYRRIDGPGHVALTADARFRDAAVAFLTD
>OLS47908.1 acyl-CoA dehydrogenase [Rhodovulum sulfidophilum]AB14

MFDDTTPMIDRVRAILPQIKANAAKAEADRMVPAENIDLLKGTGLHRAFQPKAYGGLEMPVPDFENCIALIATACGST
AW
AISLLAEHAHQIALYSKELQDEIWGEDPDTLSSSSIAPYGQAVEVEGGVRFSGEFGWSSGCDHATWAILGFLRDDAEMG
K
VYSFAIVPRADYEIKDNWFTAGMRGTGSKTLVVKDAFVPNHRIESVPALMTLTSAGGALYPDSQIYQVPFIYVFASCFSA
VSLGIAERMIELYTERTKGRVRAYTGAKVGQSIPACMRLAESTHQVAAGRAFLEKTWAEMRDHAERHEYPTPTQMAF
WRT
NQAYAVKMFVAAVDRLFEASGGSAWFDDAEAQRLFRNSHMTAAHAYTDYDICAQILGRALMGLEPDPSLF
>OLS47909.1 nitrilotriacetate monooxygenase [Rhodovulum sulfidophilum]AB14
MGSMNFPAESGFDPRAFRAALGCFATGVTVITAQADGQRVGVTANSFNSLSLTPPLILWSLLKTSSSYPVFEAASHFAV
N
ILAHDQIELSNHFARRSDDKFAEIEVTEGQGGCLLLPGTCAKIQCARENILDGGDHWILIGRVSGFETSDSAPLLYHRGQ
YSLAQPHPQVAAR
>OLS47910.1 peptidase [Rhodovulum sulfidophilum]AB14
MKTTYTLGATAVSLTMAAVPAQAIDVGPADYTIVPSGTAVAMIYYQHLSSDRLDVDGDDVPGSTFEADVAILRGLYYTE
Y
GGLPAVWHLVMPFSNIGDVDIGTLPQDTTSGIGDTTVGLTLWPVQPDSPETGTTLGLSLFATAPTGNYDPTSIGIGEGT
W
SITPQVGLIQGLGNGLYLDAALDVAFSMDHTEDGDDYERAPSWQLQTMLRKQWGPTSVTFGYNGQRGGEQKVNGV
ETGLK
THRDQLRLYGSHWIDQTTQIQGMYAKDINVEGGFEYDNVFQLRLVKVF
>OLS47911.1 transposase [Rhodovulum sulfidophilum]AB14
MAEVQIRVAILNRFTALGIPVTVAAG
>OLS47912.1 MFS transporter [Rhodovulum sulfidophilum]AB14
MADDLRCEGGTDVLGTRQWALLASLYSTQYLGLNFFVVALVAILRTEGAQLDVLGLVYMLGLIWPLKLIWAPMIDRFTI
G
RGGHFRGWLLLTQGLLVLLLLAIGRFDVIDDFPLVYALCLGVALLAATQDIAVDGLACRLLPETGRGLGNGLQIAGGLVG
NMVGGGLILMLYPQIGWEGCLTLLAVLTAIPAVQLIGFDEPAWQRYGDATGKLYRRMTVFWWQPGQRCWLGIVLLV
SASS
GMAYAVLMPLLVDQGWGPARIGLVVNVFGSIAGLVAALISGWLMHRLSRRDALVMGVGLQIAGVAALLLPAWGLTQ
AAAF
GAIAYFLCYNPVSVVMATMMMDRAAPQSAATDFTLQFTSGQFAAIAAMSAGAAMAAQIGYGGVLGVALGAGSVAL
LLAVG
FGRSRAAG
>OLS47913.1 hypothetical protein BV379_06210 [Rhodovulum sulfidophilum]AB14
MSWDAAPTGRRGRQQTYGDAVIQTCLSIEGAIRHGAQARRRLWQVYFYGRRPRQRSDRADAIDQAIDRPGRPLLYFN
NTS
IALRQP
>OLS47914.1 hypothetical protein BV379_06215 [Rhodovulum sulfidophilum]AB14
MRPGVVYLAENGLAPYASYSTSFEPTPGSDFAGEAFKPTTGEQFEIGVKYQPVGFDGFVTLSTYELTQQNVTTTDPVNT
R
FDVQTGETRVRGIELEGVAALSDSLNVKASYTYMDGKVTQSNDYLGNVPAQIPDHVASLWGDYTFPDGGPLAGLEIGA
GV
RYVGGRFGDAANRMDLPATTLVDASLRYDFENLSPDLEGLSLRVNASNLFDKTYVGQCASTRYCQYGGRRTVSASLGF
QW
>OLS47915.1 hypothetical protein BV379_06230 [Rhodovulum sulfidophilum]AB14
MDRDVWIQQMSSKGVRPLVVANLSCNASLSPGISLDGKARGLDSIFIERLWRPLIYVCNYLHACA
>OLS47916.1 alkylhydroperoxidase [Rhodovulum sulfidophilum]AB14

MTPRMNYYKAAPDTMKAMIAAEETIKDLSLPEVLCELVKMRVSQINGCAFCLDLHAPAARQAGVSQQKLDVLAAWRE
SPV
FDDRERAALAWAEALTRIEQTGAPDSDYRLLAASFDERERVELTFVITMINAWNRFAVGFRQPHTRREARDAAG
>OLS47917.1 hypothetical protein BV379_06240 [Rhodovulum sulfidophilum]AB14
MRQADRHSQIFEGERRRLAGLAYRMTGSVAETDDILQDAALKFRDADLSGIDRPARWLSTVVMRLCLDHLRSARVRRE
TY
VGAWLPDPLIADHVPGAEEEWILTEDVGIALILTLDSLTPEMRAAFILRDAFDYSFEEIGALVDKTPTACRQLVSRARRR
LAGAEPADPPSSETEHPLVTAFWEASRQGDMDRLLALFAEDIEVHTDGGGRVKAAINVLNGSRNAARLFAGLARKRRF
PP
GPCPPLRWINGSPGFVSIEGGVLQTTALSIRRGRIEAIWIMRNPHKLKHLRLQM
>OLS47918.1 ABC transporter ATP-binding protein [Rhodovulum sulfidophilum]AB14
MNVKPDFNKNANQAATAPAFLSVYDLHAYYGESYIVQGVSFNVHEGEILALLGRNGAGKTSTLRAIARLASPELTYGEI
W
LDHAALHGMAAHQASAAGMALVPEDRRIIPGLTVEENLQLAQIVPPVGWTLDRLYDLFPRLGERRKQEGVTLSGGEQ
QML
AIARALARDIKVLLLDEPYEGLAPVIVDEIEKTLVHIKEQGITTVIVEQNAIRALELSDRAVILDTGQIVFDGSAAEVLS
NAELRAEYLAI
>OLS47919.1 ABC transporter ATP-binding protein [Rhodovulum sulfidophilum]AB14
MGILEVKGVNKRFGGLQALGDVNLSVPENTVHAIIGPNGAGKSTLLNCLVGKLIPDTGSVMFDGQSVLGRKPHEINQM
GI
SRVFQTPEIFGDLTVLENVLIPCFAKRDGAFRMHAFETVAREGEIVARAEQMLEDVHMLDKAGMQAASLSRGDKRRLE
MA
MCLVQEPRLLLLDEPTAGMARADTNNTIDLLKEIKATRNITLAIIEHDMHVVFSLADRITVLAQGTPLVEDVPEKIKGHP
KVQEAYLGQAH
>OLS47920.1 branched-chain amino acid ABC transporter permease [Rhodovulum sulfidophilum]AB14
MFGLSARDTRFLLIVIALTLVTPILLQPFPEGSSLAQFNAGYPDLMQKFAIFGIFAIGFNILFGLTGYLSFGHAAFLGVG
SYTVVVWMYKLLGYNVLPGLLLSVVISAAFALLIGFISLRRSGIYFSILTLAFAQMSYNLAYSVLTPITNGETGLQVYASD
PQILAGSGAPTSPHLFGLVMRESAQLDLGGWVFTFNIGYYFCALMAILAFYISLRIFRSPFGMMLRAIKTNQTRMNYTGL
NSRPYTLAAFVISGMYAGLAGGLLAAMDPLAGAERMQWTASGEVVLMTILGGAGTLMGPVLGAGFIKYFENIFSKIND
GV
LHSWFAFLPDGLEDFVVACFHPFVGKGWHLTLGILFMLVVIFLPGGLVEGMQRLRSLFRRRGKTGGPRRKDPEHRPHT
PP
HVAE
>OLS47921.1 branched-chain amino acid ABC transporter permease [Rhodovulum sulfidophilum]AB14
MDAIILQILNGLDKGSAYALIALGLTLIFGTLGVVNFAHGALFMIGAFCAVTMQRLLSLSYEEIDPTRTDFLGNPLKVEV
PYVEHWFGPEIGGAIIDWSVPFAILLAIPVMVGLGFAMERGLIKHFYKRPHADQILVTFGLAIVLQEIIKYFFGANPIQT
GAPPAFSGSFDFGTLLGFDPNVIIYPYWRMVYFLFSLAIVGGVFAFLQFTTFGMVVRAGMADRETVGLLGIDIDRRFTL
M
FGLAAAVAGLAGVMYTPILAPNYHMGMDFLVLSFVVVVVGGMGSLPGAVLAGFMLGILQSFASMNEVKSLIPGIDQV
IIY
LVAIVILLTRPRGLMGRKGVMED
>OLS47922.1 transcriptional regulator [Rhodovulum sulfidophilum]AB14
MTRSALTGTRIRERRTMIGLRQADLARMAGISPSYLNLIEHNRRRIGGKLLVEIARNLDVEASALTEGAAAALIETLREA
AADHDAAEAELPRLEEFAGRFPGWAALIADRHRRMRELERTVEMLTDRMTHDPFLSASLHEVISAVTSIRSTAAILADTE
DIDPDWQRRFHRNLSDDSRRLAEGAQALVAYLDEGSEDPGVQSSPQEEVEAFLRARDWHLPELERSLPPDPARIVAEA
PE
LTSAPARELALAHLRRYRRDAELMPLQRFAEAARGAGLDPGALAQAFGVDPGAVFRRLAALPEERAGGPVGLVACDGS
GT

LTFRKPVEGFSLPRFGAACPLWPLYQALSRPMAPVRAVVELGGSAPRRFLTYAICQPALAAGFDGPQVLEAAMLILPEG
T
VDLPRLAPQPVGTSCRICPRRDCVARREPSILADGF
>OLS47923.1 response regulator [Rhodovulum sulfidophilum]AB14
MDKRVLLIEDEPNIIEAIRFILARDGWRVDTHAEGSTAFARVAETRPDIVILDVMLPGRSGFDILRDIRSESGLDGLPVL
MLTAKGQVKDRDLAERMGASRFMAKPFSNTEVLASVRELTGA
>OLS47924.1 hypothetical protein BV379_06285 [Rhodovulum sulfidophilum]AB14
MARAPLFLAREGYRRRRLADMARLLPVLGAALFLVPALDAGDGLGAGMLIYLFIAWFWLILASAFVSSRLVRAAPREPA
E
PSGEAP
>OLS47925.1 hypothetical protein BV379_06295 [Rhodovulum sulfidophilum]AB14
MAEDGHHAILRRILGEDRVSLGGGRGGGNNGAAALRLETARAMRDSFGLTALVEECKADRAGLPEIQAEIEPGSLPILLR
TSDGRLGLVVYSSELALALLEWRLIGFLSETAPPDRPLTATDAAVLADLTDPVLDRFGRAMKEADGVDWASGYTQGARI
D
DPRHVPLIMAAGRYRLYRLRLVAGRGRAGGLIIALPEAAPVLRPRTAENVARPWPEALRAGVLGAELTLNAVLWRSRVS
A
AAIARFRPGDLIPLPAAALGAVQLEGPGGVAIAGGRLGQAHGDRAVKLDTESGEADASGFDAGPAEIGALAASGLPGS
DL
PMGEAGGGLDDAGGFDFAAEGLPADGLDQGGEDFPFDPAGFDSDLENALENGLDGGGFGAAPLDLSEDFPSVSA
>OLS47926.1 hypothetical protein BV379_06310 [Rhodovulum sulfidophilum]AB14
MAFEQLKAGIALILEEIEKRPEDRHVLQEELRGKIAELRALGMPVPEDILRLEQELEDDGSDDLYDDMPV
>OLS47927.1 hypothetical protein BV379_06315 [Rhodovulum sulfidophilum]AB14
MAPLLAVEHPAPGVVRLVLDAPPDNALTAPLHDALDAGLTTALAAGSTRAVMIAARGAVFSAGASSAPSGTAAPLGAI
CG
RIDAAEVPVVALLPGPATGPGCELALACHYRVATRTAALCLPDIALGLPPQAGTTSRLPRLTDPVTALEMMLSARPFSAS
RARQTGLLDALVDGDLDTAGLRFVLGLLVEGAGPRPAPSRPMPEFRALQALLQRLRAGPGPDGAVRARRAVLDCVEA
AAM
MPAEAALAFEEAAVEDCRDSAVHRALCHLAEAEQALTARARELAEAGSEPRRVGIWGWGPQAAALGAALLTAGPSVR
LGA
PDAATLGRGLVEIEALLDAARLAAGWDEATRVRYRARLSAGPGPSALTGCDAVIEAGSGTMSDRQARFPDLARVAAPG
AV
LAATGGLASPSALGAPAGREADTVWFHLPEAVPGCRLAEVIRSPRTGSRPVAVFGALARRLERLVLVAERESPSLVLLLG
ALDAADAMVEAGAAPAAVDAAMEAWGMALGPYRMADRLGLSQLLALRRRQPGGDDPELRPVLILGQLVREGREGY
LAGRG
YYRYRAGGAAGPDPQVAEIVAAAREVLAVAALRDFAEAEIQARVLAGMVQAGAALLRRGGVSSAAELDLAAVHGLGV
ARW
RGGPMRAADEAGLLSVRKTLRALAAERPRGGIWDLDPLLAETIKNGRELASAVAPGRGALSPA
>OLS47928.1 hypothetical protein BV379_06320 [Rhodovulum sulfidophilum]AB14
MEALIWAGAAVSVLGLAGIFWCIRDMLGARNSGLPEPEIRARLQKAVAKNMAALALSAIGLMMVVVGIMLG
>OLS47929.1 hypothetical protein BV379_06325 [Rhodovulum sulfidophilum]AB14
MEIEDEQDDTRSADSSYRVTAGELRQFIERFERLEAEKKDLADQQKEVMAEAKARGYDTKVMRKVISLRKRDKEDIAEE
E
AILELYKEALGMA
>OLS47930.1 iron ABC transporter permease [Rhodovulum sulfidophilum]AB14
MSDQIARLSPLRRVRLRPDPWSLGALVIAALVLMPILSVLVLALTPEENIWPHLISTTLPRYLGNTVTMMAGVAVLASAV
GAGAAWLVSMYRFPGVRWLEWLLLLPLAIPAYVGAYALVDFLEYAGPVQTGLRTLFGWHNARDYWFPEIRSRGAAVL
VLS

AALYPYVYLLSRAAFREQSGASYEVARALGAGPLALFWRVGLPLARPAIAAGAAIAMMETVNDFGVVEYFAVQTLTAGIF
SVWLEGGNAGGAAQIAGVILAVILALVALEKVSRRNLRFHNLGRHHRPLVPVPLEGAAGWAATAACALPVATGFVLPVGV
ILSHALANSGGWADPGLVRALIHTLSAGGIAAVLTVAAGLFLVYGVRLTGRALPRLLLPVTTIGYAAPGAVLGVGLLIPM
AAADNALADTWVGLFGVDPGLLMTGSAFAIIYAYAVRFFAIAQGAADAAMGRIAPSLPMAARSLGRSARGTLTAVYLPLI
RTSVGTALLLVFVDCVKELPATLLLRPFNYNTLATRVYDKASLENLADAAPAALLVCLVGLVAVGLLARANR
>OLS47931.1 ATP-dependent Clp protease ATP-binding subunit ClpA [Rhodovulum sulfidophilum]AB14
MPSFSNTLEQAIHAALAQANARRHELATLEHLLLALIDEPDASKVMKACSVDLEELRKTLTEFIEDELATLVTDIEGSEA
VPTAAFQRVIQRAAIHVQSSGRTEVTGANVLVAIFAERESNAAYFLQEQEMTRYDAVNFIAHGVAKDPSFGEHRPVTGAS
EFEEEPQAGAAQPEGGEARESALAKYCVNLNAKSRKGDIDPLIGRDQEVERCIQVLCRRRKNNPLLVGDPGVGKTAIAEG
LARKIVGGETPEVLSKATIFALDMGALLAGTRYRGDFEERLKAVVTELEDHPDAILFIDEIHTVIGAGATSGGAMDASNL
LKPALQGGKLRCMGSTTYKEFRQHFEKDRALSRRFQKIDVTEPSVEDAVKILKGLKPYFEEHHEVKYTSDAIKSAVELAA
RYIHDRKLPDKAIDVIDEAGAAQHLIAESKRRKTIGPREIEAVVAKIARIPPKNVSKDDAKLLKDLETTLKRVVFGQDKA
IEALSASIKLARAGLREPEKPIGNYLFAGPTGVGKTEVAKQLASSLGVELLRFDMSEYMEKHAVSRLIGAPPGYVGFDQG
GMLTDGVDQNPHCVLLLDEIEKAHPDVFNILLQVMDHGKLTDHNGRQVDFRNVILIMTSNAGAAEQAKAAIGFGRDRREG
EDTAAIERTFTPEFRNRLDAVISFAPLPKETILQVVEKFVLQLEAQLMDRNVTIELTRPAAEWLADKGYDDKMGARPLGR
VIQETIKKPLAEELLFGKLAKGGVVKVGVRDGEIDLKIEEPEKRRLDSSKKPPLLTAE
>OLS47932.1 hydroxyacylglutathione hydrolase [Rhodovulum sulfidophilum]AB14
MPLEIVTVPCLDDNYAYLVKGPGGAVLIDAPEAGPIAAALETRGWTLDTILITHHHHDHVEGVAELRARFGAKVMGPAAE
ADRLPPLDRALSEGDRVGEGPIAADVIAVPGHTLGHVAYRFGAGDAVFTADSLMALGCGRLFEGTPAMMWDSLKKLSSLP
PETLVFSGHEYTSSNARFALTVEPGNAALRARSEEIDRLRAEGRPTVPVPLSVELETNPFLRADRPELRKALGMETEGDI
EVFAEIRARKDRF
>OLS47933.1 hypothetical protein BV379_06350 [Rhodovulum sulfidophilum]AB14
MHLDVLDLRNFYYRTNLGRVAQRAIREQVREIWPAAKGQTVAGFGFAVPLLRPLRTSARRVVALMPGQQGVMPWPAGEEN
VSLLCEETTWPLPDGFVDKLILLHGLETSEHPAAVLEESLRVLRPGGRALFIVPNRSGLWARRDGTPFGFGRPYSSGQLE
AQLRQHGFEPEAHRAALFSPPTSRRFWLKTASIWENAGQRMSAYYAGGVLMVEATKRVYRPTGLPTKDGLPRPLRILDAL
PQPGAEPA
>OLS47934.1 F0F1 ATP synthase subunit delta [Rhodovulum sulfidophilum]AB14
MSEPASISSGIAKRYATALFEITKEANAIPALEADVDALDAALLESGDFRDLISSPVYSRDQQSRAIGSIASKMGLGQVL
SNTLQLMGSKRRLFVLPQMVTELRALIAEAKGEVSAEVVSARPLSDAQRKALATALKQNAGKDVKINASVDESLIGGLIV
KLGSKMIDTSIRARLSALQNTMKEVG
>OLS47935.1 ATP synthase subunit alpha [Rhodovulum sulfidophilum]AB14
MGIQAAEISAILKEQIKNFGQEAEVAEVGRVLSVGDGIARVHGLDNVQAGEMVEFPGGIRGMALNLEVDNVGIVIFGSDR
AIKEGDVVKRTNSIVDVPCGDALLGRVVDALGNPIDGKGPIATTERRIGDVKAPGIIPRKSVHEPMATGLKSVDAMIPVG
RGQRELIIGDRQTGKTAVALDTILNQKTYNEAAGDDESKKLYCVYVAIGQKRSTVAQLVKKLEETGAMAYSIIVAATASD
PAPMQYLAPYTATAMGEFFRDNGRHALMIYDDLSKQAVAYRQMSLLLRRPPGREAYPGDVFYLHSRLLERSAKMNEDHGA
GSLTALPIIETQAGDVSAYIPTNVISITDGQIFLETDLFYQGIRPAVNTGLSVSRVGSSAQTNAMKSVAGPVKLELAQYR

EMAAFAQFGSDLDASTQKLLNRGARLTELMKQPQYSPLTNAEIVCVIFAGTQGYLDKVAVKDVTRYESDLLNFLRSKKQ
D
LLDDITNNDRKVKGELEDKIRAALDEFAKNFA
>OLS47936.1 F0F1 ATP synthase subunit gamma [Rhodovulum sulfidophilum]AB14
MPSLKDLKNRIASVKSTRKITKAMQMVAAAKLRRAQEAAENARPYAAQMEKVMSGLAASATGAANAPKLLVGTGSE
KVHL
LVVMTAERGLCGGFNSSIVRLARHKAQDLIAEGKTVKILTVGRKGREQLKRDLGDHFVGHVDLSEVKRIRYGNAREICQ
D
IIGRFEAGEFDVATLFFSRFKSVITQEPTAQQIIPAVFDEPEDSSTMYDYEPTEGEIMAELLPRGVATQIFAALLENGAS
EQGARMSAMDNATRNAGDMIDKLNLEYNRSRQAAITKELIEIISGAEAL
>OLS47937.1 ATP synthase subunit beta [Rhodovulum sulfidophilum]AB14
MANAVGKVTQVIGAVVDVQFDQHLPEILNALETENNGKRLVLEVAQHLGENTVRTIAMDATEGLVRGAKVTDLEKPIS
VP
VGDATLGRILNVIGEPVDEKGPVHATETRAIHQPAPEFAEQSTSSDILVTGIKVIDLLAPYSKGGKVGLFGGAGVGKTVL
IMELINNIAKVHSGYSVFAGVGERTREGNDLYHEMMESNVIKIDDLSQSKVALVYGQMNEPPGARARVALTGLTLAEQ
FR
DQSGTDVLFFVDNIFRFTQAGSEVSALLGRIPSAVGYQPTLATDMGALQERITSTKRGSITSVQAIYVPADDLTDPAPAT
SFAHLDATTVLSRAISELGIYPAVDPLDSTSRILDPAVVGEEHYQVARDVQGILQRYKSLQDIIAILGMDELSEEDKLTV
ARARKIQRFLSQPFDVAKVFTGSDGVQVPLEKTIASFKAVVAGEYDHLPEAAFYMVGDIDEVVAKAEKMAADAA
>OLS47938.1 ATP synthase F1 subunit epsilon [Rhodovulum sulfidophilum]AB14
MADTMQFDLVAPERRVASEKATSVVIPGFEGDLTAMPQHAPLITTLRPGLLEVFSESGSRKYIVTGGFAEITAESATVLA
ERSHPVEDVTQEMIDEMVAEARAAARDAHPDLVDAAARMLADMVAMGTHMGFEPQA
>OLS47939.1 VWA domain-containing protein [Rhodovulum sulfidophilum]AB14
MFLPFFQTLRTCGIPVSLREYLSFLAGMKAGLVTYDPEGFYYLARTALVKDERQLDRFDRAFAESFGGLEQIDPGEVLEA
LELPEDWLRKLAEGHLSPEERAGIEAMGGFDKLMEALKERLKEQKERHQGGNKWIGTAGTSPFGAYGYNPEGVRIGQ
DES
RHRRAVKVWDRREFRNLDDSVELGTRNIKVALKRLRRWARDGAREELDLDGTIRATADRGYLDVRTRPERHNAVKVLL
FL
DVGGSMDDHIRVVEELFSAARAEFKHLEHYYFHNCLYEGVWRNNRRRWDRVTPTWEVLRTYGPDYKCIFVGDASMS
PYEI
AFAGGANEHWNAEPGQVWLERARAQWPDHLWINPVPEQHWQYTQSIAMIREIFEDRMVPMTLDGIERGMKSLG
>OLS47940.1 ATP-dependent transcriptional regulator [Rhodovulum sulfidophilum]AB14
MSRHGARIGGFVLALVLAGCTATGPDLAARHPPVPTDLPPMKTFASHRVTPPGRSNAAIARDFLDLAFRMESGRPLPY
MT
RFEEPITLRVAGPVPPAAEPDLAALLARLRREAGIDIHRVAADAGAKVTIEFLPRAKLQRLVPQAACFVVPRISSWDEYR
RGRRSGGVDWTTLARRERVAIFIPGDVSPQEVRDCLHEELAQAIGPLNDLYRLPDSVFNDDNFHTVLTGFDMLILRAYY
A
PELGNGTTRQEAERVLPRLLARLNPRGERIGVSGPLPPTPRAWIDAIETALGPGASSSRRVAAARTAVEIARNQGWRDT
R
LAFSLFAFGRLSLNRNASEASLAAFLEAGKIYAANPVTRVQSAHVAMHIAAFALSGGQPESAIALIDGHLADVRSGENAA
LLASMLLMKAEALDMLGRDAEAGATRRDALGWARYGFGSEDEIRARIAEIAWLNPARSD
>OLS47941.1 GNAT family N-acetyltransferase [Rhodovulum sulfidophilum]AB14
MQASDSPLLPAKGPIVTDLLVRPLRPEDVASWRRLWTAYLDFYDSAVPAETCDITFARLLHGGDPNMGCRLALIDGRA
VG
LVHFIFHRHCWKPENVCYLQDLYADPEVRGRGVGRALIEAVYAVADSRGTPSVYWLTQEFNDTARQLYDRIGQSTAFV
KY
VRG
>OLS47942.1 hypothetical protein BV379_06405 [Rhodovulum sulfidophilum]AB14

MSLDPVLARIDADLPQALDRLMALLRIPSISTDPAHADACREAADWMVRDLASIGFDARTCPTPKHPMVVGKAGDTG
PSL
LFYGHYDVQPVDPLSLWARDPFDPAIEETPKGKVIRARGAGDDKGQLMTFIEACRAWKAVHGSLPARITMFLEGEEES
GS
PSMVPFLKENAETLKADLALICDTGMLGNATPAIVTMLRGLVGEEITITGPDKDLHSGMYGGPAINPIRVLARIIAALHD
ETGRVTVPGFYDGVPEMPEEILAQWQGTGFDPAQFLGEVGLSTPAGEDGRSVLEMLWSRPTCEVNGISGGYEGAGFK
TVL
PSKASAKISFRLVGSQDPVRIRESFREMVRGLLPPDCSVDFAPHGASPASQMSIGDPAFEKARLALSQEWPNPAAFVGS
G
GSIPVAGYFRNLLGMDSMLIGFGRDDDAIHSPNEKYDLESFHKGIRSWARILAALSA
>OLS47943.1 5-aminolevulinic acid synthase [Rhodovulum sulfidophilum]AB14
MDYTAALDASINRLHEEGRYRTFIDIERRKGAFPEAVWSRPDGENQDIVVWCGNDYLGMGQHPVVVGAMQDAIAAT
GAGS
GGTRNISGTTIHHRRLEAELADLHHKEAALVFNSAYMANDATLSTLPVLFKDLVIVSDELNHASMIQGIRRGRCEKHIFR
HNDMAHLRQVLESIEKGRPILIAFESVYSMDGDFGPIEEICDLADEFGALTYIDEVHAVGMYGARGAGVAERDNLMHRI
D
IINGTLAKAYGVFGGYVAASAKMCDAVRSYAPGYIFTTSLPPTIAAGACASVHHLKTDATLREKHQDRARVLKARFKGLG
MPVIDHGSHIVPLIVGDPVHTKRISDMLLEGYGIYVQPINFPTVPRGTERLRFTPSPVHTPEQMDALIRAMDELWSQCA
L
NRAELTA
>OLS47944.1 helix-turn-helix domain-containing protein [Rhodovulum sulfidophilum]AB14
MIRRRQPRAAEHSHHKPKGFDAFELRLGDVMRGERATLGKSLLDVQRELKIRATYIAAIENADPTAFETPGFIAGYVRSY
ARYLGLDPEWAYRRFCEEGDFATVNAMDAAASHKPQPARRKSVSEPLADPNAIFVPRGPAMFSQVQPGAIGSVLVLLS
LI
AVLGYGGWSVLKEIQQVRLAPLDDQPGLMADVDPLQVAENAPVSDRAPSAPPSVDALDRLYRPQILDRPVMVARDG
PIAT
IDPGEIGVLVSDRSQRAVERSFDSAVATALAEAGVGAQRPGLVREPRAAENGAVQVVAADATPELAILAVRPAWVRIS
AA
DGTVLLEKILDAGESYVLPRTEKPPRLRAGNSGSVYFSVNGRTYGPSAPGAKVAKNIELSPRALTERYQLADIQADPDLA
VALAQNDVPTTRAGGPSR
>OLS47945.1 4-hydroxy-3-methylbut-2-en-1-yl diphosphate synthase [Rhodovulum sulfidophilum]AB14
MSLNSVRPWRSIDRRKTRQIHVGPVAVGGDAPISVQTMTNTVTTDVKGTIGQILRAAEAGADIVRVSVPDEDSSRALK
EI
VAESPVPIVADIHFHYKRGIEAAEAGAACLRINPGNIGDAKRVKEVIKAARDHGCSMRIGVNAGSLEKHLLEKYGEPCPE
AMVESALEHIRILEDNDFFEFKISVKASDVFLAAAAYQGIAEVTEAPIHLGITEAGGFVSGTIKSAIGLGNLLWMGVGDT
LRVSLSADPVEEVKVGYEILKALGLRHRGVNIISCPSCARQGFDVIRTVEALEKRLEHIKTPMSLSIIGCVVNGPGEALM
TDVGFTGGGNGNGMVYLAGKQDHRISNEAMIDHIVEEVEKKAAEIESTETSSGVEAAE
>OLS47946.1 disulfide bond formation protein DsbA [Rhodovulum sulfidophilum]AB14
MTRMTAAALMALGLAAPLAAQAEAFDLTDMSAAERTAFRAEVRSYLMDNPEVLMEAIGVLEQRQANAQAQNDETL
VRVNA
EDIFEDGVSWVGGNPEGDVTLVEFTDYRCTYCRKSYSEVSDLVASDGNIRFVVKEFPILGEQSVLSSRFAIAVKQLAGDA
AYEKIHDALITFRGNVSDESLKRLAEENGLDAAAVLERMKAPEVEKVIADNHALAQRLQISGTPTFILGDQMIRGYVPLE
GMKQLVEDARAD
>OLS47947.1 hypothetical protein BV379_06435 [Rhodovulum sulfidophilum]AB14
MKLVLSLAVAVATATPGLALPLSDAERVAFRAELRAYLLEHPEVIREALEGAETRRMARHANADLARIEAHAETLFRAPD
DWTGGAPDGDVTLVSFVDYRAEGARRALDAAREAAERDDRLRLVVKEAPRPGDEAAERAARFALAVLRLAGPAAYEA
AQP

RLFDAGATLDAAALDRISAELGLDPAEVTTRMAGSDITAKLAETRALTEALELGPAPAQVLKRTMVRGDVPAVALLRMV
E
AIRRKK
>OLS47948.1 1-aminocyclopropane-1-carboxylate deaminase [Rhodovulum sulfidophilum]AB14
MKVSSRGEVDPFIVMDVMEAAARAESQGRRIIHMEVGQPGTPAPAAARKALARAMEESPLGYTLGLGLPALRARIAAL
YR
DWYGVDLNPERVVVTPGSSGGFTLAFTSLFDVGARVGIGAPGYPSYKAILKALGLEPVMIRTETQHRLQPVPGDLPQDL
D
GLLVASPANPSGTMLDRDALAALIGAAHARGAAFISDEIYHGIEYERKAVTALEITDETYVINSFSKYFSMTGWRVGWM
V
VPEDHIRRVERLVQNLFICAPHASQVAALAALDCGDELQANMEVYRKNRALMLEGLPKAGFTRIAPPDGAFYVYADVS
DL
TTDSRAFAAEILDKAGVAVTPGLDFDPERGAGTLRFSYARSTADIEEGLARLARFMASR
>OLS47949.1 RarD protein [Rhodovulum sulfidophilum]AB14
MSPLRPAAKGVLAMVGASAIWGLSSMYYKLLAHVPPLEVLSHRTIWSLVFFGLVLALQGRLAELWRLFGQARALWLVA
AA
AVMISTNWFFFIYAIQIGHAVEASLGYYIFPLVAVALGFAVLGERPSVLQWGAVGLAAVAVGALTWGLGVAPWMSLAL
AG
TFALYGLAKRGVPAGPVVSVTAEVVLLLPLALVWLWGVHVAGWPGLTGRALGAFGQDLGDSLLLVFSGPLTAVPLMLF
SY
ATKRVDFATVGLVMYLNPTLQVLVATLVFREPFTGWHILAFALIWTALALYSAESLRLDRASRRSSIRAGTSDTMRT
>OLS47950.1 Rossman fold protein, TIGR00730 family [Rhodovulum sulfidophilum]AB14
MTLQNQSICVFCGSRDGDRPAYAAQADALGHALAEAGWRLVYGAGDVGLMGRVARAAQGSGGQTFGVIPVHLLDR
EVGKR
DLSAFIVTENMHERKKVMYMNSDAIVVLPGGAGSLDEFFEVLTWRQLGLHAKPIYLLNTEGFWNPLSALIHHFIDEGFA
E
PSLAGYVRMVSDVPALMEDLRDALSSRRLSAE
>OLS47951.1 hypothetical protein BV379_06460 [Rhodovulum sulfidophilum]AB14
MPERARKAPIRGSVWTWIILGGEAVLVLMVAAWFLGRDDERNSDGPQAEQDAAVTSPAAAGQAADAANPDAQTPA
AGDRS
EAVPPAMPLFAPPAFDLVRVTPEGGALVSGRAGAGTVVAVLVEGREAARAKADAAGDFAVLFDLGQSDDPRRISLEM
LGA
DGRRTASEATVILAPNRSVSTATGPAGGPEDAAEGPRPSQSGAPSSAIIGPPPADAAAILISDAEGVRLLEPPNSHEAPP
VSRLTLDAISYDAQGAVRLAGRGQPGAALRIYIDNAPLAETGIDGAGRWTLRAPGIAAGRHVLRADLLAADGRVAARIE
T
PFQREPAAEIAAAMPPPAGPAGVRVMTVQPGYTLWGIADRTYGRGMQYVKIFEANRGQISDPDLIYPGQIFDLPRLE
>OLS47952.1 acriflavine resistance protein B [Rhodovulum sulfidophilum]AB14
MTGLVDWAAARARMVVAFVLMSLVAGALAYIELPKEGEPDIEIPALFISVPFPGISAEDSERLLIKPMETELSDLDGLDK
MSATASENYAGIALEFEFGWDKTKIIADVRDRMNKAEADFPSGYETYTISEINFSEFPILVVNLAGTLPERTLVRTAKDL
QDRLEALDPVLEVSMAGQRDEMLEVILDPLKLESYNVTAQELVNVVTANNQLIAADEIETAQGAFALKIPASFESPEDVY
DLPVKVNGNRVVRLGDLAEIRLTFEDRSSTARFNGEQTLALQVVKRKGFNIIDTSRLVREEVEKARAEWPPELRQAIVVT
TSNDQSTNVESMVRQLEGSVLTAIALVMVVVLAFLGTRSALLVGFAIPTSFLLCFVLLAILDVTVSNIVMFGLILAVGML
VDGAIVVVEYADRRIAEGTGPMHAFAESAKRMFWPIVSSTATTLCAFLPMLFWPGIAGEFMGLLPVTLIFVLSASLVVA
L
VYLPVVGGVAGRFSRLLSRISARLRPRPRPLRIGVMALAMATAFFGLMRMLNPSYPAAGPASSGPLEMLPGMALFTLG
LI
GCSLAANALRRERSEPVEVGHSPTLFGRLIRAIAGNPVMPLVTVAVVGAFVAGVFFYYGKHNNGVEFFVKTEPEQAIVY
V

RARGNLSIDEKDAMVREVERIVLAEPGVENVFAFAGDGGLSQNTGGASPPLDTVGQVQIELQEWRERAGNPDLNGAL
ILE
RIERRLEKIPGIRTEILNLADGPASAKPVHLRLKGDDWDDLAAATQTVLDRFEETEGLRDIEDTRPLPGIDWQIDVDTEE
AGRFGASAALAGQMVQLVTRGVLLDTMRVPSSDEEIDIRVRLPERDRLLSTLDTLKVRTELGLVPLSNFVTISPVRKIGR
IDRVDGKRYFDVKAAVTDGLTTADGQPINANERIATLTRWLETASFPQGVSWEWTGDQENQEESTAFLSKAFTAALGL
MF
IILLAQFNSFYNSVLVLLAVVLSSAGVLIGMLVMGQPFSIIMTGTGVVALAGIVVNNNIVLIDTYQDYARYMPRIEAIVR
TCEDRIRPVFLTTVTTMAGLLSMMLGVSFDFANGGYTMGSPASLWWTQLATAVVFGLGVATVLTLVFTPSLLALREW
VRA
GAYGSLRRLAALSAGPGSRAARDLALTRAAGQLDLPEIVWSEPPSPTPPGQAAQDSKDHDGKGLRAAE
>OLS47953.1 efflux transporter periplasmic adaptor subunit [Rhodovulum sulfidophilum]AB14
MRWISMLTALLVTVSFYLLMMERERVMALAGVDPASAAPDTTAGEDAPQPVSVVVIDSEARPVRGGVRLRGETAAA
RSVA
VLAEIDGRVVSEPLRKGRSVAAGDTLCRIEPGTRPTALAQARAVLAEARARLPEARAAVTQARAQLNEAEINDRAAQRL
S
REGYASDTRVAGTASAVSTAQAAVASATAGLEAAETARRTAEAQVRNAEIEIERLTITAPFAGVLDEDTAELGALLQPGS
SCATVTALDPIRLVGAVPETEVDKIRTGAAATGKLVSGHEIKGQVSFVARISDPVTRTFRVEAEAPNADGAVRAGQSVDI
RIAGPARDGHLVPQSALTLDDSGRLGLRWVDADSRARFAPVEVIRDTPDGIWVSGLPDSARVIVVGQEFVSDGTPVEV
HR
REPPQ
>OLS47954.1 hypothetical protein BV379_06480 [Rhodovulum sulfidophilum]AB14
MTNPESFIQEVTEEVRRDRLFSWMRRYGWIAALVILILVGGAAWREWSLVQRQAEAQALGDRILDALDIQDAAARSQ
ALA
GIKAEGEAGALVALLAASEAPDAAARIAATERLEAVASDSALPPYMADLAALKLVLLRGSEIPPEARIAALEPLAGPGRP
FRPLVLEQIAYAEIEAGRTEAALDRLRGLLQDAEASADLRRRASQLIVALGGSQDAG
>OLS47955.1 quinoprotein [Rhodovulum sulfidophilum]AB14
MKFGQVTSVLALIALVAGCGSDEEILPGERLGVREALGLETAQPDPSGTERVISLPAPQAVADMPQLGGNPGHLTPHA
AL
SAQPARIWSTSIGAGNSRRYRITAPPVVAGGVIYTLDARSQVSAVSADGKVLWKADLRPEGKRGKDVSGGGLAYGDGR
LF
ATTGYGTLIALDPATGDELWTQKADAAISAPPTYREGMVYFVSRDNRAWAISAADGRVQWQIPGTPAPAAMLGGAA
PAVT
ERLAIFPFSSADLVAALRQSGVRVWGTALAGQRRGAVYAAVTDITGDPVVAGNVLYAGNQSGRSVALDVNSGERIWT
AED
GAYGAMLPVGGSVFFLTDQDKLVRLDASDGSRIWSVDLPYFTKKKPKKLKAVYAHYGPILAGGRIWLASDDGWLRGFD
PA
SGALLAEIDLPGGAASRPVVAGGTLYVLSERGELEAFR
>OLS47956.1 ribosome biogenesis GTPase Der [Rhodovulum sulfidophilum]AB14
MSFTLAIVGRPNVGKSTLFNRLVGKRLALVDDQPGVTRDLREGEARLGDLRFTVIDTAGLEEATDESLQGRMRHLTERA
V
GMADACLFVIDARAGVLAADEVFAEILRKKSAHVILAANKSEGRAADAGFFEAYGLGLGEPLRLSAEHGEGMAELHSVL
A
PLIEAAEARIDTEAAPEIDVDLYEEDDDENMEVPVRMPTAAHPLQVAVVGRPNAGKSTLINAILGEDRLLTGPEAGITRD
SISLSMDWQETPVRLFDTAGMRKRARVQDKLEKLSVSDGLRAVKFAEVVVVLLDAQIPFEAQDLRIADLAEREGRAVVV
A
VNKWDLETDRQDKLIALKEEFARLLPQLRGAPLVTVSALTGRGLDRLHDAVLSAYTVWNRRVPTARLNRWLSGMLEM
HPP

PAPGGRRIKLRYMTQVKTRPPAFVVMASLPEKLPESYRRYLVNGLREDFEMPGTPIRLTFRGQGDRNPFRAKKKKTPSR
L
RKHLGKKPEG
>OLS47957.1 hypothetical protein BV379_06495 [Rhodovulum sulfidophilum]AB14
MIDPVKAILVFVATVFFVASALMPGGFSGFSPDQFPIPQDNPPVQPAGYTFAIWSVIYVWLLISAGFGLFRRARDGAWE
A
CRVPLILSLAPGAAWIGVAQLSPVWASLLLWWMAAMAIWALIETPLVDRWWLQAPVGLYAGWLTAASWVSVGLLG
AGHGI
LFGETGWAFLALLGALITGAAVLFPLDRAPAYGAALIWGLIGIAMANITAAPLVAIAAIAGAVLAGFLTVRAAT
>OLS47958.1 serine--tRNA ligase [Rhodovulum sulfidophilum]AB14
MHDIRAIRETPDAFDAAMARRGLAPVSPAILALDEARRAKITAAEEAQAARNAASKEVGAAKARGDEAEFERLRALVAE
K
KDEIARLDEEAKAEDRRLQDMLMSLPNLPLADVPDGADEADNVERHRWGTPPDPGFAAKEHYQLPGVAAGMDFETA
AKLS
GARFVVLKGAVARIHRALAQFMLDTHVTENGLTEVNAPVLVREEMMYGTGQLPKFGEDSYQTREGWWLIPTSEVTLT
NTV
NDTLLDEADLPRRLTAHSLCFRSEAGSAGRDTAGMLRQHQFEKVEMVSIVHPEQGLDELERMTGCAQGILERLGLPYR
TM
TLCTGDTGFGARRTHDLEVWLPGQGTYREISSVSYCGDFQARRMNARFRPAGGGKPAYVHTLNGSGLAVGRCLIAVLE
NG
QREDGSVALPEALAPWLGGKTLLTVEGELA
>OLS47959.1 hypothetical protein BV379_06505 [Rhodovulum sulfidophilum]AB14
MTANRARLVDWASHLWRPDFKRFTGFGWIVRLVVALIVLALAIIACGAAVLTLAQYVGALVLAQRFASVEGQYEAIRN
AG
LVLAGLVGVPFLVWRSIVAQKQVKVAEQGLITDRLNKAVEGLGAEKTVKRRITHPDGTTEIEETTEPNIEVRIGAIYALE
RISRDSDRDHVNVMEILCAYICENSHRPPSSQPLSRSEETGAAKASLSELTDLTDVQTALTVLGRRAESRRHLETAAHYR
LSLSRCNLAKCDLSDADFSHAAFAGSNLNGAFLYRSRFRDCNFTSASMKRVQGYDSIFEGAVFENANLSQAIFNRSRFLF
SPETQIGANFSRAQMEDSVVAFSGVRGPDKWALGANFVEATWKNAIMIGFESQRDTDFGFRTARTGTDTPPRPATIH
QGL
AFRDCILGAHHIGTLDLERCFGDGTVVLIEPIIRPPHWPSEALSPAAFDRECARWRADPASYVAPQDRA
>OLS47960.1 hypothetical protein BV379_06510 [Rhodovulum sulfidophilum]AB14
MGLLLGKALRRGFKWRPDPGISRAMRPSSRPPAALSLALAVTLGAATLPTAHAAGTRDRAEPLGEAQHRAWRAIGRV
NVA
GFDTGGFCTGTLIAPDRVLTAAHCLYALRGGARAQPGQVHFLAGWYKGSYAAHRTARAVRPNPGFDPGKKPDSRSLR
SDV
AILLLETPITGIAPLAPALPERIGPEVTVIGYRRDRPNALSRVRDCRVIVQSPGMAGLGCPVTEGLSGAPVLQRAGRGWQ
VVGVISAALRGAGGIRSIAVRPGAAFIAGQ
>OLS47961.1 preprotein translocase subunit YajC [Rhodovulum sulfidophilum]AB14
MFATPAFAQAAGGGAAGAFGSFLPLILIFAIMYFLLIRPQQKKAKQHKAMVDALRRGDQIVTQGGIIGKVSKVKDDAEV
E
IEVAENVKIRVVRQTISQVLNKTEPAEKA
>OLS47962.1 protein-export membrane protein SecD [Rhodovulum sulfidophilum]AB14
MLDFALWKRVAIWGVVLLGLLFAAPNMFYSRVEGHNDALKAIETQGATPELIAKRDGWPGWLPSGLVNLGLDLRGG
AHLL
AEVQVEDVYADRMDALWPDVRDALREVRDRVGPIRRIDGTPGTLQVRLGEPSGMPAALEAVRGLARPIVSLTGVGAS
DID
VSGEGAVLTVQLSEPEKQATIDRTLRQSLEIVRRRIDEVGTREPTIQRQGEDRILIEVPGIGSADELKALIGTTARLTFN

PVVSRTSNPNEAPGASNELLPSMDEPGTYYIIEKTPVVSGEELTDSQPAFDQNGEPSVTFRFNPSGARKFGQYTAEHVG
S
PFAIVLDDEVISAPVIREAIPSGSGQITGNFTVEESTQLAVLLRAGALPAELTFLEERTIGPQLGADSIEAGKIASAAAF
LLVLLFMWASYGLFGLFANVALIVNIGLIFGLLSMIGATLTLPGIAGIVLTIGMAVDANVLVFERIREELKTAKGPARAI
ELGYQRALSAILDANITTFLTAVILFVMGSGPVRGFAVTLAIGIVTSVFVAIYVTRMMIVMWMARRRPKSIVL
>OLS47963.1 protein-export membrane protein SecF [Rhodovulum sulfidophilum]AB14
MRLKLVPSNTSWDFFKFSHISLGASALAMVVSIVLVFTMGLNFGIDFKGGTTIRTESSQPVDVGAYRQAISALDLGDVSI
TEVFDPSFGPGKNVAQIRIQAQGDAESVTPETIAALEQALSAVDPSITFPSVESVGPKVSGELVTSAAIAVSLSILAMLV
YIWLRFEWQFGLGAVVALIHDVTLTMGIFAGLQLKFDLAIIAALLTIVGYSVNDTVVVFDRVRENLRKFKKMALKDVLNL
SINETLSRTTMTSFTTLLALIALIVLGGDVIRGFVFAMTWGIIVGTYSSIFVAANILLMLGVKRDWSKEGDGGAGTQFAN
AER
>OLS47964.1 hypothetical protein BV379_06530 [Rhodovulum sulfidophilum]AB14
MKLTDMTIPDGLPIDGYGPGFFRIAGEIHEGGALVAEGKLLPWQGPDDEAPLMALEGRVDILLVGTGESLTPLPNRLQS
R
LEAAGLGVELMSSPSAARSYNVLLAEGRRVAVALVAVP
>OLS47965.1 heme exporter protein CcmB [Rhodovulum sulfidophilum]AB14
MIALLQRDLRLAIRAGGGFGLGLAFFLIIVVLVPFGVGSDSGLLAAIAPGILWVGALLACLLSLDRIFALDHEDGSLDLL
ATAPIPLEGVVAMKAAAHWVTTGLPLTLAAPGLGLLLNLPAAGYGWLAASLALGTPALSMIGSFGAALTVGLKRGGLLL
S
LLVLPLYVPTLIFGAEAVARGIDGRDTGTPLLMLTAITLAAMALLPFAAAAAIRINLR
>OLS47966.1 transcriptional regulator [Rhodovulum sulfidophilum]AB14
MSSLWEYANPQKFMRTSSVLLPVFSVAAAICLGVGLVWGFFLTPEAENFGSTVKIIYIHVPAAMMAINAWVMMLVTS
LIW
LVRRHHVSALAARAAAPIGMTMTLIALITGAIWGEPMWGTYWVWDPRLTSFMILFLFYLGYIALWEAIENPDAAADLT
SV
LCIVGSVFALLSRYAVNFWSQGLHQGASLSLDKKENVADVFYIPLLFCIAGFVCLFVALVLLRTRTEIRARRVRAMLARE
RMA
>OLS47967.1 heme exporter protein CcmD [Rhodovulum sulfidophilum]AB14
MIPDLGKYAFAVLSSYAVTMGLIAVLIAVSLWRGARVRRQLAEIEARRNADV
>OLS47968.1 aconitate hydratase 1 [Rhodovulum sulfidophilum]AB14
MPITVGQDTAKTRKTLSVGSASYAYYSIPAAEAAGLGDFSRLPAALKVVLENLLRFEDGKTVTVDDIRAFSDWAANGGK
T
AREIAYRPARVLMQDFTGVPAVVDLAAMRDGILGLGGDAQKINPLNPVDLVIDHSVMIDEFGNPRAFQMNVDREYER
NIE
RYTFLKWGQKAFNNFRVVPPGTGICHQVNLEYLAQTVWTDTDQSGDQVAYPDTLVGTDSHTTMVNGLGVLGWGV
GGIEAE
AAMLGQPVSMLIPEVVGFRLTGSMVEGTTATDLVLKVVQMLRQKGVVGKFVEFYGEGLAHLPLADRSTIANMAPEYG
ATC
GFFPVDEETLRYLTQTGRDKDRIALVEAYAKANGMWRDADYDPVYTDTLHLDMGTVVPAISGPKRPQDHTPLTNAAQ
SFF
NVVSDYRGIDESEEARDMLAEAPTPDLDADPRKSAKVEGQDYELHDGSVVIASITSCTNTSNPYVMIGAGLVARKAREL
G
LNRKPWVKTSLAPGSQVVSEYLEAAGLQEDLDAIGFNLVGYGCTTCIGNSGPLAEEISKAINDNDLIATSVLSGNRNFEG
RISPDVRANYLASPPLVVAYAIAGDMNIDLTRDPIAQDKDGKDVFLKDIWPSQAEIAELVEKTVTREAFQSKYADVFKGD
EKWQAVETTDSETYDWPASSTYVQNPPYFQGMGTEPGEITDLENARPLAILGDMVTTDHISPAGSFKETTPAGKYLIER
Q
VPVREFNSYGSRRGNHEIMMRGTFANIRIRNEMLSGVEGGYTKGPDGAETSIFEAAMAWQEKGVPLVVIGGIEYGAG
SSR

DWAAKGTALLGVKAVIAESFERIHRSNLVGMGVIPFEFTGGDTRKTLGLTGEESFSIKGLAGDLKPQAEVPCTISYADGT
EKTVTLKCRIDTAIEKEYVEHGGVLHYVLRNLAKAD
>OLS47969.1 DUF1223 domain-containing protein [Rhodovulum sulfidophilum]AB14
MRAVLTVLLAGSVFFAGPARSDPSPVVVELFTSQGCSACPPADALIAELAKRDDVIALALHVDYWDYIGWKDIFADPAF
T
RRQKAYAHAAGKRSIYTPQMVVEGADHVVGYRPMDLAQLIEAHALADSHVELGLARQGDMVTVTAVSAEPFGEDVV
LQIV
RYRPEQQVDIKRGENAGKSLRYANIVEAWTPVARWNGAEPLSVSVQAAGDEPIVAILQRPGPGAILAAARLR
>OLS47970.1 lauroyl acyltransferase [Rhodovulum sulfidophilum]AB14
MGKRNKPSRRLGDWLTDRVARLLIATALALPYERRVPFGGWVTRRLIAPIAGYRDRAEANLAMIWPDMPAPGRRRIA
DQV
ADNAGRTLIENYSTEAFLARMAGTEPKGPGLAALAAARKAGRPAILVTGHFGNYEAARAALVARGYEIGGLYRPMANP
FF
NAHYVRTMEAFGGPVFPQGRRGTTGFVRHLRSGGMLVLLTDQHARRGAELRFMGQPALTALSAAELALRYDAELIPFY
AT
RCPDGLSFEIEIEAPIARATPREMMQALNDSLEARVRRHPEQWFWIHRRWKAH
>OLS47971.1 adenylosuccinate lyase [Rhodovulum sulfidophilum]AB14
MIPRYARPEMTDIWEPATKFRIWYEIEAHACDAQAELGVIPKENAEAVWTAKDVVFDVARIDEIEAVTKHDVIAFLTHL
A
EHVGADQARFVHQGMTSSDVLDTCLNVQLMRAADLLIADMDKLLAALKRRALEHKDTVRIGRSHGIHAEPTTMGLTF
ARF
YAEMERGRRRLLAAREEIATGAISGAVGTFANIDPAVEEHVCRQMGLKPETISTQVIPRDRHAMFFATLGVIASSMENIA
IEIRHMQRTEVLEAEEFFSKGQKGSSAMPHKRNPVLTENLTGLARLVRMTVVPAMENVALWHERDISHSSVERGIAPD
AT
VTLDFALNRLAGVIDKLVIYPENMLANMNKFKGLVMSQRVLLALTQAGVSREDAYRLVQRNAMKVWEQGKDFKEELL
ADA
EVTAALSPAEIEEKFDLGYHTKHVDTIFARVFGAE
>OLS47972.1 hypothetical protein BV379_06585 [Rhodovulum sulfidophilum]AB14
MPGLEDFEGSWRLERRIDDRLTGQVLRLRGRAVFAPDGDEGLVYDETGRLEIPGQAPAVATRRYLWRARQAEGLEVLF
ED
GRFFHFISPGARPEARHDCAPDLYRVCYDFSAWPCWSSSWEVRGPRKNYAMESGYRPES
>OLS47973.1 biotin transporter BioY [Rhodovulum sulfidophilum]AB14
MAYALTQKVLVDNIMPRNGAQRMVREAALVVAGIAVLALAAHIKVPMFPVPVTMTTFAVLTLGAAYGPRRGLITILGY
LG
VGALGFDVFAGSAAEASGLTYMMGSTGGYLVGYALAALTLGTFARAGWDRSMGRMALAMLVGNVVLYIPGVIWLG
QLYGW
DQPILAWGVTPFLLGDALKLALAALAVPAVWRLIGRD
>OLS47974.1 type I glutamate--ammonia ligase [Rhodovulum sulfidophilum]AB14
MSKNKVLETIKDEDVEYVDIRFTDPRGKLQHVTVMADQVDEDFLDEGFMFDGSSIAGWKSIEASDMKLMPDPDSAYI
DPF
YAEKTLCVHCSVVEPDTGEAYDRDPRGTAEKAEAYLKSSGIGDVAYFGPEAEFFLFDDVRYSVTPNKVAYAIDVEHAAW
N
TDTEYESGNLGHRPTYKGGYFPVNPSDEGQDIRSEMLSTMKQIGMKVDKHHHEVATCQHELGMIFDSLKNQADNLQ
KFKY
IIHNVALAYGKTATFMPKPIAGDNGSGMHCNMSIWKDGKPLFAGDQYADLSNEALWFIGGLLKHAKTLNAFTNPSTN
SYK
RLIPGFEAPVLRAYSARNRSGCIRIPWTESPKAKRVEARFPDPSANPYLCFSALLMAGLDGIRNKIDPGEAMDKNLYDLP
PEELDGIPTVCASLREALDSLEKDHDFLLAGDVFTKSQIQGYLELKWEEVYAFEHTPHPMEYKLYYSC

>OLS47975.1 transcriptional regulator [Rhodovulum sulfidophilum]AB14
MKKIEAIIKPFKLDEVKEALQEAGVQGLSVIEVKGFGRQKGHTELYRGAEYVVDFLPKVKIEIVLPDDQVEAAIEAIVAA
AKTEKIGDGKIFVSPVEQAIRIRTGETGEDAL
>OLS47976.1 bifunctional ADP-dependent (S)-NAD(P)H-hydrate dehydratase/NAD(P)H-hydrate epimerase [Rhodovulum sulfidophilum]AB14
MSELLTAAQMRAIEAEAMARGDADGRTLMERAGEGVVDAALAHWPDLGTVPLSALVLCGPGNNGGDGFVVARLLT
ERGWQ
VELFLFGAPDRLPPDARLNHDRWAELGRIRPWDDAELADRLDAGGCDLVIDALFGSGCSRGIEAIAGCTAAALGRFCAA
D
GRVLAIDMPSGLCTDSGRDLGGVLPATLTVTFHRARIGQFTAEGSAFCGRIVVADIGLDPGPCPGSVTLVGAPGPEIGKV
GAVGGHKYSHGHALVLAGGAGRTGAARLAARGALRVGAGLVTLAAPGEALAECAAHLTAAMLRRVDDVGDLLALLE
DPRI
SALGLGPGFGTGSREAELLRLALDSDRPLVLDADGLSLLAQLEAPFSRLHGDCVLTPHGGEFARLFPDLAEKLAGRPPHG
PGFSRIDAVRAAAARSGAVVLLKGADTAIAAPDGRAAMHAALYDRAAPWLATAGAGDVLAGIVAGLLARGRPPFEAA
SLG
AWLHVEAARVFGPGLIAEDLPESLPAVFRGLGL
>OLS47977.1 hypothetical protein BV379_06610 [Rhodovulum sulfidophilum]AB14
MGPRRAMSVMRLLALIAFALAGSGPARASAPCPMHDWLVWIYVEGETLARAGPGMFRLEEARSLSKAIASADPASLA
RVM
RKAGLEDGLPVVSGYIELVADRLRFVPSPPEAGTVVRRMQGYLRELRCRADPAWRAGLELATAGGLAKALGSGGTSGA
RP
REPTPHRTGKPPAPQPVIADTAGQDRIDLSPPETGPSDPYPPSRDRDVNTEERLRGQPDFSQEAVNDGQLGTRLPAMS
KL
LKAARAAPWLAYAAAGIGGLALAGATTVYVLRHMRRRTPRTLCHMNTYLRLPDGRTPAVAVDISPNGAKLHLKTVPEP
GQ
RGTLELSGLEVGCRVTWSTTYFVGLAFLKTIDKAEFENLLQYHRWDASSSDMPDFKGSAAAAMDHAAKA
>OLS47978.1 beta-ketoacyl-[acyl-carrier-protein] synthase II [Rhodovulum sulfidophilum]AB14
MRRVVVTGLGMVSPLACGVEETWKRLLEGQSGIGPITRFDASRLVTGYAAEIPFGDGSDGTFNPDDWMEPKERRKVD
DFI
LYAMAAASQAVADSGWTPETDAERERTGVMIGSGIGGLNSIADTAVLIQKKGPRRVSPFFIPSALINLASGQVSIRFGFK
GPNHAVVTACSTGAHAIGDAARLIQWGDADVMVAGGTESPISEIGIAGFNACKALSTKRGDAPETASRPWDAERDGF
VMG
EGAGVVILEELEHAKARGAKIYAEVLGYGLSGDAYHITAPAEDGDGGFRSMQAALKRAGLVPADIDYINAHGTSTMAD
TI
ELGAVERLLGEAAERTTMSSTKSSIGHLLGAAGAVEAIFCILALRDQIAPPTLNLDTPPEGTRIDLAPKTAVKREIGLAL
SNSFGFGGTNASLVMGRGDR
>OLS47979.1 branched-chain alpha-keto acid dehydrogenase subunit E2 [Rhodovulum sulfidophilum]AB14
MWKAVASNALTLFIVVLLFAGGLLIWAKQKYAEPGPLDTAICLRVEPGSTMVGVSRDLGAQEAVRHPLIFRLGARYAEK
D
DDLKAGSFLLPAHASMEEIVDLVTRVGRSTCGTDVNFRIGIASAELQVRTFDPATGRYEEVLAFDPGAGEVPTEYAGLM
A
EPDLRYRVTLAEGVTSWQVVDELKQADFLDGDIAAVPAEGSLAPDSYEVTPGEERETLLAEMAARQTALLDRLWETRA
EG
LPLADKEEALVLASIVEKETGVPGERRRVASVFVNRLERGIRLQTDPTVIYGLTMGKAPLGRGIRQSELARRTPYNTYQI
DGLPPTPIANPGRASIEAVLNPETTEYLYFVADGTGGHAFARSLDEHNRNVARWRAIEAGRGGN
>OLS47980.1 DNA repair protein RecN [Rhodovulum sulfidophilum]AB14

MLRSLDIRDMLIIDRLELGFRPGLNVLTGETGAGKSILLDSLGFVLGWRGRADLVRAGAAQGEVVAEFELPAGHPAHAV
L
AEAGLEAGDELILRRINTAEGRKTAWVNDRRVSGEVLRRLSDTLVELHGQQDDRGLLNPRGHRQLLDAFAGLETELDA
VR
AAWRARARAERAVAEAETRIAELRAEEDFLRHAVDELDALAPEPGEEADLDARRRTMQAAERIREDIARAHAALGPGE
GA
EGRMGDALRWLEGAADRAEGRLDAPLAALSRALVELGEAVQATEDCLQALDFDPNALETTEERLFAIRALARKHGVQP
DD
LSAFAADLHARLAALDAGAGDLAGLGRAAAEARAGYDAAAAKLGAARRAAALRLDAAMSDELAPLKMERAVFSTVIS
DAE
PGPDGRDAVAFTVATNPGAPAGPLNRIASGGELSRFLLALKVCLVRGASPLTLIFDEIDRGVGGATADAVGRRLAALARS
AQVLVVTHSPQVAARGAHHWRVEKRVEGEMTRSIVTPLDSPARVDEIARMISGDTVTDAARAAARSLLED
>OLS47981.1 outer membrane protein assembly factor BamD [Rhodovulum sulfidophilum]AB14
MATSSRRIGAVAAIMVLSLTAACDPIGSRKPPPLETFDAPTIYQMAETKLEKDRDLEEAARLYGEVERLYPYSEWAKRAL
IMQAYTYHQAKDYETARAAAQRYIDFYPADEDAAYAQYLLALSYYDQIDLVGRDQGLTFQALQALRTVIERYPDSEYAQ
S
AILKFDLAFNHLAAKEMEVGRYYLKRKHYAAAVNRFRVVVEDFQTTTHTAEALYRLIESYLALGLDNEAQTAAAILGHNF
QGTDWYADGYKLLQAKGLEPEAKGEGWLRSVYRQMIKGEWL
>OLS47982.1 UDP-3-O-[3-hydroxymyristoyl] N-acetylglucosamine deacetylase [Rhodovulum sulfidophilum]AB14
MQTTLKSPITFTGTGLHTGRPVRLTIHPASAEYGLWFRRTDIEGPDALIPARWDAVEPSRLCTKLANGRASVMTVEHLM
A
ALSGCGVHNALIEIDGPEVPVMDGSSAPFVQGILSRGLRRLAAPVRVLRILEHVEVSEGAARASLGPADTLEIDFEITFA
DAAIGHQAKRLNMANGAFVRELCDSRTFCRNADVEAMRAAGLALGGTFDNAVVIDGDKVLSPGGLRHRDEPVRHK
MLDAL
GDLALAGAPILGRYRGERAGHALTNRLLRTLFAQPGAFRLETCSTDQGAALPGFGLNSADLKAVA
>OLS47983.1 cell division protein FtsZ [Rhodovulum sulfidophilum]AB14
MALNLTMPEQEELKPRITVFGVGGAGGNAVNNMIEKRLEGVEFVVANTDAQALQQSNAQARIQMGVKVTEGLGAG
ARPSV
GASAAEESIEQIVDHLAGAHMCFITAGMGGGTGTGAAPIIAQAARELGVLTVGVVTKPFQFEGTKRMRQAEEGVENL
QKV
VDTLIIIPNQNLFRIANEKTTFTEAFSLADDVLYQGVKGVTDLMVRPGLINLDFADVRSVMDEMGKAMMGTGEADGE
DRA
IQAAEAAIANPLLDEISLNGAKGVLINITGGYDLTLFELDEAANRIRDVVDQDANIIVGSTLDPDMDGRMRVSVVATGID
AAARIDEVPLPRRPMREPLHRPAEVQEAPAAAPAPAAAPAAQAQRPAVAARETVQEPTQEPSLFSGFDPANAGFAAD
ERA
ETARDGADLPPPAYQPEENPEAETFVAPQPRNAGEPTPEALARLRAAVAKTPARQPVHTPMAATAQDDPRGDKGRF
GIGS
LINRMSGHAPEQTERNPHTFRQQPPLYSRGQVAQQEEEDLDPDQERIEIPAFLRRQAN
>OLS47984.1 cell division protein FtsA [Rhodovulum sulfidophilum]AB14
MTDLYHSQRAMRRMRKLAMDRGVIAILDVGSSKVACLVLRFDGIAPKGGVEQMGALAGQSRFRVIGAATTRSRGVRF
GEI
HGMNEAERAVRTALQAAQKMAQVRVDQVIACFSGGEPRSYGLDGQVTVLDDAVAEQDVARVLAACEMPEIGASRE
VLHAQ
PVNFALDHRSGLVDPRGQVGHRLACDMHMLTVDDGAVHNLLHCIKRCDLEPAGLASSAYASGISALVEDEQELGAAC
VDL
GGGATGISIFIKKHMIYADSVRMGGEHVTSDISKGLHVPLSTAERIKTLYGGVVATGMDDREMIGLGGDSGDYETDSRS
V

SRAELIGIMRPRVEEILEEVRARLDAAGFEHLPSQRIVLTGGGSQIPGLDGLAARILGNQVRLGRPLRVHGLPQSATGPG
FAALVGLCLFAAQPQDEWWDFEMPMDRYPARSLKRAVRWFKENW
>OLS47985.1 cell division protein FtsQ [Rhodovulum sulfidophilum]AB14
MRPVMPPYRDPAPSRWAYRAQRLWLTPMVRRAVLKGLPLVVLAAIPAIWLADADRRAALGDGLAEIRREIETRPEFM
VKL
LVIEGASDPIDREIRAIVPLNLPASSFDLDLEAMRQAVTALDAVKSAELRIRPGGVLEMKIAQREAALVWRSRDGLSLID
AGGRRVAALDSREDRADLPLIAGDGAGKAAPEAMALFAAAAPISDRVRGLVRMGERRWDVVLDRDQRILLPETGAVA
ALE
RVIALEDVSGMLARDIEAVDLRNPERPTLRLTEAAIEDFRKIRATQAGAVN
>OLS47986.1 D-alanine--D-alanine ligase [Rhodovulum sulfidophilum]AB14
MAGESSRTAPRVAVLMGGPSSEREVSLSSGRECAAALRGEGFDVIEIDAGQDLPGQLVSAAPDIVFNALHGRWGEDG
CVQ
GLLEWLKIPYTHSGVLASALAMDKQRAKDAYRDAGLPVVDSVLAAKAEVSARHVIAPPYVVKPNNEGSSVGIHIVHEAA
N
APPQLGPEMPGIVLVESYASGRELTTTVMGDRALGVTDILTEGWYDYHAKYVQGGSRHVVPAELPEEVSEACLDFALR
AH
RALGCRGLSRTDFRWDESRGTDGLILLETNTQPGMTPTSLAPEQAAHCGISFGRLCRWLVEDASCDR
>OLS47987.1 UDP-N-acetylenolpyruvoylglucosamine reductase [Rhodovulum sulfidophilum]AB14
MTQMPEPRGRLTPGKALSELTWLRVGGPADWLFQPADTEDLATFLAALPAGMPVFAMGVGSNLIVRDGGIRGVVIRL
GRG
FNAISVAGPRVTAGAAALDAHVARRAAEAGVDLSFLRTIPGAIGGAVRMNAGCYGRYVADVLVSARAVTRSGEIVTLSP
G
DLRLGYRQSHLPEGWVITEATFEGRPGAPEALAALMEEQIARRDASQPTRARSAGSTFRNPAGFSSTGRADDSHELKA
WS
LIDAAGLRGARRGGAQMSEKHSNFLINADGATAADLEGLGEEVRKRVFQSSGIELEWEILRVGEPAPGNP
>OLS47988.1 hypothetical protein BV379_06665 [Rhodovulum sulfidophilum]AB14
MTGPTLLLAYGSWAAGPLVAYAALSHGLMRNAIGFTIMFGLYTSSVWAIWGGLKLQATGNGPAVLAPSAVLLPWGA
VALV
SAVLYALGAWIGGGDG
>OLS47989.1 hypothetical protein BV379_06670 [Rhodovulum sulfidophilum]AB14
MTLSLLLACLWVLLAAVIGVLPEKLHWIAAFSLIASGIPLLGFVTYQSGPLWGLIALAAGASTLRWPLLYLGRWLNRRRR
STGE
>OLS47990.1 hypothetical protein BV379_06675 [Rhodovulum sulfidophilum]AB14
MVSCPVIAICGPAPLAGIPGLRNMRRTAPPVTAGLLLAAAAIPVPPATAQAAFGPGAGLTGPPRVSAFRPVARPAGTG
GA
PEALSRDRGAEGGEQ
>OLS47991.1 UDP-N-acetylglucosamine--N-acetylmuramyl-(pentapeptide) pyrophosphoryl-undecaprenol N-acetylglucosamine transferase [Rhodovulum sulfidophilum]AB14
MSAPLLVIAAGGTGGHMFPAQALAETMLARGWRVKLSTDARGARYAGGFPAAVEIETIAADSPDRGGPVARAVAPLR
LAA
GVAAMLWRMLRDRPAVVAGFGGYPSFPALTAATLLRRPRMIHEQNGVLGKVNRLFAPRVHAMACGVWPTELPEGV
EAFHT
GNPIRAAVRERAGAPYIPPGDYPMSLVAIGGSQGARAISESVPAAIAALPAEMRGYLRVSHQAREEDLDRVVAAYEAAG
V
AAEVRPFFDDIPARLAEAQLVISRSGASSVADIAAIGRPAILIPYPHAAGDHQTANARGLVEAGAAILIPEKALDPVALS
DQIATVLGQPKGAMQMAVAAMRCGKPDAVDGLAEIVEALGGEGK
>OLS47992.1 cell division protein FtsW [Rhodovulum sulfidophilum]AB14

MTEMVYGTAPIRAGEPVLPRWWRTVDKWAMSFVLILFGVGMLLGLAASPPLAEKNGFPPFYYVERQAVFGGLALCA
MLVC
SMLSPTMVRRLGVIGCGAALLGLVLLPVFGTDFGKGAVRWYSLGFGSVQPSEFLKPGFIILTAWLMAAAQEVEGPPGK
TM
SLALAVIVVAFLALQPDFGQAALILFAWGVMYFVAGAPFMVLVAVAGGVVFAGTIAYENSEHFARRIDGFLNPEIDPRT
Q
LGYATNAIREGGFFGVGVGEGQVKWSLPDAHTDFIIAVAAEEYGLVLVAVIIALYCAIVIRSLFRLLKERDPFIRIAGTG
LACTFGVQALINMGVAVKLLPAKGMTLPFVSYGGSSLIAGGIMVGMLLALTRNRPQGEITDLLRRRTRE
>OLS47993.1 2-hydroxy-acid oxidase [Rhodovulum sulfidophilum]AB14
MSKPDALTALGEMLGDRLSRSKSDLDLHGRNETYFPFLPPDAVAYPETTDEVAQIVRICAAHGVPLIGWGRGTSLEGHA
L
ALQGGVTIDFSRMNRVLKVNSEDMDVVVQPGLTREDLNRELRATGLFFPVDPGANASLGGMASTRASGTTAVRYGT
MRDN
VLALEVVLPDGRVIRTGTRARKSSAGYDLTGLFVGSEGTLGLITELTLRLHGQPEAISAAVCAFDEIGAAVDTVIQTIQL
GIPMARIEFVDAATAAAFNAYASADMPEKPHLMVEFHGSAAGVAEQAESFGEVVAENGGEGFKWSSRPEDRNALWT
MRHN
AYYACLGLRPGATALVTDMCVPISRLAEAVEETRADIAQASIPGPILGHVGDGNFHAILLVEPDNAAELAEAKALAHRM
V
ERALRMGGTVTGEHGIGMGKLDYMEEEHGAAWDVMGQIKRALDPMTLMNPGKMVRAN
>OLS47994.1 voltage-gated chloride channel [Rhodovulum sulfidophilum]AB14
MKRRNSRAFGTVFRWLNGVLRVLRVHGPSQVQFWFIAAAIGIAAGFAALGFRLGIDVLQRALYGVDDPHRLHSYAQTL
HW
GWILVIPACGGLVVGLLLHRFTPDARVRSVADVIEGAALNEGRVEVKAGLASALASLITLSSGGSTGREGPVVHMAGVV
A
TWVSNRIHADGITGRDLLGCAVAAAVSASFNAPIAGTLFALEVVLRHFAVHAFAPITVAAVAGTVINRIEFGGVPEFALP
QTDRLAFYGELPAFLMLGLLCGLVAVILMRAIFWAEGIGDAVQRRTRMPRWLRPAVSGLGLGALAIWFPHIIGVGYETT
S
AALTGTLVLHEAVVFCLLKSVAVSMTMAGRMGGGVFSPSLMVGALTGLSFGIIATSAFPNVSGAETLYALAGMGAVAA
AV
LGAPISTTMIVFEMTGDWQTGLAVMVAVSLSTALASRLVDRSFFLTQLERRGKRLAAGPQSYLLAALTAGVLMRPKESP
G
AASPGACAELIGQGVMFEADTTLVEALPVFEKWGHGFVPVVARAAAGREPELLGALFHVDALRAYSRALATTAAEEHS
>OLS47995.1 hypothetical protein BV379_06705 [Rhodovulum sulfidophilum]AB14
MSDHITIHKAEGAWVVRAGGAVIAESRNALELTEGGMGPVIYFPREDVAMAFLDRSDSRTACPHKGVASYFTLRAKSY
DI
ADAAWSYEEPNTGLERIAGHLAFYADKVTVEQL
>OLS47996.1 X-Pro aminopeptidase [Rhodovulum sulfidophilum]AB14
MFQNFDSTTRPEDGPVRLAQLREEMAREGLSGFLVPRADAHQGEYVAPHDDRLAWLTGFTGSAGIACILMDRAGVFI
DGR
YRVQVKAQVDLAHFTPVPWPETKPGDWLKAALPQGGAVGIDPWLHTPRDLDALTGALAGTQVAIRETANLVDRIWA
DQPA
PPQGPITPYPEELAGESHADKRARIGRLLADAGQSAAVLTLPDSIAWLLNVRGSDIPRNPVPHTFAILHDDGRVTLYADP
AKVSDALKAHLGPEITLRPAGAFGPGLRSLLGRVRVDPASAPLWVLRQLEDAGIEIALAEDPCILPKARKTAAELEGARA
AHLRDARAMIEFLAWIDAEAPKGGLTEIDVVRGLEGFRAATGKLRDISFETIAGAGPNGAIVHYRVTEASNRAVAPGDLL
LVDSGAQYLDGTTDITRTIAVGPVPEAAKAPFTRVLKGMIAISRARFPKGLAGRDLDALARNALWQAGQDYDHGTGHG
VG
AYLSVHEGPQRIARISTVPLEPGMILSNEPGYYREGAFGIRIENLIAAREAPALTGGDDRRMLSFETLTWVPIDRRLILP
ELLDAGERAWLDAYHAEVAKRAGDGLSERARAWLAEATQPL

>OLS47997.1 cobaltochelatase subunit CobT [Rhodovulum sulfidophilum]AB14
MTKPTDNPADPFKKALAEATKVLADDAELGVSYSVDPPGLTDDAVRLPQVSRRMTRDEVLLARGTADAYALRHKYHD
GAT
HARYRPSGQIACELYEAMEMARCEAVGARVMPGTAGNIDAMIAAEAARKGYAQAREASEVPLAVAAGYIVRHLATGR
PLP
KGAAQAMELWRGFIEEQAGPALDGIDASLGDQAAFARLSRQVIADLGYADQLGDDPDADQNDEEDNSAETEDEAPE
EYGE
EESQDSDEADASPEQSQDEMQDAAQAHVNPDDIDDMEMGEETELPDGEAPREPPPPAPPSEADPDYKIFSAGFDEEI
GAQ
DLAEPAELERLRAYLDQQLDPLKGAVGRLANKLQRRLQAQQNRSWEFDREEGILDAGRLARVVANPTTPLSFKVEKDT
EF
RDTVVTLLLDNSGSMRGRPISIAAICADVLARTLERCHVKVEILGFTTRAWKGGQSREQWLAEGRPQSPGRLNDLRHIV
Y
KNADAPWRRARENLGLMMKEGLLKENIDGEALEWAHKRMLMRPEARKILMVISDGAPVDDSTLSVNAANYLEKHLR
DVIA
MVERRKAVELIAIGIGHDVTRYYQRAVTITDVEQLAGAMTEQLAALFDKDPRARARIFGIRKAS
>OLS47998.1 hypothetical protein BV379_06720 [Rhodovulum sulfidophilum]AB14
MTRVTLVAATGFATAFILAAAPAHPAPPGTPWKRAEVFATCSGRLAAVSARQQAEHDPDWPRTVSQRDMFNLMLEA
TLPE
AIRFGVPRREPVLWRSAGWSEMAGLLADIAYSGDRGRAERARAALARRLSDCTGLLLGG
>OLS47999.1 hypothetical protein BV379_06725, partial [Rhodovulum sulfidophilum]AB14
MRRPDAARSRPAVALAVLFLAAPTSFAQGADPTFPAIDCAALWQATADFRTRYAIAEGSPAEAQAMARAFREAALAE
GAD
RNGVDDRIAALRPVYLLLLQRHILDGEGRARDQYVRLSGLCDDLGRAAGLKGHRHAPLYRPTE
>OLS48000.1 cobaltochelatase subunit CobS [Rhodovulum sulfidophilum]AB14
MRRAGACRGFLLRTLRICFKTASDRTSDGVMMNDVVKPTEEISVREVFGIDTDMIVKGFEAPTDRVPDSDPTYKFDPDT
T
LAILAGFTHNRRVMIQGYHGTGKSTHIEQVAARLNWPCVRVNLDSHISRIDLIGKDAIKLKDGKQVTEFQEGILPWALR
N
PCAIVFDEYDAGRADVMFVIQRVLEVDGKLTLLDQNEVITPHPSFRLFATANTVGLGDTTGLYHGTQQINQGQMDRW
SLV
ATLNYLSHDAETAIVLAKCPHYNTDKGRKTINHMVTVAELTRTAFMNGDLSTVMSPRTVIAWAQNAEIFRNVGFAFRL
TF
LNKCDELERQTVAEFYQRLFDEELPESAASMSLG
>OLS48001.1 molecular chaperone DnaJ [Rhodovulum sulfidophilum]AB14
MTKTDPFGFDISVSADKKRRTRGRRGMSGAFETSRRKCEHPGCDLDGQYRAPKSPDQLDEYFWFCKDHVREYNLKW
NFFN
GVTEEEFEAQIDKDRVWDRQTQPFGNKDEQRAWARLGVDDPHQVLGENATRNPGKTGTPARRLPATERKALELLEA
QDNW
TKAEIRKQYKSLVKDLHPDMNGGNRADEERLQEVVWAWDQIKDSRSFK
>OLS48002.1 BolA family transcriptional regulator [Rhodovulum sulfidophilum]AB14
MGRVEQIREKLEQAFQPTTLEVLDESEKHRGHAGYQEGGESHFQVRIAAPAFATMSRVARERAVHKALGPELIGQIHAL
A
IEIET
>OLS48003.1 hypothetical protein BV379_06745 [Rhodovulum sulfidophilum]AB14
MIMPAKYEYRVLPAPERGRRGRGLKGPAERFAHALEETMNEMAAEGWIYLRAESLPSEDRQGLFGGRSRALRNVLVF
RRA
REVEANRDAGRKEGRDPAVHHGSPEEIAASAAASVQALPDRTYPPLGAPSRGSVPPRPEHALGGAPPRSDED

>OLS48004.1 protease [Rhodovulum sulfidophilum]AB14
MTSIAKSKILIMATDGFEQSELEVPLTELRAKGAQVDIASPGGAPITGWKNRNWGTTQETTKSLTDVRVQEYDALVLPG
G
QMNPDSLRVAPEAVRLVRDFVSEGRIVAAICHGPVLLVEAGAVEGRKMTSCRSIRTDLRNAGAIWVDETVVTSNGIVTS
R
AAQDLGAFVAKIVEEIEEGRHSRRAA
>OLS48005.1 lytic transglycosylase [Rhodovulum sulfidophilum]AB14
MHRLALVLAFSLLTLGCSQADMADRGRGLPTMRWDHMPEATDWTRASLDALSLYGDGLTETVPADIETWCPAYEKA
RPAQ
RRAFWAGLFSALAKHESTWNEAAVGGGGRWFGLLQISPATARYHGCAATSGSALTDGKANLSCAVRIASSAVARDGV
VAA
RGRGVAADWGPMTVASKRAEIAAWTRAQSYCRG
>OLS48006.1 TetR family transcriptional regulator [Rhodovulum sulfidophilum]AB14
MTRPRGRPPRADAPVSRDEMLTAALTLLDDEGAEAFTMRALAKRLNVNPMTIHHHFGGRDGLIGAMSERLHARISAP
QEG
TATERIRGLLKAYHAQVLLHPGLTMLIFSRPAVFPEQAKRITEELSALLAKAGLPDRKRRLWLDILVDVTHGAALATAMA
GRKAVDDPAGDQGYGATLDELLNCLGDEAALRTESG
>OLS48007.1 deaminase [Rhodovulum sulfidophilum]AB14
MITGHVFIATSLDGFIARADGDIAWLLERDEPDEDHGYDTFIASIDAILMGRGTYEKIRDIRPWPYTRPVVVLSATMTED
DLPEELGSKVRVTDRSPTEAMRMLQSEGHRRVYVDGGRVIQSFLRAGLIADMVITTVPVLLGEGRRLFGSLAGDIRLQH
E
KTKSYPSGLVQSRYRIAA
>OLS48008.1 DUF1413 domain-containing protein [Rhodovulum sulfidophilum]AB14
MTADQEAMIREALAALPAGEFHLPDAVGPRWDELWIGEKVGLGNDFLKHVRQGRFEGVEDTGRKEHGLHVYLKTG
>OLS48009.1 glycerol-3-phosphate dehydrogenase [Rhodovulum sulfidophilum]AB14
MPTRIAEGTATDTAEDKLADLLIVGGGINGCGIARDAAGRGLSVTLAEMDDLASATSSASTKLFHGGLRYLEYFEFRLVR
EALIERETLLSAMPHISWPMRFVLPLDREMRFDSSTPASRLLSRAMPWMTGRRPAWLIRLGLFLYDHLGGRKILPPTRSL
DLTTDPAGRPLKPRFRRAFEYSDCWVEDARLVALNARDAAERGAEILTRTRVVKARREDGLWTVTLRDCETGAETTRR
AR
ILVNAAGPWTADLIAGALQQAATDRVRLVRGSHIVTRRLFEHDRCYFFQGQDGRIVFAIPYETDFTLIGTTDAEHAAPDT
PPRCTEEERDYLIGFVNRYLAKPVSAEDIVWTYSGVRPLHDEGEGSASAASRDYVIRLDRAGAPLVNIFGGKITTYRRLA
ETALEKIAEIRPDLPGPWTAGVALPGGDVAVDGIDGLIATLRQDYPFLTEPWARRLVRAYGTEARGVLGDAQAAADLG
RD
FGATLTEAELIWLMDREFARRAEDVVWRRSKLGLRLSPDQIARIDDWMKDRRQRPAPE
>OLS48010.1 DeoR family transcriptional regulator [Rhodovulum sulfidophilum]AB14
MSQTFRAPDILDIARREGRVTVDGLAEHFGVTVQTIRRDLAELAEAGKLERVHGGAILPSGVTNIGYEARRGLNAEAKA
A
IAARCAASIPEEASVFLNIGTSTEAVARALLTHRNLLVVTNNMNVANILAENPGCEVVVAGGTLRRSDGGLVGALTMRA
I
QAFKVDYAVIGCSALDAEGDLLDFDIQEVSVSQTILAQARRAFLVADAAKFERSAPVRIASLRDLDEVFTDRPLPAPLAE
RCRGWNTRVDVAA
>OLS48011.1 hemolysin D [Rhodovulum sulfidophilum]AB14
MSRQDLPSAQDRASVDAPSQDGPARSGGQRRALLAGIAALVLAGAGYGGWYYWTEGRFFEDTDDAYVQADIARISS
RVQG
YVASLPVEENAHVRAGEVLVRLEDGDYRTALATAQSRVATADRRLARIDAQIDAARAAVRQAEADRDAAQAQLRNAG
NTL
ERVTGLAERNFAAQAQLDSATEGYDTAQASLAKAVAAVASAHAEVAVLVAQRAEAEGDKHELELAVDQAERDLKRTV
LRA

PADGILANLGLEIGDLVTAGTQLAALVPDDALYVEANFKETQMQGITPGARVEVTLDALPGRSFEGRVTSAAPATGSVF
S
VLPADNATGNFTKVVQRVPVRIALPPEAAATGQLRAGLSAVVAVDTRSGGAPAARLAAAR
>OLS48012.1 MFS transporter [Rhodovulum sulfidophilum]AB14
MTADEPQLTPRRVFAFVVMVFGMFMAILDIQIVSASLSEIQAGLAASPDEISWVQTSYLIAEVVMIPLSGFLARMMSTR
Y
LFVLSAAGFTASSFLCATSGSIEEMILWRALQGFLGGGMIPSVFAAAFTIFPASKRSVVSPVIGLIATLAPTVGPTVGGY
LSYTLSWHWLFLVNVLPGIGVTLGAFLLIDFDRPDWQLFDRFDWWGLLALAAFLGGLEYVLEEGPGNDWLADETVAA
FVV
VTVLGAIVSFWRAFTRDEPIVDLSAFGNVNFALGSAFSFVMGIGLYGLTYLYPLYLASIRGYDSLMIGETVFVSGLAMFV
SAPIAGFLSARIDLRLMLLAGFAGFAVSTWMLTGMTAEWDFNELLVPQILRGVSLMLSMVPINNLALGTLPRDKMKGG
SG
LFNLTRNLGGAVGLAVINTQLSDRGDLHYERLREAVSWTNDAAMRELSVMAANLSARGLDGQTGALVQMAARLRQ
QATVM
SFIDVFLMLALLFGGLAASAFFMRAPDPSGGGGAGH
>OLS48013.1 phosphodiesterase [Rhodovulum sulfidophilum]AB14
MIRIAHISDLHFGRTAPELLDPLIGALNAAGADLVAVTGDLTQRARDSQFRAARAFLNRLDPPWISVPGNHDVPLDNP
WM
RLLRPYARYRRHVSADLAPRARIGALTVQGFNTVDPLRWQRGKVRRRDLEQACQAFAEAPGPRLAMAHHPFEQAPGT
PKS
LMKHAGPSLEALARCGADIVLSGHLHRWRAEPFLTREAGRRVLQVHVGTGLSTRLRGQENDFALLAIEGDQIELTRMV
AK
EGAFRPASLAVFRKGLAGWTGV
>OLS48014.1 diacylglycerol kinase [Rhodovulum sulfidophilum]AB14
MPRSSAFCLIVNDRAGDHSGSGSRSDMLRGLCLQAGLKAEIWRPRRGQSLTGLAHEALDRGCGTLVAAGGDGTICAV
AEA
CHARDAPLGVIPQGTFNYFARGLGIPLEPEAAVAALAGGTKAEVHLGEVNSRIFLNNASLGLYPAILERRETTYRRWGRS
RVAAYWSVAQTLVGARRAMRLKMTLDGQRQELHTPLAFIANSAYQLRQFNLEGADALEDGGFALFTAPAGSQVEIM
RATL
GLALGTAQEGQDFDLHRAARIEIDPGRPRVLVARDGERQTMRTPLTIRRRDRPLRVLVPEAPA
>OLS48015.1 hypothetical protein BV379_06825 [Rhodovulum sulfidophilum]AB14
MTQIAEIAPVPFAWTRGLVSARAGALLADLFPRARDARAGECIGLVKRFIAFGRRSLTGHPAEALVVMGEDGRPVCLSA
R
HLDAEARQDLFRLEEILSAPGLLAYGALTPHASELHRATTPPRALELILGADVHLLRTLDWRADIAGRRRAGEPVARALA
ANLRLLSEHPGSPEAVEQLRDRLQVRAGLMLGLLSFDEPVLPAMAVIAA
>OLS48016.1 hypothetical protein BV379_06830 [Rhodovulum sulfidophilum]AB14
MAQAIISQDLTIEGDLVAKDSAVIVNGQVKGNITAKAIEISATGQVTGALNAEAITISGTISGRVTCTDLTLSASARVQA
DLTAESLTSSKGAKVSGRVEIAGR
>OLS48017.1 hypothetical protein BV379_06835 [Rhodovulum sulfidophilum]AB14
MIPAPNLQPNFDRELPDDLRRRAEILPVGPAAGFNGKRYGWIKMQFLRTDAETGEHRDFDAGHPCGE
>OLS48018.1 hypothetical protein BV379_06840 [Rhodovulum sulfidophilum]AB14
MILMPQELRKGAILAAHFTFFADESGHVTQEPGISDEVGPSQYLCMGGALIPKSRESEVAERLDEIQRKFKKKKFLHAAD
INHVQKIYLIREILALDIDVFGVVSDKNTLGGFRTRSRGNPQQYYNRTALYLLNMLGRHLRDRNIPGNAVEIVFERREHD
YEQLQRYIKKVAGNPTGDDVDSIPHISASSIKARKKEDSHIFSVPDIIAHAVFSAFDRNRNNFGITESRYLRELLTSSLH
ESSGLELYLIQSSKMKDICQPTSDLLRARGIFVKTRKPVQPSENLEATL
>OLS48019.1 energy-dependent translational throttle protein EttA [Rhodovulum sulfidophilum]AB14
MASYQYVYHMDGVSKTYPGGKKCFENIRLNFLPGVKIGVVGVNGAGKSTLLRIMAGWDKDFSGEAWAAKGARVGYL
PQEP

ELDPKLSVRENVMLGVAEKKAKIDRFNEVAMKVAEDYSDELMEEMTTLQDQIDAENLWDLDSQIDVAMEALRCPPD
DAPV
ETLSGGERRRVALCKLLLEAPEMLLLDEPTNHLDAETVAWLQQHLIEYKGTILIVTHDRYFLDDITGWILELDRGRGIPY
EGNYSSWLEQKAKRLAQEAREDKAKQKQLERELDWIRAGQKARQAKSKARIQAYEEMAGQSEREKLSRAQIIIPNGPR
LG
GKVIEIEGLKKGFGDRLLIEDLSFALPPGGIVGVIGPNGAGKSTFFRMLTGQETPDEGTIEFGDTVKLSYVDQSRDALDP
DKTVWEEVSDGLDIVVLGDAEVNSRAYCSSFNFKGGDQQKKVGLLSGGERNRVHMAKLLKSGGNVLLLDEPTNDLDV
ETL
QALEAALEDFAGCAVIISHDRFFLDRLCTHILAFEGEAHVEWFEGNFEAYEEDKIRRLGPDAVEPKRVKYKKFAR
>OLS48020.1 nicotinic acid mononucleotide adenylyltransferase [Rhodovulum sulfidophilum]AB14
MRAGFPKARAGMRIGLLGGSFDPAHEGHAHITRTALKRFGLDRVWWLVSPGNPLKAEGPAPMARRLAAAEAVMQH
PRVAI
TDLEARIGTRYTAETLERLMRLYPGVRFVWLMGADNLISFHRWERWRWILDNVPVGVLARPGQGVAARRARAALTH
ERYR
LDPTDAALLADSQPPAWCYLNMPMVDLSSSEIRARGDWARRG
>OLS48021.1 D-alanyl-D-alanine carboxypeptidase/D-alanyl-D-alanine-endopeptidase [Rhodovulum sulfidophilum]AB14
MKTSKTSFTRRLFLGGLLAAPASSALGAPLVRSLVPRPRPEQSGGIADPAQLVERARLTGKVAFAIADLETGALLEAHRP
QLGLPPASVAKSVTALYALDALGPEFRFTTRLIATGPIMGGRLEGDLVLVGSGDPTLDTDQLGELAAQLRANGVTEVAG
R
FLYHAGALPKIAAIDPDQPPQVGYNPALSGLNLNFNRVHFEWRRASDGYAVTMDARARRYSPEVGVARMLVVDRKAP
VYT
YASKGGIDEWTVARGALGAEGARWLPVRRPALYCAEVFQTLASAQGIAMARPEALAGSAEAFQIGRIDSAPLAEILRG
ML
KYSTNVTAEAVGLAATIARGGQPQDLAQSGQAMTEWMAARMGAQKARFVDHSGLGDASRLSPLELVRTLAIAAPEG
QLRP
LLKEVPLRDASGRPMRAHPVKVEAKTGTLNFVSALAGYIGQPGGRQMAFAIFSADEERRARIRPEEMERPQGGRAWL
TRA
RMLQHELLRRWAETYA
>OLS48022.1 hypothetical protein BV379_06860 [Rhodovulum sulfidophilum]AB14
MVPVDLPARFWPFADRFVASARIVLDRPKGSAHPRFPEIVYPLDYGYLDGTRAIDGDGVDLWRGSLPEPRVTGVIASM
DL
TKHDAELKLLLGCTEAEMARIDAFHNQAGFVAGLLIARPEGV
>OLS48023.1 2-dehydro-3-deoxyphosphooctonate aldolase [Rhodovulum sulfidophilum]AB14
MTDTETPLSAQDALVAIMVAVSASDETIRTAELVTIERMVNHLPIFGAYDQDRIRIVAQTVFDLFSEYDGLDALFGLVNE
ALPEKLNETAYVLACDVAAADGRLGETELRLLEEIRYELNIDRLAGAAIERAARARHMVL
>OLS48024.1 lysine--tRNA ligase [Rhodovulum sulfidophilum]AB14
MSNLRDAAMSSKAWPFEEARKLLKRAEKGAADKGYILFETGYGPSGLPHMGTFGEVLRTTMVRRAFEAISDIPTRLICFS
DDMDGMRKVPGNVPNQDMLQEHLQRPLTSVPDPFGEDDSFGDHNNAMLRRFLDTFGFEYEFYSATEFYRSGRFDDT
LKLA
TERYDDIMKVMLKSLRDERQQTYSIFLPIHPETGRVLYVPMKHVDAANHTITFDDEDGREWTLPVTGGKVKLQWKPDF
GA
RWAALGVDFEMYGKDHSTNTPIYDRICEILGGAKPEHFTYELFLDENGEKISKSKGNGLTIDEWLSYAATESLSYFMYQK
PKTAKRLWWDVIPKAVDEYHQQLRAYPGQEPKAQIDNPVWHIHGGNPPESRMVVPFAMLLNLASVAGATDKDGLW
GFIKR
YAPEASPETHPDLDQAAAFAVRYFHDFVAPLRQFRAPDAKERAAMEDLVARLKAWEGGQDAEELQSLVFAVGKDHG
FEPL
RDWFKALYEVLLGASQGPRFGGFIALYGVDETVRLIEDALARG

>OLS48025.1 pyridoxamine 5-phosphate oxidase [Rhodovulum sulfidophilum]AB14
MATRDPFRPADATACALARKLIAEARFGALAVLRDGAPFVTRIALAPGADGLPVTLISDLAPHTAALRAQPDCSLLVGEP
GPKGDPLTHPRLTLQARAAFATRDAERSALRETYLAQQPKARLYVDFADFHFVRLVPLSGHLNGGFGQAFRLTVEDLGA
>OLS48026.1 polyphenol oxidase [Rhodovulum sulfidophilum]AB14
MTLEILTSDALAPLRHGFFTRRGGASSGVFAALNCGGGSSDLSEAVAINRSRVAGAMGVQSDRLVTVHQVHSAEVATI
ET
PTATPMRADAMVTRQPDLALGVLTADCQPVLFADLSAGVVGAAHAGWRGALGGILEATLEAMEALGARREDTAAVI
GPTI
SQRAYEVGPEFLDAFLSEDPDSARFFAAGQGDRMQFDLPGFSLARLRGAGIGQAAWTRHCTYSDPERFFSYRRSVHVG
EA
DYGRLISTIRL
>OLS48027.1 methyltransferase [Rhodovulum sulfidophilum]AB14
MTETPLARLLARRIAATGPISIAEFMADCLMHPEHGYYSTRDPFGQSGDFTTAPEISQMFGELLGLWIAQVWLDQGAP
AP
FTLAELGPGRGTLMADLWRATRGVPGFHAAARLHLVETSPTLRARQRAALPDADIAWLDSVEALPDAPLFLIANEFFDA
L
PIRQFQRDDAGWCERLVGLDGARLALGLSAPLPLAALEHRLADTKPGDIVETCAVAEAITASLSARISTHGGAALIVDYG
GWHSLGDTLQALRTHRPEPVLDHPGEADLTAHVDFEALARAATGLRISGPVPQGTFLERLGIATRTEVLARNLAGDALA
G
HLAAHRRLTHPEEMGTLFKTLALTPPRTPDPPGLHS
>OLS48028.1 prolipoprotein diacylglyceryl transferase [Rhodovulum sulfidophilum]AB14
MQAVLSFPDLSPEIFSISVFGLDFALRWYALAYIVGIVLGWRLVVALMRRDRIWAGACPPMRPEQVEELLTWIVLGVILG
GRLGFVLFYKPFEYLQAPWEIPMLWHGGMSFHGGLLGVMIALVIFTRKQAIPLLPAADALALAVPIGLFFGRIANFINAE
LWGRPSDLPWAVVFPGPAAQYCPGWNSLPCARHPSQLYEAGLEGLLLGALLLWLALSRGALRTPGRIAGTFFTGYGLA
RF
AVEFLRQPDAQFAGPGNPLGLALHWQGYGLTMGQLLSLPMIAFGLGLILHAARRRP
>OLS48029.1 pyrroline-5-carboxylate reductase [Rhodovulum sulfidophilum]AB14
MQTRNKFLDDMSKLMTNAMGVAQGAKDEAETAMKSMLDRWLADRDFVTREEFDAVRSMAQKAREENEALKARIA
ALEAK
>OLS48030.1 diacylglyceryl transferase [Rhodovulum sulfidophilum]AB14
MSYTEDYLLSDEIHPIDLVETLASHHDWEFDRVADDQIAMAVVGQWRTYSITLAWSAFDETLRLVCTFEMEPPPERLPV
L
YETLNATNDLCWAGAFTFWNDQQLMVWRHGLMLSGGQTASPEQVDQLIRAAVSSCERFYPAFQLVVWADRTPAN
ALQVAI
ADAYGRA
>OLS48031.1 pyrroline-5-carboxylate reductase [Rhodovulum sulfidophilum]AB14
MEFNDLSARGLVLLGCGKMGSALLEGWLKQGLPAGAVWVLDPHPSDWLRARAAEGLHLNEDLPAAPALVLLAVKPQ
LMDE
ALPQVAGFADGQTLFVSIAAGKTLGYFAAHLGTGAPVVRSIPNTPAAVGRGITALIGNDTVDEAGLALAETLLSAVGQTV
RLDDETQMDAVTAVSGSGPAYVFHLIETLAKAGEAAGLPPDMAMKLARATVAGAGQLAEEAAEDPAQLRINVTSPGG
TTA
AALAVLMDPETGFPPLLEKAVAAATARGKELGA
>OLS48032.1 tRNA-binding protein [Rhodovulum sulfidophilum]AB14
MSEISFDDFLKVDIRVGQITRAEPYPEARKPAIKLWVDFGPEIGEKKSSAQITAHYTPEDLPGRRVLGVVNFPPRQIGKV
RSEVLVLGLPDENGEVVLIVPDKDVPLGGRMF
>OLS48033.1 D-glycerate dehydrogenase [Rhodovulum sulfidophilum]AB14
MARILVTRPMPERVVEAVRTLGETEIRQSTLPLSPDELRAALTDYDIVLPTLGDAFSAEVFADVPAPRARLLANFGVGFN

HIDVAAARAAGVAVTNTPGTVTDATADIGMMLILMTCRRAGEGERLVRAGHWQGWHPTQMLGLHVTGRTVGILG
MGNIGQ
AIARRCHFGFGMEVLYHTRSPKALDFPARALGSPEALAAEADILVSAVPGGAGTRHLIGANVLAAMKPTAHLVNISRGE
V
VDEAALIAALEAGKIAGAGLDVYEFEPEVPEALRSRENVTLLPHLGTATLEVRTAMGLMAVENIRAHLDGRPLPDPVG
>OLS48034.1 phosphotyrosine protein phosphatase [Rhodovulum sulfidophilum]AB14
MTTRILFVCLGNICRSPTAHGVFQTLALAEGLDIEVESAGTGGWHVGDPPDPRAIRAAAGRGYDLTRLRACQVKAADF
AT
YDLILAMDRSNLTALERMRPIDSETPVRLFLDFADTPRDEVPDPYYDGSFDLVLDLVEDASRGLIRHLAG
>OLS48035.1 NAD-dependent protein deacylase [Rhodovulum sulfidophilum]AB14
MTKIVILTGAGISAESGLGTFRDVDGLWTRYDLNDVATPEGFARDPGLVMEFYNARRRNAAEAAPNPAHLALARLEAA
LP
PGSVRIVTQNVDDLHERAGSRAVWHMHGELARARCASCGAGWQAPAEMRVADACPSCGVAATRPDIVWFGEMPY
RMEEIW
EALRAADLFVSIGTSGSVYPAAAFVQDAARAGAETLELNLEPSEIARDFDRGLHGPASEIVPVWVEEYLGARR
>OLS48036.1 copper-binding protein [Rhodovulum sulfidophilum]AB14
MSLKTTLYASLIALAPALPAIAGDIAVEGAYARASSAMAQSGAAFMTLVNTGAEDDRLIAAASDAAERVELHTHVSDAN
G
VMRMIEVEDGFPVPAHGHHDLRRGGDHVMLLGLTRQLTEGDIVGLTLTFEKAGTVEVDAPVESVRSSMGNETDMPM
KGHM
AH
>OLS48037.1 hypothetical protein BV379_06935 [Rhodovulum sulfidophilum]AB14
MTGLRSLIGICLALLLALTSLSLAAARAAPRPVDRIVLCTGFGLQAVEVDASGRPAGPPHICPDGIAALAALAPAPPALP
VRLLRARPLGHRDRRRAACGRRPPQPRSRAPPGRID
>OLS48038.1 50S ribosomal protein L28 [Rhodovulum sulfidophilum]AB14
MSRVCELTGKGPMTGNNVSHAHNKTRRRFLPNLNEVTLISDVLGRSFKFRVSAAGLRTVDHRGGLDNFLAKASEAELS
DK
ALKVKKDIEKAQATA
>OLS48039.1 glutamine amidotransferase [Rhodovulum sulfidophilum]AB14
MKPFLILQLRPEAEAADDEYAAFLRKGGIAPENARRLRLDCEPLPADLSLDAFSGVIVGGGPGCVSDPEAAKSATDRRIE
AAILGLLPDITARDIPFLGCCYGIGLLAHHLGAPVARGRFAEPVGTTTCRLTEAGRAEPLTRGLPETFPAFVGHKEAVEA
LPPGCAHLLASDTCPFQMIRHGTNVYATQFHPEADSAGFELRIRIYRDRGYFPPEAADDLIALCRARKVDIPETILARFV
ARHART
>OLS48040.1 ATPase/protein kinase [Rhodovulum sulfidophilum]AB14
MTPDELAPRIKSGERRALARAITLIESGREDHRAQAQELLGLLTDAPGQALRIGLSGTPGVGKSTLIESFGRMLTDEGHK
VAVLAVDPSSARSGGSILGDKTRMERLSRDPNAFIRPSPSQSALGGVARRTREAVALCEAAGFGIVLIETVGVGQSETM
V
AEMTDLFVLLMAPAGGDELQGVKRGIMEMADLILVNKADGDLKPAAERTRADYAGALHLLRRRPQDPEGFPKAVAVS
AVE
ERGLREAWEDMQALADWRREHGHFDARRAAQARYWFAEEVRRGLLARLDRDPAMRARMADLGAKVAGGELTVA
HASTEML
TALGVSA
>OLS48041.1 ATP-dependent helicase HrpB [Rhodovulum sulfidophilum]AB14
MTQLPIDAVLPDLTAALARAGRAVLQAPPGAGKTTRVPLAMLEAGLTGGRIVMLEPRRLAARAAAERMAEALGEPVG
QTV
GYRIRGEARVGRATRIEVVTEGILTRMIQSDPGLEGIGAILFDEFHERSLTADLGLALAWEVRSALREDLILVVMSATLD
AGPVAALMEDAPVVSSEGRSFPVTTRWLPRPLPKGTRLPAATADLVVEAAAETEGGLLVFLPGEGEIRRCAAALEGRLP
E

DCRIRPLYGALPFAEQRAAILPAREGRKVVLATSIAETSLTIEDVRVVVDAGRARRSRFDPNSGMARLVTERVTRAEADQ
RRGRAGRVAEGVCYRLWSKGEDGALAAYPPAEIEAGDLAGLALELAAWGASGPDDLAFLTPPNPGAFAEARALLAGLG
AL
DAAGRITGHGRALAALPLHPRLGHMLLSAGRQAAPLAALLSERDPLRGAGADLALRLRCLSDPKAPSPVPPDRGALARI
R
AEAKRLAGRAPGAPRPMSPGEMAALAYPDRVGLRRRGAAPRFVLSGGKGAVLDEADPLAGQRLIVALDLDGDAREAK
VRL
AAPLSEAELRAVHGAAIAWTDICEWSRRESRVTARRQERFGALALDDRSWPDPPPETVARAMLEGVRALGLPWSDAA
RRF
RARVELLRAEGAELPDFSDAGLMAGLEDWLLPHLGGVRSAEELKRLDLMPALTARLDWEARQSVDRLAPPAFETPLGR
KI
PIDYGAEAPGIELRLQELFGVTEHPTVGPNRLPLRITLLSPAGRPVQVTMDLPGFWATSYADVRKDMRGRYPRHPWPE
DP
TAAAPTLRAKRRS
>OLS48042.1 ornithine carbamoyltransferase [Rhodovulum sulfidophilum]AB14
MTHFLDIDSTAPSDLRAILDCAHAMKQARAGRTKATPDDGRPLDGRMVALIFEKPSTRTRISFDVGVRQMGGQSMVL
SGK
DMQLGHGETIADTARVLSRYVDLIMIRTFEEATLQEMADHATVPVINGLTNRTHPCQIMADLLTFEEHRGPIAGRKVV
WS
GDGNNVCASFIQAAGQFGFDLTFTGPQPLDPEPVFVEAARAKGADIVIERDPLKAVEGADLVVTDTWVSMHDPQSAR
ERR
HNQLRPYQVNERLMAAAKPDALFMHCLPAHRDEEATNAVMDGPHSVVFDEAENRLHAQKAIMRHCLGV
>OLS48043.1 acetylornithine transaminase [Rhodovulum sulfidophilum]AB14
MVPSVLPIYNRAPLSFETGEGSWLVATDGTRYLDFGAGIAVNALGHANPALVAALTAQANRIWHLSNLYHIPEQEALA
DA
LVEKTFADTVFFTNSGTEACELAVKMARKYWYEKGQPERVNIVTFSGAFHGRSSAAIAAVGTEKMTKGFGPLLPGFIHL
P
FGAHGSVLQNAIDETVAAIMVEPVQGEGGLRPLPDPCLKGLRDLCDETGTLLIYDEVQCGMGRTGKLFAHEWAGAAP
DIM
MIAKGIGGGFPLGAVLATEAAASGMSAGTHGSTYGGNPLACAVGKTVIDIISDPAFLDEVNRKAGLLRQRLEGLVASHP
E
VFEAVRGSGLMLGLKCRAANTDVVQAGYDQHLLTVPAGDNVVRILPPLTISDEEIAEAVARLDAAATAVAAKEARA
>OLS48044.1 GNAT family N-acetyltransferase [Rhodovulum sulfidophilum]AB14
MTQDLPLGAPLPGWSPPPRPDRDSLVGRYCRLEPLSRDHAAELHAAQLLDGAIWTYLPYGPFPDEAALADWIARDAS
GAD
PLFFAVREAVSGRALGVLSYLRIAPGAGSIEIGHVNFSAPLQRRPAATEAVTLLIGRAFALGYRRLEWKCNALNRASRRA
AERYGLSYEGVFRQAAVVKGRNRDTAWFAAIDAEWPALKAAYETWLDPANFDAGGRQIVPLSELTRPILAARDPALAS
GP
EAGLASRDGRPDNPVEHHQEDQG
>OLS48045.1 methylmalonyl-CoA epimerase [Rhodovulum sulfidophilum]AB14
MIGRLNHVAIAVPDLEAASAQYAETLGAKVGAPQDEPDHGVTVVFIELPNTKIELLSPLGEASPIKGFLDKNPSGGIHHI
CYEVEDILAARDRLKEQGARVLGSGEPKIGAHGKPVLFLHPKDFNGTLIELEQV
>OLS48046.1 acyl-phosphate glycerol 3-phosphate acyltransferase [Rhodovulum sulfidophilum]AB14
MPMIDTPLLLLALFGLCGYLLGAVPFGIVMARLFGLGDLRKIGSGNIGATNVLRTGNKLAAFLTLVLDAGKGGIAVLIAR
ALAGEDAAQIAGGAAFLGHLFPVYLGFRGGKGVATFLGTLLALAWPVGIAACATWAAFAAAFRYSSLSALAAAALAPV
WA
IFLGRGETVLLGAVLAVLIFIRHHANIRRLLTGTEPKIGRKTG
>OLS48047.1 dihydroorotase [Rhodovulum sulfidophilum]AB14

MRTVFSNARLIDPEAGTDSAGWLLTGGGTVLDKGHDAPPDCDQVIDCKGACLAPGIVDIGVKVCEPGERHKESFRSAG
LA
AAAGGVTTMVIRPDTLPAIDAPEVLEFVSRRAREVSPVRIKPMAALTKGRAGREMVEIGFLLDAGAVAFTDGDHVVSDS
K
VLSRAMSYARSLGALVMAHPQDPGLSTGAAATSGKFASLRGLPSVPALAERMGFDRDMGLVELTGVRYHADQITTAR
ALP
ALDRAKANGRDVTAGVSIHHLTLNEFDVGDYRTFFKVKPPLRAEADRVALVEAVASGLIDVISSMHTPQDEESKRLPYEE
AASGAVALETLLPAAMRLYHAGAITLPQLWRALALNPSTRLGLPTGRLARGAPADLVLFDPDAPFVLDRFALKSKSKNTP
FDGARMEGRVLGTWVAGTRVHGGD
>OLS48048.1 hypothetical protein BV379_06990 [Rhodovulum sulfidophilum]AB14
MFDPKTEAPLEDGETVQIQFRADRGAYLKAHAIIAVLAGIGATVLLAATGNPYPWAGIVAAVLAIAVRGAFLMSDELGT
R
WVLTDRALLGPGGRRVRLSSIARVRTIGAATQVITLGGDKHLIKFLADPAGVKTRIEAARMAAGGPEA
>OLS48049.1 aspartate carbamoyltransferase [Rhodovulum sulfidophilum]AB14
MEFRARHLLGIEHLSPWDIRIILDLAESYVDLSRGATKHADVLSGLTQINMFFEASTRTQSSFEIAGKRLGADVMNMAM
Q
ASSIKKGETLIDTALTLNAMHPDLLVVRHPHSGAVDLLAQKVNCAVINAGDGRHEHPTQALLDALTIRRAKGRIQRLTVA
ICGDIAHSRVARSNLLLLGKMENRVRLIGPPTLIPSGAADLGVEIYDDMKEGLEGADVVMMLRLQKERMDGGFIPSERE
Y
YHRFGLDAEKLAYAREDAIVMHPGPMNRGVEIDGTLADDINRSVIQDQVEMGVAVRMACMDLLARNLRAAGEAAH
V
>OLS48050.1 uracil-DNA glycosylase [Rhodovulum sulfidophilum]AB14
MDPALTPHDALALLAWQVDLGATEAICETPVNRYEEVRPAPAPAPAAAARPGPSAPVPPVRPAAVDPVAVAQGAAA
AATD
LEGLAAAMAAYELCELKKGARNLVFSDGNPKARVMVIGEAPGRDEDLQGKPFVGRAGHLLDLMFAQIGLGRGDPAAE
RAL
YITNVLPWRPPQNRDPEAGEVAMMLPFLERHVDLVDPDILVLMGNHSCGALLGRKGITRLRGRWVEALGRPALPMF
HPAY
LLRTQLAKRDAWHDLLMLQARLRGG
>OLS48051.1 molybdenum cofactor biosynthesis protein B [Rhodovulum sulfidophilum]AB14
MAGIDDSKAFVPVRIAVLTVSDTRSLDEDRSGKTLADRIEAAGHVLADRKILRDERAEIAEQLRAWCADPGIDVVISTGG
TGLTGRDVTVEAHRDVYEKEIEAFGTLFTLVSYGKIGTSAVQSRATGGVAQGTYLFALPGSTGACKDAWDEILVHQLDY
R
HPPCNFVEIFPRLDEHRRRK
>OLS48052.1 efflux transporter periplasmic adaptor subunit [Rhodovulum sulfidophilum]AB14
MRFLRRSLTGLFLLALTLGLLAYAAATLRSAIDARLAARPKAPPSAERVFSVNVIPLHPRTVTPELTAFGEIRSRRSLEL
RAEASGRVVYLHPAFEEGGRVAAGDLLLRVDPADATSARDLARADLSQSEAELRDAERRLELAHDELAATELQAGLRQK
A
LRRQKDLRGRGVGTDAAVEEAELTAAAADQAVVSRRQALAQAEIGLETATTALARARIALADAERALADTELRAAFSGT
L
IDVSLVEGGLVGANERVADLIDVSELEAGVRVSTAEFSRLLDDGGALRPLPVTVTLDVYGANLTARGVLSRESGAVPEDQ
VGRLVYARLEAAPGFRPGDFVTLRVEEPALDDVMVLPASALDAAGEILVLDAGDRLVSVPVTLLRREGDRVIIRAPELAG
REAVAERSPLLGAGIKVRPLRPGAVAGPDASTEATVELSPERRAALIAFVETSPALPQEARARIRAQLDRPFVPAQLVER
LEIRMGG
>OLS48053.1 acriflavine resistance protein B [Rhodovulum sulfidophilum]AB14
MPGRVASAAGGLLSYFTRHRTAANLLLLLLIVAGLFSAPRMRAQFFPDVIVETVSVSVIWEGAGPEEVDAGIVQLLEPEL
RAVEGVEETTSQSLEGLATITLDFEPGWDMARATNDVQAAVDAETDLPEETEEPEVTRGAWRDRVTDVVITGPVAVD
QLG

RLTDELVDRLFAAGITRTTIRGLAAPETLVEVPSIELMRHDITMAEIAAVLAAEAETAPAGDVSGGNTRVRTGVEKRSAA
SIAGVVLRSYPDGSKLTVGDLAHVRVEGADRERAYFVGPHPAISLRVDRSDRGDAIEMEAGVRAVVAGMTPSLPEGVRI
D
LIQTQADYIAGRLDILLGNGLMGLGLVLGLLFLFLNARTAFWVAAGIPVAMLATAALMFAAGLTINMISLFALIITLGIV
VDDAIVVGEHADFRARHLGEPPILAAENAARRMAAPVFSATLTTIVAFYALVVIGGRMGHLIADIPFTVIAVLTASLLEC
FLILPNHMVHALAHSAREHWYDWPSRQFNRGFRRFREGPFRGLMRLVIRGRYPVMAGCLLLLAVQVAAVIRGDVPW
RFFN
APEERSVSANFMMLSGAERADTAAMMAELQRAAEAVGARYAARYGTDPVTYALAEMGGNAGRGLAGAEIKDPDLL
GAISL
ELIDADARPYSSFDFVRALQDEVRPHPKVESLSFRSWRAGPGGDALDVEFFGAEARVLKAAAERLKTELSRFGEVSALED
SLAYDKEEAVLDLTAQGAALGFTIEDLGRVLRHRINGIEAATYPEGPRSATIRVELPEAELTADFLDRLQVRSPEGVYLP
LADLVTVERRTGFSSVRRENGLRVVTVTGDLSEDDPARAAEISAELKEAILPGIERDLGVSWRLGGLSEQERDFLADARL
GFGLCLVGIYLVLAWIFSSWSRPLAVMAIVPFGLVGAIWGHAHWDVPLSMFTVVGLIGMTGIIINDSIVLVGTVDEYAE
R
RALVPAIVDAAADRLRPVLLTTLTTVLGLAPLLFETSQQAQFLKPTVITLVYGLGFGVGLVLLVVPSLLAIGQDVGQALR
ALRRALGRRGRRALPVPAVMALALAAVFAATLLPVLATGRLPAILLAVLPPLGAVSAAAAGLILFLAGGGLVALAAWALS
RPLRLPGAPDR
>OLS48054.1 DUF179 domain-containing protein [Rhodovulum sulfidophilum]AB14
MQLGGKLLIAMPGMGDPRFERSVVYLCAHSDEGAMGLIVNKPLSEISFSDLLDQLEIVRSPESQDIRVHFGGPVEHARG
F
VLHSGDYRTNRDTTLRVDSEFGMTATLDILEAIAQGDGPAESLLALGYAGWGPGQLEGEILQNGWLTCDAKPGLVFDG
AD
ALKWERALRAMGIDPLVLSSAAGRA
>OLS48055.1 threonylcarbamoyl-AMP synthase [Rhodovulum sulfidophilum]AB14
MSAELLRPDTGGLARAATLLAEGRLVALPTETVYGLGADAHNDRAVAQIFEAKGRPRFNPLIVHLPDLAAARHLAVFSD
A
ALRLAEAFWPGALTLVLPLRPGHGLSPLVSAGLPTVALRVPDHPVAQGVLSAFGGPVAAPSANVSGAVSPTRSDHVIEG
L
GQRIAAVVEGGACPVGVESTILGLSADDGAAPVLLRPGGLPTEAIEACLGQPLAARDESAAISAPGQLGSHYAPGARVV
L
NAATPPEQGLWLGFGPDCPGAALNLSPSGDLVEAAAALFHALRQLDARAAPSEVIAVAPIPEKGLGRAINDRLRRAAAP
R
PGRGRE
>OLS48056.1 acyl-CoA dehydrogenase [Rhodovulum sulfidophilum]AB14
MAYRAPVAEFRFILEHVAGLPDVVATERFAEATDDVTAAILDGAGRLCDEVLAPLQRPGDLHPARLENGVVRTSPGYAE
G
YRAIAEGGWVALSAPPEHGGMGLPTALSTAVNDMMSGACLSLQLNPLLTQGQIEALCHHASDEIQALYLPRLIAGDWS
GT
MNLTEAQAGSDVGALKTRAVPNGDGTYAVTGQKVYITWGDADFMENVIHLVLARLPDAPEGTRGISLFLVPRNLPDA
DGR
PGVANGVKVVSLEHKLGLHGSPTALMEFDGATGWMVGAPNKGMAGMFTMMNNARLGVGAQGVGVAEAAFQQ
ALAYAGERR
QGPTPMGDGPIIGHADVRRMLAGMKAEIFAARAIALSCAVAADMAEASGEPDWAARAAFLTPIIKAWGTEVGMEVA
QQGV
QVHGGMGFIEETGAAQYLRDVRVTAIYEGTNGIQAMDLVGRKLGDGGEAAWRLLEEVEQTAEAAREAFPDLAGDLW
TAAE
ALREATEWMLTQEMTDRFAGAVPFLRAWARVLGAHLHLTAALAEGGEGPRSALARVFIRRMLPEHASALAAARAGA
GDLY

ELSDAALSA
>OLS48057.1 MBL fold hydrolase [Rhodovulum sulfidophilum]AB14
MRDGRFAPVRTPFDLPPEEGEAVEIAEGVLWMRLPLPSPLKHVNVFALDEGQGWTLVDTGLSTGRSREIWAALLAGPLGG
RPVERVILTHHHPDHVGLAGWFQTEAGAELWTSRTAWLFARMLTLDEQARPRPETLDFYRGAGMDPALLAERAAERPFNF
ADVVAPMPLGFRGLAEGDRIRAGGRDWDVRLGDGHAPDHVTLWSRDDGLVIGGDQLLSSISPNLGVYATEPLNDPVAGWL
DSCTRLAAHAREDHLVLPGHKLPFTGLPTRLVQLIENHHGALARLLEALAEERTACDCFGVLYRREIGTAEYGLALAEAV
GHLNHLYLTGRATRRLRPDGAWLYRRSGAV
>OLS48058.1 hypothetical protein BV379_07050 [Rhodovulum sulfidophilum]AB14
MFRFLIACLLSLFLLGCGAQPVWAPDEDVARAAYRDKAPSSITLYTMVNNRSGAGGHSALLINASQRVIFDPAGSWWHRM
APERNDVIYGITPTMLDFYIDYHARETYHVVEQTLQVSPELAEQALRIVEGYGAVPKAMCARSTSDVLNRLPGFESIPHT
LRPIKVMEAFGKLPGVTSRKIYDDDPDEHEELLREQQRARFDKLVEGIAEGR
>OLS48059.1 hypothetical protein BV379_07055 [Rhodovulum sulfidophilum]AB14
MDWSIAETWAIIVALGIGTFLLRFSFLGAMGGRQLPDWLLRHLRYTPVAVLPALVAPAVAWPEATGGDPDPARLAAAFVT
VAVGWWRKSVIAAIGAGAATLYAMLFLLG
>OLS48060.1 branched-chain amino acid transporter AzlC [Rhodovulum sulfidophilum]AB14
MPASTRKSAYLTGLTQGTPFLLIIVPFALLFGVVATEAGLALPEVMGFSVVVVAGAAQFSAVQLMAENAHAAVIILTALA
VNMRMAMYSASLTPYLGPAPLWQRALVAYSLFDQTYAVAHARYEARPEMGVTERIAFYAGAATPLLPTWYGMTLVGALVG
RSIPPEFALDFALPITFLAMVAPALRTPAHVAAAGSSVALALVFAGLPYNLGLMIAAVAGMAVGAEIERRMGAR
>OLS48061.1 molybdenum cofactor guanylyltransferase [Rhodovulum sulfidophilum]AB14
MRIFGTILAGGQGRRLGGVDKALLRLGGETLLERAVGRLGPQVERLALSANGPEAAYRAAGLAREDAVLPDPPPGDLGPL
AGVLAGLDWAGAGGADWLVTAAVDTPFLPCDLVPRLLLAVETAGTAGMAMAASGGRRHPTAALWPVGLRDELAAELAGGL
RKLGAFGAARGAALAEFATGPGPDPFFNINTAEDLAAAGAALR
>OLS48062.1 hypothetical protein BV379_07070 [Rhodovulum sulfidophilum]AB14
MSGLDTVIMADWSARSGRSPARPSSDAIWIAVARRGWTTATAYFRTRATAEAALADLLEGERRAGRRALLGADFAFGYPA
GFAEALTDNASARAVWAWLAARLEDRPDNANNRFELADAINAALPGGGPFWGCPAACAVRLADLTMTRPPEPGFARHRRV
ERALRDLGGPGRAVKSVWQLYGAGAVGSQVLVGLPMLHRLAQRFGADLAVWPFDTEEAPLTLAEIYPAMIGPEVAEALAA
RSGQIPDEVQVRLMAGAVLAAEDTDALLGVPPVPDLAEEGWILGTAAPGLLRAGAALSRTCR
>OLS48063.1 hypothetical protein BV379_07075 [Rhodovulum sulfidophilum]AB14
MAEKDTKKPFDIGLLAAGRRGGLSRIEIGAGLLSLVWLGLSVAVLAGSDTAGEDGALDTVVAVLAVTMPVGLIWVVAQGA
RAARTLREEAARLQAAADALRLAYLHQQQGPGPDSRPSMEKRIEDLARAQRETQDAIATFATRRDRAAETADAPVSVEAQ
PPGQPAARPARTASDPQPSLALDGPVEDVPPLTTEEFVRALNFPENTADRAGFRALRRALDDRIAAKLVRSAQDILTLLS
EEGIYMDDLQPDRARPEIWRRFAQGERGLPVSALGGVRDRSCLALTAARMKKDPIFRDVAHHFLRQFDRVLVEFEQAASD
QDLNDLAETRTARAFMLLGRVTGMFG
>OLS48064.1 transcription elongation factor GreA [Rhodovulum sulfidophilum]AB14

MEKIPMTRAGCTALDEELKTLKSVERPAVIRAISEAREHGDLSENAEYHAAREKQSFIEGRIKEIEGLLSRAEVIDPAKL
SGPIKFGATVTLADEDTDEEKTYQIVGEAEADIEKCLLNIKSPLARALIGKEEGDSVEVRTPGGERSYEVLSIRYI
>OLS48065.1 electron transfer flavoprotein-ubiquinone oxidoreductase [Rhodovulum sulfidophilum]AB14
MAEIERESMEYDVVIVGAGPSGLSAAIRLKELDPELSVVVLEKGSEVGAHILSGAVLDPIGLKALFPDWKDRGAPVSVPV
KEDNFAFLGKSGSLRFPNWLMPPLMSNHGKYIVSMGNVCRWLAEQAEALGVEIFPGMACSELVWGEDGAVRGVVA
GEFGR
QADGTPGPNYEPGMELHGKYVFIAEGVRGSLAKQIIARHELSKDRDPQKYGLGMKEVWEIDPKKHRLGTVTHTMGW
PLNG
NGGGGSFIYHAENNQIYIGFVVHLNYRNPHLSPYMEFQRFKHHPMVAELLEGGKRVAYGARAISEGGYQSIPQAAFPG
GV
LLGCSAGLVNVPRIKGNHNAMMSGIEAAKAAVAAIKAGRAGDVLSDYDEALHAGDIARDLKKVRNVKPLWSKYGGFL
ATM
LGGFDMWVAHLTGWNPLGTIHHDKTDAAATGKAADFPKIDYPKPDGKISFDRLTNVAFSFTNHEESQPCHLRLTDPD
VPV
RVNLPDYAEPAQRYCPAGVYEVVDEGQDARFVINFQNCVHCKTCDIKDPSQNITWVTPQGGDGPNYPNM
>OLS48066.1 hypothetical protein BV379_07090 [Rhodovulum sulfidophilum]AB14
MANSLPRLAALAALGLAALTPAAPAHADQGVSGAYLAARQASFLSDYRAAAVYFTEALAADPANPRLLENAMTAFVGL
GE
VEKAVPVARRIATQGNPDQLASLVLMADQIRQGQYDRVLADFEAGRRTAPLVDGLIRAWALFGQGRMGDALEAFDA
AAAQ
TGLKAFGLYHKALALAAVGDFEGADRIFSGEAEGPLRATRRGVLAHVEVLSQLERNDDAIELIDAAFGSEVDPGVAEIRA
RLAEGDVLPFDAIDGAAGGAAEVFFTVASALNGDALDGYTLLYARLAQFLAPGNSDAILLAAGLLEAQDRYELATEAYAA
VPADDPAFYAAELGRAEAMRHAGDAEGAVETLRGLADTYPDIAIVQVTLGDALRGQERFDEALQAYDAAIALFDETTR
GQ
WIIYYARGISYERLGRWPEAEADFRKALELEPDQPQVLNYLGYSYLEMNTNLDEAMTMIKRAVAARPDDGYITDSLGW
GL
YRLGRYSEAVPHMERATALMPVDPVINDHLGDVYWAVGRKVEARFQWHRALSFGPGEAEAERIRRKLEVGLDTVLKE
EGA
PPLRAANDG
>OLS48067.1 4-(cytidine 5'-diphospho)-2-C-methyl-D-erythritol kinase [Rhodovulum sulfidophilum]AB14
MTVEAFAPAKINLTLHVTGQREDGYHLLDSLVVFADIGDRLTLDPAEDISLSVTGPRAEGVPVDGRNLVLKAAALFGPG
Q
GAGIRLDKRLPAAAGIGGGSSDAAAALRGLAALWGRDLPGAEAVLSLGADVPVCLAPGPVRMRGVGEEILPAPALPEF
GI
LLVNPGVEVPTPAVFRGLARKTNAPMPDLPGWGSASGLADWLAGQRNDLEPPARAEAPVIGEVLAAIAATPGCLLAR
MSG
SGATCFGIYADRAAAERAAVLLHETRPDWWAEAGAPYSL
>OLS48068.1 farnesyltranstransferase [Rhodovulum sulfidophilum]AB14
MGLDTAVTKPHERLAALLTEEMAAVDRLIRDRMTSEYAPRIDEVAAHLVGAGGKRLRPMLTIAAARMCGYDGPYHIYL
AG
TVEFIHTATLLHDDVVDESGQRRGRPTANLLWDNKSSVLVGDYLFARSFQLMVAPGNMQVLDILSNAAAVIAEGEVLQ
LT
AAQDLGTTEEIYLKVVRGKTAALFAAATEVGGVIAGMPEEQVKALHTYGDALGVGFQIVDDLLDVAGDAASTGKNIGD
DF
RERKLTLPIIKTIAKADDTERTFWARTIEKGDQRDGDLEHALELMAKHGAIEATRQEALAWTARAKEALSLLPEHEIRDM
LSDLADYVVARIS
>OLS48069.1 hypothetical protein BV379_07110 [Rhodovulum sulfidophilum]AB14

MKELVRTNDPTVIAYASALLQGEEIDCFHMDVHMSVLEGSLGVLPRRIMVADRDLFRARAVLRDNGIPCGDEG
>OLS48070.1 methyltransferase [Rhodovulum sulfidophilum]AB14
MRAEPMTALRQDGFLGGRLALWQPVAGYRAGIDAVLLAAATPARAGQDVLELGCGAGVASLCLAARVPGLSLTGVEL
QPA
YAALAGRNAAETGLPLEVVTADLTALPPGVKARSYDHVIMNPPYFRRDCGSAAPDPGRETAMGEATPLADWVAVAAR
RLR
PGGRLTAIQRAERLPELLCAAAAVFGAIELLPLAPRPGRAAKLVLLAARKGRQGPFRLHPPLVLHEGAAHLGDGDSYTAE
LTDILRHGEALSWRG
>OLS48071.1 DUF465 domain-containing protein [Rhodovulum sulfidophilum]AB14
MNLSSHLQELRKKHKALSDEVERAQQTPAADNIQVAELKKQKLKLKEQIERLSQA
>OLS48072.1 EamA family transporter [Rhodovulum sulfidophilum]AB14
MTRPRATATGFIAVLLWALLALFTVGSAPVPPLQLNAICFAIGGTLGLVWVLARGEAALLRRISWKVYVFGTVGLFGYHA
LYFSALRLAPAAEAGLIAYLWPLLIVLMSGLLPGERLRPAHVLGALAGFAGAALIVARGAMTGFEAAALPGYGLALLCAL
TWSSYSVLSRRLGTVPTASVAVFCLATAALSWPLHLIFEETVWPQAAQGWLSVAALGLGPVGLAFYVWDIGVKRGDIQ
LL
GVASYAAPLLSTLVLVGAGVAVPTPTLFAAALLITGGAALAARASRRAADQA
>OLS48073.1 beta-ketoacyl-ACP reductase [Rhodovulum sulfidophilum]AB14
MARVALVTGGTRGIGEAISKALKAAGYTVAATYGGNDEAAKKFTDETGISTYKWSVADYEASKEGLAKVEADLGPIDVV
V
ANAGITRDAPFHKMTPTQWQEVIDTNLTGVFNTVHPIWPGMRERKFGRVIVISSINGQKGQFGQVNYAATKAGDLGI
VKS
LAQEGARSGITANAICPGYIATEMVMAVPEKVREAIISQIPTGRLGEPEEIARCVAFLASDDAGFINGSTISANGAQFFA
>OLS48074.1 acetyl-CoA acetyltransferase [Rhodovulum sulfidophilum]AB14
MTNVVIVSAARTAVGSFNGAFATVPAHELGRTMLEALVARAGIDKSDISETILGQVLTAGQGQNPARQAHVNAGFPIE
SA
AWSINQVCGSGLRSVALAAQHIQLGDAAIIAAGGQENMTLSPHVAHLRAGHKMGDVKFIDSMIRDGLWDAFNGYH
MGQTA
ENVAEQWQISREMQDEFALASQNKAEAAQKDGKFADEIVPVTIKGRKGDTIVEADEYIRHGATLESMQKLRPAFAKDG
SV
TAGNASGINDGAAGVLLMTADEAEKRGLEPLARIASYATAGVDPSIMGTGPIPASRKALEKAGWKVEDLDLVEANEAF
AA
QACAVNKDMGWDPAIVNVNGGAIAIGHPIGASGARILNTLLFEMKRRDAAKGLATLCIGGGMGVAMCLERP
>OLS48075.1 diguanylate phosphodiesterase [Rhodovulum sulfidophilum]AB14
MPDDSHENDVGSTEGAENPLARAGTEDDRNVMEMVRAALRRRGMVLAFQPVVQNMENGKIAFYEGLIRILDRGGRI
IPAA
EFIETVEPTELGRRIDCLALEMGLAALADCPDLRLAINMSARSIGHPPWIRTLNRAIETDPTLAERLILEITESSAMVTP
GLVIAFMRELQAKGIAFALDDFGTGYTAIRYLREFYFDILKIDGQFIRGIADDPDNQVLTSALIAIARHFDMFTVAENVE
TARDAAWLAAAGIDCMQGYAFGAPTLIPPWRAQTQRRTA
>OLS48076.1 DNA-3-methyladenine glycosylase [Rhodovulum sulfidophilum]AB14
MAERCGWCGSEPIYVTYHDTEWGVPEYDSRALFEKLILDGFQAGLSWLTILRKREGFRTAFAGFDPEAIAGWGEADVA
RL
LADPGIVRHRGKIEGAIGNARAWCEIEAREGFSDYLWRFVDGAPLQNDFASMAEVPAQTSRSQAMSKALKADGFKFC
GPT
IAYAFMQAVGMVNDHVLTCPRHAEVAALR
>OLS48077.1 hypothetical protein BV379_07160 [Rhodovulum sulfidophilum]AB14
MTRGARLSALLALLALSACARPHGGVAVSDEGDVSGAVSAGGVAVGAGPDGARASARVVDTGTTAVRVGTDGASVG
LRPR
GAPVRVILGTGGVGLGF

>OLS48078.1 K+-transporting ATPase, A chain [Rhodovulum sulfidophilum]AB14
MKYLRYVFLALAIWGAIHPMFYFMRWILANDWDWGGMIDAWYVNDSATGLTWDLTISAIALTLWIVIDSARRRDAL
GLVC
IPLTFGIGLSCGLPLYIFLRSRPSQVV
>OLS48079.1 diaminopimelate decarboxylase [Rhodovulum sulfidophilum]AB14
MDHFLYRDGTLHAEDVPLSEIAAAVGTPFYCYSVATLTRHFRLFDEALAGTDHLVCYAMKANSNLAVLKLMGDLGAGI
DV
VSGGEYRKARAAGVPGERIVFSGVGKTREEMRLALEGGIRQFNVESEPEMAALDEVARSMGVVAPIAVRVNPDVDAK
THE
KIATGKSENKFGIPIARAREVYARAAAMPGLEVLGIDVHIGSQLTELAPFELAYGKIAELTRVLRADGHDIRRLDLGGGL
GIPYERSNSAPPLPVEYGAMIKRVLGDLDVEIEIEPGRLIAGNAGLLVSRVIYVKEGEGRTFLILDAAMNDLVRPSMYGA
HHDIVPLTEPAAGTGYAPHDVVGPVCETGDTFARARDLPPLAEGDLVAFRSAGAYGAVMASEYNTRPLVPEVLVGGD
HFA
VIRQRPSFDEIINRDTIPEWL
>OLS48080.1 TIGR02302 family protein [Rhodovulum sulfidophilum]AB14
MTDSTPRPDDPLAELRWPLRLTWAGLLAERLSRAFWPLASLVLLVAAALAFGAHDLLSPAAAFAIGGALFAALLGTLVY
G
LRGFRRPGRAEALDRLDRSLPGRPIAALSDSQAIGAGDPASEAVWRAHVTRMAARARAARAVAPDLRLSSRDPYGLRH
VA
LIAFTVALIFGSFLRVASVADLGKGGPALASGPSWEGWIEPPAYTAKPAIYLNDISDTRLAVPQGSRVTLRLYGAVGALD
VTETVSGREPGAEGAAAAPAGTDAMARSFEVIRDGRVEIDGSGGRSWQIVLSPDIRPEVRLTGEIDVTADGEMRQPFS
AS
DDYGVTAGEATITLDLAAVERRYGLAAEPDPRAPLVLDLPMPISGDRRQFTETLVENLSQHPWAGLPVQVELAVEDAL
GQ
RGVSAPAGMVLPGRRFFDPLARAVVEQRQALLWARSNAREVAQVLRAISYRPDEIFRNESAYLQLRVAIRRLERAAPGL
T
AESQAELAGALWDIAVLIEDGDLSDAAERLRRAQERLSEAMRNGASDAEIAQLMDELREATRDYMRQLAEQQKDEDR
QQA
QNGEMQELTGDQLQQMLDRLQQLMEEGRMAEAQQLLQMLQQMMENMQVTQGPGGDSPGQQAMEGLAETLR
DQQGLSDDAF
RDLQEQFNPGQQGQGEGREGEGEQDRADPGQGGEGGQQSLAERQEALRRELERQQQTLPGLGASPESEGTREALD
RAGRA
MDEAERSLRDNDLAGALDDQARAMEALREGMRNLGEALAQNRQQRGEDGQGRGRADAGGERRDPLGRAPGSTG
QVGTEDS
MLQGEDVYRRARDLLDEIRRRSGEQNRPDLELDYLRRLLERF
>OLS48081.1 hypothetical protein BV379_07180 [Rhodovulum sulfidophilum]AB14
MKIVDWNLEWMNRWFSGNARARWGSGELTPDEARICARKAAAVIDALAPDLLCLQEGPSAEAEMALFIEEFLSDAD
GPRY
EALIGSDGGAQKLYVLRRRCGLVRAVERATDAATRALAEPWAADVNGDMLLEGYEFTRLPLVVDIDPVGGAPVRVVVL
HT
KSKYVHRGAALWRDPARRQEFVVAALLARRRISAEGFRLRAYLDALVAEDPAARIVVTGDWNDGPGTDFFERSYLTHN
VA
DIVLGSTFLPGLIFHHPLLAHVPAAALFTARFDDFVDEVTDRPLLLDHFAISPGLTPWVQAAGIGHDAYEAGLTGAGAAR
VERPSDHRPIWVEIGRPLTG
>OLS48082.1 D-amino acid aminotransferase [Rhodovulum sulfidophilum]AB14
MTRTVYVNGDYLPETEARVSIFDRGFLFADGVYEVASVVGGKLLDFHAHAARLQRSLAELEMRSPVDMDALLQIHRELI
A

KNDVDQGMVYMQITRGAPADRDFHFPDPDQVAPSLVMFTQSKEIIESKTAETGIKVISIDDLRWARRDIKTVQLLYAS
MA
KMAAEKAGADDAWMVEDGFVTEGSSNNAYIVKNGKIITRQLGTEILPGITRAAVLKFAAEAQMEIEERAFTIEEAKQAD
E
AFFTSASAFVMPVVSVDGVSLGDGLPGPVSRRLREIYIDEAVKTAI
>OLS48083.1 dipeptide epimerase [Rhodovulum sulfidophilum]AB14
MQISVTPESFRLKEAFTISRGSRTEARVLTVRVASDGATGWGECVPYARYDETLDSVTAQIGALPPSPSRAALQDLLPPG
AARNAVDCALWDLEAKEARTRVWDLAGLPAPGPEITAFTLSLDTPENMRAAAARNAHRPLLKIKLGTDADMPRLEAV
RAG
APKARIIVDANEGWTAEVYADLAPHLVRLGVALVEQPLPAGQDDMLAEIARPLPVCADESCHDRASLPALKGKYDLVNI
K
LDKTGGLTEALALKQQALAEGYGIMVGCMVGSSLAMAPAVLLAQGAAVTDLDGPLLLAEDRPDPLCFDEAGVHAPAA
ALW
G
>OLS48084.1 EBNA-1 nuclear protein [Rhodovulum sulfidophilum]AB14
MIETPYLLFLGDAPDPLAAKVAQGIRDWRPENAVGQFRMDGCKADLKLPDLDLAAAKAEGAKTLVVGVANRGGVISP
AWK
KVLVAALEEGFDLASGLHNLLCKEPDLAGVAEATGRTLHDVRIPTVAYPIANGKKRSGKRCLAVGTDCSVGKMYTALAM
D
REMKARGLKSSFRATGQTGILITGEGVPLDAVIADFMAGAIEWLTPDNDDDHWDMIEGQGSLFHVSYSGVTMALIHG
GQP
DALILSHEPTRTHMRGLPDYGLPSLETLRDTALGLARVANPKAEVVGISVNTAAMGEDEALSCLEGIEKRMGLPTVDPFR
QGAGRLADALDAI
>OLS48085.1 CoA ester lyase [Rhodovulum sulfidophilum]AB14
MSFRIQPTPVSRPNRCQLFGPGSRPAIFEKMAASAADVINLDLEDSVAPDDKSSARANVIQAIGDIDWGRKTLSVRING
L
DTPYWYRDVVELLEQASDRLDLIMIPKVGCGADLYAVDALVTAVERATGRGKRLGFEVIIESAAGIAHVEEIAAASPRLQ
AMSLGAADFAASMGMQTTGIGGTQENYYMIREGAKYWSDPWHWAQTAIVAACRTHGVLPVDGPFGDFSDEEGFR
AQALRS
ATLGMVGKWAIHPRQVALANEVFTPSGAAVDEAREILAAMEKAKAEGSGATVYKGRLVDIASIKQAEVIVRQAEMIAA
G
>OLS48086.1 hypothetical protein BV379_07205 [Rhodovulum sulfidophilum]AB14
MDNLDLLLALGAFALVASITPGPNNLMLMASGANFGLRRTLPHILGVALGFVAMIVLVGLGAMRAFEAWPMLRVLLT
WFG
AAYLVWLAWKIARAAPPEPGAAPGRPMTFLQAAAFQWVNPKCWTMALAALTLYAPGQKPAAVLLVAGAFAAASVP
STGLW
TILGTRIRLLLSRPARLRLFNRAMALLLLASLVPVFLP
>OLS48087.1 acetyl-CoA carboxylase carboxyltransferase subunit alpha [Rhodovulum
sulfidophilum]AB14
MNYLEFEKPLAEIEGKAEELRALARANSEMDVAQEASALDRKAADMLKELYKNLTPWRKCQVARHPDRPHCKDYIEAL
FR
EYTPLAGDRNFAEDNAVMGGLARFNDQPVVVIGHEKGFDTKTRLERNFGMARPEGYRKAARLMDMADRFGLPVITL
VDTP
GAYPGKGAEQRGQSEAIARSTEKCLQIGVPLISIVIGEGGSGGAVAFATANKVAMLEHSVYSVISPEGCASILWKDAEKM
REAAEALRLTAQDLLKIGVIDRIIPEPIGGAQRSREETIDRVGRNLATMLGELVGKEPAALVAERRRKFLDIGSKGLAA
>OLS48088.1 1-acyl-sn-glycerol-3-phosphate acyltransferase [Rhodovulum sulfidophilum]AB14
MRSPLQYLRSLFFVGQMYLAMPVVGLLYLPLALASPRGATMAARHYCHWVRWTARWMVGLKSEVRGTPPEGEVLIA
AKHQ

SFFDIILLVGSLPRPRFIMKKSLTWAPVLGWYALRIGCIPVDRGKRGQAIATMRRQVAEGRQIPGQLIIYPQGTRVAPGV
TRPYKIGAGVLYQQLGQPCVPAATNVGLFWPRTGIYRKPGLAVLEYLPPIAPAQPIDVFMAQLEETVETASDRLMAEAG
F
TTGA
>OLS48089.1 cell division protein FtsE [Rhodovulum sulfidophilum]AB14
MIELENVSHSYGGSDLLCDMSLRLAPASFHFLTGPSGAGKTTFLKICYAELQPSQGRMRLFGADLAGLDRDGVARMRR
RI
GVVHQDCRFLDHLPLSENITLPLMAAGRDIAAEERNLSELMTWVGLKAQAGALPPQLSGGERQRAALARAVILSPDVIL
A
DEPTGNVDWEMSLRILQLLIELNRMGKTILIATHDLNLIRAAKTQVAARVLRIKDRQLHLAGADL
>OLS48090.1 hypothetical protein BV379_07235 [Rhodovulum sulfidophilum]AB14
MRLTCPKCGAQYEVDEAVIPDEGRDVQCSNCNHIWFQEPSGPAPRDPLAGFAVPRAPEPVASGPEGADAPPRRGLDE
DVL
SILREEAEREQRAREEEATAETFSEQADLGLASPPPPVRAKAPEAAPSPEPEPDPAPAEPSRGAARRDLLPDVEKITSTL
SASTDRGAESIPRAAAARNGLSGAFLAGFLVAVLVAAAALAAYLKAPAIAALNPALEGQVAAYVETVDAARAWMAGLV
PA
DPVDREPGR
>OLS48091.1 sodium-independent anion transporter [Rhodovulum sulfidophilum]AB14
MIPASGPMQQFTPKLLTAMAEGYGLRAFRADLVAGLTVAVVALPLSLAIAIASGVPPDRGLITAIIGGFLVSALGGSRFQ
IGGPAGAFIVLVMATVQRHGLDGLALATFLSGIFLAAAGALRLGSFVKFIPYPVTVGFTAGIGVIILASQMRPFLGLTLD
GPEPGPFLEKIPALWAALPTLGPIDLAMGIGTFGFILVQRRYAPRFPGMLMAIALAGLTVWLFKLPVPTIGSAYGGIASS
LPAPHLPPLGLEKIRAVLPDALAFTLLGAIESLLSAVVADSMTGRRHRSNAELIGQGAANIASACFGGFCVTGTIARTAT
NVRAGAHGPVAGMIHSLALLGMVLLLAPLASAIPLAALAGVLVSVSLHMIDIRAIRTLARTARAEVVVLLATLAMTVLRD
LTEAIVTGLALGALVFLHRLAQMSGVDAMLDESDTESRPEDRGLNRQTVVLRLTGAYFFGSAPVIENVLDRIAARPRHLI
LDMADVPLLDMTGAHSLTGFARKALDQGMAVSLAGARPQHLQLLRAAGLPQGIHITADVAEARASADTA
>OLS48092.1 alkaline phosphatase [Rhodovulum sulfidophilum]AB14
MDDATRTEIEAAAFRRLRRHLMEDRPEVQNIDLMTLAGFCRNCLSRWMAEAAAERGVAMTEAEAREDFYGMPYKD
WKAMH
QTEADPEQKAAFEAAMAARPEADKT
>OLS48093.1 N-formylglutamate amidohydrolase [Rhodovulum sulfidophilum]AB14
MTEQPFETVGETRGGRWLVTCDHATNIVPPCVNGGSLGITPADMARHIAWDPGAAGVARALAERLDAPLVLSRFSRL
VID
PNRSEDDPTLVMRLYDGTVIPANRAIDEAETDRRIRTFWRPYHTEVARLATRRSDTVLLAIHSFTPQLKGRPPRPWQIGI
LHAADDRLARPLIARLRAEGDLCVGENEPYGGHLPGDSVDRHAEAFGRPNVLVELRNDVIAAPADQTAWAARLAPILE
AA
LADTGA
>OLS48094.1 pyruvate kinase [Rhodovulum sulfidophilum]AB14
MRRLRNLKIVATLGPSSTDYATIRALFEAGADVFRLNMSHGTHDEIAQRHATIRKIEADTGRPIAILADLQGPKLRCGQF
ANGAAELEDGQIFRFDLDPAPGEAHRVQLPHKEIFDALEPGATLLVNDGKLRLRVKEHGPDFAECEVIVGGTISNRKGV
N
VPDVVLPVAALSEKDREDLHFALDLGVDWVALSFVQRPEDVIEAREIVGTRAAILSKIEKPAAVKAFDDILEVSDGIMVA
RGDLGVELPVQSVPPIQKRLVRSCRAAAKPVIVATQMLESMIESPMPTRAEVSDVASAIYEGADAVMLSAESAAGRYPV
E
AVKMMDNVAVEVESDPTYRSVIEASRQAERSSVADGIVSAAREIAETTDIKAICCFTESGTTALKTSRERPRVPIVALTP
FPATGRRLCLSWGINCYYNEKVERFKMAVVSAARAARQGGYGEGDDKIIVIAGLPFNLPGSTNILRVAPCEERLIFNLEP
E
>OLS48095.1 50S ribosomal protein L35 [Rhodovulum sulfidophilum]AB14
MPKMKTKSSAKKRFKVTASGRVKAGQAGKRHGMIKRTTKFIRDARGTTMLSAPDEKLAKSYMPYSR

>OLS48096.1 50S ribosomal protein L20 [Rhodovulum sulfidophilum]AB14
MSRVKGGTTTHARHKKVIKAAKGYYGRRGNAFRTATQAVDKANQYATRDRKQRKRNFRALWIQRINAAVRAHDESL
TYSR
FINALAVAGIEVDRKVLADLAVNEPEAFGAIVEKAKAALA
>OLS48097.1 LysR family transcriptional regulator [Rhodovulum sulfidophilum]AB14
MTITLRQLSYFLALAEHRHFGRAAETVHVSQPALSVQIRELEAELGVQLVERQPRGVILTPAGHDLLRHARRVMQEVRA
L
KDAARWQRGLGGRLSLGLIPTVAPYLLPVALPLIRAENLTLEIGVREARTDRLLADLDEGRLDAALLALPTGRDDLVEAP
LFEDRFLLAGSARQIAALGAGAEGVRPDALDPDRLLLLDEGHCLADQALEVCGMKRDETRVDLGASSLATLCGLAAEGF
G
LTFLPEIALRSERAASPALAVHRFAMPEPSRRLGLVRRRISREDGWFVALAAILTEAGERLTRAARDG
>OLS48098.1 lipoyl synthase [Rhodovulum sulfidophilum]AB14
MRDLKNPAERHPEKARRPDTVQPKKPAWIRVRAPGGDGYRDTHRVIREHGLATVCEEAGCPNVGECWSQGHATM
MIMGEI
CTRGCSFCNVATGRPDTLDIFEPGRVADAVQKLGLKHVVITSVDRDDLDDGGAEHFAQTIRAVRHRAPGTTIEVLTPDF
L
KCGPDALETVVAARPDVFNHNLETVPGLYPEVRPGARYFHSLRLLQRVKEIDPSMFTKSGIMVGLGEDRQAVHQVMD
DLR
AADVDFLTIGQYLQPTPKHHAVARFVTPEEFAAYEKAAYGKGFLMVSATPLTRSSYHAGDDFARMRMAREARLAQG
>OLS48099.1 single-stranded DNA-binding protein [Rhodovulum sulfidophilum]AB14
MAGSVNKVILIGNLGRDPEVRSFPSGGKVCNLRIATSETWKDRNTGERRERTEWHSVAIYSEPLVRVAEQYLRKGSKVYI
EGQLETRKWQDQSGQDRYSTEVALRPYRSELTMLDGRDGGGGGGRGGDYGGGSGGGGYDQGGSRGGDYGGPQQ
PASDFDD
EIPF
>OLS48100.1 lytic transglycosylase [Rhodovulum sulfidophilum]AB14
MKRQLAAIALAALAVSGAHPEPVLADTSASSKSRLTLFRSQTRVLDGRAAQQYSNSVRLQPDRVRTPTQGMLLYSGRY
RG
EFVDVARAAARRHGVPEDLFLRLVQQESGWNSRAVSHKGAIGLAQLMPETARRLGVDPTDPRQNLEGGARYLRRQF
DRFR
SWRLALAAYNAGPAAVERHGGVPPYDETRTYVRRIWGS
>OLS48101.1 hypothetical protein BV379_07290 [Rhodovulum sulfidophilum]AB14
MFSIEHDFDATVITLVDEGPEHLREDVTITAFEDCVTVEQLDPRTDRVQKITLSLAQLHELGAALDLPEGVYRIAFD
>OLS48102.1 hypothetical protein BV379_07295 [Rhodovulum sulfidophilum]AB14
MSGQTKYDEHAHLVEKMAEVLGVDLDEETMAGRWTPEDMQDSVSRCLGCTDPSQCRSWLGTHDAGAGETPDYCR
NKDLLE
AMRARLVSA
>OLS48103.1 hypothetical protein BV379_07300 [Rhodovulum sulfidophilum]AB14
MEDQGRLSKHFWLTQGMARTIGVNLNHALRSGQLGRGEYSELVARCCHCGRAEACMGWMGHQVSGADCPPDWC
AVRCSLK
ALKKPA
>OLS48104.1 hypothetical protein BV379_07310 [Rhodovulum sulfidophilum]AB14
MPKVNSVVRISFAGGLIGLVFGSTGGKVDRILQGQNRMGWNMAEVIPDTPILVPRLLSLVATPGLWTISSGDLSITEKSR
>OLS48105.1 ribosomal protein S12 methylthiotransferase [Rhodovulum sulfidophilum]AB14
MSDPRSPNPPDLRPDLAPRARIADPSRPGQPTIGMVSLGCPKALVDSERILTRLRAEGYAISPDYAGADAVIVNTCGFLD
SAKAESLDAIGEALKENGRVIVTGCLGAEPGYITGAHPTVMAVTGPHQYEQVLDAVHAAVPPAPDPFVDLLPASGVSLT
P
RHYSYLKISEGCNHHCRFCIIPDMRGRLASRPAHAVIREASKLAEAGVKELLVISQDTSAYGVDLKHATEKDHRAHITDL

ARDLGALGIWVRLHYVYPYPHVRALIPLMAEGLILPYLDIPFQHAHPDTLKRMARPAAAARTLDEIAAWRAVCPDLALR
S
TFIVGYPGETEAEFRTLLDWLDEAQLDRVGCFQYENVSGAAANALPDHVPAEIKQERWERFMEKAQAISEAKLAAKVG
RR
LEVIVDELDADGIATCRTKADAPEIDGCLFIDEGAEGLAPGDIVTVEVEEAGEYDLWGRLES
>OLS48106.1 hypothetical protein BV379_07320 [Rhodovulum sulfidophilum]AB14
MRWMIRGLLVVVVLVGLGGALLLLMPTDRVARLAADQVEKATGRVLTISGGVRPSLWPEIGVETGAVSLANADWSD
QGPM
LSAEGLAVGIDLARLLRGEIAVREVAVIAPHILLERRADGRGNWQFDRAESAPAPEAAPSSATDEPRQGALADFTLDRGR
ISDGALVYIDHGSDARTELADVDAELTLPDRGGPGDLSLKALLNGQEVGLSARIARATALVDGEQTRVTLDAAVGQASL
A
FDGEAGLAPPSAEGKLFADVSDLRAISAAAGIAPPDLPAGVGQRLVLEGRVALTPDGAIRLDRASFRQDGNTISGDAEFV
PGARPHLEARLSAGTLDLSPFVASGGGGAGKGGSGAASQASPAGWSTAPIDVGWMGALDAEIGLDAEALDFGKVRL
GPVR
LDARLEDSTLSGDLHELQAYAGNVTGSFEVDGRDGLSLDADLDIAGVAAEALLRDMAGFDRLQTSADLQLALQASGRS
VA
ALMGSLSGGGAFDFGRGEFRGLDLAGMLVNLDPSYMGAGAKTIFDSISASFTLDGGVLSNSDLRIAAPLVAAEGRGRV
DI
GARDLDYTLVPSALRGNGKDGISVPLRITGPWAAPSVRLDAGAMAEERLKQERETLERKARERAADELGVKADEGESLE
D
AAKRRLGEEAAKGLKNLFGD
>OLS48107.1 multidrug ABC transporter permease [Rhodovulum sulfidophilum]AB14
MNATGMGVRRFGQVNWLGLYTLSGREIQRFLAVWSQTLAAPLATAGLFLGVFSLAIGPSRGDVMGVPFVQFLAPGIL
MMT
VIQNAFANTSSSILISKVQGNIVDTLMPPLSPGELLAGYTAGGVARGLLVAVAVGLLMLTVLGMGVAHPVWALVFVLLG
S
AFLSALGIMAAILANKFDQMAAITNFIITPLSFLSGTFYSIERLPLGMQIATHANPVFYLIDGVRYGIIGRSDSSPWLGL
GVCTLATALVMALCWHWFRTGYRMKA
>OLS48108.1 tryptophan synthase subunit alpha [Rhodovulum sulfidophilum]AB14
MTRIDAKFAALKAENRKAFVAYVMAGDPDYATSLDIVKGLPAAGVDIIELGLPFTDPMADGPTIQLAGQRALDGGQTL
QK
TLELARAFRAGDDSTPIVMMGYFNPIHSRGVERFLTEAKEAGIDGLIVVDLPPEEDTELCLPAQAAGLDFIRLATPTTDD
RRLPRVLENTGGFVYYVSITGITGAAAAEATDVAPEIARIKAKTDLPVIVGFGIRSPENARAMAAVADGAVVGSAIVKLV
EEKKPVAEILAYVKTLADGAHSA
>OLS48109.1 GTP-binding protein YchF [Rhodovulum sulfidophilum]AB14
MGFKTGIVGLPNVGKSTLFNALTRTAAAQAANFPFCTIEPNVGEVAVPDPRLDRLAEIAGSKQTIPTRMTFVDIAGLVKG
ASKGEGLGNQFLANIRECDAVAHVLRCFEDGDVTHVEGRVDPVADAEVIETELMIADMESIEKRLQGLVRKVRGGDKE
AV
QQERLLKVALAALNEGRPARSVEIAEDDEKAWSMLQLLTSKPVLYVCNVSEEEAASGNAFSRAVAAMAEAQGAASVVI
SA
KIEEEISQLDAEEAEVFLGEMGLEEAGLDRLIKAGYTLLDLETYFTVGPKEARAWTIPVGTSAPKAAGVIHGDFERGFIR
AETVAFDDYIACGGEQGAKDAGKMRAEGKGYVVQDGDVMHFLFNA
>OLS48110.1 type II 3-dehydroquinate dehydratase [Rhodovulum sulfidophilum]AB14
MKTIYVLNGPNLNLLGQREPEIYGRETLADVEASCARVAAGAGMEIRFLQSNHEGALVDWIHEARQAAAGIVINPAAF
TH
SSVAILDALNAFEGPVIEVHISNVHRRERFRHRSYVSLRADGVIAGCGVEGYELALKRIATLLG
>OLS48111.1 sodium:proton antiporter [Rhodovulum sulfidophilum]AB14
MDIILLTTIVACLFLVIGASEPLAARLRLPYAVILAALGILVGGGAIFFLRTDLTDALNPVAEAILGLPIRSNVFLYVFL

PTLLFQVTLGMNLRRMLDDWVPILVLAVVAVVVATLSVGYALAFASALPIAVCFLVGAIVSTTDPSAVVGIFRSISAPRR
LARIIEGESLLNDAAAIALFGLFIGFVMLGVPDPTLSDALTQVPVLVAGGVLAGWIAARVALWIMALVARFELAQITVSV
ALPYLAYIVAEQIVGASGVIAVVTAGLTLNLTGPGRLTPVAMTKLREVWDLLAHWAGALIFVLAALLIPRLLEELRLEDL
KLVAVVVAAAIAARAVILFGLMPLLSALKLSPMVERPYRVAILWGGLRGAVTLALALAVTESLRVPGEAKRVVGILATGF
TLFTLIVQGTTLRWVIGRLGLARLSPLDQALSNQVVAVSLQTVREDVARTTGNYNLSHDIVRSEAKRFGERLDEAVKAAE
AEAPILDRDRVTLGLIALAGAERDAILDRIRDRTMSTGLTDRILSNVDRLIEGARSGGRTGYVRASKASTGYGRTFRLAV
VLSNRLGVSAPLARMTADRFEILLSQRLILRDLSGFIDGRIRRIHGRRVADLLHELLARRSEAVETALDGLRLQYPGYAE
ELERRFIRRTALRLEEREYLAMREDGLIGAELHAALMQDIARRRTEAEERPRLDLALRKADLVRRLPLFADLDEPALRRL
ARSLLTRHVNPGEVIFRKDMPAKSVFFIATGAVEMETAGQTVRLGRGEMFGQMSMLLQKPRRAEARAIAPSTLLFLDE
LR
FRKLLSRSPTIRAAVRDSALRRGISPDTILPGEAQAG
>OLS48112.1 hypothetical protein BV379_07355 [Rhodovulum sulfidophilum]AB14
MTRCLSLLALCIAVIASPATAQTLSQANAQMLREMQSARGVSAAAIARIEAIFARSAVIGQGNPAITRHAMNREACNAR
V
GGNALAHYADKRFERICGARYMAPLYDPRSERPEDAKACIDQFEYPGIPCSYPVVWVKAKEAAEICAAEGKRLCDAHE
WE
GACAGALEPPDYRFDLARGQSASGAVGRMRTAHNAKYKGSASWSYGPAFRSGICAQNSVKSPSCPGGGFGNCGSNT
YPTG
SFANCKSPLQVVYDINGNAAEHMNLPLAPDQMASAGSTRLGVTEMKGSWFIWDRYRAHPDWCRWRAPFWHGTRV
MSPASHE
NYHLGFRCCKTLN
>OLS48113.1 hypothetical protein BV379_07360 [Rhodovulum sulfidophilum]AB14
MTAFIQIATQSFRALAWAVCLALPPASLPGQTIPHPGAVRLALQNADARAPRPKSVIELQGLRASQRALSRDGTRLTLTS
LNPHANGWFLLEIERPGRQPETYHLENPTPLTRDVSLSDDPAPAIMLTEGQAQIKCRPWAVATPELSDARASGLPFAPL
C
DGRLYLRNPVSGARTNLERTAEFLRDNLWFGESLVGLVKDTLYKDRYLASGQLLAGADPGAVDRLLGRAALRDFPVMY
TT
MTFDLVGATPKRMEMGAWYAIGGAPGIYASALQPRMIAPEILRRRGEANGLDGIEGRADVYLVAFDMTHFDIGYETG
TEH
PRLDWSSRPSGPGRNWSLPGPDGFDTARPLATVGMINPEEARRVAATFTGGFKRDHGAFRFGPMARSNNGHHYGF
VVHGT
VLSSLQPDLSTLYVLDDGTIGMKTWTEADNALLPQIRFARQNGVPIIERDTATGRGVVGPLVRQWGAGNWSGSANAD
LRT
LRAGACMKTAAGRTFLIYGWFSTATPSAMARTFQAYGCDYAMLLDMNALEHTYMALYTPGKAGGLDTRHLVAGMS
RIDEK
SRDGRPIPRFIGFPDNRDFFYLMRKESPR
>OLS48114.1 UDP-glucuronate 5-epimerase [Rhodovulum sulfidophilum]AB14
MTRVFITGTAGFIGFHLADLLLSEGMIVHGFDGMTDYYDISLKRRRLALLEGRPGFTMTEAMLEDDPALSAAVDACDPD
I
IVHLAAQAGVRYSLENPRAYIDSNLVGTFNLMEAARRHHVSHLMMASTSSVYGANIDMPFTETQKADLPLTIYAATKK
AT
EAMGHAYAHLHDLPTTMFRFFTVYGPWGRPDMAPIKFASRIVKGEPIDIYNHGDMYRDFTYVLDLVRGIRLLMDAVPE
RP
ADGQVPEGDSLSPVAPFRVVNIGNSEKVRLLDFIDALESELGVKAIRNYMEMQPGDVPATWADASLLQRLTGYRPQTP
MR
DGVAGFVSWFRSHYAT
>OLS48115.1 transcriptional regulator [Rhodovulum sulfidophilum]AB14
MAKDLETLTLKELKDLRKDIDSAIASFHDREKAKAKAVLEATAKEMGFSLDEIIGKKSRRVKPVAKYRNPENPTETWSGR

GRRPRWLEEALSKGASLEDFAIA
>OLS48116.1 LuxR family transcriptional regulator [Rhodovulum sulfidophilum]AB14
MDLRKKLTDRLSALAPAGYYVGLRLGFTYPAQEINRLPGDWTDHYARNGLIVQDPVFRWVYARTGVISWAALAFDDH
ADV
FGQAASFGLIHGAVVSVMRPGESGRRSYGTFARSDRTYAESEMAELLQIVTRLHRAGSERTGLTHAETEALRLQADGLR
L
KEISAELEISMSAVKARLANAKRKLGAATTSQATSMARSRGVI
>OLS48117.1 hypothetical protein BV379_07380 [Rhodovulum sulfidophilum]AB14
MNRVNKVAELLEMLQRTCDTGFVLALHVRFTRPLVLYRTYPQSWCDHYDLNGLVMLDPAVRWSFCNTGAIQWDDPS
LDDP
GGVVEAARAHGVANGVSVSVGSATSRSLGGFSRSSGRFDAADMDRLSDLVETLHLVLDGVSEDEQGPELRALRALSTS
VL
QP
>OLS48118.1 enoyl-[acyl-carrier-protein] reductase [Rhodovulum sulfidophilum]AB14
MFSLEGQTALIVGIANDKSIAWGAARALRAQGARLAITYLNEKAEAFVRPLADEVDAEIFMPLDVCNEAEMDAVFAEIG
A
RWGRLDTVLHSIAFCPRDDLHGRVVDCSAEGFSLAMDVSVHSFLRMIRKAEPLMPDGGTCMTVSFAGATRVVEHYNI
MGP
VKAALESTVRYVAAELGPKGISVHALSPGPLATRAASGIAEFDELLDAAAERAPIHHLATIEDVGAYCAFLASREAKNVT
GGVHAIDGGYSIIG
>OLS48119.1 alpha/beta hydrolase [Rhodovulum sulfidophilum]AB14
MDMHVERRSDDDPADVMRALIWGSGGMAQDRLALMSALAGGSLENADLIREVAGRHGNRMLDTHSTRTRELLAQ
MRRLME
AARNSKKEMTPGQLVADYRDYLRDAGERWLLTMDALRSRGDIFREHEAEGCPPVLAYRYEVVIDGTDLPHPCNYQLLR
IE
PPEGVELRPDKRPYVIIDPRAGHGGGIGGFKPDSQVGVAFKGGHPVYFVAFGRDPLPGQTLADVCRAEAEFVREVTRR
HP
AAAKPVVMGNCQGGWATLLLAASNPDLTGPIVLNGAPVATWSGEVGRHPMRYNAGILGGAMQPMLWSDLGGGR
FDGANLV
MNFELLNPSRNYFRKYYDIFRDIDQPGMRQRFLDFERWWGGFFTLNEAEIRWVVEQLFVGNRLTRNTAMLEPGRPVD
IKA
IRAPIIVFASQGDNITPPQQALNWILDAYAGVEEIRIRGQRIVYMVHEEVGHLGIFVSSKIARKEHTEVSSTLRTIEALA
PGLYEMRIDAAEGRGEDREFLVSFAERSFADLEQIAGGREEEAAFAAVSRMSTIQSEIYDTAVRPLVRASVNAQAAETTR
RLHPLRVQRTAMSSVNPLLAPVAAAAGRLREERHPAAIDNPFLWAERFAADMTEQWIDFGRDMRDAWVEMSFFAM
WNSPW
ARAFGAPEAVQRTRKTTAELRAMPIVQTVLSHVDQGGFVEAVIRMLILLAESRGSVRRDRLERSAWVLNHDEPFASLTA
E
CRARILHEQTLIATHDPEGAIGTLPCLLRDPEDRELAARVVQYVPGPIAEMAPHTLEMLQRFRAVLGLSPLAEDVVEDPL
ASHGAVVHGADMARSGSAATGVPGAEAGIGDAPKTVSERKTSE
>OLS48120.1 enoyl-CoA hydratase [Rhodovulum sulfidophilum]AB14
MKITENRTFDEISVGDTAQIVRICSEDDFYVFASASGNYNPMHLARLDGDGDGVDEALAPAIWEASLISAVLGNLLPGP
G
TLYRSQSLVFHERAQAGDELLTRVTVVEKRPPNRIVLETRVERCSDGALVLDGEAEVKAPPKKLVFNDLEIPGLVIERHR
HFDELIDRAEPLAPLPTAVVCPEEPRALGGALLGARHTIIAPILVGDPDKIAAAAAEIGASLAGIEIVEAADHVTAAHLA
VDLVAGGRAAALMKGHLHTDVLLRAALRKENGLRAGRRFTHVFVMDVPGVPHPLMVTDAAINIAPDLRTKVDIVQNA
IDL
AISIGIEVPKVGVLSAVETVNPDIPSSLDAALLSKMAERGQIRGGMVDGPLAMDNAVDLTAARTKGITSQVAGRAEVLV
V

PNLDAGNMLAKQLAYISHAEGAGVVMGAKVPIILNSRSDDDKARLASCAVATLHNARLNGLIPAQRAAE
>OLS48121.1 acetate kinase [Rhodovulum sulfidophilum]AB14
MSHVLTLNAGSSSLKFGFFRLNGATDDPDPRIVGSVERIGTGERARLKAKRQDGMVLVDREFGRNHVADHETALARAV
EF
LKEQRPQAELDAVGHRVVHGGPDYDMPVELTGEVMARLAQFEPLAPLHQPHNLAGVRAALKLFPEAVQLACFDTAF
HRNH
PWVNDTFAIPRKYYEQGVRRYGFHGLSYQYISETLRETAPHLHMGRTVVAHLGNGASMCGLMGGQSRASTMGFTAL
DGLP
MGSRCGQLDPGVLLYMLRSEGMTPAEIEDELYRKSGLLGLSGLSNDMRTLEAAGTEEARQAIDYFVFRIRRELGGLVAA
L
GGISTMVFCGGIGENSVLIREKVCTGMGWIGMELDETRNMRNERIISTDFSRVRILVIPTDEEIVIARAARDMLARRAAD
AA
>OLS48122.1 acetyl-CoA--acetoacetyl-CoA transferase subunit alpha [Rhodovulum sulfidophilum]AB14
MKRALKPAEAAEMIPEGAVLLIGGFMGVGAPHRLIDALVARGVGGFTVVANDTVRPGQDIGKLIDARLVTKCIVSHIGL
N
PETQRQMIAGDLEVELVPQGTLVERIRAGGVGLGGVLTATGLGTVVAEGKPTVEVDGKTYLLEKPIRGDFALIGARQCD
Y
VGNLEYTLTAHNFNPVMAMAADTVIAEPHHVMPVGMIPPDAVKTPGVLVDHILERLV
>OLS48123.1 succinyl-CoA--3-ketoacid-CoA transferase [Rhodovulum sulfidophilum]AB14
MDAKEVIARRVAQEVREGMLVNLGIGLPSMVANFVPDGMHVFFQAENGVIGLGQRPPDGMEDRDLTDAGGGFVTA
VPGAA
SIDSAMSFGLIRGGHLDMTVLGGLQVDEKGHLANWMVPGRMVPGMGGAMDLVTGARRVIVAMVHAAKGAPKIVP
ECTLPL
TATRRVSLIVTEMAVIEPTDEGLVLRERGPGVTVDDILAATSAALIVPDDVAEMVLD
>OLS48124.1 hypothetical protein BV379_07415 [Rhodovulum sulfidophilum]AB14
MTDDLSPAAGDAAAWAVLEEAEEHYRRMIRALNDLIEEIQAGNSERARMLRGALNDLGKAAQTAFDERQRVERRLNA
ESG
SDGGIQALDLDQARLEVRRRLDRLRSARDADSVSGGDG
>OLS48125.1 ATP-binding protein [Rhodovulum sulfidophilum]AB14
MDEAVLLALPYLFEFWALPHQLAPEGDWRSWVIMGGRGSGKTRAGAEWVRLQVEGARPLDEGRARRVALVGETIDE
AREV
MVFGESGILACSPPDRRPNWEATRRRLVWPNGAVAQVFSAHDPDSLRGPQFDAAWSDELAKWKRAEETWDMLQF
GLRLGG
DPQQCVTTTPRNVPVLKAILSNPMTVVTHAPTEANRANLAASFLEAVRTRYSGTRLGRQELDGILLEEAESALWTLARLE
ALRVDAAPELDRIVVAVDPPVSSHAGSDECGIVVAGVRMQGPPQSWSAYVLEDVSVAGAAPTAWAKIALAAMERHS
ADRM
VAEVNQGGDLVETMIRQLDAMVPFRAVRATRGKIARAEPVAALYEQGRVRHLRGVQVLEEQMCHMTAHGYEGRGS
PDRVD
ALVWAINDLMISPAAHWRRPRVRGL
>OLS48126.1 phage portal protein [Rhodovulum sulfidophilum]AB14
MKFNLFRRATETLPETKASATGPVIAWAGAGRAAWTPRDTGSLIRAGFNANPVGFRAVKLIAESAAALPLICQDEDRRY
E
THPVIELLRRPNPAQGRAELMEALYGQLLLSGNGYLEAVGGETGLPFELHVLRSDRMRLVPGTDGWPVAYDYMVGAR
KHR
FDMTGPVPPICHIKNFHPQDDHYGLSPMQAAATAVDVHNAASRWSKALLDNAARPSGAIIYTGSDGQSMLSEEQYSR
LQD
EMLSYHQGAANAGRPMLLEGGLDWKPMGFSPSDMEFQKTKEAAAREIATAFGVPPMLLGIPGEATYANYQEANRAF
YRLT

VVPLVAKVTGALAEFLAHFGSSAVSLKPDLDQVPALAAERDTQWKRISEAEFLTEDEKRALLGLPRRPEGG
>OLS48127.1 hypothetical protein BV379_07430 [Rhodovulum sulfidophilum]AB14
MPQPRSGSRFLYAPFDATHIRIEASERVMEERWSALEYRLKRIEEMLDQIEKRVWLTVYGIVAVILAQGVASILSAISAP
>OLS48128.1 peptidase U35 [Rhodovulum sulfidophilum]AB14
MKEYDVGSALEHKFCKNDALPLALKEGAVIEGYASVFGAADFGGDIVQGGAYAASLRQIAAEGRRVKMLWQHDPSEPI
GV
WDEVREDARGLYVKGRILTDVTRGREAAALVAAGALDGLSIGYRTKRASKDASGHRLLAELELWEVSLVTFPMLADARV
G
TKAEESAEALLRDLADAFAGARRALAEG
>OLS48129.1 capsid protein [Rhodovulum sulfidophilum]AB14
MTETEAKTGRVRGPAVEIREALEDFMKDFSSFQSEIKSRLKQQEERLTMFDRKSQVTAGRPVLSRSAEIEVPHKKAFGAY
LRTGDDDALRGLEVEAKAMSTAVAGDGGYLIDPETASSIQSVLTSTASIRSISQVVTVEASSYDVLVDHSDTGSGWSSET
GSQVETGTPQIERVTIPLHELSAMPKISQRLLDDSAFDIEGWLAGRIADKFARAEAAAFISGDGADKPRGFLNHPKVPEA
SWSWGSLGYVATGADGDFDVGAPSDAILDLVYSLGAVYRANATFVMNSKTAGAVRKMKDADGRFLWSDGLAAAEP
ARLLG
YPVLIAEDMPDIAADAAAIAFGDFQAGYAVAERPDLRVLRDPFSAKPHVLFYATKRVGGDVSDFAAIKVLKFSVS
>OLS48130.1 hypothetical protein BV379_07445 [Rhodovulum sulfidophilum]AB14
MMLIEQTRVPGAALPLAQFKAQLRLGTGFAGEENQDAYLEALLRAAIAAVEARTGKALIARSFSYELQGWRQSDRQVL
PV
APVQAVTGIRLVDRNGTAVPVPPGRYRLVADTHAPQLRGTGGTMPGIPFEGSVRIDFEAGFGADWAAVPEDLGHAVL
LLA
AHYYENRLEQPTGKGAMPFGVAALTDRWRPLRLTPGAPR
>OLS48131.1 phage tail protein [Rhodovulum sulfidophilum]AB14
MSRVPILNRQLVLEVPVPVADGAGGFSETWAARGTLWAEMSPRMAGERAGEELALSRVLYRVVVRGAPPGAPSRPV
AGQR
FREGTRYFHIRAVTEHDARGLYLACYAEEEIGA
>OLS48132.1 hypothetical protein BV379_07455 [Rhodovulum sulfidophilum]AB14
MSYGMAAALQAAIFERISGHAAIAAIVGDAIFDAVPAGRVPPIYVSLGPEEVRDASDKGADGARHLVTISVVSEEAGFAR
AKAAAGAISDALTGAPLALARGRVVGLWFDRARARRVGAKDQRRIDLRFSVRVEDD
>OLS48133.1 phage major tail protein, TP901-1 family [Rhodovulum sulfidophilum]AB14
MTAQNGKDLLIKVDISGAGDFSTFAGLRASRISFNAEPVDVTSLESQGGWRELLVGAGVKSAAISGSGVFKDAETDARA
R
QVFFDGLMPLFQLVIPDFGVVEGRFQVSSLEYAGSHNGEATYEMTLASAGVLSFTAM
>OLS48134.1 hypothetical protein BV379_07465 [Rhodovulum sulfidophilum]AB14
MPNPHAGEVALMVNAERRVLKLTLGALAELEAALGADTLLALIERYEEGHFATRDVVALLLAGLRGGGWKGTTADLAE
AD
IGGGPIAAAQAAAQLLARAFTLPEARQDGL
>OLS48135.1 phage tail assembly chaperone [Rhodovulum sulfidophilum]AB14
MGFDWGTLMRAGIRGLGLRPAEFWALTPAELMLMLGADGGPAPMSRARLEELARAFPDKPRDEG
>OLS48136.1 phage tail protein [Rhodovulum sulfidophilum]AB14
MADADDIESFEAQLEALETTLGGASGTAAAFEVELVRMRDTLTLTNREVSSLSNAIGRGLRGAFDGLVFDGMKLSDALK
S
VANSVANATYAAAVKPVQGQLGGLLAGGIEGLLGAALPFAKGASFSQGRVIPFASGGIVGQATYFPMRGGTGLMGEA
GPE
AILPLSRGADGRLGVRSESGGSARPVQIVMNINTPDVQGFARSQSQIAAEMSRLLSRAQRNR
>OLS48137.1 glycoside hydrolase family 24 [Rhodovulum sulfidophilum]AB14
MGFHEVRFPANLSFGSVGGPERRTEIVTLANGFEERNSPWEHARRRYDAGVAMSSLDDVEQLIAFFESRRGQLYGFR
WKD

WSDYKSCRPSAEIAPGDQVVGFGDGGTVQFQLQKTYASGGQSYVRPIAKPVAGTVRLAVDGTEIFAPTDYMIDLASGL
VT
LAEAPPTGAVVSAGFEFDVPVRFDTDRIQSSVESFRAGDVPSVPVVELRI
>OLS48138.1 hypothetical protein BV379_07485 [Rhodovulum sulfidophilum]AB14
MPLSGTFLAHLESGATTLARAWALVRRDGVVLGFTDHDRDLVFAGVTFRADTGMTARALSQTTGLSVDNSAAVSALS
SEA
ITEEDISAGRYDGAGLRIWLVNWRDPEQHALIFTGTLGEMTRTAGAFEAELRGLAEGLNQPQGQIYQAPCGAVLGDGA
CR
FDTGRAGFSAVLAAVAVTGHRAFRFEGLSGFASRWFEQGRLEVMSGPAAGLAGAIKSDREENGLRVIELWSEIRGPVA
AG
DTLRLTAGCDRRAETCRSKFDNFLNFRGFPHIPGEDWLMSYPTRTGVNNGGSLTS
>OLS48139.1 peptidase [Rhodovulum sulfidophilum]AB14
MNDMGERAAAEARRWIGTPYRHQGTVRGAGADCLGLLRGVWRALYGREPEEVPPYTPDWSEPQGEEVLWRAAGR
HLIAKP
LEQAAEGDVILFRMRDDTIAKHLGLQGRVSGVPSFIHAYSGHGVVESSLSEPWTRRIVARFAFPERI
>OLS48140.1 host specificity protein [Rhodovulum sulfidophilum]AB14
MATIVLSAAGAAAGSALGGSVLGLSSAVLGRAVGATLGRVIDQQLLGSGSQAVETGKVERFRLTGASEGAAVGRLWGR
SR
IAGQVIWASRFRETVSSQGGGKGAPSRPKVKQFSYSVSLGIALCEGRIARVGRIWADGTEIEPQSLTMRVYDGAEDQLP
D
ALIEAIEGEGRAPAYRGTAYVVIADMDLARFGNRVPQLTFEVVRRAAGAVDDLPGLVQGMALIPGTGEYALATTPVHFS
Y
GPGQAASANVNTPSGKTDIATSLDMLQGELPNVRSVSLVVSWFASDLRAPVARIRPKVEQTKFDGQPMPWQVAGLS
RRMA
EVVPHVDGKPIYGGTPTDASVVEAIRALRQRGQEAVFYPFILMDQLEGNSLIDPYTGVAGQPPLPWRGRITLNFAPNHA
F
TTDGSRFAEDEVALFFGTARPEDFELSGQDILYSGPDDWSYRRFILHYAHLCVLAGGVSAFCIGSELRGLTTIRGAEGRF
PAVEALRALAADVRRIVGPSCKIGYAADWSEYFGFHPEDGTGDVLFHLDPLWADPAIDFVGIDNYMPLSDWRDGDEH
ADA
GWRTIHDRDYLRSNIEGGEGFDWYYGSQAARDAQIRTPITDGAHGEPWVWRYKDLRSWWQNAHHDRIDGVRSAVP
TAWVP
QSKPIWFTELGCPAVDKGTNAPNLFSDPKSSENALPPYSTGARDDLIQHQYLRAMLGYWGDPAINPVSELYGAPMVD
MSR
AHVWAWDARPYPQFPANRALWSDGDNYSRGHWLNGRASAQALDAVIREICTEAGVFDPDVSDVYGLVRGYAVGDL
AGARA
ALQPLMLAYGIEAAERDGRIVFFNRDGRERVAIDAADLARTGELAGDIEHSRTPEAETAGRVRLSFVEADGAYETRAAEA
IFPDEASRTVSASEMPLVLTGAEGQAIVERWLAESRVARDRVRLALPLSSLSVGAGDVMHLSSGDYRIDRVDLGDTRRIE
AVRVERGLYLPPRIDDRGVLQPAFAAPLPVHALFMDLPLLTGDEVPHAPYLAAAAVPWPGSVDLYAAVEDAGYTLNTT
LE
RSAVIGITETPLHAAAPGRWDRGPALRVKLASGTLSSVREADLLAGANLAAIGTGDAAGWELFQFAEAELVEAGTYELR
M
RLRGQAGTEADMPELWPVGSTVVLIDARLSQIALPASARGLERHYRYGPGTRAFDDPSYSHARLAFDGIGLRPYAPCHL
R
SRKGPGGDLTLSWIRRTRIDGDSWASVEVPLGEAREVYALRVMNEAGDELRAETVTAPVWTYTTAMQMVDGAAAPF
AVEV
AQLSDQFGPGPYRRIEIDG
>OLS48141.1 hypothetical protein BV379_07500 [Rhodovulum sulfidophilum]AB14

MANTANLSLSLLQAAQAQKHVIVNEALIRLDALVQLRLQSLTVSAPPLGVPDGLAWGVPVGGAGEWEGQDGRIALRD
NGG
WVFVTPLTGWRAFVVDAGTDMRWTGGGWTMVGGAVAPSGAGIGFRVLEFDHPVATGGVQNTAVAIPSHATVFGV
TGRVIE
EITGTLTSWRLGVDGSDNRFGSGLGLGLNSYVRGVLGRPLTGYAAMPLRLSPEGGSFSGGMVRLAVHFTELSLPASV
>OLS48142.1 hypothetical protein BV379_07505 [Rhodovulum sulfidophilum]AB14
MRPVLHGDAVAAARALYRLPATERERMIGSMLLRVEAADCYRKRFGRGHPRWGDGSLMALAQNLDLPPEPSLDDTD
YCRC
LAQVLGALATWRDWKAALSAPG
>OLS48143.1 serine O-acetyltransferase [Rhodovulum sulfidophilum]AB14
MPEIKPRLAEIDPVWKRICAEAEEAVSAEPLLGGLMHSSLLHHAGFEDALAYRFAMKLSSGEMSEQLLREIADQAYADD
P
ALGRAARVDLTAVHDRDPACHRYIQPILFFKGYQALQAYRVGHWLWQQGRQDMAYLVQMRVSESFGVDIHPAAKL
GQGIM
IDHAHSIVIGETAVVGDNVSMLHSVTLGGTGKDDGDRHPKIGDGVLIGAGAKVLGNIHVGHCSRIAAGSVVLTDVPPCS
T
VAGVPAKIVGEAGCDQPSVMMDHRLGGCDGD
>OLS48144.1 pyruvate dehydrogenase complex dihydrolipoamide acetyltransferase [Rhodovulum sulfidophilum]AB14
MATEILMPALSPTMEEGTLAKWLVKEGDTVSAGDLLAEIETDKATMEFEAVDEGTVGRILIEAGTEGVKVNTPIAVLLEE
GESADDIDSAAKAPAPTPAPEAPQADAGGKAAPEGGTGGDSAPAAAPPAPSTGSGERIFASPLARRIAAEKGLDLAAIE
G
SGPRGRIVRADVEKASAAPKAPAASAAPAASPAAAMPAGASADAVAKIYENREHEVVTLDGMRKTIAARLTEAKQTIP
HF
YLRRDIRLDALLEFRKTLNDQLGARGIKLSVNDFIIKACALALQQVPKANAVWAGDRILQLKPSDVAVAVAIEGGLFTPV
LMDAETKSLSQLSKEMKDLATRARDRKLAPQEYQGGSFAISNLGMFGIDNFDAVINPPHGAILAVGAGVKKPVVNDAG
EI
EVATVMSVTLSVDHRVIDGALGAELLQAIRDNLENPLAMLA
>OLS48145.1 pyruvate dehydrogenase complex E1 component subunit beta [Rhodovulum sulfidophilum]AB14
MATEILMPALSPTMEEGTLAKWLVKEGDTVTAGDILAEIETDKATMEFEAVDEGIMGKILIAAGTEGVKVNTPIAVLVEE
GEDADAAAKAAQEAAPAADAAGKAASDGGSAPDAAPSAPAKAKAPAADPEIPSGTKMKSTTVREALRDAMAEEMR
RDGDV
FLMGEEVAEYQGAYKISQGLLDEFGSKRVIDTPITEHGFAGLGVGAGFGGLRPIVEFMTFNFAMQAMDQIINSAAKTLY
M
SGGQMGCPIVFRGPNGAAARVAAQHSQDYAAWYAHIPGLKVVQPYSAADAKGLLKTAIRDPNPVMFLENEILYGRSF
DVP
VMDDFTVPFGKARIWREGKDVTLVSFGIGMTHTLEAADALAKDGIEAEVIDLRTLRPLDTATVIESVKKTNRCVTVEEGF
PVCSIGNHISAVLMQEAFDWLDAPVINCTGKDVPMPYAANLEKLALVTTKEVIEAARAVTYR
>OLS48146.1 pyruvate dehydrogenase (acetyl-transferring) E1 component subunit alpha [Rhodovulum sulfidophilum]AB14
MATRKPPAKPNISKEELLTYYREMLLIRRFEEKAGQLYGMGLIGGFCHLYIGQEAVVVGLEAAAEEGDKRVTSYRDHGH
M
LACGMDPKGVMAELTGRQDGYSKGKGGSMHMFSKERHFYGGHGIVGAQVPIGAGLAFSDKYKDNGRVTFTYFGDG
AANQG
QVAETYNMAQLWDLPVIFVIENNQYAMGTSVKRSTKSPDFWERGAAYGISGESVDGMDVLAVKAAGEKAVAHCRA
GKGPY
ILEVMTYRYRGHSMSDPAKYRSREEVQKIREERDAIENVRELILQGKHATEDELKEIDKEIKAVVNEAAEFAKESPEPPV

DELYTDIYAETAPQNA
>OLS48147.1 septum formation initiator precursor [Rhodovulum sulfidophilum]AB14
MATTRLRPAWGALVYIGVSFSLAMYFTFAAVQGDYGLFRRIQIEADAAKLEARKAELEAELEAISNKTRRLSDSYLDLDL
LDERARSVLGLIRADEIVVR
>OLS48148.1 phosphoglycerate kinase [Rhodovulum sulfidophilum]AB14
MAWKTLDDMDLAGKVVLVRVDINVPVEDGRVTDATRIERIKPTVDDILKAGGKPVLLAHFGRPKGQVVPEMSLKITLP
AL
EEVFGRGVIFADDCIGLPAKQAVAALSEGDILLLENTRFHEGEEKNDPALAAAMAALGDIYVNDAFSAAHRAHASTEGI
A
KLLPCCAGRLMEAELKALEGALGNPARPLVAVVGGAKVSTKLDLLGNLVGRVDYLVIGGGMANTFLAAQEINVGKSLCE
H
DMTGTARDIKDKADTAGCQIVLPVDVVVAREFRAGAVNETVAADACPADAMILDAGPQSVELIEEVFDKCKTLIWNG
PLG
AFEIDPFDAATNAAAKKAAELTEAGQLVSVAGGGDTVAALNGAGAAEKFSYISTAGGAFLEWMEGKTLPGVAALED
>OLS48149.1 peptidyl-prolyl cis-trans isomerase [Rhodovulum sulfidophilum]AB14
MAEIKDPENTIIMELKGGRVTIALLPEVAPKHVERMKALARAGAYDNVVFHRVIEGFMAQTGDVAHGNAEKDYNPRR
AGT
GGSDLPDLPAEFSKLPHDRGTLGAARSANPNSANSQFFINFSDNHFLNGQYTVYGRVLEGMEHVDAIVRGEPPADPDR
MI
SVKVAADA
>OLS48150.1 peptidylprolyl isomerase [Rhodovulum sulfidophilum]AB14
MRKLLVALLCTLAGPALATGLDIEIAGEGANGTVHVELMDDVAPDHVQRITELAREGAYDDVVFHRVIDGFMAQTGD
VEF
GKFGMDMRRAGTGGSDRPDLKAEFSDESFDRGVVGMARSANPDSANSQFFIMFAPAPHLNGQYTVVGRVTEGMD
VVDAIK
RGTTGRNGEVMGQPDRMVKVSVTD
>OLS48151.1 tyrosine--tRNA ligase [Rhodovulum sulfidophilum]AB14
MTYHPKSDFLRVMIERGFLADCTDYQGLDEALSTGVVTTYIGYDATAASLHVGHLLNIMMLRWLQKTGHRPITLMGG
GTT
KVGDPSFRSDERPLLTPEQIDANIAGLKDVFAKYLDYGEGPTDALMLNNAEWLDGLNYLDFLRDIGRHFSVNRMLSFDS
V
KSRLDREQSLSFLEFNYMILQAYDFLELNRRYGCRLQMGGSDQWGNIVNGIDLTRRILESEVYGLTSPLLTTSDGRKMGK
SQGGAIWLNGDMLSPYEFWQFWRNTTDADVGRFLKLYTELPVEECERLGALAGSEINDAKIVLANEVTALAHGRAAAE
AA
EATAREVFEKGGTGEDLPTVALGAGELAEGISVAQLLTRTGLAKSGKEAKRLIAEGGARLNDAPLTDAGLMVDAAMLAE
P
IKLSAGKKRHALVRLDG
>OLS48152.1 anhydro-N-acetylmuramic acid kinase [Rhodovulum sulfidophilum]AB14
MMQTGPVRALGTMSGTSLDGVDVALVETDGETLAGFGATGYRPYAPAERAVLAQALGRWPGEPGVAEAARVVEAA
HRAAM
APFAGAELAGMHGQTLSHEPGGRGTHQAGDGAGLARALGMPVVWDFRSADVAAGGQGAPLAPFFHFACARRAGL
SAPVAF
LNLGGVGNLTLVDPTRDRPEAEGALLAFDTGPANAPIDDLLRSRCGQSCDRDGALAGGGRIDEAVLTEFLAHPYFRKPP
P
KSLDRNDFAGLLDAVAGCADADAAATLTAAVAATVAVGVGLCPRRPARLLVTGGGRRNPVLMRMIAERTGLAAEPVE
ALG
LDGDMLEAQAFAFLAVRVARGLPTSCPGTTGVARPVSGGRISQP
>OLS48153.1 cupin [Rhodovulum sulfidophilum]AB14

MTAEEIIATLGLAPHPEGGWYRETWVAPAGPGGRSSGTAIYYLLTEGQRSHWHRVDADEIWLYYAGDPLELSLAATEA
GP
ARDLVLGPDLAAGARPQAVVPAGQWQAARPLGAWTLVSCTVSPGFRFEGFEMAPPDFDIPRA
>OLS48154.1 hypothetical protein BV379_07565 [Rhodovulum sulfidophilum]AB14
MTQEDTAPSQRETLSSDDIVSRILGFTLFCMGFSRAPADQVARGRGWLDEAGQPTADGKALIEALMSRDSAYGVYKIV
Y
>OLS48155.1 phosphopyruvate hydratase [Rhodovulum sulfidophilum]AB14
MSTIIDIIGREILDSRGNPTVEVDVILEDGTMGRAAVPSGASTGAHEAVERRDGDKTRYMGKGVLDAVAAVNGEIAEAL
V
GFDVTEQVAIDGTMIELDGTQNKGRLGANAILGVSLAAAKAAADFTAQPLYRYVGGTSARVLPVPMMNIINGGEHAD
NPI
DIQEFMIMPVSAGNIRDAVRMGSEVFHTLKKELAAAGHNTAIGDEGGFAPALNSTRDALDFILKSIEKAGYTPGEDICLA
LDCASTEYFENGKYEMKGEGLSLTAAENVEYLGKLLADYPIISIEDGCAEDDWEGWKLLTSELGGKVQLVGDDLFVTNPE
RLARGIKDGIANSLLVKVNQIGSLTETLKAVDMAHRARYTNVMSHRSGETEDATIADLAVATNCGQIKTGSLSRSDRLAK
YNQLIRIEEMLGETAEYAGTSILRG
>OLS48156.1 EamA family transporter [Rhodovulum sulfidophilum]AB14
MDNMRGIMLMVAAMAAFALEDMFVKKVAATLPIGQILTLLGMGGGIIFALMALRRGQRLWSRALLSRPVLLRNSGEI
VGT
LCYVTAIALTPLASASAILQATPLAVTLGAALFMGAQVGWRRWTAISVGFVGVLIVVRPGLDGFEPASIFAVVAVFGLAL
RDLATRATPRNVGSLQLSAYGFFMLVPIGLVMMTFVQGPVAIDRTDALSLLGALVMGVSGYYAIVEAMRVGDVAVVT
PFR
YTRLIFALVIAMAAFGEHPDTMTLVGAAVIISSGLYTLWRERALSLRRAGR
>OLS48157.1 zinc transporter ZntB [Rhodovulum sulfidophilum]AB14
MSTAPLQPLAAFDIGPDGSTTPVADPWPDAAPGPGAAWRWLHFALTDPALVDWTEAHLPVVAAAAIVQTETRPRCT
SLEG
GLVVNMRGVNLNPGAEPEDMVSLRLWVGPGLVVSVRRSKVFALDDLRRAAESGTAPASPGAFLAEIAEGLTDRLEAVS
VE
LEDQTDALEEAILEREEDDAADGLASLRRKAIRLRRHAGPQREALVRLSEGSSGPGLIDAEARILLGESANRAARTVEEL
DAVTARLVALHDHLEGQRGTAMARNGYVLSVVAAIFLPLGFLTGLFGVNVAGMPGTASPWAFALLSAANVALALGLL
ALF
RLLRWL
>OLS48158.1 sodium:proton antiporter [Rhodovulum sulfidophilum]AB14
MSLLQIASLLIVLAALFGTINYFLLRLPQAIGILLVALIVSLSAMALDALMPGLGIGDSLTGLVRGIDFSDTLLEGMLGL
LLFAGALHVKVEDLRAQAPSVFIMATIGVALSVAVVGVGFSWLTGMPLLLAVLFGAIVSPTDPVAVLGVLRDARLSRSLE
TTIAGESLFNDGVAYVVFLILVGLAFPSAHDDHGAAAGDSAAAAAAILFLREAVGGAVLGGVLGWLTFRVMRRIDDYAL
E
VLITLALAFGGYELAVAAGVSAPIMAVVAGLMIGHVGMKYGMSEETRKYVDAFWKMIDEILNAVLFLLIGIEVFAVAFA
L
DGIATLVGAVALALLARLVSVAVPVLLIGRRQGFDRGTIPIMTWGGLKGGISVALALSLPESDWKPLILTATYVIVVFSI
LVQGLTVGALARRFSREPELV
>OLS48159.1 hypothetical protein BV379_07590 [Rhodovulum sulfidophilum]AB14
MTLSIRFDNSYARLPERFYSRQPPQPVRAPRLIALNSALAETLGLDPAALASEDGIRMLAGNRLPDGADPLAQVYAGHQ
F
GGFVPRLGDGRAILLGEILAPDGSRHDIQLKGAGRTPYSRMGDGRAWLGPVLREYLVSEAMHALGIPTTRALAAVETGE
T
VIRETVRPGAVLTRVAASHIRVGTFQYFAARQDTDALEALARHVMARHYPETETVLDMLRAVLAAQARLVAQWMGV
GFIH

GVMNTDNTSVAGETIDYGPCAFMDGYRPEQVYSSIDAYGRYAYARQADIVAWNLAQLASALLPLIDPDRDRAIEEATA
AM
HGFPDLFKAAWLDVFRPKIGLAGAEPEDAELIHGLLDLMAAERADFTNSFRDLAEGRPGAGLGDRAGFEAWAGRWQ
ARLA
RENTPQAEAEARMRAANPAYIPRNHRIEAAISAATGGDLGPFQRLQRVLERPFDSQTGADDLRDPPAAGEEVLQTFCG
T
>OLS48160.1 hypothetical protein BV379_07595 [Rhodovulum sulfidophilum]AB14
MFKTSYAAAAAVMLIGGSASAATFDFTSSSLDSNNQLTVGGVTVTAYAGVYRDFPNPDKILHTDCSTTFSCDRSVYQGS
H
GLGVAGFLDGPYIDGALGNDLLTLSFNQTVDFSEVAFSFWDSWDSFDLFIDGELVSPEERSGPNPYPLSGLSGTSISFGA
DAWNDSYKLASITVAPVPVPAAGLMLLGGLGGLAALRRKKKS
>OLS48161.1 nucleoside hydrolase [Rhodovulum sulfidophilum]AB14
MPRKIIIDTDPGQDDAVAILLALASPDEIEVLGLTAVAGNVPLALTQKNARMVCELAGRRDIRIFAGCDRPLAGQLVTAE
HVHGRTGLDGPTLPEPTMPLQEAHAVAFLIETLRAAPEGEITLCPLGPLTNIATALREAPEIAPRIREIVLMGGAYFEVG
NITPAAEFNIFVDPEAAAIVFGSGIPITVMPLDVTHKALVTAPRNAAFRALGTPVGRAVAAMTDFFERYDREKYGSEGAP
LHDPCVTAYLIRPELFRGRHVNVEIETEGRFTRGMTVADWWGVSGRAPNATFMGDLDADGFFGLLTDRLGRL
>OLS48162.1 nucleoside triphosphate pyrophosphohydrolase [Rhodovulum sulfidophilum]AB14
MTEDSDRLIRDPKGGLPRLLEIMRRLRDPKTGCPWDIEQDFRSIAPYTIEEAYEVADAIEREAWDELEGELGDLLLQSVY
HAQMGAEAGLFDFHSVADRVSDKMVARHPHVFGTEDREKSAEQQTRDWETIKAAERAGKEARGVLDDVALALPALT
RAVK
LQKRAARVGFDWPETAQVIDKIVEEAQELVEARETLTQAEVFEEFGDLLFVVANLARHLKIEPEEALRAANAKFTRRFNG
IEARLAAMGKRPEDSDLAEMDALWDAVKAEEKAPDQTGR
>OLS48163.1 agmatinase [Rhodovulum sulfidophilum]AB14
MALEDAKSEVDAAITRKGHRGLAYENAFAGVTSFLRRSLTKDLAGVDLAVTGIPFDQAVTNRPGTRFGPRAIREASTLQ
A
FDRPYGWGYNPFDAFDIVDYGDMAFDYASVAEFPARAEAHVAGILEAGAACIALGGDHSVSLPLLRAHAARHGPLALV
QF
DAHTDTWADDDMARIDHGTMFGKAVKAGLIDPARSVQIGIRTDNPDRMGITVLDAPFVHDHGPAAIAARVREIVGE
APAY
LSFDIDALDPAFAPGTGTPVWGGLTSAQAAAILRGLRGLNMVGGDVVEVSPPYDPAGVTAIAGAHVAVELICLWGWT
RRP
DAEDGA
>OLS48164.1 hypothetical protein BV379_07620 [Rhodovulum sulfidophilum]AB14
MKILPLVLLVLGLAALGGAVWFRTVPDDPAVWHVDPLTATKPATPNAALVRPEGGDRAAPVYPVTPEALAEAFDATAL
AQ
PRTARIAGRPDELFMTYVQRSALWGFPDYISVRILPAEGGATWAAFSRSRYGHSDLGVNAKRLSDWQDALEARLARPA
S
>OLS48165.1 peptide chain release factor 1 [Rhodovulum sulfidophilum]AB14
MVPLDKLEQIQRRFGYLEARLSEGASGDFARIGREYAELKPVVAEIEAYRRLLSDIDTAEAMLTDPEMRALAEDELPELR
ARLPELEQALRIALLPKDAADARPAIVEIRPGTGGEEAALFAGDLLRMYQRYAETRGWRFEILEESQTELGGIKEVTATV
QGEGVFARLKYESGVHRVQRVPATESGGRIHTSAATVAVLPEAQEVDIDIPAADIRIDTMRASGAGGQHVNTTDSAVRI
T
HLPTGIVVTSSEKSQHQNRAIAMAVLRARLFDLERQKAADERAATRKAQVGSGDRSERIRTYNFPQGRMTDHRINLTLY
R
LEQILAGDLDEVIDALIADDQAGMLAEMEG
>OLS48166.1 protein-(glutamine-N5) methyltransferase, release factor-specific [Rhodovulum sulfidophilum]AB14
MSAATGSVLLAGAVARLRKAGIPDPARDARRLLAHALEIAPGRLTLVLPEPVPVPAAARFEAALDRRAAHEPVSHIVGLR

AFWGRDFKVTRDVLDPRPETEILIEAALSAPFSNMLDLGTGSGCILLTLLAERPGAAGLGTDISDAALSVARGNAGRLGV
SAARFLQSDWYEAVEGRFDLIVSNPPYIAEAEMPGLAPDLAHEPRGALTDGGDGLSAYRAIADGARARLAPGGRLLVEI
G
PTQAAAVSRIFAAAGLEIVAVRPDLDGRHRVVETRVAASGT
>OLS48167.1 hypothetical protein BV379_07635 [Rhodovulum sulfidophilum]AB14
MRPSKSRSRSKSNRSRSMGNIVNRVFDSSGPEGKVRGTPQQIIEKYNQLARDAQLANDRVAAENFQQHAEHYARLLG
EAQ
REQEARREQQEAQQPRNRDRDDSSQNSNDESDQPTSQTSSGSSSSGGADVIDMSGDDGRESTLVETPEEKPAPKPRR
PRK
PRAVKPKGEAAEADQASGGEGKSGGRKSGGTKSDEGKRDGGDASAEAQPGPDPQREAPAAE
>OLS48168.1 16S rRNA (adenine(1518)-N(6)/adenine(1519)-N(6))-dimethyltransferase [Rhodovulum sulfidophilum]AB14
MSTIDGLPPLREVIAAHELSAKKALGQNFLLDLNLTAKIARVAGDLGGTDVLEIGPGPGGLTRGLLAEGARRVLAVEKDA
RCLAALAEIAAVYPGRLEVVNGDALAVDPLAHLAPPVKIVANLPYNVGTELLVRWLTPADWPPFWSSLTLMFQKEVAE
RI
VALPGSKAYGRLAILAQWRAEARIALRLPPEAFTPPPKVSSAVVHLTALPRPRFEADPAVLERVVAAAFGQRRKMLRSSL
KGLAPEIEDLLRMAGIAPTERAEQIPLEGFCALARALAAAERP
>OLS48169.1 4-hydroxythreonine-4-phosphate dehydrogenase PdxA [Rhodovulum sulfidophilum]AB14
MTGLPIALSCGEPAGIGPEIALRAWQSLGARLPFFWIGDPRHLPEGTPHAEIHAPAEAMHVAATALPVLRLPFPAPAEP
G
RPDPANAAAVIEAIARGVALVQEGKAAALCTAPIHKKALQDGAGFPHPGHTEYLAHLAGVGDVVMMLAAPALKVVPV
TVH
IALSEVPRALSTGLLERSLRITEAALIRDFGIESPRLAVAGLNPHAGEGGAMGHEEIALIGPVLERLRAEGMRIEGPLSA
DTMFHAEARARYDVAVAMYHDQALIPIKTLDFAGGVNVTLGLPFIRTSPDHGTAFDIAGTGRADPSSLIAALKLAREMA
L
TRAAGRH
>OLS48170.1 hypothetical protein BV379_07650 [Rhodovulum sulfidophilum]AB14
MIRRVGLAVAAAAILVLTGADDGRAQQGGPFAPRLYVNDTAITNYEFNQRVQFLRLLGNPPRIEKVALDGLIDDRLRM
DE
AKRLGVSVSDEALQEGMEEFAARGSLDLDQFVALIGQAGVSKESFRDFVKAGLAWREVVRTRYGPRTQVTDAEIDRAV
QS
AASGSGAAGTQVLLSEIVLRADTPDYRRDAMALARQLSDSIDSAGAFAAAARKYSVSRSRDQGGRMGWMPLSKLPPA
IAG
QVLALGPGKVSAPIEMDNAIVLFQLRSVREGKAASQKALAVDYAIFTIPGGTLAEAMKVRMQADTCDDLYGVARGLPP
ER
LLRETRPVADLPPDLAQELAKLDDNEVSYGLSTPAAAEVLMLCGRTPDLEDRGGIDREAVRRQLVGQRLAGYADSYLAE
L
KADAIIRKP
>OLS48171.1 organic solvent tolerance protein [Rhodovulum sulfidophilum]AB14
MMRALLALALTTALTTASLSLPGRLCAEETARPPATLVADRIGVGDLRQLVADGHVEVLYGDTRLSASRIVYDRDGETLA
IDGPITLTSGTDTMILADSAELSADMTEGILRSARLVLDQQLQIAAADIQRVAGRYNRLGETVASTCKICRDGEVPLWQI
RASRIIQDELEGQIYFDNARLEVAGVPVFYLPRLRIPDPSRKRATGFLTPSTRITDQLGSGIKIPFFIELGPSQDLTLTP
YISTSRTRTLELRYRRAFTRGEIEINGAVSRDDILKNETRHYLFAEGSFELPRDFKLDFDIETVSDRAYLLDYGYSEQDR
LESSVEISRARRDELIRAEIGYLETLRDSESNATIPSLVGDVDYRRRFAPALLGGMATLGFSADALHRSSDEATDGPDFD
SYGDGMDMARLTIEADWRRDWTLSNGMVLGALGGITLDHYEIGDDTVHGGSQTRLTPGAAVELRWPLIKAAADGSA
QVLE
PVIQLAWSHEDDDEIPNEDSVLVEFDEGNLFSMSRFAGEDARETGLRLNAGLGWTRYAASGWALGLTVGRIWRAEDS
TQF

AKGSGLDGSSSDWLTAFKLDFGSEVSLTNRAVFDDAFDLTKNDLRIDWEKDAYDIGSSLLWIEANPSEERLSDRSEWLV
D
AAYDFHRNWTGKANWRYDFVADEAAKAGVGLEYSNECISVDLSLSRRFTSSTNVNPSTDFDLTVSLAGFGSRGKSGGA
RR
CMRR
>OLS48172.1 LPS export ABC transporter permease LptG [Rhodovulum sulfidophilum]AB14
MTLHFYIARKFTLYLLGILGIFGAIMALIGMVEELRDFDLGQIGVGGALHLALLKVPGDLYTILPMVTVMATVALFLALA
RSSELVVARASGRSAMRSLVAPVAVALVYGVIAVAIFNPIIAATSREYDSVSSGYKGQEASLLSVSSEGLWLRQGSLSGQ
TVIRAARADARATRFYDVTFLAFDAEGNPAWRIEAEEARLRPQGWDIRNAKRWSFGDGIDNPEARAVVSPQLVLPSDL
TL
ARIRDSFARPAEVAIWDLPTFISGLERAGFSALPQRVWFQMELAMPAVMAAMVLLGAAFTMRHVRFGRTGTMVM
MALGLG
LGLFFLRNFAQVLGENGQIPVALAAWSPPIAGILLSLGLLLHLEDG
>OLS48173.1 leucyl aminopeptidase [Rhodovulum sulfidophilum]AB14
MSTPVAIAFAETDLDAVAGSEGRVAVLVDAEGKLDPGARRVNRLSRGALARVVESEAFSRMKPAEIVTLAWPAGLAAE
AV
DLVRLDRKASQEEARRAGAELAKRQGKADVTLLAGNHPRAADIAFGFALRAYSFDAHRSEEPVIPGAVKVMVTKPEEIA
S
AAGPMAALAEGVFFTRDLVNEPANVLTTDDFAGRLAAMQELGLDVEILDEDELKALGMRALLAVGQGSESPSKVVVM
RWN
GGAADQAPLALVGKGVVFDTGGISIKPGQGMEEMTMDMGGAAVVAGVMRTLALRKSRANVVGVVGLVENMPDG
RAQRPGD
IVRSMKGDTIEVINTDAEGRLVLADVLWYAQDRFKPAGVIDLATLTGAIIVALGNANAGVFSNDDAFCADFLKAATREG
E
GAWRMPLGASYDKLIKSRLADIRNSAGRPAGSITAAQFLQRFIAEDTPWIHLDIAGVALAQEETALAPKGATGWGVLAL
D
RLVADRFEK
>OLS48174.1 hypothetical protein BV379_07675 [Rhodovulum sulfidophilum]AB14
MIIPAIRYRDPEAALLFLVGVLGLDEHAVRRDDDGAFLHAELRLGDGLVSIRGLAGQGRPAAPPADPHGGAGPGSMTIY
A
VVPDPAHLHARVLESGAQIVAPLSGEGPGGSAFSVADPEGHVWTFGSYNPLSRD
>OLS48175.1 DNA polymerase III subunit chi [Rhodovulum sulfidophilum]AB14
MGQALFYHLTRHPLEDTLPMLLDKALERDMRVAVRGSDRDRLARLDERLWMGDGFLPHGLAGTGFDADQPVLLTTG
PEMP
NGARCLMAVDGAEVSPDETRALDRVCILFDGNDPAAVERARGQWRALTGAGLAAQYWSEETGRWQKKAESGG
>OLS48176.1 hypothetical protein BV379_07685 [Rhodovulum sulfidophilum]AB14
MTDEPRATDSNATPADDRIAEIAAAQPPFARLLGLRLVSAAPERVVADLVVTEDLTNRNGTLHGGAIMAVADNLGGTA
TF
LNIDLEAGEGTSTIESKTNFFRTVALGDTARCETVPLHRGRSTMVWQTTITRGDGTLAANVIQTQMVLRPRG
>OLS48177.1 aspartyl protease [Rhodovulum sulfidophilum]AB14
MTGDDFGRLIYLVLLGSVIAGYFLLSNRHRLGHVAQQAAIWGLIFIGAIAGFGLWSDIRHQVAPSQTVFGEERRIEVPRA
PDGHYYMTVEVNGTPLHFVVDTGATDVVLTRKDAARVGLDPEDLQYFGEAHTANGTVRTAPVRLDTVAIGPIVDRNL
RAF
VNAGEMHDSLLGMAYLNRFERIEFVRDRMILTR
>OLS48178.1 MarC family transcriptional regulator [Rhodovulum sulfidophilum]AB14
MELGFYISALVTLFVIIDPIGLTPVFVALTQGMSPRQRRAIGLRAAAISVAILTLFGIFGEQVLVFAGISMPAFRIAGGI
LLFLTALEMLFERRSKKREDRAHEELPDPSVFPLATPLMAGPGAIATMVLLTGQVDRAPLDMLLVHLAMIAVIGICLGLF
MTASLLERALGHTGIVVVSRLLGMLLAALSVQFILDGLRDFGLAPV

>OLS48179.1 glycosyl transferase family 1 [Rhodovulum sulfidophilum]AB14
MLRIDDLRYSIEGRPLFEGASAVIPDGHKVGLIGRNGAGKTTLFRLIRGELTLDSGAISFPPQARIGGVAQEVPSSATSL
LDTVLEADTERASLMAEAETATDPHRIAEIQTRLTDIDAWSAEARAASILKGLGFSEAEQHMPCSDFSGGWRMRVALA
GV
LFARPDLLLLDEPTNYLDLEGALWLENYLARYPHTVILISHDRGLLNRAVGHILHLENCKLTLYAGGYDAFARARAEQRA
VQAAEAKKQDLRRAHLQSFVDRFRYKASKAKQAQARIKMLERMPPVAAPEEMARVAFSFPQPEELSPPIVAMEGVSV
GYD
GTPVLQRLSLRIDQDDRIALLGRNGEGKSTFSKLLAGKLDRLSGRVTTANKLKIGYFAQHQVDELHLDETPLEHLRRERP
DDAPAKLRARLAGFGLGADQADTEVGRLSGGQKARLSLLLATLDAPHLLILDEPTNHLDIESREALSEALNDYSGAVILV
SHDMHLLSMVADRLWLVEGGRVAPYDDDLDAYRKMLLSADTPERAQTVRAAAPKRPGRDELLALRAEVRKCEARVA
KLEE
MRAKLQEKLADPDMYDPARADERMLWQKKFAEAEEAMERAEALWMEALEKLDKAEA
>OLS48180.1 sodium:alanine symporter family protein [Rhodovulum sulfidophilum]AB14
MTVIVDFLNTVLWGYVLIWGLIGAGLYLTIRLGGIQFRHFPEFIRVLVSTGESDRSGITPLQALTVSLASRVGTGNLAGV
AVAIYLGGPGAVFWMWIVALVGMATAYAESTLAQLYKVRNADRQYRGGPAYYIWHGLGAKWLGYVFCFFFLCSFGLI
FNA
VQSNSIADAADGAFGIPKLATGIAIGTLTGIVIFGGIRSIASVAERVVPFMAGIYLLVAAYVLVVHAAEIPGLLALIVKS
AFGIDQAAGGFAGGFAAAALNGIKRGLFSNEAGMGSAPHIAAVAVPDPHHPSSQGMVQGLGVFIDTLLICTATALMIL
LS
GVVDYGPDGITGTELTQNALSAHIGAVGPAFIAVAILFFAFTSIIGNYAYSENVMEFLGLGRGVPLAILKAAQVGMVIWG
AVQPVTRVFNFADASMATMATINLIALILLSGTVIRLTRDYFEQRKTGTEPCFRLSQHPEIDPRGIDRTIWNKDIAAERS
A
>OLS48181.1 nucleoside-diphosphate kinase [Rhodovulum sulfidophilum]AB14
MAIQRTFSIIKPDATKRNLTGAINAKLEAAGLRIVAQKRIHLSKAQAGVFYAVHKERPFYDELCEFMASEPIVVQVLEGE
DAIAKNREVMGATNPAEAAEGTIRKEFALSIGENSVHGSDAEDTAKEEIAYFFSGLELVG
>OLS48182.1 hypothetical protein BV379_07715 [Rhodovulum sulfidophilum]AB14
MKYIWDDWMPKLSSKQRLLTDVAVEGEGTTTPFSDALGFKCLSENSTSKTELMTDNPTPLSRQMNWPLLGGMGEGH
VELQ
TSNPTPLSSRMGWPLLSKRAYYGVAAK
>OLS48183.1 light-harvesting protein B-800/850 subunit beta [Rhodovulum sulfidophilum]AB14
MTDDMDKVWPTGLTLAEAEEVHKQLIDGTRVFGAIALFAHFLAAIATPWLG
>OLS48184.1 light-harvesting protein B-800/850 alpha chain [Rhodovulum sulfidophilum]AB14
MNNAKMWLVVKPTVGIPLFLVACAIASFLVHLMLVLTTGWMGDYYSGSFEAASLVSNATTLLS
>OLS48185.1 MFS transporter [Rhodovulum sulfidophilum]AB14
MNRLSKMAVNRIATVGPRFLPFAEAASEDLPLSRLLRLSMFQVSVGMAMVLLVGTLNRVMIVELEVPASIVGIMISLPLL
FAPFRALIGFKSDTHKSALGWRRVPYIWKGTLLQWGGFAIMPFALIVLSGQESAAGAPEWIGILSAAVSFLLVGAGVHT
V
QTVGLALATDLAPREDQPNVVGLMYVMLLVGMIVSALLFGMWLEDFYHAKLIKVIQGAAVATMVFNVIALWKMEAR
DRVR
ARQRLDGDPEPSFREAWGLFTRGPNARRLLWVIGLGTLGFGLSDVLLEPFGGQVLDMSVAATTKLTAAVAGGTLVGFA
WA
SRVLSRGYDPMAMAGWGAVVGLPAFAIITFSATLQSEPVFVFGTMMAGFGAGLFSHGTLTATMRSAPKAQVGLALG
AWGA
VQATSAGAGIALGGVFRDAVLHSPIGDLFGAATPYFFVYAIELALLVATIIVGYRLMLSRAFGIENAPD
>OLS48186.1 hypothetical protein BV379_07740 [Rhodovulum sulfidophilum]AB14
MRVFLGFLLLLLTALPLAAQEDDRGVLVSFLEDKLSSAGREIRIEGFEGALSSRATLKELTIADEDGVWLTVRGAVLDWS
RAALLRGEVKVDELSADEILLPRLPRGGGASVEKSEATPFALPDLPVSIDIGRVRTPRLVLGEALIGQEADLALDGALSL
AGGAGRADIEATRSDAEGDFAIRAAYANDSQELSLDVSLTEGAGGIVSSLAGLPGAPPLALTVKGDGPLSDFAADLALAT

EDEPRVTGRLTIVAPADAPEDRAFAATLSGDLAPLVAEPLRPFFGPATDLEIDGTRGADRRLDISRLEVSTGALQLGGTL
SLAATGLPEKVDLTGRLSGPVLLPVSGLDTTLDGATLEASFDAATGEAWQAAITLDGLASGPLTLAHADLTGTGSIRPQD
PALAADIAFRATGLGHAEPALARALGQAAEGHARLSWQAGVPLELTDLQIASGDARLTAKGTLGSAAKGLPFDGRIGA
AL
DDLSRFAALAGRGLGGAARADLSGRYRLLDGIFDLSLDAETTDLKTGTPRLDQLLAGAGQVRLAAARGPEGTSLKQLHV
E
TPGLRLDGQGDLSSAAGGLTLTGQVPDLALVDPKLTGPGRLDTAFGWEAGGRLRLDRFEATAAGAEIAAEGALTPGDP
AL
PAEGKVTLSAPKLSVFSGLAGRPLAGKLDATLSGQGAVKGDFDATLSAEGQGLAIGIAQIDRLIGGKTTIAAAGARKAGD
LTVGRLDIETPRLTVKAEDSAGDGTLDLTASLSDLALLVEGFPGPVALKGQARPEGADWRLTLNATGPSGTTAAIGGRLA
RDASSADLSLKGRAPLSLANPFIAPRSLDGMAAYDLTLKGPLGLSALSGRATTSGARLSAPTLGAALTDIGGRVDLSGGR
ARLDLTGQLGGGQVAVSGPITLSGAFPAELAIRLTRARLQDPTLYETSVDGSVTVTGPLTGGARIAGQLDVGRTEITVPS
TGLGGGGELPDIDHLGTPAAVQATLRRAGLVATEGEGGGGRPYPLDLTVNAPSQVFVRGRGIDAELGGRLRLGGSTAD
VV
ASGQFELIRGRLDILAQRFTLDEGRVTLSGSFDPRLYFVAATQSGDVTARIVVEGPASAPEIKFSSSPELPQEEVISRIL
FGRGLDTLSPLQAAELASAVATLMGKGGQGILGALRGATGLDDLDVNSTEEGGTELSAGKYLSDKVYSQVTVNQKGQS
EI
NLNLDVSPYVTLKGHAGAEGDTGIGIFYERDY
>OLS48187.1 anthranilate synthase component I [Rhodovulum sulfidophilum]AB14
MSRIIPEFTAFEAGYEAGRTQVVYARLAADLDTPVSLMLKLAGARTDSFMLESVTGGEVRGRYSVVGLKPDLIWECRGE
T
CRVNRMARFDPDAFEPMEGHPLASLRQILAESAIDMPEELPSIAAGLYGYLGYDMIRLVERLPNVNPDPLGLPDALMLR
P
SVVAVLDGVKGEVTVVSPARPEPGLSARAAYAQAAERVMDALRDLDRAVPAETRDFGEAREIGEPVSNFTRESYMAAV
ET
AKDYIRAGDIFQVVPAQRWTQDFPLPPFALYRSLRRTNPSPFMFFFNFGGFQVVGASPEILVRLRGGEVTIRPIAGTRPR
GATPSEDKALEADLLGDQKELAEHLMLLDLGRNDVGRVAKIGTVHPTEKFIIERYSHVMHIVSNVVGEIAEGEDALSALL
AGLPAGTVSGAPKVRAMEIIDELEPEKRGVYGGGVGYFAAGGDMDMCIALRTAVVKDETLYIQAGGGVVYDSDPAAE
WQE
TVNKSNALRAAAAEAARFVRRGNS
>OLS48188.1 hypothetical protein BV379_07750 [Rhodovulum sulfidophilum]AB14
MSKSKGKQFSKTLVWMMLIFVVLGLGGFGVSNFGGRVESIGSVGDTEISTDSYARALQRELRARAAATGQNMSLAEA
QAS
GLVDAVRAQEISNAALDNEMHRIGVSVGDDQVAREVMRLDAFKGPDGSFDREAYRYVLSQNGMTETQFETSLRKEA
ARSL
LQTAVVAGLDAPETYVDTLYTYIAERRSVSLIHLTADALDAPVPDPTEDQIKAQYEATPAAYTAPMTRKITYAWLSPEAM
ADTIEPDEALLRQLYEQHIDEFVQPEKRLVERLVFSSMDEAQTAMDRIEAGEESFDDAVAARGLAIDDIDMGDVSEVQI
G
GEAGRAVFALTEPGLAGPLMSDLGPAIFRVNAILAKQEIPFEEARETLAPEAVHSRAVRMIADKINDYDDLLAAGATLED
LARETEMQLGQIDYTADSDQGIAAYQDFRAAADAAVEGDFPEIKELDDGGIFALRLDREIPPTLRPLEAVQDQVASDW
RR
AETARRLADKAEALKARIDGGEEMTALGVDVTSETALSRGGLMPQPLSDAMFRLADIGDSAVVPIGAEVWLIRLDEILP
P
DADAPGAEMLRRNIAAQAGQGLAQDLYAYFAQAVTNAAGLSLDQAAINAVHAQFR
>OLS48189.1 hypothetical protein BV379_07755 [Rhodovulum sulfidophilum]AB14
MAASQPPRGGTAATFAPPVMQARRWLEGVSFPADRPLLNVSQAAPMDPPPEALRQAIADAALNDPGAHLYGPVLGL
PELR

AEIAAEWSRAYGGDIRAERVAITQGCNQAFCAAIASIAAAGDEVILPTPWYFNHKMWLDMAGMRAVPLATGTNLLPE
VQR
AGALITPRTRAIVLVTPNNPTGLEYPAELVADFLALCRRHGLALILDETYRDFDSRDGAPHQLFADPDWGDTLIHLYSFS
KAYRLTGHRVGALIAGEAVVAEVEKFLDTVAICPSQLGQRAALWGLRHLGAWKADERAEILARRDALSRNMSALPGW
ELM
GAGAFFAWLSHPFDIGSGALARRLVAEAGVLLLPGGMFLPETDPGARDQLRIAFANIDRPGLQALFGRLAAFRP
>OLS48190.1 xanthine phosphoribosyltransferase [Rhodovulum sulfidophilum]AB14
MDPDRLPHEKGFHVSWDQLHRDARALAWRLDGQGPDNGGWRAVVAITRGGMAPAMIVSRELDIRMVDTISVKSY
NHQSQS
APVIIKMPDRELVGDGTGVVIVDDLVDTGRTLEVVRATMPKAHVATVYAKPKGKPQVDTFITEVSQDTWIFFPWDMA
LQY
VKPYRAGE
>OLS48191.1 enoyl-[acyl-carrier-protein] reductase [Rhodovulum sulfidophilum]AB14
MSTKLMEGKRGLIMGLANDKSIAWGIARQCAEAGAELAFSYQGEALKKRVMPLAASLGSDIVLECDVASEESLDGLFA
AL
EARWGRLDFIVHAIGFSDKNELRGRYVDTSRSNFLMSMDISVYSFTAAVQRAARMMTEGGAALTLTYYGAERVMPHY
NVM
GVAKAALEASVRYMAEDLGKDGIRVNAISAGPIKTLAASGIGDFRYILKWNELNSPLRRNVTQEEVGKSALYLLSDLGSA
VTGEVHHVDAGYHGVGMKAVDAPDIDAVTGRKDS
>OLS48192.1 pyridoxamine 5'-phosphate oxidase [Rhodovulum sulfidophilum]AB14
MTEREGIFAGDDPFAIARSWLAEAEKTEPNDPNAIALATVDGQGLPNVRMVLLKEIEADAFMFYTNYESAKAGEIVLTN
K
AAFVLHWKSLRRQVRVRGITEKEEGEKADAYYASRALQSRLGAWASRQSRPLASRGELMAEVAMVTARRGVAPARPP
FWG
GFRIRPLEIEFWADGAFRLHDRFRWCRDTLDGEWQIDRLYP
>OLS48193.1 cold-shock protein [Rhodovulum sulfidophilum]AB14
MDKDSDTALRVEGRVKWFDPTKGFGFVVADEGGPDILLHANVLRNFGQNSVSDGAGIVLVVHRTARGLQATEVLRIE
PAV
STGAPALEDAMHLTAEEVAALPLEPARVKWFDKAKGFGFANVFGRDDDVFIHIEVLRRSGLADLQPGEAIAVRVTEGQ
RG
RLAISVGPWESALGPGGSSAG
>OLS48194.1 ribonucleoside-diphosphate reductase, adenosylcobalamin-dependent [Rhodovulum sulfidophilum]AB14
MRGAGKTGRIKSMKIERKFTTEETGAYGSLEFTATTSEIRNPDGKVVFRLDEVEVPTGWSQVASDVIAQKYFRKAGVPA
R
LAKLPEEGVPEFLWRSVPDTGALEALPEEERFGGETSARQVFDRMAGAWTYWGWKGGYFSAEEDARAYYDEMRFM
LASQM
AAPNSPQWFNTGLHWAYGIDGPGQGHYYVDYRSGKLTKSASAYEHPQPHACFIQSVKDDLVNEGGIMDLWVREARL
FKYG
SGTGTNFSSLRAEGEALSGGGKSSGLMGFLRIGDRAAGAIKSGGTTRRAAKMVICDMDHPDIESFINWKVIEEQKVASL
V
AGSKTHERHLNALFEAIRAWDGKQEDAYDPKANAGLKAAIRAAKKALIPEGYVKRVLDYARQGYGSIEFPAYDTDWDS
EA
YSTVSGQNSNNSVRVTDAFLKAVKADADWELVRRTDGKVAKTVKARELWAQVGHAAWSCADPGIQFHDTINAWHT
CPADG
EIRASNPCSEYMFLDDTACNLASMNLLAFWNDGAFRSEDYVHATRLWTLTLEISVMMAQFPSKEIAQLSYDFRTLGLG
YA

NIGGLLMNMGLGYDSDEGRALCGALSAVMTGIAYATSAEIAGELGAFPGHERNAEAMLRVIRNHRNAAWGKAVGYD
GLAV
PPVPLDHANCPEEGLVEMAISAWDEALALGEKHGFRNAQVSVIAPTGTIGLVMDCDTTGIEPDFALVKFKKLAGGGYF
KI
INRSVPAALEALGYGSAQIEEIIGHAVGHGTLGNCPGINHTSLIGHGFGQAEIDKIEAALPSAFDIRFVFNPWTLGEEFC
TKVLGIPASKMADPGFELLRHLGFSRAEIEAANDHVCGTMTLEGAPHLKPEHYPVFDCANPCGKKGKRFLSVDSHIRM
MA
AAQSFVSGAISKTINMPNDATIEDCLSAYELSWSLGVKANALYRDGSKLSQPLAAALVEDDEEAEEILETGTPAEKAQVL
AEKVVEKVVVKEIVRSHREKLPTRRKGYTQKAIIGGHKVYLRTGEYQDGTLGEIFIDMHKEGAGFRAMMNNFAIAVSVG
L
QYGVPLEEFVEAFTFTRFEPAGVVQGNDSIKNATSILDYIFRELAVSYLDRTDLAHVKPKGAAFEELGHGHEEGKANVTP
VSESAASKSLEVLRQISSTGYLRKRLPQELVVLQGGMNAVDFAGSATPVAAVPGSASAPAPTVSAATAIASGTVPMDAR
V
KAKMQGYEGDPCGDCGNYTLVRNGTCMKCNTCGATSGCS
>OLS48195.1 hypothetical protein BV379_07795 [Rhodovulum sulfidophilum]AB14
MQLIIGSQIVANARLRRIPGGIEAQLAGDALKRLIDATFIGGSIEVLDGTHRARGLDVTDIRMGGASTTVTLKTTGARSM
H
>OLS48196.1 hypothetical protein BV379_07800 [Rhodovulum sulfidophilum]AB14
MTETRRSPGRIVALDLARSAALLGMAVFHFSYDLEMFGHLPPGTVVTGGFRLLSVGVAAAFLALAGASLHLAHGQGLR
PR
AFLRRLLRIAAAAALVSLGTWAVFPASFVYFGILHAIATASLLGLVLVRLPPLVPAVLALGMLLMPRPAPLPDLGWLDWT
GLTATPRPSVDFEPLFPWAAAFLAGMALAGFASRAGLWHRLSGPPGRLSGLLAWPGRHSLAIYLLHQPVLIALVWAAT
RF
APV
>OLS48197.1 YeeE/YedE family protein [Rhodovulum sulfidophilum]AB14
METIPPGAIAALIGLATGCVLGLAARLGDFCTLGALETAIWGADQRRIRMWGVSLGVAIAATALLAAVGQIDTGATIYH
Q
IRWNPAASVLGGLAFGYGMALAGNCGFGALARFGGGDLRSLVIVVVISIFGFVTLSGPLAPLRDLAFPQQPSDGPQSLA
L
TGAALTGLPPLALALGLAALAAGWGLWHRPLRAEPHRIGWAVAAGLAVSAALWGTSALHEATLGAVDVEGHTFTAPL
GRT
LMWMMTSSAGGLSFSVGSVLGVLAGAWLGSWRRRSFRWEACEDPRELGRQVAGAALMGIGGVVALGCTIGQGLT
AFATLA
WSAPVTLAAIAAGAVFGLRRLIAGFQPD
>OLS48198.1 cytochrome C biogenesis protein CcdA [Rhodovulum sulfidophilum]AB14
MLEISYGGAALAGLLSFLSPCILPIVPFYLCYMAGISMTELRGSDRIPPGAVRRLVVSAIAFALGVTSIFVLLGMGATAL
GQGFREWKDELSYVAALMLFLFGLHFLGILRIPLLYREARIETRAEPSTLVGAYLMGLAFGFGWTPCVGPALAAILMVAS
GMGDIGRGGLLLLVYGLAMTLPFVLAAAFARPFLAWMQRNRRHLGHVEKAMGAMLVVFAILIATNSVGYLAQVLIDN
VPW
FTTLG
>OLS48199.1 thioredoxin [Rhodovulum sulfidophilum]AB14
MKLTTLLAAGFAALAVPALASPMGDDGLHKPDWLRQTFRDMREDLAEAEAENRRMLVIWEQRGCIYCTRMHEEVFP
DPEI
EALIRERYFVVQMNLFGDVEVTDFDGTVLPEKEMAGRWGVMFTPTLMFMPETPPEGGTAAEAAVASMPGAFGKGT
TRALL
QWVLDRGYEGDEHFQKYVARTMADPGN
>OLS48200.1 sulfur oxidation c-type cytochrome SoxX [Rhodovulum sulfidophilum]AB14

MGLTTACIALWASAGPGPAAEVAPGDVAIDGQGHVARPLTDAPGDPVEGRRLMTDRSVGNCIACHEVTEMADAQF
PGTVG
PSLDGVAARYPEAMIRGILVNSKNVFPETVMPAYYRVEGFNRPGIAFTSKPIEGEIRPLMTAGQIEDVVAYLMTLTQ
>OLS48201.1 thiosulfate oxidation carrier protein SoxY [Rhodovulum sulfidophilum]AB14
MEFTRRGAMALSAGLAAALMLPASAVRAAVEDKIAEFTGGADAGADGITLTTPEIAENGNTVPIEVEAPGAVEIMVVA
SA
NPTPDVARVSFGPLAGAQRLSTRIRLGGTQDVIAVAKMGDGSFRRAANTVKVTIGGCGG
>OLS48202.1 thiosulfate oxidation carrier complex protein SoxZ [Rhodovulum sulfidophilum]AB14
MAEGVKARVKAPRTVAAGETVVIKTLISHQMESGQRKDSDGSLIPRSIINRFAVAYNGQNVIDVALAPAISTNPYFEFEA
VIPEAGDMVFTWYDDDGSVYEEVKSIAIG
>OLS48203.1 sulfur oxidation c-type cytochrome SoxA [Rhodovulum sulfidophilum]AB14
MKTMTGRLVAAALVCGGAFSGAAVSAGPDDPLVINGEIEIVTRAPPPAHLADRFDEIRSGWTFRTDDTQALEMDDFE
NSG
MVFVEEARAVWDRPEGTEGKACADCHGAVDDGMYGLRAVYPKYVESAGKVRTLEQMINACRTSRMGAPEWDYIGP
DMTAM
VALIASVSRGMPVSVAIDGPAQSTWEKGREIYYTRYGQLDLSCASCHEQYFDHYIRADHLSQGQINGFPSYRLKNARLN
A
VHDRFRGCIRDTRGVPFAVGSPEFVALELYVASRGNGLSVEGPSVRN
>OLS48204.1 thiosulfohydrolase SoxB [Rhodovulum sulfidophilum]AB14
MIARRDFLQAALAASAIWGASGMGNWARLAARQALSQDRLLEFDSFGNVTLIHVTDIHGQLVPVWFREPEVNIGVGA
AEG
QPPHVTGADFLKLYGIAPGTPEAYALSYVDFVSLARSYGRMGGLDRVATVIKAIRADRPEALLLDGGDTWHGSYTCLRS
D
AQDMVNAMNLLKPDAMTSHWEFTLGLDRVNEIVEGLDFAFLGANIFDAEWDEPAYEPYRIFERGGAKIAVIGQAFPYL
PI
ANPNWMFPGLSFGVREERMAEMVREVRGKGADLVVVLSHNGFDVDRKMAGNVPGIDVILTGHTHDALPEPVMVG
QTLLIA
SGSNGKFVTRLDLDVREGRMMGFRHKLIPVFSDAIAPDPEMAALIERERAPYRDEMAEVLGTTDGLLYRRGNFNGTW
DDL
ICNALIDEREADIALSPGFRWGPSLLPGEPITREDLYNATAMTYPNAYRSEMTGEMLHTILEDVADNLFNPDPYYQQGG
D
MVRVGGMGYRIDVTRPQGSRITGMTLLKTGEPIDPARSYVVSGWASVNEGTEGPPIWQVVEDYVKRQGTVRVDPNK
SVVV
SGA
>OLS48205.1 cytochrome C [Rhodovulum sulfidophilum]AB14
MSKFRNLSAAALLLTLAVPAQAGDFGLGRPALPEEIAAWDLDVAPDGKGLPEGSGSVEDGEEIFSANCAICHGEFAEGV
D
NYPKLAGGEDTLDRDDPVKTVGSYWPYLSTVWDYVHRSMPFGAAQVLTVDEVYAITAYILYSNYLVEDDFVLSNENFLE
V
AMPNTDGFIVDDRETTELPLFSKPPCMTDCKQNVEITMRAAVLDVTPDEAAAN
>OLS48206.1 cytochrome C [Rhodovulum sulfidophilum]AB14
MREELCLILALALGGAGAGRAETHVEAIGDPVRGAEIWVYCSGCHEIGPGARASIGPPLNGLFGRRAASVADFPYSKSM
R
RAGDDGLTWTLETLDAYLENPVVLVSGTRMSFPGLEDAAERADLLAFLRRYSDSPSDIPEAAPTARAEDPVLPEATLALA
GDRAWGEYLSSECTTCHQRDGSDRGIPSITLWPERKFVLAMHAYKQKLRPHPVMRMMAGRLSDEEIAALAAYFGGLG
P
>OLS48207.1 flavocytochrome C [Rhodovulum sulfidophilum]AB14
MSLNRRSFLGTGLALGAALSAPCAIGQARPRVVVLGGGAGGATAARYIAKDSDGAIEVVLIEPGKTYYTCFFSNLYLGGF

RDFASIAHDYGTLASRYGINVIHDLAVDIDRAARSVTLASGHALGYDRLIVAPGIDFVDGAVPGWDMTAQNRMPHAWKAG
SQTQLLKAQIGAMRAGGVFCMVAPPDPFRCPPGPYERVSMVAHVLKESNPTAKILIVDPKENFSKQALFEDGWARHYPGM
IQRIGPDFGGANVDVRPDSMEVVIDGMTETVDVCNVIPAQKAGHIAGVAGLADDSGFCPILPGTMQSRFDENVYVLGDAT
IAGDMPKSAFSANSQAKVAAMQVRAALTGARAFPAKYSNTCWSLIDSEDGVKVGASYQVGEDRIAKVDGFVSQIGEDAAL
RKATYEESLGWYAGITADIFG
>OLS48208.1 hypothetical protein BV379_07875 [Rhodovulum sulfidophilum]AB14
MRPRLHPVFGKLGPMPAASLRAMVGYGLNEAEIGRYFGVTPSSVRRLKRSLGVCDSAAE
>OLS48209.1 flavin oxidoreductase [Rhodovulum sulfidophilum]AB14
MERFIPGPDTRRLYRDALGSFATGVTVVTAAGPEGPVGMTANSFSSVSLDPPLVLWSPARASARYPAMTGAERFAIHVLG
AGEEDIAARFARSGDDFEGLDCAPDADPPLLEQGWIARFLCRRVALHDAGDHAIVIGHVEECARREGPPLVFAGGAYGDF
SPVD
>OLS48210.1 alkylphosphonate utilization protein [Rhodovulum sulfidophilum]AB14
MPQFLHPLRFTGARILRDGEMQRRSIAIAEGRITKGPLPEVDLTGYLILPGIVDLHADTVDRHLPRMASPLAPDLALAAS
DRDAAAHGVTTGWLAQDWSWEGGHRAPDRAEALMAALAAYRPRMLTDLRLQLRCEIHTVGTAERLLEAIGRYRIDYVVFH
NRLDAAAERAQDRPWPPPCRADRDGLAAAQAAMPDVPRYLCRLAEAFDAMGVLYGSHGDPDGETRERFSMIGARIAEFPT
TRRAAAAAKAMNDPVLLGAPDVMRDGSQPGQVPATDLIAQGLCDALVSDFYYPALTHAVWRLVDLGLKTLPRAWAMVSQR
PAEILRLPDRGRLDIGKRADLCVINERTRAVEMTIAGGRLTYLAGEAAHRFVHRIHALDMAAE
>OLS48211.1 phosphonate metabolism protein [Rhodovulum sulfidophilum]AB14
MEGYRRYAIYYAPEEGALGDFGKAWLGWDAEAGTPRAHPRLRGLPRPVEELTATPRKYGFHATVKAPFRLARGSDIGLLH
ASAVALSGQLRPVLIEGLRLSLLGGFLALTPRGDTRLLAAMAGSVVEALDGFRAEPTPVELARRDPDRLSIRQRKFLEKW
GYPYVMEEYRFHLTLTGPLKVAEAEAARMVLGPVLEPLIDRPIRIRSLCLFGEAEDGRFHNLHRYRLAG
>OLS48212.1 phosphonate metabolism protein PhnM [Rhodovulum sulfidophilum]AB14
MLPTAETVLANATLVLPGEIRVGSVRIAGGRIDAIDTGAAVPAGALDCGADLVMPGLIELHTDNLERHIQPRPAVDWPHA
SAIIAHDAELAGVGITTVFDAMRVGSVPSRKARYRPYARGLASELLELRAGGALRISHFLHLRAEICSETLPEEMAAFGP
EDRVGIVSLMDHTPGQRQFRDEARLKAYLMGKNNFTEAEFRDHVAALKDLRAQNGARHEVLALAEARRFGAILASHDDTT
AAHVAVSAGQGIRLAEFPTTLEAAEACRAEGIAVMMGAPNLIRGGSHSGNVAAADLAGPGLLDILSSDYVPSALLMGALR
LGDLWGDLARGIATVTQAPADAVGLADRGRIAPGARADLIRVARIGGAPVVRGAWVQGARVA
>OLS48213.1 hypothetical protein BV379_07905 [Rhodovulum sulfidophilum]AB14
MSKTPATPSATAALEAALRAQAEGWGMMAWMGATMADHVRRTGSEMAAFARDEARRNAEAMQRLAACRTPESLTDWQGDY
LGETVAVCRDEAERLARMQAEVCDVTLNRMTGWRD
>OLS48214.1 hypothetical protein BV379_07910 [Rhodovulum sulfidophilum]AB14
MDAFSFPVWPEVPRGPFGYFFPAAPEMPGGADIAAALDDLASAMADPLPAGPGEAGRPAIYACFAQLIAADIAEGVGLAE

AAATPDIGRFTPLPRGRAEATLCNRHTGRLALDSLYGGVTPEAPDRTPAALVRLRTDADSPAPPGIAGFGGQDRNGRPA
E
ACASPFRIVRTKVGTEIGTEIGAGADIAQARPDGDRSVAWFHLALARFHQAVMAHAVGIGGPRPEIGEPAFAFARGEV
RK
HFQWLVVNDYLRRICLPEVLEAVLTEEAPVYRDLLMRCPGLPEGRLPLPLEFQAAALGCAETMARSGDDWLARLGSGR
PL
PGGLAVFPRLIDGRVSPPDRRARGANARLDTWFAPDLLCSGEKLPVPNARRRGLAERRLHRGLRLNLPSGQGCVAGM
ARL
GVRIAPLSPAELAEGPGGAVLARAGMIEATPLWYYLLREAKARSGGRRLGPLGSRILAETLVGLCVCDPDSYWHHLGSD
H
GRWHPRDGARPAGEPITTVAALIRAAGMRA
>OLS48215.1 hypothetical protein BV379_07915 [Rhodovulum sulfidophilum]AB14
MRGAFLGVGDEIGFLGFVRWHDMRIPLPVTGAPRGADGSGPPARHLPGERSGTAEHRGRVWSPCRLRPEHAF
>OLS48216.1 aldehyde dehydrogenase [Rhodovulum sulfidophilum]AB14
MTTLTCISPIDGSVLAEREALSSEAARAAVARARAAQADWAARPLSERIRLVLKGVANVGDMNDEIVPELARQMGRPI
RY
GGEFRGFEERAKYMAAIAETALAPIEAEDSGSFKRYISRDPHGVVLVIAPWNYPYMTAINTIAPALIAGNAVMLKHATQT
LLVGERMARAFHAAGVPEDVFQNVFLDHGTASALISERAFGFVNFTGSVAGGRAIERAAAGTFTPLGLELGGKDPGYVL
E
DANLDMAVEVLIDGAMFNSGQCCCGIERIYVAESLYDSFLEKAVRLVESYRLGDPLDPETTIGPMAQARFADLVRAQTA
E
AVASGARAMIDPALFPQDGGTYLMPQILTGVTHEMRVMREESFGPVVSILPVKDDEEAIRLMNDSDYGLTCSIWTND
VTR
AERIGQRIETGTVFLNRADYLDPALCWTGCKDTGRGGGLSVIGYQNLTRPKSYHLRKL
>OLS48217.1 hypothetical protein BV379_07925 [Rhodovulum sulfidophilum]AB14
MLREEMADVTLDQDTRTAEIERKTFDQAAPGRELTRGYERAQGARTAFLRKPAPGSYGRCGYRLLSQRKTPR
>OLS48218.1 hypothetical protein BV379_07930 [Rhodovulum sulfidophilum]AB14
MSCPAPRRGASHARARRGRASFRQHGQRELDASGGAAVSRLGHSDRMVTEAIRARLDRVAYAHTGFFTPEPAGPLAD
LPV
AHAPEAIARVCSVSGGAKAMKAALTLARHDMLESGQPGRPPGDRAAPKLSRQHAGGASDRRQQGTAQPLRATHDR
DLVYR
ALRCLSRPGRGRGGPWPAHDRRRAGRAGADHGRRARGGPARGPWRAPMSATSAARTCFAASSRPKTAPAAPFPPG
RRAHA
RIEAAAFEAGRLCHPMGGTIDGRQGDQVLLAPPLTPDGTHALEITDKLQFVLTALR
>OLS48219.1 hypothetical protein BV379_07935 [Rhodovulum sulfidophilum]AB14
MSDQPSIEDRIASGYDGLSARLRDAADYVAAHPVDVATRSLRSISATSGLSPATFSRLARALGFASYEEMRELSRNAVGR
RYVSLSEKVRRLQAEAERAEQPPFLLRHSGACMANLEAATRLVDPERLEAAVERLHRARHVALFGALGSTGIVEYMAYL
G
NYFASNWSIAARMGASLASSLADLGPEDALVILTKLPHARRAVLAAEMGRERGAFVMVVTDTHACPALKSADAGFILP
SD
SPQFFSSYAASLVFLETVIGMLATRSGSVAKARIEKIESRNHRLGEFWAG
>OLS48220.1 C4-dicarboxylate ABC transporter substrate-binding protein [Rhodovulum
sulfidophilum]AB14
MSFPLKPLAAAAALTAFAAPAAQAEEFITIGTGGVTGVYYPTGGAICRLVNKGRKDHGIRCSVESTGGSVYNLKTIRAGE
LEFGVVQSDFQYHAYHGSSPSFEGDGPFEGLRAVFSLHPEPFTVVARADSGIRELDDLKGKRVNIGNPGSGQRGNMEV
VM
AAKGWTMDDFALASELKPAEQSAALCDNQIDAMIYTVGHPSGAIQEATTACDSVLVNIRGPEIDKLVEDNSYYRKAIIP
G

GMYRGNDEDTETFGVGATLVTSDAVSEEAVYQLVKAVFDNFDDFKALHPAFANLKAEEMIKDGLSVPLHDGAARYYREKG
WIE
>OLS48221.1 C4-dicarboxylate ABC transporter [Rhodovulum sulfidophilum]AB14
MADKAAGGRALSEDELQDLVAASDAGARSPAGAVGLFLAGVALSWSLFQVVLASPLANVILPGDIINNSRQIHLAFAIFL
AFCAYPAFRSSPRRRVPLQDWVFAVAGALIALYGYFFYDKIVQSGGLADDTDKWVALVGLLLLFEAARRALGPAMAIIAT
IFLVYVFFGAAPWVPEVIRWKGASLQKAMSHMWTTSEGVFGIALGVSAKFVFLFVLFGALLEKAGAGNYFIKMAFGALGH
LRGGPAKAAVVGSAATGLISGSSIANVVTTGTFTIPLMKRVGFTSEQAGSVEVASSVNGQIMPPVMGAAAFLMVEYVGIS
YLEVITHAFLPAVISYIALVYIVHLEAVKRNMPTLGHGVTSLGRTIGGMFAFFAGFALLCYTVQYPVGWIVAALPEGAGW
VLAALVFVAYLALLALAATVPDLEPDDPNAAQVKLPVISEIYKSGLYFLLPIVVLVYFLMIEQKSPGLSAFWATALLFVI
LITQRPLKALFRGGSGLVASAREGGLDLVSGLIAGARNMIGIGLATATAGVIVGTVTLTGIGQVMADLVEFVSGGNLIVM
LIFVGILSLILGMGLPTTANYIVVSSLMAGVVVQLGAQSGLIVPLIAVHLFVFYFGIMADVTPPVGLASFAAAAVSGGDA
IRTGFIAFFYSLRTVVLPFIFIFNTDLLLIDVTWLQGILVFIVSTVAILVFTAGTMGWFVTRSRIYESIALIGIAFLLFR
PDFVMDRLQPPFADLPPAQLEQALAGAEPGDTLRLRVAGPDFLTLEPKETTLVVTVGTEQGGPARLAATGLLPDEVDGRV
VLEEPMFGTPFSETLQGFDFYGDDPVTLTALRVPQHQWPQELVFLPALALLAGVVWLQRSRARREGVPA
>OLS48222.1 universal stress protein [Rhodovulum sulfidophilum]AB14
MIRAILCAIDVSQPEHDREVLTVATRLAAMDGARLDVITVLPDFGMSVVGQFFDTSYHEKALSRARSLLNDFVSDVIGAE
ANAAVRHIVSTGKAYEEILHAAREAGSDLIVVGAHKPDLSDYLLGPNAARVVRHSDCSVYVVRPSS
>OLS48223.1 methionine--tRNA ligase [Rhodovulum sulfidophilum]AB14
MARILITSAIPYINGIKHLGNLVGSQLPADLYARYCRARGHEVMFLCATDEHGTPAELAAAKAGKPVAEYCAEMHEVQAE
LARGFRLSFDHFGRSSSPQNHRLTQYFAGRLAENGLIEEVNERQVYSNADGRFLPDRYIEGTCPNCGYEKARGDQCENCT
KQLDPTDLIEPRSAISGSTDLEVRETKHLYLRQSKLRDDLGAWIDGKTDWPVLTTSIARKWLNDGDGLRDRGITRDLNWG
IPVKKGEEDWPGMEGKVFYVWFDAPIEYIAASAEWAEKQGRPEADWRRWWRLDEGAEDVRYVQFMGKDNVPFHTLSFPAT
IMGSGDPWKLVDYIKSFNYLNYDGGQFSTSQGRGVFMDQALSILPADYWRWWLLSHAPENSDSEFTWENFQTSVNKDLAD
VLGNFVSRVTKFCRSKFGEVVPEGGAYGDREQELNDDLYIRIRAYDAHMEAMEIRKAAAELRAIWVAGNEYLQEAAPWSV
YKEDPDKAAAIIRFSLNLIRLYAVLSAPFIPDAAKAMMEAMQTGDDTWPEQMTHAMERLAPGQAFTTPEMLFRKILDEER
EEWQARFAGQRG
>OLS48224.1 short-chain dehydrogenase [Rhodovulum sulfidophilum]AB14
MDIRFDGKTALITGASTGIGAAIAHELAASGATVCLAARTASKLTPVVEDIAAAGGKAYAMPCDVSDPGAVEALVARTVE
TTGGLHLLVNNAGISGPAADAGAYPLDGWREVIDTNLNGVFYGLRFGLPAIEASGGGAVVNMSSILGSVGMATSPAYVAA
KHALLGLTKSAALAYAQRGIRINAVGPAFVRTPMIDDGLDAETQARVTALHPVGRMGRSDEISGLVAFLLSDRASFVTGS
YHLADGGFTAQ
>OLS48225.1 aldo/keto reductase [Rhodovulum sulfidophilum]AB14

MRKVSLGRTGLEVSCLCLGTMTWGSQNDQDEAHAQIDLACERGVQFMDTAEMYPVAPVLAETVGRTERIIGNWFRD
SGRR
KDWIVATKMTGEGMKTVREGEPVCRDSLLRAVEGSLRRLRTDYIDIYQLHWPNRGSYHFRKNWAYDPSHQDRAATLA
NME
EVLETLDELVKAGKIRHFGLSNETAWGMAQWLRLAEEKGLPRAQTLQNEYSLLCRLYDTDLAELGVNENLPLLAYSPLA
A
GLLSGKYAGDVTPDGSRRSRSPELGGRITPRVWEAVSGYLAIAKAHGLDPVQMALAFVASRPFPVIPIVGATSTAQLETA
LGAADLTLSPEIQAEIAAMHKAYPMPF
>OLS48226.1 carnitine 3-dehydrogenase [Rhodovulum sulfidophilum]AB14
MTGSVAILGAGTAGAGWAARFALMGWSVRVFDPDPGAAARVASVLGNARRALPGLSDRPLPPEGDIVHAATISQAV
SGAD
WIQESVPERLDLKRTLYQKVQEHAADDAIIASSTAGFTPTALYGQGARRCQIVVAHPFNPVYLLPLVELVMAEDAPGW
AL
ERAEQVLRGIGMMPLPVRREIDGHIADRLMEAVWREALWLVHDGVATTAEIDEAVRMGVGLNWAQMGPFESALLS
GGAAG
VDQVVSRIGPGLDLPRTHLTEMPEATEALARTVAEQAGTQAGGRSLDELERIRDRNLVAVLRALKWAGWGAGAHLRD
VDA
LLENGAAEIAGPIRSVARTVPLDWVDHNGHMTEARYMQVFSDATDRVVELMGCDAAYIAGGASFFTVESHIRHLAEA
KPG
AALHVESLCLAAEGRKFHLFHRLFAEGREVATAEALLVHVSLETRQAAEPGAELARRMAEIARAHAALPRPAGVGRSVG
Q
KPL
>OLS48227.1 DNA mismatch repair protein MutT [Rhodovulum sulfidophilum]AB14
MGETDARLRPTGGIDDPPIRDAATAIVLRDAATTPRVLMGQRGAGAAFMPNKVVFPGGAVDPVDATVPLACPLAAA
CAAR
LQAEADPAMADALAAAAIRELWEETGLILGRPGSWPGPAPKGWRGFAASGHLPSPEGFHFIFRAITPPGRPRRFDARF
FL
VEAEALASDPDDFSRAEDELSQLQWIPLAEVRRFDLPFITEVVLAEVTQLIRGGLPPASVPFFDNRAPVSKFQRLA
>OLS48228.1 hypothetical protein BV379_07980 [Rhodovulum sulfidophilum]AB14
MIGSHVRPVGAAVMRGWCKRCPNCGAGPMLSGYLAVREHCPVCMEELHHHHSDDGPAYLTVLIVGLLSAPLVAGSI
AVFR
PDPLVLTSVFSIGCVALALYLLPRLKGVMVALQWAKRMHGFGQPAE
>OLS48229.1 hypothetical protein BV379_07985 [Rhodovulum sulfidophilum]AB14
MARNATKMSSAALAVGAAAILSLAAPAMAQQGPGAGPAAGAARQGMLFDRMDADGDGAISLDEFTARARARFAG
LDTDGD
GMLSPEEVQKLGPAGRAGRAQGRDKGGWRQGGQGCGMQARHGMQRGGGMGQGMGQGMMGQMGPGMG
GACGGGPGGMGPRG
AMQGQGPMGGGYGMFGGLDGSGPMGYGGFGDPSLSPEDRAARAERLIEMLDGDGDGQLSAEELATRPGPEMMF
NRIDADG
DGSLSQDEFEAAIRTFRGMMRQQGPAAQR
>OLS48230.1 RNA polymerase subunit sigma [Rhodovulum sulfidophilum]AB14
MDMPFDAGDGEADEALLLACGRGDARAARLLTLRMTPWVLSYARRLLRDAAEAEDVAQEAMLRLWRIAPDWRPGE
ARVTT
WLYRVVTNLCTDRLRRRRGAALDEVPEPPDDSPGAEARLQQAARVAALDAALARLPERQRQAVILRHIEGLSNPEIAAV
L
EIGVEAVESLTARGKRALARTLAGQREALGFDDD
>OLS48231.1 hypothetical protein BV379_07995 [Rhodovulum sulfidophilum]AB14

MKDEALEALFEAARQDRSEPSAALMARIMDDAMVTAAAGRSAGRSLPRRARMRRGVPALLLAAIGGWPALAGVAA
AGVFG
LWIGYAGPGDVGRLSTEILGSPYGPDDLMSSLDTYLTEG
>OLS48232.1 hypothetical protein BV379_08000 [Rhodovulum sulfidophilum]AB14
MADPAETAGPRRGRNRFSLALGLSLAVNVLLLGLIGGAVLGHRSGVGRDHAVTMMALGPYGRALADTDRAAIRKALR
AEA
PRFREERREVRQGFRDMLTALRGETFDADRIAAVLDRQEARVQAHAGMVRKLMLDRLSAMSPDERSAFADRLERVLR
HGP
PGRNRCEAPPHAR
>OLS48233.1 diguanylate cyclase response regulator [Rhodovulum sulfidophilum]AB14
MSRRILIVDDVATNRIVMKVRLRNAHYQVLQASDGRTALELARTERPDLILLDMCLPDIEGTDLCRRLKAGRETASIPLI
VVTAGRAPETKMRALEAGADEFLSKPLDEMILLARVRSLLRARETAEELALRNGTHQVLGFAEAAPNFAPPGLVALIASD
PRRARAWRAALVPLISDSLTVLSRAEALNPQPGAAQPDAYVIDADLTAEGAGLRMVSELRAHPGSRDAAILIVVPEAAR
E
TAAMALDLGANDLIADPMDAQELALRIKMQIRRKTQSDRLRATLEDGLRLAVTDPLTGLFNRRYALPHVARVAERAAE
TG
RSFALMILDIDRFKLVNDHYGHASGDAVLVEVARRLKENLRAVDLIARIGGEEFMVALPETDAERACGTAERLRRAIERR
PVALPGGAGEIPVTISVGVAIGDGCAFVTGQVDLEAKVRELMEHADRALYGAKASGRNTVEMSRSAA
>OLS48234.1 hypothetical protein BV379_08010 [Rhodovulum sulfidophilum]AB14
MQDFAETVALEALAWLVGDEELLPVFLGATGASAGDLRDRASEPEFLASVLDFLLLDDAWVLRFCAACGRPGEAVMA
ARA
GLPGGADPHWT
>OLS48235.1 phosphatase [Rhodovulum sulfidophilum]AB14
MTRISAILFDKDGTLFDFQRSWAGWALDLLTELAQGNPARRAALASALGFDLCAARFRPDSPVVAGTTDDVVRRLLPH
LP
EWRAEALARHLTEASVAAPMVPAVALGPLLEGLAARGLVLGVATNDAEAPALAQLEAAGVRGAFDFIAGFDSGHGAK
PGP
GQVLAFAAATGRSPDEVLMVGDSRFDLAAGRAAGAATAGVLTGTARAEDLSDLAGVVLPDVGALPDYLDGIAAGRG
>OLS48236.1 hypothetical protein BV379_08020 [Rhodovulum sulfidophilum]AB14
MADGTQGLPGRWSYGALGGGAGLAAWLLFELLPGPMAGMTRLHLLLSSFGTAFFIAALAMAGALRLGRALAAAALLA
LPM
AGLMTWASLRFDTVAGALDDIVEPAAFAALLVLAPPFLIAGFSPGGRWLDYGALFTRSWEIVVRFSAAWLFVGLAWGI
LF
LSNALFGLVGLEVIEWLLELEPVPYLLTGLTLGLALAVVGERSEYLSPELMLGLFRLLLPVVLAVVAVFLLALPLRGIGH
LFGGLSAASILLATAFGAVTLISSAADAGEACRPRGALAESCARLLALLLPVLAALAGTAVAMRLGQYGATPERVLAAVL
ALLAMGYGLGYAVAALGRRRWATGVRRVNVAMALTAMVLAAGLLTPLLDPQRISAADQVARYADGRVPADKLDLW
TLGHD
WGRAGQAALERLAAMEDGSGAEAMERQLAALKGAASRADFELARRGRALADDRNSLALTLPVRPEGATLPEGFVAG
LRPW
EVRQISEACAARTAGGNPSCVAVLADLSGARPGDEILILAQRPGEGPLLRAYFPEAGGYTMRSPDYLGGADPYRDAGAA
I
DALIAGRYDLRPMRLNALEVEGRTLFFGR
>OLS48237.1 hypothetical protein BV379_08025 [Rhodovulum sulfidophilum]AB14
MQGLINRAIHFFLADTYGQQVWADIVRDAGLERHAFEAMLNYEDATTEALLVSASARLALPQAMLLEDLGTYLVSQSA
LE
PVRRLLRFGGETFLEFLFSLEDLPDRARLAVPDLVLPELRLSEDAADGYRLDCRGDWPAAFYILQGLLRAMADDYGALVL
LEPLEERPDGGLLGVRLLDTAFSAGRSFSLKGPLA
>OLS48238.1 multidrug efflux RND transporter permease [Rhodovulum sulfidophilum]AB14

MARFFIDRPVFAWVISILIMGIGLLSISTLPVAQYPQIAPPNVTVQASYPGASAETVANTVTQVIEQQMTGLDGLRYISS
SSSSEGGASIDLTFETGTDPDIAQVQVQNKLAQATALLPETVQRQGVTVRKSSASFLMVIALISKDGTLDQVDLSDYMEA
NLVDEFARVEGIGEVQVFGSQYAMRIWLDPSKLAAYDMTPSDVVSAVSAENAQISAGAFGSLPAPDGQQLNATITAQ
SLL
TKPEDFEQIVLRAETDGGLVLLKDVARVEIGAENYSTIARRNGQPAAGMALRLAPGANALDTADRVKAKIDEFAEFFPD
N
VDYVIPYDTTPFVKISIDEVVKTLFEAIILVFLVMLLFLQNLRATLIPTLAVPVVLLGTFGVLAVFGYSINTLTMLAMVL
AIGLLVDDAIVVVENVERIIEEEGLSPREATRKSMGQITGALVGIALVLSAVFIPMAFFGGSTGVIYQQFSVTIVSAMAL
SVLVALTLTPALCASILRSKDAVHTHRGPFGWFNILFEKVAGGYASTVGWIVRRPLRVGVIYIALAAVMALLFIRTPTGF
LPEEDQGILFTLIQGPTGASAERTQAVADEIQNYYLTTEAENVNSVFTVVGFSFAGQGQNMGLGFVQLKDWDEREDPS
QS
VQAIAGRAFGAFSQIQDAMVFPIVPPSVIELGNVSGFDFYLQARGGQSHQELLAARNQLLGMASQSPLIGSIRPNGLED
A
AQFNLDIDWRAAGAMGLSATEVGNLLSVAWAGKYVNDFVDRGRIKRVYVQGEAEARATPQDIELWRIRNDSGGLVP
FSNI
STANWEFGAQGLTRYNGMPSMSLQGSPAGGVTTGEAMAEMERLASQLPPGFAIAWTGLSLEERESGSQAPLLYALSL
AAV
FLCLAALYESWAIPFAVMLAMPIGVLGALLAAWLGGFENGVFFQVALLTVIGLTGKNAILIVEFARDRQEAGSSLFEAVV
HAAQQRFRPIIMTSMAFSLGVLPLVLSSGAGSGGRNAIGSGVLGGTLSATILGVLFVPLFYTAIRKIAGGRMAK
>OLS48239.1 efflux transporter periplasmic adaptor subunit [Rhodovulum sulfidophilum]AB14
MPRSCPRPARAVLPVLVAALTLTAAAAFAQAQGGEKPPTAVTVVTLNAEDVTLKTTLPGRVVASGMAEVRPQVAGIIT
DR
LFEEGAEVEIGDALYKIDPASYEAQVAAARASVAQAEANLKAAGKEATRVQELLERNVASEQAVDDAIAARDAAGAAL
QV
AEAQLLSAEIDLERTTIKAQLSGTVGRSLTTRGALVTAGQPEPLAVIRTLDPVYVDVTMSAAELVRWRRGHTERQLANA
D
VSVDLNLPDGSGYEHKGTLTAAEPHVDEQTGVIVLRMNFPNTEGLLLPGMYVQADMPQGVIEGAFLVPQEAVGRDSR
GRP
TAMVVNADDVVESRVLTVLRDSGNAWIVTDGIADGDRVIVAGLQKVQPGAKVAPQERAAAAADGSSGGGKDQPAQ
GN
>OLS48240.1 TetR family transcriptional regulator [Rhodovulum sulfidophilum]AB14
MTAAEREAVILDATEKVLLASGLSGTTMSAVAREAGMSKRTLYAVFDGRTALFGGVLRRLGARVVRPLDPEERELSLSV
R
LKRLLGPDLAPGICAAPLELVRAVLAEAPRAPELVELLIEEGPRSYGRAVRHELDRAVARQEIRLSDTGRAADLLLDMAL
PCSLENLLRGTEGDIAGLRDRIELAVDVFLSGADGRV
>OLS48241.1 glycerol kinase [Rhodovulum sulfidophilum]AB14
MQHILAIDQGTTSSRAILFDKDLGVVATAQEEFEQHYPASGWVEHDADDLWTSTAGTSREVIERAGLVSEDIAAIGITN
Q
RETTLVWDRATGRPVHNAIVWQDRRTGDLCRRLREEGCEAQVTEITGLLLDPYFSATKLKWILDNVEGARARAEAGEL
AF
GTVDSWLIWRLTGGKVHVTDATNAARTMLFDIRKGVWSEDMCRMLDIPMSMLPEVHDCASDFGMTRPDLFGREIPI
MGIA
GDQQAATIGQACFRPGMLKSTYGTGCFALLNTGDAPVASKNRLLTTIAYQMDGKRTYALEGSIFIAGAVVQWLRDGLQ
II
RAAAETQGLAERADPGQDLVLVPAFTGLGAPYWNAECRGGVFGLTRNSGPAEFARAALESVGYQTRDLLEAMRADW
SAEG
DTALRVDGGMSASDWAMQFLADILGAPVDRPKVLETTAMGAAWLAGQRAGLYPTMEEFGKTWALERSFAPAMES
DARDAR

YERWKRAVAAVQAV
>OLS48242.1 alcohol dehydrogenase [Rhodovulum sulfidophilum]AB14
MAGFDFAAPGRIAFGRGRAAEVLPALLGWGRRILLVRGGSVAFADRLAADLADAGAVVCQIRGRGEPDLPMLEAALA
EAR
AFAPEAVVAVGGGAVIDLGKALAALLPSDRPVLDHLEVVGAGLPLPRAPLPFAALPSTAGTGSEATRNAVIGVPEAGRK
V
SLRDIRMLPDLALVDPALTDGCPRAVTLASGLDAIVQVIEPYLSCRATPLTDALCAAAIPEGLAALPRLMAAEDPAARDA
MARVSLTGGLALANAGLGAVHGLAGVIGGRTGAPHGAICGRLLVPVLRANRAALADAGGDPARIERVLDWIGAAFGG
QGA
EALDRFGDWIAASGLPPLPGMDHAAVAAEAGASSSMKGNPVPLSAATLAEVLAEA
>OLS48243.1 hypothetical protein BV379_08060 [Rhodovulum sulfidophilum]AB14
MLGLVLVGMAVGYPCAAIALFITQSWLAALVTLSLSGLLAMLAFGLRLAVIPDDRASSAG
>OLS48244.1 urea ABC transporter ATP-binding subunit UrtE [Rhodovulum sulfidophilum]AB14
MLKTTGLTLHYGGSQILHGVDIEAGTGEVSCVMGTNGVGKTSLLKAISGCHPRSGGEIELDGAAIGRVPAQAMAHRGV
GY
VPQGRMIFPLLTVRENLETGFACLPRPERMVPDEIYELFPVLKDMAQRRGGDLSGGQQQQLAIARAMIARPKLLLLDEP
T
EGIQPNIIQQIGRVIAYLRDQGRMAIVLVEQYFDFAYGLADRFYVMERGRVTLSGTKAELAKDRLIRAVSV
>OLS48245.1 urea ABC transporter ATP-binding protein UrtD [Rhodovulum sulfidophilum]AB14
MTALLEVSGVSVSFDGFRAIDNLSFEIGPRELRAIIGPNGAGKTTFMDIVTGKTRPDTGRVLWGERSVSLLKLSEARIAR
EGVGRKFQRPTVFEDQSVFDNLLMALRKDRTPLAVLFYRQNARDRARIGELAAEIGLKDQLYRKAGELSHGQKQWLEI
GM
LLAQEPRLLLVDEPAAGMTLAERAHTAELLKEAAKTRAVVVVEHDMDFVRRLGCKVTVLHEGSVLAEGSLDHVTKNRQ
VI
DVYLGR
>OLS48246.1 urea ABC transporter permease subunit UrtC [Rhodovulum sulfidophilum]AB14
MKDSLFARNPSVLIFLGVLALFVAAVTVLGEGVGMGLISTSFVKTLGKTLCLCLVAIAMDLVWGYTGILSLGHFAFFGLG
GYMIGMWLMYARTETIVAASLSQSPIPPTPQEIRDAIGSQIFGVVGGADFPAIWAFAQSLPAQLALVVLVPGLLALIFG
W
FAFRSRVTGVYLSILTQAMTLALSLYLFQNDSGLRGNNGLSGLQNIPGAADVPQSLLSIRFFWASALALGLGYLLAAFVV
SGKFGSVIRGIRDDEARVRFLGYPVEAYKLFVFTLTAVVAGIAGALYYPQAGIINPAEMAPIASIYLAVWVAIGGRGRLY
GAVIGAGLVSLLSSWFTGGQLPDLPLGVYTVKWVDWWQVLLGLGFVLVTLFAPKGIGGLFDLIPRRTSPARHGADPGP
DD
GSFRDREASE
>OLS48247.1 hypothetical protein BV379_08080 [Rhodovulum sulfidophilum]AB14
MALCITLADLSDPAIRGLLASHAAFCEATSPPESRHFLPLEGLDVPEVTVWAAWDGPELAGIGALKALSPEAGEIKSMHT
AAARRGQGVGAALLAAIRTEAKRRGYRALWLETGTSPPFAAARALYESQGFEPCGPFGDYRIDPHSAFYTKRLAPQEAS
L
>OLS48248.1 urea ABC transporter substrate-binding protein [Rhodovulum sulfidophilum]AB14
MTSMKKILLGAVALGTISMPAWADHTIKVGVLHSLSGTMAISEMTLKDTVLMLIDEQNAKGGLLGEQIEAVVVDPASD
WP
LFAEKARELLTVQDVDVIFGCWTSVSRKSVLPVIEELNGLLFYPVQYEGEESSKNVFYTGAAPNQQAIPATDYFLDELGV
EKFALLGTDYVYPRTTNNILQAYLKSKGIPESDIFVNYTPFGHSDWSKIVSDVKALGADGKKVGVISTINGDANIGFYKE
LAAQGISAEDIPVVAFSVGEEELSGLDTSNLVGHLAAWNYFESAESPENRAFVAKWKERMGQDRVTNDPMEATYIGF
NMW
VDAVEAAGTTDVDAVSAAMIGQTFPNLTGSTAEMLPNHHLTKPVLIGEIRADGQFDIISETDPVPGDAWTDYLPASAV
LR
SDWKDLGCGMYNTETETCVQIKSNY

>OLS48249.1 urease accessory protein [Rhodovulum sulfidophilum]AB14
MEAAAVAPLGQPRAIGEVRLSVRNGPRGTAIAGLRQSGAFKAVFPRVTGAGSVPPLEAVLVNTAGGITGGDRFSCSLQ
AE
AGSHLRLTTQAAERAYRAQPGETGQVANRLTVAAGARLDWLPQETILFEGCRLERSLVAELAPGARLLLVEPLVFGRAL
M
GESLHAARFRDRIEIRREGVPLYLDALRFEGDVAAQLARPAVAQGAGALASLVLVAPDAAAHLAPLRAILPAMGGASLL
A
EDVLALRLLAPDSYHLRKVLIPVLSRLTQALPRSWMT
>OLS48250.1 urease subunit gamma [Rhodovulum sulfidophilum]AB14
MQLTPREKDKLLIAMAAEVARRRLARGVSLNHPEAIALITDAVVEGARDGRSVADMMEAGAQVVTRDQCMEGVAE
MIHEV
QVEATFPDGTKLVTVHNPIR
>OLS48251.1 urease subunit beta [Rhodovulum sulfidophilum]AB14
MIPGELFVADDNLTLNAGRESHTFMVANTGDRPVQVGSHFHFAETNPALDFDRDAARGLRLDIAAGTAVRFEPGQRR
EVR
LIPISGQRRIFGFNQKVMGAL
>OLS48252.1 hypothetical protein BV379_08110 [Rhodovulum sulfidophilum]AB14
MTFTSPAFLAANPFWAANVMFDQQMRLFQASTDFMLRANPWALMMRAGGFDPLTADSGLSRADCDDEIPLDAGV
ASGGGE
EAEPSNVSALSPTRPEPAAEPSADPAVEAAAETVAEPVVDAPARTSGQPSIGAAPAPAASNAPVGSPPVTAPNRASEAS
P
APGSEPEPEPAMAAAAPAAATASGKARPRGAAARKGSSPRAARSGASGRGRRKPASPQILPGDGSGPEDDR
>OLS48253.1 urease subunit alpha [Rhodovulum sulfidophilum]AB14
MPVTISRADYAAMFGPTTGDRLRLADTDLIVEVERDLTAERAGAGGNGPVYGEEVKFGGGKVIRDGMGQSQATRAE
GAVD
TVITNALILDWTGIYKADVGLRDGRIAKIGKAGNPDTQPGVDIVVGPGTEVIAGEGKILTAGGFDSHIHYICPQQIEDAL
HSGLTTMLGGGTGPAHGTLATTCTPGAWHLGRMLQSADAFPMNLAFAGKGNASLPAALEEQVKAGACALKLHEDW
GTTPA
AIDCCLSVADAMDVQVMIHTDTLNESGFVEQTVAAMKGRTIHAFHTEGAGGGHAPDIIKVCGEEFVLPSSTNPTRPFT
VN
TLEEHLDMLMVCHHLDKSIPEDVAFAESRIRRETIAAEDILHDMGAFSIIASDSQAMGRVGEVIIRTWQTAHKMKLQRG
R
LADETGANDNLRVRRYVAKYTINPAIAHGLSHEIGSVEEGKRADLVLWNPAFFGVKPEMVLIGGTIVCAQMGDPNASIP
T
PQPVYTRPMWGAYGRSVENSAVTFVSAAAEADGIRARLGLAKQTVAVRQTRGIGKRDLRLNDAMPKVEVNPETYEVR
ADG
ELLTCAPAEVLPMAQRYFMF
>OLS48254.1 urease accessory protein UreG [Rhodovulum sulfidophilum]AB14
MSSPHGPLRIGIGGPVGAGKTTLTAALARAFSKTHSVAVVTNDIYTQEDAEALMRMQVLPSERIKGVETGGCPHTAIRE
D
ASINLAAIAELTARFPDLDIVLIESGGDNLSATFSPELADVTVYVIDVAAGEEIPRKGGPAITRSDLLVINKTDLAPHVG
ADLGVMDRDAKRMRGALPTVFAALKQGEGVEAAIGLLCEIGGLTEPA
>OLS48255.1 hypothetical protein BV379_08145 [Rhodovulum sulfidophilum]AB14
MTDAVPEIRPRGRGRNTPLTAIATGRLVRDIWLSPHRPFFALSALWAVLAVMWWRFGDRIGLPVPALGTTTLWHAHE
MTV
GVGGAAMAAYFLTAMPNWTGARRVTGRGLMLLAGLWTLARLAAALGQVLPLAVVLAPGLAFYALFTLVYLRAVVSG
RRGD
RWAIPAAIAFLGLADAAFLATALGYLNSPGDAAMTRVLVLFFAIKVSIIAGNMAPALIAGWVGRQGKALPRIDPLASRIG

LGLMLAALALTLGGAVTAGSALLIAAGAAQLWRMRHWRGLSALGYAPALMLVLAFAWAPLGLALTGLAALLPLPWRE
ADA
IHVLMMGAMSGLAFSIAARAAARREGGLLRIGPLHKTGFALLWGAVWLRLAAPAIPSLYEPLLDATAAAFCTGWALFL
AG
YLPSLRGAVINPVFNVGGHGHGHGHGHGKGHGHGCGCGGHGGGGGHGQHGGPGRGGGPFRMPEPS
>OLS48256.1 ModD protein [Rhodovulum sulfidophilum]AB14
MLPLDDATLIGLLKEDAPYGDLTTRSLGFGAAPGRLTMRARGPMTVCGTEEAARILTLLGARAEIAAPSGTAVPAGTVL
A
EATGPAEPLFLGWKVTQTLTEWASGLATSAAAIVGAARAVAPGIGVACTRKAVPGTRALSLKAVLAGGAEIHRTGLSDT
V
LLFPEHRAFGGPEALAAQIGMLRLACPERRVVVEVKTLAEAETAAEAGADVLQLEKFPPEEAAAAARALAGWTGCLAA
AG
GITPENAAAYAATGMQVLVTSAPYYAKPADVAVTLAPA
>OLS48257.1 hypothetical protein BV379_08155 [Rhodovulum sulfidophilum]AB14
MPALRLERIGNAVLGPLDLELAAGEAVALLGPSGAGKSTLLKIVAGLLPHRGRVIFEGRDLTGLPPHRRGIGYMSQDLHL
FPHLSVAGNLALPLVFAGLDRQARARRVADTLALCAIAHRATRRPAQLSGGERQRAALARVLSLRPRLLLLDEPFANLDR
DTRAGLWAELDALRRGRGMTALIVTHDPSEARALADRSVAINAGRLNERTLLPCCPSTTRPSLAF
>OLS48258.1 ABC transporter [Rhodovulum sulfidophilum]AB14
MRGLAILLWAPLVLIVATILGALVSLASQLPLAGLAEALAAEETRFAIAMSLRSSLIALALALFLGLPTAWLLARRRFVG
KAVVETVLDLPMVTPPLVAGIGLLLLLGREAPVGGALAGLGIEILFSPAGVILAQTYIASSVVIRSARAAIGAVDPGYAA
NAATLGLPPFWAASLVELPMAGRGLMAAAVLGWARALGEFGATLMLAGATRMRTETLPMAVYLNIASGRTDIAIACA
LIL
LALAFALLLAIRLLTADRPDPMTEAQHARPQA
>OLS48259.1 molybdate ABC transporter substrate-binding protein [Rhodovulum sulfidophilum]AB14
MRLSIAFAPILGLGLSAAAPADTLHVYSGAGLRPAVEPLAEAFEARTGAELAIEYAGTGQILARARTTGRGDVFIAGTRF
FSDQLEAEGRISGVVALGIHGAAIGVSKAAAAKVADIADLGRPGLRLALGDPQAMALGRTADEIMARCGDPEAIRANT
VV
RATTLQQLAMYVATGDVDAAIMARSAALPHGERIALVPIPPECYTAEEITAGLLDSAEEPELATAFIALLASPEGRAAFE
AAGFGAE
>OLS48260.1 molybdenum ABC transporter ATP-binding protein [Rhodovulum sulfidophilum]AB14
MIEADFMGRLGGFELDAGFTAPGEGITALFGPSGCGKTTVLRAIAGLLRLPGSRLSVNGEIWQQRRQFRPPHRRAVGY
VF
QEASLFPHLSVRANLTFGLRRVKGPVRIGEDEVAELLGIAPLFDRPTRMLSGGERQRVAIGRALLSQPELLLMDEPLSAL
DRFSKDDILPYLERLHAALSIPVLYVSHDIAEVERLADTLVLMEAGRVRAAGPLAEMLADPALPLIHMPAPAAVIDGEVV
GRDPRYGLSEVRVPGGVVVVPGDLGPNGMRRRLRIPASDVSLGRHAPLDTTILNALPARIEGAEPAGAHQITVRLRLGP
D
GRGAALLARVSLKSWDRLALKPGELVVARLKAVALAEPAQSPAQPVPALARIR
>OLS48261.1 molybdenum ABC transporter permease subunit [Rhodovulum sulfidophilum]AB14
MTASPLFDAAMIETILLTLKLAGITTVVLLLVGTPLAWWLAQGGGLWKEIVATVVALPIVLPPTVLGFYLLIAMGPQSPL
TGLLGRQLSFTFTGLVLGSVIYSLPFVVNPIRNAFEAMGPRPLEAAATLRAAPLDAFVSVALPLASPGIMTGAILGFAHT
VGEFGVVLMIGGGIPGRTKVLSVTIFDYVETLQWPKAHVLAGGMLAMSFVVILAMMLIERRLNGGRR
>OLS48262.1 molybdate ABC transporter substrate-binding protein [Rhodovulum sulfidophilum]AB14
MTFRPTALRSGIAALLAALALSTPARAAETVVAVAANFTEAATEIGEAFEKATGDRVTYSFGSTGQLYAQITQDAPFEVF
LAADQARPEKAEAEGFAVPGSRFTYAVGKLVLYSTDAGAIDGSSDALGQPGFAHVAIANPVTAPYGAAAVEVMKALGL
YE
TLEPKIVQGKSISQTHQFVATGNAELGFVALSQVVADDSGSKWMIPQELYTPIRQDAVLLEKGAGNAAARAYLDFLKSP
E
ALAIIGKYGYGTED

>OLS48263.1 molybdenum-dependent transcriptional regulator [Rhodovulum sulfidophilum]AB14
MPNPSLKAALTLQREGAPRVGGDRIRLLQAIDARGSIAGAAREIGLSYKAAWDAVGSMNNLFAHPLVEAAPGGRAGG
GAR
VTRTGHDLIACFSTLEGALGKTFAAIDTGLKAAPGGAVNTLWSLMMQTSTRNTFRCTVTRVTPGAVSTEIDMALTDGH
RL
TAIITERSATEMGLAEGSEVFALIKATFVMLAAGEAPAQISACNRLTGTVSAREDGPVNSEVTLDLGEGKTITAILTRKS
AEALGFGIGSTATALFKASHVIVAMP
>OLS48264.1 hypothetical protein BV379_08190 [Rhodovulum sulfidophilum]AB14
MIDLPRPEAPMIQTRCLLDDADGDVAALTFVAGETPGPVIAAFLARLCRQGRRVLRLPQSGCDQPTGAGVPDLLALTLC
G
GVAAERHWRPALVAARAVRRPVLVPVPATRFGAWLDLTGGCAVRLACDPAALEAWWRSLDRPFPLERGLTGGVCAL
WK
>OLS48265.1 hypothetical protein BV379_08195 [Rhodovulum sulfidophilum]AB14
MDPASPSALWTDRAERLRADDPVPVVMADPDIAPRGTVLLIHGRNGAPDQVQIAVIAEAYLARGWRVVAPELPNSIA
LPA
SGPAERLTMNGLRRAAAEVMGWTRARWPEAPLALAGHSLGGFAIGHLGAELGAAHHLLAASPVLSGPRLLAARVAM
GPPA
VAALEREVPLLRAEMEAEDAAPALARVACPLGVITGAEDGIVPLSDARAYFNAAPDGRFFGALPGQNHCPSGPDVAA
MLD
VALAALGA
>OLS48266.1 hypothetical protein BV379_08200 [Rhodovulum sulfidophilum]AB14
MRRAVSATGLSRHRAGFGWTVVPAPGSILASTRVASWDPEPDYFHHWTRDAAIALAAIPAAMAAEPEAAAFWRQAV
VDHI
RFSLAISDPDRRGPSVNPLKATATGAFRRYLRPDAELAALGGPAWLAEPRCAADGGPDLERWGRPQFDGPALRAAALI
RL
GELMPEAQGPEAARLIARDLDFTLAMAGAPCLGPWEEEPARRSSFTLIAQWDALDLGARHLGRADLAEAAARLAGLIA
GA
ADPVTGGWRESLEAAPGALDAATVLALVQAGRMDGPFALTAPRSRATMAALEALFAGLYPLNRGRAAPALGRWAGD
VFFG
GNPWFPVTLGFAEAHYRIAAATGEAAEFDRAEALMAVVEDLAPEGDDLPEQADRETGAPSSCLGLSWSAAAYLAAAA
ARD
QALASR
>OLS48267.1 hypothetical protein BV379_08205 [Rhodovulum sulfidophilum]AB14
MAGVIRGLDDAALRDRIAQATAGYFLDWSHPVSGMARERSAGAFGYDIEDTVCTGGTGFGLLAQIVAAERGWRPRRE
ILD
RVARLVGFLETADRFDGVFPHFLHGATGRVLPFMAGDDGGDLVETAFLMTGLLGAAAFFTEAPEIGARIEALFGAVNW
AA
HLRADGAVMWHRHPERPWPGNALPIRGWNEAMPVFVLGAGSETFPLPPASYHDSWAKTPSFLNGRSYGGTVLPLG
PDWGG
PLFLSQYPFLGIDPRGLRDAYADYGLQARAHALVNRAHCLANPKGHAGYGPDCWGLTASDDPKGYAAHSPTHDTGVI
TPT
AALSSFPYAPAETMPVLRHLHDDLGDRIWGEAGFVDAFCPNGDWVAESRLAIDQGPIVVGLENHRSGLIWRLVSNRPE
IR
RGLLRLGFAAPYLDASA
>OLS48268.1 hypothetical protein BV379_08210 [Rhodovulum sulfidophilum]AB14
MGNRSNEDAVARLMAEMTLAEKIGQLNLLAAGEGLTTGAATDTPLAKRLDAGEVGAIFGTKSLASAQALQERALAGSR
LS

IPLFFAEDVIHGHRTVFPLPLALAASWDMALIEATAGFAAAEAAAEGLHQVYAPMIDVTRDARWGRMAESPGEDPHL
AGA
IAAAMTRGLQGTAPAAPGRVAACLKHFVAYGAPLSGRDYDNASLAWEDLLEIYLPPFADGVAAGAASVMAAFNAVNK
LPM
HAHGPLIEGWLRGRAGFDGLVVSDYTGIRELVAHGLGAPATVVLRALAAGIDMDMVGEDYLIHLPALAEAGLEAPEAG
LT
LSAERVRALIDRACARVLGFKAGLGLLDDPWRGLTGSPAAPDRAEGRKLARKAAARSAVLLKNDGLLPLSPDTRLALIGP
LADDRANMLGTWSVSGRAEDVTPLLEAVGALAGPPPSHAAGCNLVEDDWLIERLNVHGTTVTRDPRSEANMLAEAV
AAAK
AADVVLVALGEAKEHAGESSSRLCPDLPAPQRRLLDALATTGTPLVVAVFAGRALALGGIEQQCGALLYAWHGGIAGPE
G
VADLVFGRAEPSGRLPVTLPAAPGQTPLCHAEDPTGRPWTGRFAKFRTGWLDLADDSQPYPFGFGLSYTEVSYGPPRL
SA
TEARPGEQVSLSVTLTNTGARPAIETVQLYLSDPEARITRPARRLVAFRQVELAPGEEREVTFEIGAEMAQYPLAPSLEA
AEWVRDPGRILLHVGPNSRDTLSETLTWLA
>OLS48269.1 hypothetical protein BV379_08215 [Rhodovulum sulfidophilum]AB14
MRASEARAVVRKLTGFWGLVTAYWVSSRWAEAWALTIVVFGLTTLLSKASVWVATSSADFLAALANFHRAEAGVDPA
EVL
LMAAAALVGIYAARACGVALRHFLSSTLHRRARAWLVGRFDAAILADERIAFDLMSDRSETARGARLPDAIDQRLDECSI
GLYGGLIGLTMGLWGAVTSVWFVSAAILERSQPVPQLDRWGASANAWIEAEFGPGLAARIDLVPGDYGTALLVAGLIL
LY
VPAITWIAWRLGRIIERLKLQRQRRDGAWRGELGGLLHRVGQIAASRGERAQRRINTRLYGALDRTWSRQNGLGAGM
MLF
TDLYNFLSHRMLAYLPALPAFMAGHMSFKTFAASSELTAGLIGDLSWFINVMPEIATLRANAGRLTELAAAVERVRARQ
D
FYAETGISRFERSRDAKDALVEIGGLALCHRGHDATPFVEVPRLRLGRGDRIYLRGANGSGKSSILKAVAGLWPYGEGRV
ALAGGARMFFAGQEPDLPERMSLKTLVCYPDPAEKHADLVVARVLFRAGLQDFVTEMHHDLYHGKTWRTVFSGGQK
QRLV
LARILLMRPDILLLDEATSALDVNASVDFHDALCEELPKTAVIAVLHGETLPYDPDGRPFYSAVLDIAQGVGRVRPVMPP
SLAAARHAAE
>OLS48270.1 hypothetical protein BV379_08220 [Rhodovulum sulfidophilum]AB14
MNEKITPILFWEESMFTRSVIFSLLFSGAAAPVAAQTVFVDQGDSWSPSLRAAFYTQDQGSRIMPLAWMEALAQPDG
TPF
LADGLARYGYLPMPDRDDGATLPVGFTTNEGMGGTAIGMTCAACHTRQIEVRGTAYRIDGGPAIVDFQSFLADLDAA
MQA
VLDDDATFDAFADKVAGGTGDRAGLRAELETWSLRFHTLIDGSLPDPGWGPVRLDAVSMIFNRLTGLDLGRPEDAYLI
PE
NVMLADAPTRYPFLWNAARQDMTQWPGFAHNGNDLLGLARNLGEVYGVFGEFHPIKKPGLLFNRDYVTNNSANW
GGLKAL
EEWIWDIGAPRWPWGLDARLVEQGGAIFARPTDQGGCVECHGKRQGAFRSIFHATWATPILPVGTDERECEILTRTA
RTG
VLEGAKVPLSSNALGAEAPAFDLLGASVIGAIIQHAVGFRVTGGLEATGAAAALQKDDKFMAEFDDLRGAFPRSGDGS
AL
ETAAEPCAYEARVLDGIWAAAPYLHNGSVPTLRELLTPPDERVADFTPGPAYDLEAVGMAVEQTAFDHVIETTGCDDLS
S
GNSRCGHDYGTALSDDDKRALLEYLKSI
>OLS48271.1 cobyric acid synthase CobQ [Rhodovulum sulfidophilum]AB14

MTARRLMVLGTGSDVGKSLLVAGLARAYARRGLKVAPFKAQNMSNNAAVTADGGEIGRAQALQARAAGAPLSVDM
NPVLL
KPETDIDCQVVVRGQVRGRYSASAYQALKPDLRAEVVASLNRLSASHDLVLIEGAGSGAERYLRPQDISNMGLAEAADL
P
VVLLGDESRGGVTAAAIGHHRLWTETERARVRGVIVTKFRGDPEVFSPAARDIKAETGWPVLGLLRWSEAARALPEEDS
L
GLGRRKGAGGRLVIAVPELPRIANFDDLDPLAAEPGVDLRWLRPGQPVPGDADAAILLGSKTTRAALDAIRAEGWDIDL
Q
ALLRRGGRVIGLCAGYQMLGRVVRDPEGIEGPPGETPGLGLLDIETVMTGDKRLARIEATDRISAQPVTGYEIHMGRVA
G
PATARPWLDLDGTPEGAVSADGRVMGSHVHGLFGADGFRRHWLAGIGADLSPDAMAADPVERALDRLAEEIEADLD
LDAL
LTLAR
>OLS48272.1 cob(I)yrinic acid a,c-diamide adenosyltransferase [Rhodovulum sulfidophilum]AB14
MTARDPVSDDDARHAAKMAKKKAARDKIMAGKSDEKGLILVHTGTGKGKSSSGFGMILRCIAHQMPCAVVQFVKGA
WDTG
ERRLLTSHFGDLCQFHAMGEGFTWETQDRARDIAAAEKGWEKAKELIRDPEIRMVLLDEINIALRYGYLDPAEVVAFLQ
A
EKPALTHVVLTGRNAPEEVIEAADLVTEMTLVKHPFRSGIKGQPGVEF
>OLS48273.1 cobalamin biosynthesis protein CbiM [Rhodovulum sulfidophilum]AB14
MHIVDGALAAPVVIGGAVLAAGGLARGLRDLTLEKIPAAGMLSASFFVASLIHVPIGPSSVHLILNGLAGLVLGWAAFPA
LFVGLLLQAVFFGFGGLTVLGVNTLDIALPAVIVHYICRHGIATGSPRVAAIWGGIGGALAIALTAACVALALALSGAAF
IPAAKLVLLAHVPVMAIEGLLSAAAVYLAIQVKPDLFLSLQGPVR
>OLS48274.1 cobalt ECF transporter T component CbiQ [Rhodovulum sulfidophilum]AB14
MSEILTAGRSGPGAALDGIDPRARVIGAGAFACVTVACSWLPVLLAALMLALGLMALSGRPVGATLRRMAAMDGFIFL
LV
AMLPFTTPGAPVFTLWGFDASREGLVHAARILLTANAVVLALLSLVGTLEPVTMGQALHRLGAPDRLVHLLLFTIRYIEV
LRAEYLRLRRAMKVRCFRPGTSWHSYRSLGYLVGMMLVRAVERSERVLAAMKCRGFSGRMPVLSSLAYGRRDAVFGA
GLG
ALLLAMILAELAHALV
>OLS48275.1 energy-coupling factor ABC transporter ATP-binding protein [Rhodovulum sulfidophilum]AB14
MPLFEIDGLVFGYPGRPAILQGAGLTLEAGQRLSIAGANGSGKSTLLHLMVGLLQPQAGHIHAFGQVRRAEADFHEVR
RR
AGLVFQDPDDQLFCPTVAEDVAFGPLNLGLGREAAMETVETVLTRLDLMHLKDRITHRLSGGEKRLVSLAAVLAMEPE
VL
LLDEPTNALDEENAERLVAILQGLPQALLLVTHDAEMRARIAPGGLRLQAGRLVPA
>OLS48276.1 precorrin-6A synthase (deacetylating) [Rhodovulum sulfidophilum]AB14
MNELFLIGIGTGNPDHVTFQAAEAIRGADLILIPRKGADKADLADLRRQICARVLGEDASERIAEFDLPVRDASGDYRAG
VLDWHDAIAGVWTDTVAAASPAPRKVALLIWGDPSLYDSSLRIAARLSPAPRITVIPGITAIQALCAAHRIPLNEIGAPV
VITTGRQIRDHGWPEGADTVVAMLDGGCAFQSLAPEGIRIWWGAYLGMAEEIAIAGPLAEAGPEILKTREEARARHG
WLM
DIYLMRRG
>OLS48277.1 uroporphyrinogen-III C-methyltransferase [Rhodovulum sulfidophilum]AB14
MSGFVSFVSSGPGDPELLTLKAVDRLSKADAVLFDDLSSGPILGHVRPGADLVGVGKRAGRASPRQDHVSRLLVDYAAS
G
QRVVRLKSGDSGLFGRLEEEITALRAAGIGYEVIPGVTSACAAAAAAGIPLTRRLTARRIQFVTGHDVTGHLPRDIDIAA
LADEHATTVVYMGKRTFPDLAETLIARGLPADTPALLAEGVGTPEQALTRTTVAGLAALLTEGPGSAAPGLILFGPLVEE

PYDE
>OLS48278.1 cobyrinic acid a,c-diamide synthase [Rhodovulum sulfidophilum]AB14
MAERPSGLVIAAPASGSGKTTVTLGLLRAFRAAGVAVQPFKNGPDYIDPAFHARASGRASYNLDPWAMGEGLLATLAA
EG
HGADLVIAEGAMGLYDGTLTRGVAGHGSSAELARRFGWPVVLVIDVSGQAQSAAAVAHGFARHPEAPPVAGVILNRV
AGA
RHEAMIRPEMDRLGIAVLGALPRRPDLTLPERHLGLVQAGEQADLEARIEAIGRFVAEHVELDALRAAARPGPEALPGP
W
PAPPAQRIALARDAAFSFAYPHLLEGWRRAGAEILPFSPLADEAPDEGADLVWLPGGYPELHAGRIAAATRFLDGLRAH
A
ETRPVHGECGGYMVLGEALIDKDGSRHQMAGLLGLATSFESRRLHLGYRRAELLAPMPGHAPGTRLLGHEFHYSTILA
QP
DAALARVTDANGTEVAETGSRRGPVTGSFFHMIAEDPR
>OLS48279.1 precorrin-4 C(11)-methyltransferase [Rhodovulum sulfidophilum]AB14
MTVHFIGAGPGAPDLLTLRGRDLIAACPVCLYAGSLVPEEILGHCPEGARIVNTAPLDLDAIIAECKRAHEAGQDVARLH
SGDLSVWSAMGEQIRRLKALGIPVSVTPGVPSFAAAAAALGTELTLPGLAQSVVLTRTPGRASSMPEAESLANFAATGA
T
LAIHLSIHNLAKVVADLVPHYGADCPAAIVYRASWPDQQILRGTLGTLEAALPEAIERTALILVGRSLGAEGFDESCLYD
PDYDRRFRPRSARAGSDG
>OLS48280.1 cobalamin biosynthesis bifunctional protein CbiET [Rhodovulum sulfidophilum]AB14
MAETPWLTIVGVGEDGPDGLPPASRRALEAAEIVMGPARHLALVPDLAAERIEWPVPFAGGLPILRGLRGRRVAVLAS
GD
PFWFGAGSVIARHFAPGDWHALPGPSVFSLAAARLGWRLETTRCAGLHAAPFARLRPDLAPGGRAIVTLKDGAAVAEF
AA
FLTGAGFGDSRLHVLEALGGPRERIRETTARAMDLTGIAHPVAVALELAGEGPAIPAASGWPDAFFDNDGQITKRPMR
AL
TLSALAPLPGEHLWDIGGGSGSISIEWLARHPRMQATAIEADPERAGRIAANAAALGVERLSVVTGRAPEALDGLAAPD
A
VFVGGGLSQELLQTLWDRLAPGTRIVANAVTLESESLLALWHAAHGGELLRIELAEARPLGRKRGWRAAYPVVQWTAR
R
>OLS48281.1 cobalt-precorrin-6A reductase [Rhodovulum sulfidophilum]AB14
MHPTLLILGGTTEATALAARLAGTGIRATLSLAGRVENPRAQPIPMRVGGFGGVEGLIAYLRAEGVTHVIDATHPFAAQ
M
SRNAACACAALGLPLVALTRPAWTPQPGDDWRHVADIAGAVAALAGPARRVFLAVGRMHLQAFAAQPQHRYLLRLV
DAPQ
SPLPLPQAEVIVSRGPFTEADDRALMEGHGTELVVSKNAGGTGARAKIDAARALGLPVLMIDRPALPARRELARPDEVL
G
WLAHSGADLGV
>OLS48282.1 precorrin-3B C(17)-methyltransferase [Rhodovulum sulfidophilum]AB14
MTGWVAIAGLGPGAEALVTPEVTAALAEATDVVGYIPYVARVAERPGLTLHASDNRVELDRARHALEMAAGGRRVVV
VSS
GDPGVFAMAAAVFEALEAGAPEWRDLDIRVLPGITAMLAAAARAGAPLGHDFCAINLSDNLKPWALIEKRLRLAAEAD
FA
MAFYNPRSKARPEGFARVLELLREACGDDRPVIFARAVSTPEERLTVVPLTEARPEMADMRTVVLVGSSLTRVLRDGAR
P
LVYTPRSAPE
>OLS48283.1 precorrin-2 C(20)-methyltransferase [Rhodovulum sulfidophilum]AB14

MTMATVYGLGLGPGDPDLMTVRAERLLRGARHVAFFRKAGCEGKARSIVAELIPEGATEHPMEYPVTTEIPFDDPRYN
EI
LAAFYADSTARLKALAEAGEDVVVLCEGDPFFYGSFMHLYTRLKDDVAVEVVPGITGMSAAWTASGAPITWGDDVMS
VLP
GTLPEEVLVRGIKAADAVVVMKMGRHLGKLRRALLLAGRAEDAWIVEYAAMPGQTVTRLADYPGRTAPYFSIVLVHG
QGR
RP
>OLS48284.1 precorrin-8X methylmutase [Rhodovulum sulfidophilum]AB14
MPHEYEKDGAAIYLQSFATIRAEADLARFDADEEQVAVRMIHAAGMVELAPHIRFSPGFVPAARAALEGGAPILCDAR
MV
SEGVTRKRLPANNEILCTLQAEGIAARAAAMGNTRSAAALELWRPHLAGAVVAIGNAPTALFHLLNMLEDPACPRPAA
II
GCPVGFVGAAESKDALWAGQPVPCCIVEGRLGGSAITVAAINAIASRQE
>OLS48285.1 cobaltochelatase subunit CobN [Rhodovulum sulfidophilum]AB14
MHVVFRESHGLEETAVPTDLGQDPADLAVLSFSDSDLGAFAAGWHRGGGSEGRMPTLRLANLAALLHPLSVDTYAER
TLS
GAKGILIRLIGGRAYWEYGLTQIEALARARGIALAVLPADGRPDPRLDAASTLPVSTLRRLSELCDEGGAVAAQAALAQL
ALAAGLYAAPVIGTKTVPEMGAWNPEGGAVPAPDLSPTDAPRIVLTFYRAYVTAADLGPVEALFEAFRARGCAVTGLFV
P
SLKAPEAARWLAGQIAALAPAAIVNATAFSGKGEDGTSPLDAAEAPVFQVALSSAPETAWAEAERGLSPADLAMHVVL
PE
VDGRIFAGVASFKEAGARDPGLQFSRIAHRPHPARIAAIADRVMAWLGLARTPLAGRRPALVLSTYPGRPWQMAHAV
GLD
ALASAEAVLEDLARAGLPGTPAEELTGDLADTLGRETLSWPLPEYEAALAALPAPLRDALSDVWGAPEADPACRDGAFH
F
AATRRGAALVALQPERGTPEAREGDYHDLSRVPRHGYVAFYLWLRRAMGCDALIHMGAHGTLEWLPGKAVALSAAC
WPEA
LIGDMPVIYPFIVNDPGEAAQAKRRIGAVTLGHLPPPQRQAGGSERLARLETLLDEFSNADGLDPRRRDRLMADIRAEA
Q
ALGVEADLGLEPGLSPAEAITRIDRFVCDVKESQFGDGLHVFGRPPETPAEAGSGAGTEAGSGFDTAPSVAAESAALLG
A
LQGAPIAPGPSGSPWRGRQDVLPTGRNLFTTDPRAVPTRSAHAQGVRLAEDLVRRHLQDHGDWPRQLVVDLWGSA
TMRTA
GEDFAMALHLLGVKPVWDEGTERVSGIEVLPVAMLERPRLDVTLRVSGLFRDVFPSLSALYQQAIRALSQRDEAADWN
PY
AGRPETPRVYGPAPGIYGIGMDMPEPTEAGRQAAGAAWLAASAWALDGAEARRDAQGIRDRVAGAQAFVHAQDL
PETDLL
LAVDYARHEAGFAAAQDVTGSEAGGRAALYHLDNTDPARPRTRTLTEEIARVVRARATHPAWLAGMQRHGFRGAAEI
AAT
LDHMAAFARLAGVVPDHLFDLYFDATLGDPGLTTFLEEANPQALAAMRAEFAALARDGLWQTRRNSIAAALTDLETP
NAS
>OLS48286.1 cobalamin biosynthesis protein CobW [Rhodovulum sulfidophilum]AB14
MTDLAKLPVTVVTGFLGSGKTTLIRHLMQNAGGRRLAVLVNEFGDVGVDGEILKGCALPDCPAENVVELANGCICCTV
AD
EFIPTMEALLQLEPRPDHILIETSGLALPKPLLKAFEWPAIRSRITVDGVIAVADAEAVAAGRFAPNVAAVDAQRAADDS
LDHETPLSEVFEDQIACADIVLLTKPDLAGPEGVAAARAAVEAEAPRKLPVVEVTEGAVDPRVILGLGAAAEDDLDARPS
HHDNGAEHDHDEFESVVIELPEQDAPEALAARIEALARSQNILRVKGHVAVAGKPMRLLVQAVGARVRHQFDRPWAP
DET

RSTRLVVIAEHDDIDEGAIRAALSA
>OLS48287.1 metal-binding protein [Rhodovulum sulfidophilum]AB14
MSAPEPAAVELLVCTTCKRRQPADPDAPRPGSLLHDAVRTHGLPEGVRLVPVECLSACSSGCTVALRGPGRWTYVYGN
LD
ATTGAPIIAEGARAYRDAPDGLVPWRQRPEHFRKNCIARIPPMEALK
>OLS48288.1 hypothetical protein BV379_08335 [Rhodovulum sulfidophilum]AB14
MPDPRPVPPVPGPSALPGATGVLVAGAGFAGLTAAIEARLCGAEVTLIDPAPMPLWGGNTRHSRNIRLAHDSETRWQ
RGG
YPAAEFAADIERLVPSDNGLAGVLARGSADLGSWLAARGVAFEPWDEGNLPFSRRTAFFRGGGQALSHALLRAAGQL
GIR
IASGRIEALPPDWAEDRPLCLTVHTADGARRLQAEALVLATGGYGANPDEMIRHLGPGAAAFANRGTPHQDGAPLLW
LMA
HGAATVGRPGDGHLVAVDARAPASDAGIVSRADGMQFGLVVDAAGQRFRDEGADVGSARYSVWGRALAERADPR
AWLILD
AEGAARLPPLLYPPLTAETPADLARLCGIEPGGLAATLDALAAHLDRGTPFEPPRSRPEGAPFAPPLRPPLAAIPLRPGL
TFTRHGVAVDATARVRLADSGSAHRVFAAGASMAGALLGQGYLSGSALAIGGVFGRIAGAGAASLADHGVGQAGPA
TDKL
RQASAVPALQPAPPQPAAPAPEPEPEPEPWDEAARALNICNTCGFCTGLCAVFPAARLRPALAAGDLRHLAHLCHDCR
SC
HYDCQYAPPHALAVNLPAALAEARAADYAARLPARLPQAVFLLCLLGLPLMALLAIPPERLFAAGAAPGAFYEVIPHGL
M
AGGAGLAFGGAALVLAIRLWRFWRQTAGPAAQPGIRPLARGLGAALRLTNLGADRGCDGPRGPTGPARRIFHHLLAG
GFV
LTGLATLAGAVLHAGGKIAPYPLASAPVALGTLGGIAMLAGLAGLSHRRKRADRRPASARMEAADRDLRRALGIVAGS
GL
ALLALRDGPAMGLMLALHLGAVLGLALALPFGKLSHGAYRSLALIRHAAERAARPGPRR
>OLS48289.1 multifunctional 2',3'-cyclic-nucleotide 2'-phosphodiesterase/5'-nucleotidase/3'-
nucleotidase [Rhodovulum sulfidophilum]AB14
MTMRILFPAAALALLAGTAQADFALTLLHTNDVHARLEPISKYDSDCPEEDNLAGACFGGTARLATAIAAARAEGGPTLL
LDAGDQFQGTLFYSYYKGKAVAEMVNRLGYDAMAVGNHEFDDGPEGLADFAARVTMPLLMANADLSAEPRLRDAIL
KSTV
IEVAGERIGVIGLTPQGNPDLASPGPDIVFTDPVGAAQAEADRLTAAGIDKIVLLSHSGHETDKRIAAETTGIDVIVGGH
SHTLLSNTDAAAAGPYPTMLGRTAIVQAYAYGRYLGRIDLVFDDAGGLVSAGGDPILLDASVAEDAEIKARIAELAKPLE
EIRQNVVAETTAPIDGDRGSCRAGECAMGNLVAEAMLDRVRSQGIQVAIQNGGGLRASIDEGPVTMGEVLTVLPFRN
TLS
TFHVSGATLRAALEHGLGLAEEGAGRFPQVAGLRFIWAPGAEPGQRLRKVEVKEGTDWEPLDPDKSYGLVSNNYVRT
GGD
GYTMFLDAREVYDFGPDLADVTAEYLARNTPYAPEIDGRIAAE
>OLS48290.1 hypothetical protein BV379_08345 [Rhodovulum sulfidophilum]AB14
MLIYKIFRRAEWDALRERGASRGAPVDLADGFIHLSTAGQVAGTAEKHFAGESDLVLVAVEADRLGADLKWEASRGGA
LF
PHLYRELRLADLVWDKSLPLGATGHIFPEGVI
>OLS48291.1 dihydroorotate dehydrogenase (quinone) [Rhodovulum sulfidophilum]AB14
MTPLERVGLAVFHRIEPERAHALSLTALRRGLVPLPGRLTSSRLATDLDGLSLPNPVGLAAGYDKNAVALGPLMRAGFG
F
LEVGAATPRPQPGNPRPRLFRLIEDRAAINRFGFNNDGMEAIRARLAARPAGVPVGLNLGANKDSPDRAADFATVLRA
CG
AHVDFATVNVSSPNTEKLRDLQGHEALAALLAGVMAARAELSRPVPVFLKIAPDLTDAEIDDIAAVALEAGVSGIVATNT

TLARTGLKSAGAGQAGGLSGAPLFARSTRVLARLSAATGGQLPLIGVGGVGSAEEAYAKIRAGASALQLYTALVYGGMS
L
VGDIVEGLDRLLARDGFAHVSEAVGTGRKDWE
>OLS48292.1 arsenate reductase (glutaredoxin) [Rhodovulum sulfidophilum]AB14
MTAIAIWHNPRCSKSRAALALLQERGLEPQVRRYLEVPPNEKELREVLDMLGGPAIGMMRTGEPLFSELELTKDAPDEV
L
IAAMAAHPKLIERPLAMADGRAVLGRPPERVLELLED
>OLS48293.1 carbamoyl phosphate synthase small subunit [Rhodovulum sulfidophilum]AB14
MPAATPHPTACLTLADGTVFYGHGFGAVGETVAELCFNTAMTGYQEIMTDPSYAGQVVTFTFPHVGNVGVNPDDDE
TADP
VAAGMVVKWDPTEPSNWRAADALKDWLARRGRIGIGGIDTRRLTRAIRQQGAPHVALAHDPEGKFDIEKLVAKARA
WSGL
VGLDLAKDVTCAQSYRWDEMRWSWPEGHAKRTEPGHRVVAIDYGAKRNILRCLASAGCDVTVLPASATAEDVLALEP
EGV
FLSNGPGDPAATGEYAVPMIRKILDKDVPLFGICLGHQMLALALGAKTVKMNHGHHGANHPVKDLETGKVEITSMNH
GFT
VDSQTLPEGVKETHVSLFDGSNCGIRMTDRPVFSVQYHPEASPGPQDSYYLFERFVTAMADRKQA
>OLS48294.1 glutamyl-tRNA amidotransferase [Rhodovulum sulfidophilum]AB14
MDLRTRISAALKAAMKDREANRLGTLRLINAAIKDRDIAARTGDTEEGVSEAEVLAILARMVKQRQESARAYEEGGRLD
L
AERERAEIAVIEEYLPRQLTADEVDEAIGTAIVETEANSIRDMGRVMAQLKARYAGRMDFAAVGPMVRQRLS
>OLS48295.1 hypothetical protein BV379_08370 [Rhodovulum sulfidophilum]AB14
MLRYLKNVKPEDWPALLLRAGALALITVHLVTFLFIYQHPLFILTAVGMGLLLAREMYEDLSQRFFRLKAEEFERELQDL
KGTAPTAAEGGRRSLLIEETGALKDISARAFRS
>OLS48296.1 lipoate-protein ligase B [Rhodovulum sulfidophilum]AB14
MEPEWLELKELSPYEETVAAMEARAGQIAAGQAREAIWLLEHPPLYTAGTSARPEDLTDPGRFPVYETRRGGQFTYHG
PG
QRVVYVMLDVGRRGRDVRRFVWQLEQWVIETLAEFNVRGERRAGRVGVWVVRPDLPPGPSGAPREDKIAAIGVRLR
RWVS
FHGLSINVEPDLGHFDGIVPCGIRGHGVTSLVDLGLPVTMADVDVALRRSFDRVFGPLPPEAS
>OLS48297.1 polyamine ABC transporter substrate-binding protein [Rhodovulum sulfidophilum]AB14
MTIRPILVSTVALIAGSTAWAADPDLLVFDYSGFEIPAFHADYAEKYGQEPSFTFFGDEEEALQKLRSGFRTDVSHICAG
SVTKWIESGILEPWDTSRITAWEHINADLKGTDVADTGAEVYFVPTDFGSTAIAYNTEDVPAGDVASLDVFKNPEYAGR
V
TLPDNVDDAFALAYLATGTTNWSTVTDAQFDAAVAWLREVHPNLRTYWTDPAELAQLLASGEVLVSWAWNETLPT
MVDQG
FPVGFQREAKEGSSLWVCGYVDLKDGPGSEQKAYDFLNATLAPSSTKALLDAGYGQANAKAMAAIGNEALTAIGLGPI
DV
PVLAQLPMPGELREKQSAAFEKIKAGF
>OLS48298.1 oxidoreductase [Rhodovulum sulfidophilum]AB14
MFKALVVEKDEEGKTHAAVQNLDEDRLPEGDVTVAVEYSTLNYKDGLCLGSGGGLVRQWPHVPGIDFAGVVQQSAD
PRYK
PGDRVVLTGWRVGEVHWGGYAQRARVKADWLVPLPDGLSTHSAMAVGTAGMAAMLAVIALEDHGLKPGDGPVLV
TGASGG
VGSVATAVLAKLGHEVAAVTGRPGSEDYLRALGASQVVQREDLSEVTRKPLESELWAGCVDPVAGAMLGRVLKQMKY
GSS
VAAVGLAGGAAIEGALITPFILRGVNLLGIDSVMQPYEARLRAWKRIATDLPMDRLKGMIRSATLEDLPGLGKDILKGQV
QGRVVVDLNA

>OLS48299.1 universal stress protein UspA [Rhodovulum sulfidophilum]AB14
MFKKILVPVDLAHAEALKRSCDVAGDLARHYGIGVTYVAVTAPQPGRLARSPEEYRKKLEAFARAEAETHGHLADARVI
V
CSDPAAELDAALMDALKEVGADLVVMASHLPGVAERIWPSHGGRLAGHARVSVLLVRPESGSADSRS
>OLS48300.1 BCCT transporter [Rhodovulum sulfidophilum]AB14
MSDQTTEQLTDQGIPAPEGEVDLIETDYEIGQDNIEGSLGPFGFDIHNPVFLVSGLCIMAFVFYALALPEQAGAVFGWL
R
PFLTSTFDWFFLSAANVFVLFCLALIVLPVGRVRLGGVDATPDYSYSGWFAMLFAAGMGIGLMFFGVLEPVYHMAISQ
PL
GTPSPFAADGSLIPENVDAAREMGLAATIYHWGLHPWAIYAIVALALALFTYNKGLPLTIRSAFYPIFGERIWGWTGHFI
DILAVFATLFGLATSLGFGAQQANAGLSHVFGVPEDLTVQILLITGITAIALISVLRGLDGGVKVLSEINMIVAALLLVF
VLVAGPTGTILTDFGEGMLAYVRDLPALSNPFGREDTDYLQGWTAFYWAWWISWSPFVGMFIARVSRGRTVREFVIC
VLV
IPSLVCVLWMAVFGGAAIDQVLSDPAGSAVKAQVIDAYNPPMSLFAMLDALPWASITSVIGIVLVIVFFVTSSDSGSLVI
DTITAGGKIDAPLPQRVFWCTFEGAVAIVLLVGGGLSALQAMVISTGLPFTLVLLVMCFSIFRGLQTESR
>OLS48301.1 nitrogen fixation protein NifS [Rhodovulum sulfidophilum]AB14
MVALDIEFVRNQFPAFGYKDLRDKVFCENAGGSYACRYVIWRLHRFYQERKVQPHGAFGVSRLAGEEMDEAHTRLAA
HLG
IGRDELHFGPSTSANTYVLSQAFGDWIGAGQAIVVTDQDHEANSGVWRRLAARGIEIREWKVDPDTGHLDPERLEPLL
DE
TVRLVCFPHASNILGEVNDVARITAMVRRAGAFTCVDGVSFAPHGLPDLPALGADIYLFSTYKTFGPHQGVMLVRRELA
Y
QLPNQGHWFNGDDAVRRFDPAGPDHAQVAATAGISDYIDALYDHHVRGGRDAHARAGIVAGMIRAQETRVLEPLLD
YLAT
RPDLRVLGPRSAGPTRLPTVSIALERSAAEVAGRLAARGVLAGGGHFYAQRLLGAMGIDPKQGVLRLSLLHYTLPEEVDR
LIAALDSEL
>OLS48302.1 deoxyribodipyrimidine photolyase [Rhodovulum sulfidophilum]AB14
MSDQAPVIWWVRRDLRLGDNAALAAAAGSGRPVVPLFIHDETVEALGAAAKWRLGEGVAAFARALGARGSRLILRR
GAAL
GVLRAVAAETGAAAIIWGRAYDPAGRDSDNAVEDGLDGTGIELRAVAGALLFEPWTVETGQGGFYKVYTPYWKAVRD
RDL
PEPAPAPGLIPRPDFWPASDEIEDWRMGAALGRGASVLARHASVGEEAAQGRLGAFAAQKIADYAERRDFPAAGATS
RLS
EHLSWGEISPRSCWHAGLRAMEAGKAGAETFLKELVWREFAWHLLWHSPHIAERNWREGWDDFPWSTDGDSPEA
TAWKRG
RTGIPFVDAAMREMYVTGTMHNRARMVAASWLTKNLMTHWRVGMEWFADHLTDWDPASNAMGWQWVAGS
GPDAAPYFRIF
NPETQAAKFDPDGAYRDAWIAEGRQSPGQGALDYFDAVPRAWGLSPDAAYPAPAVTPEAGRRRALAAYQAREG
>OLS48303.1 SAM-dependent methyltransferase [Rhodovulum sulfidophilum]AB14
MTVLTSTRGQADLPRYFTQVFDIAGRMGHGRLDFVLPDGRRFRAEGRGAGPVAQLDIHDADVFARLIREGDLGFCEAY
VE
GGWSTPDLMAFMDLVHADNDELFDGFPGLGLLRAYERLRFWLQSNSKTQARKNISHHYDLGNEFYGLWLDETMTYS
SALF
RTGQESLEAAQELKYAAMVDQMGVAPGDHVLEIGCGWGGFAEYAAGQRGLKVTGLTISREQHDYAVARIARAGLAD
RVEI
KLQDYRDETGRYDGIASIEMFEAVGEKYWPTYFRQVNDCLKPGGQATLQVITVQERRYEIYRKGVDFIQKYIFPGGMLP
S

PGVLRAQASAAGLEIRRSMEFGESYALTLRRWHETFNDRWDEVARLGFDARFRRMWNLYLASCAATFHSGNCDVTQ
LTVA
RPA
>OLS48304.1 hypothetical protein BV379_08430 [Rhodovulum sulfidophilum]AB14
MTALPGTLWGPIHSGGRARGRAIMGRFCDPVAALPGWGGLAGGYGPWLGGLLAGIGPLPVGRGPAGLAAGAISHF
WT
>OLS48305.1 hypothetical protein BV379_08435 [Rhodovulum sulfidophilum]AB14
MERMFLYGTLCHLPLLARVLNRPAGTLDTLPARLPDHAVRLADGEGFPLILPEPGAEAEGLLVSGLTAEDRARLDYYEGG
YGFAHDVVTVETAAGPVAAGIYARARSGRAPTAAWDPDAWRARWGDLVTEAAGEVMLGYPDRPPEVNARRDRM
MLARAAS
TLRARAPGPASLRKPTRPGDVVETGRAQPYANFFAVEEYTFRHTRFDGRMSDQMVRAAFLTGDAATVLPYDPARDAV
LLI
EQLRVGPYARGDSQVWSVEAIAGLIDPGESPEETVRREAVEEAGLTLGALHRCYGYYPSPGAKAEYIYSFVGEADLGAEA
GGLGGLAAEGEDIRGHVVAFDRAMELIGTGEIGNAPLILSLLWLARHRETNQRPA
>OLS48306.1 cysteine synthase A [Rhodovulum sulfidophilum]AB14
MAAIRSDLADLIGNTPLVRLKGPSEATGCEILGKAEFMNPGQSVKDRAALSIIRDAVARGALAPGGTIVEGTAGNTGIGL
ALVGASMGFRTVIVIPETQSREKKDMLRLAGAELVEVPAKPYRDPDNYVRYSGRLAEALAKTEPNGAIWANQFDNTAN
RQ
AHAEGTGPEIWEQTGGRVDGFICAVGSGGTLAGTAMALQPKGVKIGLADPHGAALYSHYTTGEMKSEGSSITEGIGQ
GRI
TANLEGLAPDFCCRIGDDEALPVLFDLLAEEGLCLGGSSGVNVAGAMRMAREMGPGHIIVTVLCDYGTRYQSKLFDPD
FL
RGKGLPVPPWMDRAAMRNDGNGCRPALPEVFAG
>OLS48307.1 Ala-tRNA(Pro) hydrolase [Rhodovulum sulfidophilum]AB14
MTDPLYRSDPYLREAAALVTAHADGGVVLSAPLFYPRGGGQPGDGGRLVWEGGEMAVTETVKGEAGAELLLPPEGA
VLPP
VGTELIQQLDWDRRYGHMRVHTALHLLSVVIPLPVSGGAIAAGKGRLDFDMPEAPEDKEALEVALNDLIARDLPVTED
WI
TEAELDADPGLVKTMSVAPPRGAGHVRLVRIGAGAAQVDLQPCGGTHVARTGEIGPVRIGKIEKKGRRNRRVTVLLGA
>OLS48308.1 hypothetical protein BV379_08465 [Rhodovulum sulfidophilum]AB14
MQIRELALECGYNAASIRERIYADVEDGRDQAGFLIYTAAADSDGTLGGLVELGKPENLGRLLRQALDRAHICASDPLSA
GHDPSKDQSLHGAACHACSFVSETSCERENRHLDRSLVVPSRQGDSPILPELLSQIPATEDIETVTADGACDTRRCHAAI
LQHGPEPIIPIRRNGRAWEPDCPAAISRNEILRATRCLGRALWKKWAGYHVRSRVEAQMNRLKLFGERIMSRDPDRQT
AE
IQIRIIRHCRSDQWRDIAHS
>OLS48309.1 chromosome partitioning protein ParB [Rhodovulum sulfidophilum]AB14
MTKKVQLGFEQDCVMIPVADILPVRALSKSIKSSRKYTQITASIREIGLVEPPVITRNGYADGTWLLLDGHVRIEVLKDL
GREQVECLVSTDDEAFTYNKRVSRIAPIQEHRMILKAIERGVPEEKIAAALDLNPRSIQLKVKLLDGICPEAVAILKDKA
CTAAVFKTLRKMVPLRQIEAAELMVNANNYSVAYISAILAGTPQPQLVEANKPKRIKGVTPQAVARMEKELARLQAGLT
Q
IQETYGQDHLQLTVLRGYVAKLLGNARVVRYLMQTRPEFLSEFQTIAEMESVRLVALLGAEVELPLSP
>OLS48310.1 recombinase family protein [Rhodovulum sulfidophilum]AB14
MNGRWATHSDTSPAPPEKDVPAVAYVRMSTDHQKYSTENQLDIIRSYAEARGLTILRMFEDSGRSGLRLDGREALQNL
MA
EVQSGQADFKAILVYDVSRWGRFQDADEGAYHEHVCSRAGIRVHYCGEQFENDGSIGSNLLKTVKRVMAGEYSRELSV
KV
FAGQCRLVELGYRQGGAAGFGLRRLLLDEHGNSKGELSRGEQKSLQTDRVILVPGPEEEQRVVQRMYEMFVNEGRPE
REI

AEVLNAEGHRTDLDRPWTRATVHQVLTNEKYIGNNVFNKVSFKLKQRRVVNPRDMWIRAEGAYPTIVDKALFLRAREI
VD
ARSRHFTDEELLDALRAILKRQGTLSGLIIDEENDLPPSSAYRSRFGSLLRAYRLIGYEPERDYRYIEINKVLRAAHPQV
VEEIAEGVAAQGGSVLQAPDTQLLRVNDEFTVSVVLARCQATASGSLRWHIRLDTGLVPDITIAVRMNETNDAARDFYL
L
PSIDMTDARLKMAEQNGLWLDAYRTETLDDFYALAGRAKVTEVA
>OLS48311.1 hypothetical protein BV379_08495 [Rhodovulum sulfidophilum]AB14
MEHDVQDVIDTFIPGISGDILAIECCVGVTFVDHMAECLNGGNFSILIWPMCGYIAISDLVVSVIRKEKMANMDQSPPVI
DDTYHLASTIQKYQLIDLVLRVEIEVRDPQVTDHGIL
>OLS48312.1 hypothetical protein BV379_08505 [Rhodovulum sulfidophilum]AB14
MVMVTMSELDLRKNVYGALKAFQDSLRNYRRETGSPPTFLINGEAPAIPGELLKDCKVLPRREDIIADLPKGGIGVEIGT
QTGRFARKLLDIAEPEKLFLLDLDYTPFQRDILANDIEAERVVTMQGSSLELMEGFDDDYFDFAYIDAGHGYDMVAKDL
A
VSLRKVKPGGAIICNDFVVWSALEAIPYGVHKAVTETIAERGLRVTHFAFHPFGYSDIAFCNT
>OLS48313.1 hypothetical protein BV379_08510 [Rhodovulum sulfidophilum]AB14
MAVVLEGHNIVYFPVPKTACTSIKLLLFELNNGKKFEMYEEDGNVMHIHNAVYGTPSFLEVDRNPYADMIRIAVIRDPV
S
RMLSAFSNRVAFHRELSEDRIDTDLAERLGVHPDPSPDHFFNNIEKFRILSDSIRHHTDPVSSFLGPDLAYYHRVFRIER
LDELTTFLGEKVGREIQLGREQVGGEKTSFEQLGARAQKNLLQYCSGDYALMRGFYFNRNG
>OLS48314.1 hypothetical protein BV379_08515 [Rhodovulum sulfidophilum]AB14
MKLNHIDRATSARADAPEPGQTTAWDFDPARIAGYPMVTFDVFETLLERRGILRPLDLFLELGRDFAPSLGLTQEDFAD
L
RIRAERDARAWLARRHGREVRLAEIYDRFGTLMGALGRPAPTPCALSELADAEMALELSYLTPVPSVAPIYREALSSGKK
VALISDFYAPASFVAQALAKNGFDGDHRLFVSSDLDKTKNHGELYAHVCQEMGVSPRDVVHFGDNPWSDGARALQA
GITH
VRVVNPAGALLSRLRMDWRKPHPLPASTLFAACARAEYGTGLTKRQPQDLPVSERVGIEALAPLLLGMASWLYHEAQR
NE
LKALHFCSRDGLVMKQAFDMYQERFGKCTDSRYLMVSRQAIYRARAVYERDAAEALFVQNWSRLTPGEALARWGLA
AADH
ASEIAAAGFADADQEIALGDGQGMAQFARLFHACRDALQAANEDHAELFADYLAQEGVTGAARTMLVDIGWHGSL
QHGLT
RVLDGSGWTGRLAGRYLGLFLPNGPAPADDMQGYLFSKDRTPRTMALRASPSLVELLHTAGHGSTVGYQRDGGRIVP
QFE
NRPDETAQYDLMIRPIQETALRFMAEVLDTPRLRPEILPPEEAFRGLDRLLNRPDPDEVRTLGQLQIAANYGANAVSIAL
AEKSAQGYRLWNYEK
>OLS48315.1 hypothetical protein BV379_08530 [Rhodovulum sulfidophilum]AB14
MHRRIVSIWFPRLASDRALRLRPVEAPFALSLTRARVERIHCLNLAADSRGLHRGMGLADARALCPDLLSRPADPAADA
R
FLRALCRWATRYCPWAGIEGEDGLVLDITGAAHLFGGEAEMLADMRARLLF
>OLS48316.1 hypothetical protein BV379_08535 [Rhodovulum sulfidophilum]AB14
MNTGSLPFPPLRRGRAHEVCGPGAVFFALAQAARIGGPLLWVAESWQTERINPAGIAALIDPGRLLLAEPRDQTEALAV
T
EEALRSGTVPLVVTELTKPLGLTPGRRLQLAARDGNAIALSIIAEGAGSNAAETRWHCAPVFDAVFDAAGSTLQRWSLIK
NKSGTLEGWHVRWDAQTHRLDLVSPAGERPGPEAAPG
>OLS48317.1 QacE family quaternary ammonium compound efflux SMR transporter [Rhodovulum
sulfidophilum]AB14
MAWICLFAAGILEVVWAFSMKKSAGFTLLTPTAITIITMLASFGLLSVAMKSLPLGTAYTIWTGIGAVGAFVVGITVLGE
AITPARIIATLLIISGIVTMKLASAD

>OLS48318.1 drug:proton antiporter [Rhodovulum sulfidophilum]AB14
MKQDDPLVVFTPSGRRGRVPSGTSVLDAARRLGVDLDSVCGGRGICSKCQISLGTGSFPKHGITVTKEALSAKNAVEQR
Y
DEKRGLPPARRLGCQARILADAVIDVPPESQIHRQVVRKDAELRAIEMDPATRLCLVDVAEPDLQAPSGDLERLNSALA
A
QWQIGPARCPLPVLARLQATLRRGDWQVTVALHRAPDGLEILDIWPGFFEGPLYGLAIDIGSTTIAGHLTDLSTGTVIAS
SGRMNPQIRFGEDLMSRVSHAMLTPGGAAEMTAALHAGLDRLAAEIATEAGVAPRQIVETVLVGNPVMHHLALGLD
PVEL
GQAPFALASSGALWLAAGDLGIGAINPEARAYLLPLIAGHVGADAAAVALSEAPEEADELTLIVDVGTNAEILLGDRSGV
LACSSPTGPAFEGAQISAGQRAAPGAIERVRIDPATKEPRFKVIGCELWSDDPDFAEAVGPAGVTGICGSGIIEAVAEMR
LAGLVDMSGLIGSAAQTGSERCRPEGRTQSYLLHDGSSAGGPVIAVTQTDIRAIQLAKAALYAGARLLMDRRGVDRVER
V
VLAGAFGAHISPLHAMVLGMIPDVPLDRVRSAGNAAGTGARIALCSVAARRKIEEVVRRIEKVETATEPRFQEHFVAAN
A
LPHASDPFPELARQRHLPEVVFNADGGGNRRRRRPR
>OLS48319.1 N-(5'-phosphoribosyl)anthranilate isomerase [Rhodovulum sulfidophilum]AB14
MEFVSRPTPSDSWLALVFSAQAVAKGGVVRRSRHWVEREIGMERFVAEVRRRGFHMVECGGQLIVICNPHGLRVIC
>OLS48320.1 methyltetrahydrofolate--corrinoid methyltransferase [Rhodovulum sulfidophilum]AB14
MTRTVLESKTRTVTIGFDEPFCVIGERINPTGRKKLAAELEAGDFSTVEKDALEQVACGAMVLDVNAGVVYNSNPDPN
QT
EPPLMRQVIELVQGLVDVPLCIDSSVPAALEAGLAAAQGRPLLNSVTGEEERLERVLPLVRKYNVPVVAISNDDTGISED
PDVRFAVAKKIVERAADFGIPACDIVVDPLVMPVGAMASAGRQVFALVRRLRDELGVNTTCGASNISFGLPQRHGVNA
AF
LPMAIAAGMTSAIMNPVRPVEMEAIRAANLLMNHDANGADWIRFSRVLDGIAAGASFAEASTAVTAAGSGRRRRRG
>OLS48321.1 5,10-methylenetetrahydrofolate reductase [Rhodovulum sulfidophilum]AB14
MALLNFRKCDRATGRTAGQTSGKTVDQTGPMADFVRGYSIEVMPKTAAKVADFRALLPAGTRVYIAHLDGTPIEEMV
ATA
RRLTEEGFVPMPHFAARSIADRATLADWIARYAGEAGVREGLILAGGIPTPKGEFHSSMQLLETGLFDRAGFVRLHVAG
H
PEGSRDIDPDGSERNMMEALGWKQAFAARTDAEMAVVTQFAFEAAPVIAWADRVRDRGIDLPIHIGVAGPAKLQTL
VKYA
IACGVGPSLAVLQRRARDVSKLVLPFTPDGFLNDLAEAARRRADFPIAGAHFFPLGGIATNTRWLAAHGLAPAQDS
>OLS48322.1 hypothetical protein BV379_08570 [Rhodovulum sulfidophilum]AB14
MPELVIVYWRDIPAQVIVGRGRRAAKRVLPVRFEQAIDRCAMRVGAKDSDAYLAEWRRSAPVEREGNPTMLATAEAE
RIE
AEYDAARLGKLIAASGWQQS
>OLS48323.1 hypothetical protein BV379_08575 [Rhodovulum sulfidophilum]AB14
MNAHLGRAMTMMAGLTAAMAWGPAEATTMPPGTPRLVPNPVFQSRASADDGTTADVAISLGVSVCRHAAAGVTP
ACALMF
PAAIASDDGARAWPRPDLLAALDDDRGPDGGAPFWPASAGGRGAGGGGALPWAPPSAVSGGSKRRQGDGDGDG
GEDPGNR
WPVEPNAPLPVPLMPSAAFLLLAVAGFGSFARRRRS
>OLS48324.1 23S rRNA methyltransferase [Rhodovulum sulfidophilum]AB14
MTEKKGSSGRGQRDLKVKVKTARGRRLSSTRWLERQLNDPYVKRARAEGYRGRAAFKIIELDDKYRFLVPGARVVDLG
CA
PGGWCQVAVKRVNALGEKTGKARGTVLGVDLQEVEPIAGAELHQLDFLADDADELVKGWLGGSADVVMSDMAAAS
SGHKQ
TDHLRIIGLCEAAAALAFDVLDEGGTFVAKVLAGGAEAGLQTLLKQRFAKVANVKPPASRSDSSEKFVVATGFRG

>OLS48325.1 quinone oxidoreductase [Rhodovulum sulfidophilum]AB14
MEKAIIARAPGGPETLDWVDWTPPAPGPGEVLVRHGAVGLNFIDTYFRSGLYPWPGEILIPGSEAAGRIEALGEGVEGL
S
VGQRVAYTTQTGACTTARVLPADRLVPLPDAIPDEIAASIMLKGLTAQYLMRSSFPATEGQTVLVHAAAGGVGLLLGQ
WL
AAKGVRAIGTAGGPEKTALARANGYAEVIDYRAGDFVSPVMAATDGLGVDAVYDSVGQDTWRGSLACLKPRGMFVS
FGQS
SGLPDGFRLADLAKGSLSATRPVLFDFIADPAELRTRAAELFAAVADGTLTPRIGRSWPLAETAEAHRALEARATTGATV
LLP
>OLS48326.1 alkylhydroperoxidase [Rhodovulum sulfidophilum]AB14
MGTVKIWTDAEAEADPRVKAVFDDIRATRKTDFVNNVWRALAGQPAVLETTWAQVKAVIATESALDPLTKELIYMAV
SVA
NGCSYCAHSHTASARAKGMTEAQYQDFLAVIGLASQTNALVTARQVPVDPEFEV
>OLS48327.1 hypothetical protein BV379_08605 [Rhodovulum sulfidophilum]AB14
MPIVFVLTSIMGGVFSATVVSLIMETGWGTILLSYWVGGMIWGGAAVSFVALRSDEDSSGSEDHEYETSEYGASEYGA
SE
YGGAEYEDAGPGALGARSRTARSRAPEDVEEADRCGHFF
>OLS48328.1 ABC transporter permease [Rhodovulum sulfidophilum]AB14
MIKFVLADLKRFRLGSAVIVLLIALAVALGTTITLQERAFRLGSARAAEKFDLIVGAPGSATQLVLSSVFLQPAALPLMP
GDTLIALSEDPRVAWAAPIGFGDFVSGYPVIGTTTTLVGNTTTGFAEGRAFVAEGEAVIGSAVDLALGDSLVPTHGQVA
E
GGHAHAGIAYRVTGRLEPTGTPWDRAILVPIQAVWHVHGLGGNASHDDHDHDAHDEDGHGHATDGHDHDALAAT
SADHDH
DHDHDHDHDHDHDHDHDHDHDAEGHEAHKAHGEAAFDPDAPLDESWAAGEPPGLPAILVKPNSIADAYRLRQEYR
ATGDT
LAVFPAEILTGLYAMLGDAKRVLVAVAIGAQVLVAAALLLVTVMQVGQRRRQIGALRAFGAPRGAVFGIVWLEMALLF
GT
GILCGFGLGYVAADALSRSFALARGFPLPVAFTRADLWSLALLALAAALLSALPAWLAYRQPPVSALRD
>OLS48329.1 ABC transporter [Rhodovulum sulfidophilum]AB14
MPDLRLSSVSVAYPGLAAPVLDIPALDIPAGSRIAVTGPSGSGKSTLVNVLAGLVLPRAGRVVWDGTDIAALSEGARDR
W
RGANLGLVMQEFHLFPGLSALDNVLLPARLSRVADQAMTTRAGMLLAEVGLSRPDQQVETMSRGEMQRVAVARAV
LRAPG
IIVADEPTASLDPESGAAIAGLLLALAESSGATLIVVSHDRRLSARLDRVLSLAEGAILSDDVREGASP
>OLS48330.1 hypothetical protein BV379_08620 [Rhodovulum sulfidophilum]AB14
MTDPAAPDRTRRLCLALAPGLLLPGLFLTPTARPAAAAGTPLIGFDELYSAFSPLGLTFSDKVIALTGEKVRIRGFMAPP
LKAESDFFVLTAIPMALCPFCSTDTDWPDNIVVVYLDRSQTFVQPNRTIEVTGRLDRGSWVDPETGFVSQLRLRDARYR
T
V
>OLS48331.1 alkaline phosphatase [Rhodovulum sulfidophilum]AB14
MRTLLSALAATTLLAGAAQATAIYPLDRATILVSSPFDFKIELDAVHPEAEIAVTVNGKPYAETFGAKADYVAEEKGKDD
AVLGSALILRDLALAEPGSYTVEVRAGDETRTVTWEVYATPAAPKAKNIIFFVGDGLSVAHRTAARIMSKGMTEGKADG
R
LAMDDLDHMAFIGTSSTHSVATDSANTMSAYMTGHKSRVNALGVYADRTPATQDDPKVETIAEALRRTTGKSIGVVS
TAE
IEDATPAAVVAHTRKRGEKADIVGMFHDIRPEVMLGGGSAYFLKSDIPGSKRKDDSDYVAMFREAGYTLATNAAELDA
AR

GSDAQRLLGLFHTGNMDTKLDRSFLHQGTVGKFPNQPGLVEMTEAALDQLSKNEEGFFLMVEGASIDKMSHPMDW
DRAVI
DTIEFDQAIASARAFAETHPDTLIVVTGDHTHGVSVIGTIDDDKPGDEMREKVGTYDAAGFPNYTDADGDGYPDRIDVS
R
RLAIFSSNFPDHYETWRPKMDGPFEPSVQNEKGEYVANEAYKDIPGAVLRVGNLPKSADTAVHAVDDVVLQATGPGA
EEF
KGYMEQSDVYRVLVDAFALGAPRN
>OLS48332.1 hypothetical protein BV379_08630 [Rhodovulum sulfidophilum]AB14
MARAYASAVIPAPIEKVWEVVRDFNGLPNWHPAIERSEIEDGRSADAVGAVRSFHLAAGGGHVREKLLSLDDTAFTLSY
S
FVKPAFPVENYIARIALRPVSDSGETFAEWEAVFDEAPEDRGKYGPVISEGVFAEGWAALKRHFA
>OLS48333.1 hypothetical protein BV379_08635 [Rhodovulum sulfidophilum]AB14
MARFERKDLLEKFRGMIAEGRPIIGGGAGTGLSAKCEEAGGIDLIVIYNSGRYRMAGRGSLAGLLAYGNANEIVVEMAR
E
VLPVVKKTPVLAGVNGTDPFCDFDSFLDELKRIGFAGIQNFPTVGLIDGTFRQNLEETGMGYGLEVDLVALARTKDMLT
T
PYVFDTDQAKAMAEAGADIVVAHMGLTTGGAIGADTAISLEDSAVKVQSIAEAALSVRKDIIVLCHGGPISMPDDATYV
L
SNAPDCHGFYGASSMERLPTETALIDQTRRFKTITRG
>OLS48334.1 hypothetical protein BV379_08640 [Rhodovulum sulfidophilum]AB14
MTKKVFVVGTFDTKGRELAHVAGLVADQGLGVVRVDLSTSSDHRSSADVPPAEVAACHPEGAGAVMTGDRGSAVAG
MAAA
FEAFVATRDDIGAMISLGGSGGTALVAPGMQALPVGVPKLIVSTLASGNVAPYVGPADITMMPSVTDVAGLNRISRVIL
G
NAAHAIAGMAKAAREAAGDDRPAVGLTMFGVTTPCVTHLTERLEAEVDPIVFHATGTGGQAMEKLVDSGMISSVIDT
TTT
EICDLLFGGILAATEDRLGAIARSGIPYVGSCGALDMVNFGARATVPERYAHRLFYQHNPQVTLMRTTPDECRQMGEW
IG
ARLNACTGPVRFLLPGGGLSMLDAPGMPFELIEARNALFEALEATVEQTETRRLVRLPHNINDAAFSDALYTHWKEIA
>OLS48335.1 dipeptide ABC transporter ATP-binding protein [Rhodovulum sulfidophilum]AB14
MTAPVVAADHLARHYEVGGGLFSKPGLVRALSEASFSIEAGKTLAVVGESGCGKSTLARLITLIEEPSAGALTLDGLPAT
PENWRRLRSTVQIVFQDPYGSLNLRHRIGTILEEPLKINRPDMTAKDRAEKAREMMRLVGLRPEHYDRFPHMFSGGQR
QR
IAVARALMLEPKLLVLDEPVSALDLSIQSQVLNLLLDLQERMDLAYLFISHDLSVVRHVADDVMVMYLGRPVEFGTKED
V
FANPRHPYTKALLSATPSTEPDPARQRIKLQGELPSPLAIPDGCPFAPRCWKAEARCRASRPELEGAPHAAACFFPEGA
>OLS48336.1 dipeptide ABC transporter ATP-binding protein DppD [Rhodovulum sulfidophilum]AB14
MALLSIRNLSVEFSTAGGPFRAVDGVDVTVDKGEILAIVGESGSGKSVSMMALMGLLPWTARITADEMSFDGTDLRSL
TP
RQRRRVIGRDISMIFQEPMTSLNPCFTAGFQIKETLRQHLGLTSKQRHARAIELMEMVGIPDPERRLNAFPHQLSGGMS
Q
RVMIAMALACEPRLLIADEPTTALDVTIQAQILDLLRGLQGRTGLALILITHDMGVVAETAERVQVQYAGQKVEEQPVR
A
LFETPHHPYTAALLAALPERATERRLPTIPGVVPGQFDRPEGCLFAPRCRFADDRCHRAAAPVQPPGLGLARCHYPLETE
AEETAR
>OLS48337.1 dipeptide ABC transporter permease DppC [Rhodovulum sulfidophilum]AB14
MADATAEAPRPAAPQRPGRLQEFWRYFRENRGAVIGLYVFAFFVLLALGADIIAPHDPTQQFRDYTLLPPFWTAEGSW
SF

PLGTDPLGRDILSRLLHGARYSFFVGLVVVVLAASTGILIGLIAGFAPKWLDTVIMRLMDIILSFPSLLLALVLVAILGP
TLLNAMIAIAVVLQPHYVRLTRAAVMSERTRDYVTSAKVAGAGLGRLMFITVLPNCLAPIIVQMALSFSNAILDVAALGF
LGMGAQPPTPEWGTMLAEAREFILRAWWVVTFPGLAILITVLSINLMGDGLRDALDPKLKRS
>OLS48338.1 peptide ABC transporter permease [Rhodovulum sulfidophilum]AB14
MFRFFLTRLLTFVPTFVGVTLISFAFIRVLPGDPIIVMAGERGMSEARYQELREQFGFDQPVLVQFWDYLTGILHGDLGM
SYVTKKPVWDEFFALFPATVELSLCAIVLAICLGLPAGVIAAVNRGKFFDRALMSTALVGYSMPIFWWALLLIIVFSGTL
GWTPVSGRIDLLYYFPDATGFMLIDSLMSGQKGAFASAVSHLILPSIVLATIPLAVIARQTRSAMLEVLGEDYIRTARAK
GLSPMRVSGLHALRNALIPVVTVIGLSVGVLLAGAILTETIFSWPGIGKWMVDSIYRRDYPVVQGGLLLIAGMVMIVNLI
VDLLYGVINPKIRKR
>OLS48339.1 ABC transporter substrate-binding protein [Rhodovulum sulfidophilum]AB14
MTLHTFLTTSALALSLASAAAAQQTLVYCSEGSPEGFDPALYTSGTTFDAAGQAVYARLVEFKTGTTEVVPGLAESYDIS
EDGKTYTFHLRPGTKFQTTDFFTPTRDLNADDVIFSFMRQLDPENPFHNASGGTFEYFNAMSMPELIERIEKVDDLTVA
F
HLTRPEAPFIANMAMDFASIMSKEYADQLLEAGRPDMLNQQPLGAGPFAFVAYQKDAVIRYRKHEGFYREGLPKLDN
LIF
AITPDASVRYQKLKAGECHVMPFPNPADIEEMKSEDGIVVKEQEGLNVGYMAYNTQQPPFDNPKVRKALNMAMDK
QAIID
VVFQGAGEIAKNPIPPTMWSYNDAVEDDPYDPEAAKAMLEAEGVTDLSMKLWAMPVQRPYNPNARRMAEMMQA
DFEKVGV
NAEIVSYEWGEYLDRSKEVDRDGAVLLGWTGDNGDPDNFLATLLSCDGVGGANRSQWCYEPFEELIQKAKTLPTQAE
RAK
LYEEAQVIFKEQAPWATIAHSKVFMPMREEVKGYKVHPLGLHIFTEVSLDK
>OLS48340.1 acetyl-CoA carboxylase biotin carboxyl carrier protein subunit [Rhodovulum
sulfidophilum]AB14
MKRLRITVDGTPYDVTVEEIETGPAPTPAAPRTASAAPRPAPAAPSVAAAAPVPAAPAAPGAVLSPLAGTTVSVAVAVG
Q
SVAAGEPLMVLEAMKMNTDIRAPQAGTVTAINVAPGATVTEGQVLLILS
>OLS48341.1 hypothetical protein BV379_08680 [Rhodovulum sulfidophilum]AB14
MLENLALIGTGFAVVILVLASLWGACALMGWGFTRAARKTTAAAPQAPVAPGVPPHHLAAIAAAVAETLGPGHRISRV
AA
PAHLVDGWPLEGRIETFAAHRVRTGWGPTRPRPGARTPEITKGPKP
>OLS48342.1 methylmalonyl-CoA carboxyltransferase [Rhodovulum sulfidophilum]AB14
MAISRSLAEELDKRRAAALEGGGPAKAADRHAKGRMTARERIEALYSAGTFQETGLHAQHATRHFGMEKKSIPADGVI
TG
TGFVDGRPVAAFSQDFGVVGGTLGEMHSRKICLVLDHALKAGVPVVGFNDSGGARIQEGVGALSAYGQVFYRNVQLS
GVV
PQISVIAGPCAGGAAYSPALTDFVIMTREHAQMFICGPEVIRAVTGQSTTMDEIGSAAAHGSVSGNIHFIAEDDADAVA
I
VHRLLSFLPSNNMMDPPHRIEPDLAIEDDPALDALLPEDPKSAYDVRQVIARLADEGDFLEVMEGFAANLVVGFGRVG
GV
VTGFVANQPMVKAGALDIDASDKGARFVRLCNVFNIPLVTLVDVPGFLPGVAEERRGIIRHGAKMLFAYASATVPKITVI
MRKAYGGAYLAMCSQDMGADRVIAWPSAEIAVMGAEGAVNILYRKDLAEAGDRAARAQELAEEYRAEFASPYLSAG
RLFV
SDIVQPCQTRSAIALTLRGLLSKRETRPPKKHGNIPL
>OLS48343.1 1,4-alpha-glucan branching enzyme [Rhodovulum sulfidophilum]AB14
MHSDITSRPGQGVPKDVLQQIAHGTFDDPFAVLGPHGQGEFRHVTALMPGAETLEALLGHEADAGAHALAPVPDCP
GLFT

GPVPGEGAYRLRASAGGTVWETDDPYRFGPVLGEMDEHLIAKGAHRRLWEALGAHAMTHEGVAGTHFAVWAPNA
RRVSVV
GPFNQWDGRRHVMRRRGTTGVWEIFLPGMGENASYKYEILGPDGALQPLKADPLGFGSEHPPANASVVHDLRGYG
WSDGD
WMRDRAGCNARTAPISIYEVHLASWKRRADGRAISYVEAAEELVDYAAAMGFTHIELMPVSEYPFDGSWGYQPVGLY
APT
VRHGPPHEFRDLVDAAHRKGLGVILDWVGAHFPTDAHGLGKFDGTALYEYADPREGFHQDWNTLIYNFGRTEVANYL
TAN
ALYWMEEYHVDGLRADAVASMLYRDYSRKAGEWVPNKDGGRENYEAIALLQQMNTLSYGTHPGTLTAAEESTAYPG
VTRP
VDQGGLGFGYKWNMGWMHDTLGYMAEAPVNRPHHHNRMTFGLTYAFSENFILPISHDEVVYGKGSMLTKMPGDR
AEKFAN
LRAFYGFMWAQPGKKLLFMGQDFAQEAEWAYAGSLDWAALERPDHAGMQRLVRDLNTLYRGTPALHAQDCSPEG
FQWIEA
DDARHSVYAWLRRGGPEDPNAVAICNFSPVEQPACRLGLPAPGRWREALNTDAAIYGGQGRGNMGAVMAEPRPSH
GQAAS
AEICLPPLSTVILVQEDG
>OLS48344.1 acetone carboxylase subunit gamma [Rhodovulum sulfidophilum]AB14
MAYTEAKIRDLVDGTIDQDTLHQMLSMPKDRERFEIYLKILQDQVPWDDRIILPLGPKLFIVQRAEDRKWVIRSWAGHD
F
CDWTENWKLHARVRVRDTPEAMEKIYPKLMAPSTEWQVIREYFCPLSGDLLDVEAPTPWYPVIHDFEPDIDSFYRDWL
GL
EVPERA
>OLS48345.1 acetone carboxylase subunit alpha [Rhodovulum sulfidophilum]AB14
MNIQSDIKGIVRGGETLKQHRDKIMEATKSSGHYAGLRHMELRDDHPIKYNKLFSRLRAGVVDARETAKKIAASPIVEQE
GELCFSLYNAAGDCILTSTGIIIHVGTMGAAIKYMIENGWESNPGIRDRDIFCNNDSLIGNVHPCDIHTIVPIFHEGELI
GWVGGVTHVIDTGAVGPGSMTTGQVQRFGDGYSVTCRKIGENDTLFRDWLHESQRAVRTTRYWMLDERTRVAGCH
MIRKL
VEEVIADEGIESYWQFAYEAIEHGRLGLQNRIKAMTIPGTYRQVGFVDVPYSHEDVRVPSDFAKVDTIMHTPSEMTIRK
D
GTWRLDFEGSSRWGWHTYNAHSVSFTSGIWVMMTQSLIPSEMINDGAAYGTEFRLPKGTWMNPDDRRVAFAYSW
HFLVSS
WTALWRGLSRSYFGRGYLEEVNAGNANTSNWLQGGGFNQYDEIHAVNSFECAANGIGASAVRDGISHAAAIWNPEG
DMGD
MEIWELAEPLVYLGRQIKASSGGAGKYRGGCGFESLRMVWNAKDWTMFFMGNGHISSDWGLMGGYPAASGYRFA
AHDTGL
KEKIARGEPIPWGGDTDPENPVWDGMLEGARIKRDKQAITTEEMFDDYDLYLNYMRGGPGFGDPLDRKPEAVAADV
NGGY
LLARLAGDVYGVVLSEDARGQAVADLAATEARRAEIRQERLAQSVPTGEWMKAEREKILAKEAGTQVQQMFAASFKL
GPK
FERDFRAFWDLPESWQLKEADLPIPSYGKDYSMDLSELPDVTIVQLVEE
>OLS48346.1 acetone carboxylase subunit beta [Rhodovulum sulfidophilum]AB14
MTQTGRNARSVQVLGIDAGGTMTDTFFVDAEGDFVVGKAQTTPANEAQGLLESSREGLEHWGLTLEDALRNIQTGV
YSGT
AMLNRVVQRKGLRVGLIVNAGMEDFHRMGRAIQAYLGFAYEDRIHINTHYYDDPLVPRDLTRGVAERIDMMGTVVIP
LNE
ETARTAARELIAEDVEGIVISLLHSYKNPTHERRVRDIVEEEIRASGKTIPVFASTDYYPVRKETHRTNTTVLEAYAAEP
SRQTLHGISGAFKDHGSRFDFRVMATHGGTISWKAKELARTIVSGPIGGVIGAKYLGEKLGYRNIACSDIGGTSFDVALI

TQGELTIRNDPDMARLVLSLPLVAMDSVGAGAGSFVRLDPYTKAIKLGPDSAGYRVGVCWAESGIDTVSVSDCHVVLG
YL
NPDNFLGGQVKLDRQRAWDAVKVQLADPLGLSVEDAAAGVIELLDAELRDYMRAMISGKGYSPGSFTCFSYGGAGPV
HTY
GYTEGLGFEDVIVPAWAAGFSAFGCAAADFEYRYDKSLDINIDTDAHEADKAAQAEMLQAAWDELTERVLEEFERNGY
AA
DKVALQPGFRMQYRGQLNDLEIESPIGAAGNADDWDRLVDTFNDTYGRVYAASARSPELGYSVTGAIMRGTVPIPKPK
IP
EEELQDAAPPREARIGTRQFYRKGRWVEARLYQMEALLPGNQIVGPAIIESDATTFVVPDGFETWLDEHRLFHLKEL
>OLS48347.1 hypothetical protein BV379_08720 [Rhodovulum sulfidophilum]AB14
MVARGHFVRKADWARFLETGLVPGDLDPDLAASWLRSRDCGVLPDRISAPQVANEILSRTRKETAELRRLARTALLRAR
A
MLKDTGVMMILASPDCVITETEGDRGVIDTGRENRLQIGGDWQETSIGTNAIGTAAASGRPALVHGIQHFCEEIQRWS
CA
AVPILDPVDGTVRGIVDISGQTDMFQPQSMALAMAVGDEISQALARLIGEEREALYQQFMDRRARWQNTHMILIDRR
GAV
LHDTRAVWSHQAIRGGAIGARGEEDWAGEMSDRFSGMDSEVLRRDGLPIGMVLTAYRGHRRPVETPSGDPFDAFLG
TDPA
ILKLLEHARCVTASNVPVLISGETGTGKELLARAIHDASSRRHGHYVPVNCGALRADSAAGVLFEPPDRPVDRTMDQTG
L
RGGGAVAEAHGGTLCLDEIGEMPEDVQPFLLRVLEDGLTRAGGAAASDRSDFRVIALTHRDLPRDVAENRFRRDLFHRI
S
TIQFRLPPLRDRTEDIPLLAQHFLAEAAAEAGRQAPALTRAAREALMAYPWPGNARELRNDMQALVLFCGRGVIDTGD
LP
DKIRDPAAPPAPAEPDERTRILEAMAASDGNMSATARRLGIARSTLYTKIAAYGLARPGN
>OLS48348.1 acetolactate synthase small subunit [Rhodovulum sulfidophilum]AB14
MAQLNIQQGTSKHSAYELRDPNAEVSERHTLAVSVDNEAGVLARVIGLFSGRGYNIDSLTVAEVDHAGHTSRITIVTTGT
PAVIEQIKAQLGRIVPVHEVHDLTVEGPSVERELALFKVASTGEKRVEALRLADIFRANVVDSTLESFVFEITGTPQKVD
AFGELMRPLGLTEMARTGVAALARGT
>OLS48349.1 hypothetical protein BV379_08730 [Rhodovulum sulfidophilum]AB14
MRKDLLFSAFPATCWQMGRDSAGPGWPPRLRRSGRQGPAGGTSALAGGRSRDVRGLLSDRSALIRSIRTQDQGAVS
ENTI
RGSTYVPLGPDGRAPV
>OLS48350.1 ABC transporter substrate-binding protein [Rhodovulum sulfidophilum]AB14
MDRRSFLKTSALGGSAAAASATLAAPALAQGRRVLTMVTSWPNGMAGVHDSAMFCKNHIEALSDGQIKVEVKAAGE
LVGA
FEVFDAVSSGQADMYHSSDYYFVNQHPAFAFFSTVPFGMTPQELINWYYHGDGAAFHDEMGEIFGMKMFLVGNTG
AQSGG
WFRKEINAPEDFQGLKFRMPGLGGVALGKLGASIQNIPGGEVYQALSTGALDGTEWVGPWADEKAGFYEITKIYYTAG
FH
EPGAGLTLALNRDLFDSLTPFQQQVMESTAAECTQWTLSMFLSQNSAALARLASEGVRVMSFPDPVWDAFGKASQE
ALEE
SRDGDIYDRMLDSYMASMRTSSSWIQASEGTYRAQRDRVIG
>OLS48351.1 ABC transporter substrate-binding protein [Rhodovulum sulfidophilum]AB14
MDRRSFLRSSALGGSAAAAGALAAPAYAQGKRTLTLVTTWGRGLAGVHDAAQYCADQITAVTDGQLTVDLKAAGELV
GAF
EVFDAVTSGQADMYHGVDYYFVGQHPGYQFFASLPFGMTAQELHNWYYHSGGHELHNELGQIFGLKSFLAGNTGPQ
SGGW

FRKEIRSPEDFNGLKFRMPGQGGEILGKLGASVQNLPGSEVYQALSSGAIDGTEWIGPWADEKAGFQEITKIYYTAGFH
E
PGPGLSLAMNLDVFESLTPAQQATVEQVTASANQWAHALFMKNNASALRRLQAGGVQVREFGDDVWDAFGAAAQ
EVFAQY
MDDDLYKKIYDSYIASMRDSAAWIEISEGVFTRQRNRVLG
>OLS48352.1 C4-dicarboxylate ABC transporter permease [Rhodovulum sulfidophilum]AB14
MLDGILWVCRNILLAFYNFGYALSHPGLWLDWSDKQAIMRFVYYGGSVEFFFVIFTAFLVLTGIGLWRRRVMWGTVRL
LE
GMANGIGRAAAWFGLLMVLQQIMIVFLQRIFRVSEIGVGPFGYVFTKDLSWWSEELKLYNAAIVALCATYTFVQSGHV
RV
DLVYSEVSFRAKRVIDMLGALFFMMPVAVLIWMYSWFFMWRHLITPKPSASDTLERLLMKARAVRWNVETIGFSPNG
FNG
YFLFKVLLVAFTGLVFLQAIAFFYRSFLECVEGEESAGKYLDRDSLGAGEEAYEGTH
>OLS48353.1 C4-dicarboxylate ABC transporter permease [Rhodovulum sulfidophilum]AB14
MLFGLDGVEIGLIIVILCLFGGILSGFPVAFAIGGAAVISFGIIAALDSAGILVAQAIDTGSAAYDALLAQGIHPDAISH
FRYPDLPTVTTALFPQGWETAMDRNISFIVNRMNERVFAGQSIETLLAVLMFVLMGITLERSKIANDLLTTMARVFGP
MP
GGLAVSVVVVGAFLAASTGIVGATVVTMGLLSLPTMLRNKYSPELATGVIAASGTLGQIIPPSIIIVLLGTLAGDIYATA
QETRAQLAGCPDALTYLGQAAVVSVGTLFQAAILPGVLLALLYAAYAFGYALLNPDKAPPVIDAGARGRRKGSALTWFL
A
APAGVIGLALLLEATGIAGSQDVGVSRYTSTAETASLRTNVSDQCKASMIELHGQSKWDSAVAQQKEIQAGGGRIGTQ
EL
SDEALAEARAAKVAAAPPISGGILLGFTLLGLALVTARGVVPSARPAPLLIGGAGIVLGLLVDALFIGPLASPGTTLLLL
AVPVALTLYGCRYAVACLSRSDLLRVVFPPLVLIVAVLGSILGGITNPTPAAALGAGGALMLAAYRKLQEEHRTGKLILW
AAYAIIIMLLVGTNFDLRLGRESVPVEDWIAFLVAQGTYHFAMFGILYACWVLLRSGVLGPVVRETAKVTSMVFTILIGS
QLLNLVVISFGGEHYIQQFLRSFDNEMKVFLLVMLVLFILGFVLDFLEIIYIVIPIVGPVIYGGTFDPKWVTIMIAVNLQ
TSFLTPPFGFALFYLRGVAPKEVNTWHIYRGVAPFVVIQVIGLAILWFMPGIVTIVPNLLPNG
>OLS48354.1 arginyltransferase [Rhodovulum sulfidophilum]AB14
MRHTLPLAPQFYVTAPQPCPYLPRRMERKLFTALQGDTAETLNDSLSKQGFRRSQNVLYRPACADCTACLSARIRVADF
Q
PTRSQRKAQNRNADLKRQASSPWATEEQYTLFRRYLDSRHADGGMADMDIFEFAAMIEETPVRSRVVEYTAEPAIGG
RHR
PLAAVCLTDILDDGLSMVYSFYEPERTKRSVGTHVILDHVEIAREAGLPYVYLGYWVPGSPKMGYKSNFSALEIYKNDCW
QDIGSPTAHDGETHPLSVAPIAEQVARINLPDSRPTHD
>OLS48355.1 RDD family protein [Rhodovulum sulfidophilum]AB14
MTEQTEPSADRLWGLPDPALQPDFYAGVPSKRLAAWVVDVVLILVISLLALPFTAFTALFFLPLFFTLVGFAYRTVTLAR
GSATWGMRIMAIEIRTARGDKLDLSTAALHTLAYSACITFVLPQVASVILMLTGARAQGLPDFILGTAAINRPANP
>OLS48356.1 methylmalonate-semialdehyde dehydrogenase (CoA acylating) [Rhodovulum
sulfidophilum]AB14
MKDLTHFVNGQHVAGGSGRFADVYNPALGEVQSRVPLASKAEVDAAIAAAAEAQPKWAATNPQRRARVLMEFVRLL
NRDM
QKLAEALSAEHGKTVPDAKGDVQRGLEVIEFCIGAPHLLKGEFSDSAGPGIDMYSMRQPLGVVAGITPFNFPAMIPMW
KL
GPALACGNAFILKPSERAPTVPLMLAALLQEAGLPDGVLQVVNGDKEAVDAILDNPEIQAIGFVGSTPIAQYIYGRGCSN
GKRVQCFGGAKNHMIVMPDADMDQAADALVGAGYGAAGERCMAISVAVPVGEETADRLIEKLLPRIEKLKVGPYTA
GADV
DYGPVVTAEAKARVLGLIDKGVAEGAELVLDGRNFSLQGYEKGFFVGPTLFDRVTTDMEIYRTEIFGPVLSTVRAKTYEE

ALGYAMDHEYGNGTAIFTRDGDTARDFASRINIGMVGINVPIPVPLAYHTFGGWKKSGFGDLNQHGPDAFRFYTRTKT
VT
SRWPSGMKEGGEFHFKQMD
>OLS48357.1 LysR family transcriptional regulator [Rhodovulum sulfidophilum]AB14
MNWDDMRLFLAVARAESLTGAGKALRLDPATLGRRIARLEEGLGAALFARSPRGYGLTEAGERLMARASEAEQALTEG
IE
ELRGQEGQLTGAVRIGAPDGVANYLLPQVAAAICDRHPGLEVQIVALARVFNLSRREADMAIAVSPPKTGRLTVQRIAD
Y
QLHLAAHRDYLAAHPPIRDLADLAGHRIVGYIPDMIFDRELDYLAATGVERVALGSNSVSVQLNWLRTGAGLGIVHDFA
L
PAAPGLVRVLPDALALRRSFYLIRHGDDRKHPRLTRFAGLLLEGLREELLRLEGLA
>OLS48358.1 histidine kinase [Rhodovulum sulfidophilum]AB14
MQVHQILREKLVPGVATIPPETSVADAAAELSARRIGSLVVSGSDSGERVEGIVSERDIVRELGRCGPKCLKDPVSTIMT
RRIQFCALEDGADVVLARMTEGRFRHMPVLEDGRMVAIISIGDVVKARLSELSMEKEALEGMIKGF
>OLS48359.1 pantetheine-phosphate adenylyltransferase [Rhodovulum sulfidophilum]AB14
MRIGLYPGTFDPVTLGHIDIITRACALVDRLVIGVAINRDKGPLFPLEERVEMIESECAALGDRTGTEIVVHPFENLLID
CARDVGATMILRGLRAVADFEYEFQMVGMNRALDASVETVFLMADARRQAIASKLVKEIARLGGDVSSFVTPGVRDR
LVA
KFD
>OLS48360.1 hypothetical protein BV379_08790 [Rhodovulum sulfidophilum]AB14
MSDIVELERRIVAALERIGQGLDALGSGGGAEDGTDPAELDKLREALETERGVNAQLNERVKAIQERQETQVARLEQRA
A
DLTARAEAAEADVDRLRAVNAKLRETSVALREANAQGLGDPAAIDAALLAELEALTALRASDRAEIDSILAELMPAAEEG
VAHA
>OLS48361.1 cell division protein ZapA [Rhodovulum sulfidophilum]AB14
MPNIEIIIGGRSFEVACQPGEEPYLAAAARTLDAEASVLSEQNGRLPETRLLLMSGLMLADKTVGLEERLKGLEAKLAEA
SARIEAMETAPRPEPERVEVPVIPASVGESLADLAARAEALASQVEEKLAGT
>OLS48362.1 hypothetical protein BV379_08800 [Rhodovulum sulfidophilum]AB14
MIRARLWYGPAGDRLPPERVAQYLRGPLACTLGLRECNLDGAWHSEIQLTAPIKARLFLERGPEVSGEAADLVSRLPAS
A
PPALARRLARCTARLVVSDPAPDTHFAPGVPLSRSVLLPLAFAIDAIVEDTEAGRLSFYAPPAAPRTPLTARIGRILSEI
SGLMRPRRHPS
>OLS48363.1 monothiol glutaredoxin, Grx4 family [Rhodovulum sulfidophilum]AB14
MTDAAKDRIQQTISENDVVLYMKGTKAAPQCGFSSRVAGVLNFMGVDYADVNVLADEEIRQGIKDFSDWPTIPQLYV
KGE
FVGGCDIITEMTLSGELDKLFDDKGVSYSRDAADKIRAANA
>OLS48364.1 BolA family transcriptional regulator [Rhodovulum sulfidophilum]AB14
MAITPEEIESLIREKFPSAKITITDIAGDGNHYAAEVIDESFRGMNRVQQHRAVNAALKAQMEGSNGALHALALTTKAP
D
>OLS48365.1 phosphoribosylformylglycinamidine synthase II [Rhodovulum sulfidophilum]AB14
MDEPQITEDLIAAHGIKPDEYDRILQLLGREPSFTELGIFSAMWNEHCSYKSSKKWLRTLPTEGPQVICGPGENAGVVDI
GDGQAVVFKMESHNHPSYIEPYQGAATGMGGILRDVFTMGARPVAAMNALSFGQLAHPRTRQVVHGVIEGVGGYG
NCFGV
PTVGGEVRFHPAYDGNCLVNAFAAGLADADKIFYSAASGVGMPVVYLGAKTGRDGVGGATMASAEFDEGIEEKRPTV
QVG
DPFTEKRLMEACLELMATGAVISIQDMGAAGLTCSAVEMGDKGDLGVRLDLDLVPCREERMTAYEMMLSESQERML
MVLR
PEKEAEAKAIFDKWDLDFAIVGETIAEDRFLILHGGEVRADLPLKALSGNAPEYDRPWVATPAAEPMDDVPGIDPIDALK

ALISSVNYCSRQWVYEQYDSQVMADTVVRPGLGAGVIRVHGTEKALAFTSDVTPRYVRANPVEGGKQAVAEAYRNLC
AVG
ARPLATTDNMNFGNPEKPEIMGQFVGAIEGIGAACIALDMPIVSGNVSLYNETDGEPILPTPTIGAVGLLDRLDDLIAGV
PAEGHLALLIGESAGHLGQSALLYEVFNREDGDAPAVDLEAERRNGEFLRANRALISAATDLADGGLALAAFELAEASGI
GVSLDMADTPALFGEDQGRYLIAASFDAAEALMVAAGKAGVPLATVGRFGGDQISFGGQSAPLSDLTALYRSAFAEAL
G
>OLS48366.1 LysR family transcriptional regulator [Rhodovulum sulfidophilum]AB14
MDWDKLRIFYAVADAGSLTHAGESLHLSQSAVSRQIRALEESLGSTLFHRHARGLILTEQGELLFDATSAMARRLDTAVA
RIRDSVEEVFGELRVTTTTGFGSLWLAKRLPILYQRYPELKIDLMLEEQVLDLPMREADVAIRMKEPSQADLIRLRLMNI
KMRLYATRSYLEARGTPATPEDLSDHRLICQNTSSAQVAAGARLVRELMTYNIPSTLTVNNYFGVLQAVLNGLGIGVLP
D
YVTEDHPHLVRVLDTIESHDVPVFLAYPEELRASRRIAAFREFITEQIMEKRQRDKDTPQM
>OLS48367.1 indolepyruvate ferredoxin oxidoreductase [Rhodovulum sulfidophilum]AB14
MTRADIRLQDRYDLSKPSILLNGTQALVRLMLMQAARDRALGLNTGGYVTGYRGSPLGAVDQQMMRARAELEAAGV
LFQP
GLNEDLALTALWGTQQAELRGEGARDGVFGLWYGKGPGVDRSGDAIRHANLAGTSARGGVLLAMGDDHTGESSTT
CHQSD
WAMVDAYVPVLSPAGVQEILDYGIYGYALSRFAGVLVGLKVMKDTVEVTSVVDGRPGRMQVTAPDYPLPPGGLNIRL
NDH
WVPQEARMIDHKRFAAEAFAHANRIDRRVWGGPGAKIGLVAAGKTWLDLVHALELLGIGEAEAERLGIATYKVGQV
WPLD
MKSFAEFSEGLELIVVVEEKRKLIEVQAKEAIFHDRRGRRIYGWKNDRGEELFPTRYALDPVMIAETLGRILIEEGRGSD
RLSAALDRIREARRADNAEDMAARLPYFCAGCPHNSSTRVPEGGRAYAGIGCHIMALWMDRDTSGFTHMGAEGAN
WIGEA
PFSTRGHVFQNLGDGTYNHSGRLAIHAAIAAGVNITYKILYNDAVAMTGGQGNDGGLTARQIVDELLAAGVARVEVVY
DE
KEDLDPGGFPAAVACHPRADLMAVQKRLSGIGGVTALVYIQTCAAEKRRRRKKGAFPDPDRRVFINTDICEGCGDCGV
QS
NCVAIVPVETELGRKRAIDQSACNKDFSCLNGFCPSFVTLKGARPRKEASAEVEIGALPAPALPEIEGTWNLVIAGVGGT
GAVTIGAILAMAAHIDGKGAGMMEMAGLAQKGGAVHIHCRIAARPEDISAIRVAVGEADAVIGGDLVVTAGAKTVGL
MTQ
GRTGAVVNSHDIVTGAFTRDPEFRVPSDRLELALKARLKDRLQLFDATELARRLMGDTIYANMIVTGAAWQQGLIPLSH
E
AIDRAIELNGAGVAGNRRAFEIGRWAAAEPGAAARVLASETVEKPRSLRERIAFREAHLRAYQSGRLARRYRRLVERFED
PFLRAAVAKGYHKLLAYKDEYEVARLHLDTAARARAEFEGDLEITWHLAPPFLKGRGADGRPRKRAFGPWMERGFRLL
AA
LRRLRGTPFDPFGWQEERRRERAAIRRYEADMEEILARLSPATLTAATALAELPLTVRGFGPVRAEAERAADDKRAALLA
EIRAGGAALDTAAE
>OLS48368.1 glutamate racemase [Rhodovulum sulfidophilum]AB14
MAVGVFDSGLGGLTVLDAVARRLPEVPFVYFGDNAHAPYGIRDAEDVYRLTTDAVTRLFDAGCDLAILACNTASAAALR
R
MQESWVPKDKRVLGVFVPLIEALTERQWGDNSPPREVAVKHVALFATPATVSSRAFQRELAFRAIGVDVEAQPCGGL
VDA
IEAGDEMLAEALVRSHVEALKRRMPHPEAAILGCTHYPLLQDAFQEALGAGVKVYSQANLVAESLADYLGRHPEMQGP
GN
EAMFLTTGDPGFVSDKATQFLRRRIEFSAA
>OLS48369.1 hypothetical protein BV379_08835 [Rhodovulum sulfidophilum]AB14

MLDGSKEIRLTLNDVSDLALSFSLDDAAAGYTNWGGYIKWSSREDGRQDWDGPWGTCLECGYSGGGWALSHWANK
TSTDL
KLDFGKTLKTVYVWIRPTWGKGSVSYEDGSAGAPDIAPVPLPAGGLLLVSALGAALLLRKRGRKAA
>OLS48370.1 N-acetyl-gamma-glutamyl-phosphate reductase [Rhodovulum sulfidophilum]AB14
MTTSVAILGASGYTGAELIRLIATHPDLRITALSGERKAGMAMAEVFPHLRHLDLPDLVKIDEIDFASVDLVFCALPHAT
SQAVINKLPRDLKIVDLSADFRLRDPAAYEKWYGKPHAAVELQQEAVYGLTEFYRDEIRRARLVACTGCNAAAGQYALR
P
LIEAGLIDLDEIVIDLKAGVSGAGRALKENLLHAELSEGFHAYSAGGIHRHLGEFDQEFSKIAGRPVEVQFTPHLIPANR
GILATVYVRGEARDIHATLAKAYADETFIEVLPFGEHPSTRHVRGSNFAHVGVVADRRQGRAMVIVAIDNLTKGSSGQA
L
QNANLMLGIAEETGLMLAPCFP
>OLS48371.1 cytochrome c biogenesis protein CcmE [Rhodovulum sulfidophilum]AB14
MRGLKKRRRIQIIVLAFVALGLSTGLIGYAMRDGINFFRSPSQVAEAPPPPTETFRIGGLVEAGTLKRGQGETIRFSVTD
GGASVPVTYTGVLPDLFKENQGMVGTGRLVDGVFEAREILAKHDESYMPKEVVDALKEQGVYRDPEVEGANGS
>OLS48372.1 peptidoglycan-binding protein [Rhodovulum sulfidophilum]AB14
MKTVEELAREIVLREGGFVDDPDDPGGPTKHGVTIGTMRRLGLDLTGDGQIDSADVRGLDPGGAVAIYVEHYFRRPRL
DA
LPEALHASVFDMYVNAGANAVRILQRLLNEMDQPCAVDGAVGPETVRAARAAAEAAPGHIADAYGIARRNYYYALAD
RRP
ASRKYACRRDGGKGGWILRAEEFISPRYRLSEAEHRERVARWG
>OLS48373.1 carboxylesterase [Rhodovulum sulfidophilum]AB14
MGVMKGALGMLFSGGPTAIREAAEVFRVNAEAADMRDLELRRAVLEEFAHEFAQPRATLFDRLIDGANRLPRPLMAL
GTV
GLAISAMVDPVWFAARMQGMALVPEPLWWLMGAIISFYFGARYQAKGQDFQRSIAGTMAMAPVVARNLQQLRAL
SDAAPA
EPDGPQDSGTVPRSQGPNPALDDWRAGL
>OLS48374.1 c-type cytochrome biogenesis protein CcmF [Rhodovulum sulfidophilum]AB14
MVIELGHFALALAFAIALVQAIVPLIGAHKGWSGWMAMAEPAAMAQFLLTAFSFGVLTYAFVVSDFSLRVVVENSHTL
KP
MLYKVSGVWGNHEGSMLLWVLIVSLYGALAAWFGGGLPPGLKARVLAVQAAIGAAFYAFILFTSNPFERLAEAPFDGQ
DL
NPLLQDPGLAFHPPFLYLGYVGLSICFSFAVAALIEGRVDAAWGRWVRPWTLAAWICLTVGIALGSWWAYYELGWGG
FWF
WDPVENASFMPWLIAAALLHSAIVVEKREALKSWTILLSIVAFGFSLVGAFITRSGIITSVHAFANDPERGVFLLMILLV
FMGGALTLFAARASAMEAKGVFGAVSRESALVFNNILLAVAALVVFVGTIWPLVAEMAFGRKLSVGAPFFDMAFTPF
MVA
LALLLPLGSILPWKRARLGRAAKTVLPAALLAVALGALIWAMQTGRSALGPVGVALGVWLIAGALVDLGLRAGRGKGL
AA
RASRLARLPRADWGKAVAHAGLGITMIGISAMLAWNQEDIRVAQIGERYELGSYGFQLDRVQKLEGPNYISTMATVSV
WR
GDRQITEMHPEKRVYPVAGMPTTEAAIDSGLLRDLYLAIGDPQDNGGWAVRTYIKPFANWIWMGAIVMALGGLLSLS
DRR
VRVAAGARKERGPTLPQPAE
>OLS48375.1 2-(1,2-epoxy-1,2-dihydrophenyl)acetyl-CoA isomerase [Rhodovulum sulfidophilum]AB14
MTDYTTIGYDCADGVAVVTLNRPEVMNALSARMRAEITHAMRAAGQAARVVVLTGAGRAFCSGQDLGDGANIAEID
IERT
LRDEYEPMLSAIIDCPVPVIAAVNGAAAGAGANLALAADVVIASEAAVFVQAFTRIGLIPDAGGTYWLPRQVGFARAM
GA

ALFAEPVPARQAVEWGMIWEAVPEEGFEAHWRARAAQLAAGPTVAYANLKTALRRSGSNDFASQLSLEAELQGRCG
ASRD
FKEGVLAFLEKRMAKFEGR
>OLS48376.1 hypothetical protein BV379_08875 [Rhodovulum sulfidophilum]AB14
MTRLHTITAVSLLGAALLAGCGDSYGERALTGAGIGAGVGAATGNDIGTSAAVGAGAGILTK
>OLS48377.1 citrate (Si)-synthase [Rhodovulum sulfidophilum]AB14
MDDSKKSATLTLGETSYELPVLSPSAGPDVVDIRKLYGQMGIFTHDPGFTSTSSCESKITFIDGDKGELLHRGYPIDQLA
EKSHFLEVCYLLLYGELPTATQLEDFENRVTRHTMVHEQMHRFFTGFRRDAHPMAVMVAVVGAMSAFYHDSTDISDP
WQR
EVASIRLIAKVPTIAAMAYKYSIGQPFVYPRNDLDYASNFLRMCFAVPCEEYEVDPILTRAMDRILTLHADHEQNASTST
VRLASSSGANPFACIAAGIACLWGPAHGGANQACLEMLREIGTVDRIPEFIARAKDKNDPFRLMGFGHRVYKNFDPRA
KV
MKQSADEVLELLGIENNETLKVAMELEKIALEDEYFVSKKLYPNVDFYSGIILDAMGFPTSMFTPIFAVSRTVGWISQWK
EMIGDPAMKIGRPRQLYTGATRRDYVDVEDRG
>OLS48378.1 glutamate--tRNA ligase [Rhodovulum sulfidophilum]AB14
MTDMSDGPVITRFAPSPTGFLHIGGARTALFNWLYARGRGGRFLLRIEDTDRARSTPEATAAILKGLTWLGLDWDGEA
VS
QFERAPRHAEVAREMLANGTAYKCFATQDEIEAFREAARAEGRSTLYQSPWREADPATHPDAPYVIRLKAPREGATVI
GD
KVQGDVKIGNDQLDDMVLLRSDGTPTYMLAVVVDDHDMGVTHVIRGDDHLNNAARQMQIYHAMGWPVPVWAHI
PLIHGPD
GKKLSKRHGALGVEEYQAMGYPAAAMRNYLARLGWSHGDDEFFTDDQAKAWFALEGIGRSPARLDFKKLENLSGRH
LAAA
EDAALLQEVTDYLAATTQPPLSPAQKDGFARALPCLKQSAKTIPQLIEKAHFVLGERPFEPDQKAAKSLDDVSRGILAEL
TPQLQSASWTRDALEAAVTGVAEAHGLGLGKLAQPLRAALAGRTVTPSIFDMMLVIGREETLARLADTQG
>OLS48379.1 molybdopterin molybdenumtransferase MoeA [Rhodovulum sulfidophilum]AB14
MIPVDEALGHVFDLIAPLPVEEVPLAEAAGRVLARPVAARRSQPPFAASAMDGYAIREDDARPGAALRVEGEAAAGSG
WT
GTVAPGQAVRIFTGAPLPEGADRVVIQEDVTREGDRIVLGTGFDHGTNIRAAGGDFREGEPLSAPRRLGPADLALAAA
MN
LPALPVHRRPEVALIATGDELVMPGDTPGPDQIVAANGFGLKALIEAEGGIARLLPIARDSEASLRSVFALAEGADLVVT
IGGASVGDHDIVGKVAADLGMAPAFYKVAMRPGKPVMAGRLGAAAMIGLPGNPVSAMVCGHIFLRPALRAFQGLPK
APLP
TARAPLAAAVEANGPRTHYLRAQLTPDGLLPFDRQDSALLTVLAEADALLIRPAGDGPHPAGTLAEYLTLR
>OLS48380.1 cyclic pyranopterin monophosphate synthase accessory protein [Rhodovulum sulfidophilum]AB14
MAELTHFDAEGRAHMVDVSEKPETARLAVATGWVRMQPETLALVVAGTARKGDVIGVARLAGIMGAKKTSDLIPLCH
PLP
ITRVAVDLTPDDSLPGLRIEATVRTGGRTGVEMEALTAVTTAALTVYDMLKAAEKGMEIGGIRLKLKEGGKSGRYEAGE
>OLS48381.1 indole-3-glycerol phosphate synthase [Rhodovulum sulfidophilum]AB14
MTGTILDRIKAYKLEEVAADKAARPLSEIEAEARAAPAVRGFAQALHRAALTGYGLIAEIKKASPSKGLIRPDFDPPALA
HAYEAGGAACLSVLTDTPSFQGDKAYLTQARAAVKLPVLRKDFLYDTYQVAEARALGADCILIIMASVSDAQAAELEEA
A
TSWGMDALIEVHDAAELDRAAGLKSPLIGINNRNLKTFETSLETTKQLAKRVPEGSQIVSESGLHSPADLAQMAMYGVR
S
FLIGESLMRQDDVTAATRTLLSNPSMGAH
>OLS48382.1 anthranilate phosphoribosyltransferase [Rhodovulum sulfidophilum]AB14
MSDSLKPLIGLAADRPLTRDEAETAFEALFEGEATPSQIGGLLMALRTRGETVDEYAAAAKVMRSKCRKVRAPADAIDIV

GTGGDGKGTLNISTATAFVVAGAGVTVAKHGNRNLSSKSGAADAMTHLGINVMVGADVVEKALKEVGIGFMMAPM
HHPAM
AHVGPTRAELGTRTIFNILGPLTNPAGVTRQLTGTFSQALLRPMAETLDRLGTEVAWLVHGGDGTDELSIAGISHVAML
E
QGTITERELHPEEAGLPVHPFEAIIGGTPEHNARAFAALLDGEPSAYRDAVLLNAAAALVVAGRVTTLTEGAEQARASID
SGAARAKVEGLAQISSEKAA
>OLS48383.1 glutamine amidotransferase [Rhodovulum sulfidophilum]AB14
MLLLIDNYDSFTYNLVHYLGELGAETRVLRNDTLNVQEAMALRPSAILLSPGPCDPAQAGICLPLARAAAETGTPLMGV
C
LGHQTIGEAFGGKVVRAPEIVHGKMGRIEHDGSGVFSGLPSPFEATRYHSLIVERSSLPDCLIANAWLEDGTIMGLRHRD
LPIHGVQFHPESIASEHGHALLRTFLDTVKEPA
>OLS48384.1 hypothetical protein BV379_08925 [Rhodovulum sulfidophilum]AB14
MGRGFFGGVLWGAATSGLALAALAVVVPPGTEPLDEARGEAPAPRFSAVPERAGPAAPEPDSVPAPVSAPATRPEISA
AP
AAGPATDIELPPGSEFNRSRPGTEARPPEAEETPREAPTMGAPAPVDVTEAPLPDTAPAAAPAPVSAMPETMAPPMG
SDA
ALDLGGAASPAAEDPPPAARAMHGPSAPEAAAAPVPEPAAPENPREAAPATSPDASPAMPTGTSPDGSAATFPESSP
EAA
AGPAPETDPETVPDTSPETAPETALDSETVPEIAADPETGPEAEPAAAPETVPEVETVPEAGSEAPSVLPEVLPPPGPAD
AAMPGRAAGPLRPDAPLSMVGEKAAPVDAAPEATRSATPATPGALALNAVPFRAPPGAPLLSLVLIDIGAEGLDRQTLL
T
FPFPVTFVLDPAAPDAEAAMAAYREAGYEVAVLGSGLADLAPEAAAARLGEALDRIDGSIALVTPEEGGFEGDRDLSGA
V
LGVLEASGHGLVTADKGLNGALRAAEQAGLREAGIFQTLDDAGQDAETIRRMLNRAVLEARQTGHAVIVGHSHPETVT
AI
YAWALLSGPADAVLAPLSAVLRQSRP
>OLS48385.1 hypothetical protein BV379_08930 [Rhodovulum sulfidophilum]AB14
MSTAILVTDAGFAPAGPAEDWLSPADLDGPVRPEPVRVALTPETDPDLLGPHLARIAAIRVVFPAVADGRGFTLARRLR
R
MGFAGRLRAAGQLIPEQYPMARACGFDEVEIDADRALRQPEADWRRAAGRQGYLARLRRAVA
>OLS48386.1 phosphoadenosine phosphosulfate reductase [Rhodovulum sulfidophilum]AB14
MRPETGFERVEPQLAALNRRLDGATPQEVLAEMAERHAGRLALVSSFGAESVVLLHMVAELAPGLPVLFLETEMLFPET
L
AYQREVAAGLGLTGIRSLRPDAEALELHDPAGDLHRSDPDLCCHLRKTEPLERALAGFGAWVTGRKRHQTRERQTMH
VCE
TDEAGRVKINPLHGWGRDEVAAYIERHDLPRHPLVSRGYPSIGCAPCTSRVAPGEDPRAGRWRGQDKQECGIHLVNG
RFV
RMGAEGVA
>OLS48387.1 sulfite reductase [Rhodovulum sulfidophilum]AB14
MYAYSDFDESFVRDRVRQFRDQVARRIDGRLTEDEFKPLRLMNGLYLQLHAYMLRVAIPYGTLDARQMRQLAAISER
WDR
GYGHFTTRQNIQFNWPKLRDVPDMLDALADVGMHAIQTSGNCVRNVTSDHFAGAAADEIADPRPYAELLRQWSSD
HPEFQ
YLPRKFKIAITGATADRAVTRAHDIGLRVVARDGAQGFQVSVGGGLGRTPMIGRVIRDFLPEADLVPYIEAVLGVYNQL
G
RRDNKYKARIKIAVHEMGLDTIRDLVEARFAETRAEFPGAEAALADIRAAFRAPGFRAGGTAIPSGSPAFRAWADTNLA
A

HRREDHAIVTVSLKTPGQQPGDASADQMRVIADLAETYGYDEIRISHRQNVILPHIRKEDLPAVFEALAAAGLAAANAG
L
ISDIIACPGMDYCALATARSIPVAQEIAVLFETLDLAHEVGPLRLNVSGCINACGHHHVADIGILGLERAGVETYQITLG
GDPTDRIAIGERTGPGFAAGEIVGAVERIVMAYLERRDGPAEGFADAYRRLGLAPFQKALYNEGSKEDAA
>OLS48388.1 hypothetical protein BV379_08945 [Rhodovulum sulfidophilum]AB14
MKRRFNPSVVTANDLFEGHPVWRTAQGRWSATIREAEVLDDPATAQDRLAEAEAETGRVVGPYLAEVATAPQGPAP
LHFR
EVFRVRGPAHASDRPDQRLDQRPGE
>OLS48389.1 uroporphyrinogen-III C-methyltransferase [Rhodovulum sulfidophilum]AB14
MQFFPIFLRSEGARIVVSGGGETALAKLRLLLKTRARIAVHAAEADPKILDWQAEGRLSWVARPVEAADLDGARLVYAA
T
DDAAEDARVASLAQAAGVLFNIVDNREDSAFITPAMVDRDPVVVAIGTEGAAPVLARKIKAGLEAWLPAGLGARVRA
ANA
FRPEAEALPQGRARRGFWAAVFDGALDGRAPEPLDRLLEAHKAARPAVGEVLFVGAGPGDPDDLTMRARRALDRAD
VVIH
DRLVPAPILELARREAEIVEAGKTGFGPSTPQEEINRLIVGRAAEGLVVVRLKAGDPSVFGRLDEEIEACEAAGIAWSVV
PGLTAASAAAASLGQSLTRRGRNSELRVLTGHDVKGLADHDWATLARPGAVAAIYMGKRAARFLQGRLMMHGAEAA
TPVS
VVENAGRPEERILAATLGTLATEIASAELTGPAVILLGLAPRAAARSLPLLQEAAR
>OLS48390.1 hypothetical protein BV379_08955 [Rhodovulum sulfidophilum]AB14
MRLRRLVLAAIPVLATLGGVPETAALAGPHGEMLGEEAVRLNLDPASGALTLDARLPAPQGDVALPRQDWLSVETLLIG
G
EPSPLPASGVIRAARHMGRSVTVRLSGTLPELSPGIEAAGRGETASYLLGSGWLPVDAAPRARFELSLEVPPGWRPAAT
G
RLSGERVHPDGRRADFVFTGRAGDLGVFAGRYAVNEAMHRGLRLRTYFEPRDAAFSPAYIAAAAGYIDRYSDAIGPYPY
D
GFSVVSAPIPVGLGLPGMTYVSQAILGHGYMRGRSLAHEVLHSWWGNAVGVDYRRGNWSEGLTTYMADYALAEEA
GAAEA
REMRLGWLRDLAALPAAEDRPLTEFRAAAHGARQAVGYGKAAFLFHMLRAELGEADWQAGLRRFYSERYDRVAAW
HHLRA
GFEAASGRDLRWFFDQWLTRSGLPALRIEAAEPEGRKLHLVVGQSAPFYRLRVPVTVETEAGTERHIVALDGARAEVTL
A
LAATPRAVAVDPDFELARELAPGEVPPMLGDLFAGADPALLALGEGAGAAAAALGPRLLHGAPRLVDAVAAEAAPVLV
VL
GSGAEVAAWRRAHLSAPAPWSMDAGRARAWVERDARGRLIGFLSADRTEDLLPELGGLRYQAGQSYAAFADGARV
AAGDW
PTGASGLRRRFD
>OLS48391.1 thiamine-phosphate diphosphorylase [Rhodovulum sulfidophilum]AB14
MIRSRLPVYFVTPDGASPELVLAAARGGATMVQLRDKQAGDDALIELVRALKPELARRGVPLIVNDRLEVVLASGADGL
H
VGQSDGSPLALRRALGPGRLLGLSVEAMAHLGAIPAGVVDYLGVGPVRATATKPDHAAPLGFGGLARIAAASALPCVAI
G
GVGAGDARALKAAGAAGMAVVSAISAASDPEAAARALAGAWRNA
>OLS48392.1 hydroxyethylthiazole kinase [Rhodovulum sulfidophilum]AB14
MHPPADHPIRTPAEHLAAMRERAPLVQCITNFVAMNVAANVLLAAGASPAMVHAAEETGEFTPLAQALSINIGTPSP
GWA
TGMRAAASAARAAGLPWVLDPVAVGATSYRQALCGDLMAFEPTVIRGNASEILALAGVEARGRGADSVDSVEAAEAA
ARA

LAERSWAVVAVTGPVDYVTDGARAWRIANGHPLMPKVTALGCSLTALVAAFLAGGGDRAEATVAALACFGLAGEQA
AEGA
AGPGSFAVALIDALHAMTPETLERGARVSAA
>OLS48393.1 SAM-dependent methyltransferase [Rhodovulum sulfidophilum]AB14
MTPAARIAAAAELLDAWRAGAPAEKLLTTWARQNRFAGSKDRAAIRDHVFDAIRCLRSFAALGGAATGRGLMLGMLR
AAG
TDPDTVFTGLGHAPAPLGPEERAPGPALADLPEPVACDCPEALERLLRESLGADFRPVMERLRQRAPVFLRVNTRLGDP
A
AVAAQLAGEGIETRPHALSPTALEVVAGARRVHLSRAFAEGLVELQDAASQAVADMMPLAEGARVLDYCAGGGGKTL
AMA
ARGGARHFAHDAAPQRMRDLPARAERAGVAVQCLDGTALGEAAPFDLVLCDVPCSGSGAWRRSPEAKWRTTPADL
DRLTA
LQEEILGRAAGLVAPGGHLAYATCSLLAAENGARIGRFLDAFPGWVLRSERRLTPLDGGDGFYAAVMRRPG
>OLS48394.1 hypothetical protein BV379_08975 [Rhodovulum sulfidophilum]AB14
MPHFMPVLLSVRRSAAAFADRAWVALALGILLAVAAWTSPWPWLFAPLLTAGVTLGCLAALLRILGRPVSDIPSDRLRR
L
CACTPGATLVTDAEGRVLIASPEAQRRVAAMRGQPVADALQALFADPRRAVGDLIAAAARGLPASARRSLASGPLQVG
VE
RAGSGLFLWRFDSSAADGPEIALPMVRLDPAGRLSVINAAARALVGQSHEAVAALLAGASDETEEVVVETPEGPSRRRL
L
QLRMADGGREFYILPQGSASRVAEAPEGFLDALPVALLQLDTDGTVRLSNARAVRLLGGEPGEALNLCDRLEGLGRPVS
D
WLADAAQGRAPNSAEVLRVARDDREVFVQVALARMAEGALVAVLHDVTEMKTLEAQFTQSQKMQAIGQLAGGVA
HDFNNL
LTAISGHCDLLMLRHDAGDPDYGDLVQIHQNANRAASLVGQLLAFSRKQTLSPEVLDLRDTMGELAHLLDRLVGEKVQ
LR
MRHAEALPPIRADKRQLEQVVMNLVVNARDAMPEGGEIRIETEAVQLSTELQRDRAVVPPGPYAMIRVRDEGQGIPP
DRL
SKIFEPFYTSKRPGEGTGLGLSTVYGIVKQTGGFVFVDSTVGQGSCFSVYLPTHSGKVGPVAPLQESGAVEEMPGSAVVL
LVEDEAPVRSFAARAMRMRGHTVLEAESGEEALETLADPELKVDVFVTDVVMPGLDGPGWVSKALAARPGTRVIFVS
GYA
EDSFGEIKARIPNSVYLPKPFSLADLTATVQRQLRAAAEPGDTEGARDKDKLPEQVP
>OLS48395.1 DNA recombination/repair protein RecA [Rhodovulum sulfidophilum]AB14
MATANLLDMADKKASDKQKALDSALAQIERQFGKGSIMRLGADNPVQEIESTSTGSLGLDIALGIGGLPKGRIIEIYGPE
SSGKTTLTLHCVAEEQKKGGVCAFVDAEHALDPVYARKLGVNLDELLISQPDTGEQALEIVDTLVRSGAVSMVVVDSVA
A
LTPKSELEGDMGDSNVGVHARLMSQAMRKLTGSISRSKCMVIFINQIRMKIGVMFGSPETTTGGNALKFYSSVRLDIRR
I
GSIKDRDEVVGNATKVKVVKNKVAPPFKQVEFDIMYGEGISKMGEILDLGVKAGVVDKSGSWYSYGDERIGQGRENA
KNF
LRENSAVALQIEDKIRAAHGLDFHISGDENDEMIED
>OLS48396.1 alanine--tRNA ligase [Rhodovulum sulfidophilum]AB14
MPSLNEIRSTFLSYFERNGHEVVDSSPLVPRNDPTLMFANSGMVQFKNLFTGVEKRDYTRATTAQKCVRAGGKHNDL
DNV
GYTARHHTFFEMLGNFSFGDYFKSEAIPFAWELLTREFGIPKDRLLVTVYHTDDDAAAIWKKVAGLSDDRIIRIPTSDNF
WQMGDTGPCGPCTEIFYDHGDHIWGGPPGSADEDGDRFIEIWNLVFMQYEQFEGGTRRELAAQSIDTGMGLERIGA
LLQG

KHDNYDTDLMRDLIEASAHATSTEPDGPGNVHHRVIADHLRSTSFLIADGVMPSNDGRGYVLRRIMRRAMRHAHLLG
SQD
PVMYRLVPALVTQMGAAYPELHRAQALIEETLKLEETRFIQTLDRGLRLLDEELARLPEDGDLPGAAAFKLYDTYGFPLD
LTQDALREKGRQVDVAGFDAAMAEQRAKARASWSGSGETKESTIWFEIAERVAATEFLGYDTETAEGQVMALVAEGA
EVT
ELAPGTTGWMIVNQTPFYGESGGQVGDTGTYLKPEGRGEILDTQKMPGGLFAHKVTPAKAALAVGDAVELRVDHAR
RSAI
RANHSATHLLHEALRQTLGDHVAQKGSLNAPDRLRFDFSHAKAMAADELEAVESQVNGFIRQNSAVETRIMTPDDAR
AIG
AQALFGEKYGDEVRVVSMGRAATGKGHDGQTWSIELCGGTHVARTGDIGLCVVLGDSASSAGIRRIEALTGQAALDHL
IA
QERRLAEVAEAVKAPASEVVARIRTLVEDRRALEHEVAQLRRELALAGPAGAAPEAREIGGVPFLAQVLHGISGKDLPPI
IDEHKARLGTGAILLIAEADGKASVAAGVTPDLTDKVSAVDLVRAAVVELGGKGGGGRPDMAQGGAKDAANAEAAIR
AAE
AVLEA
>OLS48397.1 hypothetical protein BV379_08990 [Rhodovulum sulfidophilum]AB14
MPALWIAHVTVTDAEAYGRYAELAGPAIARHGGAFIARGGRFVQLEGRERPRNVVARFPSVEAAVACYESPDYQAALD
HA
RGASERELMVVEISE
>OLS48398.1 GTP-binding protein TypA [Rhodovulum sulfidophilum]AB14
MDLRNIAIIAHVDHGKTTLVDQLLKQSGAFRENQSVAERAMDSNDLERERGITILAKCTSVEWDGHRLNIVDTPGHAD
FG
GEVERILSMVDGVVLLVDAAEGPMPQTKFVTAKALALGLRPIVVLNKVDKPEAEPDRALNEVFDLFASLGANDEQLDFP
H
MYASGRAGWADMELDGPRKDLHALFDLVLEHVPAPKQLAKADGPFSMLATTLSADPFIGRILTGRVESGAVKPGDTV
KAL
SRTGELLESFRVSKVLAFRGLSQQPIDSAEAGDIVTLAGMSKATVADTLCAPQVTEALPAQPIDPPTISVSFGINDSPLA
GREGKKVQSRVIRERLMREAELNVAIQVTDTPGGEAFEVAGRGELQMGVLIENMRREGFELSISRPQVLMREENGQKL
EP
IEEVTVDVDDEFSGVVIEKLTGTRRGDLVEMRPGGTGKTRIIAHVPSRGLIGYQGEFLTDTRGTGVLNRVFHDWAPYKG
A
IPGRRAGVLISMEDGVSVAYALFNLEDRGKLFIGAQEQVYQGMIIGEHSRDNDLEVNPLKGKKLTNVRASGTDEAVRLT
T
PVRMSLEEAIAYIDDDELVEVTPNSIRLRKRYLDPHERKRQARSAG
>OLS48399.1 QacE family quaternary ammonium compound efflux SMR transporter [Rhodovulum
sulfidophilum]AB14
MPKHYLYLLLAVVTETMATSSLQASQQFTRFWPSALVVLGYALSFYFMAQTLKVMPVGIVYAIWSGLGIVLISGIGWVV
F
GQRIDLPGALGLGMIIAGIIVIHLFSKTAAH
>OLS48400.1 membrane protein [Rhodovulum sulfidophilum]AB14
MPTDMPPLPAPRHTLPDDIQSLVVGTLLVALSVSLLKSAGLVTGQIAGLSLVGAYASGIGFGPVFFLLNLPFYWLGYRRM
GKAFVLKTFAAVALLSVFSELLPHVLQIGPVHPLAASLLAGVTAGAGLLALFRHGASLGGVGIMALWLQESRGIRAGWV
Q
LGFDAVVFALAFAVIPPLTVLWSMAGAAALNVIIATNHRRDRYIAR
>OLS48401.1 isocitrate dehydrogenase [Rhodovulum sulfidophilum]AB14
MTKIKVDNPVVELDGDEMTRIIWDFIKKKLILPYLDLDLLYYDLGIEERDRTDDQITVDAAEKIKEVGVGVKCATITPDE
ARVEEFGLKQMWKSPNGTIRNILGGVVFRAPIICQNVPRLVPGWTKPIVIGRHAFGDQYRATDFRFPGKGQLTMKFVG
ED

GQEIEKVVYDAPAAGVAMGMYNLDESIRDFARASMNYGLSLGWPVYLSTKNTILKAYDGRFKDLFQEVYEQEFEDRFK
KA
GITYEHRLIDDMVACCMKWNGGFVWACKNYDGDVQSDTVAQGFGSLGLMTSQLMTPDGKIVEAEAAHGTVTRHYR
QHQKG
EQTSTNSVASIYAWTGGLKHRAKLDGNAQLAHFAETLERTVVETVESGFMTKDLALLVGPNQKWLTTMGFLEKIDENL
NK
ALAG
>OLS48402.1 hypothetical protein BV379_09015 [Rhodovulum sulfidophilum]AB14
MSAIDDLADQLARDALDAAARLGDDRLIDDISKAIGASSPVSQEAFVSAVRIRQALARGRAVMDSRLTQAASQKAGSEK
S
GS
>OLS48403.1 alpha/beta hydrolase [Rhodovulum sulfidophilum]AB14
MPEVIFPGPEGRLEGRYHPQKDRDAPIAIVLHPHPQFGGTMNNKVVYNLHYAFHNMGFTVLRFNFRGVGRSQGEYD
QGVG
ELSDAASALDYLQSMNQNAKHCWVAGFSFGAWIGMQLLMRRPEITGFVSVSPPANMYDFSFLAPCPSSGLIINGSAD
RVA
PPADTKTLVDKLHEQKGITVTHEEIEGADHFFQDPHMDPMIENVTTYVRRRLTEVTR
>OLS48404.1 Rrf2 family transcriptional regulator [Rhodovulum sulfidophilum]AB14
MKLSTKGRYAMVALADLSLQPEDCLVSLSEISKRQHISLPYLEQLFVKLRRAGLVESVRGPGGGYKLARPASEIRVADVL
GAVDETVSAMHTGAGVSGGTSGTRAQSLTNRLWEGLSAHVYVFLHQTRLSDVIKNDLAPCPAVPALFHVVDDE
>OLS48405.1 aminotransferase [Rhodovulum sulfidophilum]AB14
MPGRVYLDWNATAPLRPEARAAMIAAMDAVGNPSSVHAEGRAARALVEKARAQIAAALGAEGAEIVFTSGASEAAA
LACS
GRWLQGSEIEHDAVAAWLDPCLRPDGDGRVVPRLPAQSALQLANSETGVIQEIPEGLAVCDMTQGFGKLPVAFNWL
GCQM
GLVSAHKIGGPKGIGALVMRRGCDIEAQIRGGGQELGRRAGTENVIGIAGFGAAAEAAAADLEAGKWAEIARLRSVLE
NL
IAERANKTIFVGKDVPRLPNTLCLATPGWKGETQVMQMDLAGFAVSAGSACSSGKLRPSRVLTAMGLGDEVASSAIRV
SM
GPGTTEEDVRRFAESWLAAETKFRARTG
>OLS48406.1 Fe-S cluster assembly protein SufB [Rhodovulum sulfidophilum]AB14
MAALDKADMKNVKEGVDRETVEAVQSMAGKYKYGWDTEIEMDYAPMGLSEEIVRLISAKNEEPEWMTEWRLEAFR
RWQKM
EEPDWAMVDYPEIDFQKQYYYARPKSMAEKPKSLDEVDPKLLETYQKLGIPLKEQMILAGVEGAEAAPAEGRKVAVDA
VF
DSVSVGTTFQKELESAGVIFCSISEAIRNHPELVKKYLGSVVPQSDNFYATLNSAVFSDGSFVYVPPGVRCPMELSTYFR
INAENTGQFERTLIIADKEAYVSYLEGCTAPQRDIAQLHAAVVEIVILDDAEVKYSTVQNWFPGDEDGKGGIYNFVTKRA
DCRGPRSKVMWTQVETGSAVTWKYPSCILRGDDSQGEFYSIAITNNWQQADTGTKMVHLGKNTRSRIVSKGISAGHA
QNT
YRGLVSMHPKAKNSRNYTQCDSLLIGDKCGAHTVPYIEVRNNSSRVEHEATTSKVDDDQLFYCRSRGMDEEEAVALVI
NG
FCKEVLQALPMEFAMEAQQLVAISLEGSVG
>OLS48407.1 hypothetical protein BV379_09040 [Rhodovulum sulfidophilum]AB14
MPKTMAAPGAGRGAGRHPPELARGPGARLRRLSLPSIPGHGGRLVLTTRMRLLIIAQGLIFYASLQDEETIDWLGTLVPL
SKAQAALAEGLFGLVLFLLWGLCMIAWMRVFTQDRRDADLRDRGPGDQVLGDRGLRDQDRFDKDRRDKEDRT
>OLS48408.1 hypothetical protein BV379_09045 [Rhodovulum sulfidophilum]AB14
MIVTTTPSVEGHSILSYHGVVVGEAILGANVFRDIFAGITDIIGGRSGAYEASLGEARETALAELQQRARAAGGNAVVGV
DLDYEVINNMLMVSASGTAVTLS

>OLS48409.1 Fe-S cluster assembly ATPase SufC [Rhodovulum sulfidophilum]AB14
MLEIKNLRASLEEEDKEILKGVNLSVGAGEVHAIMGPNGSGKSTLSYVLSGKGGYEVTEGAATLNGVDLLAMEAEERAA
A
GLFLAFQYPVEIPGVGNMTFLRTAVNAQRKARGQEEMDAATFLKEVRTKAKELKIDAEMLKRPVNMGFSGGEKKRNEI
LQ
MAMLEPKMCILDETDSGLDVDAMKLVSDGVNALRAPDRGFLVITHYQRLLDHIVPDVVHIMANGRIIKSGGADLALEV
EH
NGYADILAEVA
>OLS48410.1 Fe-S cluster assembly protein SufD [Rhodovulum sulfidophilum]AB14
MGLPQAKLTATEARLAALAMPVQAGWTRPLREAALARVQQMGLPGRRDEYWRFTRPDELNSAAPVPAGVRPPAEN
ATFEE
LDRVRIVFVDGVFDPEASDDPAQAGLEISRLAEAEGQDLSWAADLYGTLEARGQTPVPRPLAALNTAFATDGVLIRATG
R
VGKPVNLVYRHCDETSDAILHHVIRVESGADLTVLETGPAAARFNKVMEVDVADGGAFHHVRSQGRDHDRRAATHIF
ARL
GEESIFKSFTLTMNGALTRNEVVIELTGDEGVAHVAGACMGDGAFHHDDTVFITHDAVNCESRQVFKKVLRKGATGVF
QG
KILVKEGAQKTDGYQISQSLLLDEEANFLAKPELEIYADDVACSHGSTSGAIDEDALFYLRSRGVPKAEAQDMMVLSFLA
EAIEEIESEAVRDEVSASLAAWLERHRR
>OLS48411.1 YIP1 family protein [Rhodovulum sulfidophilum]AB14
MAVTTEMVATWRGPRAALRRQLRMGQREDRALVYLMIACLIIFVGQWPRLAREAYMSPEIPLEALLGAALFGWMAL
APLF
FYGLAALSHLVARAVGGKGSWFSARLALFWTLLCVSPLWLLHGLVAGFIGGGAALMLVSTVLAVAFLAIWLLSLVEAEA
>OLS48412.1 cysteine desulfurase [Rhodovulum sulfidophilum]AB14
MYDVNAIRADFPILSRQVNGKPLVYLDNGASAQKPQVVIDAVTQAYAMEYANVHRGLHTLSTIATEKYEAVRGTIARFL
G
AADPDEIVFTSGTTEGINLVSYSWAAPRLQPGDEIVLSVMEHHANIVPWHFLRERQGAVLKWVDVDAGGNLDPQAVI
DAI
GPRTKLVAVTQMSNVLGTVVDVAAIVKGAHDKGVPVLVDGSQGAVHQPVNVDALGADFYAITGHKLYGPSGSGAIHI
RKE
RLAEMRPFMGGGDMIREVRRDGITYADAPMKFEAGTPGIVQQIGLGVALDYMMGVGMDNIAAHEKAITAYAMDRL
AGLNW
LQVQGKADHRGAIFSFTLEGPAHPHDISTVLDKKGVAVRAGHHCAGPLMEHLGLSATCRASFAMYNTTDEVDRLIEAL
EL
CHDLFA
>OLS48413.1 tryptophanase [Rhodovulum sulfidophilum]AB14
MPGVRFFSGETVPLEMHKVRVVQKLTLPDIEARLAAITAAGNNTFLLQNRDVFMDMLTDSGVNAMSDRQVAAMMV
ADDSY
AGSATYTRLETRLREIFGMEFILPAHQGRACENIVSQVMVTPGSIVPMNYHFTTTKAHIVLNGGSVEEIIHARGLEVTSD
EPFKGDMDVAALERLVAEHGAERIAFVRVEAGTNLIGGQPVSLANLQEVRAACDRHGLTLVYDASLMADNLYFLKTRE
AA
CRDLSIREITRKIADLCDVIYFSARKLGCARGGAICVRDEALYNRMRGLVPLYEGFLTYGGMSVREIEALTVGLDETMDE
DMISQGPQFIAYMVDELDRRGIPVITPAGGLGCHIDAMRFLDHVPQPQYSAGALASALYIASGVRGMERGTMSEQRA
PDG
TEPLANMELVRLAMPRRVFTLSQVKYAIDRIDWLWQNRRLVGGLTFVEEPEILRFFYGRLAPVSDWQQKLVAKFRADF
GD
SL
>OLS48414.1 nickel responsive regulator [Rhodovulum sulfidophilum]AB14

MQRITITIEDELLQALDAVMDRSGASNRSEALRDLIRRGLAKDAPEEAECVGVVTYALDPAQRDLGRLVPESRQQHHDA
A
IAALSVPLDHHAAVEVAVMRGTVGSVQAYANSLFLQRGVRHGRTTLIPVRTEVEFHLHGERAHTHDHLRVLDRF
>OLS48415.1 ASCH domain-containing protein [Rhodovulum sulfidophilum]AB14
MFKISAALKQRYPAAEAFRFGDSAASSEALLSLVRSGKKTATCSALRDIQNGEPMPTIGRRDIALNWDGTPALVIETVEL
VQCRFDEVTEAMALAEGEDDSLDAWRKGHGSYFKRNGGFSPDMQIIWERFILIEDLGREDDVLGHSSSLTDPAA
>OLS48416.1 hypothetical protein BV379_09090 [Rhodovulum sulfidophilum]AB14
MLSELLDKIPPEQEIGSVTADGAYDRRGHGCDEDCWLWRSRRAAESGSARDAGSRPFPGRQDRIRPVGPGARWRRA
RP
>OLS48417.1 hypothetical protein BV379_09095 [Rhodovulum sulfidophilum]AB14
MTGSGHLRNGPDRSPFTELSSPYATYGRGFRAAGAERRRFRCAGAAKVRKADRAIIYSLQTSECQLVRSERGPDFSTLSR
RQKALPLTIQYRGSKGPLNLVIDSTGSKLEGEGAWGLRPFRTEMIPRIVSGTPLTPQARWSQTARLAKDPPRDRQGNAG
G
SGRRVHQQWHRRRPHAVGTAR
>OLS48418.1 EamA family transporter [Rhodovulum sulfidophilum]AB14
MPRPLDLLLTALAPTVWGSTYIVTTELLPEGYPITAAMLRALPAGLLLLLLVRSLPHGRWILRALVLGALNFSLFWWLLF
VSAYRLPGGVAATVGAVQPLIVLVLARQMMGQPITGNGLVASIVGVVGVGLLVLTPEATLDTMGIVAALGGATSMAL
GTV
LTKAWQPPVSPLTFTAWQLTAGGLLLLPAAFLFEPPLPPLSASNVAGFIYLGLLGGAITYILWFRGISLLGPSAVSPLGR
LSPVAAVLLGWVILNQTLSPLQIVGMVLALASAWFGQKGERTPTLVAQPAQQTQGTPDSAERAVTARASQRSRLRP
>OLS48419.1 hypothetical protein BV379_09110 [Rhodovulum sulfidophilum]AB14
MRKENKKTGVLLAQPLYKRAEIEMTNRIATGKWEAGAQLPNEFALADEFGVSQGTIRKALMAMEHRGLLTRRPGRGT
VVC
KTTSEESLYSFFRLRDADGRMVVPSPVVEAVEFGEPTSEETRILGESCPKVVRLARVRENNGRPFVVERMSFDARDCDGI
ESDLPLPSSLYPYLHERFSLVIMKVDESITAALADPELAKSLQVEVGTPLLRIQRIARDLADRQVELRESYYRTDFASYQ
IQLTRSDGADA
>OLS48420.1 succinate-semialdehyde dehydrogenase (NADP(+)) [Rhodovulum sulfidophilum]AB14
MTDFSKYLVEANLIGGDWRLPEEGASIAVSNPASGVTLGHVPRFGAAETSEAIAAAAAAFPSFAASDLNTRVALLRRLH
D
ALMDNQDSLAALLTAEQGKPLAEARGEIGIGAAYVLWFAEEVRRSKGEILPSPVNGRHMMVTQHPVGVVAAITPWNF
PSS
MLARKLGPALAAGCTAVVKPASLTPFSALAWGKLAEEAGFPPGVVNIVTGSAREIGATLTTDPRVRKVTFTGSTEVGREL
IRQSATTVKKLSMELGGNAPFIVFDDADIDRAIEGAMIAKYRNSGQTCVCANRIYVQSGIHDAFVRRLSEATAALKVGD
G
AEQGVEQGPLVDQAAFDKVAELVNDATANGAKIETGGGRHMLGGTWFQPTVLTGITSQMKIAREEIFGPVSAVVRFD
SED
EVVDDANDTEFGLAAYVYTRDLGRAFRMNERLQYGLIGINEGLITAVEAPFGGLKSSGIGKEGGSQGLSDYLDTKYVCIG
GL
>OLS48421.1 tagatose-bisphosphate aldolase [Rhodovulum sulfidophilum]AB14
MRRMADAAGRFKMTAVDQRPPIKSAIARHYGTAEAPFDDVARFKTLLIETLQDRSTALLLDPHFAVPRGLPSLSPTKGLI
VTLEDSLFEDTGMGRRSSQIDDWSVGKIKRMGGDAVKVLAWYRPDADPVINEHQKAFTREIGEACAKYDIPFLFELLVF
P
LETDAHQTQDYVEMQGKRSDDVLRSVEEFAKPEYGVDVFKLESPIAADAVPGVGEDGWEDAQAVFDEMGRLAGRP
WVMLS
AGASKPAFHKIMTHAYKAGASGYLAGRAIWLDAFGHFPNWDAIRAELTGEGCAYMDDLNALTDAEAMPWHEHPVF
DGKGA
VFTPSDASFRHVYQDI
>OLS48422.1 transposase [Rhodovulum sulfidophilum]AB14

MKLLGPRLMARDFDRQVAELQVRIAVLNRYTALGIPVTEAVG
>OLS48423.1 chemotaxis protein [Rhodovulum sulfidophilum]AB14
MPAFLYRIPFRIYSIVIVALALSALISFLLLSRAVENAYDMRKTELHGMTDAAISLLSGLQARVAAGEIDKATAEARGRE
LLETLRFGASGYVFALTNDLVITAHPMLPDWVGTDKSDYADVNGLKLFQEMRKVALRDGSGPVEYWFKKPGETEPEM
KLG
YVQEFKPWGWFVGTGAYVSDIHGEMASMRHASAITQIVGMLILVAISFVLVRSVTRPLASFVLSMNDVADARYETAIP
EA
RRRDEIGDLGRNLAAFRDKLAESERLAAAREEDRREQERVVADLASGLRSLSEGDLTSTIDTKFADDYEQLRQDFNATVE
TLNALMGEIVENATEITTRAEEISGASDDLSRRTENQAATLEQTAAAMDEMTASVRAAAEGAAKVEDVVREARGNAE
QSG
LVVKEAIGAMSEIKKSSDGINQIIGVIDDIAFQTNLLALNAGVEAARAGEAGRGFAVVASEVRALAQRSSEAAKEIKTLI
SASSDQVETGVSLVNRGGAALTDIVERVGNIATLIADIATGAQEQSVGLGEINVGVNELDKVTQQNAAMVEEANAAAV
TM
KQEAGNLQTLVARFRLKGPAATVSVRPRTETAIPAAAPAPAAEFEPPKRAVNDAGWQDF
>OLS48424.1 phosphatidylglycerophosphate synthase [Rhodovulum sulfidophilum]AB14
MLDGVMRRLIDPPLDRLGGRLAARGVTADAVTLAGLGAGLLAAAMIAFGAPGLALVPLLAGRLADGLDGAVARAGRK
TDF
GGYLDIVADFLVYGAVPLGFVLMDPEANGAAGAFLLTSFYVNGATFLCFAILAERRKMETRARGTKSLYFTGGLLEGTET
IAFFVAICLWPGLFAPLAWGFGTLCFVTALSRVLLARRVFPEG
>OLS48425.1 imidazole glycerol phosphate synthase subunit HisH [Rhodovulum sulfidophilum]AB14
MLTAIVDYESGNLHSAQKAFERVARETGAGEVAITSDPDTIRRADRVVLPGDGAYPACHRALFDHRGVFEAIEEAVISG
G
RPFLGICIGMQMMASRGLEYEETPGFDWIGGEVVRIAPGDPHLKVPHMGWNDLVIETPHPVLDGIATGDHAYFVHSY
HFR
VADPAHRLAHCDYGEPVTAIVGRGTTIGTQFHPEKSQATGLRLIANFLAWAP
>OLS48426.1 imidazoleglycerol-phosphate dehydratase [Rhodovulum sulfidophilum]AB14
MRTAKITRKTAETEISVELGLDGSGRYDMATGVGFFDHMLDQLARHSLIDMTVRCQGDTHIDDHHSVEDTGIAIGQAL
AQ
ALGDKRGIRRYGSCLLPMDDALLRAALDLSGRPYLVWNVDFPTEKIGGFDTELVREFFQALATHGGITLHVDALHGLNS
H
HIAEAAFKAVARALREAVETDPRKADAIPSTKGAL
>OLS48427.1 AsnC family transcriptional regulator [Rhodovulum sulfidophilum]AB14
MTACVFVQLRCRPGKTYEVAEEIVLRELHSELYSTSGDFDLLMKLYIPEDQDVGKFINDNLLDIPGIDRSLTTLTFKAF
>OLS48428.1 acyl-CoA thioesterase [Rhodovulum sulfidophilum]AB14
MPELSPERGEPVIRTIAMPADTNPAGDIFGGWLMSQMDLAAGSVAARVSRGRAATIAVEGMQFLKPVKVGDEVTLY
AHLV
KRGRSSMTIEVRAWHRPRCQEWSEQVTRATFTFVAIDGNGRSRPLPDPVQAG
>OLS48429.1 hypothetical protein BV379_09165 [Rhodovulum sulfidophilum]AB14
MTKTESGERPARLHLLGALCLTDTEGRDFTPKSRKAQALLALLALSPRGTRTRIWLRDKLWSESDEGRASGSLRQSVYEL
KGALKGIDAGILTTERNALGLDLDRLWVDLRAIREGGGALPAGVDPDADLLEGFDISDEEFEDWLTMERSAWERLRENL
T
TAPPAAADAPAPPEAPAPRRMPRVIEIGLLPGIVHGGLRSGAFIGDYIIESVARALLDYHPVQIVDLREAGEAEPRPGEP
DFLIRTRTLVVGDTASVSFLVYENGTNTLVLSQSMQTRQKDFYEDEFALTNQFVAQNVDHLAKIIGGTRDGPSGIRNDY
S
ALIGYGVLAGMFDLDPGRMRATTEALDRLNERTENSLFPSLGAYASSFALGENIGGWDRARRERTEALAMDLLQANPF
NS
IALACIGHVMGFVLDRHDIAADIFGRAVRLNPMQAFVWDHLALHELYCGDLDRARAASDRAVNLGKYSPISYTYETTAC
M

VATLSGDSARAVALGRRALLKQPRFNAAMRYTLAALGHLGERAAAQALGAELLRLDPGFGDRETREMRFRLPCREASA
HV
LEGIERAGFRGGRT
>OLS48430.1 hypothetical protein BV379_09175 [Rhodovulum sulfidophilum]AB14
MFGDETGRPQGREIYDDYAALWHLESIGVLGLLRDGPRPCKGRKSRVAMIDTSVAVDHPCLATAIDRDLAIDFFSNRLG
A
FPCHPGDHRLRLDPPNLATAVADDLPETRALLGELVDRLSPGAVAHVNGVAPATSPDFSNHGTAIAGLVGARPALVEIA
A
EYRGAGVQALSVPLPYAGVDPFCELVPISTSFDTDPEMLILAFLYAEIIGADVVLLPRSIPDPGRLVPELGGVDLDGEDL
GTLATQVEIPPRERALWDELARLIVAVSMRRPVVCAAGNSQESGGIYPANLASEHNGVIAVGAANAKGMVAGYSQAA
GAT
VLAPGNDVETFDRVSVRLDEQQARFADRLCPPRNDNRKFSHFEIVTTDVPGRHGYSYSPYASDEPAEGLREFGSYFCRF
G
GSSAASAIVAGFVSLGRSLGEIDDCADGPAARNWLLSRCVPITTDEGSFLYPSWTGKVSFPDLYGL
>OLS48431.1 50S ribosomal protein L9 [Rhodovulum sulfidophilum]AB14
MQVILLERVAKLGQMGEVVNVKDGYARNFLLPQSKALRANEANIARFEADKAQLEARNLETKKEAEALAEKLDGQQFV
VI
RSASDGGALYGSVSPRDIADAATEAGFSVDRKQVVLAGPIKELGLHTVHVLLHPEVDVTVSVNVARSTEEAELQASGKSI
QDLAAEEEAAAEFEIQELFDDIGSAASDDEDDEQA
>OLS48432.1 30S ribosomal protein S18 [Rhodovulum sulfidophilum]AB14
MAAKPFFRRRKVCPFSGDNAPKIDYKDTRLLQRYISERGKIVPSRITAVSSKKQRELARAIKRARFLALLPYAVK
>OLS48433.1 30S ribosomal protein S6 [Rhodovulum sulfidophilum]AB14
MPLYEHVFIARQDLSNAQAEGLVEHFSTVLADNGGKVVDSEYWGVKTMSYKINKNRKGHYAYLRTDAPAPAVQEME
RLMR
LHEDVMRVLTIKVDAHEEGPSVQMQKREERDHRRDRR
>OLS48434.1 hypothetical protein BV379_09200 [Rhodovulum sulfidophilum]AB14
MNLIPAAATAALMAATALPALAEPVDYSLDPSHSQVLFSYEHLGFSTTWNLFSGWEGTVSFDAEDPAASRVSVSIPTEA
L
FTGWEDRFQHFMSDAFFDAAENDLVTFTSTDIELTGEETAKITGELTLNGITKPVVLDARLTQHGEHPMEKRDWLGFY
AT
TTLSRSEFDMGEYVPAVSDEVEVQISIEAGKAAD
>OLS48435.1 hypothetical protein BV379_09205 [Rhodovulum sulfidophilum]AB14
MRGLFNRPITKLSFAPPAPRPGRSGLAIVLIVKNEARHIGEWARFHRAAGAAHVYAYDNGSTDGTAAELAAALGDRLTL
L
PWDQKLFDGRSGREIHNQVLAYAHALRNFGGAHRWMACIDADEFLVPKRAESLEAALAHLDDIPSVTLPWHNFGRSG
HDA
PPAGGIVANYTRRAADPLSGARGVTNFKTLVDATRVTALKVHSVWVDGQAVTWNDRGERTDHKGRADPAFYSADHL
QLNH
YYTRSEQELAAKIARGSNQSVEARRHADRIRRNVANIEASEVEDRSAPDFLARKG
>OLS48436.1 malonyl CoA-acyl carrier protein transacylase [Rhodovulum sulfidophilum]AB14
MRAFVFPGQGAQTIGMGKALAEAYPQARAVFEEVDDALGESLSELIWQGDQAELTLTRNAQPALMATSMAAMAAL
GAEGV
SVTAASFVAGHSLGEYSALCAAGAISLADTARLLRLRGEAMQAAVPVGEGAMAALLGLDLAAVREIAAEAAAGQVVQA
AN
DNDPAQVVISGHAAAVERAVELAKTRGARRAVMLPVSAPFHCALMAPAARAMAAALEEVEIEVPLVPVVANVRACGE
TDP
ATIRTLLVDQVTGAVRWRESVAWMAGQGVSEIWEIGAGKALSGMVKRIAREVATRQVGTPDEVTAAVDGMNAAA
>OLS48437.1 beta-ketoacyl-ACP reductase [Rhodovulum sulfidophilum]AB14

MFDLSGKTALVTGASGGIGGAIARGLHAQGATVGLSGTREAPLAELAAELGERAHVLPCNLSDPEAVEALPKAATEAM
GG
LDILVNNAGITRDMLFMRLKDEDWQSVLDVNLTAAMRLCRGAMRGMMKARWGRIVNITSVVGTTGNPGQANYCA
AKAGLV
GLSKSLAHELASRGVTVNCVAPGFIATAMTDKLTDDQKGGILGAVPMGRMGTPEEIAAAVVYLASPEAGYVTGATVHV
NG
GMAMV
>OLS48438.1 acyl carrier protein [Rhodovulum sulfidophilum]AB14
MSDIADRVKKIVVEHLGVEEDKVTENASFIDDLGADSLDTVELVMAFEEEFGIEIPDDAAETIQTFGDAVKFIQEAV
>OLS48439.1 hypothetical protein BV379_09225 [Rhodovulum sulfidophilum]AB14
MTERASCYGRPGCQPVFSDVAIKLCLSVKVLFKLPLRQTAGMLANRLARLIRPAAIEVQIGDKRWPEKYGGKPSALVDN
T
RDRQFDMATPDTVWGTGITYICTQQGFACLAVVIDLLSRHVFGWAMQS
>OLS48440.1 ferrichrome ABC transporter [Rhodovulum sulfidophilum]AB14
MTGLLNLSKVSVSFGKRPILQDISFGPLKSGSVTALLGSNAAGKSTLLRRIAGELKGPGRVEIAGRPVDSWPAYHRNRPA
HVPQDISMGSSLRVFEAVLLANKQGGGWSVDDEELDVVTHMLDTLGINSLSDRELAALSGGQRQLVSIAQALIREPRVL
L
LDEPTSALDLQNQFEVLDLLRRLAREWSMCVVVAIHDVNHALRFADCAVVLHDRHVEANGSPVDILTPALLGKVYGVE
AR
METSSQGHPFLIVERSIRKPLGNDRGHD
>OLS48441.1 iron-siderophore ABC transporter permease [Rhodovulum sulfidophilum]AB14
METPPVSPLQSRNLYLRLTRRRLTLLTGIGLLTGICFLIDLSIGQAHYSSLEVLSALWDTDAPLSVRVILWDIRLPVALA
ALVVGASLAVAGVQMQTVLGNPLASPFTLGLSAAASFGAALGLILGVSLLPASAVAFAVPANAFLISMGAALFIHRLSQK
RGITTEMIVLLGTTLVFTFTALLQALQYVAPDQALSAVVFWTMGSLSRANWLKLGIMSITLALVSLAFAKQAWALTALRL
GESRATSLGVPVARLRLRGILLGSLLASVCVSFVGTIGFVGLVAPHIARMLVGEDQRFLLPTSALCGALMLSAASVLSKS
LIAGQTLPIGIVTSLVGVPLFFTLIMRAKR
>OLS48442.1 ABC transporter substrate-binding protein [Rhodovulum sulfidophilum]AB14
MVRGGLAIAALCLGSAPRAARSDRQRGSSVSITDIDGRTVAITRPIRRLLLGNGPLAYVMAVLQPEAPFANVVGWGNN
FR
AADLDGYNAYLRRFPDIDRIPSFSSGTLESIGAELAISLEPDVVVMNLSSKHAALASGLMARLAQVEIPVLFVDFRTRMF
ENTTRSIRILGELLERQDRAARFLQFRQEQIDRVIEPLAKVRSRPRVMVERAAGMYDDCCLTYGDGNFGELIRAAGGDN
L
GAHFLHGSFGTLHPEQVIESDPDVVLVTGANWSLYSPAGDWVNLGPGADPVEGQSRLHRLMQRPAYRSLRAVTAGR
VHAI
WHPFYDNPYYFIALQRVAKWLHPEVFAALDPEDTFRELHARFLPVPYESGYWLSLEGGAS
>OLS48443.1 TonB-dependent siderophore receptor [Rhodovulum sulfidophilum]AB14
MASYDQSVPSGTSRPPRPAINYYLGTSVLCLLLASPALSQETKPRKDGNASDLKAFTLEPIVIDVQDGDAGVVAKTSRVG
MKADASILETPQSVSVITRDQMDTQNPANTSSALRYTAGATSEAFGAFGSYVDITRIRGIKADYYLDGLRIISNSGSWLP
QVDPFTLDSVEVLRGPSSVLYGQGTGGGIVNQVSRRPQDEAAHEVSLQFGNFARKHIGIDTTGPLNEDRTLLFRFTASG
L
DTNGQIDDTKHRRLYLAPALTWRPDDKTSWTVMGTYSYEPEIPDYISLPAVLLGLNNSPYREIKRRRNYNDMNFADSTR
K
QTSVSSFFEHEFDNGWKFTSNSRYMHVDSNLKRSTVYGFSDVGGVPFLKGYYEDAPAESTTFSTDNFLSGEFDIGPTQH
K
ALVGFDYSFGTIKNELYTDGPILFDPYSDDHNPGKIPDFSASRAAPWKVDRDFNRGGIYLQDQISWDNWLLTLGGRRD
WF
TSDDETWSYSLTSRVTNYDDSAWSRRIGLSYQFDSGLAPYVSYSTAYDPLLENDWQGNPFKPVETKQTEIGVKYSPEGS
N

MLLSAAIFELKQTNVKTADALHLGFNTQTGEVRTRGIDLQASMEVTPNINVIAGYTYLDNEILEDTRYEGNSLTQVPKHS
ASAWVDYRFNEGALAGLKIGGGVRYQGSTFGDPTNSFKVPAETLVDLGLSYDFAQVSPKFSGALLNVNIGNLFDKDYVS
S
CTSQLYCFAGERRTVSASLSYRW
>OLS48444.1 MFS transporter [Rhodovulum sulfidophilum]AB14
MSVSRRAVAVAAVYLGTFMATLAISIVSVALPAIQASLETSLSGLQWVVGAYTLCLSAFMLSAGPLADRYGRKRTWLIG
V
AFFTLGSAICATADSLATLIAGCAFQGLAGALVIPGALSILTQAFPDPVKRAHVIGGWSSFNAISLILGPMMGGLLVDYA
GWPSIFLVNLPLGIVALGLGLVGIEESSHPDHAALDPVGQILSIVFLGTLTLSLINAGHSGWSSPVTTVPMVISALALLA
FIAVEMRTARPVLPVDLLRQKAFASANFASFVLGFSGYTSLFLFSLFLQRGQGWTATETGWRMAPVFAAMLAGSSVFG
KL
TRRFGMNRVMIAGYVLLGGSMLVMVSFTPSTPYGIVAPVFALLGIGLGLAVPSTGAAAMGSAPRERTGAASATMNAL
RQG
GMTIGIALLGTIMSARAVASLQGVLAQSGIDDVAAIAAVAVQRHEVPSTLPVASGAFHEMLAGAFTQGFSLAAGFAGIL
G
LVAAGVVALSSRRQ
>OLS48445.1 hypothetical protein BV379_09280 [Rhodovulum sulfidophilum]AB14
MVLFWAAATTVQAAGGGTGMGPSAIEVAPGAGALVLAVRTGPLPAGAELVLETSGGRRIGSVSPFGATAATSSAVQT
HLV
PVPAGTLPEGRIEIRAKLVLHGSERAATAHEFLGAAIIGH
>OLS48446.1 hypothetical protein BV379_09285 [Rhodovulum sulfidophilum]AB14
MTGTISTNRRTVLKGGAVLLAVPFLARSAAGQASHLRVSMTSEEGIAHLRTYRKAVAAMWALEPSDPHNWFRYAMIH
LMD
CPHGNWWFFVWHRPYVAHLERIVRIYGEDENFAMPYWDWTADPFVPDTFFASSTYPDNALDPTSSHYPDWDSFESA
YHDP
IEAFWNGLSDAQKQQETLRGFPDFESFWTTGVQPNFTAAPANGRSKTLAAPDLTPPASDAVTLPIIEAGLAPEIFTTVVA
TDDARTGFNSPESQNHNQGVGAAIIEGQPHNKVHNNLGSVAGGWMPSLLSSVDPIFFMHHCNVDRLWDVWTTKER
NAGRT
ALPTAEEAPRFDPEAFLFYIDDAGQPAPSTAGASMAIEPWGYAYTPGSGSASAAGLQSKRGIAPNAIALANKDFGLNEG
A
TTSLSLPEGFAAAGKEETRQFAQVTFKPPRELKGLSFDVFLSPGGEMPELSEDGPGFAGSFEFFGMLHGHDHDFTAVID
V
TDAVARLREAGTLRESGAIDVTLAASGTAIKGRMLESAPLEGVLKSVVFESF
>OLS48447.1 hypothetical protein BV379_09290 [Rhodovulum sulfidophilum]AB14
MRPSAIRMADLQDADDLRALMRDVSCGDDNMLYEVDEIPQAWRLGRIISENSLHDIFVSHIDDVLVGYAGIWRKNTR
RIQ
HVAGIYIGVSKDFRRRRVGTSLIEHLLGYAQRNGLMRLELRVSVRNLAAINLYRQVGFEFEGLARDAVVVSGRKIDKFFM
AKITQ
>OLS48448.1 hypothetical protein BV379_09295 [Rhodovulum sulfidophilum]AB14
MDVAINSDWTSRKEELARDAALLGRIMDYDHPALKERMIAKVGWSEDFTDELFNEMKRFLYLCATNEGSMAPPEDID
EIW
HNFILFTVDYAEFCRDKVGTFLHHQPLTVKQRAESDGSMVERTLSAARASFGDTLSKNWEFSKIPGSCGPGTCGASTNC
Q
DVSGCFSGPKK
>OLS48449.1 hypothetical protein BV379_09305 [Rhodovulum sulfidophilum]AB14
MSSVDGPELAERLSRIEGAFVLSINDRPEIRDLFGAFVIEEVRLNYSVSKSGATAARELIIANREVRMGLV
>OLS48450.1 hypothetical protein BV379_09310 [Rhodovulum sulfidophilum]AB14

MVTEAGDLDIDHIVPLRYAWGHGAASWSAQKRARFARDPVNLLPVSASANRSKGARGPLDWLPPDPGFRCQYVLRF
RRIA
ASYGLVQSAAEERELVALTGRVCGA
>OLS48451.1 hypothetical protein BV379_09315 [Rhodovulum sulfidophilum]AB14
MAEELAAGQGIEIDLTMPDGKGGRKYITPHVLKHTAITWALQRGASIWDAAGYFSTSPETIQRVYGHHAPDHMQSAV
EAM
DRRI
>OLS48452.1 hypothetical protein BV379_09325 [Rhodovulum sulfidophilum]AB14
MDAREQAEGEKRVRDLLVEPLLRRGLVKPGAMTKAQFEEMLADLAKRLAYMSGPNLAALEEMAAAQPGGNQGSE
>OLS48453.1 hypothetical protein BV379_09335 [Rhodovulum sulfidophilum]AB14
MATRVHTNLTIRGTTYPDAASAARALGVTAEAIRSAARKGRLDRVGTGRKGLAPMPVRIRGEVFTDAHAAAARFGVTP
QA
VWRALADGDPDRIGRPQRRPGRAPKPFEIGGLRFASQRKASRALGFSDDYLSHALTRGGRAARERILAAAMALAAKGV
TP
>OLS48454.1 hypothetical protein BV379_09350 [Rhodovulum sulfidophilum]AB14
MQKSFQKFRDPQERERKWFASLLWRSFPEATSEAQLADLVAEALNTAQRPVNPRTVRNWLRSENAPGLHYVVAVLAL
AGA
ETVFELFDPEQTA
>OLS48455.1 hypothetical protein BV379_09355 [Rhodovulum sulfidophilum]AB14
MTSNSIESREKLTLLGDTSPEAISIRLRAAREAAGYAQQKDFAAEAGLSATTYNSQEKRGAPSITVMKILYRQHRIDFNF
VLHGDFAQLPGDVQERLFEALSSSRP
>OLS48456.1 hypothetical protein BV379_09360 [Rhodovulum sulfidophilum]AB14
MKTILTTAALALLAAPVLAATGTTEDQATAVSPASAPDCFAPINGTNAFQPSNAPGCVYYTAPTGNDRDPAASGEGEG
EG
GEGEGGESSDT
>OLS48457.1 hypothetical protein BV379_09365 [Rhodovulum sulfidophilum]AB14
MAAILTIAPQIADRLAAEAAAARIEAQTARVAAKSNARDGKPLLARAAFARAERLQGRAVTLGALAAQARAGGAA
>OLS48458.1 hypothetical protein BV379_09370 [Rhodovulum sulfidophilum]AB14
MSIMDSIAPPPDALAAFVVEWRGASPEVRLAAMRGALRRHGIEIPALPGRVAFYEIQLFGLVATGCDEAEAAQNWME
AAI
RLCPFD
>OLS48459.1 hypothetical protein BV379_09375 [Rhodovulum sulfidophilum]AB14
MHPLRILIGCETSGVMRRAFAARGHDVWSCDLLPAEDATTRRRWNSSSSASATTSGSSPESVIDSPGMPSPEVACASP
TE
SICPAPR
>OLS48460.1 hypothetical protein BV379_09380 [Rhodovulum sulfidophilum]AB14
MREIARRQPGGTLPLLRLGLAATARAQALVLQLARAGDRALPGDYLTLGRHLHIVTAAEDPTAAFRQSVTLWPRLRRPV
A
PGDPVAMRAPWGSWALSGPETVLSVSQARVRTRTLQIMEAL
>OLS48461.1 hypothetical protein BV379_09385 [Rhodovulum sulfidophilum]AB14
MVQDCDGYRVGDIRKGWARAVRLAEELAAGQGIEIDLTMPDGKGGRKYITPHVLKHTAITWAVQRGAFLPDVASYFS
TSL
ETIERVYWHHSPDHQRSAVEAMDRRK
>OLS48462.1 branched-chain amino acid ABC transporter permease [Rhodovulum sulfidophilum]AB14
MEFGPHLMIAVLEGAVTAAVLALMASGLSLVFGVMRVVNVAHGQFFMLGAVLAWAVSSALGGGAWGFVAALVLAP
LIAGA
LAVAADLTVLKRVGYDPERTIVATIGLLYILQQAALMTYGPEARPVAPPFNTRLALPWVEFGPDGPGLIWPWGLSITSYK

LFVMAAAAAVLAGLWLLMSRTRIGLVMRATQLDGETAQAFGIPVGRVYAGVFGLGAALAALAGVLIVPIQQAHYLMG
GEA
LLLSFIVVIIGGLGSLGGTVIAALLIGLSDGIVSVFFSPTLAKILATLLVALVLVFRPEGLMGKAAR
>OLS48463.1 branched-chain amino acid ABC transporter permease [Rhodovulum sulfidophilum]AB14
MRGGRIIGLHLGLVALLFALNFVLPAYSHGNIARVMVLATYAMGYNILFGYTGLLSLGHAMFFAAGMYGLAMPMTYL
GLG
AAPAFLSGLGAAALLALGVGLLALRTTGVAFMIVTLMFSQAAWLTILLAGRWTRGDEGVVIPQAGRVLLGLDLSSPQAR
Y
LAALALFSAVLFGTLWLMRQPLGRVLIAIRENEGRAELLGYDIFRHKLGAVVLSGTVSGAAGAAYGLLFGYAGASFAEVQ
YSIFPLLWVLLGGAGTVIGPFLGTLFMFYLIDLSSGLTSAYMLVAGVALVLLTLFAPQGLAGEIRRRWWRGLP
>OLS48464.1 ABC transporter ATP-binding protein [Rhodovulum sulfidophilum]AB14
MSALLSTRALSRSFGGLRAVHEVDLDLGPGEVRALIGPNGAGKTTFVSMLIGRVAPSSGRIVFDGQDITRLPAHRRVRLG
LAYTFQITSVFARLSVAENVALAARRRFGRDRAAVAAEVARTLSRVGLTGRDDSAAGDLSYGHQRLLELAMGLAQAPRL
L
ILDEPTQGLAEAEIEAFKALVRDLAGETAILLIEHNMGVVMELADRITVLDAGLVLAEGAPAAIRADARVQAAYLGTAID
A
>OLS48465.1 ABC transporter ATP-binding protein [Rhodovulum sulfidophilum]AB14
MLELRDVAAGYGPVRVLRGLSLTVRPGEITCLLGRNGAGKTTAMKAIMGLLPLSAGRVLMDGEDIGRLPAHEVPRRGI
GY
IPQGRRLFSELTVAQNLEIGLMCRRSPPELRAHVLEMFPRLADRMGQRAGTLSGGEQQMLATARALCLDPRVLLLDEP
TE
GLQPSMIALLRDRVLALREAGVAILLVEQRLEAVQAAADRVVILENGEVRAEADAARLRAEPGFLRQELGV
>OLS48466.1 hypothetical protein BV379_09420 [Rhodovulum sulfidophilum]AB14
MTDPIRTTLDVLLELLSPLGGRRVLDIGCGRGGLAVPFRAAGADWRGLEPCPQAGGAPGIDVAEAQAMPYPDAVFDIA
VI
VNALHHIPVEAMAHALAEAARVIAPEAVLVVIEPRAEGDLSQVIATVDDETKIRYAAQAALDAAVAAGVLAETRAFDYV
R
REIYDDFEAFMGRISSFAPERGALPADLAEALRAAFLARAGREGAAFTLTQPMSARLFRRGPAAG
>OLS48467.1 acyl-CoA dehydrogenase [Rhodovulum sulfidophilum]AB14
MQTDPHQDIRDAIRALCATFPPEYHRRIDEERGYPEAFVQALTEAGWMAALIPEDYGGSGLGLTEASVIMEEINRSGG
NS
GACHGQMYNMTTLVRHGSEEQRRRYLPKIAAGELRLQSMGVTEPTTGTDTTRIKTTAVKKGDRYVINGQKVWISRVQ
HSD
LMILLARTTPLAEVAKKSEGLSIFLVDIREAMGAGMTVRPIPNMVNHETNELFFDNLEIPAENLIGEEGQGFKYILTGLN
AERTLIAAECIGDGYWFTDKVSAYVRERQVFGRPIGQNQGVQFPIAEAFIEVEAASLMRFEACRLYDAGAPCGAQANM
AK
YLAAKASWEAANACLQFHGGFGFACEYDVERKFRETRLYQVAPISTNLILSYVAEHVLGLPRSF
>OLS48468.1 CoA transferase [Rhodovulum sulfidophilum]AB14
MRPLEGIEVVAVEQAVAAPFATARLADAGAHVVKIERPGGDFARGYDDAAAGQSSYFVWLNRGKETRTLDLASEPGR
AAL
ADLLAGADVLVQNLKPGALARLGFSDAVLARDYPRLISCAISGFGTEGPLAGRKAYDLLIQAESGLSSITGGPEEPARVG
VSVVDIATGATAHAAILEALIRRGATGQGARISISMFDVMAEWLTVPLLNHEGGRSPGRVGLAHPSIAPYGAFQPRDGR
P
ILISVQSDREWRQLAEGFLGAPGLGTDPRFASNVARVRNRAETDRIVAAAFARHSAAEAEAALVAADIAFASVNDMAG
LS
AHPHLRRIEVDSPNGPVAMPAPAARFDGEPRRYGPVPALKPREA
>OLS48469.1 protein dehydratase [Rhodovulum sulfidophilum]AB14

MDLDIDHLRGWIGREDSATERLSADLVHRFAATFDRSWSLRQGAEAPPLIHLCLAQPTVAGAGLGEDGHPARGGFLPP
VP
LPRRMWAGGAFRFHAPLRIGDAVTRRSTITDVALKHGRSGPLLFVTVEHKIRARGRLALTERQDLVYRAAAPFKPSTASP
AAEGGEVRRLTPGPVLLFRYSALTFNGHRIHYDRPYACEVEGYPGLVVHGPLQATLLVQFAAELKGTCPSRFAFRSVSTV
FDTADMELHASCEGHSLRLWTAQAGGPVAMEATASW
>OLS48470.1 ABC transporter substrate-binding protein [Rhodovulum sulfidophilum]AB14
MLMLSAGLVSAQGALAQGTLRIGMTAADIPLTTGQTDQGGEGMRFMGYTVYDSLVEWDLTSADKPSTLIPGLATEW
AVDA
EDKTKWIFRIREGVTFHDGSDFTAESVVWNLDKLLDDSSEQYDPRQAAQGKSRIPAVASYRAIDPLTLEITTKTPDATLP
YQLAWILMSSKANWEAHGKDWEQVAMEPSGTGPWKLEEFVPRERAEMVPFAEYWDETRVPKLDKLVLIPLPEPNAR
AAAL
LSGQVDWIEAPAPDTIPAMTANGFVISSNAYPHNWTWHLSRLEGSPWNDIRVRKAANLAIDREGMGALLGGLMVPA
KGFL
PPGSQWFGKPSFDVRYDPEEAKRLLAEAGYGPDKPLKVKALISPSGSGQMLPLPMNEYIQQNLAEVGIDVEFMVVEW
NQM
INLWRAGAADPAVEGSQSINFTYFIQDPFTGLIRHLSCDLIAPNGTNWGHYCDPEMEDLFSQIRNTFDPAEQDEVMRR
TH
EKFVNDALFLMVTHDVAPRAMSPRVKGFVQAQNWYQNFSSITME
>OLS48471.1 ABC transporter permease [Rhodovulum sulfidophilum]AB14
MFSYLLRRLILVLPVAFGVSVICFLLVQIAPGDPLTAIMPVDATAEEQAAMRAAYGLDKPLPLQYAIWVGNLAQGDMG
RS
IATGRPVAAEVFGAVQNSLLLAVSAAVIAFPIGIFLGFLAGYFRDSALDKAVTAVSIAGVSVPNYWLGIVLVIIFSVNLG
WLPSMGAGPGGSRDWSWNWEHMRHLVLPAVTLAVVPIGIVSRTVRALSGDILSMDFVQALYAKGMTRFDVLIHVG
RNAAS
TALSVMGLQLGYLLGGSILIETVFNWPGSGFLLSNAIFQRDLPLLQGTILVLALFFVAMNLIVDVAQAAIDPRVKRS
>OLS48472.1 ABC transporter permease [Rhodovulum sulfidophilum]AB14
MTLTETLDPVQAPEARKGYWAGVAGRLARDPVSMLCLAVVAALILAAIFAPWLGLADPYKGSMIARLKPIGTPGHILGT
D
EQGRDMLARLIHGGRLTLFIGLMPVVFAFFIGSTLGVVAGYVGGRLNTLIMRVVDVFFAFPSVLLAIAISGALGAGIVNS
LVSLTIVFVPPITRVAEAVTSGVRSLDYIDAARATGASALTVIRVHVIGNVLSPIFVYATSLTSVCMILAAGLSFLGIGV
RPPEPEWGLMLNTLRSAIYVDPWLSALPGVMIFVTSLCLNLLSDGVRSAMNVRD
>OLS48473.1 dipeptide/oligopeptide/nickel ABC transporter ATP-binding protein [Rhodovulum
sulfidophilum]AB14
MSATELYDLLPDRGGPAQPLLTVRGLRKYFPVRGGILGRTQAMVQAVDGVSFEVRKGETLGIVGESGCGKSTTARLAV
GL
IERDEGDIIFDGDALGDTLSLRELRRGIQMVFQDSYASLNPRLTIEESVAFGARVQGLSDPMGRAHGLLARVGLDPARFA
QRYPHELSGGQRQRVNIARSLAMSPRLVVLDEAVSALDKSVEAQVLNLLADLRAEFGLTYIFISHDLNVVRYISDRVLVM
YLGEVVEVAPVAAIWARQAHPYTRSLFSAMPSMDPDNRTEEPPLSGDPPNPIDPPEGCRFHTRCPFAATLCGRKAPAL
RA
LDDSGHLAACHLHDPASGHPKAGKIA
>OLS48474.1 dipeptide ABC transporter ATP-binding protein DppD [Rhodovulum sulfidophilum]AB14
MTDKLLDIRNLTVRFRGARTVHAINDLSLSLGQGETLALLGESGSGKSVTLKALLGLLPPKRTELEGDIRVGGTDVLRLS
GRALQRYRGGDVSMVFQEPALALDPVYTVGAQIAETVMRHKRVRRAEAMEVALDMLRRVRIPSPERRLQNYPHELSG
GMR
QRVMIALALACRPRLLLADEPTTALDATVQIQILLLLRELQKEFGMGVIFVTHDIGVAVEISERIAVMYAGQIVEEGTTR
EIIRDPRHPYTRGLLAANLHDVARGERLAAIPGAPPALETRPVSCSFAPRCALAGPDCRAALPPVMTPAPRRRVACLRAD
EAVPAE
>OLS48475.1 amidase [Rhodovulum sulfidophilum]AB14

MLIEKDAYHAFMPYPAIEVENATEGPLAGLTLAVKDLFDVRGYRTGCGCPLKLAAATDAETSAPAVQRLLDAGARFVGK
T
HTDELAWALYGINAHFGTPVNPRAPDRIPGGSSSGSAVAVAAGLADIALGTDTGGSIRAPSSFCGIWGLRPTHGRVPLA
G
AMALAPSYDTAGILARDGATLLQAAGALMEPDPVPLPEIPDLLWPADMVAQLDEAPRAVLEAAFGTMPSREVEVYPE
GAA
AAYAAYLVTNGADARDAVLPFIRGSGMPLVRGIDGRAAAAEALDEAEIDKARATRHAFTAHLEEMLVDAVLLAPAVHA
AP
FALDAPVEVFDGFRHDAMRLLCVAGLAGLPQVVLPAGVVEGAPYGVSLIGPRGSDLSLIALARRLVPEPQQ
>OLS48476.1 hypothetical protein BV379_09480 [Rhodovulum sulfidophilum]AB14
MTVCPVESRLYDLGQRLPDSAPSRALFLPGKISGNLLFLSGQICEWEGVPRYFGPARDDFDLAEAQEAARICALNLLFSV
RSLLGSLTRVREVVRVGGFVSAPPGFEGGPMIVDGASQLFVDIWGEAGKHARTAVCVSSLPANALVEIDAVLEIG
>OLS48477.1 ABC transporter ATP-binding protein [Rhodovulum sulfidophilum]AB14
MPEPLLEIRGLTRRYRPRRSLLARSPEVVALDDVSLSVGRGESLGLVGESGSGKTALTRAILRLEAPDAGQILYRGQDVA
KFGPRALRAFRARVQIVFQNPQAALNPRMTAEQIVTEPMLVHREIVGLDRRGRRDRAAELMAMVGLRTGHLRRFPHE
FSG
GQQQRIGIARALAAGPEMLLLDEPTSALDVSVQAQVLNLLIDLQDRLGLTYLFISHDLGVVRYLCDRVALIRHGRLIETG
PAAQIFDAPQSAYARMLIAAAPELRQPG
>OLS48478.1 ABC transporter ATP-binding protein [Rhodovulum sulfidophilum]AB14
MVPPLLEIRDLHLRMTTSDGPAHVLNGVGLSVARGEILGLVGESGCGKSLTGLSVSRLVPSPPAHYPSGEIRLDGQDLMT
LSERRMRALRGRRIGMIFQDPGTNLNPTMRIGDQLVDAALAAGRHAPDLLGPGPARPAARALAADMLERVGIASARQ
RLN
AWPHEFSGGMRQRVLIAMALIGRPDLLIADEPTTALDVSVQAQILTLLRGMVADRGLGVILITHNMGVVARTCSHVAV
MY
AGNVVETGPVAEVLERPSHPYTRALMAAIPGRNTRRGTLKGLPGTVPDLMEPPPGCRFAPRCPRAKAACRAALPATRR
IG
PGHEVACLDPVAEAADA
>OLS48479.1 D-ala-D-ala transporter subunit [Rhodovulum sulfidophilum]AB14
MNAVPDTPLRPRREGLRRALSRFRQSRLSTVGAGLVALVLATAALGPFLAPYPDHVAGGIDTAARFLPPSARHLFGTNEL
GQDVLSLTLAGASVSVLAGFAVVLIGSAIGTLLGGIAGFAGGWIDEILMRLTDLVLTVPNLILAMAIAAALGPGFQNMVL
AISLGWWPGYARLVRGEVLSAREEVYVTAARAMGASRARVLFCHVLPNCISPVIVKMSLDMGFAILAVAALGFIGIGVR
P
PVPEWGTMLATARSYMPDVWWTAMAPGLAIFVSVFGFNLLGDGLRDLLDPRGGR
>OLS48480.1 peptide ABC transporter [Rhodovulum sulfidophilum]AB14
MLSFLLRRLLMLVPVLVGLTLIMFAIARLLPGDPVGLAAGPNATLAERAALAQEFGLDRPVWEQYLSYVAGLARGDLGR
S
LMTRRPVAEDIATYLPATLELVLAAMTIALAVGLPLGLLTALWRNRWPDYLSQVLAIGAISMPRFFLGLLLQLVFAMWL
M
WLPLGGQLPIVTFPPPRVTGFVTLDAALAGEWQTLGAACRHLALPALALSLSPLAAIMRTMRAAAIEVMAQDYVTTAR
AL
GLPRRKILLKTVGRNAISSTLTVTGLYIGWLLGGTVLVETVFDWPGLGLYATRAILSQDMMPVIGVALVSGLIFVFVNLA
IDLAYGLVDPKVRYG
>OLS48481.1 ABC transporter substrate-binding protein [Rhodovulum sulfidophilum]AB14
MPNSPQAPTRRHVLALAGAGLAAGTIPASLLASDQRLMIIASGQDFPNIDPHTSSGSAPAFFMRNVYDPLLRSSGTPPE
P
VPGLAQSWEASEDGMVFTFRLDPAARFHSGQPVTARDVVYSLARALRLAKGNSWMLRNIVTPEGLEAVDPATVRITL
ATP

FAAFLSVLPWFPVVEKAAVEAHLGEDDGQSWLMSNTAGSGPFELWQSRPGALYRFIRSPNAWQPGGGNLDGVIWR
VSREA
ASERLMLQRGEVHAALDLTSEDMDALEGAPGVVRVIEPEYRTFSIKMNTRHGPLADINMRRAISHAVNYRALLDSAGY
AE
LMVGPLPNGILGHDPGLEVPRHDLEAARAHLARTATPEGGITLKMVYVSHLDQERRYAMVLLDSLKALGIGLEIQPLSW
P
DMVAACARPETFPDFFPVYQTANYGDPDNIAYAAYHSASNGNWQNAVYSNPEVDRLIEAGRAEMDPDKRRQIYGAF
QRAV
VADAPDIFGVLEKRKLGLRASIRNYAFTPVASNAIECWPLSLT
>OLS48482.1 methylhydantoinase [Rhodovulum sulfidophilum]AB14
MTLPSGKWRLGFDIGGTFTDFVLHDGATGALTLHKRLTTPQDPSEAALAGLDEILAMQGIGPGDLSEIVHGTTLVTNAV
I
ERTGARTGLIATEGFGDILEMGTEQRYDIYDLFLPFPEPLVPRDLRLEVPERIDASGRVLAGLDEAALERAGAELVGAGC
EAVAIAFLHSYANPAHERRAAAILRSAFPDLAVSVSSDVVAGIGEYRRTATAAANAYVQPLMHRYLGRIESALAARGFAG
ALRLMHSAGGLIPLETARDFPVRLLESGPAGGALATGLFGRAAGLSDMISFDMGGTTAKAALIEDGQAAIAPEIEAARV
R
RFTRGSGLPIRTPVIEMIEIGAGGGSIAAIDEVGLLKVGPRSAGSDPGPACYGLGGTEPTVTDAALVLGYYDPGFFLGGR
MALDPEAARRAVARVADPLGLSVEEAALGIHKVVVENMAAAARVHIVERGRDPRDFAMVGFGGAGPAHAVDVARA
LGIAR
VVVPPASGAASALGFLAAPLSFEAARTLRVALGDRIDAAAINRALDAIEAEGRAHLARAGIGSTGITVERSAEMRLRGQ
M
NEISVPLPEGRLSPEDGPRLRTAFEAAYAARYTSPFEGAEIEVIALRARVSGPAPALGTGGASGGATPGKGDRSASLKGT
RSCLFEQGRFETAIHDRYALVPGERIAGPAIVEEREATTLIGPEDVARVDAGGALHIEVARPAAAAALVTAGMSRAEAVA
RIEADPIGLEIMWARLVNVVEEMWLTVCRTAFSLIIAEAQDFACELLDPEGETLAHSPRAMPVFNLTLPRAVKALLARYP
AETLKPGDVLITNDPWLCAGHLFDIALVSPAFRDGRLVGLMGTVGHVSDIGGTRDSLHAREIYEEGLQIPPMKLFDGGR
P
NETLIELIRQNVRHPDQVLGDIFSFAAANALGAERLDAFMADYGMHDLRALAEVVQGLSERAMRAAIRALPDGEYRSTI
V
SAPQGEALEFPVRVAVEGDAMLVDFEGAPPQLPQGGLNSTLNYTAAHATYPLKCMLTPGVRGNAGCYRPFRVTAPEG
SVL
NPTFPASVNMRTRTGWYLAPNIFRAMADAAARGVQAHTGLPSSVHIYGRDARGDLYSDLLLLGGGQGASAHGDGHS
ALLW
PTSAANTSVEMIESRTPLLVLCKRFAPDSGGPGRHRGGLGQILRFRKREADGTPMQVLLHPEGTARPIPGLFGGQPGCK
A
SGRVLDPAGRVLRDVGHGALLTLTGTEEIVELTLAGGAGYGPAEERAAAALARDLALGLVSPEAAATQYRMPHAVPDD
ET
VG
>OLS48483.1 lysine 2,3-aminomutase [Rhodovulum sulfidophilum]AB14
MDETHAPDAKPLRNGTAQPRALPSARPPGAARRRAAFPSSPEARAFRRRFFPEASAADWSDWRWQLRARIRSLDGLA
QVF
SLSEDERAAVARRQSGLPVGITPYYASLMGLDDPAEPLRRTHIPVGQEYLRLPGESDDPLGETPDSKVPGLVHRYPDRVL
FLATGTCSTYCRYCTRSRMVGQPGGEEGISTRNWERAFAYIAAHPQIRDVLVSGGDPLTLSDDKLDYLLGRLRAIPHVEF
IRIGTKVPLVLPMRITGALTRMLRRHHPLWMSLHCTHPAELAPEATEALTRLADAGIPLGAQTVLLRGINDTVETMRTLC
RGLLKRRVRPYYLLQCDPITGSGQFRTPIETGLAIIDGLRGHLTGYAVPHFAIDAPGGGGKVALVPDHVAGREGDDLLLR
NFEGRIYRYPDPGGRTGARD
>OLS48484.1 hypothetical protein BV379_09525 [Rhodovulum sulfidophilum]AB14
MREVDLAVVGAGPAGMAAAAEAAQAGLRVALLDAQPWPGGQVYRDIYRAGQEQEADLGAAPLEGGRLIAGLDRPG
ISLIG

GAEVSAIEPGIAPGYRLSYRQDEAAETLQARRVLLATGGLERPMPVPGRTLPGVLTVGAGQILLRQAGLLPRRGVLLGTG
PLLYLFAVQMMHAGVTPTALIETQTRGDLARAMCHLGGIFGGWSYMSEGWRMLRLLHRAGVPRITGAHEVVIEGEQ
RAEA
VRFLRRGAQHRLPCDAVLLHHGVVPNPHPARALGLTHRWMPRQHCFTPVCDDWGEAAAAEGGVEGIFVAGDGAGI
VGPRA
AELQGRISALRIAETLGRIDLGTRDALAAPLRRQLRRELAVRPFLDAAYPPFAEADGQDRDATGLGSPLPAFGTALPGGV
PGILAETGENGRETVADAAGWLPVGGADEGLRDAPEAEKTATGAACHAGEA
>OLS48485.1 hypothetical protein BV379_09535 [Rhodovulum sulfidophilum]AB14
MADASTSTRCYWIRDQDGAEILIPMCWGAAIGGPAECTCNVPESRIETAERGRAEAERQVLRLMDERDRRLYEQARN
WRR
NKLLRQRIEDLETQLAGKTN
>OLS48486.1 RNA-binding protein [Rhodovulum sulfidophilum]AB14
MATLHLPLKGAYFDQIKSGEKIEEYRLVTPFWTKRLEGRAYDRIELAKGYPPKGDTSRRITRLWRGARKTTITHSHSISA
PAPAVPGSRLSS
>OLS48487.1 hypothetical protein BV379_09550 [Rhodovulum sulfidophilum]AB14
MIGPVTSPSVMPVRKEQTEQQAATQAEFRPEFANIKPGDEVTHLFKLHKASDEFMHMDKFMAMIAEAEAERQWNES
AEGK
AWHAEQLAKQDAQPVELAVYKGGEMVGFMAGGFTAQSSIAGFLPRDWDSMSIDDLSQAFRSHGLKVVAAKDGLSM
NHGQA
ANIMNLL
>OLS48488.1 hypothetical protein BV379_09555 [Rhodovulum sulfidophilum]AB14
MLHELPPGERDYVSIRAPDFALGETANRIVGNDLSNTITGNKYDDYISGGKGNDTLKGMDGDDVLSGGAGNDRLEGG
NGD
DALYGNTGTNRLEGGAGNDTYYGGDQDDLFIFDSGLDVVSSMYGADPGHDVLQFANVAGADLRFYAVEDGASLYITS
VQD
AADGSLDSGVIVEDYFAAQHLGLVDAVVGYDWIGYDLA
>OLS48489.1 anion permease [Rhodovulum sulfidophilum]AB14
MPDTPTLLSMLLLLLSIGAIAGVLAGLLGVGGGIVLVTAFFYAFQALGYDSAYLMQICLATSLATIIVTSTSRSVLSHAKK
GAVDWQVLKGWAPGIAVGAVIGVLVAAALRSVVLQGIFGGLAMAVGLYMAFGRNDWRLGDSMPRGLKRAVLSPVV
GFLSV
LMGIGGGSFGVPLMTLHGAPIHRAVATASGFGVLIAVPSVLGFFFVQIPDGARPPLTLGAVNIPAFLVIIAMTLITTPLG
VRLAHAMDPKPLKRVFAVFLILVAGNMLRKAAGL
>OLS48490.1 penicillin-binding protein [Rhodovulum sulfidophilum]AB14
MIRFFLSLLGALFSFVTLGVAAAVLMIGAVFWMYARDLPDHEDLASYQPKTISRIYSGEGRLIDEFAEERRLFVPAEEIP
DLVKAAFISAEDKNFYTHHGYDARGMAAALFDAVKSRGRNVRGASTITQQVMKNFLLGGERTGERKIKELILATRIEETL
SKDKILELYLNEIFLGQNSYGVAAAAQSYFDKTLSELRPEEAAYLAALPKAPSNYHPVRNHDAAIGRRNYVLHEMHDNGY
LDDATYQAALAAPLRTVQNGDYPPFSASLPPRDYFTDEIRRQLSQDFGQEEFFGGGLTIRATLDPELQTEAAEALRRGLE
KYDRSRGVWRGTGKSIDPAELDTEDHWRAALAASDVPRDIRLDGQWLPAVVLTVEDQQMRLGIEGVEDGEAGPALV
PRKD
ISWLRGDFHDNFKVGDVVLVRRMTGEDGGFIRWSLRQVPEIQGGFVAMDVNTGRVLAMQGGFSYQASVFNRATQA
ARQPG
SSFKPFVYAAALDSGFTPATIVIDAPIEVDTGGGAIWRPKNSSNKYYGPTPLRTGIERSRNLMTVRLAREVGMETVARYA
EKFGVYDRLEPYLANALGSQETTLYRMVAAYAMFANGGERVEPTLVDRVQDRRGRTIYRHDSRICTDCGAVSIPEGQG
PT
IVSDRERVMNAITAYQLTSMMRGVVERGTAAGRVRLGVPTAGKTGTTNDSRDVWFVGFTSNIVAGCYMGFDRPRGL
GRGS
YGGSMCAPVFNEFMKVATAKYGGGPFKVPPGGRFIKIDRYTGERLADDASGENVVAEYFREGEEPVFGIGAMVDGGF
AMG

SNLPLFGRGEEETYEDTPSEEVTTSTGRKAVVGPKANFGTLSSGGLY
>OLS48491.1 peptide chain release factor 2 [Rhodovulum sulfidophilum]AB14
MRAEAQNTVEDIRKSLGLLAQRLDWETAQHRLEEFNAMAEDPELWNDPERAQKLMRERQALVDAIDTYKGISQELED
NIE
LIELGEAEDDQEVVREAETALKTLRETAAAKELEALLDGEADGNDTFLEINSGAGGTESCDWASMLARMYVRWAEKHG
YD
VELQSESAGEEAGIKSATYKITGRNAYGWLKSESGVHRLVRISPYDSAARRHTSFSSVWVYPVVDDNIEIEVNPSDIRID
TYRSSGAGGQHVNTTDSAVRITHMPTGIVVTSSEKSQHQNRDIAMKALKSRLYQQELDKRNAEINAQHEAKGDAGW
GNQI
RSYVLQPYQMVKDLRTGVETSDTQGVLDGDLDQFMAATLALKVSGKSRAEAQAED
>OLS48492.1 DUF1289 domain-containing protein [Rhodovulum sulfidophilum]AB14
MTDDDTAPIETPCIRTCRVDPASRLCVGCLRSMEEIGHWGGMSPAERRRIMAELPARRGRISPDAAALGALRRKRG
>OLS48493.1 Holliday junction resolvase RuvX [Rhodovulum sulfidophilum]AB14
MHDDLEEFAAALRPPTALIGLDFGEKTIGVAVSDPFWSIATPLETVRRKKFTLDAGRVLEIAAGREAGGIVLGLPRNMDG
TEGPRCQSTRAFARNLSRLTELPIGFWDERLSTVAAERALLEADTSRRRRAEVIDHVAAGYILQGLIDRLRNLTATP
>OLS48494.1 c-type cytochrome biogenesis protein CcmI [Rhodovulum sulfidophilum]AB14
MTFIVVAIGMAAVIAAVLTLTLVRGRRGDEPAAAYDLRVYRDQLDEVERDLGRGVITEDEAARIRTEVSRRVLEADRKLA
RQSGSGQAPKALSLAAGVAVLAVLMGGTWITYSANGAPGYADQPLDLRKTEAADYRATRMKQAEAEEQAPPMPGLP
TPDA
EHLELVKKLREVVAQRGDDPQGLALLAHNEAALGNFRAAYEAQKRLIALKGGSATAADYATEADMMVLAASGYVSPE
AEA
ALEQALNRNPQNGTALYYWGLMHIQTGRPDIAFRVWRELLSLSHAGDPWLPIVREQMPNLAWYAGDERYQLPPMP
AEAGA
PGSATGPSAEDIAAAAEMTPEARDEMVRGMVARLSARMADQGGSPEDWARLIGALGVLGDSEQAGAIWAEARQTF
AADPE
ALATIRAAAQSAGVAE
>OLS48495.1 sarcosine oxidase subunit beta [Rhodovulum sulfidophilum]AB14
MKRYSAFAIAREAASYHAGWERAWTSPEPKDRYDVIVVGAGGHGLATAYYLGKNHGVRNVAVIEKGWLGGGNTGR
NTTII
RSNYLQDPSAAIFEKARSLYDGLSQDLNYNVMFSPRGVLMLAQSEHEARGWKRTAAANVLQGIATEYVDAKRVKEIVP
II
NIDGPRYPVLGGLWQKRGGTARHDAVAWGYARACSAMGMDILQQCEVTAIRQKAGKVTGVATTKGEIGCGKLAIVV
AGHS
GVLAEMAGFRLPIESVALQALVSEPIKPVIDTVVMANTVHGYMSQSDKGELVIGGGADGFNNYSQRGSFQHVEDTIRA
LI
ETFPIISRLKMLRQWGGIVDMTGDRSPIISPTPLGDCFINCGWGTGGFKAIPGSGWAMAELVAKGRPGPLSAEFGLDRF
A
EGRFVDESVAAGVAH
>OLS48496.1 sarcosine oxidase subunit delta [Rhodovulum sulfidophilum]AB14
MLVLTCPCCGATAEETELAPGGEAHIARVGPGADASAFEAYLFHRKNPRGVHFERWRHAHGCGKWFHAARNTVTLE
VYGT
YPAQTPEPPRELLEKIAAKVPGFDPEART
>OLS48497.1 sarcosine oxidase subunit alpha [Rhodovulum sulfidophilum]AB14
MSFRLPSGGRLIDRSARLGFTFDGAECDGFAGDTLASALLANDRMLVGRSFKYHRPRGIVASGAEEPNALVGLGSRGRY
E
PNQRVTTTELFDGLEAESQNRFPSLAFDIGAINSRLARFLPAGFYYKTFLKPAAFWEHVYEPLIRRSAGLGRAPAEPDPD
SYEHFHVHTDVLIVGGGVAGLQAALVAGRAGARVLLVEQSPHWGGRAAVDGVEIEGHPAENWVAETVAALKRLSNV
TLRL

RCQGAGIYDHGFALAHERLSDHAPGSAGPRHRLWKIRTRQVIVAAGAIERPLAFPGNDVPGVMLASAMRDYVENWA
VAPG
RRSVVLTNNDDAYRTALCLLEAGLEVPAILDTRAEATGPLPEIARKAGIQVRTGMTVVDVAGGKRVRSVGIGPVDGKGY
P
TDRIACDAVAMSGGWSPVVHLWSQAGGKLFWDPKQSSFRPDADRAPKSHDGQTFVHAAGSANGALLAKRCQLDA
GRAGAA
AALAAGFDAEPVDAVAAAPEQQPIEPAWILPRRAGPDLRMKMFLDYQNDVKVSDVQLAAQEGYESVEHTKRYTTLG
MATD
QGKISNVNGLAVLAEALGEDIPAVGTTTFRPPYTPLTFGAIAGPGIGATFQPERRTPLQDWHEAQGAYFEPVGHWRRP
YC
YPARGESHGKAVRREIRAVRDAVGLLDASTLGKILVKGPDAGRFLDRVYTNMMSSLKVGRCRYGLMCSENGFLMDDG
VVV
RLSEDSFLCHTTSVGAEHVHQHMEEWLQTEWWDLKVHVVNQTEQFAQIGIAGPKAREVLERLGGMDLSKEALPFMA
WAEG
ELGGFPVRVYRISFSGELSFEVAIRAGQGRAFWDACLEAGADLGIAPYGTEALHVLRAEKGFIMIGDESDGTVIPQDLGL
DWAVSKKKDDFIGKRAQERSHMTDPERWKLVGLETLDGSVLPDGVYAAASGRTANGQRNTQGRVTSSYYSPTLRRGI
ALG
LVLHGPERMDEVIEFTCADGSTVRARIVSPVFYDPDGEKQNV
>OLS48498.1 hypothetical protein BV379_09640 [Rhodovulum sulfidophilum]AB14
MSEPVTALGGACIEGLVRIVEQPLQGMIALKGDLADAKIRNAATGVTGIDLPGPLEGRIEGERGLAWMAPDELLLFVPY
A
EVARTMDTIGRALKGRHATLADMSDARAVFRLDGAAGREVLARLTPADMAPEAFGPGRFRRSRLGQVPAAIWCESPD
RFG
LICFRSVAVYVRDLLTAAAAAPPVGYFPAGG
>OLS48499.1 superoxide dismutase [Rhodovulum sulfidophilum]AB14
MAFELPALPYAHDALADSGMSKETLEYHHDIHHKAYVDNGNKLIEGTEWASKSVEDIVKGTYQSGAVAQSGVFNNAS
QHW
NHSQFWEMMTPKPVAMPSELEARLKDAFGTVEKFKEDFAAAGAGQFGSGWAWLVVDTDGTLKVTKTENGVNPLCF
GQTAL
LGCDVWEHSYYIDFRNKRPAYLTNFLEKLVNWENVAERLSKA
>OLS48500.1 acetyltransferase [Rhodovulum sulfidophilum]AB14
MENLRPSTAKDMPRVLYIWRRAVDATHEFLAPEDRAAIERELAVFLPTVPLCLAVDDADVPQGFMLLHEGHMEALFV
DPA
CHGRGIGSALIRFALKAHPGLTTDVNEANRRALAFYEARGFEKTGRSALDGQGRPYPLVHLRHRSAGSAASDT
>OLS48501.1 hypothetical protein BV379_09655 [Rhodovulum sulfidophilum]AB14
MFSKSRINEPGPKAGEAEKAKAFEAPTAPRSGDAPIPSAPRAKPPASMLSSDLVVTGNLKTTGDIQIEGTIDGDISAHLL
TVGENATVRGEVVADDVVINGRVIGRVRGLKVRLTSTARVEGDIIHKTIAIESGAHFEGSVQRQDDPLNTGKRAQQAPA
A
GQAQQTRPQQGSAAAAPTAHGKPAEGAPKA
>OLS48502.1 peptidase M23 [Rhodovulum sulfidophilum]AB14
MRQTLTARFLHRIDVALERYLPEKRLFLRSEQSTRFVRLSPLTQAAALSGTTALVAWTMVASSILLIDQVGSGNIRDQAR
REQALYDSRLNAIAAERDRRAEAVRQAQDRFNLALDEVSKMQSALLASEERRRELETGMTVLQATLRRTLAERDAAQD
RS
IELAAQLAGETGPADGPRPADLEQTLTFLTAALDETSAERDKVAEDAASAYEMVQAIAYERELDAERNDQIFTSLEEALT
VSMTPLDKMFKAAGLNTKSILNRVRQGYSGVGGPLTPLSVSSKGSATELSPDEIRARAIMGRLDEINMYRIAAEKLPFAQ
PLKSSYRLTSPFGRRTHPTLGGSRMHEGTDMASAYGTPIYSTADGVVVRAQWVSGYGRMVEIRHEFGIVTRYGHMSKI
RV
KAGQRVSRGDRIGDMGNSGRSTGTHLHYEVRVDGKPVNPMTYIKAARNVF

>OLS48503.1 peroxiredoxin [Rhodovulum sulfidophilum]AB14
MPDTLTETGQTAPDFTLPRDDGAPVTLSAQRPAPVVLYFYPKDDTSGCTKEAIAFSGLLAEFEAAGARVFGISKDSLAKH
GKFRSKHELSVPLLSDEDGDVCERYGVWVEKSMYGKRFMGIERATVLIDGSGRIARIWRKVKVPGHAEEVLEAVRAL
>OLS48504.1 hypothetical protein BV379_09670 [Rhodovulum sulfidophilum]AB14
MSERESPSDGSGRPPHQRLGLGLLLGLAMLAGALGLAALVLTERMLVLPDWAVARVEARVNAEISPLMLGLDSVGLRL
SR
SEAPLVRLGGVTLTDAAGREVLRLPRAEARLDARALLGRRLEVSHLALTGAEVTLRRGADGRIDLALGGTGAPLGIGGSL
AEILDQIDRSFDAPALAPLRSVSVEGLALTYVDARADRTWRVENGLMTLDQTAESVVAQAFFSLRGAGAVASEFAFGFT
S
FRGSPAARLSAGFSDVPSADIATQSPALAPLALIEAPISGAMRSEIDESGRIGDLTAALEIGQGALTPPGGAAPIRFDSA
QSQFTYDAREGRIELSRISLDTAALRLAGKGHADLVAPGGGWPEALVAQLRFGEVGLDPEGLFETPAAFPGGALDIKIAL
DPFEAQIGQLVLSDEEGSYRASGRVRALPGGWDLTMSADLDSIDHQRLLALWPVGMAKGARDWLSANLQGGVLYDA
HLGL
RRSPGGPSRLALGARIRDAVVRFLPEFPPLRDGRGYLAIEDRRLTVSAEGGRVEAPEGGPVTVAGSTMEIPDLAADPVKA
VFALKTEGTIPAALSLIDLPPLTLARRAKLPAHPAEGRARMAVGLRLPLIRDLPIEKVGYRVAGVLEDVVSETLVPGRRL
SAPRLDLRADDEEVSVSGAAALDGVPLRARWSRAMTGSAASAITGTVELSERANKAFGLGLPEGSLSGATRGTFTLDLA
P
GAPPKLDLSSDLTGLGLRLDAVGWSKPAARAGRLTLEARLGDTPAVERLSLEAPGLSAEGQVSLRPGGGLDTARFSQVR
L
GGWLDATVTLTGQGADRPPAVAVTGGSADLAAMPQRFGAGGGGAVDGTVAVTLDRVRVGNGLQLTGVRGQLSGS
SGGFEA
RVAGRAPISVSLMPARSGMAVRVRSADAGAVLGAAGIYGRASGGTLDLVLNPTGKAGHYDGNARIANLRVGGTPVTV
ELL
SAISVIGILELLDGEGLSFSDVEAQFRLVPGAVEIREGSAIGPSLGVSLQGVYLTGSKRMDFRGVLSPVYMFNGIGSVLT
RKGEGLLGFNFSLKGDPADPEVGVNPLSILTPGMFRELFRSPAPRLKEAP
>OLS48505.1 tRNA preQ1(34) S-adenosylmethionine ribosyltransferase-isomerase QueA [Rhodovulum sulfidophilum]AB14
MKLSDFDFDLPEALIATRPVRPRSAARLLVAEGDAIRDLHVRDLADLLGPGDRLVLNDTRVIPARLSGLRRRETGQGPAE
ARVEVTLMEPAAGGQWRAMARPLKKLRPGDRIRFSDALSAEVVARGPEDATLAFDCEGAAFDAALAEAGAMPLPPYI
AAR
RPADAQDREDYQTVWAARPGAVAAPTASLHFDAALLAALEARGVAFTHVTLHVGAGTFLPVKVDDVSEHRMHAEW
GEVSE
AAAAEINATRAAGGRIIPVGTTALRLIESAADAEDRIAPWRGETDIFIRPGYRFRLTDALITNFHLPRSTLLMLVSALMG
RARIRKIYLHAVEHGYRFFSYGDASLLIPPGRDRRDSSRPKAPPSGPTA
>OLS48506.1 hypothetical protein BV379_09685 [Rhodovulum sulfidophilum]AB14
MPRREQIISFWMEEVGPAGWYKADPEVDAAIVARFKGDWEAAHSGELHEWVTNPAGAFAFLILTDQFPRNMFRGD
WRAFA
TDALARAASKQAIEHNFDMRYPEPERQFFYMPLMHSECLMDQDRCVRLMLTRMPETGADNLLHAKAHRELIRRFGRF
PFR
NAALHRGSSEEEIAFLESGGYGACVEELRASA
>OLS48507.1 dihydrolipoyl dehydrogenase [Rhodovulum sulfidophilum]AB14
MAARNFDMIVIGAGPGGYVAAIRGAQLGLSVAVVEREHLGGICLNWGCIPTKALLRSAEVFHLMHRAKEFGLKAEGIG
YD
LSAVVKRSRGVAKQLSSGVGHLLKKNKVTVVMGEATLPGKGRVSVKTDKGTEELTAKAIVLATGARARNLPGLEADGK
RV
WSYKHALQPPHEPKKLLVIGSGAIGIEFASFFNTLGAETTVVEVMDRILPVEDAEISAFAKKQFEKQGMTIREKATVKQL
DRSADKVVAHVEQNGKVEKIEVDTVISAVGIVGNVKGLGLEDLGVRIDRTHVVTDDYCRTGVEGLYAIGDLAGAPWLA
HK

ASHEGVMVAELIAGQKPHPIKPGSIAGCTYCHPQVASVGLTEARAKEQGHEVKVGRFPFIGNGKAIALGEAEGMVKTV
FD
ARTGELLGAHMIGAEVTELIQGYVVGRQLETTEADLMETVFPHPTLSEMMHESVLDAWDRAIHF
>OLS48508.1 hypothetical protein BV379_09705 [Rhodovulum sulfidophilum]AB14
MTTQSLLAKNVGGIDRALRIIIGALMVIAALVGYSNWLLIGIVPLATGLMSSCPLYSLIGVNTCKKD
>OLS48509.1 Crp/Fnr family transcriptional regulator [Rhodovulum sulfidophilum]AB14
MSWLARAEGLGGIEPEIAARLDMLPVSPLPKGQVLFCPGEAARGFTILLTGRVEVFLIGPTGREILLYAIEPGQSCVQST
LGLLGGEDYTGEAVTATDCEAVLVPKDRFLSLMDDSPVFRRFVFGAFAQRLQSVMALLERVAFQKVEGRLAADLLDRA
AG
GAVHATHQEIATRIGTAREVVSRRLDALARAGMVRLDRGCVTIEDAQGLARIAAADGIR
>OLS48510.1 3-hydroxyisobutyrate dehydrogenase [Rhodovulum sulfidophilum]AB14
MKIGFIGLGNMGAPMAANLVQAGHEVTGFDPSAPCPEGVTMAESAAGAARGREVVITMLPNGQILRAVAAEILPAM
AGGA
VLLDCSTVDVDSARAVAAEAGAAGMLAVDAPVSGGVGGATAGTLTFMAGGSDEAFAKVRPLFDIMGQKAVHCGPS
GNGQA
AKICNNMILGVTMIATCEAFALADKLGLARDKMFDVVSTSSGYSWSMNSYCPAPGIGPASPADNGYKPGFAANLMLK
DLR
LSQQAAETADADTPMGARAAELYATFVEQEDGSGLDFSAMLPRFEKRGRG
>OLS48511.1 enoyl-CoA hydratase [Rhodovulum sulfidophilum]AB14
MSDIDIRKQGRIGRITLTRPKALNALSYEMSLVIEAALDAWAPDPEVRMILIDAEGDKAFCAGGDIAVMYETGTRGDYA
Y
GQKFWRDEYRMNAKLFEFPKPVVSLMQGFVMGGGVGVGCHGSHRVVGESSQVAMPECGIGLVPDVGGSLILARAP
GRMGE
YLGVTGRRMGPADAIYAGFADYFLPEADWPALRDELIATGDWEAVDRAARTPPPAGELEALQPRIDALFAGERLLDIVN
A
LSAEGSGFALSALKVMERNSPLAMACAIEMQHRLKGSDDIRRALALEYRFTYRAPEHSDFLEGIRAAIIDKDRKPRWKHP
GLDAVPGADVARMLMPLGEYGLKLEEELQ
>OLS48512.1 acyl-CoA dehydrogenase [Rhodovulum sulfidophilum]AB14
MDFAPSEEQRAIFDLAREFGAEHIAPHARTWEAEGTIPRTLWPELGALGFGGLYVRDSSGGSGLSRLDATLVFEALSMA
C
PSVAAFLSIHNMCAKMIDAFGSGEMRARVLPEALSMQTVLSYCLTEPGSGSDAAALKTRAVRGAGGWALIGTKAFISG
GG
YSDAYVVMARSGAAGPKGISAFLVEDGRPGLSFGGLEDKMGWRAQPTRQVQFDNCAIPAANLLGEEGHGFRYAMA
GLDGG
RLNIAACSLGAAQAALDATLSYMGERQAFGQSLDRFQALQFRLADMEIELQAARIFLRQAAWKLDRGAPDATRFCAM
AKA
FVTETGSKVADACLQLHGGYGYLADYGIEKIVRDLRVHQILEGTNEIMRLIVARQLLEARG
>OLS48513.1 hypothetical protein BV379_09730 [Rhodovulum sulfidophilum]AB14
MKTTVTLEDAAGSVRIGYRVAAAVNQGDIAAGRLDPGGDGAVNSILWSEAGPLPDFETDMRNLQADTIGARFDFGVT
IGA
VG
>OLS48514.1 hypothetical protein BV379_09735 [Rhodovulum sulfidophilum]AB14
MPELVPVPAQVPLPAADGLLLAGLVEFDARAAEGVSSWLKRRRASFGVLDCVSSWLRCHSGALSK
>OLS48515.1 carboxylate-amine ligase [Rhodovulum sulfidophilum]AB14
MSASEPDFTLGIEEEYLLVDRETCALAQAPKDLMAACAADLGDQVTPEFLQCQIEVGTRVCRSVAEAREELRRLRATVA
H
HAAQFGLAPIAASCHPSADWLDQNHTDKDRYNDLDRALAAVARRMLICGMHVHVGIPDPDQRIDLMTQFSYFLPHLL
AMS

TSSPFWKGVDTGLASYRLTVFDNLPRTGLPPRFESWSEYDRSVGTLIRLGLIEDATKIWWDLRPSARFPTLETRICDMSP
RLDHTLSLAALIQCTLRMLWRLAGENLRWRIYERFLVGENRWRAQRYGVSEGLVDFGRKELVPFADLLEELIQLVEADA
A
VLGCLDEIHAARDLIAHGTSAERQRNAHAAALATGADSEEAMRSVVRLLIEEFGADL
>OLS48516.1 aminoacyl-tRNA deacylase [Rhodovulum sulfidophilum]AB14
MSKSLKRVRAALEAAGITPDIRELGQTRTAQEAATAVGCDLDQIAKSIVFRGEQSNSAILFLTAGGNRVSDEKASALAGE
PLGKADAALIRAQTGFAIGGVAPVGHLSPIRTFLDPRLLEFDTVWAAAGTPRHVFPLDAHALPGLTGAATADFTQ
>OLS48517.1 glutamine-synthetase adenylyltransferase [Rhodovulum sulfidophilum]AB14
MSFASRLTRAPLAHEPDRAAEAADRFAAQPPEIRALLEGTAGCSPYLWGLMTREADWLEDLLTGAPEPVFAALMDEV
RGL
SLEALKPGLRQAKRRAALLIALADLGGLWPLEAVTGALTDFADLCVDVTIRRLVAAEIARGRLPGLSEADAETAGGMVAL
AMGKQGAHELNYSSDIDLICLFDQDRFDPADFHEARSAFVKITRRMTAILSEATGEGYVFRTDLRLRPDASVTPVCMSM
E
AAERYYESLGRTWERAAHIKARPCAGDIEAGWAYLDRLRPFVWRKHLDFAAIQDAHDMRLRIRSHKGLHGGLVLEGH
DMK
LGQGGIREIEFFTQTRQIIAGGRDPELRVRGTVEGLARLAAKGWVPEEVAETLTADYRAHREVEHRLQMVNDAQTHLL
PN
SAEGIVRIAHFCGAPEAGPWRDALAARLRRVAGLTEGFFAPGKPAEDLPEIPKTAAEIVERWPRYPCLRTTRAQEIFNRL
KPDLFSRLTKAPRPDEAFAAFDGFLAGLPAGVQLFSLFEANPHLIDLLIDITSVSPELAQYLSRNARVLDAVIGGSFFED
WPGRAALTGALDDRLGALGDYEAQLDAARAWVREWHFRIGVHFLRGLIDARTAGAEYADLAEAVLAALWPRVVAQF
AAKH
GDPPGKGAVVLGMGSLGAGALSARSDLDLIVIYDADGVEASDGRRPLASRAYYAKLTQALVTALTAPMAEGRLYEVDM
RL
RPSGNQGPVATSLQSFRNYQRDEAWTWEHLALTRARPVAGDAALGAEVESFRRALIADKADVARTVAGIVDMRRRLA
GAK
PAKSTWDGKQGPGRGQDIELIATAAALIAASPATSVAEQMGAGVEAGWLRQDEADVLGASYRLQRQIQGAAKLISDT
PLD
AARLGESGQAFLLRGTGAEDVAALEDLLEIQRAAAATAIEAVIARSPAEEQADAD
>OLS48518.1 hypothetical protein BV379_09755 [Rhodovulum sulfidophilum]AB14
MRIELADPRGVIRESYRIEGIGAPECRSIFLDWAIGLPEGIDPQRALRRLLAEHAAAAPDHPMTAVLRAGLNAGPQARRR
GGAHGRRS
>OLS48519.1 methionine synthase [Rhodovulum sulfidophilum]AB14
MTQTAFSPTRLALSSVFDTLRAAARERVLVLDGAMGTQIQKLGLSEDDFTGHGAGCSCHLHGDKPQKGNNDLLNLTN
PEV
IEEIHYRYAMVGADIVETNTFSSTTIAQADYGLEDKVFALNEAGARLARRAMDRAEAEDGRRRFVAGALGPTNRTASIS
P
DVNDPGYRAVTFDDLRIAYAQQLRGLIGGGIDLVLIETIFDTLNAKAAIFACEEVFTEQGLRLPVMISGTITDLSGRTLS
GQTPTAFWHSVRHAQPLTIGLNCALGADAMRPHLAELSGVADTLICAYPNAGLPNEMGEYDQTPDEMAALVEGFAR
DGLV
NVVGGCCGSTYDHIAAVAQAVKGYAPRALPQPARHLRLSGLEPFTLTDDIPFVNVGERTNVTGSARFRRLIKEQDYATA
L
DVARDQVENGAQIIDVNMDEGLIDSKQAMIDFLNLVAAEPDIARVPVMIDSSKWEVIEAGLKCVQGKPVVNSISMKEG
EA
AFLHHARLCRVYGAAVIVMAFDEQGQADTEDRKVEICARAYRLLTEEVGFPPEDIIFDPNVFAVATGIEEHDNYGVDFIE
ATRRITTDCPHVHISGGVSNLSFSFRGNEPVREAMHAVFLYHAIQVGMDMGIVNAGQLAVYDQIEPELREACEDVVLN
RR
RDATERMLELAEKYRGQGGSQAREKDMSWRELPVGKRLEHALVNGITEFIEADTEEARLAAERPLHVIEGPLMAGMN
VVG

DLFGAGKMFLPQVVKSARVMKQAVAVLLPHMEAEKEGRREAAGKVLMATVKGDVHDIGKNIVGVVLACNNYEIVDL
GVMV
PPEKIVAAAKEHEVDIIGLSGLITPSLDEMVHMASEMERQGFDIPLLIGGATTSKVHTAVKIAPHYSRGQAVYVTDASRA
VGVVSSLLSDNQRAGYVEGIREEYREIAEKHARGEAAKKRLPLAAARANPVPVDWAAYRPVRPGFLGTRVYDRWDLAD
LA
AHIDWTPFFHAWDMRGVFPRILEDEKKGEAARNLYDDARRMLDRIVQGAWFAPKGVVGFWPANAVGDDVRLYTGE
DRTGT
LATFYTLRQQTAKRPGVANAALSDFVAPEGVAPDYLGGFAVTAGPEEVAIAERMAMENDDYGAIMVKALADRIAEAF
AER
MHAEVRRTLWAYAPDERFTPEELIAEPYAGIRPAPGYPAQPDHTEKKTLFRLLDAERVVGIRLTESMAMWPGSSVSGLY
F
AHPDSYYFGIGKVEADQVADYAARKGISLSEAERHLAPVLNYTPRKEGDAAAA
>OLS48520.1 paraquat-inducible membrane protein A [Rhodovulum sulfidophilum]AB14
MRALRLANLALLVLFPIAWAAPLLRAGLLPLFGLSEISVLSGLASLWQTDIFLALLVTLLALVAPYAKTVGLALIQYGLA
SPRLLPAIHVMGKLAMADVFLIAIYVVLVKGIGMGRVETAWGLYLFTFCVLASLALSSLTARILKRG
>OLS48521.1 ABC transporter ATP-binding protein [Rhodovulum sulfidophilum]AB14
MIELRHIEKAFGRNRVLRGVNLTVPKGASMVIIGGSGTGKSVLLKCILGLVRPDAGQILIEGEDASKAERDAFLARFGML
FQGGALFDSLPVWQNVAFRLLRGTGKRPKAEAREIAIEKLRRVGLKPDVADLFPAELSGGMQKRVGLARAIAADPEIIFF
DEPTTGLDPIMAGVINELIREIVVEMGATAMTITHDMTSVRAIADNVAMLHGGVIQWTGPVSDMDQSGDPYLDQFIH
GRA
EGPIEAVR
>OLS48522.1 ABC transporter permease [Rhodovulum sulfidophilum]AB14
MILLAPLGALGRTVLGALAAIGRIALYALATLYAMVRPPFYLRELGHAIWVIGYNSLPVVGLTAVFTGAALALQIYEGGS
RFNAGQVVPSIVAIGMVRELGPVLGGLMVAARVASSIAAEIGTMKVTEQIDALATLSTDPMRYLTVPRVLAATLTVPVL
V
AVGDSIGILGGYLVGTQSLGLNAAAYLKNTADYLVLWDVTSGLIKGAVFGFLIALVGCYFGMNSGRGAQGVGAATKSA
VV
AASILILASNYLLTTLFFAA
>OLS48523.1 alanine racemase [Rhodovulum sulfidophilum]AB14
MATGTLTIDLDALARNWKALDRASGAGVETGAVVKADGYGLGGDRVARALAQAGARRFFVAVAEEGAQIRNALGPG
PQIC
VFSGHMPGDTDMIGDLDLVPMLNSVEQLTRHMEGLPGRPFGIQLDSGMNRLGLEPAEWAAVRDIVLAAKPVLIMSH
LACA
DEPDHPANLQQLTAFREMTEGCDVPRSLSATGGVLLGPDYHFDVTRPGMGVYGGMPFAEAEPVVGLSFPVIQTREVE
PGE
SIGYGYTHTAGDRRVVATVAAGYADGLHRTLSNRSKLWFEGTPCPIVGRVSMDLVTADITDLGRIPPALDLLCPEQGVD
A
VADSAGTIGYEIMTALGRRYARRFLGGAGA
>OLS48524.1 orotate phosphoribosyltransferase [Rhodovulum sulfidophilum]AB14
MIPSTFPDQSEIARLSARMLLEIGAVHFNAEEPFTLASGLPSPTYIDCRKLISFPRIRATLMDFLTVTVLREAGFEAFDN
IAGGETAGIPFAALIAERMALPMTYIRKKPKGYGRNARIEGVMAEGDRVLLVEDLTTDGGSKLSFVDAIRDTGAVCGHT
A
VIFYYGIFPETEKTLGDHGIRLHHLCTWRDVLAEAKRQGAFDATTLAEVEAFLDTPRAWQEARRQQ
>OLS48525.1 dihydroorotase [Rhodovulum sulfidophilum]AB14
MTQSLTIRRPDDWHLHLRDGAMMAGVLPESARDFARAIVMPNLVPPVVTATEAAAYRDRILAALPEGADFTPLMTLY
LTE
ETDPEDVARAHAAGLVTAVKLYPAGATTNSASGVSDFDKVRGVLDKMAEIGCPLCVHGEVVDPEIDIFDREAVFIDRVL
D

PIRRATPGLKVVMEHITTADGVDYVRSATAGLGATITTHHLIITRNHILVGGIKPHYYCLPVAKRARHRDALVAAATSGD
ARFFLGTDSAPHVDPAKECACGCAGIFSATNTVSCLAEVFEREGALDRLEAFASLNGPAFYGLPPNETRITLTRDASPVA
YPARIETGAGPVTLFDPGFDLHWRVER
>OLS48526.1 hypothetical protein BV379_09800 [Rhodovulum sulfidophilum]AB14
MALANTHVAGPRGSFFSSVIAAMVRMVENHPRTRALDRLNAISDEELAARGLTRQDVIRRIFSDRFYL
>OLS48527.1 hypothetical protein BV379_09805 [Rhodovulum sulfidophilum]AB14
MKDRLASEDNTGRWQALLDRSGSGAWLKALGPDHAAVWAPGGDTLLVTFEQARKILTGETAGQPLAAQLAEPAGW
SRLTL
VAHGATWFRDPAVFRFFDERIDSEFFDGFDRVVFYGAGMCGYAACTYSVAAPGAAVVALAPQATLDPARLDWDRRFL
IAR
RRDFASRYGYAPDMIEAASCAAILYDPAQTLDAMHAALFDGPNVLRLAAPCCGDALEAHLADMGILPELIDAAGEGQL
SA
EVYHRLFRARRRHAPYLAALIDRLDRSGRKDLALRVCRHARRIAESETVSGHIAGITGARADLDRRLRRHRPADPA
>OLS48528.1 lysine transporter LysE [Rhodovulum sulfidophilum]AB14
MPVDLWLAFAAASAVLLVLPGPTILLVLSYALSQGRRVAVAMALGVALGDLIAMTASLAGLGALVLASATLFTALKWIG
A
AYLVWLGIRLFRSPPVLAEDVGPAPRPARAVFGHAALVTALNPKSIAFFIAFVPQFLRPEAPLAPQFAVLIATFVSLAAL
NALAYALLADRLRAQIRRAAVLRWVTRAGGGALIGMGALTASAARS
>OLS48529.1 acyl-CoA dehydrogenase [Rhodovulum sulfidophilum]AB14
METPLKPKDAPELARFDWEDPLRLEDQLDEDERMLRDAARAFALDRLQPRVTEAFRNETVEPGLFREMGEAGLLGVTL
PE
AYGGLGASYVTYGLVAREIERVDSGYRSMMSVQSSLVIYPIYAYGSEAQRQRYLPGLAQGTLIGCFGLTEPEAGSDPGSL
KTRAEKTATGYRLTGRKTWISNAPMADVFVVWAKSEAHGGKIRGFVLEKGMAGLSAPKIEGKLSLRASPTGEIAMDGV
EV
GEEALLPGVEGLKGPFGCLNRARYGIAWGVMGAAEACWHAARSYGLDRRQFGRPLAATQLFQKKLADMQTEIALGL
AACL
QVGRLMDEARAAPEAISLIKRNNCGKALEIARAARDMHGGNGISESYPAMRHLVNLETVNTYEGTHDVHALILGRAQT
GL
QAFF
>OLS48530.1 ATPase [Rhodovulum sulfidophilum]AB14
MAEEIGSIDAVQAMLAGQNYVCGRALGTVAFLALRLGRPLFLEGEPGTGKTEIARALASGLGRRLIRLQCYEGLDAASAV
CEWNFAAQMIAIRTAEAAGAADRSALEAELFDERFLIERPLLQAMRPQAGGPPVLLIDELDRTDEPFEAFLLEALGDFQV
TVPELGTIRAAEPPIVILTSNRTREVHDALKRRCLYHWVDYPDFVRELEILQARAPEAAETLSREIVAFVQKLRAEDLFK
KPGVAETIDWAKCLLALDVIALSPDVIADTIGALLKYQDDIQRLEGSEARRLLDAVRDELATA
>OLS48531.1 VWA domain-containing protein [Rhodovulum sulfidophilum]AB14
MADLPALGIPDEGRLSHNILHFARALRKAGLPVGPGRAIDAIRAVEAAGFTDRADFHHTLAACFVSRPEHRAVFDQVFR
L
YWRDPRFLEHMMSLMLPALRGVAEERRARPAERRAAEALLDAVASDRPDPETGEEIEIDAAGTQSARDRLKSLDFEQ
MSP
AEAAEAKRMLAGLRLPVAPLISRRSRADRLGPLPDWRGTMRAALRAGGEMRDFSTKNRRRRWPDLVVLCDISGSMS
QYSR
MVLHFLHAVSNAKGAGWARVHAFTFGTRLTNITRHLATRDVDAALAAAGAEAPDWEGGTRIGAALHAFNRDWSRR
VLVTG
AVVLVITDGLDRDETDILAREIERLHLSARRLIWINPLLRWDGFAPKARGIRAMLPYVDCFRAGHSIRSLEDLARAVSEA
RDTGERDRMLAAMRHSGPI
>OLS48532.1 hypothetical protein BV379_09830 [Rhodovulum sulfidophilum]AB14
MPLNWKIGGPLLGIVFFLAVLLARPIGVSTQFVLFDAFLWNLQQPDAGIVTRTAEGVTSTNAYLAKSDGALAAGAADLL
T

YPMVFVLAMMVGALLSSLIRSTVPRGERNVPAMYRANIGGFWPLRWVMAFAGGVLVLYGARLAGGCTSGHMMSG
MMQSSI
SGFVFAFGAFLTAFPTAMMLLKKEG
>OLS48533.1 molybdopterin biosynthesis protein [Rhodovulum sulfidophilum]AB14
MRFGSVPPAEARGAILAHSMALPGRHLPKGSRLGPAEIAALREAGIAEVTVARLDPGDVAEDAAAARLANALVPDPEA
AA
LRIGRAARGRVNLHAVCAGVVGLDLAAVQAVNRVDPGITLATLAPLGRVLPDMLVGTVKILPYAVPGAALEAACATGR
GA
LRVHPVRRRSAGLILSEVPGQKPALTGKGRSAVETRLSALGITLEAVRVVPHEARAMAAALAALPGEIGLILTGSATSDL
RDVGPEAVRLAGGTIARFGMPVDPGNLLFLGEQDGRPVIGLPGCARAPALNGADWVLERTACGIEVDAEALAAMAV
GGLL
KEIPSRPQPREA
>OLS48534.1 hypothetical protein BV379_09850 [Rhodovulum sulfidophilum]AB14
MHLYAICFTQGIAAFKKRDGGLLRDQVPSDVLIGGAARRTLACGRLPFLRLCLAKHMVAG
>OLS48535.1 hypothetical protein BV379_09855 [Rhodovulum sulfidophilum]AB14
MAENRKRSARLQAYGLQGDTRDRVKEVGKILLPHLDEVLSDFYEEISDTPDWNAFFPTQALRDHARQKQKQHWVLML
TSD
FSQQYFEGAEKIGKVHNRIGLPISFYTAAYARVGLALQGIIMNEVGRNAFFRRRDPTPLMDTLSRVLMLDTELANSAFFA
AQAAQYENRLNEISRNFEDEIGRVVDVLSTSMHQLDDAANGMTTEISRARQGADSLSDNASRIATSAQSVASAIEELSA
S
ITSITQDVNSAATASANAMEEATASSEQLESLQAAAEEIDEVVNMIAQIAKQTNLLAVNATVEATRAGDAGKGFAVVAF
E
IKALAERISQATSSINDRIRRIQSETRLMSTRISGIGDCVERVRSFSDSIRVAVTEQTQAAQHISQHADSTASSTTEIAQ
MIRDVSHRVERSGDTSLTLKDAVGTVSDEAGTLSQRVRAFLTALRAA
>OLS48536.1 translation initiation factor IF-3 [Rhodovulum sulfidophilum]AB14
MARRPHNAPPTRDTGPRINGRIRCPEIRLIGAEGENLGVVTPARGVELAEQAGLDLVEISPNAAPPVCKIMDFGKFKYE
Q
QKRESEARKKQKVIEVKEIKFRPNTDSHDYEVKMRSVVKFLENGDKVKITLRFRGREMAHQNLGRELLERVAEEIKEIGK
VENMPKLEGRQMVMMIGPAK
>OLS48537.1 hypothetical protein BV379_09865 [Rhodovulum sulfidophilum]AB14
MKKVLVAIVILAAFGLGGVLWYLGNMTGSGAESGTGATAEEAMPEEGRAPGGTGQDAPAAESQSDAEMPRSEGDTR
PDTG
PEAGSKPDAGPETEAPGSGDGSVKAMAPEEETSSAPGAMQEAPAAEAPEAAPSDAAGAEPDPEMRDAGAAAKGAV
VPEAE
E
>OLS48538.1 triose-phosphate isomerase [Rhodovulum sulfidophilum]AB14
MRRKLAAGNWKMNGLSGSLGEAVALAATHSAPDVDILLCPPTTLLDRMADAVHGSKVAVGGQDCHPAVSGAHTGD
ISAEM
LADAGAGYVILGHSERRADHGESDALIRAKTEAALAAGLVAVVCVGETEAQRDAGETLAVVGAQLAGSLPDDGVTGDS
VV
VAYEPVWAIGTGRVPTLEQIAEVHDSLRAALNARYGAAADAIRLLYGGSVKPGNAAEIFATSNVDGALVGGASLTAADF
S
AIIAALEAAPAA
>OLS48539.1 Fe-S cluster assembly protein HesB [Rhodovulum sulfidophilum]AB14
MFAIPGKQAVSLTPAAAAEVARLMHQERAQGLRIGVKKGGCAGMEYTMDYVAELDPMDEIVEQDGARVAIAPMAQ
MFLFG
TEIDYEVSLLESGFKFRNPNVVDACGCGESIKFRDADDPQEGPATA
>OLS48540.1 SUF system Fe-S cluster assembly protein [Rhodovulum sulfidophilum]AB14

MTEPQGKLEGTPLIEPSTTDHPLYDQVVEACRSVYDPEIPVNVYDLGLIYTITIDTENVVHLTMTLTAPGCPVAGDMPG
W
LQEAVEPLPGVKRVDVDITWDPPWGMDMMSDEARLELGFM
>OLS48541.1 phosphoribosylformylglycinamidine cyclo-ligase [Rhodovulum sulfidophilum]AB14
MSERKNGLTYAGAGVDIDAGNALVERIKPAAKRTGRPGTLAGLGGFGALFDLKAAGYLDPVLVAATDGVGTKLRIAIDT
G
HLDGVGIDLVAMCVNDLVCQGAEPLFFLDYFATGKLEIDEAARVIEGIAEGCLRSGCALIGGETAEMPGMYHGGDFDLA
G
FAVGAMERGHDLPAHVGEGDVLLGLASDGVHSNGYSLVRKVVEHAGLGWDAAAPFADGPLGAALLAPTRLYVKPAL
AAIR
AGGVHGLAHITGGGLTENLPRVLPEGLGATVDLSSWDLPPVFRWLAETAGLAEAELLKTFNAGIGMVLVVAGDRAEAL
TA
LLEEAGETVARIGRVGPGQQGVAYEGRLL
>OLS48542.1 phosphoribosylglycinamide formyltransferase [Rhodovulum sulfidophilum]AB14
MSEAKRVAILISGSGSNMVALAESMTGDHPARPVLVMSNDPVAKGLDRARALGIAAEGIDHRPFGKDRAGFEAALMA
RLS
EARPDILCLAGFMRVLTPGFTEAWAGRMLNIHPSLLPKYRGLHTHARALEAGDAEAGCTVHEVTAELDGGPILGQARV
PV
LAGDTADSLAARVLVQEHALYPAVLRRFASGDRTPLYLP
>OLS48543.1 ribonuclease D [Rhodovulum sulfidophilum]AB14
MKTLTTTEALAAFCEKAASAPYITIDTEFLRERTYYAKLCLVQLALPGRDGPDGGEAVLVDPLAGDMSLEPLYALFRNSA
VVKVFHAARQDLEIFFTEGKVFPEPLFDTQVAAMVCGFGEQVGYETLVRKIARSSLDKTSRFTDWSRRPLSEAQKSYALA
DVTHLRVIYEYLAAELEKTGRAKWLQEELGVLTDPETYIVRPGEAWHRVKTRTHSGKFLVAVKELARFRETYAQSRNIPR
NRVYKDDALLELASTKPQSLEDLSRSRLLLREARRGDIAEGIIAAIKAATETPQADYPKMPETDDKLQVNPALADLLRVL
LKAKSEQAGVAQKLIATASELDAIAAGRRDGPALRGWRAEVFGDDALRLCAGKIALAAKGSNVAVVPL
>OLS48544.1 hypothetical protein BV379_09900 [Rhodovulum sulfidophilum]AB14
MTPRPVSLILCATALVAACQSTTTAREAAQPAPQGRSAETETETPPEYLPCRDLEQALSMSDARMRRLNSRDMSSEATY
L
RDRNKRLIARAYECRRPS
>OLS48545.1 cysteine desufuration protein SufE [Rhodovulum sulfidophilum]AB14
MASEAFEEIVETFEFLEDWEDRYRHVIELGKAMEPLPEAFKVPATKVDGCASQVWLMADVEGEGREAIYHFKGDSDA
MIV
KGLIAVLRALYDGLNVVAVLDTDAGAEMARLGLDEHLSSQRSNGLRAMVERIRTQAAGIAEGV
>OLS48546.1 hypoxanthine phosphoribosyltransferase [Rhodovulum sulfidophilum]AB14
MAERPYVIDELLSAKTIAARVESLATEITAHFADSEKLVVVGLLRGSFIFIADLVRELDLPVEVDFIETSSYGDSTSSSR
EVRILKDLRGEIEGRDVLVVEDIVDTGHTLKHVLHLLGGRKPARLECCALLDKPARREVDVCARWVGFEIPDRFVVGLGI
DYAQRNRNLPYIGAVRFTD
>OLS48547.1 ubiquinone-binding protein [Rhodovulum sulfidophilum]AB14
MPRHSENREMPYTARQMYDLVADVGSYPQFLPWTAAARIRSRKDFGDHELMEADLVISFKVFRERFGSRVTLWPGDF
RID
TEYLDGPFRYLKSQWRFEDVAENRCKVEFFVDFEFKNRVLQGVIGAVFNEAMQRIVRAFEDRARALYGSRP
>OLS48548.1 damage-inducible protein CinA [Rhodovulum sulfidophilum]AB14
MSDKAAPLIAGARAAGLAIATAESCTGGMISAAITDVPGSSTVFDRGFVTYSNAAKQAMLGVLAETLDAHGAVSEQVA
AE
MAAGALAHSVADLAVAVTGIAGPGGSEFKPEGRVCFGLARRGADGRIETMTETVEFGALGRDRVRSATRDHALTLLM
AAL
EPRPSAN
>OLS48549.1 phosphatidylglycerophosphatase A [Rhodovulum sulfidophilum]AB14

MSLAINIATVFGVGSLRPAPGTWGSLVAMPAFWALDVTGGVVLAALATLAVIPLGIWAVREAAAGAPDPDHSEYVIDE
VA
GMWIALLPVSFGAAAAGVDATALYPGWIAAFLGFRLFDIWKPGPIGRADRMKTPLGVMLDDVLAGVAAAVVTLALAA
LSH
IFLM
>OLS48550.1 bifunctional 2-C-methyl-D-erythritol 4-phosphate cytidylyltransferase/2-C-methyl-D-erythritol 2,4-cyclodiphosphate synthase [Rhodovulum sulfidophilum]AB14
MTVTAIIVAAGRGTRVGGDKPKQYQDLAGRPVLARTVEAFAAHPRIGAIVAVLHPEDDDLFETALADSAAEVTTVPGG
AT
RDASVMAGLRAAPEGTTRVLIHDGARPMVPAALIDRVIDALDENPGAAPALPVTDALWQGREGKVAAPHPRDGLYRA
QTP
QGFHLRAILAAHEAHPGGAADDVEVALAAGLDVAIVEGDEDNLKITHKVDFARAERVLRERNMDIRTGNGFDVHAFCE
GD
GVILCGIKVPHDKALKGHSDADVGMHTVTDAIYGALAEGDIGRHFPPSDPQWKGAASEIFLKHAVALAAERGFRIANV
DL
TLICERPKIGPVAGAMRTEMARLMGMEEARVSVKATTSERLGFTGREEGIAALATVTLVAP
>OLS48551.1 tRNA dihydrouridine synthase DusB [Rhodovulum sulfidophilum]AB14
MAIRVAGISLDPPVLLAPMAGITDLPFRRLVASFGAGLVVSEMVASQEMVQAKPGIRERAALGLGEAATAVQLAGRDP
DW
MARAARMVEEQGARIIDINMGCPARKVVGGLSGSALMRDLDRALRLIEAVVKAVSVPVTLKTRLGWDDDLRNAPELA
RRA
EAAGVRMITIHGRTRCQFYKGRADWRAIREVREAVSVPVVANGDIVCSETASAALAQSGADGVMVGRGAQGRPWLL
AEIG
ARIAGRPAPAVPEGAALADMVARHYEDMLGFYGTGLGGRVARKHLGWYMDRAGTPGALRRRVLTAPAGAAVLALLP
EALA
AEGRVAA
>OLS48552.1 PAS domain-containing sensor histidine kinase [Rhodovulum sulfidophilum]AB14
MTLDQDAIWTALPIPAFLLDAEDRITNINPASELFVNATIKTLSGVPVFDRLAVDAPLDEAFARVRANRAPLFINDVDVS
AGNKPPVLCNVQVAPMNDTEEHLLLLVTPREIAGRLGRVHSVKSAARSAIGMAEMLAHEIKNPLAGITGAAQLLSMNL
SN
GDLEMTDLIVEESRRIVKLLEQVEQFGNLRPPACKPVNIHDVLDRVRRSATVGYAANMAILEEYDPSLPATWADPDQL
MQ
VFTNLITNAAQAAGPRGGTIRLRTFYDLSLRLRHPDGSGVPVPLQVEITDDGPGLPAEISAEIFEPFVSGRENGTGLGLA
LVSKIIGDHGGWIAVDSVPGRTVFRVSLPVAPKPEEDES
>OLS48553.1 nitrogen regulation protein NR(I) [Rhodovulum sulfidophilum]AB14
MDGTVLVADDDRTIRTVLTQALTRAGCKVHATSSLTTLMRWVGEGKGDLVISDVIMPDGNGLEMLPKIGQERPGLPVI
VI
SAQNTIMTAIQAAEAEAYDYLPKPFDLPDLMKRAARALETKRRVGRPRETAPPTGGDTQDDLPLVGRTPQMQALYRLV
AR
VMNADLPVLITGESGTGKSLIARAIHDFSDRRSLPFVIATAADMEGIDGPSALVSRTRGGSILFDEVADLSEEAQGRIVR
MLDSFGDNAPRVMATSQSDLMARMEAGQFRHDLFYRLGGVSVAVPSLRERVDDIPLLAEHFLGRTERDGTATRRLAE
EAM
DLIRAYSWPGNVRQLEHTIRRLVVTSPEEVITRAEVEAVLGNQPEIEPLVGGGDGEKLSASIAKHLRRYFDLHGGVLPPP
GLYARILREVEMPLIEIALDATGGNQAKCADLLGINRNTLRKKITDLDIRVTRRRKLM
>OLS48554.1 sigma-54-dependent Fis family transcriptional regulator [Rhodovulum sulfidophilum]AB14
MGDILIVDDERDIRELVSDILQDEGYTTRLAANSDACMAEINAAEPALMVLDIWLKDSTMDGIDILSRVKRDNPDIPIVI
ISGHGNIEIAVAAIKQGAYDFIEKPFNIDQLLVVIRRAMEASRLRRENQELRRKDVTQFDMIGSSPAFKMLKSNLEKVTR

SNGRVMLRGPSGCGKEVAARYIHANSGRASGPFVVVNSASIEPERMEEVLFGRESSTRGIEKGLLEQAHGGVLYFDEVA
D
MPLGTQSKILRVLVDQQFQRAGGTDKVRVDIRVISSTTRDLTAEIQAGTFREELYHRLNVVPIDVPALDERREDIPELAR
HFIAGFNKTQGLPLRALGEEAEALLQTMSWPGNVRQLKNVIERVLILGEGTDPILATELPMQADGPAEEGRIVLVGALA
T
LPLREARELFEREYLLTQINRFGGNISRTANFVGMERSALHRKLKSLGVVTTARAGGRVAHVEEEEQTAAD
>OLS48555.1 potassium transporter TrkH [Rhodovulum sulfidophilum]AB14
MRRLADLPFLVILMGIGALAMFPPVVLALAERDWETARAFFNSGLLFFVLAVLIGLSTINRPVQNQARSHLLAMLGFYTL
LPLMLAVPVSEAVGDTSFLNAYFEMVSCLTTTGATLFDSPERLSPGVHLWRATVGWLGGFFVWVTAIAILAPMNLGGF
EV
TAGTGGIAGRIGSERGEVSPAHDPRERLLRFAHQLAPVYIGLTATLWVLMVVAGEGPLVAACHAMSVLSTSGISPVGGV
A
GGQSGVPGEIMLWGFMVFAISRQMFMFDFRPESWRRLKADLELRMGVGIAWVVTVFLFARHWVGAYEVAAEENG
MAALRA
LWGAIFTVTSYLTTTGFASVEWIDARQWSGLATPGLILVGLAVFGGGVATTAGGVKLLRIYALYKHGIREMEKLVHPSSV
GGAGAFARRIRRQGAQIAFVFFMLFALSVAVVMVTLGLLGLDFEASMVLTVAALTTTGPLAAAATDPPLSYAALGDGP
KM
VLAATMVLGRLETLAIIALFNPEFWRK
>OLS48556.1 RNA-binding protein Hfq [Rhodovulum sulfidophilum]AB14
MAADKQNLQDAFLNHVRKTKVPVTIFLINGVKLQGVITWFDNFCVLLRRDGQSQLVYKHAISTIMPSQPINLYEGED
>OLS48557.1 GTPase HflX [Rhodovulum sulfidophilum]AB14
MSSSEAEGSEPRITRAWVLHPDIASDRERRDAGPALEEAVALAGALPDLEVVGAETVPLARPRAGHLFGSGKIDELKERL
GEAEVDLVLIDGPVSPVQQRNLEKAWKVKILDRTGLILEIFADRARTREGVLQVELAALSYQRTRLVRAWTHLERQRGG
L
GFVGGPGETQIEADRRAIDEAITRIRRQLSKVVKTRDLHRAARRKVPFPIVALVGYTNAGKSTLFNRLTGAEVLAKDMLF
ATLDPTMRAVALPTGLKVILSDTVGFISDLPTQLVAAFRATLEEVLEADLVLHVRDIAHPDSEAQAEDVRAILADLGVAR
ETPQIEVWNKTDLLDPERRAATEAAAARHDEVAAISAWTGQGVGPLLEAVTERLDAARQRRDLHLAHDDGRRRAWL
YEQG
IVEGEVPDDAGSTLTVNWTARQEQRFRSL
>OLS48558.1 hypothetical protein BV379_09985 [Rhodovulum sulfidophilum]AB14
MTLLRNRASARVLAFGFWLALVCFLRVAAAEPVSLIGAGGLLQREGASGPLLTQMRGPLLGPGPEGAPGRVSGASGAS
LF
AGRGGTSLFATYPVRAQQMAAPGTAAQFAPLRPGRGNAALAEGIRLLIGRAEAGRMDYDAVQHGAKIRPPGPPTRLTI
AE
IYKWIAETPGQPHAIGRYQFIPDTLRRLVAELGLDEKTRFSAPVQDRLADLLLEEAGLSAFGAGEMKREAFMHNLSRIWA
GLPTASGRSYYHGVAGNRATMTWMQFEAAMSRIAPGPGGAPKDG
>OLS48559.1 penicillin acylase family protein [Rhodovulum sulfidophilum]AB14
MPHLFRWLLRIFTAVVILAALGIGGVYYLASRSLTDYSATHEVDGLSAPVEIVRDHSSVPHIFGKTDEDVFFGLGYAHAQ
DRLWQMTVLRRTVQGRLSELFGARTMKIDELMRRLDLYRLAQDSVDAQDPQTLAALEAYSRGVNAWIETVNTEARG
RGAP
EFFLFRPEIAYWQPADSIALVKLMGVRLSSHLDAEVLRARVSLLMEPERVKDILPDAPGPGLAALPDYADLAPGVIPSSA
PLARYAEGPLSPFLGPDFAGASNAWAAAPKRSAAGGTLLANDPHLGFSAPSIWYLARLELSTGGVIGGTIPGIPTILSGR
SAKLGWGLTTSYLDDQDVFLEQLNPDNPQEYRTPDGFRPFETRRSIIKVADAEPVTITLRWTENGPVLPGTHYDLASITP
PGHVAALGWTLLSGADTSISAAMQVMRSQSVDEAIEAGRFFVAPSLNLTLVDADNIALQMIGAMPRRMAGNQSKGR
LPQP
GWKEENRWQGWLNYAANPSFRDPAGGILGNTNNKLVDRPFPLHVSFDWGDSQRIQRWKRLMETRKVHTRESFIEA
QLDTV

SFTARSLLPLVGKDLWFTGEAAPAGSPERRRQRALELLAGWNGEMNEHLPEPLIYAAWMRFLQRDLIEDDLGPLAREF
TH
VEPLFIERVFRDIDGASVWCDIRQSAAIETCTEIARKSLDEALLYLSETYGGSLESLRWGQAHQAWHDHPVLGETPLLKW
IVNIRQPTSGGDNTLNRGLTKGTDPFPFANLHGAGYRAVYDFADPDSSVFIISTGQSGHPLSRHYDDLGELWRRGEYIP
M
TLDPALARAAAVGITNLIPKSR
>OLS48560.1 oxidoreductase [Rhodovulum sulfidophilum]AB14
MARVAFLGLGVMGYPMAGHLAAAGHEVAVYNRTAAKAEDWVARHGGRSAPSPAEAAEGADLVMACVGNDDDLR
AVCEGES
GAFAGMAAGAVFVDHTTVSSMVTEEMAALAETRGIAFVDAPVSGGQAGAENGQLVIMCGGPEEAYAKAEPVMAAY
AKLSK
RMGGTGAGQLTKMVNQICIAGLVQGLSEGLHFAEKAGLDGRAVVEVIGGGAAGSWQMMNRYQTMLDDEFDHGFA
VDWMRK
DLGICLATADRTGASLPVTALVDQFYKDVQKLGGARWDTSSLIKRLRAMG
>OLS48561.1 spermidine/putrescine ABC transporter permease [Rhodovulum sulfidophilum]AB14
MNETKARPRLGRSEAAQGYFLVAPPFLYALLLLAVPLAAILLFSFWSQDYLTLDTTLTLKNYREAFSDPLYGQLLLRSLL
ISGAVTAATVLTAFPIAYFISFSVPPSQKSMWIFLITIPFWSSYLMRVFLWKVILGYNGVLNTTLSGLGIIDEPLSFILY
NANAVVITLAHAYAPFAILPIFVALEKIDRSLLEASRDLGESRAMTFLRVTLPLAMPGVVAAVLIVFIPTIGDYVTPRLV
GGPDGLMIANMIQTQFLKLNNAPMGAALAVLAMAMVTAVAVLFLVLNRRYLRGRK
>OLS48562.1 spermidine/putrescine ABC transporter [Rhodovulum sulfidophilum]AB14
MKGLRLYAIFYLLFLYAPIALLPVFAFNNGTVIAFPLRGFTLEWFRELMTIPALHAAVLNSLAIAVSSAVIATCLGICAA
RAGTRFAFPGKKGILGLIMLPLVLPEIIVAVSLLVVLLQLGLPLGSWTVVLGHVLMCTPFSIAILNSAFQSLDVSLEEAA
IDLGESRWSSFRLITLPLVMPGIVSSLLIAFTISLDEFIIAFFLTGTNPTLPVYIWGQLRFPQKIPVIMALGTLLVLTSV
ILLTVAEYVRRRGAARTGKADAGGFL
>OLS48563.1 ABC transporter ATP-binding protein [Rhodovulum sulfidophilum]AB14
MKDTLGVERPIIALENVEKYYGEFHALRGISAEIRQGEFFSLLGPSGCGKTTLLRTIAGFEELSGGRLTIDGRDMKRVPA
NVRPTNMVFQSYAIFPHLSVAENVGFGLRREALSKERKAAKVAEALEMVGLRGFGARAAHALSGGQRQRVALARALIL
EP
KVLLLDEPLSALDKKMREQMQIELRRLQRQVGITFILVTHDQEEALIMSDRIAVMFEGQIVQLDTPQQLYRRPNTRQVG
E
FIGVMNFLPATATVGDKADIEVEAAGLGRAVIGPDQLPGGWHPGQTQSVGIRPETMSILFPGETTERRVATGTVEDLSY
Y
GDMTYYVVRLDGVDRPMTISMKNLVGRPVLERGTAARVAWDARSLVLFA
>OLS48564.1 polyphosphate kinase 2 [Rhodovulum sulfidophilum]AB14
MTDLPFDGAITQFYEQDSPDEIRRAIADGGKHDILADDYPYDRRLRHAAYAADLQALQIELAKLQADVKATGKRIAVLFE
GRDAAGKGGTIKRFRQNMNPRVARLVALPAPSNREKSEWYFQRYVEQLPAGGEIVLFDRSWYNRGVVEHVFDFCTPE
ERE
RFFGQLPAFERMLVDEGILLVKFWLTVGRAEQLRRFLQREKDPLKQWKLSWVDIEGLKRWDAYSAAIRETFARSHNGH
AP
WTVIRSDDKRRARLAAIRHVLSLVDYKGRSDKVVTAPDPLICGGPEIWTP
>OLS48565.1 alpha/beta hydrolase [Rhodovulum sulfidophilum]AB14
MAEDYAPTGRLIEIDGRQVHAHQEGSGPDVILLHGAGGNTRDFTFDLLDRLSADFRVTAFDRPGLGHTDPLHPNGESP
AE
QAAVLEAASRRLGLGPAVLVGHSFGGAVALAWALDHPDRAAAIVSLAGAAMPWPGGLGPYYELVSSDFGSMFMVPL
ISAF
VPRFVAEQTIARIFEPDPVPEGYGDHIGIALSLRPETLRANTRQVNGLKPHIRAMADRYDDLKMPIEILHGTADRIVPIE
VHSRPLAERLANVHLTELDGIGHMPHHADPEAAVAAIRRAAKRAGLRATA
>OLS48566.1 homoserine O-succinyltransferase [Rhodovulum sulfidophilum]AB14

MPIKFPSDLPAYDVLSREGVMVMGEDEASRQDIRPLKIALLNLMPKKIQTETQFARLIGATPLQIELSLVRMTEHKTRNT
AAAHMETFYRPFAEVEASGEKFDGFLITGAPIEHLPFTEVTYWDELTRVFDWTQSHVHSTFGVCWGGMAMIYHFHGV
QKH
MLPAKAFGCFRHRNLAPASPYLRGFSDDCVIPVSRWTEMRQDEIDAAPGLVTLLGSDESGPCLVEDSGHRALYIFNHFE
Y
DSDTLKQEYDRDIANGTSINVPQNYYPDNDPSQAPQNRWRSHAHLLYGNWINQIYQTTPFDQNRIGAY
>OLS48567.1 hypothetical protein BV379_10030 [Rhodovulum sulfidophilum]AB14
MAAAGAVRAQQEGPEWDLQRCVWSCLSAFGPADNPAYAACVSRNCSEPTAPAPAPRWSSGATSDGRGAYAGVRA
EGDQDM
IFYVFCARDGERLLQLDGVGGDAGPNVLSLIIDGQGFPVTFGGGHDAGMLARLPPSAPVLGALQRGQMLEIRSGAGQ
GQG
LGRFPLEGAEAAISGALALCR
>OLS48568.1 ATPase [Rhodovulum sulfidophilum]AB14
MIYDTAEAWRQAEKRQVLLFGMSGLGKTHVSNMLRAEGGWFHYSVDYRIGTRYMGEFIADNFKREAMKVPLLRELL
RTDS
VFIASNITFDNLAPLSTYLGKPGASDKGGLPFDDYMRRQEQHREAEISALLDTPRFIDRAAELYGYDDFVCDSGGSICEV
VDPEDPADPVLSALAARLLLVWIEGSEAHTAELVRRFDRAPKPMYYQPAFLHEAWADYLSRTGLSEAAVDPDAFVRWT
YA
RALAHRQPRYEAMARNWGVRVTAEEVAALRGPEDFTDLIAEAIDRAPARR
>OLS48569.1 hypothetical protein BV379_10040 [Rhodovulum sulfidophilum]AB14
MKYAVGAAALAAVTTGPALAGGVERSNQSVSILFEQGTYVELGFSRFDPDVSGSSNSSGLGSGEMAPGFSSYSLAYKQ
DL
GENIAVALIVDEPVGADVSYSGTNNYPLGSTPPTLTLDGANADVNSTGVTALLKYEFPSHFSLIGGVRAIKTSGTVSLPG
VFNYALDSSTETNFGYIVGVAWEKPEIAARVALTYSSSVTHDFEAHESFYNPSTMSVVDDMTDLETEVPQSLTLEFQTGI
AADTLIFGSVRWVDWSAFNISPEYYTGTPNGLGLDPLVSYEHDVITYNLGLGRKFNENWSAAILVGYEKHEGTYTGNLG
P
TDGFRSIGLAATYTTGKVKVTGGIRYVEIGDAKTEFGPDTTRFKGNDGVGVGLRVGYYF
>OLS48570.1 EamA family transporter [Rhodovulum sulfidophilum]AB14
MSPLLRAALWMTGAITAFSSMAVAGRMVSVELDTFELMLYRSLIGLGLVLTIGGAFGRLGEISHRQLGLHFIRNLCHFTG
QNLWFLALGLIPMAQVFALEFTSPIWVAVLAPAILGERLTRMRALAAFVGFLGILIVARPDPTALEPGAIAAAGAAIGFA
GSALFTRRLTRTQSIISILFWLTAMQAAMGLICAGLDGEIAWPSPAAWVPLGVIGLAGLTAHFCLTNALSVAPASIVMPL
DFLRLPVIAVVGMLLYGEPLDPNVMIGAAIILVANFLNLRGESRQARHARRKTGAPPPAGRVND
>OLS48571.1 hypothetical protein BV379_10050 [Rhodovulum sulfidophilum]AB14
MGVTVTGWIEATPERLDALRAAAAEHVRLTRAEPGCLRFEMREDPDRPGRFLLDEEFTDAAAFAAHRARKEGTAWEL
AAA
GLPREIEIAGLPE
>OLS48572.1 glutamine-hydrolyzing GMP synthase [Rhodovulum sulfidophilum]AB14
MSTHDRLLIIDFGSQVTQLIARRLRELNVYCEIHPYQNVTDAFLRDFAPKAVIFSGGPASVLDEGSPRPPQSVFDLGVPI
LGICYGQQVMMQMLGGRVERGHGTAEFGRAYVTPAEDRIDLLKGWFSENREQVWMSHGDHVAEIAPGFAVYGTSP
NAPFA
ITADLSRNFFAVQFHPEVHHTPNGKTLYENFVRLAGFTGDWTMGAYREEAIRKIREQVGDARVICALSGGVDSSVAAAL
L
HEAVGDQLTCVFVDHGLLRLNEADEVVAMFRDHMNLHVIHAQEQDLFLGELEGVSDPETKRKTIGRLFIDVFQKYADQ
IE
GARFLAQGTLYPDVIESVSFSGGPSVTIKSHHNVGGLPEKMGLELVEPLRELFKDEVRALGHELGLPASFIGRHPFPGPG
LAIRCPGEITREKLEILRKADAVYIDQIRKHGLYDEIWQAFVAILPVRTVGVMGDGRTYDYACALRAVTSVDGMTADYYP
FTHDFLGETATRIINEVKGINRVTYDITSKPPGTIEWE
>OLS48573.1 trimethylamine methyltransferase [Rhodovulum sulfidophilum]AB14

MSEAEATTGRRGRGGGGAARRAARSNPGIETARFITRNIPDFEILTPEALEIIETNAETVLAEIGINFVDNPAGLARWRE
AGAELEGERVRIPRGLARKLCATAPARFTQHARNPARNVEIGGRNLVLAPVYGPPFVRDIEGGRRYATMEDFRRFVQL
GQ
MSKWLHHSGGTVCEPTDIPVNKRHLDMLHAHMTLSDKPFMGSVTDPSRARDSVAMCEHLFGADFVAENTVMTSLV
NLNSP
LTFDATMMGALEVYAAANQACIISPFIVGGAMAPVTVAGTLTQVLAEVMAGVAYSQLVRPGAPVIFGAFVTSIDMNS
GAP
TFGTPEASQILSGAGQLARRLGLPFRSGGALCGSKLPDAQAAYETANTLNAALLGGVNFMLHAAGWLEGGLVASYEKF
VM
DADQLGALHRMAAGVATDEAAQAMDALREVGPGGHFLGCAHTQAHFREAFWRTDLLDYKPFETWDEEGARDTATL
ASDRV
RKLLASFEAPALDPARREAVEDYMARRKAEMPDAFV
>OLS48574.1 hypothetical protein BV379_10070 [Rhodovulum sulfidophilum]AB14
MNMLPATLATLCRPRLLVRAARFGLADYRRDRDLVRLMQGRMPGSARTVLNWLIEQEERMEEIRQSGDAGYSIARHV
ELL
IALVAEARLICDIDSPRSSG
>OLS48575.1 helix-turn-helix domain containing protein [Rhodovulum sulfidophilum]AB14
MVTHRQLQSAPAWLPQAARLYLAHTEGGVSLRALARIEGCHPSTVMRQVRRVETRRDDPLIDDALSRLGGQVAQPES
HKE
TPYMSSTMRSCRPVADEATISREALRILRRLNEPGSFMAVARDMEKAAILRESAEGQTTRTAVVDRSVAEAFALKDWIV
C
RNRGRVTIYDISTAGRAALKRLQAREAARRDGATEPASAFAEQHRDWDEGKTARAERIRFNRAESPLSILARRKEKDGK
P
FLTHDLVAAGERLREDFEVAQLGPRVAQNWNRFLTGGDRGAFFGHDGPAEGASAARGRVNAALRELGPGLGDVALR
CCCF
LEGLEQAEKRMGWSARSGKIVLRIALQRLKRHYETLGDHGGMIG
>OLS48576.1 spermidine synthase [Rhodovulum sulfidophilum]AB14
MTDTPIPGWTLERLHKGYGQALQETEVFYDSQTDHQRLRVFDNPSFGRVMTLDGVVQVTEADNFIYHEMLTHVPILA
HGA
ARRVLIVGGGDGGMAREALRHASVEEVTMVEIDAGVVEFCKTYLPGISQGAFDDPRLNLVIDDGAAFMRNTQQKYDV
IVI
DSTDPVGPGEVLFTDSFYGHAKRALTEGGILVTQNGVPFVQGEELTNSMRAFKALFADWGCYLATVPTYAGGPMAFG
WGT
DGPGRAVTLAELEARAAAAGLSTEYYTPEVHKAAFALPGYVRRLMP
>OLS48577.1 hypothetical protein BV379_10085 [Rhodovulum sulfidophilum]AB14
MKDGPDISLIIAARIDAPRRASIPTAHLAGRVLSAAGITPAVASGHLPPQAEAGPIRPRKQNRHRHFAQARNAADRLRRP
ERGRHLHPTPAGAHAPAGAFQT
>OLS48578.1 S-adenosylmethionine decarboxylase proenzyme [Rhodovulum sulfidophilum]AB14
MSDANLFRLGIGLETGAHAEDHHGHPAVDATRDVFDAARDDHFIRREGRIFAGTHLIIEVVDGDGLDDEDRIQKAFRD
CV
DECGATLLHIHTHKFSPQGVSGVAVLAESHISVHTWPEIGYGAFDVFMCGDAEPWRAVEVLRAAFDAGDVRVKELLRG
ED
VVAARVAA
>OLS48579.1 hypothetical protein BV379_10095 [Rhodovulum sulfidophilum]AB14
MKTEFLLGAALAVSLGAAAPAATLSASEILSQFSIVTAGDLDTNQDNYGRAYVGGNLETSGKADFANRDMPVSAYPDVI
V
EGNARGPGSIRIGSGGTTLAVRGDVSVDTVQFGVERTGEVLVGGTLSESTRQNINNKETPTFDGVRVESGLAGDPGFEA
L

FPTDMVDTLNRASEDLSTLAATGTATVSGGNTLNIDATSGGLNVYNFDISDFGGAIGQIDFSLGATDTLILNVSGSSILF
EDNLLGLQAADGQRIIWNFYEATAVDIDRMVYGTVLAGNAALTNSTAIEGNVFAASAVLRGQVHLQPFAGELPPTDPA
PV
PLPATLPMLAAGLGLVGLMRRRRS
>OLS48580.1 50S ribosomal protein L13 [Rhodovulum sulfidophilum]AB14
MKTFTATPADIEKKWIVIDAEGVVLGRLASIIAMRLRGKHKPSFTPHMDMGDNVIVINAEKVQMTGKKRSDKKYYWH
TGH
PGGIKHRTAAQILEGAHPERVVTAAVKRMLPGNRLSRQQMTNLRVYAGAEHPHEAQNPEVLDVKSMNSKNTRSA
>OLS48581.1 30S ribosomal protein S9 [Rhodovulum sulfidophilum]AB14
MAEEIKSLDELQSVVSGTPAASADEDTPREPVRDSLGRSYATGKRKDAVARVWIKPGSGKVTVNGKDINAYFARPVLQ
MI
LRQPFTVAGVEDQFDVMATVAGGGLSGQAGAVKHGISKALQLYDPSLRGALKAAGFLTRDSRVVERKKYGRRKARRSF
QF
SKR
>OLS48582.1 ABC transporter ATP-binding protein [Rhodovulum sulfidophilum]AB14
MTEPLLVIDRLSIGFSSRKGDIPAVEAFSLSISAGEAVGLVGESGCGKSTVALGILRDLGPSGRITGGTIRFRGRDLGQL
PAAELRRIRGNEIAMVYQEPMASLNPAMRIGRQLMEVPMLHRRASRAEARALAEAMVADVGLPDPTRILNAYPHQLS
GGQ
QQRIVIAMALMSRPDLLILDEPTTALDVTVEAAIIDLIRSLGRKYGTALLFISHNLGLIGEVCDRICVMYSGEAVERGPV
RQVFEQMRHPYTQALFRAIPRPGSDKTTGRLETIPGSFLMPQDRPRGCNFAPRCTHAVAGLCDATEIAMRQVPDPTTR
QS
GHETRCLRFEEIDWQSRASRPANAPQGGVGPPVLTVTDLRKHYEVSSGGLFGPRRRALLRANETVSFEARAAETLAIVG
E
SGCGKSTLARVLLGLETATAGQVLLDGHELQNTPVQKRKRRTVADLQMVFQNPFDTLNPAQRVGRQILRALEVFGIGK
TG
RARRERMFELLDLVGLPRDFADRMPRQLSGGQKQRVGIARAFAGNARIVVADEPVSALDVSVQAAVTDLLMEIQRAH
RTT
LLFISHDLSIVRYLADRVMVMYLGHVVEIGPTAEVFAPPYHPYTEALLSAVPVPDTRIEKTRIVLEGELPSAMNPPPGCP
FQTRCRWAQRVPGNLCSRELPPMVELGPGHSILCHLPPETLDEMEPVFRSAAE
>OLS48583.1 peptide ABC transporter permease [Rhodovulum sulfidophilum]AB14
MERLSWTGALGHWLVPALEAALVVLALAALAHPVALLAGGRIAPGSEATRSDASKAARVTAGLFRLSLGAVAAVILAFLI
GGMLAPPEAKGILGRIATRLWPVWLSLGASLALIALFRRRLGLLGHLPGNPAGAVGLGLVLFWIFAALFAGQIAPFDPVT
DVIAGMKNKPPGTPLPGHDGTAYGYYLLGGDALGRDVFSRAVSGARVVLAIAPAATVFAFMVGITLGLPAGYLGGRLD
AG
LTFLANLVLAFPVILLFFLLVTPEIVAMGVPRYMAGGLFLFPLVFLTILWKSRFRTRPPLCAGLIATTLVLGGWLYAGLA
WDADPLGLVALPPGALIVFVSVVFVNAPSVFRIVRGLTLDLRSRDYVAAAQTRGEEAWYIMLWEILPNARGPLLVDFCL
R
IGYTTILLGTLGFFGLGLPPESPDWGTSINAGRGLLQFVPFPPLVPAIAIMSLVLGLNLLADGLREESLKD
>OLS48584.1 peptide ABC transporter permease [Rhodovulum sulfidophilum]AB14
MIGFILGRLGAMVLSALCMSFVVFMLTNLAPNLEKLAKTQAGARITDAEVSRWLEKNGYAEPMLRRYGEWLGVLPAA
GAT
DAAGKVLGGRCLRGRPDPEPATAPRFCGVLQGDWGYSTKFRQPVAGVIAERLKRTGVLMGWAFAVMVPLALAIGVL
AGMR
EGSALDRGLSTISIATTSTPEYVSGVIFVALFASSMVGLKWFTGSAKSAVDGVTFHNFTLPVVTVALYGMGYIARMTRAS
MAEVMTAQYIRTARLKGLSFRAVVLRHALRNALIAPFTVIMLQFPWLLTGVVIVEALFVYPGFGDALVQGAQNNDIDLL
L
AVSVVAVIVVLATQLLSDIGYAALNPRIRVA
>OLS48585.1 diguanylate cyclase [Rhodovulum sulfidophilum]AB14

MTTPSDRPDRPHPAAELYAREYRAGLMDRREFLGRATALGLSAGAAYGLIGRAAPARAQPAPRRGGTLRIQMDCRELSDP
RTYDWSEKANESRGIVEYLAELKRDGTLRPMLLEGWQANGDATEYTLRLRPGVTWNTGEAFTAEDVAANLAGWCDSSVEG
NSMPSRMAAMVENGQAAEGAIEIVDDLTVRLHLLRPDGTLMFGMTDYPAAVMHRDRIGTSVADHGIGTGAYMFEEFSVGE
RIVLVRNPEHSYWGDGAWLDRIEFLDYGTDPAAWLAGADAGEFDMTYETVSDFIDIFTALGWVESTVGTSSTVVIRANQE
AEIEGIRPYADPRVRRALALAVDNAVPLELGISGRGRESRNNFHVAPGVHPDYAEIAPHRQDIPEALRLMREAGMMEYEH
ELISIDDDYRKNTTDAVAAQLRDAGFKVRRTVLPGSTFWNDWTRYPFSSTNWSHRETGIQMLNLAYRSTAAWNETGFRNA
EFDALLDQATAIADDTARREVMARLEQMMVDDGVAIIPYFRSLYRHAREGVLNAGMHPKYEINVHHLGWA
>OLS48586.1 AraC family transcriptional regulator [Rhodovulum sulfidophilum]AB14
MSLFSELRLVPFSRLTNGSRWRVEAMRSYSAPQLLWITKGQGRITVGGVTRGYGAHNAVFIPAHTMHGFEMTSQVYGTGI
VFAGLPDIELPEGPHHLRIRDGAVQAELTFLVESLQRELEGKRPEARRAIRHLAGLVSVWLDRQIALDEAGTGRPDPARR
LASGFAALVEKDFRSARSVSDYAAALGVTPTHLTRVCNRTCGRSASELLAERKISEARRLLADTRAPIKEIASALGYASP
AYFTRAFQARAGASPSRFRKGH
>OLS48587.1 tRNA (adenosine(37)-N6)-dimethylallyltransferase MiaA [Rhodovulum sulfidophilum]AB14
MSEKRDGFNAQQIGTGATGLPDLDPDRPVLIAGPTASGKSALALAIAEAQGGMIVNADALQVFEGWQVLTARPGPEETAR
VPHALYGHVPFTADYSVGHWLRDLAPLLEGPERPILVGGTGLYFTALTEGLAEIPPTPPEIRAEADRRLATEGHEALLDA
LDADTLARIDRRNPVRVQRAWEVQQATGRGLAAWQDETPPPLLPPGAAQPVVIEAGRDWLNARIDARFERMMAEGALEEV
RANLPRWDPALLSAKAIGAPDLVAYLRGEMPLAEAIAAAKTASRQYAKRQRSWFRSRLGSWPRWPAG
>OLS48588.1 UMP kinase [Rhodovulum sulfidophilum]AB14
MSDGSADQPKPAYSRVLLKISGEALMGDQGYGLHPPTVERIAREVKAVRDLGVEICMVIGGGNIFRGLQGSAQGMERTTA
DYMGMLATVMNALAMQSALEEMGVFCRVVSAIRMDEVAEPYIRRRAVRHLEKKRVIIFAAGTGNPYFTTDTAATLRANEM
ACEAIFKGTKVDGVYDKDPVKHDDAVRYDEISYDDCLVKNLKVMDASAIALARDNDLPIIVFSLDEPGGFRGILAGQGTYTIVHG
>OLS48589.1 ribosome-recycling factor [Rhodovulum sulfidophilum]AB14
MAEDELDIDLDDLTRRMDGAMASLKHEFSSLRTGRASASMLDPVTVEAYGTPTPINQVGTVNVPEPRMVTINVWDKSMVN
KVEKAIMESGLGINPQTNGTIIMLPIPELNEERRKELTKVAAQYAEQARVAVRNVRRDGMEQLKKAKAAGMGEDDNKIWH
DEIQTLTDKYIAQVDKALETKQQEIMQV
>OLS48590.1 phosphatidate cytidylyltransferase [Rhodovulum sulfidophilum]AB14
MASGGSWEDLLPRLGSAAVMATIGVLAVWLGGAWFTGLAALVSGTMVWELTRMMVPGASGRLPIQMALVSGATVFVAWLL
PPYAAFPLLAIPCVIGAWQLPRDRGIYLAFAMAMLVTGYGLASFRADHGIVWLVWLILVVIATDVAGYFAGRFIGGPKFW
PRVSPKKTWSGILGGWFAAMLIGLIFLSFTTAGPDLPWISMALSFASQMGDAAESALKRRVGVKDSSSLLPGHGGLFDRF
DGLLGASLFMLLVAQLVYVPEVRF
>OLS48591.1 1-deoxy-D-xylulose-5-phosphate reductoisomerase [Rhodovulum sulfidophilum]AB14

MQRRRITVFGATGSIGENTIDLLTRQGGAGTYDIVALTGGRNVKRLAEQAIALKADLAVTAFDDCLDDLRAALAGSEVE
A
AAGPAALIEAAARPADWFMSAIVGFAGLAPGLRALEHGTTLALANKESLVAAGPLLLGTAAKHGARVLPVDSEHSAVF
QA
LVGEDMAAVERVIITASGGAFRDWPIEDLAGATPEQASSHPNWAMGRRITIDSASMFNKGMELIETKEYFGIAPDKIET
L
VHPESLIHAMVGYRDGAIMAHVGPHDMRHAIGYALNWPERAALPVERLDLTRIGQLTFRPACETRWPALRIAREVME
AGG
MAGAIFNAGKEAALDAFLAREIGFTRMSDVVAEVLDRLSADTALMNSALDLENVLRVDHLAREEAARTVRSMPH
>OLS48592.1 RIP metalloprotease RseP [Rhodovulum sulfidophilum]AB14
MDIASLIPSFGNAAFTVAAFVVALSVIVTVHEYGHYIVGRWTGIRAEVFSLGFGPVLASRVDRRGTRWQIAALPLGGYVR
FLGDSDGASGKDAAAMAGMDADDRRHTMHGAPLWARVLTVAAGPVFNFVLSIAVFAMLAVVSGVAIEPPTVGKLQ
SLPAA
QSALREGDTILALDGRATPDYSAFYDAVADLPVDGETAYLVRRDGVEQEVAGPYPFPPVVSAIQPKSAAMEVGLKEGD
VV
TAIDGKPIRAFSQLQDVVTASEGAPIDLTVWRDGETLNLSLTPRRMDMPNADGGFETRWLIGMTGGLAFEPATRMAG
PLE
ALSYGADQLGFIVQSSLSGLYHMIAGKISSCNLSGPLGIAESSGAAASQGLESFVWFIAMLSTAVGLLNLFPIPVLDGGH
LVFYAYEAVVGRPPSDKALRVLMAAGFALLIGLMVFALSNDIFCP
>OLS48593.1 hypothetical protein BV379_10175 [Rhodovulum sulfidophilum]AB14
MRRSLTAPVLAAMLGLAASGASAQEGRTVFVSPILTLDQDRLFEDSKAGQAVDEQLEAATAALAAENRRIEAELVAEER
A
LTERRATLPPDQFRPLAEAFDHKVETIRATQDRKARELGQKREEKRQDFFRKALPLLAGIVRERGAVAVLARDAVVLSAG
QVDITDEAIKLLDERVVFTPDDGQAPDAPATGDEPSSDPAPETGGALLRPMPAPAPLRGATEGGD
>OLS48594.1 3-hydroxyacyl-[acyl-carrier-protein] dehydratase FabZ [Rhodovulum sulfidophilum]AB14
MSDAQAPDADIDEIQKILPHRYPFLMVDRVTQIIPGQSAVGIKNVSINEPHFQGHFPSKPIMPGVLIVEAMGQTGGVLV
G
RSVDLADDDLLVYFMGMDKCKFRRMVVPGDVLELHVKVRRGGSKKVKIWKFEGVAKVGDEIAAEAEFTAMMDINPE
>OLS48595.1 acyl-[acyl-carrier-protein]--UDP-N-acetylglucosamine O-acyltransferase [Rhodovulum sulfidophilum]AB14
MAIDPSATIHPSAVVETGAQIGPGADIGPFSVIGPEVRLGPGVVVKSHAIVTGLTEIGEGTVIFPFANIGDIPQDLKFRG
EQTRLVIGKRNRIREAVTMNTGTEGGGGITRVGDDCLFMTGSHVGHDAQVGNGVIMANHAALAGHCIIGDNVVIGG
LSGI
HQWVRVGEGAIIGAVTMVTNDVIPFGLVQGPRGGLDGLNLVGLKRRGVSREEISALRAAFHELGTGEGTFHDRAERLA
AS
TDSDHVRQMVDFVLAETDRSFLTPKS
>OLS48596.1 phosphatidate cytidylyltransferase [Rhodovulum sulfidophilum]AB14
MTGIPTALIAGSGALPAHLARAMEAAGTPFLIAQVEGYEMAGAGHWPVETFALERLALLFDLLHARGMHRVVLAGTV
HRP
RLEPERIDPRTAQMLPRILPLLAQGDDGALRAVIALFEEDGFEVVGADAVAPGLLPPPGVPTAAQPSEADRRDAARAAA
I
VAALGASDLGQGAVVAQGLCLAVETLPGTDAMLAWVADVAGACRPDPKGAKGVLCKAPKPSQDRRVDLPAIGPATV
EAAH
RAGLAGIAVEAGGVLVLEPEETLARADALGLFLWVREP
>OLS48597.1 lipid-A-disaccharide synthase [Rhodovulum sulfidophilum]AB14
MTRIFLIAGEASGDALGAALMAGLRQLDPEVAFFGVGGPQMAAEGLESLFPMDELSVMGIAEVLPRYSALMGRIKQC
AAE

VVRLSPDALITIDSPDFCLRVARRVKAQRRVRTVHYVAPTVWAWRPGRAARMAEVIDQVLALFPFEPPYMEEVGLRCD
FV
GHPVVAQPRASAEEALAFRDRHGIAREAPLCLALPGSRRGEVMRMAPVFGRALQTVLAQHPDLRIVLPAASGVAALVT
EL
TAAWPVAPLVIDPRGDPEGAAGDKRAAFAAADLALAASGTVSLELAASDTPMVIAHDMNWLSRQIIGRMLRLPTVTL
VNI
VSDTFAVPEFLGANCQPGPIAAALCLLLEDPTYRAPQLEAMRLTMDRLGQGGERPGLRAARAVMDGLGAGR
>OLS48598.1 tRNA 2-thiouridine(34) synthase MnmA [Rhodovulum sulfidophilum]AB14
MPLDGAARPLNSLGFAKPESETRVLVAMSGGVDSSVVAALLAEQGYDVVGITLQLYDHGAALAKKGACCAGRDIHDA
RRV
AESVGFPHYVLDYENVFRDAVIEEFADSYLAGATPVPCIRCNERVKFRDLLATAKDLEADCMATGHYIQRKIGPNGPELH
CAADPARDQSYFLFSTTPDQLDYLRFPLGHLPNKDATRALAVKYGLPVADKPDSQDICFVPEGNYASVIEKLRPGAAEPG
DIVHADGRVLGSHKGVIHYTVGQRRGLGIGGLEEPLYVVRLDVETRQVVVGPKEMLSTRIVPVREINWLGDRPLSAQSE
W
EVSVKVRSTRPPRSAILRPLSETEAEVELLTPEEGVSPGQACVLYETGNSRVLGGGWIWRGNNRG
>OLS48599.1 hypothetical protein BV379_10210 [Rhodovulum sulfidophilum]AB14
MFLKRIDGPRTVTLPDGSTMSRADLPPADTRRWVASRKAAVVKAVAAGLISEEEALERYRLSSEEFASWKKAVRDHGIA
G
LKVTSLKSTRRSEL
>OLS48600.1 DNA-binding response regulator [Rhodovulum sulfidophilum]AB14
MRVLLVEDDPTTAKSIELMLTHANLNVYTTDLGEEGIDLAKLYDYDLILLDLNLPDMNGHEVLRQLRLARIDTPILILSG
AADTENKIKGFGFGADDYMTKPFHREELVARIHAIIRRSKGHSQSVIRTGKIEVNLDAKTVEVEGKPVHLTGKEYQMLEL
LSLRKGTTLTKEMFLNHLYGGMDEPELKIIDVFICKLRKKLAQATGGETYIETVWGRGYVLRDPEPDQAMSGSFALGA
>OLS48601.1 DNA ligase (NAD(+)) LigA [Rhodovulum sulfidophilum]AB14
MTDERPVEALSESEARAELARLAEAVAKADAAYHQEDAPTLSDAAYDALKRRNAAIEARFPALKRPDSPSDRVGAAPAE
G
FAKVAHVRRMLSLANAFDGTDVAEFDARIRRYLGLATEARLLYTAEPKIDGLSLSLRYEGGRLVQAATRGDGELGENVTA
NARTIADIPGTLAGAPDILEVRGEVYMAHADFEALNARQAETGAKTFANPRNAAAGSLRQLDPAITAARPLKFFAYAW
GE
VSIPLAATQMEAIERLKALGFQVNPLTVLCDGPEALAAHYAGIEQRRATLGYDIDGVVYKVNDLALQERLGFRATTPRW
A
IAHKFPAELAWTRLDRIEIQVGRTGALSPVARLHPVTVGGVVVSNATLHNEDYIAGRDSKGNEIRGGKDIREGDWVQV
YR
AGDVIPKVADVDLAKRPDDTLPYRFPSLCPECGSEAIREEGDAVRRCSGGLICPAQAVERLKHFVSRGAFDIEGLGAKQV
EALYKDGWITEPADIFELEARYGEGVRQLKNREGWGEKSATNLFAAIETRRLIPLDRLIFALGIRHLGEAAADLLARNYQ
TWAAFEAAVTQATVGEGPKWEALLSIDGVGTVMATSLITAFHQEAERASIDRLVAHLTVEPVAPRAAVDSPVAGKTVV
FT
GTLEKMSRAEAKARAEALGAKVAGSVSKKTDLVVLGPGAGSKAKKAEELGIETVDEDGWLALIEGA
>OLS48602.1 ATP-dependent DNA helicase RecG [Rhodovulum sulfidophilum]AB14
MSGRPDILFPLFGALDTLDGIGPKSARLLAQLGVEKPRDLIFTLPHSAIDRRPVETVADIAPGEVATVEVLVGAHMPAPR
KGRPYRIQVQDAGTAFQLVFFHARGEYLAKILPEGARRIVSGKLEVFDGMAQIVHPDHVLPPEEAGEIPEFEPVYPLTAG
LTLKTMTRAVASALTRAPDLPEWIDPTLLADEGWQGWRAALTAAHAPQSARDIAATAPARQRLAYDELLAHQLTLALA
RA
DQRRGKGRASRGDGRLRQKALAALPFRPTDAQARAMEEIAEDMGSEARMTRLLQGDVGAGKTLVAWMALLVAVEA
GGQGA
MMAPTEILARQHLESLQPLAESAGVVIEILTGRDKGKDRAAKLAALAAGDIQVLVGTHAVFQKDVAFRDLRLAIVDEQH
R

FGVAQRLELGAKGRGVDMLVMTATPIPRSLALAGYGDLDVSVLDEKPPGRTPVKTALVSVERMEEVVAHLQKAVAEG
RQA
YWVCPLVEESEVSDLTAAEERFKRLRAVFGDKVGLVHGQLPPAEKDAAMRAFQQGETSVLVATTVIEVGVNVPNASIM
VI
ERAEGFGLAQLHQLRGRVGRGAAASTCLLMYQAPLGEAGRRRLEILRETEDGFRIAEEDLAMRGAGDLIGTAQSGLPRF
R
IADLERQAALMAVAQSDARRLLAEDPGLSGPRGQAARVLLWLMERDKAIRLLKVG
>OLS48603.1 ferredoxin--NADP(+) reductase [Rhodovulum sulfidophilum]AB14
MNQQTAVTEPAKKTLPDAQTVTEVRHYTDRLFYFRTSRPQSLRFRSGEFVMIGLPGDNGKPIMRAYSIASPSWDDGLD
FY
SIKVQDGPLTSKLQHIQPGDEIILRPKPVGTLVLDALLPGKRLWFLATGTGIAPFASLMRDPDTYERYDEVIMMHTCREV
AELEFGRQLVESLPEDPLIGEFVGNKLKYYPTTTRESSKWMGRVTDNLASGKVFADLGLEGTQMDPATDRAMVCGSLD
FN
KDVMAIMEGWGLNEGANSEPAQYVVEKAFVG
>OLS48604.1 hypothetical protein BV379_10235 [Rhodovulum sulfidophilum]AB14
MQPQNVIPGAIAFWVSGIWPSMKIEQTKNRIRQIGWLRSLPVPFVDMLLAGARQRHLCRGEALFHMADPSHDMIGI
ADGV
VDVLLEPGIDGPRLGFVAKAGNWLGGVGMPGTSLRRISAIARRDVTILSVSAQHIETVALADGETWRHIASNIVNHFDN
L
GLLLHANVHRDNVVRILITLRRLHDFNDGATEFPLTQTDLAEMAGLSRNSANRVVRVLVEDGLIETGYGWIRILDPSRLS
ARLVRAHLPDWIDVHRQPVEA
>OLS48605.1 C4-dicarboxylate ABC transporter [Rhodovulum sulfidophilum]AB14
MKALTTAATAVALTLSAGAAFADCDPGEAVIKFSHVTSSTSPKGLAADLLAERVNKEMDGKVCMEVYPNTTLYDDDKV
LE
AMLNGDVQMAAPSLSKFEKYTKKLRIFDLPFMFKNMEAINAFQTSETGEAMKQSMMRRGLLGLAFWHNGLKQFSA
DKPLL
LPEDAKGLKFRVQASDVLVAQMEALDASPQPMAFSEVYGALQTGVVDGQENTWSNIYTQKFFEVQDGITESNHGVID
YMV
VTSVEWWEGLDPEIRDQLATILQEVTVTRNTESTAVNQAAKQAIIDAGTTVRTLTPEQRQAWVDVMKPVWAEFEAD
VGAD
AIAVAQEINEQY
>OLS48606.1 TRAP transporter small permease protein [Rhodovulum sulfidophilum]AB14
MSGAYRPRTALGRAVHSVEETVIALLMGLMVLVTFVNVVLRYVFNDSLVWSLELTLTLFAWLVLFGIAYGFKITMHLGV
D
AVLNMVSSRSQRLLSLVAVAICLVYAALMMKGAWDYWAPFANMAPTTGHWFPTGFKEMKPWDYRGYVPTERIPLP
EVLRA
PLEYLFLPEGEEPYEKLPIAVPYAIIPFGVAMMLYRLIEAAFRIVTGRQQGLIVSHEAEDAVEEAAREKDA
>OLS48607.1 C4-dicarboxylate ABC transporter permease [Rhodovulum sulfidophilum]AB14
MHVVLLFVMVVGFLLIGVPIAVSLGMASIIFLLLFSDTSLASVAQSLYQAMAGHYTLLAIPFFILASSFMSTGGVARRII
RFAIACVGHLRGGLAIAGVFACMLFAALSGSSPATVVAIGSIVIAAMRKVGYSRDFAAGVICNAGTLGILIPPSIVMVVY
ASATDVSVGRMFLAGVVPGTLAGLMLMTAIYVMAHVKGMPKGEWLGWGEVWESFRDAFWGLMLIAIIMLGIYGIP
GVASA
IFTPTEAAAVASVYAFLIATFVVYRDMGPLKAQGGQGPVALWQRPVALLTGFIHRDTRRTLLDAAKLTIMLMFIIANALIL
KHVLTDEQVPQKIAGAMLDAGFGKIMFLILVNVILLIGGQFMEPSGLIVIVAPLVFPIAIELGVDPIHLGIIMVVNMEIG
MITPPVGLNLFVTSGVAGMPMMRVVRAAMPFLAVLFVFLILVTYVPVLSTWLPTSLMGPELITN
>OLS48608.1 iron-sulfur cluster assembly scaffold protein [Rhodovulum sulfidophilum]AB14
MQAQNDLIKLYSQKILALAAEIPHLDRLEAPMGTARKRSPLCGSTVTVDLDVKDGRIVRFGQDVKACALGQAAASVVG
SA

ILGRTRAEIEAARDQLRAMLKEEGPVPSAPFEGLEVLLPARDYKNRHASIMLSLEATLDAFDAAEAAASCA
>OLS48609.1 phosphoribosyl-AMP cyclohydrolase [Rhodovulum sulfidophilum]AB14
MAFDPASLTYNADGLIPAIAQDAAGTVLMMAWMNAEAVARTLETGRVTYWSRSRKAFWVKGETSGHVQRLIEMRV
DCDRD
CLLMLVEQAGPACHTNRRSCFYTAIRDGAEVEILTPMAD
>OLS48610.1 tRNA glutamyl-Q(34) synthetase GluQRS [Rhodovulum sulfidophilum]AB14
MRTRFAPSPTGPLHLGHAFAALTAFARAGDGEMLLRIEDIDSARSRPEWEAQIYDDLVWLGLDWPRPVLRQSDRLAAY
RD
ALDRLGAMGLTYPCRCRRADIRAALSAPQAGAPVIGPDGLVYPGTCRGRSLAEAGPEDAIRLDMARALDRLGPALPAFT
E
TGPAHAGRHVLEAQALIGTVGDVVLARRDLGTSYHLAVVVDDAFQQIGEVTRGEDLFEATPIHVLLQALLGLPTPAYHH
H
RLILDETGRRLAKRDDARAIAAYRAEGASPADIRRMIGL
>OLS48611.1 hypothetical protein BV379_10270 [Rhodovulum sulfidophilum]AB14
MLYVLALFLPPLSILMIGRWFVALITLLIWIPAIFFSGGLTHPMFIILAWILIFQRGADRRAGL
>OLS48612.1 methylenetetrahydrofolate--tRNA-(uracil(54)-C(5))-methyltransferase (FADH(2)-oxidizing) TrmFO [Rhodovulum sulfidophilum]AB14
MTETLHIVGGGMAGAEAAWQAAQAGIDVVIHEMRPKTGTFAHKSGDLAEMVCSNSFRSDDDEQNAVGLLHWEMR
AAGSLI
MEMADTHRLPAGGALAVDREAFSQGVTNRLMSHPKIKREDTEITSLPEDGHWIFATGPLTSDALGQAIRAETGAEALA
FF
DAIAPIVHFDSIDLTKAWFQSRYDKGETEAERTAYLNCPMDRAQYEAFIDALLAAEKTAFHEGETAGYFDGCLPIEVMAE
RGRETLRHGPMKPVGLTNPHAPEVKSHAIVQLRRDNALGTLYNIVGFQTKMKYGAQTDVFRMIPGLENARFARLGGIH
RN
TFLNSPTLLDAELRLKSRPHIRFAGQITGVEGYVESSAMGLLAGRLSAATILGRALPAVPATTATGALVTHITGGAEAKT
FQPMNVNFGLFPPVEGLKGGRRGRRDRYKGYTDRAKADWTGWLGAAG
>OLS48613.1 DNA gyrase subunit A [Rhodovulum sulfidophilum]AB14
MNDTPETPENEDENGSGKGPERPAYDGPVIAIEKEMQTSYLDYAMSVIVSRAIPDLRDGLKPVHRRILFAMHETGNTH
DK
PYRKSARAVGDTMGKYHPHGDSAIYDALVRMAQPFSMSLPLLDGQGNFGSMDGDSAAAMRYTEVRMAKAAQALLT
DIEKD
TVDFQDNYDGKDKEPTVLPARFPNMLVNGAGGIAVGMATNIPPHNLGEVVDATLALIEDPDLTSEQLMQYVPAPDFP
TGG
LILGRSGARKAYLEGRGSVILRAKTRIEEIRKDRHAIIIDEIPYQVNKSTMIERIAEAAREKRIEGIAHVQDESDRVGVR
VVIELKRDATPDVVLNQLFRFTPMQTSFGCNMLALNGGRPETLTLRDFLSHFVAFREEVVARRTAFELRKARERSHILCG
LAVAVSNVDEVVATIRASADAAEARVKLMERRWPAREIADYIQLIDDPTHKMNEDGTYNLSETQARAILDLRLQRLTQI
G
VKEVTDELEELAAKIKDYLDILRSRERIMAIIAAELGEVRAQFAVPRRTEIADWSGDMEDEDLIEREDMVVTVTQSGYIK
RTPLAEFRSQNRGGKGLSSMATKEDDVVTTLFVANTHTQLLFFTTDGMVYELKCWRLPQAGRTARGKAIVNILPIGTGI
S
IAALMKIDEPEDEWENLQVVFATSAGDVRRNRLSDFTNIKRNGKIAMKLPEAVSLVNVSICDAEDDVMLVTATGRAIRF
A
TTDVRVFSGRDSTGVRGIRLAEGDSVVSMSVIRHFAADPAERAAYLKQRRLMAGVTDENGEADEDEEAVEAGQLSPA
RYA
ELSAAEDLILTITARGQGKISSSHDYPVRGRGGQGVQAIDKALRGGPLVASFPVEMDDQVMLATSRGQSIRVPVDQISF
R
SRSAGGVRVLDAATGEEVVSVARIADHGENGDEDEVSAE
>OLS48614.1 aspartate-semialdehyde dehydrogenase [Rhodovulum sulfidophilum]AB14

MPLSETELMLRGYGLTTAEFYYRMPDFRNVLNSFIWQDYDLAPDHPRLFRFIQFWQESIEGPLHSVRFTHRKRISSGEW
R
NVVAEFRLN
>OLS48615.1 disulfide bond formation protein B [Rhodovulum sulfidophilum]AB14
MTATRNQLILLAAGASAATLLAGFGFEHLGGLAPCKLCLWQRWPHAVAPILGLLAVAIGGRMLPLSGMVTMAVSTGL
AIY
HSGVERHWWAGPSSCTGSGPSLSQMTPEQILTPSLVEPVVLCDRIAWELFGLTMANYNVLLSAFLVVVWAMALARGR
G
>OLS48616.1 cytochrome B [Rhodovulum sulfidophilum]AB14
MIRRLYDWTLSLADSRNAMWALFIVAFVESSIFPIPPDVLMVPMILARPSRAFAIAGVALAGSVLGGLAGYGIGWAFFE
Q
IGRPVLEFYGKEAYFEQFGARYNDYGAWAVLIAGVTPFPYKVITILSGSTGLNLGVFMLASFIARGIRFFLVAALLWKFG
TPVRRFIEKYLGLLFTLFVLLLIGGFYAVRFL
>OLS48617.1 hypothetical protein BV379_10305, partial [Rhodovulum sulfidophilum]AB14
MLGGMLTVREIKAAPAGRYGDAGGLSLIKTSQDSGKWNFRYSFLRTRRNMGLGSWPEVGLADARRERDRWAAVLRA
GRDP
ITERKEERAATKAQRDNTDPTFAEMAQLVFEARKATLRGGGERGRWFSPLERHVLPHIGKMRVSEITPQDIKGALAPIW
R
TKFPTAEKALQRTRIVLHEGRLMGFGCDPFSAEAAQRMLGAVIHTPKHIPATPWQDMPALYARLNAGTVGHECLRFM
MLT
LVRYMGCAGARAAEVEGDVWTVPPERMKGTLKHVREFRVPLTDPALAIVQRQKEAFGDILFTGGRSAP
>OLS48618.1 MFS transporter [Rhodovulum sulfidophilum]AB14
MFRRPPLALVILLLSFPQIVETIYSPALPLIAVAYRATPEEAAQTLSLWFVAFAFGVVSWGRICDLIGRRPALLAGLALY
ALGAGWAMIALDFPQLLLARLISAFGAAVGSIVTQTSLRDSYRGTDLSRVFALLGLALAVSPAIGMFLGQAISSHAGHQG
VFAGLGLLAVLLFVLSLAFWPETRPPEIQPQHFLPTLQDMLRDGHIWRNAVLIAAFNLAIFGYYQLGPFLFERQEDRWLE
FGESGLVLAAASLLGAWANSLLLRRGTPPRGLVGYGVLLLAIGALVLSLLASGPAFVIGMAVVALAYAMAIPNILAEALQ
NYTDRLGTAGAILSMLYYTLLGLGLVVAGWGQRLDLLVLACAMLGALAFRGGQLTRR
>OLS48619.1 AraC family transcriptional regulator [Rhodovulum sulfidophilum]AB14
MVWLAPSDPFDADQLARPVVGIASRMVQHDSGWHSHARGQLLFAYAGSIRIMLPDATSILPPIRMVWIPPGTPHRVT
IRS
EVEYRSIYLDETRVAAPFAGTAIVAMTPLLREVFERMSLQPFDTDWSAGAARNLLAVCLDELATARHEPMLLPVPSDPR
L
ARLDIEELPPGLEDLSCRIGVTTRTISRIFRRETGMSYQAWRQSWRLLRAVDLLASGLGVTRVASDLGFSSDSAFIAFFR
QMTGETPKRYIDKRG
>OLS48620.1 hypothetical protein BV379_10330 [Rhodovulum sulfidophilum]AB14
MRLPLRTFAVAAAFAATTSPSSAQDALSEDQCFAVLGAMSKLELSMVGKVPLEDARAELSGLQSMLPESVSPRIDDLVA
I
AEAARGIAVGDPAHPMATGEFQEASKSYREALAPYCPGFNLDY
>OLS48621.1 hypothetical protein BV379_10335 [Rhodovulum sulfidophilum]AB14
MRTDTGHYDWLQASKREVTADEHALRIEVISGFEPLDAQFGVLAPKSVPDKLHAPLFGQPEPNPAEIAPEADISAAPSL
G
TFAVLDAARLPNLPEILSASGLEYACLFKGGAADELQDVAPWIVRLEEESRFTRSLFTQGDAPWAMWNQAPGIYLRSRD
G
LDDVRNHLRKFIRARDDEGTWFYLRLYDPRIMRAFLQGAPRFAHRLLARPFPWSNLTILTCFEDSAQIASPAENVPADM
R
PIRIGSEEKAVFRHLTFEARAAALMARLGKSNATSMSTDPNVRLAQKSAIISAFHRMYDYGFQQPLQQDRWGLWELFY
GA
GFEMNDPILKRICDTSDAPSGVRFAAFQRRLEEIYS

>OLS48622.1 hypothetical protein BV379_10340 [Rhodovulum sulfidophilum]AB14
MQNSPVVECAASPLSKFDSAEQLRNIRGVQSHVPPDFVNEGNCLALICNRSARKSGHSLTKFTRPFLHLSSTSAAQTQQ
Y
THITIESREAHSPDPRKVNDCATSRRQRRVVPDETIAEPVPLNLGDSKPCFIELASHNRADVLARGCDTGGAGGGIADD
H
RRAEARQYLGHGPAPVEGLCRFFTAKARRSVMHQIADPAALTGMAVWRPEHLGALGQGIETDDRQGHATDCSKTAA
ADRD
GNSAEHFNRAQPHKRDASGVTGQPIAKELFFTACSRIAVAGHPVAHDRIEPIRAFLIRWKRVDTVISARHIFSEGRALYI
PVDRCVDADDAGAVVDIPPTEAGKLGFEVANTVAKRDCLIFKVLPFGHLCAGMTSHHELTANRPSQLLLARGDLVVSVL
R
RPKRLPHRSGLRCIVQKLQPVAKREVAFIGEKLKQKARPLRVMPGKAVEQAILGQERAIEDDGQNLITQWQVFDVGKP
EH
LIKSDIALVERENDAFSTAALTFGAGHIGIVFPFARANRASGNCCQACSSWGGRDILRHQL
>OLS48623.1 hypothetical protein BV379_10345 [Rhodovulum sulfidophilum]AB14
MRDLCEPGKDFSASLLAADYLIGISALVVKSLIACIDIHEVRKRILFRRQVVVIQMRIFQRIYQGARLIPVDGHIRYLLL
ERPAIMRVQPLCWINILVLFVDTFRQDSLKIFMIFALRLVAFFIVPAFFLHLQCSFGFPR
>OLS48624.1 hypothetical protein BV379_10350 [Rhodovulum sulfidophilum]AB14
MRLAAFAIVLETMAYQWLILPGRGDDPRPVDRNVELLEQLVEHIGLDQRLPDKGEFLSKMVAGGIMSSDEARDKLNLA
RR
GGAADELRAQTALPPPHRSRKGTAMSKRKLPKVALGARPGVQGDLSPRALQRWSPDVRAADPGEASISVLAPIGADP
WGE
GVTARRISAALRAIGERAVTVNINMAE
>OLS48625.1 hypothetical protein BV379_10355 [Rhodovulum sulfidophilum]AB14
MARRLPSRGGRTAQEIRPDKPAKAAQKDTTARWTIKHSKAKTRAGGSRPVDFAIPVCGYKNHISIDRQNGVRSARSDQ
WR
SGSHSGVKSSPTRPGMTAPADT
>OLS48626.1 hypothetical protein BV379_10360 [Rhodovulum sulfidophilum]AB14
MEGLRAFFALGLQAGTARLSNHRNRPMLPTMLASVIVVLARARPMVRMISPIRSFWWPKTCSTFERTTDLRPFAQDVL
VY
IGRPPGFLRWISLTMPLAASQASFSTDR
>OLS48627.1 hypothetical protein BV379_10365 [Rhodovulum sulfidophilum]AB14
MQRHYPQLMEFMRLQIASRRAFERLRPCGPAALICQERAACFLSLKRQAFGGRPSGVFGVQPGCGPRFSLA
>OLS48628.1 acetylornithine deacetylase [Rhodovulum sulfidophilum]AB14
MTRLDDTCAILADLVAFPTVSSESNLEMIVHIAALLGDLGARVDLFHDATGHKANLFATLGPRNTDGGIVLSGHSDVVP
V
ADQDWSTDPFALHEAGGRLYGRGTCDMKGFIAACLAMAPVYAGLELTRPLHFAFTHDEEVGCLGGQALVAELAARGL
RPA
IALIGEPTEMRIVEAHKGCCEYTVEFHGREGHGSNPDAGVNAAEAAVRYVTRLMRLAEELKTRAPEDSPFEPPWTTVNL
G
RISGGQVHNVIPGKAEVAWEMRPVRSDDIGFVKEAMARHVAELLPAMRAVAPEASILTHVIGEVVGLEPLTENAARRL
LS
ELTGQSRAETVPFSTEAGLFQSLGCAAAVCGPGAIAQAHKPDEYVTRDQLAQCLAMLEGLAGVLTA
>OLS48629.1 ectoine hydrolase DoeA [Rhodovulum sulfidophilum]AB14
MTEEIVQFGYAAEGRAPFSLAEYERRLGLVRRAMDEKGIDILFVEDPSNMAWLTGYDGWSFYVHQGVLVFHDADPM
WWGR
QMDANGAVRTVWMAGAHITHYSDAFVQSTERHPMQNLAGLLHDLGYGARRIGVEMENYYFSAKAYVTLRDALPEA
HLVDA

TALVNWQRTVKSDEELDFMRRAASISERMIDGVLERIEPGLPKNELVADLYRDALAGNGDWGDYPAIVPLLPSGKDAS
AS
HLTWDGRPFESGEATFFEISGCYRRYHAPFCRTIFLGEPPDYMRTAEAALIEGIEAGLWAAKAGNRACDIANALAAPLER
AGIRRGARCGYPVGLSYPPDWGERTISLREEDETVLEEGMTFHFMPGLWMENWGLEITESILIREEGPAETLCNRPRKL
Y
VKS
>OLS48630.1 hypothetical protein BV379_10380 [Rhodovulum sulfidophilum]AB14
MLKNDMLEQWDRESFFHPSTHLAQFARGEAPERVISGGQGVFIEDRDGNRLLDAFAGLYCVNVGYGRSEISEAIAEQA
RQ
LSYYHAYVGHGTEASITLAKMVMDRAPDHMSKVYFGLGGSDANETNIKLVWYYNNVRGRPAKKKIISRWRGYHGSGL
MTG
SLTGLAGFHNKFDLPLAPILHTEAPYYYRRPDLDMTEADFVAHCAAELEALIEREGPDTIAAFIGEPVLGTGGIVPPPAG
YWEAIQPILEKHDILLIVDEVVTGFGRLGEMFGSSKYGLKPDLITIAKGLTSAYAPLSGSIVSDRMWKVLEEGTDEFGPI
GHGWTYSAHPIGAAAGVANLKLIDDLGLVRNAAETGGYFVNAMKTALSEHAHVGDIRGEGMLCAVELVEDKGSRTFY
DPA
GKVSGAVVAEMATSGVIARAMPQGDIIGFAPPLCLSKEEADRVVEVTAKAVKAVLG
>OLS48631.1 hypothetical protein BV379_10385 [Rhodovulum sulfidophilum]AB14
MPRAKASRRHCRIPYGYRSSNGPGGLRCTVPCRTGRLYDISICAADMGPANFVYGYRGASRPASNRVPQWVLVDGFG
DRH
AITARTYSRRIRLRGMDSRFRKGKKVRGLRWCPEARQFEFEDDDRPAT
>OLS48632.1 hypothetical protein BV379_10395 [Rhodovulum sulfidophilum]AB14
MPGLFMPGLGMAGLLLALAGCNAAMAPGGEASQRPDQTAPGAPLSVLEALPPEPGDPASATDGTAGATATAPAGL
DEASA
PVFGFERDLGVTTASLGDPTDAGFWALTPLTDEIRRGRLADPVSGKSVQVELRPSGGATGAGTRVSLSAMRALEVPLISL
TNLKVYVSE
>OLS48633.1 hypothetical protein BV379_10400 [Rhodovulum sulfidophilum]AB14
MSDESEAWHMMPVAILAILWNLAGCADYVMTQYGVAPYLQLFTQSQAEYFTSVPAFVDGAWAVAVWGGLLGAVLL
FLRVG
VAPWILGFSALGMTVAAVWLIVFATPGLTEVAGYFGDAMIAGAAAMAILFYVYARQMRVAGEI
>OLS48634.1 hypothetical protein BV379_10405 [Rhodovulum sulfidophilum]AB14
MHGRDKLLECIGGEALLRRQVRAAGASGAQVLVLVTLPRHAAGPRSEQLDALPRLKVAYVDPAEGMAASLRAGAAAA
RAA
GARGLMVLLPDMPDIETGDVVRMIEAFARAPDRCLRATTEDGRPGHPVLFPARLFPALEAVAGDTGGRDMLAAETVC
PVV
LPGERAVTDLDTPEAWAAWRARSGH
>OLS48635.1 branched-chain amino acid ABC transporter permease [Rhodovulum sulfidophilum]AB14
MTKRDIGLFVLVAVLLVGTGLLQSWNAALYILNYGLISAVMALGVNMQWGYAGLFNIGVMGFTALGGLAVVLVAMA
PVGA
AWSAGGSHVLLALAVGLGVILGAIQIWRRVGRGAIRGLAMVVWLVAGFFLYRAIFDPAVSAIEAIDPAATGYLGGLGLP
V
LLAWPVGALFAAGAAWVIGKTALGLRADYLAIATLGIAEIIIAVLKNEDWLSRGVKNVNGIPRPVPYEVDLQASAAFVEK
AQALGLDPTTASTIFVKLLYAGLFAVVLIALLWLSEKALKSPWGRMMRAIRDNETAAAAMGKDVTRRHLQIFVLGSAIC
G
LAGAMMITYDSQLTPGSFQPLRFTFLIWVMVIVGGSGNNWGAVLGGFLIWFLWVQVEPLGAWFMGAITSGMADGS
ALKTH
LLDSVAHMRLLTMGVVLLVVLRFSPRGLIPER
>OLS48636.1 branched-chain amino acid ABC transporter permease [Rhodovulum sulfidophilum]AB14

MDFINALVTFSNFVLVPATAYGAQLALGALGVTLIYGILRFSNFAHGDTMAFGTMMTILATWALQAAGVSFGPLPTALL
A
LPFGIAATALLVLGTDRLVYRFYRAQRAKPVILVIVSMGVMFILNGVVRIVIGPGEQRFADGERFIITAREFKTLTGLDE
GLAIRTTQGLTIVTAVIVVALLFWFLNRTRTGKSMRAFSDNEDLALLSGINPERVVAITWIIVAALATIAGVLYGLDKSF
KPFTYFQLLLPIFAAAIVGGLGSPLGAIAGGFLIAFSEVTVTYAWKKVLTYILPDTLAPDGLVQLMSTDYKFAVSFVILV
IVLLFRPTGLFRGKTA
>OLS48637.1 hypothetical protein BV379_10420 [Rhodovulum sulfidophilum]AB14
MRFFFMMAGLLAVSAGAAEARDLRLDCVARQVCVSNIEPGRASCQQTELRYRLEVGRKTGAKVVMTPEGDDAGMFY
EFRR
LPGRAGIRLQASGGALEAGQGAGALTVFETLDFVLTRHNALQLDPEDGALQAIAVNVHGSCAESE
>OLS48638.1 ABC transporter ATP-binding protein [Rhodovulum sulfidophilum]AB14
MADPFLIGDRMTGGYGGADILHGCTVAVEKGEIAVIVGPNGAGKSTAMKAVFGMLDIHSGAVRLNGEDITRLSPQAR
VAK
GMAFVPQTQNIFTSMTVEENLEMGAFLRRDDISATIEEVYQLFPILREKRRQAAGELSGGQRQQVAVGRALMTRPSVL
ML
DEPTAGVSPIVMDELFDRIIEVARTGISILMVEQNARQALEIADKGYVLVQGRNAYTDTGRALLDDPEVRRTFLGG
>OLS48639.1 ABC transporter ATP-binding protein [Rhodovulum sulfidophilum]AB14
MIRVENLHRHFGGFHAVDGASIEIATGSITGLIGPNGAGKSTLFNVIAGVLPPTSGRVMMNGEDITGLKPHELFHKGLLR
TFQIAHEFSSMTVRENLMMVPAGQSGESLWSACFRRGRIAEEERALARKADEVLDFLTISHVADEKAGNLSGGQKKLLE
L
GRTMMVDARIVFLDEVGAGVNRTLLNTIGDAIVRLNRERGYTFCVIEHDMDFIGRLCDPVIVMAEGKVLAEGKADEIM
QN
EAVIEAYLGTGLKNRIKAGPVSEPVSEAGA
>OLS48640.1 branched-chain amino acid ABC transporter substrate-binding protein [Rhodovulum sulfidophilum]AB14
MKKLLLASAATLGCAGGANAEDIKIGVLLGFTGPIESMSVAMGAGADLAIKEVNESGRLLDGDTVVAVRGDATCIDSSA
A
TAAAERMITSDGVKGIMGAACSGVTGAVLQNVARPNGMVMISPSATSPALSDVEDDGLFFRTAPSDARQGQVMANI
LKDD
GVDSVALTYTNNDYGKGLANSFETAFTAAGGTVTISAAHEDDKGDYSAEVGALAAAGGDLLLVAGYADRGGAGILRSA
LD
LGAFDRFHFPDGMVSDATTDAFGDEIDGSTGQLPGNDTPGAEKYAEIVDGAFDSTAIFSGESYDATALILLAMQAAGST
D
PQVYKDKIMDVANAPGEEIYPGELDKALQILADGGDIDYVGATALEFIDGGESRGNYRVVKVEDGKFVATGYR
>OLS48641.1 AraC family transcriptional regulator [Rhodovulum sulfidophilum]AB14
MKAVPERSGPSFGAAAEGRTQDLVFVLLDRFTMISFAGAIEPFRIANRMAGRTLYRWRLAAEGGEEVTCSNGTRIRPDI
G
FEPLGRDEMVIICGGLDVQSETTRGLVAWLRREARKGLVMAGLCTAAHALAKAGLLDGKRATIHWENQDGFLEDFPEI
EL
TRSVFVIDGNRMTTAGGTASIDLALTLIARTHGQDLANAVADQLVYSSIRTDQDTQRLSIPTRIGVRHPKLSQVIQVMEK
NLEDPISPATLARDVGMSTRQLERLFRRYLNRSPKRYYMELRLSKARNLLMQTDMSVINVALACGFASPSHFSKCYRAH
Y
DTTPYRERGSRSGRTEA
>OLS48642.1 3-deoxy-7-phosphoheptulonate synthase [Rhodovulum sulfidophilum]AB14
MTTWQKSDWRARPRVQMPDYTDAAALAAVEAQLAKYPPLVFAGEARNLTAKLGQAARGKAFLLQGGDCAESFSEFS
ADTI
RDTFKVLLQMAIVLTWGAKVPVVKVGRMAGQFAKPRSTATETVNGTELPSYRGDIVNGFDFTPEARIPDPSRMLHAYT
QA

AASLNLLRAFSTGGFADIHRVHAWTLGFLERDEAEKYRDMANRISDTLAFMNAAGVNGETMNGLSSVDFFTSHEALLL
EY
EEALTRIDSTSGHWIAGSGHMIWIGDRTRQIDGAHVEFCRGVMNPIGLKCGPSMSEDDLKILIGKLNPDNVPGRLTLISR
FGAGKVGDHLPRLIRVVREMGAEVLWVCDPMHGNTIKSASGFKTRPFDAVLREVQEFFAVHKAEGSIPGGVHFEMTG
KDV
TECTGGVRAVTDEDLSSRYHTACDPRLNASQALELAFLVAEELSARNDAAAAAAAI
>OLS48643.1 hypothetical protein BV379_10450 [Rhodovulum sulfidophilum]AB14
MVESETHPRAAVIALSGYRTGGALSALDDIERYWTQLRDDQGSLPSRAQLDPRGFSRALSNCFVAEAMAPRHLRLRLT
GQ
HMTDLMGMDMRGMPLSAVILPESRPMLEDKIEALMTGDGTGVRLTLISPRSYNRQALFAGMLLLPLARENGTPAKVI
GAL
ATVGTVGWTPRRFQILGETANPARRPDLAEGDAESRRPSLTVIQGGVVEPAC
>OLS48644.1 YicC family protein [Rhodovulum sulfidophilum]AB14
MLQSMTAFAARSGQTETHAWTWELRGVNSKGLDLRLRLPDWVPGLEPVVRAAVQSRVARGSVTLGLRISARDSGHG
GLQI
DPVALRSVLAMLGRVEAEAAAQGLSLAPSSAVEILALRGLADSAAPETETAPLLAALSADLTPLLEEFMAMRASEGAALA
TVLCDHLATISTLTSRAAEVAEARRDQIEETLRASLVRVMETAAGVDPDRVAQELALIAVKADVTEELDRLRAHVAAAET
LLASGEPVGRRLDFLMQEFNREANTLCSKAQSTGLTRVGLDLKATIDQMREQVQNVE
>OLS48645.1 guanylate kinase [Rhodovulum sulfidophilum]AB14
MAIRRGLLIILSSPSGAGKSTLARRLRAWDPEIAFSVSATTRAPRPGEVDGKDYHFLTEPAFKAQVARGEMLEHAFVFGN
FYGSPEAPVRAAIEAGQDVLFDIDWQGAQQIRASALGQNTLSVFLLPPSIAELRRRLVERGQDTAETIAGRMQKSWDEI
S
HWDGYDFVLVNDDLDATEAKLKTILTATRLRRSQQPGLTEHVRALQAEFEALP
>OLS48646.1 gamma carbonic anhydrase family protein [Rhodovulum sulfidophilum]AB14
MTLYTLDGVAPTLPEDGDVWIAPDANVIGKVILEDGVSVWFGATLRGDNEPITVGEGTNIQENTVCHTDMGFPLTIGA
GC
TIGHKAMLHGCTIGENTLIGMGATVLNGARIGRNCLIGAGALVTEGKEIPDNSLVMGAPAKFVRQLDAEAIEGLRQSAR
H
YRENAARFRTGLTAG
>OLS48647.1 hypothetical protein BV379_10470 [Rhodovulum sulfidophilum]AB14
MGNTGTDRSAQMAPGVDRNGMALLIGFDRVDQPQRGMPFIGARCEHLPYDLVQAALSSLQGVEMIVSPLLTDQFD
AMDLA
QQLQLGGYRGLYVVVTPVLPNSEIVRREIAALCPGMTVQMIPRTRH
>OLS48648.1 two-component sensor histidine kinase [Rhodovulum sulfidophilum]AB14
MNDSLGLSIIAGVPVPVLLIGADERVSGMNGAAEALFGPSGAGRHYITVLRHPALLDCVEEALRLRQSRRARYPRSAFGR
DATYDVTASPLEGVAAGGVVLSFTDISDLEQAGQIRRDFVANVSHELKTPLTALMGFIETLRGPARNDEAARDRFLDIM
A
REAERMNRLVSDLLSLSRVEAEERMRPGARVDVAALLRSAVQTLRPLAEEAGVALTLSGAEAEAHVLGDEDQLTQVFH
NL
IENGIKYGGSGGVLRLEMTRSERELAFRGPGLRIDVIDEGEGFDPVHIPRLTERFYRVDNHRSREMGGTGLGLAIVKHIV
NRHRGRFRIDSAPGKGSTFSVTLPTV
>OLS48649.1 phosphonate ABC transporter substrate-binding protein [Rhodovulum sulfidophilum]AB14
MSLAKLTASALAIAAVSATAAAARDNVQVAGSSTVLPYASIVAEAFGENFDFPTPVVESGGSSAGLKRFCEGVGETSIDI
ANASRAIKDKEVAACKANGVSDIIEVRIGYDGIVFASQLNGPAFTAFTPADIYRALAAELPVDGKMVANPNATWADAN
PE
LPAVDIAAFIPGTKHGTREVFEEKVLIQGCEETGAMAEMEAIGMDEKAAEKACKSVRTDGKSVDIDGDYTETLARIDSN
P
NGIGVFGLAFYENNTDTLKVATMSGVAPSTETIASGDYPVSRPLYFYIKKAHIGVIPGLKEFAEFFVSDEMAGPDGPLAA

YGLVSDPELAATQQKVANEETMSN
>OLS48650.1 phosphate ABC transporter permease subunit PstC [Rhodovulum sulfidophilum]AB14
MPVLWLVLILFALGALGFVLGRRRALGGAGGDIRLLHSLPAYYGTNVALVTMVPAFFLLGIWLLAQPVAIDARVSALLPD
AAISEGMTRDLVMSDVRRVAEGLDKAVAEGEMTRREARDIRAGFTDIRNRLGAVGVALGSNVGAPELYAAQRYRTLS
GEG
RVAMTAAVLALALGGFALSLIRTTPAYRARNSVEAGIRYALIFAATVAILTTIGIVLSLIFNTIDFFRLYPARDFFFGTT
WSPSFGGGSELGILPLLWGTFYISVIALAVSVPIGLFAALYLSEYASSHVRAIAKPLLEVLAGIPTIVYGLFALLTVGPL
LLKVFGQDGVGLMQAGTAVMTAGLVMGIMLIPFVSSLSDDIINAVPQSMRDGSLGLGATRSETVKQVVLPAALPGIVG
AI
LLAASRAIGETMIVVLGAGAAAKLSMNPFDAMTTVTAKIVSQLTGDADFASPEALVAFALGMTLFVITLGLNILALYIVR
KYREQYE
>OLS48651.1 phosphate ABC transporter, permease protein PstA [Rhodovulum sulfidophilum]AB14
MTDIPVSDARPVPAAPARTGSLLTADARTRRRNAAESRFRLYGIVAVGIGLFFLAALLYAIVTNGIGAFQQTGYTLNIEF
REDVLDKSGTRDPEALKSVRTFAYSPLLREAMLREIETHGIETPLAKAKVKGFSSPLDKLFSDGARSTLRDMVLADPSLI
GQTVKVRILASSRVDGYLKGRVSRESIERDKNLDAVHLDLIDKLRDAGVATKAFNLAFITGPDASDARPEQAGIGVAMLG
SFYMMIVVLALALPIGVGASIYLEEFAPQNRITDLIEVNISNLAAVPSIVFGILGLAVFIQVVHLPQSAPLVGGLVLSLM
TLPTIIISTRAALKAVPPSIRDAALGVGASKMQAVFHHVLPLAMPGILTGTILGLAQALGETAPLLLIGMVGFVASNYPD
GAVAGFMAPNSAMPAQIYEWAKRADPAFYERAWGGIILLLVFLMTMNIIAILLRRRFERRW
>OLS48652.1 phosphate ABC transporter ATP-binding protein [Rhodovulum sulfidophilum]AB14
MYDNSDLARATVTGETKIAAHGCQVYYGDSQAIRDVDIDIADNTVTAFIGPSGCGKSTFLRCLNRMNDTIDICRVIGDIR
LDGEDIYDPRVDPVQLRAKVGMVFQKPNPFPKSIYDNVAYGPRIHGLARNKADLDEIVERSLRRAAIWDEVKDRLQAP
GT
GLSGGQQQRLCIARAVATEPEVLLMDEPCSALDPIATAQVEELIDELRAQYSVVIVTHSMQQAARVSQKTAFFHLGSLV
E
YGDTAKIFTNPSDPRTESYITGRIG
>OLS48653.1 DNA-binding response regulator [Rhodovulum sulfidophilum]AB14
MSPSERPTVLIVEDEPAQREVLAYNLQAEGFRVSKAENGEEALLLVEEQAPDIIVLDWMLPSVSGIEICRRLKTRAETRG
VPIIMLSARSEEVDRVRGLETGADDYVVKPYSVVELLARVRAQLRRTRPATVGQRLEFEDILLDAETHKVFRSDKPLKLG
PTEFRLLSTFMEKPGRVWSREQLLDRVWGRDIYVDTRTVDVHIGRLRKALCQHGGPDPVRTVRGAGYALG
>OLS48654.1 methylamine utilization protein MauG [Rhodovulum sulfidophilum]AB14
MTRTGTATGLTLAAALLACGAASADPEEGAYSSPEAFGEALFFDVNLSKTRTQACATCHDPGHAFTDPRPTLAGRAVSL
G
ADGQSLGDRNAPTAAYARFSPPFGQRADGTYVGGQFHDGRAAGLAEQAAGPPLNPIEMGLADVDEAAARILENPRY
DATL
SALYGAATPGDPEAVFRAATSAIAAFEGTDSFAPFDSKYDRYLRGEAQLTDEEELGRILFFSQQFTNCNLCHQLRKSAIA
ADETFTDYSYHNIGVPANAAARAVNGAAGPDLGLLANPAVEDPAAAGKFKVPTLRNVAVTGPYMHNGVFEDLRTVIL
FYN
KYNSKAAARQIDPETGEAWAAPEVPGTLSMDKLTTGPALDDERIDALVAFLETLTDSRYEYLLDRDTASAGTGGTDTDR
>OLS48655.1 LuxR family transcriptional regulator [Rhodovulum sulfidophilum]AB14
MSLEYLSALIAARSLEELWALHTRKMADFGFDRLIYGFTRFRAANSFGDRDDVIVLSNHSPEYVRNFVDKGLYKNAPMV
R
WAAKHVGTSSWRHVSERLARGELTPEELDVLKFNRGMGVVAGYTISFSEVSVRAKGAIGLTARPGLSQDDVERIWARH
GD
VIQLMNDVMHLKVHNLPYAAQRPPLTPRQREVLEWVGDGKTTLDIATILGVTQATVEKHLRLAREALDVETTAQAVLK
AW
FQNQIFRANTP
>OLS48656.1 hypothetical protein BV379_10520 [Rhodovulum sulfidophilum]AB14
MTVFTPTASPARPPLPQALRLLCLALLVTAVFGALHYILVAPSRHVPVLHPAGAFGFVLFALSVLLRGSKAWGLRLWTAV

GGGVALLAGLRLIDGLLPGGPYLSGAVPLWPDRSPGPAALDLSTTYALMTLHLCLALQPFHRGAAMAVLVAAWTGIW
WSA
MTDLFEATIWGRPVSAFSLSCLGLAGLAMTHILRAEWLLRPLFSRERHGPRMRILLVLALIGPWLVGIATLHAVPETVSA
RLLIAPLLGLFSLAQLALVLTVGALLERAARADARAELFDPVAGRASKFRPDRDLLDGKGPVGVILCAPRTGVNGADPEA
DERAMRSALQRTVRHVRAEDNIGRWRRHMLMVVAHVPSEADLARIGERLERKLRPQPDLPVAAIGISMSAFDEPNLE
AAL
QRAESALYVAERRGGGTLVRVSNLAETGQGAEARNSPRDMGGAARPP
>OLS48657.1 hypothetical protein BV379_10525 [Rhodovulum sulfidophilum]AB14
MPSADQRPRKTLAIRVLGLTILMAIGLGLLQNLLDPPADTAAALHLLPATGFAVFGLLFLLRRQPSGLRGPVRMLACIVA
LLAIERLFEASVPGWIRIVGHPRIEHALESTGLQGVLSVETAFVLAMLNLALLAEPHQRQTSLLFLAAAWVGTTMGVLKL
AFNLTLWRGEMSPYALAIMILVCSAQTLKLRDQPFLRPLFARTSYGTYTRALVTGALLVPWMSGFLYFLNVRPDPASHL
A
LELAFGFIGWIMLALVLAIGHLMEKSSLALEYAARHDPLTGALNRRGLAMALGNRDEARGVVLFDLDHFKAVNDALGH
DA
GDRLLCDIAQIANRNIRESDLLVRWGGEEFLLVMASSAEVLLVQMAERLRQGIAAHAPMRIGAMPHPVSASFGVSMV
QPG
EAGIGCAIRRADTALYAAKIGGRNRVMSAAALCEAEAPVGPFARRTPPRLVS
>OLS48658.1 translation elongation factor Ts [Rhodovulum sulfidophilum]AB14
MAITAALVKELRDKTGAGMMDAKKALTENDGNMDAAIDWLRTKGLAKAAKKSGRTAAEGLVAVVVEGGKGVAIEV
NSETD
FVAKNAEFQAMVSDIAKVALGVDDVEALKAADLGGKSVADTLTDKIATVGENMTLRRMARIEGEQVVAYVHNAAAE
GMGK
IGVLVAMTGGDAAFGRQVAMHVAAVNPAALSEADMDPAVLEKELAVQTAKALDENATAAKPKPEAVITNNIIPGRM
KKFI
SESTLMGQAFVVNPDMTVEAAAEAAGATITGFVRLEVGEGIEKAEEDFAAEVAKVVQG
>OLS48659.1 30S ribosomal protein S2 [Rhodovulum sulfidophilum]AB14
MALPDFSMRQLLEAGVHFGHQTQRWNPRMAPFIYGDRNGIHIVDLTQTVPMLDQALNVIRDTVARGGRILFVGTKR
QAQK
SVADAAEKCAQYYMNHRWLGGTLTNWKTVSQSIKRLNEIDERMATGAEGLTKKERLHMEREQTKLQASLGGIREMG
GLPD
LLFVIDVRKEDLAILEARKLGIPVVAVVDTNCSPEGVDYIIPGNDDAARAIQLYCDLASRAALDGMSAQMDAAGVDLGA
L
EEAPVEELLADARSEGEA
>OLS48660.1 hypothetical protein BV379_10540 [Rhodovulum sulfidophilum]AB14
MFESKEPTAEPSDPQEPHQRRFWRGVLARLRNNFLAGLVVIAPVGLTVWLIWTVTGWIDGFVLPFVPQKFQPEQYIGI
DI
RGLGVIFFLVFTLVVGWMAKGMVGRSFIGWGERVVGQMPVIRSVYNGLKQIAETVFAQTETSFDRVVLIQYPRPGIW
AIA
FVSTNAKGEIRRLVPADDPILSVFLPTTPNPTSGFLLFVPQSDVIELDMSVEEAAKLVISAGLVAPEDPQVLAAAAAAAP
RPIQQAARRARDREKIGA
>OLS48661.1 amino acid transporter [Rhodovulum sulfidophilum]AB14
MTLLAPALTGFATSLSLILAIGAQNAFVLRQGLLQSHVFALCLFCAMSDALLISAGVAGFGAITGALPWLPRAFGLGGAA
FLIVYGALRARAAWQGGHRLEAGGRAGSVRAVLGTAAALTWLNPHVYLDTLGLIGAISTDFSPGAARMAFGLGAVAA
SFC
FFFALGYGARLLAPAMARPLAWRLLDALIAVVMWVIAARLLLGG
>OLS48662.1 3-hydroxybutyrate dehydrogenase [Rhodovulum sulfidophilum]AB14
MSLSGKTAIVTGSNSGIGLGVAHELARAGADVVLNSFTDRDEDHKLAEDMAAQHGVKVRYIKADMSKGDECRALIETA
GA

CDILVNNAGIQHVARIEEFPVGKWDAIIAINLSSTFHTSAAALPMMRKAGWGRIVNIASAHGLTASPFKSAYVAAKHGI
V
GFTKVTALETAEEPITCNAICPGYVLTPLVEAQIPDQMKTHDMDRETVIREVMLDRQPSKEFATVEEIGGTAVFLCSDAA
KQITGTTISVDGGWTAL
>OLS48663.1 ABC transporter substrate-binding protein [Rhodovulum sulfidophilum]AB14
MTGILQRAARVAGFAFTTILPAILSAGQAPAGTPTHGIAMYGAPALPAGFAALPYVNPDAPKGGRIVLGEPGSFDSLNP
F
ILKGTPPWALAHESYESLMGRSWDEPFTLYGLLAETVETGPDRDWVEFTLRPEARFSDGSPVTVEDVLWSVETLGRIGH
P
RYRNGHDKIARAEAVGPRSVRFTFNAPDRELPLILGLKPILKKAQWQGRDFAQSGLEPPIGSGPYVVASAEPGRQVTLR
R
NPDYWGRDLPFNRGKHNFDEIRHDYYADGAALFSALKAGQVDVFRETNEAKWQTVYDFPAIRSGAVVKADIPHGRPS
GIT
GLAMNTRRALFADWRVRDAMILAFNFEFINQTMTGGVQPRITSYFSNSELGMRPGPAEGKVRALLAPFADTLLPGALE
GY
ALPVSDGRPQNRRNLRAALARLAEAGWTVGRDGVLRNAEGQPFRFEILLSQGATDSTRIVTLFADALQRLGAEVHVTE
VD
TAQYVQRTTSYDFDMTFYTRALSLSPGNEQWLYWGSAGVDTPGTGNWMGIDSPAAEAMIRRLLSADERADFVAATR
ALDR
VLTSGRYVIPIWYAPQSHLAHSARLHYPARIPVYGDWVGFLPDVWWSEEN
>OLS48664.1 transcriptional regulator [Rhodovulum sulfidophilum]AB14
MAEEREHSLIRIIRAEREAEAAEPLDLGRRVRELRSEKGWTLEQAARHAGLARSTLSKIENDQMSPTYDALKKLATGLGV
SVPQLFTPMRGARAGGRMAVTRTGEGAAHATATYEHELMAGALARKRMMPYRTRIRARDIGEFDGWVRHEGEEFL
YVLTG
VARLYTEFYAPVDMRRGDSAYYDATMGHNLVSISQEDATILWVTSVE
>OLS48665.1 benzoate--CoA ligase [Rhodovulum sulfidophilum]AB14
MHAIFDEGAPAPCPAPFNLAAYVLAPATHSPEKVALAIVGPHGAERWSYARLEAAVRGLAAGLQAMGLPEGGRVLLRL
GN
GVEFPIGYLAAIAAGLVPVPTSAMLTRTEIGKLAAEIEPVAILAGPNVALPDDCPAPVLDIKTQRGFYHDAPADYVLGDP
DRPAYIVYTSGTSGQPRGVIHAHRAVWARRMMWEGWYGLRADDRLLHAGAFNWTYTLGTGLLDPWTIGATALIPAP
GVRP
DDLPLLLRRFDATLFAAAPGVYRQMLKKHPQIVLPKLRHGLSAGEKMPETTRSAWREATGTFVYEALGMSECSTFVSGS
P
ARPAPLPASGYPQAGRRIAVLGPDNAPVARGEPGQLAISSRDPGLMLGYLKAEAETRARFDGEWFLTGDMVEMAED
GAIA
YLGRGDDMLNAGGVRVSPLEVERALMEHPGIHEVAATEIAVKADTTVIAAFYTGTPQDEAALAAFAAERLARYKQPRLY
V
HLDELPKNPNGKLNRRLIRQTHEAAHDPA
>OLS48666.1 polyketide biosynthesis protein [Rhodovulum sulfidophilum]AB14
MIRLDIFADPICPWCLIGKRLLEAALAERPDHPFTIAWQPFQLNPEMPRAGMDRRTYLEAKFGGQTEAVRAYAEIDQR
AR
AAGIELNLGAIARTPNTLDAQRLIHWAGIEHRQSMVVEALFQAYFIDGQDIGDAAVLTGIAASAGLDGALVARLLASDA
D
LDGIRERDAHARARGVTGAPTFIVADTHVLVGAQPTALWLQVIAELVGAAPKAGLQ
>OLS48667.1 multidrug MFS transporter [Rhodovulum sulfidophilum]AB14
MGACADVFRFLKGPAMAARRLMRVEFVAMTALLFATVAFSIDAMLPALPEIGAELTPDNLNQAQFVVTSFMLGMGA
GTLI

MGPLSDCFGRKPVILGTAAAYMLGAAIAGLGQSLEWLLIGRVIQGFGAAGVRVVALAVVRDLYSGRDMAKIVSFVMTI
FT
ILPALAPSVGAVVMAGFGWRGIFGSFLVFSLVSMGWFAFRQAETLPQENRRPMRPGPLLAAAREVVASPVARNATLV
QTF
IFAGFFGFLTSTQQIYDIYFDRADSFPLWFGVVALCAGSASFLNAMLVGRLGMRFMIRSALIGQFMLSGLMMAAFWLH
LL
PGWAEFPAFVLWSITFFGMNALTIGNLNALAMEPLGHVAGMAASIIGAFATGVSALLAAPIGLLFDGTPQPLAIGCFLS
V
GVALAVMLAVPRR
>OLS48668.1 transcription-repair coupling factor [Rhodovulum sulfidophilum]AB14
MEELRHITAGGAPEGYDARLLARELTRSGRPILHVARDDKRLAAMRAALAFFAPEAVVLDFPAWDCLPYDRISPNADVE
A
ARMATLAGLAAGLPGPFILLTTLNAATQRVPARALLQGASFTARVGMRIDEAALRAFLVRMGFTQAPTVMEPGDYAIR
GG
IVDIYPPGEAGPVRLDLFGDVLDGARRFDPATQRTTEKLKTVELAPVSEVILDEAAITRFRQNYRIEFGAAGTDDPLYEA
VSAGRKHAGVEHWLPFFHDRLETLFDYLPGAAVYLDDQVTPARLARWETIEDQYQSRREAMTQKGRIDTVYKPCPPGL
MY
LDDAAWEAVLADHRLVQLAPLQQGLGPGVIDAGGRIGRNFAPERKLEQFSLFSALKDHIHARREEGAVVVASYSEGAR
ER
LKGLLEDEELTDATLVSDIREVPEGRGLHLAVWALEHGFEGPGLTVISEQDVLGERLIRGPKKRRRAENFLTETQSLSPG
DLVVHVEHGVGRYMGLETVAVPDPATGKPGAPHEFLLLVYAEDGKLYVPVENIELLSRYGHDEGLLDRLGGGAWQAR
KAR
LKERIREMADKLIRVAAERALRQAPILEPGHHMWEAFSARFPYQETDDQLAAIEDVLTDMASGTPMDRLICGDVGFGK
TE
VAMRAAFVAALSGVQVAVVAPTTLLARQHAKGFAERFRGFPVEVRQLSRFVPAKEATATRAGLAEGTVDICIGTHALLA
K
SVKFRNLGLLIIDEEQHFGVAHKERLKQMRSDVHVLTLTATPIPRTLQMSLTGVRDLSIIGTPPVDRLSIRTYVSEFDSV
TVREALLREHYRGGQSFFVVPRVKDLPDMEHFLREQVPEVSFITAHGQLAAGDLDERMNAFYDGKYDVLLATTIVESGL
D
IPTANTMIVHRADMFGLAQLYQIRGRVGRSKARAYAYLTTKPRAPLTPQAQKRLRVLGSLDTLGAGFTLASQDLDIRGA
G
NLIGEEQSGHVREVGFELYQSMLEEAIAKIKSGELEGMADTDSDWSPQINLGVPVLIPDDYVPDLDVRLGLYRRLSTLTT
KVELEGFAAELIDRFGKLPKEVNTLLLVVRIKAMCKRAGIARLDGGPKGATVQFHNDKFANPSGLVAFIQDQQGLAKVK
D
NKIILRRDWKKDSDKIKGAFAIARDLAEKLREAA
>OLS48669.1 component of SufBCD complex [Rhodovulum sulfidophilum]AB14
MDLYGKLFELIDMRSFSNLWYWIALALLWSGASHWVLGVPFGLIQRAQRQGGQAARDVTTLVGVNARRYLAVATEA
GLWI
AALGGFVLTMLAVLGFGYRVEFCQAVFLLVAPMTLVGLWSVRTARLYAARGPEGEALYGMLRGHRLGVQAMGIVSVF
VTA
LWGMWMNMLVTGL
>OLS48670.1 delta-aminolevulinic acid dehydratase [Rhodovulum sulfidophilum]AB14
MRPTVAPFPTTRLRRTRRSAALRALTSENTLSVGDLIWPIFVRAGENIEEPVPSMPGVMRRSVDLSVEAVKEAAALGIPA
VCIFPSPDISLKTELCEEAWNPDNLSNTLIRAIKKEVPEIAVMTDIALDPYNANGHDGIVRDGEIVNDETVEALVKMALA
QADAGADILGPSDMMDGRIGAIRKALEENGHQDVTIMSYSAKYASAFYGPFRDAVGAAGALTGDKKTYQMDPANTD
EAVR
LIERDLIEGADMVMVKPGMPYLDICRRVKTEFGVPTYAYQVSGEYAMLMAAIQNGWLDHDKVMMESLMAFKRAGC
DGILT

YFAPAAAKILNAR
>OLS48671.1 twin-arginine translocation pathway signal [Rhodovulum sulfidophilum]AB14
MFKDETTNLISRRGFVVSAGAAATLAACGNGVGSNGAAALDARVDKTRSYLFNRYPATRDLSRKAQGILWMPLMTKA
GLW
FGGAYGRGALRIDDVTVDYYSATQASFGLQIGAQQYAHALFFMTPEALRQFRSGMGWSAGADLEYAVTNDGGMVS
ADTIT
ALDPVIAVVFGQAGLIFGATLEGTKYTRIIP
>OLS48672.1 exodeoxyribonuclease III [Rhodovulum sulfidophilum]AB14
MQIATFNINGVKARLQTVLDWLDAAAPDVALLQEIKSVDEAFPRAPFEDRGYNLETHGQKGFNGVAILSKYPLEDVRR
GL
PGDDEDSQARWIEATVIDKRAVRLCGLYLPNGNPAPGPKYDYKLAWMARLEARARELLATEEPLVLAGDYNVIPQDED
AA
RPEAWARDALALPETRAAFRRLLNLGFTEAFRARVQGPGHYSFWDYQAGAWNRNDGIRIDHMLLSPQAADLMTGCR
IETE
LRAGEKPSDHVPVWLSLDA
>OLS48673.1 monooxygenase [Rhodovulum sulfidophilum]AB14
MSLGNRKITVLGAGIAGLAVATALARQGADVVVLERADAIREVGAGLQISPNGVAVLRALGLVEALAARSGRARAVVLR
D
FHHGNRVLRLDLERQAKDYFFVHRADLVDILARAARAAGVQIRLLHEVTRLDFDGARPALITAQGARASSDIVIGADGLH
SVVRAALNGAVAPGFTGQVAWRAVIPARGHVPAEAHIHMGPHRHLVHYPLRGGELINIVAVEEREAWVEEGWNRPG
DPDR
MRAVFADFGGEVPRLLEQVHSVNLWGLFRHPVAERWYRGSAAILGDAAHPTLPFMAQGANMALEDAWVLAAALTR
HPEPE
AAFASYQAERRPRTQRIVEMASRNARNYHLSFPPLRVAAQTALRIGGMMAPGAALRRFDWLYDYDATLRYPAAS
>OLS48674.1 RNA polymerase-binding protein DksA [Rhodovulum sulfidophilum]AB14
MKTEVFLPDDYRPAEDEPFMNERQLEYFRRKLIAWKNDLMNESRETLEGLQDSSRNIPDIADRASEETDRSLELRTRDR
Q
RKLVAKIDAALRRIEEGEYGYCEETGEPISLKRLDARPIATLSLEAQERHERREKVHRDD
>OLS48675.1 ATP-binding protein [Rhodovulum sulfidophilum]AB14
MKFGSTDAYVAADDLSVAVNAAVTLERPLLVKGEPGTGKTELARQVAQSLGMPLIEWHIKSTTRAQQGLYEYDAVSRL
RD
SQLGDARVHDVANYIRRGKLWQAFAAPGRVVLLIDEIDKADIEFPNDLLQELDRMEFHVYETGETVKALERPVVIITSNN
EKELPDAFLRRCFFHYIRFPDLDTMKKIVEVHHPGIKQDLLHVALTRFYEIRETDGLKKRPSTSEVLDWLKLLLAEDMSP
EDLRKEGRSALPKLHGALLKNEQDVQLFERLAFMARRGI
>OLS48676.1 3-dehydroquinate synthase [Rhodovulum sulfidophilum]AB14
MSATIETVHVALPGREYDIRIGEGLLARAGAEIGPLLQRPRVAILTDETVAARHLEAFRAGLGPIEAPALALPAGEATKC
WEQFGRAVEWLIEEKIERRDIVVALGGGVIGDLAGFAAAVLRRGVRFVQVPTTLLAQVDSSVGGKTGINSPRGKNLIGA
F
HQPALVLADTGVLETLAPRDFLAGYGEVVKYGLLGDATFFDWLEAQGPALAAGDTAARIAAVKRSCEMKARIVERDET
EQ
GDRALLNLGHTFGHALEAATGYSDRLLHGEGVAIGCALAFELSARMGLCSQEDPSRVRAHLDAMGMKKDLADIPGDL
PDA
EALIALMAQDKKVLDGKLRFVLARGIGAAFVAPEVDAGQLRDLLDEALAGR
>OLS48677.1 shikimate kinase [Rhodovulum sulfidophilum]AB14
MGTQLKKTVVLVGLMGAGKTAVGTALARRLGAPFLDSDAEIVKAANMTIPEIFARDGEAFFRAREAEVIARLLRAAPGV
L
STGGGAFMGAANREAISELGVSVWLDAGLDLLWQRVRHKDTRPLLRTPDPYATLAGFYHERRPVYAQADLAVRADPG
YSV

EDMAGRVIEALKTRPDVLEAA
>OLS48678.1 hypothetical protein BV379_10635 [Rhodovulum sulfidophilum]AB14
MSTSARTAALVAGALAWAAPALAERPLSAIDWLSDSVAATGPRRPPPTITPVPGAEPITVMPLGTTLLDAVGLLPPQVS
G
LPRTFWGPGETQAIARAVRAERVDTLPAVQALLVKLLLAELDPPTGGGPDGQLLLARLDKLLDLGALDQAQALVERAGP
T
APSLFRRWFDISLLTGQEDRACSAMRANPGIAPTFPARIFCLARGGDWNAAALTLETGLALGFVKPEEDALLVRFLDPAL
AEESEALRLPTRPSPLIFRMREAIGEPLSTTTLPVAFARADMRPTAGWKARIEAAERLARTGALAPNELLGVWTERKPAA
SGGLWDRVAALQAFDLAILSGEPEAVTETLAPAWKAMASAELEVPFAQLYGPQLVRMNLPGATGALAFRIGLLSDEYE
AL
AQGREPQTNEEAFLIGIARGGDLQGVPPLGSLAAALRDGFDRPGLGTGPSRLIKEGRLGEAVLAAIDLLTDGAQGDILDV
TRGLGLLRRLGLEDAARKSALQLMLLERRG
>OLS48679.1 recombinase XerD [Rhodovulum sulfidophilum]AB14
MTPDAARWISTFLEAKAAEQDATANTQLAYGRDLKDFAGFLDRRGGRFETADEAVITAYLVACEAEGLAKTTRARRLSA
I
KQLYRFAYEEGWRPDNPALQIRGPGRTRRLPMTLSHDEVERLIAAAGQSPRDATRNACLMQVLYATGMRVSELVTLP
VAA
ARGDPRMLLVRGKGGKERMVPLTPGAREALTAWLAERDAAEAAAEARGLPASRYLFPGRGRDGHLTRHRFYVLIKEFA
LA
GELDPARVTPHTLRHAFATHLLAGGADLRAIQTLLGHADVATTEIYTHVLDERLRELVLTHHPLARDADPS
>OLS48680.1 hypothetical protein BV379_10645 [Rhodovulum sulfidophilum]AB14
MDSPTAVADAALFISASAIIALLLISAFFSGSETALTAASRGKLRARADKGEIGAQTALNITEDNERLIGSVLLGNNFVN
ILATSLATSLFTRIFGDSGVALATLVMTLVVLVFAEVLPKTYAITNPETAASRVSPLIRLIILILSPVVSAVRVLVRQIL
ALFGVRADPDSQIMAVRDEIAGALQLGHSEGVVEKEDRDRILGALDLGDRTVEEIMLHRSQIEVVDADMSPEAILSQCL
E
SSHTRLPIYKDDPENIIGVIHAKDLLRAVERLMRGPEGAPEKLSEMRVLELARKPYFVPDTTTLDDQMREFLRRHTHFAL
VVDEYGALRGLITLEDILEEIVGEITDEFDVDSERPLRRSEDGAVLVDGAMTIRDLNRAMDWTLPDDEANTIAGLVIHEA
QSIPTPGQVFSFHGFRFEVLQRQANRITRLKIRPL
>OLS48681.1 hypothetical protein BV379_10650 [Rhodovulum sulfidophilum]AB14
MTMREFIGPLGLAMMAAIAAPALAQEAPAQPEGNAAPAQVDSTPAGDLQMGEKEAQQPPQAFVKDTFKDWRVVC
VEINER
ESCNMQQLMRDSDDNAVAQVSVAPMPPAAAPRVAAVEIATPLETLLSEDLRIQIDSSQAKRYRFSFCTPQACVARFAL
SA
EDLAAFKGGKEASVTIVPLAAPDQQADIKMSLSGFTAAFDDALSTMSGN
>OLS48682.1 beta-ACP synthase [Rhodovulum sulfidophilum]AB14
MKRVVITGQGTINALGHSVSETLAAFREGRCGIGELDIRDIDRLSIRIGGQVRGYEPEAHFNRQQIALYDRFTQFTLLAA
RQAVAQSGLTFTDDLSEEAGVVLGTSGGGMNTLDENYRSVYEEGKNRVHPFVVPKLMNNAAASHVSMAYNLKGPSF
TVAT
ACASSNHAMGQAFQMIRAGLSRVMVTGGSESMLCFGGIKAWEGLRVMSKDGCRPFSANRNGMVQGEGAAVFVFE
EYEHAK
ARGAEILAEVIGFAMTSDASDIVMPSRQGAARAIAGALRDARLAPGDVGYINAHGTGTAANDKTECAAVADVFGADA
DRL
MMSSTKSMHGHLIGGTGAVELLACIMALREGVIPPTVGYQEPDPECALDVVPNVAREAEVSAVLSNAFAFGGLNAVLA
LR
KV
>OLS48683.1 phosphopantetheine-binding protein [Rhodovulum sulfidophilum]AB14
MSDSVKDKVIEIIAEQAVLEPSDVKMDSTLEDLGIDSLGLVESIFAIEEAFDIQVPFNANEPTESEFDISSVEAIVKAVE
GLAAEKQA

>OLS48684.1 UDP-3-O-(3-hydroxymyristoyl)glucosamine N-acyltransferase [Rhodovulum sulfidophilum]AB14
MSFTIQEIATALGAPAEGDLDFKVTGAAEPTMAGGDQLALAMDPKYADGLAEGGAQAALLWSGADWRAMGLKAAI
FAPRP
RVAMAGLTRMMDPGPEIGPDIHPTAIIDPSAVIGPGAAIGPYAVIGARARIGANARIAAHAVVAEDVRIGDDVLLMYG
VR
ICARVRIGDRFIAQPGAAVGGDGFSFVTPEKSAVEAVRETLGETGSARQQHYTRIHSLGSVTIGDDVEIGANSTIDRGTI
RDTSIGSGTKIDNLVQIGHNCQVGEDCLMCGLVGVAGSARIGNRVVLAGQVGVNDNIFVGDDVIAGGATKIFSNAPA
GRV
LLGYPAMRMETHIEAYKGLRRLPRLFAQVAELQKAVSKLADRD
>OLS48685.1 murein L,D-transpeptidase [Rhodovulum sulfidophilum]AB14
MPISFLRGAAFAGLAAACIGVAAPALAETELTAFKQAVAVAAAQDDAIAAFYRDRDYAPFWTSADGEARRRALVTVLQ
EA
PAQGLPAARYDVGRLVETFQDAHTQRGIGRAEVAAMRMYLDYAHDASRGVLTPGAVVSDIKRKLDRPAPATLIAGLAE
AS
SPLAYLRGLSPQNPEYARLMREKIRLERLIAEGGWGAEIGAGGKIEPGQSGPRVVALRNRLIAMGYLPRIATRTYDATVQ
KAVQAFQFDHGLNADGIVGPSTREELDVSAERRLRSVLVAMERERWLGNARGDRHVLVNIADFHASIVDHGETVFETR
SV
VGRNARDYRTPEFSDMMNHMVVNPTWNVPRSIAVREYLPKFQNNPNAAGHLMLVDGAGRVVTRAAVDFSQYTAS
NFPFDL
KQPPSNGNALGRVKFMFPNRYNIYLHDTPSKSLFNRETRAFSHGCIRLSDPFDFAYALLAGQTDDPKGLFQSKLRSGRET
RVNLDPPLPVHLIYRTAMGKPKGRMQYRRDIYGRDGAIFDALQAAGVALPEVQG
>OLS48686.1 Tat pathway signal protein [Rhodovulum sulfidophilum]AB14
MTTPRHCLTRRGVLAAFAATAAVAAPTYSNAFGLMRGAGDVRRLRMYSGRTGESIDTVYWIDGEYIGEAVKEINHFM
RDW
RSGSVIPMDLRNFDILAAAHGLMDLQEPYMLLSGYRTAQTNAMLRSRSSGVAKKSLHIKGQAADVRLKSRSVDQIARA
AG
ACHAGGVGRYSRSNFVHMDCGPVRSWGR
>OLS48687.1 glycosyl transferase [Rhodovulum sulfidophilum]AB14
MRPARPAGSPNAYVTLVTNADFALGARALLRSLKRTDGRASDICVLHTGGVAEADLAPLADLGARLVETDLLPTSDTFN
A
AHARDRLHRDAPFTKGTKPAFHTPLDNFCKLRLWQLDYERVIFLDADTLVLQPIDRLFDYPEFCAAPNVYESLADFRRM
N
SGVFTARPDPATFEAMMARLDAPGAFWRRTDQTFLQAFFPDWHGLPVFCNMLQYVWFNLPGLWSWPDIRVLHFQ
YEKPWQ
DHAKADRLKPLIELWRAHAEGAAIPDTTTLPGPPSP
>OLS48688.1 hypothetical protein BV379_10690 [Rhodovulum sulfidophilum]AB14
MSLAVTGATGLVGRFFVEEALAAGEPVTVLGRTPPPPGFFSTPTGFRPYALGDRPDMSGIDALIHCAFSHLPGRYRGGE
G
SDPEGFSRANLDGSVALFETARAAGTGTVLFLSSRAVYGRYPPGTDLTETLTPYPDTLYGKVKAEAERSLFALASDSFTP
VALRATGIYGPAGPGRAHKWQSLFDDFRQGRPISPRRGTELHGDDLAAAARILRDTGARGIFNASDILLDRHDLLALMA
R
AERIERPLPPRSDSPVSAMRCDRLASLGWEPGGIARLHATLPALCRPCPAG
>OLS48689.1 hypothetical protein BV379_10695 [Rhodovulum sulfidophilum]AB14
MIDITEETAEGLLEIRATGRVTEQDYETILIPALTRALDWGAPVRLLYQVGPDFEGYSAGALWADARLGLKHWRGFGKIA
VVTDSDKIEAGVRMFGFTLPCPVRTFGLDEIDAARLWLRESLGTIHFEEIGADTLQVQLIGRLDSASYKTRSPDLDAYAA
AHDRFRLMVDLRRFEGWQGPSALGEHVNLVRHHRHQPYRVAVVGDKSWMKLAETLLPLMLDAKAKYFDEDDYAAG
RAWLL

T
>OLS48690.1 hypothetical protein BV379_10700 [Rhodovulum sulfidophilum]AB14
MYVAFFGLAHRPFGPAPCQGSLHWTDPHWHDFERLRLALIDGAPLALVLGRPGTGKTTFLRALLRDETLTDIFRPAFHT
N
PTGDGPTLLRWILSAFGAEAGAADPDRLLARVGTVLAEIRAAGRLPLLAIDEAQGLSAAGLQMLDRVARPPQGARQGE
DT
ALQIVLAGLPDLEPHLEQSHPSLLGLSRDSTVRLQPMTADETADYIRSRIVAAGGDADALFDAGALALIHDQAHGLPRLV
NTICDRSLNAAFGAGRPRLDGAFIREVLAEWQDSGDALCLLERPASSPPRAPQTAPPGARPRPVAPPPPPDAAAVASTP
P
PPISEPLSASSSVSVSGAVSAPGAPPVPPRPRRRGRRLLAASTGAGLGLAAVFVGGALIVGLPSGPGTAIDATSPRLAGA
GAVPESRRAARLSDRSQAAETVPGAESAETNSTETGAAAERLARTRIEAAETRLADTLSELDRLAARLRDATQETGAAEA
RRDAARDAAKRAEADFAQLQDRIAEAELRLDALDRRLDEGDADRLTGLQATAAPPAPKPRSDPAPAPLPATSPLELDAA
S
ADPRVAQRQYRHALGSTDPLDIAIAYSRAAVNGQSRAAYYLGQIYEVGDGVERDIDTARQWYRIAAAEVAAAQVRLED
LP
EAPADPDRATARPLASSRSDGVIELVWQGSGPFVVELAAEPGPPAARFATALTAARLTLPAHPSLLWWRIRAGRSAPT
DW
QRIDPR
>OLS48691.1 hypothetical protein BV379_10710 [Rhodovulum sulfidophilum]AB14
MRYAWLAAAAAVLSVSGTIGNCQETETPGEAGVGALSEQAASYRRIIAALSEESAQAEAVLEKTQERLRKALNALAEAE
A
ELEQTTRHQSEAEAAGEARLAELTARIEAARAELAGLEAEHSALTARIETATEAAETAELAADEARAARDAAGTEAADLA
ETVERAKAAAETAQTERRETEAALTDMQARLEAETARMAEARAAFETIRAQTADTRAALETAETLREENEAAARQAGS
DR
SAAESRLADLEAAQEKAAEAERAALERAATASAAADAAEARAAAQVQEIAEAEARQEALLAEGQETETALAALRKELEA
A
TGARDAAEAARQAAETAAEDGRARAATLVEEARVMAEERERAAEAKVAALEARAAQLAADIEAARRLLPATGADTTE
APE
AAPPEPPVPAARAPGARPAFRAGPAPSAEAVARAVSEAPGLGEASAAERAELERLLGEGMCAPDALRTVFGGINRQTL
VS
LIRGLGRC
>OLS48692.1 hypothetical protein BV379_10715 [Rhodovulum sulfidophilum]AB14
MTRLDVDRRFLLLTAAGYLTALAAIAAALWLVAIPTLHKLHRAALETQAQTRAAGVQDELTLVLADARTLAEAPATAAYL
AGSGRVRPTQMRRDLERLVDGMTAQALLFDRRGNVRLRVPGRIGHPVFGLAETRQGLSLLESGRLGGAPIVSYRPGKT
PV
ESAFLISLPILSDGQPRGMLVIEKHLDLSRVLTGDAVSAATRLATGFQVRMYPDWTGDRDGLIAAPVAGTDFYVMIARN
T
GLTARLAWEMGSEVFLFTGLALLLPFMAMGLAGHRFIVAPHRALEDSRRRLTAQKAELAELAQIARMSHDSIMVSDRD
RR
IVWTNPAFLTLTGYAAEEVLGRDADALLQGPGSDPAVSHTLREAIAEKRPVRAEILNYRRDGTPFWAYLSITPIFDNHGQ
VTRFAAISSDITRRKAYEAQLEAARHKLEQQALQDSLTGLPNRRALENLFEELASEADPRTIIRIDLDHFKNVNDTHGHA
AGDHVLIRIAEILREGIAPGDVAARIGGDEFVILLGPGRDALEAQAMAERLRDEILRDIPFQNKTCRIGASFGICAADKR
LVDNSALLVSADAALYASKDGGRNAITLYTPSLHRDVQQKRRLALEIEHAVLREEFLPYFQPQFDAATHALVGIETLARW
RHPLRGILTPAAFLGLAGQMSVLPEIDRIIYRKGLEEVAAMNRAGFSIPKISFNAGVAQLRDTDLPGIAATIDIGPTRIA
LEVLESVLIEEQSDEFGFHIDLLREQGFGIEIDDFGSGHASVVGLMQLAPDVMKIDQRLVVPVTRTERARRLVRSIVDIG
RTFGIGVTAEGVETSAHAQILAGLGCDTLQGFHFARPLAADDFCARLAAGRFGDRRPGASSVA
>OLS48693.1 hypothetical protein BV379_10720 [Rhodovulum sulfidophilum]AB14

MSPASESSGPDSAAAAADATVEPRPTHLLTMSRGAPEAVPASAMRVLRAMGVEAVMMPGEGVGLTFRREIPNFLRL
DPFR
LTDLAPAPAAEDEAEVGPDDSGLEAGLPEEAAEAAPVGDADESAEMPDEIAADATAEDELSDPGLPMPEEAEDETAAE
TV
VAEEDAADLDSAEGQDVDAPDTEVDLGEAMPGAAAEEELPNVPAVPEPETGDETATGLAGLPEEAGPGPDIGLGEDDI
PP
DMPEAEPAAEDELAATDSSDIAALDETGNESPDDTAELEEASDETFGAALDRVMDRLDLIEFMLGSPRDDLGDADPGE
SL
GTRIATIEAAMARVEDSQQRLAEGLERVEAAVARIEAALVSLAQPSTAPEDDTDSSQAAIEAIEERIEAIAERLGDVQSS
HSGEIAALLERIERLAEAQPEGSVSADDIAARAIEIDALSSLVTALSEQLMAGRGEERQGNLDAVLDRLNATQESIAAMV
SQLLEGSSAAERRFGA
>OLS48694.1 carbon monoxide dehydrogenase [Rhodovulum sulfidophilum]AB14
MYAFEFDRPTTLDAAVAALGQEEAQALAGGQTLIPALKQRLASPARLVSLTGIEELKGVRTTADGVLCIGAATPHAVVA
A
EAAAIYPALADLAGHIGDPAVRNRGTIGGSLANNDPSACYPAAVLASDATIRTHTREIAAGDYFDGMFATALEEGEIITE
IRFPVPRQAAYLKFVQPASRFALVGVFVARFADGVRVAVTGAAEEGVFRWGDAEEALSADFRPEAVSELTVAAEGMIG
DL
HGSPDYRAHLVRVLAGRAVAGAS
>OLS48695.1 carbon monoxide dehydrogenase [Rhodovulum sulfidophilum]AB14
MPKDHGIGASSKRREDKRFLTGKGRYTDDINLPGQAHAWFVRSQVAHGRLTSVDTAAAEAMPGVLRVFTGTDFEGV
GGLP
CGWLITDRFGQPMQEPGHPVLARDKVRHVGDPVAVVVAETAEQARDAAEAVEVEIDELPAVLDMRAALEADAPLVH
DGLK
NNLCYDWGFVEENREAVNAAFETAAHVTTLDLVNQRLVPNAMEPRVALGDFNPATEDSTLYTTSQNPHVIRLLMGAF
VLG
IPEHKLRVVAPDVGGGFGSKIFHYAEEAFCTFAAKQLRRPVKWTCTRSEAFISDAHGRDHVTRIELALDAEGHFQALRTE
THANMGAYLSTFAPSVPTWLHGTLMAGNYRTPLIYVNVKAVFTNTVPVDAYRGAGRPEATFQLERVIDKAAREMGID
PVE
IRRRNFVTEFPYATPVAVEYDTGDYHATLDKLLEIADVAGFEARRADSKSRGRLRGLGLNCYIEACGIAPSQLVGQLGAR
AGLYESATVRVNATGGLVVMTGSHSHGQGHETTFAQVVADMIGIGEDMVEIVHGDTANTPMGMGTYGSRSLAVGG
SAMVR
ATNKIIAKAKKIAGHMMEAAPEDIELKDGRFTVAGTDKSVAWGDVTLAAYVPHNYPLEELEPGLEETAFYDPNNFTYPA
G
AYACEVEVDPETGKVTICSFAAADDFGNIVNPMIVSGQVHGGVAQGIGQALMEAAAYDEYGQLLSGSYMDYAMPRA
DDVP
FYRVDHSCATPCTHNPLGVKGCGEAGAIGAPPAVVNAVIDALHRGGFTDVAHIDMPLTPSRVWAAMQG
>OLS48696.1 carbon monoxide dehydrogenase [Rhodovulum sulfidophilum]AB14
MTKVSMKVNGKPVSGEIEGRTLLVSFLRDNLGLTGTHVGCDTSQCGACVVHVDGKAVKSCTMFAAEAEGRDVTTIEG
MAN
ADGSLSRIQQAFQDHHGLQCGFCTPGMVMSAAALLAETPKPTEAEVRHYLEGNICRCTGYHNIVKAILAASGQDVSGI
GG
DAIAAE
>OLS48697.1 carbon monoxide dehydrogenase [Rhodovulum sulfidophilum]AB14
MKFAATHDIAAPRATVWEALLSEAVLEECLPGCRELSGTAEDGFEATVVQKIGPVKATFKSHVSLSEMEAPSALTLSGEG
KGGAAGFAKGSARVTLEEIAEGTRLAYDVDVEIGGKLAQLGSRMIHGVAHKLSDQFFTRFKEVVEGPEEAEDPAAEEGA
E
APKGKGWLGRMMTRKAEAE
>OLS48698.1 DNA-binding response regulator [Rhodovulum sulfidophilum]AB14

MSPDPIRPTVPSRFASAMVIDDHPLFCDALSMTLKSAVGIGKVETSDHLEDALSRLDRAPRPDIVLLDLNLPDVSGLDGL
IRLKTAVGDIPVIVVSSMADPRIIGSAIRAGAIGFVPKHSPRDVFRAAFAAIAEGTVYVPEGIELGDPGQTAATGRDDAI
RRLALLTPQQGRILQAICAGKLNKQIAFDLSIAETTVKAHVTAIMRKLGVQSRTQAVLIAQEASFSGVMSDPG
>OLS48699.1 GfdT protein [Rhodovulum sulfidophilum]AB14
MDRNATPARTLSRQPPLVRDAVADSRDPGAVGHLRRGLGPGPFALITLFVSPDADFAAVMAGAQQAYPDVSVTGCTT
AGE
IGPEGYAEGAIVALALPAAHFRAETMVIENLAALDPQTLIGEMIRRRVALARAAPGWPQDFAFLMVDGLSRCEDALTSA
M
LAGLGTAPLIGGSAGDGTRFGRTLVGAGGHAFRSAAVLSFVRTDCPVRAFSHDHLMPGRVRMVVTAADPKRRIVERIN
AE
PAAREYARLLGKDPNQLSPLTFAAHPVVVRIGGRHHVRAIQRVAENGDLVFFSAIDEGLVLTLAEALPMADRIEAELAGL
ARHKPPDVILGCDCVLRRIEAEQRQLRPALSRILARHRVRGFSTYGEQISGLHVNQTLTGLAIYPPPASGPDPEQAPPCP
TA
>OLS48700.1 hybrid sensor histidine kinase/response regulator [Rhodovulum sulfidophilum]AB14
MSDSLIDPTDPPERQAEKLRQIAEVLMRRVEQATDDGGAAYAQFQRAVLLEDQVRARTRDLEHALDLLNESNARLAEA
NR
ATETARADLANAIETLQEGFALFDQSDHLVLFNSRFVMQMPDVRDRLCPGLRFHDYVEAVSRSRYLTLPEGETPEIWAS
R
RRARHRDNPHTVINISLLGHRWVQVSDHRTEDGKTVILQTDITDIMRSERRERSRMLDDQARLVRATLDHINQGVCIFD
S
AHRLVGWNRKVGAMLSIPVAQFRLGADFDRLFGQLAADRALRFDIAPTELLDWVHHTDRRPPLRFGLGRGETLMLDV
LAE
EMPDRGFVISVSDITAERRAAGALAEAKAHLEQRALARTLALEDALSVAERANASKSRFVAAASHDLLQPLSAARLYVSS
IAEDATLPAHREVLGRAETALESVEQILEALLDISTLDSGQAAVAPGPVPLRELYTRLQTDFAPHAALKGLELRVLPRDL
TLRTDPGYIRRILQNLVSNAIRYTGCGRVLVGARRQGQSLRLEVWDTGPGIAEEDQEAIFREFHRLNARASASEGMGLG
L
AIVERACARLGHPLGLWSRPGHGSGFFVTVPLAETAAPKPAPKAPAAEPSPGLVALLIEADPEIRRALPMLVGKWGVDV
I
DVADIDEALTLLAEIDLKPDAALIEHRHGAQTGPGHGLGALSLIETRYGPIPACIVSAERSPDLAAACRARGAELLTKPL
SPAALRAFFAEIAQSERT
>OLS48701.1 2-dehydropantoate 2-reductase [Rhodovulum sulfidophilum]AB14
MKICVFGAGAIGGYMGAKLAQAGAEVSLVARGPHLAAMRARGLRLIEEESGTDETVPVRVAEDAAELGEQDYVIVTLK
AH
SVPPVVGKMQPLIGAGTTVVSGVNGVPWWYFHKIGGPLEGTRLASVDPGDAQWTGFGPDRVLGCVVYPAAEVIEPG
VVRH
IEGNRFSLGEPDGSRSERALRLSRALTSAGLKAPVRPKLRDEIWVKLWGNLSFNPISALTHATLDVLCTDPGTRGVARAM
MLEAQAIAEKLGVKFPIDVERRIEGGAAVGSHRTSMLQDLDLGRPMEIDALVGSVQELGRITGTPTPAIDTVLALVALRA
RVAGLYGR
>OLS48702.1 AMP-dependent synthetase [Rhodovulum sulfidophilum]AB14
MTLQTISDLLQGQDGNRPAIGAPGRDWLDFAGLRDQARRVAEALHGAGIGRGDRVAIVLPNGPEMAAAFVTLAQAA
VTAP
LNPAYREEEFAFYLEDLKARAIVLPEGYDGPAAAAAERLGLIVLRLSVNPSEPAGRFSLKAGGAGGDCDAGAPGPGDVAL
ILHTSGTTSRPKIVPLSQANVAASAVHIRDSLALRPEDRCLNVMPLFHIHGLLAAVTASLSAGASIWCAPGFDALKFFGW
MSEARPSWYTAVPTMHQAILSRAARNRDVIQAVPLRFLRSSSASLPAQVMTELVETFDAPLIEAYGMTEAAHQMCCN
PLP
RGSQKPGSVGVAAGPEVRIAHEAENRLIEGTGEVVISGPNVTPGYEGNPEANARSFFEAEGRRWFRTGDQGAFDAEG
YLH

LTGRLKEIINRGGEKVSPLEVDGVLMDHPAIAQCVSFALPHPKLGEEVAAAVVLKEGAEADEREIREFAAGRMADFKVP
R
KVVILDEIPKGATGKLQRIGLAEKLGLSEPA
>OLS48703.1 tricarboxylate transporter [Rhodovulum sulfidophilum]AB14
MDVLALLADGFAASISPLNLMIVLIGVTVGIFIGAMPGLGSVNGVAIVLPLTFIVPPGSAIILLAAIYYGAMYGGAISSI
LLGIPGASTAVATTFDGRPMAQKGEAGLALIVAAVASFIGGTISVLLFTIFAVPLADMALAFGPAEEFALVLLAFTTFVG
LGGDDILKTIVMICLGLVLSTVGLDIISGEPRLIFGDLPGFYSGISFLVLAIGVYGVGEVLYTIETSRNAPMVTPARLTF
SELRAGLSKMRGMWRTMSLGSVMGFFVGILPAAGATPAALMSYGVAKSISKRPESFGRGNPEGVAAPETANNAAST
GSLL
PMLTLGIPGSPTTALLLGGMVMWGLTPGPMLFVEQKEFVWGLISSLYTANVAAVLVNIALIPVFVWALRMPLTVLCA
MVL
VLCIVGGYAPGQRLHDVWMIAGFGVAGYLLRKADYPLAPLVLALVLGPLMEKSFRQTMIAEHGDVTVFVERPIAAVFV
AI
SALLFALPLIRALRQGRNGSARPAPAE
>OLS48704.1 hypothetical protein BV379_10775 [Rhodovulum sulfidophilum]AB14
MLAADRLIALALMGFSVIVMWYARRLPVGWEAETGPGAGAFPFWLGLGMLLASGAILARSFVTRPHSGETFIPDADAI
PQ
VLGVVGLLLGAIALIPLLGAYIALMLFLLAYLRIMGGHGWLLTGILTLTTPVFLFFFFEVTLKILLPKGVTEPLFYPLYA
MFF
>OLS48705.1 hypothetical protein BV379_10780 [Rhodovulum sulfidophilum]AB14
MTQHLTRRASLALMSAAALTAAAPALADDWKPIKPVEMVIMAGQGGGADQLARLFQAIIQKQGLSRMPILPVNKGG
GSGA
EALRYLKDREGDAHTIMATLNSYYTTPLRTDIGVDIAEFTPIARMALDSFVLWVSADSDIHSLDDYVAAVKEAGGSWKM
G
GTGTGQEDSLVTAMLEKEFGIDVTYVPYKGGGDVATALIGGHVSSTVNNPAEQMGFYRAGKSRPIAALTAERLTRFPDL
P
TFTELGHPLVYTMQRSFVGPKDMPAGAVDYYTEMFRQLSQTEQWRSYTEEQALLADFLTGEALQDYFLEERDKHAEIL
RG
IASSS
>OLS48706.1 XRE family transcriptional regulator [Rhodovulum sulfidophilum]AB14
MRKALIGSKLRQLRRDHGQTQAEMARQLNISPAYVNLLENNQRSLSVKVLMALAEAYGIEIRELLSDRNAGRLADLRAA
V
ADPAFGRDRPDLQELRGAVDHAPRLVDLFLQLYRSHRTAVDRISRIGGSGIEPALLVTSPETAVHDFFRDNRNYFDALET
AAENVRGQFAGDADDIYAQLKRYLRQKLGIEVRLAPADDMPDALRVFDRQAGEVRLSQALDHANRAFQLAHVLALAD
IPY
LLDSMIGEGGIDGDLAAARCRVELANYFAAALLMPYAPFLALAEATGYDVDRLGAAFGTSFEQVSHRLTALQRDGARG
VG
FFLIRVDKAGNVTKRVNATPFALAEKGGSCPVWNVHNAFATPGVILPQLVEMPDGSQFFTLSRTVKRPVFSPHLQDNR
LA
VALGCERGEAHRIGYAQAVNLGAEGPVAKIGVSCQLCPRQACSQRAHQPLHMDLPMDADRRGKTRYES
>OLS48707.1 hypothetical protein BV379_10790 [Rhodovulum sulfidophilum]AB14
MSIAVLQAPDAEAGAISTDGLGGYRGKRALDLLAAALLLALTAPLMLPIWLLLRAETGQGFARRLRIGRGGRPIRCLVFH
VERCGGPVALWVLRAGLSGLPLLWSVLRGEMSLVGPRPATRADLARYGLAQSGYLAMRPGLTGLADARDGGRFCDR
VHDD
LRYGRIASPRVDLGLIAAALMRRWRSGRPQPGLAILSGTVPLIVSSHSGSCRAGRHPSAGPCAAAGGRAANRPGAGRA
GN
>OLS48708.1 IMP dehydrogenase [Rhodovulum sulfidophilum]AB14
MEIREALTFDDVLLVPAASSVLPSTADTSTLVSRKIRLNIPLLSSAMDTVTEGRMAIAMAQAGGIGVIHRNLTVEEQARE

VSRVKRFESGVVYNPITLRPDQTLGDAKAMIERYNFTGFPVVDERGHVVGILTNRDMRFASSDAMPVSAMMTGSELA
VLR
EPVDRSEALNLMKARRIEKLLITDDKGKLTGLLTLKDTEKSVLNPQAVKDELGRLRVAAATTVGDAGFERSLALVDAGVD
MIVIDTAHGHSEGVAKAVERLRKASNDVQIMAGNVATAAATRALIDAGADAVKVGIGPGSICTTRMVAGVGVPQLTAI
MD
CATAAGDVPVIADGGIKYSGDFAKAIAAGASCAMVGSMIAGTDESPGEVVLYQGRSFKEYRGMGSLGAMARGSADRY
FQK
DAASDKLVPEGIEGQVPYKGPAGTVVHQLVGGLRAAMGYTGCATVEEMRRNCNFVRITGAGLKESHVHDVTITRESP
NYR
VM
>OLS48709.1 Crp/Fnr family transcriptional regulator [Rhodovulum sulfidophilum]AB14
MQDAATCPHSAWIGELPGGLRRRFEPGTAIARPGAVDGYGEGKGPDRLFVLDEGLARICLNGAARALTIGYLRPGGAY
VT
HTRAWVEAVSPCTVVSWPVGEMLALLTRQPALGLAAFREVGQLLHGALNLIEDLAFRPVESRFARFLLNERALQNSDLI
R
QIDSTESLAAALGTSRQTLSTLINRLIREGVLARPDRRHLRLLDIARLEAMVDLSAG
>OLS48710.1 hypothetical protein BV379_10810 [Rhodovulum sulfidophilum]AB14
MAHRSRTTRRDGPMFAPRPRFGSQTPPAIFPPVLGALGLGLAWRRAADPFGMPSAAGDLILGAITMLFLFMMVAYGL
KLW
RRPKVVAEDLRVLPGRAGLSAAVLSVYLLAAALVPLIPALAEILLWAGLLLQTGLVGLLLWLFATGPAEQRRVTPVWHLH
FVGFILACLSAVPLGHTGLATVLLYATTGVALAIWAASLVQMLRQRLPAPLRPLLAIHLAPACLIGTVAASLGLMPLALT
AAAVAVALLAALLVSARWLLAAGVSPLWGALSFPMAAFASLMLALAAALEGQAPAEAFRIAGGIALVAATLAIPPILVFV
MQLWAKGRLAAQTNAARA
>OLS48711.1 C4-dicarboxylate ABC transporter substrate-binding protein [Rhodovulum sulfidophilum]AB14
MKAAIGAMTLGAFGCCLAGEALAETWNVSLWGKRRAFTENVEKLAELVSEKTDGEFTLSLSYGGLSKSTENLDGIEIGA
F
EMAQICAGYHRDKNPTLTVLELPFLGVDSLEQERDLSMALYRHPAVEADLARWNATLLMPTPMPQYNFVGVGAPPRS
LAD
FKGMTVRATGGIGEAMEIVGAVPTSMDATEVRQALDSGVVKAVSFAPHAHMSFGTIDTGSWWTTNLNPGTVNCPV
VVNSD
ALAALSEDHRAALLGSVDEALAHYIDYYNSETMSRWGPALDERGIERITFAPEAMAEFREAAAGPVAAAWIEEMNARG
LP
AQELYDSVTGQIGQ
>OLS48712.1 C4-dicarboxylate ABC transporter permease [Rhodovulum sulfidophilum]AB14
MAIHAPVLEDDSPLSRLDRGLYRLERQLALVAGLTVFVLMLLAVISVGGRHFFNQPLPGSVDWIQQGMPLIAFLGIAYT
Q
RDGGHIRMDILVSRLSGRALWAAETVTTLAMLVVMALLVWGSFAHFQRSFDWGAPLFSRDSSIDINLPLWPAKLLAPV
AF
SVLCLRLALQLWGYGRAFLLGLTAPVAVPLVVSAADQAATEATHVSGADD
>OLS48713.1 C4-dicarboxylate ABC transporter permease [Rhodovulum sulfidophilum]AB14
MDPIEIGLAVTGFLFVTVLLGMRVGFAAALAGLVGLIWIFWSKKAYDPEAFGWALTVAVKTAGQVPHSKVASQALSLIP
T
FILIGYLAYYAGLTRALFDAAKKWFGWLPGGLAVSTVFATAGFAAVSGASVATSAVFARIAIPEMLKIGYDKRFAAGVVA
AGGTLASLIPPSAILVIYAIIVEQDVGKLLLAGFVPGMVSACIYALLIVAMALALPRFGPAVRGFGWGERLRALPKALPI
LFVVWTIVFFVYNPFGGDAWGTPTEGGAIGAFVVFLMALAHGMRWPQFKDALLETAKLSAMIFTIIWGVLIYVRFLGF
AD

LPGAFADWIAGLSHPPLLTLVMILLAYAVLGMFMDAIGMLLLTLPVVFPAVMALNGGEYVSAADSAFGMSGPMCAIW
FGI
LVVKMAELCLITPPIGLNCFVVAGVRDDLTVQDVFRGVAPFFLADAITIAVLIALPGIVLWLPGLA
>OLS48714.1 hypothetical protein BV379_10830 [Rhodovulum sulfidophilum]AB14
MPMIRPFGPLALCAVLAACAHGADETAMVPAAIAPLSPGNPHRGAISDIEIAGGKETDPALASQIADADFKAALCSALLL
SGALSASGRYLLRAEIEEITQPLFGVDMLVGLTVRYRLQDRAGKTRRERRIVTRHTARLGEAFLGSERLRYANEGAAREN
IAAFLRALGAEARAGGVAGVS
>OLS48715.1 hypothetical protein BV379_10835 [Rhodovulum sulfidophilum]AB14
MPRSMARRSLLAALPLLFLGACVQTGPMGSMAPGVIGPVAPGSIWAGRIGRVTGTDGMVDNPLWTAEFSEPGFRSD
LASS
LTRAGILTPGGTNRLNADVESVTQPQTGTKVQVTMVVHYTIHDATGRTLFDQTVTTSYTSPLGQAVRGIDRLRIASENA
A
KDNIALLLRALGASAEGAPTGGPF
>OLS48716.1 ABC transporter ATP-binding protein [Rhodovulum sulfidophilum]AB14
MAGLTLSGVGKSYGAVEVLKGIDLEIETGELVVFVGPSGCGKSTLLRMIAGLERITAGRLTIDGVPMNDVPPAQRGIAM
V
FQSYALYPHMSVRDNMAFALKIAGQSRDEIDAALTRAARMLQLEPYLDRLPKALSGGQRQRVAIGRAIVRDPKVYLFDE
P
LSNLDAALRVATRIEIAQLKEAMPDSTMIYVTHDQVEAMTLASRIVVLADKGVAQVGPPLELYEKPRSEFVAQFIGSPA
M
NLLEGAISGTGETTGVRLTTGGGTIRAQVPTEPGDKGRKVRVGIRPEDMAATDGADYAFEGRVEILEALGEVTQLYFERE
DPEADPVIAKLSGVHAGLRGKTLRMTADPGRVHLFAGGRSLLWR
>OLS48717.1 ABC transporter permease [Rhodovulum sulfidophilum]AB14
MAGHGGRKSGLLWAVHLSVAALVVIWLLPTVGLLVSSFRDRDQISASGWWEAPLPVEQTYRVRTDPEAAVERHGRVI
LSG
NVFDREDSAGAGTVTGFGVTGRAPAEFAPGESAPLRGGGRLTVARDGSFTAEAGSPFGRRGPTIYFEAASPPKVTLDNY
R
MVLGADGMDRAFINTLTVTIPATVIPILIAAFAAYALSWMEFPGRGLLIAAVVGLLVVPLQLALVPLLRLHETVGIGQSF
MGIWFAHTGFGLPLAVYLLRNYMAGLPRDIIESARVDGATDFQVFTRIVLPLSFPALASFAIFQFLWTWNDLLVAKVFLP
AASDTKVMTVKIADDLLGSYGGDWGILAAAAFISMAVPLVVFFALQRYLVRGLLAGSVK
>OLS48718.1 alpha-glucoside ABC transporter permease [Rhodovulum sulfidophilum]AB14
MSPLLQGIATILIGVGGCVGYFYGANLLLDRVIFPPSGRDPGANISRANAVRPWLFMTPAMAALGLYLVYPVLGSFWRS
L
FNRDGGTFIGLGNYTDLAADPAFRTAVLNNLLWVLVVPAVSTFLGLVAAQLTDRIRWGNIAKSIIFMPMAISFVGASLI
W
KFVYANEADIGLLNAIRAALGFDDPIDVIQIPFWNNFFLMAILIWIQTGFAMVILSAALRGIPDETIEAAILDGANPFQI
FFRIMVPQITGTILVVWTTITILMLKVFDIVYTMTGGNFGTQILPSYMMSYMFKDDGRATAVAFVIMLIVLPVMVWNIL
Q
ARKEMR
>OLS48719.1 alpha-glucoside ABC transporter substrate-binding protein [Rhodovulum
sulfidophilum]AB14
MQLIAMAGPVAAALTLAAGAAHAQGLVFTPGEDDRFSWPSYETFAESHDLSGQTLDISGVWTGPDKDLVESVLAYFE
AAT
GARVRYSGSESFEQDIVIAAQAGSAPNIAVFPQPGLAADMARRGFLSPLGADTADWVRANYAAGESWVDLGTYEDET
GAE
ALYGFFYKIDVKSLVWYVPDAFSEAGYEVPDTLEDLKALTERIAEEGGTPWCIGLGSGAATGWPATDWVEDLMLRREA
PG

TYDAWVSHEIPFNDPAVVGAIEDYGWFARNDAYVEGGSQAVATTDFRESPAGLFTFPPGCYMHRQASFIDTYFPEGSEIG
LDVDFFYFPAYAERDLGRPVLGAGTLWAITRESEAAHVFIDFLKTPIAHEVWMAQSGFLTPFTGASPEAYATDSMRAQGE
ILTSATIFRFDGSDLMPGEIGADAFWKGMVAYTTGRSDAETAAQEIERRWNAIR
>OLS48720.1 beta-glucosidase [Rhodovulum sulfidophilum]AB14
MIPTRADFPPGFLFGTATSAYQIEGAAQGGAGPCHWDSFAATPGNVAHGATGARACDHLTRYEADLDLVAGAGFDIYRFS
PSWPRVLPEGAGAPNAAGLDFYDRLVDATLARGLKPALTLYHWDLPSALADLGGWANRDTALRLADFAALMADRLGDRVW
SWATVNEPWCAGWLAHFEGGHAPGLRDIRATARAMHHLALGHGLSLAALRASGVGEIGIVLNFEPAFPAEPGEAARAASL
RYDAVFNRFFLGGVTRGHYPEPALEGLGPHLPPGWQADMAAISAPIDWLGVNYYTVKRIDYAPGPWPNWAERPGPGRKTD
MGWEIAPMALPDLLRWISGSHTGDLPLYVTENGMAAPDAIVMGEDGPEIHDEDRIAYLSSHLAAAREAIAGGVPLKGYII
WSLLDNFEWAFGYDRRFGLVHVDFESLQRTPKASYHALRRMLEDAAP
>OLS48721.1 hypothetical protein BV379_10870 [Rhodovulum sulfidophilum]AB14
MTSLVADIGGTNTRVALAEGPGLMPDTVRRYRNAEHADLTDVLTDYLREAGSPEIAGACAALAGPVRHGRGRLTNLDWTI
EEAAIARVSGTPRVKLLNDLAAQGHALDQLAPEALVPVLDGPAQPGATRLVIGIGTGFNAAAVHALPGGLMVGEAEAGQV
SLPVADAEALSLARFLAGADGFAAVEEALSGRGLGRLYDWATAGAGTPRKLDGHGVLAALDTDPEALRAAETFVRLLGTV
AGDLALQHLPYGGIYLIGGMARAVTPHAGAMGFAGTFRRKGRFSALMAEFRVSTVIDDFAALTGCASFLARTE
>OLS48722.1 S-methyl-5-thioribose-1-phosphate isomerase [Rhodovulum sulfidophilum]AB14
MKIDGTHYRSIWREADGTVKIIDQRWLPHELRIVALTNRQDFASAIRDMWVRGAPLIGATAAYGVAVQMAHDASDAALAE
TWEVLHATRPTAINLRWALDEMTRVLKPLAPADRAEAAFRRAAEICDEDVEINRQIGLNGLELIRAIAERKGGRRVNVLT
HCNAGWLATVDWGTATSPIYHALEAGIDVHVFVDETRPRNQGAQLTAWEMNHHGVSHDLIVDNAGGHLMQHGEIDLCIVG
TDRTTSQGDVCNKIGTYLKALAAKDNGVPFYVALPSPTIDWTVRDGVKEIPIEERDGGEVTHVQGRMPGGVVGLVQISPD
GTPARNPAFDVTPARLVSGLITERGVAEASAEGLARLFPERAALKVG
>OLS48723.1 hypothetical protein BV379_10880 [Rhodovulum sulfidophilum]AB14
MNADPNRRGSAAVGFLDDLDPVEAGAVRCLRLWADGDDAQERLRSEFSDRLGPRQGSDAADSLHMICSLCAMYGRRPLMR
HSASCPCLGADESCFATFVAAAGAGDREDAMLMATMLVRADMAPGLVDPAARLAMALRRLALAAVSARRAPDSAPNVRPS
RRATLH
>OLS48724.1 peptidase [Rhodovulum sulfidophilum]AB14
MTKPTLATCTLAMGLAGTAALADAPAPDAVLSTYADIAAATYGDALTGAERLQKAVDALVAEPSPQALDAARRAWLAARV
PYQQSEAFRFGNPIVDDWEGKVNAWPLDEGLIDYVDAGYGGATDENPLAAINVIASPSFEIAGETVDASEITPELLAETL
HEADGIEANVATGYHAIEFLLWGQDLNGTDAGAGQRSWTDYAKGEACTNDNCDRRGAYLKAATDLLVSDLGWMVAQWQEG
GEAREAVMADSAAGLSAILTGMGSLSYGEQAGERMRLGLMLNDPEEEHDCFSDNTHNSHYYDGLGIRNVYLGSYTRIDGT

EVSGPSVSDLVAAKDADLDAEMKMKLDTTMAALARLKTAAEAGFAYDQMLARGNEAGEALIMGGVNGLVAQTASIE
RVVE
ALGVGGVSVEGSDSLDNPTAVFQ
>OLS48725.1 (2Fe-2S)-binding protein [Rhodovulum sulfidophilum]AB14
MIVCHCQCITDHDINAAIDWMRASDPKTIVTPGKVFRSLGKRADCGDCMTLFLDTMRANANLEVPPDLQNLRPQHRK
DKT
SCKATPRSSNT
>OLS48726.1 bacterioferritin [Rhodovulum sulfidophilum]AB14
MQGDPKVIEYLNSALRSELTAISQYWLHYRLQDDWGLGHMAKKSREESIEEMNHADKLMARILFLEGHPNLQKLDPLR
IG
QTPKETLECDLAAEQDARALYKEAREYCAEVGDHVTKELFDELLADEEGHIDFLETQLDLYGRVGDENYALLNASKMDE
A
G
>OLS48727.1 peptidase M75 [Rhodovulum sulfidophilum]AB14
MIRRTVLAFALALGAAPLAAAPADIADAVIDRHVLPGFARFEAATARLQAAAEADCDPGSEPLRAAWNEAFDAWLAVS
HL
RLGPTETDNRAFALAFWPDSRGMTPRTLSGLIADADPVVESEADFATVSVAARGFYALEYLLYDPDFAEASPYGCALIRA
EARDIHALASAILADWRDGYADLMRNPGPGTPYKTDKEVLKAFFTALHEGLQVDVDLRLGRPLGTFERPRPSRAESRRS
G
RSLHNVTVTLAAMRELAMLMASEDPASAAALTADFDRADRVAADLDDPVFAGVTTPQGRLRVEILQQAIHEIAEESRA
SL
GPALGVAAGFNSRDGD
>OLS48728.1 twin-arginine translocation pathway signal [Rhodovulum sulfidophilum]AB14
MADRRSFLTGALAASLCPRVGWADAGSPAFLSAAQESDGRQMLVGLDAAGAERFRLPLPQRGHAAAAHPIRPEATAF
ARR
PGTFALVIDCREGRVNAVLDAPEERHFYGHGAYSADGRYMYTAENDYDTARGVIGVWDTEAGYLRVGEFESHGIGPH
DIK
LMPDGKSLVIANGGIETHPESGRTKLNIPFMEPNLAYVSTEGTLLETIAPPADWHMNSIRHLALGPGGTVAFAMQWQ
GPR
AELPPLLGLHRRGAAPVWLDAPGAEHAAMQGYAGSVAISEDGAEAAISSPRGGRVHVFDIAGARFAGAIEARDVCGLA
PA
AGGGFVATTGTGAVMGLRDLHPVWTAQQARAFDNHLVTVGAA
>OLS48729.1 GNAT family N-acetyltransferase [Rhodovulum sulfidophilum]AB14
MKLRRMTAEELPAVLDWAAEEGWNPGLDDAAAFYATDPGGFFVAEVAGRIVAAISVVNHTEALAFLGLYLCRPDWR
GQGI
GHSLWTHALAHAGARTVGLDGVEAQQGNYRKSGFEPAGRTVRYAGVLPGRAHPGIGPVLRDDLGTLIAIEAGATGFEK
RA
FLSAWASDTETRRTLVCRAAGEIAGFATIRACREGWKIGPLVAGDGAVAAALIETCGRVVHGAPVMIDVPEPSRWLAG
HC
AGLGLAPVFGTARMYRGPAPQPGPVIAAVATLELG
>OLS48730.1 GntR family transcriptional regulator [Rhodovulum sulfidophilum]AB14
MTDRSPTPRHESRRASRARDLAEHLTRAIHERRLGPGMKLSEDEVGEIFGVSRTIVRAALQQLAHDNLVAIEPHRGAFV
A
QPTVREAREVFEARALVEPRTARSAALRATPQDIGLLSHHIAQEHAALAAGRSGQALRLSGQFHIEIARIADQAIIAGFI
AQLISRSSLVIALYWRRPQAMCESHAHHALMRAMEDRDGDRAEEVMKGHLLDLLSLLDLHDPVPVPLTLREALTGE
>OLS48731.1 Fe3+/spermidine/putrescine ABC transporter ATP-binding protein [Rhodovulum sulfidophilum]AB14
MRTELDLELVALTKRYGETVAVDGIQHLFRGGTYACLLGPSGCGKSSTLRMIAGHEEVSDGAIVLAGQDISHLPPARRGT

AMMFQNYALFPHLSVADNVAFSLKMKRVERETRRRRAMALLELVHMTDYAERLPAQLSGGQQQRVALARALITDPQ
VLLL
DEPLSALDPFLRIRMRSELKKLQRELGITFVHVTHGQDEALALADEIVVMNDAVIEQAGPAREVFNSPRTEFVARFMGG
H
NVIALPRGHFAIRADQIAVTEAAGGKLDGHVTGIEYQGTSVALTLMTAGDQELTAIIPEDRYFARPIQPGDGVGLNWDE
D
HLHALNA
>OLS48732.1 ABC transporter substrate-binding protein [Rhodovulum sulfidophilum]AB14
MTKATRTGLSRRRLLKGAAAGVAASAFPAPMIWAQNIKDVTLRQFGTGVSNINEVAQKVKEDLGFTLEMTALDSDAV
TQR
AATQPKSFDIADIEYWICKKVWPTGNLQGMDVGRIKNYDKIVGIFKDGRLTPGSTIAQGTSPNSVGFTDGPDGTSFADH
E
TGWMTLIPTIYNADTLGIRPDLIDHPVESWAELLNPAFRGKASILDISAIGIMDAAMVCEAMGEIQYGDKGNMTRDEID
R
TLAIMTEAKKAGQFRAFWKTFDESVNLMASGEVVIQSMWSPAITTVKSRGIPCVYQPLKEGYRAWGGGIGLSKNLSGI
EL
DAAYDYINWYLEGWVGGFLMRQGYYSAVPETSKNYMTENEWGYWFEGKPATEVITNPYGDAMAQPGETRDGGSFE
ERMGH
IACWNAVMDENRYMIRKWNEFIAA
>OLS48733.1 ABC transporter permease [Rhodovulum sulfidophilum]AB14
MSAHPTTSPAAPGSLPGALLKNRHVSGWLLASPLGLVLAVFLLLPIAMIAIVSFWGATEFSIYPDFQWDNYAFLLSSPVT
YKVFLNTFKYAVLTWVFTLGIGFTVAYFLAFHIRSLTWQIALFLLCTIPFWTSNIIRMISWIPFLGRNGIANSTLMSMGV
IDAPLDWLLYSDFSVILAFVHLYTLFMVVPIFNSMMRIDRALIEAAYDAGATGVQTLTNVIIPLCKPGIMIGSIFVVTLV
MGDFITVRFMSGSQSANVGRLISNDIALLQYPSAAATAVILLITVLTVIGILLRFVDIRKEL
>OLS48734.1 ABC transporter permease [Rhodovulum sulfidophilum]AB14
MDRRPRSFYLLAAFFALFLLFLYGPTFTIAILSFQGPEGGLTFPMRGVSLHWFRDLFEQQAVGDIWGSFGRSFTLGLMV
M
VTTVIVSVMGGLAFRKRFAGSGVLFYMVVTSLVIPSILVSLGVGLIFSQSGLDVHWATSGFGAQLTWTLPFGLLIMFAVF
NRFDKSYEEAARDQGATAWQTIRHVVLPIIAPSLIGVALFGFTLSYDEFARTLLTAGSHNTLPLEIYGMTTNVTTPVIFA
LGTLTTAFSLAVIGVFLATVWTMNRRRARQGSDAGQGMV
>OLS48735.1 HyuE hydantoin racemase [Rhodovulum sulfidophilum]AB14
MTVIVINPNSTAAMTGSILAMAETAAPGVAFEGWTSTLAPPAIQGPEDVAAATPPLLELVAQAARRGAGGVILACFDD
PA
LAEARACAPCPVIGVGQAAFHHAALRGWRFSVVTTLPAAIPAIEANIRNYGLSQHLARVRASDVPVLALEDDTEAACARI
AEEAERAVAEDDIEAVILGCAGMAKVTGHLRTRLPVPVIDGVETAARFVSVL
>OLS48736.1 4-alpha-glucanotransferase [Rhodovulum sulfidophilum]AB14
MSSLAALARASGILPGYRDLTGTERPTAPETAQALLAAMGLAAATEAEAAASLDALEAARAARHLPDWLVLDEGRAPG
LA
VPAAWRIELETGRVIEGRAEDPLPALPLGIHRLRVGAEETALLSAPPALALPRRGWGVVLPLYGLRDAGRGGLGDYADL
R
QALEGFAGRGAGFVGVNPIHAGFLADPHAISPYSPSHRRRFNVAHLAVPGEAASPGGELVDYPPAIAARLAALKAAFAG
L
DAASLSELAAFRAARGPELELFAIHQALSDRFGPYWPDWPAEYRTCAAPAVADFAAANGGAVAFHAWLQCLAETQLG
AIR
EAGAAAGMAQGLYLDLAVGTHPAGAETWADPGLFAEGVSLGAPPDAFAPQGQSWGLAPMLPGVLAARGFRPLAEIL
RAQL
RFAGLLRIDHILGFERTFWVPEGGAPGGYVKMPRAALLAVARIEAAREGAVIVGEDLGNVPEGLREDLAASGLLGCRLA
M

FDPGIASWPEPVLGSFGSHDLPPFAAWGAGHDIDWHRRLGHIPPERAEALAAERAGQAQALGVRIGGAGVDALHAAL
AAS
PARLIALQTEDMLGATEQANLPGTVDSHPNWRRRIGPAAADLADCEPVRRAARIMVRAGR
>OLS48737.1 glycogen debranching enzyme GlgX [Rhodovulum sulfidophilum]AB14
MLPTTIAAGCPNTLGARYDGEGVNFAIFSEHASRVSLCLFDETGHTELANIDLPERTHHVWHGRIDGLEPGQHYGYRIS
G
PYNPDQGHRFNPNKLLLDPYARQISGQPRWHDALMGYEVGAAQADLSFDKRDSAPYVPRSVVVAPDIDWGDDAPP
ATPMA
ETVIYEAHVKGLTCRHPRLHAPGHFRALASDPMLEHLQRLGITAIELLPVQAFLNDSFLVEKGLTNYWGYQTLAFFAPDP
RYMETGDISEFQRMVARLHSAGIEVLLDVVYNHTCEGNEMGPTLSFRGIDNASYYRLSEDRRYYVNDTGCGNTLNTGH
PM
VLRMVMDSLRYWVETMHVDGFRFDLAATLGRGADGFRPNAAIFQAMRQDPVLSRVKLIAEPWDIGPGGYQLGAFPP
PFSE
WNDKFRDGVRRFWRGDPGRVPDLAARLVGSSPQFDHSGRPATSSVNLLTAHDGFTLLDVVSYNDKHNEANGEDNR
DGHSA
NFSDNMGAEGPSPEPAIIAARAQRRRSMMATLLLSQGVPMILAGDEIGHTQHGNNNAYCQDNATAWVDWQGADE
AFLDFT
AKCIAFRKAHPILRQKLFLHSRERALDGIEDLFWRRADGQPMCRSDWEDPDLRLLAVEIRTASGTPRYAALEYAIFAVFN
AGGAVEVTVPDAPEGQHWALHLDSSRPELVPEPVEGSFGMPAEAVAVFVLEEEPPV
>OLS48738.1 starch synthase [Rhodovulum sulfidophilum]AB14
MSLNVLSVASECVPLVKTGGLADVAGALPGALKAEGVEMRVLIPGYPAVLAKVGTGETLWESDDFFGGPARLCFATHG
GL
RLYVLEAAHLFDRPGGPYLDGGGCDWPDNDIRFGALSWMGAQIGAEGAGGWRPDLIQCHDWQAGLVPLYLKARSV
DLPTV
MTVHNIAFHGLMPAGRMAALGLPGWAFHPGGMEFYGQVSALKAGLVYAWKVTTVSPTYARELRTPEFGGGLDGVIR
ARAG
DVLGILNGIDEADWDPATDPHALRFKQPRGKAKARAALLEEMGLTEGPGPLCVVVSRLTGQKGLDLLLQALPDLVARG
GR
LALLGSGEPPLEQAWRSAAQRHEGVAVRIGYDEALAHRLIAGGDAILLPSRFEPCGLTQLYGLRYGTIPVVARTGGLADT
VIDANPAALAAGVATGIQFAPVNAEALSVALDRLCDLHADPELFAKLQRNAMRAPVGWQGSAAAYARLFREISPPS
>OLS48739.1 glucose-1-phosphate adenylyltransferase [Rhodovulum sulfidophilum]AB14
METRYSRLANRSMAFVLAGGRGSRLKELTDMRAKPAVYFGGKTRIIDFALSNALNSGIRKMAIATQYKAHSLIRHCQRG
W
NFFRAERNEYLDILPASQRVSESQWYQGTADAVTQNIDIVDSYGVDYVLILAGDHIYKMDYEIMLRRHVESGAEVTVGC
L
TVPREEGSAFGVMKVDETDRIVEFIEKPQDPPGMPDDPDQCLVSMGIYVFDWKFLRALLIEDAGNPASSHDFGSDLIPD
I
VRGGKAMAHRFTSSCVKDRAESPAYWRDVGTIDAFWKANIDLTDFTPDLNLWDKSWPIWTYSESVPPAKFVHDEED
RRGS
AVSSMISGGCIISGTEVRNSLLFTMVRTNSYAVLDHAVVLPYVDVGRHARLSNVVIDRGVVIPPGLVVGEDPEEDAKWF
R
VSEGGITLITQSMLDRRAEA
>OLS48740.1 glycogen phosphorylase [Rhodovulum sulfidophilum]AB14
MEAYSLTAADMRDSILRHLAYELGTDPGHATLHDWRIALSLAVRDRLVDRWFAANRATRAAQPKRVYYLSMEFLIGRL
LE
DAIVNLRLEEQAGTALADLGLDFDAVIQNEPDAALGNGGLGRLAACFMESLATIGCPAFGYGIRYENGLFRQSFQDGR
QI

EEPEDWLSQFHAWEFERRDVAQEIRFGGEVTQKDGRAVWTGAEAVIAAAFDTPIIGWKGRWANTLRLWSAQPLHGF
DLAR
FNRGEYAAAAEPEALARTISRVLYPDDTTGQGKELRLKQEYFFTAASLRDILRRFEAEGGALADLPKKAAIQLNDTHPAI
AGPELVRLLHDERGMALDQAIATARGCLSYTNHTLMPEALERWSEDLMSRLLPRHMQLIERIDEAHAAEMPGRRVTLR
EN
GEVKMGEIAFTMAHKVNGVSALHTDLMKETVFADLHRLHPDRIVNETNGVTPRRWLLSANPRLSRLITETIGEGWVDD
LE
QLDRLEASISDPGFLDAYAKAKRENKQDFAEWLAAEQGVRVDPEALFDVQIKRIHEYKRQHLNILETVALWREIKANPG
A
GWVPRVKIFAGKAAPGYVFAKEIIHLINDVAEVLNADPVTSPYLKVLFLPNYNVSLAERLIPAADLSEQISTAGKEASGT
GNMKFALNGAPTIGTLDGANVEIRDRVGARNFFLFGMTAQEVEARRAVPDHARQAIQADPRLGGALKAISQGAFSPS
DKH
RYRGVVANLTGQDYFLVCSDFTAYWDAQRRAEAAFADPAVWHRIAACNTARAGWFSSDRTIRGYMTDIWGTVPLAA
E
>OLS48741.1 NAD-dependent dehydratase [Rhodovulum sulfidophilum]AB14
MTQFPKRILVTGGAGFIGSHLCRALLAMGHAVTCLDNFSTGREANIAPLRGRPGFTLLRHDVTQPLDLDVDEIYNLACP
A
SPAHYSRDPVQTAKTSVIGTLNLLELARRSGARLLQCSTSEVYGDPLVHPQTEDYWGHVNPIGPRACYDEGKRCAETLIF
DFRRRYDLPVKVARLFNVYGPGMRPDDGRVVSNFAMQALTGAPLTVYGDGSQTRSFCYVSDMVEALVRLMATPPAV
TGPI
NLGNPAEMQVIDLARTVLRLTGARAPLVFRPLPCDDPVQRRPDIARARAVLGWSPAVGLETGLRATLDRFAAEGLSPA
PD
ACPVLPAIPAAPLDIGLALG
>OLS48742.1 hypothetical protein BV379_10985 [Rhodovulum sulfidophilum]AB14
MPADPPIRRSTLSTARLLQARRLLPRSRIEALAAAAALVVMLGAIAVIAFDIRDREAHLHDITAETAQSELTRLEMEHAE
LHTAIYMADRGVMPLDRLRLDFANFRAQAKLVTETASFAEPLRRSGLGGQADRIADALRQMTDAFAGTDADLRARLP
DLS
RTIDSIDTDVRRYSAAGSGLLVSSHLSEHAHVERLETLLALIGLALIVLLLAGMAALGRLNHRMRSRSRELHATSSRLNA
VVTTALDAVIVTDEAGTVLEFNAAAEAIFGYARDEVLGRPLEQTLALSAAIRRGGEATGRIELPAQRKSGTGFPAEISFG
RAVTDDGTVFVIFLRDISERKRVETELTAARDRALAGEKAKARLLAVMSHEMRTPLNGLLGTMELLQRSPLDDAQRQYL
D
IMSCSGEILLHHVEDVLEVSRIDAGRSKTRITDFDLTAKLGRLVALHQPLAAERDTRLALEIDAGLGAGRGMVRGDAQRL
RQIVINLISNAVKFTRGGRVTVSARRRSREIIEIRVADTGIGIAPEDQTRIFDEFFTADATYRREEGGTGLGLAIVARQV
GLLGGRIEVSSQPGQGSTFTVTLPLPEATAAAEAAAPHPAAPCPARPLDVLVVEDNRVNRFIARRLLEGLGHRVTEAVD
G
LDGVETAAARQFDVILMDISMPRLDGIEAARRIRAAEGPPPRIVAMTAHALPEDLARFQAAGMEDVALKPVSRGRLSEI
L
GEVPGAATQCKTAPGAMVPEPERDAPADKPHRAAS
>OLS48743.1 ABC transporter [Rhodovulum sulfidophilum]AB14
MSEPVLSLEAIEKCYNRGLPGEVTVLRGATLRVVPGEVVALVAPSGAGKSTLLHIAGLLDTPDSGAVRIAGADMTGQSD
H
RRTAARRADIGFVYQFHHLLPEFTALENIVLPQLAAGMPHGAAEARAMDLLTRVGIAPRAGHRPAALSGGEQQRVAFC
RA
LANQPRLLLADEPTGNLDPGTSDTVFGALMELVRETGLAALIATHNLDLAARMDRTLHLEAGRLV
>OLS48744.1 ABC transporter substrate-binding protein [Rhodovulum sulfidophilum]AB14
MTAVPFARFEWMIAWRYLRARKAEGGVSTMTWISLAGITLAVFALIATLAVRSGFREEFVDTILGANAHVTVYAASEV
NA
SGVRSRTIADYEPIAERLRAVKGVTRVAPLIKGQVMAAAPGGTAGVEVFGIAPADLATIPRIAHPEAHAGTLQRFGEGIA

IGSGVARELGVGIGDRVRLISPDGVKTPFGTSPRVKAYEVTYIFTAGRYDIDRTRVYMPFAEAQSYFNREGVADEFEVMV
EDPENVDELTPALLRAAGERALIWSWRDASGSFLRALDVEDNVMFIILSILVLIATMNIVSGLIMLVKNKGRDIGILRTM
GLSEGSILRVFFICGSSVGLIGTAFGVALGCLFAIYIDPIFSFVNYVAGGGVWDPSIRGIYNLPAKLELKDVLSAVILSL
GLSFVVTLFPARRAARMNPVEALRYE
>OLS48745.1 proline--tRNA ligase [Rhodovulum sulfidophilum]AB14
MRLSRYFLPVLKETPSEAQIASHRYMLRAGMIRQQAAGIYSWLPLGFKVLKKIEAIVHEEQIRAGHIPMLMPTLQSADL
W
RESGRYDAYGPEMLRITDRHGRDMLYGPTNEEMITDIFRTHVASYKDLPLTLYHIQWKFRDEVRPRFGVMRGREFYMK
DG
YNFDIDRDSAIHAYNRHMVSYLRTYERMGLKALPMRADSGPIGGDDTHEFLVLAETGESEVFYDKAILDLTFGDRQVDY
D
DRAECAQIVTEWTTPYARTDETHDEALYSAIPEDRRKTSRGIEVGQIFYFGTKYSEAMGATVVNDKGERVPVHMGSHGI
G
VSRLVGALIEANHDDRGIIWPEGVTPFHCGIVNLKQGDAEADAACKGLYQALSDKGLEPLYDDREERAGAKFATMDLIG
L
PWRITVGPRGLKTGVVELTSRRTGESEEMSPEAAVARVAEIYAPHRTGSHWEPMAKSSFHTWL
>OLS48746.1 AI-2E family transporter [Rhodovulum sulfidophilum]AB14
MGLPVRAQAKYWGIAAAVIVLILMLLGDVMMPFLVGMAVAYFLDPVADRLERAGLSRVGATVTITLCALVIFVLGALLV
L
PTLIDQAVALANAAPDIAGHFHAFLTERFPSLADNGSTIRKTLTGIGEAVQAKGGELLNGLLTSVVGIVDLVVLIVLVPV
VTFYLLMDWDRMVAEVDKLLPLDHAPVIRRLAGEVDRTLAGFIRGQGTVCLILGVFYAVALGLLGLNYGLIVGFVAGMI
S
FVPYVGAVVGGTLALGLALFQFWGEWWMIAIAGGIFIAGQQVEGHVLTPKLVGGSVGLHPVWLIFALSAFGSLFGFVG
LL
AAVPVAASLGVLTRFSIAQYKDSRLYRGTSGQTGPEAASEERASNEDRD
>OLS48747.1 chromosomal replication initiator DnaA [Rhodovulum sulfidophilum]AB14
MARQLAFDLPVRPARGRAAFFVAPANALAVQQIEGWQDWPARKLVLIGSEGSGKTHLVHVWADLAAAEVIHARDLP
QAEL
PALAAHGAIAVEDADRIAGRMQAEDALFHLHNLLAAEGGTLLMTARRAPLEWGLQLPDLASRVQAAATAHLAPPDDA
LLA
AVLVKLFADRQIAVGPDLIGYLVGRMERSFSAAAETVAAIDREALASGRKPGLRLAREVLDKDPGRGD
>OLS48748.1 RNA degradosome polyphosphate kinase [Rhodovulum sulfidophilum]AB14
MTQADFLVAPFPDAVEIPEPQRLGPKRFYNRELSWLGFNWRVLEEASNPRVPLLERVRFLSISATNLDEFYTVRVAGLRE
LAKKGSIVPAADGLTPAEQLVQIDRNARKLLQSQQTAWNKLKREMEDAGIHILTRARLTAADRAHLEEYFLQKVFPVLS
P
LAIDPAHPFPFIPNTGFCLALQLERERDGHPLQALLPIPHQIQRFARLPAEDGQQRFLPMEELLLVHLNSLFPGYREVGS
CTFRVLRDSDLEVEDEAEDLVREFEVALKRRRRGEVVRMKMSAGAPEELRSVIIRELHITEEEVIEVRGIIGVADLKELV
IDERPDLLWVPFTPRVPERVQDHDGDMFAAIRQKDMLLHHPYETFDMVVRFLQQAARDPDVVAIKQTLYRTSKNSPI
VEA
LCEAAEDGKSVTALVELKARFDEAANIRQSRRLERSGAHVIYGFINYKTHAKISTVVRREGDRLVTYTHYGTGNYHPITA
RIYTDLSLFTCDEALGRDATKVFNYVGGYAEPAHLENLSISPLSLKSTLMQRIAEEAEHARNGRPAHIWAKMNSLIESDV
IDALYAASNAGVKIELVVRGICGIRPGIKGLSENIRVKSIIGRFLEHSRIVCFGNGHGLPSPDARVYISSADWMGRNLNR
RVETLVECTNPTVKAQITDQIMTANLADEAQSWVMQPDGSFLRAKAGPNAFNCHHFFMENASLSGRGSAGAGDVR
QLAKV
KD
>OLS48749.1 exopolyphosphatase [Rhodovulum sulfidophilum]AB14
MDGQAEDRPNAWDPFGRPLFGGPEARALSRVGVVDIGSNSVRMVVFDGAARSPAYFFNEKVMCALGAGLSETNRLS
PEGR

SRALAALRRFAILAEAMDATPISVVATAAMRDAEDGPEFRAEIERETGLKPWVIDGTEEARLSAQGVLLGWPGAEGIVC
D
IGGSSMELAELKGGMVGLRGTSPLGPLKLREVSGGKKGLDRYIAQEIGRLVAPMPIAPPRLYLVGGSWRAIARLDMERR
G
YPLTVLHEYRMTRAALLETIDWIAGSDPDALRQKTGTSPDRMALVPYAAQVLRKLVAHLRPHEIAVSSYGIREGLLYEQ
M
PADLRARDPLIEACRFAEQRAARQPGFGNRLYNFILPLFGAAGPDKRRLIRAACLLHDTAWRAHPDYRHEICFDSATQA
N
LGGLEHWERVFLGVSLLHRYKNSRSGSRFEPLFSLLDTDRMRQAEVLGKAMRFGAMFSLHAPDSLAELRHYPKKKQLEL
I
LAEAAVPLFGEVAAARFASLAKALGVETSVRVRRR
>OLS48750.1 hypothetical protein BV379_11035 [Rhodovulum sulfidophilum]AB14
MRHRLALIPLLALLLTAAPASAQDRGGAGDDGGMSEGLGLLGEGMQMFLKGLGDELEPHMRDFAEAAEPALARLME
LIDD
LDAYQLPERLPNGDIIIRRKPDAPPLPDPEARPEGEIEI
>OLS48751.1 endonuclease [Rhodovulum sulfidophilum]AB14
MRIATYNVEWFNALFDDEGQLLEGEGPSGRHGVARQARIAALGIVFSALDADAVLVVEAPDENGRRSTVRALESFARV
FG
LRARRALIGFGNDTQQEIALLYDPERLRARHDPQGAPTGRKGAPDAPRFDGVFRWDIDVDAAPDLIRFSKPPLELAVET
A
AGLRFRMIGVHAKSKAPHGARSPDEEIRISIQNRRKQLAQCIWLRQRIETHLDAGEPLIVLGDFNDGPGLDEYEKLFGRS
GVEIVLGQEAPPERGLFDPHAREALQGLLAAQPASARFFIPATGRYLSALLDYVMISAELRARGPRWRIWHPFDDPDLY
R
IPELREALLTASDHFPVSLDIAI
>OLS48752.1 molecular chaperone DjlA [Rhodovulum sulfidophilum]AB14
MSLWSRIADAVSALAKGESLAAVFDRLRTPPERSVAFTIAVIALGAKMAKADGRVTRDEVAAFREVFSIPPGEEANAAR
V
FDLARQDVAGFEDYARRIRRMFRETRGPLSDLMEGLFFIALADGDYHPDEDAFLARVAEIFGLSERQFRTLRARFVPEAV
PDPYTVLGVDPTDDLDTIRKVWRAEVRETHPDRMQARGVPEEAIKLAERRLVAINQAWETIVADHAA
>OLS48753.1 Na+/H+ antiporter NhaA [Rhodovulum sulfidophilum]AB14
MLLRALDKFFGHEASGGILLMLAALAALIVANSPLQPFYDDTLGATFSVLLNGGGLEKPLILWINDGLMAVFFFLIGLEL
KREMMEGKLKKPADVVLPGMAAVGGMVVPALIFAAFNWGSPETLSGWAIPAATDIAFALGVLALLGDRVPASLKVFL
LTL
AILDDLGAIVIIALFYTADLKVDYLLMALLPLAGLIWLNLKGAHRVAPMVLLGTIMWFFVLKSGVHATIAGVATAFCIPL
RDKWGKSPLHALEHALSPYVLYLILPIFAFGNAGVVLTGISFSDLLQPLPLGVALGLVLGKQVGVFAITFAMVKSGLARL
PDGVGWGHIYGLSCLAGIGFTMSLFIGSLSYADPVLMNDVRLGVLSGSAISALLGYAALRLLAAPKGSKRATAAE
>OLS48754.1 methylmalonyl-CoA mutase [Rhodovulum sulfidophilum]AB14
MTDKTTRWAALAEKELRGKPLDGLTWETPEGIPVKPLYTEADTEGLAHMGSVPGEAPFTRGPKATMYAGRPWTIRQY
AGF
STAEESNAFYRKALAAGQQGVSVAFDLATHRGYDSDHPRVVGDVGKAGVAIDSVEDMKILFDGIPLGEISVSMTMNG
AVI
PILANFIVAGEEQGVERKQLSGTIQNDVLKEFMVRNTYIYPPEPSMRIIADIIEFTSTDMPRFNSISISGYHMQEAGANL
VQELAFTLADGKEYVKTALARGLDVDAFAGRLSFFFAIGTNFFMEIAKLRAARFLWHRIMSEFGPRNPKSLMLRTHCQT
S
GVSLAEQDPYNNVVRTAYEAMSAVLGGTQSLHTNAFDEAIALPTEFSARIARNTQLILQNETKVTKVVDPLAGSYYVEKL
TADLADAAWEIICEIDEMGGMTKAVASGMPKLRIEEAAARWQARVDRGEEVIVGVNKFRLEKEDEIDILDVDNVKVRQ
SQ

VARIEKIRASRDQAACDHALAALEEAARSGEGNLLRLAVEAARARATVGEISMAMEKVFGRHRAEVKTLSGVYGAAYE
GD
EGFAAIQQSVEDFAEEEGRRPRMLVVKMGQDGHDRGAKVIATAFADIGFDVDVGTLFQTPEEAAQDAVDNDVHVIGI
SSL
AAGHKTLAPKLIEALRAQGADDIIVICGGVIPHQDYDFLRQAGVKAIFGPGTNIPAAANDILRLIREARG
>OLS48755.1 hypothetical protein BV379_11060 [Rhodovulum sulfidophilum]AB14
MKKHLTIMAWVALMPLAGTADAVSEPEPLARWEADRTVVLEAEGVSLSDFHWMARPVVVFADSPANPQYRTQMQ
LLTERI
DELAERDVVVIVDTDPDARSELRMKLRPRGFMLVLVGKDGEIKFRKPMPWNVREISRSIDKIPLRQQEVRDRR
>OLS48756.1 restriction endonuclease [Rhodovulum sulfidophilum]AB14
MIPDFQTLMRPVLQSALGGERRVSDVVEEMSALFELTPEDRAEVLPSGRQGRMANRVHWARSYLKQAGLVRSPRRG
HFEL
TEEGRRALKEAPERITKAWLERYPSYLEFKNRTKGRAAEGGAAQSAPADPIASDATPEEILASAHEEIQTGLAAELLGRL
REGSPRFFEETVVKLLVAMGYGGPGGRSAVLGGSGDNGVDGVIDKDPLGVDQVYVQAKRYGEGATVGSGEIRDFFGA
LSL
KDVTRGVFVTTARFSPSAHATAEKLGARIVLVDGPRLADLMIAYEIGCQVRQTFRLHEIDEDFFE
>OLS48757.1 acetyl/propionyl-CoA carboxylase subuit alpha [Rhodovulum sulfidophilum]AB14
MFDKILIANRGEIACRVIKSARKMGIKTVAVYSDADRHALHVQMADEAIHIGPPPANQSYIVIDKIMAAIEQSGAQAVH
P
GYGFLSENKLFAEALEKAGVAFIGPPASAIEAMGDKITSKKLAAEAGVSTVPGYMGLIEDAAEAVKISREIGYPVMIKAS
AGGGGKGMRIAWNDQEAREGFQASKNEAANSFGDDRIFIEKFVTQPRHIEIQVLADRHGNCVYLHERECSIQRRNQK
VIE
EAPSPFLDEATRRAMGEQAVALSRAVGYCSAGTVEFIVDGDRNFYFLEMNTRLQVEHPVTELITGVDLVEQMINIAAG
QK
LPFEQKDLKINGWALESRLYAEDPYRNFLPSIGRLTRYRPPAESAHDAAVVRNDTGVFEGGEISMYYDPMIAKLCTWAP
T
RAQAIDEMRTALDAFEVEGIGHNLPFLSAVMDHPKFCAGDITTAFIAEEYPDGFAGVSLPEPELRKVAAAAAAMFRVVE
I
RRARISGRMDNHERMVGGDWVVVAQRESFALSVSADREGATIQFQGGDHHRVTSDWTPGQPLAVLHIDGEPLVLKV
AQSS
HGFVIRHRGAEMKVQVFTPRQAELAALMPEKLPPDTSKFLLCPMPGLIVTIDVEEGQEVQEGQALCVVEAMKMQNTL
RAE
KKGIVARINAGPGESLAVDEVIMEFE
>OLS48758.1 hypothetical protein BV379_11075 [Rhodovulum sulfidophilum]AB14
MPSPKFLAVLAAAVLLAGCNMSNDSQRAVAGGLGGALVADVLDTNVAAGAAIGALGGALCDDAGACR
>OLS48759.1 hypothetical protein BV379_11080 [Rhodovulum sulfidophilum]AB14
MLKHKGFPSRLPGTDFQFTIRRANKDGATRLVPRERYADRRPADRRADAGFLAALIEQFGDAPFERGNLDAGRLSWLL
GR
EVLPAEEPFDPESYEALLRVDIDCARTAFPELFAGGPLA
>OLS48760.1 hypothetical protein BV379_11085 [Rhodovulum sulfidophilum]AB14
MILRAVFLAGAALVLGASGEVIDVPSGQSVTHLDTITGEPGPAGLTVRFRFLAPDIAQDEGSMPATLAQGDMDYLCAEF
A
LPRLAGTGPEPAQVVITLMDRPVPFGQSDPEATQFFEAYRPEAGRCIWEGF
>OLS48761.1 hypothetical protein BV379_11090 [Rhodovulum sulfidophilum]AB14
MLREKGALTMARQLPVRFDLCASAAFPPASKGRLALQIRQDLWRMLQDLRGFSPVVTVEEEGQGLRVRAGGRAAAPF
PRS
DCERKIAALLTSPSHRARWLAQAGSEPRT
>OLS48762.1 hypothetical protein BV379_11095 [Rhodovulum sulfidophilum]AB14

MEHIEDWAKVQKHEFENMIVLCANCHARVTTGEIRKDAVRAYKRNLAIINGRYSLYEYRLMEAFYTKMREHPGEPLQA
QV
AENDYLHIKGMVDDDLLVLRRNPGGMWSFGLPISPMQALLTEAGKKLINSFFNGRDIEN
>OLS48763.1 methylmalonyl-CoA carboxyltransferase [Rhodovulum sulfidophilum]AB14
MKDILNELERRRSIARLGGGQRRIDSQHAKGKLTARERIELLLDEGSFEEFDMFVAHRCTDFGMEDSRPYGDGVVTGW
GT
INGRMVYVFSQDFTVFGGSLSETHAQKICKIMDMAVQNGAPVIGLNDSGGARIQEGVASLAGYAEVFQRNIEASGVIP
QI
SVIMGPCAGGAVYSPAMTDFIFMVRDSSYMFVTGPDVVKTVTNEVVTAEELGGASTHTKKSSVADGAFENDVEALYE
VRR
LVDFLPLNNREKPPVRPFFDKPGRVDASLDTLIPDNPNTPYDMKELILKVADEGDFYEIQADFAKNIITGFVRLEGQTVG
VVANQPMVLAGCLDIDSSRKAARFVRFCDCFEIPILTFVDVPGFLPGTGQEYGGVIKHGAKLLFAYGEATVPKVTVITRK
AYGGAYDVMASKHLRGDFNYAWPTAEIAVMGAKGATEILYRSELGDAEKIAGRTKDYEDRFANPFVAAERGFIDEVIQ
PH
STRRRVSRAFASLRGKRRETPWKKHGNIPL
>OLS48764.1 hypothetical protein BV379_11105 [Rhodovulum sulfidophilum]AB14
MPALPVPVVTIGLLVLSNLFMTFAWYGHLKYKAAPLVAVILVSWGIAFFEYLLQVPANRIGYGHFSAAQLKTIQEVITLT
VFCAFSVLYLKEPLHWNHALGFGFIALGAFFIFHRW
>OLS48765.1 Bcr/CflA family drug resistance efflux transporter [Rhodovulum sulfidophilum]AB14
MSTHPRVRFLDRTTPPHVMTLVALAGLPALTINIFLPSLPNMAVHFDTDYAVMQLSVATYLAANAVMQLFVGPISDRY
GR
RPVLLISTVLFLLATLGCIFAPSAEAFLGFRMAQAVVVTAMVLSRAVVRDMVADTQAASMIGYVTMGMALVPMIGPSI
GG
VLDEIWGWQATFLALLLAGLGVLALTWADLGETSTPRDVSLMDQFRQYPELFRSRRFWGYALTAALSSGAFFAYLGGA
PF
VGSHVFALSPSVLGLYFGIVAVGYMTGNFISGRYSVRVGINGMIYWGALITLAAPLLSMAFFAAGATQPIAFFGAMALL
G
VGNGMVLPNAIAGTLSVRPHLAGTASGLGGAIMIGGGAAFSALAGALLKPGTGAWPLLLIMATTSALAVVSVLYVRWV
DR
RAGPILAMPPKDGA
>OLS48766.1 Cro/Cl family transcriptional regulator [Rhodovulum sulfidophilum]AB14
MATRKLYAGVKLRETRSRLGLTQKDFAIRLGISLPYLNQMENNNRPVSSTVLLALVSEFGFDVTELSEGDAERLVGDMRE
ALADPVFGDLAPPLADLRLAASNAPALARAFLELHRAYRQSHERLASLDEALGREDAPLGPSPWEDVRDFFHYCDNYID
A
VDRAAERFARASDTAADPEQRALAALEAQGISLSFKDMPGLREYDEDTRTLTLSSHLRGPTRRFQLLHQLALLTQSQLLE
ATLDLARFQSPAARAIARIGMANYFAGAALLPYNRFLEAAQETRHDLELLADRFGASIEQVAHRLSTLQRPGAKGLPFYF
VRVDQAGTITKRHSTTRLQFARFGGACPLWNVHRAFETPGRFLRQLAETPDGLRYISLARDVSKPGGAWRAPVRRYAI
GL
GCEVRHADQIIYADDLDIDNAAAYEPIGISCRICERRDCHQRAVPPLERRLTVDANRRGLLPYEVAD
>OLS48767.1 hypothetical protein BV379_11120 [Rhodovulum sulfidophilum]AB14
MTHTPHELAEDLPERAALIHALRESDAHFARLVDEYHDINAAVHRAETDVEPTEDLHLNDMRKTRMHLKDEIARILDQA
S
A
>OLS48768.1 hypothetical protein BV379_11125 [Rhodovulum sulfidophilum]AB14
MHDIYALDPRGDLPDALRDLLDDCPREVWAGRLPEDESTRFWLDRHRMFRMLLARMEEDVRAMADFGTLADASPE
AAIRA
VRRDAAMLLNEVHGHRKIEDLHYFPRLAAFEPGLWAAFEMLDADHQALGLHLRELADAVSELVRGEPAGRDACIGRL
QLR

LQRFGSCLDRHLIDEEDIVLPILLRHGATLTD
>OLS48769.1 EamA family transporter [Rhodovulum sulfidophilum]AB14
MAVQSVPEAGQNPVAGILWMLATGISFVAVTALVKLSAQGLPAAQSAFLRYLLGLVFLLPILGQLSRARISRRRLALYGG
RGLAHALGVICWFFAMTRIPVAEVTAMNYLNPIYVTLGAALMFGERLAARRILAIAAAFLGVLVILRPGFREISEGHVAM
LFTAALFGISYLTAKLLSERDEASVVVAMMSVTVTIVLAPLALAVWVPPSWQQVVGLFFVAFFATAGHYGMTRAFRAA
PL
TVTQPITFLQLVWATLVGWLAFGEAVDGWVLLGGGLIVGSVVFITWREAVIKRRVVTPPAGAPET
>OLS48770.1 histidine kinase [Rhodovulum sulfidophilum]AB14
MGERTSHAIGRFDKSFLIHMIKDFFLVLLAVTVLEFALKAALVYYKFETSGAEEAQGVAEDLAESVRAIMRNEGGPVAAR
TMYPILKRNWDDLGYKIAIEPAPVTVRSIEALFGFTPAGIPAGTWPEGRFREAEVEIAAETFCLTCHAEAEVGQVLGRVT
VRDYLSRDIAAWLDEMTLSAGLALGKIVLHSVLLFLILRVRMEPLLRLRGTVAGLARAFGTLSERAEVRSADEFGALAHD
LNLFLDRINRLINELNAVLGKVVRVNDDIVSVQGDLRGQVDRLIRGMRRLEREAMLSAKREPLLSQAWFEAVRGAVAEL
D
VALERAGEAPQASGLVEHLSAVIANAEAQIAASQRLFEGLAALGDETEALKGAIAEMVRMEERMKLIVEAGGTLVQRLR
P
DLADPPQPAATAKTA
>OLS48771.1 transcriptional regulator [Rhodovulum sulfidophilum]AB14
MAGHSKWANIQHRKGRQDAARSKLFSKFSKEITVAAKMGDPDPDKNPRLRLAIKEAKAQSMPKDNIERAIKKAMGGE
GDN
YEEIRYEGYGPGGVAVIVEAMTDNRNRTASSVRSTFTKFGGNLGETGSVAFMFERVGEIVYPVSAGDADTVMMAAIEA
GA
QDVESSDDEHVIYTADTDLNEVATKMEADLGESDSTKLIWKPSNTTEMDLEAMQKLMKLIDALEDDDDVQHVTSNFS
ASD
EVLEQL
>OLS48772.1 SLC13 family permease [Rhodovulum sulfidophilum]AB14
MTQDQFILFALFATVFVALIWGRWRYDLVAFGALMAAVLLGVVDPDRAFEGFGHPATLVVALVLVVSAGLVRSGAVYL
IT
RTLVNSARGLGAHIAIMGGIGGLLSAFMNNVAALALLMPVDIQTARKAGRAPGLSLMPLSFATILGGMVTLIGTPPNIII
AAFRQDATGQSFRMFDFAPVGGVAALAGIVFVALVGWRLIPKSAGLDASADPMSDYSSYIAELTVPEGSKLIGRRLADL
D
PEAEANDVALLGLVRGGKRLYGLQRGTALAAGDSLVIEASPEALDEFRAALSLDFAEGANQERLKAEGAGLTVIEVVVPE
QARIAGRSAQLVGLAWRRRTVLLGISRQGRPVRQQIRKTKVRPGDILLLLTPQDVAQDVVEWLGCLTLADRGLAVTDT
RK
TWIAIGLFAAAVAAASFGLVDLTIAIGLVVVGYVAARIIPINEVYSHVEWPVVVLLGSMIPLGVALEESGGTRLIAETLL
GLTGDMPPWVILTALMVATMCLSDVLNNTATTVVAAPVGIQMAQALGVSPDPFLMAVAVAASCAFLTPIGHKNNTLI
LGP
GGYRFGDYWRMGLPLEIIVIAVSVPMILLVWPFQAGAA
>OLS48773.1 hypothetical protein BV379_11150 [Rhodovulum sulfidophilum]AB14
MAATLALVAGAGARFVPSSSAAGPIARILDALAPWLLAGALILALGATVLGLCRLGPGLVAAALVALAGLGALHLRQSLP
AQPGRAPDLRILFFNVCIENDLPAETIVRAALRQDPDIIVFAEAEAVAGGLPLLKDRYDFVSPCRPGACQIVLAARSRPK
RFWTMSLNPAWPDRYAVAEIETASGKRVFLAGLQLLKPWMSGLADTELERLTAQLGWLPGPAVAIGDFNAAPWSRT
LSQI
HRDTGMATARLPTATWPVGAGRFGVPIDQVLVGGGARLVRLSPFGAELGSNHRGLLAEIALP
>OLS48774.1 hypothetical protein BV379_11155 [Rhodovulum sulfidophilum]AB14
MFDQLKTRFAAIERRMTERHGADLSAPGARRAALWHFHLMDHAFLRVLWTNLVEIAPGVWRSNQPSPARLRRYRD
MGIRT
VISLRGGNRPHSHWLLEEEACAALGLRLEAVPVRARHLVPRETLLALLDIFDTAERPMVMHCKSGSDRAGFAAALYLIHA
EGAPVARARKMLSWRFLHSRRSKAGVLDLVLDRYEADTARSPMTLRDWISTRYDPAAIETEFKAGRS

>OLS48775.1 ABC transporter ATP-binding protein [Rhodovulum sulfidophilum]AB14
MAGGAQIFGWLWRGYLRRHSLTIGIALTLMTIEGASLGLLSYSFKPMFDRIFVAGERSAIPLVAGGVLAIFLARALSGFG
QRVLMARAGLSITGELQKDMVDHLLGLDGSYFQRTPPGGLIERVRGDTTAAANVWNTVLTASGRDFVSLVSLIAVALSI
D
WAWTLIAMAGIPLLVVPIIVLQRWIRRTARLARVQAAEIAGRLDEMFHGIVTIKLNRMEQREKGRFGETVASFVTSQLR
A
RAGQAGMPALVDIVGGLGFAAVLVFGGLQIIEGDKTVGDFMAFFTAIALVFEPLRRLGNVSGAWQAALASLERIHGVF
EE
RATILAPARPAQLPAPAERCDLALEDVHLAYGETPVLRGLSLTAKAGEMTALVGPSGAGKSTVFNLLTRLVEPQSGRVAI
GGVAVADLALSELRDLFSVVTQDAPMFDDSLRDNICLGRDVPEAHLREVIEAAHIDEFLPGLPAGLDSPAGPRGSNLSG
G
QRQRVAIARALLRDAPILLMDEATSALDAQSERAVQEALSHLAKGRTTLVIAHRLATVRDADRIVVMEEGRVREEGRHD
Q
LLARDGLYAGLYRLQFTAES
>OLS48776.1 folate-binding protein YgfZ [Rhodovulum sulfidophilum]AB14
MTGEEAADRTVLAITGADREKFLQGLVTNDVKALEGRLVYAAMLTPQGKYLADFLLTERDETILLDVKADIAEALARRLS
LYKLRADVRIAPAGLHVHRGLGEPPEGALPDPRHAALGWRAYAETPGTAPAIDWDRIRVAECIPETGIELIPEDSYILEA
GFDRLHGVDFRKGCYVGQEVTARMKHKTELRKGLATVGVEGAAPTGTPITAGSKEAGRLFTQAGDLAIAYLRFDRVLP
RM
TAGAATVLWPDG
>OLS48777.1 aminoglycoside phosphotransferase [Rhodovulum sulfidophilum]AB14
MADRAALEALVGAAIVRSQPLRGGDLSEVIRVDLEDGRRMVAKAGPRVAAEARMLRAIARAGVPAPAVIAEGGGWLL
IEH
LDERAASGPGWAALGTALLRLHGTTGAAYGWDEDHGFGPAAIPNAACADWPDFWAARRLLAWPEALPAGIARRVE
TLAAR
LDGLIPRHPPAALLHGDLWTGNVLFTPEGAALIDPACYHGDAEVDLAMLELFGTPGPAFRERYGAPAPGWPARRAVY
QLW
PALVHLRLFGSGYRGLVERCLATLGA
>OLS48778.1 elongation factor P [Rhodovulum sulfidophilum]AB14
MAKINGNEIRPGYILDHDGGLWAAVKVDHVKPGKGGAFAQVELRNLRSGSKLNERFRSADKVDRVELERRDQQFLYE
TDG
MLVVMDTDSYEQTELPADLLGERRPFLQDGMTVVVEYYEAEALNATLPAKVTCRIAETEPVVNGQTAAKSFKPAMLEN
GI
RVMVPPFIGPGEDIIINTETLDYSARA
>OLS48779.1 elongation factor P [Rhodovulum sulfidophilum]AB14
MVDFVDGTAFNHEQGHRARKLFAAVVLAALDDAIADDKKYGNGPEQIARWARSRDGREVLSCAGIDPNERVVRGLM
EFVG
RGVRTSVALSREESERRHSQEQAEAA
>OLS48780.1 hypothetical protein BV379_11185 [Rhodovulum sulfidophilum]AB14
MVEMRSIVDTAGVRSAGPAPEPVPSRTEPRLARLVLTPNLRVRPDPETRSAPARAMPVLLRGPLPQEPGAPALPVARE
AA
PSGRRTASVADLAEANRRDAVTLERRIAELEAAMAGVDADWDPECEEGFPAAPWVGKAQAMSAGPAAALGDEAAL
RLMVS
DIVREVVRQELRGTLGEGIGRNLRKMVRRELGRALAERDLD
>OLS48781.1 transporter [Rhodovulum sulfidophilum]AB14
MSGFLRYFAGTALSATLALGAVGAAQAETLTDALIAAYKNSHLLDQNRALLRAADEDVAQALATLRPVIAFAMTGNYT
DP

ASAANGLENTSAMAELTASLTVYDGGQNRMALDATKETVLATRAALIGVEQDVLLSAVQAYMDVFAALQTVSLAQNN
NRL
IAEELRATEDRFEVGEVTRTDVSLAQSALAEARSNLVAANGQLATARESYKLAVGHYPDTLSGIPNLPSIPTSPEGARSV
AVKTHPDIVQAQHNVTVSELNLARAKAAQGPSLTASVGYQRDDNSEEGYQAQLKLSQTIYAGGKLYAAERQAMANM
QATR
AGLLQTVRLVELDVGNAWSSLAVARAQVSASDQRIRAAQLAFDGTKEEARLGARTTLDVLDAEQDLLDARTARVEAEA
TQ
YTAIYSVLASMGLLTAEHLGLGIPTYDPNAYYNAVSSAPPKSLQGGKLDRVLQSIGRN
>OLS48782.1 protein-L-isoaspartate O-methyltransferase [Rhodovulum sulfidophilum]AB14
MADYAARREAMVDTQVRPSDVTRLPIIEALLSVPREAFVPARLREAAYIGENLPLAHDRVLLEPRSFSKMLEALDLGPGD
MVLDLGAGLGYSAAVMARMADAVVALEEDGSMAREAEATFGEHDVDNVAVEVGALTEGAPRHGPYDAIMVEGAIE
ELPQA
IADQLKEGGRIVALFMEGTLGVARIGWKVEGVLNWRFLFNASAPVLPGFARATEFEF
>OLS48783.1 hypothetical protein BV379_11200 [Rhodovulum sulfidophilum]AB14
MLSYRILLSLAAPGLALLLLRDRPFARERLGLSGASNGPGRVLQPTIWLHGASNGELASARPLIERLLARRPDLCIVATS
NSRTGRTLVESWALPRVSARLAPYDYRWALRLFIGRWQPRALILIESELWPNRIFEMARRDRPVLLLGARLSARSAARW
Q
RMPALARRLTGAIAYLSAQDPASMDRFATLGLAPERRGPVIDLKAFAPAARPDADDLNALAPLLPRARTLLAASTHEGE
E
APILEAFARRWRADPQARLVLAPRHPRRAAQIAALIERAGLGHALRSNGGLPGPERPVLLADTMGEMALWYALAGVSF
IG
GSLVDKGGHTPFEPAAAGSAILHGPHVANFAAAYGALDRAGAAESVAGADGLSAALAALDGAEQTRRAACAREVLAG
LRA
EAPGPDMLIDALECLMGAPAPTPPAEKEPGNG
>OLS48784.1 hypothetical protein BV379_11205 [Rhodovulum sulfidophilum]AB14
MAETQFSLDELEEAARLVHARMPPTPQYAWPLLAEAIGTEVWVKHENHGPTGAFKLRGALTFIDWLTRSHPDCPGIVT
AT
RGNHGQGQALAARAAGMSARIYAPEGNSAEKNAAMRAFGADLRIFGTDFDSAREEAVRVAEAEGLYLVPPFHRELVR
GVA
SYALELFTAQPDLHSVYVPIGCGSGICGTIAARDALGLSTRIVGVVSERADCVLRSMAAGDLVETPTAQTFADGLAVRVP
VAEALAIYSAGAERIVSVSDAEIAEAIRLYFSATHNVAEGAGAAPLAALMQESEMMAGRRVGVILCGGNIDSARFATVL
G
GGVPDP
>OLS48785.1 hypothetical protein BV379_11210 [Rhodovulum sulfidophilum]AB14
MIWTVHPGRGLGALSFGMTPEDVQSHPDLGRPGHVYRGSRGRVMEYRGLSVPICEYGPNGLNCIIACRKVMGVRYQ
GASL
FEADPKAVVKLMETQLGPARLCQEQLCFPGAGLLLGGFFDPHEQCFFHPESDYQDERSITVCIPGACGTACVEELEMVS
F
L
>OLS48786.1 2'-5' RNA ligase [Rhodovulum sulfidophilum]AB14
MIRAFAAIALPPEIRERLVQVQAGLPEGRPVPPENLHLTLVFLGELPEPLLDDVHLAFAGIRAGRFQLTVSGLGLFGADR
PHNLHAALAESAPLRHLQAKLETAARRAGVVLDRRRFVPHVTLARLGRDRSARRRVELAVAQAADFRAGPFEVGFFGL
FR
SEIGRNAARYTELMRYTLFASQDASPGA
>OLS48787.1 glutathione peroxidase [Rhodovulum sulfidophilum]AB14
MRPATLPVVAALLLGAGSATAAESFRFPSIDGGEIDLAAFRGRPVLVVNTASQCGFTPQYDGLQALQDSYGPRGLVVLA
V

PSDDFNQELADETAVKDFCEVNFGLTLPMTEITHVKGAEAHPFYRWMDTEHDFSPSWNFNKVLLDGSGNPVATFGSF
TKP
QSAKLTAKIETLLPQ
>OLS48788.1 hypothetical protein BV379_11225 [Rhodovulum sulfidophilum]AB14
MIRYALTCADGHRFESWFQSASAFDTLLKGGHVSCSLCGSRDVSKALMAPSVATDRPEGSGAPAPDEGSENAAAPAG
RPL
STPTGPAEQALTALRKRIEENSEHVGLRFAQEARDIHAGLRPDRPIHGEARPDEAIRLIEDGIPVAPLPFLPGRKRN
>OLS48789.1 aspartate kinase [Rhodovulum sulfidophilum]AB14
MPVLVMKFGGTSVADLARIRNAAEKIRREVERGYDVIVVVSAMSGKTNELVGWVEQTSPLFDAREYDAVVSSGENVT
AGL
MALTLQEMDVPARSWQGWQVPLRTSSAHSTARIEEIPRANIDQKFAEGMRVAVVAGFQGISHEGRITTLGRGGSDTT
AVA
FAAAFGAERCDIYTDVDGVYTTDPRIEDKARKLDRIAFEEMLELASLGAKVLQTRSVELAMRYKVKLRVLSSFEDTDETS
GTLVCDEEDIVEQNVVSGVAYSRDEAKVTLISVADRPGIAAAIFGPLAKAGVNVDMIVQNISEEGRTDMTFSCPVNQVP
R
AREAMETAMDAGEVNFREVVADTDVAKVSVVGIGMRSHAGVASTMFDALAAEGINIRVITTSEIKISVLIDRKYMELAV
Q
ALHDAFGLENAA
>OLS48790.1 phosphoenolpyruvate--protein phosphotransferase [Rhodovulum sulfidophilum]AB14
MPDPTESSSRRLLVSLRETLAEPAAGQERLDRITSLIAEQMGTHVCSIYLFRDADTLELCATEGLNPDSVHKTRLRVGEG
LVGRVAKTAHPINSGNAPQERGFRYMPETGEEIFSAFLGVPIQRLGERLGVLVVQSKDARQYSDDELYALEVVAMVLAE
M
TELGAFVGEGEALAALHQRPVMFQGTVGQEGVAVGHVWLHEPRVVVTNPIADDYETEMRRLHEAVDRLRVTVDEM
LSAAP
GGDKDQLQVLEAYRMFANSRGWKRRMEEDIARGLSAEAAVEKEQSAARARMQTVPDPYLRERLHDLDDLSNRLLRIL
TGQ
GEHTGAERPSDPILIARNIGPGELLDYGRSLRGIVLEEGSVGSHAAVVARALAIPLIVNAQRITTEALNGDPILVDGEQG
IVHLRPEETVAVAFNDKIAMQAKAQQRYATIRKEPALTLCGQRVALHMNAGLMADLPSLESSGAEGVGLFRTELQFLIR
S
KVPKRSEVAALYARVMDAADGRRVVFRTLDIGSDKVLSYMKRQDEPNPALGWRAIRVGLDRPGVLRMQLQALIRAAN
GRP
LSVMFPFVAQLDEFRTARDHLMREIDREGRLGRALPEKLEIGAMLETPSMAFSPRQFYEEADFISIGGNDLKQFFFAADR
ENERVRRRYDTLNVSFLSFLEQIVKRCEDTGTHLSFCGEDAGRPVEALCFAAIGLRCLSMRPASIGPVKHMLRRSNLAEA
RAVIDTARASGAQSVRPAIIDWLRRRD
>OLS48791.1 GNAT family N-acetyltransferase [Rhodovulum sulfidophilum]AB14
MIRVAEETAGDWWEVEALYDLCFAPGRTALSSYRLRDGVPPVAELCLTARDSEGILAAAIRYWPIRVGEAEALLLGPVA
V
HPTRQGEGLGGLLIRNSLARAEALGVARVMLVGDAPYYARFGFSRLTGVEMPPPTNPDRVLGIELRPGAWAGITGAVT
RA
RPD
>OLS48792.1 flavin reductase [Rhodovulum sulfidophilum]AB14
MFYRPEDGHGLPHNPFGAIVAPRPIGWISTRDDAGRDNLAPYSFFNAVAYEPPQVMFASTSTKPDRDGTKDSVANIRE
TG
VFCVNIVEEAATNAMNATSAPAPCGEDEFALAGIGRTPCETIACSRVADAPAALECRLTRILQLPGAANLVVFGAVTGIH
LREDCLIEGRFDVTRFRPLARLGYRDYAVVGGIFELQRP
>OLS48793.1 methylthioadenosine phosphorylase [Rhodovulum sulfidophilum]AB14
MERMIAVIGGSGVYDIDGLEDARWQAVETPWGTPSDEILTGTLDGMKMAFLPRHGRGHVHSPTSVPYRANIDALKRI
GAT

DVVSVSACGSFREDFAPGEFVVVDQFIDRTFAREKSFFGEGCVAHVSMAHPTCARLSAACLQAARDEGITVHDGGTYL
AM
EGPQFSTLAESKLYRENWGCDVIGMTNMPEAKLAREAELCYASIAMVTDYDCWHPDHDAVEVADIVKTLIGNAEKAR
GMV
SRLPRLLGAHKPCEKGCEAALEHALITAPEKRDPALIARLDAVAGRVLGR
>OLS48794.1 hypothetical protein BV379_11255 [Rhodovulum sulfidophilum]AB14
MGKKGGKKDSQLVLRLDKEQRDEFVALCKTLDTSAAREIRAFIRTFVKQHGGEAGSGKKK
>OLS48795.1 adenine phosphoribosyltransferase [Rhodovulum sulfidophilum]AB14
MIASSSVKDYIRTIVDFPHEGIMFRDVTTLFADPRGFRMAIDQLLNPYAGAQIDKVVGLEARGFILGGAIAHQLSKGFVP
IRKKGKLPAATIAEEYKLEYGEAVVEIHDDALAPGEKVLIVDDLLATGGTCEAGIKLCERLGAEVIGCAFIVDLPELGGR
KRLEEMGMDVHTLCEYAGA
>OLS48796.1 hydroxyacid dehydrogenase [Rhodovulum sulfidophilum]AB14
MLNPVTDAFAADLRARLPAAVFRELTPAYLEEPRGRFRGQGALLLAPGRVEEAAEIVRACAAARVGIVPYGGGTGLVG
GQ
IMETGPAPVIVTLDRMRAIRAVHADENVLVAEAGCILAEVQAAAEAAGRLFPLSLASEGSARIGGLLSTNAGGLNVLRYG
NARDLCLGIEAVLPDGSILKGLKRLRKDNTGYDLRHLLIGAEGTLGLITAATLKLAPRPAATGAALLAVRDPAAALALLA
MARDRVGEGVSAFELIHRAGLDFLAETMPDLRRPFADPPDWMVLIELGLGAGQDPGAALEDLFAGAFEAGLVADGLV
AQS
EAQRQDFWTMRETIPLANRRIGAIASHDIALPLSAIPDFIAAAGPALARIGCFRINCFGHLGDGNLHYNVFPLPGETRAD
HEAQRDAITRCVHDLVAGFEGSISAEHGIGRLKVAELGRYGDPARLAAMRAIKAALDPACILNPGAVIPPP
>OLS48797.1 Zn-dependent hydrolase [Rhodovulum sulfidophilum]AB14
MLRSCLILLAVLTALPAFAEGRRLSHCIAIADAAPGMRFLHRAAFGTSLDENTVRLTFVDHATFLLETPGGLVAATDYTG
YLGPNPVTPDLVTMNNAHETHFTAFPDPAIPHVLEGWAKGGEAADHHLDLGEILVRNVPTDIRGRDGTSVRQNGNSIF
VF
EVAGLCIGHLGHLHHVPDEAQFAALGRLDVVMAPVDGRLTLDLPAMIAVLTRLKSAVVIPMHWFGPRTLDRFLEGMA
GEF
EASRPGGSSLEVSLRTLPSRPTVVVLEPLSD
>OLS48798.1 GNAT family N-acetyltransferase [Rhodovulum sulfidophilum]AB14
MLGLRRKVRIETERMTLRPPAHADFQPWTMLRAESMAFLTPWEPTWSPDHLARKAFTNRVYWAQRALGSGSAVPLF
LFRR
SDDALLGAITLDNIRRGPSQSGTLGYWVGECHARRGYMREAIEAVVHYAFTQKDLSRIEAACLPENAASRGVLEKCGFK
Y
EGVAQSYLQINGRWRNHVLYANLRRDRRGRTETGLA
>OLS48799.1 peptidase M16 [Rhodovulum sulfidophilum]AB14
MSVELHRLPNGFRIVTERMPGLKSAAIGIWIMAGGRDEQPEENGIAHFLEHMAFKGTEKRTTLEIAEAIEDVGGYINAY
T
SREMTAYYARVLEADVPLALDVLADILINPLFAEDEIEVERGVILQEIGQVLDTPDDVIFDWLQEVAYPGQALGRSILGP
AERVRGFTREDLFRFIGKHYSPDRMILSAAGAVDHAEIVRLAEALFSDREPAARGALEPARFASGERREIKPLEQAHMAL
AFECPGYRTPGIYTAQVFSTALGGGMSSRLFQELREKRGLCYTVFAQTGAYADTGMMTLYAGTSAEQVGPMAELTMA
ELA
RAARDMSPAEVARAQVQMKAGLLMGLESPSNRAERLARLVSIWDRVPGVEETVAQIDAVTTEAVREFASRLTLDGRP
ALA
LYGPVSGAPALDALQGRLAA
>OLS48800.1 threonine synthase [Rhodovulum sulfidophilum]AB14
MRYVSTRGQAPVLSFEEAMLTGLARDGGLYVPETIPAMSADEIAALAGLSYEEAAFRVMRPFIGEAFTDAEFEAIIARAY
RGFNHAARAPLAQIGPNHFLLELFHGPTLAFKDFAMQLIGQLFQAALERSGQRVTIVGATSGDTGSAAIEAFRGLPNVD
V

FILFPEGRVSEVQRRQMTTPAEDNVHALALKGDFDDCQARLKDMFNHFEFRDAVGLAGVNSINWARVLAQVVYYFTS
AVS
LGAPHRPVRFTVPTGNFGDIFAGYIAGRMGLPIEKLVIATNQNDILHRALTGGAYETKGVSPSISPSMDIQVSSNFERAL
FEAYGRDGAAVAGQMEDLKAQGGFGISQGALEFLRETFASGRASEAETTETIRATFAATGLTICPHTAVGVKVAAEQPA
S
AAPMVTLATAHPAKFPDAVEAATGLRPPLPKSMADLYDRPERVSPVENDLAALEALINERRKR
>OLS48801.1 metalloprotease TldD [Rhodovulum sulfidophilum]AB14
MQHPPFRPFETALDRDRALLLLQAATLGAEDGELFLERRRSEMLSFDDGRLRTASYDASEGFGLRAVRGEAAGYAHSSD
I
SEAALKRAVETARLAVAEGGGTLALGPQPTNRRLYTDADPLAEAPFALKIDTLREIDAFARELDPRVVQVTASLAASLQE
VVILRPDGTLLSDIRPMTRLNVSVIVEENGHRESGSDGGGGRTGLAGRIERSTWEASTREALRIALVNLEAEPAPAGTM
D
VVLGPGWPGILLHEAVGHGLEGDFNRKGSSAFAGLMGQQVAAKGVTVIDDGTIPDRRGSISFDDEGTPSNRTVLIEDG
VL
VGYMQDRQNARLMGLSPTGNGRRESHAHPPMPRMTNTCMLGGDTAPADILAGLKDGIYAVGFGGGQVDITNGKF
VFSCTE
AYRVKNGKVGAPVKGATLIGDGPAAMKQIHAIGNDMALDPGIGNCGKAGQWVPVGVGQPTLLIGGLTVGGAGA
>OLS48802.1 DNA topoisomerase I [Rhodovulum sulfidophilum]AB14
MPVVVVESPAKAKTINKYLGSDYTVLASYGHVRDLPPKDGSVDTEQGFEMTWEVASDSKKHVKAIADALKEDNALILA
TD
PDREGEAISWHLEETLRARRAIKKDTAVSRVVFNAITKSAVTEAMKNPRDIDEPLVHAYLARRALDYLVGFNLSPVLWRK
LPGAKSAGRVQSVCLRLIVEREMEIEAFRAREYWTVSAQFATPRGQIYEARLITLGGQKLDKFDLGTATDAELAVRAIES
RAFTVAAVEAKPGTRNPAPPFMTSTLQQEASRKFGFGARQTMQAAQRLYEAGIITYMRTDGIDMAPEAVMAARDAI
KDRY
GAEYVPKSPRMYKNKAKNAQEAHECIRPTDMTRDPSKLRVTDEDQRKLYDLVWKRTIASQMEAARLERTTVDVESAD
REV
VLRATGQVVLFDGFLKVYEEGRDDTEDGDDRRLPQIAQGEPARPASADRFAADYATLAGTGGAARDPELIEAPGDEPV
LR
CDRAILDATKAILAQQHFTQPPPRYTEATLVKRMEELGIGRPSTYASIVTTIQDRGYVLKDKNRLVPEDKGRLVTAFLSN
YFSRYVGYEFTANLEDELDDVSAGARDYHEVLERFWKDFSAAIAETADLRISEVLDKIDDVLAPHLYPPREDGSDPRVCP
KCGAGRLNLKTARSGGAFIGCTNYPECRYTRSLSAVNGDDSSELDGKVLGHDGEDEISLRIGRFGPYVQRGEPTEEEPKP
PRASLPKGWKPEEIDLEKALMLLSLPREIGPHPEDGETVEAGIGRYGPFVKHGRKYANLPDVEEVFTIGMNRAVEVLAQ
K
KTRGGGTAAKPLRQLGEHPEKGGAIDVMAGRYGPYVKWEKVNATLPKDVEPEAVTLEQAVELIDAKAAKGGKTKAKA
KAK
PAAAKTGKTAAKKTTAKTTGTRAKATKTTGTAKTTKAKTSDSAG
>OLS48803.1 L,D-transpeptidase [Rhodovulum sulfidophilum]AB14
MTDRSMSRRKALLAFGATFGTGLAAPALLRAEPAPMTRRNISSFSQQSWREHFDTLGKAAIVADTASRALHFWSGDG
EDY
RIYPTSVPISEDLTKRGYTEVVRKRVGPDWTPTASMVERNPDLHYMPPGPDNPLGTHAMYLSWPAYLIHGTHDTRKIG
RP
SSDGCIGLYNEMIAELYAITPVGTQVRII
>OLS48804.1 hypothetical protein BV379_11310 [Rhodovulum sulfidophilum]AB14
MPGEIGMAVETCESDATVRSRTVKTPAGPLILPAGKDAWARAHWNGFRRKDRNRPPHWRGGLRKTGRPIGAPP
>OLS48805.1 hypothetical protein BV379_11315 [Rhodovulum sulfidophilum]AB14
MFAADLDRDLALVIRHGDLTGAEWGSVTRGTRDAMAVRNPFGISTTLASLSASDPTSRTIVVDGDRLGMTAEDLVAGS
ID
ALADSQITAIRTSCQMDATEGLSALT

>OLS48806.1 succinyl-CoA--3-ketoacid-CoA transferase [Rhodovulum sulfidophilum]AB14
MKKIYGTAAEALDGLLADGMLIAAGGFGLCGIPELLIQAIREAGTKDLTFASNNAGVDDFGIGLLLQTRQVRKMISSYVG
ENAEFMRQYLSGELELEFNPQGTLAERMRAGGAGIPGFYTKTGVGTVIAEGKEHKEFDGETYILERGIVADLSIVKAWKA
DETGNLVFRKTARNFNPPAAMCGKVCVCEVEEIVPVGSLEPDQIHLPGIYVHRLIQGEHEKRIEQRTTRAA
>OLS48807.1 succinyl-CoA--3-ketoacid-CoA transferase [Rhodovulum sulfidophilum]AB14
MAWDRNRMAARAAEELKDGMYVNLGIGIPTLVANYIPEGVNVTLQSENGMLGIGPFPIEGEEDPDLINAGKQTITELP
HS
AYFDSATSFGMIRGGKIAMAILGAMEVAGNGDLANWMIPGKLVKGMGGAMDLVAGVGRVVVVMDHTNKHGDSK
LLKECTL
PLTGTGVVDRIITNLGVLDVVEGGLKIVECAEGVTEDEIRAATEAHIVD
>OLS48808.1 hypothetical protein BV379_11330 [Rhodovulum sulfidophilum]AB14
MKTLALTAAAFVALSAPVFAQTQLEQDLGVQPGTYSASQLIELKAAAEKDGPLSRVWFSPASSTTMSSSGYANARVAKI
V
ASLAAESDNGYVAAHGHRDNGPAAQTIANPRVAAIAAYNAANAD
>OLS48809.1 hypothetical protein BV379_11335 [Rhodovulum sulfidophilum]AB14
MTGTSLSTALPASRRLRDTLLMAGLLVLVVVGLAGLAAASGWDHTMAQLSRLGLIQGAALLGLSLINYLLRALRWHAFG
R
QIGLPPGLWRDIRHYLGGFLMIATPGRLGELVRVRWIRRETGLPLERSAPLFVMDRAADLVAMALILCLALALSAGHIAG
AIPVAVLALAAAAVATNGRLMAALVTRGYRIFGRWPRLFGRLRGMARTLGRFADARLLVVSILLGGLGWMAEGFAFH
QLL
IWLGADIGLWKAIGIFVFATLAGGLTGAPGGLGGAEAAMIALLALEGLPLETSVAATAVIRVTTLWFAIGIGALVFPMAE
RMSKRTVNAVENR
>OLS48810.1 FAD-linked oxidase [Rhodovulum sulfidophilum]AB14
MQWKTADYAGWGRALRAHGRIARPERVSALTGALAEGGPAIGNRRSYGDGALNSGGAATEMTRMDRLLAFDPETG
VIEVE
AGATIGALLKMLAPRGWMPRVMPGTGFATIGGCIANDVHGKNHHGAGSFGQHVLAVTLMMPEGPREVTPGADPA
LFRATM
GGQGQTGAILSARLQLMPIPGQRMKVVETRMRDFDSFIAALDASDAEFTVGWIDATAKRKRLGRGILEEGMLVEGTR
PGP
ERKAKPVPFDAPKALLSRPVVKIFNRWYGSGVPDSGRKRTRALDAFFFPLDALTDWNRLYGKRGFHQFQCVVPADRVA
AL
KEILNTIARGGVASPLAVLKRMGPGRAGHLSFPMDGYTLAVDLPARAKVPALYARLEDQVLAAGGRIYLAKDALARPES
M
AAMYPELEAWRAEADKADPGRRLATDLVRRLQLRSA
>OLS48811.1 short-chain dehydrogenase [Rhodovulum sulfidophilum]AB14
MAETWIILGASSSMARALARRLAARGDGLILAGRELADLERDAADARVRGAAFAETIRFDARDPACLAPILARAEACEG
T
VSAAVFVGSMPPQAVIDADPGLIEGVVTDSFTGPARLLQGLAPIIEARGAGTVIGVGSVAGDRGRIGNYVYGAAKAGFA
T
YLSGLRNRLGRAGGHVLTVKPGFTDTAMTWGIEGMFLVASPDKVADDILTALEKRRNVIYTPFFWMGIMGIIRLIPERV
F
KKLSV
>OLS48812.1 hypothetical protein BV379_11350 [Rhodovulum sulfidophilum]AB14
MKLFDIVKTFAKSDSGAVTVDWVVLTAAIVGLGIAVLSTVRDGVNDLGDNISDALSDATVASPGTLGWSN
>OLS48813.1 Flp pilus assembly protein CpaB [Rhodovulum sulfidophilum]AB14
MRLIFGLVLLVGVGLAGCAVYLAQGYFNQTKRDLAAARAQQVALETVVVAREPLRYGQQITLADLRPVLWPEESMPD
GVF

RTKEEIFPEGQPQFRTVLRAMEAGEPLLQVKVTDPGADAGITSRLTKGMRAFAIRVDVSSGVSGFLRPGDRVDIYWSGE
S
HSKHFTKLIEPGIRLIAIDQIAHDDTTSPVIARTVTVEVSPQQVAALAQAQSIGRLSLALVGATDDVVAKAVEVDHKTLL
GIEDAPTAPPEEKRICTIRTRRGSEVMEIAIPCTN
>OLS48814.1 general secretion pathway protein [Rhodovulum sulfidophilum]AB14
MNFRIILAVALLGLATAPGALRDAGAQTLRTLGGLATGKLTVPMNRAVVVESEQVFAELSISNPQIADISTLSDRTIYVL
GKSPGRTTLTLLGPDGDLIANVEVQVTPDMSELKERLRDVLPGEPIEVRAANDGLVLSGTVSSKLVIDRALDLASRYAPE
RISNLLTVGGTQQVMLKVRFAEMNRSVSKSLGTSLGVSGLTSGGDLSFSGGTNTIAEGNNLGNLFDGNPATGLTRSRGS
N
GVFSIGIGAGDVEFAVLLEALETKGFVRTLAEPNLTALSGQSASFLAGGEYPIPVVGSDGEVTVEFKPFGISLGFIPNVV
GTNINLEMTAQVSNIDPSVTYNNNGIIISGFSTRSTATTVELHDGESFAVAGLLQDDFASLKGQVPWLGDVPVLGALFRS
SEYQRDQSELVIIVTAHLVSPIRGEALALPTDRVAPPSERDLFLYGRLSSPRTPRSGAAGEVARQDFGGSYGYVME
>OLS48815.1 hypothetical protein BV379_11365 [Rhodovulum sulfidophilum]AB14
MRASLVLLTLGTALALTGCSPQQREEFDRPAGETLDDGSFGKATTTNRMMMTADRAMAIDMTRRFAAEVPNTVTFA
FDSA
VLDRTAQGALDRQAGWILRHPFVVFRVYGHADKVGSDAYNKALGLRRAEAVIGYLAGRDVSRARLQAVVSHGETRPL
VPT
AGPERQNRRTVTEVSGVIRPRPMAIDGKYMERSYKAYLDSAQPKPGLIPEPLQAEE
>OLS48816.1 pilus assembly protein CpaE [Rhodovulum sulfidophilum]AB14
MTSMPAPIPDQVPIRACTVSRDIQDFDLLIEDMEAELGGGWGDIAFAEARDFLEQPEARHLEFIAVAVGDADAANIAAI
G
EIVEAAKRRGLRVLVIAEELSPIALHQLMRKGADDFVPYPLPEGALHDAVARLRRAGSPAPAPDPTCPRLSRSNRDGVVL
PVQGLAGGVGASTFAVNLAWELSLIAGEVGGRVCLLDLDLQFGSAATYLDLPRKEAVFELLSDIAAMDGEGFLQALQVY
R
KELHVLTAPTDILPLDIVGPDEINALIDMARSQFDFVVIDMPKTIVNWTEAVINRSHFYFALLALDMRSAQNALRVIRAL
KSEGLPRERLRFALNRAPKFTDLSGRGRVRRLADSLDIEIGLMLPDGGRSVTESGDHGLLLAETAARSPLRKEIRKLALS
LHDVGTAAAAAAQ
>OLS48817.1 pilus assembly protein TadB [Rhodovulum sulfidophilum]AB14
MQIGTELLIYGLIFLSVLALVEGLYLTLFGKSISLNARVNRRLEMLEKGLDRDQVLEQLRKEMTQHMRARGIPLYALLAE
RAQKANIAFTPLQLIAVMVVLSVLAFLGLTVGTEAALPVRALASVAIGVGGVCTWVNGKASKRLGLIEEQLPDAVELMV
R
ALRVGHPFVAAINIVASEVPDPLGSEFGVIADEVAYGRDIGEALRDLAERVGMQDLRFLAVAVTIQQQSGGNLAEVLAG
L
AKVIRSRFKLFRRVKAITAEAKWSGMFLSGFPVAAILMISILKPDYYDAVKETAIFLPAAIAVAVFLVVNVIFMRVMINI
KV
>OLS48818.1 pilus assembly protein TadC [Rhodovulum sulfidophilum]AB14
MAFVDLVNGWLTDRLGPLGPLIAMGLLGLGLIALSLPVFLRRRSDPLDKLRRQVRAAETAGARDPQAALRQVSGADRL
DR
FSTLLEPRDEKALSAARLRLIQAGYRSKSAVRLFHFAQFALGLLFLAAGLVHAFVLNAGGNIGTSGVILSVLLPGAVGYY
LPKYWVERRLQSRQEDLTNGFPDSLDMMLVCVEAGQSLDQSILRVARELRPGFPALAAEYEIVSNELKAGKDRVQVLR
DM
SERCGITDISFFITVLIQSATFGTSIADALRVFSAEMRDKRVMRAEEKANKLPTKLTLGTMMFTVPPLLIILIGPSIYEI
YDTLQNADF
>OLS48819.1 hypothetical protein BV379_11395 [Rhodovulum sulfidophilum]AB14
MRHPVLAPICVALAMTVAACQKPGDTDVDRAVQDVLASDENSLTGIMMTVADPDEAIRYFRRLLEEDPDKISARRGLA
QS
LIRAQRPAEAATAWAAVLRHPDAGNEDRVNYADALIRIGEWDKAEAELDRIPPTFETYRRYRLEAMVADGNGEWAKA
DSF

YETAAGLTTKPAGVFNNWGYSKLTRGDFKGAERLFVNALTYDPGLFTAKNNLVLARGAQRKYELPVVRMTQTERAQLY
HT
LALTAIKQGDVAIGKGLLEQAIETHPQHFEAAVQARRALEAQG
>OLS48820.1 hypothetical protein BV379_11400 [Rhodovulum sulfidophilum]AB14
MTAALWFLPFATPIALWAALSDLRTMTIPNRAVLALVAVYALVGFAALPLSPWAWRWLHLAVVLVLGFVLNLTGAVG
AGD
AKFAAAMAPFIAPPDIGAFLLLLAATIPVSFVAHRLARRVPALRQALSGWASWEARDFPMALALGPALILYLGLAAIGA
M
PSAI
>OLS48821.1 ATPase [Rhodovulum sulfidophilum]AB14
MPLQAPKLSGPPAPRSLAEMRLPMVMMRDILLKTVFRKALDAVSGIARAICLPVPVTQELVDLARSQKLLEATGTLHAN
S
GGEMGYKLTEAGKACALDALSQSEYYGAMPVPLDVYAEQVRRQSIRNIRITRSRLTAAMGHLILPDALLDQLGPAVVAG
R
SILMYGPPGNGKSSISNGICDALEDLIHVPRAIEYAGQVITVYDPIMHSAAEAQEEDPTALRRATGRTDSRYVLCRRPTV
ITGGELSLSMLDLIYNPTARTYQAPLQLKATGGVFIVDDLGRQAEPPQALINRWIVPLEESRDILALQSGEKFEVPFDTL
VIFSTNFHPNEIFDRAALRRIFFKIKLDGPCQEDFLKIFASVARRKEMPIDEDALIYLLRKKYPTIDNVYANYQPVFLID
QMIAICDFEGLPRQMSPELIDRAWANMFVRDDPILH
>OLS48822.1 peptidase M19 [Rhodovulum sulfidophilum]AB14
MPETSVDTNTLPAVFDGHNDVLLRLYRAGGPKAAESFVTGGPGHIDLPKAREGGLGGGFFAVYVPSDEGDTDHDEMA
RDS
YDLPLPEPVPQDRALQVAMTQIATLERLQELGALKICRSAAEIHISMVEHKLAAVLHMEGAEAIDPELITLDVLHGAGLR
SLGPVWSRPTIFGEGVPFRYPSDGDTGGGLTDLGVALVRRCNALKIMLDLSHMTMAGFWDVAKHSDAPLVATHSNA
HAIC
PHARNLTDAQLDAIAASDGMVGLNYATAFLRPDGRMVDTGLDDMLRHLDHLLDRLGESRVGLGSDFDGALIPSAIGT
AAG
LPALRRAMVAHGYGPELIARICQGNWLRVLEKTWGH
>OLS48823.1 ABC transporter substrate-binding protein [Rhodovulum sulfidophilum]AB14
MNAFARLLSGAALTATLAATAPALWAETPANMLVVANRIDDITTLDPAENFEFAGGDVLRNVYGRLVNFDPLDYEAGY
QP
DLAESWEVSEDGRSITFTMREGVTFTSGNPVTAEDAAFSLRRAVLLNLTPSFLLTQFGFTPDNIDETIRAEGNKLIVTTD
KAYATSFVLNCLTATIGSILDRKTVLEHEVDGDMGHDWLKTHSAGSAAYSLVAWKPNESVTLQANPDFYQGAPAMKR
VIV
RHIAESATQRLLLERGDIDVARNLNPQDVQGLDGAEGVRIQEELKGYLMYASFNQKIPELQDPKVLEALKYLVDYEGMA
N
SFLKGQWTIQQNFLPQTYLGAVKENPFFFDVGKAKELLSEAGVDGLELTIGVRDVQERMEIAQSMQNTFAQAGIDLNI
EV
GTGKQILAKYRARDLDVYLGTWGPDYPDPQTNASTFAYNPDNSAEAQNGGILAWRNSWDADGLTEMTEAAVTERD
TAKRI
EMYHEIQARFRETSPFVIMFQAIEQDGLRENVEGFTTGQAIADASYWKVTK
>OLS48824.1 peptide ABC transporter permease [Rhodovulum sulfidophilum]AB14
MAATGKAQEGRARAPFPPRWVKRTGMTLGSIAITMLGLLFVTFLIGRVMPIDPVLAIVGERASPEVYARVHQELGLDRP
L
MVQFWFYLQDVLRGDFGTSLLTARPVSEDIARVFPATLELATLGTLIGVLLGVPLGVLAAVRRGSWIDQVARVVALVGY
S
MPIFWLGLMGLLIFYGILGWVGGPGRLSIFYDGMIEPVTGMILIDSALAGDWTVFRDAVNHIVLPASLLGYYSLAYISRM
TRSFMLEQMSAEYVTTARVKGLSERAVIWNHAFRNIRVQLITVIALSYAGLLEGSVLTEIIFAWPGIGSYITNALLSADM
SAVLGGTVVVGLAFVLLNVFSDLLYKVFDPRAK

>OLS48825.1 D-ala-D-ala transporter subunit [Rhodovulum sulfidophilum]AB14
MTDMTGLRAWLLTDTPQSRTQARMASLYQGWLAFRRNPMAMFGLAILIALLAIAALAPWIAPHDPFHQDLAARLQP
LGTP
GHPLGTDSLGRDILSRLIYGSRITLYIVTLVALIAPLAGLIMGTVSGYLGGWVDMVLMRITDIFLAFPRLVLALAFVAAL
GAGIENAVLAIALTAWPPYARIARAETLTLRNADFISAVRLQGAGPVRIITRHIWPLCISSLIVRVTLDMAGIILAAAGL
GFLGLGAQPPSPEWGAMISEGRRFILDHWWVATMPGLAIFTVSLAFNLLGDGLRDVLDPKGEK
>OLS48826.1 peptide ABC transporter ATP-binding protein [Rhodovulum sulfidophilum]AB14
MTDLLDVEDLRVRFPTRTGMFEAVRGVSFKLRRERLGIVGESGSGKSMTGRSILRLIRPPGQVTAGHIKLDGEDLMQKS
E
ARMRKVRGERISMVMQDPKFSLNPVMSVGEQLIEAWRVHKKGSRAEARRRALDMLEAVSIRDPERVMRAHPHELSG
GMGQ
RIMIAMMLIPDPELLIADEPTSALDVSVQAQVLQIMDKLVQERGMGLIFISHDLNLVADFCDRVLIMYAGRVVETCDAD
K
LHEARHPYTRGLLNSLPRLDAPQKRLEVLRRDPAWAEAPSVRPA
>OLS48827.1 ABC transporter ATP-binding protein [Rhodovulum sulfidophilum]AB14
MSALYFDDVSVWFGEGRDRVDAVRGASFRVGHGESFGLVGESGSGKSTIMRALSGLAPDWSGRMAVNGNPVRSGH
RRDRQ
FYKSVQMVFQDPYASLHPRQSVDQVLSETLHLHGFRDVEPRIARLLDDVGLGSAFRFRYPHQLSGGQRQRVAIARALA
PE
PSILLLDEPTSALDVSVQAEILNLLTDLRSEHDLTFLMVSHDLAVVAHICERIAVMRAGEIVEVMGVDEMRAMTPSHPYT
RALLQASTGYSRD
>OLS48828.1 ABC transporter substrate-binding protein [Rhodovulum sulfidophilum]AB14
MRLALAIAPALAIALAAGLLGPPSAASGTDRPPFRFAQVAALEGPAAALGQGMRLGLLAAFEEANRAGGVHGRKVML
DSV
DDGYEPARSAAAVERIISEDHHLALIGQVGTPTAKATQPLSARAGMPVIGPLTGADFLRTPWIDNVTNIRASYDAETEA
W
IAYLVDRLGMDRIAILYQDDGFGQAGLEGVRRAMQKRGLTLVAEGNYTRNTLAVKVALLNIREAHPDAVVMVAAYKP
VAE
FVRVGRRLDFTPTVVNISFVGSRVLSEELGADGAGIIVSQVVPFPGDTALPVVAEYQAALKEVDAEARPDFVSLEGYLAG
RVALRALQKAGPFANRSRMLEALDQLGTFDLGGLNFSYGPGDNQGLDEVFLTRIGADGAFEPLPPAAVDPKL
>OLS48829.1 hypothetical protein BV379_11445 [Rhodovulum sulfidophilum]AB14
MLKFLSTVFGSIALKFSSILVLMGAMTAAAILIATMVFNTLSASLTGLMTDALPGISTSVTVIENTGEIRDSLSEMLMAR
DIQEVDEGYGDFISKSKDIVAAVNRLPGPEKATFLPMLTALSDEVGQMRAALDRRFARQNRMQEQLTDFSATVEEIRE
AL
AGMTEDAVRDMNIAGERTIESVSDTLETLVHTDFDSTALILQARAEINLLSGVALAIAGQKDPAMIAILRDLAAASLRKL
DTVLAELDAAGTIPQYLPILTDTRAAFANVANTGFRARAGYIEDVMKLRDDSNIALSSATDDLTFKLMIGAEETAAFNRD
TIHRLLGNEVQQIRDTADIDLAVKTLVATSYLGATARDVADADAAQTQIVDARDRLTELTGHVYLTDTLRGMIDRLAAY
A
DPNTGIVATRRAAILARDEAEAGSRSAYERLREIAGAASAQDNAAMADANAAGAALLADADAARDRLHLVTLGSGAIFI
L
APVLTWILIIWPMGRLARVTGRLAQGELTEVRGFGIFGGEIARMGAALVVFRDRLVERDRMQRLEREAEQDRQARAA
QQN
AVVSELAAALQSLSAGDLTARLENRFPEDYERLRTDFNATIDTLNELIGSVVENATEIHSRAEEIAGAAGDLSHRTENQA
ATLEQTAAALDEMTSSVRSAAEGAAKVEDVVREARGNAEQSGLVVKDAIGAMAEIKKSSDGINQIIGVIDDIAFQTNLL
A
LNAGVEAARAGEAGRGFAVVASEVRALAQRSSEAAKEIKTLISASSDHVASGVSLVNRGAAALSDIVERVSHIAELIGGI
ATGAKEQSVGLGEINVGVSELDKATQQNAAMVEETTAASTTLKQEASSLQRLVSRFRLRGVPGAARATRRAEPAPSPD
EG

RMPIEPRKSAVNDGGWQDF
>OLS48830.1 hypothetical protein BV379_11450 [Rhodovulum sulfidophilum]AB14
MPVRTIVLTTAISGLALTLPAERLRADDLGKVIVGGAILCAVTGCLKGRSQGGNQHRTTRRAGDPTVRADQQALNYFGY
D
AGGADGVTGKRTRTAISRYQGYMGYPVTGQLDTYQRQTLVGAYNWAQSGGNATYPGIQGQELLRAYASHSRGGNY
CQETG
RCMGGGYPAQNNYGGAPGYPGGNGLPPQTGYGANGTMPNAAPAYPQPVAPQGAAQLPTQVMTTGGALAGFELP
DSSGNRS
IADHCQTVKMVSSANGGAVRSPAAIVNPTQALDEQFCSASTYAVSSTESMLSGAQVTDDQLTQNCGKVVEFMAAET
DALG
TRPAADLTAAAQQKIRQASPDGNTQGLVQTGEVCLGYGYRTDDSEVVLAASMLLVGAGARPYAELLGHHLRDGLGVQ
ADA
DRARGWYDTAFSALDDGAAPVFLPSQSAQRVAIMRAALTGGAPAPELTGASTAGAASAPAALPSFNIGN
>OLS48831.1 hypothetical protein BV379_11455 [Rhodovulum sulfidophilum]AB14
MKARGFLIAATAVALTMPGPVRADTGDVVKLGIAAGAFFCMANPGRCGLGGNRNAQPSAAPQRTASPPPRQQPRA
ADPAI
RTDQQALNYFGYEAGSADGIMGRRTRDAISRYQGYMGYPSTGRLDAYQRQTLVGAYNWAQAGGGSAYPGIYGEELL
RAYA
SQMRGGNYCQETGRCPVRNSDTGQTPIPDLPQANASMANACTSSQMVTNANGPVLSPADITDPDRARQALNEQFC
SASSY
ASSRSENLLAGSGTTDATLARNCRWVVERMEDQIVRLDSQSAVEVAAAADRRVREIGGDPRQISDAATICLGYGYRTD
DP
QMALASALMLVGSGARPYAELIGHHLRSGFGTQRDPARAQGWYDIAFDALDRGSEPAVRPSQSGERVAIMRTALGG
QGPA
GSGPETSFSGSGVPSFNLQ
>OLS48832.1 hypothetical protein BV379_11460 [Rhodovulum sulfidophilum]AB14
MLRAAIFGMALGLLVIVGASGYAALAACRVEFPLLDRWLAQSCRSEAETAALARLDRLRSRNLALLRDIRRTENELAALQ
CEAVPPAPTPEPAAPPDSAGIDPGIWNRGDIRVLDGCWELDSDFRTRDIQTGRITHYTQWEMCFDREGRGRETMTAT
NGT
TCRGAVSGRFLDRDAAGSLQRLEVREPGNLRCSDGSYIHQRIITCELAGDGSASCVNFQPETGGRGEVRLRRRTEDQ
>OLS48833.1 serine protease [Rhodovulum sulfidophilum]AB14
MAEHFLTKCQIDLSQCLEIGGGLALESHGALYQALRERVSPEAAALFAEPLLSRGNETAPASVAWYTEYPGEGRPLRALD
EADRSRVEAVLSERLRAIRAHLADPEDGPLIGAALHLANHPDGDIWVVDGQPVIVNWGMLPVGMSRDPETRGAHVA
ATMG
RFLPLAAAPPLSASEAMARRADLPTDTPADTDGSAPPAAGATVAPGPVPPEPAARSWPGAIAWVPLLALLLIAGGLLV
WL
LLPGTRIFPPRNPDPAIDRAAAAAAVAEINASLEDRLARLQAALDGAQCRPDGTLTMPDGRTIEGLLPPDPANPADAPG
S
RAAADPHPILPPDPARVQVPSDGASAMDLTSLLELIEARTVMVVRYLADADARPGEQVRAERGSGFFVGPDLVVTNN
HVV
AGAQDDRIFVINRSLGQQYPVTVIKTVGPFGSTGRDFALLRVQGVNAPYFTLLKPDHSLKLQSVIAAGYPDDLLGDWRG
G
TDPVPDLAVTDGTVNAEQVLAQGTPAIVHSAPISQGNSGGPLVDMCGRVVGVNTFVRQGALRNLNVALASGDLLEFL
ESA
ALTPATVTRPCQPQLLRPVAPPASAGTPAEAAAGVPDFNLRPSSNE
>OLS48834.1 hypothetical protein BV379_11470 [Rhodovulum sulfidophilum]AB14
MFEALIVTTSSAGLTAIGSREQRSYELIADTLRDRLGPDHAALFAEPVATEQGDRYDWYAPIPGAVRRLDALDPEQRAEV

MARLEVLSDEIAALADEIAAEGSPSSERLSEALRNALQVPGEDYVYAIGDGPGPQPVLVNWAWTGDRQRSERGRLTGA
AR
VLPASAPAATESAPETGAAPADPIAAASARPGMALWPLLLWLGWLILALMLAAIVALLVPACGLRGVGLFSFCPGPPPE
A
SAADAGTLILQDRIDRIERQIAIADRACQPAPPPPPPPAAPVEESRYDRALDDAGARRGDLNFSLFWSGPSDIDLAVTCP
TGARIWYRAKNVCNGRLDVDSNVDRIRPDPVENIYFEGPASGTYRATVTLFKDRTRGPDSFRLLIRDAGSEVLQEGSLTR
QGESWTTTINYPGLP
>OLS48835.1 virulence factor SrfB [Rhodovulum sulfidophilum]AB14
MISDRKEQLRPLVNWHEDITLVPFSGIQMLDFGFAIDQAAQRPWRFIERTLDDTPDERMLIPLTGNEELDAPLESAAKP
D
DDPYSIRPAAALEPFLSKWVPVPILRMKSERGPAGEERFDPGPSTWARMRVVELAAPDPETGHTHRVQIALDTALTAQ
DQ
SAHYVAPQIADAENPRDFRFVSDPAQMDWFLRRIEEGEDGQKRDPQLWVSDWLKELFLGFKRAERPGRNISEDTLPH
QFE
HWARYLACLQFIDRAVKIPKIRFVNTVSERDAVTPVDVDLVLDVGNSRTCGILIERFPGEARVDLVRSFPLEIRDLSRPE
LYYSGLFESRVEFADLKFGDDRFASRSGRRNAFMWPGFVRVGPEALRLIQREEGTETMSGLSSPKRYLWDDETRQQD
WRF
HNHFDPNNLPKSVRAAMRQLNEAGDVIEQVRHEEKQRLRRRGRTPSDRAIRPRFSRSSLFGFMLAEIFSHALVQINDPA
S
RARRAQSDLPRRLNRIILTLPTATSVQEQAIMRSRAEGALKMVWAMLGMDEALDRYKIKPAVTVEWDEASCTQLVYLY
SE
ITQKFDGNIEAFLRLKGRDRVRPGETAPSPSLRLACIDIGGGTTDLMISTYWSEANRVLHPVQTFREGFRVAGDDMLQR
V
VSSIVLPRLQSSIEAAGGRYVGEKLRELFAGDIGGQDQQVVQKRRQFALRALMPLAVAILSHSETAAEYDRFDLSVAETL
GLPRPGPAPDEDGVAPPRAGPAEPLISDEILAYIEQAARDVGAPDWRFADVVLTTSREDVDAIAREAFQKVLGNMAEVI
D
HLGVDVVLLTGRPSRLPAVRSIVEELLVVPPYRLISMHSYKTGRWYPFRDPITQRIGDPKSTVAVGGMLIALSENRIPNF
KVTTGAFQMKSTARFVGEMDNNGQILDARLMFSDLDLDTKGGAQEATVDMFSPVHIGSRQLPIERWTTTPLFRLDFA
NPN
AQRRPTPIRVTFEKADYDDDDREDSEAKLRREALREAFEITLVEDGAGDGMKNTDVVLKLHTLGFDDEYWIDTGVFRY
>OLS48836.1 hypothetical protein BV379_11480 [Rhodovulum sulfidophilum]AB14
MTTKDQLAERCARVETAARKALDWVSDPENAETVGLDRKSLAQALRKGARRAQKLGRAARTKMSVSVFGPSQAGKS
FLVS
VLARPEDGRLVADFQGPGGQLDYIREINPEGEGESTGLVTRFTMTKAPCPEGFPIRLVLLSEADVARTIVNSFYMDGDQ
S
EPVPEPEAITAHLDAFRAKTGGADVPGLSHDDVLEIADYVNRSFGRAAYAAGLKSFWEDAALIAPKLGVADRAAFFAIL
W
GGHDALTDLYIRLGQALQQIGNAEEVHAPLAALTPRETSIIDVKTLHGLDSEPGDSLPLCLPSGARVELPRALVCALAAE
LVMPMQTRPYELFEETDLLDFPGARNRFEQPLSKTLAEPEKTLTQLLLRGKVAYLFDRYVENQEITSMLLCVPDSNMETL
DLPGLVETWIAMTHGASAKQRAASRCILFFVLTKFDKHLGESAAAGGAETRFERRMQASLLEKFGRGKDPWVDSWTP
GRP
FDNCYWLRNPNFYVDGLIDYDEAKREIRIRPDKEARLAELRTGCLEAASVQRHFADPAAAWDAALGLNDGGVSYLIGRL
A
EVCTPDSKTGQIGAQLDRLAGDLAADLAPFHVSDDIGRRIEEKREAAAVLIDELEIALQRHRFGAVLAALMVDQDLVQD
R
ISRVPSSIRISQAVSSAATARPAAETSRPAAPARPGRPLRPGRPAPAADLALAEPPSAETGANRTATGNGADIRTMSAEA
FQAESAVEVWIEGLKRFREDVGTAGHFGLSPGAVATLSAELTHALRRIGVVERIRTQLKAMAFGLTVDKQAEPAAIVCA
E

HINRFVSVLGSDALPEAERARVELPEGGSRPVFAPRPGADDADGLPPEPRPTAEETWTDWVFALDAMFVANARDGDA
GSV
NVAQNQKLGEILAEFGAGGA
>OLS48837.1 hypothetical protein BV379_11485 [Rhodovulum sulfidophilum]AB14
MALKIRPDPRRPSGGYAELSAEAEALPETEATVSVFDAFSERFLGLDGWQSDRAEFGPYEVVRDGDRARLVIGPEIVNRI
EEYAALKIRVNRIEAELSWPDDVVPMPGAARIGGLAVARAAPAEATTEATADTKGLLTGKLPDPGPEAPPPAAQAEDP
AP
EEKHRSRLPLIAGLSALALLICGGALAWFLMQPPSPPVVAERPEAEPTPPDPCRPEALAGLDGLPFAEGRALMARCGAR
V
TPQQALAALEAGADAGDADALLLFGEIYDEGANDDPVETEIGLSLTPSASKAAEYYARARAAGSAEAAKRLERLCPDLRA
ASDTLSRSAAEEYCRP
>OLS48838.1 hypothetical protein BV379_11490 [Rhodovulum sulfidophilum]AB14
MSPFRLRPALSAFAIMLSVAQSGAALAHPLLVEDTSTVYQRVLTRPGAPLHEAPDGPETRRYPAFQPLYVFARQEGWIE
V
GPSATAAPAGWVAADTVVDWKQNIVASFTNPADRARSLMFRTEDDLRGFMEDEALVATQARLIEKAETGMLAPEDG
VVAI
EPENYVNIRDRFYILPILDFVEDLHPMTYDANLLLKVASVPLRDELPVAAAEASSSAGDFDAGVVFVFDTTKSMQPYIER
TRKALARIIFDINGTDIGERINFGIEAFRDNPQAAPGLGYRTREVIPLERRINQLPVLEAIDNTRTATVSSPGFNEDSLA
GVEDAIRRTDWDRTGSGDPFDARYVILVTDAGPKDPRDPNARSEIGPVEIQRDAEGRNIVIMTLHLKTDAGAANHDYA
AE
RYRQLSRFAGRQFYYPIEGGSEQAFEDTVTALVTALTDHVRMALGQAQVLAPEDRDEDLEFLGHALQLAWLGAREGTR
AP
DVFESWVTEKAAEKPRALALEPRLLVTKNEMATMAELLDNLVRLAEGTRGEEEARGFFRQVRGVIAQMAQNPDRLVA
TDA
DSLGGALEYLERLPYRSQLLQTDEARWMSGPMVRREIIDGMRQKLTQYRKWLYDPSVWTALYEGAPDGEQVFAMPF
DILP
>OLS48839.1 ABC transporter [Rhodovulum sulfidophilum]AB14
MEAALDIRDLGVSLSDGDRGFALRVPELRIAAGETVGLTGPSGTGKTLLLELLGLLRAPEPGGIYRLRPADGPARDLAAL
WTGRGGLAPLRGRLFGFVPQTGGLLPFLTLAENVALPQRICARPDPGLVSDLIARLGLGPVAGLRPERLSIGQRQRAAIA
RALAHRPRFVIADEPTAALDPENAAEAMSLLFETAARSGAAVIVSSHDLDLLKRFSLTRYRLEIDPDHAPGRPVSRLIAA
ADGQEAA
>OLS48840.1 ABC transporter permease [Rhodovulum sulfidophilum]AB14
MRLLVTLALRALFRDWIFLLCNVAVLAGVIVPLLVLYGVRNGVYDALIGDLMSNPATLQIDTAGNQSFTPTDLAEVQGW
E
DVGFATLKTRSIFDYVNVRATGGRGRQEALIAPSGTGDPMIAPLPGLGADEVAVSPALAAQLGLAPGDRVEIFTQAENR
P
RQLVLPARLAGVLPEGRLDGRVVMADIAMLDLIEAFYDGYALPEHGIDSGKPLADRAESFEGMRVYASALDRLAPLQDR
L
EARFGIRTQARTAQVTSVLALGRNLGLALALTVAVAAAGLAASLIFGFWGEVVRTRRTLANLGLLGLPQWQLSLFPVIQ
A
LVTAVAGLFVSFLLFHLTAALAERLFETGLTDRGGLVSVSAGQAATIAAAVIAFVGCASLAAARAALATDPATVLREET
>OLS48841.1 hypothetical protein BV379_11505 [Rhodovulum sulfidophilum]AB14
MRSAALCLSLFLAAPVAAAPVTWDKAQYDPAAGTAPADLILPMPCGGAMAFQRVVVPVEADDPLADRRIRVGQSDP
ETGY
SDYLRTAYLRGAFPGPAPGDSVYYIARYEMTRAQGRALTGDCSPPKRIDRTALGGLSWFRAVELSQLYSEWLLAEASEAL
PPETDGRAYLRLPTETEWEFAARGGAAVDPTVFPSRRFFGEGDLFDYAKVVGSARGALLPVGLARPNPLGLYDIYGNAE
E

LMLEPYRLNAVGREHGQAGGLVTRGGSVLSAPAQVYSAERTEYPLYSAADGTATAGKTFGLRLVLSRSVAWSDPVLREI
K
AGWIARTEKTARGGPDQTPLGTLAEMIEEEIDPRRKAALQDLQLEVRRSDEEATAAFGEAAKSTLLNGAVLVGAMRAG
QT
EEARMSSELIGIADQTRLCARAEQCSALRSAGSRLMTKLDMLRDVQKTYLLSLRSAVESLTEGADPKLTEDAMRLLQGEL
SASGQRQILDNLARFAALLEAYRDKPDMSEEELRLLVLSH
>OLS48842.1 potassium transporter TrkH [Rhodovulum sulfidophilum]AB14
MNVVVFANGLVLGLLALMSAVVAAIYPETRPIFGEAAFQLGLIGTLVAMASRPSFETLRLPHAFMLTASVWLMAAAGG
AV
PLYLWKLSAADAFFEAMSGITTTGSTVMTGLDGTPRGILMWRALLQALGGVGFIGTGIALLPVLKVGGMQLFRTESSD
KG
EKELRNATQFAAATLLVYVSLMIFCGVLYRAGGMSVFDAATHAMTTLSTGGYSTHDASFGFFASPFLQWTCTLFMVLG
SV
PFSWYIRSVYHGKFRSEQVSAMLKTLAAVILPLSLWLMLSTDLPLPAALRLVAFNVVSVVSTTGYATTDYTTWGPVAVT
A
FVILTAVGGCTGSTSGGAKAMRWVIMYRLLREGIRSVRYPHGVYRIRYEGRVIDEDIVSSVIGFFSIYALSFAVIASLLT
LVGLDAATAFSGAITALANVGPGVGDLIGPAGTFQSLSDKAKTILAIGMYLGRLEMLTVLVLLTPRFWRSLE
>OLS48843.1 epimerase [Rhodovulum sulfidophilum]AB14
MTDTALSPRPRRIFLLGATGTIGRATLDALLARGHEVVCFLRPRAGDDPGRGLPGAARVRVGDVTDPASLAADGFRGE
RF
DALVSCLASRTGTPRDAWAIDHEAHSHALAAARAAGADQAVLLSAICVQKPLLAFQKAKLAFEAELMASGLTWSIVRPT
A
FFKSLSGQVERVRAGKPFLVFGDGRLTACKPISDADLGRFMALCLDDPGLQNRVLPIGGPGPAITPRDQAEELFRLLGRP
PKIKQVPVGVMDGIVALLSALGRVSPKLRDKAEFARIGRYYGTESMLLLDPETGRYDADATPEYGSDRLFDYYARLVSGA
ERAELGAHAAF
>OLS48844.1 dihydroxy-acid dehydratase [Rhodovulum sulfidophilum]AB14
MPAYRSRTSTHGRNMAGARGLWRATGVKDGDFGKPIIAIVNSFTQFVPGHVHLKDLGQMVAREVEAAGGIAKEFDTI
AVD
DGIAMGHDGMLYSLPSRELIADSVEYMVNAHCADAMICISNCDKITPGMLMAAMRLNIPAVFVSGGPMEAGKVTLG
DGRT
VSMDLIDAMVAAADDKVSDDDLARIEAAACPTCGSCSGMFTANSMNCLTEALGLSLPGNGSTLATHSARKELFLEAGR
RA
VELCRRWYEQDDASVLPRSIATKEAFRNAMALDIAMGGSTNTVLHILAAAQEGDVDFTMDDIDALSRKVPCLCKVAPN
TA
NVHVEDVHRAGGIMSILGELDRGGLIHRDCSTVHAPTLGAAIDKWDIGRSNSDEARNLFLAAPGGVPTQEAFSQSSSW
PS
LDTDRKAGVIRSVEAPFSKDGGLAVLKGNIALDGCIVKTAGVDDSILVFSGPARVYESQDAAVSGILTGKVQAGEVVVIR
YEGPKGGPGMQEMLYPTSYLKSKGLGKACALITDGRFSGGTSGLSIGHASPEAASGGAIGLVQDGDLIEIDIPNRSINLA
VSEEELAARRAAQDAKGWKPAAPRKRNVSQALKAYAHFAASADKGAVRIVPE
>OLS48845.1 hypothetical protein BV379_11530 [Rhodovulum sulfidophilum]AB14
MLLLDDTALACLLKRDGAVAAAAFSASGRRARMVSVALRARGSGIVCGTEEAARMFALLGCAADTAIASGTRAEPGAL
LI
AARGPAEALVGGAQAVQTLLEWASGVADCAARILRATRAVDPAVAVACPRKAIPGARPLALKAVAAGGAAIYPALGAE
TA
LVLSDCRALGGRAAPGERIAGLRQAAPDGRVTVEVRDGDEALAAARLGADRLLLARMAPDAVAATMRALGPGWPGE
ALAA
GPITEGAAPAYAQSGVHALVSSAPYHALPAEIDVAFEPG
>OLS48846.1 hypothetical protein BV379_11535 [Rhodovulum sulfidophilum]AB14

MRSTGIFSENEYRLRRLVRAGLVWLLAALVLMLGQPPEARAQPSGSSPPAIVTGWLDNPPFWKMTPTGELVGFTPRLI
AE
IQTLTGLRIEPRRYDDRQSLIAAQITGESQLMFATARLPALEATHVFTEPIARAHVHLFVLTENANRPDYIAPVGKRIGK
MRQYAGSDPSNLLTGNRTVFFDEPATMLLKLLSGEIDAALMSEDWLMGLARSVRLDHRIAAVGPPVLEFDRVIAIHNSR
A
DLLEPLNRAIATLEADGTLSALRQQWLIEADPPPPDILTVGVVNLPPFSEVREDGTMTGFSVEVMRDLGEILGIELRFQT
VSAEDWARGPQSPIDILPLIGITPERELRMDFALPIQAIDLGVFVRSGRQDTFDGLSGLRVGTWRAGVANTVAKRNASD
I
RHFDDYRDLLTALFEDRIDAIVAERGMLSRIAGEMKRGDDIAEAPFPRQRIFLAPALRPGLPTVREALNLVIPGYLASQR
YQDLRAAWLGTAPFWTDRRILLTLGGFWLLMVLAIGYIPVQHHRIRQERLRFTADMVERIPLGLLMVASNGRIEFVNSA
A
KAISPSGPDLIREGADYRQAVRALIKADARHWGTNSPAALAADLSGPSRRDGHSHEFQTAGGEIFRWSSFALPGNTILV
M
VLNVTRERRHLAEIEDLNAELRAQIAIVQRTNAELRDFAYATSHDLKSPINTLMLIADALTEASTDRLPDEDLALIEDMH
EILDRMARMIRDVLDYTRAVGVGGEQTSDTIELSGVVGDVMKDLAADIVASGARLHVGDLPCLTGNPRQLHQLIQNLI
SN
AIKFRLPGTTPEIDIETVAAPQGMVAFAVSDRGIGIEPKFHDRIFHLFTHLNDTDLYPGSGLGLAICQRVALNHGGRIMV
QSTPGAGSRFTVFLKEKQGDKHIDDGRRLSVRTQDGQAPHSQDRPRGNDAGIRNGRGGH
>OLS48847.1 hypothetical protein BV379_11540 [Rhodovulum sulfidophilum]AB14
MMVDDCRFERKMVKRLIRKTGLVETMLEFGMAEEAIDYLRDPSCPKIDVVILDINMPRMNGFEFLERICEDLPGHLRNS
V
VVILTSSECAADMRQAERFPMVRSFLRKPIGENEISHLDKLVNA
>OLS48848.1 response regulator [Rhodovulum sulfidophilum]AB14
MAQSAPTCLVVEDTAFDRMIMERKLAAYAQPLQVRFVSTLAEARNCLSEMPISIIFLDNSLPDGKGANFALELAAHRN
WM
KIPVVMVSDWPSPFMPAKAKAANVVAIWTKTEFTALTVAEVLKSRGLTA
>OLS48849.1 hypothetical protein BV379_11550 [Rhodovulum sulfidophilum]AB14
MRDLDVMIIDDDAGDRKLIRRFLERSGMCRTICEAELVHSALDHVAETFDAVFLDYVMPGENGLDGLETLRQKWPTAA
FI
LMTGQGDEQIAKSSILAGAADYTPKSRINQTGLERMLVRAVEVVRLAQRVEEQRQELLTFSNVLVHDFRAPIRSIRFICE
QLDEELGGAQSSELRKLMELMKKTATQMSQLVEALASHIRTDVDPVFECISVSDLVENALSFLDHDIRESGACIDLDLPD
IDVFCSPAMMAQLIQNLVQNAIKYSGGGLPNVRIVARRLDNGDVLFEIADRGVGVPEEYRQRIFEPFQRLAQASDMPG
TG
LGLATCQKIVKRHGGRIWCDANPYGGTLMRFRMPTGRKPFDSVTSKVWKTRSDGAAAIR
>OLS48850.1 response regulator [Rhodovulum sulfidophilum]AB14
MNQRPNLSTARAPLNLLMVEDDDGDVKAVRRALYRAGIEATMIRAVDGVEALSIMRGRARPHPSIPYIVLADIQMPR
MNG
LEFLNELRKDCSLKQVIVFVLTTSNNDQDKRKAYEAGISGYILKQNAGQDYRPLIETLDRFWNIVSFPDVRPSEE
>OLS48851.1 hypothetical protein BV379_11560 [Rhodovulum sulfidophilum]AB14
MEFAGAFFSERVLRELELMASDAGNLSVLPEIGAVADGTTRGANGTGSDDEGQVYWRKRLEAVFESVLLTRPHYTQVR
YI
GRGDNWREIVRVHRVGDRIEVVAPGGMQSKGNEPYLADRKALNAGLPFFSRITPNHEHGSEVGPPTIRFVVPVLGTAG
TV
GGALVINADFEWLLRAANLHPSDGFSVTVMTSSNDYMIFDPVTGPGRLRFHDTPEWNPAELPDLAGVGGGDMADTT
EIGW
RRAVNVTSLPSGSPNRGDRLSLRTDIFVVTELPTRLLFAPLRQQLFCGLAAAIVLSVGAAWVAWIIGKQLTAPLHRLAQG
LRKCDLGEDMSDLIPTTNDEVGELARALVSLNDRLYQKSTKLNAIFSEAADGLITISEAGVIEEVNAATARIFGYDAADM

VGRNVSILMSEEDAERHNDMLRRRIGRREQKRLIAGREVAGVRKDGTQIIVEISVASARQTGGSRYIGVIRDVTSRHEQ
Q
ARSEALVKALERSNADLDAFAYFASHDLKAPLRVIDNASAWLEEDLAPYLNEDTRDSMDLLRSRVARMERLLDDLLNHS
R
IGRIEEKAQIVEGRDLFDELRGLLQIPDGFSFEATQEFMDLRIPNMPIKTVLLNLLGNSLKHHDRETGFVRLSGYETDTA
YHLSVTDDGPGIPPKYHAKVFEMFQTLRPRDEVEASGMGLAMVLKHIQLAGGEVRIDSDGSRGTTINLVWPKAARAD
KAT
GRAA
>OLS48852.1 hypothetical protein BV379_11565 [Rhodovulum sulfidophilum]AB14
MLRRPAERTFGVLMLDDNEIDRMRLKQLCLRAGLSFRVREAADLDDFRRALDEAPYDMVFLDYDLKMDTGLDALELLK
AH
PAQGEAIAIMITSTDAPATVVEAMRSGCSDFLVKDELSISMIRRAVTAAIEREIYRAAILREHEARVSVERMLERFSAVC
AADIRGMLSGMLRKIRGVTQSVEGHAQISQEVAEAERACMELFEYLENMNGILALEVDPGRERLSR
>OLS48853.1 catalase/peroxidase HPI [Rhodovulum sulfidophilum]AB14
MDGNDRPAGTCPVLHATFGGQSNRDWWPNQLNLRILHQNPSAANPMQPGFSYAEAFGALDLNAVKSDLRALMTES
QDWWP
ADYGHYGGLFIRMAWHSAGTYRTADGRGGGGTGNQRFAPLNSWPDNVNLDKARRLLWPVKKKYGNAISWADLMIL
AGNVA
LETMGLRTFGYAGGREDIYEPEEDIYWGSEREWLATSDKPQSRYSGDRDLESPLAAVQMGLIYVNPEGPDGNPDPLAS
GR
DIRETFARMAMNDYETVALTAGGHTFGKCHGAGDAALLDAEPEAAPLQAMGLGWLSRHGSGKGADAITSGIEGAWT
PNPT
TWDMGYFDVLFGYDWELTKSPAGAWQWTPKDLKPEDHAPDPETGERNRKIIMTTADMAMRMDPIYEPIARHFHEN
PDEFA
DAFARAWFKLTHRDMGPKSRYLGAEVPSEDLIWQDPVPPVDHPLIEAADIDALKAEILGSGLTVAELVATAWASASTFR
G
SDKRGGANGARIRLAPQKDWEVNEPERLTRVLEVLERIKAGFDARATGGKRVSLADLIVLGGSAAVEKAAAEAGTPVEV
P
FAPGRTDASAEQTDIASFEVLEPLADGFRNYQKGACSVTAEELLLDRAQLLTLSAPEMTVLVGGLRVLGANVGQSPHGA
L
TERPGQLTTDFFVNLLDMGTAWAPTDADETLFEGRDRTSGAVKWTGTRVDLVFGSNSQLRALAEVYAQDDAGPAFA
ADFA
AAWAKVMNADRFDLV
>OLS48854.1 peptidase M48 [Rhodovulum sulfidophilum]AB14
MLKLTPILLATLYAIAMYRFSAWRTGRELDAQSSELADARLKALTDRMAKALDLQRIRVNIYEIEPVNGLAAPDGRIFIT
RGFHDRYHAGEVSAEELASVIAHELGHVALGHSRRRMIDFSGQNAMRTALAMVLSRFLPGIGVYIANALASLLAARLSR
Q
DEYEADAYAAALLTKAGIGTAPQKTLFAKLDRVTQSRGGAMPAWLMSHPKTADRIAAIEALEARWAVPETP
>OLS48855.1 hypothetical protein BV379_11580 [Rhodovulum sulfidophilum]AB14
MSLDTQCLDFVETLRAQTSFDALWRVFNDHLRSHGVSRIIYAQTPAVRSRTISQEMLYRSTFPADFTHLYAEKGMIDHD
P
RAHYCDSGAEAPLYWAHPDARLDADPETREVLDLCEDFGVSFGLTLPLKGASALNRSGAGLAFSARNGDEAADILAAN
GS
AICAATRLFHALAQTPAIAPGSVRLSLRERECLLWSATGLRNKEIAFRLSISEKTVEHHMASAMTKLNAMNRTHAVARAL
IFGLIVP
>OLS48856.1 PIN domain-containing protein [Rhodovulum sulfidophilum]AB14
MKVLLDACVLYPTVLREILLGVARRGGFTPLWSARILEEWARAARKIGPTGEAQARAEIALLRAAWPGAEVAPRAGLEA
R

LWLPDPNDVHVLAAAIAGGADLLLTFNARDFPGHLLAAEALRRAAPDALLMDLWLADPEPVEAAVAEVHGAAERIAA
ETL
DRRALLKRARLPRLGKALDTPRG
>OLS48857.1 SAM-dependent methyltransferase [Rhodovulum sulfidophilum]AB14
MSIDYPTIRLMPKAEARAIRHGFPWVYSDELVMDRRTRALAPGSLAVLEDTERRPLGLAAVNPLSRIVARVIDRDPEAEI
DTAWFAARIGQASGLRERLYQQPFYRLVHAEADGLPGVVIDRFGDTAVVQPNAAWAELRLEALVEALRQVTGVGTVV
KNG
SGRVRGLEGLAEETLVLTGALEGPVPVPMNGATYMADLTGGQKTGLFYDQRPNHAFAAGLAQGARVLDVFAHVGGF
ALAA
LAAGAESALAVDGSEPALALAEAGARASGLDARFATRKGDAFDTLAALDAEGAQFNLVICDPPAFAPSKKALDAGLRAY
E
RVARLAATLVAPGGYLVLCSCSHAADLAKFRAASARGIGKAGRRGPIVYTGFAGPDHPVLPQLAESGYLKALAFRLTE
>OLS48858.1 phosphogluconate dehydratase [Rhodovulum sulfidophilum]AB14
MPLHPVLEEVTQAIASRSAETRAAYLDRIGRAADQGPARAHLTCGNQAHAYAASGEAKDRLAEGRAPNIGIVTAYND
MLS
AHQPFERFPALIRDAARAAGATAQVAGGVPAMCDGVTQGQTGMELSLFSRDVIALSAGVALSHNCFDAAVFMGVCD
KIVP
GLVIAAASFGHIPAVFLPAGPMTSGLPNDEKSKVRQKYATGEIGRDELMQAEMASYHGPGTCTFYGTANSNQMLMEL
MGL
HLPGASFVTPNTDLRDALTVAGAQRALEITALGNRYTPAGRVLDERAFANGIVGLMATGGSTNLVLHLPAMARAAGIIL
D
IEDFARLSEITPLMARVYPNGLADVNHFHAAGGLGYMIGELLEAGLLHPDTLTVAGEGLARYTQEPKLVDGRLTWSEGA
G
ESLNDKILRTAADPFQKTGGLRALGGNLGRGIIKTSAVAPERHVIEAPARIFQNQEEVKAAFRRGEFTSDTIVVVRFQGP
RANGMPELHALTPTLSVLQDRGLKVALVTDGRMSGASGKVPAAIHVSPEAADGGPLARLRDGDVIRLDAPAGRLEVLT
EG
ALDRAPVTPDLSANETGLGRELFAAFRANAKSAAEGAGIVV
>OLS48859.1 keto-deoxy-phosphogluconate aldolase [Rhodovulum sulfidophilum]AB14
MTPNAASEITEEICRRAPIIPVLVIEDAALARPLAEALIAGGLPVLEVTLRTPAALDAIREMAGVEGGVVGAGTLLTPAD
VEAAAAAGATFGVSPGATPALLDAARAGDLPLLPGAATATEAMILLERGYTVQKFFPAEAAGGAPALKALASPLPQIRF
C
PTGGVSPTNAASYLSLPNVICAGGSWIAPKDKVAAGDWDAITALAREAAALPR
>OLS48860.1 adenine deaminase [Rhodovulum sulfidophilum]AB14
MVQPLSDLIRQGRGLVPADLVLKGGQVLDVVTGETVPGDVAICGETIVGIGAEYDGRETIDVSGLTLVPGFIDTHLHIES
SLVTPLEFDRCVCPRGVTTAICDPHEIANVIGVEGIEYFLEAATRTVMDIRVNLSSCVPSTGMETAGARIEAADLLPLRD
HPQVIGLAEIMNYPGVLMQEPGVLAKLEAFRGGHVDGHAPLLSGRELNAYIAAGIRTEHEATTAAEAREKLQKGMRVLI
R
EGSVSKDMAALRPLLTELSSAYMCLCTDDRNPLDIAEEGHLDHMIRWMIAQGTPVLAAYRAASLSAAEAFGLKDRGQI
AP
GKRADIVAVANLAECRAQLVLSGGRAASASAFAAREAVAPVGRGSVKAPKVTAASFRAASNAAETHVIGILPGKILTEHL
VEDMPPEGGEKRADPERDLAKVAVIERHGKNGNVAVGFVRGFGMQRGAIASTVCHDHHNIVAVGVSEADMALAAT
RLSEI
EGGFVVAEGGKVLAELALPVAGLMSLETFETVEARLKDLRAAAKSLGVVLEEPFLQLAFIALPVIPALKITDRGLVDVEK
FEIIG
>OLS48861.1 hypothetical protein BV379_11610 [Rhodovulum sulfidophilum]AB14
MPVGFDVIVRGGLPGEGERRAVTLLAALLVLGLFARRFNGWPNGARGPAGFPEHGPRYFTTRFRFCAWALLYGSVIAG
AF

LVVVFFPTLADQAVALLFPVMPGNPFLSQIDKVLPSAPPGGGTQWSLAAAVGIAAAFYTSDWERMLRERFQNNALIPR
QA
EQLFEEFTRFEAVVPVESHFGDRFMMQFRERDDLPTYHRIDLDPEQAELAQMRLERLARVEFMLWRMTQGDLPAEV
AARL
AEHVPLRQRLLDKLAAIRDEALAIDLQLVQVTNLLLCHLVEAQRPDFRRRLGQAAGLDCEQDRIETFPVSRLDDVIPVLE
DELRRIAEAEPVPTGWQGEGDGAAGQAIEALQEQVASLRALVPLCVTGLEVQRGRLRDLGTRLEDLWCETLRFAICIGL
A
APYASGKRFFCALGLRARMGYDVNLSIVLAVPMGLCFFCAVALLSGQSSDHASGANLEGAELWQRVRMLLGGMKIAA
TER
PLWLILGGFFIGWPLLQGCYVGSALAHGRQTEGGFGRPGALRPLDYLISFVMIWSVLVPGVAALIYVLGFSAAFRDVYV
G
ALAYAPVSALWGVMVSITSMRAFDTQAEGPAIRIWESVIFLLLVAGGVVLAEIWIGSVDPESDTPFNAGRFWSLLFLAS
A
LTMVLIGAILLATSSKVSGKLRRMQGARLSALRPAYPA
>OLS48862.1 hypothetical protein BV379_11615 [Rhodovulum sulfidophilum]AB14
MIRNCFIAIFIFTVSGQPGPAQSRDRAADGVCSATIRVGLREDAPPFSFRINPEARAPDLSASCGYEEKGLLGGYAGYTV
GLCRDYLLDLLSRCRGAGPQVEAVPVAVDKRHDALAAEAPPFDMLCGATTATVTLAASVPHSPYTFLTSTSALVNARFA
Q
AEGTSCRVGVVNGTTSAPGMQDRVLDPRRIETWQEFIRRNKSCSLDGLERAVGFDSTAEAILALRAPGPEGIDVLVGDH
H
ILAWYWQHLDKLHRRMVLPEAIAARFEALDDSETESTASSLARFKDELRLIPAAISIEPYAVVAPPGKAALIADFSRFLT
ARQQDRNGEYTRRLRQCFGRQVDRSLWFLMEFQSKVRDGNPLK
>OLS48863.1 ABC transporter substrate-binding protein [Rhodovulum sulfidophilum]AB14
MLATISRRDVLTGTAALLGSTALWGRAHAAGGGRLIVAADSEPRQLNPAIVASNGVFFITSKVIEPLAEASYAPGGLRPL
LATGWEGSEDGHSITFTLREGVLWHDGTPFSSADVAFSAMEVWKPLQNIGRALFANLEAVETPDPLTAVFRFSQPMPL
QL
IANALPVVSSVLPKHIYAGTDIATNPANAAPIGTGPFRWGTHRPGEYYLLSRNDSYWGEGLPLLDDIVFRVLPDRAAAAS
SLEAGEVHLAAFSAVPLSDLDRIGRVPGLEVVTKGYEALTYQLSIEINHRTEALSKLKVRQAIAHAIDRDFVLKTIFLGY
AEAATGPVPANDPTFYTPDVATYPFDIETAKALLDEAGYPEGPDGTRFGLRLLPAPYFSETRQFGDYLRQALAAIGIDAR
IVPHDAAGHIRAVYTDHAFDLAIAPTVFRADPAISTTILLRSGMPPGVPFSSQGGYADPELDAVIDEALVTLDPAARVAL
YKKLQRMVAADLPIINVADWSFTSVVSDKVTGVATNPRWVVSNWADTGLSQ
>OLS48864.1 ABC transporter permease [Rhodovulum sulfidophilum]AB14
MPRMIRIVLARLAGSLPVLLIVLVGVFLLLDRAGGDAVDAYLLSMGGGDAQMAAGLRAEYGLDGPAWKRLAFTLGQY
AQG
DLGWSLALNRPIGALIAERLPNTLWLMGSATALSFLLGSGLGVLAGRRPGAMLDRLLSGGSLVLYSIPSFWLGLVLAIVF
AVQLRWLPVSGIETIASGKAGLARAADIARHLVLPVAALGMIYLALFLRIMRAGMAEAWGEDYTLAARARGLSDWRITL
N
HAARNAILPLVTMLGLQSAAMLGGSVVIESVFAIPGFGRLAQEAVAGRDAPLLLAVILTSALMVVIANLMVDLAYGWL
DP
RIGAGGGSDRG
>OLS48865.1 ABC transporter ATP-binding protein [Rhodovulum sulfidophilum]AB14
MTPLLTLTALGIRYPGARTPALSDLGLALFAGEKLAVIGESGSGKSTLARALAGLLPADTEVSGRIDWTEPLPRPGRDIG
YVFQDPGGSLNPLLSVGRHLDEVLRTHLPLSRKAARTRAAELLERVRIPDPDRALAAYPHQFSGGQRQRIAIALAIAAGP
RLLIADEATSALDVLVQAEIAELLRGLVEAEGMTLLFITHDMALAGGLADRIAVLHDGRLIETGPAARVLHRPRETYTRT
LLAAHLDLSSPRLVGDAP
>OLS48866.1 peptide ABC transporter ATP-binding protein [Rhodovulum sulfidophilum]AB14
MSPLLDARDLAKTYPGPRRIRALDGVSLTIAPGETLGLIGPSGCGKSTLARVLTRLTEPDAGEIRFDGRDWGALRGRALR

RARGAMQMVFQDPAAAFHPRATVAGALSEPLRLHRIAPRPDWPEHVARLLSEVGLSPDLAHRPVAALSGGQRQRVAI
ARA
IAPRPKLVLLDEAVSALDASLRFHVLELLARLQRERGLAYLFVSHDLAVARALSHRIAVMEAGRIVETGPTEALLATPRS
EILRRLIAAVPEIPAEEALP
>OLS48867.1 aspartate aminotransferase [Rhodovulum sulfidophilum]AB14
MTAPPAPFWLPLPDAPDFPAEGFAPLADAIGRLMGTRNDVLLVQGEAMVALEAVASSIGAPDRKVLNIATSPYGQWF
GAW
LRRAGAEVTEIAGRAPGRPVAAAAVAEALAADRFDLVALVHAESATGILNPLAEIAALTRAHGALLAVDAVASLGGHPL
A
LDALGVDIAVMGPQKALGGPAGTSAIAVSPAAWERIAPPGAAPSILSLGDLRAHWLETGRGALPGTPSALEFHALAQTL
A
RVEAEGPEAIRTRHARAAAAARAGARALTGTEWVPPAEASALVTALALPDGLSAAGVLAHVAGDTLSAGTGAAGARVI
RL
NHTGPRARLENVLGDLARLGAALAAAGWPAPTDKALAAAETAFREAAKA
>OLS48868.1 phosphoglycolate phosphatase [Rhodovulum sulfidophilum]AB14
MRPPRTLLLDLDDTLLTAYRQPERTWAAIIAEHADALGEHSTRWVTAEVLDRVLSFLSDEEGRKLWRLEGDATRRRVVR
S
AFHRLNLARPAGSDPLHGVDADRIADRFETYLEEAITLKPGARGMLEELRARGLRLGLLTNGSGARQRAKLARFGLAPFF
GAIQIEEEAGFGKPDPRAYLRLLARLDTAPGDAWMLGDDPVWDVTAPAALGLTAIFYDETGRAPLPRGAAARIARLDA
MA
GLLGRCPVRAGPPRRIRP
>OLS48869.1 hypothetical protein BV379_11655 [Rhodovulum sulfidophilum]AB14
MDHTAGRCPFAAQRETHSGFHPLHLRRSILNREVLRPVLRQLGLETMPHDPHVTLAHSTVPVDWGLRVFEPDPHRIAI
TP
QKPRFERGAAGGVDLVFGAPELEARWQALRDAGAVWDLPPFVPRVAFGPAPAELPSICFFTGPVLFGPEWRATPDCV
KAD
APQSSVKA
>OLS48870.1 hydroxymethylglutaryl-CoA lyase [Rhodovulum sulfidophilum]AB14
MPDRVEIFEVGPRDGLQNEPRAIPVAEKIALIDLLSGAGFRRIEAASFVRPERVPQMADGAEVLAGIRRAPGLRYAALTP
NIRGYDRARAAGADEIAIFASASEGFSRANINASVDESLRRFAPVAVAARADGLPMRGYISCVTDCPYDGPTPPGAVAA
L
AERLLALGCYEISLGDTLGSATPETVGAVLRAVLAVAPPERFAGHFHDTGGRAMANIEVALEAGLRVFDAAAGGLGGCP
F
APGAPGNVATERVAARLAALGLRTGLDPDRLAEAAGFARRMRGGPSGAGSFA
>OLS48871.1 3-methylcrotonyl-CoA carboxylase [Rhodovulum sulfidophilum]AB14
MFRKILIANRGEIACRIIATARRLGIRTVAVHSTADARAAHVAMADEAVAIGPPAPADSYLRADRIIEAALKTGAEAIHP
GYGFLSENPDFVTAVEAAGLTFIGPSAEAIRSMGLKDAAKALMDQAGVPVVPGYHGADQDDAHLEGAAEAIGWPVLI
KAV
AGGGGKGMRAVARPADFAAALSSARSEARAAFGNEAVLIEKLIERPRHLELQIFGDGTDAVHLYERDCSLQRRHQKVIE
E
APAPGMTPEMRAAMGEAAVRAARAIGYRGAGTVEFIVDASDGLRPDRFWFMEMNTRLQVEHPVTEAVTGLDLVEW
QLRIA
AGEGLPLTQERIPLTGHAFEARLYAEDVPAGFLPATGTLAHLEFPETVRADTGVRTGDTISPWYDPMIAKIVAHGPTRAV
ALNRLARALEDTRVAGTVTNLAFLGRLARLPGFARGEVDTGLIDREIDLLAASPAPARAVEAAALLEAAAPPAGPLAGFT
LWAPLRRSVTLRRDGEEVTGTLLFEGPARATVEIGETVLEARLGPEGWRIDGALPGAVLRAGGAIHVFAGADGSFSFTP
V
DPLDRAAAAGAGADVIEAPMPGLLKTLAVSEGEHVAMGQPLAVLEAMKMEHMLAAPRAGIVAEVLAAPGAQVEAG
AALIR

LTPEEEEDA
>OLS48872.1 methylcrotonoyl-CoA carboxylase [Rhodovulum sulfidophilum]AB14
MRLRSAVLPTSDSFRANRTAHLEALREVSEAAALAAAGGGEAARVRHVARGKMLPRERVANLLDPGSPFLEIGATAAH
GL
YDGAAPCAGAIAGIGRVEGQEVMVLANDATVKGGTYYPMTVKKHLRAQEIAAECHLPCLYLVDSGGANLPNQDEVFP
DRD
HFGRIFYNQAQMSAAGIPQIAVVMGSCTAGGAYVPAMSDVTIIVRDQGTIFLAGPPLVKAATGEVVSAEDLGGGDVH
TRL
SGVADYLAEDDAHALALARRAVRHLNRERPATVRWETPEDPACDPEEILGVVPADLRTPYDIREVIARLTDGSRFDEFKP
RFGETLVTGFAHVMGCPVGIVANNGVIFSEAAQKGTHFIELCAQRGTPLVFLQNVTGFMVGRKYENGGIARHGAKMV
TAV
ATAAVPKITMLVGGSFGAGNYGMAGRAYSPRFLWSWPNSRISVMGGEQAAGVLATVKRDAIERAGGTWSPEEEAAF
KRPT
IEMFEAQSHPLYASARLWDDGIIDPRKSREVLALSLSAALNAPIPDTRFGLFRM
>OLS48873.1 hypothetical protein BV379_11675 [Rhodovulum sulfidophilum]AB14
MKTSLLAIALTATALAGSAADAQSKGDWTFGLGLGYVSPKSDNGTLAGGESHVGDNARPTITFEYFFADNWGVEVLGA
LP
FKHNINIDGVGKADTLQLPPVVSLQYHFVNSSSVTPFLGAGLNYTTFWDTDSALGDVDIEDSWGLALHAGLDYAIDERH
S
IRTDVRWIDIDSDVKLNGGKIGTAHIDPWVFGVSYIWKF
>OLS48874.1 hypothetical protein BV379_11680 [Rhodovulum sulfidophilum]AB14
MIQMRLPLILAAALAVATPALAQQYDFTPDATLACMEKQKMHGADIACVGNSARACFQKMKSPSVSDIAACQQAEA
EYWK
ARMDSAYEHLLGLAEAADADFAKAQGEPQHATMVQDLRDEQEQWNSWQKTRCWVEAMMRRGTPYRTTAAAGC
TMKQIAAR
AMFMESAVSYMENK
>OLS48875.1 acyl-CoA dehydrogenase [Rhodovulum sulfidophilum]AB14
MFTATMEFDLDDDTRALRDMVHRWAQERVRPMAAAIDRDNVFPAELWREMGDLGLLGITVPEAYGGAGMSYLAH
AIAIEE
IARASASVSLSYGAHSNLCVNQIKLNGSEGQKRKYLPGLISGAQVGALAMSEAGAGSDVVSMQLRAERRNDHYSLNGT
KY
WITNGPDADTLVVYAKTDPEAGPKGITAFIVEKTMTGFSTSPHFDKLGMRGSNTAELIFEDVEVPFENVLGEEGKGVRV
L
MSGLDYERVVLAGIGTGIMAACLDEVMPYMRDRRQFGQPVGSFQLMQGKIADMYTAMNSARAYVYAVARSCDRG
RVTRQD
AAACVLYASEEAMKQAHQAVQAMGGAGFLADSAVARLFRDAKLMEIGAGTSEIRRMLVGRELMKAMV
>OLS48876.1 CREA protein [Rhodovulum sulfidophilum]AB14
MPRPALLAALLAVLPLTAPAEEVGRVGVDWTGNDIVIEAIQDPDVAGVTCHVAYFSRSMLDRLSQGNWFEDPSNASIA
CR
QTGPIEIGDIDRSPDGEEVFRQSRSLVLKSIRIKRIFDAENQTLIYLAHARELSEGSAKMSISTVPLFNSAPR
>OLS48877.1 hypothetical protein BV379_11695 [Rhodovulum sulfidophilum]AB14
MTSKHPQKGKAWPSAYRRASAALSFTRRVAQGQAPRHAVPVLLVAGAALTALVTLLAVGAVPSASGRSPDATLTLGR
DAV
PVDGAVLLAGEGPARAAPLVPVARPASFARPDAGPLSADGLGAVAEARALQGRQAGRALLAAYPASPGGTLLPNGIHV
FA
GAPDLSPPARPDSDAAPEGNALAQLRPPRRPLAAADPKRPRPRPAAIGNSAAPTGPAAAATADLVVAASDRPRSRPAL
AS

AAMPSSAPDLAGLRPVSRPLVLSEAEPDALRPSARPPARAGAASVASLVPSARTGHSMATVPVVPPAPEAAETAPGLR
AS
LRPQTRPAALARLARGALVPDGRQAVAPRAEPFAHTDIARGAHGCSTRLARGIPGRPRGALSGNAIGARMSGLHGGD
RDA
LIVQQALAGNIPDFLRELTPVRVSGRLPGGRRADVTICVTPDYLALGSDADFVRVPMGLPAAARIADRFGFLLPTTRMV
D
AIYGQARMRLAPRPMQPGAQMSSTRYFLQHNRTVEGQTGGREGQLTAGQKKDLVLTNRLRSHPGRVAIYGWHKPD
GHPIQ
PLSTVHGAYYSDYSHGVRLVSQTAFVDGRPVSLASLLANPSYAQMLTGEGPIAAPERLMASLYR
>OLS48878.1 peptidase [Rhodovulum sulfidophilum]AB14
MTYCVGLMLERGLVMMSDTRTNAGVDNVSTFGKMFTWEKPGERALVLMTAGNLATTQSVVSILDERTKAHGERDP
SLFEA
PSMFQAARLVAATLKDVIAAHADVGQRADASFNATLILGGQIKGGAPRLFLIYPEGNFIEAGVDTPFFQIGETKYGRPIL
VRAYDAAMSFEKAVKVMLVSFDSTLKANLSVGMPFDLQIYETDSLQLGRRIRIDPQDAYFERISTGWGEALKSALDSLP
D
FEF
>OLS48879.1 transglutaminase [Rhodovulum sulfidophilum]AB14
MRLKIRHSTRYAFDDPVIYGLQQLRKTPKNGPEQRVIAWKTIVEGGRKELSFEDHHRNTVELISFERDVTELVVRSEGEV
EVLDTSGVIGPHRGPSPLWLFRQPTVRTRAGPGCRALLREVKGESDLDRLHALLRVVHGAVGYEIGVSRPDWTAEEALA
A
GKGVCQDHAHVFVACAREMGFPARYVSGYLMTDGVQYEASHAWAEAHVEGLGWVGFDVSNEMSPDGRYVRVAT
GLDYAEA
APVTGSRMGGSGEALSVEIEVAQQ
>OLS48880.1 A alpha-helical domain with a conserved ER moti [Rhodovulum sulfidophilum]AB14
MLGKTANGLFWMYRYLERAENTSRLVETGQRIALTRLDSPDDEWHSVMQTGGVAKAYRRHHEAVTKEGAIDWMLR
AKENP
SSVLSAISSARQNARVVRTALTHDVWGAMNGCYMEVKAALSRRVNERDLPAVIDLIKDRTALVRGTTQGTLLRNDIFN
FA
RIGTFLERADNIARILDVKYYVLLPSTYSVGSSIDNVQWETILRAVSARGGFRMVHGGQVGPRDIAQFLVLDRKMPRSLD
FSVTKIRDNLEYLAFDYGVRHPSHDLAEALKQRFTAHDIDAIFDYGLHEFLQDVLGALSELGRQIEIDYRFYE
>OLS48881.1 hypothetical protein BV379_11715 [Rhodovulum sulfidophilum]AB14
MNESQTFDEMWGREQVLRSPYERFHEWFEGEDIARLRAKQREAEEVFRLTGITFNVYGRAEAEERLIPFDIIPRIISGRE
WQRLSRGIEQRVKAINAFLYDIYHRQEIVKAGRIPEQMIARNEAFLPQMVGVKPPGGVYTHIVGIDLVRTGDNQFYVLE
D
NARTPSGVSYMLENRETMLQMFPELFSRNRVQPVQTYPLDLRRSLAACAPSDVDEKPTVAVLTPGIYNSAYFEHAFLAD
Q
MGVELVEGQDLRIVGGKVAMRTTEGYRPIDVLYRRVDDDFLDPLNFNPDSALGIPGIMDVYRAGRITIANAPGTGIADD
K
AIYSYMPEIVEFYTGEKAILENVPTWRCSEPEALAYVLENLHELVVKEVHGSGGYGMLIGPTSTRKEIAAFRDKLKARPG
NYIAQPTLSLSTVPIFTRNGLAPRHVDLRPFVLVSPAGIKITPGGLTRVALKKGSLVVNSSQGGGTKDTWVLED
>OLS48882.1 uracil permease [Rhodovulum sulfidophilum]AB14
MAVDSSIGTPDQLRDPNYTPALHKAIPLGIQHVLAMFVSNVTPAIIVAGAAGFGFGSDAGAQGFPDMTYMIQMSMLF
AGI
ATLFQTIGLGPVGARLPIVQGTSFAFIPIMIPLVAGRGVEALPVLFTGVFVGGLFHALLGTVIGRIRFALPPLVTGLVVT
MIGLALVKVGIQYAAGGVAAIDSPEYGSLLNWSAALVVVFVTLGLKFFARGILSVSGVAVGILAGYLFALATGMVTSGGI
GDSWQRAAVLEVPLPFKYGVEFSFAAIIGFCLMAVVSAVETVGDVSGITKGGAGREATDREIAGATYADGVGTAIAGAF
G

ALPNTSFSQNVGLIAMTGVMSRHVVTCGALFLILCGLVPKVGALISTIPIEVLGGGVIVMFGMVVAAGISMLSDVTWNR
R
NMVIFAIALSVGLGLQLEPKAVQYLPDTLQILMTSGLLPAALIAVVLNLVLPGELASEATEEVSGGMSGHGRGSLGQD
>OLS48883.1 Ureidoglycolate hydrolase [Rhodovulum sulfidophilum]AB14
MLEALPITPEAFAPFGEVVETGGDFKLINEGCCRRYSDLATLDILDGQPGISLFQAEIRTLPHDCTLMERHPLGSQCFIP
MQNAEFLVIVAPDEAGHPGTPSAFIAGPGQGVNIGRNVWHGVLCPISGSGLFAVIDRIGAGANLEEVRLKTTLRIRRAR
P
G
>OLS48884.1 (S)-ureidoglycine aminohydrolase [Rhodovulum sulfidophilum]AB14
MSQYYAPTGGLPGQEVLHTGRAVFTEAYAVIPKGVMRDIVTSNLPHWRGTRAWIIARPMTGFAETFAQYVMEVAPG
GGSD
RPEPDPGAEGVIFVTGGALDLRLGGLAHLMTAGGYAYLPPGADWTVENPGETPARFHWLRKRYQAVEGIDRPEAFFV
NEA
DIAPNAMPDTEGRWATTRFVDPDDLRHDMHVNIVTFEPGGVIPFEETHVMEHGLYVLEGKAVYKLNRDWVEVEAGD
FMWL
RAYCPQACYAGGPGRFRYLLYKDVNRHPALALGAL
>OLS48885.1 OHCU decarboxylase [Rhodovulum sulfidophilum]AB14
MTRYPRDLRGYGATPPHPQWPNGAKIAVQFVINYEEGGENCILHGDAASEAFLSEVVGAVPWPGQRHWNIETMYEY
GARA
GFWRLHRMFTERDMPVTVYGVATALARGPEQVAAMQDAGWEIASHGFKWIEYKDFSEEDERAHMAEAKRLHAEVT
GEAPR
GWYTGRCSAQTVRLAAEDGGFAYVSDTYDDDLPYWTAAGDRHQLVIPYTLDANDMRFSVAAGYGSGDEFYAYLRDS
FDTL
YAEGMAGAPKMMSVGLHCRLAGRPGRATALKRFLDYIRGFDGVWVARRIDIAEHWASVNPPVLRERPTAMDRDSFV
ARFG
GIFEQSPWIAEGAFGLELGPAHDSALGLHNALARVFRSAGPGKRLEVLKAHPDLAGRLAAAKRLTEASTAEQASAGLDA
L
SDAERARFEDLNRRYTERFGFPFIIAVREHDKAGILAAFERRLENTADAEFAEACRQVERIALLRLKEILP
>OLS48886.1 5-hydroxyisourate hydrolase [Rhodovulum sulfidophilum]AB14
MPGYLTTHVLDTATGAPAAGLRIALYRVSGNSHRKIAEVETNADGRTDAPILPPEAFETGTYELVFFAGDYLERTGLATG
EVKFLDQIPIRFGMEDAEAHYHVPLLLSPYGYSTYRGS
>OLS48887.1 LysR family transcriptional regulator [Rhodovulum sulfidophilum]AB14
MASINTIRMFVRVYELGNMSAAARDLQVSAAVASSRIADLERHLGIRLFTRTTRSIQPTEQGRIYYPKAVKVLEALEEAE
AAVQEITHHPRGSLYVSAPLGVGRRLIAPNVVLFQDRYPDIHVRLRLSDRRVDLAGEGLDAGFLLGDLPDSGLKVRALGD
CTRVLCAAPTYVARRGMPCCGADLIDQGHDCLLLRFPGSREFRWTLQTPEGPQSFNVKGPYACDDGDVLTAWALDGR
GVV
LKPVFDVAEHLASGALIPVCEQTPPPDARLACLFPHKKNQDPKSRLFIEFMAERIKAALRRRAQIYQLPR
>OLS48888.1 cysteine desulfurase [Rhodovulum sulfidophilum]AB14
MFDLSLVWDWLEFAVRWLHVIAGIAWIGSSFYFIALDLGLTRSRPLPEGVLGEEWQVHGGGFYHIQKYMVAPDHMP
DHLT
WFKWEAYWTFISGLILLALVYYWMGADLFLVDYDKIQWAPWQAIAISAGSLTIGWICYDLLCRSPLGRRPTVLMLILFAII
VAMAWGYTQVFTGRAALLHLGAFTGTIMVANVFLVIMPNQRIVVADLKAGRQPDPKYGAIAKLRSTHNNYLTLPVIFL
ML
SNHYPLAFASPYNWLIASLVFLMGVTIRHFFNSMHARKGRPWWTWAATAALFAAIVALSLWPTRDIAAPETEAALPAP
LA
RIVASTGFGEVQEIVESRCAMCHTAEPLWPGLGQAPKGVRLDSAPTVARHARQIYRQAGLTHAMPPGNVMMMPPE
ERARI
VEWYRKLRSGAS

>OLS48889.1 hypothetical protein BV379_11760 [Rhodovulum sulfidophilum]AB14
MIRLFGRDLTRSSLLGRSWMPDIERALRWGLDQIEAELGENAIAIVQGKTGQRLWVACPADLREYLDALPRTGRFILAK
N
LTQPLGKRTVQRLVEGVRKKIGATAYVIHGWRYTAAMQLAEAGCSDSEIAAVTGHRTLQMVQKYRAAANQRRLSRQA
QDR
RGRK
>OLS48890.1 hypothetical protein BV379_11765 [Rhodovulum sulfidophilum]AB14
MDLATDLFLENGFAATSMSRVAEAAGITKASLYHHFPGKEDLFAACVAHGYAAAIERLRGIATDQALPHTERMARVISS
S
YDTIIFSRVGRMSPLIAEVSRSFPKVARGFHRDFILPQENIVLSLIDDGVEAGAFVDLDRAVFEHLLFGPIVTLSMSREM
FATFDDLDTLYPVTELRDGHIKRMIAWLTDGLPHSE
>OLS48891.1 hypothetical protein BV379_11770 [Rhodovulum sulfidophilum]AB14
MQNHPVDFKRFLAMSLTALSLLTSSAQVSNAEDVAAPPPVPVLTMRPGEDEQAPDRMFYGRVAARATVDLAFEVGG
RIVT
LDAVEGQRIAAGTVLAALDPDPYERAVARAELSLDQAERAFRRADTLAARDAASRARADDAETARDLAAVDLREARDA
RS
DTRLVAPFDAILAKRIASVGALVSTGEPVVRLQDMSELRIDVELPERLLGRIGDPQEARFEAEIHGIDGPLDLAFREVRT
ETGEIGQSYTVSLAVEKADMHPWLLPGRSATVRAYLPKAETGVAVPATAVVTRGEGRHFVVAVEPDGDALVARHLPVE
VT
IPDGSTVRVEGLPANAEIVAVGGHLLEDGDPVSRYDGLIAKAE
>OLS48892.1 MFS transporter [Rhodovulum sulfidophilum]AB14
MQIARNAIERPLYTWLLILLCLFGGAAGYLGVGKLEDPVFTLKSALVVTSYPGASAAEVATEVSDVLEAELQQMNEVDYV
TSTNTPGLSVIEVTIRDTFGGAELPQVWDDLRDRVANATPDLPAGARAPIVNDDFGDVFGLLYAVSAPGYADAEIRDIA
T
FLRREMSTVAGVANVEVKGLPEEAIFIEPDSATLDALGIPPDALSGAIAGADAVTPTGALPSGDANLRIEAPRAEDSTGK
IAALNFGYRGEVINLADIASVHRGRIDEPSQILRHNGTEAFTLGIAGLTSANIVTVGARVEARLDEIAHVLPAGVAIEPI
YEQHRVVAEANDAFLLNLAVSVGVVVAVLALFMGWRAAAVVGGTLLLTVTATFFFMYLFGIEVERISLGAMIIAMGML
VD
NAIVVAEGMQTDMRRGRPAADAADEVARSTQMPLLIATIIGIMAFAGIALSPGDAGEFLFSLFAVISISLLLSWCLAVTV
TPYLASRLFPTGSGTDSRDPYDSAFFRGYGRIVGLAIRFRWLVIAGLVGATAAGVASMGFVRQQFFPPATTPLVYLNYKA
AQGSTVQATDKDLIRLADWLATRPEVVSVTSTAGAPMSRFFLTYAPADSNPSYGQLVIRVAEPEQIPTIREALSAAAAQI
VPWAETRVEQIIYGPPVTADVEIRLSGPDADVLRGLAGTMQEILETRTALLQVERTDWREREIVAQPVFGSARASALGIG
RGDVANAIALVSDGVQAGVVREGDRLIPLILRQPRIDGAPQTRLIDAQVYAPATESYISLGQVLDGFEPRSRDTLIRRRS
RQPTITVQAFTPPDVLPPEAFAEVQDTVEAIPMPSGYRMEWGGEYESAGDAQASLARQMPLSFGTMLLLTILLFGKFR
QT
AVVWTVVPMAVTGVGIGLLLTGLPFSFTALLGLLSLSGMLIKNAIVLVEEIDARKDEAGLPQREAIVTACVSRLRPVVLA
AGTTILGMAPLLIDPFFASMAVTIMAGLGFASILTLIGVPAIYHTYVSERKADSGENSRKASSSKVGSARSIPSDPALAA
H
>OLS48893.1 hypothetical protein BV379_11780 [Rhodovulum sulfidophilum]AB14
MLVVAARMEGRHLSGKVRGDWSGREACSQGWPDRPTIGINANSFGRRQRETSGEAAVGGGASDPYGRASLPRVFPK
EVVA
ALYAFDAIMALQNLRDSRDGASFEPKQCIGTGLDMGTALKVIPVPKVALEGSVEVFLPVTLALTGIFADAGISGSMTGR
>OLS48894.1 MFS transporter [Rhodovulum sulfidophilum]AB14
MPLPRPDPNRWLVLLAVMLAFLPVVLDMTILHVAVPTLTQALGASNTEVLWIIDIYPLLMAGLLVPMGTLADRVGTRRI
L
LAGLAVFGLASALAAYAPSPALLIAARMLLAVGGAMITPCVLGVIRRTFEDPAERGLALGLWGTVGAAGAAVGPLIGGA
L

LGHFWWGSVFLINLPVMLFVLPVCWLLLPRQEELSPGRWAIGQAVLLILGMIATVYAIKAGFGAKQPVAVVLPVLVLGV
A
LLSVFARLQLLSAEPMLDLSLFARPAILAGIIMAIVASGALAGVELTLAQELQYVLDKTPLQAGVFMIPIMVAAGLGGPV
AGWLSERFGLRPVACLSLAISAGALAGLAATDFHHPGLVVPVLLVLLGLALSIGLTASSIAIMGAADAREAGAAGSLEAT
GYELGSGLGITLFGVFMSVVFGRHLVLPEGVPEPLADQAARSIGDVYIAARQLDADQGAAVIAAGKAAFSATHSVLLTTA
ALLMGLLAVLVFFLLAQGRRAVAFQH
>OLS48895.1 multidrug ABC transporter ATP-binding protein [Rhodovulum sulfidophilum]AB14
MTDDPGHYDDLAARHVSIRRVLRLFAPYRARIGAVVALMILASAIGLAGPFLLRAIIDIALPQQDLSLLTWLAGGMIAAA
LASAGIGALQVVLSARIGQAILHDLRVRLYAHLQTLSLRFFTGTRTGEVQSRIAGDIAGLQSLMTETANELGRSLSAVAM
TAVAIVILDWRLALFVLLIVPVTVLISARVARLREALTFQQQARAADMSAAVEQTLSAAGIILSRTMGRGPWLTQRFARI
SRDLAQLEVRSHTAGEWQWSLIGFALATLPALTLLAGGLLMQTGTPASIGTLVAMIALQEQLLWPFEQLLEVARDARKT
R
ALFARVFEYLDKPVEITERADPVTIPRSDMRGAVRLDHVSFAYAGQSLRAVDDVTLTIPAGSHVAIVGPTGSGKTTLGYL
LARLYDVDAGAIRFDGVDLRDFGFATLSQMLGVVSQEPYLLNASVAENLRFACAEATGAEMIAAAKVAQIHDHIAALPE
G
YDTPVGEGGFRFSGGEKQRLALARTILRDPPILLLDEATSALDTRTERAMSQALDALAKGRTTITIAHRLSTVRDADMIV
VMQHGRMVETGIHAALAAKGGVYAGLLAGA
>OLS48896.1 two-component sensor histidine kinase [Rhodovulum sulfidophilum]AB14
MSGNLNARITKTMVALTLIATLVVSLGLMAYFLFIYDWIYPDSDDLAGWRTADTVMVLLLLAFGLLTASGIGWRLARRIV
EPLKSVAAAARRIATGDFSARADLPSGSFREAADLLADFNAMAERLERAEAELAYSNSAIAHELRTPLTILRGRLQGMSD
GVFDPTPQLYTRLIEHVDHLSALVEELRTLALSNAGQLELNRDRFDLAGEVGAALNGLEADLTAAGISVTRALAPARIEA
DRTRLRQACVAVLENCRRYAPQSHVHVETRADGDEVVFRCSDTGPGLSAEDRARAFDRFWRADASRGRSLGGSGLGL
PIV
RAIARAHGGEARIIPQPPPGLTVELRLPKSALPAGQTDD
>OLS48897.1 DNA-binding response regulator [Rhodovulum sulfidophilum]AB14
MTDALILIVEDEPEIAEILETYFAREGFRVICARDGTTGLAHHQRLRPDLVVLDIKLPGQDGYEVLAAIRRRGETPVIMV
TALAEDLDKLQALRIGADDYVVKPFNPLEVVARARAVLRRTMGRGPDHLLRLGPLTVDPQAYRAVIDAAAGPLPLELTP
T
EFRILAHMARFPGRAFRRAELVDACLPEGAALDRTVDSHVSNLRRKLAAAGADGMLEAVRGVGYRLDMPHVR
>OLS48898.1 multidrug efflux RND transporter permease subunit [Rhodovulum sulfidophilum]AB14
MSQFFILRPVFAWVIAIFIMLAGIVAIPRLPVARFPDIAPPSVSIFTTYAGADAQTVSNSVVRPIEKELSSVKNVLYYES
SIDSTGGASITVTFKPGTDPEMAQVEVQNRLKNAEALLPAPVRREGLGVEAAESGFLMVITLRSLSGATDELSLGDYLSR
NLAEELKRVPGVGRVQQFGSERAMRVWVDPAKLSAYGLSMADVTGAIARENAQATPGRVGDEPTVPGTRVSTPLTIR
GQL
TTPEAFAGITLRANTDGARLVLGDVARLELGAQTMAFSVSSNGKGAAAAAIQMSPGANAVSTSAAIEKRLAELRLAMP
ED
MEVSISYNTAPFVKVSITKVIHTLIEAMVLVFLVILLFLQKVRYTLIPTIVAPVALLGTFAVMALAGYSINMLTMFGMVL
AIGIIVDDAIVVVENVERIMARQGLSPRAATQQAMREISGAIIGITLVLSAVFIPMGLAGGSVGAIYRQFTLSMAVSILF
SAFLALTLTPALCATLLRPVTGHGEARGFFGRFNRGFDRMTRRYTGWVGWTLRRGGRMLVLYALLIGVMAAGYMRLP
TSF
VPDEDQGSFMAMFELPAGATAERTRAVIAAYEAHTATRPDITENTVILGFGFSGSGPNAAQAFTSMKDWSARKTSVD
EEI
AAAEAAMADIPEGMAMIMKPPAIESLGTTSGFSLRLEDRANAGPAALKAAEERLIALAQASPLLSGVMSEGLPDGMSI
AL
KIDREKAQALGLRFDTISETIGTAIGSTYVNDFPNQGRMQQVIVQADAPARMHVEDVLRLEMRNLEGGMVPLSEVATP
VW
ESTALQLDRYNGYPAAQISGEAAGGVSSGAAMDEMERLAGALPQGFAVEWSGQSLQERQSASQAPMLLAASMLVV
FLVLA

ALYESWSVPFAVMLVVPLGLLGAVTAVLLRGADNDVFFKVGLVTIIGLSAKNAILMVEYARHLRAGGMGLNRAILQAAR
L
RLRPILMTSMAFILGVVPLMLAQGAASEIQNAIGTGVFGGMVSATMLAVFFVPVLYVTVSRLTRGLAREKLRKTAP
>OLS48899.1 hypothetical protein BV379_11820 [Rhodovulum sulfidophilum]AB14
MADHGRRYATGVLLLVPGFCSLAVASRVITGRFLEQLRLLAIPVFEGAYGRPALRCDLRDLPFVERR
>OLS48900.1 arsenate reductase (glutaredoxin) [Rhodovulum sulfidophilum]AB14
MIVIHHNPDCGTSRNVLAFLRASGAEPVVIEYLKEGWTRPQLLALFAAAGLTPREALRETKSPAREIGLLEKDVPEDALI
NAMLAHPVLVNRPIVCTAKGTRLCRPSEVVFDLLDRLPAGPLFKEDGQMVIDENGNRI
>OLS48901.1 IS110 family transposase [Rhodovulum sulfidophilum]AB14
MQVTTVGLDLAKNIFHVHGVTETGEVAFNRPLRRAQVLAFFERLPPCLIGIEACASSHHWGRELTKLGHTVRLMPPMY
VK
PYVKRGKSDAVDAEAICEAVTRPTMRFVEIKSEDQQALLSLHRARDFVVRQRTQLINMLRSLAAEFGIAIARGVARAIDF
AKGIIEGKQSGFPELAQDVLRVLSRQLVELHNRLRWYEITMRIQARLSRQAQLLQSIPGVGPVTASAVAATIGSGHQFKS
GREFAAWLGLTPRNHSSGGKERLGKITKMGDRYLRQLIVVGMTSRVRQVTNHPERADPWLTKLLQRKPARFATVAM
ANKT
ARIMWAVLTRNEPYRPHTA
>OLS48902.1 nucleoside-diphosphate kinase [Rhodovulum sulfidophilum]AB14
MSTSTEATQKPFKRNPKIAISADDLARLQGLADSMALRQPALADRLLEEIGRARIVAPAKIRDTVVTMGSKVTYRDEISG
QEKEVTLVYPEEADITNLCVSVMTPIGVALLGLSEGASFHWETRDDQRRTLTVLSVTPPPENTKY
>OLS48903.1 hypothetical protein BV379_11840 [Rhodovulum sulfidophilum]AB14
MAYFAFAWLASRVVSVLLDAASLRGRRYPRLLRDLIAALLFFVAFAAATSLLLGQGAIAALAGSGLLLAMLGFAIRNVVA
DTLSGIALGIEGPFRIGDWIDIDGLTRGKVIEIGWRTTRILTRDSTYMIIPNSQIARQRVTNYSAPRPQYRAQVAITLDH
AMPVDRACEVMLKAVMNAKLIQQDPAPDVRVLAYEEGGITYALRFWLSRFDRDIDCRDEVYRLVDDALRSKGYLAPH
RRI
ELVRAPPTAS
>OLS48904.1 hypothetical protein BV379_11850 [Rhodovulum sulfidophilum]AB14
MTENLARITSRREFERLSWAHRLALRVVNFSLLPSWWPRRTQKMIRRHLEIRAAEAREALRRSANGMD
>OLS48905.1 DNA mismatch repair protein MutT [Rhodovulum sulfidophilum]AB14
MNTWRPASGIRFKALGLHWRGNRLLASEVYEDSGRLKGVRPLGGTVEFGETAQAAVIREFQEELGITVKIVGSPTFIENI
YVHEGSLGHEMLAIFDVTFPPEAFAGQTRIAFREDSGVECFAEWFALEELDGPGQPQLYPTGLKALLKSKR
>OLS48906.1 NYN domain-containing protein [Rhodovulum sulfidophilum]AB14
MMSRLAVLVDGDNISGCHAERILSIASRQGSPTVVRVYTNARRTSEWHDACGFRMIHAGTGKNASDVLLVIDAMELAL
SG
GIDQFVIASSDGDFTHLAHRLREHGAMVTGVGEGKAPSGFRGACSDFVLIDPGPSIRLVPSAPAVDDLDLKIRAIIAAAS
TKGAGMRLAALGATMHSKHGIRIGALPEQTWRAYLAARPHLYDLGPRGPDSMVRFKPEGFPAAA
>OLS48907.1 hypothetical protein BV379_11865 [Rhodovulum sulfidophilum]AB14
MARYCDRAKVQRDDDVREAEFASRLMHAADYEFGLLAQFIVEALTRALRADGLEACLSSRKHFAEVYHMRTAVGPGL
NPF
LAEDFRRIDPRQCFDDGDEDA
>OLS48908.1 hypothetical protein BV379_11870 [Rhodovulum sulfidophilum]AB14
MNRIVRSALFKRQLLDITASYRERAGTAIALKFVDQIESSIGFIAATPLACAIYTQLEGKDFRKWGLKDFPVSVFFRLDE
SGTIILEALYAHRMDIGQRLPKDIE
>OLS48909.1 hypothetical protein BV379_11875 [Rhodovulum sulfidophilum]AB14
MENASENKMVKRLNTTLPDPLAHYVGEITGKGALYETPSEFIRDLIRRHMEKAQDVERGQINAMLAQSLSENDYSDWT
NS
DLADARKAARE
>OLS48910.1 hypothetical protein BV379_11880 [Rhodovulum sulfidophilum]AB14

MEQHNITWRGIEIAITFTPEKFGMADHVELRSAGKVALPVTETGYRSHFLPVGIVAEHGGAVPFVSAWLEHEAARMG
WND
AQLSLF
>OLS48911.1 serine dehydratase [Rhodovulum sulfidophilum]AB14
MDDWITPGAVTAAAARIKDDVLRTPTVFSPGLSEALGRSVHLKLENLQIGGSFKARGVLNKFAALDSTSSQVKFVAVSG
G
NFGIAISEAARAAGADITVVMPRSAPSSSADRIRASGAMLILEPDVRSAFARADALAADGYEVIDDCRDTLVAEGHGTLA
LELLEDCPSLTDVFIAIGGGAMLAGAATIAKAMKPEIRIWGVETAGASSMHQALRAGKPIEVEIRSIVSTLGVPVIDQKM
LCHARRYVEDVIIVSDNDAIDGMIQFGEVAKQWVELASGTLIPAAAAAIPRLPDDAVVGLVVCGGNISHADMNRWSE
HRT
SRA
>OLS48912.1 TetR family transcriptional regulator [Rhodovulum sulfidophilum]AB14
MRYKAKKIPPETSASSRTKPAEVRLDELMSAAENLFLSQGFSATTVNEIVEAASVAKGTFYHYFSSKNEMLEALAKRYTE
RFLESVQEAIDGCSADDWEERLRVWIHANIETYVKTYHIHDIVYTKHHHHDRANQAKSALLDQLLEIIEGGRLAGVWAP
N
QPKIVALLIYSGVHGATDDIIASKTADSTSFARNVSDACLYMLPKLNAPTK
>OLS48913.1 hypothetical protein BV379_11915 [Rhodovulum sulfidophilum]AB14
MHEQTHPPPQPTGPGIPAPDTAIPSAPDDPDDGADPPHPPDGTAPEDPLGPIRAAAGQPLNDYGNGQRFCIHFGQDL
TFV
PRVGWFVWTGTHWEKDPDELAVRRKAQRIWALIEQEVEVLEPTKSEKKVIAEERRLRERFDRLDALPEAERAEDHDDE
LG
TIRARLRSIEAILKDRKSLIGRRLTHAKNAGNSGPIGNMIEQAQPGLARAYEDLDADPLTVNTLSGTLRFSTVAAEGMSP
VAELRVQRKHLRGDLLSKIMPVAYDRAAKAPRWDAFLEEILPDATVRDFVQRWMGLSMTGIKMQRLAFFYGGGANG
KSVL
VDTIARVLGPYAATARIESLTGTNRRGGGDATPDLIPLMGARFVRTSEPDEGQRLQEGLIKELTGGEPLMIRALHSDFLD
FLPYFKLTMSGNHKPEIRGTDDGIWRRVLLVPFDVQIPEERRDPELANKLWEEAPGILNWLIAGLTDFLEGGLRPPDRVL
EATAEYREESDPVGAFLTESAIVTGAEADFIYSRELIEAFNFWLEDRGETRWGGRTVSNRLKQKASRWRHPHTGKSFIPA
KRDATGYRGLRFTDEFAARMADRRDHGGAGAPVDEPF
>OLS48914.1 endonuclease [Rhodovulum sulfidophilum]AB14
MPPRLTAMPPRLASPAPRIGPAQGDARAQDRERERVKPWRAWYRTARWRKLRRKILARAGHVCEQTGVPLLGRAPA
PDSA
VIDHIRPHRGDPALFWDEDNLQAVSKAWHDGAKQRAERAAGD
>OLS48915.1 hypothetical protein BV379_11935 [Rhodovulum sulfidophilum]AB14
MTDDFSPEPGAVDLFGNPVEPLRDRRGRPCFRKDKANQDFVAARRAAGWSHEMIAVELGCDEKTLRKYFSRELQHGR
LMV
EGMCLDVLMKRAREGHTPSVRQLQERIDRVAPPAPKPKPEREAPKPEPKGKKQARLEAAARPAEDYGSLYDRIPRQ
>OLS48916.1 terminase [Rhodovulum sulfidophilum]AB14
MAPAASWAACPDWWEKLQAGATPIPDLALDANLADVAVQLFDKLVVPDVPGQPTMAEAAGEWMRDIVRAAFGSV
DPATGA
RKVGEIFNLVPKKNGKTTNAAAIGLVALQMNTVPNIKGVVVGPTQSVADTCFGQMQAMIAADDWLSDRFKVNEHKK
EITD
IYPDPATGRPMNAKLKVTSFDPAVTTGGIPAFAILDELHVMAERHFATRVIGQIRGGMITNPGSLLVIITTQSEIPPQGV
FKAELEYARKVRDGVITQDVRMLPVLYELPEAIQGAKGEPWKDPALWAPVLPNLGRSITVERLIPEFRKAVETSPEEIAR
WSSQHLNIQIGLGHHLGGWTGAHHWLAATEPTLDLDALIASSEVATIGIDGGGMDDLLGLAVLGRHAATKRWQLWC
AAWA
HEVVLSRRKSIAEKLRDLAAEDALTICAEGEATRDIEELVALVVKIHEAGLLPRQGGVGLDPEGVAAIVDALQLAGVPHE
TLASVTQGYKLNGAIKGTERKLFDGSLIHGGQPLMSWCVGNAKTEPRGNAVIVTKAVSGAGKIDPLMAAFNAVYLMSL
NP

EPARRDLSGFLDNPVMAI
>OLS48917.1 hypothetical protein BV379_11945 [Rhodovulum sulfidophilum]AB14
MIGRAMQGAMRGAWQGLRQALREGESGWEVVSTAGAHPFAGLGRQSKAARIRAEQEARAWLAWHVEALRRCPSL
PSFRSF
LGGRSGPEAAQPATEMQAMFDTVATAWARSPAARG
>OLS48918.1 hypothetical protein BV379_11950 [Rhodovulum sulfidophilum]AB14
MPLSAVTNDIQNIGREIANIGVGGNADRALKRLGLAAADLDGLDADAKVATIADAVKGLGLSAGAATAVLRDLGVRNR
EM
ALVMIQGGAAIRAAREDVEDYGLSLDAVEAGKIEAANDRIARLGLISRYAGQQLALRLVPALGRAAQAMTEALREGGLL
R
RVIDGLTENLARISTWVATAVTAFGVRYVGALAAARLATLSMAGSLAMLRGALIRSGIGIAVIAAGELVYQLGRLVTATG
GFGEALDLMKDVAAEVWERIVMGPKLMRLRMEELGARMKAIWGEVMVYLQEQWTDFLGKFAELPDFVPGAKAMK
AAADGA
ADGLTAAKEAAAAAGDEAQRLKLEANGIADAMIRTPLASMKALKETLAGVSEEAGNTATNLARIGDAAGGDGGTSAV
TQA
TGQLADTAKTASDSFKTFFKDVAKGSGDAGDAISALADRMLDDLLDRAMSPLSDALGSMFDGLFSGLFGGGRASGGG
GTV
TQLFPSGGLPGFARGTDSAPGGLAWVGEAGPELVNLPRGAQVIPNHRLGSLGGGSVRMGDIHVNVQGSNASPEQIA
AAVR
AESRRQASEIYDRRDRMARAGGAS
>OLS48919.1 hypothetical protein BV379_11955 [Rhodovulum sulfidophilum]AB14
MAFPPDAVRVASLTLKQITSSIRVQSPYSFAQTAYDFMGGMWAAELVLAAVDARESAAISGWIASLNGPAGSFELDM
AAM
EYDGPHGMISADPLVAVAATARAQALVLQLARAGDRALPGDYLTLGRHLHIVTGTEAPTPAFRQSVTLWPRLRRPVAP
GD
PVAMRAPFGTWALAGPETVLSVSQSRIRTRILQVREAL
>OLS48920.1 hypothetical protein BV379_11960 [Rhodovulum sulfidophilum]AB14
MDASGDRMLGLLVRCRFDTGDLAMFTGHGTVDWEGVTYLGAGQMLSVGEAQATSGDGIPGLSITLSGLDPEVVALA
ELEE
FQRRRVTVFLALFDETGQIETADVLFDGVADTMESDDGPDTATTTLALEPRSMALGRKFPCYYLPEDQKKRFPGDQGF
DL
VQAIQNREDTWGRT
>OLS48921.1 hypothetical protein BV379_11965 [Rhodovulum sulfidophilum]AB14
MRRAGWERRLSAAVRDWEDRPFAWGTADCLGFCRAVARSMTWEDPIPGLPEYGSEYGAAKVLVSLGFGSIEALVDA
HLAR
LPVAGARRGDWVMTACEGAMPGAMGVVTGRYAQHMGRNGLVRRPVLTAEAAWRID
>OLS48922.1 hypothetical protein BV379_11970, partial [Rhodovulum sulfidophilum]AB14
MTAALPLVLLASLALILPATPAAAMPPVVAALGVAAGGWMAGLTATAIMGQAALAAVLTGIQMAMAKKPKAKTRDE
ITLN
RIQPVTTGRILYGERMLGGSIVARATTEAGGKPHRRYHSIMPLACHEIDGAVEIWLGETLVWTEAQYRRDEAAGSGDPY
H
WGQVGSDYRGRVRLRVHNGGEDQAAEARYVAAAREWTEAHRLRGVAYVYFEADYDRDLFPSGAPQIRVRCRGKRVY
DPRE
WTSSALASATAASTVPNWAGTDDGAIGQVGGGGYPKGETATPWTGGAIAARFYFGRRYPGGLDPEAGLSPDLTATR
GETL
EASRMERGADLFAAFDVAFPDAPEGVIWEQGGAVYGAYLGVTGSDLVFRAFSGEATGAEVARASIPVADFANRRGTIY
VE

ISVADKSVALWFRGRKYSTNPALCLRDYMLTPQLRGGPGWRPGDLDEDTILALANLAEEQVPLASGRTENRYAFNGVL
ET
EATAAENLNDLSSSWGGWWTCERGRLTVGGAAWEEPAFTVTEDMLVGGIQVTARKPFEEQFNTVKAQYADPENEH
VVTDL
PVLDSATYIAADNGEPLVLDMGELPGETGFARGQRLMKLALLKGRRQKQVTLPCSLAAWPVRLGDNIRVSLPRRGWT
AKT
FEVTGRTVHIGEDGVRVTLSCIETGPAIFDWRTSEEMPKPAGGVPTLPSPTAKPVVSAPTMTEELYETRGGGGVKTRVR
L
AATTDNPFVDAWQFAWRPVSAPEPTLRALTDTPEDMIDDVAPGTYAFGVRGRNARGIWSDWAWAGAAAVQGENA
PPGAIT
GLSVQASGGAVAMLRWDRHPSLDVRQGGRIEFRHATAQAGASWQSSTGIGKSVSGGVTEATLPLKSGTYLARPYDA
MGTP
GPVSGIAVRAASIIPTTTVATLAEAPGFAGTATGCRAAGGVLAMAPGETRASYAFAGRIDLGAVQPVRLISDIELLISEA
RDLFWRPSGTAMWTPPDARLWLGSTDAYGDVDLQVSVTDDAPDTDPAAARWGPWQSFDAADFSGRAFRFRAVLG
VESPDY
TIGITGL
>OLS48923.1 hypothetical protein BV379_11975 [Rhodovulum sulfidophilum]AB14
MQELTTQIRDWWAVMLSLLGLAVWSVRLEARARTNTAALDRETARLAEEIRALEARWQRQRAEDLAARQRDREETN
ALLR
ELRADIKTVLQRVPR
>OLS48924.1 hypothetical protein BV379_11980 [Rhodovulum sulfidophilum]AB14
MLGGMLTVREIKAAPAGRYGDAGGLSLIKTRQDSGKWNFRYSFLGTRRNMGLGSWPEVGLADARRERDRWAAVLR
AGRDP
ITERKEERAATKAQRDKTDPTFAEMAQLVFEARKATLRGGGERGRWFSPLERHVLPHIGKMRVSEITPQDIKGALAPIW
R
TKFPTAEKALQRTRIVLHEGRLMGFGCDPFSAEAAQRMLGAVIHTPKHIPATPWQDMPALYARLNAGTVGHECLRFM
MLT
LVRYMGCAGARAAEVDGDVWTVPPERMKGTLKHVREFRVPLTDPALAIVQRQKEAFGDILFTGGRSAPITSAAIEKALR
V
LKEDGRPHGFRSSFRMWVQDTDACSFDVAETILGHTVGGRIERTYARSDLLERRRPVMEAWARYVTGSAENVVSSGS
AAD
G
>OLS48925.1 proline dehydrogenase transcriptional activator [Rhodovulum sulfidophilum]AB14
MPDATFLPDRYDRAILRIIAAEGRIPVAELGRRIGLSKTPTQARLRRLEAEGIVTGYRAMLSPIRLGLAHVAFVELRLSD
TREAALRAFNAAVRAIPEIEECHMIAGGFDYLLKVRTADMAAYREVMGERISALPHVANSSTFVAMEAVKEVAFATVPE
P
>OLS48926.1 bifunctional proline dehydrogenase/L-glutamate gamma-semialdehyde dehydrogenase [Rhodovulum sulfidophilum]AB14
MQDDTVPLRDRIEAATYAPQTQVLEALLEATGLDDADRRRIEAAAVRLVQAIRAGERPGLMEVFLAEYGLSTNEGIALM
C
LAEALLRVPDIETMDALIEDKIAPSDWGRHLGRSASPMVNASTWALLVSGRVLERRDPGLIATLKGAVKRLGEPVIRSAV
ARAMREMGRQFVLGESIEAALERARDLEALGYTHSYDMLGEAARCEADAERYYQAYSHAIAKIGLAATRNDVRSNPGI
SI
KLSALHPRYEVAQRDRVMAELVPRLLELALKARSMGMGLNIDAEEADRLVLSLDVIAAVLADPRLAGWDGFGVVVQA
YSP
RAGLVIDALYALAERHDRRLMVRLVKGAYWDSEIKRAQEAGVEGFPVFTRKAATDVSYIANARKLLRMTDRIYPQFATH
N

AHTVAAILDMAPDDIDSFEFQRLHGMGEALHRIVMEKGLGTRCRIYAPVGAHRELLAYLVRRLLENGANSSFVNRIVDE
T
VPPEAVATDPLSELEAQLDAEAPAGLRPGPALFEPDRRNSRGWDLAHGPHLAQIETARAPSRSAHFNAAPRLAGPVRA
ET
AAPRHSPSDPTDLVGWVQEASARDVATALAHARPWNIPERRCAEVLRQAADLYEAHAGEIFALLAREAGKTLPDAVGE
LR
EAVDFLRYYAARIDGLAAPARGLFVCISPWNFPLAIFTGQIAAALAAGNAVIAKPAEQTPLIAHLATGLLHEAGVPPECL
QLLPGDGRIGAQLTSDRRVTGVAFTGSTETARLIQRAMATHMAPGTPLIAETGGLNAMIVDSTALPEQAVRDVIASAF
QS
AGQRCSALRCLYVQEDIAPGFLAMLQGAMAELSPGAPWELSSDLGPLIDAEAHAAIASHVATARAEGRVIWEGSAPRE
GH
FQAPVAIRVPGIEAIEREIFGPVLHVATFAGGNLDRVVEAINATGYGLTFGLHSRIDDRVERIAGRLAVGNVYVNRNQIG
AVVGSQPFGGEGLSGTGPKAGGPHYLPRFTERPAPEAHVSTDPAGGAQAAPEAVAAAVRTAQPARGPETLDLPGPTG
EAN
RLTTLARPPLLCLGPGPGASAAQAEAVRAKGGAAVAVEGLLEPAALKDIEGISGALWWGDAETGRAYALALAERDGPIL
P
LIAGMPDPAHVRLERHLCIDTTAAGGNAALLAEAGSA
>OLS48927.1 rhomboid family intramembrane serine protease [Rhodovulum sulfidophilum]AB14
MFPLRDHNPSSRFPFVTLALIAANVVVFLFCLPLFSNPKALHALFMEWGMVPALITQSGDWSGMLTSMFLHGGVLHL
AGN
MLFLWIFGDNMEDRFGHLGFLLFYLAGGVAAALLQYAVAPSSPIPVVGASGAIAAVMGGYLLLFPKARVDLLIILIVIFR
VIPVPAWLMLGLWFGLQLYSGASAAADEGGVAYWAHSGGFIAGLALTLPLWLKLGGPAFWNRTAGSPPHPVGVYRA
TSVP
RVRRRR
>OLS48928.1 hypothetical protein BV379_12005 [Rhodovulum sulfidophilum]AB14
MTEDARGAGDRAPLRLRRFLNDTSGAVTVDWIVLTAAIIGTCIAIFASIQSGAVGMGDSVRAALDAAGVVELGDFGRD
D
>OLS48929.1 inositol monophosphatase [Rhodovulum sulfidophilum]AB14
MQGSANLNIMIKAARKAGRSLVKDFREVENLQVSMKGAGDFVSRADTAAEEILRQELRGARPTYGWLGEETGGEDG
DDPT
RRWIVDPLDGTTNFLHGLPHWAVSIALEHKGEIVSGVVFDAAKDELFFAEKGAGAWMNERRLRVSSRARMIEAIFATG
VP
FAAKRTLPATLQDLARLMPHCAGVRRWGSAALDLAYVAAGRYDGYWERELNAWDVAAGLLLVREAGGLVGPIHEGR
DPLE
HGEVLVANEAIYDHFAKAVRNT
>OLS48930.1 LysR family transcriptional regulator [Rhodovulum sulfidophilum]AB14
MHLELRHLRTIKAIHDAGGLARAADRLNITQSALSHQVKGLEEQVGMELFVRRTKPLRLSSAGLKLLRTAERVLPEIAAL
QAEFEGMTSGRAGRLHIALECHACYDWLLPVLDSFRKAWPEIDVDIRPGLSFEALPALAREEVDLVISSDPETIDGIGFA
PLFDYEPVFLCAASHPLAGRAFIRAEDFRGETLITYPVERSRLDVFTGLLGPARVEPLAQRQVELTAVILMLVASGRGVT
VLPDWVVREARSNADFAVLPLTEGGLTRRMYAATRSEDTAKPFMAHFVRLARSEPVKLQRGTAG
>OLS48931.1 methylenetetrahydrofolate reductase [NAD(P)H] [Rhodovulum sulfidophilum]AB14
MTAPRVSFEFFPPQTLEASFRLWEAAQALAALKPAFVSVTYGAGGTTRKLTHEAVQTIHNHYGLNVAAHLTCVDATRA
ET
LEIAEAYSAAGVSEIVALRGDPPKGQGAFRPHPDGFANSCELIEALAATGRFRLRVGAYPDPHPEAGAPDADVRWLKRK
F
EAGADTAITQFFFEAETFFRFRDACEKQGIDGAKIIPGILPIEKWAGVRRFAQRCGAHVPAHVDEAFETAIRDDREELLG
KAMATELCTELMEGGVRDFHFYTLNKPHLTRDICHALGVLPDAHLEKVA
>OLS48932.1 xanthine dehydrogenase [Rhodovulum sulfidophilum]AB14

MPLRSPSGRGGPQMERTFGKSQTGPRLEDRRFLTGKGRYVDDIAPASALHAVFLRSPVAHAEIRAIDLEDARAAPGVV
AV
LAAGDLRAAGIGAAMGATVVTNRDGSAGAAPARPLLAEGRVRFVGEAMAVVIAETLLAAKDAAERILFDLHDLDPHLT
LT
VGGPQIHPEAPLNLAYDWATGDEAAVHEAFRTAARTVRLEVADTRVIVNSMEPRGAWAEWAEGRLHLCVNGQGV
WDQKAK
LAAMLDLPETAVRVTNPDVGGGFGMKAMSYPEYYVIAHAARALGRPVRWMSDRTEAMLSDNAGRDLVSMVEIAFD
AGHRI
TGYRVETLSNLGAYNSEYAQPIQSELFAKVLTGAYDIPAAFLAVKGVYTNTTQVDAYRGAGRPEAIYALERTMDLAAREL
GLDPWELRRRNFIAPSAFPYRTPTGESYDVGDFARVLARVETMADRAGFAARKAESTARGRLRGLGLCYYIESILGDPTE
GAEIAFPEDGTVALYVGTQSNGQGHETVYARFLAEQAGLPVEAIRVIQGDSDLIARGGGTGGSRSVTTQASATLAAIEV
M
VAAFTPFLAAEMGVEMSDLGFDEGRFRAPGSNLTPGLIEAAAMARRAGRTDLLRHRADYTLPGRSYPNGAHVAEVEID
PE
TGETRVLRYTVADDFGTLINPRLAEGQVHGGVAQGLGQALCERVVHDDYGQLLTASFMDYAMPRAADLPFVQFGTEP
VPS
TANPLGMKGCGEAGTVGAMAAVGNAVLDALWSAGVRRADMPFTPERVWRLLLAAA
>OLS48933.1 valine--tRNA ligase [Rhodovulum sulfidophilum]AB14
MPMEKTFDAAAAEQRLYEAWEKAGCFTAGANASRPETFCIMIPPPNVTGSLHMGHAFNNTLQDILVRWHRMRGFD
TLWQP
GQDHAGIATQMVVERELAKEGNPGRREMGREKFLEKVWDWKGQSGGTIITQLKRLGASCDWSRNAFTMSGAPGAP
AGEEG
NFHDAVIKVFVEMYNKGLIYRGKRLVNWDPHFETAISDLEVEQVELDGNMWRLRYPLEDGATYEHPVEFDEEGTPTAF
ET
RDYLVVATTRPETMLGDTGVAVHPEDDRYRHLIGKTVRLPLCGRSIPIVADDYADPTKGTGAVKITPAHDFNDWGVGQ
RT
GLRAINIMSTRAAMFLKDNADFAEGCDDALVADCIARFDGLDRYEARKAIVADAEAQGWLDGIDSDRHMVPHGDRS
KTAI
EPYLTDQWFVDTQKIVGPALDTVRKGMAAQEAGQFDETAGYTRILPERDAKTYFHWLENIEPWCISRQLWWGHQIP
VWYG
LDLDASHFTDDEGDNALDDVELYRLLGEGFVQRGARHHCAADFEAVKAQFQDVLAGLPAPLHAMRVVEVSGRAAAE
EALA
ESLAEYNLSQDPTGLIYPIWRDPDVLDTWFSSGLWPIGTLGWPEETPELAKYFPTDVLITGFDIIFFWVARMMMMQLA
VV
DQVPFHTVYVHALVRDEKGKKMSKSLGNVLDPLELIDEYGADAVRFTLTAMAAMGRDLKLSTSRIAGYRNFGTKLWN
ATR
FAEMNGVFEGYTPTGEIPAPTETVNTWIVGETARVREAVDEALSHYRFNDAANALYAFVWGKVCDWYVEFSKPLLMD
GTE
AQKAETRATMAWVIDQCLILLHPIMPYITEELWGTTAERAKMLVHADWPAYGADLIDLQADREMNWVIALIEEIRSAR
AQ
MNVPAGAKIPMLYTELDAAGQDAWARNEALIRRMARIDSLSAAETLPKGCVTIAVEGGSFALPLAGIIDVAAERARLEK
T
LSKLEKDLKGLEGRLKNPKFVESAPEDVVEETRELVEAKTGEAAKLRGALERLAELG
>OLS48934.1 threonine--tRNA ligase [Rhodovulum sulfidophilum]AB14
MGQITLTFPDGNQRDFEAGITPAEIAAGISNSLAKKAISAQVNGRHFDLQWPITEDASIAINTMKDDAPALELIRHDLAH
IMARAVQALWPDVKVTIGPVIENGWYYDFDREEPFTPEDLGRIEAKMKEIINARDPVRTEIWDRARAVKHYEDNDEPY
KV

ELVGMIPEDQQIRMYWHGDWQDLCRGPHLQHTGQVPADAFKLMKVAGAYWRGDSDRPMLQRIYGVGFRSRQDL
KDYLTFL
EEAEKRDHRKLGREMDLFHLQEEAPGQIFWHPNGWKIYTTLQDYMRRQQERDGYVEVNTPQVVDRKLWEKSGHW
DNYQEN
MFIVEVDEEHARQKAVNALKPMNCPCHVQIFNQGLKSYRDLPLRMAEFGSCNRYEPSGALHGIMRVRGFTQDDAHIF
CSE
DQIEAEAKKFIDFLAKIYADLGFEDWTVKLSTRPEKRIGSEESWDRTEEALGSACRAAGYDYAVNEGEGAFYGPKLEFVL
TDAIGRDWQCGTLQVDPNLPERLDATYIGADGEKHRPIMLHRAVVGSFERFIGILIENYAGKLPFWIAPRQVVVASIVSD
ADDYVAEIVATLKAAGVRAEADIRNEKINYKVREHSLAKVPVILAIGQREVEERTVSVRRLGENRTETAALDAIVAALTA
EAKAPDLA
>OLS48935.1 arsenate reductase [Rhodovulum sulfidophilum]AB14
MIIYGLKTCDSCRKAKSALERLGKAVDFHDMRDDPLSREELARFLAALGPDLVNRRSTTWRGLDEAARAGDPLDLLEAH
P
TLMKRPVIEAGGQLHLGWNAKTEAALAG
>OLS48936.1 cold-shock protein [Rhodovulum sulfidophilum]AB14
MPTGTVKWFNTTKGYGFIAPDDGGKDVFVHISAVERAGMTGLSDNQKVAYEMIEGRDGRKSAGDLRAI
>OLS48937.1 hypothetical protein BV379_12050 [Rhodovulum sulfidophilum]AB14
MSVIIAVILYLATVLVFLALDALMLRHVMQPLFARYLGDWMRDRPRMGAAAVFYMAYVLGLVWLVSWPALVRGNPG
QALI
EGMVVGAMAYGTYEFTNFATLKRWSPLQVTLDTCWGTLLTGISACIGVFVARIFV
>OLS48938.1 thymidylate synthase (FAD) [Rhodovulum sulfidophilum]AB14
MPVTEDQLAEIEAQRAETAPTRRVVAEGMEARLYSVHPVLDHGFVRVVDYMGDDAAICQAARVSYGRGTKAVSNDE
GLIR
YLMRHWHSTPFEMCEVKFHVKLPVFVARQWIRHRTANVNEYSARYSILDREFYIPAPDALAAQSTVNNQGRGEVLEGE
EA
ARVLDILKTDAGRCYDNYEAMLGQDGQKGLARELARMNLPANIYTQWYWKCDLHNLFHFLRLRADSHAQYEIRVYAQ
TIC
EIVADWVPLAYKAFEDYRIGGVNLSGPAMECIRRMLKGEAVTQETSGMSKGEWREFEAALKND
>OLS48939.1 lactoylglutathione lyase [Rhodovulum sulfidophilum]AB14
MKIRYLHTMVRVKDLEASMAFYRLLGLTETRRVENDKGRFTLVFLAPPGQEDCPIELTYNWDGDEGLPSDSRHFGHLA
YG
VENIYDICARLQEAGVTINRPPRDGHMAFVRSPDNISVELLQIGEALAPAEPWASMPNTGHW
>OLS48940.1 hypothetical protein BV379_12065 [Rhodovulum sulfidophilum]AB14
MVRLAGLLALAPFKAEAGCRLALLLALDISSSVDPGEDALQRAGLAAALAAPEVQAEFLSVPDLPVALAVYEWSGRYQQ
R
VVLPWQILDSPEAILGAAEAIAGSRRSYAAFPTAMGFGLGFGARLFDSAPDCAARTLDISGDGINNDGFGPELAYRNFPF
QGITVNGLAIGGGSPSDTEVFAFYRDRVIRGPGAFVEIARDFADFERAIRRKLMREVAARVYGGTVAIPPGGG
>OLS48941.1 hypothetical protein BV379_12070 [Rhodovulum sulfidophilum]AB14
MIDPHRGTDTNGQRIDRDLADRDFPRPGPVIRGAVAFRSSVACQPAPPSASNLSSQPSAVLMAVLRAAECGPVRGSLS
AA
PATAAGLSISVQERNSLWSSMPLHSKIASATGAEGMRRKAACT
>OLS48942.1 hypothetical protein BV379_12075 [Rhodovulum sulfidophilum]AB14
MLIVLGANEFDDNTRCKAFFSGLPGELADHFRAELARGPGAFVNTASGFSDVAETIERKLPRELQAIAVGQVSPEPGSPG
AADLSRCSGSDPTSSAPSA
>OLS48943.1 MarR family transcriptional regulator [Rhodovulum sulfidophilum]AB14
MSIHSPIPEGDHREVMTDYLEALSLVERLHRLLLDVIKDEFERLGVLELNSVQALLLFNIGENEVTAGELKSRGYYQGSN
VSYNLKKLVEKGYMHHQRCEIDRRAVRVRLTPKGREIREIVGGLFARHAEGLVARNVVDDNGLRDITAALKRMERYWS
DQ

IRYIY
>OLS48944.1 succinate dehydrogenase assembly factor 2 [Rhodovulum sulfidophilum]AB14
MTESTESRRKRMHIRAWRRGTKEMDLILGGYADDRLAAMDEAELEMFDALLAQDDHALYQWVTGQDAAPDAFAPL
VAELA
ARPAPS
>OLS48945.1 transcriptional regulator [Rhodovulum sulfidophilum]AB14
MGTSETDTAEWYSEDAATFGDRVVAAREALSMTQTELAKRLGVRLETVKDWEDDVSEPRSNKLQTLAGVLNVSIMW
LLNG
HGDGLEGPGDEAAIPGEVRTILGEIRAVRADMVRLGNRLGVLEKRLRGALKEHG
>OLS48946.1 aspartate aminotransferase [Rhodovulum sulfidophilum]AB14
MSFLSATLARVKPSPTIAVSTKARELKAAGKDVIGLGAGEPDFDTPQNIKDAAKAAIDRGETKYTAVDGLPELKKAICAK
FKADNNLDYEPAQVSVGAGGKQILYNALMATLNPGDEVIIPAPYWVSYPDMVLLAGGEPVIVEGKMETGFRITPEALE
AA
ITPKTKWFIFNSPSNPTGAGYTRDQLKALTEVLMRHPHVHVMSDDMYEKLTYGDFEFCTPAEVEPGLYDRTLTCNGVS
KA
YAMTGWRIGYAAGPADLIKAMGKIQSQSTSNPSSISQWAALEALTGPQDFIAANNAVFQRRRDMVVEMLNAAEGID
CPVP
EGAFYVYPSIAGCIGKTSPGGVVIENDEVFATALLEEKGVAVVFGAAFGLSPNFRVSYATSDEALKEACTRIQDFCASLN
>OLS48947.1 MATE family efflux transporter [Rhodovulum sulfidophilum]AB14
MTSAQSLSLSAHLRATLALGLPLVGSHLAQFGISLTDAVMLGWYDVGALAAEVLATSLFFVLFVVISGFAWAVMPMIAT
A
AAAGDETQVRRVTRMALWICLGFGLLCLPAFFLSERVFLALGQTADLSAMAQDYLAIAGPGLLPALVVMVLKSYLSALE
R
ARVVLWITLGGVAVNAFGNYLLIFGKFGLPEMGLQGAALSSLIVNLVSMAAVVLYALWATPEHELFRRIWRPDWEAF
WRV
FRLGWPIGLTSLAEVGLFVASSVMMGWIGEVALAAHGIALQITSAIFMVHLGLSNAATIRAGAALGRRDRTGLYRGAV
AA
FGVSGVAVAVSLVAFLGWPEALVGAFIDPGSPDRAAVLAVGTGLLAAAALFQLVDAAQVMALGLLRGVQDTRVPMV
MAGI
SYWLVGMPVSYGLGFGLGWGGIGIWLGLALGLALAGVLMMARFWSRDWVPAAP
>OLS48948.1 SAM-dependent methyltransferase [Rhodovulum sulfidophilum]AB14
MWNERYAGDDYLFGTEPSAFLRREAARLPPGAQVLSVAEGEGRNAVWLAGQGHEVTAVEAAPNALAKAWRLAEAH
GVSVA
LCEADIERWDWAPDAFDAVLGIFIQFGDRACQDRIFAGMRQTLRPGGLVLLQGYAPRQVEYGTGGPGDPEKMYTEPL
LRE
RFAGFEILMLRDYDTELADGRGHSGRSALVEMVARKPRPEA
>OLS48949.1 DNA topoisomerase IV subunit B [Rhodovulum sulfidophilum]AB14
MPDDLLSGASRDTYDASSIEVLEGLEPVRKRPGMYIGGTDERALHHLVAEVLDNAMDEAVAGHATRIEVELHVDHSVTI
R
DNGRGIPIDPHPKFPDKSALEVILCTLHAGGKFSGKAYETSGGLHGVGVSVVNALSDRLRVEVARNRELFAQDFSRGVPL
GPVEKLGAAPNRRGTAVIFHADEQIFGQHRFRPARLLKMVRSKAYLFSGVEIRWKTAIADGDTPMEAVFHFPGGLADY
LS
ETLEGASTYAERPFSGKVGFQEKFGQPGSVEWAVNWTPSRDGFIQSYCNTIPTPEGGTHEAGFWSAILKGLRAYGELA
NN
RKAAQITRDDLMTGGCAIVSTFIREPEFVGQTKDRLATTEAQRMVEGAVRDHFDNWLASDTKAAGAILDFLVLRSEERL
R
RRAEKETQRKSATKRLRLPGKLVDCSATNRDGTELFIVEGDSAGGSAKMARNRKTQALLPLRGKILNVLGAASSKLGSN
A

EINDLTQALGTGLGTRFKIDDLRYDKIIIMTDADVDGAHIASLLMTFFFTQMRPLIDQGHLYLACPPLFRLTQGAKRLYA
LDEAEKEMWLEKGLGGRGKIDVSRFKGLGEMDAKDLKETTMDPASRKLIRVSIDEDHPGETADLVERLMGKKPELRFQ
YI
QENARFVQEVDI
>OLS48950.1 HNH endonuclease [Rhodovulum sulfidophilum]AB14
MDGDFRTSFVREPGSLKHHPALVLNADYRPLCYYPLSLWPWQEAIKAAFLHRVDIVAEYDQVVRSPTRTIRIPSVVVLKD
YVKPQKRVAFTRFNLFLRDEFTCQYCGCKGDLTFDHVVPRARGGITSWENVVAACSRCNLRKGSKSLRQAGMSLRRAP
RR
PAATDLRDVGRKFPPNYLHASWLDFLYWDAELEA
>OLS48951.1 metal ABC transporter substrate-binding protein [Rhodovulum sulfidophilum]AB14
MLRLIPLVSVLALSAGAALADDIRVGVSPGEHAEIMEQVAKVAAADGLTIDLVEFSDYVVPNQALADGDLDANSFQHRP
Y
LENQIKDRGFDLVEVATTITTPMGVYSDKIEDLAELPEKARVAIPNDPTNGGRALLLLQELGLVKLAEGTGLVPSPLDIE
DNPKGLKFLELDAAQLPRALADAEIAVINTNYAIAAGLSPKDDAIEMEAADSPYVNIIVVRDGDQDQPWVEALVEAYHS
P
EVKAFIDEKYDGAVLTSW
>OLS48952.1 DL-methionine transporter permease subunit [Rhodovulum sulfidophilum]AB14
MSANLIALLIEATLQTLYMVSAAALLGTLFGLPLGLFLATSQRGELLSAPLVNKVLGLVVNAARSVPFIILVVAIIPFTR
ALVGTSIGTDAAIVPLTIAATPFIARLVENAIREVDAGLIEAARAMGATPLQIIRKVLLREALPGIVLGLTLAVVSLIGY
SAMVGAVGGEGLGDLGIRYGYQRFMPDVMLAVVVILIVMVQLVQSFGEWIAARVDRRAPRNRGH
>OLS48953.1 methionine ABC transporter ATP-binding protein [Rhodovulum sulfidophilum]AB14
MTGATISFENVGKFFAGGAKGRVTALQDVSLTVTPGEICGIIGRSGAGKSTLLRMVNGLERPSEGTVSVGGKDVGRAQ
GA
ALRAIRRDVGMIFQHFNLLASRTVHGNIAMPLEIAGVPAAEIRSRVGDLIARVGLEAQADRYPAELSGGQKQRVGIARA
L
ATRPKVLLSDEATSALDPETTQTVLALLRDINRDLGLTILLITHEMAVVRDIASHVAVIDQGRIVEAGPTYDVFVAPRHP
TTRSFLSGVTGITLPRFVSGGLVETRPGGAAEEVLRVTFAGAHATDPMLARMTAERGISVNILAGAIEEIGPHPFGNLLI
SVDAARGAEARDYLNRHGLSTEVLGYVR
>OLS48954.1 hypothetical protein BV379_12145 [Rhodovulum sulfidophilum]AB14
MQKDWPDILGGLVLATLGAAAAGWALAHYDIGSLRRMGPGFFPVVLGALLAGLGLAIALPALGREAGSRPGIEPRAAV
AV
LAAILVFGFGLFRLGLVGATAAAVLIAALPAPHDGRIWRLVLALAISALTVLVFGLGLRMNVPLWPRLS
>OLS48955.1 hypothetical protein BV379_12150 [Rhodovulum sulfidophilum]AB14
MTTWEGLLHGIGFALQPGVLVYCVLGVVLGTFVGVLPGIGAMAAISLLLPITYYITPEAALIMLAGVYYGAQYGGSVASI
LMRLPGTPQSAVTSLDGYPLAREGRAGVALFTAMASSFAGSLIGIAVLVTLAGWLARAATAFGAADYAAMMILGLVAA
ST
IGSSSPVKGFAMVVLGLLLGCVGTDVNSGVQRFTFGRTELLDGINLVALAMGLFGVAELIANVRDPDRQGVTARIGLRG
L
MPRRDDLRRLPGPILRGGALGSFFGALPGTGSTLSSFLAYALERRVSRRPERFGKGALEGISAPEAANNAASISAFVPTL
TLGIPGDPIMALMLGALVIHGVQPGPLMLEARPEMFWGLVASFGIGNLLLLILNLPLIGIWVSMLRIPFRWLYPAILVFL
CLGVYSVRGLTFDIAAVAAIGLAGYLLALARFSPALLLLGFVLGPLIETNLRRAMLIARGDPMVFLERPIAAGFMLATVG
LLALALWQAWRRRAAARTG
>OLS48956.1 SMC-Scp complex subunit ScpB [Rhodovulum sulfidophilum]AB14
MSRPPETGDDAEGSLFEAPPAAEQERMVEAILFASAEPVRVAEMNARMPHGCDAGRALEALVRRYEGRGVHLVRVG
EAWA
FRTAPDLGFLMQKETVEMRKLSRAAIETLAIIAYHQPVTRAEIEEIRGVSVSRGTVDQLLDLEWIRFGRRRMTPGRPVTY
VVTEAFLDHFGLESARDLPGLRELRAAGLLDNRPMPGSEDEDAAEAQGEMFEDES
>OLS48957.1 segregation/condensation protein A [Rhodovulum sulfidophilum]AB14

MAEQGTDPVDAEAEDVAARQAAEALIVDVDGFEGPLDMLLTLARTQKVDLRKISVLHLAEQYLAFVETAKSLRIELAAD
Y
LVMAAWLAFLKSRLLLPPDPTEDGPSAEDLAAHLAFQLERLEAMRGVAARLMGRDQKGRDFFVRGLPEGVERVRRVQ
YTA
TLLDLMQAYARIRTKDDFRPYALDREAVYTMEEALERMRGLIGYAGDWIDLSSWLPEGWQADPARRRSATAAHFAAS
LEL
VKQGQLELRQAETFAPIQIRRKPH
>OLS48958.1 beta-N-acetylhexosaminidase [Rhodovulum sulfidophilum]AB14
MSHGVGAFIAGCSGLVLTPAERAFFAETRPFGFILFARNIADPGQVRRLTGDLRQAVGRKAPILIDQEGGRVQRLRPPH
W
RAWAPALDQIVAAGPEMAARSMYLRARLIASELSELGIDVDCAPVADIARPETHEILKNRCYGTDPSAVIDCARATAEGL
LAGGVLPVVKHIPGHGRASADSHLHLPRVTTGAPELEASDFAVFRALCDQPMAMTAHVVYDALDPDRPATVSPLAIAA
IR
EDIGFTGFLMSDDVSMQALSGSIGDRARAAIAAGCDAVLHCNGDMDEMTEVAGACGTLKGAAAERAARALACRRPP
EPFD
AAAAATALDGMLGKVAHG
>OLS48959.1 hypothetical protein BV379_12170 [Rhodovulum sulfidophilum]AB14
MAEIDFDAMRGAGGHDAAPSLKTMVNWAGGLVSLTLVAGLFVWGYQIVVRDVSGVPVIRALEGPMRIAPDDPGGR
QAEHQ
GLSVNRVAAEGEAAPPPDMLRLAPAPVDLAGDDRPVIAPQPAAGAAADPSQPVLAALNLADTPATDAAGGAPFDRA
EPAA
EPVAPEAEPTEEEALALPAGALRQSPRPAARPQEDLATRAAIASARAAMDAPAPAEIAADSVLPGTALVQLGAFDSGDE
A
RGVWDSLSGQRSFAGFFSDKTRVIQTVDRGGRTFFRLRASGFADLAAARRFCAAVSAEGADCIPVVAR
>OLS48960.1 arginine--tRNA ligase [Rhodovulum sulfidophilum]AB14
MNLFNDLRALVIDCLDAMVAEGALPAGLDFSNVAVEPPRDAAHGDMATNAAMVLARPAGQKPRDIAETLAGRLAAD
PRIA
SAEVAGPGFLNLRLAPAVWQGLVAKVLGQGAGFGRSSLGAGQKVNIEFVSANPTGPMHVGHVRGAVVGDALASLLD
FAGW
DVTREYYINDGGAQVDVLARSAYERYREANGLEPEIREGLYPGDYLIPVGAALKEKYGASLLDKGEEHWLADVRLFATD
M
MMEMIREDLAALGVTMDVFSSEKALYGTGKIEAALDRLKGMGLIYEGVLEPPKGKTPDDWEPREQTLFRSTAHGDDV
DRP
VMKSDGSWTYFAPDIAYHYDKVGRGFDQLIDILGADHGGYVKRMKAAVAALSEGRVPLDIKLIQLVKLWKNGEPFKM
SKR
AGTYVTLRDVVEQVGRDVTRFVMLTRKNDAPLDFDFDKVLEQSKDNPVFYVQYAHARISSVLRKAAGAGIAVDDATLA
GA
DLTKLDHEAEIAVARKLAEWPRLIEIAARSNEPHRVAFYLYELASELHALWNRGNELPELRFLQEGDAATSQAKIALARS
VAVVISAGLGILGVTPVDEMR
>OLS48961.1 deoxyguanosinetriphosphate triphosphohydrolase [Rhodovulum sulfidophilum]AB14
MLAPYACKPSESRGRLHPEEESAFRSCFQRDRDRIIHCSAFRRLMHKTQVFVVHEGDSFRTRLTHTIEVGQVARTMANA
L
GLNIELAEAVALAHDLGHTPFGHTGEDALAELMAPFGGFDHNAQAIRIVTRLERHYAGFDGLNLTWETLEGIAKHNGP
VP
RPLPYALAEYDARHDLELHTHAGAEAQVAAIADDVAYNNHDLHDGLRAGLFTEAELTGLPLLGACFAEVDAAAPGIDA
KR
RRHEALRRFFGVMVSDVIDGARATLAEAEPASAAEIRALGRPVIGFSPALYEGLREIRSFLFHRMYRAPSVVEMRAEVTD
IVRELFPLYLTHPEYLPRKWREDVAAATDETALARIVADYIAGMTDRFAMEEHARLTGAAPPVRRV

>OLS48962.1 gamma-glutamyl-gamma-aminobutyrate hydrolase [Rhodovulum sulfidophilum]AB14
MWQRRQDRPLIAVTTSARSGWRVFPLVAFSLWLAGGRGVRWGTGRSADIDGVDGLIIGGGDDISPDLYGMQLVTSA
RLDP
DRDALERRLVEAAIARGKPVLGICRGAQMLNVALGGTLDQDAYGTYTASQRVWTILPRKAVTVCPGTRLAEIAGPDAM
RV
NALHSQAVARLGDDLVVAAHDRGGMVQAVERRRDPFALGVQWHPEHLFYARRQRAIFAALVAAAAAGRRSHGQLS
AVDTM
LEKTTL
>OLS48963.1 cytochrome C [Rhodovulum sulfidophilum]AB14
MRKILLTAALAALPLAASAADVKDDVVEARQGYYSLLGANMGTLVSMVKGETEYDGAKAQTAADNMLILTKYNIGHLY
MP
GTSSEDMPGKTAALPKIWTDMGGVQEKGMAFVKAVENLQEVAGLDKGELAKGIQQVGGSCKGCHDNYRAKH
>OLS48964.1 hypothetical protein BV379_12210 [Rhodovulum sulfidophilum]AB14
MEYDAIILGAGAAGLMAAATAGQAGARVLVLDHADKAGKKILISGGGRCNFTNTGAEAACFLSGNPHFPKSALARYTQ
WD
FLALVERHGIAWHEKTLGQLFCDGSARQILAMLLDECARGGVDIRLATQIGEIGHGDGRFRVAGASAANLVIATGGPSIP
KMGATGLAYRIAERFGLAVVPPRPALVPLTLGPRDGAFRAIAGLSCPVTARVGPASFKEAALFTHRGLSGPAILQVSSYW
TPGMPVELTLLDNAPELLREARRQMPRARLRAALSEHLPNRLAELLATQIGLEKQLANCSDADLERAADPLARLTLHPTG
TEGYAKAEVTAGGVSTAALSSRTMAAKALPGLHVIGEAVDVTGWLGGYNFQWAWSSGWAAGMAIAERRAG
>OLS48965.1 EamA family transporter [Rhodovulum sulfidophilum]AB14
MRSSSRQASGLFPGIGAISAAVLLLSLSDALVKLQNGGLSLGQVLMVRSGTAVLCLAGMLWLFAQAGGAASGVTRWV
WAR
SLCLALMWGCYYAGLPGLPFSIAAACYYTAPIWMAVLSFLLLGTRIGARGGWAILLGFAGVLTILRPGFDALSPVIALPL
LAGFLYALAAVITQGRCRDVPPISMALNLNLVLVLAGAAHLAVLLWLGAGDEAGFLMSVWPGLGMHGWVLLIGLGVA
LAV
ITALVAFAYQRAPVPVIGLFDNGYLVFALVWSVLLLGERPDLVDLLGIVLIGCGALLASRRLPGGEEGGGEDARAPRAQP
RRV
>OLS48966.1 LysR family transcriptional regulator [Rhodovulum sulfidophilum]AB14
MQTDEFQLHRLRPFEAAARLESFTHAAEELGLTQTAVSRQIGQLEAELGLRLFERRNRAVFLTEEGRRLAQVVGDALAAI
RAEIAAIRGSRDPDAIVLRCQLCEAFYWLMPRLSRFHARHPEIQLQLVSALAPLTEATSPFDVALQTTGRASGSTRLLFT
AADTIFPVCAPSLLGDLRTPLDPEALLERPLLSHRVTPQDWFDWPDWFAAAGLAGRAKPRIVCFDSYPLVLQAAVAGQ
GI
ALGWNRTVRGLIDDGKLTPACRFTVERPNEISVFRGTRRSNYAAVDRLIAWLRSELS
>OLS48967.1 ADP-ribose pyrophosphatase [Rhodovulum sulfidophilum]AB14
MPAAAYEAALPSHSIPERPIPAAIAVVIRANQVLLVRRANPPDAGCWGFPGGKIDCGETLTEAALRELAEETGISAEPLR
VFTAVDAFDREASGALRRHFILIAILCRWTAGTPVAGDDALEARWVDLDTLADTGLALSLDVAEVAAQAAMLMQEMQ
S
>OLS48968.1 bifunctional hydroxymethylpyrimidine kinase/phosphomethylpyrimidine kinase
[Rhodovulum sulfidophilum]AB14
MIANILTIAGTDPSGGAGIQADIKTFSALGAYSCSAITAVVAQNTQGVRSFVALEPGFVAEQIDAVLDDVRIDAVKIGMI
ANAAIAEAVADRLRHHAVKNIVLDPVMVAKSGHALLAPEAVAAVRERLLPLARIMTPNLPEAAVLLGRGDDWTAEAM
QAA
LPELLALGPEWVLLKGGHLADSAESTDLLAGPGGVTPFAAPRLVTTNDHGTGCTLSSAIAALLPGRDMVEAVGTAKTYL
H
DALAASEALDVGHGHGPVHHFHALWPDHPAM
>OLS48969.1 phenazine biosynthesis protein PhzF family [Rhodovulum sulfidophilum]AB14
MIMYQVDAFAEEVLSGNPAAVLLLPDWLDEALMQAIARENNLAETAFAVARAEGGWELRWFTPTQEVDFCGHATLA
TAHV

LIAHEGQAAPLSFHTRVGVLHVTEGPSGYRLDLPRLDPEPLDALPAEVAAQFPGGVIAAFRNFENVFVELDGADRVRDY
V
PDLAAIAGLGPVGLVITGREGPGGRADFVSRYFVPGAGIPEDPVTGSIHATLTPYWAARLGRSRLTAYQASARGGWLDC
E
LTGTRVMVAGAAVTFMKAEISLPGVERFRA
>OLS48970.1 excinuclease ABC subunit B [Rhodovulum sulfidophilum]AB14
MPQSTEPHAPGGAAIAIPPREKLEGGKRFVMHSDFAPAGDQPTAIAELAAGVRQGDRDQVLLGATGTGKTFTMAKVI
EET
QRPAIILAPNKTLAAQLYGEFKGFFPENAVEYFVSYYDYYQPEAYVPRSDTYIEKESQINEQIDRMRHSATRALLERDDV
IIVASVSCIYGLGSPELYMAMTLDLEAGKTYDQRQIMADLVQQQYRRNDAAFDRGSFRVRGDSLEIWPAHLEDRAWKL
SF
FGDELESITEFDPLTGKKTDGFDKVRVYANSHYVTPKPTIRQAITGIRKELKQRLAQLTDEGKLLEAQRLEQRTQFDLEM
LEASGFCNGIENYSRYLTGRAPGEPPPTLFEFIPDNAIVFADESHVSVPQIGGMYRGDYRRKFTLAEHGFRLPSCMDNRP
LKFEEWDAMRPQSVFVSATPSAWEMEQTGGVFTEQVIRPTGLLDPQVEIRPVEMQVDDLLDEVRKMAANGFRTLCT
TLTK
RMAEDLTEYLHEQGIRVRYMHSDIDTIERIEILRDLRLGAFDVLVGINLLREGLDIPECGLVAILDADKEGFLRSETSLI
QTIGRAARNAEGRVIMYADRITGSMERALRETERRREKQIAYNEAHGITPETVKKNVDDILAGLYQGDTDMSRVTATVE
K
PMHGGNLEAHLDGLRTSMRKAAENLEFEEAARLRDEIKRLEAVDLAIADDPLARQSAVEEAVEASARSKGRSSAGRAG
QR
GGVRRRHS
>OLS48971.1 ETC complex I subunit [Rhodovulum sulfidophilum]AB14
MRARIYQPARTAMQSGQAKTHHWVLDFGPQSPRELDPLMGWTSSDDTQSQVRLRFETREAAEEYARRHGIETVVLR
PKKR
APNVRPMGYAENFSVTRRGAWTH
>OLS48972.1 hypothetical protein BV379_12270 [Rhodovulum sulfidophilum]AB14
MQWTLEQICAILKVSEEGLGAAIAEVLNELTAALGATRAQLLRPSADGRWLILEMVWQREPGPFTGLVRIEASALPAA
WW
AAFQAEAPLVCDDLANCDPALFPANSAALAGIGAVLAVPFSLDGRFAGALLLSAPAGRLLPEAVPTEALAALAQTIGSRL
GCADLAMPRTSRPLSDEAGGVAPAADAVSTADTVLTFDGDGRVTGCCSRDDRAVADGLEGLAGMMPEDFLLPEQTA
RLRA
AFDHCRIGGASSPFPLRLPTRAGPREMRAMVFERAAPETATEGASAGLGLLLQDVSGAGPDRRHAGMCARAMEAMS
VPAV
MTDASCRIAWMNAAFERRTGFGFFEALGSTPAELLRIEMPVPGDGDRLSAARLARRPIRIEIQCRTKSGAAILLDVSIVP
LFDAAGTCLGTFATAIDITERRAHAAALAGVRTAWDALVNALEALPDGFACFDAEDRLVICNARFREMLPMAGTPIVP
GL
GFRELFESALRQGMRRGDPEQRARWIAGREKARCGGARILEGERCDGGWLRLSDLPTSDGGRVVLCSDITALKRDARR
TR
EKLVAAMDASVDGVAITDSAGAYLYMNRACREMLGIGAGADIRAHVWPEFHSEEEVARIRAVSERPLQTRGIWTGLV
VAR
RTDGGSVEQEVSVTRQADGCKLCISRDVSERRRVERNALRLREELQLAQRRQVIAQVAAGLMHDFNNLLATVSGSATL
LR
NGDDAESNIARILAASAQAGALVNRLASLEQRAHRPDLIDLRDPLEEAVNLVRAGAGSGLSLTLRLPSEPMLAVADPTD
V
IQVFLNLLLNARDAICRRTQQACRGEIAVTAELAHGPALRRKPRIGQLLPDRRYFRICVSDNGSGIGWAVRRRLFEPYFS
TKGNGGTGLGLSIVEGAVTENGGAIGLQSRRGCGSRFSVFWPEAGEEPHEDLCAAGPPCGRLAGRRILLSDGDQEALRI
L

SVHLETAGAEVAASNNPEDILEAVRSDPENWDLLVTDFDMPRLTGADLAAAVRTYAPGLPALLVTAQPDRHLRSGAARRD
LFSGVLGKPVASVDLVAGAVAAIGGRQTER
>OLS48973.1 hypothetical protein BV379_12275 [Rhodovulum sulfidophilum]AB14
MKRFALGWTDRGADRIEVVGQAGMSDRDKRPGPAFALMILCVPAGWLTLIAALLLEYSLLTAVVAMFAVMWGIFVLGLIA
FLAVDALRARCRGSRREADAMVE
>OLS48974.1 hypothetical protein BV379_12280 [Rhodovulum sulfidophilum]AB14
MQDLLDEARELVDALRIYVLSDHVSDAASAVQGKAGRIGGDTGLIGRPPVRGAPQCEAARLLSLPIPEPAGDTDLGFLIV
ENRPSDRMPSSEELRCLHRLARRLGEILSARGLRLAVGAAQGAAGHVARRTGLGAAPSKDRAPAGIGADEGAQDQVPVVR
ARSGGERQAQMIERFVHDMANLVAVIDGSARMLSEEFGRSAHLHRILEANRQILAMMETLRAAGHHEIRHRWVDLGHLVR
AAADLLQAGWPEGIRLDLDLPGHEALALADPVQVSRIVLNLLCNARDGVARKAPGRAPEGRVLLSLRRQCDRPGAEGRVW
YEFYIVDDGIGMDADTRRNMFLPHFTRKGAAGTGLGIAFLDCQIRELGGRISVETALGQGTTIRILWPEVFK
>OLS48975.1 xylulokinase [Rhodovulum sulfidophilum]AB14
MVFSLGLDLGTTGLRALLLDDEGQPAGSAGRPCAVAHPHPGWSEQDPADWIAALEGAMAELADRFPKALARVRGIAVSGQ
MHGATLLDAGLAPIRPCILWNDTRSHAEAARLDALPGMREISGNIVFPGFTAPKLDWVRRHEPEAFARIAKVLLPAGFLN
LYLTGGAVADMSDSAGTSWFDTGRRDWSDALLEVGHMRRDQMPELVEGSAPAGRLRPELAARWGIAPGAVVAGGAGDNAA
AACGTGALAEGQGFVSLGTSGVLLAARDGYRPEAGKALHTFCHAVPGRWYQMGVMLSATDSLNWLSRITGQGPAELTAAL
GETLRPPGRVRFLPYLSGERTPHNDAAIRAAFTGLGAETGRDDLVRAVLEGVAFGLRDSAEVLRGAGAAPAQLLAIGGGA
RSRLWLRMIATVLGVPLEVPEGGELGAALGAARLGRVAATGAPPETVMTPPVIAATLAPDETLAPAFDEAYRQFRAAYPG
LRAIQ
>OLS48976.1 hypothetical protein BV379_12305 [Rhodovulum sulfidophilum]AB14
MARDQKTGPGHVPRPRMPDMSRPPPPTPRAGKSPIAREAAAAGPLGLGADVSAFLDTGVPGQLHREAIRRHWHSDPMLAS
LGACDPDKDKP
>OLS48977.1 mannonate dehydratase [Rhodovulum sulfidophilum]AB14
MRQTWRWFGPRDLVSIDDVRQAGAEGIVSALHHVPTGAVWTPEEIARRKAEIGTMADGTASGLTWDVVESLPVSEDIKKQ
TGDWRAHIEAYRDSMRNLAAAGIEVICYNFMPVLDWTRTDLAWKQPSGATCMRFDLTDFVAFDLFILGRPGAAEAYDDAL
REAARARFAEMPEAARDALARNVVFGLPGAAERFTLEDVRAHLAEYADMSEATLRGHLVDFLSEIVPLAEELGLRFCCHP
DDPPVPLLGLPRIMSTEAHYAEVLAAVDSPANGVTLCSGSLGARSDNDLPGMMERLGDKVHFLHLRNVTRETGATFGSFH
EAEHLGGDTDMVALVEAALREEARRRAAGRADWSIPFRPDHGQDILDDLGRHAQPGYPAIGRLKGLAELRGIIAALGPRI
G
>OLS48978.1 GntR family transcriptional regulator [Rhodovulum sulfidophilum]AB14
MDQHIDRDMRCVLDPSRPITPQLHHFLRDRIIRNVLKPGDRLSEAEIARSCEVSRQPVREAFIKLSEQGLLSIRPQRGTI

VTTIAFSTVLDARFVREAIEADIVRLLARRPADAALIARLRAQIERQRAVAGTAPRDFIQLDEQFHRTLAEAAGKSGAWT
LMEGLKSQMDRVRFLALGRFPVDKLIAQHEAIVEGIARADVSAADAATRAHLREILNDLPTIMADNPGFFDGAWADTD
TD
TDNETTGGDPI
>OLS48979.1 C4-dicarboxylate ABC transporter [Rhodovulum sulfidophilum]AB14
MTSAWKLPGLAAAALIAGLAATPTLAQEITLKLGHLANEDNPWHKASLKFGEELSALTDGRIAVEVYPNESLGKEIDLIN
GMQLGTVDMTITGESLQNWAPMAALLALPYAYKSIEHMDAVASGEIGQQIEAQIVERAQIRPITYFARGARELTSNRPI
E
SPDDLDGLKMRVPNVPLFVDVWKSLGAQPTPMAFSEVFTSLQNGTIDGQENPLALIRSANFNEVQDYVNLTDHVRSW
IYL
TISELTWAKLSPEDQAAVMEAASRAQAYERELFEQSLADDRAWLEEHGMTFVEVDNAAFAAKAKDAVLANVGADIRP
VVE
QIFAE
>OLS48980.1 TRAP transporter small permease protein [Rhodovulum sulfidophilum]AB14
MPKTERFIATVTALARIGAGLSFAVLIGAVLLQVIGRSLGNSPVWTEELTRFALLFLIAFGAGLSLRSGDMVNVDIVCEA
LPGRWPWRLRLLSALATGGMALYLLPHAWRFVSIGRMQSSPALGLKMSYVHVTVFLMLALLVLFAALRVLGMLTGTE
DGT
PEKAEDI
>OLS48981.1 C4-dicarboxylate ABC transporter permease [Rhodovulum sulfidophilum]AB14
MEVWVLFGLFVGGLVIGLPVAVTLGLSSLGYLLLAGIPPVVMPQKMYAGMDVFVLLSIPGFILAGNLMNQGGITNRIVR
F
ANALVGWIRGGLGLTNIAASMLFGGITGTAVADAASIGGVMIPGMKKAGYPADYSAAVTAASSTVGPIIPPSVPMIIVG
A
LSGISVGKMFLAGAIPGILMGFAMMVTAYVIARRKSFPRQPWQGAGEVVRSFGGAIWALAMTFLIIYGLLSGLSTPTET
A
IVASVYAVFVGCFIYRDLPFRRVPKVIIDSAVSSAGILALVGFANVFGWILVSERIPQAIAEGVLSITDNKFLVILIINL
LLLFVGMFMETIAALIILFVPLLSLAQAVGIEPLHFATFAVLNLMIGLTTPPVGVCLFVCAGIARQPLTQVVIAVLPFLA
TNIAVLLLVSYVPAIATWLPSVLIP
>OLS48982.1 L-idonate 5-dehydrogenase [Rhodovulum sulfidophilum]AB14
MDTRVCRLHAPHDIRIETLPLDGPAPGEVLVAVGAGGICGSDLHYYHDGGFGPVRVREPIVLGHEAAGTVIALGAGVSG
L
TEGQMVALNPSQPCGTCLYCQQGMFNHCLEMRFRGSAMRVPHEQGLFRDRIAIGAAQCVPVGPGISLGQAACSEPL
AVCL
HARRIAGDLTGKTVLVTGAGPIGALCAAVATEGGAAEVVVTDLQDVPLEVALQMGASRIVNIARDPAALEAYAADKGR
FD
VVFECSAAAPAIAQAIATLRPRGIFVQVGVAGETPVPLNLIVGKEIQVRGTHRFHPEFAEAVAAISSGRIDVRPMISASF
PLEDAEAAFEAAGDRTRAVKVHLSFEAP
>OLS48983.1 hypothetical protein BV379_12345 [Rhodovulum sulfidophilum]AB14
MGMAGVHWLAADWGPASLRVWAMTGHEVQAEGAADLSMEHLGPADFEPALLALADSMLPPGEVTPVLACGVAG
ARRGWIE
VPYARLPCRPSARRPVRAPSTDPRLSVGILPGLCQTEPPDVMRGEETRVAGFLTIRPDFDGTLCLPGTHPKWVRLCDGS
V
LAFRTAITGELYRLLVRGSILRRAFDDIWNDRLFHAAVDEAFCHPERIAGTLFSLRAGALLDPPTTGQIRGRLSGLLIGS
ELAAMRDMWQGRPVAVMGCERLSHLYATALDEQGAEVETIAHDRMTLAGLIATFEQTGAAVLTAPDAQPSML
>OLS48984.1 hypothetical protein BV379_12350 [Rhodovulum sulfidophilum]AB14
MAANRPAATPPSRRPSSRKHGGPKQRVWRMVHLGIDKETLEALAVEITGSHIGDAPMLPELLDKIPPEQEIGSVTADG
AY
DRGLFMTPQRGDGARIWRTPRCRRCPGDVRGEVAGLRRQ

>OLS48985.1 hypothetical protein BV379_12360 [Rhodovulum sulfidophilum]AB14
MPLLSFLSSERYVFDIFLRLSGALAVEKVGAIAKAICRYDRILVQKLKGGPFRQDISAKLQSEGTQAFSVSSVALSLCTE
HGDKALRDRLAIQLQAWLSPEQMPDRDM
>OLS48986.1 hypothetical protein BV379_12365 [Rhodovulum sulfidophilum]AB14
MRVNLRMALLNPKMFNLYVKEWHGRRACEGYKAGVAALVAGGIVVLMLNDAEFAASMAPLVKWGMIAVPAGFSIY
SIMAI
FFPSALFWLTHQHEDVKRALADKRHQ
>OLS48987.1 IS110 family transposase [Rhodovulum sulfidophilum]AB14
MQVTTVGLDLAKNIFQVHGVTETGEVAFNRPLRRAQVLAFDERLSRCLIGIEACASSHHWARELSMLGHTVRLMPPM
YVN
PYVKRSKSDAVDAEAICEAVTRPTMRFVEIKSEEQQALLSLHRARDFVVGQCTQLINMIRSLAAEFGITIARGVQRAIEF
AKTVIDGKQPGLPELAQDVLRVLSRQLLYLHNRLGWYEAMLRIQARLSRQAQLLQTIPGVGPVTASAVAATIGSGRQVR
S
GREFAAWLGLTPRNHSSGGKGRLGKIRKMGERYLRQLIVVGMTSRVRKVATHPERADPWLTKLLQRKPARLATVAMA
NNT
ARIMWAVLTGDEPYKAHAA
>OLS48988.1 hypothetical protein BV379_12375 [Rhodovulum sulfidophilum]AB14
MLCDLALPFPLRPARVHEVCGPVASSFAAGLAATVQGQVLWLRESWQGDTRNPLGFLPVLDPASVLLACPANQTDAL
AVT
EEALKDGAVALVVLEITRPLDLREGRRLQLAAGTGGTIGLCLIPEGMGSNAAETRWRATPVFDPEREDSTLMRWEITKN
K
MGTFGAWNVFWNAQAHRLDLVSPAGE
>OLS48989.1 nucleotidyltransferase [Rhodovulum sulfidophilum]AB14
MFSGTHRRIVSIWFPRLASDRVLRARPVEGPFALSQRIGSNVTIYCLNTDAERAGLHPGMRMADARSFCPSLISQPARP
D
QDARFLRLLRRWAERYCPWVGLEGADGLVLDITGSAHLFGGEAAMVADMQARLARVGVTAQLGLALSRGAAWAVA
HFGGS
EETLLSLPVAALRLPADTVIALQRLGLRKICDLTTSQRAPLARRFGQDLLTRLDQCLGHAPEPVTPEAEPPQFATRMTLP
EPIGLQADVQAALERLLTPLCQKLATQEKGARRLCLTMRRVDQASQHVELRLAAPMRDPERILPLFARRLEDVDAGFGI
D
QMRLEAVQVEKRAPPQSNALYKTAPGHATPEQLVDLITRIGTRIGLENIQRFLPAESHIPERAFLLAPAAFSAPQDGFTT
PRPRPLRLFPPEPIAAQGTDPPRAFRWRGMRLSLGRAIGPERIAPEWWSEEENWRRGLRDYWRMETREGRRLWMFY
TPQD
PGWFVQGEFA
>OLS48990.1 error-prone DNA polymerase [Rhodovulum sulfidophilum]AB14
MDVLHPARSGLVCAGGIRMSYAELCVTSNFTFLTGASHPEELVARAAELGLAALAITDRNSLAGVVRAYSALKTLREEAA
GLRIRSSAQVDTSSRQAVGRPQDLARPETLALPRLIVGARLVLRDCPLDWLALPIDRAAYERLSRLLTLGKRRATKGECH
LDLSDLAAGCGGMILIALPPLGAPETLRIEVIRAPLQTLQRAFPGHVFLGAAPRYDGSDQAWFRACADLALRANTPMVA
V
GDVLMHRAHRRPLADVLTCLREGLTIDTIGTRALPNGERRLKSAGEMARLFRHHPAAIRRTLEIAARCAFDLSELSYEYP
DEASNGEPAQIRLDRLAREGLARRYPDGASERVYKLMAKELGLVAELGYAPYFLTVHDIVAEARRRGILCQGRGSAANSI
LCYLLGITDVAPEMIGMVFERFISRHRGEPPDIDVDFEHERREEIIQWIYERYGRARAGLCATVIHFRARAAIREVGKVM
GLSADVTAALSSQIWGQSNQGADPARLREMGLNPEDRRLALTIRLIGETIGFPRHLSQHVGGFVITRGRLDDLCPIENAA
MEGRSCIEWDKNDIDTLGILKVDILSLGMLTCLRKSFDLLEAHKGERFTLATVPQEDRATYAMLTRADAVGVFQVESRA
Q
MNFLPRMKPETFYDLVIEVAIVRPGPIQGGMVKPYIRRRQGLEAPEPFGLGPALAAVTAKTLGVPLFQEQAMQIATVG
AG

YSAEEADQLRRSLASFRRMGTIEQHRERFIGGMRANGYAQEIAEACFAQIEGFADYGFPESHAAAFAMLTYVSSWLKC
HH
PEVFACALLNAQPMGFYAPAQIVRDVREHGVEVRPICLNHSTWDNSLERRADGRLALRLGFRQIKGLRAEDAGWIMA
ARG
NGYPDIESLWLRAGLAPATLERLAEADAFAGMGLTRRDALWAARAIRAPKPLPLFANPLDGEKLAEPAVPLPPMHLGE
EV
VEDYIALRLTLRAHPMELLRPGLPGITCHDQLPTAALGRTSVCGLVITRQRPGTASGVIFLTLEDETGVANVVVWPKLYE
RYRRAVMGGRLLRVSGRLEREGIVVHLIAEQIEDLSHRLSELGHPMPEKIGSTLPQADDAPRPIRPAPRARHPREQAKRL
FPSRDFH
>OLS48991.1 iron-sulfur cluster assembly scaffold protein [Rhodovulum sulfidophilum]AB14
MNEAELAALYRPRIRELAARVRSDRRLKDPEVTVSRRSQVCGSMITLDLMCEQGRITALGWRSRACTLGMASLGLVTE
VA
VGQCRAEVAAAGAALWDLLQGQEQIFAAPWEELSVFSAARGFPPRFGSIMLPFAAIDEGFRQMG
>OLS48992.1 pyridine nucleotide-disulfide oxidoreductase [Rhodovulum sulfidophilum]AB14
MQKHILVVGGGIGGTMTANSLVTKFQREIANGKVKVTMLSDNPWHYYKPAFMYVAFDMYFKGELRRKQQSLLRPEID
FQL
DKVEAFDFKGSKVKTASGKTISYDYIVVSTGCLPAPERIEGLKEGGDYFYQYEPARKLSDKLLTLEKGRVFVTVNFPETP
NVPHQCGIAPMETTLMLDEYLRRKGVRDQIEIVYTYPTVSQLLRNCLFMQEEVCSVLPPIFEERDIKYKRSFTLAKVDPE
RKVAISSEGEEEEFDILMSTPPIRAVDAVINSGLSQAANNEGWLPTDRDTLQLEGYPNAYVMGDTVDLPISKAGGSCHN
Q
CPVIVNNIAGDMRLGRIVEHYDGKVQAVAQMGLEAGMPLFYNYDKDVYPTPPTKVGGLLRKAFNRGIYWSVARGII
>OLS48993.1 hypothetical protein BV379_12415 [Rhodovulum sulfidophilum]AB14
MNDAIDSQQEPDAVRAAVQAQIIECLNRFYAMESGMWGKPLDSLIIRTVVQSRMQGKLYDFSALSEALDLPISTLHRKI
N
SLVESGYLRREPVGKSIYIAPTERTSVKFDESFEAMVTTLRRLYRGEYNFDGAPNLCDEP
>OLS48994.1 hypothetical protein BV379_12420 [Rhodovulum sulfidophilum]AB14
MATQIMGNVGTEETDDFNAFRKAVIPGRRQHVLLSSDQDNAITVGTRQGRPVVLRIDAAGMHSDGFVFWRADNGV
WLTLS
VPPQNTWFSEIPSTVLERVPSPSP
>OLS48995.1 benzoate transporter [Rhodovulum sulfidophilum]AB14
MSSHSSPSSVRLNDLTHPIVAGLISVIVNYGGTFILVFQAAKIAGLSPELTASWVWSVSIGVGLTGLFLSLLYREPIITA
WSTPAAAFLVTALATTPYAEAVGAYMISAAAFVLLGMSGYFEKVIRLIPPGIASGLLAGILLQFGIGAFGGASVDPLLVG
LLILAYVSLKRFSARYAVVGILVLGLAFLLTQGRVDLSGLRLEFAAPVFTMPEFSLNALLSVALPLFLITLTGQYMPGML
VLRNDGFKTSANPIVAVTGLGSLIMAPFGSHAFNVAAITAAIATGEEAHEDPSKRWIAGIAAGCFYVLVGVFGVTLAAVF
MAFPATFITTLAGLALLGTIGGSLAGAMAEPTSREAALITFLASAANIELLGIGGAFWGLLIGLVAYLVLNGRLPHRWHS
DLSVSEGAAK
>OLS48996.1 GntR family transcriptional regulator [Rhodovulum sulfidophilum]AB14
MFRHSQLESVKAWIAHPAHAAMPLHARIQRAIRQLIVDGALGPGKPLPASRALAKSLGVSRDTIEAAYTQLHAEGFIER
R
VGSGSFVAEMTEFTPGRRLTQRDALLRNQAPNLSKRGAAMFRSSGVREMLTPRPFAHGVPETRTFPLRLWERLERQV
RKE
VGAEALLHGDPQGTGPLRRAIADYVNLERGARATADRVLVLTSSQQALSLCANMLLDPGDRIFIEDPAYYGARKAFDAA
G
LECVPIRVDRQGILVDQVMAEPRRTKAVFLTPSHQFPTGATLALDRRLALIEWAARHQTWIIEDDYDSEFHYAGKPTAC
V
QGLDPHDRTIYIGTFTKALFPGLRIGYVVLPPQLVKPMTVARTLLDGHTAPMAQLTLARFMEGGHFGAHVRTMRGVYA
ER
LDLLAGLVRKHLSDFIEPRVPIGGLQMPCVLTCGLSERTAIDAARRVGIELLGLSALHAAGDGKAGFLMGFAAYTHLEIE

VAVRKLENALRAVTKP
>OLS48997.1 hypothetical protein BV379_12445 [Rhodovulum sulfidophilum]AB14
MDAHMLTALILTPFVLAFGYAGVHEYLRFKSDGRATYGLVFDEETGTSYVTGIGEEEEAYDPEEFNPDDYNDPVTAEEPE
ESRA
>OLS48998.1 YeeE/YedE [Rhodovulum sulfidophilum]AB14
MTDSSQAMPVSSRLLPRTAGLGLLALAVLAVAAMAGPRSGALLAIGLGFGLVLEGLRFGFTGPWRRMILQRDASGLVA
QM
ACLALVAAAAFPMLASHPGELLGAHAPVGFAMLGGAFVFGACMQIVLGCGSGTLVNAGSGNLVSVVALPFFCLGSFF
GAY
HLVWWTGLGALPVAVLAGSDGLALTLVLLASVAGLALALADPGKRAIPGRYWAAAILLAGLAILHLAVAGQPWGVVYG
LG
LWVAKGASALGADLSGSAFWSAPGNVERVRESVLTDVTSLTDLGLIAGAALAAFWRDGLSGRIADHPARAWVAVVIA
GFL
LGYSSRLAFGCNIGAFFSGISTGSLHGWAWFAAAFTGSWYGIKLRPWLGLEGRS
>OLS48999.1 sulfurtransferase [Rhodovulum sulfidophilum]AB14
MTRHAPSAAFALALLAGPALSAPDFGPLIEPRELADALDAPAPLVLDIRTGPDGAAPVFEAGHVPGAISAPYGLFRGPAE
NPGQLIREDRLTEVLRSLGVTRDRPTVIVHQGSGASDFGAAARVYWTLKSSGVSALAILNGGMAGWNAAGLRVETGP
ALA
PAPSDIEVSFSHEWLATTEDVEAIVAGKAEGTLIDARPEPFWAGNAKHPAAFRPGTLPQSRPLPHSGWFEADDPARIAP
E
AAAGLAAANHLGADDAPVSFCNTGHWAATNWFALSELAGIEGVRLYPESMVGWTRSGHEAANVPGPLRNLWNRLR
GRY
>OLS49000.1 cytochrome C biogenesis protein [Rhodovulum sulfidophilum]AB14
MFGIELIDAGLMPAMLVALAAGLLSFLSPCVLPIVPPYLAYMGGISMGELTGAGQVRRRATVAALFFVLGLSTVFLLLGF
TASAFGAFFLQNQTLFARVSGAVVVVFGLHFLHVIRIPVLDREVRVDAGDRGGSALGAYLLGLAFAFGWTPCIGPQLGA
I
LSLAASEASLSRGTILLAVYAAGLGIPFLLAALFIQRAMGIMNRLKRHMALIEKAMGLLLLAVGLALLTGAFSSFSYWLL
ETFPALARLG
>OLS49001.1 preprotein translocase subunit TatB [Rhodovulum sulfidophilum]AB14
MGPEIEIDLRGLKCPLPVLRARKALAALAPGNLLRLWADDPVAVIDIPHFCREAGHELVGTEETGTATLYLIRKG
>OLS49002.1 ribonuclease [Rhodovulum sulfidophilum]AB14
MAKKMLIDATHAEETRVVVVDGNKVDEFDFETVNKRQLAGNIYLAKVTRVEPSLQAAFVDYGGNRHGFLAFSEIHPDY
YQ
IPVADREALLAEERALAEEMDDDDGDSDKPKRRSRSRRKPRADKSASAASDSASGEDGAGRNVDGAEDREDHVAPAA
SDD
DTAPTDAEPADGVPAAAETEAAEAEDAAPAEDADEDATDRAGKDDEPPTTVMAMAAHSDDPVETREPSAGIPGMD
VVDLG
DDEESEDPAEEDNSAETRKSSSRRRRRSPRKKTDEAEDTASSAEEAGEDGPEEIHHDDQIESVAEEDVHEEIRPRKPRAR
RYKIQEVIKVRQIMLVQVVKEERGNKGAALTTYLSLAGRYCVLMPNTARGGGISRKITNAADRKKLKEIAAEMDVPEGA
G
LIIRTAGAKRTRSEVRRDYEYLQRLWEQIRELTLKSIAPAPIYEEGDLIKRSIRDLYSREIDEVLVEGEGGYRLAKDFMK
MIMPSHAKNVKNYTDPMPLFARYQVESYLTSMFNPVVQLKSGGYLVIGVTEALVAIDVNSGRATKEGSIEETALKTNLE
A
AEEVARQLRLRDLAGLIVIDFIDMEERKNNAAVEKRIKDKLKTDRARIQVGRISGFGLLEMSRQRLRPGMIEATTQPCPH
CHGTGLIRSDDNLALSILRQIEEEGVRGRSREVLLKAPIGIINFLMNQKREHVAQIEARYGLSVRVEADPFLISPDFSIE
RFKTATRAVPEVVSPVVSLDMSDMPDAVEEEDEEEAPVAVAAPAPAPEAEDKTSKKRRRRRRRKKPSQGGDDQGYEA
GQD

ESDDADSDGDTDAEAPEAVTAEASNEAASAEEKPAEEKPRRSRRSRSRGRKTERPEAGAEARAEESVAPKAEEPAAVEA
Q
VSAEAAAPVEAPAVEEALVEEAAVEDVAVEAAEASPETVELPEAELAAEAPVAEAAVEVEEKAVPEAEPAPKPEPEPETA
EASKDSAPKRQGWWSVGR
>OLS49003.1 DNA-binding response regulator [Rhodovulum sulfidophilum]AB14
MPQAMKIAIVDDEQDMRQSISQWLALSGFDTDTYARAEDALKEIGPDYPGVVVTDIKMPGMDGITFLKRLMSQDSSL
PVI
MITGHGDVPMAVEAMRIGAFDFMEKPFNPDRMTELAKRASQTRRLALDNRALRRELGDGSVLVRKLIGSSAVMERLR
EDI
LDLGQADGHVLIDGETGTGKTLVAHALHAVGPRAGRKFVQISCAAFAPEALAARLFGPIEDGSNTLPAVEEARGGTLCL
E
DVEALSPALQARLLTFINEQGSPAETRIIAVSNLQEQDKTVEDVLRPDLFYRLAAMRITPPPLRNRGEDMLTLFNRLSEQ
FSEEYGCDAPEVTAQEAAQLMQAPWPGNVRQLINIAERAVLQNRRGSGTIASLLMADNEASQPVMTTEGKPLKEYVE
AFE
RMLIDNTMRRHKGSIAAVMEELCLPRRTLNEKMAKYGLSRADYL
>OLS49004.1 two-component system sensor histidine kinase [Rhodovulum sulfidophilum]AB14
MSNARRSPYRSAFPLRGGWFLRLAIFALISTAVATVWTTNAWLTERFTESTRSRAQVRMALYSGNVVSELQRASVVPLL
L
SRDPTLIRALENVDFSQSSQLLISYVDEIGAASLQLLDDSGRVVAATDRSKIGTNLRSAPFFVEALRSNDTVFITSRAEA
GNFTFTYSRAIMVDRRKAGVIVVGVDLAKFEQSWAGISDAVIVTDSEGQIILATEPRWRGHKVEEALEARSAPSAIARAI
QATADWGALPPDAYVRGEAVMRNEARIPFRGWRIISFTPYVSIRDKVNAVLALEIMGFAMLLALSFYLLSRRAQMKSL
GY
QRETVELRELNARLQREIAERERAEKNLEVAEQTLEQSSKLAALGEMSAAISHELNQPLAAMKTYLAGARLLLQRKRPEE
ALSSFQRIDDLIERMGAITRQLKSYARKGGDAFEPLDMRFCVSAALSMMEPQLKRRKVTITRSVPREPVMVMADRIRLE
Q
VIINLLRNALDATRESPEPRVDLILSAGETATLTVRDNGHGIEDIDKLFEPFYTTKQAGDGVGLGLAISSSIVADLGGRL
TARNAREEGAVFEVQLPVLNHDKEAAE
>OLS49005.1 phosphoribosylformylglycinamidine synthase I [Rhodovulum sulfidophilum]AB14
MKAAVLVFPGSNCDRDLAVAFRNAGAEVSMVWHKESSLPEGVDVVGIPGGFSFGDYLRCGAIAARSPITRAVIEHAAR
GG
YALGICNGFQVLTETGVLPGALMRNAGLKFVCRSVPLTVGTADSAFTSGYAQGQRIEIPVAHHDGNYVADADLVARLK
DE
DRIAFSYAEPVNGATADIAGILSENRRVLGLMPHPERAAEPAHGGTDGAALFRSLLGALVPA
>OLS49006.1 alcohol dehydrogenase [Rhodovulum sulfidophilum]AB14
MRAAVLTGWRKPLEVSKVDDPDCPRDGVVLKVLACGICRSDWHSWTGADPDVALPLIPGHEYCGVVEEVGPAVTRW
QVGD
RVIAPFILACGHCSDCASGHQTICAHQLLPGFTQAGAFAELIAVPFADANLTSLPETMEPALAAALGCRVTSAWQALTG
R
AELRPGEWLAVFGGGGVGISALLLGRALGARVAVVDVVPDKLDFARALGADATIDARQGDAAEAIREITGGGAHVALE
AL
GIPETTIGAMTSLRKLGRMVQVGMPTGKNASMELPMDTVYARQLAVFGTRGMATWRYPSLLSLIDGGHVDLTPLIGR
HVK
LTEASAELAAFDAPAPPGVAVITNFLA
>OLS49007.1 phosphoribosylformylglycinamidine synthase [Rhodovulum sulfidophilum]AB14
MKARVHVMLKDGVLDPQGEAVRHALGALGFEGVGGVRQGKVIDLDIEATDRPEAEAQVREMCEKLLANTVIERYDIEI
V
>OLS49008.1 phosphoribosylaminoimidazolesuccinocarboxamide synthase [Rhodovulum sulfidophilum]AB14

MARRKKIYEGKAKVLYEGPEPGTLVQYFKDDATAFNAEKHDVIEGKGVLNNRLSEFFMSGLNAIGVPTHFIKRINMREQ
L
IRQVEIIPLEVVVRNIAAGSMSKRLGIEEGTQLPRPIIEFYYKDDALGDPLVTEEHVIAFGWASQQDLDDMVALALRVND
FLSGIMVAVGIKLVDFKIEIGRIWDGDFMRLVVADEISPDSCRLWDVKTGQHLDKDVFRRDLGSLTDAYTEVAKRLGVL
P
TNVTHASKPTLIN
>OLS49009.1 aldolase [Rhodovulum sulfidophilum]AB14
MTTFDDRELAFENKFAHDSELQFKAEARRNKLLGLWAAELMGLSEEASLDYAKQVVKADFEEAGDEDVFRKVSGDLG
DRT
DEPTLRAKMAELMAVAKAQIMSES
>OLS49010.1 methionine synthase I [Rhodovulum sulfidophilum]AB14
MTDALSRLLAERDWLMADGATGTTLFNMGLGSGDAPEFWNADHPDRIRALYRGAVEAGSDIFLTNSFGGNAARLRL
HNAQ
GRVGELNRLAAELGREIADAADRPVVVAGSVGPTGDIMEPMGPLSYARAVEIFHEQAEALKAGGADLLWVETISATEE
FR
AAGEAARLAGMPWCGTMSFDTAGRTMMGVTSSGLADLVEKLPCPPLAFGANCGVGASDLLRTVLGFAASGSERPVI
AKGN
AGIPRYVDGHIHYDGTPELMADYAVLARDCGATIIGGCCGTTPEHLKAMRAALETRPRGARPSLDEITERLGGFSSASDG
TGESESPARAPRRRRRS
>OLS49011.1 5-methyltetrahydrofolate--homocysteine methyltransferase [Rhodovulum
sulfidophilum]AB14
MANEDDDIVLAELPDEELVPQMQDDLYDGLREEVVEGVEILLSRGWAPYRVLTEALVSGMTVVGRDFRDGILFVPEVL
LA
ANAMKGGMGILKPLLAETGAPRMGKMVIGTVKGDIHDIGKNLVAMMMEGAGFEVVDIGINTDVDGYLAALETEQP
DILGM
SALLTTTMPYMKTVIDTLAAKGIREDYIVLVGGAPLNEEFGRAIGADAYCRDAAVAVETAKTFVARKHNRAVAS
>OLS49012.1 hypothetical protein BV379_12530 [Rhodovulum sulfidophilum]AB14
MNAPDDHRLTETGLPPEGRGRVLVIGCGALAHEILALKRINHWDHLDLTCLPAIWHNHPDRIAPGLRALVERHRAAYPT
I
LVAYADCGTGGDLQRTCAELGVEMMEGPHCYAFFEGAAASADRAGDEIDAFYLTDFLTRQFDAFVWRPLGLDRHPEL
ATV
YFGNYRRLVYLAQTEDAALDRQAEACAARLGLAYERRPTGYGDLAGFLAGRG
>OLS49013.1 enoyl-CoA hydratase [Rhodovulum sulfidophilum]AB14
MTEILLRDDTRGVAILTMNAPERLNALSDAMLAALQAQIDALAEDRSVRAVILKGTGKAFCAGHDLKEMQAGRQALD
GGR
AYFADLFERCARVMTGLMRLPQPVIAAPHGIATAAGCQLVASCDLAVAAEGTRFGVNGVNIGLFCSTPMVALSRNIPR
KH
AFEMLTTGQFIDAARAEALGLINRAVPEDMLEDAAWRLAETLAGKLSPAIRVGKGLFYDQLDCGIDEAYARAGAAMVE
NM
MLEETQEGIGAFLEKRAPAWQG
>OLS49014.1 hypothetical protein BV379_12545 [Rhodovulum sulfidophilum]AB14
MTWHDPLWQRIWPPARRVAFAIVGRLWWRRPPEEIAARLEARPLREQALIVGTVLAGLFLTSLLFAHAGIPGLLSFLLLV
ILLIR
>OLS49015.1 carboxypeptidase M32 [Rhodovulum sulfidophilum]AB14
MTAFDELMSFQRETEALGQVAMRLGWDQETVMPRGAAQQRAEEMGALETVLHRRRSDPRLGDWLGAIDDDLLDE
TGRAQM
RHIRRSYARATRLPETLSAEIARVTSRAQGIWAEARAADDVAHFLPTLREVVRLKREESAALAMGGDLYDALLDDYEPGA

TAAGLAAMFDRMRPRLVELRERIMGSGRAPAPLEGHFPEAVQMALSREVATHFGYDWRHGRLDFSVHPFTSGAGSD
VRIT
TRVDEAEPLGALYSTLHETGHGVYEQGIDPAYGLTPLGAGVSMGVHESQSRIFENQLGRGRAFCGWLHGRMNEVFGS
AGA
ADAEAFYAAVNRVHSGHIRTESDEVHYNLHIMLRFDLERDLIAGRLEVEDLEEAWNTRFEADFGVAVDRPSQGLLQDV
HW
SVGLFGYFPTYALGNVYAGCLYQALRAAVPSLDADLARGHAKPATAWLKAEVQRHGGLYEPAETIRRACGFEPDEGPLL
D
YLDAKFGAIYGL
>OLS49016.1 hypothetical protein BV379_12555 [Rhodovulum sulfidophilum]AB14
MSKTRSKPKTRSIFEEVGTDAPPDRPVVQPGAIDRAARRGARGAVAWWLISIFVLIAAMLISAGVARLTGHPASLPGFV
P
PYGQLPPLDASGWSAAFDRFAAAGGTADLAGFQRLYWWDWGVRQVGTLAALVWALGALGFLIFGKLPKGWAVRLL
PLGGT
ILGIGAADWAMGPAGLLPAPFDAASLRLAAMTGFGFAGMGLAAWGAMQLRRTEAQLMQARRARDGRAFGMATG
VMHLAFL
QILFGAMLGALDQGRSFPDWPLMAGGILPPEPLALEPVWRNIWDNPGLVQFIHRGLGYLVVLLGVAAWLRGRKSPHA
RTR
RAFDWLGVMIFGQAVLGVGSVLYAGQWHMALTHLVGAVLLWVLVLRARFMAQYPVQQSIRG
>OLS49017.1 RNA methyltransferase [Rhodovulum sulfidophilum]AB14
MTAAQPVFVLVRPQMGENIGAAARAMWNFGLDRMRLVAPRDGWPNPRAVALASGAGRLLDNAGIHATTAEAVAD
CDYVFA
TTARPRGLTKPVVTPEQAMTETRALLAEGRRVAVLFGPERAGLENDDIARANAIVNVPVNPDFASLNLGQCVLLSAYE
WR
RQTADILPERLELAGADPASQIEVEKLAEHYEERLSEAGFFFPETKAPGMKIALRNMWSRMPLTRPDVQILHGVLRQLTR
ARSRGE
>OLS49018.1 thiamine phosphate synthase [Rhodovulum sulfidophilum]AB14
MAEPERPQIYLITPPAFDPDSFAPRLAAVLDAHEIACLRLSMASHDEDLVARAADALREIAHARDIPLVVEAHVGLVERL
GLDGVHLTDGQRSVRKVRKLLGADAIVGAFCGALRHDGIGAAEAGADYIAFGPAGESGLGDGSRAEADLFGWWSEMI
EIP
VVAEGALDATTVAALSPLTDFFALGEEIWREEDPVSALGALIAAMG
>OLS49019.1 1-acyl-sn-glycerol-3-phosphate acyltransferase [Rhodovulum sulfidophilum]AB14
MPRIGVAGWLRVALRGTALVGLLAIGMALKLTLRLAERPLFGLRRPVTPWITQGVSRAALAVLGLRCRMRGRPMRAR
GAW
VANHASWLDIFALNARGRIYFVSKAEVASWPGIGWLARATGTVFIARDKREARAQADLFEARLKAGHRLVFFPEGTSTD
G
RRVLPFKSTLFAAFFGAGLREILWVQPVTVIYTAPEGEAAAFYGWWGDMGFGPHALKLLASARPGRVELVFHPPLKVA
DF
PDRKALALACEEAVRSAAPKL
>OLS49020.1 ornithine-acyl-ACP acyltransferase [Rhodovulum sulfidophilum]AB14
MQPPEDFFQLRLARTEADLMAAQRLRYEVFVAELGADGPLVDHEARLERDDLDPHFDHLLLIDSRRDAATLDHVVGVY
RL
LTSERAAALGKFYSEDEYDLSALKASGRRLLELGRSCVHPDYRGGTALYHLWNGLADYVLDQQIEVLFGVASFHGTDAE
A
LAEPLSYLHHRHLAPPELRVRVLPDHVQNMDLMPEAAIDRKRAMLAMPALIKAYLRLGGFVGEGAFIDRAFNTTDVCLL
M
DTARMNAKSRSRYMRGQE
>OLS49021.1 DUF3553 domain-containing protein [Rhodovulum sulfidophilum]AB14

MRPMNAILEPGMLVRHPGEPDWGLGQVQSVIGARITVNFTDAGKVVIDGTRIELMPVFSS
>OLS49022.1 hypothetical protein BV379_12585 [Rhodovulum sulfidophilum]AB14
MSDLTALVGSRICHDLISPIGAIGNGMELLAMAAAETHGEAPGEELALISDSVARANARIRMFRLAFGAAGEDTAVAGR
E
VCEILADYFGGSRLSVTADPPASLSRSAAKTALLSVLCAETALPRGGRMALSWRDGRITLSAEAERMTLDPRLWAPLTD
G
QPSGDDLRPAEVQFALLPIALADLGRSLSLGHDETSLTLAF
>OLS49023.1 glutamate-5-semialdehyde dehydrogenase [Rhodovulum sulfidophilum]AB14
MKDITEIHALMADLGARARAASAELAFASSEAKRHALEAAADAVIDRKPQILADNATDMEYGRDKDLSPAMLDRLML
DEA
RIEAIAAGLRAVAAQPDPVGQVMAEWDRPTGLHIRRVRTPLGVIGVIYESRPNVTADAGALCLKAGNAVILRGGSESFH
S
SGALHECLVAGLAKAGLPEDAILRVPTRDRAAVSEMLTMTQYIDVIVPRGGKGLVGLVQREARVPVFAHLEGIVHIYVD
K
AADPAKALSVVLNAKTRRTGICGAAECLLIHKDVAATLGADLVAALVKAGVRVHADEALSRLPGTQPATAEDWGREYL
DM
DIAARVVDDIDGAIEHVRAFGSGHTDAILTEDDAAAARFFERLDSAILMRNASTQFADGGEFGMGAEIGIATGKLHARG
P
VGAEQLTSFKYLVEGDGTLRA
>OLS49024.1 glutamate 5-kinase [Rhodovulum sulfidophilum]AB14
MASLIATRRLVVKIGSALLVDRSTGNLRADWLKGLAEDVAMLKARGTDVVLVSSGSIALGRGVLGLGLGPLALEQSQAA
A
AVGQIRLARAYEDCLEPYGITTAQVLVTLEDTEDRRRYLNTRSTMETLLGFGAVPIVNENDTIATDEIRFGDNDRLAAQV
AVTVGADVAVLLSDVDGFYTANPMQDPEAHRFDLIEEITPEIEAMAGDAGSGLSKGGMKTKLMAARTATGAGCALAI
TEG
SRLRPLKALEEGAACTWFRPKGDPRAARKGWIAAMKPHGFIRVDAGAAGALTRGKSLLPAGVTEVTGDFGRGEPVAIL
GP
KGEGLGAGLSRYTSAEARLILGCRSGDIEAILGYPGRAALIHRDDMAI
>OLS49025.1 GTPase ObgE [Rhodovulum sulfidophilum]AB14
MKFLDLAKVYIRSGSGGNGCTSFRREKYVEFGGPDGGDGGRGGDVWVEAVEGLNTLIDFRYQQHFFAQNGQGGMG
RQKSG
ASGRDMVLKVPVGTEILDEDEETVVADLTELGQRVLLAQGGNGGWGNLHFKTSTNQAPRRANPGQEGVERTLWLRL
KLIA
DAGLLGLPNAGKSTFLAATSNARPKIADYPFTTLHPNLGVVGVDGAEFVIADIPGLIEGAHEGRGIGDRFLGHVERCAVL
LHLIDGTSEDVVADCGTVLTELELYGGDLADKPRVTVLNKIDSLDEDERAEKRAALEAASGGPVMLMSGVAREGVTEVL
R
ALRAEIDEDRLRLKREEGEAWRP
>OLS49026.1 hypothetical protein BV379_12605 [Rhodovulum sulfidophilum]AB14
MRGAADGFRIETPRLVLRAFRPADLDALIRIGRDPRVAPMMCSLPVRWTRRQAATWVARSRFRGEPAFRLAIDGRDE
GLL
GVAGLGGAPASLAYFLDPAVWGRGYATEAMRGFLAAVFGRFPGLGVLAADHFADNPGSGRVLGKLGFERLGPGTGR
SLAR
LEPAPNIHYRLMRANFEALHEIP
>OLS49027.1 N-acetyltransferase [Rhodovulum sulfidophilum]AB14
MKLDLIPPQPPIAAERFVLRPVRRSDAGLLSLYTSDLRVARFTRSIPHPLPPGATEAFIHRAQDPERSEDVWVMDGAEQ
G
LSEVLGVIALDRMERDQSEIGYWVAPAFWNTGIASEAVGALLAANPQKAKTVFAEVFQDNPGSARVLTNAGFEYIGDA
EA

YSVAREAKVATWTYLKKLD
>OLS49028.1 50S ribosomal protein L27 [Rhodovulum sulfidophilum]AB14
MAHKKAGGSSRNGRDSAGRRLGVKIYGGQAVASGNIIVRQRGTKFWPGEGVGMGKDHTIFATCEGAVTFKKGLKGR
TFIS
VLPVAEAAE
>OLS49029.1 50S ribosomal protein L21 [Rhodovulum sulfidophilum]AB14
MFAVLKTGGKQYKVQSGDVLRVEKLAAEAGETVQFNEILMLDGQVGAPIVDGAAVQATVIDQIKGDKVIHFVKRRRK
HGS
QRTKGHRQQLTLLRVTEILASGGAETGVKAAIGAGSVAGAPVAEPKAAKAPKAAEAAAPSAGDDLKKLPGVGPAMEKK
LH
EAGVTRFAQIAAWTDEQVAAMDTRLGLKGKIVNDGWVALAKDLAGKA
>OLS49030.1 hypothetical protein BV379_12625 [Rhodovulum sulfidophilum]AB14
MLRLVAATALIAVLAGGAARADEAAETASLQVLVEALALPELVEVMREEGLVYGEGLEDELFPGRGGARWEAAVDRIY
DG
DRIEAEMRAVLDSRLGAAEIAPLVAFFTSPRGARIVALELSARKAMLDPEIDRASRERLEEMQADGDPKLDLVGRFIAVN
GLIEANVAGALNANYAFYTGLVDGRAFDYPVTEDQILDDVRRQAPEIREDTRTWLFSYLTMAYAPLDAATMEAYIGLSE
S
RPGRDLNDALFEGVEAVFEDISHELGLAAARFIAGQDL
>OLS49031.1 hypothetical protein BV379_12635 [Rhodovulum sulfidophilum]AB14
MIKWRDMKGMDRPVEGNVTVTLRHTKGRKVRTITLRQQAIDILLQIPRSNRSPHVFWNRTEEGYYKAAANLFRGYAQ
ETE
FGARLHDIRHKFAIERLKEGWSVHRVQRYIGHGAVKTTERYYFRYLSQEEQEIDNADGNVGL
>OLS49032.1 transposase [Rhodovulum sulfidophilum]AB14
MKRSRFTEEQIIGMLKEQEAGMTTADVCRKHGVSTATFYKYKARFGGLEVSDARRLRALEDENAKLKKLLAEAMLYNAI
L
KDVASRMRAA
>OLS49033.1 IS5 family transposase [Rhodovulum sulfidophilum]AB14
MDSFARKRNTKLLAIADAEGQPLRLFMTAGQVSEDTGARALSSSLPKAQWLLGDRGCDADWFREGLKDKGMRVCIP
GRKG
RKTAVRYDRRRYKRRNRIEIMFSWLKDLRRVATRYD
>OLS49034.1 hypothetical protein BV379_12655 [Rhodovulum sulfidophilum]AB14
MVARVLMGRKARIGREALMPGLVEERHCEEGAWLTVGQGCPNLGIDPVAVLWVRRLGPMRRGKAAGRNGARCSV
AMCRDD
GSDDGYVLMEVGLMEIGFTENGRADAGMTIVARLRGQRPGRHCECCDRSLARWAVGELKMRHGPPPRGRALRQLPI
W
>OLS49035.1 hydroxyacid dehydrogenase [Rhodovulum sulfidophilum]AB14
MTDTPLLICGAAFTEDERARLSTAVSSVSVESPAALEALAPGLRERLRVVAYKGQAPFGAREMAALPGLGLIANFGVGY
D
AIDLAAATARDIRVTNTPDVLNDDVADMAVGLILAQARCLLRGDDWVRSGRWGKYGEMPLARKVSGARAGIVGLGRI
GRE
IADRLAAFKMEIHYHSRQQKETPGWRYHADPVSLAGAVDFLVVALVGGEETRNYVSAEAIAALGPTGVLINISRGTTVD
E
GALLAALEQRRIAGAGLDVYLDEPAIDPRFQGLDNVVLQPHAGSATVETRRAMAELQLANIRAFLDGEPLPSPVN
>OLS49036.1 2-dehydro-3-deoxygluconokinase [Rhodovulum sulfidophilum]AB14
MPEHSFLAIGECMVELAPDRDGLFRMGFAGDTFNCAWYARRLLPETWSVGYATAVGADAVSDDMLAFMAVEGIAT
QAIRR
IEGRTVGLYMIALKDGERSFSYWRGQAAAKAMADDPDWLDRAFAGRDLLHLSGITLAILAPEARERLCAALGRARAAG
AR

VSFDTNVRPRLWRDPEEMRAGLMLGASVADTVLPSFDEEQAAFGDASPGATVARYREAGATTVVVKNGAAPIRLWA
EGAE
AEIAPVPVAQVVDSTAAGDSFAAGFLAARATGASPPEAVAQAAALAARVIGQRGALAPGLFADGAEP
>OLS49037.1 isomerase [Rhodovulum sulfidophilum]AB14
MRFSANLGFLWRELELPDAIRAAAGAGFSAVECHWPYDAPRSAIRAALDETGLPMLSLNTHPGDLAVGEFGLCALPGR
AA
EARAAIDQSIAWASALGVPQIHAMAGCARGPEAEAALAATLAYACLEAAPYGIGILIESINPHDVPGYALASTAEAVAMI
ERVAAPNLRLIFDCYHVQRAEGDVSARLRALMPLIGHVQAASVPDRGPPDHGELDYGHVFGLLERLGHAAPVGAEYRP
DG
PTGDSLGWMARFAANAG
>OLS49038.1 RNA methyltransferase [Rhodovulum sulfidophilum]AB14
MTAAAPFEIFLAALPGLEPLLAEEARAAGFATPAAVPGGVTVTGGWPEVWRANLVLRGASRVLVRIGAFRAFHLAQLD
KR
ARKVDWAAVLRPDRPVRVEVQCRASKIYHARAAAQRIERAIAETLGAPIGGEEAIALKVRIDDNLVTLSLDSSGEPLHRR
GHKQAVGKAPLRETMAALFLRACGFDGQEPVLDPMCGSGTFPIEAAEIAAGLAPGRDRSFAFEDFASFNAEVWATMK
APA
PAIPAEARFFGSDRDDGAIRMATANAARAGVADRTDFRRQAVSDLIRPEGPAGLVMVNPPYGARIGDRKLLFALYGAL
GA
TLRDRFAGWRVGLVTSDEGLARATALPFLPPAPPVAHGGLKVRLFRTAPLP
>OLS49039.1 hypothetical protein BV379_12680 [Rhodovulum sulfidophilum]AB14
MGLILLRHTRPDVAEGTCYGRTDYPPASSFAEEAAEVLAALPVVDRILTSPLMRCTRLAEHIGAARGLPVAADPRLIEID
FGAWEGIPWAEVPREGLDHWAGHFYESRPHGGESVGMFFARVVPFLEEMSGPGRVLAVSHAGLMRAALHHAGREG
AWQMK
FPYGSFIELP
>OLS49040.1 adenosylcobinamide-GDP ribazoletransferase [Rhodovulum sulfidophilum]AB14
MTPAASLREEARLVVLALQFVTRLPLPADAGYTPDRMRRATRYFPLCGALIGALLGLVFAAAAAVFPPTVAAVLTLAAG
V
RLTGALHEDGLADMADGLGGGLTRERALEIMRDSRIGSYGAVTLMLALVLKLAALAGLGAAAGGWAALGALVAAHGL
SRL
ASVIVMVRLPYARDEGKAAFAAAGPGRDGMAIALATGGALMLGLWIGAGLAAALTVLALTVAATLLMARMMMRRL
GGHTG
DGLGAVQQVAEIAVLLGLLAWA
>OLS49041.1 nicotinate-nucleotide--dimethylbenzimidazole phosphoribosyltransferase [Rhodovulum sulfidophilum]AB14
MSTDTDFAAALQHKIDFKTKPLGSLGRIEDLATLIATVQGTLSPQMKTCELTIFAADHGIAVEGVSKFPQEVTRQMVLNY
LADGAGANAFARAVGAEMKVVDAGVAGAPFGVAELIERRIGAGTKSFLHEPAMSAAECDRALEEGRRLGAESLADAV
AYG
EMGIANTASAAALGHKLTGVGLDVLVGRGTGLDDDGLTRKRKVLEAAVARTGALGPKEALAEYGGFEVAMMTGAMI
GAAA
AKKVVIVDGFIATAAALAAMRIAPATRAAMVFSHRSEEQGHGALLDALGAEPLLRLGLRLGEGTGALLAWPLLKAAGE
ML
NTMASFDDAGVTGPQ
>OLS49042.1 hypothetical protein BV379_12695 [Rhodovulum sulfidophilum]AB14
MTPATGYFGKIPSAGNVVTQGVPGLVRIALERWMTAHLTTRAAWPGCWPRTGLRATLDLEKGTLTALILPSRDRSRRP
FP
LACCRMPGLDWEAADRWCDGALPTAQAATAGALSPASLGAALAALPVPSGDAPEPGLWVANPPDEEDRPVAQILTD
LMGP
IGAV

>OLS49043.1 cytochrome C peroxidase [Rhodovulum sulfidophilum]AB14
MTRLIATALLSTALAVPAAASDLRDMALDYFEALPSTVPQIADNRVTPEKIELGKALFFDPRLSASGVFSCYSCHNLTTG
GDDNLETSIGHGWQKGPRNAPTVLNAVLNIAQFWDGRAEDLKAQAKGPVQAGVEMANTPDNVVATLTSMGQYID
WFEAAF
PGEADAVSFDNMARAIEAFEATLITPAPFDAFLNGDEMALDETQKEGLQLFVDKGCVTCHNGVNLGGNGYFPFGLVEK
PG
ADILPEGDKGRYSVTATASDEYVFRAAPLRNVALTAPYFHSGKVWDLKTAVQVMGTAQLGEDVSDTEAERIVAFLDSLT
G
TMPAITTPVLPPETATTPRPTAEVLQR
>OLS49044.1 nif-specific transcriptional activator NifA [Rhodovulum sulfidophilum]AB14
MPSSIIPQSGSPALPQFDIHERFPLDTVCEAAKILTGASDPVAAMPSVLNVLSSFMGLRMGALALLDDSGSGAGGNGV
NP
FAIAATTQGAASAPATSRAMPVGATRAVFRTGVALVSCDVASELGPDALPPQAPHDRFAFIAVPIREQVHSPYVIGVLG
A
WRDLTEDLYPNIDEDQRVMTMVAAILEQSVKFRRLVARDRERLMEEARLALRQANEKAAAEAPLPAEPIKGIIGDSPAI
K
SVIAQIRKVATTRTPVLLRGESGTGKELFARAVHALSDRKDKPFVKVNCAALSQTLLESELFGHEKGSFTGAMAQKKGRF
ELADKGTLFLDEIGEIGPEFQVKLLRILQEGEFERVGGTRTLRVDVRLVTATNRDLEAAVARGAFRADLYFRICVVPILL
PPLRDRLEDVPQLAQAFLDRFNQQNGMNKRMTPQAVSALARCQFPGNVRELENCISRVAALSSGTEIGAQELACHQG
TCL
SAELWRMQTGEQSPIGGLAAAVRTPLPVIDSGTGQRPPAAACPAGFGEPPVAPPAGRPPAAFAPEPQDERARLIDAM
ERA
GWVQAKAARLLGLTPRQIGYALKKHDIPLRKF
>OLS49045.1 nitrogenase cofactor biosynthesis protein NifB [Rhodovulum sulfidophilum]AB14
MSATLIPISGLQVASRKDMGAALAKGGGCSSAGSCGSSQGPADMDPETWAKVKDHPCYSEEAHHYFARMHVAVAP
ACNIQ
CNYCNRKYDCANESRPGVVSERLTPEQAARKVLAVAASVPQLSVLGIAGPGDAAYDWRRTRATFELVAKDLPDIKLCLS
S
NGLALPDHVDEIVAMNIDHVTITINAIDPEIGQHIYPWIFHDAKRRSGIEAATILRDRQLQSLEMLHERGVLVKVNSVLI
PGLNDEHLIEVNKAVKARGAFLHNIMPLISDPAHGTHFGLTGQRGPTGAELKKVQDACAGGANLMRHCRQCRADAV
GLLG
EDRGQDFTLDKLPEEIEDATEARSAYRDWVAEERRDRAAATAEAEAAVAETAKPALFAVCTRGSGRINLHFGKATEFQI
Y
EADARGIRFVGHRRADNYCLGGHGDTARMDEILRVLGDVPYLLCSKIGDGPKKRLAEAGIEAIDGLAQDYIETAIAAVLH
SKQTARPGLSAERA
>OLS49046.1 ferredoxin [Rhodovulum sulfidophilum]AB14
MALKIVKSQCTVCGACEFECPNAAIKFKGEGYIIDAEKCTECEGQFDTPQCAAVCPVPNTCVPA
>OLS49047.1 hypothetical protein BV379_12720 [Rhodovulum sulfidophilum]AB14
MAGGPDPVDWQGEALDCAACGFAGLLARRRCGPGWSCAFDRYAPRIARFFMMNPEAAESCLDDPYPETRAHAARV
ANIFR
LRPLLGDPDPGVRAVAAIRLPAAQSKAALEDPDREVRIALAWRMPFADLVPLLRDPDPFVRQVAARRADPGVLPILAH
DP
APEIRKLVAERVAPRWLETLIADPDPEVRRAVARRIAPGRLAPLARDADLRVRYAVAERAGADLARGLLDDPEEPVRQA
A
AERLAELATPPPAERNSDVDR
>OLS49048.1 nitrogen fixation protein NifZ [Rhodovulum sulfidophilum]AB14
MSTDDREIEIYRPPAFQPGEKVRARKHIRNDGTVPGREIGEFVVRKGEVGYVRDIGTFLQQFYVYAVEFIDSHAIVGMRR
DELVSLDTPAKETPA

>OLS49049.1 putative nitrogen fixation protein NifT [Rhodovulum sulfidophilum]AB14
MKITVRDTAKGMEVYVAKKDLEELIVEAEHPGIWGGWARLTNGWVFDMPAYDTPPALPVTIDARRLSTE
>OLS49050.1 SIR2 family protein [Rhodovulum sulfidophilum]AB14
MGAADRLSDLLDEVASGGRVPYLGAGVAAMADGGPPASPEALCAAIEARVRAPKRASGNLWSVAQYVEGRRFRRTL
DAIV
QEAFAARPEPGPLHRWIAQVRPPLVVDAWYDGALLDAAGAEGKGDWGLVQGVSRHGEWSEIWTRAYDAAGNLIGE
EPDPA
WQMLIYKPHGLIREGHAYLISDNDYVEVLTEIDIQSPIPEEVQARRTGRPFLFLGCRFDDQMLRTFARQIAKRSAPGHAA
VIEGKLTRMETRFLEELGIERLDLPLAGLAEAFATTA
>OLS49051.1 nitrogenase iron protein [Rhodovulum sulfidophilum]AB14
MGSLRQIAFYGKGGIGKSTTSQNTLAALVEMGQKILIVGCDPKADSTRLILNTKLQDTVLHLAAEAGSVEDLELEDVMKI
GYKGIKCTEAGGPEPGVGCAGRGVITAINFLEENGAYEDVDYVSYDVLGDVVCGGFAMPIRENKAQEIYIVMSGEMM
ALY
AANNISKGILKYANSGGVRLGGLICNERQTDRELELAEALAGKLGCKMIHFVPRDNIVQHAELRRETVIQYAPESQQAEE
YRQLATKIHGNSGQGVVPTPITMEELEEMLMDFGIMQSEEDRLAAIAEAEA
>OLS49052.1 nitrogenase molybdenum-iron protein alpha chain [Rhodovulum sulfidophilum]AB14
MAKDIPDPIAPEKLIEEVLSAYPAKAQKKRAKHLNVAAPAPEGETEIASKCDTVKSNIKSVPGVMTIRGCAYAGSKGVVW
GPVKDMVHISHGPVGCGHYSWSQRRNYYTGTTGVDSFVTFQFTTDFQEKDIVFGGDKKLEKTIDEINALFPLSKGITVQ
S
ECPIGLIGDDIEAVARKKKKDVNKTIVPVRCEGFRGVSQSLGHHIANDAIRDWVFNGGEESSYEPGPYDVNIIGDYNIGG
DAWASRMLLEEIGLNVVGSWSGDATLAEIENAPKARLNLIHCYRSMNYICRYMEENYGVGWMEYNFFGPSQIAASLR
AIA
AKFDKTVQDKAEEVIAKYQPLVDAVLAKYKPRLEGKKVMLYVGGLRPRHVVDAYHDLGMEICGTGYEFAHGDDYKRT
GEY
IKEGTLIYDDVTGYELEKFIEGMRPDLVGSGIKEKYPVQKMGIPFRQMHSWDYSGPYHGYDGFAIFARDMDLAINNPV
WG
LFDAPWKKSA
>OLS49053.1 nitrogenase molybdenum-iron protein subunit beta [Rhodovulum sulfidophilum]AB14
MPQSADKVLDHNKLFHEPEYKEMFKNKRATFENAPTAPQVTEILDWTKSWDYREKNLAREALVINPAKACQPLGAVF
AAA
GYEGTMSFVHGSQGCVAYYRSHLSRHFKEPASAVSSSMTEDAAVFGGLVNMVDGLANTYALYQPKMIAVSTTCMAE
VIGD
DLNSFIIQAKEKGSVPEEFDVPFAHTPAFVGSHVDGYDNMQKGILTHFWKGAERSETDSINIIPGFDGFAVANIREMNR
M
LGEMGVDYTVLCDVSDVYDTPSDGNFQMYAGGTKLDDVKAAVNAKATLSLQEYCTKRTLEFCEEVGQKTAALHYPMG
VAG
TDEFLMTVSELTGKEVPASLTLERGRLVDAMADSQAYLHGKTYAIYGDPDFVYAMAKFVMETGGEPTHCLATNGSKA
WAK
KMQALLDSSPFGKGCQVWAGKDLWHLRSILATEPADLLIGNSYGKYIERDLNIPLIRLTFPIFDRHHHHRFPTFGYQGGL
TVLVKILDTIFDKLDTNTGTLGETDISFDLTR
>OLS49054.1 cadmium-translocating P-type ATPase [Rhodovulum sulfidophilum]AB14
MPTRSEDARRLILTGVTLAGMLTAAALHWGPFPGTGWPHMAAMAALALVYTAGGIPAGARALHALWTERALDIDLL
MVIA
ALAAAAVGATAEGAVLLTLFSLSTTLEHRAIGRARRAIEALMELRPDRTLRRRVSGDTEEVAVADLAPGDIVILRPGARV
PVDGTIREGEGAIDESTITGESVPVHKQPGAQVFEATVNLHGVLAVEVTRPLAESTVARMIALVTEAQAARAPSERFSE
W
FGQRYTIAVLAGAVLAFAAFLWLGLGWHAALYKAATLLVAASPCAIVISVPAAILSALSASARGGVLFKGGAALETLARV

DSFAFDKTGTLTTGRQEVVELACEGDPDIFLARLAGLEAHSEHPIADAIRRAATARGLSPLAVREARAVPGEGMVGVDD
E
GVLWAGNERLAARMGAKDAPSERSAPIGDLDRRAQTLVLLGRGARYLGAVTVEDQPRPTARPGLDALRARGIARLAM
LTG
DRRAVAERIGAELGIAPAEIHAELRPEDKLRIVAGLARSGRVAFVGDGVNDAAALARADIGIAMGAAGSEVALQAADVA
L
LSEDLGRLAAAHALARRTARIIRQNLIFAMGAMAVLVASGILFDLPLPVAVVGHEGGTVLVVLNGLRLLADPIRHRSAA
>OLS49055.1 hypothetical protein BV379_12760 [Rhodovulum sulfidophilum]AB14
MAIRTLYSSALSVVGVVFAPILRFFAGASRADEPAGFQARHPEIAGDGLDEAVIGGRAGP
>OLS49056.1 hypothetical protein BV379_12765 [Rhodovulum sulfidophilum]AB14
MSTCRECGKVLSVFGSDGDALCELCAMVQDAELLFRQIRSLEDDGKTRAEIAEAVWDRWAAEERAAGMGEVAPKAG
LAAP
APVDPRLRR
>OLS49057.1 glucose-6-phosphate isomerase [Rhodovulum sulfidophilum]AB14
MQRSIGRSNDVTLSTIWERLDAQAGRAATRPLTALFEDRGRAAAFSVRFAGQSAAAAATDAEEMLFDFSKTNIDEETLE
L
LIELAIAAGVQARRDAMFAGRRINETEGRAVLHSALRNPEGKVALDGRDVMPGLRETLLRMRGFAREIRGGGICGQGG
PI
TDVVNIGIGGSDLGPAMACLALAPYHDGPRCHFISNVDGADAADTLRGLDPETTLVIVASKTFTTIETMTNAETVRGW
MA
ARVSEPGAQFVALSSALEKTADFGIAPERVFGFEDWVGGRYSVWGPIGLSLMIAIGPERFDEFLAGGAAMDAHFRSAP
LR
ANLPVLLALVGIWHAQVQGHATRAVLPYDQRLARLPAYLQQLEMESNGKSVTMSGQAVSRPTGPVVWGEPGTNGQ
HAFYQ
LIHQGTQIVPCEFLVAARGHEPELARHHRLLLANVLAQSKALMEGRGIEAARGIAAKAGFQGDELERQARHRVFPGNR
PS
TTLIYPQLTPRMLGQIVALYEHRVFTEGVILGINSFDQWGVELGKELANSLEPLLAGGPRPETLDGSTAQLLDFIAAAG
>OLS49058.1 6-phosphogluconolactonase [Rhodovulum sulfidophilum]AB14
MKLHTYPDLEMMMIALANVLAGELNNALFHEDRVTVALPGGTTPGPLYDDLCASSLDWARVRVMPTDERLVAPDHP
RSNE
GMIRARLLTGKAAEARFVGLRPGADGMEGLTTRVRRALPLNVLVLGMGEDMHTASLFPGAPELDAALSPEAPPVMQI
TPP
GQPEPRISLTAPVLQGALSTHILITGAAKRAALEQAADLADPHLAPVCTVLKDATVHWTE
>OLS49059.1 glucose-6-phosphate dehydrogenase [Rhodovulum sulfidophilum]AB14
MVSRVIPVDPFDLVIFGATGDLSTGKILPGLYRRDADGQLPEAARIVGAARSPMGDEDFRTLVRAAIHAAIPEKKRDREA
IERFLTRLSYVAVDATGEDGWPELAAKMRPDVVQAYYFSVGPGLFGALAERLHAHGMNAETCRIVVEKPFGHDLASAR
AL
NATLAEHFDEHQIYRIDHYLGKETVQNLMAVRFANMLFEPLWKAQYVDHIQITVAETVGVAGRGGYYDKSGAMRDM
VQNH
MMQLLCLIAMEPPYAFEPDAVRDEKLKVIRALDPVRPADLVRGQYRAGGGMGSYHDDVEAPDSRTESFVALKAHVSN
WRW
KGTPFYLRTGKRLRARTSEIAVTFKDPPHSIFGDQEGHRANLLVIRLQPNEGIKLKVTIKEPGPGGMRLIEVPLDMSFAE
ALGPGAQEIPDAYERLIMDVIRGNQTLFMRGDEVEAAWAWTDPIIAAWEARGDRPQPYDPGGSGPEDALMLLHRDG
RRWR
EIRP
>OLS49060.1 hypothetical protein BV379_12785 [Rhodovulum sulfidophilum]AB14
MAFDSFADARSFADDPGLEADICIMGAGAAGITLASALADSGLRVCLLEAGGMDMDPVVEDASIVPDIGRPYDVTANR
LR

FFGGATNHWGGHCVRLEPDDFEPKDWIPMSGWPFGADTLAPYYRQAHAFLGLGEYEYDPAPVARALGTRLMPFDG
PMLKS
QLSRYRRVFYGDEYGPRLGRTPNVRVVLYADVCDIRLGEASGETVSHVVVRSPAGNRFDVRARYFVLAAGGIEIPRMLL
V
ANGQRPAGLGNENDLVGRYFQEHLMFKSGLLLPRDPADVLPLYLSEVPYGDIAVRCHLVLTAEAVRARRIGKFRSEFYSL
PLRLRDLRALRSGRIDAADIAGLVSDPFGTARAYRCGSNARHDTYGLFNNIEQRPNPASRVSLRADKDAYGRPQAQLD
WQ
ITRQDHDCILAAHQVIAQEAGRIGFGRMRIEIDPEPEVFLDGVGGGGHHMGTVRMAEDPRRGVTDGDGRLHSVENLY
VAG
SALFPTGGYPNPTLTITAMAFRLSDHLKARAEREGLL
>OLS49061.1 hypothetical protein BV379_12790 [Rhodovulum sulfidophilum]AB14
MKRRHLILGGLGLGAAGYMGTILTGLARPCGTGRPRLATLAGPRRIARALLRQGYVGRNKAPEAKSPEALATAAQRDFA
A
GDTVICDGWVLARSEANFALACLRDDTA
>OLS49062.1 GntR family transcriptional regulator [Rhodovulum sulfidophilum]AB14
MVRAPLALDIDRADGGPLFLVIAEAITRDITRGRLRPGARLPGTRALARELGVHRNTVDAAYQELRMQGWLQAAPARG
TF
VAEDLPQTMTAPPAAPVLAEPAVRRPALAFSDGAPDPGLVPDKALARAFRRALLSPAFRNGADDGDARGTRALREALA
DY
LASGRGVVADPGRLLIARGSQMALYLAARATLAPGAAVAVEEPGYPLAWAAFRAAGVEPRGVPVDAGGLSVVALEAA
LDA
DPRIGAVCVTPHHQYPTTVTMGAARRLQLLDLAQRRGLVVIEDDYDHDYRFEGRPVLPLAARAPDDLPLIYVGSLSKLLS
PGIRLGYALAPEPLLSRMAEARAAIDRQGDAPLEAALAEMIRDGDLGRHARKARRIYRARRDLLAGALAERLGERVVFD
L
PAGGLALWMRSAQGGADLWAMRAGQAGLALLPGARFMLGAPAPQAFRLGYAALDAAQIVRAVEILAQSCPQA
>OLS49063.1 MOSC domain-containing protein [Rhodovulum sulfidophilum]AB14
MKQIQNAGVTLLTGAIAALPDSGRPSAIVKTPVERPLALGPEGLEGDAQADRRVHGGVEKAVHHYPAGHYIRWRAAL
GDL
PALSAPGAFGENISVGGPTETEVAVGDVFRLGTALLQVSQGRQPCWKLNHRFGLPDMARRVQQTGRTGWYYRVLEP
GIVA
PGDRLKLTDRVAPDWTLNRLWHALHVDRLNRDDLEGIAALEVLAESWRRLAQRRLDSGRVEDQRARLEGQA
>OLS49064.1 pyridoxal kinase [Rhodovulum sulfidophilum]AB14
MTRPPLVIAIQSQVVFGHVGNSAALFPMQAAGLEVAAIPTVVFSNTPDYPTLRGRALPPEFFSDLLQGARERSLRERAD
Y
ILTGYIGSLEVALMVADFIAEARARNPGLTYLCDPVMGDSGPGLYVPETIADVMRERLLPMADIATPNPFELSWLTGREI
ARIADLEAARTELGIAPGAHLIATGCALEDTPQGHIESVILGPGGAGDIGRHPTEHLPIALPGTGDLFAALILAGIARGA
PLAAAVKTAQTLTAEALRDARALGAGEVVLSGPGFRRALLTLGRAEMSCAAGRRRIRH
>OLS49065.1 phospholipase [Rhodovulum sulfidophilum]AB14
MTRVLETGRAGAAEGRAERLVIFLHGYGANGADLLGLAQPLGPHLPGTAFLAPDAPESCEGVPFGYQWFPIPWIDGSS
EE
QSEAGMRRAVDDLNAWLDAVMAEEGVGPENTVLFGFSQGTMMALHVAPRRAVPFAGVVGFSGRLLAPETLEDETV
SRPPV
LLVHGDADEVVPVGALPEAGNALTAAGFEVYAHVMKGTGHGIAPDGLSLALAFMRERFGIEGAE
>OLS49066.1 3-methyladenine DNA glycosylase [Rhodovulum sulfidophilum]AB14
MVGRIIETEACLAEGAAHLAAVEPRFLHALSLTGPLPLRRRKDGFEALLSAIVSQQVSVAAADAIWGRMKAARLTGPRKI
IWASDEDLRACGLSRQKIRYAKALASAGIDFAALRGVPTEEVVATLVAVPGIGRWTAEIYAMFSLGRADVFAPADLALQ
E
SARLLFDLPERPKERALRQMAEPWAPWRGVAARALWAYYRVAKDREGIR

>OLS49067.1 MFS transporter [Rhodovulum sulfidophilum]AB14
MTDTLPSPAVSSRARRNVTVLILAQALLGAQMPMIFTVGGLAGQTLAPNACLATLPISLIVLGSMLTAGPLSALMQRW
GR
RAGFVLGVAGGAAGAGASALGLYLGSFPLFLLGALLTGIYMSAQGFYRFAAADTAEGTFQPKAISYVLAGGLAAAILGPQ
LVKVTAETMVIPFLGTYLTAIAINLGGGLLFAFLDIPRPPPRDPAHHAGRTRAELLRDPVIAVAIICGTVSYALMNLVMT
STPLAVVGCGFDTGTAADIVTAHVLAMYVPSFFTGHLIARFGARPIVALGLAILAGAGTVALTGVEIEQFFAALVLLGIG
WNFGFIGATAMLSSAHGPEERGAVQGMNDILVFGGVTMASLASGGLMNCSGGVPQEGWVAVNLAMVPFLALAGG
ALIWLV
LRPGDKD
>OLS49068.1 phytoene synthase [Rhodovulum sulfidophilum]AB14
MSLEACAEILRRGDPDRFLAAMSAPVAARARLLPLYAFNVEVARAPWVTEEPLIAEMRLQWWLDVLDEIAEGRPVRSH
EV
ATELAGVLDAEAARALVPLVEARRWDAYREPFADAAAFEAHLTAGSGALMRASARVLGARDGTAALSDLGFAMGLAG
WFR
AVPELVARGRQPLPHDRPEAVAELARSGLARLASARRAGVPAQAVPAARAAWRTGAILSRAAADPGRVLEGGLEDAEI
AR
RAGLLWRALAGRW
>OLS49069.1 citramalate synthase [Rhodovulum sulfidophilum]AB14
MKDRLYLYDTTLRDGQQTQGVQFSTDEKIRIAHVLDALGLDYIEGGWPGANPTDSAFFEARPETRATFTAFGMTKRAG
RS
AENDEVLAGVLNAGTDAVCLVGKTHDFHVTTALGISLEENLENIAKSVAHLVARGREALFDAEHFFDGYKDNPAYALSC
C
QAALEAGARWIVLCDTNGGTLPPEIGRITAEVIAAGIPGERLGIHTHDDTGNAVAGSLAAIDAGARQVQGTLNGLERC
G
NANLTTLIPTLLLKEPYASRFETAVPGEALAGLTRASRLLDDILNRVPLKSAPYVGSSAFAHKAGLHASAILKDPSTYEH
VAPGSVGNARIIPMSNQAGQSNLRRRLAEAGLELGRDDPRLAEILDEVKAREGQGYSYDTAQASFELVARRVLGDLPAF
F
RIKRYRVTVERRRVEPARALTLSEAVVVLDVDGERKLSVSESLDAEGNDRGPVNALWKALAKDLGPYQAFIDDMKLVDF
K
VRITNGGIEAVTRVIIDSEDGAGRRWSTVGVSPNIVDASFEALLDAVVWKLVRDGAPDYDAGAHVA
>OLS49070.1 cysteine--tRNA ligase [Rhodovulum sulfidophilum]AB14
MTDIQLYNTRTRRKERFEPLDPANLRMYVCGPTVYDRAHLGNARPVVTFDLLFRLLRHVYGADRVTYVRNFTDVDDKI
NA
AALARKQAGDARPLEELIRERTEETIGWYHADMDALGALRPSQEPRATEFIGQMIAMIGDLIEKGHAYAAEGHVMFRV
RS
YKDYGALSGRSVDDMIAGARVEVAPFKEDPMDFVLWKPSDDDLPGWDSPWGRGRPGWHIECSAMSHELLGDSFDI
HGGGI
DLQFPHHENEIAQSCCAHPEGGFAKVWMHNEMLQVEGRKMSKSLGNFFTVRDLLEQGVPGEVIRFVFLSTHYRKPM
DWTA
DKVRLAESTLERWRNCTAHLEPSSENDFEADPGVMEALCDDLNTHLAITRLNALRSEPQKLLDNARILGFLSDQFRNGK
P
VGAIPNSAGGEDDLFLGNDYIVLYEMRDRLVELRNLAMKTKDFSEVDSLKKKLIDAGVEVRMSKAGVELLPGAGFDPAK
L
EGAE
>OLS49071.1 hypothetical protein BV379_12845 [Rhodovulum sulfidophilum]AB14
MTSYFAPAAIALGLTAAAAAPAMAGSYSPAPAEPVIVPAVPAAAPDGDWGGAYLGLSYGYIDVSTDSGAPDDDSDAL
GIH

AGYRWDFGKTVAGLEVEYGEPDLDFGSDEVDSVWRIKGQYGYDFGRTLAYATAGIAPTDTSLGDETGYLVGVGVDYQ
MTQ
NWVMGAEVLYHDFDEFGSSGVGADATTAAVRLSYRF
>OLS49072.1 3-hydroxyacyl-CoA dehydrogenase [Rhodovulum sulfidophilum]AB14
MQISETRAIVTGAASGLGAATARRLCAAEAAVTILDRDTERGMELAAEIGAGFQPVDVTDEPSVEQAIGEASAQMGGI
TA
CINCAGIATAERTLDRSGPHGLESFRRTVEINLIGSFNVLRLAAAEMAQNAPDDEGCRGVIVNTASVAAYDGQEGQAAY
A
ASKGAIAAMTLPLARDLAGLRIRVMTIAPGLFRTPMQEGPGEADMDGLAANVPNPPRLGDPAEYAALVLHILQNGYLN
GE
VIRLDAGLRMG
>OLS49073.1 hypothetical protein BV379_12855 [Rhodovulum sulfidophilum]AB14
MSPRQNRVTPLGQILADPARGLFMGNRGCLHHAEGRLGRARWRHKAWICCLTEFRGQHRPIMAPGRYTELFFLDEA
VALA
AGHRPCAACRRADFRRFAKGFGATTAARLDALLHAARICPASGRQRHHTAPAAALPDGSFVLLEEAPHLILGAAALPW
RP
AGYGAPRARPAGKVTVLTPAPTVAALAAGYRPVLHPSAGLPG
>OLS49074.1 ornithine-acyl-ACP acyltransferase [Rhodovulum sulfidophilum]AB14
MLSLRKGRYAARLAETPADILAAQRLRYRAFRATGAHPEGADADAFDTLCRHVLVEETRGGTLVCCFRLLPLKGGAEIG
Q
SYSAQYYELSALAAFDGPMVEMGRFCIHPDWRDPDILRVAWGAMTRFVDEEGVEMLFGCSSFRGTEAETYMDAFALL
QDR
HLAPKRWLPRVKAPNVFRFGQMLRRRRPDARRAMLGMPPLLRSYLAMGGWVSDHAVVDRDLNTLHVFTGLEIRAIP
PSRK
RLLRAAAG
>OLS49075.1 peptide-methionine (R)-S-oxide reductase [Rhodovulum sulfidophilum]AB14
MTQKKSETDPDWRDRLPELAYRVTREHATERPFSHDDFPKTPGTFACLCCGAPLFDQADKFESGTGWPSFTRPAAPD
ALG
ERTDRRLLMTRTEVHCASCEAHLGHVFPDGPAPTGLRYCINGVALKFKPEDTAEGDT
>OLS49076.1 outer membrane protein assembly factor BamE [Rhodovulum sulfidophilum]AB14
MARPRAVHARLALAAVLSAGLLACSPIHRSHGYAPSEEDLSAISVGVDTRDSVAELVGPPTMGGVMRDEAWYYVQSD
WET
VGFREPKETERQVVAISFDGSGTVENVERFGLEDGRVIALNRRVTEANVKGISFLRQLLGNIGQFSAEDILGDR
>OLS49077.1 hypothetical protein BV379_12880 [Rhodovulum sulfidophilum]AB14
MTAPRSLRLAGRRGREPVDFVLTPDPEERAALAGRLGITSLKKLRFAGALRPEGQADWRLEASLGATVVQPCVVTLAPV
S
TRIDTDVLRLYRADMPARPEAEEIEMPEDENEEPLPDRLDLAQVMEEALALALPLYPRAEGADLGEAVFAAPGVAPMQ
DA
ELKPFAALARLRPSRDDAEGGGTPE
>OLS49078.1 50S ribosomal protein L32 [Rhodovulum sulfidophilum]AB14
MAVPQNKVTRSRRNQRRSHDALVAGNPDECSNCGELKRPHHVCAACGHYDDREVVAQADEIDLDEDDAA
>OLS49079.1 phosphate acyltransferase [Rhodovulum sulfidophilum]AB14
MTEEHPQELRAAPRTVISVDAMGGDRGAAAVVAGISLSATKNPDIRFILHGQRDELERLVARRKPLRGRVEIRHADGVV
T
MDAKPSHVMRHGQDTSMWSAIDSVRNGEATVAVSCGNTGALMLLSIMRLRKLPGVNRPAIACLWPSRNPGGFNV
MLDVGA
DVRADERDLLQYALMGTSYARNGLGLERPRVGLLNVGTEENKGRAELKAAHDLIDAAAESANYSFVGFVEGGDIPSAK
VD

VIVTDGFTGNVALKTGEGTAKLLSDFMREAFTATPLSKMAALLAYTSLRRLKKRMDPRRANGGVFLGLNGTVVKSHGA
SD
ATGVSAAVKLAFELAQSGFTERLAARVAASNATEPESVRELAGGSKR
>OLS49080.1 ketoacyl-ACP synthase III [Rhodovulum sulfidophilum]AB14
MSTRAIVRGVGHYLPAHVVENAEFEATLDTSDEWIRTRSGIERRHFAAEGETTSQMAVEAAKAALEDAGLEADDLDAI
VL
ATSTADLTFPSAATMVQAGIGMTHGFAYDVQAVCAGFIFALANANSMISSGLARRVLVIGAETFSRIMNWTDRGTCVL
FG
DGAGALILEASEGTGTTADRGILSVDLNSDGRHRDILYVDGGVSTGTSGHLVMQGKEVFRHAVEKLSDTAHSALAKVGL
D
GTDVDWLVPHQANLRIIKATAQRLHLPMEKVIVTVQDHGNTSAASIPLALSVGRHEGRIVPGNLVVTEAIGGGLAWGS
VV
LRW
>OLS49081.1 integration host factor subunit alpha [Rhodovulum sulfidophilum]AB14
MGERMAGNTLTRMDLTEAVFREVGLSRNESADLVESVLQHMSDALVRGETVKISSFGTFSVRSKAARIGRNPKTGEEV
PI
HPRRVLSFRPSHLMKDRVAAGNRG
>OLS49082.1 hypothetical protein BV379_12905 [Rhodovulum sulfidophilum]AB14
MATDKSPEAFRTISEVSTWLDTPAHVLRFWESRFTQVKPVKRAGGRRYYRPSDMLLLGGIKRLLHEDGLTIRGVQKILRE
EGVRHVAALSQPLDDADAADLADATDPDIEAPMALDAQAEAAEILPMRRRPAPEPDEAILPGPDCGPPWQQAPSAPT
PTP
ASPNGAASTDDDEVLAPPPSDAPAFDADPEQTAPEPSAARPPVAQAPTPARLRQSPALDEGTPAPETAPDSMPPEAE
DLP
SGMTEPAEAAPTVAPPEQPPIPQPLGADLPGTDPADDDPAFVPTGLPTRSRLLKHGFQRLLDADPDAAGRAAERLARV
VL
SIETAARD
>OLS49083.1 2'-deoxycytidine 5'-triphosphate deaminase [Rhodovulum sulfidophilum]AB14
MTAPGVLPAQDIRRLIDTGAIGLSAPLAEGQVQPASLDLRLGPVAYRVRASFLAGEGRSVAERIADFRMHAIDLSQGAV
L
EKGCVYVVPLMERLALPPGIQAVANAKSSTGRLDLLTRTITDGGVEFDRIRGGYDGPLYAEICPRSFSVLVRPGQRLNQI
RFRAGQAVLSDDALRALDASAGLVSGGPAVISEGLGFSVDLKPSGDDLVGYRAKPHSGVVDLDGIAAHDPAEFWEEIRS
S
AGRIILDPGAFYILVSREAVHIPPDYAAEMAPYLAMVGEFRVHYAGFFDPGFGHDDAGGAGSRGVLEVRCHESPFVLEH
G
QIVGRLVYERMSARPDALYGQGIASNYQGQGLKLSKHFRM
>OLS49084.1 nitrogenase iron-molybdenum cofactor biosynthesis protein NifN [Rhodovulum sulfidophilum]AB14
MARLTRPRRALSTNPLKSSAPLGAAMAYLGIEGAVPLFHGAQGCTAFAMVHMVRHFKEAIPLQTTAMNEVSTILGGA
EQI
EEAIANIRKRAHPRFIGIASTALTETRGEDVPGELREVLTRRRDFGDLKVVYASTPDFEGAMEDGWAAAVTAIVEALVPE
TDPRQRNTDSRQVNVLPSACVTPAEIEEIDRLIRAFGLTPIFLPDLSTALDGHLAEDWSGRSLGGTRLGDIPAMARSAAT
FAIGESMQGAARLLGARGGMPVQVFPSLTGLRAVDGFVQALMALAGTADAPPAIRRDRARLQDAMLDAHFQTGGL
RYAIA
ADPDLAYALSSALAGMGAEAVAVVTTSGTNPIVELIPAEEAVLGDLGDLEAGARKAGASLLIAHAHGRHAAQALGLPLL
R
AGFPINDRLGAQDICRVGYRGTRAFLFEVANAVLSHPHDAARPEDFGAAPIEPEFEHDRPQTAPH
>OLS49085.1 nitrogen fixation protein NifX [Rhodovulum sulfidophilum]AB14
MTARRLRLIEPETDMTDSEIPLKIAIATNDLEHLDAHFGSAAKFAVYEVTTASSRLVEVLEFDAVTDQKGGHDSLDDRIT

PKVEALSGCALLFVLAIGGPSAARVVRAGIHPIKRKAPEPIAEVLARTQTMLKGAPPPFLRKVLGRPRTDFATDPAGEYS
E
>OLS49086.1 hypothetical protein BV379_12935 [Rhodovulum sulfidophilum]AB14
MSTEAETLPRGGALPVSDYLSQLIAVIRAEDTHGSWEGKPDAEILADFIVTREERREIPIICDPDPDLLWRVERFYDAVA
LLLERRTGAQAAQMSKITHEGFGRIVLISGRLVVLSRHVRDIHRFGFETFAKLADTGEAMTRDAAEMIARFPEAARA
>OLS49087.1 iron-sulfur cluster assembly accessory protein [Rhodovulum sulfidophilum]AB14
MIQITEAARDAMASAIQGAGRPIAGLRLMVQSGGCAGLQYAMALELTREATDAVVETEGGVTVLIDPESQGYLLGTTI
DF
VTGLEGSGFVFDNPNAKGGCGCGKSFC
>OLS49088.1 hypothetical protein BV379_12950 [Rhodovulum sulfidophilum]AB14
MLDYSEILTDHFLNPRNRGTLDSANALGEAGSVRSGNAFRLTLKIEAGQIAAAMFQSTGGGPEIAAGSALTELVTGKTLA
EARAIDADAIEAALGGLPAEDRSAAEVAAEGLAAALKDYAQPPAFRAAQGLRVAQPLGVAPIRPALPSKTDAPRLSAEEE
RARVEAVLEEMRPRFRADGGDVAFHDILGPRVRVHLKGSCSGCQLAGLTLGGLQKRLAEELGRPVFVVPAPSR
>OLS49089.1 homocitrate synthase [Rhodovulum sulfidophilum]AB14
MTAPYVPDDFTPAPPPARICDTTLRDGEQAAGVAFSAAEKIEIATALDLAGVAEIELGVPAMGMAEVAAIREAGARLTR
A
RGVAWCRLRDSDLELAAMTALSRLHLTVPASDRQLAGKLGRDRDWALAEVARLVRLAAQAGFEVSVGAEDASRADIG
FLA
QLAQVAAEAGAIRLRLADTLGLMDPFAAHTLVSILATGPLPLEFHAHDDLGLATANTLAAHRGGAGFLSVTVNGLGERA
G
NAALEQVVAALAVQGRPTGVALDRLCGLSQIVARASNRPLSPTRPIVGANVFSHESGIHVDGLLKDPGTYEAQALSPAR
F
GRAREIVLGKHSGLAAVTRALSAAGLPSDEASARALLPVLRDWADREKRAATAADLILLTARAEALARHPGPTEPQERSL
T
>OLS49090.1 hypothetical protein BV379_12960 [Rhodovulum sulfidophilum]AB14
MTRPAPRPSMLDQLYALSSAEDLFAFLDLPFDQAVLNRARLHVMKRMGQYLDETDLDRLDDDGVRAAAREALQKAH
ADFE
NSTPRAQKALKIYTQPRGNVVPMEGLRLSVK
>OLS49091.1 electron transfer flavoprotein subunit alpha [Rhodovulum sulfidophilum]AB14
MAQAPQPPAGSRAAMQMQLAEKYKDYKHVWVFIEIERGKVHPVSWELMGEGRKLADDLGVDLCALVLGGRDEDTK
QAIRQ
TYHYGADVCYICEDPLLADYRNEPFTQAMTDLVNKYQPEILLLGATPLGRDLAGSVATTLQTGLTADATELKIADDKSLA
ATRPTFGGTLLCTIHTLNHRPQMATVRPRVMAMPEPDTSRSGRMVWHSLRMQEDDIVTKVLEFVSDAQVEKANLAY
ADIV
VAGGLGLQQPQNLALVQRLAEVLGAEWGVSRPLVQKGWAPAERQIGQTGNTIRPKLYIAAGISGAIQHRVGVEGADLI
IA
INTDPEAKIFEFAHLGVVADALTVLPALTDAFARRLSARAMAS
>OLS49092.1 nitrogen fixation protein FixC [Rhodovulum sulfidophilum]AB14
MPAEHFDAIVVGAGPSGNAAAYTMGKAGMNVLQIDRGEYPGSKNVQGAILYADALERIIPDFRETAPLERHVVEQRF
WML
SENSHTGTHHRSDTFNEEKPNRYTILRAQFDKWFSKQVREAGVTLITETTVTELVKNEAGKVIGVLTDREGGPVYADVV
V
LGEGVNGLVGQRSGFRPELKPQHVALAVKETHFLPEETIRERFNLADEEEGVVIEVLGSVSSGMVGTGFLYTNKESISIG
VGCLVADFAREKIPPYELLDRFKNHPSVKPLLKGAEMKEYVAHLIPEGGYDAIPRLTGDGWLIVGDAGQFLNAVHREGS
N
LAMTSGRLAGESLAALKAQGLPASEENLKLYRDALEESFVIKDLRKYRKIPRLLEKNRQLFTTYPAILNRSMNAFLRVDG
RDKRAKEAEILKGLKTSRGNWLNVAVDMLKVARAWR
>OLS49093.1 ferredoxin family protein [Rhodovulum sulfidophilum]AB14

MNDKTNGTKSPRMEERLYQNRYQVDEGRPHVKINHQPGGDSEELKALTKICPAGCYSINEEGQVEVAADGCMECGTC
RIV
CQATGELEWSYPRGGYGVLFKFG
>OLS49094.1 RNA polymerase sigma-54 factor [Rhodovulum sulfidophilum]AB14
MEMMVSQRQTQGLAMTAKMQAALRILQMTAVDLSAYLAEEALENPCLEVSVPQALRAVPSGVAGSGAGSDFDAVA
ALASD
KPSLYDHVLRQIALAFPDPRLARVAETFAEALEPTGWLGSPPETVAAAAGVPISVAEAVLARLQQFEPAGLFARGLADCL
RLQAEDRDMLTWELSVILDNLGMVAEGRIDALAELCDGTPQDVRNALRAIRGLDPKPGLAFAATEPPILPPDLRVIRKD
G
TWAVELNRSALPAIKVTEAPETGRDTAARTYATRARSRAQWLARAVERRHSTLLAAAVCLVRRQGDYLDRGPRHLHPL
GT
EDVAEELGLHASTVSRAVQHRMIETPRGTVPLRTFFSRAFTPGSGEDGPSQDALIALVGDIVAKENPARPLSDAAIAAEA
KAAGFALARRTVAKYRDVLGIPSSYDRRQNARISA
>OLS49095.1 4-hydroxy-2-oxo-heptane-1,7-dioate aldolase [Rhodovulum sulfidophilum]AB14
MENPVNRFKAGLRDGRHQLGVWNAIPDATVAECLAACGFDWIVIDGEHGAMEVTDALPALQAVAGYPAASAVVRP
AINDW
VLIKRHLDQGAQTLMLPYVQSRDEAEAAVRAMRYPPRGLRGMAGMTRASRYGLVADYLRRAEEELCLILQIETVAALE
RL
EEIATVEGVDGIFIGPADLSASMGYPGQPEHPAVVAAIEGAIGRLKAVGVPSGILALDPDFARHCIALGTGFTAVGVDM
V
TLQRGMTALVETFASG
>OLS49096.1 HlyD family type I secretion periplasmic adaptor subunit [Rhodovulum sulfidophilum]AB14
MTARSPESARLPLAVGLVAILCLVGGIGGWSVATSIAGAVVAPGTIKVETERQVVQHADGGVVGEILAKDGDAVAAGQ
VL
VRLDGTFLRSEQMIVERQLLEIQARKARLVAERDGLDSPDFAVLDSFDNIDPAWVLDHVEGQTRLFAARKTALAQELDQ
L
HEQTVQIDNQIEGTRAQQDALQKQLVLIEKELADKMTLYRQSLVPASQVLALQREEAGLEGNIGRLASQEAELRAKVSE
L
VVRGLRLRESRREDAITTLRDLQYSEIELQERRLSLAERLSRLDIRSPVDGVVFGSKVFTVGSVVQAAEPMMYVVPGGQA
LLVSARVETTDIDEVFPGQPVSLKFTTFDSRTTPNVSGQVLRVSADALTDEATRATFYEVVIQPDIDEMADLPDIELVPG
MPVESFIRTRDRSPLSYLLHPLAVYFERAFRET
>OLS49097.1 type I secretion system permease/ATPase [Rhodovulum sulfidophilum]AB14
MIRRGPKKELTLGRARSRGLLLAVLVFSVFTNLLMLTGPIFMLQVYDRVLGSRSEETLVALFILVAALYLFYWLLDYARG
RVMARIGLRLQAALARRVFSAMVERAALRGGQGPGATAIGDLDAIRNLFAAPVLLALFDMPWTPVFLAAIFLFHPLLGF
A
AIGGGAVLILATLVNRWVTGGALERSQSRSSRAARLARQAEEGGGLIWSQGMASVMSERWARMQDEANAEGMSA
NDRSGS
FASFSRAFRFFLQSAILALGAYLVLQHAVTPGAMIASSILLGRALAPIEQVLGQWALIQRARSGWRALTDFLQAVPERIR
PTRLPDPVAHLSVTNIGLRASAPGAPPVLQGISFEITPGEALGVIGKSGVGKTTLARIIQGLLAPSAGEVRLDGATLDQY
GPERLGQLMGCLPQEVRFFDGTVAENIAHMELNPDPARVVQAATLARVHDIILRLPQGYDTMLIEGSGLLSGGQRQRL
AL
ARALYHDPVLLVLDEPNSALDADGSEALNAVVTDMKRAGKSVLIMTHRPTAIAVCDRLMVIDGGQQKALGPRDEVLRS
VV
RNAPEVQRAMGAVRSTAAPRREARG
>OLS49098.1 LysR family transcriptional regulator [Rhodovulum sulfidophilum]AB14
MIDKLEMFIVLARERHFGRAAEACGVTQPTLSAAIKQLEEQLDVLLVWRGSRFGGLTPEGARVLERARVIVADARALRE
E
MRAVKRGLSGDLRLAAVPTALAAITALTGPLTLRHPNLRITIFSRNSTEIVQMLDDLQVDAGLTYLENEPLGRVATVPLY

AEHYTLVMRADHPLAGRAAVPWAEAAAEPLCLLSPDMQNRRIVDRHMAEAGAVATARLESNSLLALIAHVQTSGWV
TILP
LRTAQPFLGAGSLVAVPLTEPEAEHVVGLIAPHREPMTPVLDALMEAARQAKFQ
>OLS49099.1 formate dehydrogenase subunit gamma [Rhodovulum sulfidophilum]AB14
MTTDIIARCRSIAAAHAHLDGPLLPVLHAVQDQIGHVPMTAYPALAEELGLSTAEIHGVVSFYPDFRTEPGGRRVLRVCR
AEACQAMGAAALGEDLKARLGVEWHGTTEDGAVTLEPAYCLGLCACAPAALVDDRPVGRVTADRLERLAREGSA
>OLS49100.1 formate dehydrogenase [Rhodovulum sulfidophilum]AB14
MSLTLYLPLDAAALALGADEVARALQTEAEARGLDLVLKRTGTRGMVWLEPLLEIEENGLRMGFGPLEPADVPALFEGA
F
EDHPKALGPVEQFPFFARQTRLAFARCGIAEPLDLEAYRAQGGLKGLERAMTMTPEEIVAEITTSGLRGRGGAGFPTGIK
WKTVLEAGGARKAVVCNADEGDSGTFADRMLIEGDPFCLIEGMAICARAVGADRGYVYMRSEYPQAIATLDASLRIAR
EA
GILGEHFDIEDRVGAGSYVCGEETALLNSMEGKRGTVRPKPPLPAHKGFMGRPTVVNNLITLASVPAILSEGAEHYRSFG
LGRSLGTVPLQIAGNVRRGGLFEAAFGMTIGELVEGVCGGTASGRPVKAVQIGGPLGAYFPRSLFDTPLGYEECDEAGG
L
IGHAGLVVFDDTAEMGAMARFAMEFCAVESCGTCTPCRIGAVRGMETIDRILAGDPAAPAILTDLCETMRAGSLCALG
GF
TPYPVESALRHFPEDFAPAREAAE
>OLS49101.1 formate dehydrogenase subunit alpha [Rhodovulum sulfidophilum]AB14
MKDFIIPDADFGTPARRSDKTVTLTVDGMEVTVPEGTSVMRAAAEAGIEIPKLCATDSLEPIGSCRLCMVEIEGRRGTPA
SCTTPAAEGMQVQTQTPRLAKLRRGVMELYISDHPLDCLTCAANGDCQLQDMAGAVGLREVRYTPGANHFEPRQNG
EANP
RYEARDSSNPYFTYDPSKCIACFRCVRACEEVQGTFALTVEGMGFEARIASGTTDFLNSDCVSCGACVQACPTATLQEN
A
VIEMGTPDRAIVTTCAYCGVGCSFRAEMKGDQLVRMVPWKDGKANRGHSCVKGRFAFGYAAHPDRIQSPMIRETID
QPWR
EVSWEEALAFTARRLRAIQAESGTGAIGGITSSRCTNEETFLVQKLIRQVFGNNNVDTCARVCHSPTGYGLKTTFGTSAG
TQDFDSVEAADVVLVIGANPTDGHPVFASRLKKRLRQGAKLIVADPRRIDLVRGPHVQAAHHLALRPGTNVALLTAMA
HV
IVTEGLTDRAFIAERCDREAYEDWAAFVAEEHLGPEATEALTGVPAAEVRRAARLYATGGNAAIYYGLGVTEHSQGSTT
V
MAIANLAMATGNIGRAGVGVNPLRGQNNVQGSCDMGSFPHELPGYRHVHDAGTRAIFERLWKVTISDEPGLRIPNM
LDAA
VDGSFRGLYVQGEDILQSDPDTKHVAAGLAAMDCVIVHDLFLNETANYAHVFLPGSSFLEKDGTFTNAERRLNRVRRV
MA
PMTGLADWEVTMALANAMGAGWDYDHPSEIMDEIAATTPSFAGVSYDLLEREGSVQWPCNEAAPRGTPVMHEAG
FVRGKG
LFVITDYVPTDERTGPRFPLLLTTGRILSQYNVGAQTRRTANTAWHGEDRLEIHPHDAETRGIAEGDWVRLASRSGETSL
RATLTDRVSPGVVYTTFHHPETQANVVTSDYSDWATNCPEYKVTAVQVALSNGPSDWQEDYAAHARAARRILPAAE
>OLS49102.1 hypothetical protein BV379_13025 [Rhodovulum sulfidophilum]AB14
MRPWATSQVQSVGPDGAAPDIRRLAEEVPVALCYDGSTHAVMLASPADLDDFALGFSLSEGIVERPDQIARIERADHD
EG
IELRIWLAPGLGARLAGRRRSMVGPVGCGLCGVESLAAATRPLPVLPPGGLCLTPPEIAEAMTALGLGQELHGATRAAH
A
AGFWTRAEGLVALCEDVGRHNALDKLVGRLARLGRDASGGAILLTSRVSIELVQKAVLARAPALIAVSAPTLRAVAAAEA
AGLTLVARARGQSLDIHSHARRIAAAEIPHDP
>OLS49103.1 formate dehydrogenase [Rhodovulum sulfidophilum]AB14
MTPEKLAYMANQIAIAFARLPGEEAEAAIADHIDQFWDPRMRARLLVLAETPDADLSDRVRGAAALIRRPETA

>OLS49104.1 hypothetical protein BV379_13035, partial [Rhodovulum sulfidophilum]AB14
GATGGAPTLSPARASALRAEWGARILARVDRRHRYPRGTRASGRALVELVLARDGRLLSARLVRSAGDPRLDDAALAA
VR
RAGAVRRAGRFPPAPEGLGAPRYSFTLPLRFEWKG
>OLS49105.1 indolepyruvate ferredoxin oxidoreductase [Rhodovulum sulfidophilum]AB14
MFSFDTPRRPRRPSLTPMIDVVFLLLVFFMIAARFGPEAGIEIVAAQAGGAGAWQGPPRLIEVRPDGLSLNGQAMVPD
QI
AAELARITPAPGDPVLLRARDGADLQALVDAMEALGQAGVTGLMLVE
>OLS49106.1 flagellar motor protein MotA [Rhodovulum sulfidophilum]AB14
MIDPILSFLQTGGAAMWAIAALSVATLSLILWKLWRLALLGAFAGARAERSVALWQEGARAAAAEAVSGRVGLRARLV
RA
AIGALSLGLPEAKAREETTRVARRLLVELRSGLRALELIAVIAPLLGLLGTVLGMIDAFQALQSAGAAADPADLAGGIWE
ALLTTAAGMAVAIPAAVALSWFESVADSARADMEDLATRLFLATEAG
>OLS49107.1 ABC transporter [Rhodovulum sulfidophilum]AB14
MTGLELSHLTVRRGTRPVVEDVTATLSPGDCVGLVGPNGAGKTTLMRAALGLVPHKGSSSLARLAPSERARIAAWLPQ
MR
EIAWPVSVETLVGLGRTPHLGPGQGPGPRDRAAIAGALDRLGLAGFTGRRATELSGGEQARVLIARALAQEAPVLMAD
EP
IAGLDPAHQFTTMELFAGLAAEGRLVMISLHDLGLAVRFCTRLLLMHRGRLVADGPPRAVLSEANLAEVFGLRAYLADT
P
DGPVFQPLEVVR
>OLS49108.1 Cu(I)-responsive transcriptional regulator [Rhodovulum sulfidophilum]AB14
MNIGDISKATGLPAKTIRYYEEIGLIRPARGANGYRHFSETDRHNLTFLGRARSLGFSIEECRALLALYTDKERASANVK
QIARVHLAQIEAKIAELQAMRATLAELVSACAGDQRPDCPILKGLAGEG
>OLS49109.1 copper-translocating P-type ATPase [Rhodovulum sulfidophilum]AB14
MTAMTTTRLKLEGLHCAGCVRRVERALTELPDVGAARVNLATGSAEIKHSGPVAPLIGAVEGVGFGVGQTEIALAVEG
AS
CASCTGRIERTLMAQPGVLSASMNLASGQARVTLAEGLDDAAALARAVTEAGYPATPLRAEARPEPGAAQAAEARAL
ARR
AWTAGLLALPVVLLEMGGHVAPGAREALAGIVSETGLRIVSFLLTTAILAGPGRAFFVKGVPLLLKGAPDMNALVALGT
A
AAWGYSTLATFAPGLLPEGTAHVYFEAAAVIVTLILVGRSLEARARGRTGEAIRHLLGLQPRSARVVTETGLEERPVEQL
APGDLIEIRPGERIPTDGEIVEGQGPVDESMLTGEPMPADKAPGDAVTGGTVNGASSLRFRATRVGRDTALAQIVAMV
ET
AQGAKLPVQALADRITGIFVPAVLAAAALTVAIWLVFGPDPALGLALVAGVSVLIVACPCAMGLATPTSVMVGTGRAA
EM
GVLFRKGDALQGLSAVATVAFDKTGTLTEGHPELTGIRLADGFEKGDVLAAAAALEARSEHPLAQAILAAAPDHPEAEG
V
EALPGYGVSGRVGGRDVLVGAQRLMVREGIAPGPLEAQAATLAATGATPVFVAIDGAMAGVLALADPIKPGARDAIA
GLH
RMGVATALITGDTEPTARAVAAELGIDHVVAGVLPGGKVQALEGLRADGPVAFVGDGLNDAPALASADVGLAIGTGT
DVA
IEAGDVVLMSGDPQGVVNALHVSRRTLANIRQNLFWAFAYNVALIPVAAGVLYPLNGLLLSPMLAAGAMACSSLFVLS
NA
LRLRGLGPLEARS
>OLS49110.1 LysR family transcriptional regulator [Rhodovulum sulfidophilum]AB14
MSTLRPEAITFKQLRALCAVAEHGTLTAAAESIHLTTPAVHTQLRQLEENFGARLVDRGPQGVARLTLQGEAALVGARAI

ETALEACVDHVRALNDGMEGMVTLGVVATAKYFAPRLVAELHRAFAEIDVILRVGSREMVVSALQAASVDIAIMGRPP
RL
PAVHATVLGPHPHVLIAPPDHPLAGQGRIAAERVLAETFLAREAESGTRVLMERFIERLGKGRPQRVVELESNETIKQAV
MAGLGVALISRHTVAGELAAGRLVELDAPSLPLVRQWFLVHRSDTALSAAGRRIHGYIVGRQGAFLPR
>OLS49111.1 fructose-bisphosphatase [Rhodovulum sulfidophilum]AB14
MQTENPIRDLQHIPAALQPIMNALCEVGIDLSRVIQRGPLGGALGAAVGENTGGDTQKALDLMADDAFCERLKAANV
RHY
ASEEQDTVAVLDPEGAYALAIDPLDGSSNIDVNVSIGTIFSIFEAAEDPDASFLRPTDQQVAGGYIIYGPQTMLVCTFGN
GTQEYLLDPDSKQFVLVAEAIKIPDTSREFAINASNYRHWSRPIRAYIDDCLAGEEGPRASNFNMRWVASLVAETHRILT
RGGVFLYPGDARKGYARGRLRMVYECAPIAFVIQQAGGLATDAQDPILGKTAEKLHARTPFVFGSTDKVSRIAAYHDLP
D
EEVNALFGNRGLFRV
>OLS49112.1 phosphoribulokinase [Rhodovulum sulfidophilum]AB14
MSKKHPIISVTGSSGAGTSTVKHTFDQIFRREGVNAVSIEGDAFHRFNRKDMKAELDRHYAEGDAGFSHFSYEANELKIL
EEVFRQYGETGTGRTRHYVHDAEESAKRGVAPGEFTDWAPFAEDSDLLFYEGLHGSVVNDEVDIASHADLKIGVVPVIN
L
EWIQKIHRDKEKRGYTTEAIRDVILRRMYAYVHCICPQFTETDINFQRVPVVDTSNPFVARWIPTADESLVVIRFKNPRG
IDFSYLVSMIHGSWMSRANSIVIPGGKLDLAMQLILTPMINRLVSESKRA
>OLS49113.1 transketolase [Rhodovulum sulfidophilum]AB14
MNAPTKIDTSAEALMATAIRVLTMDAVQAANSGHPGMPMGMADVAAVLFNRFMQIDPSCPKWADRDRFVLSAGH
GSMLVY
SINHMLGYADMDMDQIRSFRQWGARTAGHPEYGHAEGIETTTGPLGQGITTAVGMALAERMHNARFGDDLVDHYT
YVIAG
DGCLMEGISHEAIDMAGHWQLGRMIVLWDDNKITIDGSTELSTSVDQKARFAAAGWHVAEVDGHDREAVAAAIEDA
RKDP
RPSLIDCKTVIGQGAPNKAGSHKVHGAPLGADEIAATRAALGWTHEAFTLPKEVYTAWHAVAARGREAREGWEARLL
CAE
HGGAFEKSLAGPDKAAMAEAITAYKQQLSADAPKVATRKASEMALAVVNATLPNAIGGSADLTGSNLTLTKGMSPVSK
DN
YGGNYIHYGIREFGMSAAMNGIALHGGFFPYGGTFLVFADYARPAIRLSALMGLPVAYVMTHDSIGLGEDGPTHQPVE
HL
ASLRAMPNLNVFRPADVVETAEAWEIAATSDKTPSLLALSRQGLPCLRTEHRTENLTAKGAYVLREPEGGRDVTLIATGS
EVEIAMTAADELARQDVRAAVVSAPCFELFAAQDDAYRASVLGDAPRVGVEAAVEQGWAPLLGAGSAFVGMTGFGA
SAPA
DDLYKHFGITAEAVAAAAKKLIK
>OLS49114.1 type I glyceraldehyde-3-phosphate dehydrogenase [Rhodovulum sulfidophilum]AB14
MTVKVAINGFGRIGRNVLRAIVESGRTDIEVVAINDLGPVETNAHLMRYDSVHGRFPGTVTVDGDTIDVGRGPIKVTAI
R
DPKELPWGDVDVALECTGIFTARDKAAIHLENGSKRVLISAPGAGADKTIVYGVNHDVLTKDDLVVSNASCTTNCLSPV
A
KALNDAIGITRGFMTTIHSYTGDQPTLDTMHKDLYRARAAAMSMIPTSTGAAKAVGLVLPELNGKLDGVAIRVPTPNV
SV
VDLVFEAGRDTTVEEVNAAIRAAADGPMKGVLGYTDEKLVSSDFNHDQHSSVFHMDQTKVMDGNMVRILSWYDNE
WGFSN
RMADTAVAMGALI
>OLS49115.1 phosphoglycerate kinase [Rhodovulum sulfidophilum]AB14
MAGLPLTSLDVAGRRLAVRADLNVPLGPAGVTDATRITRFASGMKPLLARGARLVVLSHLGRPGGEMTPALTLERVQG
AL

ADALEAPVRFVDTCAGPMAERLGQDLAPGEVLLCENLRFERGEERNDPRLGAELARLGDIYVNDAFSCCHRLHASTTA
AI
DAAGQVAAGPLLLEELAQLGRALDAPPSPSVALMGGQSMTERLGLMKRLVRRVGTILLGGGLANSFLFARGYSIGRSFC
E
PELADDILEIAALAEVAGCRIVMPRDFVVDGLQHAGGHAYPVPLGGIRPNRRVYDLGPDTVELYRGILRSARTIVWNGP
L
GTCETPPFDAATRALAETVAEQTRAGLCVSVAGGGDTLVALNRSGHAHDFTYVSTAGGAFLEWLEGRPLPGLEALERA
KR
AD
>OLS49116.1 fructose-1,6-bisphosphate aldolase [Rhodovulum sulfidophilum]AB14
MALITLRQLLDHAAEHGYGVPAFNINNMEQGLSIMEAAKACDAPVIIQASRGARQYANDIMLAKMIEALAEIYPGIPLC
M
HQDHGNNEATCLGAIKHGFTSVMMDGSLEGDGKTPASYEYNVDITKRVADMAHWVGASVEGELGVLGSLETGKGEA
EDGH
GAEGELSRDQLLTDPDQAADFVSKTKVDALAIACGTSHGAYKFTQKPTGETLAMSVIEKIHAKLPDTHLVMHGSSSVPQ
Y
LQDLINENGGAMPQTYGVPVEEIERGIKMGVRKVNIDTDNRMAMTGTFRRIAKENPAEFDPRKFLKPAMAEMTKLCK
DRF
ERFGTAGNASRIKSIPMDDMAKRYAGGSLDPKTK
>OLS49117.1 ribulose 1,5-bisphosphate carboxylase [Rhodovulum sulfidophilum]AB14
MDQSNRYARLDLDEKALIAGGRHVLCAYTMTPKGDGDYLATAAHFAAESSTGTNVEVHTTDDFTKGVDALVYEIDPEN
GV
TKIAYPVELFDRNIIDGRAMLASFLTLTIGNNQGMGDVAHAKMRDFYVPPAYLRLFDGPAMNIADMWRVLGRSVTDG
GMV
VGTIIKPKLGLRPQPFADACYEFWLGGDFIKNDEPQGNQVFAPLKETIRLVSDAMKRAQDKTGEAKLFSANITADDHYE
M
LARGEYILETFGENADHVAFLVDGYVAGPAAITTARRAFPHQFLHYHRAGHGAITSPESQRGYTAFVLSKMSRLQGASG
I
HTGTMGFGKMEGDPSDKIMAFMLTEDVAEGPFYRQEWLGMKPTTPIISGGMNALRLPGFFDNLGHSNVIQTSGGGA
FGHV
DGGTAGAISLHQAHDAWKAGVNLVDYAKEHKELARAFESFPFDADKLFPGWREKLGVTEAAE
>OLS49118.1 ribulose-phosphate 3-epimerase [Rhodovulum sulfidophilum]AB14
MFDRSIKIAPSILSADFANFGAEIRAIEAEGADWVHVDVMDGHFVPNLTFGPPAVKAFRPHVKTFMDVHLMISPVDAYI
E
AYAEAGADMITAHFEAGPHIHRTLQAIRAQGVKAGVVLNPATPAESVEHILDLCDMVLIMTVNPGFGGQKFIPSCLDKI
R
RVRAMIGDRPVHIQIDGGVTTETAPLVVEAGANVLVAGSAVFKGGSVEAPEVYGRNIRAIREAAEGAQKAA
>OLS49119.1 thioredoxin family protein [Rhodovulum sulfidophilum]AB14
MAVSPPICDFGWQAPDFSLPGTDGRLHTLSDVHGPMGTLIMFICNHCPYVLAVRDRILRDARDLQAMGVGICAICSN
DAK
AYPEDSFEGMKKVAAELDLPFPYLHDEDQSVARAYNAICTPDFFGFNSLLELQYRGRLDASRKQAGPADLRRDLYEAML
Q
VAHTGHGPHEQIPSMGCSIKWKDAA
>OLS49120.1 phosphoglycolate phosphatase, bacterial [Rhodovulum sulfidophilum]AB14
MEGRRVKAIIFDLDGTLIDSAPDIHAAVNRTLAGLDREALDLDTIRAFIGDGVPVLMERVIKARGLEPGQRDAMVEAFLG
HYEADPATLTTLYPGVEDALEQFRDEGWAMGVCTNKPEAPSRAILKTFGIDGFFSAVVGGDTVEARKPDPKPLHAVCE
QL
GAKQAVFVGDSEIDAATAQATGMRFALFTEGYHRAEDVDDIPQDARFSDYDVLVAIVARLADELR

>OLS49121.1 phosphatase [Rhodovulum sulfidophilum]AB14
MTIRALIFDLDGTLAETEETHRAAFNASFAARGLDWHWSREDYAVLLKTGGGKERLRAFQAGLPEAARLPEADLVRLH
AG
KTETYGRMIAEGAVGLRPGVEDLILHAREAEMAIAMATTSSRPSVEALVRACWGKPADRVFDVIAAGDEVARKKPDPG
IY
RLALDRLGLPARDCLAFEDSHIGLQSARAAGLGVVITPSTYTAEDDFTGAAYCAPTLEQHNWPLILKAMLLSC
>OLS49122.1 hypothetical protein BV379_13135 [Rhodovulum sulfidophilum]AB14
MIATYDARRSLGDLRHAAANLFAPGSAQRDLQDGAEALTSEAAKAYRYYETHRETASLARRSLADCMPTAKALFNADL
EQ
AIGELMKQFDIVFADVGWRAELSPDARQAKWEKLTPGVPSEDRNTMDATIATQVARIEDICLPQFRTAGRKRRDDRPS
V
>OLS49123.1 hypothetical protein BV379_13140 [Rhodovulum sulfidophilum]AB14
MTGVNDERFPKSLIQADRIVTDTKWNTAENGSTPFSDFFGVAMLSNGAVTAALTTRRATPFSGALHLPLVFRRRSKTRL
W
TGGRLLSWG
>OLS49124.1 amidohydrolase [Rhodovulum sulfidophilum]AB14
MPVRNRLAELQPEIAAWRRDIHENPELMYDLPRTSALVAEKLRDFGCDEVVEGIGRSGVVGVIRGRSDGQGRAIGLRA
DM
DALPILEATGLPYASRTPGRMHACGHDGHTAMLLGAARYLAETRNFEGRAVVIFQPAEEGGAGGLAMCEDGVMERF
GIAE
VYGLHNMPGIPVGSFAIRPGPIMAATDEFDITVTGRGGHAAKPHDCIDSTLAASHIVVALQSIAARNADPLKQIVVSVCS
FRTDSEAYNVIPQTVRLKGTVRTLEEEMRDLGEARVLAVAEATAAAYGATAELCYRRGYPVTANAPDQTDFAAAMAEK
VS
GRVDRATAPLMGGEDFSYMLLERPGAYIFLGNGDTASVHHPAYDFDDEAIPFGASWFVELVETRLAAG
>OLS49125.1 acetylornithine deacetylase [Rhodovulum sulfidophilum]AB14
MSSLLTPRQIFEKLVSFPTVSRDGNLALVDWVEGYLAEAGIAASRVWNPARSKAGLYAHAGPQVAGGVMLSGHSDVV
PVD
GQDWSSDPWELTERGGRLYGRGACDMKGFDALAIWAMVEAQRAEVARPLQLALTYDEEFGCLGAPPLIAQMAREG
FPKAA
AVVVGEPSMMQVVTGHKGNIGFGIRLRGHEVHSSLQHRGVSAVMEAGRLIQWANEANDRNRARAPEGDDGLFDPP
WTSVH
VGEIAGGTAQNITAADCRMGLDYRCVPSDSLEACRAALLAEVARIEAGMKAVHPEAGIAVEETFSVPALAPEQDGAAE
AL
ARRLTGDNATHVVSYATEAGQFQEGGFSTVVCGPGDIAQAHQPDEYITLEQFRQGEAFMTRLLAHLQE
>OLS49126.1 hypothetical protein BV379_13155 [Rhodovulum sulfidophilum]AB14
MPIQDHPLRYALTNELHTRPFPSLQAPCHAAFLAIKPPLDAAIRDRDADRAHVIDLLDRFGAQHPKPGDTHFFGQMGR
HK
LKWESHTEFSTYTIFTPGVADRPFDPTAFEVFPDDWLETAPGARLTSALIRIEQISDGRAEICDRFGEWFAPDSLSAAAI
LDGAAVMATDFRIDTGGHMRMAIFVQPEIGQRRIGRIVQRLTEIETYKAMSMLGLPRARQINARLAELEPQLSALVGE
MT
GGQRQPEETLRNLLAVSAELESLLAQSAFRFSATTAYEALVHQRVEVLREERFQHRQTFQEFMMRRYDPSMRTVKASE
AQ
LDSMTRRAVRAANLLRTQVDVERSAQNQKLLESMDRRADLQLRLQRTVEGLSVVAISYYAVSLVSYMIAPGAEALAVSK
A
LATALVTPVVVLAVWLMIRQLRKGMEH
>OLS49127.1 NAD(P) transhydrogenase subunit beta [Rhodovulum sulfidophilum]AB14
MSIGIVSAAYIAAAVLFILALGGLSNQEKAKRAVWYGIVGMGLAVLGTVFGPGIGHYWLLVPAVAIGGYAGYEVARRVE
M

TEMPQLVAALHSFVGLAAVFIGLNAQLELNHVMALKAADDGAAVEALRGFASVLAHKTPVEISILKVEVFLGIFIGAVTF
TGSVVAFAKLAGRIDGKPHKLPGGHMLNLAALLLSIALGYLYFTGAGIWTMVLMAILAGFIGWHLIMGIGGADMPVVV
SM
LNSYSGWAAAAIGFTLGNDLLIVTGALVGSSGAILSYIMCRAMNRHFVSVILGGFGGTGGPAAEVEGEMVAIDADGVA
AA
LEDADSVIIVPGYGMAVAQAQNSVSVLTEHLRKQGKTVRFAIHPVAGRLPGHMNVLLAEAKVPYDIVMEMDEINEDFS
QT
DVAIVIGSNDIVNPAAQDDPNSPIAGMPVLEVWKAKQVIVSKRGQGTGYSGIENPLFYKENTRMFYGDAKQSLDKLLTL
I
R
>OLS49128.1 NAD(P) transhydrogenase subunit alpha [Rhodovulum sulfidophilum]AB14
MKIGAPKEILEGEARVAMTPDSAVQLRKLGYDCLIETGAGVAAGFADAAYEEAGVKVVRSAEALWQGADIVIKVREPA
AE
EVARLRKGQTLISFFWPAQNEALLEQCKAKGANVIAMDMVPRISRAQKMDALSSMANIAGYRAVIEAGSNFGRFFTG
QVT
AAGKVPPAKVLVVGAGVAGLAAIGTATSLGAMVYAFDVRPEVAEQISSMGAEFVYLDFEEDAADGSETGGYAKPSSPE
FR
EKQLAKFRELAPDMDIVITTALIPGRPAPKLWLEDMVAAMKPGSVIVDLAAEKGGNCDLTRPGEKIVSDNGVTVVGYT
DF
ASRMATQSSTLYATNIRHMMTDLTPDKDGQVVHNMEDDVIRGATVTHEGEITFPPPPPKVKAIAAAPKKDKPKELTPE
EK
KAQEAAAFRKQTITQIGMLGVGALLMLLVGAFAPASFMSHFIVFALSCFVGFQVIWNVSHSLHTPLMAVTNAISGIIILG
ALLQIGSGNWLVVILATISVLIATINIVGGFLVTRRMLAMFQKS
>OLS49129.1 hypothetical protein BV379_13170 [Rhodovulum sulfidophilum]AB14
MARLLHYLDLPPVWLALFAGLAWAQSLALPFAFLGRAGDVAGGLALAFGLGLSVWAALGVVLARTSLIPRQRPDRLVV
TG
PYRFSRNPIYLADAVILLGLILIWDALPSLILVPLFPKLIEWRFIRREEALIHEAFGPEYDAYCRQVGRWI
>OLS49130.1 kynureninase [Rhodovulum sulfidophilum]AB14
MTDFAATRAQFHIPEGVIYLDGNSLGPMPVAAEARAAQAVREEWGEMLIGGWNKAGWMAMPRVLGRHVGRLIGA
EPGTVV
IGDTLSIKVYQALAAALAMRPERRVVLSDSGNFPTDLYMAQGLIKSLGQDHELRIVAPEEVEAAIDEDIAVVMLTEVDYR
TGRLHDMAAISARARTVGALTVWDLAHSAGALPIDLAGSGADFAAGCTYKYLNAGPGAPAFIYVAPRHSDMALPALSG
WL
GHAAPFAFEQGYRPGEGIDRMRVGTPPVLQMAALEAALGVWDGVSMTDIRARSIELSELFIAEVESRCPQLELASPRDP
L
QRGSQVSFRFEESYAAVQALIARGVVGDFRAPDIMRFGIAPLYIGEDEIRRAALILQDVMENRLWDDPTYRARAAVT
>OLS49131.1 hypothetical protein BV379_13180 [Rhodovulum sulfidophilum]AB14
MRLRRARPGDGAPTRALFHDAVHRGAALSYSPAERDAWSPSPEAPPDWEDKLLSRHTVIAEIRGLPAGFMTLGRDGYL
DF
AYVSPDWTGRGVGRALLQAILREARRSGLDHLSTEASLVARPFFARHGWRETARQSVIRHGVALTNFRMELEL
>OLS49132.1 hypothetical protein BV379_13185 [Rhodovulum sulfidophilum]AB14
MKRPFLIGVAGGAAVAAGLGGWLWLQRPGADVEAGGSRALVQVEMPRLSPQAEAGQAVFSDNCAQCHGTRADGR
EGLGPP
LIHPIYEPDHHGDYAFLRAARQGVRAHHWGFGDMAPVPGVSTADIASVVRFVREVQRANGIR
>OLS49133.1 gamma-glutamyl-gamma-aminobutyrate hydrolase [Rhodovulum sulfidophilum]AB14
MARPRVGIIGNSFLINDEYPAHAGGTMNSEAVAEVAGALPLLIPADPRFVSVPELVECFDGFLFTGGRPNVHPEEYGEA
A

TEAHGAFDRARDAIALPLIRALVERGQPFFAICRGFQEVNVAMGGTLHPEIRDLPGRINHRMPPDGTLDEKFALRHMV
TL
TEGGLFHRILGATEVMTNTLHGQGIKRPGPRIVIEGVAPDGTPEAIHVADAPGFALAVQWHPEYRAARDPVSRPLFEAF
G
KAVADWARGDRPAVLKTA
>OLS49134.1 hypothetical protein BV379_13195 [Rhodovulum sulfidophilum]AB14
MAAPPDPAADARLAAWARGETGLPAVDAAMRALIHDGMLDAGRRGLLAAVGCGQFGLDPGACCRVLARLSADYDP
RLHGA
LFRAAAARRVDPLRWGRAADPEGVFLRRWLPELADLPSDALHAPWTCPAAARRLGRRYPHPLVAPGRCRPAGSRSAS
PAA
RRQDSRQLAFDL
>OLS49135.1 hypothetical protein BV379_13200 [Rhodovulum sulfidophilum]AB14
MGVTILSFGRDLRVEDHPALARAGQEAGAEGGAVLPVFIVDPAVWSAPEASARQWRVVAEGLARRRAPAPGAAARR
TDRA
ARAGARADPRGPRSGARLRSLSGAAARWPVRGRAGAGRGAGWRRSRCRVVGASGLGRAVGGRGGPGGPPCARGG
RRAPPR
FLVGPARPVSAGPGRRSRPCGRAPGPRGRCATGGLGARRDRAACGRCRDARADP
>OLS49136.1 aminopeptidase N [Rhodovulum sulfidophilum]AB14
MTDATPQPVFLKDYTPFGFLVDRVELTFRLAPTETRVRARIAFRPNPAATDRRFFLHGEELTLISAAIDGHPVTPEITAA
GLSCEVPDAPFTWDCEVEIAPKENTALEGLYMSNGMYCTQCEAEGFRKITFYPDRPDVMAPFRVRIESELPVLLSNGNP
V
ARGTGFAEWDDPWPKPSYLFALVAGELVATSERYTTSEGREVDLNIWVRPADQGKADYALDSLIRSMRWDEEVYGRA
YDL
DVFNIVAVDDFNMGAMENKGLNIFNSRYVLASPETATDRDYENIERIIAHEYFHNWTGDRITCRDWFQLSLKEGLTVFR
D
SQFSADMRSAAVKRIEDVMTLRARQFREDNGPLAHPVRPESYTEINNFYTATVYEKGAELIGMLRRLVGPEGYRKALDL
Y
FSRHDGQACTIEDWLQVFEDATGRDLTGFKRWYSQAGTPRVKVTEDFKNGTYTLHLEQETAATPGQDKKAPQVIPLAL
GL
LSPNGDEVLPTTVIELTEARQEAVFEGLGAKPVPSLLRGFSAPVILERDSSSEERAFLLAHDTDPFNKWEAGRSLAREIL
IEMVTEGAAPAPAWLEALASVARDEALDPAFRALALRLPGEDELAEALAGRGTVPDPLAIHAARETARTAIARHLEPLW
P
GLYRQMTCPGPYSPGAADAGRRALRLVALGYMARLDGGAQARAHFAAADNMTDEIGALSVLVETGGAEEALDRFAK
RWGD
DRLVMDKWFGLQASLAAPERAVAVTRALTEHPDFTWKNPNRFRAVIGGLTANAAGFHDPSGAGYDLLADWLIRLDG
VNPQ
TAARMSTAFETWARYDAGRRAKSKAAMERIAAREGLSPDMGEMIGRLLAAGQSAD
>OLS49137.1 hypothetical protein BV379_13215 [Rhodovulum sulfidophilum]AB14
MLRRLLRGLRGSTRAKGAAPQAAGARAAPLPDDTPFGEGLARRAVAFLEATSAVWGEAAIVRTHRDHTGHGLFHDRA
TGR
FFLSRSLDGWPCAPEYLSFADAAGFRRWLAAQSDASLSGQVPSDIPGFERDPGNQRITRSFVEDYLRRHGNGGASGRQ
GS
>OLS49138.1 short-chain dehydrogenase [Rhodovulum sulfidophilum]AB14
MDLGLAGKRALVCAASKGLGRGCAEALAEAGVDLVINARGAEALLATSEEIRRAHGVAVTPVAADIVSEEGRATVLEAA
G
QVDILVTNAGGPPPGLWSDWERDDFIAALDANMLTPIALMKALLPGMIERGWGRVVNITSQSVKAPIPVLGLSNSAR
AGL

TGYVAGTARQVAPHGVTINNLLPGIHATDRAVALDRGVAEAEGIALAEARTRREATIPARRYGTPGEFGAACAFLCSRQ
A
GFIVGQNILLDGGATNATL
>OLS49139.1 chromosome partitioning protein ParA [Rhodovulum sulfidophilum]AB14
MKTVLIANPKGGCGKTTIAVTLAAALADGGWKVALADADPQKSALRWLRRRPAAASAIRGLDWSGGRGAGKPGKGI
GWLI
VDAPGALPDAAAAGLVDEARAVVVPVLPSVFDAEATRRFLSEIAEFKRIRKGKVGVDLVANRVRPRGRDAAWIRDMAE
RV
GQAPVAEISDRVAYGDLAEQGLTVFDRPQRAYAPLRAQWTPLLAALTARG
>OLS49140.1 hypothetical protein BV379_13230 [Rhodovulum sulfidophilum]AB14
MTATFLVATDLSPRADRAVNRAFRLASWTGADILLAAVIDSDLPEDVARPLAEAAETRLARFAASIPHSAEVRHDTRILT
GDPSAAIPLLAEEIDAALIVLGLHRDRPFLDLIRETTMERIVRHAARPVLLVRDPVDHDYAALLAALDFAPASTAAVKIA
ADLAPEATIHGVHALHIPYRGFVAPHGSSIAIAPFRRSAEMRLAEWRSDPDLPERLAEIDIVEGAAHVVLNREIEDHAPD
LLVLGAHGRAGDAPSILGSLANDLMRHPPCDLLIAR
>OLS49141.1 hypothetical protein BV379_13235 [Rhodovulum sulfidophilum]AB14
MFEPYTRALTQAQLDIMLGDTGTILPDGLHHPAHWPDPEPPGPHSHGVKRDDSSAAASSSPSIRAKVQERPVAELRHA
LQ
ASYPETAVLAEYIAGQIQNEIAAHRMTAIPNDAEALSDYNAKLNFLNETLIAVQRLHEAIPIAEPDQKVTPEEARRIKEI
LIDIAGKTDLAVRYLDNDTGTYGNFWKIGLIGACSGLFGCFGIPVNLGAPIAAGVIGASTIRLIVEGRRGP
>OLS49142.1 peptide chain release factor 3 [Rhodovulum sulfidophilum]AB14
MLDAPSNRPALPPEIARRRTFAIISHPDAGKTTLTEKFLLYGGAIQMAGQVRAKGEARRTRSDFMKMEQDRGISVSASA
M
SFDYDRFRFNLVDTPGHSDFSEDTYRTLTAVDAAVMVIDGAKGVESQTRKLFEVCRLRDLPILTFCNKMDRESRDTFEII
DEIQEMLAIHVTPASWPIGVGREFLGCYDMLRNRLELMDRADRNKVAESIKIEGLDDPKLAEHVPQDLRQKLIEEVEMA
K
ELLPALDPQAVLEGQMTPIWFGSAINSFGVKELMDGIANYGPEPQIQPAESRQIAPEEPKVSGFVFKVQANMDPKHRD
RV
AFVRLASGHFQRGMKLTHVRSKKPITVSSPVLFLAADRELAEEAWAGDIIGIPNHGQLRIGDTLTEGEALKVTGIPSFAP
ELLKNCRAGDPMKAKHLDKALMQFAEEGAAKVFKPMVGSGFIVGVVGALQFEVLASRIELEYGLPVRFEPSQFTSARW
VH
GEKADVDGFVQKNKQHMATDNDGDVVYMTRLQWDIDRVLRDHPELTLSATKEMQV
>OLS49143.1 hypothetical protein BV379_13245 [Rhodovulum sulfidophilum]AB14
MIESAISRPYSGYHRSIWRKAAALTQSMVGNHGFVDGNKRTALLLVETLIERSGYYLVLSGQDRFDDLIVGVASGEIDFD
ALVVWFEERVARR
>OLS49144.1 L-lactate dehydrogenase [Rhodovulum sulfidophilum]AB14
MHTNKLVVVGAGHVGSYVLADAMKIGLFAEIGVIDILGNVAFGEALDQAQATALPYMSNIKVTSGGYEQCADADVIVV
AA
GPSVIRDPENSKAEPDRTLLTSTNCKVIREVMAGIVKHTTEAIVILITNPLDTMVYIAENEFGYPRGRVFGTGTMLDSAR
LRKVVADAYGIDPKSVTGFMMGEHGSTAFPVLSHVNVAGIPFAELDRHFEALGDIRSPETVKARVISAAYDVFNGKGW
TN
AGVAQSAVTMARAVLLDERSVFPACTTLRGQYGHDGDVALSMPCLLGRDGIVKQLPVGLNAWEAEKLAESIAFIQAT
MRD
AGTGPDKAV
>OLS49145.1 hypothetical protein BV379_13255 [Rhodovulum sulfidophilum]AB14
MVGHCRDVDLILANLGTADIAYNKPDSMIRGKPGIISFVVDLSPQEVPSDAFDGLPGQIVRADVPVSRYISAELWGAED
V
FKISPAGPVRKQIFASSENRWDWTVKPLLGGAQVLKLKTYVHLTENGAPRGAIGWKTFEATIPVKVTTSDRIRDMAAEI
Q

PIHAIVLFVMSLAGGAWANLRRRKKTEPQEALTTGQAGKPFRPDAREEA
>OLS49146.1 hypothetical protein BV379_13260 [Rhodovulum sulfidophilum]AB14
MKNSMFLLSAAALTLAAPAFAEDVFPAKLAGHAVLPAMTMVAPPADAPRDAWISGKFTGGARNMAPMSAMGDTG
GLHGHR
ETGIALPFLGQPLQGFSGFAMARAEDGSVYALTDNGFGSKANSPDAMLFFHRILPDFETGKVDVKETVFLRDPGFKVPF
R
IAYEGSEGRYLTGADFDLESIQLVDGSLWIGEEFGPYLLNVSLDGVVLGVYPTMADGKELRGADNPAVSATSVPGRDW
KS
QRSGGYEGMALQPGTGTLWAMLEKPILGDDGKTEGDFLRVMAFDTVSKEWTGEMFRFALDEGATAIGDFNFIDDTH
ALVI
ERDNGEGDPSLKCDGEAAPDCFPEPAMHKWIVLIDTSDVDAEGYVRRIGHIDLMDIADPDGVARLQTDAARDLSGRFT
FP
FFTIEDVMRVDDTHILAANDNNLPFSSGRKLDAAADNEVILLEVPELLSAQ
>OLS49147.1 hypothetical protein BV379_13265 [Rhodovulum sulfidophilum]AB14
MNSQDDWIISSLLEDLAALALANDRISIADLVEALGARGFGPLLVMLSAFLILPVGMLPGLPGVVAIFMILIGGRMMTGE
TTLWLPARLRRVHISGHVLAVSVARAQPAALWLRPLLTPRLVALIEGRIATRLIALILIGTGVLIFLLGFIPGLPFVLSL
HVLMLGLALSTRDGLVAALAYALMGPEIIFIWRVFT
>OLS49148.1 DEAD/DEAH box helicase [Rhodovulum sulfidophilum]AB14
MTTFAELGLDPKVLSAVSDAGYTAPTPIQAGAIPPALEGRDVLGIAQTGTGKTASFVLPMITLLGRGRARARMPRSLVLA
PTRELAAQCAENFDIYAKKTKLTKALLIGGVSFKEQDALIDKGVDVLIATPGRLLDHFERGKLLLTGVQIMVVDEADRML
DMGFIPDIERIFQLTPFTRQTLFFSATMAPEIERVTNTFLSAPAKIEVARRASAAENIEQAVICYTASRKDRQAKEKREL
LRALIEREGEGCKNAIIFCNRKTEVDIVAKSLKAHGYDAAPIHGDLDQSQRTRTLESFRDGKLRFLIASDVAARGLDVPA
VSHVFNYDVPSHAEDYVHRIGRTGRAGRDGKALTICVPYDEKWLTAIEDLVEKKIPRVESPLKSGGEDRGNRSRRNKRH
E
GDAEAAAAPAVQAAPESPERSEEPRESARPPRNERRPERSERTERSESQDRNKPQTRHQSPRRSGGRGHRDEPVVGM
GDH
MPAFLLRSFKPRTEAEPAAADEPVEQDAE
>OLS49149.1 MBL fold hydrolase [Rhodovulum sulfidophilum]AB14
MSKNRLIYLPLGGAGEIGMNAYVYGYGAPGKERLILVDLGVTFPNMETSPGVDLIFADMAWLEERRDRLEAIFITHAHE
D
HVGALGHLWPRLKAPVYARPFTGTLARLKMEDQGQDPETVRFVEPWPNRIEAGPFQVSYLPVSHSIPESSGLVIDTPVG
R
IVHTGDFKLDRTPIVGDPFDPVLWGEVAAEGVKVLTCDSTNVFSPNPGRSETTVGPAIRQLVERAEGMVVATTFASNIA
R
LKTLAEAGRDAGRSVCLLGRAMRRMVEAGIESGVLTDFPKVVSPEEASDLPRDKVMLIVTGSQGERRAASAQLSRGKY
MG
LTLAAGDLFLFSSKTIPGNEREVIRIINALSEMGVDVVSDDTDLYHVSGHANRPDLSRIHTLMKPDMVVPMHGEHRHLR
E
HVKLAAENGLPAVVAVNGTMLDLTGERPQPAEFIETGRTYLDGAVQIGALDGIVRDRIRMALRGLVVVTAILDEEDEPL
G
EPWVDLSGLPETGRSRAALSEVIEEDLDQFLRRADDKTLADDDKLEEAMRRIVRQTAQGEIGKKPEVVVVISRLSA
>OLS49150.1 type III pantothenate kinase [Rhodovulum sulfidophilum]AB14
MLLCIDCGNTNTVFSLWDGTRFLCTLRTATEHTRTADQYYVWFSTLLDRYGIEPEIADVIISSTVPRVVFNLRVFTDRFF
GLRPLVVGKPECLLPHEARVDLGTNVGPDRLVNTAGAFDRHGGDLILVDFGTATTFDVVAHDGAYVGGVIAPGVNLSL
EA
LHMAAAALPHVDVTQPTRVIGTNTITCMQSGVFWGYIGLVREICGRIRAEYDRPMRIIGTGGLAPLFAQGDVLFDRIED
D
LTMHGLTVIHAYNKENGAT

>OLS49151.1 biotin--[acetyl-CoA-carboxylase] ligase [Rhodovulum sulfidophilum]AB14
MSTEGEGWHWPAGHDLILLDEVDSTMAEARRRRDLDRPTWIVARRQTAGVGRRGRAWGAPGGNLAATLVFAPGC
PPATAA
LYSFVTSFALYATCLAYLDDRDAITLKWPNDVLLNGGKLAGILLESEGTMTGVGRLAIGLGVNLNALPEAAMIEPRALMP
ATLAEEIGEPVDPLEFLAFFAEHFDHQDRFFRENGFGPLRSAWLERAARLGDTITARMGAEEVSGRFLTVDEDGQLVIET
PGGRRAIAAADVFF
>OLS49152.1 NADH-quinone oxidoreductase subunit N [Rhodovulum sulfidophilum]AB14
MISDDLSVVLPEIILSVYAMLALVGAVYTGKDRAAPVLVWTTSALMLALAVWIGVTAGPTETAFGGALIDDGFARFAKV
V
VLASAALVMLMSQDYMRRQKTLLFEFPVLITLGVVGMMLMVSAGDLMMLYIGLELQSLALYVVAAIRRDNVRSTEAG
LKY
FVLGALSSGLLLYGASLTYGYAGTTLFSGILASIGDGQIGVGLLFGLVFMITGLAFKISAVPFHMWTPDVYEGAPTPVTA
FFATAPKMAAIALFARVLFDAFGTVTGDWGQVLAVLALLSMFLGAFAGIGQRNFKRLMAYSSIAHMGFALMGLVAGT
ALG
VQAMLVYMAIYVTMSIGTFAFIMGMEKDGQPVTDIRSLNMYASSEPLKALALMMLMFSLAGVPPFVGFFGKFYVIRA
AVD
AGYVWLAVAGVIASVISAFYYLRIVFYMYFGEEGDRRLDGSMPPVLWGIMMVASFAMILGVVNLFGVEGLALAAAQT
LVN
>OLS49153.1 NADH-quinone oxidoreductase subunit M [Rhodovulum sulfidophilum]AB14
MDNLLSIITFTPLIAALILGVFLKGDSASDQANAKWVALIATTATFLISLFVLFQFEPSDPGFQFVEDRSWLLGLRYKMG
VDGISVLFVMLTTFLMPITIGACWTVTYRVKEYMIAFLVLETLMLGVFCALDLVLFYLFFEGGLIPMFLIIGIWGGKDRI
YATFKFFLYTFLGSVLMLVAMLAMYIDAGTTDIPTLLTHQFSAGDIVIGGKTVVGGMQTFLWLAFFASFAVKMPMWPV
HT
WLPDAHVQAPTAGSVVLAAIMLKMGGYGFLRFSLPMFPVASDIWAPLVFWLSAIAIVYTSLVALAQKDMKKLIAYSSV
AH
MGYVTIGIFAANRQGLDGAIFQMLSHGFVSGALFLCVGVIYDRMHTREIEAYGGLVNRMPAYALIFMLFTMANVGLPG
TS
GFIGEFLTLAGIFQVNTWVAAVAATGVILSASYALWLYRRVVFGDLIKESLRTISDMNLRERAIFAPLVAMTLLLGVYPA
LVTDIIGPSVEALISNYQTALAETPVSTQLAGN
>OLS49154.1 NADH-quinone oxidoreductase subunit L [Rhodovulum sulfidophilum]AB14
MEKIILFAPLIGSLLCGFGYRMFGEKAAMWITTAMVFLSMLFSWVVFTGFDGEMRQVEIFRWIDSGSLVADWSIRIDRL
T
AIMLIVVTTVSAFVHLYSFGYMAHDPEWREGESYKPRFFAYLSFFTFTMLALVTADNLVQLFFGWEGVGVASYLLIGFYY
RKPSANAAAIKAFVVNRVGDFGFLLAMVSLFFLLDSVSFDDIFDKAPELATTQLSFLSGSWSAANVVGVLLFIGAMGKSA
QLFLHTWLPDAMEGPTPVSALIHAATMVTAGVFLVCRMSPVLEFAPDAKAMIVFIGALTAFFAATIGLVQNDIKRVIAY
S
TCSQLGYMFVAAGVGVYSAAMFHLLTHAFFKALLFLGAGSVIHAMHHEQDMRNYGGLRTKIPYTFGAMMIGTLAITG
VGI
PLTHIGFAGFLSKDAIIESAWGGGANLAFWMLVIAAAFTSFYSWRLIFLTFFGKARGDKHAHEHAHESPWTMLVPLGVL
S
VGAVLAGMLWYGDFFGDHDQVNRFYGVPSHEVAAAAGEHGGTAAEGAAALPGQGAIYTAPDNHAMENAHEAPK
WVKASPF
LAMLGGFAVAWLFYISSPELPARTARAARPLYLFLLNKWYFDELYDAIIVQPARNIGRALWKGGDGGIIDGGINGVAMG
I
VPFFTRLAGRLQSGYVYHYAFAMVIGIAVLVTWMALTGGAQ
>OLS49155.1 NADH-quinone oxidoreductase subunit K [Rhodovulum sulfidophilum]AB14
MIGLEHYLTVAAALFVIAIFGIFVNRKNVIVILMSIELMLLSVNINLVAFSAYLGDLVGQIFTMFVLTVAAAEAAIGLAI
LVCFYRNRGSIAVDDVNVMKG

>OLS49156.1 NADH:ubiquinone oxidoreductase subunit J [Rhodovulum sulfidophilum]AB14
MTLGEFAFYAFSVVTVVSGLFVVLARNPVHSVLWLILAFLSSAGLFVLLGAEFVAMLMVIVYVGAVAVLFLFVVMMLDV
D
FTELKAKMAAYFPIAGLIGAVFLAQLVLVYGAWKSADGAAALRGAPTPDPAEIHNTLAIGQLIYVDYIHLFQAAGLVLLV
AMVGAIVLTLRHRPNVKRQNVLAQMYRDPAKAMELKDVKPGQGL
>OLS49157.1 hypothetical protein BV379_13315 [Rhodovulum sulfidophilum]AB14
MSGYAKLFEQMLEQSHKMARTFNPALESFQVHGFDKLIPTMPKDFMDMMWGNTFNPDGLDSKTRLLVVVAGLTVL
GAQAD
AQFKIAVRHALEAGATEREIAEVIYQMAMLGGIPAMSRALDLAQTVFDETDEEAGK
>OLS49158.1 NADH-quinone oxidoreductase subunit I [Rhodovulum sulfidophilum]AB14
MAAIDWQRAGKYFLLMDFLQGFRLGMKYFFAPKATLSYPHEKGPLSPRFRGEHALRRYPSGEERCIACKLCEAICPAQAI
TIDAEPREDGSRRTTRYDIDMTKCIYCGFCQEACPVDAIVEGPNFEFATETREELFYDKQKLLENGDRWEAEIARNLELD
APYR
>OLS49159.1 NADH-quinone oxidoreductase subunit H [Rhodovulum sulfidophilum]AB14
MADFMDTGIGLALTIMGHGLMVIAFVMISLLFLVYGDRKIWAAVQMRRGPNVVGVFGLLQTVADALKYVVKEVIIPA
GAD
RPVFLIAPLIAFVMPLLCWAVIPFSDRLVMADINVAVLYILAISSLEVYGVIMGGWASNSKYAFLGSLRSAAQMISYEVS
IGLIIIGVILSTGSLNFGDIVAAQDGRFGFLSWYWLPHFPMVFLFFISALAETNRPPFDLPEAESELVAGYQVEYGSTPF
LLFMACEYIAMFLMCALMSILFFGGWLSPVPGLPDGAIWMVGKMAVFFFLFAMVKAIVPRYRYDQLMRLGWKVFLP
MSLI
WVVAVAFFAQFELFGGAYARWAVGG
>OLS49160.1 NADH-quinone oxidoreductase subunit G [Rhodovulum sulfidophilum]AB14
MAELRKIIIDGTEIEVDPAMTVLQACEVAGIEIPRFCYHERLSIAGNCRMCLVEIVGGPPKPAASCAMQVKDLRPGPEGA
PPVVKTNSPMVKKAREGVMEFLLINHPLDCPICDQGGECDLQDQAMAYGVDFSRYREPKRASIDPDLGPLVGTCMTR
CIS
CTRCVRFTTEVAGITQMGQTGRGEDAEITSYLGQTLDSELQGNIIDLCPVGALTSKPYAFTARPWELTKTETIDVMDALG
SNIRVDTKGREVMRILPRNHDGVNEEWISDKTRFVWDGLKRQRLDRPYIRENGKLRPASWAEALRAAAAAMTGRKVT
GLI
GDLAPVEAIFALKSLVEGLGGTVECRTDGARLPAGNRSAYVGTTAIEDIDSARQIYLIGSNPRAEAPVLNARIRKAWMHG
AAVHRVGPAVDLTYDVIEEGADRAALVRLAGSAEKTENALVIVGQGALTEADGEAVLSQAMALAGRLGGGLLVLHSAA
SR
VGAMDLGAVTEGGIEAATGGAEVIYNLGADEVEIGAGPFVIYQGSHGDRGAHRADVILPGAAYTEEAGLFVNTEGRPQ
LA
VRAGFPPGEAKENWAILRALSAELEARLPFDSLTQLRRKLTEAHPDFRLVDEVIENPWRPLDLAEPGRAEFRYAVRDFYL
TNPIARASALMAELSANAKARRAAPMAAE
>OLS49161.1 NADH-quinone oxidoreductase subunit F [Rhodovulum sulfidophilum]AB14
MLTDQDRIFTNLYGMSDRTLSGARARGHWDGTAQIIAKGRDWIIDEMKKSGLRGRGGAGFPTGLKWSFMPKESDGR
PHYL
VINADESEPGTCKDREIMRHDPHTLIEGALIASFAMQAHACYIYIRGEYIREREALQAAIDEAYDAGLLGRNAAKTGWDF
DLYLAHGAGAYICGEETALLESLEGKKGMPRMKPPFPAGAGLYGCPTTVNNVESIAVVPTILRRGGDWFSSFGRPNNA
GT
KLFAISGHVNRPCVVEESMSIGLRELLDRHCGGVRGGWENLKAVIPGGSSVPLLPAQICEDAVMDFDWLREQKSGLGT
AA
VIVMDQSTDVIKAIWRLASFYKHESCGQCTPCREGTGWMMRVMDRLVRGEAEIEEIDMLFDVTRQVEGHTICALGDA
AAW
PIQGLIRHFRDEIEDRIRARKTGRTSAVAAE
>OLS49162.1 NADH dehydrogenase [Rhodovulum sulfidophilum]AB14

MKDNAFQDVRTGEQKIRNFNINFGPQHPAAHGVLRLVLELDGEIVERADPHIGLLHRGTEKLMESRTYLQNLPYLDRLD
Y
VAPMNQEHAWCLAVEKLTGTEVPRRAQLIRVLFSEIGRVLNHLLNVTTQAMDVGALTPPLWGFEEREKLMVFYERAC
GAR
LHAAYFRPGGVHQDLTPQLIEDIDAWAEAFPRVLEDINGLLTENRIFKQRNVDIGVVTEQDILDWGFSGVMVRGSGLA
WD
LRRSQPYECYDEFEFDIPVGKNGDCYDRYLVRMEEMRQSTRIIRQACAKLRKEPGEVLARGKITPPKRGDMKTSMEALI
H
HFKLYTEGFHVPAGEVYAAVEAPKGEFGVYMVADGTNKPYRAKLRAPGFLHMQAMDFISKGHMLADVAAIIGTLDIVF
GE
VDR
>OLS49163.1 NADH-quinone oxidoreductase subunit C [Rhodovulum sulfidophilum]AB14
MDESLKELGALIAAKRPDCVIGWEVAHRELNIDAAPAAIVAFTEFLRSDAGCRFSSLVDITAVDHPEREARFVVVYHFLS
MYQNQRIRIRCALREDEMIPSIVSVHPSANWFEREVFDMFGIIFSGHPDLRRLLTDYGFRGHPLRKDFPTTGYTEVRYDE
AQKRVVYEPVNLVQEFRQFDFMSPWEGADYILPGDEKAKA
>OLS49164.1 NADH-quinone oxidoreductase subunit A [Rhodovulum sulfidophilum]AB14
MDQLLSNYLPIAIFFGMSIALGLILMLSAVIVAVRHPDPEKLSAYECGFNAFDDARMTFDVRFYLVAILFIIFDLEIAFL
FPWAVAFGTIGLVGFWSMMVFLAVLTIGFAYEWKKGALEWA
>OLS49165.1 enoyl-CoA hydratase [Rhodovulum sulfidophilum]AB14
MQDDLEIGIDARGVARVTLRREDKHNALSADLIAGLDGAARRLGADPAVRAVVLTGAGESFCAGGDLAWMTAQMQ
ADAAT
RAAEARKLAGMLRALNEMPKPLIGRVQGQAFGGGIGLISVCDVAVGVEAARFALTETRLGLIPATIGPYVVARLGEAGA
R
RVFMSGRRFGAAEAVSLGLLARAVPGESLEAAVEAEVAPYLACAPGAVARAKAQVLSLGPRIGEAEIEASIARLVAAWE
T
EEAAEGIAAFFARRKPAWSAPAEP
>OLS49166.1 DNA repair protein RadA [Rhodovulum sulfidophilum]AB14
MAKPTTVFTCTACGATQNKWSGRCDACGAWNTITEDAPLSAGPASKSLGAARGRRLALSDLATSEPEPPRAASGLSEF
DR
VLGGGLVPASAVLVGGDPGIGKSTLLLQAAAAFANQGLKCLYISGEEAGAQVRMRARRLGLAEAPVKLATETDLRAILTT
LEAEKPDLAILDSIQTLWADMVDSAPGSVAQVRASAHELTVFAKRHGTAVMLVGHVTKEGQIAGPRVVEHMVDTVLY
FEG
ERGHQFRILRSVKNRFGPADEIGVFEMTGGGLAEVANPSALFLSERGTPAPGAAVFAGIEGTRPVLTEIQALVAPSPLGT
PRRSAVGLDAGRLSTTLAVLEARCGIPFAGFDVYLNVAGGLKVTEPAADLAVAAALLSAREDVALPPDCVLFGEISLSGA
LRPVAQTENRLKEALKLGFSSAIAPERSKLGAPGGMKVRQMPDLTAFVGEIFGAG
>OLS49167.1 colicin V production CvpA [Rhodovulum sulfidophilum]AB14
MESFTVVDGIVAAVILVSAILAYSRGMVREIMAIAGWIIAAIMAFTFAPQAEPLVREIPYLGDFIGSSCEISIMASFAAV
FAVALVIVSLFTPLLSSMIQGSVLGGVDQSLGFLFGAARGALLVAVAFFVYGMVITDHSFPAVENSRSSAVFASLTAKIA
ETDPEQALGWLTTQYEQLVGTCGAPQ
>OLS49168.1 amidophosphoribosyltransferase [Rhodovulum sulfidophilum]AB14
MPPSHPFDDDKLKEECGVFGVIGLADAANFVALGLHALQHRGQEAGGIVSYAPESGFQSARRFGYVRDNFTKASLMET
LP
GSLAIGHVRYSTAGSKGQTAIRDVQPFFGEFAMGGAAIAHNGNITNAVALRRELIQHGSIFQSSSDSECIIHLMARSFQK
TIPERMKDALRRVEGAFSVVAMTRTKLIGVRDPLGVRPLVLGRIGEGWVLSSETCALDIIGAEFVREIEPGEMVLISPEK
GVESFQPFERRASRFCIFEHVYFSRPDSILGGRSVYETRRQIGIELAREAPVEADLVCPVPDSGTPAAIGYSHESGIPYG
MGIIRNQYMGRTFIEPTEQIRNMGVRLKLNVNRALIAGKRIILVDDSVVRGTTSQKIREMILDAGAAEVHFRIASPPTAW
PCFYGVDTPQRDKLLASRMSEDEMCEHLGVDSLKFISLDGLYRAVGEAGGRNDRCPQYCDACFSGDYPVAPSDMIQK
GFQ

TKAAE
>OLS49169.1 hypothetical protein BV379_13400 [Rhodovulum sulfidophilum]AB14
MTDQKLALVTGASRGFGAAVAEELARAGYHVVAVGRTTGALEELDDRIKALGGSATLAPMDITETDAMRTLCRGIYDR
WG
HVDLWVHAAIQSPPFSPAEQIDLPFWTKAVKADVEALGVLVSFIAPLLRVAPGGGSAVLIDDQAEGPFLGAYAACKAAQ
R
ALFQAWEAEQARLQDVHVRRFVPAPMPTNHRNRLYPGENTETLARPADEARRMAAELGLA
>OLS49170.1 5'/3'-nucleotidase SurE [Rhodovulum sulfidophilum]AB14
MRILITNDDGINAPGLAVLEAIAAEVAGPTGEVWVVAPAFEQSGVGHMISYTRPMMIAELGPRRFAAEGSPADCVLAG
LY
DVMTDARPDLVLSGVNRGNNAAENVLYSGTIGGAMEAALQGLPAIALSQYLGPLNYDDDLQFEAAAAHGAATVNALL
DRG
TWDGADYRLFYNVNFPPVPADQVKGLKVGPQGYRRDASFGVEPHLSPSGRKFLWIRGGAQNLPTAPGSDAALNLDG
YIAV
TPLRADLTAHDCIADVKARLG
>OLS49171.1 protein-L-isoaspartate O-methyltransferase [Rhodovulum sulfidophilum]AB14
MTDDPTAERKMQFLFALRSRGVTDRRVLTAMEEIDRGQFVTGHFAQHAYDDTPLPIACGQTISQPSVVAIMTQALDL
QPR
NKVLEIGTGSGYQAAVLSRLSRRVYTLERHRRLAAAAQKRFEALGLTNVTAMAADGSRGLPDQAPFDRILVTAAAEDPP
G
PLLAQLRVGGIMIVPVGQSDTVQRLIKVTRGENGFDYDELRQVRFVPLLEGLGQD
>OLS49172.1 hypothetical protein BV379_13415 [Rhodovulum sulfidophilum]AB14
MDLIFPARRLPARLLALGLGMTLVAGCESGWDYDFRNLGNNPTPPRVEAAPRPEPDRRGVISYPTYQVAVARHGDRLA
DV
AGRVGLPASELASYNGISETARLNNGEIIALPRRVAESGSGVAAAPAGNGTITPAGQVDIATLAGDALDRAGGTAPAAS
K
PTGQEPVRHKVARGETAYSIARLYNVTPRALADWNGLGPDLTVREGQYLLIPVASEPRRQNGTATAGASAAAAAQTTE
KP
GQGSAVSPPPSAATPLPRPETTAKPAVPASPQMAKEATAATARFAMPVSGSIIRGYQKGKNDGVDISAKAGTAVKAAA
DG
TVAAITRDTDQVPILVVRHADNMLSVYANIDGIAVQKGAKVTRGQTVAQVRSGNPAFLHFELRKGFESVDPMPYLTP
>OLS49173.1 AAA family ATPase [Rhodovulum sulfidophilum]AB14
MTGQTQDPRFGPEAADSLKRIAEALERRFPAPAAAPDFASAEAFLWHVAPDRLSPVPEVNRVDLTLLQGIDRSRDTLLE
N
TRHFARGLPANNALLWGARGMGKSSLVKAVHAAVAAEGHPLKLVEVQREDLPSVGRLLGLLRGADERFVLFCDDLSFS
HD
DQHYKSLKAVLDGGIEGRPANVVFYATSNRRHLMPRDMIENERSTAISPSEATEEKVSLSDRFGLWLGFHPCSQDEYLA
M
IGAYCAAHGIEIDPETLRAEAIEWQATRGARSGRVAWQFFTDLAARRGVRI
>OLS49174.1 twin arginine-targeting protein translocase TatC [Rhodovulum sulfidophilum]AB14
MTRDDIDDTSAPLIEHLAELRTRLIYALAAFVAGMVACFMVWNPLFNVLTHPICSALAERGQECGLYLIKLQEGFFVAIQ
ISLVGGFALAFPFIAFQLWRFVAPGLYRSEKRAFLPFLLASPFMFILGAAFAYFIITPLAFDFFLGFQQAGSLGAEGGGQ
SETAGITFHGSVQEYLSLTIKFILAFGLCFQLPVLLTLMGKAELVSAAGLGSVRKYAVVGILVLAAVVTPPDVITQVILF
TVVYGLYEISILLVRRGEKTREKELRAQGLWFDDDEDEPEEGPATEPEPRS
>OLS49175.1 twin arginine-targeting protein translocase TatB [Rhodovulum sulfidophilum]AB14
MFDIGWTELLLIGIVALIVVGPKDLPGMFRTLGRFTAKARNMAREFQRAMDDAADQSGVRETVDDLKDIADPRKLGLD
RL
NEAADRFEKWEPKLPKPGESASPATDARAAKAEKIRRAMAETGAANRAAPEAAEGDVPAPEDIPAKTPDRTSDA

>OLS49176.1 Sec-independent protein translocase TatA [Rhodovulum sulfidophilum]AB14
MLNNIGLPGLLLIAVVVLVLFGRGKISSLMGEVGKGITAFKKGVKEETEEAQKSLESTRDVTPETEKDKA
>OLS49177.1 sulfurtransferase [Rhodovulum sulfidophilum]AB14
MHSEDVNGKVLEHWDCDDLEAAMAAGEVVTIDVRTPQEYLFEHIRGALLLPLAGFDPAFLPEGGDKRLVFHCGSGVRS
RK
VAEAWLAAGHDRVAHLEGGFAAWKAALKPYTGTDMASGAPIRVNAKTD
>OLS49178.1 transposase [Rhodovulum sulfidophilum]AB14
MARGFDRQVAELQIRIAVLNHYSALGIPVAEAVG
>OLS49179.1 hypothetical protein BV379_13450 [Rhodovulum sulfidophilum]AB14
MTQQSPSRTPDKDAWPSRVHSVVQGEYHVSRDPDIVLSTVLGSCVAACIFDPRVGLGGMNHFLLPGDAASGSTELKY
GAM
AMELLINELFKAGARRADMKVKLFGGARVSAAFRDIGARNVDFARSYIRREGFEVVSESLGGMQARRLHFRPVTGAAR
LV
LLPPTEAPPEKRIPLPKVKTDIDITLF
>OLS49180.1 hypothetical protein BV379_13455 [Rhodovulum sulfidophilum]AB14
MRVMIADERTGTRNRLAAAVEQMRAVSLGACVSNLSEAYHIAESRPPQAVIVAEALARLAEFEVLLMLFKALGIRCVVV
S
ETAAGMARLPPSLIREGVAVTHSDASVEDLRQCLLRVHLGRTAQKASRRQSDSAMSFQHGRLILLGASTGGVDALVKVL
E
AFPPNCPPTLIVQHTSESFSAGLARLLDRNTAATVEEAQDQEPLVPGRVLLAPGKTHHLEVGVAGALRCRLREGPLQAG
H
RPSVDALFMSAVPVASRVCAAILTGMGRDGAEGMLALHRAGAHTIGQDEKTSLVYGMPRAAYELGGVRDRLPLDSIG
RAL
LENCSRRSAA
>OLS49181.1 hypothetical protein BV379_13460 [Rhodovulum sulfidophilum]AB14
MDVFGHELQHAAELLRRYEKAVLERQVSQAVRQGGASELQLVDLVIQILEDMAPFSQDLAKLMPEETRVPESLIDRLRL
D
QLRAVMGGSEIRDAPHSGGDIDVF
>OLS49182.1 response regulator [Rhodovulum sulfidophilum]AB14
MSLKDSLRIMVVDDMTVSRGLVTQALEELGIFHVATEASGRAALSRLAADPVHLVLADYNMPGMDGLQLLEGLRRNR
STQ
RIGYILVTGTPTPELVQKGQQLGLNNMVRKPFTTATMKKAIEAVVGRL
>OLS49183.1 chemotaxis protein [Rhodovulum sulfidophilum]AB14
MNAPSPSMTPRRRSDIVFEESDFQRIAAFAKSEYGLHLEPSKKAMIHSRLGKRISALNLGGFAEYDKYLKANIEAESEHF
ISALTTNVTNFYREKHHFEQLQADVLPTLIERARSGGRVRLWSAGCSSGPEPYSLAGSVILLCPEAPKLNIRILATDIDP
FVLRRAEAAVYSDEEAKIPPGAWADQVFEKAGGKRTEFRVRSDVHSLVSFRRLNINGPWPMSGLFDVIMCRNMAIYF
DSQ
TQQTLWRRLAEQLVPGGMLFIGHSERVSGPATALVEAAGVTAYRKRSAS
>OLS49184.1 chemotaxis protein CheW [Rhodovulum sulfidophilum]AB14
MPRPQQWGDDDLLEFVTLKAAGQSFCIEITQIREIRRWTPVTALPHAEDAVLGVMNLRGAVIPIIDLAARLGLGECEAS
P
RHVIIVVAIGSKTIGLLVDSVSEILTVEGSQICDAPTMGGGDIPNSILGLISIEEGMSRILAPEILVPRGLTDAA
>OLS49185.1 chemotaxis protein CheA [Rhodovulum sulfidophilum]AB14
MSRDDLKATFFAECEDLLDQLSEGTGELEDGTATEETVHAIFRAVHSIKGAAGAFALDTLVKFAHRLETVLDRLRSGSLV
LDPDSLQVILRSGDMLAELVELAQAESDEEPPQMAELIESLVALSGAEEGGPSAPEPDEAFEFAPVAFMPMSFDAPDEE
P
EDVYKIRVPAIADLFRNGHDPFLLFSELVALGQLEVSCDASALPVSEAFDPDHCYLCWELTLTGTMTEASIRDAFVFLDD

ICEIEQVSGGSAALGGDLPGLQIEEGPMSVSEPENPSLPQVAAEEAAAEPATAASATSEPAAAEKSKAAKPAASMPQTTL
RVDPERVDRLINTVGELIINQAVITQKLLASGMASNSDIMSDLDDYQYLARELQEGVMAIRAQPVKPLFQRMQRIVREAA
YGLGKDVALISEGESTEVDKTLVERLAEPLTHMIRNAVDHGIESSEKRTAAGKPAQGTIKLSAAHCSGDVLIEISDDGAG
LNRKRIFEKAASQGLIAADAKMTDAEIDNLLFMAGFSTAEKVTNLSGRGVGMDVVKTAITSLGGRISISSRPGLGTKFSI
ALPLTLAVMDGMVIHAAGETLVAPLSSVVETIRPKPKDILPFGREGRLLSIRGTYVPIVNLAKLLGFPTDAEITSDNILI
LIRSEQSGLVALAVDDISDQRQVVIKSLEGNYGAIEGISAATILGDGRIALIIDTDAILSLAGHFGRDMASREEMDYAQTATMG
>OLS49186.1 response regulator [Rhodovulum sulfidophilum]AB14
MINILAIDDSLTIREMVRETLSSAGFAVTTANDGQDGVDQFSKDRYDAVITDINMPKMDGFGVIEHIRGGGTNARVPILV
LTTESGASLKERARAAGATGWIVKPFQDEGLISVIRRVTGAR
>OLS49187.1 hypothetical protein BV379_13490 [Rhodovulum sulfidophilum]AB14
MESECQYDLDITDPAWSEDAGGVMKSVLEAAPKKLLVQAAEAPAQSALSAQLLLFLRQYVESSGGTFSVADASPAFSEGL
SLLGLHDMILGTAEAQQ
>OLS49188.1 hypothetical protein BV379_13500 [Rhodovulum sulfidophilum]AB14
MTAPEDTAEDPVLALLQGYSPPNGVADELLRPDGSLRPVWRPLIELLARQSPEQMAERFARGDQYLHDAGVYYRQHTQNG
SALRDWPLSHVPVVIDGAEWQGLVEGIVQRADLLERVMADLYGTGDLVANGLLPAELIARNPEWLRPVVGIRPPSGHFLH
FLAFEVGRSPDGSWLVLGDRTQAPSGSGFALENRMATSRVFQDLFPAANVERLAGFFRAHRNAMTALKAGGEGKVAILTP
GHHSDTYFEHAYIARYLGLLLLECEDLKLERGRLMVRTIHGPQPVSVLWRRLDSRFADPLELDENSALGTPGMLAALRAQ
DVTMLNCLGSGVLEARALMAFLPRICEKLMGAALKLPNIATWWCGQDWERSYVYENLHRMMIGRALSSDLPFEVDASTAL
GGDFRGTARRPIGEWLERDGPDLVGQEAVTLSTTPAMIDGRLLPRPALIRVFATRTPGGWVVMPGGYARIGRSGDPTALG
MKNGGSVADVWVTSERPVEPETLTGPGEGPFLRRAPSLLPSRAADNLFWLGRYIERAEARVRELRAYHLRLSEGGDPDLP
LVARLGAFIEGFGVDIGEPVPAAVLELFEAAQSCAGKVRDRFSTDGWHALTDLSFTARGHFSRVPPGDPAVRAMGELLRK
LAGFSGLVHENMFRSSGWRFLTMGRALERADQMISLLTVFADPAQPAGGFDVAIEVGDSVMTHRRRYSVHTDRNTLIDLL
GLDIGNPRSVLFQLDLVREQERMLARSAPGEPMSDLARAILLLHTELSAAVPEDIGPDRLYRFQQDVRDISTLLTQRYLS
>OLS49189.1 IMP dehydrogenase [Rhodovulum sulfidophilum]AB14
MAITATIRHLTHYRYDRLVTLGPQVIRLRPAPHSRTRVISHSLKVSPGGHFVNHQQDPYGNWLSSFVFPEPVRELKIEVD
LVADMTVYNPFDFFVDETAEDWPFDYPADLAEDLSIYRSPEPAGARMQAFLASVPREKIRTIDFLVALNARLEREIDYLI
RMEPGVQSPEETLRLASGSCRDTSWLLVQVLRHLGFAARFVSGYLIQLKPDLVALDGPAGTDHDFTDLHAWTEVFLPGAG
WVGLDPTSGLLSGESHIPLAATPHYRNAAPISGMASPAEVDFRFHMEVHRTAEHPRITRPFPEDAWQALDVLGRKVDARI
AAQDMRLTMGGEPTFVSIDDFEAEEWNTGAVGPMKRGLADRLIRRLRDRFAPGGFLHYGQGKWYPGETLPRWTFSLYWRR
DGVPVWNNPDLIAEETHTGATAEDAGRFLSGLAEAIGVGAEYVQAAYEDPVEWILREADLPVNVTPEDAKLKDPETRARI

ARVFSRGLDTPAGYALPIQRWQSKAAAGRWRSERWKTRRGHLFLIPGDSPVGFRLPLGSLPFVPPSAYPYVYPADPSAA
S
GPLPDFHAGREDRRLALMQEFERIAREQETAAMPAAMPAATPGRTQPVSEPQAAGPRQTVIEQGVDPKADAVRTAI
AVEP
RDGRLCLFMPPVEALEDYLELLAAAEDTAAALGLPIHVEGYTPPQDARMNVIRVAPDPGVLEVNVQPAQSWEDCKAIT
FG
VHEEARQCRLGAEKFMIDGRHCGTGGGNHVVVGGATPMDSPFLRRPDLLRSLILYWQRHPSLSYLFSGLFIGPTSQAP
RI
DEARHDSLYELEIALSQVPDPGEGTAPPPWLVDRLLRNILIDVTGNTHRTEICIDKLFSPDGPTGRLGLVEFRGFEMPPD
ARMSLAQQVLIRAILSRLWDDPVKGAPIRWGTALHDRFMLPHFVWADFMEVLRDLRDHGFAFRPDWYEAQAEFRFP
FCGE
VDYEGVHLELRQALEPWHVLGERGAIGGTVRHTDSSVERMQVRLTTTNPGRYSVTCNRRAVPLAETGTSGLRVGGVR
FKA
WQPAEALHPVLPVNAPLVFDIYDAWSGRALGGCTYHVAHPGGRSYDTFPVNANEAEARRLARFEKMGHHAGQYRPV
PDPV
HPEFPMTLDLRRPPGP
>OLS49190.1 nucleotide-disulfide oxidoreductase [Rhodovulum sulfidophilum]AB14
MATRIVVLGGGFGGMYAARALRRQFGRKAEIEVINGQNYFVFQPLLPEVAGGSIMPAHAVSPLRFILGGIFVRKAVVDS
V
DFDRKVVTVFQGIQRRPTEIAYDHLVIALGQGTDFSRMPGLEEHALKMKTLEDARRLRGHVIEQLEHAQVTQLPDTKRG
A
LTFTVVGGGFSGVETVGEMKEMIDRSLRFYPKIDPSEVRVQLIEFAPRILGEMPKELADYAVGHLERHGIDIKLGTGVKS
ATHRQLVTTTGEVIDTRTIVATIGNAPLPVVQRMGLPLDKGRIPVDRMLRVPGVEGVWALGDCALIPLKEGASERADFA
P
PTAQFAVREAKRVAQNIAATVKGRAPKPFAYASRGALASLGAKRGVANVFGRNITGFPAWFIWRSYYLALLPGIGTRIR
V
MINWSLDMLGARSLVQLKFYGKPPLRYVYYRAGDRIYNAGDRSDGFYTVITGAVEMERPDSESGAMTTRIIGPGGHFG
ER
LILGATRRRTTVRAKEDCKVLVMNREEFMMLAEGFSAFRDYFQPYMEERGVALPGGDEKDGR
>OLS49191.1 C4-dicarboxylate ABC transporter substrate-binding protein [Rhodovulum
sulfidophilum]AB14
MTRATALTAYLRLCHWLDRATLAFCVVAALFLTGATLAIVILRYGFGTGSIQFQNAADYAFASLLVFSIPVTLARGGHVR
VEVFSERLSPRYIRIADIVAVLVFLIPVFGLMIRAFWPDLSYSWSIREGAIETGGLGGLYIVKSALPLASALMILQGIAM
ILKPSGKPSERGKDMQETL
>OLS49192.1 tripartite transporter [Rhodovulum sulfidophilum]AB14
MTGQEGVLLALLLGLFGGILSGIPAMLAIAGVPLVTALIASAFGLFDLSFLNFFPGRVWGVMSNRLLMAVPLFVLMGVLL
ERAQLAERMLGVLGRLTRGSPRGMGLSVLAFSALIAASTGIIGATVVMLVLISLPAMRSAGIDKRLAAGLICASGTLGQV
IPPSIILVLLGDQIGNVYLEAQQAAGNFAPDPVSVGDLFAGALLPGLMLVAIYAIYVALRLGPAPEPRAPREPVPASEIL
FTFLPPILLILAVLGSILAGVATTTEAAALGAIGAMLMAAQRAEGGAFGRWLIASAGLAALVLVGMGMAGLGRAALGTL
P
GLLALGAAALIAAGTLMAGLRTWRAGLLQGALGDTVRISGMVFGIVVAASLLSLVFRGFGGDHLVEELMASLPGGGM
AAI
FLVMAVIFVLGFILEAVEIIYIVVPLLGPPILGTDISPVWFAILIAMNLQTSFLTPPFGFALFYFRSVAPPDITTLDTYR
GVLPFVALQLVALGVLVALPELATWLPQAIFR
>OLS49193.1 UTP--glucose-1-phosphate uridylyltransferase [Rhodovulum sulfidophilum]AB14
MIPSPVRTAIFPVAGLGTRFLPATKATPKELLPVIDTPLIQFAIDEARAAGVERMVFVSHPSKTAIERHVRADAGLIETL
RARGKARLAAELAAAALDEDEHEVEFVMQHEQRGLGHAVLCARSQVLPGPVAVLLPDDLILSEPGCLAQMVEAYGRA
GAG

HMVAAMDVTPETVSRYGVLDPTGQEGRITRARRMVEKPAAAEAPSMKAVVGRYILDAAIFDTLASQGPGANDEIQLTDAI
ARDAARLGLAGFGFDGVRYDCGSKSGMLDATLALASRDPDCAEVLARWSDDSPTAIAAE
>OLS49194.1 hypothetical protein BV379_13535 [Rhodovulum sulfidophilum]AB14
MFFLAVWLLSLTAPALAQKAGIGAWENSRYTTIRWIEDHPALGWYYTWRTDQLWTGRPVSRSVEFVPMIHSERDLAAAIR
SDRPVRALLAFNEPDGHGAHQGGLSVARALALWPQVEAKGRGLRIGSPAVTRDGTLGPGSWLRRFMTEADRRGYRIDFMA
VHYYSTDGDVAAFRRWLQAVHRAYRRPIWVTEFALVDWDRPGAGSYAASAAFLEGAVRMMDGLPFVERHAWFAANPYPWK
GAVPKINLVDDHLRPTPAGQAFRRVLSELGGREIARSD
>OLS49195.1 paraquat-inducible membrane protein A [Rhodovulum sulfidophilum]AB14
MTAPSPTEPDLSAPDLSAQDLSELIACPQCDAVYRAVQPAPRARAVCARCHTVLIAPQRQAGQRLIAVALTVVVLICAAT
LTPFLEVEAGGMSNATSVLDAALAFAGRPLLLVLTLAVAALIVFVPVLRALLILYVLTPIVFDRPPARHARAAFRLSETL
RPWSMAEVFAIGCAVALVKISDLAHVAFGPAFWMFSALVILIVVQDTFMCRYSVWRSLDR
>OLS49196.1 paraquat-inducible membrane protein A [Rhodovulum sulfidophilum]AB14
MGLVACTRCTRIWPAGTRHCARCGQKLSQRAPLSLQRVWAWWCLGLIAYVPANIYPMLETRTLVTTQDNTIIGGAVEIAR
HGNWGIAAVILTASVLIPIGKFLAVAGLALGITRGSRLSRVRRQQLYEVVEYIGRWSMIDVFVVAILSSLVQLGAVVSIS
PGTASLFFAVSVIATMLSAQSFDSRLLWDKTDPPPAPRSHMA
>OLS49197.1 paraquat-inducible protein B [Rhodovulum sulfidophilum]AB14
MTDLPPEAPVEPAGRGLLDRISIVWVIPAAALAIALGVAWHSYVERGPLLELTFETASGIKAGETELHYRDVTVGLVEKV
GFTDNLDEVLVSVRLDKAVAPYVDRDADFWVVQPEVSARGVTGLSTVLSGVFIQGIWDDTPDGFVERHRGKSAAPLNREG
RPGLRLRLRAASDAGLTEEAPILYRGIEVGRIGKAEVSRDGSTVEADAIVFAPHDRLISSSTRFWDSSGFSFSLGPGGAR
VDFSSVAALLSGGVTFQSVVSGGKRVEPDTRFEVYPDESTARASVFAAKDGPALTLTAYFAENVEGLSIDAPVMLGGLRI
GRVTALNGTVDADRFGDDRVRLAATLAIQPARLGLEAPASTEDALDYLAGRVRDGLRARLATASILTRRLKVELIETAGT
APAELDRDADPNPVIPTTASDISDVSATAEGLYNRINDLPIEDLMQSAIDALNSVSTLAGSDEIRAVPGEVRALLGEARG
IVGSDEMQALPGRIDGTVAAIETLARQLAEADAASRLTAALDAAAGAAETIDTAAAGLPDLVARLNAVADQAATLPLDEL
AGRISALLDSTDRLIDTDQARDLPTDLSAALRELAAMLAELREGGVVGNANATLASARSAADDISTAARDLPDLLARAQA
VLGQASSTIEGYDASRGMGREIDTALREIQRAAQAVTALSRALERNPNSILFGR
>OLS49198.1 hypothetical protein BV379_13555 [Rhodovulum sulfidophilum]AB14
MKHRLLPAALVPLALALTACSGSPERYTVPDTAVDGPRLPVAWRSVAIRDVSLPTYAASEEIYSRSASGALTSSSAVLWA
DDPSRAITRDLARLLARMTGAQVAAEPWPFFDRAQATLEVRVADMLAESDGTFRLTGQFFVAPDAGGRNRAVPFAISAPI
AAPAGPAEIAAARGVALRELATRIARTGLR
>OLS49199.1 aspartate aminotransferase family protein [Rhodovulum sulfidophilum]AB14
MPDTAPASWEARAESSSFYGFTDLPSVKARGTVVITHGEGPYVIDTNGRRYLDGNSGLWNMVAGFDHAGLTEAAKAQYDR
FPGYHAFFGRMSDQTVMLSEKLIEVSPYERGRVFYTNSGSEANDTLVKMLWFLARAEDQPRRRKILTRKNAYHGVTAVSA
SMTGKPYNELFGLPLPDFIHLTCPHYWREGRPGETEAEFTARMATELEEVIAREGAETIAGFFAEPVMGAGGAIPPSAGY
FQAIQPILKKHGIPLISDEVICGFGRTGHVWGCETYDFIPDAIISSKALTAGYFPMGAVILGPDLADRLHRASEAVEEFP
HGFTASGHPVGCAIALKAIDVILNEGLLENVRRLTPLFEEGMNRLAENPNIGEFRGKGLMGALEAVADKPTKTPFPSTLS

VSERIANTCTDHGLICRPLGQSIVLCPPFILTEGQMDEMFTKLEAALGEVFAGLGAEA
>OLS49200.1 aminoacyl-tRNA deacylase [Rhodovulum sulfidophilum]AB14
MSTATPATQALKRAGVAFRPIEYDYVAGQDRIGLHAAEAIGAEPGRVLKTLMVEIDGKPACAVIPSDRTLSMKKVAAAF
G
AKSAQMMPPDKAERLTGFHTGGISPFGQKRRAPLAFEADALNVPEIVLNGGRRGLMVALAPADALAVAGAQAADLC
AERG
>OLS49201.1 FmdB family transcriptional regulator [Rhodovulum sulfidophilum]AB14
MPIYNYRCTACEAEFELLRSLSDDSAPACPDCGSPEVKRLLSRVAASGESAKLLKGARSLARKEGHFSNYSKSERKKSGV
>OLS49202.1 hypothetical protein BV379_13575 [Rhodovulum sulfidophilum]AB14
MDKDSKQPSTGRIAWGVGIAVTLVTLLMTDWGYLTAGPVHTVSSIAAAVFFGFLAAQSAIFGIEFREKRRREKD
>OLS49203.1 phosphoribosyl-ATP diphosphatase [Rhodovulum sulfidophilum]AB14
MTGPELEALARLATTVEARKGADPDTSWTAKLFSKGPEKCAEKFGEEAVEAIIEAVRGDRARLTAEAADVVYHLLVMLA
A
RDVSLADVAAELARREGVSGIAEKASR
>OLS49204.1 imidazole glycerol phosphate synthase subunit HisF [Rhodovulum sulfidophilum]AB14
MLKIRIIPCLDVAEGRVVKGVNFVNLVDAGDPVEAAKAYDAAGADELCFLDIHATHENRGTMFDVVRRTAEQCFMPLT
VG
GGVRTAEDVRALLLAGADKVSFNSAAVADPDVVHQAADQFGSQCIVVAIDAKTVSPGKWEIFTHGGRKPTGIDAVDF
ART
VVAKGAGEILLTSMDRDGTRAGFNLPLTRAISDAVDVPVIASGGVGTLDHLVEGVREGGASAVLAASIFHFGEFTIGEAK
AHMAAAGIPVRLT
>OLS49205.1 1-(5-phosphoribosyl)-5-[(5-phosphoribosylamino)methylideneamino]imidazole-4-carboxamide isomerase [Rhodovulum sulfidophilum]AB14
MILYPAIDLKDGRCVRLLRGDMDKATVFGEDPAAQALAFQKAGCDWLHLVDLNGAFAGAPVNADAVEAILAAVEVPA
QLG
GGIRDMATIEMWLGRGLARVILGTVAVENPDLVREAARAFPGRIAVGIDARNGRVATKGWAEETDVMATDLAKSFED
AGV
SALIYTDIDRDGAMAGPNVAATEALARAVSIPVIASGGVSSLADLMALRATGAISGAISGRALYDGAIDLAGALAALKG
>OLS49206.1 imidazoleglycerol-phosphate dehydratase [Rhodovulum sulfidophilum]AB14
MRIAIPIAALTLAATAALADPVEGVWQTPLDDTGHFGHIRIAPCGASLCGTLIRAYDSKGRQIETDTIGRQIVWDMKPQ
G
GGRYGKGKVWAPDRDKTYNSKMALEGDVLSVSGCVIGICRDGGRWKRVK
>OLS49207.1 hypothetical protein BV379_13600 [Rhodovulum sulfidophilum]AB14
MDDKPEPEALPPDLKLLRMLVTLLMVTMIGGFLVIVALFVIRMPGQDETLPLPPALTLPDGAEAEAVTRGRDWVAIVTR
D
DRILIYDAATGALRQTVEIRH
>OLS49208.1 RNA pseudouridine synthase [Rhodovulum sulfidophilum]AB14
MSTPLSSPRRLAVIIGPAPPGRLDKALARDVPEQAGLTRSRLAKLIAAGAVRRGGAALTDARAKVAEGERLEIELAEPAE
IATLAEDIPLNVVYEDDALIVIDKPAGLVVHPAPGSPSGTLVNALLAHCGDSLAGIGGERRPGIVHRIDKDTSGLLVAAK
TDKAHAALAAQFAAHTVERQYSAIVHGLPDASDPRLRGVAGASFEPGGVLRIATRIGRHRTDRQRQAVLAAGGRHAVT
RV
RVVTPFGTPPAAALVSCWLETGRTHQIRVHMTHAGHALIGDPVYGGRRRLSEKAMGRDAAAAAAAFPRQALHAATL
GFVH
PETGETLRFAAPLPADMAELMRRLEG
>OLS49209.1 RNA polymerase factor sigma-32 [Rhodovulum sulfidophilum]AB14
MSNYANLPAPSPEQGLNRYLQEIRKFPLLEPEEEYMLAKSWVDHHDTEAAHRLVTSHLRLAAKIAMGYRGYGLPQAEVI
S

EANVGLMQAVKRFDPEKGFRLATYAMWWIRAAIQEYILRSWSLVKLGTTSAQKKLFFNLRKAKARIGALEDGDLRPEN
VQ
RIAHDLNVTETEVIAMNRRLSGGDASLNAMVGSDGDTTTEWQDWLEDEDANQAEAYAEKDELDSRRALLTEAMDVL
NDRE
KDILMQRRLQEQPVTLEDLSTVYNVSRERIRQIEVRAFEKLQKRMKELASQKGLLAQA
>OLS49210.1 hypothetical protein BV379_13615 [Rhodovulum sulfidophilum]AB14
MVSGIQRDLDRLAEAATRADIDSFEAISAELRNRAVADGGLQPDLLREIAAERCRLTEGPAFRDLNARLRAAHAAAERC
G
DTGRLQTLSAALRRQNAILLAALRTPEARHELLPGPAQALFDAIATGNLARLGAALRGTDPNARHGSRGETALGRALSVE
HRSPAVLRQLIAAGADPNAGSAGGHAPLHELGVYPRPWEPPEITAKLARLLVEAGARIEAETGAYRWTPLHRAVLEGSP
P
VLEALLALGADPNRPFDERSEPWFTPGRLPLQIAAADHAMVDCLLRHGAAPQALDARGEGVLPYLEGQLGHHLAFDGL
GE
LYRGEVEIAMRTIRNWPPRS
>OLS49211.1 malonate transporter [Rhodovulum sulfidophilum]AB14
MEALIEVILPVFLVLGFGYVAVWRGLFSDAGVDGLMKFTQHFAIPCLLFRAVSTLDLGQNFDWRLLLSFYTGALTGFAA
G
LLGARLMFRRTWEDSVAIGFIGLFSNSVLLGIPITERAFGVEALTPNYAIIAVHSPICYGVGLTAMEIVRNRGAGLVRTS
GAIFRAMFRNALVIGIALGLAVNLGGIPVPLVVQNAVDLMVKAALPAALFGLGGVLARYRPEGDLRTVLFACAVSLVLH
P
AVVWGMGTMLGLSTGQFRSAVLTAAMAPGVNAYIFANMYGAARRVAASAVLFATLGSILTVWVWLALMP
>OLS49212.1 phosphoribosyl 1,2-cyclic phosphodiesterase [Rhodovulum sulfidophilum]AB14
MSAELRFTILGCGSSGGVPRLGGHWGACNPDNPKNRRQRCSMLVERETAAGVTRVLIDTSPDLRAQLLQAGVGEIDA
VVY
THAHADHVHGIDDLRQIVFNMRRRLPVWADGPTQEALIARFGYAFIQPEGSPYPPILDLHPIDGPVTVMGAGGPVTL
MPF
RARHGAIDALGFRIKGLAYLPDVAELYEESWAMLEGLEILVLDALRYEPHPTHLNLETALGYIDRAGPRRAVLTNMHIDL
DYDRLEAETPDSVTPAYDGMVLRLAD
>OLS49213.1 LuxR family transcriptional regulator [Rhodovulum sulfidophilum]AB14
MSDTETPQITDSHCHLDFPDFANELDQVLARAEAAGVTRMVTICTRLRQEPQVREIAERYAPVFYAAGTHPMSAAEEP
MA
TLDELLALTKHPKFVGIGETGLDYHYSADSAEVQKQSLRIHIAAAREAGLPLIIHSRAADDDMARILAEEYRNGAYSCVM
HCFSSSPELARATLDLGFYLSMSGIATFPKSAEIREIFAAAPLERILVETDSPYLAPPPHRGKRNEPAFAAHTARVGAEV
FGLEPAEFAARTQANFDRLFAKAAGWRDAA
>OLS49214.1 DNA polymerase III subunit delta' [Rhodovulum sulfidophilum]AB14
MSGTELPEPDRIEGAPHPRDTVRLIGQGAAEAAFLDAFTGGRMHHGWLITGPRGVGKATLAWSVARFLLTRPAAAEG
AGL
FGAAPAPDSLAVPADHPVARRMAAQSEPGLFVLRRGPNDKGNALSAEIRVDDVRRLKSFFALSSAEGGRRVVIVDTAD
EM
NASAANALLKLLEEPPANTTLLLVSHSPARLLPTIRSRCRELRLGPLGPAEMAEALAQAGQDDSQGAALAELSGGSVGEA
IRLARNDGLALYADLVALLAGHPRLDRMRLLKFAESTVGRGAEGRFDLVLRLIELALARLARTGATGTPPPEAAAGEAAL
LTRLAPDAAAARLWAEAAQDLIARARQGRAVNLDPASLILDTVLRLDETAGRLAA
>OLS49215.1 dTMP kinase [Rhodovulum sulfidophilum]AB14
MDRAARFISFEGIDGSGKSTQARLLAERLRGQGCEVVLTREPGGSPGAEQIRRLVLEGDPDRWSPETELLLFTAARRDHL
ERTVRPALGRGAVVICDRFADSTRIYQGITRGDLRATVDRLHELMIGCEPDLTFILDMAPATGLARARGRNGSEERFEDF
GEALQARGRQGFLDLAAEFPGRCRVVAADRAPEEVARDIARLADEALA
>OLS49216.1 hypothetical protein BV379_13650 [Rhodovulum sulfidophilum]AB14

MQAYRIARVVLLLGVAGGLSACQGAGFGLFKKDDTETMPVRAESSAAAEAEGEEAEAPEVFHVEEAGLWDGRPSLGG
IWV
AHPDAADPERVLIRNSETGQSVTGALFRRERENPGPRLQVSSEAAARIGLLAGQPTEISVTALRRAPVPEAGGRKAEPAE
ADAGGRFVQVATLDSADGAEATAARLDKAGLSAEIRAPDPGGQGLWRVVAGPAKTEADRAVLIEAVRGLGYDDAYAV
NR
>OLS49217.1 hypothetical protein BV379_13660 [Rhodovulum sulfidophilum]AB14
MSNGPWTDAENDLIVADYFAMLADDIAGRRYSKAAHRRALLPLLNDRSEGAVEFKHQNISAVLKALGEDWIPGYKPAF
NF
QMTLVDAVARRLTPHPDWLGRLPGPRPANGMGEAAQLWIGPPPTLSNQPPPQELDQMLRIARKFDVAGRDERNRA
LGRAG
EERVLAHEQATLKAAGRDDLARKVRWVSEEDGDGAGYDIASYAPDGRSRLIEVKTTNGWERTPFHISRNELAVAGERR
AE
WCLFRMWNFSRAPKAFELYPPLDAHVSLTPTSFQASFH
>OLS49218.1 hypothetical protein BV379_13665 [Rhodovulum sulfidophilum]AB14
MARGSFLANGFKPSIAVSISSPDQIRVLPMIRTGEGFERYVNRLLRRASLAGHAPRGLRDPAPCFLAALPMDAPSAGAA
E
AQ
>OLS49219.1 daunorubicin resistance protein DrrC [Rhodovulum sulfidophilum]AB14
MTDKPMRILRARTNNLRNLSVEIPKGRIVVFTGVSGSGKSSLLFDTIAAESQRQLAETYPSFVRNRLPHHGQPDADRLEN
LPASIVVDQRRLGGNRRSTVGTATEIYALLRLLFSRIGKPHVGESSLFSFNNPDGMCPLCQGLGTVRRIDEAALFDRDRT
LDEGAILFPGFEPGSWRWKRYALSGFFDTSKPLSAYSDEEWRRLTVEQGTPVTDPKPGWLKSTVYEGVLPRLRRSFLDR
D
EADNSEAERDALTRVTTSGPCDACGGGRLNPAVLSCRIAGRNIADCAGMEVGDLLEFIRGIDVPEMRPVISSLTEKLESM
IDIGLDYLSLDRGTSGLSGGESQRIKMIRHLGSSLADIAYIFDEPSVGLHARDVDHLGQLLVRLRDKGNSVLLVEHDPDL
IAIADHVIDMGPGAGTSGGSIVYQGALAGLRQADTPTGRGFRKSRSLNHMPRRPSGWIGVRNQSMFNLHDLSVDIPL
GVM
TVIAGVAGSGKTTLASRILPGLRSDVVVIDQAELRGSSRSTPASYLGIMDDIRREFARSSRKPVGLFSTNAAGACPVCKG
RGTVRTDLAFLDAVEATCEACGGSGYGVEARSVRVAGCSIDQVMGMRAAEARALFRRHGALSDALARLEQVGLAYM
QIGQ
RLSTLSGGERQRLKLAAELGRAGRIYVIDEPTSGLHMADVDRLTELFRALTGRGITLIVVEHNLDMIAAADHIIEMGPGA
GRRGGNIIYQGGPAGLIADSLSVTGPYLARAFGCGPATAVPSRT
>OLS49220.1 MarR family transcriptional regulator [Rhodovulum sulfidophilum]AB14
METKNLDELEGALSLLQCELVARRNRFTPETVTWAQYDVLDVLRIEGALRPSVMSERLGVARPQLSKALRVLKDLGLVD
Q
FPDEEDRRVQATTLSAHGKAFMERAALQRRDAAQRVASAMTPAEQAMFAELCRKAVRGLQAEVNGND
>OLS49221.1 aminotransferase [Rhodovulum sulfidophilum]AB14
MARRFSRSFTQQEPLSDAAIEAAVAVLRHGRLHRYNLTEGEAGEVALLEEAFAAFTGARFALAVASGGYALATALRALQ
V
APGEPVLTNAFTLAPVPGAIASLGARPVLVEVTPDLVIDLGHLEARIAGTGARVLLLSHMRGHICDMDRLMAICDGAGV
A
VIEDCAHTMGAAWNAVASGRHGAMACYSTQTYKHLNSGEGGLLISDDPELMARAILLSGSYMMYPRHRAAPGPEHF
EGLL
AEMPNVSGRMDALRAAILRPQLAELPDRVARWAELYRALEAGLRGCPGITLIERPAAERFVGSSFQFRLPGWPPERIAD
F
LGRTAARGVELKWFGAEQPQGFTSRYVHWGYAAPEPLPQTDRVLAGLIDLRLPLTFSPADCHEIAAILVEEIAADRA
>OLS49222.1 haloacid dehalogenase [Rhodovulum sulfidophilum]AB14
MRTVIFDLDGTLADTSRDLIDAANACFEALGQGRPLDPEADAATAFLGGRAMLRLGFERLGRIGEDAPVAEQFPLLLDH
Y

AAGLDRHTRLYPGAVAAVERLRAAGIGVGICTNKPAALAEELMTRLGVRGLFASLVGADTLPVRKPDPAPYALAVEQVG
G
QVGRSMLVGDSGTDRATARAAGVPCVLVSFGPAADEMPALEPEALMADYAELDGLVAKLLPAA
>OLS49223.1 UDP-N-acetylglucosamine diphosphorylase/glucosamine-1-phosphate N-acetyltransferase [Rhodovulum sulfidophilum]AB14
MPTALILLAAGQGNRMMSDRAKVLHEVAGAPLLAHAMAAGRALDPARTVVVTGHQAEAVRAAALDIDPEVAVAEQ
TERLG
TGHAVAQARPALEGFSGDAIVLYGDTPFIRPETLEAMAAARARHAVVVLGFHAADPKRYGRLVMAGETLDRIVEYNDA
TE
DERALTLCNSGVIAADCATLFELIGALSNDNAAGEYYLTDVVGLARARGLSAGVVVCDEAETLGVNSRTDLAAAEAAFQ
A
RARTGALENGVTLVAPETVFFAFDTVIGRDAVIEPNVIFGPDVTVESGARIRAFSHLEGCHVSRGAVVGPFARLRPGAEL
AEDVRVGNFVEIKNAQVAEGAKVNHLTYIGDATIGEQANIGAGTVTCNYDGVFKHRTEIGAHAFIGSDTMLVAPVRIG
AH
AMTASGSVITSDVPEDALALGRARQQVKPGLARRLMDRLRAEKAKRKG
>OLS49224.1 glutamine--fructose-6-phosphate transaminase (isomerizing) [Rhodovulum sulfidophilum]AB14
MCGIVGVLGDHEAAPLLVAALGRLEYRGYDSAGIATVNAGRLDRRRAVGKLVNLSDLLVHEPLPGKAGIGHTRWATHG
AP
TLANAHPQRAGRVAVVHNGIIENFRTLRDELGARGIGFESDTDTETVAHLTQTYLDEGLAPREAAARALDRLEGAFALLF
LFEGEDDLMIAARKGSPLAIGHGEGEMFVGSDAIALAPLTNRITYLEEGDRAVVTRAGAEIFDAAGDLVLREIRTIEIDA
TRIEKGGYKHFMAKEIAEQPAVLGDALHHYLGEEGTEITLPGGGIDFSGVTRITMVACGTAYLACLTAKYWIERLARLPV
DVDVASEFRYREPPIPEGTLAIFVSQSGETADTLAALRYCTGRADRIVSVVNVPESSIARESDLALPILAGAEIGVASTK
AFTCQLAVLALVALDAARQRGRLAPADLARHLASLQAMPARFNAALALHEPIGRLARRLAEATDVLFLGRGAMYPLALE
G
ALKLKEISYIHAEGYASGELKHGPIALVEKTVPVIVMAPHDRLFDKTVSNMQEVMARGGPVLLITDARGAAEAGSGVWE
T
LIMPEASDLFAPMLYALPVQLLAYHTAVAKGTDVDQPRNLAKSVTVE
>OLS49225.1 hypothetical protein BV379_13700 [Rhodovulum sulfidophilum]AB14
MTVFARICRARHGRSPPHRDRKLQGQRFRIEGVFFMRAVPDRQPPGQMRRIAICVAATVMFR
>OLS49226.1 hypothetical protein BV379_13705 [Rhodovulum sulfidophilum]AB14
MRSLLNTLRLMLKELRAIRRDRVMAVLILYVFTVATWMVANAASTEIRDLAVAVVDEDRSPLSHRLTDAIRPPLFQEAP
V
LSPEAAAAAQLEGRYVLVLSIPPDFERDLRSGHAPKLMLLVDATAMAQAGNGATFLQQLLADELQDYADPGGDGTAP
VDV
VFRNRFNPNLTATWFTSVMQLMNNVTILTLILSGAAMIREREQGTIEHVLVMPVRPHEIVLSKILASGLVILLASVASLV
TVIHWGLGVPLTNSLGLYVLGAAVYVVAVGSIGLLLATFTRNMGQFGLLVIPVIIVMLLLSGGVTPLESMPDWMQAVIR
T
VSPAPHFVAFAQSVLYRDAGFAQVAGELLRMAAMAALALSVVLVRFRKVLSG
>OLS49227.1 multidrug ABC transporter ATP-binding protein [Rhodovulum sulfidophilum]AB14
MAPVPEHSPGAGGIAAEIAGLTHRYGGKAALSGVGLAVPAGCMTGLIGPDGVGKSTLLGLVAGVRRLQEGQLRVLGR
DMS
DPAARRALAPRIAYMPQGLGRNLYPTLSVRENLEFFGRLFGQGAAERGHRIEMLLEATGLAPFPDRPAGKLSGGMKQK
LG
LCSALIHDPDLLLLDEPTTGVDPLSRRQFWELIDRIRAEMPGMSVLVATAYMEEADRFEWLAAMNAGQVLASGSPAEL
KA
KAGAATLEEAFVRLLPDRPEAPPLVSRPLGEGSGRIAIGARHLTRRFGDFTAVDDVSFTIPEGEIFGFLGSNGCGKSTTM
KMLTGLLDPSEGEAELFGRKLRASDIESRMRIGYMSQGFSLYSELTVRQNLELHADLYHLPKERRGPRVDEVLAEFELDG

LADRLPWDLPLGQRQRLQLAVATLHAPQVLILDEPTSGVDPVARDAFWRTLMSLSRDKGVTIFISTHFMNEAERCDRIS
L
MHAGRVLDVGTPAEIVARRGAASLEEAFISCLEAELAPDQTARAAPVAAPQTETVPRFGGLTRLWAYCWRETLELMRD
PI
RVFFALAGPAILLLTMGYGISFDVDRLSFAVRDQDRSPESRELVEQFTSIRQFAETENLGGPSDLGDRMARGDLTVALEI
PDEYGRDLRRGDVPEVSLWIDGAMPFRAETARSYTLGLMSDYAARRAPGAGPAVGIESRFLFNQAFKSAHAMVPSM
MMVI
LMLIPAIMSAISVVREKETGTIANFRSTPVTRIEFLLGKQLPYLAIAWASFWMLYAIGRTVFGVPFTGSLPALAASSFLY
VFATTGFGQLISAFTRSQVAAVFATSVLSIIPAVNFSGLILPVASLDTPGRLVGQSFPSAWYQAVTNGTFLKGFGWAEIA
PNAAVLAGFCMIYLSLAILGLRKQEA
>OLS49228.1 MFS transporter [Rhodovulum sulfidophilum]AB14
MPDRPSALAPFRKATYRNLWLASNISNFGGLVQTVGAGWMMTELTHSPVMVAMVQASNTLPIMLFSLIAGALADSF
DRRR
IMLTAQIFMLAVSAVLAAAAFQGALTPWLLLAFTFLIGLGRALYNPSWQASVGDIVPRSDLPAAVALNSMGFNLMRSV
GP
AVGGLIVAAGGAAAAFAVNALSYLPLWLSLFLWRPALAERRLPREALGPAISAGLRYVSMSPVLLRVLFRGFLFGLSAVA
LLALLPIVAHELLDGGAFGYGVLLGCFGTGAIGGAVSSARLRDRFPNEAIVRGGFLGFAAALTGLAFSHELWISGLLLLP
AGASWVLSLSLFNVSVQLSTPRWVVGRALALYQTCTFGGMAAGSFLWGQVAAGAGLAEALWAAAAVLVLGALVGLR
LPLP
NFGTEDLAPLGRFVEPQLGVDLRARSGPIAITVEYDIAPGDVDAFLAAMTERRRIRIRDGARQWVLLRDLERPTVWAES
Y
HVATWAEYLRHHERRTVADAEVSDRLLALHRGAGKPRVRRMIERQTVPRRDELPLKPTDIA
>OLS49229.1 hypothetical protein BV379_13725 [Rhodovulum sulfidophilum]AB14
MRSPFTTGPFKSGSRLLIGFGAVLLALGLGGAAAMHFGLVPVSARPPHNALTTAVLHDTFRASVERRAREVEPAPDLAD
P
GLIALGARHYGQVCSSCHGGPGLGQSPVALSMRPRPQSLPAVVDGFSASELYVILRDGVRFTAMPSWPSDGNFNEIW
AVV
AFLRQLPEMTPEAYRAATVADVAGAGAPPAMAWTRRGPQMPVKLHDEAAPIEDYLYAAPSTGWRPIGLQDVPVAYC
AACH
GADGTGAPTGGQAPNLAILDADTIAARLRAYAEGTRESGIMATVASSLSGPQIEALAAYYADLPDRTLGAPDRGDPERG
A
VLARRGDFARALPACGACHGGNAGASGLGVPRLAGQGAAFLERRLDSFAAEDGPDRQGWNPMPAIAKALSPDERAA
VATY
LAGLPADDPLIPARPPVPAEAGADDEAAELLLRCTACHDPGPALGADRDLSPNLTLQSAAYLDHQMALFDAGRRPVGS
MQ
QAAHEMTAEQIGRLSAALGAAPAARSGLDPERARRPDFELAEAGKLAEKGDPARGLPACLSCHGARPTAAVGLLPRLD
GQ
RARYLEDRLDYFAGDAAPEVLAPMTEIAARLTGDERAALADWFAARPPLAK
>OLS49230.1 hypothetical protein BV379_13730 [Rhodovulum sulfidophilum]AB14
MRRLGCPSCGAEIWFHNTACLSCGAELGFQPGRGLVPLGTCGGTACANRAVIGCNWVAAAPGGLCLSCAHSTVVPDL
SVP
GNDRRWARIERDKRMLMRLLDALGLPLRDAAGAPAPVFELRGDPIGPAPGPKVITGHAHGTITLNIAEADDAERERIRA
E
MGEPYRTLAGHLRHEVAHHYWDLLVAENPAALDRLRPVFGDERADYGAALERHYREGPPPGWEATHVTAYATAHP
WEDFA
DTWAHLFHILDSMETALAYDLLRDDGLQGLEVAALAALPVERLAEPWIALSVALNAVNEAMGHGRFYPFVLSPAVLEKL
E
AIRRLLPVAR

>OLS49231.1 hypothetical protein BV379_13735 [Rhodovulum sulfidophilum]AB14
MNQFFRRLFPLLLATLFLSSLPAQAQTAPAAEDAPGAEARALIDILRNDSAREALIAELEQAAGRGAADRDRAAAVPSEA
DGQGLGRRVAEATSAAVEDIAGRIAAGWGALGRAGQAFEGLDAGTFEAFGAAMRNLLAVVAATVLVQFALRRAVRP
LHRR
LGRTAAGQGLLRRLAPAALAALLDLGALVLAWAAGYALAAAAIGSAGTVGLNQSLYLNAFLVVGVVRLALDILLAPGAA
P
LRLLPVSDPGARRTAGLGMAIAGLLAYGHLLAVPVVARDVSALAAASLGALVTVLALLSLGALVVLYRRPVAQWMLTRL
A
PPVSSAPGTEDAPEPQTAPETGAPRRHGRLALALAQNWHWAALAYLAVLAAIVLTGPDGVVLAALAGSARLLAALLLA
GI
VSGALARAAARGVRLPERISDRLPLLEPRLNRVIPVAMVVLRYLIVLSVALFALDTLGVLAVGSWMESRFGLRVTGALSA
VLLILAVAYGLWLALTSWVDYRLAPAPGRAASPREATLLSLLRNALTIVIVVLAAMFCLSEIGLDIGPLLASAGVLGLAI
GFGAQKLVQDVITGVFIQLENAMNVGDVVSLGGTTGTVEKLTIRSVSLRDVSGTYHIIPFSSVDMVSNLTRDFSYYVCD
M
GVAYREDVDEVKVAMEDAFAELGRDPEARDAIRGDLEWFGLNSFGDSAVVVRARIRTLPGRQWGIGRTYNGILKRLFD
ER
NIEIPFPHQTITFAEAKDGRTQPVRVINGEAQAAEQGA
>OLS49232.1 hypothetical protein BV379_13740 [Rhodovulum sulfidophilum]AB14
MPAPTSPRSRPLRLAIAGLGPRGLGALEALAARLPQAGSSVAIDIFEPGQWPGAGPNFAPGQSPLMLLNIPIRAVDIDPP
GPAPARISSFGTWLMSPSDDERFPARAELGAYLAARVRTLAATLPEGVALSRHETAVLRLERSGTGWYLESAAGRFGPY
D
EVLLAPGQPRSAPDPQLARWQDHAGRTGAAVLPAYPADRLLKAAEDWAGASVAIRGLGLSTLDVVRLLTLGRGGRFE
DGR
YLPSGREPARILPFSLDGLPPWPKPASAGIDARFAPLPAESRSFEAALERALAAAPEAALEAISAALEPAARRVLEAAGA
DPGGVAAWLAAERAAPASQESRDAAELLREGLAMARGTRPPATGYAIGQIWRHWQNALRRAVNPGTASPETVAALI
GFDE
GLKRYSYGPPAETAEELSILIAAGIVDLRTVDDPDILLTPEGWQLVEEDDSARVSVMIDAVLPSPALERLEEPVLAGLRD
AGRVAAVGEGLGIRTRPDAQVVDSRGQVQAGLCLLGRAALGSVIAVDSIHDCFGASAHRWAEGVLDRAALGAARAAS
PG
>OLS49233.1 hypothetical protein BV379_13745 [Rhodovulum sulfidophilum]AB14
MGPRDLTGRAMQEVAAGSAGRFSSPEDGAADRGRQIIPSRLSCSVGRNPLIRFKCSVYTVDLKNYSTVVRSMTA
>OLS49234.1 TetR family transcriptional regulator [Rhodovulum sulfidophilum]AB14
MRSDKRDELVRKALDVFYRDGFHATGLDRLSDETGISKTTMFRHFRSKDDLILATMQRRDDGVRAWLRHRMDAAGP
PRAR
LMALYDALGEWVAEPGFRSCLFLRAAAEYPDPANPIHRLATEHKRRLARQVEAVAAEAGAADPGGLSRALLLLKDGAA
VA
AHLGHAENPVADARAGAEAILAATLPVARQGGLPSARTGAPP
>OLS49235.1 C4-dicarboxylate ABC transporter [Rhodovulum sulfidophilum]AB14
MTFSTRILAFSAAAFALGAGMAHADTWRYAFEEAMDEVQGIYAQKFKEEIEANTDHEIQLFPYGTLGESADIMEQAQD
GI
LQFVDQSPGFTGSLIPEAQVFFVPYLLPQETETLASFFKNSKAINEMFPALYADQGLELLTMFPEGEVAITTKEPFTTPA
DLDEVKIRTMTNPLLVESYKAFGATPTPLPWGEVYGALQTNIIQGQENPMFYVESTKMYEVTDYVTRIGHNNFTTAVM
AN
KDFFDGLPDEDKKAIRAAAEAAFAHIIDYQKGLTEASVEKIKEAKPSMTFTTLSEEERQPFKDAAGEVEARFLEMTGDSG
KRVLDQMKEDLEAAQQASQS
>OLS49236.1 C4-dicarboxylate ABC transporter permease [Rhodovulum sulfidophilum]AB14
MAAAIFSIMIVLLLLGFPMMIPLIAGALVGFVTLFGGLGQLETMIQQMMAGIRPASLIAVPMFIFAADIMTRGQSAGRLI
DMVMAFVGHLKGGLAISTAAACTMFGAVSGSTQATVVAIGGPLRPRMQKAGYGDSFILALIVNASDIAFLIPPSIGMIIY

GVVSNTSIAELFIAGIGPGLLILLLFALYSWAYAVRQGVPTEPRVSWGARLRAVRAALWPLGFPVIIIGGIYGGIFSPTE
AAAACVLYALILETIVFREMGLRDIYRTAKSTGLITGVVFILVAAGAAFSWVISFAQIPQAILSAVGIDQMGQIGVLFAI
SVAFFIGCMFVDPIVVILILVPIFAPVVSDVGLDPVLVGTLITLQVAIGSATPPFGCDIFTAIAVFKRPYMEVIRGTPPF
ILILLGVSVALIFFPQIALFLRDLAFHGGAG
>OLS49237.1 universal stress protein UspA [Rhodovulum sulfidophilum]AB14
MFKSILVPYDGSAGAERALRQAVELAGLCDAQLSVLTVFRHHSMLEASLSMVRRDEPGPLDDAMRGYAREVAEYAKRL
AR
EAGAEGARAFIKAGPPARTIIRFAREHENDLIVIGSRGLGSVENYLLGSVSHKVTGLADCPVLVV
>OLS49238.1 hypothetical protein BV379_13775 [Rhodovulum sulfidophilum]AB14
MAGDRAASWQSIRDTIMLRIRDRTYPPGAFIPNEADIAAEFGCARATVNRALRDLAEEGFLDRRRKAGTRVIEAPQRKA
T
LEIPQIRREVEARGQVYGYRLLARETGPAPGELRARLKLPEAEATLYVESLHLADGAAHMFERRWVVLRTVPGIAAADLA
VVSPNEWLVREAPYTHGGMEVHAEPAGQAVATALDCAPADPVLVMERQTWLHEAPITYTREHFAQSYRLCLGL
>OLS49239.1 formimidoylglutamate deiminase [Rhodovulum sulfidophilum]AB14
MQRVWARHALVMNSWAKDVEIAVESGGKIAELRENVACPPGATVVETLLPAPANLHSHAFQRAMAGLTESRGPDPR
DSFW
TWRRLMYRFLDRIGPEDVEAIAAFVQMEMLEAGYGTNVEFHYLHHGPGGAAYADPAEMSGRIAAAAAVSGIGLTLLP
VLY
TYGGLDRRALVAGQDRFGNDPDRFARLFEGAAAHVEALSPDCRMGVAPHSLRAVDAAGLAAAQALAPEGPIHIHIAE
QQA
EVDEVRAATGLRPVEWLLENAPVDARWCPIHATQMEPAETLALAATGAVAGLCPITESSLGDGIFDGVRWAQAGGRF
GVG
SDSNIRISLSEELRTLDYSQRLRDRTRAALATGDRSTGRVLFQGAAEGGAQAAGRASGGLAVGNWADLLALGDMGPD
GIG
RQGDLLLDTWIFARDDRAVSDVWAAGRHVVRGGRHIRRDEIVAGYAAAMRRLRDGL
>OLS49240.1 imidazolonepropionase [Rhodovulum sulfidophilum]AB14
MTDLTLANATILPLGEDAPRRIERGWLRIEGDRIAALGDGPAPEGGPVEDLGGRLVTPALVDCHTHLVYGGSRAREFEM
R
LEGADYEEIARAGGGILSTVTATRAASEDDLVASALPRLDCLLAEGAGTIEVKSGYGLTVEDELKMLRAARRLGRERPVR
IVTSWLAAHALPPEYKGRAEAYIDEIAIAGLRAAHAEGLVDAVDGFCEGIAFSVPQMARIFDVADELGLPVKLHAEQLSH
LGGSLLVVERGGLSADHVEHADAADAAALARAGAAAVLLPGAFYTLRETQMPPIAAFREAGTAMALATDCNPGTSPLT
SL
LLTMNMAATLFRLTPDECLRGVTANGARALGLTDCGRLAPGLRADLAVWDIAEPAELTYRIGFNPLHARYFEGCKC
>OLS49241.1 histidine ammonia-lyase [Rhodovulum sulfidophilum]AB14
MLTLTPGETTLAQLETLWREEAPARLAETTGPAIAASAARIAAAAQGDQAVYGVNTGFGKLASVRIPPEDTATLQRNLIL
SHCCGVGAPLDIATARLVMALKLLSLGRGASGVRPEIVALIEAMLEKGVTPAIPEQGSVGASGDLAPLAHMAAVMIGA
GE
AIYQGETLPGAEALARAGLTPVTLGAKEGLALINGTQVSTALALTGWFRAMACLRAAIVTGAMSTDAIMGSTAPTRAEI
H
ALRGHRGQIDVAAAMRGLLKGSEIRESHREDDARVQDPYCIRCQPQVTGAALDLLRFAGQTLETEANAVTDNPLVLSD
GS
IVSGGNFHAEPVAFAADQIALAVAEIGAIAQRRVALMVDPALSFGLPPFLTPEPGLNSGLMIAEVTTAALMSENKHLAA
P
CSVDSTPTSANQEDHVSMAAHAARRLGGMTRNLAVILGVEALGAGQGIEFRAPLATSEPLKRALEAIRAEVATMGEDR
YL
APDLARAQALIETGVLVAAAGLEMAE
>OLS49242.1 N-formylglutamate deformylase [Rhodovulum sulfidophilum]AB14
MTPVEISRGDSPVILGLPHTGTHVPEDILARLNARGRELTDTDWHVHELYDGLLPGATTVRATFHRYVIDANRPPEDESL

YPGQNTTGLVPLTDFDGEPIWDTTPDAAETARRLAEFHAPYHAALAAEIARVRASHGIAILYDCHSIRSVIPYLFEGTLP
DFNIGTANGTTCAPAIAEAAAGVCAGAEGYSHVQNGRFKGGWTTRHYGRPETGVHAIQMELAQSTHLTAEAAPFALD
PDK
AARLRPHLAAILARIAALAPELSS
>OLS49243.1 CoA transferase [Rhodovulum sulfidophilum]AB14
MKPLDGLRILDLTRVLAGPFATAILADLGAEVIKIEPPRGDEYRHIGPFREGESALFSLTNRGKQSVVLDLKAPEGQALA
RKLAAGCDVVVENFRPGVAARLGLGAEDLRRDNPGLVHCAISGFGQSGPAADLPAYDIVVQAMSGWMDATGEEDG
APLKV
GEALGDVAAGLYAVIAILAALVGRGRTGKGATLDVAMLDCLVAMLPTSHALHLYANAPVARVGNRHPFSTPFGAFRTA
DG
HVVIAVLSPRQFEGLCTAIGRPEAADDPHFATDELRTAHEPEIRAMIEAWTGLRPTAQAVTAFAAAKVPAAPILTLADQL
GSDHANARGLVSELPHPRLGQAPVVGQPVRFDGQAPLAETGAPALGEGTRAVLERLGLDQGQIAGLVAAGIVRECED
A
>OLS49244.1 acyl-CoA dehydrogenase [Rhodovulum sulfidophilum]AB14
MREFDHLLSAEECQFLDMLDRFAAEVLSPQAAETDRTGAFVHDQLAALAETGMMGANLPERWGGADISAHALFEAV
AAVA
GGCGSTVSALTAHFLATDSLLLGGDDAIRARFLPAAAEGRMLGAFGLTEPNAGSDPADMRSRATRDGDSWHIKGRKC
FIS
NGGVADFVVVFCVTDPEAGHRGISAFVVEKGTPGLVPGRVEQTMGLKGGHVWELDLDVTVPDANRVGPEGSGFKTA
MKVL
DNGRTEVAAMAVGIARAALSEARDWAKSRIIGGEPLASRQGIQWMLADMATELSAAELLGHEAARQRQAGLRFTTA
ASKA
KLYASEAAGRIADTALQIHGGYGFTHDFPLERHARDLRIMRIYEGSSEIQRNIIAGRLLA
>OLS49245.1 urocanate hydratase [Rhodovulum sulfidophilum]AB14
MTDPRHNTRDIFPPTGPEITAKSWQTEAPMRMLMNNLHPDVAENPHELVVYGGIGRAARTWQDFDLMVETLKTLE
DDQTM
LVQSGKPVGVFRTHRDAPRVLIANSNLVPHWATWEHFNELDRKGLAMYGQMTAGSWIYIGAQGIVQGTYETFAEAG
RQHY
DGNLRGKWILTGGLGGMGGAQPLAAVMAGACCLAVECDEDHADFRIRTRYCDEKTHSLDEALAMIGRWTEAGEAKS
VALI
GNAADVFPEILRRMQAGEPLPNGRPDIVTDQTSAHDPFHGYLPKGWTVAEWRARQESDPKGVATAARASMKDHVA
AMVGF
WNAGIPTLDYGNNIRQMALEEGLEDAFAFPGFVPAYIRPLFCRGVGPFRWAALSGDPEDIYKTDAKMKELFPENAGLH
RW
LDMARERIAFQGLPARIMWIGLGDRHRAGLAINEMVRNGELKAPVVIGRDHLDSGSVASPNRETEAMKDGSDAVSD
WPLL
NALLNTASGATWVSLHHGGGVGMGFSQHAGMVICCDGSEEADRRLERVLWNDPATGVMRHADAGYEIARDCARE
YGLTLP
GILK
>OLS49246.1 hypothetical protein BV379_13815 [Rhodovulum sulfidophilum]AB14
MRPIRRNDLEPRPWKNGGGLTWELAAFPEGAGFSDMLWRISLAEVGSDGPFSAFPGIDRTLTVLAGRGMILDFGAGE
VKL
GEDSAPLSFPGEAPVTARLSAGAILDINVMTRRGRAAHAVHALSAGEPAPRDTRVLVARQNPVTVDGFALAPGEALMS
DP
GRDLGGAVGDAPMLAVSLRLVP
>OLS49247.1 amino acid ABC transporter [Rhodovulum sulfidophilum]AB14
MTLRLSMKSALFGLSALALSASGALADQLSDIQAAGKIVAATEMHYAPFDMLEKGEYVGFDRDLFDEVAKELGVTPEYQ
D

LPWTSILPGLEVDKFDFVLAPVTMTAERAERYAFTLPISDSTVAFVQRTGGDMETPEDAKGKTVGVQKGTAQQAQLEA
YA
ETIGGIKIKEYGTPDEAYADLMTRRLDAVAGSAPLLDYLAKSRPEQFSVVKPPFGKPTYFAWVARQGEGDTLVAAINEAL
IKIQEDGRMAKIQEKWFGTATELPQEMPEL
>OLS49248.1 amino acid ABC transporter [Rhodovulum sulfidophilum]AB14
MFSFQTIVENLPVLLGAARYTLLISVLGCLQGLILGAILCAAALSKRKSLRRAAGLYVSFFRGVPLLIQLLVAYYLLPVI
GINVPPLAAAVLTVGLCAAAYLAEVLRGSINAIPKGQAEAALAIGMSPGNLWVRVLLPQALKIGLPAIVNELILLVKASS
LVTVVGIVEITRMSQSIASSTYRYLEIYLAAAFLYLAINLVIAAAGRLLERRLTV
>OLS49249.1 amino acid ABC transporter [Rhodovulum sulfidophilum]AB14
MLEMLHRYGPDLLTGFGLTLLTWVLGTAGGLVLGFLMEMARRFGPRIWVGWPIRAYVEFIRGTPFLVQLFVLYYGGPLI
G
LRLDALPAGVLGLSLYGAAYFTEILRAGFDAVPKGQIEAARAIGLPGRTIVWRIMLPVMMVSALPALVNFAIILTKETVI
LSMITVPEIMFQTQTMMSETFAFVLPILILALFFWGFVEVISRFGRRLERRFTHYLLEES
>OLS49250.1 ATP-binding protein [Rhodovulum sulfidophilum]AB14
MSLEPTATTPASAHLRAVNKFYGDFQALKDITLDIPPGKVTCLIGPSGSGKSTLLRCINFLEEYDSGEVRIDDRLIGYET
PGGRRMSGRRLREMRRDIGMVFQQFNLWPHMTALQNVAEGLERVRGLKHAEATERAREALAKVGLADKEANHPSR
LSGGQ
QQRVAIARAIAMQPRIMLFDEPTSALDPELVGEVLAVMKQLAAEGMTMVVVTHEMGFAAHVADRVVFMERGELVA
AGAPR
EIFQNPTDQRLASFLQGYRERNSLDF
>OLS49251.1 alpha/beta hydrolase [Rhodovulum sulfidophilum]AB14
MRLFALLSLFCPTVALAGPVQIEGPAGPLEAEALAAPGARAAVVIVPGSGPVDRDGNAPALGLHSDSYRLLAEALQTAGI
ASLRIDKRGFGGSAGAIADPNDVTIAAYAGDLRRWVARAAGLAPCVWIAGHSEGGLVALVAAEEPPEALCGLILMATP
GR
PVGQLMVEQMRALPGNAPLMPEIEAMVATLEAGDRRDPETLPPPLRPLFGAGLQDYMADLFAYDPAAVAERWHGP
VLILQ
GGADMQVRPEDAGLLAAAMPQARRADLPGVTHMLKADLPGRPYATYTDPALPLAPGVVPAILEAVEGR
>OLS49252.1 carbonate dehydratase [Rhodovulum sulfidophilum]AB14
MPDRSDRPFHALTAEETLAELSAGPGGLAPAEAAERLALHGPNRLPEPPGRNAVLRFLAQFHNVLIYVLIVAAGVTAAL
Q
HWVDTGVILAVVLVNAVIGFVQEGRAEQAMAAIRDMLAPRSAVLRGGRRVTVEAADLVPGDVVLIEAGDRVPADLRL
IEA
RGLRVEEAILTGESVPVEKAVAAVAPEAALAERLPMLFSGTLAAAGTGRGVVVATGPATQIGRISGMLASVETLTTPLVR
QMDLFARWLTVFILLVAGSLLAYGFYVGHMSFGELFMAVVGLSVAAIPEGLPAVLTITLAVGVQAMARRNAIVRRLPAI
E
TLGSVSVICSDKTGTLTRNEMMAARLALGEAELAVAGEGYAPEGEVCPVDVAPGPGSEAGTDPGLLAALARAAALCND
AV
LHEEGGVWRVEGDPMEGALLALAGKIGAPGGDRTRRDAIPFDAAHRYMAVLVEAPEGGAELLVKGAPEAVLALCADR
MAP
GGGTVPIVPADWHRKVEELAADGLRVLALARRAMPAGQERFSHDDLAGRLSLIGLVGLIDPPRPEAIAAVADCHSAGIR
V
KMITGDHAATARAIAAEIGLKNTDRVLTGADVEALDDAELAAAATGTDIFARTSPAHKLRLVTALQARGLTVAMTGDG
VN
DAPALKRADAGIAMGLKGSAAAREAAELVLADDNFASIAAAVREGRTVYDNIKKVISWTLPTNAGEALTIVVALFAGM
ML
PITAVQILWVNLITAVTLGLALAFEPSEPGTMRRPPRARTEPLLTGGLIWHIVLVSGLFVLAVFGIFAYATERGHDPALA
QTMAMNTLVVLEIFHLFFIRNIHGTSLTWTGLRGTRIVWACVLTVTGAQVAVTYLPLLQPVFGTRPVPLEDGLLIVGIGV
IFFALIETEKQMRLAFRRGQPGR

>OLS49253.1 hypothetical protein BV379_13855 [Rhodovulum sulfidophilum]AB14
MKTVLVGTDFSERSDRALRRATLLARQTGAALKLVHVVDDDLPVRIVEAEREAATGLLQQQAATLRAHDGLDCSAHVV
LA
DPFAGLIEAVPQLGADLLVIGPHRRQLLRDVFVGTTAERTIRAAACPVLMANAPPVGPYRHALLATDLSPGSRGALRRF
G
GLGFGHRLRGSVLHVFDATEAAMMRIHATREDDREAHLREAGAEAARALNAFLRDLPQGPMVELVRHRNGSAASEIL
AAA
AETEADLIVVGTQSRGAFETMVLGSVARQVLRNSTADVLAVPPGAA
>OLS49254.1 cobalamine-related hypothetical metal-binding protein CrdX [Rhodovulum sulfidophilum]AB14
MQPKDLTDNEAFKGFTNSACPFLPCHKGVAREFNCLFCYCPLIAYECPGPYQTYTDANGLTRKDCSACTLPHDGYLQS
WN
FIQRWLEYPQPWSGKPQTDPPVRRPRPPKPTDADEIHRRRREAGAAGDGGD
>OLS49255.1 succinylglutamate desuccinylase [Rhodovulum sulfidophilum]AB14
MTARAEFTLGRETVPPGSRRTVDLPVSVLSDHTPVTLSAHVVHGRRPGPAMFVTAGVHGDEVIGVEIVRRLLSLPVLGA
L
RGTLIAIPIVNAFGFLNLSRYLPDRRDLNRCFPGSSGGSMAARLAHLLVEEVVGRCQIGIDLHSAAVHRTNLPQVRVMPD
DPELARLARVFAAPVVLSSEARQGSLRETAARMGVQMLLYEGGEGLRFDEVAVRAGLAGVLRVMFDRGMIPAKGVPK
ARR
APLDCRVSRWLRAPQGGLLRTYRSEGDVVAEGELLAVVSDPFGEIETEIRATEPGIIVGRAMLPVVNEGNAVFHLAAIAP
ADRAEDRIGAWASEVAAGQLFDEDEIL
>OLS49256.1 MATE family efflux transporter [Rhodovulum sulfidophilum]AB14
MTEAVARPDMTPPLRLGHRRVLAIALPIVLSNVTVPILGAVDTGVVGQIGEAAPIGAVGVGAVVLAALYWMFGFLRM
GTT
GLTAQARGAGDADEVAAMLTRVLMIAAAAGLGLIVLQGPLFTAALGIAPASAEVEGLASQYMAVRIWSAPATIALYGL
TG
WLIGQERTRAVLAIQLWMNGLNVALDLVFVLALGWGVPGVAAATVLAEWSGLILALWLCRAAFRDVAWRAWARVF
DRARL
RHIAGVNTDILIRSMLLQGIMLSFLFVGAGFGDVPLAANQVLLQFLEITAYALDGFAFAAEALVGQAMGARSVAALRRG
A
VLTSGWGLVSVIGLAAAFAFGGGAIVDLMTTAPEVRAEARVYLPYMVAAPVAGLAAWMLDGIFIGATRTADMRNM
MALSA
AIYALAVALLVPVFGNHGLWLALLVAFVARGVTLGLRYPALEAAARSGQGG
>OLS49257.1 thioesterase [Rhodovulum sulfidophilum]AB14
MSRRPPEPVQIVKQRRDAALAALVEGLPYARFLGISFDRRGDELTGVMSFHEALIGNPALPALHGGATAAFLELTAIVGL
SWAMIWDDLESGRLEAATLTPETLPRLPKTIDFTVDYLRPGLPRDAYARARVNRSGRRYASVHVEAWQDNRARLFAQ
ATG
HFLMPT
>OLS49258.1 thioesterase [Rhodovulum sulfidophilum]AB14
MSDPKTDLARRFIEAIPHSHALSMQVDALGDGRAELSMPYDSRLVGDPHGGVISGGAVSALLDTCGGTAVISHPEGPG
QT
ATISLRIDYMRAATPGQRLVARAECYHVTRSVAFVRATATDDDRDHPVATATGTFTADGGAA
>OLS49259.1 MerR family transcriptional regulator [Rhodovulum sulfidophilum]AB14
MSDVRLNFKEMCARFDVTPRTLRYYEYIELLSPTKSGRSRFYGPREVARMTLILRARRFGFSLEEIRQWLLIYDEQGREA
QMAALVGLADRQLKVLRDQRTQLDASIEELQTLRDAATGQA
>OLS49260.1 MerR family transcriptional regulator [Rhodovulum sulfidophilum]AB14
MTDELMTIRQMCDAFDVTARTLRFYESKELISPLREGQRRLYGRRDRARLKLILRGKRFGFSLEEIRQMLDLYDLDDGQE
TQFRRTCELARERLAAMERQRVELDDAISELRRQLAWGEAKLEHFEKRKPAAE

>OLS49261.1 acyl-CoA dehydrogenase [Rhodovulum sulfidophilum]AB14
MPIYTPPTEDMQYLLHDVLKIGGSGIPGYAELDREFTGAILGEAGKLAAEVLAPLNASGDAEGCQLENGVVRTPGGFKA
A
YDQICAGGWIGLDMPEEVGGQNMPYLINSAVGEMFVSANMALNMYQGLSHGAMSAILAHGSDEQKATYVPKMVA
GTWSGT
MNLTEPQCGTDLGLIRTRAEPQEDGTYAITGTKIWISAGEHDLTENIVHLVLARIPGGPEGIKGISLFIVPKFLPDENGA
PGRRNGVACGGLEQKMGIHANATCVMNYDGATGFLIGEEHKGMRAMFTMMNEARLGVGLQGYAQGAVAYQNAL
AFAQERL
QGRALGDPANPAGPADPILVHPDVRRNLMDQKSFVEGARALTFWGAQMIDRAHRMQDPEAEAMISLLTPVIKGFLT
DKGF
ETTVLAQQTYGGTGFTRAAGAEQFVRDARIAMIYEGTNGVQSLDLVGRKLGLNGGKAVMAFFDLIKTFIAEHEDDAAL
KE
DFLDPLKSASKDLQAAATYFMAEGMKTPENALAGSYDFMHLFGHVCLGLMWSRMAVASRAALAAAEGNAAFHEAK
LVTGR
YYMARQLPATALHLARIRSGAEPVMALDAANF
>OLS49262.1 glutathione S-transferase [Rhodovulum sulfidophilum]AB14
MTIRLYCFGESGNAYKAALTLTLAGLDWEPVYVDFFNGGARSPDFLALNPMGEVPVMIAEGVTFTQSGTMQLWAAE
QSGR
FGGRDAAETREVLRWMFWDNHKMSGQAGSLRFLMNFLPEEKRPQEAIRWLSGRVKAALLTLERVLQDRDWLVGDG
PTTAD
FACCGYLFYPEPFGFDRKAFPAIDAWLGRIEALPGWKHPYDMMPGSPADRA
>OLS49263.1 acetyl-CoA acetyltransferase [Rhodovulum sulfidophilum]AB14
MTDAFIYDAVRTPRGKGRKDGSLHEVTSLRLSALVLDALKDRNGLEGHAVEDVIWGNATQVGEQGGCLARSAVLASGL
DE
SIPGLSINRFCASGLEAVNLAANQVRAGAGRAYIAGGVEMMGRVAMGSDGAAIAVDPSLAMKTYFVPQGISADIIATE
YG
FSRDDVDGLAVESQKRAATSWEEERFARSIVPVRDVNGLTILGRDEYMRPGTDMQTLGALKPSFKEMGETMPGFDQL
ALM
KYPHLERIEHVHHAGNSSGIVDGAAGILIGDAEFGKAHGLTPRARIRASCKIGTDPTIMLTGPVPATEKILKDAGMQISD
IDLFEVNEAFAAVVLRFMQAFDVDPAVVNPCGGAIALGHPLGATGAIILGTLLDELERTGKGTGLATLCVASGMGAATII
ERV
>OLS49264.1 3-hydroxyacyl-CoA dehydrogenase [Rhodovulum sulfidophilum]AB14
MAEFHYKTDADGIATITWDVPEKSMNVLSLTGIEELNAHIDSALADDAVTGVVITSAKKDFAGGMDLNIIARMKEEAG
DD
PARGLFEGTMRLHGLLRKIERAGMDDRNKGGKPIAAALPGTALGIGLELPLACHRIFAADNPKAKIGLPEIMVGIFPGGG
GTTRLVRKLGAMGASSFLLEGKLSDPKKAKAAGLIDEVCEDPVAAARAWVLSEPNIVKPWDERGYKMPGGAPYHPAG
FMT
FLGASAMVHGKTKGVYPAAKALLSAVYEGALVPFDTALKIEARWFTHVLMNPSSSAMIRSLFINKEALEKGAVRPRDVP
D
QTVKKLGILGAGMMGAGIALVSAQAGIEVVLVDQSQEAADKGRAYTAAYMDKGIKRGKASPEKKAEILGRITATTDYG
AL
KGADLIVEAVFEDPGIKAEVTKKVQAVVDGNCIIATNTSTLPITDLAKAADRPEDFIGIHFFSPVEKMLLVEIIRGKATG
DRAVAKALDFVRQIRKTPIVVNDARFFYANRCIIPYINEGIRMVAECVAPALIENAAKLVGMPLGPLQLVDETSIDLGVK
IAKATKAAMGEAYPDDAVDEVIFWLADEGRLGRKAKAGFYAYDEAGKRSGLWDGLAAKFPPAADQPDLHEVQHRLLF
AQV
LEAVRALEDGVLMDIREGDVGAILGWGFAPWSGGPLSWLDMIGAGRAVALCEDLTARHGDRFAPPALLREMADKGE
SFYA
RFAPDAQAA

>OLS49265.1 hypothetical protein BV379_13915 [Rhodovulum sulfidophilum]AB14
MVFRIDFLGTRPVSELVAAAGDAAGALPPVGLARGFRAGTRVATREGCRPVELLEPGDCVLTVERGAQPVRRIERGCL
WR
GPSACPPPLWPLEVPAGALGNRRRLTLLPEQRVLIDAAHAAETHAERFVLLPARMLAGVMGIRRVAPPAEVCVVSIAFD
A
PATVYANGAARVHCRSWQEEGAGATDRPVDLRPEMPCCGITPGLDEARRIVAALEGAHAGAAIPPRLPMVQTVPLLD
ALL
RHAA
>OLS49266.1 acyl-CoA synthetase [Rhodovulum sulfidophilum]AB14
MGWLSDETGLEKCAANYVPLTPLSFLKRAVRVFPDRTALVYGKRRYSYRQYHDRVTRLASALAAMGVKPGDVVATLLP
NV
PAQAEATWAVPACGGVLNTINIRLDANTVSYILDHGGAKVLLVDPQFLPLAADALEDLEGERPVIIEVGDDPEGVHDFG
D
FPEYEDVLAGADPDFEWIMPEDEWESLALNYTSGTTGRPKGVVYHHRGAYLNAMGQIVSWRLVMHPTYLTIVPLFHC
NGW
CHTWMIAALGGTVVCCRDITARAIYDAIADEGVTHFGGAPIVMNMIVNARDEERRDFDHIVEVFTAGAPPAAATLAAI
EP
LGFNITHVYGLTETYGPATECTWRAEDWDGLPTEERARMKARQGVAMPFMEDVTVLDPKTMEQVPMDGQHQGEI
MFRGNG
VMKGYLKNPRATRESFKGGWFHSGDLAHQWEDGYFEIADRAKDIIISGGENVSSVEIEGVLMHHPDVLLCAVVAKPDE
KW
GEVPCAFVELKDGHKVDGDALIAFARERLAGFKTPKRVVFQDLPKTSTGKIQKFELRKVAKAL
>OLS49267.1 hypothetical protein BV379_13925 [Rhodovulum sulfidophilum]AB14
MTALKKFQRLESPGLWRESPEAQRRDVILSFGNASLMVSDSAERVLTHWSLAALERLNPGQMPALYSPGADSGETLEIE
D
DLMVDALETVRKAIGRTRPRPGRLRIWIVAATAAGIAALALFWLPGALVRQTVAVAPPEIRAEIGQAVLARMARAAGTP
C
ERAGGQRALDRLSARLLPGSGKRVRVLPGGLPRALHLPGGLILLNRALVEDHEDVAAVAGFILAEDLRATRSDPLADLLR
AAGPLATFRLLTTGTLPEAALSDYAREVLIAPEAPLPEAELLARFREAGLPSTPYALALDVTGESQRGLIEGDPLRGRET
DPPISDGDWLRLQAICGG
>OLS49268.1 hypothetical protein BV379_13930 [Rhodovulum sulfidophilum]AB14
MRFPRYCQAVTVALALCAGGTGAVRADPVAAGAVPLQPPHGYREVWEDDRLNPLRGPRTEWGDAQMRRVWTEQ
VPQRLVD
PTTRRRAVAEAPVAGVAAPGRRYIQVGAFTEAGAAAEAVADLRRLGLPARQGRMMAGDALVTVIFAGPFGDRDSMG
RARG
ALVGAGYAPVVTGR
>OLS49269.1 uracil phosphoribosyltransferase [Rhodovulum sulfidophilum]AB14
MYDHLTVVTHPLVQHKLTLMRDKATSTAGFRQLLREISQLLAYEITRELEMTTRRIETPLREMDAPVLAGKKMALISILR
AGNGLLDGVLELVPSARVGFVGLYRDEETLQPVQYYFKVPQDLKDRLVIVVDPMLATGNSSAAAVDLLKQAGANDIRF
MC
LLAAPEGVARMKEAHPDVPIVTASLDERLDEQGYIMPGLGDAGDRMFGTR
>OLS49270.1 adenosine deaminase [Rhodovulum sulfidophilum]AB14
MTELPKIELHHHLEGAAPPALIRDMARARNLDISRIFDADGGYVTGDFLQFLDTYEAASSALRTPEDYARLTSAVLEETA
AEGVIYLETFLAPDFCGGCDLSAWVDHLEAIREAAGRAEGQGILLRANVTCIRHFGPDRARAAALCAAETAGDFVVGFG
M
AGDERRGRPRDFAYAFDMAREAGLGLTVHAGEWSGPASVREALADLRVDRIGHGVRAIEDPGLVDHLVEAGTVLEICP
GS

NVALGLYPSLAAHPVERLRRAGVKLTVSTDDPPFFHTTMRREYARLAEIFGWEDSDFAAMNRVALDSAFCDAETRAKL
KT
RLEAADV
>OLS49271.1 phosphopentomutase [Rhodovulum sulfidophilum]AB14
MTRAFLAVMDSVGCGGAPDAAEFFNAGQPDTGANTLVHIARACAEGRAETGRSGALKLPHLDALGLGAAIRLASGEG
APG
LCAEPQGLWGAATEVSRGKDTPSGHWELAGVPLPWDWSYFPREVPAFPEDLLAEIRRIAGTGGTLGNCHASGTEILDR
LG
AEHMRTGWPILYTSSDSVFQIAAHEEVFGRARLQALCAAIAPRLHAMRIGRVIARPFIGTPETGFVRTAHRHDYAMAPP
A
PTLCDWAQAAERRVQAIGKIGDIFSMRGIDEVRTGPDADLMTHWADLMEEAPEGSLTFANFVEFDSLYGHRRDVAGY
ARA
LEWFDAALPRILGRMRPGDLVLITADHGNDPTWRGTDHTRERVPVIGAGRGTRALGLRAFADTAASIATHLGIPPQGP
GR
SFL
>OLS49272.1 thymidine phosphorylase [Rhodovulum sulfidophilum]AB14
MDARSIIARVRDDEALTDEDLRWFAEGLATGAVTDAQAGAFAMAVLLNGLSDPGRVGLTRAMRDSGKVIEWNLPGP
VLDK
HSTGGIGDCVSLALAPALAACGAFVPMISGRGLGHTGGTLDKLESIPGYRGEVSLAGLKSITAQVGCAIVGASDDIAPAD
RRLYAIRDVSGTVESIDLITASILSKKLAAGLEALVLDVKVGSGAFMKDMDEAEDLARALVATAQGAGCMTAALITDMS
Q
PLVGAAGNALEVIEVMETLTGTGVTPALWDLTCALGGEVLALGGLAADAADGAQRIGRAIESGDAVLKFGEMVAELG
GPA
DFIERWPDRLPAAPVLCEVASPAGGIVQAIDGAALGRAVVALGGGRRREGDRINPAVGLAEMAALGDRIAPGEPLAIIH
A
ASETDADAVAGQVRAAFTLGDETPGIPPLVQRRLLR
>OLS49273.1 cytidine deaminase [Rhodovulum sulfidophilum]AB14
MPLLTAARAVRENAHAPYSRFKVGAAILGADGRIHVGCNVENAAYPEGTCAEAGAIAAMVAAGCREIREVAVIADSPD
PV
PPCGGCRQKLAEFAAPGVPVTLATTGGASRVVTVAELLPGAFAPAHMGD
>OLS49274.1 propionyl-CoA synthetase [Rhodovulum sulfidophilum]AB14
MSYAEVYEQWKADPNGFWMEAAEAIDWVEKPSKALFDENAPNYEWFNDGMVNTCWNAVDRHVEQGRGEQVAII
YDSPITH
TKREITYVELRNRVATLAGALRDKGVTKGDRVIIYMPMIVEALEAMLACARLGAVHSVVFGGFAAHELAVRIDDAKPKCI
MAASCGIEPGRLVHYKPLLDGAIDQATHKPEFCVIYQREQEVAQLTEGRDVNWHGFQYGVQPAECVPVEGNHPAYILY
TS
GTTGQPKGVVRPTGGHLVALNWTMKNIYNLDPGDVFWAASDVGWVVGHSYICYAPLIHGNTTIVFEGKPVGTPDAG
VFWR
VISEHKVKSFFTAPTAFRAIKREDPNGEFVNKYDLTGLKALYLAGERADPDTIKWAQRMLGVPVIDHWWQTETGWAIA
GN
PLGIEKLPVKVGSPSVPMPGYDIHILDEGGHEVGPNTLGAIAVKLPLPPGTLPTLWNAHERFEKSYLAHFPGYYETGDAG
YKDDDGYLYIMARTDDVINVAGHRLSTGAMEEVLASHPEVAECAVIGVADELKGQLPMGFLCLNAGCSRDHGEIVREC
VR
LVRDQIGPVAAFKLACIVDRLPKTRSGKILRGTMSKIADGMDFKMPATIDDPAILDEIADAVKRLGYPQAA
>OLS49275.1 hypothetical protein BV379_13965 [Rhodovulum sulfidophilum]AB14
MAERGFRAELTESGHGAQKRAAMLGHDMRAAISDILGGLSLAELGPLDPDSRLQLKRVQSAAEQLARLTDETLALVSG
DE
LDQIEAPIHTTLQPFLDRITGRWQAHAEEHGLAFTLDTKPDLPGIIGTDPVALERILSNLIGNAMKYSSAGTVRLKAHMG

PRESLCFRVRDTGPGFSDAAMARLFEFAGRPAESSQPGTGLGLHIVRDLARRVRGQLQLANLAEGGAEVSVILPRAAW
AP
GVQSTGDLHDLPDLSCCEVLLAEDNPTNQLLMRQMLETLGARCTLVKDGQAAAEMLAAHRFDLVLIDIEMPRLSGIDV
IR
RLRTAEGDDPGTAVLAVTAFVLAANRDQIYAAGADGILAKPIMSLDALGEAIMGVLNRRPCNCTPLSDSPPPFDALHLD
R
LLALAGPEDGRELLIRMHDDLETVRGGLLEAFERGDRTLLLSRAHVLISLAGAVGNGALQALAENLNAAARRQDNTEVG
R
LCPRIVEHIEALCTALKAEFGSRYNKAGA
>OLS49276.1 sigma-54-dependent Fis family transcriptional regulator [Rhodovulum sulfidophilum]AB14
MSVPPLLLIEDTPSLQMIYETVLRNAGYKVVSASTAAEGLAAFRELKPQVVLLDLMLPDRPGQELLQEFLALAPETRVIV
VTANGSINRAVEAMRAGAYEFLLKPFNEQRFLHAVETALAERAAADPAPAPAPPRTTPEPPSGFVGNSPQMAAVYDRI
AS
VGRSKATVFITGESGTGKEICAQAVHAASARADGPFVPMNCAAIPRDLLESEVFGHLKGAFTGALADKPGAAAVADGG
TL
FLDEICEMDMGLQTKLLRFLQTSTVQPIGASHPRKVNVRIVCATNRDPLEEVRLGRFREDLYYRLHVVPIHLPPLRERGE
DVVEIAQSALEKLSAEEEHNFDRLAPEVADLFRRLPWPGNVRQLLNVLRHAVVLNQGPEITLDMLPIALQQEHDRITAS
G
HRMPEEVGGAAKPIQVEDLIGRTLAEIEQMVIEGTIAREGGSLPRAARVLDVSPSTLYRKREAWARKDRDDPADEPEKD
L
HATH
>OLS49277.1 NAD kinase [Rhodovulum sulfidophilum]AB14
MAGPTRIAFLASETASAEGALDRLTAQYGQCAPEQAEVIVALGGDGFMLQTLHATQGFDLPVYGMNCGTIGFLMNEY
HED
GLADRLAAAEEEIINPLRLKATRPDGTVVEALAINEVSLLRAGPQAAKLRISVDGKVRLEELVCDGALVATPAGSTAYNY
SAHGQILPIGSDVLALTAVAPFRPRRWRGALLPKRATVTIEVLEPAKRPVMTEGDSRGAGVVSMVEIRSEATIRHRILFD
PGHGLEERLCREQFL
>OLS49278.1 serine hydroxymethyltransferase [Rhodovulum sulfidophilum]AB14
MNVHPQDSGFFKEDLATRDPEIARAIGLELGRQRDEIELIASENIVSRAVMEAQGSVMTNKYAEGYPGRRYYGGCQFV
DI
AENLAIERACKLFDVAFANVQPNSGSQANQGVFAALLKPGDTILGMSLDAGGHLTHGAAPNQSGKWFNPVQYGVRE
SDLE
VDYDQIEALAREHQPKMIIAGGSAIPRILDFKRFREIADMVGAWLMVDMAHFAGLVAAGLYPSPFPHAHVATTTTHKT
LR
GPRGGMILTNDEALAKKFNSAIFPGIQGGPLMHVIAGKAVAFGEALRPDFKTYQEQVIKNAKALGAALVEGGLSLVTGG
T
DSHVLLVDLRPKGVKGNATEKALGRAHITCNKNGIPFDSEKPTITSGIRLGSPAGTTRGFGEAEFAQIGRWITEVVDGLA
ANGEEGNAEVEARVKAEVEALCARFPIYPGL
>OLS49279.1 hypothetical protein BV379_13990 [Rhodovulum sulfidophilum]AB14
MSVTGYSRMQIRLHWVVFGLVVVQFLFAGGIGQAFGRYIETGEKGVTLLVALHILLGLAILVLAFWRLGLRFLHGVPAP
D
PSHTPAQVMLARVIHGALYLLMVALPASGMLAWSLGSELAAGVHAAFRVILVLLLLLHVAAALMGQYIKKDGTLTRML
RP
EG
>OLS49280.1 hypothetical protein BV379_13995 [Rhodovulum sulfidophilum]AB14
MTLTKTFGKTLTMAALGLTLAFAATPAAAKDVRRLVTVLTDAAPQTQLMAMVLTLQALERGVETEILLCGPAGDIALAE
P
PEAALAPQPPAGLSPQAALRTAIGKGARAEVCAIYLPGQGAGPEVLIEGVGVARPGAMARDLLDRRARVWSF

>OLS49281.1 hypothetical protein BV379_14000 [Rhodovulum sulfidophilum]AB14
MISLDDIEDMTDLSRDEITAIAEHENLPDADASLLGDYLMHLHHGPQRVQAMICEDIRAALHRDDTGRARELYAVLHCF
L
TTHPEAARGTP
>OLS49282.1 hypothetical protein BV379_14005 [Rhodovulum sulfidophilum]AB14
MARRLISTGSPFEARIGYSRAVVDDGWVFVAGTTGYDYATMTMPDRVEAQCANALATISAALAEAGASLDDVVRVRYI
LP
DPAEFPACWPALSEAFARARPAATMISAGLMEPEMKIEVTARLSA
>OLS49283.1 ATP-dependent Clp protease ATP-binding subunit ClpX [Rhodovulum sulfidophilum]AB14
MATNSGSDSKNTLYCSFCGKSQHEVRKLIAGPTVFICDECVELCMDIIREETKSSGLKSTDGVPTPRDICEVLDDYVIGQ
MHAKRVLSVAVHNHYKRLNHGAKSADIELAKSNILLIGPTGCGKTLLAQTLARILDVPFTMADATTLTEAGYVGEDVENI
ILKLLQASEYNVERAQRGIVYIDEVDKITRKSDNPSITRDVSGEGVQQALLKIMEGTVASVPPQGGRKHPQQEFLQVDTT
NILFICGGAFAGLEKIIAQRGKGSAIGFGADVKDNDSRGIGEVFTELEPEDLLKFGLIPEFVGRLPVIATLTDLDESALV
QILTAPKNALVKQYQRLFELEDTQLTFTDDALSAIAKRAIARKTGARGLRSIMEDILLDTMFDLPGMEAVQEVVVNEEAV
NSDAAPLMIYADQKKEGKTAG
>OLS49284.1 ATP-dependent Clp protease proteolytic subunit [Rhodovulum sulfidophilum]AB14
MTDMIEHYMNNLVPMVVEQTSRGERAYDIFSRLLKERIIFITGPVHDGMSSLIVAQLLHLEAENPTKEISMYINSPGGVV
TSGLSIYDTMQYIKPKVSTLVVGQAASMGSLLLTAGAEGMRFSLPNSRIMVHQPSGGYQGQATDIMIHARETQKLKDR
LN
QIYVKHTGQDLETVIDALERDNFMDAETAKEWGLIDEIVESRSALEDAK
>OLS49285.1 MarR family transcriptional regulator [Rhodovulum sulfidophilum]AB14
MKRSELSLVALRRILRATELYGRELARAAGLTAVQIRVLQIVAETGQSTPKTIATRMGVSQATMTTLIDRLVAKGLVDRR
RSEADRRQMIIAITALGRESIDRAPDPLHDRYVIAFEKLPDWEQAMLVAALERVAALLDAGNLDAAPVLDLGDIRKTLPE
>OLS49286.1 diaminobutyrate acetyltransferase [Rhodovulum sulfidophilum]AB14
MAITIQSSSRRASAPITFRPPTGEDGAAVWALVRACKPLDENSMYCNLIQCDHFADTCILAEMDGEAVGWISAYRLPD
DP
ETLFVWQVAVSDKARGRGLGRRMLFDLLERDACDGVMRLQTTITRDNDASWGLFRGFARRLGARIEDTPHFTRSEHF
DGR
HATEHMVTIRLVEALSRAA
>OLS49287.1 diaminobutyrate--2-oxoglutarate transaminase [Rhodovulum sulfidophilum]AB14
MSTVETAETTIYDRRESEVRSYCRNFPVSFASAEGATLTDADGNTYIDFLAGCSSLNYGHNDPDMKAALIAHLEGNGLA
H
ALDLHTEEKAAFLTAFEHHILHPRGMEHRVMMTGPTGTNAVEAALKLARKVTGRRNVIAFTNGFHGMTLGALALTGN
GGK
RGGAGVPLPDVTHMPFENSMGDLDTLEHLDAMLSNPSSGIDAPAAFIVELVQGEGGLNAASCEWAQGLERLAKKHG
ALFI
VDDIQAGIGRTGSFFSFDHMGIEPDMVTLAKSLSGFGLPFACALIRPEHDIWKPAEHNGTFRGNSHAFVTARVALEKFW
A
DDDLRNEVALKSQVLEERLSQIGNRIGGATLKGRGMMRGIDLGDGALAGRICAECFRRGLIIETSGAHDEVVKVLAPLTI
PMEQFRKGLDILDAAVEACVPFAMAAE
>OLS49288.1 L-ectoine synthase [Rhodovulum sulfidophilum]AB14
MIVRDFHKLKDTDRAVSDARWTSVRMLLADDGMGFSFHITVLQAGSEHTFHYKHHFESVYCISGKGSITDLASGETYQI
R
PGVMYALDQHDHHTLRAEEELVMACCFNPPVTGTEVHREDGSYAPVEELAD
>OLS49289.1 aspartate kinase [Rhodovulum sulfidophilum]AB14
MTGRPRGNPAGATTVTKRHHTVEKIGGTSMARVHDLVDTLFTRGREAPDYGRIFVVSAFGGITDLLLEQKKTGEPGVY
GL

FANADNDHAWSDALNRVAGAMREAHGKVLDHPADIAAADDFVRERIEGARSCLIDLQRLCSYGHFRLAHHMGLIREL
LSG
LGEAQSAFVTAALLRRRGIDARFVDLSGWRDETECTLEERIVEGLRDVDIFHEMPIVTGYAQCSEGLMAEFDRGYSEVTF
ARIAALTGAGEAIIHKEFHLSSADPKLVGEGAVVKMGHTNYDVADQLSNMGMEAVHPKAAKMLRQAGIPLRVTNAFE
PED
PGTLIDDAEGEAPRVEMVTGLGVYALELFEQDMVGVKGYDSAILDVLTRHQVRIVAKTSNANTITHYLDAPLKLVRRVE
N
DLEARFPGATLTARNLGVVAAIGRDLKGLKVLARGLAALDAAGIEPVAVQQTTRDVDAQFVVPREDMEPAIRALHAAL
VE
EDSRQEAMAA
>OLS49290.1 ABC transporter substrate-binding protein [Rhodovulum sulfidophilum]AB14
MRRFVPLAVTLGLLAGPAVASDPADWPALLSEAQGQTVYWNAWGGAAATNDFIRWAASEVEERYGVTVEHVKLAD
TADAV
SRVLAEKTAGKDTGGAIDLIWLNGPNFAAMKEAGLLYGPWAEDLPNWRFVDTEGKPAVTADFTVPTEGFEAPWAMA
QVVF
YYDGARLAEPPRTMPALLDWARAHPGRFTYPQPPDFLGTTFLKQALYELAPDPSVLQKPTDEADYDAVTAPLWAFLDE
LT
PHLWRQGRAYPQSGPQQLQLMADGEIDLAISFSPGEASTAIANGQLPDTVRTFVLDGGTIGNASFVAIPYNASAKAGA
MV
LANFLLSPEAQAHAQDPDILGYGTVLAMDKLAPKDRARFEALDLGIATLSPAELGPALPEPHPSWATRLETDWAARYG
MA
P
>OLS49291.1 ABC transporter permease [Rhodovulum sulfidophilum]AB14
MRPLRAAPALTLALMLGPVLAGLAGTLAPALGIFPALGGRSLSLEPARALADWPGLPRAIGLSVATGLAATLIALGTVTL
FTAGWAGTRGMALLERLLSPLLAVPHAAAALGLAFLIAPSGWILRALSPWATGWQRPPDLLILQDPMGLAMIAGLAVK
EI
PFLFLMTLAALPQAAPARRLTIARALGYGRVTGWLKAVFPAVYAQIRLPVYAVLAYSMSVVDVAIVLGPATPPPLSVQV
V
RWMSDPDLSLRFQAAAGAILQLGLVLAVLGLWRLGELATARLGRRWIEGGARRLAEPLLRAAGLGLAALAAAAVILGLA
G
LALWSVAGFWGFPDALPEGIVLRSWVRHGPALIAPARETLVLAALATALALALTLACLEAEHRFGLAPGSRSLWLLYLPL
LVPQVAFLPGLQTLALSLGAGTGRGTVVLAHLVFVLPYVFLALSAPWRAWDPRHATVAAALGAGPGRIFRTVRLPMLL
AP
VLTAAAVGFAVSVGQYLPTLLPGGGRIRTLPTEAVALAAGGDRRIIGVTVLVQTAVVAAPFALALAAPRLIFRRRRGMRG
A
>OLS49292.1 ABC transporter ATP-binding protein [Rhodovulum sulfidophilum]AB14
MKGLRLQDIAIARGTEPLIAVDAHVAPGEVLSVMGPSGSGKSTLLAAVIGTLPPGFAMTGRVLLNGREVTGLPPEDRRI
G
ILYQDELLFPHLSVGGNLAFALPRSLRGRKARRARVEAALEEIGLPGFAKRDPATLSGGQKARVALMRMLLAEPEALLLD
EPFSRLDATLRDQIRALVFARARDRGLPVLLVTHDRADAEAAGGPVIGLGA
>OLS49293.1 ornithine decarboxylase [Rhodovulum sulfidophilum]AB14
MELSGHIWPGLLEMTMYATQADFGGGPDLLSNSRVETYIRTNRFERPTLVIDTDRVADRFHALRAGLGRAAIHYAVKA
NP
AREIVERLVGLGCHFDAASRGEIELCLGLGAAPHQISFGNTVKRVADIAFAHRAGLTLFAADAEEELDKIAEHAPGAEVY
IRVLVTATEADWPLSRKFGCAPGKVMPLMARAVALGLKPVGLSFHVGSQTRRADMWLPVLNEVAEIWQAARAEGFD
LDLL
NIGGGFPAFYGEEVQPPAMYAAEVMRLVTERFGAVPRVMAEPGRGMVAEAGAIAAEVMLVSQKSEDDTYRWVYLDI
GKFS

GLAETMDEAIRYQFATDRDHEPTAPCILAGPSCDSADVLYEKRPVQLPVGLRSGDKVIIRNCGAYTSTYSSVGFNGFPPL
DVVVI
>OLS49294.1 L-lactate dehydrogenase [Rhodovulum sulfidophilum]AB14
MKVGIVGAGMVGSAAGYALALTGAASRIVLVDRSEALALAQAQDIAHATPFTAAVTVEAGGYEALAGADVVILAAGV
GQK
PGESRLALLERNAAVFEDVIRAVLRAAPDPILLVATNPVDVMTHVTARIAGLAPGRVFGSGTILDTARFRSLVAHHLGLA
PQSIHGYVLGEHGDSEVLGWSGARVGSEPLTSVAAQLGSAITADVRARIDDGVRRAAYRIIEGKGATNYGIGAGLARIV
Q
AIRRDEGAVMSLSLVTPEVTGVHNVALSLPRVVGASGLRSTLMPTLDAQEVAALTASARMLRETSDALGL
>OLS49295.1 UDP-N-acetylmuramoyl-L-alanine--D-glutamate ligase [Rhodovulum sulfidophilum]AB14
MIPVRGVEGQKLAVLGLGRSGLSAARALRAGGAEVLPWDDNEAARAAAEAEGFVPVDPTRAGALDGVASLIVSPGIPH
LY
PGPNRAIVAAWAAGVPVDNDIGLFFRSLATPDWDSFDVAPKVIAVTGSNGKSTTSALIHHVLEHCGRPSQLAGNIGRG
VL
DIDPPGEGGAVVLELSSYQTDLARALTPDIAVFTNLSPDHLDRHGGRGGYFAAKRRLFAEGGPDRAIIGVDEDEGMFLA
N
QLAEGAADDRVIRISSGQKLAGPGWLVFARKGFLSEWRKGRQVASIDLRAIAGLPGAHNHQNACAAYAAARCLGIGPK
QI
EEALGSFAGLPHRSQLVAEADGVRFVNDSKATNAASAAMALAAFPKIRWICGGLEKEGGLGPLLPHLGHVAKAYVIGR
EA
AAFALHLKDAPHEVCTTMETAVARAADEALPGEVVLLAPAAASFDQYDSFERRGEDFIARVRARLGG
>OLS49296.1 hypothetical protein BV379_14075 [Rhodovulum sulfidophilum]AB14
MQMNPVEDTYNYLSPAARLGSPSLHAVTPDVKLKSWNGVPPYAVWAGLGYLLVLAVIFSDQIVPYCEANFAQPQNCA
PIF
GKIIPIFVAGIILAPALAGAVQRMGGNKATGLAFSFAVAVVPPVMLGWSYLLG
>OLS49297.1 phospho-N-acetylmuramoyl-pentapeptide-transferase [Rhodovulum sulfidophilum]AB14
MLYWLADLSDGGGVFNLFRYITFRAGGAFFTALLFGFLFGRPLINLLRKRQRNGQPIRTDGPEGHIVSKAGTPTMGGVLI
LGGLIVSTLLWGRLDNPYIWMLLFVTLSFGAIGFADDYAKVSKNNTKGVPGKIRFGLGLLIAAIAGYWAACAHPADLSYN
LALPVFKDTLIHMGVLFVPFAMIVIVGSANAVNLTDGLDGLAIMPVMIAAGALGVIAYAVGRVDFTDYLEVHYVPGTAE
I
LIFSAGLIGAGLGFLWYNAPPAAVFMGDTGSLALGGALGAIAVATKHEIVLAIVGGLFVVEALSVIIQVGYFKATGKRVF
LMAPIHHHFEKKGWAEPQIVIRFWIISLILALIGLATLKVR
>OLS49298.1 UDP-N-acetylmuramoyl-tripeptide--D-alanyl-D-alanine ligase [Rhodovulum
sulfidophilum]AB14
MSALWTAAEALGATGGRVTRDWSVSGVSIDTRSLKPGDLFVALKDQRDGHDFVAEALARGAGAALVSRLPEGVDESA
PLL
IVPDVLAALEALGRAGRARSGARVVAVTGSVGKTSTKEMLRAVLAGQGRVHAAEASFNNHWGVPLTLARLPREADFA
VLE
IGMNHPGEIAPLARLARPHVALITTIAPAHLEAFDGLAGIAAEKAMIFEGLEPGGIAVYPGDLETTPVLAAGAAGRASTD
ISFGAEDGCDFRMVRADLHADSTVVRAEARGRDLLFNIHAPGRHFANNALGVLAVAEALGVDLVVAACDLGQWRPP
GGRG
ARLRIALDPVEESLSVEVIDDAFNANPASLAAALEVLAAARPTDGLGRVARGRRIAILGDMLELGPDEQAMHAGLADLD
A
MARVDLVHCVGPRMRALYFALPLSRRGQWFDEADLLAAKVHSLVDAGDVVLVKGSKGSRVSVVVDALKRLGQGGPG
SDEE
LS
>OLS49299.1 UDP-N-acetylmuramoyl-L-alanyl-D-glutamate--2,6-diaminopimelate ligase [Rhodovulum
sulfidophilum]AB14

MTIRTETTLGDLGLTAQGGRNTHITGLSVDSRHTKPGHLFAAMPGTRSHGAEFVPFALRMGAAAVLTDRAGARIAAEA
LA
DSEAALVVAEDPRQALAGAAALFYGAQPATMVAVTGTAGKTSVANFTRQIWAGLGHAACNLGTMGVQGAYEAPLA
HTTPE
PLTLHRLLAEMARAGVTHAAMEASSHGLDQRRLDGVALKAAAFTNFSQDHLDYHAGFEDYFAAKAGLFDRVLPEDGT
AVI
NIDDARGAQMLGHARARGQTILTVGRAEEADLRILAQRYDATGQDLRFAWEGRSQQIRLDLIGGFQAENVLAAAGLVI
AA
GEAPGQVFAALPELATVRGRMELAATRANGSTVFVDYSHKPGALAAAIASLRPHVMGRIVVVFGAGGDRDRGKRPLM
GQV
ACEQADVVIVTDDNPRSEDPAAIRAEILQGCAEAIEVGDRAEAILRGVDALEKGDALLIAGKGHETGQIVGDAVYPFDDA
EQASVAVAALDGRLA
>OLS49300.1 cell division protein FtsI [Rhodovulum sulfidophilum]AB14
MIRTPLRPLARILEARAKGENPDAIERENKRLRHEKMRDQARVRAEGRLLVLGVVFCAAFAVVGVRMGLLAGSVPSEPR
A
TAPGASIVAQRADIVDRNGNILATNLATHSLYAQPPLMIDPKAVARELARIFPDLDEARMVKDFTGSRKFVWIKKKISPE
QMQLVHDIGDPGLSFGPREMRLYPNGALAAHILGGAGFGREGVQSAEVIGVAGVERYFDDYLRDPANGGQPLRLSLD
LTV
QAAVERVLAGGMKLMNAKGAAAILMDVHSGEILSMVSLPDFDPNERPRQLVKGDPSDSPLFNRAVQGVYELGSTFKT
LTL
SQALEEGIVTPNTMIDTKGPLVWGRFRIHDFHNYGSSLSVTTVLVKSSNIGTARIAQRIGGERQRAFLSRLGLLDPSPVE
LVEAAGAKPLLPANWSELSTMTISYGHGVSTSPLNLAAAYSTIANGGRLVRPTLRAGGGRLGERVISEETSRQIRKMLRE
VVVHGTASFGEVPGYRVGGKTGTADKPKPTGGYYDKKVIATFASMFPAEAPQYVLIVTLDEPQDTTGPKPRRTAGWTA
VP
VAAEIIRRAAPLLGLRPEIEQAPGFGVTLTAN
>OLS49301.1 hypothetical protein BV379_14100 [Rhodovulum sulfidophilum]AB14
MRSLVYVLTAMAVMALAFWAYRENYRTQAALDNVQRLNRAIGSLREQLAMQNAEWAYLNRPERLRELAALNYDRLG
LGPL
APEQFGQIDQVSYPEMVLPDIVLPDELFQEIMAEADRPAQTAAPTLPVEDAAAAPAVPEALDAQDAQDAEAGQ
>OLS49302.1 16S rRNA (cytosine(1402)-N(4))-methyltransferase [Rhodovulum sulfidophilum]AB14
MAAADNSAPAEPPHIPVLLRPLLEAVAPVSGLWLDGTFGAGGYSRGLLEAGADRVIGVDRDPSVADLAASWAAGWG
DRLQ
LVEGVFSELDRHAEAPLDGVVLDLGVSSMQLDQAERGFSFAKDGPLDMRMGAEGPSAADLVASASDTALADILFHYG
EER
ASRRIARAIVKARAIAPIVTTAQLAEVVARCLPPHRPGQTHPATRSFQAIRIAVNDELGELVRGLEAAERALKPGGKLAV
VTFHSLEDRIVKRYLQVRSGQAAGGSRYAPEARPDPARFELVTRRAVAPDPVELEANPRARSAKLRVAIRTGEPAGPVD
R
ARLGLPKIMED
>OLS49303.1 cell division/cell wall cluster transcriptional repressor MraZ [Rhodovulum sulfidophilum]AB14
MARRFRGESQHKVDAKGRVSIPALFRRVLEACDPDWTEGLRPQLVIVYGDHRRKYLECYTIEAIDEVDEKIAALPRGSM
Q
RRMLERMFHGQSHPTEVDNDGRLTLPQKLRDKIGLEGEAFFIASGDTFQIWKPETYETEELAKTEAWLDELPDDVDPLS
F
LDGAVAEPAGGG
>OLS49304.1 sodium:proton antiporter [Rhodovulum sulfidophilum]AB14
MPLSNDVILAELSRIAVPGGGDLVSRDLVRALSVDGETVRFVIEAETAEAARAMEPVRAAAQAAVEAVPGVAQASVVL
TA

HGGAAAAPQPGPRIGRHPTPDDGPRNPSGVDRILAVASGKGGVGKSTVASNLAVALARQGRRVGLLDADIYGPSLPR
MMG
ATRRPASPDGKIIEPVSVHGVAMMSIGLLLKEGEAVVWRGPMLMGALQQLLGQVNWGRLDVLVVDLPPGTGDIQLT
LCQR
TQLTGALVVSTPQDVALLDARKALDMFAKLQAPVLGLIENMSTYICPNCGHEAHIFGHGGVEAEARKLGVPFLGALPLA
L
EVREAGDAGRPVALGEGAMAEAYAQIAGRLIGGGMA
>OLS49305.1 DNA helicase II [Rhodovulum sulfidophilum]AB14
MCPIDDSDAFETAAEAPLSVRASVGAMAARSAPYLDGLNPSQRQAVETLDGPVLMLAGAGTGKTRALTARIAHLLFTG
AA
RPNEILAVTFTNKAAREMKERVGRIMGETVEGMPWLGTFHAICVRLLRRHAELVGLKSNFTILDTDDQIRLLKQLIASAN
IDEKRWPARQLAGLIDHWKNRAWTPEAVPASEASGFDGRGVKLYAAYQERLKTLNAADFGDLLLHVVTIFQAHPDLLE
QY
RRWFRYILVDEYQDTNVAQYLWLRLLAGGHKNICCVGDDDQSIYGWRGAEVGNILRFEKDFPGAAVIRLEQNYRSTPH
IL
AAASHLIAANKGRLGKTLWTEAEGGEQVRLIGHWDGEEEARWIGEEAEALQGGSRGLRLFSLDDMAILVRASHQMRA
FED
RFLTIGLPYRVIGGPRFYERMEIRDAMAYFRLAVSPADDLAFERIVNTPKRGLGDKAQQKIQATARAEGVPLVEGARLLL
EAGGLGGKGATELRKLVDGIARWHEATLGPLVRGAVDDEFAVIDDEVGVSVAEFRRGYPEIGHVELAERILDESGYTAM
W
QADKTPEAPGRLENLKELVKALEEFDNLQGFLEHVALIMDNESEASGAKISVMTLHAAKGLEFPVVFLPGWEDGLFPSQ
R
AMDETGLKGLEEERRLAYVGLTRAEELCTVSFAANRRVYGKWQSQLPSRFIDELPPSHVDVLTPPGLYGGGYGAAAPQ
AA
VSGIEARASRADVYNSPGWQRLQSRNAGRAPATPSRGARGGGVVIDAEALPVFELGARVFHQKFGYGTVTGIEGDKL
DIA
FDKAGAKKVVARFVVAAASADDVPF
>OLS49306.1 hypothetical protein BV379_14125 [Rhodovulum sulfidophilum]AB14
MSRYRIILSGLALAALAACGTPERPETGVPKVVTPEMLPQAPETSAPACSAEMAARFHQPEAGIFQVGTGPTAAGFWI
DM
KMAGRDGTWRCQTTRDGRVVTVTPISST
>OLS49307.1 pyruvate carboxylase [Rhodovulum sulfidophilum]AB14
MTEFTKVLVANRGEIAIRVMRAANELGKRTVAVYAEEDKLGLHRFKADEAYKIGEGLGPVQAYLSIEEIIRVAKMSGADA
IHPGYGLLSENPAFVEACAAEGIAFIGPKAETMRALGDKASARKVAIKAGVPVIPATEVLGDDMDAIAKEAEEIGYPLML
KASWGGGGRGMRPIHGPEELAEKVREGRREAEAAFGNGEGYLEKMITRARHVEVQILGDKHGTIYHLWERDCSVQRR
NQK
VVERAPAPYLSPAQRETLCNLGKKIAEAVNYECAGTVEFLMDMETGNFYFIEVNPRVQVEHTVTEEVTGIDIVRAQILIA
EGKSLVEATGVASQYDVDLSGHALQCRITTEDPLNNFIPDYGRITAYRGATGMGIRLDGGTAYSGAVITRYYDSLLEKVT
AWAPTPEAAIARMDRALREFRIRGVSTNIAFVENLLKHPTFLNNEYSTRFIDETPELFSFEKRRDRATRILTYIADITVN
GHPETTGRAKPPAEARDPRPPLARHETYAPGTRQILEQSGPEALAEWMAAQKQLLITDTTMRDGHQSLLATRMRSID
MIR
VAPAYAHNLPQLFSVECWGGATFDVAYRFLQECPWQRLRDLRAAMPNVMTQMLLRGANGVGYTIYPDNVVQGFVR
QAAET
GVDVFRVFDSLNWVENMRVAMDAVLETGKVCEGTICYTGDILDPARAKYDLSYYVAMGKDLKAAGAHVLGLKDMAG
LLKP
ASATVLIKALKDEVGLPIHFHTHDTSGIAAATILAAAEAGVDAVDAAMDGFSGGTSQPCLGSIVEALRNTPRETGIDMG
A

VREISAYWEAVRQQYAAFESGLMAPASEVYLHEMPGGQFTNLKAQARSLGLEERWHEVAETYAEVNQMFGDIVKVT
PSSK
VVGDMALMMVSQGLTRAEVEDPSKDVAFPDSVIDMMRGNLGQPPGGFPEAIRNKILKGEKPFLDRPGKHVPAADID
RIRG
ELEAQFEDLTFDNEDLNGYLMYPKVFTDYATRHLQYGPVRTLPTRTFFYGMEPGEEIEAEIDPGKTLEIRLQAVGETAED
GEARVFFELNGQPRVIRVPNRKAKASVARRPKVELGNPAHVGAPMPGAVATVAVAAGQEVKEGDLLLTIEAMKMET
GLHA
ERDGMIKAVHVAPGQQIDAKDLLIEFE
>OLS49308.1 hypothetical protein BV379_14135 [Rhodovulum sulfidophilum]AB14
MEHFLRTMGRKPLPGELGCALSHRRILEWLTRSEFPLILIFEDDALPADPRSIDVLPELLAPFLDHGRKSAAFLLHLGVR
RRQRPQNINRVLAGCGVGFWHRWILRDAGKDHSIWRTHAYVISRKAARRTVAAETSIRNVADGFAQRGKEGVHDHF
LYSE
PGLFLPAPEAGSMINPNGEIRRANRKKRKSWKKPEQPNAMGKLKIMDRLRRLSSKTFHL
>OLS49309.1 multidrug transporter AcrB [Rhodovulum sulfidophilum]AB14
MLQEALREAESGGTSLFVRRPILALVLSALIVLAGLAALFGVEIRELPNVDRPVVTVTTYFTGAAPETIDQEITSRIEGA
VGRVSGIRSIESNSRFGRSRVTLEFAEDVDIDVAATDTRDAVARIQRELPDEADDPRVVKADADAQPVLRVAVTSSRRSA
EALTEIVEDRIEDRLVSLPGVADVHVYGDRAPIFRIDIDQARLASRGLTLADLAETLSDAGFNAAAGDLEGGRQSLSVRT
TAMVVSAEAFEDLRIAPNVFLRDVARVDLGPEPNTSVLRANGRTGIGMGVIRQAGSNTLDISREVRRTVAELDRSLPEDI
SIFVTSDDAVFIKGAIAEVLKTLGLAVAIVVLVIFLFLRDLRATLIPALTMPVSLIGTLAAIYLAGFSVNILTLLALVLA
TGMVVDDAIVVLENIVRRRAEGMGPRAAAVLGAKQVFFAVVTTTATLAAVFVPLSFLPGQAGGLFREFGFTLAIAVGIS
S
VVALTLCPVLASRMLARAPGTGAGTGARIGAALAGLYRRSLAGALALPLVVVTAALLFAATALLVAGGVRQELTPREDRS
VALLSIQAPQGVSLDYTQAKMAAIEDAIAPLRASGEIVNIFSIAGSGGSDSRGFMVFTLAPWQARDRSQDEIVTDINTRL
REVIGVRAYAIQPNSLGIRGAGSGISVALTGESYDRLARTAQALVERMQDDGAFGEVRLDFERTQPELFVEVDRTRAAD
L
GIDIAGLGDALRAFLNGRSVVSVFVGDQNYDVQLVSSAEPVDDPGDLENIFVKSTGGRMVPMSTFVRLEERPVAPDLD
RL
DQSRAVSISADLGPGQSLGGAFERIVQLSDGLLSDDNRILPKGAAATMDETNSGLLLIFGFAIAVVFLVLAAQFESFISA
IVVMATVPLGLACAIFALLVTGQSLNVYSQIGLVMLVGIMAKNGILIVEFANQLRDGGATVRDAIFDAATIRLRPVMMT
M
VSTVLGGVPLILSSGAGAEAREALGWVIVGGLGLATLSTLYLTPVAYLLLARFSPPRAEEKQRLVRELEEALAG
>OLS49310.1 hypothetical protein BV379_14160 [Rhodovulum sulfidophilum]AB14
MARQIGLSVLLLAGTLALWVAFVPGARPLLDRIGIGAVLGLAPAGSDVGSGPLGPRGGPVAVTVGEVAQGRIRDRVAAI
G
DGRALRAVTLRSKVTGEVVGIGIRDGERVAAGDLIVQLDDEAERIALDRAQLVLEDARDEAERVTRLETTGAVTEVRRRE
ADLALRTAELELRQAEFDLSERTVSAPFAGWAGVLDIDVGDRVSSQDALAVIADRSRILIDFHVPERVVGQVTEGLALTA
RPLGMPGRLLEGRVHAVDNAVDPASRTLRVQGELDNADDLLRGGMSFEVVLNFEGEPLPMVDPLAVQWSSAGAFV
WAVRD
GRATRVPVAIRQRNADSVLVEADLAVGEPVVVEGLQTLREGTELRVVGPQAGRALPRPARDAG
>OLS49311.1 cold-shock protein [Rhodovulum sulfidophilum]AB14
MATGTVKWFNTTKGFGFIAPDGGKKDVFVHISAVERSGLAGLQDGQKVSFEIEAGRDGRESATKLAEI
>OLS49312.1 TRAP transporter small permease protein [Rhodovulum sulfidophilum]AB14
MARRALAAAVAFWAILGGLVLAGVVGLNLASILGGMVGHPVPGDFELTEIGTAIAVFAFLPWCQLTRANVTADVFTM
GAS
PIWTARMSLAGSLAALAVAGILFWRMSLGLADQHAYGYTSAILQIPLWPCFVPVLVSLALLALAAAATLAEDATAARGR
G
NA
>OLS49313.1 C4-dicarboxylate ABC transporter permease [Rhodovulum sulfidophilum]AB14

MPDPMMLGLIGLGALVLLIAGRMPIAYAMILVGGAGIAALNGPALLLNQLKTLAWGQFSIYDLSVVPLFVLMGALAGH
AG
LSAALFRAGAAWLGWMRGGTAMAAIAACAGFGAVCGSSLATASTMGKVALPELRRQNYSGALATGTLAAGGTLGILI
PPS
VVLILYAILVEANVVTMFMAALLPAALAVLLFLLTIAGYVALRPGAGPAHAPAGRAEFRAATLGVLPVLGIFALVLGGIY
LGLYNPTPGAAVGVALVWAYGTLRGRLGLGALRASVLETAATSGMIYLILLGAELMKIFMSRAGLPQAGAEWIAGAGL
AP
LAVLVLLLAALIVLGCLMDSLSMILLVIPFFWPVLVEVNGGLYQMAEGSGFGMGTEELKIWFGILALIVVELGLITPPVG
MNVFVISSLARDVPMAETFRGVLPFFLAELFRVGLLLAFPALVLWLPRVLGG
>OLS49314.1 OpgC protein [Rhodovulum sulfidophilum]AB14
MSRPDPVSLPGKRPRDPRIDAFRGLALVMIFIDHVPGNPYENWTIRNFGFSDAAEAFFVMSGIAAGLAYSGRFAAPQIR
L
NGLWAAIRPIWSRSWTLYVVHVLLSAWAIAIFAWGAMALDVPELLTRINLRQVFENTAQALIGIPLLTHQFGYVNILPAY
SVLLLFAPVAIFVGMRAPLLMVAASVAIWFAAGMGRIDLPNYPNPGGWFFNPLSWQIIFVLGILTGIHMRRGERFVAR
RR
WLFWSALAFLGFVFLWAQIPALGQPLHALMRQAGEAGLPFHVVGFDKTYVSLPRMLHVLALFYVLSCLPVVVRLAAS
WAA
APLRLMGQHGLLVFSFGTAVSLLFQVLLAGYPDLALLPWLLPPLGVAAMLVAAMLGQAARRDRRRVPLPVVDPVTAP
AGP
LPQAAAVAAAVPCPDCSADPLPTTTLPSAGRAPGLQPAE
>OLS49315.1 glucan biosynthesis glucosyltransferase H [Rhodovulum sulfidophilum]AB14
MSRILASGFRTRLLRGLAIGFSLAAASVAASLLAEASAQDGVDPWDMARTTLIFITTAWLAWGAAGAFLGLLGHLRRAP
E
PQLRLPHQPTVVLVPICNEDPVTTFARIAAMDGSIQAAGLRCDIAVLSDTRDPENAARERDVFARLLAETGGAGRIFYRR
RTDNRGRKAGNIEDFIQSSGGAYEFALILDADSLMEGVTIRRMIARMQADPRLGLLQTLPKIIGAGSLFGRAMQFSASF
H
SPVFARGLARLQGNTGPFWGHNAMVRVRAFAESCGLPALEGKPPFGGHILSHDYVEAALLARAGWRVKLDETIEGSFE
EG
PDNILAHAKRDRRWCQGNLQHIRLVLAPGLAGWSRFVFLQGIFAYLVSLLWGAFLIASLLATVFAPAPDYFPEPYQLFPV
FPSDRTKEIITLLIGICGLLLLPKLSILLGAVLSRRAEGFGGGKRAAASVLAEILLTSLLAPLMLMYQSRAVLQVLSGRD
GGWPANQRGEGKLTLLQGLRAGLWITLTGAAALALVTRIAPDLTVWLLPVCLPMLIAPLLIAWTSRPLTHSLFAVPQEA
E
AAPVIRAYRDLVRRWMAEDARKEAVDVAA
>OLS49316.1 glucan biosynthesis protein D [Rhodovulum sulfidophilum]AB14
MFPDSSSSSVRTGESGPSRRRLLAALAGSAAVAALGLPGLAAAQDAAPEAEPSAPAPEVAPEAVPEPAPEPVPFSFDVLS
DRMQALSKEPYKAAALPDGPLSGLDYDAYRAIRFRPDHSRWADEGTFFRLQAFHPGWLYKEPVEIYELVDGIAEPMHF
SA
ADFEYDGKAADLIPADFEMPGVAGFRLHAPFNRADTFDEVVVFQGASYFRALGRGNVYGLSARGLAVNTASGKTEEFP
RF
SAFWLERPAPGATSMTIYAALDSKSLTGAYRFIVTPGANTVMDVTARLFMRENVDQLGIAPLTSMFLLGDNDRGDFDD
FR
HSVHDSEMLIMNTRDGETCVRPLNNPPRLASSYLAAQDPVSFGLIQRNRSFDDYLDAGAHYERRPSLMVEPVGNWGR
GTV
RLVEIPSDLEANDNIVAFWVPEQRARKGDAMEYDYRLHWGTNPPGAVGTRAHVVRTLVGRGGVAGAAGSPGLRKFV
VDFE
GGTLGELPDDADVTAEVYASTGRMEKPVFSRIPDTNTWRLVIDITADTGALVELKAEITGYGQNLTETWLYQWINE
>OLS49317.1 hypothetical protein BV379_14200 [Rhodovulum sulfidophilum]AB14

MTHRSPNGGAVVPLRHEAVLEAVAAIRAAGVLGRSPRRARLLEYLVDRELSGHGDEIKPHAIAVEVFDRDPRLETGRAG
V
VRSEIGRLREALARFYALADPELPVIEIPDGSCRATLRPGPGEAGPPPSPGWWRVALLAGLVLLLILPVLWQAHLRHDRD
EIIAAAARPIEQAPYQVLRVAVLPLRAEGRLAGLEDLAFGLAAEIATDLATRPWIAVSAPSDAARAAETADYLLRGRLMW
RSQALLAQLELISFPERRTVWTTFQRVGDSAEAVRAVERDAVGHILRRLGAAHRMGPDQVGSPPPRQGAGGLPELSCL
LG
VHRYLATPAAPAHAELGLCLGRLVERHPGLGEAWAALALVRLDEARFGRNLRPGPDAVAAAWSDAARASAQALRLAP
KGL
MTLQAAAMLAIEGPARDPESFRFHAARLLELYPHHPPSLALIGSRIAAFEGRWDEGLALVAQALRLEPDPPGWFHVAP
AL
HALMGADAVAARDAVRPLDPSGSRVERLLVGLAEARNRSAPPEAQAGLTGLAAWTAPGGLAAADALGDALGAGAAA
FVRS
RGYAPALQAELLRQIAAAGPVAPAP
>OLS49318.1 hypothetical protein BV379_14205 [Rhodovulum sulfidophilum]AB14
MDDQNPRSLLVEDDGLIRMDLAIMLEEMGYSLTEASSADQALDLLNESTGFELLVTDIDMPGAMNGLALAIAVRDRLP
QC
QILIVSGGRPPRPDEMPAGSRFLPKPVTSSALEETIRALNAA
>OLS49319.1 transglutaminase [Rhodovulum sulfidophilum]AB14
MPSVTILHATDYRYRTEVPLGAHRLMLRPRETRDLSLTSFELEITPEARIDWSNDVAGNAVATAQFDHTTTMLSIRARTR
LELRAPVWPVFPIAAAAASYPFAYSPDDWTDLGALAMPQYADDTGRLSKWVERFVMSRPTDTLSLLKDVSNGITALISY
Q
SRETEGTQGPLETLDRGWGSCRDFAVLLAEAVRTLGFGARLVSGYLFDPDGDRTGSAATGSTHAWVEIFVPGAGWVA
FDP
TNRAMGSANLIPVAVARRIAQVSPVSGSFHGRPSDLLALNVEVSVCQDGAPR
>OLS49320.1 hypothetical protein BV379_14215 [Rhodovulum sulfidophilum]AB14
MIDAPIDLDGHRSAAGQHDAEMRRRPVNTEPESGQSRQPHLEDLEDRMLAEPARSWVEVMEKWRFLLDRYATTPEA
GDER
VQMLIKRALGDMERLRKREERK
>OLS49321.1 hypothetical protein BV379_14220 [Rhodovulum sulfidophilum]AB14
MTTSIKTTAVFLRPFRLPSFNETLPEGEYEIETELSQPVDRIEPASWTSSVLVRLHPRASHPGLSRTLTVPLAELEHAVA
KDKLTGKALIDFFLEEMLADPMIRLVMQADGVAEADLRDLYARQPDRQHAGPDGRRPAREAGNTGPGAEGRHAGIG
K
>OLS49322.1 hypothetical protein BV379_14235 [Rhodovulum sulfidophilum]AB14
MTETALCRQLTATRTGFHSEAALSPQEDISMGMLEWLEPGIYGAVIAAAILATGGFTRAGRATLSGVPASDRDLARARP
V
APGVAASPRRPDVTGENA
>OLS49323.1 DEAD/DEAH box helicase [Rhodovulum sulfidophilum]AB14
MTFTELGLNARMVSRLGALGLTKPTPIQEKAIPQALQGRDILGIAQTGTGKTAAFGIPLIVALSKQQERPAPHSARGLVL
APTRELAGQIVQSLQGMTADLRITLVVGGKSINVQAEKLARGTDILVATPGRLIDLMDRRAIDLGRTRFLVLDEADQML
D
IGFIHALRRIAPKLSRDRQTMMFSATMPKAMAELSRDYLDKPVRVEVSPPGKVADKVSHSVHFVAKDRKQDLLIELLRG
H
RDDRALVFSRTKHGAERLMKHLDKAGFAATSIHGNKSQGQRDRAIAAFKAGSVRVLVATDVAARGIDIPDVGHVYNYD
LP
NVPENYVHRIGRTARAGHSGRAIAFCAADEVAELKAIEKVLGTRVEIAGGRPWEAMKAAPKSPQKRRTRQRSAGRSDR
LA
A
>OLS49324.1 cold-shock protein [Rhodovulum sulfidophilum]AB14

MANGTVKFFNTQKGFGFIETETGGKDVFVHISAVERAGLTGLSDAQKVTFDVETGRDGRESATNLALA
>OLS49325.1 hypothetical protein BV379_14250 [Rhodovulum sulfidophilum]AB14
MSKAKKPTKKPKEAPAIQLGEHSRYQIKSGWLAGTFVARAFPKPPTKARGMIAEATGATEEAAITALHEIIDAREDRRTD
KRRKDTQNGVDVPSVEEYVEAIRQIALSGPQCAMLTALSLAEEDGLKDAQMANAAGYKSKASANRSLVAAGLLIAEYLS
V
ESQADDAPGAPEGAALLGFRGTTANEEETGNWILHPELRQAVRQVL
>OLS49326.1 hypothetical protein BV379_14255, partial [Rhodovulum sulfidophilum]AB14
MAKDVLVSEIDHAAALKHKAWWQKKIAAENLSMESANKDFANMAGMLRRYYESIAQPDPPYPYPYPYHRISLKDRHK
AES
RKLEIPVDWITEKWFAPGAFDGTNDEARDILLISVETGCRQSEICDLPPEAIVLDHPIPHLRLRFEEGENRRECIDPLK
>OLS49327.1 hypothetical protein BV379_14260 [Rhodovulum sulfidophilum]AB14
MTWDAAPTGRRGRQRTYSDAAIQSCLTMKVLFGMALWPKISGGHHLHMQTTMFQPKGGMDMIARGFEGEVGDLI
TYGAKV
TRIENTETGVRATWIPSEGGGAETVEEADYCICTIPFSVLGQIKTNFSSRMTGIIQNMPYYSATKVGLEFKRRFWEEDEH
IYGGISYTNLPISQISYPSTDYFSDGPGVLLGAYVWGAYSYQFNALPGEQRVEKALEYGAQIHARWQGRGRRRDVSRVG
R
Q
>OLS49328.1 hypothetical protein BV379_14265 [Rhodovulum sulfidophilum]AB14
MPDGKGAVGAGMYPALAGNENLLGADYTIHVILNGLRAMPALDGQLDHDQVAAVVNYIRTGFGNDWSENPATAET
VAASR
>OLS49329.1 hypothetical protein BV379_14270 [Rhodovulum sulfidophilum]AB14
MKLAATLAAACVAFGTIAHADEVIRHPIPNSDFPILQAVEVPGDATTVYLSGVVPAKKADAGDATGIEAYGNMEEQTVS
V
LTRIEATLASLDLDMGDVIKMQAFLVAEDGQPVDFSGFMAGYTQFFATEAQPNTPTRSAMVVDALASPAWLVEIEVT
AVR
P
>OLS49330.1 hypothetical protein BV379_14280 [Rhodovulum sulfidophilum]AB14
MNRIVRSALFKRQLLDITASYRERAGTAIALKFVDQIGNSIGFIAATPLACAIYTRLEGKDFRKCGLKDFPVSVFFRLDE
SGTIILEALYAHRMDIGQRLPRDIE
>OLS49331.1 hypothetical protein BV379_14285 [Rhodovulum sulfidophilum]AB14
MENAYENKMVKRLNTTLPDPLARYVGEITGKGALYETPSEFIRDLIRRHMEKAQDVERGQINAMLAQSLRENDYSDWT
NS
DLADARKVARE
>OLS49332.1 hypothetical protein BV379_14290 [Rhodovulum sulfidophilum]AB14
MEYIRDILGDIDIKATQQVVFAFDWASVPELRPGPTKLPDGREIWLTSFFWGVILRNGNASALRMMLSATVSWPSQGR
AR
LLPSM
>OLS49333.1 hypothetical protein BV379_14305 [Rhodovulum sulfidophilum]AB14
MGPSPPAHAPKRRGSLTIRFAPEMSWDAAPTGRRGRQQIYSDAAIPTTCPSESWPRR
>OLS49334.1 hypothetical protein BV379_14320 [Rhodovulum sulfidophilum]AB14
MKSLRLWQVLRLSRRLWVRTSLIAVLGIVAAGAAALLGPVIPQELADRLGEDAVMPVLDVLASSMLAVATFSLSIMVTT
H
RQASSQVTPRSHRLLLEDTTTQTVLATFIGAFIFALVSIILFRAGAYTGAAPFVLFAFTVLVVVLVVVAILRWIDHLSRL
GSMDETIAQAAARARDTLTAARNAPSLLATPLPDGAVPPAGARPVPAPRGGYVQLVDMAALSVLAEKAGAEIWVAAV
PGS
FLVAGRALAHVNPPGDIDRDAICAAFEIEAVRSFEQDARFALAVLSEIASRALSPGLNDPGTAIDILGRLEQLLAECPPA
GDEIRYPRLHAPPLRADDLVEDAFAATARDGASMREVARRLIHALNTLRQGEDPALARAAEAMTRRALAHAEAALVLD
ED

KAGLRQLAGL
>OLS49335.1 glycine dehydrogenase (aminomethyl-transferring) [Rhodovulum sulfidophilum]AB14
MPYTPSTYLPYDFANRRHIGPSPEEMTEMLEVLGLESLEELIGQTVPDSILQAEPLDFGKPKSEREMLWFMRQIAKKNR
A
FTTLIGQGYYGTVTPPAIQRNILENPAWYTAYTPYQPEISQGRLEALLNYQTMVSDLTGLEIANASLLDEATACAEAMTM
AQRVAKSKAGAIFVDEACHPQNISVIRTRAEPLGIEVIVGDPFADLDPEKVFAGIFQYPGTYGHIHDFTGPIADLHAAQA
LGIVVADPMALVVLKEPGAMGADIAVGSTQRFGVPMGFGGPHAAYMACRGAYKRAMPGRIVGVSVDARGNKAYRL
ALQTR
EQHIRREKATSNVCTAQALLAVMAGFYAVFHGPKGLKAIAQRIHRKAVRTARGLQEAGFAVEPAEFFDTFTVEVGPMQ
GV
IYNAALAEKINLRRIGKTRLGIALDETTRPASIEALWRAFGVTRKDKGGDGEYRFPEGMSRESDFLTHPVFQMNRAETE
M
MRYMRRLADRDLALDRAMIPLGSCTMKLNAAVEMMPITWPEFSNVHPLAPADQVRGFAEMLDDLADKLCRITGYDA
MSFQ
PNSGAAGEYAGLMTIAAYHRARGEGHRRICLIPTSAHGTNPASAHMAGMKVVAVKSAENGDIDLEDFRAKAQAAGG
DLAA
CMITYPSTHGVFEETVREVCEITHAHGGQVYLDGANLNALVGLVRPGEIGSDVSHLNLHKTFCIPHGGGGPGMGPIGV
KA
HLAPYLPADPVAGGTGAVSGAMHGSASILPISYAYVLLMGGAGLTQATKVAILNANYIAKRLEGAYDVLFKGRNGRVA
HE
CIIDTRDFVETAGVTVDDIAKRLVDCGFHAPTMSWPVAGTLMIEPTESETKAELDRFVTAMLAIRDEIREIAEGRIEKGN
NPLKKAPHTVEDLVADWDRPYSRERGCFPPGAFRVDKYWPPVGRVDNAYGDRNLICTCPPVEDVADDAVE
>OLS49336.1 glycine cleavage system protein H [Rhodovulum sulfidophilum]AB14
MKFTEQHEWLRVEDDLVVVGITEHAAEQLGDVVFVDLPEEGTTVSKDDEVVVIESVKAASDILAPLDGEIVEVNQALTD
A
PAKVNEDPAGDGWFFKMKLEDLSALDEFMDEAAYRAFID
>OLS49337.1 glycine cleavage system protein T [Rhodovulum sulfidophilum]AB14
MSALQRTALYDLHLELGAKMVPFAGYEMPVQYGLGVMKEHLHCRARAGLFDVSHMGQVSLRPKSGMVEHAAIALE
HLVPV
DLVGLKLGRQRYAMFTNEAGGIRDDLMVARRCDDLFVVLNAACKTEDIAHLHATIGCDCDIVELTDRALLALQGPEAA
AV
LETVAPGVSDMRFMDVAIIDTADGPLWVSRSGYTGEDGFEISVPVERAEALARQLLAHEAVEAIGLGARDSLRLEAGLC
L
YGHDIDTGTSPVEADLAWAIQKVRRTGGAREGGFPGAARILGELDTGAARQRVGLRPEGRAPMREGTALYAAETGGE
PVG
TVTSGGFGPSVGVPVSMGYVPVDLALEGAKLWGEVRGKRLPAEIVRLPFRPSTYKR
>OLS49338.1 hypothetical protein BV379_14345 [Rhodovulum sulfidophilum]AB14
MFLHFETQKDAMACAAARPGLLRSLAGVINRDGGSDLPVGGVFAGKLPEEDRMPHPEQAGPGAGAQDRLEAARMR
LFVEP
GMSSASLGIDGPVCRGTERQVPIGLRYRPW
>OLS49339.1 hypothetical protein BV379_14350 [Rhodovulum sulfidophilum]AB14
MRVKQTLGAMAAYVRSFAPEAVNASQRILSDRIGIFEIAAEPTGREAQRTARLFNRFEGTL
>OLS49340.1 glutamate--tRNA ligase [Rhodovulum sulfidophilum]AB14
MPMTTVTRFAPSPTGYIHVGNLRTALFNYMIARKAGGTFILRFDDTDPERSKQEYIDGIREDLEWLGFSWDREEQQSAR
L
DRYSEVAAELRASGRLYECFETPTELDLKRKKQLNMGKPPVYDRAALELSEDEKAALRAERPGHWRFLLDRERIEWTDGI
LGDISIDAASVSDPVLIRGDGQVLYTFASVVDDVDMGVTHIVRGSDHVTNTATQIQIIRAIGGTPPTFAHHSLLTGPQGE
ALSKRLGHLSLRDLRKQGVEPMALLSLMARLGSSQPVELAGSLDELIEGFDISTFGAAPTKFDEHDLFPLTSRHLHGLPF

AAVREEIAALGVPDDRAERFWQAVRENIVTRHDLAGWWALCRDGAEPLVDEEDRDFIAEALTMLPEPPYDENSWSA
WTQA
VKEKTGRKGRKLFMPLRKAVTGRERGPELGDLLPLFQKKPG
>OLS49341.1 neutral zinc metallopeptidase [Rhodovulum sulfidophilum]AB14
MTDPTRHGLPPSLDEIADLAAAARDALPEPFRGPARAVAIRVEDLASDEMLRELDIESPFELTGLYDGIPLTEKSVMDQP
ERPDTVWLFRRAILDEWVDREEVTLTELVTHILIHEIAHHFGWSDDDIASIDPWWE
>OLS49342.1 MBL fold metallo-hydrolase [Rhodovulum sulfidophilum]AB14
MTKMTRRHALLAGAALPLAAAVRPARAAAPMMGPARPPFNRFALGSFEVTTILAGSRTVPDPQTIFGLNVAPERFVAV
SE
AAMIPADKAQFFFTPTVVNTGAELILFDTGLAPRGTVAALEAAGYTPDQIDTVIITHMHGDHIGGLMENGTETFSNARY
V
TGAVEFDAWDMSGDESFETKMRPLAEKTRFIEDGDEAAPGVTAVAAFGHTPGHMAYQLESNGKALLLGADFANHYI
WSLA
HPDWEVLYDRDKEAAAATRRRLLDMLAAERMPFIGYHMPFPALGYVEKQETGYRYLPASYQMMLE
>OLS49343.1 hypothetical protein BV379_14370 [Rhodovulum sulfidophilum]AB14
MHDILTVTLNPTVDLSTSVAHVVPGDKLRCAPPVTDPGGGGINVSRAIRLLGGESLALVALGGHNGDKLRALLEHEGIRL
IPLVAPGETRMSLAVTDSGSGEQFRFVLPGPIWNEAGLKATLAAVTTAVPKSGYVVISGSLPRGLPEDFTERVCTKIGAR
GARVIADTSGPALFRLARARDAVPYVLRMDGLEAEELAGRALPDRKDSVDFAAELVARGVAEVVVIARGAEGNVLAAE
GL
RLHVGAARVPVRSLVGAGDSFVGAFVLALARDEGLARALQWGAAAASAAVMTDATALCTREDTEALLERCPVTEL
>OLS49344.1 NAD(P)H:quinone oxidoreductase, type IV [Rhodovulum sulfidophilum]AB14
MTRPKLAVIFYSTYGTNHQIAEAAAAAATEAGAEVRLRRVAETAPEDVVKGQAAWAEQLEKMADIPVATPEDMAWA
DAYF
FSAPTRFGVVASQMRAFIDTLGPVWQDGKLAGKAATATTSAMNAHGGQEATLLTLYPTFMHWGSILVPPGYTDPAIF
EAG
GNPYGYSAKAGEFDDKGRAAVAHQARLLVEIAGKLIA
>OLS49345.1 NAD+ synthase [Rhodovulum sulfidophilum]AB14
MADDRFRLTLAQYDPTVGALEDNAAKARAAWAAGRDAGADMVALPEMFLTGYQLQDLAQRPAFLADVAARLEALA
RDCAD
GPALGIGAPVVEDGRHHNAYFVIHRGRVLARVCKHHLPNDEVFDEKRVFDGGPIVGPVRVGPLRIGMPVCEDAWYED
VAE
ALAETGAEILLVPNGSPYHRGKHDSRMNIMVSRVVETGLPLVYLNLVGGQDDQVFDGGSFVLNPGGALALQMPVLEE
GLA
HVDFRRTPEGWRADRGPRAVLPDAWEQDYRVMVTALGAYVEKSGFSKVVLGLSGGIDSALVAAIAADALGPENVHC
VMLP
SDFTSAGSLEDAAAVAGALGCRLDEVPISAPQAAATEVLAPLFAGTEPGVTEENLQSRLRGLLLMAISNKFGEMLLTTGN
KSEVAVGYATIYGDMAGGYNPIKDLYKTRVFETCRWRNAHHRPWIKGPAGEVIPSRVIEKPPSAELRADQKDEDSLPPY
P
VLDRILEGLVDDARSVEDLVAEGLERDMVKRVEHLIYISEYKRFQSAPGARLTQRAFWLDRRYPIVNRWRDPS
>OLS49346.1 2-isopropylmalate synthase [Rhodovulum sulfidophilum]AB14
MTARLALVAALAAGGAALAQDDGNVVTKQYDNGGIYEGTFKNGKQDGTGTYRLPNGYEYSGQWVDGRIEGKGIARL
VNGS
VYEGEFTDGKPDGTGKITFADGGTYEGEWQDGKITGTGVAVYANGVRYEGEFRNAVHHGIGRMTSPNGYVVYEGSWV
NGVK
DGKGKIVYPGGRVYEGEMKQNQRSGKGTLTTPEGLSYSGSWKDGEMNGLGTLTQPNGDIYEGQFVNGERHGSGKVT
FANG
DVYEGEYRNDKRHGKGTLIGADGYRYDGDWVAGRIEGEGRLTYPDGAVYEGQFRADMPDGTGKVTYADGSTYEGE
WTGGV

IGGAGKATFPDGLVYEGQFKDAAYNGKGTMTHAGGYSYTGDWKGGLRHGEGTATYADGSVYSGGFVEGQREGQGT
ITMPD
GFSYTGGWKAGKFDGKGRATYANGDVYEGMFSDGRYEGKGVMKYADGEIRSGTWTNGMLTTAPEDTTDRPAAPN
APLPAD
>OLS49347.1 hypothetical protein BV379_14390 [Rhodovulum sulfidophilum]AB14
MRIAILDLNNFSRYPTLSVGQIAAILRRAGHEVAVISPFAVGVGSYRRVTRPHALGYYGSVLRHATAVSGARGVRDLRRK
VVAARSPASARAQTATLAEVQNALDRGTDLLLISAYSMYREVCGAIAAEAARCSVPVVVGGPMFGMPEIAAHWLDLP
GIS
GVFAGEAEGHLLDLVDSVTGSGRAVPGLATPDRPELVPAAPFADFDALPFPDYSDFPWDSYPNRIVPMMTGRGCGW
GGCT
FCSDVQTTSGRTFRSRSFAHVRDEMAWQSRRHRTGIFTFLDLKLNSDLAVWHGLIDEAQSVAPGCEWTASVHVQSNG
ENG
LGPDALKAARRAGLSRITTGLESAAQGVLNAMGRGTNAELLSRFVRHASEAGISVRMTSILGYPGETAEDVLCTARFLAE
HRPYIDRVVVNRLAIVGHTPLGRAIRRNPDRYPQIGALVQNLGTGLMDHENETFRSAGHLSAAMRMLREVHRINRKPL
KG
ASLTFEGVM
>OLS49348.1 peptide-methionine (S)-S-oxide reductase [Rhodovulum sulfidophilum]AB14
MFSLLGRKTDMVREAEALKGRPEPIATAETHHIFRRPLKAPVPEGMEEIVFGMGCFWGVERLFWKLPGVWMTAAGY
AGGF
TPNPTYEEICTGRTGHAEVVRVVYDPAEIPLERLLRTFWEGHDPTQGMRQGNDRGTQYRSVIFTQTEAQTATAAASLA
AY
QDRLRAAGHRAITTEIRAAPPFFHAEAYHQQYLAKNPGGYCGLGGTGVACPGGEDT
>OLS49349.1 hypothetical protein BV379_14400 [Rhodovulum sulfidophilum]AB14
MGMRIHITGASGAGCSTLARGLAIRLASQCFDTDDFYWHPSDPPFTRKRRPEERLALMKEVFLPRPDWILAGSLLGWG
DP
LVPWFSHVVFLTLPPARRLAQLRRREHLRYGDAILPGGPRAEAHQAFMDYAMGYDFPDFTGRTRLKHELWLEEMPCP
VIR
LDASAPPAALVETAIAALDQTVGAA
>OLS49350.1 ribosomal protein L11 methyltransferase [Rhodovulum sulfidophilum]AB14
MPTYSALTTLTAKEAAEALAEAIEERLDPEPMGVGVFEMEDGSGLWELGAFFTEAPDEAGLALLATLHGARPFTVSEVP
E
TDWVAKVRRDLAPVEAGRFFLYGSHDADRVPEGPVPLLIEASMAFGTGHHGTTLGCLRAVDRLAEAGFAPRSVADIGC
GT
AVLAMAAAHLWQVPILASDIDPVAVEVAATNVAANGLSERIRCLEATGFDHPALAAEAPFGLIFANILKAPLIDLAPEIA
AGLAPGGVAILSGLLNEQAGDVAAAYTACALELKDSEQIGDWTTLCMQKPAA
>OLS49351.1 hypothetical protein BV379_14410 [Rhodovulum sulfidophilum]AB14
MSLSFRPIRLDDAGTLPLWRRPVSLLFVMAFAMPVAFATWSALLNNFVIEAAGFTGVEIGWLHTVREVPGFLAIGVIALI
MFMREQVLGVVALLLLGAATAVTAWFPSLGGILTITMLSSIGFHYYETVNQSLQLQWIEKDKAPQTLGWLMAAGSAAS
LL
AYGLLVVTWKSFDLSYNFVYLASGGFTAAAALFCLLAYPQFEAPNPQLKRLILRRRYWLYYLLQFMSGARRQIFTVFAAF
MMVERFGFEVHEVTALFLINFVAGMIFAPLMGRAVARWGERRALAFEYVGLICVFLAYGGIYYLGWGVVLAATLYVID
HM
FFALAFALKTYFQKIADPGDIAPTAAVAFTINHIAAVFLPAALGYLWVTSPGGVFLLAAGMAAASLSLALMIPRHPAPGN
ETVFSRLMPAPAE
>OLS49352.1 lipocalin [Rhodovulum sulfidophilum]AB14
MDIVQLVDRFGEGPTATLFGLIAGGLFGVAAQRSAFCLRAAAVEVARGQLGARLAVWLLCFSTALVWVQAAELSGLM
RSG

DARIMAVPGSWSGAIAGGLLFGIGMVLARGCAGRMLVLAATGNLRSVVSGLIFAVVGQMSLHGWLAPLRDRIAALW
VTAG
GRNSDLLRELGLPREGGLGIGLVIAVLALYLARRNRISFRKLVFGAGVGFAVAVGWVLTFGLSQVAFDPVQVQSLTFTGP
SANTLMFFLERPALLQFDIGLVPGVFLGAFAAAWIGGDLRLQGFESEVNMRKAMIGAALMGFGGMLAGGCSIGAGVT
GAS
IFAGTAWLALFSMWLGAMATDYLLDRPTGAARPV
>OLS49353.1 transaldolase [Rhodovulum sulfidophilum]AB14
MKFFVDTADIEAIRELHDLGIVDGVTTNPSLIQKSGRDILEVTKEICDLVEGPVSAEVVATKAPAMIEEGRKLAQIASNI
AVKVPLTWDGLKACKTLSGEGHMVNVTLCFSAAQAILAAKAGATFVSPFIGRLDDINLDGLDLIADIRTIYNNYDFRTEI
LAASIRSVNHISEAAKIGADVVTAPPAVLQSMAAHPLTDKGLAAFMADWQKTGQKIL
>OLS49354.1 recombinase XerC [Rhodovulum sulfidophilum]AB14
MSSTAAIADDLRARIIDEPTVILDDPDLMRALVAANDKMMGDNIVDLRGIAMERLEARLDRLEETHRSVIAAAYENLAG
T
NQIHRAVLRLLEAPDFETLLSLLEDDLAATLRVDCVRLVLETDGDRAEAARAAPGDAVRIMSAGFARSYAGNGTNRPPR
R
VVLRRTAPHCEQVYGAGADALRSEAVLTLDLGEARQPALLALGSADAQQFTPNQGTDLMDFFAGAVERVLRRWLA
>OLS49355.1 recombinase XerC [Rhodovulum sulfidophilum]AB14
MSLALSPALRDALEAWLAEERALDGASEATIRAYATDLRRFLGFLAEYDGEPAGPARLKRVELRDLRAWMAHERGRGL
GP
RSLARALSAVKGFFRWFGKREGFEPTAVLSVRAPKFTRKLPRPLAEEAAREVIADLGTTPAAPWIAARDAAVATLLYGSG
LRISEALGLKGRDAPLPEVLRIRGKGGKERLVPALPVARQAVDAYLDLCPFEKALDAPLFRGARGGALNPRLVARAMEG
A
RTRLGLPASATPHALRHSFATHLLAAGGDLRTIQELLGHASLSTTQAYTAVDTVRLMEIYEAAHPKA
>OLS49356.1 hypothetical protein BV379_14440 [Rhodovulum sulfidophilum]AB14
MNISMTSSALTGLGAARQSPPPPSPPSEVADTTSRRAEEKRLDRLEIEDSKDSASPTLSLRNSDDTSETTSDTDAASALK
LRTEVADDTPPPPPPPSQTESIMQGVISSLSNTGSSADQATTQAEALYTAMQQMLTAQDA
>OLS49357.1 isoleucine--tRNA ligase [Rhodovulum sulfidophilum]AB14
MCAETPDYKDTLFLPETDFPMRAGLPKREPDWLERWARIGIYDRLREKEGRAPFTLHDGPPYANGHLHIGHGLNKILKD
F
IVRSQQMMGRDARYVPGWDCHGLPIEWKIEEQYRAKGLDKDKVDVVDFRQECRRFAEGWVDVQREEFKRLGVTGN
WADPY
LTMDFHAEAVIAEEFMKFLMNGTLYQGSKPVMWSPVEKTALAEAEVEYHDHQSHTIWVKFPIVSGAAEALDGAQVVI
WTT
TPWTIPQNRAVCFGPGIGYGLYEITGTPEECWAKVGDRFILADALAESVLGSARLTPGMWTRVADVSADELGALTLAH
PF
RGIEGGNGEWDYDVPMLPGDHVTDDAGTGFVHTAPSHGDDDYQIGLKFRLPMTHNVLEDGSYRADLPLFAGALIIRP
DGK
EGDANRRVIDKLVEAGALIARGRLKHSYPHSWRSKAPLIYRNTPQWFAAIDHKLDDGMDTYGDTIRARALASIDQLVK
WT
PQSGRNRIHSMIESRPDWVLSRQRAWGVPLTCFVKTGARPDDPDFLLKDPAVNARITAAFEAEGADAWYAPGAKERF
LGS
DYNAEDYTQVFDVLDVWFDSGSTHAFVLRDRADGSEDGIADLYLEGTDQHRGWFHSSMLQACGTLGRAPYRGVLTH
GFTL
DEKGNKMSKSLGNTIVPEEVVKQYGADILRLWVAQTDYTNDQRIGPEILKGVADSYRRLRNTMRFMLGNLAGFSEAEK
VA
PAEMPELERWVLHRLAELDVKVREGYDAYDFQGVFQALFNFCTVDLSAFYFDIRKDALYCDAADGLTRRAARTVLDILF
H

RLTTWLAPVLVFTMEDVWLSRFPEDEGSVHLQDIPETPADWRDETLAAKWADIRQVRRVVTAALELQRKDKVIGASLE
AA
PVVHVRDAEVLKALKSVDFADICITSAVQLSGDPRPQEAFRLPDVDGVGVVFEKAEGEKCGRCWKILPDVGQHAHPQT
CA
RCSAVLG
>OLS49358.1 hypothetical protein BV379_14450 [Rhodovulum sulfidophilum]AB14
MTHAVPAALLGAALVLAAEGAAASTVYGIDFPGGDMSFADRVLRFSPGFGFGGDVAPCNDASLALGVAWAAPDRYL
GECS
GYVSLGEGGELVLGFVDNFLVPSGDDAPDLQIFAGGDHDEHFMAWIGAGTEESGASWHQLTGPAGPVLFGGSLGFDI
DSL
AGVAPGMRFSLVAIYDMPRYCNRETGRMVYSVGEACGTDNQNLPYDGPWAGVDIYGLGAISTVPAPVPLPATGLML
AAAL
GGLAALRRRNG
>OLS49359.1 TIGR01620 family protein [Rhodovulum sulfidophilum]AB14
MTRRPVVFDLEDEEKVSARPARNAAPRPDPFSDPQEDEPPRGPADVPPVPDDEDEMAPEGRAMKTVALLASRRSSRL
ARF
FWTSFVTLLGFVISVAAWNFVIGLFQRNEILGAVALGLVGLVVLAVLLISLREMAAFARLRRLDTLHREAEAAIAANDVK
AARALVDRLTTFYRAREDTRWGRARLAEKRDELFDADGLMGLAERELLAPLDAVAAREIEAAARQVAAITAIVPLALAD
L
LTALTANLRMIRRIAEIYGGRGGVLGSIRLARAVMTHLVATGAVAVGDDMIGSIAGGGMLAKVSRRFGEGVVNGALTA
RV
GVAAMEVCRPLPFREARRPSVSALVKRALTGVFSRADG
>OLS49360.1 hypothetical protein BV379_14460 [Rhodovulum sulfidophilum]AB14
MRIGDLADGMTRGVEGIAASVSETFMEPVLRLGVTGLSRAGKTVFITSLVANLLDRGRMPQLRAAASGAIVSAYLRPQP
D
NTVPRFDYEAHLAAMTGSEPRWPDSTRAVSELRLSLKVRPGGLGSIFGAVTGPRTIHVDIVDYPGEWLLDLALMDKDY
AE
WAAETLGRLDRRPGSDGFVAALAGIDPGAPFDEATAQLLAAHFTEALNTAREAGFSDCTPGRFLLPGEMAGSPVLTFA
PL
PVPGGSTPRGSLWREMERRYEAYKREVVKPFFRDHFSRIDRQVVLVDALGAIHAGPRAVEDLRRAMTEILGAFRPGRN
AW
LTRFLTGKRVEKILFAATKADHLHHSQHARLTSIMQALMKEARRRADFAGADTAAMAIAALRATVEETREQRGESYDC
VR
GTLMSTGRQAALYPGELPEDPALLLEPAREGAEDWLDGDYQIMHFAPARLTMKPGEGPPHIRLDKAAEFLLGDRLR
>OLS49361.1 tRNA pseudouridine(38-40) synthase TruA [Rhodovulum sulfidophilum]AB14
MPRYALKLEYDGRPFSGWQRQADRPSVQGAVEAALARLEHDLPSIAAAGRTDAGVHALGQVAHCDMAKDWDPFRL
SEALN
HHLKPHPVAVVACAAVSDDFHARFSTQERRYLFRLVSRRAPLVHDKGQIWRVGHPLALAPMQEAARHLLGRHDFTTF
RAS
LCQAASPVKTLDALDIEELDYPGGREFRFHVRARSFLHNQVRSFVGTLERVGAGAWAPEDVKAALEACDRAACGPVCP
PD
GLYLAAARYDPSPFDT
>OLS49362.1 IS5 family transposase [Rhodovulum sulfidophilum]AB14
MHAICDSKGRPISFFVSAGQVSDYIGAPALLSSLPSFKWLLGDCGYDADWFREALKDKGISACIPARTQRKTTIKYDKHR
DKLRNRIELMFGRLKDWRRVATRYDRCPKPFFSAIALAATVIYSL
>OLS49363.1 hypothetical protein BV379_14475 [Rhodovulum sulfidophilum]AB14
MPSESAFLPRHRQIFFTFLSIFLVMLGCGVTIWKVVFADGWSRHLDTAWLIAGTDCLFKGMSP
>OLS49364.1 hypothetical protein BV379_14480 [Rhodovulum sulfidophilum]AB14

MTVPLLALALWQIARVTAVDGPARLLRACWVTLGVSIGGTAGALYVGQPSVLVALGICLVHLGLRDSRAAIFLTGAALCS
LKPHLSLIWVVAALLMPPARLARAKGLLAGFVLLAAAALTLADPQVFSDYLHALKVLAGLSRAELLYGLPGLLDWGRSHP
RGIAILAVALVTSAAAGRALARRRPAQCDWSIPMLAGACLAEAFVSAHKGYDFSVYALVLALAARLPRTVQTLLLVPALL
DLLPVVRGAANHVVLLDAVLACSAGFLILYGLGQRARSQQSRAAAPVVPRI
>OLS49365.1 hypothetical protein BV379_14490 [Rhodovulum sulfidophilum]AB14
MDEIARHKRVAPPRIRFEGIDPRLPGLAQRQRGSAPPFVDMTWLGAERNLASLFARHCATGIPDEPPQPMTERNTHS
GDR
QHGLYRSVSAAC
>OLS49366.1 potassium transporter KtrA [Rhodovulum sulfidophilum]AB14
MLGSAPDLRAKEGRPVKRRTFAVIGLGTFGSTVATELARFGNPVMGIDIDEKIVGRYADVFSEAVIADGRDELALREAGV
GNYDVALVAIGEDLEANILCSMSVKMLGVKTIWVKALSKTHHRILSKIGADRIVLPEQEVGQHIAQMLHNPLVRDYVSL
G
NGFHVVDLTVPEPLEGRAVGEVKLLSGYDLRALGAIREGEFLSCESGELELKTGDKLLVLGRRADLRRFGDDLLDEA
>OLS49367.1 histidine phosphatase family protein [Rhodovulum sulfidophilum]AB14
MRLILLRHGETLWNAEKRLQGHDNSPLSPRGIEQARAIVPRLGALSPARVVTSDLGRVRQTAEIVGHGDAPSDDRLREL
D
MGEWTGLRKDVLIAEKAELYGAWRAGRYTPAGGETWDGFVSRITSALSDWLYAGQGDLLAIVHSGVVRAACFGFLGL
KPA
HLLPVTPGHLTILDFEDVRDTAPPRLEAYNLGEFAPDMDVAD
>OLS49368.1 permease [Rhodovulum sulfidophilum]AB14
MPETRTETAPGPSPKGPGPDKDRKDTLLRRYGIFLAAAVFLALYLMPNPAGLEPEGQKALAVFCGALTLWVAGTIPIYLT
SMIAIIALALTGTVGEKTAFGTLGFNVIWLMVAAFVLTSAMVKSDLARRFALWMVTRFGGTPTRTLAILVLINFVIVFFI
PSTTARASLMVPICIILLEIYRAVPGKSNFGKLMMLLGVQADALATSGVMTGTAANMIAVQFIYDQAGGEIGYMDWLL
AG
MPMAMMSMCLTFLVGLRLFRFSDEIDFSHGMSKLEEQHRSLGPISLAEKKAMAIFALTVTLWATADYQEIWFDGFSVS
IY
VTAVIAAVLCLMPRVGLLEWKEANIKWDLMIFAAGAYAAGNALESTGGAEWMIGGLVRGLGIETMSTAAVYVVVIFLS
MF
SHMVFTSKTVRTTILIPSIIALAQTLGLNPVTLALAASFTLTYTITLPPHSKVNTLYFATGYFSVLEQLKYGIVTCLIGG
TVISVSVFTWFKLLGYGL
>OLS49369.1 hypothetical protein BV379_14515 [Rhodovulum sulfidophilum]AB14
MNIAQLETFLELSDCLNVTETAARMNCTQPAVSARIRGLEQSLDAPLFDRIGKRMHLTANGILFLDYARKAVGALQEAS
E
HLRQMDDPLAGTIRFGASNFTGIYLMPAFLGRYRQAAPDLEFELEIASSAQLLAELDTAKLEFLILSDHIAVDERRYQCR
HICNDEMVLVVPPAHPLAKARTATLTDLSQDVFLMKPPPSASRSFLLDRIQGDDSSLGRVMHISSTEAIKQGVLHGLGIS
VLSRFVVAQELADGRLKEVPMALRPFRRGIRAINLRDKLLTPAASEFIKRLVESGGRAEALA
>OLS49370.1 acyl dehydratase [Rhodovulum sulfidophilum]AB14
MSGQAVRLGRGFYWQDMTVGDRYRTYGRTVTPSDISAFVNLAGMIEVLFTNAEYRRSEAAIKGNPSPGALVFALAEGL
AL
NATAQETGLAFLGTTMTIKGPVLEGDTIDVEIEVLEVRQESKGTRGLVKTRNTVRNQRGETVMVYEPLRLMQGR
>OLS49371.1 CoA ester lyase [Rhodovulum sulfidophilum]AB14
MRSWLFVPGNRPDRIAKARGLSADVLILDLEDSVAISEKAAARSCVLEALRAPRGPNRLWVRVNPLESSDFEADLDTAL
A
GGADGIVLPKAESGDCIRALGAACAARGQACPPVLAIATETARAIFGLGSYAGLSSSLAGLTWGAEDLSAALGSARSRGE
DGTLTGPYALARNLTLFGAVAAEVQPVDTVWTDIRDLAGLEAECLAAVRDGFTGKMAIHPAQVPIINRCFRPSEEEIEAA
RRIVEAFATDPTAGVVAIDGEMIDMPHLKRSRLILQRAGEPA
>OLS49372.1 dehydratase [Rhodovulum sulfidophilum]AB14

MAGRWLEEFRAGEVIHHAITRTVTEADNVGFSVQTMNPQPLHIDRHFAEATEWGQPLVNSLLTLGIMIGISVHETTLG
TT
LGNLGMTDVSFPAPVFHGDTLRVETEVISARPSRSRPGQGVVVFEHRARKQDGTLVAQCRRSALMMSSLDKEAV
>OLS49373.1 hypothetical protein BV379_14535 [Rhodovulum sulfidophilum]AB14
MLDLLKDVRVVELTTIVLGPMAGQMLGDFGADVVKVEAPNGDLARYVDPRSQAGVSSMFANNNRNKKSIALDLKTA
EGRE
ILLKLIDGADVFLHNMRLQAVERLGLGPAELMARNPRLIYCSAIGFGSDGPYAARPAYDDVIQAASGIASLPTYTGNDPS
YVPGVIADKIAALYAASAISAALVGRARSGRGCEIEVPMFEVMTSFVMAEHMAAASFDEAAPAGYQRLLNRHRRPFPT
SD
GWIAVLPYTERHWRRTFEELGRGDVLAQPWFADATERSQHVGEMYEILGQEMPKRATDTWIATFERLDVPHSRVSGL
DDL
LGDPHLQAVGFFAPTDDLENRVRSVPQPVRFRAQPPVPDRRAPELGEDGPALLAELGFGPEAIRGLEARGVLGRMAGS
DK
EEA
>OLS49374.1 hypothetical protein BV379_14540 [Rhodovulum sulfidophilum]AB14
MKSTISILSICAALASASTVAAQEVTLKIADSLPADHIITRNTTDVFTARLKEEFGDRIEIEHYPAQQLAKAKDILSITQ
AGIADIGYIVPSYVSEKMPLGGVVELPGEIASSCEGTLAFADLIQPGGTLDRLEFQPNGVRVLYNVALSPYQAVFSKRAD
VTSLEGFKGMKMRSNPGAMELSLQSAGATAVRMTPPEIFDAMTKGTVDGALLPFTSTYSYGLDQIVGSATRNANFGS
VGI
TYAISQRKWDSLPADIQEAMTRIGREVTESACKAFDTAEAELKDKLSGAGATVFALDEEEAARLNADFASVADDWAAA
LD
KRGKDGSEILAEFRAEVAALRENGN
>OLS49375.1 C4-dicarboxylate ABC transporter permease [Rhodovulum sulfidophilum]AB14
MTSLLVVIGLLGLLLLGVPVGFALAFTGLVGLMTIVGFDSALSVLSTTPLSTTNGFELIAVPLFILMAEFVIVSGIADRM
FKAITIWVGRLPGGLAVATALAGAGFGAISGSSTAAAAALSSTSIPAMRRAGYDVKFASGVVAVSGTLAMLIPPSIALVL
YGIIVEVNIASLLIGGVIPGIIVTLAIIATIYVLMARDPSIAPAARPWPMRDKLRALRAIGPMLLLLLCVTGSIYLGIAT
PTEAAGLGAFGAFLIAAAFRTLTLKTTWGALARAVRATCMIFMIIIGAHIFGYVLTLGQITPDFVNWITGLEVSPYLVMA
GIIAFYLVMGCFMDQMAILILTVPVMVPAITSLGFDPVWFGVLVVVAAEVGMITPPLGMNIFVVSRYAERPLSEIFRGV
A
PHVVSHIIVLALLVAFPQLVLWLPGTTQ
>OLS49376.1 TRAP transporter small permease protein [Rhodovulum sulfidophilum]AB14
MIRKAMSFVDHAFTAVAVLALAVMTLLIVADVALRYGAGTPIFFAHDLVVLYLTPAVFFFGFGPTYWRSEHLSVDLLTLN
LPRRLRELTDVLSSAIGLWIFGLLTWVAWERAAGSFANDEVIASIVPWPAWASYALVPLGSAAMVLVCAARILISLGNAF
GRVSGEAAQ
>OLS49377.1 hypothetical protein BV379_14560 [Rhodovulum sulfidophilum]AB14
MAVRQADNVLRFFEYFAASQRPTTLTELSEALKLPLSSTSNLVSTLRERGFLFEVRKRGGFYPTRRMYDLTLSILDGDPV
LGLVREHMEALREETGETILLAAREGDRVIYLEALESRKGVRYSARPGETRPIYAISSGKAILAALDDAALRREIDRLDY
SEAAEASITDRAALFDDIVSGRARGWFLNASEYTPEVSAVGMLLDIAEQKYGLSVAGPNYRMQGRYEEIAAALGRVVA
AI
KSQIHETGAGR
>OLS49378.1 hypothetical protein BV379_14570 [Rhodovulum sulfidophilum]AB14
MGIDKQSDFAANLQIGPTSLGMVRIYIEGDGLEIPMDFDPEEAEDIAEELRAAAAQARTLAEKKKVKGSKPKG
>OLS49379.1 hypothetical protein BV379_14575 [Rhodovulum sulfidophilum]AB14
MPDPVSQPLSPSLAPGQLLARGVCRHLRDLGFASVEEFVPAPGLRVDVMALGAAGEIWVVECKSSRADFTSDRKWSG
YLD
WCDRFFWAVGPDFPSDLLPPETGLILADGYGAEILREPAERRLAPARRKVLTRSFARHAALRLQALRDPGLSPSAWSP
>OLS49380.1 hypothetical protein BV379_14580 [Rhodovulum sulfidophilum]AB14

MPKLVTLYIRQATMGFALSGVFVGLLMYFDVANLWHLVTNVSGGWLAAALLFLFNGIVFGGVQFGIAVMAMARDDD
PDSG
RRRPEPVRGLADVLVPIPVPVKDERRMRR
>OLS49381.1 transcription antitermination factor NusB [Rhodovulum sulfidophilum]AB14
MTGAHKSNRNQMRSAARLYAVQALFQMEQSGQNVDTVRREFETWRFGVGYEDGEELAEGDVELFRATLEGAVSW
QAKIDQ
MTDRALVAKWPIARIDPTLRALFRAAGAELVAAKAPPKVVITEYVDVAKAFFPEGREPKFVNAVLDHMAREARPEAF
>OLS49382.1 6,7-dimethyl-8-ribityllumazine synthase [Rhodovulum sulfidophilum]AB14
MAEQDPHHALELPAFDRPAKLLVVVAPFYRDIADMLIAGAVATLDAAGVTHELVEMPGALEIPSAIALAHRLSNYDGYV
A
LGCVIRGETTHYETVCNDSSRGLTLLGLQGALIGNGILTVEDRDQALRRADPADMDKGGGAAAAALHLIALARRWSGE
TK
GVGFRPSGGIQIAEGSDRA
>OLS49383.1 3,4-dihydroxy-2-butanone-4-phosphate synthase [Rhodovulum sulfidophilum]AB14
MHDIGEPNYQDAISPIEEIIEDARNGRMFILVDHEDRENEGDLVIPAQMATPEAINFMATHGRGLICLTLTGERIDALGL
PLMASQNSSRHETAFTTSIEAREGVTTGISAHDRARTVAVAIDAGKGAADIATPGHIFPLRARDGGVLVRAGHTEAAVD
V
SRLAGLNPAGVICEIMNEDGTMARLPDLVAFAQRHNLKIGTISDLIAYRRRHDNLVNERDVRHVTSAHGGDWVMRIFT
DE
TQGAEHVVLTKGDISTDAPVLVRMHALDPLQDVLGLGPHPAGELATAMEMIAAEGRGVVVLLRDTTMKLVLDGAASP
QTL
RQYGLGAQILAALGLHRLVLLTNSPLPRVVGLDAYGLSIEGTRSIAMEG
>OLS49384.1 riboflavin synthase [Rhodovulum sulfidophilum]AB14
MFTGIITDIGTVRALTMKGDLTARIGCAYAADSIDIGASIACNGVCLTVTDRGTSGDGAWFDVSISAETVSKTNIAGWAE
GARINLERALKVGDELGGHIVSGHVDGVAEIVAMAEEGDSTRMTFEAPEALARFIAPKGSVALNGTSLTVNEVDGPRFG
I
NFIPHTKEATTWGLVRVGDRINLEIDTMARYVARLREYDAA
>OLS49385.1 sugar ABC transporter substrate-binding protein [Rhodovulum sulfidophilum]AB14
MLAATAACGLPRPGPSKPEIFDGSSLRDGDAFIVEVNDRVNRTTAVVPESGFPPAFLNAAPIGSDTIRAGDTLGLTVFEN
VDDGLLAPSESNAATLTQLQVDSTGFIFIPYAGRIRAAGSTPEGLRKKITEKLDVQTPDPQVMVTRLAGDGATVAVSGG
V
GKQGVFPIERPNRTLSGMLATAGGIVIPPEIARITVIRGKTRGQIWFEDLYDDPTLDIALRNGDRILIEEDTRDFTALGA
TGRQRNVRFITQSMSAVEALAQVGGLNSALADPTGIFVLRNEPEEIARQVLGRDDLTGEQRVAYVLNLTEPAGLFLARD
F
AIRDGDTVYVTEAPYVRWVKLLSVITGTAGAARNVSASVDN
>OLS49386.1 transcriptional regulator NrdR [Rhodovulum sulfidophilum]AB14
MRCPFCGNIDTQVKDSRPAEDHVAIRRRRFCPACGGRFTTYERVQLRDLVVIKSSGRREDFDRDKLERSIRIALQKRPVD
PDRVDQMVSGIVRRLESMGETDIVSKVIGEIVMEALARIDTVAYVRFASVYKNFQEAGDFDKFVSELRPSAAPDE
>OLS49387.1 hypothetical protein BV379_14620 [Rhodovulum sulfidophilum]AB14
MTDLTHLAVPGTEIAVRVTPKASRARIVAEEGQIRVYVTVVPEDGKANAAVTKLLSKAVGVPKSRLELVRGQTARDKLFR
VL
>OLS49388.1 nitroreductase [Rhodovulum sulfidophilum]AB14
MPDPRPEVLDFLLTRRSRPAKTLAAPAPDREVLTRILTAAARSPDHGKLEPWRFIVLEKPALTRLADMVRAQGPALGQP
P
EAVEKAASAYANSPLAVVVVSSPKASSKVPEIEQLLSAGAVCLGLLNAASAAGWGANWLTGWPAHERAVLPEALGLG
DAE
TIAAIIHIGTETVAPPERPRPDIAAITEWVAE
>OLS49389.1 hypothetical protein BV379_14630 [Rhodovulum sulfidophilum]AB14

MGGRMILTAFLKTLGQIGDRAFRRVLLLGIGLSVLLLAAIYAVIFFLIGWLVPDTLTLPWIGEIHWVDNILSGASFLLMI
VMSVFLMVPVASAFTGIFLDDVAEAVEARHYPDLPPATPIPLLDQIRDSIGFLGVVLGVNLVALVLYFFVGPVAPFLFWA
VNGFLLGREYFQMAAMRRLGRQGARDLRRRHGATIFLAGLLMAVPLSLPLVNLVIPILGAAVFTHLVNRLIEARRAPA
>OLS49390.1 alpha-hydroxy-acid oxidizing enzyme [Rhodovulum sulfidophilum]AB14
MPVITTIEDLHRIYRRRVPKMFYDYCETGSWTEQTFRDNCSDFAKIRLRQRVAVDMEGRSTATRMIGQDVAMPVALA
PVG
MTGMQAADGEIKAARAAEKFGVPFTLSTMSICSIEDVAAHTEKPFWFQLYVMRDQDYLANLIQRAKAANCSALVLTLD
LQ
ILGQRHKDIKNGLSAPPKMTIPTMANLATKWGWGLEMLQTKRRFFGNIIGHAKGVTDASSLVDWTASNFDPRLDWS
QVKK
LKEDWGGKLILKGILDAEDARRALDVGADAIIVSNHGGRQLDGALSSIRALPSILRAVGDQIEVHVDGGIRSGQDVLKAV
ALGATGTYIGRAFVYGLGAMGEAGVTRALEVIHKELDTTMALCGRKSIDGLDRDILLVPKDFEGDWA
>OLS49391.1 50S ribosomal protein L25/general stress protein Ctc [Rhodovulum sulfidophilum]AB14
MAGEIPDLNATVRTGTGKGAARQARRDHLVPGIVYGGGAEPLPINIKYNELLKRLKAGRFLSTLFNLKVDGQEDVRVICR
GVQRDVVKDLPTHVDLMRLRRTSRINLFIPVEFVGEETSPGLKRGGVLTVVRPEVELVVTAGDIPEKLTVDVSALNVGDI
VHISDITLPQGTTPTITGRDFVIANISAPSGLRSADNEEEEEAGEE
>OLS49392.1 hypothetical protein BV379_14645 [Rhodovulum sulfidophilum]AB14
MRYPVLSSFPTPLGLAVLLIVVLGLSNGPARGGSGVPPVDSGLAAAVAGDWEAEPGDLSDEVATGRLRAGILCTSWAR
DL
ISQHWRRLDRLVGPGTWSSDRQTLWLQLGWVDAFERRHLRPRIDADSQEALAAAWWPDGVSTPAGSAGATEMHR
FCMRLP
GVFGSIDPDILWREEEAMLR
>OLS49393.1 antibiotic biosynthesis monooxygenase [Rhodovulum sulfidophilum]AB14
MYLTMNRFKVKPGSEEAFEAVWKNRDSHLKTVPGFVGFHLLKGPAGETYRLYASHTFWESEAAFTAWTRSEAFRAAH
KGA
KGPSDMYLEPPELEVFESVQSLV
>OLS49394.1 aminoacyl-tRNA hydrolase [Rhodovulum sulfidophilum]AB14
MRLFVGLGNPGAQYAGNRHNIGFMAVERIAEDHHFPPWRAKFKGQTAEGRLGSEKVILLKPETYMNLSGDAVQAAV
QFYK
LTPADVTVFHDELDLAPGKCRLKSGGGHAGHNGLRSIHQHLGPDYERVRLGIGHPGHKDRVSGYVLSDFAKADRDWL
DDL
LRGISDGAADLARGDGARFLNAVALRVAPPRNGATATGAAAPKAPAGPAAPRPGQAEAAEDSRTPLQKLADRFR
>OLS49395.1 hypothetical protein BV379_14660 [Rhodovulum sulfidophilum]AB14
MTDTKAPSVNVLGGPLAPCSTTPLTGFFRNGRCDTCREDSGSHTVCAVMTAEFLAYSKYVGNDLTTPRPEFGFVGLKP
GD
HWCLCAARFLQAHDEGCAPKVRLAATHRDALEVVPLDVLRHHAEEA
>OLS49396.1 tryptophan synthase subunit beta [Rhodovulum sulfidophilum]AB14
MPDDILNSFKTGPDENGRFGIFGGRFVSETLMPLILELEAQYEHAKTDDSFWAEMDFLWKHYVGRPSPLYFAERLTERL
G
GAKVYFKRDELNHTGAHKINNVLGQIILARRMGKTRIIAETGAGQHGVATATVCAKFGLKCVVYMGATDVERQKPNVF
RM
RLLGAEVVPVTSGRGTLKDAMNDALRDWVTNVRDTFYCIGTVAGPHPYPAMVRDFQSIIGTEAKAQMLEAEGRLPDT
LIA
AIGGGSNAMGLFYPFLDDKEVRIIGVEAGGHGVDQKMEHCASLTGGRPGVLHGNRTYLLQDEDGQILEGHSISAGLDY
PG
IGPEHAWLHDTGRAEYVSITDREALAAFQLSCETEGIIPALEPSHALAHVMKIAPELPADHLICMNMCGRGDKDIFAVAR
HLGFDMGEA
>OLS49397.1 hypothetical protein BV379_14670 [Rhodovulum sulfidophilum]AB14

MRGLLGLIALLAVAGLGLAFTKPTEAAFEAALEARLLDRIDRADPEAENNPASAVLLATCKLGRAQCAELIGSMISLDYA
DKVLFSQADVSLGGRSYGVCYGAATQIFCRETANPR
>OLS49398.1 N-(5'-phosphoribosyl)anthranilate isomerase [Rhodovulum sulfidophilum]AB14
MSDAIRVKICGLTSPAAVDAAARLGASYLGFVFFPGSPRHIDPETARGLAFAAPPGLAKVALTVDADDAALDALLDLVPI
DMLQLHGRESPERVAGIRARFGLPVMKAVGVATEADLPALTDYARVADQILVDAKPPAEAALPGGNGLAFDWRLIAG
RRW
PVPWMLAGGLTPANVAEAIALTGARQVDVSSGVERAPGVKDEALMGRFIAAAGGRERP
>OLS49399.1 DUF1049 domain-containing protein [Rhodovulum sulfidophilum]AB14
MRLIRYLFLVLLAICLVVVATANLGPVSVTLLPNDLATYSGFQVTAEVPLYVVGFGGMLLGLLVGFFWEWLRESKHRSVA
SRQRREAARMQRELDRLKADKRRNDGHDEVLALIEQNGSAR
>OLS49400.1 integration host factor subunit beta [Rhodovulum sulfidophilum]AB14
MIRSELIQKIAEENPHLYQRDVERIVNTIFEEVISAMARGDRVELRGFGAFSVKKRDSRVGRNPRTGESVEVDEKCVPFF
KTGKLLRDRLNGK
>OLS49401.1 30S ribosomal protein S1 [Rhodovulum sulfidophilum]AB14
MAQSASMEDFEALLNESFEIDTPSEGSVVRGKIIAIEAGQAIIDVGYKMEGRVDLKEFSNPGESPEVTVGDEVEVYLDRV
ENARGEAVISRDKARREEAWDRLEKAYADDQRVEGAIFGRVKGGFTVDLGGAVAFLPGSQVDVRPVRDAGPLMGLK
QPFQ
ILKMDRRRGNIVVSRRAILEESRAEQRAEIISKLNEGDAVDGVVKNITEYGAFVDLGGVDGLLHVTDMAWRRVNHPSEI
L
SIGETVKVQVIKINKETHRISLGMKQLQADPWDTVEAKFPLGTVHKGRVTNITDYGAFVELEPGVEGLVHVSEMSWTK
KN
VHPGKIVSTSQEVDVMVLEIDTAKRRVSLGLKQTMRNPWEVFSETHPVGTEVEGEVKNITEFGLFIGLDNDIDGMVHLS
D
LSWDQRGEEAIQQYRKGDVVRAVVTEVDTDKERISLSVKSLDGDPFAEAVGGVKRGSIITVAVTAIEDGGIEVEYEGMK
S
FIRRSDLSRERSEQRPERFQVGDHIDVRVTNIDTKTRRLGLSIKAREIAEEKEAVEQYGSSDSGASLGDILGAALKGEDR
>OLS49402.1 hypothetical protein BV379_14695 [Rhodovulum sulfidophilum]AB14
MNTLLDRRAGRIEPPIPVAVVIEQHGAWRVLFAAIAALFDARGRSGELRPGELWPGDLDNHLRRDIGLPPLPEPWSEPP
R
WM
>OLS49403.1 cytidylate kinase [Rhodovulum sulfidophilum]AB14
MSFTVAIDGPAAAGKGTISRAVAAHFGFAHLDTGLLYRAVGARMLDGLDPVEAARTLEPSVLERGDLRAPEVAQAASK
VA
AIPDVRAALVDFQRAFAARAGGAVLDGRDIGTVICPEAPAKLFVTASDEVRAERRFLELRQKGVETSFEEVLAEMRERD
A
RDSARATAPLRPAEDAVILDTSRLSIEAAVGAAIETIRTRHPA
>OLS49404.1 hypothetical protein BV379_14705 [Rhodovulum sulfidophilum]AB14
MTVTGSCHCSQCCKASGHHVAATTVLRDDLVIEGEQCGFASSAGAQRGFCPVCGANLFWDGGRDNLAVFAGTRDG
DPGIR
MAAHIFCADKGAYYEITDGLPQAPGLPEDIA
>OLS49405.1 3-phosphoshikimate 1-carboxyvinyltransferase [Rhodovulum sulfidophilum]AB14
MSAHGDPIPMTARRSGPLKGEAVVPGDKSISHRALILGALAVGETRISGLLEGQDVLDTARAMRAFGAEVTHEGPGA
WTV
QGVGVGGFAEPPDVIDCGNSGTGVRLIMGAMATSPVTATFTGDASLRKRPMGRVTEPLALFGTRAFGRQGGRLPMT
LIGA
AEPVPVRYAVPVPSAQVKSAVLLAGLNAPGETVVIEREATRDHTERMLAGFGATLRVEDGAEGRAIVLTGQPELRPQTI
A

VPRDPSSAAFPVAAGLIVEGSDVLVPGIGLNPTRAGLFTTLREMGADLSYENAREEGGEPVADLRARFSPDMTGIEVPP
E
RAPSMIDEYPILAVVAAFADGATVMRGIGELRVKECDRIAAMVTGLRACGAEVEEGEDWMIVHGHGPGSLPGGATCA
THL
DHRIAMSFLCAGLASQRPVLIDDGGPIATSFPIFEGLMAALGAAFARGTE
>OLS49406.1 hypothetical protein BV379_14715 [Rhodovulum sulfidophilum]AB14
MSDIRITNCHIHTFTAAHAPLCYPAPPVAALRAWPGLLRGLRGAARLLPWEGAYEALLRLEHFRDTGCRDSQRAVFLEV
L
RHYPDSARFVVLPMDLAFCGYGPVPVGLEAQHRELCELSRDPQVGHRVIPFATIHPDRPGAVAELRRCLDEYGFRGVKI
Y
PRLGYAPSHEILMREVYPLCVERGLPVVSHCSRGGVRNRRWSRARADAVTDPVAHVPVLRAFPDLRLCLAHFGGDRD
WLS
YLGEGLDPLDPEARRRNWAISIADMIESGAYPNLFTDISYTLFRFAACMPLLKLFLERERLASRILFGSDFYMTRQQDLS
EKAVAIRLRAALGEASFRRIAETNPAIWLGEA
>OLS49407.1 methionine adenosyltransferase [Rhodovulum sulfidophilum]AB14
MTRQNYVFTSESVSEGHPDKVCDRISDAVLDAFLAEEPNARVACETFATSSLVVIGGEVGLTDKVRLKQLMGSIGEIARE
CIRDIGYEQDKFHWNTCHVLNFLHEQSAHIAQGVDKDGAGDQGIMFGYATDETPELMPAPIQYAHAILRRLTEARKSG
AE
PTLRPDAKSQLTVRYEHGRPVAVDSIVLSTQHAREDQNSDDIRAIVEPYIREVLPEGWLHEGTEWWVNPTGTFVIGGP
DG
DAGLTGRKIIVDTYGGAAPHGGGAFSGKDPTKVDRSAAYAARYLAKNVVAAGLASKCTLQLSYAIGVAKPLSIYVDAHG
T
GKVPETEIEKAVEQAMDLTPRGIREHLGLNRPIYARTAAYGHFGRAPEEDGGFSWERTDLAEALKKPF
>OLS49408.1 apolipoprotein N-acyltransferase [Rhodovulum sulfidophilum]AB14
MRPARFRRVFARPAWPTWAAFGLGLGLALGQAPWSLWPLALCALAGLIWLVARAGTPGRSARIAWAGGAGHFALA
LSWIV
EPFLVDIRHDGWMAPFALILMAAGLALFWGAAGAFAGWAGRGPRGRALAFVLALSAAELARGYVLTGFPWALPGHV
WIGA
PQMQLAALTGQYGLTLVTLLVAALPVLLPVGPGRSLGAGLAVAAVAGLGLWGAVREAARVPAEPDPPQVRLIQPNAA
QHL
KWRRDMIPVFWERQLAYTAEPGDPPPDLIVWPETAVPFWLENAGDGLGQIARAAGGVPVAAGIQRSDGPLAYNSLA
VIGA
GGRLLGLYDKHHLVPFGEYIPGAALLGRFGIRGLAANDVYGYAPGPGPRLLDFGARLGKALPLICYEAVFPQDLRGTARP
RWILQVTNDAWFGTRTGPYQHLALARLRAVETGLPFLRAANTGVSAVIDARGRVLASLPLGRAGQITAPLPPALDETPY
A
RTGDAPVLILLCLGLAFFVATRAMK
>OLS49409.1 magnesium/cobalt efflux protein [Rhodovulum sulfidophilum]AB14
MGDETDGSSRAAQGALDTDNLDSRGLLGRLVAAILPAGAEHSEEGGDHALGNGIARPALTHGIGNLAKMRVEDVAVP
KAE
IDAVPLDVSMEDLVQVFRESGLTRLPVYDGTLDSPVGMVHLKDFALRHGFNGKDTPFVLKDMIRPLLYAPPSMPIGVLL
Q
KMQSQRMHMALVIDEYGGVDGLVTIEDLIEQVVGEIEDEHDIDEGSLWIVEKPGVYLVQARAPLDAFEDEIGMRLLDA
EE
EEEVDSLGGLIFMMIGRVPARGEVIRHASGAEFEVVDADPRRIKRLRVRLPEATAAAAATA
>OLS49410.1 rRNA maturation RNase YbeY [Rhodovulum sulfidophilum]AB14
MTIEILFEDARWAEAGLDRLAETACRETLAHLGFDPDAHEISLLACDDMRIAGLNASFRGKSAPTNVLSWPTEDLAAEA
D

GDAPLPPEPEIEGAPVELGDIAIAWETCLREAREQGKPFNDHVTHLMVHGCLHLLGYDHVRSRDAALMEGIEVEILAKL
G
LTDPYD
>OLS49411.1 phosphate starvation-inducible protein PhoH [Rhodovulum sulfidophilum]AB14
MGISALTPPPQTEDVHEMLLEFPDNRLLIDLCGEFDRNLAQIEHKLGVHIPRRGNRLAIVGDPEARGQAAMVLQQLYAR
L
ETGQPVEAGDIDGALRLGETAGPEAAAGDQLEMFQGGRIEIHTRKKVVEPRTEAQKAYVRALQENELCFGIGPAGTGK
TY
IAVAVAVNMFIGGHVDRIILSRPAVEAGERLGFLPGDMKEKVDPYMQPLYDALHDFLPGRQLAKLIEEKRIEIAPLAFMR
GRTLANAFVVLDEAQNATTMQMKMFLTRLGEGSRMVITGDRSQVDLPRGVSSGLADAERLLKPIDNISFSYFTARDVV
RH
PLVARIIEAYDADDAKT
>OLS49412.1 hypothetical protein BV379_14750 [Rhodovulum sulfidophilum]AB14
MSTDPEPQNSAELLSEEKMPLIAYALYIIGVIFPLLTLAGLILAYLKRGEAPDWHKGHYTYLIRTFWIGLLISFVGLILT
FVGIGVLVLIACGVWYLVRTIKGLLRFNSRRPIDNPTTWLI
>OLS49413.1 tRNA (N6-isopentenyl adenosine(37)-C2)-methylthiotransferase MiaB [Rhodovulum sulfidophilum]AB14
MTSSTSPTRKLFIKTYGCQMNVYDSERMAEALAPSGYEIADSAEGADLILLNTCHIREKAAEKVYSELGRLKALKAENPD
LKIGVAGCVAQAEGDEILRRQPLVDLVVGPQSYHRLPELEARARQGAKALDTDFPEEDKFDRLGGRARARRGPTAFLTV
Q
EGCDKFCAFCVVPYTRGAEVSRPAEKVLAEARDLVERGVREITLLGQNVNAYHGAGTDGDWGLARLIRELAAIDGLDRL
R
YTTSHPNDMEDDLIAAHGEVPELMPYLHLPVQSGSDRVLKAMNRKHTAESYLRLIDRIRAARPDILMSGDFIVGFPGET
E
ADFEDTLALVREVGYGQCYSFKYSPRPGTPAAERAHVAEEVKAERLALLQALLAAQQEAAQQAMVGKSVGVLFEKAGR
MP
GQLVGKSDHLQAVHVEAPETLLGRIARVDITAAAPNSLAGRLSVE
>OLS49414.1 hypothetical protein BV379_14760 [Rhodovulum sulfidophilum]AB14
MAGRYKGLADFLARAGKALGPRPVGLIFVEDEVEVESTIDHLDALGFGALVLFTPPGLKLSPASEERIHHVAYDTRAEGA
LPRAVSAVIAAAPGIWLHYCYNAEYLFFPFCESRSIGEMLDFHGEDGREAMPTHVVDLYAADLDRHPGGVSRDSALLDR
A
GYFSRPRTDPATGRVLERQSDIFGGLRWRFEEHVPAHRRRIDRIGLFRARRGLRLLDGYLMSDPEMNTCAGDRGNTLT
AA
ICSFRAAKALRINPASRLEIADFRWPMSEEFRWETQQLMDLGLMEPGTWF
>OLS49415.1 transcriptional repressor [Rhodovulum sulfidophilum]AB14
MAQLNENPNHPDAIERSGRWLSKAGLRPTRQRVSLAALLIGDGNNRHVTAETLYAASCATGEKVSLATVYNTLRAFCD
AG
LMQEVMVDGTKSYFDTRVDDHPHFFWEDDHRLSDAPAGSLEIARLPEAPEGAEISKIDVVIRLRRV
>OLS49416.1 3-hydroxyacyl-[acyl-carrier-protein] dehydratase FabA [Rhodovulum sulfidophilum]AB14
MTDFPTSFGKDELLKCARGELFGPGNAQLPEPPMLMMDRITEISADGGPNGKGHVVAEFDIHPDQWFFACHFPGNPI
MPG
CLGLDGLWQLTGFNLGWRGWKGRGYALGVGEVKLTGMVRPDRKMIRYFVDFTKAVQTRRLTMGVADGRVEADGE
TIYHVK
DMKVALSES
>OLS49417.1 beta-ketoacyl-[acyl-carrier-protein] synthase I [Rhodovulum sulfidophilum]AB14
MRRVVITGMGIISPIGNSTAEVEASLRAGRSGIRFEPVYAENGFRSQVAGIPQIDLEGSIDKRQLRFMGAGAAYNYLAM
E

QAIADAGLEADEVSNPRTGLIVGSGGPSTANFFVAHSTVKEKGSPKRMGPFMVTRCMSSTNSACLATPFRIKGVNYSIT
S
ACSTSAHCIGNAAELIQMGKQDVIFAGGGEEVDWTLSCLFDAMNAMSSKYNDTPETASRPFDATRDGFVIAGGGGVV
VLE
ELEHAKARGATIYAEVTGYGATSDGYDMVAPSGEGGERSMELALATLPEGRRVDYINAHGTSTVAGDVTEVKGVRNVF
GE
DAMPPIASTKSLTGHSLGATGVHEAIYSLIMMRKGFIAASANVTELDPEIRPGEIATELREGVEIDSVLSNSFGFGGTNA
TLLMSKYHG
>OLS49418.1 enoyl-ACP reductase [Rhodovulum sulfidophilum]AB14
MPELMKGKRGLVMGVANDRSIAWGIARAMAAEGAELAFSYQGENFGRRVQPLAESVGSSLLVDVDVTDDASLDAAF
GRIE
KEWGRLDFLVHAIAYSDKTELTGRFINTSRENFRNSLTISCYSLIEVARRAQPLMPDGGTILTLTYQGSNRVTPFYNVMG
VAKAALESTMRYLASDLGPEGIRVNAISPGPMKTLAGAAIAGGRKTFRHTEMNAPLGHNATLEAIGGTAVYLASDMGA
CT
TGEIITVDGGYHVLGMPRFENI
>OLS49419.1 peptidylprolyl isomerase [Rhodovulum sulfidophilum]AB14
MTQVKPGDKVRIHYTGTLADGSTFDSSQGRDPLEFTVGAGQIIPGLDAALPGMSEGDTKTVDVPCAEAYGAHNPDAT
QAI
PREQVPDTVPLEVGTRLNVQTAEGEVLPVTVADVTEEVVLLDGNHPLAGKDLTFEIELVSIT
>OLS49420.1 hypothetical protein BV379_14790 [Rhodovulum sulfidophilum]AB14
MSQNHGQVWWTELMTRDVPGALKYYKAVCGWHFQPVPMGAGVYQVGHRGARPVVGVMDLAGLPDMDQVPPH
WFSYFAVDD
LKKALNETERGGGAVIRQPFEVPDIGRIAIVSDPTGAALGLITPAR
>OLS49421.1 haloacid dehalogenase, type II [Rhodovulum sulfidophilum]AB14
MPITTCIFDAYGTLFDVSAAARRAAAEPDHACLAGRWAEFAVTWREKQLDYTWLRAITGAHADFRQVTGEALDWALE
AHG
LAGEAGLRARLMALYRDLDAYPEAPAMLGALRRAGKATAILSNGSPGMLADAVAAAGMDGLFDDLLSVETVGIYKPH
PSV
YDLVGQRFGCDRSEVLFVSSNGWDAAAAAGYGFVTAWIDRNRAPVERLPWRPAHVLHDLAEIPSLVEALS
>OLS49422.1 alpha/beta hydrolase [Rhodovulum sulfidophilum]AB14
MTRFAADDGRMLAYDDRGRGTPLLCLAGLTRNMDDFEPVLDAFVGRARVIRLDTRGRGGSDRDPDFMNYNLIREGR
DALN
LMDHLGLDRVAILGTSRGGLIAMMLAASHKDRLSGVCLNDIGPLVEPRGMANIMSYLGLPPGYADYDDAAAQLALKM
RDR
FPGVGRDQWQAYARRLWDATPDGLALRYDPALRHAVMEQSATDALPDLWPLFDALAGLPLALIRGAHSDILSPETAS
EMQ
RRRPDMIFAEIPDRGHTPFLDEPAAVAAIATLLERVS
>OLS49423.1 threonine ammonia-lyase [Rhodovulum sulfidophilum]AB14
MTIDQIEAAAARLSGKARKTPLLSSPFLDEIAGRRVLVKAECLQHTGSFKFRGGWSALSVLPPEARARGVIAYSSGNHAQ
GVALAARMLDAPAVILMPSDAPAPKIANTRALGAEVVTYDRATGDRDAIGAELAEARGLTLVRPFDDPQVIAGQASCG
LE
IAAQAADEGIAAAEVLVPCGGGGLTSGVALALEARAPGFRVRPVEPEGFDDVARSLASGRIETNTRRAGSLCDAIQTPA
P
GALTFPVMARLCGPGIAVTEDEALRAMAAAFERLKIVLEPGGAVALAAALYHPEAVAGEAVIAIGTGGNVSTETFRMAL
D
RYGNVA
>OLS49424.1 3-oxoadipate enol-lactonase [Rhodovulum sulfidophilum]AB14

MKIADLGDVALHYREDGPPDGAPVVFANALGCDLRIWDDVIERLPAGLRAIRYDMRGHGLSSCPPGPYSMGALIRDAE
RL
LESLAVRDCVFVGLSIGGMVAQGLAVKRLDLVRAMVLSNTAAKIGTPEIWQDRIEAVRKGSIAALSEATLARWFSRRYR
Q
STDCTLWQNMLNRQPVEGYLGCCAAISGTDFYTPTASLTLPTLVLAGSEDGSTPPDLVRETAGLIKGARFHLIRGAGHLP
CIDRPEDYAAVLTTFLREIGHVESCPESAG
>OLS49425.1 esterase [Rhodovulum sulfidophilum]AB14
MSRFLLIHGSCHGAWCWRDVLTALHRSGAEARAIDLPSHGSDPTPIADVTLDLYADAILDAIDEPVILVGHSMAGFPISA
AAEKAPGKIRALVYVCAYAPVAGRSLVDMRRAAPRQPLLEAIVKSADGLSFTFDPDLVREKLFHDCSDAAVAYAKARLD
P
QPILPQATPIALSERYASVPKHYIRCLQDRAIPSEYQATMTSDWPAERVDTLDCSHSPFLAQPEALAALLLKAAAMS
>OLS49426.1 hypothetical protein BV379_14820 [Rhodovulum sulfidophilum]AB14
MQKYDVQPEIEAFDLDHIFQAMKRNRHGRLKAPLYVRFVMGSGTPCPSTGRASNSMSRR
>OLS49427.1 tetracycline resistance MFS efflux pump [Rhodovulum sulfidophilum]AB14
MPQPSTRLPIVFILVTLVLDAIGIGLILPVMPDLIRDIDGGTGLGQAAVWGGILSTVFAVMQFLCGPTVGSLSDRFGRRP
VLLISLATMALDYVVMALAGSIWLLLAARVVGGVTASTQATASAFLADISKPEEKAARFGMVGASFGIGFVIGPLIGGFL
GELGPRAPFWAAAGLATLNLIFGAMVLPETVTDRIRRPFEWRRANPLGAFRAIGRLPGIRPLLLLFFLYEFAMFIYPAIW
AYFTQYRFGWSTGTVGISLAMFGIGVAIVQGGLIRIVLRVMGERIAVWYGLGYNFIAFLAIGLIGNGSVVLVLTPMIALG
AVVTPALLGMMSKAADDNQQGELQGMVSSLRSVAMIASPLVMTQTFFLFSRPDAPLHLPGAPFLLASGVMVICALFF
ALR
RAATADTGIRRRA
>OLS49428.1 hypothetical protein BV379_14835 [Rhodovulum sulfidophilum]AB14
MLITSLQGIRILGALPLLFASAPAGADKPLSVEILRIEARPAEITYTLTGTVQAVDDYPASFRDGGRIIEVAVETGDALE
RDDVIARVDATEAEARLRSAKAALQAAEAALVQARQARDRAANLLDEGNGTQSDLDAAVQAFLSARSDRDQAAARR
AVAR
RAAEDTVLRAIEPSIVIRRSAEPGQVVPAGQAVVTLANRGPRDAVFQVPDIPELDAFLGSDVRLSPLGGGRGCDAAVTEL
APVVSGNGTVQAKVRIEGEAAGRFSIGQPLVGIVTLGRPTALVVPWTALTATADGPAVWTVGAEDNRVRLTPVTIDGY
SD
SGIRIAEGLSEGEAVVGAGSQYLFPGREVRPLETAK
>OLS49429.1 ACR family transporter [Rhodovulum sulfidophilum]AB14
MTRRFNLSDWALGHRSLVWFLMIVSLVAGLLAYLSMGREEDPSFAIKVMVVSAALPGATAEETVTQVTDRIEKKLQEID
Q
LKFTRSVTYPGRSVVYVELRDDTRAEAVDATWLRVRNMMSDIRGEFPSEFAGFSFDDDFGDVFGSIYAFVGDGFTPREI
E
DRVEDVKAEILRLDQAGKVELVGVRAPEIHIEFSNRRLAALGLDREAVLQTLAQENAIVPSGTIRTAGERILIRVTGQYA
SAEDLAATPLRTGDVFYSLSDVADVTPGYEDPPAELFRYNGHEGIALLVGMRKGGNILQFGAELEALMDRVAERLPVGI
E
LHRVADQPKVVEESVGHFTRALIEAVLIVLAVSFISLGLRAGMVVTLSIPLVLAMTFVLLDVMGITLQRVSLGALIIALG
LLVDDAMIAIETMISRLEIGESLHKAASYAWTSIAFPMLTGTLVTAAGFIPIAFNSSQAGEYTRSLFYVIAISLLLSWIV
AVLFAPILGTTFLPKKLRHAHKGPGPLRRGFRRVLLQAMRFRWLTIGLTALLFAGSVFGLRHVERQFFPTSERPEIVVEV
TLRQNGAFAATDAAIAAFEAWLGQQPEAGYTTAYVGRGVPRFVLTLDQPTPSENIGQLAIMTPDLTARDRLREKIAAYD
R
AHPEADFYTRLIELGPPVGKPVQYRLSGPDIAPLRDRARDLAALLDRDPRLTSISLDWSEPARVVRVVLDQARLRQLGLT
QSDVARTLYGLFDGSGVTEFRDGRKLVDVLARGVAADRSTVAALETLQFGNAAGNPIPLLSIARLEWTTEQPEIHQRDR
V
PTLTVGAAIRGSVQPATVASDLAPAIADFAAGLPPDYRIALGGSAESSAESQAPILKVVPVMVLCILTLVMVQMQSFRLL
FIVLAVAPLGLIGVVAALLPSGAPLGFVALLGVLALVGILIRNSVILIHEVSVLSRTGRSPWEAVYEASDSRARPILLTA
AAASLALIPIAREVFWGPMAFAMMGGIIAGTGITLLFAPALYCVVFRIRPPAPEARPEA

>OLS49430.1 propionyl-CoA--succinate CoA transferase [Rhodovulum sulfidophilum]AB14
MTVAQPIASRIADPTLRGRVMSAEDAAALIAPGSTIGMSGFTGSGYPKSVPLALANRIEAEHAAGNPFQVKVWTGAST
GP
ELDGALAKAGGIEFRLPYNSDPIAREKINAGEMNYFDMHLSQVAPMAWQGFLGKLDTAIVEISGITSDGQLIPSSSIGNN
KTWLDQADQVILEVNRWQSEALRGMHDVYYGTALPPHRRPIPLASTNDRIGQPYFRCDPKKIAAIVETDAPDRNAPFK
AP
DAVAQAIAGHLLDFFLHEVAKGRLPQNLLPLQSGVGNVANAVLTGLMNSPFEQMTAYTEVIQDGMLELLDAGKLRCA
SAT
ALSLSPSAAEWFNDRAYRFRDKLILRPQEISNHPELVRRLGCIAMNGLIEADIYGAVNSTHIMGSRIQNGIGGSGDFARN
GYVSIFMTPSTAKNGKISAIVPKAAHVDHIAQDVQILVTEQGLADLRGLSPKQRAAQIIENCAHPDYRPALRDYYKRAME
SSFGKHAPHLMDEALSWHARFVRTGSMQED
>OLS49431.1 trypsin [Rhodovulum sulfidophilum]AB14
MSNKAKRNAVSAVALAALLGATAGIGLDHAVPAANAATAPAGGYVDLVSRVAPAVVTIEVEKTAPRAQMQNFQFPD
DFPF
EEFARRFGMPMPGYPQGPHGQAPRLKGVGTGFIISQDGKVVTNAHVVDGADSVTVTLADGRSFGGKVLGADKATDI
ALID
IDGQNLPHVDFGSSDALQVGQSVVAMGNPFGLGQTVTSGIVSALGRDINSGPFDNFIQTDAAINKGNSGGPLFNEEG
DVV
GINTAILSPTGGSVGIGFAVPSDMAKSVVADLEKGGQVDRGFLGVQIGGVSEEVAAALGFDSPRGTMIVDVTADSPAA
KA
GLKKGDIVLAVDGQEVSGPRDLTRFIATDAPGTKVSLNLLRAGKSMDVSVTLGNRADRPA
>OLS49432.1 DNA-binding response regulator [Rhodovulum sulfidophilum]AB14
MKLLVVEDDPTTAQFIARGLGEEGHSVDCLDNGRDALTRALATAYDVMVVDRMLPELDGLSLVRALRAAHVTTPAIFL
TS
MSGVDDRIDGLRAGGDDYLVKPFAFGELSARIEALGRRPPMQETETVLKAADLEMDLVRRSVTRAGQRIELLPREFALL
E
HLLRHKGRIQTRTMLLESVWDINFDPQTNVVETHISRLRAKVDKPFDRELIATVRGAGYRIGD
>OLS49433.1 hypothetical protein BV379_14865 [Rhodovulum sulfidophilum]AB14
MATRAVSLRHLIRSTPVRQALVLVAVVALANLMSLGGAYLTLRADLVRAIRADLAGDLASFEISATPAALSAIVQARARA
TDPAKKVTVFLPPGGRQVGNARAILREGGDIRLVPGTHPLGAAGYLHETRRLAGGLLIVAESLGPIAELRRTFLSLLAFS
LGPTVLISLGVGVLLARRSARRVDGIEDTLDRLSVGDLSARLPPDPAPADDLSRIGAGVNRLAARQEAATEALRQVTADI
AHDLRTPLQRISVLIQDLRARLPDESEPAELADRASAEVDRAVSVFRAMLEIAQIEGGQASARVQPFDLGLTVRQVVELY
QPSVEDAGHCLTLDCPGTGPTVRGEPSLIAQALANLIENALRHTPEGSRIDIAVSADPPGLSVADDGPGVPEDERGKVLR
RLYRLERSRTTPGHGLGLSLVAAIAGLHDASLTLEDNAPGLRVRLVFPPEPGGLAATGSSDPTR
>OLS49434.1 short-chain fatty acid transporter [Rhodovulum sulfidophilum]AB14
MLSVLARPLVRVFERYLPDPYVFVIILTIVVMAAAVLFEGRSPVAVIEMWGDGFWTLLSFSMQMLLVLVTGYMMASTP
AV
RGILNRIADTARAPARAILLVTLVSLLASWINWGFGLVVGALLAKAVARRVAVDYRLLVASAYSGFLVWHGGLAGSIPLA
IATPGHTFEDQIGLIGTSETIFAGFNLIIVAALFIAVPLVNWLMLPKAGHEMIVDPAKLEEPEFGGGPTHRPSDRIENSP
ILSWIIGGAGLAWLVIDLLDGGGLTLNYINFLFLFVAIILHGTPRRLLDALAEGVKSGAGIVIQFPFYSGIMAIMAGSGL
AVSISDGLTSFASAESLPFWSFISAGVVNFFVPSGGGQWVVQAPVMLPAAEALGVDQARVAMAVAWGDAWTNMV
QPFWAL
PILAIAGLNAKDIMGFCVVQLVVSGVIIALGLSFL
>OLS49435.1 hypothetical protein BV379_14880 [Rhodovulum sulfidophilum]AB14
MGIEEMHRTRGRVVERAGDAMAALRACASEWDLAVSETAEGVIMDLPGARLVLQPTGTGLGIEIAAPDDVHLRAAQ
DNLT
EVLAGGGIEIGWEDVAAGALAPGLSIGEVISCGQSAPGFLRLRVGLADAGRFASAGLHFRLLLPPLDRDPIWPQIGKTAR

VDWPEGADALHRAVYTVADLGADWIDMDIFDHPDSPTCTWVRDAAPGAPVGIMGPGGGWVPEGDPVLLFGDETA
LPAILR
ILRLSENEVRAWVTASPEDLSALNADPRVERVDDLLAALAGTDPGEAGFVWVAAGERIAREARRHLTDRGLPRDRFSAI
A
YWR
>OLS49436.1 glutamate--cysteine ligase [Rhodovulum sulfidophilum]AB14
MSIPQSGGGPIESHEQLVEYLASGCKPKPDWRIGTEHEKFGYCRDALKPIPYDGPRSVKAVLEGLRDRHGWAPVMEGE
NL
IGLQKDGANVSLEPGGQLELSGAPLETIHQTCDEVTEHLREVKGVADEIGVGFIGLGAAPIWSHEDMPMMPKGRYRL
MTD
YMDRVGTMGKTMMYRTCTVQVNLDFGSEADMVQKLRVALALQPVATALFANSPFLDGKPNGMKSWRSRVWRDL
DAARTGM
LPFVFEDGFGFERYVDYALDVPMYFVYRDGRYIDALGQSFRDFMAGRLPALPGEVPTLSDWADHLTTIFPEARMKKFIE
M
RGADGGPWRRLCALPALWVGLTYDQGALDAAWDLVKDWDAETRDAWRVAAATDGLDAEVNGVKMLGLARQVV
DIARGGLK
ARARPGLGGMVQDERHFLHALEDSIETGKTPADELLEHYHGDWNGDLSRIYADFSY
>OLS49437.1 hypothetical protein BV379_14890 [Rhodovulum sulfidophilum]AB14
MSFLRPEAVAALSRWRELIASGAVALGGLWLFGLGGYFFQALGLLALGLGLALGFVAIRRARFRIAGDGPGLVRVDEGQ
I
TYLAPQGGGFVALSEIRAIEMVFDETGARGWRLTQTRFPAVTIPASARGAEALFDVFMALPCANPRTILSALDRSPEDG
P
VTVWSRIVRPALT
>OLS49438.1 16S rRNA (uracil(1498)-N(3))-methyltransferase [Rhodovulum sulfidophilum]AB14
MSDVRSARIRLYVEQPLGEGQTVPLTGDQAHYLFAVMRLVPGDPVLVFDGKSGEWLAEVVEARKRAGVLICREATRPL
QL
PPDLWLCFAPIKKTRTDFIVEKAAELGAARICPVQTDFTNSERIRRDRLQAHAVEAAEQCGGTFVPEVTELQKLSALLDA
WPEGRRLMFCDEALVGAGQVLGAAPKGPWAILIGPEGGFSETERARLRALPLAHPVSLGPRVLRADTAAVAALTVWQ
QAL
GDWG
>OLS49439.1 4-hydroxybenzoate polyprenyltransferase [Rhodovulum sulfidophilum]AB14
MSDTTETPEARRQTDGQVADAVTGNWVDRLAPAWTRPYLRLSRADRPVGTWLLYLPCLWGVTLAAASTGFRWFDL
WIVLG
CGIGAFLMRGAGCTWNDISDRNIDGEVARTRSRPIPSGQVTLAQAVFWMVVQALAAFLILITFNMNAILMGVGSLALV
AV
YPFAKRFTWWPQIFLGLAFNWGAMLAWVAHTGSLTLAPVLLYLAGIAWTVFYDTIYAHQDKEDDALIGVKSTARLFAE
DT
PLRLRQFLLGSAALMVLAALLALLRPVGGAAAFMGLAGALGFALHMAWQLSQLDTGDNDRCLSLFRSNRDAGLIPVLC
FG
LAILA
>OLS49440.1 hypothetical protein BV379_14905 [Rhodovulum sulfidophilum]AB14
MRRLLHKLIVPGVFALAALVCIGAALLAVERIESQATEATREALLARGIDWASPRTDGMLMHLSGTAPDEAARFHALAA
A
GSVIDSTHLIDEIEIAAPNPVDGPRFSVELLRNDTGISVIGLIPQSSRTEALADELSRIAGRLPVTDMMDVADDPAPQGW
DGALGFGLTALERLKRSKVSITAERVAVTAISNSTEDKARLEGELRAAAPKGLDLVLEISAPRPIIAPFTLRFLMTEEGA
RFDACAADTEGAAALIVAAAREAGAPGDTTCTLGLGAPSAEWGPAAASGIEALAKLGAGSLTFSDADVTLVAAAGTEP
AL

FDKVSAALKADLPEAFKLHAVLPAPVDTDATAGPAEFIATLSPEGLVQLRGRATDEMVRDATESYARSRFGSARVHSAL
R
LDPALPEGWPVRMLAGLEALAQLRNGSAIVQPDFVQISGLTEDETARARVAQILSDRLGNAQNFDIDITYRAPVAPPGS
V
HPDPAACVAQIDAALDERKITFDPGSATIDGASLRVVDEIADILRGCPRAKMEIAGYTDSQGRDEMNLRLSQARADAVV
D
ALMARRVLVGNLTAKGYGEEAPVATNDTEEGREANRRIEFHLIDDSAPDEAAAEDAEGTEAPEAEAEAGADGATEQAP
PD
ADATEEAATEDDESGEAAPAAAEDETPAEAGAEDRTDGQD
>OLS49441.1 hypothetical protein BV379_14910 [Rhodovulum sulfidophilum]AB14
MDRTEFILVTSVMLFVAFALGWVACWLVRRLGRTTTADMGELDRMAHALHEAEEARDQAMGYLEQREAELTNQLN
QTEAE
LNAAMDGLQHSRQEAEELRAYIERINAR
>OLS49442.1 anion permease [Rhodovulum sulfidophilum]AB14
MIFGIDIYLFLAAVGVALVAGVVKGAVGFALPMILISGLSSFLPAELALAGLVLATLLTNLAQALRQGGAAAWASVMRYR
LLIGCVVLAICVSAPLVPVLSDRVLYLLIGVPITIFTVSQLAGRHLILPARHRGRAEIAMGLTGGFFGGISGVWGPPVIA
YLLSFNTEKTEMIRVQGVVFLIGSVMLLFAHLGSGVLNAQSLPFSAALVLPAAIGMWAGLRLQDRLDPVRFRRATLVVL
A
VAGLNLIRKGLMG
>OLS49443.1 acyl-CoA dehydrogenase [Rhodovulum sulfidophilum]AB14
MPHDGQDRPAETALLADLLTLTEAALGPVEAVTEAARTALAARVTEEGRVSGRLVEAEQHAAHGLAWLATYARALHQ
MRG
WADRLTEQGKFGEIEQLILQIAFGEYLAQIAGGIPMSQGEIVRLPELGVSSEAHHAFYSDAVSALIRGGNTPAARARLVA
LMREQVANVTLGATGLDDELEMIREQFRRFALDRVEPNAHDWHLNDTLIPIEIIEEMAELGVFGLTIPEEFGGFGLSKAS
MCVVSEELSRGYIGVGSLGTRSEIAAELILCGGTDEQKAHWLPKIASGEILPTAVFTEPNTGSDLGALRTRAVKAGETYE
VTGNKTWITHAARAHVMTLLARTDPETTDYKGLSMFLAEKTPGTEDDPFPSAGMTGGEIEVLGYRGMKEYELGFDRFE
VP
AGNLLGGVEGQGFKQLMQTFESARIQTAARAIGVAQSALDVSMRYAEDRKQFGKPLIAFPRVADKLAMMAVEIMVA
RQLT
YFAAWEKDHDRRCDLEAGMAKLLGARVAWAAADNGLQIHGGNGFALEYTISRILCDARILNIFEGAAEIQAQVIARRVL
G
>OLS49444.1 diphosphomevalonate decarboxylase [Rhodovulum sulfidophilum]AB14
MTDRARAIFDAVLPARLPEPVPFSADAPSNIALSKYWGKRDAGLNLPLNSSLSISLAEWGTTTRVGPAEDGTDAVWLN
GE
RLAPETGFARKVVAFADRFRRAQTLPLHVRTENTIPTAAGLASSASGFAALTRALAGSFGLDLAEDRLSMLARFGSGSAT
RSLWHGFVRWDRGAREDGTDSFARPLPETWSDFRIAIIRVDTGPKSQSSRDGMGHTVATSPLFGQWPEQAEADCAAI
EAA
IEARDFLRLGALTEANALAMHATMIAARPALCYLKPESWQVLDRLWAARRDGLEAYATMDAGANVKLIFLEDSAPAIA
EL
FPDAAIVEPFGPAAQR
>OLS49445.1 hypothetical protein BV379_14930 [Rhodovulum sulfidophilum]AB14
MRVSAPGSIMITGEHAVVYGHPAIVAAIEQRIAIEVSPLPERRLEIVSDIAEPASVELDRMEVGGAYRFVLAAAARFAGR
LPCGARLEIRSEIDPTLGLGSSAAVTVAALGAFARLSGAPTGDLHADALAVVRQIQGRGSGADLAASLGGGMSAYCLGP
G
MLSGTPPGDARARIEPLPMPPALGLRYAGYKTPTAEVLARIARAMEGREAEFAALYDRMGECAVAAIAAARAQDWTG
FGA
ALDRYQGQMAELGVSDEMLDRIIADARAMPGLLAAKISGSGLGDCVLALGARPEGFTPVAVAEEGLVIDD
>OLS49446.1 hypothetical protein BV379_14935 [Rhodovulum sulfidophilum]AB14

MSAEPVPATLPEDASRAHAPGKIILSGEHAVVYGAPALAVAVAIYTEVWFRPIHPSDGLKTVFEQVSQGAFYPLSRLGHF
KQSLDRRFDSFLRGELKVQQILQRPDDLIVYTLMSLAQRLPMPGKSPGHCLPVPGQLGGTSALPLGAGMGSSASIIAAT
M
VLYETLLDRLHSTEERFEWVRFCERLQHGKGGAIDAAAVTHGGAVRVQGDSRHAPAIAPGHGIWRGDGWYWVLHGK
PDSS
TGECVAAVREAHGDDQALWQGFEACTDAFQAALETDSSPLESLRENHRLLSRIGVVPEAAGRFVEEVELAGGAAKICG
AG
SIRGDHGGVILVHQPDAPAMARLMEGHPDQRWAPLHVAPAGAAFGVPQ
>OLS49447.1 TRAP transporter small permease protein [Rhodovulum sulfidophilum]AB14
MTTLARYITALNRGLFGLAKWLVYAIVALMLWEVVQRYFLSHPTSWAPELATLVFGPFFLLGGPYMLHTGGHVAVDILS
S
RASGGMARALHLAGLALAAAFGLILLWFSLPLVLQSISYGETSYSAWNPVVWPAKAFLPLAAGLLVLQALADAILAFRAE
DAA
>OLS49448.1 C4-dicarboxylate ABC transporter substrate-binding protein [Rhodovulum
sulfidophilum]AB14
MTNTTRRRMVQLGAAALAAPAFLRTGAARADGVTRWRMQALWGGGTTPMRYEEKFCARVAELTGGAFEITPFSGG
QIVPS
AQAFDAVRGGAFEMMKTFDGYTAGKIPAHGFSSTVPFGFPEADQYEAWFYERDGLELARESYAPAGLTYIAPTVYGEE
PI
HSRVEIRRIADMNGLKGRFVGLASAVMARFGVSVSPLPTSEVYSALDKGLIDIADRGDIKANYEEGLHEVAPFLILPGVH
QPTTATSYVANTRAYEALDDQKKAALAVAAREISGTLRQNIIVSNGEYLAKFEEAGVEILTLDPEDVVEARGRAIEYWSE
AATDDLSRRMMESQIALMKELRLL
>OLS49449.1 hydroxymethylglutaryl-CoA reductase, degradative [Rhodovulum sulfidophilum]AB14
MTDSASEPEASVLSRIEGLRDKSPAERHAAVAAAAGLAPEALEALSDRPALSVSRADGMIENVIGTFELPLGVATNFTVN
GRDYLVPMAVEEPSVVAAASYMAKLARAGGGFTASSTAPIMRAQIQILGLGDPFGARALLLAHRDELIALANSRDSML
VE
LGGGCKDIEIEVFATSPIGPMTVMHLLVDVRDSMGANAVNSMAERLAPRVEEISGGQVRLRILSNLADRRVVTASVRV
PA
EALSTKAHDGADVARRIVEACALALIDPYRAATHNKGIMNGIDPVVLATGNDWRAIEAGAHAYAARSGRYTSLSTWEI
GA
GGALVGTLELPMALGIVGGATRTHPAAQAALKLMGIGSAQELAEVTAAVGLAQNMAALRALATEGIQRGHMALHAR
NIAI
TAGATGGEVDRVAKAMVAAGDVSVAHARLVLKGA
>OLS49450.1 hypothetical protein BV379_14960 [Rhodovulum sulfidophilum]AB14
MSTLSNLSTAQIAALTTATIASLTSSDIASISSAQMGAMSTAQIGALTPSAIRGIGSVQASGLSTAQMANFSTDQIRQLS
SRAINGLTTDSIVALTTAQAAELSSRQISALSSTQVAAMQTADLVTLSTVAMRGLGSSQVAGLSTAQVAALTTAQAGV
MS
SVTLSGLSSTQMAALTTAQVGALSSLAIRGLTSTQTAGLTTAQLAGLSTGQIRALGSSAMAGLGTANIVALSTAQAAEIS
STQVAALSSTQIAAMQTADLVALSTMAMRGLGSTQVAGLSTAQVAALTTAQAAVMSSRTLSGLSSTQMAALTTAQV
DALG
SLAIRGLTSTQTEGLTTAQLARLSTDQIRALGSSAMAGLGTANIVALSTAQTAELSSTQIAALSSTQVAAMQTADLVTVS
TASMRGFGGDQIEGLSTAQVAAFTTAQAAVMSSTLLGGLSSAQLGALTTAQVDALGSIAIRGLTSTQTAGLTTAQLARL
S
TDQIRALGSSAMAGLGTANIVALSTAQAAELSSTQVAALSSTQVAAMETGDLVRLSTLAVKGLGSEQVGGLTTAQVAA
LT
TAQAAVLSDTALGGLSSTQMAAMTTAQIGALSSRAIRGLGATQTAGLTTAQLAKLSTDQIKGLGVSAIEGLGTANIVAIS
TAQAAELSSLQIRALSSTQMAAMETADLVRLSTAAIRGFSGDQIDGLTTAQVAAITTAQTAVLSSAMLGELSSTQMAAL
T

TAQIDALSTLAMKGLGATQTEGLTTTQLARLSTDQIRVLGSSAISGLGTANIVAISTAQAAELSSTQVGALSSTQVAAME
TADLVRLGTSAMRGLGVDQVAGLTTAQVAALTTTQTAVMSNITLSGLSSAQMAALTTAQVGSLSTRSLRGLSSTQTEG
LT
TAQMVALSTDQIRSLGGSAMSGLGIASIVALSTAQAGELSSIQIAGLGSTQIAAMETADLVRLDTSAIRGFGADQVAGLT
TAQMAAITTAQSAGLSSTVLGNLSSTQLAAMTTAQIGALSTRAINGLGATQTAGLTTAQMAALSTDQIRRLGVSAVEGL
G
TASIVALSTAQAAELSSVQIRGVSSVQMAAMETADLVRLDTSAIRGLTSEGISGLTSAQAAALTTAQVTALSTLQIGNIS
TSSIGGFGTASIQAFTTRQMGGFNSQQIAALSTTQVAALQTDDIAALSDAQTEAFTSTQLAAMSTAQLNALFL
>OLS49451.1 SAM-dependent methyltransferase [Rhodovulum sulfidophilum]AB14
MDSLTLATDGATSSLKTVLHVGCGMADPAKLPADAFPAENWRELRLDIDASVMPDIVASITDMRVLSEGAVDAVWSS
HNL
EHLRAHEVPLALSEFFRVLRPGGFALMTMPDLQRVAALVAQGLLEEPAYISSAGPIAPIDILYGYRPSIEGGNDFMAHRT
GFTVGSLSAHLIRTGFVDVQVESDGRFALWARGVRPA
>OLS49452.1 secretion protein HlyD [Rhodovulum sulfidophilum]AB14
MSPVARDALPFQPPLVQVRDAPLPWLLRLWPFGSAALLVALVTLAAFVPIDVVVTAEGRIVTDDPPVVLKPLGSAVLDR
L
LVHPGDAVEPGQLLARLDARDPATDVAVLAAERAALEAEIARIEAELGDRPLTGEGEEFALQARIQRERAAAEAAQRDQ
L
VSALAQIDSGIEAALEGVPALAEAVATAREIETMHETLLARQSGTRLAVNQARLLRLDAETDSRENMARLLSLRQERETA
AARLAAFESDLRQSRLEDLAGLRPRLAVIERQLSQATDRARLYDLRAPQAGVVLSVAEGGPGSLMTIADPVVVIAPTGG
A
MHAEIGLLSRDVGLVHPGDPVRIKVDAFPWRRHGLIAGRLSDVAPSSSLPSGGGPARHAARLAFDGDAALPEGLMPG
MTL
TSDIATGHRTLLTYFLDPVLNGLSESLREPRS
>OLS49453.1 sodium:alanine symporter family protein [Rhodovulum sulfidophilum]AB14
MTDFLNAVSSFVWGAPVLVLLLGTGLYLCIGLRFLPQRKLGYGIRMMLEGRKKGEGHEGEITPFQALTTALSATIGTGNI
AGVATAIFLGGPGAVFWMWLTALVGMATKYAEAVLAVHYREVDAKGRYVGGPMYYIKNGLGPRFAWLAWLFAFFA
TIAAF
GIGNTVQANSVADAVETTFGVPHLWTGIALAALTFVVIVGGVKRIGAVAEWLVPLMAVLYIIGALTILALNFQQVPGAIA
LIFDSAFNGAAATGGFAGAAVMAAIRFGVARGIFSNEAGLGSAPIAHAAALTDDPVRQGNVAMLGTFIDTILVCSMTA
LV
IITTGVWSSGETGAALSALAFNTGLPGGDLIVSFGLIVFAFTTILGWSYYGERAAEYLFGPKVILPFRLLWVAGILIGAV
ANLGVIWIVADIMNALMAIPNLIALIALSGTVFAVSRAYWRKQAE
>OLS49454.1 U32 family peptidase [Rhodovulum sulfidophilum]AB14
MEVSPMAAELTLGPNLFHWPAEMKLDFYNRIADESPVSTVYLGEVICSKRTPFFDDHHMDKVAERLERAGKKVVFSSF
SE
VVIKRERNLLRQWAEQEGRDLEVNNTAALLYLKNKPHRIGALLNVYNEDTMAYLHEQGANHFALLPELPRKAVAELGK
KA
GELGVGVEVQVFGRASLALSARCYHARAHARTKDNCQFVCGEDCDGMILNTMDEKPFLVVNGIQTLSYGYLDLLPDIA
DM
KALGVTHFRVMPHSCDMIAVTRLFDDVLAERIGVEEGEAKLRELGIEAPFVNGFYHGKAGHDWTARAAAELA
>OLS49455.1 protease [Rhodovulum sulfidophilum]AB14
MIKPELICPAGTPAALRSAVDAGADAVYCGYQNATNARNFVGLNFTPEEMEKSIKYAHDGGTKVLITLNTFPPAGNVQL
W
KDAADLSAKLGADACIVADIAVAAYIQKTHPQMRIHLSVQAAASSPEAIRYYVQEFGVKRIVLPRIMTVSEIKGLVQEIP
CEVETFIFGNHGLMVEGRCSLTNYVTGQSTNMDGACSPASCVKYERAEDGSMSTILGDYMIDRFKPDETAGYPTICKG
RY

KCDARDDGYYAFEEPVSLNASTMLPDLINAGVHAFKIEGRQRSRAYVKKVVAAFREAVDSIMAGQEPKLADLVALTEG
QK
HTEGALKTKKWR
>OLS49456.1 hypothetical protein BV379_15005 [Rhodovulum sulfidophilum]AB14
MRLSICLAALAATAALAACVPVDTRPHPILTDRGNRVQPTGNPGEFEVLTRPGDAGAQLWCAAGEYAESQTDARPSD
RIY
LVHPRGPSASHPGRKSAIFTVRPDAELLAQARALPDQIVLGVSRVGSNWARIHAQQVCRPLFNLPD
>OLS49457.1 mismatch-specific DNA-glycosylase [Rhodovulum sulfidophilum]AB14
MGSEDILPDYVAPDLRSVFCGMAAGPRSAELGLYYAGHGNRFWPVLHRAGLVPEPLAVGDEARLLAQGIGLTDLAKG
AWG
VDAAVPAAAHDPDRLARFVETWRPRSLAFNGKRAAQIFLGRKRVDYGAQAFRGLPVWVLPSTSGAAARYWDETVWR
AFGA
ALRRGG
>OLS49458.1 RNA polymerase sigma factor RpoD [Rhodovulum sulfidophilum]AB14
MAAKDTDDSKTDEQDADLALDMSQAAVKKMIAEAKERGYITYDQLNQVLPPEQVSSEQIEDVMSMLSEMGINIIEAE
ESA
EEGEEKSTEVVTTTSTSREVAVSSTTSETLDRTDDPVRMYLREMGSVELLSREGEIAIAKRIEAGRNTMIAGLCESPLTF
QAITIWRDELLNEDILLRDVIDLDATFGRALEEDEGDEPVVDGIEVETSNTTARKEDDSPELDADGNPIRRDDEDDEEEQ
ANMSLAAMEAALKPRVLETLDQIAGDYSKLSEIQDKRIQATLSKTSRFSDRDEAEYQKLRTEIVLLVNELHLHNNRIEAL
IDQLYGINRKIMSIDSALVKLADQARINRREFIEEYRGHELDPNWMEQMARKGGRGWSAFTERSGAKVAELRADMAQ
VGQ
YVGVDIQEFRRIVNQVQKGEKEARQAKKEMVEANLRLVISIAKKYTNRGLQFLDLIQEGNIGLMKAVDKFEYRRGYKFST
YATWWIRQAITRSIADQARTIRIPVHMIETINKLVRTGRQMLHEIGREPTPEELAEKLQMPLEKVRKVMKIAKEPISLET
PIGDEEDSQLGDFIEDKNAVLPLDSAIQENLKETTTRVLASLTPREERVLRMRFGIGMNTDHTLEEVGQQFSVTRERIRQ
IEAKALRKLKHPSRSRKLRSFLDQ
>OLS49459.1 hypothetical protein BV379_15025 [Rhodovulum sulfidophilum]AB14
MDAGMPDELATLKVSQIRRVIGLAVLVMLAGLLIQIGVMRPPEGIGYRVLLLALGAGALTLAELMRRATARRLILTPAGL
FDDAGREIARIDRISGLDRGMLAFKPSNGFSLSLTEPLPRAWAPGVWWRLGRRVGVGGVTSAGEAKAMAEILTALLQT
RG
ERS
>OLS49460.1 hypothetical protein BV379_15030 [Rhodovulum sulfidophilum]AB14
MNWPLLSSSNSKTELKTDDPTPLSSQLRWPLLSGRKSQVELTTGSTPISDMMKWPLLSSKSTMFDTELPSAKGGNGKS
TG
DSPVSKFIRGIVTGEPQK
>OLS49461.1 TetR family transcriptional regulator [Rhodovulum sulfidophilum]AB14
MARTSGSHSEITGPRLRAAAQALFARHGYAAVSMRRIAAEVGVQAGALYNYIPDKQGLLFELMKSHLDELLAAWEALP
KG
RGPRDALEAFTRFHIRFHLDRPDAVFIAYMELRNLEPGNFRTIEALRRRYETVLEDILREGGARGVFDVADSKHAAFAVI
AMLTGVTTWYRDEGRLSRAEVEDLYWNMVKKAVSA
>OLS49462.1 hypothetical protein BV379_15040 [Rhodovulum sulfidophilum]AB14
MARLPLCLAAAVLPMLAACAEFPEIDAAERAAAGPTLVPEIAPIDPLLARAHSLRDSSAAPDSLESRAARLRARAARLRA
RDI
>OLS49463.1 homoserine dehydrogenase [Rhodovulum sulfidophilum]AB14
MTTPLRLGIAGLGTVGAGVVKIVQSHAGLLAQRGGRPLVISAVSARSRGKDRGVDLSDYAWEDDPVALARRDDVDVL
VEL
MGGSDGPAKAATEAALATGKDVVTANKAMLAIHGQALAETAEAAGRVIRFEAAVAGGIPVVKALTEGLAGNRIDRIM
GVM
NGTCNYILTRMEREGLPYETVFEEAQALGYLEADPTLDVGGIDAGHKLALLTAIGFGARVDFDAVELEGIERISIVDIRY

AADMGFRIKLLGVAQTTGRGLEQRMSPCLVPANSPLGQLEGATNMVVLEGDAVGQIVLRGAGAGEGPTASAVMSDI
LDIA
RGFRVPTFGQPAAGLETARPARATIAAPYYLRIELQDKPGAMAKVAAVLGEAGVSIDRMRQTRHSETTAPVLFVTHKTT
S
DALDQALKGFAATGVVVGEPVSIRIEDV
>OLS49464.1 hypothetical protein BV379_15050 [Rhodovulum sulfidophilum]AB14
MPQVISLASARSRRASVSESAAPRRPSDLRLGELLLASGALSPETLSRALAERARMEIRLADLLIAEGLVPPDEIDATLC
RLHDTGRADFDSYSPDPRLIDLLGPARCLRDGLLPWRRVGGATLIASSRPERFEELRADLPAGLQPAVMVICRETELQEA
LATCRAPALARAAETRVNDWDSCRFWYHAGPRAAVVLGLSGLGLATLAAPLAVLMLLTLWTMLWMTLGSGLKLAAA
VAQI
RALHRARRKPPDPPPTIARLPAVSILVPLHRETELAPRLIARLARLDYPRALLDICLVTEADDLDTRAMLEAVRLPPWMR
ALTVPAGQIKTKPRALNYALEFCRGSIIGIYDAEDAPAPDQLRRMVAHFHSCAPEVACLQGVLDFYNARANWLARCFAA
E
YAAWFRVVLPGLARIGLPVPLGGTTLFFRRPALEALGGWDAHNVTEDADLGIRLARHGYRTEFLDSVTEEEANCSAWP
WV
RQRSRWLKGYAMTWTTHSRRPGLLLRQLGPWATLGMQVLFLGSLTQFVFAPLLWSFWLLPLGLPHPLAASLGPAGL
WLVF
KLFLACEAINLAVGAVGLWRAGKPGLIALLPSLHVYFPLASLGSYKALWEMATRPFYWDKTRHGLGERDAAPRTTENRT
A
GKPASGSPADKGAAEARNAGPDAQPSASIASAS
>OLS49465.1 GntR family transcriptional regulator [Rhodovulum sulfidophilum]AB14
MKDIRPAQKDAYELILEAIDVGVYRPGDRLVESELAERFGVSRTPIREALQRLETQSLLSRDGRSLIVSSLTHDQMAELY
VVRAELEGLAARLASRHATAEEIRVLRSMVEDDFKLLNDPAALARANRRFHKQIHLASHNRYLVQQLDLVHRTMALMA
TT
SLAVDGRGRDALSEHDALVCAIEAGDGDAAYDALKRHISIAFETRLRHDADAMLAEG
>OLS49466.1 pyrimidine 5'-nucleotidase [Rhodovulum sulfidophilum]AB14
MMVKDLSNLGGWVFDLDNTLYPPEARLFELIEQRMTGYVMSTLGVGEDEANRLRKSYWARFGTTLTGLMELHGVEP
GPYM
THVHDIALDRLSPDPELRARIAALPGRKIVHTNGSAPYAERVLAARGLDGIFDAVFGVEDAGYVPKPRRESFETIAALGG
IIPGEAAIFEDDARNLAAPHEMGMRTVHVAPAPEAAPHIHHHTADLTAFLGRL
>OLS49467.1 hypothetical protein BV379_15065 [Rhodovulum sulfidophilum]AB14
MSSETSEAPVAETPRWLRIVYTLPVIGWVLKDVNEGDADNIWYLLGGFGCLWIVAILQWGVLGLYLPAVAATWVCLG
MLI
WISRG
>OLS49468.1 2-octaprenyl-6-methoxyphenyl hydroxylase [Rhodovulum sulfidophilum]AB14
MTRISTDILISGGGVAGLTAAALFGSEGYSVLCVDPAAPVTEETAEGADLRTTAFLQPSLQVLKTAGLWDRLAPFATDLQ
VMRIVDAGGKIPSPRVTRNFNARDVSDLPFGWNLPNWLLRREMVRRLEDLPNVDFRPGTGTASVLTRDSAALVDLSD
GTR
VSARLLIGADGRNSPVREALGIGVRTMRYGQKALAFAVTHPEPHGNVSTEIHRSGGPFTLVPLPDRDGRPCSAIVWME
RG
PEALRLAALPEAEFNAEMTARSGAIYGPLRLETRRTVWPIISQVAHRMSGERTALIAEAAHVMPPIGAQGLNMSLADLR
V
LLDLCRAAPNRIGEEAMLDLYHRRRHPEVLARVTGIDLLNRASMQSAQPLRDLRAAGVASLYALKPIRKTLMRLGMGT
GA
>OLS49469.1 AsnC family transcriptional regulator [Rhodovulum sulfidophilum]AB14
MIELDETDRRILRALAEDATQSAGALGRRFGLSQPACWRRIRRLEEAGAIAGRRVDLDAEALGFSVSVFLGVKLATKGRV
GLEQFERAVTAIPEVQTVQHVLGLFDYRLKVVARDLPDFERVLRRRIMTLPGVGNVEANVILSEERLPGPLG
>OLS49470.1 AsnC family transcriptional regulator [Rhodovulum sulfidophilum]AB14

MTIPLDDADRRLLRYLQAEPSLSTADLAERAGLTAATCWRRMEKLRAKGVLRGIRAVIDWRALGYSVEVSLRFSLDKTD
P
RAFDEFIAAARLVPEVREIQSFLGRVDVRLNILARDMAHYQELYRTRILTLPHIADIEALMHVADIKRAEALPL
>OLS49471.1 ketol-acid reductoisomerase [Rhodovulum sulfidophilum]AB14
MRVYYDRDCDINLIKDKKVAILGYGSQGHAHALNLRDSGAKNIVVALREGSASAKKAEAEGLQVMGIAEAAAWCDLIM
FT
MPDELQAETYKKYVHDNLKEGAAIAFAHGLNVHFGLIEPKPGVDVIMMAPKGPGHTVRGEYVKGGGVPCLVAVAND
ATGK
ALELGLSYCSAIGGGRSGIIETSFKEECETDLFGEQAVLCGGLVELIRMGFETLVEAGYAPEMAYFECLHEVKLIVDLIY
EGGIANMNYSISNTAEYGEYVSGPRVLPYDETKARMKAVLNDIQSGVFVRNWMQECAVGQPSFKATRRNNDAHRIE
QVGE
KLRDMMPWITAGKMVDKSKN
>OLS49472.1 hypothetical protein BV379_15090 [Rhodovulum sulfidophilum]AB14
MRWSWSRHAVFAFHVLLANAAMFVSLSEAPRLLREMVVLASPVCLLLPAYLHARYRNKSQRLRLSGAKP
>OLS49473.1 phosphoglucosamine mutase [Rhodovulum sulfidophilum]AB14
MSRQIFGTDGVRGRANSHPMTPDMALRLGQAAGRYFRTDGLNRHRVVIGKDTRRSGYMLENALTAGLTSTGMNVLL
LGPV
PTPAVGFLTQSMRADLGIMISASHNPHEDNGIKFFGPSGFKLSDEAEAEIEAILEAEIALSQPANIGSAKRIDDGLWRYG
EFLKTTFPRGLRLDGLKVVVDCANGAAYRVAPSVLWELGAEVVPVGTEPNGFNINRNCGSTHTDIAAAAVVAHGADLG
IC
LDGDADRVMIIDETGAVADGDQIMALFAERWAAQGRLRHGTLVATVMSNLGLERHLAAQGLKLERTAVGDRYVVEA
MRKG
GWNLGGEQSGHIVMTDYCTTGDGLLAGLQFLAAMVETGRPASDLARRFERVPQLLKNVRYAAGADPLGTASVQEAIA
AAE
KRLNGHGRLLIRKSGTEPLIRVMAECEDDAQLIEVVGRVVEAVEQVA
>OLS49474.1 dihydropteroate synthase [Rhodovulum sulfidophilum]AB14
MTSYYRPIVQTDPARPSEALTLAGGWGWFAMAEMLERDEPPRLVPARAIPEDWRIALTAPRPAIAGLTMDRPRLMGI
LNV
TPDSFSDGGQHDRPETALAHGQALAVDADILDIGGESTRPGSADVSVPEEIARTAPVIAALRAAGVSRPISIDTRKAAVA
EAALAAGADMVNDVAALGFDPDLAGATARAGAAVCLMHAQGDPATMQDDPHYDHVLLDVYDFLVERVRAALAAGI
PASRI
VVDPGIGFGKTLAHNLALLRGISLFHGLGCPVLLGASRKRFIGAIGGAETAAARGPGSVAVALAAIAQGVQFVRVHDAA
E
TRQAMRLDAAVKGLELGHE
>OLS49475.1 cell wall hydrolase [Rhodovulum sulfidophilum]AB14
MGRVSIRVAGIAAATALLLVAPAGQSEMTGSQSNDPTAVLGDNLARLLGAERAGLQRLGQRRINRLSTSPAVRDARRY
DP
KWLDAQPVAKGGEAWTCLSEALYFEARGETVKGQFAVAEVILNRVASPLYPDSVCGVVHQGTGKLNRCQFSYTCDGR
AEH
ISDQRTYRRMGKIARMMLDGAPRALTGGATHYHNRSVDPGWARRLPRTASIGQHLFYRQPAAVAAN
>OLS49476.1 pyruvate, phosphate dikinase [Rhodovulum sulfidophilum]AB14
MRKLTDIVDFTEITPDAAIAADRHGGRAKCLQRLVRIDLPVPRTVALSFEAVHRIALGQMPDAEALLAVFGAAPLVSVRP
SSADPDWGGPASILNIGMNDARHATLCKTHGAAAANALYLRFIQAYAIHVARLDPDLFESEDYSAEAVAAALAAYAEET
E
EPFPQDPVRQLLEVLRSMARAWEGTTARLLRQAKGAPAEAGLGLVVQQMALGLGPEESGSGVIQFVDGVTGQRRITG
RYL
GQSQGRDAVSRQKQALFLTRDPRGPALEDRCPELFARLKRYGDTCRINLCEEMQIEFTIEAGRLWVLDAVRVQRSARSA
V

RIAVMLADEGIIAREEALARIEPRAVSELLHSQVDPTGRRQVIAQGIAASPGAAVGRLVFGSAAAQASAARGEPCILVRR
ETTPEDIRGMHVAAGVMTERGGMTSHAAVIARGLGVPCVVGVSDMRLNRRDGVLITADGRKLHEGDIVTLDGTNGQ
ALLG
EAKMLPPALDDAFHALMGWAEEACDIGIRANADTPADAETARRFGADGIGLCRTEHMFFEHDRLTVMHEMIFAAGP
EDRG
TVLERLLPMQRGDFIRLFEIMQGLPVCIRLFDPPLHEFLPQSREGIRELAEALGLPVSDVTRRIQALSEFNPMLGMRGVR
LGIAVPEIYDMQARAIFEATIEASRKGWPIVPEIMIPLVSAMREVELVKTRIDSLAAAVRTERGADFAYRLGVMVETPRA
ALRAGDIAQHAAFLSFGTNDLTQMTYGLSRDDAGRFMGSYVKQGVFAEDPFHVLDTEGVGELLSIGADRGRLARPEIV
LS
ICGEHGGNADSIAFCREAGFDYVSCSPFRVPVARLAAAQLALGAALRPKHGRY
>OLS49477.1 glycine--tRNA ligase subunit beta [Rhodovulum sulfidophilum]AB14
MPDLLIELFSEEIPARMQAGAREALRQRVTDGLVEAGLTYGHARAYSTPRRLVLAVEGLSPRSPDLKEERKGPRVDAPEK
ALEGFLRSTGLTKDRLEARDDKKGRVWVAVIDKPGREAAAIVAEVLEATIRNFPWPKSMRWGAGSLRWVRPLHSILCLL
T
TEAGAEVVPLDIDGIRAGDTTRGHRFMAPEPFRVTGFEDYAVRLKRARVMLDQDERADQIWHDATNAAFAQGLEVV
EDKS
LLTEVAGLVEWPVVLMGAIGEAFLDLPPEVLQTSMKEHQKFFSVRDPKTGRIVRFVTVANRETADNGETILKGNGKVLS
A
RLSDAAFFWGNDLAVAKAGMEDWREALTHVTFQSALGSQADRIARIAALAREIAPMVGADPDLAEQAAKVAKLDLAS
QMV
YEFPELQGLMGRYYAAAAGLPAEVSEACALHYKPLGPSDEVPTAPVSVAVALADKIDTLTGFWAIDEKPTGSKDPFALRR
AALGVIRLVLTNGLKVSLSELIQEARETSIQKWSTRKTAELTTQLLNDYSAATQTIAELRALRRGEITHINDIWAGIEEA
EEETSVTSQNADMIAGAYIARAMQVELHAVWIIRDDLLAFFHDRLKVHLRDQGIRHDIIDACLAMPGNDDLTLLVRRAE
A
LQAVIGTEDGENLVQGFKRANNILSQAEEKDGVEYSFGADRKFADDPAEAALFDALDAAEAAIGPAMEAEDFAKAMA
AMA
ALRAPIDAFFEAVQVNSDNEIIRRNRLNLLHRIRATCGSVADLTRIDG
>OLS49478.1 glycine--tRNA ligase subunit alpha [Rhodovulum sulfidophilum]AB14
MSDGKPRSFQEIILRLQTYWASKGCAILQPYDMEVGAGTFHPATTLRSLGTRPWAAAYVQPSRRPTDGRYGENPNRM
QHY
YQYQVLIKPSPPDLQELYLGSLAAIGIDFDYHDIRFVEDDWESPTLGAWGLGWEVWCDGMEVSQFTYFQQVGGHDCH
PVS
GELTYGLERLAMYVLGIDHVMDMPFNDPDAPIALKYGDVFRQTEEEYSRWNFDVADTDKLLHFFEAAEAECARILDQP
AE
DPRSGKRIIMVHPAYDQCIKASHFFNLLDARGVISVTERQAYIGRVRALAKRCADAFVETKAGGWTGDAPEAAN
>OLS49479.1 hypothetical protein BV379_15130 [Rhodovulum sulfidophilum]AB14
MMRFLATIAFVLLAAMPAAAQDRAWVQVEAHPTLRETEERARAYAATLKDVGAYRLNSGWYSIAVGPYSPPVAEAEL
ARY
KAAGLVPADTFIADGTSYERRVWPAGARMQDPASETVTPPTTQPDPATAAPAATAPVALPDETPLEARRSESRLDRQA
RM
EIQTALKWEGVYSSTIDAAFGPGTRRAMADWQSAQGYEPTGVLTSRQRAELVDGYKAQIARLGMTPVDEPEAGIRIE
MPM
GLVRFDRYEPPFVHYASRGDSGVTALLISERGDQSTLFGLYDIMQTLEIVPLEGPRERSARSFTLIGKNDRLQSHTYAQL
TGGMVKGFSLIWPPSADPKLMQRAVAMMKDSLDSVPNVSLDETLGEATAEQRADMLSGLEVRKPDATHSGFFVDPE
GAVL
TTTNGLDSCRRITIGPELEVTLAATDAAQGLALLRPGRTLAPIGFAEFAPGVPRLRSDIAVAGFSYGEVLDLPVLTYGVL
ADLKGLSGEENVERLELDALPGDTGGPVLDASGAVLGMLLAASGGARQLPEQVRYAADASAVGAFLTANGIEPRTSSA
TG

ALPPAELSRRAADMTVPVSCWN
>OLS49480.1 potassium transporter TrkH [Rhodovulum sulfidophilum]AB14
MLDLRPVGYVIGLLVAVLGATMLFPMALDLWDGNPNWSAFLESALLTGLTGGLVALSCASGSDGGLSIRQTFLLTSAV
WL
ALPLFAGIPFVLGLPEARPVDAFFEAMSGLTTTGATVFTGLDTLPRGTLLWRSMLQWFGGIGIIVVAMVFLPELKVGGM
Q
IFRSEAFETSGKVLPRAVEIAAQISGIYLFLTVACMLCFVVVGMPLFDALNHALTTMSTGGFSTHDASFSLYQGVPEYVA
SVFMVLASLPFVRYVQLLAGQARPILRDSQIWTYLGVIAAFVLIIAAFRVIANGDHYEHAFREGVFNVTSIISGTGFSST
DYQLWGALPVILFFFIGLIGGCAGSTACSIKIFRYQLLFSAIRVQIKSIHAPHGMFQPRYDGRRVDEDVLSSVMSFFVYF
VVALGVVSVLLGATGLDATTALSGAATTLGNIGPGLGDKIGPSGNFAGLNDIAKWILSMAMFVGRLELLVVFAIFTVQF
W
RA
>OLS49481.1 GTP cyclohydrolase [Rhodovulum sulfidophilum]AB14
MNVFNRDIDREPTRAEAERALALLRDWAGAASDGEIETLDPALSALCPGRAVSDYPTLSRTYPDGFEADAAYKASLPDL
Q
NGPASLIRGARRQIQHVGISNFRLPIRFHTRENGDLTLETSVTGTVSLEAEKKGINMSRIMRSFYAHAERTFSFQVIEAA
LASYKADLDSFDARIMMCFSFPMKMRSLRSGLDGFQYYDFALELVETAGLPLKIMHLDYVYSSTCPCSLELSEHARRERG
QLATPHSQRSVARISVALEPGGDCLWFEDLIDLCRKAVPTETQVMVKREDEQAFAELNAANPIFVEDAARLFAEALLTD
P
RIGDFRVIASHQESLHSHDAVSVLTEGATFAADSLDPKLFSTLVHGR
>OLS49482.1 O-succinylhomoserine sulfhydrylase [Rhodovulum sulfidophilum]AB14
MERDWTKRTRAVHEGARRSQYAELSEALFLTQGFDYPSAEAAEARFEKAGRDEFIYARYGNPTVAIFEDRLAMIEGAED
A
FACASGMAAVNGALMALLKAGDRVVAARALFGSCIYILEEVLGRFGVEVVLVDGTDLEAWRRAVTPGTAAVFFEMVS
NPV
LEVIDLPAVAGIAHAAGAKVIVDNVFATPVFSRAFEQGADLVVYSATKHIDGQGRCLGGAILGPREVIRGPVQTYMKHS
G
GAISPFNAWIMLKGLETLDLRVRAQAAGARLIAEALENDPRFARVIYPGLASHPGHALFPEGGTLLALDLAGGKEAAFRL
LNGLDIWRLSNNLGDAKSIVTHPATTTHQSLSDETKAALGIGPGLLRLSVGLEDPADLLADLRAALDRI
>OLS49483.1 hypothetical protein BV379_15150 [Rhodovulum sulfidophilum]AB14
MPLIQVNAAGERPEPQGGAARLGPLLDRALEGLAPRAPVAVLIHGLKFSLSDPAQSPHRHILALEPEDRCWKAVSWPR
AL
GFTGQGRAEGLCIAFGWEAGRNVWRAYDEAPRAGRALARLIEAIRVRAPGRRVDLLAHSLGARVALSAMGALDEAAV
GHT
VLMAPAEFASAARPALCSAAGRQARVLNVTSGENDLYDWLLERFVARRGGGPTLGRGLGRTEANWTDLRIDDADTLA
ALA
RLGHPVAPPERRICHWSPYARPGMLGLYAALIRGELPYDLLRARLPEAQAARWSRLLGLPRVELPLPFAGNAPS
>OLS49484.1 glutathione S-transferase [Rhodovulum sulfidophilum]AB14
MTSEIELYYWPTPNGWKISIALEEMELPYRLTLVDIGAGDQFKPDFLKIAPNNRMPAIIDPDGPDGAPVSIFESGAILQY
LARKTGRFCGRTERERISVDQWLMWQMGGLGPMAGQAHHFLKYAPSLEPPQDLPYAKDRYRSEVARLYGVLDRQLA
QAEF
VAGDFFSIADMAIWPWASNWEGQEQNLDDKPHMARWLENVAARPGVQRGRALAAERKADLSTDRKAQAMLFRR
G
>OLS49485.1 intracellular septation protein A [Rhodovulum sulfidophilum]AB14
MAEKRKINPGLKLALELGPIVAFFVAYLRLRDRTFTIAGTDYQGFVLATAGFIPLLLVTTAILWVLTRHVSKMQIATAVL
VVVFGGLTVWLNDERFFKMKPTAIYALFAAVLGFGLLRGKSYLQYVLDEAVPLERAGWMILTRRLMLFFVGLAVLNEAI
W
RTMSTDAWVNFKTFGLTIAIFGFFMAQGRLFKRYGLEETGRDG

>OLS49486.1 multidrug transporter [Rhodovulum sulfidophilum]AB14
MTDWIASVEGTSAGHDLALILALSAAVLHAVFGALQKGRHDPWLSRGAIDFSYGIMAAPVALFVVPWPEPHMWPIFA
GAF
AIHLAYKLAQGYTYTKGAYTVVYPVVRGTGPLFTVIGAWALFGEVFQPLQWLGVAVLLAGIYGLAVYNLRHWTTDRETL
K
PALAFAVLTGGFVAAYTTFDAYGIRATADPFTFLAWFFFIDGLSTMPWLALMRWRQMAERPAPGPLMLRGVAGGLV
AFMS
FGAVMMATRLDKVGEAAVLRETSTVFAALIGWLVLGEKVGPRRLALMALIAAGAVIVEMGG
>OLS49487.1 hypothetical protein BV379_15170 [Rhodovulum sulfidophilum]AB14
MLDNLAEEFSNPFGGLPTEVAILRLVAAMTLGGLIGFERERRGKPAGLRTHMLIALAACLFTLIALRMIDAASMGDVTLR
IDPLRLIEAVTAGVAFLAAGAIITNRGGVRGLTTGAGMWLAGAIGLACGTGALPLAGLATLVALIVLEVVRLLE
>OLS49488.1 signal recognition particle-docking protein FtsY [Rhodovulum sulfidophilum]AB14
MAFFSKLRQRLGKSSSKIEQGLEEIVRDGGREEEVASSAAARADETATAPVLPADPEPAESAPERPPEPTPAPEPAAPRE
QDRPTPLVLGAAEAVTHAAEPETEARRDETGSEPEPSPAPAPEPEPDPEPTPAPEEPVPTPEPEPAPEPLPMPDPEPQP
A
PAPVPDPEPLNIPPGPSERPPEPPQESPDWPGEVPPSTPDEFPADIPTELSSDMAPASSDAFRGGLPEAGDESGEEVSEE
TGQETGQETGQETGQETGADTPRRGFFGRVFGRSEQKASEPKKTVIRRELDDAMLESLEELLISADMGVDTAMRVTA
NMA
EGRFGKRLSVDEIKELLAQEITRIMEPVARPMPLYPKKPQVVLVVGVNGSGKTTTIGKLASQFRDAGKSVIVAAGDTFRA
AAVEQLQVWGERAGVPVMTAPQGADPASLAFDAMARAEAEGTDLLMIDTAGRLQNRQDLMQELAKIVRVIRKRDE
TAPHN
TLLVLDATTGQNALSQVEIFRNLADVSGLVMTKLDGTAKGGVLVALADKFGLPIHAIGLGEQIDDLAPFEPEDFANALAG
LDE
>OLS49489.1 hypothetical protein BV379_15180 [Rhodovulum sulfidophilum]AB14
MRRAPIAFLLAAALPALAAGLTAAEAAEPMSAAAFDRYATGKTLFYASGGRAYGAEQYLSGRRVIWTFLDGDCSEGVW
FE
ADGQICFRYDHDPEDPQCWSFFESGQGLMARFENDPDQTELIEVNQSREPLICSGPRVGA
>OLS49490.1 exodeoxyribonuclease VII large subunit [Rhodovulum sulfidophilum]AB14
MSDLIDDDGPGGPNSPEFTVSEISGAVKRTVEGAFSHVRVRGEVGRVSLPRSGHIYLDLKDDRNVLASVIWKGTAQRLA
T
RPEEGMEVIATGRLTTFGGQSRYQMVIEDIAPAGVGALMAMLEKRKAALAAEGLFAAERKRALPYLPRVIGVVTSPSGA
V
IRDILHRLGDRFPRHVLIWPVAVQGAACAPEVARAIQGFNALPEGGAIPRPDLLIVARGGGSLEDLWGFNEEIVVRAAA
A
SHIPLISAVGHETDTTLIDHAADRRAPTPTAAAEMAVPVRIELIAALDGLEARRSAALGRLLSQRRQRLADLARALPRPE
TLLNFAAQRLDRAADRLPAALSARVARERLRLSEAGAALRPASLRRRLVEERRRADERVARVAPALARLRRLRASELEKA
ARRVRPELLQRRVETGREALARLDARLSAALGAGFERRRARLEALERMRAGLSYRETLRRGFAVVRAGGQVVTSRAAAE
Q
AGALEIEFRDGRLSTAGRPPRRSRKTDEPPEQGTLL
>OLS49491.1 hypothetical protein BV379_15190 [Rhodovulum sulfidophilum]AB14
MNRPAIAFAPTAIPGALLAASYRADLLETGFQRLGPDQRRAVQAELRELGLYAARIDGAFGPETEAALVRGADYLVWSS
R
GAMRVDLGTPRGVAVYLRGLALGQVAA
>OLS49492.1 phosphoribosylamine--glycine ligase [Rhodovulum sulfidophilum]AB14
MNILILGGGGREHALAWAILQNPKCDRLIVAPGNAGIAAIAECATLDIEDGGAVAAFAEENAVDFVVIGPEAPLAAGVA
D
RLEAAGILCFGPSRAASALESSKSFTKAICDASNAPTAGYGHFTDAGAAKAHVRKAGAPIVVKADGLAAGKGVIVAMTL
A

EAEAAIDDMFGGAFGGAGAEVVIEEFMEGEEASFFVLCDGTDVLAIGTAQDHKRVGEGDTGPNTGGMGAYSPAPVM
TDAV
CAKAMEEIVKPTVAEMARRGTPYKGVLYAGLMVRDGQPRLVEYNVRFGDPEAQVLMMRLGGQALDLMLACAEGRL
AEAKV
NWADDHAMTVVMAAKGYPGAYEKGSVIGGLDALPADSFRMVFHAGTRAEDGKVTAAGGRVLNVTARGASLAEARE
KAYAM
IDRIDWPEGFYRRDIGWRAL
>OLS49493.1 hypothetical protein BV379_15200 [Rhodovulum sulfidophilum]AB14
MDLWVIVTIGAAAVQTVRFMLQKRLRGLGLSTAGATFSRFVFAAPLAALGLAGLMAVRGEALPGIAPAFWTHAATGG
LAQ
IIATFATVALFSERSFAVGVAFTKTEVIQVAAFSALLLGEPVSAPGMAAIAIGVLGVIALSRPPGGWGGMSWRALGLGLL
AGGFFGLSAIGYRGATLQVASEDALMRALLALSCVTMMQTLAMAAWMAWREPGELARVARKWRAVLPVGATGML
GSAGWF
TAFALQNAAYVRSLGQIELIFSILASVLVFHEKLRAAEIFGMALLLASILMLVLLI
>OLS49494.1 2Fe-2S ferredoxin [Rhodovulum sulfidophilum]AB14
MAKITYIEHNGTEHVIDVANGLTVMEGARDNGVPGIDADCGGACACSTCHAYVHPDWVDRLPPVEDMEADMLDFA
YATDP
KRSRLTCQIKVTEALDGLVVQMPEKQI
>OLS49495.1 GNAT family N-acetyltransferase [Rhodovulum sulfidophilum]AB14
MTNQPAPPAPLTLGDCAQADLPGVQVIYAEAVRTGTASFELAPPDLSEMRARWEKVTEAGFPWLVARAGNRIAGYAY
ANT
YRPRPAYRGTVENSVYVDAAFRGQGVGRALLGALLARCEAAGFRQMVAVIGDSQNTGSIALHRALGFRDAGVLRSVG
WKH
GRWLDSVEMQKPLGPGDTTPFDGLPGDRPQGQHSGGAAPDDPPNRP
>OLS49496.1 C4-dicarboxylate ABC transporter [Rhodovulum sulfidophilum]AB14
MTDMSNMNRRSFLRRSALAGGVAGTGALAAPAVLAQAPIVLKMQTSWGASNIWDEFARDYARRVDEMSGGRLRID
VLPAG
AVVSAFQVMDAVNDGVLDASHSVCAYWYGKNKAASLFGTGPVFGGSATTMLGWFYAGGGDELFRELTQDVMGLD
VVGFLG
FPMFAQPFGWFKDEVDTVADLQGFKYRTVGLAADLTARMGMSVAQLPGGELVPAMERGVIDAFEFNNPSSDKDFG
AQDVA
RHYYLASYHQASESFEFLFNRPFFEDLEPDLQAILRYGVEAASTANTAKAMDRYSADLQWLQDEAGVSVHRTSKEILDA
Q
LAAWDELIPELEEDAFMKKCLDSQRAWVERVTFYEVMNAPDYTLAYDHYFPGRLRL
>OLS49497.1 TRAP dicarboxylate transporter subunit DctQ [Rhodovulum sulfidophilum]AB14
MVGFIRFADALSAWFGKAFAWLVVLMALGTGYEVFVRYVLNSPTAWALDVSFIMYGTLFMMGGAYTLSRGGHVRG
DFLYR
LWQPRTQAMVDLVLYIIAFFPGVLALIFAGWKYAARSWRYGEVSVNSPAGIPIYQFKTVIVVAGVLLFIQGIAQVMRCLL
AIRSNEWLEAEEDIFETEDILMREADKAERGPHI
>OLS49498.1 C4-dicarboxylate ABC transporter [Rhodovulum sulfidophilum]AB14
MTDPEVALIQLGLFIVFVFLGFPIAFTLMAMGLGFGYFAYFDAGRMWRAFDRLDETAGMWTRITSWVVGLFNNRIFD
LFV
NQTYTVMSNEVLTAVPLFLFMGYVVERANIVDRLFSTLNIASKKIPGSMGVAALITCALFATATGIVGAVVTLMGLLALP
AMLRARYDPAFASGIICAGGTLGILIPPSIMLIVYAAATNVSIVRLYAGALLPGLTLVGLYLVYVVGRSILQPSAAPRPR
ADEIPDMPFGRLLLQIATSFLPLAVLIAAVLGSILFGLATPTEAASVGALGGLALAVIYRALNWQRLRESVYLTVRTTAM
VCWLFVGSYTFSAVFSYLGGEQLIGQFVEGLNLTPMQFLILAQIIIFLLGWPLEWSEIIIIFVPIFLPLLAYFQIDPLFF
GILVALNLQTSFLTPPMAMSAYYLKGIAPPEVRLTQIFRGVMPFLVCVLISMVLMYVFPQIVYFLPELVYG
>OLS49499.1 amidase [Rhodovulum sulfidophilum]AB14

MKDLETALDLGAVELRDRLAGGALSALDLVEACLARIAAREPGIGAWAWIDPDYAREQARRLDAHRASGRTLGPLHGL
PV
GLKDMIDTARIPTGNGCPLDAGRVPARDAFVVERLRAAGALILGKTVTTELAFLHPGKTRNPHNPAHTPGGSSSGSAAA
V
ADAMVPLALGTQTGGSVIRPAAFCGVTGFKPSFGAIPRRGVLMQSHSLDTVGVFAADPLGAALVAETLFGFDETDPAS
SL
APPPRLLDTARGAPPLPPVFAFVRPPGWDRADPDTHAAFAELTAALGDQVFALDLPPAFEEAAPQRAKINMAEMARH
YYR
YDRDGRDLLGAETRAAIDEGANMPARDYLAALDWPRVLNAALDEIFTRCDAILCPAAPGPAPEGLGTTGDPIFNGLWT
LC
GTPAVTVPILTAGNGLPMGVQLVGPRGGDARLLRSAQWLYDWAAGA
>OLS49500.1 sorbosone dehydrogenase [Rhodovulum sulfidophilum]AB14
MKTTLIGAAIWVALALPAVAQSTDVPDNLEKLGNFRTTGTTDFTYVEQDGPYAEGIRKTLERITLPPGFRIGLYAVVPDA
RHMAVGPQGIATFVGTRKDKVWAVTDRNKDRIADEVKDFAPSLTFAIPNGPCFSKDGFLYIAEQNRVLLFPAAEFFYES
P
DVAAFSLVPQGALIPPGEESYNHTARVCRIGPDGRIYISLGQPFNVTPRDKLELYREIGIGGIIRMKTDGTGREVYTTGI
RNSVGHDFDPATGDLWFTDNQVDGMGDDIPPGEINHQTEMGQHFGYPWYGGGDVRTDDYADEEIPLEVVMPAVE
TVAHAA
DLGMTFYTGSLFPEEYRGAIFSAQHGSWNRTEPIGARVMVTRIAEDGSASTEPFAEGWIDANGEYLGRPVDVAQLRDG
SL
LVSDDMAGALYRIWYDGM
>OLS49501.1 hypothetical protein BV379_15240 [Rhodovulum sulfidophilum]AB14
MSDTKASPPATEGGARAAPETYSFRQKLAHWGVVVLVAAQFLVFDAIGRDFGRGMKTGDWVYSPTATGHILFGLAIL
ALM
IWRLKLRRDEGVPALPAAEPGWAATLARLTHAAFYLLLLAMPVVGAVAWFLQSGNAAEMHEAAANLLIALIAIHVAAV
AL
HKFRWKSNILSRMM
>OLS49502.1 hypothetical protein BV379_15245 [Rhodovulum sulfidophilum]AB14
MPVCLPSASVRRPATTLRPGARVAGHFGEFLQGRLGVDGPLALVTVPCPALELRAVRHPGRIPGLWQGKRPVLSHAQF
MT
LAARLGTRPGRMRLHAGMPPGGGAGASTAALLAAIRCLARHALSAEEEAALCLALEGATDPLMHPAPGRLLWAPRAA
RVL
GRLPPEPAFEVVGGFCGKGQRTDPADTAFADISDLVEAWRATPDPAGRARLATESTRRCHALRGGPDPAPLLELGRNL
GA
LGLVAAHTGAAQGLLFAPGTAPAGTEAALRAAGLGQVLRFRTGGGGR
>OLS49503.1 cobalamin biosynthesis protein CobD [Rhodovulum sulfidophilum]AB14
MSAAAMLVALAIEGMAGWPDRLHARIGHPVTWIGALIAALEARLNRSGDAPATRRRLGALAVAVTLAAAVLPACALV
SIL
PGGWAGAVLTGILAAPLVASRSLFDHVARVAKPLAAGDIAGAREAVSMIVGRDPARLDQAGLARAALESLAENASDGI
VA
PVFWGVLLGLPGIAGYKAVNTLDSMIGHRNDRYEDFGRVAARLDDLVNLLPARLTGALFCLVAGHPLRAARTMMRDA
GAH
RSPNAGWPEAAMAGGLGLRLSGPRIYGDRVSDEPWLNAGAPDPAPGDLWRGLWLYVRAMAGLALGLAALVWIFG
G
>OLS49504.1 threonine-phosphate decarboxylase [Rhodovulum sulfidophilum]AB14
MADGQDRGRDHGGDLSGAMARFGGTEDDWIDLSTGINRVPYPAPPASAAALCDLPGRAAINRLSAAAQGAYGTDW
PLLPL

AGAQAAIQLLPVVAPEGAVRVLGPTYNEYAGAFEAVGRAVETVVAAEALAGAGLAVIVNPNNPDGRRLAPEALRAIAA
TV
GVLVVDESFADPHPELSLLPGTCPKNVLVLRSFGKFYGLAGLRLGFVLGSKALLGPLAGRAGPWAVSGPALEAGALALA
D
AGWRADSRARLSAETARADALAEAAGWRPLGGTALFRLYETEDAAEAQARLARSWIWSRIFPYSAGWLRLGLPGSEA
EWD
RLEEAL
>OLS49505.1 gamma-glutamyltransferase [Rhodovulum sulfidophilum]AB14
MPNWPIALLLTAFAGTAAAQEAADAVAPESATSATEAFAGLSPEARAAWDSREAGRPTEARDWMVAAANPLAVEAG
AEIL
AQGGSAADAMVAVQTVLGLVEPQSSGLGGGAFLLWYDAGTGKLTTLDGRETAPLAATPQLFQTPEGEPLAFYDAVVG
GRS
VGTPGTPMLMEEAHRRWGRIAWPDLFDAGIGLAEGGFQVSPRLAELVAGDEDRLRRHPATEAYFFPGGVPVAAGTEL
VNP
AYADTLRQIARDGSRAFYTGAIAEDIVATVRGAEDNPGLLSTVDLALYRVKERAPVCAPYRGHEICGMGPPSSGGLTVG
Q
ILGLLAPYDLAALGPEAAESWRLIGDASRLAFADRARYMADSDFVPVPAKGLLDPAYLAGRARLLEGDDALPETAPGKP
G
WDHARLWADDRSIEFPSTSHISIVDAQGNALSMTTTIENGFGSRLFVRGFLLNNELTDFSFRTHEDGRPIANRLEPGKRP
RSSMAPTIVLTDGKPSLVIGSPGGSRIIGYVAQAIVAHLDWGMDVQQAVAMPHLLNRFGTYDLEAGTSAEDLAEPLAA
LG
FEVGTRELNSGLHAISVGETLQGGADPRREGIALGR
>OLS49506.1 2-isopropylmalate synthase [Rhodovulum sulfidophilum]AB14
MSESSHQDRVLIFDTTLRDGEQSPGATMTHDEKLEIAAMLDEMGVDIIEAGFPIASEGDFEAVRDIAKLSKNAVICGLAR
AQLPDIDRAWEAVQHAARPRIHTFIGTSPLHRDITALTQDEMVERIEATVAHARNLCDDVQWSPMDATRTERDYLCR
VVE
TAIRAGATTINIPDTVGYTAPRESAKLIEMLLERVPGADEIIFATHCHNDLGMATANALAAVEAGARQIECTINGLGERA
GNTALEEVVMALKVRNDIMPFRTGIDTTKIMGLSRKVATVSGFPVQFNKAIVGKNAFAHESGIHQDGVLKNAETFEIM
RP
ADIGLNATNLVMGKHSGRAALRSKMKELGFDLGDNQLKDVFVRFKALADRKKEVYDDDLLALMRDSDLDAEHDRIQV
KFL
RVICGTEAPQSADLILSVDGVEHRVTAQGDGPVDATFAAVKQLIEHDAHLELYQVHAVTGGTDAQATVSVRLSEDGKIA
T
GQASDTDTVVASAKAYVNALNRLMVRREKTKPEGNLAAI
>OLS49507.1 rod shape-determining protein [Rhodovulum sulfidophilum]AB14
MVGFGGLFSSDMAIDLGTANTLVYVKGRGIILNEPSVVAFHVKDGRKQVLAVGEDAKLMLGRTPGSIEAIRPMREGVIA
D
FDVAEEMIKYFIRKVHKRTTFTKPKIIVCVPHGATPVEKRAIRQSVLSAGARRAGLIAEPIAAAIGAGMPITDPTGSMVV
DIGGGTTEVAVLSLGDIVYARSVRVGGDRMDDGIINYLRRQQNILIGEATAERIKTSIGSARMPDDGRGAVMHIRGRDL
L
NGVPKEIEINQAQIAEALAEPVQQICEAVMTALEATPPDLAADIVDRGVMLTGGGALLGELDLALREQTGLGISVADESL
NCVALGTGKALEYEKQLRHVIDYES
>OLS49508.1 rod shape-determining protein MreC [Rhodovulum sulfidophilum]AB14
MAADRNAQIDYARPIRRILIGGTVFVLVALFVLWRIDSPRVERMRAALIDRVVPGFDWAMAPITKGAELIEGFQSYARL
A
EQNQELRRELRQMKAWKEAALQLEQENAKLLDLNNVRLDPSLTYVTGVVMADSGSPFRQSVLLNVGARDGILDGWA
TTDG

LGLVGRVSGVGKTTARVILLTDSNSRLPVTIQPSGQRAVLSGDNSSAPVLDFVEDPALVRPGDRVVSSGDGGVFPAGLL
V
GHLALGRDQRLRVRLAAEYDRLEFLRVLRSHANERISDPGALVIPGPVGPPPIPAPAAGTGGDPGNG
>OLS49509.1 hypothetical protein BV379_15285 [Rhodovulum sulfidophilum]AB14
MVDPTTLDRWVYRLVFLGIAVMTVFVMILPLETRPGRLPAPDWMLCLTLAWLQRRPDYLPPWLIAGVFLMLDMLLM
QPPG
LMTALVLVGSEFLRARQHVSVETAFGSEWLVTAATIFAIFLTEGVVLALFSVPEADFGRLMFLALATALAYPLVAAVSRY
ALGVRRVTPGDLDPRGIGR
>OLS49510.1 penicillin-binding protein 2 [Rhodovulum sulfidophilum]AB14
MKRSQREAEESNRRIGRRTLIMGGAQALFAGVLALRMRQLQVTQAAEFRLLAEENRINIRLIPPARGILYDRQGHVIAE
N
DQNYRIVIVREDAGDPEQALARLTRLVQLDDETVERALKEIRRHSPFVPVTIADRMRWEEVASVAVNAPVLPGIAPEVG
Q
SRHYPMGPDFAHIVGYVGPVSDYDLSKLEDPDPLLQIPRFQIGKTGVEAKEEELLRGKAGTRRIEVNAAGRVIRELDRID
GQPGSDLQLTIDAGLQNFMQARLGEESAAAVVLDLQHGDVLGIASSPSFDPNKFVQGISVADYRALTETPFRPLSNKAV
Q
GAYPPGSTFKLVTALAALEAGAITPDETVWCPGFLKLGDRRFHCWRRGGHGHVNLAESLQQSCDVYYYDIGQRVGIDN
IA
AMARKLGLGVHHDLPLSAVGEGLAPDRAWKRARYGQDWQVGDTLNASIGQGYVLATPLQLAVMSARIATGRAVTPR
LLRS
IDGVEQPSGAGAPLDIKPVNLERIRKGMDAVVNSRRGTGRRSRVVAEGMAMAGKTGTSQVRNITAAERAAGVFRNE
DLPW
ERRDHALFVAFAPVEAPRVAIAVVVEHGGGGSAVAAPIARDLTLRALYGRVPPGDAYPESDRWEAERRLRDLPLRDPGS
R
SSGGRGRA
>OLS49511.1 rod shape-determining protein RodA [Rhodovulum sulfidophilum]AB14
MSYLEYNVKRVPTGLSKVLYVNWALVLLLTAVAGIGFMMLYSVAGGRLEPWADPQMKRFALGMAVMFTVAFVPIWF
WRNM
AALAYGISVLLLIAVDFFGSVGMGAQRWIDLGVVRLQPSELSKITLVMMLAAYYDWLDIRNVSKPQFVALPVLLILLPTA
LVLKQPDLGTALLLIAGGGIIMFLAGVHWAYFAVVIAAGTGLVTAVFASRGTPWQLLKDYQFRRIDTFLDPSTDPLGAGY
HITQSKIALGSGGWAGRGFMQGTQSRLNFLPEKHTDFIFTTLAEEFGFIGGMSLLALYVLIIVFCISSAMQNRDRFSSLL
TLGIAATFFLFFAVNMSMVMGLAPVVGVPLPLVSYGGSAMLVLLAAFGLVQSAHIHRPRGR
>OLS49512.1 glyoxylate/hydroxypyruvate reductase A [Rhodovulum sulfidophilum]AB14
MVRILFSAPPGLWAEYRAPLARALDEAGIEAEIVTGTGDPAAIDYVVFAPGGGLSDFTPFTGAKAVLSLWAGVETVVGN
P
TLTQPLCRMVDAGLEEGMVEWVTGHVLRHHLGFDRYICATEPHWDQRVPPLARDRTVAVLGLGALGRACAEALAAL
RFRV
LGWSRTPRAVPGIDCHHGAEGLRAVLEQAGIVVLLLPRTPETEGLIDAERLSWLPEGAVLLNPGRGALIDEAAVLAALDA
GRLGHATLDVFRTEPLPPDHPFWRHPRVTVTPHVASETRPASAARVIAENVRRAEAGEALLFRVDRARGY
>OLS49513.1 hypothetical protein BV379_15305 [Rhodovulum sulfidophilum]AB14
MTAETPPAAPQPVSPLDRAHAAMTAAPEDPALRLVYYGRLAATELVLLLDREAEDDDRIAPSVFDLEEGRVVLAFADEG
R
LTDFTGAPSPYAAMPGRRLAEMLTAEGLGLGIDLGGETAELLPPEAVAWWAEMLAATPETLDLVPEALTPPGDLPAPL
LA
ALDTCLAGMGGLAARAWLAGLRHPDGATSLLLAFLDPAEGAEPALARAVSEAVRFSGLETAALDLAFFPASGPMAAHL
AR
LGLRIDLPAPEPMTETPLIDPDAPPRLR
>OLS49514.1 uracil-DNA glycosylase [Rhodovulum sulfidophilum]AB14

MTVAPDLLRSRIAACRLCAPRFAATATGHAPRPVPWFGPAPRILVCGQAPGLRVHKAGRPFFDPSGDRLRSWMGIDSE
TF
YDLSRVSVLPMAFCFPGYDDAGADLPPPALCAETWHRAVLAALGPVPLTLLIGGYAQRWHLGAAARRGVTATVQGW
RAHA
PQVFPLPHPSWRNTAWLKRNPWFEADLLPVLRQRVAEVLA
>OLS49515.1 sodium:proton antiporter [Rhodovulum sulfidophilum]AB14
MYRVWNFVTAYSLLLIGGAVTALIWVNLHPNSYHDFVEYVLIDDFFIGHAHEVGGHVSRTLTVHYLVNDLLMAFFFAIA
A
KEVWEAIILRNGSLRGRKAATPLFATLGGMLGPIAVYLGLAAILGSSTFDAVHRGWAIPTATDIAFSYLVGRIVFGAGHP
AVRFLLLLAIADDAAGLIILAIFYPSGDLQPLWLLLSVGAALATFLLFNWLPRRMDRGRQMRPCSSWVRRYLGFWPYAL
A
GAASWFGFQEAGLHPALGLLPVVPAIPHADRAFGIFSEAEQYLTDLLNHIEHNLKVPVEIVLFFFGLCNAGVEFSSIGVP
TWLVLAGLLIGKPVGVLLFGWLAARPLRLGLPQGMRTADLLVVGCVAAIGFTVSLFIASVAFNSGVTLDGIDVQEAAKM
G
ALFSFVAALIAFGAGKLMRVRKQS
>OLS49516.1 HNH endonuclease [Rhodovulum sulfidophilum]AB14
MVKLVLLHKADSIYEDAPDVVYDFPRSYLKAVEQAVGDWIVYYEPVKAGPRGYFAVAKINRVIPKPCAEGRFLAMIEPG
S
FLPFDREVPRLLDGRPLEAALTEPDGTPKKGGAVQLAVRRLPEADFARIVNFGLPQDLERIEATRYDPQPAELAEGAELF
ERPVLERLTRRPYRDVVFRRKVRAAYGYRCAMSGLMLRNGGGRPEVQAAHIRPVESQGNDTVRNGLALSGTLHWMF
DRGL
ISVAEDCETILVSHNKVPGDVVGRLLAPDGKLVRPEDARDAPHPENLRWHRENVFGRSLPGGPAPSLPV
>OLS49517.1 3-deoxy-8-phosphooctulonate synthase [Rhodovulum sulfidophilum]AB14
MKHIEIGGLPVGNTRPLTVIAGPCQLESLDHALMIGRAMKEACAAAGAGYIFKASFDKANRTSLSGRRGLGIDKGLEVLA
ELRRQLDVPVLTDVHSPEQCAPAAEAVDVLQIPAFLCRQTDLLLAAGETGAAINVKKGQFLAPWDMGNAVTKIESTGN
RR
ILLTERGVSFGYNTLVTDMRSLPIMAGLGYPVVMDATHAVQQPGGQGGSSGGQREFAPVLARAAVSLGLAAVFIETHE
DP
DSAPSDGPNMIPLDQMPDLIATLMAFDRLAKAHPLSI
>OLS49518.1 hypothetical protein BV379_15335 [Rhodovulum sulfidophilum]AB14
MANWYSADLHFGHHRIIDFCKRPFASPEELEQMQRVARHFDVAGRDERNRALRQKRRPCQRLSGLSKPTATFKCQTV
AP
>OLS49519.1 hypothetical protein BV379_15345, partial [Rhodovulum sulfidophilum]AB14
FQWVDTKEREDILLALKYGLPVHPKLLAEFNLEAVPNPKAPDIDAGRAPFGKDDDDGDGIPV
>OLS49520.1 hypothetical protein BV379_15350 [Rhodovulum sulfidophilum]AB14
MFCNHAPFNILCISSLSQIFPNALVIVMIRNEKWVIQGLEHSFIAGYKWAGETLEERAEVWANYYLHLSGVDLPNVVTLD
YDELCLHPRKVLERLTKTACSFLGCRPEDFDKSVFAKSWANDRTSGRSTLSRIEEKDAITFNPLKGFDETQWKDGWSERI
QDILRPTIEVLDLFKRRHLAV
>OLS49521.1 hypothetical protein BV379_15355 [Rhodovulum sulfidophilum]AB14
MGKEAVVLLSGGPDSSTLAFWAFKQGYDIRALFVNSGQYYADHELAGARAVANKLSIHLEVAELANFRQVLIGFIPHPY
T
AMGGGAEVWKRGGAFLPTAVACSYAAITGCEEVLVAMIAEDIELWPDARSFIENSAPNFRLITKNKDANISLPFASKRKS
EVLQLGSELGVPFERTRTCQNTTINHCGECVRCKKRASAFAEAGIPDPTTYEVLPTVLADSSISAA
>OLS49522.1 hypothetical protein BV379_15360 [Rhodovulum sulfidophilum]AB14
MFRDFQRRERAVLLVRIVLGDATRDMTVAEFIRQLINELPRIDALMLSASQQRKSRAGYSYEHHIEAMLSGGKIPFEKQV
VIEAKKRPDFLLPSLAFISSGEATAATGLILSAKTTLRERWKQVEREKGERRLYLTTVDENIAGNAIQDMEGIGVQLVIP
ESLMDAKETEYAGRANVLTFRRFCDEVIQPNLKVWL
>OLS49523.1 ABC transporter [Rhodovulum sulfidophilum]AB14

MTPLVETRGLAVGHGGRVLLEEIGLGLHRGRILCLLGPNGVGKTTLFRTLLGLMPPLGGEVFLGGRRLAELGRAEVAQRL
AYVPQTLTTPFAWRAVDLVLMGAAVRLGPLARPGRAEIARAEAALALLGIDDLGPVEITRLSGGQRQMVQIARAIAQG
AE
AVAMDEPTASLDFANRIRVGRAIQGLASRGIGVLLCTHDPDQAADLGHEALLIERGGILAAGPTAEVMTAARMTRLYDI
P
VRRDALPGGRLHFSGPSGREDEGPVAGASVGQNV
>OLS49524.1 iron ABC transporter permease [Rhodovulum sulfidophilum]AB14
MSERAAAPLLALGLAGALAAGAMLGPYPLSPVDVADTLIGRPPDPQAAIVLWNIRLPRVVAAALIGAALAAAGAAYQT
VF
RNPLVAPDILGVSQGAGFGAVLGILLGLPVLAIQLMGFAFGIGTIALVMAIALALRGMGQVLVVVLCGMAVAAIAGAGI
S
LVKLLADPDDQLPAITFWLMGSLAGIKQADVAAALPAIVAGLLPLIALRWRIGLLALGDDEARTLGVEAGRLRLLVILSA
TLVTAAAVSAAGVIGWVGLMVPHMARLITGPRFDRLLPVALAMGAGLMVLVDAAARCIAQQEVPLGILTAVLGGPLFL
WL
LARGGRTWAE
>OLS49525.1 iron ABC transporter substrate-binding protein [Rhodovulum sulfidophilum]AB14
MMPLRLTALMAGGLVMGLAALTAAAETRTITDATGRPIEVPADPRRVFAAGPPAATLLYTLKPDAMVGWPRAPEDED
RPF
LRPETRDLPALGRLTGKGDTVNLEVLLSARPDLIVDYGTVNATYRDLARRVHAQTGTPYALIDGSLPTIPATLRTLGELL
AETQRGETLAAYAEATFAGIDAILAAIPEDRRPTVYLARGPEGIETAARGSINAEIIDRVGGRNVVAAGPDGLATVSPEQ
VQAWAPEVIVTIDADFAAHVGTMPEWQGIPAVRDGRVYLAPAAPFGFIDSPPSVNRLIGLKWLADKLYPEEARTDIEAE
V
ARFYTLFYGVTPDRAALDGLLGAGHPGE
>OLS49526.1 tRNA (N6-threonylcarbamoyladenosine(37)-N6)-methyltransferase TrmO [Rhodovulum sulfidophilum]AB14
MTLAASETLRPHEECVPVPDAFDAGLWFIGRIRAPWAQRAECPRRGDPVNGPDCRIVLDARWLSALEGVAGKDRMQ
VLYW
MHLSRRDVVRQNPFFGDGSLGTFALRSPLRPNPIASSLVRLLRVEGNVLTVRGLDCIDGTPLVDLKPEFGLLP
>OLS49527.1 hypothetical protein BV379_15390 [Rhodovulum sulfidophilum]AB14
MRNINQGHLKFYKLVLQRLPKSYGKSPKDADRTIDELLARAEDLPEKKVGLSPRTINGHLDRFKLIFQFAKSESITISDT
IELGLLRVREEKRNRDKREAFNPFWRAERR
>OLS49528.1 hypothetical protein BV379_15395 [Rhodovulum sulfidophilum]AB14
MVDQRVPKLRKDDDAAKFTALIEQNRWLVEPDALDVLREAFEREAWIDDEGVMPEGHVTLEPGDDAVDIGQLARDA
RPVW
PWQI
>OLS49529.1 hypothetical protein BV379_15400 [Rhodovulum sulfidophilum]AB14
MRKLKIFLSHSSRDKDFVRRINKDLKSGGFDTWLDEDDIPFAASISEEIQSGLKNSDILFLFLSKSSVASRWVATEWQSK
FFKQISSGNTIVVPVLIEDCSIPEFLNDRKYIDFRKSSNYSKRLSETLGFLQIEKSKISGKVPAEFDRKLGIRSHVTEIL
SELEGEMIQLPQVRRIPIVNTLKKIKRSGKLIRLQGFKPDLQVRTVYDHIISLAHLADCFLPSLSRNLSSVELRDFSLLI
AYHDINEVILGDIPAYTSLSRSKRASIKVYAEQRLKTVDPEELERISNEFVWLFLSEQHRQAFRAAMDLSKDTESSTFRI
FKALDKIDPIVAVWRYLNHYRGKIGDDPRLFNSRMKDFYENPDVAGFFRDFQIDTKLVDTLAFLQDRSNSWDYYVDKD
FL
KNRSQDFLSIPAERLQSAIEDVPLFSS
>OLS49530.1 hypothetical protein BV379_15405 [Rhodovulum sulfidophilum]AB14
MRITFTENGDEACTLSQKSNSSSTAFSIPVSKPALQAAFRDLLLDPEKREVMIGSVGVDRYRDGLRVHAGGGRFELPFRH
LFPLVMEVAE
>OLS49531.1 hypothetical protein BV379_15410 [Rhodovulum sulfidophilum]AB14

MTRARWDRLQAQYHLIRDCISGEDAVKDQGQLYVPKPDGMTTDNYNHYLDRGCFYGAPAMTLRALVGLALRKDPVI
NLPP
RLEAMRLNATYENAPMQVLVEDMVREVAAMGRFGILLDFPAEGATANTVPHISTFRAENIEDFETGFVRGAESADPRA
SR
I
>OLS49532.1 hypothetical protein BV379_15425 [Rhodovulum sulfidophilum]AB14
MTRDERKHVFISYSWKDASAAEQVRNSIPREFEVWFDKERIRPGESISHAIQEGLNASDYYLLLISENSNQSD
>OLS49533.1 hypothetical protein BV379_15435 [Rhodovulum sulfidophilum]AB14
MPTRLFISHSWSYSERYNAMVNLLNNRPYFNFTNYSVPEAKAFGPMARYKMEDELRGQIRPVNCVIIGGMWANHSD
WIK
FEMDFANSINKPVLGVRPRGAQVMPVAVAQASDLVVNWNSDSIVAGIRQIT
>OLS49534.1 hypothetical protein BV379_15440 [Rhodovulum sulfidophilum]AB14
MAVEQYEEFLNLTDEDASDAYDAWAILGARLDDFPNGARQYFKRLCEKIEEFDPHTDDELALTEHLEMPIIMPKNRKVL
G
GKSPHDHAHVFDWVVAWQKTEGVSLDQAFWDYVEIHGLDLDQRETIKGIYHKVRRARLKSMGQERPNFRKPLGPSE
V
>OLS49535.1 hypothetical protein BV379_15445 [Rhodovulum sulfidophilum]AB14
MTLELRIITGRYKDGVALAEVEAAQRLINHYVDEGFVKELHCRDDGYLGFVYHASHFEERARQSDAD
>OLS49536.1 two-component sensor histidine kinase [Rhodovulum sulfidophilum]AB14
MVERWIKRMLPRGLYGRAALILIVPIVTIQLVVSVVFIQRHFADVTQQMTKALVLELAYLTRIVDAAPDLAAARSEAGAV
ARPLELALTLPTDAPQEISRRFYDLSGKTVTATLRQGLPGFTGIDLATDTARVWVGIVTDKGPMEVSFNRRRVSATNPH
Q
LLVLMMFTSALMTLIAFLFLRNQLRPIRRLARAADAFGKGRPIPYRPTGATEVRAAGRAFLDMRARMERQIEQRTLMLS
G
VSHDLRTPLTRLKLELSMAEDSPETRAMLRDINDMGRMIDTFLDFSRADALDDPEELDPAALLMRVAERARAGGGAV
DIG
ALPPAEPASLRPMAVERALQNLIDNGLRHGNRVALSLYLRPGALVFTVEDDGPGIPEAQRELAMQPFQRLDAARNQN
RGT
GVGLGLAIAADIARGHGGALHLGTSDRLGGLRADLVLAR
>OLS49537.1 sulfate ABC transporter ATP-binding protein [Rhodovulum sulfidophilum]AB14
MHIDIDEIAKEFGTARALHPVSLSIPNGALVALLGPSGSGKTTLLRILGGLDYPSSGRIFFDGQDTTGLSVQDRRAGFVF
QNYALFRHMNVFENIAYGLRARRRRDRPAEAEITRRVNRLLALIQLPDIGARYPGQLSGGQRQRVALARALAIEPRMLLL
DEPFGALDAKVRKELRQGLREIHDATGLTTLFVTHDQEEAMDLADLVVVMSMGRIEQVGKPAEIRARPACAFVRDFVA
A
>OLS49538.1 sulfate ABC transporter permease subunit CysW [Rhodovulum sulfidophilum]AB14
MAEIAFPRREPVTTEPRLARWSLIAAVLALVGILLFAPLATVFAEALAGGGAAALAALGSPDARAAIGLTLTVAALSVPL
NAAIGIAAAWAIARFEFRGKALLVTLIDLPFSVSPVVAGLALMLLFGVNGPLGDWLLAHDLKIVFALPGIVLATLFVTFP
FVARELIPVMIEQGRTEEEAALTLGASGWRTFLTVTLPNIRWALLYGLLLCNARAMGEFGAVAVVSGKIRGETTTMPIMI
EMLYNEYLSVAAFSLAALLALLALLTLTLKTLLEWRCAELLSATRRY
>OLS49539.1 sulfate ABC transporter permease subunit CysT [Rhodovulum sulfidophilum]AB14
MRAGPFRSPSPLPGFGLSFGIAMAGLSIVILLPLGALLGRGLAIGPVELLDMVNSRRIRAALGLSFRAALIASLFNLAFG
LALAWVLVRYRFWGRRLIDAAVDLPFALPTAVAGIALTALYAPTGPFGHALAPLGIEIAYSEAGIWLALIFIGLPFVVRT
VQPVIEEIDREAEEVSATLGAGRLYTLRRVVLPTLTPALMTGFALALARSVGEYGSVIFIAGNLPFRTEIAPLLIVIRLE
EYNHDAAAAIGIAMLMISFAVLLTLNTIQFWSRRRIGLG
>OLS49540.1 transcriptional regulator [Rhodovulum sulfidophilum]AB14
MKLDRIDLKILDLLQRDATIPLARIADRVGLSQTPCWKRIQKHEEAGVIRERVAILDPEVLGLALTAFVMIEALDHTADW
RESFIEAVERFEEVRDLYRLAGRYDFLLRVVVRDMADFDRFYADLTAGVRLRSVDSFFALERMKVSTALPLAGMLA
>OLS49541.1 transcriptional regulator, partial [Rhodovulum sulfidophilum]AB14

IFRLLVQRGPMGTPAMEVGRLLDLKPSTLSGHLATLKRAGLVETERQHREVLYSPRFDAVNGLVRFLLEDCCGGDEDAC
A
GLELPRT
>OLS49542.1 ferredoxin [Rhodovulum sulfidophilum]AB14
MTYVVTENCIACKYMDCVEVCPVDCFYEGENTLVIHPDECIDCGVCEPECPADAIRPDTEPDSERWVEFNRKYAEIWPV
I
TAAGEPPANADEMDGIPNKLQTHFSPEPGQKEA
>OLS49543.1 superoxide dismutase [Rhodovulum sulfidophilum]AB14
MAFDLPALNFAHSALEDRGMSTETLELHHDKHHQAYVTALNGFVEKNADLQGMSLEDIVTATYGVAGREGIFNQAG
QHWN
HIHFWNALSPEGGSIPGALEKKLTADFGSVDDFKQAFKAAATGQFGSGWAWLIQKSDGSLGVTKTPNGVNPLATGEG
TAL
LGLDVWEHSYYVDFRNRRPDYVDNFLGRLANYDFAEANLA
>OLS49544.1 MarR family transcriptional regulator [Rhodovulum sulfidophilum]AB14
MSTDNLEKEGRPPRSAAERILMALKMHGALTSAEVGARLGTTGEAARQQLVKLAEDDLVADERRPSGRGRPAVRWHL
TEK
GQSRFPDTHAALTVEILRSISDVLGAEALDRIIHAREATTQALYEEAMADCVTLGDRVGKLVELRNAGGYMAAAEEGED
G
GLLLVENHCPICAAASFCQGFCRAEKAVFENVLGDGISVERVEHIVGGGRRCTYVIREAS
>OLS49545.1 preprotein translocase subunit TatC [Rhodovulum sulfidophilum]AB14
MDARPSRPVARFPEAVAEGLDEAMIREVVHHFYAAAREDPVIGPVFRARVPDDRWQAHLDTIEDFWCSSLLGTRRYE
GRP
MPKHLAISELNDDHFRRWLALFRHTVTQICPQKTAELFVERSERIAEAFRINIGMARGEDLMFRPKLTREPYP
>OLS49546.1 UDP-N-acetylenolpyruvoylglucosamine reductase [Rhodovulum sulfidophilum]AB14
MRAPPAPLVPQFDLSGRNTLGLRSRASYGGVLADEGEMIAAAQFADRVGLPFHLLGGGSNCLLAEEIDAVVGIAGARG
RR
LQRVGGGVRVTARAGETWDDIVRWTVGQGFGGLENLAGIPGTAGAAPIQNIGAFGVELSDVAESVDVIDTEDWTQR
RLAA
AECGFGYRQSRFKEDPGRFLVTGITLYLPAAWRPVLSHEGLSRGGLSRGGLSGGASLTGAAQVMGAVLARRGARLPD
WRL
TGNAGSFFHNPVVPVAAAARIRDVCGHPAPGGVKLPAGKLLDLCGFRGRRRGGAAFSEINALILVNAGGATLRDVDAL
AA
RAQDAVRLRFGVDLVQEPSRVGD
>OLS49547.1 short-chain dehydrogenase [Rhodovulum sulfidophilum]AB14
MLLEGKTIIITGASSGIGAAAAKIFAAEGARLVLGARRAELLERTVNEISADGGEADFIVGDVEESGYAEELVKRAEERF
GGLDGAFNNAGTTGNMGPIPDMDESNWHKVMAVNLNSGFYAAKHQIPAMRKRGGGSIVFTSSFVGHTIGLPGMG
AYAAAK
AGLIGMTQVLAAEHGPENIRVNALLPGGTMTPMAGEDAAVHEVVRGFHALKRMADPSEIAQAALFLLSDHASFVTGT
AMI
ADGGNSINKL
>OLS49548.1 co-chaperone GroES [Rhodovulum sulfidophilum]AB14
MSFIPLHDRVLVRRIEGDAKTSGGLIIPDTAKEKPQEGEIVAVGAGAKDEDGDRVAMDVKAGDRNLFGKWSGTEIKLD
GE
DLMIMKESDILGVMT
>OLS49549.1 hypothetical protein BV379_15550 [Rhodovulum sulfidophilum]AB14
MSTASEHAGQAALSICEALLLAMNDRGLLTEHEIVGVLRDAAATHENAVGTDHETENHRAVADLINAIIAGGNSVRRL
>OLS49550.1 Crp/Fnr family transcriptional regulator [Rhodovulum sulfidophilum]AB14

MKSVELSPLTRKLSAFVALSDVELAVLERLHQRRRSFVAGRDMVHQGQTTQAAYILSAGWVCSYKLQADGTRQIVDFQ
VP
GDFLGLRSVLLRTSDHSFEPIVTIEAAEVLTNDLLDAFAQTPRLATAILWAASRDEAMVVEHLVGIGRRDADARMAHFLL
ELGSRLALVGMGSKEGFDCPVTQYHLADTLGLSAVHVNRVLRQLRESGLVTFRDGHVTFHDYGGLVELAEFDPAYLDQ
TG
PLLA
>OLS49551.1 DUF3309 domain-containing protein [Rhodovulum sulfidophilum]AB14
MSLGTILIIILVIFLLGGFSGRFGGYGYGFGHAGTGVLGTILIIVVILMLLGRI
>OLS49552.1 CsbD family protein [Rhodovulum sulfidophilum]AB14
MDKDRVIGSAKVVKGKAKVAVGMAIGDAKLEAEGEADKIEGKIQNAVGGINDTIRDA
>OLS49553.1 hypothetical protein BV379_15570 [Rhodovulum sulfidophilum]AB14
MSPEQTKTADKMKSVKATWDKAPAGPKKDAALKHYQAAEKAHAAKNDAETNKELDAATHALA
>OLS49554.1 hypothetical protein BV379_15575 [Rhodovulum sulfidophilum]AB14
MEEHFWTSGADNARATTANNTVMVFPYPPGILQGDQIWTHLKQSTGWRSVVMAERRVTRCQDIAILTYRVSAEKPD
VPIY
KALCASTYLNDDDTWLRIFHQQTIVA
>OLS49555.1 hypothetical protein BV379_15580 [Rhodovulum sulfidophilum]AB14
MTMAQPDVLNPHPPEFERFLYASVGEDRNGYVVTVLSTLARLGLDPWAETADLVTLGRDAARTRLGILLARSRDVPAL
AS
DYGRVARALSQLLPDGQTSGSLKRVASTVADGRPGTNGAIWAVLAIIFLLFQMLMVGGSGSGE
>OLS49556.1 hypothetical protein BV379_15585 [Rhodovulum sulfidophilum]AB14
MNVRSSRSTLTFSNPFTLPGYPGELPAGDYALLVEEELLQGLSFEAYRRTATYLTVRGRGRHAGRSELRAISDSDLKEAL
NRDQAATEDRKNSSDAALSPQEDSK
>OLS49557.1 hypothetical protein BV379_15590 [Rhodovulum sulfidophilum]AB14
MNMPEWLKPGIYGALIGAVFVGVVGFTWGGWVTGGTANDRAMAMSRDDVVASMVPVCLDMARSDPSRTDKLAT
IRAASSY
QRRDALMDAGLATMPGTDAPDRDIAQACLAALEL
>OLS49558.1 hypothetical protein BV379_15600 [Rhodovulum sulfidophilum]AB14
MAPAASLSKTRPEATDLERRVLAHERILQSPIADMARTEPRFVDHLRERFVEPMSMARHEHDHRETDDYAEESIRAVM
LL
GEVRAPKSTETDVTGKQPMPARSEGKKEPCMDRPAQRGGVEVRERSGIWEVQVDGKFRGDYHQKEHALAAAGLHRL
SLR
>OLS49559.1 hypothetical protein BV379_15605 [Rhodovulum sulfidophilum]AB14
MATPGQPDLLHVVNERGQPQPLRTQKRGRQSGHDQAGEAQTLRACRQKSFGGRVDVGQRFARPLPRLARSDRAW
GRQRGL
DRAQQQVLRGTCCLDAQIPMAEQHPGSCGIEFGDSRKIDAGRGSGPMETGAHVLERIQRQRPGQGQNWSPRKRHR
RRRE
>OLS49560.1 arsenical-resistance protein [Rhodovulum sulfidophilum]AB14
MSDTFTPAPMGRFERLLSLWVALAMAVGIAVGNLAPGAFAAIAETEVASINLVVAVLIWAMVYPMMVSVDFGAVAG
LARQ
PKGLLVTLVVNWLIKPFTMALLAVLFFDHIFAPWIAPEDASQYIAGLVLLGAAPCTAMVFVWSQLTRGDATYTLVQVSV
N
DLIMVVAFAPIVAFLLGVTEITVPWSTLVLATLLYVVLPLLAGLLTRRALGTETAISAFTARIKPVSMGGLVATVAILFG
LQGDTILSRPGVIALIAVPILIQSYAIFALAYGAAWLLEVPHRIAAPCAMIGTSNFFELAVAVAISLFGLHSGAALATVV
GVLVEVPVMLSLVAFANRTRARFPEAERGA
>OLS49561.1 hypothetical protein BV379_15620, partial [Rhodovulum sulfidophilum]AB14
MASWASGFTQRSMNKSGTHRRLLLSRAEEIAPGHNFLGTRRGGKLASGFDVPQGRTLASYRDVYHRFEQNPVVLADR
DRM

KGRREPRLCEWQERFIDYVLNRWHDPRQPKLASVFRLATKVFHRSPREMAQEINFPSITTVRTRAKAISDVVAVLGRGG
T
RHGTNTKGAGSTDVRALAFGEKFEWDQCLLSIFTSGDGVVRAEVINPKEAPQELADNEIRRCWLHVILDVATREVLGWI
R
LRARL
>OLS49562.1 hypothetical protein BV379_15625 [Rhodovulum sulfidophilum]AB14
MSLWRELRTWGHLNAPTIGDELNLPALRYSLRMVDEDTLEVQRFDNGWLRLGAWFCFFLMMYFVVLSWWEQNILW
DNIQI
WLNSEAYFRATLERLVKRSPEVYGNEDFTSFYTRMMEMHGDRWVGGAIFLTLPTFFFIAACIWPRYRPIRFNRKLGIAYT
WSWGRFFLSRSDPSLGLGRGVGDFDLMLRRFTIVRAGVGSPESHSIGALVLRLRHHRWTRYKESWTLGVFPPACFYQN
SQ
ISISISDFLTNPVRPDWCEALELQPSPLSWAHRLALRVVNFSLLPSWWPRRTQKMIRHHLEIRAAEEKAAAEAREALRRR
ANGMD
>OLS49563.1 hypothetical protein BV379_15635 [Rhodovulum sulfidophilum]AB14
MHAEQSTHTFAILDAAKIPDLPDTLANSGLTYLNLFRGTAAQELADVSPYIVKLEIENDFVRKLFTDTGKKTDLWSNEVG
IYVRSKDTIENLLRLFRKFTRVRDEHDNWSYFRFWEPDVAEVHFSYLSRRADLATGWFYTDNENPVGYVIVRRDGRAAK
Y
IPNRPFAGISSSNFRMEVEEKEALKVRRVQASFLKIQSRLATLLPGKTEFEIRDALNRVAERFSQYGFVNTGSIFALCWC
ELTQGPHFERKLKVSALKDLEQKQVAEDLRILKLQSALGQ
>OLS49564.1 hypothetical protein BV379_15640 [Rhodovulum sulfidophilum]AB14
MDDLSFPKDDFFDLQQKGSISAVTTSCPLIDLEIAFFFDGTGNNLANVEEGNRVRAEGGVVSQASYANDLSNVAKLYRS
Y
DSFVPKTAENNTDGAKIRRFSEIFDGIGTTAGQDDSTIGFALGWGRSGVEERVKDAFLVFLRHLNKAKSSGEIKSIKVDV
FGFSRGAAAARHFLNCVKRGHAGHGYGGPFYELREEDKSKLEIRFLGIFDTVVSWGLVANDSSWGNLRITLPDDIATKIV
HITALDEFRENFPLTEAPDSAETIRMPGAHSDIGGGYPEFYKECVEVKRARSQFDNFNPRPAETGRVFGYSDFTAEDLAI
FNQLVSDGWINKSDHNALTREYETIGSNRYDPYWNRWLLKRKWIKDPGLSHVALHVMFRRAIDAGVPFTALISGRNYD
LP
ENDEVLEHLKDRLLSGEIITPAESIQYRRNYVHHSASWTPSGPFFPLQPHEGASRRTVYNNDGSTKES
>OLS49565.1 hypothetical protein BV379_15645 [Rhodovulum sulfidophilum]AB14
MSVLRNLLSVRLVLESAPECWDGKLRSAALLGAVVFGVFYFAALLQINDPLDMFRPEAVSWRAWVSWAAICLPATIFLS
S
WLLVVLRRAVGYIVFSVSMIYLGPMMTAVAISLVARSGPAAAQHGAGAPRRPLGRLLPIADVEPHAANTVDETRKWTS
AW
AIDQLSIGDEGRSSLRGG
>OLS49566.1 ribonucleoside-diphosphate reductase, adenosylcobalamin-dependent [Rhodovulum sulfidophilum]AB14
MSRFAAPIAEQIWDMKYRLKGSDGTPVDMTVEDSWRRIARSLAEAEPEAERDAREAEFFAALEDFKFLPAGRITAGAG
TG
RAVTLFNCFVMGTIPDSMAGIFDMLKEAALTMQQGGGIGYDFSTIRPKGADVKGVAADASGPLSFMDVWDAMCRTI
MSAG
SRRGAMMATMRCDHPDIEQFITAKQDPARLRNFNLSVLVSDAFMEAVKAGGTWELQFGGKVYHTLEARDLWNRIM
QATYD
YAEPGVIFIDRINQMNNLAYCETIAATNPCGEQPLPPYGACLLGSINLARLVANPFEKGAALDEAALEALVATAVRMMD
N
VVDISRFALDAQRHEAKAKRRIGLGVTGLADALLMVGQRYGSEEAARQTSRWLHRIARAAYLASVQLAKEKGAFPLFD
AE
QYLGSGALAQMDADVRAAIAEHGIRNALLTSIAPTGTISLYAGNVSSGIEPVFAYSYKRKVLQRDGSRTEEEVVDYAVQL

WRDKFGDAELPEYFVNAQTLPPSEHVRMQAAAQTWVDSSISKTINCPEDIAFDAFKDVYMQAYETGCKGCTTYRPND
VTG
SVLSVSENSEKTPEAETGADVVYLSDPLDRPHELEGNTYKLKWPDSEHAIYITINDIVIAGRRRPFEVFINSKNMEHFAW
TVALTRMISAVFRRGGDVSFVVEELKAVFDPRGGAWMQGKYIPSILAAIGGVIERHLVTIGFIEAEGKGLKIDPKYDEMA
VGDAPRGPSCPSCGAYEIRMVEGCKTCMSCGYSKCQ
>OLS49567.1 lysophospholipase [Rhodovulum sulfidophilum]AB14
MARGHVNLVKLCVGAERVEDLIDWQAERRAALPDGLPRHVTRMWPKRAAELLDGGSLYWVFKGQILARQSVLRLDE
VTGE
DGITRCAIVLDPEVIRTEAVPRRPFQGWRYLEADAAPRDLPAASAREDDLPPQLMAALAEIGVR
>OLS49568.1 ATP phosphoribosyltransferase catalytic subunit HisG [Rhodovulum sulfidophilum]AB14
MTLKLGVPSKGRLMEKTFDWFAERGISIARTGSDREYSGAVEGVDGIELVLLSAGEIPRELASGRIHLGVTGSDLVQERI
ADWEHRVLELEPMGFGFADLIIAVPRCWADVDTLDDLDAAAAAFRAAHGHRLRIATKYHRLVRVFLRDAGVADYQLV
DSQ
GATEGTVKNLTAEAIADITSTGETLRANHLKILSDGLIHRSQATLFKSRLAAYSDTDRTNLRALLDRLGLTA
>OLS49569.1 ATP phosphoribosyltransferase regulatory subunit [Rhodovulum sulfidophilum]AB14
MEDKGRILAQAERLHVFFRGAGAEPVQTAILQPAETLLDLYGEDIRARAYVTSDALRGEQMLRPDFTVPVVQMHMDS
AAD
PARYTYCGEVFRRQERDPSRPNEYIQVGYELFGGESPAEADAEVFALFSEALSPLDLRAATGDIGLILAAVDGLSASDKR
KAALRRHVWRPRRFRALLERFAGRMPLPESRKRLLAEIEAHGVEAVLKAAAPEIGLRSRADVACRLHALVQDAAEPPVS
T
GETELLEDLLSIRETSVNALAHLRDIAVDLRTIGPAVDRMQARLDALEGQGIDVDLLDFEASYGRTTMEYYDGFVFGFYA
EGQPGLPPVATGGRYDALTRILGRGREVPAVGGVVRPELVLSLTEALA
>OLS49570.1 histidine--tRNA ligase [Rhodovulum sulfidophilum]AB14
MAKDKKPRRPRAETPKGFRDYFGVDVTERKAMLDRIAEVYHRYGYDPLETSAVETVEALGKFLPDVDRPNEGVFAWQ
EED
SDWVALRYDLTAPLARVAAQFRNDLPNPYRRYAMGPVWRNEKPGPGRFRQFYQCDADTVGAASVAADAEICAMLS
DALET
VGIPRGDYVVRVNNRKVLNGVMEVAGVLDPADPLKFEAERGIVLRAIDKLDRLGEAGVRALLGEGRKDDSGDFTDGAK
LS
EAQAEVVMGFMAARRETGAETCARLRELVAGSAMGAQGVDELETIATLLDAQGYGPDRIILDPSVVRGLGYYTGPVFE
AE
LTFEILDEKGRPRQFGSVAGGGRYDDLVKRFTGQEVPATGVSIGVDRLLAALRAKGRAGAGMDGPVIVTVMDRDRMA
EYQ
TMVGELRSAGIRAEVYLGNPKNFGNQLKYADKRQSPVAIIQGSDEAARGVVQIKDLVLGAKIAESASLEEWKSQPAQFE
A
ARADLVAEVRRLLAQGEG
>OLS49571.1 hypothetical protein BV379_15675 [Rhodovulum sulfidophilum]AB14
MDRELTILDNRIARLSGELDGLSRDIARQGKEIDRFRRQVTATLARAAERDRTETAEVAP
>OLS49572.1 DNA polymerase III subunit alpha [Rhodovulum sulfidophilum]AB14
MSAPRFIHLRLHTEYSLLEGAVRVKKLPGLCAAAGMPAVAVTDTNNMFCALEFSSYATGAGIQPILGCQVDLTYDPAAP
G
ERPRAPAPVVLLAQNEAGYGNLMKLNSCLYLGETGELPQVSPDELIAHADGLICLTGGPDGPVGRLIRDGQRAKAEALV
T
RLAAAFPTRLYVELQRHPGEGGQPTEAERETERAHVEMAYAMDLPLVATNDVYFPKSDMYEAHDALLCIADGAYVDQ
AEP
RRRLTPQHYFKTPEEMAALFADLPEALENTVEIARRCAVKSEKRKPILPRFADDEVEELRRQAWEGLEARLAVIPHAAPV
EDYEKRLKFELGIIEGMGFPGYFLIVADFIKWGKEHGIPVGPGRGSGAGSLVAYALTITDLDPLRYSLLFERFLNPERVS

MPDFDIDFCMDRREEVIHYVQKKYGREKVAQIITFGALLSKAAVRDVGRVLQMPYGQVDKLSKLIPVEGVKPVSISQALT
DEPRLREAAKNEEVVDRLLTYGQQVEGLLRNASTHAAGVVIGDRPLDDLVPLYQDPRSDMPATQFNMKWVEQAGLVKFDF
LGLKTLTVVRNAIDLILKSGRPLHQAADGTVLYEPAAGAENDIGHIPLDDPKTYELYSTAHTVAVFQVESSGMMDALRRM
KPTCIEDIVALVALYRPGPMENIPTYCEVKQGLKPLESIHPTIDHILAETQGIIVYQEQVMQIAQVMAGYSLGGADLLRR
AMGKKIKEAMDAERPKFVEGAKKNGVDAKKAAEVFDLLEKFANYGFNKSHAAAYAVVSYQTAWLKANHPVEFMAGVMNCD
IHLTDKLAVYAEEVRRGLDLEIVAPCVNRSFPTFDVQEGCLVYALGALKNVGIEAMKLIVAARGDRPFDSLFDFARRVDM
KHVGKRPLEMLARAGAFDALDGNRRRVFESLDALVGYSAAIHDQRASAQVSLFGEAGEDLPEPRPAQVPDWLPNERLAEE
QKAIGFYLSGHPLDDYMPALRRKKVMTKAEVEEKTKAGPAVLRMAGAVAARQERKSAKGNRFAFVQMSDPTGLFEVTLFS
DVLEVARDQLEPGMNVVVQVEANLENDQLKLLGRSVQPVDTVVADAGAIGLKVVVETEAAVEAVATLLDRARGESGARGR
GPIEFSLVAPDLPGEVRMTLNDRYPVNPQIKGALRSLPGVAMVEEV
>OLS49573.1 glyoxalase [Rhodovulum sulfidophilum]AB14
MYPHALHHVQLAMPEGREDEARAFYGGLLGLEEEEKPEALAARGGVWFRRGELRLHLGVETPFAAARKAHPALEVRGLGA
LADRLERAGLAPRWDGALPGMRRFYVDDPFGNRIEFLEQV
>OLS49574.1 xanthine dehydrogenase small subunit [Rhodovulum sulfidophilum]AB14
MDIAFLLNGESVALTGVAPTATLLDWLRETRGLTGTKEACREGDCGACTVMVTDAEGTRALNACILFLPQIHGKAIRTVE
GLAAPDGRLHPVQQALIDHHASQCGFCTPGFAVSMATAQQTGDTDLEAALAGNLCRCTGYAPILRAAEAARDAPVPDWIG
EDANFFLAEIPSGGADPQDRWGADSPPAFRPATADALAAWYETHPDATLIAGATDIGLWVTKGLQDLSQVAFLSGCEDLT
RIEITETEIRLGAGVTIARLREMMRAYHPHFARMLTRFGSAQVRAAATLGGNIANGSPIGDSPPALIALGATLHLRRGAR
RRSLPLEDFFLDYRKQDRAPGEFVEAVTIPRQPDNLRCYKLSKRRDQDISAVLGCFNLTVRAGRVETARIAFGGMAGIPK
RARAVEAALTGQPFAQATIEAALPAFASDFQPMSDMRASAGYRLRAAENMLWRLWHESRNDPTNIDELAP
>OLS49575.1 xanthine dehydrogenase molybdopterin binding subunit [Rhodovulum sulfidophilum]AB14
MSVTRPLPHDAARLHVTGAARYLDDIPAPGNTLHLAFGLSQIAHGRILTMDLSAVRAAPGVVAVLGPDDLDPMPDCSPSA
HDEPLLAPGEISYCGQPLFLVIAESHLAARKAARLAEIRHEEWPAILTIDQALAANSRFEDGPQIWERGDAAAAIAGAAH
RVEGAIEIGGQEHFYLEGQAALALPQEGGDMVLHASTQHPSEIQHKVADALGLPMHAVRVQVRRMGGAFGGKESQGNALA
IACALAARATGRPCRMRYDRDDDMIITGKRHDFRIRYRAGADGEGRLTGVEFVQYARCGWARDLSLPVADRAMLHADNAY
FLPAVRIESHRLKTHTQSATAFRGFGGPQGMVGIERVMDHIAHALGRDPLEIRRPNYYDGAAEAGGLSAPRRSSGSAPLE
GISAKKKTPFGMEVEDFILHEMTEALARSADYAARRNAVAEWNARHAVLKKGIALTAVKFGISFTLTHLNQAGALVHVYQ
DGSVQVNHGGTEMGQGLYQKVAQVAAHAFGLPMAAVKITATDTGKVPNTSATAASSGSDLNGMAVKAACDEIRGRIAAHL
AATRQTDPAQVRFVGGRVFAGGDEMSFAEAARHAYQARVSLSATGFYRTPKLSWDRIAGRGRPFLYFAYGAAVSEVVLDT

LTGENRILRTDILHDAGTSLNPALDIGQIEGGFVQGAGWLTMEELVWDAKGRLSTHAPSTYKIPAVGDRPQVFNVALW
DG
ANREETIHRSKAVGEPPFMLGISVLMALSDAVAACGPGYPALDAPATPERLLRAIDRVRA
>OLS49576.1 xanthine dehydrogenase accessory protein XdhC [Rhodovulum sulfidophilum]AB14
MSLDRDALAQAVRARGRVARVAVAETRGSTPREAGASMLVWADGQQGTIGGGTLEYEASADARRLLASGGTRVARI
ALGP
GLGQCCGGAVLLVTETFVPGDLAGLPGDAHARRVEGHAEMPLSIRRALTETRSGLGPPPVLLREGWFLEPVAPPARPL
WI
WGAGHVGRALMAVLAPLPGIGITWIDTAAQRFPSDLPEGVLPRVAPDPAALVPQAPANAHHLVLTHSHALDFALCHA
LLG
HGFASAGLIGSDSKWARFRTRLGQLGHSPAEIARICCPIGQKSLGKHPQAIAIGVAAALLSEGVEGIAARDIAG
>OLS49577.1 ABC transporter [Rhodovulum sulfidophilum]AB14
MTALLTVEGLSKAYPGVVANDQVGFAIDEGEVHALLGENGAGKSTLVKMIYGLVQPDSGRMTLRGAPYAPSEPSAARR
AG
VAMVFQHFSLFDALSVAENITLGMETPPPMRRLADQIREVSETYGLPLKPDRLVGDLSAGERQRVEIIRCLLQDPRLLIM
DEPTSVLTPQEVEILFATLRKLAAEGTSILYISHKLEEIRSLCDRATILRLGRVVESCNPREKSAAELAEMMVGAKLATP
ARAGGTPGAAVLEVRALNLPSPSPFGTSLRDISFTVRAGEILGIGGVAGNGQDELLDALSGEAPVGPDTVLLHGQPIGAL
GPSLRRRIAVLAAPEERLGHAAAPDMSLTENAMLTAAIRQGLASRGFLKWPAARAFAERVIETFDVRTPGPQNAARSLS
G
GNLQKFVIGREVLQRPEVLVVNQPTWGVDASAAAAIRQSLLDLAASGTAVIVISQDLDELMEISDRFTALNEGRMSAPR
P
ARGLSLEEIGLMMGGAHGMEVAHVAE
>OLS49578.1 sugar ABC transporter permease [Rhodovulum sulfidophilum]AB14
MIRLEKRPSPSRVWTVATPVLAVALTMVAGGILFAMLGKDPVAAIRTIFWDPLFNAQFASYSRPQLLVKAGPLILIATGL
SLGFRAGIWNIGAEGQYIIGALCGAGVALALYPLDSFIVFPLMVLAGGLGGWAWAMIPALLKTRFRTNEILVSLLLVYVA
EQILAAMALGLMRNPEGGGFPGSRNLQQYPAAANTELIAGTGMHWGVVTAFIAVIAAYVLLNRHMLGFQIRLTGQSP
RAA
RFAGVNPNRLVTICLGASGALAGLAGLFEVSGPAGQVSIDFNQGYGFTAIIVAFLGRLHPVGILLAGLLMALTYIGGELA
QFTLGVPAAAIQAFQGMLLFFLLGVDVLSNYRIRLGRRGVA
>OLS49579.1 ABC transporter permease [Rhodovulum sulfidophilum]AB14
MDLSAINPVVLIASLMVAATPILLAAIGELVVEKAGVLNLGVEGMMITGAICGFAAAVETGSPALGFLAAMLGAALLSLV
FGLLTQVLMSNQVATGLALTLFGLGLSALIGQRYVGLKPPATARLDLGPLSDIPVLGRILFSHDAVVYLSLALVAAVWAF
LRFTRTGLILRAVGENHDAAHALGYKVVRIRLAAIAFGGACAGLGGAYLSLVRVPQWTEGMTAGAGWIALAIVVFASW
RP
TRVLLGAYLFGGVAVLQLNLQAAGVAVPVELLSASPYLVTILVLVIMSADRGRAALNAPASLGRVFHAST
>OLS49580.1 BMP family ABC transporter substrate-binding protein [Rhodovulum sulfidophilum]AB14
MKLNAILAGAALALGLAGAAQAEDKTKIGFVFVGPIGDGGWTYEHNQGRLAVEREFGDKIETVYQESVPEGADAERVL
TQ
MALSGAKLIFTTSFGYMDPTINVARKFPDVKFEHATGYKRAENVGTYSARFYEGRAVQGLIAGRMTESNIVGYIASYPIP
EVVRGINAAYLHAKKVNPDVQFKVIWAYTWFDPAKEADAATALIEQGADVILQHTDSTAPQAAAEKAGNVITFGQAS
DMS
EYAPSPRVSSIIDNWTPYYIERIQAVLDGTWESDDTWDGIGPGMVKIGEISSAVPEDVKAEALTLKAALASGDYHPFTGP
LNKQDGSPWLAEGEVADDATLLGMNFFVEGLTGEIPQ
>OLS49581.1 glycerol-3-phosphate dehydrogenase [Rhodovulum sulfidophilum]AB14
METDFIVIGGGIAGVSVGARLSHLGRVTLFEAEKALGCHASGRSAALFDECLGAPEIVTLNRAGRAYHETANGGVLTPRD
LMVIARAEDRAAFERDCRHMALAQISPAEARARVPILAPGPLAFAGLTDRAWDIDTDRLLQNFAREIRAQGSRIETGRR
V

TAIARVATGWQVRTGDDITEGRILVNAAGAWADEVARMAGLTPLGLTPMRRSMAQLPAPAGHDIRTWPMVIGAGE
RWYCK
PEAGKLLVSPAEEDPMPPHDAYADDMVLAEGLDRYQEMVTEPVTRIEANWAGLRTFAPDRLLVIGPDPRDPDFFWH
AGQG
GFGIQTSPAASELAADILGDRASELRRSLAPAFSPARFG
>OLS49582.1 methyltransferase [Rhodovulum sulfidophilum]AB14
MPKRLTLPDSASLATPDAGGRLSAPSALRNAEAICATLERVAPARGRALELASGTGEHAVRLAARLPGLVWQPTEIDPQ
R
RASIDAWAAEATLPNLRPALALDACQPGWGASHAGQDLIFASNILHLVSNAQAETLIAEAALALAPGGMLAIYGPFLRD
E
GFASPGDAAFHASLIAQDPGIGYKPVAWVADRGKAAGLRLEAQISMPANNLTIIFRQPSVIAQRNH
>OLS49583.1 alkylhydroperoxidase [Rhodovulum sulfidophilum]AB14
MDWTAKRDGTKDQLRRLNKALPDQTKAFGALGKAVKEGGELDFKTKEFIALGIAITQRCQACIVLHMEALVRVGATRE
EV
GDVLAMAIQMGGGPAMMYAAEAMECYDHLIEEKAQAAE
>OLS49584.1 hypothetical protein BV379_15740 [Rhodovulum sulfidophilum]AB14
MRRPTLSVLALSGFVLALAAGAASADTQRAPILYEKWDADRSGTISRFELQSQAPRIFAEFDTNGDRHLDAAEAAGFDR
A
RGPLAYFRGQEAEERKVVVKGQKLMRNDLDGNDLIELAEFRLAAYEWFAMHDRDGDGRLTLWDVSAR
>OLS49585.1 diguanylate cyclase [Rhodovulum sulfidophilum]AB14
MKNHVKALVVGGGAVGTSIAYHLARAGWDTMLLERDELTSGSTWHAAGLLPYFNMSFATTHIHDYSIRFYKTLEAETG
LN
PGFSVVGNLRMAQSQARMDEYRLYASTAETCGVPYEWLTPAQMKERWPLMRTEDLVGAIFHPTDGYINPADVTQA
MAKGA
RQFGATIERKWQVDGYQWTGSEWRVTLTKMAEKGGNLVPTEEQTVVTAEHVVTATGNHAQRTARLLGLKIPAIPVE
HQYI
VTEPDPALVEWRKAGNPEHPVLRDADAKWYVREERGGWILGPYERNAPARFAYGCPDSFRADLFPLDLDRIEEEYMS
MIH
RLPSSETVGLKDDYNGPICYTPDGNPLLGPAPGLRNMWLAEGFSFGITAAGGSGYYLAQLMTEGEAEIDMGALDPRRY
GS
WMTTDYAVAKNEEAYEHVYILHHPDEERPAARPLRTAPCYDRMKALGAQFGQVNGWERPNYFAPEGFDDHAARSFR
RGGW
WEYTLAEAKAIRETAGLIDATAFAKHVVRGPGATAFLDWFTCNKLPKVGRVNLTYALTSAGTVRTEYTIVRQAEDVYYLV
SAGGWHAYDEDFLLKAVEDKAAEFGPVIVEDVTTRHGVFALAGPNARAILAGLIRDADPATALSNKRFPWLSARQIELG
M
VPVNALRVAYTGELGWELHHPIEMQNHLFDRLMEAGAAHGLKPVGARAQNWLRQEKSYRAFGTELGRDATPLEAGL
DRFV
DLSKEFHGKAAMEAKGIRSKCVTLLIDGPEDADPWGREALYDGETRVGRLTSGGYSVAFGKSIGMGYVRPDLAVPGTR
LS
VRMFDALWTAEVVGDSPHDPSNARIRADG
>OLS49586.1 hypothetical protein BV379_15750 [Rhodovulum sulfidophilum]AB14
MTEATASRPAFLFLLTLAAYFAAQVGLRVALGGSFEGDEAEMVVLARDWHFTSGSQPPLYNWYQTVFFDLFGVNTLGL
VL
AKNLMLFATYATMFLALRKLAGDRPAMVGALALGLIPNLAWWAQRTGSHSIALAAMTALTVAAFLALVRRPGLSRYLL
LG
LAVGLGGLAKPNYWMVPPALILAALSTATYRAAILDRRIWLSAALALGVVAVPYAAMLAEPLATFADTWEFRSGSGAG
QA

AAMWPDGLRHVAKATMAELIPALLAVALALGFGGKAMRRIPVPHPEAALLMRAALIGLGLVALGVVATDVGFFRSRW
LLP
LFVLGLPPLMMTLFQQASARAWRNFLRGMACLALVILAAIADTRLRGAGSDSLRVDLLAEAIEEAVPDVPPLLGPHYFT
G
NLLLNRPDWTAFPPFPTSRLEHPTGRVLVIEEKRREGEIIGAMREQGYRGEAPPAPVLSLDVLVPYRFDEGEPRLIGLSL
YDFGPGSD
>OLS49587.1 hypothetical protein BV379_15755 [Rhodovulum sulfidophilum]AB14
MAMDDAADRRATAIFLLAISVYFAAQVGLRLALGGAYETDEAEMLVMTPGLRLGYGPQLPLYNWLQLGFFELFGRGLL
AL
SLLKNLLLWLTAFLAFLGLRIWLPASGAALAALSLFLLPDYAWEAQRATTHSNMLLVTSAATLAAFLWAVKGGRLIAWA
L
LGIAIGLGGLAKYNFYLVPLGLVLAGLTLAPLRAQILRRRALIAPAIAAAIVALPYRWMIANRDLAFSSVGKLQLEAEPV
RQMLPAGLLRLPEGLLALLGLALIVAAGLWLFGRSRGKPVGPGPVAALLLRAAIVATLLTAVGVWLAQAGTLTARWLLP
L
AFLSVLGIFAALAPRLGRGGRVGLVGFLGLVALLVFTGLTYDRFKEGARRDVDFSLLPERLAAIEGSEGLPVIAEFYTAG
NLAARQPLRPVAPYLPFAARRFEGETVLFLLRETVPPSFEVGLERAGWPAFARFDILSEGDFTLPYMHSDEDMPFRYVLA
RPVAQP
>OLS49588.1 D-glycerate dehydrogenase [Rhodovulum sulfidophilum]AB14
MPAQRLSVVVTRRLPAPVETRMKELFDVELNEADVKMSREALVDAMRRADVLVPCVTDMIDQAMLAQAGDRLKLIA
NFGA
GVDHLDVQTARQRGILVSNTPGVVTEDTADMTMALMLAVTRRIPEGLHVMQAGEWRGWAPTAFMGGRIGGRRLG
ILGMGR
IGQAVARRARAFGMQIHYHNRKRLRPEIEEEFEATYWESLDQMVSRMDVISVNCPHTPSTFHLMNARRLKLMKPDAV
IVN
TSRGEVIDENALTRMLRAGEIAGAGLDVFEHGHEINPRLRELSNVVLLPHMGSATIEGRGEMGEKVIINIKTFADGHRPP
DLVVPSML
>OLS49589.1 cation-efflux pump [Rhodovulum sulfidophilum]AB14
MPETSRLNLSAGLASVATAILLVALKLWALGQTGALSIAASLADSALDLMVSLGALAAIFYAARPPDDDHAFGHSAAEDL
AALGQAVFILASASIIAFAAMTRLLSADPEPLSQEGPGIVAMALSITLTALLVLYQRWVARKTGNRVVAADSLHYLGDLL
PNIGAIFALWASANLGLMRIDAVVALAAAGMLAVGALGIGKGAWDALMDRAAPPETIARIEALTKGCTGVRGFHDLK
TRR
SGQRLFVNLHIELDGAQSLNDAHDIGARLKHAILDEFAGADVIIHFDPVR
>OLS49590.1 molybdopterin biosynthesis protein [Rhodovulum sulfidophilum]AB14
MGLVLGIALVLWSVGMLLRLSRQLRWGMIAGLYLAVLIVQIVMPEGHALRHAIGGTPEGWAALGVVGLLVVAYRGVL
GAL
KRRVAREVVPMAPKGPLKPVELDRYARHIMLREIGGPGQRKLKDARVLVIGAGGLGSPALLYLGAAGVGTIGIVDDDYV
D
LSNLQRQVIHTDTRLGMPKVFSAQKAILAQNPFVTVRPYRRRLDEATAEALFPDYDLILDGTDNLETRYLVNRMAARLG
K
PLIAGAITQWEGQISLYDPAHGTPCYRCIFPEAPAPGLAPTCAEAGVIGALPGVIGAMMAVEAIKTVTGAGEGLGGRML
I
YDALYGESRMITLHPRSGCPDCGAA
>OLS49591.1 deoxyuridine 5'-triphosphate nucleotidohydrolase [Rhodovulum sulfidophilum]AB14
MSPRLEIVWEDWADRDLPLPAYQTPGSAGADIRANLPPEIRDEGMTLAPMERRIVPTGFRMAFPVGFEVQIRPRSGLA
LK
HGITLPNTPGTIDSDYRGPLGVLLVNFGAEPFTIRHGDRIAQMIVAPVVQAAFEVVEDLDETLRGAGGFGSTGRG
>OLS49592.1 bifunctional phosphopantothenoylcysteine decarboxylase/phosphopantothenate synthase
[Rhodovulum sulfidophilum]AB14

MVSGKRILLIIAGGIAAYKTLDLIRRLRERGAAVTPVMTAAAEGFVTPLSVSALAGEAVHRDLFDLTREAEMGHIQLSRA
ADLVVVAPATANLMAKMAGGLADDLASTLLLATDKAVLLAPAMNVRMWTHPATRRNLATLQSDGIRFVGPTEGDM
ACGEY
GPGRMVEVPEILAAIETALGAGPLAGRRIVVTSGPTHEPIDPVRYIANRSSGAQGTALARALVALGAKVVFVTGPASVPP
PAGVDLVRVETAREMLAAVEAALPADAAVFAAAVADWHVANAADRKMKKDGSGRAPTLDFAENPDILARIAGRAEG
RPRL
VVGFAAETHDVEAHAEAKRARKGCDWILANDVSPATGIMGGAENEVSLITAAGVETWPRLPKDEVAARLARRIAEALA
>OLS49593.1 RNA polymerase factor sigma-32 [Rhodovulum sulfidophilum]AB14
MAFDGFDANSLSRQAMKAELLDAETELRLAYAWRDERDEAALHRLITAYMRLAISMAAKFKRYGAPMNDLIQEASLGL
MK
AADKFDPDRGVRFSTYAVWWIKASIQDYVMRNWSMVRTGSTSSQKSLFFNMRRVQARLEREAAGRGETLDRHQLR
QMIAS
EVGVPLHDVEMMEGRLSGSDFSLNATQSTDDEGREWIETIEDDSAQAADLVEDGRDRAQLRGWLVHAMGQLNDRE
RFIVK
ERKLRDDPRTLESLGNELGLSKERVRQLEAAAFGKMRKSLEAQSPEVFHFLA
>OLS49594.1 glutathione S-transferase [Rhodovulum sulfidophilum]AB14
MTYVLFIGDRTFSSWSLRGWLMLEKFGLPFRVEEVGLYTGTLAADLTPVAPARFVPALMMPGGEPLGDTLAMAETLAE
RH
PEAGLWPDDPAARMRARWLVAEMHSGFAALRSHCPMALIRSYDGFRPPPEVLAELDRLQALWNEAQTRFGAGGPW
LFGRY
CLADVFFAPVAARIAAYGLPVGDAAAAYVAQHLADPAFRRWRAEGLTFRYAPEPHAMDLPHLPWPGPEPVPARAVA
HGPA
INAACPVTGRPPTHFLEINGAVIGFATLSARDMVANDPEAFPEVAAIYHSVTKQA
>OLS49595.1 AAA family ATPase [Rhodovulum sulfidophilum]AB14
MVARAYTVAFDGIEARIVEVQCAVAAGLPAFSVVGLPDKAVSEARDRLRAALVAMGVALPSKRITVNLSPADLPKEGSH
F
DLPIAVALLAALDVIPRDMAEGMVALGELSLDGRLVPVIGALPAAMAAAEAGRALLCPKACGAEAAWVGATQVLAAP
DLA
SVVRHFTGQAPLAPAEPGEIAPEADPLDLSDVKGQERAKRALEIAAAGRHHLLLVGEPGSGKSMLAARIPGILPPLSPAE
ALETSMIHSLAGLLDEGGISHQRPFRYLHHTASATAIVGGGRDAWPGEISLAHNGVLFMDEFPEFPSKVLDTLRQPIETG
EITVSRANAHSRYPCRFLLVAAANPCKCGYLADPARACARVPHCGEAYLGRISGPLMDRFDLRIEVPPVAFRDLDLPASG
ESSAQVAKRVAAARALQNARFEHAPSVRVNAEAEGALLEDVAAPDAEGRALLLRAAERFRLTARGYHRVLRVARTIADL
D
GADRVARPHVAEAVNFRLAIGNL
>OLS49596.1 hypothetical protein BV379_15810 [Rhodovulum sulfidophilum]AB14
MIKTRGLAALLVLSTFNLTLMVPGGFVETRSFPGYSVTVLAAFNIFLTLLGLGSLILAYRVVKSGRVGIFPALAGAAFMA
VYLLDLATIFPVAAAPMSTTLATMEWIGTFLGLALLATGGWLAVSQDKRPSGSSRLPLGLLVAMGVVTVAIVTFATLSA
M
>OLS49597.1 hypothetical protein BV379_15815 [Rhodovulum sulfidophilum]AB14
MEFCEHGFENASLNRIIAEAGESKGRTYHYFADKGDLFRATLERRLARLGTLDGTTEAIAGAERQAYWTRIAELCRRLAG
ALQEDERLASLLRTLHQEAAAQRAFAEPLAQLRAQIERVLASGQSVGAVRDDLPLGLLADVALNLLVTVDRWFAINASD
L
AEGEEARFAERAFSLLMTPLLPPDYSGKTDT
>OLS49598.1 glutathione synthase [Rhodovulum sulfidophilum]AB14
MPLKVAIQMDPVGAINIDADSTFRIAEEAQARGHSLFYYTPDRLFWNDGRVMARGWPLEVRRVRGDHFTLGEEQEV
DLGD
FDVVWLRQDPPFDMGYITTTHLLEMVMPGTLVVNDPFWVRNFPEKLLVLRFPDLIPPTVIARDLATLSAFKDLHGDIILK

PLYGNGGAGIFRLGPDDRNLASLHEVFAGMNREPLIAQKFLPAVRSGDKRVILVDGAPVGAINRVPADGETRSNMHV
GGR
AERADLTPRDLEICAAIGPTLKEAGQVFVGIDVIGEHLTEINVTSPTGIQELERFDKTNIAARIWEAIEARRAG
>OLS49599.1 hypothetical protein BV379_15825 [Rhodovulum sulfidophilum]AB14
MSGTVSYHAGRSAEDCVARGYEGRGMAIAARRWRGAGGEIDLVARDGDALVFVEVKQSRSFSSAAERVTRRQMDRV
MMAA
QEYLAGEPGGQLTDIRFDVALVNGFGEVDILENAFCA
>OLS49600.1 16S rRNA (cytidine(1402)-2'-O)-methyltransferase [Rhodovulum sulfidophilum]AB14
MEILRNPLAPGLYFVATPIGAARDITLKALDVLASADVIAAEDTRTARHLMQIHGIALGERSLIAYHDHSAASVRDALVE
MAAAGKSVACVSEAGTPMVSDPGFPLARAAIAAGVAVHAAPGPSAMLAALVVAGLPSDRVLFAGFPPAQSGARSRM
LEEL
SNVPATLVFYESPKRISRLLAEMAVSLGGDRDAAVCRELTKRFEEVSRGSLVELAEVFRGREVRGEIVLLVGRAPVRAVE
QNDLEATLDEALVRLSVKDAVAEVAAALDLPRRRVYQAALLREKRDAGPEEAE
>OLS49601.1 penicillin-binding protein activator [Rhodovulum sulfidophilum]AB14
MFAVFRACRKRLPSVRPRVLASVLGLGALALVAACQPGGIGGGPSIDGSKPVPVALLVPGGSANAGDQVLARSLENAA
RL
AISDLKGVKVDLRVYQTAGNPDIAAKVAAEAVSDGAKIILGPLYAQSANAAGLAVARRGVNVLSFSNNPQIAGGNVFVL
G
PTFENTADRLVRYAATQGRTNVMIVHDPDVAGQAGRAAIASAVARNGGSVAATGSYELSQNGVVQAIPDLASKARGS
GAS
AIFFTASTAGALPLLAQLLPENSVDPAKVQFIGLTRWDIPPSTLALPGLQGGWFALPDPGVSDRFERRYTAAYGEPAHPA
AGLAYDGIAAIGALASQGRSDALSVSGLTQPSGFVGVNGVFRLRTDGTNERALAVAEIRDNQVTVIDPAPRNFGGAGF
>OLS49602.1 murein biosynthesis integral membrane protein MurJ [Rhodovulum sulfidophilum]AB14
MRPIRLVSGFLTVGVWTLASRILGFARDILIAAYLGTGPVAEAFLVAFSLPNMFRRFFAEGAFNMAFIPLFSKKLERGEG
ALDFARDAFTGLATILIGFTLLAQIAMPALVLAMAGGFAADQRFDIAVVYGRIAFPYILFISLAALLSGVLNATGRFVAA
AAAPVLLNILLVGAMLLAAAMGWPVGDALVWTVPLAGIAQMALVWTAAAKAGFRLTFRRPRLTPELRRLAIIAAPAAL
AG
GVVQVNLLIGRQVASFFEGAVAWLSYADRLYQLPLGVVGIAIGVVLLPDLSRRLGAGDTAGGRDAFNRAGEFALALTLP
A
AVALATIPGPIVAVLFQRGAFDQADVAATALAVAVYGAGLPAFVMQKVLQPLFFAREDTRRPFHYALGALAVNAGIAIG
L
APLCGYIAAAWGTTLAGWAMVALLWRGSRRFGSAAEADARFLHRTGRIALASALMGAILWLAARGAQAWLTAEGLR
YPAL
AGLVALGILSYFGLGHLLGAFRLSEFRRAMRRQR
>OLS49603.1 rhomboid family intramembrane serine protease [Rhodovulum sulfidophilum]AB14
MQSDPNAPPINPLPAVVWLLVLPVIAMEVVLSLGARGLVGGPGAVGWRLQAIRDYGFSGQVFDWMLQSGQFPADQ
MIRFV
TYPFVHASLTHAVFVVVFILALGKMVGEVFRAWAVLLVFFGAAIVGALAYGLLLDTPTPLIGGYPAVYGLIGAFTFLMWV
RLAGTGPTQYRAFTLIGVLLGIQLLFGLLFGGGFDWVADLAGFCTGFVLSFVVSPGGWARVRAKIRQR
>OLS49604.1 tryptophan--tRNA ligase [Rhodovulum sulfidophilum]AB14
MAEVVHTPRVFSGIQPSGGLTLGNYLGALKRFGETQDQGEMPTIYCIVDMHAITVWQDPEKLRHATRALTAGFIASGI
DP
TKSILFNQSAVPGHAQLGWIFNCVARLGWMQRMTQFKDKAGKNSENVSLGLFAYPALMAADILVYHATHVPVGEDQ
KQHV
ELTRDIAIKFNNDYGTDFFPVPEPVIEGAATRVMSLRDGTKKMSKSDPSDMSRINLTDDADTIAKKIRKAKTDPEPLPET
LDGLEGRPEARNLVNIYAALSGKAAAEVVAEYAGQQFGTFKPALADLAVERLSPISSEMSRLMQDPAEIDRILAHGAEQ
A
NAIAAPIVRRARDIVGMVGA

>OLS49605.1 glyoxalase [Rhodovulum sulfidophilum]AB14
MTPVPAETRIGHIHLKVADLDRAIAFYSGILGFELTQRYGPGAAFLSAGGYHHHIGLNTWESLGATPPPPGHTGLYHTAI
LFPDRASLGAALARVLAAGISLTGAADHGVSEAVYLNDPDGNGVELYRDRPEADWPRDAEGRLRMVNHPLDLDALLA
EAG
PQADTEFTEPTQA
>OLS49606.1 branched chain amino acid aminotransferase [Rhodovulum sulfidophilum]AB14
MATGTNIRTYYNGTWHRGDIPVMCAADHGAWLGTTVFDGARYFEGIAPDLQAHCERINRSAEALMITPTTDGADMA
EIVR
EGLKTYPADAAVYIRPMYWALEGDSLGVAPKPGATGFAICLEEIPMAAPGSATTMTTTRFRRPTLDCAVVNAKAGCLYP
N
NARMLAEARAKGFGNALVADALGNVAETATANVFIVKDGEFFTPIANGTFLAGLTRKRHISNLTAAGYKVHEAVLSFED
V
RQADEVFLSGNMMKVTPVKAFDDVQYQAGPLTAKVREIYWDWAHSAA
>OLS49607.1 universal stress protein [Rhodovulum sulfidophilum]AB14
MRKFLVVLDDSRECLNAMRFAALRAAKTGGGVQILSVIAPEEFNHWIGVGDLMREEARERIEAHYEVFAKWMRDRQ
GIDP
ELVIREGEAVPEILDQIREDPEIGVLVLGAGTDSKGPGPLVTQLARSSGSLPIPVTIVPGGLSKERLEAVC
>OLS49608.1 NifU family protein [Rhodovulum sulfidophilum]AB14
MFIQTESTPNPATLKFLPGQAVLEAGTADFPTADSSATSPLATRIFGVEGVRGVFFGTDFVTVTKADAVEWDHIKPSILG
AIMEHFQSGQPVMTGENADAGHAEHDGPDEDIVRQIKQLLDTRVRPAVAQDGGDITFHGFDRGVVYLHMQGACAG
CPSST
LTLKMGIENLLRHYIPEVTEVRPVADAM
>OLS49609.1 tRNA (adenosine(37)-N6)-threonylcarbamoyltransferase complex dimerization subunit type 1 TsaB [Rhodovulum sulfidophilum]AB14
MGRGIAPILAFDTSGPHCSAALLRDGEIVLSRHLEMARGQAEALMPLLGEVLDEAGLDWSDLGGLGVGVGPGNFTGIR
IS
VAAARGLALAAGLPAIGVSMFQVLRRSAPGPETDLLSIEAPRGLAYVQPLRAGKLGAARMIDPEAPVPLAEIGADGIAH
V
TGFAARRLGTAFGTGWHEAVPAEIAPAIAREAAALLSGGTASPRPAPLYVRPADAAPPADPPPAILP
>OLS49610.1 ribosomal-protein-alanine acetyltransferase [Rhodovulum sulfidophilum]AB14
MTPAVLAALHARCFTTPRPWSAAEFEALLATPGTFLCGDAQAMALGRVAAGEAELLTIAVAPECQRTGLGRARLAAFE
AG
ARARAAEEAFLEVATDNAAACALYRAAGFELAGCRPRYYTAPDGRGIDALVMRKALSSQG
>OLS49611.1 BMP family ABC transporter substrate-binding protein [Rhodovulum sulfidophilum]AB14
MTKMQKALAAATALALTAGNALADPALIYDVGGKYDKSFNEAAFTGAERWKAETGGSYREIELQSEAQREQALRRFAE
AG
LNPIAMTGFAFKAVLEEVAPDYPDTKFEIIDETVEAPNVRSIVFSEHEGSYLVGMMAVMASETNTVGFVGGMDIPLIRK
F
ACGYAQGAKAVGADVTVLQNMTGTTADAWADPVKGSELARAQIGQGADVIFAAAGTTGLGVLQAAADEGILSIGVD
SNQN
HLHPGQVLTSMVKRVDLAVYEAFSAGPEMKTGSFRLGLADDAVGYAMDDNNRELVTPEMIAAVEAAKAKIISGEIAVH
DY
MSDNTCPVN
>OLS49612.1 heme ABC transporter ATP-binding protein [Rhodovulum sulfidophilum]AB14
MAGAALSGADAAVPALELRGISKAFGPVQANRDISLRVRRGTIHGIIGENGAGKSTLMSILYGFYRADAGEILIGGRPVR
IADSQDAIRAGIGMVFQHFKLVEPFTVLENIILGSEEGPLLSRSLGKARRALRKLAADYGLEVDPDARIEDLGVGMRQRV
EILKALYRQADILILDEPTGVLTPAEADHLFRILENLKSQGKTIILITHKLREIMAATDTVSVMRRGEMTATLATSQTSP
AELAERMVGRKVLLRTDKSPARPGRIVLEIEGLCVTDAEGVRRLSDVSLQLRAGEILGIAGIAGNGQSHLLEVLGGIRPA

TGHIRIDGKELDLSGPRGDARARRAVGIAHVPEDRQREGLIMPFHAWEDSVFGYHTDPALRTRLRLMDNARIRDRAGA
QM
ARFDIRPPDPDLTAQSFSGGNQQKLVLAREIDRNPDLLLIGQPTRGVDIGAIELIHRRILELRDAGKAVLLVSVELDEIL
NLSDRIAVICGGRIMGERRADETDETELGLMMAGLGGRAA
>OLS49613.1 sugar ABC transporter permease [Rhodovulum sulfidophilum]AB14
MSAMPKWADLVLIPLISLAIAGLLSALVILGIGEDPVAAVKMMVSGALGSTYGWGYTLYYATNFIFTGLAVAIAFHAGLF
NIGGEGQATLGGLGVALVCLALPWPHWALALPAAALGAALFGAAWAAIPAWLQARRGSHIVITTIMFNFIAASLLNYL
LV
NVLRPAGAMDPSSARFGDGTHLPTLHELLAPFGIAFSKAAPANISFLLALAACLAVWLLIWRTRLGYEIRAFGHSEPAAI
YAGIHPLRTVMWAMLISGALAGLMAVNTVMGEAERLVMNSVEGAGFIGIAVALMGRSHPLGVLLAAILFGFLYQGGA
ELA
LWTDIPRELIVVIQALVILFTGALDNMVRMPLERLFVALRKGSA
>OLS49614.1 sugar ABC transporter permease [Rhodovulum sulfidophilum]AB14
MDFPTLLQVLDSTVRLATPLLLACLAGLFSERSGIYDIGLEGKMLAAAFGSAATAAVTGSVWLGLLAGIGSSMALAGLHG
LSAITFRGNQLISGVAINFLAAGLTVVLAQDWFHQGGRTPTLPAGARFEAVTLPFAETLRPVPGLGPVYAELISGHTLLV
YVALALVPVSWWGLYRTRFGLRLRAVGENPEAVDTAGVSVPATRYAAVAICGALCGLAGAYLATSLQAGFSRDMTAG
RGY
IALAALIFAKWRPWQALAATFLFGLLQAMALRYQNIELAGVAFPAQVMDALPYILTVVILAGFVGRAIPPRAGGEPYVKE
RKI
>OLS49615.1 anion permease [Rhodovulum sulfidophilum]AB14
MQIYLPIAEVSVNAFLLLGLGGLVGILSGMFGVGGGFLLTPLLFFIGIPPAVAVATQAAQIVASSISGVLAHFRRKTVDL
RMGSVLLAGGMAGALLGVWIFNLMRGIGQVDLLVKLCYVVFLGIIGALMFVESLAALNRTRKGVVRKRKQRTWFAAL
PFK
MKFRTSGLYLSAIPPLIVGAFVGVLSAIMGVGGGFIMVPAMIYLLGMPTKVVIGTSLFQIIFVSGFTTILHATTNHTVDL
GLALLLLIGGVVGAQIGTGIGLKMKAEQLRILLAALVLAVCAKLALDLLIEPTELYSIADRVR
>OLS49616.1 hypothetical protein BV379_15915 [Rhodovulum sulfidophilum]AB14
MARRLALLLPLLLLAALPARAESVVAGLSQTRVSITTNFAGSEILIFGAVKRDAPAPEAPPLEVVVTVAGPSTPATVRKK
DRRGGIWINTESVRISAAPSFYAVAATGPLERALSQTEDLRHQVSIPRAIRAVGVTQETPEAQSFIDALIRIRSETGLYQ
ELPGAVELDEQTLFRTSIALPSNLTEGEYATRIFLTRGGEVVDAYETTIGVHKVGLERWTYLLAHEHPLAYAILSLAIAI
AAGWGASAAFRIMKS
>OLS49617.1 short-chain dehydrogenase [Rhodovulum sulfidophilum]AB14
MTGRTILITGASSGIGRATAELFLDHGWRVGLLARRADLLDEIAAGREAALALPADVTDAGAVEAAFDAFMEWAGRLD
VL
FNNAGTFGPSGPIDEIDPADWAGVLSVNVGGMFLAARAAFARMRGQDPQGGRIVNNGSLSAHVPREGSVCYTTTKH
AVTG
LTKTLSLDGRPFGIACGQIDIGNARTALVEDLTARARAGERPVQPTMDVAHAARSVLHMAELPPEANVQFMTVMATT
MPY
IGRG
>OLS49618.1 ATPase [Rhodovulum sulfidophilum]AB14
MTLPELRFSDDQAEAHDRVTELLRGFGVDLDEGSHSARTEGKTGVLAITGKAGSGKTLLLAELVRAAVEAGVEIVSGDYE
SKRRKERRTLAVLAPTNKAASVLRQRGVPATTIHRIIYTPVYDPEYEKIAEWLTGECDRPEIEGLTEAALDRAQAFYASV
ASVPGALAAAGLKGSDFIKGWKRREDPLDIGFVDEASMLDERQFDDLRELFPALVVFGDPAQLAPVNQSGQMVFDRL
AGP
QTMELHRIHRQETDNPILDLAHALADPEVDFERFERMVEEAAARDPRVELAPRVDSDLMARSPVLVWRNATRIRLIHA
FR
HAHGAPADALLPGEPLICDGIELPLKHRKKRIDLEARGLIKGAQVIYVGPGRKPGFSIMHVIGAEDPRLSVASIVKIEMP
EEEEPFIPYAATMGAAFLHGAAVTIHKAQGSQWPAVQVFAPDLYAASRAGRVEAGQPLWKRLAYVAITRAEERLLWV
TRY

RLARPAGALSADDLTAAPAPLTLTAEEARP
>OLS49619.1 autoinducer synthase [Rhodovulum sulfidophilum]AB14
MLRYIYGVDLHKFPLLRDTMFRDRARQFHDRLGWAVTVNDRGEERDQYDGLNPLYVIWEQANGRHGGSMRFLPTT
GRTMV
NEHFLHVTGGVRIESPLIWECTRFCLAPDAGGEVTAGLVLGAGEVMAQFAVEHFVAVFDLRLARIYRVMGVEPDVIGA
AG
NGRNWIGLGLWEMQEAAWPRTLARLGIGRETSLGWFRASFSAPTAAPRELAVA
>OLS49620.1 protein meaA [Rhodovulum sulfidophilum]AB14
MTETTKDRPWLFRTYAGHSTAEASNALYRANLAKGQTGLSVAFDLPTQTGYDSDHELARGEVGKVGVPIDHLGDMRT
LFD
RIPLEQMNTSMTINAPAPWLLALYIAVAEEQGADVSKLQGTVQNDLIKEYLSRGTYICPPKPSLRMITDVAAYTREHLPK
WNPMNVCSYHLQEAGATPEQELSYALATATAVLDDLRGKVAPEDFPGMVGRISFFVNAGIRFVTEMCKMRAFVELW
DEIC
QERYGVEDPKYRRFRYGVQVNSLGLTEQQPENNVYRILIEMLAVTLSKSARARAVQLPAWNEALGLPRPFDQQWSIR
MQQ
ILAYETDLLEYEDLFEGNPAVAARVEKLKHDARAELAQIDAMGGAVAAIEHMKSRLVESNSDRIGGVETGATVVVGVN
AW
TEGEPSPLTSGEDAIMVVDASVEADQIARLAAWRETRDGAAVEAALAALREAAATGRNIMPPSIEAARAGATTGEWG
DVM
RAVFGQYRGPTGVSKNPSNRTEGLDEIRAEVDALAARLGRRPKFLVGKPGLDGHSNGAEQIAARARDCGMDISYEGIRL
T
PSEIVAAARAEGVDIVGLSILSGSHIPLMESLMRLMKEEDLTHIPVVVGGIIPEEDARRLRAMGVAKVYTPKDFELNRIM
FDVVALISPEQVAAE
>OLS49621.1 purine-nucleoside phosphorylase [Rhodovulum sulfidophilum]AB14
MSLHIGAQPGEIAETVLLPGDPLRAEWAARTFLDDPLCINRVRGMLGFTGFWRGHRVTIHGSGMGMPSLSIYANELIR
EH
GAKTLIRIGSAGAMQAHVALRDIVLAMTSSTLSTPSRGTFRELNFAPCADFGLLAAAHRAAVSKGASPHVGGIYSADAFY
DERPDLNAELTRHGVLAVEMEAAELYILAARHGARALAVLTVSDHLLTGAALSSDDRQSGFGEMVEIALEAAFS
>OLS49622.1 hypothetical protein BV379_15955 [Rhodovulum sulfidophilum]AB14
MPTEPRPEPADAAERRAVPPVVCYPPESLPAPPMALYDGLRAGMTRVAQLEIPARDARAFRVPAGHVLRISCTQGPQV
GD
LNLWNAHDLSERFYSGKTRALHGTHVTTGDRLWSGFPHLRPMVTVIADSLGWYGFDADGAGVHDVIGTRCDPYTNA
RLGG
APFHRCCHSNLIRALAAETGLAPDAAEPLVHDVLNVFMCTGFTRDSHRYFTKASPVRPGDHLDLFAEIDLLCALSACPW
G
DCSGNSTDHARCHPLGVEIFAPAPGALDGWTPPEPAPYDRSHGL
>OLS49623.1 hypothetical protein BV379_15960 [Rhodovulum sulfidophilum]AB14
MLRDILILATAAAGLVLILSPPMRRWRFWQAMVTPLASIIGSGFLVLGPILNQSLGVWSPAAMALLALIAFGFGGAIRYS
IAAGAHGSPPGRLGALAETAAGWMLAFSYVISVAYYLNLLGAFAVRLTPFDTPLAARAVTTAVFLLILATGLTRGFKALE
GLEKYAVSLNLAAICGLLVGLGLFFGDTATTTGPVLTPASVEPWAALTLFFGLIVTVQGFETSRYLGAEYDADLRIRSMR
AAQYVSTAIYLVYVTLLVLCFAPGKGALDDTAIIGMTAAVSPLLPLVLTVAALAAQFSASVADTTGAGGLVAERSKGLIG
NRAAYAVLVMAGIALTWTADIFAIIAYASRAFALYYALEAGIAALAARRAGQGGRAAGFAALAVLGLAIALLGRPVE
>OLS49624.1 succinate dehydrogenase iron-sulfur subunit [Rhodovulum sulfidophilum]AB14
MVQLTLPKNSTVRKGKTWPKPEGAKNLRTFRIYRWDPDTGENPRVDTYFVDLDSCGPMILDALIKIKNEIDPTLTFRRSC
REGICGSCAMNIDGVNTLACTRGMDEIKGDVKIYPLPHMPVVKDLVPDLTHFYAQHASVMPWLETQSNTPAKEWRQ
SIED
RKKLDGLYECIMCASCSTACPSYWWNGDKYLGPAALLHSYRWIIDSRDEATGERLDALEDPFKLYRCHTIMNCTKTCPK
G

LNPAKAIASIKKMMVERSV
>OLS49625.1 hypothetical protein BV379_15970 [Rhodovulum sulfidophilum]AB14
MTRDIRLCLPCPDPRPGKIWGDWFFAQSLARALERAGRGVGLATAKRRSGGFWMRAVPQRWPWQDEIDLVIRGGK
AWPKL
GRRPLFIWLISRPDSLTDREIREAEHIFVASELYMAELERRGARAVSFLPQCTDPDLFSPDRAEKTLASEILFVGNRRKS
FPRAVVERALATGRSLSVWGRGWHDALPPGTLRGVEIPNAELGRHYASAGVVLNDHHPDMLAHGFVSNRVYDVLAS
GRPV
LTEDMPGLPEDLRPAVFAYSDGTFAERLGTALDDDRQDRSEIAAHVRSRHGFDNRVRSILEVIGHA
>OLS49626.1 hypothetical protein BV379_15975 [Rhodovulum sulfidophilum]AB14
MIVGTMATYPPRQRSMLATVARIAPQLDVLNVVLNQYASVPPELLSFGNVNAIRPEEDTKDTGKFLPDHRGAGWVFLL
DD
DIAYPETYVKDSVAAMQALGPGRLVGGYCGAVYRKPQMRQKTLSGLNRYRKELFRRRGEVRDSLKLDAGFRQAIVADE
LG
TGVMIARPEHLPDFESVRSAQKFIDVRMARWCFEKGITQVCLPRPAGYLRALEHEESIHTGFTAKRHAAVTREIQEYALK
NPRAGQPFEDGR
>OLS49627.1 succinate dehydrogenase flavoprotein subunit [Rhodovulum sulfidophilum]AB14
MAAYEYETHEYDVVVVGAGGAGLRATLGMAEQGLRTACVTKVFPTRSHTVAAQGGIAASLGNMGPDHWQWHMY
DTVKGSD
WLGDTDAMEYLAREAPKAVYELEHYGVPFSRTEAGRIYQRPFGGHTTEFGEGPPVQRTCAAADRTGHAILHTLYGQSL
KQ
KAEFFIEYFAIDLLMSEDGQCQGVLAWKLDDGTMHVFSAKMVVLATGGYGRVFFSATSAHTCTGDGGGMIARAGLPL
QDM
EFVQFHPTGIYGAGCLITEGARGEGGYLTNSEGERFMERYAPTYKDLASRDVVSRCMTIEIREGRGVGPNKDHIHLNLSH
LPPETLAERLPGISESARIFAGVDVTKEPIPVLPTVHYNMGGIPTNYWGEVLNPVEGDFDRVAPGLMAVGEAGCASVH
GA
NRLGSNSLIDLVVFGRAAAIRAGQIVDPETAVPVPPKASIDQALERFDGLRHATGSVATAELRLEMQKTMQADAAVFRT
D
KSLADGVTKMTAVAGKLGDIKVTDRSLIWNSDLMETLELANLMPNALATVVAAEARKESRGAHAHEDYPDRDDANW
RKHS
LAKVEGAGVELAYRPVHLDPLSTTDEGGIDMKKIAPKARVY
>OLS49628.1 succinate dehydrogenase, hydrophobic membrane anchor protein [Rhodovulum sulfidophilum]AB14
MRYLTDRKRAEGLGAAKAGTHHHWSMNVSGVGLTLLVPFFVFFIGTSLGASHDEVIATFSRPFPAVVALLTIAAGMLHF
R
RGAQMLIEDYCDGMTRKALLWGATCLSYAVMAAGMYAVVRIAI
>OLS49629.1 succinate dehydrogenase, cytochrome b556 subunit [Rhodovulum sulfidophilum]AB14
MADIERGNRPLSPHLQIYRPQLTSVTSILTRVTGNALILGAVLIVWWLLAAGAGPEAFARADWVLTSWFGNLVMFGSL
WA
LWYHTLAGIRHIIWDNGYGLEIETAEKLGWICVIGSAVLSVLTVLILWIF
>OLS49630.1 hypothetical protein BV379_15995 [Rhodovulum sulfidophilum]AB14
MLNRLASIGTFVGGAATMGLALLGFYAFLHDPMPRTAEEAVAQLTRIADGMGGIVGDRTDTATRAAIVSEAARVAAE
AAR
PLDDATAVIAHVSFGVFDLPVLETWEVEASDGRVIPLTVLRGGSGYVDLAFDGKDYRLGVGKFLSYESGADLCRLLLVRK
VDGGVYRLRWRCIPDHKDQL
>OLS49631.1 hypothetical protein BV379_16000 [Rhodovulum sulfidophilum]AB14
MAKTNPGRFFEDYAVGQVIPHAVPRTLSGGERALYHALYPARHALYSSDAFAAACGLGTAPLDDLIAFHTVFGKTVPDI
S

LNAVANLGYAEGRWHRPVRPGDTLRSASEVIGLKQNSNGKSGVVWVRTRGLDQRGELVLDYVRWVMVRKRDLEAP
APETV
VPRLAGVVAAGDLVLPEGLDFTGYDFTLAGEPHRMADYEIGEVIDHVDGVTVEEAEHMQATRLWQNTAKVHFDATFR
EDG
RRLIYGGHVISLARALSFNGLANAQMIVGLNGGAHANPCFAGDTVRAWSEVLDKAETSAPGVGAIRLRLVAVKAGGVP
GE
LRGEDGKYRPEVLLDLDYWALMPV
>OLS49632.1 hypothetical protein BV379_16005 [Rhodovulum sulfidophilum]AB14
MQAYRLLTGEDSAAFCHKVTAALAQGWALYGAPAYAHDPASGKMRCAQAVVKDVEGDYAPDLKLGEL
>OLS49633.1 hypothetical protein BV379_16010 [Rhodovulum sulfidophilum]AB14
MFLLILGVALWSFAHLFKRLFPGIRAMLDDKLGMASKALFAVPLVVSVVLMVIGYKTAGMPYRFAGPSWLVHVNNTL
MLG
AVALFGVGSSKSHARQWFRHPMLYGFITWAVAHLLVNWDLASIILFGGLGAWAVAEIVLINRSEKGVTRYTGGTLKGD
LR
LGGIAIALYVGIILVHFWAGVPPLPMTS
>OLS49634.1 CoA ester lyase [Rhodovulum sulfidophilum]AB14
MDPRARPFRSVLYIPGSKDRALDKARGLPVDAIIFDLEDAVAVEEKPAARTLLAETLKAGGYGGRFRIVRVNGFDTPWG
R
DDVAAIAGAGADAVLLPKVETTAQLDDLAAAIPDVPLWAMMETPKGILNAEALAAHPRLEGFVMGTNDLAKELGCRF
RPD
RLALTASLTHCLLAARVHGIVAVDGVYNAFKDEAGLKAECEQGRDLGFDGKTLIHPAQIAIANAAFAPTEAEIDLARRQI
AAFEEAERAGQGVAVVDGRIVENLHIVTARETLAKAAAIAELEAV
>OLS49635.1 hypothetical protein BV379_16025 [Rhodovulum sulfidophilum]AB14
MIDFGPFFFAVAIPAILFAGVSKGGFGGGVAFAGSAVMVVILGPAVALGVTLPLLMLMDVTALRPYWRRWDGASARV
LIL
GAVPGIAAAAAVFHLTDPDVFRVLIGLVALGFVGFQLAVRLRLIPARQRPFGRRAGAISGAVAGFTSFVSHAGGPPVAV
Y
LLSQRLDKTSFQATTVLVFWAINLLKFGPYAFLGIFTADTWLAVLLLAPVAVLGVWLGVRAHRLVPERWFFGITYAALAL
TGAKLLWDGLS
>OLS49636.1 malate dehydrogenase [Rhodovulum sulfidophilum]AB14
MARPKIALIGAGQIGGTLAHLAAIKELGDVVLFDIADGVPQGKALDIAESGPSEGFDAGLKGTNDYADIAGADVCIVTAG
VPRKPGMSRDDLLGINLKVMKSVGEGIRDNAPNAFVICITNPLDAMVWALQQYSGLPAEKVVGMAGVLDSARFRHFL
SLE
FNVSMKDVTAFVLGGHGDTMVPLTRYSTVGGIPLPDLVHMGWTTQDKLDAIVQRTRDGGAEIVGLLKTGSAFYAPAT
SAI
EMAEAYLKDQKRLLPCAAWVDGALGLKGLYVGVPTVIGAGGIERVVDIKLDKPEQEMFDKSVGAVRGLVEACKAIDNS
LA
>OLS49637.1 hypothetical protein BV379_16035 [Rhodovulum sulfidophilum]AB14
MTSNAEASQDTADFETALAQALGAQILDEAHVLSFDMETRSLVSASEPTIFLLELSEDALGVHDFDSLIASPGQTSDDLW
NRAVAGTGPVWSGSFTASRSGSSHPMQMRSVVSVCADGSLRLSVVGEPACAAPPATEKAPAEVDSALASLRYDAEGH
ILQ
VSAPMCALLGQTSEDLIGQHMRRQVDAEFGRGTAWAEAWAGLLRGERTVLDIPHRHSGHGTVWTRSHLLPLTDAEG
GFAG
ALEIATDITRDRRDLDRMRARHTALDSRLAIAEFRTDGTILEASGGFCKLLGLARADLDGASHKTLMPAEFAGSRRYDDF
WDRLSRGEPVSGLFRHEGPDGRIVWLSSCYMPQAAAETGEVQSVLFVGHDMTEARKAETAAEGRLDALDRAMMVA
EYDSA
GRILSANGMLLDRMGHRIEDIRGSSHGLFLEKGRDKDGTEARFWEDLRDNGGFSGEIMWVDADGREVWLHTVYHPV
ADPE

GHSERIIQIAVDITDTKRLMLDLDGKWKAALNNQAVVEFDSDGKILQANEGFLRLVGHSLREVRGQSHSSFCHGDDIRS
S
AYTDFWMALRKGEAQSGRYRHVARFDRDLFMLAHYTPLTDFRGEVDRVVMCGYDISSQVALERATAARAQALTAEL
GSLH
EVNDMLRGDADLLARTNEKLQDSARNGDRLLDGALSEMRTAREAADKVTEIVDLVGDIAVQTNLLAFNAAIEAARAGE
HG
AGFSIVADEVRKLAERNGEAAREISRHIERANDSILRSADGTEQTIERIRAIGSMAEEAIGGLTQVVAHAGVGEAAAATL
ARLAEELGQAAGPNGQ
>OLS49638.1 hypothetical protein BV379_16040 [Rhodovulum sulfidophilum]AB14
MMQTASETRREATYALMEVDGGVIGIDIEHLSEVAPIDALSPLLVAHPALMGAIRLRGHIIPVVDPRSVSGIAQTGAPPG
IAAILNQGDRVLALAVNAISGLARLPDTAVQPLAAGDAETPFFVGGFFHEGQLVNAVDPAALFALGGLPVAARFRRDER
P
RNGSGGEGRAYLTFRAGGATFAAPAVEVYRTLPRQTVERNALTGGDCLGSVTVNGLRVPVLDATGLLGLGGTRERPDP
EI
VVFGFGDDRLIGLAVDVISRIATFRTDDLQPPPALLGSGAGAIASLAIAEDGAQTYVIGVERLREMKGLQAIAEMSAPVS
PKPAAHTSAPARPAAAAMPARPSGVGAEPVPDTIPASAPGAPSDHDAGEAPALSPDLLIRDRRRYLTFQAGIAHAAPIE
A
VIRIIEPPSAITPMMHPVPGVLGFFSVGGRATTLVCLSRFLGEDPPRKGQPRRILLVGPVERRIGFQVSSVDGIEPANWR
ALNTAPAPFREEMVSLGRGTAPRLLPCLDICRLAADIAERFHDHNIYA
>OLS49639.1 hypothetical protein BV379_16045 [Rhodovulum sulfidophilum]AB14
MRPVRLTLQAFGPFPGREVVDFRAAVAAGLFGIYGRTGSGKSTIFSAMTFALFGEAARAEQDAPSLRADHADPDMPTE
VE
FVFDLGARRYVLLRRPDQSRPKTRGSGETRDPHEAYLFDATGLPLDRIGAEGRGKILAEKKVGAVSAAVTELLGYGSHQF
RQIVLLPQGRFETFLAARTRERLEILRELFDVSIYRRLAATMKTEAEMLERTLREARAVCEGRLAAEGFEGMEALETGLA
GAEARLAEARTAETAATREAVASRAELSAAEQTEARFLAAAAADQALAGIREAGPAMAELAAQVRRADRARLLLDLEA
RV
VEAAREAEAASGRMDAAEARLETAKTAATRAAARQEAETARGPETEALRREVETLKRHAQTLAAAAELRQRAEEAQA
GAE
AARAEHSAAQGALAQRMAQRGKAAEAVQAARQTSANRALLSARLQALETEMKAALSVEAATRDVSETTEAVARLVR
AQEH
AAHRTRAAHAEMEAAERRLARSQALHLAAGLQPGAPCPVCGATDHPAPAAGAGEASASEAALRTARAAWEEADRA
QREAD
RALASERGVLAGRQERLAGLDRPATDTATLKTRIRATHEALAALGAETEIAVAEGLLARLDAAVRAAEDTREQCREVIAE
RESEALREAARIEAMLAPVPEALRSETALTAALSARQAALEARDAARQGAMKTATEAREAALAARAGHEAAVRAMAE
ARA
RHEGAREAFARRLGETGLSADDLAALKPAIVRLEEDRAAVEAHGRALAVAEERARATAEAVEGRTRPDLPALREALAAA
E
ARHAEATERRAAAAHASDHLASLRDDLAGTLQRLEAEEAASAPLRRLSALFDGRNPQGLDLETYAIGAMFDRVLAAAN
RR
LGPMSGGRYRLEREAEGGGGRGRRGLGLQVFDLFTGKARPTATLSGGETFIAALALALGLADAVESASGKVRLDTIFIDE
GFGSLDAEDGAGTLDRVLQALGALVGHTRAVGLISHVPLVQEAIPNGFYVHKEPAGSRIETRGPV
>OLS49640.1 exonuclease sbcCD subunit D [Rhodovulum sulfidophilum]AB14
MRFLHTADLHLGRQFNGLSLEADHAAILDQIAAAALAHEVDALVIAGDVFDRAAPPASAVRQFNAFLTRIRAETGAAVV
M
IAGNHDSGDRIEAMSVMTDTARALIRGTVAAEEPPLILADAAGPVAFSALPFAWEYAAREAFGDETLTSPEDVLRAQIG
A
ARAALPEGARWVVVAHGTVAGASVSEAERPLARIGGVETVGAELFDGAHYVALGHLHRPQSAGRPEIRYAGAPLAFGF
DE

AGEEKSMSLVEIDGAGEVRIAPIPFRPIRGVRVVEGRHEDLLRLPPSEDFLRAVLTDEAPVIDAMRRLRAVFPNACDLCY
AREARAPELKALGPVPRGLDDPMAVIGGFLDQVRGERADAEELAAIAATLDGLRGGEEGA
>OLS49641.1 spermidine/putrescine ABC transporter permease [Rhodovulum sulfidophilum]AB14
MTRQGSGLVLRLYMGLFFLYMFLPLAIMVAAGFNDYSPPSVTVWKGVTLRWFAELWQDQRMWSGLMNSLLIAGAV
IGLAI
PMGLAGALLLGRLGGRAAGLLYTAMVSPLLTPGIILGISTAIFWNGLGVAAGLFTAILAQTTFIASYTMLMFMARLQRQ
D
AALEEAALDLGATPFQAFRRITLPFLKPTILTAAVIAFLQSFENYNTTMFSIGGRHTLVTEIGSRMRFGLSPAVNALGIL
FILATIAFALAWGALKERERRAAARL
>OLS49642.1 spermidine/putrescine ABC transporter [Rhodovulum sulfidophilum]AB14
MTRLRRSFGAPLAMLLVVLAGTWILGLIAAPQAIMIERSLWKMERPAGAAEISVEIDRLYNRLDVLALDRAAAGTTSDS
P
GRLAAIDAERAALSARIAKLEEQESTPVRTWTLSNYAQMGRAHLWIFAKTILAALSVTAIAFAVCYPIAFAIAKLEPPRR
AALIMLALVIPYAINELLRVFAWQMILNYGGPLNALLGLAGIGPVPFLESGAGVFVAMVYAYILFMVFPLYNVLETLDMN
QIEAARDLGAKSWQIHRRIVLPHARPGIAVGCIMTFMLSAGSYSVPYIMTRGTAPPWFAQLVYNRFFQASEWNTGAA
YAV
SLLAVCTGFVFLMMRLLKVRLRDIAK
>OLS49643.1 ABC transporter ATP-binding protein [Rhodovulum sulfidophilum]AB14
MDIALAGAAVRFGSFQAVHPTDLAIEAGAFFSILGPSGCGKTTILRLLSGFLDPSEGEVRIGGRPMAGIGPSERPTALIF
QNLALFPLMSVWENVAFGLEMRGWARRRRRDRAFELLEMVALGGQGDKLPSELSGGQRQRVAIARALAVEPAVLLL
DEPL
SALDLKLRQHMRAELKQLQVATGITFVYITHDQGEALTMSDRVAVMNRGRIVQVGSADDIYDRPANAFVATFVGEMN
RLP
GRIEQAGGATARIATAGGSFLAQNPGGLGMGEEAVLFLRPERIHLLGPGETAANSLGGRLARRDMEGAFSTLIVETPAG
A
LTVQRVNAGAGGLPEGALRLGFSEAAGVILPEGETADDAA
>OLS49644.1 spermidine/putrescine ABC transporter substrate-binding protein [Rhodovulum sulfidophilum]AB14
MTKTVPSLSRRAVLATGAATLAAPLIARRALASSGEVNVFAWGDYFRTGEILEAFTSETGIKVNVSTYGSNEEATSKLRS
SGGKGFDILFPSVDTRPDYDDGNLLAEIDETRLKVERVQPALWRSSLQLGAAHRGKRYLVPFDWGTEGITWDASVHDI
AP
DALSYGHLWAEGLDRQVALRQKSVLTSLAIYLDAIGEVPSNRAMDIYTSEAENRRVFDACIAFVKKHRGNIGAFWNNAT
E
ATGAFTDGGCSIGQTWDTTGIRLHTDTDPKWRYGAPKEGALAWMDTAAIPSGAENVDQAYEFLNFLLTPEIGGMFSN
ATG
YNSAAVGAADHMTEAKKAAFNFAYQGDAIDNLWWWPMFTPWISPLREEYTEKLTNA
>OLS49645.1 murein transglycosylase [Rhodovulum sulfidophilum]AB14
MTIARRPLLMGLVASAVSACNMGAPRPVANPGFEAWRRSFRPRAEAAGITPATLDRALGRAGYLPQVIEKDRNQSEFT
RS
LEDYLAIAASDDRVAAGRAMLARHARRFSGIEARYGVAAPVVAAIWGLESRYGARRGDIPVVSALATLAYDGRRGRFFE
G
QLIAALKILDRGDITPARMTGSWAGAMGHTQFIPTSYLAYAVDFDGDGRRDIWSDDPTDALASTAAYLQRSGWQPG
QPWG
MEVRLPDTLDLARTGRKRRQDAADWSALGVTRPDGRPLPRAGAAAILAPAGRQGPAFALFGNFEVILRYNNAVNYGI
GIG
HLSDRLAGGPPLSKAFPPDAAGMTLDDRKALQRRLTAAGYDTEGADGVIGPKTEEAIRRYQARNGLTVTGRPSRALLTR
L
GRT

>OLS49646.1 hypothetical protein BV379_16080 [Rhodovulum sulfidophilum]AB14
MHRLHLRWQIVLCAGLMQLAALALAALVLFHKAREAVDVEMAAAEAGARAQVIAAMNAALRNLPPEQVMAELSATL
VEPR
HVRIALADARSGALPVARAESRQAHDAAPQRRRAPGWFRDLVMPAERETRLSVLANGTDYGFVSITAAPQDEIDEVW
EDA
AALLWALGLGFGLTGVLLVLLIRRALAPLDMLRHGLGALRGGALTTRLPEVATPDFRPLVAGFNALARSLATSEADRAAL
ARKIVEIGDAERRTIAMELHDEFGPGLFDLKVRVGALQRAAARTGDAALQRDAATMSAIVDQIQTANTRLLTTLRPMT
AS
QLPLAEALGDMLDGFRSTHGDLDWTIDLPEHLPETETIVDLTAYRVLQEGVTNALRHGKPDRLRVEVQSQDLPGQAEIL
C
LTVEDDGVGLPARAVEGGGLTAMRDRVGALGGTLELGRRPGGGTRLSAILPLTRNTSPQSEPEPAG
>OLS49647.1 class V aminotransferase [Rhodovulum sulfidophilum]AB14
MPGLRPDVDPDGLSEFSVVFTDRSLNHMSAKFQTAMRDISEMLREVYGAAEVALVPGGGTFAMEAVARQFATGRRA
LIVR
NGWFSYRWTQIFEAGDFAGETTVLMARPAGNAAKAPFAPAPIEDVTAKIRETRPDVVFAPHVETSAGIILPVDYIAAMA
A
AAHEVGALMVLDCIASGCIWVDMQATGVDVLISAPQKGWSASPSAGMVMLSARAAERLADTVSNSFAMDLKKWRA
IMKAY
EDGGHAYHATMPTDALIGLRDAMAETRTMGFEAAKQAQAELGRKVREMLAARGFASVAAPGFAAPGVVVCYTDDP
EIQSG
ARFRAEGMQIASGTPLQVGEGSDFRTFRIGLFGLDKLVDTDATLARLQAVVDRVV
>OLS49648.1 malate synthase G [Rhodovulum sulfidophilum]AB14
MTERRAGAGYVSKAGLEVAADLAAFVEEAALPETGISPDRFWTGFAELLHDLTPKNRALLETREKLQLQIDAWHQARR
GA
PHDHEAYKAFLEEIGYLRPEGEDFEIETAGLDPEIASVPGPQLVVPITNARYALNAANARWGSLYDALYGTDAMGGPAP
A
GGYDRGRGARVVARARVFLDEAFPVAGTSHADVRRYHVQGGALLIDDHPLEDPSKFAGYRGHPRAPDAVLLKNNNLH
VEL
VFDRTHPVGARDQAGLADIRLESAVSAIMDCEDSVACVDAADKIVAYRNWLGLMRGELSTDFVKGGERVTRALSPDL
DFV
APDGTPCTVKGRALMLVRNVGHLMTNPAIRDGAGQEVFEGLMDAAITALIAIHDLKKTEGARNSVAGSIYVVKPKMH
GPE
EVAFADETFTRVEELLGLPRYTVKLGIMDEERRTSVNLKDCIRAAKHRVAFINTGFLDRTGDEIHTSMEAGPFSRKEFIK
RKSWITAYENLNVDIGLECGLSGKAQIGKGMWAMPDRMAAMLSSKIEHPKAGATCAWVPSPTAATLHALHYHQVDV
FAVQ
AGLKAGGRRAHVDGILDIPLAAFRKWSRAQILREVENNAQGILGYVVRWIDQGVGCSKVPDINDIGLMEDRATCRISA
QH
IANWLHHGVVSEDEVAQVMRRMAEVVDRQNADDPAYLSMAPDFDGVAFRAACDLVFEGRRQPSGYTEPVLHARRL
ERKAE
LAAEG
>OLS49649.1 plasma-membrane proton-efflux P-type ATPase [Rhodovulum sulfidophilum]AB14
MDGQDQPEGEKLPPTDPQQGLSTAEAQARLGRFGANTLAEEHRSLFKRIFGYFWGPIPWLIEIAAILSLILSDWADFGVI
LAMLLVNAGVGFFEENKADSAIALLKQKLAPAARVLRDGRWQEIAAKDLVPGDLVRLEIGNIIPADLRLTEGQYLSADEA
ALTGESLPVDKAVGDIAYSGSVVRLGEMVGEVTATGMNTYFGRTAALVQGPERKSHFQQAVMRIGNFLIVSALVLVSII
L
IVSIWRENPLWETVQFALILTVAAIPVALPTVLSVTMAVGAERLARMKAIVSRLVSIEEMAGVDVLCSDKTGTLTLNQLT
VETPRPAAGATRDDLLLAAALACNANAPDAIDQAVLAAADGIEARRDYKLGTFTPFDPVSKRTLAEARRGDETWRFAK
GA

PQVILDLAGLEGAARTALEDEVDTLAAQGFRAIGAARAEGEGAWQFLGLIPIFDPPRPDAAETIAKAGEMGVTVKMITG
D
HEAIARQIAGQLGLGQAMYPAGQLLEGDPAEVEARVRAADGFARVLPEHKFRIVEVLQQGGSIVAMTGDGVNDAPAL
KRA
DAGIAVSGATDAARAAADLVLTGTGLSIITEAIAEARRIFERMTSYATFRIAETIRVLLFMSLAILAYDVYPVTAIMIVL
LAVLNDFPIMMIAFDNVAVSPKPVRWDMRRVITIASTLGVMGVAETFLLFWYVDSVLHLPRETIQTLIFLKLLVSGHMTL
YVTRNQDWFWTRPWPNWKLFCTTEATQVIGTLIAVYGVLVTPIGWTAALLVWAYCLVWLPIEGAIAMGIRRLYAARL
QG
>OLS49650.1 esterase [Rhodovulum sulfidophilum]AB14
MADTPHEIEQSVPLFWPMAAVLGWQEAGYQAMADGLRFVQEAAEISAPPEPSWATPNEVLLDLDTMRLRDFGGSG
SGTGL
PVIIDAPYAGHSATIADFAPGQSLVATLLAAGLGRVLVTDWKSAGPEMRDFDIDKYLAEINVAIDEVGGRAILVGLCQGG
WMSAMVAARFPSKVAALVLAGSPIDTDAGDGPIRRMAHETPLSAYRDMVASHHGRMSGSLMLTGWKNMHAEKQY
LGKFLD
LYAHIEDRNYIERTETFERWYENPLDLPGRYYLQAIDLLFKQNLFAKGEFVGLGRRLSLKDVTCPAYLLAGDSDDITTRE
QVFAAEDLLGTPKTQIRKLLAKGGHIGLFMGRHTLADVWPGIAQWIAEQGGK
>OLS49651.1 CDP-diacylglycerol--serine O-phosphatidyltransferase [Rhodovulum sulfidophilum]AB14
MKKFIRRDPQHRDQVPFLQLLPNLVTLSGMCLGLTSIRFSMDGRYDGAALLLLLAALMDGLDGLLARRLNATSNFGAEL
D
SLSDFLCFGVAPGLLIYRFFLEPSNNFGWIFVLVFAAACCLRLARFNVMRDENAAETSHHFLGVPAPAAAMLGLLPLFVS
LAGVVDLRPVPHLVAFWLGFVGVLMISRLRTFSPKAMRIPRALVGLMMFATVVAVGLMFTRIWLLLTALDLIYLAMLG
HA
ALQARRPGAE
>OLS49652.1 phosphatidylserine decarboxylase [Rhodovulum sulfidophilum]AB14
MSVSMLSTFVKPMHPEGRRFVAIAAGLTVLFFLIWPPLGWIGLGLTVWVYYFFRDPERVTPTRPGLMVSPADGIVSLLE
P
AVPPAELGLGDAPMTRVSVFMSVFNCHVNRLPAAGRVTAVAYRPGKFLNASLDKASEDNERNGLAVELADGRRYGVV
QIA
GLVARRILCWTAEGQSLATGERFGLIRFGSRLDIYLPEGVEPLVAIGQTMVAGETVIADLGA
>OLS49653.1 MarR family transcriptional regulator [Rhodovulum sulfidophilum]AB14
MISDPDPDFDLGEFLPYLLNQAAQETSAGFHAIYRAEYGMLRTEWRVMVHLGRYGEMSASEIGRLSMTHKAKISRAVR
AL
EEKRFLHRSPDADDRRAEILSLTPEGHAALARLSARARAYGADIEARFTDEERALLKSFLRRLATETA
>OLS49654.1 hypothetical protein BV379_16125 [Rhodovulum sulfidophilum]AB14
MARKDVNARRTVGYSKAKSHADGSKPVNRALPTFGDTSHISIAPQSAITRRQIIANPTRHDDARPREGPVQRTRTALKA
R
ADPA
>OLS49655.1 hypothetical protein BV379_16135 [Rhodovulum sulfidophilum]AB14
MKRDLPKHVYRKGTAIYWQRRGEPTVRMENQELTPAFWAEYALRLKGPVRAPSKRTFNGLIDSYRRSERYTSLAPRTRK
D
YERVLSVIEAKWGALDPVKAQRKHFIALRDANADAARFANYVVQVSRVLMEHAIDIGMVSENPAKGIKQVKGETIDRR
PW
PADLVKAYRDAAEGRALLVFELCLGTGQRIGDVLKMRWSDLENGGINVRQNKTGKTLWVPFTKTLRATLEAADRRSVF
IL
TNERATGPWSYRGAAQAVRKVREEIGAEAYDIHALRHTAASELCTLGLTDEEIASVTGQSMAMVAHYTAHVRQKVRAL
KA
QERRL
>OLS49656.1 hypothetical protein BV379_16140 [Rhodovulum sulfidophilum]AB14

MPSEILTRSFGRFEAEATPAEARLIIRDDCKDVIRLFGRDLIRSALLGGSWTPDIERALRWGLELIEAELGEDLRYRAGV
MAAVKLCWRLRLTNDPRRAERLLLGDPEAGQPPVEPRICPACGELVTDSLGCGGGICWASAAFPKPVEDPADKAFREA
VA
ELVDCAMKEGKPDA
>OLS49657.1 DNA methyltransferase [Rhodovulum sulfidophilum]AB14
MDSIAPPPDAPFEPATPMIIDSFAGGGGASTGIEMALGRSPDVAINHSGPALALHQANHPETLHLNSNIWDADPLSVT
GG
RPVGLLWASPDCKHFSKAKGAALCDRNIRDLAWVVVRWAEEARPDVICMENVEEFVTWGPVGEDGRPIREFAGMTY
ELWV
KRLRKAGYKVKWRELRACDYGAPTIRKRWFCVARRDGRPIAWPAPTHGDPASPAVRKGRLLPWRAAADCIDWSLPCP
SIF
DSSAEIMARHGLRAVRPLAKNTLARIARGMRRYVLDAERPFIVNLTHGARTEDLAQPLRTITGANRGEKALVAPSLTRFN
GGATGADLRDPMPTVTANSWIKRPGGAMPLGIVTPHLLSLKGSARRDGAATAPHPTVLAGGGHSAVVAPVLTYAQQ
GGGN
RSAEAPHHTICASKKDQNSLLAATMVHVGNGERPGQAPRALDIGAPLNTVVAGGVKHYPVAAFLAQQNGGPRMATH
AGHD
PRAPLSTVAASGSHQTPVAAWFAKYYGTGDGARMDAPCHTVTVKDRMGHMQADLAAPPFAPEHEARAREVAEFLR
AHGVW
DGGAFVTLEIDGQSFVVIDIGMRMLSPRELFNAQGFPPDYVIEGAWEGLDGDAPTFRPFSKDVQISCCGNSVCPPVAA
AI
IAANCPHLTASRQEQEAHDAV
>OLS49658.1 hypothetical protein BV379_16150 [Rhodovulum sulfidophilum]AB14
MAAILTIAPQIADRLAAEAAAARIEANTARVAAKSNARDGKPLLARAAFARAERLQGRAVTLGALAAQARAGGAA
>OLS49659.1 hypothetical protein BV379_16155 [Rhodovulum sulfidophilum]AB14
MKTILTTAAALALLAAPALAATETTEDLRAIRQPSADSQGCFAPIPGTNAFQRSNAPGCIYYVAPTGNDHDRAASDPSEG
GEGEGGESSDP
>OLS49660.1 hypothetical protein BV379_16160 [Rhodovulum sulfidophilum]AB14
MFKRYKDRGNRKKKSKEAGFAFTESRLNFGKRESLIFQIHNQKVDIIFFHGGGAFAGEPLSLVWAGKPLAEKGIRLILPS
YRVLSRDRSSVFDAISDAESFVHKFLKKLPHDRRLIVGGASFGGLLALHILRAMPEKTFNGILLVNPVCDTTRGGFSNKQ
IPTSGLDDISPNLYCDSLPQVPCLILHPADDQVVALRQTTDFLSRWRNGKAAMEVWPRSGGHGAFNLVGNQGKFVEK
CSA
FSEKCDA
>OLS49661.1 phage repressor protein [Rhodovulum sulfidophilum]AB14
MDRDRFKSRLSQIVEERGLEVAPLARAAGVKRDVVRDLLRGKKESLVVENAAMIASALGMSLDEMLGPATGSPTIAIAG
K
VGAGARVPVFDAYEKGGGPQVECPPGLSPHNIVAVEIEGDSMEPVYSEGDLLFYTRWSEGSVPSEAIGRRCVCECEEGF
G
WVKVVRQGREPGLFDLHSFNDQTPPMYGVRLKWAAPIKLHWPAELARRV
>OLS49662.1 hypothetical protein BV379_16175 [Rhodovulum sulfidophilum]AB14
MQKSFQKFRDPQERERKWFASLLWRSFPEATSEAQLADLVAEALNTAQRPVNPRTVRNWLRSENAPGLHYVVAVLAL
AGA
ETVFELFDPEQTA
>OLS49663.1 hypothetical protein BV379_16180 [Rhodovulum sulfidophilum]AB14
MTVRAEVAAEFQATALAMQSRQRPVSIRDRLRLALAGLGSGDEELRAAIARFLADHDTAPAAAGAALQETILRRCTAAP
T
RHAWQDRADLDG
>OLS49664.1 hypothetical protein BV379_16185 [Rhodovulum sulfidophilum]AB14
MAEAPLTRADQVLIAAAAALAVPPVMDRDLTARRMAMALDVIPHVAPDGPTCGLAFEIEAMDRARRAGDGPTWI

>OLS49665.1 hypothetical protein BV379_16190 [Rhodovulum sulfidophilum]AB14
MDLTDASCAVIRFAMNPNVLRSGEGLQIRMSTDGGASWNAWTELVFYPSRGPATLQGQVYISFDDIEAAWRRTMM
VSYGN
STATWGHEASSVSGSIDLVGRPSCMQIRLGRNRNRSFAAGTVEIFSGPTDSFVIGRDVS
>OLS49666.1 hypothetical protein BV379_16195 [Rhodovulum sulfidophilum]AB14
MQELTTQIRDWWAVMLSLLGLAVWSVRLEARARTNTAALDRETARLAEEIRALEARWQRQRAEDLTARQRDREETN
ALLR
ELRADIKTVLQRVPR
>OLS49667.1 hypothetical protein BV379_16200 [Rhodovulum sulfidophilum]AB14
MHLVPQWRALIRRAWSIRLILIAGLLSGAEAGLSLASPDATGLPRGLFAGLSALVTAAAFAARLIAQRME
>OLS49668.1 hypothetical protein BV379_16205 [Rhodovulum sulfidophilum]AB14
MRRILKRGAAATALALSFIGGNEGLKTEAYLDIVGVPTVCFGETRGVELGDSYTPAECRAMFAGRLAEFEAGLDRLIADP
VEALIPDRSYVGILDWAYNVGLGAAGRSTLIRKLNAGDLRGACDELPRWRFAGGKGIRGLLIRRNKARQLCHEGLDGVP
A
DVPFRWDGA
>OLS49669.1 hypothetical protein BV379_16210 [Rhodovulum sulfidophilum]AB14
MRALLAALLLAGCGGLPLPLGPNIAANVQAGAENVQGQKVENAPSIVRPRAREIRQEQSENRLRADRVDTVIVNVIPP
WI
VLVALIGWIAPSPGEIGRRIGEAVRGWRA
>OLS49670.1 hypothetical protein BV379_16215 [Rhodovulum sulfidophilum]AB14
MNLPQKQRSALIETIQSGLPTWSGRAAGALSVPDVLQACERFFFDTFDGCQYPVMIYDRMGQRLVHPLTQGDEPAFP
ESL
TALEDYAKAPTFVTSKDALIFIFDRLDRWPLAPAEVAFVAVLIEREQFNAATGMSLTPSEYLLVSLLLVGHNLQSAADYT
GANYDTKRKQARTVLEKAGLSGQAALLREVSLAVSSDVLSDLLQPNQRRPEVELAQHTYGQDVVIHSIAVGASWDVPV
WE
FGARRGQPILYFHSMLAPIMFTTNMVETLKERNLRVLMIPRHFFQAEPGGRGAQSQILQAASDIADYFCGEPVICMGES
A
GCAWAAQFTRAFPDQVSELVLVATPQAVRPKDAERFLPHSLTLLLEVSNRIRKDDRVIAGLTRVYNAIGRVPALSRRSLD
FLFRQAPSDVASIEESYAKLFLGDWLRLIANKATRASMDEVAHLHSDWVKDLQHLTLPMRFFHGTEDTLCPIEDAKAM
VE
GLPTATCQAFEGAGHLVLGQRLDDVLASLAADRKAAEPLR
>OLS49671.1 hypothetical protein BV379_16220 [Rhodovulum sulfidophilum]AB14
MDRNIFLIGPGGIGKTSSGRNLAELLGREFIDLDEKFMSGPGHIGQYISEHGYAKYVRENSNLFFEHINQSAEPFVAALS
SGFLIAEVEIETVQKNRFAVRNGHSVLLLPHADPDECADIIVSRQVQRGLNLREEIERPKFLERLPVYRSLADQVTVIQG
TPEESAKAIFVQLRGVEKTG
>OLS49672.1 hypothetical protein BV379_16230 [Rhodovulum sulfidophilum]AB14
MSDVRFHGANGQFGFLSNFSDHGFMHEGIRWATVEHFYQASKFNDPVSIINISQCETPALAKHLGGRTAGIRADWHD
VRL
SVMRTGVLLKFQQNKEIAEKLCKITGDIVEVSKDDAFWGAGIDGNGENNLGKILMEARQRICDQ
>OLS49673.1 hypothetical protein BV379_16235 [Rhodovulum sulfidophilum]AB14
MIWVFSIAADITTPNPPEGYLRADTEAEAFALIGHPDTNLYRLCDDVEWPGGELDSSSLWWDRR
>OLS49674.1 hypothetical protein BV379_16240 [Rhodovulum sulfidophilum]AB14
MTKRELPKYVYRQRNGLYFQRRGWPSQKIGSDFGSPEFWAEYAAILKGEGQAKVLRRNFSALIRSYHQSPRYRNLKPRT
A
LDYDKFTAFIDERFGPLNPANLQRRDVIRLRDENAGKPYFANYAVKVIRILMEHSIDLGWRTDNPAKGVSLLKADADPRL
PWPQSLIDAFRAAVPLGARERLLMELCLGTGQRIGDVLEMQWGDIQDGGITVKQNKTGKRLWVPIVPSLQEALDRAP
RRS

LFMLTNQSGTSRWSYRGASQAIRKIREDIGALDFDIHSWRYNAACELVEAGCTDELVAAVTGQSPKMVAHYTQQIRQR
VR
AIEAQARRKKPSTGEGLATKRRTEQKRNV
>OLS49675.1 hypothetical protein BV379_16245 [Rhodovulum sulfidophilum]AB14
MAEILTRSFGRFHAEVTPAEARLIIRDDCKDVIRLFGRDLIRSSLLGGSWAPDIERALRWGLDRIEAELGEDLRYRAGVM
AAVKLCWRLRLANDPRRAERLLLGDPEAGQPPVEPRICPACGELVTEGQGCGGGICRASDAFPKRVEDPADKAFREAV
AE
LVDCAMKEGKPDA
>OLS49676.1 hypothetical protein BV379_16250 [Rhodovulum sulfidophilum]AB14
MSHPLRILIGCETSGVMRRAFAARGHDVWSCDLLPAEDATNRHMICDIREVLHLGWDLLAVMHPPCTRLCNSGVRW
LHVP
PTNAPAEARPDERAAWPDLPEDTRRAIMWRLLDEGAALFSACWRAPIPRVAVENPVMHRHGRARLPADLPKPQIVQ
PWWF
GEPVFKATGLYLRGLAPLSATNRLTPPAPGTEAHKRWSAVHRAPPGPDRWRIRSRTFEGLAAAAADQWGENARKEAA
>OLS49677.1 hypothetical protein BV379_16255 [Rhodovulum sulfidophilum]AB14
MADETIARDIARDAEPEWILKFSIKGGDTVEWIAAKSTVFGALEELNKRECETGPVGWVFLPMGAINMSEVRYVSIHPR
P
RETKPKPARRARFTWA
>OLS49678.1 hypothetical protein BV379_16260 [Rhodovulum sulfidophilum]AB14
MSVFDSIAPPPDALAEFVVEWRGASPEVRLAAIRGALRRHGIEIPALPGRVAFYEIQLFGLTATGRDEAEAAQNWMEAA
I
RLCPFD
>OLS49679.1 hypothetical protein BV379_16265 [Rhodovulum sulfidophilum]AB14
MAAILAIAPQIADRLAAEAAAARIEAQTARVAAKSKARDGKPLLARAAFARAERLQGRAVTLGALALQARAGGAA
>OLS49680.1 hypothetical protein BV379_16270 [Rhodovulum sulfidophilum]AB14
MKTILAASALALLAAPALAATDTTEDQATAMSPASAPNCFAPINGTNAFQRSNAPGCVYYTAPTGNDRDPAASGEGEG
GE
GEGGESSDT
>OLS49681.1 SIR2 family protein [Rhodovulum sulfidophilum]AB14
MINFRKGSKDEFAKLPPEAGFDERAEVIRRHLAEVFGSKNLSFLIGSGCSSYTHENNELGIPTMGPLAAEFQTILKTMPG
LPGFNSFVTVEQRAALNDTLGIELTNGDFGKNLERMMEALMTAQQFCKTSTNTDFQEALKTVEAVISGVKGFILQKCSE
G
MFAKGDETVVTLYRRFYQSLATRSRGLAPPWVFTTNYDLFNEKAMDRSGIPYSNGFSGTVERRFNPATYRRALAEQLDI
S
SKRWAAVDGYVYFCKLHGSVNWTEEETGLFPIRESAGRLDPTEDRVMIYPTPSKQTASFGSPYSDMFREFQRQIVQDQ
SV
LFVMGFSFGDEHINNIIFQGLTLPGFRLIAFLNPEDTNNPVTKELAALGDPRIWFIWGSDEETGKKAHYFDSIVQDLMPT
SADQKEEQAIEKVFKKLLSKQQDGNNGER
>OLS49682.1 ATPase [Rhodovulum sulfidophilum]AB14
MARDDASRAIGKIVSVSADRLVVELHRGSDNFTVVGFDDVHYVATLGSYLMVPTQSEYVVLEVIGLREKDFSPAYGNGP
E
MDKATAAKFLDVVPVGSMPVQGGSFKFGVSTFPPLYADVLYARDEDLDRIFNVEQPADRQTKIDAEGAEKEGAVPHTI
EI
GTSAVFKDYPVKAQIDALFGGHVAVLGNTGSGKSCTVASIFQSVFMKPDAFGALGASFVIFDVNGEYRQALDNLTLSIN
S
SFLKATDPGEEIGVSRRSLLGNEEVSRFRLPHWFMTVDEWALLLRASERSQQPALRTALGLTTLFAEQESGIVEDAAGIS
EIRNHVLAKTLLAILQTDASSPSKSDRLVAILASFRTDEISIPQIRHQLGVNYGSLADAIGLSEFLSEFVQDEPVLPTYA
NKPFSFERLQAALDLALHYEEANGNRQIRDYCSQMVTRLKSISDNPEFEFLRASVEELEEHDREPTHFVDRLLGIARDGG

RYRKERQLCIIDLNDASDEIIELASAVVARLIFDRMRRATPRNKMPVHLILEEAHRYISERPSRFAIDAGITFQRIAKEG
RKYGAFLIVASQRPSELSKTVLSQCNNFIVHRIQNPDDLSQIKQMTPFISETVLKRLPSLPKQHALIFGSAVKIPTTFRV
RDADPTPNSDDTQVRDLWYVPQNPSLHWKV
>OLS49683.1 hypothetical protein BV379_16290 [Rhodovulum sulfidophilum]AB14
MLRAFFARSDYLDYMVLRPLSHITANWEATWDDDHYQLETDSFTGDLNEIIDQIAIATRPDRYHDNEDRLAERVLTELK
W
PIQKKGGRWLGADYQSILEQGAFRDFGQKELATAAAGRVQMALDFGQPHFDEMDDAHMTMLAALMTIMIYHRDC
DGASVR
VPEQIDG
>OLS49684.1 transcriptional regulator [Rhodovulum sulfidophilum]AB14
MTDETFDLGEALKEGLEEARAWKRGEISLEARNVDPMPPSRIREIRKKVARSAAAFEKRFGIPAATINNWEQGRRRPDP
A
ARLLLSVIDNNPDTVEAVARQQAAPFNPS
>OLS49685.1 hypothetical protein BV379_16305 [Rhodovulum sulfidophilum]AB14
MHLVPQWRALIRRAWSIRLILIAGLLSGCEAGLSLASPDAIGLPRGLFAGLSALVTAAAFAARLIAQRME
>OLS49686.1 hypothetical protein BV379_16310 [Rhodovulum sulfidophilum]AB14
MRRILKRGAAATALALSFIGGNEGLKTEAYLDIVGVPTVCFGETRGVELGDSYTPAECRAMFADRLAEVEAGLDRLIADP
VEPLIPARSYVAILDWAYNVGLGAAARSTLIRKLNAGDLRGACDELPRWRFAGGKGVRGLLIRRNKARQLCHEGLDGIP
A
ELPFRWDGA
>OLS49687.1 hypothetical protein BV379_16315 [Rhodovulum sulfidophilum]AB14
MRALLAALLLAGCGGLPAPLGPNIAANVQAGAENVQGQKVENAPSIVRPRAREIRQEQSENRLRADRVDTVIVNVIPP
WI
VLVALIGWIAPSPGEIGRRIGEAVTRRRA
>OLS49688.1 hypothetical protein BV379_16325 [Rhodovulum sulfidophilum]AB14
MQELTTQIRDWWGVMLSLLGLAVWSVRLEARARTNTAALDRETARLAEEIRALEARWQRQRTEDLAARQRDREETN
ALLR
ELRADIKTVLQRVPR
>OLS49689.1 hypothetical protein BV379_16330 [Rhodovulum sulfidophilum]AB14
MLSLLGLAVWSVRLEARARANTAALDRETARLAEEIRALEARWQRQRAEDLAARQRDREETNALLRELRADIKTVLQR
VP
R
>OLS49690.1 hypothetical protein BV379_16340 [Rhodovulum sulfidophilum]AB14
MRALLAALLLAGCGGLPVPLGPNLAANVQAGAENVQGHKVENAPSILRPRAREIRQEQSENRLRADRVDTVIVNVIPP
WI
VLVALIGWITPSPGEIARRVREAVTRRRA
>OLS49691.1 hypothetical protein BV379_16355 [Rhodovulum sulfidophilum]AB14
MARRPRKTLEKWEVAIVKAMLQKGCIPQDVHAYFSRPTRSVNQARISQIKDGKMHAAIKPASDDELNAFLNAWPDVD
PAT
GLHLKGDELLIKAREAMIAAVHTFNSAGLHFRAELFIVTSVIAWTYLMHAYYKREGVDYRYKQAGVVKKTKHGADMYW
EL
GQCLAHGGCPIEKGAIDNLNFLTEIRHEIEHQSTDRIDDVISAKLQACCINFNDAIKTLFGKEFALEKRLPIALQFVTFD
GAQRASLVGRDLPPNLAAAMDDFHNSLTDAEQADPKFRYRVAFVPKVAGKASKSDLAIEFVKAGTPEAVNVERVLLKE
TE
RPKYRPTEIVAKAKASGFPQFAIFDHSLLVKQLDARNPGKGYGVEIAGQWYWYDGWFDKVMEKLNEGWTR
>OLS49692.1 hypothetical protein BV379_16360 [Rhodovulum sulfidophilum]AB14
MGTIIALSVGGMDIDECKNFAGTDHGSLFQESDRKRIRCDQVDYDYYEDPNDPDLAAMEIGFVKPLRDVIQRLELLGYT
Y

ESIRVEYLRAAEIAAEQSREYNVDLGAGDDVQSEPKNFPAFEDFYKFASKQAISDLRGDFVEGFLPEADKEFQGRFFGAG
IRHNVPLNLLHDSPTLSERSCFSNLLDFLDPYSVAWLLGQNPDNLDQMVTWQYGPLVNNGWANESEFVACPGRREKF
LIA
TEGSLDTKILKHAIELLRPEISDFFRFIDVEEGHPFSGTGNLHKFAEGLAKIDVQNQTIFLYDNDAEGRATFKRTSALNL
PDNMRVQILPDLNCFKEFSTLGPSGKGFDDINGRAAAIECYLDLEQSGLPDPVVRWSNYRSDTDSYQGSLDGKTQFAK
RF
FKLKAETVGASDYDTSKIAAVLDVIVSECSNLAGTMRAKRYQR
>OLS49693.1 GNAT family N-acetyltransferase [Rhodovulum sulfidophilum]AB14
MSIVTLTPGSLSNEHICCAISDKKCVTGYKAKKRWLSDQYEHGYRFSRLDERGKVFIEYGPGEHTWMPVEAPDWMVM
GCF
WISGRFKQQGHGKALLQTALDEARAAGLAGLVSVVGKKKMHFQSDGKWLLRQGFREVDETESGFSLLALGHEDDGA
VGIP
RFSESAKRGLPSDTKGITVFYSERCPFTDFHIGSSLRETCKKRGLSATVVKLDSLDKAQNAPTPATIFSLFVDDRFVTTD
LSVCMDSRFDKIIEKAGEGKALGL
>OLS49694.1 glutamyl-tRNA amidotransferase [Rhodovulum sulfidophilum]AB14
MSTTSSPDPCNALVEALDLPPAKDGPLSGARFTVKDNIDIAGHRTSFGSPAWRDTHPAPVHNALCVDQLLAAGAHCV
GKA
VADEFTYSLDGESQFFGTPQNAKAPGRVPGGSSSGSAASVANGLADFSIGTDSGGSVRVPASLCGIWGMRPSLHRISE
AG
VLPFMPSVSTVGILAESLPLLEAVARVLLHSQTTPVTAPRRLLVLSDAVKLALPAVQQQLRAALDEITRRTGLVAEPIRF
AELIGEDVALSDCNLKALRDLQTAEFQSTVGNWIEATRPELGATFTLAYGNVQRFDRIAALESLARSERLFQQINAALPP
GTIICFPTTPTTAPLKGSLDTLEAVTAFYDPTMTLTAFSGVARLPEISAPLLTVEGCPVGMSFAAGHYQDESLLVALSEL
LGPQGS
>OLS49695.1 hypothetical protein BV379_16375 [Rhodovulum sulfidophilum]AB14
MAIFDIEKDDLLRLSDERLEELIARLAEAEVAAHGHSPASVSWSGSIKAPDEGIDIHVQVDTTNLETGFLTRPDTVLQSK
KDPMPKSAIAAEMLKNGEPNPTIANQAKISGSYIIVSLADDCSPPMKKDRLDAMRAALASDPNKDNIHLDFFDRSKLVQ
W
VRQHPSVLLWLKGKLGQSYSGWQPYGAWSNPPRESPDTLISAPGVTVHVPTERGEKLSIEDAIEPMRRLIRTSNKAIRIT
GLSGVGKTRIVQALFDETAGDDPLDRTIAVYGDTGQDPDPSASAMLDRLIAEGRRAVMVLDNCPSDLHSALAAKVSAA
SG
DVRLITVEYDIRDDKPQTTEVIHIEANGPEVAEKLLVRRFPEIGQGNARRVAEFADGNARVALAIAERVEQGESLAQLSD
AELFDRLFQQRKGADGDLREQAEALSLVYSFSVSEPEVGQNELEVLGSISGHSKAQLFKATKKLADRHVVQKRAHWRAI
L
PHAVANRLAESALESIPVETLRSTFEAPGNSRLLMSFAHRLGLMHDNQIAREIVEAWLQPDGLLGRISNLNENGSRILYY
IGPVAPDALLDRIEAELTAADFQGMDPRHNPRRTTILNLLQSMAYEPKAFNRCVNLLIRVAESEDEGSSYDSIRDKIIRF
FQAYLSGTHAALEQRVYLVEQCIQSENQTRRSLGLRMLSSALEGSHWTGSGLSDFGARPRDYGYRPDNDQLASWRKA
FVD
IAVQLGTGDDPGLADSARSILAQNFRGLWRQQAIHNDLVDAARALHAKEPWGEGWRAIRSTIYFDYKKKKPDADPRPI
PN
SLLALEADLEPHDLLPMIKTYVLDKGRNNWALDEEFDHDDPEKYSKAEERLQAKALQLGQDFSHSDNDLEDLGPALFLT
D
WMPHRTAFGRGLARGADDLQQTWRSLVSQLEAPKTENFDCSVLCGFLEEVDNFEPQLAQRLLDRCADHNLLRQVIVG
LHP
ARKFDEADLDRCLAVLGDPNINGRMYNNILWRDTYASLPSHRIIEVAERILAKPNGDDAVLEGLTMKLHGEDSQLDTLG
P
ELRRLGLVAATQRMLRDHNDPGGSTNHRMECVVRAALRFQGNEKEKNAWLDAIFKVVDERYGYLHAFEGAVRNTAA
EMPN

DFLNRVFAGDEETRERRRFFIESGGMDRLPLAQVDVHTLVSWCKRQEANEVWSFIGSNLELWKVDKEAGAVVLTDAA
MRF
LEAAPNPRDVLNGYASRMEPRSGWSGNQADTMQPRADAILALASHADAEIAEAAKVVAAEANEHIERIRRRERQRDE
ARE
QTFE
>OLS49696.1 hypothetical protein BV379_16385 [Rhodovulum sulfidophilum]AB14
MRALLVALLLAGCGGLPAPLGPNVAANIQAGAENVQGQKVENAPSIVRPRAREIRQEQSQNRLRADRVETVIVNVIPP
WI
VLVALIGWIAPSPGEIGRRIGEAVGRR
>OLS49697.1 very short patch repair endonuclease [Rhodovulum sulfidophilum]AB14
MDTRLPEQRRRIMQAVKSKDTGPELCVRRLLHGMGYRFRLHRKDLPGTPDIVLPRHLKAIFVHGCFWHAHGCSKGRP
PKS
NLDYWLPKLEQNVYRDRTKMEQLDTLGWKALVVWQCETKNLEALSAKLRSFVDGC
>OLS49698.1 endonuclease [Rhodovulum sulfidophilum]AB14
MAKKVRNRYQALIEKIFFDHYSEGATELGFSRQEIKDAARDLGIELPDNLGDVIYSFRFRTELPPRVLETQAEGMEWVIE
LAGRSQYRFSLVKINRILPRDDLARIDIPDATPELIRAYALDDEQALLAIVRYNRLIDTFLSLTTYSLQNHLRTTVKGIG
QIEIDELYIGLDKRGCHYVIPVQAKGGKDQIGIVQTTQDIRFVEQKFPGMRCRAIAAQFMEDNIVALFELTLQDGDMKV
V
EERQYRLVPACDLDQSAIRDYRD
>OLS49699.1 hypothetical protein BV379_16405 [Rhodovulum sulfidophilum]AB14
MSHERLPAGVRAIDIADEFERLQISQQLPVDGALQIILSIIKRSGDVKLLESLAVELEERGYKQLAAAIRNG
>OLS49700.1 hypothetical protein BV379_16410 [Rhodovulum sulfidophilum]AB14
MARPALAAALATGLAAMLALAAAAGLAAATARAVACLLRAVAARAAAGPPPGRPGEGVEDLGLDLGRSGVGCLRLRL
GVC
GRVGLGLSWVDTMVCSLLPMRCC
>OLS49701.1 RNA-binding protein S4 [Rhodovulum sulfidophilum]AB14
MTDEAPRRTVRLDKWLWQARFFKTRTLAAKVVAGAHVRVNGTKTDKPAQKIGPGDTLTFPQGRDIRVVRVLALTERR
GPA
TEARTLYEDLTPIKAPAPASPRYDGGGRPTKKERRDLDRSRKPPLE
>OLS49702.1 ferredoxin [Rhodovulum sulfidophilum]AB14
MTYVVTDNCIACKYTDCVEVCPVDCFYEGENMLVIHPDECIDCGVCEPECPADAIRPDTEPDMDEWVEFNRKYAETW
PCI
ITRKDPLPEAEERDGEEGKRAKYFSETPGAGG
>OLS49703.1 CarD family transcriptional regulator [Rhodovulum sulfidophilum]AB14
MTKTKKSEFRPNDYVVYPAHGVGQIVSIEEQEIAGIPLELFVISFEKDKMTLRVPTNKATEVGMRSLSTPDVVSKAMATL
KGKAKVKRAMWSRRAQEYEQKINSGDLISIAEVVRDLHRADDQREQSYSERQLYEAALERLTREVAAVAGMDEAGAQ
KQV
DEVLISRAAA
>OLS49704.1 hypothetical protein BV379_16430 [Rhodovulum sulfidophilum]AB14
MDTETDPRSCLFGSPQKDAAAIIGIGASAGGSAALETFFENCPTESGAAFVVIQDIPTKHESLTAGLLAQRTRMSVEMIE
TDTRVRPDHVYLNPPGAAMRIEKGMLRLTPCDEGPSLPIDSFFTSLAEECGDRASGILLSGTGSDGTRGASAIHAVGGLV
LIQHPGDPHFDGTPMSLRRAGIVDAELPVTDLARRALAHRQRSPQAPFGAAPTTEDARPDAKNGALPEEIEAILQTLEE
A
TGTDFRQYKSSTLLRRIGRRMQLLELPDLQSYVRLMQSAPGEVAALTRELLISVTRFFRDEDAFRVVEDSVTPRIVETLP
PGQTARVWVAGCSTGEEAYSIAILLHEAFHKAGRQPMVKVFATDINPDSLQFASAGSYPASVGAEIGTERLKHYFHTDG
D
RLQVTNALRQSLAFARHDLLCNPPFTRMDLVSCRNTLIYFRQDAQKDALLRLQYAIRKSGYLFLGSSETLSSLQNGFQPI

DARHKVFIRVAARPSARFDLSALKSSPPFDQGQKARHLSTDKEIRPSMQASAPRSHGPLRESPGPENSPKGTAQVDLAT
T
QARIDSLESELDATRADLEATIQELEASNEELQSVNEELIASIEALQGSNQELQSVNEEINTVNAEYQEKMALMNRLDAE
LNSMIQSVGVATVFLDEALLITRFSPEACSAFKLRESDIGRPLGDIAHKLKYPALQEDIRDTIRTGVSLERDATAEDGAL
YRVRITPYAIRSSGHRGAVVTLRDVTVHRDLARLQGAIDALPDHVAALDVGATILLTNKA
>OLS49705.1 hypothetical protein BV379_16435 [Rhodovulum sulfidophilum]AB14
MGRHDAPAPLPAAEDGRTSDIGQASPRPSGQSFELELQAFTRQLAACGPDTSATRSDRLDLIQVELDLQNRELREAHA
AS
ERERRKYLTLFERLPLPAAVLDRGGVVREANEAAAAVFGFATTEQLCNRSVYRMLDETARSRLHAYLAAPPSGTEEGPA
T
AIPVHVGRPRDGVYLSYDAHLARLPACSEEDGRVHMLLVERHSDDRQEHERHCHQMLSESTSTLIHAFDLTGNLIFANR
A
ASAYLDGAETLSVEQRRAFDGLLGSRALDIEVLIAGKPTSNQGRHATAPDDRRSFLIQKFPLRDDSGKIVAVGGTSTDVS
TLMAGHSDLHAAFSLANDLANRDPLTGLANRPGFLERLRDALSRLDPMGKGLALGFLDIDAFKDINDGMGHEVGDAIL
SA
FARRLEDIMGERACLGRFGGDEFLFFIADSTPEEAERLLQRTLGDIRSPYEVAGTRVLLTCSAGLSHYPRDAERAEDLLR
AADMALYSAKSNGRDRISHFKESLRFTSERRLRVFSALRKALCEDSFRLVFQPKFDISDRRRIIGAEALLRWHDPQLGDV
SPAEFVPLAEANGLYNALDLRVLKLFARQQGDWVRRGLRVPVSVNISARSLQGTELVPSVLALLDYHEIPPELLLLEITE
TGLMNCTGEATRNLADLADAGVRISIDDFGTGYSSLAYLQKLHPSELKIDRSFIAHLDAGDGDGDGASERIVRAMLALAE
SLQLSTVAEGIETEEQLAWLVENGCEFGQGFLVSPGLEIAAFETLIGA
>OLS49706.1 hypothetical protein BV379_16440 [Rhodovulum sulfidophilum]AB14
MSAQACSSCTFFQDHALNSGKSAEAAGLCRYNPPVTQPEADSRGLWPVVKTDDWCGHFEQENA
>OLS49707.1 putative heme utilization radical SAM enzyme HutW [Rhodovulum sulfidophilum]AB14
MTVQNVTPSDESRSDPFFARISDDPLAGAFSGGIRAHAHGRVSAVPAEDVPALWDRLCETPRRGPTVAYVHVPFCENH
CL
FCGFYQNPWRREAGAPYVDAVLAQMQAGAAMAVQQGHPIRALYLGGGTPSALSARDLARLVGGLRETLPLSPDCEIT
LEG
RIVSFGLEKARAAFDAGVNRVSLGVQSFDDRVRRRLGRKSTRSETLRFLEGLVAADCGAVVIDLIYGLPGQDRATFREDL
RMAAALGLDGLDLYALKTIPGTPLLLAQEKGKLLPAAPHSLGHYYADGCEEMDRAGWEPISTTHWRRGLRERNLYNAD
VK
SGAACLAFGAGAGGNLHGHGYRLQPDLDAYAEATRQGGSALLAGLMRPSALAAVHNAIKAGMERGRLDPALVDRAL
ADCG
HARRFEDLAAPLLAQWQEAGLMTAEPGALPLTLAGRFWQVAMTARLIAWTDQAVREHTSAKENAG
>OLS49708.1 hemin uptake protein HemP [Rhodovulum sulfidophilum]AB14
MSRVEYSFRTGATPVHDARALTDGEVTTQILLDGVAYTLRITRAGKLILTK
>OLS49709.1 hypothetical protein BV379_16460 [Rhodovulum sulfidophilum]AB14
MTPDRGSRGAAPVATLDELPPLERAAIRCLRGWSDGPMTRARMRRNFGQILGPRAEAQEELLDALTDLVRGGGRRP
MASH
RPAHREVGGDENAFGQMVAAALGGEREDAFLFALALLGPQAASQAVAIAEELGLALLGLVRAHPANAPVSSPVLH
>OLS49710.1 hypothetical protein BV379_16465 [Rhodovulum sulfidophilum]AB14
MSGSGCERRLGPLDRPLAEAGYDPSEHGVLTVARYYFQSFAFPNSQAWINAFAKAESLFPPGMARAGASEIAVAVLAA
VQ
QMRAARGSGFRFSNPDCPGCARILSPHERQFMEVLVALRRGRRSRAHTAAMLICEGNASETFLVAMSDLAALTGALSP
GQ
VHPVGAQCDRGPAVS
>OLS49711.1 heat-shock protein Hsp20 [Rhodovulum sulfidophilum]AB14
MLYPSTFAADPFALMRQMGRDLDRALGPRPGAGAGAAAFPAVNVWQNDEAVAITAELPGIAPSDIDITIKDNVLTLSG
ER

RAPDLGDTAIWHRRDRAYGRFSRAIRLPFPARDDQVEARFINGVLRVVVARPEEDKPRRIEIKAV
>OLS49712.1 heat-shock protein Hsp20 [Rhodovulum sulfidophilum]AB14
MTTEMTAPDKRPADAPETARGGRIYRPLADIVERDGTVTLLLEMPGVAAPDVEIALENRVLTISGHAGTARPGRLQLVHA
EYGEGDYERAFTLSEDIDPDRIAAEMKDGLLRVTLPRAEAAQPKKIAVRSA
>OLS49713.1 heat-shock protein Hsp20 [Rhodovulum sulfidophilum]AB14
MQIRDLIPWARKEHSPEPKTRTETGEDNPIATLQRDMNRVFEGFWNRLGALDWPLGGDAKSDVVETANAIEVSIELPGME
MKDIEVSVTDDTLTIKGEKRVERQEEKTGYYLSERSYGAIYRSLPLPPGVDGSKAEASFRNGVLTIRLPQTAEAQSRVRRIEVQGA
>OLS49714.1 acetate kinase [Rhodovulum sulfidophilum]AB14
MILTINCGSSSLKYQLYTTDLSEVVAKGLIARIGETGSYSDHKAGCRGEKVALPVPDHKTALELMLASLMHADYGVIDGP
AAISAIGHRAVHGGDVFVDSVVVDDEVMAQLRACIGFAPLHNPCNIAGIDAAKRLFPGVPNVVVFDTAFHQTVPPEAHVY
ALPYELYSKHGVRKYGFHGTSCRYVSQAAADLLNWPIGDMKMVICHLGNGVTLTAVDGGKSVDTSIGFGTVGGVPMGTRS
GDFDPTIIFHLVNQLGMDLKDVEKLCYHKSGLLGVSGVSNDMREVTMRAEHGDERCALAVEIFVYQIKKTIGAYAAAMNG
LDALVFTAGIGENSAEIRSRVCAGLGVLGVRIDEASNEVARDGYPNISAPDASVATLVVPTDEERMIAMDTLRLTPEHLLTTPRGVSSRGYEQARPI
>OLS49715.1 permease [Rhodovulum sulfidophilum]AB14
MTALGLLVAFGGGIFGAAIGALPAFAFVGILTIAGVAIQLAVAPAATDFFGIPFGVFGPHVGGFASGVAAAAYAASKGKL
DSGRNIVTAGMGLNSPDVLLVGGIFGIVGYVICWGVAMVPDVGPGLPWTDYPGMSVVISAFIVRFIWGTCGLCGKAPKGE
RFFHPTDATKWLVFQSSFGQIAMIGVGVGLLGGWLGITYGMPGALLAFGIAAASLLFLTVGVLTPVTHHIALPAAIVGVA
SGSIIWAGIAGLVCAFVGEFFARIFLDRGDTHIDPPACTIAAMTLVFNVLTSIGFWALLPMPV
>OLS49716.1 protease [Rhodovulum sulfidophilum]AB14
MFGSSKYKVLMIVGDYSEDYEVMVPFQALDMVGIAVDAVCPGKCKGDTIRTAIHDFEGDQTYSEKPGHLFRLTAAFGLIE
PDSYHGLYLAGGRACEYLRLDDKVLGLVRTFMNDDRPVAAICHGPQILAAAGVLAGRNLTAYPAVAPEIRAVGGTYVAVA
PEDTVIDGNLATSPAWPGHPGLLRAFLRRIAPDAVN
>OLS49717.1 glycyl radical enzyme [Rhodovulum sulfidophilum]AB14
MIEKGFTKPTDRVLRLKKQIIDAKPYVESERAVLATEAYKATEGLPAILRRAKVVEKIFNELPVTIREDELIVGAVTKNP
RSTEICPEFSFEWVEAEFDTMATRMADPFEIPKETAAELHEAFKYWPGKTTQALAASYMSKKTHDCMNNGVFTVGNYYFG
GVGHVCADYGKILKIGFRGVIEQAIKSIECLDEKDPDFIKKKQFYNATVIAYNAAINFAHRYAAKAEELAAQESNPARKA
ELEQIAKNCRRVPENPASTFWEACQTMWFIQCMLQIESSGHSISPGRFDQYMNPYFLADKSISKEFAQELVDCVWIKLND
VNKTRDEISAQAFAGYAVFQNLCVGGQTEDGLDATNEMSYMCMEATAHVKLPQPSFSIRVWQGTPDEFLFRAAEVVRLGL
GVPAVYNDEVIIPALQNRGVSLAHARDYGLVGCVEPQSIHRTEGWHDAAFFNVAKVLEITMNNGRVGDTQLGPKTGEMTE
WKSLDDFYAAFKAQMAFFVKHLAEADNCVDIAHAERAPLPFLSAMVDDCLGRGKSVQEGGAIYNFTGPQAFGVADSGDSV
YAMEKFVFDEKKVSMADLKEAMDANFGVALGSSAPADNSKGPSEEDVYAAVRKVLSTNSSVDVATLKDEVYRTLATNSAP

TSDSSKYENIRRLMDSAECFGNDNDDVDLIARKCARIYCLEVEKYTNPRGGQFQAGIYPVSANVLFGKDVGALPDGRLA
K
EPLSDGVSPRQGKDAKGPTAAANSVAKLDHFIASNGTLYNQKFLPSALAGDQGLQNFASLVRSYFDHKGMHVQFNVI
DRE
TLLDAQKHPEAHNDLVVRVAGYSAQFVVLAKEVQDDIISRTEQTF
>OLS49718.1 alcohol dehydrogenase [Rhodovulum sulfidophilum]AB14
MTRYTSFQTRTEIVFGPGLLDRLHAYRGQKVGIVTDAFLAQSGALDRVRKALPDCQIRVFDEVVPDTPIDAVSAGARHL
G
GFGPEVIIALGGGSPIDGAKAILAIVREMHPDRYIPFVAIPTTSGTGSEVTTFAVISDPANGRKFPLISEELVPDVALLD
PEFVRTAPPKVTADTGMDVITHALEAVVARGANHFTDAFADKALSLAFAHLPRAYADGNDMEARDAMHQASCLAGF
AFAA
AGLGVNHSLAHALGGKLHVVHGRTNSVLLPVVIPWNAVEPRAAAAYARIARLIGLDAPDTGAAVTALIAKIREFNTLFGI
PATIPEIGADKEEYRRVRSVVAAAALADACTTSNPRQPSQADLEDLLAQVGG
>OLS49719.1 ethanolamine utilization protein EutM [Rhodovulum sulfidophilum]AB14
MQQEALGMVETKGLVGAIEAADAMVKSANVTMIGYEKIGSGLVTVMVRGDVGAVKAATDAGAVAAGKVGTVTSVH
VIPRP
HTEVERILPHPAPAAE
>OLS49720.1 microcompartment protein PduB [Rhodovulum sulfidophilum]AB14
MKDDLVEQLMGEVIRKMGGSESSSSSEPQPETKAAPAKAPKACQLTEFVGTAIGDTIGMVIANVDPSLHEAMKIDKKY
RS
IGIVGARTGAGPHIFAADEAVKATNSEVLVIELPRDTKGGGGHGSLVIFGAADVSDARRAVEVMLGELDRTFGDVYGN
DA
GHLEFQYTARASYALNKAFGAPVGQAFGITVGAPAAIGVLMADTAAKAATVETIGYASPGNGGTSYSNEAIYTFSGDSG
A
VRQAIHAARDVGKQLLGAMDPAPLESTTTPYI
>OLS49721.1 hypothetical protein BV379_16520 [Rhodovulum sulfidophilum]AB14
MTVMTEQSLGLIETIGLAAAVEAADAAVKSANVQLVGYELTNGDGMTVVKLRGEVGAIKAAVAAGAAAAERVNRVVS
TQV
IARPARDVSLLVDSPATVGTLPPTDPDPDPDGGLAAASGPAAEAGPSPVEAAQPASAEVDTAEAETRAETDAAAASDA
TG
TLASDDTPNTPAETPTEAAPATGAETAPETGPETQTQTETPAETEAPAETGSEATAEAAPSETAADASDETAAAGAGTS
N
AARRSRRRSTPARKPGRSGK
>OLS49722.1 phosphate propanoyltransferase [Rhodovulum sulfidophilum]AB14
MLETGNMIDRIVSEVLAQGRAAARDTAGTNAGSDPALVPVGVSNRHIHLSRQDMDALFGPGVMLTRKKAMKQPGQ
YAAEE
TVILRGPKGEIGKVRVLGPLRKDTQVEVSTADGFALGVRPPLRMSGKLEDTPGLEIVGPHGSVRKSSGVIVAMRHIHML
P
ETAALLGLANGDEVSVLVGGPRGGIMHHVAIRAAEASATEMHIDVEEANAFGLKNDDLVRILRD
>OLS49723.1 ethanolamine utilization protein EutJ [Rhodovulum sulfidophilum]AB14
MSTDRTLRDFAELIRSGDVLPAERRAPGPLKIGVDLGTANLVLAVLDAANRPVAGTTYRSTVVRDGIVVDYIGAVRAVRE
MKAALEERLGEPLIRAAAAIPPGIHSGSTKAIGNVVEAADFELAEIVDEPAAAARFLGIADGAVVDVGGGTTGISVLKGG
EVLASFDEATGGTHMTLVLAGARGVSFEEAEAEKLDPAAERDVFAVIRPVADKMASIVARFLEAYPDLDSVHIVGGACS
F
SDFEQVFAKQTGRRIVKPAEPLLVTPLGIAMFPQATGEK
>OLS49724.1 hypothetical protein BV379_16535 [Rhodovulum sulfidophilum]AB14
MDRAVLDTLLDRALLDLLSDRIVARLKARERSALLLVSGTDLGLDAAIRSLSPLNAAGWSFEIRRSPDAAGLLTPERLRL

LGGARAIPPMAAPGETDIDGVLARHGLVVVPALSAALAARASLGMAEDEVSALLTGALERGRRVVAARDGLCPAGRER
KA
RGLTGNEAYREMLTGHLLRLQSYGVELAWAAKLGDAIASPRPGDRGPGPGRGTATPPVPGSRVFGWSQAKTFDSAEL
KLD
ADVLITPLAAEELRARHVRVVRA
>OLS49725.1 ethanolamine utilization protein EutN [Rhodovulum sulfidophilum]AB14
MHLGRVIGTVVSTSKDPSLTGSKLLIVARLTPQLEPTGFTEIAVDTVGAGWGETVIVAKGSGARMAGTLGKSVSDAAIV
G
IVDTVETH
>OLS49726.1 aldehyde dehydrogenase EutE [Rhodovulum sulfidophilum]AB14
MNDKDISDAVAQVLSGYGSTAAEAGLPPAKPKSGEPMSDLVARILAESSSEAPAADTKPAYVPGSKRCCWTKSDPNGD
PI
DDIVNDLVSQALGDKKPCSVCPHSDSSEGVAVSDDEATTLGDGVFATMDEAIAAAELAQRQYLFCTMAARKQFIDGIR
EV
ILSNGTLERISHMAAEETGMGNPAHKIIKNRLACEKTPGIEDLTTAAVSGDDGLTLVEYSPFGVVGAITPTTNPTETIIC
NSIGMLAAGNSAVFSVHPRATKVSLLTIKLINRKLATLGAPANLVVTVQTPSIDNTNAMMAHPKVRMLVATGGPGIVR
TV
MSTGKKAIGAGAGNPPVVVDETADIEKAARDIVNGCSFDNNLPCIAEKEVIAVNQIADFLISEMQKCGAYLIKDPAVIKQ
LEGVVINEKGGPKTSCVGKSAAHLLGLIGITVGDDVKVILTEADKDHVFVREELMMPVLPVVRAPSVDDAIDLAVELENG
NRHTAMMHSTNVRKLTKMAKLIQTTIFVKNGPSYAGLAVGGEGYATFTIAGPTGEGLTSPKSFARKRKCVMVEALNVR
>OLS49727.1 hypothetical protein BV379_16555 [Rhodovulum sulfidophilum]AB14
MMRVINAPQENIMDMLTRRIAPHTRKWIEANPISAIGFVQTSVPDLFYYADLAGKTANVYTVELFGSCPQTTTTLAVLG
E
TSAVRMAMAAIEAAAQPAAF
>OLS49728.1 glycyl-radical enzyme activating protein [Rhodovulum sulfidophilum]AB14
MTDIDYSCEGTVFDIQRYSIHDGPGLRTIVFLKGCPLRCRWCSNPESQNPEPELFFRSSSCIKCGQCVPVCPTQALAPTN
PGFVDRSKCVECGTCTTVCPTQALSMSGKKMSVTQVMQELRKDAIHYRRSGGGVTLSGGEPLFQHEFAKQLLMACHE
QGW
NTAMETTGYTTREIVRDVIPHVDHVLLDMKSIDPDAHVANTGVDNRIILENALCISSLTHTVVRVPVVPGVNDSIEAAEA
VARFAKLMRGVDTVHLLPYHTYGENKYSLLGRSYPMGDTPNMKPETVTPLVEAIESFGLTCVVGG
>OLS49729.1 methionine adenosyltransferase [Rhodovulum sulfidophilum]AB14
MTTCISSESVTEGHPDKVCDQISDAILDAYVAKDPMARVAAEVLASGNTLHIAGEITSTAHVDVPAVARGVLRRIGYSDP
ALGFDAEGCFILTDLHEQSPDIAQGVSRDAEIGAGDQGIFYGFACDETPTLMPATIHYAHRLTETLAAVRHSGRLPWLRP
DGKAQVTFEIGTDGRPAVLRSLVLSAQHDPEIDREDMLRGLIGEVIAPVMGQWLRAETRVLINPTGRFVLGGPAADTG
LT
GRKLMVDTYGGIGRHGGGAFSGKDSTKVDRTAAYMCRYIAKNIVAARIAQSCEVSLAFAIGQLLPEMVGITLPSGCEAD
P
DKLEKAVRSVFPLSVAGMIETLELRRPVFERTAAYGHFGRDTEGFPWERTDRAVALQKAFN
>OLS49730.1 DNA-binding response regulator [Rhodovulum sulfidophilum]AB14
MFDIAIIEDEELERRALRSILEKNLDSCRIVGEARNGTEAMALIDSQHIDLMLVDIKIPRPTGLEVIQSVRDRGLATKVM
ILTAYDYFELMQSAIHLKADNFLLKPIRTADLLKAVRECLPEHEPVAARAADAWTVTAPAPDSIRAPEPPVADRIAELVD
QSAYRECLALVRRHLETIYAQREAAPRRAVMEFMRIMLQMVERRGLALPATLARHVEALETQRLDLHSHFQVQEILCQI
T
DVLFEANESGGAHASDRIQDVLNYIERNLHKGVTLEDAADFAQISPCYLSRLFRKEMDVTFISYLKAQRIERAKQLLQDS
DLPITNVSLDLSFQDANYFGKAFKKEVGLSPSEYRRRFR
>OLS49731.1 acetamidase [Rhodovulum sulfidophilum]AB14
MADGSRTGGACRSVIINGFTNSVLDPDAPMLGPVENGGTVIANTAPGCWGPMITPSLRGGHEVTRPVAVEGAEPGD
GIAI

RIRDVTVTSLATASGHDSSPEGFCLGDPYVAARCPTCDALWPETHLEGIGQEAVRCNVCGNPVTPFSIVHGYTVTFDDT
R
SVGVTLPSDAAEAIARDAPHYAALPDASHQHSILVYAPSDMPGALVRMRPFLGQLGTCPSKAMPDSHNAGDFGAFLV
GAP
HPYALTPEELAEHKTDGHMDIDAVRPGAILVCPVKVQGGGLYMGDMHAGQGDGEIAGHTMDVAGSVTLQVEVVKD
YPIDG
PVLFPLAEDLPPMARPFSDVERARGERLARRWHVNALEEVAPVSVIGTGANLNIAIENGLERAAKLLDLSVAEIRNRATI
NGAIEIGRAPGVIQVTFLAPLDRLDAVGLGGYAREQYGL
>OLS49732.1 hypothetical protein BV379_16585 [Rhodovulum sulfidophilum]AB14
MAASTQKKYEDRIAEHELEAIDAKAPIRALALFESIRREGAHELNRPASALALSGLTAGLAIGFSVLSEALLRTHLPDAP
WRPLIENIGYSVGFVLVILGQLQLFTENTITAVCPVLDDPRPRVFASLARLWSLVLATNLIGAAAFGWVLHAGTVYEPEL
RAAVVALSDHATGYGWWETAVRGIGAGWLIAALVWVKPNARNSELLLIVVVTYLIALADFSHVIAGTTEAAVMVYSGG
MS
VFEALGGFVLPALVGNILGGTVFFTLLTYAQIRSELVGDPESGRTRY
>OLS49733.1 hypothetical protein BV379_16590 [Rhodovulum sulfidophilum]AB14
MILDDQTEVTAFLRAQKRGPDPLREIETHISRIFLTGDRAWKLKRAVRLPYADFSTPEIRLRTCEKEFALNRPTAPGIYL
GVRRITRQADGLAFDGDGPLVDAVVEMVRFSQDDILDRVAEAGDLTEADAECLARTVAAMHARAPIRRHPGGAARM
DAVL
DINRAGFATSRVFAPAEVAALDESFRRIFRRHRGQLDRRGARGLVRLCHGDLHLANIVRHDGRPELFDAIDFNDALATV
D
VAYDLAFLLMDLWHRGLPEIANRVANRYFDAAGAEDAHGLLPFLMAVRAEVRAHVTATAASGDPARATQARVYFELA
REL
LTPAPGRVVVLGGLSGSGKTTLAERLAPHLGRAPGARIVESDRARKALFGVEATEHLPREAYAPGISDEVYARIAARSRA
LAEGGATVILGAVFDTAAHRAQIEGRLAGLRVNTLWLDAAPERLRERIATRGESASDATLDVLETQLVHGTEGISWPRID
TNGGLETTLVRLRAALS
>OLS49734.1 hypothetical protein BV379_16595 [Rhodovulum sulfidophilum]AB14
MARVLIVSSYVASGHVGLSAATAVLQALGHEVTALPSVVLSNHLGWPHAAGGPLPAGELCEMTAALRENGLLAGHDA
VLI
GYLPSPEHVAFAADLVAELRAAGPAPRIVIDPILGDAPGGLYVPVAVAEAVRARLVPLADTMTPNHFELSWLAGRALSD
L
EATAGAAAALAPEVLVTSPPLGPETTGVLAVRGARRELFRTLRRGSVPHGVGDAFAAMIAAGLAPGEALGRLDALVLCS
I
GAPCLAIASAAPRWISAPAIGPEPHP
>OLS49735.1 hypothetical protein BV379_16600 [Rhodovulum sulfidophilum]AB14
MGCAGGRVGGGTLGGSPILHSDAFRHRADIEIVGILHLTAFLFPSFPLRILSGGFVQHHCHSAFAGLHAFLPRPKPRGAI
ETPGVTPVLCGRASTVLRLSPVRPASIGIISLRPRLAGPLGRIVTQRFAHGTGLVPHAADRARRQMATALQAVHSRIRSL
VPMLSSPLRPRWVMRGSVA
>OLS49736.1 hypothetical protein BV379_16605 [Rhodovulum sulfidophilum]AB14
MPDARPCIALMGEIGAGKTTLAHLLVGRPLMPRTTTATQLPPVRVTYGTAPPSREDNRGRTAPVDLADLSRLSPADTRL
I
RIQAEAEAEILRHCDLIDTPAISDPEMGPDVWEYATGLADGVLWCSHAPEAWRPSEAAIWDMFAPRLGGSSLLLLTRF
DR
LTSERDRERVLRRVRRETAGLFAGVLPIALTEALEAPSGWAASDAEALGHALMRILRGLGGGTGQDAGPMRLDPAWR
VAD
PPDCGPPDCGTHGTVRPRRVRVDASRTRSPRPTGTEPPGHERSGHEPHPF
>OLS49737.1 hypothetical protein BV379_16610 [Rhodovulum sulfidophilum]AB14
MSPVPGLSRAYCRGFSFARMVAGGLSVMSTASAAPRVVPGVARRDLDDFGDGPAHLRQRGFDPGIRSDQGAKRLVG
IARR

PQENSIPPSRMGTASRGGWSMPNTSVEAAAAWPPRSRAPQRQSAS
>OLS49738.1 hypothetical protein BV379_16615 [Rhodovulum sulfidophilum]AB14
MAEISETIPPRDMTLGDGKGQETRLLVARPHLLRVGPPHPAARAAVPLPPDMQGDRAAPLIAAFQDRVRRFVATAADL
SI
TAARPACRVEPTEPGCPIDALRVIACHEGATSESPA
>OLS49739.1 hypothetical protein BV379_16620 [Rhodovulum sulfidophilum]AB14
MTRSSFCLGLGLATLAPLHALAEDLDDSLLLDEITASASLTPVATNRTGATVETLDRDQIAAGGQNLGETLATLPGVTIA
QNGGLGQTATLRIRGLSNGYIATRIDGMDFSDPSSTPTALQFGTLTPGLASRIEVLKGSQSALYGANAIGGVIDMTSWR
P
DRPGLSGRAEVEAGTYGTYSGTLSFGNMTDRGQIALTMSHVETHGFSSQDGNRESDGFDQDLILLSAEFDATEQLTLGF
S
ALYSDSETDYDDNATDTSPVTASTETRKGVRVFAGFDIGTVENELAFAWTQTERGYNSPSWGRSSFTGKRRTLSYLGST
D
LTEAARLSFGADWSEQEADISSGNVDTESRAVFGELQYALSNAADLSFSLRHDDYSDFDNQTTGRLALAWRLEDDLILR
A
VLGTGYLTPSLYQLFDSAYGNPDLKPESSRSAELGLEKRFGRDGFVKATLFHTEIDDLIGYDDDTFVTKQVPGTTTSKGI
ELSARHALSQTAHIFGNYTYTDSRGSEGRSTHVPRHDLMLGIDTALGDRANARLTLQRSTDSVDRDGALDDYTLAGAM
VS
YALTDSSEAYLRVENLFNDDYQTFRGYNTSGRALYVGLRASF
>OLS49740.1 hypothetical protein BV379_16625 [Rhodovulum sulfidophilum]AB14
MWACVPPSSAIRLAAALALAGTAALADPPGRVVSMNLCTDQLAMLVAGPGQLVSVSDLAANPMNSAMAEAARAYP
ANTGG
AEEIFLMRPDLVLAGTYSNPATLAMLERLGIRVARIGLTTRLDEVPDRLRQTGRLLGREAYAEAMARAFEQDLARLSQSG
PPRPLAAFFYANGYTQGEGTLADDILTRAGFANLARTDQAAQGRHGGFDLALEELVIADPELLILSRPYPKTSRAEEILA
HPALRALPAAAAPAFSGPDWVCGTPYALRALAKMTRRRDSDAATAPD
>OLS49741.1 cobyric acid synthase CobQ [Rhodovulum sulfidophilum]AB14
MTRALMLQGTGSNVGKSLLVAGLCRAALRRGLSVAPFKPQNMSNNAAVTADGGEIGRAQALQARAAGLEPLTDMN
PILLK
PETDTGAQVVVQGRARGALRARAFAAEKPKLMAPVLESFHRLATGRDLIIVEGAGSPAEVNLRPGDIANMGFARAAKV
PV
VLIGDIDRGGVIAQIVGTQAVLDPGDLAMVRGFAINKFRGDPRLFDDGYRLIEARTGWPGFGVIPWFPEACRLPAEDA
VD
LGGTGRGSFHVVCPMLSRIANFDDLDPLKLEPGIRLTMVPPGRALPGDADLVILPGTKSTRGDLAFLRDQGWDIDLAAH
L
RRGGRVLGLCGGYQMLGRRIADPEGLEGPPGVTEGLGLLDVETVMHADKTLTRIEASHAATGAAFSGYEIHIGATDGP
DR
ARPFAHIGDRPEGAISADGRVSGSYLHGLFSEDGFRRAWLGRLGAETGPEGYDAKVEATLDALALHLETHLDIDALFALA
GELPAP
>OLS49742.1 mannosyl-3-phosphoglycerate phosphatase [Rhodovulum sulfidophilum]AB14
MPRPALLIFTDLDGTLLDHETYSHAEAAPALTALRAAGVPVILASSKTAAEIAPLRAELGLSAHPAIVENGAGRLEPGAE
APRTGGRYRALRTALSQVPAPLRSRFRGFGDGGADWIAEVTGLAPEAAALAAQRLYSEPGIWSGSETEKAGFLAALQA
QG
ISGREGGRFLTLSFGASKAGQMAEIAARYSTPFVVALGDAPNDREMLEQADQGFIIANPHRRPMPPLDGEETGRIQRIA
A
AGPKGWNLALRQVISDFGI
>OLS49743.1 glycosyl transferase [Rhodovulum sulfidophilum]AB14
MADFHQNGNVATLHNLRSAPAELLVRELANFAQTRKISLILPSLFSELEGDALPRILDELAEVPFLHRIVIGLDQADEAQ

FAHALQFFDRLPQNHVVIWQDSPRMQAIHARLEAHNLQPGDPGKGRNVWFCMGYLMACKDSSVMALHDCDIVTYS
REMLA
RLVYPVANPNFPYQLAKGFYPRIGANKLNGRVSRLLVSPILLALKKTIGDRDYLDYLRSFRYPLSGEFALRTATLPDLRI
PSDWGLEIGVLSEAWRNLAPRSVCQVEISDAYDHKHQPVSQEDASKGLSRMSVDICKSIFRKLAADGTVFSEEIFRTLKA
TYYREALDLLDCYYNDARMNGLSLDRHAEENIIELFAQNITEAGQVFLDNPAETPFIPTWNRVNSADPTLMDDMRAAV
AA
DSTDYRSATK
>OLS49744.1 alpha-amylase [Rhodovulum sulfidophilum]AB14
MTDTTQTLRIKLAGLVGQIYPDQDRELLIDRIVEAFWPDGTRPRRRARMPSNRLWSCEDSMVITYGNSFTDGQHKPLD
LL
NDFLHRHLAGTVGGVHILPFFPFTSDDGFAVTDYRMVNSNLGDWEDIRRIAGSFRVMSDLVLNHVSSQSHWFSEYRQ
GHP
PYDRFFFEASPEDDLAPVVRPRTSPLLREVETANGPRHVWCTFSHDQVDLDFRNPEVLLEFLRIMRLHVENGVRIVRLD
A
VAFLWKEPGTPSIHMPETHAIVQLMRVLCDFAVEPIVLLTETNVPNHENLSYFGNRNEAHAVYNFSLPPLVLHALLSGTA
EYLHRWQATMPPAQLGCAYLNFTASHDGIGMRPVEGLLPEEQIGDIIGAVRSFGGEVSMRSTVGGGETAYELNITWFD
AM
KGTLAGEDGWQVERFLCSQSIVMALEGFPAFYVHSLLATPNDHAGVEKTGIKRAINRHRWDYPALLALLENPESMQAL
VL
RQMKRRIGIRRRQPAFHPNATQFTLHLERRLFGLWRQSLDRDQSIFALHNVSAEELSVSPLSINLIGGEDWVDLLSGERI
SPQGTDIPFAPYQCRWISNRDGAGREPG
>OLS49745.1 glycerate kinase [Rhodovulum sulfidophilum]AB14
MTLQAPAPPSAPLAASPADPPAFLRALFETAVAAADPMRTVAPALPPRPAGRVVVIGAGKASARMAEAVEAAWGPC
EGLV
ITRYGHARPCAGIEIVEAAHPVPDAAGEVATRRVMHLLEGLSAEDLVLALISGGGSSLLVQPAGQITLEEKRLVTSGLLA
SGAPIGEMNGIRKELSAVKGGRLAAAAFPARVLALLISDVPGDAPADIASGPTVGHRGDAARALAALARWGVVPPPSV
QS
FLEAGGDPVRPDDPRLARVANVVIAAPARSLEAAAEAARGAGCAVEILGDALEGEARDLAAAHADLARARQAAMAPG
DPP
LLLLSGGECTVTRRGDGVGGPNAEYALALAVALDGAEGIDAIACDTDGVDGAAEVAGAMIGPSTLAEAQRLGSDPARA
LS
TNDSHGFFAALGAQVVPGPTLTNVNDFRAILIRPQP
>OLS49746.1 Rrf2 family transcriptional regulator [Rhodovulum sulfidophilum]AB14
MRLTTRTNIAMRALMYCAVNGGRTVRKSDIAGACNTSENHMAQVINTLAHHGLVKTTRGRNGGLTLKRTPEDITVGE
VFR
HLESGVPFAECFDPATNTCPISCCCLLKGALQRALAEFYATLDGITLADLVVGNRDLEEALALAS
>OLS49747.1 thiol reductant ABC exporter subunit CydD [Rhodovulum sulfidophilum]AB14
MTAPVMSLLERAGAMATAASLLWLIQAAAVALALGQMLQPDTAWGPTWAFALVFVAVGLGRALLDHRAGALSFRA
ADIVL
ALERARLVTRESRRATDDPARPPAAVTATLAAEKLAALSPFLTRYAPAKRRSTFVPLVILGVAFYFSWAIGLVLLFAGPL
IPIFMALVGIAAKKASERQMEELSNMNVLLLERLTALVDIRLLDATERTVESFRSAADRLRGRTMEVLRVAFLSSTVLEL
FSAIGIAMVAVYVGFSLLGELNFGTWGTPLTATEGIFLLLLAPDYFQPLRDLAAAWHDKADANAVARELAELEARDETTF
LGLGAAAAPLETTEALSVRGLAYRPPGGRLIRFPDFDLHPGETLAITGPSGIGKSSLLRLLAGMAVPAEGEIRIGGTALS
PETADAWRARLGWLPQQPRFVSGSLRKNLTLALRPGRELPLDRALKLAAADGVVAQLPRGLNTRLGETGVGVSGGEA
RRL
IVARAAHAHPDLLLADEPTADLDEETATLVTEGLLGLAQGGVALIVATHDPALAARMDRQIAIKPEHAA
>OLS49748.1 hypothetical protein BV379_16670 [Rhodovulum sulfidophilum]AB14

MKDILQILRLTWQAQRGPLIRGTALAVIVVAMGVALLGLAGWFITAAAAAGLAGIGIAFNVFQPSAAVRFLALSRTAAR
Y
GERLLTHDATLKALTDIRVALLRGVLALPFQTLSRLRGAETMNRLVADVDALDGVVLRLFIPAVTGVILYVAVFPLLWWM
TDFTLAIWIFASFTLGCGLVLIGAGRHALAPSRRAEKALQAFRIRLIDLLRARDDLVVYGRLPEQTEAVQLAEARMRDNL
GAIDRLERRAGLALSLTTTLATGTALALGGVLVARGAITPAQAALGFFVALALSETVQPLRRALAELGRMLDAARRVTRL
LPEEDGTPAQAAEQAHPAAAAEGAPALEMTGIAYRHPGAEAPVLKDFSLRLDPGACIALTGASGSGKSTVLALAAGLIQ
P
EAGSIRLAGCPIETLSETRLRAGMAYLPQRSMLLGQSFFDMLALADPGLTEREALELIEAVALSSTVAARGGLHASIKEN
GAGLSGGEQRRLALARALLRRPRLLLLDEPTEGLDRPTATRVLGAIREILPEAAILTASHRKAERDWADRLIAMT
>OLS49749.1 cytochrome d terminal oxidase subunit 1 [Rhodovulum sulfidophilum]AB14
MELDIVELSRLQFAMTAMYHFLFVPLTLGLSVIVAIMETVYVMTNRPVWRQMTKFWATLFGINFVLGVATGITMEFQF
GM
NWSYYSHYVGDIFGAPLAIEGLMAFFLEATFVGLMFFGWGKLSKVGHCVVTWLVAIGSNFSALWILIANGWMQNPV
GAEF
NPMTMRMEMTSFFDVMFNEVAQAKFVHTVSAGYVTASIFVLGVSAWYLLKGRHIELARRSIAVAASFGLAGALSAVV
LGD
ESGYNATHTQKMKLAAIEGMWETHAAPAPFNLFGIPDQADRDTQFALKIPVVMGLIGSRSLTEELPGINQLVDEADARI
R
NGLIAYDALMQIRAERGTATPELAAQFEEHSGDLGFAFLLKRYLEDPRQATDAQIAQAAQDTVPEVLPLFWAFRVMVIL
G
FGFIAMMLYFFIRANFYGMRFPRWALRAAVVAIPTPWIAAEMGWFVAEFGRQPWTVDGVLPTALSVSHLSVTQVALT
LAG
FVTFYSVLFVIEIGLMRKYILKGPYMDVAETDDWTRRHTDRLTGGRGYEPAE
>OLS49750.1 cytochrome d ubiquinol oxidase subunit II [Rhodovulum sulfidophilum]AB14
MILHEFVDFDILRVIWWGLLGVLLIGFALTDGFDLGVGTLLPFVARTDIERRVAINTVGPVWEGNQVWFILGGGAIFAA
W
PPLYAVSFSGFYLAMFLILAALILRPVGFKYRSKREDERWRSAWDWALFVGGFVPALVFGVAVGNVLQGVPFRLTDDLL
S
IYEGSFFGLLNPYALLAGLVSLSMLVMHGAAWLQLKTEGMVADRARSYGAWAALATIVTYALAGLWLAGGIEAYSFVG
TV
APDGPSNPLMNEVARGGSWMAAYEARPWIAIAPALGFLGAALAFLGFKTDKEGWTMLASKLSILGIIASVGLTMFPFIL
P
SSLDPSSSLTVWDSSSSHLTLFVMLVSAVIFVPLILAYTAWVYKVLWGKVTASDVVDNSDSVY
>OLS49751.1 cyd operon protein YbgT [Rhodovulum sulfidophilum]AB14
MWYFAWILGLPLAALFAVVNAMWLELHADRTMMDGKPRDDR
>OLS49752.1 C4-dicarboxylate ABC transporter [Rhodovulum sulfidophilum]AB14
MSTTAETDPGPRDSRLAHFPVTFFATVMGMMGLTLAWHAGEQALGPRLGLSLAVSPVLLYASCALLVAVAALYALKLA
TH
FPMAAAEWQHPVRLAFFPTASISLILLSVALMGDYPGLARPVWIAGTALQGLLSLMVISDWIGQRHFQPIHLNAAWFIP
P
VGNILVPVAGVPLGHPEISWLFFSGGALFWVVLLTLVMNRLTFHDPLPGRLQPTLVILIAPPAVGFISWMRLHGGLDPL
A
MFFLNAAYVFALIVATQLPRILRLPFALSFWALSFPLAALTIATFLYAETSGSAFHLWLGGALLAFLSAVIAGLVGRTLL
AIARNEICHPE
>OLS49753.1 hypothetical protein BV379_16695 [Rhodovulum sulfidophilum]AB14
MLDDFLVRAALAGVAVALAAGPLGAFVVWRRMAYFGEATAHAAILGVAIALGFSISIFAGTLGAALAMALAVTSLSGR
GL

AMDTALGVAAHAALAVGLVSVSFLSGVRVDLSAYLFGDILAVGRSDLAVIWAGALAVLALIVWRWTALLTVTVSEELAT
A
SGLDPGRERLVLTVALALVVAVAIQVVGALLIVAMLIVPAAAARPWSRTPEAMALIASGIGALSALAGLWGSYHFDTPA
G
PSIVVAASCAFALSLAVPRRA
>OLS49754.1 zinc ABC transporter ATP-binding protein ZnuC [Rhodovulum sulfidophilum]AB14
MTLVAARGLTVRYGGVAALTGVDFVIDGGEIVTLVGPNGSGKSTLVRAVLGAVRPSAGTVTRRPGLRIGYVPQKLAVD
DR
MPLTVRRFLSLPERRGAAEIAAMLERVGAAGLETRQMTALSGGQFQRVLLARALLGGPELLILDEATQGLDQPGTAAFY
Q
LIADLRRETGTAVLMVSHDLHVVMSATDRVLCLNGHVCCQGQPHVVTAAPEYRALFGHGTQGTMALYQHQHDHAH
DGCHG
QGHAHGPEPGRGGAGHKTEPEAADA
>OLS49755.1 Fur family transcriptional regulator [Rhodovulum sulfidophilum]AB14
MRIDAERVFSAHDHAACRQATLERAEEIVAAEGLRLTPVRRRTLEILLESHQALGAYEVLDRLVASGFARQPPVAYRALD
FLVDHGLAHRIRRLNAFAACMRPGEPHRPAFLICDTCHAVAEAPGTAVADAMLAAAGTVGFEIERMSLEAVGRCPACR
EA
RA
>OLS49756.1 ornithine cyclodeaminase [Rhodovulum sulfidophilum]AB14
MFIVPEKEIAGLVSADQCFEAVEAVFAAMARGDAYNFPVIREAIGHAEALYGFKSGFDRAGAVLGLKSGGYWPGNAAK
GL
TNHQSTVVLFDPDSGRPAALVGGNLLTALRTAAASAVSIRYLARSDARVLGMLGAGHQSAFQLRAAVAQRRFEKIVG
WNY
HPEMLPRLAETAAELGLPFEAVSLEDLGAQADVIVTISSSFSAQLMDAHVRPGTHLACMGTDTKGKQEIDPAIFARARV
F
ADEIAQSVEIGEAQHAVAAGLIAAEDIVEIGAVINGTAPGRQSDDEITVFDGTGVGLQDLAVAARAVAAARARGVAVEV
D
L
>OLS49757.1 alanine racemase [Rhodovulum sulfidophilum]AB14
MKDVTDFDGLEVGYDIPALPGMAESEIQTPCLVLDLDALERNIKKMGDWARAHGMRHRVHGKMHKSVDVARLQER
LGGAC
GVCCQKVSEAEVFARGGIKDVLVSNQVRDPAKIDRLARLPKLGARTLVCVDDPENVAELSEAAVRHGTQIECLVEIDCG
A
GRCGVTTTPEVVAIARAIDAAPGLRFAGLQAYQGAMQHLDTYEDRKAKIEIAVAMVREAVEALKAEGLACDIVGGGGT
GS
YYFEGSSGVYNELQCGSYAFMDADYGRILDAEGKRIDRGEWENALFLLTSVMSHAKADKAICDAGLKAQSVDSGLPVIF
G
REDVTYAKCSDEHGVIADPEGVLKVGEKLKLVPGHCDPTCNVHDWYVGVRNGKVECLWPVSARGKAY
>OLS49758.1 serine dehydratase [Rhodovulum sulfidophilum]AB14
MKDHGPLYIPTYDDVIRAHERIAPHIHRTPVLTSSCIDGMAGASLFFKCENFQKAGAFKVRGACNAVFGLDEERAAAGV
A
THSSGNHALSLSYAAGRRGIPCHVVMPRTAPQAKKDAVRGYGGEITECEPSTSSREAVFAEVEARTGAEFVHPYNDWR
VI
AGQGTCARELNDQIRDLDAVIAPIGGGGMVSGTCLTLSSLAPHIEIYAAEPEQADDAYRSFKAGHIIADDAPETVADGLK
VPLKDLTWHFVSSNVTDILTVSEAEIVEAMKLIWKRMKIVMEPSSAVPLAAVLKNRELFAGKRVGVIVTGGNVDLDRLP
W
MRA
>OLS49759.1 serine--glyoxylate aminotransferase [Rhodovulum sulfidophilum]AB14

MHASNPIFLPGPTNLPEPLRRACDIPTMDHRSAAFGPILAAAKEGVARILGLSGGEVAIFPATGTGGWEAAVTNALWP
GD
KVLAARHGMFSRKWIDLCQRHGLEVEIVERPWGEGVPVEEFAAALAADSGHQIRAVLVTHNETATGVTSDVAAVRAA
MDA
AGHPALLMVDGVSSIGSIEFRMDDWGVDLAVAGSQKGFMLPAGLAIIGISAKARQAMETAGLPRCFFDIRDMADGNY
PYT
PPVGLINGLAVSTGLLLDEGMENVRARHHRIAEGVRRAVAAWDMTTCAAAPELCSDTVTAIVTPEGFDAGRIVRHAAE
AY
GVAFGGGLGEVAGKVFRIGHLGLMSDVMALSGIATAEMAMVDLGLPVRLGSGVAAAQEHYRNSRTAPVRAAA
>OLS49760.1 IclR family transcriptional regulator [Rhodovulum sulfidophilum]AB14
MTQPRPRGRPRAFHDKTDQNTVRALDRAMGLLTALSETKGLTLSELAALSNQSPATVYRVLITLQGHDIVELEEEAQR
WH
VGPGAFRIGSGFLRRTNVAERSRGAMQALMRATGETANLGVEDRDEVLFLTQVETHETIRAFFSPGTRGPMHCSGIGK
AL
LAFLPAARVAAILETQGLPGFTPRSITSETGLHADLDRIRARGYAIDDQERAEGMRCIAAPIFNAHGEPIAGLSVSGPAF
RIPLDAADGIGAEVRAAADRVTAATGGISPTEKSA
>OLS49761.1 5-oxoprolinase [Rhodovulum sulfidophilum]AB14
MAADRWDFWIDRGGTFTDLVARRPDGRLLTHKLLSENPERYDDAAVQGIRELLGLGPDEPLPRGAIGSVRMGTTVAT
NAL
LERKGERVLLLITEGFGDLLRIGYQARPRLFDLEIRRPELLYEEVAEIRERLDAEGEVLVPLDEDAARAALTAAHARGIR
AVAIALMHGYLNPVHEDRLAELARETGFTQVSVSHRASRLIKLVGRGDTTVVDAYLSPILRRYVDRVAEALDLGQGGCE
R
LLFMQSNGGLTDAALFQGKDAILSGPAGGIVGMVKTAEAAGFERLIGFDMGGTSTDVSHYAGSYERSFETEVAGVRM
RAP
MMDIHTVAAGGGSICKFEDGRFQVGPESAGADPGPACYRRGGPLTVTDCNVMLGKLTPKHFPAVFGPGGDQPLDVE
AVRM
KFAVLAREVAAETGTPERSPEDMAEGFLRIAVDNMANAIRKISVQRGHDVTGYTLQCFGGAGGQHACLVADALGMR
RVFL
HPFAGVLSAYGMGLAEIRAMEEGQLDLPLADEAGAEAALADLTARAEAALRRQGVEPTAIRTLARAHLRYEGSHQTLE
VP
FGPAAAMQAGFEELHRARFGFASPDRALIVDMLDVEAIAETGAVPAAMAPEARAEINDRVPVHLGGETVEVPLYARA
TLG
PGTRIEGPAIISEPTGTDMVEPGWVAEADAMGNLILTRNAAARHAPAMGTEADPVLLEVMSNLFMSVADQMGATLA
NTSW
SVNIKERLDFSCAIFDAQGDLVANAPHVPVHLGSMSESIRTVMRGHAGAIRPGDAFMLNSPYNGGTHLPDVTVVTPVF
LG
DEILFWLGSRGHHADIGGRTPGSSPPDSRRIEEEGVLIDNLRLVDRGRLLEAEAEAVLRSGPWPCRNVSQNMADLKAQI
A
ANETGRRALLEVVERYGRDVVVAYMGHVQDNAEESVRRVIGTLKDGQFRYPMDHGAVIEVALRVDRAAREAVIDFTG
TSP
QHEGNYNAPFAVCRAVVLYVFRTMVGADIPLNEGCLKPLRIVAPEGCLLNPTYPAAVIAGNTEVSQAACNALYGALGV
VA
GSQATMNNFVWGNDRFQNYETIAGGTGAGPGFAGCDAVQSHMTNTRMTDPEVLERRFPVRLEAFAIRKGSGGAGR
WRGGD
GAIRRLRFLEPVTVTTLCAHREIPPFGASGGAPGSVGENWVEQADGRRERRRGNDEIALEAGALFEIRTPGGGGWGRP
>OLS49762.1 ArsR family transcriptional regulator [Rhodovulum sulfidophilum]AB14
MAHDDPQPKLDALDRRILGELQTDSGQSLDEIARKVGSSKTPVWNRIRKMREAGVIRQQTVIVDPKALDRGTCFFVLIR
T

SEHEADWQRRFLDALMKRPEVLEAHRLAGEIDYILKVRVADAAAYDVFYQALISDVRIFNVTALLSMEEIKATTVLPV
>OLS49763.1 AraC family transcriptional regulator [Rhodovulum sulfidophilum]AB14
MDDRLTGLRASVLSLWRAHGRQTPIAGLVLASAEVPTGPFHGVYRPSLCAVLQGAKVSRLGGQAFRYDAGKCLIASLD
VP
IRAEIVEARPDAPYLALALEIDTAVVAELMLGQGNVADPPPGAAIEVHDMGEALIDPLERLLALAERPQDIPVLAPMIRR
EIVWHLLTGPQGGRLRQIGLADSRLARIGRAIGWIRTHYAEPLRVAHLAALAGMSPATFHRHFKAATAMTPVQFQKSL
RL
QEARRLLLTRPGDVAGIGFRIGYDSPSQFSREYRRLFGEPPGRDGAELRRGLAEAGP
>OLS49764.1 aldo/keto reductase [Rhodovulum sulfidophilum]AB14
MRYRRLGPSGLFVSELCLGTMTFGGSEGMWGQIGQLGQAEVDSLVGTALEAGINFIDTANVYAGGESERLLGRALKNL
AV
PREEVIVATKVMGPMGEGVNARGASRHHIMDQCKASLTRLGLDHIDLYQIHGFDPATPVVETLEALDTLVRQGHVRYL
GL
SNWAAWQIVRAVGIAEARRLAPILSLQAYYTLAGRDLEREIVPMLKETGIGLMVWSPLAGGFLSGKYDREGKAADGRR
AS
FDFPPVDKDRAHDAIEAMRGMAEARGASVAQIALAWLLHQEVVTSVIIGAKRADQLTDNIGATQVALSEDELARLDAV
TR
LPAEYPGWMLERQGEYRSQS
>OLS49765.1 phosphomethylpyrimidine synthase ThiC [Rhodovulum sulfidophilum]AB14
MNRPDTVPQITLGPLPASEKTYMPGRLHPIRVPLRRIATGEGAVTVYDSSGPYTEPDARIDIAAGLAPVRLPFARDRGEA
ETYPARAVTAADNGFAEGARLVPAFPLRPEPLRATAGRAITQLAYARAGIVTPEMEFAAIRENEGRTAAHSRDGEPFGA
E
IPDLVTPEFVRDEIAAGRAIIPANINHPELEPMVIGRAFKVKVNANIGNSAITSSMEEEVEKMVWAIRWGADTVMDLST
G
RNIHNIRDWILRNAPVPIGTVPLYQALEKVGGVAEDLSWEVYRDTLIEQAEQGVDYFTIHAGLRRGMIGLTARRVTGIVS
RGGSIMAKWCLHHGRESFLYERFEEICDICRAYDVSFSLGDGLRPGSIADANDEAQFAELRTLGELAQVAWAKDCQVM
IE
GPGHVPMHKIKANMDEQLKHCHEAPFYTLGPLTTDIAPGYDHITSAIGAAMIGWFGTAMLCYVTPKEHLGLPDRDDV
KTG
VVTYKLAAHAADLAKGHPGAQARDDALSRARFGFRWEDQFNLGLDPDTARAFHDETMPKAAHKEAQFCSMCGPKF
CSMKI
SQDIRDAAAAEAGTCPNGLSPDD
>OLS49766.1 GntR family transcriptional regulator [Rhodovulum sulfidophilum]AB14
MVPQLREDPMETPKETRIVEQILDAIADQRLPAGTRLGEQALSDHFSCNRTQVRRALAALTAYQVVEHLPHRGASVAT
PT
AEDARDVFQARRAVETAICRNALRRATAEDLAALRAHLAEEDRAKDDGDRSEAIRLSRGFHNLLARIAGNAVLARYVQE
L
TMRSSLIIGLYGTQARTLCAKDEHLSLVEAIAARDEAGALRLMDAHLRHIEAGISFSESRAPAGTLSSMLGGGSPRP
>OLS49767.1 hypothetical protein BV379_16780 [Rhodovulum sulfidophilum]AB14
MPPGFASSLVDVAIDVGADFLTIDFTNSAPFSLFATGFKNTYVFTFASSVALNITDAIIDTNVTTLGLTDGRVTFSGNEL
FVNVQSLPFDTSTFVRINLTSEEAVAAVPLPAGLPLLLLGLLGIGLQARRKKAA
>OLS49768.1 NrdH-redoxin [Rhodovulum sulfidophilum]AB14
MSIIVYSKPACVQCTATTRALEARGLPYELIDLTEDDKAFAHVSELGYRQLPVVVAGKDHWAGFRPDLIGRLA
>OLS49769.1 ribonucleotide reductase assembly protein NrdI [Rhodovulum sulfidophilum]AB14
MAGLVYFSSRSGNTARFVERLGLPALRIPIAGDDLPRPDTPFALICPTYADGEGRGAVPKQVIRFLNDPARRALLRGVIA
SGNRNFGATYALSGEVISRKCKVPVLYRFELAGTDTDIARVRDGLDKFWETECLTV
>OLS49770.1 hypothetical protein BV379_16805 [Rhodovulum sulfidophilum]AB14
MDSKGVWHDNVCVERLWPGITYEAVHLRAYASASEARAGICRYMDFDNSRRSRSALDGRMPDQALLNPLPPDLAAA

>OLS49771.1 glycosyl hydrolase [Rhodovulum sulfidophilum]AB14
MSGPAPAEAALSRRNLLRGKLDGGPAVAGWQDMAGAPTVPGDVLWAVHACGETLFVGGDEGTILQFDGALWRRS
RLSARV
PVHAIAGDPETALLAVGWMGAVWRFDGTDWRLERGCEVGADGKYAACAENTPLFGLAADGEGRFWAVGDDGTIL
HFDGEG
WHAETSGTRFHLRAVTMLGDGRVMAAGANGTVLIRGHDGAWSSEDCPVATTIHSALALAPDDVLLAGGRYFLDAGG
FRGD
LLRWDGKGFRKLDPGPGVTRLRALAALSGGALAVGDRGQSFRISGERVTRLADGTSHDLLGLAVTASGAAVAAGDFGT
LL
AGDGATAPAPVVRTRPAPCWDRMTTGTDRQIWAVWQHPETGTAYACGEEGLVLRREGDGWEALPPVGTLGLHALA
ASEDG
GVLAAGQMGEIHHFDGTRWRRHFDLKLDVTILALWSDGAGTMIAAGDEGLVLRWTGESWTRMVSGTQSALYALW
GPDPEH
LLAVGDSGLVLRWNGRNWQEFTCGSEAFLFGVWGTGLDNIFVTGLSGTIRHFDGQGWRAAPTRMRNDLLAISGKGA
EAVA
VGTAGAALRHDGKAWRSEPTGFDGGLRAVSATGPAILAAGDGGAILRRRGP
>OLS49772.1 chromosome partitioning protein ParA [Rhodovulum sulfidophilum]AB14
MAHPNLKIPDGSGEECGRGHACGFCPQEQTCTLDKRAHNKTLVEARLGEIGQIVLVLGNKGGVGKSTVSANLAAGLAA
RG
FRVGMADADIHGPNQSRYFGFAGEHIRVDARGLAPKHFTDPALRHGLRVGSLAFMLQDDDAPVVWRDAYKHDFVH
HLMGS
FDWGALDFLIVDMPPGTGNELITLTDLLEGCDVGALLVTTPQAVAQMDSLKAARFCLERGLPVIGCVENMAGVVCPHC
AG
AFHLFPEAGLGEKLTALGIRRLARLPLDPAIAAGSDDGRPVALDPDAAPPVAEALAAIVGACAALGSAGFQDAALRSLDA
AFAENLEDEDLARALAALPEAEREQIGRALAEALTEESGRLHSAASAPGGKA
>OLS49773.1 hypothetical protein BV379_16820 [Rhodovulum sulfidophilum]AB14
MPGFRFFLAATAAFLATSPALPLSADSLNAGNIRLIEPGETVPVIKIPDGIYLRTPNDPDDIIWARVPEFRVEMVMAPPV
HPSVGLRYLDEYPEQDLVVQLARTSERFYVRLRWVDPTRDMSTLRDRFRDGAAIEFSESDDSVSYMMGTDAESPVNIW
YW
HPDGDRVESLAAGSPGSLTRLDRQPVTGASEYRTGHGPDDSQWIVVMSRPLASEGDHQVSFERDTIPVAFALWQGA
DAQR
DGLKLVSLNWIFARMTPDAAPAPGN
>OLS49774.1 protein DdhD [Rhodovulum sulfidophilum]AB14
MTAQTCRPPIHDDALARARAGESLWRLGALAFGHPVEEFHRALLDGSFHEAVDAAWSALTGRPWPREAPPARFADLE
AGY
IAAFEHGRGGKRLASLLAGEHKDLLAGQARPAFMLNVAAFYRHFGLRQATADEGRVDEPDHLACLLEFMAVLSHLEAA
AI
TGGRDPGPVRRAARDFLVRYVEPMLALIAAALRRPSEPPLDATLVRLIGDLHGFAESRIAELAAQVGPYRSAEARSDAAP
DAAAHQNLWG
>OLS49775.1 selenate reductase subunit beta [Rhodovulum sulfidophilum]AB14
MVKRQISMVLDLNKCIGCQTCTSACKLQWTNRNGREYMYWNNVETHPGPGYPRNYEHSGGGFDEEGALKIGITPSA
EDYG
IPWEYNYEEALMTGTDPWLRPNVKPTWGANWNEDEGRGEYPNSYYFYLPRICNHCANPGCLAACARNAIYKRQEDGI
VLV
DQERCRGYRYCITACPYKKVYFNEQISKAEKCIFCYPRIEKGLPTACAKQCVGRIRFIGYLDDEAGPVHLLVERYKVAIP
LHPEWGTKPSVFYVPPLAPPRIGDDGEPTEETRVPLAYLKELFGEAVVPALATLKTERAKKQSGAESELMDTLIGYRHPE
MFKLS
>OLS49776.1 dimethylsulfide dehydrogenase [Rhodovulum sulfidophilum]AB14

MLRTTRRTLMQGASLVGAGLFAAGRGWALNRLEPIGDTLAEEYPYRDWEDLYRNEFTWDYVGKAAHCINCLGNCAF
DIYV
KDGIVIREEQLAKYPQISPDIPDANPRGCQKGAIHSTSMYEADRLRYPMKRVGARGEGKWQRISWDQATEEIADKIIDIY
EKYGPGKLMTHTGSGNMSMMRMAAPYRFASLVGGVQLDIFTDVGDLNTGAHLAYGNALESFTSDAWFGADYIMFL
LFNPV
ATRIPDAHFLWEAKWNGARVVSVAPDYNPSSIHSDLWMPIKQGADPFLAMSMVNVIIEGKLYNEAFMKEQTDLPILV
RSD
NGMLLREADLEEGGSDQVFYHWDSRTGAAVKVKGSMGSEEKTLVLGDVDPALEGSFEVGGIPVTTVFEKVRTEAAKYP
PE
ETAAITGIGPGVVRAEAETFARAKKALLMTGFNIGRYSNGIYTSWALTLMLALTGHGGRTGGLDTSWIAWNQPALLEL
AF
FDFKKLPRLEAGGLGEFVRGGMMEHSRQHYDNDKLKARTGFDLDELQEMIDESIDAGWMPYYGDMKGLISIADNKFR
RNK
NAEAYRERILEEVEELFVDINVRMDSTAQWADYLLPAAAHYEAWDLRSIAFHRFVNVFSRPVPPIGEAKSDWEIMEILT
R
KIQERAIARGITGYEDGDVTRDFATIHDDYTMDGTLMTDHDVVSWLVENGPEFAGATLEEGVERGFFVMGEDAGPTQ
KLR
PSEPYHAFLQQTEGKEPYKTMTGRITFFVDHPRFVRLGATVPTARHHAGRDASNYPLNFFSPHTRWGIHSNWRSNKF
MLR
LQRGEPNIYISPQLAAAKGIADGAQVRVFNELSFFFAQAKFYPSLPPDTIMMEHGWEPHQFPNWRPMNVCMATLLQ
PLEL
VGGWGHLNFSLWHWNANQLAHESSVDIEPA
>OLS49777.1 hypothetical protein BV379_16840 [Rhodovulum sulfidophilum]AB14
MPREIGPTPRTDQLCADFAQISESLVDAAIRRARAKGYARFTTTIRAAWEEAVAAVSESLGAYLPEGQAASDGPHARIDY
AADPRFARLREVAIRHRAHGVSLQHYLGFLKHFREIYLEVLGADANTAGAVELADRVRGFFDLAELGIAAHWADCGDD
TR
LRDMQSRNRAVTLEKDRYFAVFESLRDPAFLLDRERRLITGNQAAAELFVGEAGAGEIVYLSTMRARKAPLERALRDLDL
WSCENPEGCDALWIDTRMGRLCFDVRVRQIHDAVQNLRLGYVAILHDVTEHRRATDESMRAQRAMSQFLATMSHEI
RTPL
HGVLGAAELLRDSDPERIGIYRDAIQVTGRHLLQTLNKVLDYSRLEARPPAPKPRVVDLRAVLDDYARFATTLARNAGVD
FRIETTTRLPAWIDIDWEMAQQVLTNLISNAVRHTRTDITLAIRCRAATGRRPGLRFEVRDGGAGIAPDVAQTLFEPFGA
TTPGQGETSGSGLGLAICRRLVTAMEGEIGFCNRRTGATFWFEIPLVAARRPSVPAALASDDPADAIAGLSCLIVDDDAI
SRFVTVEHLRRLGAEPVEAASIRTALDRVADREFDVFLIDYHLPDGDGVDLLAKLAQRGRMGAGRRAVALTANAELISG
G
RSAFDAVVAKPATSDALRKALTGAGQGQDSGPPRQAAHDLDGVSPGILRTMVSAFETQWGSDRAALLRAFDTANGQ
EAAE
IAHRMASSCTTLGFVDLAEELAACETRCRRHPPAADLGLWRRRLMPLLDTAPARASGLAKAHPC
>OLS49778.1 hypothetical protein BV379_16850 [Rhodovulum sulfidophilum]AB14
MRGQKPKLGNVVPRKADATAPVPEMRQGRAARSRDMELAELAARLAAGVSRRLLVSVPLVEAETGASQRNSDHLGD
PLLR
HEGKQCTRLAPTGRRNDVRTSKLVKAAKARTARQGCDI
>OLS49779.1 hypothetical protein BV379_16860 [Rhodovulum sulfidophilum]AB14
MQTRPRPSRPRLALLVGIGVWPIITTLLYLIRPVSQGWPVWLTTLALTPAMVIIMVWGLLPAIHRYFGKFLMRG
>OLS49780.1 ABC transporter [Rhodovulum sulfidophilum]AB14
MTLFAADTIGLHYPGRPDPVFHDLSLTIRKGESLGLSGPSGTGKTSLMRVIAGLERPTSGRIPVRPRRIGMAFAEPRLLP
RLSAMENLSFIAPKARAEGCAMLTALKMAALQDASADSLSKGQAQRVALIRALLIRPEILLLDEALGGLDMATWQSARD
L
ILAKRDRDGFALVEISHDPARRIMPEARAISFP

>OLS49781.1 ABC transporter permease [Rhodovulum sulfidophilum]AB14
MTRKLAFLTGVAAIVLFWSLAAQANLPAILPGPLAVARALGLLLGDPGFWSGTLGPSLGRASGGLALAFAIGAPLGLAS
W
RWPVVAALLAPLRLILMGLPAPVLAILCILWFDGSTTTTILTVAALLVPVFQIAVAEGMGAIDPQLDEMARLFHVPPIRR
LRRIILPALWTALGPALRIAVANAIRVTLLTELLSGAEGLGAAVQRAQSWLQTDRLFALVLLILALIGLAEVALAALTRE
RKRP
>OLS49782.1 taurine ABC transporter substrate-binding protein [Rhodovulum sulfidophilum]AB14
MRLLPLFLAVLPLPALAEEILLSGPPIWESAPLIALAEDQPVEGITFSFRPWASPEQLRKRVIADRPLMAVAPSPTAAIF
DANGMALRVVSATITEGSLSIVGRGTEITELADLQGASLALPFKGYLPDLMMRRIAEPGAETWQPHYTGSLVAGMQLLL
A
ERGVDAALLAEPMATLALAQDRDLTRQADLCALWRGATALADCPPAGVIIVNPAFAERPEIRAAYHTAFAKLAADPAS
AA
GLLAAHFPEMAQAGPGFARISAIDLPMPEQADVLADFYAAILEIEPAAIGGRLPEGDFYGK
>OLS49783.1 methyltransferase type 12 [Rhodovulum sulfidophilum]AB14
MVSPDRSSGAARLLQTLEGPIRYALLDWALESGVLGLCESHIAPDLLGRRMELPVGPLTLALRALVAAGFMEAGEAGFR
T
APDILPFVKTGSPRNMVETLRMMAGTRHAGLGQFGALVAGKAAPSGSRLFDEAHWDAHHRSLAGFHRAVGSDATAP
CLTG
LPEWPHARNLLDIGPGSSALAARLLAMRPDLRITLFDLPPVAERIGAEAESLPLAIMPGNYNRTLPDGPFDIIWCSMSLY
FHDGGLPALIARLAERLAPGGVLVSFHEALSDDRSAPPEHVLGRLLPALRQGDVSFAEGEIADAMAAAGLVRPTSQHLS
T
AFGRFRLDAARKEV
>OLS49784.1 TonB-dependent receptor [Rhodovulum sulfidophilum]AB14
MSRHSSSRPVPGRIALFALSTALTGSLIPGPAQAGEDMTYLGEITIFANYWEELARKSAVTATVLGGGDLARPVSPDLDA
MAGRSANTVFQRANSQERLVVRGMSAFDNALSDPVGYMVNGVALPMGTIQLPHFFGADSVTLLKGPQGTTFGRNSE
AGLL
AFDTIRPGSREGGEINLSVSGSDAGASPLGGSGSVLWSGRARNGPALAFGLEYARDPGVISNPVTGADDGGKDRRATG
FA
AAEWDLQDGGYLRLTTLAENQKFNKEQFRSISGPFATGRYESVYSDPSWERRRTSVTALEYGTGFDGFDLTAITGFTTFD
RDFVLDFDGSPLTLGVTELDVRDRTISQEIRLTSTGAGPLKWVLGVNAFKQSTDADFNLGAMSTDRHTEIDQNGVALYG
F
TEYAISERFRLGGGLRLDHVSSEAWQSFTSPMARLRYKGNESSTTVLPKLTLAYDLSEATTIHASYARGYMPAGYNYGFA
NSADSLTYGAEYSWNAEIGVKHAFANGAALTLAAFHTTVKDKQITETIPGAAQYISNAGEAKSYGIEAELTAPLGSGWEI
AANAGWQRARASAFRTTSMDLGTGSLTPVDWSGNALPMAPETTWGLALSHQGEVWRARGSLHGSGSYWFDPGNT
LKQKGF
MTVDAEISRSLGKGELTLWATNLLDEEYFSTAASTMRGVVVEDGSPRTIGMNWKIAW
>OLS49785.1 DNA-binding protein [Rhodovulum sulfidophilum]AB14
MNLIEPHRFTPFEAGSPTSVTLPGLHRNTHVDVMRINTGLVLAHSHFSAATRHEGDIRRPEDQLILAFNLSGRMLLIDPQ
GAAHEISGGEGWIIRSGGRRLQRIVEKGDGCSNLVIALSVSRLPPALAEGLEPLLPQAAPFRRLCLPVPGKSMLETLLDG
RTDPAALLRKEGQCLALIGHALEEIAAAPDHGPASGDPGLLIARVTAFLSDNLANPITISGIAQEMRMSHVTLNQIYRAA
TGMTVFQHLRSLRLEAAERLIRHTDRSFTEIAGECGFSDASHLSNAFRKRHDMTASDWRRQVRKKT
>OLS49786.1 hypothetical protein BV379_16910 [Rhodovulum sulfidophilum]AB14
MTDAIHGAKAPAASARSGPVSSLYRAVWRWHFYAGLLILPFLITLSITGAIYLFREEVDQLVHADFMTVTPVAEQRLAPS
EIIAAALAAEPGRAVKYTDPATDTRSAEVTVQPETGGRMAVYVNPYSGKVLEVRGDRSTFAWTVRYLHSFRYFGATPRK
V
IEIVGGWSILMVLTGIYLWWPRGQGGGVVTVRGKPGRRVFWRDLHAVSGIVVGGCLVFLAITGMPWSSVWGAKVN
QWANG

NNFGYPSGVRVDVPMSGTRLDDVAKTAWSLEQARIPETALPAAGMTPLGLDAAVAEFDRLGLHRGYTVALPSGPTGV
YSG
SVYPDDLSQQRVVHLDQYTGKPLLDMGYADYGPLGRWLEFGINTHMGQTFGMLNQIVLVLVCAGIVALSVSAAVMW
WKRR
PSGSLGVPPLPSDKKVFVGLFVLLGVGGILFPLTGLSLVAMLVLDLAWQGISARRAI
>OLS49787.1 hypothetical protein BV379_16915 [Rhodovulum sulfidophilum]AB14
MLHLRGRHLRWVAVWAMLLPFALASLVAQGVMPAKAGDGTVSLIICTGDSFVEMAFDPVTMEPVSENKRRDERGN
APSHC
AWAAAHPVCAEPAAMEMKAPVSQTLTVLSPPCRTVLRVAAATGLPPSTGPPSSV
>OLS49788.1 hypothetical protein BV379_16920 [Rhodovulum sulfidophilum]AB14
MPLDAKNFPLVWMSYDEEPDHDHDEDFEALEACFGRGAPFVILTDNAPAEDHEHSQEEKKRTALWMKTHKAELRAL
VLAM
IVIEPSATKRLAFRSFGVAFAKFWGHPLRLAPSREEAMEIAGKLLSERAG
>OLS49789.1 AraC family transcriptional regulator [Rhodovulum sulfidophilum]AB14
MYLDVRSDWLSHVDEVPRALVAASAVANLGALEQSPRHHHKKAQLLFTVRGVINCEVEDAVWIVPPQCAVWIPGGLP
HTV
FGSGEVECISLFIDPPEAPNLPTECCTVTVSNFLRQLLMRANVLPDLYDIDGPDGRIVSVIFDELAVAPVEDLRLPIPGD
PRLKKLTDLLIAAPADHATVAEWASRIALSERSLSRLLAKEVGMSFGRWRRQLHIVLALRQLSTGRTVQAVAMDLGYES
A
SSFVTMFRKMVGKPPSRYLLERRRPVLMASRRRAKASGVSDRSLLK
>OLS49790.1 transposase [Rhodovulum sulfidophilum]AB14
MKSLGQRLMARGPDRQLGELQLRVAVVNGFIALCMPVTKALG
>OLS49791.1 integrase [Rhodovulum sulfidophilum]AB14
MTAANLPAIRACRPAWNRGRIIGQKRPLMPKHVWAIRVRLELAGRIRDLALFNLAIDSKLRGCDLVKLKVADIYAAGQIK
ERTSITQSKTRQSVRFEITDGTRKSLAAWLDDPFMVGSEHLWPGRFHERRHISTRQYARLVRDWITSIGLEPSSYGTHS
M
RRTKVAQIYRKTGNLRAVQLLLGHTKMDSTVRYLGVELEDALAISEAVEI
>OLS49792.1 MATE family efflux transporter [Rhodovulum sulfidophilum]AB14
MSVGPAPNRIENPFLTRPIGRLFLSNALPMAVVMSMGGLLNVADGIFVGRFVGPQALAAVSLAFPVVMLLTALTTLAG
GG
MSSLIARHLGAGSRPEAGAVFAGAHGLALAISAVLIAGALVVGPATVLALAAGNATVAEAAQSYLLILILGAPIQFGLGL
HADALRNEGRAGQIAMLSVLVNLLNIGTNYVAIVLLGLGTAGSALGTVAAQSLGLALLLLLRTRDRALLPLGSLWRSSWL
SGWGRILSLGLPLCLNFIGMALVAGMVLLLIGTAAADHATYIAAYGVTTRLLGLAFLPQMAIALTTQSITGNNVGAGRM
D
RADRSLRLAMGSAFLWCLGVALTGAFAGAPLGGLFSGDPAITEAVAAILRPMTALYAITGPVLVLALHFQAMGYPIRTA
A
LTLIKPWLLTPALLVVMNALWGIEGLWFAFPVADAILLVLAMTLVLRTRPIAPVPAPCL
>OLS49793.1 hypothetical protein BV379_16950 [Rhodovulum sulfidophilum]AB14
MTQPSLETVKTQAKALRQALQAQGTAISHAQALELIARQHGARDWNTLHARLGQRNAPAELALGSKVTGHYLGQAYS
GVI
VALSGPSGNRHVEIALDQPIDTVRFDSFSNWRHRIRGTIGEDGRSSRKTSDGSPHLTVNIAPT
>OLS49794.1 2-nitropropane dioxygenase [Rhodovulum sulfidophilum]AB14
MLDKLGISLPILQAPMAGVSTPELAAAVSNAGGLGALGIGASDVEGASRMIAETRQRTLRPFNVNVFTHATPQADLECE
R
AWLQSMRPLFAEFGAEPPTKLRTIYKSFNETSDLLALLLKMRPAVVSFHFGLPLPEAVASLHEAGITLLATATNLEEARL
IAGAGIDGIVAQGIEAGGHRGVFDCTAHDPQLGTLALTRLLVTEIELPIIAAGGIMDGAGIAAAINLGATAAQLGTAFID
CPESGADAAYREALRGPAAYGTRLTSVISGRPARALDSRFTRAEGRYLTGRPPAYPAAYDAGKALHRVAKEKGDHGFGA
Y

WAGQGAPLARSLPAAELVSALTQELHCAMH
>OLS49795.1 hypothetical protein BV379_16970 [Rhodovulum sulfidophilum]AB14
MTDPKPRPGRGRPRTITRERVADAGIALGLAQLSVVGVAAALGVTQMALYKRVGGLEELRGLVAEEAFLRWPLPAPEE
DA
DLEAYLHRFSLSLWRLVHRHAGIAPYLLRRDWITDAMLRHTQEHQARVAECFGLTAAQSSRLVFTVAYHCCAIADTSRS
E
APSEAPDGEKIEPLHEFGLKALIAGAIALSGDDG
>OLS49796.1 ABC transporter ATP-binding protein [Rhodovulum sulfidophilum]AB14
MTTQPNGPAGPSTPPITRLLAPIRTQLRLAAALAGTGQILTLVPLAGIALIGRQALGLPEAAATVPVLTVATICIAALVV
GLSLLTLAEYLAHLADNSLTDTLRNRLTERLTQVPLGWFTSRSSGSVKRALQDDMNTLHELSAHYFTTRARCFGAIAAAA
AFLALVDWRMAIVSLAPFPLYYLIFGATRRSIGPDRMASYVSAQTAIGDAVGEFGRAMPVLRTFGKTGEAHEAYSEAVH
R
FLDAFLRFTKPLVAPLATANALITPVTVVSIVLASGALFIGAGWMPPADLLPFLLIAPGVSAPLLLLGFMAHGLAHATAA
ADRIDAVLNTPTLPVAAPGEGVTPDGNRLCFEAVGYAYEDGTETLSGIELTLEPGTMTAIVGASGAGKSTIARLALRFFD
PTEGRITLGGADLRDIAPAELYRRVGFVLQDVQMIHASLHDNIALGRPDATREEVSAAARAAQIHDRILALPQGYDSILG
RDAMLSGGEAQRVSIARALLLDPPLLVLDEVTSAMDAEGELALQKALSELARGRTLLVIAHRLETIVGADRIVVLQDGRT
AETGTHEALLARNGRYATLWAQSGSQLPAPTQDPETAR
>OLS49797.1 ABC transporter ATP-binding protein [Rhodovulum sulfidophilum]AB14
MMRMFLKLLGDDVKIFRRYAAMALASGALNTITIALTVPVLLRLLQGDAAAALPWLAALLAGALACLILRRRVEQAGIKV
GIAVLEGVRLRLGNHVARLPIGWFDSAATGRLNHALTQGVMEVAQLPAHVFTPVLTGLVVPPGLALVLFWLEPRIGLIA
L
AALPLMVAVFALAARLGRDADRGYHAAAARTGERAVEFARTQAVLRAFSGDGASARFLMDAFAGQRQAVRRLIRLST
ASV
VLNGWALQAVFAAMLLVALRLLDADLSPAQGLSLVVALVLAQRFIEPMTELAGYGEALRGARNHLAALTEIAEARPLPR
A
EHPQPPRDSSVALEDVTFRYSPDTAPVLEGLSLRVAPGSMTALVGASGSGKSTVVRLVARFFDVERGQVSIGGVDVRD
MS
EEVLSAQISQIFQDTYLLEGTIAENIRMGRPDAGAEEIAEAARLAGVDEIVRRLPQGLDSPTGEGGVRLSGGERQRIAIA
RALLKDAPVLLVDEATAALDAENRALVTETLARLRGKRTLIVIAHQLSTIRMADRIAVLEAGRIVEQGPPEALAQRGGPY
ARLLRLREAAQAWRMDSPE
>OLS49798.1 iron ABC transporter permease [Rhodovulum sulfidophilum]AB14
MPLSPATPSLARRAALPAAFAVLSLASLLIGAKDIGIGWIFDPSPEALLVLTASRLPRLIALVLTGMGLAISGVILQQIL
RNRFAEPATTGGMEAAKLGILVALTLFPAAAPLLRMGVAMLFCLAANLIFVAILQRIRFRDAVLVPVIGLMFGAVLGALA
EFYAFSRNMLQSMQGWMLGDFSRIVQGHYEMIYLVLPVVALTYVFARRFTVLAMGQDMAESLGLGYRGMVALGLTL
VSAT
VAATVITAGAVPFVGLVVPNLVTLWRGDNLRQTLPTVALSGGCLLLFCDILGRIVIYPYEVPLGLTVGAVGGAIFLLLIL
RRPT
>OLS49799.1 iron ABC transporter permease [Rhodovulum sulfidophilum]AB14
MRHGLVLAALAALALLYLGWGTGFAPGFLLSRRLIRLGAMAVGGTGIAMASILFQTVARNRILTPAIMGYEAAYLLLQAL
LVLALGTASLRLLGDLGNMALSLALMLGWSLALHGLLFRDERNSMHRMLLIGLVLTLTLTSITQFVQLQISPGEFAVLQS
FALISLDGISPNRLAISAVAVLAAGAIALRRAAIWDVMALGRDQALSLGLDHPREVRLCLALIAVFAAATTSLIGPSAFM
GIFIANIAYALTGGFRHRRTLPAGSLVAVAMLLIAELMVQHLFRFGTTIGILINLICGGYFLFLILRRAGRPA
>OLS49800.1 iron ABC transporter ATP-binding protein [Rhodovulum sulfidophilum]AB14
MITVTDLSKTYGDKPVLSGVTAEIPRGRLTSLIGPNAAGKSTLLMLMARLLRPSAGQVRLAGTPVGEIAPDRYARQVATL
RQAPGLDLRLSVEDLVAFGRFPYSRGRLSREDRAAIDRALAFLSLDELRHRNIDELSGGQRQLAYLAMTVAQETECLLLD
EPLNNLDMHHAVEIMRVLRQLCDDLGRTVVLVIHDINFAANYSDHILALKDGRLACSGPVAEIVTEDRLRALYDLDFQVL
RNGDSVLCNYFTPRHRR
>OLS49801.1 ferric anguibactin-binding protein [Rhodovulum sulfidophilum]AB14

MIMPIPSLATRLGLPLALAALIFGAAAPAEADTAASTPFAPMTLVHDLGETVLEHRPERVAALGMNDLDFLDSLGVPVA
G
IPKDFVPHFLSAYADDPQIRDLGFIVKPNIEQIYALQPDLVLMSPLQAEHYREISSFAPVLYFDVDYRNSATGHLDAVKD
HFLTLGKIFGKEEAAREGVAALDRQVARLRDEIADRPEKALILLYNNGAFRSFGQRSRYGFVFSDLGVTPAGEASETELH
GRPVTSEFIEAADPDILFIIDRTAVMEHRPVLTRDQIANPLLESTPAWANDRVIFVDPEAWYVTAAGPTSLSIIIDELRE
GYRRR
>OLS49802.1 type B chloramphenicol O-acetyltransferase [Rhodovulum sulfidophilum]AB14
MQNFFESPFRGVTLDEQVGNPNILVGRYSYYSGYYHGHSFDDCARYLLPDEGVDRLVIGSFCSIGSGAAFVMAGNQGH
RN
DWISTFPFYWMSEVPAFEGAENGYRPAGDTVIGNDVWIGSEAIIMSGVTIGDGAVIGTRAVVTRDVEPYAIVGGNPAR
II
RKRFGNDRIALLLDLRWWEWSDDQLRVAMPILTSGNVERLHEHWETHIRSR
>OLS49803.1 hypothetical protein BV379_17010 [Rhodovulum sulfidophilum]AB14
MPLVLLIVFAVFAGAAVPFQGGANAALGRALGHPLWATLASLAVSVLCIVPILMALRVPLPSLGGLAGQPKWIWIGGIA
G
AVYVTSAILLMPKLGAASFMIAVIAGQVVAALLIDWIGGVGLASRPIDASRLVGTVLVLIGAVVVQWPALSR
>OLS49804.1 LysR family transcriptional regulator [Rhodovulum sulfidophilum]AB14
MHSMHNLRNVDLNLLVVLDALLAERHLSRAAERLNMSQPAVSHALARLRVLLDDPLFHRRRREMEPTLRALELSEPLA
EA
LAQIRTVLAPEGFDPTGRHVFRLAMSDFGAALVLPGLMASLRRDAPGIDLVVTQQSRESMIGAVADGQIDMAFGVFPE
LS
SRCAADILFEDEYACLLDPSAQPQPYRGLTESRYLSAPHVLVAVHGEAATEIDLHLRSHRKARRIALVLPHWSVAPQVIR
GTDLILTVARRCLSEVAPGLEVTAAPVDLPTIPFAAIYAKRRRSDPALRWLMGRTKQLLSPQDGSCSI
>OLS49805.1 hypothetical protein BV379_17020 [Rhodovulum sulfidophilum]AB14
MGPVLDVGLFAATAGKLRHCAAILRAIGELYALRHCHPDQWHSNFLIDEGNMDAISQCRGGISEALRGRQIFGLPVTPD
E
SVFRALAIATNRWDCPSPERISVMSM
>OLS49806.1 hypothetical protein BV379_17025 [Rhodovulum sulfidophilum]AB14
MISRQTSGSEILTDSETCVTGYSAILPGADGLDAVWQVLSEGRCTISDMPEDRWSAARWLSPEPGLAGHSYTRRAGIVA
G
AFDFDAGCFGITPREAEQMDPQQRLLIEVTARAFDHAGIDPAAVEPDRVGVFIGAASLDHSTTMLQDPQMAGPQYM
LGNT
GSIMANRISHQWNLQGPSYIVDTACSSGLFALDHARRAIGSGQIDMAIVGAVNLLMSPMPFVGFSQAGMLSPQGLCR
AFA
KGADGYVRSEGAVAFVLERLDSAQAAGRRVRSVLVATSVNSAGRTPGIALPSADRQAALIEAALTEGDYDPEALAFVEA
H
GTGTAVGDPREADAIGRVYGRRRALPLPIGSAKSNFGHLEPASGLVGLLKAQLALERQTIPATLHCDEPNPDIDFAGLNL
DVARSARALPAADRPWLAAVNSFGFGGANAHAVIRQPRPDENVPVAASGELPPALLLSAANETTLANLARSWLTLAET
AP
ADLGARIATANWRRARHRHRLCLAAGRADALAQGLADWLEDPRQAAPGAAAETALRDAPVAFVYAGNGAMWAGA
ARTLIE
RDPTFRSVFNDIAEAFAGEGIAGLAEALADPDLEARLGEAELAQPLTFAIQVALTEALAEAGLRPDAVLGHSMGEVAAAI
AAGRIAREDGIRIIAQRSRAFEPLRGKGGMAAFAGSRSQIEALIETERIAAEISAENAPDSVTVSGSDDALKALTRIARA
HRIAGRILPIPYPYHSAAVETLRPALTRGLARIAPRDGSVPFYSGWDGSRYDAPLDGVYWWRNARLPVAFRAGVQAMA
ED
GIRVFVEISPRTVLRSYLRDTLAELDCETRVLESLDSTRAEARDAGTIARAALAAGGAADRARILGPEQPVADGLPDYPF
DRQTYRLDSPVATDIFHRRPQHPFLGTRLRPDSAEWQGEIALARTPWLGDHRVGGRVLLPATAIVEMFAHAGCEILKTE
E

IELRDVEFLRPLELPEKGIVEVRLVHEPTARRMRIEAGGPRGWTPVAVARLFSATTARPAPVTAPEAMGEAEPLYAGLRA
AGLDYGPDFARVTVLDRDGTAVRTTLAPRDGTEGFRFDPALADAGLHGAVLLFGGRDGDDRLRVPARIAKVRLCGRGR
IV
CARLTAAGGWDESLAFDTVLADATGTALLSFEGMRLRPLPAGTATPHDFWDERLVPLAGDGAATALRAAKALCGTTET
AS
DADVLRAALGSRLAWDLLAEGAAPSDPRQEAARRWMVAAGHDRNSDVRAPDLPSDCPWPTADALLTMLPEVAPGA
SAELK
SALAAAAGENRPADPLPQAARTLDTLLDSAAPLGRIALAGLPDLGLVAAALRAAGHVTVLAEDKDALDRLRARLDTGLP
L
RFLALTEAGAADAVDLVLGCCLSQGFAQRESAGRLAALVAPGGALIAAEEAPDLFALMTRRHRYADALDRLEHALHAA
GL
DTASAPASGSEALRLRIRATRPARRAAPRPELRITGNGPLAEALRALPQGSETAVALHCAVPEEDDPLAAARIFRALPDAD
GTLWLAVAETGRWEELTGWRRVVANETGRDIRLICAAPGVEAERIAALAATSPETEIRLTATGAAAPRVVPVRPLSAET
G
PRRLMADRRGVALDGLVWQTPPARALQPSEIEIEAVASGLNFRDVMWAQGLLPPEALEAGFSGPCLGMECAGTVLRA
GVQ
AGLKPGDRVIAMAPHAMASRVITRAEAAMPLPDTVDPEAAAGLPVVFVTADYALTEVARLAPGETVLVHGGAGGVGL
AAL
QVARRLGARVFATAGSPAKRRLVAAMGAEAVFDSRSLGFEDEVMAATGGRGIDVVLNSLAGEAMERSLACLAPFGRF
VEL
GKRDLYENNTIALHAMRNNIGFHAVDADQLLLHRPDAARRTLARLRDAFAEGVYQPLPVTAYPAEDVAEAIRSMQRA
AHV
GKIVLRAPQEHGAARPAAAPIRGGWLVVGGTRGFGLETARWLATQGAEHLWLMSKSGTVAPGTLDGLGTAAEALAC
DVAD
AAALDAALTRIRAAEVPLRGVIHAAAIFDDAPLADLDEARLSAVLTPKLVGGQVLDRLTRTDALDHFWVFSSIAARFGNP
AQAPYVAANRGLEGLARARREAGLPGLAIAWGPIADAGYLARETGLREALARRLPPMTTAAALDRLGRALAAGACGPT
LT
AAPMDWSRMAGTLPVLDTPLFDLIDIRASASGPGLFDLDELLRSKGPKEARKVLTRLLVEEAAQLMRAAPGDIDPRQPL
E
LMGFDSLMAINLRMAVEERLGANVPVMSIDDGLTLAGLVNRIIEAGGAPGAEAGDAMVEEMARRHLADGTLGEDRL
EQIR
TLSQGRTGGR
>OLS49807.1 hypothetical protein BV379_17030 [Rhodovulum sulfidophilum]AB14
MPGTRDTLDRLRALHVGTPRNVAAPKPAAAGSGRADFTTHPAYRQVAVAQSAARTLELENPFFRETHAIEGTRVRIGE
RW
VENFSGYDYLSINGDPRLAAAVGAAVAEFGVSARASRLVGGDLSLHRRLETGLARFLGTEAALATVSGHATNLAAIRTLT
GPGDVVLVDSFAHNSVFEGLRASGADHVSLPHNDSAWLEDWLSRNRERYDRVLIVVEGLYSMDGDIPDLAALVRIKDR
FE
AWLMVDEAHSVGVLGATGRGLCEETGIAPGQVDLLMGTLSKTFCSCGGFLAGSEAAIEILRHTAPGFVYSVGLSVPNTA
A
ALTALEILEAEPERVARLKARSARFLAASEAEGLDCGLGQGFAVAPLIVGDSLKATLASNRVFEDGFQAFPIIAPAVPNQ
KARLRFFINADHPEDAIDAAVASAAARIREVGGARF
>OLS49808.1 hypothetical protein BV379_17035 [Rhodovulum sulfidophilum]AB14
MTDLIRSVVLTGASGGLGRALAAELAAPGRRMLLMGRDAQRLAEAAGAARARGAEVETAAVPLTETSALSRCLQEFDA
AH
PVDLLIANAGVKTGNRGGIEPAVQAERVIAVNLTATIHAVQALLPAMCARRRGRIAIVGSLAAVAPHGDLLSYSATKAGL
QAYATALRRSLAGSGVGVTTVIPGFMDTPMTDRQKGPAPMLMPPARAARIVARGLERGRATIAFPLPLIIGAWLGERL
PA

PLADRIMARYRAEILPDPDEAAGG
>OLS49809.1 hypothetical protein BV379_17040 [Rhodovulum sulfidophilum]AB14
MADRFAEAFADIVARERPTDLVMLGDGRRYHVNAVSVAKVAVGITPWVVEQGYLRPGLISVEAWGTGGRSAIPAAFA
ATT
DTRPELPVPTPAPGSFLRYAAYDVVYHLANVLLGPVFYPRYRHYALDSPWAEWRGWVGKGLRARSRARQTEAALARIA
AH
AGPVFLVPLQLDTDFQIRDHGTGRTQDQDLAAVMASFAAHAPAGALLAVKEHPLDNGLRRWDRRAERLARAAGIGD
RVAV
FTGGRVEALYPKLAGIVTINSTVGLSALLAGVPVKVLGRAIYDLPGLTDPDGLDAFWAAPARPDPAEAERFRRFLRHDYH
VPGAFDGPDAQRGAEALARFIAGERPQ
>OLS49810.1 hypothetical protein BV379_17045 [Rhodovulum sulfidophilum]AB14
MAFDHRACKGRVFLMLQGHPSGFWRDLAAGLEADGHRVIKVNFGLADRIFWGSRPAIDFQGGLKRWRRFVFRLIERE
GVT
DILYYADRLPYHAIALEEGRRLRRRCWAIEFGYLRPDWLTLEPEGMGALSTFPRTRSEIEAMAEGVEAPDMRNLYPFGF
G
VEAFNEVSFALLQSFGRPFYPLHVSDRPNWPAIEYLSWLPELLRERRRKRAAVALIERLKARATPFNLIAMQLEVDYQIR
ASSDYPDLLTFLDETMASFAAHAPADRHLVVKIHPLDSGLGRWFSRVPRLARRHGLAKRVHVIRGGDLGALIKGSQGVV
L
VNSTVGIHALRMGIPVCAMGQAVYDLPGLTHHRGIDRFWTAPEPVDSDYFHSFERALSTIQVKGSFYNADGRAAAIEEI
R
RRFIL
>OLS49811.1 hypothetical protein BV379_17050 [Rhodovulum sulfidophilum]AB14
MSRPVQGSRGEAAASLFGEVADLVALMAAPAWATGPDGTVLAANPAAAALLDRPVADWVGLGDAALLVARQVARL
RCARA
GIGRDGAVRVRFRGCFLTGGGHPRLLDCSLTALAETRGGAVLVQVDPVAPSRVGTACSAAGTGHLSPPRAVFPRAAEL
AG
FAEWSLDPDCGRIRVAEGMLAMLGHAPDGGEIDSAWLRARVHPEDRPAMRAVAARLIRGDDESIRIDCRLRHRDGRY
LWV
EASARRVEPGPGQRPVFCGVLSDVTHHKEHEAQLQQALFEARRVRTEAAKTEGIMRIAAEGAGFGHWTFDPVIETYW
LDE
VFSRTLGYRPGAIDTSRSGFRELLHPDDAEAARAAMDALVSGGSERMHIDCRLRAADGTWRWFETSARLVSLSEVGLP
PL
VCGISRDITPRKTYEQELAHALELARQARDEAEAARVKQQLQATILRVSTEYSNVVPWHAVPSLGLFRADDHLAAMLGY
P
PGRRFTAAEFRSLIHSEDASEALGRVDALYRRKLEAIAVEFRVRRGDGGWCWVSSKARWLDRAASGLPDMICGTLTEV
TA
LKANETRLAEALAAAETARIDSQTREEMLRTSALCSGIAHWVVCPATGEGWIPDGTYGLLGYAPGAFPPQKGCWRGLI
HE
EDSVEAVALMRELLEGRSETYAHEHRLLHADGSYHWYRAVARRVRRAASGQPDLIAGALVSIDHVKENEARLAEAAEA
AR
LAGERLNTLADNAPGALFIFLISPDGRFELPYFSAKLPALMGLSRAEIEADASALMTRVAPDHIRALRRAIATSQRGLTP
FEMRLRVCRPEGGFRSVLVASLPFAREDGTVVWYGNVFDITDRLDAERRAAEAAEEGRRVHERLSSVAMIAPVGLYEFR
R
QADGSIDFPYTTPKFEDIMGLSREEIGMRSCDVLRRIDPEDLPHVLASIEDSARDLTLWSQRFRIRHPERGLIWLSGASA
PRRQEDGTILWAGALHDVTGDVQREAELRQAHRLAEAMRAENERQALHDGLTGLPNRRFYDRVLSSRIEAARAGQGP
RDC
VLIRIDLDHFKYVNDTLGHEAGDRVLIRVAEVLRQMLRAVDFVARIGGDEFSILLGPDMNQDNARELVERVQGALSRPL
T

YEGRLCRFGASFGIAHTGDITELGAELQLFADAALYRAKEGGRNRMEFFTAELHRNILNDRRIAVELHEALERDEFEPFF
QPQIAAEDGRLVGAETLLRWRHPEKGLLSPDDFMHVAEQLRIVPEIDRIMMRKTRSALARWQDQGLVLPKISFNVSSG
RM
HDPDVVSLARSMAGGGTKVTFELLESILVEEESDAFRFHLDQIREAGIDIEIDDFGSGHASIIGVMEIAPKALKIDRRIV
RPVACDMRSRNLVRAIIEIAETLGIATVAEGVETEEQAKILRGVGCQVLQGYLFSHPLDEGDFLAFTRADLKKRA
>OLS49812.1 hypothetical protein BV379_17055 [Rhodovulum sulfidophilum]AB14
MASVLRGGESSILNGLGLAAATFLTLILGLGALSLGQIGSVTAVSLEMRDVALPKLLAVNEIEIALDAHALLARRRVQVT
DFRQAADIDREMRRAAARFLTGIERLDQLTLGARALEVEQSLEAAWLEYLAALDRVGRLYERGALADAEAAMQDSVRV
AE
ADVRRSVSDLVDVVQAEAAGISAAAEREWQRARVLTLGALLAGLAATLLTMRWVVRRVSRPLLAISAAMRRLNRGEE
AAI
LPVLPGRTDEIGILAEAAAVFRDSVLEARALAADLELQREILAASVRNMPLGLCMLDELGRVAVSNRALAEIFDLSPADC
APGVAQRDLLDRLAARVDRGRKSAGEFSTGIAATVARGDQMTETWQLMAGGSVSVIIQPTPDGWMMIAEDITERVS
HEAN
IRHLARHDPLTGLPNRRAFSEEIDRAIAEAGAECPAAVLFVDLDRFKNVNDTLGHGIGDALLRQVAARLSWAVRDADTV
A
RFGGDEFAVIQRGCVQPEGARGLGERIIALLFSPFEIEGNSLRIGGSVGVALAPGDGDCVADVLRNADLALYAAKAAGR
G
QCLFFDPEMNARQRALQKLEHDLRQAVLSDAFVMEYQPLFDIASGDLVGAEALMRWIDPVRGRVSPAEFVPMAEELG
LIV
PLGARALEMACADAATWPAHLKVAVNLSPVQVHRVGVIEDVRAALAASGLDPRRLEVEITEGVLLDHGPEVLGTLDAL
HE
MGVQIALDDFGTGYSSLGYLRALRLDKVKIDISFVRELENDPSAAAIVRAVCSLCASLDIGVTAEGVETAEQLASVAAAG
CIEAQGFYLGRPGAAEIIAGLAGGALQGGRGVA
>OLS49813.1 entericidin EcnAB [Rhodovulum sulfidophilum]AB14
MTRIVVFCLSAAALLGVAACETVEGAGKDIQSAGGAISQEAREAQN
>OLS49814.1 hypothetical protein BV379_17075 [Rhodovulum sulfidophilum]AB14
MTDTLKARISTRRLTKSLALWLIGALTVAMLPLGLISVSQTSKVLHETERLSGVALMDRTQRAAVRTQALIQEAFGAARS
VAAANVVFEGGGLSCDAVMARLVKDNPTYLFAGFVEPDGRITCSSNGEHVSLVDQAYFLNLLRDPQPRVDTRFSVPLE
GA
RVVRVSVPVFEEGELQGFVLISMPYAAAYYTLDDGGAEVDLLVFDHEGEFLTAEAATREEGAPPETFEEVLPRNHTLEAL
AAAGRQSFRGTNRLGETRDFAVVPVIDDRIFVMGSWAPDRNGTVPTTGRALALYFPILMWVAGIVVAYFAVHRLVIRH
IK
RLQSWMRLYASGHTDFGQARLDHAPHELEVVAEAFRSMTRRLAEQDRALEEDLAEKTILLKEVHHRVKNNLQLITSLM
NM
QIRNARSAEARRLLKRVQDRVMALAAIHRYLYMARKLSLVRADRLLDDIIRQLVVVSDIGGAEKSVKIATQFSPVEVNPD
QSVPLSLLATEAAINAVKYCGTPPEGGQAWITMALQTLEDGRICLSVVNSRSEDPEPDPGAETGALTDSSGMGRQLISS
F
AAQLDAEFEVHSEPDRYELHVTFAPLGASLEEDEAEPEEEAASGGVSEGT
>OLS49815.1 hypothetical protein BV379_17085 [Rhodovulum sulfidophilum]AB14
MEHKDRRGRMEEQIDDNLRRVYAQAVEEQVPDRFLKLLEQLRGAGAEASNGSSGKGSDT
>OLS49816.1 response regulator [Rhodovulum sulfidophilum]AB14
MTHAPHSDDLSRVIGSELPYLRRYARALTGSQTSGDAYAIATLEAILSDREVFEQGLPPKVALFKAFNRIWGSSGAPLSE
SQPASAIEAQAQTRLAGLTRNTREALLLQSIEGFTTEEVGTIMGLPGTEAEQLIRIARDEMARSIAGRVLIIEDEAIIAI
DISAIVQGLGHEVTGIGRTRRGAVDLAKKDPPDLILADIQLADKSSGIDAVNDILGDLGDRPVVFITAFPERLLTGERPE
PAFLISKPYTEDQVRSAVSQAMFFSSTETLGA
>OLS49817.1 DUF1328 domain-containing protein [Rhodovulum sulfidophilum]AB14
MLYWALVFLVVALIAAAFGFGGIASASAGIAQILFFVFLVLFVVALVARALRGRMP

>OLS49818.1 hypothetical protein BV379_17100 [Rhodovulum sulfidophilum]AB14
MAQSAEKRNDSSASVQDLEVQIEALKDDIAQIARTLGDLSEAKKNDMRDTVQRRASELRERGEATVAGVQDRGAELG
EQA
ADAVRRQPAAAMGIAVGLGFLVGLMTGRR
>OLS49819.1 hypothetical protein BV379_17105 [Rhodovulum sulfidophilum]AB14
MFSLPLDEAERRARAFSHRLALGTAGSLCAGVGAAFLTVAAWLVLSAGHGAVFAATVIGSAYLGAGLILWALSLRPRQP
E
RTAPPADPYTPFIHMAEGFAAGLEAGRAARKPGSSDRARD
>OLS49820.1 5-(carboxyamino)imidazole ribonucleotide synthase [Rhodovulum sulfidophilum]AB14
MSEPRVSARLAPGATIGILGGGQLGRMLSVAAARLGFRCHVFEPAAAPPAGQVAERVTTAPYEDTGALARFAASVDVV
TY
EFENIPTAALDAIEAVVPIRPGRKALAVSQDRLVEKSFLNGQGLATAPFAAVDDEVDLAEALEEIGAPAILKTRRFGYDG
KGQARIMAPEDGPAALAGMTGAPAILEGFVEFSSEISVIAARGVSGEVACFDPGANIHRDGILHTTTVPAPLSVAQRTD
A
VLLAGRILNALDYVGVMGVELFVTKAGLLVNEIAPRVHNSGHWTQNGCVIDQFEQHIRAVAGWPLGDGARHSDIVM
ENLI
GEDMDRVPDLAADPKVALHLYGKAETRVGRKMGHANRWTGPAAS
>OLS49821.1 5-(carboxyamino)imidazole ribonucleotide mutase [Rhodovulum sulfidophilum]AB14
MGDVKVGIIMGSQSDWSTMRQAADILDALEVPYETRIVSAHRTPDRLWDYGKSAVGRGLQVIVAGAGGAAHLPGM
MASKT
RVPVIGVPVQTRALSGVDSLYSILQMPKGFPVATMAIGAAGAANAGLMAAGILALNDPALAGRLDRWRADLSASIPEV
PV
DE
>OLS49822.1 hypothetical protein BV379_17120 [Rhodovulum sulfidophilum]AB14
MNIASEMKSDEVLRVKLEVLRREHRDLDDAIHALQEKSTGDSFTLKRLKKQKLSLKDRIAQIEDQLTPDIIA
>OLS49823.1 heat-shock protein Hsp20 [Rhodovulum sulfidophilum]AB14
MTKLTLGSHPFLLGFEQLERLVERTAKSGNEGYPPFNIEQTSETGYRITLAVAGFREGDLAITVEDRQLVIRGRQVEDGA
ERIFLHRGIAARQFQRSFVLADGVEVSGATMENGLLHVDLSRAVPESVVQTIRISTA
>OLS49824.1 hypothetical protein BV379_17130 [Rhodovulum sulfidophilum]AB14
MNVKYDPLPDTGERIVYVRPVKVAELPEELRRQAPDLDELYAVHDSNGDRLALVRDRKLAFLLARQNDMAPVNVH
>OLS49825.1 lysophospholipase [Rhodovulum sulfidophilum]AB14
MDRAPLYADVADGPADGSAWWLRTEDGVRIRIGVWGQEETGGTVLLFCGRTEYAEKYGSAAAALRARGFATLAVDW
RGQG
LADRPLGDASLGHVARFGDFQADVRAVRRAAEALGLPRPYHLLAHSMGGAIALRALNEGLDVASAAFSAPMWGIRLG
PVA
APLVWALSGVGRGAARVPGTGARAYVLATDFEANDLTGDRAMYEKLRRQVTVHPDLGIAGPTVTWLRESLSEAARLS
RLP
APPVPALTFLGGRERIVSPAAIRRRMANWPGGTLDLVEDGRHEILMDRPEIRERAYDAAAAHFRAHGG
>OLS49826.1 sterol carrier family protein [Rhodovulum sulfidophilum]AB14
MSDTLDKAVEALNDKLSGGFNGIAKFSIKDTGDIIVDEDGAHAGDAEADVTLSASLETFRAMMEGDLNPTTAFMTGKL
VI
EGDMGKAMQLAQIFT
>OLS49827.1 hypothetical protein BV379_17145 [Rhodovulum sulfidophilum]AB14
MRGMSPFSACCAILSSVMANTLRFLNLALAVFSGAALIAGQGIADSGRAEDLLEELQRPDLPNWQRVEDALWGEWSK
SGS
AAMDLLLRRGRDAIEAEDYGAAIGHLTALVDHAPDFAEGYNSRALAYYRSGLYGPALADLARTLDLNPRHFGALTGLGT
L
LEETGQDAAALGAYRAAHAIHPHDPDLEDAVRRLEIVTSGRTL

>OLS49828.1 hypothetical protein BV379_17160 [Rhodovulum sulfidophilum]AB14
MQERAMMLDRQMQRQGQRNGPIRRAMPVRAALEWAFGTECARLDHDEIEAVGGTGWRPFGMEYVALERAQLGT
RVDTSRG
RSRPHDDAELIATVVRNVLPWYAATLVADLARAGRTPDWMPDARPRLRPVEWQQNQHRAYGRACDSAELPDGWQ
PIPRRN
RKGVIVQDRARYTPCVWEPSPARIAAARRAYLDWWGYLQDVQAALGATNLAQICVSGDMPPMTPWR
>OLS49829.1 hypothetical protein BV379_17165 [Rhodovulum sulfidophilum]AB14
MRKRDSYRARKSALKNKTRPVRSEAGASVGRIDALTRNARNTWFALLGLLVFVGITLMSVEHIDFYGIDRATRLPLVNV
D
VPTRYFFVAAPILTAAFYIYFHLHLIRLWDALGAADNRIDDQPLGDAVMPWLVTDAALHLRNRWRADECCTPRALDRP
AT
VLNLVLAWGFGLIVLGLLWWMSMPARTFWMTAIACAALLAATGVGVSSLKCIFEKMSPDKRIWQRRRSAPIILIVVALI
A
FSVSGLRTRGLVGLWTPPIRLLAPIDMTGLAIVEKPTGWLPRDIAKSDFFGDWCKREGTDCTRKELSEEQLTLFEREFRR
RREAAIAELRKPQWHQPGHQKPDFRGAPLELEAAIAELRKPQWHQPGHQKPDFTGAQLADTFLAGANLSGAQLESA
DLSG
AQLEGADFGGAQLEGADLSGAQLEWADLTGAQLEGADLSWAQLEGANLGGAQLEGADLRGAHLEGAELAGAHLEW
ADLTG
AQLEGADLSWAHLEWADLTGAQLEGANLSLVQLEGADLSMSFLTGVRFELLIIQSLNLRAVTNDGGALRFADFRSVTFN
E
KTDFRNAFLDGSVQITDGFRKQMGDPCQWHADVLSDEEFFGRWRGWVEASENISLYWQGRSPEVWSDVAAIPPAD
GCTWK
TGSISDTSQ
>OLS49830.1 endonuclease [Rhodovulum sulfidophilum]AB14
MPPRLTAMPPRLASPAPRIGPAQGDARAQDRERERVKPWRAWYRTARWRKLRRKILARAGHVCEQTGVPLLGRAPA
PDSA
VIDHIRPHRGDPALFWDEDNLQAVSKAWHDGAKQRAERAAGD
>OLS49831.1 hypothetical protein BV379_17175 [Rhodovulum sulfidophilum]AB14
MTDDFSPEPGAVDLFGNPVEPLRDRRGRPCFRKDKANQDFVAARRAAGWSHEMIAVELGCDEKTLRKYFSRELQHGR
LMV
EGMCLDVLMKRAREGHTPSVRQLQERIDRVAPPAPKPKPEREAPKPEPKGKKQARLEAAARPAEDYGSLYDRIPRQ
>OLS49832.1 hypothetical protein BV379_17185 [Rhodovulum sulfidophilum]AB14
MSWCVGNAKTEPRGNAVIVTKAVSGAGKIDPLMAAFNAVYLMSLNPEPARRDLSGFLDNPVMAI
>OLS49833.1 phage portal protein [Rhodovulum sulfidophilum]AB14
MIGRAMQGAVKGAWQGLRQALREGESGWEVVSAAGAHPFAGLGRQSKAGQRVSASSALTVSAAWACVKGNSELI
GALPVA
LYERGANGSRARIEADLAEILTLSPSAGQTAMEFWEAQVAHLLLQGNAYSEKLTVGSRLVGLRPLFNVMPVRRDDGRF
DY
RVTEDGRSHVLPPGKVFHVRGFGGGDGLGMSAIRFGVQSFGSALAADETAARVFSNAMMPSGVIESDQTLTPEQRA
QLGR
MLEAYVSSDRAGKTLALEAGLKYRQLQMNPEDAQLLETRRFQVEDVCRWFGTPPVVIGHAAQGQTMWGTGVEAIM
MAWLR
TGINPLLRRLESRILKDLIPAAKRGRWFAEWNREAMLQMDSKAKGEFLSKMVAGGIMSSDEARDKLNLARRGGAADEL
RA
QTALAPLTDLGKEQP
>OLS49834.1 peptidase [Rhodovulum sulfidophilum]AB14
MSKRKLPKVALGARPGVQGDLSPRALQRWSPDVRAADPGEASISVLDPIGADPWGEGVTARRISAALRAIGERPVTVN
IN

SPGGDFFEGLAIYNLLREHAGTVTVNILGIAASAASVIAMAGDEVRIARAGFLMIHNTWIMAAGDRHALAEVCEWLAP
FD
ATAIDIYAARTGIERDALAAMLDRETWIGGQAALDQGFADSFLPADQLETAPGDGAPAASMRAERRFDLLARRAGLTN
SA
SRALLRELRTGRPGAAHDGLQPGAAADLEQGLAALLEDLRTR
>OLS49835.1 hypothetical protein BV379_17200, partial [Rhodovulum sulfidophilum]AB14
MKIMMPGAAQAALSQVMPKGTTAPLPLSVPKAVIGTPRMEAAGSVDRLLADVKAELGRIGDDVRRTAEDALSQARAS
GDV
SAEVKASADKLLAEFHTLSGAQQKLEGKLEALETRNLDLEQAVAEGGRGRASAAMTVG
>OLS49836.1 phage portal protein, partial [Rhodovulum sulfidophilum]AB14
RPLFNVVPVRRDDGRFDYRVTEDGRSHVLPPGKVFHVRGFGGGDGLGMSAIRFGVQSFGSALAADETAARVFSNAM
MPSG
VIESDQTLTPEQRAQLGRMLEAYVSSDRAGKTLALEAGLKYRQLQMNPEDAQLLETRRFQVEDVCRWFGTPPVVIGHA
AQ
GQTMWGTGVEAIMMAWLRTGINPLLRRLESRILKDLIPAAKRGRWFAEWNREAMLQMDSKAKGEFLSKMVAGGIM
SSDEA
RDKLNLARRGGAADELRAQTALAPLTDLGKEQP
>OLS49837.1 peptidase [Rhodovulum sulfidophilum]AB14
MSKRKLPKVALGARPGVQGDLSPRALQRWSPDVRAADPGEASISVLDPIGADPWGEGVTARRISAALRAIGERAVTVN
IN
SPGGDFFEGLAIYNLLREHAGTVTVNILGIAASAASVIAMAGDEVRIARAGFLMIHNTWIMAAGDRHALAEVCEWLAP
FD
ATAIDIYAARTGIGRDALAAMLDRETWIGGQAALDQGFADSFLPADQLETATGDGAPAASMRAERRFDLLARRAGLT
NSA
SRALLRELRTGRPGAAHDGLQPGAAADLEQGLAALLEDLRTR
>OLS49838.1 hypothetical protein BV379_17215 [Rhodovulum sulfidophilum]AB14
MKIMMPGAAQAALSQVMPKGTAAPLPLSVPKAVIGTPRMEAAGSVDRLLADVKAELGRIGDDVRRTAEDALSQARA
SGDV
SAEVKASADKLLSEFHTLSGAQQKLEGKLEALETRNLDLEQAVAEGGRGRASAAMTVGHRPSARTVRP
>OLS49839.1 capsid protein, partial [Rhodovulum sulfidophilum]AB14
MTAEGAPAPVSSYGWSQADAPVRKIIHVTHISDEALADAGQLQAEIDGELRYGLDLEEEAQILAGDGAGQNLTGLITVA
T
AYAAPEGLPDETRIDRLRLGLLQVALANFAA
>OLS49840.1 capsid protein, partial [Rhodovulum sulfidophilum]AB14
QTKRENGAAMTAEGAAAPVSSYGWSQADAPVRKITHVTHISDEALADAGQLQAEIDGELRYGLDLEEEAQILAGDGA
GQN
LTGLITVATAYAAPEGLPDETRIDRLRLGLLQVALANFAA
>OLS49841.1 capsid protein, partial [Rhodovulum sulfidophilum]AB14
AAMTVGQEVAADDSLRAYVAGGLQGSVVLRPKNAITTATGSAGGLIWPTEERGAIGLPRQRLPVRALLMQATTDSNM
VRY
ARQTKRENG
>OLS49842.1 hypothetical protein BV379_17235 [Rhodovulum sulfidophilum]AB14
MRLTLTRTQKTALGLLQRGIPYEFDLGKPAERAVAEDLRARNVATETPAEAPAPKRKGKPAPAPDPDPAP
>OLS49843.1 hypothetical protein BV379_17240 [Rhodovulum sulfidophilum]AB14
MTRIPLEELKRHCQAEGFEDDDALLAGLGEAAERFVADYTRRDLDAAFPAGWPAPCAMAAKLLVAHWYRSREAVAEG
ASA
EVPLGVRDLLAPYRDLG
>OLS49844.1 phage head-tail adapter protein [Rhodovulum sulfidophilum]AB14

MAAPRAGELTRVVRFERATLVDDGLGMVERWAPHGRSQRAKRTDLSDGERWRAAEIGASVTTRFVLKRTAFTADLTP
KDR
LTCDGLSFGISGIRELAADPRFLELTCTARTDR
>OLS49845.1 hypothetical protein BV379_17250 [Rhodovulum sulfidophilum]AB14
MSVTMKLEGFAELEAELDRLSQAAGKGALRRALRTAAEPLAERMRANAPTDEHELEESIAVSTKLSPRQRKAHRKLVRD
D
KASVEMFVGAGPLPQAHLQEFGTVSTPPQPWARPAWEAEKDPILDRLRAEMKAEIDKSVRRAEARAARAAARG
>OLS49846.1 hypothetical protein BV379_17255 [Rhodovulum sulfidophilum]AB14
MERLLRARLRADPGVAALAGERVNWDAHPQGAPRPALVLHLIAGAPGRTLTGPDGLWTGRIQIDAWARERAGAGAL
ARAT
IACLNGLSESVIRGLWLDSQRRDRAADPDDRTWLYRVSMDFSATWRER
>OLS49847.1 hypothetical protein BV379_17260 [Rhodovulum sulfidophilum]AB14
MSDDITEADIGHGARLGLQAEDGSFEDLAMVTRIKPVGWTRDEEEATHLQSPGRYKQFLPGLKEQTPCEADLNWIPSV
PD
RLIAAFEAGRQTWQITAPNGVRMQFPGFITTYEPGEFSNGKMTASVTIRPAGQAKILEAE
>OLS49848.1 hypothetical protein BV379_17265 [Rhodovulum sulfidophilum]AB14
MIPTLSHSAGEWMCGNFQMAATLYDRQEHEVLISSEHGNNFIEGMKTMKGSKRLALANKRPAALVTGGFTFA
>OLS49849.1 hypothetical protein BV379_17270 [Rhodovulum sulfidophilum]AB14
MRLTLTRTQKTALGLLQRGIPYEFDLSKPAERAVAEDLRARNVATETPAATPAPKRKGKPNPAPAPDPAP
>OLS49850.1 hypothetical protein BV379_17275 [Rhodovulum sulfidophilum]AB14
MTRIPLEELKRHCQAEGFEDDDALLAGLGEVAERFVADYTRRDLDAAFPEGWPAPCAMAARLLVAHWYRSREAVAEG
ASA
EVPLGVRDLLAPYRDLG
>OLS49851.1 phage head-tail adapter protein [Rhodovulum sulfidophilum]AB14
MAAPHAGELTRVVRFERATLVDDGLGMVERWAPHGRPQRAKRTDLSDGERWRAAEIGASVTTRFVLKRTAFTADLTP
KDR
LSCDGLSFGISGIRELAADPRFLELTCTARTDR
>OLS49852.1 hypothetical protein BV379_17285 [Rhodovulum sulfidophilum]AB14
MSVTMKLEGFAELEAELDRLSQAAGKGALRRALRTAAEPLAERMRADAPTDEHELQESIAVSTKLSPRQRKTHRKLVRD
D
KASVEMFVGAGPLPQAHLQEFGTVSNPPQPWARPAWEAEKGPLLDRLRDELKAEIDKSLRRAEARAARAAARG
>OLS49853.1 hypothetical protein BV379_17290 [Rhodovulum sulfidophilum]AB14
MEEILRARLRADPGVAALAGERVNWDAHPQGAPRPALVLHLIAGAPGRTLTGPDGLWTGRIQIDAWAREVTEAGALA
RAT
IACLNGLSESVIRGLWLDSQRRDRAADPDDRTWLYRVSMDFSATWRER
>OLS49854.1 hypothetical protein BV379_17295 [Rhodovulum sulfidophilum]AB14
MSDDITEADIGHGARLGLQAEDGSFQDLAMVTRIKPVGWTRDEEEATHLQSPGRYKQFLPGLKEQTPCDCEGSRYTLR
MD
MLALAAFEDATGENALSWAEAAEGGQAAVGDMIEMIRCTLARHHPEADYRLAGDILSEDPGVLLRLFRAASPELAQDE
PE
DGPEDGPGKPLPAPAAG
>OLS49855.1 hypothetical protein BV379_17300 [Rhodovulum sulfidophilum]AB14
MTLESLFEAHVAAGFDPAAFQDLSLKEYGLAMRGARARIRAEHEARAWLAWHVEALRRCPSLPSFRSFLGGRSAPEAA
QP
ATEMQAMFDTVATAWARSPAARG
>OLS49856.1 hypothetical protein BV379_17305, partial [Rhodovulum sulfidophilum]AB14
MSQSVIGALRVNLGLDSAKFERGAKRVKDPLRVMRSQFRAVAAAASAMGAALSAAALKGAGEIDRAAKAARRLDASI
GAF

RALELAAGEAGVPLSA
>OLS49857.1 hypothetical protein BV379_17310, partial [Rhodovulum sulfidophilum]AB14
IEMIRCTLARHHPEADYRLAGDILSEDPGVLLRLFRAASPELAETEDDSEDGPGKPLPAPAAG
>OLS49858.1 hypothetical protein BV379_17315 [Rhodovulum sulfidophilum]AB14
MTLESLFEAHVAAGFDPAAFQDLSLKEYGLAMRGARARIRAEHEARAWLAWHVEALRRCPSLPSFRSFLGGRSGPEAA
QP
ATEMQAMFDTVATAWARSPAARG
>OLS49859.1 hypothetical protein BV379_17320 [Rhodovulum sulfidophilum]AB14
MSQSVIGALRVNLGLDSAKFERGAKRVKDPLRVMRSQFRAVAAAASAMGAALSAAALKGAGEIDRAAKAARRLDASI
GAF
RALELAAGEAGVPLSAVTNDIQNIGREIANIGVGGNADRALKRLGLAAADLDGLDADVKVATIADAVKGLGLSAGEATA
V
LRDLGVRNREMALVMIQGGAAIRAAREDVEDYGLSLDAVEAGKIEAANDRIARLGLISRYAGQQLALRLVPALGRAAQ
AM
TEALREGGLLRRVIDGLTENLARISTWIGTAVTAFGVRYVGALAAARLATISLAGSLAMLRGALIRSGIGIAVIAAGELV
YQLGRLVTATGGFGEALDLMKDVAAEVWERIAEGPHLVTLAVRQMGWAIRADFLEAMADIQARWSGFIGNIATVGK
LIPG
FGASFRTLAASVTGSAESMRALREESRLAAQYATNYGTQVEGLFTNMTRPLETLKALREALAETPPGGPGRPRLPEDED
D
QTGLGGIGTATERLSETARKTGDSFKSFFKDVISGSGDAGDAISALADRMLDDLLDRALSPLTDAMGSLFDGLFSGLFGG
GGASGGGGTVTQLFPSGGLPGFARGTDSAPGGLAWVGEAGPELVNLPRGAQVIPNHRLGSLGGGSVRMGDVHVSV
AQTNA
DPRQIAEAVRRVSREEAQRVYARQRREGR
>OLS49860.1 hypothetical protein BV379_17325 [Rhodovulum sulfidophilum]AB14
MAFPPDAVRVASLTLKQITSSIRVQSPYSFAQTAYDFMGGMWAAELVLAAVDARESAAISGWIASLNGPVGTFELDM
AAM
DYEGPHDKISADPVVAVAATARAQALVLQLAGAGDRALPGDYLTLGRHLHIVTAAEAPTAAFRQRVTLWPRLRRPVAP
GD
PVAMRVPFGTWALAGPETVLSVSQARVRTRTLQIMEAL
>OLS49861.1 hypothetical protein BV379_17330 [Rhodovulum sulfidophilum]AB14
MDASGDRMLGLLVRCRFDTGDLAMFTGHGTVEWEGVTYLGAGQMLSVGEAQATSGEGIPGLSITLSGLDPEVVALAE
LEE
FQRRRVTVFLALFDETGQIETADVLFDGVADTMESDDGPDTATTTLALEPRSMALGRKFPFYYLPEDQKKRFPGDQGF
DL
VQAIQNREDTWGRT
>OLS49862.1 hypothetical protein BV379_17335 [Rhodovulum sulfidophilum]AB14
MRRAGWERRLSAAVRDWEDRPFAWGTADCLGFCRAVARSMTWEDPIPGLPDYGSEYGAAKVLVSLGFGSIEALVDA
HLER
LPVAGARRGDWVMTACEGAMPGAMGVVTGRYAQHMGRNGLVRRPVLTAEAAWRID
>OLS49863.1 hypothetical protein BV379_17340 [Rhodovulum sulfidophilum]AB14
MAYRLILPALLILLAGPAAAMPPVVAALGAAAGGWMAGLTATAIMGQAALAAVLTGIQMAMAKKPKAKTRDEITLNR
IQP
VTTGRILYGERMLGGSIVARATTEAGGKPHRRYHSIMPLACHEIDGAVEIWLGETLVWTEAQYRRDEAAGSGDPYHW
GQV
GSDYRGRVRLCVHNGGEDQAAEARYVAAAREWTEAHRLRGVAYVYFEADYDRDLFPSGAPQIRVRCRGKRVYDPRE
WIST
ALASDSAITRSRAWAGGDSGAIGETAGGSYPIGESAAAWNGGAISARFYFDSLYPGGTAPEAGRTPNVTATEGDVLDR
AK

MAIGANLFAAFDVSFPAAPEGVIWEQGGSGRGAYLGVTGSDLVFRAFSGEATGDEVARASIPVADFANRRGTIYVEISA
D
DKSIALWFRGRKYSTNPALCLRDYMLTPQLRGGPGWRPGDLDEDTILALANLAEEQVPLASGRTEDRYAFNGVLETEAT
A
AANLNDLSSSWGGWWTCERGRLTVGGAAWEEPAFTVTEDMLTGGIQVTARKPFEEQFNTVKAQYADPENEHVVTD
LPVLD
SATYIAADNGEPLVLDMGELPGETGFARGQRLMKQALLKGRRQKQVTLPCSLAAWPVRLGDNIRVSLPRRGWNAKTF
EVT
GRTVHIGEDGVRVTLSCIETGPAIFDWRTSEEMPKPAGGVPTLPSPTAKPVVSAPTMTEELYETRGGGGVKTRVRLSAT
T
DNPFVDAWQFAWRPVSAPEPTLRGLTDTPEDMIDDVAPGPYAFGVRGRNARGIWSDWAWAGAAAVQGENAPPG
AITGLSV
QASGGAVAMLRWDRHPSLDVRQGGRIEFRHATAQAGASWQSSTGIGKSVSGGVTEATLPLKSGTYLARPHDAMGTP
GPVS
GIAVRAASIIPTTTVATLAEAPDFAGAAEGCHAAGGVLAMAPGETRARYAFAGRIDLGAVQSVRLISDIELLISEARDLF
WQPSGTAMWTPPDARLWLGSTDAYGDVDLQVSVTDDDPETARWGPWQSFDAADFSGRAFRFRAVLGVESPDYTI
GITGLT
VTALQAA
>OLS49864.1 hypothetical protein BV379_17345, partial [Rhodovulum sulfidophilum]AB14
MTQATYLLDTGHTKAQAFAMLQGALSAIQSGNAGASPPAETAAGMLWVDTDGRALKMRNIANNGWITLGSLAGEA
FTPAG
VLQLTLAQATDPTGTEFGALSGQILKAVVEAFAPPQIRPRVYSYHLLDTSVPTGVMDLTDA
>OLS49865.1 hypothetical protein BV379_17350, partial [Rhodovulum sulfidophilum]AB14
SCIETGPAIFDWRTSEEMPKPAGGVPTLPSPTAKPVVSAPTMTEELYETRGGGGVKTRVRLAATTDNPFIDAWQFAWR
PV
SAPEPTLRALTDTPEDMIDDVAPGPYAFGVRGRNARGIWSDWAWAGAAAVQGENAPPGAITGLSVQASGGAVAML
RWDRH
PSLDVRQGGRIEFRHAGAQAGASWQSSTAGVPLSAVTNDIQNIGREIANIGVGGNADRALKRLGLAAADLDGLDADA
KVA
TIADAVKGLGLSAGEATAVLRDLGVRNREMALVMIQGGEAIRAAREDVEDYGLSLDAVEAGKIETANDRIARLGLISRYA
GQQLALRLVPALGRAAQAMTEALREGGLLRRVIDGLTENLARISTWIGTAVTAFGVRYVGALAAARLATLSLAGSLAML
R
GALIRSGIGIAVIAAGELVYQLGRLVTATGGFGEALDLMKDVAAEVWERIVMGPKLMRLRMEELGARMKAIWGEVMV
YLQ
EQWTDFLGKFAELPDFVPGAKAMKEAADGAADGLTAAKEAAAAAGDEAQRLKLEANGIADAMIRTPLASMQALKET
LAGV
SEEAGNTATNLARIGDAAGGDGGTSAVTQATGQLADTAKTASDSFKTFFKDVARGSGDAGDAISALADRMLDDLLDR
ALS
PLTDAMGSLFDGLFSGLFGGGGASGGGGTVTQLFPSGGLPGFARGTDSAPGGLAWVGEAGPELVNLPRGAQVIPNH
RLGS
LGGGSVRMGDVHVSVAQTNADPRQIAEAVRQVSREEAQRVYARQRREGR
>OLS49866.1 hypothetical protein BV379_17355 [Rhodovulum sulfidophilum]AB14
MAFPPDAVRVASLTLKQITSSLRVQSPYSFAQTAYDFMGGMWAAELVLAAFDARESAAISGWIASLNGPVGRFELDM
AAM
EYDGPHGMISADPVVAVAATARAQALVLQLARAGDRVLPGDYLTLGRHLHIVTAAEAPTPAFRQSVTLWPRLRRPVAP
GD
PVAMRVPFGSWALAGTETVLSVSQARVRTRTLQIREAL
>OLS49867.1 hypothetical protein BV379_17360 [Rhodovulum sulfidophilum]AB14

MDASGDRILGLLVRCRFDTGDLAMFTGHGTVDWEGVTYLGAGQMLSVGEAQATSGDGIPGLSITLAGLDPEVVALAE
LEE
FQRRRVTVFLALFDETGQIETADVLFDGVADTMESDDGPDTATTTLALEPRSMALGRKFPCYYLPEDQEKRFPGDQGF
GL
VQAIQNREDTWGRT
>OLS49868.1 hypothetical protein BV379_17365 [Rhodovulum sulfidophilum]AB14
MRRAGWERRLSAAVRDWEDRPFAWGTADCLGFCRAVARSMTWEDPIPGLPDYGSEYGAAKVLVSLGFQSIEALVDA
HLER
LPVAGARRGDWVMTACEGAMPGAMGVVTGRYAQHMGRNGLVRRPVLTAEAAWRID
>OLS49869.1 hypothetical protein BV379_17370, partial [Rhodovulum sulfidophilum]AB14
MAYRLILCALALILLAGPAQAMPPVVAALGVAAGGWMAGLTATAIMGQAALAAVLTGIQMAMAKKPKAKTRDEITL
NRIQ
PVTTGRILYGERMLGGSIVARATTEAGGKPHRRYHSIMPLACHEIDGAVEIWLGETLVWTEAQYRRDEAAGTGDPYH
WGQ
VGSDYRGRVRLRVHNGGEDQAAEARYVAAAREWTEAHRLRGVAYVYFEADYDRDLFPSGAPQIRVRCRGKRVYDPRE
WTS
SALASATAASTVPNWAGTDDGAIGQVGGGGYPKGETAAPWTGGAIAARFYFGRRYPGGLDPEAGLSPDLTATRGEILE
AS
RMERGADLFAAFDVAFPDAPEGVIWEQGGAVYGAYLGVTGDDLVFRAFSGEASGPKLARATLPVSAVAGRRGTIYVEI
DR
TTGTVSIWFRGRKYSTNPALCLRDYMLTPQLRGGPGWRPGDLDEDTILALANLAEEQVPLASGKTENRYAFNGVLETK
AT
AAANLNDLSSSWGGWWTCERGRLTVGGAAWEEPAFTVTEDMLVGGIQVTARKPFEEQFNTVKAQYADPENEHVVT
DLPVL
DSATYIAADNGEPLVLDMGELPGETGFARGQRLMKLALLKGRRQKQVTLPCSLAAWPVRLGDNIRVSLPRRGWTAKT
FEV
TGRTVHIGEDGVRVTLSCIETGP
>OLS49870.1 hypothetical protein BV379_17375, partial [Rhodovulum sulfidophilum]AB14
MTQATYLLDTGHTKAQAFAMLQGALSAIQSGNAGATPPAETAAGMLWVDTATRALKMRNVPNNGWITLGSFASGS
FVPAG
VNQLTEAQAKDAFSDIYGAVTGAILRAAILAHAPDAVIPGWHPFDMLCPGDGATGLIWSYDKAGTIQNVETPEFEDGY
EY
ALVFEAVATNKTTSALQISVQPEITGTWSAFTSFPGAGFASGDICSGMLRLPMPRQPRLVHGCIWDAPLKAGLEDLAVP
T
GAPEASALSAVRVRWAEGNR
>OLS49871.1 hypothetical protein BV379_17380, partial [Rhodovulum sulfidophilum]AB14
IEFRHAGAQAGASWQSSTGIGKSVSGGVTEATLPLKSGTYLARPHDAMGTPGPVSGIAVRAASIIPTTTVASLAEAPGFA
GTATGCSAAGGVLAMAPGEIRARYAFAGRIDLGAVQPVRLISDIELLISEARDLFWQPSGTAMWTPPDARLWLGSTDA
YG
DVDLQVSVTDDAPAAARWGPWQSFDAADFSGRAFRFRAVLGVESPDYTIGITGLTVTALQAA
>OLS49872.1 hypothetical protein BV379_17385 [Rhodovulum sulfidophilum]AB14
MTQGTYLLDTGHTKAQAFAMLQGALSAIQSGNAGATPPAETAAGMFWVDTAARALKMRNVPNNGWITLGSFASGS
FVPAG
VNRLTEAQAKDAFSDIYGAVTGAILRAAILAHAPDAVIPGWHPFDMLRPGDGATGLIWSYDKAGSIQNVETPEFEDGY
EY
ALVFEAVATNKTTSTLQISVQPEITGTWSAFTSFPGAGFASGDICSGMLRLPMPRQPRLVHGFIWDAPLKAGLEDLAITT
GASEASALSAVRVRWAEGNRSAGSIHTDCRIKETGNIGELNRGFRYRYRSRK
>OLS49873.1 hypothetical protein BV379_17395 [Rhodovulum sulfidophilum]AB14

MTSMADEADTLLAQRVESSRQLGGNPELAMHGGGNTSVKVMREGRRVLHVKGSGWDLAVMEPQGMPGLWLDPL
FEVKDGA
RLSDSEMVRFLRAHLLDASAPNPSVETLLHAYLPARFVDHGHASAVLALASRPEAEQREMLRALFGDGLVFLPYVFPGF
D
LSIEGARAAEAEPQAWGMWLAQHGLFTWGETAEESLTRFRDAVAACEAHLAARGAALMPPTARQGNEALEALVPHL
HAAL
PGLAFTDARGADPLAALSGRAGMAEAVSRGTVTPDHVIRIKPWPLILAPGTDAAGIRAALAGFGAAYERYFQDHSDGT
QI
MLDTLPRVAFVPGAGVIGLGRSASEASINADLAEQNLRVAASAEALGGYQPLPRPEQFLIEYWELEQAKLRK
>OLS49874.1 autoinducer 2 aldolase [Rhodovulum sulfidophilum]AB14
MADLDDIIDGKDFGLTTPPKNEPFFLKGAGAYDWGMQNRLARIFRPDTGRTVMLAFDHGYFQGPTTGLERVDLNIAP
LFD
HADVLMCTRGMLRTTVPASTRKPIVMRASGGNSIMSELSNETIAVDMEDALRMNVAAMAAQVYIGAEFEHKSITNIIN
LI
DRGNRVGMPTLAVTGVGKDMARDARYFGLATRIAAEIGAQYVKSYYVDEGFEKVCAGCPVPIVIAGGKKIPEAEALEVA
Y
KAIDQGAAGVDMGRNIFRAGDPVAMIQAVSAVVHDLLPAAQAFEMYNDLAARKAA
>OLS49875.1 hypothetical protein BV379_17405 [Rhodovulum sulfidophilum]AB14
MTGYLIGVDAGNTMVKAALFDTEGTTLAVASRGSETLQPAPGFVERDPDGLWQAASGAIRDCLAKAGIDGAEVLAVG
AAG
HGNGLYLLDAEGRGLIGIQSMDNRAETLASQIAAEHGDEILSRAQSRPWPAMTPVLLRWIAENRPEIMARAHKALLCK
DV
IVHGLTGRMGSEVSDLAGAGLLALPALDYDDALLRLYGIEGLKHVLPEPAQSDAVVGHVTAAAARVTGLAEGTPVVGGL
F
DVLASTLGAGTSHAGEASIVAGSWSINQVFAERLPAEPRALLASVLGRDTWVSCECSPTSAANLEWFVQTFLADEARAT
G
EDPFALCNRLAVGADPEKDLPVFHPFLYAGPVPGARGGFSAMGAWHSRGDMVRALYEGVAFGHRAHFEILDPDRRF
RFAT
LSGGAAKSELWAQIFADALGMTLRLSDCAETGARGAAIAAAIGAGLFADFDDATQAMARHVEEITPCPAAQALLDARY
RR
WCAYRDALGAAWAPPKLPGFDSRETQERTSHG
>OLS49876.1 hypothetical protein BV379_17415 [Rhodovulum sulfidophilum]AB14
MMDQLQDIAQIRRIYTALTLHYVDGLTQAQVAERMGQSHSTVNRMIKAGHDMGMVEISIRSPFQDHLALERELAEVS
GLR
EVVIETTAAGNPEAVLARVGITAAELLVSKLKPGMTVCLTGGKGVSACVAGLRQVVPLRGMRAVPATGLVQGKHYTDV
NH
AAATLARAFGGEAMQMLSPMFAETEEQHAMICGMKTVSRVMEAARAADVAVVGIGGLREKATSYFDLHRDVPAEEE
ALYA
SGAVAELLAHVLDARGAVTDYVRNRLVVAISPSDLARVPLSIGVAAGQEKAEAICAVVRGGYINTLVTDDATARCVLARL
KEAA
>OLS49877.1 ABC transporter permease [Rhodovulum sulfidophilum]AB14
MSETSLSGGMTQTRRDPGPLRRLAGMREMGLLCIILALCVVMSFASPYFLTWSNFRTIFLSFSIEGIVVIGMTLLLIVGG
LDLSVGSVVCLAMVVAGKLFLMGLDPWLASAAGVAAAACVGAAMGLFVVRVGLSHFIASLAAMVIVRGLCLLITKGTP
MS
LFSLPPEFKFVGQGSLGGLPVVVVIFVVLAVVFDFLLRRSARFRAIIYTGSNEQAAEYSGIRTKRVIFWVTVLCSTLTGL
AGVIFMARFGSATPQFGVGMELNVIAAAVIGGASLKGGQGSIFGAVLGVALLSLVTSSLILLDVSVYMQDMIKGCILLAA
VSFDHLMNKRGKRG
>OLS49878.1 D-xylose ABC transporter ATP-binding protein [Rhodovulum sulfidophilum]AB14

MPPLLHLSNIAKSFGPVRALRNVFFELRAGEIHALAGENGAGKSTTMKIVDGILQPDAGEIRIDGEPRRIRSPLEAQALG
IGFVHQEIALCPDVSVAENIFMSATATRRGLMDFAALERKARTVLAELCDVDPAAKVGDLSISDQQLVEIAKALTLDARI
LILDEPTSALTETETEKLFRIVHRLRERGLGIIHISHRMAEIFEHCDRVTVFRDGAYVTCLDIPQTTPQEVVNNMVGREI
SHLYPDKATTPPGRPLLEVEGVTCDVLRDVSLTLREGEILGIGGLIGSGRSELAKAICGLRRSAKTVRLNGEPVEIPDFS
AALERGMVYVSEDRKGEGLFLDLPISQNVSSLRLSQVTRAGLVDRAAELAQAETLGRRLRLKAGRPEDPPSTLSGGNQQ
K
VALARMLSVNPRVVFLDEPTRGVDVGAKSEIHAILRELCDQGVGIVVISSELPELIGLSDRIVVLHEGGMTGELGSEEMT
EEAIIHLASGLHIQGDAA
>OLS49879.1 transporter [Rhodovulum sulfidophilum]AB14
MKTRYLAAAAASFVVASAAGALAQDADAPWEQSPFHCEPGETYVMNVMVSGVEYWFPVYEMFKQAAHQFGCETD
YTGTPE
YDVNKQIATFEQALARQPAGILLHPMSPDPFVEPINRAIEQGTAIVTFAADSPNSKRVSYVTSDNFREGRVAAQQIAEA
M
GGKGQYAVLENPGQDNHDRRVAAFVEEMETNYPDMELVGRASTGQDPNKAYQAVLSLAQAHPDLGAVFMPEASSA
MGAAQ
AAKEAGGTIRVMSVDVNASVLDMIKAGDMFGAINPNQGAQGYYGFLLLWLAKHPELIDPMNDHAEAGFNPMGIPM
LDNGY
SVVTQENADSFYWDKYLKKRGTRGIEG
>OLS49880.1 hypothetical protein BV379_17435 [Rhodovulum sulfidophilum]AB14
MTWIIHVANASGQLDGLVSPIRAAIDRARACAEAVTNPVNLDVVVQAWPGRVIAHLGHAGYTPTGDMIQLTFDPGN
CSCA
QNLGEPLERVIVHELHHALRWRGPGYGCTLGEALVTEGLAGQFAQQLYGGPPEKWEVSLAADDLAHAAQDAMTTW
DDRNY
NHAAWFFGTDPAWRGYALGYALIGRYLAEHPAETPATLVHADAESFRGALKAMVD
>OLS49881.1 endopeptidase La [Rhodovulum sulfidophilum]AB14
MPEHLSPSYPVLPLRDIVVFPHMIVPLFVGREKSVRALEEVMQDDKQILLSAQVDPAMDEPSAEGIYRTGVLANVLQLL
K
LPDGTVKVLVEGRTRVRITGYIDNPSFFEASCEPLDEEIGDEEVVEALLRSVGTEFERYAKVKKNIPEEALSAVADTREP
AKLADLVAGHLGVEVEQKQDLLETLVVAQRLEKVYGLMQGEMSVLQVEKKIKTRVKSQMERTQREYYLNEQMKAIQR
ELG
DGDEGQNEIAELEERIAGTKLSKEASEKVAAEVKKLKSMSPMSAEATVVRNYLDWMLSIPWGVKSRVKKNLGAAEKVL
DN
DHYGLEKVKERIVEYLAVQRRSQKLKGPILCLVGPPGVGKTSLGKSVARATGREFIRISLGGVRDESEIRGHRRTYIGSM
PGKIIQAMKKAKTTNPLILLDEIDKMGQDFRGDPASAMLEVLDPEQNASFVDHYLEVEYDLSNVMFLTTANSYNMPGP
LL
DRMEIISLAGYTEDEKHEIAMRHLVPKQVKNHGLKSKEFELTPEALTEIVRRYTREAGVRNLEREIAKLGRKAVTKLIKK
ETDKVTITPDALEDYLGVKKFRYGLAEKEDQIGVVTGLAWTSVGGDLLSIEALKLPGKGRMKTTGKLGDVMKESIDAASS
YVRSIAPEIGFKPPRFDKIDIHVHVPEGATPKDGPSAGVAMVTSIVSVLTQIPVRKDIAMTGEVTLRGNVLAIGGLKEKL
LAALRGGIKTVLIPEENEKDLREIPDNVKEGLTIIPVSHVREVLKQALVRAPEPIEWDEAAEEAAAAAAAKASGGETAGQ
VAH
>OLS49882.1 alpha-L-glutamate ligase [Rhodovulum sulfidophilum]AB14
MTEHTDPAPPGFRLGWEEWVSLPDLGLPAMKAKVDTGAKTSALHAFDIEPFGPASRPKVRFAVHPIPGREDLSIPCSAA
I
VDRREVTSSNGDSEWRYVIAAEIVVGGRRWPIDLTLTHRGGMAYRMLLGRQALGEDVIVSPGDSCCQPILSYDVYHSQ
RV
REVAPARVLRIAVLSREPHNYSTARLVAEGEARGHVVEVIDTARCYMLLDALAPDVHYDGTRLPRFDAVIPRIGASVTAY
GTAVIRQFETIGTYCVNGSEGITASRDKLHAHQILARHRIGMPTTAFAASPKDTGNLISLVGGAPLIVKLLESTQGKGVV
LAETTNAASSVISAFRGLKASFLVQRFIKEAAGEDIRCLVVGSKVVAAMKRTGAEGDFRSNLHQGGHAITVKITPQEREI

AVRAARAFKLNLAGVDLLRAHDGPRVLEVNSSPGLEGIEKSSGKNIVGLLYDEIEKRVRPLPVPKGRRRR
>OLS49883.1 flagellar motor protein [Rhodovulum sulfidophilum]AB14
MALTRRSGGRFQAMIWPGFVDAMTALLLVLMFVLSIFMLVQEMLRETITGQESELTELNTELAALADALGMERRRAS
GLE
EQVGELGAALTTARSDAEAQAALIASLTDRLSTREADLAAAETRIGDIEDRVARLVTERDAARARGETLEGELAELEGSR
DRLEAALASTRQEVDARAEAARLAAARREALEALVADLRARNTEAEAARATLEGQVESAEAALSQAEKDRLAEAAAAA
AL
RDKLAGSEDELSAMSLALEQARQRAEDTLTLLAAAEKAREQAATEAEGALSEADRRAALLAVANERLETEEAKSAESQR
K
VALLNRQMAALRGQLGELQGLLDDAQERDAAARVQIETLGSKLNAALARVASEQRERAKLEEAARRKAEEEAARLEAE
KK
TLEGYRSEFFGRMRALLAGREGVRIVGDRFVFSSEVLFETGKADLSGDGEAQIANVVAILRGVADEIPPDIDWILRIDGH
TDDVPLARGADFADNWELSQARALSVVRYMIDDLGFPPDRLAAAGFGQYQPVAAGKSPEARAQNRRIELKLTER
>OLS49884.1 biopolymer transporter ExbB [Rhodovulum sulfidophilum]AB14
MDKTPREAEPQFSRPRRQVVLMTSACLFFATGAYLAYPQVAPVFLASPYLNGAIVFVFLFGVATCFLQVIQLGASVRWI
E
DFAGGIPGHGLTRAPKLLAPLAALLRKRSDKRMQINTASARTILDSVATRIEEQRDITRYLSSLLIFLGLLGTFYGLATV
VPAVVDTIRSLAPRDGEGGVEVFGRLMVGLESQLGGMGTAFASSLLGLAGSLVVGLLELFAGHGQNRFYQELEDWLSS
IT
RLGFSTGESEGEGGGAEVGLLAATLDRLADQMEGLHTLFAESEGERVALESRVGTLVEAVNTLGSAMERQLDDTRAVT
DA
LSRVAEGQARATAAIEARMGEEGALADAEARMRLRSIDVQLLRILEEISAGRQETVTDLRGDLAALGRTLSRLAPEGER
W
G
>OLS49885.1 gamma-glutamylcyclotransferase [Rhodovulum sulfidophilum]AB14
MQDGLWVFGYGSLLWKPGFEVAERRLARLEGWHRSFCMRSIHHRGSEAEPGLVLALDAAPGASCQGVALRAAPGTE
AETL
AALRERELISAAYFEVELDLVPAGGGTIRAVCYVIDPDHVQYCGGLPLEEQAQIIARAQGGMGPNDEYLFNTVAHLAEL
G
LSDPDLDWLADRVRALKSD
>OLS49886.1 hypothetical protein BV379_17475 [Rhodovulum sulfidophilum]AB14
MRFLLSLALLAALAWSGYWALAARGIERGLAGWLDTRRAEGWAADAGRIDVTGFPTRFDSRIEDLSLADPGTGLAWS
MPE
FSFSAQSHRPQRILARWPETQQIATPLERIAVTAETMTGTLAFRPGLAFELASARLSVGAFGLVSDRGWRASMEDAEIAI
KAVTDRPGAQHLQLSASGMRPPAGLVATLDPAGLLPPVFQRVSFEAVAVFDAPWDRRAIEERRPQVQRVEIARLDAQ
WGD
LALQAAGSFDVDAEGQPSGSLTLRATNWREMIAIARSSGQLPAALADRLEDALGLLAGLSGRPEMLDVPLRFALGQTW
LG
PVPLGPAPNFRLR
>OLS49887.1 hypothetical protein BV379_17480 [Rhodovulum sulfidophilum]AB14
MRRPGLVLALVLVAGTGSAASFDSIPRPKPRPALAWGALPAEAVAVRPEARPATVLSRVMDGSRRARPEAVAGRGLD
RSL
RPVPRPSDLLAKLAAAGLGRQPSAVSTPRRGVVCGDPAIAGEMIAPIVSRVAGCGVARPVRVSAVDGVALSQPVTVDC
AT
ARALRSWVSNGIKPAVGRRGGGVASLRVFAHYVCRTRNNRKGAKISEHGRGHAVDIGAVTLRNGTSLTVLGGWKDPA
QGR
VLKQIHRAACGPFGTVLGPGADRFHQDHIHVDTARYRGGAYCR
>OLS49888.1 cyclohexadienyl dehydrogenase [Rhodovulum sulfidophilum]AB14

MKSPLYDRVALIGLGLIAGSMALAMRRAGLAGEIVGTARSAETRAVAAEIGLVDRVVETAAEAAEGADLVVLAVPVGA
MG
AVAAEIAPHLAPGATLTDVGSVKRAVIETVAPHVPEGVHFIPAHPLAGTEHSGPRSGFAELFDNRWSLIVPVPGTDPAA
T
ARLKRFWEGMGANVEEMDPDHHDLVLAVTSHVPHLIAYTMVGVADDLRRVTDGEVIKYSAAGFRDFTRIAASDPTM
WRDV
FLTNKEATLEILGRFTEELFALQRAIRTGQGDELFDYFTRTRAIRRGIIEAGQDTEAPDFGRGAKART
>OLS49889.1 histidinol-phosphate transaminase [Rhodovulum sulfidophilum]AB14
MTRIPPQPGILEIALYQGGQSKIEGMTDVVKLSSNENPFGTSEKAKAAFSRCVHSLHRYPSTDHAELRAAIGEQHGLDPE
RVICGVGSDEIIAFLCQAYAGPGDEVIHTRHGFAMYRISALAAGATPVEVEEVERTVDVDAILAGVTDRTRIVFLANPGN
PTGTMIPEAETRRLAESLPESVVLVLDAAYAEYVEGFDGGRALVDRHHNVVMTRTFSKIYGLGGLRVGWAYAAPEVIGV
L
NRVRSPFNLGQAQLETAEAAVRDQAFVARCRAENIRLAAWLVEALNEAGVPTDPTFSNFVLPRFADAAEAEACDAYLK
SQ
GLIVRRVGGYKLPHALRITVGDEAACRRVAHAVRAFKEGAR
>OLS49890.1 hypothetical protein BV379_17495 [Rhodovulum sulfidophilum]AB14
MADKPTAELVFERGLFASRWLMAPMYLGLVVSLAMLVVVFLREIAYYAPQVLTMSSETAILAVLTLIDLTLAANLLLIVL
FSGYENFVSKLDIGDHSDRPDWMGTVDFSGLKMKLIASIVAISGIHLLKRFMEIGQAKADAVYGNNELFWLVLIHMVFV
L
SGVLLAAMDWLSARAKKH
>OLS49891.1 30S ribosomal protein S4 [Rhodovulum sulfidophilum]AB14
MTKRTSAKYKIDRRMGENIWGRAKSPVNRREYGPGQHGQRRKGKLSDFGIQLRAKQKLKGYYGDMTEKQFRRIFAEA
ERQ
RGDTGELLVGLLERRLDTVVYRAKFVPTIFAARQFVNHGHVTVNGQRVNIPSYRVKEGDVIEVREKSKQMTALLEAVQL
A
ERDVPDYIEADHSKMTATFVRIPGLGDVPYPVVMEPNLVIEFYAQN
>OLS49892.1 hypothetical protein BV379_17505 [Rhodovulum sulfidophilum]AB14
MADVHVTEPIHPDALALLASAGLSVSCGWKDADPAAGLATARAWILRTFVVSPEMIAGAPGLEAICKHGVGTDNIPLA
EA
ERRGIPVFNTPGANANAVAEHTMMMLLALTRQAVAMDRVARAGFAGFERLAPLELSGMRMLILGFGPIGQRVAQLA
RAFD
IEVTLWHRRLSAEEAGVPVVRDLAAALPAAQILTLHLPLNAGTRGLIGAAELAALPEGAILLNTGRGGVVDEAALVAAAP
RLGGIGLDVFETEPPPPSDPLLALPNALLSPHAAALGERSVRRMGMMAAEAAIRALGA
>OLS49893.1 ATP-dependent DNA ligase [Rhodovulum sulfidophilum]AB14
MKRFAALFTALDRTTTRTTAKVEALAAYLAEAPEPDRLWTVALLSGRRPRRAVTATRLREWAAERAGLPLWLFEECYPVV
G
DLAETIALVLPPPASDSDRSLSARMTDILAMARMDEPARKAGILKAWDSLGGAERFLFTKLLTGGFRVGVSQTLMTRAL
A
RATGQDEAELAHRLMGDWTPATTSWQALIEAPDPAAALSRPYPFYLAYQIEGPPEALGAPADWIAEYKWDGIRGQMI
ARG
GVHFLWSRGEDLITGRFPELAVARDFLPAGTVLDGEILAWKDGRPLPFAALQTRIGRKTLSKRLLAEAPAAFLAYDLLED
GGTDIRPRPLAERRARLEQIIAGMPPGGPLRLSQALPFDDWEDLARTRGAARAAEAEGVMLKRADAPYLAGRRKGDW
WKW
KLDPLTVDAVMIYAQAGHGRRATLYTDFTFAVWDGDRLVPVTKAYSGLTDAEFREITAWVRKNTLQRFGPVRQVAPEL
VF
EIAFEGIRASPRHKSGLALRFPRMLRWRRDKPATEADRLDTLKALLGAVGGETTPGA
>OLS49894.1 oligoendopeptidase F [Rhodovulum sulfidophilum]AB14

MRIASPRPVFDANASAGGALGDLPEWDLSDLYAAPDAPEIARDMGWLEQECAAFAADYEGRLAGLDAAGMLSCITRY
EAI
DRVAGRIMSYAGLRYYQNTMDAGRAKFLSDCQDRITNYTTPLVFFSLEFNRIADDAYEALFAADAALTRYKPVFDRMR
AT
RPYQLSDELEKFLHDQSVVGASAWNKLFDETIAGLEFEVEGEDAPLGIEATLNLLTDPARDRREAGARALAEVFGANIKT
FARVHNTLAKEKEIEDRWRKMPTPQTGRHLSNHVEPEVVEALRNAVVAAYPKLSHRYYALKAKWLGLDKLQVWDRN
APLP
IEEQKTVGWEAAKATVMEAYSEFSPKMAEIAEPFFDRGWIDAGVKPGKAPGAFAHPTVTDVHPYVMLNYLGKPRDV
MTLA
HELGHGVHQVLAAGQGELLASTPLTLAETASVFGEMLTFRKLLAGATTQAQKKTLLAGKVEDMINTVVRQIAFYDFECK
L
HEARRGGELTPEDINDIWMSVQGESLGPVFEFMPGYETFWSYVPHFVHSPFYVYAYAFGDGLVNALYAVYEESGAGF
QDK
YFEMLKAGGSKHHKDLLAPFGLDASDPAFWDKGLSMISGMIDELEAMED
>OLS49895.1 hypothetical protein BV379_17525 [Rhodovulum sulfidophilum]AB14
MTDTNSTLNEGAQASDAQDKGYRPALIRGAWMLLMLALFYAAQMIMIIAAILQFGWLLFAKEENERIAEFGDKLANW
MAI
AARYLTVAGDAKPFPWTDWK
>OLS49896.1 hypothetical protein BV379_17530 [Rhodovulum sulfidophilum]AB14
MKPFKTLVLVVNEREARLLVNRGTGKGLAEVTRFSKAHEIEYADGRGRDQGGATAGRHAMEPPTSLRVQNRESFAAD
VLG
LTAEVWAKGDYDRFVMAAPPKMLGELRNRIEGSLERALAVDIDKDIVGVAAADLPRHFEDVIVF
>OLS49897.1 polyamine ABC transporter permease [Rhodovulum sulfidophilum]AB14
MDLPPYATLGQRIWHYAYYVICGLIFFFLIAPILVIIPLSFNAENFFTFTPKMLALDPEGYSLKHYQDFFTSNAWQHALW
NSVKIAPAATFLAVSLGTLAAVGLSQEHVPFRRTIMAILISPMIVPLIISAAGMYFFYSRIGLQGTYVGVVLAHAALGIP
FVIITVTATLVGFDTSLTRASASLGANPVTTFFKVQMPLILPGVVSGGLFAFITSFDEVVVVLFVGSANQQTLPWQMFTG
LREQISPTILAVATILVVISIGLLTTVEILRRRSEKMRGLSPG
>OLS49898.1 polyamine ABC transporter permease [Rhodovulum sulfidophilum]AB14
MTGAATDTAASGPVLAADGAPLKRSLRRALRAQKLRALMLIAPLLVFVLVTFIAPIADMLFRSVENQIVSDTLPRTAREI
RDYDPDSGQMPSDRVLAAFTYDLVAAAERKQHTRLGSRLNYEYSGISSLFRKTGRRVDDFGESYLDQFVAVDPAWEEP
AT
WVALMADPDWTASYRAWSAARDAAKAAGEPFALAQPLFALRDGVGEVLPETASAYAEFARIVQVEDDDDPAAEEP
WAPVY
LALYRDLLHIGPGAVARLGPEAEVMLDAAHDAVEDFAPVDLSGLYLKADRDWADPGVWGTIRAFSPAYTPGYFLNAV
DLR
LTADGIAAQPEDRQIYIMLFERTLFMSLAIMGSCVLLGYPIAYLLSNLPLRTSNLLMILVLLPFWTSLLVRTSAWKVLLQ
QQGVINDILVWLGIVSNSGRLILINNQTGTIIAMTHILLPFMILPLYSVMKTIPPSYVRAAKSLGATNWTAFWRVYFPQS
VPGIGAGCILVFILAIGYYITPELVGGTSGTFISNRIAYHISSSLNWGLAAALGAILLGVVLILYWAYDKLVGIDNVSLG
>OLS49899.1 hypothetical protein BV379_17545 [Rhodovulum sulfidophilum]AB14
MPKGYIIAHITVTDPEAYRDYVDRDTPILESFGGRFLVRGGASEAPEGPMKDRHVVIEFPNFEAAKRAFHSPEYQEVAAI
RHATATSDIVLVEGVA
>OLS49900.1 spermidine/putrescine ABC transporter substrate-binding protein [Rhodovulum sulfidophilum]AB14
MTIRHLLSATALAALPAGFAFAEDLPPCEGCADSLTVVSWGGAYQASQQKAYSEPYAEMTGTDFTWDESSNEGVAKL
RAM
NEAGNITWDLVDVEGPDSQRLCDEGLAMEVDLDEMLAPAPDGTPASEDFGDSVVTDCFVPQIVFSTTFGYRTDVAA
WNGK

EPEGLCALFDTETFPGKRSLEKRPKKNLEWALLCDGVEKDDLYDVLDTPEGVDRALAKLDTIKDDVIWWSAGAETPQRL
A
DGEVVLGSTYNGRLFSLIVEQKQPVKMLWDWQVFDYDGWAIPAGLPEDRLNRVKHFLYFATDTQRLADQARYISYGP
SRA
SSQPLVSTHAELGIEMAPFMPTNPANQKNYLVNNIEWWADNQDDVEAKFQAWLAK
>OLS49901.1 polyamine ABC transporter ATP-binding protein [Rhodovulum sulfidophilum]AB14
MTGTNDGFVVFDRVQKSYDGEILVVKDLNLSIGKGEFLTMLGPSGSGKTTCLMMLAGFETATHGDILLDGNPINNIPPH
K
RGIGMVFQNYALFPHMTVAENLAFPLEVRKIGRAEREEKVQRALDMVQMGAFGGRRPAQLSGGQQQRIALARALVF
EPEL
VLMDEPLGALDKQLREHMQFEITRLAHNLGITTVYVTHDQTEALTMSDRVAVFDDGRIQQLARPDELYEKPVNSFVAQ
FI
GENNTLLGRVKQIDGNALIELDDGEVIDAVPVNVSQPGERTLVSIRPERVEIDPKRLTPGAHLIHAEVLEFIYMGDIYRT
RLRVAGNDDFVIKTRNAPDQRRLKPGEHIEIGWLPEDCRALDAP
>OLS49902.1 hypothetical protein BV379_17560 [Rhodovulum sulfidophilum]AB14
MGSGLKLSHIVAGFIAVLLGYTSSVAIIFQALEVLGASQAEANSWMLALGGGMGASGLILSLRYRMPILTAWSTPGAALL
VVGLDGVTMPEAIGAFLFCGLLLTLTGLSGWFEKLSHLIPDAIGNALLAGILFKFGLGIFVSTEENLPLVAVMCAVYLAG
RIWFARFAIPAVLLAGCLFCAATGAFEQGALDLSLARPVAVLPEFSLPVLIGVGLPLFIVTMASQNTPGVVTLRAAGYAP
PVSACLTVTGLTTLVLAPFGGYAFNLAAITAAICAGPEADDNPDTRYRAVVVAGLLYMLVGILGAAVVGLFTIAPKALVM
TVAGLALLGTIGTSLQAALAAPERREAALVTFMTTVSGIGFLGIGAPLWAVLLGMGTGAALSLSARARRPAAARP
>OLS49903.1 hypothetical protein BV379_17575 [Rhodovulum sulfidophilum]AB14
MVETMLIVFGSIYGLLVLVGLPALIFAHFGLRSENALLRIRVEDLLKDFEALTREVAALRRGAPPQTSPEPAPEQAAEAL
SPEPAPPPVASPAPDPVTAPVARRGEDTAEDAPPVEAAPKAPPPEPKGPALTDRLAAWLRENWTLALAALSLILGGLFM
V
QYGVEQGLLTPPLRVLGALVLGAALIAGGEAIRRRHGDSATPNLRHLPSTFSGAGTVVLFIAILSAHALYGLIGSVPALA
GMAAVSLVAVLLGGLYGPVLSAIGLLGATAAPFFVGGSSDKAVLLYPYFAMVGLAGLGIDTFRRWAWVSGLALGLPAG
AL
GLLWLHEPDARGLALAALALGLGAVLIPARSLRPVQDGPMTGQGLAGWAAPAFPTWIAAAGVALASGAGLVLMLDG
ASPA
AAALGYALLLLVFAGVALGLARAPAFEDLALLPGVAALVGLGLHAALDGPLYSAFRAGLDRLPESPAPTTVWWLVAGGA
A
LSVIAFLRLARQIGTDHRGLWALGAAVLLPAAILVLEFLWVPGEVTGAYPWALAVMAGAGLMVVLAERRSAMGGEAR
GFE
TGLFAASALILIGLALFLVLTKTALTLALSAMMVAAVALDRQRPMRPLGWLAQLGAAVIGYRLLVDPGLDWAIHGAGTL
D
VLLAHLGPLAAFAAILVLARPERAALRALASSSLVTVMALAVTVALRRGLGAEGFETHWAQGLTATVWILAAFAQIWRL
P
HDPSLLRPLRIIFAVVMALAACGAVAVMIDDLSWLLATGRGRVAGPPVLDSLALAILPLAAVFGAGAALLGRPGRPGAR
W
LRPGLGGLSVLALGVWGWLEIRRIWRGPDLSLPGPSDGELYSYTLALLLASGLGLLLAVLTRSHALRRLAMAGVALTVAK
VFLIDMSGLSGLVRVASFVGLGLALAGLAWLDRAVSRIWARG
>OLS49904.1 RNA-binding transcriptional accessory protein [Rhodovulum sulfidophilum]AB14
MTADLAPRIARTIATEIGAQPQQVTAAVELLDGGATVPFVARYRKEATGGLDDTQLRDLAERLAYLRELEARRATILDTV
REQGKLTETLEADIRKAATKAALEDLYLPYKPKRRTRAMIARENGLGPLAEAIMADRTAAPETLAEGYLSEAVPDVKSAL
TGARDIVAEGLSENADLIGRLRSFLQDNALVTAKVVEGQEQAGAKFSDYFDHREPWARVPSHRALAMMRGSKEGVLT
VDI
GPDPEEGVARVQTMVAGALDVRGDGPGDRWLREAAGWTWRTKLSLSMMLDLMGDLRTRAQEEAIRVFARNLKDL
LLAAPA

GAKVTMGIDPGIRTGCKVAVVDATGKLLDTATIYPFQPRNDLRGAQATLAALIRKHGVGLIAIGNGTASRESERMVAELL
GDLPAPKPTKVVVSEAGASVYSASELAAKEFPGLDVSLRGAVSIARRLQDPLAELVKIEPKAIGVGQYQHDVDQHRLARA
LDGVVEDAVNAVGVDLNMASASLLARVSGLGGSLAEAIVAHRDANGAFAKRKDLLKVSRLGPRAFEQCAGFLRITAGD
EP
LDASAVHPEAYDVARRIVAACGRDLRTVMGDEARLGALDPMDFVDDRFGLPTVKDILSELAKPGRDPRPEFVTASFAD
GI
EEITDLRPGMLLEGTVTNVAAFGAFVDIGVHQDGLVHVSQLADRFVQDPHEVVKAGDVVKVRVVEVDVPRKRIGLSM
RKD
GGAEAASRDGATGKGPGRGAGGKGAQGKAPGKGSGKGTGKGSGQKSGRKPGQGTERQGPGGGKGGGASGAETG
ALGAALL
EAMKRR
>OLS49905.1 hypothetical protein BV379_17585 [Rhodovulum sulfidophilum]AB14
MQEKPLESVELDLLAVIRQIHAEEGPFGRDLLLVRARGRGLEVSRPLGRLKALGLVEEIERKPSVLRRLFGAKPEALLRP
TATGLALGSEATASETTAAVEAEPEAGTGPDTGTAPKTGTGTEAEPATRAPETPRETVDETTREAAARAPATAVPSPAP
A
PAPSPAPAPVAPASRAEPPAPPAPPAEPPVSAPPVKVAPPTPRVEPTAPATPVADAAPLPMPRRAKPRNVRGYTEELG
GS
PVAAKTVPLRHDVDPAMMENLREALAVIGIEMTFAGEALIADRMGRGASIGEALSQVVLFAFAHAVAEDARAGEQLQ
ALG
LTDYAVDVIAEFERLRDAGEIDGDRLEEDMRRLWTLVGDSPDRLRLAEELLQDPVGGLTPPALLPEDLRPVEES
>OLS49906.1 hypothetical protein BV379_17590 [Rhodovulum sulfidophilum]AB14
MPVAKAHPTYVVLERDALISADLIQAIESLGPCRVMHFSSVDEMASGLEGVGSVNAAFLELRLDEALASGLATNLGKLG
A
RLVLTLGEDVERVMSEGCRLLLRPFTERMVLDALTETAPA
>OLS49907.1 CsbD family protein [Rhodovulum sulfidophilum]AB14
MNTDIFQGKWKQIKGRAREAWGALTDDDLDKAEGDREQLVGLIQERYGKAREDAEREVDEFLAKFR
>OLS49908.1 hypothetical protein BV379_17600 [Rhodovulum sulfidophilum]AB14
MTDPTIRLDPMSRVALLVLAVVAGFTALKLAEDIFAPLCLALVAGIILSPLADTLERRLRLPPMSVAAALPMLGIIAVGA
LAFAVEPLVGRMVDQWPTIRWELRGVVNDFRGFVQNVGQVGDEVQRALGGTGNGNDAEAAAPSVPSLTDALFVAP
RVAAQ
ILIFIAALFFFLLTRADIYAWMARHAGQRLGGEALLTRIRTAERLVAHYFMTISLINAGLGIALAIALGVLGMPGAIAWG
AGAALLNFVLYVGPATMVAGLLLGGLIAFDGWTSIFPAVIYLVLNGIESQVVTPTFVGRRIAVNPLAIFVSLVFWLWFWG
PLGGVIAIPVLVIVLAMLDVFDDPAPQADEPPLPAPKPAETA
>OLS49909.1 phospholipase [Rhodovulum sulfidophilum]AB14
MHGFTENSAALAAATGPVEELELLLTAAEAYPALERAFLGARHSISASFRIFDLATRLRSPEARAIGEDWFDLLVHTLER
GVSVRLVLTDFDPIGAADLHRLSWNARRRLIAAREVARGGRLEVLSALHSAQAGIGARLALWPGAHAKLRSVLDDLNA
RS
EPDRRRFLEEAPGIARITRERPDGRIVAVPGLPDLTPATHHQKMAVFDGRQLYVGGLDLNERRYDSPRHCRAPERTWH
DV
HAMVRGPVVTAAAQHLDGFLDIVAGRRPPGPAAPGFLRTLSRPARRAPFRFSPRPLVTEIERAHLSRIGAAERLIYLETQ
FFRHLPLARAMARRAEARPDLRAILVLPAAPEDVAFENSSGLDVRYGEHLQSRCLSLLYDAFGPDRVLVLSPVQPRQLAV
EGRAELEDAPIIYVHSKVSIFDGDAAILSSANLNGRSMRWDTEAGLELTAPAHVDLIRRRVMGHWLPREGDGAVDPAA
LD
PRTAFEAWRVLADLNSRRSPERREGFLVHYDSDPARKLGLPVPGVPPEIV
>OLS49910.1 hypothetical protein BV379_17610 [Rhodovulum sulfidophilum]AB14
MEVTGIGGLIVLVLDIWAIVSVLNSGTGTGNKVLWILLVLLLPLVGFIIWLIAGPRSASVR
>OLS49911.1 hypothetical protein BV379_17615 [Rhodovulum sulfidophilum]AB14

MKRILATTALATLVAMPVLAQSQGAASAPDVEVAGQSMSADTLIGKRIYVATGEADGLSMTDLTDAPDSWEDVGEIG
DVY
IDDSGTVNAVIADIGGFLGMGEREVALQMDSLEFAPDTDDQGEYFVLFQGDPSTLEGKEAYDRTAMDTEGDFLGSGN
QHA
SSESRIEPGAEGSDMAAAGDTDDSMNLGTPLTESERAELTAEDLQGHTLIGANGERIGKIGQLLLTDDGRIDKVIVDVGG
FLGIGSKQVQLAYGELELSHDEDGALKVSTVQTKDTLKSLPEWTG
>OLS49912.1 hypothetical protein BV379_17620 [Rhodovulum sulfidophilum]AB14
MNIHGNTAFAQRIATYSREADMAGIRSPDFEKEVAETGKMGPRARPHGAAAIADIFVRHDMTSISPREIDTMAEELRD
AG
FDDVDFLLGLETYGEKFQSHLVEAIQSAGIEARHPDYTQPMDLISVNRDQLAHARRAGAPTEWREAFLARLEAFQAAA
HP
APTGLSTAESLVLARAT
>OLS49913.1 hypothetical protein BV379_17625 [Rhodovulum sulfidophilum]AB14
MDSFAERAPAKLPVNQNVAARLGLQALTLLAVGQFVRQIRRQATRPDPRETAPHLTRTGLILCATGTLFAALREIPLAEA
TATFFIGPFILSVPGAFSLEEAVSWRRILASAIGFPGAMPVIWSGFAAFGSLALLPLATALCLTPYMALTRQIARRMPRV
AQ
>OLS49914.1 hypothetical protein BV379_17635 [Rhodovulum sulfidophilum]AB14
MVGPPKVLTVSYGAFSCKLEGFEDSFGMMKKITEYFRDLAAEDRYFGAEPPPPDAETLQRIAEREVRHRVETRVELDGM
V
LRSGALEGRASSPAPRTQPPSVQPPRIDEPDSVAAKLARIRAAVETARAADAELADAGFEDETDESDTHGATREPFPPD
G
PPEPRKTEITASEEAPAPSSCDRESRDRAAESETTAHPGAGTSPPSPPRARVLKLRRDDLSATRPPDEDAEQSAPGLRPA
ETDEAGAAAGAPVRLTDPAPAAPARSRSADLADHELALQRLLDEANTKLAGAEQRRRQASIAHLKAAVAATVAERRVD
KR
LRRDDDSHMRPYRQALAEVVRGDRKTPPTGEGNRLAPLILVTEQRIDRPAPETGAKTGLATDPSGKDKASPEALRLDAP
I
LPMTEARLKGFAAATAGHDPADLTGLLAAAAAYLTRQADGQEGAPRFTRPQLIELARSVAGDGFEREEMLRAFGRHLR
DG
RFRRVGRGQFELDEAAAPPQAAGANRTA
>OLS49915.1 hypothetical protein BV379_17640 [Rhodovulum sulfidophilum]AB14
MALSWKARRRWSLVILLIGLPAYIVVAVSVMNALGRPSFLVELLVYVALGIVWALPFRFVFRGIGQPDPEAKQDDRDGR
D
>OLS49916.1 adenylosuccinate synthase [Rhodovulum sulfidophilum]AB14
MANVVVVGAQWGDEGKGKIVDWLSERADVVARFQGGHNAGHTLVIDGTIYKLNALPSGVVRGGKLSVIGNGVVLDP
WHLV
KEIETIRGQGVEVTPETLMVAENTPLILPFHGELDRAREGQNAMAKIGTTGRGIGPCYEDKVGRRVIRVADLADDATLEL
RVDRALVHHNALRAGLGLPEIDRDALLASLREIAPEILKYAAPVWKVLNEKRRAGNRILFEGAQGALLDIDFGTYPFVTS
SNTIAGQAATGTGIGPGSIDFVLGIVKAYTTRVGEGPFPTELDDADGERLGTRGREFGTVTGRKRRCGWFDAVLVRQTC
A
TSGMNGIALTKLDVLDGFETLKVCVGYELDGERLDHLPTAADQQARCTPVYEELPGWQESTEGARSWADLPANAIKYV
RR
VEELIDCPVALLSTSPERDDTILVTDPFAD
>OLS49917.1 hypothetical protein BV379_17650 [Rhodovulum sulfidophilum]AB14
MAALFLRWLVAFLLLALTYNPTSWNYLRWAMSGWDSQTPMVVLAGLVLFTGYVIYLRATLRSIGPFGMALVTALVGA
LLW
VLVDWKVLRLDNPGLNTWIGLLALSFILGVGLAWSILRRRLSGQADIDDVDE
>OLS49918.1 hypothetical protein BV379_17655 [Rhodovulum sulfidophilum]AB14
MSGFLLRWLVAFLLLAATYNPTEYNYIAWAQANFATSKPLVIGLGVMLALALLIYVVAAVRTLGLLGLFLLVVIMALLGY

ILVGNGTLQLELNSFNIWGCAILLSLILGATMSWRSPSKIRAQAKKAQKARSTAKPANA
>OLS49919.1 preprotein translocase subunit SecG [Rhodovulum sulfidophilum]AB14
MENVILIVHLILAVCLIGIVLLQRNEGGGLGMGGGGTITGRAPTTPLGKLTWFFAICFIASSIALTIIAAENAAGSSVVD
RLGGPAPAAEQEGAAQEAPATEPGLLPPAPVVPSDTPAAPPRAD
>OLS49920.1 CTP synthetase [Rhodovulum sulfidophilum]AB14
MARFIFITGGVVSSLGKGLASAALGALLQARGFTVRLRKLDPYLNVDPGTMSPFEHGEVFVTDDGAETDLDLGHYERFT
G
VAARATDSISSGRIYSNVLERERRGDYLGKTIQVIPHVTNEIKDFIATGEDEVDFMLCEIGGTVGDIEGLPFFEAIRQFS
QDKPRGQCIFMHLTLLPYIKASGELKTKPTQHSVKELRSIGIAPDVLVCRSEGPIPAKEREKLALFCNVRPDSVIAAQDL
KSIYEAPLAYHREGLDQAVLDAFQINPAPKPDLSKWEDVSDRVFNAEGEVRVAIVGKYTQLEDAYKSIAEALTHGGMAN
R
VKVKAEWIDAEIFEREDPGPWLEGFNAILVPGGFGERGTEGKMKAAQFARTRGVPYLGICLGMQMAVIEAARNVAEL
PDA
GSEEFDHEAGKKRFTPVVYHLKEWVQGNAMVKRKIGDDKGGTMRLGSYTAHLLKDSKVAGIYGSTVIEERHRHRYEVD
IK
YRKQLEACGMVFSGMSPDGRLPEIVEWKDHPWFIGVQFHPELKSKPFAPHPLFADFVRAAVEVSRLV
>OLS49921.1 23S rRNA (adenine(2030)-N(6))-methyltransferase RlmJ [Rhodovulum sulfidophilum]AB14
MLSYQHIYHAGNLADVHKHALLCAILDYLTRKDKPLSYIETHAGRGLYDLAAPEAVRTGEAAAGIGTMEGRLPGDHPYL
R
RLAEVRARFGDSAYPGSPLLAALSLRPGDALHLAELHPQERTALETAMAPFRAHVMARDGFEAAQALTPPLPRRGLMLI
D
PSYEVKADYAAIPRHMAALHRKWNVGTLVLWYPLLPAAPHRPMIAALRAAFPEALCHEVRFPPAREGHGLIGSGLFVV
NP
PWGLDAEAAALGRLFGTVPGHAG
>OLS49922.1 hypothetical protein BV379_17675 [Rhodovulum sulfidophilum]AB14
MFADFLNRLIGPDPAQLDDPDARLALAALLVRIARTDGDYASEEAERISRILATRYGLSPEQALELRDRAETLEAQAPDT
VRFTRAIKDAVPYEDREGVVEALWEVVLADGIRDEEEDGLLRLVANLLGVNDRDSALARQRAEARLAG
>OLS49923.1 ATP-binding protein [Rhodovulum sulfidophilum]AB14
MTDTTPPVLDIRDLHKSYGDLEVLKGVSISAPKGHVVSLIGSSGSGKSTLLRCANLLEDSQQGEILFEGEPVKWRGRGPG
RHPSDRHQVIRIRTNLSMVFQQFNLWAHMTILQNVMEAPVTVLGEPRQEVEARARALLDKVGIGDKCGAYPAQLSGG
QQQ
RAAIARALAMEPRALLFDEPTSALDPELQQEVVKVIKVLAEEGRTMMLVTHDMRLAADVSDHVVFLHQGLIEEQGPPG
QV
FGAPASERLKQFLSATAA
>OLS49924.1 amino acid ABC transporter [Rhodovulum sulfidophilum]AB14
MKKLILGTAALALSAGMALAQDVVRLATEGAYPPYNFINDAGDVDGFERDLGDELCKRAELNCTWVTNDWDSIIPNLV
SG
NYDAILAGMSISPERQEVVAFTQPYTPPSLSAYAALSDDVDLENSVISAQTGTIQANHVVDTGATLLEYPTSDECVDAVR
NGEAEAVLADKDVLVPIVNEGDLVFVGEDTALSNGIGMAFRKSDPELKDKFDTAVQSMKDDGTLNELIIKWMGEDSPR
W
>OLS49925.1 ABC transporter permease [Rhodovulum sulfidophilum]AB14
MFASCADPSTLEGLNWLACYLTSGKHMAFYQSFLVVLALLAVTAPAALLFGFGGAMAARSRIWPVRILGRIYTAMVRG
VP
DIVFFLFVPIAMDQGFEWLRHKTKCPDWTEPVRQGSDFVVCPAAKLPLSTSPQWVHETYGFALAVLAFAVVFGAFAA
NVL
YGAMRAVPRAQIETAEAYGMTRRQAFWRVLVPQMWIYALPGLSNLWMILIKATPLLFLLGVQDIVYWARELGGSKTSI
FA
YPHPDWRIYYFLGLLIFYLLLTRVSEAVLSRLTARLSHGQATLAGEAQRKAAQ

>OLS49926.1 amino acid ABC transporter permease [Rhodovulum sulfidophilum]AB14
MSCAQTFHDYAFRSLGYGERLLPRSDFTLCEQFTLIGSGLVWNIYFGVLALFFGFFLATAVAVARDSRLLAVRKPAEWFI
FVFRGSPLFIQFFLAYQAFVLLPRNGIELNLVFTQITADTHLLTRAWAGALIVLFLNTAAYSGEIFYGALRSVPKGDLEA
ADAYGMAGWTRFRRIVWPTMLRLAWPAYTNEAIFLFHATTLVFFSGFPAWQQKGDALYYANYFADKTFNPFIPYPIVA
FY
FILVTLAIVGLFGLINRRLNRHLPAENRPRLRVRPQVIR
>OLS49927.1 glutamine synthetase [Rhodovulum sulfidophilum]AB14
MSTWLRDNPEIRTIRMAACDLNGQARGKRVPSRFAGKIEEEGTRFPLSVLNLDIWGEDIEGSPLVFETGDADGVLRPTG
R
GFVPMPWLDAPTALLPLWMFDERGRPFEGCPRHALAVVLEEYEKRGWAPQVATELEFYLIDDSAAVLHPPRSPRSGKR
RE
AGEILSLRTLDAFDNFFTDLYDACEAMDIPADAAISEAGTGQFEINMMHGPAMKAADDAWLFKLLVRGMARRHGFA
ASFM
AKPYADDVGNGMHTHFSVLDAHGHNIFDDASPEGSGFLRQAVAGCLAALPDMTLILAPHANSYTRLVPGNHAPTGIC
WAH
ENRTAAIRIPGGSPRARRIEHRVAGGDINPYLMIAAILGAALQGVEEQLEPPPPITGNAYELDLPQIPTCWQSAIDSFEQ
SPRMARIFRRGLIRNLAMTKRQEARRIAELDEEAQMALYLDTV
>OLS49928.1 glutamine amidotransferase [Rhodovulum sulfidophilum]AB14
MQIGILQTGHAPDALRAAHGDYPDMFVRLLEGHGLEFRTWPVLDMAFPPSVHAAEGWLITGSRFGAYEDHAFIHPLE
EFI
RQAHAEGVPLVGICFGHQIIAQALGGRVEKFAGGWSVGPRDYRIEDRDYRLNAWHQDQVVVPPAGAETVGSTPGCA
HAAL
IYGDRAFTVQPHPEFAPAFERGLIEERGPGVVPEDALAEARARLDLPTDSAAMAGRIARFFTERR
>OLS49929.1 glutamine synthetase [Rhodovulum sulfidophilum]AB14
MQDWRDQLPEAALTYIEGRRLDEVECIVADLAGVARGKAMPAGKFATQTRFYLPNSIFQQTITGDWADIEGRPFTEPD
MI
LTPDFSTASAAPWTADYTLQVIHDVFDQQGEMVPTAPRNVLKRIVALYNDRGWIPIVAPEMEFFLVARNTNPSEPVQP
PM
GRSGRRAAARQAYSLSAVDEYGPVIDDIYDFAEAQGLEIDGILQEGGAGQVELNLRHGDPVKLADEIFYFKRLIREAAMR
HECYATFMAKPIEHEPGSAMHIHHSVVDATTGLNIFSNADGSESPAFLHFIAGLQTHMPSVVALLAPYVNSYRRYVPDF
A
APINLLWGRDNRTTGLRVPVSEPEARRVENRLAGMDCNPYLGIAASLACGYLGLIEGQDPRPEFTGDAYSGDEDIPRNV
A
EALDLFEAASPMRDILGEEFCTVYSAVKELEYKQFLQVISPWEREHLLLNV
>OLS49930.1 oxidoreductase [Rhodovulum sulfidophilum]AB14
MNLLFANDRRGTYPPSWYAETTPPLPPFAPLDGDRRADICIVGGGYTGLSAALHLAEAGRDVVLVEAHRVGFGASGRN
GG
QVGSGQRLHQDALERMLGDGPATRLWHLAEDARTLVSDLIARHDIACDLRPGIVYAESTARGTRDAHAYAEKLRSDYD
YD
LLEPLTRDALQAIVGAPCYRGGVLDWGGSHLNPLAYALGLARAAERAGATIHEATLVERVDAGAVVVTDRGRIIADRVIL
AANGYLGGLERHVAARVMPINNYMVATEPLGDRAASVLARDVAVADSRFVVNYFRLSPDGRLLFGGGESYGYRFPEDI
RA
LVRKPLLKVFPQLSDVALTHAWGGTLAITRSRLPCLATPAPGIFSASGFSGHGVAMATLAGRVLAEAAQGDSTDFETFA
R
IPSRPFPGGGAFRAPLLALAMAWYALRDTAGI
>OLS49931.1 aminotransferase [Rhodovulum sulfidophilum]AB14
MTCSRNVYDAEPIPEGAGAEIERLLQSGDLFRYTAASDAPVTLLERDFAAMMGLPYALAVSSCSAALFLALKALDLPRGA
RVLVPAFTFAAVPSAVVHADCVPVLVEVGSDFRIDLEDFAARIDEDIAAVLISHMRGHTSDMDVIAAACAARGLPLIEDA

AHSLGTLWHGRKVGTLGRIGCFSFQSYKLVNGGEGGMLLTDDPDLVARAVIMSGAYEHNWKKHDGLAASFARWQN
RLPLY
NMRMQNLSAAVIRPQLPEVPRRVRDGRAGHDHVAERLNRSPWLSVPPPLPPETRAPDSIQFNLVGFDSDDEARAFQT
AAA
ERGVQVQVFGLAADNARAFWNWGFLDELPGGPRRDLPKTRAMLMRACDARLPAHLPRAELDRIADMLVAAAEAVK
GEEAR
AYGT
>OLS49932.1 23S rRNA (guanosine(2251)-2'-O)-methyltransferase RlmB [Rhodovulum sulfidophilum]AB14
MAKKPTWVIEKERARRAAAAETVWLFGLHAVRDALENPLREKLRLVVTRNAADRLAPAIAAAGIEPEISDPRRFAAPLD
P
DSVHQGAALEVKPLDWGKLGDVCDPGRGAALTVLLDRVTDPHNVGAILRSAEVFGARAVIAPHRHSAPETGALAKTAS
GA
LERQPYLRVPNLARTMEELKGMGYTLLGLDGEAGQTLGEGLAGLGAAPVGLVLGAEGPGLRELTRKTCDGLVRIPAAG
GF
GSLNVSNAAAVALYAAAQEQALGR
>OLS49933.1 hypothetical protein BV379_17730 [Rhodovulum sulfidophilum]AB14
MSMVIVFEGVEKNERLTWTQVANVELLLDAFTISKRSCWPCSYQVGAPDATFGAVVDLMLARTA
>OLS49934.1 C4-dicarboxylate ABC transporter permease [Rhodovulum sulfidophilum]AB14
MSDLIPGLGAGSEMVILFFVLMALRVPVAFSLGLSAFYAMGQIGFGFDLAGDLIAAGVTKFSLLAIPFFILAGTLMGTLG
IAERMIRFFRVMIGSMPGGMGVVGTVVCLFWGAVSGSGPASVAAIGPMIVKGMEEDGYSRASAAGLVCTGAALSIVI
PPS
IGLVIYGVIAETSISQLFLAAVVPGLFMGLVMLATLPFAARRGAGREALDALSPAPHADLPYGRRLAASFREAFWGLLTP
LVILGGIYSGIFTPTEAALVATVYALGVGAIVYRTLTIRGLYEALADAAASSAVVMLVVAYAGIFGWVVTVEDLVGTYSG
ALLGLSDNQWVILLAIMVILLVAGMFMDAITVMFISLPIFLPVMREMGWDPVWFGVVVMVNLAIGLFTPPVGINLYVA
AN
VTKLSIEQIARGAAPFLLTSLLGLAAVAAFPELSLKLVELLR
>OLS49935.1 C4-dicarboxylate ABC transporter permease [Rhodovulum sulfidophilum]AB14
MRFLLREAEKIVCAVLFLGMTAIGFANVVVRYGTNYSLAASEELLTNGFLLLTVFGAAIAARRGEHLAVTLVHDLLPRPL
RKPLFVLAAGLSALLLGLSAWVSWDVLANLHANGMRSYALGIPAWYYQAAVPFGFALILIRYLQHVREVLGGGPAELPP
E
TPHV
>OLS49936.1 C4-dicarboxylate ABC transporter [Rhodovulum sulfidophilum]AB14
MKAVRLGGGALAALIAGAMMLGTASAETLRLSHNVGDTTTWHKGAEKFGELLAEATDGELKVRVFPNAQLAGGDQ
MKMTE
MVGRGALDLVVTSAINVTPLVPEMAVFSLPYLYGSYDEVDATTQGAPGEMMAEILLDKGIVVLAWGENGFRELTNSVR
PV
KTPEDMQGLNMRVSGPMYIDVMNALGANPTQMQWSETLTALQQGVVDGQENPIGAVIIPQQVYEMQKYLTAWH
YSYDPVF
LGVSQSKWDSLDAETQEKMRKAAVEAMAWQRQISREATASGVDFLREKGMEVYEPTPEELEAFRAATKPAFDKWSE
KVGP
EIVEAFQSSIAGSN
>OLS49937.1 ribokinase [Rhodovulum sulfidophilum]AB14
MIVTFGSLNADLIFSVETLPRPGQTLMAKGLATEAGGKGANQALAAALDGAEVAMAGAVGQDALAEVALAGLKGRV
DLSR
VARLEASTGCAAIHRDAEGRNEIVVAGGANLAASSGSVEDALLDRASVLLLQMENDPAQIERLIRRCRPAKAMSILNLAP
ACRLDPEVLGLCDLIVVNEDEAAAMAGWLGCAPEPRALALASGAGILRTLGGEGAELCWQGQEISIPALSCRVKDTTAA
G

DCFVGVFAAALDRGETPEAALRRAAAAAAIACTRIGSQSSLPEARDTDAVLAGAAV
>OLS49938.1 glutathione ABC transporter ATP-binding protein GsiA [Rhodovulum sulfidophilum]AB14
MDTPVLSVEELSAAFRVEGQWRTVVEGVSFDIGPQETVAVVGESGSGKSVTAMSIMRLLSPANSRTEGRIVFEGQDLL
AM
PLDDMQAIRGNRIGMIFQEPMTSLNPVLKIGDQITEVLRRHRGMDGAAAEAEAVRLLDRVRIPSAAARLGEYPMNFSG
GM
RQRVVIAIALACAPRLLIADEPTTALDVTIQAQILELIKTLQEEEGMSVLFITHDMGVVAEISDRTVVMYRGEVVEKATT
EAIFRAAHHPYSRALLSAVPQLGAMAGQDRPLRFPRVDMATGEIAPPPPTADTVRADEPPVLKVDNLVTRFPVRKGLL
RR
VVGRIHAVEDISFELRQGETLSLVGESGCGKSTTGRSLIRLEDPSGGSVAMDGHDVRGAGHSELTKIRRDVQMIFQDPF
E
SLNPRIRIGEAIAEPMVVHGLVSRREAPARVADLLERVGLSGEMMQRFPHEFSGGQRQRICIARALALEPKLIVADESVS
ALDVSVKAQVINLMLDLQERYGLGYLFISHDMAVVERISHRVAVMYLGEIVEIGPRAAVFENPRHPYTRKLIAAVPVADP
SRRRRRVVSGDEIRSPYRPADYVAPKRLYREIGEGHFIQIPESEAAA
>OLS49939.1 peptide ABC transporter permease [Rhodovulum sulfidophilum]AB14
MAAPSETARSVHASAAAPASVSPFRQTMGKLLRDPLALTGLVIVTLLMIGAVFAPLIAPQDPYDLAQLDIMDGRLGPGS
Q
SMTGMVYLLGTDDQGRDLFSAILFGLRTSILVGLASAAIALVIGASVGLFSAYAGGRTDAFLMRIVDIQLSFPAILIALI
FLAILGQGVDKIILALVVTQWAYYARTIRGSALVERKRAYVDAARSMALPDRRILFRHILPNCLAPLIVVATMRVAYAIM
LEATLSFLGIGLPVTEPSLGLLVSNGFEYLLSGDYWISFFPGLALLVLIVSINLIGDALRDILNPRREG
>OLS49940.1 ABC transporter permease [Rhodovulum sulfidophilum]AB14
MSGYLFSRLLQTLVTLFVMSVLVFGGLHMVGNPVDVLLSPTATPAERLEVIRSFGLDQPVWVQYWIFLKNALSGDLGN
SF
VYNQPALHLILQRMPATLELAAAALVMALAIGLPLGVYAGLRPNGVLAKSIMAFSILGFSLPTFWIGLVMIMIFAVQLG
W
LPSSGRGDTVDVFGVPLSLFTLDGLAHLALPAFNLALFKISLVIRITRAGVLETMQLDFVRFARAKGLPERRIVRVHVLK
NTLIPLITVIGLELGSLIAFAVVTETIFAWPGMGKLIIDSIAVLDRPVILAYLMITVVMFSLINLTVDILYSLVDPRVRL
EGN
>OLS49941.1 nucleoside hydrolase [Rhodovulum sulfidophilum]AB14
MRRIPVIIDTDPGVDDAAAILMALGSPELDVLGITTVAGNVGLSATLTNACRIVGLTGRTDLPVMAGARGPLMRDQVF
GK
YAAIGAFPVPLLGGPAVAPSSENAVRFIARTARAAAEAGTPLTICAIGPLTNIALALGMHPDVGRGIRRIVCMGGAIATQ
GHRTPWAEFNIYADPHAAEIVFGSGVPLVLFPLDVTTQALFTEAHVAEFRARGGAAGRALANLLTEYDRSDPERYGRPG
G
PLHDPMTIAWLLRPELFTGRAARIGVQVGGATSGSTFADFKAPSPRSAVITGVDEAAYVALVLDRIAAHGAGRAAASQK
V
ETE
>OLS49942.1 nucleoside hydrolase [Rhodovulum sulfidophilum]AB14
MTTPIPVIFDCDPGIDDAIALLGAFAAPELDIRAILAVCGNQPLERTLRNALQICELGGREDIPVHAGAHRPLCRDPIHG
KFHGENGLGDTLLPDPLKRPEPVGAVEALIDHLGQAARSAGPKITLCCLGPLTDLALALRLAPDIAAGIERVVMMGGAFR
EPGNRSLTSEFNMLADPHAAAIVFAAGLPMTVLGLDATHQVMLRPEHVASFAAVSGRISRSVAGLMAFWDRNDPQR
YGSR
GGPLHDPLVTAFLLAPELFETRPARVFVETESPLCLGQTVADWFGKTGAPANADIAIGVDAGGVIELYLELLARYGTGAA
A
>OLS49943.1 dihydrodipicolinate synthase family protein [Rhodovulum sulfidophilum]AB14
MSASREPGFHGVFPYLVSPLDASGGVREGVMVALVERLIAAGVHGLAPLGSTGEFAYLSNDQRLAVARAVIRAAKGRV
PV
VPGVASTSTADAVAQTRAMVAAGADGILAILDAYFPVGEAGVVAYFTAIAEAAEGLPVVLYTNPNFQRSDLTLPVIDRLS

RVPNIRYIKDASSNTGRLLSILNRCGDRIEVFAASAHIPACVMMIGGVGWMAGPACIVPEQSIALYEAARAGDWERAM
DL
QRPLWRINELFARYSLAACIKAALEVQGFEVGDPVAPQTPLDAVARGELAQVLGAIGAL
>OLS49944.1 ABC transporter substrate-binding protein [Rhodovulum sulfidophilum]AB14
MKRFASVAILAASAACLPSVVSAQSLTVGLAASTTSMDPQFYVVGPNSAMARNIFDGLVNQDDKQQIQPALAVSWSV
IDD
TTWEFRLREGVTFHDGSDFTAEDVVASLNRVPMASKNSPSSFMPYVKAITSVEAVDPLTVRITTAAPTPLLPNDLSRIAI
LPAEDAEVTTEEMNAGKGVIGTGPFEFVSWVPDSEVKVARNDAYWAGPAAWEEVTFKVFKNNSARVAAMLSGDVD
MIESI
PTADTRRLKSADDLKVVSVAGNRVMYLHMDQGREVSPFATGPDGKNPLRDARVRRALSLSLNREAIVDRIMDGQGVP
AGQ
LVPEGYFGFDPSIKVDPYDPAKAKQLLAEAGYPDGFKLTFHASNDRYPNDSRVAQAIGQFFSRIGIDTSVETLPASVYFS
RASNLEFSFIMGGAAVETGEASGVLGPLLETYGDSAGRGNRGRYSNPDFDAALQQARQTLDAPTRRDLLQKATRIAMT
DL
GVIPVFYLANTWALEDGLSYPGRSDGYTLAYEVTAE
>OLS49945.1 LysR family transcriptional regulator [Rhodovulum sulfidophilum]AB14
MRLRHRQVEAFRSVMMTGGITSAAAAMHVTQPAVSRLIRDLEETIGLSLFQRRGARLDPTPEASLLYREVERLYLGLDQI
AQAAEDIRDHKNIVIRIGTVTSLVRPYLQRAIRDVIGDRRDVPLVIDVENSRHVWEMVENAQYDIGFVYRRQRMDDRA
RP
LRQMTAVAAVPPGHPLAGRAVCTPDDLAGERLLIPGRNSPARLALDSALAQAAGAPASLMESSMLNCCHFAADGMG
VAIV
DEISIRAARANAGAALVAIPFRPEIGVGYYAIRPPGAHRIAILDAIVAHLEAQLNAPAQV
>OLS49946.1 universal stress protein [Rhodovulum sulfidophilum]AB14
MYANILVPVAIDHAPDLGDVLEIARRLRAEAGTITVLTVAEAIPPYVSQYLPEGQEEETRAAIRQELAGALAGVSDIGIR
VVTGHAGITIVDYAERHGVDLIVMHSHRPDLTDYFLGSTAARVVRHAPCAVHVVR
>OLS49947.1 antibiotic biosynthesis monooxygenase [Rhodovulum sulfidophilum]AB14
MPQITPQADLQTVITTFEVTPGTCQDLLDELQDTFAAFISKQPGFIGAALHVNDAQTRIANYSQWRRREDFQAMLRSD
EM
RGHTRRFAELCKSFEPVMYEVAASYD
>OLS49948.1 DUF1992 domain-containing protein [Rhodovulum sulfidophilum]AB14
MSFDKLAERQILKAQAEGALDHLEGEGKPLSVRPHEDAVGIGMRIMAEAGMLPREFELKKAVDKQFLVLQATTGALER
KA
EMKKLADLTLRLEIEREARRSFFK
>OLS49949.1 hypothetical protein BV379_17810 [Rhodovulum sulfidophilum]AB14
MPPGDTLYGGVRLIRPLLRRITARVERDLQGTGITVGQRAILEALSEAKSATAPMLTDWLDVKRQFVARELKALLEAGMI
EKTPNPDRARSFLYRLTPESAELIGAIREREVLTFAAFSDQFTEDELLAFRKIMEALYANMLAGENRDTQQEDGSPS
>OLS49950.1 hypothetical protein BV379_17820 [Rhodovulum sulfidophilum]AB14
MLAGTNVINANPASAHGAIPLRPEARPATLTAHMHEASGALAPVPPRPKRDAVRVAWMPRNALKRVVASMRSEIAR
LRPS
KIWCVPFARNASGIDIHGDAWTWWRQADGENYAKGLVPVQGAVLNFRPTHVMPLGHVAMVSKVISQRKIMVDQA
NWIDNE
ITIDRLCCTNRVMGGVTPSPDRLIPQPP
>OLS49951.1 hypothetical protein BV379_17825 [Rhodovulum sulfidophilum]AB14
MSNVISVLTFKSIETILAAGGTQSWALDRNRAARCDYVVICRNAKTRDPEGPEEHGAAFMVGKVKDVVPSTETEGRWL
IQ
ISDYALVDWPDQWEGRNPVAYWKDDDYEDIDFKALDYQPLRKLPRGLTIPEAKAGLALGLNVPESAIEIVVRS
>OLS49952.1 hypothetical protein BV379_17830 [Rhodovulum sulfidophilum]AB14

MYPPFTHDQEHHLPTVLSAPRFATYLRSAGNDRTRALNLYQWNLLVSSALLVPLHVLEVALRNAVVEAIEAVHSGTWP
WT
QGFIRSLPNPGPPAYNPKRDLSNCANNQPTAGKVVAELKFAFWESMLTKRHQNRLWDKHFFHVFPDAPRGVTSDQR
RSEL
RADIEEIRKLRNRIAHHEPIFPRALQDDFDRILRCIQWRNAATATWVRDIETVQAFFPLRP
>OLS49953.1 hypothetical protein BV379_17835 [Rhodovulum sulfidophilum]AB14
MKLSQYPLSQGLPGSMNAVFATMAWRGHQTLQEAERYTCAAQLKKLVMGDEHIENIVSLEIRDTKTAKA
>OLS49954.1 hypothetical protein BV379_17840 [Rhodovulum sulfidophilum]AB14
MRVLAVLAALAAATPSSAIEADTQPTPGILIGDRLLNVATHLRIVPQAYSIRQTDSALDTLLESAGSLPLGTPAHLVDEN
PCAILVALLETEAPRIVVESSDPFDVTDPAQDPQRLMPLITQADLVEHMGTAIETVDFTIGADLAIFQIGEMFGRSAEAA
ALLDEREARIASVEALLPLARSMRATVLHGADGGDMAVTVERPGGPTDLALLDRIGVETVGAPTAVPDAGGAPMRVD
WHA
IAAAEPDVIVATGDADQVRAALRRATASVPALSGIPAVAADAILPLPAYSDMDVVGYPYTLEAWTRAFSEIAMAAVSAP
A
ASR
>OLS49955.1 hypothetical protein BV379_17845 [Rhodovulum sulfidophilum]AB14
MTFCGRQGLVLATTILSGSLPALADEDARLGIIYLEEEIDWGALAPLTMDREDTDTFGDTSGDVNEAIATLPNVQNETQR
TVGGVSDNGVSSDSMLDLQPSKLSISGAAVTENNIMINGIGINSIVAAEPFAVGSLDKNTGSTGIDTIYGLHPQTQYVPE
AFIDGVEVQTSNVSARYGGFQGGVVNYLLREPTGESRGLLSFGFSSDDLTTYNLETESGTNPLNRAKPEWTRKEVGVEY
E
TPLTDTTSVLLSFSRNTAWGRKPIGHQYIGDRVESESSSDFGRVVLRHELQDGGVFKLSGAATEYEQEWQSNYSYGYQL
D
TKTSGRTLSGEYTNIFDAWDLSARLTYSRNKVANDSNVDYYANWIGRNTDDTLYDGALDWCNVSPLTTGYVSCMEGG
FTG
NKRYDDKTLHADLDLSRDLAGGRLNFGASVIKSQAKRARLSDTTAYAAFMPNALGICPPGDLFCNADQFWFVRVIYPA
YA
LEVDAMKYESFLEWERSWERFDLRAGLRLDRNDVMDNTDIAPRLSLGYRLSDTVRADLGLNRYYSDDYMAYAIHDKTP
RG
INETRPVPTMDPVPYDFGEYRYTQQGLKTPYTDEVALGLTAADPWTGGTWRMTLLNRHGRDQFAHSSVPSGGTYPD
NVLT
NDGWNRYRSATLEYARVWDRPGPRLLDSVGLRVSGVVADRETSSSGYYNASDELNEVDLIWYNDKSYTNEQFDQLTG
NYD
IPVRAAVEVQTSWDRGRYGLGLGADINFGYEGVRDTDEDETHVNPTYGSRPHSVYEDYRFKTYARLNLSAQARVYSAA
GR
DVHLKLKVANLLNKSLGGSTDMHPWVEGRSYWLGTEVTW
>OLS49956.1 NADPH-dependent 7-cyano-7-deazaguanine reductase QueF [Rhodovulum
sulfidophilum]AB14
MSEDIYSNLKQLGGATRIPTSPEEAELERVANPQADVTYNVRFTAPEFTSLCPMTGQPDFAHLVIDYVPGLWLIESKSLK
LFLTSYRNHGAFHEDCTISIARRLVEFLDPQWLRIGGYWYPRGGIPIDVFWQTGPLPKGAWIPDQAVPPYRGRG
>OLS49957.1 7-carboxy-7-deazaguanine synthase QueE [Rhodovulum sulfidophilum]AB14
MTLRIAEIFGPTIQGEGALIGEPTVFVRAGGCDYRCSWCDSLHAVESRYRHSWATMTSETVWEQVRRLSGDRPLTVSLS
G
GNPAIQDFGALIAMGRAAGYRFACETQGSVARPWFADLDTLVLSPKPPSSGEEVDWDAFDACRKLGVGARRQVMKI
VIFD
ETDYQWARDVHARHSDLPLYLQPGNTEVNPAAPVDLQALADRLGWLTGKALADGWFAPRILPQLHILIWGNKRGV
>OLS49958.1 6-carboxytetrahydropterin synthase QueD [Rhodovulum sulfidophilum]AB14
MFRIRKEFHFSASHQLPHLPEDHQCARLHGHNYILVVELAAETLNADGFVRDYHDLKPLKTYVDDHFDHRHLNDVMEG
PS

TAENMARHFYDWCAARWPETSAVLVSETPKTWAEYRP
>OLS49959.1 7-cyano-7-deazaguanine synthase QueC [Rhodovulum sulfidophilum]AB14
MKTLVICSGGLDSVSLAHITANEHQLTRLVSFDYGQRHRKEVDFAEAASRRLGVPFHLIDMRGIGAVLSGSALTDDIDVP
DGHYAEETMRITVVPNRNAIMLSIGFGIAAAQGDEAVATAVHGGDHFIYPDCRPAFTRAFDAMQQAALDGYAEIRLH
TPF
VEHSKAEIVRQGARHGTPFAQTWSCYKGGEVHCGRCGTCVERREAFHLAGVSDPTVYADPDYWTEALAARGQS
>OLS49960.1 peptide chain release factor-like protein [Rhodovulum sulfidophilum]AB14
MSDREALLLSSGNGPGECRQAVGHLLSWLSTKATQYGVDLDVSTRDAAHGPASAVVVLSGARAGDLVRSVEGVILWR
CQS
ELRPGHKRKNWFVQAFRLPAATDVVRIDPAAVEMQAIRAGGPGGQHQNKTSSAIRARWTSEDGKVYAVVVRDNRS
QHQNR
RLALERLAALAAAEKAEADAAREGETWVLHGQLQRGEPRRIFEGRQFKEVK
>OLS49961.1 RNA ligase RtcB family protein [Rhodovulum sulfidophilum]AB14
MGTPISGERSAARARISKFYNRATWIDGRAEEQLEQVAGWPGMMAVAGFPDLHPGRFGPVGAAFLADRIWPQLVG
PDIGC
GMALFRLDLPRRRFKLDKAARRLAVLEVGADPERALAALDDAGLTGSIGPEALGTIGGGNHFCEVQVVDAVFDPSRTDL
R
KDDLCLLVHSGSRGRGAGIFQGLDDSWKQGFAAESDAAVRYLALHDAAICWARVNRSLIASTASEALGSEARLICDAVH
N
HVVPHSGVWLHRKGAACPEGGLVPLAGSREAPSFLLEVPDLVPEALGSVSHGAGRRYDRASMHGRIRKTRSELAALTR
TR
FGGRVICGDRDLLLEEAGRAYKDPEQVLHDLSASGLASAVARLLPLVTFKTTGVAI
>OLS49962.1 ABC transporter [Rhodovulum sulfidophilum]AB14
MPASVSLSSLSWSAPDGTSLFTDLDLTFGAERTGLVGRNGTGKSTLLHLIAGHSSSASGRISVTGSVAMMRQEALAHPE
E
TVADLLGVRPALELMDRAERGRATSEELAEADWTLPARIEAALARCDLSVAPDTRLACLSGGQRSRAALAALVLAEPDF
L
LMDEPTNNLDRAGRRAVIDLLRGWSGGAIVASHDRELLEEMDAIVELTSLGAARYGGNYSAFRQQRESARAAAAQDL
AHA
QKLRAEAAHRAQQAAERKVRKDSAGRKSRARGDQPKILMDAAKGRAEASGGAGARLRDVRRDAAEDALTAAREKVE
VLTP
IHMDIAPSGLPAGRNVLTLDRVTAGYDPAAPILEDLSFTLTGPERVVIAGPNGSGKTTLLKLITGALRPMAGEAQLCVPW
ALLDQHVGLLAPDRTLREVFRDRNPQASMQMAHAALARFGFRASDAQRLAEGLSGGEKLRAGLACVLGGTPPPQLLL
LDE
PTNHLDMEGVEALEAALASYDGAMVVVSHDRAFLASLRADRIIFVKR
>OLS49963.1 hypothetical protein BV379_17895 [Rhodovulum sulfidophilum]AB14
MSRAFLVLGAAMREAGRPGPALIRRAEHAAALWRAALETLVVTSGAGGEAEAAAAICRAAGVPPERLICESAARSTAE
NI
AFCRPILARSGICRVTLVTDGYHAPRALFLARRAGLSADASCPARPPGPPLRRALALLREAAAIAKALVLSTTGRP
>OLS49964.1 cobyrinic acid a,c-diamide synthase [Rhodovulum sulfidophilum]AB14
MAGTIITIAQQKGGSGKTTLAANLAVAFRRRGLAVALIDTDPQGSLGRWFMTRIEGREAPDMEFSTASAWGVGYETG
KLA
RDHDVVLIDTPPKADSDLRPALRAADLVLVPVAASHVDLWATEGVLDLARREDKPAIVVLNRLKAKTRLAAEIAEIAGG
M
DADLATVRLANRVSYAETLGRGLGVQEAGRRGPGADEIEALAEEVLAALAD
>OLS49965.1 hypothetical protein BV379_17905 [Rhodovulum sulfidophilum]AB14
MAGAWLQEKGSGLLAFSHEANRDRTWDSRWGYNSLYVEYGMTPKLTLGLDAGQGDDADDWKAIAFLRFATELGW
MPGRLA

TEFGAGAAGAPGGLTEPVIRTGLSWGTGFTTPDGTGWINLDARTDTRPQSALVDYKLDITLGVMPNPRTQLTLELWGEDS
ATEARRVKLSPAVSRRIGKSTWLRFGGIFGIHNDRSIGLVIGSRIEF
>OLS49966.1 AsnC family transcriptional regulator [Rhodovulum sulfidophilum]AB14
MPGSRLDAIDRKILAELQADGRMTNVELARRVGISAPPCLRRVRTLEEAGFIRGYHADINTRELGFEVQVFAMVGLQSQA
EADLSRFEARCRAWPLVRECHMLNGEVDFILKCVAPDLSSFQSFLTEELTAAENVASVKTSLVIRCAKDEPGVPFEVLEARLSRSA
>OLS49967.1 hypothetical protein BV379_17920 [Rhodovulum sulfidophilum]AB14
MTMNTTLRGATAAICILFGAGTASAAVIDLGFVLDGSGSVTSYDFDSATAALSAALSKIPTSGDNEYRVAVTSYGTSSYT
VVPPTVVTAANIASIQSTVLTAYKDGGGTDTAGAITYITDLFLDDGLGDTTLINITTDGTPNSQYLTENAALAANTAGVD
GISLEAVGSGVSSPYALSNMARIAGLGTSGDADAGVIAADLDSLPNATETGFVIPVADFDAYGAAIKAKIDQVIDDTGGD
TGVVPLPAAMPMLLAGLGVFGFVRRRQTAG
>OLS49968.1 hypothetical protein BV379_17925 [Rhodovulum sulfidophilum]AB14
MFRYVLAAVAGLMTAPGLQAQEVAQQTVSDEMPPHCAVVDSSRIVSVVVCDGPRDEAAFAEAGRAACGARRPCGAWFWPS
REAAPETAPANHDGLSQAQVVSSYGVWVAERGLFVRIERAE
>OLS49969.1 thioredoxin-disulfide reductase [Rhodovulum sulfidophilum]AB14
MTKQRHVRVLIIGSGPAGYTAAVYASRAMLEPVLIQGIQPGGQLTITTEVENWPGETEIQGPELMQKMEAHARAMGAEIV
SDYISRLDLSKRPFTAIADSGTTYTCDALILATGAQAKWLGLPSEEKFKGFGVSACATCDGFFYRGQEVAVIGGGNAAVE
EALFLTRFASKVTLIHRRGELRAEKVLQKRLFANPKVEILWDHNLDEVTGSENPLGVEGIRVKHVQTGETREIACKGVFI
AIGHAPASELVSDQLETHMGGYVVTAPDSTATSIPGVFAAGDLTDHKYRQAVTSAGMGCMAALEVERFLAEAGLDEGKDI
SLPLGYGAEVPQDA
>OLS49970.1 hypothetical protein BV379_17935 [Rhodovulum sulfidophilum]AB14
MIAFRGLVWRLLPAERAGAPCARVASPEGRFHRPGEAVLYASLTAEGAGVAIARYLTPDVPARALVPLAVAADRIVDLRA
LPAPNPASIVWQDLRASGAPAPTWAFSDAARAEGAEGLLYPSRSRPELSHLVLFDPAPPLVQPAGPARPWP
>OLS49971.1 adenylyltransferase [Rhodovulum sulfidophilum]AB14
MSISNLAPIPELYVSHESAQKLKVEAGDLPSWDLTPRQICDLELLMNGGFNPLKGFLDEADYDSVVERMRLEDGTLWPMP
VTLDVSTEFADKIEIGQDIALRDQEGVILATMTVTSRWEPDKHREAEKVFGADDLAHPAVYYLHHSAGRIYLGGPITGIQ
QPVHYDFRARRDTPNELRAYFRKLGWRRIVAFQTRNPLHRAHQELTFRAAREAQANLLIHPVVGMTKPGDVDHFTRVRCY
EAVLDKYPQATTTMSLLNLAMRMAGPREAVWHGLIRKNHGCTHFIVGRDHAGPGKNSAGEDFYGPYDAQELFRKHQDEIG
IEMVDFRHMVYVQERAQYEPADEIEDGVTVLNISGTELRRRLAEGLEIPEWFSFPEVVSELRKTRPARSKQGFTVFFTGL
SGSGKSTIANAVMVKLMEMGGRPVTLLDGDVVRKHLSSELGFSKEHRDINIRRIGYVASEITKNGGIAICAPIAPYSATR
RGVREMIEAYGAFCEVHVATPLEECEKRDRKGLYKLAREGKIKEFTGISDPYEAPETPELAVDTTGLDVDNCAQQVILKLEQMGLIAGR
>OLS49972.1 O-acetylhomoserine aminocarboxypropyltransferase [Rhodovulum sulfidophilum]AB14
MSEDRPYGFDTLQIHAGAKPDPATGARQVPIYQTTSYVFRDAEHAAALFGLKEVGFIYSRLTNPTVQALAERVAALEGGA
GAVCCSSGHAAQIMALFPLMAPGRNVVASTRLYGGTVTQFSQTIKRFGWSARFVDFDDLDAVKAAIDDDTRAVFCESVAN
PGGYVTDLDAISKIADTAGLPLIVDNTSATPYLCRPIEHGATLVVHSATKYLTGNGTVTGGVVVDSGKFDWSASDKFPSL

SEPEPAYHGLNFHQTFGPAAFTFHSIAVGLRDLGMTMNPQGAHYTLMGIETLSLRMERHCANAIKVAGWLEGHPKVA
RVT
YAGLPSSPYADRARRICPKGTGGLFTFALKDGYDACVKFVDSVRLFSHVANLGDTRSLVIHSASTTHAQLTEAQQIAAGA
NPEVVRVSIGIEDADDLIADLEQALAQI
>OLS49973.1 hypothetical protein BV379_17955 [Rhodovulum sulfidophilum]AB14
MKPDFALTLAEDGIGLQHRAKAGWRSLGEVSLHDPGLAETLRLLRATAGNLAEGRPLVALVIPDSQILYTELPVEGESRA
EKHASLLRGLDGLTPYPVGELVFDWQETGGRARLAVVARETLAEAEAFAQAHQFDPVSFVARPPAAQFEGEASFGPTA
AA
ATLLAEDKPAAPTPGARKSPRPATEAKPAPQTEPEAEPEPEAKTVPEARPAPKAEPAAKAEPAPKADPAPAPMPERKPA
A
ADAVAPPSPPPAADPAPTATAAPAAPAARPVSSAPASPVPAAPSAARPPRPETPAGTGQPATAPPRAGTARRASGSLR
PE
PQPMRGLDLSAAARPSTGPEPMQILKLGGAVIAVVAAIAAGAILLPRDETPAPDPDATAGLVAPSGANLSQAEAPDPQ
PP
FDAAANNQPRAEGSGSAPLAGTATGTGTPASPVPAPLSEAEALEAYASTGMWQRAPDPMSAPPQDRIEDLYLAGIDQ
AVR
PPEAGLLPDPQIARSDDRPLPMQADPPGPDQTFELDERGLVEATPEGALTPDGVTVFRGAPAVVPSARPADLVPEPEP
MT
VPAPGNTADGAEITAEGVPVYGVRPETTPPGRPVDLAPPAGTAADAPDDRAEAPAGEDATAAPGETDPRPSARPAPR
TAE
ASPPAVVPADPEVAARIASHTPRARPATVLARAGAPATAVPGTHPQVWPKNLAPPPTHDTDAAVTAALEDLADGEGE
AAS
DLPENALSASLRPASRPSDFEDRVQSAMAIAVQPAIPTAASVARQATLPDAINLRDLNLIGVYGTSADRRALLRLPSGRF
VKVKVGDKIDGGQVAAIGDTQLRYVKRGRNIVLDLPGG
>OLS49974.1 potassium transporter [Rhodovulum sulfidophilum]AB14
MDSFLLPALICLAAAVIAVPLAARLGLGSVLGYLAAGIAIGPALALLDAEVEDIRHVAEFGVVLMLFLIGLELEPKTLWR
MRTRLVGLGGLQVTLTTLAIMAAAIPALGLDWRPALAVGLVFALSSTAIVLQTLSEKGLMPTNGGRASFSVLLAQDIAVI
PMLVALPLLVAAPKAKIAPDGSVIRPALEAAPHGGSFSLVAGLPGWGVALVTLGAVAAVILAGHYLIRPVFRFVNMAHL
R
EMYTALALLLVIGIAVLMGMVGLSPALGTFLAGVVLADSEFRHDLESNIEPFKGLLLGLFFITVGAGIDFQVLLADPAGI
FALALGVMVVKGLVLAALGRLFGLKGRDRWLFTLGLAQAGEFGFVILGLAVQQDVLSQRLTQTLLLVIALTMLLTPLFFF
VYEMLSKRFAVAGIGTDRAPDRIDERNPILIAGIGRFGQVVQRMLQMSGYPATMLDDDLPTINLMRKFGIKGFFGNPT
RP
ETLMAAGLGTARVFVVALDDPAATTRVVRYARGVRPDLTIIARARDRAHVYALYRAGADKIVRELFDSSLRVARYSLEA
M
GLSATEARDRESAYYDFDRRMVRELADLWKPGVPTARNEPYMRRARELNGELDTALLSRRARRRAAEEAAAEEAAAE
AAG
EASAEVRD
>OLS49975.1 phosphoribosylpyrophosphate synthetase [Rhodovulum sulfidophilum]AB14
MHNIHEPKLISGNANRPLASAIARRMSMHRGISVDLVDARVERFNDQEIFVEVYENVRGEDMFVIQPTSNPANDNLM
ELM
IMADALRRSSAARITAVIPYFGYARQDRRTKARTPISAKLVANLLTESGIERVLTMDLHAAQIQGFFDIPVDNLYASPIF
ALDIAHEFNGLASKTTVVSPDVGGVTRARELAQRIGSGLAIVDKRRVRPGEVAEMTVIGDVEGQICFIVDDLCDTAGTLC
KAADKLIEAGAEEVHAYITHGVFSGPAVERIRNSKLKSVVVTDSIQPTEAILATPNIRIVPTAPLFAQAIMNIWQGTSVS
SLFEHETLVPIYEGLYAPRRG
>OLS49976.1 2-hydroxychromene-2-carboxylate isomerase [Rhodovulum sulfidophilum]AB14
MAHIDYFFTVISPYAYLAGNRLEEIAARHGATVTYKPVDMMALFARTGGTPLPQRSEARKAYRLQELRRQSVKTGLKLSL

APAHFPTNPAPAAYAIIAAQATGEGDPGQLVHALTRACWSEDRNVAEDEVIRDCLQAAGFDPGLADRGLLAGAETYA
ANL
EEATARGVFGAPFYITDGDERFWGQDRLDDLDQHLAGKL
>OLS49977.1 low specificity L-threonine aldolase [Rhodovulum sulfidophilum]AB14
MHFASDNTSPVPPQILDALIAANHGYAMPYGSDAIMAEVRTTIREIFGAPEAEVYLVATGSAANALALSVLCPPWATVF
C
HRNAHIEEDECGAPEFFTGGAKLTLVEGADAKVTPEALREAIVHAARAGVHNVQRGAVSITNATEQGTVYSAAEVSALA
D
VAADYGLPVHMDGARFANALMRSGASPAEMTWKAGIDILSFGGTKNGCMGVEAVVLFDPAHAWEFELRRKRGGHL
FSKHR
YLSAQMAAYLKDGLWLKLAARANDAATRLSEGLAAHDSARLLHPTEANAVFAAFPREAHRRAHAAGVQYYLWPMDC
SLDG
PGDELLSARLVCSWCTTDEEVDALLAIVRGD
>OLS49978.1 (R)-hydratase [Rhodovulum sulfidophilum]AB14
MLDNMPRGTICIEELEIGMRRHLRKQVTDRDIEMFAEVSTDCNPVHLDDDYAMETVFEGRIAHGMLTAGLISAVIGEQ
LP
GHGTIYLGQTLKFIAPVRPGDTVLAEVEVKSIDHSRRRVTLDCRCLVDGKPVLKGEAVVLAPSKKFD
>OLS49979.1 HAD family hydrolase [Rhodovulum sulfidophilum]AB14
MTPIIESLDEVSSRYKALFVDLWGCVHNGREPFAEAVAALQRYRARGGKVVLITNAPRHRKAVEGQLEEIGVARDSWD
TI
ATSGDSARAAMFRGIVGKRVYFMGIDFDLTFFDPIGIVRDPVEIERVPLEEAEGIVCTGPFDSHADPDVLRPQLLYAKQK
GLKLLCANPDVVVDRGETREWCAGAIAELYTKMGGESLYFGKPQPPIYDLARQRLAEIADVSDAEILCIGDGIRTDIAGA
IGEDLDSLFISGGLAAAETGTRNQPDPEQLEAFLKQHMASPTYAIGFLR
>OLS49980.1 manganese-dependent inorganic pyrophosphatase [Rhodovulum sulfidophilum]AB14
MTIKVLGHKAPDTDSTGSPLIWAWYLSEIKGQPAEAKLLGEPNTEAKFVLNRWGFEQPEIIADVAPGEDVVIVDTNNLA
E
LPSRINEANVIAIIDHHLLQGGLKTRSPIEITVRPVACTATIMFDLMGEDAAKMPDNIKGAMLSCILSDTLEFRSPTTTP
ADTAVAGRLAAELDVSIPDYAAEMFAAKSDVSAFSDAELLRMDSKQYEVAGKKFRVSVLETTSPGTVLDRKDSLMQAM
TG
VAEEDGADQVLLFVVDILKQEATLLVPNDLVKAVAEKSFAVTVSGDTVVLPGIVSRKKQIIPNLQL
>OLS49981.1 co-chaperone GroES [Rhodovulum sulfidophilum]AB14
MAFKPLHDRVLVRRVESDEKTKGGLIIPDSAKEKPAEGEVIACGEGARKDSGELIEMAVKAGDRVLFGKWSGTEVTIEGE
ELLIMKESDILGIIA
>OLS49982.1 chaperonin GroL [Rhodovulum sulfidophilum]AB14
MAAKDVKFDTDARNRMLKGVNILADAVKVTLGPKGRNVVIEKSFGAPRITKDGVTVAKEIELEDKFENMGAQMVKEV
ASR
TNDEAGDGTTTATVLAQAIVKEGTKAVAAGMNPMDLKRGIDMATAKVVEAIKAAARPVNDSDEVAQVGTISANGEA
NIGR
FIADAMQKVGNDGVITVEENKGMETEVEVVEGMQFDRGYLSPYFVTNPDKMVADLEDCLILLHEKKLSSLQPMVPLLE
SV
IQSQKPLLIIAEDVEGEALATLVVNKLRGGLKIAAVKAPGFGDRRKAMLQDIAILTGGQVISEDLGMKLENVTMDMLGQ
A
KKVSITKDETTIIDGHGDKAEIEARVSQIRQNIEETSSDYDREKLQERVAKLAGGVAVIRVGGLTEVEVKERKDRVDDAL
NATRAAVQEGVIVGGGVALVQGAKVLEGLTGDNSDQNAGIAIVRRALESPLRQIAENAGVDGAVVAGKIRESDDTHFG
FN
AQTEEYGDMFKFGVIDPAKVTRTALEDAASVAGLLITTEAMVADKPEPKGAPAAGGGMPDMGGMGGMM
>OLS49983.1 hypothetical protein BV379_18020 [Rhodovulum sulfidophilum]AB14
MFTNGNDHETIVPSGSAQTFDALGGFDTVTIDATSLLAGLSGTLLGTTWSSTYNSVSLSIANVEQLVVIGGAGNDAITGD

DGNDTLLGGTGNDYLNGEGGTDLINGGVGNDEIWAHGGDTVIGGAGTDMLHLVLGPGAGQSIDLTTGAGTGVTWS
GFERI
GASLSGGERDVYAGFLLADIDGAGCLHLDYSGTDLGGRTALSVNLDVYGGTIHLSDSSTQSFGIYGFDDFDILGSAGNDL
IDISEAGGGTVDGGAGNDTIWGSFESSVLDGGDGHDQIHGCSDYDILHGGNGNDTIYGYLYSLSELWGDDGDDYLDS
GDS
DGELHGGAGNDTLIAGEGAPLLDGGSGDDYIYTQLGQDRIYGGTGNDTLVIMAPYYPIDYDLDISQGGDIRGIEYADITL
AEGNDTIRIGIASAYIDGTGGTDHLILDYSETDENGRTATAVVFDLAANASIATLTDSGTISCNLDLDLFTVIGSGGHDW
IDGSIATQACTFEGGKGADTLLGGSGNDTITGGGGNDTIEGGAGNDRLEGGYGNDRIDAGTGNDLLIGGAWDDVLNG
GSG
NDTFLYWAGANGYDMVDGGAGAADRILATADNVTIGLRGLAGVEIVDADGHVGVVLQGSAEHNLLDFSGTTLSGIEH
IDG
GSGSDTIIGSAGDDVIIGGKGNDNLSGGAGGDILTGMAGADIFDFNVASHSNGANIDRITDFVRGEDLLDLSGIDANSG
L
AGDQDFDFIGTAAFSGTAGELRADLTAVPGMTVILADINGNGAVDLEIRLDGLYSLAATDFLL
>OLS49984.1 hypothetical protein BV379_18025 [Rhodovulum sulfidophilum]AB14
MEKADRKTAREVLKYRLTKPDHPPSGEKNRSEAVFPCGGQAERMGRSVREVGGKCFYHDPRDQLRRQLAAVMAAEN
FDRR
LKTLGGVMPREEICKIRFSEPVRSLLVPIRQMPGSDT
>OLS49985.1 hypothetical protein BV379_18030 [Rhodovulum sulfidophilum]AB14
MVDRPDRLLRLHLADPRVGRPQALEWGRLHSTICSTKPGAGEAIEGMAVSDPADPDRTLNPLGGSPLALAN
>OLS49986.1 transcriptional regulator [Rhodovulum sulfidophilum]AB14
MITQKTKYALKAMIALAEEAAGAGEALTIEQISQRSGAPKRFLEHILLDLKRTGYLGSRRGRSGGYMLIKEPRQVSIGEL
LREVDGPIAPLPCLSRRAYQPCDDCEDEESCRIRRLFGQVFYAYLLLIESLTLADLLESPERIERLVADAALPPV
>OLS49987.1 dATP pyrophosphohydrolase [Rhodovulum sulfidophilum]AB14
MSLDQIVVLALLAIVFLSFIKETYPPEVTALAASAALLATGIIGTGDFLTVFGSSAPITIAMMFIISAALERTGVLATLG
EFMTARAGGSYLRALFLMMIVTVLASAFMNNTPVVILLTPVMISVAASVGVAPSRLLIPLSFSAIFGGTLTLVGTSTNIL
MSGVARETGQPPISMFEMTLPGMIFVLVGMAYLAIAGRFLLPDRQSLAHLVGRETRRRFLARLLIPAGSRYIGKRLDDLP
FNTAETRILDVIRGEVSMRRRLDDLELERGDRLVLKTGTGEILGLKENGQVAFRDLSDRGVEPVTADQTVTLEASIGPNS
ILRGRPLGELRLRRNYGVYVMAVHRADRNLSERTDDIRLQFADTLLLEGPAEGLRRLVEDGGVVNLSAPSERPMRRAKA
P
VAIATMMAVVGLAAFDVMPIAGLAVIGAVAVMLTRCVDPEEAFNAIDWRILFLIFGMLGLSKGMEETGTAQLIVDWV
VGL
LGGFGPLAILAAVYVLTSALTEMISNNAVAVLVGPIVIGLAVQLGVDPRPFIMAVMFAASASFATPIGYQTNTFVYGAGG
YRFRDFLKVGLPLNIIFAVVAVGVIPLFFPF
>OLS49988.1 transcriptional regulator [Rhodovulum sulfidophilum]AB14
MSHLTKDKPKLLARVRRLKGQIEAIERAIEAEKDCGAILQLVASVRGATAGLTQELIEDHLRHHVILVEDETGREQGGAE
LASVLRTYLK
>OLS49989.1 sulfurtransferase [Rhodovulum sulfidophilum]AB14
MTLDRAVMMFAGCMILLSLGLGTWVSSLWYLLTAFVGLNMMQASVTGFCPAAILFRRLGIRPGTVFD
>OLS49990.1 ABC transporter ATP-binding protein [Rhodovulum sulfidophilum]AB14
MLLSLSDIGKGYETGGARVEVLGGVDLALAPGETVALTGESGSGKSTLLHLCAGLDRADRGRIEVAGQDLTHASELRLAA
LRRETVGLVFQQFNLIPSLDVAANLAFQARLAGRHDPDRAAKLADRLGLAGLLSRYPEQLSGGQQQRVAIGRALAIRPA
L
LLADEPTGNLDEATGDAVMELMLELVAEAGAGLLMVTHSPRLAARLGRRVHLAGGRLAA
>OLS49991.1 iron ABC transporter permease [Rhodovulum sulfidophilum]AB14
MNVKALICALLLPLPALAQGFAGLGGTAEGYALSDPAARLEFPRDHGAHPGFRIEWWYVTANLEGEDGKEYGIQWTLF
RS

ALAPGDPGTGWQNRTAWMGHAAVTSADTHKSAERLARGGIGQAGVTLAPFRAEIDDWHLVSTAAPGADPLSAARL
GASGA
GFAYDLDLVATGPLVLQGARGYSVKSRDGQASHYYSQPFYRAEGRLIFPDRTVRVTGRAWLDREWSSQPLAADQTGW
DWL
SLHLDDGAKLMVFRLRGAGDDFLTGTWIAPDGTPAPLPPGAVEMIPKAETELDGRSIPTLWQIRLPARGLDITVTALQP
G
AWNATRVPYWEGPVTARGSHPGEGYLEMTGY
>OLS49992.1 pyridine nucleotide-disulfide oxidoreductase [Rhodovulum sulfidophilum]AB14
MSPGPQLVLIGGGHAHALVLRGQPLPSGLSVTLIDPSPKAAYSGMLPGFIAGHYPRAALEIDLARLASAAGARFVIGRAE
GLDRARRHVLVAGQAPVPYDILSLDIGITSDLPDLAGFADHAVPAKPLGHFADRWPGFLAGVAAGHNAPRVAVIGAGV
AG
VELALAARYRLARDGAAPEVTLIDAADEILRELRPGARAALLSAIGRAGIALRTGGRIARIGAEGVTLATGETIPAAFVI
GAAGARPQGWLAETGLGLTDGFVTVDRTLRSVSDPRIFAAGDCAHLSHAPRPKAGVFAVREAPILRRNLIAALTGGRM
RA
YRPQRHYLKLVSMGEKRAAADRLALRIEGRWVWRWKDRIDRGFMRRFQDLPPAGRP
>OLS49993.1 hypothetical protein BV379_18080 [Rhodovulum sulfidophilum]AB14
MSRSAAGVSGSGAKALGGDAFFVASRLGWALLRPDTLILVSLGFGLFLLWRGRFGAGRAVLGLVLIALLVLSLTPLAPRA
MARLEAAYPVPAIEGRPVAGIVVLGGGEEPAISVARGEPTVNEAGDRFLAALALARRFPEAPVLFTGGSGRIRPAAIPEA
NVAAAFLLRAGLDPSLLILEDASRSTAENARLAAELAPDRPGDWVLVTSAFHMRRAVATFCAAGWRDILPYPVDFRTAP
G
FRASPGLPENLGLLTLALHEALGFAVYRASGLAADPLPEGCLAG
>OLS49994.1 UDP-glucose 6-dehydrogenase [Rhodovulum sulfidophilum]AB14
MKIAMVGTGYVGLVSGVCFSDFGHEVVCIDKDPRKIEMLMRGEVPIYEPGLEAVMARNVAAGRLSFSGDLAAAVAGA
DAV
FIAVGTPTRRGDGHADLSYVMAAAEEIANALTGYCVIVTKSTVPVGTNRQVAEVVSKANPAAEFDVASNPEFLREGAAI
D
DFMRPDRVVVGVESERARAVMADIYRPLFLREFPIVYTDLESAEMIKYAANAFLAAKITFINEMAMLCEKVGADIKAVSK
GMGLDGRIGNKFLHAGPGYGGSCFPKDTNALARIGQDHASPCAITETVIEVNDRVKRRMVDKLVDLCGGSVNGKTVA
VLG
VTFKPNTDDMREAPALTIVPALVGGGAKVRVIDPQGRREGEHLLPGVDWVEDPYAAATGADALVILTEWNEFRALDL
AQL
AQAMATPRLADLRNIYPPEDVRQAGFEAYVAVGRRGFGLGS
>OLS49995.1 hypothetical protein BV379_18090 [Rhodovulum sulfidophilum]AB14
MTSTDPPLGPALLTLAIGAAGAALAAFLHMPAPFIIGPALSVTIAALAGLRAVLPRGFRDTCFVVMGVNLGASVTPEAID
TAIRWPLSLVVLTISVLTIIGLGARLLRRLFGFDPMSALLTATPGHLSFVLSLSTDLRADLPRVTMIQTVRVLALILLVP
FLAPLLGHGDLPALPPQTAPAMSLLVLVPLLALAGALGLLLDRLRVPAGLLLGGMGLSALGHATGTVSGAVPAWLSVPA
F
IAMGTLIGTRFSGVTPRMLLQSLGAASLLTGFAALVSLVAAALVAKLLGLPMLSALIAFAPGGLETMMAMSLLLNADPA
Y
VTTHHVFRLLMLTVALPLLLARTRARLAREAAAAPPPG
>OLS49996.1 drug:proton antiporter [Rhodovulum sulfidophilum]AB14
MKLAVAARIARRELRGGLRGFYVFLACLALGVAAIAAVGSVRESIEAGLTREGAALLGGDAEMTLTYRFASDEERAWME
G
VSDALSEVVDFRSMVAVRHDGETERGLTQVKAVDDAYPLYGQVVLDPPMPLDRAIAGNGAVMERVLADRLGLEPGD
SFRL
GTADYTLSAVLLREPDGAGAGFGLGPRLILRKAALEPSGLLAPGTLYEVKYRLKLPPGHDIAALERAAESRFADRGVRWR
DARNGAPGMTRFVDRMASFLVLVGLAGLAVGGVGVSAAVRAYIDGKTEVVAILKTLGAEGSTILAVYLMQIGALAGLGI
A

IGLGLGALLPLAAGPFLAAQLPVPAEFTVHAGPLAEAALYGVLTALLFTLWPLARTEEMRAAALFRDASGRVRAWPRLR
Y
VAATLGLAAALIGTAAWFSGVPKLAFWAAAGILGALGLLVLAAAALRRIARAAARVGAVGGPGGETASVVLSLGLGLSIL
AAIGQIDSNLRGAIQQELPERAPSYFVVDIQPGQLEGFLARLGRDPGVSRVETAPMLRGVLTRINGRPAREVAGDHWV
VR
GDRGVTYADRPPENTVVTDGLWWPSGYDGPPQVSFAREEADEIGLKLGDEITVNILGRDITATVTSFREVDFSTAGIGF
V
MTMDPAAVQGAPHTHIATIYAEPSAEAAILRDLASAYPNITAIRIKDALDRVAEVLSGLAAAIAYGASATLVTGVIVLIG
AAAAGQRARVFEAAVLKTVGAVRGQILAYFALRSAMLGAAAGTVAILAGGVAGWAVMRFVMEGEYRFEPVSALAIVL
GGA
LATLLAGLAFAWRPLAARPARVLRARE
>OLS49997.1 ABC transporter [Rhodovulum sulfidophilum]AB14
MADPILSLSEVKLTLAGNAGPVEILHGISLDVRPGETLGLVGPSGSGKSSLLMLMGGLERASAGRVTALGRDLTAMNED
Q
LARFRREHMGVVFQSFHLIPTMTALENVATPLELAGHRDAFERARDELCAVGLGARIDHYPSQMSGGEQQRVALARA
AAP
RPHLLLADEPTGNLDGSNGAAIMELLFGLRDRHGATLVLVTHAADLAARCDRVIELADGRISGDAARRAAE
>OLS49998.1 FAD-binding oxidoreductase [Rhodovulum sulfidophilum]AB14
MDPIPYLTDLLGPAHVLTGEDTRRHAHDWTGKYKGTPRALLRPADRDQLAAIVTYAARHRLPVVPVAGNTGLAGGAY
CDG
ALMVSVERLNRIREINPRARSATVEAGVILSQLHDAAEAQDLVFPLSFGARGSAMIGGVLSTNAGGSNVVRYGSTRGLC
L
GLEAVLPDGRVMDVMSPLHKDNTGLDLKDLMIGAEGTLGIVTAAVLRLFPRPRAYATAMVAVSDLDAALDLLNRLQEA
TG
GAVEAFEYMPDDYMRQLFRLRPEMRPPFDRAYPISIMVEIGATAPRDATPGPDGRLPVVQLLEDTLGEGFERGEVLDA
VI
AQNETQREEIWARREISAEICFSAAHVVDNDIALPLDRVGTFLERMTARLPQIDPAAESITVGHLGDGNLHYTVWASSG
S
ADLKETIREAVEDEVAALDGSFSAEHGIGLSKLSTMARRKNPVALDAMRRIKAALDPEGIMNPGKLVPPAD
>OLS49999.1 hydroxymethylbilane synthase [Rhodovulum sulfidophilum]AB14
MAITLPSPASPLKIGTRGSPLALAQAYETRERLMKAFDLPEDAFEIVVINTIADRVLDRALKEIGGKGLFTKEIEDAMLA
GKIDIAVHSTKDMAVEQPEGLVLDTFLPRENYRDAFISKSVASLKDLAEGAVVGTSSLRRRSQLLNYRPDLKVVEFRGNV
QTRLKKLEDGVAEATFLAMAGLNRLGMEDVAKSTIEPEEMLPAVAQGAIGIERRSDDAMAGHLLEALHDKATGEMM
ACER
AFLDALDGSCETPIGGLALHEGGNLWLRGEILRPDGSERIAGEDRAPIGDGAEMGFALGKKLREEAGEGFFDWD
>OLS50000.1 uroporphyrinogen decarboxylase [Rhodovulum sulfidophilum]AB14
MAETKKLLRALSGETMDVPPVWLMRQAGRYLPEYKATRAEAGDFLSLCYNSDLAAEVTLQPIRRYGFDAAILFADILLLP
QALGAELWFETGEGPRMSTITGAEGLKALKGKDDIHDHMAPVYETCRILSRELPKETTLIGFAGAPWTVATYMIAGRGT
K
DQAPAHALRQEDEATFAALIELLTEATIEYLSKQVEAGCEVVKIFDSWAGSLKGEAFDKYALEPAKRIISEMKARHPGLP
CIAFPREAGDKYIGFAKATGADCVALDNSVSTEWAVENVQKDGCVQGNMAPKLMVTGGQELIDETKRIKEAFSKGPHI
FN
LGHGITPDADPENVTIMLEALRG
>OLS50001.1 hypothetical protein BV379_18125 [Rhodovulum sulfidophilum]AB14
MALPSRDHSVSDLRLRAVDAAIALMQARGDADFAMAELAAAAGLTEARLAVAFPDRDALLAELRRVSYDRLADRRSAI
YE
APGGDPATRLMRGGQDYLDFARANPALYRLMFCDPADGGIARIIAEAMPAEALDALTEGVRRCQLQRGRRTDRARDL
AFL

LWTTLHGVVVLALDTDDAANADAKWEKVAATVARLTELICAASDA
>OLS50002.1 coproporphyrinogen III oxidase [Rhodovulum sulfidophilum]AB14
MTEMFDDRKAQAATWFRNLRDEIVAAFEALEDGPAAEPEAGRAPGRFEVTETRRKSEDGGDAGGGLMSVMRGGRIF
EKVG
VNVSTVHGTLNPKAQAAMAARKGLEGMREDPRFWASGISLVAHMQNPHSPAVHLNTRMFWTPHGWWFGGGTDL
NPCLPRD
EDTAAFHGVLKAHCDRHDVAHYPRFKAWADEYFHVPHRGRARGVGGIFFDDLNSGDWAADFAFTQDVGRAFLPAFL
PLVE
RRATTPWTEADRAAQLAHRGLYAEYNLLYDRGTRFGLETGHDPEAVLMSMPPLASWP
>OLS50003.1 hypothetical protein BV379_18135 [Rhodovulum sulfidophilum]AB14
MRAIALLLLLSLAACATPRQSCERAALYDLRVLDKLIRQSEQTIARGFAYRREPYERSRLDFCYGSWLGRNPAAGFTFCD
WPEIRVREVPVAVDLAAERRNLAELKAKRAETERRAARALADCAARYPAQAEAPAGAKK
>OLS50004.1 oxidoreductase [Rhodovulum sulfidophilum]AB14
MSFSIQGKTAIVTGAANGLGLAIARHFADRGANVMFADRDEPRLVDECGRNAEGDSAVRYFAGDLRERLTIANLLSATI
D
AFDRIDILVNASRQMVLSDPLDPDNDGFDVMMEQNVMTSLRLSQIVSRRMIRQAGEAGQTEGQAGAIVNMSSIAAT
RTHG
ELLAYSVSCAALEQLTRSMAVALAPHRIRVNGVAFGSVMSASLQNILKDQPDYRSEIRNHTPLGRIAKASELSETVQFLS
SDAAQFMTGQILAVDGGRTLIDPVATPAH
>OLS50005.1 MFS transporter [Rhodovulum sulfidophilum]AB14
MTVSRLTLALQSGAVSVPPDGRIAVFDAPAGADLSALPKDRVQIVQGFAPDHDAWAARGWDVAVTPEGAFAAALVV
LPRA
KAAAQARLAEALALTGGGPVLVDGAKTDGVDSLLKALRKGGAEVSAPFAKAHGKTFSFGAEPAALAHWLPGEGGLIDG
RY
RTLPGVFSADGIDRGSALLVGALQAPLKGRVADLGAGWGYVAAEALAAHPGIAEMHLIEADHAALSCARENVTDPRAR
FH
WADATVFKADRGFDTVLTNPPFHVSRTADPGLGRAFLAAAARLLAPHGQALIVANRHLPYERDLAGLFGETREIGAEA
GF
KLLLARRPSRRPR
>OLS50006.1 HAD family hydrolase [Rhodovulum sulfidophilum]AB14
MSGRITTIGFDADDTLWQNERFFRLTQSRFAELLADYAPSEALMARLLEAERRNVGHYGFGIKGFVLSMIETAIEVTEER
VPAAVIRELLAAGQEMLRHPIELMPDAREAILALSESHRILLITKGDLLDQERKLAQSGLGDLFDGVEIVSDKTAPRYGA
IFARHGDGAAQAMMVGNSLKSDVIPAIEAGAWGVFVPHDLTWELEHADPPRTHARFAELQHLGELPDLIARLG
>OLS50007.1 D-alanyl-D-alanine carboxypeptidase [Rhodovulum sulfidophilum]AB14
MPERDRSKQKSGRPEAVKRQTHVRRASLCVRRALLFAILAIGMTVTASGLRAAPSAAFVVDARTGEVLHAKNADTRLH
PA
SLTKMMTLYLAFEAIEHGEISPETMVKISSHAASEPPSKLGLRAGQRIKFKYLIRAAAIKSANDAATAIGEALEGSEAAF
ARRMNRTARAMGMNRTTFRNANGLTEKGHLSTARDMTILARHIVYDYPGYYNIFSRTNDDAGIRRVYSTNRRFLGSYS
GA
DGIKTGYTNAAGYNLVGSARRGQERIIATVMGGRSTAARNAEVAKLLDLGFRKAPTRVAMRRPQKPPYLTGPARLAPV
PP
VGKAPSVALLARSLRPQARPGLPAPSEEMIAALAADVSTAVSTAKAELTAAPAASAEALAASILPRARPGRPSSSATPED
PPSGPAAQIAADTPPTLELASADAAVQTEIVSRLSSSGGRYWGINVGNYGSRYQAERVLLRTALQEMGILDNALRKVVQ
G
RRGFEANFVGMTEAEADQACRRLQSHGAACNTFGPPS
>OLS50008.1 fumarylacetoacetate hydrolase [Rhodovulum sulfidophilum]AB14
MTAYALPPPPVPSLAIAGDTARFPVRRIFCVGRNYAEHAREMGADGREPPFFFTKPADALVGDGVDCPYPPATAALHH
EA

ELVIALGQGGADIPAASALSHVWGYAPGNDLTRRDLQAEAKAARRPWDMAKGFDASAVCGALVPALRIGHPTRGRIL
CHV
ESQLRQEGDLSDMIWSVPEIVSHLSRLVALAPGDLIYTGTPAGVGPVGRNQTCSVEIEGLGRVTTRFS
>OLS50009.1 cytochrome oxidase maturation protein, cbb3-type [Rhodovulum sulfidophilum]AB14
MQIIAYLIPVSLFLGGLGLGAFFWALRSHQFEDPEGDSRRILNDDWDEHPKP
>OLS50010.1 ATPase [Rhodovulum sulfidophilum]AB14
MSAEMQMRPAACPACAVAPSAETLAAAGAAAPKEARLFLSLPTIHCAACISAVERALNGTAGVLDARVNLTLKRVAVE
AG
PEVTADRLIEVLAAIGYEAHELDPGVIAATATDRAGRDILMRLAVSGFAMMNVMLLSVAVWSGAADATRDLFHWVSA
MIA
LPTIVFAAQPFFRNAFAVLRHGRLNMDVPISLAIVLAAGMSLYETAQHGPHAYFDAALSLTFFLLAGRYLDHRTRAVARS
AAEELAALEVPRAQRRTGAGEETVPIAEIAVGDVVLVRPGGRVPVDGVVAEGQSEIDRSLLTGETLPVFAGPGMALSAG
E
VNLTGPLAVTVTAAGQDSSLHRMADLVAVAESSRNRYTSLADRAARIYAPGVHLLSLLAFLVWLWISGGDLRLALNIAV
A
VLIITCPCALGLAVPAVTTAASGRLFRRGLLIKDGTALERLAEIDAVVFDKTGTLTLGAPEPTNLGDHSRRDLEIALALA
EGSAHPLARALAEAARQAGLHPARVGDIREVPGYGVEGALGGETVRFGRAAWVGAGAAPGSETASWLRVGARAPVA
FTFA
DRLRPGAETAVAELVALGMQVRLVSGDAPGPVADLAARLGISDWQAEALPEDKAAAVQALGDEGRRVLMVGDGLN
DTAAL
AAAHVSISPASALDAARVASDIVLLGRDLAPLGGAVATARRATRRIRENFAISALYNLIAVPVALVGLATPLAAALAMSA
SSITVSLNALRLR
>OLS50011.1 nitrogen fixation protein FixH [Rhodovulum sulfidophilum]AB14
MGKELTGRKVLIFTVSAFAVVVGVNLTLAYQAVHTFPGLEVANSYVASQEFNTHLHEQQALGWTTSIDYDEAEKVLALR
F
EDEAGKPVEVRDLDVLVGRATSVRDDRTPRMVYYNGAFSSPLDLGGGFWTIRVTAAAEDGTPFRQRLQLHVDG
>OLS50012.1 cytochrome c oxidase accessory protein CcoG [Rhodovulum sulfidophilum]AB14
MSSTDQNPDRLYSAREPIFPRRVSGKFRSLKWWLLAICLGIYYLLPWMRWDRGPNLPDQAVLIDLANRRFFFFTIEIWP
H
EFYFVAGLLIMAGLGLFLFTSALGRVWCGYFCPQTVWTDLFILVERWVEGDRNKRVKLWNARWDTRKWRLRLTKWA
LWLT
IAVATGGAWVFYFADAPTLLWNLVTFQAHAVAYSTILVLTLTTLILGGFMREQVCVYMCPWPRIQAAMMDEDTLTIGY
RE
WRGEPRGKHRKAEGAAQLGDCIDCNACVNVCPMGIDIRDGQQLACITCGLCIDACDDVMDKVGKPRGLIDYMALSD
EARE
RAGTPAKAVWRHVFRVRTIFYTLLWSAVGVGLMVALFIRPDIGMTVAPVRNPIFVTMSDGSIRNTYDVRVRNMHGEA
RDF
QLSVTPAEAFTISVEGAEDATFTVPADETLLKRVYIVAPAGSAPASASTSEVRIWLEGLAAEERTYDDTVFNGRGMK
>OLS50013.1 cytochrome-c oxidase, cbb3-type subunit III [Rhodovulum sulfidophilum]AB14
MSSSDHHTKPGEVGTTGHSWDGIEELNNPLPRWWLWTFYACIAFALIYSVLYPAWPLVKGATPGLLGYSTRAAVEVEI
KE
YEDRNAELSSQLAAASMEDPAAIEAEPALYNYAVQGGASVFRTNCAQCHGSGAAGVQGQGYPSLLDDDWLWGGTLE
DIYY
TVTNGVRNEDNMDARWSQMPSFGTDGLLTSGEIDQVVQYVRQISGQEHDAALASQGETVFLDNCAACHGEDGRGD
RNVGA
PNLTDAIWLYGGDVATLTETVTKARFGVMPTWKQRLSETERKAVAVYVHQLGGGE
>OLS50014.1 cbb3-type cytochrome C oxidase subunit 3 [Rhodovulum sulfidophilum]AB14
METYSFMREFADSWMLLVLTLFFVGVVVWAFRPGSAKEHSDTANIPFRHEDQPAGTEDGATCHKEART

>OLS50015.1 cytochrome-c oxidase, cbb3-type subunit II [Rhodovulum sulfidophilum]AB14
MAILEKHKILEKNASLLLVFSFLVVTVGGLVEIAPLFYLQNTIEKVEGMRPYSPMELEGRDIYVREGCYVCHSQMIRPMR
DEVERYGHYSLAAESMYDHPFQWGSKRTGPDLARVGGRYSDAWHVNHLRNPQSVVPESVMPKYAFLERRMIDPEHI
ADKL
RTHRAVGVPYTDEMIENAVADFNAQIDPYGDGADALLERYPGAQLRNFDGNEGISEMDALIAYLQMLGTLVDFSTFEP
DA
SR
>OLS50016.1 cytochrome c oxidase, cbb3-type subunit I [Rhodovulum sulfidophilum]AB14
MWDYVKIAALGVIAVLAAVAAFMARDMAYMVHAVIILLVAGGLVALTVRGLGAPKPVVDKTGYMDDVIRAGVIATCF
WGV
VGFAAGTFIASQLAFPGLNFEWASGYMNFGRLRPLHTSAVIFAFGGNALIATSFYVVQRTSAARLWGGNLAWFVFWG
YQL
FIVLAATGYLLGVTQSKEYAEPEWYVDIWLTIIWLVYLAIFLGTVFKRKEPHIYVANWFYLSFIVTVAMLHLINNLSLPV
SIWGSKSVQVFSGVQDAMTQWWYGHNAVGFFLTAGFLGMMYYFVPKQAERPVYSYKLSIIHFWALIFLYIWAGPHHL
HYT
ALPDWAGTLGMVFSIMLWMPSWGGMINGLMTLSGAWDKIRTDPIIRMMVVAVGFYGMSTFEGPMMSIRAVNSLS
HYTDWT
IGHVHSGALGWNGMITFGALYFLAPRLWNKERLYSTTLVSWHFWLATIGIVLYAASMWVTGIMEGLMWREVDAQGF
LVNS
FADTVAAKHPMYIVRALGGALYVAGGVIMCYNIWKTATSAPAKQPAAVAAPAE
>OLS50017.1 universal stress protein [Rhodovulum sulfidophilum]AB14
MGYKTILTIITETDIADSGLEPAITLAAREEAHLDILCLGVDRTQTGYYYAGTNAFIQQETLHKAQEDASAIEAAVKARM
ARETVTWSSEAAVAQIAGLSHLVAQRARFSDLVIMPRPYGEGRGIEDEAATEAVLFDGHAPLLVVPAGADTAHIGRKIVI
AWNQSQEAMAAIKEALPLMQAADVVNIAVVDPPKHGPDRSDPGGALSQMLSRHGVKTEISVLAKTMPRVSDVLQR
HATDI
EADMMIMGAYGHSRFREAILGGATRGMLEKTAIPVLMAH
>OLS50018.1 transcriptional regulator [Rhodovulum sulfidophilum]AB14
MAVTDTSVVHTECFECPIRHRAVCARCEADELEALEAVKYYRTYDAGQSVIWSGDRMDFVGSVVEGIATLTQTMEDG
RTQ
MVGLLLPSDFVGRPGRDLAAYDVTATTQLTMCCFRRKPFEELMVRTPHIGQRLLEMTLDELDAAREWMLLLGRKTAR
EKI
ASLLAIIARRDAVLRLGPTRGRIVFDLPLTRESMADYLGLTLETVSRQISALKRDGVIVLEGKRHVTVPDFDRLLGETGD
DNDGGMPA
>OLS50019.1 oxygen-independent coproporphyrinogen III oxidase [Rhodovulum sulfidophilum]AB14
MSTESQLTRLGLFDAKVPRYTSYPTAPHFNASVDDGDFAGWIGQIEPGSQISLYLHVPFCRRLCWFCACRTQGTRSDSP
V
EAYLETLKTEIAMLGKRLPEGVTLSRLHWGGGTPTLLSPDMMKQLTEAIFAVAPMHEEGEFSVEIDPNEIDEARLDVLA
G
AGMNRASIGIQDFDPQIQSVIGRIQSYDITRDAVDLIRSKGITSLNADILYGLPHQSNERIAESVQKLMSLSPDRVALYG
YAHVPWMARRQSMIPTEALPTPENRLKLYETAKELFLWDGYREIGIDHFARPEDGLARALEAGELRRNFQGYTDDTAA
AL
IGLGASSISRFPQGYAQNLSGTSDYTARIRDGRFATGRGHVFTDEDRMRSRMIEALMCDFHISTAEITESFGIPKAELNA
MFQEVADRFAGFIDLTNDGLTVPEHGKPLARMIARGFDAYDLSKAGHSSAI
>OLS50020.1 microcin ABC transporter ATP-binding protein [Rhodovulum sulfidophilum]AB14
MSPVLKVDNLQVSFQQDGRQQVAVRGVSFEVGRGETVALVGESGSGKSVTALSTVGLLPGAATVSGSVIYEGEELAKA
PE
KRLRQVRGNDISFIFQEPMTSLNPLHTLEKQITESLALHQGLTGAAARARVLELLERVGIRDPESRLGAYPHQLSGGQRQ
RVMIAMALANGPDLLIADEPTTALDVTIEAQILELLAELKREETMSLLFITHDLGVVRRIADRVCVMKDGEIVETGPTAE

IFRAPQHPYTRKLLAAATVGGPDPLKTEAPEIVRTEGLKVWFPIQRGFMRRTVDHVKAVNGADISVRAGETLGVVGESG
S
GKTTLALAIMRLISSEGRIVFRGSAIDGLKSRQIRPLRRDMQIVFQDPFGSLSPRMTVEQIVAEGLGVHGSQSGRSARED
VAQILTEVGLDPAMMHRYPHEFSGGQRQRIAIARAMILRPRMVVLDEPTSALDMTVQVQIVDLLRDLQRRYDLAYLFIS
H
DLRVVRAMSHKVAVMRLGEIVEAGEVHEVFDAPKTEYTRTLMRAAFGAPGESPGAA
>OLS50021.1 peptide ABC transporter permease [Rhodovulum sulfidophilum]AB14
MAWLSPLNQRRWRNFKRNRRAHWSLMIFAVIFGLSLFAELLANDKPIYVNYRGESFFPVYNFYPETDFGGDLRTEAVY
RD
IEIQCLIVSGGLEACWDDPEGVIADAEDGVVDGETIEKGWMLWPPIPYSYDTVNNIDGTAPSAPDAQHWLGTDDTTR
DVL
ARVIYGFRLSILFTLMVTGIASVIGVVTGAVQGFFGGLTDLIFQRFIEIWTSTPQLYIIIILFAVLGRSFWLLVFLTALF
GWPALVGVVRAEFLRARNFEYVRAARALGVSNWKIMFRHMLPNAMVATLTMLPFIITGTIGTLASLDFLGFGLPASAP
SL
GEMTLQAKENLQAPWLGFTAFFTFAIMLSLLVFIFEGVRDAFDPRKTFQ
>OLS50022.1 microcin ABC transporter permease [Rhodovulum sulfidophilum]AB14
MGAYILRRLALIVPTLLGIMIINFTLTQFVPGGPVEQVIAKIEGQGDVFSGFAGGGGDAGAALGAGGGSGSPTDKYIGAR
GLPPDFIAELEKEFGFDKPPLERFLTMIWNYARFDFGQSYFRSVSVVDLVLEKMPVSISLGLWSTLIAYMVSIPLGIRKA
VRDGTSFDTWTSAVIIIGYAIPGFLFAVLLLVLFAGGSYLQWFPLRGLVSENWAELSWPAKILDYFWHITLPVVATTISS
FATLTLLTKNSFLDEIKKQYVVTAKAKGLSEGRVLYGHVFRNAMLIVIAGFPGMFIGVFFGASIIIETIFSLDGLGRLGF
EAAVARDYPVIFATLYVFGLIGLLVGILSDLTYVLVDPRIDFESRET
>OLS50023.1 ABC transporter substrate-binding protein [Rhodovulum sulfidophilum]AB14
MTGSSPMPTPRSHPDSAACAAAARPFPTTPLMAPVLALFLAFGAGPAPAEEAATGMSDGKVIESFAYSALGAPKYGPD
MA
HLDYVNPDAPKGGEISIWAPGTFDSFNQYSRMGVPAALNTIGTESLMTSTADDPYAMYCYLCTSIEYPEDLSWVIFNLR
D
DVTFQDGTPMTAEDIAFTQKLFLEQGIIEYRRLVEAYFGPIEVLGPHRIKFTFNDVTPMRDRIGLAGGTPAFSKAWFEET
GARLDQSTKTPFMATGAYVLDSFDYNRRVVYRRNPDFWGADQPFNIGRHNFDRIRVEYFADSNAAFEAFKSGAYTFRT
EN
SSKTWATGYDFPGIERGDVVREAIPDGSVGTRLSWVFNLDRAKWQDIRVRRAVEMMFNFEWSNKTLFYGYFTQPVSF
WSG
TDLAASGTPGPDEAAILQPLVDEGLLEPEILTEEVASPPDHDADTYRPSRRIIRAASKLLDEAGWVAGDDGIRRKDGQPL
ELVIIQRDPQFDRAINPFLENLRMLGVSGRLERVDTAQYVERRRAGMFDLANQGFVMGFEPSSGLSQWFGSKTADNS
SRN
LMRLRSPAVDRLIEDVIAADTLDGMKTRVHALDRVLRSLHFDIPLWFNDKTWVAYYDMYRHPETLPPLDVGELDFWW
FDQ
EAADRLHASGALR
>OLS50024.1 recombination protein RecR [Rhodovulum sulfidophilum]AB14
MADRPDEIDRLIDLMARLPGLGPRSARRAVLHLVKKRAVLLLPLAEAMRKVGEAVHECAVCGNVTTADTCEICTDPVR
AN
GEICVVEDVADLWAMERGGAFRGRYHVLGGTLSALDSVGPEDLRIPELVERVGREGISEVILALNATVDGQTTAHYIVD
E
LAETGIRLSSLAQGVPIGGELDYLDDGTISAALKARRGL
>OLS50025.1 nucleoid-associated protein, YbaB/EbfC family [Rhodovulum sulfidophilum]AB14
MLKGMGGLGDMAKMMKAAGEMQGKMAQLQETLAATLVEGESGAGLVKATATAKGTLTALSIDPSIFNADEKEVVE
DLILA
AIKDAQEKAMEKTKEETRKLTEDMGLPPGLGLPV
>OLS50026.1 DNA polymerase III subunit gamma/tau [Rhodovulum sulfidophilum]AB14

MAETEGTAYQVLARKYRPATFADLIGQDAMVRTLKNAFAADRIAQAFIMTGIRGTGKTTTARIIAKGMNCIGPDGQGG
PT
TEPCGECEHCRAIAEGRHVDVMEMDAASRTGVGDIREIIDSVAYRAASARYKIYIIDEVHMLSTSAFNALLKTLEEPPAH
VKFIFATTEIRKVPVTVLSRCQRFDLRRIEPEVMIAHLRRIAGLENAPISDEALGLITRAAEGSVRDAMSLLDQAISHGA
GETGVDQVRAMLGLADRGRVLDLFDMIMAGDAAAALGELAGQYADGADPLAVLRDLAEISHWLSVIKITPEAADDPT
VGP
DERARGRTMAERLSMRVLTRMWQMLLKALEEVSMAPNAMMAAEMAVIRLTHVSTLPSPEDLVRKLQENPPTGPAP
SGPAP
GGPAPSGGGGNGAGVQGQARGAPVHAGPSGGGAVTALAHAPEQTLAHYPSFEAVVDLIRANRDMVLLVEVEAGVR
LVSYS
PGRIEFEPAPDAPADLAQRLGHRLQTWTGARWAVSVVAKGGAPSIDETRNAAEAARRAEAAEHPLVRAVFAAFPKAEI
ID
IKPPEVLAAEAAAEALPEVEEEWDPFEDDD
>OLS50027.1 hypothetical protein BV379_18260 [Rhodovulum sulfidophilum]AB14
MRHGKTAYLDNVAIWFDADQNHIHIAVADPEYFHSTVGPDPASKRGHPHLFPTLFKALRDAGVSHPPIPENQNT
>OLS50028.1 prephenate dehydratase [Rhodovulum sulfidophilum]AB14
MTSRIAFQGEPGAYSHQACHDARPDMEALPCRTFEDVIEAVRRGEAEMAMLPVENSTYGRVADIHQLLPDSGLHIVG
EAF
VRVHINLLAVPGAALAGIRRAMSHTVLLGQCRDFLQTHDIHRVTAADTAGSARQVAETGDPAVAALAGDLAAEIYGLE
VL
AHHIEDHDKNTTRFLLMSRAPDRSRRGDLGMMTTFVFRVRNIPAALYKAMGGFATNGVNMTKLESYMVDGSFTATQ
FYAD
IEGHPDDAHVARALEELDYFTSHIDILGVYPADRKRS
>OLS50029.1 cytochrome c family protein [Rhodovulum sulfidophilum]AB14
MLKKVSFTKLLGSASGALLVFVLVDRAGGLYFSNPGLDQPAYVVIEEEPATPAEPEPELTFEERMAQADAAAGKRVFNQ
C
KSCHRVEEGANAVGPSLYGVVGRPVHSAEGFSYSDAFAGLEGSWTAERINAFITSPRDVVPGTAMTFAGLKDPQDRA
NVI
AYLESVVE
>OLS50030.1 cytoplasmic protein [Rhodovulum sulfidophilum]AB14
MNKPIMAKATAVWLVDNTTLSFRQIADFCGLHELEVQGIADGDVAAGVRGFDPVANSQLDQSEIDKAEKDPLYKLRLK
FN
PAAVGEEKRRGPRYTPLSKRQDRPASILWLVKFHPELSDGQIAKLVGTTKPTIQSIRERTHWNISNIQPIDPVALGLCKQ
SELDAAVQKAARKRAAEGDLMTDDERRKLVSTEQSLEMGTEARLPTAISGLETFSLTRPEPEEEEDRSVPDADSFFNLPD
HDEDEEEDDDPRR
>OLS50031.1 ribonuclease T [Rhodovulum sulfidophilum]AB14
MRCLAILLLSATLARAEEDVAGRFDYFLLSLSWAPSWCAAGEAPRGAARQSPHCAPGGGAGWVLHGLWPQYERGWP
SYCR
STEADPSRSQIARLAELTGSPRAAWHQWKKHGRCSGLSAGDYAAAARRAYEAVSRPAAFGKLTAPVRLPAGVVEEAFL
QA
NSGLLPDMLTVTCKAGRIREVRICLTKALRPRPCAPDTVEDCDLRDALMPPPR
>OLS50032.1 ubiquinone biosynthesis protein [Rhodovulum sulfidophilum]AB14
MTDTPRGMDSITQELLDAALIHVPFDGWSEAAFRAAVADSGVDPALARAACPRGAADLAVAFHLRGDDEMVRRLAE
TDLS
EMRFRDRVALAVRYRLDAVDDKEAVRRGATLFALPLYAAEGSRLVWGTADRIWTALGDSSEDVNWYTKRASLAAVYG
ATV
LYWLGDESPDHAETSAFLDRRIGDVMQVEKVKAQVNKSPLLKPFMLGPNWLARQIRPPQGPKRKGGLPGALHARMT
GRT

>OLS50033.1 30S ribosomal protein S21 [Rhodovulum sulfidophilum]AB14
MQVSVRDNNVDQALRALKKKLQREGVFREMKLKQHFEKPSEKKAREKAEAVRRARKLARKKAQREGLL
>OLS50034.1 hypothetical protein BV379_18305 [Rhodovulum sulfidophilum]AB14
MILTCQEDISAPIERVFSRLTDFTRFERAIETRGGDVTRHAGPPDTPAPGMAWTIRLHFHGAHREIEAELTDYAPPSAMC
LGAASSGLGAEGRIELAALAEGLTRLSVAIDLGPKTLGGRVLMQTLKLARGKLAEEMEAGLARFARRIEAEAAG
>OLS50035.1 NADH pyrophosphatase [Rhodovulum sulfidophilum]AB14
MRLAERVTFGGGGLDRAAHRRGDAAWLAEAWAGAQVLPLWRGKPLFGVEGLGWVASAHPLMAGRSTEAVFLGLS
AGRPKF
AIELSDWVPAEMPETLGQFRDPSEQRHPDLPGDLSFRELRREMTGLLPVEAELAATAKGLFDWHRGHGFCARCGQPS
RPA
EAGWQRRCPGCGAPHFPRTDPVVIMLVRRGNAVLLGRAPGWAEGMFSLLAGFVEPGETVEAAVRREVAEETGVRVG
QVGY
LASQPWPFPASLMLGCWAEAETDRITPDPAEIEEARWVGREDMLAVLAGERTDMQPPRPGAIAGFLIRMWLADRLD
>OLS50036.1 2',3'-cyclic-nucleotide 2'-phosphodiesterase [Rhodovulum sulfidophilum]AB14
MIAPHPFSVPPAADRVHLRLLETSDLHVHIFPYDYYTDRPIDTAGLARTATLIAALRRDVANALLFDNGDFLQGNPMGD
Y
IAHERGLRPGELHPVIAAMNAIGYDAATLGNHDFNYGLDFLLDAIAGARFPVVSANLLPGPLAPPGLTRLPRHALLDRSV
TDGAGRRHRLRIGVIGFLPPQIAIWDRRHLEGRAEGGDIVSAARELVPRMRRDGADLVVALAHSGIGEPTHVARQENA
CL
PLARIEGIDAILSGHQHQALPGPDFAGIAGVDTETGSIHGKPAVMPGFWGSHLGVIDLLLERAATGWCVAEHRSSIHAI
A
TRRSDRSAEPLVGSDPAVLSAAARAHAETLAYTRRPVGRTEIPLHSYFALVAENASVKIVADAQRWYVARKLKGTRHEG
L
PLLSAAAPFKAGGHGGPDYYTDVPAGDIAIRNVADLYLYPNMVRAVRVSGAELRGWLERSAGIFNRIRPGAADQLLLD
PD
FPCYNFDVIDGLEYRIDPSQPPRYGPHGRLLNPHASRIADLSWNGRAVTDGMEFVIATNSFRAGGGGGFPGTGPDAVI
LE
APVTNRDVILRYIARRGSVTPRTVPTWRFADLPGTTVLFDTGPAARRHLGAVERLAPDPAGTSPDGFARFRISL
>OLS50037.1 class II aldolase [Rhodovulum sulfidophilum]AB14
MTDEKDKRAAIVAACRSMNALGLNQGTSGNISLRHGAGLLITPSSIPYDVLAPEDIVFMEMDGTAHGARRPSSEWRFH
LD
ILRSRTDLNAVVHAHAPYCTMLAILHKPIPAVHYMVAAAGGGDIRCAPYATFGTQALSDLVLEALEGRKACLMAHHGM
IA
AEAGLDKAMWLAVEVEALARQYHGALAIGTPPLLSETELAEAIAQFTGGYGQGDPTAT
>OLS50038.1 AsnC family transcriptional regulator [Rhodovulum sulfidophilum]AB14
MALDETDLRLLRVLQKQGRISNAELSERVNLSASACHRRVQRLEEGGFIRDYVALLDARKMGRQTTVFVEITLSGQADE
T
LAAFEKAVARVPDVLECHLMAGNADYLLKVVAEDSEDFARIHRRYLTRLPGVARMQSFFALRTVFKTTALPL
>OLS50039.1 alanine dehydrogenase [Rhodovulum sulfidophilum]AB14
MHIGCPREIKPQEFRVGLTPDAAREAAAHGHAVLVETGAGLGAGFEDADYIAAGAEIAATAEDVFARAELVVKVKEPLA
E
ERRRLREGQVLFTYLHLAPDPAQTRDLLDSGVTAIAYETVTDDRGGLPLLAPMSEVAGRLAPQMGAWALQKANGGRG
VLM
GGVPGVGPAEVLVIGGGVVGTHAARIAAGMGAHVTVLDKSLPRLRQLDEIFDGRVTTRHSSEATLAALLDRADMIVGA
VL
IPGATAPRLLSRADLARMKPGAVLVDVAIDQGGCFETSRPTTHEAPVFSVDGIQHYCVANMPGAVPRSATLALGNAT
MPF
LLALADKGWRQACTDDPHLLAGLNVHAGQLTCYAVGKAQDIDVLSPQLALTA

>OLS50040.1 gamma-glutamyltransferase [Rhodovulum sulfidophilum]AB14
MRDFQLPGRSAVFATNGMCATSHPLAAKVAVQMLEAGGNAVDAALAGAVLLGICEPQMTGLGGDCFVLIKPAGEDR
IVAL
NGSGRAPKGLEAARLRERGLDAMPTDSADAVTVPGAVDAFCRLSKDWGRIGLKASLAPAIHYAETGVPVAPRTAFDW
ARA
ADHLQGAARDFYLPDGEAPRPGQVFRAPKQAEVLRRIAMDGRAGFYEGEVAEDMVSSLSALGGHHTLEDFAATACDY
GSP
VSGSYKGVELVEHPPNGQGATAILMANILAKFDLPSMDPLGAERAHVEAEAAKLAYDARNRFVADPAHMTRLDHML
APGT
AAKLAALIDPDRAMASPAPLSEAVHKETIYITVVDRDRMAASLIYSIFHSFGAGLASTKFGINFQNRGAGFSLTEGHPNE
AAGGKRPMHTIIPAMLKKDGKLIMPFGVMGGAYQPNGHARVLTNIVDYGMHPQQALDAPRSFADQGELLVERGYAE
EVCA
ALAAKGHAVAMPPSAIGGAQAILIDDETGVLQGASDPRKDGCALGY
>OLS50041.1 DNA-binding protein [Rhodovulum sulfidophilum]AB14
MLKAHAKYHLGQVVRHRKHPFRGVVFDVDAMFSNTEDWYAAIPEETRPPKDQPFYHLLAENDHSYYVAYVSEQNLVA
DET
GEPVDHPDLSDLFGEFEDGQYPLQFQLN
>OLS50042.1 transporter [Rhodovulum sulfidophilum]AB14
MNLLLTVTEIVAPVFLLAAVGFLWVRFGNEYRVQFVTQLTMTLSVPCLIFTALMKTEIEPQALTALSLAAAAAYGAVTIL
CLAVVRLARLDRRTYLSPIIFGNTGNIGLPLALFAFGERGFDYAVVVFAVMAIYSFTFGVWLVAGGGSPLKALKEPLVWA
TALGGLFLWQGWQTPAFLTNALSLIGQIAVPLMLITMGVAIARLHPGHLPRAVWLSLVKLAICLGVAAGIGLWFGLDRV
A
LAVLVLQISTPVAVTSYLLAEKYGADADAVAGLVVVSTLLSMLALPLTLAVLL
>OLS50043.1 lytic transglycosylase [Rhodovulum sulfidophilum]AB14
MGRFLLIAACLFLTACGDKSPPRNLEDACAIARERPKYMRAMERSERRWGVPVPVQMAVIHQESKFVGNARTPHRFV
LGI
IPMGRQSSAYGYAQAIDGTWDMYRNGTANRGARRDDIFDATDFMGWYMAESRKKLGIPLSDARSQYLAYHEGHAG
YARRS
YRSKAWLMAVANRVEDRAQLYYAQLITCRR
>OLS50044.1 cell envelope biogenesis protein LolA [Rhodovulum sulfidophilum]AB14
MKPLRLSLALAALLAATPVLADPIPLSSLSAYLNSFQTAQADFTQINADGSISTGTLSIKRPGRARFEYAPPEKALVLAG
GGQVAVYDDKSNQPPEQYPLKRTPLNLILERNVDLGRARMVVGHAEDGTSTRVVAQDPEHPDYGTITLVFTNDPVELR
QW
IITDEAGSQTTVVLGQLKTGMPLGARMFNIIQETADRMKRR
>OLS50045.1 aspartate aminotransferase [Rhodovulum sulfidophilum]AB14
MTFPERFSNLPDYAFPRLRRLLDVHEPGGVPVHMTIGEPRHAFPDWVAGIVAEHAHEFGRYPPNEGTPELRTAIAGWV
GR
RYGVTLDPDTQVTTLNGTREGLFNAALALCPETKAGARPAILTPNPFYQVYAVAALAVAAEPVYVPATAETGFLPDYGA
L
PPELLDRVAIAYLCSPSNPQGAVADRGYWADLIRLAEKHDFRIFADECYSEIYRDSPPPGALSVAAELGADPERVVVFHS
LSKRSNLPGLRSGFAAGGPGSIGRIKQLRSYAGAPLPLPLQRVAERAWSEESHVAASRTRYQGKYAIADEIFGGVAGYRA
PEAGFFLWLPVADGEAAALKLWRETGVRVLPGAYLARDVEGTNPGAGYIRVAMVAAEDEMRAALVRLRGCLYD
>OLS50046.1 amidase [Rhodovulum sulfidophilum]AB14
MASDWLGMSAGDLGRGIETGKIDPVDLATGFLDAIAGHPLCDRIYARPTRERALAEAEAARSRARSGLRLGPLDGVPIS
W
KDLFDSAGTATEAGAALLEGRVPGCDAEVLATATAGGLVCLGKTHMSELAFSGLGVNPVTATSPCINAPNWAPGGSSS
GA

AASVAFGLAPGAIGSDTGGSVRIPAVWNDLVGLKTTAGRISLKGVVPLCPKFDTVGPLTRNVEDAGLLLAALEGGRPAD
L
GGARLEGMRFMVLETAAFDGLRDAPRAGFNHALERLAEEGATIVREQAPEVAEALALAGVLFATEAYGTWRDRIEAEP
DK
MYPPILDRFRGGRAFSGADYVAAWQTLGLLRGTWARRVSGFDAVLLPTCPILPPDVDRLLADPDFFMAENLLALRNTRI
G
NLMGSAVLTLPTGVPATGISFMGLPMSEERLLRIGAAAERALT
>OLS50047.1 2-octaprenyl-6-methoxyphenyl hydroxylase [Rhodovulum sulfidophilum]AB14
MTYDSEILIVGGGLNGPALALALAQAGLSVTVIDALPRTDRDSDLFDGRSYALALGSTRLLSAIGIWPGLVHRGQPILEI
VASDGRAGEGAAPFFLDFHHGEIEEGPMGHMIEDRYLRRAMLEALEAEPRITTLSGETVVAQETGPTGVTVTLASGRQL
C
ARLLVGCDGRRSGTAQRAGIGRTGHDYGQTALVCAIAHERPHGGTAHQFFMPPGPLAILPLPGNRSSIVWSERHAQA
AAI
QALDDAAYLEMLRPRFGDFLGEIALEGARFTYPLSLSLANAFCAPRVALLGDAAHGVHPIAGQGLNLGLRDVGALAQVL
V
EAKRRGEDIGAIDVLERYQTWRRFDTTLMAAGTDTVNALFSNDNPLLRLGRDLGLGAVASLPALRRRFIREAAGLTGGL
P
RLMTGRPL
>OLS50048.1 phytoene synthase [Rhodovulum sulfidophilum]AB14
MIDPADLKHCEDSIRTGSYSFHAASKLLPARVRDPSLALYAFCRLTDDAVDLTHDKSAAVLMLGERLDLIYRGQPRNAPC
DRAFAAVVDEFEMPRTLPEALLEGMAWDAMGRRYETLSGVYAYSARVAAAVGVMMAVLMRVRDPHALARACDLG
VAMQLT
NIARDVGEDAAEGRFFLPLEWMNEAGLDVEEFLARPQPSPSIRRLTQKLLADANRLYFRSEPGVAELPFACRPGIFAARN
IYAAIGREVRRADYDNITRRAYTNRRQKLGWLIWSSIQALTVSAMPRSAVLYGKPLAECAYLVEATATRRPSDNPWGEG
R
AGEIIAILAQLEARERKRRGATLEQYRRKAI
>OLS50049.1 phytoene dehydrogenase [Rhodovulum sulfidophilum]AB14
MMTRVDPALADEAVRAGRPHAVVIGAGLGGLAAAMRLGAKGYRVTVVDKLDMPGGRGSAIWQDGHRFDLGPTIVT
VPQVF
RELWAACGRDFDQDVTLAPMDPFYEIVFADGERFRAQQDTEKMRAEVARISPDDLAGYDRFLKDAEKRYWFGFEGLG
RRP
MNEWKDLVKVLHIFGMLRADRSVYAHAASRVKDERLRMALSFHPLFIGGDPFNVTSMYILVSYLEKEFGVHYAMGGV
AAL
AEAMAGVIEGQGGTLRMKTEVDEILVKKHRATGVRLASGEVLNADVVVSNADAGFTYSHLLRNTRRWRWTDESLAKK
RWS
MGLFVWYFGTKGTRGKWADVGHHTVLNGPRYKGLVKDIFFRPEELAEDMSLYIHRPTVTDPSAAPEGDDTFYALSPVP
NL
HCDNPVDWKAHAESYRQSVQAVMEEKLLPGLGEHVSTSRVFTPESFQARYLSPYGAGFSLEPRIFQSANFRPHNVSEE
VE
GLYLVGAGTHPGAGLPSVVTSAEVMAKLVPDADSVAAG
>OLS50050.1 hypothetical protein BV379_18395 [Rhodovulum sulfidophilum]AB14
MQVVTISFFRYDNWASRLEALSQMARGRFALKSVPGLEFFKLLGTGSDEGFNPRPNVDVNAILCVWPDMATAKAGLK
GSI
THRTLRDHSCETYTIYLTPTSARGMWSGVEPLQVTEKAEEGPLAVLTRATVKLPVALQFWKHVPQIQKEVAADPTCVFH
M
GLAEVPWVQQVTFSLWQGTGTMAGFAHGQTPHGRAIRAAYGDHWFKEDLYARFKILEEEGTWEGGSPMAKLKSDS
RPAAP
APAEPAATEKPAAQAPAKPKKAPRKSRGSKKSKVSA

>OLS50051.1 magnesium chelatase ATPase subunit I [Rhodovulum sulfidophilum]AB14
MTQPFPFSAIVGQEEMKQAMILTAIDPGIGGVLVFGDRGTGKSTAVRALAALLPPIQAVASCPVNSPSLDRCPDWAHL
TS
TEIVTRPTPVVDLPLGVTEDRVVGALDIEKALTNGEKAFEPGLLARANRGYLYIDEVNLLEDHIVDLLLDVAQSGENVVE
REGLSIRHPARFVLVGSGNPEEGELRPQLLDRFGLSVEVSSPKDIDTRIEVIRRRDSYEMDYEGFMETWRPADADLREKI
IAARNHLKTMKSDEAVLRDCAELCLALGSDGLRGELTLLKASRALAAFEGADAVSRAHVRQVAPSALRHRLRRDPLDEA
G
STTRVGRVVEEVLGEPA
>OLS50052.1 magnesium chelatase [Rhodovulum sulfidophilum]AB14
MDWERDGYDWPNREFSRFVRHRPHYWHLQEAGDGPVLLLLHGTGGATHSWRDVFPILAERYHVVALDLPGQGFTK
LGARQ
RCSLNAMSRDISALLDGEGLQPDAIIGHSAGAALALRMVRDLKRPPRALVGINAALEAFKGAAGFLFPMMARMMALN
PFS
ASEISRWLGNAAGVEYMIAGIGSRISPEGLALYRRLVADRHHTDATLAMVAQWSLDGLWHDLPGIDLPTLLIVGEDDR
AV
PVAGSVRAAARLPQAELARFPGLGHLAHEEASEAVTARIETFLAQHL
>OLS50053.1 hypothetical protein BV379_18415 [Rhodovulum sulfidophilum]AB14
MISKFAIAATAAMIAGPALALDVTGDAAAGEKVFNKVCQTCHVVKNDAGEVVAGRNAKVGPNLFQMPGRHAAAID
GFRY
SKLMKEAGEKGLVWTEEELVKYVPNPTDFLRNYTEDPKGRGLMTNQRVKEQEIRDVFAYIASFGIHE
>OLS50054.1 hypothetical protein BV379_18420 [Rhodovulum sulfidophilum]AB14
MIATAWFAALAAFFIWWFSTGAILIVVRRAEHRGRLAHLRATVMALPLLGGGVWLFEVTRHVETVATAYLAFLSAIAIW
G
WVELAFLTGVITGPNTTRCHPHARGWERFMRAYGTIAYHETLLVVILFAMLVTSHGSANGVGVWTFIVLFFARISAKLN
L
FLGVPKINIEFLPTPLSHLPSHFKISRLNWLFPISISALTFAVACFMERLFAVDAPHDKVGFGLLTTITALALLEHWLMV
LPLPDERLWRWMLPERKASAEKDKIPSNDLSRT
>OLS50055.1 magnesium-protoporphyrin IX monomethyl ester (oxidative) cyclase [Rhodovulum sulfidophilum]AB14
MNAHTADATTVEESLAIQEQQAVLDSETATAVAMQDTLLTPRFYTTDFEALDAIDVTPVRADWDALIAEMQADPNKG
HFR
KDETWDHVDWDGMEPELRKEFIDFLISSCTAEFSGCVLYKEMKRRGSNHDVTELFQLMARDEARHAGFINDALREAGI
AV
NLGFLTQKKKYTYFRPKFIYYATYLSEKIGYARYITIFRHLEEHPERRFHPIFKWFREWCNDEFRHGEAFALLMKTDPKL
TSGVNVLWIKFFLTAVFATMHVRDHQRPEFHKALGVDTDWYGQEVFRKTSELSRQIFPITLDIDHPRWARGLAALQEA
SV
AIDTARKRGGVAGKLAQWAGSARAAGAFLSLLTIPARRHEVPASPRLEPVY
>OLS50056.1 hypothetical protein BV379_18430 [Rhodovulum sulfidophilum]AB14
MGIFTRRKETAPCTVEVSHRFESLHAHVRFNNGAVVYPGDEVLVQGPEIMAPYGEVVREDRTAIITRATPLERLWTRLT
G
DLEFMELCEFSFSEEVKL
>OLS50057.1 hypothetical protein BV379_18435 [Rhodovulum sulfidophilum]AB14
MARNNRHTGPYNHGEKIPPILIKGMFGVALFALILVSYARLTDRPLDALPDAGAPVAVERQIVISAKIDGSAKVYDTEGN
QVFEFAPNEGGFVAGVWRALELKRKQAGVPLDAPVRLVRFEDGRISLYDDQTGWRAELVGFGADNTAAFARLLD
>OLS50058.1 hypothetical protein BV379_18440 [Rhodovulum sulfidophilum]AB14
MSDDDFAAEPIKGLPERLPEGEHILWQGRPDWWALARESLALFWVGGYFVVLFLWRMIVAAETMPWSQAAVASSFF
LVLG
AVVCLLLLITAFVQARATVYTVTNRRVALRIGAALDMTLNLPYTQIGNANLDLRKSGSGTIAFELMGTTKFSYLLCWPHI

RPWRMARTEPAFRCIPDAAKIARLIADAAETRVSEPRLTRVDADDTVAAE
>OLS50059.1 photosynthetic reaction center subunit H [Rhodovulum sulfidophilum]AB14
MVNAFFGNFDIASLAIWSFWLFFAGLIFYLQRMNMHEGYPLEDEVGNAAPNQGMFPLPAAKTFKLPHGQGEKTVPD
MQTD
PRNADLALQKVTKSNGYPLEPTGDPMVDGVGPAAWCARKDEPELDGRGHPKIQPLSVLKTFKVSAGRDPRGMPVIAG
DGE
AVGTIVDMWVDEPEQLVRYLELELDEAHGGGRRLLPMQLAKIGWFKPEVSVHSIYGKHFAAVPTIKSAKQITKLEEDKV
C
AYYAGGKLYADPAERLEPQF
>OLS50060.1 MFS transporter [Rhodovulum sulfidophilum]AB14
MSKKPHPLTRMSAKFLPFADAASDGLPMNELLRLSLFQVSVGMATVMLLGTLNRVMIVELSVPAIVVAMMIAVPVLV
APF
RALMGFRSDNHRSAIGWKRVPYMWFGTLWQMGGLAIMPFSLLLLSGDQTLGPTWAGEVFAAIAFLMTGLGLHMTQ
TAGLA
LASDRATDETRPRVVALLYVMFLLGMGISSIVIGWLLRDFSSIRLIRVVQGAAVVTILLNLVALWKQERMCPMSKEEREA
PQPGFGDAWRDFLKGGSAGRLLAVVFVGTLAFSMQDVLLEPYGGQILGLSVSSTTLLTALWAFGALIGFGLAARWLGQ
GM
NSHRMGALGILGGIVAFTLVIFAGPLHSGLLFFFGAMLIGFGGGLFSVSTLTAAMALPVRDFAGRGLALGAWGAAQAT
AS
GLAVALGGALRDGIGHLATAGVLGEGLNTPATGYAAVYHIEIVLLFVTLMVLGPLVRPQDRNHPNRDSSAKIGLADFPT
>OLS50061.1 magnesium protoporphyrin IX methyltransferase [Rhodovulum sulfidophilum]AB14
MDSYARTRERLETYFGRTAAKTWENLTSDAPVSRIRQTVREGRDQMRAAMLARLPEDLTGCRVLDAGCGAGPMTLEL
VKR
GAEVVACDISQSLLDVAKSRIPDKYHRQITFVAGDMLSERLGRFDYVMAMDSLIHYQARDIAEALGKLAPRTRGKIVFTV
APKTPLLSVMHVTGKLFPRSDRSPRIIPHSEARIASALRKALVKGRLRAVARVAKGFYISQAMELER
>OLS50062.1 ferredoxin:protochlorophyllide reductase (ATP-dependent) iron-sulfur ATP-binding protein [Rhodovulum sulfidophilum]AB14
MSPRDDIPVLKGEDGEGSVQVHQDETMKIEGAKVFSIYGKGGIGKSTTSSNLSAAFSMLGKRVLQIGCDPKHDSTFTLT
G
HLQSTVIDVLKEVDFHPEELRPEDFMVEGFNGVMCVEAGGPPAGTGCGGYVVGQTVKLLKQHHMLEDTDVVIFDVLG
DVV
CGGFAAPLQHADRAVIVTANDFDSIYAMNRIIAAVQAKSKNYNVRLAGCIANRSEDTDQVDKYCNRVGFDRIAHMPLV
DA
IRRSRLMKKTLFEMPDDEDIVQCRAEYVRLAELLLAGTEPLAPHPLPDREIFELLGFD
>OLS50063.1 magnesium chelatase [Rhodovulum sulfidophilum]AB14
MRDERHYPGYRVVIVTLDSHSAGPCERVTERLAEDFPGLRVTVHAAATWSENPGALVRAKDDVMNADIILVNLLFIEEH
V
NAILPELRARRDDCDAMIGIIADKQIVQLTRMGDLDMSKPASGPMKLLKKLRGNVRDDNGQVKTGGEKQMAMLRRL
PKML
KYLPGKAQDLRAWFLVMQYWLGGSDDNVEQMVRFLISRYAKDASWRGGASEAPIEYPETGLYHPDIPNRITTDIDDLP
RP
EAPVATVGILLMRSYILASDTAHYDAVIHALEDRGIRVIAAFAGGLDARPAIAAYFTGEESVHIDTLVSLTGFSLIGGPA
YNDSAAAVEVLSGLDVPYMAAHPLEFQTLGQWSDSSGGLGPVETTMLIALPELDGAISPTVFGGRHGAQGCKGCPKD
CSY
KGESKQMSACSERVAMLAARVERLARLRRSETESRKVGIILFGFPPNAGAIGTAAYLSVFESLFNTLHRMKAEGYDTGEL
PANVDELRAAILEGNAKTYGQEGNVATHVPADDLVRGTPWLAEIESVWGPAPGKIRSDGRGVFVLGREFGNVFVGVQ
PTF

GYEGDPMRLLFERGFAPTHAFSAFYLWLKNTFRADVLLHFGMHGALEFMPGKQAGMGFRDWPDRLIGDMPNVYLY
ASNNP
SEATLAKRRSNAVTVTHLTPPLAASGLYKGLQELKDSLTRWRQTEPGSPEQADLEELIAAQADAVDMTGTPPEKLWLKL
L
ETEDALIPDGLHVVGRTVTPEERTEHLRVMTDTDDETREKVDFWLQQDSELPAIMRALGGRYIKPVPGGDLIRSADVLP
T
GRNIHAFDPFRMPTQFAMQEGAKQAQKLLDTHASLPRSVAMVLWGSDNIKSDGGPIAQAMALMGTKPRFDNYGRL
AGADL
VPLEELGRPRIDVVMTLSGIFRDLLPLQTRMLAEAAWKAATADEPVEQNFIRAHSLAHAEKMGVDMETAALRVFSNAE
GA
YGSNVNGLVDSGAWGDEDELADAYEARKSFAYGRDGKSKANAALLQQTLKDVDVTYQNLESVELGVTTVDHYFDTLG
GIS
RAVKRARGGSDAAVYIGDQTRGEGKVRTLQDQVALETRSRSLNPKWFEGMLKHGHEGVRQIEAQVTNTLGWSATTG
QVEP
WVYQRLSETFVLDEDMRKRLASLNPAASARMANRLLEANDRNYWQPDAETLAALQAAADELEDRLEGIVAAE
>OLS50064.1 ferredoxin:protochlorophyllide reductase (ATP-dependent) subunit B [Rhodovulum sulfidophilum]AB14
MKLTVWTYEGPPHVGAMRVATAMKGLHYVLHAPQGDTYADLLFTMIERRNHRPPVSYTTFQARDLGADTANLFKTA
LHDA
FDRFHPQAMIVGSSCTAELIQDDPEGMAATLDLGIPVIPLELPSYQRKENFGSDETFYQIVKTIARPMERTAHVSANLIG
PVGLGFRHRDDIVEVTALLEEMGISVNVVAPMDATPADIARMGAAHFNVLMYPEHAETAARWMEKTLGQPFTKTVPI
GVG
ATRDFIAEVAQIAGVAPRADDGRLRMPWYSQSVDSTYLTGKRVFLFGDATHVKAMARVARDEMGFDVVGLGCYNRE
FARD
IRKLAREFGVEALISDDYLEVEQTIEALAPEMILGTQMERHIGKRLGIPCAVISAPVHVQDFPARYSPQMGWEGANVIFD
TLVHPLVMGLEEHLLHMFREDFEFHDDAGASHHGGAAKARAVEAEPDTDQEAAAAAAPAEGEIVWLSAAEKELKKIP
FFV
RGKAKRNTEKFALEKGVHEISVETLYEAKAHYAR
>OLS50065.1 ferredoxin:protochlorophyllide reductase (ATP-dependent) subunit N [Rhodovulum sulfidophilum]AB14
MTGFVPAPPAAGCRNAPVLKERGQREVFCGLTGIIWLHRKMQDAFFLVVGSRTCAHLLQSAAGVMIFAEPRFGTAILE
EK
DLAGLADANAELDREVDRLLARRPDIKQLFLVGSCPSEVIKLDLHRAAERLTERHAPHVRVMNYSGSGIETTFTEGEDAC
LATMVPTLPATEERQLLMVGALPDVVEDQALGLLEAMGVKPVRCLPAHRAAETPGVGPNTVVALTQPFLNETAAHLE
RRG
ARILSAPFPFGEEGTTAWLWAVAQEFGVSPEVFERVTEAPRARARKAVAAAAETLRGKSIFFFPDSQLEIPLARFLTREC
GMTAVEVGAPFINKQIVGPDLDLLAEGPVIAEGQDVDLQLDRCRAARPDLTVCGLGLANPLEAEGLSTKWAIELVFTPV
H
FYEQAGDLAGLFARPVRRRAILAGLEGAA
>OLS50066.1 2-vinyl bacteriochlorophyllide hydratase [Rhodovulum sulfidophilum]AB14
MPEHVGTAARAVQLYTPEERRRRDETKWTLVQGVLAPLQFLVFLVSLVLVLRYLVAGAGYEAATISIVVKTFVLYTIMVT
GAIWEKVVFGKYLFAPAFFWEDVFSMAVISLHTLYLWATLSGTVGPIGQMLIALTAYAAYVVNAGQFLWKLRMARLQ
GDA
AEGAA
>OLS50067.1 hypothetical protein BV379_18485 [Rhodovulum sulfidophilum]AB14
MAELQKSIASRAQAGPQTAQQGDVETLAAQVLSALAHRSGSGSASLDPVLLAEFCADVLGGGRSIGTRALATLRRRGIP
P

TAIIDGYIPAAARELGERWVRDDLSFADVTIGAARLQAMLRELGAANAADMLAASDAPNVMMIVPHDEYHTLGGMT
AANQ
MRRMGASVCLALGQTEEEILQKAMPRQFDLVAISVSCRQKLDSARRLVAALRRVLGDGVPVVIGGRVADGAEDICALT
GA
DAVTTDAHEALRLCRIGALQAPGAARTGKDQ
>OLS50068.1 transcriptional regulator PpsR [Rhodovulum sulfidophilum]AB14
MTSRGTKYWNSGAIPLIAPEMLGEIIATACDLAIVISPEGRILSVVVNKDHHAHGRIDHWEGRDIRDVLTPDSRPKLEAR
LELFAQDRDISGAVELNHADGAGWSFPVRYSFHAIGPDGGTLLMLGRDLRPIAEMQMQLIEAQMALERDYETQREYD
TRY
RVLTEATRDAFVFIGTKSGRVVDANPVACDLLDVERAELIGNAFAQEFEGRRRAEFLEALTRATMSPEAHPIALETRRSR
VRIKLHPVAFRAAGEPLILCRLDPAEAREPNGGEMGGTLLAFYRQGVDALVFTDPQGVILAANDSFLGLADAPHAGAVK
G
RSLGDYLVRGSVDLRVLVENATRSGQMRMFATKLSGEYGTQVSVEISATYLKDQASPCIVFVIRDASRMESVRKPGVAV
S
DDAMRSVMELVGSANLKDIVAETTDVVEKMCIETAVELTRNNRVAAAEMLGLSRQSLYVKLRKYGLLNRDGGDH
>OLS50069.1 bacteriochlorophyll/chlorophyll a synthase [Rhodovulum sulfidophilum]AB14
MTDISNMPNRRLPEPAAMVTLIKPITWFPPMWAYLCGTVSSGVSPLSKPGLVLLGIVLAGPVVCGMSQAANDWCDR
HVDA
INEPYRPIPSGRIPGRWGFYIACAMSLLALVIGWQLGPWGFGATILGVASAWAYSAEPVRLKKSGWWGPGLVGISYESL
P
WFTGAAVLSAGAPSVYVVVIALLYGIGAHGIMTLNDFKALEGDRQMGVNSLPVTMGPEKAAWVASVVMAVPQAVVI
GLLA
LWDRPFHAAGVTAVLLVQFWAMSVMMKDPKGKAPWYNGVGVVLYVSGMMISAFAIRSLGGA
>OLS50070.1 MFS transporter [Rhodovulum sulfidophilum]AB14
MTLSWLQIVRLGLVQMCLGAVVVLTSSTLNRLMVVELALPAVLPGLLVALHYAIQITRPSWGFLSDTRGQRTRFIILGMI
ILALGGYLAATGVVVMEDHYGAGLMLSILAYALIGIGVAACGTSLLALLATATAPERRAAAATITWLMMIAGIGITAGTV
GAFLDPYTPALLLEIVTVVTLGAVVLTTLAVWGIEKRVVAHREPDEMPFWKGIGEVWAEPRARIFTLFVFLSMVAYFMQ
E
LILEPFSGLVFDFTPGQSTSLSGAQHGGVFLGMVVVGIGSSVLRIWALRSWVILGCLGSAAALASIAALGHFGPHLPLTP
AVVTMGFFNGIFTVAAIGAMMALAGEGRDAREGTRMGLWGAAQAIAAGFGGLVGAGAADGLRQIMDDAPAFGVV
FLTEAI
LFLLAAAMALRIMDGHRPTTENSGLVPGE
>OLS50071.1 geranylgeranyl diphosphate reductase [Rhodovulum sulfidophilum]AB14
MIYDVVVVGGGPAGATAAEDLARSGKKVALLDREGRIKPCGGAIPPRLMRDFNIADEQLVAKIDTARMISPSGRFVDIPI
ENGFVGMVDRKDFDPFLRARAERAGATRFTGTFIRVEREEEKTFLVFRDKQSKEEVRLQTKFIIGADGARSDVARAEVP
G
GDKIPYVIAYHEIIDAPMAVGNYKPNRCDVIYDGRISPDFYGWVFPHGHTCSVGMGTGVDGFDLKQATADLRAMAGL
DQA
ETIRKEGAPIPLRPMDKWDNGRDVVVVGDAAGTVAPSSGEGIYYAMYGGRVGAAAVSAALASGRASDLKLARQMFM
KDHK
NTYKALQAMQDAYYHSDERRERFVSLCHDVDVQTLTFESYMNKKLTRKRPLAHIKIGIKNVAHLTGLVSPQYT
>OLS50072.1 isopentenyl-diphosphate Delta-isomerase [Rhodovulum sulfidophilum]AB14
MSEMIPAWVNGELTPVEKLEAHQKGLRHKAISVFVMDGDRVLIQRRALGKYHTPGFWANTCCTHPRWDEDSKTCAN
RRLA
EELGITGLTLEFREQVEYRAEVGNGLIEHEVVDIFIAAAGPDLKWDLNPEEVMDARWIALDDLARETRETEGVYTPWLRI
YLEKHSAAIFGALASA
>OLS50073.1 ABC transporter [Rhodovulum sulfidophilum]AB14
MSGLLALLDDVAAIAKVASASVDDVVGQAVKASSKAAGAVIDDAAVTPKYLRGFSPARELPIVWSIARGSLFNKLVILLP

LALLLSQFAPWAIAPLLMLGGCYLCFEGVEKVLHALAPQEAHAKAGADKPLSATRLEEAKVKGAIKTDFILSAEIMTIIL
AAMPDHLTLWMEAGAMALAGALITLAVYGAVGLIVKADDIGLAMAEKGRLAATRSLGVCIVRAMPKVMTLLLVVGTA
AMI
WVGGSILVHGAHQLGWHLPQEMIHHVAVAAASVLSLGGAVEWTVTAALDGAVGIVVGLVLVPVIQHAILPAAGFLRK
E
>OLS50074.1 hypothetical protein BV379_18520 [Rhodovulum sulfidophilum]AB14
MDSDQRQPAEAFPVPIGDVISRPLPEANLRRVAQEALMRVARLPGRRAPLPEPPAPAEIEALCTALATGSLEQARRIVD
D
HLARGRDKTWIAEGLLPEAARLMGRRWESDEVSFIEVGQAVGRLQRLLRLLRAEAGPRRPDPVRRAIFATPETETHTFG
V
IVAADAFRERGWEIDLSLSEPPDRLLADLTRSQVPLLGLSVSSRRSAPPLMQLLADLRTALPRLRVLLCGTILSTSPEVA
RQIPVDDRAADLPSAFEAADRLLLESLA
>OLS50075.1 hypothetical protein BV379_18525 [Rhodovulum sulfidophilum]AB14
MTGGQLEAFARSDLAAAVLMERIVRGAYDTRNSSEIQPLQWSILRYLDGFAEDRCTLTLITSFLGLTHAPVSRAVRTMR
D
RGLVRQRSHPRDARSAIISLTDAGRSALELDPIRKIALLLGTLPDADRSAIKRALRSIEIELAS
>OLS50076.1 hypothetical protein BV379_18530 [Rhodovulum sulfidophilum]AB14
MDTAAIRIALRGRNRRLPEKGHKRRQGQPARHPGQGPKAHGGPPRPQFPENRIGRAIQRMEPVDLAAAIHPFPEQAR
RPA
DPAKASPDDRQSPSTLDFAAALTEA
>OLS50077.1 hypothetical protein BV379_18535 [Rhodovulum sulfidophilum]AB14
MPSVLFAHENFPAQFGGIAGYLAARGWEVVFATAAEAFPRDRLVRLPETGLRVMGYARHRDVAPATHRYLHGTERAV
LNG
QGLARAAIRLRRQGFVPDLVVAHSGWGSGSFARAVWPEARFVPYLEWWYNYPPVDMAAETGESAEDRHAAALCRN
LPFLL
DLQGADAILVPTAFQRDQLPDWLAARATVIHDGVDIDALIHPPPDMPPFTLDGLPETAPLVTYATRGMEPMRGFPQF
MAD
LAKLQAEDARVHAVVAGEDSVHYGGRLPEGESWKARALAAHDFDMARLHFTGRLPPKRYRALLRRSDVHVYLTRPFVL
SW
SLLDAMAAACPLVVSDTAPVREAVPPDAARFVDFETPGEIARGIAAVLAEPAASRAMGGRARDHAAAHYAQSVLHPR
REA
FFRAVIEGRGLPGQ
>OLS50078.1 exonuclease [Rhodovulum sulfidophilum]AB14
MHERLGLRLRFALFFAALALGGMAVLVLGLWFGHTRAGGPLDGYVVAGMVAGCGLLGLAAWIGLLFDANVARPIQAL
AAD
LQTRAGVDVAADIDEAPARYLGALAPAANAIHDALAATRDAQARAVAERTAALNREKAIFEALLRDLAEGVVVATPDH
RI
MLYNRAALGLLGDLGLDRPLTAVLRPEPLTHALDRMGARAARGEAEAERFLAATASGERFLMARVSPVAVAGRRAGY
VLI
FHDATEDLEAHAELDHLFNTMLEGIRRPTAAIGALFDAIEADPDMGDEMRSAFFARLRGEQAMLVDRMNQVADHYG
TATT
RRWPMPAVASDDIFDALTARLPGDLRFEGARQFVRCDGFAISELLARVIEGLRGDDRRDGFVLRAVPEGREVCLELHW
RG
PPVSDGQIDRWLSAPLSAGYGEYLGRDALEGHGTELWAEPTETGHRLLLPLAAAAAPLLTPADPRPEFYDFSLPPPAAG
A
LADRPLGDLSFVVFDTETTGLAPRQGDEIVQIAGVRIVNGRILRGEVFDTLVNPGRHIPAASTAVHGIDDATVRGSPDVI
AAGRRFHAFCEGAVLVAHNAAFDMTFLKMKEDRLGLRFDHPVLCTVLLSAALYPHAEDHTLDALACRFGVRLEAEHRH
TA

LGDAMATAEVFRQMLGMLQVSGVRTLGEAVARSNEMHGIRRAQSY
>OLS50079.1 response regulator [Rhodovulum sulfidophilum]AB14
MGEKSVLLVEDEDGIALALEFLISRQGYDTRRVTDGDAALRAMEEDRPDLVVLDVMLPERSGYEVCQIMRQDEGLKHV
KV
LMMTARGGEVERRKGLALGADAFLTKPFATADLTAQIRALLGGDMDA
>OLS50080.1 hypothetical protein BV379_18550 [Rhodovulum sulfidophilum]AB14
MYIELIAIIVAGIGAGGLVLVVNRLTGRRLPRFAVPLAAAVAMFAYALWSEYTWGARTVASLPEGVVEVTHVDQAIPWK
P
WTLLAPVTTRVMAANRIGVQSRPDAPEVRLVELYLLARWHSARTVMVLIDCAAPARADATDAALTDPSVADWQPLG
PADP
LLRAVCVPPE
>OLS50081.1 histidine kinase [Rhodovulum sulfidophilum]AB14
MSDPEADILASLTRYAPFDMVPAAALEVLGASLTRIAAEAGQTLSYEGERLEALLVIESGSVEIETDGSGPLALRGPGDV
IGERGLLRLGRATLTARVAEDAVLIRLPKERFYDLIDEVPAFAAWFQRASPARPADSGADAAGLTALKVSDLMTKGPVTC
PVGAGVRDVARLMRDNGISSVILMEGEVPAGIVTVRDLANKVLAEGLDGTTPVARVMTSDLITIAPDALGLDALMRLA
DH
NISHLPVAEGGRIVGLIGRTDLFRQQAATASHMIADIVDAGSAAEMARVVAQVPSLLTQLVSAGAGASAITRRITDITDA
ITRRLLVLAEAELGPPPVPYLWLACGSQGRREQTGQSDQDNCLILDDAAEPGHDAYFQALAAFVSDGLHACGFVYCPG
DM
MATNPRWRQPRRVWRAYFAGWIAQPDNQAQMLASVMFDLRPIHGETALFRDLQAETLAMARRNSIFVAHMVSNS
LKHTPP
LGLLRGFALIRSGEHKDMVDLKLSGVVPVVDLGRVYALRGELMAVNTRERLEAAREAGVISKAGAHDLIDAYDFIAETRL
RHQAAQIRAGERPDNFMPPAQLSELERNHLRDAFMVIKTMQSSAGQGRGVMG
>OLS50082.1 cation acetate symporter [Rhodovulum sulfidophilum]AB14
MDQFTLNLIFVGLTFAIYIGIAIWARAGSTAEFYAAGRGVHPVLNGMATGADWMSAASFISMAGIIALAPAGGYTQSA
FL
MGWTGGYVLLALLLAPYLRKFGKFTVPEFIGDRFYSGGARLLAVICLIVISITYVIGQMRGVGVTFGRFLEVSTDTGLYI
GAAIVFAYAVFGGMKGITYTQVAQYVVLIIAYTIPAIFISLALTDSFLPQLGLIGSYAPGGGDQAFLAKLDEVVTELGFT
AYTADTPNMLNMFLFTMSLMIGTAGLPHVIIRFFTVPKVADARTSAGWALVFIALLYTVAPAVGSMARLNLTTTMWP
NAV
AGNALDGDAVSLEAIQSDPRLNWVKTWEQTGLLHMEDLNGDGRIQYYNDKNPEMQALAAERGWQGNEVTKIDNDI
MVLAN
PEIAALPSWVIALVAAGAVAAALSTAAGLLLAISSAISHDLIKGAINPKISEKGELLAARISMTGAILIATWLGLNPPGF
AAQVVALAFGLAAATIFPVLMMGIFSKRVNKEGAIAGMLVGLVFTAIYIFLYKGWFFIPGTATFADVPENYLFGISPLSI
GAVGAILNFAVAFGVSSVTRTPPREVVELVESIRVPRGAGAATGH
>OLS50083.1 hypothetical protein BV379_18565 [Rhodovulum sulfidophilum]AB14
MSGQDSAYWRANVRIILVCLVIWAVSSFGLGIVLRPALEGIMIGGADLGFWFAQQGSIYIFLVLIFVYAWLMNGLDRKH
G
VDE
>OLS50084.1 hypothetical protein BV379_18580 [Rhodovulum sulfidophilum]AB14
MRGGLEVLAFGGLALVLHLAVWPGDEAPGISAAGGGGEDLATLAAATAAIARRVSEWDSPPAPPQALPAPEAPPPPE
APP
ELAPPELLPELTDPAPRRPDLPRLAALAAPEPPPEPERRPDPRPEVRQARADTPRPVERPDPKPEPTPAPKPAPASQQPV
RAAASQSSDMQQAEKAAGAGGAARGRAGEASAATRDPGRVATLMAQWGAAIRARIQRRVPEGAGRGAALVELTVS
ARGTL
VSVRVVRSSGNARLDGLALAAVRQAGRFPAAPAGLGDGARSFSLEVRSR
>OLS50085.1 hypothetical protein BV379_18585 [Rhodovulum sulfidophilum]AB14
MRLPMPAPRPARESVVPMINVVFLLLIFFLIGARLAPPDPIEIDPPEAASDRPPEAEAALLVAADGMLAYGDARDEAVYA

ALAEAPREGPLSVRADRAAEAAVVARVLARLAAEGVSPVELVAAAR
>OLS50086.1 indolepyruvate ferredoxin oxidoreductase [Rhodovulum sulfidophilum]AB14
MSLGLQPAAARRRPSLTPMVDVVFLLLVFFMLAARMGAEVALPVSGGAGGTAEWSGPPRLIDIRPGTLALNGVETPLE
GL
AEALAPLVSEPSDPVVLRPREGAELQRVIDVIEALSAAGYSQPVLVE
>OLS50087.1 flagellar motor protein MotA [Rhodovulum sulfidophilum]AB14
MDLTHKTAAIAEFLDRGGPAIWAIAGLSVMTVALILWKIWRLALAGAWSRAAVQGAVAGWLEGDVWRARAALDGR
RGLRA
VAVRAAIEARLDPELDATTAREEVARVATRELTRARSGLRALDLIATIAPLIGLLGTVLGMIAAFKTLQETGARADPSAL
AGGIWEALLTTAAGMAVAIPASVALSWFESVTEAARVDIEDAATRVLTRGGPGRTALRAAAE
>OLS50088.1 heme ABC transporter ATP-binding protein [Rhodovulum sulfidophilum]AB14
MLEATGITVRLGRREILTGVDFRAQPGQITAIVGPNGSGKTTLLRALTGELSHDGRIRLNHHDPASLRPWVLAGLRGVLP
QATPMAFPFTAAEVVRLGLMGGIEAAEPDLVPKALARVGLAGFEPRPYQELSGGEQQRVQLARVLAQVWHPTAHGQ
PRWL
FLDEPVSALDIGHQLQVMAIVRDFADRGGGIVTVMHDLNLTAMFADSVALMAGGGLMAQGAPAEVLCDELLSRAY
MCPLR
VNALPSDGPYLLPQASGPIAAE
>OLS50089.1 iron ABC transporter [Rhodovulum sulfidophilum]AB14
MVAISHDVPGSHDVPGPTPHWDRTPRAWRLTVLLALALVAISAASLAVGAAGTMPWTVLSDMAAGREIALADRVVL
FEIR
LPRLVMGALVGAALAVSGAVMQGLFRNPLADPGLVGVSSGAGLGAIAAIVLGGLLPAGIADALGYHLVPFAAFAGGW
ATT
LVLYGIATRRGQTSVATMLLGGIALGALAGALSGILVYMADDAQLRDLTFWGLGSLAGATWAKLAAAAPLMILALVAA
PM
LGRGLNGLALGEAAAAHVGIPVQRLKTVAILTVAGATGAAVAVSGGIGFVGIVVPHLLRLATGPDHRPLLVNAALLGAA
L
LIGADMIARSLIAPAELPIGIVTAILGGPVFLWILLRRRGLLGI
>OLS50090.1 hemin ABC transporter substrate-binding protein [Rhodovulum sulfidophilum]AB14
MIRPLVALTLAALAALPAQAADRVVSLGGAVTEIVAALGEADRLVARDTSSSWPEAITALPDVGYVRRLSPEGVMSVAP
D
LILAEEGAGPPEAVDLLKSAAIPFVVVPDGYDRDAVIAKIEVVAEALGVPEKGATLAADLGRALDAAAAQAAEGPARRVL
FVLSVQGGRLLAGGRDTAADGIIRLAGGVNAIDDFEGYKPLTDEAVTRAAPDVILMMSRAGAEDLAPEVLALPAVRTTP
A
AAHGALVSMDGLYLLGFGPRTAQAVRDLSDALDRAGS
>OLS50091.1 hemin-degrading factor [Rhodovulum sulfidophilum]AB14
MGDGKAMSPEQIRSMRQEKSTARSRDLADRLGLAEAAILAAEAGRGVTRIAAHPDALMQAMPGLGEVMALTRNDS
AVIEK
VGTYGDYTPGDHAAMVLSPAIDLRIFPRHWVHGFAVEEQKGGKARRSIQIFDAAGDAVHKIHLRDASDTGAWQDLVA
ALR
LEDQSDSLALEPRSPVEPALIRPEAEATLRAQWDRLTDTHQFLQMVRKLKMNRLGAYRIAGAPYVRPLAPFCLRGLLNA
L
ADEAVPFMMFVGNRGCIEIHSGPIETVKPTGPWLNILDPGFNLHLRADHVAEVWAVTKPTRRGPAISVEAFDADGMLI
LQ
AFGMRVGDVDHTGPWGEIVATLPGLEVAA
>OLS50092.1 ligand-gated channel [Rhodovulum sulfidophilum]AB14
MTVGLLACGTAIGLAWPATAQESFSLDPVMLGQSKREVRTDTAISETVVDEDEIEDRQATTIAELVDSVPGVSLINGNTP
QGSGINIRGFGANSTNGSDQKVAIQVDGASVGSEELYRIGTQLFTDPALYKQVTVIRGTAGTFEYGSGIIGGLIRLDTKD

ASDFTGGEPGFKLRQTLQYGSNGDAWASSTIFAWQPDENLEFLGNYTYRSQGVQKDGDGNGIGSDGFDLPSWLVKA
KYRF
GEAREHYVKLSYSDTSSDQKDVPYDTFGAVGDSFGNVDRKMRSKTAVMEYGFNPAGNDLINLTATLSYAEQKIDQDYV
PG
SSSCETTFCGIPFPPGGFAATNADHRYETTKLTVKNQALFATGQLGHDLRLGVEYIRKERLDAASAPGGTDRRWALFAV
D
DISFGGLTLTPALRYETQDIGGDSYADYDNDALMGGLSARYAFGNGWAVFGSAAYTENLPIIDDLGTPVYMTRSEKART
Y
ELGFSYDRGTVFADGDDLALKLTAYHTTVWDITSYTTDMMVPITDARMKGLELEAAYSLPSGHYVDLNADIARGEITTP
G
VPDDWEGTPADRIRMTLGRKWGETLDLSWEVAHAAEMNRSAKPSGSNTVHNLRATWRPQDGVFKGTEIRAGIENA
FDLDY
TPHLATRPAPGRTFKLTLARTF
>OLS50093.1 hypothetical protein BV379_18625 [Rhodovulum sulfidophilum]AB14
MTALRPARFLRREEGSLTVEFVLVLPLLLWALVALHVFVGAFELRNTATKATYTLADMLSRQTRPVDQPFLDSADAVLRF
LAGPTARLRVTVIRCSAACDADSTRELALDWSGASEGMQALTASDIARSGFRGRIPAMELGDRMIVVETEVDYAPIAGY
G
LPGMTFDTLMATPPRFAPQLCWQSCG
>OLS50094.1 hypothetical protein BV379_18630 [Rhodovulum sulfidophilum]AB14
MIGATGRFARDRRGSVTVEMVILFPILFAVFLMAFELGILNVRQMALARATDIAVRDVRLGAPGVADFDGFRDRICETA
A
IIRNCRDVLRVEMRSLPAAAWTTMTAAPKCVDRAETIDASLDFRNGAGNELMFIRVCALFQPIFPGTGLGRRLPRTAEG
D
YALIVTGAFVNEPAT
>OLS50095.1 hypothetical protein BV379_18635 [Rhodovulum sulfidophilum]AB14
MHQRGDGILRSVGNRFRSFGGDERGALVVFFLFAFVGMLIAGGMAVDFLRADEERTRLQATLDRAVLAAASLDSRQD
PEG
LVHDYFRREGLEDRISNVVVQSDLDGRVVGATARFDIDTIFLQFTGIETLTSGASGVAREHVPDSEISLVLDISGSMAWD
RRMAMMRPAARGFIRSVLGNDPTRVSINLVPYAGQTNPGPVMFEYLGGQWRADAGNHFEEWPQDISHMTVYFDS
DGDGMP
DTVVKVDEFPDAGRPGHVSNDPDDFFAGMVDFIRSAVPALAPATVIGARVKGGIQTTQYYAVAANANGDTPDPDPLV
SGD
GENGGKKNSEAKADHELMWKDFDFQAVPIPRPGACLELGSDDFGQSGLPADGPYEQTPLFMYRAIDRAVMDWGW
CPEDDT
AIQYAQNDAAALDAFLDDLRLHDGTGTHYAMKYALGLLDPSSNAAFRHLAAEGLVPAEFVDRPAAWDDPETAKYIVLL
TD
GQITEQYRPTEPDAAVNATVELERQALSKRRMITSDSTNLASFQETCRLARANGVMIFTIAYLAPDEARQQMQSCASTP
L
YFFDATPANIEEVFSAISGKINTLRLIH
>OLS50096.1 hypothetical protein BV379_18640 [Rhodovulum sulfidophilum]AB14
MIRTSLLAALLGATIAAPALADDIPMIKSIDVETSLTDLKNPQAAAYWKTLDEDLEAAIASQLVGQLGETGMDIKIDMNE
VELANFFQEQIGAADNQLSGTVNVIDMEDNSNFETFDLTVKMNQIMPMLPEGTDVTVITPTSEEVYVALVATFARAVV
DH
IDV
>OLS50097.1 hypothetical protein BV379_18645 [Rhodovulum sulfidophilum]AB14
MSAVPTHLRLVEPSAPPAAEPPVAETPVTETIAAETLAEIASGRFGAGIAGPRETIEQAFLAMGSGLMAGARLLSDISDA
HRDMALEFEGEAFLSGVAQIGLLRDEVGRVLAGIASGDAEIARLDETTRQVRVPLEQMEASIRLLGLVAVNARIVAAGIG

SDQNDLNAFTSEMAGIAAEALGTVRSIAEVHDRVCHGVAETRRSQAAFSRDHWRIMTEIRDRLDADLQSLEAQRAQA
AEH
AGKSAALAAGIGAEVGTAVAAVQAGDMTRQRLEHVEAILARLGAAAERDGAPRLAALLGPLAAEQLRATHEDFTAELA
QF
VAALEAMRVDADAVLRAGTERAGRTLAASGAALAALVRDLQSIGPLLEDDARNREAARRLGERAADAMQQMMSRID
VLER
TEHDVRLLGLNMAIRCSGLGDRASGLRVIARELGGIAAETSAAALRIRALLVGAQQTVADTRRRAAEGRGVEGDPAVAA
G
QLETVLDRLQAHGATLAEACPRTIALLDQTAGQAHRISASGEGWPGLVARLGLSRGAPAPEEVAAFGDLLANLRRLYT
MQ
KERDLHDAVLGLPRPADEGDEAPAGAADEGEIDGFLF
>OLS50098.1 chemotaxis response regulator protein-glutamate methylesterase [Rhodovulum sulfidophilum]AB14
MTFQSGFTGQSSLPGRKWRVLIVDDSASVRRVLSDIIAGDPCLEVMGTAADPYVAAERIARELPDVIFLDIEMPRMDGL
T
FLRKIMAQRPLPVVICSSLTEEGSAALMAAMEAGAVDVVTKPRMGSVEFLQHSRMMICDTARAAVQARLRPQRARR
MQVE
KKLSADAVLPARSGRGPSRATAERIVCLGASTGGTEAIREVLEALPADCPPLVIVQHMPEGFTAAFARRLDGICAMTVRE
AGDGDPILPGQVLIAPGNRHMTVVRSGAHARVTILDGPPVCRHRPSVDVLFRSAAQQLGRSAAGILLTGMGEDGAQG
LWE
MREAGAATFAQDEESSVVFGMPREAIERGAAGRVLPLARIAPAILQFGAAPATGGAGK
>OLS50099.1 chemotaxis protein CheW [Rhodovulum sulfidophilum]AB14
MADQTQYVTLGAADSLFAVPVERVQQILETQPVAAMPHAPADFLGMIDVRGQSVPVMDLRRRLALAAAEDSHSTRI
MVLD
ARIGEVPRRIGLKTDRVFEVTELDTGSMEAPPDLGASWRTEFVAGVGRRNGAFVTVLDLDRLFAAEDLPAAARI
>OLS50100.1 hypothetical protein BV379_18665 [Rhodovulum sulfidophilum]AB14
MKLSLRAKLSLAFGTMLLLIGLLGGVALLQMNRINAESTIIAENWLPSVDEVRQLDTIMARYRVGEYAHVLAASESEFER
IEAYIDGVRDQLDASRAAYEALIVAPEERRLYAEFSDRLAGYLENSGRILRYSRSNQKELAGRQMSDSLGLFDTVTSDLT
QLVAFNSTGAKSASAEGDIAYATSQKVVFGVIALALLIGIGTAVFLIRNITRALGGEPDYARAVIREIAGGNLEVDVATR
AGDSDSLLAAARDMVAKLKEVISEVAVSARNVGSGSQEMSAAAEELGQGATEQASSTEEVSSSMEEMAANIKQNAD
NAAQ
TETIARQSAGDAKSSGEAVGKAVKAMETIAEKILIVQEIARQTDLLALNAAVEAARAGEHGRGFAVVASEVRKLAERSQA
AAQEISGLSGDTVKAAQQAGEMLTRLVPDIQRTAELVAEISNASQEQNAGAAQINVAIQQLDKVTQQNTSASEQMAS
TAE
ELESQSGQLQQVIGYFRLALDDLAGRTGQHHAGALRGGRTDSSPVEEAIRKTVRHSAPARGAATGGFTLDMGGDDEID
GQ
FLRNGAA
>OLS50101.1 chemotaxis protein CheW [Rhodovulum sulfidophilum]AB14
MSGAGKDSAPAAMRALTIGLRGEVFALPAETVSEILDMGPVTEVPNARAFVNGVINVRGKVVPLADLRVMFGMERTP
PDA
DTRIVVTEIDLDGEPTLVGIIADKVHDVTEISEASVEETPKVGMRWPAEFLRGIGKHGDDFIIIPDMGRIFGSKGDRDPS
FATDERTVK
>OLS50102.1 chemotaxis protein CheA [Rhodovulum sulfidophilum]AB14
MTQSADPAETFRQEARDLLDGLEQTLLDLEQDPGDGALVDASFRALHTLKGSGAMFGYDEVAGFVHEFETAFDRVRK
GKA
AISPGLVRIALAAGDHIADLIAAPGRHAAEGAPILEALRETVDTGSGAASGEAAAAAEASAESVRLRFRLPPDAIAFGTD
PLLLLDELRELGADSIVALTDRLPGLEEMDPNGTYLGWEACFEAGVSPAQIDDVFLFVRDGMELTLEPLRPPKAAEEISD

TAAPADAARRGREAEGRAASSLRVPAERLDELMDRVGELVIAQSRLAQIASAGDDLNLKAVVEELERLSSGLRDTTMGI
R
MVPIGTLFSRFRRLVHDLSGELGKEIDFVTSGEETELDKTMIERLADPLVHLIRNAIDHGVEAPVARGHAGKPARGRVRL
SAAHTGAEVAITIADDGAGLDPARIRAKAEQNGLLMPEQKLTDQELYQLVFHPGFSTASEVTSISGRGVGMDVVKRTIE
G
LRGSIELTSAPGQGTVATLRLPLTLAIIESMLVQVGAGRYAIPLSAVEECVELPAAPEGRETGRNFLNIRGRLVPFLRLR
ELFGTRAERAEHQKVVIVSAGEARVGMVVDQIIGNSQTVIKSLSKLHADVETFSGATILGDGTVAMILDVAALVGFGQA
Y
ESRLRGEQLERVA
>OLS50103.1 response regulator [Rhodovulum sulfidophilum]AB14
MSKTILAIDDSPSILQMVSHTLTTAGYRVIEAVDGADGYDKAVGNRIDAVITDLNMPRLNGLDFIRKYRSHGASAGVPIV
FLTTETDDGLKREAREAGATGWIAKPFRQDQLLAVVKKVAGA
>OLS50104.1 hypothetical protein BV379_18685 [Rhodovulum sulfidophilum]AB14
MDPEKTDGIGPGKLCLAHELGLRDAAGIRDRLLAALNGEGAVVVDTSPATGMHTAILQILAAAARDAARQGRSFTLIAP
P
SGACATAFERAGLDLPGQAPAA
>OLS50105.1 C4-dicarboxylate ABC transporter substrate-binding protein [Rhodovulum
sulfidophilum]AB14
MLRRSFLSAAAAALALGGPALAADLDGMKPVTLRLAHVVNEQDAYQVAAVAFRDLVAERSDGKITVEIVPNATLGDER
TL
LEGMQIGSVDMGLITNGPVANFVEEMAVFELPFLFPSDEAAYKVLDGEIGQELLDRLADVNLKGLAYAERGFRNLTNSR
R
PVATPADLDGLRIRVMENPVYTDSFRALGANAIPMAWTEALTAMQQGTIDGQENPVGVVHAFKLNETQTYMTMTR
HTYAP
AIFVMGMPAWSKLPEAAQEIVLQSARDAAAAERAFNAETEAEQLAELKAAGMEVNETPDLAAFKQAVAPVYEKYGDK
FGD
ILTRIQAELN
>OLS50106.1 C4-dicarboxylate ABC transporter permease [Rhodovulum sulfidophilum]AB14
MLSFLGRIDRAIGAVLAPVIAAGMAALIAVITLQIVSRVAFTAVGWTEEVARFLLIGLTFLGAALAWQRHRHIAVGVMV
D
RLPDRAQRIATALALVIAVAFLISLAWVGQTYMTMQSYQKSAALRLPMSYVYAIVPVSAGLMAWLAATDLMRLLLTGA
RP
AAGEPVE
>OLS50107.1 C4-dicarboxylate ABC transporter permease [Rhodovulum sulfidophilum]AB14
MSLLLFGLFFLFLILGLPVALAIGGATMVAMQAGGVPLLVLPQQMFSGINSFALVAVPMFILAGDIMAQGKVSARLVAL
A
DALIGFLKGGLSIVSVLAAMFFAAISGSGAATTAAVSSTLVPELTRKGYDPASAASLIAAGGTIGVVIPPSVPMIIYAVI
AQESVSALFLNGFLPGIAMGAGLMIVALVQGHKRAYPKGAPFSLKTIWRSFVSAFWGLMTPVIILGGIFSGVFTPSEAAV
IAVNYAILVSLFVYRDMGLKDIYRLMVRSGVTTAVIMFVISASAALSWALSNWQVPAQIGALVLSVSDNPMVVLAMML
VL
ILVTGIFIETASALIIILTPLLLPLALQMGIDAVHFGVILVVGLAIGMITPPVAINLYVASSVTGQGLERIAKAVLPYLAV
LVAVLILVTYLPVLAG
>OLS50108.1 glycine/betaine ABC transporter substrate-binding protein [Rhodovulum
sulfidophilum]AB14
MTRVTAFGAILAALAVPAMAEADCGEVSIAEMNWSSSAVVTNVEKFLMEQGYGCSVTVVPSDTVPAVTSIAENGEPD
TIS
ELWLNSAGEAYTKLEAQGKVERLGKALNPGGVEGWWIPDYLAEEHPELKTIEGVLAHPEWVGGTFNNCPDGWGCRI
VNDN

LIPALDLEGHGFKIFNHGSGETLATSMASAYEKKEPWFGYYWAPTAVLGKYNMVKVDLGGIDEAAHEANQNPNNPDP
KVS
DFPAAPVLTAVTADFADREPEIAEMLSKVSFDVDDMSAVIAWMDENNASAEEGAVHYLTEHPDVWSTWINDAAREK
LSAL
LNR
>OLS50109.1 glycine/betaine ABC transporter permease [Rhodovulum sulfidophilum]AB14
MASYDFIFDGLGLRDWCDGGASSAPMSMADLLGKARGSAEPAASVWDLPFPSMDALNHACPAFPQARELTKGLETG
FLSI
KDGLSVVLDPLTQPLSWALEGALWLFQTSPWWIMIPLILLVVRLVARSWGLVAFVGACIGFLAFINHYEYAMQTLAIIFV
CAFICVLVGVPIGIAMSRSDTFQRIAIPILDMLQTLPPFVYLIPLIFLFSVTEPKLYGIAVIVYAIVPVVRLTNLGIRLV
DKEVIEAADAFGMTKRQKLWGVQIPLALPNIMAGVNQTIMMSLAMVVIASMVSAPGLGVLVLRGIRNLELGVGLVSG
LGI
VLLAVMLDRVTKAALARVNAAQKQ
>OLS50110.1 glycine/betaine ABC transporter [Rhodovulum sulfidophilum]AB14
MSDDVKIAIRRLYKIFGADPQSVLPMARSGMDKAELLETHHHVLGLRDINVDMRAGEVTVIMGLSGSGKSTLIRHLNRL
I
EPTAGEVIVDGEDVLAYDEASLRELRRVRMSMVFQKFALLPHHTVLENAGTPLEVRGQPRSAWEGEARQWLNRVGLE
GQD
NQYPHQLSGGMQQRVGIARALTSNADIMLMDEAFSALDPLIRTDMQDLLLELQAELHKTVVFITHDLDEALKLADHLVI
L
KDGEIVQQGEPQEILMHPNDPYIIDFISDINRARVLRVRSVMQRSEAPQSGLVGEVDADDNLESVIALSGGDTSLSYRV
M
RGGKQVGILSVKDLVRALVPTEASDAGVRARAG
>OLS50111.1 hypothetical protein BV379_18720 [Rhodovulum sulfidophilum]AB14
MSARPIAGRALRLGLCLAAAGLALAVMTAPLRLGPEDLRAAYAGPPETWPLPEIDPGVDYVELAPRALPSRPVPGSPAA
A
RRALGQALFVDPALSASGQIACESCHNPRLGWGDGLPRSKGHDRQEGPRNAPALHVAGAGLPLFWDGRAETLEAQA
LGPL
GNPLEMANADLGAIAPRLAGRAEYAPMFRAAFGSQEVTLERIVAALAEFQKYLDRPTRLDRFLSGDRRALSDLELRGLH
L
FRTRARCANCHFGPLLSDGQFHDLGLSYLGRRFEDLGRYVVTGRPGDAGRFRTPSLRHVSDTAPYMHNGLFPHLPGLIR
L
YAIGGGRSARLPADPDAVETWAATASPLLRRLDLDESDVEALAAFLGAV
>OLS50112.1 hypothetical protein BV379_18725 [Rhodovulum sulfidophilum]AB14
MTAKTNRPALRAGAALLSLAALPAVASAQEAFTLDPVTLAETEETGTEIATGDPNDSGRTALGKGSVEVRGDGSGDAN
TA
LTSLPNVQYRNDTDTDAGENGDDILDLKPLEFSISGGRVTENNIMLNGIGINSVTGNESPYNSTELSREDSAPNMYAFYG
LHSQTQFVPSAMVESVEVYDSNVSAEYGGFQGGVVDYKLAEASTRRASGKLSFRFQSDDLVRYHLGTDSGDNPNGTA
KPD
FTKREISLILNQPLGENTALIFGFGRRDAQTTKDKHPQYLSGRADNDSRSDFWRLGLAHDFDNGDRIELNGYLTDYNQD
W
DSPYSDDLHIDVKTRSLSTDLAYTRHLGAFDLGPAALSNVTLKLTALHQDNEASNVTEQNEFYSWYGSYFSEKRGKSFYT
DAFDDWCDTPKAARSAEDYYGCRKGGYGSRYYSDLRNRLGMLVTGDVWRGSFKLGAHVERIEADRSGDGFVSYTGP
VMHL
GGPDYICPAGDPACLPDQYLKNLHTQNPYDIEVDANVAEAFFELDQRFGDFDLRAGARFSYDDVLENAVLAPRLSLGW
QP
SSDFSVVLGANRYYSDDYMAYAIHDALPRGVRYRRDHDDTTGEVGDWEFITDFGSYSYNQSGLKTPYVDEASLSFLYRD
A

WTGGAWRLKLIDRQGKDQFARSSDSTKFKNSLTNDGTNEYQSVSLEFQRDWKTPRGALDGLSFYASGVWARRESSN
NTYF
GDLGEVDDFIWYKGKSYTGAEFGAVTGNLDIPIRTTVELNSSWKNGRYALGIGADVAFGYTAALDTDKDGTYENPEYGS
H
SHDIYEDYEFDAAVSTFLTARVRMARLSSGDLDLNIKVANLFDDIGNRRATDKNPWMAGRSFYVGTTYSW
>OLS50113.1 hypothetical protein BV379_18730 [Rhodovulum sulfidophilum]AB14
MVLSDIRSYCAETGQVSLQELANRFDTDPEAIRGMVDVLVQKGMLRCLTETPEGGCGKGCCGCTNSCAPLAGMSDAV
YQW
VGRSRRPLH
>OLS50114.1 ferrous iron transport protein B [Rhodovulum sulfidophilum]AB14
MSSFTIATAGNPNCGKTTLFNALTGSRQRVGNWAGVTVDRKEGVFTHKGAEVAVVDLPGTYSLGSISETGLDESIARDY
I
LSGEPDLVINIVDASNLERNLYLTAQLVEMRVPTILALNMMDMAEENGLRIDPAALAKRLGCPVVPLVASRKKGVEALK
D
AIVAAAAAPKPPTATVTHPSAVEEALTGLAPATAPVAAEKGVDPRWLAVKLLDGDAAAQAMVPPALAAEAEAARLKV
EDL
LDEDIDILLADSRYGFANGLARSAVRRTGRISRSLSDRIDTVVLNRVLGIPIFLLIMYVMFLFAINFASAFIDFFDILAG
TLLVDWPSEVMADIGAPAWAIAVLPGGVGAGIQTVATFIPVIGFLFLFLTFLEDSGYMARAAFVMDRSMRAIGLPGKSF
I
PMILGFGCTVPAVMATRTLDNRRDRIMTVMMSPFMSCGARLPVYALFAAAFFPMGGQNIVFLLYVIGILAAILTGLVLK
N
TLLRGTTSPLIMELPPYHIPTLKSVLRTSWDRLKAFLFKAGKMIVAVVVVLGFLNSLGTDGSFGNEDTDASVLSSIGKGI
TPVVEPMGLREENWPATVGLFTGIFAKEAVVGTLNALYGQMADDGEADEPFSVMAGIGEALASVPANIADAAGSLAD
PLG
LNIGDVEDQSLVAEEQGVSTGIFGEMAARFDGTAGAMAYLLVILLYAPCVAATSAIWRETGTKWTLFALSWTTGLAYG
TG
VVTYQSSRLASHPAEASAWIIGILAIFVAVLVGMRIASTPRGPDDAAARTA
>OLS50115.1 ferrous iron transport protein A [Rhodovulum sulfidophilum]AB14
MDSILPTTGTTARVKGYVRGDRAGRERLLTMGLTPGTELTVTRVAPMGDPIEIHVRGFALSLRKDEFALLTLEAADQ
>OLS50116.1 hypothetical protein BV379_18745 [Rhodovulum sulfidophilum]AB14
MAGPDACPLALADEGVWLRVVALRPGRGVHHRLIDLGLTVGARVRILQRGGGCPMLIGLGDARLALGQGLAGKIMVT
PAN
EDPQDERDF
>OLS50117.1 hypothetical protein BV379_18750 [Rhodovulum sulfidophilum]AB14
MTAFAMRMVMPPGLAMSVPPPMAMAVIVHLLGPVAGTGSEAGRVRGLQTIRLQVVMGFPVVSGWLHVLRPF
>OLS50118.1 hypothetical protein BV379_18755 [Rhodovulum sulfidophilum]AB14
MRIHLIAPAPDDKTDTAAETGMAGVKTPFTLSAAAGIVSVAAWLPRGTDIRICDEAVQEVDLGAEVDPVGLSINVSQVA
R
GLDLARAFRRRGVPVVLGGAHVSLAPEILRGRADCLVIGEFETAAPDVAADLLAGALKPEYRCGKADMAAMPLPRRDP
YP
NDRAIAGVVQTSRGCPFDGTFCDVIQYVGRKQRHKPPEKVLAEPDQLYRLGDREVSLSDDNFTVYRKRPRALPEALIGW
N
GSAGRDPVRFSPQISIDTDLLALCHRAGLRQVFVGLETSAPAALAEANKRQNLHVGLIGECRKLVAAGLTIRGGLITGFD
HDDLGCFERQFAFAQALPVVGFNVSARVAPYATPLYAEMKRAGRSVDADTHRGSAAGARLTNMLPAQVTREELAEG
RAWL
KAALLVPDAALVRFEAYARTLAPPSQDMGRTAWVKPGARRHPLHELLPLMMKEKGTRRVILAVREMIAERPETGYDLT
ST
LTLYPSDCARAANRAEAPLEAAQ

>OLS50119.1 hypothetical protein BV379_18760 [Rhodovulum sulfidophilum]AB14
MIPPPPNPLAAALLRAFEARASAEQIAIRTRHGDREVFTGTRLGTEAGALAETFRGWLGPPPRTLMLALPAGSDFILALL
GGMLAGLTLVPVPLPRAGSHSDRFRHIAADSGAAALLCLPAHADAVRAALCLDGPAPCPVATLPLDPAGLPAPAPGRP
PE
GAAPALVQYTSGSTRLPKGVRVMPRNILANAALVARAWGMDETARFVNWLPHYHDMGLMGGILYPLLCGGVSAQ
MSPLYF
IRDPASWIAAIAAERAGFSGGPAFAFSDCLSRIGPEQAAAYDLSCWRRAFIGAEPVPAGLPAAFHARFAPAGLRREAVFA
CYGMAEMTLFAAGLPGTGDGSGGVAPCGLTPDLAAGLAIVDPETGARRPDGAEGEIWLCGASVGAGYIGPPEHGAGS
FGA
RLDGHAGWLRSGDLGRIEDGALYVTSRIKDVVFCNGRTLSAPEIEWLACGLSPALNPMAAALFMPDPEASGRAVLIAET
R
TARLPPDAEALARAIRHAVAGEWGLTLDRILFLPRGRLPRTSSGKIRRRAAAELWRAGKWRAGEMAPCLP
>OLS50120.1 hypothetical protein BV379_18765 [Rhodovulum sulfidophilum]AB14
MARGQMAGRGDGAMPAVNSSAWTDPDRFLTDPDDVAAVLRDPHFTVSQLTAHLDEIVARGGPRFPHLEAMARHA
FAFQSG
EPHLAARRATAPFFAAPALAAWTPVAERAIAAALASLETSRSPDLMRDYCEAAFLDFIRAFCGCPGGADDRVLDLIRLAN
ETTRPMLSLKALGRIDAALGELLDYLSTAPVPGLEPGLVSFPAFLGHRADRLADPEAAPYIALSALVGSHTVAQSLALAL
HGMLLGDLGAWQAAARPGWTDTGLDRAISLYPSTLTLVRIAGDGARIGGCPCHKGEAAVMDVVGANAALRRRVPDG
SAHL
SFGAGPHKCPGAPLSRMFLARAIPTLARRFPRLALHRDGVRFHVTPLVQYPVALPVTRDARSRRLTGRMVEIRDLDEAR
A
IVNDDDTWSPPRMEPYLDALAERSGRDLGLARLVARNAMFFMSGPRHAAIRRAVADSLGGNRLACWQPLIAAETRA
ALTR
LASVPAPDLIADYAEPLFRGIAGPVLGLAPADPERFDALAPILQDVLEPWLPIRELDRLQEVIGEILAGLGPPATEGLPG
TPLLTRLMEEDLPETDPEDLKALVLVMYGASFNMAHTLGNILHHLLSLPPEDRRGAADPAWIDAELERLISLCASPKYIY
RMARGPARIGEIEIRTDETLRLQLLSIDRGIGTGNLAFGHGLHRCVGAALSKRMLRTALPALFARFPGLALRPQRHRYFD
MTQTVALATLPCRLGPSGKDRA
>OLS50121.1 hypothetical protein BV379_18770 [Rhodovulum sulfidophilum]AB14
MTTTLTSPAPEIVNFLTAGIARFTRQDPAAIRPDSALVDLGLQSIDAVLLSGEVEDHFGIELDPATIFEHDTLASFASDI
TARLEAR
>OLS50122.1 carbamoyl transferase [Rhodovulum sulfidophilum]AB14
MSSWYLGLSTSGHDPALALVDATGRVVFAEATERFMQDKRAWGVVPDHVPHLASALAAVGARPGDRIVPATSWAS
VKADL
GLKVHDALLPATDTLWMQGLQARMQHSAGAALLRLGYASESPEPLRFDHHLCHAVAACHFTPLEEAACLVIDGEGDV
GAV
SHFRLAGRRLARGWRSWGPGSLGTFYGWLTGLCGFDWREGEEWKVMGLAACGRANPALVETLTGLLAVDRGRLRF
ADEAA
IAAIRAAVAPLARAPGAPVTDAADLAAAGQAAYAVWADRVLAAIAEDGPALILSGGCALNSSYNGTIAGRTGFESVFVP
P
CPADDGNAIGAALLAWMTDTGAGRIPFGGGSAFLGSAPRAKTLETARHAAAAGHAVTDLAGRSAEAAAGLLAQGRIL
GVM
RGRAEFGPRALGNRSILADPRPGDMKDAINARVKGREPYRPFAPVLAEADLAEWFERPQPSPYMSMTLPWRHGVRD
RVPA
VVHDDGTGRLQTVGPDSAPWMADLVAAFGRETGVPVVLNTSFNIMGKPILHSVEDALGMLATTGLDAVMIEDILIEKP
VP
S
>OLS50123.1 hypothetical protein BV379_18780 [Rhodovulum sulfidophilum]AB14

MQDEIYRFGNGVRVFRRHVLDHQVNRYVKAQAGNLHEPVEEHWFRTLLGGIAAPDPLVYDIGGGIGYYTLLLLRDRPG
AE
AVVFEPLPEHAAAIRENLALNGIAPDRLRLIEAAAHVDEGEVCFEAADFSSHVAAEGGLRVAAHDIAAMLAAEARPVDL
A
KLDVQGAELALLRWIIEAGQAERVRRWIVGTHGDALFAECRALLDRHWRVVHSDPAPAHQPDGLIVAVRD
>OLS50124.1 hypothetical protein BV379_18785 [Rhodovulum sulfidophilum]AB14
MEETLERTELERLFAEAGIDYDFYRNKQNPQSIAFMAELLPLLERLYPARYHRKTVLDVGARTGSGSALLATMLDPASYA
RLKCTVTALDIDARMARISHALRPEMEYLVADIFEIDDRQWDIVICSHCIEHVPEPEPFLTQLRRLAREHVVISTPFREN
PETRIPAHLHSFDETFLDAVGAENITIYNGFHWPHSEIVTFSLPPL
>OLS50125.1 hypothetical protein BV379_18790 [Rhodovulum sulfidophilum]AB14
MTPPTETPASGSARSAVPFEAGSAPVAAPLAAFRDWIETRALPFQAQAVRDPAGGFHERIAPDGHPVAMPRRARVAA
RQL
YAFSRLAGLGLGDTVGPGLAGHARDMLLGAHIRPDGRIVPLAGGPEGYDLYDIAFCLFALAAAPEAVLPRAGACALAAQ
I
LTRLETGWRHPEMGFEEAMPRRLPLRSNPHMHLFEASLEWLATGPCDPRFEALADEIAGLCLARFLDPATGALRELFD
GG
WQIAPGAENAVEPGHQYEWGWLLLRWGALRDRAEARRAGLGLIDLAEARGLNADGLAASLLDPALAMADPTARLW
PQTER
IKAWALRARLATRRADAALTEAKAVEACQGLMRFFQHPLRGSWWENLAEGGAPRREPARTSSLYHIVGAYHELAALE
ARR
SLPIAKEETRASSTA
>OLS50126.1 hypothetical protein BV379_18795 [Rhodovulum sulfidophilum]AB14
MPALTAPLPRQAAILCGGLGTRLGPLTAQCPKPLLPVGDTPFLSVLIAELARQGIERVLLLAAFEADRIEDFARDLPGRL
PRPVGIEVAREARPAGTSGALWQARDRLEDRFLMLNGDSWFDILLADLAAAQAARPGLLGALALRRVPEADRYGTVRL
ED
GLLRAFEPRGAGRGAKTGPVLINGGVYCFSRGIVDHLVEDGSLEAEVLPRLAADGRLAGIERAGFFLDIGVPGDFEAAQS
LVPAQRRRPAIVFDRDGVLNLDHGHVGHPDRLDWIEGAIAAVRRVNEAGWYAFVATNQAGIAKGYYSEADYLALRAQ
MYR
DLAAAGAHIDDERHCPTHPDGTVSALAVSSDRRKPGPGMLLELKARWPVDWSRSAMIGDKDSDMRAAAAAGLDGIL
FTGG
NLDLLVEQVLQSRGGAP
>OLS50127.1 hypothetical protein BV379_18805 [Rhodovulum sulfidophilum]AB14
MIRRLILHIGRHKTGTSAVQRYLADHRAAYAAEGIVVPDLGGDERPDAEPADRAAHHRIALDCGTGPAAEARRRAEAA
DG
WRARLAAAAEGGHTLVLSSEAFQNCADFAALRALCRDHYVEAVCYLREYLDYALSAYAQEVKKVGLYAGFFEFERGFDP
Q
LGRFVARWEEFANRCHWRLYDRTLLAEGDVVQDFLATAGLPDFGGDRFQSENPRLGGSLLGFKLMANGAGLHSLRLA
RAL
EPLAAARSDFRAPWRIEPGRQAQLRAGRNYNAALEGLVGEVRLADAATGTLPFESPGFAADLTGIVAALEGFPALRAHP
L
FAVCQEAGPCPIPP
>OLS50128.1 hypothetical protein BV379_18810 [Rhodovulum sulfidophilum]AB14
MDEMTDLADTLADEMTARLKDDVLFIETDRSVEETEAFCTRHAPWRSVYRFSNGVTTESFPRSEHVPNPRCLAKLRRF
HA
RIDLSRFAGKRVLDLGFNEGYQSIFMAKYLGCQVDGIEAYQVAVDRTSAIAEFCGAPCNFWVADANSFVAPETYDVILH
C
GLLYHLHDVWSAVKNTCRSLKPGGEVLLETTSYEGADKFDCKFMNWHAKGNHNYWALSPETIRYLFAEFGCTEGREV
DSF

EVGALAGTGMKRTMMWFRKPA
>OLS50129.1 hypothetical protein BV379_18815 [Rhodovulum sulfidophilum]AB14
MSPNDSQHILSGRGERAVTKPVILVHYHVYKNAGSSVDLANRAAVGAANFIELDKHRGFRKAPAFNAALVRQVLRAHP
DM
RCFSCHNFVPTVHRCPDFTALPIVFLRHPLLRLASVYRFEAISEAHKHTPAGSLAQKVDFPEWLEAFLHMPNGKNYQTCV
LSRTEDGGHSHFTNDHPRHIGDIRVAAERLDEVNALTGVGIVEDFEASAARIEAKVVRHMPGFRLSGAAANQTKKIGN
WR
EELAALERSLPADLLARTVMANASDYALYDRYR
>OLS50130.1 hypothetical protein BV379_18820 [Rhodovulum sulfidophilum]AB14
MKMTRRVILHIGRHKTGTTGLQLFLESRRADLTKAGFCYPLSGRNQSPGQSREDQSAHHGLARSLIGPRDIVRQSLKALR
PAFLDEVRDAHTVILSSEGFQNVEHLDLLAEFLDGFEIETICYLREYLSYVASAYAHEVKGSGFVSDFVAFEERFYLDLP
DFIARWRHVVRRCEWRLYDRARLVDGNVIADFVATAGLPLEPSRVVVDSNPSISGNLLGFKMLVNMLGLHRPELGPP
MND
LALAHARFRGPIYVDLPTQKRMRDRNSCKRTLRNLFGDVQEFDFEAGNRVFDPFFLEQDMKTILQEFAQFEAVANHPLI
A
ALERGQMVAGEMPR
>OLS50131.1 hypothetical protein BV379_18825 [Rhodovulum sulfidophilum]AB14
MHFMFANIKGCYGPREGKEYSFFAKDRDFPYERNFDVDGDDADALRFEFSPRYTTVTAPSWHKTVVERIRNGVDNPF
VIF
TIREPVSRSISAYRHFLHPYARFGKERHLAEQRCLSDPYRKTFDQAVRSDQLVGCLLSDIVGLYVDSFGKDNVMFFFLEE
DAASFDRFYDFVLDRTGLHVPNTRRGAEIPQSSKAKGHPRYYYSQDHDICIRNREGREAYLPAGQFYICNERGHEIISDM
DPEVAASCMDAQKLWTREFRAAYQSDIFDELFREDFQKLSDYWVPGSYSGAVPDYFNWSYSDKVFPCAEPDFDLLND
ASP
ELKKASLSEM
>OLS50132.1 GDP-mannose 4,6-dehydratase [Rhodovulum sulfidophilum]AB14
MPKRALITGITGMVGSHLTDFLLAETDWDIIGMARWRSPMDNIAHLTDRINQRDRLRLVHGDLRDSLSLLHAVEEARP
DY
VFHLAAQSYPRTSFEAPLETYDTNVEGTSRLLEAIKRTVPEAVIHVCASSEVFGRVPREKLPIDEECTFHPASPYAISKV
GTDLIGRFYGEAYGMTVMTTRMFTHTGPRRGDVFAESSFAKQIALIEANAIPPVIRVGNLDSLRTFADVRDAVRAYYLLV
TVKPQPAAYYNIGGAHSCTIREMLDHLIGLSSARDSIRVETDPERLRPIDADLQVPDTAKFRAHTGWAPEIPFETTMLDL
LDYWRGRVAEGAVRLVR
>OLS50133.1 hypothetical protein BV379_18835 [Rhodovulum sulfidophilum]AB14
MSAGGGKRLCAVTGPGGFVGGHLMPVLSARGWQILPMGRAAADFSDPEALAACLARHGVTDIVHLAAESNPAGGD
ARAFY
ETNAFLTERLLQAAATAGLPGRFLLVSATSVYGDSGPAPQREDDPKQPPNHYGASKLLAEVFAGWYRDRLDVTIARPSN
C
MGAGQNPRYLVPKLVRAFAERAPEIAMGDTAIARDFVDIRDAADILARALEAPARSLDAINVSSGRATAIAEILETLTRI
TGHAPEIRRDDRFIRKGDMRFQACDSARARSLGHVPRHDLADTLSWMLAAAPAVPEPVEMRLP
>OLS50134.1 hypothetical protein BV379_18840 [Rhodovulum sulfidophilum]AB14
MNDGTMIAPYLRVVPENRSALRFFTICARNFLPGAQILCDSVRRAHPGAVFTVYLCDRDMGYDAEALGMDIEPLEALGI
P
ALERMIRTYNITELCTAIKPYCFLREFARGPEAGARTEAGAAEQSPHVVYLDPDIELFSPLVEVAEALDAGASAVLTPHV
LKPAERAEFADDHFLRYGIYNLGFIALRATEAVADVVRWWARRMVDQCIIDLPQGIFVDQKWMDLLPAFLDGVHVLR
HPG
YNVAYWNLHERRIHGPDTALQCNGQPLRFVHYSGIILEDVEQLSRHSGMFYVSNSFGYGPLVRRYRARLTAPENRRLRQ
L
PYAFFWNGAGDSNAHTPGGGGAPAWRRPDSFLFARQAFSLEQYLGYLSAEAREIESQRATEAALTPKGRREAFEFTCY
CA

VCGGRHPMVTSFMYSCATDGEGNPIPNWREHIACRSCGLPNRVRASVHLFLQEFDPAPDSRIYITEQKTALYTLLSGRFS
RLTGSEYFGNRVPKGAMFDGVRNENFEALSFQSESFDYLLSFDVLEHVPHPERAFAEAFRVLEPGGSLIFSVPAHLDRYP
STIRAELAPDGQIVHHLPAEIHGNPVDEKGGALCFRHFGWDVMDMLREAGFRRPFLYHYWSREFGYFGPGQALFVAE
KPR
>OLS50135.1 hypothetical protein BV379_18845 [Rhodovulum sulfidophilum]AB14
MRNSKIMRAIARDFDARWYLAQYPDVAESGMKPLLHYVRYGAREGRNPRPDFDGTSYLIINPDVRDAGMNPFYHWV
LYGR
AEGRVISHDQGSAGGSSGEMPVLEFSGLLTPLDCLASSQLRRLDLSALPDKLMGRGFPYGMAPGPVSFPLVLFRDGPGE
V
PADRIQQALALTTGAVHVLSRNAGRRRGKGRDRLRHWSLTGDAALADLKAVLKGLDSEFVGLWDAAFSPAISAWHFL
EAA
LRQDPVLAHAGASLVRRDGRLWRSGFRERAGEAGLAALGRDAHFLDPYQFRLKSSAMLDPCFGLARVAALRDAAAHV
GRR
DGPCMTEAILRLAAPLARLGQPVFAAQGCALALSEDEPYQPLKPAKADDGVLPNDPGPVEAVVFVDSVPPMPDQDAG
SVT
ASNFVDIFLKRGAEVVFYSTAQRNWNNPYALALAARGVVCLTDPVVRNYTQACEHIAAAGYERLSFLLTRIYAGGELVE
Q
TRARFPQARLIFNTVDLHGLRELREARIAGSPAREFSAQTTFARERDIIQRCDATILLSEAEMTELSPTLGHANLHLIPM
VNEFSPPKARYSQRRGLMFIGGFAHHPNVDAVEYILDELWAPIRARDPDMVLQIVGPHFPDRLRDRLPEGVEALGFVP
DL
GAALEKVRLTLAPLRYGAGIKGKIGTSLAHGVPCVATSLAAEGMGLQAGRDILVADTPEAFAEAVISLHGDEMRWTRM
SR
AGYDFCEARYSRRVVAAKLNALLDGVPA
>OLS50136.1 hypothetical protein BV379_18850 [Rhodovulum sulfidophilum]AB14
MRGRRKGVALPFDHVRPGQLDLRTANGVIRIAATSARIARVGSEAATALLAPDGGGGIRHDLPGMRFLIWHDVRSGRE
IR
VPLYRAHDYVLGGLRAALSLILFPFRHNADLIGYFLKGDSAAGDRLERALLPQLAAADHPPDAATGLFGASPMPPLTEPV
DIILPVYNAADVLALCLERIAANTPEPHRLILIDDASPDPRVRPLLEDVVRRHPQARLLVNPQNLGFIGTVNRGLAEARG
HVVLLNSDALVPPGWLTRLMAPILDDASIASVTPLSNDAEIFDVPVECRARPLGPGEAEAADRAAARLNWRSALCDAP
VG
VGFCMAMARHWLELLPDLDTAFGRGYGEEVDWCRKAAALGARHVCAAHLFVEHRSGSSFGAEKAERVRANNQLISN
RYPG
YDLMVQQFRDKDPLVGPRLAVGIGMLGQGAPLRIYLAHRLGGGSEFWLQTRLEEVVTENGAGLVVRDAPGPDRLQVE
LYC
REGVTRSVLDASDLAAMLAGLDARHLVYSNLVAAEDPLGLMERALSWLAPGDRLSVLFHDFLPLCPSYTLIGNKGRFCG
L
PGPETCQACYARLAVTSGKRPAAIDTWRAGWRKVMDRADELIVFSEDSRSQLIRVWPDLAGRIELQPHRPQHLPRRVA
PP
KGGSLTVGVLGAIGHNKGAGVLRDLAERAAPDLGIVVIGKIDPAFAHRRIRVHGTYGRDEIADLAEKYRIRCWLIPSIWP
ETFCYTAHETLATGLPVFVVYDIGAQAGAAAEAINGHLLPLGCEGEALDRHLRRHLGASTAMPAARLA
>OLS50137.1 transferase [Rhodovulum sulfidophilum]AB14
MSSITVAIGDLNAKGVEVLSRGSSKLRMPRRTRLEAPCSLKWTQYEFSLELGAFSYQVSGYCFGARIGRYCSFGEEVQIG
RQDHPLTWASTSPMFYLHDRVFDLGNGFAGAAEYHDYRFANGKPPTKAKITTIGNDVWIGHGAMIRAGVTIGDGAM
VAAR
SVVVRDVPPYAVVAGNPAEIKKFRLPPDQIEMMQRARWWRFAPWQLTHLDATDPAAFAEGALQMTDTPEFRPDVI
DLGTG
AP
>OLS50138.1 hypothetical protein BV379_18860 [Rhodovulum sulfidophilum]AB14

MSPIVFLHIPKTAGQGIHAALETLVGDPGRVSPVRVHTQCPTGSQMPPGYDLYSGHIDWTELDTLPPDRFAFTVLRDPRE
RIASFYFFLLKEARALSPEALARPENTGKRAVLELSADDYFFRGTPGWQTFVRDHYDNFYTAYLATRRMRGRQRLRGLSE
TERLDRARQGAAALQGIYEVDRLQRLEQDLSARFGRPVALAGRVVNAGEHVRGERRWPKLLARFESDASAARLAEFTSLD
DALMADLLSSDGS
>OLS50139.1 hypothetical protein BV379_18865 [Rhodovulum sulfidophilum]AB14
MQDELAEIIQRVRVLEASGASGPALEMLAHALARRPDDLSLRLRQGVLLREAGRVEDSLTHLRGLAAEAPRALWVGIELA
ASLRRAGRHAEAYETLQELLTADPRQRQALVARIEAAAQELRYDLALEATEAALAALPGDLPLRLRQARLMLQRGHVLAA
RDRLEALAAECPEDPETDLARAQSLLDEDPEAALQLSEAVIARAPDRAGAWPLRIAAARTLWRFDAAESWGAEAADRFPG
RIGITQARFATRLALDGPEAALALLPDAPLAPALLIARGNGLLRLGRPEEADAAFAAALETGGLLPAAAGGRIAAAQELD
RVGPVHRDLYGLVARHCAAGHRNGDRIRAEIEAQIGDWPRTERICVRLLDRQGADTSLLHLQARAGLALGRTAAAETSLR
QLLALDPAHCRGNELMDTLCLASGRPEDYLARRLKQQARPDSTRARRDVELIRDLDLTGRHDTARRLLDATLRIARGTPN
LFLANELTARGRTEDATAILPRVVRSRRNMIGAPDRATTGAARLARLFDFETGPPLAPSVIPPSGPLAWHLARDRSHGQL
EWTRRAERAGAVWRWLAAAPADAAALEPWLRDGEIDRLRALPRPCILAATHFGPLVFSILHELYDDFHYLGARSAANPAT
DNLGRLIPAASHPKQAAADAMRALRKGSIVLMTHDMPGPAMRRGPGGGLARGRLFGQPIGLLDTMPKLAAGLKLPSLMLQ
PLWRDGRISFDIAPLPRPETGEDGQAWLDRWAAAYLERIEWMMRGAPENQNLQAMLWRWLLFTPLSGKLRDTPAAKASTA
>OLS50140.1 glyoxalase [Rhodovulum sulfidophilum]AB14
MNREAGLGVLFVAGFGPITTDAESSRELYVETLGLPLEPYGEGDYLLTEDGALKGCRHFALWPLAQAAQSCFGCDEWPAD
LPRPQAWLEFDVENIAVATEGLLARGCQLLVADRQEPWGQRVTRFLSPEGLLLAVSETPWLRGS
>OLS50141.1 hypothetical protein BV379_18875 [Rhodovulum sulfidophilum]AB14
MSRGPVFGQPRFLAGPQSPTADLYLGFSLAGAIAAGRIRRLAPTVFLAGDALIVMRHLAGLGLLPRYRRLIYLIDDDIPA
GLRDRALPRAYRAKLAALELPALLRCGAAADAVVATSPALVAQWRARYPGKTVIGTAPAWPVAEMPVPRPAAPLRRIALI
MGRSHARDAAPLWAPLLALLDRRPALGLTVSGNLDLPAEARSHPRVDILPVRGWDDYLGWLRTARADIGLVPHFASRDFN
AARSGSKLGEYAMTGAAVLGAEAWAEGRAAAAAGRCLALPPDPAAWVAAIERLAEAPDEARALAARNRSALIAEDACGRQ
QRLWSDLLGMDLMDLRGVPAEPG
>OLS50142.1 ABC transporter ATP-binding protein [Rhodovulum sulfidophilum]AB14
MLEFRAVEKAFRLPGGGRRRILSGLTLDLPQKYRVAIIGRNGAGKSTFLRMVAGTLQPDHGEIRRLGRISWPMGFSGGFH
PALTGRQNARFVARIYGADTDDLVEEVQAFCDLGPYFDARLQTYSSGMKARLALGVSLATNFDCYLVDEITAVGDAAFRK
KAQRTFAERLDRVQVIMVSHSEATLREYCNSALFLQDGEGWFFEDLDEGLAAYRKSIAS
>OLS50143.1 hypothetical protein BV379_18885 [Rhodovulum sulfidophilum]AB14
MRFGFLGTVVLPTLLTAAYLWLFAADQYHSEAAFSVRSEDFSNPLEALSAFTQVGSSSSSDSQILYDFIRSQPLIQKVDA
SLDLRRIYGKPSLDPVFSLEDDASREDKLDYWRRMVKVAVDPSTGVLSLQVRAFTPEDAQAVAKEILTESGFMVDDLSRI
ARQDAIRFALEDVEEAEAMLRDIRRNVRVFRLENDIIDPTEVAESQMGVIAALESQLASALVERETIAGFAGQEDQRVKR

IDRRIEAVRSQIDQERRRIGKSTSGPTSLVDVIGAYEELLVDLEFAQQAYTAALASEEQARAEARRTSRYLAVHIPPTLA
EDSIYPQRWLLILLVLICSLAAYATVLLVYYNIRDRG
>OLS50144.1 hypothetical protein BV379_18890 [Rhodovulum sulfidophilum]AB14
MSLFRPQPGPGWRLPSLRVFGALVIREMITRYGRSPGGYIWAVLEPAGMIAILALAFSQFIHTPPLGESFILFYATGYLP
FHAYSEIAGGVSNSVTVNRPLMHFPIVTPLDAVLARFFLSFLTIVIVCVLVFSTLLVITDDPVRLQLGPLFAAIGSAALL
GLGVGTLNAVIFAFVPLWQRVWTIINRPLFILSAVFFTYESLPRNAREILFWNPIVHCVGEARKAFYPVYEGNYVVLGFP
LGVAAFCFLLGGFLLMRNRSAVIDA
>OLS50145.1 amidohydrolase [Rhodovulum sulfidophilum]AB14
MAVENWVAKHIPELVAFRHDMHRNPELLYDLPRTSARVAEALRAAGVDEVHEGIGRTGVVGVIRGQSNVSGRTIGLR
SDM
DALPIHEETGAEWASTVPGKMHACGHDGHMTMLLGAARHLADSRAFDGTVIVIFQPAEEGGAGAKAMIDDGLFAR
WSCDE
VYGMHNRPGLPVGQFTIAPGPIMASVDEIRITVSGRGGHAGRPHLSVDPMPAAAAVIQAVQAITARSVDPIDSATISLC
T
IQGGNAFNVIPTEVTLTGTVRALREEVRDHIEARLAETVAGVAAGFGATGTLDYIRHYPVTVNHEAQTDLAAAAARVVA
G
DEAVTTDMPPTLGGEDFSFMLNEVPGALINIGNGPSQGLHHPRYDFDDEAIGWGCSYWTTLVRQRLPL
>OLS50146.1 2-amino-thiazoline-4-carboxylic acid hydrolase [Rhodovulum sulfidophilum]AB14
MKDLPILERRRIEAMILKHVFDVLSERSGRAEAEAVIGETCSRSAIEQGKALAADLDHPPTLEDFAAILPNWTKEDALSI
EPIETSAEKMDFNVTRCRYAEMYREMGLGEIGHLLSCNRDGDFCIGYNPEIEMTRTQTIMKGASHCDFRYRMKKD
>OLS50147.1 oxidoreductase [Rhodovulum sulfidophilum]AB14
MTDPVTFPPSLWADTAPDRVPAPPLQGTIEADTVIIGGGFTGLSAALHLAREGRKVVLLEGQAVGWGASGRNNGQVI
PTL
TAAEPDAWVARFGETGRRFAELIGGSAGLLFDIVRREGLKAEAEQSGWFQPAHSPGRVRLSEKRVEAWRRFGFDVSLK
DA
RETAELLGSEFWHGGMYAPSGGHINPLALARELARAAEGHGAVIHEQSPVKWFERQGAEWVAATAAGKVRARALILA
SNA
YTGKLAGGLAPKIARSFIPVHSWQMATEPLGEDLRRRILPGRQAVSDTRGDLRFFRYDARNRLITGAAIIPGWNDAGRA
E
AKAAQNLGEAFPELGMPKMTHVWSGYLSMNWDRFPRVHNLGPDGWAWAGCNGRGVALGTALGREMARAVAGE
DPRGLALP
VTEPQAFPYHALLTRLAPAYLAWLRRKDLHEPDL
>OLS50148.1 aminotransferase [Rhodovulum sulfidophilum]AB14
MSLQFDDIRRALIGEGTAIPGPFGPRALIYADYVASGRALGFVEDAIRQHVLPYYGNTHTETSFTGRRTTELRELARASV
REGMGADDGHAVVFCGAGSTAATDKLVRALQMGVPAGRLGPDAVVFVGPYEHHSNDLPWRESGAKLVRIPLDAAG
TLCLE
TLKREIAAHAGAGLILGAFSAASNVTGIRTDIGAVARLLHEAGGWCVIDFAAAAPYVAIRLAESAPGAGDRIDAALISPH
KFPGGPGASGLLIADCALLAAARPTVVGGGTVSFVSAAGQCYIDGVEAREEAGTPSIVGNIRAGMVMALKTELGAEAIE
T
REGELRARLDAALSAEPGIELLGPRNAPRLGIFSFNIRIGARHLHHGFVVAVLNDLFGIQARGGCSCAGPYGHHLLQIDD
DRSQRFSEAVARGHTAFRPGWVRLGVNYFFSDEDVDRLARALVFIARNGLALLPLYRLDVEHGVWRALDGARSPAPES
LA
ALWAETPETPEPPDFDTCLAEAARLAETGRARADAVAGTPLHPEDEALRWFWLPEEAAAALTDEETPA
>OLS50149.1 microcin ABC transporter ATP-binding protein [Rhodovulum sulfidophilum]AB14
MTDPVLDIRGLKIALPKGADRPFALAGLDLSVRPGEIVCLVGESGSGKSLTAGAIMRLLPEPHVRVAEGTIRFEGQDLLA
LSEREMKQIRGDRIAQIFQEPMTALNPQKTVGWQIDEVLRLHTRLRRKARRARVLELLDLVHIPDPPSVFNAYPHQVSG
G
QRQRVMIAMALACEPKLIIADEPTTALDVTTQLQILKLIRELQEKEGAGILFITHDFGVVAEIADRVVVLSKGEMVEQGP

AAEILNRPRHPYTRALIGAIPALVPPAPRDFDAAPVVLKAEGLVKTFAARGSLISGKRAPVKAVDNLSFTLRRGETLGVV
GESGSGKTTVSRIVTRLLESDCGAVEVDGTDLLACSPRELREMRRHIQMVFQDPMASLNPRKRVEDLIAQGPIVHGAEP
R
AARARARELLELVELSPAAATRYPHEFSGGQRQRIGIARALAMEPKVIVADEPVSALDVSVQAQVLRLLADLRDRLHLSL
LFVTHDLRVAAQLCDRVIVMQKGRIVETGITAELFADPRHPYTQQLLASIPGRGWTPPSLETDAA
>OLS50150.1 ABC transporter permease [Rhodovulum sulfidophilum]AB14
MTAATTDLPRPEVHPAVALWRRFARNRAALLGLVLFGAVLVMAATAGLIEPVDPLRRAGDPLTKPFIDWAVPLGTDQL
GR
DILAELFHGARISLLVGVLATLLSIGIGVVVGAVSGFFGGWVDDVLMRVTEAFQTIPNFVLLLALVAILGSSIEYITLAI
AIVSWTAPARMVRAEFLSLRSREFVDAARNLGVSNGALIFREILPNALPPVIVYASVIMALSILLESALAFLGLGDPNYA
SWGNMIGQGRAVLRTDWYCAVIPGIAIVLTVLSFSLLGEGLNDALNPRQKKQ
>OLS50151.1 ABC transporter permease [Rhodovulum sulfidophilum]AB14
MQLTQVLTYAMRRLLQAVPVVILIMVGTFLLLKLAPGDTVDALVGDMGGADAAYIARLRAEYGLDQPVWVQLGRYM
AKLI
QFDFGWSFVYEQPVSAILWERLGTTLMLMGTSLSLAFSIGVGLGAIAARRAYSLTDNAISTLGLVFYATPSFFLSLMMIL
LFSVKLGWLPVGGLTDVAAFYTGWDHVLDVALHLIMPTAALSLIYLSFYLRLMRASVLEVADLDFVRTARAKGAGETRL
M
VHHIMRNALLPVVTLLGLQFSTVLGGSVVVETIFALPGLGQLAYQSVIQRDMNMLMGIIFLCAIIVVIVNFLTDLLYARL
DSRIALK
>OLS50152.1 peptide ABC transporter substrate-binding protein [Rhodovulum sulfidophilum]AB14
MITTCRSAGRPGWGWLLAGAALATMAGPAAAEEPQRGGTAIVHMVSEQRVLNPALRASTGVYNITGKIMEPLIDKSY
DGP
VGVLATDWSSTDDGLHITVNLREGVNWHDGEPFTCDDVAFSAMELWKPLLNYSSTLQQYLESVDCTDAHTAVFNYSQ
PMP
LGLFVAAMPDLGHPVPKHLYEGTDIMRNPHNTAPVGTGPFKFVEYKRGQYVIAERNDDYWRGEEYPYLDRIVWRFIPD
KS
AAAAAMEAGEIHESGTNSLSMADIERFSKMDGFDVGTKGYENNVAHSTVEFNHRNPILADLKVRQAIYHGLDIDYAIKT
I
MRGYAKPGRGPIPSTGGANYTDDVTTYDYDPELAKQMLDEAGYPAGEDGIRFKLRHRPAPWGEYTQLWAEYFAQAM
GEIG
IDVEILTNDAPGFLNGVYRDHDFDTANGWHQFRSDPAVSTMVWLRSGSPKGTPWSNQFGWKSDEMDRMIDEAQA
ELDPAE
RAADYHAIQAKAMEELPLIFAIEHPFISVTSQKLHNHHNNPRWISSSWYDLWIEH
>OLS50153.1 oxidoreductase [Rhodovulum sulfidophilum]AB14
MQNCVWITGAGSGIGAAMARAFSAAGYRVALTARSEDRLTALADELGGAVRVFAGDVTDRARILAIAEEIVQWAGRI
DVL
CNNAGLNVPLRRWDELDWDSWDRVIATNLTGALNVIAAALGPMRRQQSGVMIHTSSWAGRFHAPVAGVPYGASK
HALTDL
SASLNAQEGENGIRSTALCPGEVATPLLNGRPGFDPARAAEMIQPEDMAALALHVAGMNPNVAIHEITLAPVQRATR
>OLS50154.1 hypothetical protein BV379_18940 [Rhodovulum sulfidophilum]AB14
MTHPVKGVDHVFLMVEDLDRAAEAYRRLGFTLSPKGLHSAAKGTANYTLMFPGDYMELLGIVTPTEGNAPRRAALAR
EGE
GLRAVACRIDDAEAAKTALEALGIATGEVGHFERPVPLPDGGTGRAAFSTMSFADSELPFGIVFMCQHRTRETVWLPEL
L
THPNRACGLAEIVAQSADPEAAAQRLARLYASGRVTPAAGGYRVETGPASAPILCLSGAALAEYFPGLDLSAAPAEGFA
G
LALHSEDMAATAACLAASGVSPVETARGIALAPSDCAGAVLEFRQR
>OLS50155.1 aldehyde dehydrogenase family protein [Rhodovulum sulfidophilum]AB14

MTLRSETECLMSRLGVAPDALSGGSMACVSPLTGERIADISPASVADAAAAIAAAKAAFGDWRMVPAPRRGELVRLLG
EE
LRAAKEDLGRLVSVEAGKSPSEGAGEVQEMIDICDFAVGLSRQLYGLTIATERPGHRMMETWHPLGPVGVITAFNFPV
AV
WAWNAALALVCGDPVIWKPSEKTPLTALACGAVLGRALARFGAAPEALVQVLPGGPELGNALVESPDVALVSATGST
RMG
RIVGPKVAARFGRSILELGGNNAGIVCPSADTAMTLRAVAFGAMGTAGQRCTTMRRLFVHDDIYDALVPGLIRAYASV
SV
GNPLETDALVGPLIDGAAYRAMQEALEAARAAGGTVHGGTREDIGPDSAYYARPALVEMPAQTGPVLEETFAPILYVM
RY
SDFDAVLEAHNAVGAGLSSSIFTTDLREMELFLSARGSDCGIANVNIGTSGAEIGGAFGGEKETGGGRESGSDAWKSY
MR
RATNTINYSRELPLAQGVVFDI
>OLS50156.1 hemolysin secretion protein D [Rhodovulum sulfidophilum]AB14
MHLLKHHVPTVLIVLIGVLGVVVVLFAWHLPPFGGSEIRTENAYVRGNVATLSPQIAGYVTEVRVTDFQTVSTGDILVRL
DDRIARQTVARAEAELDSARAALKANDQDINSADATLGSRRAAAEAARAAVDTARAEWDRHDRLRRKGVISASDAED
AEL
RLRQAEAALTEALSDVAVAREDLATAKVRTDTLSASVTSALAAVELAKIELEHTVIRAPQNGRLGQVNVRVGQYVTAGT
A
LVSLVSSRVWVIANIKETDLAGLHSGQKVRFAVDALGGQPFTGKVALLSPATASEFSILGNSTATGNFTKIAQRLPVRIE
VDPGQEGQDRLAPGMSVVVHIPRDRG
>OLS50157.1 MFS transporter [Rhodovulum sulfidophilum]AB14
MPAAVPSDAPAPAPQPQPTGLTRPLPLAAAYMLASMFIALSQSLGQGFLAANIPTLAGELGATQSQTAWLMVAFMA
PRAS
LPLMLIKIRSQYGLRRFAEVSIALYAAVALLSLLTRDLRSALLVELLSGISAAPLATLAFLYALEPLPQRLKLTVGLPFA
LSFIALGMPLARAISPTVLTDGGWTDILLLKLGMAMVCLFLVFLLPLPHGERTRSIRGLDLISFALIAASFGGLVACFTT
GYIYWWTAAAWMGPVLALSIAGLVLALVIELHRTAPLLDVRWLATPEMLHLTAALLIFRVILSEQTAGAPGLFRTLGFAP
EQIAPLFWGISLATLAGGAACALVMKLDRVPAIHVAALLLIALGSAMDAHANTDVSPTLMIASQAMVGFAAAMFLPSA
MA
AGLVKALSKGPQYLLSFVIVFLSSQILGGTVGAGLFRTFVAVRTTAHSQALAAGLQTGDPLVAARLAGIAAQLSTTVTDP
VRLKAEAVAQLAADTARQATVSAYNDVFALISALALCALAALLGHIARDWLRARMARRVPADG
>OLS50158.1 hypothetical protein BV379_18965 [Rhodovulum sulfidophilum]AB14
MHVVQKTFLPEVFGLVRAIRKRFDARATSIDMTYARAQALMSIASCEGLTQVDLAERLDIRTPSMNRTLDNLEAAGLIE
R
RASAEDKRVRRLYLTPAARARAEAVVDFTDELRREVYRGIDPAELDRALATIRKIQANLAAMDR
>OLS50159.1 hypothetical protein BV379_18970 [Rhodovulum sulfidophilum]AB14
MGRSGVTGNASSRLRRRRRSCPMAAHDRLPAPARAWVARAVLPWSATSVARIWTRAMAETGSEAAALDRLAAAER
ATLAR
EAALACRGGTG
>OLS50160.1 4-hydroxytetrahydrobiopterin dehydratase [Rhodovulum sulfidophilum]AB14
MSDRLPVTVLSGFLGAGKTTVLNHVLGNREGRRVAVIVNDMSEVNIDADLVRAGSTLERGEERLVEMTNGCICCTLRD
DL
LVEVRRLAEEGRFDSLLIESTGVSEPLPVAATFEFRDEEGRSLSDIARLDTMVTVVDAANLLRDFSSRDFLADRGEELGP
EDERSLVDLLTDQIEFADVIVLNKVSEASPADLAAARKILRALNADARIVETDYGRVDASGILDTGLFSFEAAHRHPTWV
KELYGFADHVPEDAEYGITSFVYRADRPFDPTRLASFFETPLPGVIRAKGHFWIATRPHWAGEFSMAGSMVEVRGLGL
WW
AAVPEAHWPPEARERMAERVAGEFGDRRQEMVFIGTLGSMNRDRISAMLDRCLVPETRFCPELWSGLPDPFPQWG
EAA

>OLS50161.1 YggS family pyridoxal phosphate enzyme [Rhodovulum sulfidophilum]AB14
MGLEDIRARLANAEREAGRAPGSVTLVAVSKVQPLARVEAVLEQGQRIFGENKVQEAAGKWPGFRDRFDGVELHLIG
PLQ
SNKSRQAVELFDAIHSLDRPKLARRLADHAQEGGACPDLFIQVNTGEEPQKAGVLPAETDAFVAECRGMDLPVRGLM
CIP
PVGETPSLHFALLAKIAARNGLAGLSMGMSDDFEQAVALGATHVRVGSAIFGTRSNG
>OLS50162.1 hypothetical protein BV379_18985 [Rhodovulum sulfidophilum]AB14
MLPLSQTETRRRLDAAQRLQRAGRIEAAEAAYRSLLDGQPGLAAAAFQLGRIALGRGAAEEAAARFEAARKARPHEAPI
W
QMEAEALARLGDGPRNTRFLKEARKAGLPPALLSAIEARLAPEKIPEKARAKGTRRAPLGSAPPKAAEAVVADLRAGRAE
AAEAKALALTRAHPDSALAFNLLATARTERGRTAEARASFEAGLALAPDYAELRVGLGRLLVETGETAAGVRHLEAALAR
LPAHPPGQLALGKGLAQLGRKDEAEAALRRAVAADGRYLEAHLALAHHLMETGNPAAAEEVLESALAAGHDRAIIRVR
LA
EAQAKQGRTEAARAGFDAAIAAEPGFALAYSLRGMLLQTLDEFAAAEADFRQAIALEPRNGEHYRTMLATYKVTPEDP
LL
AEMEALFDDPTLGDESRMHLGFALSRAMEQIKAHDRVFTYLRPANDLMRARHPYDISERRAEIDAVMAAFEGTDFSLR
KI
EGAPDYAPIFVTGMPRSGTTLVEQIVASHSRVTGGGEIGYVAGEGLALLHEAGGGLRSLERVGDAEIAALGGRIEATFRE
RFPGSDLVSDKSIQTYMMAGLVRLALPQAKIVVVHRDPRDICLSIYKNMFAEGTHRYSTDLRDLGLYYRMFLEMIAFWR
D
KLPGGFHEIRYEDLIADPEAEARALVAACGLDWQDNCLNFHKTERRVATLSLAQVRQPIYASSMRAWQRHADELAELT
EA
LGDAIGGENGA
>OLS50163.1 hypothetical protein BV379_18990 [Rhodovulum sulfidophilum]AB14
MKKVLFATTALVASAGFAAADITFSGSAEMGIVGGGKNAPGVSSYKGNGDDSTTEFNNDFTLTVVGSGQTDSGLSFGI
SV
DFSTDDEPDANGNVSLDNEAVFISGDFGTLTLGEIDGAVDKRLTEVYNAIGQYSIGDEETVHAGFQGSYGDGAYDNQIL
R
YDYAFGDFGFSASMELNDDRSGADNADNGYALALSYNTDMGGVGLGFGLGYQYLETTASGWAPGNLEDYTGLTWN
AGTEV
DIIAATVSADFNNGFTAGLEYSNFDPDGPSGSNYGDIDHYSIGLGYSVNALSLGVSYGKYDADNLGDADGYALTAAYDL
G
GGATIVAAYSDSDVDGSGSADDFETYSLGVVMNF
>OLS50164.1 leucine--tRNA ligase [Rhodovulum sulfidophilum]AB14
MARYTPAEIEPKWQSAWEEGGTFTATRDAARPKYYVLEMFPYPSGRIHMGHVRNYTMGDVVARYKMSCGFSVLHP
MGWDA
FGMPAENAAMERGGHPKDWTYGNIADMRAQMKPLGLSIDWSREFATCDPEYYGQQQAMFIDMLGAGLVYRKNAT
VNWDPV
DMTVLANEQVIEGKGWRSGAAVERRELTQWFFKISDFAEELLEALDGLDNWPEKVKLMQRNWIGKSRGLQMAFDLV
DAPE
GFDRLEVYTTRPDTLLGASFLGISPDHPLAKSLEADNPEMAAFCAECRRTGTSEEDLEKAEKKGFATGLTVRHPFDETWE
LPVYIANFILMDYGTGAIFGCPAHDQRDLDFARKYGLPVIDTFEALEGGTPVGTEAFTPPKSEKVRWLKAYGWDETATG
D
EAVDAAIAHCEAKGVGQGVTKFRLRDWGLSRQRYWGCPIPVVHCPDCGVVPEKKENLPVRLPDDVTFDQPGNPLDR
HPTW
RDCACPSCGKPARRETDTMDTFVDSSWYYARFTAPHAATPTQMADAEYWMNVDQYIGGIEHAILHLLYSRFFARAM
QITG

HLPEKAIEPFDALFTQGMVTHEIYMTRDARGRPVYHLPEEVENGALKATGAAVEIVPSAKMSKSKKNVVDPVNIIQAFG
A
DTARWFVLSDSPPERDVEWTAAGAEAAFRHLGRVWRIAEAIAEEMNGGGIAAYEEEAPAEAARSANGPGAAERGGD
DLAL
RRALHKTIHDVTMGIESFGFNSSVAKLYAFTNTLAKSSASLAARRDAAMAMAQLMAPMTPHLAEEIWEMLGGDGLV
ADIP
WPEADPAMLVEDSVTLPIQINGKRRSELRVAKDLPKEEVEKLVLADDAVVKALEGGAPKKLIVVPGRIVNVVI
>OLS50165.1 hypothetical protein BV379_19005 [Rhodovulum sulfidophilum]AB14
MSSSDRRTFLVSGLSAAGLSLAGCGFRPAYGPGGTAEGLRGRIAVDAPDSIDDFTLVEQLETRLGRASAPRWRLSYRVA
T
ESEGLGVTAEQDTTRYNITGRAAFQLRDIDSGSVVQSGEVDSFTAYSATGSTVATRTAERDAYRRLMVILADEMVTRLL
A
GSGAWAK
>OLS50166.1 DNA polymerase III subunit delta [Rhodovulum sulfidophilum]AB14
MKLSPRDAAGYFARPDPDRPGLLIYGNDAMRVALKRQQVIAALLGPNAEEEMRLTRLSAADLRADTAALTDAVKARGF
FP
GPRVVFLEDATDALSDKILGALADWQKGDAQIVLTARQLPPKSALRKAFEAHPEAYAVGIYDDPPGRPEIESMLTQAGL
G
QADRAAMGTLLDLSTALDPGDFRQTVEKIALYKLDDPAPLSPEDILACAPRSTEAEADALIHAVAEAKTVEIGPLMQRLQ
AQGMTPVTLCIAATRHFRQLHAAAADPDGAASGINRLRPPVHFKARDRMARQAQLWGMHRLEQALGLLIDTDLKLR
SAQR
APQMALVERSFIRLAVMASRRT
>OLS50167.1 glutathione S-transferase [Rhodovulum sulfidophilum]AB14
MYRVIGSGNSRARRVLWMLEELGLGYDHVPALPQTEDVVAINPGGKVPVLIADGVPIPDSTAILTFLADRHDRFSHPAG
T
IERALQDSMTQFLLDEFDSALWMAARHSFVLPEELRQSAIKDSLKWEFIRSQEALVGRMGTGPFLMGAEMTVPDIILTH
C
GDWAETAHFPVSQPRLAAYLERMRARPAYRRAQAA
>OLS50168.1 NAD(FAD)-utilizing dehydrogenase [Rhodovulum sulfidophilum]AB14
MGNQNEAGAFARQEAGRLPALVVGAGPAGLMAAEALGAAGVPVVIAEALAAPARKFLMAGKSGLNLTRAEPFDRFL
AAYA
EAAPHLRPMLADFGPDQVCGWAEGLGQPVFTGSTGRVFPTAMKASPLLRAWLGRLAGQGASLRRRWHWAGWDG
AASVFDT
PDGPRRVEAEVTILAFGGASWARLGSNGAWAGPLADAGLPLAPFRPANVGLKVDWSAPMARHYGAPVKGVALRAG
SGGWH
RGEFVISARGLEGGGIYALSRPLREGAPLLADLLPDRDAAWVAERLARPRGKASLSNHLRKALGLDPARLALLMEFGRPL
PGGPALAALIKALPIAHAGPRPLDEAISTAGGLGWKALDSDLMLEARPGTYAAGEMLDWEAPTGGYLLTACLATGLWA
GR
RAAARLGRPV
>OLS50169.1 HNH endonuclease [Rhodovulum sulfidophilum]AB14
MTRPPICPLCDRPIPPGSRQSLHHLTPKLRGGKGGPTVLLHQICHDAIHVRLTETDLARDFASIEALRAHPGLAKFLAWV
ARRPPGFHSRTPGPRRRR
>OLS50170.1 enoyl-CoA hydratase [Rhodovulum sulfidophilum]AB14
MIGLRREGGLWTVTLNRPEKANSLTRDMLVRLEEIALEAQAEGVAALVLTGEGKVFSAGADLDEARAGLATDGVWER
VSA
AIAGLDALTVAALNGTLAGGAFGMALACDFRIAVPEARFFYPVMKLGFLPQPSDPGRLAALVGPSRAKLVLMGGAKLD
AD
EALAFGLVDRLVPRDELMATAAGLAADALAAAPEHRVAIKALCLGRRVAF

>OLS50171.1 short-chain dehydrogenase [Rhodovulum sulfidophilum]AB14
MTGMTGKSVLITGASRGIGAAAAWAFAEAGAHVALAARSQAAIGTIAQEIADSGPRAIALPCDVAEFAQVADAVRATV
QA
FGRIDVLVNNAAVVDPIARLDAGDPEDWSTAIDINVKGVFHGIRAALPVMAAMGGGTILNIGSGAATSPQEGWSAYC
ASK
AAVAMLTKCADHEGRGQGVRALALSPGTVATDMQKTIRDSGVNPVSRLDWSEHVPPAWPAQALLWMCSADADEF
LGQQVS
LRERAIRRRAGLPT
>OLS50172.1 hypothetical protein BV379_19040 [Rhodovulum sulfidophilum]AB14
MTLLKSASALAIATSFGLALPAAADVTADEVWSSWQALAQGSGQSMSGTESRSGDRLTVKDLLITSTDEQGGSVQVGF
DS
IVFAERGDGSVAIEVPENYPMSVEIRNAKGEVTELDFTVTQPDFEMIASGTPEALVQDFNSPEVTLTLDRVSGDGPADV
E
ASMKIDGMSGHYAQTEAEPRVFESDLAIDRVHLELTAEGRNEDTDVKLVGTVHDVSSTSDSRMPVGIDPTELAEMMR
AGL
MSQGELEHGAMAYAFDLIEDGNKTHAEVSVNSGRIAFDLGGTGVSYDASGNETRMALYDPQLPVPEIEAKMAESGIH
FEM
PLIKADEPSDFGLGIRLKDLTVNDEVWATFDPTEQLPRDPATLLVDLAGKTRLLTDFTDADVTESDGIPAELDALNVKDM
QLKLAGADLTGSGAFTFDNSDKVTFDGMPLPEGSLSLRLEGANALLDKLVAMGLVPEDQVMGLRMMMGMFARPGD
GPDTM
VSEITVSDGGQVRANGQRLR
>OLS50173.1 modulator protein [Rhodovulum sulfidophilum]AB14
MSDDPIPSLTQALLDAARQAGADAADALVTAGTSLSIDVRDGRLEQAERSEGIELGLRVLIGRRQACVSASDSRPETLAE
MASRAVAMAREAPEDATVGLADPSDLARNRDAGFLELADPSPEPDPAALEDDARRAEAAALAVPGVAQVQSASAGY
GHRR
IMLAASNGFSGSYARTSRSTSCVAISGEGSGMERDWCAESRTFQAELPSADEIGRIAGARAAALAGARRPKSGAFPVLF
D
ERVAASLIGHLLSAINGTAIVRGASWARDLIGRDVLPAELSLIEEPHRPRIAGSRPFDSEGLATTRRALVENGVLTGWVL
DLASARKLGLSSTGNAVRPVSGPPSPAAGNVTLSPGSLDRDALIAEMGTGLLVTSLIGSTINPTTGDYSRGASGFWVENG
RIAYPVNECTIAGHLPEMLRRLQPANDARDHLSRRVPSLLVEGMTLAGA
>OLS50174.1 3'(2'),5'-bisphosphate nucleotidase CysQ [Rhodovulum sulfidophilum]AB14
MPARDLALLTTAALEAGKLARRYWCRDPQTWEKPDHQGPVSEADLAIDRMLRQMLTAARPGYGWLSEETEDDPARL
GAER
VFIVDPIDGTRTFLEGDRSFAHALAVAKAGRVVAAVVFLPLRDKLYSAIRGGGAYLNGAALRTGSRSALDGASVLAPRPT
LDPGHWTGPVPHLHRALRASLAYRLCLVAEGRYDAALALRDSWDWDTAAGSLILEEAGARISDRLGDPLAFNTPVPLSR
G
IVAGAPLLHAEILSRLA
>OLS50175.1 inositol monophosphatase [Rhodovulum sulfidophilum]AB14
MTQRLHLVFGGELVDPTHTVFKDVDDIHIVGMFPDYETAYNAWKAEAHRTVDNAHMRYFIAHIHRLRDEEVEAGPTE
ELG
A
>OLS50176.1 hypothetical protein BV379_19060 [Rhodovulum sulfidophilum]AB14
MTANRAERDAEPGGRSDAGPLLWLHNPPPEDRSTAAELIRELAQEQPDLRVLVTGRDAAPDWPARAPGLPEVIVDIVP
SD
GHRASAAFLDCWRPDLGVFYPDHLPSTLISLANTRGTALFLICRDLPRSWRSRWRVGLAGTRRLLRRFDRIFAQTPGTA
G
ELRALGLPRWQISPCGPLSEGSAALSCSEAERDALAAVIGGRPAWLAAATRPEEDAAVVAAHAWTTRHAHRLLLILCPD
Q

PGRGPALAARLRADGWDVALRSDDEEPSTETQIYVADTEDEAGLWLRLAPVCFIGGTLSANAGPDPYRAAALGSAILH
GP
ATGAHPDHYRRLSNALATRTVQDGASMAEVLADVLAPDRAAAMARAAWEVSTQGTMATERVVRRMIEALDMTETV
>OLS50177.1 tetraacyldisaccharide 4'-kinase [Rhodovulum sulfidophilum]AB14
MQAPAFWYAPPGRPGLMARALTPLGRLYARATAKRLAQGEGARVGVPVICIGNINAGGTGKTPTAIALTMRLIARGV
AVH
VVSRGHGGRLTGPVRVEERRHSAADVGDEPLLIAAFAPVWVARDRAEGARAAVAAGAQAILLDDGFQNPALVRDLSL
VVV
DAAKGFGNGRTIPAGPLREPVAAGMRRADLLLSVGPEPAQRQFTESWGGQVPVPRITGALRPLPTGMDWQGLRALA
FAGI
GHPEKFFATLRGLGAELLRAEALDDHQPLTSALMTRLETEAKLLGAQLVTTEKDAVRLPASFRRKVLTLPVRLELDDWSA
IDAAFDRLGLGSGR
>OLS50178.1 CoA transferase [Rhodovulum sulfidophilum]AB14
MPAPLSGLKVVELARILAGPWAGQILADLGAEVIKVEAPEGDDTRRWGPPFVTRDDGTREAAYFHACNRGKRSVTADF
RT
PEGQALVRGLVAGADIVIENFKVGGLAKYGLDYAGLSAVNPRLIYCSITGFGQDGPYAHRAGYDYIIQGMSGLMSITGET
DGQPQRVGVAVADVFTGIYAATAILAAVHQRAETGRGQQVDMALFDVATAITANQAMNYLTTGTAPGRLGNTHPNL
APYQ
VFDCIDGHAIVAVGNDAQFGRLCGLLGLPELAADPRFASNADRVAHREALTEALTEATRVRSKADLLAACEAQGVPAG
PI
NRMDEVFADPQIRHRGLVVENDGLPGVRTPIRFSEAGLVLDRAAPRLDQDGAEIRAEFSAPSPAPAGRTPRR
>OLS50179.1 hypothetical protein BV379_19075 [Rhodovulum sulfidophilum]AB14
MIRKLTLSLGLAALSTLGLGLAPAPLLSSAAWAEETPDVAPFTLGDPNAPIELEEFASFTCGHCAHFHEDVFKRLKADYI
DTGKVHFTYREVYWDPYAVWAGLLARCGGEMRFFGIVSMLYEKQKDWIDPKDPQKTSENLRRIGQTAGLSEDAMNQ
CLTD
AALAKALSENSDKTVDAAGVTGTPSMVIDGQLYKNMSYRDLKEILDEKLAG
>OLS50180.1 RNA-binding protein [Rhodovulum sulfidophilum]AB14
MTGMQRKAEKDSRRMRGFERASMLLSGRIRRAGEKRGFAVARLLTHWPEVVGDEIARVCRPVEVRYGRKEGFGATLT
LLT
TGAHAPLLEMQKEDIREKVNACYGYAAISRIRLTQTAPTGFAEGQAAFAPAPRPAAPPPDPGTVAVASDLTVEVHDEGL
R
AALQDLGTRVLSRPKR
>OLS50181.1 A/G-specific adenine glycosylase [Rhodovulum sulfidophilum]AB14
MRDETLGAELLAWYDRHARDLPWRVGPAARAEGAQPDPYRVWLSEVMLQQTTVAAVKDYFHRFTTRWPDVGALA
AAEDAD
VMGEWAGLGYYARARNLLKCARAVASEHGGRFPDSHAALLTLPGIGPYTAAAIAAIAFDRPETVVDGNVERVMSRLYA
VE
TPLPTAKPELTAHAARLTPVERPGDHAQAVMDLGATICTPRNPACGICPWQAPCRARTAGIAAELPRKLPKKPKPIRFGI
AYLGRRSDGAWLLERRPASGLLGGMLGWPGTDWAETAPAPAPPVEADWHPLGAEVRHTFTHFHLRLALCVAELGPD
CAPG
RGEFLPPDAFRPSDLPTVMRKAFDLARTASATA
>OLS50182.1 epimerase [Rhodovulum sulfidophilum]AB14
MKLLVIGASKGIGLETVRYALGRGHDIRAFAPSAQSIEIDDPKLEKLTGDARNAAEVKAAVEGVDAVIVALGLPKTLPAM
MRKTTLFSDTTRVLLSAMTAAGVRRLLAVTGFGAGDSKAKMSTVEKLGFKAVLARAYADKTAQERLIRDSGLDWTIVR
PG
ILTSTAPSHDYEVHVEPETWHNGLISRGDVAHFLVHAAEDGSYLRDAPVLTR
>OLS50183.1 DNA modification methylase [Rhodovulum sulfidophilum]AB14

MTRTKGAPELPLNTILSGDCIAEMNSLPEGSVDLIFADPPYNLQLKGALHRPDNSKVDAVDNDWDRFDSFAAYDRFTH
DW
LAAARRVLKPNGAIWVIGSYHNVFRMGAELQNLGFWILNDVVWRKSNPMPNFRGKRLTNAHETLIWASKSEGAKYTF
NYE
ALKALNEGVQMRSDWVLPICTGHERLKDDKGDKAHPTQKPESLLHRVLLATTNPGDVVLDPFFGTGTTGAVAKMLGR
DFI
GIEREEAYRKVAERRLAKVRKFDREALVVSASKRAEPRVPFGQLVERGLLRPGEVLMSPRGKTAKVRADGTLVGEAVTG
S
IHQVGAAFEGAPSCNGWTYWHYHREGQMVPIDVLRQQVRAQMHD
>OLS50184.1 ribonuclease HII [Rhodovulum sulfidophilum]AB14
MTAVRPDFSLETEAFARGVRLVAGVDEVGRGPLAGPVMAAAVCLVADDIPDGLRDSKTLSARRREGLAAEIHARALVG
LG
EASTGEIEEMNILQASHLAMCRALAALPAMPDLALIDGNRMPRALPCDGLCLVKGDARCLSIAAASIVAKVARDAVMT
EL
AQHWPGYGWERNAGYPTRQHLQALRDLGVSPHHRRSFRPVHNILWQEKSVNP
>OLS50185.1 hypothetical protein BV379_19110 [Rhodovulum sulfidophilum]AB14
MRSALDPRARLGQDRFMHIRTGLLCLLLCLVPLRVAAEDVLLRSSSLLPAAYDSYLALNLKLPPQNRTLPLVGDRGLRSG
GQVLRHLAGQQAVNGFDGVLYDNRDHGHSSLPEELFPRLDRLVYDKTLRKARADYGLAGKILLPAVVFGNSSTSVTTGP
A
KRSLPRLAMTSPGGPERSAQLYENNHLYVYPEHRDHDETDRFPANWPYMLISQGSSGSDQPFLAALAMTLAAFPPDTF
AA
LRDNRLIAPTLQMILRRGYAPVRSRAAYFTAAAHPVVFEGDALRPGRMMGEAAAMRPDAIPPMVRLRVEQDGFGEA
EGLA
RLSERLFDTAGAIARIWRNFDWSREITVSAAATRDPNGRALTFTWALLQGDPARVRIEPLDPEGSRARIRLAWHDPKTV
L
TRDRTGYHNRSLSRVEIGVFASNGIHDSAPAFVTVFFPDHQKRVYATSGAGGMRLVSVDYDAIGRDAYYDPVLFWSAP
WT
DRAEYDGAGARTGWTRIGHDGNTRTLPPEIGEGVYQIERLQPERPYLIMP
>OLS50186.1 lipid A biosynthesis protein [Rhodovulum sulfidophilum]AB14
MAIETVFGWLHVHSWVEFWWVVVGLGGQLMFTARFLVQWIASERARNSVVPVAFWYFSMAGGLILLAYAIYRKDP
VFILG
QVTGVFIYTRNLWLIHAHRRRNA
>OLS50187.1 dolichol-phosphate mannosyltransferase [Rhodovulum sulfidophilum]AB14
MPDVDKPSAIRFKRGQSLRCRKEACMPAVSIVIPMKNEAENVAFLLGEIAEACAALDSHEVIVVDDGSTDATAAIVADM
M
KTDPSLRLLRHPNSGGQSAAVHSGVLAARAPVVCTLDGDGQNPPAELPKLWAPLLADDTGRLGLVAGQRVGRQDTAS
KKW
ASRAANAIRSRLLKDGTRDTGCGLKGFRRDLFLTLPYFDHMHRYLPALFRRDGWEIALVDVSHRERHAGTSNYSNLQRA
L
VGAHDLIGVSWLIRRRKKARAAEELPHGN
>OLS50188.1 LacI family transcriptional regulator [Rhodovulum sulfidophilum]AB14
MTADPPDRPRRPLTLRDVSEASGVSEMTVSRVLRQSGDVSAKTRARVLEAAKRLGYVPNRIAGGLASQRVNLVGVIIPS
L
SNMVFPEVLAGINEVLEDTPLQPVFGLSRYDPPHEEKVLFEMLSWRPSGLIVTGLEHSEATRAMLRAAGIPVVEILDLDG
EPIDSVVGISHRRAGAEMARAIAAAGYRRIGFLGTKMPMDHRARKRFEGFTGALARAGLEIVDQEFYSGGSGLEKGRE
MT
RALLDRHPDLDFLYFSNDMIAAGGLLYCLERGFDVPGKLGLAGFNGIELLSGLPLRPATMDIQRREIGRRAAKIIADCVE
KGLPGKGERVALSPRIDPGDTLRADLARCGQAVRDQV

>OLS50189.1 hypothetical protein BV379_19140 [Rhodovulum sulfidophilum]AB14
MRRVLPTEGHLPWSRDDIAKFRARWPHATSQRLAFELLFWTGARMSDAVRLGPGMVDADGWLSYRQSKTGGKVDV
PFTAA
APEWAEPSDRVHLFSALREQADRHAVWMVTAQGRPRSIKAASQWFSAAARAAGITGGRSAHGLRKTRAQIMAERG
ATPDQ
RAAWLGHESLSEVQHYSRGADRRRMLAGTDPEPQSSNSSDRVPTWMRK
>OLS50190.1 hypothetical protein BV379_19145 [Rhodovulum sulfidophilum]AB14
MHRTCAPVAQAPELASPATPYTRAAFGTGWVDSDGDCLDTRAELLADLSTVPVTLAPSGCSVRHGRWIAPYTGQVVT
EAG
DLDIDHIVPLRYAWGHGADRWSAAKRARFALDPVNLLPVTASANRSTGCRPIKDSAASMCCGSGGSPRPMGWRSRP
RKNG
IWWR
>OLS50191.1 hypothetical protein BV379_19150 [Rhodovulum sulfidophilum]AB14
MRGRRQALYRPDFALVVSGAVRCLVEVKEPGANLDDHEWQVRAYAVLLNGEALDDRPVRYYLLTNGTETRVYHTDRN
RPL
FTVPFTGFAEGTARFDEMRGLLGRNSIVTNVGRSTETLEMERPTISEVNAAFSWCHQHIYRKDNISQSDAFSEFVKLISL
KLLSDRRIRDTNPEVLELDTFDIAASDVQFSQAWIKQNEPNAPNPVSDILFRRFMDDMERDIARRTRKRIFERDSRINLR
PETIHGVVKRLEKLFLFGIDADLNGRLFETFLNATMRGKDLGQFFTPRSLVKLGLRLSQLKVDVPRPDGTRHTDLVLDAC
CGTGGFLIDVLADMWEKVDARADLADDQKLEIKHSVANEAIVGVDIANAPILARIARLNMYLHGDGGTRIFHLNALDP
RL
ADADTDSPETINEKAELRRLFLRSQFDVVITNPPFAKALDRSTPEEVRILDSYEIGRDRINLGGAVRSVLLFLERYAQLL
KPGGRMITIVDDGILSGDDYRWFRDKLRSWFLIKAVVSLPGDAFQRSNARVKTSYIVFEKRSTAEQENPPVFMYPCQFV
G
NDDPKRQRPRAGDAELRELANREIESVMRAFDEFQQGCGDPKFTVEASRIQDRLDVKNCLMSPGRQEAVWVDQGH
AIYPL
HEMLEERLYDDDEVVTRDSADPVTVMVVRYEGIAEAADEMNPADSSYAKLFPVHAGDVVISNIAASHGSIAVVPNELD
GC
VVSSEYTVLQARPEFDPVVLQMILRSPEIRSDILLSSSGANRTRTRWDLIRDLKAPYPSDVMANTIRQMADRAEEAKREA
ARLLSDARASIQEQLQLETGDAEMVLTAFKPPK
>OLS50192.1 hydrolase TatD [Rhodovulum sulfidophilum]AB14
MIDLHCHIDLYPEPASILREVDARGTYVLAVTTTPKAWRGTGKLVGARKRVRVALGLHPELVALRYSEVALLCELLPEAR
FVGEIGLDGSPAHRSSLGLQQEVFERILEASASLGGRIMSIHSRGAATLVLDALERHRGAGVPILHWFTGTVSELERAIA
MGCWFSIGPAMFQSKKGRDLALRMPLKRVLTETDGPFARNGQNAIMPWQAYDCLVELASLSGGEPDELRQQVQEN
FRSLS
TAR
>OLS50193.1 hypothetical protein BV379_19165 [Rhodovulum sulfidophilum]AB14
MTSVACLPQDLPDIPDTDIRFDIYTNSRTPGRGRVGDLVPGRLRRLGLFPSGAAWDFMSFAMAVVAADEAVSRVRSPD
GW
TRKIDLTVAVSDPAFWNSESERLTQALRFLSGDMWSVQFIAGGGMPFPRGPRRPRLEDMICLLSGGMDSLIGAIDAVS
EG
RQPLLVSQMAKGDTQDQRTFASSVAPRNLHLQLNHHARPPCASERSQRARSIAFLGFGVMAATCLQSHADGEIVELR
MPE
NGFISQNVPLTPLRTGSLSTRTTHPYFLRLIQETLDAADLRVHIRNPYEFQTKGEMLKGCQDPALLAALVDASTSCGRYS
RTGFQHCGRCVPCQVRRAAYVAWGVPDRTVKGYKYEHLGQRDARHAGFDDVRSVAIAIETVRLQGIAALIGGAMNV
QLLG
DPTPYRHVVKNGIAELAVFQAKLGVT
>OLS50194.1 hypothetical protein BV379_19170 [Rhodovulum sulfidophilum]AB14

MGTSASSRGPGAGPDIPIVPPWVDDPPGQPNQPQPQPQPQPGTPVQPAPGGPGHAPQQPPASPQQPQVPPQQPP
TPSQPG
RWRGARTSLGKFAGGGDGRDKHLKRGLGHYSRSGMGGSRNAARRMGGSAKAASVLHGALTSLASGQPLPQELGIDP
QTLA
GRTPVELADILVDAIRPIDGTQDAEVTRDSVARALSDILAQHNDLTNLTTEQVDQVTASTLGYDVAHRIELDVGKAIIDK
APTKGEGLERLQEMKDYIREVVAAEYAAERARGSVVGHSSIERISREAIQQTFEVFEEEAGL
>OLS50195.1 hypothetical protein BV379_19175 [Rhodovulum sulfidophilum]AB14
MWSDNETLRDLVNFSHVAEIAAERIVGAKGEPLSLGISGDWGVGKSSMMKLLRTSLEERDDVNIRFVEFNAWLYQNY
DDA
RAALMETITAAVVDQALAKKDTLGEKALKRAASLLGRVKKLRVLGIAGSALFDYMTQGSVTPFVAGGISAVSGLLDGEVT
GEDLRKVKELAAEGIKDGKELIKEGDEAEVGAETPRKAIQAFRDDLESTLADLDITLVVMIDDLDRCLPKTAIATLEAMR
LFLFLKHTAFVIAADERMIRQAVRSHFEEVALNDDLVTNYFDKLIQLPIRVPPLGTHEVRAYLMMLFVDAEASLELEQKE
DIRLAVCRRLGRTWKGERVDKSFVSGLMQNCPPGLLNRLSLAERIAPLLTTSPNIQGNPRLIKRFLNTLSLRSSMARIQE
VVVDEAVLVKLLLFERCGDKDAYAELLRAVNESENGLAEFLADKEASARGDAEAPPLEKPWNDAFHQNWLALDPPLAE
KD
LRGALYVGRESHPIISRADELSSEALETLDMLLLMTSASATVDAEVARLTNQEIGRLTDKVIAKARQETSWGAPKILNAL
QSLAGASPSAEASIVAFFSEVPSSQVKPSIVPRLSQVEWGKRVLAEFFTRPDLPTPAKNAIGKVGKSK
>OLS50196.1 hypothetical protein BV379_19185 [Rhodovulum sulfidophilum]AB14
MTKIKFIDARFADPDENEFALKRRFEWQLRKLRALQDGVSPKVPGDFEDELRWNEDLIAMPLSEKDRNKVLRRARRVM
GA
RAKASGLSHLSSDDRRALEAFRGGADLVGIASEHRADEIAAAIHAEMPWMAAATDFLWKAMRRSVRVGEPGFRLPPV
LLD
GPPGIGKSVWSRELGRHLRVPRCGIEGTAEQASFVVNGSQRGWGSAFPGRPLQTIVQSLCANPIVVIDEIEKAGTPTSTK
GQAYGLAEGLLPLLERSSAVAWKCPYYQVGFDMSWISWLLTSNSLSTLPAPFLSRLEILRLAGPGKGDLISFAEREGARR
GLSEAALGAICEVIDQVAEAHELNLRHVSRMLSRAEAMASSPLSH
>OLS50197.1 hypothetical protein BV379_19190 [Rhodovulum sulfidophilum]AB14
MAGALAVPERHGDTAALAAAWADQTVALRLAGAGHAPPEIGAHLRRSFLGAMGRAASPAAEAGRPCDWDPPCALD
VFRRE
QLRGPKGDGLPKPYVLAAWSDGPDLAVTLRIFGMAIDWAMAAAEALVAGIGTILPWGRVLPGAGGTPVIAGREIGLYR
IV
ADPPPNAARLVFLSPMDATGTDAMAQPWTILSRALRRADALSRWNGMALDDEVAAAIAAGLRDLEYDVTGLRPGRY
ASPN
RHGQARQDPTLAGQLIVRGDLSDIWPVLRIAERAHIGRHAVEGLGRIRLETVEAPPPCTKEE
>OLS50198.1 hypothetical protein BV379_19195 [Rhodovulum sulfidophilum]AB14
MTSLKQSRRDDRRSGSLRLAGVTLVTLTLAGLFVYFVEQTAAALLPDLKTSPDFFSAAGLIAFIIAGVFYWQHPRIMRQI
NAKVRTVDASGLTEQPYLICGYSPLKVDFPCLETLTRDLDEACAPTGPGYVLNSWQQNLRVLRKIPTIQHVYVLENDKYQ
FEEFRKVLRHFFPEDRLKITLIEAEQGGTTFKDDVKATLAPDYEDLRYVRRGIDRAIEMIARARREKEDDVEKLCIIDVT
GGLKTFSIAAAIASLNRDLVFVYAGTGERTGDVLGYDATIELALRFEF
>OLS50199.1 putative CRISPR-associated protein [Rhodovulum sulfidophilum]AB14
MTAYFVTRHSGALDWAKAQGIDATPLSHLNPASIARGDTVIGSLPVHIAAEVTARGARYLHLSLDLPAGARGRELTAEE
M
ARYGARLTEYRIKEADP
>OLS50200.1 CRISPR-associated RAMP family protein [Rhodovulum sulfidophilum]AB14
MTLSAPYSFVPLSQKVFLPDEAKLGVPSQDAPVEGGVSGTIAFTLTSDTPLLVGGANDKKGEKTFMKAPGDCPVIPGSSL
RGMIRNVMEIATFGKMQLVDDQRVTVRDLQHGAKEDYRDRMSGTIDGAYAPRSFAGFLKFVDGRPTLFSCEFGRIDH
RDL
EDLIDRSRLPKVEVDDAGTMRRKTLHEVFETISDGDSPAENGIAITQTEARFVEGRFLELGGKLEHKDLWVQTGRAHHL
H

RDDKKLAYRRVAASMESATRETNMPAQKIPGRLVFTGMPSDKKHMEFFFFGRRDDLACEISETVWRKFLNAHEEQEK
ESI
TWKWRKLALNRGEAIPVFWLGIRDERSISVEHLGLAMMFKLPADNSIHDMIGRESPHLDEPRPDLATRLFGRIADTTKE
T
GMAATQPGFKTRLSFSWAILTTPREQYSELEKRTPRARPKPGYVPSYVRQRDLQANGQLRQIGMRKKKPIYAPYRSYM
NK
KDGQADRIRGWKRYPARPVPEGAPVPDVPEGAEGNAVILRPIQARQGELTFKGKIRFHNLHPAELGALLWVLTWGGN
SEL
VHSLGSGRDFGWGQVRFRIGGISYAYGPPSTDPVMEFRQMMDEWAGRAWEDLREVRQLKAMASPAIGAEQMAEL
QQMKLT
VGEKDESPFVRAKRSGQALPEYPMWRDGKPEPAPFRSPVTNPSNTSTRKAFYFGEPVQILRQEGDKAWIRYEGDEDGE
PE
RVLLKHLTFR
>OLS50201.1 hypothetical protein BV379_19210 [Rhodovulum sulfidophilum]AB14
MKAEETINALKGLVQVNGLAAVDLDVPLSEHLTDPTDVEARIASWTGTGWIARQSGVRTIVSGAGDDTALGQPVSAEI
VD
ASGVSRQLRRVVDGWNITTVKETAGTSHLAETVRHVALDHRIVVYRRYWSLPDDGAAEVVAWRFVGFEEFKS
>OLS50202.1 hypothetical protein BV379_19215 [Rhodovulum sulfidophilum]AB14
MTSERFFHVRRFTLEAASPAVISSGGDDPLFDNLLARDANGLPMIPATTLAGALRARLEEADARRVFGFQERESGQRSA
L
TFTDALAHWEDDRPRDGWAVLPGSDAIVALLTRESPVTRQHVRLNANGVPDGEGKFERSAVPRGTRFTFEISCWGDD
ADL
KRIAAMVRKGLSLGGAKRSGYGAFLCIAEGQQSFDLKNPSEGAGYRNYASAPLGASAGFVMDPAAQEESGPTGWVID
GKI
EGPLLVGAPRSDGRGGRRPYGEISIDWQSKPAMVKHAEAVLPGSAVKGPLRHRVLFHLTRCGVGSAQDIVEDVFGAAA
AG
PRGGAGKLRFHDGQISGGTVIPVAHVSLDRFTGGARDKSGALFTDDMIWSPRLEIRIDRLEALSPEAENAFALALEDLKA
GLLGLGAEWGEGAGVFGFETCTIKVPEDGAAIP
>OLS50203.1 hypothetical protein BV379_19220 [Rhodovulum sulfidophilum]AB14
MSVLGVVVTLRDPVVIPAQAATATRPQSLSYIPGATLLGIAAGRLYGELATQDAFAAFHAGAVRFGDAFALADSGHICLP
VPISLHEPKTRGDKAPSATDERVRDFAAAERGQDFVQIRGRDCVMDGCARFHELGREASMRTAIAAETGLADEGQLY
GYE
ALSAGQRFRARLDGDVALLARIGKALKGDHFIGRSRTAEFGRVRIEIEEASYWQLASGAAARGRRFVWLLSDAWFFDRY
G
FPTARPDADMFCPGCAIDWAHSFIRTRRVAPYNTAWRARAEERTLVTRGSVITLENCSLVPGLQSFGMGQERGLGMA
FVS
SHPIGEVLCTDAGAVTPPAPACPPLPAPTAFTNWLKGKQAGIQSRATAAATSDDDLQTLRGLYRRARHIAGEPVGPTAS
Q
WSGLATFLASDIQTGSLLGTREDRDSRLWGARYGLGEGDTGTFRDFVTTILKAKDGRTRLRLLAQKARRAIEEEEWLDP
Q
RDERGAVNDQ
>OLS50204.1 hypothetical protein BV379_19225 [Rhodovulum sulfidophilum]AB14
MTVILKLRILSYWRAGTGRNRPGALDALCQRDHDGLPMIPGKHLKGLLRDAVRWAHGVGWTPSLDETTLFGGLDAQA
DDG
LNGTRGGCIRVDSALLPDAERAVLRENPDLIALLFDTRRSTAIGPNGTAEPGSLRFEEVAVPMTLEARLCAVGAVPDGW
E
SELEKALTLVRAVGAQRTRGLGRCILTLEKRGAA
>OLS50205.1 hypothetical protein BV379_19230 [Rhodovulum sulfidophilum]AB14

MRSAFLFEIAGIQSYVFATGKLRDASGASELISSLAHEDLSDGLVSEAGLFAETIRAASLTGKIRVFRCAGGVVDFTCDT
GQTHALYRFRALWRLALSLSAPGLAFRDAVSEGADDAAARGAARRAILQAPPPAHADAPPSVPIVRPAALTDAAPAIW
RT
ANRADGRCRITGDYACAPTLAKRTHLDQGSDDLARLFTAGDAGNYDWPLTFEPDPGRPQDPVFPFPGKVKRVALVHI
DGN
GVGRFFAGLQGKLSDVRPVSAALAQATKDAAAEAMIAVLKASVPPPGGGKPVVPARPILLGGDDLTVLLRADLALDFAR
G
FSAAFERKTEELFGQIEGLQGHEGLTAKTGIVFMGPKQPFVQAYELCEDLAKEARAETQSRVSFWRLTGAMIPRDADEI
K
ALTSRDGMTFWRKSWAWDEFAKLEDLVEVMSSDEIGRGPLRRVPELAQAGRTGSGPAPEEVYRRALNVLRNRAPAIH
AGF
LNAMRELGVDHAETRFDADGYVPLLDAHVLGKLAEDQAA
>OLS50206.1 CRISPR-associated endonuclease Cas2 [Rhodovulum sulfidophilum]AB14
MSRGEMLTVFSYDVSSDKRRRKIARLLEDAATRVQYSVFETRMSRARAEALCQRLAAHLGEGDSLRLYVIGHDGEQRSR
V
FGDGAPFESDEGYWLF
>OLS50207.1 CRISPR-associated endonuclease Cas1 [Rhodovulum sulfidophilum]AB14
MPLSPKYDLWQSLLSLRRLDRAWEKVRANAGAAGGDGVTVAVFQAGAGPRLGRLRLALKRRDWRPLPYRMVDIPKK
KGGH
RRLMIPSVADRVVHTALAMVLTPVLDPMFEEASYAYRPGRSVQQAVREIGRLRDAGYWHVIEADIVGYFDAVAHGPLL
DK
LAAALDGWPGAEDILDLVALILEDQGQETGRMGRGLAQGSALSPLLANLYLDELDARIDGRGVRIVRFADDFIVLCKKRE
TAEDALREVAEVMGELGLELHEDGTRVLDFDRGFEFLGQLFVRSLTLPQPNDPEEEVVDLLRQLGTEDMRQTESREAER
K
AGYDRGARVLYLTVPGRRLGMANLSFCVTGEGGRELGAIAHRRVDRIELGPGTAADSPVIEHALATETDLAFVNRFGET
L
GVLTARPGQSARLHLAQASAALDSGLAAGFARALTEARIRNQRTQLFRLNRTRDDAEVVAALARMQRQLRKLADLAEV
AE
IRGIEGACGAEYWPALGRLCEDARQPFRRQRPAEDPLNAAINYLSAILERDTRAAVLAAGLHPGFGLLHRPQDRAEACV
Y
DLMEPFRAPLTEGLAVFLFNARRLRPQMFAGVGGGVRISTEGRRALIAGYETAVARRVNVPDRSHKLAWRPMMRRQ
AQDL
AQALRAGDPALFRPYRMEA
>OLS50208.1 hypothetical protein BV379_19245 [Rhodovulum sulfidophilum]AB14
MIRRLPILVALVPGETPASFASRLAWANGLSFASEFCADQGFALQSLADGEERALSILAQLGGVDRAKLLRGLVTKTSTG
QYLVAGQLIEKRWITRRRLRFCPKCVAEDIAASAVGGAAQRCVWHIPSIRACPQHGCRLHEDDSYSHSRGPHDFAGRIR
D
LGLEPEQLEQLVVPQAPTGLESYLARRMAGERQDRWLDDLGFGAVALGAEILGIVDLFGVGAKPKFLSEGDKSQAGAR
GF
EILSRGERGLRDLLEAIHRDAGSPVSLSRRHYGCLFNWLEQSHADQYEPLRTIVRDFMAETYPIGEGDMLFGKPCAARRV
YSVEALRRDLGLGYRQTLELLQANGFLTSGGDAGKSALLDARALKPIVERFTGAICQVAAQRRVNAPRAQFDALVAGGI
L
SPIAGSPVGRPYYDEGQLDAFLASIKPRVELGDGDCGAERLVGIQTTCRKAVAGAADVVRLIQKGALKTVAKSSKQSGYL
SVRLDPQEVAEALRLLKPEGYLRSTLARYLGINESAVRYLVETGYLTQVRARHPLSRKSIKVVTYESVDHFLERYVPCKH
LAEFEGEGTRSVGDQLEAIGLKPLALSPAGKGRVFLRADVSCGREEREKGPRFRHHDEIAWPDL
>OLS50209.1 hypothetical protein BV379_19250 [Rhodovulum sulfidophilum]AB14
MTRISREKVSHIVADLESRFIAHDAYRDLQDQIDDILYRRKAHMAAGRMPAERGIVVIGNAGAGKTVSTKHALEQHPKI
S

YGSPLREALTVRLETGATIKGVGVDTLDAFGYESKSERNAHSIWQQVMRQARAHKTLFFHFDEAQHILRSKSKHDLDQVM
LVWKSCLEYATWPVSLILSGTCELLDLINRDDQLKRRFEPVHFAPMTYAAHGHEVVGVLEAYLSAAKLGRGQSFQAASSV
QRLLHASRYEFGLTTRLIIEAIKIALLEGSASVEQAHFATAYRKQKGCVDALNPFIVENFRAINTGQLLGGADLEAPPVKTRGAA
>OLS50210.1 hypothetical protein BV379_19255 [Rhodovulum sulfidophilum]AB14
MNEIIRLEPYSQIRHGGKASLVVDQGPLGVTVTREADGKIAFDELTYDEINDGLKTGHIVVLDGYHSEEAARQRTKFGEQ
VVSQLPRKQRGKVFFRQTWCDFFLDAEAREEIGRDWRDVDAWILKVSPQIARHIPQPEGPAQKRKYCGKKRSRNEIDPPS
SSALLRWVRKYQDAGRSVLALIDKRHLAGRTKVKFGPESAALLRKCADRYASFKRPSKKSVIRQTKRAFRLANAFRVDRG
LPALEVPSESTIYREINRLEPYYVMCQRQGLEASNRHFAIIGEGLNVLRPGERLEVDEWKVDLITFFAEAGVFGHLTEEQ
IKTLPKGRRWIYVVIDVRTRLILSVKVCAAPNTEDAIAALDLATRDKTELAKLFGCKLPYWPGTGFETVASDQGVAFANF
DFRAAVTDLRGTVMFPPAGVPKLRGHVERCFRTFGVELMQCLSARTYSNIVEKGDFEPEDYAALADDELTRILVRFVVDV
YNNKPHGGLGGQTPLNCWRELCSTVGKPRQPGRFERLGAFGRKYTRTISGRGVRFAGIFYTCQAVEDYFLSNPDLEVEIR
VDHEDLGLIEVHLDGVWVVARTRAKGFDGLSLDLWTEACTNLRRRFEAEAELTQDVIDQALADFSEEDRAASQRLGISAL
APTRADVERNRDQLWLGLDVVAGHSPAVSRKKRGLLSGVIPKAETALPAPLTVLPSEEDVVSAPSSQRPRWRMEEGE
>OLS50211.1 hypothetical protein BV379_19260 [Rhodovulum sulfidophilum]AB14
MEKESFVAPRASRSTRNLSERSKARTYVETGAFRPYGGESQKVKFDSLGEFDAFVTTTAIYRVADMVEQPSAIEYVDAEG
RPRRKVIDQLYTFADGTKVAAEVKPAARAKAQNLEEQLSFVAAAMPPEFAETLVLITEENYDQWEALNARRLLAYRREDD
PEAASIIAGLAAELELPVTIRELIARAAIGGRAWPEVFKAIYAGVLRPLEDGIIDLEMLVEGASK
>OLS50212.1 hypothetical protein BV379_19265 [Rhodovulum sulfidophilum]AB14
MDANKMRQQIVKLHLGVSGTTITEFAESIGYSRPAVSNVLAGRKRSPRIEAAIAERTGFRADDLWPAPGVRKTGGQT
>OLS50213.1 hypothetical protein BV379_19275 [Rhodovulum sulfidophilum]AB14
MTASEARPDGAALAAAGLAVLMWGAVPVGTRYLVAPGGEALPPTLLVALRMVISTVVLLPVLLRARPWAWPSEDKRLAAL
SAGLGIVGYNLPVTLGQVEASAGVTALVIATEPLWILLLWSLKRREQPSPAQVVGALSGLTGVILLVAPSFAGFGSGRGV
AWVFLGAFFWSAYSVVVVPLVQKYGASAVSATTVSIGSLPLTILAIPEAHGMARISGSGALVILLLALGSTVVASLAWNS
AVSRLPGPVSGQFLFGIPLVGVVAGHVLLSEAFFANTWFAVGFIAFGLWVSRHRSRSQGSYTTS
>OLS50214.1 hypothetical protein BV379_19280 [Rhodovulum sulfidophilum]AB14
MDRIFSSSPWEKRSGYARAVAGKDIICIAATAATDEAGKVLCKGDLEGQTRVILEKMSGILDQAGSDMEHVLHTRLYITD
ISSAAEAGRAHAEAFSAAPPAMSLVHVLPFVDPDMLVEIELMAERRR
>OLS50215.1 hypothetical protein BV379_19285 [Rhodovulum sulfidophilum]AB14
MPFDKPLLSALTALGFTQYEAKAYCALQSGGPGNGNEVAKVSGVPPSKVYETLARLLEKGAVLKTQQEPVRYMARPWQEV
ASKLLQHLGSAAKTVETELGKLQHPESPKAIWTLPSRDAVLETARSFITDADVNIFASIWDDEIAELSPELEAAARRGVQ
VHVAVYGQTRLSSARGYDLTACGRSAMERLAGRRLMTLVAGTGQALVAELHPDGGVEAIRTNAPVIGLLVSEYVKADVLG
RLLIDDMGDARFEHLRRDPGLIEELLRG
>OLS50216.1 hypothetical protein BV379_19290 [Rhodovulum sulfidophilum]AB14
MRMPNEIDWERVRVILDQLYEATENLEDLFPGRKFTLDGHLVGSVGEVVASYMFGLNLNPASTKGHDAVSSSGRPVEIKL
TQGKSVAIRHRPEHLLVLCRPKGSKISVVFNGPGAIAWEAAGSMQKNGQRSIGIKKLAELDALLDDAARLPLLRQVPV
>OLS50217.1 lysine transporter LysE [Rhodovulum sulfidophilum]AB14

MDLTYWLIFSGTVIGVSLIPGPSTFIAFAHGASHGWLRSLATACGNASASTLQAIAASAGLGLILTSSALLFAIVKYAGA
LYLIYVGIQLWRSAARRVSLGNAEVASAGSIRKLYSGGFAVAVSNPKAIAFFTALFPQFLSPDGNDIGQLMGMVLVVCGS
AFAVAAAYGGLGAYVRGLELSRLVMTRVYKTTGGLFVASGVGLAASRA
>OLS50218.1 hypothetical protein BV379_19300 [Rhodovulum sulfidophilum]AB14
MLAYPIDLTPDDNGTFLVTCPDLPEVTSFSDTEAEALRVANGAISEALASRLSGFEDIPRPSQGLRRAAVPLQLQIKILL
FWALSDAGKSRADLVRELGWHRPQVDRLFDPNHATRLEQYDAAFAALGQAPGFALEAAA
>OLS50219.1 addiction module toxin, HicA family [Rhodovulum sulfidophilum]AB14
MNSKELEKLLRSKGCTFETHKGGSGHKTVRLGDRKSQIPMHGGRKELGTGLVNKILKDLGLT
>OLS50220.1 RNA-binding protein [Rhodovulum sulfidophilum]AB14
MATLHLPLKGAYFDQIKSGEKIEEYRLVTPFWTKRLEGRAYDRIELAKGYPPKGDTSRRITRLWRGARKTTITHSHFGPD
PVEVYAIRVN
>OLS50221.1 hypothetical protein BV379_19315 [Rhodovulum sulfidophilum]AB14
MREEDGIEILIPMCIGAAIGGPAECTCNVPESRIETTERGRTEAEAQVLRLMDERDRRLYEQARNWRRNKLLRQRIEDLE
TQLAGKTN
>OLS50222.1 helix-turn-helix domain-containing protein [Rhodovulum sulfidophilum]AB14
MGDTPQPIYFRPRDAKALFGVSANTVRRWLERAGPAVRTIKMGNTRLIHREEMEVWLEANGEKA
>OLS50223.1 hypothetical protein BV379_19330 [Rhodovulum sulfidophilum]AB14
MMLDVFRTLWAIAGAFGFAALARIGWECGGRIIRALTDTDPLVNEATVRGGPRIRWFTDRTGQIVHIERVEGRDDG
>OLS50224.1 hypothetical protein BV379_19335 [Rhodovulum sulfidophilum]AB14
MPDRFTRLEERLSVRRAYWPGDLIQLLRDNRRRAARSTLRPMEGAMTDMSQGYRIKPLVWGEWAHVMSCHTWNEL
FAMRT
AKSVLGPYYIGRKRILSRRGFGEHMSDDWWVEAPRQNCLLGPFKNDIEARTAAQADYEARIAPAIEPTGEMARLREEN
ER
LREGRYDA
>OLS50225.1 hypothetical protein BV379_19340 [Rhodovulum sulfidophilum]AB14
MAEILTRSFGRFNAEVTPAEARLIIRDDCKDMIRLFGRDLVRSSLLGGSWAPDIKRALRWGLDRIEAELGEDLRYRAGVM
AAVKLCWRLRLTNDPRRAERLLLGNPEAGQPPVEPRICPGCGELVTEGQGCGGGICWASAAFPKPVEAPADKAFREAV
AE
LVDCAMKEGKPDA
>OLS50226.1 hypothetical protein BV379_19345 [Rhodovulum sulfidophilum]AB14
MHPLRILIGCETSGVMRRAFAARGHDVWSCDLLPAEDATNRHMICDIREVLHLGWDLLAVMHPPCTRLCNSGVRWL
HVPP
TNAPAEARPDERTAWPDLPEATRRAIMWRLLDEGAALFSACWRAPIPRVAVENPVMHRHGRARLPADLPKPQIVQP
WWFG
EPVFKATGLYLRGLAPLSATNRLTPPAPGTEAHKRWSAVHRAPPGPDRWKIRSRTFEGLAAAAADQWGENARKEAA
>OLS50227.1 hypothetical protein BV379_19350 [Rhodovulum sulfidophilum]AB14
MSIMDSIALPPDALAEFVVEWRGASPEVRLAAIRGALRRHGIEIPALPGRVAFYEIQLFGLVATGRDEAEAAQNWIEAAI
RLCPFD
>OLS50228.1 hypothetical protein BV379_19355 [Rhodovulum sulfidophilum]AB14
MAAILTIAPQIADRLAAEAAAARIEAQTARVAAKSSARDGKLLLARAAFARAERLQGRAVTLGALAAQARAGGAA
>OLS50229.1 hypothetical protein BV379_19360 [Rhodovulum sulfidophilum]AB14
MPEQIDSTSDGRTTNNAVRHKYRALTDAEKTQMQDVKDMGAVFIAKLHEIGGTDPEGDRFGSRDLSLANTHAEDAV
MRAC
RHITA
>OLS50230.1 hypothetical protein BV379_19365 [Rhodovulum sulfidophilum]AB14
MKTILTTVSALALLAAPVLAATDTTEDQATAVSPASAPDCFAPIQGTNAFQRSRAPGCVYYAAPTGNDRDRAASDDGE
GS
GEGEGGESSDP

>OLS50231.1 transcriptional regulator [Rhodovulum sulfidophilum]AB14
MEREAITMLVASNIEAAMAKRGTNAAEVARKAGLNPTAVYDILKGKSRNPRLDTLYKIATQGLEVPFSSLFSEPEDHDLD
NEIMEAIRCLPTSERSRILAVVRAFSVDV
>OLS50232.1 hypothetical protein BV379_19375 [Rhodovulum sulfidophilum]AB14
MQKSFQKFRDPQERERKWFASLLWRSFPEATSEAQLADLVAEALNTAQRPVNPRTVRNWLRSENAPGLHYVVAVLAL
AGA
ETVFELFDPEQTA
>OLS50233.1 hypothetical protein BV379_19380 [Rhodovulum sulfidophilum]AB14
MTVRAEVAAEFQATALAMQSRQRPVSIRDRLRLALAGLGSGDEELRAAIARFLADHDTAPAAAGAALQETILRRCTAAP
T
RHAWQDRADLDG
>OLS50234.1 hypothetical protein BV379_19385 [Rhodovulum sulfidophilum]AB14
MAEAPLTRADQVLIAAAAALAVPPVMDRDLTARRMAMALDVIPHVAPDGPTCGLAFEIEAMDRARRAGDGPTFSDC
HWRL
RSAVARFFETRAAHAHERWRRETGKA
>OLS50235.1 hypothetical protein BV379_19395, partial [Rhodovulum sulfidophilum]AB14
MTDDPLDRVRATFAAAEDVDMGDIPAPDTAAPDDPGSAPGGGAEPPHPPDGTAPEDPLGPIRAAAGQPLNDYGNG
QRFCI
HFGQDLTFVPRVGWFVWTGTHWEKDPDELAVRRKAQRIWALIEQEVEVLEPTKSEKKVIAEECRLRERFDRLDALPEA
ER
AEDHDDELGTIRARLRSIEAILKDRKSLIGRRLTHAKNAGNSGPIGNMIEQAQPGLARAYEDLDADPLTVNTLSGTLRFS
TVAAEGMSPVAELRVQPEHLRGDL
>OLS50236.1 hypothetical protein BV379_19400, partial [Rhodovulum sulfidophilum]AB14
LGSVKGGAIRLARSSGTRAMVMGEGIETTFSALAARALPGAAFWAGISLGNMAGRRITRGKGMRFAGVPDLDDLQA
WLPP
AGVEHLVFIQDGDSEPRSTRAQLLSGLRRARARVPSVTRISIVHAGEGRDLNDVLMGEGEE
>OLS50237.1 hypothetical protein BV379_19405 [Rhodovulum sulfidophilum]AB14
MTDDPLDRVRATFAAAEDVDMGDIPAPDDAPNDPGDAPGDGADPPHPPDGTAAKDPLGPIRAAAGQPLNDYGNG
QRFCIH
FGQDLTFVPRVGWFVWTGTHWEKDPDELAVRRKAQRIWALIEQEVKVLEPTKSEKKVIAEERRLRERFDRLDALPEAER
A
EDHDDELGTIRARLRSIEAILKDRKSLIGRRLTHAKNAGNSGPIGNMIEQAQPGLARAYEDLDADPLTVNTLSGTLRFST
VAAEGMSPVAELRVQRKHLRGDLSICAAICCRRSCRSPMTAPRKPRAGTPSSRRSCPTPPCATSSSAGWGSR
>OLS50238.1 hypothetical protein BV379_19410 [Rhodovulum sulfidophilum]AB14
MPVAYDRTAKAPRWDAFLEEILPDATVRDFVQRWMGLSMTGIKMQRLAFFYGGGANGKSVLVDTIARVLGPYAATA
RIES
LTGTNRRGGGDATPDLIPLMGARFVRTSEPDEGQRLQEGLIKELTGGEPLMIRALHSDFLDFLPYFKLTMSGNHKPEIRG
TDDGIWRRVLLVPFDVQIPEERRDPELANKLWEEAPGILNWLIAGLTDFLEGGLRPPDRVLEATAEYREESDPVGAFLTE
SAIVTGAEADFIYSRELIEAFNFWLEDRGETRWGGRTVSNRLKQKASRWRHPHTGKSFIPAKRDATGYRGLRFTDEFAA
R
MADRRDHGGAGAPVDEPF
>OLS50239.1 hypothetical protein BV379_19415 [Rhodovulum sulfidophilum]AB14
MEEILRARLRADPGVAALAGERVNWGAHPQGAPRPALVLHLIAGAPGRTLTGPDGLWTGRIQIDAWARERAGAGAL
ARAT
IACLNGLSESVIRGLWLDSQRRDRAADPDDKTWLYRVSMDFSATWRER
>OLS50240.1 hypothetical protein BV379_19420 [Rhodovulum sulfidophilum]AB14
MSDDITEADIGHGARLGLQAEDGSFEDLAMVTRIKPVGWTRDEEEGTHLQSPGRYKQFLPGLKEQTPCEADLNWVPS
VPD

RLIAAFEAGRQTWQITAPNGVRMQFPGFITTYEPGEFSNGKMTASVTIRPAGQATILEAE
>OLS50241.1 hypothetical protein BV379_00020 [Rhodovulum sulfidophilum]AB14
MALVLLAGMLVLELAFALMGGTLLGMGGSPDGPDLPEMDLPDPGALGLDPGIDPGEFELADPDGFSGASPADGGTG
ALGG
VAALLGLGKMPLMIWLASVFMAFGVAGIAIQTAAYGLWAGLLPGGLVAIPSAALAILFTRSFGAVFARLLPRTETQSVR
A
RHLGRRMGVVTQGTASRGRPAEVRVRDRYGNTHYLRAEPLRDEDSIPAGCEVLVMRHRPTGSFRLISLDN
>OLS50242.1 efflux transporter periplasmic adaptor subunit [Rhodovulum sulfidophilum]AB14
MRRHAGGVALTTVLAVCALLLGHNLWKYYVDAPWTRDGRVTADVVRIAPEISGTISAVHVIENQFVHEGDVLFQISPE
RF
QLAVEAAQADLDSALEAMKLDISTAERNTKLEEKGSLSAEAAERSQREAAAARAEARSAEVALDVAKLNLKRTAVRAPV
D
GYVTNLHLRKGDYAVTGDATVTLVDAGSFRVTGYFRETQLARIKPGDPVRIGLMGVSGELQGHVESFGRGIANSNAAS
DD
LGLPLVEPVFSWVRLAQRIPVRIAIDGVPSGIELAAGMTASVSVVTR
>OLS50243.1 C4-dicarboxylate ABC transporter permease [Rhodovulum sulfidophilum]AB14
MTTVVALLGFQVVARFGMGRSFSWLEEVSRFAFVWAVYFGFVIAAEGDRHIRVSLHLGLLPTKLRLCMLTLADLLWLG
FN
AVVIWYGYQFTVSMFEFPYISQTTGINLVWVQMIVPLGFAFMSIRILQVMVRRWQTGEGPADARIGD
>OLS50244.1 PAN domain-containing protein [Rhodovulum sulfidophilum]AB14
MALAITVLATLAGGAAAQSVAASADNPLPDRRAVLWRDMDFPGGDLRTLLDTDLPGCLRACLSDTRCAAFTFNARSN
ACF
PKARAEAPTPYAGAVSARIVATDPATLAAAPTRAAELDFLRDRDLAGARAQAGKLPQNHPVDGATLDELRRAAASARA
AN
RPAEARRFLGAAVALSDDAAAWADYAAATAALSPEDRAARRRARAAALSASVNAYLRGGPPALRARALDVMAGALE
DLGR
GRDMIPALRLAQDLAPRDELQARLEDAIGKYGFRITDTVVESDAARPRICAEFSEDLVKAGTDYAPFVQLPDPGLAVTAE
DRRLCVEGLSHGESYRLTLRAGLPAASGETLAKPVTLTQYVRDRSPSVRFPGRAYVLPKAGGTALPVVTVNSGRLDLTLY
RMSDRNLLRSVQDGYFGQAISPWQEEAVSTDLARKVWSGHAEVGSDLNRDVTTRLPLDEALAGQPAGVYALSAAIPG
EDP
YEMPAATQWFVLSDLGLASMSGTDGLHVFVRALGSAEPKAGIKVSLLSRANAVLGTAMTDAQGYARFEPGLTRGRAG
AAP
AMLTAEEGTEDIAFLSLTDPEFDLSDRGVEGREPAPPIDVFLTTDRGAYRTGETVHATALARDDRAEAIADLPLTAILSR
PDGLEHARQLSPGGIAGGHVFDLDIAGTAPRGTWTLDLYADPEAPALASAKVLVEDFLPERIDFELGLPEAPIDPAAPPL
LSVEARYLFGAPGADLPIEGEVRLAAAAGLPDYPGFVFGRYDTEIAPAMALIVGAGRTDAAGRAQIGLTLPALKAEGKPV
EAWITLRLSEGSGRPVERSLTRPVKPSGPMVGIKPRFDGDLPEGAEAGFDLIVLGADGQPENTPLHWALNRVETRYQW
YR
VDGDWKWEPITSRERISEGETRPENGRASIAVPVRWGRYELVAERREGAWAEAAVAFDAGWYAPADATTTPDMLEL
SLDK
PAYRPGETATLRIVPRQPGKGLVTVMSNRLIDMVPVDLTEGENVIRLPVTDDWGAGAYVAATLIRPMDLPAGHNPAR
ALG
LAHATVDPGPHRLAAAFEVPAEAEPRQPLDIALKVGGIAPGETAYATIAAVDLGILNVTGFASPDPDGHYFGQRKLGMG
L
RDVYGRLIDGLNGAMGTVRSGGDATAGIRRQAPPPTEELVALFSGPVTVGADGIAHARLDIPAFNGTLRLMAVAWSPT
GV
GQAEADILIRDPVVVTASLPRFLAPGDESRLLLELVHAEGPAGRMPLSVTAEGVTLDPAAIPEAVELAQGGKTALSIPIA
AGAPGVQHVKVALSTPDGKRLTRTLTLPVEVTDPEIMRRSRFTLADGQSFTADDALFADMRPGTGRATLALGPVARLD
AA

GLLASLDRYPYGCTEQVTSQALPLLYLDQLAEALGLGTRAHLADRLDRAVTEVLANQSPSGGFGLWAPGSGDFWLDAY
VT
DFLSRARAQGVAVPDRAFGMALDNLRNRVNYAPDFDSGGEDIAYALMVLAREGRAAMADLRYYADTRAGAFATPLA
AGQL
GAALAAYGDPTRADRMFARAARMMAGRMGRDEPPVWRSDYGTDLRDAAGLLTLAVAAGSTAVDRDALTGYLAAT
DRPLST
QEASWMLLATHELIDRPGTGGEGFTLNGAPLTGSLVRLLAPGDAPAVLGNDSGRPETLTVTSFGVPEVPEPAGGNGYAI
S
RRHYTLEGDPVTLDAVPQGTRIVTVLEVTPFSDDRARLMVADPIPAGFEIDNPNLLRAGDIRALDWLGTTAEVRHSEAR
Q
DRFLAAIDWGSADPFRLAYILRATAPGSYRAPAASVEDMYRPDRRAWSETSRASVTP
>OLS50245.1 hypothetical protein BV379_00340 [Rhodovulum sulfidophilum]AB14
MLKFPKWFFKWSEENPTDLMGPGILVGTVGGAVAVAAIIVAFGNPNATIDQQTGPRGIGMAVSKFVKDNPQFDVYE
AEYQ
VFDRVEAPEGTPTAAEAYGDSVVAFGDMDQANFDQLTKAMSAWVGMDVVLYDDGEVDETTLAITKNCIEATQYLND
SWDT
HNLATEGKGVNCYTCHRGQPTPPGSWMKSGNVNSAMEGWSGVQNRLLVGRKYTDSQYTSLPVDALEKLLLDGDSIK
VTDT
ESRVDQQKGDPTWQDAERTFSLMNHQANSLNVGCVYCHNTRAFYDPTQVTPQWSVTTLAQQMSIDINQTFYEPRS
EILGH
ESAKVDCMTCHMGVISPLNGHDMVAEWPELAAPAE
>OLS50246.1 carotenoid 1,2-hydratase [Rhodovulum sulfidophilum]AB14
MSHDGERSISIISFIGSVFSPWYRWSGRKSPDNHCCINVATYGPGGRWTMTDRGRDALRQTRDTFEVGPSKLTWAGG
KLV
FDIDEMTWPHMGRLKGRVTVTPSAVTGVELPLKADGSHVWRPFAPSSEIEVDLNQPGWQWKGHGYFDANFGTAAL
EDDFS
YWTWGRFPTKANGAWCFYDASMRDGSELAVGVHFDADGRAEVAEAPPRTRFPRSKWLVRRETRADPGYKPRQVHD
MLDAP
FYCRSAIRTKVFGEEVVGVHEALDLDRFASPLIKPMLAVKVPRKPGWTFPA
>OLS50247.1 dienelactone hydrolase [Rhodovulum sulfidophilum]AB14
MLAIIGPWGPALAAPYHAGVARSAFTTASGQANAVIWYPTDLPEISWQAGPFEIDATREAPVAEGRFPVVLLSHGHNG
GP
LSHRELASQLARAGFIVIAPAHKGDTAGQPPRASQDRILKNRPAQAIAALDAALRNERLAPHADPARIGMIGYSAGGYT
G
LILAGAKPDFDHAAAYCAKEGRDDTGSCGEARFIGISEKLATWSAPSEPRLKALVLLDPLAVMFDAPGLADVGNPVLLFR
PQEDIYMRSAANAVALARDLPRAPQEIVLPGTHFTFVDPCPEDITAEAAMVCNDPPGIDRPAIHRSLEDDVIAFLNETL
>OLS50248.1 terminase [Rhodovulum sulfidophilum]AB14
MPEGPNAGKPVSLAPFQRQFIEGAMAPETAAAVLSIGRGNGKSAITAGLALGGLVGVWDRQPRREIVAAARTRDQGR
IIW
DFVSGFAASLPLELQRRLIYRRAPRLEIEFEGDGGGHVLRVIAADGKSALGGAPTMAILDERGHWALDRGDELEHAILSG
LGKRNGRAFLISTSARDDTHSFSRWIDDPVPGSYVQEHRPPPALPADDRDSLLIANPGAPHGIGSSLDWLEGQARRSIA
R
GGSTLANFRLYNRNERVSGETLDMLITPDEWLNCETDTLPPREGGVVIGIDLGGSASMTAAAFYWPATGRLEAMGTFP
SA
PSLLDRGQSDGVAGRYVEMRERGELTTLGDRTVPVAAWLTEVMSRIEGETVLALTGDRYKGAELGEAIARAGIRAPVV
WR
GQGFRDGGEDCERFRRAAFEGRVKARPSLLLRSAFADAVCKRDDANNLKLTKARAPGRIDAASASVLAVAQGARISAQ
PK

AKVRFHFL
>OLS50249.1 amidohydrolase [Rhodovulum sulfidophilum]AB14
MKIAAAAYPIDRLDSWAGYEAKLTAWVAEAADAGADLLVFPEYGAMELAMLDGAAAAADLEASLVAVSARCEQASAL
HAR
LAREHGVHILGASAPVWQGTRPVNRARLFGPRGEAGHQDKQIMTRFEREDWNVHPGGPLALFDTALGRIGVLICYDC
EFP
LLARALVEAGAEILLVPSCTDTLAGYWRVRIGAMARALEGQCVVVQSPTLGEAPWCPAVDVNIGAAAIYGPPDTGFPP
TG
ILAEGALNAPGWVHAEIDRAEIARVRTAGQVLNRAHWPEQSLFSDTVVRVPFHAFTT
>OLS50250.1 hypothetical protein BV379_00995 [Rhodovulum sulfidophilum]AB14
MTARLCHVELDDSNMPPPTPEIEQERKVALFDLMEDNSFALPGREDRPAPAGPFRLTLSIREKRLVFDVRTETSDPAAEF
HLSLSPFRQAVKDYWQICESYFDAVKRLPPSQIEAIDMARRGIHNEGARLLQERLEGKADLDIDTARRLFTLICVLHHGG
>OLS50251.1 MBL fold metallo-hydrolase [Rhodovulum sulfidophilum]AB14
MTASPVVTSFHDEATGSLQYVIHDPATLDGAVIDPVLDFDPAEARVTTANADRILDHVRAEGLRIGWVLDTHPHADHF
SA
AHSLGRALGARRGIGARVIEVQALWRDLYNLPDAFPAGGRDWDRLFRDGDEIALGNLTLRVLATPGHTLASVSYLAGD
AA
FVADTFMRPDAGTARADFPGGSASVLYDSLGRLLSLPDETRIFIGHDYPEEGAAVQPMATVAEQRRNVHLDGATREDF
LK
TRQARDATLSLPDRMLAALQVNLRGGRLPEPEDDGRSYLKIPLNRF
>OLS50252.1 hypothetical protein BV379_01105 [Rhodovulum sulfidophilum]AB14
MDWTRLPPLTALRAFEATARLESFSAAARSLNVTHAAVAQQVRALEDHLQLALVRRDSRGMTATPEGARLAAALGEA
FRK
IQEGVDEVRASEDAAPLKISLTPAFAENWLMPRLGSFWATHPEIELALIPSTALADLRAGGYDMAIRYGSGAWPGLEAE
L
LTSTRFVVVGTPELTGDPRPGSFAELTRFTWLTAQDSPEQALWAESTGLSWTRVRATSFPTTGLSLAATRAGYGLSIQPI
TLVEDDLRAGRLAALFEQESLGLGYHIVTRPGHRPPRLRAFIGWLRQAAGPQAGI
>OLS50253.1 murein transglycosylase [Rhodovulum sulfidophilum]AB14
MRGFGAAVCLALALGGGASAQAAPSVELLDFDDLKGWKTDNHAEALAAFRETCPDLPDPPWTALCALSERQSNARSF
FEL
FFRPVLIGGDSPALFTGYFEPVLKAARAPDARFRYPIYRLPPDLPTDRPWFSRREIAEDGHLDGQGLEIAWLEDPVDVFF
LQVQGSGRLSLPDGTLLRVGYGGKNGHDYRSIGRELVRRGIYQPHQVSARVIRSWVRRHPEAGAELLLHNPSFVFFREL
D
SLPPGKGPLGAMNRPVTPMRSLAVDPSLVPLGAPVWIEKGGRHPLHRLMVAQDTGSAIKGPQRADIFYGTGPGAGRL
AGA
VRDPGRMIVLLPIELALALLPGG
>OLS50254.1 preprotein translocase subunit Tim44 [Rhodovulum sulfidophilum]AB14
MSSAIIQLLVLAGIAVFLILRLRSVLGTREGFEKPPVQMPDPAARRADNRPSFEVIEGGPDTDITDHVEDGSPAAKALAA
MKLAEPGFSVSEFLHGARGAYEMILMSFEHGTLDEVAGFLAPDVRDSFAEVIRLREEQGLSVDANFIGVRELVLKDATY
D
RDSGEAELTVRFVGELTSVVRNQDGEIVEGSPSEIKRQRDVWSFARVMGSDDPNWQLVATDA
>OLS50255.1 16S rRNA (guanine(527)-N(7))-methyltransferase RsmG [Rhodovulum sulfidophilum]AB14
MSAEEFSDVSRETSERLASYAALLRKWNPAINLVSRATLDELETRHFADSAQLFDLAPAEARHWVDLGSGGGFPGLVIAI
IGEEQAPGLRVTLIESDQRKATFLRTVIRELGLKRVEVIDARIEAADPQEADVLSARALAPLDRLLGFAERHLASGGVAL
FPKGARYADEVNRALASWRFEVQNHPSKTDPQAVVLKLGGIARV
>OLS50256.1 heat-inducible transcriptional repressor HrcA [Rhodovulum sulfidophilum]AB14
MLEEMNDRSREVFRRVVEGYLESGDPVGSRTLTRSMSEKVSAATIRNVMQDLEFLGLLNSPHVSAGRVPTQLGLRMF
VDG

LLEVRDLKEEDREKIDATLGANTSDVGALLDRVGAALSGATHGASLVLAPKHEAPIRHIEFVSLGADRALTVLVFADGHV
ENRLFHPAPGQTPSSMREAANFLNALAEGLTLSELREAMKVEIARRRQELDALARTMVEAGLAVWENEGHDQARLIV
RGR
ANLLSDPAEAEDLDRIRSLFDDLERKRDIVEFLELAEEGEGVRIFIGSENKLFSLSGSSLVVSPYMNADRKIIGAVGVIG
PTRLNYGRIVPIVDYTAQLVGKLIADRGKG
>OLS50257.1 DNA mismatch repair protein MutS [Rhodovulum sulfidophilum]AB14
MMAQYLEIKSANPGALLFYRMGDFYEMFFDDAVAAAQALDIALTKRGKHQGEDIPMCGVPVHAAESYLLTLIRKGFRV
AV
CEQMEDPAEAKKRGSKSVVRRDVVRLVTPGTLTEESLLEARRHNFLAAFAEVRGEAALAWVDISTGAFRAMGCPGVRL
GA
ELARLAPREVLLNEATERDWAEIVSESGAAVTPLSRASFDSSSAETRLTELFSVGTLEGFGTFSRAEMSAMGALVDYLEI
TQKGKLPLLRPPVREAMGGTMQIDAATRRNLELTASLSGGREGSLLAAIDRTVTAAGARLLERRLSSPSRDLPTIHARLA
AVRALVGDTGLCAALRDSLRKCPDIDRALSRLGLDRGGPRDLAAIRNGLAQAGDVAGLLGDDLPQTLTGARAALTGHD
EL
CDLLDRALVAEPPLLVRDGGFIATGFDEDLDEARTLRDEGRGVIARMQAGFVEETGISSLKIKHNNVLGYFIEVTATHAE
RMLSPPLSDTFIHRQTTANALRFTTVELSELETRILNAGNRALEIEKRLYDSLKTAVLNHAGPAGQAAAALAEIDLATGL
ADLARGEDWCEPEVDDSRAFAVTGGRHPVVERALRRQGGTPFIANDCDLAEAGSGAAIWLLTGPNMSGKSTFLRQN
ALIA
LLAQAGSFVPAASAHIGLVSQLFSRVGAADDLARGRSTFMVEMVETAAILNQADDRALVILDEIGRGTATYDGLSIAWA
T
LEHLHDANRCRALFATHYHEMTALTQKLSGVENATVTVKEWEGDVIFLHEVRKGAADRSYGVQVARLAGLPDSVVER
ARV
VLDALERGEREGGRSPSALIDDLPLFSAAAIPQPSQSAPAGPAPLDERLSAIHPDELSPREALDLIYELKALARPE
>OLS50258.1 threonine dehydratase [Rhodovulum sulfidophilum]AB14
MTDFAAKARAAEKAMRALFPPTPLQRNLHLSERYKADIWLKREDLSPVRSYKLRGAFNAMRKVLAAAAEPPVFVCASA
GN
HAQGVAFACRHFGVRGVIFMPVTTPQQKIGKTRLFGGDNIEIRLTGDYFDDTLASAQGYCREVGGHFLAPFDDEDVIEG
Q
ASVAVELMSVLYHTPDMVILPVGGGGLAAGVVSFLRETAPGIGFRFVEPVGGASLTAALAEGAPVTLPGVDTFVDGAA
VA
RIGARTFDRLKGIDPAHVLCAPEDRVCTTILELLNVEGIVLEPAGAMSIDVLPELADEIAGKSVICVTSGGNFDFERLPE
VKERAQRFSGLKKYFILRMPQRPGALRDFLDLLGPQDDIARFEYLKKSARNFGTVLIGIETAEARNFSALFARMDAQGLT
YRDITDDPTLADFLI
>OLS50259.1 hypothetical protein BV379_01365 [Rhodovulum sulfidophilum]AB14
MDRRAFLTLTAMAALAGCGSKFKTYRGPEVTRVVVMKGQRMMYLLHHDQVLKAYPISLGYAPAGPKQVEGDGKTPE
GRYH
IDRRNPDSRYHLSVGISYPNEADIETAKALGMKPGGDIFIHGRAKENRRRTDDWTAGCIAVSDREMEDIYAMVRDGTVI
D
IFP
>OLS50260.1 hypothetical protein BV379_01375 [Rhodovulum sulfidophilum]AB14
MTTRSTRGLSRRNFLAGTALAVTAGPALANTTEIEPELTDMVRRNISSFRALDWRPYFASLKNGAILVDVKSRALHFWSE
DGTIYKLYPTSVPLSEDLTRLGRTSVVRKVEGPSWRPTPAMKKRNPEWPDFVPPGPENPLGTHALYLGWTYYRIHGTH
DT
RKIGRRSSNGCIGLFNEDIAELFGLAKVGTQVLLI
>OLS50261.1 ferrochelatase [Rhodovulum sulfidophilum]AB14
MTGARPEHGPEDHPPVPPERIGVLLANLGTPDGYDYWSMRRYLNEFLSDKRVIDYSRWIWQPLLQLVVLTKRPFASGE
NY
RSIWNHEADESPLMTITKAQTAKLEAELKAAYGEKIVVDFCMRYGNPSTRAKVRSMLKKGCQKILFFPLYPQYSGTTSAT

ACDSFFEALMDEKWQPDARTVSTYFDNPHYISALAASVERAYAAAEQEPDILVCSYHGVPQRYLTEGDPYHCHCLKTTR
L
LKERLGWDDSRITTTFQSRFGPEEWLQPYTVEEVARLAKSGKKRIAVIAPAFSADCIETLEEINEEICESFEEAGGEKFT
YIPCLNDDDAHIRALSEIVKVNLRGWAD
>OLS50262.1 ribosome maturation factor RimP [Rhodovulum sulfidophilum]AB14
MSELIAKTAIDRRLAEILTPAIEGMGFELVRVRLMGGKTKTLQVMAERPDGGIEVDDCAKISTAISALLDVEDPVEEAYT
LEVSSPGIDRPLTRLKDFGTWEGYEAKLETAELIDGRRRFKGMLQGVEDDEVLIEIDGPEGDPVIIGLKYDWLSDAKLVL
TEELIREMLKARKDAGLIDESDFDEIEADDGSAPQED
>OLS50263.1 bifunctional ornithine acetyltransferase/N-acetylglutamate synthase [Rhodovulum sulfidophilum]AB14
MRSQIVTLSEKVPAKTPVISPLAPAAGFPALPVIEGAEFAAGEAGVRYRGRPDVMLARLAPGTTIAGVFTRSATRSAPVL
DCEAKLAATPAPEAGAAIVVNSGNSNAFTGASGAEAVKAISTGLAEAAGVPEARVFTASTGVIGEPLPHERITAVLGDLT
ERLSPEALPMAARAIMTTDTFPKGAAAVIEVEGQPIRIAGIAKGSGMIAPDMATMLAYVFTDAKIAQPLLQRMLGKLSD
E
TFNSITVDGDTSTSDTLILAATGKSEAPEITDLRSAAGRAFQEALRGVMLDLAQQVVKDGEGATKFVEVAVTGAASPGD
A
RKVAMAIANSPLVKTAVAGEDPNWGRIVMAIGKSGAKADRDLISIRFGDILVAEKGWVAESYAEEVGAAYMKREELAI
RV
DLGLGKGSSTVWTCDFTKRYIEINADYRS
>OLS50264.1 hypothetical protein BV379_01475 [Rhodovulum sulfidophilum]AB14
MPLFAEAPVRALAPGERLPSYLRDHRRRLRDRFMAGGAAAMPDYELLEMVLFRALPRQDVKPLARLLLDRFRDFNGVL
SA
PVARLQEVRGVGPSVIVELKLVEAAAQRLARTRVMHRPVLSSWDALLDYCRTAMAHRETEQFRVLFLDRKNVLIADEE
QA
SGTVDHVPVYPREVVKRALELNASALILVHNHPSGDPTPSQSDIVMTTQIEAACAALGLVLHDHLIVGKARDLSFRSEGY
L
>OLS50265.1 LPS export ABC transporter ATP-binding protein [Rhodovulum sulfidophilum]AB14
MPGPAGGLRIVDLRKSYRRRPVIRDVSLRLDRGEVVALLGPNGSGKTTCFYAVAGLLTPDGGRVMLDGRDVTTLPMYR
RA
RLGIGYLPQEVSIFRGLDVEDNIKAVLEIVEPDRHKRRERLEELLSEFSIEHLRRAPALSLSGGERRRVEIARCLAADPS
YLLLDEPFAGVDPIAVGEIRALVADLKTRGIGVLITDHNVRETLEIVDRAYILHDGKVLMSGTTQEVIRDENVRRVYLGQ
SFRIT
>OLS50266.1 monofunctional biosynthetic peptidoglycan transglycosylase [Rhodovulum sulfidophilum]AB14
MAKPTKSTGTRRSGTKTTGTRNAGAKAAGKKPARRDSVPLPLARRLRRAARRGALAVLALVALVLIAPSLVNPPTTPTM
M
SERMRLGDIDQQWAPMREIAPAMQRAAVAAEDANFCRHWGFDMGAIRGALEAGGRYGASTISQQTVKNVYLWQ
GRSWPRK
ALEAVLTPMVELTWSKRRILEVYLNVAEFGEGVFGVDAAAWHYYGIPPSKLSRSQAARLAVLLPNPKDRDPNHLSPTLL
R
RADQVEEGAATILADGRSDCFDH
>OLS50267.1 DNA-binding response regulator [Rhodovulum sulfidophilum]AB14
MGEAELHLLIVDDDERIRELLRKFLMRQGFWISTARDADHARKLLQGLEFDLIVLDVMMPGEDGIALTRALRQEIATPIL
LLTARGETSDRIRGLEAGADDYLPKPFEPKELLLRINAILRRVPQVRSEIELPQVLQLGPVRYDVGRGELWQGEERVRLT
ATEAQLMKIFSANPGEAVSRTRLVEELGRDGGQAQERAVDVQITRLRRKIENDPKQPRYLQTVRGEGYMLAPD
>OLS50268.1 choline/ethanolamine kinase--aminoglycoside phosphotransferase [Rhodovulum sulfidophilum]AB14

MKTLGCASHPMEEAIEAVIRKAPLFRGGAGMVYGPVSGGISNENWRVRATDGSGDWFVKIPGNGTEMFIDRKAAFE
ASQK
AAAAGLGPGVYGDLAAEGVEINDFMADRRPATHSDFADPAKRRAAIAAYRKLHGMPGLAATKTVFDMIDEHAAQAA
DLGA
PRFPDHDWIMLNERLAREALMASGLDLVPCFNDPMPGNFMVGEDGSILLIDYEYASMNDRCYDFGLLFGEMFFTPEQ
ELE
LIEAYFGEVRPEIVSRVILHKALADVKWALWSMVQLRVSRLAFDFHKYGMWKLMRFRQITGHQDWPKHLRTV
>OLS50269.1 nickel ABC transporter, nickel/metallophore periplasmic binding protein [Rhodovulum sulfidophilum]AB14
MTTPLTALAAGRSGETVLRFAWPLNAGPLNPHLYSPNQMFAQNMLYDPLVRYRADGRVVPWLARSWTVSEDGREYS
FALR
EDVVFSNGEIFDAAAAKANFDAVLANRSRHAWLELANQIVAAEVVAPFTLKLRLKDAYYPTLQELALPRPFRFIAPSQFV
DGGTRDGILAPIGTGPWVLTETSLGEHDVFRRNDRYWGARPGFAGVTVKVIPDPNTRAIALRTGQIDMVYGIDGPLSP
DT
FAELARSGDFKAGLSAPMETSSLALNTARGGTRDLSVRRAINHAVDKDALIAAVLHGTEKRADTLFAPNVPYADIGLEPY
RFDPAAAAGLLEADGWVATRAGQPRQKDGQPLILDLCFVGTDAVAKAKAEILQADLAAVGIGVSLIGEEESSIYARQRD
G
RFHMIFNRTWGAPYDPHAFLSAMRVPAHADYQAQLGLPDKAEIDRRIGEVLISTDEATRQAMYRDLLTRFHEAAVYLP
LT
HLTAMAVARPGIGPLRFGAMSSEIPFEMLTPDA
>OLS50270.1 hypothetical protein BV379_01880 [Rhodovulum sulfidophilum]AB14
MIWAGLLVLMLLAAWPLAEALRRPMAARARSEAPGEIAALPRGRTHLVWEGPAEGPVVVLLHGLTIGSHMLDRLAGG
LAA
RGYRVLRYDLYGRGFSDRPRGPQDRAFFLSQLEALLEARGVGGDLTLVGYSMGGAILTAFAAAHPGRVRQLVLLAPAGL
G
QTPPRLADMAVRLPGVGDWLMAVIGAVVLRRMARAADRAYGLPGTLIAAQGAETRYRGFLPAVLSSMRHMLAEDLS
EAHR
RLAEAGVPVLAIWGGQDEVIAAGAGAGLAAINPAARQLLLPEAGHGLPVTHADAILAEIPAAPAQ
>OLS50271.1 ornithine cyclodeaminase [Rhodovulum sulfidophilum]AB14
MPENIGFEEGERVLTWPGLCAALARGHALPRAEIADSFLYRGPDTLLNRAAWIDGLGLAVKTATIFPGNGASGRPAVN
GG
VCLYSDADGTLEAMVDFHLVTKWKTAGDSLLAATRLARPDSRRILVLGAGNVARSLVEAYRSAFPEAEFTIWSRSGDSA
R
RLADPLGLAVATDLEAAVKAADIVTSATMATEPLIRGDWLRPGQHIDLIGAFRPDMREADDRALQRARVFVDSRETVL
AH
IGELKIPLASGAIGPEDVLGDYYDLAAGGVRREDRDEITLFKNGGGAHLDLMTARHILDAWRGA
>OLS50272.1 GNAT family N-acetyltransferase [Rhodovulum sulfidophilum]AB14
MTSIDRIEATDWDGCACPEASGGGRPEDPFTTHRFLAALEDSASVGPGTGWQPHHLVARDASGIFAVAPLYAKSHSQ
GEY
VFDHGWADGYERAGGRYYPKLQIAVPFTPATGRRFLARPGHETRGRAALIEGAVRLAAENALSSVHVTFCTEAEAEAG
RE
IGLLHRVTQQFHWENRDYPDFETFLATLSSRKRKNIRKERATARAFGGTIRALTGDEIRPDHWDAFWRFYQDTGARKW
GM
PYLTRDCFDRLHETMRDDMLLVLAERDGRPVAGALNFIGRSRLYGRYWGCIEDHPCLHFELCYYRAIDFAIAHGLDTVE
A
GAQGVHKLSRGYLPTPTHSLHWIADPGLRAAVDHFLRAEREAVAEEIEVITAYGPFRKTAKDQEEEQQ
>OLS50273.1 hypothetical protein BV379_02000 [Rhodovulum sulfidophilum]AB14
MSDWIQIVSSLRKATLWTGRLAVLSTILFLSACAHPEAPGTIRDPYEARNRQIHEANKTLDRSVFRPVSTAYGKSVPRPV

RSGVSNFASNLSLPGAVINSLLQFRPGDAAQNTTRFLINTTIGLGGIFDPASRMGATKVNTDFGETLYVWGMREGAYVE
F
PVLGPMTERAMVGRVVDFAMNPLRPLADSEERNVMAGTGVLRGANARYENSDLVDSVLYDSADSYAQSRQIYLDSR
RHKL
SRNQETDYFDPYEDPYADSNAE
>OLS50274.1 SsrA-binding protein [Rhodovulum sulfidophilum]AB14
MAKAKTDPNYKVIAENRRARYDYAIEDDLECGIMLQGSEVKSLRENGANIAESYASVEDGELWLVNGYVPPFEQARTW
GH
DERRRRKLLVSRKEMARLWSDTQRKGFTLVPLVLYFNHKGLVKLKIGIAKGKKTQDKRDTEAKRDWQRQKARLLKDHG
>OLS50275.1 FAD-dependent oxidoreductase [Rhodovulum sulfidophilum]AB14
MDWREDGVPVSTRFDDPYFSIADGLAETRHVFLDGNGLPGRFAPGFHIAELGFGTGLNLLAALIAWRGAGVKGPLRFT
SF
EAFPMSAEAMDRALSAFPEARAVADPLIAAWAQGARRIETDGLIAEIVEGDARETLPHWQGRADAWFLDGFSPAKNP
ELW
SPELMAEVARHTAPGGTAATYTAAGFVRRGLAEAGFEVTRQPGFGRKRHMSVARLP
>OLS50276.1 HAD family hydrolase [Rhodovulum sulfidophilum]AB14
MPDATEAGGRDAAWAFAAYQAIRPRLPDAQAGGAAREVASLSEAAEDVDLVLLDAYGVLNVGETPIPGAAARIAALR
AAG
KRVAVVSNSAGYPKRHMMARWQRLGFDFSAAEVTSSREALLETLAGDTRHWGIMLGDAHGNEDLGDLGVSFLGDD
PGTYA
RVDGFLLIGASGWSEARQSLLARALATRPRPVFCGNPDLVAPREAGLSLEPGYWAHRLADATGQAPAFCGKPFPSVFD
LA
RLRAGAGIPPNRVLMVGDTLHTDILGAQALGYRSALVTGHGALKSLSCQSCCREAGIHPDFVMASI
>OLS50277.1 phosphonate ABC transporter substrate-binding protein [Rhodovulum sulfidophilum]AB14
MFDTTFRGLAAASAIAMIAGAAQAQAQDCPHGDLDPMYCDRDGDLVADAPAEGLQDPGTLVFAYTPVEDPAIYADI
WEPF
IEHLEEATGKDVRFFAVQSNSAEVEAMRSGRLHIAGFSTGPTPFAVNLAGAVPFAIMGSDEGEFGYKLQVYTRKDSGID
S
MEDLAGKRVAHTSPTSNSGNLAPRALFPNLGVTPGEDYEVVYSGSHDQSMLGVVAGDYDAAPVASEVVDRMAERGL
YDPE
EVRIVWESKPFPTTSFTYAHDLDPALVEKIRQAFFDFDFSGTALGEEFSGVSKFVPVTYQEDWAVIREIQAANGVQYTPE
GLAAE
>OLS50278.1 glutathione-dependent reductase [Rhodovulum sulfidophilum]AB14
MGQLIDGRWDSGWYDTESHGGRFVRSTAGFRNWITADGAPGPEGEGGFRAESGRYHLYVSYACPWAHRALIFRAIK
GLAP
HIGVSVVHPDMLEDGWSFATDFPGATGDGLYGLPFLRDIYIKAKPDVSGRVTVPVLWDRQRETIVSNESSEIIRMFNSA
F
DGITGNRDDYWPEELREGIEAVNARVYDTVNNGVYKAGFATSQDAYDEAVRALFDSLDWLEERLSTRRYLMGERVTE
ADW
RLFTTLLRFDPVYHLHFKCNRNRLIDMPNLWGYARELYQWPGVAETVHFDHIVRHYHYSHDTINPNRIIPINPRIDWTG
P
HGRDRLKAA
>OLS50279.1 hypothetical protein BV379_02185 [Rhodovulum sulfidophilum]AB14
MVALFVALGLDGKPPGHYDPLPPYFHQVYFWDVHRPDELGRDGLPHIGGDGFIPDLGLPRRMWAGGRLTFYGPLRQ
GLPA
QKTSTIEKVQLKKGRSGPLAFVTLRHDIVEGGMLRVTEYEDLVYLPDPDRGRSRPPAPVAPETWEDAVELTFTPTLLFRY
SALTLNGHRIHYDREYCQRVEGYPGLVVQGQLLAQFLMLRANAHGTIRHFSMRAYAPVFDFETVQLCRAGDRYWIRG
EDG

RLAVMGEAVFAPGAL
>OLS50280.1 ribonuclease III [Rhodovulum sulfidophilum]AB14
MKLAADLIAFSRRLGHDFARPELLLRAVTHSSLSSPTRPDNQRLEFLGDRVLGLVMAEALLKADRAASEGQLAPRFNALV
RREACADVAREIGLGDVLKLGRSEMMSGGRRKEALLADAMEAVIAAVYRDAGFETARDMVLRLWGARIEQVKPDAR
DAKT
ALQEWAQARGQQPPRYVEIAREGPDHAPVFTIAARLETGEEAKATAGAKRQAEQAAAAALLKALHRSPS
>OLS50281.1 hypothetical protein BV379_02300 [Rhodovulum sulfidophilum]AB14
MLRSLLHIGVIVFLTLLTQIGGIAWLMALAFRRTLLAFVLFYVGLSVAAVWIAPGFGRVALSCAERGPLAAQSWISCALN
RNYVTPELADVLSDTAEEMDRRHPGTTTLFLDANFPFFDGFPLLPHLSHDDGEKADLAFYYADPSGYRPGAVRSYIGYFA
FEQGPTDCPSAWPTLRWDFTALQPLWRDFRLEPDRNRAVLDVLSKDPRVGRIFVEPHLAESLGATGDVIGFQGCRAAR
HD
DHIHLQLR
>OLS50282.1 hypothetical protein BV379_02410 [Rhodovulum sulfidophilum]AB14
MRGSAVFSPCGRYRTRLERDWTPEGQPGRTILFCGMNPSTADALVQDNTCAKETRRAAALGFTRYLKGNVLDYRVTN
PKD
LPKDPDLACSPGNLPAILAMAEEAELVVMAYGRLHKRYAGVVGGIIAALRETGKPLYCFGLNQDGSAKHPLYLRNDAPL
L
PF
>OLS50283.1 hypothetical protein BV379_02430 [Rhodovulum sulfidophilum]AB14
MTQVASRQWVNSGPGSVLPGLLLAALIAALAFGLRHIPGIGVLSPLILAILIGMAFHNLIGTPAIARPGTSFSVKRVLRA
GIILLGLQLTAQQVIAVGGVGVALIVATLLATFAFTTWLGRMLRVEARLTQLIAAGSSICGASAVIATNTVTRARDEDVA
YAVACVTIFGSLSMVLMPLVGGLLDMGPHAFGLWTGASIHEIAQVVAAAYARGQEAGEFGTIAKLTRVMMLAPMVL
GLGV
LASRRLRRDGGEADHAVPPVPWFVLGFVAMVGLASTGWVPEATVHVTTDITQFLLATALAAMGLETDMRKLAAEGL
RPAL
LGAGAWVFISLFSLALILFLA
>OLS50284.1 hypothetical protein BV379_02445 [Rhodovulum sulfidophilum]AB14
MRIRAALRLLAVLLPLPAAAEPSCTPLTHEGRGYVICSADPATDDLRLFLRTEQGGIYGSFGRVNQALAAGGETLAFAMN
AGMYHPDRRPVGLYLENGKQEMRAVAPAGPGNFGMLPNGVLCLEDGRAAVIETRAYLADPPDCRYATQSGPMLVID
GALH
PRFLPDSTSRHVRNGVGVDAAGRVHFAISDAPVSFHEFARLFRDVLETPNALYFDGKVSKLYAPALGRHDAGLPMGPIV
G
AVVPAGD
>OLS50285.1 4-hydroxy-tetrahydrodipicolinate reductase [Rhodovulum sulfidophilum]AB14
MDSLPGIVVMGASGRMGQMLIGAVLQSGKARLAGCVERPGHPWIGRDVGEAMGGPAVGVKVTDDALAVIAKAQA
VIDFTT
PEATVATATLTAQARAVHVIGTTGFEPEHLQKIKAAARHAVIVRAGNMSLGVNLLMQLTRRVAAALDDDFDIEIVEAH
HR
HKVDAPSGTALMLGQAAAEGRHIALDECSERGRDGITGERAKGAIGFSAIRGGDIVGEHDVIFAGEGERIVLRHVATDR
R
IFARGALKAALWGQDQKPGEYDMLDVLGL
>OLS50286.1 peptidase M16 [Rhodovulum sulfidophilum]AB14
MPIRLAVLALIATAGIARAEKVTDFTLQNGMEVVVIEDHRAPVVVQMVWYKVGAADEPPGKSGIAHFLEHLMFKGTD
AVG
PGDFSKIVEENGGSDNAFTSWDYTAYFQRVAADRLDLMMQIEADRMTGLKLSEEDVATERNVILEERNQRTENDPGA
LFS
EQRRAAQYLNHPYGIPIIGWRHEMEGLSRADALSYYRTYYAPNNAVLVVAGDVEPDAVRAMAEAHYGPLAPSETLPPR
LR

PQEPPQLAARHLTYDDPRVAQPYVIRTYLAPERDPGDQKQAAALTYLAEILGGSGTNSVLSDKLQFDSRTAIYSSAFYNG
LSLDKTTFGFVVMPAEGVSLGEAEAGLDRALAEFLDQGVDPALFESIKTQLRASEIYARDNIQGLARRYGAALAVGMSV
E
DVQSWPEVLQEVTPEDVMEAARAVLDPRTSVTGWLETPPGVAPSDAPATIPTGSQEVTQ
>OLS50287.1 RNA polymerase subunit sigma [Rhodovulum sulfidophilum]AB14
MTERQTADTQWVTCVDRIGASRDQAAFARLFAHFAPRVKAFLMKSGADEALAEECAQEVMATVWHKAHLFDPERA
SVATW
IFTIARNRRIDVLRKQRRPEPEDLPWGPDHAPDQSETVALQQETEQLGRALEALPEAQRVLIERAYFGELSHREIAAETG
LPLGTIKSRIRLALDRLRHEMT
>OLS50288.1 peptidase S41 [Rhodovulum sulfidophilum]AB14
MKKFVVAALGGVVAGTLVATQVAGPLLADEAKSNSSVYEQLDLFGDVFERIRSQYVEEVDPTKLIEAAINGMLTSLDPH
S
SYLPPKDFDDMQVQTRGEFGGLGIEVTQEDGFVKVVSPIDGTPAEEAGIESGDFITHVNGESVLGLTLDDAVETMRGPV
G
SEIVITVVRKGVDEPFDVTITRDTIKLTAVRSRVEGNTVVLRVTTFNDQTYSNLKEEWKKSVEELDGIENVNGVVLDLRN
NPGGLLPQAIKVSDAFLDAGEIVSTRGREPQDSDRYNATKGDLTGGLPMVVLINGGSASASEIVAGALKDHRRAIVVGT
R
SFGKGSVQTVMPMKGEGAMRLTTARYYTPSGRSIQALGVSPDIVVEQPRRDRTAAEETEDQPSSALHMRSEADLRGA
LDN
DSMTEDERKQAEEEQAKAEAAAKLREEDYQLAYAVDILKGLSALDPEHK
>OLS50289.1 peptidase M23 [Rhodovulum sulfidophilum]AB14
MAGLPGPFSSGPALAASASADPAETAREAATALRAAAERLRAAGGARDRVAALTGTVRAYEEGLTALREGLRRAALRE
QT
IAAVLDARREEIARLVGVLAGIERNPETVVLLHPTGPLGAARAGMMLGEVTPALQAEAEALRRQMDEVALIRALQESAA
D
DLAAGLAGVQTARTELSQAIAERRDLPQRFAADPERLAHLALSSETLDGFAEGLAQVGGMAPEGSAPPGFEALRGTLPL
P
VEGTLLRGFDEADAAGIERPGLVLATRPLALVTTPVAATIRYRGPLLDYGNVMVLEPAEGYLLVLAGMAQVYGGEGQVL
P
GGAPVGLMGGADPNRAAFLDAAAEGGGNDRPETLYMELRQGGSPVDPAPWFARQ
>OLS50290.1 23S rRNA (pseudouridine(1915)-N(3))-methyltransferase RlmH [Rhodovulum
sulfidophilum]AB14
MRVHVCAVGRLRKGPERDLIEDYTARFDRTGRGLGLGPLSFAEVEDRKGGGPAAEAALLTRALPQGAAICALDERGRTL
G
SPDLAALLARWRDAGRGDAAFVIGGADGLDPALVSRAETVLSFGPMVWPHMLARTMLSEQLYRAATILAGLPYHRA
>OLS50291.1 mechanosensitive ion channel protein [Rhodovulum sulfidophilum]AB14
MLAEQARGFGQSLTRPGWRMYQIGILLGLFALAQIGAWMFYRRLYGWLRAREGWPIWRFRTALILLRRLRIILLVILAW
I
TVWAMRELTWPSRSYLVAIGATLASAWLFVGLAARLVRNPFLRRLVTWGLWVYVTLYVIGLDDEAAAVLDSLRLGMG
DLH
LSALTLIRAAVVTGVLFTGARLIATTSAARIRRDDDISPSMRVLIVKILQMSLYGAAVFLGLKLAGFDLTGLAVLSGAIG
VGLGFGLQKVVSNLVSGVIILLDKSIKPGDVISLGDTFGWINALGARYVSVVTREGKEYLIPNEDLITSQVVNWSHSNEF
VRLDIFFGISYGDDPHLVRRLAVEAAAGVDRVLAERPPVCHVVGFGDSSVDFILRFWIVDPSEGLTNVRGAVFLALWDA
F
KANGVTIPFPQREVTVLPGSRLAAGPLPDTVSDAPLPEKEP
>OLS50292.1 dihydropyrimidinase [Rhodovulum sulfidophilum]AB14
MLDLAIRNAQIVTAADRYVADIGIRDGRIVEIAERVGEAREEIDATGLWAMPGGIDAHVHLAQPQGGGVVMADDFET
GTR

SAAAGGNTTVLPFALQQKGQTLREAVQAYHEQAEGKCHVDVSFHLIITDPTPQVLGQELPALIKDGYTSFKVFMTYDD
MI
LNDAELLEVFDTAQREKALVMVHAEGHDAIKFMTRKLEETGRTAPYYHAISHSTVAEREATHRAISHAELTDVPIVIVHV
SGREPMEQIQWAKRRGRRIFAETCPQYIALTRDDLKGLNMDFSGAKYVCSPPPRDTDSQAAIWEGLRDGTFDIFSSDH
CP
FRYEDPAGKDVKGARTSFKWVPNGIPGVAARLPVLFSEGVSKGRISAERFVALTASNPAKLYGLYPKKGTIAIGSDADIA
LWDPEIETTITQDKMHHGCDYTPYEGMAVKGWPVRTILRGRTVAADFEVTGSAADGQYLPRKISSEAFPG
>OLS50293.1 aldolase [Rhodovulum sulfidophilum]AB14
MAFCLAGRAEAVLLAKGAGFDFAVMDMEHGPIGLSELGQAAAVGQAAGFPVHARVTGPGSPDLARVLDCGAAGVIV
PHVD
SADEARRVVRACRFAPVGRRALPGPLPVADYVPLPADALVGAAGGGKVMAMIESAEALAAVEEIAAVPGLDALIVGTN
DL
ADGMGLRGRVGHPGIQAAFGRIAAAARARGLAFGAMGLPPELVESHALALGASILVATNETNLIVEGGAALLAALRGP
TG
S
>OLS50294.1 cadmium-translocating P-type ATPase [Rhodovulum sulfidophilum]AB14
MTALTSPRTFEWTVTGMDCGSCAGKVRGAVERLPGVDEVEVSLMREKLRLRLEAGTSSPSEVEAAVRAVGFGIGPAGP
QA
GQGAGQAAGGDCDCHCGCGGSGTAADAGPADRAAAPDQEGRSWYRTGRGRFVLATGALLAASWAVGLALPASLA
GLPFVL
ACLIGLAPVARRAWSLARAGMPFTIEMLMTLAATGALVIGAAQEAAMVVFLFAVGELLEAVAADRARAGIRALSALVP
RS
ARIEEADGRVREIAAADLVPGQRMLVRPGDRIPADGTIFEGVSGIDESPVTGESLPVTRGPGDTVRAGSIATEAALRVEV
TRAGEDSTIARIVRLVEEAEAARAPTERFIDRFSRYYMPVIVGLAVLVAVVPPLAFGQDWGTFIYRALALLLIGCPCALV
ISVPATIASALSSGARRGLLIKGGAVIETLAKVQNIAFDKTGTLTEGRPQVTGIVPLEGRAADEVLRLAGAVETGSSHPL
ARAILNAAEARGLDLPQARGARSLPGRGAEAEVAGASIWVGAPAHAPEGIGAGQEMAAAVAEIEERGGSAVLVVAEG
RAL
GVIGLADAPRADAAEAVAELAGMGLRAVMLTGDNPRAARAIAEPLGLEPRAELLPEDKLAAIAEMADRGGVAMIGDG
IND
APALKRAGVGIAMGAGSDVALETADAALLRNRVTDAPAMIRLSRAAMHTIHQNVALALGLKALFLVTSVTGLTGLWM
AVL
ADTGATVLVTANALRLLAFDPRRAARD
>OLS50295.1 2-methylcitrate dehydratase [Rhodovulum sulfidophilum]AB14
MELHLLRMHKSAEALAREEQLAWKLAELAADPVSVPDDTAEMIVNRVIDNAAVAAASVARRPVASARAQALCHPYKR
GAT
VFGMPKDTRVSPEWAAWANGVAVRELDFHDTFLAAEYSHPGDNIPPILAVAQHCGLSGQALLRGIATGYEIQVNLVK
AIC
LHEHKIDHIAHLGVSAAAGIGTALNLPKEVIYQAIQQALHVTTTTRQSRKGEISSWKAYAPAFAGKMAIEAVDRAMRGE
G
APSPAWEGEDGFIAWMLSGPEAVYEVPLPGKGAPKRAILDTYTKEHSAEYQSQALIDLARRLRGEIGDLRQVESIVIHTS
HHTHYVIGTGANDPQKMDPTASRETLDHSIMYIFAVALEDGAWHHEKSYAPERAARPSTVDLWHRISTREDPEWTRR
YHS
RDPQEKAFGGRVVVTLKNGRVIEDEIALADAHPHGARPFERPNYVKKFLTLSEGVLDPKEQDRFLNAAENLADIPEAGL
G
ILNFTVAPEKLGAPRPRGLFDRS
>OLS50296.1 hypothetical protein BV379_03110 [Rhodovulum sulfidophilum]AB14
MVEATPLGVRAALLEMRARFGPSMPFDGSLGMAEIVLAEVLNNIVEHAYSGGRSGPIELRLVRDGIWLRVDVRDYGRA
MP

GESLPDAEKTNADMTGAEFRPETAPEGGFGWFLIRAFARDLDYSRNGNQNRLRFTVPLNEDC
>OLS50297.1 DNA repair protein RecO [Rhodovulum sulfidophilum]AB14
MEWRDEGVVLAMRPHGESSAIVELFTPGHGRHLGVVRGGAGRRLAPVLQPGAQLSAVWKARLEDHLGAFAVEPLRS
RLGH
VMGDPAALAGLDAVTSLLAFALPERAPHPGLYARSVALLDLLGADGDWPLAYLRWEMALLEEMGFGLDLGRCAVTGA
RDD
LVFVSPKSGRAVSRAGAGDWADRLLPLPPCLMGQGAALPGEIAGGLRLTGHFLSKWLAPALGDRPLPGARARLVRLLA
RV
PG
>OLS50298.1 EamA family transporter [Rhodovulum sulfidophilum]AB14
MGRAEWAMLLALSVLWGGSFLFVGIAVAELPTLTLVWLRVALAALVLWAAVAATGRRLPRAPRVWAAFLGMGLLNN
VIPF
SLIVAGQHSIGSGLASILNATTPLFTVVVAGLLLADERPGRLQLAGVAAGLAGVAVMIGPDALAGLGSNALAQLAVLGG
A
LSYACAAVFGRRFRHMGVDPLVTAAGQVGAATLLLSPAMLIADRPWTLPMPGVGTCAAVLGLAVLSTALAYLLYFRILA
R
AGATSLSLVTFLIPVSAILLGGLVLGERLVPTDFAGMACIALGLAAVDGRLFRR
>OLS50299.1 LysR family transcriptional regulator [Rhodovulum sulfidophilum]AB14
MEFRTLRVFVEVVRAGGFSAAAGRVHATQSTISKAVRQLEDEIGMPLLIRGPAGIALTAAGEIVFQRAQTLLAGRETLKT
ELQELAGLKRGLLRIGLPPIGSSELFAPVFTLYRQRYPGVDIKLVEHGSDRLEELLRASEIDLAASLLPVSEEFDWLAMR
RDPLVALMATDAPLAQAPGPLTLDDLRSMPFILFDSGFALNRIIRAACARHGFEPEVTARSSQIDFIIRLAAAGLGVAFL
PRLIARARVHVPVAQVPLDEPGTDWAMATLWRRGAYLPQAARAWLDIAAGDPARTAEGPEQD
>OLS50300.1 glycerol-3-phosphate dehydrogenase [Rhodovulum sulfidophilum]AB14
MLGAGSWGTALARVLVRNGRNVRLWARRPALAREIAATGRNSQYLPGIELGDALRATASLTEALEGAEAVLIVTPSATL
R
EMCRAAAPHMPAGIPIALCAKGIELGSGLLLSEVAAQELPGHPIGAISGPTFARETALGHPTAATVAFPFAYADRLAPHD
SPAARMAMSVGGHGFRPYVSDDLVGVEVGGAVKNVIAIACGMMSGAGFAENTRAALIARGVDEMKVLAESLGGQR
ETVTG
LSGAGDLTLTCSSTTSRNMALGTQLGQGIARADCFGGRPVVVEGEVNARSVIDLARRIEVPMPICETVNAVLHEGADLE
R
SFSDLWTRPIEAEPRALTLSLDHPAPTVPLPFGGSS
>OLS50301.1 hypothetical protein BV379_03365 [Rhodovulum sulfidophilum]AB14
MGPTLPTDRDARLARLREAAEKLEARFLSEMLASAGLGNVSGELSSGTFGGGIGETQFTSFLRDAQATEMARAGGIGLA
E
SIFESLKERMDDPD
>OLS50302.1 flagellar biosynthesis regulator FlhF [Rhodovulum sulfidophilum]AB14
MNALQMAQSAYSAPNQNKTRSPRATEYAAFVRITSQIKQAHEAGRCAFPALAAALHKNRKLWRTLAIDVADKDNVLP
AEL
RARIFYLYEFTDHHTDEVLAGRAKPTALIEINTAIMRGLAERERVS
>OLS50303.1 histidine kinase [Rhodovulum sulfidophilum]AB14
MRDSKRRADDAAGPGDAPCGRSGADRRSRGTWRGFVSLKRSPLARRIIAANLVAQIVLISGVLYLNPARDSLLSLRESG
M
VAETNLVADMFEAQIRRDDPLERLASNSLDVDAMLNGLELAQGAEVFVFDTAGNLVGHARGLVRTDLPVEEYEGDSW
ITV
ITDALYRAWLGWVSLFGREEGNDAEGDPQRRLTDQVAITLTDGQQVATGVDSSGHTTFSVTAQILRDRQVVGVVAM
VSAS
GEIDRLVRIQREQVLQVFVIALLVSVGLGLVLASTIANPLSELAAAAEIGQNRNARSMRPGRVRIPDLTARPDEIGRLSG
ALRGMVAALYDRIDANEQFAADVAHEIKNPLASLRSATGTLRVARREDQRAKLLDVIEHDVRRLDRLVSDISNASRLDSE

LVKEEETTVDLVRMLRGLADHLGQQARERGIDFIADLPPDPIEIQGLEARLAQVFVNLITNAISFCEAGDAVRVWARKRD
NRVLVVVEDTGPGIPGEALTKIFNRFYSERPAGQFGNHSGLGLAISKQIVEAHRGVIWAENIRPTEADVTSEQLGARFVV
GLPI
>OLS50304.1 transcription termination/antitermination factor NusG [Rhodovulum sulfidophilum]AB14
MAKRWYSVSVLSNFEKKVAEQIKQAVADAGLEDEIEEVLVPTEEVIEIRRGKKVTSERRFMPGYVLVRMEMTDRGYHLV
T
QINRVTGFLGPQGKPMPMRDEEVNQVLNRAEEGEAQPRSLITFEIGENVSVTDGPFEGFAGMVEEVDEDNQRLKVTV
SIF
GRATPVELEFAQVSKGQL
>OLS50305.1 30S ribosomal protein S8 [Rhodovulum sulfidophilum]AB14
MNDPLGDMLTRIRNAQMRGKSTVRTPASRLRAWVLDVLKDEGYIRGYEATTGKTGLPELEVSLKYYEGQPVIREVKRV
ST
PGRRVYMSVKDLPSVRQGLGVSIVSTSKGVMSDASARSANVGGEVLCTVF
>OLS50306.1 GNAT family N-acetyltransferase [Rhodovulum sulfidophilum]AB14
MTQSDILIRAPRPGDHARLSAIWYAASREAHGFLGEDRLRAQRKLIETQYLPGSELWLACRQDRPMGFIGLLGAFIGGLF
VAPEQQGQGIGRALVAHAAGLKGWLELEVYSANARACAFYRGLGFVEVSRRPDDDEGLPFETLRLRREA
>OLS50307.1 phosphoglycerate mutase [Rhodovulum sulfidophilum]AB14
MSLRLILVRHAKSSWDDPSQDDHDRPLNARGREAAPKIGRWLAERDHVPAEVVSSTSCRTLETWDRMAPLLGESSVM
RRD
PGLYHAPAERMLETLNNCAASPVLLLGHNPGIADFAARLLEAPPELPRFANFPTCATLVAEFDLGDWTEVDFGTGRLVD
F
ITPKQLDG
>OLS50308.1 ribonuclease P protein component [Rhodovulum sulfidophilum]AB14
MNRASETSESVKASPAFSSCLTVLSRRADFLLAARARRQSAPAFTLQARRRTEAPEGGIRIGFTCSKKVGNAVARNRAKR
RLREIARTELPVHGRPGWDYVLIGRPVTTASRDFAALRADLRRALERLHANRS
>OLS50309.1 hypothetical protein BV379_04030 [Rhodovulum sulfidophilum]AB14
MAKEIKAAEKAVSTARDAATKPLHAAWQAEIARWKPTLEDLDRIKKGLAALVSAFKVRLKAEQDEAARKARAEADRKR
RE
AEEATRTAAAGDIEAQRAAAQAQAEAKAARKAASAAGKDRVKGVRTVTRYEIESHKAALHDIAKNDRDALTDFVEAYV
RR
HHKDRVIAGVRVWEEKEAY
>OLS50310.1 hypothetical protein BV379_04250 [Rhodovulum sulfidophilum]AB14
MTKALILVDVQNEYFDGGKLPLPDMDEALTHIEALLTCFRSNRAPIFHIQHFALTADGPFTLGSPGAELHDRVKPQLTM
Q
PPEVVIPKYYTNAFRMTWLAAALQGSGVSEVVVVGAMVQNCIDATVRGADDFGFKVTVASDACAAAPITLKDGRVIE
AAT
VHDLFLEALNNVADVLPTTEVIAVIEQERDPAADHVSWGG
>OLS50311.1 hypothetical protein BV379_04445 [Rhodovulum sulfidophilum]AB14
MVTGDAAKGEKAFIQCATCHSVVSPSGETLAGRGLKVGPNLYGMPGRTAGTLEGFRYSKSMKAAGEAGLVWDEDKV
SIYA
EDPTAYLRDYLDDPRARGLMTYNARDPQEVKDVYAYISKLSEE
>OLS50312.1 GNAT family N-acetyltransferase [Rhodovulum sulfidophilum]AB14
MPVIPALMTERLALRGPEPRDFEALRAFYASERSGFVGGPKSPEGAWRQLAAEIGHWALRGYGRWIVTERASGRTVG
MVG
LWNPEGWPEPEVAWDLFEGFEGKGYATEAARAARDHAYGALGWPTAISLVDPANAASARVAERLGAVYQGEVSHET
YGAI
GVYRHPAPEACR
>OLS50313.1 transcriptional regulator [Rhodovulum sulfidophilum]AB14

MQDRLDIVFSALADPTRRAILAMLLEDDMAVTDVAEPFEMSLAAISKHLAILTRAGLISQEKRGRVKWCKLEPDALKDA
S
IWMQGFGQFEAVNLDAFERFLAAELNG
>OLS50314.1 trypsin [Rhodovulum sulfidophilum]AB14
MVALAAGSGGSAGTGADGPLHPLVTGDQSRGWKAVGRLELGAGGFCTAALVAPDLVLTAAHCLFDGTTGGRLPLAG
LEFR
AGWRNGRAEAFRGIRRGAVHPGYAHGKAASVGRVRHDLALLQLDRAIRLPGLVPFGIASQPGRGDAVGVVSYARDRS
DVA
SLQELCHVLAGIDGVVMLSCEVDFGASGAPVFRIEDGVPRILSVVSAKAELGSRQVALGTVIHDGLEVLRTRLAPDAPLG
ATPRFGSGTAPSAGAKFLRP
>OLS50315.1 microcin ABC transporter ATP-binding protein [Rhodovulum sulfidophilum]AB14
MPVLRQVSLGIGRGEILGLAGESGSGKSMTALALMRLLPEGARAEGRIRLDGRDLMDLSERQMCRRRGRDLGMIFQE
PMT
ALNPVQRIGDQVAETLIVHGEATRAEARRAAARLLHRVGLPPERFPPGRFPHELSGGQRQRVCIAMAIALRPQLLIADEP
TTALDVTTQARILDLLKGLVAETGMGLLLITHDLAVISGLADRLAVMRAGEIAEEGPTATVLTARRHPYTSALVAASAHS
PATVPPPPGPPLLEVRDAVRTYPGPRRGLIGRSPPQRAVDRVSFGIARGESLGLVGESGCGKSTLARAVLGLEPLQGGEI
RLDGTAIRTGSPVPGQVRRRMNAVFQDPYGSFNPRHSVARLIAEPFHLTGRPADAAARIDEALLAVGLDPTDRTRFIHE
F
SGGQRQRIAIARALIARPDLIVLDEAVSALDVRVRAQILDLLADLRARFGLSYLFISHDLSVVRSITERVLVMKAGRIVE
EGATEKVLHDPAHAYTKALLAAAPKLKEPD
>OLS50316.1 transporter [Rhodovulum sulfidophilum]AB14
MLGNQPIAFILLALTVGTFIGRFRFKGISLGSTAGTLLVGVVLSMIAEAAFGITYSIPGILSSFMLLLFMYALGLKVGPQ
FFSGLRRGGLAFVGIGVMVWSLNWVICYFGAAMAGLAPGYATGLISGSYTITAILGVGQSALDSGAFTPPDGISAAEVG
A
NMAAAYAISYVLSSVGIILLIRYLPRLFGRDATVDAKQAEAEYSGGSTDPVPGAPGALVMNFSPYDLRAFTVTHESLLGR
SVAELAADFPEAPILRLVRDGVIVNLEDNPTIAPGDIVTVRANVHDLILEDGELIGPESDEPLARNIPMEVADIHIGSHA
ISGKTLAELDTQAHGGVEVQAHFRAGNELPLGPNSDVRFGDVLRLTGPDAALKAVARQFGGRAILPSFKSEVLYLALAM
L
VGYLVGIATVRIAGIPFALGTSAGCILAGVAVSYFRSRNPEFGGPVHEGARSFLQDFGLNTFVAVLSANVGPKVISALGG
DTILWLALIGTAGALIPPLIAFWVGIKVFGLNSVISDGATTGGRNSTPGLNAIIEESGSSVAAVPYPVSYALTTVLALIG
GYFSMILS
>OLS50317.1 carbamoyltransferase HypF [Rhodovulum sulfidophilum]AB14
MEVVPGREIRVRGQVQGVGFRPFVWQLAREFGLGGEVFNDAEGVLIRAAGGDLDGFQAALSARAPGLSRIDALEARD
CRF
DAVPETFAIAASRGGAARTRVTPDAATCPDCLAEIAGGGRRQGYAFTNCTRCGPRFTILRGLPYDRAQTTMAGFSMCP
SC
RAEYGDPADRRFHAQPIACPDCGPRLWLERAGGVVAAGDPLALAADLLRQGGILAVKGLGGFHLACDAGNPATLDRL
RRA
KRRPAKPFALMLREADLTAVCTPSAADLARLRDPAAPIVLVPGTGVLPEAVAPGMTRLGVMLPYTPLHHLLLEAVARPL
V
MTSGNLSGAPQAVGNDEARALLDGLADAVLLHDREIARRLDDSVERADPPMILRRARGRVPGTLPLPEGFGGAPQVL
ALG
GQMKSAICLVKGGQALLSHHLGDLDEADSWDAFLGAIADYGALFDHRPALVACDAHPGYRATQHAGTLGLPVIEVQH
HHA
HLAACLAENGWPRAGGPVAGVVLDGTGYGPDGTVWGGELLLGDYRGVERVGHLAPAPLVGGDAAAREPWRNLLVR
LDAAG
LADSADRLLTEYPLAALRAAARAGLNAPASSSAGRLFDAVAAALGLCVGRQSYEGEAAMRLEALAEGCPADRPYRFAGL
D

PAPAFREMMGDIEAGVPHPLISARFHAGLARAFADPARALVETGRARAVALSGGCFQNARLLAETLAALRGVKVLIHRE
T
PANDGGLALGQAVIAAAGELPQMESF
>OLS50318.1 hypothetical protein BV379_05570 [Rhodovulum sulfidophilum]AB14
MHDQSADLRAEALALAQKVHSAQVDKLGNPYIEHVCAVASAVAHLGPEFEIVGLLHDSLEDCAVRSLVSMDILIGLFGP
D
IADAVTAMTKQPHEDYETQYLPRVMSHPIAAEVKRADVAHNRSRLHLLDSETRSRLEAKYRSFSERYRSDLNFIYR
>OLS50319.1 gamma-glutamyltransferase [Rhodovulum sulfidophilum]AB14
MAALSRTMEIRKHVVETDRGVVAAQHRQAAEAGAEALRAGGDAVDAAIACSFVLGVLEPWMSGPAGGGAALHWR
ADTGRA
HALNFGMRAPASLRIEDFPLSGEGLAGDLFPWDRVVEDRNVLGAKAVAVPALVDGLEAAHRRWGRLPWAELVAPAIA
AAR
RGLAIDWYAALIIASATRDLASDADAAALFLTDGQWPRSFSWAADSDDRLDQSRMADSLDILAREGAKALHGGDLGA
ALA
RDVRDKGGYLGLDDLAGNRARFETPLAVPYRDALFHVMPGLTAGPTFARAMEGLAGETLSGPDEACPAYARALIAAYR
DR
LSGMGADGEISAAPGSTTHFSVVDRDGNMVAHTQTLLSLFGARVVSPSTGLLLNNGIMWFDPVQGRPNSLGPDRPCL
MNV
CPALGHAGGRRFALGAAGGRKIVGAVTQLASFLTDFGMDLEAAFAQPRIDVSGTDRIVADDRIPDRICDALKAVAPVTV
T
RKSILPYPFACPSGVLQEGSRRSGMTEPTSAWGDAVAEQG
>OLS50320.1 IclR family transcriptional regulator [Rhodovulum sulfidophilum]AB14
MRAVGRTMRVLSAFHHASGPLSLSEIAARAELDRSAVQRMVHTLVRLGYMRRSLDDRGYLPGVRLLDHTLDLLRLDPV
VQ
KATPVLLELRKTVRERVDFSLYDETRLVYALRMQSKRETFYATLVGHSVPTFCTAGGRAVLSALSDDAAREVVMRSQLH
K
FTSKTVNDPEGILALIAQARSDGYAVVVDEFVQGEVAIGVAVAGRDGVPLGAIHVAGSLSEWTRDAFVRHAAPVATEA
AR
AINGSL
>OLS50321.1 hypothetical protein BV379_05645 [Rhodovulum sulfidophilum]AB14
MFEVSHPQRAGLLADAPRFGLVTAFFARLRNRDEVRRTRDALSLLTDRELDDIGLCRGDIDRLLH
>OLS50322.1 tRNA lysidine(34) synthetase TilS [Rhodovulum sulfidophilum]AB14
MAAALERACPGEAPLVVAVSGGGDSLALLLAAAARAEASGPKVSAVTVDHGLRPEAAAEARRVAAICATRGISHDTLV
WQ
GWDGRGNLQDAARQARLRLIAGWARERGIGRVALAHTRDDQAETVLMRLARGAGVDGLAGMAPERRAGGVTWLR
PFLDIS
RADLRLYLEACGQDWIDDPSNDDRRFERVRVRQALDLLRPLGIGAAGLAATATRLASARAALEAATVEAAARLVREEAG
D
LLIEAGGLAALPAEIARRLMLAGLGWVSGAAYPPRARALEATLAALTRGPRATLHGCLLQRARGRIRIGREPAAVAGLSC
APGSLWDGRWRLSGPEGAERQEALEIRALGAAGLAACPDWRAAGLPRTTLIASPAVWRGGGLIAAPMAGHGKDWS
AAVNC
SFTAYLLSH
>OLS50323.1 hypothetical protein BV379_05765 [Rhodovulum sulfidophilum]AB14
MRRLFQSGVLLGVLAGCQLAPAPPETRPVARPDGAETRPRSADSEIIARHYARVQNDLLARGLLRTDGGEADVPFTRH
AL
VEDFIRVALFDEYVTRDGHLVARETQSRLRRWEQPVRFGLVFGDTIPPGQRAIDRAEIAEYVGRLSRLTGLSMRITAPEQ
ANFQIFVVNEDERRRIGPELRRLIPGIDIAAESTIAVMPRSTFCLVLAFSEGDASTYSSAVAVIRGEHPDRLRQSCIHEE
LAQGLGLANDSPAARPSIFNDDEEFALLTPHDELLLKILYDRRLRPGMRPVEARPIVEDIVAELMAGES

>OLS50324.1 hypothetical protein BV379_05965 [Rhodovulum sulfidophilum]AB14
MEWQGLLARSRHGEPQIVDAPERPAFLVDLDRVIFSQPFRRLANKTQVHPLYDNDHLHHRMIHSLETSSVGRSLGFEV
GQ
ALAARGILPEGAGHDLSYILQAACAAHDIGNPPFGHAGEDAISEWFATRRPFGEDLSDGQRREFEHFEGNAQGFRILTR
M
EMYRLEGGMRLTHAVLGAFTKYPCLAKDGALGRDAPTIGLKKYGAFEAEAAHLAEMAESTGLIPQEAGWRRHPLAFLV
EA
ADNICYNILDLEDAYTTGVLSHGEVRPLLGALIGKEVAPVPGGTPAEHVGHLRARAVHGAIRASVAAFLDHHDEILAGRF
STELTDVFALKEAFAAISEVANDRIFPSSRKIELEVAGRDLIFALLDRFHAVIEGLADHGWDAAAMAEKKPYLRKLVTSA
GMDLRDVCDPYSGLHALTDFVSGMTDRYAVKMARPLGL
>OLS50325.1 hypothetical protein BV379_05975 [Rhodovulum sulfidophilum]AB14
MPRRGRLWGEFVLFYVCTPLAIAVFMPPQALFPVLLSLTAVGLGLLSHTEGFRWSELIRGLRGIDPWLVLGFAVVTTAIV
GTLTVLVVPQDALALVRGNPLLLASILLFYPPLSALPQEIVFRVLFFRRYGPILPGLRSAIVMNAALFSLAHLMYWNAVV
CGLTFLGGLVFAWACEARRNFPLAVLLHAVAGWILFTGGLGVLFHAGNAVRPF
>OLS50326.1 Hly-III family protein [Rhodovulum sulfidophilum]AB14
MQRVPEHPRDYSPTERFSDAIVHVTGLAAVAIGVPTLVVLAILIRGDTPAILGITVYGVALCAMIGFSALYHMVRHPNWT
PIFRRLDHSAIYWKIAGTYTPFTLLSGHGAALLAGLWAAAMAGTGLRVLAPYRFRVVNLVLYPMMGWAGVVAGWSM
FASL
SAPVLGLAIAGGALYSVGMIFYLWERLPYHNTIWHVFVLIASMLFFAAITTHVVQTG
>OLS50327.1 elongation factor 4 [Rhodovulum sulfidophilum]AB14
MTELRYIRNFSIVAHIDHGKSTLADRLIQLTGTVAERDMKEQMLDAMDIERERGITIKANTVRIEYPAQDGHTYVLNLID
TPGHVDFAYEVSRSMRAVEGSLLVVDATQGVEAQTLANVYTAIDADHEIVPVLNKIDLPAAEPDRVKEQIEDVIGIDASE
AIEISAKTGLGIPDVLEAIVKHLPQPHEGERDKPLKALLVDSYYDPYLGVVVLVRIIDGVLKKGDRIRMMRTGGVYPVDR
IGVFRPAMQNVDELGPGEIGFLTASIKQVRDTRVGDTVTTEKKGCEAPLPGFKPSQPVVFCGLFPVDTNDFDDLRDAIE
K
LALNDASFTYEMETSAALGFGFRCGFLGLLHLEVVRDRLEREYGIDLITTAPSVIYKIHMKDGEVRELHNPADMPDLTYV
DHIEEPRIKATILVPDEYLGDVLKLCQDRRGEQLDLTYAGSRAMAVYDLPLNEVVFDFYDRLKSVTKGYASFDYQISGYR
TDFLVKMQILVNDEPVDALSVMVHRDRAEMRGRAMCEKLKELIPRHMFKIPIQAAIGGRVVARETLAALRKDVTAKCY
GG
DATRKKKLLEKQKAGKKKMRQFGKVEIPQQAFINALKMDD
>OLS50328.1 hypothetical protein BV379_06030 [Rhodovulum sulfidophilum]AB14
MQEHRTETRSAAVTATAPQGAVLRGLDKLSGDTVDLTLAPGETGQVWRLKVTLDECRFPADNPASDAFARLDISDPSR
GS
HLFSGWMIASSPALNPLDNARYDVWVLRCRSS
>OLS50329.1 TonB-dependent receptor [Rhodovulum sulfidophilum]AB14
MNGQDDPLNFSSVHEGEDIEKGRLRNFEDVLRATPGIGVNSSGGTNLSTIYIRGVGSMYPMSLDDSAATFTLDGSPLSA
R
HISLGTLDAERIEVLKGPQGTKSSAAALAGTVNVITAKPSRVQEGYLRSEIGQEGQYLLEGAVGGPLSETLSGRVALRYS
GSEHWVENLQTGGPVSDPKEFAFRGSLLWEGQQGTSALLTYEHQKIEALPNLIMLKPYDRYGKLDLPPGLYDQVEKSID
R
TSLQITHDLGGSRITSLTSYLTGDNTEVAAYDRILYEALYGTPGEFWNIDEAREKVFNQDIRWASSEGAPFDWVIGASFE
HFDGSYDTPRNTYGTSSATFRDFESERYALYGNVTIPLGGEVDLSAGLRQNWTESSYDGAYHGGSSIVYESRSRSEDFLT
GQLGLSWDVSPATTLHAKIARGHVPGGYNIYATQPADSDPYHGATNDTVEVGFATAFADGALELNGALFASQVDNNH
LLS
YDSVTYVVSTLNADTRSRGLELQGTWHASPAFSISAGVSYVDAEITSNVFGIGDGDVNSGNKVPDVAPWTAHLSTNYR
RT
VQSFLGLANPILDARLDYSFVGSRPADPQNHFDLDPYHKLDMRLGLTQGQTEYYVAVENLLDEQIELYGYYAPDSGASY
G

GIARGRAVTVGLTHRF
>OLS50330.1 hypothetical protein BV379_06195 [Rhodovulum sulfidophilum]AB14
MPVSKALRDTGLFHPTQGRQCRAVFLKLQDGVDLLYWAGSFQSGMELPLKDDSDRVSLSFTTNLKGTASRSFTDKPDA
DL
PVRCNTGSIEYRPGRSGIYRQKGALENLTIMVENNLFNQWVDDDDTELVELTKHGGSSGGYLGGELSAVIHLLNRELAF
H
DRDQPSQRHPLWFQAQALSIAGLFLECRTPSRVESVNPDLRRQLLHARDLLLADLSAAPSIPALAHEAGLSAPSLTRGFK
KLFGTSPYKLFQRERMHAAHHRLSIGRASVTTVAADLGYINLSHFSAAFRKEFGVLPNKMNRKG
>OLS50331.1 branched-chain amino acid ABC transporter substrate-binding protein [Rhodovulum sulfidophilum]AB14
MTRPTLTRRGVIKTGAYAGAGLALPTIFTSRARAFTNAPTGSTVKFGFNVPQTGPYADEGLDELRAQELAIEHLNGMGD
G
GMLNTFSSKALDGTGILGKQVDYVTGDTQTKSDAARASAKAMIEKDGVVMINGGSSSGVAVAVQGLCQEAGIIFMAG
LTH
SNDTTGKDRKKNGFRHFFNAYMSGAALAPVLEKAYGTDRRAYHLTADYTWGYTQQESIQASTEAIGWETVNNVLTPV
GAG
DFSSYIAPVLNSGADVLVLNHYGGDMINSITQAVQFGLRDKQVNGKNFEIVVPLYSELMAEGAGDNIKGVYGSMNWN
WQL
SDPGTKAFVKSFGEKYGRPPSNSAHTCYVQTLLYADAVARAGSFDPCAVAEALEGFEFDGMGNGPVLYRAEDHQCFK
DVL
VMKGKDNPSTKYDILEIVEVTPVDHVTYPPDHPMFGGAEATLGQCNNGA
>OLS50332.1 sodium:solute symporter [Rhodovulum sulfidophilum]AB14
MAFDMLVAICLVYVALLFGVAFLAERRGARGSNGWLRSPVIYTLSLSVYCTAWTFYGAVGYAARSGLEFVTIYLGPTLVL
IGWWWTLRKLVRIGKTQRITSIADLISSRYGKSNLLGVIVTLIAVVGTTPYIALQLQSVTLSFAVFAAAGTNGPWQSTDL
KAAAFSVAVGLAVFTVIFGTRNLDANERHHGVVTAIAVEAAVKLVALLAVGLFVVWGIGGGAQATLARIEASPLAGMP
LA
PGRWIGLTFLAGAAILTLPRMFQVLVVENEDERHLATAAWAFPAYVFLMSLFVVPIAAVGLAEMPPGANPDLFVLTLPL
A
RGQDGLATLAFLGGFSSATSMVIVAAIALSTMVSNHIVMPLFLHWTRAGATMSGDVRNLVLRSRRLSIGGVLLLGYLYY
R
LTGGSEALAEIGLISFAGVAQFLPALLGGILWRGATRAGALAGLTLGFALWAYTLFLPSFGEGAVMSASLLADGPWGIG
W
LRPQGLFGLDGLDPLVHAVVFSMGANALGFTLVSMLDFPGPMERLQAAQFVNVFDHSPSGQGWSQGRAEAEDLLL
MAQRI
LGADDAQRLFQRAAVDQGKQGYLPDTTPEFLQRLERRLAGSVGAATAHAMVGQIVGRSSVSVEDLMKVADETAQM
MEYSS
QLEAKSEELSRTARQLREVNEKLTELSVQKDAFLSQISHELRTPMTSIRAFSEILMENGDLPPERLVDYSRIIHQESMRL
TRLLDDLLDLSVLENGQVSLHPQPVSLSDLLDRSVAAAGGGSGRAPMTIRRDPAAERVALVTDGDRLSQVFINLVTNAR
K
YCDAAEPELTISVTEEEGAVAVDFVDNGSGIPERSRSIIFEKFSRLTDPRKAGGAGLGLAICREIMGRLGGAIVYLPGQG
GAAFRVTLPRGCAEVA
>OLS50333.1 TIGR01244 family protein [Rhodovulum sulfidophilum]AB14
MMWLDKEFAAAPQPGIETLAALAQAGFRSVICNRPDEEVAADACAAAMEAAARAAGLGFAAIPVRHAPITPAMIAA
QTEA
MGALPGPVFAYCRAGFRSSVIWALAQAGRKPTADIMAALERAGFAMPGLEAQIEELATAR
>OLS50334.1 5-aminolevulic acid synthase [Rhodovulum sulfidophilum]AB14
MGLALGLGAALTASAAPVDGKDARKLVFKPDGAEVAVVDLPFLSETDRKTLVVVGAQQPYYGAIAVAPTEGLRSKATL
AA

ANYHDVDAARVAALRGCDAARKPEGAPCVIAAEIRPKGWEPRALQLSADATDGLRSEYGRRGGPRAMAISPSTGRWA
VIK
GQGAASRALEACADQSGATDCRIAVEDR
>OLS50335.1 peptidase M48 [Rhodovulum sulfidophilum]AB14
MTGPARGLIGLALALILSFAPAASRAQSLIRDAEIEYALKRLTQPLAVAAGLSPERVRVMVLQDSSLNAFVLDSQTVMVH
SGLLLRLESAAELQAVISHELAHIANGHITRRLSNMRSAGSAAAMGMLMSLAVAAAGHADAGAGIAMGSSSTAQRLFF
AH
TRAEEASADQAGARYMARAGISPQAMVKVLEIFRGQEALSQSRQDPYIRTHPLNADRLRNARGYASAYSGAVQSDPE
ADY
WFARARGKLGAFLQNPRYTLRRAEKDKTAVGMMRRAVAWHRTPDARRAMAEIARLEAARGSDPFVQELKGQILLES
RQFG
PAVQAYARAVQMAPKEPLILAGYGRALLALDRPAENRKALAALEKARARDPRDPRMLRDLAVAYARTGNNGMASVA
TAER
YAVAGRMKTAAVHARRAEGLLPRGSPGWLRADDILAAAEKLKQR
>OLS50336.1 metal ABC transporter permease [Rhodovulum sulfidophilum]AB14
MRTIRKVAPYLWPQGQGWVKRRVVLALAALLLAKVVSVGTPFLYKAAVDSLTGEARNDTLLLAVGAVGLTVAYGMAR
LMN
TGFQQLRDAIFSRVGQRALRRLALETFTHIHRLSLRYHISRKTGGLSRVMERGVKGVEFLLRFLLFSIAPLVLELAMVGV
VFALAFDLWYLAVVAAVIMVYVKFTFVVTDWRVKIRRRMNDQDTDANQKAIDSLLNFETVKYFGAEAREAERYDRAM
EGY
EEAAIKTSYSLALLNFGQALIITAGMIAVMVMAALGVQAGKLTVGDFVMVNAYMIQITMPLNFLGAVYREIRQSLIDM
GD
MFALLEQPAEVTDRPGAAELKVTGGAVEFRDVRFGYDPGRPILKGVSLSVPAGRKVAIVGPSGAGKSTIGRLLFRFYDVT
GGAVAIDGQDLREVTQDSLHRAIGVVPQDTVLFNDTILYNIAYGRPGASPAEIEAAARAAKIHDFVTSLPKGYQTLVGER
GLKLSGGEKQRVGIARTLLKNPPILLLDEATSALDSETEAGIQSELKAMGEGRTVIAIAHRLSTIVDADEIVVLEDGQVV
ERGTHEALLAHGGRYAAMWQRQQAGEDGDIAAE
>OLS50337.1 heme ABC exporter, ATP-binding protein CcmA [Rhodovulum sulfidophilum]AB14
MELVARDLACARGSVPVLEGVSFTLAAGQALMLRGPNGSGKTTLLRTLAGLQPPLEGSVSAPPESIAYAAHADGLKATL
T
VSENLTFWARIFGQSDIGPALEAFDLGDLADRPAQNLSAGQKRRLGLARLLVTGRPIWMLDEPTVSLDAASVAQFGRA
VR
GHLDRGGAALIATHIDLGFEAALLDISTFRARPRRIDSFDEAFA
>OLS50338.1 thiol:disulfide interchange protein [Rhodovulum sulfidophilum]AB14
MLLPPVLFAGLAALFFIGMTRDDPDALPSMLIGKPAPALALSDLAGQGPFDAATLADGNIKLVNFWASWCAPCRQEHP
EL
ETLAGTGVEIYGINYKDDPTKAMRFLDELGNPFVALGADPSGRTAIEWGVYGVPETFVIDGEGTVLLRHPGPLTPAIVEA
EIRPLLDNPPTTSN
>OLS50339.1 hypothetical protein BV379_06575 [Rhodovulum sulfidophilum]AB14
MKKTETHVQSFGAPIAPHGLTSRQKAAIIVRLVLNEGIDLPLHSLPASVQTDLADEIANMRYIDGNTLQGVIEEFLMEIE
QFGLSFPGGIDEAMRLLGDHLSPEAAAAIRARFGSGRGGDPWATLSELPPDRMTELLLAESPEIAAVALSKLPSPIAATI
LGRMPGPEARRIAFAISRTAEVPPAAVTRIGRALADRINSAPEPAFELAPETRMGAILDVAPAATREDVLQGLRETDAEL
AEKVRQAVFTFADIPDRLDERDVPALTRAIEQTKLITAMAGADGRTGPAVEYILANMSQRMANQIRDEINERDAPGAE
EA
EAAMSEVIGAIRNMEAAGEIKLRKPSKTA
>OLS50340.1 UDP-N-acetylmuramate--L-alanine ligase [Rhodovulum sulfidophilum]AB14
MNAATKLPTDVGPIHFVGIGGIGMSGIAEVLLNLGYRVQGSDLKASKITDRLAGLGATVFEGQREENLENAEVVVISSAI
KPGNPELDAARRRGLPIVRRAEMLAELMRLKSNVAVAGTHGKTTTTTMVATLLDAGGLDPTVINGGIIHAYGSNARM
GAG

EWMVVEADESDGTFNRLPATIAIVTNIDPEHMEHWGSFEALRQGFYDFVSNIPFYGLAVCCTDHAEVQALVGRVTDR
KVI
TFGFNAQADVRAINLRYETGKAHFDIALQAEEMVIEDCTLPMPGDHNVSNALAAVAVARHLGMKADEIRAALEGFAG
VNR
RFTRVGEVNGIAVIDDYGHHPVEIAAVLKAARQATEGRVIAVHQPHRYSRLHSLFDDFCTCFNEADVVGIAEVYAAGEE
P
IQGATRDDLVAGLIRHGHRHARAILSEDDLVRLVREQAGPGDMVVCLGAGTISAWANNLPARLSE
>OLS50341.1 aspartyl/glutamyl-tRNA amidotransferase subunit B [Rhodovulum sulfidophilum]AB14
MLDLTFEPPKPKVISGAKADWELVIGLEVHAQVASKSKLFSGASTGFGAEPNSHVAFVDAAMPGMLPVINEFCVAQAV
RT
GLGLKAEINLVSAFDRKNYFYPDLPQGYQISQLYHPIVGEGEVIVDMGPGVARRVRIERIHIEQDAGKSIHDMDPNMSF
V
DLNRTGVALMEIVSRPDIRGPEEAAAYVGKLRQILRYLGTCDGNMQNGNLRADVNVSICRPGAYEKFRETGDASVLGT
RC
EIKNMNSMRFIQAAIEYEARRQIAIVEDGGSIDQETRLYDPDKGETRSMRSKEEAHDYRYFPCPDLLPLEIEQAWVDEIA
ASLPELPDEKKARFVADFGLTEYDASVLTADVESATYFEAVAQGRDGKAAANWVINELFGRLKKDDRDITDSPVSPDQL
G
GILDLIAAGDISGKIAKDLFEIVYTEGGNPAEIVAERGMKQVTDLGAIEKAVDEIIAANPAQVEKAKANPKLAGWFVGQV
LKATGGKANPQAVNEIVARKLG
>OLS50342.1 Fe3+/spermidine/putrescine ABC transporter ATP-binding protein [Rhodovulum sulfidophilum]AB14
MGNSARPRVTLQTSDRNPRLEIRHIDRIFGGRKVVDGVTLQVLPGQVTCLLGPSGCGKSTTLRIIAGVDRQDAGEIRIDG
QLVCDTVFRLPPERRSVGLMFQDFALFPHLSVAENVAFGLTGPKAARRARVEALLDRVALGRFIDAFPHELSGGEQQRV
A
LARALAPRPKIMLMDEPFSGLDNRLRDEIRDETLQVLKEEGAAVLLVTHEPDEAMRMADEIALMRGGRIVQQGAPYNL
YN
APVDRAAAAFFSDINVIRGRVRNLLTETPFGKFLTPGMEDGAEVDIIIRPQHLRIDFDRNGRGPNPTPEEGVAARARVQ
R
ARFMGSESLVEFRMDHDGSVLKATVPAVFLPAPGTAMWLTIRRDRCFVFPASGQDGDGRQI
>OLS50343.1 transcriptional regulator [Rhodovulum sulfidophilum]AB14
MAQNCDSPRRPCRRHAAGEDPAKRDQIMAGAMKAFLDKGFDATSVNDICRAAGVSKGTLYVYFADKQDLFVALVAE
QREK
MFGGVDAVLRSDLPLAEKLTRFGRQLAEIVTSDHVVQWQRTIAGIVDRMPELGVRFYDAGALRTHTDLTDLLRREVAA
GR
LDLPDPVLASAQFIDLSGAGIWRARLFGKRPTPPGPDEIERTVDSAVRLFLAAYARPGARRED
>OLS50344.1 ribonuclease BN [Rhodovulum sulfidophilum]AB14
MFRVWRGMDERNVALIAAGVAFYSFLSIFPALAALIAIWGFLADPTVIQDQLSMASELLPAGAYEVVQRQVEALVGTSS
S
TLGWTSLVSILLAIWSARAAVAGLIRGLNAIYGEKNRGNPIMRIALAVALTAVLIFVALLAFSAVVLIPAVLAFVKLPAA
IELPITLLKWVLMLGVAFLAIALVYRYGPNRRSARLQWVTPGAVFAVGAWALGSVALAAYMRSFDRLNEVYGSLGAVV
AL
LFWLYLSAAVTLIGAALNAELELLTRCDSTTGPERAEGERGAHVADHVRDETGTLNGKAPSSEAGRGAEEASGMT
>OLS50345.1 threonine transporter RhtB [Rhodovulum sulfidophilum]AB14
MGPVDLLTLAAFVPAALALNLTPGADMMFCLGQGLRGGPRAALAADLGIALGGFVHVTLAGLGLGAVVAAVPGAFEA
IRW
AGVAYLLWLAWRAVRQGALAPDLPAVSPRRAFREGLTVNLTNPKVILFVLAFLPQFVVVERGAVLAQFLILGAVLSLGG
L
LVNGAVGLFAGQARRLLGGSRAVQRGLGLVSATIFTALAVRLALLQKG

>OLS50346.1 hypothetical protein BV379_07025 [Rhodovulum sulfidophilum]AB14
MLAAFAATTTATAPAPVAAGTAARQDGVLQAEILPGWQTAHGTYMAGLHLRLAPGWKTYWRAPGDAGIPPSFDWR
GSSNL
AAVKIHWPRPRVFSSNGIRSIGYVADVVLPIELTPADPERPMELRARIDLGVCQDICMPMELRLDRRLPDAGPAEPIRAA
LASQPLDARTARVGRVTCEIAPISDGLRLTARIEVPPLGDTETAVFELPDPTIWVSQTELARQGGTLVARSDMVPADGTP
FALDRSALRITLLGRDRAIDIRGCDAAR
>OLS50347.1 GNAT family N-acetyltransferase [Rhodovulum sulfidophilum]AB14
MNIRDFRPGDSAMLASIFHEAVHAVGSRDYSPEQVEAWSPAPVPAEQFLTRISDGRSVFVAVTGSDAPITFIELEEDGHI
DCFYCSPEFVGRGVGKALYQRLETAALAMGLSRLHVEASEAARRFFLGQGFTLVKRREFERNAVRIHNYLMEKTLR
>OLS50348.1 redoxin [Rhodovulum sulfidophilum]AB14
MLRSLVLYIALGLCANAAHAADIATLEAMREGSMKKLVLHAEPRALPDAVLLDETDAERSLGEYRGRWVVLNFWGTW
CPP
CLAEMPTLDSLAATMGSDRFAVVTVATGRNPPQAIDRFFDDKQIAHLPKWRDRSSALARGMGIMALPVTVILDPEGR
EIG
RLMGEADWNSPGAKALLGRLIEGS
>OLS50349.1 argininosuccinate lyase [Rhodovulum sulfidophilum]AB14
MWGGRFAAGPDAIMEAINASISFDKRLAPQDIAGSRAHAAMLAATGILTDTDAETIREGLLTVLSEIEDGSFAFSTALED
IHMNVEARLKEIVGEPAGRLHTARSRNDQVATDFRLWVRDQADAAIDGIGRLIRALLVQAEAGADWVMPGFTHLQTA
QPV
TWGHHMMAYVEMFARDLSRFTDARERMNESPLGAAALAGTSFPIDRHMTAEALGFARPMANSLDAVSDRDFALEFL
AAAS
ICAMHLSRFAEELVIWSSAQFRFVALSDRFSTGSSIMPQKKNPDAAELIRAKIGRIFGANVALMTVMKGLPLAYSKDMQ
E
DKEQVFDAADNLMLALAAMEGMVRDMTANRPALEAAAASGFSTATDLADWLVRELGLPFREAHHVTGTLVAMAEK
KGCDL
PDLSLAEMQSVHEGIREDVFGVLGVHNSVASRMSHGGTAPEQVRAQIARWKDRLG
>OLS50350.1 cysteine synthase A [Rhodovulum sulfidophilum]AB14
MAEDSIRKTTGRGRRFDSVLDTIGDTPVIRINNLGPNHVTLYVKAEFFNPGASVKDRLALNIIEAAERSGALKPGQTVVE
ATSGNTGIGLALVCAQKGYPLVITMAESFSVERRRLMRMLGAKVVLTPKADKAMGMYRKAQELAEKNGWFLAHQFE
TDAN
ADIHETTTAREILADFEGERLDYVVSGYGTGGTVTGIARALRRERPETKIILSEPADAALIASGRAQKRNAEGEADESHP
AFHPHPIQGWTPDFIPLVLQEAIDQGFYDELIPVEGPEAQKQARALAAKEGILTGVSGGATVAVALQVAETAEPGSVILA
MLPDTGERYLSTPLFEAVGAEMNDEEKAISASTPGFQIG
>OLS50351.1 cell division protein FtsX [Rhodovulum sulfidophilum]AB14
MKQRARLLVSALSSDPEASRVVPPTGFTARLTLFTAATMAFLAVFALALSMASGRLADRWADALARTATVRISAPPGQ
MG
TQSEAVLQILRTTPGIASARAMSDIEQRQLLEPWFGPDLPVETLPLPRLIEVTETRAGIDAEALRLRLAAEAPGAVLDDH
TRWRQPLVRAAGRLRLLGWTAMLLIGASMAAMIALAANAALAANAQVIGVLRLVGARDAFIARAFVRRFTRRTLEGA
ALG
TLAGMGAVALLPGDGPDGFLTGLGFSGPQWIVPLLLPVLAGAIGFAATRWTAMRMLRRIT
>OLS50352.1 iron ABC transporter substrate-binding protein [Rhodovulum sulfidophilum]AB14
MRTALAATAAAATLAPTAQADVNIYSYRQPELIQPILDAFTEDTGIATNIAFLNKGLVERLQAEGARSPADIILTVDVAR
LAAAVDAGVTQAVDSDTLVEKIPAAFRDADNHWFGVTTRARIVYAAKDRVDPSEITTYEELADPKWKGRICTRSGTHA
YT
LALVAAMIHHHGEEAAKTWLEGVKANLARKPQGNDRAQVKAIWAGECDLSLGNTYYMGQMLKDPEQEAWAKSVN
VLFPTF
EGGGTHVNISGIAMTKAAPNREEALQFMEYLTSDHAQEIYAEVNYEYPVVPGTEPSDLVKSWGSFTADDVNLIALSRDR
A

AALRLVEEVDFDG
>OLS50353.1 LPS export ABC transporter permease LptF [Rhodovulum sulfidophilum]AB14
MPRFDRYLLAQLTVLFGFFALVLVSVYWVNRGVSLFDDIIGDGQSALVFLEFAALTLPNVIRLVLPIAAFAATVYVINRL
TNESELVVMQALGCSPWRLARPVAIFGLLVAIPICILTHFLVPASRAQLDLRQAEISSDFTARFLSAGQFLHPAGGITVF
IRDITRSGELKQIYLSDATSPGTRVTYTANRAFLVQSDDGPKLVMVDGMAQWLREEDRRLSTATFDDFAYDISALIGSAR
IRRPSLDVMSTRDLLFPSPELIEAAGKPKAVFLYEGHARTAQGLLAVAGALIGYGALMVGGFSRFGLGRQIGLAIALLIA
VNFLNNTAAGFAQLDESYWPVTYLPPLAGIGIGLGLIWLSAMRHRRRRPRQERPA
>OLS50354.1 hypothetical protein BV379_07735 [Rhodovulum sulfidophilum]AB14
MAVLTAVFLASGPHPAAATEAVLSAPGASEELRQALEASALTLEAARAPDASAQDIFAAARADYARLVGVLYARGYYGP
E
VHVTLDGREAADLSPLSVPARIGRVEIRVASGRPFAFSQALIEPLAPGTELPDGFAPGKPAEAATVRAAAKAGVDGWRA
A
GHAKAKVAGQSLIADHPDATLAARLRLAPGPAIRFGDLVIAPGSGVREDRLRTIAGLPMGEPFDPEALDRSAERLRRTGA
FRSVRLTEAETLGPGNSMDIGLEVVDQKKRRAGAGVEFSSLEGATVSGFWMHRNLMGGAERLRFDFEIAGIGGQDDG
GED
LSLSARFDRPAVIDADTGLYLLGAVEDLDEPDYTETNARAGGGLTRIFSPHLKGEAGVLLRYADVSDDLGDRKMTHLTFP
VSLTWDRRDDPLDAHKGFYLDGSVTPLLAVGGAAESGALIKADGRIYQQLGSRLVLAGRAQIGSVVGAASDGVPPALLF
F
SGGGGTVRGQPYQSLAVDLPNGDRIGGRSFIGLSGEARVGVTRAIQMVAFYDAGFVGPDSWVSDGDWQSGAGLGLR
YDTG
IGPIRFDVAAPVDGDTGDGVQFYIGIGQAF
>OLS50355.1 hypothetical protein BV379_07780 [Rhodovulum sulfidophilum]AB14
MTGAAAAAGLFASVAVFASVAAGSAQAAECAENRVDLRGDWGTARFTVELADDEAERALGLMNREHLAGSAGMLFI
YPGP
RRVAFWMENTLIPLDMIFLDAAGRVRRVAADAVPLDRTPIDGGPGILAVLEINGGLAARLGIVPGTELRHPAFGPDAA
WP
CN
>OLS50356.1 transcriptional regulator [Rhodovulum sulfidophilum]AB14
MDVIQAENMLRHAESASSFLKALSHEGRLMILCHLSTGEKSVTELETLLSSRQAAVSQQLARLRLEGLVSYRREGKAIYY
SLADEKARRLLELMHDMFCNARAPAQASAARPGD
>OLS50357.1 hypothetical protein BV379_07815 [Rhodovulum sulfidophilum]AB14
MAGQPARAELELGMVERVGCAYCVLWDREIAPIWPKTREGRTAPLRRIDLDANLPGDVRFASRPRFTPTFVLLRDGLEI
G
RLEGYPGEDFFWGLIGAMLQQQPEWAQTDEDEGG
>OLS50358.1 sulfite dehydrogenase [Rhodovulum sulfidophilum]AB14
MTDRPGAPTRRGFLKAGLAGGGALVAGKAAAEGPDPLITETQPWAQMWGDGVDVTPYGMPIEYESDVIRRNVDWL
TADTI
SSVNFTPIHALDGTITPQGCAFERHHSGAIDLPKEDYRLMINGLVETPLIFTYADLERFPRVNHTYFLECAANSGMEWAG
AQLNGAQFTHGMIHNMEYSGVPLRTLLEEAGVKPEGKWIYVEGADASSNGRSIPLDKALDDCLVAFKANGEALRKEH
GYP
VRLCVPGWEGNMWIKWLRRVEVTTEAIQSREETSKYTDTLADGTSRKWTWAMDAKSVITSPSPQSPITHGPGPLVITG
LA
WSGRGAIERVDVSLDGGKSWQTARLARPGQKMALTRFYLDIDWDGSEMLLQSRAMDDTGYVQPTKNALREIRGLNS
VYHN
NGIQTWWIKTSGEAENVEVS
>OLS50359.1 phosphonate metabolism transcriptional regulator PhnF [Rhodovulum sulfidophilum]AB14
MTPTALWRSIAATLRNEIAAGSYRPGDKLPSEADLARRFGVNRHTVRRALADLAGARLVQARRGAGVYVAARPTDYPL
GR

RVRFQQNLAAAGQSAERKALLHATRAADRREAEALGLSDGAPVHVYEGISLADGSPLALFRSVFPAERFPDLPGALAETA
SVTAALARAGVTDYTRASTRITAKRASPTQALHLELRPDDPILRSVSVNVDTAGRPVEYGRTWFAGDRVSLIVTDDPPED
>OLS50360.1 hypothetical protein BV379_08030 [Rhodovulum sulfidophilum]AB14
MSGHEVGPALSGAALDRLMPMHLWIDASGDVIRAGPTLQRLAGAPLALRAWTEIVTLRRPRATRRLEELLQMQGGALKFT
LNARPEIALKGLVVPLPGGAALLNLSLGISLVDAVGRFDLTSSDFAPTDLAIELLYLNEAKTAVVGELRRLALRLNGARL
TAEVEAATDMLTGLANRRALDDALGRLSASSLPFALMQLDLDLFKAVNDSHGHGMGDAVLRMVAAVLRSHFRSRDVIARV
GGDEFVVLMVDFVDRDHLLSHASRLIERIEQPMEFQGVDCRISASIGIAVATLARRPPCDVLLQEADIALYESKRRGRGR
ATFYDDWQRRRHGPGDTAF
>OLS50361.1 urea ABC transporter permease subunit UrtB [Rhodovulum sulfidophilum]AB14
MAQSGPAVPGQGQPSPIQNILQENRELIEKSSRRTIGPAIAALRDSGLPAARNVLEKWQDKALYQRKSDGLFFFAEKAGE
DSYRLIDIATGTDADAGTANRQDLDQLKPNSGVRALIGAALVPYQLTDPDPAVRSGALDAISRNGEAAHLGPLRGAIASE
TDPGLKARKERLERLLTIAYDPDPSARIAAIESFAGDLGVDVRAALNPLVATTRAVTTGAPPETENIARRLIPGSDALPE
AEAYRLLVAAGLAPPRISAADRRNALAAHIEDGAVAGIPLEALNSDAARERAYAALADRGLVPPTATTEEVQAALEGHGF
YDRYAEPDPAVTAAAGAALDAIAAKLGLNQAVNLSLDALSLASIYFLAAIGLAITFGVMGVINMAHGEFIMMGAYTGYVV
QQIVPNYTVSILIALPLAFAVTFAAGVAMERLVIRWLYNRPLETLLATFGISIALQQLARNIFGTQARPLTSPGWLDGAL
HLNDVVSISYIRIAIFVLALLFLALFLFIMRRTRLGLEVRAVTQNPRMAASMGIDPDRINMLTFGLGSGIAGVAGVAIGL
YAKVTSELGQDYIVQSFMTVVVGGVGNIWGTLLGASMIGFLQKGIEWLNPSNTLAAQTYMILFIILFIQIRPRGIVALRG
RAAGD
>OLS50362.1 hypothetical protein BV379_08115 [Rhodovulum sulfidophilum]AB14
MLACGGLPGTALAACPGNTQLEINDCAASAYRDADAQLNAVWPAARAAAAASGADGLLLDAQRKWIAFRDAACAAEAAQY
RGGSIQPLVHADCLRRLTDRRTADLRALLPQ
>OLS50363.1 hypothetical protein BV379_08125 [Rhodovulum sulfidophilum]AB14
MTVKSVPGGMSERLDLFFAGLGLGVNPYGLRRARMREIQNLNACSDAQLAKLGITRDDIPGYVFRDLFS
>OLS50364.1 urease accessory protein UreF [Rhodovulum sulfidophilum]AB14
MATDGQILRLAQWLSPAYPVGAFAYSHGLEAAVASGAVRDGAALQDWLADILRFGAGRSDTIFLKAAHEARPEDLPRIDA
LARAFQPSAERRTETTAQGAAFAQVTSAVWPGDLPAFAYPVALGRAARLHDLPLGLTARMYLQAFVSNLVSAGIRLIPIG
QTEGQRVLTALQPLCEEVAEVALTETLDDLAGTTFLADIASMTHETQYSRLFRT
>OLS50365.1 cobyric acid synthase CobQ [Rhodovulum sulfidophilum]AB14
MLDPAPPRRARSLMLQGTSSDVGKSLLVAGLARAYARRGLKVAPFKAQNMSNNAAVAADSDLPPGPDGTAPKGEIGRAQA
LQAQAAGLPPSIHMNPVLLKPQAMVGSQVVLRGRPLGNWPARHYHKLKPQLLPAVMDSFNRVAAEADLVLVEGAGAGTET
YLRKSDIANMRFAEAADLPVVLVTDNDRGGAMAAVVGSWCLHSEAERARIAGYLVNKFRGYFSLYEPACETITATTGWPF
LGVARWFEGAGRLPAEDALALERPASGAGQGLVIAVPHIARMANFDDLDPLSAEPGVDLRWVRPGEPIPAEADLVLLPGS
KTTRPALDSIRANGWDIDILAHLRRGGRVLGICAGFQMLGHRVHDPEGIEGPPGETEGLGLLDIETTIGGDKRLVEIAAE

DAISGTRVTGYEMHMGVTRGPGLERPWLRLDGRAEGAVSADGRVMGGYLHGLFGADGFRRHWLTAMGGRASGLD
HAARVE
AALDALADQLEHDLDLDALLALAR
>OLS50366.1 nickel transporter [Rhodovulum sulfidophilum]AB14
MFHGNLVTAALALTLVPGVAAAHFQLAYTEESLIDAPGDVPVKLIFWHPQESGPVMDMGEPLEFYAIHRGERIDLKPTL
S
QTVFHGPLNEAVAWDGSLPVKRMGDYVLVTVPAPYYEESEDIFIQQITKTYVNRGQMPTDWDGPQGLKAEILPLVKPY
NV
IAGSTFTGRVLSEGAPVAGAEIEIEYMAAEPDMETDAPKPAIAGPMPGGAIVAVSDENGYFTFGVPKAGYWGFAALGV
GP
DTEYEGKELSQDAVLWIRAFDLK
>OLS50367.1 hypothetical protein BV379_08255 [Rhodovulum sulfidophilum]AB14
MRPVFLTLALVLAPLPALAHKVIAAVYADGALIEGEVGFSNGDMAADATVAVSDPEGNPLGETATDADGMFTFRPSVA
VA
HVFHADLGAGHVAEVTMSAEDVAAILDSPAARAAAEAAAGTGADTGADTGGIIGAGQAEAASDGGNPSTFPLDAAS
GTAA
VGSSAVPLTAALGLSDADKAAVAKIVRDEMRPLRREIAAYREKNDLQSILGGIGYIVGIFGLGFYVAARRRMKG
>OLS50368.1 precorrin methylase [Rhodovulum sulfidophilum]AB14
MRVAGFGFRAAATEASLADALDRAGGPVGLTALAVPADKAGAAGIAALAARLALPLCPVAGAELTRAATPTRSAHSLA
HR
GTGSVAEAAALAACGEGARLLAPRRVSNDRMSTCAIATKDLP
>OLS50369.1 damage-inducible protein DinB [Rhodovulum sulfidophilum]AB14
MARYNRWQNRAMMEEVARLGAAERMAPRGAHWGSIQGTMAHLLWADHVWMSRLDGWEAPHARLEDSAGFV
ADWSEYRSKR
QITDARISRWSQGLSEADLAEDLSWYSGAAGREMTVPRAICIVHMFNHQTHHRGQISAMLTAAGVLTAPTDLFLMPD
LVG
>OLS50370.1 mechanosensitive ion channel protein MscS [Rhodovulum sulfidophilum]AB14
MAAAVFSHGGAAPAFAQAQSQQEGIDYAQWDRDASRAEKVLAEGKATTPAFDTLRSELVDWRTRFLDAQNINSSRIK
TLR
DQISALGDPPAEGESEPREIAQRRAELTKQLNQAQAPSLKAVEAYSRADGLIREIDQTIRERQADQLLKLGPSPVNPVN
W
PAGLIAIRGTLVEVGNEIAANWDNPNARASLRSGLPVTLLFLTLAGLLTVRGRRWMEILSDFLAQRGRGRGRTLFSGIAS
LGQIVVPTLGIVLLAGALKSSAMLGLRSERLVQMLPMLGLTVFTAGWLGGRVFAQLGVSHAILTLPPERRSEGRFNSWIL
GLILALAMLAREAGKVEEYPAEAVAVLTFPILLLAGLMLSRMGQLLLVHVRADTAEGPEERSYRNRVAAFFGRAAIGAGL
IGPLLGAVGYAEAGDFFVYPSIMTLALVGFLLLLQRFVTDIYAVLMGRDVEDPENGGDLIPVLIGMILALLALPVLALIW
GARVADLTELWARFTAGYSIGGTRISPTSFLTFVVVFTLGYALTRLLQGMLRSTVLPKTKIDPGGQTALISGVGYIGIFL
AGVVSVTTAGIDLSSLAIVAGALSVGIGFGLQTIVQNFVSGIILLIERPVSQGDWIEVGGVMGIVKDISVRSTRIQTFDR
TDVIVPNADLIAGQVTNWTRQNLTGRVILKVGVAYGSDTRQVEKILMEIGESHPLVMVNPAPLVVFQGFGPDALEFEL
RV
ILRDINYGLSVRTELNHEIARRFAEEGFEIPFAQRDIWLRNPETLGDRPAARTQQAPGPFLPRQSAEPQPEPLDDLGGDA
DGDGGDGPR
>OLS50371.1 chromosome partitioning protein ParB [Rhodovulum sulfidophilum]AB14
MTKSTEKTHQTEVTLVPTDSIRVLNPRVRNPRTFADMVENIAKIGLKRPITITRRTGTDPQEYDLVCGQGRLEAFIELKQ
KAIPAIVIHADESDCLVMSLVENCARRQHRPIDLMREIGTLRERGYNDRQIGDKIGVSPDYVGMIAGLLERGEERLVTAV
ETGLLPLNLAIDISKTDAEGAQRALMDTYTQKKLRGKKLAAARRLIEQREARGKKIHPKQYGRDGRNRRPLTSEALVRAY
QKEADRQKLLIKKAELTQGRLLFICQAFRTLLEDDHFVTLLRAEGLETMPAYLQDSITEGAAP
>OLS50372.1 hypothetical protein BV379_08485 [Rhodovulum sulfidophilum]AB14

MAAALKWSLGDSRAGVKTVAAWTGANEKTVKNWFSGTYGPSGAHLIALARHSDEVLSTFLTMAGREDLMVAIKLAA
AEEA
VEELLEAVRQLNRKKSGDRTI
>OLS50373.1 hypothetical protein BV379_08520 [Rhodovulum sulfidophilum]AB14
MHKPARPGLAVARRLAYGRVAVVMRTKDRAILLERAIDSVLQQTFTDWALVVVNDGGDPRPVQELLDSRKDQLQGRS
LLI
NNPTSTGMENASNAGITASASEFIVIHDDDDAWAPTFLERCVDRLDGSPPEIGGVVTQTTVVIEDIEDGRVIERERFPFA
NIEAVELARLAVENQFPPISFLFRRSALETVGSFDGRLPVLGDWDFHLRMAQHYPISVLHETLAFYHHRTAGVTNQYGN
T
VVAQKETHRRQRAEYINRHIRDTVGQCGQGSEGTMLLAGEMHRVVSEKLTSMHDHLQWLEKLLNDEREHMLYLEKLI
DDR
QK
>OLS50374.1 exopolyphosphatase [Rhodovulum sulfidophilum]AB14
MAPRRPNGAGAFPKAVETPAPVPPDPATLYAALDLGTNSCRMLIAQPKGSQFHVVDSFSKSVQLGTGLEGSGRLSRAS
MA
RTVQALRVCKGKLETHAVERMRLIATEACRRAENAREFIKMVRRETGLRLEIIQPEEEARLAVVSCAPLVSTKTEQLLVV
DIGGGSTELVWIDLSMVPRPERPRAIMRLHAGFHSETGPFPAARVVDWISVPLGVATLKEQFDDVEDDHARFALMSW
FFE
ENLAEFSPYAAEQTKDGFQIIGTSGTVTTVAATHLGLRRYDRTKVDGLRMTSGQIDEVIRKYLELGPEGRRADPRIGRDR
HALIMSGAAILQALMRVWPTDRMSVADRGLREGLLYAQMSADGVLEEGPF
>OLS50375.1 cyclase [Rhodovulum sulfidophilum]AB14
MCDICVMTAVKGRMLSRRGFFAGAAGAGLTAAAVLTASAPPAMAQGHAAVTDMTHELSADFPTFFGERQFFWDQK
FNFEE
HGFNLAEYRVNEHTGTHIDAPLHFSADGAAVNEIAVETLVAPLCVIDIAARAAGDPDTTLTPDDIRAWIAAHGEIPENAC
VALFSGWADKASGPGFRNADAGGVQHYPGFHAEAAEMLIEETAAASIGSDTLSLDPGNSPDFAAHYAWLPSGRFGIE
CLA
NLDRMPAAGATLVIGAPKHKGGTGGPARIFALV
>OLS50376.1 glutaconyl-CoA decarboxylase subunit beta [Rhodovulum sulfidophilum]AB14
MTALPDVTWQMAVMWLIVAGLFYLAIYRKFEPLLLVPIAFGALLANLPTEGLVNPPGDGVAGGLFHYISLGVDLELFPPL
IFLGVGALTDFGPLIANPRTLLLGGAAQFGVFATFLGATLLGFSPQEAGAIGIIGGADGPTSIFLANKMAPHLLAPIAVA
AYSYMALVPLIQPPIMRALTTEAERRIRMQSLRPVSRLEKLIFAGLVTVIVILLVPAASALIGMLMLGNVLRESLVTDRL
AKAAQNEVINVVTILLGTSVGITMTGERFLNADTLKILGLGVVAFGIATAAGVLMARAMNLVSRSKINPLIGSAGVSAVP
MAARVSQIEGQRADPGNYLLMHAMGPNVAGVIGTAVVAGYFIAQYAG
>OLS50377.1 hypothetical protein BV379_08695 [Rhodovulum sulfidophilum]AB14
MPLRDRIEFDEAAARAEAEAAREALTTAETAFEAQGRIARRLGGRVEEGRAVFGFWTPEILDARVPSGDVFLEVLAPLEP
MDLTRAHQEIRFERAWVPVIVEEAHCFTAVTGMRAGSRETLGDFYALAWRDPEGGWHRILDPLAMSLPFGVMAPAE
LYDV
EAMLAAREDTAYFQAFRGEAPHKFGPPSNILQVHVPTATAGGTLASLTRHYHRLAERVARELPLEPADKLFLGYDAVQL
L
PVEPTTVHEAGPDFWQEVDGDEEGVTVSLLRPDTTNWGYDIVISGMATVNPVLLETGRPDELVDFAAQLHNFPGMPK
KLI
LDVVFGHADNQGLGALNSHFFAGPNMYGQNLDYQNPAVRAILLEMQRRKVNFGADGVRVDGAQDFKWWDASTQ
TLRHDDD
YLQSMSDLVQEAAGTRYRPWFIFEDGRPWPEEDWELSSDYRAVIEAQTDEDVFQWGPLTFAHNTPFLYTFWLSKFWR
IRE
IVELGAHWISGTSNHDTLRRGTQVSPKLNVNTRLGRTRMEILDKAYDNPANHVLTYVALPGVPMDFLNAMARASWG
FIRN
QDDRYGVKVVAEEAISLKWQVDEYSYSVPANFRRLKEMGFETRSDLVRFFEFLPALVEVTEYDLDEIARLLNKAEPPLAG

PEVYTVAALKHMARAWMDDMHEYCNVSTSLPALDADQAAFCLALRGFRRKRPWLAANYAGRDSFDYLRPIAGRTLF
SALR
HGPDGEQIYAVMHMEGKPTAEIDPLSLPVPGIGGGGWEVALRTPSIGPDYHGGPITLSDSMALVFSRHLPE
>OLS50378.1 hypothetical protein BV379_08700 [Rhodovulum sulfidophilum]AB14
MGQALDSLQDDVNLAVTGKALTMPVAILHGDADWIVPKSEWDTPFGAIATDRKRMYLSQTDTHGCPGHFANHEQA
TDDTS
FFRPLLSLLLLDGVGAEDTLDWRYLWSALDRVIREGARADQLDFDMGQWSDGRPVRPVEVYLSGS
>OLS50379.1 acetolactate synthase 3 large subunit [Rhodovulum sulfidophilum]AB14
MTQAMTGAKMVVQALKDQGVDVIFGYPGGAVLPIYDELFQQNDIRHILVRHEQGAVHAAEGYARSTGKPGVVLVTS
GPGA
TNAVTGITDALMDSIPLVVLTGQVPTFLVGNDAFQEADTVGITRPCTKHNWLVKDPDHLAQTIHQGFHVATSGRPGPV
LI
DIPKDVQFAEGTYVGPVKAGHGHYNPKVKGDREMITLLVEAMETAERPIFYTGGGVVNSGEAATQLLRELVEATGFPIT
S
TLMGLGAYPASGDKWLGMLGMHGLYEANLAMHDCDLMICIGARFDDRITGRVADFSPGSLKAHIDIDPSSINKTIHVD
LP
IIGDVAHVLEDLLRIWKSRGRKVNKEALKPWWAQIDEWKKVDCLRYGTSDTIIKPQYALQRLEALTKARKDRFICTEVGQ
HQMWAAQFLGFEAPKHWMTSGGLGTMGYGLPASVGVQVAHPEALVVNVAGEGSWLMNMQEMGTAVQYHLPV
KQFILNNER
LGMVRQWQTLLHGGRHSHSWSEALPDFVKLAEAFGAKGLRCSDPADLDDAIAEMLAYDGPVIFDCLVEKHEDCFPMI
PSG
RAHNDMLLAADAEAFKEGAALV
>OLS50380.1 cytochrome C biogenesis protein CcdA [Rhodovulum sulfidophilum]AB14
MPRRALIALLLALSLTPVLSGPLRAVEPNEVLADPAEEARAREISKEIRCLVCRNESIDDSNADLARDLRLLVRERIEAG
DSNTEVVDYLVARYGEYVLLKPTATGSNLVLYIAGPAMLLLGLGVAAFYIRSRRGGRDTGSEALNAEEEARLKEILGK
>OLS50381.1 competence protein [Rhodovulum sulfidophilum]AB14
MPARAMSALLTAVEARRGHLFPWTPVFLSLGIGLYFTRATEPPVWALWAFAGAAAVLALAARRLPELWAPVLIALALV
AG
GAALAGARAHMVAAPKLDHRVYGALEGRVLVLDRSSSDAPRITLDRVWIERVPPDETPRRLRVALHGAGGVTAPPPGA
RI
AMMAHLSPPSAPAEPGGFDFRRHAWFKQIGAVGYANSPAVTLSPPEGGATLLVHRMRMALSEALRARIPGDQGAFA
AAIL
TGDRSAMSRDTIQALRDSNLAHLLAISGLHMGLLTGVIFAALRFALALWPRLALRLPVKKIAAAGALAVGAVYLALSGGN
VATERAFIMVAAVLGAVMADRRALTLRAVALAALIVLVLTPESLTQAGFQMSFAATTALIAVFAALRDRPEGGWRPPR
WA
QGVLAVVISSAVAGAATAPFGAAHFNQVSQFGLLANLASVPLMGLLVMPAAVAAACLTPFGLAGLGLEPMRWGIAWI
LWV
AERVAGLDGAVWPVPAPGPAVLPLIALGALWVMLWTGRARALGLLPILAAFALWSAAQRPALLIAESGGLMGAMGP
EGRA
LSKPSGEGFVALSWLENDGDRADQATAFARPGLTGARGEQEAMVAGQRVTLITGRGWAERIAPACARGWVVVPQY
VDDPP
AGCRIFERAALARTGALALFPGADGPRLVTARALAGARLWAQ
>OLS50382.1 repressor LexA [Rhodovulum sulfidophilum]AB14
MLTRKQLDLLEFIHKRMQRDGVPPSFDEMKEALDLRSKSGIHRLITALEERGFIRRLAHRARAIEIVKLPEALEAKGFRP
VVIEGDRSETPPPSGAMDVTPVHALELPVMGRIAAGTPIEAISEVSHTVAVPGAMLSGRGRHYALEVKGDSMIEAGIND
G
DVVVIREQNTAENGEIVVALVEGFEATLKRFRRKGEMIALEAANPAYETRILRSDQVKVQGRLVGLIRSY
>OLS50383.1 YIP1 family protein [Rhodovulum sulfidophilum]AB14

MTHFRPSLLSLVRDTVTDPRGSAGRVLSLAPPRAVLWEALGLVVILSVLLTELGELISPAPVDPMMPAFMHYPLAAAAV
Q
GLLLIVTVHAIAVIGRVFGGTGDLDGALALTVWLQFVMVCLQVVQLVLLVVLPPAAGLLGIFGIGLFFWLLSHFVAVLHG
FPSVMKTFGMILFSMIGIVFGMSLILSILGITLTGAMPDV
>OLS50384.1 hypothetical protein BV379_09170 [Rhodovulum sulfidophilum]AB14
MDILKKAEFVVTTAGLVLKGTGGSLGDAGFPGFDGAEAASGGALSTLLAMNRSSLNRNRNRNRNRNRNRNRNRTTRE
NRE
SGADTDGARLTLEPWRERAALYGMEAQWIDLPVALPPALDQLRAEAIELLKKQVRLARVDQTMRREEILRESRLELSAY
L
RPYGIEGRSGYSATIGLVLLVLLLCDEIGLRYKERFDVLRPNTVEPRLRPFLPIPAHASYPSNHSFQSFSVAFVLSRIVP
EHPASSEVFVAARRVAENREWAGLHYPSDTRAGHDLARMILPVLEVVCRDQMLAAQKEWI
>OLS50385.1 trigger factor [Rhodovulum sulfidophilum]AB14
MQVTETLKDGLKRGYTITVTAAELEAKVNEKLAEAQPQIEMKGFRKGKVPMPLLKKQFGERLMGEAMQETIDGAMSS
HFE
ETGDRPAMQPEVKMTNEDWKPGEDVIVEMSYEALPEIPEADLKAIKLEKLVVEPAEAEITEALEKLAGTAQTYADRRKG
S
KAKDGDQVIIDFVGKLGDEPFEGGSAEDYPLVLGSESFIPGFEAQLVGAKAEDDITVTVTFPENYGAENLKGQEATFDVK
VKAVKAPKPAEIDDELAKQFGAEDLDGLKTQVAERLKAEFGGAARAVMKRALLDELDTAVSFEVPPSLLDAEAGQIAHQ
L
YHEEHPDDHGHDHGEIEPSEEHLKLAERRVRLGLLLAEIGRKAEVTVSDNEMTQAVMAQARQFPGQERQFFEYVRQN
QAL
QQQIRAPLFEDKVVDYVFELAEVTEKPVSKDELQAAVEKLEEE
>OLS50386.1 amidohydrolase [Rhodovulum sulfidophilum]AB14
MGRRNFIRTGAAAASLAALMPKEGRTQAISTEETTIFTNANVLTVDDAFSEAEAIAIRGNRILAVGSNGEVRTVAGKNA
A
VIDMQGKTVLPGFIDAHAHVISGAVADDVMEYVGMNSYATVDDVLQQIAQRASETPAGEWLVFRNFDPAVQEGAET
LTFS
DLDPISSEHPIFVLNASGHIAYANSKAFEVSGITNDVSDPPGGEFVRDAEGNLTGMMKNNVAFIQIAENYPEMKKVEPV
D
ALLRLLENWAQYGLTTVSELSLGVLKHPPGDVQIMTEAAQSGRMKARIRAYPFYTLGAEAWDRADVMQGNGNALARI
AGY
KLVADGSNQGFTGWQREPYLGSDNRGLRYMEPDEMTAAALERAGKGWHLAIHGNGDAAIDNILDTFQALVDAGVD
MSRVR
PRIEHCSILHDDQIARMKKLGVSASFLIGHVYYWGIAMRDKVFGEEKAQLLDRCRSAEKAGIRFTLHSDFMVTDPNPLH
M
IEMAVTRRTWKEPDYVLAPQETISVETAIRAMTSEAAWQLFSDHELGSLEPGKLADLVILEQDPRDVRQDEIKNIRILET
WMDGRQVYSA
>OLS50387.1 transposase [Rhodovulum sulfidophilum]AB14
MSRPIPPNYETKKWSAYNFVRRLKTLGGLTPYEYTCKIWISEPDRFILNPIHQMPGLNT
>OLS50388.1 hypothetical protein BV379_09300 [Rhodovulum sulfidophilum]AB14
MPYERAAFGSGWTDPDGDCLDTRAELLADLSTVPVRLAPSGCSVRHGRWFAPYTGQVVTEAGDLDIDHIVPLRYAWG
HGA
ASWPAAKRARFARDPVNLLPVSASANRSKGARGPLEWLPPDPGFRCQYVLRFRRIAASYGLVQSAAEERELVALTGRLC
G
A
>OLS50389.1 IS5 family transposase, partial [Rhodovulum sulfidophilum]AB14
MRQTTGFVESLLRLVGLDWSVPDFSTLSRRQQTLAVNITYPGSKGPLHLLIESTGIKVEGEGEWHARKHGGPKRRVWR
KI

HLGGDEETLEIRAVEITGSHIG
>OLS50390.1 hypothetical protein BV379_09340 [Rhodovulum sulfidophilum]AB14
MKETPQDEAVREKTYRVAADELRGFIERFETLAEEKAAIGDQQTAVMAEAKGRGYDTKALRRLIALRKRHAEDIAEEEA
V
LQLYREALGM
>OLS50391.1 branched-chain amino acid ABC transporter substrate-binding protein [Rhodovulum sulfidophilum]AB14
MPSKPASGTELSLASGVDEPPRSPARRSALKTLAAAAGGASLPLWARYAEAQSAAPIRIGFQMHATGIGAAYGRWYNR
TT
EAALRLINEAGGINGRPVELVTEDDGTDPKRGAEVVEKFATQHRCDVGYGTLFSHVVMSSAPRAGELKLPYFVVSEGH
HV
ASGALNRWTLQPGITDVKSQVLAMAPFVRENLGRKVTMIFPDYAFGHDHRDFFTQAIEAQGGEVVAKLAIPPSETSFT
KY
FPKIPRETEVIYHVMVGPSVLTFVKEMGEFFGPSRPEIFGFIDSLEAVDLQSPGLEFLEGTYFWEGMPRYAQPDQTDFDR
HYRAAIGIDENGALAADPRDVSAYAHMFGCWETLYVIKAGMEACGYRGPDDRAALVEAVEAMSAMPLSNEHPQGA
KIFNG
KTHQVFGPQYISKVENGRLTVLEETSIEAGFYPDEVDYTTQPL
>OLS50392.1 hypothetical protein BV379_09425 [Rhodovulum sulfidophilum]AB14
MDLRQLRYFLEIVERGSITGAAATLNVAQPALSLHVRNLEARLGTRLLLRRPTGVVPTEAGEILARRARSILAEVARTEA
EIRGLETDPSGRVVIGLTGTIGGLLAVPLIEALRERYPQITLQIAEAMSGFVAGWLSEGRVDLAVLYEAPADPGLACDPL
LEEELVVLWPSDWTAPPEIAMAALDGVPLVLPSRAHGLRRMIDAELAGDGIAPELAVEIDSYSNIKNLVAAGFGASVLP
W
HAVAPEAEAGRLAVSRIVAPGLWRGAWLIRPGARPVTRAQEAVAETLRMVVGDLLARGVWAAARAPGTAPSSGASP
PDAN
S
>OLS50393.1 hypothetical protein BV379_09445 [Rhodovulum sulfidophilum]AB14
MTEDARPETASARHWGRLRAVFPYVLAAGLFVLGLLALYRLLATVDFADVVAQVRATPWSTVGLALAATAASYVSLVG
YD
WSALRFIGKPLNFPVVLAGGFMAYAFGNTIGLSAVSGGAVRWRIYSALGLDGYDVAAVSTFAAVSFGVAATLVGLGAL
AV
HPAALAAMLPLAPGTVQVLALGGMAVIALPLLWASVRQRRVRLGRFTLRAPVPRDLAAQLIFCLGDIGFAAATLYLLLP
A
NDLGFATFLALFAAATMAGVLSHVPGGVGVFEAVVIAAMPAATPISAVAASLLLYRLIYFLVPFFLALLALAGYELFRSA
GGHLPRGQWGRALGAVDPAFRAVERIAPMVLGTMILGAGLWMSVASILPPATEAARAAEALFPLPFIEGSALASSVIGS
A
LVLISFGVMRRALAAFWLAVAVMEAGAAVALVQGVDLDRAVLLATGALLLLPFRHAFHRHTMLSHATLTPAWAALIVA
LI
AGFGFMLFFAHKETPYAHELWWQFAVNERAPRALRAGLLGSLVLGLGALLILLRAPRVAAEPPAREDLDRAARIAAGG
DN
PDAGFALTGDKSILVSDDGRAFVMFAVSGRSWIAYGGPVGPADAAEDVAFAFVDAARRAGARPAFYEVRAADVPVM
LDLG
LSLHKMGEEAVVDLTAFSLEGSARKKLRAAHARALRDGLSLGWAEPPHAPAFLAELGVVSEAWLSAKRAREKGFSVGR
FD
PDWLNRWPVATVRQDGRVVAFANVLVTDSRAQAAIDLMRHVEHAPSGTMEFLFTALMLRLKEEGHAAFSLGMAPLS
GLSP
ERSRRLWDRFGALIYRHGRSFYNFEGLRAFKAKFDPDWRPRYLAAPSALPPLLTLADAARLIGGRNERRQTLRRKADRG
E
ET

>OLS50394.1 transcriptional regulator [Rhodovulum sulfidophilum]AB14
MTILENAAHVLRCFGNDCTDLTVSEVSDRLGLPKASASRLLKTMRETGMLEEIGGTRRHRPGRMMLNIAAAYRYSSSVF
G
RARAVVHETSQQFGHTGYVSIRDGRSVMAVADFEGTNALRVGSAVGRRLPAEICATGRSLLARLSDDEIARLYGDEPTA
D
RLARDLEPVRKRGYAFVSQQAMPGVDGLAVAVGDPVTGEAVSLCLVYPHNLVSDSERDDMIGTLVEGAGGIAEALGD
SLL
LHAAGDRAPA
>OLS50395.1 tRNA dihydrouridine(20/20a) synthase DusA [Rhodovulum sulfidophilum]AB14
MSGNERNCRCLYVIENNKKLEISTTARLSVAPMMDWTDRHCRYFHRLMSRSALLYTEMVTAPALVRGGALHLLDHDP
AEH
PVAVQLGGSDPDELAEATRLCCAAGYDEVNLNVGCPSDRVQSGAFGAVLMKSPGLVGDCLAAMRAVSSVEVTVKCRI
GVD
DQEPREVLPAFLETVAATGVSRVIIHARKAWLQGLSPKENRDVPPLDYALVAEMKRAFPVMHVSVNGGISTLAEAQSLL
A
MGLDGVMIGRAAYHDPAAILLGADEDLFGLPGRKTARQVAEEMRPYIARHLAAGGRLHNVTRHMLGLFAGAPGARA
WRRV
LSEGAAGQGAGLEVLDAALAALPRAA
>OLS50396.1 hypothetical protein BV379_09590 [Rhodovulum sulfidophilum]AB14
MKAVSVVLRAAVALWFGFGPGSAGALGAEPLTALARPDLAASSLDDLKGGGIALSLGLSQPVPYRVFTLNAPPRLVVDF
R
EVDWRGSDPAALKGSAQVRSVLAGAIRPGWSRLVLELSAPMALVSAEMPRDPATGRARLALQLKPADTANGGAVRP
PAQT
ALWGLPEPEKLAPPPRRHDGSRALRVVIDPGHGGIDPGAESAGIREADLMLTFARELAEALARAGIEPILTRDADVFVPL
ETRIDIARAARADVFLSLHADALADGYASGATVYTLSETASDSASARLAERHDRDALLAGVDLSDQDDVIAGVLMDLAR
T
DTQPRSDRLAEAIVAGLRGHVGHLYKTPHMSAAFSVLKSPDIPSVLIETGFLSSADDRERLISPEWRARAAAGIVEALRG
WAEADAAAAALLRR
>OLS50397.1 hypothetical protein BV379_09595 [Rhodovulum sulfidophilum]AB14
MAQPLDRAAVEAQFRDWLGGTIWPRARAAGVSRRTFDAALGGIRPDWDLPGLVPPGSRPGPQHQAEFGAPGAYFG
EAALR
EAAAVGRRMAAREAPALAAAEQATGVPARIVLAIWSRESAYGRAPIRHDLFRVLATRGFMSPRADYFAEELVAALRIAE
A
GPVPRDLMKSSWAGAMGQPQFMPSSYLAHATDGDGDGRADIWRSRPDTIASIAAYLAQHGWVAGRDWGFEVRLP
ARVSCT
LEGPDQGRSIADWEALGVTRVSGRPFFPAHERRGEGFLMMPAGRHGPAFLVTPNFYVLKAYNMSDLYALHVGHLGDRI
AFG
SGAFARGWAPLGGLDRSGIVAIQRWLEARGRDVGGVDGLPGYKTRREIGRWQEASGRAATCFPDPGMAAAMAR
>OLS50398.1 hypothetical protein BV379_09695 [Rhodovulum sulfidophilum]AB14
MARVYPHIEEIRPKDASIHAEKGELVLIDVREAHEVARSGKARGAIHIPLMMIRHKANARGPNFDPALDPAKSFALYCEN
GSKAIQAARMMKKMGYEDVYNMGAFREWLNAKLPVE
>OLS50399.1 excinuclease ABC subunit A [Rhodovulum sulfidophilum]AB14
MAELKRIEVRGAREHNLKDVDVDIPRDALVVITGLSGSGKSSLAFDTIYAEGQRRYVESLSAYARQFLDMMEKPDVDHIT
GLSPAISIEQKTTSKNPRSTVGTVTEIYDYMRLLFARAGTPYSPATGLPIEAQQVQDMVDRVMAMEEGTRAYLLAPIVR
D
RKGEYRKEFVELRKQGFQRVKVDGQFHDLDEPPTLDKKFRHDIDVVVDRIVVKEGLETRLADSFRTALDLADGIAVLETA
PKEGDPDRIVFSENFACPVSGFTIPEIEPRLFSFNAPFGACPDCDGLGVELFFDERLVVPDAALTVYDGAIAPWRKGKSP

YFRQTIEAIARHYGFDKSTPWKDLPRKVQQVFLRGSGEEEIQFRYDEGGRVYQVTRAFEGVIPNMERRYRETDSAWVR
EE
FENYQNNRPCQSCGGHRLRPEALAVKIAGLHVGQVVQMSIREAHAWVQTAPEHLSKQKNEIARAILKEIRERLGFLNN
VG
LDYLTLARNAGTLSGGESQRIRLASQIGSGLTGVLYVLDEPSIGLHQRDNGRLLTTLKNLRDQGNSVLVVEHDEEAIREA
DYVLDIGPGAGVHGGRIVAKGTPEEIAADPASVTGQYLSGTREIAVPADRRPGSGKAVTVVKASGNNLQDVTARFPLG
KF
VCVTGVSGGGKSTLTIETLFKTASMRLNGARQTPAPCETIKGLEHLDKVIDIDQRPIGRTPRSNPATYTGAFTPIREWFA
GLPEAKARGYKPGRFSFNVKGGRCEACQGDGLIKIEMHFLPDVYVTCETCHGARYNRETLEIRFKGKSIADVLDMTVED
A
QEFFKAVPSIREKMDALMHVGLGYIKVGQQATTLSGGEAQRVKLSKELSKRATGRTLYILDEPTTGLHFEDVKKLLEVLH
ELVDQGNTVVVIEHNLDVIKTADWIIDIGPEGGDGGGRIVAEGTPEEVAACEASHTGRYLKDMLKPRRVAAE
>OLS50400.1 replicative DNA helicase [Rhodovulum sulfidophilum]AB14
MNTVRTIRPEGEAAEDTGGLPHNIEAEQQLLGAILTNNEIFDRIASIVQPPHFYDPVHSRIFEIAANRIQKNQLASPVTL
KAFLEDDEGLAELGGPAYLARLAGAAISSFAARDYAQMIYDFWLRRELMKLGAEISDKAAKVDIASEPATQIVEAEQGLY
KLAEKGQSETGFQSFLRAVTDAVKVANAAYQRDGGLAGVSTGLTDLDKKLGGLHRSDLLILAGRPSMGKTSLATNIAFN
I
ARAYKRGAMPDGSVGAVNGGVVGFYSLEMSAEQLAARVLSEASEVPSEQIRRGDMTEAEFRRFVNAAKELEACPLYID
DT
PALPISQLAARARRLKRTHGLDALFVDYLQLVRPATAKDSRVNEVSEITQGLKAIAKELDIPVVALSQLSRQVESRDDKR
PQLSDLRESGSIEQDADVVMFVFREEYYKEREKPSDHDLEGMARWQEAMESLHGKAEVIIGKQRHGPIGSVELSFEGRF
T
RFGNLVQPWQQGQAVEGF
>OLS50401.1 hypothetical protein BV379_09835 [Rhodovulum sulfidophilum]AB14
MTLVIAGLLGAAFGAMLDRIGATNPNWIGRMLTLTDTNLMKTILVAIGTAAILIFAGQIYGLVDVGHMSVKAAHVGVLL
G
GAILGFGWAIAGYCPGTGLCALAVGRIDALFFVAGGLVGAAGFMISYPFWELIGFLNGEKWTLGAIPGADYPAAIPALP
G
DLAGIAMGAVFILIAILLPRRITGRNAARAAEAAAAGRADPAE
>OLS50402.1 XdhC/CoxI family protein [Rhodovulum sulfidophilum]AB14
MAEHDAIPETALDWHRTGQGAALATVVETWGSAPRPVGSQLAISGAGAMAGSVSGGCVEGAVIAEALEALGDGQPR
LLEF
GVSDGDAFAVGLACGGRIRVLVEPVGPVGRALPEDLLADLVAARAARRAVACLVDTESWARRLAGPEDPVLGQAIGA
RMR
ADRSGFEGTVFVGLHNPPLRLVVVGAVHIAQPLVQMARLAGFAPVVIDPRPAFGAAARFPGEVVIDAWPDAALTDLGL
DA
RSAVVTLTHDPKLDDPAIMAALDSDVFYLGCLGSSRTHAGRVARLEAAGLAPGRIAAIHAPVGLGIGARTPGEIAVAILA
EILQRLRRG
>OLS50403.1 hypothetical protein BV379_09905 [Rhodovulum sulfidophilum]AB14
MKSSLSAALCAALAVSSCGTVRESRLNPFNWFSSSTETETAAEQPLRRNEVADPRPLVDQVTGMTVDRAPGGAILTVT
GL
PARQGFWDAALVPVPAAEDEAPADTLVFDFRIARPGTATPVGTEPSRLITAGRFISEDDLKGIRMLVVRGAQNQRQSR
R
>OLS50404.1 PAS domain-containing sensor histidine kinase [Rhodovulum sulfidophilum]AB14
MALERLLRLRRSRQAGNAATLSVVVLGPVLAVLTFLTLGPLDQGASSHGLRIVLLADLVYALVVATLVVQRIARMVAARR
RHSAGSRLHLRLTGVFALIALIPTVSVAIFAGLTINIGLEGWFSERVRNVVGASLAAAEAYEEEHRRDLVSDAHTLVGYL
AAARQATIFLSDGDMRQILTQAQARVQRGLKEAYVIDGSGEIRARGERSYLFNYERPDDAQIARAGTGEAVVIQDWDN
NE

FRALVKLDGYLDRYLYISRTVDGDILSLLDDTQQSVRLYQQLEHERGRVLFEFGLLYLGFAMILILAAVWLGLWFAERLS
RPVGRLAGAAQRVGAGDFDVRVPEETSDDEIAMLGRMFNQMTRQIKLQRDALLDNTRQIERRRRLFDSVLSSVTAGV
MGL
DAEGRVEVMNRSALALLRLDAAEAEGQELCVAVPEFADLLDKLKSSRRGTAQEEVRLLRGGNMEHLLVRMAMRKRAD
GGA
EGYVVAFDDVTDLVTAQRMAAWGDVARRIAHEIKNPLTPIQLSAERIKRKFRGAVGDQAEALDQMTDVIVRQTNDLR
RIV
DEFSKFARMPEPDRKPVDLAALLRDAVMLQEAGQPGVAFRADIPPEPTPAELDATMIAQALTNIMKNAGEAIESLREK
GD
PEGYRPEIRVTFSVEEGNAVILIADNGIGLPQDRSRLFEPYVTTRDHGTGLGLPIVKKIIEEHGGRLDLRDAEPFAEGAH
RGALAEITLPVSLVQAPAQGAAEPARSDTTSATAATGE
>OLS50405.1 Trk system potassium transport protein TrkA [Rhodovulum sulfidophilum]AB14
MDPPCRFCEGLAQFSLTDTGRMKVIICGAGQVGWQIARHLSGEKNDVTIVDRNADLIRRATDALDVQGIAGHASYPD
VLE
RAGARDADMIIAATQSDEVNMVTCQVAHSIFAVPRKIARLRAQSYLTAIYSDLYRRDHMPIDVVISPEREVAEAALQRLA
APAAFDTESFLRGKAQLLGITLEEDCPVLRTPLRQLTDLFSTLRALVVGVRREGTLFAPEPGDQLFPEDQIYVFAHVEDL
NRTLEIFGKSTKKQERVVIIGGGNVGLSVAKALEAHEQRIRAKVIEKDRRRAEVAADALERTIVLNGDGLDINLLAEANI
KRADAVLAVTDDDKTNLLAAVRAKMAGCPMAICLVNDPSLVSLMGALEVDAYINPRATTVSSILRHVRHGRVRGVYSI
GD
AEAEVLEAQVLSTSPMAGKRVQDIDFPEGALVGAVMKSGKVILPRGDTRIEEGDMVVIFALSADVPEVERLLQVSIDFF
>OLS50406.1 cadmium carbonic anhydrase [Rhodovulum sulfidophilum]AB14
MNLCNIHTHTNAEHAGPGFALGAGSGTHGGFRCNETARLTEKELKDPAAGLGAFHGAKPGDTIEVHWVFSSCEVAPG
KGL
GSCMSERCQNPQLRVEAQAFLLVNDHTALDFKDYIYRGTQVNGLHQPRALPAGTGTPVIYGGSTTGPRYNQAICSPLQ
VT
WSVRPDCARLDINSLYEWAALGNVFEEDHSHGVRPLVTAEELLSQIR
>OLS50407.1 di-trans,poly-cis-decaprenylcistransferase [Rhodovulum sulfidophilum]AB14
MDGNGRWAQMRGRPRLFGHQAGARRVREIVEACPDLGIRYLTIFAFSTENWRRTQVEVTGLMTLFRRYIQKETNALL
QKG
VRVRFIGDRDRLDPKLVGMMHALEEITAGNEMVNLTVAINYGGRDEVARATKRLAREVAAGRLDPEDVDAETLAHFL
DTC
LLPDPDLVIRTSGEARISNFLLWQSAYAEYEFTPVLWPDFTSAHFAEIVGNYSARERRYGAVLS
>OLS50408.1 outer membrane protein assembly factor BamA [Rhodovulum sulfidophilum]AB14
MAVGLGAGVPQLALAQSYSFSNVRVEGNERVDASTVATYADIGRGETVSAARLNDAYQAIVDSGLFESVELVPQGGTL
VI
KVTEYPTVNRISFEGNSRIKDEDLQRIIQSQSRRVYSPATAEADVAAIIAAYEQSGRIAATVTPKIIERSENRVDLVFEI
VEGAVVEIERISFVGNRAYSEGRLRRALGTKQAGILRRVIRSDTFVADRIEFDKQVLRDFYMSRGYVDFQVLSVTPELTR
NRDAYLLTYNIREGQKFHFGSVTTSSDLSEIDPDEFMAAAKVSEGDTYTPLAIENTVARMERLAVRKGLNFIRITPRITR
NDRDLTLDVEFVVEKGPRVFVERIDIEGNATTLDRVVRRQFRTVEGDPFNPRQIRQTADRLRALGFFANAEVSAREGTS
P
DQMIVDVNVEEQPTGSLSFGLSYSQIDGAGAALSFSENNFLGRGQYLSFRLDTGTDNATSRFVFVEPAFLDRNVRFRFA
A
TYAQTDSGNYDSEYDTQKIEISPSLEFPISENGRFELRYAVGENTFKNYEGSSSYLQAEAEDPGQTYSKIGYGFTYDTRR
SGLDPNSGIVLKFNQDYAGLGGDLEFVETTALAGAQQMVWNEEVTLRAELEGGVLSMLGNSKSRVTERYFLNGKIRGF
DG
NGLGPRDGADKEALGGNMYAVARFEAEFPLGLPAEYGITGGLFADFGSVWGLDDGGAIDDSFHLRSAVGFSVFWKTP
IGP
LRFNFSNAVIKEDYDKEQNFDFTVSTKF

>OLS50409.1 hypothetical protein BV379_10200 [Rhodovulum sulfidophilum]AB14
MRPCLALVLVGTLLAPPLWADASRDERLEAAHDYVQRAVADMDLEEIVRSMWRPTVASIEASGTQLTPSQRRRIETLY
YD
IYTDRLRDLMLSQDELMADLMTLDEIQALRDFYATPEGRSVMQKLPKILNAQQPQIIELVKSTMPEVLSKMQGIVDTP
>OLS50410.1 cyclic pyranopterin phosphate synthase [Rhodovulum sulfidophilum]AB14
MQQTPLVDPFARAITYLRVSVTDRCDFRCVYCMAENMTFLPKRELLTLEELDRLCSTFIGLGVKKLRITGGEPLVRRDIL
TFFGAMGRHLESGALDELTVTTNGSQLEKHAAALYAAGVRRVNLSLDTLNEEKFARVTRWGRLAQVMRGIDAAQAAG
LRI
KINTVALKGFNEDELFDLVDWCASRDMDLTFIEVMPMGDLGNDYRVGQYWSVGEVRAQLEERFTTVDLAERTGGPA
RYVR
LAETGQKIGFITPLSHNFCESCNRVRLTCTGELYMCLGQEDRADLRAPLREHPDSNAPLIGAIRGAIAAKPEGHDFDYSR
QRADGQVSRHMSHTGG
>OLS50411.1 phosphate transport system regulatory protein PhoU [Rhodovulum sulfidophilum]AB14
MMKDPHIVKSYDRDLEGIQALIMKMGGLVEIAILEAATSLEARDEELAERVRAGDKAIDALEEKVNEEAAQLLALRQPSA
GDLRTVLSVMKISANLERCGDYAKNMAKRTGVLVQMAPMEGAAGAIRRMAKAVELMLKDALDAYIQRDAELAEDVR
QRDL
EVDQMYNALFREFLTFMMEDPRNITACMHLHFIAKNIERMGDHVTSIAEQVIYLATGHMPEDERPKADRTSVEALRS
>OLS50412.1 patatin [Rhodovulum sulfidophilum]AB14
MTTLRINLALQGGGAHGAFTWGVLDRLLRDPEIEIAAISGASAGAMNGAAVKSGLVSGGCETARENLDWFWQQVGA
VGDM
RLTSWMMAFLPGAGAVAQFMEQSMPISLIDTASRSVSPYALGPFYRNPLERVVRRLDFERVCARAEPLLFVGATNVRT
GK
VRIFKGDEIDSDVLLASACLPTLFQAVEIDDPDTGRTEAYWDGGYTGNPALFPLFAPELPEDIVVVSINPMERDEVPTTP
QDIQNRINEISFNSSLLRELRTISQLEQLIAEGRIDPGSMKLPRIHMISDDGLMNSLSIVTKLMPSPIVLAELKAAGEAA
AERFLCETKPNLQKASSVDLRRICADG
>OLS50413.1 NAD-dependent dehydratase [Rhodovulum sulfidophilum]AB14
MKIAVLGGDGFCGWPTSLHLSSLGHEIHIIDNLSRRWIDTELGVQSLTPMDSIQERCRIWKETSGEQIHFHLIDLAKEYY
RLRDWLSENRPDAVIHFAEQRAAPYSMISDRHKIYTVDNNINATHNLLVAMVETGLDAHLVHLGTMGVYGYSTVGAPI
PE
GYLDVSIDTPDGSKEMEILYPTRPGSVYHMTKSLDQILFQFYAQNDGVRVTDLHQGIVWGTNTDQTRRHPQLVNRFDY
DG
DYGTVLNRFLAQAAIGYPLTVHGTGGQTRAFIHIQDTVRCIELALKDAPAKGERVKIFNQMTETHRVRDLAELVAKMTG
A
EIAYLPNPRKEAAENELIVKNDNFLGLGLDPITLQEGLLEEVVEIAKKFAHRVDRKRVPCISAWTRDLASRVERDPEGKR
LKSVS
>OLS50414.1 C4-dicarboxylate ABC transporter substrate-binding protein [Rhodovulum
sulfidophilum]AB14
MFTAIAGLVTATSVPAQTFEKNILTGGPTGTYIQFGRDIAGIAAECGITLNVRESAGSLENFVGVRQRSFTQFGIVQSDV
LEYLRTYAANDPEMARTIAGVRIAFPLYNEEVHLLARADIGSLADLAGRRVAVGVENSGTHVTATLVLDLAEVAAERVQI
GPDESLRQLLAGEIDAFFYVAGAPTKLFQTPGIAAGQFHLLALTDPVLQAVYTPATIPDGTYPFQSGPVEAVAVKAVLMT
YDYRLRKNRYHRASCDAVRSISHLVLRRFDALKETGHPKWSQVDLTDIPPGWEVAACVNMGIDPEFSLPCTREDATAG
PG
ANEANALYRRRVCAAIGGC
>OLS50415.1 nitrogen fixation protein CowN [Rhodovulum sulfidophilum]AB14
MTDQTPDRYVSFCGIECDRKADRLMAMLAAHMAESQSRWVGYFEQKLAEKARMGNDNLHFVGSQVNSLSAFFEEL
GDSAA
EDLLYDLEQTCC
>OLS50416.1 alpha-glucosidase [Rhodovulum sulfidophilum]AB14

MSEDPDWWRGAVIYQIYPRSFQDSNGDGIGDLGGILARLPHVVSLGVDAIWISPFFPSPMKDFGYDISEYCDVDPMFG
SL
ADFDALVAAAHGLGLRVLIDLVLSHSSDRHHWFVESRSSRDNPRAEWYVWSDPRPDGTPPNNWLSIFGGSAWSWDP
GREQ
YYLHNFLASQPDLNLHCEAVQQAIFETARFWLDRGVDGFRLDTINFYFCDKELRSNPPLPVEDRNDNIAPRVNPYNHQL
H
LYDKNQPENIEFLKKFRSVLDEQGAVALGEVGDAQSDLEILGQYTSGGDKVQMCYAFDFLASTPLTAARIAAVYERLDR
V
AGDGWVCWALSNHDVIRHPTRWNLSPAALRGFLTLTLCLKGTVCLYQGEELGLPEAEVPFEALQDPYGIAFWPEFKGR
DG
CRTPMVWAAEDLHAGFSRAPRTWLPIPETHIGMAVAAQEHAPEAPIHHYRRAIAFRHANPVLARGRMEQVRAEGNV
LSFR
RCDDAAEMFVAINLDENPAAMPAPDGDWRPLGAEINAARPGADGLVHLGPWQPSLMIRRKEAGWRV
>OLS50417.1 thiol oxidoreductase [Rhodovulum sulfidophilum]AB14
MPSLPFTALPFTAAEQARIAQATALPRDFTRPQPFEALAGGAGTVAGAAAFSSPLANLPPGEGLDFRGGQALFDRGWV
SA
PASTIASDGLGPLFNARSCLACHPGGGRGAAPAGPGDLPAALMLQLGVPDPAAIEGYLTAHPDPVYGRQIQPLGLPGQ
TA
EGRPRVAYDEIAVPLSEGESATLRRPRYELAEPGFGPLDEATVISPRIAPGLAGLGLLEAIPEQAILALADPDDADGDGI
SGRASLLPGPEGQPVLGRFGWTAAQPTLQAQIAHAFAHDIGLSSPLYPEGHGDCTPVQTGCRAAPDGGDPEQDGFEIS
AT
ALDLVTRHARHTAVPARRDADAPEVLAGKKLFHDTGCAACHRPAFVTRRLPEAPALSFQLIWPHTDLLLHDMGAGLAG
GG
AGASGQEWRTPPLWGLGLARALEPQAGYLHDGRARTLLEAVLWHGGEAAPARDRVATMPASDRAALIRFLESL
>OLS50418.1 1,4-alpha-glucan branching enzyme [Rhodovulum sulfidophilum]AB14
MQAGLHDDPFGVLGPHGQGRRRIVAAFLPGAATLEAVSGGKTHPLARNPEAPDLFAGPVPGKAPYVLRARDGSGNC
REFE
DPYRFGPVIGELDEYLLGEGTHQRLWQVLGAHVRIHEGIAGTHFAVWAPNARRVSVVGPFNAWDGRRHPMRRRGA
TGVWE
IFLPGIGEGECYKYEILGADGSVQPLKADPVGFGAEHPPKTASVVRDIRGYGWHDDAWMTYRAGNNARTAPISIYEVHL
G
SWRRRAREGNRPLSYREAAEELVDYVADMGFTHLEFLPLSEHPFDGSWGYQPVGLFAPTVRHGPPHEFRDLVDAAHR
KGL
GLILDWVPGHFPTDAHGLARFDGTALYEHADPREGFHHDWNTLIYNFGRTEVANFLYSNALYWLEEYHVDGLRVDAV
ASM
LYRDYSRKEGEWVPNVHGGRENLEAIAMMRKMNALCYGEVPGIMTAAEESTSFPGVSRPVDAGGLGFGYKWNMG
WMNDTL
SYIAHEPVHRKFHHHQMTFGLVYAFSENFILPISHDEVVHGKGSMLAKMPGPDWEKFANLRAYYGFMWGHPGKKLLF
MGQ
EFAQRAEWDRAAELDWGVLEAEPHRGMQRLVRDLNTLYRATPALHVMDCEPEGFRWVEANAADISVYAWLRQGQ
AGDPPV
AVVANLTPVERRGVRLGLPQAGRWREVLNTDAALYGGGNRGNLGGVVAGGVAHHGLPASAELTLPPLSVLYLMPGE
D
>OLS50419.1 DNA polymerase IV [Rhodovulum sulfidophilum]AB14
MDAFFASVEQRDRPELRDRPVAVGHGRARGVVAAASYEAREFGVRSAMPSVTALRKCPELIFVPPRFEVYRGVSADIH
AI
FAEYTDLVEPLSLDEAYLDVSQACADGRPASEIAEEIRARIRAATGLTASAGVSYNKFLAKLASDQNKPDGLTVIPPHRA
AAFVAGLPVGRFHGVGPVTEAKMLRLGIRSGADLRAKSLAFLQAHFGSSAAHYHAISRGIDPRPVRPDRPRKSIGAETTL

SEDLSAFEAATEVLARLAAKLSRPCAEKGLAGRTVTVKVRFTDFEQVTRRRTLSRPLTETAEMLEVARQLLAGLHPFPRP
VRLLGITLSGLEPRAEAAAEKQLDLFDESSRNSG
>OLS50420.1 chemotaxis protein [Rhodovulum sulfidophilum]AB14
MFGRPRKSAQEKEAHALAEVFMAVIDRTQATIQFAPDGTILTANANFLDVLGYKLDEVVGKHHSMFVDPAYVKTEAYR
NF
WKALAAGKSFTDQFPRVHKNGEVIWIQATYAAYVDENGQTTRVLKIATDVTERQHGVDAIAKGLERLQERDLSHRVPH
SN
LPDIEILGKAYNTAVAQLASALSAVKHLSDKVERTAREVGDASSHLSQRTETQAATLEQTAAAIEELTSNARSTAALAKE
VEASASNAQNTAEAGGKIVEDAVGAMSNIETSSRKISQILGVIDDIAFQTNLLALNAGVEAARAGDAGRGFAVVASEVR
A
LAQRASDAAGEIKGLVHESSESVKQGVGQVSSAGEQLKKIIAAVAGINSHIGQIATGATDQSFTLSEINTGVSQLDTVTQ
QNAAMVEESTAASQVLASDAQDLGRQVSLFQTEEGPRFNASGHRPNRNAA
>OLS50421.1 DNA protecting protein DprA [Rhodovulum sulfidophilum]AB14
MRSRRVGATTFFRLLEDHGSAAAALEALPEIAREAGVRDYTPFPLDLAEAEIARARKLGAVALLHGDPSYPPALHDLADA
PPVLWALGRTDLTRRPMVALVGARNASSLGLRMARRLAEGLAEAGYVIVSGLARGIDRAAHIAAIETGTVAVQAGGID
VP
YPVENTGLAEEIGQKGLRLSEQPPGLEPQARHFPQRNRIVSGLARAVVVVEAASRSGSLITARAALDQGREVLAVPGHP
F
DARASGCNMLIRDGAVLVRHPEDVLDALGPAPRPAAAAPAKPPPRMTEVPETPAEARRAAPPDTAALHARILDRLGPS
PL
AEDQLIRDLGLPAGTVTSELLTLELDGRIARAPGGLLSRPV
>OLS50422.1 type II secretion system protein E [Rhodovulum sulfidophilum]AB14
MFSRYRKPAPGKVPPAPPQPQAKTPPEIPAEASVPALPALPLQAATNEKDRKRKERLGELKAELHKTLLENLNLAALEHA
EEKELRTEIQAIASETLEEMGVVLNKDDRNQLYQELFDEVTGLGPLEPLLKDDSVNDILVNGPRQIFVERGGKLQLTDIA
FRDEKHLLRIIDRIVSAVGRRVDESTPYVDARLADGSRFNAMVPPVAVDGSLVSIRKFRKDKLGIDDLVKFGAFTEEMAA
YLQAAVATRLNIIVSGGTGSGKTTTLNALSSFIDNSERILTIEDTAELQLQQTHVGRMESRPPNVEGKGAVSQRDCLKNA
LRMRPDRIIVGETRGNEVIDMLQAMNTGHDGSMTTIHANNARDAVSRLENMIAMAGIEMPLRAVRAQIASAVNLIV
QASR
LQDGSRRLVSITEVTGMEGEVISMQEVFRYERLGLDPGGKIAGRFTATGMRSHFSERFRQWGYDLPTAIFEPGRGG
>OLS50423.1 tetratricopeptide repeat protein [Rhodovulum sulfidophilum]AB14
MRRGAVLVAVFCTACSASGTLSPAYRGLPAPADAAHPATAVDGLVVGHRLMAAGEYDLALKAYYRAAAKHGLSADVL
SGI
GSANLRLGRLGQAETQLRRAISANARFVPAWNNLGVVLMEKGEPGEARRVFETAFALDSGRSEDIRRNLRLAIAKTENP
D
YDSPRNQGTTFELVRRGEGRYLLLAPP
>OLS50424.1 peptide ABC transporter permease [Rhodovulum sulfidophilum]AB14
MLGRPEALFGAAILLALILVAVFAPLIAPGDPLAIAGRPLIPPFTDPALPLGTDRLGRDLLAELIHGARTSVSVGLSAAL
VAVLLGMSIGTAAGFAGGLTEAALMRLVDAFQIVPGFLLALAFVSAAGPSLGIVVLAIALGAWTGPARLARARVLSLRET
DFVAAARVMGMSPAEIAFRQILPLALPPVLAVASVIVAAAVLTEAALSFLGLGDPNSITWGAMIAEGRNQLRSAAYLSII
PGIALVLTVISTYLLGEALTDILSGRRNR
>OLS50425.1 efflux transporter periplasmic adaptor subunit [Rhodovulum sulfidophilum]AB14
MAGTLALFQLAGRPDAAPAEQSGTEMAVQVTVETIRPEPIVLATEYFGRVSAARRVEIRPQIGGLILERRVEEGTQISAG
EVLFRIDPAPLKADLALAEAALTRAEAAEAHARRAVERSDALLARKAISQQSNDTAHTDLRLAVAGIAEARAAVEHKRLD
LDFATLRSPIGGYVAAGLADVGGLVTVGGEKALAVVQDLDKVYVDLRLPADDLDAVLMAAEEGLGPVRILRERDGAAP
LT
GVLKSSDVIVDPGTGYVSVRIETANPKLALLPGMFVRAKLPRGAVPDALLVPEDAVLHTAGGGAQLVVVSPEGKARRRD
V
VPGARIGNRIIIASGLAPGEVVAIRGQNRVPEGGAVSVDARPAET

>OLS50426.1 hypothetical protein BV379_11920 [Rhodovulum sulfidophilum]AB14
MMDRQMQMQGQRKHPARRAMSVRAALEWAFGTECARLDHDEIEAVGGTGWRPFSMEYVALERAQLGTRVDTSR
GRSRPHD
DAELIATVVRNTLPWYAATLVADLARAGRTPDWMPDARPRLRPAEWQQNQHRAYGRACDSAELKDGWQPILRRNR
KGVIV
HDRARYTPCVWEPSPARIAAARRAYLDWWGYLLEVQAALGAADLARICITRDMPPMTPWR
>OLS50427.1 hypothetical protein BV379_11925 [Rhodovulum sulfidophilum]AB14
MESYVGQPVTEAMLDYGAPSNVMTLEDGRRAYQWTINKSRTVAVPTTSTTTVRGPSGTASVHGRSMTHIPRSQTCVY
TLT
AAQRGGQWIVDGFRKPRSACK
>OLS50428.1 polyphosphate kinase 2 [Rhodovulum sulfidophilum]AB14
MPPVDKPAASVSAPLGSAPEAAPPVASPANIAHAFETGEYPYRSKMSRRSYEAQKARLQAELLKVQIWAQETGQKFVIL
F
EGRDAAGKGGTIKRFMEHLNPRSARVVALNKPTEEEKGQWFFQRYVRELPTAGEMVFYDRSWYNRAGVERVMGFCT
PNEY
LEFMRQTPELERMLVRSGVRLFKYWFSVTREEQRRRFEARETDPLKRWKLSPIDKASLDKWEDYTEAKEAMFFYTDTA
DA
PWTIIKSNDKKRARLNCMLHFLHGLDYPGKTPRIAHAPDPLIVGRAEHVIHKAEHMLGTSLHPDLRRGRED
>OLS50429.1 hypothetical protein BV379_12140 [Rhodovulum sulfidophilum]AB14
MTRRTALAGALALAALPAAAEDAWPTAPLHVYVGFPAGSSPDTLTRIITEPLAERLGQPVVVENKPGAGGVIGVQQML
AN
AGDGHSFATTINGPLTTAARLMTSTGYDAATDIAPVTLIATSPLVLAVSARSDFTDLESFVAAAGAEPEALSYGSVGQGS
GAHLTAELFADAAGVSLLHIPFTSYAEVTTSIVGGEIDAGFMAPSAALPQVEAGRMRLLGITSAEPFAQAPDVPVLAGRA
GLPEEFRAELWNAFIAPAGTAPAVIERLNTEIGEILAEPDVQEKLLAIGWQAAPGTPADLADRISADTAMWGAVIDRVE
A
KP
>OLS50430.1 iron-sulfur cluster assembly accessory protein [Rhodovulum sulfidophilum]AB14
MTLPPRVTERAYARLSEIGAAKQGKALRIAVEGGGCSGFQYAITLDDPAEDDLVLEGAGEKVVVDSVSLPFLQNATIDFS
KELIGAKFVIENPNATSSCGCGTSFSI
>OLS50431.1 hypothetical protein BV379_12190 [Rhodovulum sulfidophilum]AB14
MTQLVPRDRFLPLRPRETHAGEPRRAGIEIEFGELTEAEAADVVARTLGGTPREIAAHDFEVETPDLGRVLICLDTSFRD
KAGSALTDLGLQLGRAVVPVEVVTPPLHPDQLPEAERLRGALRAAGARGSREGVFLGFGLHLNPEIPGQDAADLVPIVR
A
FALIEDWLRKADPIDPSRRLLPFVDPYPRGLIDRLASAGPDWTLEAFADAYLDETPTRNRGLDMLPCLAHLAKDRVAQA
L
GPGAASIGARPTFHYRLPDCRIDEPGWRLAYEWNRWVMVERLAADTAALKGLAADWLDYRAALTTTRSDWYAHLDR
VLCK
LELWDGSCGKDGKTAP
>OLS50432.1 cytochrome B [Rhodovulum sulfidophilum]AB14
MEETGDTRPGAETLQTVQIWDPLLRLFKWSLTISVAAAWGLGMFGPDIMTLHFWFGYAVLGLLAFRLVWGLWGPEH
ARFK
SFAYGPVAILKYVFGMFRRKPSYWPGHNPLGGLFVFVLLAVLLLQAVSGLFVDPDDYINVGPLAKYVGSETTRAALSFHV
QLGWVVLGLVGLHVAAILFYSKWKGENLVTPMLTGRKKVRKKS
>OLS50433.1 IS1182 family transposase, partial [Rhodovulum sulfidophilum]AB14
MAGFIDGIDRDQVTLFPDRLEDWIGEDHLVRVVALFVEELDLPALGFERCVPARTGRPGYHPSVLLKLFIYGYLNRLPSS
RRLEREAGRNVELMWLTGRLAPDHKTIADFRRDNGSAIRRTCAQFVELCRRIGVLKGDCVAIDGSKFKAVNNRDRNFT
KN

KIASRLAHLETDVERYIKEMVRIDRQEDGEARADKVAHLARRYRQEITRLKAMDQALTDAPDGQISLTDPDARAMATS
AR
HSGLVGDNVQTAVDAETHLIVAHDVIHQGFDRDQLSPMAAAAREALQRDDLHAIADKGYFSGSQILACHQAGITTTM
PRP
ETSGNRATGMYVKANFAYDPDRDVYVCPAGEELTYCYTREEDGLRVGRYWTNECQHCPVRHRCTTSKERWITRWDH
EHLV
DAMRKRLGGHA
>OLS50434.1 DNA-binding response regulator [Rhodovulum sulfidophilum]AB14
MKVLVADDHELLRDTLELYFQQSDGVDADFVGDYPSALEKMQGETEYDLVLLDYSMRGMNGLEGLKAALDVAGSNR
VALM
SGIAPRDVAAEALNLGAAGFVPKTLSAKSLLSAVRFMAEGERYAPIDFLMGEDQTSLANPLAEKLLSREVQVLRRLRAG
M
SNKEIARELGVTEPTVKLYLKSLFRKINVNNRTQAALFAQENGLF
>OLS50435.1 hypothetical protein BV379_12295 [Rhodovulum sulfidophilum]AB14
MREQETHRLAEAERALLEGERNRMRAVFDLLPDTVIELDAERRYTHIHSHSRDSVRGRIDGLLGFRPEDCLPPATAEAIH
ALLDRAEAAGFAALPELVHPPVPAAGPKIYTLSAATHATAPAQGSPGYVLVIRDVTEESAQRRERDRLGRVVEHMTREV
F
LTDCSHRLTWANRATEEHIGRPVDRLVGLSPRQAFGAVWSDATRLDRVCEQLEAGNGFAGEAAFLDHGGTKIWLDIA
VTP
LCDGSGGREGWMWILSDITERRRDKDRLAQIARRAREAEERVAQAIETLPDAFAFFDSQDRLILFNDRYRQFYPHSAPA
I
ELGQTFEAILRYGLAHGEYPGDSRSDEAMLAERLARHRRPYDESEQHLADGRWLRVIERRTPDGGYVGLRSDITELKQL
A
QRAQADRLAAMDLSRDAIAIAGPDGHFLYMNPAHRDMFGLDATEGCRERTWTELYDGETPELMTGTALPALDARGN
WRGE
VWYRRPDGSRVLHELSLTRRHAGGILCIARDLSERVRANRELARLRDDLQVARQRELIGHLSIGLTHDFNNLLAAISGSA
SLIADSGIVGPNVQRIQLAVGQASGLLRRLSDFGMRPPLRETVDMRGPVRAAVDLLLADGSGAVELQLDLPARPVMVD
AV
QTDMMQVALNLLTNARDSILSEEYPTGRGRIRIALCIASDAELKPSLAFGSLDPARAYCLLEVEDDGPGLDADARSQVFA
PHLFSPSAVDCGSGLAILPSIVGKNGGAIALTSDGSSGARFSVFWPLDSDRNAPPAAPHGSRRRARPLSGRLDGHMVLV
T
DDNPDVLRVISAILENAGAEVASCTMAEDALEAVSEDPRHWDLLVTDYAMPSMSGARLAREVHAHVPDLPVLLMTAH
TDW
RTHGEGHSGREFAAVLGKPISSADLLAAAEAAILDR
>OLS50436.1 galactose mutarotase [Rhodovulum sulfidophilum]AB14
MGRAPDGSPVEALWIGQAGTRARIMTWGATLMDLRLDGIGHSLVLGSPDLAPYLGPMRDFGAVVGPVANRIAGASA
PLGG
RRLALGANEAGRTMLHGGAAGCSVRNWRIESESATACRLGLSLPEGTGGLPGPLTLRASYSIEDEGALEILLEGESGALT
FCNLAPHGYWCLDGSGTLDAHLLEIAARTYLPVDAALIPLGSPAPVAGTRFDFRRPRPVRAPGDAALDHNFCLGDASGR
L
RPACRLSVPGIALDLSTTEPGLQVYDGGGIDTGPFPGHQGRLYRAHAGLALEPQRWPDAPNRPDYPSVLLRPGETCRQ
LS
RFHPRRIGAPGPG
>OLS50437.1 IS5 family transposase, partial [Rhodovulum sulfidophilum]AB14
MALRQRTGFVESLLQLVGLDWTVPDFSTLSRRQKTLTVNIPYRRS
>OLS50438.1 hypothetical protein BV379_12405 [Rhodovulum sulfidophilum]AB14
MLERINQESAFSDAATEEGLIDLANKLAPLIQGRRLHNVIDLMSLASDGVDMADDAMIQKMMTAYEDITSTAWALGN
AAR

YAANQAATAPVPSRLGLLRAAGDEDVRRGLHFALQFLAVLGRQATPEPDVD
>OLS50439.1 thioesterase [Rhodovulum sulfidophilum]AB14
MDASALTAFLRSAFPQIGEEFVVEEVAPMQVRVRLAVTERHLRPGGTVSGPSMFALADVGIYLAVLAMIGPEALAVTT
NC
SIDFMRKPASGRDLICEARLLKLGRVLAVGDAMIRSEGMEGVVARASLTYSIPPK
>OLS50440.1 dihydroxy-acid dehydratase [Rhodovulum sulfidophilum]AB14
MGDTRANRRFRSQEWFDNPNNPGMTALYVERYQNQTFTRGELQSERPVIGIANSGSDLVPCNKIHIFLMDRIKAGIRE
AG
GVPMEFPVHPLQETGKRPTAALDRNLAYLGLVEILHGYPLDGVVLTTGCDKTTPAMLMGAATVDIPAIALNGGPMLDG
WW
QGKRAGSGTIVWESRRLLAEGRIDYDEFMDRVCSSAPSLGHCNTMGTASTMNAMAEALGMSLTGNAAIPAPFRERM
AMAY
ETGRRIVQMVLDDLKPSDILTRAAFENAITVNSAIGGSTNAPPHLQAIARHAGVELDVTDWERIGHDVPLLVNMQPAG
EY
LGESFFRAGGVPAVMAELARAGRLHVGAMTATGRTLAENLERVRSADPDVIATYDAPLRAQAGFKVLSGNLFSSALMK
TS
VISDEFRARFLSEPGQEGVHEARAVVFEGPEDYHARINDPELGIDESAMLFIRNVGCVGYPGSAEVVNMQPPDALVKQ
GV
RHLPTVGDGRQSGTSESPSILNASPEAAVGGGLALLRTGDRVRLDLNASRLDALVDDAEWDLRREAWEPPEIVSQTPW
EE
IYRANVGQLAEGGCLELATAYRRIARDLPRDNH
>OLS50441.1 hypothetical protein BV379_12840 [Rhodovulum sulfidophilum]AB14
MTEDALCAFCDALPGATRSQPFGPGVEVWKIGGKIFALMAPGSGRISVKCADPEFATMLIETGRAEKAPYLPRGGWIS
VG
FEAQNADEMAHRLRESHATVRASLPRRVREGLD
>OLS50442.1 elongation factor 3 [Rhodovulum sulfidophilum]AB14
MARAPLLQLSSISLTFGGDPVFSGLDLVIQENDRVALVGRNGSGKSTLMKVMAGLVEADSGGRVLSPGTSVGYMEQEP
DL
SAFATLGDFAASALAPGEGYRAEIAAEGLKLDLSAPAATASGGERRRAALARLLAAAPDLMLLDEPTNHLDIEAIGWLET
QLAETRSAYVLISHDRAFLRNLTRATLWIDRGGVRRQDRGFAGFEDWRDKVWADEDEARHKLDRKIKAESRWAVEGI
SAR
RKRNQGRLRALHAMRDDRSAMIRRQGTAAMALEAGPKSGKRVIEASGISKSFGDRVILRPFSLRIQRGERVALVGPNG
VG
KTTLLKMLIGELAPDTGEVRLGTNLEMAVFDQNRAALDPEMTLWESLAGDPEMRVSGKADQVLVRGQPKHVVGYLK
EFLF
DDVQARAPVRSLSGGERARLLLARIMARSSNLLVLDEPTNDLDVETLDLLQELVSDFDGTVLLVSHDRDFLDRVATTTVA
MEGDGRAVVYAGGWSDYRAQRAEAAGPESGADKPAGKSSGGRSKPAASADAGAGTKKAAGLSYTEKHRFDALPGE
MERLE
AEIARLEALLEDPELFTREPVKFRKATEALMARQEALAKAEEDWLELAEKAENAG
>OLS50443.1 ferredoxin III, nif-specific [Rhodovulum sulfidophilum]AB14
MTATTRGGTEYIPEFLMAIDKGKCIGCGRCFKVCARNVLTLKGVTDEDEIVDLDDEEFEDEIERRIMVIADGDDCIGCGA
CARVCPSSCQTHAPA
>OLS50444.1 nitrogen fixation protein FixA [Rhodovulum sulfidophilum]AB14
MDAPEAPVETKKGLNIVVCIKQVPDSAQIRVHPVTNTIMRQGVPTIINPYDLFALEEAMRLRDDYGGTVTVLTMGPPM
AE
DALRRALGLGADKAVLITDRRFAGSDTLATSFALSAALAKLAEDGPIDIVFTGKQTIDGDTAQVGPGIARRMGLNQLTYV
SAIDSLESDSTTITVRRRSEGGVQVLQSRLPCLITMLEDTNLLRRGSLQDMLRAARAPLTIWSAEDAGIEDPMKCGLRGS
PTVVRRVFAPQPRAEKAEMVAVEGKAPAQIGDAILDVLFAKSPKLEADLSAKTPA

>OLS50445.1 ABC transporter permease [Rhodovulum sulfidophilum]AB14
MRGQGLFWALALLVAALFLLSLLIGPAGLSVRESFSALLAGRGEAAVLVMREIRLPRAILAVLVGASLGLSGAALQGYMR
NPLAEPGLIGVSSSAALGAVLALQTGLASAHVLALPASALTGALIAVSLLLLLAGPRGGAFTLILAGIALSALAGALTQL
VLNLSPNPYATADIVFWLMGSLADRSFTHVWLALPLTLLGWILLAGLGRGLDALTLGEDAAAALGIRLGRLRLALVIGVA
ASVGAGVAVAGAIGFVGLIVPHLLRPLVGARPARLLPASALGGAALLLAADIGVRLVLPDRDLKLGVVTALIGAPLFIRL
IYRTRAEAGA
>OLS50446.1 glycerol acyltransferase [Rhodovulum sulfidophilum]AB14
MTRRMKERIDPLIEERAPWLFADHPWSVPSRRVLNGLLGYDRTVALGEVLQDHPSPVIMDEMARLLARDVRGTGIENI
PA
TGPAMIVANHPTGIADGVMLWSLLRQRRPDLFFYANRDILRVLPQMEEIIAPVEWREEKRSHAKTRETMAYTRAAIEK
GR
LAVIFPAGRLAKRRGLSLHERPWMASAAMIARKFDLPVIPVNIRSRNSVLFYLFDLLHPTLRDITLFHETLNKNRQPYRM
TVGRPIPPGALPVRSEEGIEMLRAATLALGGRYAPRVSLLQNSRKPSWIKSTALG
>OLS50447.1 hypothetical protein BV379_13330 [Rhodovulum sulfidophilum]AB14
MAGLVLAVATGSAAGCSPLPGASAPGRTPAYLGAETMALKDGLVEVALRMRAPDGVGGLDDYGRCAVARHALDNGY
GFAR
HLRTTLGREGGGVWTADAVYMMTRTFPRGVSRVDARQEIRDCATRGIPTV
>OLS50448.1 hypothetical protein BV379_13340 [Rhodovulum sulfidophilum]AB14
MGRAAAFAAGLGLAATVSAAGAEPRPALGEQTEIVRGLQVIATADMIRKKCPTIGARMLTAFGYMQRLKSKASRLGYP
DA
EIRAFVEDKTEKARVETAARNWLTARGVRLDEPPSYCPVGLAEIRSRTEIGRLLRARQ
>OLS50449.1 NADH dehydrogenase [Rhodovulum sulfidophilum]AB14
MTGPNTAGGDREVATQALNAELQDRGFLVTSTADIINWARTGSLHWMTFGLACCAVEMMQVSMPRYDLERFGTAP
RASPR
QSDLMIVAGTLTNKMAPALRKVYDQMPEPRYVISMGSCANGGGYYHYSYSVVRGCDRVVPVDIYVPGCPPTAEALLY
GLL
QLQRKIRRTGTIVR
>OLS50450.1 transglutaminase [Rhodovulum sulfidophilum]AB14
MEYDIRLEIGYVYDSPAASTRTLLRMLPLTNGEQTLISGRVSADPDPDFRRDGTDFFGNATTEIAHDAGQTEFRFRFKGR
VRRIPRGTTLDLSCGLGALGAELQSIHSILPMSPHHFLGASERIRHEPEIAAFAEELTGSDMSVLAALKAVSHAIHERFD
FDPEATEVSTLPIEAFRNRRGVCQDISQVTIASLRAIGIPAGYVSGFLRTVPPPGQSRLEGADAMHAWVRAWCGVETG
WV
EIDPTNDMLAGPDHIAVAIGRDYADVAPVKGSSRTSGSHRTDHKVDVIPL
>OLS50451.1 ABC transporter substrate-binding protein [Rhodovulum sulfidophilum]AB14
MHRRTFLKTGAGIAATGTLASPALASGKIHWKLPTAFPKMAPAVGTNATNFAARVAAMSDGRLSFTVYGAGELVPAF
GVE
DAVQAGNAPVGHGPTYYAAGKNPALHWFTGVPFGMTSVEHMAWLRWGGGQEVWDEIYAERNLKPLYSGNSNTQ
SAGWFKK
PIESVEDLKGLNMRIAGLGGEVFRKLGVNSVLMPPAEIFQALQSGAIDAAEWVSPHLDQAFGLQKITDICYLPAFTEPGA
GLSIVFNQDAWDELPADLQAICEAAALAAAYEVQGQFDHFNAKSLAELKAQGVRFLNFPEDVITAQRDAWNEVQDAL
AAE
HPDVAKVRASYDPALAEARDYAKAMTLPLLAGRS
>OLS50452.1 D-alanyl-D-alanine carboxypeptidase [Rhodovulum sulfidophilum]AB14
MTMRPTFLSALAGALSLLALAAPAQANFETRAGAAYVVDQSTGTVLLSKNADQPVPPASMSKLMTLNMLFEALRDGR
VTL
ETQFSVSSRAMAMGGSTMFLNERDRPTVEDLIQGIIVQSGNDACVVVAEGLAGTEEAFARLMTERAQVLGMTNSTFA
NAS

GWPHPNQRMSMHDLGILAMRLIDEFPEYYGFFGEQEYDYDGRAPQNRFNRNPLLKLGIGADGLKTGHTQEAGYGLV
GSAL
QNGRRVTFVVSGLDSAEQRAEEGERIINWAFRQFVQRDVLRRGERLAETDVWMGEIEKVGLVARADLEVLLPAIVRDT
VE
AEVHYHDPVLAPIEAGQELGELVIQRPDMEPLRVPLVAERAVARGGFVPRLRTATEVILRWAEEKAADL
>OLS50453.1 efflux transporter periplasmic adaptor subunit [Rhodovulum sulfidophilum]AB14
MSDRQIFSEFSDSPFPRDALRGLFGPAFLALTLAVAVPVPAQADGGLAGLWQRFFGPAETGGISSSNGRLEAQSVDVAT
K
YAGRVAAVEVEEGATVEAGAVIARIDDRDTRAQLLGARAAVLRAKAARELAEASVMQAQSALDVARTNRARVERLHA
DGH
ASDSANDDARNAFTSAEAALAMAHAQVSDAEALMASSEAEVDRLEIALDDLTIRAPIRGRVLYRLHEPGEVVAAGAPV
VT
LLDLTDVYMNIYLPAPTVGLISANDEARLILDPIPQYVVPARVTFISPESQFTPKSVETIAEREDLVFRVKLTIPRNLLE
RFEDQVKTGVRGRGFVRTAPETPWPADLQVQLPD
>OLS50454.1 C4-dicarboxylate ABC transporter permease [Rhodovulum sulfidophilum]AB14
MQAKSQNQSQTRSQTDTTAADDPDEVAYVSTLPGVLGKIDAAIARVESVLLATGVLLMALNTVANVVGRYLFQSSLFF
TE
ELNRVLIVVITFAGISYAARQGRHIRMSAIYDALPVRARKILMIVIALTTAVLMFGLCWFAVAYLLTQAGRGRVLPALQI
PVWTTLVIVPVGLFMTGMQYLLTAVKNAISRDVYLSTAVLEGYDDDETEI
>OLS50455.1 sodium:proton exchanger [Rhodovulum sulfidophilum]AB14
MMEALLHSPFGEVATLLVLAAVLGFAGLLLRQPLIVSFIAVGLVAGPSALDIVRSSDQIDLLSQLGIAVLLFLVGIKLDV
KLIRSLGAVSLLTGLGQVAFTSVFGYLIGLGLGLGHVTSLYVAVALTFSSTIIIVKLLSDKREIDALHGQIALGFLIVQD
LVVVLAMIVLSAIGIGSGEGGGDGTGNVLSVLGFGLAMVGAVVLFVRHVADPLSERLARAPELLVIFAIALAAIFAALAD
FVGLGMEVGGLLAGVALASTPYRETIAARLAPLRDFLLLFFFIALGAALDLSLLGAHVPGAILFSLFVLIGNPLIVLAIM
GAMGYRKRTGFLAGLTVAQISEFSLIFVAMGVSLGHVAPEALGLVTMVGLVTIAASTYMITYSHRLYDLCEPVLGIFERR
GTPREPEAGATSGAARAGVLIFGLGRFGTAIGMRLQKRGIDVLGVDFNPQAVRRARALGIEACYGDASDPEFLAELPLA
E
AAWMVSSVPILPGGLTQEDTRRTLIQLARGAGFRGRIAVASHNAADTQDLLGAGADLVLEPFQDAADRAVELLTGAPE
TE
RTAIPGIDSEAPQPA
>OLS50456.1 alpha/beta hydrolase [Rhodovulum sulfidophilum]AB14
MLATQIHGDRSAAPPLLIVHGLFGSARNWGAIARRLSDSRRVVAVDMRNHAGSPWTETHRYPDLASDLAEVIAAEGG
QAD
VIGHSMGGKAAMMLALTRPETVGRLIVADIAPVAYGHSQSGLIRAMRGLDLSAVSRRGEADRALAADIADPSTRAFLL
QS
LDLRAEPPRWRLNLDTLDAEMPYITGWPDDIEGRFEGPALMLTGALSDYVGPGDRDRIKALFPAARFARIPGAGHWLH
AD
KPREFEAAVRAFLELAPSPRA
>OLS50457.1 NnrU family protein [Rhodovulum sulfidophilum]AB14
MGGWGEFAAAYALFLLSHALPVRPGVQAGLRRWFGRRGYLAAYVAVSLAALGWLIVAAGRAPFIPLWAPLPWQLWL
PVLV
MPFVCLLIAATLGSPNPLSFGGAGNARFDPERPGIVGLTRHPLPVALTLWALAHAAANPDLAHLMMFGGFAVFAVAG
SRA
IDRRNRRLMGAEWDRLAARTATLPFAALVTGRWRPSGPPPLWRLVLGLALWAGLLAVHRPVIGVSPLPF
>OLS50458.1 C4-dicarboxylate ABC transporter substrate-binding protein [Rhodovulum
sulfidophilum]AB14
MVGAFAAATAAIWTAGAALAQDYTFKLHHFLPATSPTQTETLEPWARAVEARSGGRVRIEIYPSMTLGGRPPELVTQA
RD

GVVDLIWVVNGYTPGLFPRTEVMELPNVYVNDPGAANLALADLFETDLKPDYKGLEVMFLHVHAGNGLALRGPEIHA
PAD
LAGKKMRIPSRTGAWVLEALGAVPVAMPVPELPQALQKGVVDGALVPFEIVPALKLQDQTDRLVEGDSHTRFGTTTFQ
MS
MNKARWDSLPPEIQAAFREASGPEWLAELGRIWRGTEETGVAITTAAGRSHQVLSPEETEAFRTALAPVVERWIEDAE
RR
GIDGAALVARARAAIAAHESR
>OLS50459.1 transglutaminase [Rhodovulum sulfidophilum]AB14
MRMRIGCRLKYRLTQPTPLIAMLNVHYSRFGDLERADYLVTSPSVPLESYRDGFGNWCTRMVAPAGEFTLSTDGIFRDC
G
LPDPVAPDVPQQAVQDLPFDMLVYLLGSRYCDTDLLSQEAWRLFEHTPPGWARVQAICDFVHSSVSFDYMQASATRT
ASE
TLAGRQGVCRDFAHLAIALCRCMNIPARYCTGYLSDIGQPEPHPPGDFAAWMEVYLGGRWWVFDPRNNTRRMGRIL
IARG
RDAADVPLTQTFGPSILTQFDVWTWDAEADPTEAVPVFRV
>OLS50460.1 hypothetical protein BV379_14230 [Rhodovulum sulfidophilum]AB14
MAAMLAVGMVASLAFSALAQDGTGTVPENAVARNYDGGWDCSPGYRVAGAECVAIELPQNAYPNGRSYGPGWGC
LRGYEE
VGDTSCGQIPVPENAFLRASGYDWQCNRGYRQDRKACVPIVLPEHAYLTDDRSGSGWVCDRGFTVSSGACVPIAVPE
NGY
LTNADYGDEWACERGFFANEGRCDPVALPKNAFLDQDVYGPGWRCERGFEPVGNACVAIDLPANAHLDRSGNSWR
CDRSF
QLSGGGCVLGR
>OLS50461.1 hypothetical protein BV379_14300, partial [Rhodovulum sulfidophilum]AB14
MGQKRHKPEEIVAKPRQIDVLVSQGRSVADAVRSIGVTQFTYSRWRKERAIVRHRSEDNGKCGLKTDQVKSLKDLEKE
NE
RLRKAVSELTLEKLILREA
>OLS50462.1 gamma-glutamylcyclotransferase [Rhodovulum sulfidophilum]AB14
MQDPFFFGYGSLVNRATHGYRDAVRARISGWRRVWRHTPLRPVAYLTAEPAPGVTLDGLIAAVPGGDWTALDQREY
AYDR
HAVGKVVDHGLAARIDVHVYAIPEGLAEPPDIRHPILLSYLDTVVQGYLREFGPAGAEAFFATTEGWDAPVLNDRDAPR
Y
PRAQLLNTAERNTVDAGLEARGVRQFLPG
>OLS50463.1 primosomal protein N' [Rhodovulum sulfidophilum]AB14
MREVFPEGARVGVLTAQPLGHVLDYLAPEGGCGLGDYVEVPLGPRRVMGVVWGPGEGSFDAAKLRRVGRVLDARP
MRDEM
RAFLTRAADYTLTPLSLMLRLATRTPGLGEAPGALRVYRRGEGQPERMTVPRERVLEVLEAFGGLSFTLSELATEAGVST
GVVKGLVPSGAVLEEDQPRDRPYPRLDPARPGKALAGDQAEAAQTLCAGLASGAYGTTLLKGVTGSGKTEVYLEAVAE
CL
RQGRQALVLLPEIALTAEFLTRVEARFGARPAEWHSGVTSVERRRLWRMAGEGGAQLVVGARSALFLPFQDLGLIVVD
EE
HDSSYKQEEGVLYNARDMAVLRASLCSAQVVLASATPSLESWANADAGKYARLDLSSRFGTATLPEMQAIDMRAEET
APG
RWISPALEAAVRARLGVSRQSLLFLNRRGYAPLTVCRACGHMIGCEHCDARMVEHRFQRRLVCHQCGESRPIPEACPS
CG
VEGKLAPVGPGVERLAEEAVALFPEARVAVLSSDLFGSARALKAQIEAIAGGEADIIIGTQLVAKGHNFPLLTLVGVIDA
DLGLQGSDLRAAERTFQLMRQVAGRAGRGSERGLALLQTYQPEHPVIRAILTGDEEGFWRAEAEERRAAGVPPYGRM
AGI

ILSAPEARPAFDLGTWLARNDGALRRVGAQVFGPAPAPVARIRGRHRVRLLVKASKGVPLQAAISDWIAPVKLPPNLRL
A
VDIDPQSFM
>OLS50464.1 chemotaxis protein [Rhodovulum sulfidophilum]AB14
MELASEKAGLLAQKVAGQITEATSVGSAMGGALAGYIETGRATRPDIIAMIGGVPGQYDNIFGAWMAGIPDGPTNQL
LSG
EGGLNPEGIFAPWWHKSDSGGLIYATFTVNTDNEWYTLPLSTGRSSITEPYMSEGGFLMTSVTLPVRVDGQIVGIAGVD
I
GLGDLTATMNDMTAFEGGRSMLVSQGGNWLASTDPADLNQPYDENGGKLISAALADGRMRVISDMPDGATRLVYP
FTAPG
MNTTWAALVDVPAEVFSAPVRAEFVRTMTGGALILAMALGTLYLASMAMVRRPLARMLVAVDELATGDYARPVPGR
ERRD
EIGAMAGSVEALREGLLEKNELEKEQEQLRGEQERVVADLASGLRSLSEGDLTSTIDTKFADDYEQLRQDFNATVETLNA
LMGEIVENATEITTRAEEISGASDDLSRRTENQAATLEQTAAAMDEMTASVRAAAEGAAKVEDVVREARGNAEQSGL
VVR
EAIGAMSEIKKSSDGINQIIGVIDDIAFQTNLLALNAGVEAARAGEAGRGFAVVASEVRALAQRSSEAAKEIKTLISASS
DQVETGVSLVNRGGAALTDIVERVGNIATLIADIATGAQEQSVGLGEINVGVNELDKVTQQNAAMVEEANAAAVTMK
QEA
GNLQTLVARFRLKGPAAAVAPRPRTETAIPAAAPPPTAEFEPPKRAVNDAGWQDF
>OLS50465.1 Ktr system potassium transporter B [Rhodovulum sulfidophilum]AB14
MLIYAALIAAGTLLLKLPVARTVAMSWSDLAFTATSAVTVTGLAVRDTGSEFTLFGQAVILVLIQLGGLGLMTFAVLILT
MLGLPVGITHRQYLREDLNQTSMSGLIRLTRTILRFVLVCEAAGAALLAVVFVPEFGWGAGLWASLFHAVSAFNNAGF
AL
YPDSLSAWVGHPVVNLTVPALLILGGLGFTVIADMQRRTGWRGYTVHTKLMLVGTLALLVWSTASFAALEWHNPATL
GQF
DRLSTRLWASWFQAATTRTAGFNTVDIGGLTQATTLIFMTLMVVGAGSTSTGGGIKVTTFIMLLLATYSFFRRHTAIDVF
GRRLGAEQIMKVLALSMVSMLTVLVALFVLMLFHEGDFLDIAFETVSAFGTVGLSRGITGDLDTGGRAVLMLVMFLGR
VG
PLTLGFLLAVRHPRRVSYPAGTVYLG
>OLS50466.1 cupin [Rhodovulum sulfidophilum]AB14
MYFMTEAKAAPYAPRGHSGTVNRRLVSPETGATQIEVLIGRIEPGEGASPHFHPGIDQFCWMLEGRARVEIGGFTREIG
P
EESCFFPADMPHTFTAIGETPVKVLICYGPPYGEGARVEC
>OLS50467.1 riboflavin biosynthesis protein RibD [Rhodovulum sulfidophilum]AB14
MALALALGARGKGRTWPNPAVGCVLVREGRIVGRGWTAPGGRPHAEPQALAQAGRQARGATAYVTLEPCSHHGKT
PPCAE
ALIAAGVARVVTALEDPDPRVAGRGHALLRAAGIAVETGVLAEAATRDHRGFLLSRCAGRPMVTLKLASSFDGRIATAA
G
ESRWITGPAARRHVHALRAAHDAVMVGGGTARADDPVLTVRGLGIAHQPVRVICASGLDLPQTGALAASVGDAPLW
LCHG
AGADPARRSAWQARGARLIEVATGAGGGLDPAALLSALGAAGLTRVFCEGGGSLAAALLGAGFVDELIGFTAGLALGA
EG
RPSIGPLELAHLAEAPRFALTETRPLGPDLLHVWSRG
>OLS50468.1 tRNA (guanosine(46)-N7)-methyltransferase TrmB [Rhodovulum sulfidophilum]AB14
MPDTEGGAPARAWRNFYGRRHGKTLRPSQKDYLDSDLAALAPGAVDWDENPDRVPIDLEARFGGREVWLEVGFGG
GEHMV
HQAATHRDAAIIGCEPFINGVAMLLGKIRAAGVDNLAVHPGDVRDLFDVLPEASISRAFLLYPDPWPKTRHHRRRFVTP
E

FLEPLARVLKPGSQFRVATDIPDYVRQTLVEVPKAGFDWLAEGADDWRRPWGDWISTRYEQKALREGRVPHYLTFRR
R
>OLS50469.1 hypothetical protein BV379_14840 [Rhodovulum sulfidophilum]AB14
MTASADSHACSEGSAQIRPVVSEKVSADPTRQRAFAGVVAAAHSADLAFQTIGRVARVAVDPGDRVSEGDVLASLDR
VNL
QEDLASARAALGSAKAEAAYARQSHERVRILAERGIAPIAQLEKAQAARDAAEARAEAASADLARAREALGHGTLRAPN
D
GIVLSTAIEAGTVVAAGTPILTLADLRGREAVIDVPGEFLQVLPPDARFDVVIHAEDATSHPARLRLVEPMLGDNIRTRR
LRLTITEGAEDFRIGSLVSATLARLDLPVMTLPLTALSGPAEAPSVWRVAPKDRRLARVPVETGRRLADRIEITRGLAVG
DEIVVKGTACLGEGQTVGPMVAP
>OLS50470.1 TRAP dicarboxylate transporter subunit DctM [Rhodovulum sulfidophilum]AB14
MESSLVLVFMFTALVAFMLSGAGLAFVLGAIAFISTILLWGPSALIVAVLNTFETMTSEALMAIPLYVLMASILEKSGII
EALYEAMEIWFSRLNGGLAVGTIAICTILAAMTGVVGAAVAAMGILALPSMLARGYHPPLALGAICAGGTLGILIPPSVV
TIVYAITAQMSIGQMFAAGIVPGLILAGSYSAYVLIRTWLNPALAPKPADAPQLPLGEKLARLKSLVLPGIVVLGVLGSI
YAGLATPTEAAAVGVLGAAASALVTRKLSLPMLSEAAGDTLRVTAMILWITIGARAFVSVFAATGGADSLLGFVEELETS
RWVVLGTMVLVLIFLGLFLDEIGTILLCVPVFLPIVKALGFDPLWFGVLFMITAQMAYITPPFGYTLFYLKGVLPPGLGI
GQVYRGIVPFFLIQLGVLVLFALFPGLVTWLPETLLPSLK
>OLS50471.1 acetylglucosamine transferase [Rhodovulum sulfidophilum]AB14
MPPDLDTLTRQAAACGDAAAVVELAARTPEAAARLYRIWLELNGARPDAWAAWFNLSVLLNAAGDLAGAYEAAAQA
HRIK
PDLWQAALAAGQAAEALGDRAAAIAFLRQALPPAEGRVALHKQLGRMLELDKRLDASAEELRASLLIDPHQPDVIQHL
VH
TRQKMAAWPVTDLRVPGLTATEAELWCGPLATLALHDDPAMQRRVAADWIARNVPDPGQRLAPAAGYRHDRVRL
GYLSSD
FCRHAMSYLIAELLERHDRDRFEITGYCGSPEDGSDIRARILAALDRHVPIGALGDEEAARRIRADEIDILIDLNGLTSG
GRPGILRWKPAPVQATYLGYIGSVPLPELDWLLCDGITVPEAETTSYAPAPLRIEGCYQANDGRAPELPEVSRASEGLPE
DRFVFVCVSHHYKITAPVFAAWCRIAAAAPEALFWLIEDTPESVAALSERWQAAGLAPDRLIFAPRVDPARYRARLALG
D
LFLDTTPYNAGTIASDALRMGLPVLTLAGRSFSARMGASLLTAVGLTECIATDPEDYLRRAVAMATDPSRRPGLRGPALA
AHWRATLGDGTAFARRFEAALISVLKRP
>OLS50472.1 ABC transporter [Rhodovulum sulfidophilum]AB14
MSTLRGAFLIAAHNGIVLSPDELPDLGAGDLAPGLMDLFATKGLRCRLIERAGWRLASRLGQGLPVLAEVEQGRWIVLV
H
AGLVEKLRNAAILDPRHEAEGVRNMPWEEFARRWTGRLILIGRPDAAAAESRAFGLRWFLPALRRERGLFAGVAVAVV
TG
NLIAFSLPLLLQVMIDRVIAHQAWYTLMSVTAVYVVLVLFDAGFGYIRQRLSQIAGGRIDADIGSQSVAHLLRLPMTVFE
TTPAGVLTRNIQQTDKIRHFLTGRLLQTLLDAALLPLLLTTLALLSGTLTLIITLYAIVIAGCIGLMLPILQSRLNALYG
VEAERQALLVETLHNMRAVKSLAVEPARQRLWEDSLAQSVRRQWDLGRVSALTGTLTGLLERMMQVTVVAVGVGLA
LQGA
LTVGTLVAILLLSGRVSGPLVQIVGLINEWQEAALSVRMLRKIMDHPPERQDDDKLAQPMLSGTLELREVGFAYPGAAT
P
ALDRVSFAVAPGQVVGIVGRSGSGKTTLTRLIQGIETPQQGLILFDGIDIRNIALTALRRQVGIVLQDNLLFRGTVRDNI
AVAVPGAPLEAVLRAATMAGADEFIRRLPKGFDTMVEEGGANLSGGQRQRIAIARALLADPRILILDEATSALDPESEAL
VNRNLTAIAHGRTMIVVSHRLSSLVRADQIMVLDQGRLLDMAPHETLLTRCETYAALWRQQTEHIA
>OLS50473.1 hypothetical protein BV379_15000 [Rhodovulum sulfidophilum]AB14
MPPLPLFLLQPVLKRIVTKIAADNPDMFDRLGHHRHAYFLVDPVDMPFMLYLRPDPEALDFKALNRRRALPPHCARISG
K

FLDLLTLMDTEQDGDALFFSRDLIVTGDTEAVVCLRNALDDVEEPIAESVADMFGAPGRGALNMLRRAAENAQKRKGS
HS
>OLS50474.1 DNA primase [Rhodovulum sulfidophilum]AB14
MSLPPGFLDELRTRTSIAQVVGRKVMWDPRKSNQGKGDLWAPCPFHQEKTASFHVDDRQGFYYCFGCHAKGDAIKF
VQDT
ENVGFMEAVEILAREAGMPMPVRDPADKVRVDRQTRLLEVMEVAQRHYRMQLQTGAAAEARAYLARRGLSPEAQA
RWDIG
FAPPGWQGLWDHLTGCGLDPDLILAAGLARASDKGRKPYDVFRNRILFPIRDGRGRVIAFGGRAMDPNDNAKYLNSP
QTE
IFDKGHTLFNLARARETAGKGQPLIVAEGYMDVIALSEAGFGAAVAPLGTAVTEHQLRLLWRIHPEPVIALDGDRAGIG
A
ARRVMDLALPLLEAGQALRFAIMPPGQDPDEVIRAGGAGAMRKILDEARPMVAILWERAIEDRVFDSPERRAALERDL
AA
AVEKIRDPTLRHHYDEDLKRLRWELFRPARGGGSGGGGRKGGWTREPRRRPVSSTKSSELVQAMPRFEEELREAVILAT
L
IVHPALIARFAGELEALVCATPDHAALQQALLVLPPPEEGTSFRDALEARVGPAPLEKLFSLSHVRIAPPVHDPADHEKA
ALCIAHEVDRLMRLRGAERDIEEGMRDLEGLADEGVTWRLSQAARARYGAGPSQEEDGADFELAPNGAHVDRDERD
AFAD
LLGRINYGKGRT
>OLS50475.1 diguanylate cyclase [Rhodovulum sulfidophilum]AB14
MTDDIRLAFAHPSERAEAMDPARPLRDRISLRDHVAEVEIGAFQAERGSTQRVRFNVVVEVRPHPAPLDDDVDRVLSY
DM
IAEAIAAELSSERLNLLETLAERVAERILAAPQAMRAYIRIEKLDRGPGALGVEIVRDRAPDLPRPVPVAAEPEPLVAPL
VVFLTNAALAAPELPGWLDRWQAGSRPLVLCVGASETPAPQATAPLVQRRIDLLAIEQNAWVLAGRDPRCVVRGSRT
EID
WAMKHDQISVWAPSKIVLDAVESPSADPHDALELALWFAAELHAETVLVIGENVPEHPARGLDLRYVSPDAAGTAI
>OLS50476.1 TonB-dependent receptor [Rhodovulum sulfidophilum]AB14
MVRSSDETTQGGTSSTISAEEIKRTNRATLDDALRTLPGVSVGNTGGSRNERLIFVRGFDRFQVPLYVDGIRVYLPADNR
LDFGRFLTPDLAETQIQKGYVSVLNGPGGMGGAINLVTRQPTEVFEGEARVGLEAGNRGDVTTRTGYLSLGSRMDRFF
VQ
GSYLTRDNNGYYLSRDYTPTEVQGAGRRDYSDTEDSRLNLKFGFTPNATDEYVLSYTRQEGSKNAPYNVDQPVRGITPA
P
LPVGASYQRDWEWPEWDLEGLTFYSTTDLGGGAWLKTKLFHHQFDNVLSAYDDYTHSGQTVGRAFDSTYDDTAWG
GTLEL
GSDIGPRNTLTSAFHYRYDRHRDIQLSRPDINSIPDPTETSRERTLSFALEDRFQLREDLSLVGGLSFDRAEVLSASRTS
TDYGDPVGSSDAVNWQLAAIWTPGAGGEYHASLSSRTRFPTLFERYSTRFGTAVPNPDLDAERALTAEIGYRGDLGPV
AV
ESALFYSEIDDTIQSLNVGVGLTQNQNVGDGTYVGFEIGARTDLTGSLALSANYTYLDRDITDPIRPGLKATDTPRHSAY
LRLDWQATASFSIAPSLELSSSRLSDSAIQPADPTDVSYTRNAGFGLVNLDMEWKASDRASIVFGVRNALDKNYELVEG
F
PEAGRTFFLTTAYTF
>OLS50477.1 hypothetical protein BV379_15420 [Rhodovulum sulfidophilum]AB14
MPFLLCERERVPFYDANGRQELTKRRREIVRADHLPAMSYADLAIVTRNLMMDPSIAGRAYLVVDSSGVGRAFCDLLD
TK
SVQHTRVQMVAGENETETTERGRTFNNVGRTRLLSALNSAIHTGDLSIGNFEARDLMRQELESFEADVGASGRVKIEG
GT
KFGHADLAVSAALALWLSDHRSVGAHIGQVPLKGYW
>OLS50478.1 hypothetical protein BV379_15430 [Rhodovulum sulfidophilum]AB14

MEYPALYDSANNGSIKAQSRFLRTIRLEYFLLFCVSVASATRGMSGINPLVITLLLVVLAGLFVFKIFKRLDQDWYRCRA
LAESVKTSTWRFSMRAHPFEDTSSIEIPKANFRNLLRDILQANRHVAANLETSVAEQVTDSMVEVRKLTLDQRMDYYVK
N
RIDNQRTWYSKKSKANKNALTFWVAATIVLYIIAAISLNAEQLGLPWATLAFGPLIVIVTSALGWLQMKRHGELMASYN
L
TAHEIGIIRGNSELVKTEEELSDFINEAELAFSREHTQWVARRDAS
>OLS50479.1 MBL fold metallo-hydrolase [Rhodovulum sulfidophilum]AB14
MDEPAAFDPMPGRVDRLAPGLRRVLAPNPSPMTFRGTNSYILGEGEVAVVDPGPALRPHLDALLAALAPGERVTRILV
TH
SHLDHSGLARPLAEATGAPVFAFGDSSAGRRADLAGLAGLGGGEGVDAAFRPDERLADGAVLVTPDWRLTALHTPGH
MGN
HLCLAWSGLDPEGAVLFTGDHVMGWASSMVSPPDGDLGAFMSSLERLAGRPERCFLPAHGAPVADGPGRVATLAA
HRRAR
GARLLALLSEAPATPAALARTIYTDTPPALLPAATRNVLAHLIELKDKSLAVAEDGAVTEAVWRLV
>OLS50480.1 sulfate transporter subunit [Rhodovulum sulfidophilum]AB14
MPAATPLRRSWHPFRWRNGILTVLAAFGLALGTPEAAFAAATDAPQILNVSYDPTREFYRDYNELFQAWWEAGDHA
PVTV
QQSHGGAGAQARAVIDGLPATVVTLALSADIDAIAEHTGKIPADWQSRLPHNSAPYTSTIVFLVRKGNPKAIENWDDLI
R
DDVQVVTPNPKTSGGARWNFLAAYGYALDRFDGDEARAQEFMTRLYRNVPVLDTGARGATTTFSQRGIGDVLLAWE
NEAF
LALDALGAEKFDIVVPSISILAEPPVTLVDGNIGSDAQRAVAEAYLEHLYSPEAQALALRHFYRAWDSSAAAPEDVARFP
ELRLVTIDDFGGWARAQPVFFGDGGLFDKIYED
>OLS50481.1 IS1182 family transposase [Rhodovulum sulfidophilum]AB14
MPDHKTIADFRRDNGAAIRRTCAQFVELCRRIGVLKGACVAIDGSKFKAVNNRDRNFTKNKIASRLAHLETDVERHIKE
M
VRIDRLEDGEARADKVAHLARRYGRIRQEITRLKAMDQALADTPDGQISLTDPDARAMATSARHGGLVGDNVQAAVD
TET
HLIVAHDVTNQGFDRDQLSPMAAAAREALQCDDLHAIADKGYFSGSQILACHQAGITTTMPRPETSGNRRKGMYVKA
DFA
YDTARDVYVCPAGEELTYCYTREEDGLRVGRYWTNECQHCPVRHRCATSKERRITRWDHEHLVDAMRERLGGHADP
MTLR
RCTIEHPFGTLKAWMGHTHFLIRRLKNVRTEMALNVLAYNTKRVVALIGIRGLMRAIPA
>OLS50482.1 hypothetical protein BV379_15595 [Rhodovulum sulfidophilum]AB14
MDKASLEDIHPSDRSGNADISPPEGESGFGETLLARGLFHAIERLLLVIIVAMTLTAVSFELLAIYRLGEISLADILLMF
LYLEVIGMVSVYYARRNSVFVYPIFIAITAVGRLVVLQGKEMEPVNILIEAAAILLLSISALVIIWAIGQQAARLHPS
>OLS50483.1 helicase IV [Rhodovulum sulfidophilum]AB14
MKLVSRPLPAFLSDPFRKNPRALALQDDTIAASDGAHARIHLSEIAASPVVQRGRVSARLTLDLGNQSFVLIPGVQEAAA
RAFAQAVETAWSGYNLAQLSKEDDAIASLLTAIEGLKAPTMYPAACLVAPAASQAADLDARVLKKLNSAALGKETMAR
LA
PILELARNPEQLRDAAIAAFIDQQLTDWKAFFDTVESMPLTPEQRLSVVVDEDATLVLAGAGSGKTSVITAKAAYLVKAG
IRQPHELLLLAFAKDAATEMSERIEARCGVPIMARTFHALAYEIIGTVEGEKPPLAPTATDDKAFLQLIKDILRDIVSRM
ADIAGTVVGWFAGFFDDIPNAWDYDSAHKWYSEVESRNLRSLKGDTVASFEELMIANWLYLNGIAYEYEPTYEHKLTG
TG
KRAYTPDFRLTESGVYLEHFGVRKTTGKDGTEELTTAPFIDRDAYLESMTWKREVHAEHETILIETYSWENEEGRLLEAL
AEKVAPHVTLRPRPALEIYDSVTSLGVVDGFTSLIATFLRHFKNGGYHLDDCETKAATLKMGKRGEAFLKIFGAVYSEYQ
DRLGERIDFEDMVNRATVLVESGRYDSPFRHILVDEFQDISTGRARLIQALKNQHAEARMFAVGDDWQSIYRFAGSDI
HI

MRNFGREFGGVFAGHTGVHRTVDLGRTFRSVDKIALAARRFVLCNPAQITKTVVPAGEAEHPAIRIAWTRRETGDQVLDD
SLKALAAEPAPPGRKATVLLLGRYRFVEPDMRGLRRRHPNLTITFKTIHASKGLEADHVVLLGADSARMGFPSMIVDDPL
LALVSPEAEPFANAEERRVMYVAMTRARRTLTILASEARPSAFVTELLKDPAYDVASPHEAQERTHTCGQCGGRLLFMPG
GDGPGWYRCEHIKHCGNRMPACPACGTGLPVHQEPEGEKICGECGAVQEPCPECTDGWLVERKGRYGTFLGCVRFPDCTG
KSKKERQVGKRTSGELDAGKRQRPLGKPGRR
>OLS50484.1 aspartyl-trna synthetase [Rhodovulum sulfidophilum]AB14
MFIIGSFPWAGPVQATERGRVTNLPLPRYVSLKATLGNVRRGPSLSHRIDWVYTRRGVPLQITAEFGHWRQVRDRDGAGG
WMHYSLLSGVRTVLVDQDMLPLLRAPRTDSEVLAKAEIGVIARLGECTPDWCRIEADGGAGWVPKTAIWGVDPDEIRE
>OLS50485.1 hypothetical protein BV379_15790 [Rhodovulum sulfidophilum]AB14
MAALAAALTATTAGAEEIGPADLSSLPAADVVVLGETHDNPEQHANQAAAVRALAPKALVFEMLTQGQAAKVTPGLRLDG
VALGQALGWAESGWPDFAMYFPIFSAAPEAQIYGAALPRDEVRRAVTEGAAAVFGDAAALYGLDTPLPAAELDQRIDDQR
EAHCNALPEHLLPGMVEAQRLRDASLARSARQALDETGGPVAVIAGNGHARTDWGLPAAFARVAQDARLISVGQIEGAAT
GAPPYDYWLVTGVAERDDPCAALTGG
>OLS50486.1 [protein-PII] uridylyltransferase [Rhodovulum sulfidophilum]AB14
MTPDSLICPASDIFDQTAFEAELTRLTDQGIGSKALRDAVVQYLSEARQKGRSAIAAAFANRPFDAGPTVRAYTWLTDCL
VRATLKVSCEVLHPRPNPTEGERLAVFSVGGYGRSEMAPHSDVDLLFLIPYKLTPWAESVIESMLYMLWDLRLKVGHSSR
TVKDCLRLGREDYTIRTALLENRLLGGYMPLAKTLDSALWDDLFKGTAAEFIEAKLAERSERHRKQGGQRYVLEPNVKEG
KGGLRDLQSLYWIAKYVYRVDRASQLVKLGFFTRDEFTGFVEAERFLWAVRCHLHLIANRAIDQLTFDMQVEVAARMGYT
DHGGRRAVEHFMQDYFRHATTVGDLTRIFLTKLEAAHVKKEPALIGLFRRRRKVPKGYEIVQNRMNVSDPKAFLADKLNL
LKIFEEALRTGTLLHPDAMRLIQANLRLIDDDVRNAPEAQRIFLDLLLKHGNPERSLRRMNELGVLSALIPEFEPIVAMM
QFNVYHSYTVDEHTIQCVSTLAQIERGELIEELPIASRILKDGVNRRVLYVALLLHDIGKGRPEDHSVLGAQIARKVAPR
LGLRPEECETVEWLVRYHLLMSDMAQKRDLSDPRTVRDFAKAVKTRKRLDLLTVLTVCDIIGVGPGTWNNWKAQLIRSLY
RETVRALEGGLEDVNRDRREAESKRALREALSGWDAKELRAEIGRHYGPYWQGLPTGTHVVMARLLRGISDDEIRIDLAE
DADRDATRVCFALSDHPGIFARLAGALALVGANVVDARTYTTKDGYATAAFWIQDGDGSPYDASRLSRLSQMIEKTLMGE
VRPREKLADRDKLKKRERSFRFPTSITFDNDGSEIYTIIEVDTRDRPGLLYDLTRVFATSNIYIASAMIATYGAQVVDSF
YVKDSFGLKLYARSRQESLERKLREAIVKGTERANK
>OLS50487.1 LuxR family transcriptional regulator [Rhodovulum sulfidophilum]AB14
MQGGLELFLDRLQRATTLEDLYCVTEDLRDRYGVLHIVYHWVNSVGERYGAGTYSTEWVDRYLECDYLMIDPVIFGCYSR
FHPVDWKELDWSSKQARVFLRDAMSYGLGNQGVSIPLHGPKGQFALFTVNDSCSDAAWEMFKQINLRDLILIAHDFNKKA
LEFEQWSDPAPRAALSPRELSAMTLLAKGMSRAQAAQELSISEHTLRVYVETARHKLGALNTTHAVARALSTGLIVV
>OLS50488.1 crotonyl-CoA carboxylase/reductase [Rhodovulum sulfidophilum]AB14

MALDTNPSIAPYEAPEKELYEIGEMPPLGYVPSKMYAWAIRRERHGEPDKSFQAEVVETPAIDSHEVLVLVMAAGVNY
NG
IWAGLGIPVSPFDGHGAPYHIAGSDAAGIVWAVGSAVKNWKVGDEVVIHCNQDDGDDEECNGGDPMFSPSQRIWG
YETPD
GSFAQFTRVQAQQLLPRPRHLTWEESACYTLTLATAYRMLFGHHPHELKPGQNVLVWGASGGLGSFAIQLINTAGAN
AIG
VISDESKRDFVMDLGAKGVINRKDFNCWGQLPKVNTPEYKAWFGEVRKFGKAIWETTGKGVNVDMVFEHPGEATFP
VSTF
VVKRGGMVVICAGTSGFNLTMDARYVWMHQKRIQGSHFAHLKQAAAANRLMCQRRLDPCMSEVFPWAEIPQAHL
KMLRNE
HKPGNMAVLVQAPRTGLRTFADVLEAGPSA
>OLS50489.1 hypothetical protein BV379_16020 [Rhodovulum sulfidophilum]AB14
MALPQNQGQNQRQNQGQNQGQNQAIGRQCGPRDGILDQLRNRYGESRQAMGLAGNNVVVEIFANTRTGTWTVT
GTFANGL
TCLIASGEAYDPVAEPPGQAL
>OLS50490.1 hemin uptake protein HemP [Rhodovulum sulfidophilum]AB14
MTLLQTIRHSTGETTQHDARILTGGGNTAVIVLDGVAYTLRITRSGKLILTK
>OLS50491.1 hypothetical protein BV379_16170 [Rhodovulum sulfidophilum]AB14
MEQLIAEVEAYAAACSKTPQKVLRDAIGASWGQWDSWKSGDGSPTMKVVDRLRDYMRTHPTPSGEAA
>OLS50492.1 phosphohydrolase [Rhodovulum sulfidophilum]AB14
MQDQFPKPHVAKALLPHAFDLRDGAHDEAHLLRVWRNVERIASEEGGDIEILLAATLLHDCIWIDKGSSQRHMASRLA
AA
KARDVLTGMAWSDDRINQVCHAIESHSFSACVTPASLEAKILQDADRLDAIGFIGVARCFYIAGLRKASICDAVDPAAND
RPLDEDLFALDHFQTKLMKLERGFQTMTGARLAADRSAHVKSFYNGLLAELS
>OLS50493.1 phage repressor protein [Rhodovulum sulfidophilum]AB14
MGEIGDDFLAGLERIMDARGLRAAPLSEAAGLSKSFIRDLRRGKAASPKLDTAMKVARALGISVEEILAAANGSVAGVPT
ISIAGRVGAGAKVPVFEAYERGAGPQVECPVGLSPQGIAAVEIVGESMEPVYSAGDLLFYSRPTHEGVPSEAIGRRCVCE
CERGFGWIKLVKLGSQPGYFHLIALNPDAENQHDVRLKWAAPVRLHWPADLAKRV
>OLS50494.1 hypothetical protein BV379_16300 [Rhodovulum sulfidophilum]AB14
MKIVRTKRYLKDLKRIRATEAEAEAVEQMIATNPEAGDVIQGLRGVRKIRFGLRNKGKSGGGRAIYFLMVNDDVAIMIT
A
YMKKEQSDLTTTQRKAILKMLEDLHDD
>OLS50495.1 DNA methyltransferase [Rhodovulum sulfidophilum]AB14
MRKTVPATPVAPWKGGKKALHRRIIERIEAIPHRTYVEPFLGMGGVFLRRSWRPRLEVANDLNGEITNLFRVLQRHYPQ
L
MEVMRFQITSRREFERLRACDPATLTDLERAARFLYLQRLAFGGQLRGVFGISPNSGPLFSLARIGPLLDAAHTRLDGVV
FENLPWQEVLARYDGPQALFYLDPPYWGGEDDYGKGLFDRDQFAELAERLGRIEGAFVLSINDRPEIRDLFGAFRIEEVR
LNYSVSKSGATAARELIIANREVRVGLV
>OLS50496.1 hypothetical protein BV379_16350 [Rhodovulum sulfidophilum]AB14
MPYQRAAFGSGWTDPDGDCLDTRAELLADLSTVPVRLAPSGCSVRHGRWFAPYTGQVVTEAGDLDIDHIVPLRYAW
GHGA
ASWSAQKRARFARDPVNLLPVSASANRSKGARGPLDWLPPDPGFRCQYVLRFRRIAASYGLVHSAAEERELVALTGRL
CG
T
>OLS50497.1 DNA (cytosine-5-)-methyltransferase [Rhodovulum sulfidophilum]AB14
MRPIGIDLFAGAGGMSLGFEQAGFDVAAAVEIDPVHCAVHKYNFPDTAIIPRSVEGLSGAAIRRAARIGDRRVDCVFGG
A
PCQGFSLIGHRVLDDPRNRLVLDFVRIVSELEARTFVFENVKGLTVGKHKAFLQEFVAAFDAAGYEVRIPWKVLNAGHFE

TPQSRERLILFGCRKGERLPDYPQTRTNLSGRSRVIDGLPFGPTCADALGDLPDADRFQTLTASDSVRTTAFGEPTPYAA
ELRCLTNAAWHFGYVRKWDPSSLTSSARTAHTYISRRRFAETEPGDVERISRFFKLPETGVSNTLRAGTDGARGAFTSPR
PIHYRFDRCVTVREMARLHGFPDWFRFHVTKWHGARQIGNSVPPPLARAIAGQVVEALGIDPQRPEEAICLGDPSLLSF
D
LTAAAAHFGVEAPPSRRNQKSGSRKRKQVDIERERIAGQVMYG
>OLS50498.1 hypothetical protein BV379_16450 [Rhodovulum sulfidophilum]AB14
MPSSTEPTPTARPLAGRHLFVFGAGFCAGLVALRAQAAGARVSATTRDPLRAAELIEQGIGMHLFDTAASGRRAALAE
ML
EGVTDLLISIPPGEGGCPALSALTAAGALPSGLGWIGYLSSTGVYGDCGGAWIDETRSPAPQTADARARLIAERDWAAL
A
AEQGAALDILRLSGLYGPGRRNALDRMRSPGIQAVLRHGQVFNRIHVDDAAGAILAAMSTPAGLRRLNLTDDLPAPAH
AL
LDHAARLLGRPPAPRVPFDAAGLPPGAAAFWAENRRIRNDRLKALPGFALRYPTYREGLAAILADERASATTAAE
>OLS50499.1 hypothetical protein BV379_16545 [Rhodovulum sulfidophilum]AB14
MNRDETQLIERLVTAALERRGVAPAPATGLSLAEAEAIAARAQAASVAMGVPVVIAVADATGDQILFHRMEGSLPASA
AL
APGKAWTAAAFRMGTDELGRLAQPGGMLFGVESTHDGRVVAFGGGLPVRRGGTVVGAVGISGGTVDEDMAIARQ
ALNGIS
NE
>OLS50500.1 histidine kinase [Rhodovulum sulfidophilum]AB14
MSIDMLQKVQDNFSAAVGVAMVIVDPEGEPVTRPSGFSSFCDTIRFHPKWRERCYHCDAVGGRTALSTGDPSIYKCHA
GL
VDFAAPIIMRGEYLGAVICGQVKMRPGEMAPDLIDFSGLFPVEASWREDPRLMALYHDIGEISYGRLRSAAYSLFYIASH
IVEESYSSSVTQELYAKNLRLMEESKHRAELERSLREAELQALSYQVNPHFLFNVLNTIGRLALIEDAEQTESTVHAFAD
MMRYILKKSGTQFVPLATEVEHVRNYLFLLQLRLGDRFSFSIDLPEELGEVSCPFMVLQPIVENCINYAVEPRERDGLVE
IRAYRDGQDVIIDITDNGDGISTERRIEVLAGTADRRGRKSIGIHNVDSRLRHYFGDGHGLEIVSPFRDGRGTLVRLRLP
TGFDEATAQARQD
>OLS50501.1 phosphoadenosine phosphosulfate reductase [Rhodovulum sulfidophilum]AB14
MPDLGHVDMAGLLHDPRLGTRALVSSFGTESAVLLHYVTRILPRIEVLFIDTGRHFDETLAYRDRLVRLLGLNLVEVRPD
PAAIAAEDPDHRLAERDPNMCCLLRKTFPLADALAGVDTWISGRKRYQGATRAALPAIERDGTHLKVNPLAFWSHADL
DA
YFAAHDLPRHPLEGRGFRSVGCAPCTRPVRPGEDARAGRWADNPDKTECGIHLGPDGRFVRRRSG
>OLS50502.1 zinc ABC transporter substrate-binding protein [Rhodovulum sulfidophilum]AB14
MFFPVFLALAAPAAAQPPRVVTDIPPVQSLVAEVMGDLGSPEILLDQGGDPHHFQLKPSQARAISEAGLVFWVGPELT
PW
LDRALNGLGPDAQAVALIEAPGTYRRSYDGEAHDHDHDHDHDHDHDGEPEEHHHEAAMPDEDHDHDDHAGEEGHHH
HNGIDP
HAWLDPENARVWVLRISGALAEADPDNTAAYEANAHAALDRIAAAETEARQLLVPVGDRPIMVFHDAYGYFAEHFDL
TVA
GSIAMGDAAEPGAARLAGLRDELRHEGVACLFPEAQHDPAYAEAIAEGTDVRVGAPLDPSGSTLAYGPDLYPALLTGLA
R
TIAACVTGTEG
>OLS50503.1 Asp/Glu/hydantoin racemase [Rhodovulum sulfidophilum]AB14
MKLVVINPNSTVSMTEKIVAAARAVAAPGVEIEGRTATGAPASIQGHHDEVMCAPHLLREVREAEEAGADAIVLACFD
DP
ALGACREVATGPVIGICEAGVKAASMIATSFSVVTTLPRSVPVIQDLVRGYGLAHRCRRVRSAAIPVLALEEPGSDARAR
VRDEILRAIEEDGCEAVVLGCAGMADLVAWLSEETGIPVIDGVAVATKFAEALVGAGLRTSKSGAYAQPETH
>OLS50504.1 transporter [Rhodovulum sulfidophilum]AB14

MPETAAAAPGQIPGQVPHVIEEKGLGEESLAPQETRIMDPWSYLFAWLGGCVSIGTFTIGSGLVGTLNLLQTFLAIAIGC
TVIGVALMINGRAGHKYGIPFMVQARSAFGFSGTRLPALVRSVPAIVWFGFQSWIGAGALNLVSETLFDYSNLWAFFIG
F
QFLQILLSVLGFHGIKWLENIGSVFIIGSLIYMFFSVIGKYGDAIMTNLVNVEGTWGAPFWGATMLFLGIYSTMMLNVS
D
YSRELRHGVGPMRQVTIYANSILPATLFMGMIGLMVSGATGQVDPIKVFSSAVDNKLLLVTTLLFIAFAQVTTNILNNVV
PPAYALMDVFKLKFKTSAVLVGLLAFATFPWKLVQDESAAGLNIFIQTYSAFLGPIFAILVVDYYILRRQQLDLGKLYDA
AGPYRGINPAALIATAIGVVAAFAFSNVSWYASLVPAGLSYYLLMLWLPSARRFCDD
>OLS50505.1 hypothetical protein BV379_16845 [Rhodovulum sulfidophilum]AB14
MTPARSRILIVDDDAVTRAMLRGILGEAGHHVGEARTSDEARAAIRNAAWDLVLLDRRLPDGDGLLLIRRVQESCRCPII
VLSVMRDEQDRMLGIGLGAVDYISKPFNANEIRLRIRNLLASGRRSLDVAMDGPITRGPFRLHPATRTLSLGNRTLRLSP
SETRLLAIFLTHAGEVLERERLTPRICGRDWSYEDRAIDVLVARLRKRIEDDPKNPRWIVTVHGDGYCFCDAEEETGDQT
ASTRPS
>OLS50506.1 hypothetical protein BV379_16925 [Rhodovulum sulfidophilum]AB14
MIDNTPVKLAFAWLIPAVGAAIFVTIQCFSYLNGYVSSGGTMQAMTFGPVALWGVSIFYGGWVVPPLLALAGRRSTD
WAM
LVLGGLLFLMSTLAGVSDGLRDGGHLVGLELLAVTLPGAVALIMSWRHIRSN
>OLS50507.1 MFS transporter [Rhodovulum sulfidophilum]AB14
MAGGSRGSGAYRGLLGILHKDHIVAPEGFNRWLVPPASIAIHLCIGSVYAWSVFNPALSRQLGVVAPAAEDWSLGAVV
WI
FSVAIVFLGLSAAFAGKWLEQVGPRMVGVVAALLWGGGFIVGSAGIATHQLWLLYLGYGVLGGCGLGLGYVSPVSTLI
RW
FPDRRGMATGMAIMGFGGGAMIAAPLKGWLLNLFERAPAYLGPREAVATVTEGGRLFAETASGRLEVVIASAAQAAE
LPG
GGEAGVYVVGTGDTGAAATFLVLGVAYLVVMMIAAFSYRVPREGWTPEGWQPKPVASGMITQKNVHIDQALKTPQF
WKLW
VMLCFNVTAGIGVIGVAKTMMSDIYGTTMPWIVTGAFASTYVLMISVFNMLGRFFWASASDYIGRKLTYATFFLLGTIL
Y
LSIPFFATAGAANPSILYLAGFYIATMIIFSMYGGGFATIPAYLADMFGTMHVGGIHGRLLTAWSTAGVLGPLAITSLRQ
MSVGNAIEDLAAKVDPEAFRTAFGAPVAQLQELVAAKTVTIAKLMEIAPPGTVDPTPGLYNTTMYCMAALLVVAFFAN
LA
IRPVRERHHHNEPELAAVPVE
>OLS50508.1 hypothetical protein BV379_16965 [Rhodovulum sulfidophilum]AB14
MQIRLSSRAALMASALVAPVCIAPGTALAQEGPDTGYYLGDIIIRGNIAERSLKDTSSSVSVLGDEEIEEKSASGTVNEV
IADTPNVLIPGSTSAPIIRGVDTDGPLIAGNAYLAKPLPRATMSIDGRYVSSVELGAGGGTTWDVESIEVFRGPQTTSQG
ANAIAGAVVVTTKDPTFEPEYSAQAVYGSRNKRRLSFAASGPLSQDFAARLALDYTGRDTFVDYTNPDFDYDFASLNGR
L
KLLWEPADMPGLRVKLTYSHTDLHRPSNEQVTKPYRDLDNTSEYQDYMDVIADGVVLDTQYDFDNGIRLSNQLQYSE
ADY
DFTFALPYSGTAARDSATLSNELKLTLGQGEDIWSGVLGVYFSDEDTDADFANSYKGAPLGSADLSYRTRSLALFGEATW
RFASDWALTGGLRLQQDRIEIEGKTSYVVGERLDYDETFTEILPALTLSHDLNSDWTLGATVSKGYLPGGSGTNFHGKEY
YEFDPETALNSEIFLRGSALDGALDVSANLFYTKYRDIQRSVINCLNADCSVTQGSMIVNGERAHAYGFELSADYQASST
LRLSGDIGALQTEVTEFSDHDGTKYEGNEFARSPGYSVGLGAAWDITPDVTLQGDMRYVDGYYSDDTNEPAAEVDGY
ALA
NMRLSYRPRDGVEYFAYVNNVFDNRAELSVSTNDAAEIAEPRELGLGLRVTF
>OLS50509.1 hypothetical protein BV379_17060 [Rhodovulum sulfidophilum]AB14
MPVLGRAALDLAERVERMSGGTLRLSIASPSAAAPALDIIVEMNHGAIDGVWAAAGWYAQRDSAFGLLTGMPFGPTP
DEY

LAWYFYGGGRELAQDLYRPYDVHNILCGIIPSEASGWFPREMTSVDDLVGLRMRFFGLGAKVMQRFGVEVVLLGPEQ
MFE
AVESGRIDATEFSLPSMDVPLGLHKILKYYYFPGWHQPATLINLYLPRPVWVALPDRHKAVLEAGCGDMIRQTLAEGEA
V
QAQALRAIAAEGVELHRWPAEMLVAFENAWQAVVAEESRLNPNFARVYASLAKFRADRQQWRHFRFLQ
>OLS50510.1 hypothetical protein BV379_17065 [Rhodovulum sulfidophilum]AB14
MREAPVSAVLDAVEQAAQGDRLSVRRMFEALGSVSFLPLMLAPALAVVSPLSGIPGFSSLCGIGIALIAGQMLVRRRHL
W
APDWLMRREIRADRARTAIAWLRRPAGWLEWAARPRLKFLVTPPLVAVLQLLCILAGLAMPFLELVPFSSSLLGMTVVT
L
SVAMLARDGLLALIGMAPFAAALAVIVGVLELG
>OLS50511.1 RNA polymerase subunit sigma [Rhodovulum sulfidophilum]AB14
MASDPREDLVTHLPAMRAFALSLTRNSATADDLVQDAIVKAWSNFDKFEPGTNLRAWLFTILRNTYYSMHRKRRREVE
DP
DGALAAHLSEKPHHDGRLALADFKVAFAKLPDEQREALILVGAEGFSYEEAAATCGCAVGTIKSRVNRARVRLAELMHL
N
DADDIAMTDPVTLAGLSSKRPA
>OLS50512.1 terminase, partial [Rhodovulum sulfidophilum]AB14
MTPAASWAACPDWWEKLQAGATPIPDLALDANLADVAVQLFDKLVVPDVPGQPTMAEAAGEWMRDIVRAAFGSV
DPATGA
RQVGEIFNLVPKKNGKTTNAAAIGLVALQMNTVPNIKGVVVGPTQSVADTCFGQMQAMIAADDWLSDRFKVNEHKK
EITD
IYPDPATGRPMNAKLKVTSFDPAVTTGGIPAFAILDELHVMAERHFATRVIGQIRGGMITNPGSLLVIITTQSEIPPQGV
FKAELEYARKVRDGVITQDVRMLPVLYELPEAIQGAKGEPWKDPALWAPVLPNLGRSITVERLIPEFRKAVETSPEEIAR
WSSQHLNIQIGLGHHLGGWTGAHHWLAATEPALDLDALIASSEVATIGIDGGGMDDLLGLAVLGRHAA
>OLS50513.1 glycerol-3-phosphate dehydrogenase [Rhodovulum sulfidophilum]AB14
MSRAARLDAMDGAAPKVLVIGGGINGASVFRELALNGVPVMLAEKSDFCAGASSALSRMVHGGLRYLENGEFDLVRE
ALD
ERNRLLRNAPHLVGPLRTIVPIDSWVSGALNAPLRFLGLSKAPSRRGALPVKLGLSFYDIYTRGKGGLPPHAFWSAKTAQ
ARFPFRPGLRCAATYYDGQVSHPERVVTEIFEDVARAGCDAVALNHLEVTKIGPEVALTDRIGGREITLCPEIVVNATGA
WIDRTNARAGATEDMVQGTKGAHLVLDHPELRDMLGDDMVYYENVDGRICIMFCLHGMVLIGSTDIKVPDPDKVRC
EADE
QAYILEGLSWVFPGITVRPDQVRHVFAGVRPLPVSGAGTTGQIPRGHFNRWTEGGIPMLSMIGGKWTTFRAFGELAA
DDV
MARLGLTRRTGTGDLPIGGGAGYPADPEAWCAEQAAASGLSQARVATLLARYGTLAGALAQSFAGERMLPGLPGYSD
AEI
DYLIAHEQVETLADLALRRTVVALAGGLDAAALEALAGRLAAARGWSAVRREEELHRFRVKLATDHGADPERIGLHRKG
E
VAAQ
>OLS50514.1 tRNA guanosine(34) transglycosylase Tgt [Rhodovulum sulfidophilum]AB14
MTTFGFTLHATDGAARSGAISTPRGQIRTPAFMPVGTAGTVKAMLPESVAATGADILLGNTYHLMLRPGAERVARLGG
LH
GFMHWDKPILTDSGGFQVMSLAGLRKMSEEGVRFKSHIDGSTHVLSPERSMEIQRLLGSDIVMCFDECPALPASEAEV
AR
AMRLSMRWAERSKQAFGDRPGHALFGIMQGGVTRELREESALALTDIGFDGYALGGLAVGEGQEAMFGVLDYATGF
LPED
RPRYLMGVGKPDDIVGAVARGVDMMDCVLPSRSGRTGQVFTRRGVVNIKNARHADDPRPLDEACQCPACRNYSRA
YLHHV

FRAQEIISSMLLTWHNLHYFQELMAEMRAAIAGGRFQDWQAGFHAARAEGDIEPL
>OLS50515.1 DNA ligase-associated DEXH box helicase [Rhodovulum sulfidophilum]AB14
MSVLTVTGHGLYCPAGDFHIDPWAPVDRALITHGHADHARAGHRHYLATDIAAPVIRHRLGTIALDTIRYGEPRRIGAA
T
VSFHPAGHVPGSAQIRVEHKGEVWVVSGDYKPEPDGLSDPFEPVRCHTFISECTFGLPIFRWRPQAEVMAEIRDWWA
ENA
ALGRASLLGAYALGKAQRLLAGLCDGAEPGPGPILTHGAIEATCRVMRDQGLDLPDTVPVTPETDPRAHPGALVLAPPS
A
LGSAWARRFGRASTGLASGWMALRGVRRRRAAERGFVLSDHADWDGLNTAIRETGAERVLVTHGYTGPFRRWLAE
QGLDA
GIVATAWEGETTEGDAE
>OLS50516.1 XRE family transcriptional regulator [Rhodovulum sulfidophilum]AB14
MTDSKIRINLRAARTRAGLSLAQAAELTGVSKAMLGQIERGESSPTLATMWKLARGFRLPLTALIEDLVQTTGSFAPAER
DPVRFQGGIGVRTVFPFDPVFGSETFLLTLAPGQVHLSNPHDTGVVEDVFVTEGALGVLLDGEWQTCRAGDGLRFPAD
RP
HGYRNQTDAPARFHNTLHYPRTALLDDGPGAD
>OLS50517.1 hypothetical protein BV379_17570 [Rhodovulum sulfidophilum]AB14
MAPQTGAAHSGAASPRAGGPRLSAVDELVSKLRALISSEGLRVGDSLPTERELCDRFRASRNTVREAMGILKAYGVVTV
R
PKVGATIVDDRMERALELFSFNTLEVSRKTFSDIQGFRGLLEVTGIDLILARLARADIAEMREINDGLRRAQSSDEIAEI
DFGFHTRLIAVMGNKAILDIYKIMKPVILRIMSKGKDSPVHATATHADHLGILDALGARDRLAYQYRMRSHLQAGLGNF
D
P
>OLS50518.1 thiamine diphosphokinase [Rhodovulum sulfidophilum]AB14
MTTHIVHHSDKITLLGGATVDPAHLRRALEIAPALVAADGGADAALAAGCLPEAVIGDFDSISDAARAAIPDDRQYRVTE
QDSTDFEKCLRHVAARLVLGLGFLGDRFDHGLAALNALVRNHERRCLLIGDDDICFHCPPDLALDLPEGTRFSLFPMRPV
RGVSEGLHWPIDGLDFAPGGRIGTSNRVSGPVRLRMEAPGMAVILPVEALDTAVAALVPPVVAAASG
>OLS50519.1 hypothetical protein BV379_17910 [Rhodovulum sulfidophilum]AB14
MRRYEVWGMTPDGSPQSFIRAMPADPHVDEAFCAFAHGTLIATPQGPVAVEDLVPGMEVDTAGAGAQPLLWVGST
VLAPP
AGQRGEIAMLTRLTADSLGLGRPMPDLVLGPRARLLMRHPRCHALTGAEAAFAPATGFVDGTALIALRVARPTRVYHL
AL
NGQQVLLANGVEVESYHPGENAGLLIDNGNRPRFLSLFPHVSGFGGFGPMQVPRLTVFEIEKLNAA
>OLS50520.1 hypothetical protein BV379_17950 [Rhodovulum sulfidophilum]AB14
MRLVPLAPLALAVSALVAPVAADAAPELRVTGTGSATAAPDMATLRLGVGHEADTAAEAMALVSDDLGRVMAELQA
AGIE
ARDIQTSGLNLSPVWGDRSEDKRPRITGYSAANGVTVRVRELDGLGPLLDTVIGTGANTLDGLSFGLQEPGPVMDEAR
RA
AVAEARRKAELFASASGQSLGSLVSLVEQGANQPMPMQMKMSFESMADTMPMAEGELELDATVTMVFELGDAE
>OLS50521.1 hypothetical protein BV379_17980 [Rhodovulum sulfidophilum]AB14
MRSRFWERVALKDMTPAEWEALCDGCGKCCLNKLEDADTGEVVFTRIACRLLDDSTCRCANYAIRKSIVPECVVLSPKA
L
GEVVYWMPASCAYRRLWEGRGLADWHPLVSGDPESVHEAGMSVQGWTIPEFDVPLEDWEDHLIEEEL
>OLS50522.1 riboflavin biosynthesis protein RibF [Rhodovulum sulfidophilum]AB14
MQTFASWTGNATGDRGAAAAIGNFDGVHLGHRVVIEAARAEAAKRGAPLGILTFEPHPREFFAPDAPPFRLMNAEAR
ANR
LAKLGVDHLYELPFDARLAGLSPEAFAREVIVDGLGLSHVVVGADFCFGKGRAGTVEDLVRFGAEMGFGVTAVPLLSNH
V

GEVSSTSIRHALSEGRPGDAAGMLGHWHRIEGEVLHGDARGRALGFPTANMSIRGLHRPRFGVYAVRVDVLSGPHRG
TYD
GAASIGIRPMFGENVPNCESYLFDFEGDLYGTHLSVALVEFLRDEARFDSLNALVAQIDADCTRARAVLAGDLPLPFARD
>OLS50523.1 cation transporter [Rhodovulum sulfidophilum]AB14
MFLGADHDRNARRTWLVIGITATMMVIEIAAGTVFGSMALLADGWHMATHAAALSIAALSYMFARRHAHDPRFTFG
TGKL
GDLAAFGSAVVLGLVALLIGWESLVRLVHPVGIHFGEAILVAIVGLAVNIGSAVLLHQGPHAHGHHGHDHGHGHGAHA
HA
DHHSHGHDHHHEVDNNLRAAYLHVLADALTSVLAIAALVAGRAFGWVWLDPVIGLLGALLIARWSFGLMRDSGGVL
VDFM
PRSETLPQKIREAIETDGARVVDLHVWQLGPGHRGAIVAISDADPRPAAEYRGRLAHLGRLSHVTIEVEAV
>OLS50524.1 ABC transporter permease [Rhodovulum sulfidophilum]AB14
MRTAITALLSHWRRHPLQLAGLFLGLALATALWSGVQAINAEARQSYDRAAAVLAQDRLDSLVARDGQHIGQQVFLD
LRR
AGWLVSPVLEGRWSHRGVPLRILGVDPFTLPAGSLPEAVADESRSGAFLSSEGLAFAAPQTVEELTRIGAPGLPPLAAAP
GMAPGLLLVDIGTAQRLLRAGGEISRLLLAPEQPLRQRPLDEIAPGLIRQSPAEQADMGALTGSFHLNLTAFGFLSFAVG
LFIVHSAIGLAFEQRRPVFRTLRALGLPLRLLVGLLAAEIMGFALIAGLAGIALGYVIAAALLPDVAATLRGLYGASVSG
SLSLRPEWAVAGLGMALAGAGIAAAQSLWRLSRMPVLAPAQPRAWSRASARSLAWQTALAGLLALIALGAAVRGEGL
VGG
FVLLGALLLAAALALPGALAVALALGERLSKGVIAQWFWADTRQQLPGLSLALMALLLALSANIGVGTMVSSFRLAFTA
W
LDQRLPSELYVTGRTEDEARRLAEWLTPRTEALLPIWKIDGPLLGRPGEVYGVADHRTYPDNWPMIRGDASVWQSLAR
GE
GVIVNEQLWRRAGLRLGDPVTLPGGATRPLIGVYADYGNTKGQAILGIADLLALYPGIDRRRYAIRTDPANVPALRSAIE
DDFGLPADNMISQQEVKQVSLMVFERTFAVTAALNVLTMGVAGFAMFMSLLTLSTMRLPQLAPVWALGLTRGRLAR
LELL
RAVLLALLTMALAVPVGLLLAWVLLSVINVEAFGWRLPMQLFPKDWLWLGALALIAAGLAAAWPAWRLARLAPVNLL
KVF
ADER
>OLS50525.1 arylesterase [Rhodovulum sulfidophilum]AB14
MGASLVLALVLCAAGTARAGVTLAALGDSLTQGYGLPQEEGFVPQLEAWLNAHGAEVTVLNAGVSGDTTAGGLSRIG
WTL
TPEVDGLIVAFGGNDLLRGIDPAASRANLDAILTQADAADVPVLLVGLDAPGNYGPEFKAAFDAMYPELAKAHDALYFP
N
FLKGLSDLPDRGAAMARYMQDDGIHPSAAGVKLIVEAMGPSVLELVDRAEAAGH
>OLS50526.1 ATP-dependent Clp protease adaptor ClpS [Rhodovulum sulfidophilum]AB14
MADNSGDPEGETGVITKAKPKTKRPPLYKVLLLNDDYTPMEFVVHVLERFFGMSHAQAFEIMLVVHKKGVAVVGVFSF
EV
AETKVAQVMDFARRHQHPLQCTMEKE
>OLS50527.1 NAD(P)H-quinone oxidoreductase [Rhodovulum sulfidophilum]AB14
MPQVMRAIEIAEPGGPEMLRPTERPVPVPQPGEVLIRLAYAGVNRPDALQRAGSYAPPPQASDLPGLEGAGEVVALGE
GV
TGLKPGDPVCALLPGGGYADYVTAPAAHALPVPEGMGLKQAACLPETFFTVWSNVFMRGGLTAGERFLVHGGSSGIG
TTA
IQLASAFGARVFATAGTPAKCAACRALGADRTIDYRSEDFVDVLKSEGGADLILDMVGGDYLPRNVRALADEGRLVQIA
F
LQGAKVTLNFAHVMMRRLTITGSTLRPQSDLAKARIAAELREKVWPLLASGRVAPVMDSEFDLTEAARAHARMEQGA
HIG

KIVLKVA
>OLS50528.1 cell division protein FtsK [Rhodovulum sulfidophilum]AB14
MASYQMKRREPLLDQTTQAAIERRGKELLGIGLMLVALLLALTLGSYVTDDPSWFSATDAPARNLLGRFGASIASPVYIV
VGYGGWALVAVPAAWGLRLLTHWGEERALSRVIFAPFLVCLASVYAATNVPTPDWTHSFGMGGLFGDTVLGAVLGL
VPIG
TIVGLKLVSALVWLGTLALALFVCGVDRDEIRRFGRGVLLGLILSYAALRAATGRGVRGALQGAVRYGEDLQARRNARA
E
RRQTAFAPEAYEEDDDEAYDRDCDLHEDEAPARTGLFARLPVFRRRADPELIDEDDDEVAMTPASEERIRARIADVVRA
R
SSRGASVPTGFRPEPPVVRSPSALTVPQAPDPDMRIDVPEIDDYDDFDEETPFETAPSVAEAAPAPAIPVAEPRRVVEQS
VRKPVAPSRRARDEAQPGLKFDESEDGYQHPPLSLLTNPDIIERHYLSEEALEENARMLENVLDDYGVKGEIVSVRPGPV
VTMYELEPAPGLKASRVIGLADDIARSMSALSARVSTVPGRSVIGIELPNARREKVVLREILSARDFGDSAQRLPLALGK
DIGGDPVVANLAKMPHLLIAGTTGSGKSVAINTMILSLLYKLTPDECRLIMIDPKMLELSVYDGIPHLLSPVVTDPKKAV
VALKWVVGEMEERYRKMSKMGVRNIEGYNGRVRDAMAKGEMFKRTVQTGFDDETGEPVFETEEFLPKTLPYIVVIVD
EMA
DLMMVAGKEIEACIQRLAQMARASGIHLIMATQRPSVDVITGTIKANFPTRISFQVTGKIDSRTILGEQGAEQLLGMGD
M
LYMAGGGRLSRVHGPFVSDEEVEEIVSYLKTFGPPDYVGGVVEGPDEDKESDIDLVLGLGGNTDSEDALYDQAVAIVIK
D
RKCSTSYIQRKLAIGYNKAARLVEQMEDQGVVSAANHVGKREILVPEQA
>OLS50529.1 sensory protein TspO [Rhodovulum sulfidophilum]AB14
MDWPLFGIFLATTYAAGATGAFFSPDGWYDRIDKPKWTPPNWLFPVAWFVLYICMAYAATRVAISGSPLLVYALAIW
GLQ
VAFNTLWSPVFFGLHRLGAALVVLCGMWISVLAMAVSFYQADMIAGLLILPYVVWTSYAWALNFSIWRRNRGAVQA
AGA
>OLS50530.1 magnesium chelatase ATPase subunit D [Rhodovulum sulfidophilum]AB14
MPDGIWTRVEIGLNALAVDPAGLGGLWLRARSGPVRDRVIEALPALPLPLKRLHPSIGDEQLFGGIDLTATLSSGKLVER
AGLLAEPAILVLPMAERTQPGLAARLAGALDQGTGHCLVAMDEGAEPQETLSEALTDRLALHLDLDALPWGQTERIAM
DG
DALAAARARLPGVRAGDKVLEDLTILAVRLGIDSLRAPMLALKAAKALAALAGNDEVDQDDLSAAVQLVLAPRATQFPE
E
EPDEDDQPPQDPPPPDPDPEQNQNDQDEGESQSLDDLPLQDILLEAAKATLPADLLANLAAQKAQRAGGGISGAGAK
RKG
NRRGRPIPPRRGRLDTGARLDVISTLRAAAPWQTIRKRESSFERSLHIRTDDFRIKRFEEKSDRLLIFVVDASGSAAMTR
LAEAKGAVELLLAEAYARRDHVALVAFRGEAAELLLPPTRSLVQTKRRLAALPGGGGTPLAAGLKTALELAIQSKGRGMT
PTVALLTDGRANIALDGTANRTMAAEDATRMATAIRANGYPAMVIDMGQRPERSLKALAETLGAPYIPLPRADADRLS
TA
VSAALDA
>OLS50531.1 zinc metalloprotease HtpX [Rhodovulum sulfidophilum]AB14
MGYFRTALLMAALTAVFMGVGAMIGGSAGAMIALVVAAAMNLVTWWNSDAMVLRMNNARPATGPDGAELQRIV
TGLARGA
GMPVPKVYLIDSDQPNAFATGRSPENAAVAATTGLLARLSSEEVAGVMAHELAHIQNRDTLIMTVTATFAGAISMLAN
FA
FFFGGRREGGAGIVGTLAMALLAPMAAGLIQMAISRSREYEADRIGAGICGHPLWLASALTRIEALAARIDNPAAERNP
A
TAHMFIINPLHGGARDKLFSTHPNTANRVAALQRMAPGAAPAMTRPATPRPIRRAPRGPWG
>OLS50532.1 chemotaxis protein CheR [Rhodovulum sulfidophilum]AB14

MQTGRAENGDRIDDKSFSRIAALVNRQTGIRLPPSKRTMVEARLRRRMRACGKPDLAAYCRFLFDEGGIERETLHLIDV
V
TTNKTDFFREPDHFRFLAGPGLEAMLARRGRPCRLKLWSAASSIGAEAYTIAMVLAEAAARGPGFDFTVLGTDISTAVL
A
RARMAIYPTGFAAPVPEPMRRRYFMAARDPARRELRVVPELRARVTFARLNLMDAHYGVDADFDAIFLRNVLIYFERE
VQ
IAVVGRLIRHIRPGGFLFLGHSESSVGSELPLRQLAPATFQVL
>OLS50533.1 dehydrogenase [Rhodovulum sulfidophilum]AB14
MTTRARAPLRLGLAGGGTDLSPYCDLYGGAVLNATINRFAFASIEARSDGRIAFIANDLGLSESFGRDDDLSGARLALHR
AVYERMVAEFNDGRRFGATIQTNVEAPAGSGLGSSSALVVVLVDAFRAHLSAPLGQYDVAHLAYEIERIDLGLAGGKQ
DQ
YAAAFGGVNFIEFLAGDRVIVNPLRIPRDILNELESRLVTCFSGVSRHSADIIERQTASVQKSDRAAVEAMHSLKSDSIE
MKRSLMTGDFDGLAEILNHSWIAKKATAKTVSSPHIEEMFAHALAHGAEAGKVSGAGGGGFMFFLVDPPRRHGLIAA
LRE
IGGDADPVVFTRAGCQSWQMPCRR
>OLS50534.1 ArsR family transcriptional regulator [Rhodovulum sulfidophilum]AB14
MPDFDEIDRKILEILQRDADLAVNALADIVGLSPTPCWRRIKRLEEAGVIKGRVAIVDHARANVGMTVFIGITAPRHETA
WLNQFRALIDDLPEIVEAYRLTGATDYILKVVVPDISTYDRVYKAMIERLDFSQINSSISMEELKFTTAVPSTYL
>OLS50535.1 hypothetical protein BV379_18995 [Rhodovulum sulfidophilum]AB14
MTISSFSRVALIAVTTAGLSGCGGGWFGGGSDPDARQANLDAQYQNTPMAEMQRERRSTSTVWDLFDGDDPNVKV
EVNKY
LWQASLEVLNFLPVRSVDPFTGVIVTDYGTPPGGGRAYRATVYVQDPALDARSLKVSLQSKGGPVSQETVRAVEDAILT
R
ARQIRVRDSRL
>OLS50536.1 hypothetical protein BV379_19105 [Rhodovulum sulfidophilum]AB14
MLRAMMLRPILACLVLAALSGPAAASCYADYKAKQDNPLRLHYGVIELPDAACNSRDAATREIDRRIRRGGWQLLNV
MSI
FGASGLGERKASAGGYFLKY
>OLS50537.1 hypothetical protein BV379_19115 [Rhodovulum sulfidophilum]AB14
MGPAAAVVLAVTALRVWLLWLNRMDLFVDEAQYWLWGRELAFGYYSKPPMIGWVIRAATELAGSDAPFWVRLPAP
LFHAA
TALILGRIAAGLWGARAAIWVALGYVTLPMVAVGSILISTDTIMFPFLALALGFWIKMLSRSTAASGWALAAGVALGLA
F
LSKYAAIYYPLCAALAALLIPSARPRLRDAGLALLAFAATISPNVVWNLQNGMTTLEHTLDNADWVRAPSERAGFDISGL
TEFLGSQFAVFGPVLFAALLWGVLRWARQDAPTRLMLVFALPVVAIVSVEALLSGAYANWAAAAYLAGTLAVLPRLGR
GW
RISSFAINGTVALALPVLGVFAPVASLGGHPLLERYLGRADMSTTILDISEADGDSAIMADNRDLLADLFYTGRDRGVSI
YAPPPAGRAPNHYALRHALPDDLEGDVLYVAGGTPPACAEGVTPLADLAPETGAYRGKRIRIFRLPASCWNG
>OLS50538.1 metallophosphoesterase [Rhodovulum sulfidophilum]AB14
MANWYSADLHFGHHRIIDFCKRPFASTAEMNAALIANFQASIHHDDDLWILGDFAFGRAEDTAQFEGWFHSLPGRKH
LII
GNHDDEAVISLPWASTEYMAEIKDGDQSLVLCHYPMITWNGARRGALQLFGHVHDQWPGSRNSLNVGVDQWDFR
PVQVSD
IVSRAAKLPVNKHWQDVEHGNELS
>MCE8454993.1 helix-turn-helix domain-containing protein, partial [Rhodovulum sulfidophilum]AB15
ERLFLSREAVTMAFKFTPVDPDEYARAFEEEEEAQSQEEALAAALAVEPHANLERFRKKRGFTKTEMAEMMDITPRSYY
A
YESGKRSIPTEALVRLNMYTGVDLNEILTGRPSSEGYERVVSTTIWMLRVLLTDYKGIPLSRQEKIINETIGYAQERGLM

IDKRLVDEVVASEMVYKFHPENIPAPPDPEAYEDSQFEQYERDEATWQKHVDEGLDGRRWPR
>MCE8454994.1 M48 family metallopeptidase [Rhodovulum sulfidophilum]AB15
MTDAARHSIVFGGEEFAFLIERTTRRKTVAISVGFDGVRVLAPSDLDDDSVLGIVRRKGPWLLRKQAMYRELGGQPIAK
E
FVSGETFHYLGCQYRLKLIPDETAVMTRIIAKGSNLVAPVLPNTHKSIQRAAVRSGLKQWYRTHAAQHFPARARMIANT
L
GIPTPTLKVVDQSKRWGSCDARGCIRLNWRLVMAPMPLIDYVVAHEACHILEHNHSRRFWRSLETIMPDFEDRLRRLD
RM
GHHFTW
>MCE8454995.1 type I restriction endonuclease subunit R [Rhodovulum sulfidophilum]AB15
MSQANDQFERDWVEEPAADLLRVLFDYKSLAPDEVRHLREGLSTEPVLTVRLAECLKRLNPWLDEDAVRRTVAAVTRV
SA
VDVMEANEVAYTALTYGVTAAHAESGRRQDRNVRFFDFDDPSANTFEFARQVAIKGPRKEIIPDLIVYVNGLPIAVIECK
SPSLADPMGEAIRQFRRYESRDEFSGLGAPRLFETAQISIALARDTAKFGTTLTPVRHWAEWKDPYPLTLDALSAQLGRT
PTGQDILLAGLLAPANLLDVLRNFVIFETIDGRRIKRIARYQQFVAVGKAIERIRTAVTPQRRGGVIHHTQGSGKSLTMV
FLATKLRRLREAENPTLVIVTDRTDLDDQITGQFQRSGFANPIQAESGATLRSALSGGAGTTVLTTVHKFHSAVPKGSPI
ISAARNVFVMVDEAHRTQYGALAARMRAGLPNACMIAFTGTPIDKTDRNTREVFGDYLHRYLIDQAVKDGATVPIFYE
MR
DAQLRIDGRDLERDVRAAFPELSDEEFQKLKRGMRLHEKLAGAKVRVEAVAKDILSHYRSTIEPNGFKAQIVTVSRDVAV
SYVEALRNLGAPECALIMSTSNNDSARLQAHHLSKSERDTLIGRFKKRDDPLRILVVCDMLLTGFDAPVEQVLYLDAPLR
EHTLLQAIARVNRTAEGKTYGLVVDYWGDNRRIADALDMFSDEDGITSALRPLSEKIQLLESRHRAAIRLFEGLHSHDDA
ECVELLRPDDIRAKFELDFLRFSEAMDMVLPDPKALEDPYPSDLKWLSRIRVLARRAYHEEPFDPKDYGAKVGEIITNHL
SVTGVEQLLVKFDILDPAFRPHLDRLRSGAAKAAEIEHAIRHEIHVHRDENPAAYASLWEQLEGIVNDKREARVSAASAL
SQLEAIVGKAADIRSGAACSSGGLSGTAAAILPFIDPDLPETERSQSAAGITEALEAYAEVIDWQQKEDVQRQMRRSIKV
RLRSLGLDAQKVEATTAKIMDVARARYVG
>MCE8454996.1 restriction endonuclease subunit S [Rhodovulum sulfidophilum]AB15
MSAELPDGWTAARFRDVMRLEDRREPIAPIRSYRLLGVRWYGNGAFLREERLGDELSAQHIYRVQPGDVIYNRLFAWK
GS
FALVGDDLADCYVSNEFPLFAARLDRVDPQFLLRVLQHPRTSERADAFSTGTTSISRNRLSEDDFLHFPLALPPLAEQRA
IAEALGSVEAAIAKTEALIAALAESRVELTRDFQGSEEHSKWPARPIGSLIRKCQYGLSIPLDGDGSVPVLRMPDIRDGR
VNLDVSTLKSTDVSTTEVTSCEIKEGDILFNRTNSQALVGKTGIVRSTPCAQIVFASYLIRLVVNSDINAFWLNAVLNLP
RTQERLKALATPGVSQWNINSKTLKRFEVAVPPKANQDQFANLYESIEAALETERTKLTAMNEARYALAQELLSGRIRLP
DSLIVRHRDNAGQAA
>MCE8454997.1 type I restriction-modification system subunit M [Rhodovulum sulfidophilum]AB15
MTKLTRAELENHLWRSADILRGSIDSSDYKIYIFGFLFLKRLSDRFEEEAKQAIRQGLPEDVAYSDPDEHEFFLVERARW
SSIKKLTTGIGDHLNKACAAIEDANPSIEGVLANIDFNSESRLGDAKNREGVLSRLIDHFSRIDLSNASLSEPDMLGRAY
EYLIDKFADDAGKKGGEFYTPHHVVRLIVELLDPKPGMRISDPTCGSGGMLVQVAEHVAKLEGKRLGEALNITLHGQEK
N
LGTWAIAKMNLLLHGLRDARIEKGDTIRNPRLLDQDGNLFLYDRVIANPPFSLDSWGADDVKGDTEKKGHNRFIYGIPP
K
NMGDLAFVQHMVATLNAKGVCGVVMPHGVLFRGSGDGRIRKAMLEADLFEAIVGLPENMFAGTGIPATVLILNKAK
APER
KGKVLFIHGAKEFEERPKKNILGEGNITRVVDAFVAWKDEERFCRVVNMDEIAENDFNLNISRYIDTLEPEKPIDVPAEL
AKLWHAERARDEAAARMNALFKEMGYVG
>MCE8454998.1 restriction endonuclease subunit S [Rhodovulum sulfidophilum]AB15
MRKHVDITSTPFTQARLADVADVFSGIGVSREETAERPGERMPVIGVRDLQDGDVAPRAALDTVGFSAPSKALTYAVR
TG
DVLVTGRGTVLKFGLVSTETAGAIASANIIVVRPGPEVTGGALFAILSSEVFRPKIEVLRRGATTLLSLSPKDLAKLEIN

LPSRDEQERIAALVNESQIAYRAALEAADIRRTLARRFVDARLFGGTQNT
>MCE8454999.1 hypothetical protein LZ188_00035 [Rhodovulum sulfidophilum]AB15
MSKRARPSGLKITERDAALIRGMIERGDRHHDIAAFFGLNQGRIAEVKDGKRFPKVPPASSDELPPRGPYLTPKAAWTE
N
RLVS
>MCE8455000.1 YegP family protein [Rhodovulum sulfidophilum]AB15
MFEILRANAGGYFWRLKGTNGETLCHSEVYTSKQSAQAGVNAAKRVAPLAQVHDRT
>MCE8455001.1 hypothetical protein LZ188_00045 [Rhodovulum sulfidophilum]AB15
MSEVGGKYLFGTWLSATEIANLAICTDVSEQGDPWTAEAKDAVYKLLKPTISPHISSRSRGGTGRKRSLDQAYQLLLLDL
ATHGMDAPAAVMRKLLTIWALETFGRSTALSGFDDGQVLDAVLEELELTK
>MCE8455002.1 hypothetical protein LZ188_00050 [Rhodovulum sulfidophilum]AB15
MSCDDFLSAVEQAEAGPSSGDIDGAPMIDNWYLVWRDSDAIRADGDVSGHPEISDPRVTTSPVLGFQYDFDLEAGWL
RTM
SRWYRLGRLRAVPVASPDPQAKRREALDAAQDLLRRHREAWRNQIVSGGRPVATDLPETNTAGVDGPNEPLGPVTGL
FI
>MCE8455003.1 hypothetical protein LZ188_00055 [Rhodovulum sulfidophilum]AB15
MTRELDARIEELRARRNITIRKAEAELAEMQAELDRQMTAFRKEVAAEANRIGGLMQVTGRRVVAAEKRQAKLEADLA
IL
SKESGLLLRRHRRLGLGILLGSVLLGSTFISLALWGAPLWPF
>MCE8455004.1 plasmid recombination protein [Rhodovulum sulfidophilum]AB15
MSGPQFFHLQSFSRKANPAGQSVEQVLAEAARVPRFSTHVPAPRPPRVVAGLHPARIKERHDAMVEAGRISVTLADGS
TK
ERGIRKDRHTLLTCVASHPLLSSQVEADAEARQKYETWVEYNLAHFRRMFGDKLMGVVEHLDEEHPHLHAFVLPTDDP
TC
SARDLNPAWAAKSEAEAAAREAGHDAKAAVKLGNKAYKARARELQDEYHREVGIAAALTRLGPKRERLSRAEWRARK
AAA
EQDALLLREMEDRVSALAIHEEELSATEEEITARLAEQLEHAALLDAEAAAKRQSASTESDALLEAARRDREAAVARLAT
AEAEARRLENRARKKAEEDTAVADAYLLDTKRKARQVRLDAERKMEEVRSQADAFRRDGVREAFELARKLMIGVLTGE
VV
PAPTGEGWTIHDERLKRRVGELGVASSLDRILQIVSRCWELIKRHLKPAAAEAELSDLVEDQPPAGRDPLHQSERGIEP
>MCE8455005.1 hypothetical protein LZ188_00065 [Rhodovulum sulfidophilum]AB15
MTAAELLTAVKAYALHSAGHTQSKVSFSDNWFRLGKWRPFVEAARAEAHRPQEIAERQLSDAADAIREQKDWMYRH
LPEG
RVFQAVQRGLITREEAQAAGFLR
>MCE8455006.1 helix-turn-helix domain-containing protein [Rhodovulum sulfidophilum]AB15
MSEVLPFCKIKLQYLLEMVTDHDLDDNALRVALYLALAHADHETGESRPSFETIGAAIGKHAKSVKRALNKVEAAGYMT
V
ERGTNKGKSSRYRPTKAAMRRATERRREGDKVVPLSRAKGGQSCPQSGTDLSGKGGQDRPPNREQEPRKEQGRASAP
DLP
DERQSGGTRPDLVFVPRGICFVRDWDARLAQEGMTTLERSLPLSPHEHHLGFWLPARTPAPVGSAEWQAQLRVLRDV
IRQ
SQDGTCPAPSAPLIASFAPRQMQRASRLG
>MCE8455007.1 NAD-dependent succinate-semialdehyde dehydrogenase [Rhodovulum sulfidophilum]AB15
MTGNTIADRTRLSDRGLLRDRSRIGGDWVGSETRFEVRDPADGAVLGSVAAMSAEQSRAAIDSAQTAFPGWAGTLP
QDRS
AILRRWFELVTAAREDLARIMTVEQGKPLSEARGEIDYAASFIEFYAEEAKRPNIEGVTSHLPDAEVELWLEPVGVVALI

TPWNFPAAMLTRKAAAALAAGCSVLAHPSAETPHSALALAELAARAGVPPGVFNVVTGDAATIVGPWTEDPRVRALS
FTG
STEIGRLLYRQSAATVKRLVLELGGHAPVIVFSGAELDHAVAETIKAKFATSGQDCLGANRIYVERPVYDEFCRRFTAAT
QALTVGRGMDDPDIGPLMNEAALTKQEDHVADALAKGARLACGGSRLPLGDLFYAPTVLTDVPHDAKIMREETFGPV
AAL
TPFDTEDEVIARANDSEFGLVAYVHSLNPRRIYRVSRALQYGMVAVNRTKVTGAPIPFGGMKQSGIGREGARRGMEAF
ME
IKYVCRDWH
>MCE8455008.1 tripartite tricarboxylate transporter permease [Rhodovulum sulfidophilum]AB15
MAGDLIGAMALIATPDTILSIVIASFFGFVVGALPGLTATMAVALLIPITFLLSPVAAIASIVSAAAMSIFAGDIPGALL
RIPGTPASAAYMTDANALVRKGEAGFVIGASAVFGMIGGVIGTVILIAAAPLLADFALSFSSFEFFWLGCLGLSCAVLVA
GDDLLKGMGALMIGLLIACVGMDYTTGYPRFTFGSWALLEGISFIPALIGMFALPEILRLLSDRSPRHQLPEAEPGSALM
KIARAGRRHWWPSLRGSVMGTAIGSLPGAGADIAAWISVAVTRRATREPEKWGKGHIEPIVAGGSANNAALAGAWIP
ALV
FAIPGDTITAIAIGVLYLKGVNPGPMVFVENGQIVYAVFLVFFLANLMLLPFGWIAIRMAGLVLKVPTAVIAPTLLVLAC
LGAFAINNDMSGLLVMLGLGALAVGMNAAGLPLAPAVLGIVMGRIVEQNLMQSLISTQGSLAGFFERPASAVLGALTI
GL
WSIALLRQLARMLRGARRAG
>MCE8455009.1 tripartite tricarboxylate transporter TctB family protein [Rhodovulum
sulfidophilum]AB15
MRIHDALAGALLAALGGAVIWYVAGFPRVAGQIYGPDLFPRLIGIGLVLFGAGLVLRGARAAGGLPALLDLPSNGEIRRG
LLATLYIVASVLAVVFLAERLGSQILVFAILISGLLAVYRRPVLSLALAVGLTVAFDLIFRVLLRVPLPSGPLTGVL
>MCE8455010.1 tripartite tricarboxylate transporter substrate binding protein [Rhodovulum
sulfidophilum]AB15
MTRTKALIAGLSLTLAAGAAWAEFPDRPVQLIVPYPAGGGTDIVARTLAVELEKELGQPVNVVNRAGGGGIPGQTAIA
HA
RPDGYTIGLIASDISLYKPQGLTDLTHADMTALGQTNELPGSVTVAADAPYRTMDDLVTAIRDNPATIKGTGAAPGASW
H
AGFLGLMFALDMAPDQVIWVPTQGGTKGHLDVAAGNSDFSTASLVEARALIEAGKLRPLATMAAERVAIFPEVPTLQE
QG
IDYTYGLWHGVTGPKGMDPAALEVLSAAVEKAANSDGFVATLEERGFKHVWRPGPEFEAFMAEDLAKMQAIFDRLN
D
>MCE8455011.1 amidohydrolase family protein [Rhodovulum sulfidophilum]AB15
MTRRAGAETVQIVDAHCHMWDLSLGKHPWLAEGVLHPHRYGDYSAVKRDYLPADYRAATRPWTIAAAVHMEAEW
TPEDPL
GEARWIADLHAKTGFPAAMTAQVWLDRPEAQDLIAAQAAFPLVRSLRHKPRAFARAADWHEGHALPGSMRCPEFRR
GYAG
LAAHGLHFELQTPFWHLPDAAELARDFPDTMIVINHAGVPGSRDPAVLAQWRRALSVAAARPNIRIKVSGLGVPGEA
WTT
EAQREVILTCIETFGAGRAMFASNMPVDGMFRSFPDLIADFMAVTSDLPEADRRAFFAGTAADTYGLDLPGLT
>MCE8455012.1 GntR family transcriptional regulator [Rhodovulum sulfidophilum]AB15
MGDKWQAMSEEVAAWPRGQRSILVTQFIRDLIVSGEIPPGERINERVLTERLGISRTPLREAFKILDGEGLVSIRPNAGA
TVVRLSPEDVEDSLEILIGLESIGAERAVEHATPEMIEEIAELDARMGEAFGRRALMEYFHINQAIHQKIIDAAQNEALS
RVYASESARIRRFRFAGNRDHGRWARAVQEHAQILDALQRREGPLLRELLRAHHRAGWKVARHALEAAASNH
>MCE8455013.1 flagellar biosynthesis protein FlgA [Rhodovulum sulfidophilum]AB15
MNYHTYFSAGDRMVEACVIGTGGFGRSFLAQGRKVPALSVRIGVDVSAETAAEAMRDVGIPEDRIRIVTGAAEALAAF
EA

GAHVAAGDFAHVAGLPFEVAVEATGNPVHGARHARLAIEAGKHVAMVSKEVDSVVGPGLAAMAAEKGLIVTPVDGD
QPSL
LIGLITWAETLGLEIVAAGKSSEYDFVFDRGTGTIRCNGQTYPAADFAPYARMGDMEAAALVAARAEAARTLPQRAVP
DL
CEMTVVANACAMGVDTPELHCPIARIDEVPSILCPVADGGILGRAGVLDVFHCLREPGEISFAGGVFVVIRCDHADTWE
V
LREKGHIVSRNGRTAMVFLPRHLLGLEAATSILEVGLRGVSSGTPRPTHHADLVAHADRDLAAGHVLKMGGHHHSIDA
VS
ARILPGGPLSEDSPAPFYLASDCRLLRDVPAGEPIRMRDIEIPEDSPLAALRALQDSHFAPSGQTA
>MCE8455014.1 TRAP transporter substrate-binding protein [Rhodovulum sulfidophilum]AB15
MTLNRRSFIAAAAAGAAGALLAAPSILGAAGKITLRMGHPHPETDSWQDTALWMGETLAEKSGGEIGLQVFPNGLLG
NDP
TMINSVRAGALDIMVTGNPFFTSLAPELNVLDLPYLFHDRDHVARVLDGEIGDRLRTDFEGTGLYALALWETGWRHVT
NS
RRPVETPDDIKGLKIRTTPNPAHIEAFRLLGAVPTPMAFTELFTALEMGAVDGQENPTTLILNSRFYEAQKYLSLTQHAF
TAAPLVTNAAKLDAMDEDMRALLIETAQEGARRQRALNVEREVTSLAKLREEGMEVVETPDRKAFRAIVAEPVQASFG
EE
FGPELIEQINAAA
>MCE8455015.1 cyclase family protein [Rhodovulum sulfidophilum]AB15
MTIVDLSMTVGPHFRWSPEISVTGDKSAGDQFRVTRLSTTCHGFSHVDAQAHFIENGPTIEATPLDRVVGPCRIFDLSA
V
APDEEIGPETLAAADPGGAEGEILLLAAQWDLKRDSGCREFWTESPWLNRAAAEYLLAQKPSAVAVDFPQDYPIRLLLD
G
IAVPDAEHVTHDVLLRAGVTLIEYVVNTAALKGPRTFLCAAPLKIGNADGAPARIFAIEDMA
>MCE8455016.1 TRAP transporter small permease [Rhodovulum sulfidophilum]AB15
MRPARLAVLAVRVADRVLAAVEAVLVLVLAAMTLLVFFNVVLRYGFDTGITVTDEVSRMMFVWIAFAGGIAVSRRQQ
QLG
IDVLVAALPPLPRRLAHVAGNLAVLACCLVLVSGAWTQMQLNWRNTAPVSGLPTAITYAAPWFAGIGIGCVALANALG
AA
FGLTGGIMASGKEDPGA
>MCE8455017.1 TRAP transporter large permease subunit [Rhodovulum sulfidophilum]AB15
MTTILIFVGVLLVTAIAGIPIAFGLMLAGIAMLWQLGMLDWQSIALQMTNSADSFPLLAIPFFLLAGEAMNAGGLSRRL
V
NLGLALVGHLRGGLGYVAIVTAVLLASLSGSAIADTAVLAGMLVPIMRQSGYPMGRASGLIASGGIIAPVIPPSIGYIVF
GVAGNVSITRLFLSGIVPGVAMALALTATWWLMCRRENMATLPRRTGPEIRTALVDAVVAMMLPVIIVGGLKFGIFTP
TE
AGVVACIYALLAGTVIYREITLGVLYECMLNAVRTTAAVMLLIAAAGITAYAITIAGVPQQLAVLLGPLAQHQTLLMLAI
VVIVILVGTALDFIPTLLVLTPVLLPIAKQAGIDPVYFGVIFMMSAAIGLLTPPVGAVLNVVCAVARQQYADVVRGVMPF
IVVQVLLLLLLVLFPQIVILPGRWLY
>MCE8455018.1 four-carbon acid sugar kinase family protein [Rhodovulum sulfidophilum]AB15
MILGAIADDLTGATDLSLILARNGMRVVQTIGLPDDLGAVAAEADAVVVALKSRTIPAEEAVAMSLDAARALQAAGAR
RF
FFKYCSTFDSTDEGNIGPVASALMSHFDMECSVACPSFPENGRTVYQGHLFVHDRLISDSPMKDHPLTPMRDPDLVRV
LS
RQTERPVHLVPLATMRGAPGAIAAQVTATPGIFILDAIDDSDLRAIARDLADLPLITGGSAVAMGLPDIYRARGWLSAAA
AQAPFEAPAGKGVILAGSCSEMTRKQIDAARRAGLPMLQIDAEGIAEGRITAGTVVDFVEKAAGQGPLPPVVYSSADP
GT

VARAQKALGRMRAGELVETLLGEVAASLAGRGFTRIVSAGGETSGAVAGALGASALRIGPEIDPGVPWVMTLDGERPL
CL
ALKSGNFGSEDFFLKAWELLA
>MCE8455019.1 aldolase [Rhodovulum sulfidophilum]AB15
MTAILSEATAARDRICAAGKSIFDRGLTFGSTGNISVRLSDGSMLMTPTNACLGALDPARLSRFSADGQFLDGDRPTKES
FLHMCMYCKRPHSNAVVHLHSTHSVAVSILADIDEEDVLPPLTAYYVMRVGRLPLIPYFAPGDEKLARAVEAQAGSHHA
V
LLANHGPVVAGGSLEEAQYATEELEETARLFLMLQGHRIRPLGEEEAKALRSRS
>MCE8455020.1 right-handed parallel beta-helix repeat-containing protein, partial [Rhodovulum sulfidophilum]AB15
MKRFLQLVMAFSGAAGLFGGSSAGALVLRGIATSDEEVEKAGPEAPPSGAETAELAAQSAEADDIGMEEPGTGEDPAS
AA
AGDETGDVAAGGSGATGRNASDGDAFDAAHADAVALPETTTEPGSFVASAPSEGSALSPAIVKFASLLGVDPWSLVSS
DG
GSGSGSGFQTPPDDGVGGPPPVDGGGGDGDPGDGPDDGPGEGPGGDPGGEPPDQGPDEGQDGSGTAVPLDGEV
EVTTGRV
TTLTLPDAEDAVSVEIVTGPGFGNVTVNPDLSLALVLTGETQTGSLSFDYAVTHADGTVTTHGASLSAVAGEQGGGWG
AG
DFYMLETAADGSLVIEHGDVHESVYVTASEDGLSRADIAAMEGMKVEQVNDYWLADQTTYGFDEGLAVDQDVGSAI
WRSL
TGPEPSSHWLLLERGYSYDDFKGLVDPSIRGESELHPIYVGAYGEGAPPEITEEMRSFAQTKENVVVEGLTFSDGFSMFN
SQNILLSDLTITGDSLIIKTADGFTLRNSSVYDIWREAPANDGDTWAQNLNRVSGFFLTKSEGVLVENNVFDHNGWEDG
Y
DYARSAEDGQPPSMYSHDIYMTDTNSDVTLRDNIIMRAASYGAQFRMGGVVEDNIFIDNNGAINPAAGEAGAGNYSL
VLG
NVVTSAGYKTVDHSEGALSQGIRAQAQDVSLVDNIIAHLADPNNAEEQVEKVKG
>MCE8455021.1 isopropylmalate isomerase [Rhodovulum sulfidophilum]AB15
MQDIYSCVFGTWQPGIGDPTWQGWTTVAVYLAVAALALHAVFRAVFPAPSARRERIFWSLVALVLLGLAINKQLDLQS
AL
TATGRCLAKLQGWYGQRRAVQTEFIAAIGTLSLLSLIGLLWLLRGTLRRSAPAIVGLCFVSGFVLIRAAGFHHFDHLLGM
PTLLSVRANMVLELTGPLLIALAALRLPRLRHSQGHGIAGKP
>MCE8455022.1 glycosyltransferase family 4 protein [Rhodovulum sulfidophilum]AB15
MRVVFVQHGNYAEAYRRMCAGEAETYRDQFRSVSLVAGLAAAGPVVTVALGDAHHDAQLAPNLRSVGLPRADIDRA
GSLL
DDLAPDRLVCRTPHTRLLAAAAARGIATLPCFADMMRPPSPLGLWRHWRLRNALRARTVPCVANHSLNASRSLVEAG
LVS
AGHVVPWDWSRLVPNPDPKPGMPDPQNPTILFAGKMTVEKGLGDVLAAMSVLAGQGLNARLTVAGGGDVRPWQ
AEAARLG
LAERVDFLGLVPNTEIRHRMRAYDIVVVPSRHDYPEGLPNTIYEGLASRSVLMLSDHPAFRGRVEPGRHCLMFRAGNPA
A
LGAEIRKLCAAPELYAHLSRQAPEALNSLYVGLAWGDLVTYFLDDPANLTGWVERHSLCALGLSG
>MCE8455023.1 VPLPA-CTERM-specific exosortase XrtD [Rhodovulum sulfidophilum]AB15
MSIFNEAFALRRQSRAFPGLGLFWLGLAVLGAGLFFRPGLDALLVAWQKPEYSHGPLIPLLSGLLFLRQLKTVPERPGEI
PHRWPGVLVVLLALMLGMVGRVSNIDDIVAYALIVWVGGLLLISFGWSTGKHFWPPVLHLVYMLPLPDTLYYKATTWL
QF
ISSELGVWFLGLLAVPVYLEGNIIDLGVYKLHVAEACSGLRYLFPILSFSYIFAVLYRGPVWHKAVLLLSAAPITVLMNA
VRIAIAGWMVDEFGVEWVEGFTHFFEGWVIFIICVAILFALARIMLFFHPAKMSLTEALDLDTEGLVPQLMRLRLVQPSL

ALIAAAGLMFAGVGVLQMLPDRSATAVVRDSFVTFPRRFGDWQQSGPPAVLAPDIARALQADDYHSITLTSPDTPAYV
GV
FLAWYADQSHGGVHPPEVCLPAGGWEIAWLERTDVASELGSAEPFHLNKAIIQKGEVRMMVYYWFDQKGRKVAWD
FAAKL
WLIVDALRTGRTDGALVRLTTPILPEESDADAAARLDAILEDMAPQLPRFIPED
>MCE8455024.1 WecB/TagA/CpsF family glycosyltransferase [Rhodovulum sulfidophilum]AB15
MMGWSPTGRGTDRAVTVTVASMAGLLADLEARLTRGEGFAVATLNLDHVVKLTRDPAFRAAYLAQSHVTADGNPV
VWLSR
LAGQRVELVPGSELIGPVFALAARHALPVAFLGSTAETLERAREVVEADYPGLRVVAALAPPMGFDPDGAAADEMIEEL
R
RSGARICLVALGAPKQERFAARAAPRLPGTGFLSIGASLDFVAGRQRRAPRWVRRIASEWLWRMLSDPARLGRRYLDC
LL
ILPGLTRRALCQRLRRGG
>MCE8455025.1 hypothetical protein LZ188_00175 [Rhodovulum sulfidophilum]AB15
MVPPMLALWSWVIVTVILFRVLRPAGALVACLLGAYLLLPTRTSFDLPLMPTLNKHSLPAIVATVMTATVAAGVQRPRP
A
GAEGAGRILPGWFPGNIWIRLLFLVAVLSVGLSALVNNDTLVYGPTVRRGLGPYDAGSMVLAMAVTLLPFLLGRKMLA
DE
TGRRVLLWGLCLSASAYAFLALFEVRMSPQLNMMVYGFRPSAWIQHLRGNGYRPLVFLEHGLRLGIFLTMATLVACGL
SR
IDRRRRIVCLLAAAWLFGTLVLSKNLGALAIGLLLVPVALFLSTRLQIVAAAVLAGTVLFYPLLRSADLIPVDQVVGLFE
KYSSANRIGSLNYRLKNEDILLDKAMQRPALGWSTFARNRVFDPETGRDISVTDGVWVIVIGESGLIGYMAQFGLLTVPI
LLLVRRPRQVEPSTALLALALVANLADMIPNSSLTPISWLIAGTLAGRLEQARAPAAATAGPTTTDPPRPPRLRGGQAAE
EEPPLPTMRATPYTRQTTLHSRSRRG
>MCE8455026.1 glycosyltransferase family 2 protein [Rhodovulum sulfidophilum]AB15
MTHSLVVSIINYRTADMTLTCLASVLEDLDGIDGHVVIVDNASGDGSETVLQDWLDAHPGAPVTLVRSAANTGFSGGH
NQ
GMAARPARFYLLLNSDAVLRPGFCSTILAAAEAQPEAGLFAPRILRDSGETQDSCFRFHSPMSELIRGANTGLVTRALRR
FEVSLGTEPVPAQIDWASFACILLRTEMTAAIGPMDEGYFLYYEDAEYCLRARRAGWRIARVPEAVAVHFRGGSGPVKT
L
RTARKRLPPYYYASRARFLYQAHGLMGLWAANLMWHAGRGIALLRRLAARPGRQPPLEGEARDLWINAADPLGPRLA
PHE
T
>MCE8455027.1 sulfotransferase [Rhodovulum sulfidophilum]AB15
MTHPDFIIIGAMKSGTTTLAAQLAAQPGLFLTDPKEPNFFSDDEIHARGPDWYAGLFAAAPPDALKGEASTHYTKLPTY
P
DTVRRLAAAGPAPKLIYMIRNPLERAVSHYIHEWTTGEIRTDLETALERHPTLTDYGRYGMQIAPFAETFGPERILLTSL
EGLKADPEGELGRISAFIGLETPPVWQSDLGARNVSAERFRKLPFQRLLVDNPLARSLRHALVPKALRERVRRARSITRR
PELSEAARARLIPIFAADRDALARVFPDDPALGLCYPFLAP
>MCE8455028.1 glycosyltransferase [Rhodovulum sulfidophilum]AB15
MTPPAPSAPRDLAAIVIGRNEGARLIRCLDSLAGRVARIVYVDSGSTDGSLEAARQRGAEVVELDRTRPFTAARARNAGL
ARLTETGPLPRHVQMVDGDCELREGWIETARAFLDATPAAAVACGRRRERFPEATLWNGLIDAEWDTPVGRARDCG
GDAM
MRLEALVAVGFYNPALIAGEEPELCVRLRRAGWEIWRLETEMTWHDAAMTRFGQWWTRARRAGHAYAEGAHLHG
APPERH
NVAQMRRALIWGPGVPLAAALAALVLGPWALALLLVWPLQVLRLGLRGTGWRRALFLTLGKLPEALGVAQFHLNRLR
GRN
ARLIEYK

>MCE8455029.1 capsular polysaccharide synthesis protein [Rhodovulum sulfidophilum]AB15
MPFASALRRRVRQGLGTALWALRDLPGAPTFESFARRLDRMNRAHVAEIRSRGGEPMTPRAPDTIPKIVWIYWAQGL
DEA
PPVVQRCIASWRAANPGWDLRVLDRDAAGALVDLSDLPAALPRRHEADFLRLRLLERFGGIWADATVFCHRPLEDWL
PLL
AGSGFFVFRDPGPDRWLSTWFIAAAPGHPLIAAWARQYGEYLIARRRPPGPYFMVMYAFQFAVSRSPELMREWRCCS
GLW
AVPSFRLMAVLDGQVGAEEAAVESLRRGLPVSKLNWKKPFDQVRFDAILGAAGVKRDVG
>MCE8455030.1 hypothetical protein LZ188_00200 [Rhodovulum sulfidophilum]AB15
MTIINRIELDTYDLRMACLAYNSGRAEASTVRAELDRLVQRVAGSEHPVLITDEAFFGPHVGQDGETRLFPRAHEVTQM
M
VEAFAPHPVEILLYTRDEFSWLKSVHNQSVKQDRYAGSFEEFLAEIGGGGDLDAVAARIAATIGPEQVRVFRLEDDHHH
P
FGSGGALLDHVGIPREVQQDLKRASGINQSLDARHLRIMRRLNGLALPDFLYSRLRRGVLRLQRRARS
>MCE8455031.1 oligosaccharide flippase family protein [Rhodovulum sulfidophilum]AB15
MVQNPQPETGLKKRAMRGTVFSLAEFGAANLLRLVSNLILTRLLFPEAFGLMAIVQVFMSGLQMFSDVGIRTSITLSKS
G
DDPDYLNTAWTVQILRGAILWLAAFAISGPVADFYDEPLLAQLLPVVALNALFAGFMSTNMATANRHLRLGRQTALNI
GS
QVLALALNIVLAFWLRSVWALIWGGLASTLFMVVASHTLMPGIRNRLRWHRASLGELIRFGKWIFLSTIAGFAMNQAD
RA
ILGKFITLDQLGVYTIAFFFGNLPILLSRATLGKILGPLYRMRHPSKSEANRRNVIKARRLAVGGLVAMTVPLAYGGGLL
IELLYDPRYVLAGPMVVLFAFALPPQLVSVAYGGILLAVGDSRRQFYLTALTAGLQVALMLVGVVTVGVVGVILAPALAA
MLIYPLRSRYLNLYNADDPVSDMMLLAVSLVLNGYACWLHKDGLLQLLS
>MCE8455032.1 hypothetical protein LZ188_00210 [Rhodovulum sulfidophilum]AB15
MSRIAFYRLAGLVSLLFAAGTLYRLFPIVTGQPALSEFFMTEDGYLMLTIARNMAMGLGMSVSDGTIPTNGVQPLATFL
F
TLPYLQTGGDKVTSLIGIHLIMAAWSVAGALLVRALAARILDPESQTPALPWIAALLWYLSPLLLLHTMNGLETGLYSAA
VLAVLLMFLRLLRREGPLGWGWHLGFGLMGGIAFLARIDAVFLLLAVYLVWAIDGLFLRRDPIRTVAARILPSGLISILV
AAPWLLNNQIRFGSIMPISGTAQSMAAEFGQNLGVLPVKIFEYFMPMLPVPNPLEKTLPVIVLASAVSLVVLAWFLIGVF
TRASRPVRLMVLAYLLFGAMVSGYYGLWFGAPHFMSRYLAPLAPLCIIASLWVLADLSRWLFPARGLRPAIWTGSAAIG
L
CVLLLGRLLMPGVQEQGHMQVVDWVERNVPDSAWVAAVQTGTLGYWHDRTINLDGKVNPEALEARRREGNVLAY
VVNSRI
DYIADWYGVGGWATRTDGGFAEAFELIEHRKSPSLAVMRRRVLHDPG
>MCE8455033.1 mannose-1-phosphate guanylyltransferase/mannose-6-phosphate isomerase
[Rhodovulum sulfidophilum]AB15
MIHPVILCGGSGTRLWPSSRKAYPKQFAPLIGRESLYQRTLRRFSGGGFAAPLVMTGNEFRFMATEQAAAIGLSDARVV
V
EPLGRDTAPAILAAALMLERDDPEALMLVAPSDHVIADLPAFLAAVEAGCPPARDGALVTFGVAPDRPETGYGYLELTA
P
PAGATPVPLASFREKPDLATAREMLAAGTYLWNAGIFLFRAADAIAAFETHAPELLAPCRAAIAEGQTDLGFFRLAEAPY
AGARAISFDYAVMEKASRVMAVPLKGGWSDLGSWDALWAAAGPDADGVVADGAVTAIDCRDSYLRSEDESMQLV
GLGLEG
VVAVAMRDAVLVADKSRAQEVKTVVETLRADGVAQADQYPRFHRPWGWYETLCLGTRFQVKRIMVRPGGVLSLQS
HHHRS
EHWIVVSGTAEVTIGEEARLVTENQSVYIPLGTVHRMANPGKLPMYLIEVQTGAYLGEDDIIRYQDIYDRS
>MCE8455034.1 replication initiator protein A [Rhodovulum sulfidophilum]AB15

MEAAAIGDFFVCDFLGPSPKHDIAAMEHPLFSLSTRPDRRILDYEHNGVRVTVTPSVRGRATIFDADILIFCISQLMAAL
NAGKPTSRHLTLTAHDLLLATNRETSGDSYKRLRDAFERLVGTRITTNIATGDVETTTGFGLIDSWEIVRRTRAGRMVSV
SVTLSEWLYRAVLAKSVLTLSRDYFHLRKPLERRIYELARKHCGRQPEWRISIPVLWKKTGSASPVRVFRAMVREILRED
RLPEYRLVEEPGDILCVLRRDGLIERGTGEPPPVLSPEALEAARARMPGADVYALEAQWRGVWAASGRPRIRQPDRAFL
A
WVEKRRTED
>MCE8455035.1 AAA family ATPase [Rhodovulum sulfidophilum]AB15
MSRDDIPPYLNISPDRAAELLGPPADTPGFASVAEACRRGRQDLASRGLDETGERQLRLFSTWEITRYLIPVAPAHFRRV
LRQNPDLPQGQSESAGGAKWFTLDEVLRLRAHFGAEGSKAKNYLPYRPKGLPAKIVAVANFKGGVGKTSTCAHLAMS
AAL
DGYRVLVIDLDSQGSMTSIFGGQVEDEWQTVFPLLARHYATTLRADNQRRLDRGEPPLPFDDTLTEALKLKAPDLIRKT
H
WPNIDLIGAQLNLYWSEFQIPVWRMQSRGWRLWDALTDVLAEDGVLDDYDLVFLDTPPALGYLTINGLAAADILLVPL
GA
SFLEFDSTGRFFDMLHATFASIEEGENVAARALGREELHFEWDAVRAVMTRYDAGQQAEMAGLIQAYMGQVLSPHR
QDFT
ALIGQAGEQVNGIYEADYRDFNRETYARGRATFDATYAAFKMLLYGIWRREETARTAAAE
>MCE8455036.1 ParB N-terminal domain-containing protein [Rhodovulum sulfidophilum]AB15
MARRKRLSPITGPAEDTGPVPPGPIPAIARPPVAEIAAASASQAALEQLADEVAVARREGRMVMSLPLEVIEAGHLLRD
R
MHHDAEDMAALKASLAARGQQTPIEVTDLGQGRFGLISGARRLAALSELAAETGEPRFGKVLCLLRSPEGAAEAYLAM
VE
ENEIRTDLSFYERGRLVAEAVRMGLFADPAAAVRGLFGNAPPARRSKILSFVALHRALGTVLRFPTAIPEKLGLAIVAGL
KADPKLADRLGAALAKAKPADAEAERRLIERALRKAPAAAAPRGREVAPGIRLEARKGRLVLSGAGVGPELAEALEAFLA
ARDPLE
>MCE8455037.1 glycosyltransferase family 2 protein [Rhodovulum sulfidophilum]AB15
MSVGEDLARIRINTGISVIVPTYREAENIPHLLVELDRLRREYDVTLEVLFMDDDSRDGSVEAVAASGCDWARIIVRTGD
RGLSRAVIDGFRAARHPVLICMDCDLSHPVDAIPQMILGLASGQQFAIGSRYVPGGSTDDDWGLFRWLNSVVATLLAR
PL
TKVRDPMSGFFALRKADFDAAQGLNPVGYKIALELIVKCRMENVGEVPIEFSDRRFGESKLTLKEQLKYIQHLRRLYLFR
FANAMYLLQFLAVGATGAVVNLAVLSGLQLAGLAAWICLAGGIGVSLVTNFLLNRRFTFSYARRGHAGRQFLGFLGASL
V
GLTVNYAVALGLSHKVLTGAYGLQVSAMIGIAAGMVFNFLGNRYIVFRKRYIRD
>MCE8455038.1 sulfotransferase domain-containing protein [Rhodovulum sulfidophilum]AB15
MRPDFLLIGSMKCGTSTVSAYLEDHPEVFMLPGQEPNFFSHDKVHDRGAEWYERLFDGAAGARRIGEGSNDYASGLM
YPD
TAARIAAYSPEMRLIYMVRDPVARLASAWVQNRVDQGDQVPPTLERAVREMPDLYLGQSLYWHNLQLYRTHFPDDRI
FIG
FMEDLKTDRFAFFARLCGFLGIEPMNEIPRGHINPSRGKAVPGQLYSALNALPGIGRLKALAPKGLRSTVKTRLLSRRIG
ETPEIPAALRRELTERLGPDTRAFLAHCGRPTDFWGL
>MCE8455039.1 glycosyltransferase family 2 protein [Rhodovulum sulfidophilum]AB15
MSDPSRAYLLISPCRDEAAHMRRTLDSVLAQSLRPAKWVIVDDGSTDETPQILADYAAAHPWIEIVAKPDRGRRAVGP
GV
IEAFYAGLDRVEPGAFPYLCKLDLDLDLPPRYFETLIERMEAEPRIGTCSGKPYIRRGGALVSERRGDEMSVGMTKFYRS
ACFRQIGGFVRAVMWDAIDCHKARELGWIAVSWDHPDLNFEHLRPMGSSQSSIYAGRRRHGFGQYYMGSDPLYFAA
TCVF
RGLEPPYLLGGLAMMQGYLGAWLGGAPRHPDAELVAFIRAYQRRALRVGRARAVAGIEAERAAIFDPDRAV
>MCE8455040.1 sulfotransferase domain-containing protein [Rhodovulum sulfidophilum]AB15

MTLPDFFIIGAAKAGTTSIFALLEQHPDIFMPTPKEPEFFARDDLYSAGIETYSALFAGARPGQRLGEASTLYSHAPLFP
DTAARIARHAPGAQFIYVLREPVARAYSFYVQLVKNYQNGTRDYAVHRCFEDFVLPERHALAAPREKALAPFNAHFPDT
P
ELCLAGSDYLLQIETYLACFPRDRFLFLTFEAFRDDPRATLRRITGFLGLDPLPEAAFGGGAAARNVSRAHFDQLEAAAG
AQRLRGRVGPLWALRRLLPDPLRAALRRRVLPAGAGRAHEPPPMEPATRQLLAERFHPQIPRLEQLTGLSLASWQAER
LC
>MCE8455041.1 glycosyltransferase family 4 protein [Rhodovulum sulfidophilum]AB15
MTTTDRPRVLVLAESCNPEWPSLPVVGYKYARALAGVADVTLATHIRNRENIEAAGDMGCPVVYIDNEWIAARMYRL
ARL
LRGGSEVAWSTNQIMAYPPYIAFEWQALRRFRAELDAGHFDIVHRITPMSPTQPSYIAGRIRQPFVLGPLNGNLDWPA
AF
RGEQKRERERLRGLRDLYRFLPFARSTFTGAAAILAAFPHTIADLPQRLSDRIVNFPEIGFDPDMFHSRGRRPVLAGEGP
RRFLFAGRLVPYKVPEVAVRAFIGSERLKPHLLHVVGDGPERPRLEAMVAEAGAGDRVIFEGRRSQAEVAQSMRQSDV
FV
FPSIRELGAGVVIEAMASGMVSLVTDYGAPGHLAAEGRGTRVALQPLEGLVRDYRAAMEACLEAPEAHAEMARRAEA
HAV
AHYTWAAKAGHTAEIYAALQAGRGLQGLHPYG
>MCE8455042.1 phosphomannomutase, partial [Rhodovulum sulfidophilum]AB15
MAPKFGTSGLRGLVSELTPDLVGAYVRAFLVACPAGPRVLVGRDLRASSPRIAEDAIAAIRAAWREAVDCGALPTPALAL
AAMGQGAAAIMVTGSHIPADRNGLKFYLPSGEISKADEAAIGQALERPMATPAPAAGQEGARSFAPEAGAAYVARYV
QAF
GDAALAGLRVGVYQHSSVARETLVEILGALGAEPVPLAASDSFVPVDTEAVDAGTRAMLAEWCAGQGLDALVSTDGD
ADR
PMLTDAAGRVVPGDILGAITARALGAGAVCTPVSSNSLVTRLPEFETVRLTRIGSPFVIAAMEEILAADPAARVAGFEAN
GGFLLGFAARGPAGPLAPLMTRDAMLPLVTVLA
>MCE8455043.1 2Fe-2S iron-sulfur cluster-binding protein, partial [Rhodovulum sulfidophilum]AB15
MKDFIIPDADFGTPARRSDKTVTLTVDGMEVTVPEGTSVMRAAAEAGIEIPKLCATDSLEPIGSCRLCMVEIEGRRGTPA
SCTTPAAEGMQVQTQTPRLAKLRRGVMELYISDHPLDCLTCAANGDCQLQDMAGAVGLREVRYTQGANHFEPRQN
GEANP
RYEARDSSNPYFTYDPSKCIACFRCVRACEEVQGTFALTVEGMGFEARIASGTTDFLNSDCVSCGACVQACPTATLQEN
A
VIEMGTPDRAIVTTCAYCGVGCSFRAEMKGDQLVRMVPWKDGKANRGHSCVKGRFAFGYAAHPDRIQSPMIRETID
QPWR
EVSWEEALAFTARRLRAIQAESGTGAIGGITSSRCTNEETFLVQKLIRQVFGNNNVDTCARVCHSPTGYGLKTTFGTSAG
TQDFDSVEAADVVLVIGANPTDGHPVFASRLKKRR
>MCE8455044.1 formate dehydrogenase [Rhodovulum sulfidophilum]AB15
MSLTLYLPLDAAALALGADEVARALQTEAEARGLDLMLKRTGTRGMVWLEPLLEIEENGLRMGFGPLEPADVPALFEG
AF
EDHPKALGPVEQIPFFARQTRLAFARCGIAEPLDLEAYRAQGGLKGLDRAMTMTPEEIVAEITTSGLRGRGGAGFPTGIK
WKTVLEAGGARKAVVCNADEGDSGTFADRMLIEGDPFCLIEGMAICARAVGADRGYVYMRSEYPQAIATLDASLRIAR
EA
GILGEHFDIEDRVGAGSYVCGEETALLNSMEGKRGTVRPKPPLPAHKGFMGRPTVVNNLITLASVPAILSEGAEHYRSFG
LGRSLGTVPLQIAGNVRRGGLFEAAFGMTIGELVEGVCGGTASGRPVKAVQIGGPLGAYFPRSLFDTPLGYEECDEAGG
L
IGHAGLVVFDDTAEMGAMARFAMEFCAVESCGTCTPCRIGAVRGMETIDRILAGDPAAPAILTDLCETMRAGSLCALG
GF
TPYPVESALRHFPEDFAPAREAAE
>MCE8455045.1 formate dehydrogenase subunit gamma [Rhodovulum sulfidophilum]AB15

MTTDIIARCRSIAAAHAHLDGPLLPVLHAVQDHIGHVPMAAYPALAEELGLSTAEIHGVVSFYPDFRTEPGGRRVLRVCR
AEACQAMGAAALGEDLKARLGVEWHGTTEDGAVTLEPAYCLGLCACAPAALVDDRPVGRVTAERLERLAREGSA
>MCE8455046.1 LysR family transcriptional regulator [Rhodovulum sulfidophilum]AB15
MIDKLEMFIVLARERHFGRAAEACGVTQPTLSAAIKQLEEQLDVLLVWRGSRFGGLTPEGARVLERARVIVADARALRE
E
MRAVKRGLSGDLRLAAVPTALAAITALTGPLTLRHPNLRITIFSRNSTEIVQMLDDLQVDAGLTYLENEPLGRVATVPLY
AEHYTLVMRADHPLAGRAAVPWAEAAAEPLCLLSPDMQNRRIVDRHMAEAGAVASARLESNSLLALIAHVQASGWV
TILP
LRTAQPFLGAGSLVAVPLTEPEAEHVVGLIAPHREPMTPVLDALMEAARQAKFQ
>MCE8455047.1 type I secretion system permease/ATPase [Rhodovulum sulfidophilum]AB15
MIRRGPKKELTLGRARSRGLLLAVLVFSVFTNLLMLTGPIFMLQVYDRVLGSRSEETLVALFILVAALYLFYWLLDYARG
RVMARIGLRLQAALARRVFSAMVERAALRGGQGPGATAIGDLDAIRNLFAAPVLLALFDMPWTPVFLAAIFLFHPLLGF
A
AIGGGAVLILATLVNRWVTGGALERSQSRSSRAARLARQAEEGGGLIWSQGMASVMSERWARMQDEANAEGMSA
NDRSGS
FASFSRAFRFFLQSAILALGAYLVLQHAVTPGAMIASSILLGRALAPIEQVLGQWALIQRARSGWRALTEFLQAVPERIR
STRLPDPVAHLSVTNIGLRASAPGAPPVLQGISFEITPGEALGVIGKSGVGKTTLARIIQGLLAPSAGEVRLDGATLDQY
GPERLGQLMGCLPQEVRFFDGTVAENIAHMELNPDPARVVQAATLARVHDIILRLPQGYDTMLIEGSGLLSGGQRQRL
AL
ARALYHDPVLLVLDEPNSALDADGSEALNAVVTDMKRAGKSVLIMTHRPTAIAVCDRLMVIDGGQQKALGPRDEVLRS
VV
RNAPEVQRAMGAVRSTAVPKREARG
>MCE8455048.1 HlyD family type I secretion periplasmic adaptor subunit [Rhodovulum
sulfidophilum]AB15
MTARSPESARLPLAVGLVAILCLVGGIGGWSVATSIAGAVVAPGTIKVETERQVVQHADGGVVGEILAKDGDAVAAGQ
VL
VRLDGTFLRSEQMIVERQLLEIQARKARLVAERDGLDSPDFAVLDSFDNIDPAWVLDHVEGQTRLFAARKTALAQELDQ
L
HEQTVQIDNQIEGTRAQQDSLQKQLGLIEKELADKMTLYRQSLVPASQVLALQREEAGLEGNIGRLASQEAELRAKVSE
L
VVRGLRLRESRREDAITTLRDLQYSEIELQERRLSLAERLSRLDIRSPVDGVVFGSKVFTVGSVVQAAEPMMYVVPGGQA
LLVSARVETTDIDEVFPGQPVSLKFTTFDSRTTPNVSGQVLRVSADALTDEATRATFYEVVIQPDIDEMAELPDIELVPG
MPVESFIRTRDRSPLSYLLHPLAVYFERAFRET
>MCE8455049.1 HpcH/HpaI aldolase/citrate lyase family protein [Rhodovulum sulfidophilum]AB15
MENPVNRFKAGLRDGRHQLGVWNAIPDATVAECLAACGFDWIVIDGEHGAMEVTDALPALQAVAGYPAASAVVRP
AINDW
VLIKRHLDQGAQTLMLPYVQSRDEAEAAVRAMRYPPRGLRGMAGMTRASRYGLVADYLRRAEEELCLILQIETVAALE
RL
EEIATVEGVDGIFIGPADLSASMGYPGQPEHPAVIAAIEGAIGRLKAVGVPSGILALDPDFARHCIALGTGFTAVGVDMV
TLQRGMTALVETFASG
>MCE8455050.1 RNA polymerase factor sigma-54 [Rhodovulum sulfidophilum]AB15
MMVSQRQTQGLAMTAKMQAALRILQMTAVDLSAYLAEEALENPCLEVSVPQALRAVPTGVAGSGAGSDFDAVAAL
ASDKP
SLYDHVLRQIALAFPDPRLARVAETFAEALEPTGWLGSPPEAVAAAAGVPISVAEAVLARLQQFEPAGLFARGLADCLRL
QAEDRDMLTWELSVILDNLGMVAEGRIDALAELCDGTPQDVRNALRAIRGLDPKPGLAFAATEPPILPPNLRVIRKDGT
W
AVELNRSALPAVKVTEAPETGRDTAARTYATRARSRAQWLARAVERRHSTLLAAAVCLVRRQGDYLDRGPRHLHPLGT
ED

VAEELGLHASTVSRAVQHRMIETPRGTVPLRTFFSRAFTPGSGEDGPSQDALIALVGDIVAKENPARPLSDAAIAAEAKA
AGFALARRTVAKYRDVLGIPSSYDRRQNARISA
>MCE8455051.1 ferredoxin family protein [Rhodovulum sulfidophilum]AB15
MNDKTNGTKSPRMEERLYQNRYQVDEGRPHVKINHQPGGDSEELKALTKICPAGCYSINEEGQVEVAADGCMECGTC
RIV
CQATGELEWSYPRGGYGVLFKFG
>MCE8455052.1 FAD-dependent oxidoreductase [Rhodovulum sulfidophilum]AB15
MPAEHFDAIVVGAGPSGNAAAYTMGKAGMNVLQIDRGEYPGSKNVQGAILYADALERIIPDFRETAPLERHVVEQRF
WML
SENSHTGTHHRSDTFNEEKPNRYTILRAQFDKWFSKQVREAGVTLITETTVTELVKNEAGKVIGVLTDREGGPVYADVV
V
LGEGVNGLVGQRSGFRPELKPQHVALAVKETHFLPEETIRERFNLADEEEGVVIEVLGSVSSGMVGTGFLYTNKESISIG
VGCLVADFAREKIPPYELLDRFKNHPSVKPLLKGAEMKEYVAHLIPEGGYDAIPRLTGDGWLIVGDAGQFLNAVHREGS
N
LAMTSGRLAGESLAALKAQGLPASEENLKLYRDALEESFVIKDLRKYRKIPRLLEKNRQLFTTYPAILNRSMNAFLRVDG
RDKRAKEAEILKGLKTSRGNWLNVAVDMLKVARAWR
>MCE8455053.1 electron transfer flavoprotein subunit alpha/FixB family protein [Rhodovulum sulfidophilum]AB15
MAQAPQPPAGSRAAMQMQLAEKYKDYKHVWVFIEIERGKVHPVSWELMGEGRKLADDLGVDLCALVLGGRDEETK
QAIRQ
TYHYGADVCYICEDPLLADYRNEPFTQAMTDLVNKYQPEILLLGATPLGRDLAGSVATTLQTGLTADATELKIADDKSLA
ATRPTFGGTLLCTIHTLNHRPQMATVRPRVMAMPEPDTSRSGRMVWHSLRMQEDDIVTKVLEFVSDAQVEKANLAY
ADIV
VAGGLGLQQPQNLALVQRLAEVLGAEWGVSRPLVQKGWAPAERQIGQTGNTIRPKLYIAAGISGAIQHRVGVEGADLI
IA
INTDPEAKIFEFAHLGVVADALTVLPALTDAFARRLSARAMAS
>MCE8455054.1 electron transfer flavoprotein subunit beta/FixA family protein [Rhodovulum sulfidophilum]AB15
MSEKLTEPDFPAAEAIDAPEAPVETKKGLNIVVCIKQVPDSAQIRVHPVTNTIMRQGVPTIINPYDLFALEEAMRLRDDY
GGTVTVLTMGPPMAEDALRRALGLGADKAVLITDRRFAGSDTLATSFALSAALAKLAEDGPIDIVFTGKQTIDGDTAQV
G
PGIARRMGLNQLTYVSAIDSLESDSTTITVRRRSEGGVQVLQSRLPCLITMLEDTNLLRRGSLQDMLRAARAPLTIWSAE
DAGIEDPMKCGLRGSPTVVRRVFAPQPRAEKAEMVAVEGKAPAQIGDAILDVLFAKSPKLEADLSAKTPA
>MCE8455055.1 nitrogenase-stabilizing/protective protein NifW [Rhodovulum sulfidophilum]AB15
MTRPAPRPSMLDQLYALSSAEDLFAFLDLPFDQAVLNRARLHVMKRMGQYLDETDLSQLDDDGVRAAARDALQKAH
ADFE
NSTPRAQKALKIYTQPRGNVVPMEGLRLSVK
>MCE8455056.1 homocitrate synthase [Rhodovulum sulfidophilum]AB15
MTAPYVPDDFTPAPPPARICDTTLRDGEQAAGVAFSAAEKIEIATALDLAGVAEIELGVPAMGMAEVAAIREAGAVLTR
A
RGVAWCRLRDSDLELAAMTALSRLHLTVPASDRQLAGKLGRDRDWALAELARLVRLAAQAGFEVSVGAEDASRANIG
FLA
QLAQVAAEAGAIRLRLADTLGLMDPFAAHTLVSILATGPLPLEFHAHDDLGLATANTLAAHRGGAGFLSVTVNGLGERA
G
NAALEQVAAALAVQGRPTDVALDRLCGLSQIVARASNRPLSPTRPIVGANVFSHESGIHVDGLLKDPGTYEAQALSPAR
F
GRAREIVLGKHSGLAAVTRALSAAGLPSDEASARALLPVLRDWADREKRAATAADLILLTARAEALARHPGPTEPQERSL
T

>MCE8455057.1 iron-sulfur cluster assembly scaffold protein [Rhodovulum sulfidophilum]AB15
MLDYSEILTDHFLNPRNRGTLDSANALGEAGSVRSGNAFRLTLKIEAGQIAAAMFQSTGGGPEIAAGSALTELVIGRSLV
EARKFDAKAIEAALGGLPAEDRSAAEVAAEGLAAALKDYAQPPAFRAAQGLRVAQPLGVAPIRPALPSKTGAPRLSAEE
E
RARVEAVLEEMRPRFRADGGDVAFHDILGPRVRVHLKGSCSGCQLAGLTLGGLQKRLAEELGRPVFVVPAPSR
>MCE8455058.1 iron-sulfur cluster assembly accessory protein [Rhodovulum sulfidophilum]AB15
MIQITEAARDAMASAIQGAGRPIAGLRLMVQSGGCAGLQYAMALELTREATDAVVETEGGVTVLIDPESQGYLLGTTI
DF
VTGLEGSGFVFDNPNAKGGCGCGKSFC
>MCE8455059.1 SseB family protein [Rhodovulum sulfidophilum]AB15
MTAETPPAAPQPVSPLDRAHAAMTAAPEDPALRLVYYGRLAATELVLLLDREAEDDDRIAPSVFELEEGRVVLAFADEG
R
LTDFTAAPSPYAAMPGRRLAEMLTAEGLGLGIDLGGETAELLPPEAVAWWAEMLAATPETLDLVPEALIPPGDLPAPLL
A
ALDTCLAGMGGLAARAWLAGLRHPDGARSLLLAFLDPAEGAEPALARAVSEAVRFSGLETAALDLAFFPASGPMATHL
AR
LGLRIDLPAPEPMTETPLIDPDAPPRLR
>MCE8455060.1 uracil-DNA glycosylase family protein [Rhodovulum sulfidophilum]AB15
MTVTPDLLRSRIAACRLCAPRFAATATGHAPRPVPWFGPAPRILVCGQAPGLRVHKAGRPFFDPSGDRLRSWMGIDSE
TF
YDLSRVSVLPMAFCFPGYDDAGADLPPPALCAETWHRAVLAALGPVPLTLLIGGYAQRWHLGAAARRGVTATVRGW
RAHA
PQVFPLPHPSWRNTAWLKRNPWFEADLLPVLRQRVAEVLA
>MCE8455061.1 Na+/H+ antiporter NhaA [Rhodovulum sulfidophilum]AB15
MYRVWNFVTAYSLLLIGGAVTALIWVNLHPNSYHDFVEYVLIDDFFIGHAHEVGGHVSRTLTVHYLVNDLLMAFFFAIA
A
KEVWEAIILRNGSLRGRKAATPLFATLGGMLGPIAVYLGLAAILGSSTFDAVHRGWAIPTATDIAFSYLVGRIVFGAGHP
AVRFLLLLAIADDAAGLIILAIFYPSGDLQPLWLLLSVGAALATFLLFNWLPRRMDRGRQMRPCSSWVRRYLGFWPYAL
A
GAASWFGFQEAGLHPALGLLPVVPAIPHADRAFGIFSEAEQYLTDLLNHIEHNLKVPVEIVLFFFGLCNAGVEFSSIGVP
TWLVLAGLLIGKPVGVLLFGWLAARPLRLGLPQGMRTADLLVVGCVAAIGFTVSLFIASVAFNSGVTLDGIDVQEAAKM
G
ALFSFVAALIAFGAGKLMRVRKQS
>MCE8455062.1 HNH endonuclease [Rhodovulum sulfidophilum]AB15
MGAAALRARNPCVLPGSGFPGRMVKLVLLHKADSIYEDEPDVVYDFPRSYLKAVEQAVDDWIVYYEPVKAGPRGYFAV
AK
ISRVIPKPCAEGRFLAMIEPGSFLPFDREVPRLLDGRPLEAALTEPDGTPKKGGAVQLAVRRLPEADFARIVNFGLPQDL
ERIEATRYDQPPAELAEGAELFERPVLERLIRRPYRDVVFRRKVRAAYGYRCAMSGLMLRNGGGRPEVQAAHIRPVESQ
G
NDTVRNGLALSGTLHWMFDRGLISVAEDCETILVSHNKVPGDVVGRLLAPDGKLVRPEDARDAPHPENLRWHRENVF
GRS
LPEGPAPSLPA
>MCE8455063.1 3-deoxy-8-phosphooctulonate synthase [Rhodovulum sulfidophilum]AB15
MKHIEIGGLPVGNTRPLTVIAGPCQLESLDHALMIGQAMKEACAAAGAGYIFKASFDKANRTSLSGRRGLGIDKGLEVL
A
ELRRQLDVPVLTDVHSPEQCAPAAEAVDVLQIPAFLCRQTDLLLAAGETGAAINVKKGQFLAPWDMGNAVAKIESTGN
RR

ILLTERGVSFGYNTLVTDMRSLPIMAGLGYPVVMDATHAVQQPGGQGGSSGGQREFAPVLARAAVSLGLAAVFIETHE
DP
DSAPSDGPNMIPLDQMPDLIATLMAFDRLAKAHPLSI
>MCE8455064.1 metallophosphoesterase [Rhodovulum sulfidophilum]AB15
MANWYSADLHFGHHRIIDFCKRPFASTAEMNAALIANFQACVAHDDDVWILGDFAFGRADDTAQFESWFHSLPGRK
HLII
GNHDDEAVISLPWASTEYMAEIQDGEHSLVLCHYPMITWNGARRGALQLFGHVHDQWAGSRNSINVGVDQWDFR
PIQIGE
IAKRAARMPVNKHWADVEHGNELP
>MCE8455065.1 AAA family ATPase [Rhodovulum sulfidophilum]AB15
MSAHFFVVTGGPGAGKTSLITELARRGFHTIPESGRAIIREEMQSGGDALPWADRAAYAERMLQRDLRSYEDAQAHSG
PV
IFDRGIPDILGYLTLCSLPVPPHVAAAAKAARYNRRVFLAPFWDEIFTQDTERKQSQTEAEATCAVMFETYIALGYEIIE
LPRTDIARRADFVCEQLAI
>MCE8455066.1 ATP-binding cassette domain-containing protein [Rhodovulum sulfidophilum]AB15
MKINGALSYVSIGSASLCINTLALAAPLFILQVYDRIIPNHSENTAALIGLGVLCALALETALKIARSKVLAPLAIDYEV
RASCGAARHLFHADLAAFERVSPGAHMERLAAVAQRRFNGTGQTLLAICDFPFLFVFMWMIWILGGPVIIAPALGAIA
LA
VAAAPANRSAREALARALSLDDLRYDFLLSTLSSLPSLKVANLERPVLRRYESLQKRRLAAQGEAFTADQRLTDLSQLVV
QLSIIGVIVLGSLLILQGRMSIGGLSASTLLAGRFLALIRNLILLASRLQTSSIGREQMDAIFKIPVRSASSGILDASQA
PPRLSLRGVVFDDNDGKRVLDGVDLDLREGHTVGLVGDNGSGKSILLALAAGLYSPTSGEVLLGGHPLSDYTDQSLRRTI
ALVSQQETLFAGSILENISMFAPERVDAALRAAERTGLTELIKPLPKGFATIVGDTAADGLPRGVVQRIALCRAMALDPQ
VLLFDDANSAIDDQGDEKVAELFADIRERCAILFVSHRPSVLGRADAIVRLANGRISTVEGTF
>MCE8455067.1 ATP-binding cassette domain-containing protein, partial [Rhodovulum
sulfidophilum]AB15
MAGTGTENRSGGANGRLARLEAEPAVHGQGQVWNAVDETLAVCLYDALRDLGWQGELRDLRLSFALDLSGDRVLDT
LNAA
ARLGFAGHVRRGRAFSDKDLARTALGPGGTTQGLLFLPLRGEPRFLRMRAADTRPSFRLSAGSLIRFERIETPASEIGKS
WLGQRLRAQLPLVLRALLLTFFANLAAVANPLFVMAVYDKVVVSESTALLAYLAAGVVAAAWMEMLMRRLRGRIMN
RASL
RLGYIVGNAVFGRILTLPSRTTERAGLSSQIARVRDVDRIRDLLRGPLEQALLDTPFTLLFVAAVAIVGGWLALIPLCFM
ILFAALAAIGERHMRGRGAEVAKASAQRQLVTLEMMHRMRAIRAAGGETVWLERYRDSAAKAARASERNTTSAQILA
AIG
QSLGKGAALATLFAGIFLVLADAMTTGGLIASMMLVWRLIAPIQNAFLAATRIGQLVASARQIDRLMETEAEIAPGEGD
G
ERPPIQGEIAFNRVTFRYGRETEPILGGVSFTAQPGEIVAIVGRNGSGKSTLLKLVAGLYEAQAGNIRLDGRDLRQFDPT
HLRRAISYVPQLPSFHYGTLRDNLLMAEPEAEEQDIAEALQLADAATAIAALPEGLDTFYDAQLVTLPVGLLSRLSLARM
YLRKAPVVLLDEPATGIDFMGEFAFIGGLELLRARGATVL
>MCE8455068.1 double-strand break repair helicase AddA, partial [Rhodovulum sulfidophilum]AB15
ILEDLMIRVETARPRRLALDAAERTLALHRFAEAFLPAYAARKQLQGWLDFDDLILKARDLLTDPGVAQWVLFRLDGGL
D
HILVDEAQDTSPVQWQVIELLAQEFTAGEGARSGVDRTIFVVGDKKQSIYSFQGADPEGFDRMRALFRDRLENVDRGL
LN
LTLEYSFRSSEAILGAVDRTFPEGARAGLDREMRHRAFHADLPGRVDIWPVVEDSEAADPGHWADPVDMLGASHHSV
RLA
RQVASEIRAMIDRGETIAVKGGGRRTIHEGDILILVQSRTDLFHEIIRACKAERLEVAGADRLKIGGELAVRDLTALLSF
LALPEDDLSLASALRSPLFGWSEDALFRLAAPRKGYLWQALRHADRAGTETVPMLQALLDEADFLRPYDLLERILTRHDG
RRRLVARLGQEAEDGIDALLTQALAYETTEVPSLTGFLAWLSSGEVEIKRQLDAAGRRIRVMTVHGAKGLEAPVVILPHS

AQRTVTIRDQIYTLEDGMPAWKTGSDARPEALAAAHAARVQREEEERSRLLYVAMTRAEQWLIVAAAGKLAKDGSD
WYSR
IRRGTEALGPVPRDFGFGEGLRYERGLWPEDRVLAAPETAPPDTALPDWALRDAPRPDRPPQPLSPSDLGGAKALPGD
PL
APAEAEDVRARGTWMHLLLEHLPGTDPADRPALARALLTAADPPAPEAAIPALAAEAEALMARPDLAPVFAPGSLAEV
PL
TAALGQGRLSGTVDRLIVSPTRITAVDFKTNAVVPDRPEDTPEGILRQLGAYAMALAQIYPGHEIDTAILWTRTGALMTI
PRELGHAALARAGLA
>MCE8455069.1 thioredoxin [Rhodovulum sulfidophilum]AB15
MPTHAVTDDTFDAEVRQSDIPVVVDFWAEWCGPCKQIGPALEELSAELEGKVKIVKINVDDNPTSPAQLGVRGIPALFL
F
KDGQVISSKIGAAPKAALQNWIQESV
>MCE8455070.1 ATP-dependent protease subunit HslV [Rhodovulum sulfidophilum]AB15
MSKSEFPGWHGTTIIGVRRGGQVVIAGDGQVSLGQTVIKGSARKVRRLSPGGYDVVAGFAGSTADAFTLLERLEAKLQ
AT
PGQLQRAAVELAKDWRTDKYLQKLEAMLIVSDGAELYVITGAGDVLAPEHDVAAIGSGGNYALAAARGLIENTELSAG
AI
ARKAMAIAADICVYTNGNLTVESITR
>MCE8455071.1 ATP-dependent protease ATPase subunit HslU [Rhodovulum sulfidophilum]AB15
MTDLTPREIVSELDRFIIGQAEAKRAVAVALRNRWRRKQLADDIREEVYPKNILMIGPTGVGKTEISRRLARLARAPFIK
VEATKFTEVGYVGRDVEQIVRDLVDTALVQTRDWMREDVKAAAYKAAEDRVIAAIAGADAREQTREMFRAKLKKGEL
DDT
LIELEIADTSNPLQGMEIPGMAPGQGQQGMMSLGDLFGKAFGGRTVKKRLTVADSYEILISEEADKLLDDETVNRTALE
A
VQENGIVFLDEIDKVCARSDMRGADVSREGVQRDLLPLIEGTTVSTKYGPVKTDHILFIASGAFHIAKPSDLLPELQGRL
PIRVELRALTEDDFVRILTETDNALTRQYTALMGTEEVAVEFTDDGIAALARVAAGVNESIENIGARRLYTVMERVFEEL
SFTAPDRAGDTVRVDADFVDQNLGELTKSADVTRYIL
>MCE8455072.1 Smr/MutS family protein [Rhodovulum sulfidophilum]AB15
MRRRRLSREDRALWDEVARRVRPMRDAPPDAVSGPPPEKTLPEKTTPAPVPAAPSPLKPFRLGAKAAAAVSPTPSAPP
TI
RMDRKAFLRMKGGKLDPEARIDLHGMTLAEAHPALIGFILRSQAEARRLVLVITGKGSGAEWDPAFADRRGVLRRQVP
HW
LHQAPLSGAVMQVAPAHRRHGGEGAFYVYLRRPR
>MCE8455073.1 MltA domain-containing protein [Rhodovulum sulfidophilum]AB15
MRGFGAAVCLALALGGGASVQAAPSVELLDFDDLKGWKTDNHAEALAAFRETCPDLPDPPWTALCALSERQSNARSF
FEL
FFRPVLIGGDSPALFTGYFEPVLKAARAPDARFRYPIYRLPPDLPTDRPWFSRREIAEDGHLDGQGLEIAWLEDPVDVFF
LQVQGSGRLSLPDGTLLRVGYGGKNGHDYRSIGRELVRRGVYQPHQVSARVIRSWVRRHPEAGAELLLHNPSFVFFREL
D
SLPPGKGPLGAMNRPVTPMRSLAVDPSLVPLGAPVWIEKGGRHPLHRLMVAQDTGSAIKGPQRADIFYGTGPGAGRL
AGA
VRDPGRMIVLLPIELALALLPGG
>MCE8455074.1 hypothetical protein LZ188_00435 [Rhodovulum sulfidophilum]AB15
MYDGMSCAKARKTLIDVNAEIQELSRRQSGAATADAIGVFPTLVPVSMVTGGDRAGRIAAAKGRKLALEARLESCG
>MCE8455075.1 EF-P lysine aminoacylase GenX [Rhodovulum sulfidophilum]AB15
MTDTPWWSPERHADRRPALLARGRIQAAIRGWFAEEDFVEVDPPALVTSPGNEAHLHAFATDAIGNDGVARRMYLH
TSPE

FAMKKLLAAGEPRIAAFCHVWRNRERGPRHSPEFTMLEWYRAGEDYAVLMEDTARMLQLACEAAGTRHLGHAGQH
CDPFA
PVERVSVAEAFAAHAGIDLLSTLAPGGAPDRAALAVQAEAAGIGFAADDGWSDIFSRVLVERVEPHLGRGRITVLDRYP
A
AEAALARTCADDPREAERFELYACGVELANGFGELTDPVEQRRRFEAEMDEKERLYGERYPLDEEFLAALAFMPPASGI
A
LGFDRLAMLATAAPRIEDVLWVPVA
>MCE8455076.1 elongation factor P [Rhodovulum sulfidophilum]AB15
MKVIASSLRKGNVVEKDGQLYVVLKAENFHPGKGTPTTSVDMRRISDGVKTTERWKTTEQVEKANVDEREYDFLYDDA
EG
FHFMNPESFDQVAAQPDVVGDAAAYLEPGMRVFLQTFEGNPISIELPQKVVVEVTETEPVVKGQTASSSYKPAMTNIG
VR
VMVPPHIDVGTRIVINTADNTYVERSKD
>MCE8455077.1 Tim44/TimA family putative adaptor protein [Rhodovulum sulfidophilum]AB15
MSSAIIQLLVLAGIAVFLILRLRSVLGTREGFEKPPVQMPDPAARRADNRPSFEVIEGGPDTDITDHVEDGSPAAKALAA
MKLAEPGFSVSEFLHGARGAYEMILMSFEHGTLDEVAGFLAPDVRDSFAEVIRLREEQGLSVDANFIGVRELVLKDATY
D
RDSGEAELTVRFVGELTSVVRNQDGEIVEGSPSEIKRQRDVWSFARVMGSDDPNWQLVATDA
>MCE8455078.1 FxsA family protein [Rhodovulum sulfidophilum]AB15
MWLFLLFLSIPLIEIALFIQVGGLIGLWPTLCIVLATAAAGTWLMRQQGALALSQVRSSFNELRDPTEPLAHGAMILFAG
AMLLTPGFFTDTMGLLLLIQPVRAAAYRWLSKRVVVHSAGFGPAGGPTRQARPARDDIIDGDFEDLTSRNGHGDEDG
TLP
PSGWTRH
>MCE8455079.1 protein-export chaperone SecB [Rhodovulum sulfidophilum]AB15
MSETEGNGAAAQQPQVRMQILGQYIRDMSFENILVQKGASGEVTPDVTVQVALDARKRPADHQYEIITKFTVSSKNK
ADQ
SQLFLMELDYAGLFHIENVPEDQLHPFLLIECPRMLFPYVRRIVSDVTRDGGFPALNLEPVDFVALYRQEIARRAADIQK
QETPSGTA
>MCE8455080.1 DNA polymerase III subunit epsilon [Rhodovulum sulfidophilum]AB15
MREIVLDTETTGFEPETGDRIVEIGAVVLWNHLPTGETFHVYTNPGRDMPDEAFGVHGIGPDILANPRPAKPGEVTLRD
K
PPFSQVGQKFLDFVQDSKLVIHNASFDMKFLNAELGWLGLPKLPMSQAIDTLAMARKKFPGSPATLDALCRRFGIDNT
MR
DKHGALLDSEILADVYLELIGGRQPDFMLATRSRASRGTSGGETEAWRPRPRPTPLPSRLTEEEAAAHRAFVETMGDDA
L
WKSFD
>MCE8455081.1 dephospho-CoA kinase [Rhodovulum sulfidophilum]AB15
MSRPRLLGLTGSIGMGKSTTAALFREKGVPVWDADAAVHALYAEGGGAVRPLAALCPEAVRHGAVDRDALKAWIARE
PEA
LMRIEAVVHPLVAADRQAFIDRHQNAPLLVLDIPLLFETGADKWLDAVLVVTAAPEVQRARVLDRPGMTEAQLDAILA
RQ
MPDAEKRARADHVIETLTPDHAGAEVAALIESLGTANA
>MCE8455082.1 shikimate dehydrogenase [Rhodovulum sulfidophilum]AB15
MSDRIPLAGVIGSPIAHSKSPLLHRHWLERYGLRGFYVPMDVAHADLKQVLEALPRMGFVGVNVTIPHKETILSLADSVS
DRAALIGSANTLIFRADGKVYADNTDGIGFIENLRQGAPGWDPTSGPAAVLGAGGAARAVVAALSEQGVREIRVSNRT
RS
RAEALRAEFGPKIVIHDWVQAGAMIDGAATVVNTTSLGMVGKAEFKVPLDALSPDAVVSDLVYVPLETPFLRAARARG
CV

AVDGLGMLLHQAAPGFERWFGRRPEVDEAARAVLLEP
>MCE8455083.1 Maf family protein [Rhodovulum sulfidophilum]AB15
MSDRVILASGSAIRRQLLTRAGLVFEAMPGRVDEDAIRAALEAEGAEPGDIAEELAAFKAKRVAEKSPGALVIGCDQVLA
HRGKVLAKAETREEARSQLRALRGGQHQLLSAVVVYDDMRPLWRHVGKVSLTMRPFSDRYLDEYLDRNWPAMRDA
VGAYK
LEEEGVRLFSAIEGDYFDVLGLPLLPLLTFLTTRGVLMA
>MCE8455084.1 CopD family protein [Rhodovulum sulfidophilum]AB15
MSEFLANMYPWTKSLHIIAVLTWMAGLFYLPRIFVYHTEKVTPGHETDALFQVMEMKLYRYIMNPSIIAVWIFGLALVL
T
PNVVYWDTIWPYTKGAAVIAMTWFHMWLGARRKDFVAGRNTRTGRTFRMMNELPTILMLVIVFSVVVKF
>MCE8455085.1 transcription termination factor Rho [Rhodovulum sulfidophilum]AB15
MTQECLNLADLKAQSPKALLSMAEELEIENASAMRKGEMMFSILKERAEDGWEISGDGVLEVLQDGFGFLRSPEANYL
PG
PDDIYVSPDMIRQHALRTGDTVEGVMQAPRENERYFAITKVTKINFQDPEQARHKINFDNLTPLYPDERLKMEIEDPTIR
DRSARIIDLVSPIGKGQRALIVAPPRTGKTVILQNIANSIETNHPETYLIVLLIDERPEEVTDMQRSVKGEVISSTFDEP
ATRHVAVAEMVIEKAKRLVEHKRDVVILLDSITRLGRAYNTVVPSSGKVLTGGVDANALQRPKRFFGAARNIEEGGSLTI
IATALIDTGSRMDEVIFEEFKGTGNSEIVLDRKVADKRVFPAMDILKSGTRKEELLVDKSDLQKTFVLRRILNPMGTTDA
IEFLISKLKQTKTNSEFFDSMNT
>MCE8455086.1 tRNA uridine-5-carboxymethylaminomethyl(34) synthesis GTPase MnmE [Rhodovulum sulfidophilum]AB15
MDTIFAQATARGKAGVAVIRISGPRAHSAARALAGSLPAPRQAGLRRLADADGVPLDEALVLCFAKGASFTGEDVVEF
QC
HGSPATVAALLSALGRIEGLRLAEPGEFTRRALENGVLDLAQVEGLGDLLEAETEAQRRQALRVLSGALGAKVDRWRDA
L
IRAAALLEATIDFADEDVPVDVTPEVTGLLDEVAASLRSEIDGVSVAERIRDGFEVAIVGPPNAGKSTLLNHLAGREAAI
TSEHAGTTRDVIEVRMDLDGLPVTFLDTAGLRESADEVETIGIARAVARAEAADLRVFLLDDTGKTQGLAPGPEDIVLRG
KADLADGAAHGVSGLTGEGVADLVAQISTTLEGRASRTVTATRERHAEAMRRAGGAIAEAQNQLSLGMETAELAAED
LRR
AVRALETLLGRVDVENLLDEIFSRFCLGK
>MCE8455087.1 tRNA uridine-5-carboxymethylaminomethyl(34) synthesis enzyme MnmG [Rhodovulum sulfidophilum]AB15
MFHVKHRAFDVIVVGGGHAGAEAAHAAARLGARTALVTLRRDGIGVMSCNPAIGGLGKGHLVREVDALDGVMGRA
ADRAG
IQFRLLNRRKGPAVQGPRAQADRKLYRQAVQTALSAEPGLEIVEGEAADFLQRDGRIAGVVLADGSELSAGAVVLTTGT
F
LRGVIHIGDRQSPGGRIGDRPSVRLAERIDEFGLPLGRLKTGTPPRLDGRTIDWDRLDRQPGDDEPVLFSFLSSHVEARQ
IACGITHTNEQTHDIIRKNLSRSAMYGGHIGGVGPRYCPSIEDKVVRFAEKESHQIFLEPEGLDDPTVYPNGISTSLPEE
VQEAYVHSIKGLENATILQPGYAIEYDYVDPRALRPTLELRDVPGLFLAGQINGTTGYEEAAAQGLVAGLNAARDVAGQ
G
NVIFSRTTSYIGVMIDDLITRGVSEPYRMFTSRAEFRLSLRADNADQRLTPLGLEIGCVGERRRTSFESKMERLSAGRAR
LEAMSVTPKQVAAAGIKVNADGTRRSGIDLLAFPDVNFEDLIPLDPELEEVDHESRVQLARDALYANYIARQTLDVEAM
R
RDEAQRIPSGFDYEKLEGLSAELKGKLSRVQPETLGQAARIDGMTPAAATLILARLRQMERQSA
>MCE8455088.1 16S rRNA (guanine(527)-N(7))-methyltransferase RsmG [Rhodovulum sulfidophilum]AB15
MIISAEEFSDVSRETSERLASYAALLRKWNPAINLVSRATLDELETRHFADSAQLFDLAPAEARHWVDLGSGGGFPGLVI
AIIVEEQAPGLRVTLIESDQRKATFLRTVIRELGLKRVEVIDARIEAADPQEADVLSARALAPLDRLLGFAERHLASGGV
ALFPKGARYADEVNRALASWRFEVQNHPSKTDPQAVVLKLGGIARV

>MCE8455089.1 AAA family ATPase [Rhodovulum sulfidophilum]AB15
MSDPTRPKGPKIIAVANQKGGVGKTTTAINLGAALAELGKTVLIVDIDPQGNASTGLGVAPEARVRTTYDLILDDAPLAD
IVVKSPKGNLYIAPSTTDLSSADIEMVANEKRSFLLHDALRQPAMDAYGFDFVLIDCPPALSLLTINAMVAAHSVLVPLQ
SEFFALEGLSQLMLTIRELRQTANSSLRIEGVVLTMADRRNNLSQAVENDARAYLGDLVFRTVIPRNVRVSEAPSYAMP
V
LDYDPNSRGAEAYRALAQEMLEPKVPVGA
>MCE8455090.1 ParB/RepB/Spo0J family partition protein [Rhodovulum sulfidophilum]AB15
MKQPEKRGLGRGLSALMADVRIEPEAERDETPRSKADQMIALDLLEPNPDQPRRLFSEEALKELSDSIREKGVIQPLIVR
PSKRSEGRYEIVAGERRWRASQLAQKHEVPVIVRELDDTEVLEIAIIENIQRADLNPLEEAMGYRQLMDRFGHTQDKVA
E
ALGKSRSHIANLLRLLQLPDEVQGYLREGRLSAGHARALITTTDPATMARQVIKGRLSVRETEKLAKSGSTTKKRRPPPP
VAKDADTRALEQDLAANLGMDVSIEHRTDGSGDLVVSYRSLDDLDRLCAVLSAPSGGGED
>MCE8455091.1 radical SAM family heme chaperone HemW [Rhodovulum sulfidophilum]AB15
MSAEDWQEGGFGLYVHWPFCLSKCPYCDFNSHVSAEIDTARWQEAYLSEIRRLGAEVGPRVLNTVFFGGGTPSLMPP
ELV
GAILDTARSVWTPANDIEVTLEANPTSVEAGRFTGFAEAGVNRVSLGIQALNDADLRRLGRTHSVAEARAAFDIARSRF
P
RVSFDLIYARQSQSLAAWRDELGEALAMAVDHLSLYQLTVEAGTAFGDRLARGGLKGLPDEDLGADLYELTQELCETAG
M
PAYEISNHARPGAESRHNLIYWQAGDYAGIGPGAHGRLTLGGRRTATDTPLPPMSWLERVERQGTGDLSRSTVAPAD
QGA
EYLMMGLRLVQGLSLRRYERIANSQIDGSKITELSDLGLIETEADHLRTTAAGRAVLNAVIRALLPD
>MCE8455092.1 RdgB/HAM1 family non-canonical purine NTP pyrophosphatase [Rhodovulum
sulfidophilum]AB15
MRRFDGKTLLVATHNSGKLEEIADLLKPFGISVTGAAEHDLPEPDETEDTFVGNARIKAHAAAQATGLPALADDSGLAV
D
ALDGAPGVYTADWAETGSGRDFPMAMEKVWTRLEEAKAPEPRTAQFCCTFVLAWPDGHDEVFEGRMPGRIVWPM
RGDQGH
GFDPIFQPDGHDITFGEMDRWEKNRISHRARAFEKLVSLFER
>MCE8455093.1 ribonuclease PH [Rhodovulum sulfidophilum]AB15
MRPSRRDVSELRPVSIETGVTKHAEGSCLIRMGDTHVLCTATLEERVPPFLKNSGLGWVTAEYGMLPRATTTRMRREA
AQ
GKQGGRTVEIQRLIGRSLRAGVDRVALGERQITIDCDVIQADGGTRCASITGGWVALRLAVNKLMKTGEVISDPLVDPV
A
AVSCGIYAGQPVLDLDYPEDSEAGVDGNFVMTGSGKLIEVQMSAEGSTYTRAQMNALLDLAEKGVAELVAAQKAAIG
A
>MCE8455094.1 heat-inducible transcriptional repressor HrcA [Rhodovulum sulfidophilum]AB15
MNDRSREVFRRVVEGYLESGDPVGSRTLTRSMSEKVSAATIRNVMQDLEFLGLLNSPHVSAGRVPTQLGLRMFVDGLL
EV
RDLKEEDREKIDATLGANTSDVGALLDRVGAALSGATHGASLVLAPKHEAPIRHIEFVSLGADRALTVLVFADGHVENRL
FHPAPGQTPSSMREAANFLNALAEGLTLSELREAMKVEIARRRQELDELARTMVEAGLAVWENEGHDQARLIVRGRA
NLL
SDPAEAEDLDRIRSLFDDLERKRDIVEFLELAEEGEGVRIFIGSENKLFSLSGSSLVVSPYMNADRKIIGAVGVIGPTRL
NYGRIVPIVDYTAQLVGKLIADRGKG
>MCE8455095.1 nucleotide exchange factor GrpE [Rhodovulum sulfidophilum]AB15
MAEPEKTEFDDVATETAAETETPEITPEEVEALKAERDELRDRFMRALADAENARKRADRDRREAEDYGGSKLARDML
PV

YDNLSRALEVVTDEQREANAALLEGVELTLRELLNVFAKHGIERVAPEAGDKFDPQLHQAMFEAPVPETRAGDIIQVMAV
GFMLHDRLLRPAQVGVSSTPAA
>MCE8455096.1 hypothetical protein LZ188_00545, partial [Rhodovulum sulfidophilum]AB15
MTLVKNGGGSVPDGGSTPDGGNPVETPNDGPVVVDPVEATPDQGAGNYSLVLGNVVTSAGYKTVDHSEGALSQGIRAQAQ
DVSLVDNIIAHLADPNNAEEQVEKVKG
>MCE8455097.1 acyltransferase [Rhodovulum sulfidophilum]AB15
MPIEALRVLAVLLLVAYHVIGAGPDAGLNIEYPHPLRLFAEFLVDARMPLFAFVAGFVYAFRPPVLADYGGFLTGKIRRL
IIPGWIAALLFSVAGMVVGNVYSLTVQGPVRVLFHHYAHYWFLQSIMVIFVVFGLVDALLRHRHTALLFLAAVAMTLAGI
TLRTSVLSIDGAFYLLPYFLFGTLCHRHADQLLRHRTWLAALALGAILAAGWWNIGLYLETGRLSGYRGDLQSLGFGLGI
ALAMLLLMPRIGWLDALRPFSFTIYLYHVLATSATRRALLALDVTDVWVLFGCGLSTGLLVPVLLHLVIDRIPALRQPVL
GIRRSRRPKPAAARSAADTQSS
>MCE8455098.1 glycosyltransferase [Rhodovulum sulfidophilum]AB15
MTPTAIAYLVPEFPGQTHAFFWREIQALEEAGLRVRTLSTRRPAPGACPHDFAAAAAARTVYLFPPPLASVARLAGRPVR
TLRALAYVLGLSETPWRRRLALLGLIASASALGRICRRERLGHVHIHSCATAAHLGALAAILDDLSYSLTLHGDLPVYGT
DHAAKMARARFVAAVTRPLAAQIAAVSPGTPAPVIWMGVDADRFCPPASARPGGEALEVVTVARLNHTKGHVHLLNAMAR
LRAEGVAIRYRMAGDGPERAALEAHVARLGLQDEVRFLGPLSEAGVIDLLHSADVLALTSFGAGEAAPVTVMEAMATGLP
TVVSAIGGTPDMIEDGVDGLLVPQRDEEAIAAALRRLAADPALRARIGQAARKTALARFDYRAQARALLEALTSDAVA
>MCE8455099.1 rhomboid family intramembrane serine protease [Rhodovulum sulfidophilum]AB15
MAVLRRGRAVLIGLIALCTLLELTLSLSDAGLLAPRLRTLAYDYGGFWPGLLGNWKPNYPAQPYAMFVTYSILHGGPAHL
AVNMITLWSLGSAVIDRVGAWRFGLLYGASILGGAAGFALLAPDLRPMVGASGALFGLAGGLIAWASLDRVTYRETLWPV
IRAVLLLLGLNVVLWWAMHGMLAWQTHLGGFLVGALCALALDRRALGPEAF
>MCE8455100.1 hypothetical protein LZ188_00575 [Rhodovulum sulfidophilum]AB15
MRRRRTYRLTGGLAAAGLVLLGIPPAAAQEQGGLETQLRFSQRLTQEDGTLSTTTGLGVTLSSRTRTEAFLFSVDSGLEQ
DFDGSALQQKDPRLILSYSRESLSSALAADFSYRQSDVDALSEDDDISDGVLRLDEGLLKRYSGNLSLQLGRDAPFGATV
DLGYSAKTYSETSDPDLLDEESVNATVALRFDLDPRISAGLRSSFSETDRDDPGQDTDSASIGGTLDFGVTKTLSTNAYL
GYSRVTKSTPGFPEDVQKGLAYTLSFTELRPNGDLSGSLGSDIDEAGRRTTARLDRSLELPSGALRAGVGLSTSEDDDLR
PLLSFSYDRGLKRGTFGVKLDQAFSTDSDGEEALNSDFRVTYRQELTPLSSLQAGVVLRDTDFVDDANTDSRKLQLDLVY
RHALAEDWNLVGGYSHSISDKSDEDRDHSSKVFIGLEKATRWRF
>MCE8455101.1 polysaccharide export protein [Rhodovulum sulfidophilum]AB15
MLYFKRILSGIVLILAATAAMAQQEYRVRPGDTLAIEVLEDTTLNRTAVVLPDGRFSFPFAGTIPAAGRTVGQIQNAVTQ
AIASNFAVSPNVFVSVQPGLASPYEGTMTDAIDVFFMGEVNTPGLVQVATGTTFLQALAQSGGLTRFAATKRIQLRRTDP
RTNVQKVYQIDYKALSRGAVLAKDIRLLDGDVILVPERRLFE
>MCE8455102.1 tetratricopeptide repeat protein [Rhodovulum sulfidophilum]AB15
MPAPIRLVATAALLSASLMLSACQSSEEKAEAHYQNALTLLQEGDVDRAIVELRNVFHEDGRHRDARQKLAELLLQKGNR
QQAYSQYLRLAEQYPDDLEVRIALAEIAFSSGRWDEVDRHGKAAEKLAPDDPRVKAISIARAYRDAAASDDASARREAAA
RAEALLAEQPDNALLHVTLLDNAVRDQDTTRALAETDWLIAHDPSDIRPYQQRLQILVSLGDMKAAEALLREMVAKFPKD
KENKAALIRFYLAQKNLDGAEAFLRELAAASAPDNPGPTIDLIRFLAELRGPEAARAAIDEAITERPDPLPFQVIGAGLD

FEAGRRDQAISTLETILNGAEPSEQTRNIKVALARMLLATGNEVGARQRVEEVLAEDAAQPEALKMQAAWQIQSDETDAA
IAGLRTALDHAPNDSQAMTLMAEAYIRAGRPELANDYLSLAVDASGNAPAETLRYARKLIDDDRLRPAEDLLLAALRRAP
DNSDLLNTLGRLYLQMQDYGRARHVADTLRRLGDEASVAAADRIEAERLNLQSGTAEALSYLEEIAGTADASLSAKIALI
RARLATGDVDAALAQAQELRQDHPDDDTVAVVLAATYGAQGALDRAAEIYQGLLRSDPNRPGIWLELSNLQMRQGDRAAA
AASIGKGLEQTPDDPRLLWAKASMLERDGDIDGAIGIYESLYARNSSSLVVANNLASLLATYRDDAESLDRAWTVARRFR
DTKVPAMQDTYGWIALRRGESAEAVTYLEAAAKGLPEDPLVHYHLGKAYMALSRQADALEQFRKAVEIAGPADRRPQIDE
ARAQIEALATAAPAEN
>MCE8455103.1 sugar transferase [Rhodovulum sulfidophilum]AB15
MTLYLTDLNADASARPVLGAFSVRSAAAGWGAYRKVFKRVVDLTLVLLSAPFSVPLIALLALLVALDGHNPFYTQLRVGR
NGRYFRIWKLRSMVPDADRILEAYLASNPAARREWNATQKLRHDPRITRIGRVLRKLSLDELPQLFNVANGTMSLVGPRP
MMVKQQLFYPGQAYYRLRPGITGLWQVSDRNDCDFVGRVDYDEVYDRDMSLRTDLDVLFKTVSVVVRATGC
>MCE8455104.1 AAA family ATPase [Rhodovulum sulfidophilum]AB15
MNTSLYNAHFGFHERPFSLSPDPDFLFWSKAHTRAFAVLEFGLVSRAPLTVVTGEVGTGKTTLIQALLRQVEDDATIGLI
SNAQGGRGDLLRWVLNALDVEAPEGADYVSLFQTFQSFVLQEYAAGRHVILIVDEAQNLGSETLEELRMLTNINSGKDDL
LQIVLVGQPELRDLIRHPSLRQFAQRVSAVYHLEPMDLPTTRAYILHRLKHAGGSGEEFSASAIRHIHEEAGGIPRMVNK
LCDLALVYASSADAPAVDEATIRELIDDGLILKPFRDPLLLSGAIDPFGKAAE
>MCE8455105.1 chain length-determining protein [Rhodovulum sulfidophilum]AB15
MTIDLPLYWRLFLRRLPVMLIFIICGTAAGVVAALRMPETYSTSARLLVEAPQIPDSMVSSMVQTDAVEQLDIIEQRLMT
RANMIDIANRFKVFPNIRQMEPDEVVAAMRSGTRIRRSAGRNQATLMTISFSSEYPQVAANVVNEYVTLVLQTNAEFRKS
RAENTLQFFEQEVERLGQELDRQSSAIASFKSQNAEALPDDQSYRMGRQNLLQERLGGLMRDLKAGQSQRADVVRIFEAT
GRVKQGAFAERRTPEEERLNAARAELDNAKAIYSETSPRVISLQAVVDRLEQVVTEQKEDGTAAAEAGDVSPEQAMLDAT
LSEMDSRLTTLQEEIDTTNSEIDKLQGAIAKSAANGIQLASLERDYEIIQTRYNAAVSNLNQARMSERIETTSKGQRISV
IENASVPQLPSGPGRTKVMALGAAAGLGLAGAYFVLLELLNRTIRSPAELSNRFDITPITTIPYMKPPKERGSRLRRRNR
KEPPPSGSIMPG
>MCE8455106.1 CpsD/CapB family tyrosine-protein kinase [Rhodovulum sulfidophilum]AB15
MEKLQAALEKARQTRAVRSSTVPTRQQLRVTPASDADGAWQALAPADLDEAALSQHLIVTREAGVEATPFDILRTKALLQ
MRQNGWKRLAITSPVPNSGKTTVACNLALALGRQRDLRAMLFDLDLRDPSVHDFLNVTPEHGIGEMLSGRVPFAAQGLRH
GENVAFSISLHPEPDPTRLLLAEETAERLDEIEAVYKPDVMIFDLPSVLVNDDTRAFLKNVDCALIVIRAETTRHAQFDT
CEREIAEHTNVLGVVLNAYRQADAKG
>MCE8455107.1 glycosyltransferase family 4 protein [Rhodovulum sulfidophilum]AB15
MVATYARLLKARGHEVTVVSRPWPRRTLLNRLRRALRGQPIRPHPSSPLFEALGDRHRVLDRRRPVAADVPDGDAVVAT
WWETAEWVAALPVSKGRKFYLLQDVELFDHLPVDRVRATYALPLHKLAVSGYIRDRVRKEQPGAEIGLLPNAVDHALFTA
PPRHRGRPFTVGFLYSSAPRKRAELAFATVEAARVRLPDLQVLAFGSKGDPSEIPPGIVYRRAPPQQEIPKLYAACDLWL

FTSAHEGFGLPLLEAMACRTPVLATRAGAAPDLIDGRNGRLLPPDPEAFSAEIETISRMDPAEWRAMSDAAHATAAAHSW
EAATDRLLGYLGEEARS
>MCE8455108.1 hypothetical protein LZ188_00615 [Rhodovulum sulfidophilum]AB15
MADLFKLHTQGLDSPPSRLTEVTPDDSAELPFASRALNVASSGTVRVTTVDGDTGTVFVASGIALPLRVRRVWATGTSAT
GIVALF
>MCE8455109.1 hypothetical protein LZ188_00620 [Rhodovulum sulfidophilum]AB15
MIGLSISIGSGQNGGQRSGNRSLTLSGLIDGAARPGSASGITAAFSDGFPIQSLSWGMTPGGTEFGTGASPQDFSSADTG
LFDAPGLLHVTARAAGRSYSLSVPIRTAPPVFLTPPAFAAPLYKIGEAVEVSLGTVDAAPATLTVASFTLDGEDRQSGLA
GLAWSSAGETAWGRAGVLRLQARAESSGGVVLSAPVTARLGDAPRAFSAADWSVDETGVRIAALPDDGNVPITNIRYRID
GGPDRSVNGVTDFALADYGDIQIRAVNALGIGAWSDTKTRIAQTPTLSIEAAPLLAEVAAVEGNTLTATAGPVSGGPSSR
TWQWYRGTAAISGATGESYMLQPADIGQRIKVRQIETPSGGTPVTRDSAVSPVVVALNFQAQDNGDGTFGITYADGARST
VEFVLSQPTVYAGSFNLDLAALETGPICLVQPALSGTPAVGAALSATPFLWVYPEALGALSVSSGWRRNETVLAGESGST
HTVTAADAGTDLQYIETGTQSGAAAPVTAASPSVRIGEAAAVLSGVSGTRSAAGQITVTLSTDWGEGTIFYVVTGGAAPS
AAQIAAGTGADGTPLAVPYRGSMPVVAAGPQTLTISGLAEPISATVHLVQQTGSGVQSVPVSTARIASPVTSGYSPQSSA
SALAWWDASDEATITHAAGAVSQIADKIGTAHLTGTGTTGVGASDVNGLNTIVMDGTSDYLATAGVYPFPADGNWVLTII
ADIGAVNDYMDSLFSAEFGSVNAYLRASSSVYFAGKLYVGAANQAVSPQIGAGLHTVQIVADKENNVARCFVDGEEVFTL
AYPTAVDSPVVQFFRFGTSKNLGGKFLEVFIDSDTGTQAGALAFATKKWAL
>MCE8455110.1 hypothetical protein LZ188_00625 [Rhodovulum sulfidophilum]AB15
MAKMKFKGIPVGEKVTMTVRDSAGNTLVTAQSPAWAQSTTPAEVPGQVNTAADLGIVSSIPFNAIAANIEAVEVLTQPSY
GHVSVVGTSVLVNTVDVPEIRAVTTEPVVLRTPENPLSKMVNNLVKSTDPITFTLRVTQAGQSPQDVTVVCRIDAHLSLS
GWSCGRGMKLAVDNNDETVLEPGEKHFDLYVSLSPAAYTRADIMTEIGGNTISGKVDDYIVAQLTPGGATISGVTIAYDP
INYYGSQAKPVRPIAAAGLVTELLEEHGFDTVHIHYERGYDYSTDRGAANFGKAGLLHPNAVLAWGEGAKPIVPRARFNG
NTKKHGMVFRGVQFPLGQGLGGSGGTNVIVENCLVPAMIYFYGQDAFGYPARFTVRNSILPHSFAVEDVRDGTPDYWAGD
YRPACIKPGGGSNGIIIEGNFLALGAFAPDFALDNGADGLPGPDDVYPFIDVGSDSRWGLNGFSTPGPNLLAHPMYCDTY
TRHIQIRKNFIFGSGGSLIQLRGGAVMYDCAFTWGNQTFSVGPGTFNNYSSEVFDDRNDGHKSWLHDVVLTHAGYHDGPQ
AGNALSEGIVVGSALTSLDRVVILHDIDPNNPADADRLVSSWNGASHGRKSGFKYSAAYEAGGKFENYHGRERDLVCANW
GSVENPDYNPGSLDLSNINNATLFRWYDAQRGNAPDTTTDPLDIYWWLRGHEGPEIKALVRSFVTFMQAPLGIVPTWRTS
ASGCSFVPDLAEDGCRWDNTNNWGRDLIPGSFAGDTAALNGHKVFYQLDTLTVAGLDLGAGGHLHAVNGRLNVSAAPVCT

GSGAITVDESGQVWVNGYTDTDPLTVTVKGGRFANTGTFSGPADIHVDGSTDPYGKAEFLCAYGAASMTVTSGRKME
IVG
SGPRVGFDGTGGTQARLSFESGAVLSFVAKEGRLATIREFRSGVNGTATPDVVSTVALGGAALMLDLTGVAVGSHVLV
SV
DNVIGTFGSITGKIDPRLDATVTVTAKGVSLSIGSGSGQIRLT
>MCE8455111.1 DUF2029 domain-containing protein [Rhodovulum sulfidophilum]AB15
MLLLGLQVAMVGYRLDFYQYDFRAFYIAANLFAVGGEPYSFEELVRVSKGLGLSDNNHPFLYHPLTLFIFEPLSDLSYRA
AYSLWLFLQVSSLAIIAWVCIRFFKIPAPWLLPMMAVGLNGAIAANLRAGQLTLMTLAMILLASAMVMRNRAAAATVF
IV
LCAIPKLWVTPALGLLLNRISSGRMVLVASGALAILAALGLSYYLAPDDFRKFLTVAERFSGGSEPGGASNGSSRNMLAS
LAGTAGFDAAQFKLIWVLWIVSVLGISLLAWTRLLRDGATPYPELFSLVLLSICLVMPRMATYQWTIAIPAVAHIAATRP
YPVGIPLTAIAVMPTLYIGRYCLDRPIEARADTFLEIAFYFANLWSVLGAWLILCVSVLLLGSRGRRRENYPVSAVSISS
EPLAQRPR
>MCE8455112.1 transposase, partial [Rhodovulum sulfidophilum]AB15
MKRKRFTEEQIIGVLKESEAGAKTDDICRRHGISSATFYSWRKKYGGMEAG
>MCE8455113.1 translation initiation factor IF-2, partial [Rhodovulum sulfidophilum]AB15
MDDTDGKKTLGLRGGSRSGQVKQSFSHGRTKNVVVETKRKRVVVPKPGAAKSGGAGGGRAGGDPSRRPAGISDSEM
ERRL
KALQAAKLREADETRQREEDERQREAERQRRREEAEAKEREQREIEEAARRKAEEEERKKREEEDAAKKKAAAAERAAS
A
PAKPAPSVGPEETRGPAKTSSPRKVDREREGGADRPNRGKTSPGKGDRRSGKLTVSQALTGGEGGRQRSLAAMKRKQ
ERM
RQKAMGGADKHEKVVRDVQVPETILVQELANRMAERSADVVKSLMQMGMMVTQNQPIDADTAELIVEEFGHKVV
RVSDAD
VEQVIDSVEDKAEDLKPRPPVITIMGHVDHGKTSLLDAIRNAKVVAGEAGGITQHIGAYQVKTDSGAVLSFLDTPGHAA
F
TSMRARGAQVTDIVVLVVAADDSVMPQTIEAINHAKAAGVPMIVAINKCDKPAADAQRVRTELLQHEVVVEAMSGE
VQDV
EVSAKTGQGLDTLLEAIALQAEILELKANPERAAMGAVIEAQLDVGRGPVATVLVQNGTLKQGDIFVVGEQWGKVRAL
VN
DKGERVQSAGPSVPVEVLGLNGTPEAGDVLNVVDTEAQAREIAEYREHAAKDKRAAAGAATTLDQLLAKAKEDANVA
ELP
ILMKADVQGSAEAIVQAMEKIGNDEVRVRVLHAGVGAITETDVGLAEASGAPIIGFNVRANAPARNAANQKGVEIRYY
SV
IYDLVDDVKAAASGLLSNEIRENFIGYAQIKEVFKVSGVGKVAGCLVTEGVARRSAGVRLLRDDVVVHEGTLKTLKRFKD
EVAEVQSGQECGMAFENYDDIRAGDVIEIF
>MCE8455114.1 RNA-binding protein [Rhodovulum sulfidophilum]AB15
MARGGAGRPADEPERRCIATGEVTPKAGLIRFVVGPDDAAVPDLLGKLPGRGMWVAADRDALEKAVKKRLFSRAAKR
PVT
VPEDLLEQVEAGLLRRVVDSLSLARKAGRAVVGYEKTKGWLDSGQAAVLIQASDGSERGKTKLRPPREDDGFIGCLTAR
E
LGLAFGREHVIHGALAAGGLTERVVEDAAKLSGVRKKDGGGTPGKG
>MCE8455115.1 transcription termination factor NusA [Rhodovulum sulfidophilum]AB15
MAITSANQLELLQTAEAVAREKMIDPDLVVQAMEESLARAAKSRYGAEMDIRVAIDRKTGRATFTRVRTVVEDELLENY
Q
AELTVKQAKQYLADPQIGDTVVDEVPPVELGRIAAQSAKQVILQKVREAERDRQYEEFKDRAGTIINGQVKREEYGNVI
V
DIGRGEGILRRNEKIGRESYRPNDRIRCYIKDVRRETRGPQIFLSRTAPEFMAELFKMEVPEIYDGIIEIKAVARDPGSR

AKIAVISYDNSIDPVGACVGMRGSRVQAVVNELQGEKIDIIPWNEDQATFLVNALQPAEVSKVVIDEEAGKIEVVVPDE
Q
LSLAIGRRGQNVRLASQLTALDIDIMTEAEESQRRQAEFEQRTKLFMDTLDLDEFFAQLLVSEGFTNLEEVAYVDLDELL
VIDGVDEDTAGELQARARDHIEAMNRKALEHARELGVEDSLVDFGGLTPQMIEALAEDGVKTLEDFATCADWELAGG
WTT
VNGERQKDEGLLEKFDVSLEEAQTLVMTARVQLGWVDPSELEPEAEPEEDEGGEEEAGA
>MCE8455116.1 ribosome maturation factor RimP [Rhodovulum sulfidophilum]AB15
MSELIAKTAIDRRLAEILTPAIEGMGFELVRVRLMGGKTKTLQVMAERPDGGIEVDDCAKISTAISALLDVEDPVEEAYT
LEVSSPGIDRPLTRLKDFGTWEGYEAKLETAELIDGRRRFKGMLQGVEEDEVLIEIDGPEGDPVIIGLKYDWLSDAKLVL
TEELIREMLKARKDAGLIDESDFDEIEADDGSAPQED
>MCE8455117.1 class I SAM-dependent methyltransferase [Rhodovulum sulfidophilum]AB15
MHVNARFWDRLAPRYARMQLRDPEAYEKKLAMTRAWLGPEAQVFEFGCGTGTTALKHAPHAGHVTAIDCAPAMIG
IARQK
AAEAGIGNVAFRVGDLADMPDSSAAFDMVMAHNVLHLLPDRAEALARVHRMLKPGGVFVSSTACAAGAPRLFRWV
LAAGH
ALRLLPLFNPLTADALRAEIAATGLGIVEDWRPEGGRVPVLFLIAQKPG
>MCE8455118.1 prolyl aminopeptidase [Rhodovulum sulfidophilum]AB15
MDKSAGQKRASQHLYPPIDPFDQRMLDVGDGHRIYVEQCGHPQGIPVIVLHGGPGGGCSPAMRRYFDPTEYRIVLFD
QRG
CGRSRPHASVENNTTWHLIADIEKIREMLGIGRWVLFGGSWGATLALAYAETHPDRVAYMALRGVFLMTGAELDWFY
GGG
AGQFWPDLWARFSAPIPADERSDMIAAYHRRLFSGDLLTETRHARAWAAWENALASIQHEGPAGEAPADYARAFAR
LENH
YFMNRGFLERDGQLIEDVPRISHVPATIVQGRYDMVCPPGSAYRLAERWGRAELKMVPMAGHALSEPGISAELVRTM
RDV
QRLAVALGL
>MCE8455119.1 bifunctional 2-polyprenyl-6-hydroxyphenol methylase/3-demethylubiquinol 3-O-methyltransferase UbiG [Rhodovulum sulfidophilum]AB15
MQAAETTVDPSEVAKFEAMAAEWWDPAGKFKPLHMLNPCRLDYITRQIAAEFDRDLAAEAPFKGLRLLDIGCGGGLL
SEP
MARLGAEVVGADAAERNIPVARIHAEQSGLQIDYRHTTAEALAEAGEQFDVVLNMEVVEHVADPLAYLTACRALLKPG
GL
HLCSTINRNPKAFMMTILGAEYVMRWLPKGTHEYQKFITPDELCSLLEQAGLKLLDRSGFVFNPLTWDWSISSRDLSVN
Y
VTAARKPA
>MCE8455120.1 MarR family transcriptional regulator [Rhodovulum sulfidophilum]AB15
MNEISESLLVTLFSEIFMADQLARNRISKALPKGMEISHFSVLNHLARLNEERSPAQLAASFHVTRGAMTNTLTRLEWAG
HVHIRPDWDDARRKMVSISPSGRSAREVAIQAVAPVLAEAVADIGEDRLKAALTVLRMLRQKLEG
>MCE8455121.1 carbon-nitrogen hydrolase family protein [Rhodovulum sulfidophilum]AB15
MRAALIQMTSSDLPEENIGQVRELVREAAAGGAGMVLTPEVTNCLSTSRDWQRNVLTEEAGDPTLAALRQEAASLGI
WLL
IGSLGLKTTDPDGRFANRSILIAPGGGIVARYDKIHMFDVEISATETFRESDGYRPGARAVLAPTPFGAVGMAICYDLRF
AHLFRALAKAGAEILTVPAAFSPVTGAAHWEPLLRARAIETGCYVLAPAQCGLHPASQGQSRRTHGHSLAVSPWGEVL
AD
GGEAPGISYVELDREEVARARARVPSITHDRGFEGP
>MCE8455122.1 glutaredoxin 3 [Rhodovulum sulfidophilum]AB15
MQTVEIYTTSTCGYCHAAKRLLDGKGVCYAEFDVTADPGLRQQMMERADGGYTVPQIFIGETHVGGCDDLYDLERAG
KLD

PLLAG
>MCE8455123.1 double zinc ribbon domain-containing protein [Rhodovulum sulfidophilum]AB15
MRLIYPPRCVACGEPVERDHALCARCWGELDFVTGLVCDACGLPLPGEEADDPVWCDDCLSAPRPWARGRAALLYRG
RAR
GLVLAFKHGDRTDLARAASGWMLRAGRPILVPGMLVAPVPLHRWRLLRRRYNQSALLSRGVAEGAGAEHCPDLLVRL
RAT
RPQEGMTAEARAANQAGAIAVGPRHRGRVAGRTVLLIDDVMTSGATLGAAAGACLAAGAAEVRVLVLARVAKAD
>MCE8455124.1 methyltransferase domain-containing protein [Rhodovulum sulfidophilum]AB15
MTRPPDLTDRQALALHRARALKAGPALFLHEAAADEIKERLIEVNRTFTAPAVVTPFAEAWQGLLPGARMVPAEETLDL
A
PGAHDLVIHALALHWANDPVGQLIQCRRALKPDGLFLGVMFGGRSLQELRAVLAEAEAATLGGLSPRVLPMAEIRDLG
AL
LQRAGLALPVADCAEFSVTYQSAYHLMRDLRAMGETNALAARHRRTAPRRLFSEAARRYAEFHTMPDGRIPATVEMIY
LT
GWAPAETQPQALKPGSARMRLADALNRAAPQPGERNDGGSD
>MCE8455125.1 ferrochelatase [Rhodovulum sulfidophilum]AB15
MFDSKEGGASSATSGLTGARPEHGPEDHPPVPPERIGVLLANLGTPDGYDYWSMRRYLNEFLSDKRVIDYSRWIWQPL
LQ
LVVLTKRPFASGENYRSIWNHEADESPLMTITKAQTAKLEAELKAAYGEKIVVDFCMRYGNPSTRAKVRSMLKKGCQKI
L
FFPLYPQYSGTTSATACDSFFEALMDEKWQPDARTVSTYFDNPHYISALAASVERAYAAAEQEPDILVCSYHGVPQRYLT
EGDPYHCHCLKTTRLLKERLGWDDSRITTTFQSRFGPEEWLQPYTVEEVARLAKSGKKRIAVIAPAFSADCIETLEEINE
EICESFEEAGGEKFTYIPCLNDDDAHIRALSEIVKVNLRGWAD
>MCE8455126.1 hypothetical protein LZ188_00705 [Rhodovulum sulfidophilum]AB15
MKKLALAAAFAATASTAFAGSYAEPMIEPEVIVQDTSSSAQGVIVPLLALILVAAVASN
>MCE8455127.1 L,D-transpeptidase [Rhodovulum sulfidophilum]AB15
MTTRSTRGLSRRNFLAGTALAVTAGPALANTTEIEPELTDMVRRNISSFRALGWRPYFASLKNGAILVDVKSRALHFWSE
DGTIYKLYPTSVPLSEDLTRLGRTSVVRKVEGPSWRPTPAMKKRNPEWPDFVPPGPENPLGTHALYLGWTYYRIHGTH
DT
RKIGRRSSNGCIGLFNEDIAELFGLAKVGTQVLLI
>MCE8455128.1 CAP domain-containing protein [Rhodovulum sulfidophilum]AB15
MIRSALGLLAVLFALSACSTGNEVRIGPDGKPLPQVYRISRADQGKIQYRMLDSVNALRQARGIAPLELDSRLNAAAATH
SRDMSVQNRPWHFGSDGSSPLERVARAGYQGHLRGENISETYETELETLAAWMEESDTRNIILDPQATDLGFAFYQE
MNG
KIWWTLVTGQRDAAVPDA
>MCE8455129.1 L,D-transpeptidase family protein [Rhodovulum sulfidophilum]AB15
MIDRRAFLTLTAMAALAGCGSKFKTYRGPEVTRVVVMKGQRMMYLLHHDQVLKAYPISLGYAPAGPKQVEGDGKTP
EGRY
HIDRRNPDSQYHLSVGISYPNEADIETAKALGMEPGGDIFIHGRAKENRRRTDDWTAGCIAVSDSEMEDIYAMVRDGT
VI
DIFP
>MCE8455130.1 class I SAM-dependent RNA methyltransferase [Rhodovulum sulfidophilum]AB15
MSHLVERLSLRGEGVTGDGLTVALALPGEEVEGAPEEGRIARPRIVTPVVERVRPPCRHFASCGGCALQHGSDAFVAG
WK
TQVVERALTGQGLGASVRPILTSPSASRRRAVLAGRRTKKGALVGFHARASEVLVEIPDCRLLHPDLMAALPACEAAILL
GASRKGALSIALTRSEDGVDMAVEEARPLDTGLRMDLAALAETHDLARLSWNGEQLAERRPPRQAMGRAHVVPPAG
AFLQ

ATEAGQAALTDAVGAALGGVRSVADLFAGCGTFTLPLAATAEVHAVEGSAPMLAALDRGWRGTPGLKRVTTEARDLF
RRP
LLASELARFEAVAIDPPRAGAEAQCAEIARSDLARVAMVSCNPVTFARDAKRLCSGGFRIDWVQPVDQFRWSGHVELV
AQ
FSR
>MCE8455131.1 ABC transporter ATP-binding protein/permease [Rhodovulum sulfidophilum]AB15
MFRYFENLIDPYAPHAQTDTPPNRLWPFLAAYMRPFRKVFAIAAVLSLLSAFVEIWLIDVMGQLVDILSSGAPEQVWHE
H
GAALIGLALFILILRPLIQALDAAVLNNAIMPNFGALIRWRAHSHVLRQSVGWFENDFAGRIANRQMQTPPAAGEAVF
QV
FDAIAYALAYFGGALVLLGQADPRLMLPLLIWLGLYAALVRWTMKNVGVASKASSDARSTLTGRVVDAYTNIHSVKMF
AH
HDRELTYAREAIEGLRKAFGTEMRIITTMDVALVVLNGLLIVGVVGWAIALWMQGQASVGVVAAATALTLRLNSMTG
WIM
WAMSSFFRSLGVVAEGMETIAQPIALVDAPDATDLEFREGRVEFRSLTHHYGRGSGGLEGVELALKPGEKVGLVGRSG
AG
KSTLVKLLLRFYDPEAGQILIDGQDIARVTQESLRRQIGMVGQDASLMHRSVRENILYGRPEAGEEEMIAAAKRAEAHD
F
ILDLKDPEGRRGYDAHVGERGVKLSGGQRQRVALARAILKDAPILVLDEATSALDSEVEAVIQQTLYRVMEGKTVIAIAH
RLSTIAHMDRIVVLEDGRIAEDGSHSELLAKDGLYARFWARQSGGFIGTEAAE
>MCE8455132.1 CCA tRNA nucleotidyltransferase [Rhodovulum sulfidophilum]AB15
MTRVSGDPFFADHTQAVCALLTEAGYRALFVGGCVRNALLGVAVSDIDIATDARPGKVMELAEAAGLKAVPTGFDHG
TVT
VVAAHVPHEVTTFRRDVETFGRRAVVAFADSPEEDARRRDFTMNALYATPAGEVLDPLGGMPDLRARRLRFIDDADA
RIR
EDYLRILRFFRFHAWYGDPSNGLDPEGLAACAANSAGIETLSRERIGAELKKLLAAADPGPSIAAMAATGVLARTLPGAD
PRVLPVMIHLEAALGLAPDPIRRLAALGGEDVAGRLRLGKAEARRLWRLAEGAAAGEGPAVLAYRVGPEEARDSVLLRA
A
SLGADLPEGLGAEIARGAHARFPVAAADLMPRFSGPALGQMLKRLEADWIASDFRLDRAALIALAGRGD
>MCE8455133.1 CoA pyrophosphatase [Rhodovulum sulfidophilum]AB15
MPSDPFDALAAALARPGCETSDHDLNPGFVLPEGRVLRPAAVLVAFWPGPGGTRLVLTKRSSRLKHHPGQIAFPGGKL
EP
GEDAPRAALREAREEIGLPEAGVEILGTMPPHETVTGFTVTPVLGRIAEDFPRIPEAGEVDEVFDVPFAHVSDPARFSIQ
SRMWRGQRRYYYTVPYGPYYIWGATARILRALAERVSA
>MCE8455134.1 Hsp33 family molecular chaperone HslO [Rhodovulum sulfidophilum]AB15
MSIGSQIAWDDTVLPFQLDRSDVRGRVARLDGVLDRVLAQHQYPPAIEALVAETALLTALIGQTIKLRWKLSLQVRGDG
P
ARLIATDYYAPTEDGAPARIRAYASFDEDRLDAGADPFSQIGQGYFAVLLDQGAGTVPYQGITPISGGSLSACAEAYFAQ
SEQLPTRFALSFGRAILPGADKDEHWRAGGVMLQMMPKASIPQTPEGGSGDEGLLQAEDMLEGDQAENWSRANILL
DTVE
EIELVGPRVHPTDLLVRLFHEERPRVFDPQRVQFGCTCSEDRVRQSLSIYSAKDIGHMTTPEGIVTADCQFCGAHYELDP
ATLGFEAEKDPDAE
>MCE8455135.1 NUDIX hydrolase [Rhodovulum sulfidophilum]AB15
MIRRYGEAVQRDRRYRFRPGAYALLERDGMLLLTHQQAPVPEYQLPGGGIDPGESPIQALHREVFEETGWRIALRRRL
GA
FRRFTFMPDYEIWAEKLCTVYLGRPVRPHGAPGEPGHTAVWMPPDLALGRLGNAGDRAMLHRWLGQSR
>MCE8455136.1 serine/threonine-protein phosphatase, partial [Rhodovulum sulfidophilum]AB15

PCGQVGGDLVGFFPAGPGEVGLFSADVSGHGISSAMLTARLAGLLGSRSPDQNIALEPAPGGQYRARPPEEVAAHLNR
LF
LEDIETEHYFTLLLAIVTLDTGRMRFVQAGHPHPVLCRADGSLDCLGDGGLPLGLIPGAGFEGCEVGLRPGDRLLLVSDG
ITECPAPDGAQLGDDGLRCLIETHAATRGPAFLDAIQAGLTFFAGRDEFPDDVSAVLFEFG
>MCE8455137.1 aldolase/citrate lyase family protein [Rhodovulum sulfidophilum]AB15
MSFRDKLLAGEVLIGTFVKTPSPHVVEILGLAGLDFAVIDQEHAPIGLAEMDMMAMAGRAVGLPLLSRRWGKGTDWI
APL
MDLGMTGVMVPHVLDPEGAEAVCDAVRFARLKRGLSPSPRAGDYGGMSIPSYREKSDAESVVMVQIEDECALAHLD
AIAA
IKDVDLVFIGPADLSQSMGVGFPSPELDDAIMTIVEACKHAGCAVGLFVGDAAAIPAWVERGVGVFVCGSDQALLRKG
AA
GIMAAR
>MCE8455138.1 acetyl-CoA C-acyltransferase [Rhodovulum sulfidophilum]AB15
MQDIYILGAARTPIGSFGGALSSLGPDALGRIAAEAAIARAEVSARMIDNAVAGNVIPTQPRDMYLARAVAMDAGMP
ATS
QALTLNRLCGSGAQAIATAATMIRAGESRLALAFGAEAMSRAPHTVGGMRDGVKMGDASVTDWLTGALSDPFDIGH
MGVT
AENVAGLHGITRQEQDAFAAESQRRAGIAIDEGRFDTQICPVTIKGRRGDTVVSQDEYPKPGTDAARLAGLRPAFRKDG
T
VTAGNASGINDGAAALVLADEDAARAAHPIARLLSWSVAGVPPEVMGLGPVEAVPLALSRAGLSLADIDVIESNEAFAA
Q
AIAVNRLLGLDPEKVNPNGGAIALGHPLGATGAILAVKAIHELQRMAGRYALVTMCIGGGQGIALVIERLEDTA
>MCE8455139.1 DUF6282 family protein [Rhodovulum sulfidophilum]AB15
MGGERVKMQGTMPLEHEARISELLVGAVDPHVHSGPSIAPRALDHLELARELSEAGFSAVVTKDHDYSGVMCATMIA
HHH
PDLTTRVFSGIALNNVVGGLNPYGVEHTAAMGGKIVWLPTLAAENHLSWQATSGWSHPASTSRIRAATAVRLWDDD
GKML
PELHDILDVCAATGLALASGHVHVSETKKIFAEARKHGVERLIFTHPEDIVGASMEDTREIADMGAMVEHSLAFFLEGSK
FRNHTTEQLGEYIELVGPERTILCSDLGQVGTLTPLQGFRRAVSDCLSLGYDDAAVRAMVSGNAAQVIGLDR
>MCE8455140.1 IclR family transcriptional regulator [Rhodovulum sulfidophilum]AB15
MENDKKMGEGLQSVILTMQIVEHVSRTGKGVGVTSLATALGTSKSRIHRHLQTLVQQGYVFQHEDSERYEVGHQLVSL
AQ
AVLEGTGLIAATRDALLALRDRLGHSTVASQLTPDGMLVIATVSGRSPIEIGVRVGSLLSFHGSAQGKVAAAFSSEAFRN
RVLGGTLIAFTEATITDPDWLQAEFELIRERGWATAPDQAALGLNTLASPILDGANMVCGTVGCVNLTQSLGAEPSPEQ
I
EALLETARQVSVLMGHSGDQLLR
>MCE8455141.1 hypothetical protein LZ188_00785 [Rhodovulum sulfidophilum]AB15
MYDKSDPRAALASTPADSMIGVTGLVAEEQVATFYDSPPQLDDDTGQTWLHRGHNFLVALSHAVPGASFDRVAQVD
EYCV
LLPKGGATIEWNGSVTEVPGHSIAFVPAGDSRVTAEGGEIVRLFTTANEDLLALCGNADGYDRPRSHVKPLTAWPAPKG
G
FKVRHYSLDVAPEPGRFGRIFRCTTLMVNVLEPFEGPRDPSKMSPHHHDDFEQGSLALSGEFTHYLRWSWTSDMAD
WKDD
KVLKMAAPSLLVIPPPVIHTTRATGPGDNLLADIFSPPREDFSLKPGWVLNAEDYPMPEQA
>MCE8455142.1 TRAP transporter substrate-binding protein [Rhodovulum sulfidophilum]AB15
MTITRRRLLTRGAAFTTVLAAPNIARASGRRMRFAHAANEVHPGHIMATSFAAALEELRPGGFDVRIFPNRQLGDDKQ
LL

EGAVAGTMDVVSVSGVLVPLVTGRQAMNAWQMPFLIRDYDHFGKLAHGPVGQQILDDLRPAGLVGLSIANTGQRH
FLSID
RNVASIEDFAGLKTRIVPVPLHQQIWETVGTAPAGLPYGEVYGALETGVIDAVEINVSSMLGENLWEVGKHFTLTGHYP
W
HMTTLMSERFFEGLDEEERDVVVEAGKMSTAATLEYARQQDLEGREELAARDVEIAELSDLSEMRARVEGLTADWSSK
DP
LIAAMVEAAAKSA
>MCE8455143.1 TRAP transporter small permease [Rhodovulum sulfidophilum]AB15
MQYRSGPLARLVAGYLCLIRGLAGLSMAAIVAIMLAQVFWRYVLGGSLIWAEELCRYLLIWQTFLVLGLAFSKGEFVSLD
FMPSLLSARLRWGLRAVMAVPILAFLAVMAWYGWDFAERMGPQTIPALDFIWSAVTGEALGLSIRWVYISVSVGMVL
LAG
HILTGLVVDFHRQVILNLGEEDAALAQTSEMT
>MCE8455144.1 TRAP transporter large permease [Rhodovulum sulfidophilum]AB15
MSSFFLLFAAFLATGVPIALTLGIAGTAYLYLSGNGAMALMLPQRMIAGIDQFVLLTIPLFLLAGALMNVGGVTTRIVTF
ARAMVGHRPGGMSSVSILSSGFFAGISGSATAEASALGSILIPSMSKQGMPPAYAAALIAVSSVMGPIIPPSITMIIYGV
LSGASIGQLFLAGIMPGILIASGLLIYATWRARRDGFPVIPRMTARERLASTRRTLPALILPLIILVGIKAGIFTPTEAA
AVAVGYALIIGFIYRELTPSRVWEALIATALVSTSILFITSMASIVSFVFTLEQVPQLIADGMLSLTENPLLILLLLNIF
LLVLGMFLEPISILILTMPILLKFQDLIGMDSVQFGTVVVLNVVIGMATPPVGILLFIGAAISGQPVTRVIREALPLIAI
CLLVLAIIALVPQVALFIPRFMV
>MCE8455145.1 NAD(P)H-dependent oxidoreductase [Rhodovulum sulfidophilum]AB15
MTHVLIVIAHPDPASFNHALAFATAEALRAAGHEVTISDLAAEGFRADLSPADMTERADPARFHVQAEQANAARTGTY
AA
DIAREQARVAQADAVILQFPIWWGGPPALLKGWIDRVLSYGFGYVDGRRFDTGLFKGRRAMISVTTGGTPERFSEQG
VYG
PIDPLLMPIRKLALEYMGFEVEPLQVAYAVSRISEPERKKHIDRLIAAACAMSSRPSHRSDAWKTALDDVPEGAWARRG
>MCE8455146.1 ClbS/DfsB family four-helix bundle protein [Rhodovulum sulfidophilum]AB15
MGVPVSKAELLDAISTTFGNLIYDLERVPPELARTASMEGHAAGTMMSPADLVAYLLGWNELVLKWLDRDDRGEALEL
PE
TGFKWNQLGSLAQKFYADYQHLDWHELLTGLAATNLRLVETISSRTNDELYGHPWYGKWTKGRMVQFNTSSPYANA
RVRI
RKWLKVVS
>MCE8455147.1 hypothetical protein LZ188_00825 [Rhodovulum sulfidophilum]AB15
MRLIAVWAMLLPFALASLVAQGVMPGKASDGTLTLIICSGDSFVEMVVDPVTMEPVSDNQDRDDQRAASGYCAWA
AAHPV
CAEPVAVAIHGPVSQARAVLRPPSPTILRVAAATGLPPSTGPPYSV
>MCE8455148.1 PepSY domain-containing protein [Rhodovulum sulfidophilum]AB15
MTDAIHGAKAPAASARSGPVSSLYRAVWRWHFYAGLLILPFLITLSITGAIYLFREEVDQLVHADFMTVTPVTEQRLAPS
EIIAAALAAEPGRAVKYTDPATDTRSAEVTVQPETGGRMAVYVNPYSGKVLEVRGDRSTFAWTVRYLHSFRYFGATPRK
V
IEIVGGWSILMVLTGIYLWWPRGQGGGVITVRGKPGRRVFWRDLHAVGGIVVGGFLVFLAITGMPWSSVWGAKVNQ
WANG
NNFGYPSGVRVDVPMSGTRLDDVAKTAWSLEQARIPETAPPAAGMTPLGLDAAVAEFDRLGLHRGYTVALPSGPTGV
YSG
SVYPDDLSQQRVVHLDQYTGKPLIDMGYADYGPLGRWLEFGINTHMGQTFGVLNQIVLVLVCAGIVALSVSAAVMW
WKRR
PSGSLGVPPLPSDKKVFVGLFVLLGIGGILFPLTGMSLVAMLVLDLAWQRFRAAHAT
>MCE8455149.1 hypothetical protein LZ188_00835 [Rhodovulum sulfidophilum]AB15

MSGDEIGLCLSGDLDRPVGGTIIDNQDQTVPESGGRLSDLGNGCFQRRLFVVTGDLHDNSGVNVRGHQDKQSILGMI
SDA
SHISDEARNASLFFHLISRWRYFPHGKRIADLLLP
>MCE8455150.1 glycosyltransferase family 2 protein [Rhodovulum sulfidophilum]AB15
MRKATAAFRNCLILVVDDGSTDGTVEVARKAKADFIARHSSNRGLARAYMTGLAASLNLGADIIVNTDADNQYRAAGI
PA
LVRAISEEQADVAVGARPIEQTEHFSRLKRRLQRLGTRVVRGLSGTDVRDATSGFRAITREAALRLNTFSDYTYTLETLI
QAGRSGLRVTSVDIETNPPTRPSRLMRSMRQYVLRSIGDMLRISTIYSPLRSYLTVAAPPLAVAIFLGLRYVFLVSFVDP
SRSHAPSLILAGILTGLAFLLCGLGIIGEIMTVNRRILEELRINQRRQLAESGSLGGRVDFNLIEISHGTSK
>MCE8455151.1 hypothetical protein LZ188_00845 [Rhodovulum sulfidophilum]AB15
MTGSHPLRKRLERLGTRSLPKLLTLQDRNPHSPTYGCFDRNFWHLRITDFPSGMAQEFVLPLALAYSNRLEGNDFFGDP
M
IREWVRAGISYAARSAHPDGSCDDYYPFEKAAGAAAFSLYACIEAIPAAGLDPAPFLPFLTQRAEWLARHQESGRLSNHE
ALIANALYRLSALVGEPRFAELGRRRLDRLLTWQSEEGWFFEYQGCDPGYLSLTIANLAELAELRPELPLKAPLLRAIGF
LHSLQPPDGWLGGEWTSRNTNNFFPHGFELLGKTIPEALDINTRAVGAMDPAPEYDDDHIIGHHCWSYIKTALSWRDT
RP
EPSVSPDRDVVFPEAGLSLLRKGPMTLLTAEKKGGAFRLYRGDRLIHADTGVSLQLRRGDRTRTYVCHLWSDDNEIERC
P
ASVTVSGAMGRAKSSQMTPLKNLVLRSLMLTLGRFNPDLIRAILQRLLITGKPRSGLRFARHLALDDSGLTVRDRIEGGG
EIVDCGIGPAQTSIYTVMSRIYHPVQRQPWCDLSAEIMPGSALRLDHTRRIGEAE
>MCE8455152.1 flippase-like domain-containing protein [Rhodovulum sulfidophilum]AB15
MKRLVSVLVSLAFLAVVYSLLDVDAIIGALRRTDPGMLGLSLILLVGLIFVSALRLSILSRTGGLPLGRRKAIQATFAAN
ALNMFLPGKLGDILKASLMAEDDSAQLAKATSLGIWEKISDLAALFLMASVPLALTSPSSWAWLPLGLLGLCGLAALLAP
RILQLPFGALRATRPLARDWGAVVRSLRSRPGSLTLCLALTALLWLGHLVQISLMAASLGVSGDVSFWAHFMALMPIAI
V
AGLIPLTFAGVGTRDAAIVMLIGAQAGPETAAALGVLFWLRYLVPGLIGSPLLPRFLRTAAAHRARFAK
>MCE8455153.1 hypothetical protein LZ188_00855 [Rhodovulum sulfidophilum]AB15
MTGSAGSNGRPLPWLPIFWGVLVLAQITSCGPELWYNPWSIIQDDGRHFVAWLRQMSDPALFPDDPIATYFLALTPEL
FK
ALHLPAVWLGIDVGAWNLLVVSPLSIALFLAAANTFAARMQLARPERHVLVLLLLAAVYSDVTSGLPRGFGPTIVLGLLG
LFMARRRIALGIAMAAGANIYPIAALVGGGAIGFNAVLRSMSVRRIDRNDVILVLIAAAAGIVGLSPFLASTDGAGTTFT
LAEARDIPIFGPGGRTSFFRDSWVDMLLCSSQRSGLLPTCAGGPGWPPLVLTAAVFIMGVLLLRRRDVPRDVGTALISLA
LSGLVLTALAYVFAFKGHLPGRYSRLSLEQVYAFSWVFAVTSGVVWAARRRGLAERRLFSGLVVVLLAAALVGTRPSAEF
IRDPAPLISAALRSQPPETVVVGFSRYVDSVPALTGRKVFASLELTVPYKKPYYYRIEQKMADMGALYRGPVSSGWRALL
DRSGIDFFILDSEASARARQWYVSFPVLKGSREQSVFVSNPDAVRSCTFEAQGSLKMISASCFADRALE
>MCE8455154.1 hypothetical protein LZ188_00870 [Rhodovulum sulfidophilum]AB15
MRGLFTTLRLLLFVAGLALVAGCAGKDDLDEPPVPLGDFRLGYTIVVAKNAKDIPPSRQATAEEWEAVLKEELQRRFGRY
DGDRIYHFGIAVDGYALAVPGVPVILAPKSMLVLSANLWDDAAGKKLNAESEQLSVGEGLNGATLVGTGLIRTKDEQM
RA
LARNAAKRIERWLVEHREEWLHYDPDAVPPLDAMAGTASDEASAPASVPGAEVVTTPLDDPLDAPPGPSPAKPDEAP
SRA
N
>MCE8455155.1 DNA topoisomerase IV subunit A [Rhodovulum sulfidophilum]AB15
MSDATEDPKHDALSEPLRRAIGERYLTYALSTIMDRALPDARDGMKPVHRRILFAMRELKLTATGGFRKSAKISGDVMG
N
YHPHGDAAIYDAMARLAQDFNVRYPLVDGQGNFGNIDGDNPAASRYTEARLTAAAEALMEGLSENAVDFRPNYDGT
LEEP
AVLPAAFPNLLANGSSGIAVGMATNIPPHNIDDLVGACLHLIKAPDAQDDTLLQYVPGPDFPTGGVIVEPKDSIAQAYRT

GRGSFRLRARWEIEDLGRGQWQVVVTEIPYQVQKSKLIERIADLIQTKKIPILADVRDESAEDVRLVLEPRSRNVDPEVL
MSTLFRNSDLEIRFSLNMNVLIDGRTPKVCSLKEVLRAFLDHRREVLIRRSQHRLDKIDHRLEVLAGLLIAFLNLDRVID
IIRYDDDPKAALMAEDWARPHARAMSERDYVSPLPVAGPGGLTEVQAEAILNMRLRSLRRLEEMELTAERDRLTEERA
GL
LELLGQEEIQWKRIAEELREVRKNFGARAPGGARRTVFAEAAEVAEVPIEAMIEREPVTVVCSKMGWIRAMKGHLADG
SA
LKFKDGDEGRFLIQAETTDKLILFGANGRFYTLPVVQLPGGRGMGEPVRLMVDLPNESEIVALFIHRPGEKLLVASSAGD
GFLVAGDDVLAQTRAGKQALNVRAPAVARVCVPVHGDHVACVGQNRKVLVFPLEELPEMGRGKGVRLQKYKDGGL
ADATT
FTLADGLSWHDPAGRTRTETDLTDWLGKRAGSGRMAPRGFPRDNRFT
>MCE8455156.1 SDR family NAD(P)-dependent oxidoreductase [Rhodovulum sulfidophilum]AB15
MTGKSVLITGCASGIGHDAAHALKARGWRVFATCRQAADCDRLAAEGLESLTLDYASEDSIATALDEVLSRTGGRLDAL
Y
NNGAFACPGAVEDLPRDALREIFETNLFGVHDLTRRVIPAMRAQGHGRIVNCSSVLGLVPLKWRGAYSATKYALEGLTD
T
LRLEMRDTPIRVILIEPGPVTSKIRANSIPHFERWIDWQNSARADQYRNSLLTRLYEDRGRDFFELPPSAVSRKLIHALE
SPRPRPRYFVTAPTHLMNLARRLLPTPALDWLIDKG
>MCE8455157.1 electron transfer flavoprotein subunit beta/FixA family protein [Rhodovulum sulfidophilum]AB15
MKVIVPVKRVIDYNVKVRVKADGTGVDLANVKMSMNPFDEIAVEEAIRLKEKGQATEIVAVSIGVKQAQETLRTALAM
GA
DRAILVVAADDIHDDIEPLAVAKILKAVVDEEQPGLVLCGKQAIDNDMNATGQMLSALLGWSQGTFASKLEIDGDTAQ
VT
REVDGGLQTIKVKLPAIVSVDLRLNEPRYASLPNIMKAKKKPLDEKTPADYGVDTAPRLTVLKTTEPRERKAGVMVASVD
ELLTKLKDEAGVL
>MCE8455158.1 FAD-binding protein [Rhodovulum sulfidophilum]AB15
MAVLLLAEVTGGELQLDATAKAVSAAKTLGEVTALCVGEHARAAGEAAARIEGVAKVLVAEDAIYGHRLAEPTADLIVSL
AGAYDHIVAPATTDAKNIMPRVAALLDVMVISDVVAVVDADTFDRPIYAGNAIQTVKSSDAKKVMTIRTSTFEAAGEG
DA
APVEDIAATGNPSLSEWVGDTAAESDRPELTSAGVIVSGGRGVGSEENFKLIEALADKLGAAVGASRAAVDSGFAPND
WQ
VGQTGKVVAPELYIAVGISGAIQHLAGMKDSKVIVAINKDEEAPIFQVADYGLVADLFDAVPDLTAKL
>MCE8455159.1 DUF6473 family protein [Rhodovulum sulfidophilum]AB15
MIFDNPGSGSLAYSPCRYGNSKLEFRGPRRPLEQPYCAAVGGTETYGKFVPKPYPALIEEATGLQMVNLGCINAGLDVFL
NDASLMDVVAGAQVTIFQALGAHNMSNRFYVVHPRRNDRFLRASAMLKALYPGVDFTEFHFTRHMLSVLKQKSPSSF
EIV
VAELQMAWQARMRKLAGRVPGDKILLWLPGRPPGYGQPCDGLGEDPLFVTQHMLDTVRSYFTRVVEFEPSPEALAR
GAEG
MVHGEADEAAAASLPGPAVHEELAEVLSGTLRELHAI
>MCE8455160.1 3-hydroxybutyryl-CoA dehydrogenase [Rhodovulum sulfidophilum]AB15
MDVKTVGIVGAGQMGNGIAHVFALAGYEVRLNDISADALDRALDTIGHNLDRQVSREKITETEKRDALGRIAITTALTD
L
GACDLIIEAATEREAVKTAIFEELQPHLTPTTILTSNTSSISITRLASRTDRPEKFMGFHFMNPVPVMQLVELIRGIATD
EATYRSLLKVVETLGKTAASAEDFPAFIVNRILMPMINEAVYTLYEGVGSVRSIDESMRLGANHPMGPLELADFIGLDTC
LAIMNVLHEGLADTKYRPCPLLTKYVEAGWLGRKTHRGFYDYRGETPVPTR
>MCE8455161.1 lysophospholipid acyltransferase family protein [Rhodovulum sulfidophilum]AB15
MHGQSAAEPPHDARELSYARTFPDPLRAQTIRAIEILTGKLTLLRLVRDFEARGVPRGQAFWRQALDVMGIRVATPPEQ
V

ARIPREGPLVIVANHPHGLVDGMVLAELVGRVRRDYKILTRSLLTDVAEVAEFMIPVPFPHAEDARERNLEMRRAAMD
HL
GQGRAVVLFPAGVVASSQTFFGPAVEALWNPFTAKLIQRSGAQVLPVRFQGQNSRWYQIANRLSPTLRQGLLLHEVV
HAL
NRPQAPVVGAPIGRDEIEPWARDPRGFMAWLRARTLALT
>MCE8455162.1 HPr family phosphocarrier protein [Rhodovulum sulfidophilum]AB15
MTSNRVTRIFEIVNEKGLHARASAKFVECVEEHDATAEVLRDGMSASGDSIMGLLMLAASKGTAIEVSTHGPEALKLAD
A
LERLVATRFGEDM
>MCE8455163.1 PTS fructose transporter subunit IIA [Rhodovulum sulfidophilum]AB15
MIGIVIVAHGGLAREYLAAMEHVVGPQPGIRAIAIAPDDDRTAKQDEICRAADEVDEGGGVVVVTDMFGGSPSNLSIR
AC
APGDRKILYGANLPMLIKLAKSRRMSVSDATASALAAGRKYIDSFDAGGL
>MCE8455164.1 RNase adapter RapZ [Rhodovulum sulfidophilum]AB15
MSNPLEIDPPAPRQRIVLVTGASGAGRSTALGVLEDLGFEAIDNLPISLIPRLLEGPVPGPSLALGVDIRTRDFSARALA
DLILGLSAAPGFEADVLYLDCRPDVLLRRFSETRRRHPMAPAESPAEGVARELDILGPIRELASSLIDTSGLTIHEFRAE
MTARFAPGGAAPLAVSLHSFSYKRGLPLGIDMAFDCRFLRNPYWDPSLRALDGRDPAVAAHIAADPRHSAFLDRISDLA
A
LVLPAHRDEGRSHISIGFGCTGGQHRSVAMAEALAKRLAATEWQVSIRHRELERRAQGVSAMRTGTGA
>MCE8455165.1 HPr kinase/phosphatase C-terminal domain-containing protein [Rhodovulum sulfidophilum]AB15
MPDASSCILHASTVAHRGRAVLIRGASGAGKSALALQLMALGAELVADDRTIVTRTGSGLLASAPEAIRGLIEARRVGLL
RAEAVASAPVVLAVDLDSPETERLPPARVTEILGESLPLLRRVDRPYFAAAILQYLIAGPSD
>MCE8455166.1 sensor histidine kinase [Rhodovulum sulfidophilum]AB15
MRDSKRCADDAAGPGDAPCGRSGADRRSRGTWRGFVSLKRSPLARRIIAANLIAQIVLISGVLYLNPARDSLLSLRESG
M
VAETNLVADMFEAQIRRNDPLDPLAGNGLDLDVMLNGLELAQGAEVFVFDTAGNLVGHARGLVRTDWAVEEYEGDS
WITV
ITDALYRAWLGGVSLFGQEEGSDADGDPQRRLTDQVAITLTDGLQVATGVDSSGHTTFSVTAQILRDRQVVGVVAMV
SAS
GEIDRLVRIQREQVLQVFVIALLVSVGLGLVLASTIANPLSELAAAAEIGQNRNAPSMRPGRVRIPDLTARPDEIGRLSG
ALRGMVAALYDRIDANEQFAADVAHEIKNPLASLRSATGTLRVARREDQRAKLLDVIEHDVRRLDRLVSDISNASRLDSE
LVKEEETTVDLVRMLRGLADHLGQQARERGIDFIADLPPDPIEIQGLEARLAQVFVNLITNAISFCEAGDAVRVWARKRD
NRVLVVVEDTGPGIPGEALTKIFNRFYSERPAGQFGNHSGLGLAISKQIVEAHRGVIWAENIRPTEADVTSEQLGARFVV
GLPI
>MCE8455167.1 response regulator transcription factor [Rhodovulum sulfidophilum]AB15
MSRIALVDDDRNILTSVSMTLEAEGFEVETYNDGQAALDAFNKRMPDMAVFDIKMPRMDGMDLLQRVRRKSAMPV
IFLTS
KDDEIDEVLGLRMGADDYVKKPFSQRLLVERIRALLRRQEVIAADAGPSPEESKTMVRGQLTMDPLRHSVTWKGLDVS
LT
VTEFLLLQALAQRPGFVKSRDQLMDVAYDDQVYVDDRTIDSHIKRLRKKMRQVDPEFSAIETLYGIGYRYNEE
>MCE8455168.1 phosphoenolpyruvate carboxykinase [Rhodovulum sulfidophilum]AB15
MEFGRVNPNQRLEAQGIDGLGKVYYNLLEPALVEEALKRNEGTLGKGGSLLVATGVHTGRSPKDKHVVRTPEVVEHIW
WE
NNRPMEPEAFDLLHADMLAHLKGREVFVEDLYAGADPDNRLDVRIVTEMAWHGLFSRHLLRRPELEQLDSFAPEFTII
NC
PSFKADPERHGCRSKTVVALNFAKKLILIGDTEYAGEIKKSVFTLMNYILPSKGIMAMHCSANHAKDNPVDTAIFFGLSG
TGKTTLSADPARTLIGDDEHGWSDNGTFNFEGGCYAKTISLNPEAEPEIYATTSKFSTVIENMVFDPHTKELDFEDDSLT

ANMRCAYPLHYISNASETSLGGHPKNIIMLTCDAFGVLPPIARLTPAQAMYHFLSGFTSKVAGTEKGVTEPEPTFSTCFG
APFMPRRPEAYGNLLRDKIAKHGATCWLVNTGWTGGAYGTGSRMPIKATRALLTAALDGSLNDAEFRKDPNFGFEVP
LAL
HGVSEELLDPRRTWAEPAEYDAQAQKLVRMFSENFAQYLPFIDDDVKEAAIG
>MCE8455169.1 molybdenum cofactor biosynthesis protein MoaE [Rhodovulum sulfidophilum]AB15
MRIRVQAAPFDFGAECAAFSTGCGNAGAVVTFAGLVRDDGGLARMELEHYPGMTERALETISAEAMKRFGLLDCLVI
HRH
GSLIPGETIMMVATAAPHRADAFAAAEFLMDYLKSRAPFWKKEFGTDGAAWVAARDADEDALARWSDS
>MCE8455170.1 molybdopterin converting factor subunit 1 [Rhodovulum sulfidophilum]AB15
MIDVLYFAWVRERIGLPKERIETGAPTVAALVDELAAREERYAAAFADLSALRVAVDQELADFDAPLAGVREVAFFPPM
T
GG
>MCE8455171.1 CDP-diacylglycerol--glycerol-3-phosphate 3-phosphatidyltransferase [Rhodovulum sulfidophilum]AB15
MRWTLPNILSVLRLAAAPLFPLVYFLWDSPVADWVALVLFIGASVTDWIDGYVARRWHLTSRFGAMIDPIADKAMVL
AAL
LVVVAKFGMPTWLTLAATVIVFREVFVSGLREFLGAQAGRLKVTKLAKWKTTVQMVAISVLIAHGILPHAVIEWLGTAL
L
LAAAALTLITGADYFAKSWPYLKEEKVP
>MCE8455172.1 excinuclease ABC subunit UvrC [Rhodovulum sulfidophilum]AB15
MTPTEDSEAAPRPRGHACIQSYLATLDNSPGVYRMLDAESRVLYVGKARNLRKRVSNYAKPSGHSSRIARMISETASM
MF
LTTRTETEALLLEQNLIKQLKPRYNVLLRDDKSFPNILVAKDHRFPQIRKHRGAKTVKGAYFGPFASAGAVNRTLNQLQK
AFLLRNCSDSMFESRTRPCLLYQIKRCSGPCVGRISEAGYAAAVRDAEDFLKGKSSGLQEALAAEMQAASEAMEFERAA
G
LRDRIRALTQVQSVQGINPRGVAEADVIALHMEGGQACVQVFFIRAHQNWGNRDYYPKTGSGAEPPELLQGFLGQFY
DNK
EPPRLLLLSHDIEDRELMAEALSQKAGRKVELAVPQRGEKAELVENAARNARESLGRRLAESAAQGKLLAGLADSFGLEA
PPKRVEIYDNSHIQGSHAVGAMVVAGPEGFLKSQYRKFNIRGDSLTPGDDFGMMKEVLTRRFQRLLKEDPDRQGETW
PDL
LLIDGGAGQVGAVAEIMGDLGVEDLPVVGVAKGIDRDAGKEEFHRPGERPFALRHNDPVLYFVQRLRDEAHRFAIGTH
RA
KRAKAMGATPLDEVPGVGATRKRALLTHFGSAKAVSRAGLADLKAVEGISDSLAETIYGFFHERG
>MCE8455173.1 SDR family oxidoreductase [Rhodovulum sulfidophilum]AB15
MALHLAGRGHDVAVHYAGSAEAAAETVAEIRALGRRAVALRADLLDEDETQALVPRAAEALGGPLTVLVNNASIFEYD
TI
RTATRESWDRHLGSNLRAPFVLMQGLAAGVTEPARDAAGEALATGLVVNVIDQRVLKPTPEFMTYTLAKMGLWALTR
TAA
QALAPAIRVNAIGPGPTLKGARQSEDHFARQRASTVLERGADPGDICAALDYLLTARAVTGQLICVDGGQHLGWRTPD
VL
GVE
>MCE8455174.1 calcium/sodium antiporter [Rhodovulum sulfidophilum]AB15
MASDLLHAGLGLALLLLAGDSLVKGAVNLSLRLGVPALIVSLTIVAFGTSAPELLISIKAVLDDSPGIAMGNVVGSNIAN
VLMVLGIPALMTGIRTDECDTRRTYALMIGATLIFLFVCLIGPITWIHGIALMLVLGFVLVDAFLSARRHRRDAAEAARC
AAPDAAEDEVEGADPSLGWFKICMFLVLGLLGLPLGADLLVDASISIAREIGVSETVIGLTLVAVGTSLPELATTVMAAL
RRQADVAMGNVIGSNMFNLLAIIGMASFFGDIAVPPEMLRFDLWVMLGAALLLIPFVVWRVALGRAWGAVFFLLYA
AYAG
VLLL

>MCE8455175.1 hypothetical protein LZ188_00975 [Rhodovulum sulfidophilum]AB15
MLLKVALLFLIGMAALGMFGKLRLPRPRGGLLPGRRRPAKCPHCGRFRIGRGPCPCGKG
>MCE8455176.1 S49 family peptidase, partial [Rhodovulum sulfidophilum]AB15
RRGARLAADRDLFTGDVWVGQQSVEVGLADGIGHLVPKMKERFGDKVRFSLHAPRRGLWSRLGAPGVVGIADELEA
RLLW
SRYGL
>MCE8455177.1 LysR family transcriptional regulator, partial [Rhodovulum sulfidophilum]AB15
MKLGSENLNITLRHLRALHAIRAHGSFARAAEALGVVPSALTETVRQAEAEAGAPLFDRRMRPPQPTPLGLAFLEETAP
V
LADLDRALGRMRAQAGLSEGRLAVGAAPSAIGDLVGPALARFRAAHPGISCLLHDDVAERLAGLVSDGTLDLAVAGRA
RQ
SPDLTQTELRRDPFGLACGAAHRLAGREAVALSEIDPAEVITLSAETGTRQLLAGAAALPEALKETRLQAHSTIAQLCMI
RAGLGVALMPKAAVGLFGDPAIRFVPVADLGLWRSLYLLEPARRARSHVATAFLGQLERGAE
>MCE8455178.1 FAD-binding oxidoreductase [Rhodovulum sulfidophilum]AB15
MTDQSPPALTTALRAAGFRGEVETDRALRNAMSTDNSVYQVMPDLVVAPMDAADMVTLERVMAEPRFAHLPVTAR
GGGTG
TNGQSLSRGVIVDTRRHMTRLLDVDAAAGWADVEPGMVLDDLNERLRPHGVFFAPETSTSTRCTIGGMVSTDASGK
GSRV
YGKTSDNILGLEIARPEGLLSSLAPAPDWARPMLDAAETAARSGRAAFIAATPRLNRRFTGYDLERACPEPGGFEWWRL
F
LGAEGTLGLVSRIRVALRPLAREKRLLVAGFGSFRDALASAMPLAEFEPTAIEVMDERVQKLADGAGILTRLPPALRASE
GQPLAYVFIEFNGDDPDDLQTRLSACETAIRALPGARAIHMARDAAEIRELWAVRSAGVGLLGKVDGRARPVAFVEDC
VV
PPGNLPAFLDDFLAVLDAHGLDFGIYGHVDVGCLHVRPALNIDTDRDRLVAVSDAVFALTRKHGGIFWGEHGKGVRGA
YL
RDWIGPEAYAALQGVKAAFDPAGRCNPGKLVGLDAPVPAIDEIPFRAFNAPEGDALSRAFRCNGNAQCLSYAAATPM
CPS
FKATADLRQSPKGRADALRAWRQARLKGRVDAALEADLTATLDACLGCKACASSCPVQVDIPAMRAAFYADLYARTR
RPL
ADRLILLAERESVRVQALAPFMRPLWPLLARIGGALTGTVDLPKALAPALPRADRIGAADLARPLPEGTVILVEDWFSAL
FDGAARTDALAGLKALGYRPRMLDMRPAGKTAQSLGDPRFAAMATRFRRHLEAAAATGAPLIGLDPALVMQLRQDY
PQAG
LALPPVLLPQEFLAREIAAGRRFPDFATAKGPAPALFSHCTEATGATQAAADWRRVFAALGVEIDTPATGCCGMAGLF
GH
KTRHQTVSKRLFEMSWAPKIGTGAPVLATGFSCRCQTERLAKLPARHPLGLIADLAA
>MCE8455179.1 GntR family transcriptional regulator [Rhodovulum sulfidophilum]AB15
MNGADPAAWIRPVEKQSLSEQAYRGLRNALMRGELQPGTVLTLRPLSQRFGISATPMREALMRLVAENALRLDSRGR
TCV
PHLTRGILMEIRGIREHLEGNAAAEAARVAVPGDIDALEAMHRRMIAAQDAGDFAEAIGENTHFHLMLCEMAGQPVA
LDL
VSNLWMRCGPILSHLYDAGIPPNWEPHPHRRIVAAIRAGDPEAARDALIYDIRGNGEGLLAHVAEGLPD
>MCE8455180.1 NAD(P)H-quinone oxidoreductase [Rhodovulum sulfidophilum]AB15
MTGVLSVTAPVPGGPEALTLETRTLPAPGPGEMRVRVHGAGVNRPDAMQREGNYPPPPGASDVLGLELSGEVLECGP
GVT
RFAPGDRVMALVAGGAYAEEAIVQADVALPVPEGLSMTEAAGIPETYFTVWSNLFIRAGLQPGETALIHGGTSGIGVTA
T
LLARAIGAEVITTCGSDEKCRASEKLGARASVNYRDTDFVAAVRELTGGKGPEVIFDMVGGPYMQRNLDLVAEDGRIA
QI

AFQQGSRAELDMGPVLFKRLTVCGSTLRARPLAMKAELAREIEAKVLPLILEKGARPIIDSTFPLNRVQDAHARLEASAH
TGKIVLLTSAD
>MCE8455181.1 ABC transporter substrate-binding protein [Rhodovulum sulfidophilum]AB15
MTTRSILLGTALGLTLAGSAMADVKIGSLYPFSGPLALLGEESARGLDLAVDRINSEGGIHGEQIVLERGDAVDNNQAIG
EARRLISLEGVKAVFGTYSSSRVIAASQVTELAGIPYFELGAVADEVTGRGMTYLFRTNPTAENMGIAIIDMLADQIAPA
AGKAPEDLKIGIIYEDSSYGTSVAGHEVAHAKERGLNVVLKSGYPANIVDMSSLVLDLKGKGVDVVLQTSYQNDTVLFLQ
QANEAGYEPMAIIGGGGGYSMQPTADAVGDDVIDGIYDVDFTQYAINPDFTPGIETFLDAYRAKYGEEPRSGHSLTNY
VG
ALAVLQAIGEGEGVEPDQIVASVKAMDIPDGSTAAGYGVKFDDTNQNERARMMGMQWQDGKLVTVYPSDAAVAEI
RLPE
>MCE8455182.1 branched-chain amino acid ABC transporter permease [Rhodovulum sulfidophilum]AB15
MTTFLQILLNGLMLGGLFAIVSIGLTLIFGIIKVVNFAHGEFLMGGMFITWLITTQLGLHPYAAVIVVVPVLFVMGALTQ
RLLIQPLMSARDDHIQIFATVGLSTALINLALLIFGADIANTPPHGLRAPLEFGDLRLLSGQLMIFGAAIVLVVGLELFL
KYSQTGRAIRAVAQNRGAAELMGINVNHIYVLTFGLGAACVGLAAVLIAPLYPTSSNIGTYFVLTAFVVVVLGGLGSIPG
AFLGALIIGEVDALSGYYIGSDLREAVVFGLFLLILILRPSGLFGARQNLSHLS
>MCE8455183.1 branched-chain amino acid ABC transporter ATP-binding protein/permease [Rhodovulum sulfidophilum]AB15
MSNETTSGTSSVPAPSLPWPAIGVALVLASLLLIPLGIGNAFVTHIFVTICLMAALSSAWNLVGGFAGQLSLGHATFYGI
GAYTAVILVNLGITPWIGMFAGAGIAAIVGMAISYPCFRLKGPFFSLASIAFLEVFRVLTLHFSDFTGGATGKMIPLKLG
WTWMVFRERWPSLLIIFGMLVAVLVVVWAVRRSRLGFYLIATRERESAAKAAGVKTVQVRLVAVAISSGLCAMLGTFH
AM
YLTFIEPSAMFSLTFSIQIAMFALIGGLGTVSGPLLGAVLLVPLTEWARASLGASALGLHGFVYGVVLLLAVLFMPNGIM
GVLQKFRRAPHHDIDEDEKTTQTDSIKAPAQAATPPSPDREGIGRDVMRVQNLNKRFGGLHVTKNVNFTLREGEVLGL
IG
PNGAGKTTLFNMLSGFLTPNEGSVTVRGPDGGYHTPVNPADFAALGVGRTFQIVQPFAAMTVEENIMVGAFYRHHD
EHDA
REAARQTAWRMGLGPLLQDEARGLTIGGLKRLEVARVMAMEPRILLLDEVMAGINQTDVRRAIDLMLSIRDSGVSIIAI
E
HVMQAVMSLSDRVLVLAAGEVIAEGDPHEVVRDPHVIEAYLGKEFANAHR
>MCE8455184.1 ABC transporter ATP-binding protein [Rhodovulum sulfidophilum]AB15
MLTVENVHAGYGPTSILQDVSLDVGEGEIVTIVGANGAGKTTTLKTIAGLIAPTSGRITFCGEDITRLRGDQVVDRGITL
IPEGRQLFPDMTVRENLLMGAYRRAARDFQDERLEEVLDIFPRVRERLNQTASSLSGGEQQMVAIARGMMANPRLL
MFDE
PSLGLAPIIVSQVFEVVQQIAAKGTTVLIVEQNVYSTLKVAQRGYVLENGRIVLSESAEDLLQDDYVRQAYLGI
>MCE8455185.1 3-oxoacyl-ACP reductase FabG [Rhodovulum sulfidophilum]AB15
MTRSETVRPEFRNRLSHQGRRVLVTGGARGIGLAIAEAFAARGATVALCDLDPEAVAGATAQIAASDAAGSALPVTCD
VS
DYAALCAALEAAGGNFDTVINNAGISPKHDGVAHKVWEMDPAEFRHVLDVNLTGSFNTVRALAPGMIAAKRGWVV
NMSSV
AGKTFMPVVACHYAATKSAILGFTRHLAGELGPHGIRVNALAPGRIETPMVLGVAPEVNAAQVEMTPLARLGEPAEVA
EA
ALWLTDAESSFVTGQTIDVAGGLLMT
>MCE8455186.1 shikimate dehydrogenase [Rhodovulum sulfidophilum]AB15
MITGKTRVWSILADPIGHVKTPQEINALCAAQGIDGVMVPMQVHPENLATAVAGLKAIESFGGFVVTVPHKTKIVALC
DR
LIGDAARIGAVNVVRREADGTLTGAILDGLGFVEGLRSEGIEPRGMDACLLGAGGAGAAIAFALAEAGVRRLTIVNRSQ
D

KAEDLAARVAQAYPEVPVAAGAPETASRDLIVNATSLGLKPDDPLPLDDTAFHTGQVVADAIMDPVETRLLAAARAAG
AR
VHPGLPMLRQQIRLMAEHLGALPRPPQQD
>MCE8455187.1 GMC family oxidoreductase N-terminal domain-containing protein [Rhodovulum sulfidophilum]AB15
MPQDLPDRSRFDYVIVGGGTAGCILAETLSRSGRASVLLCEAGGEARSPWITIPAGFYKLLTNPAYNWGFWSEAEPATL
N
RRIAIPRGKGLGGSTLINGMIYVRGQPQDYDLWAERGCTGWGWDDVLPHFRALERWAGPDPDGMRGQDGPMPISE
VVEKN
PVGQAFIDAARAQGQPVNPDYNGARQEGVGWYQVNQERGERHSAARAWLARARRRPNLTVLTHARATRILLEGRR
ATGVT
LARPSGTVRVDAGEVILAAGAVQTPQLLELSGIGDAERLRGLGIEAVHDLPGIGENYLDHFCTRMNWRVSQPVTLNELT
R
FPKLLGEVAKYALNRRGVLTYGTGLAHGFLRSREGLDRPDVQYFFMHASYANAAERKLDRFPGMTLGVTQLRPQSRGS
IH
AVSPDLAVQPEIRPNFLDHPEDRRAMVDGMKLGREIVGQSPMDAFRVAELSPGPDCRDDEDWLQFARANGQTIYHA
AGTC
RMGTDIAAVVDPELKVRGIDGLRVADASVMPEMVSGNTQAAVMMIARKAAQGIISAG
>MCE8455188.1 NAD-dependent succinate-semialdehyde dehydrogenase [Rhodovulum sulfidophilum]AB15
MYPDLTLILGGEARATASEGTMTVSNPANGQDLATIPRAGRAEAEEAARLSLSGFAEWSALSPYERSKVMRRAAGLMR
ER
ADRAARIMTLEQGKPLAEARGEWMGSADLLDWFAEEGRRVYGRIVPSRAPSIHISVLKKPAGPVACFTPWNFPAWNT
MQK
VAPALGAGCSVVIKPASETPGTAWMIGQALLDAGLPPAAISVVWGKTSDISDALIAAPEIRKVSLTGSTRVGRIVAAQAG
QHLKKVTMELGGHGPVIVAGDADLDRLVPMAVQWKYRNAGQVCVSPTRFIVDDAVHDEFVARLAEGASALKLGDGL
EEGT
QMGPLTQKAQLNQVAEMVADAESRGAKVETGGARVGNIGNFFAPTVVSGMTPEMLAMNEEPFGPIALVIRSTGLEA
ALAE
ANRLPVGLGAYLFTRSMEVSQEVQDRLKSGMLGINHFALALPETPFGGVLDSGYGSEGGIEGIEAYLSTMTVTAAI
>MCE8455189.1 GFA family protein [Rhodovulum sulfidophilum]AB15
MSGVHEGGCLCGAVRFRVTGDFGHFFLCHCARCRHDSGSAFAATLFSGAARLDWLAGREQVRTFRLEGSRHGRAFCG
RCG
SALPLETEGAGLAVPAGCLDGNPGIAPEAHICWADRAVWEDALEHAPRIAGLPGAE
>MCE8455190.1 FadR family transcriptional regulator [Rhodovulum sulfidophilum]AB15
MGRDLPDDIAARLGQRLAGGEFGAGERLPSERALAAEYGVSRAVVREALSHLKSDGLVEARAGSGVFVTENREARSFR
MT
GLDLDGTEALAQVMELLEAVEVAATRSAALHRDAADLKAIRRALIGMEYAIASDELGDEQDFAFHQAIVEATHNPHFVS
L
CKHLEFGARDVIRKARANTRANLSDLLDAVQAEHQAIYRAIAEGDPEAAAAAAACHLRNAAERTQLYRHRERGA
>MCE8455191.1 SDR family oxidoreductase [Rhodovulum sulfidophilum]AB15
MPDRDSTPPARVPVTFDFTGRSLILTGANGGIGRAVARIFAEGGAHLLLADRDLAPLEDFAATLPGPGARICQQIDAAD
P
DAAQALIDEAVAEFGGIDHVVPSAGIYLAEPFAEMTDAAWRRTLSINLDGVFYLLRRAVPHLREGSSIVTLSSMAAYRGA
LTNAHYGASKGAMVSLTRSLANELGPKTRVNGVAPGIIETPMIGDYMKTRAEATLANTPLKRLGQAEEVAAAIAFLCSD
A
AGFITGETLQVNGGLHMA
>MCE8455192.1 SDR family oxidoreductase [Rhodovulum sulfidophilum]AB15

MTDFTGKRVLVTGAGRGLGAAIAEGFAEAGAETLLTDIDPETLSATAEALGARGLSARAIPLDVTDRPAVEALAATLDGL
DVLVSNAGVAGRATFDDPAQAEVWDRVIAVNLGGAFNIARALTPALIARRGNVVQLSSVAGFVAGGSTAGYVTSKGAI
RS
LTQAMARDLAPHGVRVNAVAPGIMDSEMANAQLARPGGAEWFLSRAMLGRIGTQAELVAPILFLASDRASFITGTVL
PVD
GGFLAA
>MCE8455193.1 thiamine pyrophosphate-dependent enzyme [Rhodovulum sulfidophilum]AB15
MPDITDLTPLAPWREIRATEADRTALDPETGRTMLAQLCLIRAFEEKVLDLAGHGLVHGPAHSAIGQEGGAVGSALAM
RP
TDQVNGSHRAHHQFLAKALAYVAEAGIDPEAEFDGEIRDLSKKTLAEIMGLSQGFCRGRGGSMHLRWAEAGNLGTNA
IVG
GGVPMAAGAAWAHQQAGTGDVVYTYFGDGAVNIGSVLETMNLAAAWKLPICFFIENNRYAVSTHVNEATAEPRLSA
RGLA
FGIPAFKVDGMDALSVLIASHEANRIMRSGEGPVVIEADLYRYFHQNGPLPGSAFGYRDKAEEAEWRARDPIEALAGLL
Q
TRQIIGEAAVAGLRARCETMMEEIAGELTETEGGKTRVIPSLWPEESFRDAGLRGDLSEMDGARYEEEHSFSGALDANA
K
FIDAVAAVMHRRMDTDDRVVVLGEDVHRLKGGTNGATRGLSQDFPDRCLGTPIAENAFTGLAAGMAADGRVRPVIE
FMYP
DFMWVAADQVFNQIGKARHMFGGDSPMPLVLRTKVAMGTGYGSQHSMDPAGIFATAPGWRIVAPSTPFDYVGLM
NSALRC
DDPVLVIEHVDLYASKGAGPVDDLDYCIPLGKAKVVRPGARLTILTYLAMVEKTRQVVEDLGVDAEIIDLRSLDRASLDW
ETIEASIRKTNNVLIVEQGARGTSYGGMLADELQRRVFDWLDQPVQRVTGGEASPSISKVLEAAACARPADIAEAIRAA
M
AAQGLPLAAE
>MCE8455194.1 E3 binding domain-containing protein, partial [Rhodovulum sulfidophilum]AB15
MATELTLPALSAGMEDAVIAAWLVGPGDRVEKGQPLAEVETDKATMELEAEAPGRVGRLAVAAGARADVGQVIAVL
LAEG
ETLPAPDPETPASAPAPASAPAAMAPAMGPAAEPDRPCASPLARRLAAQAGLALDELAGSAPKGLIV
>MCE8455195.1 ABC transporter ATP-binding protein, partial [Rhodovulum sulfidophilum]AB15
MTEAAMAGAPPGNEASKPPDFFASLKLLMRHGGKAGPLLSISAAMATCAVALELVPVWAVWRLVTELVSGTATPAIF
VTC
AVAALVAVILGYLLMGLALGLSHVAAFRLIHALRLALARHLARLPMGWFAGRRSGGAKKLIVDEPERLELLVAHGLPEGI
SE
>MCE8455196.1 TonB-dependent receptor [Rhodovulum sulfidophilum]AB15
MMRSLLNTACVAALAFGSLAFAARPLEARDRSADGELDLGTIFVRGAKRPQEALSFPGAVAEADASELQAREVRNIADL
D
RVFPGLTVDTRSARVYSNFTLRGQSSLDYYNPSVQVYVDGLPQDPAVLAQMFPGALENVEVLYGPQGSLYGRGAVGG
VIN
VTTTKPGEGAPFSWSAGGSDQGYQAQMRGQVQLGEGLWADLSLARDDAEDDLDVMGTGEDVGGTVEDRAQLRLR
YAPEGS
PLDIMLSAARNRIDSSEEQFVKGTDLSGRKAVPFPSDYIQDTDSYGLTADYDLGWGKLSLLTGYQDRSLDRTIFGSDTPE
WQKTLTQELRLSSATGGPVDYVVGLFYSHTKFHRDAYGASTLQKTDSYAAFGDLTWHATDRLDVTAGARWDYEKTDA
TAT
GAVSLDGSDDWSALSPKLGVSYALAPDWQAYGIVSSGFKAGGFTRTLTPSNISYTYDPATTWNGEAGLKYRSGDGSVE
AS
VAAYYSVTDDYQMFVGPAASQYLQNVGEVTAKGIDARLRVNRGAWGVTGGATWGSSRFTGYDDASGTDHDGNDV
PYAPDF

TARLAVDYTHDLGHGRGSLIPRMGLSYQGKVWFDEANSLGQGGYWLVDAGLAWDMGQDRVLDLYATNLTDETYAS
YGFDA
SGYGLGDVYQLGRGREVGIRFTQSF
>MCE8455197.1 MFS transporter [Rhodovulum sulfidophilum]AB15
MPALALVGLYAAQSITGSMVQSALPVVLRDAGVGLDRIGLLSLLFIPWVLKVFWSPLVDRFATPRRWILTCQGLLCLCFL
AGAAFAPERHLGALLPVVFAMAVLAATQDIATDAAGVYATTRRTRSLASGASTVGGYAGFLIGGGLWLWIYARLGWEI
SM
IFLAVAMLLLTLPVRALRIGRPEPAARPPSPLLSLKNPALMRGAGLLLLWQGGVRLGLAMTGPMLVDAGFSMELIGWL
RG
AGGMAAGLIAAVAGTVLCRRFGPRPVLLAAGGGIGTCCAGLALWAAAPGALWQLAALQLGLSALTAVSFVALYAEM
MNWC
APAQVATDFALLQSLDAALAVATGILAGQLAQHMGYAPVFGLAVVLLMSALPLVRRLAGTCPASEIPLKNMEIAE
>MCE8455198.1 AraC family transcriptional regulator [Rhodovulum sulfidophilum]AB15
MGLQIETRQLAHAEPHEVAVLGPQPLSSSFGGMPESGSYRILGLRKGVTISLFDIETGTGMAADMHTAPCVALSLLFEA
A
GTGWITEGEGQFGPIPLRPGIYCMIAPAGATGQDRFKPSSRLRGIDIRLEPSFWSALGGPSSIDALGAGHPHAAMASPS
V
WVGLLPLTPRLMADAQALFRSGMSGTADLTVEARSLYIIDEAIRALAAGVNMATAIAPAPLLARDRRAVQRVIALMEG
DL
GRAWTVPELAQAAGISHNRLKQSFRAETGCAVYGFLQERRLGEARRLLLEGKGSVTDIALSVGYGSLSHFTALFRRRFGQ
TPSDVLSLSKTARRTG
>MCE8455199.1 BLUF domain-containing protein [Rhodovulum sulfidophilum]AB15
MLKESPKDGTAEGRLFRIAYKSRAHPGCDLASAGAMAAEDALRNRDRKVTGVLLFERGWFLQWLEGPARTVLSLASAI
RT
DPRHGEMQILSAGATDERRFAGWPMRLAPPSGPLADRADFDAAAAALAFDRAAALYREECGGEADWRDLADGFVR
DLARA
PEEAVPDLPPSALETLSARAGFVDALCAALAEAWHASALSGFEVTLATVRLNRLLLRAGRPATSLAPKGRVLVLAPYGCT
EIAGAIAKTDLLRQAGYSVRMVPWASGAALDDAVGETGSCPVIVYAGRVGIGTKDAMRGAALAERLSARMPGRTVLL
GGR
DAGPLCDWSHRLAFLAQQLPTGADPAADPALRTRQRV
>MCE8455200.1 isocitrate lyase [Rhodovulum sulfidophilum]AB15
MSQRKTYSELRSDVLSRYPSGRSSSGVSVDDIVQLKLQNTYATHLDIAREMASVMRADMAAYDADPRNSTQSLGCWS
GFH
AQQMIKSVKRLRGSARGVYVYLSGWMVAGLRNRWGHLPDQSMHEKTAVAELIQEIYTSLRQADEVALNDLFKALDA
ARTA
GDTAAEAAAIEAIDGFESHVVPIIADIDAGFGNEHATYLLAKELIKAGACCLQIENQVSDAKQCGHQDGKVTVPREDFIE
KLRACRLAFEELGVDDGVIVARTDSLGAGLTQKVPVSRAPGDLASQYIKWLKTEPITEENPIREGELALYRDGEFMRPAR
LPNGLFPFMEGTGRARVVEDCIASLTQGGADLLWIETDTPNVDEIASMVSEIRAAVPDAKLTYNNSPSFNWTLNLRKQ
VR
AQWLAEGKISEDSYPDGNELMKPDYDETDLGREADARLRAFQTDISTRAGVFHNLITLPTFHLTAKAMDELSRGYFGED
R
MLAYVATVQREEIRRGISAVRHQHEVGSDLGDTFKEMVGGERALKAGGSANTMNQFAAE
>MCE8455201.1 malate synthase A [Rhodovulum sulfidophilum]AB15
MSLDHHVPTASTPLVLREAPGAERVLTAEALEFVAALQRRFGMNLHALMIARQRRQERNDNGRLPDYLEETQAIRQG
VWQ
AAPVPEALADRRVEITGPVDRKMMINALNSGARVFMADFEDATAPSFANIVAGHANLLDYRDGTLTHDDPKTGKSYR
VGE

NPALLIARPRGLHMEEANVLLAGRPVSAALFDFGLTLFHCGRALAATGRGPFYYLPKIESHREAAFWNSVFEFAQDRVG
L
PRGTIKATVLIETLPAAFEMDEIIWELRDHIAGLNCGRWDYIFSYIKTLRAHPDYVLPDRAEVIMDEAFLAAYAARLVKV
CHRRGVHAMGGMAAQIPVRNDPEANEAALARVRADKLREVRLGHDGTWVAHPDLVPVALAVFDAEMPGPNQIRT
PRQEYR
LLPEMLLRPHEGRITEEGVRTNISVAIEYLAQWLSGRGAVPIMNLMEDAATAEISRAQIWQWIRHGARVAARDGTERR
MD
AAWLDALVQEEIAAILGRTGPGGFHRGHYASAARIFQEAATAETLPDFITTPAYAVLNALD
>MCE8455202.1 short-chain fatty acyl-CoA regulator family protein [Rhodovulum sulfidophilum]AB15
MAKRGEKLVIGQRLKVLRKSLGLTQAQMAAELGISPSYITLIEADQRPASARLLMRLAEVYDLNVSELAPGIDAQLASDF
TAALKDPALEARGVGRAEIEAVLQSAPGIAAALVRLQAKYTHAAMQAQAEDNPLTDRNKVELLGQIARPVEIVRDWFY
ET
QNYIDRVDRAAEAVAEDMALRRTEPHLALTERLADHGIRIRILPAPVMGGSLRRFDLHRRELLLSELLGQSSRRFQIGVL
LARLEQQELIDAVIRSAGFGAGFEDGDACRLARVSLANYFAAALMMPYGRFLAACESERYDVELLSHRFGTSYEQTAHR
M
STLQRPEARGIPFFFVRVDRAGNVSKRFSAGRFPFSRFGGTCPLWNIHGAFEAPGRVHTQVIRMPEGASYFSIARTVTR
A
GGTHSAPAPQLAIGLGCDIAYAPRLVYADQLDLGRVKPVDIGLNCFLCERRDCASRAQAPINRKLAVNELERSLAVYRFE
GE
>MCE8455203.1 metal ABC transporter permease [Rhodovulum sulfidophilum]AB15
MMLETLLMPFQFGFMQSAFAIALIVSVPTALLSCFLVLKGWALMGDAVSHAVLPGIVLAYVAGLPLILGAFAAGMLTA
VA
TGFLSENSRIKQDTVMGVVFSGMFGLGIVMFVALRTNVHLDHVLFGNMLGVGPADLWTAGVISVCVSLVLVVKWKD
LMLY
AFDPAQARASGLPVGLLHYGLLAILSLTIVATLSATGLVLAVGLLISPGAIAFLIVRSFGRMLAVSVLVCALAMLGGTYA
SFFLDSAPAATIILVLTGLFVLAFLRRLHLTRSASRRAATA
>MCE8455204.1 metal ABC transporter permease [Rhodovulum sulfidophilum]AB15
MSALLEPFAYGYMTNAMWVSALVGGVCAFLSCYLMLKGWSLIGDALSHSVVPGVAGAYILGLPFALGAFLSGGLAAG
AML
FLSDRSGLKTDVVIGIIFTAFFGLGLFMASVNPMSVSIQTITMGNILAITPGDTLQLAIIGIVSLTVLVLKWKDLMVTFF
DESHARSIGLRPGLLKAVFFVLLSASVVAAMQTVGAFLVIAMVVTPGATAYLLCDRFPRLILTSVTIGASTSFAGAYISY
FLDGATGGVIVVLQTLVFLATFLMAPKHGLLAARRKAAAALTEEPA
>MCE8455205.1 manganese/iron ABC transporter ATP-binding protein [Rhodovulum
sulfidophilum]AB15
MKDELQKTGAARPDATDPGSGIEARGVTVTYRNGHTALRNASFRIPRGTVTALVGVNGAGKSTLFKAIMGFVPVAQG
DIR
LLGLGVKEALRRNLISYVPQSEEVDWSFPVLVEDVVMMGRYGHMGFLRRPAKADRAAVDAALGRVGMREFRHRQIG
ELSG
GQKKRVFLARALAQDGQVILLDEPFTGVDVKTEEQIIALLRALKQEGRVMLVSTHNLGSVPEFCDRTILVKGTVLAHGPT
ETTFTRPNLEAAFGGVLRHFTLGGPDLHEDDDMRQVSIFSDDERPLVQYGERTRKAERSQ
>MCE8455206.1 metal ABC transporter substrate-binding protein [Rhodovulum sulfidophilum]AB15
MAGPALADEDRMKVVTTFTVLADMARHVAGDAADVVSITKPGAEIHGYEPTPRDIVRAQDADLILSNGLNLELWFQQ
FLD
HLGEVPSVTLTDGIDPIPIAEGAYQGKPNPHAWMGLDNALVYIDNILEAFAEHDPRNAATYVRNASAYKDELRATLEPL
K
AEIATIPEAKRWLVTCEGAFSYLARDFGMKELYLWPMNADQMGTPQQVRAVIDGVRQNHVPVVFCESTVNTAPAEQ
VARE
TGASYGGVLYVDSLSEPGGPVPSYLDLLRVTSQTVADGLTATTN

>MCE8455207.1 transcriptional repressor [Rhodovulum sulfidophilum]AB15
MTKANHESLLDICRRHGLRITGQRRVILEVLDASGDHPDVEELHRRVAAIEPGINIATVYRTMNKLAEKGLIERHMFEDG
RLRYEPAPEQHHDHFINLETGEVVEFRSDEIERLQEEIARKHGFEIVSHKLELYVRARKPR
>MCE8455208.1 5-methyltetrahydropteroyltriglutamate--homocysteine S-methyltransferase [Rhodovulum sulfidophilum]AB15
MTTEDTSSLRPALPRADHVGSLLRPQAIAGARMQGLKGAELCAVEDREIAALVAMQKEAGLRVFTDGEARRQFWHYD
FMG
MLTGLDMKPSTRSLPFQTKHKTPPIEAHLTGPLDFPADHPMLRHFTYVRDLVAPGEGIAKISIPGPSACHYRMVPENIEH
KPYLDQELMFHDIAATYRKAVQAFYEAGCRYLQLDDIFFAYLGDEKQREERRAMGQDPDELVHKYAWMLEEAIKDRP
ADM
VIGMHMCRGNFRSSHVAEGGYHGAADAIFNTTSVDIYLMEYDTERAGGLEPLASLPKGRKRVMPGFITTKTGKLESLD
WI
RAKYDEASKYVDIDQLGVAPQCGFASTEHGNEITEDDQKRKLELVVKTAEAIWGES
>MCE8455209.1 Crp/Fnr family transcriptional regulator [Rhodovulum sulfidophilum]AB15
MVGKLDESLLQDVGPFRRLDRGQIREILDLATARRFDEGAAVFREGHPAERFHLLMDGVIRVMRLTAEGDAVTALHIPS
G
QLFGIARALRRETYPATAVCARECLTLSWPMSLWDGFVDRYDGFAEEGYRTVGTRVGEMNARLIEMATQQVEQRVA
GALL
RLIDQSGRKGQEGIEIAFPVTRQDISEMTGTTLHSVSRLLSAWEKAGILASRRRHVTVTDPRRLELIGEGR
>MCE8455210.1 NnrS family protein, partial [Rhodovulum sulfidophilum]AB15
MSTIPRPALFSYGFRPFFLLAALFAAAVIPLWLAVWAGHAALAGPFGPRDWHIHEMLFGYVPAVLAGFLFTAIPNWTG
RM
PRRGLPLVALSGLWLLARLAVAGALALPAWAVMVLDLSFLLAVAAISVVEIVAGRNWRNLAVIVPVGLLAAANATFHIE
A
MTRGAADIGMRAGFACILFLILLIGGRIVPSFTRNWLSRQGPGPLPAPFGRFDRLALMASAAVLALWSLAPGLPGLCAA
F
AGLAGLHLLRLARWRGGRCGRAPILWILHIAYLFLPLGFAALALDAPVAGLHLLGIGAIGGMTLAVMMRASMGHTGRP
LE
AGRVLTASFLLLCAAALIRALLPDQGLDGIGGPGLSGALWTAAFAGFVLKVGPWLILPSVARR
>MCE8455211.1 flotillin [Rhodovulum sulfidophilum]AB15
MSSFAIPVIMGAFVAFLLLIGLVLARLYKRATREVSLVRTGAGGKRIVLDGGCVVIPLLHEVSRVNMRTLRLEVRRSGEG
ALITQDRMRVDVGVEFYVCVAPTEDGIAKAAQTLGDRTFETEGLREMIEGKLIDGLRSVAARMTMDGLHENRADFVQ
EVQ
NAVSEDLTKNGLSLESVSLTALDQTPFEDLDENNAFNAEGMKILAEVIANSKKRRAEIEKDADVAVARSRMEGEREKISI
EQDEEQARIEQNQRLARMRAEQEAQIAADQEKSSEEKERARIVREQAIRAAEIASARELEVAEQDRQITVSRKSEEESQA
RASADTARAEAIGAEESVKTAREVAEAERAKQIALIGAAREAEERATEIRVAALAEKEAAADRAAALREQAQAEADAARL
RAEAQKAEMLAEAEGRRAMVESENGLNQSVIDMKIALARLEAFPAILAEAVRPVEKIDGIRIHQVTGLGSASFGHGAPG
T
ETAAGPESPVNQALNGLMGMAVQMPALRRLGEQAGLSMDAGLTGLVDAALSDSVPGHEDVPVNGAAARGGPAEK
AGSLAQ
>MCE8455212.1 YqiJ family protein [Rhodovulum sulfidophilum]AB15
MTGTYGFFGVFLSPQLVPFTTALVLLAGMLVLELGFALMGGTLLGMGGSPDGPDLLETDLPDPGALGLDPGIDPGEFEL
A
DPDGFSGASPADGGTGALGGVAALLGLGKMPLMIWLASVFMAFGIAGVAIQTAAYGLWAGLLPGGLVAIPSAALAILF
TR
SFGAVFARLLPRTETQSVRARHLGRRMGVVTQGTASRGRPAEVRVRDRYGNTHYLRAEPLRDEDSIPAGCEVLVMRH
RPT
GSFRLISLDN

>MCE8455213.1 sigma-70 family RNA polymerase sigma factor [Rhodovulum sulfidophilum]AB15
MTADCKDIAALYVTEHDRLERQIGRRVGCRNTASDLVHDIFLRLWERATDRNGDSRAYLTRCARNAAIDHIRAERTRSD
A
AGSILAEQYGQAPARPEEILAARQDLRSLDQVLADLPKRTRHIFILNRVHGGTLSEIASVMGVSRRTAPLLRTKPAPSRP
VRISGRIRR
>MCE8455214.1 LuxR C-terminal-related transcriptional regulator [Rhodovulum sulfidophilum]AB15
MAYDQISDADLNLTGFMDAPIGLMVLSNRVIQRINTEIERIFGWTRDELEGRSIRALYPSSVDYEKTGSRWRRWLDERP
R
YTDERFMQCRSGEIIWTRASGRTLTPGDPFRLMVWTFEHLENRPPNSATLTPREREVARYIVNGYTSKETGLAMGISPR
T
VEVHRGSVMKKLGVQNAAELVAKMIVQN
>MCE8455215.1 thiolase domain-containing protein [Rhodovulum sulfidophilum]AB15
MTQDIFITGSGHTRFGRLEDTLEGMIVEAAREALEEARIPPADIDAVFLGHFNSGLVPDGFASSLILQAHPDLRFKPATR
CENACASGAAALHAGLNAITAGRARNVLVVGAEKMTHRPTADVTAALAGAGYQNDAAEAALSFPQVFAIAAGQYAD
RYGD
PMDAMARIAAKNHENAMKNPLAHMHKPLSHEFCREVSDKNPLIAPPLRLSDCSLVTDGAAAIVLSAADAHARDATAR
VRI
AAAEHVSDYLPMSRRDFVAFEGPERAIRRAYETAGITVDDLDFAEVHDCFTPAELLIYEAMGLAPKGQGARALDDGRV
HA
DGRTPVNLSGGLKAKGHPVGATGVSMNALAFRQLTGRAGDMQRKGAEWGLTFNMGGAAVANYATILQADRA
>MCE8455216.1 AMP-binding protein [Rhodovulum sulfidophilum]AB15
MNIANWLYQTALSWPSRPALYEGDSLRQTYHALLQATCDRAAWMQAVHGIEKGDRVAIFAKNTPEYIELLHACWWL
GAVV
VPVNCKLHPREAEWIIRDSGAALTLTESGRVFADRPDIREVALGSPVAGTKEIVPPARVGDRDLAWLFYTSGTTGTPKGV
MLSHDNLRHMTLCYALDVDTARPEDHMLYAAPMSHGAGLYMFAQIRAGGAHLVPASHGFDSDEIIALAKTRGNLVFF
AAP
TMVKRLIATAKQQGHDGTGFRTIIYGGGPMYAHDIDEALACFGPRFVQIYGQGETPMTISVLPRDLVADQSHPRWRA
RRA
SVGIAQGAVTLAVLGPDGTALPPGHTGEICVQGPTVMTGYWRNEAASAQALQDGWLRTGDLGHLDEDGFLYLTDRS
KDVI
ISGGTNIYPREVEEALLAHPAVFEIAVIGEPEPDWGEQVVAYIVFEPGKSASVPELDSWCRQQIAAFKRPKRYLFVEELP
KNSYGKVLKSDLRSGKGAATATA
>MCE8455217.1 C4-dicarboxylate TRAP transporter substrate-binding protein [Rhodovulum
sulfidophilum]AB15
MKRRDFLKSTGAFAGAGLISGLPGLANAATSLKFDSYVSETAGPSWVDRWFLEELAKRSNGDIGIKYYWAGSLNRVGE
HL
GAVRDGTSEMTLIAPGYYQAELPVTRGLEWYFRMDRADELQLVCRDVYENFAPLRQEWEERHRSKVLYWTNWNYAP
LVMR
DPISSIADIKGKKIRGYGVATDVIESLGGIAVPMAAGEVYQALERGVLDGVYGFDFVTAVAYKLHEIAPNFYDIGDGPHA
PAVTIMNRRVWEGLPAEQQQICTDLADELYGGKFAEIYNKVLADYVRKAVAEGVVFASLSDEEKAQTKALVQPAQVDK
WI
ETVARPNGIDGAAMQAVVEEAIARHAGTGTMKRPIEIAADL
>MCE8455218.1 TRAP transporter small permease [Rhodovulum sulfidophilum]AB15
MLARLNRLFDGLARITSWVALGFLAFMMVAITVDVVARATFGRAVPGLFEMTEMSMVMVVFMGLGATLLDDGHIR
VTMMT
DALPGPFSRGCTALAWLFAALTFLMLAWPATQEAAYSFSIREFRWGYFQVPIWWAKIAVAAGLWFAALQAALHSLRIA
LK
QIELPHIDSLRSGPGDKPLQ

>MCE8455219.1 TRAP transporter large permease [Rhodovulum sulfidophilum]AB15
MDVTLITLLAVGLIFFLMALGAPVFAALALSGAFGIVMVENLGFLLARLKSFSFSHTANYALTVIPLFILMGAFAHHAQV
GKRLFHVANKWVGHLSGGLAMASILTSAGFAATSGSSVATAATVGAVAIPEMKEKGYDPRLSAGAVAAGGVLGVLIPP
SV
LLIFYAALTEVSAGKMLIAGFIPGLITTAAFMIGVALIGRRVQHHSTRAPKASWGERLSSTTQAWQVVVLFAIVLGGIYL
GLVTPTEAGAIGALAALFMLVFAKSARGHLGHYLSESFRSTATTTVMILFTMIGAGIFSFFLTLVQVPQMIAETITESGI
PPHLIVAVLLLLYIPLGMFLDAFSMMVITLPIVFPTVSGLGFDPIWFGILCVKMCEIGLITPPVGLNCFVIAGIDRDTPL
PDVFRGALWFVAIEMVTIVLLFAFPALITWLPDTMMAR
>MCE8455220.1 SDR family oxidoreductase [Rhodovulum sulfidophilum]AB15
MTQRTDYGTAFITGGGSGIGRASALRLAALGARVAVADLRFEAAEEVAQAIRSQGGEAIALAHDVADADVLPGVVAQA
EE
QLGPIDILVNSAGLILVEPLLDFAPESWNRIMDVNVTGTFRVSQHIARGMAARGYGRIINIASISGLRAGVGRTAYGTSK
AAIIGLTRQFALELGSRSITANAIAPGIIETPMTLAAYSEDTWRRVLEMIPARRRGQPEDIAEAVAFLASPAASYVNGEV
LTVDGGYVASGMTQTGNLET
>MCE8455221.1 hypothetical protein LZ188_01230 [Rhodovulum sulfidophilum]AB15
MEEAKETRRAFVDRIELAPAPESDGLTIDLHGALAALLRLATGGEGTDAN
>MCE8455222.1 hypothetical protein LZ188_01240, partial [Rhodovulum sulfidophilum]AB15
LGLITGFFLAALLAAIMSTVSSQLLVSSSSLAEDFYKLVLNRGASEKTLVAIGRISVVAVGAVAALIAGDPESEVLDLVA
NAWAGFGAAFGPLVILSLTWPRMNGAGAVAGLVVGAATVGLWIFTGLKAATGVYEIIPGFVLAWLAIYVVSLATKDRG
EF
RSTISAPAE
>MCE8455223.1 acyltransferase domain-containing protein, partial [Rhodovulum sulfidophilum]AB15
MIRQPRPDENIPVAAAGELPPALLLSAANETTLANLARSWLTLAETAPADLGARIATANWRRARHRHRLCLAAGRADA
LA
QGLADWLEDPRQAAPGAAAETALRDAPVAFVYAGNGAMWAGAARTLIERDPTFRSAFNDIADAFAGEGIAGLAEAL
ADPD
LEARLGEAELAQPLTFAIQVALTEALAEAGLRPDAVLGHSMGEVAAAIAAGRIAREDGIRIIAQRSRAFEPLRGKGGMAA
FAGSRSQIEALIETERIAAEISAENAPDSVTVSGSDDALKALTRIARAHRIAGRILPIPYPYHSAAVETLRPALTRGLAR
IAPRDGSVPFYSGWDGSRYDAPLDGVYWWRNARLPVAFRAGVQAMAEDGIRVFVEISPRTVLRSYLRDTLAELDCETR
VL
ESLDSTRAEARDAGTIARAALAAGGAADRARILGPEQPVADGLPDYPFDRQTYRLDSPVATDIFHRRPQHPFLGTRLRP
D
SAEWQGEIALARTPWLGDHRVGGRVILPATAMVEMFAHAGCEILKTEEIELRDVEFLRPLELPEKGVVEVRLVHEPTAR
R
MRIEAGGPRGWTPVAVARLFSATTARPAPVTAPEAMGEAEPLYAGLRAAGLDYGPDFARVTVLDRDGTAVRTTLAPR
DGT
EGFRFDPAVADAGLHGAVLLFGGRDGDDRLRVPARIAKVRLCGRGRIVCARLTAAGGWDESLAFDTVLADATGTALLS
FE
GMRLRPLPAGTATPHDFWDERLVPLTGDGAATALRAAKALCGTTETASDADVLRAALGSRLAWDLLAEGAAPSDPRQ
EAA
RRWMVAAGHDRSRDVRAPDLPSDCPWPTADALLTMLPEVAPGASAELKSALAAAAGEDRPADPLPQAARTLDTLLD
SAAP
LGRIALAGLPDLGLVAAALRAAGHVTVLAEDKDALDRLRARLDTGLPLRFLALTEAGAADAVDLVLGCCLSQGFAQRES
A
GRLAALVAPGGALIAAEEEAPDLFSLMTRRHRDADALDRLEHALHAAGLDTASAPASGSEALRLIRATRPARRAAPRPELR
ITGSGPLAEALRALPQGSEAAVALHCAVPEEGDPLAAARIFRALPDTDGTLWIAVAETGRWEELTGWRRVVANETGRD
IR
LICAAPGVAPERIAALAATSPETEIRLTATGAAAPRVVPVRPLSAETGPRRLVADRRGVALDGLVWQTPPARALQPSEIE

IEAVASGLNFRDVMWAQGLLPPEALEAGFSGPCLGMECAGTVLRAGVQAGLKPGDRVIAMAPHAMASRVITRAEAA
MPLP
DTVDPEAAAGLPVVFVTADYALTEVARLAPGETVLVHGGAGGVGLAALQVARRLGARVFR
>MCE8455224.1 polyketide synthase [Rhodovulum sulfidophilum]AB15
MISRQTSGSEILTDSETCVTGYSAILPGADGLDAVWQVLSEGRCTISDMPEDRWSAARWLSPEPGLAGHSYTRRAGIVA
G
AFDFDAGCFGITPREAEQMDPQQRLLIEVTARAFDHAGIDPAAVEPDRVGVFIGAASLDHSTTMLQDPQMAGPQYM
LGNT
GSIMANRISHQWNLQGPSYIVDTACSSGLFALDHARRAIGSGQIDMAIVGAVNLLMSPMPFVGFSQAGMLSPQGLCR
AFA
KGADGYVRSEGAVAFVLERLDSAQAAGRRVRSVLVATSVNSAGRTPGIALPSADRQAALIEAALTEGGYDPEALAFVEA
H
GTGTAVGDPREADAIGRVYGRRRALPLPIGSAKSNFGHLEPASGLVGLLKAQLALERQTIPATLHCDEPNPDIDFAGLNL
DVARSARALPPPTGRGLRRSTPSASAAPMPMR
>MCE8455225.1 hypothetical protein LZ188_01255 [Rhodovulum sulfidophilum]AB15
MGWLLWAGLALTQRHGIGCRPEVEYERPKPRLKTCPIGCFHIDITEIRSGEGQSHLFVAIAKARKTLSSGITGRPKRWRP
RYASEIPSRRCHIALIDQEIGMPFVRMTMAKGVQLTNRPQDRRAMAQFPGSSREETRVKHQPHIQPIAFAAKTQDFFQ
AP
VRSRG
>MCE8455226.1 LysR substrate-binding domain-containing protein [Rhodovulum sulfidophilum]AB15
MHNLRSVDLNLLVVLDALLAERHLSRAAERLNMSQPAVSHALARLRILLDDPLFHRRRREMEPTLRALELSEPLAEALAQ
IRTVLAPEGFDPTARHVFRLAMSDFGAALVLPGLMASLRRDAPGIDLVVTQQSRESMIGAVADGQIDMAFGVFPELSS
RC
AADILFEDEYACLLDPSAQPQPYRGLTESRYLSAPHVLVAVHGEAATEIDLHLRSHRKARRIALVLPHWSVAPQVIRGTD
LILTVARRCLSEVAPGLEVTATPVDLPTIPFAAIYAKRRRSDPALRWLMDRTKQLLPPQGRSFPI
>MCE8455227.1 DMT family transporter [Rhodovulum sulfidophilum]AB15
MPLVLLIVFAVFAGAAVPFQGGANAALGRALGHPLWATLASLAVSVLCIVPILMALRVPLPTLGGLAGQPKWIWIGGIA
G
AVYVTSAILLMPKLGAASFMIAVIAGQVVAALLIDWIGGVGLASRPIDGSRLVGTVLVLIGAVVVQWPALSR
>MCE8455228.1 type B chloramphenicol O-acetyltransferase [Rhodovulum sulfidophilum]AB15
MQNFFESPFRGVTLDEQVGNPNILVGRYSYYSGYYHGHSFDDCARYLLPDEGVDRLVIGSFCSIGSGAAFVMAGNQGH
RN
DWISTFPFYWMSEVPAFEGAENGYRPAGDTVIGNDVWIGSEAIIMPGVTIGDGAVIGTRAVVTRDVEPYAIVGGNPAR
II
RKRFGDDCIALLLDLRWWEWSDDQLRVAMPILTSGNVERLHEHWETHIRSR
>MCE8455229.1 siderophore ABC transporter substrate-binding protein [Rhodovulum sulfidophilum]AB15
MPVPSLATRLGLPSALAALIFGAAAPAQAETAASTPFAPMTLAHDLGETVLEHRPERVAALGMNDLDFLDSLGVPVAGI
P
KDFVPHFLSAYADDPQIRDLGFIVKPNIEQIYALQPDLVLMSPLQAEHYKEISSFAPVLYFDVDYRNSATGHLDAVKDHF
LTLGKIFGKEEAAREGVAALDRQVARLRDEIADRPEKALILLYNNGAFRSFGPRSRYGFVFSDLGVTPAGEASETELHGR
PVTSEFIEAADPDILFIIDRTAVMEHRPVLTRDQIANPLLESTPAWANDRVIFVDPEAWYVTAAGPTSLSIIIDELREGY
RRR
>MCE8455230.1 ATP-binding cassette domain-containing protein [Rhodovulum sulfidophilum]AB15
MITVTDLSKTYGDKPVLSGVTAEIPRGRLTSLIGPNAAGKSTLLMLMARLLRPSGGQVRLAGTPVGEIAPDSYARQVATL
RQAPGLDLRLSVEDLVAFGRFPYSRGRLSREDRAAIDRALAFLSLDELRHRNIDELSGGQRQLAYLAMTVAQETECLLLD
EPLNNLDMHHAVEIMRALRQLCDDLGRTVVLVIHDINFAANYSDHILALKDGRLACSGPVAEIVTEERLRALYDLDFQVL
RNGDSVLCNYFTPRHRR

>MCE8455231.1 iron chelate uptake ABC transporter family permease subunit [Rhodovulum sulfidophilum]AB15
MRHGLVLAALAALALLYLGWGTGFAPGFLLSRRLIRLGAMAVGGTGIAMASILFQTVARNRILTPAIMGYEAAYLLLQAL
LVLALGTASLRLLGDLGNMALSLALMLGWSLALHALLFRDERNSMHRMLLIGLVLTLTLTSITQFVQLQISPGEFAVLQS
FALISLDGISPNRLAISAVAVLAAGAIALRRAAIWDVMALGRDQALSLGLDHPREVRLCLALIAVFAAATTSLIGPSAFM
GIFIANIAYALTGGFRHRRTLPAGSLVAVAMLLIAELMVQHLFRFGTTIGILINLICGGYFLFLILRRAGRPA
>MCE8455232.1 iron chelate uptake ABC transporter family permease subunit [Rhodovulum sulfidophilum]AB15
MPLSPATPSLARRAALPAAFAALSLASLLIGAKDIGIGWIFDPSPEALLVLTASRLPRLIALVLTGMGLAISGVILQQIL
RNRFAEPATTGGMEAAKLGILVALTLFPASAPLLRMGVAMLFCLAANLIFVAILQRIRFRDAVLVPVIGLMFGAVLGALA
EFYAFSRNMLQSMQGWMLGDFSRIVQGHYEMIYLVLPVVALTYVFARRFTVLAMGQDMAESLGLGYRGMVALGLTL
VSAT
VAATVITAGAVPFVGLVVPNLVTLWRGDNLQQTLPTVALSGGCLLLFCDILGRIVIYPYEVPLGLTVGAVGGAIFLLLIL
RRPA
>MCE8455233.1 ABC transporter ATP-binding protein/permease [Rhodovulum sulfidophilum]AB15
MIRMFLKLLGDDVKIFRRYAAMALASGALNTITIALTVPVLLRLLQGDVAAALPWLAALLAGALACLILRRQVEQAGIKV
GIAVLEGVRLRLGNHVARLPIGWFDSAATGRLNHALTQGVMEVAQLPAHVFTPVLTGLVVPPGLALVLFWLEPRIGLIA
L
AALPLMVAVFALAARLGRDADRGYHAAAARTGERAVEFARTQAVLRAFSGDGASARFLMDAFAGQRQAVRRLIRLST
ASV
VLNGWALQAVFAAMLLVALRLLDADLTPAQGLSLVVALVLAQRFIEPMTELAGYGEALRGARNHLAALTEIAEARPLPR
A
EHPQPPRDSSVALEDVTFRYSPDTAPVLEGLSLRVAPGSMTALVGASGSGKSTVVRLVARFFDVERGQVSIGGVDVRD
MS
EEVLSAQISQIFQDTYLLEGTIAENIRMGRPDADAEEIAEAARLAGVDDIVRRLPQGLDSPTGEGGVRLSGGERQRIAIA
RALLKDAPILLVDEATAALDAENRALVTETLARLRGKRTLIVIAHQLSTIRMADRIAVLEAGRIVEQGAPEALAQRGGPY
ARLLRLREAAQAWRMDSPE
>MCE8455234.1 ABC transporter ATP-binding protein/permease [Rhodovulum sulfidophilum]AB15
MTTQPNGPAGPSTPPITRLLAPIRAQLRLAAALAGTGQILTLVPLAGIALIGRQALGLPEAAATVPVLTVATICIAALVV
GLSLLTLAEYLAHLADNSLTDTLRNRLTERLTQVPLGWFTSRSSGSVKRALQDDMNTLHELSAHYFTTRARCFGAIAAAA
AFLALVDWRMAIVSLAPFPLYYLIFGATRKSIGPDRMASYVSAQTAIGDAVGEFGRAMPVLRTFGKTGEAHEAYSEAVH
R
FLDAFLRFTKPLVAPLATANALITPVTVVSIVLASGALFIGAGWMAPADLLPFLLIAPGVSAPLLLLGFMAHGLAHATAA
ADRIDAVLNTPTLPVPAPGEGVTPDGNRLCFEAVGYAYEDGTETLSGIDLTLEPGTMTAIVGASGAGKSTIARLALRFFD
PTEGRITLGGADLRDIAPAELYRRVGFVLQDVQLIHASLHDNIALGRPDATREEVSAAARAAQIHDRILALPQGYDSILG
RDAMLSGGEAQRVSIARALLLDPPLLVLDEVTSAMDAEGELALQKALSELARGRTLLVIAHRLETIIGADRIVVLQDGRI
AETGPHEALLARNGRYATLWAQSGSQLPAPTQDPEIAR
>MCE8455235.1 TetR/AcrR family transcriptional regulator [Rhodovulum sulfidophilum]AB15
MTDPKPRPGRGRPRTITRERVADAGIALGLAQLSVVGVAAALGVTQMALYKRVGGLEELRSLVAEEAFLRWPLPDPEE
DA
DLEAYLHRFSLSLWRLVHRHAGIAPYLLRRDWITDAMLRRTQEHQARVAECFGLTAAQSSRLVFTVAYHCCAIADTSRS
E
APSEAPDGEKIEPLHEFGLKALIAGAIALSGDDG
>MCE8455236.1 TonB-dependent receptor [Rhodovulum sulfidophilum]AB15
MMQIRLSSRAALMASALVAPVCIAPRAALAQQGPDTGYYLGDIIIRGNIAERSLKDTSSSVSVLGDEEIEEKSASGTVNE
VIADTPNVLIPGSTSAPIIRGVDSDGPLIAGNAYLAKPLPRATMSIDGRYVSSVELGAGGGTTWDVESIEVFRGPQTTSQ
GANAIAGAVVVTTKDPTFEPEYSAQAVYGSRNKRRLSFAASGPLSQDFAARLALDYTGRDTFVDYTYPDFDYDFASLNG
R

LKLLWEPADMPGLRVKLTYSHTDLHRPSNEQVTKPYRDLDNTSDYQDYMDVIADGVVLDTQYDFDNGIRLSNQLQYSE
AD
YDFTFALPYSGTAARDSATLSNELKLTLGQGEDIWSGVLGVYFSDEDADADFANSYEGVPLGSADLSYRTRSLALFGEAT
WRFASDWALTGGLRLQQDRIEIEGKTSYVVDERLDYDETFTEILPALTLSHDLNSDWTLGATVSKGYLPGGSGTNFHGK
E
YYEFDPETALNSEIFLRGSALDGALDVSANLFYTKYRDIQRSVINCLNADCSVTQGSMIVNGERAHAYGFELSADYQASS
TLRLSGDIGALQTEVTEFSDHDGTKYEGNEFARSPGYSVGLGAAWDITPDVTLQGDMRYVDGYYSDDTNEPAAEVDG
YAL
ANMRLSYRPRDGVEYFAYVNNVFDNRAALSVSTNDAAEIAEPRELGLGLRVTF
>MCE8455237.1 IS3 family transposase, partial [Rhodovulum sulfidophilum]AB15
MGQKRHKPEEIVAKLREVDVLVSQGRSVAEAVRSIWMT
>MCE8455238.1 ASCH domain-containing protein [Rhodovulum sulfidophilum]AB15
MFKISAALKQRYPAAEAFRFGDSAASSEALLSLVRSGKKTATCSALRDIQNGEPMPTIGRRDIALNWDGTPALVIETVEL
VQCRFDEVTEAMALAEGEDDSLDAWRKGHGSYFKRNGGFSPDMQIIWERFILIEDLGREDDALGTAAA
>MCE8455239.1 nickel-responsive transcriptional regulator NikR [Rhodovulum sulfidophilum]AB15
MQRITITIEDELLQALDAVMERSGASNRSEALRDLIRRGLAKDAPEEAECVGVVTYALDPAQRDLGRLVPESRQQHHDA
A
IAALSVPLDHHAAVEVAVMRGTVGSVQAYANSLFLQRGVRHGRTTLIPVRTEVEFHLHGERAHTHDHLRVLDRF
>MCE8455240.1 tryptophanase [Rhodovulum sulfidophilum]AB15
MPGVRFFSGETVPLEMHKVRVVQKLTLPDIEARLAAITAAGNNTFLLQNRDVFMDMLTDSGVNAMSDRQVAAMMV
ADDSY
AGSATYTRLETRLREIFGMEFILPAHQGRACENIVSQVMVTPGSIVPMNYHFTTTKAHIVLNGGSVEEIIHARGLEVTSD
EPFKGDMDVAALERLVTEHGAERIAFVRVEAGTNLIGGQPVSLANLQEVRAACDRHGLTLVYDASLMADNLYFLKTRE
AA
CRDLSIREITRKIADLCDVIYFSARKLGCARGGAICVRDEALYNRMRGLVPLYEGFLTYGGMSVREIEALTVGLGETMDE
DMISQGPQFIAYMVDELDRRGIPVITPAGGLGCHIDAMRFLDHVPQPQYSAGALASALYIASGVRGMERGTMSEQRA
PDG
TEPLANMELVRLAMPRRVFTLSQVKYAIDRIDWLWQNRRLVGGLTFVEEPEILRFFYGRLAPVSDWQQKLVAKFRADF
GD
SL
>MCE8455241.1 cysteine desulfurase [Rhodovulum sulfidophilum]AB15
MYDVNAIRVDFPILSRQVNGKPLVYLDNGASAQKPQVVIDAVTQAYAMEYANVHRGLHTLSTIATEKYEAVRGTIARFL
G
AADPDEIVFTSGTTEGINLVSYSWAAPRLQPGDEIVLSVMEHHANIVPWHFLRERQGVVLKWVDVDASGNLDPQAVI
DAI
GPRTKLVAVTQMSNVLGTVVDVAAIVKGAHDKGVPVLVDGSQGAVHQPVNVDALGADFYAITGHKLYGPSGSGAIHI
RKE
RLAEMRPFMGGGDMIREVRRDGITYADAPMKFEAGTPGIVQQIGLGVALDYMMGVGMDNIAAHEKAITAYAMDRL
AGLNW
LQVQGKADHRGAIFSFTLEGPAHPHDISTVLDKKGVAVRAGHHCAGPLMEHLGLSATCRASFAMYNTTDEVDRLIEAL
EL
CHDLFA
>MCE8455242.1 YIP1 family protein [Rhodovulum sulfidophilum]AB15
MTHFRPSLLSLVRDTVTDPRGSAGRVLSLAPPRAVLWEALGLVVILSVLLTELGELISPAPVDPMMPAFMHYPLAAAAV
Q
GLLLIVTVHAIAVIGRVFGGTGDLDGALALTVWLQFVMVCLQVVQLVLLVVLPPAAGLLGIFGIGLFFWLLSHFVAVLHG
FPSVMKTFGMILFSMIGIVFGMSLILSILGITLTGAMPDV
>MCE8455243.1 YIP1 family protein [Rhodovulum sulfidophilum]AB15

MAVTTEMVATWRGPRAALRRQLRMGQREDRALVYLMIACLIIFVGQWPRLAREAYMSPEIPLEALLGAALFGWMAL
APLF
FYGLAALSHLIARAVGGKGSWFSARLALFWTLLCVSPLWLLHGLVAGFIGGGAALMLVSTVLAVAFLAIWLLSLVEAEA
>MCE8455244.1 Fe-S cluster assembly protein SufD [Rhodovulum sulfidophilum]AB15
MGLPQAKLTATEARLAALAMPVQAGWTRPLREAALARVQQMGLPGRRDEYWRFTRPDELNSAAPVPAGVRPPAEN
ATFEE
LDRVRIVFVDGVFDPEASDDPAQAGLEISRLAEAEGQDLSWAADLYGTLEARGQTPVPRPLAALNTAFATDGVLIRATG
R
VGKPVNLVYRHCDETSDAILHHVIRVESGADLTVLETGPAAARFNKVMEVDVADGGAFHHVRSQGRDHDRRAATHIF
ARL
GEESIFKSFTLTMNGALTRNEVVIELTGDEGVAHVAGACMGDGAFHHDDTVFITHDAVNCESRQVFKKVLRKGATGVF
QG
KILVKEGAQKTDGYQISQSLLLDEEANFLAKPELEIYADDVACSHGSTSGAIDEDALFYLRSRGVPKAEAQDMMVLSFLA
EAIEEIESEAVRDEVSASLAAWLERHRR
>MCE8455245.1 Fe-S cluster assembly ATPase SufC [Rhodovulum sulfidophilum]AB15
MLEIKNLRASLEEEDKEILKGVNLSVGAGEVHAIMGPNGSGKSTLSYVLSGKGGYEVTEGAATLNGVDLLAMEAEERAA
A
GLFLAFQYPVEIPGVGNMTFLRTAVNAQRKARGQEEMDAATFLKEVRAKAKELKIDAEMLKRPVNMGFSGGEKKRNE
ILQ
MAMLEPKMCILDETDSGLDVDAMKLVSDGVNALRAPDRGFLVITHYQRLLDHIVPDVVHIMANGRIIKSGGADLALEV
EH
NGYADILAEVA
>MCE8455246.1 heavy metal-binding domain-containing protein [Rhodovulum sulfidophilum]AB15
MIVTTTPSVEGHSILSYHGVVVGEAILGANVFRDIFAGITDIIGGRSGAYEASLGEARETALAELQQRARAAGGNAVVGV
DLDYEVINNMLMVSASGTAVTLS
>MCE8455247.1 hypothetical protein LZ188_01375 [Rhodovulum sulfidophilum]AB15
MSMPKTMAAPGAGRGAGRHPPELARGPGARLRRLSLPSIPGHGGRLVLTTRMRLLIIAQGLIFYASLQDEETIDWLGTL
V
PLSKAQAALAEGLFGLVLFLLWGLCMIAWMRVFTQDRRDADLRDRGPGDRDLRDQDRRDKEDRT
>MCE8455248.1 Fe-S cluster assembly protein SufB [Rhodovulum sulfidophilum]AB15
MAALDKADMKNVKEGVDRETVEAVQSMAGKYKYGWDTEIEMDYAPMGLSEEIVRLISAKNEEPEWMTEWRLEAFR
RWQKM
EEPDWAMVDYPEIDFQKQYYYARPKSMAEKPKSLDEVDPKLLETYQKLGIPLKEQMILAGVEGAEAAPAEGRKVAVDA
VF
DSVSVGTTFQKELESAGVIFCSISEAIRNHPELVKKYLGSVVPQSDNFYATLNSAVFSDGSFVYVPPGVRCPMELSTYFR
INAENTGQFERTLIIADKEAYVSYLEGCTAPQRDIAQLHAAVVEIVILDDAEVKYSTVQNWFPGDEDGKGGIYNFVTKRA
DCRGPRSKVMWTQVETGSAVTWKYPSCILRGDDSQGEFYSIAITNNWQQADTGTKMVHLGKNTRSRIVSKGISAGHA
QNT
YRGLVSMHPKAKNSRNYTQCDSLLIGDKCGAHTVPYIEVRNNSSRVEHEATTSKVDDDQLFYCRSRGMDEEEAVALVI
NG
FCKEVLQALPMEFAMEAQQLVAISLEGSVG
>MCE8455249.1 cysteine desulfurase [Rhodovulum sulfidophilum]AB15
MPGRVYLDWNATAPLRPEARAAMIAAMDAVGNPSSVHAEGRAARALVEKARAQIAAALGAEGAEIVFTSGASEAAA
LACS
GRWLQGSEIEHDAVAAWLDPCLRPDGDGRVVPRLPAQSALQLANSETGVIQEIPEGLAVCDMTQGFGKLPVAFNWL
GCQM
GLVSAHKIGGPKGIGALVMRRGCDIEAQIRGGGQELGRRAGTENVIGIAGFGAAAEAAAADLEAGKWAEIAQLRSILEN
L

IAERANKTIFVGKDVPRLPNTLCLATPGWKGETQVMQMDLAGFAVSAGSACSSGKLRPSRVLTAMGLGDEVASSAIRV
SM
GPGTTEEDVRRFAESWLAAETKFRARTG
>MCE8455250.1 Rrf2 family transcriptional regulator [Rhodovulum sulfidophilum]AB15
MKLSTKGRYAMVALADLSLQPEDCLVSLSEISKRQHISLPYLEQLFVKLRRAGLVESVRGPGGGYKLARPASEIRVADVL
GAVDETVSAMHTGAGVSGGTSGTRAQSLTNRLWEGLSAHVYVFLHQTRLSDVIKNDLAPCPAVPALFHVVDDE
>MCE8455251.1 alpha/beta hydrolase [Rhodovulum sulfidophilum]AB15
MPEVIFPGPEGRLEGRYHPQKDRDAPIAIVLHPHPQFGGTMNNKVVYNLHYAFHNMGFTVLRFNFRGVGRSQGEYD
QGVG
ELSDAASALDYLQSMNQNAKHCWVAGFSFGAWIGMQLLMRRPEITGFVSVSPPANMYDFSFLAPCPSSGLIINGSAD
RVA
PPADTKTLVDKLHEQKGITVTHEEIEGADHFFQDPHMDPMIENVTTYVRRRLTEVTR
>MCE8455252.1 hypothetical protein LZ188_01400 [Rhodovulum sulfidophilum]AB15
MSAIDDLADQLARDALDAAARLGDDRLIDDISKAIGASSPVSQEAFVSAVRIRQALARGRAVMDSRLTQAASQKAGPE
KS
GG
>MCE8455253.1 NADP-dependent isocitrate dehydrogenase [Rhodovulum sulfidophilum]AB15
MTKIKVDNPVVELDGDEMTRIIWDFIKKKLILPYLDLDLLYYDLGIEERDRTDDQITVDAAEKIKEVGVGVKCATITPDE
ARVEEFGLKQMWKSPNGTIRNILGGVVFRAPIICQNVPRLVPGWTKPIVIGRHAFGDQYRATDFRFPGKGQLTMKFVG
ED
GQEIEKVVYDAPAAGVAMGMYNLDESIRDFARASMNYGLSLGWPVYLSTKNTILKAYDGRFKDLFQEVYEQEFEDRFK
KA
GITYEHRLIDDMVACCMKWNGGFVWACKNYDGDVQSDTVAQGFGSLGLMTSQLMTPDGKIVEAEAAHGTVTRHYR
QHQKG
EQTSTNSVASIYAWTGGLKHRAKLDGNAQLAHFAETLERTVVETVESGFMTKDLALLVGPNQKWLTTMGFLEKIDENL
NK
ALAG
>MCE8455254.1 YitT family protein [Rhodovulum sulfidophilum]AB15
MPPLPAPRHTLPDDIQSLVVGTLLVALSVSLLKSAGLVTGQIAGLSLVGAYASGIGFGPVFFLLNLPFYWLGYRRMGKAF
VLKTFAAVALLSVFSELLPHVLQIGPVHPLAASLLAGVTAGAGLLALFRHGASLGGVGIMALWLQESRGIRAGWVQLGF
D
AVVFALAFAVIPPLTVLWSMAGAAALNVIIATNHRRDRYIAR
>MCE8455255.1 SMR family transporter [Rhodovulum sulfidophilum]AB15
MPKHYLYLLLAVVTETMATSSLQASQQFTRFWPSVLVVLGYALSFYFMAQTLKVMPVGIVYAIWSGLGIVLISGIGWVV
F
GQRIDLPGALGLGMIIAGIIVIHLFSKTAAH
>MCE8455256.1 translational GTPase TypA, partial [Rhodovulum sulfidophilum]AB15
MDLRNIAIIAHVDHGKTTLVDQLLKQSGAFRENQSVAERAMDSNDLERERGITILAKCTSVEWDGHRLNIVDTPGHAD
FG
GEVERILSMVDGVVLLVDAAEGPMPQTKFVTAKALALGLRPIVVLNKVDKPEAEPDRALNEVFDLFASLGANDEQLDFP
H
MYASGRAGWADMELDGPRKDLHALFDLVLEHVPAPKQLAKADGPFSMLATTLSADPFIGRILTGRVESGAVKPGDTV
KAL
SRTGELLESFRVSKVLAFRGLSQQPIDSAEAGDIVTLAGMSKATVADTLCAPQVTEALPAQPIDPPTISVSFGINDSPLA
GREGKKVQSRVIRERLMREAELNVAIQVTDTPGGEAFEVAGRGELQMGVLIENMRREGFELSISRPQVLMREENGQKL
EP
IEEVTVDVDDEFSGVVIEKLTGTRRGDLVEMRPGGTGKTRIIAHVPSRGLIGYQGEFLTDTRGTGVLNRVFHDWAPYKG
A

IPGRRAGVLISMEDGVSVAYALFNLEDRGKLFIGAQEQVYQGD
>MCE8455257.1 phenylalanine--tRNA ligase subunit beta [Rhodovulum sulfidophilum]AB15
MKFTLSWLKEHLETEATLDEILYALTDLGLEVEGVENPARALQEFTIGKVLKAEQHPDADRLRVCQVATGQGEKQIVCG
A
PNAREGITVVIAHPGMYIPGIDITIQVGKIRGVESHGMMCSEREMELSDEHDGIIELPSGEVGQSFVDWLAANDPAKVD
P
VIEIAITPNRPDALGIHGVARDLAARGVGRLKPVEVAPVAGSFPCPVDVTIDPETQSHACPVFYGRAIRGVKNGPSPQW
L
QQRLKAIGLRPISALVDITNYFTYDMNRPLHVFDIAKLEGGLKVHAAAGGEEFLALDGKTYVIEPGMTVISDATGVESLG
GVMGGEATGCTEATVDVFLEAAYFDPIRTAMTGRALKINSDARYRFERGIDPAWTPEGIEAATRMILDLCGGEASELVV
A
GVVPDMARAYRLDTDRVESLVGMEIPAETQRATLTALGFRMEGDMAHVPSWRPDVLGEADLVEEVARVASLTKLEG
RPMP
RPQPGVPTAILTPMQRRSSAARRTCAALGYNECVTYSFVDRASAVLFGGGSDAMALENPISSEMSHMRPDLLPGLLQA
AA
RNQARGFADLALFEVGPAFQGAEPGEQHIQATGLLVGAAAPRDPYGSRRPVDLYDAKADCEAVLAAIGAPAKAQILRA
AP
DWWHPGRSGRVCLGPKKVLASFGELHPKVLAEMNVKGPAVAFTVFLEETPMPRRKGATRAALTVSDLQAVERDFAFV
LEE
GVEAMTVVNAAAGADKALIEEVRVFDEFVGGALGEGKKSLAITVRLQPSDKTLTEKDIEAVGQKIVAKVEKATGGTLRR
>MCE8455258.1 STAS domain-containing protein [Rhodovulum sulfidophilum]AB15
MKLQSQLRGDALHIVVDEDRIDSAVAIRFKEAVREQAADGPGRVVLDLGRVDFLDSSGLGAVVSVMKLIGPGGKLELC
NL
TPNVDKVFRLTRMDRVFTIHDSLDSAFPERQGIA
>MCE8455259.1 ATP-binding protein [Rhodovulum sulfidophilum]AB15
MTLSRAPRGEAPPAAEDTSSRQPADNPPPVIVEATPLGVRAALLEMRARFGPSMPFDGSLGMAEIVLAEVLNNIVEHA
YS
GDRSGPIELRLVRDGIWLRVDVRDYGRAMPGESLPDADRTGADMTGAEFRPETAPEGGFGWFLIRAFARDLDYSRNG
NQN
RLRFTVPLNEDC
>MCE8455260.1 class A beta-lactamase [Rhodovulum sulfidophilum]AB15
MRIPASVLSRVAAGLSLGLCLASASLAQTPLETLADTVERVESRLGARVGVSLVDTGSGLSWTHRENERFLMNSTMKVP
L
CGAVLARRDAGDLSLQTELPVRKSDLLAYAPVTEKRAGGEMTIAELCLAAIDMSDNTAANLLIAHLGGPQAVTRFFRSA
G
DTISRLDRNEPELNTFNAADLRDTTTPAAMTETLRGLLLGDVLSRSSRGQLAEWMSHGGVTGNLLRAEAPEGWVILDK
SG
SGTHTRNLVALVTPVDGSPWIVTIFVSDVDADSQTRNRALQDVGRAAMAVMRD
>MCE8455261.1 LysR family transcriptional regulator [Rhodovulum sulfidophilum]AB15
MDRPDLPLNALRVFEVAMRQGSFTKAAIELRVTQAAVSHQIARLEDLLGVVLFLRTSQGLVPTDEGRLLFPVLEHGFDA
M
ARVLGRLGGRRDVEVLKVGVNTTFALGWLMPRLAAFAQAHPEIDLRISTNNNRVEILREGLDMAIRFGTGGWTGHDAI
PL
IEAPLTPLCAPAMAAHLLHPADLGQMTLLRSYRSAEWPGWFEAAGVACPPITGPVFDSSIALAELAASGAGTALLPITM
F
ESYIAQGRLAQPFGTTVSAGRYYLAWPSDRPATPAMRIFSSWLSGKSTL
>MCE8455262.1 ABC transporter substrate-binding protein [Rhodovulum sulfidophilum]AB15

MSKKAILALGSAVLAVSGVALQAATDYPLTIENCGQSVTFSQAPQNAVALGQNSAEIMLLLGLEDRMAATAFWPNAVL
PE
LAEANDGVEVLTVEFPTLESVLSKQPDFVAAMLTTLLGTDSKVASRSDFEELGIPTYLSPSACSTTLDAGDAYGSRDKLW
NMDLLYKEIEDLSRIFDVAERGQAVIADFKAREAALRAEFARNEDLTFLFWFSSPSPADDAFLGGGNGPSGYIADLLGGS
NAIRTEADWPALGWEGIIAADPTVFVATQVDRNRWDLDAAQNKIDFLTSDPTVSQMTAVRNGRIVVMSGAAMNPG
IRTLY
GAEQVAEQLKSLDLP
>MCE8455263.1 iron ABC transporter permease [Rhodovulum sulfidophilum]AB15
MSFEVSEEGSARLVVFLCLSLIALIFAIAAATAFGDYNIGLRTVFLSVTNELGLTGAEITPIEQSVVWNLRLSRALVAAL
SGAGLAICGAILQALLRNALAEPFVLGVSAGASTGAVCVIVLGIGAGGLSLSLGAFAGAFAAFALVALLSNGATSGPNHT
ILAGVAASQLFNALTSYIVTTSGNAQQARDVMFWLLGSFGGVRWPDVQLLLAVVIVSLAICIWMARSLDAFTFGDEDA
AA
LGVPVARIRLVLFGVTALLTATIVSMVGAIGFVGLVVPHAARYIVGPLHMRLLPACAVVGAVFMVIADIASRMIATQQT
V
PIGVVTALVGVPFFAIILYRARPQA
>MCE8455264.1 ABC transporter ATP-binding protein [Rhodovulum sulfidophilum]AB15
MTILAENLTWGVKRRTILSDVSLRVAPGETLGLIGPNGSGKSSLLRLLAGLRRPISGRVRINGQDIAHVSRKALSRQVAF
VQQSAATDTNVTVSDVVRLGRTPHRSALAGWSETDEAAVTQALERVEMAAHRKQAWQTLSGGERQRVHIARALAQ
TPQVM
FLDEPTNHLDIHHQIEILGMVRDLDLTSVIALHDLNLAAMFCDRIVVLERGVMRACGTPDAVLTREILRDVFRVTAHVCR
LQDASRPHIRFSAK
>MCE8455265.1 DMT family transporter [Rhodovulum sulfidophilum]AB15
MTARSSLEAPLHPVRNERLGIALRVLSTLAFAMMGVCVKALGDAVPLGQVVFFRSAVALLPLIAFLWWRGEWPRGLAT
SR
PMGHVGRCLMGAVAMFTSFATIRLLPLAEATMLAYLAPVMLALLGWALLGERPSAGRIGGVVLGLAGAAAFCLPAFA
GAL
PDTGALGVVLGLVTAALTAGALIQVRRLTLLGEGAGAIAFWFAVVSALIGLATLPWGWVWPGPATLLLLIGTGLAGGIA
H
ILMTLSFSHADASALAPFEYLSVLWAVALGFAFFAELPGLAFLLAGPLILTGAFIARPAKPK
>MCE8455266.1 ABC transporter substrate-binding protein [Rhodovulum sulfidophilum]AB15
MNTKTALLSAALLVCAGTAQATDYPLTLENCGQTLTFDAAPQRTVTVGQATTEILYLLGLGPTVTGTSVWFSNVLPQFA
E
LNAGIPRLADNDPSFESVAAETPDLVTVQHEWHVGPQGIVATREMFHDIGVPTYVLPSDCASKDNSRGSDGTRSEMFS
TA
SIHRSIEELARIYDVEDRGAELVDELKAREAAAIEHARALDLPEGVSAVFWYSSAEMDIDPYVAGRKGAPGYMMQELGI
E
NVVASDEEWPTVGWESIAKADPTVIVLAEMTRRRFPADDVEKKLEFLRTDPVAKEMTAVRENRIVVMDLQAMEATVR
AVY
GLEELSAALAGLDLQG
>MCE8455267.1 iron ABC transporter permease [Rhodovulum sulfidophilum]AB15
MRSRARRARWHWGWIAPPVLLAALIAGTAIGETRIPAATVLQVLGNKLFGTVYPVARIDEGIIWNYRLARAVVAAACG
AG
LALSGVVLQALLRNALADPYLLGISAGASTGAVAVTVAGFGAGALSLSTGAFAGALLAFVMVAALARAAGGGTGLQAA
GV
IVLAGIAGAQLFNAMTAFLIAKSANAEQARGIMFWLLGNLSGARWQDSLVAVPAAALGLGVCLWHVRSLDAFTFGAD
SAA
SLGINVRRVQAVLIGAAALVTALIVSLVGAIGFVGLVIPHAMRFLVGGRHRLLVPASALAGAVFLIAADIVSRILIPGQV
LPIGVITALIGAPAFAAILIRGRARK

>MCE8455268.1 ABC transporter ATP-binding protein [Rhodovulum sulfidophilum]AB15
MILSGEGLGWRVQGRAIVSDVTLSVRRGERFGLIGPNGSGKSSLLRMLAGIRAPSEGEVRLEGQPLSRLPRRDVAQRVA
L
VEQQAETTERITVRDAVELGRTPWLSALHPFSDEDETAVQAALVAVGMQDFAARYWHTLSGGERQRVHIARALAQRP
GVL
ILDEPTNHLDIGSRLSIMALVEGLPVTTIAALHDLNEAMACDRLAVLQDGQLIALGPPDEILVPPLLRKVFGVEAHLLTD
PVDGARILRFKPI
>MCE8455269.1 pseudoazurin [Rhodovulum sulfidophilum]AB15
MRRLALSVLLPAMILSAPFAAAKTVEVKMLNRNASGPMVYEPDYLVMEPGDTVRFLPTAPGHNAGTIDEMLPAGAEP
FKG
QINEEIEVTLTEPGIYGIKCSPHFAMGMVMLIQVGTQPAAEADLPAGLPTRADKRLREIIARQDTGSEQAEAHP
>MCE8455270.1 acyl-CoA thioesterase [Rhodovulum sulfidophilum]AB15
MTALIFPETVNPHGTLFAGAGLAMMSKAAFLVASRRARRTVVMAGSEKIDFRTPVLPGEALELTAVVTRIGRSSMSVEV
T
GEAETLTTGARHLAMTGRFEMVAVDENGRPTPIAIPKEEKTQ
>MCE8455271.1 MmgE/PrpD family protein [Rhodovulum sulfidophilum]AB15
MKDGAPQIELHRLRMHKSAEALPREEQLAWKLAELAADPVSVPDDTAEMIVNRVIDNAAVAAASVARRPVTSARAQA
LCH
PYKRGATVFGMPRDTRVSPEWAAWANGVAVRELDFHDTFLAAEYSHPGDNIPPILAVAQHCGLSGQALLRGIATGYEI
QV
NLVKAICLHEHKIDHIAHLGVSAAAGIGTALNLPKEVIYQAIQQALHVTTTTRQSRKGEISSWKAYAPAFAGKMAIEAVD
RAMRGEGAPSPAWEGEDGFIAWMLSGPEAVYEVPLPGKGAPKRAILDTYTKEHSAEYQSQALIDLARRLRGEIGDLRQ
VE
SIVIHTSHHTHYVIGTGANDPQKMDPTASRETLDHSIMYIFAVALEDGAWHHEKSYAPERAARPSTVDLWHRISTREDP
E
WTRRYHSRDPQEKAFGGRVVVTLKNGRVIEDEIALADAHPHGARPFERPNYVKKFLTLSEGVLDPKEQDRFLNAAENLA
D
IPEAGLGILNFTVAPEKLGAPRPRGLFDRS
>MCE8455272.1 2-methylcitrate synthase [Rhodovulum sulfidophilum]AB15
MTGIIRKPKKSVALSGIVAGNTALCSVGQTGNDLHYRGYDILDIAEACDFEEIAHLLIHGHLPSTAELSAYKAKLRRLRG
LPAAVRQTLEAIPAATHPMDVLRSGVSALGCVLPEPTDQNPAAARDIADRLIAVQGSMLLYWMHYARNGRRIEVETED
ET
VAGHFLHLLHGAAPSQEHVRAMHTTLILYAEHEFNASTFTARSIAGTGADIYSAITGAIGALRGPKHGGANEAAFEIQKR
YATPDQAEEDIRRRVAAKEVIIGFGHPVYTVSDPRNVVIKRVARHLAEHEGATRRFDIAERIEEVMADAKGMFPNLDWF
S
AVSYHLLGVPTELFTPIFVIARTAGWGAHVVEQRADGKIIRPSANYIGPENRRFLPLDQRGGLLTDQTEA
>MCE8455273.1 dihydroorotase, partial [Rhodovulum sulfidophilum]AB15
GATTNSASGVSDFDKVRGVLEKMAEIGCPLCVHGEVVDPEIDIFDREAVFIDRVLDPIRRATPGLKVVMEHITTADGVDY
VHSVPEGLGATITTHHLIITRNHILVGGIKPHYYCLPVAKRARHRDALVAAATSGDARFFLGTDSAPHVDPAKECACGCA
GIFSATNTLSCLAEVFEREGALDRLEAFASLNGPAFYGLPPNETRITLTRDASPVAYPARIETGAGPVTLFDPGFDLHWR
VER
>MCE8455274.1 orotate phosphoribosyltransferase [Rhodovulum sulfidophilum]AB15
MIPSTFPDQSEIARLSARMLLEIGAVHFNAEEPFTLASGLPSPTYIDCRKLISFPRIRATLMDFLTVTVLREAGFEAFDN
IAGGETAGIPFAALIAERMALPMTYIRKKPKGYGRNARIEGVMAEGDRVLLVEDLTTDGGSKLSFVDAIRDTGAVCGHT
A
VIFYYGIFPETEKTLGDHGIRLHHLCTWRDVLAEAKHQGAFDATTLAEVEAFLDAPRAWQEARRQQ
>MCE8455275.1 replicative DNA helicase [Rhodovulum sulfidophilum]AB15
MNTVRTIRPEGEAAEDTGGLPHNIEAEQQLLGAILTNNDIFDRIASIVQPPHFYDPVHSRIFEIAANRIQKNQLASPVTL

KAFLEDDEGLAELGGPAYLARLAGAAISSFAARDYAQMIYDFWLRRELMKLGAEISDKAAKVDIASEPATQIVEAEQGLY
KLAEKGQSETGFQSFLRAVTDAVKVANAAYQRDGGLAGVSTGLTDLDKKLGGLHRSDLLILAGRPSMGKTSLATNIAFN
I
ARAYKRGAMPDGSVGAVNGGVVGFYSLEMSAEQLAARVLSEASEVPSEQIRRGDMTEAEFRRFVNAAKELEACPLYID
DT
PALPISQLAARARRLKRTHGLDALFVDYLQLVRPATAKDSRVNEVSEITQGLKAIAKELDIPVVALSQLSRQVESRDDKR
PQLSDLRESGSIEQDADVVMFVFREEYYKEREKPSDHDLEGMARWQEAMESLHGKAEVIIGKQRHGPIGSVELSFEGRF
T
RFGNLVQPWQQGQAVEGF
>MCE8455276.1 alanine racemase [Rhodovulum sulfidophilum]AB15
MATGTLTIDLDALARNWKALDRASGAGVETGAVVKADGYGLGGDRVARALAQAGARRFFVAVAEEGAQIRNALGPG
PQIC
VFSGHMPGDTDMIGDLDLVPMLNSVEQLTRHMEGLPGRPFGIQLDSGMNRLGLEPAEWAAVRDIVLAAKPVLIMSH
LACA
DEPDHPANLQQLTAFREMTEGCDVPRSLSATGGVLLGPDYHFDVTRPGMGVYGGMPFAEADPVVGLSFPVIQTREVE
PGE
SIGYGYTHTAGDRRVVATVAAGYADGLHRTLSNRSKLWFEGTPCPIVGRVSMDLVTADITDLGRIPPALDLLCPEQGVD
A
VADSAGTIGYEIMTALGRRYARRFLGGAGA
>MCE8455277.1 ABC transporter permease [Rhodovulum sulfidophilum]AB15
MILLAPLGALGRTVLGALAAIGRIALYALATLYAMVRPPFYLRELGHAIWVIGYNSLPVVGLTAVFTGAALALQIYEGGS
RFNAGQVVPSIVAIGMVRELGPVLGGLMVAARVASSIAAEIGTMKVTEQIDALATLSTDPMRYLTVPRVLAATLTVPVL
V
AVGDSIGILGGYLVGTQSLGLNAAAYLKNTADYLVLWDVTSGLIKGAVFGFLIALVGCYFGMNSGRGAQGVGAATKSA
VV
AASILILASNYLLTTLFFAA
>MCE8455278.1 ATP-binding cassette domain-containing protein [Rhodovulum sulfidophilum]AB15
MIELRHIEKAFGRNRVLRGVNLTVPKGASMVIIGGSGTGKSVLLKCILGLVRPDAGQILIEGEDASKAERDAFLARFGML
FQGGALFDSLTVWQNVAFRLLRGTGKRPKAEAREIAIEKLRRVGLKPDVADLFPAELSGGMQKRVGLARAIAADPEIIFF
DEPTTGLDPIMAGVINELIREIVVEMGATAMTITHDMTSVRAIADNVAMLHGGVIQWTGPVSDMDQSGDPYLDQFIH
GRA
EGPIEAVR
>MCE8455279.1 paraquat-inducible protein A [Rhodovulum sulfidophilum]AB15
MRALRLANLALLVLFPIAWAAPLLRAGLLPLFGLSEISVLSGLASLWQTDIFLALLVTLLALVAPYAKTVGLALIQYDLA
SPRLLPAIHVMGKLAMADVFLIAIYVVLVKGIGMGRVETAWGLYLFTFCVLASLALSSLTARLLKRS
>MCE8455280.1 methionine synthase [Rhodovulum sulfidophilum]AB15
MTQTAFSPTRLALSSVFDTLRAAARERVLVLDGAMGTQIQKLGLSEDDFTGHGAGCSCHLHGDKPQKGNNDLLNLTN
PEV
IEEIHYRYAMVGADIVETNTFSSTTIAQADYGLEDKVLALNEAGARLARRAMDRAEAEDGRRRFVAGALGPTNRTASISP
DVNDPGYRAVTFDDLRIAYAQQLRGLIAGGIDLVLIETIFDTLNAKAAIFACEEVFTEQGLRLPVMISGTITDLSGRTLS
GQTPTAFWHSVRHAQPLTIGLNCALGADAMRPHLAELSGVADTLICAYPNAGLPNEMGEYDQTPDEMAALVEGFAR
DGLV
NVVGGCCGSTYDHIAAVAQAVKGYAPRALPQPARHLRLSGLEPFTLTDDIPFVNVGERTNVTGSARFRRLIKEQDYATA
L
DVARDQVENGAQIIDVNMDEGLIDSKQAMIDFLNLVAAEPDIARVPVMIDSSKWEVIEAGLKCVQGKPVVNSISMKEG
EA
AFLHHARLCRAYGAAVIVMAFDEQGQADTEDRKVEICARAYRLLTEEVGFPPEDIIFDPNVFAVATGIEEHDNYGVDFIE

ATRRITTDCPHVHISGGVSNLSFSFRGNEPVREAMHAVFLYHAIQVGMDMGIVNAGQLAVYDQIEPELREACEDVVLN
RR
PDATERMLELAEKYRGQGGSQAREKDMSWRELPVGKRLEHALVNGITEFIEADTEEARLAAERPLHVIEGPLMAGMN
VVG
DLFGAGKMFLPQVVKSARVMKQAVAVLLPHMEAEKEGRREAAGKVLMATVKGDVHDIGKNIVGVVLACNNYEIVDL
GVMV
PPEKIVAAAKEHEVDIIGLSGLITPSLDEMVHMASEMERQGFDIPLLIGGATTSKVHTAVKIAPHYSRGQAVYVTDASRA
VGVVSSLLSDNQRAGYVEGIREEYREIAEKHARGEAAKKRLPLAAARANPVPVDWAAYRPVRPGFLGTRVYDRWDLAD
LA
AHIDWTPFFHAWDMRGVFPRILEDEKKGEAARNLYDDARRMLDRIVQGAWFAPKGVVGFWPANAVGDDVRLYTGE
DRTGT
LATFYTLRQQTAKRPGVANAALADFVAPEGVAPDYLGGFAVTAGPEEVAIAERMAMENDDYGAIMVKALADRIAEAF
AER
MHAEVRRTLWAYAPDERFTPEELIAEPYAGIRPAPGYPAQPDHTEKKTLFRLLDAERAVGIRLTESMAMWPGSSVSGLY
F
AHPDSYYFGIGKVEADQVADYAARKGISLSEAERHLAPVLNYTPRKAGDAAAA
>MCE8455281.1 hypothetical protein LZ188_01545 [Rhodovulum sulfidophilum]AB15
MRIELADPRGVIRESYRIEGIGAPECRSIFLDWAIGLPEGIDPQRALRRLLAEHAAAAPDHPMTAVLRAGLNAGPQARRR
GGAHGRRS
>MCE8455282.1 glutamine-synthetase adenylyltransferase [Rhodovulum sulfidophilum]AB15
MSFASRLTRAPLAHEPDRAAEAADRFATQPPEIRALLEGTAGCSPYLWGLMTREADWLEDLLTGAPEPVFAALMDEVR
GL
SLEALKPGLRQAKRRAALLIALADLGGLWPLEAVTGALTDFADLCVDVTIRRLVAAEIARGRLPGLSEADAETAGGMVAL
AMGKQGAHELNYSSDIDLICLFDQDRFDPADFHEARSAFVKITRRMTAILSEATGEGYVFRTDLRLRPDASVTPVCMSM
E
AAERYYESLGRTWERAAHIKARPCAGDIEAGWAYLDRLRPFVWRKHLDFAAIQDAHDMRLRIRSHKGLHGGLVLEGH
DMK
LGQGGIREIEFFTQTRQIIAGGRDPELRVRGTVEGLTRLAAKGWVPDEVAETLTTDYRAHREVEHRLQMVNDAQTHLLP
N
SAEGIVRIAHFCGAPEAGPWRDALAARLRRVAGLTEGFFAPGKPAEDLPEIPKTAAEIVDRWPRYPCLRTTRAQEIFNRL
KPDLFSRLTKAPRPDEAFAAFDGFLAGLPAGVQLFSLFEANPHLIDLLIDITSVSPELAQYLSRNARVLDAVIGGSFFED
WPGRAALTGALDDRLGALGDYEAQLDAARAWVREWHFRIGVHFLRGLIDARTAGAEYADLAEAVLAALWPRVVAQF
AAKH
GDPPGKGAVVLGMGSLGAGALSARSDLDLIVIYDADGVEASEGRRPLASRAYYAKLTQALVTALTAPMAEGRLYEVDM
RL
RPSGNQGPVATSLQSFRNYQRDEAWTWEHLALTRARPVAGDAALGAEVESFRRALIADKADVARTVAGIVDMRRRLA
GAK
PAKSTWDGKQGPGRGQDIELIATAAALIAASPATSVAEQMGAGVEAGWLRQDEAEVLGASYRLQRQIQGAAKLISDT
PLD
AARLGESGQAFLLRGTGAEDVAALEDLLEIQRAAAATAIEAVIARSPAEEQADAD
>MCE8455283.1 YbaK/EbsC family protein [Rhodovulum sulfidophilum]AB15
MSKSLKRVRAALEAAGITPDIRELGQTRTAQEAATAVGCDLDQIAKSIVFRGEESNSAILFLTAGGNRVSDEKASALAGE
PLGKADAALIRAQTGFAIGGVAPVGHLSPIRTFLDPRLLEFDTVWAAAGTPRHVFPLDAHALPGLTGAETADFTQ
>MCE8455284.1 carboxylate-amine ligase [Rhodovulum sulfidophilum]AB15
MSASEPDFTLGIEEEYLLVDRETCALAQAPKDLMAACAADLGDQVTPEFLQCQIEVGTRVCRSVAEAREELRRLRATVA
H
HAAQFGLAPIAASCHPSADWLDQNHTDKDRYNDLDRALAAVARRMLICGMHVHVGIPDPDQRIDLMTQFSYFLPHLL
AMS

TSSPFWKGVDTGLASYRLTVFDNLPRTGLPPRFESWSEYDRSVGTLIRLGLIEDATKIWWDLRPSARFPTLETRICDMSP
RLDHTLSLAALIQCTLRMLWRLAGENLRWRIYERFLVGENRWRAQRYGVSEGLVDFGRKELVPFADLLEELIQLVEADA
A
VLGCLDEIHAARDVIAHGTSAERQRKAHAAALATGADSAEAMRSVVRLLIEEFGADL
>MCE8455285.1 hypothetical protein LZ188_01565 [Rhodovulum sulfidophilum]AB15
MPELVPVQAPVPLPEADGLLLAGLVEFDARAAEGVSSWLKRRRASFGGLDCVSSWS
>MCE8455286.1 acyl-CoA dehydrogenase family protein [Rhodovulum sulfidophilum]AB15
MDFAPSEEQRAIFDLAREFGAEHIAPHARTWEAEGTIPRTLWPELGALGFGGLYVRESSGGSGLSRLDATLVFEALSMA
C
PSVAAFLSIHNMCAKMIDAFGSEEMRARVLPEALSMQTVLSYCLTEPGSGSDAAALKTRAVRGAEGWALTGTKAFISG
GG
YSDAYVVMARSGAAGPKGISAFLVEDGRPGLSFGGLEDKMGWRAQPTRQVQFDNCAIPAANLLGEEGHGFRYAMA
GLDGG
RLNIAACSLGAAQAALDATLSYMGERQAFGQSLDRFQALQFRLADMEIELQAARIFLRQAAWKLDRGATDATRFCAM
AKA
FVTETGSKVADACLQLHGGYGYLADYGIEKIVRDLRVHQILEGTNEIMRLIVARQLLEARG
>MCE8455287.1 3-hydroxyisobutyryl-CoA hydrolase, partial [Rhodovulum sulfidophilum]AB15
MSEIDIRKQGRIGRITLTRPKALNALSYEMSLAIEAALDAWAPDPEVGMILIDAEGDKAFCAGGDIAVMYETGTRGDYA
Y
GQKFWRDEYRMNAKLFEFPKPVVSLMQGFVMGGGVGVGCHGSHRVVGESSQVAMPECGIGLVPDVGGSLILARAP
GRMGE
YLGVTGRRMGPADAIYAGFADYFLPEAEWPALRDELIATG
>MCE8455288.1 Holliday junction branch migration DNA helicase RuvB, partial [Rhodovulum sulfidophilum]AB15
MTGPDPLLRPEARPEDSDRALRPETLDAFIGQAEARANL
>MCE8455289.1 Holliday junction branch migration protein RuvA [Rhodovulum sulfidophilum]AB15
MIGRLAGRLIHKGADQVMIDVGGVGYVVHVSDRTLMGLPGLGEAVALYTDLLVREDLLQLYGFPTLHDREWHRLLMG
VQG
IGAKASMAILGTLGAEGVGRAIALGDWGAIKAAPGVGPKIAQRVVNELKEKAPAVMALGGHLSPDPAAGEVIAPADPA
PK
AGNKTRTKAPAAAPVSSSVSAQADALSALGNLGYAPSEAAQAVARAAGETPEADTTALIRAALKLLAPRAD
>MCE8455290.1 crossover junction endodeoxyribonuclease RuvC [Rhodovulum sulfidophilum]AB15
MRVLGIDPGLRNTGWGMIEQRGSRLGHIANGVCHSSGETLAERLLSLHIQLTEIVARHAPEAAAVEQTFVNRDAAGTL
KL
GQARGIALLVPAQAGLEVGEYAPNAVKKAIVGVGHADKRQIDHMVRLQFPGIEIAGPDAADALAIALCHAHHLAGGAR
LA
AALARAGA
>MCE8455291.1 DUF1127 domain-containing protein [Rhodovulum sulfidophilum]AB15
MAMFEVSHPQRAGLLADAPRFGLVTAFFARLRNRDEVRRTRDALSLLTDRELDDIGLCRGDIDRLLH
>MCE8455292.1 N-acetyltransferase [Rhodovulum sulfidophilum]AB15
MLIRNEVGSDATMIRRIVTEAMQLLPQATGAEADIIDRLRRDDALILSLVAEDRGEAVGYLAVSMSRIGAESGWGLIGPL
AVLPSRHGEGVGSALMAEAIRRLRSTCKGAALVGDPGYYGRFGFRPYPGLRVGDCPPQFVQALPFDAAEPEGELHHHA
AF
GLTQEG
>MCE8455293.1 YjhX family toxin [Rhodovulum sulfidophilum]AB15
MNISRHEQRALHVLALGGRILHEREYGSKITSVTCVTREGMILDGFDMTVFHRLRRKRLIESRCGSAYRISKRGRLSVRA
QLDNQGA
>MCE8455294.1 MATE family efflux transporter [Rhodovulum sulfidophilum]AB15

MSVGPARNRIENPFLTRPIGRLFLSNALPMAVVMSMGGLLNVADGIFVGRFVGPQALAAVSLAFPVVMLLTALTTLAG
GG
MSSLIARHLGAGSRPEAGAVFAGAHGLALAISAGLIAGALAVGPATVLALAAGNAAVAKAAQSYLLILILGAPIQFGLGL
HADALRNEGRAGQIAMLSVLVNLLNIGTNYVAIVLLGLGTAGSALGTVAAQSLGLALLLLLRTRDRHLLPLGSLWRSSWL
SGWGRILSLGLPLCLNFIGMALVAGMVLLLIGTAAADHATYIAAYGVTTRLLGLAFLPQMAIALATQSITGNNAGAGRM
D
RAGRALRLAMGSAFLWCLGVALTGVFAGAPLGGLFSEDPAITEAVAAILRPMTALYAITGPVLVLALHVQAMGYPLRTA
A
LTLIKPWLLTPALLVVMNALWGIEGLWFAFPVADAILLVLAMTLVLRTRPIAPVPAPCL
>MCE8455295.1 hypothetical protein LZ188_01615 [Rhodovulum sulfidophilum]AB15
MTQPSLGTVKTQAKALRQALQAQGTAISHAQALELIARQYGARDWNTLHARLGQRNAPAELALGTKVTGHYLGQTYS
GVI
VALSGPSGNRHVEIALDQPIDTVRFDSFSNWRHRIRGTIGEDGRSSRKTSDGSPHLTVIIAPS
>MCE8455296.1 hypothetical protein LZ188_01620 [Rhodovulum sulfidophilum]AB15
MGRAARKARRPLDHFEPIDRQVIRLSVEAKRLLADKGRDADRGRKA
>MCE8455297.1 DUF2177 family protein [Rhodovulum sulfidophilum]AB15
MILYAVTAILFLLIDAVMLTLVMEPLFTRHIGTAMRDGLMIAPASLFYLAYVAGLVFLVSAPALRDRAPERAALHGAVLG
AMAYGTYEFTSMSIMKNWSWTMVATDTIWGAVLTGFSAWAGVMILLRLRG
>MCE8455298.1 hypothetical protein LZ188_01630 [Rhodovulum sulfidophilum]AB15
MKAALVASWLADSTPISASDFEPWRVHSLLQRRWVSAFDIGGRLGIGALKTEWDTAACPSGRVALPPGMSTTSSPDPF
NT
LVEALDLPPVKDGPLNGTRFTVKDNIDIAGHRTSFGSLAWRDTHPAPVHNALCVDQLLAAGAHCVGKAVADEFTYSLD
GE
SQFFGTPQNAKAPGRVPGGSSSGSAASVANGLADFSIGTDSGGSVRVPASLCGIWGMRPSLHRISEAGVLPFMPSVST
VG
ILAASLPLLEAVARVLLHSQTTPVTAPRRLLVLSDAVELALPAVQQQLRAALDEIARRTGLVAEPIRFAELVGEDAALSD
CNLKALRDLQTAEFQSTVGNWIEATRPELGATFTLAYGNVQHFDRIAALESLARSERLFEQINAALPPGTIICFPTAPTV
APLKGSLDTLEAVTAFYDPTMTLTAFSGVARLPEISAPLLTAEGCPVGMSFAAGHYQDESLLMALSEMLGPQGS
>MCE8455299.1 helix-turn-helix transcriptional regulator [Rhodovulum sulfidophilum]AB15
MTARFSRTRHDLAEFLRRKRASVQPEDVGLPRTARRRTPGLRREEVATVAGIGLSWYTWLEQGREISVSAHLLENVARA
L
WMTPEERQHLYLLAQNRLPAAPAQSWCEMPPLIRRLLDDLTERPAFVMNLRWDVIGWNAAGDRLFAFSDYPAGSRN
MLWM
YFADHRLSRRVQDWPRQAPQIVASFRRDYAAGKDAPDMQALADHLQATSRTFRRLWHDHDVQGRCEGRRSFDMA
DRGPVA
FDHTTLIVDQDRHLRLVYYAAVERGEAGRKPMGGCSIDHAAE
>MCE8455300.1 MFS transporter [Rhodovulum sulfidophilum]AB15
MSGARKPCRHGSSSNRITSPIVTSYGMSDRLHDVAEGAVTMTVPARRTLGATPLFALLLGGFITIFDLFVVNVALPSIRA
DLRASLAGIGWVLAGYELAFGVTLIAGGRLGDRYGRRKVFMLGMAGFTLASVLCGLAVTTQSLIVARLLQGATAGIMFP
Q
IYAMLRVLLDAEGRRRAFGLLGMALGFAAIAGQVLGGWIVTADIADLGWRMIFLINLPVGIIGLLCARRLPETGQADAA
R
TDWAGTLLATIALILLLFPLLEGAHSGWPFWTWGSFIASAAALTLFVAWERRVKLQGGTPAVDPALFTNRSFAAGSIIVL
LVYSTSTSFFLGFALLVQEGLGLSAFAAGTLFAPASVAFIAASMTAPRMVARFGDRAIAGGAGIYLFGTAAVALTALSGA
HEAWLIPSLILFGFGQGLSMTPLLNFVLGFTAEREAGMAGGIISTMQQLGGAIGVAMISILFGQILGEAGYARAFAGGLT
YNIAALLFSALGISLLIRREAREAREA
>MCE8455301.1 hypothetical protein LZ188_01650 [Rhodovulum sulfidophilum]AB15

MKAARKDTNAHWTLSYSKAKANADDGKPIDLAIPTFGYAVRGRLSNRTAFMRRASWSSWLRRASRPFLPERMIPTVE
TGP
EIIVFKRISLS
>MCE8455302.1 GAF domain-containing protein [Rhodovulum sulfidophilum]AB15
MTAAEFANVRERMEPWSRAAGASLDQLFGAVGAAGCCVLLADSDGVPVERRGVAGDDATFDDWGLWPGALWSE
ATEGTNG
IGTCAIERRPVTVHRDQHFLARNGALGCIAAPIHDHDGELVGVLDVSSCRTDLTATFAGLIALSVREAARRIEAEAFRRS
FAHARILAVSGLEHNPAALVAVTADDLAIGASHSARVALGIKGNLAKAPVPASDLLSVPGVESLEEAERAVLARALARTG
GNVSAAARILGISRATFHRKLGHRKSGARRPEAVTQDPAAPCRIPATDAPSFPRGGG
>MCE8455303.1 aldehyde dehydrogenase family protein [Rhodovulum sulfidophilum]AB15
MNQLERSFDQTRPPFAARYDNYIGGKWVAPKSERYFDNVTPITGKTLCEVARSDAADIEAALDAAHAAKDAWGKTNV
AQR
AFILNRIADRMEENLDLLATAETWDNGKPIRETKAADLPLAIDHFRYFASCVRAQEGTLSEIDHETIAYHFHEPLGVVGQ
IIPWNFPILMAVWKLAPALAAGNCVVLKPAEQTPASIMVLMDVIGDLLPDGVLNVVNGFGVEAGKPLASSNRISKIAFT
G
ETSTGRLIMQYASQNLIPVTLELGGKSPNIFFDDVCAEDDEFFDKAVEGFVMFALNNGEVCTCPSRALIQHSIYDRFMER
ALERVKAIKQGNPLDPETMIGAQASSEQLEKILSYIDIGQQEGAEMLVGGARSHLGGELEGGYFVEPTVFHGNNRMRIF
Q
EEIFGPVVSVTTFKDEEEALHIANDTLYGLGSGVWSRDMNRCYHFGRNIQAGRVWTNCYHAYPAHAAFGGYKQSGIG
RET
HSMMLDHYQQTKNLLVSYNPKKLGFF
>MCE8455304.1 alcohol dehydrogenase AdhP [Rhodovulum sulfidophilum]AB15
MAKTMKAAVVREFGKPLSLDEVPVPEPGPGQIQVAIRASGICHTDLHAAEGDWPVKPTPPFIPGHEGVGYVSGVGAG
VTR
VKEGDRVGIPWLYTACGHCEHCVGGWETLCESQQNTGYSVNGGFAEYVVADPNYVGHLPDNVGFTEIAPILCAGVTV
YKG
LKTTDARPGNWVVISGIGGLGHLAVQYAKAMGFNVAAVDIDDHKLELARKLGATLTANAATEDAAAKIKKETDGGAH
GVL
VTAVSLKAFEQAENMVRRGGTIAMNGLPPGSFPISVFDMVLAGITVRGSIVGTRLDLQEALDFAGHGKVKSIINPVKLE
D
VNSVFEKMHKGQIDGRAVIEIAD
>MCE8455305.1 transposase, partial [Rhodovulum sulfidophilum]AB15
MPAPSPTRYRTINWSGYNASLRRRGSLSVWFDPDMNWYARKVGKRGHPETFSESAIRFCLKLKGEEHPGNDPGD
>MCE8455306.1 iron-sulfur cluster assembly scaffold protein, partial [Rhodovulum sulfidophilum]AB15
MITLDLMREQGRITALGWRSRACTLGMASLGLVTEVAVGQCRAEVAAAGAALWDLLQGQEQI
>MCE8455307.1 DUF1641 domain-containing protein [Rhodovulum sulfidophilum]AB15
MANPTANGAETPLLERINQESAFSDAATEEGLIDLANKLAPLIQGRRLHNVIDLMSLASDGVDMADDAMIQKMMTAY
EDI
TSTAWMLGNAARYAANQAATAPVPSRLGLLRAAGDEDVRRGLHFALQFLAVLGRQATPEPDVD
>MCE8455308.1 NAD(P)/FAD-dependent oxidoreductase [Rhodovulum sulfidophilum]AB15
MASFISWPLSRTPVFQNGKVDAWSQAKSARTWAPGPKSTALVGGGSQYSRGGIMQKHILVVGGGIGGTMTANSLVT
KFQR
EIANGKVKVTMLSDNPWHYYKPAFMYVAFDMYFKGELRRKQQSLLRPEIDFQLDKVEAFDFKGSKVKTASGKTISYDYI
V
VSTGCLPAPERIEGLKEGGDYFYQYEPARKLSDKLLTLEKGRVFVTVNFPETPNVPHQCGIAPMETTLMLDEYLRRKGVR
DQIEIVYTYPTVSQLLRNCLFMQEEVCSVLPPIFEERDIKYKRSFTLAKVDPERKVAISSEGEEEEFDILMSTPPIRAVD
AVINSGLSQAANNEGWLPTDRDTLQLEGYPNAYVMGDTVDLPISKAGGSCHNQCPVIVNNIAGDMRLGRIVEHYDGK
VQA

VAQMGLEAGMPLFYNYDKDVYPTPPTKVGGLLRKAFNRGIYWSVARGII
>MCE8455309.1 helix-turn-helix domain-containing protein [Rhodovulum sulfidophilum]AB15
MNDAIGSQQEPDAVRAAVQAQIIECLNRFYAMESGMWGKPLDSLIIRTVVQGRMQGKLYDFSALSETLDLPISTLHRKI
N
SLVESGYLRREPVGKSIYIAPTERTSVKFDESFEAMVTTLRRLYRGEYNFDGAPKLCDEP
>MCE8455310.1 tyrosine-type recombinase/integrase [Rhodovulum sulfidophilum]AB15
MTRSRSEIFKWKQHAPEVKAKAALIPITPELTEVIDTTPAGRLLILTNASGNALTPHRASEGVRQWRDKAKLSDDLRLYD
ARGTAATRLLNAGLSLAEIANHMGWSVRYAANVIEHYARVSPDETDAVLIKLAQAKGGVA
>MCE8455311.1 hypothetical protein LZ188_01705 [Rhodovulum sulfidophilum]AB15
MLGAALTLAEFDAWAVASAVWAARLSARERASLAYAALRSLEPEQAEMTAATVLRAAGAPMPPFLRGMEEARFWAS
LANR
AELKAFALASFEAMAPRDQTAFLRHISEIEVAA
>MCE8455312.1 Clp protease ClpP [Rhodovulum sulfidophilum]AB15
MTETIHIYGPIGEGENTPPRIGAFLDDNAGVPVVIAVNSYGGIASDGGAILAQLDRHGQVRVEVLGVAASAASLLVMGA
A
EIAMHRAAHLMIHEPSNLAWGTADDLRREAGMLDKISETYAQAYARATGHPVERIRAWMAAETWMTADEALALNF
CDSLF
GDEEAFQSEPVAAFDYTRFKAAPPELVRLAARNGWTGNAA
>MCE8455313.1 helicase RepA family protein [Rhodovulum sulfidophilum]AB15
MTDEPKMGTDSAVEGAVSHLYGNRARLKPRALALLSATTWAADLSPCLARPYLVKGWLDQSALSVLYGPSNSGKSFLA
LD
LAHHVAKGRSWGNRRVNKGRVLYIAAEGGGGFANRVAALDDPEFFVLSVPITLTGTDSAAGPLTEVLQHLSAVGGAGF
DM
IVIDTMARVMGGRDENAAPDIADLVRNLDLIRRVTGAHVMLVHHTGKDTGRGARGHSSLRAAIDTEIELSRDDLGQIT
AE
VTKQRDGPTGYRFCYQLRQVELGLDQDGDPVTTCLVDPAVPAEAGRAGVSEAARKALSLLNRLIEDRGEIHRKPQYPSH
A
GVSFDLWRDACMAEGELSSSENRDTRKRAFNRCREELETAREIVVRDDLVWVVQ
>MCE8455314.1 hypothetical protein LZ188_01720 [Rhodovulum sulfidophilum]AB15
MNARFLRAKAKPATSAEVIADLIEIERLIRIVCTREGNPLTLRAADELAKTHRRIRNLRYRIESEAGL
>MCE8455315.1 Mu-like prophage major head subunit gpT family protein [Rhodovulum
sulfidophilum]AB15
MIAEDIRGAFASNPAQHSAPTGRRGAGIARAEGTPSGVPASGSASAARNGQPSARHLAQMRGAAFKAGLSAEFADL
MAET
GVSAAQAEAVASGLAEFQKVSAPFVQGEQLDHAALMSIAKRAAMAGLALPETDAAAITKEIARVAANEAMALMASNE
PPI
RPCAIGIGQEAPSRADAMADGLLARMQSGHVPTHGREFANMRLDDLARLSAGSSFLRGQRHAVMWGGAMGADDF
KTALAI
ASNKVLLAAYEAAESQIKKASREVSAPDFRPINTVRVSGGVELAKVLENGEFGQGQITDAGEAFVLETYGKIFTLTRQAI
VNDDLRAFEQSSRLFGQGAALTEARIFAGLLEANGGAGPAMSDGKTLFHADHGNLAETGAALSIGSLSLARTAMRRQK
GL
AGEAIRVDPALLIVPPELETQAERLVAEISAASADDVNPFQQKLTVLADANLTDPAAWYIAALPGRPDALQHAYLDGAS
G
PQIFTRDGFETDASEFKVRLDFGAGFVDHRAWYKNPGA
>MCE8455316.1 DUF4236 domain-containing protein [Rhodovulum sulfidophilum]AB15
MQQSLRKLRSLTVKPCDVLLIVLAKRGRNVRFRKSIKVMPGVRLNVSGSGVSTSIGGRGATVNLSKRGVRSTLSVPGTGL
SWSSQKGWSEGSRSSPADEIEQLRVTASKTMDAVAKAAEKVDALGVRIDRAIVALNGGRGITASKVQTFEKRMLTEEQ
KL

SEIEEKVQENCLFLEAIDERLRAMKFGMFSAGKKRHRDGVADAVASCAVETRKISDQIAETHHAMSAKLTEVQEALEAN
G
PFNTIGNSAKPRTIS
>MCE8455317.1 helix-turn-helix domain-containing protein [Rhodovulum sulfidophilum]AB15
MGVRELAEAAGVSTNTITRLEKGEALKPRTLEAIQSALESAGVEFIPENGGGPGVRLKK
>MCE8455318.1 hypothetical protein LZ188_01740 [Rhodovulum sulfidophilum]AB15
MPNPFPAAVTALPAARLYEIHDCLALALDATERPGRYTQSEREARSYLRTALRHTLRLMEARA
>MCE8455319.1 hypothetical protein LZ188_01745 [Rhodovulum sulfidophilum]AB15
MKRRTFFAAAPLAVLPGSGVALADAPQLDQGSAVDPLVTLYREWLSARREWRELADLPGNENWDDPRSLAAEAREN
DAED
MMLALKPASLEGIAALTALAWSLVSPGNLDAEGFEAAAQSYDCRVIASIWRACTGLDGYPVT
>MCE8455320.1 hypothetical protein LZ188_01750 [Rhodovulum sulfidophilum]AB15
MKITEGTRNELDGGRRRGVLPIAQGDNFGFPDDEAVFSWADFTLSDALRMRVYFDLTRSGLKAQDVKSFVGNGFARV
EHG
QVQRPFDFHNPGPKPWLNPPRGLFDVWEEEIWIAREVWTCADIEEITKGPNFSVTWHGGTKSEIERAVADLKQGFDQ
RET
LSLQFYNLSFARQKVLDAAREINLPELDDLNGGKA
>MCE8455321.1 hypothetical protein LZ188_01755 [Rhodovulum sulfidophilum]AB15
MKQAEFRALVEAGHLPRGREIAPGLIRWPVEDLRRIASGEAAEGMGDVSW
>MCE8455322.1 tyrosine-type recombinase/integrase [Rhodovulum sulfidophilum]AB15
MSKRYLWRHPSGRIYVRLRGRLCRIMAPEGTAEFDREYWEILTGKRMQAKTSWNALMDDYRTSDRWLGLKPRTRQD
YDKV
MAYLREKIGTRDVKALTRADVIAAQKANAHRTRFANYIPQMLVILCEHAIDLGWLANNPAKGVRALKTPERRRKEHLP
WP
DWAVEKFRSEAEELPRLIFEIGVGSVQRPGDWVGFRWGDYNGESLTLRQNKTDKSMVLPCTPQLKAALDRAKDALGA
VPI
AGRPILMKRDGNPVSYRYMAQIMLKERRRLGLEAYDLHALRYRGVQELALHGCTDDEIAAYSGHATKAMIEKYAGEAR
QI
MRARQAAEKRR
>MCE8455323.1 DUF1851 domain-containing protein [Rhodovulum sulfidophilum]AB15
MADIEKNYRARAEKLGEPTAREPMPEAELAYCEGRLPPRLIRFMEELGYATFQQGGVTIAKPSALAPVLALVFKSDPDLR
HDDCTVVSYNPFGDLLVWSDRLKTFRISLAEGRISSQELAPTQFGPSLQRPKPASPPDPNLIARSAVMVRPDDFDFLDYA
GQEMLARCIAMHGPIDLGECYGFFPALALAGVESPMRRVENIRRVPALEHFAFLAQAQPFYLTKVGPAGVEKVREIG
>MCE8455324.1 polymorphic toxin type 15 domain-containing protein [Rhodovulum
sulfidophilum]AB15
MAEVPDPAEVAARSERSARAEATPPHGVCATCRGMVLVRARYDDYWRTPLTLQDLRVTDMNGVVFDGDIRTQGLLN
FGTA
DGEVSDTDIAALRDYLGSAEAADAQRGPLLVETIPDPAAAPQVADLERQILSDLRVFASRMETAIQPWIDDWERSGWF
GA
LETFLGGLRDGAAAWVDGESEFWAGMWEWVSGLPDMLGEAWDDLSAGVRALWDNRHRIVELLQSLAEGAVDAFE
RGFAAL
LDALDGLPGIEELVELLRSLVIRSAEWAGAMIEVVRQTDVLRALTATALGVLVLIPPNFWAEVAGTGVGYLIPEAIIAIL
FLIVAAFTGGTGGAGLAARLVIFANNVGQRMRAAGQAGGAIVDMLDFLGGLVSKLTELVRKVMRNRRERATGQTDRE
VPI
VRSVRRHDVPCFDLPRGADPAEFDRQLREQMDAINQMTADDMAYAHYVLDQARAEHARQMAAGLRPAGSSFTDIL
RDNGA
QRSARQDYALDLENQGFDDEEISEIMSSVDATHFLDIVAGGNPSDVGIGGSAENRGIGRQWIREGRAESLGQTARGM
RQD

GLADRRMNVNLERCR
>MCE8455325.1 DUF4123 domain-containing protein [Rhodovulum sulfidophilum]AB15
MLAALYGPEAPRHVYLLVDGTLRAEVAGLFDLDVIEAPARSLFQGEAEETMGEAGPWLVDMSISDPREPGDVSILQDLL
A
RHWPAGYSILVSTDAPFEAIRRHLRRFIQLPILEDGRSLTFRFWDPRVLGPFLATITQDRSRARRMTMTDEGTSIVYMLP
VSADATGGTSAVAVRIVPDKTLEAEPLRPMRLRYADFDTLARSRESARRDRMTDRLRADFAPELADRPATDVRATVDA
AL
DRFGAFGFQDHAHLHFFAAWSLFYGPDFEQDDPMGELEEILRSDAPETERFRAFRRRFETFTKQRVT
>MCE8455326.1 ATP-binding protein [Rhodovulum sulfidophilum]AB15
MPIYLNAIALQFYKGIGPEMQYVAPFSEMNFFIGANNSGKSIILNFISQHLPFREGVAVSTSMGSNADIYRGSTTGNYMA
AVGVPATVMASRLKQK
>MCE8455327.1 S-(hydroxymethyl)glutathione dehydrogenase/class III alcohol dehydrogenase
[Rhodovulum sulfidophilum]AB15
MKIRAAVAWEANRPLEIEEIDLDGPKEGEVLIRIVTTSLCHTDMFTLSGADPEGLFPAVLGHEAVGIVEELGAGVTSVKP
GDHVIPLYTPEDPNCPYIKSGKTNLCQTIRKTQGAGVMPDGTSRFSYKGRKIHHYMGTSTFAEYTVLPEIAVAKISKDAP
LAKASVMGCAIPTGMGAVRNTAKVEAGATVAVFGLGAVGMAAIQGAKMRGASRIIGIDTNPNKFELARSLGAMETIN
PAD
FTQPIQDVIVEITNGGVDYSFECIGNVNVMRAALECCHKGWGECTVIGVAGAGEEISTRPFQLVTGRVWRGSAFGGVL
GR
SELPGMVDEWLRGDFDVDPYITHNMTHEQINTAFDLLHAGKSIRSVIHFQPNETMLNNNLPGVIAPA
>MCE8455328.1 helix-turn-helix domain-containing protein [Rhodovulum sulfidophilum]AB15
MLEQTEGADQRRQIALVVYPGFKTLEATGPLSVLGYASQHLAAAGRPGGYDVVIAAPEAGPVPSDTLMSLEASVALRD
LG
ASDTVLIAGAARIEDALVRETALVAWCRDSGPKVPRCAALCTGSFFLAEAGLLDGKRAATHWNYADRLRQRFPRVEVD
AD
AIFVQAGRFWTSAGVTAAIDLTLAFVEQDYGRDIALAVARDMVMYLKRPGGQSQFSTLLTGQMAGHGAGPSSMKDV
LSWM
GERLDQPLTLDGIATAHGMSIRSLSRAFAAEIGASPMATLESLRCDTAKALLLDTDLPMKTVAFRAGFRTDEQMRKAFR
R
RFSLSPRDYRARFATAGT
>MCE8455329.1 DUF1826 domain-containing protein [Rhodovulum sulfidophilum]AB15
MIIDEARELKQCTQVASGPEGLAAIHQSGTAATLWERAPLDRFQAWIDALPPEQLPRTRIILRPEAVCNALIEVTRFYGT
PECPERNMLIEDASALAAIFADVMDCNYLQLRLDVIQTNACRKFHIDAVTARLICTYRGTGTQYGLSTTGDDPATVLTVP
TGSPIVLRGTRWPEDPPSGLLHRSPPIAGTGETRLLLVLDPVEDPEQERGTVYIH
>MCE8455330.1 phosphoribosyl-AMP cyclohydrolase [Rhodovulum sulfidophilum]AB15
MNTQKAPEIKITEEEIAQALVTWGNGKIAISRAFEEGGIEAARQVALTNLDAYYGYTLGPVLFKPTMASGEQTFRPTKPG
ALAYFCGHSEEYPLDNGFAIMGWRACEHITSASFIQGDIAMWMGWIRLTNKDGSVTTVDKSFGYKKDETGTLRIVLHH
SS
LPYQPPEDGA
>MCE8455331.1 His/Gly/Thr/Pro-type tRNA ligase C-terminal domain-containing protein [Rhodovulum
sulfidophilum]AB15
MIHVHLPDGAARPLAPSSSAADLASTISPSLARRTVAALIDGTLCDLSAPLPDGASVELLSRDDPRALELVRHDLAHVLA
EAVQALWPGTKTAIGPAIEHGFYYDFERDMPFTPEDLPAIEKKMREIIAAKAPFTGILIEHHAGHLPLWLAPVQVVVATV
TGAADGWAVEVAQTLKAAGLRVEADLRNEKIGYKVREHALTKTPVQIALGGHEAEERTVTIRRHGAPDTRTLSLEDAV
QM
LLTEARAPGAAEPVQSE
>MCE8455332.1 membrane protein insertion efficiency factor YidD [Rhodovulum sulfidophilum]AB15

MATGAVWLLKLPVRVYRLLWSPWVGHACRFQPTCSAYAIEALEGHGPLRGTWLTLSRLIRCHPWGGAGYDPVPPPPA
PTY
QRKTPE
>MCE8455333.1 GTP-binding protein [Rhodovulum sulfidophilum]AB15
MAWDDDRLPVTLLTGFLGAGKTTLLNHLTRDPEAGRIAVIMNEFGDVGLDHDLIEEATEETILLQSGCLCCALLGDLAET
LGALEERRRAGEVDFDRVVIETSGIADPAPIIHTLAADRLLAQTFRLDGVVTLADAATGMGTLDRQFEAVQQVAMADV
LI
LSKTDLADAVTLARFETRLAGINAGATRIHARHGRVPPGTLFGLSAMRLGADAPEIDGWLGLMEQTAKVDPLAGLSGL
KP
PSDTPAAFSPAAPIRQGRHDQRIVSASIEVSKPIPAEVFDLWLDTLIAFKGPNVLRMKGLLHVEGLQYPFVFHGVQHIFD
APVPFKAWSGDNTTSRVVVIARDMEEGELKASLGMLLLQPTKDHAAPGGLMAETTEMPF
>MCE8455334.1 phosphoribosyl-AMP cyclohydrolase [Rhodovulum sulfidophilum]AB15
MEFHERRSIEQIEEGTELAPKFGPDGLLPCITTDAASGEVLMLGWMNAEAFRLTIETGEAHYFSRARQVLWHKGATSGL
V
QKVVEARIDDDQDALWLRVAVASSGASCHVGYRSCFYRAVPHGDEAGRLLTFREDHKIFDPVAMYGDAPNPTLL
>MCE8455335.1 bifunctional diaminohydroxyphosphoribosylaminopyrimidine deaminase/5-amino-6-(5-phosphoribosylamino)uracil reductase RibD [Rhodovulum sulfidophilum]AB15
MQRALGLARGVRGQVWPNPPVGCVVVRQGQVLSEAATHPGGRLHAERAALEQAVDAEGATLYVTLEPCCHWGQT
PPCTDA
IVAAGFTRVVCAMKDSDPRVNGGGFARLKDAGIMLSVGLCAREAGLVMSGFLHRVRTGQPELCILAGPAYAIPDGVDA
LL
VTVDGRVAVRTRTQQDLTVPDSEYPITGDLLTVLGRMGLTSVAVPARDPIAAILRRDTAALAPSDRI
>MCE8455336.1 hypothetical protein LZ188_01830 [Rhodovulum sulfidophilum]AB15
MSDQSALQTAKVLWHLQRIKRLVRDRLGRGSAQLVSVRGTVCLDPGYPGPATEIRVVNLALMQTRFVIHKPADQVSA
ADV
STLL
>MCE8455337.1 DUF2083 domain-containing protein [Rhodovulum sulfidophilum]AB15
MCQRDLRQEKKDEGNGQSHGAPLEGPAGLLKPRFREPGADGRNPATAGPAGSSLAITLPHPASKALRGARIWSAQPD
LAT
PIGVGCKICDRRASPRRAFPMTGRPVRPDPGVSRLAPYSGA
>MCE8455338.1 DUF1007 family protein [Rhodovulum sulfidophilum]AB15
MFIDASAGFRFDDDGRLAKLRIVWLYDAFTTLNLYVQLGLDADGDGKLDADDLEKIARGETDWPPDYPGDTYLFQNEA
PV
ALGRPQHGTARMIGDRVEVSFDLPLVRPLAVDGQRTSLKLYDPEYFYAYTLKTVLAPGSMPRECQTRLIPFTPDAADAA
T
QKLLASLSVEQLPSNPDIGARFADELRLTCD
>MCE8455339.1 hypothetical protein LZ188_01845 [Rhodovulum sulfidophilum]AB15
MRLMLAIIGALALFAIITWLATGTQSVALFHWAAMQQRAVQEAMAGSLQAIRAGNPLALAGLCLLAATYGFLHAVGP
GHG
KVILGSTALTGEVPLRRMLAIGLAASLAQSGTAILLVGAGFGAAAMSGGSASLTEDTLADISRWAIAAIGALMLWQGGR
R
LWRLHRPPDATQHHHHGEGCSCGHAHGPTVEQVTSLGSTREAAALILSIAIRPCTGALVLLTIAFGLRIPLAGILATLAM
GAGTAALNAIAIFGGNAIGRMSALETRLGLAAAPGISGAALRIAGGLIIIVLPLGFGL
>MCE8455340.1 hypothetical protein LZ188_01850 [Rhodovulum sulfidophilum]AB15
MLIIDRDSRNGSCGLRWGRGSVGEVVKEAFARSGLPMIFNTYHGSAGVPVRLVEARETDGVEVATEAVRTPSCQTLVP
GR
ALAAFDPGKRRLKDRSQGAGR
>MCE8455341.1 metal ABC transporter permease [Rhodovulum sulfidophilum]AB15

MSLLDDFMMRAVLAGLGLAVAAAPLGCFVVWRRMAYFGDATAHAAILGVALSLTFSVSIFGGVMVVALAMALTVSSL
GGR
GFAMDTMLGVLAHSALAFGLVAASFLTGIRIDLMAYLFGDILAVMPGDLAVIWTGAVLVAGLIAWRWSALLTATLNPD
LA
RAEGVNPEREQLVLTLALAITVAVAIKVVGALLITAMLIIPAAAARPFSRSPLQMALLAMVLAAWAVIGGVRASYSFDTP
TGPTIVCVVAVLFTLSLSVSRMVRQH
>MCE8455342.1 metal ABC transporter ATP-binding protein [Rhodovulum sulfidophilum]AB15
MDLFEFADVSLRIGDITILSHVSMRMEPGEIVTIVGPNGSGKSTLLRLLIGSLTPSEGRISRDRGLRIGYVPQRLAIDPT
LPMTAADLLSLRRKVRPAQIAQALAEAGVAGLERRQISALSGGQLQRVMLARALLGEPRLLVLDEATQGLDQPGVAAF
YR
QIEQVRRRRGCGVLMVSHDLHVVMSASDRVICLNGHVCCQGAPHHVASAPEYRALFGEGTQGALALYTHHHDHDHD
RTRA
AE
>MCE8455343.1 transcriptional repressor [Rhodovulum sulfidophilum]AB15
MKAVGTVGHDREASTAERLASAESFCAENRLRFTAVRRRVFEILLDEQKALGAYDILERLDADGLGSQPPIAYRALDFLV
KHGFVRRIERLSAYVACLQPRETHDPAVMICRICDSVEETTFAPSRGMLGAAARDAGFLIEQTVVEAIGVCANCRAAGA
V
>MCE8455344.1 zinc ABC transporter substrate-binding protein [Rhodovulum sulfidophilum]AB15
MRRNLILGTVSVLGLATAANAEVPSVVADIPPVHSLVSIVMGDLGTPGLLVQPGASPHGYSLRPSEAEALDDADAVFW
VG
EALEPWLEGPLDALAADAHVVELMETNGTTELEFRENATFEPHSHDHGHKHAEAGHDHDHDHEHAEDHDHDHEGH
HHHGH
DPHAWLDPENAKAWLGAIAAELSELDPENAATYAANAKAGQAELDGLIGEISAELAPVRGKHFIVFHDAYHYFENRFDF
P
ASGSITLSDASAPSPARIEEIQNKVRELDVTCAFSEPQFNPKLVATVFRGTDAKAGVMDPLGADLAIGADLYPHLLRNLS
SSLTDCLE
>MCE8455345.1 hypothetical protein LZ188_01875 [Rhodovulum sulfidophilum]AB15
MASRRPPAARWPGIRPQREANRPFAWRQSEASHPASSDPSEASPKSRFCSPRLNLDQIEEGNDLTPPFDTDGLLPVITT
D
ADSGKVLMMGAVTRSIETGRAHCWSRSRQCLCHGRTTSGLVQHIVETRIDDDQDADWLRVRTDGDANCHVGDRSCF
HRSG
PMGRPGLLACTESTKEFYGDVPTPRGSDADRRWMERALPLARRRIWPPLRSAASLSMTKRGRQRQRPSLAIGPITRRW
CA
DGRARNCTVYVMLEPCSNWGRTPPCAEAIIATGMARVVVAMMQAP
>MCE8455346.1 hypothetical protein LZ188_01880 [Rhodovulum sulfidophilum]AB15
MRRLSSALAALLAAAPVAAADLASHRLSNPTAYIPPQCYTDTMDAEGNAHNPCFTCHVRARAPNYIIGDADLQTEYSLP
G
PALKNPWTNLFVDRSARISAIPDAEIDAYVAQDNYFDPDGTITLARKLAHPPAAWDENGDGAWAGYVPDCYYDFDAE
GFD
RAPDGTPTGWRAFAYVPLPGVFWPTNGSTDDVLIRLAAPFRSTAEGSYDPTIYKVNLAIAEALIKRADVPLDPVDETRLG
VDLDRDGTLGSATQVTFDWAPLKGRDMSWVGAAKGELLAAGLFPVGTEFLHTVRYIDVSFGEAAMAPRMKEVRYGI
KTRW
QTYADLEEGGLEEAKERYDFPDRIDLFDGGLETGIANGMGWRYQAFIEDASGDLRPQSFEETVFCIGCHSGIGLTDDATI
SFPRKLDAADAHAGGWYHWSQKSLAGTPDPIRADGRRELLTYLQENGAGDEFCNNAEVKARFIASDGSVKPEAAAAL
RDD
ITSLIMPSPERARTLNKAYREIVLEQSFAKGRDAVAEPASHVLKEVDQNQPTGIEIPVGASHW
>MCE8455347.1 PhoX family protein [Rhodovulum sulfidophilum]AB15
MTRITFLLATTALLATPAFADSFEFSSVSAPTTDAQKRAVQASDSVTIGGETVKIGYHILARSGQKIGDGTWGLLVDKTG

KPVRSEDGSEHISVDTDFSSLLPVGKKLFSISHFESRPGAMYLTELSQDPETGVLTPVSTKPVDFSDVGGLWVPCAGSVT
PWGTHIGSEEYEPDAREVADAEELADIDDYFYPMARYFGLSPSEMTLDQFRAAFNPYSFGHPTEVTVTEDGNATAAKH
YA
MGRLSIELPYVMPDSRTTYITDDGTNVGLYMFVADEAGNLDAGTLYAAKLTQTSDEGAGAFDLDWVDLGHATSDAVK
KII
DDKPGFFDIFEAADPAEGGTCGEGFTSINTTSGHECLKIRDGMELAASRLETRRYAAMMGATTELRKEEGITFDPENNR
M
FLAMSEVAKGMGRETKYDIGGPDSVQVAENKCGAVYQLSMAPDVAIGSDFVLQDMAPLVEGTPTEYGADSEFAGNT
CEID
GIANPDNLTYMTGHDTLIIGEDTGSGHQNDAIWAYNMTSGTLTRIFTTPYGSETTSPYWYPDINGFGYLMGVIQHPYG
ES
DADKLADPADAMAYAGYFGPFPASK
>MCE8455348.1 DNA-processing protein DprA, partial [Rhodovulum sulfidophilum]AB15
MVSSYHPLAPPTREDTLVTWLRLIRSRRVGATTFFRLLEDHGSAAAALEALPEIAREAGVRDYTPFPLDLAEAEIARARK
LGAVALLHGDPSYPPALHDLADAPPVLWALGRTDLTRRPMVALVGARNASSLGLRMARRLAEGLAEAGYVIVSGLARG
ID
RAAHIAAIETGTVAVQAGGIDVPYPVENTGLAEEIGQKGLRLSEQPPGLEPQARHFPQRNRIVSGLARAVVVVEAASRS
G
SLITARAALDQGREVLAVPGHPFDARASGCNMLIRDGAVLVRHPEDVLDALGPAPRPAAAAPVRPPPRMTEVPETPA
>MCE8455349.1 metalloprotease TldD [Rhodovulum sulfidophilum]AB15
MQHPPFRPFETALDRDRALLLLQAATLGAEDGELFLERRRSEMLSFDDGRLRTASYDASEGFGLRAVRGEASGYAHSSD
I
SEAAVKRAVETARLAVAEGGGTLALGPQPTNRRLYTDADPLAEAPFALKIDTLREIDAFARELDPRVVQVTASLAASLQE
VVILRPDGTLLSDIRPMTRLNVSVIVEENGHRESGSDGGGGRTGLAGRIERSSWEASTREALRIALVNLEAEPAPAGTMD
VVLGPGWPGILLHEAVGHGLEGDFNRKGSSAFAGLMGQQVAAKGVTVIDDGTIPDRRGSISFDDEGTPSNRTVLIEDG
VL
VGYMQDRQNARLMGLSPTGNGRRESHAHPPMPRMTNTCMLGGDTAPADILAGLKDGIYAVGFGGGQVDITNGKF
VFSCTE
AYRVKNGKVGAPVKGATLIGDGPAAMKQIRAIGNDMALDPGIGNCGKAGQWVPVGVGQPTLLIGGLTVGGAGA
>MCE8455350.1 threonine synthase [Rhodovulum sulfidophilum]AB15
MRYVSTRGQAPVLSFEEAMLTGLARDGGLYVPETIPAISADEIAALAGLSYEEAAFRVMRPFIGEAFTDAEFEAIIARAY
RGFNHAARAPLAQIGPNHFLLELFHGPTLAFKDFAMQLIGQLFQAALERSGQRITIVGATSGDTGSAAIEAFRGLPNVD
V
FILFPEGRVSEVQRRQMTTPAEDNVHALALKGDFDDCQARLKDMFNHFEFRDAVGLAGVNSINWARVLAQVVYYFTS
AVS
LGAPHRPVRFTVPTGNFGDIFAGYIAGRMGLPIEKLVIATNQNDILHRALTGGAYETKGVSPSISPSMDIQVSSNFERAL
FEAYGRDGAAVAGQMEDLKAQGGFGISQGALEFLRETFASGRASEAETTETIRATFAATGLTICPHTAVGVKVAAEQPA
S
AAPMVTLATAHPAKFPDAVEAATGLRPPLPKSMADLYDRPERVSPVENDLAALEALINERRKR
>MCE8455351.1 insulinase family protein [Rhodovulum sulfidophilum]AB15
MSVELHRLPNGFRIVTERMPGLKSAAIGIWIMAGGRDEQPEENGIAHFLEHMAFKGTEKRTTLEIAEAIEDVGGYINAY
T
SREMTAYYARVLEADVPLALDVLADILINPLFAEDEIEVERGVILQEIGQVLDTPDDVIFDWLQEVAYPGQALGRSILGP
AERVRGFTREDLFRFIGKHYSPDRMILSAAGAVDHAEIVRLAEALFSDREPAARGALEPARFASGERREIKPLEQAHMAL
AFECPGYRTPGIYTAQVFSTALGGGMSSRLFQELREKRGLCYTVFAQTGAYADTGMMTLYAGTSAEQVGPMAELTMA
ELA
RAARDMSPAEVARAQVQMKAGLLMGLESPSNRAERLARLVSIWDRVPGVEETVAQIDAVTTEAVREFASRLTLDGRP
ALA

LYGPVSGAPALDALQGRLAA
>MCE8455352.1 GNAT family N-acetyltransferase [Rhodovulum sulfidophilum]AB15
MLGLRRKVRIETERMTLRPPAHADFQPWTMLRAESMAFLTPWEPTWSPDHLARKAFTNRVYWAQRALGSGSAVPLF
LFRR
SDDALLGAITLDNIRRGPSQSGTLGYWVGECHARRGYMREAIEAVVHYAFTQKDLSRIEAACLPENAASRGVLEKCGFK
Y
EGVAQSYLQINGRWRNHVLYANLRRDRRGRTETGLA
>MCE8455353.1 MBL fold metallo-hydrolase [Rhodovulum sulfidophilum]AB15
MLRSCLILLAVLTALPAFAEGRRLSHCIAIADAAPGMRFLHRAAFGTPLDENTVRLTFVDHATFLLETPGGLVAATDYTG
YLGPNPVTPDVVTMNNAHETHFTALPDPAIPHVLEGWAKGGEAADHHLDLGEMMVRNVPTDIRGRDGTSVRPNGN
SIFVF
EVAGLCIGHLGHLHHVPDEAQFAALGRLDVVMAPVDGRLTLDLPAMIAVLTRLKSAVVIPMHWFGPRTLDRFLAGMA
GEF
EASRPGGSSLEVSLRTLPSRPTVVVLEPLSD
>MCE8455354.1 FAD-binding oxidoreductase [Rhodovulum sulfidophilum]AB15
MLNPVTDAFAADLRARLPAAVFRELTPAYLEEPRGRFRGQGALLLAPGRVEEAAEIVRACAAARVGIVPYGGGTGLVG
GQ
IMETGPAPVIVTLERMRAIRAVHADENVLVAEAGCILAEVQAAAEAAGRLFPLSLASEGSARIGGLLSTNAGGLNVLRYG
NARDLCLGIEAVLPDGSILKGLKRLRKDNTGYDLRHLLIGAEGTLGLITAATLKLAPRPAATGAALLAVRDPAAALALLA
MARDRVGEGVSAFELIHRAGLDFLAETMPELRRPFADPPDWMVLIELGLGAGQDPGAALEDLFAGAFEAGLVADGLV
AQS
EAQRQDFWTMRETIPLANRRIGAIASHDIALPLSAIPDFIAAAGPALARIGRFRINCFGHLGDGNLHYNVFPLRGETRAD
HEAQRDAITRCVHDLVAGFEGSISAEHGIGRLKVAELGRYGDPARLAAMRAIKAALDPAGILNPGAVIPPP
>MCE8455355.1 adenine phosphoribosyltransferase [Rhodovulum sulfidophilum]AB15
MIASSSVKDYIRTIVDFPHEGIMFRDVTTLFADPRGFRMAIDQLLNPYAGAQIDKVVGLEARGFILGGAIAHQLSKGFVP
IRKKGKLPAATIAEEYKLEYGEAVVEIHDDALAPGEKVLIVDDLLATGGTCEAGIKLCERLGAEVIGCAFIVDLPELGGR
KRLEEMGMDVHTLCEYAGA
>MCE8455356.1 hypothetical protein LZ188_01930 [Rhodovulum sulfidophilum]AB15
MGKKGGKKDSQLVLRLDKEQRDEFVALCKMLDTSAAREIRAFIRTFVKQHGGEAGSGKKK
>MCE8455357.1 S-methyl-5'-thioadenosine phosphorylase [Rhodovulum sulfidophilum]AB15
MERMIAVIGGSGVYDIDGLEDARWQAVETPWGTPSDEILTGTLDGMKMAFLPRHGRGHVHSPTSVPYRANIDALKRI
GAT
DVVSVSACGSFREDFAPGEFVVVDQFIDRTFAREKSFFGEGCVAHVSMAHPTCARLSAACLQAARDEGITVHDGGTYL
AM
EGPQFSTLAESKLYRENWGCDVIGMTNMPEAKLAREAELCYASIAMVTDYDCWHPDHDAVEVADIVKTLIGNAEKAR
GMV
SRLPRLLGAHKPCDKGCEAALEHALITAPEKRDPALIARLDAVAGRVLGR
>MCE8455358.1 flavin reductase family protein [Rhodovulum sulfidophilum]AB15
MFYRPEDGHGLPHDPFGAIVAPRPIGWISTRDGAGRDNLAPYSFFNAVAYEPPQVMFASTSTKPDRDGTKDSVANIRD
TG
VFCVNIVEEAATNAMNATSAPAPCGEDEFALAGIGRTPCETIACSRVADAPAALECRLTRILRLPGAANLVVFGAVTGIH
LREDCLIEGRFDVTRFRPLARLGYRDYAVVGEIFELQRP
>MCE8455359.1 N-acetyltransferase [Rhodovulum sulfidophilum]AB15
MIRVAEETAGDWWEVEALYDLCFAPGRTALSSYRLRDGVPPVAELCLTARDAEGILAAAIRYWPIRIGEAEALLLGPVAV
HPTRQGEGLGGLLIRNSLARAEALGVARVMLVGDAPYYARFGFSRLTGVEMPPPTNPDRVLGIELRPGAWAGITGAVT
RA
RPD
>MCE8455360.1 hypothetical protein LZ188_01950 [Rhodovulum sulfidophilum]AB15

MANRLTKTGSPRTARFRNPHPISVGQCPVFVADSVDGNLRILEEAVGLAAARSQVHPVGGRVKHDTAAAFLEARAAPA
MT
SEPLSLPIPGMAIEVSFGMPSAVRQVAAISAPPSSV
>MCE8455361.1 phosphoenolpyruvate--protein phosphotransferase [Rhodovulum sulfidophilum]AB15
MPDPTESSSRRLLVSLRETLAEPAAGQERLDRITSLIAEQMGTHVCSIYLFRDADTLELCATEGLNPDSVHKTRLRVGEG
LVGRVAKTAHPINSGNAPQERGFRYMPETGEEIFSAFLGVPIQRLGERLGVLVVQSKDARQYSDDELYALEVVAMVLAE
M
TELGAFVGEGEALAALHQRPVMFQGTVGQEGVAVGHVWLHEPRVVVTNPIADDYETEMRRLHEAVDRLRVTVDEM
LSAAP
GGDKDQLQVLEAYRMFANSRGWKRRMEEDIARGLSAEAAVEKEQSAARARMQTVPDPYLRERLHDLDDLSNRLLRIL
TGQ
GEHTGAERPSDPILIARNIGPGELLDYGRSLRGIVLEEGSVGSHAAVVARALAIPLIVNAQRITTEALNGDPILVDGEQG
IVHLRPEETVAVAFNDKIAMQAKAQQRYATIRKEPALTLCGQRIALHMNAGLMADLPSLESSGAEGVGLFRTELQFLIRS
KVPKRSEVAALYARVMDAADGRRVVFRTLDIGSDKVLSYMKRQDEPNPALGWRAIRVGLDRPGVLRMQLQALIRAAN
GRP
LSVMFPFVAQLDEFRTARDHLMREIDREGRLGRALPEKLEIGAMLETPSMAFSPRQFYEEADFISIGGNDLKQFFFAADR
ENERVRRRYDTLNVSFLSFLEQIVKRCEDTGTHLSFCGEDAGRPVEALCFAAIGLRCLSMRPASIGPVKHMLRRSNLAEA
RAVIDTARASGAQSVRPAIIDWLRRRD
>MCE8455362.1 aspartate kinase [Rhodovulum sulfidophilum]AB15
MPVLVMKFGGTSVADLARIRNAAEKIRREVERGYDVIVVVSAMSGKTNELVGWVEQTSPLFDAREYDAVVSSGENVT
AGL
MALTLQEMDVPARSWQGWQVPLRTSSAHSTARIEEIPRANIDQKFAEGMRVAVVAGFQGISHEGRITTLGRGGSDTT
AVA
FAAAFGAERCDIYTDVDGVYTTDPRIEDKARKLDRIAFEEMLELASLGAKVLQTRSVELAMRYKVKLRVLSSFEDTDETS
GTLVCDEEDIVEQNVVSGVAYSRDEAKVTLISVADRPGIAAAIFGPLAKAGVNVDMIVQNISEEGRTDMTFSCPVNQVP
R
AREAMETAMDAGEVNFREVVADTDVAKVSVVGIGMRSHAGVASTMFDALAAEGINIRVITTSEIKISVLIDRKYMELAV
Q
ALHDAFGLENAA
>MCE8455363.1 DUF1178 family protein, partial [Rhodovulum sulfidophilum]AB15
ALRKRIEQNSEHVGLRFAQEARDIHAGLRPDRPIHGEARPDEAIRLIEDGIPVAPLPFLPGRKRN
>MCE8455364.1 S49 family peptidase, partial [Rhodovulum sulfidophilum]AB15
MKRWLPFVKSDPLVAVVRLSGTIAASSRGSALSDAGLAPVIERAFKKGKPDAVALIVNSPGGSPAQSALIAARIRRLADE
HQTPVYSFVEDVAASGGYWLACAGDEIWVDASSIMGSIGVIYAGFGFQDLIARHGVERRVHAAGESKSFLDPFKPEKPE
D
VARLMQLQEPIHAAFKDHVRER
>MCE8455365.1 S-(hydroxymethyl)glutathione dehydrogenase/class III alcohol dehydrogenase [Rhodovulum sulfidophilum]AB15
MRTRAAVAFQAGKPLEVTEVELDGPREGEVLVEIKATGICHTDEFTLSGADPEGLFPAILGHEGAGVVVDVGPGVKSVK
K
GDHVIPLYTAECRECEYCLNPKTNLCQAIRTTQGQGLMPDGTSRFRTLDGDPILHYMGCSTFSNYTVLPEIAVAKIREDA
PFDKVCYIGCGVTTGVGAVVNTAKAEPGCRAVIFGLGGIGLNVIQGLRMIGADQIVGVDLNPAKKAMAERFGMTDFV
NPS
EVEGDLVPYLVDLTKGGADYSFDCTGNVNVMRTALECAHKGWGESIIIGVAPAGAEISTRPFQLVTGRTWKGTAFGGA
RG
RTDVPKIVEWYMQGKIEIDPLITHTLSLDEINKGFDLMHSGESIRSVVLY
>MCE8455366.1 flagellar type III secretion system pore protein FliP [Rhodovulum sulfidophilum]AB15
MSLRLVPAVLFLFALALPAQAQEISISLGEGGSLAGRGVQMILLVTVLSLAPGLAIMLTCFPFIVTVLSLLRQAIGLQQS

PPNMLIVSLALFLTYFVMEPTFTQAWQVGVVPMMNETLPIGEAVPRILDPFRNFMAGRADAETFERLLSLRPQADPTA
AI
ADAPLSLLVPSFLLSEIQRAFEIGFLIFLPFLIIDLVVAAILMSMGMMMVPPAVVAMPFKLAFFVVADGWSLISEALVRG
YFPGG
>MCE8455367.1 FliM/FliN family flagellar motor switch protein [Rhodovulum sulfidophilum]AB15
MADTSSDAPGEGLGAKFLDQVPIEITVSVGKARPLLRDLLQIDRGGILPLDRRIDDPVELYIGDRLIARGELQEVEGAQA
GQIAVRLTEIIDTVSGA
>MCE8455368.1 flagellar biosynthesis protein [Rhodovulum sulfidophilum]AB15
MSRLFLEDFSDHGAGPQIDPEPPAIDTEEIRLNAYEEGYRAGWDDASAAEAAARTRIGADFEKTLQELSFGYHEARVHV
L
EALEPLLSVMVERVLPELARGGLARKVVEIAREMAETEADRPLQLQVCPENREALEELVGADPGLPLRIVDDPGLGPGQ
A
VLRGGAAEREIDIDGMLAAIRSALNDFMTTEEEARRHG
>MCE8455369.1 flagellar M-ring protein FliF [Rhodovulum sulfidophilum]AB15
MWTGLSPRRRVVVAIATVVMFAAVLALSRVATSPRMALLYSGLDTAAAGDVIRALDQSGVAYEVKGPAIYVDSSQRDA
LR
MTLASDGLPAIGSAGYELLDGLSGFGTTSQMFDAAYWRAKEGELARTIAGNSQFRSARVHIATPSSRPFARDVRPSAAV
T
VAAAGGSVSGAQAKALRYLVAASVSGMRPEDVSVIDANSGTVVADDSSLSAAGEDRAEILKARLERLLSARLGPGRSIVE
VSVDTETQRESIRERIIDPEGRVAISENNQQRQSSSSDTNGGAVTVASNLPNGDGESDRSSQHKESETQEVVNYEVSQT
E
REILRAPGAVKRLSIAVLVEGVTSTAADGTETWQPLPDADLESLRELIASAAGFDAERGDALTIKSMRFEPLDAGGSGPQ
SSLFGEFTLDPLTLIQLAVLSVVALILGLFVVRPILAGQGKAAVAGLPAPAGGGQDQTWLNGEIDPADGPQDGMEDDL
GL
PAMTMAAIDPADFSDQPLGMDGFGGGGDSPVDRLRDLIESRREESIAILRGWMEDRQEKA
>MCE8455370.1 flagellar basal body-associated FliL family protein [Rhodovulum sulfidophilum]AB15
MAATDMAEAEEPAKSSKKPLIIGAALALVLGGGAFFALYSGLILGGDPTDETAAGAHGGHGKAAESGPLDVAFVPIEKM
I
ISLGPESASRHLRFEAQLEVTPAYQEDVTLLMPRVLDVLNSYLRAVDMAELEDPTTLITLRAQMLRRVQLVTGNGRVRD
L
LITEFVLN
>MCE8455371.1 hypothetical protein LZ188_02005 [Rhodovulum sulfidophilum]AB15
MALISDILLIAGALGATLYCYVLARRLRRFNDLENGMGGAIAVLSAQVDDMTKALKGAQFSAKSSTDSLESLTGRAEDVA
RRLELLVAAMHDLPEDTVQTAASQPPKPAATAAPETAPAAQPPTATADGEPMVLFRRTAREAAE
>MCE8455372.1 hypothetical protein LZ188_02010 [Rhodovulum sulfidophilum]AB15
MKGSSRSKRASGAVLPVIAGLFLASGLIRFGDGTAQALAQELQEMTGPAGDSGTDGGHDTATDPCPPPADIADLLEAL
KT
RAAELDAREAALNDRAATLDIAGEEIAQQMASLQAAEDSLKSTLALADQAAENDVARLTAVYESMKPEEASALFAQM
DPE
FAAGFLGRMRPDLAAAVMSGLEPNVAYSISVILAGRNARAPKE
>MCE8455373.1 flagellar motor stator protein MotA [Rhodovulum sulfidophilum]AB15
MIGIVGIILIFVMVFGGYVMAGGKMGIILHSLPFEFAMIGGAAAGAFVISNDMAAIKHTLKDISKVFKGPHWKPEDYRD
L
LCLLFELIRVARQNPLELETHIEAPGDSVIFGRYPKIVADHEAVDLICDTLRAAGMNYDDPHQVEEVLEKRLEAHLHHSM
HSSHALQSVADALPALGIVAAVLGVIKTMASIDQPPEILGKMIGGALVGTFLGVFLAYGFMGPFASKLKVVVEEDAHFYL
LIREVLVANLHNHPPAICIEVGRQNTPGGSRPSFSELEEALKASKQEAA
>MCE8455374.1 hypothetical protein LZ188_02020 [Rhodovulum sulfidophilum]AB15

MNARHIFLALALFACQPALAQQVEIRSGEHPDYSRLVFETDAPQAWTLGRGPDGYLLRFDNRALRLDLGGVFDRIPRRR
L
TDAALVAPGLVSLRIGQNAHVEAFELRPGLLVLDFRTGPAPEDSPFETALQSAADTPAEEEVPPAPAEPVRPDAPSAVDI
AAQSPSPSPEPPPVRLPIDLGLKSGLNPSAGMKPPAAMVEKMLEPEIPPAETDPRVAAMRSELMKQIGRATAQGLLEP
AI
DLPTQPDPGPAETVSNAQDALPDSGQRAEDSGQAETQIAEDHTPSDWRNMRVETSMDRGLDTLSDDSTLAADGTRC
LSDD
NFDVIGWGGDRTPGDLLAEKRSALLDMRDATDPSGVIGLARAYIAMGFGAEARAVLAATATETPVVPLLREMAAIVDT
GQ
ANHPELFDSQISCPTRAALWAALARPKLGDLSDFNRTALLQSFSELPIHLRRHLGPRLAERFLAAGDQATALQIRNAVAR
TGAKGQTTDLTIVEAQIELARGSDARADRKIDEVLASGGSGTADALALRIETGLSRGQAPSEDALALAESLAFERRATAS
GTRLLTLAIRGHGARGDFETAFAMLAHHGLEGQTELSSELLSELARSGADEEFMREVYLGALTLPALSADTEARLAVADR
LTAMGFQDRAATVLQPVAPQGTPRERLVRARLALAQGSAGEAQQYIAGIDTPEADSLRALIAQRQGDLKSAADYLGAV
GD
AEAQSAMAWRARDWSGIESSGDPNQRAFVQSRRDREPADLTIEPSLAVARDALGTSEIMRERLRALLDQPSTQP
>MCE8455375.1 flagellar biosynthesis protein FlhA [Rhodovulum sulfidophilum]AB15
MPGFSVSELFRPTILMALALMAIIIMMVLPMPAWVLDIGLAASFSIAILIFTVTLFIERPLDFSSFPTILLASLMLRLSL
NVSSTKLIIGQGHTGTGAAGDVIEGFAMFVMGGNVFLGLVVFGVLMIVNFIVITKGAGRMAEVGARFALDGMPGKQL
AID
SDMAAGAIDHAEAKTRREREQAETTFFGSLDGASKFVKGDAVAGLLITLLNLLMGLAIGVIMHGMPLGGAFETYAILTV
G
DGLVSQIPAVIISIAAALLLSRGGTTGTTDLAVFTQLGKHPAALGTVSVLLCLFALVPGLPFVPFIVGSLVLGTAAFNRW
RRLQHEAEKAAQPEAAEKAEPANRMGDLLDLDDIHLEFAPDLVGMVLDPSTGIEARIVSMRRHVAESFGLILPEMRLTD
N
GALPPGTYVVRIQGVEQARDRLRPDRVLALQAGDITLLPPGEDVREPVYGAPARWVSADRREELALQGVTVVNASEVL
AT
HLLEVVKNNFARLLTNKALRQIFDELKNLSDQTRAQANRRMIDDLVPEKVPPDLLLAVLRLLLEERVSIRNMPLILEAIA
EARPSHAGAEAICEHVRRRLGFQLVAEHRREDGTLPLLQLSPEWESRFSTYQVGGEAGVSDVALPPEEFNRLASAVSEKL
AKAGENGIFPVIATSGRRRRFLQMALSAKGIAAPVLSFEEIGTNTRPALVGMVAA
>MCE8455376.1 flagellar biosynthetic protein FliR [Rhodovulum sulfidophilum]AB15
MTLELAPALEIWRPLAWAGFLVFLRVGAIMAMLPAFGERAVPIRVRLGLALAFTAVVSPAVSPGLIETPPSLALAFATET
VIGLALGLILRLMILALQMAGEIAAQATSLSQILGAASMDPQPAMGRLMLMAGLALAVMSGLHVRAAEAMILSYDLLP
AG
RFPDAGDLTDWGVGGIAKGFALAATLAMPFVIASLIYNVALGVINKAMPQLMVAFVGAPAITAGGLIILFLSLPPMLTF
W
QDRLLTLFAAPFGAGG
>MCE8455377.1 flagellar type III secretion system protein FlhB [Rhodovulum sulfidophilum]AB15
MSDEDPGDKQFEPTQKKLDDARRKGEIARSTDINTAAVYAGFALTALALGGTSLIKMGELGAGMIGQADRLAPLLLEG
HG
SAPVGGLMAQFGLVILPWLAIPALAALASVLAQRSLVVAPEKLLPKLSRINPIETAKNKFGRSGLFEFTKSTVKLALYTI
LLALYLSHHVEEMLGTMYLSPAMAAAKLVRMSAEFLTVVVAIAASIGVVDYLWQIAEHRRRHRMSLEELKEEIKQSEGD
P
HIKQQRRQRAMDIATNRMLRDVPDADVVIVNPTHYAVALKWDRARRGAPFCLAKGVDEVAARIREVAVEAGVPMRS
DPPT
ARAIYGSVEIGDEIKPEHYKAVAAAIRFAEEMRTRAKGL
>MCE8455378.1 hypothetical protein LZ188_02040 [Rhodovulum sulfidophilum]AB15
MNTRDDRPERLVRIGTILKDAALSRLRQAAQECRRIEEAIAELDDEAQRAAIDNDIAMRAVFDRSRTLRHRERRTALNAE
LARARAQEALLMREAARAFGREEAIRGLFDRKHR

>MCE8455379.1 PhzF family phenazine biosynthesis isomerase, partial [Rhodovulum sulfidophilum]AB15
MIMYQVDAFAEEVLSGNPAAVLLLPDWLDEALMQAIARENNLAETAFAVARAEGGWELRWFTPTQEVDFCGHATLATAHV
LIAHEGQAAPLSFHTRVGVLHVTQGPSGYRLDLPRLDPEPLDALPAEVAAQFPGGVIAAFRNR
>MCE8455380.1 excinuclease ABC subunit UvrB [Rhodovulum sulfidophilum]AB15
MHSDFAPAGDQPTAIAELAAGVRQGDRDQVLLGATGTGKTFTMAKVIEETQRPAIILAPNKTLAAQLYGEFKGFFPENAV
EYFVSYYDYYQPEAYVPRSDTYIEKESQINEQIDRMRHSATRALLERDDVIIVASVSCIYGLGSPELYMAMTLDLEAGKT
YDQRQIMADLVQQQYRRNDAAFDRGSFRVRGDSLEIWPAHLEDRAWKLSFFGDELESITEFDPLTGKKTDGFDKVRVYAN
SHYVTPKPTIRQAITGIRKELKQRLAQLTDEGKLLEAQRLEQRTQFDLEMLEASGFCNGIENYSRYLTGRAPGEPPPTLF
EFIPDNAIVFADESHVSVPQIGGMYRGDYRRKFTLAEHGFRLPSCMDNRPLKFEEWDAMRPQSVFVSATPSAWEMEQTGG
VFTEQVIRPTGLLDPQVEIRPVEMQVDDLLDEVRKMAANGFRTLCTTLTKRMAEDLTEYLHEQGIRVRYMHSDIDTIERI
EILRDLRLGAFDVLVGINLLREGLDIPECGLVAILDADKEGFLRSETSLIQTIGRAARNAEGRVIMYADRITGSMERALR
ETERRREKQIAYNEAHGITPETVKKNVDDILAGLYQGDTDMSRVTATVEKPMHGGNLEAHLDGLRTSMRKAAENLEFEEA
ARLRDEIKRLEAVDLAIADDPLARQSAVEEAVEASARSKGRSSAGRAGQRGGTRRRHS
>MCE8455381.1 ETC complex I subunit [Rhodovulum sulfidophilum]AB15
MRARIYQPARTAMQSGQAKTHHWVLDFGPQSPRELDPLMGWTSSDDTQSQVRLRFETREAAEEYARRHGIETVVLRPKKR
APNVRPMGYAENFSVTRRGAWTH
>MCE8455382.1 hypothetical protein LZ188_02065 [Rhodovulum sulfidophilum]AB15
MKTIKPTQAVATILSRYQRRRNCQVPVTVTDVYADTIARVCGDDVDLDPVERGLIHLKRQKVISGGRLVTLLARHQREIRPE
>MCE8455383.1 IS3 family transposase [Rhodovulum sulfidophilum]AB15
MSKRKQHAPEFKAKVALEALKGEETAAELASRFEVHPTMIHHWKRALLEGASGVFERGSRKKPEIDEEQVKELHAKIGEL
AVANFFFGTKAEALGREVRRGMIEPDHSDLSIGQQCKLLSIARSSFYDTPKGESEQNLGLMRRIDEQFLETPFFGVRQMT
WHLRNDGHLVNEKRIRRLMRLMGLMPIYQKPNTSRPAKGHKTYPYLLGGLRVERPNQVWCSDITYLPMRRGFLYLVAIMD
RHTRKVLSWRISNTLEADFCVEALNEAIHKFGPPEIMNTDQGSQFTSFAWTDRLRRSGVRISMDGKGRFLDNIFIERLWR
TLKYECVYLHAWETGSETKAAIQKWMTFYNHQRPHSALNGKPPALAYWQRNGINQPGQQVQRVA
>MCE8455384.1 hypothetical protein LZ188_02085 [Rhodovulum sulfidophilum]AB15
MSDSFPFLNAPVINAQTDGAHLIIYQNHHVISSEAFDRSTFMKALAKAGLWNQRDYHLNGIQLVSKIDGQFVTQMDPAIR
QQIVGGAAHTGGHTKYNASQKFILDLFDEEYDDLLLENADWDGYGSENAWLEAQSKKIYGYVGFLKAELVNPNSILKLHS
ADRSVSDLKSGQIWDKFSKVFFDPEMLEFTSQVTEHPAYQIGTNGSYSGPQGMVDPTKAGTLDANLDQLDASHGMGNAFD
TSAYSATTDVITALETGPLGIDPNPPSAFHKVSGSLFESMIENGANKFIIAAAFVPLQGILADHAEAHTNGDQVLALMEL
GGSLTKEDFRQIAVSATAELGISVALGPVGAAHKVFQLLLSGEDVMSVVKLYELRHPENETIEWMADQVREIESSGVWQQ
YKDGVQSIEDLIDFLISSDLEGYANIGTWEADPKTGQELRVTEYGDGTHFEETRSPDDPGNILQRNQFEDDVLVASTTYK
ADGSVQTTLFDVHDSQPWDNQTFVTDSAGNFVQKTENYGRLTVTTDADGVVESTIDGRSIDGARNLSSLVNSGEADLAIA

MGGPSLNDLGNSPQGATYDPYDTDWSNFFDYANPVLQYLDIGLSYSGYLYDFPGIGLDIYNDYFYWGGPDSIHWAGF
DSL
HGWTPESTGCSSDAGVVTVDSSGVIHLGTICIPADPIVLDLDGDGIELISGSSSPVFFDQDGDGFRERTGWTGPDDGFLV
IDLAQNQSTGADGKITHRKEMQFALWHQDAETDLEGLALAFDSNNDGVFDSNDARFSEFRVWRDANQNGETDAGE
LQTLS
DAGIQSINLASDQQAYQLNGNNVRGETTYSRTDGTSAIAADVALTFDPFGILVTDTTNQEYLPKPLTTGQVIVSEESVVP
LVNFWSMAYALYSNQLHISTSSNGIVPIGDGHWISELPHVQELPSFRTVLADVIFGSTLVYGLDYIFSLFGSPIVYEDGA
EIYYFRAPETSSGEDIALHQCPGVDFSGAQGGDGSDHFHSAQNQSVALAGGDGNDTLEGGSNNDVLMGGDGADLLK
GGGG
DDVFFFDGHDTVVGGDGFDVAIAEGAIGVTLDLGQTSIEAVYGDIGDDHFYTSTNLGSLMSGNAGNDQLYGGSGSDTL
LG
GPGEDVLQGGAGDDTLGGGTGSDIFVFRDGFGDDVILDFDLSQIGEKVDLSLVSTISDFSDLRASHLRQSGNDTIIEDGN
GNIITLNNVDMSNLVVDDFLF
>MCE8455385.1 hypothetical protein LZ188_02090 [Rhodovulum sulfidophilum]AB15
MPASRPMNYAHLISSNINDGSSVGMTWLPEMPLELFRSIYLGQATLEEFPKDQALLIRKLVKGKVPEVIGMSTGPFVLA
P
ILREFLETHEPGGHQFFPVRILTEKPENGATEHGLHWLLLPPPPVDCLDFEGTVFIDDIRGNTWSRRRDDRKYWGGGLP
S
QDPDEPCAFHGDKVQRRHVWRYAIGVTEDRFKSRYACSMEFWQFYRSHRMLGWGSDKICTVT
>MCE8455386.1 FRG domain-containing protein [Rhodovulum sulfidophilum]AB15
MTGSISSIGELIEAIRTEKGREEGTIWFRGQSDSSWKLLPGLLRQKNRMSETSLLARFKQSAALLALTKPGNSFDWVFLM
QHYGMPTRLLDWSENPLVAMYFAVTGGDPDTDAAVWMLRPNKLNTNAHISDKDEPDYIPSFDDEEVQGYSTERVRI
DKRM
QLFPIATIATRNNARIQAQLGTFTIHHNEPVPIEEVGSGDQCVRLIIPSAAKQKIREELSLLGINQLSLFPEIASISETL
KEVM
>MCE8455387.1 DUF262 domain-containing protein [Rhodovulum sulfidophilum]AB15
MAYVQTSALKNATIMLLYAERDEIKLDPEYQRSGGVWTMNKRRLLIDSILNDYDIPKLYFHDLGDGSMHEEGKSYAVID
G
RQRLETIWSFMDGGFTLSDDFEYQRDPEISMAGFTYEDIAKKFPKIRIKFDSFVLPIVLVSVTDDDIDLIEDMFSRLNEA
VPLNAAEKRNAIGGDLVAAINEVSRHKYFSEKVKFNDARYKHREVAARLLLLEDSLQERGSLIDTKREYLDGLATRLKSG
NKEKVEELKRNVIATLDVMASTFSKKDDLLMAQGIQAVYYLLFRSAKKYGASESVDRAGLIKFRKSLSENRELAASDYEK
ASFELLEFDRLNQQGTNDANSIKERYSTLCKHFEISPDPLIAT
>MCE8455388.1 HD domain-containing protein [Rhodovulum sulfidophilum]AB15
MQDQFPKPHVAKALLPHAFDLRDGAHDEAHLLRVWRNVERIASEEGGDIEILLAATLLHDCIWIDKGSSQRHMASRLA
AA
KARDVLTGMAWSDDRINQVCHAIEAHSFSACITPASLEAKILQDADRLDAIGFIGVARCFYIAGVRKASICDAVDPAAND
RPLDEDLFALDHFQTKLMKLERGFQTMTGARLAADRSAHVKSFYNGLLAELS
>MCE8455389.1 hypothetical protein LZ188_02115, partial [Rhodovulum sulfidophilum]AB15
MAAISGEPAPTMQPLDSYIATRSDEAFHQAEFEANIDDYYFTLDKSRRRGPDTHIVVMPIFTGDAAQEAKRASGVLYFD
S
EEGMKAWARSHSDEASQSRNGHWHQQVHGYEDRHPDLVGMVRDTLIDDGYTLEQPFLHVRPYSGSREAALAALAT
PDYVP
VYVLLGVGAVLLVVSFGKRISARQARDVTD
>MCE8455390.1 NUDIX hydrolase, partial [Rhodovulum sulfidophilum]AB15
MGLDADLAIERQHPLGSQIAALPLRLDRNGHLGVLMVTSRGTGRWVVPKGWTMEGHSPWRAAGIEALEEAGAEGR
VASHS
IGSYEYDKRLDDGTVLRCRVEVFPMLVCKLRRNWKERDERIRRWFTPKAAAQRVDEPELAALLRGLSQKPE
>MCE8455391.1 inorganic phosphate transporter [Rhodovulum sulfidophilum]AB15

MSETYKDRQWKTLDKDLKRLSRVEIAAQTVSRPLLGVGVSLAFIVIAALGAALVTGNTPGMLFVVAAAAFGAYMALNIG
A
NDVANNMGPAVGANALSMAGAIAIAAICESAGALIAGGDVVSTISKGIIAPESVADSATFTWAMMAALLAAAVWVNI
ATW
IGAPVSTTHSVVGGVLGAGVAAVGIAAVNWSTMGAIALSWVISPVLGGVIAAGMLAFIKWRVIYRDDKIAAARTWVP
VLI
GVMAATFATYLALKGLKHLFDVTIGHAVAIGVVVGIASGLGAVPLIRRQSRGLENRNRSLKLLFAAPLVISAGLLSFAHG
ANDVANAVGPLAAIVHAQQAGEIAGKVAIPVWVMVIGAFGISFGLVLFGPKLIRMVGSQITKLNPMRAYCVALSAAM
TVI
VASWLGLPVSSTHIAVGGVFGVGFFREWHAERRLRRANLTKPGAKRLGPEERRRRKLVRRSHFMTVVAAWGVTVPAT
AVL
SGGMFLAISALQ
>MCE8455392.1 malonyl-CoA synthase [Rhodovulum sulfidophilum]AB15
MTNPLYDRLFGRHAGRETPFLHLPDGGSLTHAAFLALAARHAHALNAFGLGPGDRLAVQVEKSPEALALYAACLQAGV
IF
LPLNTAYTPAELSYFIGDSGARLVICDGRRMGALTPVAEAAGAHIATLNTDGSGSFAVIAATKSATYPTVARDPDDLAAL
LYTSGTTGRSKGAMLSQRNLLSNAEVLTEAWRFTEDDVLLHALPIFHTHGLFVATNIMLLAGGAMILLPKFDIDAVIAAL
PRATAMMGVPTFYTRLLDDPRFDRALAAHIRLFVSGSAPLLAETHRAFEARTGHRILERYGMTETNMSTSNPYDGERIA
G
TVGLPLPGVELRIADPATRAECSPGETGQIEVRGPNVFQGYWQMPEKTREELCEDGFFLTGDLGTIDAKGYVRIVGRAK
D
LIISGGLNVYPKEIEEVLDAQPGVLESAVIGVPHPDLGEAVLAVLVPAPGETPELQAIAGSVATQLAGFKRPKRLELIEA
LPRNAMGKVQKAALRESYRDSFA
>MCE8455393.1 monovalent cation:proton antiporter-2 (CPA2) family protein [Rhodovulum sulfidophilum]AB15
MEGFLLNATLYLLCIVLAVPLASRLGLGSVLGYLLIGLMLGPVLGLVGAEMVTLRHVSEIGVVMMLFLIGLELDPRALWA
MRNKLVGMGGLQVLITVALVTAAAIAFGQTWQSALAMGMIFSLSSTAIVLQTLTEKKLMNTPGGRSAFSVLLMQDIAV
IP
MLAIMPFLASPEIRAAGTEGGHGAMHLIGSLPAWGVTLVTLGAVAGTVLAGHYLVRPLYRYVQAARLRELNTAMALVI
VA
GIASLMLMVGLSPALGTFLAGVVLANSEFRHELESSIEPFKGLLLGLFFITVGAGMDFSVFWDAPILLPVYVTGLVMIKV
AVLYGLAMAFRLDRQSRWLFTLGLAQAGEFGFVLVGFASTQRIMPPDIVTMSSLVIALSMLITPALFILHDVIAKVLQGR
GGARPAADTIDETHPVIIAGIDRFGLTVNRMIALTGGKTTAIDDDLRAVRRVRKLGFKAYYGDATRPALLASAGIDTAEV
LVVCIGDAKAATTLVRYARRIRPDLFIIARARDREHVFELYRAGADETVREVYDSALRCGRQVLERSGLSAYEAENAERT
LVTFDRTALRDLAKVWRPGVPLEDNADYIELSRRLQADLEHAFAETLLHEAPPPRSQTEATSPADILAEADGNPERQTGR
TQT
>MCE8455394.1 IS3 family transposase [Rhodovulum sulfidophilum]AB15
MMMPKEGFFGPLKNEPGQRTRFRSRREVRAALFEDVAIFHNRRRCHSSSGDRTSEQARIDMTDEMAAE
>MCE8455395.1 glycosyltransferase [Rhodovulum sulfidophilum]AB15
MSEFRDIIGASISNLRHLWTSRELGRQWTGNIDRILDGIIEGWVKNTGSDHPVRIDIMLDDAPIATNVVADRHRKDVED
A
GIGSGRFGFTCPVPADRPLEGARLEVRLAATRKILLTKRVTPQMLPAKPGSIREVSPTDHPKQDPVVKCEGRLENLTDDC
LRGWASDTLSRGRLFEVEVLVDGVFLTRIRNDRPRGDLARHGKSKGLGGFELKLFLFELEPGPHSVTVVLPDGQKLTKTV
TSTGNRRGYPLNIGATQIAISETAVIVPIYNAAEDVETCIRRLVEHTPPELEVVLIDDASPDPRIAEILADAVSLPGFRI
LRNESNLGFTRTINRGLEEIGQKHAILLNSDARVTPGWAQGMLRAAGSRPRVATVTAMSDRAGAFSAPNIGNDNRLP
DGV
DEITYARAFRRRSLGVYPVVPTGNGFCMFVNRACIDEIGPLDAEAFPRGYGEENDFCMRAGRAGWTHLIDDRTYVFHD
RS

KSFGETKTDLMTAGRRIVDERYPEYKLAIRVFGAGPDLMMARYRAAQALADCSRPDAGLPVILYVVATQTGGTPQTN
MDL
MAEVSDEMAPWLLHCDSRKMTLSRLDGSRMTEIRSHELAEPVDPLTHRSGEYDAVLQDWLDIANPRVVHIRHLAWH
SLSL
PALAKAWGSRVVFSFHDFYTLCPTVKLLDECNVFCGGTCTKTSGDCAIELWDPKSMPRIKGTWVHVWRQRFAEVLRD
CDA
YVTTSESARELILRHLHLDPRRFFVIPHGRSFTEMEKLREHPSHGKPVRILVPGNISTAKGREVIKALLEHDSAGLLEFH
ILGKISDPEELAHHKRLVMHGTYKRNEFSRRVARIRPHLGAVLSIWDETYCHTLTELWSAGVPAIVFDFPTVATRTRASG
AGWVVPHGDIPRLYERLLAISFDEEEQRRADMAIMRWQKGHGVGRTTKVMSTAYREVYRNASAQQLDRPVIAVVCP
ASPE
QDRANASTEIRIWQRTINGAGRKCLFVRMTPDSLMANLRDGIVDGVILQRDVIPATMVAPLLNEMRRSGISHILDIDDD
L
LAVPSDKDPTGKYAAYRPALEALIDSAAMVTTSTEPLRRKLMARNPHTLLLPNRISERLWRGSIPPRKKDDTVRALYMGT
ATHREDFNMIVPALEALMEADPGFRVSIIGIQGDALPSWAERIAVPDNEKSYARFVPWLKSISGRFDFGLAPLTDDPFNL
FKSNLKALDYGALGLPVLASDIPVYRSVRGRIPGLRLVKSRPGSWTHALTDMVAEVRSGAIDRAGIRATTMEGYGMAAT
L
GDFDDLLLSIARPGDMDRLAAHPEPADDNIQHLKSIA
>MCE8455396.1 ethanolamine ammonia-lyase subunit EutC [Rhodovulum sulfidophilum]AB15
MSDSVSTGFGRLRAMTPARVRLDAAAGPTPLGALLDFQEAHAGARDAIHARPDWDALAAALAPLATIRVQSQAVERS
IYL
RRPDLGRRLAEPVDLPRAGHPLVVVVGDGLSAPALAHAAPVIAALRPALPELDATPVILAEGARVALGDDIARRMGAA
MC
LMLIGERPGLSVSDSLGAYLTADPAPGTRDSARNCVSNIHRMGGLSPEAAAARIAWLIGAARKLGRTGVALKDESGTRP
A
GVLAP
>MCE8455397.1 ethanolamine ammonia-lyase subunit EutB [Rhodovulum sulfidophilum]AB15
MPRYSATAGGERFVFGSLARLLAAATPERSGDQLAGIAAESATERVAARRALADLPLAEFLTEAVVPYEEDEVTRLILDT
HDRAAFAPFAAMTLGDFRGWLLSPAADAARLAEAAPGLTPEMVAAVSKLMRNQDLIRVARKVEVVTAFRNTIGRTGT
LST
RLQPNHPADDARGIAVATLDGLLFGAGDAVIGINPASDNLENCAGLLSALDEMVASLAIPTQTCVLTHVTNALRLIEGG
A
PVDLIFQSVAGTEAANEGFGVSLSVLAEAHEAGRALGRGTVGDNVMYFETGQGAALSAEAHHGVDQQTLEARAYGV
ARAF
RPLLVNTVVGFIGPEYLYDGKQIIRAGLEDHFCGKLLGLPMGVDVCYTNHVEADQDDMDVLMTLLTRAGVTFLITVPGA
D
DVMLGYQSLAFEDIGYLREVTGLRPAPEFEAWLARMGLAQGAPALPDPAALGKRMGILT
>MCE8455398.1 phosphotransferase family protein [Rhodovulum sulfidophilum]AB15
MSMKTLGCASHPMEEAIEAVIRKAPLFRGGAGMVYGPVSGGISNENWRVTATDGSGDWFVKIPGNGTEMFIDRKAA
FEAS
QKAAAAGLGPGVYGDLAAEGVEINDFMADRRPATHSDFADPAKRRAAIAAYRKLHGMPGLAATKTVFDMIDEHAAQ
AADL
GAPRFPDHDWIMLNERLAREALMASGLDLVPCFNDPMPGNFMVGEDGSILLIDYEYASMNDRCYDFGLLFGEMFFTP
EQE
LELIEAYFGEVRPEIVSRVILHKALADVKWALWSMVQLRVSRLAFDFHKYGMWKLMRFRQITGHQDWPKHLRTV
>MCE8455399.1 phosphotransferase [Rhodovulum sulfidophilum]AB15
MTGALVTDQMMAAACAAAGLSDADARPGPAPLASPSYLALESASILTAGGVFLKVMHPEMREGFDLDAAMTLARQA
GAAG

VGPRVLWSDAGQGAIAMEALTEAGGWRRAMQKDLQEPPVLAGAMATLKALHATPALANRFDPFAQIDALIAEYRRL
GAPL
PDDIDWLRRLIGAAEPVLRAGATLAPCRNDGSASNLMLGPDGVVLLVDYDRAGMNDPLYDLGALLAETTDFERDMQA
GFA
AYAGGFDETGFARARLWSFVDDMLHALWSRLKAQTSVRGGVEWLKYGEWRLMRLRMALNHPGFEQKIRLAGGAA
>MCE8455400.1 Zn-dependent hydrolase [Rhodovulum sulfidophilum]AB15
MPEMPSLARIDADLLHGLMEKVSEFGSTGDGGVDRPVLTDAHKAARDWFRSELTARGYTVLVDAIGNLFGRIDLAGPD
AP
LVMIGSHLDSQPRGGRFDGAYGVMAALAAIESFRKGGGTPRCNYVIADWMNEEGARFQPSLLGSSVFAGLVDLDWA
LARR
DRDGKSVGEELVRTGYKGTDPAPRPDLYLEIHIEGDARMETAGARIAPFLRHWGALKVRIEVTGEQNHTGPTPMEDRK
DA
VLGAAHIIAEIRRLADIAEDTLYTSVARVDISPNSPNIVPGKAVLFCELRAPEPAMLEWSEASLRAALPELAAKAATRAE
IVSIDRRPAGKFDPRLARLTERVADDFGLPRMQLDTIGGHDAVAVNAILPSLVFAVPSVGGVIHRNDEYTSPEDLAAGG
D
VLTDMVRRIDRAGGDLDLALGANA
>MCE8455401.1 ABC transporter ATP-binding protein [Rhodovulum sulfidophilum]AB15
MALDMTATPETLLPDTLLSVRGLNVVAGDRPILTDISFDLAPRSILSVIGATGAGKTVLARALASWLSAPLRVTGGEIRF
RGRDLVADPAHARNLAGREIAYVGGNPAGALDPTMTVGAQLVEKLRAVRPDISAEAARQKAIRLLEAVHIPSAARRFH
EY
PFQYSGGMMQRAMIVDALISDPALLIADNITQPLDVTVAAQILKLMRELNSEFSTAILFICSSLPVACDASDEVMVLHEG
RVVERQTPAALVAGPEHSYTRALIAELPTLWQDGQTGAGRDIDRNRRAIMSVRGLAKAYEVRARGQAGVSRVQAVRN
VEF
DVFPGDNFGVVGESGCGKSSLMRLLTGLERPDAGSVFFEGENVAAASPRRLRELRRRFQLVLQDPYGCLPPQSAIGAILE
EPLKIHRIGNAAERRDRARAVMAEVGLDDSLYRSLPTGLSAGQRQRLNVARAMILEPRLLIMDETLSALDQTEQGKLLD
L
FDKLQAQHGFTYIFISHDLTMVRQVCSRIAVMYLGETVEVAPNERLFFDPGHPYSRALLSAAPTLEERRYRPEDCLLEGE
PPSPIDLPPGCAFASRCPQAFDRCRAENPALRARGGHALAACFLNPEPDIALPENAPPETATETPHA
>MCE8455402.1 ABC transporter permease [Rhodovulum sulfidophilum]AB15
MTSTDMPLAMTGPETGRRLSTRIWQTAFASVEFRIGLTVFVLLLLAALLYPELSGIDPTKMNIRAKLTPPVFMGEGWSW
A
HPLGTDQLGRDMLSRALVGLRYSLLIGVSTTVLMLLAGAMIGLFSGYFGGRTDTVLMRLTDAQLSIPMIILAITILGVSR
PTIPSIILVLGLAGWPVYARVMRSTVMSERKKEYVRGAMVLGATDLRIMFTLLLPLVLPPMAFVAVLDIARMMIFESVLG
FLGLGVQPPTPTFGNVIADGRKYLMNAWWIATMPGVFLVLTLTSINLMGASLERARNAIYGGA
>MCE8455403.1 ABC transporter permease [Rhodovulum sulfidophilum]AB15
MFILRFLARRLGQGAIIIFLVSALIFTLLRVVPGDPVRLMVGGMAPDTLVEEIAAEMGLRDPIYVQFGRYMGNVLKGDLG
ESFVRPASGAAVGGASFDDSTRGERAKVLDLIAETAPMTLQLALLAMVFALMIGVPIGVWGGLYPGRLPDRLALYTSSL
F
VSLPNFWLGIVLALLLSVKLNLLPAIGYKGFSYTILPALVLAVEIAPFIIRTLTVSVAGVMGQSFIDIARVRGLSRNQIV
FRHALKNSAVPLLNLLGVQFSMLLGGVLVIEFIFDYPGLGLLTINAVMQRDFPLIQGIAIVTAAVFVLINIAVDLLATAI
DPRLDY
>MCE8455404.1 ABC transporter substrate-binding protein [Rhodovulum sulfidophilum]AB15
MDRRSFLRYGALAGTALGAARINPDFFFSSAFAQEARPLVFLSAENITGNWDPTAHTTLSQTNIEGFVMGYLTRAPMR
PD
DPEKVVYELATEITELDAHRLQIKLRDGITFHDGKPFTAEDVKATFEYGAQLDRPKQVYPGGPDTFAVETPDDHTVIVDT
SKGGYGASLFIFLASYLPILSAKDVAEGPKGPLSQRLNGTGPFRFVEQRGNDTVMEAYEGYFRGAPKVTGVTFSFVGDAT
TRMLSLMNGQADVIERLEPEQVETLEARDDIKISRLVSVENKYLWFRCSKPPFDDWRVRRAACHAIDRSMIMEIMGSA
GE

ASSNFISPIKFGYIDLENYPEYDPDECQRLLAEAGYPGGEGLPELEYITSTGFYPKTKEYGELIAALLQEQGFPVKLNVM
EVAAWNERLYDRPGGGPGHMVDCGWSTGSPEPDLVLRTHFHSSAKRICGIVDPEIDAALDAERDAPSLEARKESLQTN
LM
PMLADKAPALSLFTSVLIHGMRSNVDGLFIYPDGQSDASRTTLS
>MCE8455405.1 histidinol-phosphatase [Rhodovulum sulfidophilum]AB15
MSELGDALRFANDTADEAGRVALKHFRQALDVESKADDSPVTLADRAVEALMRDRIMARFPGHGIFGEEQAPLRPDS
DHL
WVVDPIDGTKSYVTGNPLFGGLMALLKDGAPCLGQIDMPALGERWCGVEGQATTLNGRPCRTSTCTDPAEAFAYTTD
PML
FSGADSEVLEMLRRSVRMLRFGGDCYAYALLASGHCDLVLETGLQPYDYLPLVQVIRGAGGVITDWQGQALGVGSAG
EVL
AAATPELHAAMLDRIARLRAGQAA
>MCE8455406.1 DeoR/GlpR family DNA-binding transcription regulator [Rhodovulum sulfidophilum]AB15
MQPYERHAKILERLGHDRRVFTAQLARMFDVSHETVRRDLLEMEQSGSLTRVHGGAVAADREILPEPAFSERRVVHAD
KK
AAIGALAATLIPPGSTVFIDAGTTTCAFARALAREGEMRIITNSIEIAQLAGPARDCDTLLLGGRPHTDVPATYGEMTLS
EIDRFLADYAVLSPVGLHADRGATDYALHEAEVARAMMRRSRDCIMLCHSEKIGTESRVSICRLDEIDHLVTDDAADRSL
RLPRGEIHYAETRPDL
>MCE8455407.1 HU family DNA-binding protein [Rhodovulum sulfidophilum]AB15
MAKPMTKTQLVAALAEEMEADKKTASAALEAMTAIITREVAAGGSVTLPSVGKIYCRERPERMVRNPATGEQFKKDA
DKV
VKVTIAKALKDSVNG
>MCE8455408.1 AMP nucleosidase [Rhodovulum sulfidophilum]AB15
MPDQSDLPLSSPSLPEAEAFTDAAAAVGRLVELYETATGFLRARFAEGLSGSAPHARFRAVYPEIRFTTVSHALVDSRLS
FGHVAEPGTYVTTVTRPDLFANYLRQQIGLLLENHGQPVVIGPSTTPIPIHFAMGSAGVTVPQDGALEVPLRDLFDVPDL
TTTNDDIVNGTWTMPEGAARPLAPFTAQRVDYSLARLAHYTATAPEHFQNHVLFTNYQFYVEEFEALARRALADPDSG
YT
ALVGPGNAEIRAPETQLPVPLKLPQMPSYHLKRADGQGITLVNIGVGPSNAKTATDHIAVLRPHAWLMVGHCAGLRN
SQR
LGDFVLAHGYLREDHVLDNDLPLWVPIPALAEIQVALEDAVEEVTRLEGYGLKRIMRTGTVATIDNRNWELSDQTGPV
QR
LSQSRAIALDMESATIAANGFRFRVPYGTLLCVSDRPLHGELKLPGMASDFYKTQVARHLAIGVRAMELLRDMPLERIHS
RKLRSFEETAFL
>MCE8455409.1 SDR family oxidoreductase [Rhodovulum sulfidophilum]AB15
MTGTLLSIGHGYSARELAQSLEPGWRVIGTSRSAEGAEALRRTGIEARIWPGDDLGPDLAAATHLMTSVPPGASGDPVL
A
ALGETLRAARHLRWVGYLSTTGVYGDHGGDWVDETAPLTPATSRGEARVAAEAGWAALGLPLHIFRLAGIYGPGRSPL
DR
VREGRAQRIVKEGQVFSRIHVADIAQVLAASMAAPDPGAIYNLADDEPAPPDEVVAFAAEMLGLPVPPAIPFENAELSP
M
ARSFYAESKRVRNDRIKTELGVHLRHPDYRAGLAALLETERAAMIR
>MCE8455410.1 PTS sugar transporter subunit IIA [Rhodovulum sulfidophilum]AB15
MQLSTLITPEAVRVFGTMSSKKRLFQELGEVAHAAYTLPPGDAVDALQERESLGPTGVGQGVALPHARLSGIDRVRGA
FI
RLEKAIDFGAVDRQPVDLIFALFAPQDSGVDHLKALALVSRTLRDNTICAKLRANTDPATLHAVLTEAAASQAA
>MCE8455411.1 ribosome-associated translation inhibitor RaiA [Rhodovulum sulfidophilum]AB15

MLYQISGKQIDIGEALQTHVKDELTVAVSKYADRATDANVVFSRNAHQFVCEATVHLSTGLTAQASAQATEIYAAFDSC
C
DKMEKQLRRYKRRLKDHHRERSEPVELSGGSAYILASTEEGDESDSLQPMIIAEMETQIPSISVGEAVMQMELRGAPLL
V
FRNEKTGKINVVHAREDGNVGWIEP
>MCE8455412.1 LPS export ABC transporter ATP-binding protein [Rhodovulum sulfidophilum]AB15
MPEPAGGLRIVDLRKSYRRRPVIRDVSLRLDRGEVVALLGPNGSGKTTCFYAVAGLLTPDGGRVMLDGRDVTTLPMYR
RA
RLGIGYLPQEVSIFRGLDVEDNIKAVLEIVEPDRHKRRERLEELLSEFSIEHLRRAPALSLSGGERRRVEIARCLAADPS
YLLLDEPFAGVDPIAVGEIRALVADLKTRGIGVLITDHNVRETLEIVDRAYILHDGKVLMSGTTQEVIRDENVRRVYLGQ
SFRIT
>MCE8455413.1 lipopolysaccharide transport periplasmic protein LptA [Rhodovulum sulfidophilum]AB15
MSLETSGKDLVLSVRAGLAALALVLTIPAGAGAQGAEIAFGSLNHDSSLPVEVTAESLSVDQGDGTAVFTGDVVVGQG
QM
RLAAGRMLVVYGAGGAGGGRISRLEASGSVVLTLDAEAAEADKAVYDIGAATIVMTGNVLLTQDGNTIEGQRLVVDLT
RG
TGSMEGRVRTVLTPSDKP
>MCE8455414.1 LPS export ABC transporter periplasmic protein LptC [Rhodovulum sulfidophilum]AB15
MQAHDNAYSRFIAAAKIVLPLAALGLLSTLFMISRDHGGGDDGLPPRTRVEIEGLLREQRLGAPNYAGVTRDGTAISMR
A
DTARTDPDNRGRSEASNIQARLDLPDGSSAEIEARTGAIDTDDQVAVLEGDTRISTSTGYRIETDRLRSAFADTRVEADG
PVDAEGPPGRIEAGSMTLGPDPEHEGRYLLVFKDGVRLVYEPRN
>MCE8455415.1 KpsF/GutQ family sugar-phosphate isomerase [Rhodovulum sulfidophilum]AB15
MTPETCLSIGRRVLRIEADAVHRLAEGLDQEFIRAVETILGATGRVIVSGMGKSGHVARKIAATFASTGTPAHFVHPAEA
SHGDLGMVARGDVCLVLSNSGETPELADIVAYTRRFGIPLIGVAGRPRSTLLRQADVALLLPSADEACPMGLAPTTSTT
M
TLALGDALAVALMEHRQFTPEHFREFHPGGKLGARLSKVADLMHKGEDIPLVATGTGMAEALIVMSRKSFGVVGVTTP
DG
RLAGVVTDGDLRRHMAGLLERSVDEVMTANPLTISPDVLAEKAVALMNERKITCLFAVGADGRPGGILHIHDCLRAGV
A
>MCE8455416.1 ribonuclease D [Rhodovulum sulfidophilum]AB15
MANFLHKNDLPDDLDLGPMVAIDCETMGLNPHRDRLCLVQMSAGDGDAHMVQIAQGQTEAPNLTRMLADPDTLK
LFHFGR
FDIAVLEHTFGVTTAPVYCTKIASKLVRTYTDRHGLKYLLQELLAIDISKQQQSSDWGAPALSPAQLDYAASDVLYLHRL
KEVLDGMLAREGRSEIARACFDFLPTRARLDLSGWPETDIFAH
>MCE8455417.1 complex I NDUFA9 subunit family protein [Rhodovulum sulfidophilum]AB15
MSKLVTIFGGSGFVGRYVARRMARAGWRVRVAVRRPNEALFVRTYGAVGQVEPVLCNVRDRESVRRALQGAEAAVN
CVGI
LAETGRNTFGLVQHYGALHIAEEARAAGVERLVQISAIGADIESDSGYAETKGRGEAAVRESFPDAVILRPSLVFGPEDQ
FFNRFAQMARFSPVLPVVGCYTRFQPVYVDDVAAAAAKAAMGEAAPGIYELGGPEIRTFGELMEEMFTVIGRPNRIVL
PL
PLALGAAMGTVFDLVQSLSLGLLENKVVTRDQVRNLGRDNVVGEGLPGLAELGIQPTAMEAVLPEYLWRYRPAGQFY
DVR
EAAKRLKS
>MCE8455418.1 undecaprenyl-diphosphate phosphatase [Rhodovulum sulfidophilum]AB15
MTLFHLFLIAVIQGITEFLPISSSGHLILLPGLTGMADQGHIIDVAVHVGTLAAVILYFRKDAMLALGGLTRVARGQIDT

PGAFLALCLIIATIPVMAVGLVLELTGLEDMMRSIAVIGWTMLIFGLVLWVADTRFPATRKANSWTLKHALIMGLWQV
LA
LIPGTSRSGITITGARMLGYDRVGAARLSMLMSIPTILASAVLVGGKAATEADMAVLRDGAIAAGFTFVAALAALAVM
MR
LARSVSFTPYVIYRVILGLVLLWIAYT
>MCE8455419.1 NAD(P)-dependent oxidoreductase [Rhodovulum sulfidophilum]AB15
MAQQKMLQFVRVAKETPEKREADDRAQDFGEIYAEFAAEKAAEQASRCSQCGVPFCQSHCPLHNNIPDWLRLTAEG
RLEE
AYAMSQATNTFPEICGRICPQDRLCEGNCVIEQSGHGTVTIGAVEKYITDTAWEEGWVPPIAPVEERSESVAIIGAGPG
G
LAAADRLRRAGLQVTVYDRHDRAGGLLTYGIPGFKLEKDVVMRRVEQLEKGGVEFVLNCNVGTDISFDAIRGKHDAVLI
A
TGVYKGRDLTGPGAGAQGIVAALDYLTASNRVSFGDEVPEYLSGALNAEGKRVIVVGGGDTAMDCVRTAIRQGAVSV
KCL
YRRDRANMPGSQREVQNAEEEGVDFVWLSAPNGFAGDPVSSVMVQKMRLGPPDASGRQSPEVIEGADYAEPADLV
IKALG
FEPEDLPALWGEEALEVTRWGTVKADFRSHETSLPGVFAVGDIVRGASLVVWAIRDGREAADSILDFLAQPAQVAAE
>MCE8455420.1 GFA family protein [Rhodovulum sulfidophilum]AB15
MSDTTLKGHCLCGAVTVEIDLAHRVLGACHCGMCRKWTSGPLMAVHTTQPIRAEGPVKTYASSQWAERAFCSECGSS
LWY
AFTAEGAHKGHGFVSAGLFEGTLDLPLNSEVYIESKPAGYAFAGDHVRLTEAQMMASAADGMSMGSTGSEGEQQ
>MCE8455421.1 glutamate synthase large subunit [Rhodovulum sulfidophilum]AB15
MTKYDAAWVAREEAKRAFVAENGLYSPEDEHSSCGVGLVVSIDGKPSRKVVENGIDALKAVWHRGAVDADGKTGDG
AGIH
VQIPVPFFYDQIRRTGHEPHADRLIAVGQVFLPRTDFGAQEVCRTIVETEVLRMGYYIYGWRHVPVNTDVLGEKANATR
P
EIEQILISNSKDVDEETFERELYVIRRRIEKAAAAAHVPSLYICSLSCRSVIYKGMMLAEQVAEFYPDLKDDRFESAFAI
YHQRYSTNTFPQWWLAQPFRMLAHNGEINTLKGNLNWLKSHEIRMASSAFGELAEDIKPIVAQGSSDSAALDSVFEVL
VR
AGRNAPMAKTMLVPEAYSKQTTDEMKPAWRDMYAYVNSVMEPWDGPAALAMTDGRWVCAGLDRNGLRPLRYV
VTGDGLLI
AGSESGMVPTEEAAVLEKGALGPGQMLGVDMATGKLFRDSEIKDMMAASQPFGEWLDTVSEIDEVLGDLPEKVLFD
GADL
RRRQIAAGYTIEEIESILMPMAEDGKEALASMGDDTPSAVLSEKYRPLSHFFRQNFSQVTNPPIDSLREYRVMSLKTRFG
NLRNVLDEDGGQTRIVILESPFLGNAQFNELVKHFSVPMAEIDCTFPPGAGPEALRLGLQRIRNEAEDAVRSGAGHIVLT
DHHQGEDKVAMPMILATSAVHSWLTRKGLRTFCSLNVRSAECVDPHYFAVLIGCGATTVNAYLAEDTLADRIKRGLLD
CS
LTEAVSRYRKAIDLGLLKIMSKMGISVISSYRGGLNFEAVGLSRAMCAEYFPGMHSRISGIGVTGIQRKLEEVHRLGWKG
GQDVLPIGGFYKARRTGERHAWEASNMHMLQQACNTASFEVWKQYSAAMRSTPPIHLRDLLDIKSLGKPVPLEEVESI
TS
IRKRFVTPGMSLGALSPEAHKTLNVAMNRIGAKSDSGEGGEDPAHFYPEPNGDNPSAKIKQVASGRFGVTAEYLNHCE
EL
EIKVAQGAKPGEGGQLPGMKVTDLIARLRHSTKGVTLISPPPHHDIYSIEDLAQLIYDLKQINPRCKVTVKLVASSGVGT
IAAGVAKAKADVILISGHNGGTGASPATSIKYAGLPWEMGLTEAHQVLSMNNLRERVTLRTDGGLRTGRDIVMAAM
MGAE
EYGIGTAALIAMGCIMVRQCQSNTCPVGVCTQDEKLRGKFTGNADKVVNLITFYAQEVRELLSQIGARSMDEIIGRSDLL
NQISRGSAHLDDLDLNPLLIQVDANDRPTLDRTRSRTEVPDTLDAEILRDAAPFFEDGEKMQVSYAVRNTDRTIGTRVSS
LIVQRFGMRNKLQPDHLTVKLAGSAGQSLGAFGAPGLKIIVAGDANDYVGKGLSGATIVVRPPLSSPLVASENTIIGNTV

LYGATEGYLFAKGRAGERFAVRNSGARVVVEGCGSNGCEYMTGGIAVILGTIGANFGAGMTGGMAYLYDPEGAAAEK
INP
ETLVTCRIAVQHWEAELKGLISRHFDETGSPKARDLLTHWELEKGNFLQVCPKEMLPHLSHPLGIEERAVPAE
>MCE8455422.1 DUF1269 domain-containing protein [Rhodovulum sulfidophilum]AB15
MADLIAVAFDDESKAFDMRGEMVRMQKDYLLDMEDVVVVTRDAEGKVKLHQAVNLTAAGAVGGSFWGLLIGLLFL
NPLLG
LAVGAGAGAISGALSDIGIDDGFMKELGETLTPGSSALFVLVRKATGDRVIERLREVNVEGRILRTSLSKEDEATLREVL
EKSRATAE
>MCE8455423.1 monofunctional biosynthetic peptidoglycan transglycosylase [Rhodovulum sulfidophilum]AB15
MVKRRGLGYHPGMAKPTKSTGTKRSGTRNAGAKAAGKKPARRDSVPLPLARRLRRAARRGALAVLALVALVLIAPSLV
NP
PTTPTMMSERMRLGDIDQQWAPMREIAPAMQRAAVAAEDANFCRHWGFDMGAIRGALEAGGRYGASTISQQTVK
NVYLWQ
GRSWPRKAFEAVLTPMVELTWSKRRILEVYLNVAEFGEGVFGVDAAAWHYYGIPPSKLSRSQAARLAVLLPNPKDRDP
NH
LSPTLLRRADQVEEGAATILADGRSDCFDH
>MCE8455424.1 glutathione S-transferase family protein [Rhodovulum sulfidophilum]AB15
MNRLYHMPLSPFCRKVRLVLAEKRIEVELVEERYWERDADFMRRNPAGKVPVLRMDGRIMAESQAICEYLDESFPDPC
LL
PGSPAERCEARRLVFWFDDKFHHEVTSKLLYERVNKKVTKSGYPDSRNVKAGATAIKYHLDYMGWLLDQRRWLAGDV
MTL
ADFAAAAHLSCLDYISDVDWNRNAAVKDWYAKIKSRPAFRNILADQVPGFPPPRHYANLDF
>MCE8455425.1 DUF1730 domain-containing protein, partial [Rhodovulum sulfidophilum]AB15
MTAALKDRLAARAREEGLAAMGVCRPDAIPEAAGRLAEFVAKERHGQMGWMAERMSWRGNPAALWPEARSVVM
FAEPYTQ
AHDPLAALDRPDRAAISVYAQ
>MCE8455426.1 hypothetical protein LZ188_02345 [Rhodovulum sulfidophilum]AB15
MQDRAMMMDRQMQMQGQRKHPARRAMSVRAALEWAFGTECARLDHDEIEAVGGTGWRPFGMEYVALERAQL
GTRVDTSRG
RSRPHDDAELIATVVRNTLPWYAATLVADLARAGRTPDWMPDARPRLKPEDWMVNRWGRHAKTADAATLGIEGW
QPIPRR
NRKGVIVQDAVRFTPCIWDPTPARIAAARRAYLDWWGYLLAVQAALKGADMTRIEVTRDMPPMRPWG
>MCE8455427.1 hypothetical protein LZ188_02355 [Rhodovulum sulfidophilum]AB15
MIGPVTSPSLAQFFKQHAEQHEAARATFCENIKKLGGGSAEELGLTPFQAPAEFMAMMAKSDAEAAWIESAEGKAW
QAEQ
RAKQDAQPVELAVYKGGEMVGFMAGGFTAQSSITGFLPRDWDSMSIDDLSQVFRSHGLKVVTAKDGLSMNHGQAA
DIMNL
L
>MCE8455428.1 hypothetical protein LZ188_02360 [Rhodovulum sulfidophilum]AB15
MEDFAIHYCAVRFRRNAAFFSLMGLKHAMRGEIGAAVFLKLPRTLRGYCDTRRAAPETFFLTFL
>MCE8455429.1 hypothetical protein LZ188_02365 [Rhodovulum sulfidophilum]AB15
MKLTAGVSPSDLRRLVSRLREVERKQMPYATMLALNATGEAVLEENRTLMRKVFDRPTRWTLNAFFLRRATKRKPEAT
VE
RKDAPRGRHYLEVEERGGTRPKTGIERLILGNVAYEDHIEAVVPARGARLNAHGNLPAGQIQRALSNIGAQQDRAQTG
TD
GSRKRSARAARYFVPKPGQLSPGVWKRQGKRLTKFLSFTDRLPRYGARFDMEGHGRIVAAREMPDRMRAAIRKAFST
AR

>MCE8455430.1 phage terminase large subunit family protein [Rhodovulum sulfidophilum]AB15
MVVIRDARIGALRSFAPLPGLTTPEEILADALPMLDPPSRLSVTEAAERYINVQVQGAWQGFDRSVTPYMVEPSDVTQS
R
LFKVVAFMGPSQSGKTMMLQTVSLHAVTCDQNPTLIVHMSRPERDKWVEEKLNQLICNSPEVYSRLGRGRDDDTFSR
KRF
RGMRLLIGYPTPQVLSGGTYKTVLLTDLDHMPLVLGGKDNPEGAPVRMAVQRIKSYLSRGCVLAESSPAYPVTDPGWQ
PD
PAAPHALPPVKGGIARLYNDGTRARLYWECPDCGGEFEPRFDRLRYDDSLDPGTAGAGAEMECPHCGTLIAHRHKME
LNR
AILKGRGGWRHEGPEGQLLALGDAGMKRTDIASYALNGAAATFASWRNLVAQYETARRQADLLGDETDLATTFYTEI
GLP
YCPRKAGAEDEIGTDFLRAHARDTPRGVAPAWTRFVTVSVDVQGTYFAVQVTAWSETGTAQIIDRFELSAPPDGAERV
LD
PATLIGDWSVLLPLGRRVWPVEGTDYGLRALALAIDYQGRPGVSDNAEAFWRTRRKGAGPGRWFITRGHGGFHHGS
RIWY
ASPERKSGGGKGRNIKLLNMATDRLKDTVFAMLGRVGTGEGALFAGRWMTEAQIAELVAEERLSKGWDKKPGQVRN
ETLD
LSVQARALAEQKGLLRLDPAALPDWALGGRENPNAVALAKAPAERPGTTDMTDAARSAPAPEAPAPRPGQPAPRRIN
FLK
R
>MCE8455431.1 hypothetical protein LZ188_02380 [Rhodovulum sulfidophilum]AB15
MAHTQDELDALKAAFARGVTRVRFNGEEVQYDSATALLKRIRIIEAELAGHGARGLSVSYPRTPRGL
>MCE8455432.1 phage portal protein [Rhodovulum sulfidophilum]AB15
MGWLDRAILSLAPQAGLARIQARARAQVLMNYDAASKGRRTYGWKAPATAADAAGASRAQLRQLSRDMIRNRSLA
ARGQA
VVTGNVVGTGIMPSVRMAGDADGTAAMELIRAHLLSPAIDVYGVHALPGLQWQVMNAVFSDGEVLVRRRARNLAF
EPGLV
LPFQIQVMEADHLDTTITSHGRNEVIEGIEYGPTGRAEAYHVFEQHPGATHRITAGRFRSTRVPARQILHVRRTERPGQ
M
RGIPWLAPVMMTVGELSDYQEAQILKQRIASLLAFFVEASADGEVYAGAEIERLEPGAVIGLKEGQKVTPSEPPPLDGYA
EFMREGVRAIATGLGLTYESFGDLTGVNFSSGRMGRIEMDRFIQVWQQQIMIGQFCRGVGRWTLEAWQVAGASAG
LPPAP
RALEWTAPRRPMIDPGKEIDAAVKEIGAGLNSRQRKQREMGLDPDVIARERAEDAARDPAPDTTKEEDIAHGGV
>MCE8455433.1 ATP-dependent Clp protease proteolytic subunit [Rhodovulum sulfidophilum]AB15
MEGSDLILNGEIILEGDVLPHEYCSFAETGCFSARMVREALARFDADVTLRVNSPGGCPCEGEAIRAALEAHPGRVTVIV
SGFAASAASLMIMSADRIEMTAGSFLMIHNPSRGLYGTAADLRKKADDLDKLARIYASVYAARSGRAVEDVMRMMDE
ETY
FGPEDAVAAGFADAVSGTAPEIAPELAAVMSAHRGALSRLQMCARSFAAEGNPAPAAADPTGPSGSSLADPAATEEIP
TM
TTPSPAAEPTAATVQPPAQTQPQTPAPTTTMQADAEAMVARAVQAERERARSIRSMAQPSMRAGLLSEAQVEAVID
EGVS
QAAAGNRLMAIMAAAEPPIPAGGAGAARITRDETDTHVEGLIAAMMRDYSGPGEQFREMGLKDMVMHLASPNHRY
RGRKA
VADGFLSTRMMGGAHGVSDFAYITTEVMNRYLIREYDRRGATWQAVAGEPRRVSDFREVHGVRFGGDLTLKDVKEN
GEYE
QAMLEDQAEGLKLVRRGRTLTLTFDMVMNDDLNVLADLPNAFARHARRMENDMVWALIRTNAKTKSDGKALFHAD
HGNLA

AAAGQISVATVGAARKAMWEQTAFGSKDAEDFLQVTPDQLIVPPALETVALQFATATTPVKDAETNPFKGTLTPFVVP
NL
GASVSGGSDSAWYLVSSELPPISVAYLDGYETPTVQTVEGMNPDKVTMNARHIFGAAPTEFRGAYRNPGK
>MCE8455434.1 DUF2190 family protein [Rhodovulum sulfidophilum]AB15
MKNYLSPGETVTLTAPAETASGDGVLVGSIFGVAQADAAAGAPVVLVRRGRFTLPKSGAEAWAEGDPVYWDGAVCT
AEAS
GNVLIGAATAPAANPSATGTVLLDGTIR
>MCE8455435.1 hypothetical protein LZ188_02400 [Rhodovulum sulfidophilum]AB15
MASVFDGMGALLTGALGAPVTIAPSGEAPRRIRALFRDADTAILTEGGAEVLNAVPTLQARRDQLSGLCPGGLVDLAD
GR
RFRALAPMDGGNPDPGGMVTIQLEEA
>MCE8455436.1 hypothetical protein LZ188_02405 [Rhodovulum sulfidophilum]AB15
MFASKAIRHAIADLLEAAGISVDTPAGRLPVTILRSPPSGGWVKDDALPGIYMYLRREEIRPATMTRSARTITMDLILEA
RGADAEDQLDDMQRAVERSIAASANLGGLVMEIRPVDAEIHLERGSVIFGARRLSWSVRVEVPRADPSL
>MCE8455437.1 hypothetical protein LZ188_02410 [Rhodovulum sulfidophilum]AB15
MPESQALMAYDIGFAIHDGEDPGTFEDLAEVNEITPPNQQADDIDVTHQKSPNKTREYIAGMIEPGDMTVSLNWIPGS
AT
DMTIQTLKASGARRQMRIAWNNGVSWTFTGYIKGFEPSTALGDKMTGTVTIKVAGSTEISGMGG
>MCE8455438.1 hypothetical protein LZ188_02415 [Rhodovulum sulfidophilum]AB15
MANRERGEVGARFRGEDIALVLDFNAICALEDHYDEDIDTVLARLDSAGGKRRVRGRDLRRVVLELMRAARPGTTEAD
AG
DFITETGLEALPDLLGRVIAASRMVASDEAPSPEDPARPPETGAA
>MCE8455439.1 hypothetical protein LZ188_02420 [Rhodovulum sulfidophilum]AB15
MIDVGRLRVRLGLDNREFQTGLAKARGAFGKFAGRIAAAAAPVAIGAAVAAAARSSFEMVDAQAKLAQSLETSTRSVQ
LL
GRAADLSGVPMSQLQQAALALNKRLSEASSGAGPAVKALRALGLEARDLLGLDLDQRFDRINGAIAGLEDPAKKAAVA
AA
LYGDRAYAAITRLDSATLSQAARDVDQFGVALSELQADNIEAANDAMSTLGVVMRGVGNQVAASMAPAITALATGFT
DLL
REGTPLRAMLDGLGASVSGTFQVMSGTIGRLMSYAGTFASFLAGRFVTSMSLAAASVVTLNAKLLLTRAALMRTGFGA
LV
IGVGELIYRFTGLVEATGGVGQAFDALKTVAIEVWERIGAGTRIVVLAVKRMGWAIRADFYGTLEAIQSKWGGVITNM
AA
IGAIIPGFGSQFRAVAAEIRKSSAAAKDLKTATSEATGKVAENARAIEAALTGFTAPLASVEALRAKLREARAEMDNTGT
SSQGLKAALKDLNDALSGDGKGGKGSGVDEVESGLTRLGTSVNATSSSFQSFFKDIVKGSGDAGDAISALADRMLDDLL
D
RAMSPLSDALGSMFDGLFSGLFGGRASGGGGTVKPIGDLIGKVMDGGSFDGGGRTPIGPRAGGLDGKGGFLAMLHP
NESV
IDHTRDSGGFGGFGAVRGGDVYVSVQGSNASPEQIAAAVRAESRRQASEIYDRRKRMARAGGAS
>MCE8455440.1 hypothetical protein LZ188_02425 [Rhodovulum sulfidophilum]AB15
MAFPPPSVRVAAFTLTRITSSLRVQSPYSFAQTAYDFMGGMWAAELVLAAVDARESAAISGWIASLNGPVGTFELDM
AAM
EYDGPHGAISADPVVAVAANARAQALVLQLARAGDRALPGDYLTLGRHLHIVTAAEAPTPAFRQSVTLWPRLRRPVAP
GD
PVAMRAPFGSWALAGPETVLSVSQARVRTRSLQIREAL
>MCE8455441.1 hypothetical protein LZ188_02430 [Rhodovulum sulfidophilum]AB15
MDASGDRMLGLLVRCRFDTGDLAMFTGYGEVDWEGVTYLGAGQMLSVGEAQATSGEGIPGLSITLSGLDPEVVALAE
LEE

FQRRRVTVFLALFDETGQIETADVLFDGVADTMESDDGPDTATTTLALEPRSMALGRKFPCYYLPEDQEKRFPGDKGFGL
VQAIQNREDTWGRT
>MCE8455442.1 hypothetical protein LZ188_02435, partial [Rhodovulum sulfidophilum]AB15
MRRAGWERRLSAAVRDWEDRPFAWGTADCLGFCRAVARSMTWEDPIPGLPDYGSEYGAAKVLVSLGFGSIEALVDAHLER
LPVAGARRGDWVMTACEGAMPGAMGVVTGRYAQHMGRNGLVRRPVLTD
>MCE8455443.1 terminase family protein [Rhodovulum sulfidophilum]AB15
MDEAVLLALPYLFEFWALPHQLAPEGDWRSWVIMGGRGSGKTRAGAEWVRSQVEGARPLDEGRARRVALVGETIDEAREV
MVFGESGILACSPPDRRPNWEATRRRLVWPNGAVAQVFSAHDPDSLRGPQFDAAWSDELAKWKRAEETWDMLQFGLRLGG
DPQQCVTTTPRNVPVLKAILSNPMTVVTHAPTEANRANLAASFLEAVRTRYSGTRLGRQELDGILLEEAESALWTLARLE
ALRVDAAPELDRIVVAVDPPVSSHAGSDECGIVVAGVRMQGPPQSWSAYVLEDVSVAGAAPTAWAKIALAAMERHSADRM
VAEVNQGGDLVETMIRQLDAMVPFRAVRATRGKIARAEPVAALYEQGRVRHLRGVQVLEEQMCHMTAHGYEGRGSPDRVD
ALVWAINDLMISPAAHWRRPRVRGL
>MCE8455444.1 hypothetical protein LZ188_02445 [Rhodovulum sulfidophilum]AB15
MTDDLSPAAGDAAAWAVLEEAEEHYRRMIRALNDLIEEIQAGNSERARMLRGALNDLGKAAQTAFDERQRVERRLNAESG
SDGGIQALDLDQARLEVRRRLDRLRSARDADSVSGGDG
>MCE8455445.1 3-oxoacid CoA-transferase subunit B [Rhodovulum sulfidophilum]AB15
MDAKEVIARRVAQEVREGMLVNLGIGLPSMVANFVPDGMHVFFQAENGVIGLGQRPPDGMEDRDLTDAGGGFVTAVPGAA
SIDSAMSFGLIRGGHLDMTVLGGLQVDEKGHLANWMVPGRMVPGMGGAMDLVTGARRVIVAMVHATKGAPKIVPECTLPL
TATRRVSLIVTEMAVIEPTDEGLVLRERGPGVAVDDILAATSAALIVPDDVAEMVLD
>MCE8455446.1 3-oxoacid CoA-transferase subunit A [Rhodovulum sulfidophilum]AB15
MKRALKPAEAAEMIPEGAVLLIGGFMGVGAPHRLIDALVARGVGGFTVVANDTVRPGQDIGKLIDARLVAKCIVSHIGLN
PETQRQMIAGDLEVELVPQGTLVERIRAGGVGLGGVLTATGLGTVVAEGKPTVEVDGKTYLLEKPIRGDYALIGARQCDY
VGNLEYTLTAHNFNPVMAMAADTVIAEPHHVMPVGMIPPDAVKTPGVLVDHILERLV
>MCE8455447.1 acetate/propionate family kinase [Rhodovulum sulfidophilum]AB15
MSHVLTLNAGSSSLKFGFFRLNGAADDPDPRIVGSVERIGTGERARLKAKRQDGMVLVDREFGRNHVADHETALARAVEF
LKEQRPQAELDAVGHRVVHGGPDYDMPVELTGEVMARLAQFEPLAPLHQPHNLAGVRAALKLFPEAVQLACFDTAFHRNH
PWVNDTFAIPRKYYDQGVRRYGFHGLSYQYISETLRETAPHLHMGRTVVAHLGNGASMCGLMGGQSRASTMGFTALDGLP
MGSRCGQLDPGVLLYMLRSEGMTPAEIEDELYRKSGLLGLSGLSNDMRTLEAAGTEEARQAIDYFVFRIRRELGGLVAAL
GGISTMVFCGGIGENSVLIREKVCTGMGWIGMELDETRNMRNERIISTDFSRVRILVIPTDEEIVIARAARDMLARRAADAA
>MCE8455448.1 bifunctional enoyl-CoA hydratase/phosphate acetyltransferase [Rhodovulum sulfidophilum]AB15

MKITENRTFDEISVGDTAQIVRICSEDDFYVFASASGNYNPMHLARLDGDGDGVDEALAPAIWEASLISAVLGNLLPGP
G
TLYRSQSLVFHERAQAGDELLTRVTVVEKRPPNRIVLETRVERCSDGALVLDGEAEVKAPPKKLVFNDLEIPGLVIERHR
HFDELIDRAEPLAPLPTAVVCPEEPRALGGALLGARHTIIAPILVGDPDKIAAAAAEIGASLAGIEIVEAADHVTAAHLA
VDLVAGGRAAALMKGHLHTDVLLRAALRKENGLRAGRRFTHVFVMDVPGVPHPLMVTDAAINIAPDLRTKVDIVQNA
IDL
AISIGIEVPKVGVLSAVETVNPDIPSSLDAALLSKMAERGQIRGGMVDGPLAMDNAVDLTAARTKGITSQVAGRAEVLV
V
PNLDAGNMLAKQLAYISHAEGAGVVMGAKVPIILNSRSDDDKARLASCAVATLHNARLNGLIPAQRAAE
>MCE8455449.1 DUF3141 domain-containing protein [Rhodovulum sulfidophilum]AB15
MDMHVESRSDDDPADVMRALIWGSGGMAQDRLALMSALAGGSLENADLIREVAGRHGNRMLDTHSTRTRELLAQ
MRRLME
AARNSKKEMTPGQLVADYRDYLRDAGERWLLTMDALRARGDIFREHEAEGCPPVLAYRYEVVIDGTDLPHPCNYQLLR
IE
PPEGVELRPDKRPYVIIDPRAGHGGGIGGFKPDSQVGVAFKGGHPVYFVSFGRDPLPGQTLADVCRAEAEFVREVTRR
HP
AAAKPVVMGNCQGGWATLLLAATNPDLTGPIVLNGAPVATWSGEVGRHPMRYNAGILGGAIQPMLWSDLGGGRF
DGANLV
MNFELLNPSRNYFRKYYDIFRDIDQPGMRQRFLDFERWWGGFFTLNEAEIRWVVEQLFVGNRLTRNTAMLEPGRPVD
IKA
IRAPIIVFASQGDNITPPQQALNWILDAYAGVEEIRIRGQRIVYMVHEEVGHLGIFVSSKIARKEHTEVSSTLRTIEALA
PGLYEMRIDAAEGRGEDREFLVSFAERSFADLEQIAGGREEEAAFAAVSRMSTIQSEIYDTAVRPLVRASVNAQAAETTR
RLHPLRGQRTAMSSVNPLLAPVAAAAGRLREERHPAAIDNPFLWAERFAADMTEQWIDFGRDMRDAWVEMSFFA
MWNSPW
ARAFGAPEAVRRTRKTTAELRAMPIVQTVLSHVDQGGFVEAVIRMLILLAESRGSVRRDRLERSAWVLNHDEPFAALTA
E
CRARILHEQTLIATHDPEGAIETLPCLLRDPEDRELAARVVQYVPGPIAEMAPHTLEMLQRFRAVLGLSPLAEDVVEDPL
ASHGAVVHGSDMAQSRSAAAGVPGAEAGVGDAPKTVSERKTSE
>MCE8455450.1 enoyl-ACP reductase FabI [Rhodovulum sulfidophilum]AB15
MFSLEGQTALIVGIANDKSIAWGAARALRAQGARLAITYLNEKAEAFVRPLAEEVGAEIFMPLDVCNEAEMDAVFAEIG
A
RWGRLDTVLHSIAFCPRDDLHGRVVDCSAEGFSLAMDVSVHSFLRMIRKAEPLMPDGGTCMTVSFAGATRVVEHYNI
MGP
VKAALESTVRYVAAELGPKGISVHALSPGPLATRAASGIAEFDELLDAAAERAPIHHLATIEDVGAYCAFLASREAKNVT
GGVHAIDGGYSIIG
>MCE8455451.1 autoinducer binding domain-containing protein [Rhodovulum sulfidophilum]AB15
MNRVNKVAELLEMLQRTCDTGFVLALHVRFTRPLVLYRTYPQSWCDHYDLNGLVMLDPAVRWSFCNTGVIQWDDPS
LDDP
GGVVEAARAHGVANGVSVSVGSATSRSLGGFSRSSGRFDAADMDRLSDLVETLHLVLDGVSEDEQGPELRALRALSTS
VL
QP
>MCE8455452.1 LuxR family transcriptional regulator [Rhodovulum sulfidophilum]AB15
MDLRKKLTDRLSALAPAGYYVGLRLGFTYPAQEINRLPGDWTDHYARNGLIVHDPVFRWVYARTGVISWAALAFDDH
ADV
FGQAASFGLIHGAVVSVMRPGESGRRSYGTFARSDRTYAESEMAELLQIVTRLHRAGSERTGLTHAETEALRLQADGLR
L
KEISAELEISMSAVKARLANAKRKLGAATTSQATSMARSRGVI
>MCE8455453.1 H-NS histone family protein [Rhodovulum sulfidophilum]AB15

MAKDLETLTLKELKDLRKDIDSAIASFHDREKAKAKAVLEATAKEMGFSLDEIIGKKSRRVKPVAKYRNPENPTETWSGR
GRRPRWLEEALSKGASLEDFAIA
>MCE8455454.1 GDP-mannose 4,6-dehydratase [Rhodovulum sulfidophilum]AB15
MTRVFITGTAGFIGFHLADLLLSEGMIVHGFDGMTDYYDISLKRRRLALLEGRPGFTMTEAMLEDDPALSAAVDACDPD
I
IVHLAAQAGVRYSLENPRAYIDSNLVGTFNLMEAARRHRVSHLMMASTSSVYGANTDMPFTETQKADLPLTIYAATKK
AT
EAMGHAYAHLHDLPTTMFRFFTVYGPWGRPDMAPIKFASRIVKGEPIDIYNHGDMYRDFTYVLDLVRGIRLLMDAVPE
RP
ADGQVPEGDSLSPVAPFRVVNIGNSEKVRLLDFIDALESELGVKAIRNYMDMQPGDVPATWADASLLQRLTGYRPQTP
MR
DGVAGFVSWFRSHYAT
>MCE8455455.1 hypothetical protein LZ188_02500 [Rhodovulum sulfidophilum]AB15
MTAFIQIATQSFRALAWAVCLALPPASLPGQTIPHPGAVRLALQNADARAPRPKSVIELQGLRASQSALSRDGTRLTLTS
LNPHANGWFLLELARPGRQPESYHLENPAPLTRDVSLSDDPAPAILLTEGQAQIKCRPWAVATPELSDARASGLPFAPL
C
DARLYLRNPVSGARTNLERTAEFLRDNLWFGESLVGLVKDTLYKDRYLASGQVLAGADPGAVDRLMGRAALRDFPVM
YTT
MTFDLVGATPKRMEMGAWYAIGGAPGIYASALQPRMIAPEILRRRGEANGLDGIEGRADVYLVAFDMTHFDIGYETG
TEH
PRLDWSSRPSGPGRNWSLPGPDGFDTARPLATVGMINPEEARRVAATFTGGFKRDHGAFRFGPMARSNNGHHYGF
VVHGT
VLSSLQPDLSTLYVLDDGTIGMKTWTEADNALLPQIRFARQNGVPIIERDTATGRGVVGPLVRQWGAGNWSGSANAD
LRT
LRAGACMKTAAGRTFLIYGWFSTATPSAMARTFQAYGCDYAMLLDMNALEHTYMALYTPGKAGGLDTRHLVAGMS
RIDEK
SRDGRPIPRFIGFPDNRDFFYLMRKEPPR
>MCE8455456.1 hypothetical protein LZ188_02505 [Rhodovulum sulfidophilum]AB15
MTRRLGLLALCAAVIASPAMSQTLSQANAQMLREMQSARGVSTAAIARIEAIFARSAVIGQGNPAITRHAMTREACNA
RV
GGNALAHYADKRFERICGARYMAPLYDPRSERPEDAKACIDQFEYPGIPCSYPVVWVKAKEAAEICAAEGKRLCDAHE
WE
GACAGALEPPDYRFDLARGQSVSGAVGRMRTAHNAKYKGSAGWSYGPAFRSGVCAQNSVKSPSCPGGGFGNCGSN
TYPTG
SFANCKSPLQVYDINGNAAEHMNLPLAPDQMASAGSTRLGVTEMKGSWFIWDKYRAHPDWCRWRAPFWHGTRV
MSPASHE
NYHLGFRCCKTLN
>MCE8455457.1 iron chelate uptake ABC transporter family permease subunit, partial [Rhodovulum sulfidophilum]AB15
SGLSAAELWPGRWPRIVTALSAGVMLALAGVVIQGVTGNPLASPESLGISSGAGLGIILALFAGGMATLFAGAAAGAGL
A
FGLVVLIGARNRFAPGALLLTGVAFGTFASALISLMIASGDPRAFFVLAWSMGPTYRAEPLGALAGAGGAVLALLIFPLF
LRWLRVLPLGAAQARALGVRVGPARLVLMAYVAVLTGIATLAVGPISFAGLIAPHLARASGAVGPGPQGLCAAAWGG
AIL
VLADLAGRLIWFPYEIPAGALAALLGAPFFLWLIGRNR
>MCE8455458.1 ABC transporter ATP-binding protein [Rhodovulum sulfidophilum]AB15
MTLFSCHALERRDGHRTLLGPLDLNIAQGRLTAILGHNGSGKSTLLKALAGQERPDGGRIDFMGRPIGGWSGRELARRL
A

WLPQSPALAPGMTLRGLVALGRFPWHGLLGRRSARDREACDEALALTGTTALADRLVDTLSGGERQRGWIAMLLAQ
GAEV
LLLDEPVSALDIAHQVEVMELLANLNRRRGLSVIMVLHDLNLAMAWCDEAIALRGGRLAAHCPIADLHDPALLHRLYGL
E
METVTWRGSRLVMPSRLLGPGAAAE
>MCE8455459.1 siderophore-interacting protein [Rhodovulum sulfidophilum]AB15
MSGILPIAVKTVRDLSPSMMRLVFEVDGFERLRPTPHPDEWVSLWFPDETGALPRPLPRALAGPSGGRLPRARSRPYSL
R
RINPVAGTVTIDIVRHEGGLAAGWAARARPGDRLLMSQPEGRFRLPAGAGQLLAFADITGLPALARIVETLPPRVRLSAE
IALPCPGDRPDLPLGPGARLRWHPMGDAAQLPCPAEAFVPGRDYVWIAAEASVVAGLKRHFLGQGTTARDLVAIGY
WIRG
RSRR
>MCE8455460.1 peptide chain release factor-like protein [Rhodovulum sulfidophilum]AB15
MSDREALLLSSGNGPGECRQAVGHLLSWLSTKATQYGVDLDVSTRDAAHGPASAVVVLSGARAGDLVRSVEGVILWR
CQS
ELRPGHKRKNWFVQAFRLPAATDAVRIDPAAVEMQAIRAGGPGGQHQNKTSSAIRARWTSEDGKVYAVAVRDNRS
QHQNR
RLALERLAALAAAEKAEADAAREGETWVLHGQLQRGEPRRIFEGRQFKEVK
>MCE8455461.1 RNA ligase RtcB family protein [Rhodovulum sulfidophilum]AB15
MGTPISGERSAARARISKFYNRATWIDGRAEEQLEQVAGWPGIMAVAGFPDLHPGRFGPVGAAFLADRIWPQLVGP
DIGC
GMALFRLDLPRRRFKLDKAARRLAVLEDGADPEQALAALDDAGLTGAIGPEALGTIGGGNHFCEVQVVDAVFDPSRTD
LR
KDELCLLVHSGSRGRGAGIFQGLDDSWKQGFAAESDAAARYLALHDAAICWARVNRSLIASTASEALGSEARLICDAVH
N
HVVPHSGVWLHRKGAACPEGGLVPLAGSREAPSFLLEVPDLVPEALGSVSHGAGRRYDRASMHGRIRKTRSELAALTR
TR
FGGRVICGDRDLLLEEAGRAYKDPEQVLHDLSGSGLATAVARLLPLVTFKTTGVAI
>MCE8455462.1 RNA polymerase sigma factor SigJ [Rhodovulum sulfidophilum]AB15
MRQADPHSQIFEGERRRLAGLAYRMTGSVAETDDILQDAALKFRDADLPRIDRPTRWLSTVVMRLCLDHLRSARVSRE
TY
VGAWLPDPLIADHAPGAEEEWILTEDVGIALILTLDSLTPEMRAAFILRDAFDYSFEEIGRLVDKTPTACRQLVSRARRR
LAGAEPADPPSSETDHPLVTAFWEASRQGDMDRLLALFAEDIEVHTDGGGRVKAAINVLNGSRNAARLFAGLARKRRF
PA
GACPPLRWINGSPGFVSVEGGVLQTTALSIRRGRIEAIWIVRNPDKLRHVCLQM
>MCE8455463.1 carboxymuconolactone decarboxylase family protein [Rhodovulum sulfidophilum]AB15
MTPRMNYYKAAPDAMKAMRAAEETIKDLSLPESLRELVKMRVSQINGCAFCLDLHAPAARQAGLSQQKLDVLAAWR
ESPV
FDDRERAALAWAEALTRIEQTGAPDSDYRLLAASFDERERVELTFVITMINAWNRFAVGFRQVHTLREARDAAG
>MCE8455464.1 hypothetical protein LZ188_02550 [Rhodovulum sulfidophilum]AB15
MSMSNATPVSAEMPARRPIAAREASIPAFCRNMSPSPKVFWSGGRRRTSDAEASTFPRAGLTTWFGLIERGALHVCET
VL
IEGAGGVALFCLQIARAHDVRTIVVSGSAEKGAGGPVTVMASPETCVPADRRPIMHWHSNRITRGGQLRTLRALNTLLL
V
SQISILSRLVSIDKERRQR
>MCE8455465.1 ATP-binding cassette domain-containing protein [Rhodovulum sulfidophilum]AB15
MPASVSLSSLSWSAPDGTPLFTDLDLTFGAERTGLVGRNGTGKSTLLHLIAGHSSPASGRIGMRGSVAMMRQEALEHP
EE

TVADLLGVQSALELMDRAERGRATSEELAEADWTLPARIEAALARCDLSVDPDTRLACLSGGQRSRAALAALILAEPDFL
LMDEPTNNLDRAGRRAVIDLLRGWSGGAIVASHDRELLDEMDAIVELTSLGAAKYGGNYSAFRQQRESARAAAAQDL
AHA
QKVRTEAAHRAQQAAERKARKDSAGRKSRARGDQPKILMDAAKGRAEASGGAGARLRDARRDAAEDALTAAREKVE
VLTP
IHMDIAPSGLPAGRMVLTLDRVTAGYDPAAPIFEDLSLTLTGPERVVIAGPNGSGKTTLLKLITGALRPMAGLKGSTPAL
ASLGRSACHGIPSSTARPRGGTKRPWKAARSSGNKSAAERWS
>MCE8455466.1 hypothetical protein LZ188_02560 [Rhodovulum sulfidophilum]AB15
MGEPVLERKVHSVRTAARELGMDPRRLRKLLVDARTARPHLDRINARVSAKDFQDALSMSRSQFDLLRKEGNFRAAIE
GG
DHKPLWDVRAAHRHLESLLSGAEPIYLPMHAWDNIPTTAQSLKASPGTILKRLEVGRVRRIGRHMDQDEIERLLFRYQY
R
GLRPPVLSDTRCRHATCK
>MCE8455467.1 efflux RND transporter permease subunit [Rhodovulum sulfidophilum]AB15
MQIARNAIERPLYTWLLILLCLFGGAAGYLGVGKLEDPVFTLKSALVVTSYPGASAAEVATEVSDVLEAELQQMNEVDYV
TSTNTPGLSVIEVTIRDTFGGAELPQVWDDLRDRVANATPDLPAGARAPIVNDDFGDVFGLLYAVSAPGYADAEIRDIA
T
FLRREMSTVAGVANVEVKGLPEEAIFIEPDSATLDALGIPPDALSGAIAGADAVTPTGAIASGDANLRIEAPRAEDSTGK
IAALNFGYRGEVINLADIASVHRGRIDEPSQILRHNGTEAFTLGIAGLTSANIVTVGARVEARLDEIAHVLPAGVAIEPI
YEQHRVVAEANDAFLLNLAVSVGVVVAVLALFMGWRAAAVVGGTLLLTVTATFFFMYLFGIEVERISLGAMIIAMGML
VD
NAIVVAEGMQTDMRRGRPAADAADEVARSTQMPLLIATIIGIMAFAGIALSPGDAGEFLFSLFAVISISLLLSWCLAVTV
TPYLASRLFPTGSGTGSRDPYDSAFFRGYGRIVGLAIRFRWLVIAGLVGATAAGVASMGFVRQQFFPPATTPLVYLNYKA
AQGSTVQATDKDLMRLADWLATRPEVVSVTSTAGAPMSRFFLTYAPADSNPSYGQLVIRVAEPEQIPTIRDALSAAAA
QI
VPWAETRVEQIIYGPPVTADVEIRLSGPDADVLRGLAGKVQEILETRTALLQVERTDWREREIVAQPVFGSARASALGIG
RGDVANAIALVSDGVQAGVVREGDRLIPLILRQPRIDGAPQTRLIDAQIYAPATESYISLGQVLDGFEPRSRDTLIRRRS
RQPTITVQAFTPPDVLPPEAFAEVQDAVEAIPMPSGYRMEWGGEYESAGDAQASLARQMPLSFGTMLLLTILLFGKFR
QT
AVVWTVVPMAVTGVGIGLLLTGLPFSFTALLGLLSLSGMLIKNAIVLVEEVDARKDEAGLPQREAIVTACVSRLRPVVLA
AGTTILGMAPLLIDPFFASMAVTIMAGLGFASILTLIGVPAIYHTYVSERKADSGDNSRKASSSKAGSANSIPSDPALAA
H
>MCE8455468.1 efflux RND transporter periplasmic adaptor subunit [Rhodovulum sulfidophilum]AB15
MQNHPVDFKRFLAMSLTALSLLTSSAQVSNAQDRAVPPPVPVLTMRPGEDDKAPDRMFYGRVAARATVDLAFEVGG
RIVT
LDAVEGQRIAAGTVLAALDPAPYERAVARAELSLDQAERAFRRADTLAARDAASRARADDAETARDLAAVDLREARDA
RS
DTRLVAPFDAILAKRIASVGALVSTGEPVVRLQDMSELRIDVELPERLLGRIGDPQEARFEAEIHGIDGPLDLAFREVRT
ETGEIGQSYTVSLAVAKADMHPWLLPGRSATVRAYLPKAETGVAVPATAVVTRGEGRHFVVAVEPDGDALVARHLPV
EVT
IPDGSTVRVEGLPANAEIVAVGGHLLEDGDPVSRYDGLIAKAE
>MCE8455469.1 TetR/AcrR family transcriptional regulator [Rhodovulum sulfidophilum]AB15
MTRDAKKQNRERRHEIMDLATDLFLENGFAATSMSRVAEAAGITKASLYHHFPGKEDLFAACVAHGYAAAIERLRGIAT
D
QTQPHTERMARVISSSYDTIIFSRVGRMSPLIAEVSRSFPKVARGFHRDFILPQENIVLSLIDDGVEAGAFVDLDRAVFE
HLLFGPIVTLSMSREMFATFDDLDTLYPVTELRDGHIKRMIAWLTDGLPHSE
>MCE8455470.1 ATP-binding cassette domain-containing protein, partial [Rhodovulum
sulfidophilum]AB15

SLEDLSLTLTGPERVVIAGPNGSGKTTLLKLITGALRPMAGEAQLCVPWALLDQHVGLLAPDRTLREVFRDRHPQASTQ
M
AHAALARFGFRAGDAQRLADGLSGGEKLRAGLACVLGGTPPPQLLLLDEPTNHLDMEGVEALEAALASYDGAMVVVS
HDR
AFLASLRPDRIMSLKG
>MCE8455471.1 DEAD/DEAH box helicase [Rhodovulum sulfidophilum]AB15
MTTFAELGLDPKVLSAVSDAGYTAPTPIQAGAIPPALEGRDVLGIAQTGTGKTASFVLPMITLLGRGRARARMPRSLVLA
PTRELAAQCAENFDIYAKKTKLTKALLIGGVSFKEQDALIDKGVDVLIATPGRLLDHFERGKLLLTGVQIMVVDEADRML
DMGFIPDIERIFQLTPFTRQTLFFSATMAPEIERVTNTFLSAPAKIEVARRASAAENIEQAVICYTASRKDRQAKEKREL
LRALIEREGEGCKNAIIFCNRKTEVDIVAKSLKAHGYDAAPIHGDLDQSQRTRTLESFRDGKLRFLIASDVAARGLDVPA
VSHVFNYDVPSHAEDYVHRIGRTGRAGRDGKALTICVPYDEKWLTAIEDLVEKKIPRVESPLKSGSEDRGNRSRRNKRHE
GDAEAAAAPAVQAAPESPERSEEPRESARPPRNERRPERSERTERSESQDRNKPQSRHQSPRRSGGRGHRDEPVVGM
GDH
MPAFLLRSFKPRTEAEPAAADEPVEQNAE
>MCE8455472.1 exopolysaccharide biosynthesis protein [Rhodovulum sulfidophilum]AB15
MNSQDDWIISSLLEDLAALALANDRISIADLVEALGARGFGPLLVMLSAFLILPVGMLPGLPGVVAIFMILIGGRMMTGE
TTLWLPARLRRVHISGHVLAVSVARAQPAALWLRPLLTPRLVALIEGRIATRLIALILIGTGVLIFLLGFIPGLPFVLSL
HVLMLGLALSTRDGLVAALAYALMGPEIIFIWRVFT
>MCE8455473.1 esterase-like activity of phytase family protein [Rhodovulum sulfidophilum]AB15
MKNSMFLLSAAALTVAAPAFAEDVFPAKLAGHAVLPAMTMVAPPADAPRDAWISGKFTGGARNMVPMSAMGDTG
GLHGHR
ETGIALPFIGQPLQGFSGFAMARAEDGSVYALTDNGFGSKANSPDAMLFFHRILPDFETGKVDVKETVFLRDPGFKVPF
R
IAYEGSEGRYLTGADFDLESIQLVEGSLWIGEEFGPYLLNVSLDGVVLGVYPTMADGKELRGPDNPAVSATSIPGRDWKS
QRSGGYEGMALQPGTGTLWAMLEKPILGDDGKTEGDFLRVMAFDTVSKEWTGEMFRFALDEGATAIGDFNFIDDTH
ALVI
ERDNGEGDPSLKCDGEAAPDCFPEPAMHKWIVLIDTSDVDAEGYVRRIGHIDLMDIADPDGVARLQTDAARDLSGRFT
FP
FFTIEDVMRVDDTHILVANDNNLPFSSGRKLDAAADNEVILLEVPELLSAQ
>MCE8455474.1 hypothetical protein LZ188_02600 [Rhodovulum sulfidophilum]AB15
MHTNKLVVVGAGHVGSYVLADAMKIGLFAEIGVIDILGNVAFGEALDQAQATALPYMSNIKVTSGGYEQCADADVIVV
AA
GPSVIRDPENPKAEPDRTLLTTTNCKVIREVMAGIVKHTTEAIVILITNPLDTMVYIAENEFGYPRGRIFGTGTMLDSAR
LRKVVADAYGIDPKSVTGFMMGEHGSTAFPVLSHVNVAGIPFAELDRHFEALGDIRSPETVKARVISAAYDVFNGKGW
TN
AGVAQSAVTMARAVLLDERSVFPACTTLRGQYGHDGDVALSMPCLLGREGIVKQLPVGLNAWEAEKLAESIAFIQAT
MRD
AGTGPDKAVA
>MCE8455475.1 peptide chain release factor 3 [Rhodovulum sulfidophilum]AB15
MLDAPSNRPALPPEIARRRTFAIISHPDAGKTTLTEKFLLYGGAIQMAGQVRAKGEARRTRSDFMKMEQDRGISVSASA
M
SFDYERFRFNLVDTPGHSDFSEDTYRTLTAVDAAVMVIDGAKGVESQTRKLFEVCRLRDLPILTFCNKMDRESRDTFEII
DEIQEMLAIHVTPASWPIGVGREFLGCYDMLRNRLELMDRADRNKVAESIKIEGLDDPKLAEHVPQDLREKLIEEVEMA
K
ELLPALDPQAVLEGQMTPIWFGSAINSFGVKELMDGIANYGPEPQIQPAESRQIAPEEPKVAGFVFKVQANMDPKHR
DRV
AFVRLASGHFQRGMKLTHVRSKKPITVSSPVLFLAADRELAEEAWAGDIIGIPNHGQLRIGDTLTEGEALKVTGIPSFAP

ELLKNCRAGDPMKAKHLDKALMQFAEEGAAKVFKPMVGSGFIVGVVGALQFEVLASRIELEYGLPVRFEPSQFTSARW
VH
GEKADVDGFVQKNKQHMATDNDGDVVYMTRLQWDIDRVLRDHPELTLSATKEMQTG
>MCE8455476.1 pentapeptide repeat-containing protein [Rhodovulum sulfidophilum]AB15
MGNPEHLKWLLEGVEAWNARRKREDFVPDLSEMDIRAAFEKTGKLEEGRIPLLRANLREADLGGADLGRADLREANL
GGA
NLRGANLRGTNLRETNLKGANLEGANLGGANLRGVNLRGVNLRGANLGGADLGGAHLREAKLGGAYFGEADVRTVY
SGVG
TNDVGTPEYTNLSQARALTQAQLDTMRGDTGTILPDGLHHPAHWPDPEPPQVSIESDAPPPLPEGVAAEDVAEKDGR
LFL
VNIAPPPRSDLETIHGDLREDLAALLASGAFDNISKGLGTALSRYAGIVDCAYADLDQVRFGVQTKALRLKLDTHRQDLS
DAAPEKTGDLEAALLSAELIAARLPDWQTFLKETADERSPVAEHEKEITEAIESAAEEMREDPAHFAPALPERVSEYLNT
ATIEGYLAATALLNNLTFATMAAVRRFTRDVGNETRKQAVSSLAKGVVAALGATLMKFVGILPAEFDWVLPWLRYIPTL
F
G
>MCE8455477.1 universal stress protein [Rhodovulum sulfidophilum]AB15
MTATFLVATDLSPRADRAVNRAFRLASWTGADILLAAVIDSDLPEDVARPLAEAAETRLARFAASIPHSAEVRHDTRILT
GDPSAAIPLLAEEIDAALIVLGLHRDRPFLDMIRETTMERIVRHAARPVLLVRDPVDHDYAALLAALDFAPASTAAVKIA
ADLAPEATIHGVHALHIPYRGFVAPHGSSIAIAPFRRSAEMRLAEWRSDPDLPERLAEIDIVEGAAHVVLNREIEDHAPD
LLVLGAHGRAGDAPSILGSLANDLMRHPPCDLLIAR
>MCE8455478.1 ParA family protein [Rhodovulum sulfidophilum]AB15
MKTVLIANPKGGCGKTTIAVTLAAALADGGWKVALADADPQKSALRWLRRRPAAASAIRGLDWSGGRGAGKPGKGI
GWLI
VDAPGALPDAAAAGLVDEARAVVVPVLPSVFDAEATRRFLSEIAEFKRIRKGKVGVDLVANRVRPRGRDAAWIRDMAE
RV
GQAPVAEISDRVAYGDLAEQGLTVFDRPQRAYAPLRAQWTPLLAALTARG
>MCE8455479.1 SDR family oxidoreductase [Rhodovulum sulfidophilum]AB15
MDLGLAGKRALVCAASKGLGRGCAEALAEAGVDLVINARGAEALLATSEEIRRAHGVAVTPVAADIVSEEGRATVLEAA
G
QVDILVTNAGGPPPGLWSDWERDDFIAALDANMLTPIALMKALLPGMIERGWGRVVNITSQSVKAPIPVLGLSNSAR
AGL
TGYVAGTARQVAPHGVTINNLLPGIHATDRAVALDRGVAEAEGIALAEARTRREATIPARRYGTPGEFGAACAFLCSRQ
A
GFIVGQNILLDGGATNATL
>MCE8455480.1 hypothetical protein LZ188_02630 [Rhodovulum sulfidophilum]AB15
MLRRLLRGLGGGARAKGAAPQAAGARAALLPDDTPFGEDLARRAVAFLEATSAVRGEAAIVRTHRDYTGHGLFHDRA
AGR
FFLSRSLDGWPCAPAYLSFADGARFRRWLAAQSDASLSGQIPSDIPGFERDPGNQRITRSFVEDYLRRHENGGASGRQ
GG
>MCE8455481.1 lysophospholipid acyltransferase family protein [Rhodovulum sulfidophilum]AB15
MKERIDPLIEERAPWLFADHPWSAPSRRVLNGLLGYDRTVALGEVLQDHPSPVIMDEMARLLARDVRGTGIENIPATG
PA
MIVANHPTGIADGVMLWSLLRQRRPDLFFYANRDILRVLPQMEEIIAPVEWREEKRSHAKTRETMAYTRAALEKGRLA
VI
FPAGRLAKRRGLSLHERPWMASAAMIARKFDLPVIPVNIRSRNSVLFYLFDLLHPTLRDITLFHETLNKNRQPYRMTVGR
PIPPGALPVRSEEGIEMLRAATLALGGRYAPRVSLLQNSRKPSWIKSTALG
>MCE8455482.1 aminopeptidase N [Rhodovulum sulfidophilum]AB15
MTDATPQPVFLKDYTPFGFLVDRVELTFRLAPTETRVRARIAFRPNPAATDRRFFLHGEELTLISAAIDGHPVIPEITSA

GLSCEVPDAPFLWDCEVEIAPKENTALEGLYMSNGMYCTQCEAEGFRKITFYPDRPDVMAPFRVRIESDLPVLLSNGNP
V
ARGPGFAEWDDPWPKPSYLFALVAGELVATSERYTTSEGREVDLNIWVRPADQGKADYALDSLIRSMRWDEEVYGRA
YDL
DVFNIVAVDDFNMGAMENKGLNIFNSRYVLASPETATDRDYENIERIIAHEYFHNWTGDRITCRDWFQLSLKEGLTVFR
D
SQFSADMRSAAVKRIEDVMTLRARQFREDNGPLAHPVRPESYTEINNFYTATVYEKGAELIGMLRRLVGPEGYRKALDL
Y
FSRHDGQACTIEDWLQVFEDATGRDLTGFKRWYSQAGTPRVKVTEDFKNGTYTLHLEQETPPTPGQDEKAPQVIPLAL
GL
LSPNGDEVLPTTVIELTEARQEAVFEGLGAKPVPSLLRGFSAPVILERDSSSEERAFLLAHDTDPFNKWEAGRSLAREIL
IEMVTEGAAPAPAWLEALASVARDEALDPAFRALALRLPGEDELAEALAGRGTVPDPLAIHAARETARTAIARHLEPLW
P
GLYRQMTCPGPYSPGAADAGRRALRLVALGYMARLDGGAQARAHFAAADNMTDEIGALSVLVETGGAEEALDRFAR
RWGD
DRLVMDKWFGLQASLAAPERAVAATRALTEHPDFTWKNPNRFRAVIGGLTANAAGFHDPSGAGYDLLADWLIRLDG
VNPQ
TAARMSTAFETWARYDAGRRAKSKAAMERIAAREGLSPDMGEMIGRLLAAGQSAD
>MCE8455483.1 deoxyribodipyrimidine photo-lyase, partial [Rhodovulum sulfidophilum]AB15
MGVTILSFGRDLRGQDHPALARAGQEAGAEGGAVLPVFIVDPAVWSAPEASARQWRVVAEGL
>MCE8455484.1 cell division protein FtsZ, partial [Rhodovulum sulfidophilum]AB15
MALNLTMPEQEELKPRITVFGVGGAGGNAVNNMIEKRLEGVEFVVANTDAQALQQSNAQARIQMGVKVTEGLGAG
ARPSV
GASAAEESIEQIVDHLAGAHMCFITAGMGGGTGTGAAPIIAQAARELGVLTVGVVTKPFQFEGTKRMRQAEEGVENL
QKV
VDTLIIIPNQNLFRIANEKTTFTEAFSLADDVLYQGVKGVTDLMVRPGLINLDFADVRSVMDEMGKAMMGTGEADGE
DRA
IQAAEAAIANPLLDEISLNGAKGVLINITGGYDLTLFELDEAANRIRDVVDQDANIIVGSTLDPDMDGRMRVSVVATGID
AAARIDEVPLPRRPMREPLHRPAEVQR
>MCE8455485.1 cell division protein FtsA [Rhodovulum sulfidophilum]AB15
MTDLYHSQRAMRRMRKLAMDRGVIAILDVGSSKVACLVLRFDGIAPKGGVEQMGALAGQSRFRVIGAATTRSRGVRF
GEI
HGMNEAERAVRTALQAAQKMAQVRVDQVIACFSGGEPRSYGLDGQVTVLDDAVAEQDVARVLAACDMPEIGASRE
VLHAQ
PVNFALDHRSGLVDPRGQVGHRLACDMHMLTVDDGAVHNLLHCIKRCDLEPAGLASSAYASGISALVEDEQELGAAC
VDL
GGGATGISIFIKKHMIYADSVRMGGEHVTSDISKGLHVPLSTAERIKTLYGGVVATGMDDREMIGLGGDSGDYETDSRS
V
SRAELIGIMRPRVEEILEEVRARLDAAGFEHLPSQRIVLTGGGSQIPGLDGLAARILGNQVRLGRPLRVHGLPQSATGPG
FAALVGLCLFAAQPQDEWWDFEMPMDRYPARSLKRAVRWFKENW
>MCE8455486.1 cell division protein FtsQ/DivIB [Rhodovulum sulfidophilum]AB15
MRPVMPPYRDPAPSRWAYRAQRLWLTPMVRRAVLKGLPLVVLAAIPAIWLADADRRAALGDGLAEIRREIETRPEFM
VKL
LVIEGASDPIDREIRAIVPLNLPASSFDLDLEAMRQAVTALDAVKSAELRIRPGGVLEMKIAQREAALVWRSRDGLSLID
AGGRRVAALDSREDRADLPLIAGDGAGKAAPEAMALFAAAAPISDRVRGLVRMGERRWDVVLDRDQRILLPETGAVA
ALE
RVIALEDVSGMLARDIEAVDLRNPERPTLRLTEAAIEDFRKIRATQAGAVN
>MCE8455487.1 D-alanine--D-alanine ligase [Rhodovulum sulfidophilum]AB15

MGGPSSEREVSLSSGRECAAALRGEGFDVIEIDAGQDLPGQLISAAPDVVFNALHGRWGEDGCVQGLLEWLKIPYTHS
GV
LASALAMDKQRAKDAYRDAGLPVVDSVLAAKAEVSARHVIAPPYVVKPNNEGSSVGIHIVHEAANAPPQLGPEMPGIV
LV
ESYAPGRELTTTVMGDRALGVTDILTEGWYDYHAKYVQGGSRHVVPAELPEEVSEACLDFALRAHRALGCRGLSRTDF
RW
DESRGTDGLILLETNTQPGMTPTSLAPEQAAHCGISFGRLCRWLVEDASCDR
>MCE8455488.1 UDP-N-acetylmuramate dehydrogenase [Rhodovulum sulfidophilum]AB15
MTQMREPRGRLTPGKALSELTWLRVGGPADWLFQPADAEDLATFLAALPAGMPVFPMGVGSNLIVRDGGIRGVVIR
LGRG
FNAISVAGARVTAGAAALDAHVARRAAEAGVDLSFLRTIPGAIGGAVRMNAGCYGRYVADVLVSARAVTRSGEIVTLSP
E
DLRLGYRQSHLPEGWVITEATFEGRPGAPEALAAQMEEQIARRDASQPTRARSAGSTFRNPAGFSSTGRADDSHELKA
WS
LIDAAGLRGARRGGAQMSEKHSNFLINADGATAADLEGLGEEVRKRVFQSSGIELEWEILRVGEPAPGNP
>MCE8455489.1 hypothetical protein LZ188_02675 [Rhodovulum sulfidophilum]AB15
MTGPTLLLAYGSWAAGPLVAYAALSHGLMRNAIGFTIMFGLYTSSVWAIWGGLKLQATGNGPAVLAPSSVLLPWGAV
ALV
SAVLYALGAWIGGGDG
>MCE8455490.1 DUF2484 family protein [Rhodovulum sulfidophilum]AB15
MTLSLLLACLWVLLAAVIGVLPEKLHWIAAFSLIASGIPLLGFLTYQSGPLWGLIALAAGASTLRWPLFYLGRWLNRRRR
STGE
>MCE8455491.1 hypothetical protein LZ188_02685 [Rhodovulum sulfidophilum]AB15
MVSCPVIAICGPAPLAGIPGLRRAAPPVTARLLAAAVIPAPAQAAFGPGAALTGPLHISAFQPVERPVGPGGAPEALSRD
RGAEGGEQ
>MCE8455492.1 UDP-N-acetylmuramate--L-alanine ligase [Rhodovulum sulfidophilum]AB15
MNAATKLPTDVGPIHFVGIGGIGMSGIAEVLLNLGYRVQGSDLKASKITDRLAGLGATVFEGQREDNLENAEVVVISSAI
KPGNPELDAARRRGLPIVRRAEMLAELMRLKSNVAVAGTHGKTTTTTMVATLLDAGGLDPTVINGGIIHAYGSNARM
GAG
EWMVVEADESDGTFNRLPATIAIVTNIDPEHMEHWGSFEALRQGFYDFVSNIPFYGLAVCCTDHAEVQALVGRVTDR
KVI
TFGFNAQADVRAINLRYETGKAHFDIALQAEEMVIEDCTLPMPGDHNVSNALAAVAVARHLGMKADEIRAALEGFAG
VNR
RFTRVGEANGIAVIDDYGHHPVEIAAVLKAARQATEGRVIAVHQPHRYSRLHSLFDDFCTCFNEADVVGIAEVYAAGEE
P
IQGATRDDLVAGLIRHGHRHARAILSEDDLVRLVREQAGPGDMVVCLGAGTISAWANNLPARLAE
>MCE8455493.1 UDP-N-acetylglucosamine--N-acetylmuramyl-(pentapeptide) pyrophosphoryl-
undecaprenol N-acetylglucosamine transferase [Rhodovulum sulfidophilum]AB15
MNAPLLVIAAGGTGGHMFPAQALAETMLARGWRVKLSTDARGARYAGGFPAAVEIETIAADSPDRGGPVARAVAPL
RLAA
GVAAMLWRMLRDRPAVVAGFGGYPSFPALTAATLLRRPRMIHEQNGVLGKVNRLFAPRVHAMACGVWPTELPEGV
EAFHT
GNPIRAAVRERAGAPYIPPGDYPMSLVAIGGSQGARAISESVPAAIAALPAEMRGYLRVSHQAREEDLDRVVAAYEAAG
V
TAEVRPFFDDIPARLAEAQLVISRSGASSVADIAAIGRPAILIPYPHAAGDHQTANARGLVEAGAAILIPEKALDPVALS
DQIATVLGQPKGAMQMAVAAMRCGKPDAVDGLAEIVEALGGEGK
>MCE8455494.1 putative lipid II flippase FtsW [Rhodovulum sulfidophilum]AB15

MTEMVYGTAPIRAGEPVLPRWWRTVDKWAMSFVLILFGVGMLLGLAASPPLAEKNGFPPFYYVERQAVFGGLALCA
MLVC
SMLSPTMVRRLGVIGCGAALLGLVLLPVFGTDFGKGAVRWYSLGFGSVQPSEFLKPGFIILTAWLMAAAQEVEGPPGK
TM
SLALAVVVVAFLALQPDFGQAALILFAWGVMYFVAGAPFMVLVAVAGGVVFAGTIAYENSEHFARRIDGFLNPEIDPRT
Q
LGYATNAIREGGFFGVGVGEGQVKWSLPDAHTDFIIAVAAEEYGLVLVAVIIALYCAIVIRSLFRLLKERDPFIRIAGTG
LACTFGVQALINMGVAVKLLPAKGMTLPFVSYGGSSLIAGGIMVGMLLALTRNRPQGEITDLLRRRTRE
>MCE8455495.1 FAD-binding protein [Rhodovulum sulfidophilum]AB15
MSKPDALTALGEMLGDRLSRSKSDLDLHGRNETYFPFLPPDAVAYPETTDEVAQIVRICAAHGVPLIGWGRGTSLEGHA
L
ALQGGVTIDFSRMNRVLKVNSEDMDVVVQPGLTREDLNRELRATGLFFPVDPGANASLGGMASTRASGTTAVRYGT
MRDN
VLALEVVLPDGRVIRTGTRARKSSAGYDLTGLFVGSEGTLGLITELTLRLHGQPEAISAAVCAFDEIGAAVDTVIQTIQL
GIPMARIEFVDAATAAAFNAYASADMPEKPHLMVEFHGSAAGVAEQTESFGEVVAENGGEGFKWSSRPEDRNALWT
MRHN
AYYACLGLRPGATALVTDMCVPISRLAEAVEETRADIAQASIPGPILGHVGDGNFHAILLVEPDNAAELAEAKALAHRM
V
ERALRMGGTVTGEHGIGMGKLDYMEEEHGAAWDVMGQIKRALDPMTLMNPGKMVRAN
>MCE8455496.1 chloride channel protein [Rhodovulum sulfidophilum]AB15
MQFWFIAAAIGIAAGFAALGFRLGIDVLQRALYGVDDPHRLHSYAQTLHWGWILVIPACGGLVVGLLLHRFTPDARVR
SV
ADVIEGAALNEGRVEVKAGLASALASLITLSSGGSTGREGPVVHMAGVVATWVSNRIHADGITGRDLLGCAVAAAVSA
SF
NAPIAGTLFALEVVLRHFAVHAFAPITVAAVAGTVINRIEFGGVPEFALPQTDRLAFYGELPAFLMLGLLCGLVAVILMR
AIFWAEGIGDAVQRRTRMPRWLRPAVSGLGLGALAIWFPHIIGVGYETTSAALTGTLVLHEAVVFCLLKSVAVSMTMA
GR
MGGGVFSPSLMVGALTGLSFGIIATSAFPNVSGAETLYALAGMGAVAAAVLGAPISTTMIVFEMTGDWQTGLAVMV
AVSL
STALASRLVDRSFFLTQLERRGKRLAAGPQSYLLAALTAGVLMRSKESPGAASPGACAELIGQGVMFEADTTLVEALPVF
EKWGHGFVPVVARAAAGREPELLGALFHVDALRAYSRALATTAAEEHS
>MCE8455497.1 DUF427 domain-containing protein [Rhodovulum sulfidophilum]AB15
MSDHITIHKAEGAWVVRAGGAVIAESRNALELTEGGMGPVIYFPREDVAMAFLDRSDSRTACPHKGVASYFTLRAKSY
DI
ADAAWSYEEPNTGLERIAGHLAFYADKVTVEQL
>MCE8455498.1 aminopeptidase P family protein, partial [Rhodovulum sulfidophilum]AB15
TQVAIRETANLVDRIWADQPAPPQGPITPYPEELAGESHADKRARIGRLLADAGQSAAVLTLPDSIAWLLNVRGSDIPR
N
PVPHTFAILHDDGRVTLYADPAKVSDALKAHLGPEITLRPAGAFGPGLRSLLGRVRVDPASAPLWVLRQLEDAGIEIALA
EDPCILPKARKTAAELEGSRAAHLRDARAMIEFLAWIDAEAPKGGLTEIDVVRGLEGFRAATGKLRDISFETIAGAGPNG
AIVHYRVTEASNRAVAPGDLLLVDSGAQYLDGTTDITRTIAVGPVPEAAKAPFTRVLKGMIAISRARFPKGLAGRDLDAL
ARNALWQAGQDYDHGTGHGVGAYLSVHEGPQRIARSSTVPLEPGMILSNEPGYYREGAFGIRIENLIAAREAPALTGG
DD
RRMLTFETLTWVPIDRRLILPELLDAGERAWLDAYHAEVAKRAGDGLSERARAWLAEATQPL
>MCE8455499.1 penicillin acylase family protein, partial [Rhodovulum sulfidophilum]AB15
MPHLFRWLLRIFTAVVILAALGIGGVVYYLASRSLTDYSATHEVDGLSAPVEIVRDHSSVPHIFGKTDEDVFFGLGYAHAQ
DRLWQMTVLRRTVQGRLSELFGARTMKIDELMRRLDLYRLAQDSVDAQDPQTLAALEAYSRGVNAWIETVNTEARG
RGAP

EFFLFRPEIAYWQPADSIALVKLMGVRLSSHLDAEVLRARVSLLMEPERVKDILPDAPGPGLAALPDYADLAPGVIPSSA
PLARYAEGPLSPFLGPDFAGASNAWAAAPKRSAAGGTLLANDPHLGFSAPSIWYLARLELSTGGVIGGTIPGIPTILSGR
SAKLGWGLTTSYLDDQDVFLEQLNPDNPQEYRTPDGFRPFETRRSIIKVADAEPVTITLRWTENGPVLPGTHYDLASITP
PGHVAALGWTLLSGADTSISAAMQVMRSQSVDEAIEAGRFFVAPSLNLTLVDADNIALQMIGAMPRRMAGNQSKGR
LPQP
GWKEENRWQGWLNYAANPSFRDPAGGILGNTNNKLVDRPFPLHVSFDWGDSQRIQRWKRLMETRKVHTRESFIEA
QLDTV
SFTARSLLPLVGKDLWFTGEAAPAGSPERRRQRALELLAGWNGEMNEHLPEPLIYAAWMRFLQRDLIEDDLGPLAREF
TH
VEPLFIERVFRDIDGASVWCDIRQSAAIETCTEIARKSLDEALLYLSETYGGSLESLRWGQAHQAWHDHPVLGETPLLKW
IVNIRQPTSGGDNTLNRGLTKGTDPFP
>MCE8455500.1 NAD(P)-dependent oxidoreductase [Rhodovulum sulfidophilum]AB15
MARVAFLGLGVMGYPMAGHLAAAGHEVAVYNRTAAKAEDWVARHGGRSAPSPAEAAEGADLVMACVGNDDDLR
AVCEGES
GAFAGMAAGAVFVDHTTVSSMVTEEMAALAETRGIAFVDAPVSGGQAGAENGQLVIMCGGPEEAYAKAEPVMAAY
AKLSK
RMGGTGAGQLTKMVNQICIAGLVQGLSEGLHFAEKAGLDGRAVVEVIGGGAAGSWQMMNRYQTMLDDEFDHGFA
VDWMRK
DLGICLATADRTGASLPVTALVDQFYKDVQKLGGARWDTSSLIKRLRAMG
>MCE8455501.1 ABC transporter permease [Rhodovulum sulfidophilum]AB15
MNETKARPRLGRSEAAQGYFLVAPPFLYALLLLAVPLAAILLFSFWSQDYLTLDTTLTLKNYREAFSDPLYGQLLLRSLL
ISGAVTAATVLTAFPIAYFISFSVPPSQKSMWIFLITIPFWSSYLMRVFLWKVILGYNGVLNTTLSGLGIIDEPLSFILY
NANAVVITLAHAYAPFAILPIFVALEKIDRSLLEASRDLGESRAMTFLRVTLPLAMPGVVAAVLIVFIPTIGDYVTPRLV
GGPDGLMIANMIQTQFLKLNNAPMGAALAVLAMAMVTAVAVLFLVLNRRYLRGRK
>MCE8455502.1 ABC transporter permease [Rhodovulum sulfidophilum]AB15
MKGLRLYAIFYLLFLYAPIALLPVFAFNNGTVIAFPLRGFTFEWFRELMTIPALHAAVLNSLAIAVSSAVIATCLGICAA
RAGTRFAFPGKKGILGLIMLPLVLPEIIVAVSLLVVLLQLGLPLGSWTVVLGHVLMCTPFSIAILNSAFQSLDVSLEEAA
IDLGESRWSSFRLITLPLVMPGIVSSLLIAFTISLDEFIIAFFLTGTNPTLPVYIWGQLRFPQKIPVIMALGTLLVLTSV
ILLTVAEYVRRRGAARTGKADAGGFL
>MCE8455503.1 ABC transporter ATP-binding protein [Rhodovulum sulfidophilum]AB15
MKDTLGVERPIIALENVEKYYGEFHALRGISAEIRQGEFFSLLGPSGCGKTTLLRTIAGFEELSGGRLTIDGRDMKRVPA
NVRPTNMVFQSYAIFPHLSVAENVGFGLRREALSKERKAAKVAEALEMVGLRGFGARAAHALSGGQRQRVALARALIL
EP
KVLLLDEPLSALDKKMREQMQIELRRLQRQVGITFILVTHDQEEALIMSDRIAVMFEGQIVQLDTPQQLYRRPNTRQVG
E
FIGVMNFLPATATVGDKADIEVEAAGLGRAVIGPDQLPGGWHPGQTQSVGIRPETMSILFPGETTERRVATGTVEDLSY
Y
GDMTYYVVRLDGVDRPMTISMKNLVGRPVLERGTAARVAWDARSLVLFA
>MCE8455504.1 polyphosphate kinase 2 [Rhodovulum sulfidophilum]AB15
MTDLPFDGAITQFYEQDSPDEIRRAIADGGKRDILADDYPYDRRLRHAAYAADLQALQIELAKLQADVKATGKRIAVLFE
GRDAAGKGGTIKRFRQNMNPRVARLVALPAPSNREKSEWYFQRYVEQLPAGGEIVLFDRSWYNRGVVEHVFDFCTPE
ERE
RFFGQLPAFERMLVDEGILLVKFWLTVGRAEQLRRFLQREKDPLKQWKLSWVDIEGLKRWDAYSAAIRETFARSHNGH
AP
WTVIRSDDKRRARLAAIRHVLSLVDYKGRSDKVVTAPDPLICGGPEIWTP
>MCE8455505.1 alpha/beta fold hydrolase [Rhodovulum sulfidophilum]AB15
MAEDYAPTGRLIEIDGRQVHAHQEGSGPDVILLHGAGGNTRDFTFDLLHRLSADFRVTAFDRPGLGHTDPLHPNGESP
AE

QAAVLEAASRGLGLGPAVLVGHSFGGAVALAWALDHPERAAAIVSLAGAAMPWPGGLGPYYELVSSDFGSMFMVPL
ISAF
VPRFVAEQTIARIFEPDPVPQGYGDHIGIALSLRPETLRANTRQVNGLKPHIRAMADRYDDLKMPIEILHGTADRIVPIE
VHSRPLAERLANVHLTELDGIGHMPHHADPEAAVAAIRRAAKRAGLRATA
>MCE8455506.1 homoserine O-succinyltransferase [Rhodovulum sulfidophilum]AB15
MPIKFPSDLPAYDVLSREGVMVMGEDEASRQDIRPLKIALLNLMPKKIQTETQFARLIGATPLQIELSLVRMTEHKTRNT
AAAHMETFYRPFAEVEASGEKFDGFLITGAPIEHLPFTEVTYWDELTRVFDWTQSHVHSTFGVCWGGMAMIYHFHGV
RKH
MLPAKAFGCFRHRNLAPASPYLRGFSDDCVIPVSRWTEMRQDEIDAAPGLVTLLGSDESGPCLVEDAGHRALYIFNHFE
Y
DSDTLKQEYDRDIANGTSINVPQNYYPDDDPSQAPQNRWRSHAHLLYGNWINQIYQTTPFDQNRIGAY
>MCE8455507.1 hypothetical protein LZ188_02765 [Rhodovulum sulfidophilum]AB15
MRGWKSGAGWSAAAFAAGLVAAAGAVRAQQEGPEWDLQRCIWSCLSAFGPADNPAYAACVSRNCSEPTAPAPAP
RWSSGA
TSDGHGAYAGVRAEGGQDVIFYVFCARGGERLLQLDGVGGEAGPNVLSLIIDGQGFPVTFGGGHDAGMLARLPPSAP
VLG
ALQRGQTLEIRSGAGQGQGLGRFPLEGAGAAISGALALCR
>MCE8455508.1 ATPase [Rhodovulum sulfidophilum]AB15
MIYDTAEAWRQAERRHVLLFGMSGLGKTHVSNMLRAEGGWFHYSVDYRIGTRYMGEFIADNFKREAMKVPLLRELLR
TDS
VFIASNITFDNLAPLSTYLGKPGASDKGGLPFDDYMRRQEQHREAEISALLDTPRFIDRAAELYGYDDFVCDSGGSICEV
VDPEDPADPVLSALAARLLLVWIEGSEAHTAELVRRFDRAPKPMYYQPAFLHEAWADYLSRPGLSEAAVDPDAFVRWT
YA
RALAHRQPRYEAMARNWGVRITAEEVAALRGPEDFTDLIAEAIDRAPARR
>MCE8455509.1 hypothetical protein LZ188_02775 [Rhodovulum sulfidophilum]AB15
MKYAVGAAALAAVTTGPALAGGVERSNQSVSILFEQGTYVELGFSRFDPDVSGSSNSSGLASGDMLPGFNGASLAFKQ
DL
GDNATVALILDEPIGADVSYDSTNNYPLGSPTTTLDGANADVNATGVTALLRYELPSNVSLIGGLRAIRTSGTVSLPGVG
AYSLNSSTETDFGYILGIAWEKPEIAARVVLTYSSAIDHSFDTVETAGSSSFSNDLDVTVPESLTLEFQTGVAVDTLIFG
SARWVDWTSFDITPDLYTGSVVPGGSLVDYDHDVITYNLGVGRKFNENWSAAILVGYEKHEGTYTGNLGPTDGFRSIG
LA
ATYTTGKVKVTGGIRYVEIGDAKTEFGPTRTRFKGNDGVGVGLRVGYYF
>MCE8455510.1 DMT family transporter [Rhodovulum sulfidophilum]AB15
MSPLLRAALWMTGAITAFSSMAVAGRMVSVELDTFELMLYRSLIGLGLVLTIGGAFGRLGEISHRQIGLHFIRNLCHFTG
QNLWFLALGLIPMAQVFALEFTSPIWVAVLAPAILGERLTRMRALAAFVGFLGILIVARPDPTALEPGAIAAAGAAIGFA
GSALFTRRLTRSQSIISILFWLTAMQAAMGLICAGLDGEIAWPSPAAWVPLGVIGLAGLTAHFCLTNALSVAPASIVMPL
DFLRLPVIAVVGMLLYGEPLDPNVIIGAAMILVANFLNLRGESRQARHARRKTGAPPPAGRVND
>MCE8455511.1 antibiotic biosynthesis monooxygenase [Rhodovulum sulfidophilum]AB15
MGVTVTGWIEATPDRLAALRAAAAEHVRLTRAEPGCLRFEMREDPDRQGRFLLDEEFTDAAAFAAHRARKEGTAWEL
AAA
GLPREIEIAGLPE
>MCE8455512.1 glutamine-hydrolyzing GMP synthase [Rhodovulum sulfidophilum]AB15
MSTHDRLLIIDFGSQVTQLIARRLRELNVYCEIHPYQNVTDAFLRDFAPKAVIFSGGPASVLDEGSPRPPQSVFDLGVPI
LGICYGQQVMMQMLGGRVERGHGTAEFGRAYVTPEEDRIDLLKGWFTENREQVWMSHGDHVAEIAPGFAVYGTSP
NAPFA
ITADLSRNFFAVQFHPEVHHTPNGKTLYENFVRLAGFTGDWTMGAYREEAIRKIREQVGDAKVICALSGGVDSSVAAAL
L

HEAVGDQLTCVFVDHGLLRLNEADEVVAMFRDHMNLHVIHAQEQDLFLGELEGVSDPETKRKTIGRLFIDVFQKYADQ
IE
GARFLAQGTLYPDVIESVSFSGGPSVTIKSHHNVGGLPEKMGLKLVEPLRELFKDEVRALGHELGLPASFIGRHPFPGPG
LAIRCPGEITREKLEILRKADAVYIDQIRKHGLYDEIWQAFVAILPVRTVGVMGDGRTYDYACALRAVTSVDGMTADYYP
FTHDFLGETATRIINEVKGINRVTYDITSKPPGTIEWE
>MCE8455513.1 cadmium carbonic anhydrase, partial [Rhodovulum sulfidophilum]AB15
EKELKDPAAGLGAFHGAKPGDTIEVHWVFSSCEVAPGKGLGSCMSESCQNPQLRVEAQAFLLVNDHTALDFKDYIYRG
TQ
VNGLHQPRALPAGTGTPVIYGGSTTGPRYNQAICSPLQVTWSVRPDCARLDINSLYEWAALGNVFEEDHSHGVRPLVT
AE
ELLSQIR
>MCE8455514.1 hypothetical protein LZ188_02800, partial [Rhodovulum sulfidophilum]AB15
MTERRAGAGYVSKAGLEVAADLAAFVEEAALPETGISPDRFWTGFAELLHDLTPKNRALLETREKLQLQIR
>MCE8455515.1 plasma-membrane proton-efflux P-type ATPase [Rhodovulum sulfidophilum]AB15
MDGQDQPEGEKLPPTDPQQGLSTAEAQARLGRFGANTLAEEHRSLFKRIFGYFWGPIPWLIEIAAILSLILSDWADFGVI
LAMLLVNAGVGFFEENKADSAIALLKQKLAPAARVLRDGRWQEIAAKDLVPGDLVRLEIGNIIPADLRLTEGQYLSADEA
ALTGESLPVDKAVGDIAYSGSVVRLGEMVGEVTATGMNTYFGRTAALVQGPERKSHFQQAVMRIGNFLIVSALVLVSII
L
IVSIWRENPLWETVQFALILTVAAIPVALPTVLSVTMAVGAERLARMKAIVSRLVSIEEMAGVDVLCSDKTGTLTLNQLT
VETPRPAAGATRDDLLLAAALACNANAPDAIDQAVLAAADGIEARRDYKLGTFTPFDPVSKRTLAEARRGDETWRFAK
GA
PQVILDLAGLEGAARTALEDEVDTLAAQGFRAIGAARAEGEGAWQFLGLIPIFDPPRPDAAETIAKAGEMGVTVKMITG
D
HEAIARQIAGQLGLGQAMYPAGQLLEGDPAEVEARVRAADGFAQVLPEHKFRIVEVLQQGGSIVAMTGDGVNDAPA
LKRA
DAGIAVSGATDAARAAADLVLTGTGLSIITEAIAEARRIFERMTSYATFRIAETIRVLLFMSLAILAYDVYPVTAIMIVL
LAVLNDFPIMMIAFDNVAVSPKPVRWDMRRVITIASTLGVMGVAETFLLFWYVDSVLHLPRETIQTLIFLKLLVSGHMTL
YVTRNQDWFWTRPWPNWKLFCTTEATQVIGTLIAVYGVLVTPIGWTAALLVWAYCLVWLPIEGAIAMGIRRLYAARL
QG
>MCE8455516.1 alpha/beta fold hydrolase [Rhodovulum sulfidophilum]AB15
MADTPHEIEQSVPLFWPMAAVLGWQEAGYQAMADGLRFVQEAAEISAPPEPDWASPNEVLLDLDTMRLRDFGGSG
TGLPV
IIDAPYAGHSATIADFAPGQSLVATLLAAGLGRVLVTDWKSAGPEMRDFDIDKYLAEINVAIDEVGGRAILVGLCQGGW
M
SAMVAARFPSKVAALVLAGSPIDTDAGDGPIRRMAHETPLSAYRDMVASHHGRMSGSLMLTGWKNMHAEKQYLGK
FLDLY
AHIEDRNYIERTETFERWYENPLDLPGRYYLQAIDLLFKQNLFAKGEFLGLGRRLSLKDVTCPAYLLAGDSDDITTREQV
FAAEDLLGTPKTQIRKLLAKGGHIGLFMGRHTLADVWPGIAQWIAEQGGK
>MCE8455517.1 hemin uptake protein HemP [Rhodovulum sulfidophilum]AB15
MTLLQTIRHSTGETTQHDARILTGGGNTAVIVLDGVAYTLRITRSGKLILTK
>MCE8455518.1 phosphatidylcholine/phosphatidylserine synthase [Rhodovulum sulfidophilum]AB15
MKKFIRRDPQHRDQVPFLQLLPNLVTLSGMCLGLTSIRFSMDGRYDGAALLLLLAALMDGLDGLLARRLNATSNFGAEL
D
SLSDFLCFGVAPGLLIYRFFLEPSNNFGWIFVLVFAAACCLRLARFNVMRDENAAETSHHFLGVPAPAAAMLGLLPLFVS
LAGVVDLRPVPHLVAFWLGFVGVLMISRLRTFSPKAMRIPRALVGLMMFATVVAVGLMFTRIWLLLTALDLIYLAMLG
HA
ALQARRPGDE
>MCE8455519.1 phosphatidylserine decarboxylase [Rhodovulum sulfidophilum]AB15

MSVSMLSTFVKPMHPEGRRFVAIAAGLTVLFFLIWPPLGWIGLGLTVWVYYFFRDPERVTPTRPGLMVSPADGIVSLLE
P
AVPPAELGLGDAPMTRVSVFMSVFNCHVNRLPAAGRVTAVAYRPGKFLNASLDKASEDNERNGLAVELADGRRYGVV
QIA
GLVARRILCWTAEGQSLATGERFGLIRFGSRLDIYLPEGVEPLVAIGQTMVAGETVIADLGA
>MCE8455520.1 MarR family transcriptional regulator [Rhodovulum sulfidophilum]AB15
MISDPDPDFDLGEFLPYLLNQAAQETSAGFHAIYRAEYGMLRTEWRVMVHLGRYGEMSASEIGRLSMTHKAKISRAVR
AL
EEKRFLHRSPDADDRRAEILSLTPEGHAALARLSARARAYGADIEARFTDEERALLKSFLRRLATETA
>MCE8455521.1 hypothetical protein LZ188_02835 [Rhodovulum sulfidophilum]AB15
MSGRVGDDLAAGDGALRGNGNVAGVAEGRERAVDRIAAIRVAFASEYQAVRRVFMFFRAMFASAIAQISVWSHPP
>MCE8455522.1 tyrosine-type recombinase/integrase [Rhodovulum sulfidophilum]AB15
MKRDLPKHVYRKGTAIYWQRRGEPTVRMENQELTPAFWAEYALRLKGPVRAPSKRTFNGLIDSYRRSERYTSLAPRTRK
D
YERVLSVIEAKWGALDPVKAQRKHFIALRDANADAARFANYVVQVSRVLMEHAIDIGMVSENPAKGIKQVKGETIDRR
PW
PADLVKAYRDAAEGRALLVFELCLGTGQRIGDVLKMRWSDLENGGINVRQNKTGKTLWVPFTKTLRATLDAADRRSVF
IL
TNERATGPWSYRGAAQAVRKVREEIGAEAYDIHALRHTAASELCTLGLTDEEIASVTGQSMAMVAHYTAHVRQKVRAL
KA
QERRL
>MCE8455523.1 hypothetical protein LZ188_02850 [Rhodovulum sulfidophilum]AB15
MNKHLRTDDRVRRLVSAVEACGKTVQRVIVRGREVELILLRDQDEIDEFEAMDLRR
>MCE8455524.1 hypothetical protein LZ188_02855 [Rhodovulum sulfidophilum]AB15
MAETFTRSFGRFHAEVTSAEARLIIRDDGKDMIRLFGRDLIRSSLLGGSWAPDIECALRWGLDRIEAELGEDLRYRAGVM
AAVKLCWRLRLANDPRRAERLLLGDPEAGQPPAEPRICPACGELVTEGQGCGGGICRASDAFPKPVEDPADKAFREAV
AE
LVDCAMKEGKPDV
>MCE8455525.1 hypothetical protein LZ188_02860 [Rhodovulum sulfidophilum]AB15
MSHPLRILIGCETSGVMRRAFAARGHDVWSCDLLPAEDRSNRHMICDIREVLHLGWDLLAVMHPPCTRLCNSGVRWL
HVA
PTNAPAEARPGERAAWPDLPEDTRRAIMWRLLDEGAALFSACWRAPIPRVAVENPVMHRHGRARLPADLPKPQIVQ
PWWF
GEPVFKATGLYLRGLAPLSATNRLTPPAPGTEAHKRWSAVHRAPPGPDRWKIRSRTFEGLAAAAADQWGENARKEAA
>MCE8455526.1 hypothetical protein LZ188_02865 [Rhodovulum sulfidophilum]AB15
MADETIARDIARDAEPEWILKFSIKGGDTVEWIAAKSTVFGTLEELNKRECETGSVGWVFLPMGAINMSEVRYVSIHPR
P
RETKPKPARRARFTWA
>MCE8455527.1 hypothetical protein LZ188_02870 [Rhodovulum sulfidophilum]AB15
MSIMEAIAPPPDALAEFVAEWRGAWPEVRLAAIRGALRRHGIEIPALPGRVAFYEIQLFGLVATGRDEAEAAQNWMEA
AI
RLCPFD
>MCE8455528.1 hypothetical protein LZ188_02875 [Rhodovulum sulfidophilum]AB15
MVAILTIAPQIADRLATEAAAARIEANTARVAAKSSARDGKPLLARAAFARAERLQGRAVTLGALAAQARAGGAA
>MCE8455529.1 hypothetical protein LZ188_02880 [Rhodovulum sulfidophilum]AB15
MPRIPKIPDLPATYGFGTWRDALLGLFISFAFIGSAWGGLMLARIAKASL
>MCE8455530.1 hypothetical protein LZ188_02885 [Rhodovulum sulfidophilum]AB15

MKTILAASALALLAAPALAATDTTEDQATAVSPASAQDCFAPINGTNAFQPSNAPGCIYYIAPTGNDRDPAASDDGEGS
G
EGEGGESSDT
>MCE8455531.1 helix-turn-helix domain-containing protein [Rhodovulum sulfidophilum]AB15
MQQETSPYFRPVSLCNLSSMGEIGDDFLAGLERIMDARGLRAAPLSEAAGLSKSFIRDLRRGKAASPKLDTAMKVARAL
G
ISVEEILAAANGSVAGVPTISIAGRVGAGAKVPVFEAYERGAGPQVECPVGLSPQGIAAVEIVGESMEPVYSAGDLLFYS
RPTHEGVPSEAIGRRCVCECERGFGWIKLVKLGSQPGYFHLIALNPDAENQHDVRLKWAAPVRLHWPADLAKRV
>MCE8455532.1 hypothetical protein LZ188_02895 [Rhodovulum sulfidophilum]AB15
MEQLIAEVRAYAEATNKSPEKVLREAIGASWGAWAAWQAGTSGPTMRSADRLRAFMTANPAPAKQEDTAC
>MCE8455533.1 hypothetical protein LZ188_02900 [Rhodovulum sulfidophilum]AB15
MQKSFQKFRDPQERERKWFASLLWRSFPEATSEAQLADLVAEALNTAHRPVNPRTVRNWLRSENAPGLHYVVAVLAL
AGA
ETVFELFDPEQTA
>MCE8455534.1 hypothetical protein LZ188_02905 [Rhodovulum sulfidophilum]AB15
MTLAIRIYWRIAGRFNQVRAGRARAAARVFEARAEKFLARFKGGRT
>MCE8455535.1 hypothetical protein LZ188_02910 [Rhodovulum sulfidophilum]AB15
MLALILGIQVSFGLIFLGALIWGMRHMAETRRRLEAEAADFDRQFDRAYTRIRRTQLQ
>MCE8455536.1 DUF2312 domain-containing protein [Rhodovulum sulfidophilum]AB15
MKETPQDEAVREKTYRVVADELRGFIERFEALAEEKAAIGDQQKEVMAAAKARGYDTKALRRLIALRKRHTEDIAEEEA
V
LQLYREALGM
>MCE8455537.1 hypothetical protein LZ188_02920 [Rhodovulum sulfidophilum]AB15
MATRVHTDLTIRGTTYPDAASAARALGVTPEAIRSAARKGRLDRVGTGRKGLAPMPVRIRGEVFTDAHAAAARFGVTP
QA
VWRALADGDPDRIGRPQRRPGRAPKPFEIGGLRFASQRKASRALGFSDDYLSHALTRGGHAARERILAAAMALSARQA
RT
RKSLPNGPACPEPMEELHHG
>MCE8455538.1 extracellular solute-binding protein, partial [Rhodovulum sulfidophilum]AB15
MRKTTLLLTTALCAAAGLARAQDQDMSCKGKLEALNVIGQGMPAVTALDAYLPEFGEKWDTEVKVTMLGENERRAK
ARLD
ASTGAGAYQVLYIDEANLAEYVSADWVYPLADVVPESYDIADFRTDLSNVATVDGVQYFAPFVGGGDVMMYRKDLLE
AAE
IERPTTLEEMVTAIKTLDEADNGAHGWSARGQRGAGMNVWRWTPFFRAMGGEWLDGDMPAFNSEAGVKATELYI
ELMQSA
PPGVATFNWSDSVEAFRGGQVAILIESDVFGPWMEDPAKSSVVGKVGYAPPPDPLPSAGWAHGWAVSRTGAADDC
ARAIA
GDFVGWATSKEMEQRRLEAGIASDIGRVSTLQSDAYKALVPAEYIDALLATGDKTNLLIMKSPVWPEIGDSLGLTLEELF
AGTRDDVQEALDEAAFFAEDSLMR
>MCE8455539.1 carbohydrate kinase [Rhodovulum sulfidophilum]AB15
MLIAIDAGGTAVKVVAFDLSGTEIAAEAADVPTQHHPDGRVERDLDIFWAGTVSALRRLAADCAGREILGVGCTGFGN
GI
FLVDAQGEATRPGIVSVDHRAQPLVDTLCASQAAREIAAVTGNRIWGGQTLMQIARLAQTEPEIMACTRWALACKDV
IRF
RLTGAALTDPSDASGGGLMDLAGGDYARDVLERLGFGAHLHRLPPIVPSDAVAGRISRAAAEATGLPEGTPVAGSMM
DVA
ACALGAGAVDAPVLTMIAGTWSINSLECAGPPGDEAPILNLLYRGGKRLVAEGSPSSAANLGWFLDRALGRRVGVDEA
NA

LVAACPASQDRCQFLPYVFGPEPRRGGFVDLGASDDLGTMLRAIYEGVAFQARRHAEDAVALAGQPFPGTIRLAGGAA
KS
PVWTQIFADICQRPVEAVRATEVGALGAAICASVAAGAYGDLGEAAARMTGVARRHEPDRELAALYDRRFCTFRRLDA
GL
AALMTA
>MCE8455540.1 glycerol-3-phosphate dehydrogenase/oxidase [Rhodovulum sulfidophilum]AB15
MISPHTLHSRPATQRDESATRLDALAEPEVLILGGGVNGVATLRELALNDVSCVLIDTGDFCAGASGASSRMAHGGLRY
L
EGREFRLVAESARERNRLVAHAGHLVSPLEIVVPLEHFVTGFGRAILRFLGLSNRNGPISLLALEGGLLVYEIFGRREHP
LPSHSTFLRRAGFPNGLRRSTRAVTSYFDGQITQPEGLVLEMLGEALDQGPHVAALNHVSWQAENGAIHLSDRLGTGS
WT
LRPKVIINATGAWIDRVNAGLGGQTRYLRGIKGAHLVLHNPELFERMGGRAFYFDDGTGRMVISLPVDSNVLVGTTEV
ET
SDPDDRRVAGDEIDYLIRAMDGLFTDIAVDRTQIVSVTSGIRPLRRGGQGSATSAARDHALEEDRFPGCPAPVLSLVGG
K
WTTFRSFGEQSADRALALLGRTRSISTAARGYPGAAPCAPGELDLPLSQAARLIGRYGALARKVAPLCRGYGATPLAGAP
DWCRGEIRWAIRARGAATLEDLVLRRTRLSFGAGLRPETLDDLVALLSEETGRPMDELAAERQAALREPRLMGTRAGP
AQ
G
>MCE8455541.1 hypothetical protein LZ188_02940 [Rhodovulum sulfidophilum]AB15
MAKIMKDDQISPPRRGRRVPRTTDSRHNLGIAPNLLRRNFRAEEPDRIWLADISYEPTDEGWLYLAAIKHIP
>MCE8455542.1 TRAP transporter substrate-binding protein DctP [Rhodovulum sulfidophilum]AB15
MTIKAFAFACGATALTALPAAATDLRLLSSWPDNQAYTENVALPLAEKVSELSDGALTISVVGPDVVPAFEQLEPVQAG
V
FDMLFTHPAYHIGTASIGLGVEGTRADPTLRRSSGIIDTLDEYYQTLGLKALAVAPVGSVAFNFILREPITGDPSLGGRK
IRGTQTYNPVIEGTGGSPVIMPMTDIYSSLQTGVIDGAGTTLTGIAEQKWYEVSNYLSRPKFGTVGLFIFMNIDSWDSLS
PEEQDILTRAAMEIELESTDRFDTLAAEEEAFLIEQGMELTEFSAADAEQVEQVFSDGAWATARITGGDLAEKMHQQA
EE
AGLLQ
>MCE8455543.1 TRAP transporter small permease [Rhodovulum sulfidophilum]AB15
MIRERAHSRQVGGIVDLLVRICFLISCTSLVIILMITIFEITMRYFFDSPTKWVSDSVRYLLAFMIMFGLPDVTRRNEHV
SISLFLDKLRLDHPLRRLLFVISACVCLFVTYLAVDIALTQFSRNLYTQGTWRIPRVWITGSVATGFSVAALAFATLAIW
PKRKE
>MCE8455544.1 TRAP transporter large permease [Rhodovulum sulfidophilum]AB15
MEWALILLTFFSALLLLMLTGINVFVAFLTVNLTATALLIGERGFGLFVNSVTDSVSNGGYATIPLFILMGEILFRSGSV
DVLFDSVDRLVGRVRGRLYAVVIVIATIFGALSGTAVAVSAMLGRSAMPTMEARGYSPRLSATVILAGASLAPIIPPSLL
AIIIGSLADVSIAGLLIAGIIPGIIISIMTGAYVTTSVMRNPELAPPQSDDDEKDRGSALGAVLRMMPFMFIIFMVMGMI
MLGIATPGESAATGVVGSVLVAILYKRASAQMLISALASATRLSVMIMVIVASSTLFSQLVAFTGASRALVAWATDLGLS
PMVMFFILMALPLVLCMFIDQIAFLLMAVPLYEPLVSALGYDPIWFWTIFLINLTVGGVTPPFGYTLFALSAAAPNISVG
QVFRASYPVLVIFLLAMLLLTFVPGIVTWLPSVI
>MCE8455545.1 acetyl-CoA C-acetyltransferase [Rhodovulum sulfidophilum]AB15
MTDLTDAYIVAPLRTPIGKFGGALRPLTADALMVHVVHALLARTGIPAGTLDDVIVSQSYASSEAPCIGRYGALAAGLPI
EVPGYTLDRRCGSGLQAVINAAMSVQTGQADAVMVVGVESMSNIEYYSTDMRWGSRAGSVTLHDRLDRGRVRSQP
QARFG
VISGMPETADTLAREYDITRDQADAFAVRSHANAHAAWENGLFEAEVIPVDVPGSKGAVTTFERDEGIRPETTMDTLS
GL
RVIREGGVCTAGNSSQQNDAAAGCLVVSGAYLEQHGLTPMARLKGWGVAGCDPARMGIGPVPATQKALARGGLTL
DQMDL

IELNEAFASQALAALKGLGVADMDRVNVNGSGISLGHPIGATGVRILTTLLHEMDRRDARFGLETMCIGGGMGLAAVF
ER
P
>MCE8455546.1 PaaI family thioesterase [Rhodovulum sulfidophilum]AB15
MHGGVIASLLDAACGLPVRFIAPETDLVRAVTLTLSVSYIAAPKGPRVRATGRITGGGHRLVFSSGEVRDTDGRLIATAT
GSFKRLAPAAPGPTSKEPT
>MCE8455547.1 thiolase family protein [Rhodovulum sulfidophilum]AB15
MRDVIISGIGMTAFGKFMDRNLKSLSEEAVSLALADAGIAPAQVDQVIFGNAAAGVVTGQEMIRAQSSLRNTGLDGVP
MF
NVENACASSSSAFHLGWLMIASGQAECVLVMGSEKLTHEDKAVSFGAFSRAVDLEEPLPEGYQAGSGSLFMDLYAVKA
RK
WLEKTGGAPEDFGRVVVKSRHAGSLNPVAQFRKETTLDEVMASRMISDPLTLFMCSSIGDGAAAIVLTSPEFAKKLDTP
D
IRVRASAVVSAKAHGERDLVAVIASRKAYEMSGIGPEDLDVIELHDASAPAELIHYENLGLCAPGGAIEMIRNGDTAIGG
RVPVNPSGGLLSRGHPIGATGAGQIIELVQHLRGTAGARQRDGAKVALAENNGGQLAGDSAVAVVTILSL
>MCE8455548.1 SDR family oxidoreductase [Rhodovulum sulfidophilum]AB15
MSDKVAIVTGGAQGIGLGITRALLDKGHRVAVWDRDAAALAALPEGVFGQEVDVTNGAGVSAAHAAVTARWEAPSI
LINN
AGIARDKRIVNMKESDWDAVLNVNLKSQFLCCKAVIPAMVDAGFGRIVNIASRAWLGGFGQANYSASKGGVVSLTRT
LAI
EYARKGITVNAVAPGIVETPLMASYTDEQRARLEATVPMGRMGQPDDISKAVAFFADAANAYVTGQLLYVCGGRSLSS
PS
V
>MCE8455549.1 enoyl-CoA hydratase/isomerase family protein [Rhodovulum sulfidophilum]AB15
MTVNMSTADHITVLELDDAPALNALTPEGLMDLRRALRAAQDDDAVRVIILTGAGERAFCTGANLKKTFPPKNGFARN
HL
LGREAEALDGGYTRLLDLSDLQIWKPMIAAVNGYCLGGGLELALQCDLRIASTSASFALPEVKVASVPAVGGAQALLRAI
PSAHAMKMALTGDRIDAQTALDIGLVSDLTDPEALLPEARAIAARIAANGPLAVQMVKKLSQETAHLSPPEFMAQSNT
AW
GLLRDSADRIEGRTAFGEKRAPNFTGN
>MCE8455550.1 acyl-CoA/acyl-ACP dehydrogenase [Rhodovulum sulfidophilum]AB15
MLADSAKRLSEDTLMPILAREAADKPLSKPVMLEIYGHLAEFGITAARLHEDFGGSGLSMLDYGLIMEQLPPAIALSLLS
HDGSTVRFNAGADEAVRDAYLPDFREGRKIVCTANSEPNAGSDSNTISTKLEIEGDRAFITGTKMWITNASVCDCMIVS
C
SAGNDERGRPVPQRVLVDLSQSKVGLAETPLAGLKQGHLCEVTFDRTEVPLSHLIGSPGDAGKYMTIVWNANRPLIGL
MA
LNIARRAYDMALAHVTDRKQFGSPLAAKQLVQQDLSDMEAAITSARLTCLMALATLDTGARSNGISAMAKRLATENA
NRV
VHTAMQLCGGMGVTEELGLAQLWADARVFQVPDGTQGILALIHGRDITGTAAWR
>MCE8455551.1 glutathione S-transferase family protein [Rhodovulum sulfidophilum]AB15
MNILHWSPRSPYVRKVMVAIHEKGLLDSIETVRTLADPMFPPRDFFATNPLAKIPTLERDGASPIWDSRVILEWADLSSE
SGPRLFPVDPETRLQALTDEALGTGMLETGMALLIERHMRAADRQDARVFDVCAAKFGACLDYLEANAARLAARDFD
AGH
LSIGVALCYLDFRFGDLQWREGRPGLAAWHDGFAARPSVLATTFRDDPRPTE
>MCE8455552.1 ATP-binding cassette domain-containing protein [Rhodovulum sulfidophilum]AB15
MKGLRLQDIAIARGAEPLIAVDVHVAPGEVLSVMGPSGSGKSTLLAAVIGTLPPGFAMTGRVLLNGRKVTGLPPEDRRI
G
ILYQDELLFPHLSVGGNLAFALPRSLRGRKARRARVEAALEEIGLPGFAKRDPATLSGGQKARVALMRMLLAEPEALLLD

EPFSRLDATLRDQIRALVFARARDRGLPVLLVTHDRADAEAAGGPVIGLGA
>MCE8455553.1 type III PLP-dependent enzyme [Rhodovulum sulfidophilum]AB15
MHATQADFGGGPDLLSSSRVETYIRTNRFERPTLVIDTDRVADRFHALRAGLGRAAIHYAVKANPAREIVERLVGLGCH
F
DAASRGEIELCLGLGAAPHQISFGNTVKRVADIAFAHRAGLTLFAADAEEELDKIAEHAPGAEVYIRVLVTATEADWPLS
RKFGCAPGKVMPLMTRAVALGLKPVGLSFHVGSQTRRADMWLPVLNEVAEIWQAARAEGFDLDLLNIGGGFPAFYG
EEVQ
PPAMYAAEVMRLVTERFGAVPRVMAEPGRGMVAEAGAIAAEVMLVSQKSEDDTYRWVYLDIGKFSGLAETMDEAIR
YQFA
TDRDHEPTAPCILAGPSCDSADVLYEKRPVQLPVGLRSGDKVIIRNCGAYTSTYSSVGFNGFPPLDVVVI
>MCE8455554.1 L-lactate dehydrogenase [Rhodovulum sulfidophilum]AB15
MKVGIVGAGMVGSAAGYALALTGAASRIVLVDRSEALALAQAQDIAHATPFTAAVTVEAGGYEALAGADVVILAAGV
GQK
PGESRLALLERNAAVFEDVIRAVLRAAPDPILLVATNPVDVMTHVTAKIAGLAPGRVFGSGTILDTARFRSLVAHHLGLA
PQSIHGYVLGEHGDSEVLGWSGARVGSEPLTSVAAQLGSAITADVRARIDDGVRRAAYRIIEGKGATNYGIGAGLARIV
Q
AIRRDEGAVMSLSLVTPEVTGVHNVALSLPRVVGASGLRSTLMPTLDAQEVAALTASARMLRETSDALGL
>MCE8455555.1 UDP-N-acetylmuramoyl-L-alanine--D-glutamate ligase [Rhodovulum sulfidophilum]AB15
MIPVRGVEGQKMAVLGLGRSGLSAARALRAGGAEVLPWDDNEAARAAAEAEGFVPVEPTRAGALDGVASLIVSPGIP
HLY
PGPNRAIAAAWAAGVPVDNDIGLFFRSLATPDWDSFDVAPKVIAVTGSNGKSTTSALIHHVLEHCGRPSQLAGNIGRG
VL
DIDPPGEGGAVVLELSSYQTDLARALTPDIAVFTNLSPDHLDRHGGRGGYFAAKRRLFAEGGPDRAIIGVDEDEGMFLA
N
QLAEGAADDRVIRISSGQKLAGPGWLVFARKGFLSEWRKGRQVASIDLRAIAGLPGAHNHQNACAAYAAARCLGIGPK
QI
EEALGSFAGLPHRSQLVAEADGVRFVNDSKATNAASAAMALAAFPKIRWICGGLEKEGGLAPLLPHLGHVAKAYVIGRE
A
AAFALHLKDAPHEVCTTMETAVARAADEALPGEVVLLAPAAASFDQYDSFERRGEDFIARVQARLGV
>MCE8455556.1 hypothetical protein LZ188_03015 [Rhodovulum sulfidophilum]AB15
MQMNPVEDTYNYLSPAARLGSPSLHAVTPDVKLKSWNGVPPYAVWAGLGYLLVLAVIFSDQIVPYCEANFAQPQNCA
PIF
GKIIPIFVAGIILAPALAGAVQRMGGNKATGLAFSFAVAVVPPVMLGWSYLLG
>MCE8455557.1 phospho-N-acetylmuramoyl-pentapeptide-transferase [Rhodovulum sulfidophilum]AB15
MLYWLADLSDGGGVFNLFRYITFRAGGAFFTALLFGFLFGRPLINLLRKRQRNGQPIRTDGPEGHIVSKAGTPTMGGVLI
LGGLIVSTLLWGRLDNPYIWMLLFVTLSFGAIGFADDYAKVSKNNTKGVPGKIRFGLGLLIAAIAGYWAACAHPADLSYN
LALPVFKDTLIHMGVFFVPFAMIVIVGSANAVNLTDGLDGLAIMPVMIAAGALGVIAYAVGRVDFTDYLEVHYVPGTAE
I
LIFSAGLIGAGLGFLWYNAPPAAVFMGDTGSLALGGALGAIAVATKHEIVLAIVGGLFVVEALSVIIQVGYFKATGKRVF
LMAPIHHHFEKKGWAEPQIVIRFWIISLILALIGLATLKVR
>MCE8455558.1 UDP-N-acetylmuramoyl-tripeptide--D-alanyl-D-alanine ligase [Rhodovulum sulfidophilum]AB15
MSALWTATEALAATGGRVTRDWSVSGVSIDTRSLKPGDLFVALKDQRDGHDFVAEALARGAGAALVSRLPEGVDESA
PLL
IVPDVLAALEALGRAGRARSGARVVAVTGSVGKTSTKEMLRAVLAGQGRVHAAEASFNNHWGVPLTLARLPRAADFA
VLE

IGMNHPGEIAPLARLARPHVALITTIAPAHLEAFDGLAGIAAEKAMIFEGLEPGGIAVYPGDLETSPVLAAGAAGRASAD
IRFGAEDGCDFRMVRADLHADSTVVRAEARGRDLLFNIHAPGRHFASNALGVLAVAEALGVDLVVAACDLGQWRPPG
GRG
ARLRIALDPVEESLSVEVIDDAFNANPASLAAALEVLAAARPTDGLGRVARGRRIAILGDMLELGPDEQAMHAGLADLD
A
MARVDLVHCVGPRMRALYFALPLSRRGQWFDEADLLAAKVHSLVDAGDVVLVKGSKGSRVSVVVDALKRLGQGGPG
SDEE
LS
>MCE8455559.1 UDP-N-acetylmuramoyl-L-alanyl-D-glutamate--2,6-diaminopimelate ligase
[Rhodovulum sulfidophilum]AB15
MTIRTETTLGDLGLTAQGGRNTHITGLSVDSRQTRPGHLFAAMPGTRAHGAEFVPFALRMGAAAVLTDRAGARIAAE
ALA
GSEAALVVAEDPRQALAGAAALFYGAQPATMVAVTGTAGKTSVANFTRQIWAGLGHAACNLGTMGVQGAYEAPLA
HTTPE
PLTLHRLLAEMARAGVTHAAMEASSHGLDQRRLDGVALKAAAFTNFSQDHLDYHAGFEDYFAAKAGLFDRVLPEDGT
AVI
NIDDARGAQMLGHARARGQTILTVGRSEEAELRILAQRYDATGQDLRFAWEGRSQQIRLDLIGGFQAENVLAAAGLVI
AA
GEAPGQVFTALPELATVRGRMELAATRANGSTVFVDYSHKPGALAAAIASLRPHVMGRIVVVFGAGGDRDRGKRPLM
GQV
ACEQADVVIVTDDNPRSEDPAAIRAEILQGCAEAIEVGDRAEAILRGVDALEKGDALLIAGKGHETGQIVGDAVYPFDDA
EQASVAVAALDGRLA
>MCE8455560.1 penicillin-binding protein 2 [Rhodovulum sulfidophilum]AB15
MIRTPLRPLARILEARAKGENPDAIERENKRLRHEKMRDQARVRAEGRLLVLGVVFCAAFAVVGVRMGLLAGSVPSEPR
A
TAPGASIVAQRADIVDRNGNILATNLATHSLYAQPPLMIDPKAVARELARIFPDLDEARMVKDFTGSRKFVWIKKKISPE
QMQLVHDIGDPGLSFGPREMRLYPNGALAAHILGGAGFGREGVQSAEVIGVAGVERYFDDYLRDPANGGQPLRLSLD
LTV
QAAVERVLAGGMKLMNAKGAAAILMDVHSGEILSMVSLPDFDPNERPRQLVKGDPSDSPLFNRAVQGVYELGSTFKT
LTL
SQALEEGIVTPNTMIDTKGPLVWGRFRIHDFHNYGSSLSVTTVLVKSSNIGTARIAQRIGGERQRAFLSRLGLLDPSPVE
LVEAAGAKPLLPANWSELSTMTISYGHGVSTSPLNLAAAYSTIANGGRLVRPTLRAGGGRLGERVISEETSRQIRKMLRE
VVVHGTASFGEVPGYRVGGKTGTADKPKPTGGYYDKKVIATFASMFPAEAPQYVLIVTLDEPQDTTGPKPRRTAGWTA
VP
VAAEIIRRAAPLLGLRPEIEQAPGFGVTLTAN
>MCE8455561.1 16S rRNA (cytosine(1402)-N(4))-methyltransferase RsmH [Rhodovulum
sulfidophilum]AB15
MTVADNSAPAEPPHIPVLLRPLLEAVAPVSGLWLDGTFGAGGYSRGLLEAGADRVIGVDRDPSVADLAASWAAGWG
DRLQ
LVEGVFSELDRHAEAPLDGVVLDLGVSSMQLDQAERGFSFAKDGPLDMRMGAEGPSAADLVASASDTALADILFHYG
EER
ASRRIARAIVKARAIAPIVTTAQLAEVVARCLPPHRPGQTHPATRSFQAIRIAVNDELGELVRGLEAAERALKPGGKLAV
VTFHSLEDRIVKRYLQVRSGQAAGGSRYAPEARPDPARFELVTRRAVAPDPVELEANPRARSAKLRVAIRTGEPAGPVD
R
ARLGLPKIMED
>MCE8455562.1 division/cell wall cluster transcriptional repressor MraZ [Rhodovulum
sulfidophilum]AB15

MARRFRGESQHKVDAKGRVSIPALFRRVLEACDPDWTEGLRPQLVIVYGDHRRKYLECYTIEAIDEVDEKIAALPRGSM
Q
RRMLERMFHGQSHPTEVDNDGRLTLPQKLRDKIGLEGEAFFIASGDTFQIWKPETYETEELAKTEAWLDELPDDVDPLS
F
LDGAVAEPARDG
>MCE8455563.1 bifunctional NADH dehydrogenase FAD-containing subunit/selenide, water dikinase SelD, partial [Rhodovulum sulfidophilum]AB15
MSPGPQLVLIGGGHAHALVLRGQPLPSDISVTLIDPSPKAAYSGMLPGFIAGHYPRAALEIDLARLARAAGARFVIGRAE
GLDRARRHVLVAGQAPVPYDILSLDIGITSDLPDLAGFADHAVPAKPLGRFADRWAGFLAGVAAGRSAPRVAVIGAGV
AG
VELALAARYRLARNGE
>MCE8455564.1 iron ABC transporter permease [Rhodovulum sulfidophilum]AB15
MNVKALICALLLPLPALGQGFAGLGGTAEGYALPDPAARLEFPRDHGAHPGFRIEWWYVTANLEGEDGRQYGIQWTL
FRS
ALAPGDPGTGWQNRTAWMGHAAVTSADTHKSAERLARGGIGQAGVTLAPFRAEIDDWHLVSTAAPGADPLSAARL
GASGA
GFAYDLDLVATGPLVLQGAQGYSVKSRDGQASHYYSQPFYRAEGRLIFPDRTVRVTGRAWLDREWSSQPLAADQTG
WDWL
SLHLDDGAKLMVFRLRGAGDDFLTGTWIAPDGTPAPLPPGAVEMIPKAETQLDGRSIPTLWQIRLPARGLDITVTALQP
G
AWNATRVPYWEGPVTARGSHPGEGYLEMTGY
>MCE8455565.1 FtsX-like permease family protein [Rhodovulum sulfidophilum]AB15
MIRTAITALLSHWRRHPLQLAGLFLGLALATALWSGVQAINAEARQSYDRAAAVLAQDRLDSLVARDGQHIGQQVFLD
LR
RAGWMVSPVLEGRWSHRGVPLRVLGVDPFTLPAGSLPEAVADESRSGAFLSAEGLAFAAPQTVEELTRIGAPGLPPLA
AA
PGMAPGLLLVDIGTAQRLLRAGGEISRLLLAPEQPLRQRPLEEIAPGLIRQSPAEQADMGALTGSFHLNLTAFGFLSFAV
GLFIVHSAIGLAFEQRRPVFRTLRALGLPLRLLVGLLAAEIMGFALIAGLAGIALGYVIAAALLPDVAATLRGLYGASVS
GSLSLRPEWAVAGLGMALAGAGIAAAQSLWRLSRMPVLAPAQPRAWSRASARSLAWQTALAGLLALIALGAAVRGE
GLVG
GFVLLGALLLAAALALPGALAVALALGERLSKGVIAQWFWADTRQQLPGLSLALMALLLALSANIGVGTMVSSFRLAFT
A
WLDQRLPSELYVTGRTEDEARRLAEWLAPRTEALLPIWKIDGPLLGRPGEVYGVADHRTYPDNWPMIRGDASVWKSL
ARG
EGVIVNEQLWRRADLHLGDPVTLPGGAIRPLIGVYADYGNTKGQAILGIADLLALYPGIDRRRYAIRTDPANVPALRSAI
EDDFGLPADNMISQQEVKQVSLMVFERTFAVTAALNVLTMGVAGFAMFMSLLTLSTMRLPQLAPVWALGLTRGRLA
RLEL
LRAVLLALLTMALAVPVGLLLAWVLLSVINVEAFGWRLPMQLFPKDWLWLGALALIAAGQAAAWPAWRLARLAPVH
LLKV
FADER
>MCE8455566.1 ABC transporter ATP-binding protein [Rhodovulum sulfidophilum]AB15
MLLSLSDIGKGYETGGARVEVLGGVDLALAPGETVALTGESGSGKSTLLHLCAGLDRADRGRIEVAGQDLTHASERRLA
A
LRRETVGLVFQQFNLIPSLDVAANLAFQARLAGRHDPDRAAKLADRLGLSGLLSRYPEQLSGGQQQRVAIGRALAIRPAL
LLADEPTGNLDEATGDAVMELMLELVAEAGAGLLMVTHSPRLAARLGRRVHLAGGRLAA
>MCE8455567.1 DUF2892 domain-containing protein [Rhodovulum sulfidophilum]AB15
MTLDRAVMMFAGCMILLSLGLGTWVSPLWYLLTAFVGLNMMQASVTGFCPAAILFKRLGIRPGTVFD

>MCE8455568.1 metal/formaldehyde-sensitive transcriptional repressor [Rhodovulum sulfidophilum]AB15
MSHLTKDKPKLLARVRRLKGQIEAIERAIEAEKDCGAILQLVASVRGATAGLTQELIEDHLRHHVILVEDETGREQGGAE
LASVLRTYLK
>MCE8455569.1 CDF family Co(II)/Ni(II) efflux transporter DmeF [Rhodovulum sulfidophilum]AB15
MHDHAPSDSSHPPRNDTGEAAALCHPRGPGGPDHVFLGADHDRNARRTWLVIGITATMMVIEIAAGTVFGSMALLA
DGWH
MATHAAALSIAALSYMFARRHAHDPRFTFGTGKLGDLAAFGSAVVLGLVALLIGWESLVRLVHPVGIHFGEAILVAIVGL
AVNIGSAVLLHQGPHAHGHHGHDHGHAHADADHHSHGHAHHHEVDNNLRAAYLHVLADALTSVLAIAALVAGRAF
GWVWL
DPVIGLLGALLIARWSFGLMRDSGGVLVDFLPRSETLPQKIREAIETDGARVVDLHVWQLGPGHRGAIVAISDADPRPA
A
EYRGRLAHLGRLSHVTIEVEAV
>MCE8455570.1 SLC13 family permease [Rhodovulum sulfidophilum]AB15
MSLDQIVVLALLAIVFLSFIKETYPPEVTALAASAALLATGIIGTGDFLTVFGSSAPITIAMMFIISAALERTGVLATLG
EFMTARAGGSYLRALFLMMIVTVLASAFMNNTPVVILLTPVMISVAASVGVAPSRLLIPLSFSAIFGGTLTLVGTSTNIL
MSGVARETGQPPISMFEMTLPGMIFVLVGMAYLAIAGRFLLPDRQSLAHLVGRETRRRFLARLLIPAGSRYIGKRLDDLP
FNTAETRILDVIRGEVSMRRRLDDLELERGDRLVLKTGTGEILGLKENGQVAFRDLSDRGVEPVTADQTVTLEASIGPNS
ILRGRPLGELRLRRNYGVYVMAVHRADRNLSERTDDIRLQFADTLLLEGPAEGLRRLVEDGGVVNLSAPSERPMRRAKA
P
VAIATMMAVVGLAAFDVMPIAGLAVIGAVAVMLTRCVDPEEAFNAIDWRILFLIFGMLGLSKGMEETGTAQLIVDWV
VGL
LGGFGPLAILAAVYVLTSALTEMISNNAVAVLVGPIVIGLAVQLGVDPRPFIMAVMFAASASFATPIGYQTNTFVYGAGG
YRFRDFLKVGLPLNIIFAVVAVGVIPLFFPF
>MCE8455571.1 hypothetical protein LZ188_03100 [Rhodovulum sulfidophilum]AB15
MRRRNAGALIIFLTAYLGLFAFILTPESLWTVTSSAVGQSQSARH
>MCE8455572.1 Rrf2 family transcriptional regulator [Rhodovulum sulfidophilum]AB15
MITQKTKYALKAMIALAEEAAGAGEALTIEQISQRSGAPKRFLEHILLDLKRTGYLGSRRGRSGGYLLIKEPRQVSIGEL
LREVDGPIAPLPCLSRRAYQPCDDCEDEESCRIRRLFGQVFYAYLLLIESLTLADLLESPERIERLVADAALPPV
>MCE8455573.1 hypothetical protein LZ188_03115 [Rhodovulum sulfidophilum]AB15
MLLHLADPRVGRPQALEWGRFRSAICSTKSGAVEAIEGMAASGAVIPYRRPMLMTADPADPDQTLTPLDENPLALEN
>MCE8455574.1 hypothetical protein LZ188_03125 [Rhodovulum sulfidophilum]AB15
MEKAHRKTAREILEYRLTKPDNLSSGEKNGPEAVSPSGVQAERMNRTIREARGERFHHAPRDQLRRQLPAVMAADNF
ARR
LKTLGGVMPREEICKIRMSEPVRSLLIPIRQMPGSDS
>MCE8455575.1 M10 family metallopeptidase C-terminal domain-containing protein [Rhodovulum sulfidophilum]AB15
MFTNGNDHETIVPSGSAQTFDALGGFDTVTIDATSLLAGLSGTLLGTTWSSTYNSVSLSIANVEQLVVIGGAGNDAITGD
DGNDTLLGGTGNDYLNGEGGTDLINGGVGNDEIWAHGGDTVIGGAGTDMLHLVLGPGAGQSIDLTTGAGTGVTWS
GFERI
GASLSGGERDVYAGFLLADIDGAGCLHLDYSGTDLGGRTALSVDLDVYGGTIHLSDSSTQSFGIYGFDDFDILGSAGNDL
IDISEAGGGTVDGGAGNDTIWGSFESSVLDGGDGHDQIHGCSDYDILHGGNGNDTIYGYHYSLSELWGDDGDDYLDS
GDS
DGELHGGAGNDTLIAGEGAPLLDGGSGDDYIYTQLGQDRIYGGTGNDTLVITAPYYPIDYDLDISQGGDIRGIEYADITL
AEGNDTIRIGIASVYIDETGGTDHLILDYSETDENGRTATAVVFDLAANASIATLTDSSTISCNLDLDLFTVIGSGGHDW
IDGSIATQACTFKGGKGADTLLGGSGNDTITGGGGKDTIEGGAGNDRLEGGYGNDRIDAGTGNDLLIGGAWDDVLNG
GSG

NDTFLYWAGANGYDMVDGGAGAADRILTTADNVTIGLRGLAGVEIVDAGGHVGVVLQGSAEHNLLDFSGTTLSGIEH
IDG
GSGRDTIIGSAGDDVIIGGAGGDILTGMAGADIFDFNVASHSNGANIDRITDFVRGEDLLDLSGIDANSGLAGDQDFDFI
GTAAFSGTAGELRADLTAVPGMTVILADINGNGAVDLEIRLDGLYSLAATDFLL
>MCE8455576.1 bifunctional proline dehydrogenase/L-glutamate gamma-semialdehyde dehydrogenase, partial [Rhodovulum sulfidophilum]AB15
PETLDLPGPTGEANRLTTLARPPLLCLGPGPGASAAQAEAVRAQGGAAVTVEGLLEPATLKDIEGISGALWWGEAETGR
A
YALALAERDGPILPLIAGMPDPAHVRLERHLCIDTTAAGGNAALLAEAGSA
>MCE8455577.1 rhomboid family intramembrane serine protease [Rhodovulum sulfidophilum]AB15
MFPLRDHNPSSRFPFVTLALIAANVAVFLFCLPLFSNPKALHALFMEWGMVPALITQSGDWSGMLTSMFLHGGVLHL
AGN
MLFLWIFGDNMEDRFGHLGFLLFYLAGGVAAALLQYAAAPSSPVPVVGASGAIAAVMGGYLLLFPKARVDLLIILIVIFR
VIPVPAWLMLGLWFGLQLYSGASAAADEGGVAYWAHSGGFIAGLALTLPLWLKLGGPAFWNRTAGSPPHPVGIYRAT
SVP
RIRRRR
>MCE8455578.1 hypothetical protein LZ188_03145 [Rhodovulum sulfidophilum]AB15
MTEDARGAGERAPLRLRRFLNDTSGAVTVDWIVLTAAIIGTCIAIFASIQSGAVGMGDSVRAALDAVGVVELGEFGRDD
>MCE8455579.1 inositol monophosphatase [Rhodovulum sulfidophilum]AB15
MQGSANLNIMIKAARKAGRSLVKDFREVENLQVSMKGAGDFVSRADTAAEEILRQELRGARPTYGWLGEETGGEDG
DDPT
RRWIVDPLDGTTNFLHGLPHWAVSIALEHKGEIVSGVVFDAAKDELFFAEKGAGAWMNERRLRVSSRARMIEAIFATG
VP
FAAKRTLPATLQDLARLMPHCAGVRRWGSAALDLAYVAAGRYDGYWERELNAWDVAAGLLLVREAGGLVGPIHEGR
DPLE
HGEVLVANEAIYDHFAKAVRNT
>MCE8455580.1 LysR family transcriptional regulator [Rhodovulum sulfidophilum]AB15
MHLELRHLRTIKAIHDAGGLARAADRLNITQSALSHQVKGLEEQVGMELFVRRTKPLRLSSAGLKLLRTAERVLPEIAAL
QAEFEGMTSGRAGRLHIALECHACYDWLLPVLDSFRKAWPEIDVDIRPGLSFEALPALAREEVDLVISSDPETIDGIGFA
PLFDYEPVFLCAASHPLAGRAFIRAEDFRGETLITYPVERSRLDVFTGLLGPARVEPLAQRQVELTAVILMLVASGRGVT
VLPDWVVREARSNADFAVLPLTEGGLTRRMYAATRSEDTAKPFMAHFVRLARSEPVKLQRGTAG
>MCE8455581.1 methylenetetrahydrofolate reductase [NAD(P)H] [Rhodovulum sulfidophilum]AB15
MTAPRVSFEFFPPQTLEASFRLWEAAQALAALKPAFVSVTYGAGGTTRKLTHEAVQTIHNHYGLNVAAHLTCVDATRA
ET
LEIAEAYSAAGVSEIVALRGDPPKGQGAFRPHPDGFANSCELIEALAATGRFRLRVGAYPDPHPEAGAPDADVRWLKRK
F
EAGADTAITQFFFEAETFFRFRDACEKQGIDGAKIIPGVLPIEKWAGVRRFARRCGAHVPTHVDEAFETAIRDGREELLG
KAMATELCTDLMEGGVRDFHFYTLNKPHLTRDICHALGVLPDAHLEKVA
>MCE8455582.1 xanthine dehydrogenase family protein molybdopterin-binding subunit [Rhodovulum sulfidophilum]AB15
MERTFGKSQTGPRFEDWRFLTGTGRYVDDIAPASALHAVFLRSPVAHAEIRAIDLEDARAAPGVVAVLAAGDLRAAGI
GA
AMGATVVTNRDGSAGAAPARPLLAEGRVRFVGEAMAVVIAETLLAAKDAAERIVFDLHDLDPHLALTVGGPQIHPEAP
LN
LAYDWATGDEAAVHEAFRTAARTVRLEVADTRVIVNSMEPRGAWAEWADGRLHLCVNGQGVWDQKAKLAAMLDL
PETAVR
VTNPDVGGGFGMKAMSYPEYYVIAHAARALGRPVRWMSDRTEAMLSDNAGRDLVSMVEIAFDAGHRITGYRVETLS
NLGA

YNSEYAQPIQSELFAKVLTGAYDIPAAFLAVKGVYTNTTQVDAYRGAGRPEAIYALERTMDLAARELGLDPWELRRRNFI
APSAFPYRTPTGESYDVGDFARVLARVETMADRAGFAARKAESAARGRLRGLGLCYYIESILGDPTEGAEIAFPEDGTVA
LYVGTQSNGQGHETVYARFLAERAGLPVEAIRVIQGDSDLIARGGGTGGSRSVTTQASATLAAIEVMVAAFTPFLAAEM
G
VELSDLSFDEGRFRAPGSNLTPGLIEAAAMARRTGRTDLLRHRADYTLPGRSYPNGAHVAEVEIDPETGETRVLRYTVAD
DFGTLINPRLAEGQVHGGVAQGLGQALCERVVHDDYGQLLTASFMDYAMPRASDLPFVQFGTEPVPSTANPLGMKG
CGEA
GTVGAMAAVGNAVLDALWSAGVRRADMPFTPERVWRLLQAAA
>MCE8455583.1 valine--tRNA ligase [Rhodovulum sulfidophilum]AB15
MPMEKTFDAAAAEQRLYEAWEKAGCFTAGANASRPETFCIMIPPPNVTGSLHMGHAFNNTLQDILVRWHRMRGFD
TLWQP
GQDHAGIATQMVVERELAKEGNPGRREMGREKFLEKVWDWKGQSGGTIITQLKRLGASCDWSRNAFTMSGAPGAP
AGEDG
NFHDAVIKVFVEMYNKGLIYRGKRLVNWDPHFETAISDLEVEQVELDGNMWRLRYPLEDGATYEHPVEFDEEGTPTAF
ET
RDYLVVATTRPETMLGDTGVAVHPEDDRYRHLIGKTVRLPLCGRSIPIVADDYADPTKGTGAVKITPAHDFNDWGVGQ
RT
GLRAINIMSTRAAMFLKDNADFAEGCDDALVADCIAQFDGLDRYEARKAIVADAEARGWLDGIDSDRHMVPHGDRS
KTAI
EPYLTDQWFVDTQKIVGPALDTVRKGMAAQEAGQFDETAGYTRILPERDAKTYFHWLENIEPWCISRQLWWGHQIP
VWYG
LDLDASHFTDDEGDNALDDVELYRLLGEGFVQRGARHHCAADFEAVKAQFQDVLAGLPAPLHAMRVVEVSGRAAAE
EALA
ESLAEYNLSQDPTGLIYPIWRDPDVLDTWFSSGLWPIGTLGWPEETPELAKYFPTDVLITGFDIIFFWVARMMMMQLA
VV
DQVPFHTVYVHALVRDEKGKKMSKSLGNVLDPLELIDEYGADAVRFTLTAMAAMGRDLKLSTSRIAGYRNFGTKLWN
ATR
FAEMNGVFEGYTPTGEIPAPTETVNTWIVGETARVREAVDEALSHYRFNDAANALYAFVWGKVCDWYVEFSKPLLMD
GTE
AQKAETRATMAWVIDQCLILLHPIMPYITEELWGTTAERAKMLVHADWPAYGADLIDLQADREMNWVIALIEEIRSAR
AQ
MNVPAGAKIPMLHTELDAAGQDAWARNEALIRRMARIDSLSAAETLPKGCVTIAVEGGSFALPLAGIIDVAAERARLEK
T
LSKLEKDLKGLEGRLKNPKFVESAPEDVVEETRELVEAKTGEAAKLRGALERLAELG
>MCE8455584.1 threonine--tRNA ligase [Rhodovulum sulfidophilum]AB15
MGQITLTFPDGNQRDFEAGITPAEIAAGISNSLAKKAISAQVNGRHFDLQWPITEDASIAINTMKDDAPALELIRHDLAH
IMARAVQALWPDVKVTIGPVIENGWYYDFDREEPFTPEDLGRIEAKMKEIINARDPVRTEIWDRARAVKHYEDNDEPY
KV
ELVGMIPEDQQIRMYWHGDWQDLCRGPHLQHTGQVPADAFKLMKVAGAYWRGDSDRPMLQRIYGVGFRSRQDL
KDYLTFL
EEAEKRDHRKLGREMDLFHLQEEAPGQIFWHPNGWKIYTTLQDYMRRQQERDGYVEVNTPQVVDRKLWEKSGHW
DNYQEN
MFIVEVDEEHARQKAVNALKPMNCPCHVQIFNQGLKSYRDLPLRMAEFGSCNRYEPSGALHGIMRVRGFTQDDAHIF
CSE
DQIEAEAKKFIDFLAKIYADLGFEDWTVKLSTRPEKRIGSEESWDRTEEALGSACRAAGYDYAVNEGEGAFYGPKLEFVL
RDAIGRDWQCGTLQVDPNLPERLDATYIGADGEKHRPIMLHRAVVGSFERFIGILIENYAGKLPFWIAPRQVVVASIVSD
ADNYVAEIVATLKAAGVRAEADIRNEKINYKVREHSLAKVPVILAIGQREVEERTVSVRRLGENRTETAALDAIVATLTA
EAKAPDLA

>MCE8455585.1 arsenate reductase [Rhodovulum sulfidophilum]AB15
MIIYGLKTCDSCRKAKSALERMGKAVDFHDMRDDPLSREELARFLTALGPDLVNRRSTTWRGLDEAARAGDPLDLLEA
HP
TLMKRPVIEAGGQLHLGWNAKTEAALAG
>MCE8455586.1 cold-shock protein [Rhodovulum sulfidophilum]AB15
MPTGTVKWFNTTKGYGFIAPDDGGKDVFVHISAVERAGMTGLSDNQKVAYEMIEGRDGRKSAGDLRAI
>MCE8455587.1 DUF2177 family protein [Rhodovulum sulfidophilum]AB15
MIAVILYLVTVLVFLALDALMLRHVMQPLFARYLGDWMRDRPRMGAAAVFYMAYVLGLVWLVSWPALVRGNPGQA
LIEGM
VVGAMAYGTYEFTNFATLKRWSPLQVTLDTCWGTLLTGISACIGVFVARIFV
>MCE8455588.1 bifunctional DNA-binding transcriptional regulator/O6-methylguanine-DNA methyltransferase Ada [Rhodovulum sulfidophilum]AB15
MIETTIEQDARWNSVAARDAGADGNFVYAVKTTGIYCRPSCPSRTAKPQNVRFYETPAEAEAAGFRPCQRCNPNGQS
LAE
VNATLVAEACRLIEASDEMPKLDELAARIGMSPYYFHRQFKAITGLTPKQWAAAHRSRRVRAELTDQTANVTGAIYGA
GF
NSNSRFYEQSNKVLGMTPTAYKNGGKDAEIRFAVAQSSLGAILVAQSAKGICAITLGDDPDELVRGLQDLFPKANLIGG
D
AGFENLVARVVGFVEAPQIGLDLPLDIRGTAFQERVWQALRQIPPGQTASYAEIARRIGEPKAVRAVAQACGANKIAVA
I
PCHRVVRNDGALSGYRWGVERKKALLEKEAQP
>MCE8455589.1 2OG-Fe(II) oxygenase [Rhodovulum sulfidophilum]AB15
MSGKDYIPKPSAVTSARNRLDKYDWETLAGEMNGYGCAVLPGILTQKECADLAALYPREEHFRSHIHMARHGFGKGEY
RY
YKYPLPDLIGGLRTALYPHLADIANGWNERLGGDQLYPKEHADFLRQCHEAGQVRPTPLLLQYVPGDFNCLHQDLYGD
LA
FPIQVAFLLSEPGRDFTGGEFVLTEQRPRMQSRAEVVPMRQGDAVAFAVHNRPVRGTKGDYRVTLRHGVSRVRSGM
RHTV
GIIFHDAR
>MCE8455590.1 SDR family oxidoreductase [Rhodovulum sulfidophilum]AB15
MFLLEGKVAVVTGASSGIGRAVARMFAEHGAAVVLVARRQARLDEVVDEIRSKGGRAYASAGDIGLAEAHERAVADAI
RE
FGGLDIAINNAGTVGPAKPLAEIAPEDWQHTLTINLTAAYLGARSQIPAMLERGGGSIVFTSSFVGTSVGLPGMAAYGA
S
KAGLMGLVKGITADYAARGIRANALLPGGTDTEMAGDQSQKDWAAGLHAMKRIAQPEELASVALFLASPMASFVTG
SALF
ADGGNSAVK
>MCE8455591.1 LysR family transcriptional regulator [Rhodovulum sulfidophilum]AB15
MNFRHAANAQSGVSARVKALEEGLGILRFERHARGVRLTEAGHHFV
>MCE8455592.1 hypothetical protein LZ188_03215 [Rhodovulum sulfidophilum]AB15
MAGQGFGIAIVGAATALLPTTGVIFLPFTDEAEPVAFTAVWSAFATPKTCSTSRAG
>MCE8455593.1 hypothetical protein LZ188_03220 [Rhodovulum sulfidophilum]AB15
MDGVSLRLPTAAWSTMVRAGGLPVYYPPAFFWWWFSFDAYAPGIFMEAAAIATSGGILAIAAAIVMSIIRAREAANV
ATY
RSARWAEDREIHSAGLLGPDGVVLGRCDRDYHVS
>MCE8455594.1 Flp pilus assembly complex ATPase component TadA [Rhodovulum sulfidophilum]AB15
MTSNDCRAEAIQRGARPRNRPAARRPGRGRGDVEPGRPPLGGSAVRRALRHGRTAVRRRWRTHPATRCPSYWSGG
SCPQP

ARLGRPAGERRAVRGPAAARGRSPDLRDPQADQHRVKARRLCGCRDHVPRSGEPLREAVSARANILVVGGTSTGKSTL
TN
ALLAEVAQTQDRVVIIEDTREMQCAAPNLVALRSKDGVASLSDLVRSSCRLRPDRIPIGEVRGPEARGLLKALGTGHPGG
IGTIHADTGIGAPRRLEPLIQEVSSRSHAR
>MCE8455595.1 TrbC/VirB2 family protein [Rhodovulum sulfidophilum]AB15
MIRTPMRGYRFAAAAASTLAIGLMLAPAAHASGSSMPSEQPLQQILQSIEGPFAKTIAVIIIFTGLSFAFGASSFFPSFF
SLDDGRCGIPIGTIPNLSS
>MCE8455596.1 hypothetical protein LZ188_03245 [Rhodovulum sulfidophilum]AB15
MPLDAKNFPLVWMSYDEEPDHDHDDDFEALEACFGRGAPFVILTDNAPAEDHEHSQEEKRRTALWMKTHKAELRAL
VLAM
IVIEPSATKRLAFRSFGVAFAKFWGHPLRLASSREEAMEIAGKLLSERAGSATA
>MCE8455597.1 hypothetical protein LZ188_03250 [Rhodovulum sulfidophilum]AB15
MIDNTPVKLAFAWLIPAVGAAIFVTIQCFSYLNGYVSSGGTMQAMTFGPVALWGVSIFYGGWVVPPLLALAGRRATD
WAM
LVLGGLLFLMSTLAGVSDGLRDGGHLVGLELLAVTLPGAVALIMSWRHIRSN
>MCE8455598.1 helix-turn-helix transcriptional regulator [Rhodovulum sulfidophilum]AB15
MYLDVRRDWLSHVDEVPRALVAASAVANLGALEQSPRHHHKKAQLLFTVRGVINCEVEDAVWIVPPQCAVWIPGGLL
HTV
FGSGEVECISLFIDPPEAPNLPTECCTVTVSNFLRQLLMRANALPDLYDIDGPDGRIVSVIFDELAVAPVEDLRLPIPGD
PRLKKLTDLLIAAPADHATVAEWASRIALSERSLSRLLAKEVGMSFGRWRRQLHIVLALRQLSTGQTVQAVAMDLGYES
A
SSFVTMFRKMVGKPPSRYLLERRRPVLHDRREG
>MCE8455599.1 formylglycine-generating enzyme family protein [Rhodovulum sulfidophilum]AB15
MRSAALCLSLFLAAPVAAAPVTWDKAQYDPAAGTAPADLILPMPCGGAMAFQRVVVPVEADDPLADRRIRVGQSDP
ETGY
SDYLRTAYLRGAFPGPAPGDSVYYIARYEMTRAQGRALTGDCSPPKRIDRTALGGLSWFRAVELSQRYSEWLLAEAREA
L
PPDTDGRAYLRLPTETEWEFAARGGAAVDPTVFPSRRFFGEGDLFDYAKVVGSARGALLPVGLARPNPLGLYDIYGNAE
E
LMLEPYRLNAVGREHGQAGGLVTRGGSVLSAPAQVYSAERTEYPLYSAADGTATAGKTFGLRLVLSRSVAWSDPVLREI
K
AGWIARTEKTARGGPDQTPLGTLAEMIEEEIDPRRKAALQDLQLEVRRSDEEATAAFGEAAKSTLLNGAVLVGAMRAG
QT
EEARMSSELIGIADQTRLCARAEQCSALRSAGSRLMTKLDMLRDVQKTYLLSLRSAVESLTEGADPKLTKDAMRLLQGEL
SASGQRQILDNLARFAALLEAYRDKPDMSEEELRLLVLSH
>MCE8455600.1 ABC transporter permease, partial [Rhodovulum sulfidophilum]AB15
LEIFTQAENRPRQLVLAARLAGVLPEGRLDGRVVMADIAMLDLIEAFYDGYALPEHGIDSGKPLADRAESFEGMRVYAS
A
LDRLAPLQDRLEARFGIRTQARTAQVTSVLALGRNLGLALALTVAVAAAGLAASLIFGFWGEVVRTRRTLANLGLLGLPQ
WQLSLFPVIQALVTAVAGLFVSFLLFHLTAALAERLFETGLTDRGGLVSVSAGQAATIAAAVIAFVGCASLAAARAALAT
DPATVLREET
>MCE8455601.1 PIN domain-containing protein, partial [Rhodovulum sulfidophilum]AB15
SLLLTFNARDFPGHLLAAEALRRAAPDALLMDLWLADPEPVEAAVAEVHDAAERIAAETLDRRALLKRARLPRLGKALD
T
PRG
>MCE8455602.1 LuxR family transcriptional regulator [Rhodovulum sulfidophilum]AB15
MSLDTQCLDFVETLRAQTSFDALWRVFNDHLRSHGVSRIIYAETPAVRSRTISQEMLYRSTFPADFTHLYAEKGMIDHD
P

RAHYCDSGAEAPLYWAHPDARLDADPETREVLDLCEDFGVSFGLTLPLKGASALNRSGAGLAFSARNGDEAADILATN
GS
AICAATRLFHALAQTPAIAPGSVRLSLRERECLLWSATGLRNKEIAFRLSISEKTVEHHMASAMTKLHAMNRTHAVARAL
IFGLIVP
>MCE8455603.1 M48 family metalloprotease [Rhodovulum sulfidophilum]AB15
MLKLTPILLATLYAIAMYRFSAWRTGRELDAQSSELADARLKALTDRMAKALDLQRIRVNIYEIEPVNGLAAPDGRIFIT
RGFHDRYHAGEVSAEELASVIAHELGHVALGHSRRRMIDFSGQNAMRTALAMVLSRFLPGIGVYIANALASLLAARLSR
Q
DEYEADAYAAALLTKAGIGTAPQKTLFAKLDRVTQSRGGAMPAWLMSHPKTADRIAAIEALEARWAVPETP
>MCE8455604.1 catalase/peroxidase HPI [Rhodovulum sulfidophilum]AB15
MDGNDRPAGTCPVLHATFGGQSNRDWWPNQLNLRILHQNPSAANPMQPGFSYAEAFGALDLNAVKSDLRALMTES
QDWWP
ADYGHYGGLFIRMAWHSAGTYRTADGRGGGGTGNQRFAPLNSWPDNVNLDKARRLLWPVKKKYGNAISWADLMIL
AGNVA
LETMGLRTFGYAGGREDIYEPEEDIYWGSEREWLATSDKPQSRYSGDRDLETPLAAVQMGLIYVNPEGPDGNPDPLAS
GR
DIRETFARMAMNDYETVALTAGGHTFGKCHGAGDAALLDAEPEAAPLQAMGLGWLSRHGRGKGADAITSGIEGAW
TPNPT
TWDMGYFDVLFGYDWELTKSPAGAWQWTPKDLKPEDHAPDPETGERNRKIIMTTADMAMRMDPIYEPIARHFHEN
PDEFA
DAFARAWFKLTHRDMGPKSRYLGAEVPSEDLIWQDPVPPVDHPLIEAADIDALKAEILGSGLTVAELVATAWASASTFR
G
SDKRGGANGARIRLAPQKDWEVNEPERLTRVLEVLERIKAGFDARATGGKRVSLADLIVLGGSAAVEKAAAEAGTPVEV
P
FAPGRTDASAEQTDIASFEVLEPLADGFRNYQKGACSVTAEELLLDRAQLLTLSAPEMTVLVGGLRVLGANVGQSPHGA
L
TERPGQLTTDFFVNLLDMGTAWAPTDADETLFEGRDRTSGAVKWTGTRVDLVFGSNSQLRALAEVYAQDDAGPAFA
ADFA
AAWAKVMNADRFDLV
>MCE8455605.1 response regulator [Rhodovulum sulfidophilum]AB15
MTNMPTDSLPVLRRPAERTFGVLMLDDNEIDRMRLKQLCLRAGLSFRVREAADLDDFRRALDEAPYDMVFLDYDLKM
DTG
LDALELLKAHPAQAEAIAIMITSTDAPATVVEAMRSGCSDFLVKDELSISMIRRAVTAAIEREIYRAAILREHEARVSVE
RMLERFSAVCAADIRGMLSGMLRKIRGVTQSVEGHAQISQEVAEAERACMELFEYLENMNGILALEVDPGRERLSR
>MCE8455606.1 PAS domain S-box protein [Rhodovulum sulfidophilum]AB15
MAIGGIIARRRLGLSAKLALLALVVVLTATLVFSAVTLFGSQWYRQAALSRMEFAGAFFSERVLRELELMASDAGNLSVL
PEIGAVADGTTRGANGTGSDDEGQVYWRKRLEAVFESVLLTRPHYTQVRYIGRENNWREIVRVHRVGDRIEVVAPGG
MQS
KGNEPYLADRKALNAGLPFFSRITPNHEHGSEVGPPTIRFVVPVLGTAGTVGGALVINADFEWLLRAANLHPSDGFSVT
V
MTSSNDYMIFDPVTGPGRLRFHDTPEWNPAELPDLAGIGGDDMADTTEIGWRRAVNVTSLPSGSPNRGDRLSLRTDI
FVV
TELPTRLLFAPLRQQLFCGLVAAIVLSVGAASVAWIIGKQLTAPLHRLAQGLRKCDLGEDMSDLIPTTNDEVGELARALV
SLNDRLYQKSTKLNAIFSEAADGLITISEAGVIEEVNAATARIFGYDAADMVGRNVSILMSEEDAERHNDMLRRRIGRRE
QKRLIAGREVAGVRKDGTQIIVEISVASARQTGGSRYIGVIRDVTSRHEQQARSEALVKALERSNADLDAFAYFASHDLK
APLRVIDNASAWLEEDLAPYLNEDTRDSMDLLRSRVARMERLLDDLLNHSRIGRIEEKAQIVEGRDLFDELRGLLQIPDG
FSFEATPEFMDLRIPNMPIKTVLLNLLGNSLKHHDRETGFVRLSGYETDTAYHLSVTDDGPGIPPKYHAKVFEMFQTLRP
RDEVEASGMGLAMVLKHIQLAGGEVRIDSDGSRGTTINLVWPKAARADKATGRAA

>MCE8455607.1 response regulator [Rhodovulum sulfidophilum]AB15
MNQRPNLSTARAPLNLLMVEDDDGDVKAVRRALYRAGIEATMIRAVDGVEALSIMRGRARPHPSIPYIVLADIQMPR
MNG
LEFLNELRKDCSLKQVIVFVLTTSNNDQDKRKAYEAGISGYILKQNAGQDYRPLIETLDRFWNIVSFPDVRPSEE
>MCE8455608.1 ATP-binding protein [Rhodovulum sulfidophilum]AB15
MRDLDVMIIDDDAGDRKLIRRFLERSGMCRTICEAELVHSALDHVAETFDAVFLDYVMPGENGLDGLETLRQKWPTAA
FI
LMTGQGDEQIAKSSILAGAADYTPKSRINQTGLERMLVRAVEVVRLAQRVEEQRQELLTFSNVLVHDFRAPIRSIRFICE
QLDEELGGAQSSELRKLMELMKKTATQMSQLVEALASHIRTDADPVFECISVSDLVENALSFLDHDIRESGACIDLDLPD
IDVFCSPAMMAQLIQNLVQNAIKYSGDGLPNIRIVARRLDNGDVLFEIADRGVGVPEQYRQRIFEPFQRLAQASNMPG
TG
LGLATCQKIVKRHGGRIWCDANPYGGTLMRFRMPTGRKPFDSVTSKVWKTRSDGAAAIR
>MCE8455609.1 response regulator [Rhodovulum sulfidophilum]AB15
MTFSGGLHEKPDAPASKQKVERMAQSAPTCLVVEDTAFDRMIMERKLAAYAQPLQVRFVSTLAEARNCLSEMPISIIFL
D
NSLPDGKGANFALELAAHRNWMKIPVVMVSDWPSPFMPAKAKAANVVAIWTKTEFTALTVAEVLKSRGLTA
>MCE8455610.1 response regulator [Rhodovulum sulfidophilum]AB15
MVKRLIRKTGLVETMLEFGMAEEAIDYLRDPSCPKIDVVILDINMPRMNGFEFLERICEDLPGHLRNSVVVILTSSECAA
DMRQAERFPMVRSFLRKPIGENEISHLDKLVNA
>MCE8455611.1 transporter substrate-binding domain-containing protein [Rhodovulum
sulfidophilum]AB15
MRSTGIVSENEYRLRRLVRAGLVWLLAALVLMLLGQPPEARAQPSGSSPPAIVTGWLDNPPFWKMTPTGELVGFTPRL
IA
EIRNLTGLRIEPRRYDDRQSLIAAQITGESQLMFATARLPALEATHVFTEPIARAHVHLFVLTENANRPDYIAPVGKRIG
KIRQYAGSDPSNLLTGNRTVFFDEPATMLLKLLSGEIDAALMSEDWLMGLARSVRLDHRIAAVGPPVLEFDRVIAIHNSR
ADLLEPLNRAIATLEADGTLSALRQQWLIEADPPPPDILTVGVVNLPPFSEVREDGTMTGFSVEVMRDLGEILGIELRFQ
TVSAEDWARGPQSPIDILPLIGITPERELRMDFALPIQAIDLGVFVRSGRQDTFDGLSGLRVGTWRAGVANTVAKRNAS
D
IRHFDDYRDLLTALFEDRIDAIVAERGMLSRIAGEMKRGDDIAEAPFPRQRIFLAPALRPGLPTVREALNLVIPGYLASQ
RYQDLRAAWLGTAPFWTDRRILLTLGGFWLLMVLAIGYIPVQHHRIRQERLRFTADMVERIPLGLLMVASNGRIEFVNS
A
AKAISPSGPDLIREGADYRQAVRALIKADARHWGTNSPAALAADLSGPSRRDGHSHEFQTAGGEIFRWSSFALPGNTIL
V
MVLNVTRERRHLAEIEDLNAELRAQIAIVQRTNAELRDFAYATSHDLKSPINTLMLIADALTEASTDRLPDEDLALIEDM
HEILDRMARMIRDVLDYTRAVGVGGEQTSDTIELSGVVGDVMKDLAADIVASGARLHVGDLPCLTGNPRQLHQLIQNL
IS
NAIKFRLPGTTPEIDIETVAAPQGMVAFAVSDQGIGIEPKFHDRIFHLFTHLNDTDLYPGSGLGLAICQRVALNHGGRIM
VQSTPGAGSRFTVFLKEKQGDKHTDDGRRLSVRTQDGQAPHSQDRPRGNDAGIRNGRGSH
>MCE8455612.1 quinolinate phosphoribosyl transferase, partial [Rhodovulum sulfidophilum]AB15
MLLLDDTALACLLKRDGAVAAAAFSASGRRARMVSVALRARGSGMVCGTEEAARMFALLGCAADTAIASGTRAEPGA
LLI
AARGPAEALVGGAQAVQTL
>MCE8455613.1 DUF4405 domain-containing protein [Rhodovulum sulfidophilum]AB15
MRSILLRYATPSTIGLFLVSLITGVALFFHVGPSAFHGIHEWLSMVLILAFGLHLWRNWRPMLHYMKGRPLALSLVITLL
ASAAFFFLPAGGKGGGRPPAFGLAMQVMAQPPATVAPALGTTAEELTARLAAAGFAVTDPARPLSEIARASGKSPMA
LAR
VLIDRPR
>MCE8455614.1 hybrid-cluster NAD(P)-dependent oxidoreductase [Rhodovulum sulfidophilum]AB15

MNDLSTLPDRAIWSDNEMLECVSVIPEVPNTATFTFRAPSGALFDFLPGQFLTLEIPMPGGTVHRTYTISSSPSRPRSIS
ITTKAQPDSLGTRWLLDNLTPGTRVRALGPAGVFSHLNHPAEKYLFISAGSGITPVMSMTTYMFDLGLEPDIVFVNCAR
R
PGEIIFRERLEHMASRVPGIDLKWVVERPDRYAPWIGFQGQFNQLMLGLMAPDYLEREVFCCGPEPFMRAVREALQG
LGY
DMDRYHQESFQAPATTEADLTELDDVVPDESAQVELTFAASGVTVKAAETDTILAAARANGLNIPSGCTFGVCGTCKIR
K
LSGEVHMVHNGGISEDDIEAGYILACCSNPIGKVSVDV
>MCE8455615.1 aromatic ring-hydroxylating dioxygenase subunit alpha [Rhodovulum sulfidophilum]AB15
MKHDLPRLLAERKPGYALERPFYTDPAIFEADLETIWYRDWLFAIPACELEKPGSFVTHKVGAYRVILVRGQDGTIRAFH
NSCRHRGSIICKGAKGNLPKLVCPYHQWTYELDGRLLWARDMGPDFDASAHGLKPVHCRELEGLVYICLADAAPDFDA
FA
DLARPYLAVHDLTDAKVAFESTIVENGNWKLVWENNRECYHCGGNHPGLCRSFTDDPTVTGIKEGAAPPHLQAHFDR
MEA
QGYPAQFRMSSDFQYRLARMPLLPGASSFTMDLKPAVTRILGRVRDDTAGTLLKYHYPTTWNHFLPDHSILFRITPLTAT
TTEVTTKWLVHKEAVEGRDYDLTRLTEVWTVTNDEDRRVVEDNQQGIDSPAYEPGPYSPVHETGVMGFVDWYCNT
MTGRL
ASVALAAE
>MCE8455616.1 glutaredoxin-like protein NrdH [Rhodovulum sulfidophilum]AB15
MSIIVYSKPACVQCTATTRALEARGLPYELIDLTEDDKAFAHVSELGYRQLPVVVAGKDHWAGFRPDLIGRLA
>MCE8455617.1 class Ib ribonucleoside-diphosphate reductase assembly flavoprotein NrdI [Rhodovulum sulfidophilum]AB15
MAGLVYFSSRSGNTARFVERLGLPAVRIPIAGDDLPRPDTPFALICPTYADGEGRGAVPKQVIRFLNDPARRALLRGVIA
SGNRNFGATYALSGEVISRKCKVPVLYRFELAGTDTDIARVRDGLDKFWEMECLTV
>MCE8455618.1 class 1b ribonucleoside-diphosphate reductase subunit alpha [Rhodovulum sulfidophilum]AB15
MLDRVTDAKPAKPDSTLDYHALNAMLNLYDEDGTIRFEADRMAARQYFLQHVNRNTVFFHSLDEKLGYLVDEGYYEA
DVL
DQYPRAFLHRIWDAAYARKFRFPTFLGAFKYYTSYTLKTRDGQRYLERYEDRVVMVALALARGDEALAMSFMDEIISGR
F
QPATPTFLNAGKKSRGELISCFLLRVEDNMESIGRAINSALQLSKRGGGVALMLTNIREHGAPIKGIENQSSGVIPVMKL
LEDSFSYANQLGARQGAGAVYLNAHHPDILRFLDTKRENADEKIRIKTLSLGVVIPDVTFELAKQNEEMYLFSPHDVEKV
YGMPFSDISVTEKYDEMVGDKRIRKQKINAREFFQTLAEIQFESGYPYIMFEDTVNRANPVAGRIAMSNLCSEILQVNEA
SAFNDDLSYRHLGTDISCNLGSLNIAATMDGPDFGGTVEAAIRALTAVSEMSAIDSVPSIRRGNDEAHAVGLGQMNLH
GF
LARERIHYGSPEGIDFTRAYFAAVAYHAIRASNALAREKGQSFKGFEASKYADGAFFEKYVTRDWRPETDTVRDLFARFG
VDLPSREDWAGLKEAVSKDGLYNRNLQAVPPTGSISYINNSTSSIHPIVSKIEIRKEGKIGRVYYPAPFMTNETLEFYRD
AYEIGPEALIETYAAATEHVDQGLSLTLFFPAEATTRDINRAQILAWKKGIKTIYYVRLRQTALEGTEVQGCVSCTL
>MCE8455619.1 class 1b ribonucleoside-diphosphate reductase subunit beta [Rhodovulum sulfidophilum]AB15
MKDMIDQTRAVPRAINWNRLQDEKDLEVWNRLTVNFWLPEKVPLSNDVQSWSTLRPEERQLTIRVFTGLTLLDTIQG
AVG
APAMMRDALTPHEEAVLTNIAFMESVHARSYSSVFSTLCQTREVDEAFRWSEENPHLQDKARLILGKYDAAGDPLKRKI
A
SVFLESFLFYSGFYLPMYWSSRGKLTNTADLIRLIIRDEAIHGYYIGYKFQRAAEHLPEAERAALKDFAFALMFDLYEIE
AKYTEELYDGIGLTEDVKAFLHYNANKALQNLGFEALFPPAACEVNAAILAALSPDSENHDFFSGSGSSYVIGKAVATED
EDWDF

>MCE8455620.1 IS3 family transposase [Rhodovulum sulfidophilum]AB15
MKKSRFTEAQIMGVLHQAEGGMPVPELCREHGVSSATFYKWRAKYGGMDASMMSQMKALEDENRRLKRMFADLS
MQADLL
REALGKKLTRPAQRRELAEKAVATKGVSIALACRAFGVSETCFRYSPKRDDENEMIAELLVGLTNAHKTWGFGLCFLHLR
NVKGHVWNHKRVHRIYCELELNLRIKPRRRLKREKPEELAVPPSLPSIPRIDGFTLRPPNLVWSMDFMVDRLADGRQVR
L
LNVLDDFNREGLGIEVYFSLPAERVVRSLNQIIEWRGKPLAIRVDNGPEYVSSTLMTWAEQQGIALTYIQPGKPQQNAC
V
ERYDRTVRHEWLDLHIFDTIEEVQQIATEWLWTYNNERPNMGIGGVTPAMKLKMAA
>MCE8455621.1 hypothetical protein LZ188_03380 [Rhodovulum sulfidophilum]AB15
MSRQLDALSHLQDRPTAALERVFAIALQAQHDAIDPEDPTTMAPTITGIDGDIATFVATMRDLSSFAQQGTGTAAIFVV
R
RRIYGDMQTDLSARIGQWDRRLVEFDAAIAAVDADAGADEELKFAALTLAERLIATEATNPLSALSQTYRDDLVDNRRH
A
FVAQRDTLAALGNLATLSGLHNGLDAERGAVADFDPEPLDLSGHATKLVTLSADMLRRAATVSEEIIARLTEFDADVPEA
DAASDPRTRLEG
>MCE8455622.1 DDE-type integrase/transposase/recombinase [Rhodovulum sulfidophilum]AB15
MPVRLRISKADALIVGGTVHVVCGVSDSTLELRPLDGSSDLLCFSHADVKRMIDECKASIEYGYFSGAQAARRALASNWL
ISRMDAAEKAQVFRKQLYAECFLEAEANGEVNRSNDPCVVFLPRLGKRVEAKILEQIRLGGDAAELGKLLSMKHPGRTA
L
MAWVRDWEKTRDPMCFVKKSRFNGANAKRVSSQVESAIQTAIKTYLHPGRVLIPHVAEEANRVIRELNRSLALSDQPP
ER
EVSESTVRRRIADLDQFEVCAAREGVAKAKNKLGPYGQGVEIEAPLQRIEMDEWQIDLIAMLEEAGIDVRDPKFDDLRT
G
RFWVCVAIDAATRVILAIKMARTPSHETALATLWLAMRNKSEISRQLGCKKEWSQHGHIFNVAVDNGASFVNPEFKAA
LA
DLWIGYEVLPAGVPKLRGRIERVFRTLGTMFMPLLTGRTFSNPQERGDYPSDKCVVHTADSIVEVMVRFVVDAYHHRA
HR
GLEYATPAGLCRGRRSGADGRGPARQNLRAVRRRQLCPERSGGHPVCAGRKGHPLRRHARGRLCRRAGGPRTASRV
GRVP
RSLRCRGLQRRTVRRRNAAFHPDRPPNNAACHSGGHSHPLKPSYGAPTSCSTMR
>MCE8455623.1 hypothetical protein LZ188_03390 [Rhodovulum sulfidophilum]AB15
MDSGNRIAIAVKPGKLVERNGFDREFALIRAQTAKHFADHALLVTDSSFSAAEVRNAELLHMFRQVEDAEADEAVQQV
LR
GMNCEMSLGQVVAATCLQGRAFRAGFKAIFDGVVFTDQSRSITESSMLSLPEAQ
>MCE8455624.1 hypothetical protein LZ188_03395 [Rhodovulum sulfidophilum]AB15
MYDEDFGGPYVPPERTRATRMFPARSRASARGFVLAKLRSGAAWRQLIFESHLERTCLLVLLSQPGVVDIWDQPPQIT
>MCE8455625.1 helix-turn-helix domain-containing protein [Rhodovulum sulfidophilum]AB15
MRTPAKADLDAHERLKAELRIRGTSLAQISRELGVSDSALTLVGKRMCRSQRIEEALALAVGKAPEDLFPASREGDATMT
>MCE8455626.1 OadG family protein [Rhodovulum sulfidophilum]AB15
MLENLALIGTGFAVVILVLASLWGACALMGWGFTRTARKTTAAPPEAPVAPGVPPHHLAAIAAAVAETLGPGHRISRV
AA
PAHLVDGWPLEGRIETFAAHRVRTGWGPTRPRPGARTPEITKGPKP
>MCE8455627.1 acyl-CoA carboxylase subunit beta [Rhodovulum sulfidophilum]AB15
MAISRSLAEELDKRRAAALEGGGPAKAADRHAKGRMTARERIEALYSAGTFQETGLHAQHATRHFGMEKKSIPADGVI
TG
TGFVDGRPVAAFSQDFGVVGGTLGEMHSRKICLVLDHALKAGVPVVGFNDSGGARIQEGVGALSAYGQVFYRNVQLS
GVV

PQISVIAGPCAGGAAYSPALTDFVIMTREHAQMFICGPEVIRAVTGQSTTMDEIGSAAAHGSVSGNIHFIAEDDADAVA
I
VHRLLSFLPSNNMMDPPHRIEPDLAIEDDPALDALLPEDTKSAYDVRQVIARLADEGDFLEVMEGFAANLVVGFGRVG
GV
VTGFVANQPMVKAGALDIDASDKGARFVRLCNVFNIPLVTLVDVPGFLPGVAEEQRGIIRHGAKMLFAYASATVPKITV
I
MRKAYGGAYLAMCSQDMGADRVIAWPSAEIAVMGAEGAVNILYRKDLAEAEDRAARAQELAEEYRAEFASPYLSAG
RLFV
SDIVQPCQTRSAIALTLRGLLSKRETRPPKKHGNIPL
>MCE8455628.1 1,4-alpha-glucan branching protein GlgB [Rhodovulum sulfidophilum]AB15
MHSDITSRPGQGVPKDVLQQIALGTFDDPFAVLGPHGQGEFRHVTALMPGAETLEALLGHEADAGAHALAPVPDCPG
LFT
GPVPGEGAYRLRASAGGTVWETDDPYRFGPVLGEMDEHLIAKGAHRRLWEALGAHAMTHEGVAGTHFAVWAPNA
RRVSVV
GPFNQWDGRRHVMRRRGTTGVWEIFLPGIGENASYKYEILGPDGALQPLKADPLGFGSEHPPANASVVHDLRGYGW
SDGD
WMRDRAGCNARTAPISIYEVHLASWKRRADGRAISYVEAAEELVDYAAAMGFTHIELMPVSEYPFDGSWGYQPVGLY
APT
VRHGPPHEFRDLVDAAHRKGLGVILDWVGAHFPSDAHGLGKFDGTALYEYADPREGFHQDWNTLIYNFGRTEVANYL
TAN
ALYWMEEYHVDGLRADAVASMLYRDYSRKAGEWVPNKDGGRENYEAIALLQQMNTLSYGAHPGTLTAAEESTAYPG
VTRP
VDLGGLGFGYKWNMGWMHDTLGYMAEAPVNRPHHHNRMTFGLTYAFSENFILPISHDEVVYGKGSMLTKMPGER
AEKFAN
LRAFYGFMWAQPGKKLLFMGQDFAQEAEWAYAGSLDWAALERPDHAGMQRLVRDLNTLYRGTPALHAQDCSPEG
FQWIEA
DDARHSVYAWLRRGGPEDPNAVAICNFSPVEQPACRLGLPAPGRWREALNTDAAIYGGQGRGNMGAVMAEPRPSH
GQAAS
AEICLPPLSTVILVQEDG
>MCE8455629.1 hypothetical protein LZ188_03420 [Rhodovulum sulfidophilum]AB15
MRDDGEPGIDRAVDAAEKESQMPLRDRIEFDEAAARAEAEAAREALTTAETAFEAQGRIARRLGGRVEEGRAVFGFW
TPE
ILDARVPSGDVFLEVLAPLEPMDLTRAHQEIRFERAWVPVIVEEAHCFTAVTGMRAGSRETLGDFYALAWRDPDGGW
HRI
LDPLAMSLPFGVMAPAELYDVEAMLAAREDTAYFQAFRGEAPHKFGPPSNILQVHVPTATAGGTLASLTRHYHRLAER
VA
RELPLEPADKLFLGYDAVQLLPVEPTTVHEAGPDFWQEVDGDEEGVTVSLLRPDTTNWGYDIVISGMATVNPVLLETG
RP
DELVDFAAQLHNFPGMPKKLILDVVFGHADNQGLGALNSHFFAGPNMYGQNLDYQNPAVRAILLEMQRRKVNFGA
DGVRV
DGAQDFKWWDASTQTLRHDDDYLQSMSDLVQEAAGTRYRPWFIFEDGRPWPEEDWELSSDYRAVIEAQTDEDVFQ
WGPLT
FAHNTPFLYTFWLSKFWRIREIVELGAHWISGTSNHDTLRRGTQVSPKLNVNTRLGRTRMEILDKAYDNPANHVLTYVA
L
PGVPMDFLNAMARASWGFIRNQDDRYGVKVVAEEAISLKWQVDEYSYSVPANFRRLKEMGFETRSDLVRFFEFLPAL
VEV
TEYDLDEIARLLNKAEPPLAGPEVYTVAALKHMARAWMDDMHEYCNVSTSLPALDADQAAFCLALRGFRRKRPWLAA
NYA

GRDSFDYLRPIAGRTLFTALRHGPDGEQIYAVMHMEGKPTAEIDPLSLPVPGIGGGGWEVALRTPSIGPDYHGGPITLS
D
SMALVFSRHLPE
>MCE8455630.1 lysophospholipase [Rhodovulum sulfidophilum]AB15
MSMLLSDLRTTFGYLVQDGLCRLVGPLRPKAPPRPDYSAVYTSPDFTVERFGKCQAGSYIEVYRPGTPWLGQNGRPKA
VI
FLHGFVLGASQIYRAHLEHLVRQGYTVFFPNFQTGFCSFPARGLMTVAELLDEVFGDGLRPSQETWMKNALASVSDAY
AR
VGFGPDVAVDTYVYGHSLGGLFALSWPWYVTEDGLPANLMPLQVVTADPVPSSSAASAPGQIGQALDSLQDDVNLA
VTGK
ALTMPVAILHGDADWIVPKSEWDTPFGAIATDRKRMYLSQTDTHGCPGHFANHEQATDDTSFFRPLLSLLLLDGVGAE
DT
LDWRYLWSALDRVIREGARADQLDFDMGQWSDGRPVRPVEVYLSGG
>MCE8455631.1 acetone carboxylase subunit gamma [Rhodovulum sulfidophilum]AB15
MAYTEAKIRDLVDGTIDQDTLHQMLSMPKDRERFEIYLKILQDQVPWDDRIILPLGPKLFIVQRAEDRKWVIRSWAGHD
F
CDWTENWKLHARVRVRDTPEAMEKIYPKLMAPSTEWQVIREYFCPLSGDLLDVEAPTPWYPVIHDFEPDIDSFYRDWL
GL
EVPERA
>MCE8455632.1 hydantoinase B/oxoprolinase family protein [Rhodovulum sulfidophilum]AB15
MNIQSDIKGIVRGGETLKQHRDKIMEATKSSGHYAGLRHMELRDDHPIKYNKLFSRLRAGVVDARETAKKIAASPIVEQE
GELCFSLYNAAGDCILTSTGIIIHVGTMGAAIKYMIENGWESNPGIRDRDIFCNNDSLIGNVHPCDIHTIVPIFHEGELI
GWVGGVTHVIDTGAVGPGSMTTGQVQRFGDGYSVTCRKIGENDTLFRDWLHESQRAVRTTRYWMLDERTRVAGCH
MIRKL
VEEVIADEGIESYWQFAYEAIEHGRLGLQNRIKAMTIPGTYRQVGFVDVPYSHEDVRVPSDFAKVDTIMHTPSEMTIRK
D
GTWRLDFEGSSRWGWHTYNAHSVSFTSGIWVMMTQSLIPSEMINDGAAYGTEFRLPKGTWMNPDDRRVAFAYSW
HFLVSS
WTALWRGLSRSYFGRGYLEEVNAGNANTSNWLQGGGFNQYDEIHAVNSFECAANGIGASAVRDGISHAAAIWNPEG
DMGD
MEIWELAEPLVYLGRQIKASSGGAGKYRGGCGFESLRMVWNAKDWTMFFMGNGHISSDWGLMGGYPAASGYRFA
AHDTGL
KEKIARGEPIPWGGDTDPENPVWDGMLEGARIKRDKQAITTEEMFEDYDLYLNYMRGGPGFGDPLDRKPEAVAADV
NGGY
LLARLAGDVYGVVLSEDARGQAVADLAATEARRAEIRQERLAQSVPTGEWMKAEREKILAKEAGTQVQQMFAASFKL
GPK
FERDFRAFWDLPESWQLKEADLPIPSYGKDYSMDLSELPDVTIVQLVEE
>MCE8455633.1 hydantoinase/oxoprolinase family protein [Rhodovulum sulfidophilum]AB15
MTQTGRNARSVQVLGIDAGGTMTDTFFVDAEGDFVVGKAQTTPANEAQGLLESSREGLEHWGLTLEDALRNIQTGV
YSGT
AMLNRVVQRKGLRVGLIVNAGMEDFHRMGRAIQAYLGFAYEDRIHINTHYYDDPLVPRDLTRGVAERIDMMGTVVIP
LNE
ETARTVARELIAEDVEGIVISLLHSYKNPTHERRVRDIIEEEIRASGKTIPVFASTDYYPVRKETHRTNTTVLEAYAAEP
SRQTLHGISGAFKDHGSRFDFRVMATHGGTISWKAKELARTIVSGPIGGVIGAKYLGEKLGYRNIACSDIGGTSFDVALI
TQGELTIRNDPDMARLVLSLPLVAMDSVGAGAGSFVRLDPYTKAIKLGPDSAGYRVGVCWAESGIDTVSVSDCHVVLG
YL
NPDNFLGGQVKLDRQRAWDAVKVQLADPLGLSVEDAAAGVIELLDAELRDYMRAMISGKGYSPGSFTCFSYGGAGPV
HTY

GYTEGLGFEDVIVPAWAAGFSAFGCAAADFEYRYDKSLDINIDTDAHEADKAAQAEMLQAAWDELTERVLEEFERNGYAS
DKVALQPGFRMQYRGQLNDLEIESPIGSAGNADDWDRLVDTFNDTYGRVYAASARSPELGYSVTGAIMRGTVPIPKPKIP
EEELQDAAPPREARIGTRQFYRKGRWVEARLYQMEALLPGNQIVGPAIIESDATTFVVPDGFETWLDEHRLFHLKEL
>MCE8455634.1 hypothetical protein LZ188_03445, partial [Rhodovulum sulfidophilum]AB15
MRRTFDETLKARIDRECRALGRSGAEIGSGAVRERLLPGSAYTEVTVDFSYVSCRTGPRLHRDFCTEAGCARQRYLPGASAYVLIEDGDPE
>MCE8455635.1 exodeoxyribonuclease III [Rhodovulum sulfidophilum]AB15
MPFTLATWNINSVRLRAPLVARLMEEEAPDILCLQECKSPVEKLPLEVFAPLGYTHVVARGQKGYNGVAILSKLPLEDAG
SVDFAGLDQARHVAARLENGVLLHNFYVPAGGDVPDREVNVKFGQKLDFLTGMRDFFRESRPERSILVGDLNIAPGEEDV
WSHKQLLKIVSHTPVEVEHLGAAQEAGGWVDVTRAHIPEGKLYSWWSYRARDWDAADKGRRLDHLWATPDIAGAAADSRI
LRPVRGWEKPSDHVPVFARFDL
>MCE8455636.1 thioredoxin [Rhodovulum sulfidophilum]AB15
MLELGGKTTETGGAGAALIKDVTEASFMADVIEASQEVPVIVDFWATWCGPCKTLGPMLEQAVTAAGGKVRMAKVDVDQN
QTIAAQLRIQSIPTVYAFYQGQPVDGFQGALPASEIQAFVERVAAMGGDGGLAEAVAAAEELLAEGAAVEAAQTFAAILG
EDPKNAAAYGGLARAHLALGEVDQAEQFLAAVLPEIAEAPEVEAARAQVALARQAAEAGPVAELRAAVDHDPDDHAARFE
LAKALHAGGEVEAAVDELLELFRRDREWNDGAAKAQLFTIFEALRPQDPIVLKGRRKLSSMIFA
>MCE8455637.1 LON peptidase substrate-binding domain-containing protein [Rhodovulum sulfidophilum]AB15
MFSAVDLPDTLPLFPLPGALLLPRARLPLHIFEPRYLAMLDDTLKTTHRLIGMVQPREVPGSDEQKLHAIGCAGRVTAFS
ETEDGRYMLTLSGISRFRIARDQAGFMPYIRADVVWDGFGRDLGPGEDDPGFDREGFLKLLGRFFSSRGLSTDWDSLRDA
EDELLINSLSMLCPFAPEDKQALLEAPSLSTRRETLVTLIEFALRSGDDEEKMQ
>MCE8455638.1 Trm112 family protein [Rhodovulum sulfidophilum]AB15
MTDAVQADRKMLESLVCPMTQGPLAYDAERQELVSKAAGLAFPIRGGIPIMLVDEARRLD
>MCE8455639.1 carotenoid 1,2-hydratase [Rhodovulum sulfidophilum]AB15
MISFIGSVFSPWYRWSGRKSPDNHCCINVATYGPGGRWTMTDRGRDALRQTRDTFEVGPSKLTWAGGKLVFDIDEMTWPH
MGRLKGRVTVTPSAVTGVELPLKADGSHVWRPFAPSSEIEVDLNQPGWQWKGHGYFDANFGTAALEDDFSYWTWGRFPTK
ANGAWCFYDASMRDGSELAVGVHFDADGRAEVAEAPPRTRFPRSKWLVRRETRADPGYKPRQVHDMLDAPFYCRSAIRTK
VFGEEVVGVHEALDLDRFASPLIKPMLAVKVPRKPGWSFPT
>MCE8455640.1 phytoene desaturase family protein [Rhodovulum sulfidophilum]AB15
MAGSAGKVVVIGAGMGGLSAAIRLAHAGYEVEVVDRAPVPGGKMRTFASDAGPIDAGPTVMTLRPVFEELFDSVGAKLSD
YVTLHREEVLARHWWPDGSTLDLFASREASADAVGRFAGARAEKEFRAFCRRAERLYRAFDAPMMRAAEPSQGALTRHVV
THPWLALDMAPHRSLAKALGKGFSDRRLAQLFGRYATYVGGSPFQSPALLALIWHAEEQGVWRVEGGMHRLARALEQLAH
DRGVRFSFGSRATRVEIQKGAAAAVHLDSGTRLAADAVVFNGDPAALAAGLLGEGLRETVAPEAIRPRSLSAYVWSYAAV

PHGPEMVHHNVFFCDDPHTEFDPIARGAMPEDPTLYICAQDRGTGRSPEGLERFEIIMNGPPVDDGTDPTDEEQETCR
TR
TFQTLARFGLTFDPRPGVQALTTPRQFAKLFPASDGSLYGRSPHGLTAALKRPTARTKVAGLYLAGGGTHPGAGIPMAT
L
SGRHAAEAILTDRASLSMSPRTAMPGGISTA
>MCE8455641.1 polyprenyl synthetase family protein [Rhodovulum sulfidophilum]AB15
MDISARIEAAMRQAIAEGQAGAAPGKLASAFEYALTPGGARIRPTICLNVARACGDDRPALADAAAISIELIHCASLVHD
DMPCFDDADIRRGKPALHKAYGQPLALLTGDSLIVAGFEVIARGAQQDPARAVELIRILGQRTGMPYGICAGQGWESE
ET
IDLSAYHRAKTGALFIAATSMGAVAAGQPAEPWEELGDRIGEAFQVADDLKDALLDAEAMGKPAGQDEAHGRPSAVT
ELG
VEGAVQRFRDILSGAIASIPSCPGEADLAQMVRHMAERLIPEPARQPAE
>MCE8455642.1 acetylserotonin O-methyltransferase [Rhodovulum sulfidophilum]AB15
MTEILSGPGPAPRRGSGWRRWRNRKIASPGFQSWASRFFLTRRHVRREGERMFDLVSGFVYSQVLWAVVELDLLEAT
CDE
AQSLAALAHRAAIPVDRMEALMQAGTALGLFDRVGGGYQLSSLGAALMGAPGVVGMIRHHDVFYRDMQDPVAILR
GEKET
ELARFWPYVFGAGAAESPEVARRYSKLMADSQALVAEETLRSGALDGVARLMDVGGGTGAFLAAAGTARPDLKMVLF
DLP
AVVPDADERFRALGMADRVTVAGGSFRDDPLPEGADAVSLVRVCYDHADDTVLALMRKIFATLPAGGRLIVSEPMAG
GAR
PNRAGDAYFAFYCMAMRTGRARSPERLAEMMKEAGFQGVDMPRTHRPFIASVVVGGKPGAT
>MCE8455643.1 chlorophyll synthesis pathway protein BchC [Rhodovulum sulfidophilum]AB15
METTAVILRGPREIGLGKLGLSAPGPRDLVVEISHSGISTGTEKLFYTGEMPPFPGMGYPLVPGYESAGEVIEAGSETNF
KVGEHIFVPGANCYEGARGLFGGSAKRLVTDASRVTRIDPGLGPEGALLALAATARHALAGVDKKMPELIVGHGVLGRL
L
ARLTIAAGAPAPTVWEIDPDRQSGAMGYEVIHPDDDPRRDYRTIYDASGVGSLLDTLITRIARGGEVVLAGFYTAPISFA
FPPAFMKEARLRIAAEWEREDLIATRQLVESGALSLGGLITHSVPAEKAAEAYETAFNDPACLKMILNWGAVHDA
>MCE8455644.1 chlorophyllide a reductase iron protein subunit X [Rhodovulum sulfidophilum]AB15
MTLDVPNIKDIDQRLRDEANAPLDDEIPVTEPTKKTQIIAIYGKGGIGKSFTLANLSNMMAEQGKRVLLIGCDPKSDTTS
LLFGGKACPTIIETSTKKKLAGEEVAIGDVCFKRNGVFAMELGGPEVGRGCGGRGIIHGFELLEKLGFHDWDFDYVLLDF
LGDVVCGGFGLPIARDMAQKVILVGSNDLQSLYVANNVCSAVEYFRKLGGNVGVAGLVINKDDGTGEAQAFAEAVDI
PVL
AAIPQDEDLRRKSANYQIVGTHEGPWGDLFAGLASAVAEAPPIRPAPLTNDGLLGLFAPEDVGADFELTPATDADMRG
KN
AKPKKSLEVVYDDV
>MCE8455645.1 hypothetical protein LZ188_03500 [Rhodovulum sulfidophilum]AB15
MFDLNKMSRWDNVLEALSHHGEDREPTENAELQEAPAAEAAAPARELEDA
>MCE8455646.1 chlorophyllide a reductase subunit Y [Rhodovulum sulfidophilum]AB15
MTEEVKYDEAHTAEVIEGTPREAGTPPEGADPRLGDGMGCHAGAAEMEKAARAAGASEILDQYAKDYPQGPHDRPQ
SMCP
AFGSLRVGLRMRRVATVLSGSACCVYGLSFVSHFYGARRSIGYVPFSSETLVTGKLFEDIRDAVHELADPDRYDAVVVTN
LCVPTASGVPLRLLPDEINGVRIVGIDVPGFGVPTHAEAKDVLAGAMLNYARKEIEAGPVAAPEGGVSDRPTVTLLGEM
F
PADPIVIGQMLDPLGLACGPVVPAREWRELYAALDCGAVAAIHPFYTAAMREFDAAGRPIVGSAPVGHDGTAAWLSN
IGD
AFGLSADKVAKAQNMFLPTISEALAGAPVKGTITLSGYEGSELLVARLLIESGANIPYVGTACPRTKQAEADRDWLESKG

VKVVFRASLEDDKAAVDAIRPDLAVGTTPVVQHAKELGIPSLYFTNLISARPLMGSAGAGSLAQVVNAAIANKGRMAS
MK
EFFEGVGEGDTAGVWEGDPNLHPEFRALQQKKLDKKRRAEKAQEMI
>MCE8455647.1 chlorophyllide a reductase subunit Z [Rhodovulum sulfidophilum]AB15
MLVLDHDRAGGYWGAVYAFCAVKGLQVVIDGPVGCENLPVTSVLHYTDALPPHELPIVVTGLGEEELGREGTEGAMK
RAW
STLDPVLPAVVVTGSIAEMIGGGVTPQGTNIQRFLPRTIDEDQWEAADRAMTWIFTEFGMTKGRMPPEKPREEGAKP
RVN
ILGPMYGVFNMPSDLAEIRRLIEGIGAEVNMVMPLGAHVAEMRNLVNADANVVMYREFGRGLAEVLGKPYLQAPIGI
DST
TKFLRKLGEMLGLDPEPFIAREKHSTIKPVWDLWRSVTQDFFATASFAIVANETYARGVRHFLETDLGLHCAFAVARQR
G
AKTNNAEVRALMGQKRPLVVFGSINEKIYLAELSAGHGPKPAFIPAGFPGAAIRRATGTPFMGYAGATYLVQEVCNSLF
D
ALFHILPLGTEMDSASATPTRLRRDFPWDEDAQALLDRIVAEHPVLTRISAAKSLRDAAEKAALEQGAERVVLETIEALA
PAGFARPSSKGETK
>MCE8455648.1 protein pufQ [Rhodovulum sulfidophilum]AB15
MTDQTSDVHMVRGHRPPKAEFMVYFTIIFIAALPLAFIASFLAMVRQGDLKTKGPIGRAWSQARIITPMIFSA
>MCE8455649.1 light-harvesting protein [Rhodovulum sulfidophilum]AB15
MAESDVSFTGLTDEQAQEIHAVYMSGLWLFSAVAVLAHLAVYIWRPWL
>MCE8455650.1 light-harvesting protein [Rhodovulum sulfidophilum]AB15
MAKFYKIWMIFDPRRVLVAQGVFLFLLAVMIHLVLLSTDYFNWLTIAAEKAAGA
>MCE8455651.1 YbaB/EbfC family nucleoid-associated protein [Rhodovulum sulfidophilum]AB15
MLKGMGGLGDMAKMMKAAGEMQGKMAQLQETLAATLVEGESGAGLVKATATAKGTLTALSIDPSIFNADEKEVVE
DLILA
AIKDAQEKAMEKTKEETRKLTEDMGLPPGLGLPV
>MCE8455652.1 recombination mediator RecR [Rhodovulum sulfidophilum]AB15
MADRPDEIDRLIDLMARLPGLGPRSARRAVLHLVKKRAVLLLPLAEAMRKVGEAVHECAVCGNVTTADTCEICTDPVR
AN
GEICVVEDVADLWAMERGGAFRGRYHVLGGTLSALDSVGPEDLRIPELVERVGREGISEVILALNATVDGQTTAHYIVD
E
LAETGIRLSSLAQGVPIGGELDYLDDGTISAALKARRGL
>MCE8455653.1 extracellular solute-binding protein [Rhodovulum sulfidophilum]AB15
MLTPRSHPDSAACAAAARPFPTTPLMAPVLALFLALGAGPAPAEETATGMSDGKVIESFAYSALGAPKYGPDMAHLDY
VN
PDAPKGGEISIWAPGTFDSFNQYSRMGVPAALNTIGTESLMTSTADDPYAMYCYLCTSIEYPEDLSWVIFNLRDDVTFQ
D
GTPMTAEDIAFTQKLFLEQGIIEYRRLVEAYFGPIEVLGPHRIKFTFNDVTPMRDRIGLAGGTPAFSKAWFEETGARLDQ
STKTPFMATGAYVLDSFDYNRRVVYRRNPDFWGADQPFNIGRHNFDRIRVEYFADSNAAFEAFKSGAYTFRTENSSKT
WA
TGYDFPGVERGDVVREAIPDGSVGTRLSWVFNLDRAKWQDIRVRRAVEMMFNFEWSNKTLFYGYFTQPVSFWSGTD
LAAS
GTPGPDEAAILQPLVDEGLLEPEILTEEVASPPDHDADTYRPSRRIIRAASKLLDEAGWVAGDDGIRRKDGQPLELVIIQ
RDPQFDRAINPFLENLRMLGVSGRLERVDTAQYVERRRAGMFDLANQGFVMGFEPSSGLSQWFGSKTADNSSRNLM
RLRS
PAVDRLIEDVIAADTLDGMKTRVHALDRVLRSLHFDIPLWFNDKTWVAYYDMYRHPETLPPLDVGELDFWWFDQEAA
DRL
HASGALR

>MCE8455654.1 microcin C ABC transporter permease YejB [Rhodovulum sulfidophilum]AB15
MGAYILRRLALIVPTLLGIMIINFTLTQFVPGGPVEQVIAKIEGQGDVFSGFAGGSGDAGAALGAGGGGGSPTDKYIGAR
GLPPDFIAELEKEFGFDKPPLERFLTMIWNYARFDFGQSYFRSVSVVDLVLEKMPVSISLGLWSTLIAYMVSIPLGIRKA
VRDGTSFDTWTSAVIIIGYAIPGFLFAVLLLVLFAGGSYLQWFPLRGLVSENWAELSWPAKILDYFWHITLPVVATTISS
FATLTLLTKNSFLDEIKKQYVVTAKAKGLSEARVLYGHVFRNAMLIVIAGFPGMFIGVFFGASIIIETIFSLDGLGRLGF
EAAVARDYPVIFATLYVFGLIGLLVGILSDLTYVLVDPRIDFESRET
>MCE8455655.1 ABC transporter permease [Rhodovulum sulfidophilum]AB15
MAWLSPLNQRRWRNFKRNRRAHWSLMIFAVIFGLSLFAELLANDKPIYVNYRGESFFPVYSFYPETDFGGDLRTEAVYR
D
IEIQCLIVSGGLEACWDDPEGVIADAEDGVVDGETIEKGWMLWPPIPYSYDTVNNIDGTAPSAPDAQHWLGTDDTTR
DVL
ARVIYGFRLSILFTLMVTGIASVIGVVTGAVQGFFGGLTDLIFQRFIEIWTSTPQLYIIIILFAVLGRSFWLLVFLTALF
GWPALVGVVRAEFLRARNFEYVRAARALGVSNWKIMFRHMLPNAMVATLTMLPFIITGTIGTLASLDFLGFGLPASAP
SL
GEMTLQAKENLQAPWLGFTAFFTFAIMLSLLVFIFEGVRDAFDPRKTFQ
>MCE8455656.1 ABC transporter ATP-binding protein [Rhodovulum sulfidophilum]AB15
MSPVLKVDNLQVSFQQDGRQQVAVRGVSFEVGRGETVALVGESGSGKSVTALSTVGLLPGAATVSGSVIYEGEELAKA
PE
KRLRQVRGNDISFIFQEPMTSLNPLHTLEKQITESLALHQGLTGAAARARVLELLERVGIRDPESRLGAYPHQLSGGQRQ
RVMIAMALANGPDLLIADEPTTALDVTIEAQILELLAELKREETMSLLFITHDLGVVRRIADRVCVMKDGEIVETGPTAE
IFRAPQHPYTRKLLAATVGGPDPLKTEAPEIVRTEGLKVWFPIQRGFMRRTVDHVKAVNGADISVRAGETLGVVGESG
S
GKTTLALAIMRLISSEGRIVFQGSAIDGLKSRQIRPLRRDMQIVFQDPFGSLSPRMTVEQIVAEGLGVHGSQSGRSARED
VAQILTEVGLDPAMMHRYPHEFSGGQRQRIAIARAMILRPRMVVLDEPTSALDMTVQVQIVDLLRDLQRRYDLAYLFIS
H
DLRVVRAMSHKVAVMRLGEIVEAGEVHEVFDAPKTEYTRTLMRAAFGAPGESPGAA
>MCE8455657.1 oxygen-independent coproporphyrinogen III oxidase [Rhodovulum sulfidophilum]AB15
MSTESQLTRLGLFDAKVPRYTSYPTAPHFNASVDDGDFAGWIGQIEPGSQISLYLHVPFCRRLCWFCACRTQGTRSDSP
V
EAYLETLKTEIAMLGQRLPEGVTLSRLHWGGGTPTLLSPDMMKQLTEAIFAVAPMHEEGEFSVEIDPNEIDEARLDVLA
G
AGMNRASIGIQDFDPQIQSVIGRIQSYDITRDAVDLIRSKGITSLNADILYGLPHQSNERIAESVQKLMSLSPDRVALYG
YAHVPWMARRQSMIPTEALPTPENRLKLYETAKELFLWDGYREIGIDHFARPEDGLARALEAGELRRNFQGYTDDTAA
AL
IGLGASSISRFPQGYAQNLSGTSDYTARIRDGRFATGRGHVFTDEDRMRSRMIEALMCDFHISTAEITESFGIPKAELNA
MFQEVADRFAGFIDLTNDGLTVPEHGKPLARMIARGFDAYDLSKAGHSSAI
>MCE8455658.1 Crp/Fnr family transcriptional regulator [Rhodovulum sulfidophilum]AB15
MAVTDTSVVHTECFECPIRHRAVCARCEADELEALEAVKYYRTYDAGQSVIWSGDRMDFVGSVVEGIATLTQTMEDG
RTQ
MVGLLLPSDFVGRPGRDLAAYDVTATTQLTMCCFRRKPFEELMVRTPHIGQRLLEMTLDELDAAREWMLLLGRKTAR
EKI
ASLLAIIARRDAVLRLGPTRGRIVFDLPLTRESMADYLGLTLETVSRQISALKRDGVIVLEGKRHVTVPDFDRLLGETGD
DNDGGMPA
>MCE8455659.1 universal stress protein [Rhodovulum sulfidophilum]AB15
MGYKTILTIITETDIADSGLEPAITLAAREEAHLDILCLGVDRTQTGYYYAGTNAFIQQETLHKAQEDASAIEAAVKARM
ARETVTWSSEAAVAQIAGLSHLVAQRARFSDLVIMPRPYGEGRGIEDEAATEAVLFDGHAPLLVVPAGADTAHIGRKIVI
AWNQSQEAMAAIKEALPLMQAADVVNIAVVDPPKHGPDRSDPGGALSQMLSRHGVKTEISVLAKTMPRVSDVLQR
HATDI

EADMMIMGAYGHSRFREAILGGATRGMLEKTAIPVLMAH
>MCE8455660.1 cytochrome-c oxidase, cbb3-type subunit I [Rhodovulum sulfidophilum]AB15
MWDYVKIAALGVIAVLAAVAAFMARDMAYMVHAVIILLVAGGLVALTVRGLGAPKPVVDKTGYMDDVIRAGVIATCF
WGV
VGFAAGTFIASQLAFPGLNFEWASGYMNFGRLRPLHTSAVIFAFGGNALIATSFYVVQRTSAARLWGGNLAWFVFWG
YQL
FIVLAATGYLLGVTQSKEYAEPEWYVDIWLTIIWLVYLAIFLGTVFKRKEPHIYVANWFYLSFIVTVAMLHLINNLSLPV
SIWGSKSVQVFSGVQDAMTQWWYGHNAVGFFLTAGFLGMMYYFVPKQAERPVYSYKLSIIHFWALIFLYIWAGPHHL
HYT
ALPDWAGTLGMVFSIMLWMPSWGGMINGLMTLSGAWDKIRTDPIIRMMVVAVGFYGMSTFEGPMMSIRAVNSLS
HYTDWT
IGHVHSGALGWNGMITFGALYFLAPRLWNKERLYSTTLVSWHFWLATIGIVLYAASMWVTGIMEGLMWREVDAQGF
LVNS
FADTVAAKHPMYIVRALGGALYVAGGVIMCYNIWKTATSAPAKQPAAVAAPAE
>MCE8455661.1 cytochrome-c oxidase, cbb3-type subunit II [Rhodovulum sulfidophilum]AB15
MAILEKHKILEKNASLLLVFSFLVVTVGGLVEIAPLFYLQNTIEKVEGMRPYSPMELEGRDIYVREGCYVCHSQMIRPMR
DEVERYGHYSLAAESMYDHPFQWGSKRTGPDLARVGGRYSDAWHVNHLRNPQSVVPESVMPKYAFLERRMIDPEHI
ADKL
RTHRAVGVPYTDEMIENAVADFNAQIDPYDDGADALLERYPGAQLRNFDGNEGISEMDALIAYLQMLGTLVDFSTFEP
DA
SR
>MCE8455662.1 CcoQ/FixQ family Cbb3-type cytochrome c oxidase assembly chaperone [Rhodovulum sulfidophilum]AB15
METYSFMREFADSWMLLVLTLFFVGVVVWAFRPGSAKEHSDTANIPFRHEDQPAGTEDGATCHKEART
>MCE8455663.1 cytochrome-c oxidase, cbb3-type subunit III [Rhodovulum sulfidophilum]AB15
MSSSDHHTKPGEVGTTGHSWDGIEELNNPLPRWWLWTFYACIAFALIYSVLYPAWPLVKGATPGLLGYSTRAAVEVEI
KE
YEDRNAELSSQLAAASMEDPAAIEAEPALYNYAVQGGASVFRTNCAQCHGSGAAGVQGQGYPSLLDDDWLWGGTLE
DIYY
TVTNGVRNEDNMDARWSQMPSFGTDGLLTSGEIDQVVQYVRQISGQEHDAALASQGETVFLDNCAACHGEDGRGD
RNVGA
PNLTDAIWLYGGDVATLTETVTKARFGVMPTWKERLSETERKAVAVYVHQLGGGE
>MCE8455664.1 PAS domain-containing protein, partial [Rhodovulum sulfidophilum]AB15
MSRPVQGSPREAAAPLFGEVADLVALMAAPAWATGPDGTVLAANPAAAALLDRPVADWVGLGDTALLVARQVARL
RRARA
GIGRDGAVRVRFRGCFLTGGGHPRLLDCSLTALAEPRGGAVLVQVDPVAPSRVGTACSAAGTGHLSPPGAVLPRAAEL
VG
FAEWSLDPDCGRIRVAEGMLAMLGHAPDGGEIDSAWLRARVHPEDRPAMRAVAARLIRGDDESIRIDCRLRHRDGRY
LWV
EASARRVEPGPGLRPVFCGVLSDVTHHKEHEAQLQQALFEARRVRTEAAKTEGIIRIAAEGAGFGHWTFDPVIETYWLD
E
VFSRTLGYRPGAIDTSRSGFRELLHPDDAEVTRAAMDALVSGGSERMHIDCRLRAADGTWRWFETSARLVSLSEVGLP
PL
VCGISRDITPRKTYEQELAHALELARQARDEAEAARVKQQLQATILRVSTEYSNVVPWHAVPSLGLFRADDHLAAMLGY
P
PGRRFTAAEFRNLIHSEDASEALGRVDALYRRKLEAIAVEFRVRRGDGGWCWVSSKARWLDRAASGLPDMICGTLTEV
TA

LKANETRLAEALAAAETARIDSQTREEMLRTSALCSGIAHWVVCPATGEGWIPDGTYGLLGYAPGAFPPQKGCWRGLI
HE
EDSVEAVALMRDLLEGRSETYAHEHRLLHADGSYHWYRAVARRVRRAASGQPDLIAGALVSIDHVKENEARLAEAAEA
AR
LAGERLNTLADNAPGALFIFLISPDGRFELPYFSAKLPALMGLSRAEIEADASALMTRVAPDHIRALRRAIATSQRGLTP
FEMRLRVCRPEGGFRSVLVASLPFAREDGTVVWYGNVFDITDRLDAERRAAEAAEEGRRVHERLSSVAMIAPVGLYEFR
R
QADGSIDFPYTTPKFEDIMGLSREEIGMRSCDVLRRIDPEDLPHVLASIEDSARDLTLWSQRFRIRHPERGLIWLSGASA
PRRQEDGTILWAGALHDVTGDVQREAELRQAHRLAEAMRAENERQALHDGLTGLPNRRFYDRVLSSRIEAARAGQGP
RDC
VLIRIDLDHFKYVNDTLGHEAGDRVLIRVAEVLRQMLRAVDFVARIGGDEFSILLGPDMNQDNARELVERVQGALSRPL
T
YEGRLCRFGASFGIAHTGDITELGAELQLFADAALYRAKEGGRNRMEFFTAELHRNILNDRRIAVELHEALERDEFEPFF
QPQIAAEDGRLVGAETLLRWRHPEKGLLSPDDFMHVAEQLRIVPEIDRIMMRKTRSALARWQDQGLVLPKISFNVSSG
RM
HDPDVVSLARSMAGGGTRVTFELLESILVEEESDAFRFHLDQIREAGIDIEIDDFGSGHASIIGVMEIAPKALKIDRRIV
RPVACDMRSRNLVRAIIEIAETLGIATVAEGVETEEQAKILRGVGCQVLQGYLFSHPLDEGDFLAFT
>MCE8455665.1 EAL domain-containing protein [Rhodovulum sulfidophilum]AB15
MENDLRQPVAQRLRDSGARRGRIASVLRGGESSILNGLGLAAATFLTLILGLGALSLGQIGSVTAVSLEMRDVALPKLLA
VNEIEIALDAHALLARRRVQVTDFRQAADIDREMRRAAARFLTGIERLDQLTLGARALEVEQSLEAAWLEYLAALDRVG
R
LYERGALADAEAAMQDSVRVAEADVRRSVSDLVDVVQAEAAGISAAAEREWQRARVLTLGALLAGLAATLLTMRWV
VRRV
SRPLLAISAAMRRLNRGEDAAILPVLPGRTDEIGILAEAAAAFRDSVLEARALAADLELQREILAASVRNMPLGLCMLDD
LGRVAVSNRALAEIFDLSPADCAPGVAQRDLLDRLAARVDRGRKSAGEFSTGIAATVARGDQMTETWQLMAGGSVSV
IIQ
PTPDGWMMIAEDITERVSHEANIRHLARHDPLTGLPNRRAFSEEIDRAIAEAGAECPAAVLFVDLDRFKNVNDTLGHGI
G
DALLRQVAARLSWAVRDADTVARFGGDEFAVIQRGCVQPEGARGLGERIIALLFSPFEIEGNSLRIGGSVGVALAPGDG
D
CVADVLRNADLALYAAKAAGRGQCLFFDPEMNARQRALQKLEHDLRQAVLSDAFVMEYQPLFDIASGDLVGAEALM
RWID
PVRGRVSPAEFVPMAEELGLIVPLGARALEMACADAATWPAHLKVAVNLSPVQVHRVGVIEDVRAALAASGLDPRRLE
VE
ITEGVLLDHGPEVLGTLDALHEMGVQIALDDFGTGYSSLGYLRALRLDKVKIDISFVRELENDPSAAAIVRAVCSLCASL
DIGVTAEGVETAEQLASVAAAGCIEAQGFYLGRPGAAEIIAGLAGGALQGGRGVA
>MCE8455666.1 TRAP transporter substrate-binding protein [Rhodovulum sulfidophilum]AB15
MLAPGRAEAHPTGAPSANMQGGISGPDQLRIASFFPVDMPVLGRAALDLAERVERMSGGTLRLSIASPSAAAPALDIIV
E
MNHGAIDGVWAAAGWYAQRDSAFGLLTGMPFGPTPDEYLAWYFYGGGRELAQDLYRPYDVHNILCGIIPSEASGWF
PREM
TSVDDLAGLRMRFFGLGAKVMQRFGVEVVLLGPEQMFEAVGTGRIDATEFSLPSMDVPLGLHKILKYYYFPGWHQPA
TLI
NLYLPRPVWVALPDRHKAVLEAGCGDMIRQTLAEGEAVQAQALRAIAAEGVELHRWPAEMLVAFENAWQAVVAEE
SRLNP
NFARVYASLAKFRADRQQWRHFRFLQ
>MCE8455667.1 exopolysaccharide biosynthesis protein [Rhodovulum sulfidophilum]AB15
MARTEDFSQDSIGRIREAPVSAVLDAVEQAAQGDRLSVRRMFEALGSVSFLPLMLAPALAVVSPLSGIPGFSSLCGIGIA

LIAGQMLVRRRHLWAPDWLMRREIRADRARTAIAWLRRPAGWLEWAARPRLNFLVTPPLVAVLQLLCILAGLAMPFL
ELV
PFSSSLLGLTVVTLSVAMLARDGLLALIGMAPFAAALAVIVGVLELG
>MCE8455668.1 entericidin A/B family lipoprotein [Rhodovulum sulfidophilum]AB15
MTRIVVFCLSAAALLGVAACETVEGAGKDIQSAGGAISQEAREAQN
>MCE8455669.1 histidine kinase [Rhodovulum sulfidophilum]AB15
MTDTLKARISTRRLTKSLALWLIGALTVAMLPLGLISVSQTSKVLHETERLSGVALMDRTQRAAVRTQALIQEAFGAARS
VAAANVVFEGGGLSCDAVMARLVKDNPTYLFAGFVEPDGRITCSSDGEHLSLVDQAYFLNLLRDPQPRVDTRFSVPLEG
A
RVVRVSVPVFEEGELQGFVLISMPYAAAYYTLDDGGAEVDLLVFDHEGEFLTAEAATREEGAPPETFEEVLPRNHTLEAL
AAAGRQSFRGTNRLGETRDFAVVPVIDDRIFVMGSWAPDRNGTVPTTGRALALYFPILMWVAGIVVAYFAVHRLVIRH
IK
RLQSWMRLYASGHTDFGQARLDHAPHELEVVAEAFRSMTRRLAEQDRALEEDLAEKTILLKEVHHRVKNNLQLITSLM
NM
QIRNARSAEARRLLKRVQDRVMALAAIHRYLYMARKLSLVRADRLLDDIIRQLVVVSDIGGAEKSVKIATQFSPVEVNPD
QSVPLSLLATEAAINAVKYCGTPPEGGQAWITMALQTLEDGRICLSVVNSRSEDPEPDPAAENGALTDSSGMGRQLISS
F
AAQLDAEFEVHSEPDRYELHVTFAPLGASLEEDEAEPEEEAASGGVSEGT
>MCE8455670.1 RNA polymerase sigma factor [Rhodovulum sulfidophilum]AB15
MRAFALSLTRNSATADDLVQDAIVKAWSNFDKFEPGTNLRAWLFTILRNTYYSMHRKRRREVEDPDGALAAHLSEKPH
HD
GRLALADFKVAFAKLPDEQREALILVGAEGFSYEEAAATCGCAVGTIKSRVNRARVRLAELMHLNDADDIAMTDPVTLA
G
LSSKRPA
>MCE8455671.1 RNA polymerase subunit sigma-70 [Rhodovulum sulfidophilum]AB15
MEEQIDDNLRRVYAQAVEEQVPDRFLKLLEQLRGAGAEASNGSSGKGSDT
>MCE8455672.1 response regulator [Rhodovulum sulfidophilum]AB15
MTHAPHSDDLSRVIGSELPYLRRYARALTGSQTSGDAYAIATLEAILSDREVFEQGLPPKVALFKAFNRIWGSSGAPLSE
SQPASAIEAQAQTRLAGLTRNTREALLLQSIEGFTTEEVGTIMGLPGTEAEQLIRIARDEMARSIAGRVLIIEDEAIIAI
DISAIVQGLGHEVTGIGRTRRGAVDLAKKDPPDLILADIQLADKSSGIDAVNDILGDLGDRPVVFITAFPERLLTGERPE
PAFLISKPYTEDQVRSAVSQAMFFSSTETLGA
>MCE8455673.1 DUF1328 domain-containing protein [Rhodovulum sulfidophilum]AB15
MLYWALVFLVVALIAAAFGFGGIASASAGIAQILFFVFLVLFVVALVARALRGRMP
>MCE8455674.1 DUF883 family protein [Rhodovulum sulfidophilum]AB15
MAQSAEKRNDSSASVQDLEVQIEALKDDIAQIARTLGDLSEAKKNDMRDTVQRRASELRERGEATVAGVQDRGAELG
EQA
ADAVRRQPAAAMGIAVGLGFLVGLMTGRR
>MCE8455675.1 hypothetical protein LZ188_03655, partial [Rhodovulum sulfidophilum]AB15
MFSLPLDEAERRARAFSHRLALGTAGSLCAGVGAAFLTVAAWLVLSAGHGAVFAATVIGSAYLGAGL
>MCE8455676.1 potassium transporter TrkH, partial [Rhodovulum sulfidophilum]AB15
ILFNVTSIISGTVFSSTDYQLWGALPVILFFFIGLIGGCAGSTACSIKIFRYQLLFSAIRVQIKSIHAPHGMFQPRYDGR
RVDEDVLSSVMSFFVYFVVALGVVSVLLGATGLDATTALSGAATTLGNIGPGLGDKIGPSGNFAGLNDIAKWILSMAMF
V
GRLELLVVFAIFTVQFWRA
>MCE8455677.1 serine protease [Rhodovulum sulfidophilum]AB15
MRFLATIAFVLLAAMPAAAQDRAWVQVEAHPTLRETEERARAYAATLKDVGAYRLNSGWYSIAVGPYSPPVAEAELAR
YK

AAGLVPADTFIADGASYERRVWPAGARMQDPASETVPTPQPDPATAAPAAAAPVALPDETPLEARRSESRLDRQARM
EIQ
TALKWERVYSSTIDAAFGPGTRRAMADWQSAQGYEPTGVLTSRQRAELVDGYKAQIARLGMTPVDEPEAGIRIEMP
MGLV
RFDRYEPPFVHYASRGDSGVTALLISERGDQSTLFGLYDIMQTLEIVPLEGPRERSSRSFTLIGKNDRLQSHTYAQLTGG
MVKGFSLIWTPSADPKLMQRAVAMMKDSLDSVPNVSLDETLGEATAEQRADMLSGLEVRKPDATHSGFFVDPEGAV
LTTT
NGLDSCRRITIGPELEGTLAATDAAQGLALLRPGRTLAPIGFAEFAPGVPRLRSDIAVAGFSYGEVLDLPVLTYGVLADL
KGLSGEENVERLELDALPGDTGGPVLDASGAVLGMLLAASGGARQLPEQVRYAADASAVGAFLTANGIEPRTSSATGA
LP
PAELSRRAADMTVPVSCWN
>MCE8455678.1 glycine--tRNA ligase subunit alpha [Rhodovulum sulfidophilum]AB15
MSDGKPRSFQEIILRLQTYWASKGCAILQPYDMEVGAGTFHPATTLRSLGTRPWAAAYVQPSRRPTDGRYGENPNRM
QHY
YQYQVLIKPSPPDLQELYLGSLAAIGIDFDYHDIRFVEDDWESPTLGAWGLGWEVWCDGMEVSQFTYFQQVGGHDCH
PVS
GELTYGLERLAMYVLGIDHVMDMPFNDPDAPIALKYGDVFRQTEEEYSRWNFDVADTEKLLHFFEAAEAECARILDQP
AE
DPRSGKRIIMVHPAYDQCIKASHFFNLLDARGVISVTERQAYIGRVRALAKRCADAFVETKAGGWTGDAPEAAN
>MCE8455679.1 glycine--tRNA ligase subunit beta [Rhodovulum sulfidophilum]AB15
MPDLLIELFSEEIPARMQAGAREALRQRVTDGLVEAGLTYGHARAYSTPRRLVLAVEGLSPRSPDLKEERKGPRVDAPEK
ALEGFLRSTGLTKDRLEARDDKKGRVWVAVIDKPGREAAAIVAEVLEATIRNFPWPKSMRWGAGSLRWVRPLHSILCLL
T
TEAGAEVVPLDIDGIRAGDTTRGHRFMAPEPFRVTGFEDYAVRLKRARVMLDQDERADQIWHDATNAAFAQGLEVV
EDKG
LLTEVAGLVEWPVVLMGAIGEAFLDLPPEVLQTSMKEHQKFFSVRDPKTGRIVRFVTVANRETADNGETILKGNGKVLS
A
RLSDAAFFWGNDLAVAKAGMEDWREALTHVTFQSALGSQADKIGRVAALAREIAPMVGADPDLAEQAAKVAKLDLA
SQMV
YEFPELQGLMGRYYAAAAGLPAEVAEASALHYKPLGPSDEVPTAPVSVAVALADKIDTLTGFWAIDETPTGSKDPFALR
R
AALGVIRLVLGNVLRIKLLDVINSKALRFVENETYNTLAKEDVGWTTEELTYLNSEEFDELKTAHNAYQDAALSGDYLRL
IAVAKSRYLLSFFHDRLKVHLRAEGIRHDIIDACLAMPGNDDLTLLVRRAEALQAVIGTEDGENLVQGFKRANNILSQAE
EKDGVEYSFGADRKFADDPAEAALFDALDAAEAAIGPAMEAEDFAKAMAAMAALRAPIDAFFEAVQVNSDNEIIRRN
RLN
LLHRIRATCGSVADLTRIDG
>MCE8455680.1 pyruvate, phosphate dikinase [Rhodovulum sulfidophilum]AB15
MRKLTDIVDFTEITPDAAIAADRHGGRAKCLQRLVRIDLPVPRTVALSFEAVHRIALGQMPDAEALLAVFGAAPLVSVRP
SSADPDWGGPASILNIGMNDARHAALCKTHGAAAANALYLRFIQAYAIHVARLDPDLFESEDYSAEAVAAALAAYAEET
E
EPFPQDPVRQLLEVLRSMARAWEGTTARLLRQAKGAPAEAGLGLVVQQMALGLGPEESGSGVIQFVDGVTGQRRITG
RYL
GQSQGRDAVSRQKQALFLTRDPRGPALEDRCPELFARLKRYGDTCRINLCEEMQIEFTIEAGRLWVLDAVRVQRSARSA
V
RIAVMLADEGIIAREEALARIEPRAVSELLHSQVDPTGRRQVIAQGIAASPGAAVGRLVFGSAAAQASAARGEPCILVRR
ETTPEDIRGMHVAAGVMTERGGMTSHAAVIARGLGVPCVVGVSDMRLNRRDGVLITADGRKLHEGDIVTLDGTNGQ
ALLG

EAKMLPPALDDAFHALMGWAEAACDIGIRANADTPADAETARRFGADGIGLCRTEHMFFEHDRLTVMHEMIFAAGP
EDRG
TVLERLLPMQRGDFIRLFEIMQGLPVCIRLFDPPLHEFLPQSREGIRELAEALGLPVSDVTRRIQALSEFNPMLGMRGVR
LGIAVPEIYDMQARAIFEATIEASRKGWPIVPEIMIPLVSAMREVELVKTRIDSLAAAVRTERGADFAYRLGVMVETPRA
ALRAGDIAQHAAFLSFGTNDLTQMTYGLSRDDAGRFMGSYVKQGVFAEDPFHVLDTEGVGELLSIGADRGRLARPEIV
LS
ICGEHGGNADSIAFCREAGFDYVSCSPFRVPVARLAAAQLALGASLRPKHGRY
>MCE8455681.1 cell wall hydrolase [Rhodovulum sulfidophilum]AB15
MTGSQSNDPTAVLGDNLARLLGAERAGLQRLGQRRINRLSTSPAVRDARRYDPKWLDAQPVAKGGEAWTCLSEALYF
EAR
GETVKGQFAVAEVILNRVASPLYPDSVCGVVHQGTGKLNRCQFSYTCDGRDEHISDQRTYRRMGKIARMMLDGAPRA
LTG
GATHYHNRSVDPGWARRFPRTASIGQHLFYRQPAAVAAN
>MCE8455682.1 dihydroneopterin aldolase [Rhodovulum sulfidophilum]AB15
MTDDIRLAFAHPSERAEAMDPARPLRDRISLRDHVAEVEIGAFQAERGSTQRVRFNVVVEVRPHPAPLEDDVDRVLSY
DM
IAEAIAAELSSERLNLLETLAERVAERILAAPQAMRAYIRIEKLDRGPGALGVEIVRDRAPDLPRPVPVAAEPEPLVAPL
VVFLTNAALAAPELPGWLDRWQAGSRPLVLCVGASETPAPQATAPLVQRRIDLLAIEQNAWVLAGRDPRCVVRGSRT
EID
WAMKHDQISVWAPSKIVLDAVESPSADPHDALELALWFAAELHAETVLVIGENVPEHPARGLDLRYVSPGAAGTAI
>MCE8455683.1 dihydropteroate synthase [Rhodovulum sulfidophilum]AB15
MTSYYRPIVQTDPARPSEALTLAGGWGWFAMAEMLERDEPPRLVPARAIPEDWRIALTAPRPAIAGLTMDRPRLMGI
LNV
TPDSFSDGGQHDRPETALAHGQALAVDADILDIGGESTRPGSADVSVPEEIARTAPVIAALRAAGVSRPISIDTRKAAVA
EAALAAGADMVNDVAALGFDPELAGATARAGAAVCLMHAQGDPATMQDDPHYDHVLLDVYDFLVERVRAALASGI
PASRI
VVDPGIGFGKTLAHNLALLRGISLFHGLGCPVLLGASRKRFIGAIGGAETAAARGPGSVAVALAAIAQGVQFVRVHDAA
E
TRQAMRLDAAVKGLELGHE
>MCE8455684.1 phosphoglucosamine mutase [Rhodovulum sulfidophilum]AB15
MSRQIFGTDGVRGRANSHPMTPDMALRLGQAAGRYFRTDGLNRHRVVIGKDTRRSGYMLENALTAGLTSTGMNVLL
LGPV
PTPAVGFLTQSMRADLGIMISASHNPHEDNGIKFFGPSGFKLSDEAEAEIEAILEAEIALSQPANIGSAKRIDDGLWRYG
EFLKTTFPRGLRLDGLKVVVDCANGAAYRVAPSVLWELGAEVVPVGTEPNGFNINRNCGSTHTDIAAAAVVAHGADLG
IC
LDGDADRVMIIDETGAVADGDQIMALFAERWAAQGRLRHGTLVATVMSNLGLERHLAGQGLKLERTAVGDRYVVEA
MRKC
GWNLGGEQSGHIVMTDYCTTGDGLLAGLQFLAAMVETGRPASDLARRFERVPQLLKNVRYAAGADPLGTASVQDAI
AAAE
KRLNGHGRLLIRKSGTEPLIRVMAECEDDAQLIEVVGKVVEAVEQVA
>MCE8455685.1 hypothetical protein LZ188_03705 [Rhodovulum sulfidophilum]AB15
MAASSLIVTLALGTLCAVVVFSWISKQRTEQRQADPEAPKSTLAADTPDTRPDGRGAP
>MCE8455686.1 OpgC domain-containing protein [Rhodovulum sulfidophilum]AB15
MLAAGTGDRNPAVRAAARGLRWLASFRPLVFLGQHSLQVFSFHVFLVYAAMFADISEAPRLLRGIVMLASPVCFLLPAY
L
HARYRDRSQRLRLSGTNP
>MCE8455687.1 amidohydrolase [Rhodovulum sulfidophilum]AB15

MEEHELYGCPCCSGTIGCLFRVNETVRDLKEQAATGDDSFRNRDLDRASIGRRNFIRTGAAAASLAALMPKEGRTQAIS
T
EETTIFTNAKVLTVDDAFSEAEAIAIRGNRILAVGSNEEVRTVAGKNAAVIDMQGKTVLPGFIDAHAHVISGAIADDVME
YVGMNSYATVDDVLQQIAQRASETPAGEWLVFRNFDPAVQEGAETLTFSDLDPISSEHPIFVLNASGHIAYANSKAFEV
S
GITNDVSDPPGGEFVRDAEGNLTGMMKNNVAFIQIAENYPEMTKVEPVDALLRLLENWAQHGLTTVSELSLGVLCQS
PGD
VQIMTEAAQSGRMKARIRAYPFYTLGAEAWDRADVMQGNGNALARIAGYKLVADGSNQGFTGWQREPYLGSDNR
GLRYME
PDEMTAAALERAGKGWHLAIHGNGDAAIDNILDTFQALVDAGVDMSRVRPRIEHCSILHDDQIARMKKLGVSASFLIG
HV
YYWGIAMRDKVFGEEKAQLLDRCRSAEKAGIRFTLHSDFMVTDPNPLHMIEMAVTRRTWKEPDYVLAPQETISVETAI
RA
MTSEAAWQLFSDHELGSLEPGKLADLVIMEQDPRDVRQDEIKNIRILETWMDGRQVYSA
>MCE8455688.1 hypothetical protein LZ188_03730 [Rhodovulum sulfidophilum]AB15
MVLFWAAATTVLAAGGGTGMGPSAIEVAPGAGALVLAVRTGPLPAGAELVLETSGGRRIGSVSPFGATAATSSAVQT
HLV
PVPAGTLPEGRIEIRAKLVLHGFERAATAREFLGAAIIGH
>MCE8455689.1 tyrosinase family protein [Rhodovulum sulfidophilum]AB15
MTGTISTNRRTVLKGGAVLLAVPFLARSAAGQASHLRVSMTSEEGIAHLRTYRKAVAAMWALEPSDPHNWFRYAMIH
LMD
CPHGNWWFFVWHRPYVAHLERIVRIYGEDENFAMPYWDWTADPFVPDTFFASSTYPDNALDPTSTHYPDWDSFESA
YHDP
IEAFWNGLSDAQKQQETLRGFPDFESFWTTGVQPNFTAAPANGRSKTLAAPALTPPASDAVTLPIIEAGLAPEIFTTVVA
TDDARTGFNSPESQNHNQGVGAAIIEGQPHNKVHNNLGSVAGGWMPSLLSSVDPIFFMHHCNVDRLWDVWTTKER
NAGRT
ALPTAEEAPRFDPEAFLFYIDDAGQPAPSTAGASMAIEPWGYAYTPGSGSASAAGLQSKRGIAPNAIALANKDFGLNEG
A
TTSLSLPEGFAAAGKEETRQFAQVTFKPPRELKGLSFDVFLSPVGEMPELSEDGPGFAGSFEFFGMLHGHDHDFTAVID
V
TDAVARLREAGTLRESGAIDVTLAASGTALKGRMLESAPLEGVLKSVVFESF
>MCE8455690.1 NUDIX domain-containing protein [Rhodovulum sulfidophilum]AB15
MNTWRPASGIRFKALGLHWRGNRLLASEVYEDSGRLKGVRPLGGTVEFGETAQTAVIREFQEELGITVKIVGSPTFIENI
YVHEGSLGHEMLAIFDVTFPREAFAGQTRIAFREDSGVECFAEWFALEELDGPGQPQLYPTGLKALLKSKR
>MCE8455691.1 DUF4123 domain-containing protein [Rhodovulum sulfidophilum]AB15
MRTDTGHYDWLQASKREITADEHAPRIEVIAGVEPLDGQFGVLAPKSVPDKLHTPLFGQPEPNPAEIALGVDISAAPSL
G
TFAVLDAARLPNLPEILSASGLEYACLFKGGAADELQDVAPWIVRLEDESRFTRNIFTQGDAPWAMWGQAPGIYLRSR
DG
LDDVRNHLRKFIRARDDEGTWFYLRLYDPRIMRAFLQGAPRFAHRLLARPFPWSNLTILTCFEDSAQIASPAENVPADM
R
PIRIGSEEKAVFRHLTFEARAAALMARLGKSNATSMSTDPNVRLAQKSAIISAFHRMYDYGFQQPLQQDRWGLWELFY
GA
GFEMNDPILKRICDTSDAPSGVRFAAFQRRLEEIYS
>MCE8455692.1 hypothetical protein LZ188_03750 [Rhodovulum sulfidophilum]AB15
MATVATGPVSAREREYYTDMPCAEGQGCGGEGVVLPFDQGNVALDEMFGFTDVEDLPLSDEVLPVIFDGALLTEDGL
LDC

FARHYPERAGFLLQLLSDEGDLALGYRLEFLDDATQAAPMREALRSAQDRYNEVAARQQQLRGSVRSQLMMGGHPG
AEMT
EGQDLENEAITLRNRIRDLETQLSRLGRWDIDDSARVIRVYTTINSNVERAPLAEYVPGGDDGINPLPTNRDGADWLNA
V
MGDWMARNGYPGAGSEEEFFRNWLTRNAAGVALVGLGAIEMFGGVTVAIGSGGLAAVGGVALTVVGFDTLSQGAK
MLWTP
DRHSSERGWIGDLVHDTAMRFGGEETAQAFDRGWAVTQILAGLGAPVVIRYAATRATRVAASGKNIRPIVRGQLDEA
RLG
IAEVKGHRFGDGLVGDYAVLPSGRGAIIDFPGVGRMRLPTLDSDMRVLLRLRRTTKAAIQSRFARLTRHQGPRVVRDQS
T
SRELVEKAADHSGVDLARFVDEVRYGPSVDGELARFSVEGGQRIITITNRLPQENKYMQLKVAAHELNHARIYDKFIRR
M
GPIRGHEEYWGAHRKFGKPLYAREEVIVEKAAQMMIRSVFEGRMTGANLRRLTHALDDSENYIAGWRELM
>MCE8455693.1 hypothetical protein LZ188_03755 [Rhodovulum sulfidophilum]AB15
MAGYGSPEEDGVRDLVRQIDDEKIIENMKEGRRILQKVTTHDFGYSLHDWHNFLINDKKHSGHYMRASTWAGVERAV
KAE
LRNPDRARLEALADQM
>MCE8455694.1 HNH endonuclease family protein [Rhodovulum sulfidophilum]AB15
MRRLLSLLLLSLTLLTCAPVAQAPQSDAPALPYQRAAFGSGWTDPDGDCLDTRAELLAALSTVPVQLAPSGCSVRHGR
WF
APYTGQVVTEAGDLDIDHIVPLRYAWGHGAASWPAAKRARFARDPVNLLPVSASANRSKGARGPLDWLPPDPGFRC
QYVL
RFRRIAASYGLVRSAAEERELVALTGRLCGA
>MCE8455695.1 DNA adenine methylase [Rhodovulum sulfidophilum]AB15
MRKTVPATPVAPWMGGKKALHRRIIERIEAIPHRTYVEPFLGMGGVFLRRSWRPRLEVANDLNGEITNLFRVLQRHYP
QL
MEVMRFQITSRREFERLRACDPATLTDLERAARFLYLQRLAFGGQLRGVFGISPGCGPRFSLARIGPLLDAAHARLDGVV
FENLPWQEVLARYDGPQALFYLDPPYWGGEDDYGKGLFDRDQFAELAERLGRIEGAFVLSINDRPEIRDLFGAFVIEEVR
LNYSVSKGGATAARELIIANREVRVGLV
>MCE8455696.1 bacteriophage spanin2 family protein [Rhodovulum sulfidophilum]AB15
MRVLLAALLLAGCGGLPVPLGPNVAANVQAGAENVQGQKVENAPSILRPRAREIRQEQSENRLRADRVETVIVNVIPP
WI
VLVALIGWIAPSPGEIGRRIGKAVRGRRA
>MCE8455697.1 lysozyme [Rhodovulum sulfidophilum]AB15
MRRILKRGAAATALALSFIGGNEGLKTEAYLDIVGVPTVCFGETRGVELGDSYTPAECRALFADRLAEVEAGLDLLIADP
LEDRIPDRSYVGILDWAYNVGLGAAARSTLIRKLNAGDLRGACDELPRWRFAGGKGVRGLLIRRNKARQLCHEGLDGV
PA
EVPFRWGGA
>MCE8455698.1 hypothetical protein LZ188_03780 [Rhodovulum sulfidophilum]AB15
MHLVPQWRALIRRAWSIRLILIAGLLSGIEAGLSLASPDAIGLPRGLFAGLSALVTAAAFAARLIAQRME
>MCE8455699.1 type II toxin-antitoxin system HicA family toxin [Rhodovulum sulfidophilum]AB15
MNSKHRKTLLAVFTDPVSGTIEWTSIERLLVAAGAQVIEGNGSRVRFHKDGVIASFHRPHPSKEAKRYQVRDARDFLIQI
GVTP
>MCE8455700.1 type II toxin-antitoxin system HicB family antitoxin, partial [Rhodovulum
sulfidophilum]AB15
MKNVMSYKDYSARVEYDDEDGIFAGRIAGIEDVVGFHAESVTELKAAFQEAVDDYLEICAQTGK
>MCE8455701.1 IMP dehydrogenase [Rhodovulum sulfidophilum]AB15
MEIREALTFDDVLLVPAASSVLPSTADTSTLVSRKIRLNIPLLSSAMDTVTEGRMAIAMAQAGGIGVIHRNLTVEEQARE

VSRVKRFESGVVYNPITLRPDQTLGDAKAMIERYNFTGFPVVDERGHVVGILTNRDMRFASSDAMPVSAMMTGSELA
VLR
EPVDRSEALNLMKARRIEKLLITDDKGKLTGLLTLKDTEKSVLNPQAVKDELGRLRVAAATTVGDAGFERSLALVDAGVD
MIVIDTAHGHSEGVAKAVERLRKASNDVQIMAGNVATAAATRALIDAGADAVKVGIGPGSICTTRMVAGVGVPQLTAI
MD
CATAAGDVPVIADGGIKYSGDFAKAIAAGASCAMVGSMIAGTDESPGEVVLYQGRSFKEYRGMGSLGAMARGSADRY
FQK
DAASDKLVPEGIEGQVPYKGPAGTVVHQLVGGLRAAMGYTGCATVEEMRRNCNFVRITGAGLKESHVHDVTITRESP
NYR
VM
>MCE8455702.1 sugar transferase [Rhodovulum sulfidophilum]AB15
MSIAVLQAPAAEAGAISTDLLGGYRGKRALDLLAAALLLALTAPLMLPIWLLLRAETGQGFARRLRIGRGGRPIRCLVFH
VERCGGPVALWVRCAGLSGLPLLWSVLRGEMSLVGPRPATRAELARYGLAQSGYLAMRPGLTGLADARDGGRFCDR
VHDD
LRYGLIASPRVDLGLIGAALMRRWRPGRPQPGLALLSGTVPLKLSFRSGSCRAGRHPSAGPCAAAGGRAANRPGAGRA
GN
>MCE8455703.1 short-chain fatty acyl-CoA regulator family protein [Rhodovulum sulfidophilum]AB15
MRKALIGSKLRQLRRDHGQTQAEMARQLNISPAYVNLLENNQRSLSVKVLMALAEAYGIEIRELLSDRNAGRLADLRAA
V
ADPAFGRDRPDLQELRGAVDHAPRLVDLFLQLYRSHRTAVDRISRIGGSGTEPALLVTSPETAVHDFFRDNRNYFDALET
AAENVRGQFAGDADDIYAQLKRYLRQKLGIEVRLAPADDMPDALRVFDRQAGEVRLSQALDHANRAFQLAHVLALAD
IPY
LLDSMIGEGGIDGDLAAARCRVELANYFAAALLMPYAPFLALAEATGYDVDRLGAAFGTSFEQVSHRLTALQRDGARG
VG
FFLIRVDKAGNVTKRVNATPFALAEKGGSCPVWNVHNAFATPGVILPQLVEMPDGSQFFTLSRTVKRPVFSPHLQDNR
LA
VALGCERGEAHRIGYAQAVNLGAEGPVAKIGVSCQLCPRQACSQRAHQPLHMDLPMDADRRGKTRYES
>MCE8455704.1 tripartite tricarboxylate transporter substrate binding protein [Rhodovulum
sulfidophilum]AB15
MTQHLTRRASLALMSAAALTAAAPALADDWKPIKPVEMVIMAGQGGGADQLARLFQAIIQKQGLSRMPILPVNKGG
GSGA
EALRYLKDREGDAHTIMATLNSYYTTPLRTDIGVDIAEFTPIARMALDSFVLWVSADSDIHSLDDYVAAVKEAGGSWKM
G
GTGTGQEDSLVTAMLEKEFGIDVTYVPYKGGGDVATALIGGHVSSTVNNPSEQMGFYRAGKSRPIAALTAERLTRFPDL
P
TFTELGHPLVYTMQRSFVGPKDMPAGAVDYYTGMFRQLSQTEQWRTYTEEQALLADFLTGEALQDYFLEERDKHAEIL
RG
MASSS
>MCE8455705.1 tripartite tricarboxylate transporter TctB family protein [Rhodovulum
sulfidophilum]AB15
MLAADRLIALALMGFSVIVMWYAQRLPVGWEAETGPGAGAFPFWLGLGMLLASGAILARSFLSRPHSGETFIPDADAI
PQ
VLGVVGLLLGAIALIPLLGAYIALMLFLLAYLRLMGGHGWLLTGILTLTTPVFLFFFFEVTLKILLPKGVTEPLFYPLYA
MFF
>MCE8455706.1 tripartite tricarboxylate transporter permease [Rhodovulum sulfidophilum]AB15
MDVLALLADGFAASISPLNLMIVLIGVTVGIFIGAMPGLGSVNGVAIVLPLTFIVPPGSAIILLAAIYYGAMYGGAISSI
LLGIPGASTAVATTFDGRPMAQKGEAGLALIVAAVASFIGGTISVLLFTVFAVPLADMALAFGPAEEFALVLLAFTTFVG
LGGDDILKTIVMICLGLVLSTVGLDIISGEPRLIFGDLPGFYSGISFLVLAIGVYGVGEVLYTIETSRNAPMVTPARLTF

SELRAGLSKMRGMWRTMSLGSVMGFFVGILPAAGATPAALMSYGVAKSISKRPESFGRGNPEGVAAPETANNAAST
GSLL
PMLTLGIPGSPTTALLLGGMVMWGLTPGPMLFVEQKEFVWGLISSLYTANVAAVLVNIALIPVFVWALRMPLTVLCA
MVL
VLCIVGGYAPGQRLHDVWMIAGFGVAGYLLRKADYPLAPLVLALVLGPLMEKSFRQTMIAEHGDVTVFVERPIAAVFV
AI
SALLFALPLIRALRRGRNGSTRPAPAE
>MCE8455707.1 acyl--CoA ligase [Rhodovulum sulfidophilum]AB15
MTLQTISDLLQGQEGNRPAIGAPGRDWLDFAGLRDQARRVAEALHGAGIGRGDRVAIVLPNGPEMAAAFVTLAQAA
VTAP
LNPAYREEEFAFYLEDLKARAIVLPEGYDGPAAAAAERLGLVVLRLSVDPSEPAGRFSLKAGGAGGECDAGAPGPGDVA
L
ILHTSGTTSRPKIVPLSQANVATSAVHIRDSLALRPEDRCLNVMPLFHIHGLLAAVTASLSAGASIWCAPGFDALKFFGW
MSEARPSWYTAVPTMHQAILSRAARNRDVIQAVPLRFLRSSSASLPAQVMTELVETFDAPLIEAYGMTEAAHQMCCN
PLP
RGSQKPGSVGVAAGPEVRIAHEAENRLIEGTGEVVISGPNVTPGYEGNPEANARSFFEAEGRRWFRTGDQGAFDAEG
YLH
LTGRLKEIINRGGEKVSPLEVDGVLMDHPAIAQCVSFALPHPKLGEEVAAAVVLKEGAEADEREIREFAAGRIADFKVPR
KVVILDEIPKGATGKLQRIGLAEKLGLSEPA
>MCE8455708.1 2-dehydropantoate 2-reductase [Rhodovulum sulfidophilum]AB15
MKICVFGAGAIGGYMGAKLAQAGAEVSLVARGPHLAAMRARGLRLIEEESGTDETVPVRVAEDASELGEQDYVIVTLK
AH
SVPPVVGKMQPLIGAGTTVVSGVNGVPWWYFHKIGGPLEGTRLASVDPGDAQWTGFGPDRVLGCVVYPAAEVIEPG
VVRH
IEGNRFSLGEPDGSRSERALRLSRALTSAGLKAPVRPKLRDEIWVKLWGNLSFNPISALTHATLDVLCTDPGTRGVVRAM
MLEAQAIAEKLGVKFPIDVERRIEGGAAVGAHRTSMLQDLDLGRPMEIDALVGSVQELGRITGTPTPAIDTVLALVALRA
RVAGLYGG
>MCE8455709.1 PAS-domain containing protein [Rhodovulum sulfidophilum]AB15
MSDSLIDPADPPERQAEKLREIAEVLMRRVEQATDDGGAAYAQFRRAVLLEDQVRARTRDLEHALDLLNESNARLAEA
NR
ATETARADLANAIETLQEGFALFDQSDHLVLFNSRFVMQMPDVRDRLCPGLRFHDYVEAVSRSRYLTLPEGETPEIWAS
R
RRARHRDNPHTVINISLLGHRWVQVSDHRTEDGKTVILQTDITDIMRSERRERSRMLDDQARLVRATLDHINQGVCIFD
S
AHRLVGWNRKVGAMLSIPVAQFRLGADFDRLFGQLAADRALRFDIAPTELLDWVHHTDRRPPLRFGLGRGETLMLDV
LAE
EMPDRGFVISVSDITAERRAAGALAEAKAHFEQRALARTLALEDALSVAERANASKSRFVAAASHDLLQPLSAARLYVSS
IAEDATLPAHREVLGRAETALESVEQILEALLDISTLDSGQAAVAPGPVPLRELYTRLQTDFAPHAALKGLELRVLPRDL
TLRTDPGYIRRILQNLVSNAIRYTGCGRVLVGARRQGQSLRLEVWDTGPGIAEEDQEAIFREFHRLNARASASEGMGLG
L
AIVERACARLGHPLGLWSRPGHGSGFFVTVPLGETAAPKPAPKAPAAEPSPGLVALLIEADPEIRRALPMLVGKWGVDV
I
DVADIDEALTLLAEIDLKPDAALIEHRHGAQTGPGHGLGALSLIETRYGPIPACIVSAERSPDLAAACRARGAELLTKPL
SPAALRAFFAEIAQRERT
>MCE8455710.1 FIST C-terminal domain-containing protein, partial [Rhodovulum sulfidophilum]AB15
LGTAPLIGGSAGDGTRFGRTLVGAGGRAFRSAAVLSFVRTDCPVRAFSHDHLMPGRVRMVVTAADPKRRIVQRINAEP
AA
REYARLLGKDPNQLSPLTFAAHPVVVRIGGRHHVRAIQRVAENGDLVFFSAIDEGLVLTLAEALPMADRIEAELAGLARH

KPPDVILGCDCVLRRIEAEQRQLRPALSRILARHRVRGFSTYGEQISGLHVNQTLTGLAIYPPPASGHDPEQVPPCPTA
>MCE8455711.1 gamma-glutamylcyclotransferase [Rhodovulum sulfidophilum]AB15
MQDPFFFGYGSLVNRATHGYRDAVRARISGWRRVWRHTPLRPVAYLTAEPAPGVTLDGLIAAVPGGDWTALDQREY
AYDR
HAVGKVVDHGLAARIDVHVYAIPAGLAEPPDIRHPILLSYLDTVVQGYLREFGPAGAEAFFATTEGWDAPVLNDRAAPR
Y
PRAQMLDAAERDAVDAGLKARGVRQFLPG
>MCE8455712.1 glycine cleavage system aminomethyltransferase GcvT [Rhodovulum
sulfidophilum]AB15
MSALQRTALYDLHLELGAKMVPFAGYEMPVQYGLGVMKEHLHCRARAGLFDVSHMGQVSLRPKSGMVEHAAIALE
HLVPV
DLVGLKLGRQRYAMFTNEAGGIRDDLMVARRCDDLFVVLNAACKTEDIAHLHATIGCDCDIVELTDRALLALQGPEAEA
V
LETVAPGVSDMRFMDVAIIDTADGPLWVSRSGYTGEDGFEISVPVERAEALARQLLAHEAVEAIGLGARDSLRLEAGLC
L
YGHDIDTGTSPVEADLAWAIQKVRRTGGAREGGFPGAARILGELDTGAARQRVGLRPEGRAPMREGTALYAAETGGE
PVG
TVTSGGFGPSVGVPVSMGYVPVDLALEGAKLWGEVRGKRLPAEIVRLPFRPSTYKR
>MCE8455713.1 glycine cleavage system protein GcvH [Rhodovulum sulfidophilum]AB15
MKFTEQHEWLRVEDDLVVVGITEHAAEQLGDVVFVDLPEEGTTVSKDDEVVVIESVKAASDILAPLDGEIVEVNQALTD
A
PAKVNEDPAGDGWFFKMKLEDLSALDEFMDEAAYRAFID
>MCE8455714.1 aminomethyl-transferring glycine dehydrogenase [Rhodovulum sulfidophilum]AB15
MPYTPSTYLPYDFANRRHIGPSPEEMTEMLEVLGLESLEELIGQTVPDSILQAEPLDFGKPKSEREMLWFMRQIAKKNR
A
FTTLIGQGYYGTVTPPAIQRNILENPAWYTAYTPYQPEISQGRLEALLNYQTMVSDLTGLEIANASLLDEATACAEAMTM
AQRVAKSKAGAIFVDEACHPQNISVIRTRAEPLGIEVIVGDPFADLDAEKVFAGIFQYPGTYGHIHDFTGPIADLHAAQA
LGIVVADPMALVVLREPGAMGADIAVGSTQRFGVPMGFGGPHAAYMACRGAYKRAMPGRIVGVSVDARGNKAYRL
ALQTR
EQHIRREKATSNVCTAQALLAVMAGFYAVFHGPKGLKAIAQRIHRKAVRTARGLQEAGFAVEPAEFFDTFTVEVGPMQ
GV
IYNAALAEKINLRRIGKTRLGIALDETTRPASIEALWRAFGVTRKDKGGDGEYRFPEGMSRESDFLTHPVFQMNRAETE
M
MRYMRRLADRDLALDRAMIPLGSCTMKLNAAVEMMPITWPEFSNVHPLAPADQVRGFAEMLDDLADKLCRITGYDA
MSFQ
PNSGAAGEYAGLMTIAAYHRARGEGHRRICLIPTSAHGTNPASAHMAGMKVVAVKSAENGDIDLEDFRAKAQAAGG
DLAA
CMITYPSTHGVFEETVREVCEITHAHGGQVYLDGANLNALVGLVRPGEIGSDVSHLNLHKTFCIPHGGGGPGMGPIGV
KA
HLAPYLPADPVAGGTGAVSGAMHGSASILPISYAYVLLMGGAGLTQATKVAILNANYIAKRLEGAYDVLFKGRNGRVA
HE
CIIDTRDFVETAGVTVDDIAKRLVDCGFHAPTMSWPVAGTLMIEPTESETKAELDRFVTAMLAIRDEIREIAEGRIEKGN
NPLKKAPHTVEDLVADWDRPYSRERGCFPPGAFRVDKYWPPVGRVDNAYGDRNLICTCPPVEEVADAAE
>MCE8455715.1 DUF2254 domain-containing protein [Rhodovulum sulfidophilum]AB15
MKSLRLWQVLRLSRRLWVRTSLIAVLGIVAAGAAALLGPIIPQELADRLGEDAVMPVLDVLASSMLAVATFSLSIMVTTH
RQASSQVTPRSHRLLLEDTTTQTVLATFIGAFIFALVSIILFRAGAYTGAAPFVLFAFTVLVVVLVVVAILRWIDHLSRL
GSMDETIAQAAARARDTLTAARNAPSMLATPLPDGAVPPAGARPVPAPRGGYVQLVDMAALSVLAEKAGAEIWVGA
VPGS

FLVAGRALAHVNPPGDIDRDAICAAFEIEAVRSFEQDARFALAVLSEIASRALSPGLNDPGTAIDILGRLEQLLAECPAA
GDEIRYPRLHAPPLRANDLVEDAFAAIARDGASMREVARRLIHALNTLRQGDDPALARAAEAMTRRALAHAEAALVLD
ED
KAGLRQLAGL
>MCE8455716.1 ATP-binding protein [Rhodovulum sulfidophilum]AB15
MPESGRAIIREQMQSGGDALPWADRAAYAERMLEQDLRAYSALRALSAPVIFDRGILDVLGYLMLCGLPGPCFVNQLT
AA
VSLSPDTVILRPPSPGCRVP
>MCE8455717.1 rhodanese-like domain-containing protein [Rhodovulum sulfidophilum]AB15
MHSEDVNGKVLEHWDCDDLEAAMAAGEVVTIDVRTPQEYLFEHIRGALLLPLAGFDPAFLPEGGDKRLVFHCGSGVRS
RK
VAEAWLAAGHDRVAHLEGGFAAWKAALKPYTGTDMASGAPIRVNAKTD
>MCE8455718.1 twin-arginine translocase TatA/TatE family subunit [Rhodovulum sulfidophilum]AB15
MLNNIGLPGLLLIAVVVLVLFGRGKISSLMGEVGKGITAFKKGVKEETEEAQKSLESTRDVTPETEKDKA
>MCE8455719.1 Sec-independent protein translocase protein TatB [Rhodovulum sulfidophilum]AB15
MFDIGWTELLLIGIVALIVVGPKDLPGMFRTLGRFTAKARNMAREFQRAMDDAADQSGVRETVDDLKDIADPRKLGLD
RL
NEAADRFEKWEPKLPKPGESASPATDARAAKAEKIRRAMAETGAAKRAAPEAAEGDVPAPEDIPAKTPDKTSDA
>MCE8455720.1 twin-arginine translocase subunit TatC [Rhodovulum sulfidophilum]AB15
MTRDDIDDTSAPLIEHLAELRTRLIYALAAFVAGMVACFMVWNPLFNVLTHPICSALAERGQECGLYLIKLQEGFFVAIQ
ISLVGGFALAFPFIAFQLWRFVAPGLYRSEKRAFLPFLLASPFMFILGAAFAYFIITPLAFDFFLGFQQAGSLGAEGGGQ
SETAGITFHGSVQEYLSLTIKFILAFGLCFQLPVLLTLMGKAELVSAAGLGSVRKYAVVGILVLAAVVTPPDVITQVILF
TVVYGLYEISILLVRRGEKTREKELRAQGLWFDNDEDEPEEGPATEPEPRS
>MCE8455721.1 ATP-binding protein [Rhodovulum sulfidophilum]AB15
MTGQTQDPRFGPEAADSLKRIAEALERRFPAPAAAPDFASAEAFLWHVAPDRLSPVPEVNRVDLTLLQGIDRSRDTLLE
N
TRHFARGLPANNALLWGARGMGKSSLVKAVHAAVAAEGHPLKLVEVQREDLPSVGRLLGLLRGADERFVLFCDDLSFS
HD
DQHYKSLKAVLDGGIEGRPANVVFYATSNRRHLMPRDMIENERSTAISPSEATEEKVSLSDRFGLWLGFHPCSQDEYLA
M
IGAYCAAHGIEIDPETLRAEAIEWQATRGARSGRVAWQFFTDLAARRGVRI
>MCE8455722.1 hypothetical protein LZ188_03915 [Rhodovulum sulfidophilum]AB15
MSYNTNEVIRRETGNRARKRMVGLLRAVFWGVYVVGKIIDLIKWISQFFGE
>MCE8455723.1 helix-turn-helix domain-containing protein [Rhodovulum sulfidophilum]AB15
MIGEALRLIRVYHDLKQSDLADRLGISQSHLSEIERSKKAPSNELIERYSKEFRLPVSSIWFFDEHLTEGVKPSTLDRAR
GIIADKVLDFLRIVEGKRKANDIEA
>MCE8455724.1 reverse transcriptase family protein [Rhodovulum sulfidophilum]AB15
MTSRRNKSYPIHQCALYKLRTKKKLFELLQTTPTKLKELREAPKLYHPIKVAKKDGTYRDVLAPRGDLKRIQKRVGELLL
RVQTPDYLMAPVRGRSNIDNAVRHRGARSFHLLDVADFYPSCSANKVAAFYGKTLGCPPDVVKILLELTTLNGTLPPGS
P
ASPSLAFWAYQDMWDEIAEISEAAGCTLTVYVDDITISGHLVPGEVVYGIKERLKHHGHSFKEQKEAAQIDGAIEATGVI
LTPSGKVALPNTQHQSMHQLRTEVQRMPEGPEKELKASSLRGREINLRRILQQNRG
>MCE8455725.1 TraG/TraD/VirD4 family protein [Rhodovulum sulfidophilum]AB15
MGIRTTLILLTIAICGVLVYQDVHDRVEGAEAEPERQLRQTRDAEIARCTRKAAELRGLIGEEPGLDDRIETGQHRKYGV
QYMLYAQSRAAIEEIYDQEGRLDIEAVCEFIQVLATDDPGLINDLTTWSGTQTVAIREAKGGKFQFAMARFPGGRSDGA
K
EDPAFHSPERAGQGARIAHPQTTASENGAGSGRARQGRSPQSLHT
>MCE8455726.1 hypothetical protein LZ188_03935 [Rhodovulum sulfidophilum]AB15

MRIFRPGNNVAIRLAGLGLFFLLVSLGYAGARDIRHVEFVRYGQNNPDSPTKSILKTAEWIAPLLRPNFFELDRNSDVSF
ITRTTLYLDRETLDILAPNRSGDFATLISQDMAQVGDHPGLFGAPLLVCFYGPRSRNWGRPSSVPSDIPLETVLAHAGGV
KVNLYWNIIDHPPYQAIPNHPLRRIGAFRAICPGEAHAPFVRQFERLVRGTAEWVVKWGFQLHAPVRNLDRPPLSVRL
AI
GLEYRVAVMKRRPQTQRNGWPLSDPPPVLLRYNVTSVAYDTLGPNGLAEMDFVPIQFLLTERYSGLPRGMTAQVTPG
QVA
AARMKNKQQSYHICRYRQGHAAYWVWSDAFPLRYQGHEIRSSKGAPDRPVFSCPLDPGALWR
>MCE8455727.1 hypothetical protein LZ188_03940 [Rhodovulum sulfidophilum]AB15
MLWLRQYGTRILGTLIGLLVGGSLGLYYFFFAQIDCWFECSPEEARRVFWSALVIGISITAFAALMGWAYVSRAIDLLLR
IIRWFFRALRQERQTVQQEGRTTRAAVVLLLVAVLVVFQAAFLSVVIDAVDPSYLQSDRYHITADYLISISELLLAGAIA
IQAGNMLGRLPFFRSSLTTYLAGFILCFVAAAAVIDDIRDAGVDYILAERSTEDRRFAALVAINQMALVQQGLDFEEADL
GIEELATPDAKITLFLSPVIFYLAGLESLEATSDEKVEALLRTVDEEGIVRRIQEAADRETAEYCAFFEQMYADYTGSVN
GAVDKIAEVSRSGIGSARAYEEASRFYFNMVNQKLGRGWAVLPGLTREQFINHRDVQKAIKGRIQKTLPAMRERVAES
EF
PVAGARAVSAFEDIIRRKDIYVDPCEMNERIVRGVSSDIRGQLWQDVRRALDPERTTLGVGGEHERYGRTALEIAILPTF
SIAALLFGMVLNGRGVIGQFTGRVLGLPPGVGSTLALLFIGFAVLWPLSQPHEQLDEGAIADLMVAVQEERGVFTRYGI
E
WIVRGASGLYDAGTRIRDTLDFSMMGLENHIAEHLESRDLGPGLGPEGLTAR
>MCE8455728.1 hypothetical protein LZ188_03945 [Rhodovulum sulfidophilum]AB15
MPLPTSFIKRKAGASLLHSVRTAEAIGRPFNTFVTVSLWKLGLTPDDASRFFQELRERHFQRWSSYVPRGDTTPRNGPPT
YAWVIEAPRKLAHVHWMVHVRPGQERAFAAVLDRWIVRRTGIAQVPEGVIEIARIDNAEGLKLYFAKGLDPHLARLW
RIH
PVESGRVHGRRAGTSRNLGPAEWQPRKAAYQRSRRRVAA
>MCE8455729.1 recombinase family protein [Rhodovulum sulfidophilum]AB15
MIIGYARVSTDDQTLDSQTDALTVAGAERIFADKISGSKRERPELGKMLDQLRPGDVVVVAKYDRLARSLRDLLDLVEAI
KDRGAGFRSLAEDIDTTTPAGRLVFHVFASIAQFERERIAERTKEGLQAAKARGRVGGRPPALSTAQKAEVRRMRDDEQ
R
AISEIARLFKVSERTVRRA
>MCE8455730.1 hypothetical protein LZ188_03955 [Rhodovulum sulfidophilum]AB15
MARRISSSQIRSRLRQAQAKMRQAQAKRKQAINKCNQAVRTYNAKARAHNARVRANRDRLKRELQKLARTSSKPQYV
TFR
TSVETFQRSYSVLENRAEAEIYDDRYDRFLDLSEREAANSASVMNALEGEVPENENDESPPASEIDHILDAISQDVHDRW
HGALFALSPQNPDAARHFCTSARELLTEILERFARDEDVKSVLPDCEVTQQGKPTRRAKIRFFLHKQGVSDDAVTDFVEK
DMENVVQLFRVFNDGTHGSSGRFEHSQLLAIRARVEGAVSFLWGIITGDAFAPA
>MCE8455731.1 hypothetical protein LZ188_03960 [Rhodovulum sulfidophilum]AB15
MAKKAGSGGHYRSAISGRYVTSAHGKRSPDTTVFEKTDGGSTGSARSAKSGRFVTEGYAKRNPRTTVKED
>MCE8455732.1 replication initiator protein A [Rhodovulum sulfidophilum]AB15
MTNNAAASPARSPLLPDRHPTRDLFVCDIVDAVPKGDMTSMENPVFSLATKPDMRPRRYERGENFIEINPSRYGLATV
HD
RDVLIYCISQCMAAVNEGRQVHRKMRFKAHDLLVATNRQTSGRGYELLKDALRRLQGTQIETNLKQGGKEYFKVFGLID
S
AEIVKETRDGRMIDVEITLSDWVFDAIENNHVLTLNRQYFFLRKPLERRLYEIARKHCGAKPVWKIGLELLRDKCGSGST
VKEFRRLIGKIIEDDEQHDHMPDYAFTIDGDNVIVRMKKGRLQGALPLPLSSLRLDADTHERARQFATGWDMHNLESE
WR
SWVIEKGIAVKDPDKHFLAFCKKRGPYRR
>MCE8455733.1 ParA family protein [Rhodovulum sulfidophilum]AB15
MTYIIGMVSQKGGVGKSTLARMMAREFVAGGLATKIADLDTQQQTCTHWAGRRAENGVTPEIQVQSFAAVKTALED
APRF

DALILDGKPNASEQTAEIAKASDLVVIPTGQTVDDLHPGVVLAHGLRKKGISPDKIAFAMFKTTGSERENAAARQYLEQA
GYKVLEGEVSVSTAYGSASDIGKAITETSFRSLNDRASKLAQSVIDMMAELSERQVA
>MCE8455734.1 hypothetical protein LZ188_03980 [Rhodovulum sulfidophilum]AB15
MTTNIAKLKSAKGAPPPADDAPDVIADDTRPEKVELRPLQVRIPRTVFEEFSERAGREFGFSHGAKKQLFLKMWETYKA
Q
NM
>MCE8455735.1 hypothetical protein LZ188_03990 [Rhodovulum sulfidophilum]AB15
MAARPKPSQRLLNPEDGTEVFGIVPKLRLGHLQTEEAPIYLQWGRHKGPHHGHGASHIWAEHRNDMGKLGLTTSDEV
PAY
VKRVIKSGTSLYFEAERMRGNQRTSAVRSSAGTVILEYVEQIVQGEVQPYWRIITAFPRAQGIGTAVGRIK
>MCE8455736.1 YrhK family protein [Rhodovulum sulfidophilum]AB15
MTGLERTVRVYRHWHLSVAVAGNFLFLVGSVLFLPTLSSWETAGVWMFIVGSFLMLIGAFGEVAKAVYEKHERDRM
>MCE8455737.1 Stf0 sulfotransferase family protein [Rhodovulum sulfidophilum]AB15
MTGKPFRSYVICTVPRSGSTLLCGLLAATGVAGDPDSYFHAPSLEGWLTDHDLKRADFASDRDAVRAVFEAALMRGKG
DT
TMFGLRMQRGSFDFFMEQLKLLVPGEMSDAERIEAAFGPTLFIHLSRPDRLDQAISRLRAEQTGLWHRNADGSELERL
AP
PQEPRYDAAAIRRHMAAQEALDAAWEGWFRQERLNPLRITYDALSTGPQQVLAQVLSALGLDPAQARSVLPPTAKLA
DAL
SREWRCRFEGET
>MCE8455738.1 isochorismatase family protein [Rhodovulum sulfidophilum]AB15
MKALVIIDMQMEMQHRIGAGRDCVNADAPERIAALAAAFRGKGLPVVHIRHVDADPRSPLAVDAEGCRPMACAEA
QGGEP
VFVKRTSSAFASTDLAAWLHENRISELVVTGAVAGFCVNSTVRNGADLGFDMTVVRDAVIGFDLPGPGLSARGIFDVT
MA
HLEADFARLIDTPGILAMLG
>MCE8455739.1 4-hydroxy-tetrahydrodipicolinate reductase [Rhodovulum sulfidophilum]AB15
MDSLPGIVVMGASGRMGQMLIGAVLQSGKARLAGCVERPGHPWIGRDVGEAMGGPAVGVKVTDDALAVIAKAQA
VIDFTT
PEATVATATLTAQARAVHVIGTTGFEPEHLQKIKAAARHAVIVRAGNMSLGVNLLMQLTRRVAAALDDDFDIEIVEAH
HR
HKVDAPSGTALMLGQAAAEGRHIALDECSDRGRDGITGERAKGAIGFSAIRGGDIVGEHDVIFAGEGERIVLRHVATDR
R
IFARGALKAALWGQDQKPGEYDMLDVLGL
>MCE8455740.1 30S ribosome-binding factor RbfA [Rhodovulum sulfidophilum]AB15
MSRSSAPSQRQLRVGELIRRSLSEVLARGDVHDPELTALSITVGEVRASPDLKVATAFVLPLGGDHAEEALAALRRNRGE
LRRAVGKKTQLKFTPELRFTLDETFDRMDETRRLLADETVRRDLAAPDGDED
>MCE8455741.1 phosphodiester glycosidase family protein [Rhodovulum sulfidophilum]AB15
MAMRIRAALRLLAVLLPLPAAAEPACHPLTHEGRGYVICSADPATDDLRLFLRTEQGGIYGSFGRVNQALAAGGETLAF
A
MNAGMYHPDRRPVGLYLENGKQEMRAVAPAGPGNFGMLPNGVLCLEDGRAAVIETRAYLADPPECRYATQSGPML
VIDGA
LHPRFLPDSTSRHVRNGVGVDAAGRVHFAISDAPVSFHEFARLFRDVLETPNALYFDGKVSKLYAPALGRHDAGLPMG
PI
VGAVVPAGD
>MCE8455742.1 tRNA pseudouridine(55) synthase TruB [Rhodovulum sulfidophilum]AB15
MGRSRKGRDISGWLVVDKPEGLTSTAVVNKVRWAFNAKKAGHAGTLDPAATGVLAVALGEATKTVPFVTDALKAYRF
TMR

FGAATNTDDAEGEVIATSEKRPSDDEIHAALPAFVGDIEQVPPQFSAVKIDGERAYALARAGEEVEIAARPLYVESLEMV
ERPDADHAVLEMICGKGGYVRSIARDLGQALGCHGHVVSLRRVWAGPFEIEDGVTFDLIEKLARSPELDTHLRPLEEGL
A
DLPELRTTPDGATKLRNGNPGIVLASDVEYGDEAWASLDGEAVAVGIYKAGELHPSRVFVKG
>MCE8455743.1 AarF/ABC1/UbiB kinase family protein [Rhodovulum sulfidophilum]AB15
MSDSKFESRPLSVPSGRFARMAKFGGLASNLAGTAAINGMQQLAQGHRPKARDLFLTPANMRKVADRLAHMRGAA
MKVGQ
LVSMDAGDMLPPELAEIMARLRADAHYMPGGQLKQVLNANWGEGWLKRFARFDVRPVAAASIGQVHRAQTKDGR
DLAIKV
QYPGVRRSIDSDVDNVAALLRLSGAMPKEMDIAPLLDEAKRQLHEEADYAREGHYLGRFADLLAGAPDFVVPGRHEDL
TT
ENVLAMDFVEGVGIETLTEAPQAERDRVMALLVSLLFRELFEFRLMQTDPNFANYRYQPETGRVVLLDFGASREFPAA
MT
EAFRGLMRAGLAGDRAAIRQAILEIGFFAEDTAARHQQLMIDMFEMSMEPLHRPGAFDFAENDVALRMRDAGMAL
GQDRD
FWHVPPMDTLFMQRKFGGMYLLASRLRARIDLREILAPYV
>MCE8455744.1 hypothetical protein LZ188_04035 [Rhodovulum sulfidophilum]AB15
MSSREIPLFSWWSAARKDNFATTDRAWAAAPGTSIEAHDGYTMYRIEGALFAPDDPQPGGTVRVSRFYSPSRGDNFL
TTD
PRHLPGDILAPDYHHSRHEGYLYDPKRPQPRGTVPIFSWWNSDRGDNFATSNPDWSMPVGEIEWAGEHVSNGPVRE
GYRL
YRLEGFAKTFDLDTLDDFVRDRMKVCYKLIIDIHEDNNRDDPRLDLVVASFGGYYQIEAHTTGTLANHSGLDIGSGGSIL
PLPGEDVRNTYRGCFDVEHPANGADPHLALAIRAVDEDNSSDENRGKDLNYFKRGIRNALERATSGGVILTEPILQSAA
G
EELIDRFGRDDDDKIGVAVAVITDCYRQSVAVCRTAPLGNNEGVREDFPGSLPHILLVRQYTLEGDGARYLLDIVISVDH
FSE
>MCE8455745.1 MoxR family ATPase [Rhodovulum sulfidophilum]AB15
MSVNAMTVPAGEALEVLRAGWEAQKAGHITASYMLHGRPGVGKTQVVEQLAREIGARLFDLRLTTIEPQDLRGLPYY
DHE
TRKTRWYRPEDLPDDPDEPSILFLDELTAASPMLQPTVYGLLQERRVGRHVLPRTVFIVAAGNTVEDGAIAYQMNTALS
D
RLIHLHVQAAAADWLDNYAVPKGLDASVVAFIKTRPDLLDTTERAVAEGHLIATTPRSWERVSRIMGAVAERRVRNVLI
A
GTVGEAVAAEFALIADDIAATVQVQEMLEADRKARIALYPASMHGLNALIYGLIGVLDARNVEEVIDCMAEIRNLARHR
P
EEAFARMPLGELCTYGFEGLIRKGLEIGLGEHFLGNEAYRAYAAERKAAGL
>MCE8455746.1 VWA-like domain-containing protein [Rhodovulum sulfidophilum]AB15
MTSEGHSRRATRALQKLAEQDPAFAALALWCSHRDSDARADAAWTDGRAIRYGPGFEALSLPEQIGLAAHHILHVAFR
HA
ARMQAMQARFGDRFDAELFNLATDSIVNETLMLTGYILPRPAVRLTGLLKDAAREEMGGEEAVTRYDAERLYLRLFEAE
A
PPPRSRLRLVDRSPPGETGQGGGEAEGKGRGTAGDSRPRPGGAVAEAARDYGRRQGFAPDIEAEGDEGDQGAGSEE
EAEW
RQRVARAMEAGRVAGFGLGMLGHRLADLPEVHTPWERHLRALVSRAVMIRPAPQTTRPARRWIARDSDARATGAP
EPAFE
FAWSRAREIPRIVVGIDSSGSVEPVLLAKFAAEVAAISKRTGAETHVLVFDTRVHGRHVMGGGIWEGRITDVLFSREGGT
SFIEVMEEALALGPSAVVMLTDLMGPFGPPPPRRLPVIWAVPGEPPPAGAPFGQVLSLAR
>MCE8455747.1 DUF1643 domain-containing protein [Rhodovulum sulfidophilum]AB15

MTETDPSSLHDPGGKTRWSLPPGMRGSAVFSPCGRYRTRLERDWTPEGQPGRTILFCGMNPSTADALVQDNTCAKE
TRRA
AVLGFTRYLKGNVLDYRVTNPKDLPKDPDLACSPGNLPAILAMAGEAELVVMAYGRLHKRYAGVVGGIIAALRETGKPL
Y
CFGLNQDGSAKHPLYLRNDAPLLPF
>MCE8455748.1 metallophosphoesterase [Rhodovulum sulfidophilum]AB15
MSLSYAIGDIHGQRARLEAAHALIAEDRRQVGDTGAPVVHIGDYVDRGPDSRGVLDLLIEGRAAGRPWICLLGNHDRW
MQ
HFLRPGCEPEPARPDMLWLEDAVGGRATLASYGVDADPHRALGAIHAEARAAVPSAHLDFLAELLPSFRHGEAFFCHA
GI
RPGVPLEAQSVTDLIWIRGEFHRDTREHGALIVHGHTPVDRVTHFGNRVNLDTGAGYGRPLSVVAIEGRKVYLLTPGGR
V
PVPPEMPPEARCEAG
>MCE8455749.1 GNAT family N-acetyltransferase, partial [Rhodovulum sulfidophilum]AB15
MRGAADGFRIETPRLVLRAFRPADLDALIRIGRDPRVAPMMCSLPVRWTRRQAATWVARSRFRGEPTFRLA
>MCE8455750.1 GNAT family N-acetyltransferase [Rhodovulum sulfidophilum]AB15
MKLDLIPPQPPIAAERFVLRPVRRSDAGLLSLYTSDLRVARFTRSIPHPLPPGATEAFIHRAQDPERSEDVWVMDGAEQ
G
LSEVLGVIALDRMERDQSEIGYWVAPAFWNTGIASEAVGALLAANPQKAKTVFAEVFQDNPGSARVLTNAGFEYIGDA
EA
YSVAREAKVATWTYLKKLD
>MCE8455751.1 50S ribosomal protein L27 [Rhodovulum sulfidophilum]AB15
MAHKKAGGSSRNGRDSAGRRLGVKIYGGQAVASGNIIVRQRGTKFWPGEGVGMGKDHTIFATCEGAVTFKKGLKGR
TFIS
VLPVAEAAE
>MCE8455752.1 50S ribosomal protein L21 [Rhodovulum sulfidophilum]AB15
MFAVLKTGGKQYKVQSGDVLRVEKLAAEAGETVQFNEILMLDGQVGAPIVDGAAVQATVIDQIKGDKVIHFVKRRRK
HGS
QRTKGHRQQLTLLRVTEILASGGAETGVKAAIGAGSVAGAPAAEPKAAKAKAPKAAEAAPAAGDDLKKLPGVGPAME
KKL
HEAGVTRFAQIAAWTDEQVAAMDTRLGLKGKIENDGWVALAKDLAGKA
>MCE8455753.1 DUF2059 domain-containing protein [Rhodovulum sulfidophilum]AB15
MLRLVAATALIAVLAGGAARADEAAETASLQALVEALALPELVEVMREEGLVYGEGLEDELFPGRGGARWEAAVDRIY
DG
GRIEAEMRAALDRRLGAAEIAPLVAFFTSPRGARIVALELSARKAMLDPEIDRASRERLEEMQADGDPKLDLVGRFIAVN
GLIEANVAGALNANYAFYTGLVDGRAFDYPVTEDQILDDVRRQAPEIREDTRTWLFSYLTMAYAPLDAATMEAYIGLSE
S
RPGRDLNDALFEGVEAVFEDISHELGLAAARFIAGQDL
>MCE8455754.1 hypothetical protein LZ188_04100 [Rhodovulum sulfidophilum]AB15
MQTRPRPSRPRLALLVGIGVWPIITTLLYLIRPVSQGWPVWLTTLALTPAMVIIMVWGLLPAIHRYFGKFLMRG
>MCE8455755.1 ATP-binding cassette domain-containing protein [Rhodovulum sulfidophilum]AB15
MTLFAADTIGLHYPGRPGPVFHDLSLTIRKGESLGLSGPSGTGKTSLMRVIAGLERPTSGRIPIRPRRIGMAFAEPRLLP
RLSAMENLSFIAPKARAEGRAMLTALKMAALQDASADSLSKGQAQRVALIRALLIRPEILLLDEALGGLDMATWQSARD
L
ILARRDRDGFALVEISHDPARRIMPEARAISFP
>MCE8455756.1 ABC transporter permease subunit [Rhodovulum sulfidophilum]AB15
MTRKLAFLTGVAAIVLFWSLAAQANLPAILPGPLAVAKVLGLLLGDPGFWSGTLGPSLGRASGGLALAFAIGAPLGLAS
W

RWPVVAALLAPLRLILMGLPAPVLAILCILWFDGSTTTTILTVAALLVPVFQIAVAEGMGAIDPQLDEMARLFRVPPIRR
LRRIILPALWTALGPALRIAVANAIRVTLLTELLSGAEGLGAAVQRAQSWLQTDRLFALVLLILALIGLAEVALAALTRE
RKRP
>MCE8455757.1 ABC transporter substrate-binding protein [Rhodovulum sulfidophilum]AB15
MRLLPLFLAMLPLPALAEEILLSGPPIWESAPLIALAEDQPIEGITFSFRPWASPEQLRKRVIADRPLMAVAPSPTAAIF
DANGMALRVVSATITEGSLSIVGRGTEITELADLQGASLALPFKGYLPDLMMRRIAEPGAETWQPHYTGSLVAGMQLLL
A
ERGVDAALLAEPMATLALAQDRDLTRQADLCALWRGATALADCPPAGVIIVNPAFAERPEIRAAYHAAFAKLAADPAS
AA
GLLAAHFPEMAQAGAGFARISAIDLPMPEQADVLADFYAAILEIEPAAIGGRLPEGDFYGK
>MCE8455758.1 class I SAM-dependent methyltransferase [Rhodovulum sulfidophilum]AB15
MVSPERSSGAARLLQTLEGPIRYALLDWALESGVFGLCESHIAPDLLGRRMELPVGPLTLALRALVAAGFMEAGEAGFR
T
APDILPFVKTGSPRNMVETLRMMAGTRHAGLGQFGALVAGKAAPSGARLFDEAHWDAHHRSLAGFHRAVGTDATA
PCLTG
LPEWPHARNLLDIGPGSSALAARLLAMRPDLRITLFDLPPVAERIGAEAESLPLAIMPGNYNRTLPDGPFDIIWCSMSLY
FHDGGLPALIARLAERLAPGGVLVSFHEALSDHRSAPPEHVLGRLLPALRQGDVSFAEGEIADAMAAAGLVRPTSQHLS
T
AFGRFRLDAARKEV
>MCE8455759.1 TonB-dependent receptor [Rhodovulum sulfidophilum]AB15
MSRHSASRPVPGRIALFVLSTALTGSLIPGPAQAGEDMTYLGEITIFANYWEELARKSAVTATVLDEADLARPVSADLDA
MAGRSANTVFQRANSQERLVVRGMSAFDNALSDPVGYMVNGVALPMGTIQLPHFFGADSVTLLKGPQGTTFGRNSE
AGLL
AFDTIRPGSREGAEINLGVSGSDAGASPLGGSGSVLWSGRARNGPALAFGLEYARDPGVISNPVTGADDGGKDRRATG
FA
AAEWDLQDGGYLRLTTLAENQKFNKEQFRSISGPFATGRYESVYSDPSWERRRTSVTALEYGTGFDGFDLTAITGFTTFD
RDFVLDFDGSPLTLGVTELDVRDRTISQEIRLTSTGAGPLKWVLGVNAFKQSTDADFNLGAMSTDRHTEIDQNGVALYG
F
TEYAISERFRLGGGLRLDHVSSEAWQSFTSPMARLRYKGDESSTTLLPKLTLAYDLSEATTIHASYARGYMPAGYNYGFA
NSADSLTYGAEYSWNAEIGVKHAFANGAALTLAAFHTTVKDKQITETIPGAAQYISNAGEAKSYGIEAELTAPLGSGWEI
AANAGWQRARASSFRTTSMDLGTGSLTPVDWSGNALPMAPETTWGLGLSHQGEVWRARGSLHGSGSYWFDPGNT
LKQKGF
MTVDAEISRSLGKGELTLWVTNLLDEEYYSTAASTMRGVVVEDGSPRTIGMNWKIAW
>MCE8455760.1 AraC family transcriptional regulator [Rhodovulum sulfidophilum]AB15
MNLIEPHRFTPLEAGSPTSVTLPGLHRNTHVDVMRINTGLVLAHSHFSAATRHEGDIRRPEDQLILAFNLSGRMLLIDPQ
GAAHEISGGEGWIIRSGGRRLQRIVEKGDGCSNLVIALSVSRLPPALAESLEPLLPQAAPFRRLCLPVPGKSMLETLLDG
RTDPAALLRKEGQCLALIGHALEEIAAAPDHGPATGDPGLLIARVTAFLSDNLANPITISGIAQEMGMSHVTLNQIYRAA
TGMTVFQHLRSLRLEAAERLIRHTDRSFTEIAGECGFSDASHLSNAFRKRHDMTASDWRRQVRKKT
>MCE8455761.1 ABC transporter substrate-binding protein [Rhodovulum sulfidophilum]AB15
MITTCRSAGRPGWGWLLAGAALATMAGPAAAEEPQRGGTAIVHMVSEQRVLNPALRASTGVYNITGKIMEPLIDKSY
DGP
VGVLATDWSSTDDGLHITVNLREGVNWHDGEPFTCDDVAFSAMELWKPLLNYSSTLQQYLESVDCTDAHTAVFNYSQ
PMP
LGLFVAAMPDLGHPVPKHLYEGTDIMRNPHNTAPVGTGPFKFVEYKRGQYVIAERNDDYWRGEEYPYLDRIVWRFIPD
KS
AAAAAMEAGEIHESGTNSLSMADIERFSKMDGFDVGTKGYENNVAHSTVEFNHRNPILADLKVRQAIYHGLDIDYAIKT
I

MRGYAKPGRGPIPSTGGANYTDDVTTYDYDPELAKQMLDEAGYPAGEDGIRFKLRHRPAPWGEYTQLWAEYFAQAM
GEIG
IDVEILTNDAPGFLNGVYRDHDFDTANGWHQFRSDPAVSTMVWLRSGSPKGTPWSNQFGWKSDEMDRMIDEAQA
ELDPAE
RAADYHAIQAKAMEELPLIFAIEHPFISVTSQKLHNHHNNPRWISSSWYDLWIEH
>MCE8455762.1 SDR family oxidoreductase [Rhodovulum sulfidophilum]AB15
MQNCVWITGAGSGIGAAMARTFSAAGYRVALTARSEDRLTALADELGGAVRVFAGDVTDRARILAIAEEIVQWAGRI
DVL
CNNAGLNVPLRRWDELDWDSWDRVIATNLTGALNVIAAALGPMRRQQSGVMIHTSSWAGRFHAPVAGVPYGASK
HALTDL
SASLNAQEGENGIRSTALCPGEVATPLLNGRPGFDPARAAEMIQPEDMAALALHVAGMNPNVAIHEITLAPVQRAAR
>MCE8455763.1 VOC family protein [Rhodovulum sulfidophilum]AB15
MTHPVKGVDHVFLMVEDLDRAAEAYRRLGFTLSPKGLHSAVKGTANYTLMFPGDYMELLGIVTPTEGNAPRRAALAR
EGE
GLRAVACRIDDAEAAKTALEALGIATGELGHFERPVPLPDGGTGRAAFSTMPFADSELPFGIVFMCQHRTRETVWLPEL
L
THPNRACGLAEIVAQSADPEAAAQRLARLYASGRVAAAAGGYRVETGPASAPILCLTGAALAEYFPGLDLSAAPAEGFA
G
LALHSEDMAATAACLAASEVSPVETARGIALAPSDCAGAVLEFRQR
>MCE8455764.1 Lrp/AsnC family transcriptional regulator [Rhodovulum sulfidophilum]AB15
MSTGRKTRGADRKSAEMPGFDEIDRKILEILQRDADLAVNALADIVGLSPTPCWRRIKRLEEAGVIKGRVAIVDHARAN
V
GMTVFIGITAPRHETAWLNQFRALIDDLPEIVEAYRLTGATDYILKVVVPDISTYDRVYKAMIERLDFSQINSSISMEEL
KFTTAVPSTYL
>MCE8455765.1 aldehyde dehydrogenase family protein [Rhodovulum sulfidophilum]AB15
MSRLGVAADALSGGSMACVSPLTGERIADISPAGVADAAAAIAAAKAAFGDWRMVPAPRRGELVRLLGEELRAAKDD
LGR
LVSVEAGKSPSEGAGEVQEMIDICDFAVGLSRQLYGLTIATERPGHRMMETWHPLGPVGVITAFNFPVAVWAWNAAL
ALV
CGNPVIWKPSEKTPLTALACGAVLGRALARFGAAPEALVQVLPGGPELGNALVESPDVALVSATGSTRMGRIVGPKVA
AR
FGRSILELGGNNAGIVCPSADTAMTLRAVAFGAMGTAGQRCTTMRRLFVHDDIYDALVPGLIRAYASVSVGNPLETDA
LV
GPLIDGAAYRAMQEALEAARAAGGTVHGGTREDIGPDSAYYARPALVEMPAQTGPVLEETFAPILYVMRYSDFDAVLE
AH
NAVGAGLSSSIFTTDLREMELFLSARGSDCGIANVNIGTSGAEIGGAFGGEKETGGGRESGSDAWKSYMRRATNTINYS
R
ELPLAQGVVFDI
>MCE8455766.1 HlyD family secretion protein [Rhodovulum sulfidophilum]AB15
MHLLKHHVPTVLIVLIGVLGVVVVLFAWHLPPFGGSEIRTENAYVRGNVATLSPQIAGYVTEVRVTDFQTVSTGDILVRL
DDRIARQTVARAEAELDSARAALKANDQDINSADATLGSRRAAAEAARAAVDTARAEWDRHDRLRRKGVISASDAED
AEL
RLRQAEAALTEALSDVAVAREDLATAKVRTDTLSASVTSALAAVELAKIELEHTVIRAPQNGRLGQVNVRVGQYVTAGT
A
LVSLVSSRVWVIANIKETDLAGLHSGQKVRFAVDALGGQPFTGKVALLSPATASEFSILGNSTATGNFTKIAQRLPVRIE
VDPGQEGQDRLAPGMSVVVHIPRDRG
>MCE8455767.1 MFS transporter [Rhodovulum sulfidophilum]AB15

MPAAVPSDAPAPAPKAEPTGLTRSLPLAAAYMLASMFIALSQSLGQGFLAANIPTLAGELGATQSQTAWLMVAFMAP
RAS
LPLMLIKIRSQYGLRRFAEVSIALYAAVALLSLLTRDLRSALLVELLSGISAAPLATLAFLYALEPLPQRLKLTVGLPFA
LSFIALGMPLARAISPTVLTDGGWTDILLLKLGMAMVCLFLVFLLPLPHGERTRSIRGLDLISFSLIAASFGGLVACFTT
GYIYWWTAAAWMGPVLALSIAGLVLALVIELHRTAPLLDVRWLATPEMLHLTAALLIFRVILSEQTAGAPGLFRTLGFAP
EQIAPLFWGISLATLAGGAACALVMKLDRVPAIHVAALLLIALGSAMDAHANTDVSPTLMIASQAMVGFAAAMFLPSA
MA
AGLVKALSKGPQYLLSFVIVFLSSQILGGTVGAGLFRTFVAVRTTAHSQALAAGLQTGDPLVAARLAGIAAQLSTTVTDP
VRLKAEAVAQLAADTARQATVSAYNDVFALISALALCALAALLGHIARDWLRARMARRVPADG
>MCE8455768.1 MarR family transcriptional regulator [Rhodovulum sulfidophilum]AB15
MQKTFLPEVFGLVRAIRKRFDARATSIDMTYARAQALMSIASCEGLTQVDLAERLDIRTPSMNRTLDNLEAAGLIERRAS
AEDKRVRRLYLTPAARARAEAVVDFTDELRREVYRGIDPDELDRALATIRKIQANLAAMDR
>MCE8455769.1 DUF6525 family protein [Rhodovulum sulfidophilum]AB15
MAAHDRLPAPARAWVARAVLPWSATSVARIWTRAMAETGSEAAALDRLAAAERATLAREAALACRGGTG
>MCE8455770.1 GTP-binding protein [Rhodovulum sulfidophilum]AB15
MSDRLPVTVLSGFLGAGKTTVLNHVLGNREGRRVAVIVNDMSEVNIDADLVRAGSTLERGEERLVEMTNGCICCTLRD
DL
LVEVRRLAEEGRFDSLLIESTGVSEPLPVAATFEFRDEEGRSLSDIARLDTMVTVVDAANLLRDFSSRDFLADRGEELGP
EDERSLVDLLTDQIEFADVIVLNKVSEASPADLAAARKILRALNADARIVETDYGRVDASGLLDTGLFSFEAAHRHPTWV
KELYGFADHVPEDAEYGITSFVYRADRPFDPTRLASFFETPLPGVIRAKGHFWIATRPHWAGEFSMAGSMVEVRGLGL
WW
AAVPEAHWPPEARERMAERVAGEFGDRRQEMVFIGTLGSMNRDRISAMLDRCLVPETRFCPDLWSGLPDPFPQWG
EAA
>MCE8455771.1 YggS family pyridoxal phosphate-dependent enzyme [Rhodovulum sulfidophilum]AB15
MGLEDIRARLAKAEREAGRAPGSVTLVAVSKVQPLARVEAVLEQGQRIFGENKVQEAAGKWPGFRDRFDGVELHLIGP
LQ
SNKSRQAVELFDAIHSLDRPKLARRLADHAQEGGACPDLFIQVNTGEEPQKAGVLPAETDAFVAECRGMDLPIRGLMCI
P
PVGETPSLHFALLAKIAARNGLAGLSMGMSDDFEQAVALGATHVRVGSAIFGTRSNG
>MCE8455772.1 tetratricopeptide repeat protein, partial [Rhodovulum sulfidophilum]AB15
ANLRATARTERGLTADARASFEAGLALAPDYAELRVGLGRLLVETGETAAGVRHLEVALACLPAHPPGQLALGKGLAQL
G
RKDEAEAALRRAVAADGRYLEAHLALAHHLMETGNPAAAEEVLESALAAGHDRAIIRVRLAEAQAKQGRTETARAGFD
AA
IAAEPGFALAYSLRGMLLQTLGEFAEAEADFRQAIALEPRNGEHYRTMLATCKVTPDDPLLAEMEALFDDPTLGDESRM
H
LGFALSRAMEQIKAHDRVFTYLRPANDLMRARHPYDISERRAEIDAVMAAFEGTDFSARKIEGAPDYAPIFVTGMPRSG
T
TLVEQIVASHSRVTGGGEIGYVAGEGLALLHETGGGLRSLERVGDAEIAALGGRIEATFRERFPGSDLVSDKSIQTYMMA
GLVRLALPQAKIVVVHRDPRDICLSIYKNMFAEGTHRYSTDLRDLGLYYRMFLEMIAFWRDKLPGGFHEIRYEDLIADPE
TEARALVAACGLDWQDNCLNFHKTERRVATLSLAQVRQPIYASSMRAWQRHADELAELTEALGDAIGGENGA
>MCE8455773.1 hypothetical protein LZ188_04210 [Rhodovulum sulfidophilum]AB15
MKTVGNTRALKLAGLVYLAVLSLFLVLNSYTRNHHNWDVIGYTAAAYKLMGETGTDRLSARTYDDIRRATAPETFEKLT
T
GDYRAAVYQDPEALRQQLPFYSIRPLYVALSIAGSKLTGTIAGGTALVSALCGGLLLILGWTAVSPRSIALAALLPFALY
EAGLGSLARLSTPDGLAALVALAALLLCQRHPRPALLLHGRVPRLWIAAMLGVGAATVVGLNAFSGHYGYLRIFDFTLVS
GPQPYPAEMEETLGMTDILDAYARGLQRLVSKDTGMIALFIGTAGFLAAKAMRGRLLPFDKAVLAALGFVLLHFLAFPS
G

FVRHYFVAACLCLILWARLIDGWSARRQRRSPDDAERQAAS
>MCE8455774.1 AMP-binding protein [Rhodovulum sulfidophilum]AB15
MSVHFDDLETRSEDERAASLAECLPALVAEARALPGYAGALAEVNPLTLTTREALAALPVLRKSGLVAAQSAEAPFGGYT
AWEAGQFDHVFQSPGPIYDPGCVSTNWWRLGRFLHAAGIGAGDIVQNCFGYHLTPAGMMFENGARAVGAAVLPA
GTGQTE
LQVRAARDVGVTAYAGTPDFLKVLLDRADALGMDLSRLRRAVVSGGALFPSLRQDYADRGIACLQCYATADLGNIAYES
P
AMEGMIVEEGVIVEIVRPGTGDPLPDGEVGEVVVTTLNPDYPLIRFATGDLSAVLPGKSPCGRTNMRIRGWMGRADQ
TAK
IKGMFVRPEQVADLVARHPEVAKARVVAGRAGERDTMTVRLETEATDPAPFAASVAEVLKLKGTVELVAPGSLPNDGL
VI
EDTRSYD
>MCE8455775.1 ABC transporter ATP-binding protein [Rhodovulum sulfidophilum]AB15
MFDTAPQDETLLELNNIEVIYNHVILVLKGVSLSVPKGGITALLGGNGAGKTTTLKAISNLLRSERGEVTKGSILYRGAR
VQDLNPADLVKAGVIQVMEGRHCFEHLTVEENLLTGAYTRSDGRAALQADLEMVYGYFPRLKERRKSQAGYTSGGEQ
QMV
AIGRALMSRPQMILLDEPSMGLAPQLVQQIFEIVKSVNENEGVTFLLAEQNTNVALRYAHYGYILESGRIVMDGPAADL
R
ENPDVKEFYLGMSDQGRKSFRDVRSYRRRKRWLS
>MCE8455776.1 ABC transporter substrate-binding protein [Rhodovulum sulfidophilum]AB15
MTNRLAPLALGVLLAAGPAAADLVFPDLSYRTGPFAAGGVPFSDGYQDYFTLVNERDGGIGGVKARVPECETAYNTEK
GV
ECYEATKGDGALIYQPLSTGITYQLIPKVEADHIPLHTMGYGRTSAANGKVFEWVFNYPANYWDGASVIVKYLMDENG
GS
LEGKKITLLYHNSAYGKEPIPTLEALSKKHGFKLTLIPVDSPGQSQKSQWLQIRRERPDVVVMWGWGVMNQVAIQEAA
NI
NFPMDHFIGNWWAGAEHDVAPAGMAADGYKALNMNRVVDYPVFADIRSHVIDKGLAAGDGSYVGSVLYTRGMYA
AMLAVE
AAKTAQEMTGTAQIDAAMMRDGMEHLEMTDARMEALGMPGIGPEFTVSCADHGGSGMAIVQQWNAADKTWVP
LTDFIAPD
AEVIAPLIEADSTAFAAENGIAPRCD
>MCE8455777.1 branched-chain amino acid ABC transporter permease [Rhodovulum
sulfidophilum]AB15
MFYREAGDFKTSYRDDSQTFPIGFDRYRYYAVLVLAFAVVPFAIDDYWASAIFVPFLIYAIAAIGLNILVGYCGQLSLGT
GGFMAVGAYACYKLMTAFPDLNIVFCILGGGLVTAAVGVLFGLPSLRIKGFYLAVATLAAQFFLVWLFNKVPWFYNHSA
S
GQISAPARTVFGQPVTGAGVEAWAQYMICLVFAVILAWAARNLTRGTVGRSWMAIRDMDIAAEIIGVNPLKAKLTAF
AVS
SFYIGIAGALFFAVYLGAVEVGEAFGIQKSFLILFMIIIGGLGSIFGSFAGAAFLVLLPVLLKNVLVGTLGWPTDLTAHV
QLMILGALIIGFLIAEPHGLAQLWRVGKEKLRLWPFPH
>MCE8455778.1 hypothetical protein LZ188_04235 [Rhodovulum sulfidophilum]AB15
MSHRTRIGGFCIDCRTEDSGPALAFRSAALGRDGALVEDVRSARPSGPPAAPQVILHDPRVPLDIEAECAWPTGLGAVE
V
TRVRDRVVRQAPSGPRFCPVPPEGEFPKDAMEVD
>MCE8455779.1 hypothetical protein LZ188_04240 [Rhodovulum sulfidophilum]AB15
MQKLFAITCVIGWGVFYVFAYLALASVNDAQWMSVAYGLLAFAGFVAGMLSWVRLVRGKRPVIRTVPKGAARVRRPL
>MCE8455780.1 branched-chain amino acid ABC transporter permease [Rhodovulum
sulfidophilum]AB15

MTDLLYAVEVMINGLMAGVLYALVALGFVLIFKASGIFNYAQGVLALFAALTLVGLQEGQVPFAHLINAALGTDLHHFG
W
QLPAVVAIVLTVGVMVLLAIAIERLILKHLVNQAPIILFMATIGLAYFLEGFGDIMWGADIKTLDVGLPQGGSAWLEQAT
AGWGSEEFYGFFIDRLDIVATLVAAVLVISLTLFSQFSKQGRALRAVADDHQAALSVGISLRFIWVLVWSIAGIVALVAG
IMWGAKSGVQFSLSLIALKALPVLMLGGFTSIPGAIVGGLIIGVGEKLFEFTVGPMIGGATENWFAYVLALVFLVFRPQG
LFGERIIERV
>MCE8455781.1 ABC transporter ATP-binding protein [Rhodovulum sulfidophilum]AB15
MNRMAPPQPDEGLPTEDGRAIGPVLMELRNITLRFGGVVAIKDISFDIREGEIRAIIGPNGAGKSSMLNVISGFYRPSEG
EVWFEGARRPQMRPYKVARQGIARTFQNIALFSGMSTLDNIMTGRITRMKSSLLSQALWWGPAEREESEHRAAVEKII
DF
LEIQHIRKTPVGRLPYGLQKRVELGRALAAEPRLLLLDEPMAGMNVEEKEDMSRFILDVNDEFGTTIVLIEHDMGVVMD
L
SDRVVVMDYGRKIGDGPPEEVRASKDVIDAYLGVAHD
>MCE8455782.1 hypothetical protein LZ188_04255 [Rhodovulum sulfidophilum]AB15
MTENRPTIPMPSLDWQIPFGRAQKDVLWASFALLTAMVALGAGFWALAAVFSLALAVFAQIRVPQEPPTPEGGRLAP
LSH
ALRYGLANLVTMVFVYGAATVLGSGLRMAVTKLLAVV
>MCE8455783.1 AMP-binding protein [Rhodovulum sulfidophilum]AB15
MATRGVSADGLCSIPALLARNVALYGDRPAYREKEFGIWQAWTWAEASDEIRAMALGFLALGMARGDHVAVIGRNR
PAHY
WAMVAAQMCGAVPVPLYQDAVAEEMAYVLDHCGARFVVCGDQEQVDKVIEIRDRAPGVEHVLYNDKRGMRKYDH
TRLNWL
HDIGDEGRAAHRRLEPELDARIAGQSWDTTCVMLYTSGTTGKPKGVVLSNRNIIETAKASVAFDHLGQDEEVLAYLPM
AW
VGDFIFSIGQAMVAGFCVNCPESPDTMMTDLREIGPTYFFAPPRIFETMLTSVMIRMEDAGDLKHALFRHFMEFAKGT
GE
RFGMPRYRRRQSRPTLRRRLRWIYRYAMGIGFAVETLAENAVRGFFVRLRHPLEARKYFRARGPLLNLYPRPLRDYFEYR
TGEVLIYGPLKNTLGLSRIRVAYTAGEAIGPEIFEFYRSLGINLKQLYGQTEASVFITQQPDGQVRSDTVGVPSPGVEVK
IGESGEVYYRSPGTFVEYYRNPDSTASTKDPEGWVATGDAGFFEEASGHLRIIDRAKDVGRMADGALFAPKYVENKLKF
F
PDILEAVVFGAGRKICTAFINIDLTAVGNWAERNNIAYASYQELAGHPRVLETIKSHVEEVNRSLAGDEMLSGCQVRRFL
VLHKELDADDGELTRTRKVRRRIIEEKYADLVAALYDGSAEQYTETEVTYEDGRKGRIRATLKIRDARSFPVHSHDKVAA
E
>MCE8455784.1 DUF3775 domain-containing protein [Rhodovulum sulfidophilum]AB15
MEELLEISPSKVVQVIFQAREKRLGEAELRGFIAAMNDDEKASLVALTWIGRETFEADELEEAITTAKAEATSPTEDYLV
NMPLFADYLESGLEAIGISPADVEEEFFDR
>MCE8455785.1 SDR family NAD(P)-dependent oxidoreductase [Rhodovulum sulfidophilum]AB15
MTTDWIGKRYWIIGASEGIGRALAERLGALGVNLVLSARSEDRLKDLAASLPRKADVVPVDVTDAAAVKAAVEAVGEID
G
VIYVSGTYWPMTAGDWNAEQVETMLDVNLMGAARVLGHVVPWFVARDRGHIVLTGSLAGYRGLPGAIGYGASKAG
LMSLG
ESLQVDLRGTGVKVQIASPGFVKTRLTDKNDFKMPFIMSPEDVAHEYVELMMSDAMSRSFPTLFSWIMRLSQLLPDW
LYF
PLFGRKS
>MCE8455786.1 DUF3833 domain-containing protein, partial [Rhodovulum sulfidophilum]AB15
LALVAAKVRYLGFPAQKPADYSGQGPEFDLRRHLDGPILCEGVVYGPTGRVVSRFTGDFLAQWEGNSGTMTEEFRYDS
GA

TQSRRWSFKLANDGSIAADAPDLVGSGHGMQQGSGVNLRYRIRLPEDSGGHVLAVTDWMYLMENGAIINRSQFRKFGFLV
AELVATMRPKEAA
>MCE8455787.1 DNA primase [Rhodovulum sulfidophilum]AB15
MSLPPGFLDELRTRTSIAQVVGRKVMWDPRKSNQGKGDLWAPCPFHQEKTASFHVDDRQGFYYCFGCHAKGDAIKFVQDT
ENVGFMEAVEILAREAGMPMPVRDPADKVRVDRQTRLLEVMEVAQRHYRMQLQTGAAAEARAYLARRGLSPEAQARWDIG
FAPPGWQGLWDHLTGCGLDPDLILAAGLARASDKGRKPYDVFRNRILFPIRDGRGRVIAFGGRAMDPNDNAKYLNSPQTE
IFDKGHTLFNLARARETAGKGQPLIVAEGYMDVIALSEAGFGAAVAPLGTAVTEHQLRLLWRIHPEPVIALDGDRAGIGA
ARRVMDLALPLLEAGQALRFAIMPPGQDPDEVIRAGGAGAMRKILDEARPMVAILWERAIEDRVFDSPERRAALERDLAA
AVEKIRDPTLRHHYDEDLKRLRWELFRPARGGGGGGGGGGRKGGWTREPRRRPVSSTKSSELVQAMPRFEEELREAVILA
TLIVHPALIARFAGELEALVCATPDHAALQQALLVLPPPEEGTSFRDALEARVGPAPLEKLFSLSHVRIAPPVHDPADHE
KAALCIAHEVDRLMRLRGAERDIEEGMRDLEGLADEGVTWRLSQAARARYGAGPSQEEDGADFELAPNGAHVDRDERDAF
ADLLGRINYGKGRT
>MCE8455788.1 RNA polymerase sigma factor RpoD [Rhodovulum sulfidophilum]AB15
MAAKDTDDSKTDEQDADLALDMSQAAVKKMIAEAKERGYITYDQLNQVLPPEQVSSEQIEDVMSMLSEMGINIIEAEESA
EEGEEKSTEVVTTTSTSREVAVSSTTSETLDRTDDPVRMYLREMGSVELLSREGEIAIAKRIEAGRNTMIAGLCESPLTF
QAITIWRDELLNEDILLRDVIDLDATFGRALEEDEGDEPVVDGIEVETSNTTARKEDDSPELDADGNPIRRDDEDDEEEQ
ANMSLAAMEAALKPRVLETLDQIAGDYSKLSEIQDKRIQATLSKTSRFSDRDEAEYQKLRTEIVLLVNELHLHNNRIEAL
IDQLYGINRKIMSIDSALVKLADQARINRREFIEEYRGHELDPNWMEQMARKGGRGWSAFTERSGAKVAELRADMAQVGQ
YVGVDIQEFRRIVNQVQKGEKEARQAKKEMVEANLRLVISIAKKYTNRGLQFLDLIQEGNIGLMKAVDKFEYRRGYKFST
YATWWIRQAITRSIADQARTIRIPVHMIETINKLVRTGRQMLHEIGREPTPEELAEKLQMPLEKVRKVMKIAKEPISLET
PIGDEEDSQLGDFIEDKNAVLPLDSAIQENLKETTTRVLASLTPREERVLRMRFGIGMNTDHTLEEVGQQFSVTRERIRQ
IEAKALRKLKHPSRSRKLRSFLDQ
>MCE8455789.1 mismatch-specific DNA-glycosylase [Rhodovulum sulfidophilum]AB15
MGSEDILPDYVAPDLRSVFCGMAAGPRSAELGLYYAGHGNRFWPVLHRAGLVPEPLAVGDEARLLAQGIGLTDLAKGAWG
VDAAVPAAAHDPDRLARFVETWRPRSLAFNGKRAAQVFLGRKRVDYGAQAFRGLPVWVLPSTSGAAARYWDETVWRAFGA
ALRRDG
>MCE8455790.1 hypothetical protein LZ188_04295 [Rhodovulum sulfidophilum]AB15
MRLSICLAALAATAALAACVPVDTRPHPILTDRGNRVQPTGNPGEFEVLTRPGDAGAQLWCAAGEYAESQTDARPSDRIY
LVHPRGPSASHPGRKSAIFTVRPDAELLAQARALPDQIVLGVSRVGSNWTRIHAQQVCRPLFNLPD
>MCE8455791.1 sterol-binding protein [Rhodovulum sulfidophilum]AB15
MTTAPQTPRLPGGSGGFPFLPPLPLFLLQPVLKRIVTKIAADNPDMFDRLGHHRHAYFLVDPVDMPFMLYLRPDPEALDF
KALNRRRALPPHCARISGKFLDLLTLMDTEQDGDALFFSRDLIVTGDTEAVVCLRNALDDVEEPIAESVADMFGAPGRGA
LNMLRRAAENAQKRKGSHS

>MCE8455792.1 U32 family peptidase [Rhodovulum sulfidophilum]AB15
MIKPELICPAGTPAALRSAVDAGADAVYCGYQNATNARNFVGLNFTPEEMEKSIKYAHDGGTKVLITLNTFPPAGNVQL
W
KDAADLSAKLGADACIVADIAVAAYIQKTHPQMRIHLSVQAAASSPEAIRYYVQEFGVKRIVLPRIMTVSEIKGLVQEIP
CEVETFIFGNHGLMVEGRCSLTNYVTGQSTNMDGACSPASCVKYERAEDGSMSTILGDYMIDRFKPDETAGYPTICKG
RY
KCDARDDGYYAFEEPVSLNASTMLPDLINAGVHAFKIEGRQRSRAYVKKVVAAFREAVDSIMAGQEPKLADLVALTEG
QK
HTEGALKTKKWR
>MCE8455793.1 U32 family peptidase [Rhodovulum sulfidophilum]AB15
MAAELTLGPNLFHWPAEMKLDFYNRIADESPVSTVYLGEVICSKRTPFFDDHHMDKVAERLERAGKKVVFSSFSEVVIK
R
ERNLLRQWAEQEGRDLEVNNTAALLYLKNKPHRIGALLNVYNEDTMAYLHEQGANHFALLPELPRKAVAELGKKAGEL
GV
GVEVQVFGRASLALSARCYHARAHARTKDNCQFVCGEDCDGMILNTMDEKPFLVVNGIQTLSYGYLDLLPDIADMKAL
GV
THFRVMPHSCDMIAVTRLFDDVLAERIGVDEGEAKLRELGIEAPFVNGFYHGKAGHDWTARAAAELA
>MCE8455794.1 sodium:alanine symporter family protein [Rhodovulum sulfidophilum]AB15
MTDFLNAVSSFVWGAPVLVLLLGTGLYLCIGLRFLPQRKLGYGIRMMLEGRKKGEGHEGEITPFQALTTALSATIGTGNI
AGVATAIFLGGPGAVFWMWLTALVGMATKYAEAVLAVHYREVDAKGRYVGGPMYYIKNGLGPRFAWLAWLFAFFA
TIAAF
GIGNTVQANSVADAVETTFGVPHLWTGIALAALTFVVIVGGVKRIGAVAEWLVPLMAVLYIIGALTILALNFQQVPGAIA
LIFDSAFNGAAATGGFAGAAVMAAIRFGVARGIFSNEAGLGSAPIAHAAALTDDPVRQGNVAMLGTFIDTILVCSMTA
LV
IITTGVWSSGETGAALSALAFNTGLPGGDLIVSFGLIVFAFTTILGWSYYGERAAEYLFGPKVILPFRLLWVAGILIGAV
ANLGVIWIVADIMNALMAIPNLIALIALSGTVFAVSRAYWRKQAE
>MCE8455795.1 peptidase domain-containing ABC transporter [Rhodovulum sulfidophilum]AB15
MSAQDSQLSETLALDTPQLSTLRGAFLIAAHNGIVLSPDELPDLGAGDLAPGLMDLFAAKGLRCRLIERAGWRLASRLG
Q
GLPVLAEVEQGRWIVLVHAGLVEKLRNAAILDPRHEAEGVRNLPWEEFARRWTGRLILIGRPDAAAAESRAFGLRWFL
PA
LRRERGLFAGVAVAVVTGNLIAFSLPLLLQVMIDRVIAHQAWYTLMSVTAVYVVLVLFDAGFGYIRQRLSQIAGGRIDA
D
IGSQSVAHLLRLPMTVFETTPAGVLTRNIQQTDKIRHFLTGRLLQTLLDAALLPLLLTTLALLSGTLTLIITLYAVVIAG
CIGLMLPILQSRLNALYGVEAERQALLVETLHNMRAVKSLAVEPARQRLWEDSLAQSVRRQWDLGRVSALTGTLTGLLE
R
MMQVTVVAVGVGLALQGALTVGTLVAILLLSGRVSGPLVQIVGLINEWQEAALSVRMLRKIMDHPPERQDDDKLAQP
MLS
GTLELREVGFAYPGAATPALDRVSFAVAPGQVVGIVGRSGSGKTTLTRLIQGIETPQQGLILFDGIDIRNIALAALRRQV
GIVLQDNLLFRGTVRDNIAVAVPGAPLEAVLRAATMAGADEFIRRLPKGFDTMVEEGGANLSGGQRQRIAIARALLAD
PR
ILILDEATSALDPESEALVNRNLTAIAHGRTMIVVSHRLSSLVRADQIMVLDQGRLLDMAPHETLLTRCETYAALWRQQT
EHIA
>MCE8455796.1 HlyD family secretion protein, partial [Rhodovulum sulfidophilum]AB15
MSVPPMSPVAQDALPFQPPLVQVRDAPLPWLLRLWPFGSAALLVALVTLAAFVPIDVVVTAEGRIVTDDPPVVLKPLG
SA
VLDRLLVHPGDAVESGQLLARLDARDPATDVAVLAAERAALEAEIARIEAELGDRPLTGEGEEFALQARID
>MCE8455797.1 AMP-binding protein, partial [Rhodovulum sulfidophilum]AB15

MKLDRIARRDYADAHARFSIENEIASLAGDLDAGLNAAVECCDRHCGRDLVALRTVSAGGSLAEYTFEDLRALSARTAN
V
LKAHGVGPGDVVAGLLPRTLDLIATILGVWRLGAVYQPLFTAFGPKAIEHRLTCAGTRMIVTTAAQRGKLDEIPDCPPVA
LIRDADTPLSAGDIDLCAARATAPAEFVPVLRDKDAPFMVMSTSGTTGLPKALMVPQRAMLAFAVYMRDGIGLHEDD
TFW
NLADAGWAYGLYYAVCGPLLLGAATTLHEGGFSAETTIRLI
>MCE8455798.1 AraC family transcriptional regulator [Rhodovulum sulfidophilum]AB15
MQRRIVSAGFVADALEPLARRGIDPAPLLAGAGIAAADGPVTNQEYGRLWLAVAEALDDEFFGLGARPMRPGGFVFL
GHA
VLHAATLNVAFRRALRFLGVLLEDPSGSLRVADGQAEIVLSDRRGLRSAFAYRTYWLVLMGLSCWLIGRRIPLTRLDFAC
AAPPNRADYQRFFGAPVQFGQPESRLCFAARYLELPTIRDERALKLFLAGAPANILVRYRHDQGLSARIRARLRGTDPAL
WPDLEQVARELALSPATLRRRLRAEGQGWQAIRDDLRFAESRRLLAGTGESVAAIALRIGYAEPSAFHRAFLKWSGETP
A
AYRAARAG
>MCE8455799.1 siderophore-interacting protein [Rhodovulum sulfidophilum]AB15
MIDVETELAFPGAETAHAALSAHAKSHDIPPEIDDPGHLAFSFQGNRLEFLTVQGRIRIRLSAVSPNTLYFLKEAVAHHL
EEADPEAARKLRWPGDEGADSHRTPPNFRLLTVLERSRPVEGMTRLHLAGEDLEPLTRDGIHVKLMLPADRSRSPVWP
GV
AANGATRWPKGPDRLHVRYFTLLELDAEAGRVAIDFVDHPDGQISDWARAARSGDRIGIMGPGGGLPPATDRPLILM
GDA
TALPALLRILEALPASAEGCVIAPLRPDDAGYLPASPLETLLLGPERFAASAAGRFAEALAARPEARVWVGAEHALVHEA
RRIAAALPPERKEIGTYWRRGRRGDARRDDHDD
>MCE8455800.1 ABC transporter substrate-binding protein [Rhodovulum sulfidophilum]AB15
MVGRRGFLTGGAAALAAPRLARAGAGIEVRDVLGNRVALPAPPERIVLLDATDLISMAALIAEPGARLAGWASVPRLDL
G
RADWLAPEGLPVVGKLSPDTVSAEGILALSPDLVVASAYMLPPGGSVLESVLRAAGIPLAWTGGHDRALPAGDKLDRA
MG
FWGAVLDRPQAARELAAWGRARFDAVRAATSGPRPRTYMEIMSTYDTCCWAAGHAFWGGIFEIAGGHLLEASDGW
GAKLG
PEGLIAFDPEVYIATGGSFAPELQPGIAPGLDPEQGREGLARAAARPALKDSTATRAGRVHGIWSGLATAPLMAPVLAD
C
LGAWLHPETGAELDPGSTLAALNDRFASPLPGPLWLSLDAT
>MCE8455801.1 TonB-dependent siderophore receptor [Rhodovulum sulfidophilum]AB15
MDRGTLGRTTAALAAALGTAATAAAAQDDAYALEAIVLQADVADTSSSYALPDMRSATKTDTPVVETPQALTVLTRKQ
FD
DQNTQTVGQALRYTSGVLSEIDASTRYDSVFLRGFGGFGISSQFVSSLDGLRLPRGQAFAQPAVDPFLLDRVDVLKGPS
A
LLYGPSSPGGLVNMVSRAPDGSTGGEARLEYGTHDRVQAGFEQHGTLDEAGTLQYSLTAIGRSANTRYRDVDEERYAI
AP
VLVWQPSADTRLTFGGYWQDDPEGGYFNSIYPKGAAPAAYARYLDRDFNIGDPDFDSFERTQWALNAGLEHAFSADL
TLR
SKLRYGRIDADMKGLQMGAPLDASGMLARNALISDETARALNWDTNLEYRFQTGAVSHRLLAGADLLWNDSDWQY
SYTSA
APLDVTDPVYGGVSGPFTTMTDSHQTTRQAGIYLTDQLSFGRFRAVLGLRHDRIETDSLNRLSGTTTEQSSDNTSYRAAL
LYLFDNGAAPYVSYSTSLEPTSGVDAAGDPFVPTESEQWELGIKYQPPGMDALFTMAAFSIDQTDVLTPGDVPGYFIQT
G
KIRSRGVEFEGRGKLTERLELIGALTLLDTEVRASSNAAIIGNRPQAVPDYFGSLWLNRSFGGRLDGLELGGGVRFVGSS
YGDDANSLKSGSYTLADLAVRYDLGARNPSLAGLEATLNVRNLFDETYYSSCSYDYFCQYGEGRIVTGLRKTW

>MCE8455802.1 TetR/AcrR family transcriptional regulator [Rhodovulum sulfidophilum]AB15
MTTAKTPRPARAGRPTKRQSERIAAEIVARARELFIAQGFAATTMEQVIAACGIGKDTLYRRFATKEELFAAVTQKALER
TEAWFEAVAAECPERPLDRVRHLARWFLDANLDPELLALKREAFIEAMRARPLVGADPFTPKLAEAIRAAAAAGELAAP
D
TDFVADQLIAALVLGPSNAALMGDDTLADPAARDAWFDRAWRLFVEGAAPGRG
>MCE8455803.1 RNA ligase family protein [Rhodovulum sulfidophilum]AB15
MKKYGRTFHLPFSPGATSDDKVMSSLEGLMAEDLVVTEKMDGENTTIHAGGSHARSPDSRYHPSRDWLKAFAAGISP
YLA
EGERIVGENLYARHSVAYDALPSYFLGFAWIVGDEIQSWDLTQARFGELGIQPVATLYRGAYRTGLFEDLARALDLSRQE
GFVARIAGSFPESDMPRRMGKYVREGHVQSEIHWMKAELIANGLADS
>MCE8455804.1 exopolysaccharide biosynthesis protein [Rhodovulum sulfidophilum]AB15
MSLPILRTSRDQHDAGNLTLGSLLSGLGETSFGWAVVVFALMTMLPLPPGSSLVTALPVLVTTAQMMLGYRHVRLPGP
LA
RLRLDHDKLRRTVLRLRPVTRRLERVLRPRNPALFRRRNERALGAALFVIAFTLFLPVPGSGWFPAISLFVAGVGMVEHD
GRVTIWGLVMGAASVLLTVAIVVSLAASAEAVIP
>MCE8455805.1 DMT family transporter [Rhodovulum sulfidophilum]AB15
MTGTAATVAGRDPMPGIAAMCAGVACLCVNDAFAKALTEGYSPVQILFLRSLIALPFAAVIAVGTGGPRALVSRRPLAH
L
ARGGLVIVAALMFFTSFRYLGLAEATGLIFVAPIFITALSALVLREPVGWRRWLAVAAGFAGVLIVVRPGAAAFQPASVL
PVLAALFYAVLMLSARWLDARESVWTMQLWLVGSSVLLSGLAVGFVWTPVPVGDLWAFGCIALFGTLGMTLITQAFR
MAP
AVVIAPLDYTALLWATGFGWLFWQEVPDRATAVGAAVIVASGVFIMIRERRAAG
>MCE8455806.1 SDR family oxidoreductase [Rhodovulum sulfidophilum]AB15
MTRTLFITGASSGIGAATARAAVARGWSVGLMARSADKLNALATELGAAALALPGDATDPTAQEEAVARTVENFGAL
DAA
FANAGRGLERPGTEAGDPEDWRGMVDLNVMGVLFTARAALPHLRRTKGHLVLTGSAAGRTHIKGSIYSATKWFVHG
YGGN
MSEEMREWGGRCTVISPGMVDTPFFDSPKPDKIRPEDVADAVLYALEQNPRAELREIHIMPTG
>MCE8455807.1 PIN domain-containing protein [Rhodovulum sulfidophilum]AB15
MAADFFDTNVILYLLDDGPKADRAEALLAQGGMISVQVLNEALVNCLRKARMSHDEAGEFLFGIRDLCETVELSVDTH
DV
GRALAARYKLSVYDSMIVAAALLSGCTRLWSEDMQDGLTVEGRLKIVNPFADAA
>MCE8455808.1 PLP-dependent aminotransferase family protein [Rhodovulum sulfidophilum]AB15
MFRHSQLETVKAWLAHPAHTAMPLHARIQRAIRQLILDGALGPGKPLPASRALAKSLGVSRDTVESAYAQLHAEGFIER
R
VGSGSFVAEMTEFAPGHRRSRRDALLRNQAPNLSKRGNAMFRGGGVREMLMPRPFAHGVPDTRGFPLQLWERLER
QVLKE
TGTLALLHGDPQGAEPLRRAIADYVNLERGARATADRVLVLTSSQQAMTLCANVLLDPGERIFIEDPAYYGARKAFDAA
G
LECVPVRVDRQGILVEPILATPNRAKAVFLTPSHQFPTGATLALDRRLALIEWAAGHQAWIIEDDYDSEFHYAGKPTACV
QGLDPNDRTIYTGTFTKSLFPGLRIGYVVLPPQLVQPMTVARTLLDGHTASMAQLTLARFMEGGHFGAHIRTMRGIYA
QR
LAVLSDLVRRHLSDFVDPRVPIGGLQMPCVLTCEISERAAIEAARRVGVELTGLSALHAAGDGKAGFLMGFAAYTPAELE
GAVRKLATALQMVRMPA
>MCE8455809.1 benzoate/H(+) symporter BenE family transporter [Rhodovulum sulfidophilum]AB15
MNDLAHPIVAGLISVIVNYGGTFILVFQAARVAGLSPELTASWVWSVSIGVGVTGLYLSWRYREPIITAWSTPAAAFLVT
ALATTSYAEAVGAYIISAAAFVLLGLSGYFEKVIRLIPPGIASGLLAGILLQFGIGAFGGASVDPVLVGLLILAYVVLKR
FSARYAVVGILLIGLVFLLVQGRVDLSGLQLKVAMPIFTMPEFTLNATLSVALPLFLITLTGQYMPGMLVLRNDGFKTSA

NPIVTTTGLGSLVMAPFGSHAFNIAAITAAIATGREAHEDPSKRWIAGIAAGCFYVLVGVFGVTLAAVFMAFPATFITTL
AGLALLGTIGGSLAGAMAEPASREAALITFLASAANINMLGVGGAFWGLVIGLVAYAVLNGTFPHRRAVETATSGSAAE
>MCE8455810.1 hypothetical protein LZ188_04415 [Rhodovulum sulfidophilum]AB15
MIGQGIRGVQLLRALQFLDRFDDGLAERYPETRRREGFIRRALDEFATEAAQ
>MCE8455811.1 substrate-binding domain-containing protein [Rhodovulum sulfidophilum]AB15
MKTRYLAAAAASFVVASAAGALAQDADAPWEQSPFHCEPGETYVMNVMVSGVEYWFPVYEMFKQAAHQFGCETD
YTGTPE
YDVNKQIATFEQALARQPAGILLHPMSPDPFVEPINRAIEQGTAIVTFAADSPNSKRVSYVTSDNFREGRVAAQQIAEA
M
DGKGQYAVLENPGQDNHDRRVAAFVEEMETNYPDMELVGRASTGQDPNKAYQAVLSLAQAHPDLGAVFMPEASSA
MGAAQ
AAKEAGGTIRVMSVDVNASVLDMIKAGDMFGAINPNQGAQGYYGFLLLWLAKHPELIDPMNDHAEAGFNPMRIPM
LDNGY
SVVTQENADSFYWDKYLKKRGTRGIEG
>MCE8455812.1 sugar ABC transporter ATP-binding protein [Rhodovulum sulfidophilum]AB15
MPPLLHLSNIAKSFGPVRALRNVFFELRAGEIHALAGENGAGKSTTMKIVDGILQPDAGEIRIDGEPRRIRSPLEAQALG
IGFVHQEIALCPDVSVAENIFMSATATRRGLMDFVALERKARTVLAELCDVDPAAKVGDLSISDQQLVEIAKALTLDARI
LILDEPTSALTETETEKLFRIVHRLRERGLGIIHISHRMAEIFEHCDRVTVFRDGAYVTCLDIPQTTPQEVVNNMVGREI
SHLYPDKATTPPGRPLLEVEGVTCDVLRDVSLTLREGEILGIGGLIGSGRSELAKAICGLRRSAKTVRLNGEPVEIPDFS
AALERGMVYVSEDRKGEGLFLDLPISQNVSSLRLSQVTKAGLVDRAAELAQAEMLGRRLRLKAGRPEDPPSTLSGGNQ
QK
VALARMLSVNPRVVFLDEPTRGVDVGAKSEIHAILRELCDQGVGIVVISSELPELIGLSDRIVVLHEGEMTGELGSDEMT
EEAIIHLASGLHIQGDAA
>MCE8455813.1 ABC transporter permease [Rhodovulum sulfidophilum]AB15
MSETSLSGGMTQTRRDPGPLRRLAGMREMGLLCIILALCVVMSFASPYFLTWSNFRTIFLSFSIEGIVVIGMTLLLIVGG
LDLSVGSVVCLAMVVAGKLFLMGLDPWLASAAGVAAAAAVGAAMGLFVVRVGLSHFIASLAAMVIVRGLCLLITKGTP
MS
LFSLPPEFKFVGQGSLGGLPVVVVIFVVLAVVFDFLLRRSARFRTIIYTGSNEQAAEYSGIRTKRVIFWVTVLCSTLTGL
AGVIFMARFGSATPQFGVGMELNVIAAAVIGGASLKGGQGSIFGAVLGVALLSLVTSSLILLDVSVYMQDMIKGCILLAA
VSFDHLMNKRGKRG
>MCE8455814.1 hypothetical protein LZ188_04435 [Rhodovulum sulfidophilum]AB15
MMDQLQDIAQIRRIYTALTLHYVDGLTQAQVAERMGQSHSTVNRMIKAGHDMGMVEISIRSPFQDHLALERELAEVS
GLR
EVVIETTAAGNPEAVLARVGITAAELLVSKLKPGMTVCLTGGKGVSACVTGLRQVVPLRGMRAVPATGLVQGKHYTDV
NH
AAATLARAFGGEAMQMLSPMFAETEEQHAMICEMKTVSRVMEAARAADVAVVGIGGLREKATSYFDLHRDVPAEEE
ALYA
SGAVAELLAHVLDARGAVTDYVRNRLVVAISPSDLARVPLSIGVAAGQEKAEAICAVVRGGYINTLVTDDATARCVLARL
KEAA
>MCE8455815.1 glycerol-3-phosphate dehydrogenase/oxidase [Rhodovulum sulfidophilum]AB15
MTLSIVPPMSRAARLDAMDGAAPKVLVIGGGINGASVFRELALNGVPVMLAEKSDFCAGASSALSRMVHGGLRYLEN
GEF
DLVREALDERNRLLRNAPHLVGPLRTIVPIDSWVSGALNAPLRFLGLSKAPSRRGALPVKLGLSFYDIYTRGKGGLPPHA
FWSAKTAQARFPFRPGLRCAATYYDGQVSHPERVVTEIFEDVARAGCDAVALNHLEVTKIGPEVALTDRIGGREITLRPQ
IVVNATGAWIDRTNARAGATEDMVQGTKGAHLVLDHPELRDMLGDDMVYYENVDGRICIMFCLHGMVLVGSTDIK
VPDPD
KVRCEADEQAYILEGLSWVFPGITVRPDQVRHVFAGVRPLPVSGAGTTGQIPRGHFNRWTEGGIPMLSMIGGKWTTF
RAF

GELAADDVMARLGLTRRTGTGDLPIGGGAGYPADPDAWCAEQAAASGLSQARVATLLARYGTLAGALAPSFAGERM
LPGL
PGYSDAEIDYLIAHEQVETLADLALRRTVVALAGGLDATALEALADRLATARGWSAARRAEELHCFRVKLATDHGADPE
R
IGLHRKGEVAAQ
>MCE8455816.1 carbohydrate kinase, partial [Rhodovulum sulfidophilum]AB15
MTGYLIGVDAGNTMVKAALFDTEGTTLAVASRGSETLQPAPGFVERDPDGLWQAASGAIRDCLAKAGIDGAEVLAVG
AAG
HGNGLYLLDAEGRGLIGIQSMDNRAETLASQIAAEHGDEILSRAQSRPWPAMTPVLLRWIAENRPEIMARAHKALLCK
DV
IVHGLTGRMGSEVSDLAGAGLLALPALDYDDALLRLYGIEGLKHVLPEPAQSDAVVGHVTAAAAAVTGLAEGTPVVGG
LF
DVLASTLGAGTSHAGEASIVAGSWSINQVFAEHLPAEPRALLASVLGRDTWVSCECSPTSAANLEWFVQTFLADEARAT
G
EDPFALCNRLAVGADPEKDLPVFHPFLYAGPVPGARGGFSAMGAWHSRGDMVRALYEGVAFGHRAHFEILDPDRRF
RFAT
LSGGAAKS
>MCE8455817.1 SDR family oxidoreductase [Rhodovulum sulfidophilum]AB15
MPEPRHAIVTGASSGIGLAICNRLLADGRKVTGLSRRAPEATLGSAFTHRPVDLLDEDAVAAALPDLPAPDALIHAAGVL
RVGDHASMSRAEGSQMWRLHVDCAAQLIQHFAPRMPQNGRIVLIGSRVATGAKGKALYAASKAAVTGLARSIAAELA
PKG
ITVNIVAPAATDTPMLRDPARQGVGPAYPPIGRFVLPEEVAGTAAFLLSDAAASVTGQTLVICGGASL
>MCE8455818.1 4-hydroxythreonine-4-phosphate dehydrogenase PdxA [Rhodovulum
sulfidophilum]AB15
MTNPLIAITMGDPSGVGAEVTLRAAADLPADQRAGLIVIGDRAVLERARDALGLALELGDGPGGLRLRHVPVEGLPDRF
G
VLSPSCGEASFQYIRTAVEMAQAGEVSCIVTAPINKEALNAAGHHYDGHTGLLAHLTESRGSWMLLASERLNVIHVSTH
V
ALKDAITRATPERILETIRTGHSHLKRMGIEAPRIAVAGINPHCGESGLFGTEDDAQVAPAVAAAQAEGIDVQGPISADT
VYHRAYQGAFDLVVAQYHDQGHIPIKLVAFDTAVNVSLGLPIDRASVDHGTAFDIAGTGKANHVNMLSALSYARNLAQ
AK
RHA
>MCE8455819.1 dihydrodipicolinate synthase family protein [Rhodovulum sulfidophilum]AB15
MTSAEPRRAYVATVTCFNDDETLNLDQSRAQVRRQVEAGNDILCAGTNGDFTALLFDEKLSLIEAVLAEAAGRARVMV
NV
GAPSTFETVRLARAAADLGVDCVSVITPYFIACTQEGLRRHFEMVADAVDVPVYLYDIPARTQNHIEPDTALTLARHGNI
CGIKDSGGSQKTLEEYLEVSRQVEGFGVWSGPDHLVLWALKNGAAGAISGLGNVAPHLLAGIVRAFNAGDMEAAEAA
QAR
FGALRTDLYALGYPPAVAKRALYLTDPGVGASRQPALMPDAAQDAAIRDILDRHDLAATV
>MCE8455820.1 iron-containing alcohol dehydrogenase [Rhodovulum sulfidophilum]AB15
MLDQPLEILQPGCVRNGAGQAVRAASWLRDRGLTRPVVIADAFNATRLNVLGLDGAACVSVRPEPDLFDLNQAVTAA
RAA
RPDVVIGFGGGSAMDVAKLVAVLQDPVTTFDDISGPGRAGPRRAALMQIPTTAGTGSEAGTRALITEPGTLRKIATESL
H
MLADMVILDPGLTVSLPGPVTAATGVDALAHCVEAFTSRRAHPVIDHYALEGIRLIGRFLPRALADGDDLEARAALLLAS
FYGGVCLGPVNTTAGHAVAYPLGTRHHLPHGLANALIFPHVLAANQGACPDKTALVAAALDLEQGRPLLQAAHDFCA
GLG
LQMRLRDHGVPEDDLPEMAAEAHAIRRLLDWNPRDLSREDILALYRAAY

>MCE8455821.1 TAXI family TRAP transporter solute-binding subunit [Rhodovulum sulfidophilum]AB15
MRHIRTFATATTLAVLPLAAFAAPVDLRLITMEPGGSWYSYGSTFSEIIQGSEGENELNVEVLPRGGGMTNPVAVSQGA
A
DLGFVSASAAIWARDGIGEEFEGREANNALAIVGGLQLAYTTIAVRRDYVEKSGLTTFDEMVASDNPPRFVLKPAGSQV
P
ILANYMFEALGSSLEDMRDRGAITQISTAQIAQMLRDGTADVYIENAPVGQATMSEVTLTTPMVFVPASDTVLDHMSE
LG
APAANMPEGSYPGQEGAYRTSMTPTILIANADMDEEVAYQLTRALVEQREKIAEAFPALAGWDPEKGAQPDQAVIEL
HPG
AARYYRERGWIE
>MCE8455822.1 TRAP transporter fused permease subunit [Rhodovulum sulfidophilum]AB15
MRKISNFDTWWRALRAVLSVFYVAFLVAQFFHPASPLAAASFHVFMATALVFAWTPLAWEGRARYAARIIDIAGVALS
LM
VVALYAGEIFRLERRFEMIDPVLPVDILLHVLGLALIFEAVRRSVGWSLLAVVLIFLAYAYLGPWFPGLMQFPGFSLPTQ
AELIGMKTDGIFGVTASAAINFVFYFVLFGSVFTITGGGQLFIDLAMRATARLKGGAAKMSLVGSALFGMVSGSAIANTT
STGVLTIPIMTRSGYSKEQAAATEAIASTGGQLMPPIMGVAAFVMADMLGIPYITIAAAALIPAAAFYFALYIIVDLRAR
KSGVGDVAPELLEIPPVIPRLHLLAAPVAMIATLIAGYSAPYAALVGTAIALVAPVLRRSTRYNLSQLFETVLDTARQMA
WISAPLAAVGVVMVVATQSNLALKFVSLLSDMGTDNLYLSLLLAIFGCIIMGMGLPTVAAYIIGSLVFVPALMDLGVDRL
AANLFVLYYCVLSMVTPPVALCSYAAAGIAKSDANRTGLVAFGYSLVIFLVPFGFIKDPAVLWQGSAFQIATGAAGMLLA
TFCWAVFLIGWLRGNLSLPERGGFALASLALVITPTLTAGWVISVTLTCLLLVWRFALRPRLVTA
>MCE8455823.1 exo-alpha-sialidase [Rhodovulum sulfidophilum]AB15
MTEVMADTAFPATDLPTPRVQNHASFLHELPDGTVLCAWFGGTIEGKSDISILMSRFDGRNWSNPVQLSDDPARSEQ
NPV
LFTAPDGRLWLIYTAQPGGRQNEAEVRFRISDDGGQSWTEPCALWSQKGIFVRQPIIVADDGAWLLPGFRCRVEEGV
AWT
GENDDSVVMVSHDEGVSWKVYEVPGSLGCVHMNIVPLADGGIAAFYRSRWADHIWRSHSADGVRWSAPEPAGLPN
NNSSI
QAVRLDDGRIAMVYNHSSREDAEGRRVSLYDEIEEDGAAPAARPGRAFWGAPRAPLSLALSADGGQSWPDRIDLAFS
DGY
CLSNNSADQVNRELSYPSILQDRNGRVHVTWTHFRQSIRHMIVPLDEQHG
>MCE8455824.1 hypothetical protein LZ188_04485 [Rhodovulum sulfidophilum]AB15
MTPPRLLIVADDLTGALDSAGEAASLGIGTRVFLSPAALAAARECTLPPVVAVTTGSRDGTVAAAAAAMHNVCACLSW
LR
PERVMKKVDSRLKGHVAVECAILADALHRRKIIAAPALPDMGRVQRHGRLTGAGIETPLDIAARFSGLSPHVPDITDAA
Q
MKEAAELDGLPVGARSLAAALVAQLWPDATRQGTPPLPGPAVLAIGSRDPITLAQVERLDLPLYLAPDGRLPLDPPRPP
C
VMQMVSGGMDRSAAEAGADFAEGLTKMIMRDPVGTLIACGGETAAAILARLGVDRAEVFGLALPGIAVVRAALPGSR
VLT
IVTKSGGFGAADMLAQLVNLVDNRPTPTEVRKYRTRE
>MCE8455825.1 FadR family transcriptional regulator [Rhodovulum sulfidophilum]AB15
MKATRQKSRPSFADHVYNTLYGRISNGDYPPDEKLPSETTLAAEIGVSRPVLRDALERLRAEGLIVSRQGAGNFVRAHQS
RTLGYGRIETIADIQRCYEFRLTIEPQAARFAAERRNDEALANLSNALDLMRAATGSMLHREDADFTFHIAVAHAANNT
F
FEATLRALHEQISAGMKMHGQSLLREGGAGLERVLHEHQSIFDAILASDGEAAANRMTAHVRHSQERLFGGASIDLRM
PA
KK
>MCE8455826.1 GNAT family N-acetyltransferase [Rhodovulum sulfidophilum]AB15

MNAPYRLPDEFETDRYHLRRVNLDDAQAIFDSYASDAEVTRFLGWTPHQSVAETTAFLKIAAAEWDSGKGFPLVAFDR
RQ
PAELVGMFHPHLIGHRVSYGYVLRASAWGQGCASEVMGRLVDHALSHPSIHRAEAFCDVEHPASARVMEKAGMVRE
GILR
RYFRHPNISDIPRDCFIYSKVH
>MCE8455827.1 phosphate ABC transporter permease PstA [Rhodovulum sulfidophilum]AB15
MLTADARTRRRNAAESRFRLYGIVAVGIGLFFLAALLYAIVTNGIGAFQQTGYTLNIEFREDVLDKSGTRDPEALKSVRT
FAYSPLLREAMLREIEIHGIETPLAKAKVKGFSSPLDKLFSDGARSTLRDMVLADPSLIGQTVKVRILASSRVDGYLKGR
VSRESIERDKNLDAVHLDLIDKLRDAGVATKAFNLAFITGPDASDARPEQAGIGVAMLGSFYMMIVVLALALPIGVGASI
YLEEFAPQNRITDLIEVNISNLAAVPSIVFGILGLAVFIQVVHLPQSAPLVGGLVLSLMTLPTIIISTRAALKAVPPSIR
DAALGVGASKMQAVFHHVLPLAMPGILTGTILGLAQALGETAPLLLIGMVGFVASNYPDGAVAGFMAPNSAMPAQIY
EWA
KRADPAFYERAWGGIILLLVFLMTMNIIAILLRRRFERRW
>MCE8455828.1 phosphate ABC transporter ATP-binding protein PstB [Rhodovulum sulfidophilum]AB15
MYDNSDLARATVTGETKIAAHGCQVYYGDSQAIRDVDIDIADNTVTAFIGPSGCGKSTFLRCLNRMNDTIDICRVIGDIR
LDGEDIYDPRVDPVQLRAKVGMVFQKPNPFPKSIYDNVAYGPRIHGLARNKADLDEIVERSLRRAAIWDEVKDRLQAP
GT
GLSGGQQQRLCIARAVATEPEVLLMDEPCSALDPIATAQVEELIDELRAQYSVVIVTHSMQQAARVSQKTAFFHLGSLV
E
YGDTAKIFTNPSDPRTESYITGRIG
>MCE8455829.1 phosphate signaling complex protein PhoU [Rhodovulum sulfidophilum]AB15
MMKDPHIVKSYDRDLEGIQALIMKMGGLVEIAILEAATSLEARDEELAERVRAGDKAIDALEEKVNEEAAQLLALRQPSA
GDLRTVLSVMKISANLERCGDYAKNMAKRTGVLVQMAPMEGAAGAIRRMAKAVELMLKDALDAYIQRDAELAEDVR
QRDL
EVDQMYNALFREFLTFMMEDPRNITACMHLHFIAKNIERMGDHVTSIAEQVIYLATGHMPEDERPKADRTSVEALRS
>MCE8455830.1 phosphate regulon transcriptional regulator PhoB [Rhodovulum sulfidophilum]AB15
MSPSERPTVLIVEDEPAQREVLAYNLQAEGFRVSKAENGEEALLLVEEQAPDIIVLDWMLPSVSGIEICRRLKTRAETRG
VPIIMLSARSEEVDRVRGLETGADDYVVKPYSVVELLARVRAQLRRTRPATVGQRLEFEDILLDAETHKVFRSEKPLKLG
PTEFRLLSTFMEKPGRVWSREQLLDRVWGRDIYVDTRTVDVHIGRLRKALCQHGGPDPVRTVRGAGYALG
>MCE8455831.1 methylamine utilization protein MauG [Rhodovulum sulfidophilum]AB15
MKRTGTGTGLTLAAALLACGAASADPEEGAYGSAEAFGEALFFDVNLSKTRTQACATCHDPGHAFTDPRPTLAGRAVS
LG
ADGQSLGDRNAPTAAYARFSPPFGQRADGTYVGGQFHDGRAAGLAEQAAGPPLNPIEMGLADVEEAAARILENPRY
DATL
SALYGAATPGDPEAVFRAATSAIAAFEGTDSFAPFDSKYDRYLRGEAQLTDEEELGRILFFSQQFTNCNLCHQLRKSAIA
ADETFTDYSYHNIGVPANTAARAANGAAGPDLGLLANPAVEDPAAAGKFKVPTLRNVAVTGPYMHNGVFEDLRTVILF
YN
KYNSKAAARQIDPETGEAWAAPEVPGTLSMDKLTTGPALDDERIDALVAFLETLTDSRYEYLLDRDTASARTEGTDTDR
>MCE8455832.1 LuxR family transcriptional regulator [Rhodovulum sulfidophilum]AB15
MSLEYLSALIAARSLEELWALHTRKMADFGFDRLIYGFTRFRAANSFGDRDDVIVLSNHSPEYVRNFVDKGLYKNAPMV
R
WAAKHVGTSSWRQVSERLARGELTPEELDVLKFNRGMGVVAGYTISFSEVSVRAKGAIGLTARPGLSQDDVERIWARH
GD
VIQLMNDVMHLKVHNLPYAAQRPPLTPRQREVLEWVGDGKTTLDIATILGVTQATVEKHLRLAREALDVETTAQAVLK
AW
FQNQIFRANTP
>MCE8455833.1 hypothetical protein LZ188_04535 [Rhodovulum sulfidophilum]AB15

MFTPTASPAQPLLPQALRLLCLALLVTAVFGALHYIMVAPSRHVPVLHPAGAFGFVLFALSVLLRGSKAWGLRLWTAVG
G
GVVLLAGLRLIDGLLPGGPYLSEAVPLWPGRSPGPADLSTTYAILTLHLCLALQPFHRGAAMAVLVAAWAGIWWSTMT
DL
FEATIWGRPVSAFSLSCMGLAGLAMTHILRAEWLLRPLFSRERHGSRMRILLVLALIGPWLVGIAALHAVPETVSARLLI
APLLGLFGLAQLALVLTVGALLERAARADARAELFDPVAGRASKFRPDRDLLDGKGPVGVILCAPRAGVNGADPEADER
A
MRSALQRTVRHVRAEDNIGRWRRHMLMVVAHVPSEADLARIGERLEQKLRPQPDLPVAAIGISMSAFDEPNLEAALQ
RAE
SALYVAERRGGGTLVRVSDLAETGQGAEARNSPRDMGGAARPP
>MCE8455834.1 GGDEF domain-containing protein [Rhodovulum sulfidophilum]AB15
MASADQRPRKTLAIRVLGLTILVAIGLGLLQNLLDPPADTAAALHLLPATGFALFGLLFLLRRQPSGLRGPVRMLACIVA
LLAIERLFEASVPGWVLIVGHPRIEHALESTGLKGVLSVETAFVLAMLNLALLAEPHQRQTSLLFLAAAWVGTTMGVLKL
AFNLTLWRGEMSPYALAIMILVCSAQTLKLRDQPFLRPLFARTSYGTYTRALVTGALLVPWMSGFLYFLNVRPDPASHL
A
LELAFGFIGWIMLALVLAIGHLMEKSSLALEYAARHDPLTGALNRRGLAMALGNRDEARGVVLFDLDHFKAVNDALGH
DA
GDRLLCDIAEIANRNIREGDLLVRWGGEEFLLVMASSAEVLLMQMAERLRQSIAAHAPMRIGAMPHPVSASFGVSMV
QPG
EAGIGRAIRRADTALYAAKIGGRNRVMSAAALCEAEAAVGPLARRTPPRLVS
>MCE8455835.1 translation elongation factor Ts [Rhodovulum sulfidophilum]AB15
MAITAALVKELRDKTGAGMMDAKKALTENDGNMDAAIDWLRTKGLAKAAKKSGRTAAEGLVAVVVEGGKGVAIEV
NSETD
FVAKNAEFQAMVSDIAKVALGVDDVEALKAADLGGKSVADTLTDKIATVGENMTLRRMARIEGEQVVAYVHNAAAE
GMGK
IGVLVAMTGGDAAFGRQVAMHVAAVNPAALSEADMDPAVLEKELAVQTAKALDENATAAKPKPEAVITNNIIPGRM
KKFI
SESTLMGQAFVVNPDMTVEAAAEAAGATITGFVRLEVGEGIEKAEEDFAAEVAKVVQG
>MCE8455836.1 30S ribosomal protein S2 [Rhodovulum sulfidophilum]AB15
MALPDFSMRQLLEAGVHFGHQTQRWNPRMAPFIYGDRNGIHIVDLTQTVPMLDQALNVIRDTVARGGRILFVGTKR
QAQK
SVADAAEKCAQYYMNHRWLGGTLTNWKTVSQSIKRLNEIDERMATGAEGLTKKERLHMEREQTKLQASLGGIREMG
GLPD
LLFVIDVRKEDLAILEARKLGIPVVAVVDTNCSPEGVDYIIPGNDDAARAIQLYCDLASRAALDGMSAQMDAAGVDLGA
L
EEAPVEELLADARSEGEA
>MCE8455837.1 ABC transporter permease, partial [Rhodovulum sulfidophilum]AB15
IYQNIELAGVAFPAQVMDALPYILTVVILAGFVGRAIPPRAGGEPYVKERKI
>MCE8455838.1 sulfite exporter TauE/SafE family protein [Rhodovulum sulfidophilum]AB15
MQIYLPIAEVSVNAFLLLGLGGLVGILSGMFGVGGGFLLTPLLFFIGIPPAVAVATQAAQIVASSISGVLAHFRRKTVDL
RMGSVLLAGGMAGALLGVWIFNLMRGIGQVDLLVKLCYVVFLGIIGALMFVESLAALNRTRKGVVRKRKQRTWFAAL
PFK
MKFRTSGLYLSAIPPLIVGAFVGVLSAIMGVGGGFIMVPAMIYLLGMPTKVVIGTSLFQIIFVSGFTTILHATTNHTVDL
GLALLLLIGGVVGAQIGTGIGLKMKAEQLRILLAALVLAVCAKLALDLLIEPAELYSIADRVR
>MCE8455839.1 TIGR02186 family protein [Rhodovulum sulfidophilum]AB15
MSRRLALLLPLLLLAALPARAESVVAGLSQTRVSITTNFAGSEILIFGAVKRDAPAPEAPPLEVVVTVAGPSTPATVRKK
DRRGGIWINTESVRISAAPSFYAVAATGPLERALSQTEDLRHQVSIPRAIRAVGVTQETPEAQSFIDALIRIRSETGLYQ
ELPGAVELDEQTLFRTSIALPSNLTEGEYATRIFLTRGGEVVDAYETTIGVHKVGLERWTYLLAHEHPLAYAILSLAIAI

AAGWGASAAFRIMKS
>MCE8455840.1 SDR family NAD(P)-dependent oxidoreductase [Rhodovulum sulfidophilum]AB15
MTGRTILVTGASSGIGRATAELFLDHGWRVGLLARRADLLDEIAAGREAALALPADVTDAAAVEAAFDAFMGWAGRL
DVL
FNNAGTFGPSGPIDEIDPADWAQVLSVNVGGMFLAARAAFARMRGQDPQGGRIVNNGSLSAHVPREGSVCYTTTKH
AVTG
LTKTLSLDGRPFGIACGQIDIGNARTALVEDLTERARAGGQPVQPTMDVAHAARSVLHMAELPPEANVQFMTVMATT
MPY
IGRG
>MCE8455841.1 AAA family ATPase [Rhodovulum sulfidophilum]AB15
MTLPELRFSDDQAEAHDRVTELLRGFGVDLDEGSHSARTEGKTGVLAITGKAGSGKTLLLAELVRAAVEAGVEIVSGDYE
SKRRKERRTLAVLAPTNKAASVLRQRGVPATTIHRIIYTPVYDPEYEKIAEWLTGECDRPEIEGLTEAALDRAQAFYASV
ASVPGALAAAGLKGSDFIKGWKRREDPLDIGFVDEASMLDERQFDDLRELFPALVVFGDPAQLAPVNQSGQMVFDRL
AGP
QTMELHRIHRQETDNPILDLAHALADPEVDFERFERMVEEAAARDPRVELAPRVDSDLMARSPVLVWRNATRIRLIHA
FR
HAHGAPADALLPGEPLICDGIELPLKHRKKRIDLEARGLIKGAQVIYVGPGRKPGFSIMHVIGAEDPRLSVASIVKIEMP
EEEEPFIPYAATMGAAFLHGAAVTIHKAQGSQWPAVQVFAPDLYAASRAGRVEAGQPLWKRLAYVAITRAEERLLWV
TRY
RLARPAGALSADDLTAAPAPLTLTAEEARP
>MCE8455842.1 autoinducer synthase [Rhodovulum sulfidophilum]AB15
MLRYIYGVDLHKFPLLRDTMFRDRARQFHDRLGWAVTVNDRGEERDQYDGHNPLYVIWEQANGRHGGSMRFLPTT
GRTMV
NEHFLHVTGGVRIESPLIWECTRFCLAPDAGREVTAGLVLGAGEVMAQFAVEHFVAVFDLRLARIYRVMGVEPDVIGA
AG
NGRNWIGLGLWEMQEAAWPRTLARLGIGRETSLGWFRASFSAPTAAPRELAVA
>MCE8455843.1 LuxR family transcriptional regulator [Rhodovulum sulfidophilum]AB15
MRGGLELFLDRLQRATTLEDLYCVTEDLRDRYGVLHIVYHWVNSVGERYGAGTYSTEWVDRYLECDYLMIDPVIFGCYS
R
FHPVDWKELDWSSKQARVFLRDAMSYGLGNQGVSIPLHGPKGQFALFTVNDSCSDAAWEMFKQINLRDLILIAHDFN
KKA
LEFEQWSDPAPRAALSPRELSAMTLLAKGMSRAQAAQELSISEHTLRVYVETARHKLGALNTTHAVARALSTGLIVV
>MCE8455844.1 hypothetical protein LZ188_04590 [Rhodovulum sulfidophilum]AB15
MKHEWLIDILGDLKNYARKNGLTELSEELLTCQALAAVEISSLEAGRARGAVGERASSMTAWGDADSCDARRLRIVPGP
F
ATGNDA
>MCE8455845.1 crotonyl-CoA carboxylase/reductase [Rhodovulum sulfidophilum]AB15
MALDTNPSIAPYEAPEKELYEIGEMPPLGYVPSKMYAWAIRRERHGEPDKSFQAEVVETPAIDSHEVLVLVMAAGVNY
NG
IWAGLGIPVSPFDGHGAPYHIAGSDAAGIVWAVGSAVKNWKVGDEVVIHCNQDDGDDEECNGGDPMFSPSQRIWG
YETPD
GSFAQFTRVQAQQLLPRPRHLTWEESACYTLTLATAYRMLFGHHPHELKPGQNVLVWGASGGLGSFAIQLINTAGAN
AIG
VISDESKRDFVMDLGAKGVINRKDFNCWGQLPKVNTPEYKAWFGEVRKFGKAIWETTGKGVNVDMVFEHPGEATFP
VSTF
VVKRGGMVVICAGTSGFNLTMDARYVWMHQKRIQGSHFAHLKQAAAANRLMCQRRLDPCMSEVFPWAEIPQAHL
KMLRNE
HKPGNMAVLVQAPRTGLRTFADVLEAGPSA

>MCE8455846.1 protein meaA [Rhodovulum sulfidophilum]AB15
MTETTKDRPWLFRTYAGHSTAEASNALYRANLAKGQTGLSVAFDLPTQTGYDSDHELARGEVGKVGVPIDHLGDMRT
LFD
RIPLEQMNTSMTINAPAPWLLSLYIAVAEEQGADVSKLQGTVQNDLIKEYLSRGTYICPPKPSLRMITDVAAYTREHLPK
WNPMNVCSYHLQEAGATPEQELSYALATATAVLDDLRGKVAPEDFPGMVGRISFFVNAGIRFVTEMCKMRAFVELW
DEIC
QERYGVEDPKYRRFRYGVQVNSLGLTEQQPENNVYRILIEMLAVTLSKSARARAVQLPAWNEALGLPRPFDQQWSIR
MQQ
ILAYETDLLEYEDLFEGNPAVAARVEKLKHDARAELAQIDAMGGAVAAIEHMKSRLVESNSDRIGGVETGATVVVGVN
AW
TEGEPSPLTSGEDAIMVVDASVEADQIARLAAWRETRDGAAVEAALAALREAAATGRNIMPPSIEAARAGATTGEWG
DVM
RAVFGQYRGPTGVSKNPSNRTEGLDEIRAEVDALAARLGRRPKFLVGKPGLDGHSNGAEQIAARARDCGMDISYEGIRL
T
PSEIVAAARAEGVDIVGLSILSGSHIPLMESLMRLMKEEDLTHIPVVVGGIIPEEDARRLRAMGVAKVYTPKDFELNRIM
FDVVALISPEQVAAE
>MCE8455847.1 purine-nucleoside phosphorylase [Rhodovulum sulfidophilum]AB15
MSLHIGAQPGEIAETVLLPGDPLRAEWAAKTFLDDPVCINRVRGMLGFTGFWRGHRVTIHGSGMGMPSLSIYANELIR
EH
GAKTLIRIGSAGAMQAHVALRDIVLAMTSSTLSTPSRGTFRELNFAPCADFGLLAAAHRAAVSKGASPHVGGIYSADAFY
DERPDLNAELTRHGVLAVEMEAAELYILAARHGARALAVLTVSDHLLTGAALSSDDRQSGFGKMVEIALEAAFS
>MCE8455848.1 DUF1989 domain-containing protein [Rhodovulum sulfidophilum]AB15
MPTEPRPEPADAAERRAAPPVVCYPPECLPAPPMALYDGLRAGMTRVAQLEIPARDARAFRVPAGHVLRISCTQGPQ
VGD
LNLWNAHDLSERFYSGKTRALHGTHVTTGDRLWSGFPPLRPMATVIADSLGWYGFDADGAGVHDVIGTRCDPYTNA
RLGG
APFHRCCHSNLIRALVAETGLAPDAAEPHVHDVLNVFMCTGFTRDSHRYFTKASPVRPGDHLDLFAEIDLLCALSACPW
G
DCSGNSTDHARCHPLGVEIFAPAPGALDGWTPPEPAPYDRSHGL
>MCE8455849.1 hypothetical protein LZ188_04615, partial [Rhodovulum sulfidophilum]AB15
LEKYAVSLNLAAICGLLVGLGLFFGDTATTTGPVLTPASVEPWAALTLFFGLIVTVQGFETSRYLGAEYDADLRIRSMRA
AQYVSTAIYLVYVTLLVLCFAPGKGALDDTAIIGMTAAVSPLLPLVLTVAALAAQFSASVADTTGAGGLVAERSKGLIGN
RAAYAVLVAAGIALTWTADIFAIIAYASRAFALYYALEAGIAALAARRAGQGGRAAGFAGLAVLGLAIALLGRPVE
>MCE8455850.1 hypothetical protein LZ188_04620, partial [Rhodovulum sulfidophilum]AB15
MLRDILILATAAAGLVLILSPPMRRWRFWQAMVTPLASIIGSGFLVLGPILNQSLGVWSPAAMALLALIAFGFGGAIRYS
IAAGAHGSPPGRLGALAETAAGWMLAFSYVISVAYYLNLLGAFAVRLTPFDTPLAARAVTTAVFLLILATGLTKGFKAR
>MCE8455851.1 succinate dehydrogenase iron-sulfur subunit [Rhodovulum sulfidophilum]AB15
MVQLTLPKNSTVRKGKTWPKPEGATNLRTFRIYRWDPDTGENPRVDTYFVDLDSCGPMILDALIKIKNEIDPTLTFRRSC
REGICGSCAMNIDGVNTLACTRGMDEIKGDVKIYPLPHMPVVKDLVPDLTHFYAQHASVMPWLETQSNTPAKEWRQ
SIED
RKKLDGLYECIMCASCSTACPSYWWNGDKYLGPAALLHSYRWIIDSRDEATGERLDALEDPFKLYRCHTIMNCTKTCPK
G
LNPAKAIASIKKMMVERSV
>MCE8455852.1 hypothetical protein LZ188_04630 [Rhodovulum sulfidophilum]AB15
MTVVANMATYPPRRAGLAKAVAQLAPQVDRLNLVLNQYEAPLEEFAAFPNVHQIIPDTDTKDTGKFYPDVSGADYVL
MVD
DDIDYPPEFVERTLELFAPFGRGHLAGYHGSLYLSRWDLIRRGKLVRALSYDRSRIADFRQGFRFHSKLQNAIVVDQIAT
CAAIMHAEDFPPFEFMRDSQKFVDVRLARWCFERSITPIALPKTAKWLGKVDYEETIFHGFTQTNPPDVTEEILSYAFKV

KGRGLPPKPCASQT
>MCE8455853.1 hypothetical protein LZ188_04635 [Rhodovulum sulfidophilum]AB15
MTRDVRFCRPCPDPHSDEIRGDMSFAQTPEPVGHGARAVSVLSQRSEPDFVASARAENALAFGMLAASEIFFAGNRR
KSF
LGVVADASCVEAVDSAHVFLVELGYVPADVKCAGPTVSFSRKPGWMSTDEVCARIETYEGKQVRQ
>MCE8455854.1 hypothetical protein LZ188_04640 [Rhodovulum sulfidophilum]AB15
MIVGTMATYPPRQRSMLAAVARIAPQLDVLNVVLNEYVSVPPELLSFGNVNAIRPEEDTKDTGKFLPDHRGADWVFLL
DD
DIVYPENYVTDSIAAMQALGPGRLVGSYYGAVYRKPQLRNKTLSGLNRYRKELFRRRGQVRDSLKVDAGLQQAIVVDEL
G
TGVMISRPEYLPDFESVRSAQKFIDVRMARWCFEKGITQVCLPRPAGYLGLVEHEETIYTGFTTKRHPAVTREIQEYALR
NPLAGQHFQCGGDSRGNF
>MCE8455855.1 succinate dehydrogenase flavoprotein subunit [Rhodovulum sulfidophilum]AB15
MAAYEYETHEYDVVVVGAGGAGLRATLGMAEQGLRTACVTKVFPTRSHTVAAQGGIAASLGNMGPDHWQWHMY
DTVKGSD
WLGDTDAMEYLAREAPKAVYELEHYGVPFSRTEAGRIYQRPFGGHTTEFGEGPPVQRTCAAADRTGHAILHTLYGQSL
KQ
KAEFFIEYFAIDLLMSEDGQCQGVLAWKLDDGTMHVFSAKMVVLATGGYGRAFFSATSAHTCTGDGGGMIARAGLPL
QDM
EFVQFHPTGIYGAGCLITEGARGEGGYLTNSEGERFMERYAPTYKDLASRDVVSRCMTIEIREGRGVGPNKDHIHLNLSH
LPPETLAERLPGISESARIFAGVDVTKEPIPVLPTVHYNMGGIPTNYWGEVLNPVEGDFDRVAPGLMAVGEAGCASVH
GA
NRLGSNSLIDLVVFGRAAAIRAGQIVDPETAVPVPPKASIDQALDRFDGLRHATGSVATAELRLEMQKTMQADAAVFR
TD
KSLADGVTKMTAVAGKLGDIKVTDRSLIWNSDLMETLELANLMPNALATVVAAEARKESRGAHAHEDYPDRDDANW
RKHS
LAKVEGAGVELAYRPVHLDPLSTTDEGGIDMKKIAPKARVY
>MCE8455856.1 succinate dehydrogenase, hydrophobic membrane anchor protein [Rhodovulum sulfidophilum]AB15
MRYLTDRKRAEGLGAAKAGTHHHWSMNVSGVGLTLLVPFFVFFIGTSLGASHDEVIATFSRPFPAVVALLTIAAGMLHF
R
RGAQMLIEDYCDGMTRKALLWGATCLSYAVMAAGMYAVVRIAI
>MCE8455857.1 succinate dehydrogenase, cytochrome b556 subunit [Rhodovulum sulfidophilum]AB15
MADIERGNRPLSPHLQIYRPQLTSVTSILTRVTGNALILGAVLIVWWLLAAGAGPEAFARADWVLTSWFGNLVMFGSL
WA
LWYHTLAGIRHIIWDNGYGLEIETAEKLGWICVIGSAVLSVLTVLILWIF
>MCE8455858.1 hypothetical protein LZ188_04660 [Rhodovulum sulfidophilum]AB15
MGLALLGFYAFLHDPMPRTAEEAVAQLTRIADGMGGIVGDRTDTATRAAIVSEAARVAAEAARPLDDATAVIAHVSFG
VF
DLPVLETWEVEASDGRVIPLTVLRGGSGHADLAFDGKDYRLGVGKFLSYESGADLCRLLLVKKVDGGVYRLRWRCIPDH
R
DQL
>MCE8455859.1 MaoC family dehydratase [Rhodovulum sulfidophilum]AB15
MAKTNPGRFFEDYAVGQVIPHAVPRTLSGGERALYHALYPARHALYSSDAFAAACGLGAAPLDDLIAFHTVFGKTVPDI
S
LNAVANLGYAEGRWHRPVRPGDTLRSASEVIGLKQNSNGKSGVVWVRTRGLDQRGELVLDYVRWVMVRKRDLEAP
APETV

VPRLAGVVAAGDLVLPEGLDFTGYDFTLAGEPHRMADYEIGEVIDHVDGVTVEEAEHMQATRLWQNTAKVHFDATFR
EDG
RRLIYGGHVISLARALSFNGLANAQMIVGLNGGAHANPCFAGDTVRAWSEVLDKAETSAPGVGAIRLRLVAVKAGGVP
GE
LRGEDGKYRPEVLLDLDYWALMPV
>MCE8455860.1 DUF1737 domain-containing protein [Rhodovulum sulfidophilum]AB15
MQAYRLLTEEDSAAFCHKVTAALAQGWALYGAPAYAHDPASGKMRCAQAVVKDVEGDYAPDLKLGEL
>MCE8455861.1 NnrU family protein [Rhodovulum sulfidophilum]AB15
MFLLILGVALWSFAHLFKRLFPGIRAMLDDKLGMASKALFAVPLVVSVVLMVIGYKTAGMPYRFAGPSWLVHVNNTL
MLG
AVALFGVGSSKSHARQWFRHPMLYGFITWAVAHLLVNWDLASIILFGGLGAWAVAEIVLINRSEKGVTRYTGGTLKGD
LR
LGGIAIALYVGIILVHFWAGVPPLPMTS
>MCE8455862.1 CoA ester lyase [Rhodovulum sulfidophilum]AB15
MDPRARPFRSVLYIPGSKDRALDKARGLPVDAIIFDLEDAVAVEEKPAARTLLAETLKAGGYGSRFRIVRVNGFDTPWGR
DDVAAIAGAGADAVLLPKVETTAQLDDLAAAIPDVPLWAMMETPKGILNAEALAAHPRLEGFVMGTNDLAKELGCRF
RPD
RLALTASLTHCLLAARAHGIVAVDGVYNAFKDEAGLKAECEQGRDLGFDGKTLIHPAQIAIANAAFAPTEAEIDLARRQI
AAFEEAERAGQGVAVVDGRIVENLHIVTARETLAKAAAIAELEAV
>MCE8455863.1 hypothetical protein LZ188_04685 [Rhodovulum sulfidophilum]AB15
MRAPRPFRHPAILPVLAALMILLPQLALPQNQGQNQRQNQGQNQNQNQAVGRQCGPRDGILDQLRNRYGESRQA
MGLAGN
NAVVEIFANTRTGTWTVTGTFANGLTCLIASGEAYDPVAEPPGQAL
>MCE8455864.1 sulfite exporter TauE/SafE family protein [Rhodovulum sulfidophilum]AB15
MIDFGPFFFAVAIPAILFAGVSKGGFGGGVAFAGSAVMVVILGPAVALGVTLPLLMLMDVTALRPYWRRWDGASARV
LIL
GAVPGIALAAAVFHLTDPDVFRVLIGLVALGFVGFQLAVRLRLIPARQRPFGRRAGAISGAVAGFTSFVSHAGGPPVAVY
LLSQRLDKTSFQATTVLVFWAINLLKFGPYAFLGIFTADTWLAVLLLAPVAVLGVWLGVRAHRLVPERWFFGITYAALAL
TGAKLLWDGLS
>MCE8455865.1 malate dehydrogenase [Rhodovulum sulfidophilum]AB15
MARPKIALIGAGQIGGTLAHLAAIKELGDVVLFDIADGVPQGKALDIAESGPSEGFDAGLKGTNDYADIAGADVCIVTAG
VPRKPGMSRDDLLGINLKVMKSVGEGIRDNAPNAFVICITNPLDAMVWALQQYSGLPAEKVVGMAGVLDSARFRHFL
SLE
FNVSMKDVTAFVLGGHGDTMVPLTRYSTVGGIPLPDLVHMGWTTQDKLDAIVQRTRDGGAEIVGLLKTGSAFYAPAT
SAI
EMAEAYLKDQKRLLPCAAWVDGALGLKGLYVGVPTVIGAGGIERVVDIKLDKPEQEMFDKSVGAVRGLVEACKAIDNS
LA
>MCE8455866.1 DNA gyrase subunit B, partial [Rhodovulum sulfidophilum]AB15
YPAHILEQAAIAGAFRPGQIDADLQGTADAVARRLDLIALEYERGWLGRPTQDKGMRFSRVVRGVEEVRVLDGQVLRS
AE
AARLGTFTAKLQEIYQHPARLIRKDRDQPIHAPSELLQAILTEGEKGLSLQRYKGLGEMNPEQLWETTLDPEARTLLQVR
VEDVAEADNIFTKLMGDVVEPRREFIQQNALSVANLDF
>MCE8455867.1 glucosylglycerol-phosphate synthase [Rhodovulum sulfidophilum]AB15
MPSDLVIVYHRQPYEEVEEDGKIVLRENKSPNGIVPTLKSFFGRFDKGAWIAWKQAEDPANPDFERVIEVDDAFGQYTV
S
RLPLTAEQVSSFYHVTSKEAFWPILHSFKERYNYDPVDWPTFREVNWIFAEAAAAEAAPGAVVWIHDYNLWLVPGYLR
KL
RPDVKISFFHHTPFPSADMFNVLPWRKEILQSLLACDVVGFHIPRYVSNFVSAARSLLDVEVTGRQKVEPEFAAEVTALS

ERSVPTELEYEDRRITVQASPVGVDADYIDAVARREETAERAEEIRAEMGGAKLLLSVGRTDYTKGGIDQLLSYERLLEA
QPDLCGQVRLMHVSVPANRNMTVYENIQNDLEQMAGRINGRFGSLDWQPIVLISRAIPFEDLVAYYRAADVAWITPL
ADG
MNLVCKEFCAARVDGDGVLVLSEFAGAAVEMGAALLTNPFSHRSMDSAIAQALEMPKAERRDRMAALRDVVHRHD
VRFWA
NHQMEALSGSAPGAAAMPAA
>MCE8455868.1 HAD family hydrolase [Rhodovulum sulfidophilum]AB15
MNVATAPHPPLSDRRFVLATDLDGTFLGGTEAERRRLYDWIEANRASVGLIFVTGRDPDFIMEMCADRGLPWPEYVV
GDV
GTSIAEVHPGEAIRPIPALEEDISQRWGDSGARVRAALDGHPGLTLQPTEFRYRVSFDMDAETYDPSAEAKVEEMGLD
WL
ISAERYFDVLPKGVSKGPSLRRLVAHLAIPEGRVLAAGDTLNDLSMLECGLNAVAVGNSEPALVERVRDLDHLHIARAHG
AAGIMEAIAAFDLHTVPTGA
>MCE8455869.1 NAD(P)-dependent glycerol-3-phosphate dehydrogenase [Rhodovulum
sulfidophilum]AB15
MKDIAAPDRQSAPFDTVAVLGAGSWGTALARVLVRNGRNVRLWARRPALAREIVATGRNSQYLPGIELGDTLRATASL
TE
ALEGAEAVLIVTPSATLREMCRAAAPHMPAGIPIALCAKGIELGSGLLLSEVAAQELPGHPIGAISGPTFARETALGHPT
AATVAFPFAYADRLAPHDSPAARMAMSVGGHGFRPYVSDDLVGVEVGGAVKNVIAIACGMMSGAGFAENTRAALIA
RGVD
EMKVLAESLGGQRETVTGLSGAGDLTLTCSSTTSRNMALGTQLGQGIARADCFGGRPVVVEGEVNARSVIDLARRIEVP
M
PICETVNAVLHEGADLERSFSDLWTRPIEAEPRALTLSLDHPAPTVPLPFGGSS
>MCE8455870.1 LrgB family protein [Rhodovulum sulfidophilum]AB15
MDSLLHWTEPFWPVFGWSLLTVGFYLLAKRVYRRWPRWWLMPLALAPALLTITVLALHETYRDYIRGAGWLMLMLG
PATV
AFAVPIYEQRAMIRRYWPVLLIGMVTGSATAIAVSWGLAGLFGLDRSLQLSLLPRSISTPFAMEVSADIGGLPDLTAVFV
VVTGVLGAALGDLVLAWLPVRSALARGALLGVAAHGAGTARAHQIGRAEGSVAGLVMVLVGLLNVCLAPLVVSAVRV
VSG
>MCE8455871.1 CidA/LrgA family protein [Rhodovulum sulfidophilum]AB15
MQIGILVGFWLAGEAAVRLSGLSLPGGLVGMAAALALLGSGLLRAGTLRRGANWFLAEMLLFFVPAVLAVLNHREFLG
LL
GLQILLIIVLGTLAVMAVTALTVELCCRWRIGGDGQPSALD
>MCE8455872.1 LysR family transcriptional regulator [Rhodovulum sulfidophilum]AB15
MEFRTLRVFVEVVRAGGFSAAAGRVHATQSTISKAVRQLEDEIGMPLLIRGPAGIALTAAGEIVFQRAQTLLAGRETLKT
ELQELAGLKRGLLRIGLPPIGSSELFAPVFTLYRRRYPGVDIKLVEHGSDRLEELLRASEIDLAASLLPVSEEFDWLAMR
RDPLVALMATDAPLAQVPGPLTLADLRTMPFILFDSGFALNRIIRAACARHGFEPEVTARSSQIDFIIRLAAAGLGVAFL
PRLIARARVHVPVAQVPLDEPGTDWAMATLWRRGAYLPQAARAWLDIAAGDPAQTATDREPD
>MCE8455873.1 lytic murein transglycosylase [Rhodovulum sulfidophilum]AB15
MRQSPRPEIRSAVVAALARAGTPDTVADEARGFPGWIAGFRGRALAQGIAPGVFDRAFEDARFQPDVIRRDRNQSEF
VTP
IWDYLDNAVSEARVRNGRAALAQYGAVLERIEARYGVDKEIVAAIWGMETSYGSYRGSAPIISSLATLAYEGRRAAFFEA
QLVAALKIIQSGDTDARHMTGSWAGAMGHTQFMPTSYLEHAVDFTGDGRRDIWSEDPTDALASTAAYLAHFGWTR
GQPWG
VEVQLPRGFDHRLADRSVKRMPSDWARLGVRDMNGRAVRDYGRAAILQPAGARGAAFMIFYNFRVIERYNPADAYV
IGVG
HLADRLKGGGPIRGSWPRDDRALASAERKELQERLTRAGFDTGGVDGRIGPNTVSALRAYQSAVGMIPDGYASMSVL
RRL

R
>MCE8455874.1 ribonuclease R [Rhodovulum sulfidophilum]AB15
MTRIPAKTEILDWIAAHPGQTSKRDIAKAFGLKGADRIDLKRLLKELEDEGHLEKRKRSYRDPERLPPVTVLQVLAPNAD
GDLFARPLEWHGEGAAPRVLYLPRQSDAALGEGDRILAKLQEVRAEDHQYEARLIRKIGSNPKRILGIFRKATEGGRLVP
IDKGADREWTVAASLTHGAKDGELVEAELAGPRGRMGLPKARIVERLGDPTAPRAVSLIAIHQHGIPDDFPDEVIAEAD
R
AKPAGLGTREDLRALPLVTIDPADARDHDDAVLAIPDDDPANPGGHILWVAIADVAHYVTPGSRLDREARRRGNSSYF
PD
RVVPMLPDRLSGDLCSLHEGVPRACMAVRMKIDASGHKLSHRFTRGLMQSAGAFAYEEVQAAEEGKPSDRTAPLLDE
VIH
PLYAAYRALIRAREARQPLDLDLPERRIELSDEGKVASVKFRDRLDAHRLIEEFMVLANVAAAEELSRLQRPLLFRVHEE
PSPEKLDALREVAGASGFILAKGQVLHTRQLNDLLDQAMGTEFSELINLSTLRSMTQAYYFPENFGHFGLALKSYAHFTS
PIRRYADLIVHRALISAHGWGRDGLSPEDVDELEATAKHISETERRSMAAERDTTDRYLAAYLSDRVGNEFGGRISGIAR
FGVFVSLDETGADGLIPIRELGNEYFHHDRDAQTLMGSNSGLVIGIGQRVTVRLAEAVPVTGGLTFELLSIEDRALPRGA
ARGRGKPVRRKVVRAKAKSDKVKRKVKRTRR
>MCE8455875.1 succinyl-diaminopimelate desuccinylase, partial [Rhodovulum sulfidophilum]AB15
KGHEKTFGFNGHTDVVPAGDTAAWTADPFGAEIRDGQLWGRGATDMKSGVAAFVAAAIDLVTDTPPDGAVVLALT
GDEEG
DATDGTIALLDWMQTEGEHMACCLVGEPTCREAMGDMMKIGRRGSMTAYFTARGVQGHAAYPQRAQNPVPALA
RLVQRLD
AYSLDQGTRHFDPSTLAVTTFDTGNPATNVIPGQCRATVNIRFNEAHSSDSLADWLAAEAARVTEETGIEIGIEIKVSGE
SFVTPPGPLSTLVADAVEAETGRRPEFSTSGGTSDARFVKDLCPVVEFGLVGKTMHQVDERVETVQIEELKRIYARILGG
YFG
>MCE8455876.1 gamma-glutamyltransferase [Rhodovulum sulfidophilum]AB15
MPNWPIALLLTAFAGTAAAQEAADAVAPESATSATVAFAGLSPEARAAWDSREAGRPTEARDWMVAAANPLAVEA
GAEIL
AQGGSAADAMVAVQTVLGLVEPQSSGLGGGAFLLWYDAGTGELTTLDGRETAPLAATPQLFQTPEGEPLAFYDAVVG
GRS
VGTPGTPMLMEEAHRRWGRIAWPDLFDAGIGLAEGGFQVSPRLAELVASDEDRLRRHPATEAYFFPGGAPVAAGTEL
VNP
AYADTLRQIARDGSRAFYTGAIAEDIVATVRGAEDNPGLLSIVDLALYRVKERAPVCAPYRGHEICGMGPPSSGGLTVGQ
ILGLLAPYDLAGLGPEAAESWRLIGDASRLAFADRGRYMADSDFVPVPAKGLLDPAYLAGRARLLEGDDALPETAPGKP
G
WDHARLWADDRSIEFPSTSHISIVDAQGNALSMTTTIENGFGSRLFVRGFLLNNELTDFSFRTHEDGRPIANRVEPGKRP
RSSMAPTIVLTDGKPSLVIGSPGGSRIIGYVAQAILAHLDWGMDVQQAVAMPHLLNRFGTYDLEAGTSAEDLAEPLAAL
G
FEVGTRELNSGLHAISVGETLQGGADPRREGIALGR
>MCE8455877.1 2-isopropylmalate synthase [Rhodovulum sulfidophilum]AB15
MSESSHQDRVLIFDTTLRDGEQSPGATMTHDEKLEIAAMLDEMGVDIIEAGFPIASEGDFEAVRDIAKLSKNAVICGLAR
AQLPDIDIRAWEAVQHAARPRIHTFIGTSPLHRDITALTQDEMVERIEATVAHARNLCDDVQWSPMDATRTERDYLCR
VVE
TAIRAGATTINIPDTVGYTAPRESAKLIEMLLERVPGADEIIFATHCHNDLGMATANALAAVEAGARQIECTINGLGERA
GNTALEEVVMALKVRNDIMPFRTGIDTTRIMGLSRKVATVSGFPVQFNKAIVGKNAFAHESGIHQDGVLKNAETFEIM
RP
ADIGLNATNLVMGKHSGRAALRSKMKELGFDLGDNQLKDVFVRFKALADRKKEVYDDDLLALMRDSDLDAEHDRIQV
KFL
RVICGTEAPQSADLILSVDGVEHRVTAQGDGPVDATFAAVKQLIEHDAHLELYQVHAVTGGTDAQATVSVRLSEDGKIA
T

GQASDTDTVVASAKAYVNALNRLMVRREKTKPEGNLAAI
>MCE8455878.1 rod shape-determining protein [Rhodovulum sulfidophilum]AB15
MVGFGGLFSSDMAIDLGTANTLVYVKGRGIILNEPSVVAFHVKDGRKQVLAVGEDAKLMLGRTPGSIEAIRPMREGVIA
D
FDVAEEMIKYFIRKVHKRTTFTKPKIIVCVPHGATPVEKRAIRQSVLSAGARRAGLIAEPIAAAIGAGMPITDPTGSMVV
DIGGGTTEVAVLSLGDIVYARSVRVGGDRMDDGIINYLRRQQNILIGEATAERIKTSIGSARMPDDGRGAVMHIRGRDL
L
NGVPKEIEINQAQIAEALAEPVQQICEAVMTALEATPPDLAADIVDRGVMLTGGGALLGELDLALREQTGLGISVADESL
NCVALGTGKALEYEKQLRHVIDYES
>MCE8455879.1 rod shape-determining protein MreC [Rhodovulum sulfidophilum]AB15
MAADRNAQIDYARPIRRILIGGTVFVLVALFVLWRIDSPRVERMRAALIDRVVPGFDWAMAPITKGAELIEGFQSYARL
A
EQNQELRRELRQMKAWKEAALQLEQENAKLLDLNNVRLDPSLTYVTGVVMADSGSPFRQSVLLNVGARDGILDGWA
TTDG
LGLVGRVSGVGKTTARVILLTDSNSRLPVTIQPSGQRAVLSGDNSSAPVLDFVEDPALVRPGDRVVSSGDGGVFPAGLL
V
GHLALGRDQRLRVRLAAEYDRLEFLRVLRSHANERISDPGALVIPGPVGPPPIPAPAAGTGGDPGNG
>MCE8455880.1 rod shape-determining protein MreD [Rhodovulum sulfidophilum]AB15
MVDPTTLDRWVYRLVFLGIAVMTVFVMILPLETRPGRLPAPDWMLCLTLAWLQRRPDYLPPWLIAGVFLMLDMLLM
RPPG
LMTALVLVGSEFLRARQHVSVETAFGSEWLVTAATIFAIFLTEGVVLALFSVPEADFGRLMFLALATALAYPLVAAVSRY
ALGVRRVTPGDLDPRGIGR
>MCE8455881.1 penicillin-binding protein 2 [Rhodovulum sulfidophilum]AB15
MKRSQREAEESNRRIGRRTLIMGGAQALFAGVLALRMRQLQVTQAAEFRLLAEENRINIRLIPPARGILYDRQGHVIAE
N
DQNYRIVIVREDAGDPEQALARLTRLVQLDDETVERALKEIRRHSPFVPVTIADRMRWEEVASVAVNAPVLPGIAPEVG
Q
SRHYPMGPDFAHIVGYVGPVSDYDLSKLEDPDPLLQIPRFQIGKTGVEAKEEELLRGKAGTRRIEVNAAGRVIRELDRID
GQPGSDLQLTIDAGLQNFMQARLGEESAAAVVLDLQHGDVLGIASSPSFDPNKFVQGISVADYRALTETPFRPLSNKAV
Q
GAYPPGSTFKLVTALAALEAGVITPDETVWCPGFLKLGDRRFHCWRRGGHGHVNLTESLQQSCDVYYYDIGQRVGIDN
IA
AMARKLGLGVHHDLPLSAVGEGLAPDRAWKRARYGQDWQVGDTLNASIGQGYVLATPLQLAVMSARIATGRAVTPR
LLRS
IDGLEQPSGAGAPLDIKPVNLERIRKGMDAVVNSRRGTGRRSRVVAEGMAMAGKTGTSQVRNITAAERAAGVFRNED
LPW
ERRDHALFVAFAPVEAPRVAIAVVVEHGGGGSAVAAPIARDLTLRALYGRVPPGDAYPESDRWEAERRLRDLPLRDPGS
R
SSGGRGRA
>MCE8455882.1 rod shape-determining protein RodA [Rhodovulum sulfidophilum]AB15
MSYLEYNVKRVPTGLSKVLYVNWALVLLLTAVAGIGFMMLYSVAGGRLEPWADPQMKRFALGMAVMFTVAFVPIWF
WRNM
AALAYGISVLLLIAVDFFGSVGMGAQRWIDLGVVRLQPSELSKITLVMMLAAYYDWLDIRHVSKPQFVALPVLLILLPTA
LVLKQPDLGTALLLIAGGGIIMFLAGVHWAYFAVVIAAGTGLVTAVFASRGTPWQLLKDYQFRRIDTFLDPSTDPLGAGY
HITQSKIALGSGGWAGRGFMQGTQSRLNFLPEKHTDFIFTTLAEEFGFIGGMSLLALYVLIIVFCISSAMQNRDRFSSLL
TLGIAATFFLFFAVNMSMVMGLAPVVGVPLPLVSYGGSAMLVLLAAFGLVQSAHIHRPRGR
>MCE8455883.1 glyoxylate/hydroxypyruvate reductase A [Rhodovulum sulfidophilum]AB15

MVRILFSAPPGLWAEYRAPLTRALDEAGIKAEIVTGTGDPAAIDYVVFAPGGGLSDFTPFTGAKAVLSLWAGVETVVGN
P
TLTQPLCRMVDAGLEEGMVEWVTGHVLRHHLGFDRDICATEPHWDQRVPPLARDRTVAVLGLGALGRACAEALAAL
RFRV
LGWSRNPRAVPGIDCHHGAEGLRPVLEQAGIVVLLLPRTPETEGLIDAERLSWLPEGAVLLNPGRGALIDEAAVLAALDA
GRLGHATLDVFRTEPLPPDHPFWRHPRVTVTPHVASETRPASAARVIAENVRRAEAGEPLLFRVDRARGY
>MCE8455884.1 hypothetical protein LZ188_04795 [Rhodovulum sulfidophilum]AB15
MTGATRADDDSEDDDFSLIDLGFASGDLGALEYSADGTLYPDDDSETVSEYFLGFSTGTDTVTLDTLDIDDLAGVTSMRF
TFTVDGVERKVDIASDTGWDDLASFVEDFNAALDAEGVGVTVGSDGGYDENGDLVGGTGLSFTLDADETRSIQITVED
GD
TLRATTIDGLLDWVNSELSQAWEGAALELTEDGALVFVLPEATSTLVIEADDGVLFSAEDVGLSFLGDVTLSADLAGELT
AALTSSIGIDLAGLGSSLIDIASNALQGASDLGEDLFDALLDYAFIEDVTLTASASATATDITGSADLGIAAFTVGTSDP
SANFVAFDVQMLVTLVGTDSDGLASNRITMHNLYNAVTDVYDVVDGETVVTEAEGIDSLIGLLELSGGLLTDGEGRALA
S
DGSEVADSADLQTADAFDYGGDGTLAEFVAYLGDITLSVGDVAGLNEGVIDGIGVTVTDLFDLDGTTEIAVVSEDESAA
A
ALEALGNLEDGDILDALTAIAHMLVVVGEALEDYLPYLDAEIPLLNFSILDAISFSADFLEALQDIRNDMDAALDTLDSY
LEDVFGEDTVELGWDSDASTLTFALSFQFLDDYSESLPFQLDLAELLGDALADIVGEDLADTVTGLVDASGEGALVFDPL
LSLDFTFGLDLSGLLADPTDLAELSTALGALATTGAVNMRPSGGNDLRITRFDADGNSSKVEVDLDGLETLEEVIAAIDA
AVDAAFDGTVSFTYDAETGQITLSDSGNSKTEDTGLNLLFGSYRDSSENDDGLQEIALDDGFTDYESAATFDVQIGDERV
TVTIEVEAGRDQDGFVDALNAALAAASIDRGAISDTAIHGTTVALSSLMTARVTADGSLALTATGYDTALSYDTIAFSVT
GEDLSEGIGFAITDLGGANIAEVLGFESSRDAIVGDITSGTLYVADDGDAIRIYLDTEESGIKLSFSAGVEEGLNLTLAL
GPLSVSVTDGHAYISAADGSGDPAYIFLGINDIDGDEYEGQYDLSHLATLFSDTSLSVSDLFSYEVSIGIDVSLPMSDSI
GLFDPDTDGLTWQATLLSVSDATAATGGLIDIFNGNLGGLTGLDLTLPDLSDYLENLNILSLLNDPVLLLEGLDMIIEQI
QSLFDDFLSDISLPIVGDSIGVGVTFFEEFRMLVLEDALEYASTPLADGTMPTTVDLITGFLNDALNDLLGTSGETYLQA
YLNTDGGTEDSYVYAALNFGATIFSEYLDIDFDLGIPGFDLEVEEGSQILMQLYYSVNIGFGYDSNGFFLLNDTDTDEIT
LEFSVDAGSFEASMALFDVLGVSATAVTLDDDGNIISTASDEDGGTAKLTATMGADLYGDQGLAIIDPDDQGDSDAITS
D
GVYRDFSGVTPLDGLGEELTYETVVYLSQLDTSNLISFTFEAEVAIVLSIEGNILDPTTGKPIEINGVTYLPSVATELEF
VATFSIGDGLNIETLMFHNVRVDISEIYDALIAPILDPLMDVVEPLAEFFKFLNEEPMSILVDILGEIYPIIKMAGTVAE
IGEEVTVFLTYLNDSEGIVMFGDYDFSSMSDDLETGDAELSDASGSQVSISGSSSSSGYSGKFGVFGSGALQIEIPLLTN
PFSVIDLLLGNYEDVVLVELRMVLFNFDTGGRIDLVDEFLDLMDMPGWAADIVSSIFEAGVEARFVAQIEVGYDLGGIV
N
FVNTLDPERLLDGIFLSTELVDVYVGLDASLSSPFAGVTVAGYAGVTLYFNDPNHDGKLRLTEMIAIVEAAYEEISDGDI
LEALGYIFYGEASFGFELSVWVGIDLLFTKLSWDIELFDYDGSVDFGGNPVSGEVAGDMDGSGTVVLNIGSNAGASNSA
I
QEDGDDTLVITGPNSPIQVTYTQGGKKVTGEIDQDANALVIPAGSGDNEIDLSGITNDVITITYTGSGEDTITLPNQGLH
VVFAGDGDDTITVDGEATGTYIIFGEAGSDTVEIDGGNVVYIGSDDYGLRDIFIEAFSSGDVTAIEVLSLLGLNADGTVD
TNSDKANYDGLTLSGLLENYTGLTQTNADKSADTITLGSGNAIVLTGGGDDVILIDLTNDGEVRIYSGAGDDIINAGGSS
VYVEGGAGSDLIVADGDETTVWGWGAQAGESGLDGDDAIDTLALRDGMDVITGGSGSDMIYGQNGDDILQGGLGD
DMLSG
GLGDDLVSGGTLTLVTSGGTVIDLTTFDVDTNFTTNMILGSVDAADGDDTILGGKGSDIMMGGGGADSIEGGTGKDILI
G
DFAEIKLSPSLAAGAITSTYVNSRNAGQDALYGGTGDDILIAGGAGEGEAETLTDTEGSNILIGDFAVLEGSIILDAVTK
ITTLASDMGDDDILTAGNGTDILIGGEGSDVITAGLGADIVLGDLGIIDFSEGTIRGDSTAADGNDTITVGNDDGTSSVD
DLKDIVIGSLGDDTLAAGDGGLVVLGDGGTITTDPTALNALRTYVPLGPDPSDDDVAADLKARNLISALAASVVSTPNED
DGDDIVTVAGGETIAVLGGGSDVAELADGVNYVLGDDGTITIEANADYTGRKVELTSAAAAAAVNDDQITTGSGSDVIV
G

GEGSDKIRAGVGDNAILGDNGTLTRDDTGDTLSLSLESSYLRTDGDDLVFTGSGYDAAVLGGGDDTAELGDGDNIAMG
DS
GTATWIGDEIELESANPNYGGDDSITTGSGDDAILAGSGNDTVDAGNGRNAVIGDNGHLKIDEAGIELTSADPSVGGN
DK
ITTGSGNDAVLAGVGSDTVDAGNGSNAVIGDNGHLKIDEAGIELTSSDPSLGGNDKITTGSGNDAVLAGVGSDTVDAG
NG
GNAVIGDNGHLKIDEAGIELTSVDPSLGGDDKITTGSGNDVVLAGLGSDTVSAGDGENFVLGDNGWISLVDENVVETS
DE
SLGGDDVITTGKDDDVVIGGTGADTIVLGDGDNRALGDSGYYRSQGELVSAHLDSDGADKVTSGTGNDFVVIGGDAD
TAA
LGDGDNRAIGDSGRIVWSATEIGTMETTDTDTGAGDRIVTGSGSDAILGGDGGDTIDAGDGDNAVIGDQGRIELDAG
ADQ
VVARRVVTKDVAHGGGDVLTTGKGDDVVLAGAGGDTVSTGAGEDAVLGDNGEYVSSHMTGTGSLTAAVLDFGGDD
VIYTG
AGADIVMGSQGNDRIEVGEGEDVALGDDGTVTFINRTDVQTLVLTNIELGGDDIISASNQSGDSIFFGQAGADTVIGGS
D
DDIIIGDLVEITFTDASAAMAGQSAVDRVDYMIGVRPDLGFDDHLSGVDGNDFIIGGFGDDEIHGGDGQDFLIGDTIIYQ
RSYTEMADGTLFEVILLDTNFAYRTGGYDTIYGEAGSDVMIGNLGPDLFYGDTEEDVIFSDGYAGEYHGRWSSLGFEGD
T
AFRTLWKSNFAGPGAIDVVSRAQQDDSIGSPLDLILEEMDAANEGRGLLAGGEFISEGAGIDTSFFAGRLLDFLETDEVV
GTLATLVTLGADQAVLAQALEAAIAENFGDIWDLDAPELELVIKKMISFLLSEVQAEDAATGSAIAA
>MCE8455885.1 DUF3467 domain-containing protein [Rhodovulum sulfidophilum]AB15
MTDKPAKATGTINGVKISWDDSEMVSHYANIGTATANREEFFLLFGTHQHWRGTMDEKKEIEVKLANRIVMSPYAAK
RLA
VILTQSIKAYEEQFGKIEA
>MCE8455886.1 hypothetical protein LZ188_04805, partial [Rhodovulum sulfidophilum]AB15
MFGSPRAILWLGGSGELRTLFGALPEDIAQETLETLKGEKPVIRRLGDRLLALVPVMRAGTAAGHAVWLFDRVPPVTEL
P
ALLEELRGLSAAFAPAAVEGDAAPGKQLALPIALSRLGEGTKLKADGVAATVADACVEAGICSVALVVPCTASDGLAGV
G
RVSASDRGFDAHADEIRQIVASYRPEADRRPDPRRIAAADLDEAVFDAALIGEMLGAGALLLDLPPRGQAGTALILLDPA
PGADVAGLADLVSVASRRPSGRGRSRRAKVLRGGMAAIR
>MCE8455887.1 chromosomal replication initiator DnaA, partial [Rhodovulum sulfidophilum]AB15
MARQLAFDLPVRPARGRAAFFVAPANALAVQQIEGWQDWPARKLVLIGSEGSGKTHLVHVWADLAAAEVIHARDLP
QAEL
PALAAHGAIAVEDADRIAGRMQAEDALFHLHNQ
>MCE8455888.1 AI-2E family transporter [Rhodovulum sulfidophilum]AB15
MGLPVRAQAKYWGIAAAVIVLILMLLGDVMMPFLVGMAVAYFLDPVADRLERAGLSRVGATVTITLCALVIFVLGALLV
L
PTLIDQAVALANAAPDIAGHFHAFLTERFPSLADNGSTIRKTLTGIGEAVQAKGGELLNGLLTSVVGIVDLVVLIVLVPV
VTFYLLMDWDRMVAEVDKLLPLDHAPVIRRLAGEVDRTLAGFIRGQGTVCLILGVFYAVALGLLGLNYGLIVGFVAGMI
S
FVPYVGAVVGGTLALGLALFQFWGEWWMIAIAGGIFIAGQQVEGHVLTPKLVGGSVGLHPVWLIFALSAFGSLFGFVG
LL
AAVPVAASLGVLTRFSIAQYKDSRLYRGTSGQTRPEAASEERASNEDRD
>MCE8455889.1 proline--tRNA ligase [Rhodovulum sulfidophilum]AB15
MRLSRYFLPVLKETPSEAQIASHRYMLRAGMIRQQAAGIYSWLPLGFKVLKKIEAIVHEEQIRAGHIPMLMPTLQSADL
W

RESGRYDAYGPEMLRITDRHGRDMLYGPTNEEMITDIFRSHVASYKDLPLTLYHIQWKFRDEVRPRFGVMRGREFYMK
DG
YNFDIDRDSAIHAYNRHMVSYLRTYERMGLKALPMRADSGPIGGDDTHEFLVLAETGESEVFYDKAILDLTFGDRQVDY
D
DRAECAQIVTEWTTPYARTDETHDEALYSAIPEDRRKTSRGIEVGQIFYFGTKYSEAMGATVVNDKGERVPVHMGSHGI
G
VSRLVGALIEANHDDRGIIWPEGVTPFHCGIVNLKQGDAEADAACKGLYQALSDKGLEPLYDDREERAGAKFATMDLIG
L
PWRITVGPRGLKTGVVELTSRRTGESEEMSPEAAVARVAEIYAPHRTGSHWEPMAKSSFHTWL
>MCE8455890.1 lipoprotein-releasing ABC transporter permease subunit [Rhodovulum sulfidophilum]AB15
MTAVPFARFEWMIAWRYLRARKAEGGVSTMTWISLAGITLAVFALIATLAVRSGFREEFVDTILGANAHVTVYAASEV
NA
SGVRSRTIADYEPIAERLRAVNGVTRVAPLIKGQVMAAAPGGTAGVEVFGIAPADLATIPRIAHPEAHAGTLQRFGEGIA
IGSGVARELGVGIGDRVRLISPDGVKTPFGTSPRVKAYEVTYIFTAGRYDIDRTRVYMPFAEAQSYFNREGVADEFEVMV
EDPEHVDDLTPALLRAAGERALIWSWRDASGSFLRALDVEDNVMFIILSILVLIATMNIVSGLIMLVKNKGRDIGILRTM
GLSEGSILRVFFICGSSVGLIGTAFGVVLGCLFAIYIDPIFSFVNYVAGGGVWDPSIRGIYNLPAKLELKDVLSAVILSL
GLSFVVTLFPARRAARMNPVEALRYE
>MCE8455891.1 ABC transporter ATP-binding protein [Rhodovulum sulfidophilum]AB15
MSEPVLSLEAIEKCYNRGLPGEVTVLRGATLRVVPGEVVALVAPSGAGKSTLLHIAGLLDTPDSGAVRIAGADMTGQSD
R
RRTAARRADIGFVYQFHHLLPEFTALENIVLPQLAAGMPHGAAEARAMDLLTRVGIAPRAGHRPAALSGGEQQRVAFC
RA
LANQPRLLLADEPTGNLDPGTSDTVFGALMELVRETGLAALIATHNLDLAARMDRTLHLEAGRLV
>MCE8455892.1 methyl-accepting chemotaxis protein [Rhodovulum sulfidophilum]AB15
MFGRPRKSAQEKEAHALAEVFMAVIDRTQATIQFAPDGTILTANANFLDVLGYKLDEVVGKHHSMFVDPAYVKTEAYR
NF
WKALAAGKSFTDQFPRVHKNGEVIWIQATYAAYVDENGKTTRVLKIATDVTERQHGVDAIAKGLERLQERDLSHRVPH
SN
LPDIEILGKAYNTAVAQLASALSAVKHLSDKVERTAREVGDASSHLSQRTETQAATLEQTAAAIEELTSNARSTAALAKE
VEASASNAQNTAEAGGKIVEDAVGAMSNIETSSRKISQILGVIDDIAFQTNLLALNAGVEAARAGDAGRGFAVVASEVR
A
LAQRASDAAGEIKGLVHESSESVKQGVGQVSSAGEQLKKIIAAVAGINSHIGQIATGATDQSFTLSEINTGVSQLDTVTQ
QNAAMVEESTAASQVLASDAQDLGRQVSLFQTEEGPRFNASGHRPNRNAA
>MCE8455893.1 DNA polymerase IV [Rhodovulum sulfidophilum]AB15
MCGGESRENAAGPGRGPADARSDPPRRIIHVDMDAFFASVEQRDRPELRDRPVAVGHGRARGVVAAASYEAREFGV
RSAM
PSVTALRKCPELIFVPPRFEVYRGVSADIHAIFAEYTDLVEPLSLDEAYLDVSQACADGRPASEIAEEIRARIRAATGLT
ASAGVSYNKFLAKLASDQNKPDGLTVIPPHRAAAFVAGLPVGRFHGVGPVTEAKMLRLGIRSGADLRAKSLAFLQAHFG
S
SAAHYHAISRGIDPRPVRPDRPRKSIGAETTLSEDLSAFEAATEVLARLAAKLSRPCAEKGLAGRTVTVKVRFTDFEQVT
RRRTLSRPLTETAEMLEVARQLLAGLHPFPRPVRLLGITLSGLEPRAEAAAEKQLDLFDESSRNSG
>MCE8455894.1 ATP-binding protein [Rhodovulum sulfidophilum]AB15
MPADPPIRRSTLSTARLLRARRLLPRSRIEALAAAAALVVMLGAIAVIAFDIRDREAHLHDITAETAQSELTRLEMEHAE
LHAAIYMADRGVMPLDRLRLDFANFRAQAKLVTETASFAEPLRRSGLGGQADRIADALRQMTDAFAGTDADLRARLP
DLS
RTIDAIDTDVRRYSAAGSGLLVSSHLSEHAHVERLETLLALIGLALIVLLLAGMAALGRLNHRMRSRSRELHATSSRLNA
VVTTALDAVIVTDEAGTVLEFNAAAEAIFGYARDEVLGRPLEQTLALSAAVRRGGEATGRIELPAQRKSGTGFPAEISFG

RAVTDDGTVFVIFLRDISERKRVETELTAARDRALAGEKAKARLLAVMSHEMRTPLNGLLGTMELLQRSPLDDAQRQYL
D
IMSCSGEILLHHVEDVLEVSRIDAGRSKTRITDFDLTAKLGRLVALHQPLAAERDTRLALEIDAGLGAGRGMVRGDAQRL
RQIVINLISNAVKFTRGGRVTVSARRRSREMIEIRVADTGIGIAPEDQTRIFDEFFTADATYRREEGGTGLGLAIVARQV
GLLGGRIEVSSQPGQGSTFTVTLPLPEATAAAEPAAPHPAAPCPARPLDVLVVEDNRVNRFIARRLLEGLGHRVTEAVD
G
LDGVETAAARQFDVILMDISMPRLDGVEAARRIRAAEGPPPRIVAMTAHALPEDLARFHAAGMEDVALKPVSRGRLSE
IL
GEVPGASTQCKTTPAAMVPEPERDAPADKPHRAAS
>MCE8455895.1 transposase, partial [Rhodovulum sulfidophilum]AB15
MKRKRFTEEQIIGVLKESEAGAKTDDICRRHGISSATFYSWRKKYGGMEAG
>MCE8455896.1 ATP-binding protein [Rhodovulum sulfidophilum]AB15
MSTNSIGQVLSCSPEAIIVLVDGLQVFEGRKAALQVGRYLRIAQGNNDYTVATIQNVRGILTNDADGKPKWQFHIQCQ
AV
GTLVDDRTFERASVLLPVPTEPAFPAEKDTLDKLFAEDANYQFPLGMLSLNKATPLRVHGDRFFSKHITIVGSTGSGKSC
AVARILHDVVGIADGANSNLNKQNNSHVVIFDIHDEHTDAFTLAEEQSFTLNRLDIDTLRLPYWLMNSEELESMFTESN
E
QNSHNQVSQFKQAVILKKDETQPGN
>MCE8455897.1 ATP-binding protein [Rhodovulum sulfidophilum]AB15
MTYETPVYFSIEEVYRYIANMNREVIGRLEGENKPKLSDGTLINDRKDHYFDISCTFVPPSTANATKATVGSFNGEFNRF
VSRLETKLSDKRLRFLLQTTKADGSYFKTDDFEHIMKQFIGYLNQANVTIVDLSGIPFEVLSITVILVSRLIFDFCFHYS
KLRQGQEVLNDVPVMIVCEEAHNYIPQRDDAAYRSSRKSLERIAKEGRKYGLSLMVVSQRPSEVSETIFAQCNNFLALRL
TNDTDQNYVKRLFPDNSNGITNILPNLAPGECVVVGDAVLLPAVVQMSLPNPEPQPRSVCFHKEWQEPWRKFTFGDV
IAR
WRRE
>MCE8455898.1 IS1182 family transposase [Rhodovulum sulfidophilum]AB15
MAGFIDGIDRDQVTLFPDRLEDWIGEDHLVRVVDLFVEELDLPALDFERCVPARTGRPGYHPAVLLKLFIYGYLNRIPSS
RRLEREAGRNVELMWLMGRLAPDHKTIADFRRDNGSAIRRTCAQFVELCRRIGVLRGACVAIDGSKFKAVNNRDRNFT
KN
KIASRLAHLEADVLRYVDDMARIDRQEEGEARADKVAHLARRYGRIRQEIARLKAMEQALTESPDGQISLTDPDARAM
AT
SALHSGMVGYNVQTAVDTETHLIVAHDVTNQGFDRDQLSPMAAAARKALRRDDLHAIADKGYFSGSQILACHQAGIT
TTV
PRPETSGNRGKGIYVKADFAYDPARDAYLCPAGEELTYRYTREEDGLRVGRYWTNECQHCPIKQRCTTGKERRITRWEH
E
HLVDAMRGRLCGEADPMTLRRCTVEHPFGTLKAWMGHAHFLTRRLKNVSTEMALNVLAYNIKRVVALIGIRGLMRAI
AA
>MCE8455899.1 DMT family transporter [Rhodovulum sulfidophilum]AB15
MTTPVLFAATILIWGTTWIAIAAQVGAVPVAVSVFYRFALAGVLMLAVLAALDRLRSPAVWRFVVVQALCLFCLNFLGL
Y
TATSLVPSGLVSVIFSLASIFNAVNARIFFGDRISRQTVLAGALGVAGLACLFWPDIDITAGASTLRGIGWAVLGTLIFS
WGNMASRQNTHLGITPMTANAWGMGIGALCLLAILFATGQPLVLPRGGIYWTALVYLSVIGSIIGFTTYLMLVGRIGSA
K
AGYATVLFPIVALTLSTLFEGYEWTALSILGVILAGLGNVVMFRRR
>MCE8455900.1 AraC family transcriptional regulator [Rhodovulum sulfidophilum]AB15
MASVRDFLESSPVATAAATLDLGGGRCVTVWENRNDRVSCEAPRGHAFSLYLAGGTGTRRLDGAGASGWPGAVCVF
PDGQ
PSEWEITTPHRFAHLYLPDDRLRADFARTHDCDARRLDVPEITFADMPHLAAPLAALARAASAGDVLLADTALSEIVAQL

GARKVPLRGGLSPHILRRIDEWIEAHLDEAIRLSDFAALAALSPFHLHRMFRASRGIPLHAWITERRIDRAKSLLRGTDP
VLEVALACGFSSQSHFTRTFKSGTGTTPAAWRTAIKG
>MCE8455901.1 preQ(1) synthase [Rhodovulum sulfidophilum]AB15
MSEDIYSNLKQLGGATRIPTSPEEAELERVANPQADVTYNVRFTAPEFTSLCPMTGQPDFAHLVIDYVPGLWLIESKSLK
LFLTSYRNHGAFHEDCTISIARRLVEFLDPQWLRIGGYWYPRGGIPIDVFWQTGPLPKGAWIPDQAVPPSRGRG
>MCE8455902.1 7-carboxy-7-deazaguanine synthase QueE [Rhodovulum sulfidophilum]AB15
MTLRIAEIFGPTIQGEGALIGEPTVFVRAGGCDYRCSWCDSLHAVESRYRHSWATMTSETVWEQVRRLSGDRPLTVSLS
G
GNPAIQDFGSLIAMGRAAGYRFACETQGSVARPWFADLDTLVLSPKPPSSGEEVDWDAFDACRNLGVGARQQVMKI
VIFD
ETDYQWARDVHARHSDLPLYLQPGNTEVNPAAPVDLQALADRLGWLIGKALADGWFAPRILPQLHVLIWGNKRGV
>MCE8455903.1 6-carboxytetrahydropterin synthase QueD [Rhodovulum sulfidophilum]AB15
MFRIRKEFHFSASHQLPHLPEDHQCARLHGHNYILVVELAAETLNADGFVRDYHDLKPLKTYVDDHFDHRHLNDVMEG
PS
TAENMARHFYDWCAARWPETSAVLVSETPKTWAEYRP
>MCE8455904.1 7-cyano-7-deazaguanine synthase QueC [Rhodovulum sulfidophilum]AB15
MKTLVICSGGLDSVSLAHITANEHQLTRLVSFDYGQRHRKEVDFAEAASRRLGVPFHLIDMRGIGAVLSGSALTDDIDVP
DGHYAEETMRITVVPNRNAIMLSIGFGIAVAQGDEAVATAVHGGDHFIYPDCRPAFTRAFDAMQQAALDGYAEIRLH
TPF
VEHSKAEIVRQGARHGTPFAQTWSCYKGGEVHCGRCGTCVERREAFHLAGVSDPTVYADPDYWTEALAARGQS
>MCE8455905.1 recombinase family protein [Rhodovulum sulfidophilum]AB15
MPTPRRAAIYARYSTGLQRDASIEDQIRVGRTLADRQGYEVVKVYFDHAASDASLMRPGIRSLLQDAREARTFDIVLAH
A
LDRLSQNQADIASIYQQLQFVRILIETVSEGLIEEMHIGLKGTMNALFLKDLAKKTREGLRGRAIAGKSAGGLTFGTARS
GPSTRPASGSGAIARFIRRKRRGRAHSARLCPRARKRVDLECKVLDTLEHHLMDPGLAEAFCKEYTAERNRLQDAALPRS
RQPGKRTGRA
>MCE8455906.1 transposase [Rhodovulum sulfidophilum]AB15
MSRQPRRHFTAEFKEQAIARLSSPGASQTSVARELGVTPSQLKGWLLELTAAGSAEAIRRPQAEATELAELRRENRRLRK
EVEVMRKGEQANAMRSSEPVNAFFAQWAAKQ
>MCE8455907.1 hypothetical protein LZ188_04920 [Rhodovulum sulfidophilum]AB15
MNAKLEFIAAHAVSLMCRGLSVRTSRFHSWRAAAPKRAERRAAREALLAGIGAIFEASHRRYGAPRSCH
>MCE8455908.1 ABC transporter ATP-binding protein [Rhodovulum sulfidophilum]AB15
MTGTNDGFVVFDRVQKSYDGEILVVKDLNLSIGKGEFLTMLGPSGSGKTTCLMMLAGFETATHGDILLDGNPINNIPPH
K
RGIGMVFQNYALFPHMTVAENLAFPLEVRKIGRAEREEKVQRALDMVQMGAFGGRRPAQLSGGQQQRIALARALVF
EPEL
VLMDEPLGALDKQLREHMQFEITRLAHNLGITTVYVTHDQTEALTMSDRVAVFDDGRIQQLARPDELYEKPVNSFVAQ
FI
GENNTLLGRVKQIDGNALIELDDGEVIDAVPVNVSQPGERTLVSIRPERVEIDPKRLTPGAHLIHAEVLEFIYMGDIYRT
RLRVAGNDDFVIKTRNAPDQRRLKPGEHIEIGWLPEDCRALDAP
>MCE8455909.1 extracellular solute-binding protein [Rhodovulum sulfidophilum]AB15
MTIRHLLSATALAALPAGFAFAEDLPPCEGCADSLTVVSWGGAYQASQQKAYSEPYAEMTGTDFTWDESSNEGVAKL
RAM
NEAGNITWDLVDVEGPDSQRLCDEGLAMEVDLDEMLAPAPDGTPASEDFGDSVVTDCFVPQIVFSTTFGYRTDVAA
WNGK
EPEGLCALFDTETFPGKRSLEKRPKKNLEWALLCDGVEKDDLYDVLDTPEGVDRALAKLDTIKDDVIWWSAGAETPQRL
A

DGEVVLGSTYNGRLFSLIVEQKQPVKMLWDWQVFDYDGWAIPADLPEDRLNRVKHFLYFATDTQRLADQARYISYGP
SRA
SSQPLVSTHAELGIEMAPFMPTNPANQKNYLVNNIEWWADNQDDVEAKFQAWLAR
>MCE8455910.1 DUF1330 domain-containing protein [Rhodovulum sulfidophilum]AB15
MPKGYIIAHITVTDPEAYRDYVDRDTPILEGFGGRFLVRGGASEAPEGPMKDRHVVIEFPDFEAAKRAFHSPEYQEVAAI
RHATATSDIVLVEGVA
>MCE8455911.1 ABC transporter permease [Rhodovulum sulfidophilum]AB15
MTGAATDTAASGPVLAADGAPLKRSLRRALRAQKLRALMLIAPLLVFVLVTFIAPIADMLFRSVENQIVSDTLPRTAREI
RDYDPDSGEMPSDRVLAAFTYDLVAAAERKQHTRLGSRLNYEYSGISSLFRKTGRRVDDFGESYLDQFVAVDPAWEEP
AT
WVALMADPDWTASYRAWSAARDAAKAAGEPFALAQPLFALRDGVGDVLPETASAYAEFARIVQVEDDDDPAAEEP
WAPVY
LALYRDLLHIGPGAVARLGPEAEAMLDAAHDAVEDFAPVDLSGLYLKADRDWADPGVWGTIRAFSPAYTPGYFLNAV
DLR
LTADGIAAQPEDRRIYIMLFERTLFMSLAIMGSCVLLGYPIAYLLSNLPLRTSNLLMILVLLPFWTSLLVRTSAWKVLLQ
QQGVINDILVWLGIVSNSGRLILINNQTGTIIAMTHILLPFMILPLYSVMKTIPPSYVRAAKSLGATNWTAFWRVYFPQS
VPGIGAGCILVFILAIGYYITPELVGGTSGTFISNRIAYHISSSLNWGLAAALGAILLGVVLILYWAYDKLVGIDNVSLG
>MCE8455912.1 ABC transporter permease [Rhodovulum sulfidophilum]AB15
MSQMDLPPYATLGQRIWHYAYYVICGLIFFFLIAPILVIIPLSFNAENFFTFTPKMLALDPEGYSLKHYQDFFTSNAWQH
ALWNSVKIAPAATCLAVSLGTLAAVGLSQEHVPFRRTIMAILISPMIVPLIISAAGMYFFYSRIGLQGTYVGVVLAHAAL
GIPFVIITVTATLVGFDTSLTRASASLGANPVTTFFKVQMPLILPGVVSGGLFAFITSFDEVVVVLFVGSANQQTLPWQM
FTGLREQISPTILAVATILVVISIGLLTTVEILRRRSEKMRGLSPG
>MCE8455913.1 host attachment family protein [Rhodovulum sulfidophilum]AB15
MKPFKTLVLVVNEREARLLVNRGTGKGLAEVTRFSKAHEIEYADGRGRDQGGATAGRHAMEPPTSLRVQNRESFAAD
VLG
LTAEVWAKGDYDRFVMAAPPKMLGELRNRIEGSLERALAVDLDKDIVGVAAADLPRHFEDVIVF
>MCE8455914.1 DUF4389 domain-containing protein [Rhodovulum sulfidophilum]AB15
MTDTNSTLNEGAQASDAQDKGYRPALIRGAWMLLMLALFYAAQMIMIIAAILQFGWLLFAKEENERIAEFGDKLANW
MAI
AARYLTVAGDAKPFPWTDWK
>MCE8455915.1 M3 family oligoendopeptidase [Rhodovulum sulfidophilum]AB15
MRIASPRPVFDANASAGGALGDLPEWDLSDLYAAPDAPEIARDMGWLEQECAAFAADYEGRLAGLDAAGMLSCITRY
EAI
DRVAGRIMSYAGLRYYQNTMDAGRAKFLSDCQDRITNYTTPLVFFSLEFNRIADDAYEALFAADAALTRYKPVFDRMR
AT
RPYQLSDELEKFLHDQSVVGASAWNKLFDETIAGLEFEVEGEDAPLGIEATLNLLTDPARDRREAGARALAEVFGANIKT
FARVHNTLAKEKEIEDRWRKMPTPQTGRHLSNHVEPEVVEALRNAVVAAYPKLSHRYYALKAKWLGLDKLQVWDRN
APLP
IEEQKTVGWEAAKATVMEAYSEFSPKMAEIAEPFFDRGWIDAGVKPGKAPGAFAHPTVTDVHPYVMLNYLGKPRDV
MTLA
HELGHGVHQVLAAGQGELLASTPLTLAETASVFGEMLTFRKLLAGATTQAQKKTLLAGKVEDMINTVVRQIAFYDFECK
L
HEARRGGELTPEDINEIWMSVQGESLGPVFEFMPGYETFWSYVPHFVHSPFYVYAYAFGDGLVNALYAVYEESGAGFQ
DK
YFEMLKAGGSKHHKDLLAPFGLDASDPAFWDKGLSMISGMIDELEAMED
>MCE8455916.1 ATP-dependent DNA ligase [Rhodovulum sulfidophilum]AB15
MKRFAALFTALDRTTRTTAKVEALAAYLAEAPEPDRLWTVALLSGRRPRRAVTATRLREWAAERAGLPLWLFEECYPVV
G

DLAETIALVLPPPVCDSDRSLSARMTDILAMARMDEPARKAGILKAWDSLGGAERFLFTKLLTGGFRVGVSQTLMTRAL
A
RATGQDEAELAHRLMGDWTPATTSWQALIEAPDPAAALSRPYPFYLAYQIEGPPETLGAPGDWIAEYKWDGIRGQLIA
RG
GVHFLWSRGEDLITGQFPELAVARDFLPAGTVLDGEILAWKDGRPLPFAALQTRIGRKTLSKRLLAEAPAAFLAYDLLED
GGTDIRPRPLAERRARLEQIIAGMPPGGPLRLSQALPFDDWEDLARTRGAARAAEAEGVMLKRADAPYLAGRKKGDW
WKW
KLDPLTVDAVMIYAQAGHGRRATLYTDFTFAIWDGDRLVPVTKAYSGLTDAEFRAITAWVRKNTLQRFGPVRQVAPEL
VF
EIAFEGIRASPRHKSGLALRFPRMLRWRRDKPAAEADRLDTLKALLGAAGGETTPGA
>MCE8455917.1 ligase-associated DNA damage response exonuclease [Rhodovulum sulfidophilum]AB15
MSVLTVTGHGLYCPAGDFHIDPWAPVDRALITHGHADHARPGHRHYLATDIAAPVIRHRLGAIALDTIRYGETRRIGAA
T
VSFHPAGHVPGSAQIRVEHKGEVWVVSGDYKPEPDGLSDPFEPVRCDTFISECTFGLPIFRWRPQAEVMAEIRDWWA
ENA
ALGRASLLGAYALGKAQRLLAGLGDGAEPGPGPILTHGAIEATCRVMRDQGLDLPDTVPVTPETDPRAHPGALVLAPPS
A
LGSAWARRFGRASTGLASGWMALRGVRRRRAAERGFVLSDHADWDGLNTAIRETGAERVLVTHGYTGPFRRWLAE
QGYDA
GIVATAWEGETTEGGEE
>MCE8455918.1 3-phosphoglycerate dehydrogenase, partial [Rhodovulum sulfidophilum]AB15
MADVHVTEPIHPDALALLASAGLSVSCGWKDADPAAGLATARAWILRTFVVSPEMIAGAPGLEAICKHGVGTDNIPLA
EA
ERRGIPVFNTPGANANAVAEHTMMLLLALTRQAVAMDRVARAGFAGFERLR
>MCE8455919.1 hypothetical protein LZ188_04980, partial [Rhodovulum sulfidophilum]AB15
MTYKTAGYGLILALAANVAGAETLTFAPAEGSERSYRVELSVRSVEPDSQTVPLDPERVSFLARIRAAERDGDTVDLTLN
PLWLKTRLGSQTMTTAAPLPDRVRQGFEAGFGARLDLATGRLGEVRPLGETEGPAPMLGGLREQFAAAFPPVPIELAE
GW
STTVPEYGGVRDVTITVDHLTAERVFLRYEGADFIARLAGLVVVDRDEGWVERAAITTDRRLVPGTDENGSLRETLAIAP
RGYPVTVHADNSMRPAEWRDMPERIEPPAPLASEAEVFPG
>MCE8455920.1 DUF2478 domain-containing protein [Rhodovulum sulfidophilum]AB15
MRFGYLMSEERGAASLVLAETATLLSAQGLRLAGAVQFSAADPDGHPCDGDLKVLPDGPVLQLSQPLGQGARGCRM
DAGA
VETAAAEAARHLGGAALLIVNKFGKLEAGGHGFVPLIAEALERDIPVLVGVNGLNRSAFESFAAGLAEPVLAETGAVLA
W
LGYAPAIASANVPNTPRARASA
>MCE8455921.1 trimethylamine-N-oxide reductase TorA [Rhodovulum sulfidophilum]AB15
MTSTSNSSLTLSKRSFLKGSLATGLGFAGLPLFGRGAAAQAASKGVVSGCHWGVFYGMVEDGRAVEFKPWEGDPAPS
PQL
PGVLDSLYSESRIRYPMVRRAWLEQGPGADPDGRGSGDFVRVSWEKAIELVAGEITRVRETYGHPAVFAGSYGWKSSG
KL
HNCQTLLRRMLNLTGSYTNSVGDYSTGAAQVILPYVSGSIEVYEQCTTWNNLAENCELMVFWGANPLNNSEISWQVA
DHG
SFSGVDQIKEAGVESICIDPVRTETAQHLDSEWIAPAPQTDVAMMLGIMYALQDEGLHDQAFLDRYTVGFDKFLPYLLG
D
TDGTPKTPEWAAEICGVPADKLRDLARRFSSKRTMLALGYSTQRQHHGEQIHWALITLASMLGQIGLPGGGYGLSYHY
SS

GGSPTHTTPILKAVDDASGVKVEGAAWLAASGAVSIPVSRLVETLLNPGKVMQFNGHEVELPEIKLAYWAGGNPFSHH
QD
RNEMLRAWRRLDTFIVHDFQWTASARHADIVLPATTSYERNDIEQVGDYALSHIVPMKKIVEPVFEARSDYDIFADIAA
K
LGKGYEFTQGLSEMDWIRGFYEAAKVEARAKSMEMPVFDAFWDSNKPLAFPLAEAQKDFVRHADFRADPLLNALGT
ASGK
FELYSTAIEKYGYDDCPPHATWMEPIERLGGPTTLYPLAVASNHPDLRLHSQLCGTKIRETYEVQGREPCWMHPEDAEA
R
GLSDGEVVRVFNQRGQILAGLVITDVIRPGVIRVSEGGWFDPVEPRKIGSLCAYGDINNLTTGVATSRLAQANCGQTA
MA
EVERFEGPLPPVTVFSQPA
>MCE8455922.1 molecular chaperone TorD family protein [Rhodovulum sulfidophilum]AB15
MTEFLVDMSEEEAADLAFMAEFLASLLLTPPDRDCLQALRSMQGQVSLGRIGGYLGEETAVDAIRQILTAGSAEEVAVS
L
ERRHTALFEGIFRQRSLPPYASAWDGTGRLCGPAVGRMQQILRDLDIHLEGSSEMPDHIGIQLATLAEALRRGRPDIAA
R
LLDELDWCARFGAALIKADDTGFYGALARLVLAFKTLMTADEAAAQPLHDPATARI
>MCE8455923.1 c-type cytochrome [Rhodovulum sulfidophilum]AB15
MTVKARTLVKLAGSLLVLGGAGFLVVTSPWTWSAVNGSRELPALEGADLENGRTVFLASDCATCHASPDAEDETHLGG
GR
SLDTAFGIFHMPNISPDEETGIGSWTLAEFDRALREGVGPEGVMPDGQNLYPAFPYTSYQRLTGEDSRDLYAYLMSLPA
V
RSDVPDHELKFPYNLRRGVGIWRLAFLDGDALTPGEVPDGVDPDVYHRGEYLVEGAGHCAECHSPRGLMGEVIADKR
YGG
GPNPEGTGWFPNISPDPTGIGYWSTARIANYLHTGKNPIGRMADGDMAEVVANTSQLPFSDVQAMAVYLKSVPPVE
NRAP
GQPAPNYADHVVMLDQAVGKAPQLPVSAASAIAAGDSATVVETKDVWLGADMVATENQPDGKILGGAAAEVTARD
GDKVQ
LTLRGWQMEDAPTVLYQAKGQRVMMAVFDDAAAAAVTRGAPEVAAATGQSWVPAEIALWSDAGGMNTDRGAV
WAYGQDTF
QTACAACHVLPQKTHFTANQWIGTLKAMRRFTSFSDDQYRLILAYLQNHSKDLDVSKETVQ
>MCE8455924.1 ATP-binding protein [Rhodovulum sulfidophilum]AB15
MPPPPAPSSGPPCWPADILTDLLSGASDALVDAILRLGAANGFTDQTSSMRAAWVESIEIINASLIASLSAPNQESGPNG
HNDYATDPRFAGLREVAWRHRRSGVPLWLNHGLLKDYRHGYVAFFRELFRDEKRAPALDDPEALIERLGLFFDEVELA
ML
APWSEPGTEEAAMADRLRRLTRERDQLFGVLESLRGPVFIADEDNRLISANRAAMQTFLGLDETGALAYRLALQSRRSE
L
QPIVDEILGTEGSQWSAIWLQTQSGSRCFDIRLETVEDTAYKLDRWRIVLMNDVTDHQQAIEQAREAERTMSLFLAAM
SH
EIRTPLHSVLGAAELLRDVPPQDVEERTRLTELLDISARGLSATLENVLSFSRFEQQAPKPRPGPVVLAEVLENLVRSRD
ILARQQGVPLMLSLAADLPDEVGLDWSMVQQILGNLIQNAIRHDDGRGVALELSRETGDAGDELVFAVTDHGPGLPA
PIR
EMLTAAPTALRPRPTGKSGSGLGLAIAQRMTLAHGGTISGGSAAGPGGGARIEVRLPLVAAPAPAKATPRPDGALSSKS
C
LLIDDDPVGVLVTLAMLERMGLSVDHARSLRQAGALCQAHPENYDLFIVDYRLPDGNGVDFARSLRQGGTLGERPVFL
LS
ANVDLVRQTDGQAALFTALLEKPLDAGALGRAIRSGTRPIRASAMLDGLSPAAQRRMAEAFAEGWRAFGARLETCTD
RAP

DAELAALAHKLANGAAIFGRTELHAALRTFEQAFDSPAATERMRAVARARVLATAPEDMTAPNEAPDA
>MCE8455925.1 response regulator transcription factor [Rhodovulum sulfidophilum]AB15
MSATADILLVDDDILVQVMVEDIVTQLGHRFHAAGDGETAREAIGRNGFDLVILDRRLPDTDGLLLAPLLQQDRKIPFIV
FSSLDAPTDQVLGLGMGASDYVCKPVEPAVLRARIQARLTARRTPEEQTTFRLGDALRLDAVSRRLWIADRVEVLSPAE
S
RLLICLLRNIGKPRDRMQISMATCGREWVYGDRTIDVLISRLRRRLKGSEVRITTVHGLGYVLTLDN
>MCE8455926.1 LLM class flavin-dependent oxidoreductase [Rhodovulum sulfidophilum]AB15
MKKIGFLSFGHWSPAPSSGTRSAADALIQSIELAEAAEDLGADGAYFRVHHFARQLASPFPLLAAVAARTRTIEIGTGVI
DMRYENPFYMVEDAGAADLISGGRLQLGISRGSPEQVIEGWRHFGHAPEAGQGDADMGREHGRIFFDLLKGEGFAQ
PNPH
PMFPNPPGLLRIEPHSEGLRDRIWWGSGSNATAIWAAEMGMNLQSSTLKDDETGEPFHVQQARQIRAYREAWARA
GHSRK
PRVSVSRSIFALVDDRDRAYFGRGVQSRDSVGQLDETTRAIFGRSYAAEPDRLVAELEKDEAIAEADTLLLTVPNQLGVD
YNAHVIEAILTHVAPQLGWR
>MCE8455927.1 type II toxin-antitoxin system Phd/YefM family antitoxin [Rhodovulum
sulfidophilum]AB15
MWTLQDAKNRFSAVVEAALAGVPQEVTRRGKPAVVVLSADDYRRLLKGAAAARESFAEHLLAFPAPEIEASGIEATGIG
R
AEVRPRDVRF
>MCE8455928.1 type II toxin-antitoxin system VapC family toxin, partial [Rhodovulum
sulfidophilum]AB15
MYLIDTNVISAVRRPDRAPQVRAWLAARPEPELFLSVITLGEIERGIRQQETRDPGFARDLRVWLDRTMLVFADRLLPFG
AAEARIWGRLSAEIGHPGADLMIAATALQHGATVVTGNVPDFEPTGVPR
>MCE8455929.1 MFS transporter [Rhodovulum sulfidophilum]AB15
MATLAISIVSVALPAIQASLETSLSGLQWVVGAYTLCLSAFMLSAGPLADRYGRKRTWLIGVAFFTLGSAICATADSLAT
LIAGCAFQGLAGALVIPGALSILTQAFPDPVKRAHVIGGWSSFNAISLILGPMMGGLLVDYAGWPSIFLVNLPLGIVALG
LGLVGIEESSHPDHAALDPVGQILSIVFLGTLTLSLINAGHSGWSAPVTTVPMVISALALLAFIAVEIRTARPVLPVDLL
RQKAFASANFASFVLGFSGYTSLFLFSLFLQRGQGWTATETGWRMAPVFAAMLAGSSVFGKLTRRFGMNRVMIAGYV
LLG
GSMLVMVSFTPSTPYGIVAPVFALLGIGLGLAVPSTGAAAMGSAPRERTGAASATMNALRQGGMTIGIALLGTIMSAR
AV
ASLKSVLAQSGVDDVAAIAAVAVQRHEVPSTLPVASGAFHEMLAGAFTQGFSLAAGFAGILGLVAAGVVALSSRRQ
>MCE8455930.1 TonB-dependent siderophore receptor [Rhodovulum sulfidophilum]AB15
MASYDQSVPSGTSRPPRPAINYYLGTSVLCLLLASPALSQETKPRKDGNASDLKAFTLEPIVIDVQDGDAGVVAKTSRVG
MKADASILETPQSVSVITRDQMDTQNPANTSSALRYTAGATSEAFGAFGSYVDITRIRGIKADYYLDGLRIISNSGSWLP
QVDPFTLDSVEVLRGPSSVLYGQGTGGGIVNQVSRRPQDEASHEVSLQFGNFARKHIGIDTTGPLNEDRTLLFRFTASGL
DTNGQIDDTKHRRLYLAPALTWRPDDKTSWTVMGTYSYEPEIPDYISLPAVLLGLNNSPYREIKRRRNYNDMNFADSTR
K
QTSVSSFFEHEFDNGWKFTSNSRYMHVDSSLKRSTVYGFSDVGGVPFLKGYYEDAPAESTTFSTDNFLSGEFDIGPTQH
K
ALVGFDYSFGTIKNELYTDGPILFDPYSDDHNPGKIPDFSASRAAPWKVDRDFNRGGIYLQDQISWDNWLLTLGGRRD
WF
TSDDETWSYSLTSRVTNYDDSAWSRRIGLSYQFDSGLAPYVSYSTAYDPLLENDWKGNPFKPVETKQTEIGVKYSPEGS
N
MLLSAAIFELKQTNVKTADALHLGFNTQTGEVRTRGIDLQAAMEVTPNINVIAGYTYLDNEILEDTRYEGNSLTQVPKHS
ASAWVDYRFSEGALAGLKIGGGVRYQGSTFGDPTNSFKVPAETLVDLGLSYDFAQVSPKFSGALLNVNIGNLFDKDYVS
S
CTSQLYCFAGERRTVSASLSYRW

>MCE8455931.1 ABC transporter substrate-binding protein [Rhodovulum sulfidophilum]AB15
MQESYLSRRNMVRGGLAIAALCLGSAPRAARSDRQRGSSVSITDIDGRTVAVTRPIRRLLLGNGPLAYVMAVLQPEAPF
A
NVVGWGNNFRAADLDGYNAYLRRFPDIDRIPSFSSGTLESIGAELAISLEPDVVVMNLSSKHAALASGLMARLAQVGIP
V
LFVDFRTRMFENTTRSIQILGELLERQDRAARFLQFRQEQIDRVIEPLAKVRSRPRVMVERAAGMYDDCCLTYGDGNFG
E
LIRAAGGDNLGAHFLHGSFGTLHPEQVIESDPDVVLVTGANWSLYSPAGDWVNLGPGADPVEGQSRLHRLMQRPAY
RSLR
AVTAGRVHAIWHPFYDNPYYFIALQRVAKWLHPEVFAALDPEDTFRELHARFLPVPYETGYWLSLEGGAS
>MCE8455932.1 iron ABC transporter permease [Rhodovulum sulfidophilum]AB15
METPPVSPLQSRNLYLRLTRRRLTLLTGIGLLTVICFLIDLSIGQAHYSSLEVLSALWDTDAPLSVRVILWDIRLPVALA
ALVVGASLAVAGVQMQTVLGNPLASPFTLGLSAAASFGAALGLILGVSLLPASAVAFAVPVNAFLISMGAALFIHRLSQK
RGITTEMIVLLGTTLVFTFTALLQALQYVAPDQALSAVVFWTMGSLSRANWLKLGIMSVTLALVSLAFAKQAWALTALR
L
GESRATSLGVPVARLRLRGILLGSLLASVCVSFVGTIGFVGLVAPHIARMLVGEDQRFLLPTSALCGALMLSAASVLSKS
LIAGQTLPIGIVTSLVGVPLFFTLIMRAKR
>MCE8455933.1 ABC transporter ATP-binding protein [Rhodovulum sulfidophilum]AB15
MTGLLSLSEVSVSFGKRPILQDISFGPLESGSVTALLGSNAAGKSTLLRRIAGELKGPGRVEIAGQPVESWPAYHRNRPA
HVPQDISMGSSLRVFEAVLLANKQGGGWSVDDEELDVVTQMLDTLGINSLSDRELAALSGGQRQLVSIAQALIREPRVL
L
LDEPTSALDLQNQFEVLDLLRRLAREWSMCVVVAIHDVNHALRFADCAVVLHDRQVEANGSPVDVLTPTLLGKVYGVE
AR
METSSQGHPFLIVERSIRNPLGEEHD
>MCE8455934.1 TetR/AcrR family transcriptional regulator [Rhodovulum sulfidophilum]AB15
MRYKAKKRPSEISGSPRTKPAEVRLDELMSAAEKLFLSQGVEATTVNEIVESANVAKGTFYHYFSSKNEILEALAKRYTE
RFLESVQKAIDGCSPDDWEERLRVWIHANIETYVKTYRTHDIVYTAHHHHDRANQAKSAILDQLLEIIEGGRLAGIWAP
N
QPRIVALLIYSGVHGATDDIIASKTTDCTSFARNVSDACLHMLPRITAPKK
>MCE8455935.1 5-aminolevulic acid synthase, partial [Rhodovulum sulfidophilum]AB15
SSEADRKTLVVVGAQQPYYGAIAVAPTEGLRSRATLAAANYHDVDAARVAALRGCDAARKPEGAPCVIAAEIRPKGWE
PR
ALQLSADATDGLRSEYGRRGGPRAMAISPSTGRWAVIKGQGAASRALEACADQSGATDCRIAVEDR
>MCE8455936.1 5-aminolevulinate synthase [Rhodovulum sulfidophilum]AB15
MDYTAALDASINRLHEEGRYRTFIDIERRKGAFPEAVWSRPDGENQDIVVWCGNDYLGMGQHPVVVGAMQDAIAAT
GAGS
GGTRNISGTTVVHHRRLEAELADLHHKEAALVFNSAYMANDATLSTLPVLFKDLVIVSDELNHASMIQGIRRGRCEKHIFR
HNDMAHLRQVLESIEKGRPILIAFESVYSMDGDFGPIEEICDLADEFGALTYIDEVHAVGMYGARGAGVAERDNLMHRI
D
IINGTLAKAYGVFGGYVAASAKMCDAVRSYAPGYIFTTSLPPTIAAGACASVHHLKTDATLREKHQDRARVLKARFKGLG
MPVIDHGSHIVPLIVGDPVHTKRISDMLLEGYGIYVQPINFPTVPRGTERLRFTPSPVHTPEQMDALIRAMDELWSQCA
L
NRAELTA
>MCE8455937.1 DUF4115 domain-containing protein [Rhodovulum sulfidophilum]AB15
MIRRRQPRAAEDSHHKPKGFDAFELRLGDVMRGERATLGKSLLDVQRELKIRATYIAAIENADPTAFETPGFIAGYVRSY
ARYLGLDPEWAYRRFCEEGDFATVNAMDAAASHKPQPARRKSVSEPLADPNAIFVPRGPAMFSQVQPGAIGSVLVLLS
LI

AVLGYGGWSVLKEIQQVRLAPLDDQPGLMADVDPLQVAENAPVSDRAPSAPPSVDALDRLYRPQILDRPVMVARDG
PIAT
IDPGEIGVLVSDRSQRAVERSFDSAVATALAEAGAVAQRPGLVREPSAAENGSVQVVAADATPELAILAVRPAWVRISA
A
DGTVLLEKILDAGESYVLPRTEKPPRLRAGNSGSVYFSVNGRTYGPSAPGAKVAKNIELSPRALTERYQLADIQADPDLA
VALAQNDVPTTRAGGPSR
>MCE8455938.1 flavodoxin-dependent (E)-4-hydroxy-3-methylbut-2-enyl-diphosphate synthase
[Rhodovulum sulfidophilum]AB15
MSLNSVRPWRSIDRRKSRQIHVGPVAVGGDAPISVQTMTNTVTTDVKGTIGQILRAAEAGADIVRVSVPDEDSSRALKE
I
VAESPVPIVADIHFHYKRGIEAAEAGAACLRINPGNIGDAKRVKEVIKAARDHGCSMRIGVNAGSLEKHLLEKYGEPCPE
AMVESALEHIRILEDNDFFEFKISVKASDVFLAAAAYQGIAEVTEAPIHLGITEAGGFVSGTIKSAIGLGNLLWMGVGDT
LRVSLSADPVEEVKVGYEILKALGLRHRGVNIISCPSCARQGFDVIRTVEALEKRLEHIKTPMSLSIIGCVVNGPGEALM
TDVGFTGGGNGNGMVYLAGKQDHRISNEAMIDHIVEEVEKKAAEIESTETSSGVEAAE
>MCE8455939.1 DsbA family protein [Rhodovulum sulfidophilum]AB15
MTRMTAAALMALGLAAPLAAPLAAQAEAFDLTDMSAAERTAFRAEVRSYLMDNPEVLMEAIGVLEQRQANAQAQN
DETLV
RVNAEDIFEDGVSWVGGNPEGDVTLVEFTDYRCTYCRKSYSEVSDLVASDGNIRFVVKEFPILGEQSVLSSRFAIAVKQL
AGDAAYEKIHDALITFRGNVSDESLKRLAEENGLDAAAVLERMKAPEVEKVIADNHALAQRLQISGTPTFILGDQMIRGY
VPLEGMKQLVEDARAD
>MCE8455940.1 DsbA family protein [Rhodovulum sulfidophilum]AB15
MKLVLSLAVAVATATPGLALPLSDAERAAFRAELRAYLLEHPEVIREALEGAETRRMARHANADLARIEAHAETLFRAPD
DWTGGAPDGDVTLVSFVDYRAEGARRALDAAREAAERDDRLRLVVKEAPRPGDEAAERAARFALAVLRLAGPAAYEA
AQT
RLFDAGARLDAAALDRISAELGLDPAEVTTRMAGSDITAKLAETRALTEALELGPAPAQVLRRTMVRGDVPAIALLRMV
E
AIRRKK
>MCE8455941.1 M48 family metalloprotease [Rhodovulum sulfidophilum]AB15
MTGPARGMIGLALALILSFAPAASRAQSLIRDAEIEYALKRLTQPLAVAAGLSPERVRVMVLQDSSLNAFVLDSQTVMV
H
SGLLLRLESAAELQAVISHELAHIANGHITRRLSNMRSAGSAAAMGMLMSLAVAAAGHADAGAGIAMGSSSTAQRLFF
AH
TRAEEASADQAGARYMARAGISPQAMVKVLEIFRGQEALSQSRQDPYIRTHPLNADRLRNARGYASAYSGAVQSDTQ
ADY
WFARARGKLGAFLQNPRYTLRRAEKDKTAVGMMRRAVAWHRTPDARRAMAEIARLEAARGSDPFVQELKGQILLES
RQFG
PAVQAYARAVQMAPKEPLILAGYGRALLALDRPAENRKALAALEKARARDPRDPRMLRDLAVAYARTGNNGMASVA
TAER
YAVAGRMKTAAVHARRAEGLLPRGSPGWLRADDILAAAEKLKQR
>MCE8455942.1 aminotransferase class I/II-fold pyridoxal phosphate-dependent enzyme [Rhodovulum
sulfidophilum]AB15
MKVSSRGEVDPFIVMDVMEAAARAESQGRRIIHMEVGQPGTPAPAAARKALARAMEESPLGYTLGLGLPALRARIAAL
YR
DWYGVDLNPERVVVTPGSSGGFTLAFTSLFDVGARVGIGAPGYPSYKAILKALGLEPVMIRTETQHRLQPVPGDLPQDL
D
GLLVASPANPSGTMLDRDALAALIDAAHARGAAFISDEIYHGIEYERKAVTALEITDETYVINSFSKYFSMTGWRVGWM
V

VPEDHIRRVERLVQNLFICAPHASQVAALAALDCGDELQANMEVYRKNRALMLEGLPKAGFTRIAPPDGAFYVYADVS
DL
TTDSRAFAAEILDKAGVAVTPGLDFDPERGAGTLRFSYARSTADIEEGLARLARFMASR
>MCE8455943.1 EamA family transporter RarD [Rhodovulum sulfidophilum]AB15
MSPLRPAAKGVLAMVGASAIWGLSSMYYKLLAHVPPLEVLSHRTIWSLVFFGLVLALQGRLAELWRLFGQARALWLVA
AA
AVMISTNWFFFIYAIQIGHAVEASLGYYIFPLVAVALGFAVLGERPSVLQWGAVGLAAVAVGALTWGLGVAPWMSLAL
AG
TFALYGLAKRGVPAGPVVSVTAEVVLLLPLALVWLWGVHVAGWPGLTGRALGAFGQGLGDSLLLVFSGPLTAVPLMLF
SY
ATKRVDFATVGLVMYLNPTLQVLVATLVFREPFTGWHILAFVLIWTALALYSAESLRLDRASRRSSIRAGTSDTMRT
>MCE8455944.1 TIGR00730 family Rossman fold protein [Rhodovulum sulfidophilum]AB15
MTLQNPSICVFCGSRDGDRPAYAAQADALGHALAEAGWRLVYGAGDVGLMGRVARAAQGSGGQTFGVIPVHLLDR
EVGKR
DLSAFIVTENMHERKKVMYMNSDAIVVLPGGAGSLDEFFEVLTWRQLGLHAKPIYLLNTEGFWNPLSALIHHFIDEGFA
E
PSLAGYVRMVSDVPALMEDLRDALSSRRLSAE
>MCE8455945.1 Ig-like domain-containing protein, partial [Rhodovulum sulfidophilum]AB15
MPERARKAPIRGSVWTWIILGGEAVLVLMVAAWFLGRDDDRQSDGPQAEQDAAVTSPAAAGQAPDAANPEAQTPA
ADDRS
EAVPPAMPVFAPPAFDLVRVTPEGNALVSGRAGAGSVVAVLVEGREAARAKADAAGDFAVLFDLGQSDDPRRISLEM
LGA
DGRRTASEATVILAPNRSVSTATGPAGGPEDAAEGPRPSQSGAPSSAIIGPPPADAAAILISDAGGVRLLEPPNSHEAPP
VSGLTLDAISYDAQGAVRLAGRGRPGAALRIYIDNAPLSETGIDGAGRWTLRAPGIAAGRHVR
>MCE8455946.1 keto-deoxy-phosphogluconate aldolase, partial [Rhodovulum sulfidophilum]AB15
ILERGYTVQKFFPAEAAGGAPALKALASPLPQIRFCPTGGVSPANAASYLSLPNVICAGGSWIAPKDKVAAGDWDAITAL
AREAAALPR
>MCE8455947.1 adenine deaminase [Rhodovulum sulfidophilum]AB15
MVQPLSDLIRQGRGLVPADLVLKGGQVLDVVTGETVPGDVAICGETIVGIGAEYDGRETIDVSGLTLVPGFIDTHLHIES
SLVTPLEFDRCVCPRGVTTAICDPHEIANVIGVEGIEYFLEAATRTVMDIRVNLSSCVPSTGMETAGARIEAADLLPLSD
HPQVIGLAEMMNYPGVLMQEPGVLAKLEAFRGGHVDGHAPLLSGRDLNAYIAAGIRTEHEATTAAEAREKLQKGMR
VLIR
EGSVSKDMAALRPLLTELSSAYMCLCTDDRNPLDIAEEGHLDHMIRWMIAQGTPVLAAYRAASLAAAEAFGLKDRGQI
AP
GKRADIVAVADLAECRAELVLSGGRVASAAAFAAREVVAPVGRGSVKAPKVTAASFRAASNAAETHVIGIMPGKILTEH
L
VEDMPPEGGEKRADPERDLAKVAVVERHGKNGNVAVGFVRGFGMQRGAIGSTVCHDHHNIVAVGVSEADMALAAT
RLSEI
EGGFVVAEGGKVLAELALPVAGLMSLETFETVEARLKDLRAAAKSLGVVLEEPFLQLAFIALPVIPALKITDRGLVDVEK
FEIIG
>MCE8455948.1 hypothetical protein LZ188_05140 [Rhodovulum sulfidophilum]AB15
MLVKIFWSLVFAVAFSLSINSGGLADLEAQLRAMPVGFDVIVRGGLPGEGERRAVTLLAALLVLGLFARRFNGWPNGA
RG
PAGFPEHGPRYFTTRFRFCAWALLYGSVIAGAFLVVVFFPTLADQAVALLFPVMPGNPFLSQIDKVLPSAPPGGGTQWS
L
AAAVGIAAAFYTSDWERMLRERFQNNALIPRQAEQLFEEFTRFEAVVPVESHFGDRFMMQFRERDDLPTYHRIDLDPE
QA

ELAQMRLERLARVEFMLWRMTQGDLPEEVAARLAEHVPLRQRLLDKLAAIRDEALAIDLQLVQVTNLLLCHLAEAQRP
DF
RRRLGQAAGLDCEQDRIETFPVSRLDDVIPVLEDELRRIAEAEPVPTGWQGEGDGAAGQAIEALQEQVASLRALVPLCV
T
GLEVQRGRLRDLGTRLEDLWCETLRFAICIGLAAPYASGKRFFCALGLRARMGYDVNLSIVLAVPMGLCFFCAVALLSGQ
SSDHAAGANLEGAELWQRVRMLLGGMKIAATERPLWLILGGFFIGWPLLQGCYVGSALAHGRQTEGGFGRPGALRPL
DYL
ISFVMIWSVLVPGVAALIYVLGFSAAFRDVYVGALAYAPVSALWGVMVSITSMRAFDTQAEGPAIRIRESVIFLLLVAVG
VVVAEIWIGSVDPESDTPFNAGRFWSLLFLASALTMVLIGAILLATSSKVSGKLQRMQGARLTPLRPAYPA
>MCE8455949.1 transporter substrate-binding domain-containing protein [Rhodovulum sulfidophilum]AB15
MIRNCFIAIFIFTVSGQPGPAQSRDRAADGVCSATIRVGLREDAPPFSFRINPEARAPDLSATCGYEEKGLLGGYAGYTV
GLCRDYLLDLLSRCRGAGPQVEAVPVAVDKRHDALAAEAPPFDMLCGATTATVTLAASVPHSPYTFLTSTSALVNARFA
Q
AEGTSCRVGVVNGTTSAPGMQDRVLDPRRIETWQEFIRRNKSCSLDGLERAVGFDSTAEAILALRAPGSEGIDVLVGDH
H
ILAWYWQHLDKLHRRMELPEAIAARFEALDDSETESTASSLARFKDELRLIPAAISIEPYTVVAPPGKAALIADFSRFLT
ARQQDRNGEYTRRLRQCFGRQVDRSLWFLMEFQSKVRDGNPLK
>MCE8455950.1 ABC transporter substrate-binding protein [Rhodovulum sulfidophilum]AB15
MVPMLATISRRSLLTGTAALLGSTALWARAHAAGGGRLIVAADSEPRQLNPAIVASNGVFFITSKVIEPLAEASYAPGGL
RPLLATGWAGSEDGHSITFTLREGVLWHDGTPFSSADVAFSAMEVWKPLQNIGRALFANLEAVETPDPLTAVFRFSQP
MP
LQLIANALPVVSSVLPKHIYAGTDIATNPANAAPIGTGPFRWGTHRPGEYYLLSRNDSYWGEGLPLLDDIVFRVLPDRAA
AASSLEAGEVHLAAFSAVPLSDLDRIGRVPGLEVVTKGYEALTYQLTVEINHRTEALSKLKVRQAIAHAIDRDFVLKTIF
LGYAEAATGPVPANDPTFYTPDVATYPFDIETAKALLDEAGYPEGPDGTRFGLRLLPAPYFSETRQFGDYLRQALAAIGI
DARIVPHDAAGHIRAVYTDHAFDLAIAPTVFRADPAISTTILLRSGMPPGVPFSNQGGYADPELDAVIDEALVTLDPAAR
VALYKKLQRMVAADLPIINVADWSFTSVVSDKVTGVATNPRWVVSNWADTGLSQ
>MCE8455951.1 ABC transporter permease [Rhodovulum sulfidophilum]AB15
MPRMMRIVLARLAGSLPVLLIVLVGVFLLLDRAGGDAVDAYLLSMGGGDAQMAAGLRAEYGLDGPAWKRLAFTLGQ
YARG
DLGWSLALNRPIGALIAERLPNTLWLMGSATALSFLLGSGLGVLAGRRPGAMLDRLLSGGSLVLYSIPSFWLGLVLAIVF
AVQLRWLPVSGIETIASGKAGLARAADIARHLVLPVAALGMIYLALFLRIMRAGMAEAWGENYTLAARARGLSDWRITL
N
HAARNAILPLVTMLGLQSAAMLGGSVVIESVFAIPGFGRLAQEAVAGRDAPLLLAVILTSALMVVIANLMVDLAYGWL
DP
RIGAGGGSDLG
>MCE8455952.1 ABC transporter permease [Rhodovulum sulfidophilum]AB15
MAKALLGRPEALLGAAILLALILVAVFAPLIAPGDPLAIAGAPLIPPFTDPALPLGTDRLGRDLLAELIHGARTSISVGL
SAALVAVVLGMSVGTAAGFAGGLTEAMLMRLVDAFQIVPGFLLALAFVSAAGPSLGIVVLAIALGAWTGPARLARARV
LS
LRETDFVAAARVMGMSPAEIAFRQILPLALPPVLAVASVIVAAAVLTEAALSFLGLGDPNAITWGAMIAEGRNQLRSAS
Y
LSIIPGIALVLTVISTYLLGEALTDILSGRRNR
>MCE8455953.1 ABC transporter ATP-binding protein [Rhodovulum sulfidophilum]AB15
MTPLLTLTALGIRYPGARTPALSDLGLALFAGEKLAVIGESGSGKSTLARALAGLLPAGTEVSGRIDWTEPLPRPGRDIG
YVFQDPGGSLNPLLSVGRHLDEVLRTHLPLSRKAARTRAAELLERVRIPDPDRALAAYPHQFSGGQRQRIAIALAIAAGP
RLLIADEATSALDVLVQAEIAELLRGLVEAEGMTLLFITHDMALAGGLADRIAVLHDGRLLETGPAARVLHRPREAYTRT
LLAAHLDLSSPRLVGDSP

>MCE8455954.1 ATP-binding cassette domain-containing protein [Rhodovulum sulfidophilum]AB15
MSPLLDARDLAKTYPGPRRVRALDGVSLTIAPGETLGLIGPSGCGKSTLARVLTRLTEPDAGEIRFDGQDWGALRGRALR
QARGAMQMVFQDPAAAFHPRATVAGALSEPLRLHRIAPRPDWPEHVAWLLSEVGLSPDLARRPVAALSGGQRQRV
AIARA
IAPRPKLVLLDEAVSALDASLRGHVLELLARLQRERGLAYLFVSHDLAVARALSHRIAVMEAGRIVETGPTEALLAAPQS
DILRRLIAAVPEIPAEKALP
>MCE8455955.1 aminotransferase class V-fold PLP-dependent enzyme, partial [Rhodovulum sulfidophilum]AB15
MTALPAPFWLPLPDAPDFPAEGFAPLADAIGRLMGTRNDVLLVQGEAMVALEAVASSIGAPGRKVLNIATSPYGQWF
GAW
LRRAGAEVTEIAGRAPGRPVAAAAVAEALATDRFDLVALVHAESATGILNPLAEIADLTRAHGALLAVDAVASLGGHPL
A
LDALGVDIAVMGPQKALGGPAGTSAIAVSPAAWERIAPPGAAPSILSLGDLRAHWLETGRGALPGTPSALEFHALAQTL
A
RVEAEGPEA
>MCE8455956.1 hypothetical protein LZ188_05180, partial [Rhodovulum sulfidophilum]AB15
MAEDGHHAILRRILGEDRVSLGGGRGGGNNGAATLRLETARAMRDSFGLTALVEECKADRAGLPEIQAEIGPGSLPILL
R
TSDGRLGLAVYSSELALALLEWRLIGFLSETAPPDRPLTATDAAVLADLTDPVLDRFGRAMKEADGVDWASGYTQGARI
D
DPRHVPLIMAAGRYRLYRLRLVAGRGRAGGLIIALPEAAPVLRPRTAENVARPWPEALRAGVLGAELTLD
>MCE8455957.1 ATP-binding protein [Rhodovulum sulfidophilum]AB15
MIAFDMLVAICLVYVALLFGVAFLAERRGARGSNGWLRSPVIYTLSLSVYCTAWTFYGAVGYAARSGLEFVTIYLGPTLV
LIGWWWTLRKLVRIGKTQRITSIADLISSRYGKSNLLGVIVTLIAVVGTTPYIALQLQSVTLSFAVFAAAGTNGPWQPTD
LKAAAFSVAVGLAVFTVIFGTRNLDANERHHGVVTAIAVEAAVKLVALLAVGLFVVWGIGGGAQATLARIEASPLAGM
PL
APGRWIGLTFLAGAAILTLPRMFQVLVVENEDERHLATAAWAFPAYVFLMSLFVVPIAAVGLAEMPPGANPDLFVLTLP
L
ARGQDGLATLAFLGGFSSATSMVIVAAIALSTMVSNHIVMPLFLHWTRAGATMSGDVRNLVLRSRRLSIGGVLLLGYLY
Y
RLTGGSEALAEIGLISFAGVAQFLPALLGGILWRGATRAGALAGLTLGFALWAYTLFLPSFGEGAVMSAGLLADGPWGI
G
WLRPQGLFGLDGLDPLVHAVVFSMGANALAFTLVSMLDFPGPMERLQAAQFVNVFDHSPSGQGWSQGRAEAEDLL
LMAQR
ILGADDAQRLFQRAAVDQGKQGYLPDTTPEFLQRLERRLAGSVGAATAHAMVGQIVGRSSVSVEDLMKVADETAQM
MEYS
SQLEAKSEELSRTARQLREVNEKLTELSVQKDAFLSQISHELRTPMTSIRAFSEILMENGDLPPERLVDYSRIIHQESMR
LTRLLDDLLDLSVLENGQVSLHPQPVSLSDLLDRSVAAAGGGSGRAPMTIRRDPAAERVALVTDGDRLSQVFINLVTNA
R
KYCDAAEPELTISVTEEEGAVAVDFVDNGSGIPERSQSIIFEKFSRLTDPRKAGGAGLGLAICREIMVRLGGAIVYLPGQ
GGAAFRVTLPRGCAEAA
>MCE8455958.1 hypothetical protein LZ188_05190 [Rhodovulum sulfidophilum]AB15
MARAPLFLAREGYRRRRLADMARLLPVLGAALFLVPALDAEDGLGAGMLIYLFIAWFWLILASAFVSSRLVRAAPREPA
E
PSGEAP
>MCE8455959.1 response regulator [Rhodovulum sulfidophilum]AB15
MDKRVLLIEDEPNIIEAIRFILARDGWRVDTHAEGSTAFARVAETRPDIVILDVMLPGRSGFDILRDIRSESGLDGLPVL
MLTAKGQVKDRDLAERMGASRFMAKPFSNTEVLASVRELTGA

>MCE8455960.1 short-chain fatty acyl-CoA regulator family protein [Rhodovulum sulfidophilum]AB15
MSRSALTGTRIRERRTMIGLRQADLARMAGISPSYLNLIEHNRRRIGGKLLVEIARNLDVEASALTEGAAAALIETLREA
AADHDAAEAELPRLEEFAGRFPGWAALIADRHRRMRELERTVEMLTDRMTHDPFLSASLHEVISAVTSIRSTAAILADTE
DIDPDWQRRFHRNLSDDSRRLAEGAQALVAYLDEGSEDPGVQSSPQEEVEAFLRARDWHLPELERSLPPNPARIVAEA
PE
LTSAPARELALAHLRRYRRDAELMPLQRFAEAARGAGLDPGALAQAFGVDPGAVFRRLAALPEERAGGPVGLVACDGS
GT
LTFRKPVEGFSLPRFGAACPLWPLYQALSRPMAPVRAVVELGGSAPRRFLTFAICQPALAAGFDGPQVLEAAMLILPEG
T
VDLPRLAPQPVGTSCRICPRRDCVARREPSILADGF
>MCE8455961.1 substrate-binding protein [Rhodovulum sulfidophilum]AB15
MTRPTLTRRGVIKTGAYAGAGLALPTIFTSRARAFTNAPTGSTVKFGFNVPQTGPYADEGLDELRAQELAIEHLNGMGD
G
GMLNTFSSKALDGTGILGKQVDYVTGDTQTKSDAARASAKAMIEKDGVVMINGGSSSGVAVAVQGLCQEAGIIFMAG
LTH
SNDTTGKDRKKNGFRHFFNAYMSGAALAPVLEKAYGTDRRAYHLTADYTWGYTQQESIQASTEAIGWETVNNVLTPV
GAG
DFSSYIAPVLNSGADVLVLNHYGGDMINSITQAVQFGLRDKQVNGKNFEIVVPLYSELMAEGAGDNIKGVYGSMNWN
WQL
SDPGTKAFVKSFGEKYGRPPSNSAHTCYVQTLLYADAVARAGSFDPCAVAEALEGFEFDGMGNGPVLYRAEDHQCFK
DVL
VMKGKDNPSTKYDILEIVEVTPVDHVTYPPDHPMFGGAEATLGQCNNGA
>MCE8455962.1 branched-chain amino acid ABC transporter permease [Rhodovulum sulfidophilum]AB15
MDAIILQILNGLDKGSAYALIALGLTLIFGTLGVVNFAHGALFMIGAFCAVTMQRLLSLSYEEIDPTRTDFLGNPLKVEV
PYVEHWFGPEIGGAIIDWSVPFAILLAIPVMVGLGFAMERGLIKHFYKRPHADQILVTFGLAIVLQEIIKYFFGANPIQT
GAPPAFSGSFDFGTLLGFDPNVIIYPYWRMVYFLFSLAIVGGVFAFLQFTTFGMVVRAGMADRETVGLLGIDIDRRFTL
M
FGLAAAVAGLAGVMYTPILAPNYHMGMDFLVLSFVVVVVGGMGSLPGAVLAGFMLGILQSFASMNEVKSLIPGIDQV
IIY
LVAIVILLTRPRGLMGRKGVMED
>MCE8455963.1 branched-chain amino acid ABC transporter permease [Rhodovulum sulfidophilum]AB15
MFGLSARDTRFLLIVIALTLVTPILLQPFPEGSSLAQFNAGYPDLMQKFAIFGIFAIGFNILFGLTGYLSFGHAAFLGVG
SYTVVWMYKLLGYNVLPGLLLSVVISAAFALLIGFISLRRSGIYFSILTLAFAQMSYNLAYSVLTPITNGETGLQVYASD
PQILAGSGAPTSPHLFGLVMRESAQLDLGGWVFTFNIGYYFCALMAILAFYISLRIFRSPFGMMLRAIKTNQTRMNYTGL
NSRPYTLAAFVISGMYAGLAGGLLAAMDPLAGAERMQWTASGEVVLMTILGGAGTLMGPVLGAGFIKYFENIFSKIND
GV
LHSWFAFLPDGLEDFVVACFHPFVGKGWHLTLGILFMLVVIFLPGGLVEGMQRLRSLFRRRGKTGGPRRKDPEHRPHT
PP
HVAE
>MCE8455964.1 ABC transporter ATP-binding protein [Rhodovulum sulfidophilum]AB15
MGILEVKGVNKRFGGLQALGDVNLSVPENTVHAIIGPNGAGKSTLLNCLVGKLIPDTGSVMFDGQSVLGRKPHEINQM
GI
SRVFQTPEIFGDLTVLENVLIPCFAKRDGAFRMHAFETVAREGEIVARAEQMLEDVHMLDKAGMQAASLSRGDKRRLE
MA
MCLVQEPRLLLLDEPTAGMARADTNNTIDLLKEIKATRNITLAIIEHDMHVVFSLADRITVLAQGTPLVEDVPEKIKGHP
KVQEAYLGQAH

>MCE8455965.1 ABC transporter ATP-binding protein [Rhodovulum sulfidophilum]AB15
MNVKPDFNKNANQAATAPAFLSIYDLHAYYGESYIVQGVSFNVHEGEILALLGRNGAGKTSTLRAIARLASPELTYGEIW
LDHAALHGMAAHQASAAGMALVPEDRRIIPGLTVEENLQLAQIVPPVGWTLDRLYDLFPRLGERRKQEGVTLSGGEQ
QML
AIARALARDIKVLLLDEPYEGLAPVIVDEIEKTLVHIKEQGITTVIVEQNAIRALELSDRAVILDTGQIVFDGSAAEVLS
NAELRAEYLAI
>MCE8455966.1 AMP-binding protein, partial [Rhodovulum sulfidophilum]AB15
MTEHIYPPDPDFVSGAHIDSAKYEAMYRESVEDPEGFWGTHGKRLDWITPYTEVKRSTFKMGAVEIHWFRDGTLNVA
SNC
IDRHLRSRGDQDAIVFVPDDPGDPVRHISYRELHEKVNRMANVLLSQGVMRGDRVVIYMPMIPEAA
>MCE8455967.1 DctP family TRAP transporter solute-binding subunit [Rhodovulum sulfidophilum]AB15
MKAVRLGGGALAALIAGAMMLGTASAETLRLSHNVGDTTTWHKGAEKFGELLAEATDGELKVRVFPNAQLAGGDQ
MKMTE
MVGRGALDLVVTSAINVTPLVPEMAVFSLPYLYGSYDEVDATTQGAPGEMMAEILLDKGIVVLAWGENGFRELTNSVR
PV
KTPEDMQGLNMRVSGPMYIDVMNALGANPTQMQWSETLTALQQGVVDGQENPIGAVIIPQQVYEMQKYLTAWH
YSYDPVF
LGVSQSKWDSLDAETQAKMRKAAVEAMAWQRQISREATASGVDFLREKGMEVYEPTPEELEAFRAATKPAFDKWSE
KVGP
EIVEAFQSAIAGSN
>MCE8455968.1 ribokinase [Rhodovulum sulfidophilum]AB15
MIVTFGSLNADLIFSVETLPRPGQTLMAKGLATEAGGKGANQALAAALDGAEVAMAGAVGQDALAEVALAGLKGRV
DLSR
VARLEASTGCAAIHRDAEGRNEIVVAGGANLAASSGSVEDALLDRASVLLLQMENDPAQIERLIRRCRPAKAMSILNLAP
AYRLDPEVLGLCDLIVVNEDEAAAMANWLGCASEPRALALASGAGILRTLGGEGAELCWQGQEISIPALSCRVKDTTAA
G
DCFVGVFAAALDRGETPEAALRRAAAAAAIACTRIGSQSSLPEARDTDAVLAGAAV
>MCE8455969.1 ABC transporter ATP-binding protein [Rhodovulum sulfidophilum]AB15
MDTPVLSVEELSAAFRVEGQWRTVVEGVSFDIGPQETVAVVGESGSGKSVTAMSIMRLLSPANSRTEGRIVFEGQDLL
AM
PLDDMQAIRGNRIGMIFQEPMTSLNPVLKIGDQITEVLRRHRGMDGAAAEAEAVRLLDRVRIPSAAARLGEYPMNFSG
GM
RQRVVIAIALACAPRLLIADEPTTALDVTIQAQILELIKTLQEEEGMSVLFITHDMGVVAEISDRTVVMYRGEVVEKATT
EAIFRAARHPYSRALLSAVPQLGAMAGQDRPLRFPRVDMATGEIAPPPPTADTVRADEPPVLKVDNLVTRFPVRKGLLR
R
VVGRIHAVEDISFELRQGETLSLVGESGCGKSTTGRSLIRLEDPSGGSVAMDGHDVRGAGHSELTKIRRDVQMIFQDPF
E
SLNPRIRIGEAIAEPMVVHGLVSRREAPARVADLLERVGLSGEMMQRFPHEFSGGQRQRICIARALALEPKLIVADESVS
ALDVSVKAQVINLMLDLQERYGLGYLFISHDMAVVERISHRVAVMYLGEIVEIGPRAAVFENPRHPYTRKLIAAVPVADP
SRRRRRVVSGDEIRSPYRPADYVAPKRLYREIGEGHFIQIPESEAAA
>MCE8455970.1 ABC transporter permease [Rhodovulum sulfidophilum]AB15
MAAPSETARSVHTDAAAAPASVSPFRQTMGKLLRDPLALTGLVIVTLLMIGAVFAPLIAPQDPYDLAQLDIMDGRLGPG
S
QSMTGMVYLLGTDDQGRDLFSAILFGLRTSILVGLASAAIALVIGASVGLFSAYAGGRTDAFLMRIVDIQLSFPAILIAL
IFLAILGQGVDKIILALVVTQWAYYARTIRGSALVERKRAYVDAARSMALPDRRILFRHILPNCLAPLIVVATMRVAYAI
MLEATLSFLGIGLPVTEPSLGLLVSNGFEYLLSGDYWISFFPGLALLVLIVSINLIGDALRDILNPRREG
>MCE8455971.1 ABC transporter permease [Rhodovulum sulfidophilum]AB15

MSGYLFSRLLQTLVTLFVMSVLVFGGLHMVGNPVDVLLSPTATPAERLEVIRSFGLDQPVWVQYWIFLKNALSGDLGN
SF
VYNQPALHLILQRMPATLELAAAALVMALAIGLPLGVYAGLRPNGALAKSIMAFSILGFSLPTFWIGLVMIMVFAVQLG
W
LPSSGRGDTVDVFGVPLSLFTLDGLAHLALPAFNLALFKISLVIRITRAGVLETMQLDFVRFARAKGLPERRIVRVHVLK
NTLIPLITVIGLELGSLIAFAVVTETIFAWPGMGKLIIDSIAVLDRPVILAYLMITVVMFSLINLTVDILYSLVDPRVRL
EGN
>MCE8455972.1 nucleoside hydrolase [Rhodovulum sulfidophilum]AB15
MRRIPVIIDTDPGVDDAAAILMALGSPELDVLGITTVAGNVGLSATLTNACRIVGLTGRTDLPVMAGARGPLMRDQVF
GK
YAAIGAFPVPLLGGPAVVPSSENAVRFIARTARAAAEAGTPLTICAIGPLTNIALALGMHPDVGRGIRRIVCMGGAIATQ
GHRTPWAEFNIYADPHAAEIVFGSGVPLVLFPLDVTTQALFTEAHVAEFRARGGAAGRALANLLTEYDRSDPERYGRPG
G
PLHDPMTIAWLLRPELFTGRAARIGVQVGGATSGSTFADFKAPQPRSAVITGVDEAAYVALVLDRIAAHGAGRAAASQ
KV
ETE
>MCE8455973.1 nucleoside hydrolase [Rhodovulum sulfidophilum]AB15
MTTPIPVIFDCDPGIDDAIALLGAFAAPELDIRAILAVCGNQPLERTLRNALQICELGGREDIPVHAGAHRPLCRDPIHG
KFHGENGLGDTLLPDPLKRPEPVGAVEALIDHLGQAARSAGPKITLCCLGPLTDLALALRLAPDIAAGIERVVMMGGAFR
EPGNRSLTSEFNMLADPHAAAIVFAAGLPMTVLGLDATHQVMLRPEHVASFAAVSGRISRSVAGLMAFWDRNDPQR
YGSR
GGPLHDPLVTAFLLAPELFETRPARVFVETESPLCLGQTVADWFGKTGAPANADIAIGVDAGGVIQLYLELLARYGTGAA
A
>MCE8455974.1 dihydrodipicolinate synthase family protein [Rhodovulum sulfidophilum]AB15
MSASREPGFHGVFPYLVSPLDAAGGVREGVMVALVERLIAAGVHGLAPLGSTGEFAYLSNDQRLAVARAVIRAAKGRV
PV
VPGVASTSTADAVAQTRAMVAAGADGILAILDAYFPVGEAGVAAYFTAIAEAAEGLPVVLYTNPNFQRSDLTLPVIDRLS
RVPNIRYIKDASSNTGRLLSILNRCGDRIEVFAASAHIPACVMMIGGVGWMAGPACIVPEQSIALYEAARAGDWERAM
DL
QRPLWRINELFARYSLAACIKAALELQGFEVGDPVAPQTPLDAVARGELAQALGAIGAL
>MCE8455975.1 ABC transporter substrate-binding protein [Rhodovulum sulfidophilum]AB15
MKRFASVAILAASAACLPSVVSAQSLTVGLAASTTSMDPQFYVVGPNSAMARNIFDGLVNQDDKQQIQPALAVSWSV
IDD
TTWEFRLREGVTFHDGSDFTAEDVVASLNRVPMASKNSPSSFMPYVKAITSVEAVDPMTVRITTAAPTPLLPNDLSRIAI
LPAEDAEVTTEEMNAGKGVIGTGPFEFVSWVPDSEVKVTRNDAYWAGSAAWEEVTFKVFKNNSARVAAMLSGDVD
MIESI
PTADTRRLKSADDLKVVSVAGNRVMYLHMDQGREVSPFATGPDGKNPLRDARVRRALSLSLNREAIVDRIMDGQGVP
AGQ
LVPEGYFGYDPSIKVDPYDPAKAKELLAEAGYPDGFKLTFHASNDRYPNDSRVAQAIGQFFSRIGIDTSVETLPASVYFS
RASNLEFSFIMGGAAVETGEASGVLGPLLETYGDSAGRGNRGRYSNPDFDAALQQARQTLDAPTRRDLLQKATRIAMT
DL
GVIPVFYLANTWALEDGLSYPGRSDGYTLAYEVTAE
>MCE8455976.1 LysR substrate-binding domain-containing protein, partial [Rhodovulum
sulfidophilum]AB15
MRHRQVEAFRSVMMTGGITSAAAAMHVTQPAVSRLIRDLEETIGLSLFQRRGARLDPTPEASLLYREVERLYLGLDQIA
Q
AAEDIRDHKNIVIRIGTVTSLVRPYLQRAIRDVIGDRRDVPLVIDVENSRHVWEMVENAQYDIGFVYRRQRMDDRARPL
R

QMTAVAAVPPGHPLAGRAVCTPDDLAGERLLIPGRNSPARLALDSALAQAAGAPASLMESSMLNCCHFAADGMGVA
IVDE
ISIRAARANAGAALVAIPFRPEIGVGYYAIRPPGAHRIAILDAK
>MCE8455977.1 HAMP domain-containing histidine kinase [Rhodovulum sulfidophilum]AB15
MFLNSLSGRFLLLTIVFVMLAEVLIFVPSVARFRVDFLLARLERSQIASLALLASDGMIGDELERELLQNAGVYNVVLRR
DQARELALSSPIPEPVYRSFDLRDASVLALIPDALIRLVDPKTRVIRVIGQPVQRAGLLIEITMSTAELRKAMIDYGLRI
LVLSAVISVLSALLLFLAARRLLVVPIRHVVRAIKLYADAPEDNRRIITPSARVGELYEAETALRVLQEELTGALRQKDR
LAQLGAAVAKISHDLRNILTTAQLFADRMEASDDPAVKRAAPKIVGAISRAVHLCESTLAFGRAQEPPPRLERVALAPIV
NEVVDGERLAAGDYDISFSEDVPAGLTLRADVEQLMRVLTNLVRNARQAILASGKPGEIGIRADETEQDWVIQVTDTG
PG
LPPKAREHLFQPFHGGTRKDGTGLGLAISMELIRGHGGALELSRSDAEGTAFEIRLPKAAAALPGEDGIPN
>MCE8455978.1 FAD-dependent oxidoreductase [Rhodovulum sulfidophilum]AB15
MAPMKTDLCIIGGGAAGLSLAAGAARMGARVVLVEGHRMGGDCLNYGCVPSKALLAAAARAQAMREGGCGIAGVL
PKIDH
AAVMAHVRGAIAAIAPHDSVERFEGLGVRVIRDWARFVSPREIQAGGEVIRARRFVIATGSRPAVPPVPGLDRVPYLTN
E
TLFELDARPDRLLILGGGPIGIEMAQAHRRLGAEVAVIEAKRALGREDPEAAALVLDRLRAEGVEIFEHAEVRRVTGRAG
AITVETADGLLEGSHLLVAAGRQPALERLDLPAAGVEADAAGVRVDSGLRSTNRRIYAIGDAAGQGQFTHLAGYHAGL
VI
RPILLGLPARVRRDHMPRVTYSDPELAQIGSTEAEARAAHGDAIEVVRADYAGNDRAVTGGRTAGFVKVVLRRGRPLG
AT
LVGAEAGELVGPWALAIASGQRMTAMAGMILPYPTLGELNKRVAGAYFSPKLFENPWLKRGVRAIQRLLP
>MCE8455979.1 VTT domain-containing protein [Rhodovulum sulfidophilum]AB15
MSRTRPQRPRRRGLARYLLLAAIAAVALIGAVTLNDRLSFEALAAHREALIALRDANYALAVLGFMLAYVLIVAFSLPGA
TVATLTGGFLFGLFPGVFFNVTAATLGALLIFLAARWGLGDRLSARLDAGEGRIAAFRDGLRENEIPVLFLIRLVPAVPF
FVANLLPAMVGVRAGRFVFTTFFGIMPGALVYTWVGAGLGEVFARGARPDLGIIFAPYVLGPLLGLAALAALPIAIRVAR
RKG
>MCE8455980.1 50S ribosomal protein L34 [Rhodovulum sulfidophilum]AB15
MKRTYQPSNLVRKKRHGFRARMATKAGRKILNARRARGRKSLCA
>MCE8455981.1 ribonuclease P protein component [Rhodovulum sulfidophilum]AB15
MMPPGMNGASETSESVKASPAVSSCLTVLSRRADFLLAARARRQSAPAFTLQARRRTEAPEGGIRIGFTCSKKVGNAV
AR
NRAKRRLREIARTELPVHGRPGWDYVLIGRPVTTASRDFAALRADLRRALERLHADRS
>MCE8455982.1 membrane protein insertion efficiency factor YidD [Rhodovulum sulfidophilum]AB15
MSPLSRLIALPIRAYRIVLSPWIGFNCRYQPTCSAYALEALETHGALRGGALAFWRILRCNPWGGCGYDPVPPKPDPKH
>MCE8455983.1 tRNA 2-thiocytidine(32) synthetase TtcA [Rhodovulum sulfidophilum]AB15
MSDETDDIHPLFHGAPSTTEFKKLRKRIIRDTREAIEQYGMIERGARWLVCLSGGKDSYTLLAALYELKWRGLLPVELLA
CNLDQGQPGFPATVLPAFLERMGVPHRIEYQDTYSIVMDKVPQGRTYCALCSRLRRGNLYRIAREEGCSAVVLGHHRD
DI
LETFLLNLFHGGRLATMPPKLVNEEGDLFVYRPLAHVAEADCARFARAMDYPIIPCDLCGSQDGLQRMQVKAMLDQ
WETN
HPGRRQKMFRALMHVRPSHLLDPTLFDFAGLLRETGAPSDIAPGEPPGG
>MCE8455984.1 membrane protein insertase YidC [Rhodovulum sulfidophilum]AB15
MDDQNKNLILATALSFLVILGWFLLFPPEQQAPVEEQDAVTTTEQTGSGATAPDLAVAPPAPQGSGAASTPETAAPSLA
A
EAPRLSIETPRLTGSISLLGGRIDRLSLKDYHETLDPDSPLVSLLEPVGTERPYYALYGWAPAGSLGYEDVPGANTLWTL
DGNDALSVDSPVSMHWNNGKGLTFHRTISIDENYMFTVEQSVENATGEAVRLAPYGIVARHGQPEGSRFYILHEGVV
RQT

DSELEEIKYKKMPDMDYVERERANAEVTDVQENGWIGFTDKYWMTTLIPEPGQPFTSVAKYVQGPDIYQVEARLPVV
DVA
AGATESTSTMLFAGAKEWETIHHYQKDDGIFRFIDSIDWGWFFFFTKPIFRVLHWLNGAMSGLGVPGSMGWSIILLTVI
I
KAIVLPLAYKSYSSMAKMKELQPKMEELKERCGDDRQKLQQEMMTLYKKEKVNPAAGCLPLFVQIPIFFSLYKVIFVTIE
LRHAPWIGWIHDLSAPDPSSILNLFGLLPYAPPEPGSFFAILSLGVLPILLGISMWLQQKLNPTPTDATQAMVFAWLPW
V
FMFMLGNFASGLVLYWICNNTITFIQQYVIMRIHGFHPDVLGNIRSSFRKKKPEPEHK
>MCE8455985.1 MOSC domain-containing protein [Rhodovulum sulfidophilum]AB15
MTARLARIYRHPIKGHGAEALEAVDLQAGRTLPWDRTWAVAHEASRADGTGWAPCANFSRGAKAPQLMAIAAQLD
EAAGQ
VTLTHPDRPELSFSPDSEAAALIDWVAPLMPADRAASTRIVRAPGRGMTDSAFPSVSLLGLASLEALSGQLGQALSPLRF
RGNFWIEGLAPFAEFDWVGQKLRLGRAVIEVRERITRCMATAANPETGERDADTLGALQAGWGHKQFGVYAEVIEG
GRVA
LGDALEAL
>MCE8455986.1 ribosome biogenesis GTP-binding protein YihA/YsxC [Rhodovulum sulfidophilum]AB15
MTPLPFPLTPEPDAEAAERGRKLFAGPVEFLKGVVAMDGLPPADRPEICFAGRSNVGKSSLINALTGRKALARASNTPG
R
TQEINYFTLGESHYLVDLPGYGYAEAPLKVVEKWQRLLKAYLAGRQTLRRAFVLIDARHGVKPVDDEIMKLLDGAAVTF
Q
TVLTKADKVKVAERDKVLDQVRASLARHPAAFPEIILSSSEKGDGIATLRATVEGIA
>MCE8455987.1 SDR family oxidoreductase [Rhodovulum sulfidophilum]AB15
MEQRVMLLTGASRGIGHATVKRFSAEGWRVLTCSRHPFDSRCPWPGGAENHVQIDLADPAMTKRAIEVVRGKLTGG
KLHA
LVNNAGISPKTEEGGRLNTLETDMRTWGQVFNVNFFASIVLARGLQDELAAARGAIVNVTSIAGSRVHPFAGAAYSTSK
A
ALAALTREMAHDFGPLGVRVNAIAPGEVETSILSPGTEKIVEGLPLRRLGQPEEVASVIHFLCSEEASYVTGTEIEVNAG
QHV
>MCE8455988.1 acetylglutamate kinase [Rhodovulum sulfidophilum]AB15
MRRQTAMNRDWIATARTLSAALPYLQRYTGAIVVIKFGGHAMGDDAEMESFARDVVLMRQVGVNPVIVHGGGPMI
NEMLA
KLGVVSEFVKGKRVTDAATVEVVEMVLSGRVNKRIAQAINDQGGKAVGLSGKDAGLIVCDQADPSLGFVGAPTEVNP
EVL
HQLFAAGMIPVVAPLGAGRAGETFNINGDTAAGAIAAALKADRLLLLTDVSGVKDASGRVMTELTPDNIREMTASGVI
AG
GMIPKTETALSAIEGGVRAVVILDGRAPNACLLELFTDHGAGSLIRAPRP
>MCE8455989.1 histidine phosphatase family protein, partial [Rhodovulum sulfidophilum]AB15
MPPTRDKQSGLPMSLRLILVRHAKSSWDDPSQDDHDRPLNARGREAAPKIGRWLAERDHVPAEVVSSTSCRTLETWD
RMA
PLLGESSVMRRDPGLYHAPAERMLETLNNR
>MCE8455990.1 ABC transporter permease [Rhodovulum sulfidophilum]AB15
MQLTQVLTYAMRRLLQAVPVVILIMVGTFLLLKLAPGDTVDALVGDMGGADAAYIARLRAEYGLDQPVWVQLGRYM
AKLI
QFDFGWSFVYEQPVSVILWERLGTTLMLMGTSLSLAFSIGVGLGAIAARRAYSLTDNAISTLGLVFYATPSFFLSLMMIL
LFSVKLGWLPVGGLTDVAAFYTGWDHVLDVALHLIMPTAALSLIYLSFYLRLMRASVLEVADLDFVRTARAKGAGETRL
M
VHHIMRNALLPVVTLLGLQFSTVLGGSVVVETIFALPGLGQLAYQSVIQRDMNMLMGIIFLCAIIVVIVNFLTDLLYARL
DSRIALK

>MCE8455991.1 ABC transporter permease [Rhodovulum sulfidophilum]AB15
MTAATTDLPRPEVHPAVALWRRFARNRAALLGLVLFGAVLVMAATAGLIEPVDPLRRAGDPLTRPFIDWAVPLGTDQL
GR
DILAELFHGARISLLVGVLATLLSIGIGVVVGAVSGFFGGWVDDVLMRVTEAFQTIPNFVLLLALVAILGSSIEYITLAI
AIVSWTAPARMVRAEFLSLRSREFVDAARNLGVSNGALIFREILPNALPPVIVYASVIMALSILLESALAFLGLGDPNYA
SWGNMIGQGRAVLRTDWYCAVIPGIAIVLTVLSFSLLGEGLNDALNPRQKKQ
>MCE8455992.1 ABC transporter ATP-binding protein [Rhodovulum sulfidophilum]AB15
MTDPVLDIRGLKIALPKGADRPFALAGLDLSVRPGEIVCLVGESGSGKSLTAGAIMRLLPEPHVRVAEGTIRFEGQDLLA
LSEREMKQIRGDRIAQIFQEPMTALNPQKTVGWQIDEVLRLHTRLGRKARRARVLELLDLVHIPDPPSVFNAYPHQVSG
G
QRQRVMIAMALACEPKLIIADEPTTALDVTTQLQILKLIRELQEKEGAGILFITHDFGVVAEIADRVVVLSKGEMVEQGP
AAEILNRPRHPYTRALIGAIPALVPPAPRDFDAAPVVLKAEGLVKTFAARGSLISGKRAPVKAVDNLSFTLRRGETLGVV
GESGSGKTTVSRIVTRLLESDCGAVEVDGTDLLACSPRELREMRRHIQMVFQDPMASLNPRKRVEDLIAQGPIVHGAEP
R
AARARARELLELVELSPAAATRYPHEFSGGQRQRIGIARALAMEPKVIVADEPVSALDVSVQAQVLRLLADLRDRLHLSL
LFVTHDLRVAAQLCDRVIVMQKGRIVETGITAELFADPRHPYTQQLLASIPGRGWTPPSLETDAA
>MCE8455993.1 aminotransferase class V-fold PLP-dependent enzyme [Rhodovulum
sulfidophilum]AB15
MSLQFDDIRRALIGEGTAIPGPFGPRALIYADYVASGRALGFVEDAIRQHVLPYYGNTHTETSFTGRRTTELRELARASV
REGMGADDGHAVVFCGAGSTAATDKLVRALQMGVPAGRLGPDAVVFVGPYEHHSNDLPWRESGAKLVRIPLDAAG
TLCLE
TLRREIAAHAGAGLILGAFSAASNVTGIRTDIGAVARLLHEAGGWCVIDFAAAAPYVAIRLAESAPAAGDRIDAALISPH
KFPGGPGASGLLIADCALLAAARPTVVGGGTVSFVSAAGQCYIDGVEAREEAGTPSIVGNIRAGMVMALKTELGAEAIE
T
REGELRARLDAALSAEPGIELLGPRNAPRLGIFSFNIRIGARHLHHGFVVAVLNDLFGIQARGGCSCAGPYGHHLLQIDD
DHSQRFSEAVARGHTAFRPGWVRLGVNYFFSDEDVDRLARALVFIARNGLALLPLYRLDVEHGVWRALDGARSPAPES
LA
ALWAETPDTPEPPDFDTCLAEAARLAETGRARADAVAGTPLHPEDEALRWFWLPEEAAAALTDEETPA
>MCE8455994.1 FAD-binding oxidoreductase [Rhodovulum sulfidophilum]AB15
MTDPVTFPPSLWADTAPERVPAPPLQGTIEADTVIIGGGFTGLSAALHLAREGRKVVLLEGQAVGWGASGRNNGQVIP
TL
TAAEPDAWVARFGETGRRFAELIGGSAGLLFDIVRREGLKAEAEQSGWFQPAHSPGRVKLSEKRVEAWRRFGFDVSLK
DA
RETAELLGSEFWHGGMYAPSGGHINPLALARELARAAEGHGAVIHEQSPVKWFERQGAEWVAATAAGKVRARALILA
SNA
YTGKLAGGLAPKIARSFIPVHSWQMATEPLGEELRRRILPGRQAVSDTRGDLRFFRYDARNRLITGAAIIPGWNDAGRA
E
AKAAQNLGEAFPELGMPKMTHVWSGYLSMNWDRFPRVHNLGPDGWAWAGCNGRGVALGTALGREMARAVAGE
DPRGLALP
VTEPQAFPYHALLTRLAPAYLAWLRRKDLHEPDL
>MCE8455995.1 L-2-amino-thiazoline-4-carboxylic acid hydrolase [Rhodovulum sulfidophilum]AB15
MKDLPILERRRIEAMILKHVFDVLSERSGRAEAEAVIGETCSRSAIEQGKALAADLDHPPTLEDFAAILPNWTKEDALSI
EPIETSAEKMDFNVTRCRYAEMYREMGLGEIGHLLSCNRDGDFCIGYNPEIEMTRTQTIMKGASHCDFRYRMKKD
>MCE8455996.1 M20 family metallopeptidase [Rhodovulum sulfidophilum]AB15
MAVENWVAKHIPELVAFRHDMHRNPELLYDLPRTSARVAEALRAAGVDEVHEGIGRTGVVGVIRGQSNVSGRTIGLR
SDM
DALPIHEETGADWASTVPGKMHACGHDGHMTMLLGAARHLAESRAFDGTVIVIFQPAEEGGAGAKAMIDDGLFAR
WPCDE

VYGMHNRPGLPVGQFTIAPGPIMASVDEIRITVSGRGGHAGRPHLSVDPMPAAAAVIQAVQAITARSVDPIDSATISLC
T
IQGGNAFNVIPTEVTLTGTVRALREEVRDHIEARLAETVAGVASGFGATGTLDYIRHYPVTVNHEAQTDLAAAAARVVA
G
DEAVTTDMPPTLGGEDFSFMLNEVPGALINIGNGPSQGLHHPRYDFDDEAIGWGCSYWTTLVRQRLPL
>MCE8455997.1 ABC transporter permease [Rhodovulum sulfidophilum]AB15
MSLFRPQPGPGWRLPSLRVFGALVIREMITRYGRSPGGYIWAVLEPAGMIAILALAFSQFIHTPPLGESFILFYATGYLP
FHAYSEIAGGVSNSVTVNRPLMHFPIVTPLDAVLARFFLSFLTIVIVCVLVFSTLLVITDDPVRLQLGPLFAAIGSAALL
GLGVGTLNAVIFAFVPLWQRVWNIINRPLFILSAVFFTYESLPRNAREILFWNPIVHCVGEARKAFYPVYEGNYVVLGFP
LGVAAFCFLLGGFLLMRNRSAVIDA
>MCE8455998.1 hypothetical protein LZ188_05395 [Rhodovulum sulfidophilum]AB15
MQISSNIDGGQRVTGAVSDKAETRAQTRHRVMRFGFLGTVVLPTLLTAAYLWLFAADQYHSEAAFSVRSEDFSNPLEA
LS
AFTQVGSSSSSDSQILYDFIRSQPLIQKVDASLDLRRIYGKPSHDPVFSLEDDASREDKLDYWRRMVKVAVDPSTGVLSL
QVRAFTPEDAQAVAQEILTESGFMVDDLSRIARQDAIRFALEDVEEAEAMLRDIRRNVRVFRLENDIIDPTEVAESQMG
V
IAALESQLASALVERETIAGFAGQEDQRVKRIDRRIEAVRSQIDQERRRIGKSTSGPTSLVDVIGAYEELLVDLEFAQQA
YTAALASEEQARAEARRTSRYLAVHIPPTLAEDSIYPQRWLLILLVLICSLAAYATVLLVYYNIRDRG
>MCE8455999.1 ABC transporter ATP-binding protein [Rhodovulum sulfidophilum]AB15
MLEFRAVEKAFRLPGGGRRRILSGLTLDLPQKYRVAIIGRNGAGKSTFLRMVAGTLQPDHGEIRRLGRVSWPMGFSGG
FH
PALTGRQNARFVARIYGADTDDLVEEVQAFCDLGPYFDARLQTYSSGMKARLALGVSLATNFDCYLVDEITAVGDAAFR
K
KAQRTFAERLDRVQVIMVSHSEATLREYCNSALFLQDGEGWFFEDLDEGLAAYRKSIAS
>MCE8456000.1 hypothetical protein LZ188_05405, partial [Rhodovulum sulfidophilum]AB15
MSRGPVFGQPRFLAGPQSPTADLYLGFSLAGAIAAGRVRRLAPTVFLAGEALIVMRHLAGLGLLPRYRRLIYLIDDDIPA
GLRDRALPRAYRAKLAALELPALLRCGAAADAVVATR
>MCE8456001.1 uracil-DNA glycosylase, partial [Rhodovulum sulfidophilum]AB15
LKPFVGRAGHLLDLMFARIGLGRGDPAAERALYITNVLPWRPPQNRDPEVGEVAMMLPFLERHVDLVDPDILVLMGN
HSC
GALLGRKGITRLRGRWVEALGRPALPMFHPAYLLRTQLAKRDAWHDLLMLQARLRGG
>MCE8456002.1 LysE family translocator [Rhodovulum sulfidophilum]AB15
MMGPVDLLTLAAFVPAALALNLTPGADMMFCLGQGLRGGPRAALAADLGIALGGFVHVTLAGLGLGAVVAAVPGAF
EAIR
WAGVAYLLWLAWRAVRQGALAPDLPAVSPRRAFREGLTVNLTNPKVILFVLAFLPQFVVVERGAVLAQFLILGAVLSLG
G
LLVNGAVGLFAGQARRLLGGSRAVQRGLGLVSATIFTALALRLALLQKG
>MCE8456003.1 molybdenum cofactor biosynthesis protein B [Rhodovulum sulfidophilum]AB15
MAGIDDSKAFVPVRIAVLTVSDTRSLDEDRSGKTLADRIEAAGHVLADRRILRDERAEIAEQLRAWCADPEIDVVISTGG
TGLTGRDVTVEAHRDVYEKEIEAFGTLFTLVSYGKIGTSAVQSRATGGVAQGTYLFALPGSTGACKDAWDEILVHQLDY
R
HPPCNFVEIFPRLDEHRRRK
>MCE8456004.1 efflux transporter periplasmic adaptor subunit [Rhodovulum sulfidophilum]AB15
MRFLRRSLTGLFLLALTLGLLAYAAATLRSAIDARLAARPKAPPSAERVFSVNVIPLHPRTVTPELTAFGEIRSRRSLEL
RAEASGRVVYLHPAFEEGGRVAAGDLLLRVDPADATSARDLARADLSQSEAELRDAERRLELAHDELAATELQAGLRQK
A
LGRQKDLRGRGVGTDAAVEEAELTAAVADQAVVSRRQALAQAEIGLETATTALARARIALADAERALADTELRAAFSGT
L

IDVSLVEGGLVGANERVADLIDVSALEAGVRVSTAEFSRLLDERGALRPLPVTVTLDVYGANLTARGVLSRESGAVPEDQ
VGRLVYARLEAAPGFRPGDFVTLRVEEPALDDVMVLPASALDAAGELLVLDAGDRLVSVPVTLLRREGDRVIIRAPEFAG
REAVAERSPLLGAGIKVRPLRSGAVEPDADNTTEATVELSPERRAALIAFVETSPALPQEARARIRAQLDRPLVPAQLVE
RLETRMGG
>MCE8456005.1 efflux RND transporter permease subunit [Rhodovulum sulfidophilum]AB15
MPGRVASAAGGLLSYFTRHRTAANLLLLLIVAGLFSAPRMRAQFFPDVIVETVSVSVIWEGAGPEEVDAGIVQLLEPEL
RAVEGVEETTSQSLEGLATITLDFEPGWDMARATNDVQAAVDAETDLPEETEEPEVTRGAWRDRVTDVVITGPVAVD
QLG
RLTDELVDRLFAAGITRTTIRGLAAPETLVEVPSIELMRHDITMAEIAAVLAAEAETAPAGDVSGGNTRVRTGVEKRSAA
SIAGVVLRSYPDGSKLTVGDLAHVRVEGADRERAYFVGPHPAISLRVDRSDRGDAIEMEAGVRAVVAGMTPSLPEGVRI
D
LIQTQADYIAGRLDILLGNGLMGLGLVLGLLFLFLNARTAFWVAAGIPVAMLATAALMFAAGLTINMISLFALIITLGIV
VDDAIVVGEHADFRARHLGEPPVLAAENAARRMAAPVFSATLTTIVAFYALVVIGGRMGHLIADIPFTVIAVLTASLLEC
FLILPNHMVHALAHSAKEHWYDWPSRQFNRGFRRFRQGPFRRLMRLVIRGRYPVMAGCLLLLAVQVAAVIRGDVPW
RFFN
APEERSVSGNFMMLSGAERADTAAMMAELQRAAEAVGARYAARYGTDPVTYALAEMGGNAGRGLAGAEIKDPDLL
GAISL
ELIDADARPYSSFDFVRALQDEVRPHPKVESLSFRSWRAGPGGDALDVEFFGAEARVLKAAAERLKTELSRFGEVSALED
SLAYDKEEAVLDLTAQGAALGFTIEDLGRVLRHRINGIEAATYPEGPRSATIRVELPEAELTADFLDRLQVRSPQGVYLP
LADLVTVERRTGFSSVRRENGLRVVTVTGDLSEDDPARAAEISAELKDAILPGIERDLGVSWRLGGLSEQERDFLADARL
GFGLCLVGIYLVLAWIFSSWSRPLAVMAIVPFGLVGAIWGHAHWDVPLSMFTVVGLIGMTGIIINDSIVLVGTVDEYAE
R
RALVPAIVDAAADRLRPVLLTTLTTVLGLAPLLFETSQQAQFLKPTVITLVYGLGFGVGLVLLVVPSLLAIGQDVGQALR
ALRRALGRRGRRALPVPAVMALALAAVFAATLLPVLATGRLPGILLAVLPPLGAVSAAAAGLILFMAGGGLVALAAWAL
S
RPSRLPGAPDR
>MCE8456006.1 hypothetical protein LZ188_05435 [Rhodovulum sulfidophilum]AB15
MARHLFASRAAALMLAAFAATTTATAPAPVAAGTAARQDGVLQAEILPGWQTAHGTYMAGLHLRLAPGWKTYWRA
PGDAG
IPPSFDWRGSANLAAVKIHWPRPRVFSSNGIRSIGYVADVVLPIELTPADPERPMELRARIDLGVCQDICMPMELRLDRR
LPDAGPAEPIRAALASQPLDARTARVGRVTCEIAPISDGLRLTARIEVPPLGDTETAVFELPDPTIWVSQTELARQGGTL
VARSDMVPADGTPFALDRSALRITLLGQDRAIDIRGCDAAR
>MCE8456007.1 YqgE/AlgH family protein [Rhodovulum sulfidophilum]AB15
MQLGGKLLIAMPGMGDPRFERSVVYLCAHSDEGAMGLIVNKPLSEISFSDLLDQLEIVRSPESQDIRVHFGGPVEHARG
F
VLHSGDYRTNRDTTLRVDSEFGMTATLDILEAIAQGDGPAESLLALGYAGWGPGQLEGEILQNGWLTCDAKPGLVFDG
AD
ALKWERALRAMGIDPLVLSSAAGRA
>MCE8456008.1 threonylcarbamoyl-AMP synthase [Rhodovulum sulfidophilum]AB15
MSAELLRPDAEGLARAATLLAEGRLVALPTETVYGLGADARNDRAVAQIFEAKGRPRFNPLIVHLPDLAAARHLAVFSD
A
ALRLAETFWPGALTLVLPLRPGHGLSPLVSAGLPTVALRVPDHPVAQGVLSAFGGPVAAPSANVSGAVSPTRSDHVIEG
L
GQRIAAVVEGGACPVGVESTILGLSADDGAAPVLLRPGGLPTEAIEACLGQPLAARDESAAISAPGQLGSHYAPGARVV
L
NAATPPAQGLWLGFGPDCPGAALNLSPSGDLVEAAAALFHALRLLDARAAPSEVIAVAPIPEKGLGRAINDRLRRAAAP
R
PGQSAP

>MCE8456009.1 acyl-CoA dehydrogenase [Rhodovulum sulfidophilum]AB15
MAYRAPVAEFRFILEHVAGLPDVVATERFAEATDDVTAAILDGAGRLCDEVLAPLQRPGDLHPARLENGVVRTSPGYAE
G
YRAIAEGGWVALSAPPEHGGMGLPTALSTAVNDMMSGACLSLQLNPLLTQGQIEALCHHASDEIQALYLPRLIAGDWS
GT
MNLTEAQAGSDVGALKTRAVPNGDGTYAVTGQKVYITWGDADFMENVIHLVLARLPDAPEGTRGISLFLVPRNLPDA
DGR
PGVANCVKVVSLEHKLGLHGSPTALMEFDGATGWMVGAPNKGMAGMFTMMNNARLGVGAQGVGVAEAAFQQA
LAYAGERR
QGPTPMGDGPIIGHADVRRMLAGMKAEIFAARAIALSCAVAADMAEASGEPDWAARAAFLTPIIKAWGTEVGMEVA
QQGV
QVHGGMGFIEETGAAQYLRDVRVTAIYEGTNGIQAMDLVGRKLGDGGEAAWRLLEEVEQTAEAAREAFPDLAGDLW
AAAE
ALREATEWMLTQEMTDRFAGAVPFLRAWARVLGAHLHLKAALAEGGEGPRSALARVFIRRMLPEHASALAAARAGA
GDLY
ELSDAALSA
>MCE8456010.1 MBL fold metallo-hydrolase, partial [Rhodovulum sulfidophilum]AB15
MRDGRFAPVRTPFDLPPEEGEAVEIAEGVLWMRLPLPSPLEHVNVFALDEGQGWTLVDTGLSTGRSRGIWAALLAGP
LGG
RPVERVILTHHHPDHVGLAGW
>MCE8456011.1 sigma 54-interacting transcriptional regulator [Rhodovulum sulfidophilum]AB15
MVARGHFVRKADWARFLETGLVPGDLDPDLAASWLRSRDCGVLPDRISAPQVANEILSRTRKETAELRRLARNALLRA
RA
MLKDTGVMMILASPDCVITETEGDRGVIDTGRENRLQIGGDWQETSIGTNAIGTAAASGRPALVHGIQHFCEEIQRWS
CA
AVPILDPVDGTVRGIVDISGQTDMFQPQSMALAMAVGDEITQALARLIGEEREALYQQFMDRRARWQNTHMILIDRR
GAV
LHDTRAVWSNQAIRGGAIGARGEEDWAGEMSDRFSGMDSEILRRDGLPIGMVLTAYRGHRRPVETPSGDPFDAFLGT
DPA
ILKLLEHARCVTASNVPVLISGETGTGKELLARAIHDASSRRHGHYVPVNCGALRADSAAGVLFEPPDRPVDQTGPRGG
G
AVAEAHGGTLCLDEIGEMPEDVQPFLLRVLEDGLTRAGGGAASDRSDFRVIALTHRDLPRDVTENRFRRDLFHRISTIQF
RLPPLRDRAEDIPLLAQHFLAEAAAEAGRQAPALTRAAREALMAYPWPGNARELRNDMQALVLFCGRGVIDTGDLPD
KIR
DPAAPPVPAEPDERTRILEAMAASDGNMSATARRLGIARSTLYTKIAAYGLARPGN
>MCE8456012.1 acetolactate synthase small subunit [Rhodovulum sulfidophilum]AB15
MAQLNIQQGTSKHSAYELRDPNAEVSERHTLAVSVDNEAGVLARVIGLFSGRGYNIDSLTVAEVDHAGHTSRITIVTTGT
PAVIEQIKAQLGRIVPVHEVHDLTVEGPSVERELALFKVASTGEKRVEALRLADIFRANVVDSTLESFVFEITGTPQKVD
AFGELMRPLGLTEMARTGVAALARGT
>MCE8456013.1 acetolactate synthase 3 large subunit [Rhodovulum sulfidophilum]AB15
MTQAMTGAKMVVQALKDQGVDVIFGYPGGAVLPIYDELFQQNDIRHILVRHEQGAVHAAEGYARSTGKPGVVLVTS
GPGA
TNAVTGITDALMDSIPLVVLTGQVPTFLVGNDAFQEADTVGITRPCTKHNWLVKDPDHLAQTIHQGFHVATSGRPGPV
LI
DIPKDVQFAEGTYVGPVKAGHGHYNPKVKGDREMITRLVEAMEIAERPIFYTGGGVVNSGEAATQLLRELVEATGFPIT
S
TLMGLGAYPASGDKWLGMLGMHGLYEANLAMHDCDLMICIGARFDDRITGRVADFSPGSLKAHIDIDPSSINKTIHVD
LP

IIGDVAHVLEDLLRIWKSRGRKVNKEALKPWWAQIDEWKKVDCLRYGTSDTIIKPQYALQRLEALTKARKDRFICTEVGQ
HQMWAAQFLGFEAPKHWMTSGGLGTMGYGLPASVGVQVAHPEALVVNVAGEGSWLMNMQEMGTAVQYHLPV
KQFILNNER
LGMVRQWQTLLHGGRHSHSWSEALPDFVKLAEAFGAKGLRCSDPADLDDAIAEMLAYDGPVIFDCLVEKHEDCFPMI
PSG
RAHNDMLLAADAEAFKEGAALV
>MCE8456014.1 TRAP transporter substrate-binding protein [Rhodovulum sulfidophilum]AB15
MDRRSFLKTSALGGSAAAASATLAAPALAQGRRVLTMVTSWPNGMAGVHDSAMFCKNHIEALSDGQIKVEVKAAGE
LVGA
FEVFDAVSSGQADMYHSSDYYFVNQHPAFAFFSTVPFGMTPQELINWYYHGDGAAFHDEMGEIFGMKMFLVGNTG
AQSGG
WFRKEINAPEDFQGLKFRMPGLGGVALGKLGASIQNIPGGEVYQALSTGALDGTEWVGPWADEKAGFYEITKIYYTAG
FH
EPGAGLTLALNRDLFDSLTPFQQQVMESTAAECTQWTLSMFLSQNSAALARLASEGVRVMSFPDPVWDAFGKASQE
ALEE
SRDGDIYDRMLDSYMASMRTSSSWIQASEGTYRAQRDRVIG
>MCE8456015.1 TRAP transporter substrate-binding protein [Rhodovulum sulfidophilum]AB15
MDRRSFLRSSALGGSAAAAGALAAPAYAQGKRTLTLVTTWGRGLAGVHDAAQYCADQITAVTDGQLTVDLKAAGELV
GAF
EVFDAVTSGQADMYHGVDYYFVGQHPGYQFFASLPFGMTAQELHNWYYHSGGHELHNELGQIFGLKSFLAGNTGPQ
SGGW
FRKEIRSPEDFNGLKFRMPGQGGEILGKLGASVQNLPGSEVYQALSSGAIDGTEWIGPWADEKAGFQEITKIYYTAGFH
E
PGPGLSLAMNLDVFESLTPAQQATVEQVTASANQWAHALFMKNNASALRRLQAGGVQVREFGDDVWDAFGAAAQ
EVFAQY
MGDDLYKKIYDSYIASMRDSAAWIEISEGVFTRQRNRVLG
>MCE8456016.1 TRAP transporter small permease subunit [Rhodovulum sulfidophilum]AB15
MLDGILWVCRNILLAFYNFGYALSHPGLWLDWSDKQAIMRFVYYGGSVEFFFVIFTAFLVLTAIGLWRRRVMWGTVRL
LE
GMANGIGRAAAWFGLLMVLQQIMIVFLQRIFRVSEIGVGPFGYVFTKDLSWWSEELKLYNAAIVALCATYTFVQSGHV
RV
DLVYSEVSFRAKRVIDMLGALFFMMPVAVLIWMYSWFFMWRHLITPKPSASDTLERLLMKARAVRWNVETIGFSPNG
FNG
YFLFKVLLVAFTGLVFLQAIAFFYRSFLEFVEGEESAGKYLDRDSLGAGEEAYEGTH
>MCE8456017.1 TRAP transporter large permease subunit [Rhodovulum sulfidophilum]AB15
MLFGLDGVEIGLIIVILCLFGGILSGFPVAFAIGGAAVISFGIIAALDSAGILVAQAIDTGSAAYDALLAQGIHPDAISH
FRYPDLPTVTTALFPQGWETAMDRNISFIVNRMNERVFAGQSIETLLAVLMFVLMGITLERSKIANDLLTTMARVFGP
MP
GGLAVSVVVVGAFLAASTGIVGATVVTMGLLSLPTMLRNKYSPELATGVIAASGTLGQIIPPSIIIVLLGTLAGDIYATA
QETRAQLAGCPDALTYLGQAAVVSVGTLFQAAILPGVLLALLYAAYAFGYALLNPDKAPPVIDAGARGRRKGSALTWFL
A
APAAVIGLALLLEATGIAGSQDVGVSRYTSTAESASLRTNVSDQCKASMIELHGQSKWDSAVAQQKEIQAGGGRIGTQ
EL
SDEALAEARAAKVAAAPPVSGGILLGFTLLGLALVTARGVVPSARPVPLLIGGAGIVLGLLVDALFIGPLASPGTTLLLL
AVPVALTLYGCRYAVACLSRSDLLRVVFPPLVLIVAVLGSILGGITNPTPAAALGAGGALMLAAYRKLQEEHRTGKLILW
AAYAIIIMLLVGTNFDLRLGRESVPVEDWIAFLVAQGTYHFAMFGILYACWVLLRSGVLGPVVRETAKVTSMVFTILIGS
QLLNLVVISFGGEHYIQQFLRSFDNEMKVFLLVMLVLFILGFVLDFLEIIYIVIPIVGPVIYGGTFDPKWVTIMIAVNLQ
TSFLTPPFGFALFYLRGVAPKEVNTWHIYRGVAPFVVIQVIGLAILWFMPGIVTIVPNLLPNG

>MCE8456018.1 methylmalonyl-CoA mutase, partial [Rhodovulum sulfidophilum]AB15
MTDKTTRWAALAEKELRGKPLDGLTWETPEGIPVKPLYTEADTEGLAHMGSVPGEAPFTRGPKATMYAGRPWTIRQY
AGF
STAEESNAFYRKALAAGQQGVSVAFDLATHRGYDSDHPRVVGDVGKAGVAIDSVEDMKILFDGIPLGEISVSMTMNG
AVI
PILANFIVAGEEQGVERKQLSGTIQNDVLKEFMVRNTYIYPPEPSMRIIADIIEFTSTDMPRFNSISISGYHMQEAGANL
VQELAFTLADGKEYVKTALARGLDVDAFAGRLSFFFAIGTNFFMEIAKLRAARFLWHRIMSEFGPKNPKSLMLRTHCQT
S
GVSLAEQDPYNNVVRTAYEAMSAVLGGTQSLHTNAFDEAIALPTEFSARIARNTQLILQNETKVTKVVDPLAGSYYVEKL
TADLADAAWEIICEIDEMGGMTKAVASGMPKLRIEEAAARWQARVDRGEEVIVGVNKFRLEKEDEIDILDVDNVKVRQ
SQ
VARIEKIRASRDQAACDHALAALEEAARSGEGNLLRLAVEAARARATVGEISMAMEKVFGRHRAEVKTLSGVYGAAYE
GD
EGFAAIQQSVEDFAEEEGRRPRMLVVKMGQDGHDRGAKVIATAFADIGFDVDVGTLFQTPEEAAQDAVDNDVHVIGI
SSL
AAGHKTLAPKLIEALRAQGADDIIVICGGVIPHQDYDFLRQAGVKAIFGPGTNIPAAANDILRLIRER
>MCE8456019.1 DUF4174 domain-containing protein [Rhodovulum sulfidophilum]AB15
MKKHLTIMAWVALMPLAGTADAVSEPEPLARWEADRTVVLEAEGVSLSDFHWMARPVVVFADSPANPQYRTQMQ
LLTERI
EDLAERDVVVIVDTDPDARSELRMKLRPRGFMLVLVGKDGEIKFRKPMPWNVREISRSIDKIPLRQQEVRDRR
>MCE8456020.1 DUF6492 family protein [Rhodovulum sulfidophilum]AB15
MTQLPSKKLDIVTVVFSGELALLRLQARSIAREFHASALGDIFVIINDHEEAACAEAVEAMRGDYGPFAGKLKVVRPDEL
LSDCPTLRARLERFYLTHLRGVGRKLRGAGGGWRGNGGWGMQQALKLMAARLGASPYLLVLDAKNQFVAPVSGSDF
VAGD
GRPRSRVVETEEKQQGWIRASFAKLDLPVPALPAKAPPTVTPVVFARDDLRSALAMIEEKLGPIELFFARRRGKATEFML
VYAALDQGRGLWWELHAEGLPASFSLFSGGSVGRLPELVAAARCGDCKMIGTHSRLLAAMTETVRKDLVEFWRERGLI
RS
DSEIEALFHA
>MCE8456021.1 acetyl/propionyl/methylcrotonyl-CoA carboxylase subunit alpha [Rhodovulum
sulfidophilum]AB15
MFDKILIANRGEIACRVIKSARKMGIKTVAVYSDADRHALHVQMADEAIHIGPPPANQSYIVIDKIMAAIEQSGAQAVH
P
GYGFLSENKLFAEALEKAGVAFIGPPASAIEAMGDKITSKKLAAEAGVSTVPGYMGLIEDAAEAVKISREIGYPVMIKAS
AGGGGKGMRIAWNDQEAREGFQASKNEAANSFGDDRIFIEKFVTQPRHIEIQVLADRHGNCVYLHERECSIQRRNQK
VIE
EAPSPFLDEATRRAMGEQAVALSRAVGYCSAGTVEFIVDGDRNFYFLEMNTRLQVEHPVTELITGVDLVEQMINIAAG
QK
LPFEQKDLKINGWALESRLYAEDPYRNFLPSIGRLTRYRPPAESAHDAAVVRNDTGVFEGGEISMYYDPMIAKLCTWAP
T
RAQAIDEMRTALDAFEVEGIGHNLPFLSAVMDHPKFCAGDITTAFIAEEYPDGFAGVSLPEPELRKVAAAAAAMFRVVE
I
RRARISGRMDNHERMVGGDWVVVAQRESFALSVSADREGATIQFQGGDHHRVTSDWTPGQPLAVLHIDGEPLVLKV
AQSS
HGFVIRHRGAEMKVQVFTPRQAELAALMPEKLPPDTSKFLLCPMPGLIVTIDVEEGQEVQEGQALCVVEAMKMQNTL
RAE
KKGIVAKINAGPGESLAVDEVIMEFE
>MCE8456022.1 hypothetical protein LZ188_05515 [Rhodovulum sulfidophilum]AB15
MPSPKFLAVLAAAVLLAGCNMSNDSQRAVAGGLGGALVADVLDTNVAAGAAIGALGGALCDDAGACR

>MCE8456023.1 hypothetical protein LZ188_05520 [Rhodovulum sulfidophilum]AB15
MLKHKGFPSRLPGTDFQFTIRRANKDGATRLVPRERYADRRPADRRADAGFLAALIEQFGDAPFERGNLDAGRLSWLL
GR
EVLPAEEPFDPESYEALLRVDIDCARTAFPELFAGGPLA
>MCE8456024.1 DUF6497 family protein [Rhodovulum sulfidophilum]AB15
MILRAVLLAGAALVLGASGEVIDVPSGQSVTHLDTITGEPGPAGLTVRFRFLAPDIAQDEGSMPATLAQGDMDYLCAEF
A
LPRLAGTGPEPSQVVITLMDRPVPFGQSEPEATQFFEAYRPEAGRCIWEGF
>MCE8456025.1 hypothetical protein LZ188_05530 [Rhodovulum sulfidophilum]AB15
MKRGGWHMLREKGALTMARQLPVRFDLCASAAFPPASKGRLALQIRQDLWRMLQDLRGFSPVVTVEEEGQGLRVR
AGGRA
AAPFPRSDCERKIAALLTSPSHRARWLAQAGSEPRT
>MCE8456026.1 acyl-CoA carboxylase subunit beta [Rhodovulum sulfidophilum]AB15
MKDILNELERRRSIARLGGGQRRIDSQHAKGKLTARERIELLLDEGSFEEFDMFVAHRCTDFGMEDSRPYGDGVVTGW
GT
INGRMVYVFSQDFTVFGGSLSETHAQKICKIMDMAVQNGAPVIGLNDSGGARIQEGVASLAGYAEVFQRNIEASGVIP
QI
SVIMGPCAGGAVYSPAMTDFIFMVRDSSYMFVTGPDVVKTVTNEVVTAEELGGASTHTKKSSVADGAFEHDVEALYE
VRR
LVDFLPLNNREKPPVRPFFDKPGRVDASLDTLIPDNPNTPYDMKELILKVADEGDFYEIQADFAKNIITGFVRLEGQTVG
VVANQPMVLAGCLDIDSSRKAARFVRFCDCFEIPILTFVDVPGFLPGTGQEYGGVIKHGAKLLFAYGEATVPKVTVITRK
AYGGAYDVMASKHLRGDFNYAWPTAEIAVMGAKGATEILYRSELGDAEKIAGRTKDYEDRFANPFVAAERGFIDEVIQ
PH
STRRRVSRAFASLRGKRRETPWKKHGNIPL
>MCE8456027.1 DMT family protein [Rhodovulum sulfidophilum]AB15
MPALPVPVVTIGLLVLSNLFMTFAWYGHLKYKAAPLVAVILVSWGIAFFEYLLQVPANRIGYGHFSAAQLKTIQEVITLT
VFCAFSVLYLKEPLHWNHALGFGFIALGAFFIFHRW
>MCE8456028.1 multidrug effflux MFS transporter, partial [Rhodovulum sulfidophilum]AB15
MSTHPRVRFLDRTTPPHVITLVALAGLPALTINIFLPSLPNMAVHFDTDYAVMQLSVATYLAANAVMQLFVGPISDRYG
R
RPVLLISTVLFLLATLGCIFAPSAEAFLGFRMAQAVVVTAMVLSRAVVRDMVADTQAASMIGYVTMGMALVPMIGPSI
GG
VLDEIWGWQATFLALLLAGLGVLALTWADLGETSTPRDVSLMDQFRQYPELFRSRRFWGYAMTAALSSGAFFAYLGG
APF
VGSHVFALSPSVLGLYFGIVAVGYMTGNFISGRYSVRVGINGMIYWGALITLAAPLLSMAFFAAGATQPIAFFGAMALL
G
VGNGMVLPNAIAGTLSVRPHLAGTASGLGGAIMIGGGAAFSALAGALLKPGTGAWPLLLIMA
>MCE8456029.1 capsular biosynthesis protein, partial [Rhodovulum sulfidophilum]AB15
MIAYLAGWTRRKDAIFRAIAAAAGGGHRLPLIGLSFRGFPETRARTAEALAVAKRQPKGRLGRALKRALIAAQYNWSRR
Y
FSRHPDRLAVCWNGLTGSRRAFMEGAADAGAGRIFAELAPFPGRVTLDPVGVNARN
>MCE8456030.1 hypothetical protein LZ188_05555 [Rhodovulum sulfidophilum]AB15
MLLGVAVSGLAGAAPAEGVREGVRSEQAIVGASTMRWLVPEPKSKPSRSSKSGRKAAALALDRRLGRGSHICSASGFG
QR
PRCVAR
>MCE8456031.1 beta-mannosidase [Rhodovulum sulfidophilum]AB15
MRPDPAIAAPSLVAPRLAALSLAALSLATAAPAEPVEAPPGEMPFGVYDPDGAFADDPEVSIEHLFLPWEDVYLPSLIDA

DAYARARGRSVLATIEPWTWNRSERNTPDMLQEGIASGDYDRYMASICAVLNLFESPVTVRWAQEMEDDSGQFIWA
GWDP
ATYKAAYRRMVDICRETAPEVAYMWSPLGEEGLEAYYPGDDYVDIVGLSVFGLQPWETAILGEEQSFRSILAPRYARVA
A
FGKPVVVAELGYSGQEDYVAKWTSEVRQIFPEFPNLVAVIYFNQIEVYPWPDGYGLPDWKVGDRIIAP
>MCE8456032.1 glycosyltransferase [Rhodovulum sulfidophilum]AB15
MLGAPEARQGGRPPGAGNGGPFLVSLFTRGQRWRYGLAALAWLGAAIFFWSWWLQPAHDIGAIRFAICSALLGWLT
FLQA
YFLIFLLQARRPVGDPAGLKGARVAMVVTKSPSEPFDILRGTLSAMLAQDWCREWPHDTWLADEDPAPETLAWCAA
HGVQ
VSTRKGRADYHRPAWPRRTRCKEGNLAFFYDSIGYERYDFVVQLDADHVPQPGYLREMLRPFLDPRVGYVSAPSICSSN
A
ADSWSARARLHAEAAFHGVLQAGYSNGWAPLCIGSHYAVRTRALRQIGGLGPELAEDHSTSLLMNAGGWRGVHAID
AIAI
GAGPANFADMLTQEFQWSRSLVTILLRHTPRCLSDLPLRLRLQFLFTQSIYPGLAAFMLAGSLLPAVALVFDMRFVGVTY
PAFFGHSLPILGIFLGIGAMLRRDGYYRPSDAPVLGWERVLFPCAQWPWAAWGMLMALRDRLWGGFVDFRITPKGA
AAEA
RLPLRLLAPYFMLAALALAPVLAWSNVQEAAGFYLLSLLNGAIYVLLVAAVLRHHLTENRIRLRTGWRAYGPHVGILAVL
ALLNAGAVWARGVESLHFLTVGLGRFQVTSAQHVVSGAGQGGRKRLHYVLDFGRSSGARRP
>MCE8456033.1 UDP-glucose 4-epimerase GalE [Rhodovulum sulfidophilum]AB15
MSKPVPVLVTGGAGYIGSHTCRRLSDAGYLPVVFDNLSTGHADAVRWGPLVEGDTRDRAALVRAIRSHAPVAAIHFAA
AA
YVGQSMRDPGFYYDNNVGGMTGLLSACAETGLGRVIFSSSCASYGLPEALPITEATPQAPINPYGRTKLIGEQMLADFG
R
AHGLRHVALRYFNACGADPAAGLSERHDPETHLLPLALFAAAGRGPALKIYGTDYPTPDGTCIRDYIHVVDLAEGHLLAL
RHLERGGGDLQVNLGTGRGQSIREVLAAVERVTGRPVPVSLHPRRPGDPPALVADPGRARAALGFEARHRDLDGILRD
AA
LAFGLEVADAGSA
>MCE8456034.1 hypothetical protein LZ188_05575 [Rhodovulum sulfidophilum]AB15
MVSVIASSKQGIVRSRSASSKILGLRHPNAPDTGVALAGIVDGQPPV
>MCE8456035.1 alpha/beta hydrolase [Rhodovulum sulfidophilum]AB15
MACYEVKGRRNYYMEFGQGRPILLSHGISNSGRAWGPQIPALVAAGYRVIVPDHAGHGASDPLTAPFGVADIADDTE
ELL
TRLGIETLDVVGLSLGGMVALELALRRPTGIGRLVVANSFENTATPEFRAMSEGWASVFEQPHGPVTRLEQNWLVSVS
EA
FRNTPEGMRTYQVWHGIAATSHGPSLAHVARGIAGFDISGRLAELAMPTLFIAGELDRMSPPDLSRRMAEKTPQAKLC
VI
EGAAHISNADSPDDFTARLLRFLSSQPE
>MCE8456036.1 MarR family transcriptional regulator [Rhodovulum sulfidophilum]AB15
MDRARKAAGQWAREKPHLDIGPMVLLGRLGEAALVISRDRLNPVFAEFDLQPGEFDVLATLRRSGAPYAMTPTHLYE
AAM
ISSGSMTNRLNRLEQAGWVKRQPNPEDRRGTLVALTPGGLALIDKAVGSHVANMRTIVSGLTEREQDQLSQLLEKLLAS
Q
GS
>MCE8456037.1 nickel ABC transporter substrate-binding protein [Rhodovulum sulfidophilum]AB15
MLSRRSLLALPFYGACLTTPLTALAAGRSGETVLRFAWPLNAGPLNPHLYSPNQMFAQNMLYDPLVRYRTDGRVVPW
LAR

SWTVSEDGREYSFALREDVVFSNGEIFDAAAAKANFDAVLANRSRHAWLELANQIVAAEVVAPFTLRLRLKDAYYPTLQ
E
LALPRPFRFIAPSQFVDGGTRDGILAPIGTGPWVLTETSLGEHDVFRRNDRYWGARPAFAGVTVKVIPDPNTRAIALRT
G
QIDMVYGIDGPLSPDTFAELARSGDFKAGLSAPMETSSLALNTARGGTRDLSVRRAINHAVDKDALIAAVLHGTEKRAD
T
LFAPNVPYADIGLDPYRFDPAAAAGLLEADGWVATQAGQPRQKDGQPLILDLCFVGTDAVAKAKAEILQADLAAVGIG
VS
LIGEEESSIYARQRDGRFHMIFNRTWGAPYDPHAFLSAMRVPAHADYQAQLGLPDKAEIDRRIGEVLISTDEATRQAMY
R
DLLTRFHEAAVYLPLTHLTAMAVARPGIGPLRFGAMSSEIPFEMLTPDA
>MCE8456038.1 ABC transporter permease subunit, partial [Rhodovulum sulfidophilum]AB15
MKAFILRRLLLLIPMLFGASLVIFLMLRLGPSDPALDYLRLSKLPPTPQALADAREMLGLDRPLATQYLDWIGRALRGDF
GTSYALQRPVLPDILHFLPATMQLAGTALAFTLLVSVPLGIWAARYRDRWPDHAVRLVAFLGVSVPNFWLGFLLVLVFS
V
HLGWLPPMGREGPASLVMPVIAVALMSLAVNARLLRASMLEVAGQRHVRYARLRGLPPRMVERRHVLRNAWLPVIT
ATGM
HIGELLGE
>MCE8456039.1 aldo/keto reductase [Rhodovulum sulfidophilum]AB15
MRYRRLGPSGLFVSELCLGTMTFGGSEGMWGQIGQLGQAEVDSLVSTALEAGINFIDTANVYAGGESERLLGRALKNL
AV
PREEVIVATKVMGPMGEGVNARGASRHHIMDQCKASLTRLGLDHIDLYQIHGFDPATPVLETLEALDTLVRQGHVRYL
GL
SNWAAWQIVRAVGIAEARRLAPILSLQAYYTLAGRDLEREIVPMLKETGIGLMVWSPLAGGLLSGKYDREGKAADGRR
AS
FDFPPVDRDRAHDAIEAMRGMAEARGASVAQIALAWLLHQEVVTSVIIGAKRADQLTDNIGATQVALSEDELARLDAV
TR
LPAEYPGWMLERQGEYRSQS
>MCE8456040.1 GntR family transcriptional regulator [Rhodovulum sulfidophilum]AB15
METPKETRIVEQILDAIADQRLPAGTRLGEQALSDHFSCNRTQVRRALAALTAYQVVEHLPHRGASVATPTAEDARDVF
Q
ARRAVETAICRNALRRATAEDLAALRAHLTEEDRAKDDGDRSEAIRLSRGFHNLLARIAGNAVLARYVQELTMRSSLIIG
LYGTQARTLCAKDEHLSLVEAIAARDEAGALRLMDAHLRHIEAGISFSESRVPAGTLSSMLGGGSRRS
>MCE8456041.1 aspartate/glutamate racemase family protein [Rhodovulum sulfidophilum]AB15
MKLVVINPNSTVSMTEKIVAAARAVAAPGVEIEGRTATGAPASIQGHHDEAMCAPHLLREVREAEEAGADAIVLACFD
DP
ALGACREVATGPVIGICEAGVKAASMIATSFSVVTTLPRSVPVIEDLVRGYGLAHRCRRVRSAAIPVLALEEPGSDARAR
VRDEILRAIEEDGCEAVVLGCAGMADLVAWLSEETGIPVIDGVAVATKFAEALVGAGLRTSKSGAYAQPETH
>MCE8456042.1 NCS1 family transporter [Rhodovulum sulfidophilum]AB15
MTMPETAAAAPGQIPGQVPHVIEEKGLGEESLAPQETRIMDPWSYLFAWLGGCVSIGTFTIGSGLVGTLNLLQTFLAIAI
GCTVIGVALMINGRAGHKYGIPFMVQARSAFGFSGTRLPALVRSVPAIVWFGFQSWIGAGALNLVSETLFDYSNLWAF
FI
GFQFLQILLSVLGFHGIKWLENIGSVFIIGSLIYMFFSVIGKYGDAIMTNLVNVEGTWGAPFWGATMLFLGIYSTMMLN
V
SDYSRELRHGVGPMRQVTIYANSILPATLFMGMIGLMVSGATGQVDPIKVFSSAVDNKLLLVTTLLFIAFAQVTTNILNN
VVPPAYALMDVFKLKFKTSAVLVGLLAFATFPWKLVQDESAAGLNIFIQTYSAFLGPIFAILVVDYYILRRQQLDLGKLY
DAAGPYRGINPAALIATAIGVVAAFAFSNVSWYASLVPAGLSYYLLMLWLPSARRFRDD
>MCE8456043.1 glycine zipper family protein [Rhodovulum sulfidophilum]AB15

MRYLLVVSLAALSLTACAPVQPLSEYKPVVDPAQTSSRKFDRDLGECRQIAIDLQAEYKDRAAKEAGRNILVGVLAGVAV
GAIAGNNTGYQNDYILTGAAAGALGGAGSGDYGRDLVEYGPRRVVDRCMANRGYQILNDVGRG
>MCE8456044.1 class II aldolase/adducin family protein [Rhodovulum sulfidophilum]AB15
MTSMTDEADTLLAQRVESSRQLGGNPELAMHGGGNTSVKVMREGRRVLHVKGSGWDLAVMEPQGMPGLWLDPL
FEVKDGA
RLSDTEMVRFLRSHLLDASAPNPSVETLLHAYLPARFVDHGHASAVLALASRPEAEQRETLRALFGDGLVFLPYVFPGFD
LSIEGARAAEAEPQAWGMWLAQHGLFTWGETAEESLTRFRDAVAACEAHLAARGAALMLPTPRQGNEALEALVPHL
HAAL
PGLAFADPRGADPLAALSGQAGVAEAVSRGTVTPDHVIRIKPWPLILAPGTDAAGIRAALAGFGAAYERYFQDHSDGT
QI
MLDTLPRVAFVPGAGVIGLGRSASEASINADLAEQNLRVAASAEALGGYQPLPRPEQFLIEYWELEQAKLRK
>MCE8456045.1 3-hydroxy-5-phosphonooxypentane-2,4-dione thiolase [Rhodovulum sulfidophilum]AB15
MADLDDIIDGKDFGLTTPPRNEPFFLKGAGAYDWGMQNRLARIFRPDTGRTVMLAFDHGYFQGPTTGLERVDLNIVP
LFE
HADVLMCTRGMLRTTVPASTRKPIVMRASGGNSIMSELSNETIAVDMEDALRMNVAAMAAQVYIGSEFEHKSITNIIN
LI
DRGNRVGMPTLAVTGVGKDMARDARYFGLATRIAAEIGAQYVKSYYVDEGFEKVCAGCPVPIVIAGGKKIPEAEALEVA
Y
KAIDQGAAGVDMGRNIFRAGDPVAMIQAVSAVVHDLLPAAQAFEMYNDLAARKAA
>MCE8456046.1 hypothetical protein LZ188_05645, partial [Rhodovulum sulfidophilum]AB15
LIAAAIGAGLFADFDDATQAMARHVEEITPCPAAQALLDARYRRWCAYRDALGAAWAPPKLPGFDSRETQERTSHG
>MCE8456047.1 SRPBCC family protein [Rhodovulum sulfidophilum]AB15
MILTCQEDISAPIERVFSRLTDFTRFERAIETRGGDVTRHAGPPDTPAPGMAWTIRLHFHGAHREIEAELTDYAPPSAMC
LGAASSGLGAEGRIELAALAEGLTRLSVAIDLGPKTLGGRVLMQTLKLARGKLAEEMEAGLARFARRIEAEAAG
>MCE8456048.1 NAD(+) diphosphatase [Rhodovulum sulfidophilum]AB15
MRLAERVTFGGGGLDRAAHRRGDAAWLAEAWAGAQVLPLWRGKPLFGAEGLGWVASAHPLLAGRSAEAVFLGLSG
GSPKF
AIELSDWVPAEMPETLGLFLDPSEQRHPDLPGDLSFRELRREMTRLVPVEAELAATAKGLFDWHRGHGFCARCGQPSR
PA
EAGWQRRCPGCGAPHFPRTDPVVIMLVRRGNAVLLGRAPGWAEGMFSLLAGFVEPGETVEAAVRREVAEETGVRVG
QVGY
LASQPWPFPASLMLGCWAEAETDRITPDPAEIEEARWVGREDMLAVLAGERTDMQPPRPGAIAGFLIRMWLADRLD
>MCE8456049.1 bifunctional 2',3'-cyclic-nucleotide 2'-phosphodiesterase/3'-nucleotidase [Rhodovulum sulfidophilum]AB15
MIAPHPFSVPPAADRVHLRLLETSDLHVHIFPYDYYTDRPIDTAGLARTATLIAALRRDVANALLFDNGDFLQGNPMGD
Y
IAHERGLRPGEPHPVIAAMNAIGYDAATLGNHDFNYGLDFLLDAIAGARFPVVSANLLPGPLAPPGLTRLPRHALLDRSV
TDGAGRRHRLRIGVIGFLPPQIAIWDRRHLEGRAEGGDIISAARELVPRMRRDGADLVVALAHSGIGEPTHVARQENAC
L
PLARIEGIDAILSGHQHQALPGPDFAGIAGVDTETGSIHGKPAVMPGFWGSHLGVIDLLLERAATGWRVAEHRSSIHAI
A
TRRSDRSAEPLVGSDPAVLSAAARAHAETLAYTRRPVGRTEIPLHSYFALVAENASVKIVADAQRWYVARKLKGTRHEG
L
PLLSAAAPFKAGGHGGPDYYTDVPAGDIAIRNVADLYLYPNMVRAVRISGAELRGWLERSAGIFNRIRPGAADQLLLDP
D
FPCYNFDVIDGLEYRIDPSQPPRYGPHGRLLNPHASRIADLSWNGRAVTDGMEFVIATNSFRAAGGGGFPGTGPDAVIL
E

APVTNRDVILRYIARRGSVAPRTVPTWRFADLPGTTVLFDTGPAARRHLGAVERLAPDPAGTSPDGFARFRISL
>MCE8456050.1 class II aldolase/adducin family protein [Rhodovulum sulfidophilum]AB15
MTDEKDKRAAIVAACRSMNALGLNQGTSGNISVRHGAGLLITPSSIPYDVLAPEDIVFMEMDGTAHGARRPSSEWRFH
LD
ILRSRTDLNAVVHAHAPYCTMLAILHKPIPAVHYMVAAAGGGDIRCAPYATFGTQALSDLVLEALEGRKACLMAHHGM
IA
AEAGLDKAMWLAVEVEALARQYHGALAIGTPPLLSETELAEAIAQFTGGYGQGNPTAT
>MCE8456051.1 Lrp/AsnC family transcriptional regulator [Rhodovulum sulfidophilum]AB15
MALDETDLRLLRVLQKQGRISNAELSERVNLSASACHRRVQRLEEGGFIRDYVALLDARKMGRQTTVFVEITLSGQADE
T
LAAFEKAVARVPDVLECHLMAGNADYLLKVVAEDSEDFARIHRRYLTRLPGVARMQSFFALRTVFKTTALPL
>MCE8456052.1 alanine dehydrogenase [Rhodovulum sulfidophilum]AB15
MHIGCPREIKPQEFRVGLTPDAAREAAAHGHAVLVETGAGLGAGFEDADYIAAGAEIAATAEDVFARAELVVKVKEPLA
E
ERRRLREGQVLFTYLHLAPDPAQTRDLLDSGVTAIAYETVTDDRGGLPLLAPMSEVAGRLAPQMGAWALQKANGGRG
VLM
GGVPGVGPAEVLVIGGGVVGTHAARIAAGMGAHVTVLDKSLPRLRQLDEIFDGRVTTRHSSEATLAALLDRADMIVGA
VL
IPGATAPRLLSRTDLARMKPGAVLVDVAIDQGGCFETSRPTTHEAPVFSVDGIQHYCVANMPGAVPRSATLALGNATM
PF
LLALADKGWRQACTDDPHLLAGLNVHAGQLTCYAVGKAQDIDVLSPQLALTA
>MCE8456053.1 gamma-glutamyltransferase family protein [Rhodovulum sulfidophilum]AB15
MRDFQLPGRSAVFATNGMCATSHPLAAKVAVQMLEAGGNAVDAALAGAVLLGICEPQMTGLGGDCFVLIKPAGEDR
IIAL
NGSGRAPQGLEAARLRERGLDAMPTDSADAVTVPGAVDAFCRLSKDWGRIGLKASLAPAIHYAETGVPVAPRTAFDW
ARA
ADHLKGAARDFYLLDGQAPRPGQVFRAPKQAEVLRRIAMDGRAGFYEGEVAEDMVSSLSALGGHHTLEDFAATACDY
GSP
VSGSYKGVELVEHPPNGQGATAILMANILAQFDLPSMDPLGAERAHVEAEAAKLAYDARNRFVADPAHMTRLDHML
APGT
AAKLAALIDPDRAMASPAPLSEAVHKETIYITVVDRDRMAVSLIYSIFHSFGAGLASTKFGINFQNRGAGFSLTEGHPNE
AAGGKRPMHTIIPAMLKKDGKLIMPFGVMGGAYQPNGHARVLTNIVDYGMHPQQALDAPRSFADQGELLVERGYAE
EVRA
ALAAKGHAVATPPSAIGGAQAILIDDETGVLQGASDPRKDGCALGY
>MCE8456054.1 heat shock protein HspQ [Rhodovulum sulfidophilum]AB15
MLKAHAKYHLGQVVRHRKHPFRGVVFDVDAMFSNTEDWYAAIPEETRPPKDQPFYHLLAENDHSYYVAYVSEQNLVA
DET
GEPVDHPDLSDLFGEFEDGQYPLQFQLN
>MCE8456055.1 AEC family transporter [Rhodovulum sulfidophilum]AB15
MNLLLTVTEIVAPVFLLAAVGFLWVRFGNEYRVQFVTQLTMTLSVPCLIFTALMKTEIEPQALTALSLAAAAAYGAVTIL
CLAVVRLARLDRRTYLSPIIFGNTGNIGLPLALFAFGERGFDYAVVVFAVMAIYSFTFGVWLVAGGGSPLKALKEPLVWA
TALGGLFLWQGWQTPAFLTNALTLIGQIAVPLMLITMGVAIARLHPGHLPRAVWLSLVKLAICLGVAAGIGLWFGLDR
VA
LAVLVLQISTPVAVTSYLLAEKYGADADAVAGLVVVSTLLSMLALPLMLAVLL
>MCE8456056.1 lytic transglycosylase [Rhodovulum sulfidophilum]AB15
MGRFLLIAACLFLTACGDKSPPRNLEDACAIARERPKYMRAMERSERRWGVPVPVQMAVIHQESKFVGNARTPHRFV
LGI

IPMGRQSSAYGYAQAIDGTWDMYRNGTANRGARRDDIFDATDFMGWYMAESRKKLGIPLSDARSQYLAYHEGHAG
YARRS
YRSKAWLMAVANRVEDRAQLYYAQLITCRR
>MCE8456057.1 outer membrane lipoprotein carrier protein LolA, partial [Rhodovulum sulfidophilum]AB15
STLSIKRPGRARFEYAPPEKALVLAGGGQVAVYDDKSNQPPEQYPLKRTPLNLILERNVDLGRARMVVGHEEDGTSTRV
V
AQDPEHPDYGTITLVFTNDPVELRQWIITDEAGSQTTVVLGQLKTGMPLGARMFNIIQETADRMKRR
>MCE8456058.1 tRNA dihydrouridine synthase DusB [Rhodovulum sulfidophilum]AB15
MAGITDLPFRRLVASFGAGLVVSEMVASQEMVQAKPGIRERAALGLGEAATAVQLAGRDPDWMARAARMVEEQGA
RIIDI
NMGCPARKVVGGLSGSALMRDLDRALRLIEAVVKAVSVPVTLKTRLGWDDDLRNAPELARRAEAAGVRMITIHGRTR
CQF
YKGRADWRAIREVREAVSVPVVANGDIVCSETASSALAQSGADGVMVGRGAQGRPWLLAEIGARIAGRPAPAVPEGA
ALA
DMVARHYEDMLGFYGTGLGGRVARKHLGWYMDRAGTPGALRRRVLTAPAGAAVLALLPEALAAEGRAAA
>MCE8456059.1 ATP-binding protein [Rhodovulum sulfidophilum]AB15
MTLDQDAIWTALPIPAFLLDAEDRITNINPASELFVNATIKTLSGVPVFDRLAVDAPLDEAFARVRANRAPLFINDVDVS
AGNKPPVLCNVQVAPMNDTEEHLLLLVTPREIAGRLGRVHSVKSAARSAIGMAEMLAHEIKNPLAGITGAAQLLSMNL
SN
GDLEMTDLIVEESRRIVKLLEQVEQFGNLRPPACKPVNIHDVLDRVRRSATVGYAANMAILEEYDPSLPATWADPDQL
MQ
VFTNLITNAAQAAGPRGGTIRLRTFYDLSLRLRHPDGSGVPVPLQVEITDDGPGLPAEISAEIFEPFVSGRENGTGLGLA
LVSKIIGDHGGWIAVDSVPGRTVFRVSLPVAPKPEEDES
>MCE8456060.1 sigma-54-dependent Fis family transcriptional regulator [Rhodovulum sulfidophilum]AB15
MDGTVLVADDDRTIRTVLTQALTRAGCKVHATSSLTTLMRWVGEGKGDLVISDVIMPDGNGLEMLPKIGQERPGLPVI
VI
SAQNTIMTAIQAAEAEAYDYLPKPFDLPDLMKRAARALETKRRVGRPRETAPPTGGDTQDDLPLVGRTPQMQALYRLV
AR
VMNADLPVLITGESGTGKSLIARAIHDFSDRRSLPFVIATAADMEGIDGPSALVSRTRGGSILFDEVADLSEEAQGRIVR
MLDSFGDNAPRVMATSQSDLMARMEAGQFRHDLFYRLGGVSVAVPSLRERVDDIPLLAEHFLGRTERDGTAPRRLAE
EAM
DLIRAYSWPGNVRQLEHTIRRLVVTSPEEVITRAEVEAVLGNQPEIEPLVGGGDGEKLSASIAKHLRRYFDLHGGVLPPP
GLYARILREVEMPLIEIALDATGGNQAKCADLLGINRNTLRKKITDLDIRVTRRRKLM
>MCE8456061.1 PAS domain-containing sensor histidine kinase [Rhodovulum sulfidophilum]AB15
MTHAKGLLALERLLRLRRSRQAGNAATLSVVVLGPVLAVLTFLTLGPLDQGASSHGLRIVLLADLVYALVVATLVVQRIA
RMVAARRRHSAGSRLHLRLTGVFALIALIPTVSVAIFAGLTINIGLEGWFSERVRNVVGASLAAAEAYEEEHRRDLVSDA
HTLVGYLAAARQATIFLSDGDMRQILTQAQARVQRGLKEAYVIDGSGEIRARGERSYLFNYERPDDAQIARAGTGEAVV
I
QDWDNNEFRALVKLDGYLDRYLYISRTVDGDILSLLDDTQQSVRLYQQLEHERGRVLFEFGLLYLGFAMILILAAVWLGL
WFAERLSRPVGRLAGAAQRVGAGDFDVRVPEETSDDEIAMLGRMFNQMTRQIKLQRDALLDNTRQIERRRRLFDSVL
SSV
TAGVMGLDAEGRVEVMNRSALALLRLDAAEAEGQELCVAVPEFADLLEKLKSSRRGTAQEEVRLLRGGNMEHLLVRM
AMR
KRADGGAEGYVVAFDDVTDLVTAQRMAAWGDVARRIAHEIKNPLTPIQLSAERIKRKFRGAVGDQAEALDQMTDVIV
RQT

NDLRRIVDEFSKFARMPEPDRKPVDLTALLRDAVMLQEAGQPGVAFRADIPPEPTPAELDATMIAQALTNIMKNAGEA
IE
SLREKGEPEGYRPEIRVTFSVEEGNAVILIADNGIGLPQDRSRLFEPYVTTRDHGTGLGLPIVKKIIEEHGGRLDLRDAE
PFAEGAHRGALAEITLPVSLVQAPAQGAAEPARSDTTSATAATGE
>MCE8456062.1 sigma-54 dependent transcriptional regulator [Rhodovulum sulfidophilum]AB15
MGDILIVDDERDIRELVSDILQDEGYTTRLAANSDACMAEINAAEPALMVLDIWLKDSTMDGIDILSRVKRDNPDIPIVI
ISGHGNIEIAVAAIKQGAYDFIEKPFNIDQLLVVIRRAMEASRLRRENQELRRKDVTQFDMIGSSPAFKMLKSNLEKVTR
SNGRVMLRGPSGCGKEVAARYIHANSGRASGPFVVVNSASIEPERMEEVLFGRESSTRGIEKGLLEQAHGGVLYFDEVA
D
MPLGTQSKILRVLVDQQFQRAGGTDKVRVDIRVISSTTRDLTAEIQAGTFREELYHRLNVVPIDVPALDERREDIPELAR
HFIAGFNKTQGLPLRALGEEAEALLQTMSWPGNVRQLKNVIERVLILGEGTDPILATELPMQTDGPAEEGRIVLAGALAT
LPLREARELFEREYLLTQINRFGGNISRTANFVGMERSALHRKLKSLGVVTTARAGGRVAHVEEEEQTAAD
>MCE8456063.1 Trk system potassium transporter TrkA [Rhodovulum sulfidophilum]AB15
MKVIICGAGQVGWQIARHLSGEKNDVTIVDRDADLIRRATDALDVQGIAGHASYPDVLERAGARDADMIIAATQSDEV
NM
VTCQVAHSIFAVPRKIARLRAQSYLTAIYSDLYRRDHMPIDVVISPEREVAEAALQRLAAPAAFDTESFLRGKAQLLGIT
LEEDCPVLRTPLRQLTDLFSTLRALVVGVRREGTLFAPEPGDQLFPEDQIYVFAHVEDLNRTLEIFGKSTKKQERVVIIG
GGNVGLSVAKALEAHEQRIRAKVIEKDRRRAEVAADALERTIVLNGDGLDINLLAEANIKRADAVLAVTDDDKTNLLAAV
RAKMAGCPMAICLVNDPSLVSLMGALEVDAYINPRATTVSSILRHVRHGRVRGVYSIGDAEAEVLEAQVLSTSPMAGK
RV
QDIDFPEGALVGAVMKSGKVILPRGDTRIEEGDMVVIFALSADVPEVERLLQVSIDFF
>MCE8456064.1 TrkH family potassium uptake protein [Rhodovulum sulfidophilum]AB15
MRRLADLPFLVILMGIGALAMFPPVVLALAERDWETARAFFNSGLLFFVLAVLIGLSTINRPVQNQARSHLLAMLGFYTL
LPLMLAVPVSEAVGDTSFLNAYFEMVSCLTTTGATLFDSPERLSPGVHLWRATVGWLGGFFVWVTAIAILAPMNLGGF
EV
TAGTGGIAGRIGSERGEVSPAHDPRERLLRFAHQLAPVYIGLTATLWVLMVVAGEGPLVAACHAMSVLSTSGISPVGGV
A
GGQSGVPGEIMLWGFMVFAISRQMFMFDFRPESWRRLKADLELRMGVGIAWVVTVFLFARHWVGAYEVAAEENG
MAALRA
LWGAIFTVTSYLTTTGFASVEWIDARQWSGLATPGLILVGLAVFGGGVATTAGGVKLLRIYALYKHGIREMEKLVHPSSV
GGAGAFARRIRRQGAQIAFVFFMLFALSVAVVMVTLGLLGLDFEASMVLTVAALTTTGPLAAAATDPPLSYAALGDGP
KM
VLAATMVLGRLETLAIIALFNPEFWRK
>MCE8456065.1 RNA chaperone Hfq [Rhodovulum sulfidophilum]AB15
MAADKQNLQDAFLNHVRKTKVPVTIFLINGVKLQGVITWFDNFCVLLRRDGQSQLVYKHAISTIMPSQPINLYEGED
>MCE8456066.1 hypothetical protein LZ188_05745, partial [Rhodovulum sulfidophilum]AB15
MSSSEAEGSEPRITRAWVLHPDIASDRERRDAGPALEEAVALAGALPDLEVVGAETVPLARPPAGHLFGSGKIDELK
>MCE8456067.1 RIP metalloprotease RseP [Rhodovulum sulfidophilum]AB15
MDIASLIPSFGNAAFTVAAFVVALSVIVTVHEYGHYIVGRWTGIRAEVFSLGFGPVLASRVDRRGTRWQIAALPLGGYVR
FLGDSDGASGKDAAAMAGMDADDRRHTMHGAPLWARVLTVAAGPVFNFVLSIAVFAMLAVVSGVAIEPPTVGKLQ
SLPAA
QSALREGDTILALDGRATPDYSTFYDAVADLPVDGETAYLVRRDGVEQEVAGPYPFPPVVSAIQPKSAAMEVGLKEGDV
V
TAIDGKRIRAFSQLQDLVTASEGAPIDLTVWRDGETLNLSLTPRRMDMPNADGGFETRWLIGMTGGLAFEPATRMAG
PLE
ALSYGADQLGFIVQSSLSGLYHMIAGKISSCNLSGPLGIAESSGAAASQGLESFVWFIAMLSTAVGLLNLFPIPVLDGGH
LVFYAYEAVVGRPPSDKALRVLMAAGFALLIGLMVFALSNDIFCP
>MCE8456068.1 1-deoxy-D-xylulose-5-phosphate reductoisomerase [Rhodovulum sulfidophilum]AB15

MQRRRITVFGATGSIGENTIDLLTRQGGAETYDIVALTGGRNVKRLAEQAIALKADLAVTAFDDCLDDLRAALAGSEVEA
AAGPAALIEAAARPADWFMSAIVGFAGLAPGLRALEHGTTLALANKESLVAAGPLLLGTAAKHGARVLPVDSEHSAVF
QA
LVGEDMAAVERVIITASGGAFRDWPIEDLAGATPEQASSHPNWAMGRRITIDSASMFNKGMELIETKEYFGIAPDKIEA
L
VHPESLIHAMVGYRDGAIMAHVGPHDMRHAIGYALNWPERAALPVERLDLTKIARLTFRPACETRWPALRIAREVME
AGG
MAGAIFNAGKEAALDAFLAREIGFTRMSDVVAEVLDRLSADTALMNSALDLENVLRVDHLAREEAARTVRSMPH
>MCE8456069.1 phosphatidate cytidylyltransferase [Rhodovulum sulfidophilum]AB15
MATIGVLAVWLGGAWFTGLAALVSGTMVWELTRMMVPGASGRLPIQMALVSGATVFVAWLLPPYAAFPLLAIPCVI
GAWQ
LPRDRGIYLAFAMAMLVTGYGLASFRADHGIVWLVWLILVVIATDVAGYFAGRFIGGPKFWPRVSPKKTWSGILGGWF
AA
MLIGLIFLSFTTAGPDLPWISMALSFASQMGDAAESALKRRVGVKDSSSLLPGHGGLFDRFDGLLGASLFMLLVAQLVY
V
PEVRF
>MCE8456070.1 polyprenyl diphosphate synthase [Rhodovulum sulfidophilum]AB15
MDGNGRWAQMRGRPRLFGHQAGARRVREIVEACPDLGIRYLTIFAFSTENWRRTQVEVTGLMTLFRRYIQKETNALL
QKG
VRVRFIGDRDRLDPKLVGMMHALEEITAGNELVNLTVAINYGGRDEVARATKRLAREVAAGRLDPEDVDAETLAHFLD
TC
LLPDPDLVIRTSGEARISNFLLWQSAYAEYEFTPVLWPDFTSAHFAEIVGNYSARERRYGAVLS
>MCE8456071.1 ribosome recycling factor [Rhodovulum sulfidophilum]AB15
MAEDELDIDLDDLTRRMDGAMASLKHEFSSLRTGRASASMLDPVTVEAYGTPTPINQVGTVNVPEPRMVTINVWDKS
MVN
KVEKAIMESGLGINPQTNGTIIMLPIPELNEERRKELTKVAAQYAEQARVAVRNVRRDGMEQLKKAKAAGMGEDDNKI
WH
DEIQTLTDKYIAQVDKALETKQQEIMQV
>MCE8456072.1 UMP kinase [Rhodovulum sulfidophilum]AB15
MSDGSADQPKPAYNRVLLKISGEALMGDQGYGLHPPTVERIAREVKAVRDLGVEICMVIGGGNIFRGLQGSAQGMER
TTA
DYMGMLATVMNALAMQSALEEMGVFCRVVSAIRMDEVAEPYIRRRAVRHLEKKRVIIFAAGTGNPYFTTDTAATLRA
NEM
ACEAIFKGTKVDGVYDKDPVKHDDAVRYDEISYDDCLVKNLKVMDASAIALARDNDLPIIVFSLDEPGGFRGILAGQGTY
TIVHG
>MCE8456073.1 tRNA (adenosine(37)-N6)-dimethylallyltransferase MiaA [Rhodovulum
sulfidophilum]AB15
MSEKRDGFNAQQVGTGATGLPDLDPDRPVLIAGPTASGKSALALAIAEAQGGVIVNADALQVFEGWQVLTARPGPDE
TAR
VPHALYGHVPFTADYSVGHWLRDLAPLLEGPERPIVVGGTGLYFTALTEGLAEIPPTPPEIRAEADRRLASEGHEALLDA
LDADTLARIDRRNPVRVQRAWEVQQATGRGLAAWQDETPPPLLPLGAAQPVVIEAGRDWLNARIEARFERMMAEG
ALEEV
RANLPRWDPALLSARAIGAPDLVAYLRGEMPLAEAVAAAKTASRQYAKRQRSWFRSRLGNWPRWPAG
>MCE8456074.1 AraC family transcriptional regulator [Rhodovulum sulfidophilum]AB15
MSLFSELRLVPFSRLTNGSRWRVEAMRSYSAPQLLWITKGQGRITVGGITRGYGAHNAVFIPAHTMHGFEMTSQVYG
TGI
VFAGLPDIELPEGPHHLRIRDGAVQAELTFLVESLQRELEGKRPEARRAIRHLAGLVSVWLDRQIALDEAGTGRPDPARR
LASGFAALVEKDFRSARSVSDYAAALGVTPTHLTRVCNRTCGRSASELLAERKISEARRLLADTRAPIKEIASALGYASP

AYFTRAFQARAGASPSRFRKGH
>MCE8456075.1 ABC transporter substrate-binding protein [Rhodovulum sulfidophilum]AB15
MTTPSDRPDRPHPAAELYAREYRAGLMDRREFLGRATALGLSAGAAYGLIGRPAPARAQPAPRRGGTLRIQMDCRELS
DP
RTYDWSEKANESRGIVEYLAELKRDGTLRPMLLESWQANGEATEYTLKLRPGVTWNTGEAFTAEDVAANLAGWCDSS
VEG
NSMPSRMAAMVENGQAAEGAIEIVDDLTVRLHLLRPDGTLMFGMTDYPAAVMHRDRIGTSVADHGIGTGAYMFEE
FSVGE
RIVLVRNPEHSYWGDGAWLDRIEFLDYGTDPAAWLAGADAGEFDMTYETVSDFIDIFTALGWVESTVGTSSTVVIRAN
QE
AEIEGIRPYADPRVRRALALAVDNAVPLELGISGRGRESRNNFHVAPGVHPDYAEIAPHRQDIPEALRLMREAGMMEY
EH
ELISIDDDYRKNTTDAVAAQLRDAGFKVRRTVLPGSTFWNDWTRYPFSSTNWSHRETGIQMLNLAYRSTAAWNETGF
RNA
EFDALLDQATAIADDTARREVMARLEQMMVDEGVAIIPYFRSLYRHAREGVLNAGMHPKYEINVHHLGWA
>MCE8456076.1 ABC transporter permease [Rhodovulum sulfidophilum]AB15
MIGFILGRLGAMVLSALCMSFVVFMLTNLAPNLEKLAKTQAGARITDAEVARWLEKNGYAEPMLRRYGEWLGVLPAA
GAT
DAAGKVLGGRCLRGRPDPAPAPRFCGVLQGDWGYSTKFRQPVAGVIAERLKRTGVLMGWAFAVMVPLALAIGVLAG
MREG
SALDRGLSTFSIATTSTPEYVSGVIFVALFASSMVGLKWFTGSAKSAVDGVTFHNFTLPVVTVALYGMGYIARMTRASM
A
EVMTAQYIRTARLKGLSFRAVVLRHALRNALIAPFTVIMLQFPWLLTGVVIVEALFVYPGFGDALVQGAQNNDIDLLLAV
SVVAVIVVLATQLLSDIGYAALNPRIRVA
>MCE8456077.1 hypothetical protein LZ188_05800, partial [Rhodovulum sulfidophilum]AB15
MERLSWTGALGPWLVPALEAALVVLALATLAHPVAALAGGRIAPGSEAARFRPGKAARVTGGLFRLSLGAVAVVILAFL
I
GWMLAPPEAKR
>MCE8456078.1 50S ribosomal protein L33 [Rhodovulum sulfidophilum]AB15
MAKPTTIKIRLNSTAGTGHFYVTKKNARTMTEKMVVKKYDPVVRQHVEYKEGKIK
>MCE8456079.1 tellurite resistance TerB family protein [Rhodovulum sulfidophilum]AB15
MSFVRTMATLAVGFAAARGLETFQKMGGGAGIADSVKSAATRVGIGSQIGPLLERMQVPGGAAALKANAKWLGAKG
RETG
DHALVGLGGLMAALGGAAWAGSVNARDILNAMGEVAPPDVSMEKNARLLIRSMVMAAKSDGEIDAAERAAIFDVL
GDATE
EERAFVEAEMTRPIDIEALAAETGTHQRTAVYAAGVVTTRLDSPAEIAHLDRLAEALHLGRAARQRIHKAMRLPQVLA
>MCE8456080.1 N-acetylmuramoyl-L-alanine amidase [Rhodovulum sulfidophilum]AB15
MVVLHFTAMTDAASALDRLCDPAAGVSAHYLIARDGRVFQLVDEAGRAWHAGAGCWGGVEDVNSRSIGIELDNPG
DRPFS
APLMAALEALLPGILARWSIRPERVIGHSDMAPDRKWDPGPRFDWRRLARQGLAVWPEVRGSAGPAPEETAFRKAA
TAFG
YGAGWETGHELDALRQRFRPRARGPLAAADMAVIADLARRFPVDPGPAGA
>MCE8456081.1 hypothetical protein LZ188_05825 [Rhodovulum sulfidophilum]AB15
MRAVTSAIAVLALAACNTQLPDSAAGEGFDSYADYSTHREGVLQGAVSAEPITAAPSQDVVTEVTGAPISAVESSDGTE
P
PGAQAENHAGISDEQSFAAVTARETIASDRERLEQQREQYTVIPPEPLPPRPGGDGAGIIEYALSTTNPLGQQVYKRGRP
VAPETLARRCAKYGSDDHAQEAFLKNGGPERDRFGIDPDGDGFACYWDPSPFRLAER

>MCE8456082.1 Asp-tRNA(Asn)/Glu-tRNA(Gln) amidotransferase subunit GatA [Rhodovulum sulfidophilum]AB15
MSDLTRLTIAEAREALRKGETTSVALTEAFLAEIEGADALNAFVHKTPEIALEQAKAADARIAAGDAPALCGMPLGIKDL
FCTRGVASQAASKILDGFRPEYESTVSQNLWDAGAVMLGKLNMDEFAMGSSNETSTYGNAVNPWKVDDRQLTPGG
SSGGS
ASAVAADLCMAATGTDTGGSIRQPAAFTGIVGIKPTYGRCSRWGIVAFASSLDQAGPMTRTVRDAAIMLGAMSGHDP
KDS
TSVDIPVPDFEAMLTGDIRGKTIGIPKEYRMEGMPAEIEKLWSDGAEMLRDAGAEIVEISLPHTKYALPAYYVIAPAEAS
SNLARYDGVRYGRRARLGQGDGIVEMYEKTRAEGFGKEVQRRVMIGTYVLSAGFYDAYYNRARKVRALIKRDFDEVFA
GG
VDAILTPATPSAAFGLGEMADADPVAMYLNDVFTVTVNLAGLPGISVPAGLDAQGLPLGLQLIGQPWGEGDLLNTAH
VLE
RAAGFVSKPAKWW
>MCE8456083.1 Asp-tRNA(Asn)/Glu-tRNA(Gln) amidotransferase subunit GatC [Rhodovulum sulfidophilum]AB15
MSIDIDTARRVAHLARIAVQEDDLPALAQELSGILTFMEELNEVDVSGIEPMTGVTPMRLKRRADVVTDGEMQDRILS
NA
PDAREGFFAVPKVVE
>MCE8456084.1 formyltetrahydrofolate deformylase [Rhodovulum sulfidophilum]AB15
MASTDFILTLTCDDQPGIVATVANAVTALGGNIQDSAQFRDPESNLFFMRVAFHTAAPAEAEAVRARLAPAINGYGM
DAE
VVDAARRPKIILMVSKFDHALLHILYQIRVGWMEAEVAAVVSNHEDSRATAEAAGIPYYCWPVTKETKAEQEKQVLAL
VE
ETGAELVVLARYMQVLSDKLSRALEGRVINIHHSFLPSFKGAKPYHQAHERGVKLIGATAHFVTADLDEGPIIEQETERV
NHALSAEDYVAVGRDIESRVLARAVRLHLQRRVLLNGHKTVVFMPG
>MCE8456085.1 hypothetical protein LZ188_05845 [Rhodovulum sulfidophilum]AB15
MDVVEQIRGCRIPLAIIAIEADVPEAEIAAALKGSQALRFLQESRIARYLEGQDQRALACPPRPGHKARHRP
>MCE8456086.1 ceramidase [Rhodovulum sulfidophilum]AB15
MDWTRQIDAYCERTDPAFWSEPVNAATNAAFLLAAVIMWRRTGGLPLGRALAAVLGLIGIGSFLFHTLATEWASMAD
VGP
ILGFILLYIFVANRAYWGLGLWSSLGLTALFIPYAVVTGSLFARLDWLGSSAGYAPVALLILIYALALARRLPQVAAGLG
IGVGLLAISLAFRTLDQPLCAALPLGTHFLWHLLNAAMLGWMIEVYRRHEAGRRSSI
>MCE8456087.1 metal-dependent hydrolase [Rhodovulum sulfidophilum]AB15
MKIVWLGHSGFRIEIEDQVLLIDPWLAGNPLFPEERRAEAIGDATHVLVTHGHFDHAADTIAIARELSIPTVGIFDLMSW
WESREGISVIGFNKGGTVRLGNVSVTMVNATHSSSIQGPDGPVYGGTESGYMIAGEGHVIYVSGDTDVMADMEIFND
LHA
PDIGLLCAGGHFTMDMKRAAYAARTFFNFKTVIPCHYRTFPLLEQSAEALKQDLPGVRVIEPEVLVPIEI
>MCE8456088.1 nucleoside deaminase [Rhodovulum sulfidophilum]AB15
MSPFRSHMDQALAEARAAADRDEVPVGAVVVAADGRVVAAAGNRTRELSDPTAHAEILALRAACSAAGSERLPGFDL
YVT
LEPCAMCASAIGQARIARLYYGAADPKSGGVAHGAQVFRHPQSHHVPEIYDGIGASEAEALLRAFFAGRR
>MCE8456089.1 rRNA pseudouridine synthase [Rhodovulum sulfidophilum]AB15
MDKTGQEGERIAKVLARAGIASRREAERLIEAGRVTVNGRRIASPALNVGPADRIAVDGAAVAAPDRPRLWLYHKPAG
LV
TTARDEKGRETVFDKLPEDMPRVMPVGRLDLNSEGLLLLTNDGAIKRRLELPSTGWLRKYRVRVKGTPEDATLAPLRTG
L
VIAGETFRPMEVTLDRQQGANAWLTVGLREGRNREIRRTMEAVGLVVNRLIRISYGPFQLGDLKPGEIEELRGRVLRDQ
L

GLAAPEGEATAKPKPTRRKLRNRPAEAPEPRPARKPPRGTRPGPASPPRGARRGKAPPG
>MCE8456090.1 AAA family ATPase, partial [Rhodovulum sulfidophilum]AB15
MYVAFFGLAHRPFGPAPCQGSLHWTDPHWHDFERLRLALIDGAPLALVLGRPGTGKTTFLRALLRDETLTDIFRPAFHT
N
PTGDGPTLLRWILSAFGAEAGAADPDRLLARVGTVLAEIRAAGRLPLLAIDEAQGLSAAGLQMLDRVARPRQGSRQGE
GA
ALQIVLAGLPDLEPHLEQSHPPLLGLSRDSTVRLQPMTAAETADYIRSRIVAAEGDADALFDAGALALIHDQAHGLPRLV
NTICDRSLNAAFGAGRPRLDGAFIREVLAEWQDSGDALCLLECPAPSLPRAPQTAPPGARPRPVAQPPPPDAAAVPSIP
P
PPISAPLSASSSV
>MCE8456091.1 STAS/SEC14 domain-containing protein [Rhodovulum sulfidophilum]AB15
MIDITEETAEGLLEIRATGRVTEQDYETILIPALTRALDWGAPVRLLYQVGPDFEGYSAGALWADARLGLKHWRGFGKIA
VVTDSDKIEAGVRMFGFTLPCPVRTFDLDEIDAARLWLRESLGTIHFEEIGADALQVQLIGRLDSASYKTRSPDLDAYAA
AHDRFRLMVDLRRFEGWQGPSALGEHVNLVRHHRHQPYRIAVVGDKSWMKLAETLLPLMLDAKAKYFDEDDYAAG
RAWLL
T
>MCE8456092.1 NAD(P)-dependent oxidoreductase [Rhodovulum sulfidophilum]AB15
MSLAVTGATGLVGRFFVEEALAAGEPVTVLGRTPPPPGFFSAPTGFRPYALGDRPDLSGIDALIHCAFSHLPGRYRGGEG
SDPEGFTRANLDGSVALFETARAAGTGTVLFLSSRAVYGRYPPGTGLTETLTPYPDTLYGKVKAEAERRLFALASDSFTP
VALRATGIYGPAGPGRAHKWQSLFDDFRQGRPISPRRGTELHGDDLAAAARILRDTGARGIFNASDILLDRHDLLALMA
R
AERIERPLPPRSDSPVSAMRCDRLASMGWRPGGIARLHATLPALCRPCPAG
>MCE8456093.1 glycosyl transferase [Rhodovulum sulfidophilum]AB15
MTDGLRPARPAGSPNAYVTLVTNADFALGAKALLRSLKRTDGRASDICVLHTGGVAEADLAPLVDLGARLVETDLLPTS
D
AFNAAHARDRLHRDAPFTKGTKPAFHTPLDNFCKLRLWQLDYERVIFLDADTLVLQPIDRLFDYPEFCAAPNVYESLADF
RRMNSGVFTARPDPATFEAMMTRLDTPGAFWRRTDQTFLQAFFPDWHGLPVFCNMLQYVWFNLPGLWSWPDIRV
LHFQYE
KPWQDHAKADRLKLLIDLWRAHAEGAAIPDTTTLPGPPAP
>MCE8456094.1 NAD-dependent epimerase/dehydratase family protein [Rhodovulum sulfidophilum]AB15
MKIAVLGGDGFCGWPTSLHLSSLGHEIHIIDNLSRRWIDTELGVQSLTPMDSIQERCRIWKETSGEQIHFHLIDLAKEYY
RLRDWLSENRPDAVIHFAEQRAAPYSMISDRHKIYTVDNNINATHNLLVAMVETGLDAHLVHLGTMGVYGYSTVGAPI
PE
GYLDVSIDTPDGSKEMEILYPTRPGSVYHMTKSLDQILFQFYAQNDGVRVTDLHQGIVWGTNTDQTRRHPQLVNRFDY
DG
DYGTVLNRFLAQAAIGYPLTVHGTGGQTRAFIHIQDTVRCIELALKDAPAKGERVKIFNQMTETHRVRDLAELVAKMTG
A
EIAYLPNPRKEAAENELIVKNDNFLGLGLDPITLQEGLLEEVVEIAKKFAHRVDRKRVPCVSAWTRDLASRVERDPEGKR
LKSVS
>MCE8456095.1 DUF882 domain-containing protein [Rhodovulum sulfidophilum]AB15
MTTPRHCLTRRGVLAAFAATAAVAAPTYSNAFGLMRGAGDVRRLRMYSGRTGESIDTVYWIDGEYIGEAVKEINHFM
RDW
RSGSVIPMDLRNFDILAAAHGLMDLQEPYMLLSGYRTAQTNAMLRSRSSGVAKKSLHIKGQAADVRLKSRSVDQIARA
AG
ACHAGGVGRYSRSNFVHMDCGPVRSWGR
>MCE8456096.1 L,D-transpeptidase family protein [Rhodovulum sulfidophilum]AB15

MAAAQDDAIAAFYRDRDYAPFWTSADGEARRRALVTVLQEAPAQGLPAARYDVGRLVETFQDARTQRGIGRAEVAA
MRMY
LDYAHDASRGVLTPGAVVSDIKRKLDRPAPATLIAGLAEASSPLAYLRGLSPQNPEYARLMREKIRLERLIAEGGWGAEI
GAGGKIEPGQSGPRVVALRNRLIAMGYLPRIATRTYDATVQKAVQAFQFDHGLNADGIVGPSTREELDVSAERRLRSVL
V
AMERERWLGNARGDRHVLVNIADFHASIVDHGETVFETRSVVGRNARDYRTPEFSDVMNHMVVNPTWNVPRSIAV
REYLP
KFQNNPNAAGHLMLVDGAGRVVTRAAVDFSQYTASNFPFDLKQPPSNGNALGRVKFMFPNRYNIYLHDTPSKSLFNR
ETR
AFSHGCIRLSDPFDFAYALLAGQTDDPKGLFQSKLRSGRETRVNLDPPLPVHLIYRTAMGKPKGRMQYRRDIYGRDGAI
F
DALQAAGVALPEVQG
>MCE8456097.1 UDP-3-O-(3-hydroxymyristoyl)glucosamine N-acyltransferase [Rhodovulum sulfidophilum]AB15
MSFTIQEIATALGAPAEGDLDFKVTGAAEPTMAAGDQLALAMDPKYADGLAEGGAQAALLWSGADWRAMGLKAAI
FAPRP
RVAMAGLTRMMDPGPEIGPDIHPTAIVDPSAMIGPGAAIGPYAVIGARARIGANARIAAHAVVAEDVRIGDDVLLMY
GVR
ICARVRIGDRFIAQPGAAVGGDGFSFVTPEKSAVEAVRETLGETGSARQQHYTRIHSLGSVTIGDDVEIGANSTIDRGTI
RDTSIGSGTKIDNLVQIGHNCQVGEDCLMCGLVGVAGSARIGNRVVLAGQVGVNDNIFVGDDVIAGGATKIFSNAPA
GRV
LLGYPAMRMETHIEAYKGLRRLPRLFAQVAELQKAVSKLADRD
>MCE8456098.1 phosphopantetheine-binding protein [Rhodovulum sulfidophilum]AB15
MSDSVKDKVIEIIAEQAVLEPSDVKMDSTLEDLGIDSLGLVESIFAIEEAFDIQVPFNANEPTESEFDISSVEAIVKAVE
GLAAEKQA
>MCE8456099.1 beta-ketoacyl-[acyl-carrier-protein] synthase family protein [Rhodovulum sulfidophilum]AB15
MKRVVITGQGTINALGHSVSETLAAFREGRCGIGELDIRDLDRLSIRIGGQVRGYEPEAHFNRQQIALYDRFTQFTLLAA
RQAVAQSGLTFTDDLSEEAGVVLGTSGGGMNTLDENYRSVYEEGKNRVHPFVVPKLMNNAAASHVSMAYNLKGPSF
TVAT
ACASSNHAMGQAFQMIRAGLSRVMVTGGSESMLCFGGIKAWEGLRVMSKDGCRPFSANRNGMVQGEGAAVFVFE
EYEHAK
ARGAEILAEVIGFAMTSDASDIVMPSRQGAARAIAGALRDARLAPGDVGYINAHGTGTAANDKTECAAVADVFGADA
DRL
MMSSTKSMHGHLIGGTGAVELLACIMALREGVIPPTVGYQEPDPECALDVVPNVAREAEVSAVLSNAFAFGGLNAVLA
LR
KA
>MCE8456100.1 chromosome segregation protein SMC, partial [Rhodovulum sulfidophilum]AB15
LERRAGEAREARAVAQARAEAARETVAQAAARIAEDLETTPADLLEKLGDDPDRMPSAEAIEHEVTRLKRQRESLGAVN
L
RAEEDARAVQEEHDTLVAEKSDLDEAIRTLRAGIAGLNREGRERLLTAFEQVNANFALLFTNLFRGGEARLVLVESDDPL
EAGLEIMCQPPGKKLSTLSLLSGGEQTLTALALIFGVFLANPAPICVLDEVDAPLDDANVTRMCDMLDEMTRRTDTRFLI
ITHHALTMSRMDRLFGVTMAEQGVSQLVSVDLKKAEALVA
>MCE8456101.1 YbjN domain-containing protein [Rhodovulum sulfidophilum]AB15
MKRLVHALWALPLAAQAASADVIASDPDSVLKVLRDQGLAATMETDDLGDPKIVSHLTGDSNTEFVVLFYGCTDNVD
CSS
IQFYVAYDMKDPVTALKMNQWNREWLFTKATLDDEGDPGLEMDVILADPGIDRKLFKTTVDIWQQSIDAFEDYIDW
>MCE8456102.1 lytic murein transglycosylase [Rhodovulum sulfidophilum]AB15

MRLSPSLSALCLLSLALASGVSDRALAACGGNFGGFVAGLRDEAVRRGHDAGAARNFFAGVRQDPAVIRADRAQGVF
QRD
FIDFARRLISRSRLDNGQRNAGRYSAVFDEIERRYGVSRGVLLAFWALETDFGAVQGDFNTRDALVTLAHDCRRPELFRP
QIFSALTLYERGDFDPARTTGAWAGEIGQVQMLPADILENGVDGDGDGHVRLKASAPDALMSAGRMLKGLGWRAN
EPWLQ
EIVIPGNLDWAETGLDHGKRVSDWARLGVRPRSGSLGPGNLRASVLLPMGRNGPAFLAYPNFRVFFEWNQSLVYVTT
AAY
LATRFEGAPIFDPRAPDPGLAGAEMKALQRKLVGRGHDVGGVDGILGEKTREAVQKEQIRLGFPADSWPTRALLDRL
>MCE8456103.1 glutathione S-transferase family protein [Rhodovulum sulfidophilum]AB15
MGQLIDGRWDSGWYDTESHGGRFVRSTAGFRNWITADGAPGPEGEGGFRAESGRYHLYVSYACPWAHRALIFRAIK
GLAP
HIGVSVVHPDMLEDGWSFATDFPGATGDGLYGLPFLRDIYIKAKPDVSGRVTVPVLWDRQRETIVSNESSEIIRMFNSA
F
DGITGNRDDYWPEELREGIEAVNARVYDTVNNGVYKAGFATSQDAYDEAVRALFDSLDWLEERLSTRRYLMGERVTE
ADW
RLFTTLLRFDPVYHLHFKCNRNRLIDMPNLWGYARELYQWPGVAETVHFDHIVRHYHYSHDTINPNRIIPINPRIDWTG
P
HGRDRLKAA
>MCE8456104.1 pyridoxal phosphate-dependent aminotransferase [Rhodovulum sulfidophilum]AB15
MNFDEIIDRRGTHSSKWDMMEPIYGVSPEEGLAMWVADMDFRPPACVSQALQSMVDHGIYGYFGDDAAYKAAIG
WWMKSR
HGWEVDPAAIFTTHGLVNATALCIEAFTAPGDGVVLFTPVYHAFARVIRAAGREVVECPLAKRDGRYEMDFAAYDAQ
MTG
REKVVILCSPHNPGGRVWTRDELQAVADFARRHDLVLISDEIHHDLVYPGQRHTVMDLVDDRIRDRLVILSATTKSFNIA
GVHNGNVIIHDPALRAKFAARFAAMGMSANSFGMIMATAAYSPEGAEWIDALMGYLDGNRKLLDEGIAAIPGVSSM
PLEA
TYLSWVDFSGTGMAADEFTRRVEKEAKIAVNHGASFGTGGENFLRFNIATPRARISEAVERLQKAFADLQ
>MCE8456105.1 peptide deformylase [Rhodovulum sulfidophilum]AB15
MSIRPILIHPDPRLKTVADAVPDISDALQKIAEDMLETMYDAPGIGLAAPQIGVLSRLIVMDCAKDEGARPQPYAMFNP
E
VVWASDDKNIHEEGCLSIPETYAEVTRPAEVIVRWTDPSGDPKEDRFSGLWATCVQHEIDHLDGKLFIDYLKPLKRQFIT
RKMVKLKRARSRS
>MCE8456106.1 peptide deformylase [Rhodovulum sulfidophilum]AB15
MSVLPILRWPDPRLATACAPIGQVTPEIARLAQEMLDTMYEAPGRGLAAPQVGVLKRLFVMDVNGAAGPRNPRIFIDP
VI
EERASETMVGPEGCLSLPGIVAEVQRAIEITLRWTDLDGTRRREVLSGISAVCAQHEIDHLDGIVTLNRVDAGQRSELEA
AYGA
>MCE8456107.1 peptide deformylase [Rhodovulum sulfidophilum]AB15
MIRPILPWPDPRLATPAAPVEAVTDEIRAIWADMIDTMEAMPGHGVGLAANQIGVLLRLAVLDCNETRGAAIRLANPE
IV
AVSDDLSAHDEASPCLPGIDATITRPARVTLRFLNEQGQPETREFSGLWATSAQHQLDHLAGRMYFDNLSKLKRDRLLK
K
ARKLGAR
>MCE8456108.1 methionyl-tRNA formyltransferase [Rhodovulum sulfidophilum]AB15
MRVIFMGTPDFSVPVLDALVAAGHEIACVYTQPPRPAGRGRKDRPSPVQARAEELGLMVRHPKSLRAAPEQTAFAAL
DPD
VAVVVAYGLILPREVLEAPCRGCLNIHASLLPRWRGAAPIHRAIMAGDTETGVCIMEMEEGLDTGPVLLRETTAIGPEDT

TGALHDRLSTMGARLVVEALERLRDLPREPQPAEGVTYAAKIDKAEAKVDWTRPAPEVARHINGLSPFPGAWCDVAGERL
KLLRARTVAARMVEDSAAPGKVLDGLTIACGEGAVEIIEAQRQGKRPMSAAEILRGFPLPERLD
>MCE8456109.1 DeoR/GlpR family DNA-binding transcription regulator [Rhodovulum sulfidophilum]AB15
[truncated: 425,657 more chars]
