# Supplementary material for: Taxonomic Re-Evaluation and Genomic Comparison of Novel Extracellular Electron Uptake-Capable Rhodovulum visakhapatnamense and Rhodovulum sulfidophilum Isolates
Source: Microorganisms. 2022 Jun 16;10(6):1235. doi: 10.3390/microorganisms10061235 (PMC9230146; doi:10.3390/microorganisms10061235)
Supplement: Supplementary file 1 [file microorganisms-10-01235-s001.zip › Supplemental_Data_File S1.pdf]

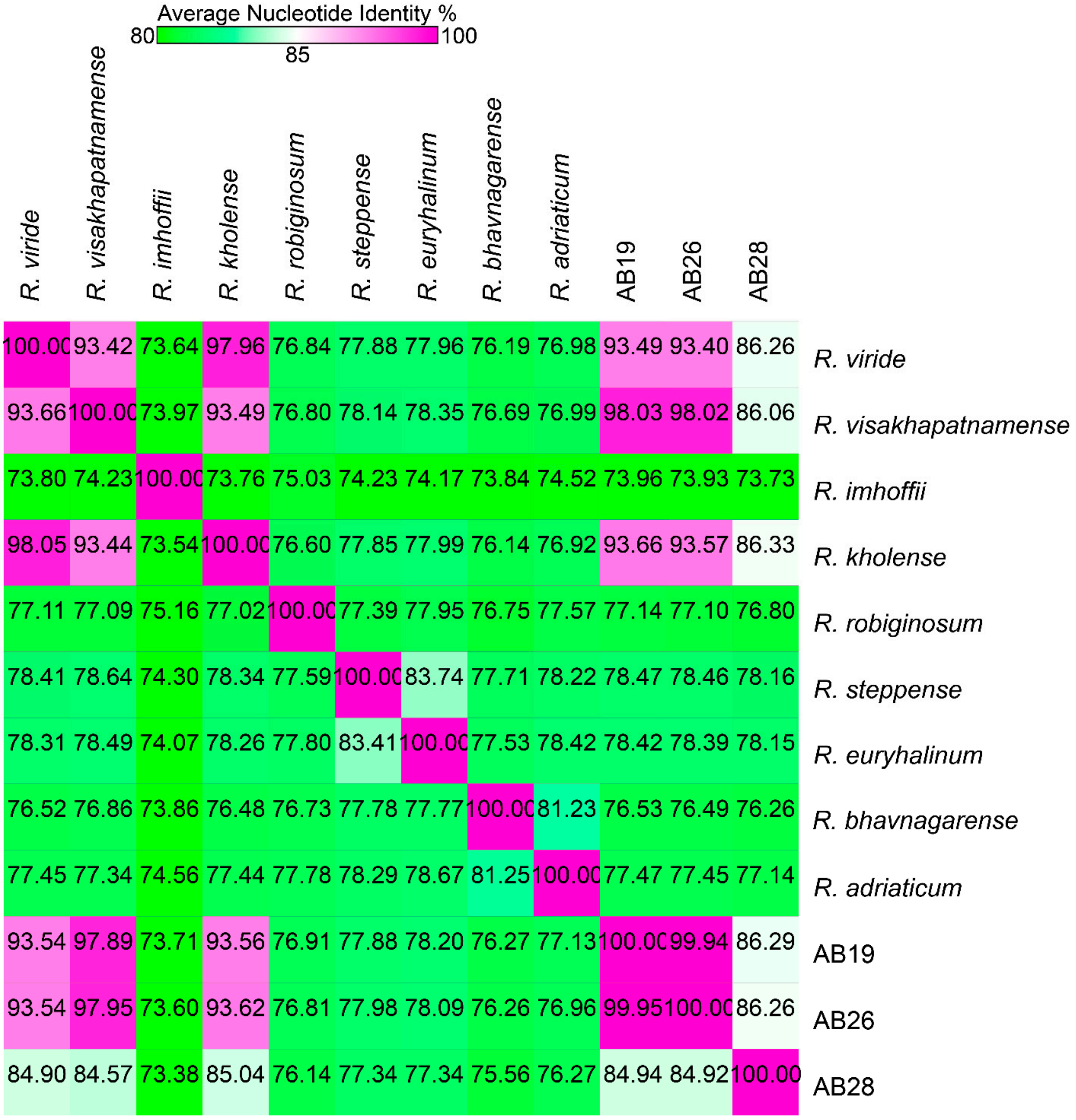

Supplemental Figure S1. Average Nucleotide Identity (ANI) heatmap comparing AB26, AB19, and AB28 to other closely related *Rhodovulum* strains. The heatmap shows AB19 and AB26 share >99% ANI to each other, and 98% ANI to *Rhodovulum visakhapatnamense*. AB28 does not show increased ANI values to any related species, despite the relatively low (~94%) ANI to the other Woods Hole isolates (**Figure 2**). ANI values were calculated using JSpeciesWSd and visualized as a heatmap using Morpheus.

Supplemental Table S1. General Genome Features of Woods Hole isolates and publicly available *Rhodovulum visakhapatnamense* and *Rhodovulum sulfidophilum* genomes from NCBI and IMG/JGI databases.

Strain	Genome size (total bases)	Assembly level	GC%	Genes (total)	CDS (protein coding)	tRNAs	rRNAs	GenBank Accession
**R. visakhapatnamense**								
**AB26**	4,380,746	Scaffold	67.9	4276	4095	43	3	GCA_001941715.1
**AB19**	4401286	Contig	67.8	4547	4214	45	3	GCA_016757185.1
**JA181**	4429538	Scaffold	67.5	4300	4243	45	3	GCA_004365965.1
**R. sulfidophilum**								
**AB14**	4351599	Scaffold	66.8	4155	3972	44	3	GCA_001941695.1
**AB15**	4536182	Contig	66.7	4837	4586	46	3	GCA_021409265.1
**AB16**	4406360	Contig	66.7	4442	4237	40	3	GCA_016757145.1
**AB17**	4527006	Contig	66.7	4731	4511	40	3	GCA_021409205.1
**AB18**	4349103	Contig	66.6	4315	4078	44	3	GCA_021409185.1
**AB20**	4234138	Contig	66.8	4637	4473	45	3	GCA_016757195.1
**AB21**	4073787	Contig	66.6	5611	5365	34	3	GCA_021409225.1
**AB22**	4569598	Contig	67	4454	4255	44	3	GCA_016757115.1
**AB23**	4454817	Contig	66.8	4330	4147	45	3	GCA_016757055.1
**AB28**	5027343	Contig	66.9	5450	5199	49	3	GCA_021409165.1
**AB30**	4256231	Contig	67	4095	3903	43	3	GCA_001941745.1
**AB33**	4429719	Scaffold	66.4	4440	4111	48	3	GCA_021568715.1
**AB35**	4642509	Contig	66.9	4501	4286	46	3	GCA_016757045.1
**DSM1374**	4347929	Complete	66.9	4143	3944	52	3	GCA_001633165.1
**DSM2351**	4732772	Complete	66.9	4376	4146	50	3	GCA_001548075.1
**IM796**	3962372	Scaffold	67.3	3747	3574	43	3	GCA_016653205.1
**SE1**	4310535	Contig	67.1	4137	3954	44	3	GCA_010119435.1
**SNK001**	4196493	Complete	66.8	4027	3849	52	3	GCA_001633145.1

Fasta Input Sequences for the 16S Phylogenetic Tree of Woods Hole Isolates

>dsm2351
CAACTTGAGAGTTTGATCCTGGCTCAGAACGAACGCTGGCGGCAGGCCTAACACA
TGCAAGTCGAGCGAACCCTTCGGGG
TTAGCGGCGGACGGGTGAGTAACGCGTGGGAACGTGCCCTTCTCTGCGGAATAG
GCTCGGGAAACTGGGTTTAATACCGC
ATACGCCCTTCGGGGGAAAGATTTATCGGAGAAGGATCGGCCCGCGTTAGATTAG
GTAGTTGGTGGGGTAATGGCCTACC
AAGCCTACGATCTATAGCTGGTTTGAGAGGATGATCAGCCACACTGGGACTGAGA
CACGGCCCAGACTCCTACGGGAGGC
AGCAGTGAGGAATCTTGGACAATGGGGGAAACCCTGATCCAGCCATGCCGCGTGA
GCGATGAAGGCCTTAGGGTTGTAAA
GCTCTTTCAGTCGTGAAGATAATGACGGTAGCGACAGAAGAAGCCCCGGCTAACT
CCGTGCCAGCAGCCGCGGTAATACG
GAGGGGGCTAGCGTTGTTCGGAATTACTGGGCGTAAAGCGCGCGTAGGCGGACT
ATTAAGTCAGGGGTGAAATCCCGGGG
CTCAACCCCGGAACTGCCTTTGATACTGGTAGTCTAGAGTTCGAGAGAGGTGAGT
GGAATTCCGAGTGTAGAGGTGAAAT
TCGTAGATATTCGGAGGAACACCAGTGGCGAAGGCGGCTCACTGGCTCGATACTG
ACGCTGAGGTGCGAAAGCGTGGGGA
GCAAACAGGATTAGATACCCTGGTAGTCCACGCCGTAAACGATGAATGCCAGTCG
TCGGCAAGCTTGCTTGTCGGTGACA
CACCTAACGGATTAAGCATTCCGCCTGGGGAGTACGGCCGCAAGGTTAAAACTCA
AAGGAATTGACGGGGGCCCGCACAA
GCGGTGGAGCATGTGGTTTAATTCGAAGCAACGCGCAGAACCTTACCAACCCTTG
ACATCCTGATCGCGGTTTTCCGAGA
GGAATTCCTTCAGTTCGGCTGGATCAGTGACAGGTGCTGCATGGCTGTCGTCAGC
TCGTGTCGTGAGATGTTCGGTTAAG
TCCGGCAACGAGCGCAACCCACACTCTTAGTTGCCAGCATTCAGTTGGGCACTCT
AGGAGAACTGCCGATGATAAGTCGG
AGGAAGGTGTGGATGACGTCAAGTCCTCATGGCCCTTACGGGTTGGGCTACACAC
GTGCTACAATGGCAGTGACAATGGG
TTAATCCCTAAAAACTGTCTCAGTTCGGATTGTTCTCTGCAACTCGAGAGCATGAA
GTCGGAATCGCTAGTAATCGCGTA
ACAGCATGACGCGGTGAATACGTTCCCGGGCCTTGTACACACCGCCCGTCACACC
ATGGGAGTTGGGTTTACCCGAAGAC
GGTGCGCCAACCCTTACGGGGGGCAGCTGGCCACGGTAAGCTCAGCGACTGGGG
TGAAGTCGTAACAAGGTAGCCGTAGG
GGAACCTGCGGCTGGATCACCTCCTTTCTAA
>dsm1374
CAACTTGAGAGTTTGATCCTGGCTCAGAACGAACGCTGGCGGCAGGCCTAACACA
TGCAAGTCGAGCGAACCCTTCGGGG
TTAGCGGCGGACGGGTGAGTAACGCGTGGGAACGTGCCCTTCTCTGCGGAATAG
GCTCGGGAAACTGGGTTTAATACCGC

ATACGCCCTTCGGGGGAAAGATTTATCGGAGAAGGATCGGCCCGCGTTAGATTAG
GTAGTTGGTGGGGTAATGGCCTACC
AAGCCTACGATCTATAGCTGGTTTGAGAGGATGATCAGCCACACTGGGACTGAGA
CACGGCCCAGACTCCTACGGGAGGC
AGCAGTGAGGAATCTTGGACAATGGGGGAAACCCTGATCCAGCCATGCCGCGTGA
GCGATGAAGGCCTTAGGGTTGTAAA
GCTCTTTCAGTCGTGAAGATAATGACGGTAGCGACAGAAGAAGCCCCGGCTAACT
CCGTGCCAGCAGCCGCGGTAATACG
GAGGGGGCTAGCGTTGTTCGGAATTACTGGGCGTAAAGCGCGCGTAGGCGGACT
ATTAAGTCAGGGGTGAAATCCCGGGG
CTCAACCCCGGAACTGCCTTTGATACTGGTAGTCTAGAGTTCGAGAGAGGTGAGT
GGAATTCCGAGTGTAGAGGTGAAAT
TCGTAGATATTCGGAGGAACACCAGTGGCGAAGGCGGCTCACTGGCTCGATACTG
ACGCTGAGGTGCGAAAGCGTGGGGA
GCAAACAGGATTAGATACCCTGGTAGTCCACGCCGTAAACGATGAATGCCAGTCG
TCGGCAAGCATGCTTGTCGGTGACA
CACCTAACGGATTAAGCATTCCGCCTGGGGAGTACGGCCGCAAGGTTAAAACTCA
AAGGAATTGACGGGGGCCCGCACAA
GCGGTGGAGCATGTGGTTTAATTCGAAGCAACGCGCAGAACCTTACCAACCCTTG
ACATCCTGATCGCGGTTTTCCGAGA
GGAATTCCTTCAGTTCGGCTGGATCAGTGACAGGTGCTGCATGGCTGTCGTCAGC
TCGTGTCGTGAGATGTTCGGTTAAG
TCCGGCAACGAGCGCAACCCACACTCTTAGTTGCCAGCATTCAGTTGGGCACTCT
AGGAGAACTGCCGATGATAAGTCGG
AGGAAGGTGTGGATGACGTCAAGTCCTCATGGCCCTTACGGGTTGGGCTACACAC
GTGCTACAATGGCAGTGACAATGGG
TTAATCCCTAAAAGCTGTCTCAGTTCGGATTGTTCTCTGCAACTCGAGAGCATGAA
GTCGGAATCGCTAGTAATCGCGTA
ACAGCATGACGCGGTGAATACGTTCCCGGGCCTTGTACACACCGCCCGTCACACC
ATGGGAGTTGGGTTTACCCGAAGAC
GGTGCGCCAACCCTTACGGGGGGCAGCTGGCCACGGTAAGCTCAGCGACTGGGG
TGAAGTCGTAACAAGGTAGCCGTAGG
GGAACCTGCGGCTGGATCACCTCCTTT
>SNK001
CAACTTGAGAGTTTGATCCTGGCTCAGAACGAACGCTGGCGGCAGGCCTAACACA
TGCAAGTCGAGCGAACCCTTCGGGG
TTAGCGGCGGACGGGTGAGTAACGCGTGGGAACGTGCCCTTCTCTGCGGAATAG
GCTCGGGAAACTGGGTTTAATACCGC
ATACGCCCTTCGGGGGAAAGATTTATCGGAGAAGGATCGGCCCGCGTTAGATTAG
GTAGTTGGTGGGGTAATGGCCTACC
AAGCCTACGATCTATAGCTGGTTTGAGAGGATGATCAGCCACACTGGGACTGAGA
CACGGCCCAGACTCCTACGGGAGGC
AGCAGTGAGGAATCTTGGACAATGGGGGAAACCCTGATCCAGCCATGCCGCGTGA
GCGATGAAGGCCTTAGGGTTGTAAA
GCTCTTTCAGTCGTGAAGATAATGACGGTAGCGACAGAAGAAGCCCCGGCTAACT
CCGTGCCAGCAGCCGCGGTAATACG

GAGGGGGCTAGCGTTGTTCGGAATTACTGGGCGTAAAGCGCGCGTAGGCGGACT
ATTAAGTCAGGGGTGAAATCCCGGGG
CTCAACCCCGGAACTGCCTTTGATACTGGTAGTCTAGAGTTCGAGAGAGGTGAGT
GGAATTCCGAGTGTAGAGGTGAAAT
TCGTAGATATTCGGAGGAACACCAGTGGCGAAGGCGGCTCACTGGCTCGATACTG
ACGCTGAGGTGCGAAAGCGTGGGGA
GCAAACAGGATTAGATACCCTGGTAGTCCACGCCGTAAACGATGAATGCCAGTCG
TCGGCAAGCATGCTTGTCGGTGACA
CACCTAACGGATTAAGCATTCCGCCTGGGGAGTACGGCCGCAAGGTTAAAACTCA
AAGGAATTGACGGGGGCCCGCACAA
GCGGTGGAGCATGTGGTTTAATTCGAAGCAACGCGCAGAACCTTACCAACCCTTG
ACATCCTGATCGCGGTTTTCCGAGA
GGAATTCCTTCAGTTCGGCTGGATCAGTGACAGGTGCTGCATGGCTGTCGTCAGC
TCGTGTCGTGAGATGTTCGGTTAAG
TCCGGCAACGAGCGCAACCCACACTCTTAGTTGCCAGCATTCAGTTGGGCACTCT
AGGAGAACTGCCGATGATAAGTCGG
AGGAAGGTGTGGATGACGTCAAGTCCTCATGGCCCTTACGGGTTGGGCTACACAC
GTGCTACAATGGCAGTGACAATGGG
TTAATCCCTAAAAGCTGTCTCAGTTCGGATTGTTCTCTGCAACTCGAGAGCATGAA
GTCGGAATCGCTAGTAATCGCGTA
ACAGCATGACGCGGTGAATACGTTCCCGGGCCTTGTACACACCGCCCGTCACACC
ATGGGAGTTGGGTTTACCCGAAGAC
GGTGCGCCAACCCTTACGGGGGGCAGCTGGCCACGGTAAGCTCAGCGACTGGGG
TGAAGTCGTAACAAGGTAGCCGTAGG
GGAACCTGCGGCTGGATCACCTCCTTT
>AB14
CAACTTGAGAGTTTGATCCTGGCTCAGAACGAACGCTGGCGGCAGGCCTAACACA
TGCAAGTCGAGCGAACCCTTCGGGG
TTAGCGGCGGACGGGTGAGTAACGCGTGGGAACGTGCCCTTCTCTGCGGAATAG
GCTCGGGAAACTGGGTTTAATACCGC
ATACGCCCTTCGGGGGAAAGATTTATCGGAGAAGGATCGGCCCGCGTTAGATTAG
GTAGTTGGTGGGGTAATGGCCTACC
AAGCCTACGATCTATAGCTGGTTTGAGAGGATGATCAGCCACACTGGGACTGAGA
CACGGCCCAGACTCCTACGGGAGGC
AGCAGTGAGGAATCTTGGACAATGGGGGAAACCCTGATCCAGCCATGCCGCGTGA
GCGATGAAGGCCTTAGGGTTGTAAA
GCTCTTTCAGTCGTGAAGATAATGACGGTAGCGACAGAAGAAGCCCCGGCTAACT
CCGTGCCAGCAGCCGCGGTAATACG
GAGGGGGCTAGCGTTGTTCGGAATTACTGGGCGTAAAGCGCGCGTAGGCGGACT
ATTAAGTCAGGGGTGAAATCCCGGGG
CTCAACCCCGGAACTGCCTTTGATACTGGTAGTCTAGAGTTCGAGAGAGGTGAGT
GGAATTCCGAGTGTAGAGGTGAAAT
TCGTAGATATTCGGAGGAACACCAGTGGCGAAGGCGGCTCACTGGCTCGATACTG
ACGCTGAGGTGCGAAAGCGTGGGGA
GCAAACAGGATTAGATACCCTGGTAGTCCACGCCGTAAACGATGAATGCCAGTCG
TCGGCAAGCATGCTTGTCGGTGACA

CACCTAACGGATTAAGCATTCCGCCTGGGGAGTACGGCCGCAAGGTTAAAACTCA
AAGGAATTGACGGGGGCCCGCACAA
GCGGTGGAGCATGTGGTTTAATTCGAAGCAACGCGCAGAACCTTACCAACCCTTG
ACATCCTGATCGCGGTTTTCCGAGA
GGAATTCCTTCAGTTCGGCTGGATCAGTGACAGGTGCTGCATGGCTGTCGTCAGC
TCGTGTCGTGAGATGTTCGGTTAAG
TCCGGCAACGAGCGCAACCCACACTCTTAGTTGCCAGCATTCAGTTGGGCACTCT
AGGAGAACTGCCGATGATAAGTCGG
AGGAAGGTGTGGATGACGTCAAGTCCTCATGGCCCTTACGGGTTGGGCTACACAC
GTGCTACAATGGCAGTGACAATGGG
TTAATCCCTAAAAACTGTCTCAGTTCGGATTGTTCTCTGCAACTCGAGAGCATGAA
GTCGGAATCGCTAGTAATCGCGTA
ACAGCATGACGCGGTGAATACGTTCCCGGGCCTTGTACACACCGCCCGTCACACC
ATGGGAGTTGGGTTTACCCGAAGAC
GGTGCGCCAACCCTTACGGGGGGCAGCTGGCCACGGTAAGCTCAGCGACTGGGG
TGAAGTCGTAACAAGGTAGCCGTAGG
GGAACCTGCGGCTGGATCACCTCCTTT
>AB26
CAACTTGAGAGTTTGATCCTGGCTCAGAACGAACGCTGGCGGCAGGCCTAACACA
TGCAAGTCGAGCGAACCCTTCGGGG
TTAGCGGCGGACGGGTGAGTAACGCGTGGGAACGTGCCCTTCTCTGCGGAATAG
GCTCGGGAAACTGGGTTTAATACCGC
ATACGCCCTTCGGGGGAAAGATTTATCGGAGAAGGATCGGCCCGCGTCTGATTAG
GTAGTTGGTGGGGTAATGGCCTACC
AAGCCTACGATCAGTAGCTGGTTTGAGAGGATGATCAGCCACACTGGGACTGAGA
CACGGCCCAGACTCCTACGGGAGGC
AGCAGTGAGGAATCTTGGACAATGGGGGAAACCCTGATCCAGCCATGCCGCGTGA
GCGATGAAGGCCTTAGGGTTGTAAA
GCTCTTTCAGTCGTGAAGATAATGACGGTAGCGACAGAAGAAGCCCCGGCTAACT
CCGTGCCAGCAGCCGCGGTAATACG
GAGGGGGCTAGCGTTGTTCGGAATTACTGGGCGTAAAGCGCGCGTAGGCGGACT
ATTAAGTCGGGGGTGAAATCCCGGGG
CTCAACCCCGGAACTGCCTCCGATACTGGTAGTCTAGAGTTCGAGAGAGGTGAGT
GGAATTCCGAGTGTAGAGGTGAAAT
TCGTAGATATTCGGAGGAACACCAGTGGCGAAGGCGGCTCACTGGCTCGATACTG
ACGCTGAGGTGCGAAAGCGTGGGGA
GCAAACAGGATTAGATACCCTGGTAGTCCACGCCGTAAACGATGAATGCCAGTCG
TCGGCAAGCATGCTTGTCGGTGACA
CACCTAACGGATTAAGCATTCCGCCTGGGGAGTACGGCCGCAAGGTTAAAACTCA
AAGGAATTGACGGGGGCCCGCACAA
GCGGTGGAGCATGTGGTTTAATTCGAAGCAACGCGCAGAACCTTACCAACCCTTG
ACATCCTGATCGCGGTTTTCCGAGA
GGAATTCCTTCAGTTCGGCTGGATCAGTGACAGGTGCTGCATGGCTGTCGTCAGC
TCGTGTCGTGAGATGTTCGGTTAAG
TCCGGCAACGAGCGCAACCCACACTCTTAGTTGCCAGCATTCAGTTGGGCACTCT
AAGAGAACTGCCGATGATAAGTCGG

AGGAAGGTGTGGATGACGTCAAGTCCTCATGGCCCTTACGGGTTGGGCTACACAC
GTGCTACAATGGCAGTGACAATGGG
TTAATCCCAAAAAACTGTCTCAGTTCGGATTGTTCTCTGCAACTCGAGAGCATGAA
GTCGGAATCGCTAGTAATCGCGTA
ACAGCATGACGCGGTGAATACGTTCCCGGGCCTTGTACACACCGCCCGTCACACC
ATGGGAGTTGGGTTTACCCGAAGAC
GGTGCGCCAACCCTTACGGGGGGCAGCTGGCCACGGTAAGCTCAGCGACTGGGG
TGAAGTCGTAACAAGGTAGCCGTAGG
GGAACCTGCGGCTGGATCACCTCCTTT
>AB30
CAACTTGAGAGTTTGATCCTGGCTCAGAACGAACGCTGGCGGCAGGCCTAACACA
TGCAAGTCGAGCGAACCCTTCGGGG
TTAGCGGCGGACGGGTGAGTAACGCGTGGGAACGTGCCCTTCTCTGCGGAATAG
GCTCGGGAAACTGGGTTTAATACCGC
ATACGCCCTTCGGGGGAAAGATTTATCGGAGAAGGATCGGCCCGCGTTAGATTAG
GTAGTTGGTGGGGTAATGGCCTACC
AAGCCTACGATCTATAGCTGGTTTGAGAGGATGATCAGCCACACTGGGACTGAGA
CACGGCCCAGACTCCTACGGGAGGC
AGCAGTGAGGAATCTTGGACAATGGGGGAAACCCTGATCCAGCCATGCCGCGTGA
GCGATGAAGGCCTTAGGGTTGTAAA
GCTCTTTCAGTCGTGAAGATAATGACGGTAGCGACAGAAGAAGCCCCGGCTAACT
CCGTGCCAGCAGCCGCGGTAATACG
GAGGGGGCTAGCGTTGTTCGGAATTACTGGGCGTAAAGCGCGCGTAGGCGGACT
ATTAAGTCAGGGGTGAAATCCCGGGG
CTCAACCCCGGAACTGCCTTTGATACTGGTAGTCTAGAGTTCGAGAGAGGTGAGT
GGAATTCCGAGTGTAGAGGTGAAAT
TCGTAGATATTCGGAGGAACACCAGTGGCGAAGGCGGCTCACTGGCTCGATACTG
ACGCTGAGGTGCGAAAGCGTGGGGA
GCAAACAGGATTAGATACCCTGGTAGTCCACGCCGTAAACGATGAATGCCAGTCG
TCGGCAAGCATGCTTGTCGGTGACA
CACCTAACGGATTAAGCATTCCGCCTGGGGAGTACGGCCGCAAGGTTAAAACTCA
AAGGAATTGACGGGGGCCCGCACAA
GCGGTGGAGCATGTGGTTTAATTCGAAGCAACGCGCAGAACCTTACCAACCCTTG
ACATCCTGATCGCGGTTTTCCGAGA
GGAATTCCTTCAGTTCGGCTGGATCAGTGACAGGTGCTGCATGGCTGTCGTCAGC
TCGTGTCGTGAGATGTTCGGTTAAG
TCCGGCAACGAGCGCAACCCACACTCTTAGTTGCCAGCATTCAGTTGGGCACTCT
AGGAGAACTGCCGATGATAAGTCGG
AGGAAGGTGTGGATGACGTCAAGTCCTCATGGCCCTTACGGGTTGGGCTACACAC
GTGCTACAATGGCAGTGACAATGGG
TTAATCCCTAAAAACTGTCTCAGTTCGGATTGTTCTCTGCAACTCGAGAGCATGAA
GTCGGAATCGCTAGTAATCGCGTA
ACAGCATGACGCGGTGAATACGTTCCCGGGCCTTGTACACACCGCCCGTCACACC
ATGGGAGTTGGGTTTACCCGAAGAC
GGTGCGCCAACCCTTACGGGGGGCAGCTGGCCACGGTAAGCTCAGCGACTGGGG
TGAAGTCGTAAC

>se1
CAACTTGAGAGTTTGATCCTGGCTCAGAACGAACGCTGGCGGCAGGCCTAACACA
TGCAAGTCGAGCGAACCCTTCGGGG
TTAGCGGCGGACGGGTGAGTAACGCGTGGGAACGTGCCCTTCTCTGCGGAATAG
GCTCGGGAAACTGGGTTTAATACCGC
ATACGCCCTTCGGGGGAAAGATTTATCGGAGAAGGATCGGCCCGCGTTAGATTAG
GTAGTTGGTGGGGTAATGGCCTACC
AAGCCTACGATCTATAGCTGGTTTGAGAGGATGATCAGCCACACTGGGACTGAGA
CACGGCCCAGACTCCTACGGGAGGC
AGCAGTGAGGAATCTTGGACAATGGGGGAAACCCTGATCCAGCCATGCCGCGTGA
GCGATGAAGGCCTTAGGGTTGTAAA
GCTCTTTCAGTCGTGAAGATAATGACGGTAGCGACAGAAGAAGCCCCGGCTAACT
CCGTGCCAGCAGCCGCGGTAATACG
GAGGGGGCTAGCGTTGTTCGGAATTACTGGGCGTAAAGCGCGCGTAGGCGGACT
ATTAAGTCAGGGGTGAAATCCCGGGG
CTCAACCCCGGAACTGCCTTTGATACTGGTAGTCTAGAGTTCGAGAGAGGTGAGT
GGAATTCCGAGTGTAGAGGTGAAAT
TCGTAGATATTCGGAGGAACACCAGTGGCGAAGGCGGCTCACTGGCTCGATACTG
ACGCTGAGGTGCGAAAGCGTGGGGA
GCAAACAGGATTAGATACCCTGGTAGTCCACGCCGTAAACGATGAATGCCAGTCG
TCGGCAAGCATGCTTGTCGGTGACA
CACCTAACGGATTAAGCATTCCGCCTGGGGAGTACGGCCGCAAGGTTAAAACTCA
AAGGAATTGACGGGGGCCCGCACAA
GCGGTGGAGCATGTGGTTTAATTCGAAGCAACGCGCAGAACCTTACCAACCCTTG
ACATCCTGATCGCGGTTTTCCGAGA
GGAATTCCTTCAGTTCGGCTGGATCAGTGACAGGTGCTGCATGGCTGTCGTCAGC
TCGTGTCGTGAGATGTTCGGTTAAG
TCCGGCAACGAGCGCAACCCACACTCTTAGTTGCCAGCATTCAGTTGGGCACTCT
AGGAGAACTGCCGATGATAAGTCGG
AGGAAGGTGTGGATGACGTCAAGTCCTCATGGCCCTTACGGGTTGGGCTACACAC
GTGCTACAATGGCAGTGACAATGGG
TTAATCCCTAAAAACTGTCTCAGTTCGGATTGTTCTCTGCAACTCGAGAGCATGAA
GTCGGAATCGCTAGTAATCGCGTA
ACAGCATGACGCGGTGAATACGTTCCCGGGCCTTGTACACACCGCCCGTCACACC
ATGGGAGTTGGGTTTACCCGAAGAC
GGTGCGCCAACCCTTACGGGGGGCAGCTGGCCACGGTAAGCTCAGCGACTGGGG
TGAAGTCGTAACAAGGTAGCCGTAGG
GGAACCTGCGGCTGGATCACCTCCTTT
>IM796
CAACTTGAGAGTTTGATCCTGGCTCAGAACGAACGCTGGCGGCAGGCCTAACACA
TGCAAGTCGAGCGAACCCTTCGGGG
TTAGCGGCGGACGGGTGAGTAACGCGTGGGAACGTGCCCTTCTCTGCGGAATAG
GCTCGGGAAACTGGGTTTAATACCGC
ATACGCCCTTCGGGGGAAAGATTTATCGGAGAAGGATCGGCCCGCGTTAGATTAG
GTAGTTGGTGGGGTAATGGCCTACC

AAGCCTACGATCTATAGCTGGTTTGAGAGGATGATCAGCCACACTGGGACTGAGA
CACGGCCCAGACTCCTACGGGAGGC
AGCAGTGAGGAATCTTGGACAATGGGGGAAACCCTGATCCAGCCATGCCGCGTGA
GCGATGAAGGCCTTAGGGTTGTAAA
GCTCTTTCAGTCGTGAAGATAATGACGGTAGCGACAGAAGAAGCCCCGGCTAACT
CCGTGCCAGCAGCCGCGGTAATACG
GAGGGGGCTAGCGTTGTTCGGAATTACTGGGCGTAAAGCGCGCGTAGGCGGACT
ATTAAGTCAGGGGTGAAATCCCGGGG
CTCAACCCCGGAACTGCCTTTGATACTGGTAGTCTAGAGTTCGAGAGAGGTGAGT
GGAATTCCGAGTGTAGAGGTGAAAT
TCGTAGATATTCGGAGGAACACCAGTGGCGAAGGCGGCTCACTGGCTCGATACTG
ACGCTGAGGTGCGAAAGCGTGGGGA
GCAAACAGGATTAGATACCCTGGTAGTCCACGCCGTAAACGATGAATGCCAGTCG
TCGGCAAGCATGCTTGTCGGTGACA
CACCTAACGGATTAAGCATTCCGCCTGGGGAGTACGGCCGCAAGGTTAAAACTCA
AAGGAATTGACGGGGGCCCGCACAA
GCGGTGGAGCATGTGGTTTAATTCGAAGCAACGCGCAGAACCTTACCAACCCTTG
ACATCCTGATCGCGGTTTTCCGAGA
GGAATTCCTTCAGTTCGGCTGGATCAGTGACAGGTGCTGCATGGCTGTCGTCAGC
TCGTGTCGTGAGATGTTCGGTTAAG
TCCGGCAACGAGCGCAACCCACACTCTTAGTTGCCAGCATTCAGTTGGGCACTCT
AGGAGAACTGCCGATGATAAGTCGG
AGGAAGGTGTGGATGACGTCAAGTCCTCATGGCCCTTACGGGTTGGGCTACACAC
GTGCTACAATGGCAGTGACAATGGG
TTAATCCCTAAAAACTGTCTCAGTTCGGATTGTTCTCTGCAACTCGAGAGCATGAA
GTCGGAATCGCTAGTAATCGCGTA
ACAGCATGACGCGGTGAATACGTTCCCGGGCCTTGTACACACCGCCCGTCACACC
ATGGGAGTTGGGTTTACCCGAAGAC
GGTGCGCCAACCCTTACGGGGGGCAGCTGGCCACGGTAAGCTCAGCGACTGGGG
TGAAGTCGTAACAAGGTAGCCGTAGG
GGAACCTGCGGCTGGATCACCTCCTTT
>AB35
CAACTTGAGAGTTTGATCCTGGCTCAGAACGAACGCTGGCGGCAGGCCTAACACA
TGCAAGTCGAGCGAACCCTTCGGGG
TTAGCGGCGGACGGGTGAGTAACGCGTGGGAACGTGCCCTTCTCTGCGGAATAG
GCTCGGGAAACTGGGTTTAATACCGC
ATACGCCCTTCGGGGGAAAGATTTATCGGAGAAGGATCGGCCCGCGTTAGATTAG
GTAGTTGGTGGGGTAATGGCCTACC
AAGCCTACGATCTATAGCTGGTTTGAGAGGATGATCAGCCACACTGGGACTGAGA
CACGGCCCAGACTCCTACGGGAGGC
AGCAGTGAGGAATCTTGGACAATGGGGGAAACCCTGATCCAGCCATGCCGCGTGA
GCGATGAAGGCCTTAGGGTTGTAAA
GCTCTTTCAGTCGTGAAGATAATGACGGTAGCGACAGAAGAAGCCCCGGCTAACT
CCGTGCCAGCAGCCGCGGTAATACG
GAGGGGGCTAGCGTTGTTCGGAATTACTGGGCGTAAAGCGCGCGTAGGCGGACT
ATTAAGTCAGGGGTGAAATCCCGGGG

CTCAACCCCGGAACTGCCTTTGATACTGGTAGTCTAGAGTTCGAGAGAGGTGAGT
GGAATTCCGAGTGTAGAGGTGAAAT
TCGTAGATATTCGGAGGAACACCAGTGGCGAAGGCGGCTCACTGGCTCGATACTG
ACGCTGAGGTGCGAAAGCGTGGGGA
GCAAACAGGATTAGATACCCTGGTAGTCCACGCCGTAAACGATGAATGCCAGTCG
TCGGCAAGCATGCTTGTCGGTGACA
CACCTAACGGATTAAGCATTCCGCCTGGGGAGTACGGCCGCAAGGTTAAAACTCA
AAGGAATTGACGGGGGCCCGCACAA
GCGGTGGAGCATGTGGTTTAATTCGAAGCAACGCGCAGAACCTTACCAACCCTTG
ACATCCTGATCGCGGTTTTCCGAGA
GGAATTCCTTCAGTTCGGCTGGATCAGTGACAGGTGCTGCATGGCTGTCGTCAGC
TCGTGTCGTGAGATGTTCGGTTAAG
TCCGGCAACGAGCGCAACCCACACTCTTAGTTGCCAGCATTCAGTTGGGCACTCT
AGGAGAACTGCCGATGATAAGTCGG
AGGAAGGTGTGGATGACGTCAAGTCCTCATGGCCCTTACGGGTTGGGCTACACAC
GTGCTACAATGGCAGTGACAATGGG
TTAATCCCTAAAAGCTGTCTCAGTTCGGATTGTTCTCTGCAACTCGAGAGCATGAA
GTCGGAATCGCTAGTAATCGCGTA
ACAGCATGACGCGGTGAATACGTTCCCGGGCCTTGTACACACCGCCCGTCACACC
ATGGGAGTTGGGTTTACCCGAAGAC
GGTGCGCCAACCTTTACGGGGGGCAGCTGGCCACGGTAAGCTCAGCGACTGGGG
TGAAGTCGTAACAAGGTAGCCGTAGG
GGAACCTGCGGCTGGATCACCTCCTTT
>AB23
CAACTTGAGAGTTTGATCCTGGCTCAGAACGAACGCTGGCGGCAGGCCTAACACA
TGCAAGTCGAGCGAACCCTTCGGGG
TTAGCGGCGGACGGGTGAGTAACGCGTGGGAACGTGCCCTTCTCTGCGGAATAG
GCTCGGGAAACTGGGTTTAATACCGC
ATACGCCCTTCGGGGGAAAGATTTATCGGAGAAGGATCGGCCCGCGTTAGATTAG
GTAGTTGGTGGGGTAATGGCCTACC
AAGCCTACGATCTATAGCTGGTTTGAGAGGATGATCAGCCACACTGGGACTGAGA
CACGGCCCAGACTCCTACGGGAGGC
AGCAGTGAGGAATCTTGGACAATGGGGGAAACCCTGATCCAGCCATGCCGCGTGA
GCGATGAAGGCCTTAGGGTTGTAAA
GCTCTTTCAGTCGTGAAGATAATGACGGTAGCGACAGAAGAAGCCCCGGCTAACT
CCGTGCCAGCAGCCGCGGTAATACG
GAGGGGGCTAGCGTTGTTCGGAATTACTGGGCGTAAAGCGCGCGTAGGCGGACT
ATTAAGTCAGGGGTGAAATCCCGGGG
CTCAACCCCGGAACTGCCTTTGATACTGGTAGTCTAGAGTTCGAGAGAGGTGAGT
GGAATTCCGAGTGTAGAGGTGAAAT
TCGTAGATATTCGGAGGAACACCAGTGGCGAAGGCGGCTCACTGGCTCGATACTG
ACGCTGAGGTGCGAAAGCGTGGGGA
GCAAACAGGATTAGATACCCTGGTAGTCCACGCCGTAAACGATGAATGCCAGTCG
TCGGCAAGCATGCTTGTCGGTGACA
CACCTAACGGATTAAGCATTCCGCCTGGGGAGTACGGCCGCAAGGTTAAAACTCA
AAGGAATTGACGGGGGCCCGCACAA

GCGGTGGAGCATGTGGTTTAATTCGAAGCAACGCGCAGAACCTTACCAACCCTTG
ACATCCTGATCGCGGTTTTCCGAGA
GGAATTCCTTCAGTTCGGCTGGATCAGTGACAGGTGCTGCATGGCTGTCGTCAGC
TCGTGTCGTGAGATGTTCGGTTAAG
TCCGGCAACGAGCGCAACCCACACTCTTAGTTGCCAGCATTCAGTTGGGCACTCT
AGGAGAACTGCCGATGATAAGTCGG
AGGAAGGTGTGGATGACGTCAAGTCCTCATGGCCCTTACGGGTTGGGCTACACAC
GTGCTACAATGGCAGTGACAATGGG
TTAATCCCTAAAAACTGTCTCAGTTCGGATTGTTCTCTGCAACTCGAGAGCATGAA
GTCGGAATCGCTAGTAATCGCGTA
ACAGCATGACGCGGTGAATACGTTCCCGGGCCTTGTACACACCGCCCGTCACACC
ATGGGAGTTGGGTTTACCCGAAGAC
GGTGCGCCAACCCTTACGGGGGGCAGCTGGCCACGGTAAGCTCAGCGACTGGGG
TGAAGTCGTAACAAGGTAGCCGTAGG
GGAACCTGCGGCTGGATCACCTCCTTT
>AB22
CAACTTGAGAGTTTGATCCTGGCTCAGAACGAACGCTGGCGGCAGGCCTAACACA
TGCAAGTCGAGCGAACCCTTCGGGG
TTAGCGGCGGACGGGTGAGTAACGCGTGGGAACGTGCCCTTCTCTGCGGAATAG
GCTCGGGAAACTGGGTTTAATACCGC
ATACGCCCTTCGGGGGAAAGATTTATCGGAGAAGGATCGGCCCGCGTTAGATTAG
GTAGTTGGTGGGGTAATGGCCTACC
AAGCCTACGATCTATAGCTGGTTTGAGAGGATGATCAGCCACACTGGGACTGAGA
CACGGCCCAGACTCCTACGGGAGGC
AGCAGTGAGGAATCTTGGACAATGGGGGAAACCCTGATCCAGCCATGCCGCGTGA
GCGATGAAGGCCTTAGGGTTGTAAA
GCTCTTTCAGTCGTGAAGATAATGACGGTAGCGACAGAAGAAGCCCCGGCTAACT
CCGTGCCAGCAGCCGCGGTAATACG
GAGGGGGCTAGCGTTGTTCGGAATTACTGGGCGTAAAGCGCGCGTAGGCGGACT
ATTAAGTCAGGGGTGAAATCCCGGGG
CTCAACCCCGGAACTGCCTTTGATACTGGTAGTCTAGAGTTCGAGAGAGGTGAGT
GGAATTCCGAGTGTAGAGGTGAAAT
TCGTAGATATTCGGAGGAACACCAGTGGCGAAGGCGGCTCACTGGCTCGATACTG
ACGCTGAGGTGCGAAAGCGTGGGGA
GCAAACAGGATTAGATACCCTGGTAGTCCACGCCGTAAACGATGAATGCCAGTCG
TCGGCAAGCATGCTTGTCGGTGACA
CACCTAACGGATTAAGCATTCCGCCTGGGGAGTACGGCCGCAAGGTTAAAACTCA
AAGGAATTGACGGGGGCCCGCACAA
GCGGTGGAGCATGTGGTTTAATTCGAAGCAACGCGCAGAACCTTACCAACCCTTG
ACATCCTGATCGCGGTTTTCCGAGA
GGAATTCCTTCAGTTCGGCTGGATCAGTGACAGGTGCTGCATGGCTGTCGTCAGC
TCGTGTCGTGAGATGTTCGGTTAAG
TCCGGCAACGAGCGCAACCCACACTCTTAGTTGCCAGCATTCAGTTGGGCACTCT
AGGAGAACTGCCGATGATAAGTCGG
AGGAAGGTGTGGATGACGTCAAGTCCTCATGGCCCTTACGGGTTGGGCTACACAC
GTGCTACAATGGCAGTGACAATGGG

TTAATCCCTAAAAACTGTCTCAGTTCGGATTGTTCTCTGCAACTCGAGAGCATGAA
GTCGGAATCGCTAGTAATCGCGTA
ACAGCATGACGCGGTGAATACGTTCCCGGGCCTTGTACACACCGCCCGTCACACC
ATGGGAGTTGGGTTTACCCGAAGAC
GGTGCGCCAACCCTTACGGGGGGCAGCTGGCCACGGTAAGCTCAGCGACTGGGG
TGAAGTCGTAACAAGGTAGCCGTAGG
GGAACCTGCGGCTGGATCACCTCCTTT
>aB16
CAACTTGAGAGTTTGATCCTGGCTCAGAACGAACGCTGGCGGCAGGCCTAACACA
TGCAAGTCGAGCGAACCCTTCGGGG
TTAGCGGCGGACGGGTGAGTAACGCGTGGGAACGTGCCCTTCTCTGCGGAATAG
GCTTGGGAAACTGGGTTTAATACCGC
ATACGCCCTTCGGGGGAAAGATTTATCGGAGAAGGATCGGCCCGCGTTAGATTAG
GTAGTTGGTGGGGTAATGGCCTACC
AAGCCTACGATCTATAGCTGGTTTGAGAGGATGATCAGCCACACTGGGACTGAGA
CACGGCCCAGACTCCTACGGGAGGC
AGCAGTGAGGAATCTTGGACAATGGGGGAAACCCTGATCCAGCCATGCCGCGTGA
GCGATGAAGGCCTTAGGGTTGTAAA
GCTCTTTCAGTCGTGAAGATAATGACGGTAGCGACAGAAGAAGCCCCGGCTAACT
CCGTGCCAGCAGCCGCGGTAATACG
GAGGGGGCTAGCGTTGTTCGGAATTACTGGGCGTAAAGCGCGCGTAGGCGGACT
ATTAAGTCAGGGGTGAAATCCCGGGG
CTCAACCCCGGAACTGCCTTTGATACTGGTAGTCTAGAGTTCGAGAGAGGTGAGT
GGAATTCCGAGTGTAGAGGTGAAAT
TCGTAGATATTCGGAGGAACACCAGTGGCGAAGGCGGCTCACTGGCTCGATACTG
ACGCTGAGGTGCGAAAGCGTGGGGA
GCAAACAGGATTAGATACCCTGGTAGTCCACGCCGTAAACGATGAATGCCAGTCG
TCGGCAAGCATGCTTGTCGGTGACA
CACCTAACGGATTAAGCATTCCGCCTGGGGAGTACGGCCGCAAGGTTAAAACTCA
AAGGAATTGACGGGGGCCCGCACAA
GCGGTGGAGCATGTGGTTTAATTCGAAGCAACGCGCAGAACCTTACCAACCCTTG
ACATCCTGATCGCGGTTTTCCGAGA
GGAATTCCTTCAGTTCGGCTGGATCAGTGACAGGTGCTGCATGGCTGTCGTCAGC
TCGTGTCGTGAGATGTTCGGTTAAG
TCCGGCAACGAGCGCAACCCACACTCTTAGTTGCCAGCATTCAGTTGGGCACTCT
AGGAGAACTGCCGATGATAAGTCGG
AGGAAGGTGTGGATGACGTCAAGTCCTCATGGCCCTTACGGGTTGGGCTACACAC
GTGCTACAATGGCAGTGACAATGGG
TTAATCCCTAAAAACTGTCTCAGTTCGGATTGTTCTCTGCAACTCGAGAGCATGAA
GTCGGAATCGCTAGTAATCGCGTA
ACAGCATGACGCGGTGAATACGTTCCCGGGCCTTGTACACACCGCCCGTCACACC
ATGGGAGTTGGGTTTACCCGAAGAC
GGTGCGCCAACCCTTGCGGGGGGCAGCTGGCCACGGTAAGCTCAGCGACTGGG
GTGAAGTCGTAACAAGGTAGCCGTAGG
GGAACCTGCGGCTGGATCACCTCCTTT
>AB20

CAACTTGAGAGTTTGATCCTGGCTCAGAACGAACGCTGGCGGCAGGCCTAACACA
TGCAAGTCGAGCGAACCCTTCGGGG
TTAGCGGCGGACGGGTGAGTAACGCGTGGGAACGTGCCCTTCTCTGCGGAATAG
GCTCGGGAAACTGGGTTTAATACCGC
ATACGCCCTTCGGGGGAAAGATTTATCGGAGAAGGATCGGCCCGCGTTAGATTAG
GTAGTTGGTGGGGTAATGGCCTACC
AAGCCTACGATCTATAGCTGGTTTGAGAGGATGATCAGCCACACTGGGACTGAGA
CACGGCCCAGACTCCTACGGGAGGC
AGCAGTGAGGAATCTTGGACAATGGGGGAAACCCTGATCCAGCCATGCCGCGTGA
GCGATGAAGGCCTTAGGGTTGTAAA
GCTCTTTCAGTCGTGAAGATAATGACGGTAGCGACAGAAGAAGCCCCGGCTAACT
CCGTGCCAGCAGCCGCGGTAATACG
GAGGGGGCTAGCGTTGTTCGGAATTACTGGGCGTAAAGCGCGCGTAGGCGGACT
ATTAAGTCAGGGGTGAAATCCCGGGG
CTCAACCCCGGAACTGCCTTTGATACTGGTAGTCTAGAGTTCGAGAGAGGTGAGT
GGAATTCCGAGTGTAGAGGTGAAAT
TCGTAGATATTCGGAGGAACACCAGTGGCGAAGGCGGCTCACTGGCTCGATACTG
ACGCTGAGGTGCGAAAGCGTGGGGA
GCAAACAGGATTAGATACCCTGGTAGTCCACGCCGTAAACGATGAATGCCAGTCG
TCGGCAAGCATGCTTGTCGGTGACA
CACCTAACGGATTAAGCATTCCGCCTGGGGAGTACGGCCGCAAGGTTAAAACTCA
AAGGAATTGACGGGGGCCCGCACAA
GCGGTGGAGCATGTGGTTTAATTCGAAGCAACGCGCAGAACCTTACCAACCCTTG
ACATCCTGATCGCGGTTTTCCGAGA
GGAATTCCTTCAGTTCGGCTGGATCAGTGACAGGTGCTGCATGGCTGTCGTCAGC
TCGTGTCGTGAGATGTTCGGTTAAG
TCCGGCAACGAGCGCAACCCACACTCTTAGTTGCCAGCATTCAGTTGGGCACTCT
AGGAGAACTGCCGATGATAAGTCGG
AGGAAGGTGTGGATGACGTCAAGTCCTCATGGCCCTTACGGGTTGGGCTACACAC
GTGCTACAATGGCAGTGACAATGGG
TTAATCCCTAAAAGCTGTCTCAGTTCGGATTGTTCTCTGCAACTCGAGAGCATGAA
GTCGGAATCGCTAGTAATCGCGTA
ACAGCATGACGCGGTGAATACGTTCCCGGGCCTTGTACACACCGCCCGTCACACC
ATGGGAGTTGGGTTTACCCGAAGAC
GGTGCGCCAACCCTTACGGGGGGCAGCTGGCCACGGTAAGCTCAGCGACTGGGG
TGAAGTCGTAACAAGGTAGCCGTAGG
GGAACCTGCGGCTGGATCACCTCCTTT
>AB28
CAACTTGAGAGTTTGATCCTGGCTCAGAACGAACGCTGGCGGCAGGCCTAACACA
TGCAAGTCGAGCGAACCCTTCGGGG
TTAGCGGCGGACGGGTGAGTAACGCGTGGGAACGTGCCCTTCTCTGCGGAATAG
GCTCGGGAAACTGGGTTTAATACCGC
ATACGCCCTTCGGGGGAAAGATTTATCGGAGAAGGATCGGCCCGCGTTAGATTAG
GTAGTTGGTGGGGTAATGGCCTACC
AAGCCTACGATCTATAGCTGGTTTGAGAGGATGATCAGCCACACTGGGACTGAGA
CACGGCCCAGACTCCTACGGGAGGC

AGCAGTGAGGAATCTTGGACAATGGGGGAAACCCTGATCCAGCCATGCCGCGTGA
GCGATGAAGGCCTTAGGGTTGTAAA
GCTCTTTCAGTCGTGAAGATAATGACGGTAGCGACAGAAGAAGCCCCGGCTAACT
CCGTGCCAGCAGCCGCGGTAATACG
GAGGGGGCTAGCGTTGTTCGGAATTACTGGGCGTAAAGCGCGCGTAGGCGGACT
ATTAAGTCAGGGGTGAAATCCCGGGG
CTCAACCCCGGAACTGCCTTTGATACTGGTAGTCTAGAGTTCGAGAGAGGTGAGT
GGAATTCCGAGTGTAGAGGTGAAAT
TCGTAGATATTCGGAGGAACACCAGTGGCGAAGGCGGCTCACTGGCTCGATACTG
ACGCTGAGGTGCGAAAGCGTGGGGA
GCAAACAGGATTAGATACCCTGGTAGTCCACGCCGTAAACGATGAATGCCAGTCG
TCGGCAAGCATGCTTGTCGGTGACA
CACCTAACGGATTAAGCATTCCGCCTGGGGAGTACGGCCGCAAGGTTAAAACTCA
AAGGAATTGACGGGGGCCCGCACAA
GCGGTGGAGCATGTGGTTTAATTCGAAGCAACGCGCAGAACCTTACCAACCCTTG
ACATCCTGATCGCGGTTTTCCGAGA
GGAATTCCTTCAGTTCGGCTGGATCAGTGACAGGTGCTGCATGGCTGTCGTCAGC
TCGTGTCGTGAGATGTTCGGTTAAG
TCCGGCAACGAGCGCAACCCACACTCTTAGTTGCCAGCATTCAGTTGGGCACTCT
AGGAGAACTGCCGATGATAAGTCGG
AGGAAGGTGTGGATGACGTCAAGTCCTCATGGCCCTTACGGGTTGGGCTACACAC
GTGCTACAATGGCAGTGACAATGGG
TTAATCCCTAAAAGCTGTCTCAGTTCGGATTGTTCTCTGCAACTCGAGAGCATGAA
GTCGGAATCGCTAGTAATCGCGTA
ACAGCATGACGCGGTGAATACGTTCCCGGGCCTTGTACACACCGCCCGTCACACC
ATGGGAGTTGGGTTTACCCGAAGAC
GGTGCGCCAACCCTTACGGGGGGCAGCTGGCCACGGTAAGCTCAGCGACTGGGG
TGAAGTCGTAACAAGGTAGCCGTAGG
GGAACCTGCGGCTGGATCACCTCCTTT
>AB19
CAACTTGAGAGTTTGATCCTGGCTCAGAACGAACGCTGGCGGCAGGCCTAACACA
TGCAACAACTTGAGAGTTTGATCCTGGCTCAGAACGAACGCTGGCGGCAGGCCTA
ACACATGCAAGTCGAGCGAACCCTTCGGGGTTAGCGGCGGACGGGTGAGTAACG
CGTGGGAACGTGCCCTGTCGAGCGAACCCTTCGGGGTTAGCGGCGGACGGGTGA
GTAACGCGTGGGAACGTGCCCTTCTCTGCGGAATAGGCTCGGGAAACTGGGTTTA
ATACCGCATACGCCCTTCGGGGGAAAGTCTCTGCGGAATAGGCTCGGGAAACTGG
GTTTAATACCGCATACGCCCTTCGGGGGAAAGATTTATCGGAGAAGGATCGGCCC
GCGTTAGATTAGGTAGTTGGTGGGGTAATGGCCTACCATTTATCGGAGAAGGATC
GGCCCGCGTCTGATTAGGTAGTTGGTGGGGTAATGGCCTACCAAGCCTACGATCT
ATAGCTGGTTTGAGAGGATGATCAGCCACACTGGGACTGAGACACGGAAGCCTAC
GATCAGTAGCTGGTTTGAGAGGATGATCAGCCACACTGGGACTGAGACACGGCCC
AGACTCCTACGGGAGGCAGCAGTGAGGAATCTTGGACAATGGGGGAAACCCTGAT
CCCCCAGACTCCTACGGGAGGCAGCAGTGAGGAATCTTGGACAATGGGGGAAAC
CCTGATCCAGCCATGCCGCGTGAGCGATGAAGGCCTTAGGGTTGTAAAGCTCTTT
CAGTCGTGAAGATAGCCATGCCGCGTGAGCGATGAAGGCCTTAGGGTTGTAAAGC
TCTTTCAGTCGTGAAGATAATGACGGTAGCGACAGAAGAAGCCCCGGCTAACTCC

GTGCCAGCAGCCGCGGTAATACGAATGACGGTAGCGACAGAAGAAGCCCCGGCT
AACTCCGTGCCAGCAGCCGCGGTAATACGGAGGGGGCTAGCGTTGTTCGGAATTA
CTGGGCGTAAAGCGCGCGTAGGCGGACTATTAAGGAGGGGGCTAGCGTTGTTCG
GAATTACTGGGCGTAAAGCGCGCGTAGGCGGACTATTAAGTCAGGGGTGAAATCC
CGGGGCTCAACCCCGGAACTGCCTTTGATACTGGTAGTCTAGAGTTCGGGGGTGA
AATCCCGGGGCTCAACCCCGGAACTGCCTCCGATACTGGTAGTCTAGAGTTCGAG
AGAGGTGAGTGGAATTCCGAGTGTAGAGGTGAAATTCGTAGATATTCGGAGGAAC
TCGAGAGAGGTGAGTGGAATTCCGAGTGTAGAGGTGAAATTCGTAGATATTCGGA
GGAACACCAGTGGCGAAGGCGGCTCACTGGCTCGATACTGACGCTGAGGTGCGA
AAGCGTGGGGAACCAGTGGCGAAGGCGGCTCACTGGCTCGATACTGACGCTGAG
GTGCGAAAGCGTGGGGAGCAAACAGGATTAGATACCCTGGTAGTCCACGCCGTAA
ACGATGAATGCCAGTCGTCGGCGCAAACAGGATTAGATACCCTGGTAGTCCACGC
CGTAAACGATGAATGCCAGTCGTCGGCAAGCTTGCTTGTCGGTGACACACCTAAC
GGATTAAGCATTCCGCCTGGGGAGTACGGCCGAAGCATGCTTGTCGGTGACACAC
CTAACGGATTAAGCATTCCGCCTGGGGAGTACGGCCGCAAGGTTAAAACTCAAAG
GAATTGACGGGGGCCCGCACAAGCGGTGGAGCATGTGGTTTACAAGGTTAAAACT
CAAAGGAATTGACGGGGGCCCGCACAAGCGGTGGAGCATGTGGTTTAATTCGAAG
CAACGCGCAGAACCTTACCAACCCTTGACATCCTGATCGCGGTTTTCCGAGAATTC
GAAGCAACGCGCAGAACCTTACCAACCCTTGACATCCTGATCGCGGTTTTCCGAG
AGGAATTCCTTCAGTTCGGCTGGATCAGTGACAGGTGCTGCATGGCTGTCGTCAG
CTCGTGGGAATTCCTTCAGTTCGGCTGGATCAGTGACAGGTGCTGCATGGCTGTC
GTCAGCTCGTGTCGTGAGATGTTCGGTTAAGTCCGGCAACGAGCGCAACCCACAC
TCTTAGTTGCCAGCATTCGTGAGATGTTCGGTTAAGTCCGGCAACGAGCGCAACC
CACACTCTTAGTTGCCAGCATTCAGTTGGGCACTCTAGGAGAACTGCCGATGATAA
GTCGGAGGAAGGTGTGGATGACGTCTCAGTTGGGCACTCTAAGAGAACTGCCGAT
GATAAGTCGGAGGAAGGTGTGGATGACGTCAAGTCCTCATGGCCCTTACGGGTTG
GGCTACACACGTGCTACAATGGCAGTGACAATGGGAAGTCCTCATGGCCCTTACG
GGTTGGGCTACACACGTGCTACAATGGCAGTGACAATGGGTTAATCCCTAAAAACT
GTCTCAGTTCGGATTGTTCTCTGCAACTCGAGAGCATGAAGTCGTTAATCCCAAAA
AACTGTCTCAGTTCGGATTGTTCTCTGCAACTCGAGAGCATGAAGTCGGAATCGCT
AGTAATCGCGTAACAGCATGACGCGGTGAATACGTTCCCGGGCCTTGTACACGAA
TCGCTAGTAATCGCGTAACAGCATGACGCGGTGAATACGTTCCCGGGCCTTGTAC
ACACCGCCCGTCACACCATGGGAGTTGGGTTTACCCGAAGACGGTGCGCCAACC
CTTACGGGACCGCCCGTCACACCATGGGAGTTGGGTTTACCCGAAGACGGTGCG
CCAACCCTTACGGGGGGCAGCTGGCCACGGTAAGCTCAGCGACTGGGGTGAAGT
CGTAACAAGGTAGCCGTAGGGGGCAGCTGGCCACGGTAAGCTCAGCGACTGGGG
TGAAGTCGTAACAAGGTAGCCGTAGGGGAACCTGCGGCTGGATCACCTCCTTTCT
AAGGAACCTGCGGCTGGATCACCTCCTTTCTAA
>AB18
CAACTTGAGAGTTTGATCCTGGCTCAGAACGAACGCTGGCGGCAGGCCTAACACA
TGCAAGTCGAGCGAACCCTTCGGGG
TTAGCGGCGGACGGGTGAGTAACGCGTGGGAACGTGCCCTTCTCTGCGGAATAG
GCTCGGGAAACTGGGTTTAATACCGC
ATACGCCCTTCGGGGGAAAGATTTATCGGAGAAGGATCGGCCCGCGTTAGATTAG
GTAGTTGGTGGGGTAATGGCCTACC

AAGCCTACGATCTATAGCTGGTTTGAGAGGATGATCAGCCACACTGGGACTGAGA
CACGGCCCAGACTCCTACGGGAGGC
AGCAGTGAGGAATCTTGGACAATGGGGGAAACCCTGATCCAGCCATGCCGCGTGA
GCGATGAAGGCCTTAGGGTTGTAAA
GCTCTTTCAGTCGTGAAGATAATGACGGTAGCGACAGAAGAAGCCCCGGCTAACT
CCGTGCCAGCAGCCGCGGTAATACG
GAGGGGGCTAGCGTTGTTCGGAATTACTGGGCGTAAAGCGCGCGTAGGCGGACT
ATTAAGTCAGGGGTGAAATCCCGGGG
CTCAACCCCGGAACTGCCTTTGATACTGGTAGTCTAGAGTTCGAGAGAGGTGAGT
GGAATTCCGAGTGTAGAGGTGAAAT
TCGTAGATATTCGGAGGAACACCAGTGGCGAAGGCGGCTCACTGGCTCGATACTG
ACGCTGAGGTGCGAAAGCGTGGGGA
GCAAACAGGATTAGATACCCTGGTAGTCCACGCCGTAAACGATGAATGCCAGTCG
TCGGCAAGCATGCTTGTCGGTGACA
CACCTAACGGATTAAGCATTCCGCCTGGGGAGTACGGCCGCAAGGTTAAAACTCA
AAGGAATTGACGGGGGCCCGCACAA
GCGGTGGAGCATGTGGTTTAATTCGAAGCAACGCGCAGAACCTTACCAACCCTTG
ACATCCTGATCGCGGTTTTCCGAGA
GGAATTCCTTCAGTTCGGCTGGATCAGTGACAGGTGCTGCATGGCTGTCGTCAGC
TCGTGTCGTGAGATGTTCGGTTAAG
TCCGGCAACGAGCGCAACCCACACTCTTAGTTGCCAGCATTCAGTTGGGCACTCT
AGGAGAACTGCCGATGATAAGTCGG
AGGAAGGTGTGGATGACGTCAAGTCCTCATGGCCCTTACGGGTTGGGCTACACAC
GTGCTACAATGGCAGTGACAATGGG
TTAATCCCTAAAAACTGTCTCAGTTCGGATTGTTCTCTGCAACTCGAGAGCATGAA
GTCGGAATCGCTAGTAATCGCGTA
ACAGCATGACGCGGTGAATACGTTCCCGGGCCTTGTACACACCGCCCGTCACACC
ATGGGAGTTGGGTTTACCCGAAGAC
GGTGCGCCAACCCTTACGGGGGGCAGCTGGCCACGGTAAGCTCAGCGACTGGGG
TGAAGTCGTAACAAGGTAGCCGTAGG
GGAACCTGCGGCTGGATCACCTCCTTT
>AB17
CAACTTGAGAGTTTGATCCTGGCTCAGAACGAACGCTGGCGGCAGGCCTAACACA
TGCAAGTCGAGCGAACCCTTCGGGG
TTAGCGGCGGACGGGTGAGTAACGCGTGGGAACGTGCCCTTCTCTGCGGAATAG
GCTCGGGAAACTGGGTTTAATACCGC
ATACGCCCTTCGGGGGAAAGATTTATCGGAGAAGGATCGGCCCGCGTTAGATTAG
GTAGTTGGTGGGGTAATGGCCTACC
AAGCCTACGATCTATAGCTGGTTTGAGAGGATGATCAGCCACACTGGGACTGAGA
CACGGCCCAGACTCCTACGGGAGGC
AGCAGTGAGGAATCTTGGACAATGGGGGAAACCCTGATCCAGCCATGCCGCGTGA
GCGATGAAGGCCTTAGGGTTGTAAA
GCTCTTTCAGTCGTGAAGATAATGACGGTAGCGACAGAAGAAGCCCCGGCTAACT
CCGTGCCAGCAGCCGCGGTAATACG
GAGGGGGCTAGCGTTGTTCGGAATTACTGGGCGTAAAGCGCGCGTAGGCGGACT
ATTAAGTCAGGGGTGAAATCCCGGGG

CTCAACCCCGGAACTGCCTTTGATACTGGTAGTCTAGAGTTCGAGAGAGGTGAGT
GGAATTCCGAGTGTAGAGGTGAAAT
TCGTAGATATTCGGAGGAACACCAGTGGCGAAGGCGGCTCACTGGCTCGATACTG
ACGCTGAGGTGCGAAAGCGTGGGGA
GCAAACAGGATTAGATACCCTGGTAGTCCACGCCGTAAACGATGAATGCCAGTCG
TCGGCAAGCATGCTTGTCGGTGACA
CACCTAACGGATTAAGCATTCCGCCTGGGGAGTACGGCCGCAAGGTTAAAACTCA
AAGGAATTGACGGGGGCCCGCACAA
GCGGTGGAGCATGTGGTTTAATTCGAAGCAACGCGCAGAACCTTACCAACCCTTG
ACATCCTGATCGCGGTTTTCCGAGA
GGAATTCCTTCAGTTCGGCTGGATCAGTGACAGGTGCTGCATGGCTGTCGTCAGC
TCGTGTCGTGAGATGTTCGGTTAAG
TCCGGCAACGAGCGCAACCCACACTCTTAGTTGCCAGCATTCAGTTGGGCACTCT
AGGAGAACTGCCGATGATAAGTCGG
AGGAAGGTGTGGATGACGTCAAGTCCTCATGGCCCTTACGGGTTGGGCTACACAC
GTGCTACAATGGCAGTGACAATGGG
TTAATCCCTAAAAACTGTCTCAGTTCGGATTGTTCTCTGCAACTCGAGAGCATGAA
GTCGGAATCGCTAGTAATCGCGTA
ACAGCATGACGCGGTGAATACGTTCCCGGGCCTTGTACACACCGCCCGTCACACC
ATGGGAGTTGGGTTTACCCGAAGAC
GGTGCGCCAACCCTTACGGGGGGCAGCTGGCCACGGTAAGCTCAGCGACTGGGG
TGAAGTCGTAACAAGGTAGCCGTAGG
GGAACCTGCGGCTGGATCACCTCCTTT
>AB15
CAACTTGAGAGTTTGATCCTGGCTCAGAACGAACGCTGGCGGCAGGCCTAACACA
TGCAAGTCGAGCGAACCCTTCGGGG
TTAGCGGCGGACGGGTGAGTAACGCGTGGGAACGTGCCCTTCTCTGCGGAATAG
GCTCGGGAAACTGGGTTTAATACCGC
ATACGCCCTTCGGGGGAAAGATTTATCGGAGAAGGATCGGCCCGCGTTAGATTAG
GTAGTTGGTGGGGTAATGGCCTACC
AAGCCTACGATCTATAGCTGGTTTGAGAGGATGATCAGCCACACTGGGACTGAGA
CACGGCCCAGACTCCTACGGGAGGC
AGCAGTGAGGAATCTTGGACAATGGGGGAAACCCTGATCCAGCCATGCCGCGTGA
GCGATGAAGGCCTTAGGGTTGTAAA
GCTCTTTCAGTCGTGAAGATAATGACGGTAGCGACAGAAGAAGCCCCGGCTAACT
CCGTGCCAGCAGCCGCGGTAATACG
GAGGGGGCTAGCGTTGTTCGGAATTACTGGGCGTAAAGCGCGCGTAGGCGGACT
ATTAAGTCAGGGGTGAAATCCCGGGG
CTCAACCCCGGAACTGCCTTTGATACTGGTAGTCTAGAGTTCGAGAGAGGTGAGT
GGAATTCCGAGTGTAGAGGTGAAAT
TCGTAGATATTCGGAGGAACACCAGTGGCGAAGGCGGCTCACTGGCTCGATACTG
ACGCTGAGGTGCGAAAGCGTGGGGA
GCAAACAGGATTAGATACCCTGGTAGTCCACGCCGTAAACGATGAATGCCAGTCG
TCGGCAAGCATGCTTGTCGGTGACA
CACCTAACGGATTAAGCATTCCGCCTGGGGAGTACGGCCGCAAGGTTAAAACTCA
AAGGAATTGACGGGGGCCCGCACAA

GCGGTGGAGCATGTGGTTTAATTCGAAGCAACGCGCAGAACCTTACCAACCCTTG
ACATCCTGATCGCGGTTTTCCGAGA
GGAATTCCTTCAGTTCGGCTGGATCAGTGACAGGTGCTGCATGGCTGTCGTCAGC
TCGTGTCGTGAGATGTTCGGTTAAG
TCCGGCAACGAGCGCAACCCACACTCTTAGTTGCCAGCATTCAGTTGGGCACTCT
AGGAGAACTGCCGATGATAAGTCGG
AGGAAGGTGTGGATGACGTCAAGTCCTCATGGCCCTTACGGGTTGGGCTACACAC
GTGCTACAATGGCAGTGACAATGGG
TTAATCCCTAAAAGCTGTCTCAGTTCGGATTGTTCTCTGCAACTCGAGAGCATGAA
GTCGGAATCGCTAGTAATCGCGTA
ACAGCATGACGCGGTGAATACGTTCCCGGGCCTTGTACACACCGCCCGTCACACC
ATGGGAGTTGGGTTTACCCGAAGAC
GGTGCGCCAACCTTTACGGGGGGCAGCTGGCCACGGTAAGCTCAGCGACTGGGG
TGAAGTCGTAACAAGGTAGCCGTAGG
GGAACCTGCGGCTGGATCACCTCCTTT
>AB33
CAACTTGAGAGTTTGATCCTGGCTCAGAACGAACGCTGGCGGCAGGCCTAACACA
TGCAAGTCGAGCGAACCCTTCGGGG
TTAGCGGCGGACGGGTGAGTAACGCGTGGGAACGTGCCCTTCTCTGCGGAATAG
GCTCGGGAAACTGGGTTTAATACCGC
ATACGCCCTTCGGGGGAAAGATTTATCGGAGAAGGATCGGCCCGCGTTAGATTAG
GTAGTTGGTGGGGTAATGGCCTACC
AAGCCTACGATCTATAGCTGGTTTGAGAGGATGATCAGCCACACTGGGACTGAGA
CACGGCCCAGACTCCTACGGGAGGC
AGCAGTGAGGAATCTTGGACAATGGGGGAAACCCTGATCCAGCCATGCCGCGTGA
GCGATGAAGGCCTTAGGGTTGTAAA
GCTCTTTCAGTCGTGAAGATAATGACGGTAGCGACAGAAGAAGCCCCGGCTAACT
CCGTGCCAGCAGCCGCGGTAATACG
GAGGGGGCTAGCGTTGTTCGGAATTACTGGGCGTAAAGCGCGCGTAGGCGGACT
ATTAAGTCAGGGGTGAAATCCCGGGG
CTCAACCCCGGAACTGCCTTTGATACTGGTAGTCTAGAGTTCGAGAGAGGTGAGT
GGAATTCCGAGTGTAGAGGTGAAAT
TCGTAGATATTCGGAGGAACACCAGTGGCGAAGGCGGCTCACTGGCTCGATACTG
ACGCTGAGGTGCGAAAGCGTGGGGA
GCAAACAGGATTAGATACCCTGGTAGTCCACGCCGTAAACGATGAATGCCAGTCG
TCGGCAAGCATGCTTGTCGGTGACA
CACCTAACGGATTAAGCATTCCGCCTGGGGAGTACGGCCGCAAGGTTAAAACTCA
AAGGAATTGACGGGGGCCCGCACAA
GCGGTGGAGCATGTGGTTTAATTCGAAGCAACGCGCAGAACCTTACCAACCCTTG
ACATCCTGATCGCGGTTTTCCGAGA
GGAATTCCTTCAGTTCGGCTGGATCAGTGACAGGTGCTGCATGGCTGTCGTCAGC
TCGTGTCGTGAGATGTTCGGTTAAG
TCCGGCAACGAGCGCAACCCACACTCTTAGTTGCCAGCATTCAGTTGGGCACTCT
AGGAGAACTGCCGATGATAAGTCGG
AGGAAGGTGTGGATGACGTCAAGTCCTCATGGCCCTTACGGGTTGGGCTACACAC
GTGCTACAATGGCAGTGACAATGGG

TTAATCCCTAAAAACTGTCTCAGTTCGGATTGTTCTCTGCAACTCGAGAGCATGAA
GTCGGAATCGCTAGTAATCGCGTA
ACAGCATGACGCGGTGAATACGTTCCCGGGCCTTGTACACACCGCCCGTCACACC
ATGGGAGTTGGGTTTACCCGAAGAC
GGTGCGCCAACCCTTACGGGGGGCAGCTGGCCACGGTAAGCTCAGCGACTGGGG
TGAAGTCGTAACAAGGTAGCCGTAGG
GGAACCTGCGGCTGGATCACCTCCTTT
>2_4_1
CAACTTGAGAGTTTGATCCTGGCTCAGAATGAACGCTGGCGGCAGGCCTAACACA
TGCAAGTCGAGCGAAGTCTTCGGAC
TTAGCGGCGGACGGGTGAGTAACGCGTGGGAACGTGCCCTTTGCTTCGGAATAG
CCCCGGGAAACTGGGAGTAATACCGA
ATGTGCCCTTTGGGGGAAAGATTTATCGGCAAAGGATCGGCCCGCGTTGGATTAG
GTAGTTGGTGGGGTAATGGCCTACC
AAGCCGACGATCCATAGCTGGTTTGAGAGGATGATCAGCCACACTGGGACTGAGA
CACGGCCCAGACTCCTACGGGAGGC
AGCAGTGGGGAATCTTAGACAATGGGCGCAAGCCTGATCTAGCCATGCCGCGTGA
TCGATGAAGGCCTTAGGGTTGTAAA
GATCTTTCAGGTGGGAAGATAATGACGGTACCACCAGAAGAAGCCCCGGCTAACT
CCGTGCCAGCAGCCGCGGTAATACG
GAGGGGGCTAGCGTTATTCGGAATTACTGGGCGTAAAGCGCACGTAGGCGGATC
GGAAAGTCAGAGGTGAAATCCCAGGG
CTCAACCCTGGAACTGCCTTTGAAACTCCCGATCTTGAGGTCGAGAGAGGTGAGT
GGAATTCCGAGTGTAGAGGTGAAAT
TCGTAGATATTCGGAGGAACACCAGTGGCGAAGGCGGCTCACTGGCTCGATACTG
ACGCTGAGGTGCGAAAGCGTGGGGA
GCAAACAGGATTAGATACCCTGGTAGTCCACGCCGTAAACGATGAATGCCAGTCG
TCGGGCAGCATGCTGTTCGGTGACA
CACCTAACGGATTAAGCATTCCGCCTGGGGAGTACGGCCGCAAGGTTAAAACTCA
AAGGAATTGACGGGGGCCCGCACAA
GCGGTGGAGCATGTGGTTTAATTCGAAGCAACGCGCAGAACCTTACCAACCCTTG
ACATGGCGATCGCGGTTCCAGAGAT
GGTTCCTTCAGTTCGGCTGGATCGCACACAGGTGCTGCATGGCTGTCGTCAGCTC
GTGTCGTGAGATGTTCGGTTAAGTC
CGGCAACGAGCGCAACCCACGTCCTTAGTTGCCAGCATTCAGTTGGGCACTCTAG
GGAAACTGCCGGTGATAAGCCGGAG
GAAGGTGTGGATGACGTCAAGTCCTCATGGCCCTTACGGGTTGGGCTACACACGT
GCTACAATGGCAGTGACAATGGGTT
AATCCCAAAAAGCTGTCTCAGTTCGGATTGGGGTCTGCAACTCGACCCCATGAAGT
CGGAATCGCTAGTAATCGCGTAAC
AGCATGACGCGGTGAATACGTTCCCGGGCCTTGTACACACCGCCCGTCACACCAT
GGGAATTGGTTCTACCCGAAGGCGG
TGCGCCAACCTCGCAAGAGGAGGCAGCCGACCACGGTAGGATCAGTGACTGGGG
TGAAGTCGTAACAAGGTAGCCGTAGG
GGAACCTGCGGCTGGATCACCTCCTTT
>TIE1

CAACTTGAGAGTTTGATCCTGGCTCAGAGCGAACGCTGGCGGCAGGCTTAACACA
TGCAAGTCGAACGGGCGTAGCAATA
CGTCAGTGGCAGACGGGTGAGTAACGCGTGGGAACGTACCTTTTGGTTCGGAACA
ACACAGGGAAACTTGTGCTAATACC
GGATAAGCCCTTACGGGGAAAGATTTATCGCCGAAAGATCGGCCCGCGTCTGATT
AGCTAGTTGGTGAGGTAATGGCTCA
CCAAGGCGACGATCAGTAGCTGGTCTGAGAGGATGATCAGCCACATTGGGACTGA
GACACGGCCCAAACTCCTACGGGAG
GCAGCAGTGGGGAATATTGGACAATGGGGGAAACCCTGATCCAGCCATGCCGCG
TGAGTGATGAAGGCCCTAGGGTTGTA
AAGCTCTTTTGTGCGGGAAGATAATGACGGTACCGCAAGAATAAGCCCCGGCTAA
CTTCGTGCCAGCAGCCGCGGTAATA
CGAAGGGGGCTAGCGTTGCTCGGAATCACTGGGCGTAAAGGGTGCGTAGGCGGG
TTTCTAAGTCAGAGGTGAAAGCCTGG
AGCTCAACTCCAGAACTGCCTTTGATACTGGAAGTCTTGAGTATGGCAGAGGTGAG
TGGAACTGCGAGTGTAGAGGTGAA
ATTCGTAGATATTCGCAAGAACACCAGTGGCGAAGGCGGCTCACTGGGCCATTAC
TGACGCTGAGGCACGAAAGCGTGGG
GAGCAAACAGGATTAGATACCCTGGTAGTCCACGCCGTAAACGATGAATGCCAGC
CGTTAGTGGGTTTACTCACTAGTGG
CGCAGCTAACGCTTTAAGCATTCCGCCTGGGGAGTACGGTCGCAAGATTAAAACT
CAAAGGAATTGACGGGGGCCCGCAC
AAGCGGTGGAGCATGTGGTTTAATTCGACGCAACGCGCAGAACCTTACCAGCCCT
TGACATGTCCAGGACCGGTCGCAGA
GACGCGACCTTCTCTTCGGAGCCTGGAGCACAGGTGCTGCATGGCTGTCGTCAGC
TCGTGTCGTGAGATGTTGGGTTAAG
TCCCGCAACGAGCGCAACCCCCGTCCTTAGTTGCTACCATTTAGTTGAGCACTCTA
AGGAGACTGCCGGTGATAAGCCGC
GAGGAAGGTGGGGATGACGTCAAGTCCTCATGGCCCTTACGGGCTGGGCTACAC
ACGTGCTACAATGGCGGTGACAATGG
GAAGCTAAGGGGCGACCCTTCGCAAATCTCAAAAAGCCGTCTCAGTTCGGATTGG
GCTCTGCAACTCGAGCCCATGAAGT
TGGAATCGCTAGTAATCGTGGATCAGCATGCCACGGTGAATACGTTCCCGGGCCT
TGTACACACCGCCCGTCACACCATG
GGAGTTGGCTTTACCTGAAGACGGTGCGCTAACCAGCAATGGGGGCAGCCGGCC
ACGGTAGGGTCAGCGACTGGGGTGAA
GTCGTAACAAGGTAGCCGTAGGGGAACCTGCGGCTGGATCACCTCCTTT
>geobactersulfurreducens
AAACTGGAGAGTTTGATCCTGGCTCAGAACGAACGCTGGCGGCGTGCCTAACACA
TGCAAGTCGAACGTGATCCATCCTT
CGGGGTGGTGAAAGTGGCGCACGGGTGAGTAACGCGTGGATAATCTGCCCGAGG
ATTTGGGATAACATCTCGAAAGGGGT
GCTAATACCGAATAAGCCCACGGGGTCTACGGATCTTGCGGGAAAGGGGGGA
CTTTCGGGCCTCCTGTCTTCGGATGA
GTCCGCGTACCATTAGCTAGTTGGTAGGGTAATGGCCTACCAAGGCGACGATGGT
TAGCTGGTCTGAGAGGATGATCAGC

CACACTGGAACTGAGACACGGTCCAGACTCCTACGGGAGGCAGCAGTGGGGAAT
TTTGCGCAATGGGGGAAACCCTGACG
CAGCAACGCCGCGTGGGTGATGAAGGCCTTCGGGTCGTAAAGCCCTGTCGGGAG
GGAAGAAATGATTGAGAGCTAATACC
TCTTGGTCTTGACGGTACCTCCGAAGGAAGCACCGGCTAACTCCGTGCCAGCAGC
CGCGGTAATACGGAGGGTGCAAGCG
TTGTTCGGAATTATTGGGCGTAAAGCGCGTGTAGGCGGTCTTTTAAGTCTGATGTG
AAAGCCCCGGGCTCAACCTGGGAA
GTGCATTGGAAACTGGGAGACTTGAGTACGGGAGAGGAGAGTGGAATTCCTAGTG
TAGGAGTGAAATCCGTAGATATTAG
GAGGAACACCGGTGGCGAAGGCGGCTCTCTGGACCGATACTGACGCTGAGACGC
GAAAGCGTGGGTAGCAAACAGGATTA
GATACCCTGGTAGTCCACGCCGTAAACGATGAGTACTAGGTGTTGCGGGTATTGA
CCCCTGCAGTGCCGTAGCTAACGCA
TTAAGTACTCCGCCTGGGAAGTACGGTCGCAAGACTAAAACTCAAAGGAATTGACG
GGGGCCCGCACAAGCGGTGGAGCA
TGTGGTTTAATTCGACGCAACGCGCAGAACCTTACCTGGGCTTGACATCCACGGA
ACCCTCCCGAAACGGAGGGGTGCCC
TTCGGGGAGCCGTGAGACAGGTGCTGCATGGCTGTCGTCAGCTCGTGTCGTGAG
ATGTTGGGTTAAGTCCCGCAACGAGC
GCAACCCTTATCATCAGTTGCCATCATTAAGTTGGGCACTCTGATGAAACTGCCGG
TGTCAAACCGGAGGAAGGTGGGGA
TGACGTCAAGTCCTCATGGCCCTTATGTCCAGGGCTACACACGTGCTACAATGGC
CGGTACAAAGAGTCGCAATGCCGCG
AGGTGGAGCCAATCTCAAAAAGCCGGTCTCAGTTCGGATTGGAGTCTGCAACTCG
ACTCCATGAAGTCGGAATCGCTAGT
AATCGCGGATCAGCATGCCGCGGTGAATACGTTCCCGGGCCTTGTACACACCGCC
CGTCACACCACGGGAGTCGACTGGT
CCCGAAGTGCGTGAGCTAACCAGCAATGGAGGCAGCGTCCTAAGGAATGGTCGG
TGACTGGGGTGAAGTCGTAACAAGGT
AGCCGTAGGGGAACCTGCGGCTGGATCACCTCCTTT
>Pseudomonas_aeruginosa
GAACTGAAGAGTTTGATCATGGCTCAGATTGAACGCTGGCAGCAGGGGCCTTCAA
CACATGCAAGTCGAG
CTTATGAAGGGAGCTTGCCTTGGATTCAGCGGCGGACGGGTGAGTAATGCCTAGG
AATCTGCCTGGTAGT
GGGGGATAACGTCCGGAAACGGCCGCTAATACCGCATACGTCCTGAGGGAGAAA
GTCGGGGATCTTCGGA
CCTCACGCTATCAGATGAGCCTAGGTCGGATTAGCTAGTTGGTGGGGTAAAGGCC
TACCAAGGCGACGAT
CCGTAACTGGTCTGAGAGGATGATCAGTCACACTGGAACTGAGACACGGTCCAGA
CTCCTACGGGAGGCA
GCAGTGGGGAATATTGGACAATGGGCGCAAGCCTGATCCAGCCATGCCGCGTGT
GTGAAGAAGGTCTTCG
GATTGTAAAGCACTTTAAGTTGGGAGGAAGGGCAGTAAGTTAATACCTTGCTGTTT
GACGTTACCAACAG

AATAAGCACCGGCTAACTTCGTGCCAGCAGCCGCGGTAATACGAAGGGTGCAAGC
GTTAATCGGAATTAC
TGGGCGTAAAGCGCGCGTAAGTGGTTCAGCAAGCTTGATGTGAAATCCCCGGGCT
CAACCTGGGAACTGC
ATCCAAAAGCTACTGAGCTAGAGTACGGTAGAGGTGGTAGAATTTCCTGTGTAGCG
GTGAAATGCGTAGA
TATAGGAAGGAACACCAGTGGCGAAGGCGACCACCTGGACTGTACTGACACTGAG
GTGCGAAAGCGTGGG
GAGCAAACAGGATTAGATACCCTGGTAGTCCACGCCGTAAACGATGTCGACTAGC
CGTTGGGATCCTTGA
GATCTTAGTGGCGCACGTAACGCGATAAGTCGACCGCCTGGGGAGTACGGCCGC
AAGGTTAAAACTCAAA
TGAATTGACGGGGGCCCGCACAAGCGGTGGAGCATGTGGTTTAATTCGAAGCAAC
GCGAAGAACCTTACC
TGGCCTTGACATGCTGAGAACTTTCCAGAGATGGATTGGTGCCTTCGGGAACAGA
GACACAGGTGCTGCA
TGGCTGTCGTCAGCTCGTGTCGTGAGATGTTGGGTTAAGTCCCGTAACGAGCGCA
ACCCTTGTCCTTAGT
TACCAGCACCTCGGGTGGGCACTCTAAGGAGACTGCCGGTGACAAACCGGAGGA
AGGTGGGGATGACGTC
AAGTCATCATGGCCCTTACGGCCAGGGCTACACACGTGCTACAATGGTCGGTACA
AAGGGTTGCCAAGCC
GCGAGTGGGAGCTAATCCCATAAAACCGATCGTAGTCCGGATCGCAGTCTGCAAC
TCGACTGCGTGAAGT
CGGAATCGCTAGTAATCGTGAATCAGAATGTCACGGTGAATACGTCCCCGGGCCT
TGTACACACCGCCCG
TCACACCATGGGAGTGGGTTGCTCCAGAAGTAGCTAGTCTAACCGCAAGGGGGAC
GGTTACCACGGAGTG
ATTCATGACTGGGGTGAAGTCGTAACAAGGTAGCCGTAGGGGAACCTGCGGCTGG
ATCACCTCCTTA
>Nitrosococcus_oceani
AGGAGGTGATCCAGCCGCAGGTTCCCCTACGGCTACCTTGTTACGACTTCACCCC
AGTCATTGACCACAC
CGTAGTAAGCGTCCTCCCGAAGGTTAAACTACCCACTTCTGGTGCAACCAACTCCC
ATGGTGTGACGGGC
GGTGTGTACAAGGCCCGGGAACGTATTCACCGCGGCATTGCTGATCCGCGATTAC
TAGCGATTCCGACTT
CATGCAGTCGAGTTGCAGACTGCAATCCGGACTACGACCGGTTTTCTGAGATTAG
CTCCGCATCGCTGCT
TGGCCACCCTCTGTACCGGCCATTGTAGCACGTGTGTAGCCCTGGCCATAAGGGC
CATGATGACTTGACG
TCATCCCCACCTTCCTCCGTTTTGTCAACGGCAGTCCCTCTAGAGTGCCCGGCCG
AACCGCTGGCAACTA
GAGATAAGGGTTGCGCTCGTTGCGGGACTTAACCCAACATCTCACGACACGAGCT
GACGACAGCCATGCA

GCACCTGTCTCCCGGCTCCCGAAGGCACCTTCGCCTCTCAGCAAAGTTCCGGGGA
TGTCAAGGCCAGGTA
AGGTTCTTCGCGTTGCATCGAATTAAACCACATGCTCCACCGCTTGTGCGGGCCC
CCGTCAATTCATTTG
AGTTTCAACCTTGCGGCCGTACTCCCCAGGCGGAGAACTTAACGCGTTAGCTGCA
ACACTGAAAGGCTAT
CCCTCCCAACGTCTAGTTCTCATCGTTTAGGGCGTGGACTACCAGGGTATCTAATC
CTGTTTGCTCCCCA
CGCTTTCGCACCTCAGCGTCAGTAGTGGTCCAGGAGGCCGCCTTCGCCACTGGTG
TTCCTTCCGATATCT
ACGCATTTCACCGCTACACCGGAAATTCCGCCTCCCTCTACCATACTCTAGCCAGG
CAGTATCAAGTGCC
ATTCCCAGGTTGAGCCCGGGGCTTTCACACCCAACTTACCAAACCGCCTACGCGC
CCTTTACGCCCAGTA
ATTCCGATTAACGCTCGCACCCTCCGTATTACCGCGGCTGCTGGCACGGAGTTAG
CCGGTGCTTCTTCTG
TAGGTAACGTCAACACGATGTGCTATTCACACATCGCTTTTCTTCCCCACTGAAAG
GGCTTTACAACCCG
AAAGCCTTCTTCACCCACGCGGCATTGCTGGATCAGGGTTGCCCCCATTGTCCAAT
ATTCCCCACTGCTG
CCTCCCGTAGGAGTCTGGGCCGTGTCTCAGTCCCAGTGTGGCTGATCGTCCTCTC
AGACCAGCTACCGAT
CATCGCCTTGGTAGGCCCTTACCCCACCAACTAGCTAATCGGACGCAGGCTCATC
CCCCAGCGCGAGGCC
TTTCGGTCCCCCGCTTTCCTCCTTAGAGATTATGCGGTATTAGCCCGAGTTTCCCC
GAGTTATCCCCCTC
TAGAGGCCAGATTCCCACGCGTTACTCACCCGTCCGCCACTCGCCAGCCACCTAA
GCAAGCTTAGGTGCT
GCTGCCGTTCGACTTGCATGTGTTAAGCATGCCGCCAGCGTTCAATCTGAGCCAT
GATCAAACTCTTCAG
TTTAA

Input Fasta file for the PufM Woods Hole Isolates Phylogenetic Tree

>AB35
MAEYQNIFTQVQVQGPSDWGMDNENNMMEERAGMGHFSILGWLG
NAQLGPIYLGYTGVVSLIAGCFAFLIIGLNMLAQVDWSLVQLLRQGFWLALEPPSPEY
GLRIPPLKEGGWYMVASFFLLISVWAWWARTYMLAVEHKMGKHIAWAFLSAIWLFM
VL
GFFRPILMGSWSEMVPYGIFPHLDWTTAFSIRYGNLYYNPFHALSIAFLYGSALLFA
M
HGGTILAVTRFGGDRELEQIYDRGTASERAALFWRWTMGFNATMEGIHRWAWWF
AVLT
PLTGGIGILLTGTVVDNWYIWAQEHNFVTEYTQPYGVDAYVGQGG
>DSM2351
MAEYQNIFTQVQVQGPSDWGMDNENNMMEERAGMGHFSILGWLG
NAQLGPIYLGYTGVVSLIAGCFAFLIIGLNMLAQVDWSLVQLLRQGFWLALEPPSPEY
GLRIPPLKEGGWYMVASFFLLVSVWAWWARTYMLAVEHKMGKHIAWAFLSAIWLF
MVL
GFFRPILMGSWSEMVPYGIFPHLDWTTAFSIRYGNLYYNPFHALSIAFLYGSALLFA
M
HGGTILAVTRFGGDRELEQIYDRGTASERAALFWRWTMGFNATMEGIHRWAWWF
AVLT
PLTGGIGILLTGTVVDNWFIWAQEHNFVTEYTQPYGVDAYVGQGG
>DSM1374
MAEYQNIFTQVQVQGPSDWGMDNENNMMEERAGMGHFSILGWLG
NAQLGPIYLGYTGVVSLIAGCFAFLIIGLNMLAQVDWSLVQLLRQGFWLALEPPSPEY
GLRIPPLKEGGWYMVASFFLLISVWAWWARTYMLAVEHKMGKHIAWAFLSAIWLFM
VL
GFFRPILMGSWSEMVPYGIFPHLDWTTAFSIRYGNLYYNPFHALSIAFLYGSALLFA
M
HGGTILAVTRFGGDRELEQIYDRGTASERAALFWRWTMGFNATMEGIHRWAWWF
AVLT
PLTGGIGILLTGTVVDNWYIWAQEHNFVTEYTQPYGVDAYVGQGG
>IM796
MAEYQNIFTQVQVQGPSDWGMDNENNMMEERAGMGHFSILGWLG
NAQLGPIYLGYTGVVSLIAGCFAFLIIGLNMLAQVDWSLVQLLRQGFWLALEPPSPEY
GLRIPPLKEGGWYMVASFFLLISVWAWWARTYMLAVEHKMGKHIAWAFLSAIWLFM
VL
GFFRPILMGSWSEMVPYGIFPHLDWTTAFSIRYGNLYYNPFHALSIAFLYGSALLFA
M
HGGTILAVTRFGGDRELEQIYDRGTASERAALFWRWTMGFNATMEGIHRWAWWF
AVLT
PLTGGIGILLTGTVVDNWYIWAQEHNFVTEYTQPYGVDAYVGQGG
>SE1
MAEYQNIFTQVQVQGPSDWGMDNENNMMEERAGMGHFSILGWLG
NAQLGPIYLGYTGVVSLIAGCFAFLIIGLNMLAQVDWSLVQLLRQGFWLALEPPSPEY

GLRIPPLKEGGWYMVASFFLLISVWAWWARTYMLAVEHKMGKHIAWAFLSAIWLFM
VL
GFFRPILMGSWSEMVPYGIFPHLDWTTAFSIRYGNLYYNPFHALSIAFLYGSALLFA
M
HGGTILAVTRFGGDRELEQIYDRGTASERAALFWRWTMGFNATMEGIHRWAWWF
AVLT
PLTGGIGILLTGTVVDNWYIWAQEHNFVTEYTQPYGVDAYVGQGG
>SNK001
MAEYQNIFTQVQVQGPSDWGMDNENNMMEERAGMGHFSILGWLG
NAQLGPIYLGYTGVVSLIAGCFAFLIIGLNMLAQVDWSLVQLLRQGFWLALEPPSPEY
GLRIPPLKEGGWYMVASFFLLISVWAWWARTYMLAVEHKMGKHIAWAFLSAIWLFM
VL
GFFRPILMGSWSEMVPYGIFPHLDWTTAFSIRYGNLYYNPFHALSIAFLYGSALLFA
M
HGGTILAVTRFGGDRELEQIYDRGTASERAALFWRWTMGFNATMEGIHRWAWWF
AVLT
PLTGGIGILLTGTVVDNWYIWAQEHNFVTEYTQPYGVDAYVGQGG
>AB14
MAEYQNIFTQVQVQGPSDWGMDNENNMMEERAGMGHFSILGWLG
NAQLGPIYLGYTGVVSLIAGCFAFLIIGLNMLAQVDWSLVQLLRQGFWLALEPPSPEY
GLRIPPLKEGGWYMVASFFLLISVWAWWARTYMLAVEHKMGKHIAWAFLSAIWLFM
VL
GFFRPILMGSWSEMVPYGIFPHLDWTTAFSIRYGNLYYNPFHALSIAFLYGSALLFA
M
HGGTILAVTRFGGDRELEQIYDRGTASERAALFWRWTMGFNATMEGIHRWAWWF
AVLT
PLTGGIGILLTGTVVDNWYIWAQEHNFVTEYTQPYGVDAYVGQGG
>AB15
MAEYQNIFTQVQVQGPSDWGMDNENNMMEERAGMGHFSILGWLG
NAQLGPIYLGYTGVVSLIAGCFAFLIIGLNMLAQVDWSLVQLLRQGFWLALEPPSPEY
GLRIPPLKEGGWYMVASFFLLISVWAWWARTYMLAVEHKMGKHIAWAFLSAIWLFM
VL
GFFRPILMGSWSEMVPYGIFPHLDWTTAFSIRYGNLYYNPFHALSIAFLYGSALLFA
M
HGGTILAVTRFGGDRELEQIYDRGTASERAALFWRWTMGFNATMEGIHRWAWWF
AVLT
PLTGGIGILLTGTVVDNWYIWAQEHNFVTEYTQPYGVDAYVGQGG
>AB16
MAEYQNIFTQVQVQGPSDWGMDNENNMMEERAGMGHFSILGWLG
NAQLGPIYLGYTGVVSLIAGCFAFLIIGLNMLAQVDWSLVQLLRQGFWLALEPPSPEY
GLRIPPLKEGGWYMVASFFLLISVWAWWARTYMLAVEHKMGKHIAWAFLSAIWLFM
VL
GFFRPILMGSWSEMVPYGIFPHLDWTTAFSIRYGNLYYNPFHALSIAFLYGSALLFA
M
HGGTILAVTRFGGDRELEQIYDRGTASERAALFWRWTMGFNATMEGIHRWAWWF
AVLT

PLTGGIGILLTGTVVDNWYIWAQEHNFVTEYTQPYGVDAYVGQGG
>AB17
MAEYQNIFTQVQVQGPSDWGMDNENNMMEERAGMGHFSILGWLG
NAQLGPIYLGYTGVVSLIAGCFAFLIIGLNMLAQVDWSLVQLLRQGFWLALEPPSPEY
GLRIPPLKEGGWYMVASFFLLISVWAWWARTYMLAVEHKMGKHIAWAFLSAIWLFM
VL
GFFRPILMGSWSEMVPYGIFPHLDWTTAFSIRYGNLYYNPFHALSIAFLYGSALLFA
M
HGGTILAVTRFGGDRELEQIYDRGTASERAALFWRWTMGFNATMEGIHRWAWWF
AVLT
PLTGGIGILLTGTVVDNWYIWAQEHNFVTEYTQPYGVDAYVGQGG
>AB18
MAEYQNIFTQVQVQGPSDWGMDNENNMMEERAGMGHFSILGWLG
NAQLGPIYLGYTGVVSLIAGCFAFLIIGLNMLAQVDWSLVQLLRQGFWLALEPPSPEY
GLRIPPLKEGGWYMVASFFLLISVWAWWARTYMLAVEHKMGKHIAWAFLSAIWLFM
VL
GFFRPILMGSWSEMVPYGIFPHLDWTTAFSIRYGNLYYNPFHALSIAFLYGSALLFA
M
HGGTILAVTRFGGDRELEQIYDRGTASERAALFWRWTMGFNATMEGIHRWAWWF
AVLT
PLTGGIGILLTGTVVDNWYIWAQEHNFVTEYTQPYGVDAYVGQGG
>AB20
MAEYQNIFTQVQVQGPSDWGMDNENNMMEERAGMGHFSILGWLG
NAQLGPIYLGYTGVVSLIAGCFAFLIIGLNMLAQVDWSLVQLLRQGFWLALEPPSPEY
GLRIPPLKEGGWYMVASFFLLISVWAWWARTYMLAVEHKMGKHIAWAFLSAIWLFM
VL
GFFRPILMGSWSEMVPYGIFPHLDWTTAFSIRYGNLYYNPFHALSIAFLYGSALLFA
M
HGGTILAVTRFGGDRELEQIYDRGTASERAALFWRWTMGFNATMEGIHRWAWWF
AVLT
PLTGGIGILLTGTVVDNWYIWAQEHNFVTEYTQPYGVDAYVGQGG
>AB21
MAEYQNIFTQVQVQGPSDWGMDNENNMMEERAGMGHFSILGWLG
NAQLGPIYLGYTGVVSLIAGCFAFLIIGLNMLAQVDWSLVQLLRQGFWLALEPPSPEY
GLRIPPLKEGGWYMVASFFLLISVWAWWARTYMLAVEHKMGKHIAWAFLSAIWLFM
VL
GFFRPILMGSWSEMVPYGIFPHLDWTTAFSIRYGNLYYNPFHALSIAFLYGSALLFA
M
HGGTILAVTRFGGDRELEQIYDRGTASERAALFWRWTMGFNATMEGIHRWAWWF
AVLT
PLTGGIGILLTGTVVDNWYIWAQEHNFVTEYTQPYGVDAYVGQGG
>AB22
MAEYQNIFTQVQVQGPSDWGMDNENNMMEERAGMGHFSILGWLG
NAQLGPIYLGYTGVVSLIAGCFAFLIIGLNMLAQVDWSLVQLLRQGFWLALEPPSPEY
GLRIPPLKEGGWYMVASFFLLISVWAWWARTYMLAVEHKMGKHIAWAFLSAIWLFM
VL

GFFRPILMGSWSEMVPYGIFPHLDWTTAFSIRYGNLYYNPFHALSIAFLYGSALLFA
M
HGGTILAVTRFGGDRELEQIYDRGTASERAALFWRWTMGFNATMEGIHRWAWWF
AVLT
PLTGGIGILLTGTVVDNWYIWAQEHNFVTEYTQPYGVDAYVGQGG
>AB23
MAEYQNIFTQVQVQGPSDWGMDNENNMMEERAGMGHFSILGWLG
NAQLGPIYLGYTGVVSLIAGCFAFLIIGLNMLAQVDWSLVQLLRQGFWLALEPPSPEY
GLRIPPLKEGGWYMVASFFLLISVWAWWARTYMLAVEHKMGKHIAWAFLSAIWLFM
VL
GFFRPILMGSWSEMVPYGIFPHLDWTTAFSIRYGNLYYNPFHALSIAFLYGSALLFA
M
HGGTILAVTRFGGDRELEQIYDRGTASERAALFWRWTMGFNATMEGIHRWAWWF
AVLT
PLTGGIGILLTGTVVDNWYIWAQEHNFVTEYTQPYGVDAYVGQGG
>AB26
MAEYQNIFTQVQVQGPGDWGMDNENNMMEERAAGTGHFSILGWL
GNAQLGPIYLGYTGVISVIAGAFAFLIIGLNMLAQVDWSLVQFLRQGFWLALEPPSPE
YGLRIPPLKEGGWYMIASFFLLIAVWAWWARTYMLAVEHKMGKHIAWAFLSAIWLF
MV
LGFFRPILMGSWSEMVPYGIFPHLDWTTAFSIRYGNLYYNPFHALSIAFLYGSALLFA
MHGGTILAVTRFGGDRELEQIYDRGTASERAALFWRWTMGFNATMEGIHRWAWW
FAVL
TPLTGGIGILLTGTVVDNWYIWAQEHNFVTEYTQPYGIDAYVGQGG
>AB28
MAEYQNIFTQVQVQGPSDWGMDNENNMMEERAGMGHFSILGWLG
NAQLGPIYLGYTGVVSLIAGCFAFLIIGLNMLAQVDWSLVQLLRQGFWLALEPPSPEY
GLRIPPLKEGGWYMVASFFLLISVWAWWARTYMLAVEHKMGKHIAWAFLSAIWLFM
VL
GFFRPILMGSWSEMVPYGIFPHLDWTTAFSIRYGNLYYNPFHALSIAFLYGSALLFA
M
HGGTILAVTRFGGDRELEQIYDRGTASERAALFWRWTMGFNATMEGIHRWAWWF
AVLT
PLTGGIGILLTGTVVDNWYIWAQEHNFVTEYTQPYGVDAYVGQGG
>AB30
MAEYQNIFTQVQVQGPSDWGMDNENNMMEERAGMGHFSILGWLG
NAQLGPIYLGYTGVVSLIAGCFAFLIIGLNMLAQVDWSLVQLLRQGFWLALEPPSPEY
GLRIPPLKEGGWYMVASFFLLISVWAWWARTYMLAVEHKMGKHIAWAFLSAIWLFM
VL
GFFRPILMGSWSEMVPYGIFPHLDWTTAFSIRYGNLYYNPFHALSIAFLYGSALLFA
M
HGGTILAVTRFGGDRELEQIYDRGTASERAALFWRWTMGFNATMEGIHRWAWWF
AVLT
PLTGGIGILLTGTVVDNWYIWAQEHNFVTEYTQPYGVDAYVGQGG
>AB33
MAEYQNIFTQVQVQGPSDWGMDNENNMMEERAGMGHFSILGWLG

NAQLGPIYLGYTGVVSLIAGCFAFLIIGLNMLAQVDWSLVQLLRQGFWLALEPPSPEY
GLRIPPLKEGGWYMVASFFLLISVWAWWARTYMLAVEHKMGKHIAWAFLSAIWLFM
VL
GFFRPILMGSWSEMVPYGIFPHLDWTTAFSIRYGNLYYNPFHALSIAFLYGSALLFA
M
HGGTILAVTRFGGDRELEQIYDRGTASERAALFWRWTMGFNATMEGIHRWAWWF
AVLT
PLTGGIGILLTGTVVDNWYIWAQEHNFVTEYTQPYGVDAYVGQGG
>TIE_1
MAQYQNIFTQVQVEGPAYAGVPLRPGSSPRETQTTFNYWLGKIGDAQVGPVYLGF
TGVCSLLCGFVAIEI
IGLNMLASVDWSPIEFLRQFCWLALEPPKPEYGLTIPPLKEGGWWLMAGFFLTVSIA
LWWVRTYRRSRAL
GMGTHVSWAFASAILLYLALGFIQPLLMGSWSEAPPFGVFPHLDWTNNFSIKYGNL
YYNPFHCLSIAFLY
GSALLFAMHGATILAVSRYGGEREIEQMLDRGTALERAALFWRWTMGFNATAESIH
RWAWWFAVLCPLTG
AIGIILTGPVVDNWFDWGVKHGLAPPR
>Chloroflexus_aggregans
MATMDMMPGDLELGRDRGRIGKPIEIPLLENFGFDSQLGPFYLGFWNTVAYISGGIF
TFIWLFVMLSQVN
FNPVAFIKYFVVLQIDPPSSRYGLSFPPLNEGGWWLIATFFLTLSIFAWYMHIYTRAK
ALGIKPYLAYGF
TGAIALYLVIYIIRPVWMGDWSEAPAHGIKALLDWTNNVSVRYGNFYYNPFHMLSIFF
LLGSTLLLAMHA
GTIWALEKYAAHEEWNEIQAPGTGTERAQLFWRWCIGFNANAYSIHLWAFWFAWL
CGITGALGVFFSMPD
FVNNWFQWGVEAGIAYPQGPTPLP

Fasta File Input for the Woods Whole EeuP Phylogenetic Tree

>AB26
MFTRSVIFSLLFSGAAAPVAAQTVFVDQGDSWSPSLRAAFYTQDQGSRIMPLAWMEALAQPD
GTPFLADGLARYGYLPMPDRDDGATLPVGFTTNEGMGGTAIGMTCAACHTRQIEVRGTAYRID
GGPAIVDFQSFLADLDAAMQAVLDDDATFDAFADKVAGGTGDRAGLRAELETWSLRFHTLIDG
SLPDPGWGPVRLDAVSMIFNRLTGLDLGRPEDAYLIPENVMLADAPTRYPFLWNAARQDMTQ
WPGFAHNGNDLLGLARNLGEVYGVFGEFHPIKKPGLLFNRDYVTNNSANWGGLKALEEWIWD
IGAPRWPWELDAGLVEQGGAIFARPTDQGGCVECHGKRQGAFRSIFHATWATPILPVGTDER
ECEILTRTAKTGVLEGAKVPLSSNTLGAEAPAFDLLGASVIGAIIQHAVGFRVTGGLEATGAAAA
LQKDDKFMAEFDDLRGAFPRSGDGSALETAAAPCKYEARVLDGIWAAAPYLHNGSVPTLRELL
TPPDERVADFTPGPAYDLEAVGMAVEQTAFDHVIETTGCDDLSSGNSRCGHDYGTALSDDDK
RALLEYLKSI
>AB33
MFTRSVIFSLLFSGAAAPVTAQTVFVDQGDSWSPSLRAAFYTQDQGSRIMPLTWMEALAQPD
GTPFLADGLARYGYLPMPDRDDGAALPVGFTTNEGMGGTAIGMTCAACHTRQIEVRGTAYRID
GGPAIVDFQSFLADLDAAMQAVLDDDATFDAFADKVAGETGDRAGLRAELETWSLRFHTLIDG
SLPDPGWGPVRLDAVSMIFNRLTGLDLGRPEDAYLIPENVMLADAPTRYPFLWNAARQDMTQ
WPGFAHNGNDLLGLARNLGEVYGVFGEFHPIKKQGLLFNRDYVTNNSANWGGLKALEEWIWD
IGAPRWPWGLDAGLVEQGGAIFSRPTDQGGCVECHGKRQGAFRSIFHATWATPILPVGTDER
ECEILTRTAKTGVLEGAKVPLSSNALGAEAPAFDLLGASVIGAIIQHAVGFRVTGGLEATGAAAA
LQQDDKFMAEFDDLRGAFPRSGDGSALETAAAPCAYEARVLDGIWAAAPYLHNGSVPTLRELL
TPPDERVADFTPGPAYNLEAVGMATEQTAFDHVIETTGCDDLSSGNSRCGHDYGTALSDDDK
RALLEYLKSI
>AB22
MFTRSVIFSLLFSGAAAPVAAQTVFVDQGDSWSPSLRAAFYTQDQGSRIMPLAWMEALAQPD
GTPFLADGLARYGYLPMPDRDDGATLPVGFTTNEGMGGTAIGMTCAACHTRQIEVRGTAYRID
GGPAIVDFQSFLADLDAAMQAVLDDDATFDAFADKVAGGTGDRAGLRAELETWSLRFHTLIDG
SLPDPGWGPVRLDAVSMIFNRLTGLDLGRPEDAYLIPENVMLADAPTRYPFLWNAARQDMTQ
WPGFAHNGNDLLGLARNLGEVYGVFGEFHPIKKPGLLFNRDYVTNNSANWGGLKALEEWIWD
IGAPRWPWELDAGLVEQGGAIFARPTDQGGCVECHGKRQGAFRSIFHATWATPILPVGTDER
ECEILTRTAKTGVLEGAKVPLSSNTLGAEAPAFDLLGASVIGAIIQHAVGFRVTGGLEATGAAAA
LQKDDKFMAEFDDLRGAFPRSGDGSALETAAAPCKYEARVLDGIWAAAPYLHNGSVPTLRELL
TPPDERVADFTPGPAYDLEAVGMAVEQTAFDHVIETTGCDDLSSGNSRCGHDYGTALSDDDK
RALLEYLKSI
>AB35
MFTRSVIFSLLFSGAAAPVAAQTVFVDQGDSWSPSLRAAFYTQD
QGSRIMPLAWMEALAQPDGTPFLADGLARYGYLPMPDRDDGAALPVGFTPNEGMGGTA
IGMTCAACHTRQIEVRGTAYRIDGGPAIVDFQSFLADLDAAMQAVLDDEATFDAFADK
VAGGTGDRAGLRAELETWSLRFHTLIDGSLPDPGWGPVRLDAVSMIFNRLTGLDLGRP
EDAYLIPENVMLADAPTRYPFLWNAARQDMTQWPGFAHNGNDLLGLARNLGEVYGVFG
EFHPIKKPGLLFNRDYVTNNSANWGGLKALEEWIWDIGAPRWPWGLDAGLVEQGGAIF
ARPTDQGGCVECHGKRQGAFRSIFHSTWATPILPVGTDERECEILTRTAKTGVLEGAK
VPLSSNTLGAEAPAFDLLGASVIGAIIQHAVGFRVTGGLEATGAAAALQKDDKFMAEF
DDLRGAFPRSGDGSALETAAEPCKYEARVLDGIWAAAPYLHNGSVPTLRELLTPPDER
VADFTPGPAYDLEAVGMAVEQTAFDHVIETTGCDDLSSGNSRCGHDYGTALSDDDKRA
LLEYLKSI
>AB20
MFTRSVIFSLLFSGAAAPVAAQTVFVDQGDSWSPSLRAAFYTQD
QGSRIMPLAWMEALAQPDGTPFLADGLARYGYLPMPDRDDGATLPVGFTTNEGMGGTA
IGMTCAACHTRQIEVRGTAYRIDGGPAIVDFQSFLADLDAAMQAVLDDEAVFDAFADK

VAGETGDRAGLRAELETWSLRFHTLIDGSLPDPGWGPVRLDAVSMIFNRLTGLDLGRP
EDAYLIPENVMLADAPTRYPFLWNAARQDMTQWPGFAHNGNDLLGLARNLGEVYGVFG
EFHPIKKPGLLFNRDYVTNNSANWGGLKALEEWIWDIGAPRWPWELDAGLVEQGGAIF
ARPTDQGGCVECHGKRQGAFRSIFHATWATPILPVGTDERECEILTRTARTGVLEGAK
VPLSSNTLGAEAPAFDLLGASVIGAIIQHAVGFRVTGGLEATGAAAALQKDDKFMAEF
DDLRGAFPRSGDGSALETAAAPCAYEARVLDGIWAAAPYLHNGSVPTLRELLTPPDER
VADFTPGPAYDLEAVGMAVEQTAFDHVIETTGCDDLSSGNSRCGHDYGTALSDDDKRA
LLEYLKSI
>IM796
MFTRSVIFSLLFSGAAAPVAAQTVFVDQGDSWSPSLRAAFYTQD
QGSRIMPLAWMEALAQPDGTPFLADGLARYGYLPMPDRDDGAALPVGFTTNEGMGGTA
IGMTCAACHTRQIEVRGTAYRIDGGPAIVDFQSFLADLDAAMQAVLDDDATFDAFADK
VAGETGDRAGLRAELETWSLRFHTLIDGSLPDPGWGPVRLDAVSMIFNRLTGLDLGRP
EDAYLIPENVMLADAPTRYPFLWNAARQDMTQWPGFAHNGNDLLGLARNLGEVYGVFG
EFHPIKKQGLLFNRDYVTNNSANWGGLKALEEWIWDIGAPRWPWGLDAGLVEQGGAIF
ARPTDQGGCVECHGKRQGAFRSIFHATWATPILPVGTDERECEILTRTAKTGVLEGAK
VPLSSNALGAEAPAFDLLGASVIGAIIQHAVGFRVTGGLEATGAAAALQKDDKFMAEF
DDLRGAFPRSGDGSALETAAAPCAYEARVLDGIWAAAPYLHNGSVPTLRELLTPPDER
VADFTPGPAYDLEAVGMAVEQTAFDHVIETTGCDDLSSGNSRCGHDYGTALSDDDKRA
LLEYLKSI
>AB30
MNEKIIPILFWEESMFTRSVIFSLLFSGAAAPVTAQTVFVDQGDSWSPSLRAAFYTQDQGSRIM
PLTWME
ALAQPDGTPFLADGLARYGYLPMPDRDDGAALPVGFTTNEGMGGTAIGMTCAACHTRQIEVR
GTAYRIDG
GPAIVDFQSFLADLDAAMQAVLDDDATFDAFADKVAGETGDRAGLRAELETWSLRFHTLIDRSL
PDPGWG
PVRLDAVSMIFNRLTGLDLGRPEDAYLIPENVMLADAPTRYPFLWNAARQDMTQWPGFAHNG
NDLLGLAR
NLGEVYGVFGEFHPIKKQGLLFNRDYVTNNSANWGGLKALEEWIWDIGAPRWPWGLDAGLVE
QGGAIFAR
PTDQGGCVECHGKRQGAFRSIFHATWATPILPVGTDERECEILTRTAKTGVLEGAKVPLSSNTL
GAEAPA
FDLLGASVIGAIIQHAVGFRVTGGLEATGAAAALQQDDKFMAEFDDLRGAFPRSGDGSALETAA
EPCAYE
ARVLDGIWAAAPYLHNGSVPTLRELLTPPDERVADFTPGPAYDLEAVGMAVEQTAFDHVIETTG
CDDLSS
GNSRCGHDYGTALSDDDKRALLEYLKSI
>AB23
MFTRSVIFSLLFSGAAAPVAAQTVFVDQGDSWSPSLRAAFYTQD
QGSRIMPLAWMEALAQPDGTPFLADGLARYGYLPMPDRDDGAALPVGFTTNEGMGGTA
IGMTCAACHTRQIEVRGTAYRIDGGPAIVDFQNFLADLDAAMQAVLDDDATFDAFADK
VAGETGDRAGLRAELETWSLRFHTLIDGSLPDPGWGPVRLDAVSMIFNRLTGLDLGRP
EDAYLIPENVMLADAPTRYPFLWNAARQDMTQWPGFAHNGNDLLGLARNLGEVYGVFG
EFHPIKKQGLLFNRDYVTNNSANWGGLKALEEWIWDIGAPRWPWELDAGLVEQGGAIF
ARPTDQGGCVECHGKRQGAFRSIFHATWATPILPVGTDERECEILTRTAKTGVLEGAK
VPLSSNTLGAEAPAFDLLGASVIGAIIQNAVGFRVTGGLEATGAAAALQKDGKFMAEF
DDLRGAFPRSGDGSALETAAEPCKYEARVLDGIWAAAPYLHNGSVPTLRELLTPPDER
VADFTPGPAYDLEAVGMAVEQTAFDHVIETTGCDDLSSGNSRCGHDYGTALSDDDKRA
LLEYLKSI
>AB16

MFTRSVIFSLLFSGAAAPVAAQTVFVDQGDSWSPSLRAAFYTQD
QGSRIMPLAWMEALAQPDGTPFLADGLARYGYLPMPDRDDGAALPVGFTTNEGMGGTA
IGMTCAACHTRQIEVSGTAYRIDGGPAIVDFQSFLADLDAAMQAVLDDEAVFDAFADK
VAGETGDRAGLRAELETWSLRFHTLIDRSLPDPGWGPVRLDAVSMIFNRLTGLDLGRP
EDAYLIPENVMLADAPTRYPFLWNAARQDMTQWPGFAHNGNDLLGLARNLGEVYGVFG
EFHPIKKQGLLFNRDYVTNNSANWGGLKALEEWIWDIGAPRWPWGLDAGLVEQGGAIF
ARPTDQGGCVECHGKRQGAFRSIFHATWATPILPVGTDERECEILTRTAKTGVLEGAK
VPLSSNALGAEAPAFDLLGASVIGAIIQHAVGFRVTGGLEATGAAAALQKDGKFMAEF
DDLRGAFPRSGDGSALETAAAPCKYEARVLDGIWAAAPYLHNGSVPTLRELLTPPDER
VADFTPGPAYDLEAVGMAVEQTAFDHVIETTGCDDLSSGNSRCGHDYGTALSDGDKRA
LLEYLKSI
>AB14
MNEKITPILFWEESMFTRSVIFSLLFSGAAAPVAAQTVFVDQGDSWSPSLRAAFYTQDQGSRIM
PLAWME
ALAQPDGTPFLADGLARYGYLPMPDRDDGATLPVGFTTNEGMGGTAIGMTCAACHTRQIEVR
GTAYRIDG
GPAIVDFQSFLADLDAAMQAVLDDDATFDAFADKVAGGTGDRAGLRAELETWSLRFHTLIDGS
LPDPGWG
PVRLDAVSMIFNRLTGLDLGRPEDAYLIPENVMLADAPTRYPFLWNAARQDMTQWPGFAHNG
NDLLGLAR
NLGEVYGVFGEFHPIKKPGLLFNRDYVTNNSANWGGLKALEEWIWDIGAPRWPWGLDARLVE
QGGAIFAR
PTDQGGCVECHGKRQGAFRSIFHATWATPILPVGTDERECEILTRTARTGVLEGAKVPLSSNAL
GAEAPA
FDLLGASVIGAIIQHAVGFRVTGGLEATGAAAALQKDDKFMAEFDDLRGAFPRSGDGSALETAA
EPCAYE
ARVLDGIWAAAPYLHNGSVPTLRELLTPPDERVADFTPGPAYDLEAVGMAVEQTAFDHVIETTG
CDDLSS
GNSRCGHDYGTALSDDDKRALLEYLKSI
>AB15
MFTRSVIFSLLFSGAAAPVAAQTVFVDQGDSWSPSLRAAFYTQDQGSRIMPLAWMEALAQPD
GTPFLADG
LARYGYLPMPDRDDGAALPVGFTTNEGMGGTAIGMTCAACHTRQIEVRGTAYRIDGGPAIVDF
QSFLADL
DAAMQAVLDDEATFDAFADKVAGGTGDRAGLRAELETWSLRFHTLIDGSLPDPGWGPVRLDA
VSMIFNRL
TGLDLGRPEDAYLIPENVMLADAPTRYPFLWNAARQDMTQWPGFAHNGNDLLGLARNLGEVY
GVFGEFHP
IKKPGLLFNRDYVTNNSANWGGLKALEEWIWDIGAPRWPWGLDAGLVEQGGAIFARPTDQGG
CVECHGKR
QGAFRSIFHSTWATPILPVGTDERECEILTRTAKTGVLEGAKVPLSSNTLGAEAPAFDLLGASVI
GAIIQ
HAVGFRVTGGLEATGAAAALQKDDKFMAEFDDLRGAFPRSGDGSALETAAEPCKYEARVLDGI
WAAAPYL
HNGSVPTLRELLTPPDERVADFTPGPAYDLEAVGMAVEQTAFDHVIETTGCDDLSSGNSRCGH
DYGTALS
DDDKRALLEYLKSI
>AB17
MFTRSVIFSLLFSGAAAPVAAQTVFVDQGDSWSPSLRAAFYTQDQGSRIMPLAWMEALAQPD
GTPFLADG

LARYGYLPMPDRDDGAALPVGFTTNEGMGGTAIGMTCAACHTRQIEVRGTAYRIDGGPAIVDF
QSFLADL
DAAMQAVLDDDATFDAFADKVAGETGDRAGLRAELETWSLRFHTLIDGSLPDPGWGPVRLDA
VSMIFNRL
TGLDLGRPEDAYLIPENVMLADAPTRYPFLWNAARQDMTQWPGFAHNGNDLLGLARNLGEVY
GVFGEFHP
IKKPGLLFNRDYVTNNSANWGGLKALEEWIWDIGAPRWPWELDAGLVEQGGAIFARPTDQGG
CVECHGKR
QGAFRSIFHATWATPILHVGTDERECEILTRTARTGVLEGAKVPLSSNALGAEAPAFDLLGASVI
GAIIQ
NAVGFRVTGGLEATGAAAALQKDDKFMAEFDDLRGAFPRSGDGSALETAAEPCKYEARVLDGI
WAAAPYL
HNGSVPTLRELLTPPDERVADFTPGPAYDLEAVGMAVEQTAFDHVIETTGCDDLSSGNSRCGH
DYGTALS
DDDKRALLEYLKSR
>AB18
MFTRSVIFSLLFSGAAAPVTAQTVFVDQGDSWSPSLRAAFYTQDQGSRIMPLTWMEALAQPD
GTPFLADG
LARYGYLPMPDRDDGAALPVGFTTNEGMGGTAIGMTCAACHTRQIEVRGTAYRIDGGPAIVDF
QSFLADL
DAAMQAVLDDDATFDAFADKVAGETGDRAGLRAELETWSLRFHTLIDGSLPDPGWGPVRLDA
VSMIFNRL
TGLDLGRPEDAYLIPENVMLADAPTRYPFLWNAARQDMTQWPGFAHNGNDLLGLARNLGEVY
GVFGEFHP
IKKQGLLFNRDYVTNNSANWGGLKALEEWIWDIGAPRWPWGLDAGLVEQGGAIFSRPTDQGG
CVECHGKR
QGAFRSIFHATWATPILPVGTDERECEILTRTAKTGVLEGAKVPLSSNALGAEAPAFDLLGASVI
GAIIQ
HAVGFRVTGGLEATGAAAALQQDDKFMAEFDDLRGAFPRSGDGSALETAAAPCAYEARVLDG
IWAAAPYL
HNGSVPTLRELLTPPDERVADFTPGPAYNLEAVGMATEQTAFDHVIETTGCDDLSSGNSRCGH
DYGTALS
DDDKRALLEYLKSI
>AB28
MFTRSVIFSLLFSGAAAPVAAQTVFVDQGDSWSPSLRAAFYTQDQGSRIMPLAWMEALAQTD
GTPFLADG
LARYGYLPMPDRDDGAALPVGFTTNEGMGGTAIGMTCAACHTRQIEVRGTAYRIDGGPAIVDF
QSFLADL
DAAMQAVLDDEAVFDAFADKLAGETGDRAGLRAELETWSLRFHTLIDGSLPDPGWGPVRLDA
VSMIFNRL
TGLDLGRPEDAYLIPENVMLADAPTRYPFLWNAARQDMTQWPGFAHNGNDLLGLARNLGEVY
GVFGEFHP
IKKQGLLFNRDYVTNNSANWGGLKALEEWIWDIGAPRWPWGLDAGLVEQGGAIFARPTDQGG
CVECHGKR
QGAFRSIFHATWATPILPVGTDERECEILTRTAKTGVLEGAKVPLSSNALGAEAPASDLLGASVI
GAIIQ
HAAGFRVTGGLEATGAAAALQKDDKFMAEFDDLRGAFPRSGDGSALETAAAPCAYEARVLDGI
WAAAPYL
HNGSVPTLRELLTPPDERVADFTPGPAYDLEAVGMAVEQTAFDHVIETTGCDDLSSGNSRCGH
DYGTALS
DDDKRALLEYLKSI

Input Fasta File for the 16S New Taxonomic Reassignment Tree

>Rhodovulum_viride_JA756
ATGCAGTCGAGCGACCCTTCGGGGTTAGCGGCGGACGGGTGAGTAACGCGTGGGAACGT
GCCCTTCTCTGCGGAATAGGC
TCGGGAAACTGGGTTTAATACCGCATACGCCCTTCGGGGGAAAGATTTATCGGAGAAGGA
TCGGCCCGCGTCTGATTAGG
TAGTTGGTGGGGTAATGGCCTACCAAGCCTACGATCAGTAGCTGGTTTGAGAGGATGATC
AGCCACACTGGGACTGAGAC
ACGGCCCAGACTCCTACGGGAGGCAGCAGTGAGGAATCTTGGACAATGGGGGAAACCCT
GATCCAGCCATGCCGCGTGAG
CGATGAAGGCCTTAGGGTTGTAAAGCTCTTTCAGTCGTGAAGATAATGACGGTAGCGACAG
AAGAAGCCCCGGCTAACTC
CGTGCCAGCAGCCGCGGTAATACGGAGGGGGCTAGCGTTGTTCGGAATTACTGGGCGTA
AAGCGCGCGTAGGCGGACTAT
TAAGTCGGGGGTGAAATCCCGGGGCTCAACCCCGGAACTGCCTCCGATACTGGTAGTCTA
GAGTTCGAGAGAGGTGAGTG
GAATTCCGAGTGTAGAGGTGAAATTCGTAGATATTCGGAGGAACACCAGTGGCGAAGGCG
GCTCACTGGCTCGATACTGA
CGCTGAGGTGCGAAAGCGTGGGGAGCAAACAGGATTAGATACCCTGGTAGTCCACGCCG
TAAACGATGAATGCCAGTCGT
CGGCAAGCATGCTTGTCGGTGACACACCTAACGGATTAAGCATTCCGCCTGGGGAGTACG
GCCGCAAGGTTAAAACTCAA
AGGAATTGACGGGGGCCCGCACAAGCGGTGGAGCATGTGGTTTAATTCGAAGCAACGCG
CAGAACCTTACCAACCCTTGA
CATCCTGATCGCGGTTTTCCGAGAGGAATTCCTTCAGTTCGGCTGGATCAGTGACAGGTG
CTGCATGGCTGTCGTCAGCT
CGTGTCGTGAGATGTTCGGTTAAGTCCGGCAACGAGCGCAACCCACACTCTTAGTTGCCA
GCATTCAGTTGGGCACTCTA
AGAGAACTGCCGATGATAAGTCGGAGGAAGGTGTGGATGACGTCAAGTCCTCATGGCCCT
TACGGGTTGGGCTACACACG
TGCTACAATGGCAGTGACAATGGGTTAATCCCAAAAAACTGTCTCAGTTCGGATTGTTCTCT
GCAACTCGAGAGCATGAA
GTCGGAATCGCTAGTAATCGCGTAACAGCATGACGCGGTGAATACGTTCCCGGGCCTTGT
ACACACCGCCCGTCACACCA
TGGGAGTTGGGTTTACCCGAAGACGGTGCGCCAACCCTACGGGGGCAGCTGCCA
>Rhodobacter_viridis_JA737
CTGCCATGCGGTCGAGCGAGACCTTCGGGTCTAGCGGCGGACGGGTGAGTAACGCGTGG
GAACGTGCCCTTTGCTACGGA
ATAGCCCCGGGAAACTGGGAGTAATACCGTATGTGCCCTTCGGGGGAAAGATTTATCGGC
AAAGGATCGGCCCGCGTTGG
ATTAGGTAGTTGGTGGGGTAATGGCCTACCAAGCCGACGATCCATAGCTGGTTTGAGAGG
AGATCAGCCACACTGGGACT
GAGACACGGCCCAGACTCCTACGGGAGGCAGCAGTGGGGAATCTTAGACAATGGGGGAA
ACCCTGATCTAGCCATGCCGC
GTGAGCGATGAAGGCCTTAGGGTTGTAAAGCTCTTTCAGGTGGGAAGATAATGACGGTAC
CACCAGAAGAAGCCCCGGCT
AACTCCGTGCCAGCAGCCGCGGTAATACGGAGGGGGCTAGCGTTGTTCGGAATTACTGG
GCGTAAAGCGCACGTAGGCGG

ATCAGAAAGTCAGAGGTGAAATCCCAGGGCTCAACCTTGGAACTGCCTTTGAAACTCCTGG
TCTTGAGGTCGAGAGAGGT
GAGTGGAATTCCGAGTGTAGAGGTGAAATTCGTAGATATTCGGAGGAACACCAGTGGCGA
AGGCGGCTCACTGGCTCGAT
ACTGACGCTGAGGTGCGAAAGCGTGGGGAGCAAACAGGATTAGATACCCTGGTAGTCCAC
GCCGTAAACGATGAATGCCA
GTCGTCGGCAGGCATGCCTGTCGGTGACACACCTAACGGATTAAGCATTCCGCCTGGGGA
GTACGGTCGCAAGATTAAAA
CTCAAAGGAATTGACGGGGGCCCGCACAAGCGGTGGAGCATGTGGTTTAATTCGAAGCAA
CGCGCAGAACCTTACCAACC
CTTGACATCGAGATCGCGGTTACCAGAGATGGTTTCCTTCAGTTCGGCTGGATCTTAGACA
GGTGCTGCATGGCTGTCGT
CAGCTCGTGTCGTGAGATGTTCGGTTAAGTCCGGCAACGAGCGCAACCCACACTTTCAGT
TGCCATCATTCAGTTGGGCA
CTCTGGAAGAACTGCCGATGATAAGTCGGAGGAAGGTGTGGATGACGTCAAGTCCTCATG
GCCCTTACGGGTTGGGCTAC
ACACGTGCTACAATGGTGGTGACAATGGGCCAATCCCAAAAAGCCATCTCAGTTCGGATTG
GGGTCTGCAACTCGACCCC
ATGAAGTCGGAATCGCTAGTAATCGCGTAACAGCATGACGCGGTGAATACGTTCCCGGGC
CTTGTACACACCGCCCGTCA
CACCATGGGAATTGGGTCTACCCTAAGATGGTGCGCCAACCTGCAAAGGAGGCAGCCAGC
CACGGTAGGCTCAGTGACTG
GGGTGAAGTCTAACCAACGTGCCCCACCCCC
>Rhodovulum_robiginosum_N2
GAACGCTGGCGGCAGGCTTAACACATGCAAGTCGAGCGAAGTCTTTCGGACTTAGCGGCG
GACGGGTGAGTAACGCGTGG
GAACGTGCCCTTCACTACGGAATAGTCCCGGGAAACTGGGTTTAATACCGTATACNCCCTT
CGGGGGAAAGAATTTCGGT
GAAGGATCGGCCCGCGTCTGATTAGGTAGTTGGTGGGGTAATGGCCTACCAAGCCTACGA
TCAGTAGCTGGTTTGAGAGG
ATGATCAACCACACTGGGACTGAGACACGGCCCAGACTCCTACGGGAGGCAGCAGTGAG
GAATCTTGGACAATGGGGGCA
ACCCTGATCCAACCATTCCGCGTGAACGATGAAGGCCTTAGGGTTGTAAAGTTCTTTCGCC
TGGGAAGATAATGACGGTA
CCAGGTAAAGAAGCCCCGGCTAACTCCGTGCCAGCAGCCGCGGTAATACGGAGGGGGCT
AGCGTTGTTCGGAATTACTGG
GCGTAAAGCGCGCGTAGGCGGACTGTTAAGTCAGAGGTGAAATCCCAGGGCTCAACCCTG
GAACGGCCTTTGATACTGGC
AGTCTAGAGGTCTGGAGAGGTGAGTGGAATTCCGAGTGTAGAGGTGAAATTCGTAGATATT
CGGAGGAACACCAGTGGCG
AAGGCGGCTCACTGGCCAGATACTGACGCTGAGGTGCGAAAGCGTGGGGAGCAAACAGG
ATTAGATACCCTGGTAGTCCA
CGCCGTAAACGATGAATGCCAGACGTCGGGCAGCATGCTGTTCGGTGTCACACCTAACGG
ATTAAGCATTCCGCCTGGGG
AGTACGGTCGCAAGATTAAAACTCAAAGGAATTGACGGGGGCCCGCACAAGCGGTGGAGC
ATGTGGTTTAATTCGAAGCA
ACGCGCAGAACCTTACCAACCCTTGACATGGGTATCGCGGTTTTCCGAGAGGGATTCCTTC
AGTTCGGCTGGATACCACA
CAGGTGCTGCATGGCTGTCGTCAGCTCGTGTCGTGAGATGTTCGGTTAAGTCCGGCAACG
ACGCGAACCCACACCCTTAG

TTGCCAGCATTCAGTTGGGCACTCTAGGGGAACTGCCGATGATAAGTCGGAGGAAGGTGT
GGATGACGTCAAGTCCTCAT
GGCCCTTACGGGTTGGGCTACACACGTGCTACAATGGCATCTACAGTGGGTTAATCCCCA
AAAGATGTCTCAGTTCGGAT
TGTCCTCTGCAACTCGAGGGCATGAAGTCGGAATCGCTAGTAATCGCGTAACAGCATGAC
GCGGTGAATACGTTCCCGGG
CCTTGTACACACCGCCCGTCACACCATGGGAGTTGGGTTCACCCGAAGGCCGTGCGCCAA
CCTTTCGAGGGGGCAGCGGA
CCACGGTGAGCTCAGCGACTGGGGTGAAGTCGTAACAAGGTAGCCGTAGGGGAA
>Rhodovulum_iodosum_N1
GAACGCTGGCGGCAGGCTTAACACATGCAAGTCGAGCGAAGACTTTCGGTCTTAGCGGCG
GACGGGTGAGTAACACGTGG
GAACATGCCCTTCACTACGGAATAGTCCTGGGAAACCGGGTTTAATACCGTATACNCCCTT
CGGGGGAAAGAATTTCGGT
GAAGGATTGGCCCGCGTCTGATTAGGTAGTTGGTGGGGTAATGGCCTACCAAGCCGACGA
TCAGTAGCTGGTTTGAGAGG
ATGATCAGCCACACTGGGACTGAGACACGGCCCAGACTCCTACGGGAGGCAGCAGTGAG
GAATCTTGGACAATGGGGGAA
ACCCTGATCCAACCATGCCGCGTGAACGATGAAGGCCTTAGGGTTGTAAAGTTCTTTCACC
GGTGAAGATAATGACGGTA
GCCGGAGAAGAAACCCCGGCTAACTCCGTGCCAGCAGCCGCGGTAATACGGAGGGGGTT
AGCGTTGTTCGGAATTACTGG
GCGTAAAGCGCGCGTAGGCGGACTATTAAGTCAGGGGTGAAATCCCGGGGCTCAACCCC
GGAACTGCCTTTGATACTGGT
AGTCTAGAGTTCGAGAGAGGTGAGTGGAATTCCGAGTGTAGAGGTGAAATTCGTAGATATT
CGGAGGAACACCAGTGGCG
AAGGCGGCTCACTGGCTCGATACTGACGCTGAGGTGCGAAAGCGTGGGGAGCAAACAGG
ATTAGATACCCTGGTAGTCCA
CGCCGTAAACGATGAATGCCAGACGTCGGGCAGCATGCTGTTCGGTGTCACACCTAACGG
ATTAAGCATTCCGCCTGGGG
AGTACGGCCGCAAGGTTAAAACTCAAAGGAATTGACGGGGGCCCGCACAAGCGGTGGAG
CATGTGGTTTAATTCGAAGCA
ACGCGCAGAACCTTACCAACCCTTGACATCCTGATCGCGGTTTCTCGAGAGAGATTCCTTC
AGTTCGGCTGGATCANCNN
CAGGGGCTGCATGGCTGTCGTCAGCTCGTGTCGTGAGATGTTCGGTTAAGTCCGGCAACG
AGCGCAACCCACACTCTTAG
TTGCCAGCAGTTCGGCTGGGCACTCTAAGAGAACTGCCGATGATAAGTCGGAGGAAGGTG
TGGATGACGTCAAGTCCTCA
TGGCCCTTACGGGTTGGGCTACACACGTGCTACAATGGCAGTGACAATGGGTTAATCCCC
AAAAACTGTCTCAGTTCGGA
TTGTCCTCTGCAACTCGAGGGCATGAAGTCGGAATCGCTAGTAATCGCGTAACAGCATGAC
GCGGTGAATACGTTCCCGG
GCCTTGTACACACCGCCCGTCACACCATGGGAGTTGGGTTTACCCGAAGACGGTGCGCCA
ACCTTTCGAGGGGGCAGCTG
GCCACGGTAAGCTCAGCGACTGGGGTGAAGTCGTAACAAGGTAGCCGTAGGGGAA
>Rhodovulum_steppense_DSM21153
TAACACATGCAAGTCGAGCGAGGACTTCGGTCCTAGCGGCGGACGGGTGAGTAACGCGT
GGGAACGTGCCCTTCTGTGCG
GAATAGCCTCGGGAAACTGAGAGTAATACCGCATACGCCCTTCGGGGGAAAGATTTATCG
CAGAAGGATCGGCCCGCGTC

TGATTAGGTAGTTGGTGGGGTAATGGCCTACCAAGCCTACGATCAGTAGCTGGTTTGAGA
GGATGATCAGCCACACTGGG
ACTGAGACACGGCCCAGACTCCTACGGGAGGCAGCAGTGAGGAATCTTGGACAATGGGG
GCAACCCTGATCCAGCCATGC
CGCGTGAACGATGAAGGCCTTAGGGTTGTAAAGTTCTTTCAGTCGTGAAGATAATGACGGT
AGCGACAGAAGAAGCCCCG
GCTAACTCCGTGCCAGCAGCCGCGGTAATACGGAGGGGGCTAGCGTTGTTCGGAATTACT
GGGCGTAAAGCGCGCGTAGG
CGGACTATTAAGTCAGGGGTGAAATCCCGGGGCTCAACCCCGGAACTGCCCTTGATACTG
GTAGTCTAGAGGTCGAGAGA
GGTGAGTGGAATTCCGAGTGTAGAGGTGAAATTCGTAGATATTCGGAGGAACACCAGTGG
CGAAGGCGGCTCACTGGCTC
GATACTGACGCTGAGGTGCGAAAGCGTGGGGAGCAAACAGGATTAGATACCCTGGTAGTC
CACGCCGTAAACGATGAATG
CCAGACGTCGGCAGGCATGCCTGTCGGTGTCACACCTAACGGATTAAGCATTCCGCCTGG
GGAGTACGGCCGCAAGGTTA
AAACTCAAAGGAATTGACGGGGGCCCGCACAAGCGGTGGAGCATGTGGTTTAATTCGAAG
CAACGCGCAGAACCTTACCA
ACCCTTGACATGTGTATCGCGGTTCCAGAGATGGTTCCTTCAGTTCGGCTGGATACAACAC
AGGTGCTGCATGGCTGTCG
TCAGCTCGTGTCGTGAGATGTTCGGTTAAGTCCGGCAACGAGCGCAACCCACACTCTTAG
TTGCCAGCATTCAGTTGGGC
ACTCTAGGAGAACTGCCGATGATAAGTCGGAGGAAGGTGTGGATGACGTCAAGTCCTCAT
GGCCCTTACGGGTTGGGCTA
CACACGTGCTACAATGGCAGTGACAATGGGTTAATCCCAAAAAGCTGTCTCAGTTCGGATT
GTCCTCTGCAACTCGAGGG
CATGAAGTCGGAATCGCTAGTAATCGCGTAACAGCATGACGCGGTGAATACGTTCCCGGG
CCTTGTACACACCGCCCGTC
ACACCATGGGAGTTGGGTTTACCCGAAGACGGTGCGCCAACCTTTCGAGGAGGCAGCTG
GCCACGGTAA
>Rhodovulum_bhavnagarense_JA738
ACCATGGCAGTCGAGCGAAGACTTCGGTCTTAGCGGCGGACGGGTGAGTAACGCGTGGG
AACGTGCCCTTTGCTACGGAA
CAGTCCCGGGAAACTGGGTTTAATACCGTATACGCCCTTCGGGGGAAAGAATTTCGGCGA
AGGATCGGCCCGCGTCTGAT
TAGGTAGTTGGTGGGGTAATGGCCTACCAAGCCTACGATCAGTAGCTGGTTTGAGAGGAT
GATCAGCCACACTGGGACTG
AGACACGGCCCAGACTCCTACGGGAGGCAGCAGTGAGGAATCTTGGACAATGGGGGCAA
CCCTGATCCAGCCATGCCGCG
TGAGCGATGAAGGCCTTAGGGTTGTAAAGCTCTTTCGCCAGGGATGATAATGACAGTACCT
GGTAAAGAAACCCCGGCTA
ACTCCGTGCCAGCAGCCGCGGTAATACGGAGGGGGTTAGCGTTGTTCGGAATTACTGGGC
GTAAAGCGCGCGTAGGCGGG
CTATTAAGTCAGGGGTGAAATCCCGGGGCTCAACCCCGGAACTGCCCTTGATACTGGTAG
TCTAGAGATCTGGAGAGGTG
AGTGGAATTCCGAGTGTAGAGGTGAAATTCGTAGATATTCGGAAGAACACCAGTGGCGAA
GGCGGCTCACTGGCCAGATA
CTGACGCTGAGGTGCGAAAGCGTGGGGAGCAAACAGGATTAGATACCCTGGTAGTCCACG
CCGTAAACGATGAATGCCAG
TCGTCGGGTAGCATGCTATTCGGTGACACACCTAACGGAGTAAGCGTTCCGCCTGGGGAG
TACGGCCGCAAGGTTAAAAC

TCACAGGAATTGACGGGGGCCCGCACAAGCGGTGGAGCATGTGGTTTAATTCGAAGCRAC
GCGCAGAACCTTACCAACCC
TTGACATCCTGATCGCGGTTTTCCGAGAGGAATTCCTTCAGTTCGGCTGGATCAGTGACAG
GTGCTGCATGGCTGTCGTC
AGCTCGTGTCGTGAGATGTTCGGTTAAGTCCGGCAACGAGCGCAACCCACACCCTTAGTT
GCCAGCATTCAGTTGGGCAC
TCTAAGGGAACTGCCGATGATAAGTCGGAGGAAGGTGTGGATGACGTCAAGTCCTCATGG
CCCTTACGGGTTGGGCTACA
CACGTGCTACAATGGCAGTGACAATGGGTTAATCCCCAAAAACTGTCTCAGTTCGGATTGT
CCTCTGCAACTCGAGGGCA
TGAAGTCGGAATCGCTAGTAATCGCGTAACAGCATGACGCGGTGAATACGTTCCCGGGCC
TTGTACACACCGCCCGTCAC
ACCATGGGAGTTGGTTACCCGAAGACGGTGCGCCAACCTTCGAGGAGGCAGCTGGCAAC
GTAA
>Rhodovulum_euryhalinum_DSM4868
AACGAACGCTGGCGGCAGGCCTAACACATGCAAGTCGAGCGAGGACTTCGGTCCTAGCG
GCGGACGGGTGAGTAACGCGT
GGGAACGTGCCCAAAGGTACGGAATAGCCTCGGGAAACTGAGAGTAATACCGTATGAGCC
CTTCGGGGGAAAGATTTATC
GCCTTTGGATCGGCCCGCGTCTGATTAGGTAGTTGGTGGGGTAATGGCCTACCAAGCCTA
CGATCAGTAGCTGGTTTNAG
AGGATGATCAGCCACACTGGGACTGAGACACGGCCCAGACTCCTACGGGAGGCAGCAGT
GAGGAATCTTGGACAATGGGG
GAAACCCTGATCCAGCCATGCCGCGTGAGCGATGAAGGCCTTAGGGTTGTAAAGCTCTTT
CAGTCGTGAAGATAATGACG
GTAGCGACAGAAGAAGCCCCGGCTAACTCCGTGCCAGCAGCCGCGGTAATACGGAGGGG
GCTAGCGTTGTTCGGAATTAC
TGGGCGTAAAGCGCGCGTAGGCGGATTGGAAAGTTGGGGGTGAAATCCCAGGGCTCAAC
CCTGGAACTGCCTCNAAAACT
ACCAGTCTAGAGGTCGAGAGAGGTGAGTGGAATTCCGAGTGTAGAGGTGAAATTCGTAGA
TATTCGGAGGAACACCAGTG
GCGAAGGCGGCTCACTGGCTCGATACTGACGCTGAGGTGCGAAAGCGTGGGGAGCAAAC
AGGATTAGATACCCTGGTAGT
CCACGCCGTAAACGATGAATGCCAGACGTCGGGCAGCATGCTGTTCGGTGTCACACCTAA
CGGATTAAGCATTCCGCCTG
GGGAGTACGGCCGCAAGGTTAAAACTCAAAGGAATTGACGGGGGCCCGCACAAGCGGTG
GAGCATGTGGTTTAATTCGAA
GCAACGCGCAGAACCTTACCAACCCTTGACATGTGTATCGCGGTTCCAGAGATGGTTCCTT
CAGTTCGGCTGGATACAAC
ACAGGTGCTGCATGGCTGTCGTCAGCTCGTGTCGTGAGATGTTCGGTTAAGTCCGGCAAC
GAGCGCAACCCACACTCTTA
GTTGCCAGANTAAGTTGGGCACTCTAAGAGAACTGCCGATGATAAGTCGGAGGAAGGTGT
GGATGACGTCAAGTCCTCAT
GGCCCTTACGGGTTGGGCTACACACGTGCTACAATGGCAGTGACAATGGGTTAATCNCCA
AAAGCTGTCTCAGTTCGGAT
TGTCCTCTGCAACTCGAGGGCATGAAGTNGGAATCGCTAGTAATCGCGTAACAGCATGAC
GCGGTGAATACGTTCCCGGG
CCTTGTACACACCGCCCGTCACACCATGGGAGTTGGGTTTACCCGAAGACGGTGCGCCAA
CCTGTTCGCAGGAGGCAGCC
GGCCACGGTAAGCTCAGCGACTGGGGTG
>Rhodobacter_azotoformans_KA25

CTGGCTCAGAATGAACGCTGGCGGCAGGCCTAACACATGCAAGTCGAGCGAAGTCTTCGG
ACTTAGCGGCGGACGGGTGA
GTAACGCGTGGGAACATGCCCAAAGGTACGGAATAGCCCCGGGAAACTGGGAGTAATACC
GTATGTGCCCTTCGGGGGAA
AGATTTATCGCCTTTGGATTGGCCCGCGTTGGATTAGGTAGTTGGTGGGGTAATGGCCTAC
CAAGCCGACGATCCATAGC
TGGTTTGAGAGGATGATCAGCCACACTGGGACTGAGACACGGCCCAGACTCCTACGGGAG
GCAGCAGTGGGGAATCTTAG
ACAATGGGCGCAAGCCTGATCTAGCCATGCCGCGTGATCGATGAAGGCCTTAGGGTTGTA
AAGATCTTTCAGGTGGGAAG
ATAATGACGGTACCACCAGAAGAAGCCCCGGCTAACTCCGTGCCAGCAGCCGCGGTAATA
CGGAGGGGGCTAGCGTTATT
CGGAATTACTGGGCGTAAAGCGCACGTAGGCGGACTGGAAAGTCAGGGGTGAAATCCCG
GGGCTCAACCCCGGAACTGCC
TTTGAAACTCCCAGTCTTGAGGTCGAGAGAGGTGAGTGGAATTCCGAGTGTAGAGGTGAA
ATTCGTAGATATTCGGAGGA
ACACCAGTGGCGAAGGCGGCTCACTGGCTCGATACTGACGCTGAGGTGCGAAAGCGTGG
GGAGCAAACAGGATTAGATAC
CCTGGTAGTCCACGCCGTAAACGATGAATGCCAGTCGTCGGGCAGCATGCTGTTCGGTGA
CACACCTAACGGATTAAGCA
TTCCGCCTGGGGAGTACGGCCGCAAGGTTAAAACTCAAAGGAATTGACGGGGGCCCGCA
CAAGCGGTGGAGCATGTGGTT
TAATTCGAAGCAACGCGCAGAACCTTACCAACCCTTGACATGGCGATCGCGGTTCCAGAG
ATGGTTCCTTCAGTTCGGCT
GGATCGCACACAGGTGCTGCATGGCTGTCGTCAGCTCGTGTCGTGAGATGTTCGGTTAAG
TCCGGCAACGAGCGCAACCC
ACGTCCTCAGTTGCCAGCATTCAGTTGGGCACTCTGGGGAAACTGCCGGTGATAAGCCGG
AGGAAGGTGTGGATGACGTC
AAGTCCTCATGGCCCTTACGGGTTGGGCTACACACGTGCTACAATGGCAGTGACAATGGG
TTAATCCCAAAAAGCTGTCT
CAGTTCGGATTGGGGTCTGCAACTCGACCCCATGAAGTCGGAATCGCTAGTAATCGCGTA
ACAGCATGACGCGGTGAATA
CGTTCCCGGGCCTTGTACACACCGCCCGTCACACCATGGGAATTGGTTCTACCCGAAGGC
GGTGCGCCAACCTCGCAAGA
GGAGGCAGCCGACCACGGTAGGATCAGTGACTGGGGTGAAGTCGTACAAAGGTAACCA
>Rhodovulum_imhoffii_DSM18064
NANCCGGCTCAGAACGAACGCTGGCGGCAGGCTTAACACATGCAAGTCGAGCGAAGGCT
TCGGCCTTAGCGGCGGACGGG
TGAGTAACGCGTGGGAACGTGCCCTTCACTACGGGATAGTCCTGGGAAACTGGGTTTAAT
ACCGTATACGCCCTTTTGGG
GAAAGAATTTCGGTGAAGGATCGGCCCGCGTCTGATTAGGTAGTTGGTGGGGTAATGGCC
TACCAAGCCGACGATCAGTA
GCTGGTTTGAGAGGATGATCAGCCACACTGGGACTGAGACACGGCCCAGAKTCCTACGGG
AGGCAGCAGTGAGGAATCTT
GGACAATGGGGGCAACCCTGATCCAGCCATGCCGCGTGAACGATGAAGGCTTTAGGGTTG
TAAAGTTCTTTCACCGGGAA
GATAATGACGGTAGCCGGAGAAGAAGCCCCGGCTAACTCCGTGCCAGCAGCCGCGGTAA
TACGGAGGGGGCTAGCGTTGT
TCGGAATTACTGGGCGTAAAGCGCACGTAGGCGGACTATTAAGTCAGGGGTGAAATCCCG
GGGCTCAACCCCGGAACTGC

CTTTGATACTGGTAGTCTAKAGTTCTGGAGAGGTGAGTGGAATTCCGAGTGTAGAGGTGAA
ATTCGTAGATATTCGGAGG
AACACCAGTGGCGAAGGCGGCTCACTGGCCAGATACTGACGCTGAGGTGCGAAAGCGTG
GGGAGCAAACAGGATTAGATA
CCCTGGTAGTCCACGCCGTAAACGATGAATGCCAGASGTCGGGCASCATGCTGTTCGGTG
TCCACCTAACGGATTAAGCA
TTCCGCCTGGGGAGTACGGTCGCAAGATTAAAACTCAAAGGAATTGACGGGGGCCCGCAC
AAGCGGTGGAGCATGTGGTT
TAATTCGAAGCAACGCGCAGAACCTTACCAACCCTTGACATCCTGATCGCGGTTTTCCGAG
AGGAATTCCTTCAGTTCGG
CTGGATCAGTGACAGGTGCTGCATGGCTGTCGTCAGCTCGTGTCGTGAGATGTTCGGTTA
AGTCCGGCAACGAGCGCAAC
CCACGTCTTTAGTTGCCAGCAGTTCGGCTGGGCACTCTAAAGAAACTGCCGATGATAAGTC
GGAGGAAGGTGTGGATGAC
GTCAAGTCCTCATGGCCCTTACGGGTTGGGCTACACACGTGCTACAATGGCGATGACAAT
GGGTTAATCCCTAAAAGTCG
TCTCAGTTCGGATTGTCCTCTGCAACTCGAGGGCATGAAGTCGGAATCGCTAGTAATCGCG
TAACAGCATGACGCGGTGA
ATACGTTCCCGGGCCTTGTACACACCGCCCGTCACACCATGGGAGTTGGGTTTACCCGAA
GACGGTGCGCCAACCTGCTC
GCAGGAGGCAGCCGGCCACGGTADGTCAGGAKGGGGGA
>Roseibaca_ekhonensis_CECT_7235
TGGCGGCAGGCTTAACACATGCAAGTCGAGCGAAGCCTTCGGGCTTAGCGGCGGACGGG
TGAGTAACGCGTGGGAACGTG
CCCTTTGCTACGGAATAGTCCCTGGAAACGGGGTTTAATACCGTATGTGCTCTTCGGAGGA
AAGAATTTCGGCAAAGGAT
CGGCCCGCGTTGGATTAGGTAGTTGGTGGGGTAAAGGCCTACCAAGCCGACGATCCATAG
CTGGTTTGAGAGGATGATCA
GCCACACTGGGACTGAGACACGGCCCAGACTCCTACGGGAGGCAGCAGTGGGGAATCTT
AGACAATGGGGGCAACCCTGA
TCTAGCCATGCCGCGTGAGCGATGAAGGCCTTAGGGTTGTAAAGCTCTTTCGCTGGGGAA
GATAATGACGGTACCCAGTA
AAGAAGCCCCGGCTAACTCCGTGCCAGCAGCCGCGGTAATACGGAGGGGGCTAGCGTTG
TTCGGAATTACTGGGCGTAAA
GCGCGCGTAGGCGGATTGGAAAGTTGGGGGTGAAATCCCGGGGCTCAACCCCGGAACTG
CCTCCAAAACTATCAGTCTTG
AGTTCGAGAGAGGTGAGTGGAATTGCGAGTGTAGAGGTGAAATTCGTAGATATTCGCAGG
AACACCAGTGGCGAAGGCGG
CTCACTGGCTCGATACTGACGCTGAGGTGCGAAAGTGTGGGGAGCAAACAGGATTAGATA
CCCTGGTAGTCCACACCGTA
AACGATGAATGCCAGACGTCGGGCAGCATGCTGTTCGGTGTCACACCTAACGGATTAAGC
ATTCCGCCTGGGGAGTACGG
TCGCAAGATTAAAACTCAAAGGAATTGACGGGGGCCCGCACAAGCGGTGGAGCATGTGGT
TTAATTCGAAGCAACGCGCA
GAACCTTACCAACCCTTGACATGGATATCGCGGGACCGGAGACGGTTCTTTCAGTTCGGC
TGGATATCACACAGGTGCTG
CATGGCTGTCGTCAGCTCGTGTCGTGAGATGTTCGGTTAAGTCCGGCAACGAGCGCAACC
CACGTCCCCAGTTGCCAGCA
TTCAGTTGGGCACTCTGAGGAGACTGCCGATGATAAGTCGGAGGAAGGTGTGGATGACGT
CAAGTCCTCATGGCCCTTAC

GGGTTGGGCTACACACGTGCTACAATGGTGGTGACAGAGGGATAATCCCTAAAAGCCATC
TCAGTTCGGATTGGGGTCTG
CAACTCGACCCCATGAAGTCGGAATCGCTAGTAATCGCGGAACAGCATGCCGCGGTGAAT
ACGTTCCCGGGCCTTGTACA
CACCGCCCGTCACACCATGGGAGTTGGGTCTACCCGAAGGTGGTGCGCTAACCAGCAATG
GAGGCAGCCAACCACGGTAG
GCTCAGCGACTGGGGTGAAGTCGTAACAAGGTAGCCGTAGGGGAACCTGCGGCTGGATC
ACCTCC
>Rhodovulum_marinum_DSM_18063
TCGAGCGAAGCCTTCGGGCTTAGCGGCGGACGGGTGAGTAACGCGTGGGAACGTGCCCT
TCTCTGCGGAATAGCCACTGG
AAACGGTGAGTAATACCGCATACGCCCTTCGGGGGAAAGATTTATCGGAGAAGGATCGGC
CCGCGTTAGATTAGGTAGTT
GGTGGGGTAATGGCCTACCAAGCCTACGATCTATAGCTGGTTTGAGAGGATGATCAGCCA
CACTGGGACTGAGACACGGC
CCAGACTCCTACGGGAGGCAGCAGTGAGGAATCTTGGACAATGGGGGCAACCCTGATCCA
GCCATGCCGCGTGAACGATG
AAGGCCTTAGGGTTGTAAAGTTCTTTCAGCKGGAAGATAAGACGGTACCAGCAGAAGAAG
CCCCGGCTAACTCCGTGCCA
GCAGCCGCGGTAATACGGAGGGGGCTAGCGTTGTTCGGAATTACTGGGCGTAAAGCGCG
CGTAGGCGGATTGGAAAGTTG
GGGGTGAAATCCCAGGGCTCAACCCTGGAACTGCCTCCAAAACTACCAGTCTAGAGTTCG
AGAGAGGTGAGTGGAATTCC
GAGTGTAGAGGTGAAATTCGTAGATATTCGGAGGAACACCAGTGGCGAAGGCGGCTCACT
GGCTCGATACTGACGCTGAG
GTGCGAAAGCGTGGGGAGCAAACAGGATTAGATACCCTGGTAGTCCACGCCGTAAACGGA
TGAATGCCAGACGTCGGGCA
GCATGCTGTTCGGTGTCCACCTAACGGATTAAGCATTCCGCCTGGGGAGTACGGCCGCAA
GGTTAAAACTCAAAGGAATT
GACGGGGGCCCGCACAAGCGGTGGAGCATGTGGTTTAATTCGAAGCAACGCGCAGAACC
TTACCAACCCTTGACATCCTG
ATCGCGGTTTCTCGAGAGAGATTCCTTCAGTTCGGCTGGATCAGTGACAGGTGCTGCATG
GCTGTCGTCAGCTCGTGTCG
TGAGATGTTCGGTTAAGTCCGGCAACGAGCGCAACCCACACTCTTAGTTGCCAGCAGTTC
GGCTGGGCACTCTAAGAGAA
CTGCCGATGATAAGTCGGAGGAAGGTGTGGATGACGTCAAGTCCTCATGGCCCTTACGGG
TTGGGCTACACACGTGCTAC
AATGGCAGTGACAATGGGTTAATCCCCAAAAGCTGTCTCAGTTCGGATTGTCCTCTGCAAC
TCGAGGGCATGAAGTCGGA
ATCGCTAGTAATCGCGTAACAGCATGACGCGGTGAATACGTTCCCGGGCCTTGTACACAC
CGCCCGTCACACCATGGGAG
TTGGGTTTACCCGAAGACGGTGCGCCAACCTTTCGAGGGGGCAGCTGGCCACGGTAAGC
TCAGCGACTGGGGTRAAGTCG
TAACAAGGGTAACCAA
>Rhodovulum_adriaticum_DSM_2781
CCTGGCTCAGAACGAACGCTGGCGGCAGGCCTAACACATGCAAGTCGAGCGAAGCCTTC
GGGCTTAGCGGCGGACGGGTG
AGTAACGCGTGGGAACGTGCCCTTCACTACGGAATAGTCCCGGGAAACTGGGTTTAATAC
CGTATACGCCCTTCGGGGGA
AAGAATTTCGGTGAAGGATCGGCCCGCGTTAGATTAGGTAGTTGGTGGGGTAATGGCCTA
CCAAGCCTACGATCTATAGC

TGGTTTGAGAGGATGATCAGCCACACTGGGACTGAGACACGGCCCAGACTCCTACGGGAG
GCAGCAGTGAGGAATCTTAG
ACTAGATGGGGGCAACCCTGATCTAGCCTGCCGCGTGAACGATGAAGGCCTTAGGGTTGT
AAAGTTCTTTCGCCTGGGAA
GATAATGACGGTACCAGGTAAAGAAGCCCCGGCTAACTCCGTGCCAGCAGCCGCGGTAAT
ACGGAGGGGGCTAGCGTTGT
TCGGAATTACTGGGCGTAAAGCGCGCGTAGGCGGCTGTTAAGTCAGAGGTGAAATCCCA
GGGCTCAACCCTGGAACTGC
CTTTGATACTGGCAGCCTGGAGGTCTGGAGAGGTGAGTGGAATTCCGAGTGTAGAGGTGA
AATTCGTAGATATTCGGAAG
AACACCAGTGGCGAAGGCGGCTCACTGGCCAGATACTGACGCTGAGGTGCGAAAGCGTG
GGGAGCAAACAGGATTAGATA
CCCTGGTAGTCCACGCCGTAAACGATGAATGCCAGTCGTCGGGCAGCATGCTGTTCGGTG
ACACACCTAACGGAGTAAGC
ATTCCGCCTGGGGAGTACGGCCGCAAGGTTAAAACTCAAAGGAATTGACGGGGGCCCGC
ACAAGCGGTGGAGCATGTGGT
TTAATTCGAAGCAACGCGCAGAACCTTACCAACCCTTGACATCCCTGTCGCGGTTTTCCGA
GAGGAATTCCTTCAGTTCG
GCTGGACAGGTGACAGGTGCTGCATGGCTGTCGTCAGCTCGTGTCGTGAGATGTTCGGTT
AAGTCCGGCAACGAGCGCAA
CCCACGTCCTTAGTTGCCAGCATTCAGTTGGGCACTCTAAGGAAACTGCCGATGATAAGTC
GGAGGAAGGTGTGGATGAC
GTCAAGTCCTCATGGCCCTTACGGGTTGGGCTACACACGTGCTACAATGGCAGTGACAAT
GGGTTAATCCCCAAAAACTG
TCTCAGTTCGGATTGTCCTCTGCAACTCGAGGGCATGAAGTCGGAATCGCTAGTAATCGCG
TAACAGCATGACGCGGTGA
ATACGTTCCCGGGCCTTGTACACACCGCCCGTCACACCATGGGAGTTGGGTTTACCCGAA
GACGGTGCGCCAACCTGTTC
GCAGGAGGCAGCTGGCCACGGTAAGCTCAGCGACTGGGGTGAAGTC
>Rhodovulum_kholense_DSM_19783
GGCCTACACATGCAAGTCGAGCGAACCCTTCGGGGTTAGCGGCGGACGGGTGAGTAACG
CGTGGGAACGTGCCCTTCTCT
GCGGAATAGGCTCGGGAAACTGGGTTTAATACCGCATACGCCCTTCGGGGGAAAGATTTA
TCGGAGAAGGATCGGCCCGC
GTCTGATTAGGTAGTTGGTGGGGTAATGGCCTACCAAGCCTACGATCAGTAGCTGGTTTGA
GAGGATGATCAGCCACACT
GGGACTGAGACACGGCCCAGACTCCTACGGGAGGCAGCAGTGAGGAATCTTGGACAATG
GGGGAAACCCTGATCCAGCCA
TGCCGCGTGAGCGATGAAGGCCTTAGGGTTGTAAAGCTCTTTCAGCTGGGAAGATAATGA
CGGTACCAGCAGAAGAAGCC
CCGGCTAACTCCGTGCCAGCAGCCGCGGTAATACGGAGGGGGCTAGCGTTGTTCGGAAT
TACTGGGCGTAAAGCGCACGT
AGGCGGATTGGAAAGTTGGGGGTGAAATCCCGGGGCTCAACCCCGGAACTGCCTCCAAA
ACTCCCAGTCTTGAGGTCGAG
AGAGGTGAGTGGAATTCCGAGTGTAGAGGTGAAATTCGTAGATATTCGGAGGAACACCAG
TGGCGAAGGCGGCTCACTGG
CTCGATACTGACGCTGAGGTGCGAAAGCGTGGGGAGCAAACAGGATTAGATACCCTGGTA
GTCCACGCCGTAAACGATGA
ATGCCAGTCGTCGGGTAGCATGCTATTCGGTGACACACCTAACGGATTAAGCATTCCGCCT
GGGGAGTACGGCCGCAAGG

TTAAAACTCAAAGGAATTGACGGGGGCCCGCACAAGCGGTGGAGCATGTGGTTTAATTCG
AAGCAACGCGCAGAACCTTA
CCAACCCTTGACATCCTGATCGCGGTTACCCGAGAGGGTTTCCTTCAGTTCGGCTGGATCA
GTGACAGGTGCTGCATGGC
TGTCGTCAGCTCATGTCGTGAGATGTTCGGTTAAGTCCGGCAACGAGCGCAACCCACACT
CTTAGTTGCCAGCATTCAGT
TGGGCACTCTAAGAGAACTGCCGATGATAAGTCGGAGGAAGGTGTGGATGACGTCAAGTC
CTCATGGCCCTTACGGGTTG
GGCTACACACGTGCTACAATGGCAGTGACAATGGGTTAATCCCAAAAAACTGTCTCAGTTC
GGATTGTTCTCTGCAACTC
GAGAGCATGAAGTCGGAATCGCTAGTAATCGCGTAACAGCATGACGCGGTGAATACGTTC
CCGGGCCTTGTACACACCGC
CCGTCACACCATGGGGAGTTGGGTTTACCCGAAGACGGTGCGCCAACCCTTACGGGGGG
CAGCTGGCCACGGTAAGCTCA
GCGACTGGGG
>Rhodobacter_capsulatus_SB1003
AGAGTTTGATCCTGGCTCAGAACGAACGCTGGCGGCAGGCCTAACACATGCAAGTCGAGC
GAGACCTTCGGGTCTAGCGG
CGGACGGGTGAGTAACGCGTGGGAACGTGCCCTTTGCTACGGAATAGCCCCGGGAAACT
GGGAGTAATACCGTATGTGCC
CTTCGGGGGAAAGATTTATCGGCAAAGGATCGGCCCGCGTTGGATTAGGTAGTTGGTGGG
GTAATGGCCTACCAAGCCGA
CGATCCATAGCTGGTTTGAGAGGATGATCAGCCACACTGGGACTGAGACACGGCCCAGAC
TCCTACGGGAGGCAGCAGTG
GGGAATCTTAGACAATGGGGGAAACCCTGATCTAGCCATGCCGCGTGAGCGATGAAGGCC
TTAGGGTTGTAAAGCTCTTT
CAGGTGGGAAGATAATGACGGTACCACCAGAAGAAGCCCCGGCTAACTCCGTGCCAGCA
GCCGCGGTAATACGGAGGGGG
CTAGCGTTGTTCGGAATTACTGGGCGTAAAGCGCACGTAGGCGGATCAGAAAGTCAGAGG
TGAAATCCCAGGGCTCAACC
TTGGAACTGCCTTTGAAACTCCTGGTCTTGAGGTCGAGAGAGGTGAGTGGAATTCCGAGT
GTAGAGGTGAAATTCGTAGA
TATTCGGAGGAACACCAGTGGCGAAGGCGGCTCACTGGCTCGATACTGACGCTGAGGTG
CGAAAGCGTGGGGAGCAAACA
GGATTAGATACCCTGGTAGTCCACGCCGTAAACGATGAATGCCAGTCGTCGGCAGGCATG
CCTGTCGGTGACACACCTAA
CGGATTAAGCATTCCGCCTGGGGAGTACGGTCGCAAGATTAAAACTCAAAGGAATTGACG
GGGGCCCGCACAAGCGGTGG
AGCATGTGGTTTAATTCGAAGCAACGCGCAGAACCTTACCAACCCTTGACATCGAGATCGC
GGTTACCAGAGATGGTTTC
CTTCAGTTCGGCTGGATCTTAGACAGGTGCTGCATGGCTGTCGTCAGCTCGTGTCGTGAG
ATGTTCGGTTAAGTCCGGCA
ACGAGCGCAACCCACACTTTCAGTTGCCATCATTCAGTTGGGCACTCTGGAAGAACTGCC
GATGATAAGTCGGAGGAAGG
TGTGGATGACGTCAAGTCCTCATGGCCCTTACGGGTTGGGCTACACACGTGCTACAATGG
TGGTGACAATGGGCCAATCC
CAAAAAGCCATCTCAGTTCGGATTGGGGTCTGCAACTCGACCCCATGAAGTCGGAATCGC
TAGTAATCGCGTAACAGCAT
GACGCGGTGAATACGTTCCCGGGCCTTGTACACACCGCCCGTCACACCATGGGAATTGGG
TCTACCCTAAGATGGTGCGC

CAACCAGCAATGGAGGCAGCCAGCCACGGTAGGCTCAGTGACTGGGGTGAAGTCGTAAC
AAGGTAGCCGTAGGGGAACCT
GCGGCTGGATCACCT
>Rhodospirillum_rubrum_ATCC_11170
AGAGTTTGATCCTGGCTCAGGACGAACGCTGGCGGCAGGCCTAACACATGCAAGTCGAAC
GCATCCTTCGGGATGAGTGG
CGCACGGGTGAGTAACACGTGGGAACGTACCTTGGAGTGCGGAATAATCTTTGGAAACGA
GGACTAATACCGCATACGCC
CTTAGGGGGAAAGATTTATCGCTCCAAGATCGGCCCGCGTCCGATTAGCTAGTTGGCGGG
GTAATGGCCCACCAAGGCGA
CGATCGGTAGCTGGTCTGAGAGGATGGCCAGCCACACTGGGACTGAGACACGGCCCAGA
CTCCTACGGGAGGCAGCAGTG
GGGAATATTGCGCAATGGGGGCAACCCTGACGCAGCCATGCCGCGTGAGTGAAGAAGGC
CTTCGGGTTGTAAAGCTCTTT
CGGGTGTGAAGATGATGACGGTAACACCAGAAGAAGCCCCGGCTAACTTCGTGCCAGCAG
CCGCGGTAATACGAAGGGGG
CAAGCGTTGTTCGGAATTACTGGGCGTAAAGAGCGCGTAGGCGGTCTGATTAGTCAGAGG
TGAAATCCCAGAGCTCAACT
TTGGAACTGCCTTTGATACTGTTAGACTAGAATCCGTGAGAGGGTGGTGGAATTCCCAGTG
TAGAGGTGAAATTCGTAGA
TATTGGGAGGAACACCAGTGGCGAAGGCGGCCACCTGGCGCGGTATTGACGCTGAGGCG
CGAAAGCGTGGGGAGCAAACA
GGATTAGATACCCTGGTAGTCCACGCCGTAAACGATGAGTGCTAGATGTCGGGGTACATG
TACCTCGGTGTCGCAGCTAA
CGCATTAAGCACTCCGCCTGGGGAGTACGGCCGCAAGGTTAAAACTCAAAGGAATTGACG
GGGGCCCGCACAAGCGGTGG
AGCATGTGGTTTAATTCGAAGCAACGCGCAGAACCTTACCAGCCCTTGACATCCCGTGACA
CTTCCAGAGATGGAAGGTT
CCCTTCGGGGACACGGTGACAGGTGCTGCATGGCTGTCGTCAGCTCGTGTCGTGAGATGT
TGGGTTAAGTCCCGCAACGA
GCGCAACCCTCATCTTCAGTTGCCAGCAAGTAACGTTGGGCACTCTGAAGAGACTGCCGG
TGACAAGCCGGAGGAAGGTG
GGGATGACGTCAAGTCCTCATGGCCCTTACGGGCTGGGCTACACACGTGCTACAATGGCG
CCTACAATGGGCAGCGACCT
CGCGAGGGGAAGCTAATCTCCAAAAGGCGTCTCAGTTCGGATTGCACTCTGCAACTCGGG
TGCATGAAGTCGGAATCGCT
AGTAATCGTGGATCAGCATGCCACGGTGAATACGTTCCCGGGCCTTGTACACACCGCCCG
TCACACCATGGGAGTTGGTT
CTACCCGAAGACGGTACGCTAACCGCAAGGAGGCAGCCGGCCACGGTAGGGTCAGCGAC
TGGGGTGAAGTCGTAACAAGG
TAGCCGTAGGGGAACCTGCGGCTGGATCACCTCCTTT
>Rhodobacter_sphaeroides_2.4.1
CAACTTGAGAGTTTGATCCTGGCTCAGAATGAACGCTGGCGGCAGGCCTAACACATGCAA
GTCGAGCGAAGTCTTCGGAC
TTAGCGGCGGACGGGTGAGTAACGCGTGGGAACGTGCCCTTTGCTTCGGAATAGCCCCG
GGAAACTGGGAGTAATACCGA
ATGTGCCCTTTGGGGGAAAGATTTATCGGCAAAGGATCGGCCCGCGTTGGATTAGGTAGT
TGGTGGGGTAATGGCCTACC
AAGCCGACGATCCATAGCTGGTTTGAGAGGATGATCAGCCACACTGGGACTGAGACACGG
CCCAGACTCCTACGGGAGGC

AGCAGTGGGGAATCTTAGACAATGGGCGCAAGCCTGATCTAGCCATGCCGCGTGATCGAT
GAAGGCCTTAGGGTTGTAAA
GATCTTTCAGGTGGGAAGATAATGACGGTACCACCAGAAGAAGCCCCGGCTAACTCCGTG
CCAGCAGCCGCGGTAATACG
GAGGGGGCTAGCGTTATTCGGAATTACTGGGCGTAAAGCGCACGTAGGCGGATCGGAAA
GTCAGAGGTGAAATCCCAGGG
CTCAACCCTGGAACTGCCTTTGAAACTCCCGATCTTGAGGTCGAGAGAGGTGAGTGGAATT
CCGAGTGTAGAGGTGAAAT
TCGTAGATATTCGGAGGAACACCAGTGGCGAAGGCGGCTCACTGGCTCGATACTGACGCT
GAGGTGCGAAAGCGTGGGGA
GCAAACAGGATTAGATACCCTGGTAGTCCACGCCGTAAACGATGAATGCCAGTCGTCGGG
CAGCATGCTGTTCGGTGACA
CACCTAACGGATTAAGCATTCCGCCTGGGGAGTACGGCCGCAAGGTTAAAACTCAAAGGA
ATTGACGGGGGCCCGCACAA
GCGGTGGAGCATGTGGTTTAATTCGAAGCAACGCGCAGAACCTTACCAACCCTTGACATG
GCGATCGCGGTTCCAGAGAT
GGTTCCTTCAGTTCGGCTGGATCGCACACAGGTGCTGCATGGCTGTCGTCAGCTCGTGTC
GTGAGATGTTCGGTTAAGTC
CGGCAACGAGCGCAACCCACGTCCTTAGTTGCCAGCATTCAGTTGGGCACTCTAGGGAAA
CTGCCGGTGATAAGCCGGAG
GAAGGTGTGGATGACGTCAAGTCCTCATGGCCCTTACGGGTTGGGCTACACACGTGCTAC
AATGGCAGTGACAATGGGTT
AATCCCAAAAAGCTGTCTCAGTTCGGATTGGGGTCTGCAACTCGACCCCATGAAGTCGGAA
TCGCTAGTAATCGCGTAAC
AGCATGACGCGGTGAATACGTTCCCGGGCCTTGTACACACCGCCCGTCACACCATGGGAA
TTGGTTCTACCCGAAGGCGG
TGCGCCAACCTCGCAAGAGGAGGCAGCCGACCACGGTAGGATCAGTGACTGGGGTGAAG
TCGTAACAAGGTAGCCGTAGG
GGAACCTGCGGCTGGATCACCTCCTTT
>Rhodobacter_sphaeroides_KD131
AGAGTTTGATCCTGGCTCAGAATGAACGCTGGCGGCAGGCCTAACACATGCAAGTCGAGC
GAAGTCTTCGGACTTAGCGG
CGGACGGGTGAGTAACGCGTGGGAACGTGCCCTTTGCTTCGGAATAGCCCCGGGAAACT
GGGAGTAATACCGAATGTGCC
CTTTGGGGGAAAGATTTATCGGCAAAGGATCGGCCCGCGTTGGATTAGGTAGTTGGTGGG
GTAATGGCCTACCAAGCCGA
CGATCCATAGCTGGTTTGAGAGGATGATCAGCCACACTGGGACTGAGACACGGCCCAGAC
TCCTACGGGAGGCAGCAGTG
GGGAATCTTAGACAATGGGCGCAAGCCTGATCTAGCCATGCCGCGTGATCGATGAAGGCC
TTAGGGTTGTAAAGATCTTT
CAGGTGGGAAGATAATGACGGTACCACCAGAAGAAGCCCCGGCTAACTCCGTGCCAGCA
GCCGCGGTAATACGGAGGGGG
CTAGCGTTATTCGGAATTACTGGGCGTAAAGCGCACGTAGGCGGATCGGAAAGTCAGAGG
TGAAATCCCAGGGCTCAACC
CTGGAACTGCCTTTGAAACTCCCGATCTTGAGGTCGAGAGAGGTGAGTGGAATTCCGAGT
GTAGAGGTGAAATTCGTAGA
TATTCGGAGGAACACCAGTGGCGAAGGCGGCTCACTGGCTCGATACTGACGCTGAGGTG
CGAAAGCGTGGGGAGCAAACA
GGATTAGATACCCTGGTAGTCCACGCCGTAAACGATGAATGCCAGTCGTCGGGCAGCATG
CTGTTCGGTGACACACCTAA

CGGATTAAGCATTCCGCCTGGGGAGTACGGCCGCAAGGTTAAAACTCAAAGGAATTGACG
GGGGCCCGCACAAGCGGTGG
AGCATGTGGTTTAATTCGAAGCAACGCGCAGAACCTTACCAACCCTTGACATGGCGATCGC
GGTTCCAGAGATGGTTCCT
TCAGTTCGGCTGGATCGCACACAGGTGCTGCATGGCTGTCGTCAGCTCGTGTCGTGAGAT
GTTCGGTTAAGTCCGGCAAC
GAGCGCAACCCACGTCCTTAGTTGCCAGCATTCAGTTGGGCACTCTAGGGAAACTGCCGG
TGATAAGCCGGAGGAAGGTG
TGGATGACGTCAAGTCCTCATGGCCCTTACGGGTTGGGCTACACACGTGCTACAATGGCA
GTGACAATGGGTTAATCCCA
AAAAGCTGTCTCAGTTCGGATTGGGGTCTGCAACTCGACCCCATGAAGTCGGAATCGCTA
GTAATCGCGTAACAGCATGA
CGCGGTGAATACGTTCCCGGGCCTTGTACACACCGCCCGTCACACCATGGGAATTGGTTC
TACCCGAAGGCGGTGCGCCA
ACCTCGCAAGAGGAGGCAGCCGACCACGGTAGGATCAGTGACTGGGGTGAAGTCGTAAC
AAGGTAGCCGTAGGGGAACCT
GCGGCTGGATCACCT
>Rhodobacter_sphaeroides_ATCC17025
AGAGTTTGATCCTGGCTCAGAATGAACGCTGGCGGCAGGCCTAACACATGCAAGTCGAGC
GAAGTCTTCGGACTTAGCGG
CGGACGGGTGAGTAACGCGTGGGAACATGCCCAAAGGTACGGAATAGCCCCGGGAAACT
GGGAGTAATACCGTATGTGCC
CTTCGGGGGAAAGATTTATCGCCTTTGGATTGGCCCGCGTTGGATTAGGTAGTTGGTGGG
GTAATGGCCTACCAAGCCGA
CGATCCATAGCTGGTTTGAGAGGATGATCAGCCACACTGGGACTGAGACACGGCCCAGAC
TCCTACGGGAGGCAGCAGTG
GGGAATCTTAGACAATGGGCGCAAGCCTGATCTAGCCATGCCGCGTGATCGATGAAGGCC
TTAGGGTTGTAAAGATCTTT
CAGGTGGGAAGATAATGACGGTACCACCAGAAGAAGCCCCGGCTAACTCCGTGCCAGCA
GCCGCGGTAATACGGAGGGGG
CTAGCGTTATTCGGAATTACTGGGCGTAAAGCGCACGTAGGCGGACTGGAAAGTCAGGGG
TGAAATCCCGGGGCTCAACC
CCGGAACTGCCTTTGAAACTCCCAGTCTTGAGGTCGAGAGAGGTGAGTGGAATTCCGAGT
GTAGAGGTGAAATTCGTAGA
TATTCGGAGGAACACCAGTGGCGAAGGCGGCTCACTGGCTCGATACTGACGCTGAGGTG
CGAAAGCGTGGGGAGCAAACA
GGATTAGATACCCTGGTAGTCCACGCCGTAAACGATGAATGCCAGTCGTCGGGCAGCATG
CTGTTCGGTGACACACCTAA
CGGATTAAGCATTCCGCCTGGGGAGTACGGCCGCAAGGTTAAAACTCAAAGGAATTGACG
GGGGCCCGCACAAGCGGTGG
AGCATGTGGTTTAATTCGAAGCAACGCGCAGAACCTTACCAACCCTTGACATGGCGATCGC
GGTTCCAGAGATGGTTCCT
TCAGTTCGGCTGGATCGCACACAGGTGCTGCATGGCTGTCGTCAGCTCGTGTCGTGAGAT
GTTCGGTTAAGTCCGGCAAC
GAGCGCAACCCACGTCCTCAGTTGCCAGCATTCAGTTGGGCACTCTGGGGAAACTGCCGG
TGATAAGCCGGAGGAAGGTG
TGGATGACGTCAAGTCCTCATGGCCCTTACGGGTTGGGCTACACACGTGCTACAATGGCA
GTGACAATGGGTTAATCCCA
AAAAGCTGTCTCAGTTCGGATTGGGGTCTGCAACTCGACCCCATGAAGTCGGAATCGCTA
GTAATCGCGTAACAGCATGA

CGCGGTGAATACGTTCCCGGGCCTTGTACACACCGCCCGTCACACCATGGGAATTGGTTC
TACCCGAAGGCGGTGCGCCA
ACCTCGCAAGAGGAGGCAGCCGACCACGGTAGGATCAGTGACTGGGGTGAAGTCGTAAC
AAGGTAGCCGTAGGGGAACCT
GCGGCTGGATCACCTCCTTT
>DSM2351
CAACTTGAGAGTTTGATCCTGGCTCAGAACGAACGCTGGCGGCAGGCCTAACACATGCAA
GTCGAGCGAACCCTTCGGGG
TTAGCGGCGGACGGGTGAGTAACGCGTGGGAACGTGCCCTTCTCTGCGGAATAGGCTCG
GGAAACTGGGTTTAATACCGC
ATACGCCCTTCGGGGGAAAGATTTATCGGAGAAGGATCGGCCCGCGTTAGATTAGGTAGT
TGGTGGGGTAATGGCCTACC
AAGCCTACGATCTATAGCTGGTTTGAGAGGATGATCAGCCACACTGGGACTGAGACACGG
CCCAGACTCCTACGGGAGGC
AGCAGTGAGGAATCTTGGACAATGGGGGAAACCCTGATCCAGCCATGCCGCGTGAGCGAT
GAAGGCCTTAGGGTTGTAAA
GCTCTTTCAGTCGTGAAGATAATGACGGTAGCGACAGAAGAAGCCCCGGCTAACTCCGTG
CCAGCAGCCGCGGTAATACG
GAGGGGGCTAGCGTTGTTCGGAATTACTGGGCGTAAAGCGCGCGTAGGCGGACTATTAAG
TCAGGGGTGAAATCCCGGGG
CTCAACCCCGGAACTGCCTTTGATACTGGTAGTCTAGAGTTCGAGAGAGGTGAGTGGAATT
CCGAGTGTAGAGGTGAAAT
TCGTAGATATTCGGAGGAACACCAGTGGCGAAGGCGGCTCACTGGCTCGATACTGACGCT
GAGGTGCGAAAGCGTGGGGA
GCAAACAGGATTAGATACCCTGGTAGTCCACGCCGTAAACGATGAATGCCAGTCGTCGGC
AAGCTTGCTTGTCGGTGACA
CACCTAACGGATTAAGCATTCCGCCTGGGGAGTACGGCCGCAAGGTTAAAACTCAAAGGA
ATTGACGGGGGCCCGCACAA
GCGGTGGAGCATGTGGTTTAATTCGAAGCAACGCGCAGAACCTTACCAACCCTTGACATC
CTGATCGCGGTTTTCCGAGA
GGAATTCCTTCAGTTCGGCTGGATCAGTGACAGGTGCTGCATGGCTGTCGTCAGCTCGTG
TCGTGAGATGTTCGGTTAAG
TCCGGCAACGAGCGCAACCCACACTCTTAGTTGCCAGCATTCAGTTGGGCACTCTAGGAG
AACTGCCGATGATAAGTCGG
AGGAAGGTGTGGATGACGTCAAGTCCTCATGGCCCTTACGGGTTGGGCTACACACGTGCT
ACAATGGCAGTGACAATGGG
TTAATCCCTAAAAACTGTCTCAGTTCGGATTGTTCTCTGCAACTCGAGAGCATGAAGTCGG
AATCGCTAGTAATCGCGTA
ACAGCATGACGCGGTGAATACGTTCCCGGGCCTTGTACACACCGCCCGTCACACCATGGG
AGTTGGGTTTACCCGAAGAC
GGTGCGCCAACCCTTACGGGGGGCAGCTGGCCACGGTAAGCTCAGCGACTGGGGTGAAG
TCGTAACAAGGTAGCCGTAGG
GGAACCTGCGGCTGGATCACCTCCTTTCTAA
>DSM1374
CAACTTGAGAGTTTGATCCTGGCTCAGAACGAACGCTGGCGGCAGGCCTAACACATGCAA
GTCGAGCGAACCCTTCGGGG
TTAGCGGCGGACGGGTGAGTAACGCGTGGGAACGTGCCCTTCTCTGCGGAATAGGCTCG
GGAAACTGGGTTTAATACCGC
ATACGCCCTTCGGGGGAAAGATTTATCGGAGAAGGATCGGCCCGCGTTAGATTAGGTAGT
TGGTGGGGTAATGGCCTACC

AAGCCTACGATCTATAGCTGGTTTGAGAGGATGATCAGCCACACTGGGACTGAGACACGG
CCCAGACTCCTACGGGAGGC
AGCAGTGAGGAATCTTGGACAATGGGGGAAACCCTGATCCAGCCATGCCGCGTGAGCGAT
GAAGGCCTTAGGGTTGTAAA
GCTCTTTCAGTCGTGAAGATAATGACGGTAGCGACAGAAGAAGCCCCGGCTAACTCCGTG
CCAGCAGCCGCGGTAATACG
GAGGGGGCTAGCGTTGTTCGGAATTACTGGGCGTAAAGCGCGCGTAGGCGGACTATTAAG
TCAGGGGTGAAATCCCGGGG
CTCAACCCCGGAACTGCCTTTGATACTGGTAGTCTAGAGTTCGAGAGAGGTGAGTGGAATT
CCGAGTGTAGAGGTGAAAT
TCGTAGATATTCGGAGGAACACCAGTGGCGAAGGCGGCTCACTGGCTCGATACTGACGCT
GAGGTGCGAAAGCGTGGGGA
GCAAACAGGATTAGATACCCTGGTAGTCCACGCCGTAAACGATGAATGCCAGTCGTCGGC
AAGCATGCTTGTCGGTGACA
CACCTAACGGATTAAGCATTCCGCCTGGGGAGTACGGCCGCAAGGTTAAAACTCAAAGGA
ATTGACGGGGGCCCGCACAA
GCGGTGGAGCATGTGGTTTAATTCGAAGCAACGCGCAGAACCTTACCAACCCTTGACATC
CTGATCGCGGTTTTCCGAGA
GGAATTCCTTCAGTTCGGCTGGATCAGTGACAGGTGCTGCATGGCTGTCGTCAGCTCGTG
TCGTGAGATGTTCGGTTAAG
TCCGGCAACGAGCGCAACCCACACTCTTAGTTGCCAGCATTCAGTTGGGCACTCTAGGAG
AACTGCCGATGATAAGTCGG
AGGAAGGTGTGGATGACGTCAAGTCCTCATGGCCCTTACGGGTTGGGCTACACACGTGCT
ACAATGGCAGTGACAATGGG
TTAATCCCTAAAAGCTGTCTCAGTTCGGATTGTTCTCTGCAACTCGAGAGCATGAAGTCGG
AATCGCTAGTAATCGCGTA
ACAGCATGACGCGGTGAATACGTTCCCGGGCCTTGTACACACCGCCCGTCACACCATGGG
AGTTGGGTTTACCCGAAGAC
GGTGCGCCAACCCTTACGGGGGGCAGCTGGCCACGGTAAGCTCAGCGACTGGGGTGAAG
TCGTAACAAGGTAGCCGTAGG
GGAACCTGCGGCTGGATCACCTCCTTT
>SNK001
CAACTTGAGAGTTTGATCCTGGCTCAGAACGAACGCTGGCGGCAGGCCTAACACATGCAA
GTCGAGCGAACCCTTCGGGG
TTAGCGGCGGACGGGTGAGTAACGCGTGGGAACGTGCCCTTCTCTGCGGAATAGGCTCG
GGAAACTGGGTTTAATACCGC
ATACGCCCTTCGGGGGAAAGATTTATCGGAGAAGGATCGGCCCGCGTTAGATTAGGTAGT
TGGTGGGGTAATGGCCTACC
AAGCCTACGATCTATAGCTGGTTTGAGAGGATGATCAGCCACACTGGGACTGAGACACGG
CCCAGACTCCTACGGGAGGC
AGCAGTGAGGAATCTTGGACAATGGGGGAAACCCTGATCCAGCCATGCCGCGTGAGCGAT
GAAGGCCTTAGGGTTGTAAA
GCTCTTTCAGTCGTGAAGATAATGACGGTAGCGACAGAAGAAGCCCCGGCTAACTCCGTG
CCAGCAGCCGCGGTAATACG
GAGGGGGCTAGCGTTGTTCGGAATTACTGGGCGTAAAGCGCGCGTAGGCGGACTATTAAG
TCAGGGGTGAAATCCCGGGG
CTCAACCCCGGAACTGCCTTTGATACTGGTAGTCTAGAGTTCGAGAGAGGTGAGTGGAATT
CCGAGTGTAGAGGTGAAAT
TCGTAGATATTCGGAGGAACACCAGTGGCGAAGGCGGCTCACTGGCTCGATACTGACGCT
GAGGTGCGAAAGCGTGGGGA

GCAAACAGGATTAGATACCCTGGTAGTCCACGCCGTAAACGATGAATGCCAGTCGTCGGC
AAGCATGCTTGTCGGTGACA
CACCTAACGGATTAAGCATTCCGCCTGGGGAGTACGGCCGCAAGGTTAAAACTCAAAGGA
ATTGACGGGGGCCCGCACAA
GCGGTGGAGCATGTGGTTTAATTCGAAGCAACGCGCAGAACCTTACCAACCCTTGACATC
CTGATCGCGGTTTTCCGAGA
GGAATTCCTTCAGTTCGGCTGGATCAGTGACAGGTGCTGCATGGCTGTCGTCAGCTCGTG
TCGTGAGATGTTCGGTTAAG
TCCGGCAACGAGCGCAACCCACACTCTTAGTTGCCAGCATTCAGTTGGGCACTCTAGGAG
AACTGCCGATGATAAGTCGG
AGGAAGGTGTGGATGACGTCAAGTCCTCATGGCCCTTACGGGTTGGGCTACACACGTGCT
ACAATGGCAGTGACAATGGG
TTAATCCCTAAAAGCTGTCTCAGTTCGGATTGTTCTCTGCAACTCGAGAGCATGAAGTCGG
AATCGCTAGTAATCGCGTA
ACAGCATGACGCGGTGAATACGTTCCCGGGCCTTGTACACACCGCCCGTCACACCATGGG
AGTTGGGTTTACCCGAAGAC
GGTGCGCCAACCCTTACGGGGGGCAGCTGGCCACGGTAAGCTCAGCGACTGGGGTGAAG
TCGTAACAAGGTAGCCGTAGG
GGAACCTGCGGCTGGATCACCTCCTTT
>AB14
CAACTTGAGAGTTTGATCCTGGCTCAGAACGAACGCTGGCGGCAGGCCTAACACATGCAA
GTCGAGCGAACCCTTCGGGG
TTAGCGGCGGACGGGTGAGTAACGCGTGGGAACGTGCCCTTCTCTGCGGAATAGGCTCG
GGAAACTGGGTTTAATACCGC
ATACGCCCTTCGGGGGAAAGATTTATCGGAGAAGGATCGGCCCGCGTTAGATTAGGTAGT
TGGTGGGGTAATGGCCTACC
AAGCCTACGATCTATAGCTGGTTTGAGAGGATGATCAGCCACACTGGGACTGAGACACGG
CCCAGACTCCTACGGGAGGC
AGCAGTGAGGAATCTTGGACAATGGGGGAAACCCTGATCCAGCCATGCCGCGTGAGCGAT
GAAGGCCTTAGGGTTGTAAA
GCTCTTTCAGTCGTGAAGATAATGACGGTAGCGACAGAAGAAGCCCCGGCTAACTCCGTG
CCAGCAGCCGCGGTAATACG
GAGGGGGCTAGCGTTGTTCGGAATTACTGGGCGTAAAGCGCGCGTAGGCGGACTATTAAG
TCAGGGGTGAAATCCCGGGG
CTCAACCCCGGAACTGCCTTTGATACTGGTAGTCTAGAGTTCGAGAGAGGTGAGTGGAATT
CCGAGTGTAGAGGTGAAAT
TCGTAGATATTCGGAGGAACACCAGTGGCGAAGGCGGCTCACTGGCTCGATACTGACGCT
GAGGTGCGAAAGCGTGGGGA
GCAAACAGGATTAGATACCCTGGTAGTCCACGCCGTAAACGATGAATGCCAGTCGTCGGC
AAGCATGCTTGTCGGTGACA
CACCTAACGGATTAAGCATTCCGCCTGGGGAGTACGGCCGCAAGGTTAAAACTCAAAGGA
ATTGACGGGGGCCCGCACAA
GCGGTGGAGCATGTGGTTTAATTCGAAGCAACGCGCAGAACCTTACCAACCCTTGACATC
CTGATCGCGGTTTTCCGAGA
GGAATTCCTTCAGTTCGGCTGGATCAGTGACAGGTGCTGCATGGCTGTCGTCAGCTCGTG
TCGTGAGATGTTCGGTTAAG
TCCGGCAACGAGCGCAACCCACACTCTTAGTTGCCAGCATTCAGTTGGGCACTCTAGGAG
AACTGCCGATGATAAGTCGG
AGGAAGGTGTGGATGACGTCAAGTCCTCATGGCCCTTACGGGTTGGGCTACACACGTGCT
ACAATGGCAGTGACAATGGG

TTAATCCCTAAAAACTGTCTCAGTTCGGATTGTTCTCTGCAACTCGAGAGCATGAAGTCGG
AATCGCTAGTAATCGCGTA
ACAGCATGACGCGGTGAATACGTTCCCGGGCCTTGTACACACCGCCCGTCACACCATGGG
AGTTGGGTTTACCCGAAGAC
GGTGCGCCAACCCTTACGGGGGGCAGCTGGCCACGGTAAGCTCAGCGACTGGGGTGAAG
TCGTAACAAGGTAGCCGTAGG
GGAACCTGCGGCTGGATCACCTCCTTT
>AB26
CAACTTGAGAGTTTGATCCTGGCTCAGAACGAACGCTGGCGGCAGGCCTAACACATGCAA
GTCGAGCGAACCCTTCGGGG
TTAGCGGCGGACGGGTGAGTAACGCGTGGGAACGTGCCCTTCTCTGCGGAATAGGCTCG
GGAAACTGGGTTTAATACCGC
ATACGCCCTTCGGGGGAAAGATTTATCGGAGAAGGATCGGCCCGCGTCTGATTAGGTAGT
TGGTGGGGTAATGGCCTACC
AAGCCTACGATCAGTAGCTGGTTTGAGAGGATGATCAGCCACACTGGGACTGAGACACGG
CCCAGACTCCTACGGGAGGC
AGCAGTGAGGAATCTTGGACAATGGGGGAAACCCTGATCCAGCCATGCCGCGTGAGCGAT
GAAGGCCTTAGGGTTGTAAA
GCTCTTTCAGTCGTGAAGATAATGACGGTAGCGACAGAAGAAGCCCCGGCTAACTCCGTG
CCAGCAGCCGCGGTAATACG
GAGGGGGCTAGCGTTGTTCGGAATTACTGGGCGTAAAGCGCGCGTAGGCGGACTATTAAG
TCGGGGGTGAAATCCCGGGG
CTCAACCCCGGAACTGCCTCCGATACTGGTAGTCTAGAGTTCGAGAGAGGTGAGTGGAAT
TCCGAGTGTAGAGGTGAAAT
TCGTAGATATTCGGAGGAACACCAGTGGCGAAGGCGGCTCACTGGCTCGATACTGACGCT
GAGGTGCGAAAGCGTGGGGA
GCAAACAGGATTAGATACCCTGGTAGTCCACGCCGTAAACGATGAATGCCAGTCGTCGGC
AAGCATGCTTGTCGGTGACA
CACCTAACGGATTAAGCATTCCGCCTGGGGAGTACGGCCGCAAGGTTAAAACTCAAAGGA
ATTGACGGGGGCCCGCACAA
GCGGTGGAGCATGTGGTTTAATTCGAAGCAACGCGCAGAACCTTACCAACCCTTGACATC
CTGATCGCGGTTTTCCGAGA
GGAATTCCTTCAGTTCGGCTGGATCAGTGACAGGTGCTGCATGGCTGTCGTCAGCTCGTG
TCGTGAGATGTTCGGTTAAG
TCCGGCAACGAGCGCAACCCACACTCTTAGTTGCCAGCATTCAGTTGGGCACTCTAAGAG
AACTGCCGATGATAAGTCGG
AGGAAGGTGTGGATGACGTCAAGTCCTCATGGCCCTTACGGGTTGGGCTACACACGTGCT
ACAATGGCAGTGACAATGGG
TTAATCCCAAAAAACTGTCTCAGTTCGGATTGTTCTCTGCAACTCGAGAGCATGAAGTCGG
AATCGCTAGTAATCGCGTA
ACAGCATGACGCGGTGAATACGTTCCCGGGCCTTGTACACACCGCCCGTCACACCATGGG
AGTTGGGTTTACCCGAAGAC
GGTGCGCCAACCCTTACGGGGGGCAGCTGGCCACGGTAAGCTCAGCGACTGGGGTGAAG
TCGTAACAAGGTAGCCGTAGG
GGAACCTGCGGCTGGATCACCTCCTTT
>AB30
CAACTTGAGAGTTTGATCCTGGCTCAGAACGAACGCTGGCGGCAGGCCTAACACATGCAA
GTCGAGCGAACCCTTCGGGG
TTAGCGGCGGACGGGTGAGTAACGCGTGGGAACGTGCCCTTCTCTGCGGAATAGGCTCG
GGAAACTGGGTTTAATACCGC

ATACGCCCTTCGGGGGAAAGATTTATCGGAGAAGGATCGGCCCGCGTTAGATTAGGTAGT
TGGTGGGGTAATGGCCTACC
AAGCCTACGATCTATAGCTGGTTTGAGAGGATGATCAGCCACACTGGGACTGAGACACGG
CCCAGACTCCTACGGGAGGC
AGCAGTGAGGAATCTTGGACAATGGGGGAAACCCTGATCCAGCCATGCCGCGTGAGCGAT
GAAGGCCTTAGGGTTGTAAA
GCTCTTTCAGTCGTGAAGATAATGACGGTAGCGACAGAAGAAGCCCCGGCTAACTCCGTG
CCAGCAGCCGCGGTAATACG
GAGGGGGCTAGCGTTGTTCGGAATTACTGGGCGTAAAGCGCGCGTAGGCGGACTATTAAG
TCAGGGGTGAAATCCCGGGG
CTCAACCCCGGAACTGCCTTTGATACTGGTAGTCTAGAGTTCGAGAGAGGTGAGTGGAATT
CCGAGTGTAGAGGTGAAAT
TCGTAGATATTCGGAGGAACACCAGTGGCGAAGGCGGCTCACTGGCTCGATACTGACGCT
GAGGTGCGAAAGCGTGGGGA
GCAAACAGGATTAGATACCCTGGTAGTCCACGCCGTAAACGATGAATGCCAGTCGTCGGC
AAGCATGCTTGTCGGTGACA
CACCTAACGGATTAAGCATTCCGCCTGGGGAGTACGGCCGCAAGGTTAAAACTCAAAGGA
ATTGACGGGGGCCCGCACAA
GCGGTGGAGCATGTGGTTTAATTCGAAGCAACGCGCAGAACCTTACCAACCCTTGACATC
CTGATCGCGGTTTTCCGAGA
GGAATTCCTTCAGTTCGGCTGGATCAGTGACAGGTGCTGCATGGCTGTCGTCAGCTCGTG
TCGTGAGATGTTCGGTTAAG
TCCGGCAACGAGCGCAACCCACACTCTTAGTTGCCAGCATTCAGTTGGGCACTCTAGGAG
AACTGCCGATGATAAGTCGG
AGGAAGGTGTGGATGACGTCAAGTCCTCATGGCCCTTACGGGTTGGGCTACACACGTGCT
ACAATGGCAGTGACAATGGG
TTAATCCCTAAAAACTGTCTCAGTTCGGATTGTTCTCTGCAACTCGAGAGCATGAAGTCGG
AATCGCTAGTAATCGCGTA
ACAGCATGACGCGGTGAATACGTTCCCGGGCCTTGTACACACCGCCCGTCACACCATGGG
AGTTGGGTTTACCCGAAGAC
GGTGCGCCAACCCTTACGGGGGGCAGCTGGCCACGGTAAGCTCAGCGACTGGGGTGAAG
TCGTAAC
>SE1
CAACTTGAGAGTTTGATCCTGGCTCAGAACGAACGCTGGCGGCAGGCCTAACACATGCAA
GTCGAGCGAACCCTTCGGGG
TTAGCGGCGGACGGGTGAGTAACGCGTGGGAACGTGCCCTTCTCTGCGGAATAGGCTCG
GGAAACTGGGTTTAATACCGC
ATACGCCCTTCGGGGGAAAGATTTATCGGAGAAGGATCGGCCCGCGTTAGATTAGGTAGT
TGGTGGGGTAATGGCCTACC
AAGCCTACGATCTATAGCTGGTTTGAGAGGATGATCAGCCACACTGGGACTGAGACACGG
CCCAGACTCCTACGGGAGGC
AGCAGTGAGGAATCTTGGACAATGGGGGAAACCCTGATCCAGCCATGCCGCGTGAGCGAT
GAAGGCCTTAGGGTTGTAAA
GCTCTTTCAGTCGTGAAGATAATGACGGTAGCGACAGAAGAAGCCCCGGCTAACTCCGTG
CCAGCAGCCGCGGTAATACG
GAGGGGGCTAGCGTTGTTCGGAATTACTGGGCGTAAAGCGCGCGTAGGCGGACTATTAAG
TCAGGGGTGAAATCCCGGGG
CTCAACCCCGGAACTGCCTTTGATACTGGTAGTCTAGAGTTCGAGAGAGGTGAGTGGAATT
CCGAGTGTAGAGGTGAAAT
TCGTAGATATTCGGAGGAACACCAGTGGCGAAGGCGGCTCACTGGCTCGATACTGACGCT
GAGGTGCGAAAGCGTGGGGA

GCAAACAGGATTAGATACCCTGGTAGTCCACGCCGTAAACGATGAATGCCAGTCGTCGGC
AAGCATGCTTGTCGGTGACA
CACCTAACGGATTAAGCATTCCGCCTGGGGAGTACGGCCGCAAGGTTAAAACTCAAAGGA
ATTGACGGGGGCCCGCACAA
GCGGTGGAGCATGTGGTTTAATTCGAAGCAACGCGCAGAACCTTACCAACCCTTGACATC
CTGATCGCGGTTTTCCGAGA
GGAATTCCTTCAGTTCGGCTGGATCAGTGACAGGTGCTGCATGGCTGTCGTCAGCTCGTG
TCGTGAGATGTTCGGTTAAG
TCCGGCAACGAGCGCAACCCACACTCTTAGTTGCCAGCATTCAGTTGGGCACTCTAGGAG
AACTGCCGATGATAAGTCGG
AGGAAGGTGTGGATGACGTCAAGTCCTCATGGCCCTTACGGGTTGGGCTACACACGTGCT
ACAATGGCAGTGACAATGGG
TTAATCCCTAAAAACTGTCTCAGTTCGGATTGTTCTCTGCAACTCGAGAGCATGAAGTCGG
AATCGCTAGTAATCGCGTA
ACAGCATGACGCGGTGAATACGTTCCCGGGCCTTGTACACACCGCCCGTCACACCATGGG
AGTTGGGTTTACCCGAAGAC
GGTGCGCCAACCCTTACGGGGGGCAGCTGGCCACGGTAAGCTCAGCGACTGGGGTGAAG
TCGTAACAAGGTAGCCGTAGG
GGAACCTGCGGCTGGATCACCTCCTTT
>IM796
CAACTTGAGAGTTTGATCCTGGCTCAGAACGAACGCTGGCGGCAGGCCTAACACATGCAA
GTCGAGCGAACCCTTCGGGG
TTAGCGGCGGACGGGTGAGTAACGCGTGGGAACGTGCCCTTCTCTGCGGAATAGGCTCG
GGAAACTGGGTTTAATACCGC
ATACGCCCTTCGGGGGAAAGATTTATCGGAGAAGGATCGGCCCGCGTTAGATTAGGTAGT
TGGTGGGGTAATGGCCTACC
AAGCCTACGATCTATAGCTGGTTTGAGAGGATGATCAGCCACACTGGGACTGAGACACGG
CCCAGACTCCTACGGGAGGC
AGCAGTGAGGAATCTTGGACAATGGGGGAAACCCTGATCCAGCCATGCCGCGTGAGCGAT
GAAGGCCTTAGGGTTGTAAA
GCTCTTTCAGTCGTGAAGATAATGACGGTAGCGACAGAAGAAGCCCCGGCTAACTCCGTG
CCAGCAGCCGCGGTAATACG
GAGGGGGCTAGCGTTGTTCGGAATTACTGGGCGTAAAGCGCGCGTAGGCGGACTATTAAG
TCAGGGGTGAAATCCCGGGG
CTCAACCCCGGAACTGCCTTTGATACTGGTAGTCTAGAGTTCGAGAGAGGTGAGTGGAATT
CCGAGTGTAGAGGTGAAAT
TCGTAGATATTCGGAGGAACACCAGTGGCGAAGGCGGCTCACTGGCTCGATACTGACGCT
GAGGTGCGAAAGCGTGGGGA
GCAAACAGGATTAGATACCCTGGTAGTCCACGCCGTAAACGATGAATGCCAGTCGTCGGC
AAGCATGCTTGTCGGTGACA
CACCTAACGGATTAAGCATTCCGCCTGGGGAGTACGGCCGCAAGGTTAAAACTCAAAGGA
ATTGACGGGGGCCCGCACAA
GCGGTGGAGCATGTGGTTTAATTCGAAGCAACGCGCAGAACCTTACCAACCCTTGACATC
CTGATCGCGGTTTTCCGAGA
GGAATTCCTTCAGTTCGGCTGGATCAGTGACAGGTGCTGCATGGCTGTCGTCAGCTCGTG
TCGTGAGATGTTCGGTTAAG
TCCGGCAACGAGCGCAACCCACACTCTTAGTTGCCAGCATTCAGTTGGGCACTCTAGGAG
AACTGCCGATGATAAGTCGG
AGGAAGGTGTGGATGACGTCAAGTCCTCATGGCCCTTACGGGTTGGGCTACACACGTGCT
ACAATGGCAGTGACAATGGG

TTAATCCCTAAAAACTGTCTCAGTTCGGATTGTTCTCTGCAACTCGAGAGCATGAAGTCGG
AATCGCTAGTAATCGCGTA
ACAGCATGACGCGGTGAATACGTTCCCGGGCCTTGTACACACCGCCCGTCACACCATGGG
AGTTGGGTTTACCCGAAGAC
GGTGCGCCAACCCTTACGGGGGGCAGCTGGCCACGGTAAGCTCAGCGACTGGGGTGAAG
TCGTAACAAGGTAGCCGTAGG
GGAACCTGCGGCTGGATCACCTCCTTT
>AB35
CAACTTGAGAGTTTGATCCTGGCTCAGAACGAACGCTGGCGGCAGGCCTAACACATGCAA
GTCGAGCGAACCCTTCGGGG
TTAGCGGCGGACGGGTGAGTAACGCGTGGGAACGTGCCCTTCTCTGCGGAATAGGCTCG
GGAAACTGGGTTTAATACCGC
ATACGCCCTTCGGGGGAAAGATTTATCGGAGAAGGATCGGCCCGCGTTAGATTAGGTAGT
TGGTGGGGTAATGGCCTACC
AAGCCTACGATCTATAGCTGGTTTGAGAGGATGATCAGCCACACTGGGACTGAGACACGG
CCCAGACTCCTACGGGAGGC
AGCAGTGAGGAATCTTGGACAATGGGGGAAACCCTGATCCAGCCATGCCGCGTGAGCGAT
GAAGGCCTTAGGGTTGTAAA
GCTCTTTCAGTCGTGAAGATAATGACGGTAGCGACAGAAGAAGCCCCGGCTAACTCCGTG
CCAGCAGCCGCGGTAATACG
GAGGGGGCTAGCGTTGTTCGGAATTACTGGGCGTAAAGCGCGCGTAGGCGGACTATTAAG
TCAGGGGTGAAATCCCGGGG
CTCAACCCCGGAACTGCCTTTGATACTGGTAGTCTAGAGTTCGAGAGAGGTGAGTGGAATT
CCGAGTGTAGAGGTGAAAT
TCGTAGATATTCGGAGGAACACCAGTGGCGAAGGCGGCTCACTGGCTCGATACTGACGCT
GAGGTGCGAAAGCGTGGGGA
GCAAACAGGATTAGATACCCTGGTAGTCCACGCCGTAAACGATGAATGCCAGTCGTCGGC
AAGCATGCTTGTCGGTGACA
CACCTAACGGATTAAGCATTCCGCCTGGGGAGTACGGCCGCAAGGTTAAAACTCAAAGGA
ATTGACGGGGGCCCGCACAA
GCGGTGGAGCATGTGGTTTAATTCGAAGCAACGCGCAGAACCTTACCAACCCTTGACATC
CTGATCGCGGTTTTCCGAGA
GGAATTCCTTCAGTTCGGCTGGATCAGTGACAGGTGCTGCATGGCTGTCGTCAGCTCGTG
TCGTGAGATGTTCGGTTAAG
TCCGGCAACGAGCGCAACCCACACTCTTAGTTGCCAGCATTCAGTTGGGCACTCTAGGAG
AACTGCCGATGATAAGTCGG
AGGAAGGTGTGGATGACGTCAAGTCCTCATGGCCCTTACGGGTTGGGCTACACACGTGCT
ACAATGGCAGTGACAATGGG
TTAATCCCTAAAAGCTGTCTCAGTTCGGATTGTTCTCTGCAACTCGAGAGCATGAAGTCGG
AATCGCTAGTAATCGCGTA
ACAGCATGACGCGGTGAATACGTTCCCGGGCCTTGTACACACCGCCCGTCACACCATGGG
AGTTGGGTTTACCCGAAGAC
GGTGCGCCAACCTTTACGGGGGGCAGCTGGCCACGGTAAGCTCAGCGACTGGGGTGAAG
TCGTAACAAGGTAGCCGTAGG
GGAACCTGCGGCTGGATCACCTCCTTT
>AB23
CAACTTGAGAGTTTGATCCTGGCTCAGAACGAACGCTGGCGGCAGGCCTAACACATGCAA
GTCGAGCGAACCCTTCGGGG
TTAGCGGCGGACGGGTGAGTAACGCGTGGGAACGTGCCCTTCTCTGCGGAATAGGCTCG
GGAAACTGGGTTTAATACCGC

ATACGCCCTTCGGGGGAAAGATTTATCGGAGAAGGATCGGCCCGCGTTAGATTAGGTAGT
TGGTGGGGTAATGGCCTACC
AAGCCTACGATCTATAGCTGGTTTGAGAGGATGATCAGCCACACTGGGACTGAGACACGG
CCCAGACTCCTACGGGAGGC
AGCAGTGAGGAATCTTGGACAATGGGGGAAACCCTGATCCAGCCATGCCGCGTGAGCGAT
GAAGGCCTTAGGGTTGTAAA
GCTCTTTCAGTCGTGAAGATAATGACGGTAGCGACAGAAGAAGCCCCGGCTAACTCCGTG
CCAGCAGCCGCGGTAATACG
GAGGGGGCTAGCGTTGTTCGGAATTACTGGGCGTAAAGCGCGCGTAGGCGGACTATTAAG
TCAGGGGTGAAATCCCGGGG
CTCAACCCCGGAACTGCCTTTGATACTGGTAGTCTAGAGTTCGAGAGAGGTGAGTGGAATT
CCGAGTGTAGAGGTGAAAT
TCGTAGATATTCGGAGGAACACCAGTGGCGAAGGCGGCTCACTGGCTCGATACTGACGCT
GAGGTGCGAAAGCGTGGGGA
GCAAACAGGATTAGATACCCTGGTAGTCCACGCCGTAAACGATGAATGCCAGTCGTCGGC
AAGCATGCTTGTCGGTGACA
CACCTAACGGATTAAGCATTCCGCCTGGGGAGTACGGCCGCAAGGTTAAAACTCAAAGGA
ATTGACGGGGGCCCGCACAA
GCGGTGGAGCATGTGGTTTAATTCGAAGCAACGCGCAGAACCTTACCAACCCTTGACATC
CTGATCGCGGTTTTCCGAGA
GGAATTCCTTCAGTTCGGCTGGATCAGTGACAGGTGCTGCATGGCTGTCGTCAGCTCGTG
TCGTGAGATGTTCGGTTAAG
TCCGGCAACGAGCGCAACCCACACTCTTAGTTGCCAGCATTCAGTTGGGCACTCTAGGAG
AACTGCCGATGATAAGTCGG
AGGAAGGTGTGGATGACGTCAAGTCCTCATGGCCCTTACGGGTTGGGCTACACACGTGCT
ACAATGGCAGTGACAATGGG
TTAATCCCTAAAAACTGTCTCAGTTCGGATTGTTCTCTGCAACTCGAGAGCATGAAGTCGG
AATCGCTAGTAATCGCGTA
ACAGCATGACGCGGTGAATACGTTCCCGGGCCTTGTACACACCGCCCGTCACACCATGGG
AGTTGGGTTTACCCGAAGAC
GGTGCGCCAACCCTTACGGGGGGCAGCTGGCCACGGTAAGCTCAGCGACTGGGGTGAAG
TCGTAACAAGGTAGCCGTAGG
GGAACCTGCGGCTGGATCACCTCCTTT
>AB22
CAACTTGAGAGTTTGATCCTGGCTCAGAACGAACGCTGGCGGCAGGCCTAACACATGCAA
GTCGAGCGAACCCTTCGGGG
TTAGCGGCGGACGGGTGAGTAACGCGTGGGAACGTGCCCTTCTCTGCGGAATAGGCTCG
GGAAACTGGGTTTAATACCGC
ATACGCCCTTCGGGGGAAAGATTTATCGGAGAAGGATCGGCCCGCGTTAGATTAGGTAGT
TGGTGGGGTAATGGCCTACC
AAGCCTACGATCTATAGCTGGTTTGAGAGGATGATCAGCCACACTGGGACTGAGACACGG
CCCAGACTCCTACGGGAGGC
AGCAGTGAGGAATCTTGGACAATGGGGGAAACCCTGATCCAGCCATGCCGCGTGAGCGAT
GAAGGCCTTAGGGTTGTAAA
GCTCTTTCAGTCGTGAAGATAATGACGGTAGCGACAGAAGAAGCCCCGGCTAACTCCGTG
CCAGCAGCCGCGGTAATACG
GAGGGGGCTAGCGTTGTTCGGAATTACTGGGCGTAAAGCGCGCGTAGGCGGACTATTAAG
TCAGGGGTGAAATCCCGGGG
CTCAACCCCGGAACTGCCTTTGATACTGGTAGTCTAGAGTTCGAGAGAGGTGAGTGGAATT
CCGAGTGTAGAGGTGAAAT

TCGTAGATATTCGGAGGAACACCAGTGGCGAAGGCGGCTCACTGGCTCGATACTGACGCT
GAGGTGCGAAAGCGTGGGGA
GCAAACAGGATTAGATACCCTGGTAGTCCACGCCGTAAACGATGAATGCCAGTCGTCGGC
AAGCATGCTTGTCGGTGACA
CACCTAACGGATTAAGCATTCCGCCTGGGGAGTACGGCCGCAAGGTTAAAACTCAAAGGA
ATTGACGGGGGCCCGCACAA
GCGGTGGAGCATGTGGTTTAATTCGAAGCAACGCGCAGAACCTTACCAACCCTTGACATC
CTGATCGCGGTTTTCCGAGA
GGAATTCCTTCAGTTCGGCTGGATCAGTGACAGGTGCTGCATGGCTGTCGTCAGCTCGTG
TCGTGAGATGTTCGGTTAAG
TCCGGCAACGAGCGCAACCCACACTCTTAGTTGCCAGCATTCAGTTGGGCACTCTAGGAG
AACTGCCGATGATAAGTCGG
AGGAAGGTGTGGATGACGTCAAGTCCTCATGGCCCTTACGGGTTGGGCTACACACGTGCT
ACAATGGCAGTGACAATGGG
TTAATCCCTAAAAACTGTCTCAGTTCGGATTGTTCTCTGCAACTCGAGAGCATGAAGTCGG
AATCGCTAGTAATCGCGTA
ACAGCATGACGCGGTGAATACGTTCCCGGGCCTTGTACACACCGCCCGTCACACCATGGG
AGTTGGGTTTACCCGAAGAC
GGTGCGCCAACCCTTACGGGGGGCAGCTGGCCACGGTAAGCTCAGCGACTGGGGTGAAG
TCGTAACAAGGTAGCCGTAGG
GGAACCTGCGGCTGGATCACCTCCTTT
>AB16
CAACTTGAGAGTTTGATCCTGGCTCAGAACGAACGCTGGCGGCAGGCCTAACACATGCAA
GTCGAGCGAACCCTTCGGGG
TTAGCGGCGGACGGGTGAGTAACGCGTGGGAACGTGCCCTTCTCTGCGGAATAGGCTTG
GGAAACTGGGTTTAATACCGC
ATACGCCCTTCGGGGGAAAGATTTATCGGAGAAGGATCGGCCCGCGTTAGATTAGGTAGT
TGGTGGGGTAATGGCCTACC
AAGCCTACGATCTATAGCTGGTTTGAGAGGATGATCAGCCACACTGGGACTGAGACACGG
CCCAGACTCCTACGGGAGGC
AGCAGTGAGGAATCTTGGACAATGGGGGAAACCCTGATCCAGCCATGCCGCGTGAGCGAT
GAAGGCCTTAGGGTTGTAAA
GCTCTTTCAGTCGTGAAGATAATGACGGTAGCGACAGAAGAAGCCCCGGCTAACTCCGTG
CCAGCAGCCGCGGTAATACG
GAGGGGGCTAGCGTTGTTCGGAATTACTGGGCGTAAAGCGCGCGTAGGCGGACTATTAAG
TCAGGGGTGAAATCCCGGGG
CTCAACCCCGGAACTGCCTTTGATACTGGTAGTCTAGAGTTCGAGAGAGGTGAGTGGAATT
CCGAGTGTAGAGGTGAAAT
TCGTAGATATTCGGAGGAACACCAGTGGCGAAGGCGGCTCACTGGCTCGATACTGACGCT
GAGGTGCGAAAGCGTGGGGA
GCAAACAGGATTAGATACCCTGGTAGTCCACGCCGTAAACGATGAATGCCAGTCGTCGGC
AAGCATGCTTGTCGGTGACA
CACCTAACGGATTAAGCATTCCGCCTGGGGAGTACGGCCGCAAGGTTAAAACTCAAAGGA
ATTGACGGGGGCCCGCACAA
GCGGTGGAGCATGTGGTTTAATTCGAAGCAACGCGCAGAACCTTACCAACCCTTGACATC
CTGATCGCGGTTTTCCGAGA
GGAATTCCTTCAGTTCGGCTGGATCAGTGACAGGTGCTGCATGGCTGTCGTCAGCTCGTG
TCGTGAGATGTTCGGTTAAG
TCCGGCAACGAGCGCAACCCACACTCTTAGTTGCCAGCATTCAGTTGGGCACTCTAGGAG
AACTGCCGATGATAAGTCGG

AGGAAGGTGTGGATGACGTCAAGTCCTCATGGCCCTTACGGGTTGGGCTACACACGTGCT
ACAATGGCAGTGACAATGGG
TTAATCCCTAAAAACTGTCTCAGTTCGGATTGTTCTCTGCAACTCGAGAGCATGAAGTCGG
AATCGCTAGTAATCGCGTA
ACAGCATGACGCGGTGAATACGTTCCCGGGCCTTGTACACACCGCCCGTCACACCATGGG
AGTTGGGTTTACCCGAAGAC
GGTGCGCCAACCCTTGCGGGGGGCAGCTGGCCACGGTAAGCTCAGCGACTGGGGTGAA
GTCGTAACAAGGTAGCCGTAGG
GGAACCTGCGGCTGGATCACCTCCTTT
>AB20
CAACTTGAGAGTTTGATCCTGGCTCAGAACGAACGCTGGCGGCAGGCCTAACACATGCAA
GTCGAGCGAACCCTTCGGGG
TTAGCGGCGGACGGGTGAGTAACGCGTGGGAACGTGCCCTTCTCTGCGGAATAGGCTCG
GGAAACTGGGTTTAATACCGC
ATACGCCCTTCGGGGGAAAGATTTATCGGAGAAGGATCGGCCCGCGTTAGATTAGGTAGT
TGGTGGGGTAATGGCCTACC
AAGCCTACGATCTATAGCTGGTTTGAGAGGATGATCAGCCACACTGGGACTGAGACACGG
CCCAGACTCCTACGGGAGGC
AGCAGTGAGGAATCTTGGACAATGGGGGAAACCCTGATCCAGCCATGCCGCGTGAGCGAT
GAAGGCCTTAGGGTTGTAAA
GCTCTTTCAGTCGTGAAGATAATGACGGTAGCGACAGAAGAAGCCCCGGCTAACTCCGTG
CCAGCAGCCGCGGTAATACG
GAGGGGGCTAGCGTTGTTCGGAATTACTGGGCGTAAAGCGCGCGTAGGCGGACTATTAAG
TCAGGGGTGAAATCCCGGGG
CTCAACCCCGGAACTGCCTTTGATACTGGTAGTCTAGAGTTCGAGAGAGGTGAGTGGAATT
CCGAGTGTAGAGGTGAAAT
TCGTAGATATTCGGAGGAACACCAGTGGCGAAGGCGGCTCACTGGCTCGATACTGACGCT
GAGGTGCGAAAGCGTGGGGA
GCAAACAGGATTAGATACCCTGGTAGTCCACGCCGTAAACGATGAATGCCAGTCGTCGGC
AAGCATGCTTGTCGGTGACA
CACCTAACGGATTAAGCATTCCGCCTGGGGAGTACGGCCGCAAGGTTAAAACTCAAAGGA
ATTGACGGGGGCCCGCACAA
GCGGTGGAGCATGTGGTTTAATTCGAAGCAACGCGCAGAACCTTACCAACCCTTGACATC
CTGATCGCGGTTTTCCGAGA
GGAATTCCTTCAGTTCGGCTGGATCAGTGACAGGTGCTGCATGGCTGTCGTCAGCTCGTG
TCGTGAGATGTTCGGTTAAG
TCCGGCAACGAGCGCAACCCACACTCTTAGTTGCCAGCATTCAGTTGGGCACTCTAGGAG
AACTGCCGATGATAAGTCGG
AGGAAGGTGTGGATGACGTCAAGTCCTCATGGCCCTTACGGGTTGGGCTACACACGTGCT
ACAATGGCAGTGACAATGGG
TTAATCCCTAAAAGCTGTCTCAGTTCGGATTGTTCTCTGCAACTCGAGAGCATGAAGTCGG
AATCGCTAGTAATCGCGTA
ACAGCATGACGCGGTGAATACGTTCCCGGGCCTTGTACACACCGCCCGTCACACCATGGG
AGTTGGGTTTACCCGAAGAC
GGTGCGCCAACCCTTACGGGGGGCAGCTGGCCACGGTAAGCTCAGCGACTGGGGTGAAG
TCGTAACAAGGTAGCCGTAGG
GGAACCTGCGGCTGGATCACCTCCTTT
>AB28
CAACTTGAGAGTTTGATCCTGGCTCAGAACGAACGCTGGCGGCAGGCCTAACACATGCAA
GTCGAGCGAACCCTTCGGGG

TTAGCGGCGGACGGGTGAGTAACGCGTGGGAACGTGCCCTTCTCTGCGGAATAGGCTCG
GGAAACTGGGTTTAATACCGC
ATACGCCCTTCGGGGGAAAGATTTATCGGAGAAGGATCGGCCCGCGTTAGATTAGGTAGT
TGGTGGGGTAATGGCCTACC
AAGCCTACGATCTATAGCTGGTTTGAGAGGATGATCAGCCACACTGGGACTGAGACACGG
CCCAGACTCCTACGGGAGGC
AGCAGTGAGGAATCTTGGACAATGGGGGAAACCCTGATCCAGCCATGCCGCGTGAGCGAT
GAAGGCCTTAGGGTTGTAAA
GCTCTTTCAGTCGTGAAGATAATGACGGTAGCGACAGAAGAAGCCCCGGCTAACTCCGTG
CCAGCAGCCGCGGTAATACG
GAGGGGGCTAGCGTTGTTCGGAATTACTGGGCGTAAAGCGCGCGTAGGCGGACTATTAAG
TCAGGGGTGAAATCCCGGGG
CTCAACCCCGGAACTGCCTTTGATACTGGTAGTCTAGAGTTCGAGAGAGGTGAGTGGAATT
CCGAGTGTAGAGGTGAAAT
TCGTAGATATTCGGAGGAACACCAGTGGCGAAGGCGGCTCACTGGCTCGATACTGACGCT
GAGGTGCGAAAGCGTGGGGA
GCAAACAGGATTAGATACCCTGGTAGTCCACGCCGTAAACGATGAATGCCAGTCGTCGGC
AAGCATGCTTGTCGGTGACA
CACCTAACGGATTAAGCATTCCGCCTGGGGAGTACGGCCGCAAGGTTAAAACTCAAAGGA
ATTGACGGGGGCCCGCACAA
GCGGTGGAGCATGTGGTTTAATTCGAAGCAACGCGCAGAACCTTACCAACCCTTGACATC
CTGATCGCGGTTTTCCGAGA
GGAATTCCTTCAGTTCGGCTGGATCAGTGACAGGTGCTGCATGGCTGTCGTCAGCTCGTG
TCGTGAGATGTTCGGTTAAG
TCCGGCAACGAGCGCAACCCACACTCTTAGTTGCCAGCATTCAGTTGGGCACTCTAGGAG
AACTGCCGATGATAAGTCGG
AGGAAGGTGTGGATGACGTCAAGTCCTCATGGCCCTTACGGGTTGGGCTACACACGTGCT
ACAATGGCAGTGACAATGGG
TTAATCCCTAAAAGCTGTCTCAGTTCGGATTGTTCTCTGCAACTCGAGAGCATGAAGTCGG
AATCGCTAGTAATCGCGTA
ACAGCATGACGCGGTGAATACGTTCCCGGGCCTTGTACACACCGCCCGTCACACCATGGG
AGTTGGGTTTACCCGAAGAC
GGTGCGCCAACCCTTACGGGGGGCAGCTGGCCACGGTAAGCTCAGCGACTGGGGTGAAG
TCGTAACAAGGTAGCCGTAGG
GGAACCTGCGGCTGGATCACCTCCTTT
>AB18
CAACTTGAGAGTTTGATCCTGGCTCAGAACGAACGCTGGCGGCAGGCCTAACACATGCAA
GTCGAGCGAACCCTTCGGGG
TTAGCGGCGGACGGGTGAGTAACGCGTGGGAACGTGCCCTTCTCTGCGGAATAGGCTCG
GGAAACTGGGTTTAATACCGC
ATACGCCCTTCGGGGGAAAGATTTATCGGAGAAGGATCGGCCCGCGTTAGATTAGGTAGT
TGGTGGGGTAATGGCCTACC
AAGCCTACGATCTATAGCTGGTTTGAGAGGATGATCAGCCACACTGGGACTGAGACACGG
CCCAGACTCCTACGGGAGGC
AGCAGTGAGGAATCTTGGACAATGGGGGAAACCCTGATCCAGCCATGCCGCGTGAGCGAT
GAAGGCCTTAGGGTTGTAAA
GCTCTTTCAGTCGTGAAGATAATGACGGTAGCGACAGAAGAAGCCCCGGCTAACTCCGTG
CCAGCAGCCGCGGTAATACG
GAGGGGGCTAGCGTTGTTCGGAATTACTGGGCGTAAAGCGCGCGTAGGCGGACTATTAAG
TCAGGGGTGAAATCCCGGGG

CTCAACCCCGGAACTGCCTTTGATACTGGTAGTCTAGAGTTCGAGAGAGGTGAGTGGAATT
CCGAGTGTAGAGGTGAAAT
TCGTAGATATTCGGAGGAACACCAGTGGCGAAGGCGGCTCACTGGCTCGATACTGACGCT
GAGGTGCGAAAGCGTGGGGA
GCAAACAGGATTAGATACCCTGGTAGTCCACGCCGTAAACGATGAATGCCAGTCGTCGGC
AAGCATGCTTGTCGGTGACA
CACCTAACGGATTAAGCATTCCGCCTGGGGAGTACGGCCGCAAGGTTAAAACTCAAAGGA
ATTGACGGGGGCCCGCACAA
GCGGTGGAGCATGTGGTTTAATTCGAAGCAACGCGCAGAACCTTACCAACCCTTGACATC
CTGATCGCGGTTTTCCGAGA
GGAATTCCTTCAGTTCGGCTGGATCAGTGACAGGTGCTGCATGGCTGTCGTCAGCTCGTG
TCGTGAGATGTTCGGTTAAG
TCCGGCAACGAGCGCAACCCACACTCTTAGTTGCCAGCATTCAGTTGGGCACTCTAGGAG
AACTGCCGATGATAAGTCGG
AGGAAGGTGTGGATGACGTCAAGTCCTCATGGCCCTTACGGGTTGGGCTACACACGTGCT
ACAATGGCAGTGACAATGGG
TTAATCCCTAAAAACTGTCTCAGTTCGGATTGTTCTCTGCAACTCGAGAGCATGAAGTCGG
AATCGCTAGTAATCGCGTA
ACAGCATGACGCGGTGAATACGTTCCCGGGCCTTGTACACACCGCCCGTCACACCATGGG
AGTTGGGTTTACCCGAAGAC
GGTGCGCCAACCCTTACGGGGGGCAGCTGGCCACGGTAAGCTCAGCGACTGGGGTGAAG
TCGTAACAAGGTAGCCGTAGG
GGAACCTGCGGCTGGATCACCTCCTTT
>AB17
CAACTTGAGAGTTTGATCCTGGCTCAGAACGAACGCTGGCGGCAGGCCTAACACATGCAA
GTCGAGCGAACCCTTCGGGG
TTAGCGGCGGACGGGTGAGTAACGCGTGGGAACGTGCCCTTCTCTGCGGAATAGGCTCG
GGAAACTGGGTTTAATACCGC
ATACGCCCTTCGGGGGAAAGATTTATCGGAGAAGGATCGGCCCGCGTTAGATTAGGTAGT
TGGTGGGGTAATGGCCTACC
AAGCCTACGATCTATAGCTGGTTTGAGAGGATGATCAGCCACACTGGGACTGAGACACGG
CCCAGACTCCTACGGGAGGC
AGCAGTGAGGAATCTTGGACAATGGGGGAAACCCTGATCCAGCCATGCCGCGTGAGCGAT
GAAGGCCTTAGGGTTGTAAA
GCTCTTTCAGTCGTGAAGATAATGACGGTAGCGACAGAAGAAGCCCCGGCTAACTCCGTG
CCAGCAGCCGCGGTAATACG
GAGGGGGCTAGCGTTGTTCGGAATTACTGGGCGTAAAGCGCGCGTAGGCGGACTATTAAG
TCAGGGGTGAAATCCCGGGG
CTCAACCCCGGAACTGCCTTTGATACTGGTAGTCTAGAGTTCGAGAGAGGTGAGTGGAATT
CCGAGTGTAGAGGTGAAAT
TCGTAGATATTCGGAGGAACACCAGTGGCGAAGGCGGCTCACTGGCTCGATACTGACGCT
GAGGTGCGAAAGCGTGGGGA
GCAAACAGGATTAGATACCCTGGTAGTCCACGCCGTAAACGATGAATGCCAGTCGTCGGC
AAGCATGCTTGTCGGTGACA
CACCTAACGGATTAAGCATTCCGCCTGGGGAGTACGGCCGCAAGGTTAAAACTCAAAGGA
ATTGACGGGGGCCCGCACAA
GCGGTGGAGCATGTGGTTTAATTCGAAGCAACGCGCAGAACCTTACCAACCCTTGACATC
CTGATCGCGGTTTTCCGAGA
GGAATTCCTTCAGTTCGGCTGGATCAGTGACAGGTGCTGCATGGCTGTCGTCAGCTCGTG
TCGTGAGATGTTCGGTTAAG

TCCGGCAACGAGCGCAACCCACACTCTTAGTTGCCAGCATTCAGTTGGGCACTCTAGGAG
AACTGCCGATGATAAGTCGG
AGGAAGGTGTGGATGACGTCAAGTCCTCATGGCCCTTACGGGTTGGGCTACACACGTGCT
ACAATGGCAGTGACAATGGG
TTAATCCCTAAAAACTGTCTCAGTTCGGATTGTTCTCTGCAACTCGAGAGCATGAAGTCGG
AATCGCTAGTAATCGCGTA
ACAGCATGACGCGGTGAATACGTTCCCGGGCCTTGTACACACCGCCCGTCACACCATGGG
AGTTGGGTTTACCCGAAGAC
GGTGCGCCAACCCTTACGGGGGGCAGCTGGCCACGGTAAGCTCAGCGACTGGGGTGAAG
TCGTAACAAGGTAGCCGTAGG
GGAACCTGCGGCTGGATCACCTCCTTT
>AB15
CAACTTGAGAGTTTGATCCTGGCTCAGAACGAACGCTGGCGGCAGGCCTAACACATGCAA
GTCGAGCGAACCCTTCGGGG
TTAGCGGCGGACGGGTGAGTAACGCGTGGGAACGTGCCCTTCTCTGCGGAATAGGCTCG
GGAAACTGGGTTTAATACCGC
ATACGCCCTTCGGGGGAAAGATTTATCGGAGAAGGATCGGCCCGCGTTAGATTAGGTAGT
TGGTGGGGTAATGGCCTACC
AAGCCTACGATCTATAGCTGGTTTGAGAGGATGATCAGCCACACTGGGACTGAGACACGG
CCCAGACTCCTACGGGAGGC
AGCAGTGAGGAATCTTGGACAATGGGGGAAACCCTGATCCAGCCATGCCGCGTGAGCGAT
GAAGGCCTTAGGGTTGTAAA
GCTCTTTCAGTCGTGAAGATAATGACGGTAGCGACAGAAGAAGCCCCGGCTAACTCCGTG
CCAGCAGCCGCGGTAATACG
GAGGGGGCTAGCGTTGTTCGGAATTACTGGGCGTAAAGCGCGCGTAGGCGGACTATTAAG
TCAGGGGTGAAATCCCGGGG
CTCAACCCCGGAACTGCCTTTGATACTGGTAGTCTAGAGTTCGAGAGAGGTGAGTGGAATT
CCGAGTGTAGAGGTGAAAT
TCGTAGATATTCGGAGGAACACCAGTGGCGAAGGCGGCTCACTGGCTCGATACTGACGCT
GAGGTGCGAAAGCGTGGGGA
GCAAACAGGATTAGATACCCTGGTAGTCCACGCCGTAAACGATGAATGCCAGTCGTCGGC
AAGCATGCTTGTCGGTGACA
CACCTAACGGATTAAGCATTCCGCCTGGGGAGTACGGCCGCAAGGTTAAAACTCAAAGGA
ATTGACGGGGGCCCGCACAA
GCGGTGGAGCATGTGGTTTAATTCGAAGCAACGCGCAGAACCTTACCAACCCTTGACATC
CTGATCGCGGTTTTCCGAGA
GGAATTCCTTCAGTTCGGCTGGATCAGTGACAGGTGCTGCATGGCTGTCGTCAGCTCGTG
TCGTGAGATGTTCGGTTAAG
TCCGGCAACGAGCGCAACCCACACTCTTAGTTGCCAGCATTCAGTTGGGCACTCTAGGAG
AACTGCCGATGATAAGTCGG
AGGAAGGTGTGGATGACGTCAAGTCCTCATGGCCCTTACGGGTTGGGCTACACACGTGCT
ACAATGGCAGTGACAATGGG
TTAATCCCTAAAAGCTGTCTCAGTTCGGATTGTTCTCTGCAACTCGAGAGCATGAAGTCGG
AATCGCTAGTAATCGCGTA
ACAGCATGACGCGGTGAATACGTTCCCGGGCCTTGTACACACCGCCCGTCACACCATGGG
AGTTGGGTTTACCCGAAGAC
GGTGCGCCAACCTTTACGGGGGGCAGCTGGCCACGGTAAGCTCAGCGACTGGGGTGAAG
TCGTAACAAGGTAGCCGTAGG
GGAACCTGCGGCTGGATCACCTCCTTT
>AB33

CAACTTGAGAGTTTGATCCTGGCTCAGAACGAACGCTGGCGGCAGGCCTAACACATGCAA
GTCGAGCGAACCCTTCGGGG
TTAGCGGCGGACGGGTGAGTAACGCGTGGGAACGTGCCCTTCTCTGCGGAATAGGCTCG
GGAAACTGGGTTTAATACCGC
ATACGCCCTTCGGGGGAAAGATTTATCGGAGAAGGATCGGCCCGCGTTAGATTAGGTAGT
TGGTGGGGTAATGGCCTACC
AAGCCTACGATCTATAGCTGGTTTGAGAGGATGATCAGCCACACTGGGACTGAGACACGG
CCCAGACTCCTACGGGAGGC
AGCAGTGAGGAATCTTGGACAATGGGGGAAACCCTGATCCAGCCATGCCGCGTGAGCGAT
GAAGGCCTTAGGGTTGTAAA
GCTCTTTCAGTCGTGAAGATAATGACGGTAGCGACAGAAGAAGCCCCGGCTAACTCCGTG
CCAGCAGCCGCGGTAATACG
GAGGGGGCTAGCGTTGTTCGGAATTACTGGGCGTAAAGCGCGCGTAGGCGGACTATTAAG
TCAGGGGTGAAATCCCGGGG
CTCAACCCCGGAACTGCCTTTGATACTGGTAGTCTAGAGTTCGAGAGAGGTGAGTGGAATT
CCGAGTGTAGAGGTGAAAT
TCGTAGATATTCGGAGGAACACCAGTGGCGAAGGCGGCTCACTGGCTCGATACTGACGCT
GAGGTGCGAAAGCGTGGGGA
GCAAACAGGATTAGATACCCTGGTAGTCCACGCCGTAAACGATGAATGCCAGTCGTCGGC
AAGCATGCTTGTCGGTGACA
CACCTAACGGATTAAGCATTCCGCCTGGGGAGTACGGCCGCAAGGTTAAAACTCAAAGGA
ATTGACGGGGGCCCGCACAA
GCGGTGGAGCATGTGGTTTAATTCGAAGCAACGCGCAGAACCTTACCAACCCTTGACATC
CTGATCGCGGTTTTCCGAGA
GGAATTCCTTCAGTTCGGCTGGATCAGTGACAGGTGCTGCATGGCTGTCGTCAGCTCGTG
TCGTGAGATGTTCGGTTAAG
TCCGGCAACGAGCGCAACCCACACTCTTAGTTGCCAGCATTCAGTTGGGCACTCTAGGAG
AACTGCCGATGATAAGTCGG
AGGAAGGTGTGGATGACGTCAAGTCCTCATGGCCCTTACGGGTTGGGCTACACACGTGCT
ACAATGGCAGTGACAATGGG
TTAATCCCTAAAAACTGTCTCAGTTCGGATTGTTCTCTGCAACTCGAGAGCATGAAGTCGG
AATCGCTAGTAATCGCGTA
ACAGCATGACGCGGTGAATACGTTCCCGGGCCTTGTACACACCGCCCGTCACACCATGGG
AGTTGGGTTTACCCGAAGAC
GGTGCGCCAACCCTTACGGGGGGCAGCTGGCCACGGTAAGCTCAGCGACTGGGGTGAAG
TCGTAACAAGGTAGCCGTAGG
GGAACCTGCGGCTGGATCACCTCCTTT
>2_4_1
CAACTTGAGAGTTTGATCCTGGCTCAGAATGAACGCTGGCGGCAGGCCTAACACATGCAA
GTCGAGCGAAGTCTTCGGAC
TTAGCGGCGGACGGGTGAGTAACGCGTGGGAACGTGCCCTTTGCTTCGGAATAGCCCCG
GGAAACTGGGAGTAATACCGA
ATGTGCCCTTTGGGGGAAAGATTTATCGGCAAAGGATCGGCCCGCGTTGGATTAGGTAGT
TGGTGGGGTAATGGCCTACC
AAGCCGACGATCCATAGCTGGTTTGAGAGGATGATCAGCCACACTGGGACTGAGACACGG
CCCAGACTCCTACGGGAGGC
AGCAGTGGGGAATCTTAGACAATGGGCGCAAGCCTGATCTAGCCATGCCGCGTGATCGAT
GAAGGCCTTAGGGTTGTAAA
GATCTTTCAGGTGGGAAGATAATGACGGTACCACCAGAAGAAGCCCCGGCTAACTCCGTG
CCAGCAGCCGCGGTAATACG

GAGGGGGCTAGCGTTATTCGGAATTACTGGGCGTAAAGCGCACGTAGGCGGATCGGAAA
GTCAGAGGTGAAATCCCAGGG
CTCAACCCTGGAACTGCCTTTGAAACTCCCGATCTTGAGGTCGAGAGAGGTGAGTGGAATT
CCGAGTGTAGAGGTGAAAT
TCGTAGATATTCGGAGGAACACCAGTGGCGAAGGCGGCTCACTGGCTCGATACTGACGCT
GAGGTGCGAAAGCGTGGGGA
GCAAACAGGATTAGATACCCTGGTAGTCCACGCCGTAAACGATGAATGCCAGTCGTCGGG
CAGCATGCTGTTCGGTGACA
CACCTAACGGATTAAGCATTCCGCCTGGGGAGTACGGCCGCAAGGTTAAAACTCAAAGGA
ATTGACGGGGGCCCGCACAA
GCGGTGGAGCATGTGGTTTAATTCGAAGCAACGCGCAGAACCTTACCAACCCTTGACATG
GCGATCGCGGTTCCAGAGAT
GGTTCCTTCAGTTCGGCTGGATCGCACACAGGTGCTGCATGGCTGTCGTCAGCTCGTGTC
GTGAGATGTTCGGTTAAGTC
CGGCAACGAGCGCAACCCACGTCCTTAGTTGCCAGCATTCAGTTGGGCACTCTAGGGAAA
CTGCCGGTGATAAGCCGGAG
GAAGGTGTGGATGACGTCAAGTCCTCATGGCCCTTACGGGTTGGGCTACACACGTGCTAC
AATGGCAGTGACAATGGGTT
AATCCCAAAAAGCTGTCTCAGTTCGGATTGGGGTCTGCAACTCGACCCCATGAAGTCGGAA
TCGCTAGTAATCGCGTAAC
AGCATGACGCGGTGAATACGTTCCCGGGCCTTGTACACACCGCCCGTCACACCATGGGAA
TTGGTTCTACCCGAAGGCGG
TGCGCCAACCTCGCAAGAGGAGGCAGCCGACCACGGTAGGATCAGTGACTGGGGTGAAG
TCGTAACAAGGTAGCCGTAGG
GGAACCTGCGGCTGGATCACCTCCTTT
>TIE1
CAACTTGAGAGTTTGATCCTGGCTCAGAGCGAACGCTGGCGGCAGGCTTAACACATGCAA
GTCGAACGGGCGTAGCAATA
CGTCAGTGGCAGACGGGTGAGTAACGCGTGGGAACGTACCTTTTGGTTCGGAACAACACA
GGGAAACTTGTGCTAATACC
GGATAAGCCCTTACGGGGAAAGATTTATCGCCGAAAGATCGGCCCGCGTCTGATTAGCTA
GTTGGTGAGGTAATGGCTCA
CCAAGGCGACGATCAGTAGCTGGTCTGAGAGGATGATCAGCCACATTGGGACTGAGACAC
GGCCCAAACTCCTACGGGAG
GCAGCAGTGGGGAATATTGGACAATGGGGGAAACCCTGATCCAGCCATGCCGCGTGAGT
GATGAAGGCCCTAGGGTTGTA
AAGCTCTTTTGTGCGGGAAGATAATGACGGTACCGCAAGAATAAGCCCCGGCTAACTTCGT
GCCAGCAGCCGCGGTAATA
CGAAGGGGGCTAGCGTTGCTCGGAATCACTGGGCGTAAAGGGTGCGTAGGCGGGTTTCT
AAGTCAGAGGTGAAAGCCTGG
AGCTCAACTCCAGAACTGCCTTTGATACTGGAAGTCTTGAGTATGGCAGAGGTGAGTGGAA
CTGCGAGTGTAGAGGTGAA
ATTCGTAGATATTCGCAAGAACACCAGTGGCGAAGGCGGCTCACTGGGCCATTACTGACG
CTGAGGCACGAAAGCGTGGG
GAGCAAACAGGATTAGATACCCTGGTAGTCCACGCCGTAAACGATGAATGCCAGCCGTTA
GTGGGTTTACTCACTAGTGG
CGCAGCTAACGCTTTAAGCATTCCGCCTGGGGAGTACGGTCGCAAGATTAAAACTCAAAG
GAATTGACGGGGGCCCGCAC
AAGCGGTGGAGCATGTGGTTTAATTCGACGCAACGCGCAGAACCTTACCAGCCCTTGACA
TGTCCAGGACCGGTCGCAGA

GACGCGACCTTCTCTTCGGAGCCTGGAGCACAGGTGCTGCATGGCTGTCGTCAGCTCGTG
TCGTGAGATGTTGGGTTAAG
TCCCGCAACGAGCGCAACCCCCGTCCTTAGTTGCTACCATTTAGTTGAGCACTCTAAGGAG
ACTGCCGGTGATAAGCCGC
GAGGAAGGTGGGGATGACGTCAAGTCCTCATGGCCCTTACGGGCTGGGCTACACACGTG
CTACAATGGCGGTGACAATGG
GAAGCTAAGGGGCGACCCTTCGCAAATCTCAAAAAGCCGTCTCAGTTCGGATTGGGCTCT
GCAACTCGAGCCCATGAAGT
TGGAATCGCTAGTAATCGTGGATCAGCATGCCACGGTGAATACGTTCCCGGGCCTTGTAC
ACACCGCCCGTCACACCATG
GGAGTTGGCTTTACCTGAAGACGGTGCGCTAACCAGCAATGGGGGCAGCCGGCCACGGT
AGGGTCAGCGACTGGGGTGAA
GTCGTAACAAGGTAGCCGTAGGGGAACCTGCGGCTGGATCACCTCCTTT
>geobactersulfurreducens
AAACTGGAGAGTTTGATCCTGGCTCAGAACGAACGCTGGCGGCGTGCCTAACACATGCAA
GTCGAACGTGATCCATCCTT
CGGGGTGGTGAAAGTGGCGCACGGGTGAGTAACGCGTGGATAATCTGCCCGAGGATTTG
GGATAACATCTCGAAAGGGGT
GCTAATACCGAATAAGCCCACGGGGTCTACGGATCTTGCGGGAAAAGGGGGGGACTTTCG
GGCCTCCTGTCTTCGGATGA
GTCCGCGTACCATTAGCTAGTTGGTAGGGTAATGGCCTACCAAGGCGACGATGGTTAGCT
GGTCTGAGAGGATGATCAGC
CACACTGGAACTGAGACACGGTCCAGACTCCTACGGGAGGCAGCAGTGGGGAATTTTGCG
CAATGGGGGAAACCCTGACG
CAGCAACGCCGCGTGGGTGATGAAGGCCTTCGGGTCGTAAAGCCCTGTCGGGAGGGAAG
AAATGATTGAGAGCTAATACC
TCTTGGTCTTGACGGTACCTCCGAAGGAAGCACCGGCTAACTCCGTGCCAGCAGCCGCGG
TAATACGGAGGGTGCAAGCG
TTGTTCGGAATTATTGGGCGTAAAGCGCGTGTAGGCGGTCTTTTAAGTCTGATGTGAAAGC
CCCGGGCTCAACCTGGGAA
GTGCATTGGAAACTGGGAGACTTGAGTACGGGAGAGGAGAGTGGAATTCCTAGTGTAGGA
GTGAAATCCGTAGATATTAG
GAGGAACACCGGTGGCGAAGGCGGCTCTCTGGACCGATACTGACGCTGAGACGCGAAAG
CGTGGGTAGCAAACAGGATTA
GATACCCTGGTAGTCCACGCCGTAAACGATGAGTACTAGGTGTTGCGGGTATTGACCCCT
GCAGTGCCGTAGCTAACGCA
TTAAGTACTCCGCCTGGGAAGTACGGTCGCAAGACTAAAACTCAAAGGAATTGACGGGGG
CCCGCACAAGCGGTGGAGCA
TGTGGTTTAATTCGACGCAACGCGCAGAACCTTACCTGGGCTTGACATCCACGGAACCCTC
CCGAAACGGAGGGGTGCCC
TTCGGGGAGCCGTGAGACAGGTGCTGCATGGCTGTCGTCAGCTCGTGTCGTGAGATGTTG
GGTTAAGTCCCGCAACGAGC
GCAACCCTTATCATCAGTTGCCATCATTAAGTTGGGCACTCTGATGAAACTGCCGGTGTCA
AACCGGAGGAAGGTGGGGA
TGACGTCAAGTCCTCATGGCCCTTATGTCCAGGGCTACACACGTGCTACAATGGCCGGTA
CAAAGAGTCGCAATGCCGCG
AGGTGGAGCCAATCTCAAAAAGCCGGTCTCAGTTCGGATTGGAGTCTGCAACTCGACTCC
ATGAAGTCGGAATCGCTAGT
AATCGCGGATCAGCATGCCGCGGTGAATACGTTCCCGGGCCTTGTACACACCGCCCGTCA
CACCACGGGAGTCGACTGGT

CCCGAAGTGCGTGAGCTAACCAGCAATGGAGGCAGCGTCCTAAGGAATGGTCGGTGACT
GGGGTGAAGTCGTAACAAGGT
AGCCGTAGGGGAACCTGCGGCTGGATCACCTCCTTT
>Pseudomonas_aeruginosa
GAACTGAAGAGTTTGATCATGGCTCAGATTGAACGCTGGCAGCAGGGCCTTCAACACAT
GCAAGTCGAG
CTTATGAAGGGAGCTTGCCTTGGATTCAGCGGCGGACGGGTGAGTAATGCCTAGGAATCT
GCCTGGTAGT
GGGGGATAACGTCCGGAAACGGCCGCTAATACCGCATACGTCCTGAGGGAGAAAGTCGG
GGATCTTCGGA
CCTCACGCTATCAGATGAGCCTAGGTCGGATTAGCTAGTTGGTGGGGTAAAGGCCTACCA
AGGCGACGAT
CCGTAACTGGTCTGAGAGGATGATCAGTCACACTGGAACTGAGACACGGTCCAGACTCCT
ACGGGAGGCA
GCAGTGGGGAATATTGGACAATGGGCGCAAGCCTGATCCAGCCATGCCGCGTGTGTGAA
GAAGGTCTTCG
GATTGTAAAGCACTTTAAGTTGGGAGGAAGGGCAGTAAGTTAATACCTTGCTGTTTGACGT
TACCAACAG
AATAAGCACCGGCTAACTTCGTGCCAGCAGCCGCGGTAATACGAAGGGTGCAAGCGTTAA
TCGGAATTAC
TGGGCGTAAAGCGCGCGTAAGTGGTTCAGCAAGCTTGATGTGAAATCCCCGGGCTCAACC
TGGGAACTGC
ATCCAAAAGCTACTGAGCTAGAGTACGGTAGAGGTGGTAGAATTTCCTGTGTAGCGGTGAA
ATGCGTAGA
TATAGGAAGGAACACCAGTGGCGAAGGCGACCACCTGGACTGTACTGACACTGAGGTGCG
AAAGCGTGGG
GAGCAAACAGGATTAGATACCCTGGTAGTCCACGCCGTAAACGATGTCGACTAGCCGTTG
GGATCCTTGA
GATCTTAGTGGCGCACGTAACGCGATAAGTCGACCGCCTGGGGAGTACGGCCGCAAGGT
TAAAACTCAAA
TGAATTGACGGGGGCCCGCACAAGCGGTGGAGCATGTGGTTTAATTCGAAGCAACGCGAA
GAACCTTACC
TGGCCTTGACATGCTGAGAACTTTCCAGAGATGGATTGGTGCCTTCGGGAACAGAGACAC
AGGTGCTGCA
TGGCTGTCGTCAGCTCGTGTCGTGAGATGTTGGGTTAAGTCCCGTAACGAGCGCAACCCT
TGTCCTTAGT
TACCAGCACCTCGGGTGGGCACTCTAAGGAGACTGCCGGTGACAAACCGGAGGAAGGTG
GGGATGACGTC
AAGTCATCATGGCCCTTACGGCCAGGGCTACACACGTGCTACAATGGTCGGTACAAAGGG
TTGCCAAGCC
GCGAGTGGGAGCTAATCCCATAAAACCGATCGTAGTCCGGATCGCAGTCTGCAACTCGAC
TGCGTGAAGT
CGGAATCGCTAGTAATCGTGAATCAGAATGTCACGGTGAATACGTCCCCGGGCCTTGTACA
CACCGCCCG
TCACACCATGGGAGTGGGTTGCTCCAGAAGTAGCTAGTCTAACCGCAAGGGGGACGGTTA
CCACGGAGTG
ATTCATGACTGGGGTGAAGTCGTAACAAGGTAGCCGTAGGGGAACCTGCGGCTGGATCAC
CTCCTTA
>Nitrosococcus_oceani
AGGAGGTGATCCAGCCGCAGGTTCCCCTACGGCTACCTTGTTACGACTTCACCCCAGTCA
TTGACCACAC

CGTAGTAAGCGTCCTCCCGAAGGTTAAACTACCCACTTCTGGTGCAACCAACTCCCATGGT
GTGACGGGC
GGTGTGTACAAGGCCCGGGAACGTATTCACCGCGGCATTGCTGATCCGCGATTACTAGCG
ATTCCGACTT
CATGCAGTCGAGTTGCAGACTGCAATCCGGACTACGACCGGTTTTCTGAGATTAGCTCCG
CATCGCTGCT
TGGCCACCCTCTGTACCGGCCATTGTAGCACGTGTGTAGCCCTGGCCATAAGGGCCATGA
TGACTTGACG
TCATCCCCACCTTCCTCCGTTTTGTCAACGGCAGTCCCTCTAGAGTGCCCGGCCGAACCG
CTGGCAACTA
GAGATAAGGGTTGCGCTCGTTGCGGGACTTAACCCAACATCTCACGACACGAGCTGACGA
CAGCCATGCA
GCACCTGTCTCCCGGCTCCCGAAGGCACCTTCGCCTCTCAGCAAAGTTCCGGGGATGTCA
AGGCCAGGTA
AGGTTCTTCGCGTTGCATCGAATTAAACCACATGCTCCACCGCTTGTGCGGGCCCCCGTC
AATTCATTTG
AGTTTCAACCTTGCGGCCGTACTCCCCAGGCGGAGAACTTAACGCGTTAGCTGCAACACT
GAAAGGCTAT
CCCTCCCAACGTCTAGTTCTCATCGTTTAGGGCGTGGACTACCAGGGTATCTAATCCTGTT
TGCTCCCCA
CGCTTTCGCACCTCAGCGTCAGTAGTGGTCCAGGAGGCCGCCTTCGCCACTGGTGTTCCT
TCCGATATCT
ACGCATTTCACCGCTACACCGGAAATTCCGCCTCCCTCTACCATACTCTAGCCAGGCAGTA
TCAAGTGCC
ATTCCCAGGTTGAGCCCGGGGCTTTCACACCCAACTTACCAAACCGCCTACGCGCCCTTTA
CGCCCAGTA
ATTCCGATTAACGCTCGCACCCTCCGTATTACCGCGGCTGCTGGCACGGAGTTAGCCGGT
GCTTCTTCTG
TAGGTAACGTCAACACGATGTGCTATTCACACATCGCTTTTCTTCCCCACTGAAAGGGCTTT
ACAACCCG
AAAGCCTTCTTCACCCACGCGGCATTGCTGGATCAGGGTTGCCCCCATTGTCCAATATTCC
CCACTGCTG
CCTCCCGTAGGAGTCTGGGCCGTGTCTCAGTCCCAGTGTGGCTGATCGTCCTCTCAGACC
AGCTACCGAT
CATCGCCTTGGTAGGCCCTTACCCCACCAACTAGCTAATCGGACGCAGGCTCATCCCCCA
GCGCGAGGCC
TTTCGGTCCCCCGCTTTCCTCCTTAGAGATTATGCGGTATTAGCCCGAGTTTCCCCGAGTT
ATCCCCCTC
TAGAGGCCAGATTCCCACGCGTTACTCACCCGTCCGCCACTCGCCAGCCACCTAAGCAAG
CTTAGGTGCT
GCTGCCGTTCGACTTGCATGTGTTAAGCATGCCGCCAGCGTTCAATCTGAGCCATGATCAA
ACTCTTCAG
TTTAA
>Rhodovulum_visakhapatnamense_JA181
GGCTCAGAACGAACGCTGGCGGCASGCCTAACCATGCAASTCGAGCGAACCCTTCGGGG
TTAGCGGCGGACGGGTGASTA
ACGCGTGGGAACGTGCCCTTCTCTGCGGGATAGGCTCGGGAAACTGGGTTTAATACCGCA
TACGCCCTTCGGGGGAAAGA
TTTATCGGAGAAGGATCGGCCCGCGTCTGATTAGGTAGTTGGTGGGGTAATGGCCTACCA
AGCCTACGATCAGTAGCTGG

TTTGAGAGGATGATCAGCCACACTGGGACTGAGACACGGCCCAGACTCCTACGGGAGGCA
GCAGTGAGGAATCTTGGACA
ATGGGGGAAACCCTGATCCAGCCATGCCGCGTGAGCGATGAAGGCCTTAGGGTTGTAAAG
CTCTTTCAGTCGTGAAGATA
ATGACGGTAGCGACAGAAGAAGCCCCGGCTAAYTCCGTGCCAGCAGCCGCGGTAATACG
GAGGGGGCTAGCGTTGTTCGG
AATTACTGGGCGTAAAGCGCGCGTAGGCGGACTATTAAGTCGGGGGTGAAATCCCGGGG
CTCAACCCCGGAACTGCCTCC
GATACTGGTAGTCTAGAGTTCGAGAGAGGTGAGTGGAATTCCGAGTGTAGAGGTGAAATT
CGTAGATATTCGGAGGAACA
CCAGTGGCGAAGGCGGCTCACTGGCTCGATACTGACGCTGAGGTGCGAAAGCGTGGGGA
GCAAACAGGATTAGATACCCT
GGTAGTCCACGCCGTAAACGGATGAATGCCAGTCGTCGGCAAGCATGCTTGTCGGTGACA
CACCTAACGGAYTAAGCRYT
CCGCCTGGGGAGTACGGCCGCAAGGTTAAAACTCAAAGGAACTGACGGGGGCCCGCACA
AGCCGGTGGAGCATGTGGTTT
AATTCGAAGCAACGCGCAGAACCTTACCAACCCTTGACATCCTGATCGCGGTTTTCCGAGA
GGAATTCCTTCAGTTCGGC
TTGGATCAGTTGACAGGTGCTTGCATGGCTTGTCGTCAGCTCGTGTCGTGAGATGTTCGGT
TAAGTCCGGCAACGAGCGC
AACCCACACTCTTAGTTGCCAGCATTCAGTTTGGGCACTCTAAGAGAACTGCCGATGATAA
GTCGGAGGAAGGTGTGGAT
GACGTCAAGTCCTCATGGCCCTTACGGGTTGGGCTACACACGTGCTACAATGGCAGTGAC
AATGGGTTAATCCCAAAAAA
CTGTCTCAGTTCGGATTGTTCTCTGCAACTCGAGAGCATGAAGTCGGAATCGCTAGTAATC
GCGTAACAGCATGACGCGG
TGAATACGTTCCCGGGCCTTGTACACACCGCCCGTCACACCATGGGAGTTGGGTTTACCC
GAAGACGGTGCGCCAACCCT
TACGGGGGGCAGGGGCCACGGTAKGTCAGCGRTGGGTGAG
